# Measuring progress from 1990 to 2017 and projecting attainment to 2030 of the health-related Sustainable Development Goals for 195 countries and territories: a systematic analysis for the Global Burden of Disease Study 2017

**DOI:** 10.1016/S0140-6736(18)32281-5

**Published:** 2018-11-10

**Authors:** GBD 2017 SDG Collaborators, Rafael Lozano, Nancy Fullman, Degu Abate, Solomon M Abay, Cristiana Abbafati, Nooshin Abbasi, Hedayat Abbastabar, Foad Abd-Allah, Jemal Abdela, Ahmed Abdelalim, Omar Abdel-Rahman, Alireza Abdi, Ibrahim Abdollahpour, Rizwan Suliankatchi Abdulkader, Nebiyu Dereje Abebe, Zegeye Abebe, Ayenew Negesse Abejie, Semaw F Abera, Olifan Zewdie Abil, Victor Aboyans, Haftom Niguse Abraha, Aklilu Roba Abrham, Laith Jamal Abu-Raddad, Niveen Me Abu-Rmeileh, Gebre Y Abyu, Manfred Mario Kokou Accrombessi, Dilaram Acharya, Pawan Acharya, Abdu A Adamu, Oladimeji M Adebayo, Isaac Akinkunmi Adedeji, Rufus Adesoji Adedoyin, Victor Adekanmbi, Olatunji O Adetokunboh, Beyene Meressa Adhena, Tara Ballav Adhikari, Mina G Adib, Arsène Kouablan Adou, Jose C Adsuar, Mohsen Afarideh, Mahdi Afshari, Ashkan Afshin, Gina Agarwal, Sargis Aghasi Aghayan, Dominic Agius, Anurag Agrawal, Sutapa Agrawal, Alireza Ahmadi, Mehdi Ahmadi, Hamid Ahmadieh, Muktar Beshir Ahmed, Sayem Ahmed, Temesgen Yihunie Akalu, Ali S Akanda, Mohammad Esmaeil Akbari, Mohammed Akibu, Rufus Olusola Akinyemi, Tomi Akinyemiju, Nadia Akseer, Fares Alahdab, Ziyad Al-Aly, Khurshid Alam, Tahiya Alam, Ammar Albujeer, Animut Alebel, Kefyalew Addis Alene, Ayman Al-Eyadhy, Samia Alhabib, Raghib Ali, Mehran Alijanzadeh, Reza Alizadeh-Navaei, Syed Mohamed Aljunid, Ala'a Alkerwi, François Alla, Peter Allebeck, Christine A Allen, Ali Almasi, Fatma Al-Maskari, Hesham M Al-Mekhlafi, Jordi Alonso, Rajaa M Al-Raddadi, Ubai Alsharif, Khalid Altirkawi, Nelson Alvis-Guzman, Azmeraw T Amare, Kebede Amenu, Erfan Amini, Walid Ammar, Nahla Hamed Anber, Jason A Anderson, Catalina Liliana Andrei, Sofia Androudi, Megbaru Debalkie Animut, Mina Anjomshoa, Hossein Ansari, Ansariadi Ansariadi, Mustafa Geleto Ansha, Carl Abelardo T Antonio, Palwasha Anwari, Lambert Tetteh Appiah, Olatunde Aremu, Habtamu Abera Areri, Johan Ärnlöv, Monika Arora, Krishna K Aryal, Hamid Asayesh, Ephrem Tsegay Asfaw, Solomon Weldegebreal Asgedom, Rana Jawad Asghar, Reza Assadi, Zerihun Ataro, Suleman Atique, Sachin R Atre, Madhu Sudhan Atteraya, Marcel Ausloos, Leticia Avila-Burgos, Euripide F G A Avokpaho, Ashish Awasthi, Beatriz Paulina Ayala Quintanilla, Henok Tadesse Ayele, Yohanes Ayele, Rakesh Ayer, Mahmoud Reza Azarpazhooh, Peter S Azzopardi, Natasha Azzopardi-Muscat, Tesleem Kayode Babalola, Arefeh Babazadeh, Hamid Badali, Alaa Badawi, Kalpana Balakrishnan, Ayele Geleto Bali, Maciej Banach, Amitava Banerjee, Joseph Adel Mattar Banoub, Amrit Banstola, Aleksandra Barac, Miguel A Barboza, Suzanne Lyn Barker-Collo, Till Winfried Bärnighausen, Lope H Barrero, Celine M Barthelemy, Quique Bassat, Arindam Basu, Sanjay Basu, Robert J Battista, Bernhard T Baune, Habtamu Wondifraw Baynes, Shahrzad Bazargan-Hejazi, Neeraj Bedi, Ettore Beghi, Masoud Behzadifar, Meysam Behzadifar, Yannick Béjot, Bayu Begashaw Bekele, Abate Bekele Belachew, Aregawi Gebreyesus Belay, Saba Abraham Belay, Yihalem Abebe Belay, Michelle L Bell, Aminu K Bello, Derrick A Bennett, Isabela M Bensenor, Habib Benzian, Adugnaw Berhane, Abadi Kidanemariam Berhe, Adam E Berman, Eduardo Bernabe, Robert S Bernstein, Gregory J Bertolacci, Mircea Beuran, Tina Beyranvand, Neeraj Bhala, Ashish Bhalla, Anil Bhansali, Suraj Bhattarai, Soumyadeep Bhaumik, Zulfiqar A Bhutta, Belete Biadgo, Molly H Biehl, Ali Bijani, Boris Bikbov, Nigus Bililign, Muhammad Shahdaat Bin Sayeed, Sait Mentes Birlik, Charles Birungi, Donal Bisanzio, Tuhin Biswas, Helen Bitew, Hailemichael Bizuneh, Espen Bjertness, Eshetu Mulisa Bobasa, Soufiane Boufous, Rupert Bourne, Kayvan Bozorgmehr, Nicola Luigi Bragazzi, Michael Brainin, Luisa C Brant, Michael Brauer, Alexandra Brazinova, Nicholas J K Breitborde, Paul Svitil Briant, Gabrielle Britton, Traolach Brugha, Gene Bukhman, Reinhard Busse, Zahid A Butt, Lucero Cahuana-Hurtado, Charlton SKH Callender, Ismael R Campos-Nonato, Julio Cesar Campuzano Rincon, Jorge Cano, Josip Car, Mate Car, Rosario Cárdenas, Juan J Carrero, Austin Carter, Félix Carvalho, Carlos A Castañeda-Orjuela, Jacqueline Castillo Rivas, Franz Castro, Kate Causey, Alanur Çavlin, Kelly M Cercy, Ester Cerin, Yazan Chaiah, Julian Chalek, Hsing-Yi Chang, Jung-Chen Chang, Aparajita Chattopadhyay, Vijay Kumar Chattu, Pankaj Chaturvedi, Peggy Pei-Chia Chiang, Ken Lee Chin, Vesper Hichilombwe Chisumpa, Abdulaal Chitheer, Jee-Young J Choi, Rajiv Chowdhury, Hanne Christensen, Devasahayam J Christopher, Sheng-Chia Chung, Flavia M Cicuttini, Liliana G Ciobanu, Massimo Cirillo, Rafael M Claro, Thomas Khaled Dwayne Claßen, Aaron J Cohen, Daniel Collado-Mateo, Cyrus Cooper, Leslie Trumbull Cooper, Leslie Cornaby, Monica Cortinovis, Megan Costa, Ewerton Cousin, Elizabeth A Cromwell, Christopher Stephen Crowe, Matthew Cunningham, Alemneh Kabeta Daba, Abel Fekadu Dadi, Lalit Dandona, Rakhi Dandona, Anh Kim Dang, Paul I Dargan, Ahmad Daryani, Siddharth K Das, Rajat Das Gupta, José das Neves, Tamirat Tesfaye Dasa, Aditya Prasad Dash, Adrian C Davis, Dragos Virgil Davitoiu, Kairat Davletov, Anand Dayama, Barbora de Courten, Diego De Leo, Jan-Walter De Neve, Hans De Steur, Meaza Girma Degefa, Louisa Degenhardt, Tizta Tilahun Degfie, Selina Deiparine, Robert P Dellavalle, Gebre Teklemariam Demoz, Balem Demtsu, Edgar Denova-Gutiérrez, Kebede Deribe, Nikolaos Dervenis, Getenet Ayalew Dessie, Subhojit Dey, Samath D Dharmaratne, Meghnath Dhimal, Daniel Dicker, Mesfin Tadese Dinberu, Eric L Ding, Shirin Djalalinia, Huyen Phuc Do, Klara Dokova, David Teye Doku, Dirk Douwes-Schultz, Tim Robert Driscoll, Leilei Duan, Manisha Dubey, Eleonora Dubljanin, Eyasu Ejeta Duken, Bruce B Duncan, Andre R Duraes, Soheil Ebrahimpour, David Edvardsson, Charbel El Bcheraoui, Erika Eldrenkamp, Ziad El-Khatib, Iqbal RF Elyazar, Ahmadali Enayati, Aman Yesuf Endries, Babak Eshrati, Sharareh Eskandarieh, Alireza Esteghamati, Sadaf Esteghamati, Kara Estep, Mahdi Fakhar, Hamed Fakhim, Jessica Fanzo, Mahbobeh Faramarzi, Mohammad Fareed, Farzaneh Farhadi, Talha A Farid, Carla Sofia e Sá Farinha, Andrea Farioli, Andre Faro, Maryam S Farvid, Farshad Farzadfar, Mohammad Hosein Farzaei, Hossein Farzam, Ali Akbar Fazaeli, Mir Sohail Fazeli, Valery L Feigin, Andrea B Feigl, Wubalem Fekadu, Rachel Feldman, Netsanet Fentahun, Seyed-Mohammad Fereshtehnejad, Eduarda Fernandes, Joao C Fernandes, Garumma Tolu Feyissa, Daniel Obadare Fijabi, Irina Filip, Samuel Finegold, Jonas David Finger, Florian Fischer, Christina Fitzmaurice, Luisa Sorio Flor, Nataliya A Foigt, Kyle J Foreman, Tahvi D Frank, Richard Charles Franklin, Takeshi Fukumoto, Kai Fukutaki, John E Fuller, Thomas Fürst, João M Furtado, Emmanuela Gakidou, Silvano Gallus, Fortune Gbetoho Gankpe, Ron T Gansevoort, Ana Cristina Garcia, Alberto L Garcia-Basteiro, Miguel A Garcia-Gordillo, William M Gardner, Abadi Kahsu Gebre, Teshome Gebre, Gebremedhin Berhe Gebregergs, Tsegaye Tewelde Gebrehiwot, Amanuel Tesfay Gebremedhin, Bereket Gebremichael, Teklu Gebrehiwo Gebremichael, Tilayie Feto Gelano, Johanna M Geleijnse, Yilma Chisha Dea Geramo, Sefonias Getachew, Peter W Gething, Kebede Embaye Gezae, Mohammad Rasoul Ghadami, Reza Ghadimi, Keyghobad Ghadiri, Maryam Ghasemi-Kasman, Hesam Ghiasvand, Mamata Ghimire, Aloke Gopal Ghoshal, Simona Giampaoli, Paramjit Singh Gill, Tiffany K Gill, Giorgia Giussani, Elena V Gnedovskaya, Ellen M Goldberg, Srinivas Goli, Philimon N Gona, Amador Goodridge, Sameer Vali Gopalani, Taren M Gorman, Atsushi Goto, Alessandra C Goulart, Bárbara Niegia Garcia Goulart, Ayman Grada, Max G Griswold, Giuseppe Grosso, Harish Chander C Gugnani, Francis Guillemin, Andre Luiz Sena Guimaraes, Yuming Guo, Prakash C Gupta, Rahul Gupta, Rajeev Gupta, Tanush Gupta, Giang Hai Ha, Juanita A Haagsma, Vladimir Hachinski, Nima Hafezi-Nejad, Hassan Haghparast Bidgoli, Tekleberhan B Hagos, Michael Tamene Haile, Tewodros Tesfa Hailegiyorgis, Gessessew Bugssa Hailu, Arvin Haj-Mirzaian, Arya Haj-Mirzaian, Randah R Hamadeh, Samer Hamidi, Graeme J Hankey, Hilda L Harb, Sivadasanpillai Harikrishnan, Hamidreza Haririan, Josep Maria Haro, Mehedi Hasan, Hadi Hassankhani, Hamid Yimam Hassen, Rasmus Havmoeller, Caitlin N Hawley, Simon I Hay, Yihua He, Akbar Hedayatizadeh-Omran, Mohamed I Hegazy, Behzad Heibati, Behnam Heidari, Mohsen Heidari, Delia Hendrie, Andualem Henok, Ileana Heredia-Pi, Claudiu Herteliu, Behzad Heydarpour, Fatemeh Heydarpour, Sousan Heydarpour, Desalegn T Hibstu, Martha Híjar, Hans W Hoek, Daniel J Hoffman, Michael K Hole, Enayatollah Homaie Rad, Praveen Hoogar, Nobuyuki Horita, H Dean Hosgood, Seyed Mostafa Hosseini, Mehdi Hosseinzadeh, Mihaela Hostiuc, Sorin Hostiuc, Peter J Hotez, Damian G Hoy, Mohamed Hsairi, Thomas Hsiao, Guoqing Hu, John J Huang, Caitlyn Hughes, Chantal K Huynh, Ehimario U Igumbor, Chad Thomas Ikeda, Olayinka Stephen Ilesanmi, Usman Iqbal, Seyed Sina Naghibi Irvani, Caleb Mackay Salpeter Irvine, Sheikh Mohammed Shariful Islam, Farhad Islami, Rebecca Q Ivers, Neda Izadi, Kathryn H Jacobsen, Leila Jahangiry, Nader Jahanmehr, Sudhir Kumar Jain, Mihajlo Jakovljevic, Moti Tolera Jalu, Amr A Jamal, Spencer L James, Simerjot K Jassal, Mehdi Javanbakht, Achala Upendra Jayatilleke, Panniyammakal Jeemon, Ravi Prakash Jha, Vivekanand Jha, John S Ji, Catherine O Johnson, Sarah C Johnson, Jost B Jonas, Jitendra Jonnagaddala, Zahra Jorjoran Shushtari, Ankur Joshi, Jacek Jerzy Jozwiak, Suresh Banayya Jungari, Mikk Jürisson, Madhanraj K, Zubair Kabir, Rajendra Kadel, Amaha Kahsay, Molla Kahssay, Rizwan Kalani, Umesh Kapil, Manoochehr Karami, Behzad Karami Matin, Marina Karanikolos, Narges Karimi, Seyed M Karimi, Hamidreza Karimi-Sari, Amir Kasaeian, Dessalegn H Kassa, Getachew Mullu Kassa, Tesfaye Dessale Kassa, Zemenu Yohannes Kassa, Nicholas J Kassebaum, Srinivasa Vittal Katikireddi, Anil Kaul, Norito Kawakami, Zhila Kazemi, Ali Kazemi Karyani, Dhruv Satish Kazi, Prakash KC, Seifu Kebede, Peter Njenga Keiyoro, Laura Kemmer, Grant Rodgers Kemp, Andre Pascal Kengne, Andre Keren, Chandrasekharan Nair Kesavachandran, Yousef Saleh Khader, Behzad Khafaei, Morteza Abdullatif Khafaie, Alireza Khajavi, Nauman Khalid, Ibrahim A Khalil, Ejaz Ahmad Khan, Muhammad Shahzeb Khan, Muhammad Ali Khan, Young-Ho Khang, Tripti Khanna, Mona M Khater, Alireza Khatony, Zahra Khazaeipour, Habibolah Khazaie, Abdullah T Khoja, Ardeshir Khosravi, Mohammad Hossein Khosravi, Jagdish Khubchandani, Aliasghar A Kiadaliri, Helen W Kiarie, Getiye D Kibret, Daniel N Kiirithio, Daniel Kim, Jun Y Kim, Young-Eun Kim, Yun Jin Kim, Ruth W Kimokoti, Yohannes Kinfu, Sanjay Kinra, Adnan Kisa, Katarzyna Kissimova-Skarbek, Niranjan Kissoon, Mika Kivimäki, Jonathan M Kocarnik, Sonali Kochhar, Yoshihiro Kokubo, Tufa Kolola, Jacek A Kopec, Margaret N Kosek, Soewarta Kosen, Parvaiz A Koul, Ai Koyanagi, Michael A Kravchenko, Kewal Krishan, Kristopher J Krohn, Barthelemy Kuate Defo, Burcu Kucuk Bicer, Andreas A Kudom, Xie Rachel Kulikoff, G Anil Kumar, Manasi Kumar, Pushpendra Kumar, Michael J Kutz, Hmwe Hmwe Kyu, Carl Lachat, Deepesh P Lad, Sheetal D Lad, Alessandra Lafranconi, Abraham K Lagat, Dharmesh Kumar Lal, Ratilal Lalloo, Hilton Lam, Faris Hasan Lami, Prabhat Lamichhane, Qing Lan, Justin J Lang, Van C Lansingh, Sonia Lansky, Heidi J Larson, Anders O Larsson, Dennis Odai Laryea, Zohra S Lassi, Arman Latifi, Kathryn Mei-Ming Lau, Avula Laxmaiah, Jeffrey V Lazarus, Janet L Leasher, Georgy Lebedev, Jorge R Ledesma, James B Lee, Paul H Lee, Andrew T Leever, James Leigh, Mall Leinsalu, Cheru Tesema Leshargie, Janni Leung, Sonia Lewycka, Shanshan Li, Xiaohong Li, Yichong Li, Juan Liang, Xiaofeng Liang, Misgan Legesse Liben, Lee-Ling Lim, Miteku Andualem Limenih, Shai Linn, Shiwei Liu, Yang Liu, Rakesh Lodha, Giancarlo Logroscino, Alan D Lopez, Stefan Lorkowski, Paulo A Lotufo, Lydia R Lucchesi, Ronan A Lyons, Erlyn Rachelle King Macarayan, Mark T Mackay, Emilie R Maddison, Fabiana Madotto, Dhaval P Maghavani, Carlos Magis-Rodriguez, Narayan Bahadur Mahotra, Marek Majdan, Reza Majdzadeh, Azeem Majeed, Reza Malekzadeh, Deborah Carvalho Malta, Abdullah A Mamun, Ana-Laura Manda, Luiz Garcia Mandarano-Filho, Srikanth Mangalam, Helena Manguerra, Mohammad Ali Mansournia, Chabila Christopher Mapoma, Joemer C Maravilla, Wagner Marcenes, Ashley Marks, Randall V Martin, Sheila C O Martins, Francisco Rogerlândio Martins-Melo, Ira Martopullo, Tivani Phosa Mashamba-Thompson, Benjamin Ballard Massenburg, Manu Raj Mathur, Pallab K Maulik, Mohsen Mazidi, Colm McAlinden, John J McGrath, Martin McKee, Brian J McMahon, Suresh Mehata, Man Mohan Mehndiratta, Ravi Mehrotra, Kala M Mehta, Varshil Mehta, Fabiola Mejia-Rodriguez, Tesfa Mekonen, Tefera C Chane Mekonnen, Hagazi Gebre Meles, Addisu Melese, Mulugeta Melku, Peter T N Memiah, Ziad A Memish, Walter Mendoza, Desalegn Tadese Mengistu, Getnet Mengistu, George A Mensah, Gert B M Mensink, Seid Tiku Mereta, Atte Meretoja, Tuomo J Meretoja, Tomislav Mestrovic, Haftay Berhane Mezgebe, Bartosz Miazgowski, Tomasz Miazgowski, Anoushka I Millear, Ted R Miller, Molly Katherine Miller-Petrie, George J Milne, G K Mini, Shawn P Minnig, Parvaneh Mirabi, Mojde Mirarefin, Erkin M Mirrakhimov, Awoke Temesgen Misganaw, Philip B Mitchell, Babak Moazen, Ali Akbar Moghadamnia, Bahram Mohajer, Karzan Abdulmuhsin Mohammad, Moslem Mohammadi, Noushin Mohammadifard, Mousa Mohammadnia-Afrouzi, Mohammed A Mohammed, Shafiu Mohammed, Murali B V Mohan, Viswanathan Mohan, Farnam Mohebi, Modhurima Moitra, Ali H Mokdad, Mariam Molokhia, Lorenzo Monasta, Julio Cesar Montañez, Mahmood Moosazadeh, Ghobad Moradi, Mahmoudreza Moradi, Maziar Moradi-Lakeh, Mehdi Moradinazar, Paula Moraga, Lidia Morawska, Joana Morgado-da-Costa, Naho Morisaki, Shane Douglas Morrison, Abbas Mosapour, Marilita M Moschos, W Cliff Mountjoy-Venning, Simin Mouodi, Seyyed Meysam Mousavi, Achenef Asmamaw Muche, Kindie Fentahun Muchie, Ulrich Otto Mueller, Oumer Sada S Muhammed, Satinath Mukhopadhyay, Erin C Mullany, Kate Muller, John Everett Mumford, Manoj Murhekar, G V S Murthy, Srinivas Murthy, Jonah Musa, Kamarul Imran Musa, Ghulam Mustafa, Saravanan Muthupandian, Ashraf F Nabhan, Jean B Nachega, Ahamarshan Jayaraman Nagarajan, Gabriele Nagel, Mohsen Naghavi, Aliya Naheed, Azin Nahvijou, Kovin Naidoo, Gurudatta Naik, Nitish Naik, Farid Najafi, Luigi Naldi, Hae Sung Nam, Vinay Nangia, Jobert Richie Nansseu, Bruno Ramos Nascimento, Haseeb Nawaz, Nahid Neamati, Ionut Negoi, Ruxandra Irina Negoi, Subas Neupane, Charles Richard James Newton, Frida N Ngalesoni, Josephine W Ngunjiri, Anh Nguyen, Grant Nguyen, Ha Nguyen, Huong Lan Thi Nguyen, Huong Thanh Nguyen, Minh Nguyen, Emma Nichols, Solomon Gedlu Nigatu, Dina Nur Anggraini Ningrum, Yirga Legesse Nirayo, Muhammad Imran Nisar, Molly R Nixon, Nomonde Nolutshungu, Marika Nomura, Ole F Norheim, Mehdi Noroozi, Bo Norrving, Jean Jacques Noubiap, Hamid Reza Nouri, Malihe Nourollahpour Shiadeh, Mohammad Reza Nowroozi, Peter S Nyasulu, Carla Makhlouf Obermeyer, Richard Ofori-Asenso, Okechukwu Samuel Ogah, Felix Akpojene Ogbo, In-Hwan Oh, Anselm Okoro, Kelechi E Oladimeji, Olanrewaju Oladimeji, Andrew T Olagunju, Tinuke O Olagunju, Pedro R Olivares, Helen Elizabeth Olsen, Bolajoko Olubukunola Olusanya, Jacob Olusegun Olusanya, Kanyin L Ong, Sok King Ong, Anu Mary Oommen, John Nelson Opio, Eyal Oren, Andrei Oros, Doris D V Ortega-Altamirano, Alberto Ortiz, Justin R Ortiz, Eduardo Ortiz-Panozo, Erika Ota, Stanislav S Otstavnov, Mayowa Ojo Owolabi, Mahesh P A, Smita Pakhale, Abhijit P Pakhare, Wen-Harn Pan, Adrian Pana, Basant Kumar Panda, Songhomitra Panda-Jonas, Jeyaraj Durai Pandian, Nikolaos Papantoniou, Eun-Kee Park, Charles D H Parry, Hadi Parsian, Shanti Patel, Sanghamitra Pati, Ajay Patle, George C Patton, Vishnupriya Rao Paturi, Deepak Paudel, Katherine R Paulson, Neil Pearce, Emmanuel K Peprah, David M Pereira, Norberto Perico, Aslam Pervaiz, Konrad Pesudovs, William A Petri, Max Petzold, Michael R Phillips, David M Pigott, Julian David Pillay, Meghdad Pirsaheb, Martin Pletcher, Constance Dimity Pond, Maarten J Postma, Akram Pourshams, Hossein Poustchi, Dorairaj Prabhakaran, Swayam Prakash, Narayan Prasad, Caroline A Purcell, Manita Pyakurel, Mostafa Qorbani, Reginald Quansah, Amir Radfar, Anwar Rafay, Alireza Rafiei, Fakher Rahim, Kazem Rahimi, Afarin Rahimi-Movaghar, Vafa Rahimi-Movaghar, Mahfuzar Rahman, Md Shafiur Rahman, Mohammad Hifz Ur Rahman, Muhammad Aziz Rahman, Sajjad ur Rahman, Rajesh Kumar Rai, Fatemeh Rajati, Sasa Rajsic, Usha Ram, Saleem M Rana, Chhabi Lal Ranabhat, Prabhat Ranjan, Davide Rasella, David Laith Rawaf, Salman Rawaf, Christian Razo-García, K Srinath Reddy, Robert C Reiner, Cesar Reis, Marissa B Reitsma, Giuseppe Remuzzi, Andre M N Renzaho, Serge Resnikoff, Luz Myriam Reynales-Shigematsu, Satar Rezaei, Shahab Rezaeian, Mohammad Sadegh Rezai, Seyed Mohammad Riahi, Antonio Luiz P Ribeiro, Maria Jesus Rios-Blancas, Kedir Teji Roba, Nicholas L S Roberts, Leonardo Roever, Luca Ronfani, Gholamreza Roshandel, Ali Rostami, Gregory A Roth, Ambuj Roy, Enrico Rubagotti, George Mugambage Ruhago, Yogesh Damodar Sabde, Perminder S Sachdev, Basema Saddik, Ehsan Sadeghi, Hosein Safari, Yahya Safari, Roya Safari-Faramani, Mahdi Safdarian, Sare Safi, Saeid Safiri, Rajesh Sagar, Amirhossein Sahebkar, Mohammad Ali Sahraian, Haniye Sadat Sajadi, Nasir Salam, Joseph S Salama, Payman Salamati, Raphael de Freitas Saldanha, Zikria Saleem, Yahya Salimi, Hamideh Salimzadeh, Joshua A Salomon, Sundeep Santosh Salvi, Inbal Salz, Evanson Zondani Sambala, Abdallah M Samy, Juan Sanabria, Maria Dolores Sanchez-Niño, Itamar S Santos, Milena M Santric Milicevic, Bruno Piassi Sao Jose, Mayank Sardana, Abdur Razzaque Sarker, Nizal Sarrafzadegan, Benn Sartorius, Shahabeddin Sarvi, Brijesh Sathian, Maheswar Satpathy, Miloje Savic, Arundhati R Sawant, Monika Sawhney, Sonia Saxena, Mete Saylan, Mehdi Sayyah, Elke Schaeffner, Maria Inês Schmidt, Ione J C Schneider, Ben Schöttker, Aletta Elisabeth Schutte, David C Schwebel, Falk Schwendicke, Soraya Seedat, Mario Sekerija, Sadaf G Sepanlou, Edson Serván-Mori, Seyedmojtaba Seyedmousavi, Hosein Shabaninejad, Katya Anne Shackelford, Azadeh Shafieesabet, Amira A Shaheen, Masood Ali Shaikh, Mehran Shams-Beyranvand, Mohammad Bagher Shamsi, Morteza Shamsizadeh, Heidar Sharafi, Kiomars Sharafi, Mehdi Sharif, Mahdi Sharif-Alhoseini, Jayendra Sharma, Rajesh Sharma, Sharad Kumar Sharma, Jun She, Aziz Sheikh, Muki Shehu Shey, Peilin Shi, Kenji Shibuya, Chloe Shields, Girma Temam Shifa, Mekonnen Sisay Shiferaw, Mika Shigematsu, Rahman Shiri, Reza Shirkoohi, Shreya Shirude, Kawkab Shishani, Ivy Shiue, Farhad Shokraneh, Haitham Shoman, Mark G Shrime, Sharvari Rahul Shukla, Si Si, Soraya Siabani, Abla Mehio Sibai, Tariq J Siddiqi, Inga Dora Sigfusdottir, Naris Silpakit, Diego Augusto Santos Silva, João Pedro Silva, Natacha Torres da Silva, Dayane Gabriele Alves Silveira, Jasvinder A Singh, Narinder Pal Singh, Om Prakash Singh, Prashant Kumar Singh, Virendra Singh, Dhirendra Narain Sinha, Eirini Skiadaresi, Karen Sliwa, Amanda E Smith, Mari Smith, Adauto Martins Soares Filho, Badr Hasan Sobaih, Soheila Sobhani, Michael Soljak, Moslem Soofi, Masoud Soosaraei, Reed J D Sorensen, Joan B Soriano, Sergey Soshnikov, Ireneous N Soyiri, Angela Spinelli, Luciano A Sposato, Chandrashekhar T Sreeramareddy, Raghavendra Guru Srinivasan, Vinay Srinivasan, Jeffrey D Stanaway, Vladimir I Starodubov, Vasiliki Stathopoulou, Nadine Steckling, Dan J Stein, Leo G Stewart, Leo Stockfelt, Mark A Stokes, Kurt Straif, Agus Sudaryanto, Mu'awiyyah Babale Sufiyan, Bruno F Sunguya, Patrick John Sur, Ipsita Sutradhar, Bryan L Sykes, P N Sylaja, Dillon O Sylte, Cassandra E I Szoeke, Rafael Tabarés-Seisdedos, Takahiro Tabuchi, Santosh Kumar Tadakamadla, Koku Sisay Tamirat, Nikhil Tandon, Frank C Tanser, Aberash Abay Tassew, Segen Gebremeskel Tassew, Mohammad Tavakkoli, Nuno Taveira, Nega Yimer Tawye, Arash Tehrani-Banihashemi, Tigist Gashaw Tekalign, Merhawi Gebremedhin Tekle, Habtamu Temesgen, Mohamad-Hani Temsah, Omar Temsah, Abdullah Sulieman Terkawi, Manaye Yihune Teshale, Destaw Fetene Teshome, Belay Tessema, Mebrahtu Teweldemedhin, Jarnail Singh Thakur, Kavumpurathu Raman Thankappan, Andrew Theis, Sathish Thirunavukkarasu, Laura Anne Thomas, Nihal Thomas, Alan J Thomson, Amanda G Thrift, Binyam Tilahun, Quyen G To, Ruoyan Tobe-Gai, Marcello Tonelli, Roman Topor-Madry, Anna E Torre, Miguel Tortajada-Girbés, Marcos Roberto Tovani-Palone, Jeffrey A Towbin, Bach Xuan Tran, Khanh Bao Tran, Tung Thanh Tran, Srikanth Prasad Tripathy, Christopher E Troeger, Thomas Clement Truelsen, Afewerki Gebremeskel Tsadik, Lorainne Tudor Car, E Murat Tuzcu, Hayley D Tymeson, Kingsley N Ukwaja, Irfan Ullah, Rachel L Updike, Muhammad Shariq Usman, Olalekan A Uthman, Muthiah Vaduganathan, Afsane Vaezi, Gaurang Vaidya, Pascual R Valdez, Aaron van Donkelaar, Elena Varavikova, Tommi Juhani Vasankari, Narayanaswamy Venketasubramanian, Ramesh Vidavalur, Santos Villafaina, Francesco S Violante, Sergey Konstantinovitch Vladimirov, Vasily Vlassov, Sebastian Vollmer, Stein Emil Vollset, Theo Vos, Kia Vosoughi, Isidora S Vujcic, Gregory R Wagner, Fasil Shiferaw Wagnew, Yasir Waheed, Judd L Walson, Yanping Wang, Yuan-Pang Wang, Molla Mesele Wassie, Elisabete Weiderpass, Robert G Weintraub, Jordan Weiss, Fitsum Weldegebreal, Kidu Gidey Weldegwergs, Andrea Werdecker, Adhena Ayaliew Werkneh, T Eoin West, Ronny Westerman, Joanna L Whisnant, Harvey A Whiteford, Justyna Widecka, Katarzyna Widecka, Tissa Wijeratne, Lauren B Wilner, Andrea Sylvia Winkler, Alison B Wiyeh, Charles Shey Wiysonge, Haileab Fekadu Wolde, Charles D A Wolfe, Shouling Wu, Denis Xavier, Gelin Xu, Rixing Xu, Ali Yadollahpour, Seyed Hossein Yahyazadeh Jabbari, Bereket Yakob, Tomohide Yamada, Lijing L Yan, Yuichiro Yano, Mehdi Yaseri, Yasin Jemal Yasin, Pengpeng Ye, Jamal A Yearwood, Alex Yeshaneh, Ebrahim M Yimer, Paul Yip, Biruck Desalegn Yirsaw, Engida Yisma, Naohiro Yonemoto, Gerald Yonga, Seok-Jun Yoon, Marcel Yotebieng, Mustafa Z Younis, Mahmoud Yousefifard, Chuanhua Yu, Sojib Bin Zaman, Mohammad Zamani, Zohreh Zare, Luis Zavala-Arciniega, Desalegn Tegabu Zegeye, Elias Asfaw Zegeye, Ayalew Jejaw Zeleke, Kazem Zendehdel, Taddese Alemu Zerfu, Anthony Lin Zhang, Xueying Zhang, Maigeng Zhou, Jun Zhu, Stephanie R M Zimsen, Sanjay Zodpey, Leo Zoeckler, Inbar Zucker, Liesel Joanna J Zuhlke, Stephen S Lim, Christopher J L Murray

## Abstract

**Background:**

Efforts to establish the 2015 baseline and monitor early implementation of the UN Sustainable Development Goals (SDGs) highlight both great potential for and threats to improving health by 2030. To fully deliver on the SDG aim of “leaving no one behind”, it is increasingly important to examine the health-related SDGs beyond national-level estimates. As part of the Global Burden of Diseases, Injuries, and Risk Factors Study 2017 (GBD 2017), we measured progress on 41 of 52 health-related SDG indicators and estimated the health-related SDG index for 195 countries and territories for the period 1990–2017, projected indicators to 2030, and analysed global attainment.

**Methods:**

We measured progress on 41 health-related SDG indicators from 1990 to 2017, an increase of four indicators since GBD 2016 (new indicators were health worker density, sexual violence by non-intimate partners, population census status, and prevalence of physical and sexual violence [reported separately]). We also improved the measurement of several previously reported indicators. We constructed national-level estimates and, for a subset of health-related SDGs, examined indicator-level differences by sex and Socio-demographic Index (SDI) quintile. We also did subnational assessments of performance for selected countries. To construct the health-related SDG index, we transformed the value for each indicator on a scale of 0–100, with 0 as the 2·5th percentile and 100 as the 97·5th percentile of 1000 draws calculated from 1990 to 2030, and took the geometric mean of the scaled indicators by target. To generate projections through 2030, we used a forecasting framework that drew estimates from the broader GBD study and used weighted averages of indicator-specific and country-specific annualised rates of change from 1990 to 2017 to inform future estimates. We assessed attainment of indicators with defined targets in two ways: first, using mean values projected for 2030, and then using the probability of attainment in 2030 calculated from 1000 draws. We also did a global attainment analysis of the feasibility of attaining SDG targets on the basis of past trends. Using 2015 global averages of indicators with defined SDG targets, we calculated the global annualised rates of change required from 2015 to 2030 to meet these targets, and then identified in what percentiles the required global annualised rates of change fell in the distribution of country-level rates of change from 1990 to 2015. We took the mean of these global percentile values across indicators and applied the past rate of change at this mean global percentile to all health-related SDG indicators, irrespective of target definition, to estimate the equivalent 2030 global average value and percentage change from 2015 to 2030 for each indicator.

**Findings:**

The global median health-related SDG index in 2017 was 59·4 (IQR 35·4–67·3), ranging from a low of 11·6 (95% uncertainty interval 9·6–14·0) to a high of 84·9 (83·1–86·7). SDG index values in countries assessed at the subnational level varied substantially, particularly in China and India, although scores in Japan and the UK were more homogeneous. Indicators also varied by SDI quintile and sex, with males having worse outcomes than females for non-communicable disease (NCD) mortality, alcohol use, and smoking, among others. Most countries were projected to have a higher health-related SDG index in 2030 than in 2017, while country-level probabilities of attainment by 2030 varied widely by indicator. Under-5 mortality, neonatal mortality, maternal mortality ratio, and malaria indicators had the most countries with at least 95% probability of target attainment. Other indicators, including NCD mortality and suicide mortality, had no countries projected to meet corresponding SDG targets on the basis of projected mean values for 2030 but showed some probability of attainment by 2030. For some indicators, including child malnutrition, several infectious diseases, and most violence measures, the annualised rates of change required to meet SDG targets far exceeded the pace of progress achieved by any country in the recent past. We found that applying the mean global annualised rate of change to indicators without defined targets would equate to about 19% and 22% reductions in global smoking and alcohol consumption, respectively; a 47% decline in adolescent birth rates; and a more than 85% increase in health worker density per 1000 population by 2030.

**Interpretation:**

The GBD study offers a unique, robust platform for monitoring the health-related SDGs across demographic and geographic dimensions. Our findings underscore the importance of increased collection and analysis of disaggregated data and highlight where more deliberate design or targeting of interventions could accelerate progress in attaining the SDGs. Current projections show that many health-related SDG indicators, NCDs, NCD-related risks, and violence-related indicators will require a concerted shift away from what might have driven past gains—curative interventions in the case of NCDs—towards multisectoral, prevention-oriented policy action and investments to achieve SDG aims. Notably, several targets, if they are to be met by 2030, demand a pace of progress that no country has achieved in the recent past. The future is fundamentally uncertain, and no model can fully predict what breakthroughs or events might alter the course of the SDGs. What is clear is that our actions—or inaction—today will ultimately dictate how close the world, collectively, can get to leaving no one behind by 2030.

**Funding:**

Bill & Melinda Gates Foundation.

**Research in context**

**Evidence before this study**

Measuring country progress on the UN's Sustainable Development Goals (SDGs) has been an important international priority since the SDGs were introduced in 2015. The UN, the Sustainable Development Solutions Network, WHO, and the World Bank also report on the SDGs, but their analyses do not consistently measure indicators for each location and year. The Global Burden of Diseases, Injuries, and Risk Factors Study (GBD) 2015 estimated 33 health-related SDG indicators and the overall health-related SDG index from 1990 to 2015 for 188 countries. In GBD 2016, the number of indicators included was expanded to 37, and projections of health-related SDG achievement in 2030 were estimated for the first time. The ability of decision makers, particularly at the national level, to adequately monitor progress on the health-related SDGs and budget and plan for the future is potentially hampered by the scarcity of disaggregated data, such as by subnational unit, sex, and socioeconomic level. Complete estimates of SDG progress at these levels are needed to identify, and target programmes to, the populations that are most at risk of falling behind.

**Added value of this study**

GBD 2017 provides consistent, comparably generated estimates of the health-related SDG indicators for 195 countries and territories from 1990 to 2017. Additionally, GBD 2017 provides, for the first time, estimates of health-related SDGs at the subnational level for select countries and by sex. Newly estimated indicators in GBD 2017 include health worker density per 1000 population (SDG indicator 3.c.1), sexual violence by non-intimate partners (SDG indicator 5.2.2), and population census status (SDG indicator 17.19.2a), as well as disaggregation of SDG indicator 16.1.3 into prevalence of physical violence (SDG indicator 16.1.3a) and sexual violence (SDG indicator 16.1.3c) following the March, 2018, refinements accepted by the UN Statistical Commission. Measurement improvements included reporting on prevalence of current smoking rather than of daily smoking to better align with the UN's definition and internally consistent, systematic estimation of adolescent birth rates within the broader GBD study. We used a forecasting platform that systematically captures the effects of independent drivers of population health into the future to generate projections through 2030. On the basis of past trends, we assessed country-level probabilities of attainment for SDG indicators with defined targets. We also calculated the rates of change required to meet defined SDG targets at the global level from 2015 to 2030, and then compared them to annualised rates of change observed at the country level from 1990 to 2015; this analysis provided a way of benchmarking the pace of progress needed to meet ambitious SDG aims with what the world has achieved in the past. We then applied the mean percentile of the global required rates of change to all SDG indicators, providing a historically grounded foundation to evaluate progress for indicators without explicit targets and the relative feasibility of current ones.

**Implications of all the available evidence**

Most countries were projected to improve their health-related SDG index scores by 2030, although our results revealed gaps in potential progress at and beyond the national level. This information is urgently needed to inform strategies for attaining SDG targets, which for many countries will require rates of progress that are faster than rates achieved in the recent past. Most countries already have national action plans in place for, and are in a better position to meet indicator targets that have origins in, the Millennium Development Goals, whereas the SDGs have not been similarly operationalised in many national policies. In the remaining years of the SDG era, it is crucial that governments and international institutions invest in and implement SDG-related programmes and continue to monitor inequalities in the health-related SDGs within populations to truly deliver on the promise of leaving no one behind.

## Introduction

During the early years of implementation of the UN's Sustainable Development Goals (SDGs), which were adopted in 2015,^1^ various international efforts have sought to galvanise faster progress towards the SDGs' bold aims. A recent example includes WHO's 13th General Programme of Work (GPW13) for 2019–23,^2^ which involves an ambitious agenda of measurable goals and interconnected strategies to ensure healthy lives and wellbeing for people of all ages. The GPW13 has three strategic priorities that will be measured by existing, or composites of existing, SDG indicators: achieving universal health coverage (UHC), addressing health emergencies, and promoting healthier populations.^3^

We are in the third year of the SDG era, and progress towards the world-changing aspirations of the SDGs remains a gradual, ongoing process. Although, for some indicators, many countries have maintained the pace of progress made during the era of the Millennium Development Goals, for other indicators, countries have seen gains slow.^4^, ^5^, ^6^, ^7^ These trends underscore the need to focus existing programmes and policies on the expanded scope of the SDG agenda. For instance, some countries in sub-Saharan Africa and Latin America will need to hasten progress against non-communicable diseases (NCDs) if corresponding SDG targets are to be met, and NCDs are a major component of the GPW13.^8^, ^9^ Although NCD prevention is a UN policy priority,^10^ and many evidence-based policies and programmes exist to target NCDs, substantial implementation gaps remain. *The Lancet* has called for 2018 to be the year for action against NCDs,^9^, ^11^, ^12^ and in the report Time to Deliver,^5^, ^6^ the WHO Independent High-Level Commission on Noncommunicable Diseases declared there is no excuse not to act.

The Global Burden of Diseases, Injuries, and Risk Factors Study (GBD) 2015 was the first GBD effort to measure the health-related SDGs, producing estimates for 33 health-related SDG indicators and generating an overall measure, the health-related SDG index, from 1990 to 2015 for 188 countries.^13^ For GBD 2016, this effort was expanded to include four additional SDG indicators, as well as projections of SDG attainment through 2030 based on past trends.^5^ GBD 2016 also improved methods for measuring UHC service coverage.^5^ Other organisations measure a subset of the health-related SDG indicators, but not consistently across locations and years.^4^, ^6^, ^7^

Although national SDG analyses can be useful for guiding health policy, the most vulnerable populations within countries are still at risk of being left behind. Country-level measures of population health likely mask disparities between and within subnational administrative divisions, particularly in low-income and middle-income countries.^14^, ^15^, ^16^, ^17^, ^18^ National governments need subnational data to inform the localisation of global SDG policies and programmes, allowing decision makers to better target resource allocation and service delivery.^19^, ^20^ In many places, males and females also experience disparate risk exposures and corresponding health outcomes,^21^ yet SDG reports do not typically provide data disaggregated by sex, with the exception of those on smoking prevalence. The Inter-agency and Expert Group on Sustainable Development Goal Indicators has requested that metadata be disaggregated by sex for 19 health-related SDG indicators,^22^ and more detailed data will need to be collected and monitored to assess progress, identify high-risk populations, and develop targeted approaches to prevention and treatment. Although valuable, even data at this level might not be sufficient to capture inequalities underlying macro-level trends. Finally, in the absence of clearly defined targets, progress on several health-related SDG indicators cannot be benchmarked against SDG aims. Target setting is a complex process that requires a delicate balance of technical and political inputs; yet, without established targets, galvanising greater political and financial commitments to address health needs during the SDG era could be challenging.

In this study, we provide updated estimates for 41 of 52 health-related SDG indicators and the overall health-related SDG index for 195 countries and territories. For ten indicators, we compare progress from 1990 to 2017 by sex and by Socio-demographic Index (SDI), a composite measure of overall development. Using past trends, we project global progress and analyse attainment of the health-related SDGs through 2030. These estimates will provide a benchmark against which the feasibility of attaining SDG targets by 2030 can be assessed on the basis of what countries have achieved in the past. Compared with GBD 2016, GBD 2017 includes four additional health-related SDG indicators and improves the measurement of some previously included indicators. This analysis can support global efforts, such as the monitoring of the GPW13, and national-level decision making by international institutions, policy makers, and national governments who implement the health-related SDGs.

## Methods

### Overview of GBD

Each year, the GBD study produces age-specific, sex-specific, and location-specific estimates of all-cause and cause-specific mortality, non-fatal outcomes, overall disease burden (ie, disability-adjusted life-years), and risk factor exposure and attributable burden from 1990 to the current study year.

This analysis of the health-related SDGs is based on GBD 2017 estimates. Broader GBD 2017 methods are described elsewhere,^21^, ^23^, ^24^, ^25^, ^26^, ^27^ while further detail on data sources and estimation approaches used for this analysis are available in appendix 1 (part 1). We used previously established GBD methods to generate indicator-specific estimates for 1990–2017, including the Cause of Death Ensemble model for causes of death,^23^, ^28^ DisMod-MR for many non-fatal causes,^26^, ^29^ and spatiotemporal Gaussian process regression for most risk factor exposures, measures of intervention coverage, and other SDG indicators (eg, well-certified death registration [SDG indicator 17.19.2c]).^21^, ^30^

Each year, GBD includes subnational analyses for a few new countries and continues to provide subnational estimates for countries that were added in previous cycles. Subnational estimation in GBD 2017 includes five new countries (Ethiopia, Iran, New Zealand, Norway, Russia) and countries previously estimated at subnational levels (GBD 2013: China, Mexico, and the UK [regional level]; GBD 2015: Brazil, India, Japan, Kenya, South Africa, Sweden, and the USA; GBD 2016: Indonesia and the UK [local government authority level]). All analyses are at the first level of administrative organisation within each country except for New Zealand (by Māori ethnicity), Sweden (by Stockholm and non-Stockholm), and the UK (by local government authorities). All subnational estimates for these countries were incorporated into model development and evaluation as part of GBD 2017. To meet data use requirements, in this publication we present all subnational estimates excluding those pending publication (Brazil, India, Japan, Kenya, Mexico, Sweden, the UK, and the USA); these results are presented in appendix tables and figures (appendix 2). Subnational estimates for countries with populations larger than 200 million (as measured with our most recent year of published estimates) that have not yet been published elsewhere are presented wherever estimates are illustrated with maps, but are not included in data tables.

The GBD study uses standardised and replicable methods that comply with the Guidelines for Accurate and Transparent Health Estimates Reporting (GATHER).^31^ Analyses were done with R version 3.4.4, Python version 2.7.14, or Stata version 13.1. The entire GBD time series is updated annually with improved methods and data sources, and thus GBD 2017 findings, including the SDG analysis presented here, supersede all previous GBD publications.

### Indicators, definitions, and measurement approaches

The health-related SDG indicators are shown in table 1. GBD 2017 assesses four more indicators than assessed in GBD 2016. The first is health worker density (SDG indicator 3.c.1), which is defined by the UN as health workers per 1000 population, by cadre of health worker. For this analysis, we report estimates for three main groups of health workers: physicians, nurses and midwives, and pharmacists. We used International Standard Classification of Occupations (ISCO) 88 to map cadres of health workers from multiple data sources and coding systems, resulting in comparable and consistently defined groupings of health workers over time and across locations (appendix 1 part 1).

**Table 1:** Health-related goals, targets, and SDG indicators

	**Health-related SDG indicator**	**Indicator definition**	**Currently measured by GBD**	**Further details**	**SDG target**	**SDG target used in this analysis**
**Goal 1: End poverty in all its forms everywhere**						
Target 1.5: By 2030, build the resilience of the poor and those in vulnerable situations and reduce their exposure and vulnerability to climate-related extreme events and other economic, social, and environmental shocks and disasters	Disaster mortality (1.5.1; same as indicators 11.5.1 and 13.1.1)	Death rate due to exposure to forces of nature, per 100 000 population	Yes	Existing datasets do not comprehensively measure missing persons and people affected by natural disasters; we thus report deaths due to exposure to forces of nature	Undefined	..
**Goal 2: End hunger, achieve food security and improved nutrition, and promote sustainable agriculture**						
Target 2.2: By 2030, end all forms of malnutrition, including achieving, by 2025, the internationally agreed targets on stunting and wasting in children younger than 5 years, and address the nutritional needs of adolescent girls, pregnant and lactating women, and older people	Child stunting (2.2.1)	Prevalence of stunting in children younger than 5 years, %	Yes	Stunting is defined as below −2 SDs from the median height-for-age of the WHO reference population. No indicator modifications are required	Eliminate by 2030	≤0·5%
Target 2.2 (as above)	Child wasting (2.2.2a)	Prevalence of wasting in children younger than 5 years, %	Yes	We have separated reporting for indicator 2.2.2 into wasting (2.2.2a) and overweight (2.2.2b). Wasting is defined as below −2 SDs from the median weight-for-height of the WHO reference population	Eliminate by 2030	≤0·5%
Target 2.2 (as above)	Child overweight (2.2.2b)	Prevalence of overweight in children aged 2–4 years, %	Yes	We have separated reporting for indicator 2.2.2 into wasting (2.2.2a) and overweight (2.2.2b). We used the IOTF thresholds because the WHO cutoff at age 5 years can lead to an artificial shift in prevalence estimates when the analysis covers more age groups. Furthermore, considerably more studies use IOTF cutoffs, which allowed us to build a larger database for estimating child overweight	Eliminate by 2030	≤0·5%
**Goal 3: Ensure healthy lives and promote wellbeing for all at all ages**						
Target 3.1: By 2030, reduce the global maternal mortality ratio to less than 70 per 100 000 livebirths	Maternal mortality ratio (3.1.1)	Maternal deaths per 100 000 livebirths in females aged 10–54 years	Yes	No indicator modifications required	Reduce to <70 deaths per 100 000 livebirths by 2030	<70 deaths per 100 000 livebirths
Target 3.1 (as above)	Skilled birth attendance (3.1.2)	Proportion of births attended by skilled health personnel (doctors, nurses, midwives, or country-specific medical staff [eg, clinical officers]), %	Yes	No indicator modifications required	Universal access (100%)	≥99%
Target 3.2: By 2030, end preventable deaths of newborns and children younger than 5 years, with all countries aiming to reduce neonatal mortality to at least as low as 12 per 1000 livebirths and under-5 mortality to at least as low as 25 per 1000 livebirths	Under-5 mortality (3.2.1)	Probability of dying before age 5 years, per 1000 livebirths	Yes	No indicator modifications required	Reduce to 25 deaths per 1000 livebirths or lower by 2030	≤25 deaths per 1000 livebirths
Target 3.2 (as above)	Neonatal mortality (3.2.2)	Probability of dying during the first 28 days of life, per 1000 livebirths	Yes	No indicator modifications required	Reduce to 12 deaths per 1000 livebirths or lower by 2030	≤12 deaths per 1000 livebirths
Target 3.3: By 2030, end the epidemics of AIDS, tuberculosis, malaria, and neglected tropical diseases and combat hepatitis, water-borne diseases, and other communicable diseases	HIV incidence (3.3.1)	Age-standardised rate of new HIV infections per 1000 population	Yes	We report HIV incidence of all populations and in terms of age-standardised rates	Eliminate by 2030	≤0·005 per 1000 population
Target 3.3 (as above)	Tuberculosis incidence (3.3.2)	Age-standardised rate of tuberculosis cases per 100 000 population	Yes	No indicator modifications required	Eliminate by 2030	≤0·5 per 100 000 population
Target 3.3 (as above)	Malaria incidence (3.3.3)	Age-standardised rate of malaria cases per 1000 population	Yes	No indicator modifications required	Eliminate by 2030	≤0·005 per 1000 population
Target 3.3 (as above)	Hepatitis B incidence (3.3.4)	Age-standardised rate of hepatitis B incidence per 100 000 population	Yes	No indicator modifications required	Undefined	..
Target 3.3 (as above)	Neglected tropical diseases prevalence (3.3.5)	Age-standardised prevalence of the sum of 15 neglected tropical diseases, %	Yes	People requiring interventions against neglected tropical diseases is not well defined; thus, this indicator is revised to the sum of the prevalence of 15 neglected tropical diseases currently measured in the GBD study: human African trypanosomiasis, Chagas disease, cystic echinococcosis, cysticercosis, dengue, food-borne trematodiases, Guinea worm disease, intestinal nematode infections, leishmaniasis, leprosy, lymphatic filariasis, onchocerciasis, rabies, schistosomiasis, and trachoma	Eliminate by 2030	≤0·5%
Target 3.4: By 2030, reduce by one-third premature mortality from NCDs through prevention and treatment and promote mental health and wellbeing	NCD mortality (3.4.1)	Age-standardised death rate due to cardiovascular disease, cancer, diabetes, and chronic respiratory disease in populations aged 30–70 years, per 100 000 population	Yes	No indicator modifications required	Reduce by one-third by 2030	Reduce by one-third
Target 3.4 (as above)	Suicide mortality (3.4.2)	Age-standardised death rate due to self-harm, per 100 000 population	Yes	No indicator modifications required	Reduce by one-third by 2030	Reduce by one-third
Target 3.5: Strengthen the prevention and treatment of substance abuse, including narcotic drug abuse and harmful use of alcohol	Substance abuse coverage (3.5.1)	Coverage of treatment interventions (pharmacological, psychosocial, and rehabilitation and aftercare services) for substance use disorders, %	No	Prevalence of specific substance use disorders (opioid, cocaine, amphetamine, and cannabis use disorders), as well as alcohol use disorders, are presently estimated as part of GBD. Efforts to extract and synthesise data on coverage of specific interventions (eg, opioid substitution therapy) are currently in progress as part of the broader GBD study	Undefined	..
Target 3.5 (as above)	Alcohol use (3.5.2)	Risk-weighted prevalence of alcohol consumption, as measured by the SEV for alcohol use, %	Yes	For this indicator, we include three categories of alcohol consumption because national alcohol consumption per capita does not capture the distribution of use. The SEV for alcohol use is based on two primary dimensions: individual-level drinking (current drinkers and lifetime abstainers, and alcohol consumption by age and sex) and population-level consumption (litre per capita of pure alcohol stock). The SEV then weights these categories with their corresponding relative risks, which translates to a risk-weighted prevalence on a scale of 0% (no risk in the population) to 100% (the entire population experiences maximum risk associated with alcohol consumption)	Undefined	..
Target 3.6: By 2020, halve the number of global deaths and injuries from road traffic accidents	Road injury mortality (3.6.1)	Age-standardised death rate due to road injuries, per 100 000 population	Yes	No indicator modifications required	Reduce by one-half by 2020	Reduce by 50%
Target 3.7: By 2030, ensure universal access to sexual and reproductive health-care services, including for family planning, information and education, and the integration of reproductive health into national strategies and programmes	Family planning need met, modern contraception methods (3.7.1)	Proportion of women of reproductive age (15–49 years) who have their need for family planning satisfied with modern methods, %	Yes	No indicator modifications required	Universal access (100%)	≥99%
Target 3.7 (as above)	Adolescent birth rate (3.7.2)	Number of livebirths per 1000 females aged 10–14 years or 15–19 years	Yes	No indicator modifications required	Undefined	..
Target 3.8: Achieve UHC, including financial risk protection, access to quality essential health-care services, and access to safe, effective, quality, and affordable essential medicines and vaccines for all	UHC service coverage index (3.8.1)	Coverage of essential health services, as defined by the UHC service coverage index of nine tracer interventions and risk-standardised death rates or mortality-to-incidence ratios from 32 causes amenable to personal health care	Yes	Tracer interventions included vaccination coverage (coverage of three doses of DPT3, one dose of measles vaccine, and three doses of the oral polio vaccine or inactivated polio vaccine), met need for family planning with modern contraception methods, antenatal care coverage (one visit and four visits), skilled birth attendance coverage, in-facility delivery rates, and coverage of antiretroviral therapy among people living with HIV. The 32 causes amenable to personal health care, which compose the Healthcare Access and Quality Index, included tuberculosis, diarrhoeal diseases, lower respiratory infections, upper respiratory infections, chronic respiratory diseases, diphtheria, whooping cough, tetanus, measles, maternal disorders, neonatal disorders, colon and rectum cancer, non-melanoma skin cancer, breast cancer, cervical cancer, uterine cancer, testicular cancer, Hodgkin lymphoma, leukaemia, rheumatic heart disease, ischaemic heart disease, cerebrovascular disease, hypertensive heart disease, peptic ulcer disease, appendicitis, hernia, gallbladder and biliary diseases, epilepsy, diabetes, chronic kidney disease, congenital heart anomalies, and adverse effects of medical treatment. We then scaled these 41 individual inputs on a scale of 0–100, with 0 reflecting the worst levels observed between 1990 and 2017 and 100 reflecting the best observed during this time. We took the arithmetic mean of these 41 scaled indicators so as to collectively capture a wide range of essential health services pertaining to reproductive, maternal, newborn, and child health; infectious diseases; NCDs; and service capacity and access	Universal access (100%)	≥99%
Target 3.8 (as above)	Financial risk protection (3.8.2)	Proportion of population with large household expenditures on health as a share of total household expenditure or income, %	No	Comprehensive and comparable datasets on household expenditures on health as a fraction of total household expenditure or income are not currently available across all locations and over time. Efforts to quantify incidence of catastrophic health spending, at both 10% and 25% of total expenditure or income, for the full time series and locations included in the GBD study are currently under way	<10% or <25% of total expenditure or income	..
Target 3.9: By 2030, substantially reduce the number of deaths and illnesses from hazardous chemicals and air, water, and soil pollution and contamination	Air pollution mortality (3.9.1)	Age-standardised death rate attributable to household air pollution and ambient air pollution, per 100 000 population	Yes	No indicator modifications required	Undefined	..
Target 3.9 (as above)	WaSH mortality (3.9.2)	Age-standardised death rate attributable to unsafe WaSH, per 100 000 population	Yes	No indicator modifications required	Undefined	..
Target 3.9 (as above)	Poisoning mortality (3.9.3)	Age-standardised death rate due to unintentional poisonings, per 100 000 population	Yes	No indicator modifications required	Undefined	..
Target 3.a: Strengthen the implementation of the WHO Framework Convention on Tobacco Control in all countries, as appropriate	Smoking prevalence (3.a.1)	Age-standardised prevalence of current smoking in populations aged 10 years and older, %	Yes	We report on populations aged 10 years and older	Undefined	..
Target 3.b: Support the research and development of vaccines and medicines for communicable and NCDs that primarily affect developing countries; provide access to affordable essential medicines and vaccines, in accordance with the Doha Declaration on the TRIPS Agreement and Public Health, which affirms the right of developing countries to use to the full the provisions in the Agreement on Trade-Related Aspects of Intellectual Property Rights regarding flexibilities to protect public health, and, in particular, provide access to medicines for all	Vaccine coverage (3.b.1)	Coverage of eight vaccines in target populations, %	Yes	Vaccines included DPT3, both doses of measles vaccine (one dose and two doses, reported separately), polio (three doses), hepatitis B (three doses), *Haemophilus influenzae* type b (three doses), pneumococcal conjugate vaccine (three doses), and rotavirus vaccine (two or three doses). We then used the arithmetic mean of coverage of these eight vaccines to calculate overall vaccine coverage of target populations. For GBD 2017, we made some methodological updates for this measure. We now assess coverage for all eight vaccines for every location-year rather than limiting the aggregate to vaccines expressly included in national vaccine schedules. This revision allows for greater comparability across locations over time and helps to avoid overly penalising countries for introducing and scaling up new vaccines. As a result, we were able to remove the 3 year lag that had previously been used for new vaccine introduction; its original utility was to provide a window in which coverage could be scaled up before it counted towards the aggregate. By replacing all location-year estimates with 0% coverage before a given vaccine's introduction, any amount of scale-up now contributes to improved overall coverage for this indicator. We also now take the arithmetic mean across the eight vaccines rather than the geometric mean to avoid over sensitivity to the 0% estimates for vaccines that have yet to be introduced in a given location-year and to provide a more easily interpretable measure of overall vaccine coverage	Coverage of all target populations (100%)	≥99%
Target 3.b (as above)	Developmental assistance for research and health (3.b.2)	Total net official development assistance to the medical research and basic health sectors	No	Development assistance for health is currently assessed within a comprehensive, comparable analytical framework by source, channel, recipient country, and health focus area from 1990 to 2017; however, funding specifically for medical research (eg, research and development of vaccines and medicines, as described in Target 3.b) is not systematically available across source and recipient countries. Additionally, the appropriate assessment of country-level performance remains unclear (eg, whether countries that receive high levels of developmental assistance for medical research are equivalent, in terms of indicator performance, to countries that disburse high levels of developmental assistance for medical research)	Undefined	..
Target 3.b (as above)	Essential medicines (3.b.3)	Proportion of health facilities that have a core set of relevant essential medicines available and affordable on a sustainable basis, %	No	Across all locations and over time, comparable data on the stocking and stock-out rates of essential medicines for all types of facility (hospitals, primary care facilities, pharmacies, and other health-care outlets) and facility ownership (public, private, and informal) are not currently available. In the absence of robust measures of stock-outs in both the public and private sectors across countries and over time, the measurement strategy for producing comparable results for this indicator is unclear. Furthermore, what should constitute a core set of relevant essential medicines is likely to vary by location based on its epidemiological profile, and thus work is needed to more precisely define what these core sets of relevant essential medicines should be given known disease burden, risk factor profiles, and health risks across countries. Lastly, the proposed indicator stipulates measurement of not simply access to a core set of essential medicines but also access to affordable medicines. No comprehensive and comparable datasets on the status of essential medicine affordability, in addition to their stocks, presently exist	Universal access (100%)	..
Target 3.c: Substantially increase health financing and the recruitment, development, training, and retention of the health workforce in developing countries, especially in least developed countries and small island developing States	Health worker density (3.c.1)	Health worker density per 1000 population, by cadre and summed across cadres	Yes	Three health worker cadres—physicians, nurses and midwives, and pharmacists—currently comprise indicator 3.c.1; they are reported separately and summed across cadres in this study. Cadres are categorised based on International Standard Classification of Occupations 88 codes, against which alternative or earlier classification schemes and codes are systematically mapped to produce comparable and consistent measures of cadres over time and across locations	Undefined	..
Target 3.d: Strengthen the capacity of all countries, in particular developing countries, for early warning, risk reduction, and management of national and global health risks	IHR capacity (3.d.1)	The WHO-recommended measure of IHR capacity and health emergency preparedness is the percentage of 13 core capacities that have been attained at a specific time (IHR core capacity index). The 13 core capacities are: national legislation, policy, and financing; coordination and national focal point communications; surveillance; response; preparedness; risk communication; human resources; laboratory; points of entry; zoonotic events; food safety; chemical events; and radionuclear emergencies	No	Comprehensive and comparable data for all components of the IHR core capacity index, for all locations and over time, are not currently openly available. Self-evaluations have been undertaken by some member states, with a subset followed up with independent assessments via the Joint External Evaluation process. To date, 23 countries have completed this process and made reports fully available out of a total of 43 completed Joint External Evaluations. An additional 30 countries are scheduled for assessment by the end of 2018. As these data become more openly available it might be possible to model regional and temporal trends to obtain estimates for outstanding countries, but this will likely necessitate creating bespoke covariates relating to policy status and types of surveillance system that are not currently reported in the GBD study	Undefined	..
**Goal 5: Achieve gender equality and empower all women and girls**						
Target 5.2: Eliminate all forms of violence against all women and girls in the public and private spheres, including trafficking and sexual and other types of exploitation	Intimate partner violence (5.2.1)	Age-standardised prevalence of ever-partnered women aged 15 years and older who experienced physical or sexual violence by a current or former intimate partner in the past 12 months, %	Yes	Data on exposure to subtypes of violence are not systematically available across locations and over time; we thus report physical or sexual violence by a current or former intimate partner	Eliminate by 2030	≤0·5%
Target 5.2 (as above)	Non-intimate partner violence (5.2.2)	Age-standardised prevalence of women aged 15 years and older who experienced physical or sexual violence by a non-intimate partner in the past 12 months, %	Yes	Data on exposure to subtypes of violence are not systematically available across locations and over time; we thus report physical or sexual violence by a non-intimate partner	Eliminate by 2030	≤0·5%
Target 5.6: Ensure universal access to sexual and reproductive health and reproductive rights as agreed in accordance with the Programme of Action of the International Conference on Population and Development and the Beijing Platform for Action and the outcome documents of their review conferences	Female informed reproductive health (5.6.1)	Proportion of women aged 15–49 years who make their own informed decisions regarding sexual relations, contraceptive use, and reproductive health care, %	No	The proportion of women who make their own informed decisions regarding all three dimensions of this indicator—sexual relations, contraceptive use, and reproductive health care—are included in the Demographic and Health Survey series. Data availability for non-Demographic and Health Survey countries is unclear. The feasibility of measuring this indicator as part of future iterations of the GBD study is being considered	Universal access (100%)	..
Target 5.6 (as above)	Reproductive health equal access (5.6.2)	Number of countries with laws and regulations that guarantee full and equal access to women and men aged 15 years and older to sexual and reproductive health care, information, and education	No	Across all locations and over time, comprehensive and comparable data documenting the status of laws and regulations regarding access to sexual and reproductive health care, information, and education currently do not exist. Compiling the past and current status of such laws and regulations might be possible; however, systematically assessing their depth or intensity, enforcement, and effectiveness in guaranteeing access to reproductive health care, information, and education might be challenging across locations and over time	Universal access (100%)	..
**Goal 6: Ensure availability and sustainable management of water and sanitation for all**						
Target 6.1: By 2030, achieve universal and equitable access to safe and affordable drinking water for all	Water (6.1.1)	Risk-weighted prevalence of populations using unsafe or unimproved water sources, as measured by the SEV for unsafe water, %	Yes	Different types of unsafe water sources have correspondingly different relative risks associated with poor health outcomes; we thus report on the SEV for water, which captures the relative risk of different types of unsafe water sources and then combines them into a risk-weighted prevalence on a scale of 0% (no risk in the population) to 100% (the entire population experiences maximum risk associated with unsafe water)	Universal access to safe water (100%); 0% on the SEV for unsafe water	≤1%
Target 6.2: By 2030, achieve access to adequate and equitable sanitation and hygiene for all and end open defecation, paying special attention to the needs of women and girls and those in vulnerable situations	Sanitation (6.2.1a)	Risk-weighted prevalence of populations using unsafe or unimproved sanitation, as measured by the SEV for unsafe sanitation, %	Yes	We have three mutually exclusive, collectively exhaustive categories for sanitation at the household level: households with piped sanitation (with a sewer connection); households with improved sanitation without sewer connection (pit latrine, ventilated improved latrine, pit latrine with slab, composting toilet), as defined by the Joint Monitoring Programme; and households without improved sanitation (flush toilet that is not piped to sewer or septic tank, pit latrine without a slab or open pit, bucket, hanging toilet or hanging latrine, shared facilities, no facilities), as defined by the Joint Monitoring Programme	Universal access to safe sanitation (100%); 0% on the SEV for unsafe sanitation	≤1%
Target 6.2 (as above)	Hygiene (6.2.1b)	Risk-weighted prevalence of populations without access to a handwashing facility, as measured by the SEV for unsafe hygiene, %	Yes	Access to a handwashing facility was defined as having an observed handwashing station with soap and water available in the household	Universal access to handwashing facility (100%); 0% on the SEV for hygiene	≤1%
Target 6.3: By 2030, improve water quality by reducing pollution, eliminating dumping, minimising the release of hazardous chemicals and materials, halving the amount of untreated wastewater, and substantially increasing recycling and safe reuse globally	Treated wastewater (6.3.1)	Proportion of wastewater safely treated, %. UN Water defines this indicator as the proportion of total wastewater generated by both households (sewage and faecal sludge) and economic activities (based on International Standard Industrial Classification categories) that is safely treated. Although the definition conceptually includes wastewater generated from all economic activities, monitoring will focus on wastewater generated from hazardous industries (as defined by relevant International Standard Industrial Classification categories).	No	Across all locations and over time, comprehensive and comparable data containing information about total wastewater, as generated by both households and non-household entities (however they are defined), and wastewater treatment status do not currently exist. UN Water suggests that there will be sufficient data to generate estimates of global and regional levels of safely treated wastewater by 2018; however, in the absence of more country-level data, it is difficult to determine the representativeness of such global and regional estimates	Halve the proportion of untreated wastewater	..
**Goal 7: Ensure access to affordable, reliable, sustainable, and modern energy for all**						
Target 7.1: By 2030, ensure universal access to affordable, reliable, and modern energy services	Household air pollution (7.1.2)	Risk-weighted prevalence of household air pollution, as measured by the SEV for household air pollution, %	Yes	Existing datasets do not comprehensively measure population use of clean fuels and technology for heating and lighting across locations; we thus report on the exposure to clean (or unclean) fuels used for cooking	Universal access to improved fuels (100%); 0% on the SEV for household air pollution	≤1%
**Goal 8: Promote sustained, inclusive, and sustainable economic growth; full and productive employment; and decent work for all**						
Target 8.8: Protect labour rights and promote safe and secure working environments for all workers, including migrant workers, in particular women migrants, and those in precarious employment	Occupational risk burden (8.8.1)	Age-standardised all-cause DALY rates attributable to occupational risks, per 100 000 population	Yes	This indicator is reported as DALY rates attributable to occupational risks because DALYs combine measures of mortality and non-fatal outcomes into a single summary measure, and occupational risks represent the full range of safety hazards that might be encountered in working environments	Undefined	..
**Goal 11: Make cities and human settlements inclusive, safe, resilient, and sustainable**						
Target 11.5: By 2030, significantly reduce the number of deaths and the number of people affected and substantially decrease the direct economic losses relative to global gross domestic product caused by disasters, including water-related disasters, with a focus on protecting the poor and people in vulnerable situations	Disaster mortality (11.5.1; same as indicators 1.5.1 and 13.1.1)	Death rate due to exposure to forces of nature, per 100 000 population	Yes	Existing datasets do not comprehensively measure missing people and people affected by natural disasters; we thus report on deaths due to exposure to forces of nature	Undefined	..
Target 11.6: By 2030, reduce the adverse per-capita environmental impact of cities, including by paying special attention to air quality and municipal and other waste management	Mean PM_2·5_ (11.6.2)	Population-weighted mean levels of fine particulate matter smaller than 2·5 μg in diameter (PM_2·5_), μg/m^3^	Yes	No indicator modifications required	Undefined	..
**Goal 13: Take urgent action to combat climate change and its impacts**						
Target 13.1: Strengthen resilience and adaptive capacity to climate-related hazards and natural disasters in all countries	Disaster mortality (13.1.1; same as indicators 1.5.1 and 11.5.1)	Death rate due to exposure to forces of nature, per 100 000 population	Yes	Existing datasets do not comprehensively measure missing people and people affected by natural disasters; we thus report on deaths due to exposure to forces of nature	Undefined	..
**Goal 16. Promote peaceful and inclusive societies for sustainable development; provide access to justice for all; and build effective, accountable, and inclusive institutions at all levels**						
Target 16.1: Significantly reduce all forms of violence and related death rates everywhere	Homicide (16.1.1)	Age-standardised death rate due to interpersonal violence, per 100 000 population	Yes	No indicator modifications required	Undefined	..
Target 16.1 (as above)	Conflict mortality (16.1.2)	Death rate due to conflict and terrorism, per 100 000 population	Yes	No indicator modifications required	Undefined	..
Target 16.1 (as above)	Physical violence (16.1.3a)	Age-standardised prevalence of physical violence experienced by populations in the past 12 months, %	Yes	No indicator modifications required	Undefined	..
Target 16.1 (as above)	Psychological violence (16.1.3b)	Age-standardised prevalence of psychological violence experienced by populations in the past 12 months, %	No	Indicator 16.1.3 involves three separate types of violence experienced by populations: physical, psychological, and sexual. Current data availability allows for reporting of physical and sexual violence as part of the GBD study, whereas substantial challenges remain for the measurement of psychological violence across locations, by sex, and over time. These include issues with self-report and recall periods; non-standard classifications and reporting of types of psychological violence; and overall minimal data availability on psychological violence, particularly among males	Undefined	..
Target 16.1 (as above)	Sexual violence (16.1.3c)	Age-standardised prevalence of sexual violence experienced by populations in the past 12 months, %	Yes	No indicator modifications required	Undefined	..
Target 16.1 (as above)	Safety walking alone (16.1.4)	Proportion of people who feel safe walking alone around the area in which they live, %	No	The Gallup World Poll, which is currently active in more than 140 countries, includes questions about reported safety while walking alone near one's residence. Pending data sharing and access to currently available data, this indicator will be included in future iterations of the GBD study	Undefined	..
Target 16.2: End abuse, exploitations, trafficking, and all forms of violence against and torture of children	Child sex abuse (16.2.3)	Age-standardised prevalence of women and men aged 18–29 years who experienced sexual violence by age 18 years, %	Yes	No indicator modifications required	Eliminate by 2030	≤0·5%
Target 16.9: By 2030, provide legal identity for all, including birth registration	Birth registration (16.9.1; same as indicator 17.19.2b)	Proportion of children younger than 5 years whose births have been registered with a civil authority, by age, %	No	Currently, birth registration data reported to WHO do not fully cover all locations or years under analysis, and supplementary data sources, such as household survey data, are often required to estimate births and birth rates outside of high-income regions. Substantive data collation efforts would be required for birth registration by location and over time	Universal coverage (100%)	..
**Goal 17: Strengthen the means of implementation and revitalise the global partnership for sustainable development**						
Target 17.19: By 2030, build on existing initiatives to develop measurements of progress on sustainable development that complement gross domestic product, and support statistical capacity building in developing countries	Population census (17.19.2a)	Population census status within the past 10 years	Yes	Indicator 17.19.2 involves three separate country-level components pertaining to demographic and health data collection and monitoring: status of conducting at least one population and housing census in the past 10 years, birth registration, and death registration. Although these data collection and monitoring systems are interconnected, their actual status or functionality at a given time can vary. Thus, we have separated reporting on 17.19.2 into three indicators. For indicator 17.19.2a, census status was ascertained according to whether a population and housing census was conducted within the past 10 years for a given location-year or a population registry had been established. Census implementation was cross-checked against the World Population and Housing Census Programme online database	Census conducted within the past 10 years	..
Target 17.19 (as above)	Birth registration (17.19.2b; same as indicator 16.9.1)	Proportion of countries that have achieved 100% birth registration, %	No	Indicator 17.19.2 involves three separate country-level components pertaining to demographic and health data collection and monitoring: status of conducting at least one population and housing census in the past 10 years, birth registration, and death registration. Currently, birth registration data reported to WHO do not fully cover all locations or years under analysis, and supplementary data sources, such as household survey data, are often required to estimate births and birth rates outside of high-income regions. Substantive data collation efforts would be required for birth registration by location and over time	Universal coverage (100%)	..
Target 17.19 (as above)	Well-certified death registration (17.19.2c)	Percentage of well-certified deaths by a vital registration system among a country's total deaths, %	Yes	Indicator 17.19.2 involves three separate country-level components pertaining to demographic and health data collection and monitoring: status of conducting at least one population and housing census in the past 10 years, birth registration, and death registration. Although these data collection and monitoring systems are interconnected, their actual status or functionality at a given time can vary. Thus, we have separated reporting on 17.19.2 into three indicators. For indicator 17.19.2c, well-certified deaths were determined by three measures: completeness of death registration, fraction of deaths not assigned to major garbage codes (ie, causes that cannot or should not be underlying causes of death), and fraction of deaths assigned to detailed GBD causes	80% of total deaths	≥80%

Detailed descriptions of the data and methods used to estimate each of the 41 health-related SDG indicators included in the GBD 2017 study are located in appendix 1. For the 11 indicators currently not measured by GBD, additional information about data and measurement needs are provided in this table. DALY=disability-adjusted life-year. DPT=diphtheria-pertussis-tetanus. GBD=Global Burden of Diseases, Injuries, and Risk Factors Study. IHR=International Health Regulations. IOTF=International Obesity Task Force. NCDs=non-communicable diseases. PM_2·5_=fine particulate matter smaller than 2·5 μm. SDG=Sustainable Development Goal. SEV=summary exposure value. TRIPS=World Trade Organization Agreement on Trade-Related Aspects of Intellectual Property Rights. UHC=universal health coverage. WaSH=water, sanitation, and hygiene.

The second new indicator is sexual violence by non-intimate partners (SDG indicator 5.2.2), which is defined as the prevalence of females aged 15 years and older who have been subjected to sexual violence by non-intimate partners in the past 12 months. The third is the separate reporting of the prevalence of physical and sexual violence (SDG indicator 16.1.3). In March, 2018, the UN Statistical Commission approved refinements to SDG indicator 16.1.3, such that the indicator is now defined as the “proportion of population subjected to (a) physical violence, (b) psychological violence, and (c) sexual violence in the previous 12 months”.^32^, ^33^ Following the GBD precedent of measuring each component of an SDG indicator (eg, reporting separately on child wasting and overweight [SDG indicators 2.2.2a and 2.2.2b] and on sanitation and access to handwashing facilities [SDG indicators 6.2.1a and 6.2.1b]),^5^, ^13^ we report the prevalence of physical violence and that of sexual violence separately. Owing to measurement challenges and data sparsity, we did not measure the prevalence of psychological violence.

The final new indicator is population census status (SDG indicator 17.19.2a), which was defined as covered if a location had conducted a population and housing census within the past 10 years or had an established population registry that routinely captured nationally representative demographic information (appendix 1 part 1). To assess population census status, we used data compiled for GBD 2017 population estimates,^24^ as well as all available data on population census implementation since 1980 and documentation of population registries.

As well as adding new indicators, we have improved the measurement of several previously reported indicators. For smoking prevalence (SDG indicator 3.a.1), we now report prevalence of current smoking (daily and occasional smokers) rather than only daily smoking to better align with the UN's definition (appendix 1 part 1). For vaccine coverage (SDG indicator 3.b.1), we include all eight vaccines in the aggregate measure for each location-year, rather than limiting the aggregate to vaccines expressly included in national vaccine schedules. Additionally, we now take the arithmetic, rather than the geometric, mean across the eight vaccines. These revisions allow better comparability across locations over time, avoid inadvertently penalising countries for introducing and scaling up new vaccines, and provide a better reflection of overall vaccine coverage for target populations.

The UHC service coverage index includes nine measures of coverage for a subset of interventions for communicable diseases and maternal and child health and the 32 causes that comprise the Healthcare Access and Quality (HAQ) Index (appendix 1 part 1). The HAQ Index is an overall measure of health-care access and quality based on risk-standardised death rates or mortality-to-incidence ratios from causes amenable to health care.^34^ Following updated HAQ methods,^34^ we used mortality-to-incidence ratios for cancers rather than risk-standardised death rates for the UHC service coverage index to better approximate access to quality cancer care. Considerable updates were made to measurement of adolescent birth rate (SDG indicator 3.7.2), which was based on comprehensive estimates of population and fertility from GBD 2017,^24^ as well as of fatal discontinuities (mortality due to natural disasters or conflict and terrorism), among other indicators. Further detail can be found in appendix 1 (part 1) and accompanying GBD 2017 papers.^21^, ^23^, ^24^, ^25^, ^26^, ^27^

We report estimates for all health-related SDG indicators with both sexes combined and sex-specific estimates for HIV incidence (SDG indicator 3.3.1), tuberculosis incidence (SDG indicator 3.3.2), hepatitis B incidence (SDG indicator 3.3.4), NCD mortality (SDG indicator 3.4.1), suicide mortality (SDG indicator 3.4.2), alcohol use (SDG indicator 3.5.2), road injury mortality (SDG indicator 3.6.1), poisoning mortality (SDG indicator 3.9.3), smoking prevalence (SDG 3.a.1), and homicide (SDG indicator 16.1.1). We selected indicators for sex-specific analysis according to the availability of GBD sex-specific data and the utility of presenting sex-specific data by indicator.

We used SDI,^35^ a composite measure of overall development based on rescaled values of fertility, education, and income, to compare performance on the health-related SDGs across quintiles of overall development. For GBD 2017, SDI was updated to include only fertility rates for females younger than 25 years rather than total fertility rates.^24^ The GBD 2017 population and fertility analysis found that total fertility demonstrates a U-shaped pattern with SDI at higher levels of development, whereas fertility in females younger than 25 years does not.^24^ Quintile breaks were generated from the distribution of SDI at the national level in countries with populations greater than 1 million applied to all 195 locations. A complete list of SDI quintiles by location are available in appendix 1.^24^

### Projection of the health-related SDG indicators to 2030

To generate projections to 2030, we used forecasting methods developed by Foreman and colleagues that produced reference forecasts and alternative health scenarios for life expectancy, all-cause mortality, and cause-specific mortality.^36^ The modelling framework was designed to account for the relationships between risk factors and other independent drivers of health outcomes (eg, gains in sociodemographic development, select interventions such as vaccine coverage, and met need for family planning), thus better capturing causal pathways of health change shown in randomised controlled trials and cohort studies.

We generated projections for independent drivers by calculating the annual change in each location and year from 1990 to 2017 in logit or natural-log space, and then computing weighted annualised rates of change. If weights were closer to zero, annual rates of change over time were more equally weighted across years; if weights were closer to higher values, recent years were more heavily weighted than were earlier years. These weights were selected through out-of-sample predictive validity tests; further details on the overarching forecasting framework and weight selection are in appendix 1 (part 3). Some causes (eg, natural disasters, conflict and terrorism, and HIV) required model modifications or alternative estimation strategies to account for either their stochastic nature or, in the case of HIV, unique sensitivity to intervention coverage (see appendix 1 part 3, and elsewhere^36^).

Some indicators were inputs or outputs of the forecasting platform; for others, we used the weighted annualised rate of change method to produce projections to 2030 (appendix 1 part 3). For the UHC service coverage index, a modified version of the overarching forecasting framework was used, modelling the relationship between total health spending per capita and the UHC service coverage index with stochastic frontier analysis.^37^ We did not generate projections of census coverage because of its binary nature and the lack of documentation about planned censuses across all countries. Additionally, we do not currently project indicators by sex or subnationally.

### Health-related SDG index

The health-related SDG index was originally developed in GBD 2015.^13^ The overall index for GBD 2017 consisted of 40 health-related SDG indicators (population census coverage was not included because of its binary status and because it does not have forecasts). To create the health-related SDG index, we used a preference-weighted approach in which we considered the SDGs as representing the expressed preferences of UN member states and thus assumed that each SDG target should be weighted equally.

Each indicator was scaled to a value from 0 to 100, reflecting worst to best performance, to enable optimal comparison across diverse indicators, with 0 being the 2·5th percentile value and 100 being the 97·5th percentile value of 1000 observed or projected draws over the period 1990–2030. This approach reduced sensitivity to extreme outliers in given location-years. Negative indicators, for which lower values were more desirable than higher values (ie, mortality, incidence or prevalence, and risk exposure), were assigned 100 for the 2·5th percentile and 0 for the 97·5th percentile. For mortality and incidence, values were scaled in log-space.

We calculated the geometric mean of scaled health-related SDG indicators by target, and then took the geometric mean across all health-related SDG targets to produce the overall health-related SDG index. We used the geometric mean to allow for partial substitutability (ie, permitting high values for some indicators to only partially compensate for indicators with very low values). We restricted indicators to a minimum value of 1 when calculating the overall index to mitigate issues with values close to 0. To generate subnational SDG indices, we used the national-level 2·5th and 97·5th percentile values for each indicator to scale indicators for each subnational location. We used the same overall index construction methods for national and subnational locations.

For health worker density (SDG indicator 3.c.1), we used a modified scaling approach to reflect the importance of each health worker cadre (physicians, nurses and midwives, and pharmacists). On the basis of logistic regressions between each cadre and the HAQ Index,^34^ we identified the values at which additional increases in health worker density resulted in diminishing returns on the HAQ Index.^34^ In per 10 000 population space, these threshold values were 30 physicians, 100 nurses and midwives, and five pharmacists (appendix 1 part 1). Although we used the 2·5th percentile value of 1000 draws observed or projected from 1990 to 2030 to set the 0 threshold for all three cadres of health workers, we used cadre-specific thresholds to set the bounds for a 100 score rather than the 97·5th percentile of 1000 draws. We then took the geometric mean of scaled scores across the three cadres to estimate overall performance on the health worker density indicator.

### Country and global SDG indicator attainment

Some health-related SDG indicators have targets explicitly defined by UN resolution 71/313,^38^ including absolute targets and targets set in relation to 2015 values, whereas some indicators have undefined targets. In this analysis, 25 indicators had defined targets, for which we applied corresponding thresholds to analyse 2030 attainment (2020 in the case of road injury mortality, as set by the UN). For indicators with targets related to universal coverage or access, we set thresholds as 99%, whereas for indicators with targets related to achieving elimination or ending epidemics, we set thresholds as an incidence of 0·5 per 100 000 population or less or a prevalence of 0·5% or less. Thresholds or relative reductions for each target are shown in table 1.

For GBD 2017, we estimated the probability of each country attaining health-related SDG indicators with defined targets. We used our indicator projections to 2030 at the draw level (1000 draws in total), calculating at each draw whether or not a country would attain a target. The total probability of attainment was the number of draws in which the country would attain the target divided by the total number of draws. We also calculated the mean estimate in 2030 (the average of 1000 draws), and used that estimate to assess whether or not a country would attain a target. Consequently, countries could have some probability of attainment for given targets despite not having projected attainment at the mean level.

We also used past rates of change observed before the SDG era (ie, 1990–2015, or the monitoring period of the Millennium Development Goals) to analyse the feasibility of attaining SDG indicator targets in 2030. For SDG indicators with defined targets, we compared country-level annualised rates of change for 1990–2015 (ie, what has been achieved in the past) with the global pace of progress required to meet targets during the SDG era (2015–30) based on 2015 global estimates for each indicator; these estimates were derived from population-weighted means. For each indicator, we compared the required global annualised rate of change for 2015–30 against the distribution of past country-level annualised rates of change, and calculated in which percentile of performance the global required annualised rate of change would fall. We took the mean of those percentiles across the 25 indicators with defined targets and found that, on average, the required global annualised rates of change for 2015–30 would be in the 90th percentile of performance compared with the country-level annualised rates of change for 1990–2015. To see what global progress could be achieved if performance on all indicators was projected at that level from 2015 to 2030, we calculated the annualised rate of change required to meet the 90th percentile for each indicator, including those without defined targets, and projected the 2030 value and corresponding percentage change from 2015 to 2030 based on these annualised rates of change.

### Uncertainty analysis

For all indicator estimates, GBD 2017 produced 1000 draws by location, age, and sex and for all years. Draws from the posterior distribution represent uncertainty in steps in the estimation process as well as in underlying data sources. For each scaled SDG indicator and the health-related SDG index, we calculated 95% uncertainty intervals (UIs) on the basis of these 1000 draws using simulation analysis. Further information about GBD uncertainty analysis is provided in related GBD publications.^21^, ^23^, ^24^, ^25^, ^26^, ^27^

### Role of the funding source

The funder of the study had no role in the study design, data collection, data analysis, data interpretation, or writing of the report. All authors had full access to all the data in the study and had final responsibility for the decision to submit for publication.

## Results

### Health-related SDGs in 2017

**Figure 1:**
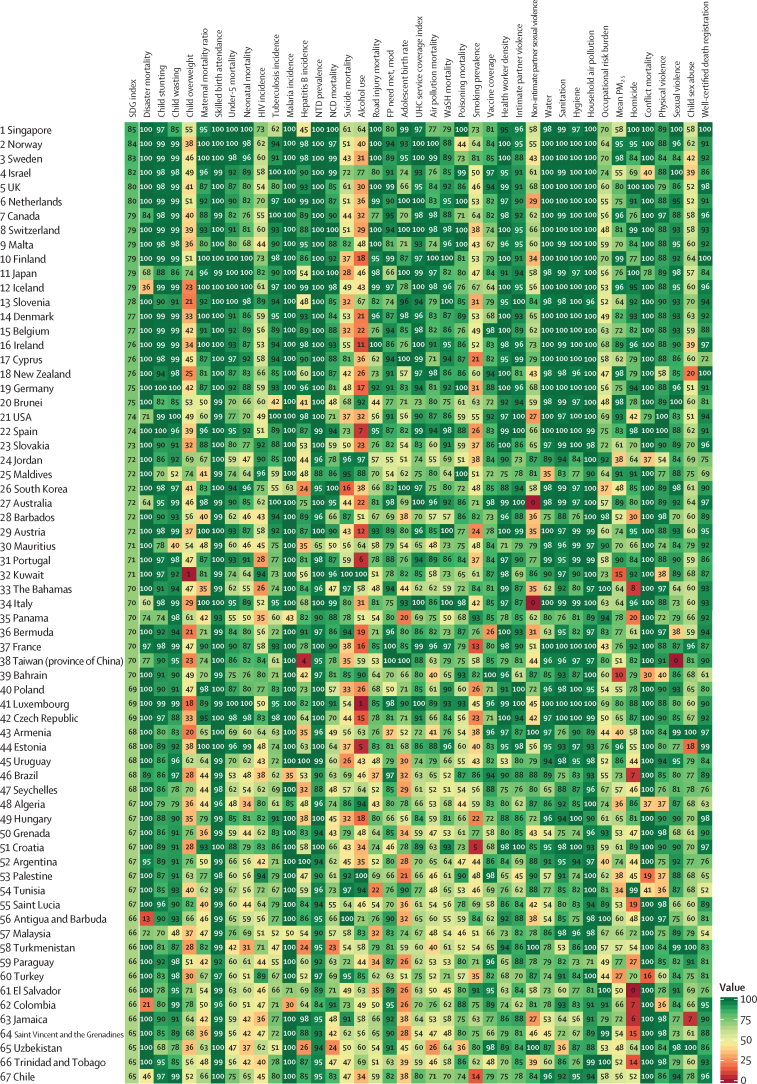

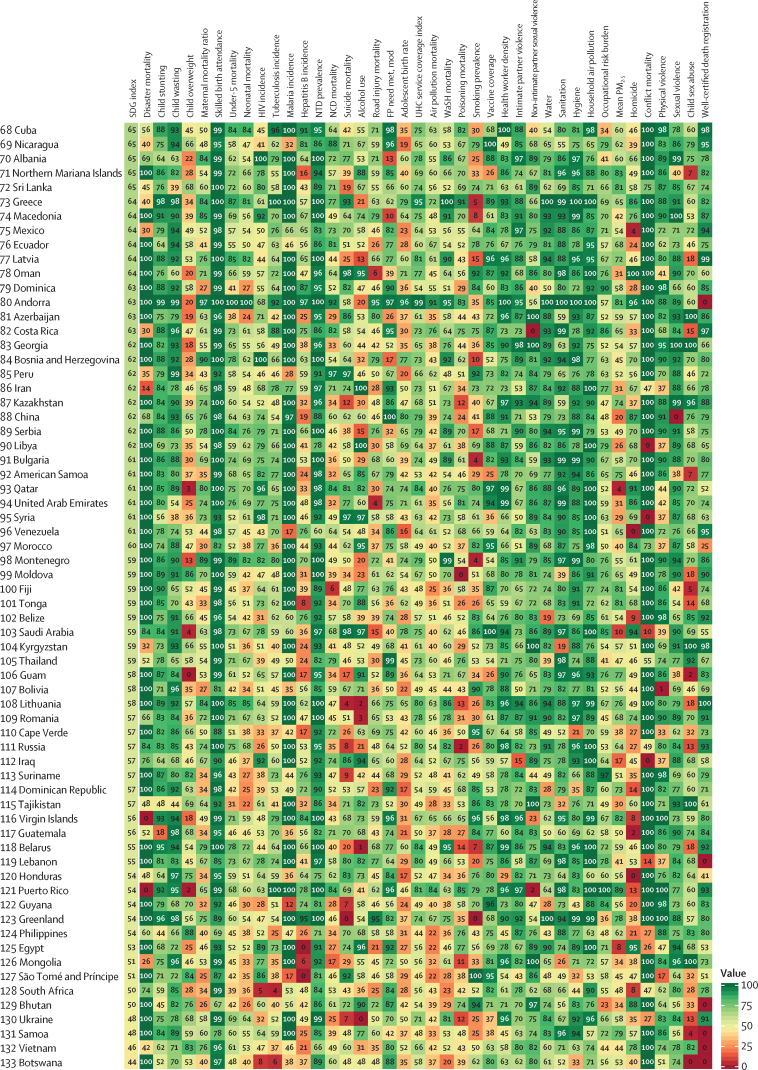

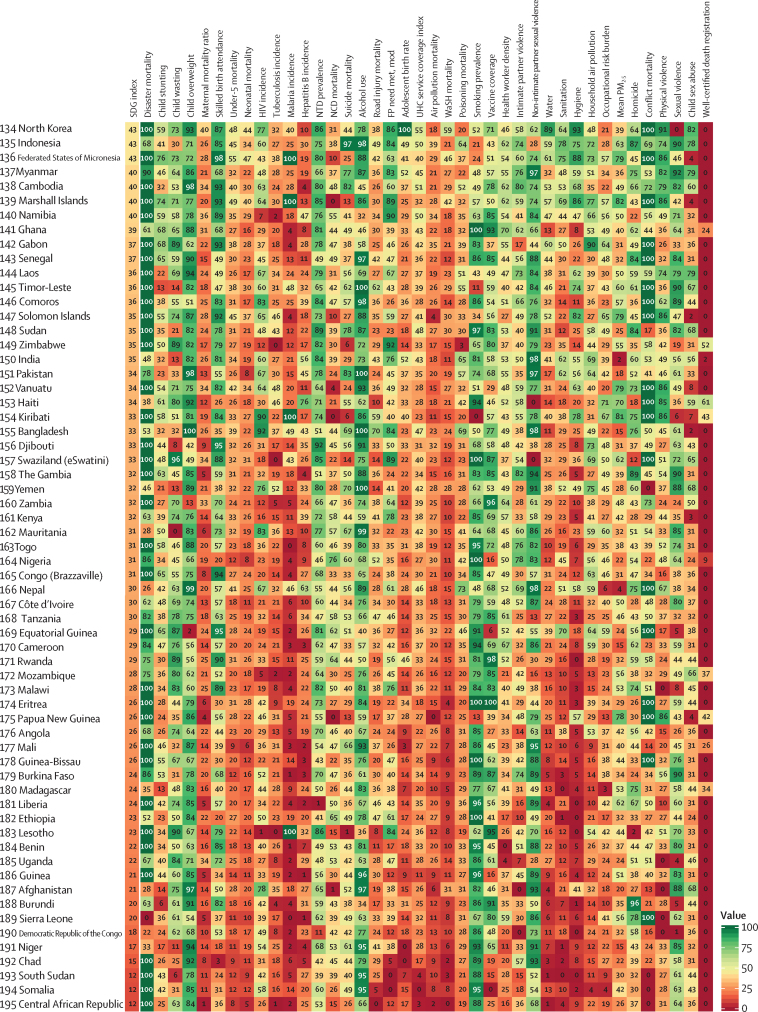
Performance on the health-related SDG index and 40 individual health-related indicators, by location, in 2017 Countries and territories are ranked by their health-related SDG index from highest to lowest in 2017. Indices and individual indicators are reported on a scale of 0 to 100, with 0 representing the worst scores from 1990 to 2030 and 100 reflecting the best during that time. SDG indicator 17.19.2a, population census status within the past 10 years, was not included in the health-related SDG index because projections were not generated for this indicator. Definitions of health-related SDG indicators are shown in table 1. FP need met, mod=family planning need met with modern contraception methods. NCD=non-communicable disease. NTD=neglected tropical disease. PM_2·5_=fine particulate matter smaller than 2·5 μm. SDG=Sustainable Development Goal. UHC=universal health coverage. WaSH=water, sanitation, and hygiene.

The global median health-related SDG index was 59·4 (IQR 35·4–67·3) in 2017, ranging from a low of 11·6 (95% UI 9·6–14·0) to a high of 84·9 (83·1–86·7; figure 1). The overall health-related SDG index masked substantial variation across indicators within countries. Many countries with low overall index scores performed reasonably well on some individual indicators and vice versa. For example, although Kenya scored only 31·7 (30·6–32·9) on the overall health-related SDG index for 2017, the country scored much better on met need for family planning (77·8, 74·6–80·9) and smoking prevalence (85·4, 82·6–88·0). By contrast, South Korea, which scored 72·2 (69·0–74·4) on the overall index, scored comparatively worse on suicide mortality (16·3, 11·5–21·1). Results for each indicator and country can be explored through the online data visualisation tool.

Scores for NCD mortality were worst in Afghanistan and in many countries in Oceania; the best scores were primarily among higher-SDI countries, with the exception of Peru (figure 1). Most countries with the best alcohol use scores were in north Africa and the Middle East, whereas countries with the worst values were generally concentrated in Europe. The worst smoking prevalence scores were found among a heterogeneous set of locations (eg, Greenland, Kiribati, and Montenegro), and the best were primarily found in sub-Saharan Africa. Suicide mortality scores were generally best in countries in the Middle East and worst in a variety of countries (eg, Greenland, Lesotho, and Lithuania).

The worst scores for health worker density were primarily in sub-Saharan African countries; by contrast, Cuba, Qatar, and many European countries recorded among the best scores for this indicator. Several Latin American countries had the worst scores for sexual violence by non-intimate partners, whereas several countries in central Asia, eastern Europe, and south Asia had the best scores for this indicator.

During 2008–17, 165 countries conducted at least one population and housing census. 30 countries had existing or had implemented population registries during this time, and eight of these countries had conducted at least one census since 2008 (appendix 2). Eight countries did not have this important source of demographic information over the full time period.

### Global and subnational variation

**Figure 2:**
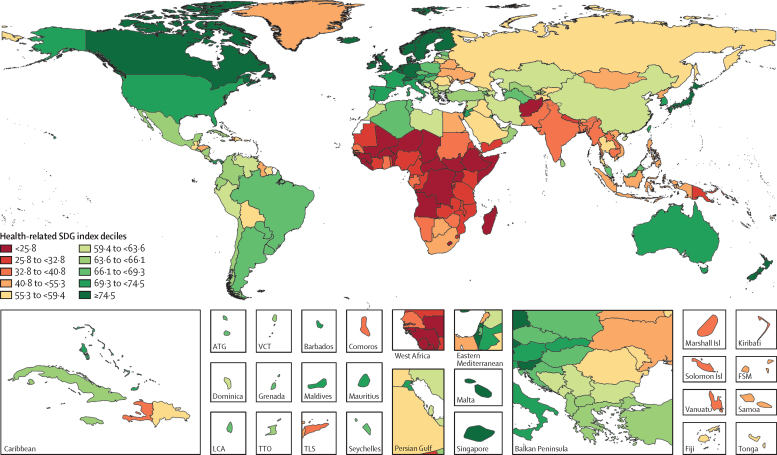
Health-related SDG index by decile, 2017 Deciles are based on the distribution of health-related SDG indices for countries and territories in 2017. ATG=Antigua and Barbuda. FSM=Federated States of Micronesia. Isl=Islands. LCA=Saint Lucia. SDG=Sustainable Development Goal. TLS=Timor-Leste. TTO=Trinidad and Tobago. VCT=Saint Vincent and the Grenadines.

**Figure 3:**
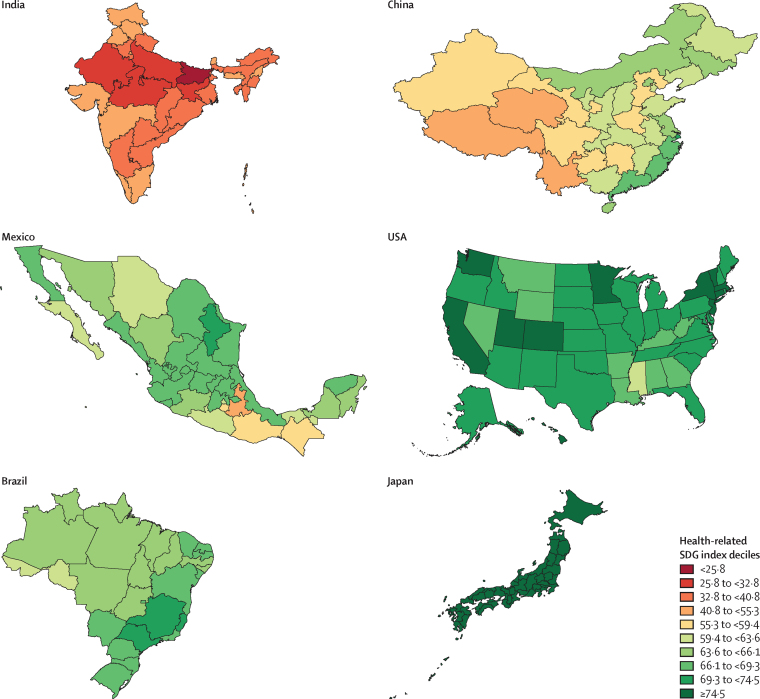
Health-related SDG index for selected subnational locations, 2017 Deciles are based on the distribution of health-related SDG indices for countries and territories in 2017, and then applied for subnational locations. SDG=Sustainable Development Goal.

Performance on the health-related SDG index in 2017 varied globally (figure 2) and at the subnational level (figure 3). Countries in the tenth decile of performance—those with the best index values—were primarily in western Europe, although Canada, Japan, and Singapore were also in this decile. Afghanistan was in the first decile of performance, which otherwise predominantly included countries in sub-Saharan Africa.

Among the countries with subnational SDG index scores (figure 3), India (which ranked in the third decile nationally) had the largest range in 2017, with a 34·9-point difference between states with the highest and lowest scores. China also had considerable subnational differences, performing in the sixth decile nationally but recording a 19·3-point difference in scores across provinces, followed by the USA (ninth decile nationally and a 14·8-point difference across states) and Mexico (seventh decile nationally and a 15·3-point difference across states). Scores were most homogeneous in Japan (tenth decile nationally and a 3·0-point difference across subnational locations), the UK (tenth decile nationally and a 3·6-point difference across regions in England), and Brazil (eighth decile nationally and an 8·0-point difference across states).

### Variation by sex and SDI

**Figure 4:**
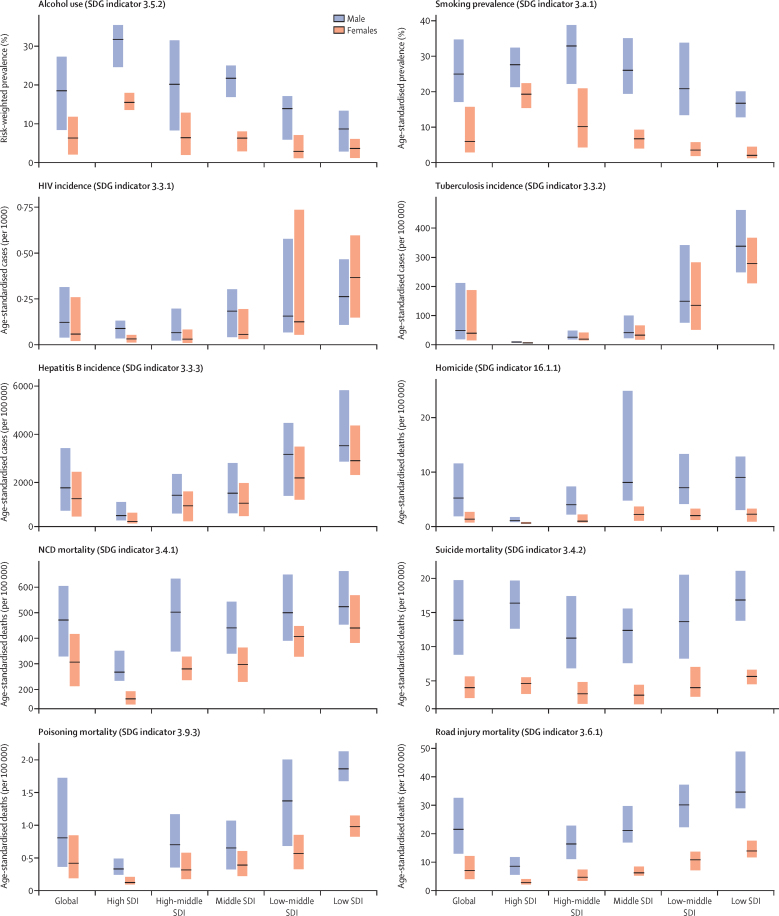
Median values for select SDG indicators, by sex, at the global level and by SDI quintile, 2017 The lengths of the coloured rectangles represent the IQRs. More detail on the SDG indicators included in this figure can be found in table 1. SDG=Sustainable Development Goal. SDI=Socio-demographic Index.

Globally, the median age-standardised NCD mortality rate, as it aligns with the UN definition, was higher for males (472·0 [IQR 330·5–604·9] per 100 000) than for females (307·8 [215·4–417·9] per 100 000; figure 4). Variation by sex was also pronounced for alcohol use (18·5% [IQR 8·4–27·3] of males *vs* 6·4% [2·2–11·8] of females), smoking prevalence (25·0% [IQR 17·2–34·7] of males *vs* 6·1% [3·0–15·8] of females), and suicide mortality (13·8 [IQR 8·8–19·7] per 100 000 males *vs* 4·0 [2·5–5·6] per 100 000 females; figure 4).

NCD mortality showed an inverse association with SDI quintile, with the lowest mortality in high-SDI countries. Males in high-middle-SDI countries were the exception, recording higher mortality rates than males in middle-SDI and low-middle-SDI countries. Disparities in NCD mortality by sex emerged across SDI quintiles, particularly among high-middle-SDI countries, for which the median age-standardised mortality rate was 512·4 (IQR 356·2–643·9) per 100 000 males and 285·9 (250·1–333·6) per 100 000 females. Alcohol use was generally higher among higher-SDI quintiles, although differences between sexes were smallest in low-SDI and low-middle-SDI countries. Smoking prevalence was also higher among higher-SDI quintiles, with the exception of males in high-SDI countries who had a lower smoking prevalence than males in high-middle-SDI countries. Differences between sexes were pronounced across SDI quintiles but were often smallest in high-SDI countries. The median age-standardised suicide mortality rate showed a U-shaped pattern, with the highest rates in high-SDI and low-SDI countries.

Overall, males had worse health outcomes—higher mortality, incidence, and risk exposure—than females for all ten disaggregated indicators globally and across SDI quintiles; the primary exception was HIV incidence among low-SDI countries, where the incidence was higher in females than in males (figure 4). In 2017, the global median age-standardised incidence of HIV was 0·14 (IQR 0·04–0·37) per 1000 males versus 0·07 (0·02–0·30) per 1000 females. For tuberculosis, the global median age-standardised incidence was 48·9 (IQR 18·6–211·2) per 100 000 males and 39·7 (14·8–187·0) per 100 000 females. Overall, age-standardised mortality rates for road injuries and poisoning were lower among higher-SDI countries. In 2017, the global median mortality rate from road injuries was 21·5 (IQR 12·9–32·5) per 100 000 males and 7·0 (4·0–12·1) per 100 000 females, while the equivalent for poisoning mortality was 0·8 (0·4–1·7) per 100 000 males and 0·4 (0·2–0·8) per 100 000 females.

### Health-related SDGs in 2030 and target attainment

**Figure 5:**
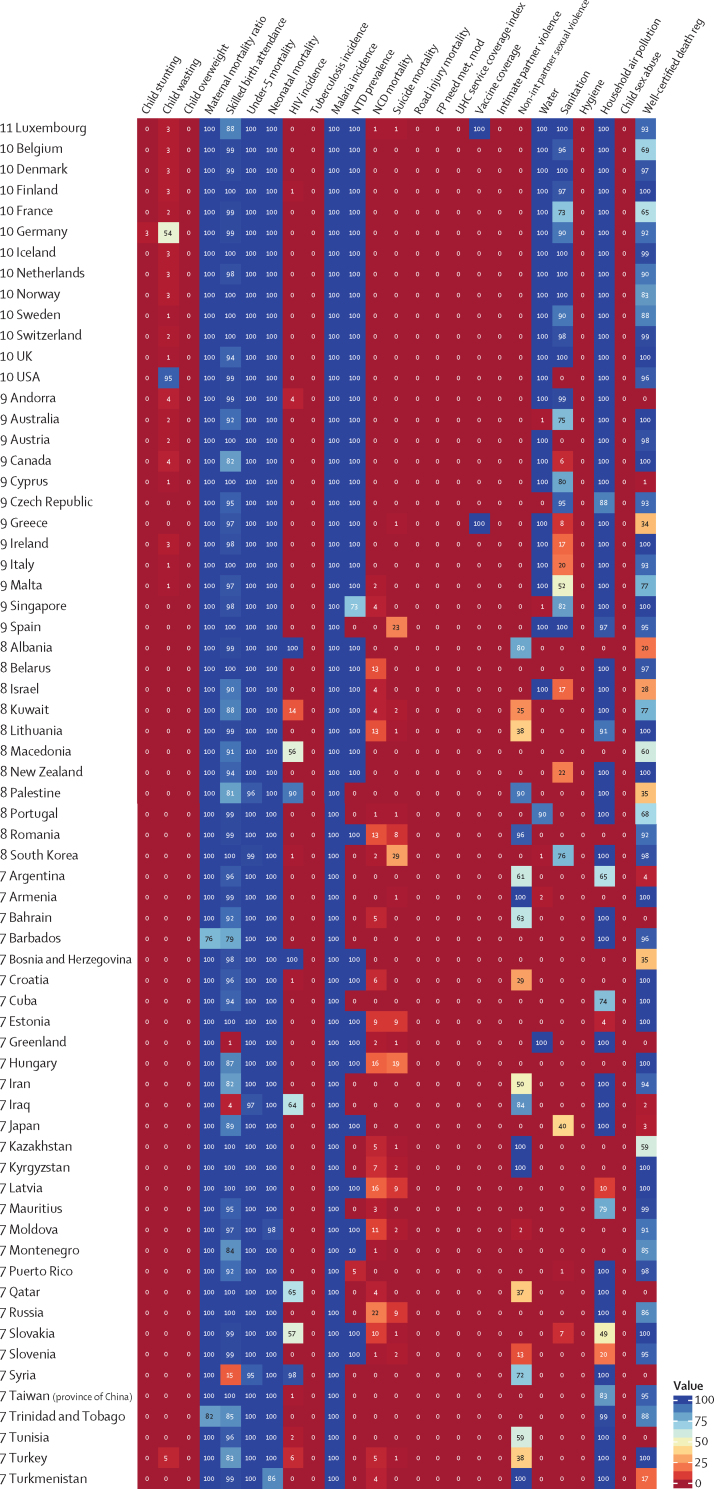

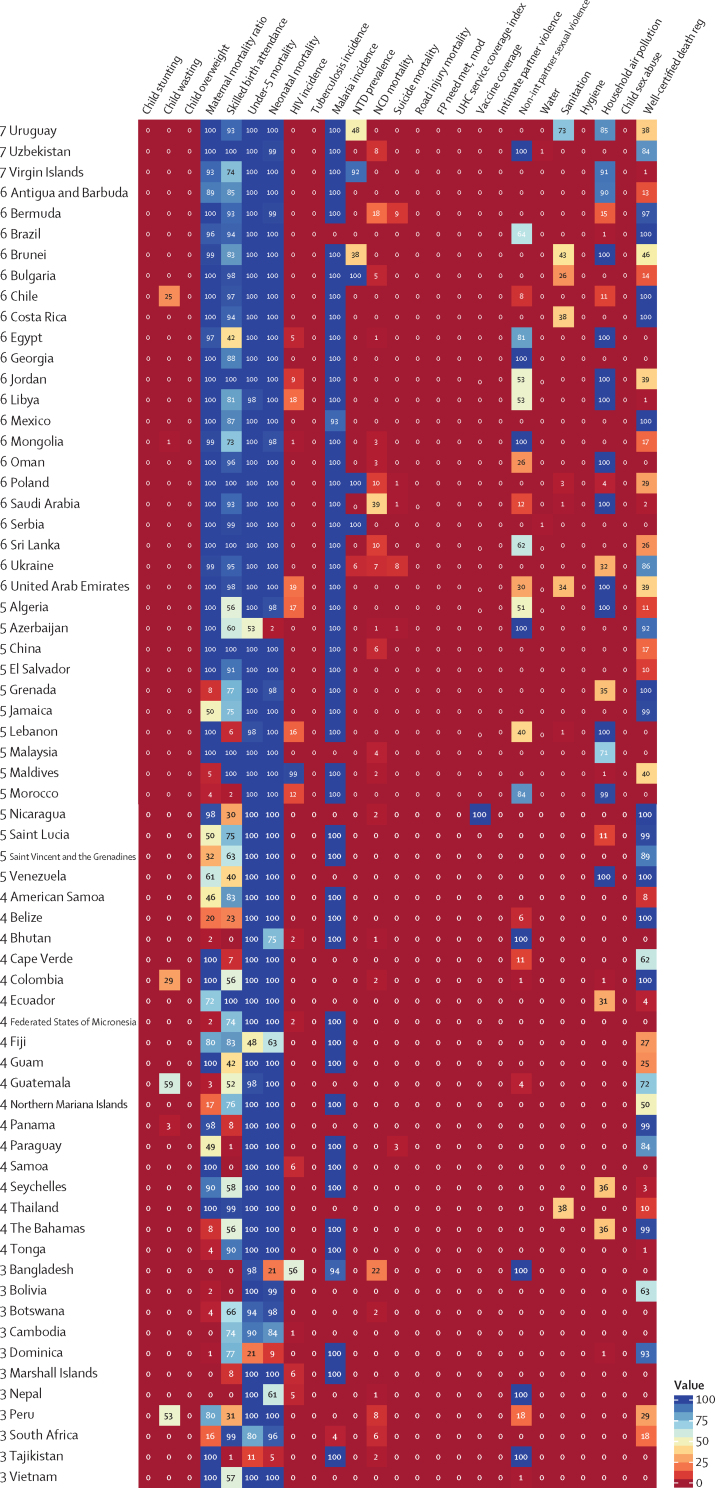

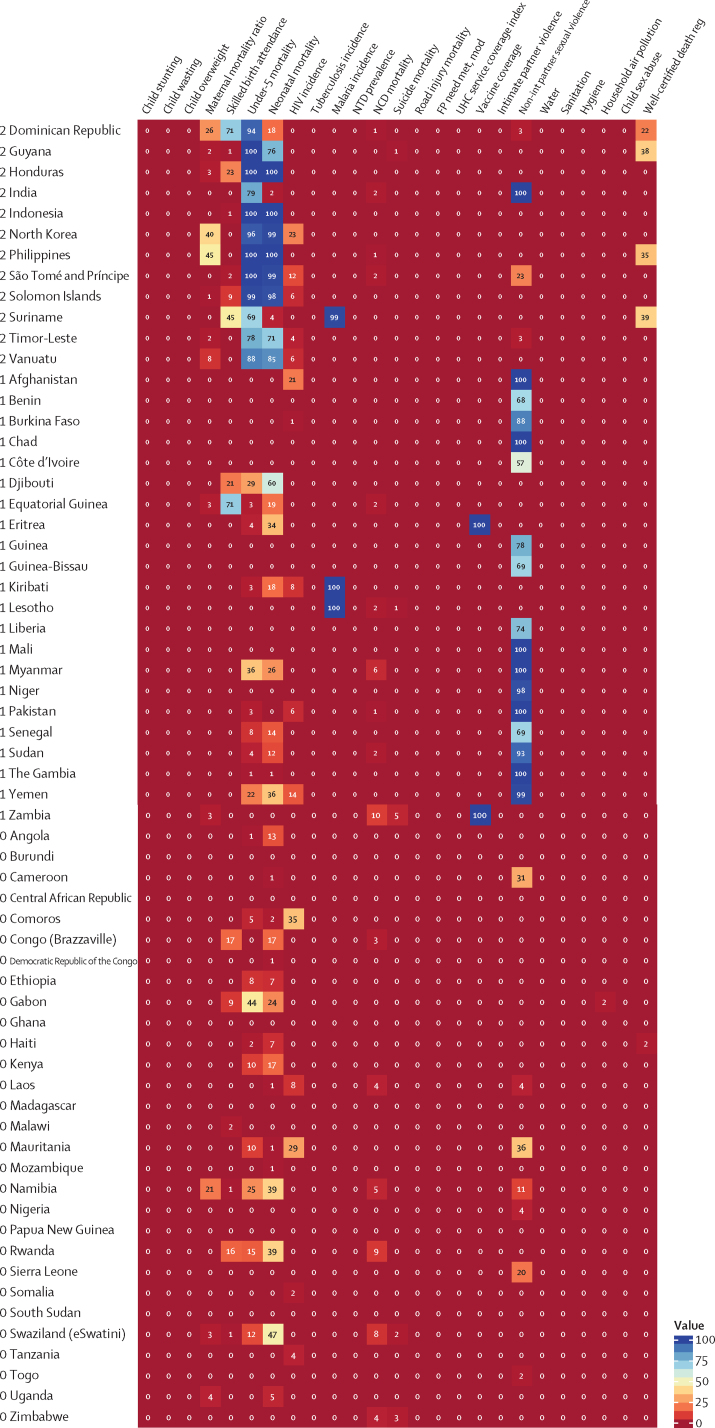
Comparing the probability of attainment for defined health-related SDG indicator targets based on past trends, by location, in 2030 Countries and territories are ranked from highest to lowest by the total number of SDG indicator targets they are projected to attain on the basis of mean estimates for 2030. Numbers preceding the country names are the numbers of targets projected to be met by each country or territory based on means. Values reported reflect the probability of projected attainment based on the percentage of draws that fell above or below defined targets in 2030. Of the 41 health-related indicators measured in this study, 25 had defined targets linked to each indicator. SDG target 3.6 aims to reduce road injury mortality by 50% between 2015 and 2020, and thus attainment for this indicator is based on estimates from 2015 to 2020 rather than 2015 to 2030. Definitions of health-related SDG indicators and targets associated with them, as well as the specific target thresholds applied, are shown in table 1. FP need met, mod=family planning need met with modern contraception methods. NCD=non-communicable disease. Non-int=non-intimate. NTD=neglected tropical disease. reg=registration. SDG=Sustainable Development Goal. UHC=universal health coverage.

On the basis of past trends, most countries were projected to have higher health-related indices in 2030 than in 2017 (appendix 2). For the health-related SDG indicators with defined targets (figure 5; appendix 2), the probability of attainment by 2030 varied substantially across locations and by indicator. Under-5 mortality, neonatal mortality, maternal mortality ratio, and malaria had at least 100 countries or territories with at least a 95% probability of attaining defined targets in 2030. Indicators including vaccine coverage, HIV incidence, neglected tropical diseases prevalence, non-intimate partner violence, well-certified death registration, and environmental risks such as sanitation and household air pollution showed substantial heterogeneity in terms of projected attainment, with many locations recording probabilities of less than 10% and others recording probabilities of 95% or higher. For nine indicators, including child overweight, road injury mortality, and tuberculosis, all 195 countries and territories had lower than 5% probability of attainment by 2030.

**Figure 6:**
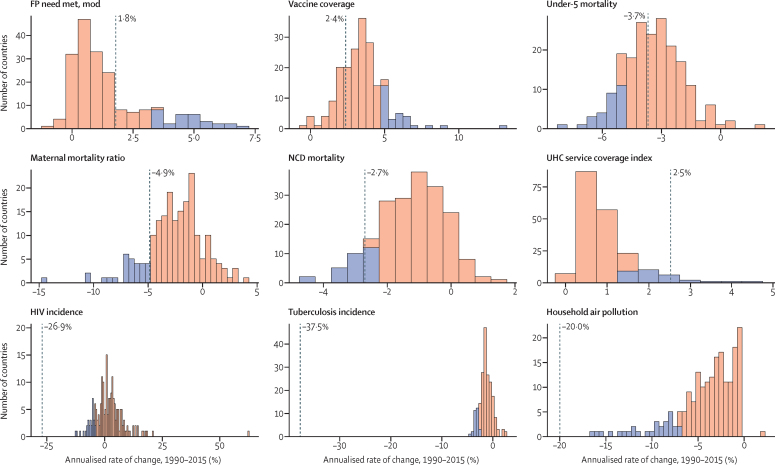
Global annualised rate of change required to meet selected SDG targets based on annualised rate of change achieved by countries or territories, 1990–2015 For the 25 SDG indicators with defined targets, we estimated the required global annualised rate of change (dotted line) required to meet each target using the global average in 2015 and specific thresholds to be met by 2030 or relative reductions to be achieved by 2030. The top performing decile (the 10th decile) is shown in blue and all other annualised rates of change are shown in red. A subset of SDG indicators with defined targets are shown here; the remaining plots can be found in appendix 2. Definitions of health-related SDG indicators and targets associated with them are shown in table 1. FP need met, mod=family planning need met with modern contraceptive methods. NCD=non-communicable disease. SDG=Sustainable Development Goal. UHC=universal health coverage.

Figure 6 shows the distribution of annualised rates of change in 195 locations for 1990–2015. For several indicators, the global required annualised rates of change were met or exceeded by many countries not in the top decile of performance from 1990 to 2015. These indicators generally had value-specific SDG targets, such as under-5 mortality (ie, ≤25 deaths per 1000 livebirths), or had targets linked to universal access or coverage of specific interventions (ie, vaccine coverage and met need for family planning). Furthermore, most of these indicators had origins in the Millennium Development Goals era. Despite substantial progress in the past, only countries in the top decile of performance for 1990–2015 met or surpassed the global rate of change required to meet the maternal mortality ratio target for 2030 (ie, <70 deaths per 100 000 livebirths), and very few countries recorded the rates of change required at the global level to meet the UHC service coverage target by 2030. A similar pattern emerged for NCD mortality. For other health-related SDG indicators, the global annualised rates of change required to meet their SDG targets far exceeded the pace of progress ever recorded by any country in the past (figure 6). These included several elimination indicators for infectious diseases, such as HIV and tuberculosis, and environmental risks such as household air pollution, among others (figure 6; appendix 1).

For NCD mortality and suicide mortality, performance at the global mean percentile of past rates of change (90th percentile) aligned with defined SDG targets of reducing rates by a third from 2015 to 2030 (ie, the equivalent of a 32·5% reduction for NCD mortality and a 31·4% reduction for suicide mortality; table 2). Yet, for several indicators, performance at the 90th percentile would not equate to meeting established SDG targets. Many indicators with elimination targets saw the widest gaps in how the 90th percentile could translate into global attainment in the SDG era. For example, indicators under SDG target 2.2, which aims to end all forms of malnutrition, would see the global average for child stunting decrease to 18·0% and wasting to 5·0% in 2030 if the 90th percentiles of past rates of change are achieved. For child overweight, the 90th percentile equated to a 0·5% decrease by 2030, from a global average of 15·9% in 2015 to 15·8% in 2030. SDG target 3.3 calls for ending the epidemics of several infectious diseases, including tuberculosis, HIV, and neglected tropical diseases; based on the 90th global mean percentile, the global percentage change from 2015 to 2030 would fall short of such aspirations for most of these causes (ie, decrease of 7·9% for hepatitis B, 35·6% for tuberculosis, 53·4% for HIV, and 48·2% for neglected tropical diseases). The exception was malaria, which would decrease by 94·0% at the 90th percentile.

**Table 2:** Predicting global attainment of health-related SDG indicators on the basis of past pace of progress observed across countries

	**2030 target used in this analysis**	**Projected attainment in 2030**	**Global average in 2015**	**Global required annualised rates of change to meet SDG target by 2030**	**Global percentile based on country-level annualised rates of change from 1990 to 2015**	**Country-level annualised rates of change from 1990 to 2015 based on the mean percentiles for defined targets (90th percentile)**	**Global average in 2030**	**Global percentage change, 2015–30**
Indicator 1.5.1: Death rate due to exposure to forces of nature	Undefined	..	0·2 per 100 000 population	..	..	−9·3%	0·05 per 100 000 population	−76·8%
Indicator 2.2.1: Prevalence of stunting in children younger than 5 years	≤0·5%	0%	28·7%	−27·0%	100	−3·1%	18·0%	−37·6%
Indicator 2.2.2a: Prevalence of wasting in children younger than 5 years	≤0·5%	1%	8·0%	−18·5%	100	−3·2%	5·0%	−38·3%
Indicator 2.2.2b: Prevalence of overweight in children aged 2–4 years	≤0·5%	0%	15·9%	−23·1%	100	−0·5%	15·8%	−0·5%
Indicator 3.1.1: Maternal mortality ratio in females aged 10–54 years	<70 deaths per 100 000 livebirths	56%	145·0 per 100 000 livebirths	−4·9%	86	−6·0%	57·0 per 100 000 livebirths	−60·7%
Indicator 3.1.2: Proportion of births attended by skilled health personnel	≥99%	54%	80·1%	1·4%	76	3·0%	>99%	>22·8%
Indicator 3.2.1: Under-5 mortality rate	≤25 deaths per 1000 livebirths	72%	43·3 per 1000 livebirths	−3·7%	56	−5·2%	19·4 per 1000 livebirths	−55·3%
Indicator 3.2.2: Neonatal mortality rate	≤12 deaths per 1000 livebirths	71%	18·6 per 1000 livebirths	−2·9%	58	−5·0%	8·6 per 1000 livebirths	−53·7%
Indicator 3.3.1: Age-standardised rate of new HIV infections	≤0·005 per 1000 population	4%	0·3 per 1000 population	−26·9%	100	−5·0%	0·1 per 1000 population	−53·4%
Indicator 3.3.2: Age-standardised rate of tuberculosis cases	≤0·5 per 100 000 population	0%	139·6 per 100 000 population	−37·6%	100	−2·9%	89·9 per 100 000 population	−35·6%
Indicator 3.3.3: Age-standardised rate of malaria cases	≤0·005 per 1000 population	61%	31·8 per 1000 population	−58·4%	97	−17·1%	1·9 per 1000 population	−94·0%
Indicator 3.3.4: Age-standardised rate of hepatitis B incidence	Undefined	..	2123·8 per 100 000 population	..	..	−0·6%	1955·2 per 100 000 population	−7·9%
Indicator 3.3.5: Age-standardised prevalence of the sum of 15 neglected tropical diseases	≤0·5%	24%	18·2%	−24·0%	100	−4·3%	9·4%	−48·2%
Indicator 3.4.1: Age-standardised death rate due to cardiovascular disease, cancer, diabetes, and chronic respiratory disease in populations aged 30–70 years	Reduce by one-third from 2015 to 2030	0%	382·7 per 100 000 population	−2·7%	91	−2·6%	258·2 per 100 000 population	−32·5%
Indicator 3.4.2: Age-standardised death rate due to self-harm	Reduce by one-third from 2015 to 2030	0%	10·0 per 100 000 population	−2·7%	93	−2·5%	6·8 per 100 000 population	−31·4%
Indicator 3.5.2: Risk-weighted prevalence of alcohol consumption, as measured by the summary exposure value or alcohol use	Undefined	..	11·6%	..	..	−1·4%	9·4%	−18·7%
Indicator 3.6.1: Age-standardised death rate due to road injuries	Reduce by 50% from 2015 to 2020	0%	16·1 per 100 000 population	−13·9%	100	−4·3%	13·0 per 100 000 population	−19·6%
Indicator 3.7.1: Proportion of females of reproductive age (15–49 years) who have their need for family planning satisfied with modern contraception methods	≥99%	0%	75·7%	1·8%	72	4·1%	>99%	>27·3%
Indicator 3.7.2: Number of livebirths per 1000 females aged 10–19 years	Undefined	..	21·7 per 1000 females	..	..	−4·2%	11·4 per 1000 females	−47·4%
Indicator 3.8.1: Coverage of essential health services, as defined by the universal health coverage index	≥99%	0%	67·7	2·5%	97	1·8%	88·3	30·4%
Indicator 3.9.1: Age-standardised death rate attributable to household air pollution and ambient air pollution	Undefined	..	55·6 per 100 000 population	..	..	−3·5%	32·5 per 100 000 population	−41·5%
Indicator 3.9.2: Age-standardised death rate attributable to unsafe water, sanitation, and hygiene	Undefined	..	35·7 per 100 000 population	..	..	−7·3%	11·4 per 100 000 population	−68·1%
Indicator 3.9.3: Age-standardised death rate due to unintentional poisonings	Undefined	..	1·0 per 100 000 population	..	..	−4·7%	0·5 per 100 000 population	−51·5%
Indicator 3.a.1: Age-standardised prevalence of current smoking in populations aged 10 years and older	Undefined	..	18·4%	..	..	−1·6%	14·4%	−21·9%
Indicator 3.b.1: Coverage of eight vaccines among target populations	≥99%	3%	69·3%	2·4%	23	5·2%	>99%	>42·7%
Indicator 3.c.1: Health worker density (physicians, nurses and midwives, and pharmacists) per 1000 population	Undefined	..	5·9 per 1000 population	..	..	4·2%	10·9 per 1000 population	85·4%
Indicator 5.2.1: Age-standardised prevalence of ever-partnered females aged 15 years and older who experienced physical or sexual violence by a current or former intimate partner in the past 12 months	≤0·5%	0%	13·4%	−21·9%	100	−1·4%	10·9%	−18·5%
Indicator 5.2.2: Age-standardised prevalence of females aged 15 years and older who experienced physical or sexual violence by non-intimate partner in the past 12 months	≤0·5%	21%	1·0%	−4·3%	100	0·2%	1·0%	3·5%
Indicator 6.1.1: Risk-weighted prevalence of populations using unsafe or unimproved water sources, as measured by the summary exposure value for unsafe water	≤1%	13%	34·3%	−23·6%	100	−4·3%	17·7%	−48·3%
Indicator 6.2.1a: Risk-weighted prevalence of populations using unsafe or unimproved sanitation, as measured by the summary exposure value for unsafe sanitation	≤1%	10%	31·7%	−23·1%	100	−6·2%	12·2%	−61·4%
Indicator 6.2.1b: Risk-weighted prevalence of populations without access to a handwashing facility, as measured by the summary exposure value for unsafe hygiene	≤1%	0%	33·2%	−23·3%	100	−2·5%	22·8%	−31·3%
Indicator 7.1.2: Risk-weighted prevalence of household air pollution, as measured by the summary exposure value for household air pollution	≤1%	34%	20·1%	−20·0%	100	−8·3%	5·5%	−72·6%
Indicator 8.8.1: Age-standardised all-cause disability-adjusted life-years attributable to occupational risks	Undefined	..	830·0 per 100 000 population	..	..	−1·7%	646·2 per 100 000 population	−22·1%
Indicator 11.6.2: Population-weighted mean levels of PM_2·5_	Undefined	..	47·5	..	..	−0·9%	41·4	−12·9%
Indicator 16.1.1: Age-standardised death rate due to interpersonal violence	Undefined	..	5·1 per 100 000 population	..	..	−3·3%	3·1 per 100 000 population	−39·1%
Indicator 16.1.2: Death rate due to conflict and terrorism (per 100 000 population)	Undefined	..	1·9 per 100 000 population	..	..	−47·5%	<0·01 per 100 000 population	−100·0%
Indicator 16.1.3a: Age-standardised prevalence of physical violence experienced by populations in the past 12 months	Undefined	..	7·8%	..	..	−0·1%	7·7%	−0·9%
Indicator 16.1.3c: Age-standardised prevalence of sexual violence experienced by populations in the past 12 months	Undefined	..	3·3%	..	..	−0·9%	2·9%	−12·1%
Indicator 16.2.3: Age-standardised prevalence of females and males aged 18–29 years who experienced sexual violence by age 18 years	≤0·5%	0%	9·8%	−19·8%	100	−0·1%	9·6%	−1·8%
Indicator 17.19.2c: Percentage of well-certified deaths by a vital registration system among a country's total population	≥80%	37%	43·5%	4·1%	98	1·9%	57·4%	32·0%

Using the global average observed in 2015 for each of the 25 health-related indicators with defined targets, we calculated the global annualised rates of change required to meet these targets by 2030 (or by 2020 in the case of road injury mortality). The global required annualised rates of change were then compared with country-level annualised rates of change calculated from 1990 to 2015. The percentiles in which the global required annualised rates of change fell among country-level annualised rates of change in the past were ascertained, and then the average of these percentiles was computed (90th percentile) to serve as a way to assess defined SDG targets and potential targets for indicators that do not currently have defined SDG targets. The 90th percentile annualised rates of change observed for each indicator was applied to the global average in 2015 to compute the equivalent average in 2030 and percentage change from 2015 to 2030 if these rates of change are achieved during the SDG era. SDG=Sustainable Development Goal.

For two leading risk factors, alcohol use (SDG indicator 3.5.2) and prevalence of current smoking (SDG indicator 3.a.1), annualised rates of change in the 90th percentile would equate with 18·7% and 21·9% reductions, respectively, at the global level from 2015 to 2030 (table 2). Global percentage declines would range from 41·5% for mortality attributable to ambient air pollution and household air pollution (SDG indicator 3.9.1) to 68·1% for mortality attributable to unsafe water, sanitation, and hygiene (SDG indicator 3.9.2), reflecting the substantive improvements in reducing mortality attributable to these risks that many countries achieved in 1990–2015. If the 90th percentile was used as an SDG target, adolescent birth rates would need to decrease by 47·4%, or to a global average of 11·4 per 1000 females aged 10–19 years in 2030, and health worker density would need to increase by 85·4%, from an average of 5·9 per 1000 population in 2015 to 10·9 per 1000 population in 2030.

## Discussion

### Summary of findings

Although nearly all countries were projected to have improved health-related SDG index scores by 2030, progress varied by country and across individual indicators. Performance on health-related SDG indicators differed subnationally for several countries, as well as by sex and across SDI quintiles, highlighting the need for disaggregated data to ensure that no one is left behind. For many indicators, the annualised rate of change required to meet defined targets far exceeded the pace of progress achieved by any country in the recent past. Yet, even for those indicators with a mean projected value that fell short of the 2030 target, there was some probability of attainment by 2030, highlighting the potential for future SDG achievements if progress can be accelerated in the coming years. These results highlight the need for more rapid yet strategic implementation of programmes and continued monitoring of inequalities in the health-related SDGs within populations.

### SDG indicator progress and challenges

Health-related SDG index scores were projected to be higher in 2030 than in 2017 in almost all countries; however, improved index performance does not inherently reflect whether or not countries will attain individual SDG targets. The composite nature of the index means that many factors have a part in determining a country's overall score, and ensuring that progress on the index score translates into progress across indicators and equitably across populations will continue to be a challenge for countries.

Countries that performed well on the health-related SDG index commonly scored worse on the individual indicators of childhood overweight and alcohol use than on other indicators. Among countries that performed worst on the health-related SDG index, well-certified death registration was a frequent challenge. As populations age, all countries will need to strengthen health information systems to ensure death registration keeps pace with increasing mortality in older populations.^23^ Although suicide mortality and alcohol use prevalence appeared to be lowest in many countries in north Africa and the Middle East, in places where these practices are deemed illegal or shameful, our results might reflect a dearth of accurate data rather than true circumstances.^39^, ^40^

Without a concerted scale-up of efforts to prevent and treat NCDs, most countries will fall short of the 2030 SDG target.^9^ The NCDs included in SDG indicator 3.4.1—cardiovascular diseases, cancer, diabetes, and chronic respiratory diseases—accounted for 82·8% of all deaths due to NCDs in 2017.^21^ Total deaths due to NCDs increased from 9·5 million in the population aged 30–69 years in 1990 to 12·9 million in 2017 (or 62·9% of all deaths in that age group).^23^ Of those 12·9 million deaths, 6·3 million were due to cardiovascular diseases, 4·9 million to cancers, 1·1 million to chronic respiratory diseases, and 0·6 million to diabetes.^23^ Although the absolute number of deaths due to NCDs is rising annually, in most countries, this increase is the result of population ageing and growth; with the exception of diabetes, age-standardised NCD mortality rates for NCDs included in the SDG indicator have generally decreased.^23^ Nonetheless, many NCD-related risk exposures have seen minimal changes over time or are increasing, portending future challenges if more deliberate action is not taken against NCDs.^21^

In many countries, reductions in mortality due to cardiovascular diseases have been driven by improved access to antihypertensives and statins for addressing high cholesterol.^41^, ^42^ Investing in programmes that promote the early diagnosis and control of such metabolic risks should be prioritised by national governments and development partners.^43^ Furthermore, prevention of other modifiable risk factors, such as smoking, harmful alcohol consumption, and obesity, should also be a priority, as advocated by WHO's best buys for NCD control, including taxation of alcohol and tobacco and reduced salt intake.^44^ Yet, national financing of NCD programmes remains low, with patients often paying out-of-pocket for related services,^45^ and surveillance and reporting of NCDs are still sparse in many regions.^46^ The lack of action against NCDs is a current paradox of global health: despite numerous high-profile commitments and robust evidence underscoring the impact of NCDs, the actual scale-up and maintenance of NCD-related interventions and programmes is lacklustre at best.^9^ Inadequate access to affordable diagnostics and treatment, poor prioritisation of NCD risk-prevention programmes, and low overall UHC are among challenges facing many low-SDI and middle-SDI countries.^42^, ^47^ Political influences and corporate interests might also affect the effectiveness of NCD programmes and policies, particularly those targeting sugar and alcohol consumption.^48^, ^49^

Our estimates from 2017 indicate that 47·2% of countries and territories have less than one physician per 1000 population and 46·2% have less than three nurses or midwives per 1000 population. The largest gaps in health worker density were found in sub-Saharan Africa, although density was also low in southeast Asia, south Asia, and some countries in Oceania. GBD 2017 provides consistently estimated time series across locations for health worker density as an SDG indicator and by cadre, which supports supplementary analyses of the types and numbers of health workers required to deliver particular sets of interventions or health programmes. For instance, the threshold of 23 physicians, nurses, or midwives per 10 000 population, which was set by WHO in 2006, was a widely referenced minimum required to provide essential maternal and child health services during the Millennium Development Goals era.^50^ 12 years later, this recommendation persists, despite it being likely that very different health workforce quantities, composition, and quality of training are needed to provide a broader range of effective health-care services—particularly as more countries aim to make progress towards UHC.

With their explicit emphasis on eliminating violence, the SDGs offer an opportunity to reduce and prevent violence against females and children.^51^ This is the first time that a global development agenda has prioritised all forms of violence, including violence against females and children, homicides, and armed conflicts. Reliable information and evidence are required to develop programmes and policies to prevent violence, as well as to demand accountability and resources from governments, civil society, and international institutions when violent acts occur. As a result, it is vital to strengthen routine reporting of violence across all ages, and to ensure that accurate, timely measures of violence are accompanied by effective support and systems for survivors of violent acts to enable long-term recovery.

### Monitoring the health-related SDGs by sex and SDI

Despite the major part sex has in determining health behaviours and outcomes, only a third of the global health organisations included in the Global Health 50/50 report produce data disaggregated by sex.^52^ Data disaggregated by sex are crucial to uncovering key sex inequalities in health from which gender inequalities can be inferred or extrapolated.

Several studies^53^, ^54^, ^55^, ^56^, ^57^, ^58^ have shown that sex differences in health outcomes vary across causes of disease and disability. However, differences in health between sexes are less clear than is often assumed.^59^ Although males generally had worse outcomes than females for most indicators disaggregated in this study, this pattern might not hold true for many of the health-related SDG indicators that are not currently disaggregated by sex. Increased sex-specific collection of data, modelling, and reporting are needed, particularly for health-related SDG indicators related to child or neonatal mortality.

We showed that SDI quintile is also related to sex-specific patterns. For example, although globally, HIV incidence is higher in males, a large portion of the global HIV incidence occurs in lower-SDI countries in sub-Saharan Africa, where incidence is higher in females.^23^ Prevalence of alcohol use and current smoking showed similar patterns by sex: on average, males were more likely than females to smoke and consume harmful levels of alcohol, and while use was often higher among people living in higher-SDI countries, sex differences in these countries were also more pronounced.^60^, ^61^ Although the global smoking prevalence decreased from 1990 to 2017, particularly in males in high-SDI countries, some low-SDI and middle-SDI countries saw increasing prevalence in females.^61^ Alcohol use did not show similar rates of decline, which is unlikely to change in the absence of effective legal instruments and taxation policies.^62^, ^63^, ^64^, ^65^ Further research is needed to understand the role of attitudes and practices associated with smoking and alcohol use by sex.^66^, ^67^, ^68^, ^69^, ^70^

### Measuring progress at the subnational level

It is well known that national averages mask subnational disparities within countries, and the results of the health-related SDG index at the subnational level showed substantial differences in performance within countries, particularly in India and China. Differences between localities were lowest in Japan and the UK. Across countries, the states with the lowest SDG index scores in the USA (Mississippi, Arkansas, West Virginia, and Nevada) had lower scores than did 17 states in Mexico and 12 states in Brazil, while ten states in the USA had lower scores than Shanghai.

Disparities on the health-related SDGs at the subnational level were particularly pronounced among low-SDI and middle-SDI countries, indicating that greater investments in targeting the most vulnerable or disadvantaged people in a country are probably required to improve the health of the entire population. Generally, we found that higher-SDI countries had less variation in their performance among first administrative levels; however, differences at more focal levels (eg, counties in the USA and municipalities in Brazil) and by age and sex might still present considerable challenges to reaching the SDG aims of leaving no one behind. Identifying such gaps is a necessary first step to focus the attention of local decision makers when targeting resources and programmes. Few reports of countries seeking to address SDGs at the local level exist, although many countries have published voluntary reports of SDG progress with national-level data.^71^, ^72^, ^73^, ^74^, ^75^, ^76^, ^77^

Owing to the broad and multisectoral nature of the health-related SDG indicators, a whole-government approach is needed to strengthen their monitoring. Data are needed to inform planning and investment. Disaggregation of data can empower local administrations and improve local health information systems—an important government need beyond reporting indicator progress—but it is unlikely that it will be sufficient to identify all health inequalities.^19^, ^20^ The increased number of national and subnational units included in GBD 2017 is in line with the recommendation of the High Level Political Forum on Sustainable Development to produce regular voluntary national reviews of progress at national and subnational levels.^78^ The production of subnational estimates for the GBD study is not intended to substitute for country-level reporting; on the contrary, we hope that the dissemination of these results might help stimulate subnational reporting in a proactive manner.^79^

### Setting and assessing SDG targets

Projected attainment differed substantially among indicators with defined SDG targets. This pattern was particularly true for indicators that had few countries projected to meet targets based on mean values but had relatively more countries that showed some probability of attaining them by 2030. For some indicators with strong links to the Millennium Development Goals era—eg, maternal mortality ratio, child mortality, malaria, and skilled birth attendance—more than 50% of countries were projected to meet 2030 targets; furthermore, of those countries not projected to achieve these targets based on their mean values, many showed some probability of attainment by 2030. These findings highlight possible trajectories for meeting targets if progress can be accelerated in the future.

Nonetheless, based on past rates of progress, no country is currently on track to meet all defined health-related SDG targets. For under-5 mortality, which has many countries on track for or already achieving the target of 25 deaths or fewer per 1000 livebirths by 2030, 31 countries or territories would need to achieve annual rates of decline from 2015 to 2030 that are two to ten times higher than what was recorded for 1990–2015. To bring such ambition closer to reality for all populations, the UN and other agencies will need to provide technical leadership and financial support, particularly for the countries with the lowest health-related SDG performance.

A subset of SDG indicators, such as NCD mortality and suicide mortality, represent both the expansion of the SDG agenda to encompass broader health priorities beyond the narrower focus of the Millennium Development Goals and the establishment of ambitious yet potentially more feasible targets to meet within a 15 year time frame. At first glance, reducing NCD or suicide mortality by a third from 2015 to 2030—on average, the equivalent of a 2·7% reduction per year—might not seem particularly ambitious. Yet, amid the rising number of total deaths due to NCDs and challenges associated with providing quality NCD services in many countries,^23^, ^34^ this rate of change ranked in the 91st to 93rd percentiles of country-level annualised rates of change achieved from 1990 to 2015. However, to truly eliminate many health challenges, as the SDGs set out to do by 2030, most—if not all—countries will need to achieve rates of change that surpass those ever achieved in the past. This is particularly true for child overweight, for which most countries have only seen rising prevalence since 1990; tuberculosis, a disease that has received comparably less international funding than have HIV and malaria;^79^ road injury mortality; and most non-fatal violence measures. All countries had less than 5% probability of attaining child overweight and road injury mortality targets, highlighting priority areas for intervention globally.

Our global attainment analysis, which is grounded in historical rates of change, offers a mechanism to assess the ambitiousness of undefined targets. For instance, SDG target 3.a calls for strengthening the implementation of the WHO Framework Convention on Tobacco Control in all countries, a target against which changes in current smoking prevalence are not easily evaluated. The 90th mean global percentile of country-level rates of change in current smoking prevalence was a 1·6% decline per year from 1990 to 2015, which equated to a 21·9% decrease from 2015 to 2030 at the global level. Notably, the GPW13 calls for a 25% reduction in current tobacco use within 5 years (ie, a 5·8% decline per year),^3^ a pace that far exceeds what countries have achieved in the recent past.

The setting of targets is both a technical and political exercise, aiming to balance important societal objectives with the reality that many bold targets might be challenging, if not impossible, to achieve within short periods of time. Instituting targets solely on the basis of past rates of change and data is unlikely to happen within global and national policy circles; even if it could occur, such an approach might be equally unhelpful given that galvanising new funds, innovations, and commitments to improving population health worldwide often requires setting sights beyond what seems possible today. Instead, we hope that the methods used in this study will be useful in providing technical underpinnings for setting realistic and achievable targets; in the long run, such targets are likely to be more effective in driving action. We view these results as an important entry point for charting possible pathways to accelerated gains by 2030, as well as identifying more tangible interim goalposts against which countries can track ongoing advances and needs. As the Inter-agency and Expert Group on Sustainable Development Goal Indicators and UN Statistical Commission prepare for their formal review and revision of the global indicator framework in 2020, these findings might serve as inputs for consideration.

The perceived feasibility of target attainment can shape how international institutions, funding agencies, and countries approach health challenges. In particular, strict interpretation of the UN's targets for elimination or universal coverage sets very high bars for success (eg, 100% coverage or elimination). Although elimination has been achieved for some diseases in various settings (eg, malaria and a subset of neglected tropical diseases), truly ending an epidemic is much more complex for others, especially in the absence of fully effective vaccines or a radical cure. For other SDG indicators, such as intimate partner violence, elimination should be wholly feasible; yet to completely end all violent acts would require societal, cultural, political, and legal structures to fully align. Without more concrete strategies and funding for elimination, setting such ambitious targets could risk setting countries up for short-term failure and longer-term obstacles to instituting sustainable programmes; such arguments have been recently made about ambitions to eliminate HIV.^80^ Nonetheless, HIV remains a massive public health threat, particularly because global financing has plateaued, domestic health spending on HIV has stayed low among high-burden countries,^79^ and its incidence has not declined as quickly in younger as in older populations.^26^ It could also be argued that aiming for elimination but falling short in 2030 would still substantially improve the lives of millions and facilitate medical breakthroughs that might not be funded without a global elimination campaign. How best to galvanise accelerated action against the world's largest health challenges is far from clear; going forward, the GBD study can offer international agencies and countries alike a platform through which different operationalisations of SDG targets can be tested.

### Comparisons with other assessments

International agencies and the GBD study began producing annual reports of country estimates for the health-related SDG indicators in 2016. Of the 52 health-related indicators, GBD 2017 reported on 41, WHO reported on 37 in its 2018 World Health Statistics report,^6^ the World Bank covered 33 in its 2018 SDG Atlas,^7^ and the Sustainable Development Solutions Network included 27.^4^ Standardisation of definitions and methods used to calculate the health-related SDG indicators could improve comparability across organisations and collaborations involved in monitoring the SDGs. The complete set of metadata for SDG indicators, provided by the UN and other international organisations, comes with instructions on how indicators should be measured.^81^ However, GBD approaches to measurement differ from WHO approaches in various ways. For example, we use age-standardised rates for indicators that include mortality or incidence (eg, NCD mortality, suicide mortality, probability of death), whereas WHO generally use all-age rates. Furthermore, we define child overweight in terms of body-mass index for age and sex to align with the definition of overweight and obesity for adults, rather than in terms of weight for height. We also include all women of reproductive age in measurement of the met need for family planning indicator rather than limiting this measure to only women who are married or in a union. GBD also offers estimates for more years and locations than other organisations currently do, supporting the overarching SDG endeavour of leaving no one behind.

### Strengths

An important strength of GBD 2017 is the increasing number of collaborators involved: participation increased by more than 44% from 2016, with collaborators from 144 countries and two territories. The collaborator network offers multiple benefits to the GBD study, and in the case of the SDGs, it provides the particular benefit of supporting international and national policy dialogue, connecting technical information to the political needs of the health-related SDGs. Health programmes and plans have a limited chance of success in the absence of robust evidence and policy dialogue. The benchmarking presented in GBD 2017 can help countries to promote and increase accountability at the national level. The bottom line is the need to enhance mutual understanding of the SDG agenda across the entire global range of stakeholders and to champion the importance of national ownership of local guidance, monitoring, and management in achieving SDG targets.

To facilitate comparisons across locations and over time of the diverse array of health-related SDG indicators, we have produced an overall SDG index since GBD 2015.^5^, ^13^ The health-related SDG index is not presented in lieu of monitoring individual indicators, which we also do here. Instead, this index provides a mechanism by which overall performance across health-related SDGs can be more easily compared. A single, robust measurement such as the health-related SDG index is a useful tool for policy makers and other decision makers to interpret the performance of a particular location. With the production of time trends for several indicators, the SDG index also facilitates the understanding of the pace of progress. While index values represent a combination of different dimensions considered together as a proxy of health-related SDG indicator performance, results reported by individual indicator allow for more nuanced analyses.

### Limitations of indicator measurement

Our measurement of the health-related SDG indicators is subject to the limitations of the broader GBD 2017 study and its estimation processes; details can be found in the accompanying GBD 2017 capstone papers^23^, ^24^, ^25^, ^26^, ^27^ and in appendix 1 (part 1). Beyond these limitations, there are other important limitations that are specific to this analysis.

First, for measurement of health worker density we used ISCO 88 codes as the base classification system instead of ISCO 08, which is a more recent system than ISCO 88 that offers greater detail and standardisation. However, few occupational data sources currently include ISCO 08 codes, and benchmarking all past surveys to ISCO 08 would have resulted in substantial information loss. In future GBD iterations, we aim to collate more recent occupational data and to further refine health worker cadre mapping. Additionally, the UN includes density of dentist personnel in health worker density estimates, but the GBD study does not because including the four health cadres leads to counterintuitive results. Finally, the measure of health worker density can reveal only the quantity, not the quality, of available care.

Second, continued data sparsity for many violence measures, particularly for males and non-intimate partners, results in comparatively high uncertainty for these SDG indicators. Data on the prevalence of any form of violence are also subject to a number of measurement biases. All data for these indicators are self-reported and subject to recall bias and varying interpretations of survey questions. Variation in case-definitions or survey questions used by different surveys might also lead to increased uncertainty. We did not estimate psychological violence because there was no good standard for how to consistently measure it. Cultural influences, legal barriers to reporting, and stigma can also lead to underreporting and make the interpretation of self-reported data challenging, particularly for sensitive topics such as violence and other self-reported SDG indicators. Suicide is another indicator that might be affected by religious, cultural, and legal barriers to reporting. Accurate monitoring of violence measures requires routine, carefully implemented data collection, experienced interviewers, and thoughtful design for data intake, as well as ensuring that adequate protections and resources are available for victims of violence.

Third, owing to overall data sparsity, many challenges remain in modelling of both temporal and age patterns for non-fatal health outcomes; for the SDGs, this challenge is particularly pronounced for hepatitis B. Our current hepatitis B vaccine coverage covariate, a key input into hepatitis B incidence modelling, is limited to infant vaccination coverage. Because the current iteration of DisMod-MR cannot accommodate age-specific covariates, the effects of vaccination in older children and young adults for countries with long-running hepatitis B vaccination programmes (eg, Taiwan [province of China]) are not well captured. A priority of future GBD iterations is to use a version of DisMod that will allow for age-specific covariates, which will benefit hepatitis B modelling. Additionally, expanding the underlying data inputs for hepatitis seroprevalence, particularly for age groups that have benefited from vaccination programmes, has the potential to substantially improve hepatitis B estimation; for instance, the Polaris Observatory has markedly increased its seroprevalence data collection efforts in recent years, and such data have yet to be incorporated into the GBD study.

Fourth, our estimates of UHC currently only capture service coverage and do not include the second dimension of financial risk protection. The addition of financial risk protection and catastrophic health spending is a priority for future iterations of the GBD study. Ongoing review by the WHO Task Force on Metrics for GPW13 will likely yield recommendations that will inform future GBD revisions of how to measure UHC service coverage.

Fifth, the UN's metadata definition for vaccine coverage includes the human papillomavirus vaccine, but we do not currently include this vaccine owing to the limited number of countries with available data. Future iterations of the GBD study will aim to estimate human papillomavirus vaccine coverage.

Sixth, conflicts and refugee populations might affect SDG indicator trends in ways not well captured by our data. Although these factors introduce additional uncertainty to our estimates, these populations cannot be ignored, and GBD strives to make the best estimates based on the available data.

### Limitations of forecasting and attainment analyses

Generating forecasts has inherent limitations: when forecasts are grounded in past rates of change, we cannot fully account for what might occur between now and 2030, including changes in health financing and global health priorities, conflict, and climate change. Positive developments, such as new medical advances, can be challenging to predict, as are negative events, such as the emergence of drug resistance. Continued improvements in our overall forecasting framework and specific approaches by cause, risk, and intervention have the potential to further advance our ability to understand how and where the largest challenges in making progress on the health-related SDGs might occur.

### Limitations of indicator scaling and index construction

Ideally, we would systematically implement a scaling approach that accounts for the lowest possible levels of avoidable mortality, non-fatal outcomes, and risk exposures given current medical technologies and population-level interventions and the highest levels of coverage conditional upon measurable scale-up constraints or system inefficiencies. Scaling these indicators to the 2·5th and 97·5th percentiles of 1000 draws over time approximates this approach and allows consistent comparison of performance across indicators and locations. Nonetheless, our scaling strategy can either mask the potential for further improvements or imply worse performance, especially in relation to more modest SDG targets. Although the addition or refinement of new health-related SDG indicators with each GBD cycle is viewed as supportive of a more comprehensive assessment of the health-related SDGs, such updates can result in changes in individual countries' overall index values and relative rankings (appendix 2). These changes reflect our efforts to collectively improve the data for and science of monitoring the health-related SDGs. Overall index scores can be affected by indicators linked to fatal discontinuities, whereby an abrupt increase in deaths one year might not be present in the following year. We continue to use the geometric mean for the health-related SDG index. This decision might result in lower overall index scores for some locations if they experience worsening performance on particular indicators; however, it provides a more direct reflection of how the SDGs have been established by the UN and UN member states.^82^

### Limitations of data availability and disaggregation

GBD data are currently only available by sex, and not by gender, which limits the scope of analyses that can be conducted. The binary application of sex does not encompass differences in transgender and other populations. We currently only present data disaggregated by sex for ten indicators. Further collection and disaggregation of data would strengthen the ability of the GBD to support additional sex-specific analyses. For some new indicators, such as violence, some countries have better data collection systems in place than do others, and increased data collection is needed to make accurate comparisons between countries. Overall, the availability and representativeness of reported numbers from each country, particularly low-SDI and middle-SDI countries, could be improved by increased data collection, surveillance, and reporting at the national level.

### Future directions

Important refinements to our estimation process are made with each GBD iteration. As discussed already, one priority is to improve our measurement of UHC service coverage (SDG indicator 3.8.1), generate comprehensive estimates of catastrophic health expenditures (SDG indicator 3.8.2), and then ultimately develop an overall measure of UHC. In theory, UHC service coverage and catastrophic health expenditures (the inverse of financial risk protection) should be linked; however, to date, their measurement has generally involved separate en—deavours. GBD and collaborators are working to update estimates of UHC in this direction in the near future, in line with the WHO/World Bank framework.^83^ We currently use the arithmetic mean to construct the UHC service coverage index; however, other methods of index construction might be considered for future analyses. Other future priorities include analysing results by age as well as by sex, generating subnational projections, decomposing potential drivers of indicator-level progress, and further advancing the quantity and quality of data used in overall GBD estimation. Two other indicators, coverage of substance use disorder treatment (SDG indicator 3.5.1) and populations who feel safe walking home alone (SDG indicator 16.1.4), also are candidates for future inclusion pending data availability and access. Furthermore, it might be worthwhile to revisit what constitutes health-related SDGs, particularly as the UN Statistical Commission prepares for its formal review of current indicators and proposed additions. Finally, in view of the reasonably short window for the SDG era, there is increasing interest in developing model-based scenarios wherein the effects of investment “X” or introduction of intervention “Y” can be explored. The forecasting framework developed by Foreman and colleagues sets the foundation for such work^36^ given that the causal relationships captured and propagated through projections can be set at different levels or altered in response to funding changes (eg, 10% budget cut and its effects on HIV).^83^

### Conclusion

International institutions are increasingly calling for disaggregated data to guide decision making in health, and the SDGs are no exception. The SDG health-related index varied greatly at the subnational level in many countries, reflecting the need to focus on subnational health inequalities in the SDG era. Globally, males generally experienced a greater toll than did females from the ten health-related indicators analysed, emphasising the importance of both health data and programmes that incorporate sex-specific dimensions. Although most countries were projected to have improved SDG index scores in 2030, progress is slower than what is needed to attain defined targets across a wide range of health domains, including NCDs, which have many recommended best buy policies that have yet to be widely implemented. Countries and supporting international agencies must move beyond commitment to implementation, with a special focus on monitoring gains and gaps on the health-related SDGs beyond national trends. As shown by past rates of country-level progress, we have the opportunity to catalyse substantial gains in the future if the right investments and focus can occur today.

Correspondence to: Prof Rafael Lozano, University of Washington, Institute for Health Metrics and Evaluation, Seattle, WA 98121, USA rlozano@uw.edu

## Data sharing
