# Department of Error

**DOI:** 10.1016/S0140-6736(19)31048-7

**Published:** 2019-06-22

*GBD 2017 SDG Collaborators. Measuring progress from 1990 to 2017 and projecting attainment to 2030 of the health-related Sustainable Development Goals for 195 countries and territories: a systematic analysis for the Global Burden of Disease Study 2017.* Lancet *2018; **392:** 2091–138—*In this Global Health Metrics paper, affiliation details have been amended for Mina Anjomshoa, Joseph Adel Mattar Banoub, Behzad Khafaei, and Yasin Jemal Yasin; and the declaration of interests statement has been amended for Boris Bikbov. Additionally, the reference numbering has been updated. These corrections have been made to the online version as of June 20, 2019.

