# Supplementary material for: Measuring progress from 1990 to 2017 and projecting attainment to 2030 of the health-related Sustainable Development Goals for 195 countries and territories: a systematic analysis for the Global Burden of Disease Study 2017
Source: Lancet. 2018 Nov 10;392(10159):2091–138. doi: 10.1016/S0140-6736(18)32281-5 (PMC6227911; doi:10.1016/S0140-6736(18)32281-5)
Supplement: Supplementary appendix 1 [file mmc1.pdf]

THE LANCET

Supplementary appendix 1

This appendix formed part of the original submission and has been peer reviewed. We post it as supplied by the authors.

Supplement to: GBD 2017 SDG Collaborators. Measuring progress from 1990 to 2017 and projecting attainment to 2030 of the health-related Sustainable Development Goals for 195 countries and territories: a systematic analysis for the Global Burden of Disease Study 2017. *Lancet* 2018; **392:** 2091–138.

Methods appendix to Measuring progress and projecting attainment on the basis of past trends of the health-related Sustainable Development Goals in 195 countries and territories: an analysis from the Global Burden of Disease Study 2017

This appendix provides further methodological detail for the health-related Sustainable Development Goals. The appendix is organized into broad sections following the structure of the main paper.

Table of Contents

Authors' Contributions i

List of Supplementary Results: Figures and Tables........ 3

Preamble 6

GATHER Statement 7

Part 1. Health-related Sustainable Development Goals (SDGs) indicators........8

Section 1. SDGs overview

Section 2. Health-related SDGs

Section 3. Indicator-specific estimation

Part 2. SDG index construction 387

Part 3. Projections for the health-related SDGs 388

Section 1. Overall projection modeling strategy

Section 2. Independent drivers

Section 3. Cause models

Section 4. HIV

Section 5. Universal Health Coverage (UHC) index

Part 4. Online tools and abbreviations 410

Section 1. Online tools

Section 2. List of abbreviations

Section 3. List of ISO3 codes and location names

Appendix Table. Socio-demographic Index groupings by geography, based on 2017 values........416

Authors' Contributions

Managing the estimation process

Ashkan Afshin, Tahiya Alam, Celine Barthelemy, Molly Biehl, Michael Brauer, Aaron Cohen, Elizabeth Cromwell, Lalit Dandona, Rakhi Dandona, Louisa Degenhardt, Samath Dharmaratne, Daniel Dicker, Kara Estep, Valery Feigin, Christina Fitzmaurice, Nancy Fullman, Emmanuela Gakidou, Caitlin Hawley, Simon Hay, Spencer James, Nicholas Kassebaum, Ibrahim A Khalil, Hmwe Kyu, Heidi Larson, Stephen Lim, Alan Lopez, Rafael Lozano, Ashley Marks, Awoke Misganaw, Ali Mokdad, Erin Mullany, Christopher Murray, Mohsen Naghavi, Helen Olsen, David Pigott, Robert Reiner, Joseph Salama, Katya Shackelford, Chloe Shields, Shreya Shirude, Amanda Smith, Mari Smith, Jeffrey Stanaway, Stein Emil Vollset, Theo Vos, and Harvey Whiteford

Writing the first draft of the manuscript

Ryan Barber, Celine Barthelemy, Julian Chalek, Leslie Cornaby, Erika Eldrenkamp, Nancy Fullman, James Lee, Rafael Lozano, Molly Miller-Petrie, John Everett Mumford, Chloe Shields, and Jamal Yearwood

Providing data or critical feedback on data sources

Solomon Mequanente Abay, Cristiana Abbafati, Alireza Abdi, Ibrahim Abdollahpour, Zegeye Abebe, Victor Aboyans, Aklilu Abrham Roba, Laith Abu-Raddad, Niveen Abu-Rmeileh, Gebre Abyu, Manfred Accrombessi, Abdu Adamu, Oladimeji Adebayo, Isaac Adedeji, Rufus A. Adedoyin, Olatunji Adetokunboh, Tara Ballav Adhikari, Mina Adib, Mohsen Afarideh, Mahdi Afshari, Sargis Aghayan, Dominic Agius, Alireza Ahmadi, Hamid Ahmadieh, Muktar Ahmed, Ali Shafqat Akanda, Mohammad Esmaeil Akbari, Rufus Akinyemi, Tomi Akinyemiju, Nadia Akseer, Fares Alahdab, Khurshid Alam, Kefyalew Addis Alene, Raghib Ali, Syed Aljunid, François Alla, Peter Allebeck, Ali Almasi, Rajaa Al-Raddadi, Ubai Alsharif, Nelson Alvis-Guzman, Azmeraw T. Amare, Walid Ammar, Jason Anderson, Hossein Ansari, Ansariadi Ansariadi, Mustafa Geleto Ansha, Carl Abelardo Antonio, Palwasha Anwari, Olatunde Aremu, Monika Arora, Krishna K Aryal, Ephrem Tsegay Asfaw, Solomon Weldegebreal Asgedom, Rana Asghar, Reza Assadi, Ashish Awasthi, Beatriz Paulina Ayala Quintanilla, Rakesh Ayer, Peter Azzopardi, Arefeh Babazadeh, Alaa Badawi, Kalpana Balakrishnan, Maciej Banach, Amitava Banerjee, Joseph Banoub, Amrit Banstola, Miguel A. Barboza, Till Bärnighausen, Lope Barrero, Sanjay Basu, Robert Battista, Habtamu Wondifraw Baynes, Neeraj Bedi, Ettore Beghi, Masoud Behzadifar, Meysam Behzadifar, Bayu Begashaw Bekele, Saba Abraham Belay, Yihalem Abebe Belay, Aminu Bello, Derrick Bennett, Isabela Bensenor, Adam Berman, Robert Bernstein, Mircea Beuran, Suraj Bhattarai, Soumyadeep Bhaumik, Zulfiqar Bhutta, Belete Biadgo, Boris Bikbov, Nigus Bililign, Muhammad Shahdaat Bin Sayeed, Sait Mentes Birlik, Charles Birungi, Donal Bisanzio, Espen Bjertness, Rupert Bourne, Nicola Luigi Bragazzi, Luisa Brant, Michael Brauer, Alexandra Brazinova, Nicholas Breitborde, Gabrielle Britton, Traolach Brugha, Julio Cesar Campuzano, Josip Car, Mate Car, Juan J Carrero, Felix Carvalho, Deborah Carvalho Malta, Carlos Castañeda-Orjuela, Franz Castro, Alanur Cavlin, Yazan Chaiah, Hsing-Yi Chang, Jung-Chen Chang, Pankaj Chaturvedi, Peggy Pei-Chia Chiang, Abdulaal Chitheer, Devasahayam Christopher, Sheng-Chia Chung, Massimo Cirillo, Rafael Claro, Cyrus Cooper, Monica Cortinovis, Lalit Dandona, Rakhi Dandona, Paul Dargan, Ahmad Daryani, Siddharth Das, José Das Neves, Anand Dayama, Louisa Degenhardt, Kebede Deribe, Nikolaos Dervenis, Getenet Dessie, Samath Dharmaratne, Meghnath Dhimal, Klara Dokova, David Teye Doku, Leilei Duan, Manisha Dubey, Eyasu Ejeta Duken, Andre Duraes, Soheil Ebrahimpour, Ziad El-Khatib, Iqbal Elyazar, Aman Endries, Babak Eshrati, Sharareh Eskandarieh, Alireza Esteghamati, Sadaf Esteghamati, Mahdi Fakhar, Hamed Fakhim, Mohammad Fareed, Carla Farinha, Andrea Farioli,

Andre Faro, Farshad Farzadfar , Mohammad Hosein Farzaei , Valery Feign, Rachel Feldman, Seyed-Mohammad Fereshtehnejad, Eduarda Fernandes, Joao Fernandes, Irina Filip, Jonas David Finger, Nataliya Foigt, Richard Franklin, Takeshi Fukumoto, Nancy Fullman, João M. Furtado, Ruoyan Gai, G Fortune Gankpe, M.A. Garcia-Gordillo, Tigist Gashaw, Teshome Gebre, Tsegaye Gebrehiwot, Amanuel Tesfay Gebremedhin, Merhawi Gebremedhin, Bereket Gebremichael, Tilayie Gelano, Johanna Geleijnse, Ayele Geleto, Peter Gething, Kebede Embaye Gezae, Reza Ghadimi, Keyghobad Ghadiri, Hesam Ghiasvand, Mamata Ghimire, Simona Giampaoli, Kidu Gidey, Giorgia Giussani, Srinivas Goli, Philimon Gona, Amador Goodridge, Sameer Gopalani, Atsushi Goto, Bárbara Goulart, Ayman Grada, Giuseppe Grosso, Andre Guimaraes, Prakash Gupta, Rahul Gupta, Rajat Das Gupta, Rajeev Gupta, Tanush Gupta, Juanita Haagsma, Nima Hafezi-Nejad, Tekleberhan Beyene Hagos, Michael Tamene Haile, Arvin Haj-Mirzaian, Arya Haj-Mirzaian, Hilda Harb, Josep Maria Haro, Mehedi Hasan, Hadi Hassankhani, Hamid Y. Hassen, Rasmus Havmoeller, Mohamed Hegazy, Behnam Heidari, Delia Hendrie, Andualem Henok, Ileana B. Heredia-Pi, Fatemeh Heydarpour, Hans Hoek, Michael Hole, Enayatollah Homaie Rad, Praveen Hoogar, H Dean Hosgood, Mehdi Hosseinzadeh, Mihaela Hostiuc, Sorin Hostiuc, Mohamed Hsairi, Guoqing Hu, Trang Huyen Nguyen, Ehimario Igumbor, Usman Iqbal, Sheikh Mohammed Shariful Islam, Farhad Islami, Kathryn H. Jacobsen, Nader Jahanmehr, Mihajlo Jakovljevic, Amr Jamal, Simerjot Jassal, Mehdi Javanbakht, Achala Jayatilleke, Panniyammakal Jeemon, Vivekanand Jha, Catherine Johnson, Sarah Johnson, Jost B. Jonas, Zahra Jorjoran Shushtari, Jacek Jozwiak, Mikk Jürisson, Zubair Kabir, Rajendra Kadel, Amaha Kahsay, Molla Kahssay, Rizwan Kalani, Manoochehr Karami, Narges Karimi, Seyed M. Karimi, Amir Kasaeian, Getachew Mullu Kassa, Nicholas J Kassebaum, Anil Kaul, Norito Kawakami, Zhila Kazemi, Dhruv S. Kazi, Peter Keiyoro, Andre Keren, Yousef Khader, Morteza Abdullatif Khafaie , Muhammad Ali Khan, Young-Ho Khang, Mona Khater, Alireza Khatony, Zahra Khazaeipour, Abdullah T. Khoja, Ardeshir Khosravi, Mohammad Hossein Khosravi, Jagdish Khubchandani, Aliasghar Kiadaliri, Helen Kiarie, Daniel Kim, Jun Kim, Young-Eun Kim, Yun Jin Kim, Anh Kim Dang, Adnan Kisa, Niranjan Kissoon, Jonathan Kocarnik, Sonali Kochhar , Soewarta Kosen, Parvaiz Koul, Ai Koyanagi, Michael Kravchenko, Kewal Krishan, Kristopher Krohn, Barthelemy Kuate Defo, Burcu Kucuk Bicer, G Anil Kumar, Manasi Kumar, Pushpendra Kumar, Michael Kutz, Carl Lachat, Deepesh Lad, Sheetal Lad, Alessandra Lafranconi, Dharmesh Lal, Hilton Lam, Prabhat Lamichhane, Huong Lan Nguyen, Van Lansingh, Anders Larsson, Dennis Laryea, Jaet Leasher, Yirga Legesse, Misgan Legesse Liben, James Leigh, Mall Leinsalu, Janni Leung, Xiaohong Li, Yichong Li, Juan Liang, Xiaofeng Liang, Lee-Ling Lim, Stephen Lim, Shiwei Liu, Alan Lopez, Stefan Lorkowski, Paulo Lotufo, Erlyn Rachelle Macarayan, Mark Mackay, Emilie Maddison, Dhaval Maghavani, Carlos Magis-Rodriguez, Narayan Mahotra, Marek Majdan, Reza Majdzadeh, Azeem Majeed, Reza Malekzadeh, Abdullah Mamun, Luiz Garcia Mandarano-Filho, Srikanth Mangalam, Mohammad Ali Mansournia, Joemer Maravilla, Randall Martin, Francisco Rogerlândio Martins-Melo, Benjamin Massenburg, Manu Mathur, Mohsen Mazidi, Colm Mcalinden, John Mcgrath, Brian Mcmahon, Man Mohan Mehndiratta, Varshil Mehta, Addisu Melese, Mulugeta Melku, Peter Memiah, Ziad Memish, Walter Mendoza, Getnet Mengistu, Gert Mensink, Atte Meretoja, Tuomo Meretoja, Tomislav Mestrovic, Haftay Berhane Mezgebe, Bartosz Miazgowski, Tomasz Miazgowski, Ted R Miller, Parvaneh Mirabi, Erkin Mirrakhimov, Babak Moazen, Karzan Mohammad, Moslem Mohammadi, Noushin Mohammadifard, Shafiu Mohammed, Murali Mohan, Farnam Mohebi, Ali Mokdad, Lorenzo Monasta, Julio Montañez, Mahmood Moosazadeh, Ghobad Moradi, Maziar Moradi-Lakeh, Mehdi Moradinazar, Lidia Morawska, Joana Morgado-Da-Costa, Naho Morisaki, Shane Morrison, Seyyed Meysam Mousavi, Achenef Muche, Ulrich Mueller, Erin Mullany, Manoj Murhekar, Srinivas Murthy, Kamarul Imran Musa, Ghulam Mustafa, Ashraf Nabhan, Gabriele Nagel, Mohsen

Naghavi, Seyed Sina Naghibi Irvani, Luigi Naldi, Hae-Sung Nam, Bruno Nascimento, Haseeb Nawaz, Ionut Negoi, Ruxandra Irina Negoi, Charles Newton, Frida Ngalesoni, Josephine Ngunjiri, Anh Nguyen, Ha Nguyen, Huong Nguyen, Marika Nomura, Mehdi Noroozi, Jean Jacques Noubiap, Hamid Reza Nouri, Malihe Nourollahpour, Okechukwu Ogah, Felix Ogbo, Anselm Okoro, Olanrewaju Oladimeji, Andrew T. Olagunju, Tinuke O. Olagunju, Bolajoko Olusanya, Jacob Olusanya, Kanyin Ong, Sok King Ong, John Nelson Opio, Alberto Ortiz, Stanislav Otstavnov, Mayowa Owolabi, Mahesh Pa, Smita Pakhale, Wen-Harn Pan, Basant Kumar Panda, Songhomitra Panda-Jonas, Jeyaraj Pandian, Shanti Patel, Vishnupriya Rao Paturi, Deepak Paudel, Neil Pearce, Emmanuel Peprah, Norberto Perico, William Petri, Max Petzold, Michael R Phillips, Meghdad Pirsaheb, Hossein Poustchi, Swayam Prakash, Narayan Prasad, Manita Pyakurel, Mostafa Qorbani, Amir Radfar, Anwar Rafay, Alireza Rafiei, Fakher Rahim, Afarin Rahimi-Movaghar, Vafa Rahimi-Movaghar, Mohammad Hifz Ur Rahman, Sajjad Rahman, Rajesh Kumar Rai, Fatemeh Rajati, Sasa Rajsic, Usha Ram, Chhabi Lal Ranabhat, Prabhat Ranjan, David Laith Rawaf, Salman Rawaf, Christian Razo, Giuseppe Remuzzi, Andre Renzaho, Luz Myriam Reynales-Shigematsu, Shahab Rezaeian, Antonio Luiz Ribeiro, María Jesús Ríos Blancas, Leonardo Roever, Luca Ronfani, Gholamreza Roshandel, Ali Rostami, Gregory Roth, Ambuj Roy, Enrico Rubagotti, Hosein Safari, Saeid Safiri, Mohammad Ali Sahraian, Nasir Salam, Joseph Salama, Payman Salamati, Yahya Salimi, Hamideh Salimzadeh, Evanson Z Sambala, Abdallah M. Samy, Juan Sanabria, Milena Santric Milicevic, Bruno Sao Jose, Muthupandian Saravanan, Mayank Sardana, Abdur Razzaque Sarker, Nizal Sarrafzadegan, Shahabeddin Sarvi, Maheswar Satpathy, Monika Sawhney, Sonia Saxena, Mete Şaylan, Mehdi Sayyah, Elke Schaeffner, David C Schwebel, Falk Schwendicke, Soraya Seedat, Mario Šekerija, Sadaf Sepanlou, Edson Serván-Mori, Seyedmojtaba Seyedmousavi, Amira Shaheen, Masood Ali Shaikh, Mehran Shams-Beyranvand, Kiomars Sharafi, Mehdi Sharif, Jayendra Sharma, Sharad Kumar Sharma, Jun She, Aziz Sheikh, Peilin Shi, Kenji Shibuya, Girma Temam Shifa, Rahman Shiri, Ivy Shiue, Farhad Shokraneh, Haitham Shoman, Soraya Siabani, Abla Sibai, Diego Augusto Santos Silva, Dayane Silveira, Jasvinde Singh, Prashant Kumar Singh, Virendra Singh, Dhirendra Narain Sinha, Mekonnen Sisay, Eirini Skiadaresi, Karen Sliwa, Amanda E Smith, Soheila Sobhani, Michael Soljak, Reed Sorensen, Luisa Sorio Flor, Angela Spinelli, Luciano Sposato, Chandrashekhar T Sreeramareddy, Vasiliki Stathopoulou, Leo Stockfelt, Mark Stokes, Kurt Straif, Agus Sudaryanto, Muawiyyah Babale Sufiyan, Rizwan Suliankatchi Abdulkader, Bruno Sunguya, Bryan L Sykes, Dillon Sylte, Cassandra Szoeke, Rafael Tabarés-Seisdedos, Mesfin Tadese, Frank Tanser, Nuno Taveira, Gebre Teklemariam Demoz, Awoke Temesgen, Mohamad-Hani Temsah, Omar Temsah, Abdullah Terkawi, Belay Tessema, Mebrahtu Teweldemedhin, Tung Thanh Tran, Laura Thomas, Nihal Thomas, Giang Thu Vu, Marcello Tonelli, Miguel Tortajada-Girbés, Marcos Roberto Tovani-Palone, Bach Tran, Khanh Bao Tran, Thomas Truelsen, E Murat Tuzcu, Kingsley N. Ukwaja, Irfan Ullah, Olalekan Uthman, Muthiah Vaduganathan, Afsane Vaezi, Gaurang Vaidya, Pascual Valdez, Aaron Van Donkelaar, Tommi Vasankari, Narayanaswamy Venketasubramanian, Sergey Vladimirov, Vasiliy Vlassov, Sebastian Vollmer, Fasil Wagnew, Yasir Waheed, Judd Walson, Yanping Wang, Ying-Wei Wang, Yuan-Pang Wang, Elisabete Weiderpass, Robert Weintraub, Jordan Weiss, Inbal Weiss Salz, Andrea Werdecker, Adhena A. Werkneh, Ronny Westerman, Joanna Whisnant, Justyna Widecka, Tissa Wijeratne, Charles Shey Wiysonge, Charles Wolfe, Shouling Wu, Denis Xavier , Gelin Xu, Ali Yadollahpour, Seysd Hossein Yahyazadeh Jabbari, Bereket Yakob, Lijing Yan, Yasin Jemal Yasin, Pengpeng Ye, Jamal Yearwood, Alex Yeshaneh, Manaye Yihune, Ebrahim M. Yimer, Biruck Desalegn Yirsaw, Zemenu Yohannes, Naohiro Yonemoto, Gerald Yonga, Marcel Yotebieng, Sojib Bin Zaman, Mohammad Zamani, Zohreh Zare, Luis Zavala-Arciniega, Desalegn Zegeye, Elias Asfaw Zegeye, Kazem Zendehdel, Xueying Zhang, Jun Zhu, Sanjay Zodpey, and Liesl Zuhlke.

Developing methods or computational machinery

Ibrahim Abdollahpour, Rufus A. Adedoyin, Mehdi Ahmadi, Mohammad Esmaeil Akbari, Mehran Alijanzadeh, Christine Allen, Catalina Liliana Andrei, Suleman Atique, Marcel Ausloos, Habtamu Wondifraw Baynes, Neeraj Bedi, Abadi Kidanemariam Berhe, Sait Mentes Birlik, Donal Bisanzio, Michael Brauer, Charlton Callender, Austin Carter, Kate Causey, Kelly Cercy, Yilma Chisha, Cyrus Cooper, Ahmad Daryani, Getenet Dessie, Daniel Dicker, Leilei Duan, Farshad Farzadfar , Samuel Finegold, Christina Fitzmaurice, Tahvi Frank, Kai Fukutaki, Nancy Fullman, Keyghobad Ghadiri, Taren Gorman, Max Griswold, Hamid Y. Hassen, Claudiu Herteliu, Mehdi Hosseinzadeh, Chantal Huynh, Spencer James, Sarah Johnson, Nicholas J Kassebaum, Laura Kemmer, Muhammad Shahzeb Khan, Adnan Kisa, Xie Rachel Kulikoff, Michael Kutz, Kathryn Lau, Misgan Legesse Liben, James Leigh, Stephen Lim, Randall Martin, Tefera Chane Mekonnen, Shawn Minnig, W Cliff Mountjoy-Venning, Haseeb Nawaz, Grant Nguyen, Bolajoko Olusanya, Jacob Olusanya, Kanyin Ong, Mayowa Owolabi, Adrian Pana, Charles Parry, David Pigott, Martin Pletcher, Alireza Rafiei, Chhabi Lal Ranabhat, Robert Reiner, Seyed Mohammad Riahi, Nicholas Roberts, Gregory Roth, Abdallah M. Samy, Maheswar Satpathy, David C Schwebel, Mehdi Sharif, Tariq Jamal Siddiqi, Naris Silpakit, Virendra Singh, Dhirendra Narain Sinha, Vinay Srinivasan, Jeffrey Stanaway, Leo Stewart, Patrick Sur, Ipsita Sutradhar, Dillon Sylte, Andrew Theis, Irfan Ullah, Rachel L. Updike, Muhammad Shariq Usman, Stein Emil Vollset, Ali Yadollahpour, Seysd Hossein Yahyazadeh Jabbari, Lijing Yan, Jamal Yearwood, Naohiro Yonemoto, and Leo Zoeckler.

Applying analytical methods to produce estimates

Rufus A. Adedoyin, Ashkan Afshin, Sutapa Agrawal, Mehdi Ahmadi, Muktar Ahmed, Sayem Ahmed, Christine Allen, Azmeraw T. Amare, Catalina Liliana Afndrei, Olatunde Aremu, Marcel Ausloos, Arindam Basu, Robert Battista, Bayu Begashaw Bekele, Gregory Bertolacci, Muhammad Shahdaat Bin Sayeed, Sait Mentes Birlik, Donal Bisanzio, Alexandra Brazinova, Charlton Callender, Austin Carter, Kate Causey, Kelly Cercy, Julian Chalek, Yilma Chisha, Devasahayam Christopher, Cyrus Cooper, Leslie Cornaby, Elizabeth Cromwell, Matthew Cunningham, Ahmad Daryani, Tamirat Dasa, Anand Dayama, Getenet Dessie, Daniel Dicker, Leilei Duan, Aman Endries, Alireza Esteghamati, Mohammad Fareed, Samuel Finegold, Christina Fitzmaurice, Tahvi Frank, Kai Fukutaki, Nancy Fullman, William Gardner, Bereket Gebremichael, Teklu Gebremichael, Ayele Geleto, Keyghobad Ghadiri, Ellen Goldberg, Max Griswold, Nima Hafezi-Nejad, Michael Tamene Haile, Gessessew Bugssa Hailu, Hamid Y. Hassen, Claudiu Herteliu, Thomas Hsiao, Trang Huyen Nguyen, Caleb Irvine, Spencer James, Catherine Johnson, Sarah Johnson, Madhanraj K, Manoochehr Karami, Nicholas J Kassebaum, Jagdish Khubchandani, Yun Jin Kim, Anh Kim Dang, Adnan Kisa, Jonathan Kocarnik, Xie Rachel Kulikoff, Hmwe Kyu, Huong Lan Nguyen, Van Lansingh, Kathryn Lau, Jorge Ledesma, James Lee, Misgan Legesse Liben, James Leigh, Xiaofeng Liang, Stephen Lim, Lydia Lucchesi, Emilie Maddison, Helena Manguerra, Randall Martin, Ira Martopullo, Mohsen Mazidi, Tefera Chane Mekonnen, Hagazi Gebre Meles, Anoushka Millear, Shawn Minnig, Shafiu Mohammed, Ali Mokdad, W Cliff Mountjoy-Venning, John Everett Mumford, Mohsen Naghavi, Haseeb Nawaz, Grant Nguyen, Emma Nichols, In-Hwan Oh, Bolajoko Olusanya, Jacob Olusanya, Kanyin Ong, Andrei Oros, Adrian Pana, Katherine Paulson, David Pereira, David Pigott, Martin Pletcher, Caroline Purcell, Fakher Rahim, Mahfuzar Rahman, Chhabi Lal Ranabhat, Robert Reiner, Marissa Reitsma, Seyed Mohammad Riahi, Abdallah M. Samy, Maheswar Satpathy, Seyedmojtaba Seyedmousavi, Masood Ali Shaikh, Mehdi Sharif, Shreya Shirude, Virendra Singh, Reed Sorensen, Luisa Sorio Flor, Chandrashekhar T Sreeramareddy, Vinay Srinivasan, Jeffrey Stanaway, Leo Stewart, Patrick Sur, Dillon Sylte, Tung Thanh Tran, Andrew Theis, Giang Thu Vu, Bach Tran, Khanh Bao Tran, Christopher Troeger, Hayley Tymeson, Kingsley N. Ukwaja, Irfan Ullah, Rachel L. Updike, Theo Vos, Ronny Westerman, Tissa Wijeratne, Lauren

Wilner, Ali Yadollahpour, Jamal Yearwood, Biruck Desalegn Yirsaw, Seok-Jun Yoon, Chuanhua Yu, and Leo Zoeckler.

Providing critical feedback on methods or results

Degu Abate, Aberash Abay, Solomon Mequanente Abay, Cristiana Abbafati, Nooshin Abbasi, Hedayat Abbastabar, Foad Abd-Allah, Omar Abdel-Rahman, Alireza Abdi, Ibrahim Abdollahpour, Zegeye Abebe, Haftom Abraha, Aklilu Abrham Roba, Niveen Abu-Rmeileh, Gebre Abyu, Dilaram Acharya, Pawan Acharya, Abdu Adamu, Oladimeji Adebayo, Isaac Adedeji, Rufus A. Adedoyin, Victor Adekanmbi, Olatunji Adetokunboh, Tara Ballav Adhikari, Mina Adib, Kouablan Arsene Adou, José C. Adsuar, Mohsen Afarideh, Ashkan Afshin, Gina Agarwal, Sargis Aghayan, Sutapa Agrawal, Anurag Agrawl, Muktar Ahmed, Sayem Ahmed, Temesgen Akalu, Ali Shafqat Akanda, Mohammad Esmaeil Akbari, Mohammed Akibu, Rufus Akinyemi, Tomi Akinyemiju, Nadia Akseer, Fares Alahdab, Ziyad Al-Aly, Khurshid Alam, Tahiya Alam, Ammar N.H. Albujeer, Kefyalew Addis Alene, Ayman Al-Eyadhy, Samia Alhabib, Raghib Ali, Mehran Alijanzadeh, Reza Alizadeh-Navaei, Syed Aljunid, Ala'a Alkerwi, Peter Allebeck, Ali Almasi, Fatma Al-Maskari, Hesham Al-Mekhlafi, Jordi Alonso, Rajaa Al-Raddadi, Ubai Alsharif, Khalid Altirkawi, Nelson Alvis-Guzman, Azmeraw T. Amare, Kebede Amenu, Erfan Amini, Walid Ammar, Catalina Liliana Andrei, Sofia Androudi, Mina Anjomshoa, Hossein Ansari, Mustafa Geleto Ansha, Carl Abelardo Antonio, Palwasha Anwari, Lambert Appiah, Olatunde Aremu, Johan Ärnlöv, Krishna K Aryal, Hamid Asayesh, Ephrem Tsegay Asfaw, Solomon Weldegebreal Asgedom, Reza Assadi, Zeihun Ataro, Suleman Atique, Sachin Atre, Madhu S. Atteraya, Marcel Ausloos, Leticia Avila-Burgos, Euripide Avokpaho, Ashish Awasthi, Beatriz Paulina Ayala Quintanilla, Henok Tadesse Ayele, Rakesh Ayer, M.Reza Azarpazhooh, Peter Azzopardi, Natasha Azzopardi Muscat, Tesleem Babalola, Arefeh Babazadeh, Alaa Badawi, Amitava Banerjee, Joseph Banoub, Amrit Banstola, Aleksandra Barac, Miguel A. Barboza, Till Bärnighausen, Lope Barrero, Quique Bassat, Arindam Basu, Sanjay Basu, Robert Battista, Bernhard Baune, Habtamu Wondifraw Baynes, Shahrzad Bazargan-Hejazi, Neeraj Bedi, Ettore Beghi, Meysam Behzadifar, Yannick Béjot, Abate Bekele, Bayu Begashaw Bekele, Saba Abraham Belay, Yihalem Abebe Belay, Michelle Bell, Aminu Bello, Derrick Bennett, Isabela Bensenor, Habib Benzian, Adugnaw Berhane, Abadi Kidanemariam Berhe, Adam Berman, Eduardo Bernabe, Robert Bernstein, Balem Demtsu Betsu, Mircea Beuran, Neeraj Bhala, Ashish Bhalla, Suraj Bhattarai, Soumyadeep Bhaumik, Zulfiqar Bhutta, Belete Biadgo, Ali Bijani, Boris Bikbov, Nigus Bililign, Muhammad Shahdaat Bin Sayeed, Sait Mentes Birlik, Charles Birungi, Tuhin Biswas, Helen Bitew, Hailemichael Bizuneh, Espen Bjertness, Eshetu Mulisa Bobasa, Soufiane Boufous, Rupert Bourne, Kayvan Bozorgmehr, Nicola Luigi Bragazzi, Luisa Brant, Michael Brauer, Nicholas Breitborde, Gabrielle Britton, Traolach Brugha, Reinhard Busse, Zahid Butt, Alessandra C Goulart, Lucero Cahuana-Hurtado, Ismael Campos-Nonato, Jorge Cano, Mate Car, Rosario Cárdenas, Juan J Carrero, Austin Carter, Felix Carvalho, Deborah Carvalho Malta, Carlos Castañeda-Orjuela, Franz Castro, Ester Cerin, Yazan Chaiah, Julian Chalek, Jung-Chen Chang, Aparajita Chattopadhyay, Vijay Kumar Chattu, Peggy Pei-Chia Chiang, Ken Chin, Yilma Chisha, Vesper Chisumpa, Jee-Young Choi, Hanne Christensen, Devasahayam Christopher, Sheng-Chia Chung, Flavia Cicuttini, Liliana G Ciobanu, Rafael Claro, Thomas Claßen, Aaron Cohen, Daniel Collado-Mateo, Cyrus Cooper, Monica Cortinovis, Megan Costa, Ewerton Cousin, Christopher Crowe, Alemneh Kabeta Daba, Abel Dadi, Lalit Dandona, Rakhi Dandona, Paul Dargan, Ahmad Daryani, José Das Neves, Tamirat Dasa, Adrian Davis, Kairat Davletov, Anand Dayama, Barbora De Courten, Jan-Walter De Neve, Hans De Steur, Megbaru Debalkie, Louisa Degenhardt, Tizta Degfie, Robert Dellavalle, Edgar Denova-Gutierrez, Nebiyu Dereje, Kebede Deribe, Nikolaos Dervenis, Getenet Dessie, Subhojit Dey, Samath Dharmaratne, Meghnath Dhimal, Eric L. Ding, Shirin Djalalinia, Klara Dokova, David Teye Doku, Tim Driscoll, Manisha Dubey, Eleonora Dubljanin, Eyasu Ejeta Duken,

Bruce Duncan, Andre Duraes, Soheil Ebrahimpour, David Edvardsson, Ziad El-Khatib, Ahmadali Enayati, Aman Endries, Babak Eshrati, Sharareh Eskandarieh, Alireza Esteghamati, Sadaf Esteghamati, Hamed Fakhim, Jessica Fanzo, Mahbobeh Faramarzi, Farzaneh Farhadi, Talha Farid, Carla Farinha, Andrea Farioli, Andre Faro, Farshad Farzadfar , Mohammad Hosein Farzaei , Hosein Farzam, Mir Sohail Fazeli, Andrea B. Feigl, Valery Feign, Wubalem Fekadu, Netsanet Fentahun, Seyed-Mohammad Fereshtehnejad, Eduarda Fernandes, Joao Fernandes, Garumma T Feyissa, Daniel Fijabi, Irina Filip, Samuel Finegold, Jonas David Finger, Florian Fischer, Christina Fitzmaurice, Nataliya Foigt, Richard Franklin, Takeshi Fukumoto, Nancy Fullman, Thomas Fürst, João M. Furtado, Ruoyan Gai, Silvano Gallus, G Fortune Gankpe, Ana Garcia, Alberto L. García-Basteiro, Tigist Gashaw, Abadi Kahsu Gebre, Teshome Gebre, Gebremedhin Berhe Gebregergs, Tsegaye Gebrehiwot, Merhawi Gebremedhin, Afewerki Gebremeskel, Bereket Gebremichael, Teklu Gebremichael, Aregawi Gebreyesus Belay, Tilayie Gelano, Johanna Geleijnse, Ayele Geleto, Sefonias Getachew , Kebede Embaye Gezae, Reza Ghadimi, Keyghobad Ghadiri, Hesam Ghiasvand, Mamata Ghimire, Kidu Gidey, Tiffany Gill, Meaza Girma, Giorgia Giussani, Srinivas Goli, Philimon Gona, Amador Goodridge, Aloke Gopal, Sameer Gopalani, Taren Gorman, Atsushi Goto, Bárbara Goulart, Ayman Grada, Giuseppe Grosso, Harish Gugnani, Francis Guillemin, Yuming Guo, Rahul Gupta, Rajat Das Gupta, Rajeev Gupta, Tanush Gupta, Juanita Haagsma, Vladimir Hachinski, Nima Hafezi-Nejad, Hassan Haghparast-Bidgoli, Tekleberhan Beyene Hagos, Dessalegn Haile, Michael Tamene Haile, Gessessew Bugssa Hailu, Arvin Haj-Mirzaian, Arya Haj-Mirzaian, Randah Hamadeh, Samer Hamidi, Graeme Hankey, Hilda Harb, Habtamu Hareri, Sivadasanpillai Harikrishnan, Hamidreza Haririan, Josep Maria Haro, Mehedi Hasan, Hadi Hassankhani, Hamid Y. Hassen, Rasmus Havmoeller, Caitlin Hawley, Simon Hay, Mohamed Hegazy, Behzad Heibati, Behnam Heidari, Mohsen Heidari, Delia Hendrie, Ileana B. Heredia-Pi, Claudiu Herteliu, Fatemeh Heydarpour, Sousan Heydarpour, Desalegn Hibstu, Daniel Hoffman, Michael Hole, Enayatollah Homaie Rad, Praveen Hoogar, Nobuyuki Horita, H Dean Hosgood, Mostafa Hosseini, Mihaela Hostiuc, Sorin Hostiuc, Peter Hotez, Guoqing Hu, John Huang, Trang Huyen Nguyen, Ehimario Igumbor, Olayinka Ilesanmi, Usman Iqbal, Sheikh Mohammed Shariful Islam, Farhad Islami, Rebecca Ivers, Neda Izadi, Kathryn H. Jacobsen, Nader Jahanmehr, Mihajlo Jakovljevic, Moti Jalu, Spencer James, Mehdi Javanbakht, Ahamarshan Jayaraman Nagarajan, Panniyammakal Jeemon, Ayalew Jejaw Zeleke, Ravi Prakash Jha, Vivekanand Jha, Sarah Johnson, Jost B. Jonas, Jitendra Jonnagaddala, Zahra Jorjoran Shushtari, Ankur Joshi, Jacek Jozwiak, Suresh Jungari, Mikk Jürisson, Madhanraj K, Zubair Kabir, Rajendra Kadel, Amaha Kahsay, Molla Kahssay, Rizwan Kalani, Manoochehr Karami, Marina Karanikolos, Seyed M. Karimi, Hamidreza Karimi-Sari, Amir Kasaeian, Getachew Mullu Kassa, Tesfaye Kassa, Nicholas J Kassebaum, Vittal Katikireddi, Zhila Kazemi, Dhruv S. Kazi, Peter Keiyoro, Andre P Kengne, Chandrasekharan Nair Kesavachandran, Yousef Khader, Behzad Khafaie, Morteza Abdullatif Khafaie , Nauman Khalid, Ibrahim Khalil, Ejaz Khan, Muhammad Ali Khan, Muhammad Shahzeb Khan, Young-Ho Khang, Tripti Khanna, Mona Khater, Zahra Khazaeipour, Habibolah Khazaie, Abdullah T. Khoja, Ardeshir Khosravi, Mohammad Hossein Khosravi, Jagdish Khubchandani, Aliasghar Kiadaliri, Getiye Kibret, Daniel Kim, Young-Eun Kim, Yun Jin Kim, Anh Kim Dang, Ruth Kimokoti, Yohannes Kinfu, Sanjay Kinra, Adnan Kisa, Katarzyna Kissimova-Skarbek, Niranjan Kissoon, Mika Kivimaki, Sonali Kochhar , Yoshihiro Kokubo, Tufa Kolola, Jacek Kopec, Margaret Kosek, Parvaiz Koul, Ai Koyanagi, Kristopher Krohn, Barthelemy Kuate Defo, Andreas Kudom, G Anil Kumar, Manasi Kumar, Pushpendra Kumar, Sudhir Kumar Jain, Deepesh Lad, Sheetal Lad, Alessandra Lafranconi, Abraham Lagat, Dharmesh Lal, Ratilal Lalloo, Hilton Lam, Faris Lami, Prabhat Lamichhane, Qing Lan, Huong Lan Nguyen, Justin Lang, Van Lansingh, Sonia Lansky, Anders Larsson, Dennis Laryea, Zohra Lassi, Arman Latifi, Avula Laxmaiah, Jeffrey Lazarus, Paul Lee, Yirga Legesse, Misgan Legesse Liben, James Leigh, Mall Leinsalu, Cheru T Leshargie,

Sonia Lewycka, Shanshan Li, Lee-Ling Lim, Stephen Lim, Miteku Limenih, Shai Linn, Yang Liu, Rakesh Lodha, Alan Lopez, Stefan Lorkowski, Paulo Lotufo, Ronan Lyons, Saleem M. , Erlyn Rachelle Macarayan, Mark Mackay, Fabiana Madotto, Marek Majdan, Reza Majdzadeh, Azeem Majeed, Reza Malekzadeh, Abdullah Mamun, Mohammad Ali Mansournia, Chabila Mapoma, Joemer Maravilla, Wagner Marcenes, Sheila Martins, Francisco Rogerlândio Martins-Melo, Tivani Mashamba, Manu Mathur, Pallab K Maulik, Mohsen Mazidi, Colm Mcalinden, John Mcgrath, Martin Mckee, Brian Mcmahon, Suresh Mehata, Kala M. Mehta, Varshil Mehta, Tesfa Mekonen, Tefera Chane Mekonnen, Hagazi Gebre Meles, Addisu Melese, Mulugeta Melku, Peter Memiah, Ziad Memish, Walter Mendoza, Desalegn Tadese Mengistu, Getnet Mengistu, George Mensah, Gert Mensink, Beyene Meressa, Seid Tiku Mereta, Atte Meretoja, Tuomo Meretoja, Tomislav Mestrovic, Haftay Berhane Mezgebe, Tomasz Miazgowski, Ted R Miller, Molly Miller-Petrie, George Milne, Shawn Minnig, Mojde Mirarefin, Erkin Mirrakhimov, Philip B Mitchell, Babak Moazen, Bahram Mohajer, Moslem Mohammadi, Mohammed Mohammed, Shafiu Mohammed, Murali Mohan, Farnam Mohebi, Ali Mokdad, Mariam Molokhia, Lorenzo Monasta, Ghobad Moradi, Mahmoudreza Moradi, Maziar Moradi-Lakeh, Mehdi Moradinazar, Paula Moraga, Lidia Morawska, Joana Morgado-Da-Costa, Shane Morrison, Seyyed Meysam Mousavi, Achenef Muche, Kindie Fentahun Muchie, Ulrich Mueller, Erin Mullany, Srinivas Murthy, Jonah Musa, Kamarul Imran Musa, Ghulam Mustafa, Ashraf Nabhan, Jean Nachega, Gabriele Nagel, Seyed Sina Naghibi Irvani, Aliya Naheed, Azin Nahvijou, Kovin Naidoo, Gurudatta Naik, Farid Najafi, Vinay Nangia, Jobert Richie Nansseu, Bruno Nascimento, Haseeb Nawaz, Ayenew Negesse, Ionut Negoi, Ruxandra Irina Negoi, Subas Neupane, Frida Ngalesoni, Josephine Ngunjiri, Grant Nguyen, Emma Nichols, Solomon Nigatu, Muhammad Imran Nisar, Nomonde Nolutshungu, Ole Norheim, Mehdi Noroozi, Bo Norrving, Jean Jacques Noubiap, Hamid Reza Nouri, Malihe Nourollahpour, Mohammad Reza Nowroozi, Dina Nur Anggraini Ningrum, Peter Nyasulu, Richard Ofori-Asenso, Okechukwu Ogah, Felix Ogbo, In-Hwan Oh, Anselm Okoro, Kelechi Oladimeji, Olanrewaju Oladimeji, Andrew T. Olagunju, Tinuke O. Olagunju, Pedro Olivares, Helen Olsen, Bolajoko Olusanya, Jacob Olusanya, Anu Oommen, John Nelson Opio, Eyal Oren, Doris V. Ortega-Altamirano, Alberto Ortiz, Justin R Ortiz, Eduardo Ortiz-Panozo, Stanislav Otstavnov, Mayowa Owolabi, Mahesh Pa, Smita Pakhale, Abhijit Pakhare, Adrian Pana, Basant Kumar Panda, Songhomitra Panda-Jonas, Jeyaraj Pandian, Eun-Kee Park, Charles Parry, Hadi Parsian, Shanti Patel, Sanghamitra Pati, Ajay Patle, George Patton, Deepak Paudel, Emmanuel Peprah, David Pereira, Norberto Perico, Aslam Pervaiz, Konrad Pesudovs, Michael R Phillips, Julian Pillay, Meghdad Pirsaheb, Martin Pletcher, Constance Pond, Maarten Postma, Akram Pourshams, Hossein Poustchi, Dorairaj Prabhakaran, Swayam Prakash, Mostafa Qorbani, Reginald Quansah, Amir Radfar, Anwar Rafay, Alireza Rafiei, Fakher Rahim, Kazem Rahimi, Afarin Rahimi-Movaghar, Vafa Rahimi-Movaghar, Mahfuzar Rahman, Md Shafiur Rahman, Muhammad Aziz Rahman, Sajjad Rahman, Rajesh Kumar Rai, Usha Ram, Chhabi Lal Ranabhat, Prabhat Ranjan, Davide Rasella, David Laith Rawaf, Salman Rawaf, Christian Razo, Robert Reiner, Cesar Reis, Marissa Reitsma, Giuseppe Remuzzi, Andre Renzaho, Serge Resnikoff, Shahab Rezaeian, Mohammad Sadegh Rezai, Seyed Mohammad Riahi, Antonio Luiz Ribeiro, María Jesús Ríos Blancas, Kedir Teji Roba, Leonardo Roever, Luca Ronfani, Gholamreza Roshandel, Ali Rostami, Ambuj Roy, George Ruhago, Yogesh Sabde, Perminder Sachdev, Basema Saddik, Hosein Safari, Yahya Safari, Roya Safari-Faramani, Mahdi Safdarian, Saeid Safiri, Amirhossein Sahebkar, Mohammad Ali Sahraian, Haniye Sadat Sajadi, Nasir Salam, Raphael Saldanha, Zikria Saleem, Yahya Salimi, Hamideh Salimzadeh, Joshua A Salomon, Evanson Z Sambala, Abdallah M. Samy, Juan Sanabria, Maria Dolores Sanchez-Niño, Itamar Santos, Milena Santric Milicevic, Bruno Sao Jose, Muthupandian Saravanan, Mayank Sardana, Abdur Razzaque Sarker, Benn Sartorius, Brijesh Sathian, Thirunavukkarasu Sathish, Maheswar Satpathy, Miloje Savic, Arundhati Sawant, Monika

Sawhney, Sonia Saxena, Mete Şaylan, Mehdi Sayyah, Elke Schaeffner, Maria Inês Schmidt, Ione Schneider, Ben Schöttker, Aletta Schutte, David C Schwebel, Falk Schwendicke, Sadaf Sepanlou, Edson Serván-Mori, Seyedmojtaba Seyedmousavi, Hosein Shabaninejad, Azadeh Shafieesabet, Amira Shaheen, Masood Ali Shaikh, Mehran Shams-Beyranvand, Mohammadbagher Shamsi, Heidar Sharafi, Kiomars Sharafi, Mehdi Sharif, Mahdi Sharif-Alhoseini, Jayendra Sharma, Rajesh Sharma, Aziz Sheikh, Muki Shey, Chloe Shields, Girma Temam Shifa, Mika Shigematsu, Rahman Shiri, Ivy Shiue, Haitham Shoman, Mark Shrime, Sharvari Shukla, Si Si, Soraya Siabani, Tariq Jamal Siddiqi, Diego Augusto Santos Silva, João Pedro Silva, Dayane Silveira, Jasvinde Singh, Narinder Pal Singh, Om Prakash Singh, Paramjit Singh, Prashant Kumar Singh, Virendra Singh, Dhirendra Narain Sinha, Mekonnen Sisay, Eirini Skiadaresi, Adauto Martins Soares Filho, Badr Sobaih, Soheila Sobhani, Michael Soljak, Moslem Soofi, Reed Sorensen, Joan B Soriano, Luisa Sorio Flor, Ireneous Soyiri, Luciano Sposato, Chandrashekhar T Sreeramareddy, Raghavendra Guru Srinivasan, Jeffrey Stanaway, Nadine Steckling, Dan J Stein, Leo Stockfelt, Mark Stokes, Muawiyyah Babale Sufiyan, Rizwan Suliankatchi Abdulkader, Bruno Sunguya, Ipsita Sutradhar, Bryan L Sykes, Pn Sylaja, Dillon Sylte, Cassandra Szoeke, Rafael Tabarés-Seisdedos, Takahiro Tabuchi, Santosh Tadakamadla, Mesfin Tadese, Koku Tamirat, Nikhil Tandon, Nuno Taveira, Arash Tehrani-Banihashemi, Gebre Teklemariam Demoz, Awoke Temesgen, Habtamu Temesgen, Mohamad-Hani Temsah, Omar Temsah, Tewodros Tesfa, Belay Tessema, Mebrahtu Teweldemedhin, Tung Thanh Tran, Kavumpurathu Thankappan, Nihal Thomas, Alan Thomson, Amanda Thrift, Giang Thu Vu, Binyam Tilahun, Quyen G. To, Marcello Tonelli, Roman Topor-Madry, Miguel Tortajada-Girbés, Marcos Roberto Tovani-Palone, Jeffrey Towbin, Bach Tran, Khanh Bao Tran, Srikanth Tripathy, Thomas Truelsen, Lorainne Tudor Car, E Murat Tuzcu, Kingsley N. Ukwaja, Irfan Ullah, Muhammad Shariq Usman, Olalekan Uthman, Muthiah Vaduganathan, Afsane Vaezi, Pascual Valdez, Tommi Vasankari, Narayanaswamy Venketasubramanian, Ramesh Vidavalur, Santos Villafaina, Francesco S Violante, Sergey Vladimirov, Vasiliy Vlassov, Sebastian Vollmer, Stein Emil Vollset, Theo Vos, Kia Vosoughi, Isidora Vujcic, Gregory Wagner, Fasil Wagnew, Yasir Waheed, Judd Walson, Yuan-Pang Wang, Molla Mesele Wassie, Elisabete Weiderpass, Robert Weintraub, Jordan Weiss, Inbal Weiss Salz, Fitsum Weldegebreal, Andrea Werdecker, Adhena A. Werkneh, T. Eoin West, Ronny Westerman, Harvey Whiteford, Tissa Wijeratne, Lauren Wilner, Andrea Sylvia Winkler, Alison Beriliy Wiyeh, Charles Shey Wiysonge, Haileab Wolde, Charles Wolfe, Denis Xavier , Gelin Xu, Ali Yadollahpour, Bereket Yakob, Tomohide Yamada, Lijing Yan, Yuichiro Yano, Mehdi Yaseri, Yasin Jemal Yasin, Pengpeng Ye, Jamal Yearwood, Alex Yeshaneh, Manaye Yihune, Ebrahim M. Yimer, Nega Yimer, Biruck Desalegn Yirsaw, Engida Yisma, Zemenu Yohannes, Naohiro Yonemoto, Seok-Jun Yoon, Marcel Yotebieng, Mustafa Younis, Mahmoud Yousefifard, Chuanhua Yu, Sojib Bin Zaman, Mohammad Zamani, Zohreh Zare, Elias Asfaw Zegeye, Kazem Zendehdel, Taddese Zerfu, Olifan Zewdie, Anthony Zhang, Inbar Zucker, and Liesl Zuhlke.

Drafting the work or revising is critically for important intellectual content

Nooshin Abbasi, Gebre Abyu, Olatunji Adetokunboh, Mohsen Afarideh, Mohammad Esmaeil Akbari, Tomi Akinyemiju, Fares Alahdab, Khurshid Alam, Animut Alebel, Syed Aljunid, Catalina Liliana Andrei, Marcel AUSLOOS, Peter Azzopardi, Hamid Badali, Suzanne Barker-Collo, Celine Barthelemy, Shahrzad Bazargan-Hejazi, Yihalem Abebe Belay, Habib Benzian, Tina Beyranvand, Neeraj Bhala, Ashish Bhalla, Molly Biehl, Sait Mentes BIRLIK, Alessandra C Goulart, Franz Castro, Julian Chalek, Leslie Cornaby, Natacha Torres da Silva, Ahmad Daryani, Dragos Davitoiu, Jan-Walter De Neve, Selina Deiparine, Getenet Dessie, David Edvardsson, Sadaf Esteghamati, Andre Faro, mohammad hosein farzaei , Seyed-Mohammad Fereshtehnejad, Joao Fernandes, Nataliya Foigt, Nancy Fullman, Bereket Gebremichael, Aregawi Gebreyesus Belay, Kebede Embaye Gezae, Keyghobad Ghadiri, Maryam Ghasemi-Kasman, Elena

Gnedovskaya, Rajat Das Gupta, Michael Tamene Haile, Mehedi Hasan, Hadi Hassankhani, Rasmus Havmoeller, Akbar Hedayatizadeh-Omran, Behnam Heidari, Claudiu Herteliu, Behzad Heydarpour, Daniel Hoffman, Enayatollah Homaie Rad, Trang Huyen Nguyen, Amr Jamal, RAVI PRAKASH JHA, Jacek Jozwiak, Mikk JÜRISSON, Umesh Kapil , Manoochehr Karami, Getachew Mullu Kassa, Grant Kemp, Chandrasekharan Nair Kesavachandran, Morteza Abdullatif Khafaie , Alireza Khajavi, Mona Khater, Yun Jin Kim, Anh Kim Dang, Niranjan Kissoon, Kristopher Krohn, Dharmesh Lal, Huong Lan Nguyen, Justin Lang, Mall Leinsalu, Stephen Lim, Alan Lopez, Azeem Majeed, Ana Laura Manda, Mohammad Ali Mansournia, Joemer Maravilla, Francisco Rogerlândio Martins-Melo, Benjamin Massenburg, Colm McAlinden, Tuomo Meretoja, Molly Miller-Petrie, Bahram Mohajer, Karzan Mohammad, Ghobad Moradi, Mehdi Moradinazar, Abbas Mosapour, Erin Mullany, Christopher Murray, Nahid Neamati, Molly Nixon, Andrew T. Olagunju, Tinuke O. Olagunju, Andrei Oros, Doris V. Ortega-Altamirano, Adrian Pana, Nikolaos Papantoniou, Emmanuel Peprah, Konrad Pesudovs, Michael R Phillips, Julian Pillay, Vafa Rahimi-Movaghar, Muhammad Aziz Rahman, Seyed Mohammad Riahi, Leonardo Roever, Ehsan Sadeghi, Sare Safi, Saeid Safiri, Juan Sanabria, Milena Santric Milicevic, BRUNO SAO JOSE, Muthupandian Saravanan, Mayank Sardana, Shahabeddin Sarvi, Maheswar Satpathy, Monika Sawhney, Seyedmojtaba Seyedmousavi, Katya Shackelford, Mehdi Sharif, Mika Shigematsu, Inga Dora Sigfusdottir, Virendra Singh, Eirini Skiadaresi, Karen Sliwa, Leo Stockfelt, Ipsita Sutradhar, Nuno Taveira, Tung Thanh Tran, Nihal Thomas, Giang Thu Vu, Marcos Roberto Tovani-Palone, Bach Tran, Thomas Truelsen, Muhammad Shariq Usman, Olalekan Uthman, Yuan-Pang Wang, Robert Weintraub, Seifu Kebede Weldegiorgis, Manaye Yihune, and Inbar Zucker.

Extracting, cleaning, or cataloging data; designing or coding figures and tables

Hedayat Abbastabar, Isaac Adedeji, Olatunji Adetokunboh, Mehdi Ahmadi, Muktar Ahmed, Christine Allen, Erfan Amini, Jason Anderson, Aleksandra Barac, Celine Barthelemy, Robert Battista, Habtamu Wondifraw Baynes, Bayu Begashaw Bekele, Adugnaw Berhane, Gregory Bertolacci, Sait Mentes Birlik, Donal Bisanzio, Paul Briant, Julio Cesar Campuzano, Kate Causey, Alanur Cavlin, Yazan Chaiah, Julian Chalek, Leslie Cornaby, Elizabeth Cromwell, Matthew Cunningham, Ahmad Daryani, Tamirat Dasa, Robert Dellavalle, Getenet Dessie, Daniel Dicker, Erika Eldrenkamp, Iqbal Elyazar, Hamed Fakhim, Andre Faro, Rachel Feldman, Takeshi Fukumoto, John Fuller, Nancy Fullman, G Fortune Gankpe, William Gardner, Ayele Geleto, Keyghobad Ghadiri, Ellen Goldberg, Taren Gorman, Atsushi Goto, Max Griswold, Yuming Guo, Michael Tamene Haile, Mehedi Hasan, Yihua He, Behzad Heibati, Sousan Heydarpour, Nobuyuki Horita, Mehdi Hosseinzadeh, Chad Ikeda, Caleb Irvine, Moti Jalu, Vivekanand Jha, Catherine Johnson, Sarah Johnson, Manoochehr Karami, Tesfaye Kassa, Nicholas J Kassebaum, Vittal Katikireddi, Norito Kawakami, Laura Kemmer, Grant Kemp, Andre Keren, Ardeshir Khosravi, Mohammad Hossein Khosravi, Jagdish Khubchandani, Jun Kim, Young-Eun Kim, Yun Jin Kim, Jonathan Kocarnik, Barthelemy Kuate Defo, Xie Rachel Kulikoff, Michael Kutz, Sonia Lansky, Kathryn Lau, Jorge Ledesma, James Lee, Andrew Leever, Misgan Legesse Liben, Janni Leung, Shanshan Li, Shiwei Liu, Emilie Maddison, Dhaval Maghavani, Narayan Mahotra, Reza Malekzadeh, Helena Manguerra, Mohammad Ali Mansournia, Wagner Marcenes, Ira Martopullo, Mohsen Mazidi, Hagazi Gebre Meles, Haftay Berhane Mezgebe, Anoushka Millear, Ted R Miller, Shawn Minnig, Modhurima Moitra, Lorenzo Monasta, Maziar Moradi-Lakeh, Naho Morisaki, Abbas Mosapour, W Cliff Mountjoy-Venning, Ghulam Mustafa, Mohsen Naghavi, Hae-Sung Nam, Minh Nguyen, Mohammad Reza Nowroozi, Kanyin Ong, Ajay Patle, Katherine Paulson, David Pereira, Hossein Poustchi, Caroline Purcell, Anwar Rafay, Alireza Rafiei, Fatemeh Rajati, Seyed Mohammad Riahi, Luca Ronfani, Enrico Rubagotti, Hosein Safari, Saeid Safiri, Payman Salamati, Abdallah M. Samy, Muthupandian Saravanan, Shahabeddin Sarvi, Maheswar Satpathy, Monika Sawhney, Sadaf

Sepanlou, Seyedmojtaba Seyedmousavi, Mehdi Sharif, Jun She, Peilin Shi, Shreya Shirude, Farhad Shokraneh, Haitham Shoman, Soraya Siabani, Virendra Singh, Karen Sliwa, Joan B Soriano, Luisa Sorio Flor, Jeffrey Stanaway, Patrick Sur, Dillon Sylte, Mohammad Tavakkoli, Awoke Temesgen, Omar Temsah, Anna Torre, Christopher Troeger, Kingsley N. Ukwaja, Irfan Ullah, Rachel L. Updike, Gaurang Vaidya, Tommi Vasankari, Yasir Waheed, Inbal Weiss Salz, Joanna Whisnant, Tissa Wijeratne, Shouling Wu, Denis Xavier , R Xu, Ali Yadollahpour, Jamal Yearwood, Ebrahim M. Yimer, Nega Yimer, Naohiro Yonemoto, Mahmoud Yousefifard, Xueying Zhang, Stephanie R M Zimsen, and Leo Zoeckler.

Managing the overall research enterprise

Ashkan Afshin, Tahiya Alam, Peter Allebeck, Celine Barthelemy, Zulfiqar Bhutta, Molly Biehl, Michael Brauer, Deborah Carvalho Malta, Aaron Cohen, Elizabeth Cromwell, Lalit Dandona, Rakhi Dandona, Louisa Degenhardt, Samath Dharmaratne, Daniel Dicker, Kara Estep, Valery Feigin, Christina Fitzmaurice, Kyle Foreman, Nancy Fullman, Thomas Fürst, Emmanuela Gakidou, Tsegaye Gebrehiwot, Caitlin Hawley, Simon Hay, Peter Hotez, Spencer James, Nicholas Kassebaum, Ibrahim Khalil, Kristopher Krohn, Hmwe Kyu, Heidi Larson, Xiaofeng Liang, Stephen Lim, Alan Lopez, Rafael Lozano, Ashley Marks, George Mensah, Molly Miller-Petrie, Awoke Misganaw, Ali Mokdad, Erin Mullany, Kate Muller, Christopher Murray, Mohsen Naghavi, Molly Nixon, Carla Makhlouf Obermeyer, Helen Olsen, David Pigott, Robert Reiner, Joseph Salama, Joshua A Salomon, Benn Sartorius, Katya Shackelford, Chloe Shields, Shreya Shirude, Amanda Smith, Mari Smith, Jeffrey Stanaway, Roman Topor-Madry, Stein Emil Vollset, Theo Vos, Andrea Werdecker, and Harvey Whiteford.

Did not provide contribution information

Dash A P, Jemal Abdela, Semaw Ferede Abera, Nahla Anber, Yohanes Ayele, Anil Bhansali, Michael Brainin, Gene Bukhman, Jacqueline Castillo Rivas, Rajiv Chowdhury, Leslie Cooper, Diego De Leo, Maryam Farvid, Ali Akbar Fazaeli, Kyle Foreman, Ron Gansevoort, Segen Gebremeskel , Mohammad Rasoul Ghadami, Damian Hoy, Caitlyn Hughes, John Ji, Prakash K.C., Behzad Karami Matin, Ali Kazemi Karyani, Daniel Kiirithio, Heidi Larson, Georgy Lebedev, Giancarlo Logroscino, Fabiola Mejia-Rodriguez, Gk Mini, Ali Akbar Moghadamnia, Mousa Mohammadnia-Afrouzi, Viswanathan Mohan, Simin Mouodi, Satinath Mukhopadhyay, Gvs Murthy, Nitish Naik, Erika Ota, K. Srinath Reddy, Satar Rezaei, Rajesh Sagar, Sundeep Salvi, Reza Shirkoohi, Masoud Soosaraei, Sergey Soshnikov, Destaw Teshome, Js Thakur, Paul Yip.

List of Supplementary Results: Figures and Tables

Supplemental figures and tables providing more detailed results can be found in the supplementary results appendix.

Supplementary figure 1 . Census or population registry status and coverage of a population and housing census within the last 10 years, by location, from 1990-2017. Per SDG indicator 17.19.2a, the target is that each country is to have conducted a population and housing census in the last 10 years, or have a population registry from which detailed population and housing statistics are derived. Census years are designated by a dark blue colouring, followed by a lighter blue colour indicating census coverage for the 9 years following a census for a total of 10 years of census coverage. A medium blue colour is used to designate the implementation of a population registry. White indicates that a given location-year is not covered by a population and housing census or population registry. Census status for 1990-2000 was informed by census conducted from 1980-1989, which are not shown here. SDG=Sustainable Development Goal.

Supplementary figure 2. Map of health-related SDG index, by decile, in 1990, 2000, and 2017 for Japan (A), Sweden (B), England (C), USA (D), Mexico (E), Brazil (F), India (G), and Kenya (H). Deciles were based on the distribution of health-related SDG index values for countries and territories in 2017, as found in figure 2 of the main text, and then were applied for subnational locations over time. SDG=Sustainable Development Goal.

Supplementary figure 3. Projected performance, based on past trends, on the health-related SDG index and 40 individual health-related indicators, by country or territory, 2030. All projections were based on past trends and rates of change observed from 1990 to 2017. Countries are ranked by their health-related SDG index from highest to lowest in 2030, which was projected based on past trends. Indices and individual indicators are reported on a scale of 0 to 100, with 0 representing the worst levels from 1990 to 2030 and 100 reflecting the best during that time. SDG indicator 17.19.2a, population census status within the last 10 years, was not included in the health-related SDG index as projections were not generated for this indicator. Definitions of health-related SDG indicators are shown in table 1 in the main manuscript. SDG=Sustainable Development Goal. SDG=Sustainable Development Goal. Mort=mortality. Mat Mort Ratio=maternal mortality ratio; Skill Birth Attend=skilled birth attendance. Incid=incidence. Prev=prevalence. TB=tuberculosis. Hep B=hepatitis B. NTD=neglected tropical diseases. NCD=non-communicable disease. Inj=injury. FP Need Met, Mod=family planning need met with modern contraception methods. Adol=adolescent. UHC Serv Cov Index=universal health coverage, service coverage index. Air Poll=air pollution. WaSH=water, sanitation, and hygiene. Cov=coverage. Int=intimate. Viol=violence. HH=household. Occ=occupational risk. PM2.5= particulate matter smaller than 2.5 microns in diameter. Cert Death Reg=well-certified death registration.

Supplementary figure 4. Comparing attainment of defined health-related SDG indicator targets in 2017 and, based on past trends, projected to be attained in 2030, by country. Countries and territories are ranked by the number of SDG indicator targets they were projected to attain based on the mean estimate for 2030. All projections were based on past trends and rates of change observed from 1990-2017. Of the 41 health-related indicators measured in this study, 25 had defined targets linked to each indicator. SDG target 3.6 aims to reduce road injury mortality by 50% between 2015 and 2020, and thus attainment for

this indicator is based on estimates from 2015 to 2020 rather than 2015 to 2030. Definitions of health-related SDG indicators and targets associated with them, as well as the specific target thresholds applied, are shown in table 1 in the main manuscript. SDG=Sustainable Development Goal. Mort=mortality. Mat Mort Ratio=maternal mortality ratio; Skill Birth Attend=skilled birth attendance. Incid=incidence. Prev=prevalence. TB=tuberculosis. NTD=neglected tropical diseases. NCD=non-communicable disease. Inj=injury. FP Need Met, Mod=family planning need met with modern contraception methods. UHC Serv Cov Index=universal health coverage, service coverage index. Air Poll=air pollution. WaSH=water, sanitation, and hygiene. Cov=coverage. Int=intimate. Viol=violence. HH=household. Cert Death Reg=well-certified death registration.

Supplementary figure 5. Global annualised rate of change required to meet selected SDG targets based on annualised rate of change achieved by countries from 1990–2015 for selected SDG indicators with defined targets: well-certified death registration, child overweight, child sex abuse, child stunting, child wasting, hygiene, intimate partner violence, malaria incidence, neonatal mortality, non-intimate partner sexual violence, NTD prevalence, road injury mortality, sanitation, skilled birth attendance, suicide mortality, and water. For the 25 SDG indicators with defined targets, the global ARC required to meet each target was computed using the global average in 2015 and specific thresholds to be met by 2030 or relative reductions to be achieved by 2030. SDG target 3.6 aims to reduce road injury mortality by 50% between 2015 and 2020, and thus the global ARC is based on the time span from 2015 to 2020 rather than 2015 to 2030. Global ARCs are compared with the ARCs achieved across countries and territories from 1990-2015. The best-performing decile of ARC is shown compared to all other deciles against the global ARC required to meet the defined SDG target. A subset of SDG indicators with defined targets are shown in figure 6 in the main manuscript; the remaining plots are shown here. Definitions of health-related SDG indicators and targets associated with them are shown in table 1 in the main manuscript. ARC=annualised rate of change. SDG=Sustainable Development Goal.

Supplementary figure 6. Comparing 2016 values on the health-related SDG index from GBD 2016 to 2017 values on the health-related SDG index from GBD 2017. Countries are colour-coded by SDI quintile, and are abbreviated according to their ISO3 codes, which are listed in the appendix. The 7 territories included for GBD 2017 are not shown since they were not included in previous GBD SDG analyses. SDG=Sustainable Development Goal. GBD=Global Burden of Disease.

Supplementary figure 7. Comparing 2030 values on the health-related SDG index from GBD 2016 to 2030 values on the health-related SDG index from GBD 2017. Countries are colour-coded by SDI quintile, and are abbreviated according to their ISO3 codes, which are listed in the appendix. The 7 territories included for GBD 2017 are not shown since they were not included in previous GBD SDG analyses. SDG=Sustainable Development Goal. GBD=Global Burden of Disease.

Supplementary figure 8. Comparing 2016 rankings on the health-related SDG index from GBD 2016 to 2017 rankings on the health-related SDG index from GBD 2017. Countries are colour-coded by SDI quintile, and are abbreviated according to their ISO3 codes, which are listed in the appendix. The 7 territories included for GBD 2017 are not shown since they were not included in previous GBD SDG analyses. SDG=Sustainable Development Goal. GBD=Global Burden of Disease.

Supplementary table 1. Health-related SDGs excluded in the present analysis, and measurement needs and strategy for future reporting, by SDG target. Definitions and descriptions of health-related SDG indicators beyond the specific indicators originate from the IAEG-SDGs compilation of metadata for each SDG (as

provided by each indicator's custodial agency). DAH=development assistance for health. DHS=Demographic and Health Survey. GBD=Global Burden of Disease. IAEG-SDGs=Inter-agency and Expert Group on SDG Indicators. IHR=International Health Regulations. ISIC=International Standard Industrial Classification. JEE=Joint External Evaluation. NCD=non-communicable diseases. SDG=Sustainable Development Goal. TRIPS=Agreement on Trade-Related Aspects of Intellectual Property Rights. UHC=universal health coverage. UN=United Nations. WHO=World Health Organization.

Supplementary table 2. Health-related SDG index in 2000, 2015, 2017, and 2030, by country or territory. The health-related SDG index is reported on a scale of 0 to 100, with 0 representing the worst levels from 1990 to 2030 and 100 reflecting the best during that time. SDG=Sustainable Development Goal.

Supplementary table 3. Unscaled values for the 40 individual health-related SDG indicators in 2000, 2015, 2017, and 2030, and projected percent change from 2015 to 2030, by country or territory. Projected estimates for 2030 were produced based on past trends and rates of change observed from 1990 to 2017. Estimates are reported for each country and territory included in this analysis, and ordered by health-related SDG indicator followed by GBD super-region and region. SDG indicator 17.19.2a, population census status within the last 10 years, is not included here as projections were not generated for this indicator. All results from 1990 to 2017 for SDG indicator 17.19.2a can be found in supplementary figure 1. SDG=Sustainable Development Goal. GBD=Global Burden of Disease.

Supplementary table 4. GATHER checklist of information that should be included in reports of global health estimates, with description of compliance and location of information for GBD 2017 SDG Capstone.

Preamble

This appendix provides methodological detail, supplemental figures and tables, and more detailed results for the health-related Sustainable Development Goals (SDGs). The appendix is organized into broad sections following the structure of the main paper. This study complies with the Guidelines for Accurate and Transparent Health Estimates Reporting (GATHER) recommendations, and thus this appendix aims to be comprehensive and encyclopedic. It includes detailed tables, figures, indicator modeling write-ups and flowcharts, and information on data sourcing in an effort to maximize transparency in our estimation processes and provide a comprehensive account of analytical steps. Components of this document are the same as described in earlier GBD 2017 Capstone appendices but much more of this appendix are new text for the SDG Capstone. We intend this to be a living document, to be updated with each annual iteration of the Global Burden of Disease and in accordance with the 15 year timeline of the SDG cycle until their conclusion in 2030.

GATHER statement

This study complies with the guidelines for Accurate and Transparent Health Estimates Reporting (GATHER) recommendations. We have documented the steps involved in our analytical procedures and detailed the data sources used in compliance with the Guidelines for Accurate and Transparent Health Estimates Reporting (GATHER).

Part 1. Health-related SDG indicators

Section 1. Sustainable Development Goals overview

In September 2015, the United Nations (UN) General Assembly established the Sustainable Development Goals (SDGs). The SDGs substantially broaden the development agenda beyond the MDGs and are expected to frame UN member state policies through 2030. In March 2018, the global SDG indicator framework was updated, now specifying 17 universal goals, 169 targets, and 232 indicators leading up to 2030. Here we provide an analysis of 41 out of the 52 health-related SDG indicators based on data used and generated by the Global Burden of Diseases, Injuries, and Risk Factors Study 2017 (GBD 2017).

Section 2. Health-related SDGs

Health is a core dimension of the SDGs; the third SDG aims to "ensure healthy lives and promote wellbeing for all at all ages." Health-related indicators are also present among ten of the other 16 goals. Across these 11 goals, there are 29 health-related targets with a total of 52 health-related indicators.

Of the 52 health-related indicators included as part of the SDGs, estimates for 41 indicators, using consistent approaches built on systematic efforts to compile all available data, are included as part of the GBD study. In this paper, while acknowledging the continued debate about the structure and choices of SDG indicators, we use the GBD study to provide an assessment of the current status of these 41 health-related SDG indicators, develop and compute a summary indicator of the health-related SDG indicators, and document historical trends. For GBD 2017, we produce projections based on past trends for the health-related SDGs through 2030 and examine projected attainment for defined SDG targets by 2030. We also conduct a global attainment benchmarking exercise, comparing the global required rates

The GBD study is an annual effort to measure the health of populations at national, and selected sub-national levels, from 1990 to the most recent year (2017 for GBD 2017). The GBD study produces estimates of mortality and morbidity by cause, age and sex as well as that attributable burden to a selected set of major risk factors. Many of the 50 health-related SDG indicators are produced as part of the GBD, and thus use the same statistical methods as those reported in the broader GBD study (ie, Cause of Death Ensemble model [CODEm] for causes of death;[1,2] DisMod-MR for many nonfatal causes;[3] spatiotemporal Gaussian process regression [ST-GPR] for many risk factor exposures[4,5]). Elsewhere in this appendix, we outline the 10 SDGs, corresponding 25 health-related targets, and 41 health-related indicators included in this iteration of the GBD. Part 1. Section 3 of this appendix also further outlines the definition of each indicator used in analysis, as well as the estimation method and data sources.

Direct outputs of the GBD study that are health-related SDG indicators include mortality rates disaggregated by age (under-5 and neonatal) and cause of death (maternal, cardiovascular diseases, cancers, diabetes, chronic respiratory diseases, road injuries, self-harm, unintentional poisonings, exposure to forces of nature, interpersonal violence, and conflict and terrorism) as well as measures of disease incidence (HIV, malaria, tuberculosis [TB], hepatitis B) and prevalence (neglected tropical diseases [NTDs], nonfatal violence measures). The GBD risk factor analysis includes measurement of exposure prevalence included as health-related SDG indicators (under-5 stunting, wasting and overweight; tobacco smoking; harmful alcohol use; intimate partner violence and non-intimate partner sexual violence; unsafe water, sanitation, and hygiene [WaSH]; household air pollution; ambient particulate matter) as well as deaths or disease burden attributable to risk factors selected as health-related SDG indicators (WaSH, household and ambient air pollution, and occupational risks). In addition, a number of measures of intervention coverage, including skilled birth attendance, antenatal care, in-facility delivery rates, met

need for family planning with modern contraception methods, antiretroviral therapy, coverage of several vaccines, and health worker density per 1,000 are produced within the GBD study. Two additional health-related SDG indicators, well-certified death registration and population census status, are derived from broader GBD estimation processes and databases (ie, causes of death and population).

As noted in Table 1 in the main manuscript, for selected SDG indicators, we made modifications to the definition for clarity and/or based on the definition used in GBD. For example, Indicator 2.2.2 proposes to measure of malnutrition that combined prevalence of wasting and overweight among children under 5. As childhood wasting and overweight have very different determinants, effects on health outcomes, and interventions, we have selected to report them separately. For childhood overweight, we report prevalence among children aged 2 to 4 years, the definition used in GBD based on thresholds set by the International Obesity Task Force (IOTF).

Three indicators have been added for the GBD 2017 analysis: health worker density per 1,000 (Indicator 3.c.1), sexual violence by non-intimate partners (SDG indicator 5.2.2), and census status (SDG indicator 17.19.2a). In addition, SDG indicator 16.1.3 was disaggregated into prevalence of physical violence (16.1.3a) and sexual violence (16.1.3c) following the March 2018 refinements accepted by the UN Statistical Commission. We thus report on these indicators separately rather than the combined prevalence of physical and sexual violence, resulting in a net gain of four health-related SDG indicators for GBD 2017.

Further details on the estimation used for all indicators, compliant with Guidelines for Accurate and Transparent Health Estimates Reporting (GATHER), are included in Appendix Part 1. Section 3. Indicator-specific estimation.

Section 3. Indicator-specific estimation

The indicator-specific modeling write-ups follow the order of the SDG goals, targets, and indicators established by the UN. In some cases, multiple indicators were addressed in a single write-up, for example mortality due to natural disasters (SDG indicators 1.5.1, 11.5.1, and 13.1.1) are included in a single write-up along with mortality due to conflict and terrorism (16.1.2). In other cases, particular measures may be present in multiple indicators (e.g., mortality due to cardiovascular diseases are included in SDG indicators 3.4.1 and 3.8.1); in these cases, we refer include these model write-ups for one indicator, and reference that indicator write-up as needed elsewhere.

The organization of this section is as follows:

Natural disaster mortality (1.5.1, 11.5.1, 13.1.1), conflict and terrorism mortality (16.1.2)
Child stunting (2.2.1) and child wasting (2.2.2a)
Child overweight (2.2.2b)
Maternal mortality ratio (3.1.1.)
Skilled birth attendance (3.1.2, also in the UHC service coverage index [3.8.1])
Under-5 mortality (3.2.1), neonatal mortality (3.2.2)
HIV incidence (3.3.1)

TB incidence (3.3.2)
Malaria incidence (3.3.3)
Hepatitis B incidence (3.3.4)
NTD prevalence (3.3.5) – includes 15 individual NTDs
NCD mortality (3.4.1) - includes cardiovascular diseases, cancers, diabetes, and chronic respiratory diseases
Self-harm mortality (3.4.2), road injury mortality (3.6.1), unintentional poisonings mortality (3.9.3), interpersonal violence mortality (homicide) (16.1.1)
Alcohol use (3.5.2)
Met need for family planning with modern methods (3.7.1, also in the UHC index [3.8.1])
Adolescent birth rate (3.7.2)
Universal health coverage (UHC) service coverage index (3.8.1) – includes coverage of three childhood vaccines, antenatal care (1 and 4 visits), in-facility delivery rate, antiretroviral therapy coverage, and risk-standardized death rates from causes amenable to healthcare (3.8.1)
Mortality attributable to household air pollution and ambient air pollution (3.9.1), household air pollution (7.1.2), and mean PM2.5 (11.6.2)
Mortality attributable to WaSH (3.9.2), water (6.1.1), sanitation (6.2.1a), access to handwashing facility (6.2.1b)
Smoking prevalence (3.a.1)
Vaccine coverage (3.b.1)
Health worker density (3.c.1)
Prevalence of intimate partner violence (5.2.1), sexual violence by non-intimate partner (5.2.2), child sexual abuse (16.2.3), prevalence of physical or sexual violence (16.1.3a and 16.1.3c)
DALY rates attributable to occupational risks (8.8.1)
Population and housing census (17.9.2a)
Well-certified death registration (17.19.2c)

References

1 Foreman KJ, Lozano R, Lopez AD, Murray CJ. Modeling causes of death: an integrated approach using CODEm. *Popul Health Metr* 2012; **10**: 1.

2 Naghavi M, Abajobir AA, Abbafati C, *et al.* Global, regional, and national age-sex specific mortality for 264 causes of death, 1980–2016: a systematic analysis for the Global Burden of Disease Study 2016. *The Lancet* 2017; **390**: 1151–210.

3 Vos T, Abajobir AA, Abate KH, *et al.* Global, regional, and national incidence, prevalence, and years lived with disability for 328 diseases and injuries for 195 countries, 1990–2016: a systematic analysis for the Global Burden of Disease Study 2016. *The Lancet* 2017; **390**: 1211–59.

4 Ng M, Freeman MK, Fleming TD, *et al.* Smoking prevalence and cigarette consumption in 187 countries, 1980-2012. *JAMA* 2014; **311**: 183–92.

5 Gakidou E, Afshin A, Abajobir AA, *et al.* Global, regional, and national comparative risk assessment of 84 behavioural, environmental and occupational, and metabolic risks or clusters of risks, 1990–2016: a systematic analysis for the Global Burden of Disease Study 2016. *The Lancet* 2017; **390**: 1345–422.

1.5.1, 11.5.1, 13.1.1, and 16.1.2 Fatal Discontinuities SDG Capstone Appendix

Input data & Methodological summary

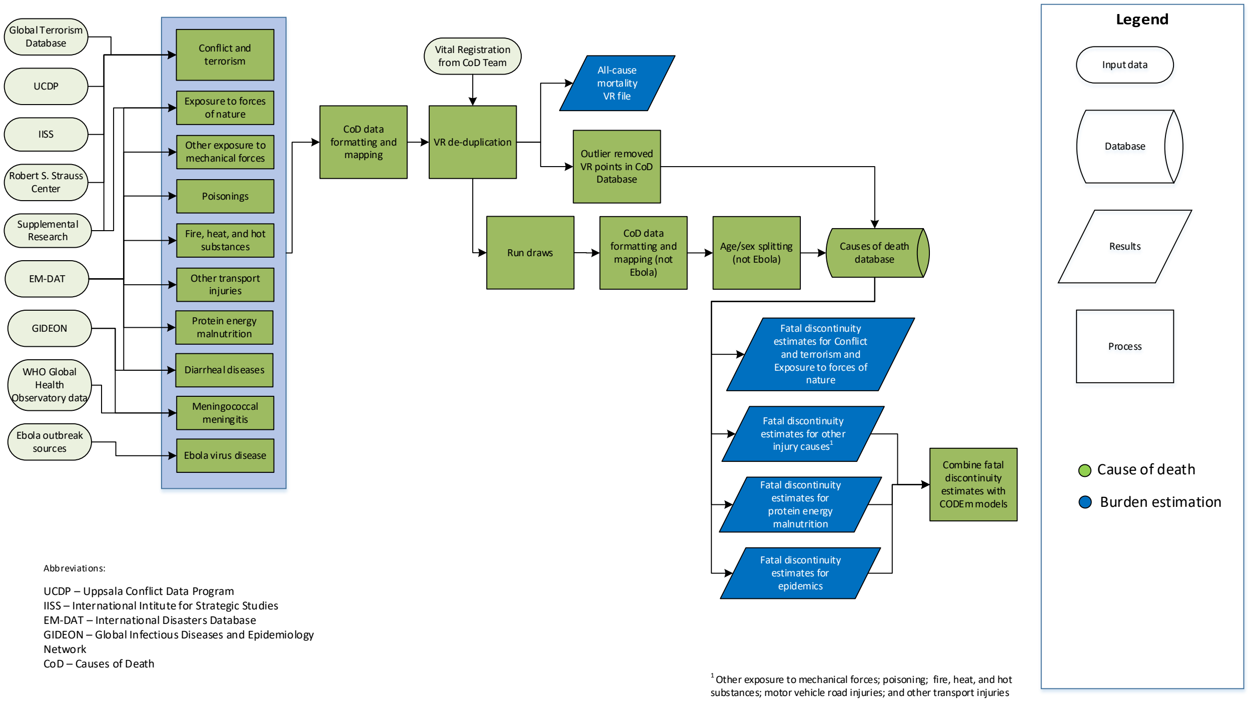

Indicator definition

This modeling strategy encompasses indicators associated with mortality due to exposure to forces of nature (natural disasters): 1.5.1, 11.5.1, 13.1.1; and mortality due to conflict and terrorism: 16.1.2.

Indicator 1.5.1

As a component of SDG Goal 1. End poverty in all its forms everywhere, SDG Target 1.5., by 2030, build the resilience of the poor and those in vulnerable situations and reduce their exposure and vulnerability to climate-related extreme events and other economic, social and environmental shocks and disasters, is measured using SDG Indicator 1.5.1, deaths due to exposure to forces of nature per 100,000.

Indicator 11.5.1

As a component of SDG Goal 11. Make cities and human settlements inclusive, safe, resilient and sustainable, SDG Target 11.5, by 2030, significantly reduce the number of deaths and the number of people affected and substantially decrease the direct economic losses relative to global gross domestic product caused by disasters, including water-related disasters, with a focus on protecting the poor and people in vulnerable situations, is measured using SDG Indicator 11.5.1, deaths due to exposure to forces of nature per 100,000.

Indicator 13.1.1

As a component of SDG Goal 13. Take urgent action to combat climate change and its impacts, SDG Target 13.1, strengthen resilience and adaptive capacity to climate-related hazards and natural disasters in all countries, is measured using SDG Health Index Indicator 13.1.1, deaths due to exposure to forces of nature per 100,000.

Indicator 16.1.2

As a component of SDG Goal 16. Promote peaceful and inclusive societies for sustainable development, provide access to justice for all and build effective, accountable and inclusive institutions at all levels, SDG Target 16.1, significantly reduce all forms of violence and related death rates everywhere, is measured using SDG Indicator 16.1.2, deaths due to conflict and terrorism per 100,000.

Input data

Overall
Input data for fatal discontinuities are compiled a range of sources, including country vital registration (VR) data; international databases that capture several cause-specific fatal discontinuities; and supplemental data in the presence of known issues with data quality or representativeness, or time lags in reporting. A systematic literature review was not used to identify input data for fatal discontinuities, though some literature sources were identified through online supplemental research. Below we provide more detail on the different input data sources by sub-causes of fatal discontinuities.

Subnational locations and population splitting
In locations where we produced estimates at the subnational level for GBD 2017, deaths due to all fatal discontinuity causes were assigned to the relevant subnational location(s) when that information could be obtained either through country data sources (eg, VR) or through additional online research. In the rare case that no subnational location could be found, the deaths were split proportionally by population across all subnational locations.

In locations that have experienced boundary changes or split from other locations that we currently estimate (eg, the former Yugoslavia, Czechoslovakia, the Soviet Union, Sudan and South Sudan), we split deaths due to events that occurred prior to boundary changes proportionally based on the populations residing within the boundaries of present-day locations unless we found documentation that clearly indicated whether the event and corresponding deaths occurred in one of the present-day GBD 2017 locations.

Choosing between multiple sources for same event
Where multiple sources reported shock deaths for the same location-year-cause, a cause-specific prioritisation scheme was followed that reflected the available detail in the cause-specific datasets. For example, the Generalized Event Dataset from UCDP was prioritised above all other non-VR sources because it included detail on how deaths were distributed between multiple actors and locations in each conflict event. In most cases, VR from 4- or 5-star locations was used where available. In some cases, VR from 4- or 5-star locations was not chosen if there were well-known data-quality issues or discrepancies in the cause of death data reporting related to a particular event (eg, supplemental death data for

Louisiana was used for Hurricane Katrina because of established data reporting issues). The process for prioritidation among various sources for location-year fatal discontinuities is described more in the Modelling strategy below.

Major data sources other than country vital registration for each fatal discontinuity cause follow.

Conflict and terrorism. In GBD 2016, data for conflict and terrorism came from the Uppsala Conflict Data Program (UCDP), International Institute for Strategic Studies, and Robert S. Strauss Center for International Security and Law. For GBD 2017, data from the Global Terrorism Database (GTD), the University of Chicago Suicide Attack Database, and the RAND Database of Worldwide Terrorism Incidents were used in addition to those used in GBD 2016. The table below provides details about the various datasets we utilised from these sources, the dates they were last accessed, and the years for which we used the data provided. Where these data sources reported deaths due to gang violence, the cause was re-mapped to physical violence by other means. Where these data sources reported deaths due to legal intervention, the cause was re-mapped to executions and police conflict.

Data source name	Date accessed	Years of data downloaded	Type of data included
**Uppsala Conflict Data Program**[1]			
Georeferenced Event Dataset, Version 17.1	1/16/2018	1989-2015	UCDP battles, non-state, and one-sided conflict deaths with the most disaggregated location information available
PRIO Battles Deaths Dataset, Version 3.1	1/16/2018	1970-1988	Armed conflict (civil wars, etc.)
**International Institute for Strategic Studies**			
Armed Conflict Dataset	11/17/2016	1997-2016	Insurgency, Inter-state, Intra-state conflict deaths
**Robert S. Strauss Center For International Security And Law**			
Armed Conflict Location and Event Dataset (ACLED)	1/16/2018	1997-2016	Actions of opposition groups, governments, and militias in selected locations in Africa, Asia, and the Middle East specifying the exact location and date of battle events, transfers of military control, headquarter establishment, civilian violence, and rioting
Social Conflict Analysis Database (SCAD)	1/16/2018	1990-2016	Protests, riots, strikes, inter-communal conflict, government violence against civilians, and other forms of social conflict (covers Africa, Latin America, and Asia)
**University of Maryland, Global Terrorism Database**			
Global Terrorism Database (GTD)	1/16/2018	1970-2016	Attacks aimed at attaining political, economic, religious, or social goal, includes evidence of intention to coerce, action was outside precepts of International Humanitarian Law.
**University of Chicago, Chicago Project on Security and Threats**			
Suicide Attack Database (CPOST SAD)	8/5/2017	1974-2016	Attacks in which an attacker kills him/herself in a deliberate attempt to kill others, includes only attacks perpetrated by non-state actors
**RAND National Security Research Division**			
RAND Database of Worldwide Terrorism Incidents	9/8/2017	1968-2009	Terrorism, defined by the nature of the act, not by the identity of the perpetrators or the nature of the cause; including violence, calculated to create fear/alarm, intended to coerce certain actions, motive is political, group, or individual

Supplemental online research was conducted for recent conflicts where the databases above were not up to date. In addition, deaths due to conflict and terrorism in Iraq from 2003 to present were estimated using a combination of supplemental sources. The source found with the lowest number of deaths, Iraq Body Count[2], was used as the lower bound of the uncertainty interval from 2003 to 2016. Estimates from the Iraq Mortality Study by Hagopian et al[3] from 2003 to 2006, the deadliest years of the war, were used to scale deaths to generate the upper uncertainty interval limits using the following formula:

$$deaths_{GBD\ 2017,\ high} = deaths_{IBC} \cdot \overline{\left[\frac{deaths_{IMS}}{deaths_{IBC}}\right]}_{2003-2006}$$

We used the average ratio between IMS and IBC reported deaths between 2003 and 2006, multiplied by the number of deaths reported by the IBC. This high estimate was carried forward through 2017 under the assumption that the Iraq Body Count similarly undercounts the number of deaths due to the ongoing civil war in Iraq. The final, best estimate for conflict and terrorism deaths in Iraq from 2003 to 2016 is the midpoint of the high and low estimates given above.

We identified four major conflicts that were not represented in these databases: 1997 civil conflict in Albania[4]; 1971 genocide in Bangladesh[5]; 1972 genocide in Burundi[6]; and 1993 genocide in Burundi[6]. In these cases, we used literature sources in order to account for these fatal discontinuities.

For country-years where multiple sources provided estimates, we prioritised sources in the following order: (1) country VR data, if death estimates were highest of all sources; (2) UCDP; (3) IISS; (4) country VR if death estimates were not the highest of all sources; (5) Robert Strauss Center; (6) Global Terrorism DB; (7) CPOST Suicide Attack Database; (8) online supplemental research.

Exposure to forces of nature, other injury causes, and protein-energy malnutrition. The Centre for Research on the Epidemiology of Disasters' International Disaster Database (EM-DAT) served as the primary non-VR source of fatal discontinuities due to exposure to forces of nature (ie, natural disasters); other transport injuries (eg, plane, train, and boat accidents); poisonings; fire, heat, and hot substances; other exposure to mechanical forces (eg, building collapse); and protein-energy malnutrition (ie, famine or severe drought). Data from EM-DAT were last accessed February 14, 2018. Supplemental online research was conducted for events where EM-DAT was not up to date.

For country-years where multiple sources provided estimates, we prioritised sources in the following order: (1) country VR data, if data quality rating is 4 or 5 stars; (2) country VR data if data quality rating is less than 4 stars and death estimates were highest of all sources; (3) EM-DAT; (4) online supplemental research. Exceptions were made where it was clear that VR systems had been compromised by the event being measured.

Meningococcal meningitis and diarrhoeal diseases. For GBD 2017, we included fatal discontinuities due to a subset of infectious diseases: meningococcal meningitis (or meningococcal infection) and diarrhoeal disease caused by cholera. These two infectious diseases were first included on the fatal discontinuity cause list for GBD 2016 because (1) their current modelling strategies with the Cause of Death Ensemble model (CODEm) do not optimally capture the potentially highly variable – or epidemic – mortality levels and trends characteristic of these two causes; and (2) they can contribute to significant total fatalities in a given location-year. Other infectious diseases for which the latter is true – high death rates in the presence of an outbreak or epidemic – are currently modelled with alternative cause of death methods (eg, natural history models for measles and yellow fever), which allow for greater variation year-over-year if or when outbreaks occur. In future iterations of the GBD, we plan to revisit the inclusion criteria for infectious diseases as fatal discontinuities and develop more of an ensemble approach to modelling causes that can be both endemic (and thus result in more uniform levels and trends over time) and epidemic (and subsequently lead to rapid increases – and decreases – in deaths for a given location-year).

The Global Infectious Diseases and Epidemiology Network (GIDEON) served as the primary data source for collating cholera and meningococcal meningitis or meningococcal infection death reports.[7,8] For any year in which cholera or meningococcal meningitis deaths were recorded in a country or territory

covered by the GBD, we directly extracted reported deaths from 1970 to 2016. When there were reporting gaps in cholera or meningococcal meningitis deaths over this period of time and the World Health Organization (WHO) annual cholera or meningitis reports had death reports for those years, we used the WHO reports. The primary exception were two major cholera outbreaks in Bangladesh – 1982 to 1983 and 1991 – which were not captured by either GIDEON or WHO. As result, we used the EM-DAT records for the 1982–1983 outbreak and literature for the 1991 outbreak.[9] For the Yemen Cholera outbreak in 2016 and 2017, we used estimates from local collaborators in the absence of other data sources.

Ebola. Since GBD 2015, outbreaks due to Ebola virus disease have been estimated using the data and methods described in the Ebola write-up of this appendix and included in GBD death estimates in the same way as other fatal discontinuity causes.

Modelling strategy

All input data for fatal discontinuity causes were run through the causes of death data formatting and mapping process.

VR de-duplication

For injury causes that also have continuous background mortality and a CODEm model, a process was established to avoid duplication of fatal discontinuity deaths in the two models. First, location-years with fatal discontinuities data from non-VR sources were identified. If these location-cause-years also had VR death estimates that were greater than 40% higher than the immediately surrounding years and could be linked to a specific fatal discontinuity event, these years were marked as outliers in the VR data and the difference between the outlier year and the average of the surrounding years was included in the relevant cause in the fatal discontinuities database. The deaths from the identified events were subtracted from the all-cause VR estimates used in the all-cause mortality estimation process.

Uncertainty analysis for input and draw-level input to age-sex splitting

Uncertainty intervals for deaths due to conflict and terrorism were generated using UCDP high and low death estimates, except in the case of Iraq 2003–2016, as explained above. In cases where low and high estimates were not included in the available data, the regional average uncertainty interval was applied to the available death estimate across all fatal discontinuity causes.

We assumed a log-normal distribution using mean death rates and standard error based on high and low estimates. In the case that standard error was less than 10e-8, the draws were set equal to the mean rate. 1,000 draws were sampled from this log-normal distribution. These 1,000 draws were then converted back to count space and used for final calculations of means and uncertainty intervals.

Age-sex splitting

All compiled data were run through the causes of death age-sex splitting process, except for where we had strong supplemental information on the age distribution of specific, large events, such as United States mortality in the Vietnam War and Iranian mortality from the Iran-Iraq conflict in the early 1980s.

Changes from GBD 2016

GBD 2017 saw an effort to systematise the collection of up-to-date fatal discontinuity data through supplemental online research. New tools included use of Twitter to identify events not covered by other sources, most notably in identifying events that occurred recently (2016, 2017). This process resulted in a more comprehensive set of conflict and terrorism data for 2017, as well as large natural disasters not contained in EM-DAT or VR.

For GBD 2017, efforts were also made to improve location tagging in raw data to the GBD location hierarchy using several approaches. Identifying the correct GBD location for each event is difficult, as reports of fatal discontinuities come in many formats, often with limited metadata. The approaches used for improving the location tagging included a) utilising the collaborator network to more accurately tag events to subnational locations when information in the data was scarce, b) automated matching with GBD location names, c) overlaying a spatial file of the most-detailed GBD geographies, d) geo-coding using precise place names, and e) for events spanning multiple GBD locations, but without detail in the raw data, deaths were split using population.

We completed a detailed review of the fatal discontinuity cause mappings for conflict and terrorism, police conflict and executions, using the text descriptions of each event when provided in the data. This exercise resulted in updating the GBD cause assigned for a number of events present in the GBD 2016 analysis, which is one contributor in the differences seen in the GBD 2016 and GBD 2017 fatal discontinuity estimates.

References

1 UCDP/PRIO Armed Conflict Dataset Codebook. Uppsala Conflict Data Program (UCDP); Centre for the Study of Civil Wars, International Peace Research Institute, Oslo (PRIO), 2013.

2 Iraq Body Count. https://www.iraqbodycount.org/database/ (accessed May 8, 2017).

3 Hagopian A, Flaxman AD, Takaro TK, *et al.* Mortality in Iraq Associated with the 2003–2011 War and Occupation: Findings from a National Cluster Sample Survey by the University Collaborative Iraq Mortality Study. *PLOS Medicine* 2013; **10**: e1001533.

4 Jarvis C. The Rise and Fall of Albania’s Pyramid Schemes. *F&D* 2000; **37**. http://www.imf.org/external/pubs/ft/fandd/2000/03/jarvis.htm.

5 Obermeyer Z, Murray CJL, Gakidou E. Fifty years of violent war deaths from Vietnam to Bosnia: analysis of data from the world health survey programme. *BMJ* 2008; **336**: 1482–6.

6 Milton L. Rwanda, 1994: International incompetence produces genocide. 1994 https://ezproxy.uwc.edu/login?url=http://search.proquest.com/docview/234405747?accountid=42411.

7 Inc GI, Berger DS. Cholera: Global Status: 2017 edition. GIDEON Informatics Inc, 2017.

8 Inc GI, Berger DS. Bacterial Meningitis: Global Status: 2017 edition. GIDEON Informatics Inc, 2017.

9 Siddique A, Zaman K, Baqui A, *et al.* Cholera Epidemics in Bangladesh: 1985-1991. *Journal of Diarrhoeal Diseases Research* 1992; **10**: 79–86.

2.2.1 and 2.2.2a Child Growth Failure Capstone Appendix

Flowchart

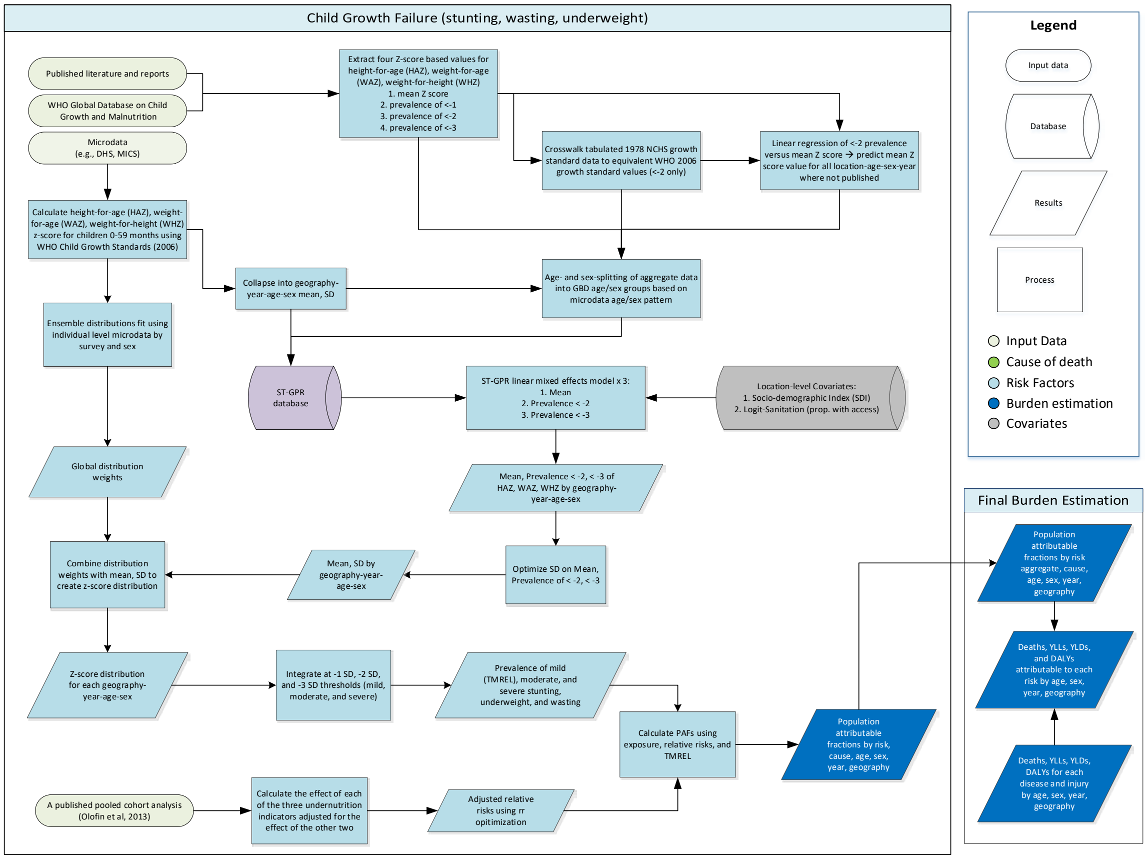

Input Data & Methodological Summary

Indicator definition

This modeling strategy encompasses indicators associated with child undernutrition: 2.2.1 and 2.2.2a.

Indicator 2.2.1

As a component of SDG Goal 2. End hunger, achieve food security, and improved nutrition, SDG Target 2.2, by 2030, end all forms of malnutrition, including achieving, by 2025, the internationally agreed targets on stunting and wasting in children under 5 years of age, and address the nutritional needs of adolescent girls, pregnant and lactating women and older persons, is measured using SDG Indicator 2.2.1, prevalence of stunting among children under 5 (lower than two standard deviations from the median height for age of the reference population).

Indicator 2.2.2a

As a component of SDG Goal 2. End hunger, achieve food security, and improved nutrition, SDG Target 2.2, by 2030, end all forms of malnutrition, including achieving, by 2025, the internationally agreed targets on

stunting and wasting in children under 5 years of age, and address the nutritional needs of adolescent girls, pregnant and lactating women and older persons, is measured using SDG Indicator 2.2.2a, prevalence of wasting among children under 5 (lower than two standard deviations from the median weight for height of the reference population).

Exposure

Case Definition

Child growth failure is estimated using three indicators, stunting, wasting, and underweight, all of which all of which are based on categorical definitions using the WHO 2006 growth standards for children 0-59 months.[1] Definitions are based on Z scores from the growth standards, which were derived from an international reference population. Mild, moderate, and severe categorical prevalences were estimated for each of the three indicators.

Input data

There are three main inputs for the GBD child growth failure models: microdata from population surveys and tabulated data from reports, published literature, and the WHO Global Database on Child Growth and Malnutrition.[1] The primary data additions in GBD 2017 for child growth failure were from population surveys that include anthropometry. Population surveys include a variety of multi-country and country-specific survey series such as Multiple Indicator Cluster Surveys (MICS), Demographic and Health Surveys (DHS), Living Standards Measurement Surveys (LSMS), and the China Health and Nutrition Survey (CHNS), as well as other one time country specific surveys such as the Indonesia Family Life Survey and the Brazil National Demographic and Health Survey of Children and Women. These microdata contain information about each individual child's age (from which age in weeks and age in months are calculated), as well as height and/or weight. From that information, a height-for-age z-score (HAZ), weight-for-age z-score (WAZ), and weight-for-height z-score (WHZ) are calculated using the WHO 2006 Child Growth Standards and the LMS method.

All available data from the WHO Global Database on Child Growth and Malnutrition was extracted for GBD 2016 – much of which is from published studies. Exclusions included examination date prior to 1985, non-population representative studies, and those based on self-report. A systematic literature review was last completed in GBD 2010. We looked for four metrics from all sources with tabulated data: mean Z score, prevalence <-1 Z score (mild), prevalence <-2 Z score (moderate), and prevalence <-3 Z score (severe). All data for each metric was extracted for each of stunting (height-for-age Z score; HAZ), wasting (weight-for-height Z score; WHZ), and underweight (weight-for-age Z score; WAZ).

To maximize internal-consistency and comprehensiveness of the modeling dataset, we performed three data transformations. First, any data that were reported using the National Center for Health Statistics (NCHS) 1978 growth standards were crosswalked to corresponding values on the WHO 2006 Growth Standards curves based on a study that evaluated growth standard concordance.[3] Crosswalks from 1978 to 2006 growth standards were performed only on <-2 (i.e. moderate) prevalence data as that is where the concordance was most consistent. Second, for any study that lacked a measure of mean Z score for any of stunting, wasting, or underweight, we predicted a mean value for that study based on an ordinary-least squares regression of mean Z score versus <-2 prevalence for that metric from all sources where both were available. Third, any data that was presented as both sexes combined or for 0-59 months

combined, we used the age and sex pattern from all data sources that included that detail to split into corresponding and age- and sex-specific data. All data was uploaded to a database and all inputs are catalogued in the Global Health Data Exchange (http://ghdx.healthdata.org). A representative dataset coverage map for moderate stunting is shown below.

Figure 1: Number of data points in moderate stunting (<-2 HAZ) in males, 1990 to 2017

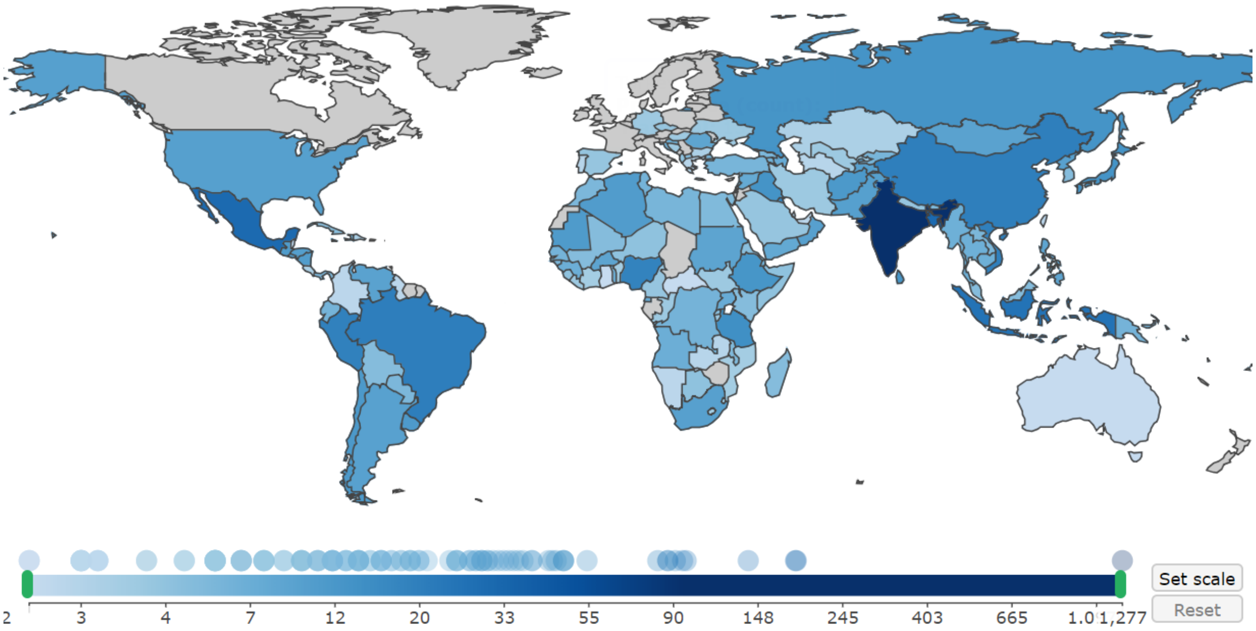

Modelling strategy

Exposure estimation

The following three-step modelling process was applied to each of stunting, wasting, and underweight.

First, all microdata was fit using an ensemble modelling process, a modelling framework developed for GBD 2016 that is described elsewhere in this appendix. A series of 12 individual distributions (normal, log normal, log logistic, exponential, gamma, mirror gamma, inverse gamma, gumbel, mirror gumbel, Weibull, inverse Weibull, and beta) were fit to the entire set of microdata (approximately 2.5 million individual z-scores) at the individual survey level. A weighting algorithm combined each distribution to find the optimal combination of these distributions for each survey, minimising the absolute prediction error across the entire distribution. Ensemble weights for each survey were then averaged across all surveys to produce a single set of global weights of the ensemble distributions. Weights were different for each sex, but invariant across geography, time, and age group. All component distributions that were used to derive weights were parameterised using “method of moments,” meaning that each corresponding probability density function (PDF) could be described as a function of the mean and variance of the quantity of interest.

Second, models were developed for mean Z scores and prevalence of moderate and severe growth failure. Individual level microdata were collapsed to calculate three metrics: mean z-score, moderate prevalence, and severe prevalence. These data were combined with that derived from literature, GHDx review, and the WHO Global Database on Child Growth and Malnutrition. Each of the three metrics was then modelled using spatiotemporal Gaussian process regression (ST-GPR), a common modelling

framework used across GBD, generating estimates for each age-group, sex, year, and location. Location-level covariates used in all models included Socio-demographic Index (SDI) and logit-transformed proportion of households with improved sanitation.

Third, we combined estimates of mean, prevalence (moderate and severe) with ensemble weights in an optimisation framework in order to derive the variance that would best correspond to the predicted mean and prevalence. This variance was then paired with the mean and, using the method of moments equation for each of the component distributions of the ensemble, PDF of the distribution of Z-scores were calculated for each location, year, age-group, and sex. PDFs were integrated to determine the prevalence between -1 and -2 Z scores (mild), between -2 and -3 Z scores (moderate), and below -3 Z scores (severe). These were categorical exposures used for subsequent attributable risk analysis.

Ad-hoc data exclusions were limited. In some cases, we identified surveys with evidence of data entry issues (e.g. weights entered in a mixture of pounds and kilograms) that could not be corrected and these data were outliered. We initially ran all models with the complete dataset. Data plausibility inspection began with examination of time trends in stunting. If a given datum was judged to have led to a change in the prevalence of moderate stunting in 1-4 year olds of 50% or greater in 5 years or fewer, and was inconsistent with data prior to and after that year (a change considered implausible), we outliered the offending datum and reran the model. We then further visually-inspected the results of moderate stunting, wasting, and underweight in parallel to look for location-age-sex-years where the results were not internally-consistent (e.g. stunting and wasting decreasing, underweight rapidly increasing). This inspection revealed very few inconsistent data.

Improvements from GBD 2015 to GBD 2016/ 2017

In GBD 2017, the primary changes from GBD 2016 were the 1) addition of a significant volume of new survey data, 2) crosswalking instead of down-weighting data based on NCHS 1978 growth standard, 3) utilization of updated versions of location-level covariates, and 4) utilization of an updated version of the ST-GPR modeling framework that empirically derives many of the modeling parameters.

There are several important differences from the GBD 2015 analysis. First, our systematic data searching efforts led to an approximately 30% increase in the number of data sources since GBD 2015, including a significant increase in data sources for Oceania, Latin America, and South Asia. Most notable was the increase in data for India through our collaboration with the India Council for Medical Research (ICMR) and Public Health Foundation of India (PHFI). Second, while GBD 2015 also used ST-GPR to model growth failure, models were completed for a single 0-5 age group, followed by application of a pooled uniform age-sex split which resulted in the implicit assumption that the age pattern of growth failure is invariant over time and geography. GBD 2016 estimates, owing to smaller sample sizes in younger age groups, do have wider uncertainty in those age groups. Third, GBD 2015, like all analyses of growth failure before it, assumed that high-income countries had zero prevalence of child growth failure. We suspended this assumption in GBD 2016 as it is not accurate and instead made explicit estimates of growth failure in all locations. Fourth, GBD 2015 did not use an ensemble approach or estimate the entire distribution of Z scores. Fifth, we changed the name of this risk factor category changed from childhood undernutrition to child growth failure to more explicitly identify the specific aspects of childhood undernutrition that are covered by the three component indicators.

Theoretical minimum-risk exposure level

Theoretical minimum risk exposure level (TMREL) for underweight, stunting, and wasting was assigned to be greater than or equal to -1 SD of the WHO 2006 standard weight-for-age, height-for-age, and weight-for-height curves respectively. This has not changed since GBD 2010.

Relative risks

The final list of outcomes paired with child growth failure risks included lower respiratory infections (LRI), diarrhea, measles, and protein energy malnutrition (PEM) as shown in Table 1. These were derived from a pooled cohort analysis by Olofin and colleagues.[5]

There is a high degree of correlation between stunting, wasting, and underweight. Failing to account for their covariance and assuming independence would overestimate the total burden significantly. This is the main reason that GBD 2010 only included childhood underweight. In GBD 2013, a method was developed to adjust observed RRs of Olofin and colleagues by simulating the joint distribution of the three indicators using the distribution of each indicator and covariance between indicators in the countries included in the meta-analysis (extracted from Demographic and Health Survey (DHS) micro-data).[4] Based on the analysis done by McDonald and colleagues, we assumed there is an interaction between the three indicators, and extracted the interaction terms from the corresponding analysis. We calculated the adjusted RRs by minimizing the error between observed crude RRs (from meta-analysis) and expected crude RRs derived from adjusted RRs.

Of historical note, URI and otitis media were included as outcomes in the GBD 2013 risk analysis, based on the "analogy" causal criterion, assuming there is similar pathway as LRI outcome. However, closer review for GBD 2015 did not find sufficient evidence to support their inclusion and they were excluded, a decision that was carried forward into GBD 2016. We also attributed 100% of PEM to childhood wasting and underweight but not stunting. To build on the existing literature base for GBD on risk-outcome pairs, a literature search was conducted for GBD 2017 searching for case-control studies published after January 1st, 1985; this search did not return any sources that were appropriate for this work.

Table 1: Adjusted RRs for each risk-outcome pair for child growth failure

Outcome	Stunting	Wasting	Underweight
Diarrhea	<-1: 1.111 (1.023-1.273)<br><-2: 1.222 (1.067-1.5)<br><-3: 1.851 (1.28-2.699)	<-1: 6.601 (2.158-11.243)<br><-2: 23.261 (9.02-35.845)<br><-3: 105.759 (42.198-157.813)	<-1: 1.088 (1.046-1.134)<br><-2: 1.23 (1.163-1.314)<br><-3: 2.332 (2.076-2.802)
Lower respiratory infections (LRI)	<-1: 1.125 (0.998-1.655)<br><-2: 1.318 (1.014-2.165)<br><-3: 2.355 (1.15-5.114)	<-1: 5.941 (1.972-11.992)<br><-2: 20.455 (70.84-37.929)<br><-3: 47.67 (15.923-94.874)	<-1: 1.145 (1.044-1.364)<br><-2: 1.365 (1.215-1.755)<br><-3: 2.593 (1.908-4.39)
Measles	<-1: 1.103 (0.861-1.719)<br><-2: 1.54 (1.029-3.222)<br><-3: 2.487 (1.129-6.528)	<-1: 1.833 (0.569-8.965)<br><-2: 8.477 (1.33-42.777)<br><-3: 37.936 (5.088-199.126)	<-1: 0.995 (0.5-1.726)<br><-2: 2.458 (1.26-5.118)<br><-3: 5.668 (1.767-12.414)
Protein-energy malnutrition	0% PAF	100% PAF	100% PAF

References

1. WHO | WHO Global Database on Child Growth and Malnutrition. WHO. http://www.who.int/nutgrowthdb/en/ (accessed July 30, 2018).

2. Wang Y, Chen H-J. Use of Percentiles and Z-Scores in Anthropometry. In: Preedy VR, ed. Handbook of Anthropometry. New York, NY: Springer New York, 2012: 29–48.

3. Uribe Á, Cecilia M, López Gaviria A, Estrada Restrepo A. Concordance between Z scores from WHO 2006 and the NCHS 1978 growth standards of children younger than five. Antioquia-Colombia. *Perspectivas en Nutrición Humana* 2008; **10**: 177–87.

4. McDonald CM, Olofin I, Flaxman S, *et al.* The effect of multiple anthropometric deficits on child mortality: meta-analysis of individual data in 10 prospective studies from developing countries. *Am J Clin Nutr* 2013; **97**: 896–901.

5. Olofin I, McDonald CM, Ezzati M, *et al.* Associations of Suboptimal Growth with All-Cause and Cause-Specific Mortality in Children under Five Years: A Pooled Analysis of Ten Prospective Studies. *PLOS ONE* 2013; **8**: e64636.

2.2.2b High Body Mass index SDG Capstone Appendix

Flowchart

Adult (Ages 20+) High Body-Mass Index: Data and Model Flow Chart

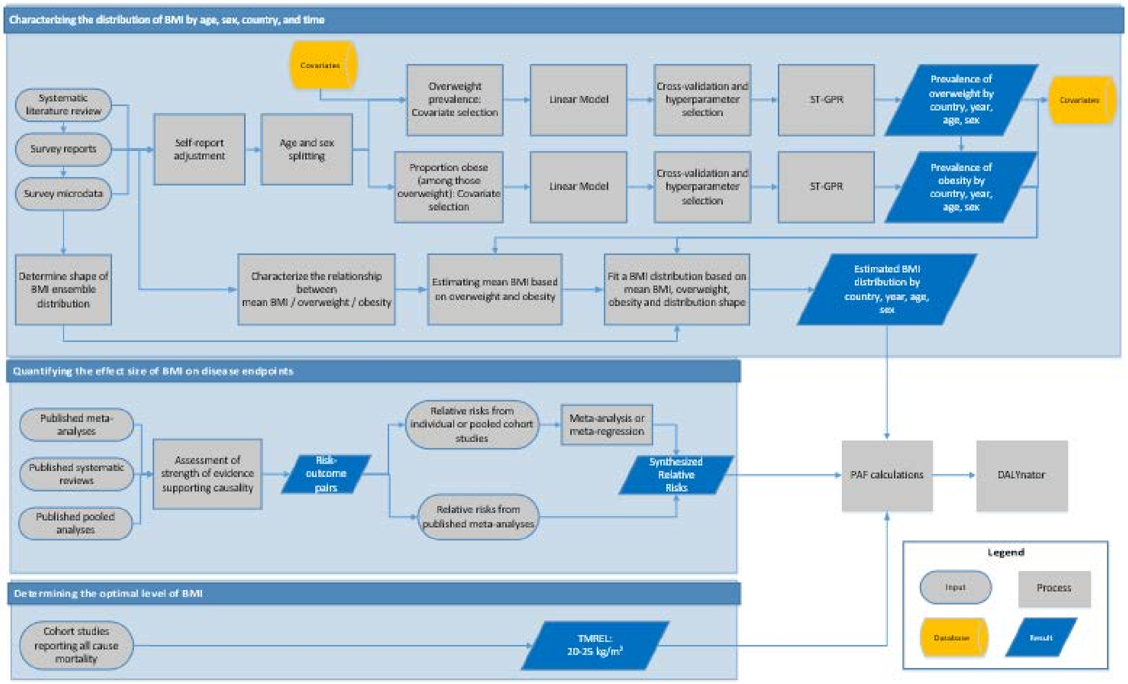

Childhood (Ages 2-19) High Body-Mass Index: Data and Model Flow Chart

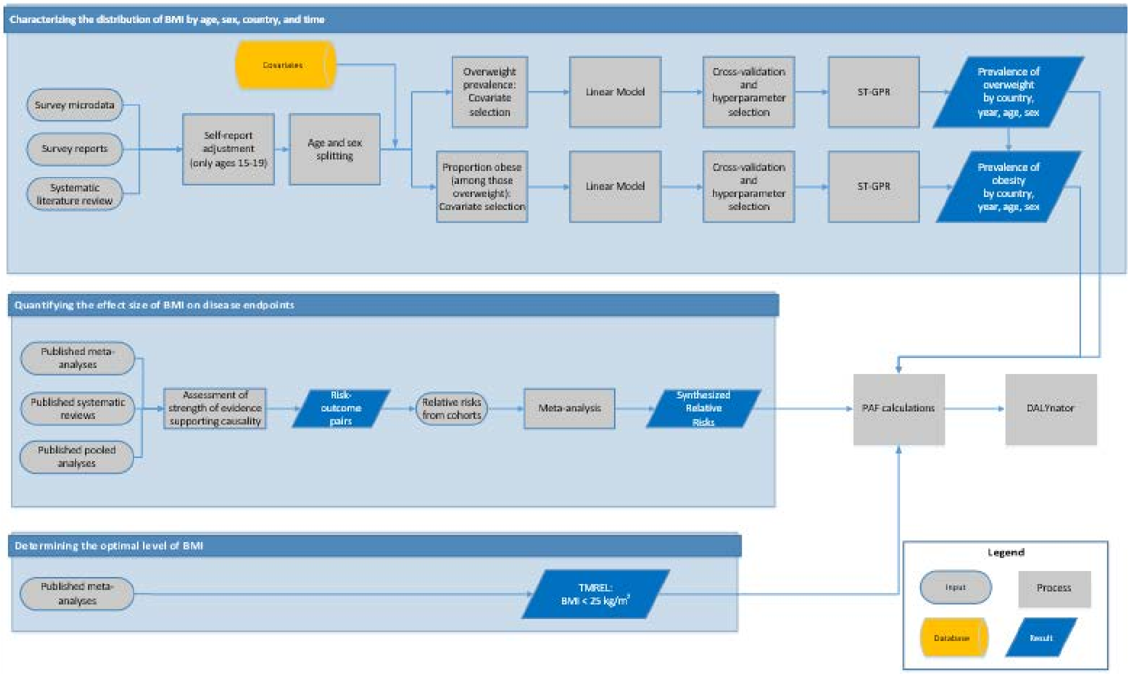

Input data and Methodological Summary

Indicator definition

This modeling strategy encompasses SDG indicator associated with childhood overweight: 2.2.2b.

Indicator 2.2.2b

As a component of SDG Goal 2. End hunger, achieve food security, and improved nutrition, SDG Target 2.2, by 2030, end all forms of malnutrition, including achieving, by 2025, the internationally agreed targets on stunting and wasting in children under 5 years of age, and address the nutritional needs of adolescent girls, pregnant and lactating women and older persons, is measured using SDG indicator 2.2.2b, prevalence of children aged 2 to 4 years with a body-mass index (BMI) exceeding the overweight cut-offs established by the International Obesity Task Force (IOTF) for each sex and by month of age.

Case definitions

High body-mass index (BMI) for adults (ages 20+) is defined as BMI greater than 20 to 25 kg/m^2. High BMI for children (ages 1-19) is defined as being overweight or obese based on IOTF cutoffs.

Input data and methodological summary

Data sources

We systematically searched Medline to identify studies providing nationally or subnationally representative estimates of overweight prevalence, obesity prevalence or mean body-mass index (BMI). We limited the search to literature published between January 1, 2016 and December 31, 2016 to update the systematic literature search previously performed as part of GBD 2015.

The search for adults was conducted on 4 January 2017 using the following terms:

((("Body Mass Index"[Mesh] OR "Overweight"[Mesh] OR "Obesity"[Mesh]) AND ("Geographic Locations"[Mesh] NOT “United States”[Mesh]) AND ("humans"[Mesh] AND "adult"[MeSH]) AND ("Data Collection"[Mesh] OR "Health Services Research"[Mesh] OR "Population Surveillance"[Mesh] OR "Vital statistics"[Mesh] OR "Population"[Mesh] OR "Epidemiology"[Mesh] OR "surve*"[TiAb]) NOT (Comment[ptyp] OR Case Reports[ptyp] OR "hospital"[TiAb])) AND ("2016/01/01"[Date - Publication] : "2016/12/31"[Date - Publication]))

The search for children was conducted on 4 August 2016 using the following terms:
((("Body Mass Index"[Mesh] OR "Overweight"[Mesh] OR "Obesity"[Mesh]) AND ("Geographic Locations"[Mesh] NOT “United States”[Mesh]) AND ("humans"[Mesh] AND "child"[MeSH]) AND ("Data Collection"[Mesh] OR "Health Services Research"[Mesh] OR "Population Surveillance"[Mesh] OR "Vital statistics"[Mesh] OR "Population"[Mesh] OR "Epidemiology"[Mesh] OR "surve*"[TiAb]) NOT (Comment[ptyp] OR Case Reports[ptyp] OR "hospital"[TiAb])) AND ("2016/01/01"[Date - Publication] : "2016/12/31"[Date - Publication]))

Our search for adult estimates identified 456 abstracts, of which 25 met inclusion criteria and were extracted. The search for childhood estimates identified 137 articles, of which 4 were extracted. Including sources from the previous GBD systematic literature searches, a total of 11,220 articles were identified, of

which 845 were included. Additionally, we searched the Global Health Data Exchange (GHDx) database for individual-level data from major multinational survey series or country-specific surveys and identified 5,385 location-year sources meeting the inclusion criteria.

Eligibility criteria

We included representative studies providing data on mean BMI or prevalence of overweight or obesity on uts or hi ren. or uts stu ies ere in u e i the e ine o er ei ht s ^{2} n obesit s ^{2}, or if estimates using those cutoffs could be back-calculated from reported categories. For children (children ages 2-18), studies were included if they used International Obesity Task Force (IOTF) standards to define overweight and obesity thresholds. We only included studies reporting data collected between January 1, 1980 and December 31, 2016. Studies were excluded if they used non-random samples (e.g. case-control studies or convenience samples), conducted among specific subpopulations (e.g. pregnant women, racial or ethnic minorities, immigrants, or individuals with specific diseases), used alternative methods to assess adiposity (e.g. waist-circumference, skin-fold thickness, or hydrodensitometry), had sample sizes of less than 20 per age-sex group, or provided inadequate information on any of the inclusion criteria. We also excluded review articles and non-English language articles.

Data collection process

Where individual-level survey data were available, we computed mean BMI using weight and height. We then used BMI to determine the prevalence of overweight and obesity. For individuals aged over 18 years, we considered them to be overweight if their BMI was greater than or equal to 25 kg/m^2, and obese if their BMI was greater than or equal to 30 kg/m^2. For individuals aged 2 to 18 years, we used monthly IOTF cutoffs[2] to determine overweight and obese status when age in months was available. When only age in years was available, we used the cutoff for the midpoint of that year. Obese individuals were also considered to be overweight. We excluded studies using the World Health Organization (WHO) standards or country-specific cutoffs to define childhood overweight and obesity. At the individual-level, we considered BMI$<$10 kg/m^2 and BMI$>$70 kg/m^2 to be biologically implausible and excluded those observations.

The rationale for choosing to use the IOTF cutoffs over the WHO standards has been described elsewhere. Briefly, the IOTF cutoffs provide consistent child-specific standards for ages 2-18 derived surveys covering multiple countries. On the other hand, the WHO growth standards apply to children under age 5 and the WHO growth reference applies to children ages 5 to 19. The WHO growth reference for children ages 5 to 19 was derived from United States data which is less representative than the multinational data used by IOTF. Additionally, the switch between references at age 5 can produce artificial discontinuities. Given that we estimate global childhood overweight and obesity for ages 2-19 (with ages 19 using standard adult cutoffs), the IOTF cutoffs were preferable. Additionally, we found that IOTF cutoffs were more commonly used in scientific literature covering childhood obesity.

From report and literature data, we extracted data on mean BMI, prevalence of overweight, and prevalence of obesity, measures of uncertainty for each, and sample size, by the most granular age and sex groups available. Additionally, we extracted the same study-level covariates as were extracted from microdata (measurement, urbanicity, and representativeness), as well as location and year.

In addition to the primary indicators described above, we extracted relevant survey-design variables, including primary sampling unit, strata, and survey weights, which were used to tabulate individual-level

microdata and produce accurate measures of uncertainty. We extracted three study-level covariates: 1) whether height and weight data were measured or self-reported; 2) whether the study was predominantly conducted in an urban area, rural area or both; and 3) the level of representativeness of the study (national or subnational).

Finally, we extracted relevant demographic indicators, including location, year, age and sex. We estimated the standard error of the mean from individual-level data, where available, and used the reported standard error of the mean for published data. When multiple data sources were available for the same country, we included all of them in our analysis. If data from the same data source were available in multiple formats such individual-level data and tabulated data, we used individual-level data.

Self-report bias adjustment

We included both measured and self-reported data. We tested for bias in self-report data compared to measured data, which is considered to be the gold-standard. There was no clear direction of bias for children ages 2 to 14, so for these age groups we only included measured data. For individuals ages 15 and above, we adjusted self-reported data for overweight prevalence, obesity prevalence and mean BMI using the following nested hierarchical mixed-effects regression models, fit using restricted maximum likelihood separately by sex:

$$\text{logit}(\text{overweight})_{c,a,t} = \beta_0 + \beta_1 m + \sum_{k=2}^{19} \beta_k I_{A[a]} + \sum_{l=20}^{55} \beta_l\, I_{A[a]} I_{M[m]} + \alpha_s + \alpha_s m + \alpha_r + \alpha_r m + \alpha_c + \alpha_c m + \alpha_t + \alpha_t m + \epsilon_{c,a,t}$$

$$\text{logit}(\text{obesity})_{c,a,t} = \beta_0 + \beta_1 m + \sum_{k=2}^{19} \beta_k I_{A[a]} + \sum_{l=20}^{55} \beta_l\, I_{A[a]} I_{M[m]} + \alpha_s + \alpha_s m + \alpha_r + \alpha_r m + \alpha_c + \alpha_c m + \alpha_t + \alpha_t m + \epsilon_{c,a,t}$$

$$\log(\text{BMI})_{c,a,t} = \beta_0 + \beta_1 m + \sum_{k=2}^{19} \beta_k I_{A[a]} + \sum_{l=20}^{55} \beta_l\, I_{A[a]} I_{M[m]} + \alpha_s + \alpha_s m + \alpha_r + \alpha_r m + \alpha_c + \alpha_c m + \alpha_t + \alpha_t m + \epsilon_{c,a,t}$$

Where m is a fixed effect on measurement (binary, either measured (1) or self-report (0)), $I_{A[a]}$ is an indicator variable for specific age group A, $I_{A[a]} I_{M[m]}$ is an interaction term between age and measurement, α_s, α_r, and α_c are random effects at the super region, region, and country, respectively, and α_t is a random effect by time-period (1980-1989, 1990-1999, 2000-2009, 2010-2017). Random effects at the country level and time-period level were used to fit the models, but were taken as noise and were not used in adjustment of self-reported data. We propagated the uncertainty in the self-report adjustment model by adding the variance of each of the regression coefficients used in adjustment to the data variance in delta-transformed space. After adjustment, regressions confirmed that self-reported data was no longer significantly different from measured data.

Age and sex splitting

Any report or literature data provided in age groups wider than the standard 5-year age groups or as both sexes combined were split using the approach used by Ng et al.[1] Briefly, age-sex patterns were identified using sources with data on multiple age-sex groups and these patterns were applied to split aggregated report and literature data. Uncertainty in the age-sex split was propagated by multiplying the standard error of the data by the square root of the number of splits performed. We did not propagate the uncertainty in the age pattern and sex pattern used to split the data as they seemed to have small effect.

Prevalence estimation for overweight and obesity

After adjusting for self-report bias and splitting aggregated data into 5-year age-sex groups, we used spatiotemporal Gaussian process regression (ST-GPR) to estimate the prevalence of overweight and obesity. This modelling approach has been described in detail elsewhere.

The linear model, which when added to the smoothed residuals forms the mean prior for GPR is as follows:

$$\mathrm{logit(overweight)}_{c,a,t} = \beta_0 + \beta_1 \mathrm{energy}_{c,t} + \beta_2 \mathrm{SDI}_{c,t} + \beta_3 \mathrm{vehicles}_{c,t} + \beta_4 \mathrm{agriculture}_{c,t} + \sum_{k=5}^{21} \beta_k I_{A[a]} + \alpha_s + \alpha_r + \alpha_c$$

$$\mathrm{logit(obesity/overweight)}_{c,a,t} = \beta_0 + \beta_1 \mathrm{energy}_{c,t} + \beta_2 \mathrm{SDI}_{c,t} + \beta_3 \mathrm{vehicles}_{c,t} + \sum_{k=4}^{21} \beta_k I_{A[a]} + \alpha_s + \alpha_r + \alpha_c$$

where $energy$ is ten-year lag-distributed energy consumption per capita, SDI is a composite index of development including lag-distributed income per capita, education, and fertility, $vehicles$ is is the number of two or four-wheel vehicles per capita, and $agriculture$ is the proportion of the population working in agriculture. $I_{A[a]}$ is a dummy variable indicating specific age group A that the prevalence point captures, and α_s, α_r, and α_c are super region, region, and country random intercepts, respectively. Random effects were used in model fitting but were not used in prediction.

We tested all combinations of the following covariates to see which performed best in terms of in-sample AIC for the overweight linear model and the obesity as a proportion of overweight linear model: ten-year lag distributed energy per capita, proportion of the population living in urban areas, SDI, lag-distributed income per capita, educational attainment (years) per capita, proportion of the population working in agriculture, grams of sugar adjusted for energy per capita, grams of sugar not adjusted for energy per capita, and the number of two or four-wheeled vehicles per capita. We selected these candidate covariates based on theory as well as reviewing covariates used in other publications. The final linear model was selected based on: 1) if the direction of covariates matched what is expected from theory, 2) all the included covariates were significant, and 3) minimising in-sample AIC. The covariate selection process was performed using the dredge package in R.

The new version of ST-GPR for GBD 2017 incorporates information about data density into the process for smoothing over space and time. Estimates in areas/years with few observations have more weight on regional observations. To specify the distribution of time weights and space weights, we used values of lambda=0.2 and zeta=0.05, respectively. We used a value of omega=1.0 for the distribution of age weights. We set the GPR scale parameter to 20, and used the default global cutoff setting for amplitude.

Estimating mean BMI

To estimate the mean BMI for adults in each country, age, sex, and time period 1980-2017, we first used the following nested hierarchical mixed-effects model, fit using restricted maximum likelihood on data from sources containing estimates of all three indicators (prevalence of overweight, prevalence of obesity, and mean BMI), in order to characterise the relationship between overweight, obesity, and mean BMI:

$$\log(\mathrm{BMI}_{c,a,s,t}) = \beta_0 + \beta_1 \mathrm{ow}_{c,a,s,t} + \beta_2 \mathrm{ob}_{c,a,s,t} + \beta_3 \mathrm{sex} + \sum_{k=4}^{20} \beta_k I_{A[a]} + \alpha_s(1 + \mathrm{ow}_{c,a,s,t} + \mathrm{ob}_{c,a,s,t}) + \alpha_r(1 + \mathrm{ow}_{c,a,s,t} + \mathrm{ob}_{c,a,s,t}) + \alpha_c(1 + \mathrm{ow}_{c,a,s,t} + \mathrm{ob}_{c,a,s,t}) + \epsilon_{c,a,s,t}$$

where $\mathrm{ow}_{c,a,s,t}$ is the prevalence of overweight in country c, age a, sex s, and year t, $\mathrm{ob}_{c,a,s,t}$ is the prevalence of obesity in country c, age a, sex s, and year t, sex is a fixed effect on sex, $I_{A[a]}$ is an indicator variable for age, and α_s, α_r, and α_c are random effects at the super region, region, and country, respectively. The model was run in Stata 13.

We applied 1,000 draws of the regression coefficients to the 1,000 draws of overweight prevalence and obesity prevalence produced through ST-GPR to estimate 1,000 draws of mean BMI for each country, year, age, and sex. This approach ensured that overweight prevalence, obesity prevalence, and mean BMI were correlated at the draw level and uncertainty was propagated.

Estimating BMI distribution

We used the ensemble distribution approach described in the manuscript. We fit ensemble weights by source and sex, with source- and sex-specific weights averaged across all sources included to produce the final global weights. The ensemble weights were fit on measured microdata. The final ensemble weights were: exponential = 0.002, gamma = 0.028, inverse gamma = 0.085, log-logistic = 0.187, Gumbel = 0.220, Weibull = 0.011, log-normal = 0.058, normal = 0.012, beta = 0.136, mirror gamma = 0.008, and mirror Gumbel = 0.113.

One thousand draws of BMI distributions for each location, year, age group, and sex estimated were produced by fitting an ensemble distribution using 1,000 draws of estimated mean BMI, 1,000 draws of estimated standard deviation, and the ensemble weights. Estimated standard deviation was produced by optimising a standard deviation to fit estimated overweight prevalence draws and estimated obesity prevalence draws.

Assessment of risk-outcome pairs

Risk-outcome pairs were defined based on strength of available evidence supporting a causal effect. We performed a systematic review of published meta-analyses, pooled analyses, and systematic reviews available through PubMed using the following search string: ("Body Mass Index"[Mesh] OR "Overweight"[Mesh] OR "Obesity"[Mesh]) AND (Meta-Analysis[ptyp] OR "systematic review"[tiab] OR "pooled analysis"[tiab]). Inclusion criteria are 1) the health outcome is included in GBD, 2) at least one prospective cohort is included, and 3) that the summary effect size is statistically significant. For outcomes meeting inclusion criteria we completed causal criteria tables to evaluate the strength of evidence supporting a causal relationship. Appendix Table 6 in the GBD2017 Risk Factors Capstone paper reports the results of our assessment for included risk-outcome pairs and Appendix Table 7 reports the supporting scientific literature.[2] Gallbladder disease, cataract, multiple myeloma, gout, non-Hodgkin lymphoma, asthma, Alzheimer disease, and atrial fibrillation were added as new outcomes in GBD 2016, resulting in a total of 38 outcomes.

Theoretical minimum risk exposure level

For adults (ages 20+), the theoretical minimum risk exposure level (TMREL) of BMI (20-25 kg/m2) was determined based on the BMI level that was associated with the lowest risk of all-cause mortality in prospective cohort studies.[3]

For children (ages 2-19), the TMREL is “normal weight”, that is, not overweight or obese, based on IOTF cutoffs.

Relative risk

The relative risk per 5-unit change in BMI for each disease endpoint was obtained from meta-analyses, and where available, pooled analyses of prospective observational studies. In cases where a relative risk per 5-unit change in BMI was not available we computed our own dose-response meta-analysis using two-step generalised least squares for time trends estimation methods.

For childhood outcomes (ages 2-19), we computed categorical relative risks for overweight and obesity using a random effects meta-analysis.

Relative risks for all 38 outcomes, by age and sex, are reported in Table 6 of the GBD 2017 Risk Factors Capstone paper appendix.[2]

References

1.) Ng M, Fleming T, Robinson M, et al. Global, regional, and national prevalence of overweight and obesity in children and adults during 1980–2013: a systematic analysis for the Global Burden of Disease Study 2013. The Lancet 2014; 384: 766–81.
2.) GBD2017 Risk Factors Collaborators. Global, regional, and national comparative risk assessment of 84 behavioural, environmental and occupational, and metabolic risks or clusters of risks, 1990–2017: a systematic analysis for the Global Burden of Disease Study 2017. The Lancet Under Review.
3.) Angelantonio ED, Bhupathiraju SN, Wormser D, et al. Body-mass index and all-cause mortality: individual-participant-data meta-analysis of 239 prospective studies in four continents. The Lancet 2016; 388: 776–86.

3.1.1 Maternal disorders SDG Capstone Appendix

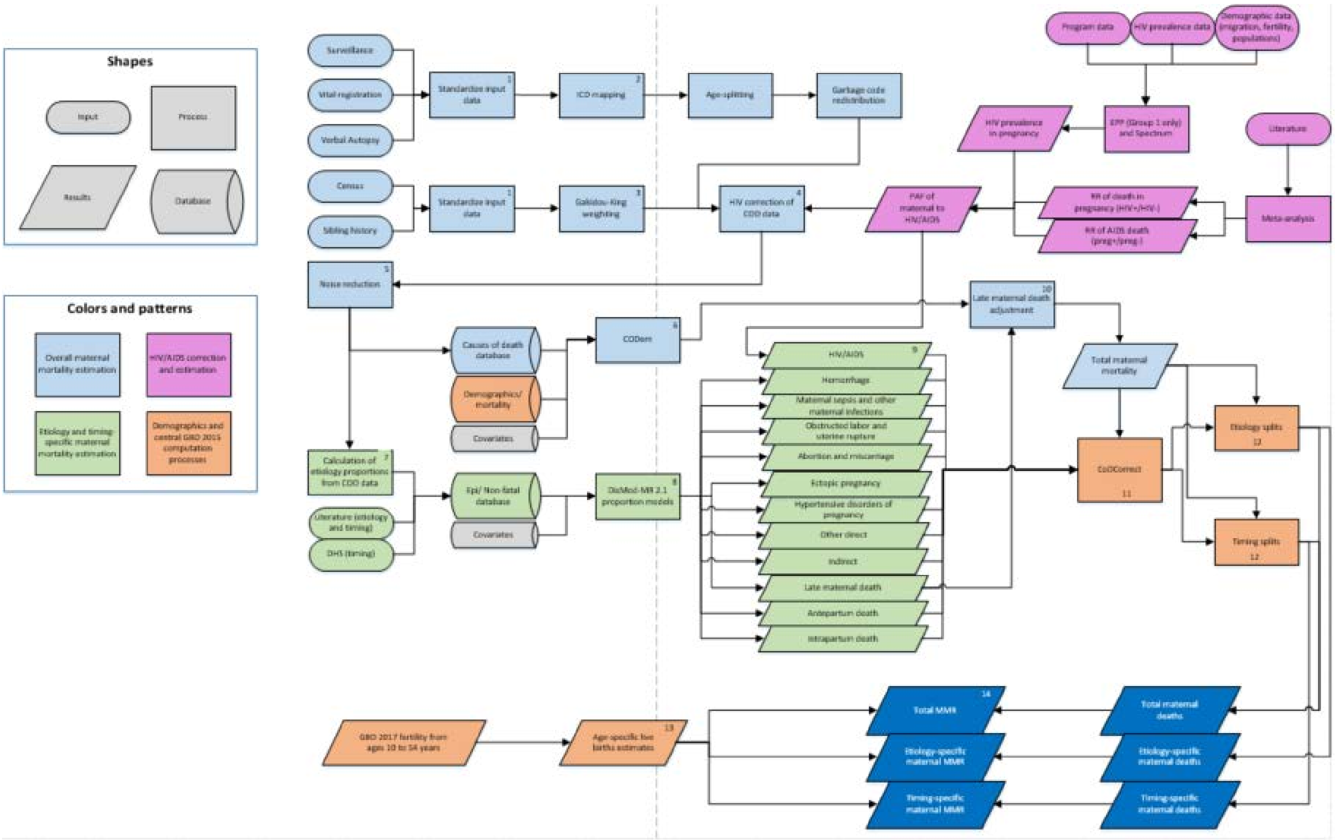

Input Data & Methodological Summary

Indicator definition

This modeling strategy encompassed the indicator associated with the maternal mortality ratio (SDG indicator 3.1.1).

Indicator 3.1.1

As a component of SDG Goal 3. Ensure healthy lives and promote well-being for all at all ages, SDG Target 3.1, by 2030, reduce the global maternal mortality ratio to less than 70 per 100,000 live births, is measured using SDG indicator 3.1.1, maternal mortality ratio (maternal deaths per 100,000 live births)

Input data

CODEm models were informed by centrally prepped data stored in the cause of death (COD) database. All data were corrected for incidental HIV deaths. Spectrum outputs of HIV prevalence in pregnancy were combined with relative risk of mortality during pregnancy (HIV-positive versus HIV-negative) to calculate population attributable fractions (PAFs). A proportion of these deaths are incidental and a proportion are maternal. PAFs were applied to all sibling history and census data to remove incidental HIV deaths. We performed an updated literature review to search for new scientific articles reporting data on maternal

mortality, morbidity, and relative risk of mortality in pregnancy in HIV-positive versus HIV-negative women. We completed this search on July 20, 2017, using the following search string:

(((((("Postpartum Hemorrhage" OR "Uterine Hemorrhage") OR (maternal[Title/Abstract] OR pregnant[Title/Abstract] OR pregnancy[Title/Abstract] OR mothers) AND (haemorrhag*[Title/Abstract] OR hemorrhag*[Title/Abstract]) NOT "case report"[All fields]) OR (("induced abortion" OR "Therapeutic abortion" OR "legal Abortion" OR "medical abortion" OR "miscarriage" OR "Abortion, Induced"[Mesh] OR "Abortion, Therapeutic"[Mesh] OR "Abortion, Legal"[Mesh] OR "ectopic Pregnancy") NOT ("case report"[Title/Abstract] OR "birth defect"[Title/Abstract] OR congenital[Title/Abstract])) OR ("obstructed labour" OR "obstructed labor" OR "labour dystocia" OR "labor dystocia" OR dystocia OR "cephalopelvic disproportion" OR "cephalo-pelvic disproportion") OR (("obstetric fistula" OR "vesicovaginal fistula") OR "rectovaginal fistula") OR (("Puerperal Infection"[Mesh] OR "Puerperal Infection" OR ((maternal[Title/Abstract] OR pregnant[Title/Abstract] OR pregnancy[Title/Abstract]) AND (Sepsis OR infection[Title/Abstract]))) NOT "case report") OR ((Pre-Eclampsia[Title/Abstract] OR preeclampsia[Title/Abstract] OR Eclampsia[Title/Abstract] OR Pre-Eclampsia[Mesh] OR Eclampsia[Mesh] OR "Hypertension, Pregnancy-Induced"[Mesh] OR "pregnancy induced hypertension"[Title/Abstract] OR "gestational hypertension"[Title/Abstract] OR "Hypertensive disorders of pregnancy"[Title/Abstract]) NOT ("case report" OR "kidney don*"[Title/Abstract] OR polymorphism*[Title/Abstract] OR endotheli*[Title/Abstract]))) AND (2016/08/01[PDat] : 2017/12/31[PDat]) NOT (animals[MeSH] NOT humans[MeSH]))) OR (((((("maternal mortality"[Title/Abstract] OR "maternal death"[Title/Abstract] OR "MM"[Title/Abstract] OR "confidential enquiry"[Title/Abstract] OR ((obstetric[Title/Abstract] OR pregnancy[Title/Abstract]) AND (etiology[Title/Abstract] OR cause[Title/Abstract] or pattern[Title/Abstract]) AND (death[Title/Abstract] OR mortality[Title/Abstract])))) NOT (fetal[Title/Abstract] OR newborns[Title/Abstract] OR newborn[Title/Abstract] OR neonatal[Title/Abstract] OR “case report”[Title/Abstract] OR “case study”[Title/Abstract] OR pathogenesis[Title/Abstract] OR thromboprophylaxis[Title/Abstract])) NOT (animals[MeSH] NOT humans[MeSH]) OR (((“maternal mortality”[Title/Abstract] OR “maternal death*”[Title/Abstract] OR “MMR”[Title/Abstract]) AND (“Afghanistan”[Title/Abstract] OR “Albania”[Title/Abstract] OR “Algeria”[Title/Abstract] OR “Andorra”[Title/Abstract] OR “Angola”[Title/Abstract] OR “Antigua and Barbuda”[Title/Abstract] OR “Argentina”[Title/Abstract] OR “Armenia”[Title/Abstract] OR “Azerbaijan”[Title/Abstract] OR “Bahrain”[Title/Abstract] OR “Bangladesh”[Title/Abstract] OR “Barbados”[Title/Abstract] OR “Belarus”[Title/Abstract] OR “Belize”[Title/Abstract] OR “Benin”[Title/Abstract] OR “Bhutan”[Title/Abstract] OR “Bolivia”[Title/Abstract] OR “Bosnia and Herzegovina”[Title/Abstract] OR “Botswana”[Title/Abstract] OR “Brazil”[Title/Abstract] OR “Brunei”[Title/Abstract] OR “Bulgaria”[Title/Abstract] OR “Burkina Faso”[Title/Abstract] OR “Burundi”[Title/Abstract] OR “Cambodia”[Title/Abstract] OR “Cameroon”[Title/Abstract] OR “Cape Verde”[Title/Abstract] OR “Central African Republic”[Title/Abstract] OR “Chad”[Title/Abstract] OR “China”[Title/Abstract] OR “Colombia”[Title/Abstract] OR “Comoros”[Title/Abstract] OR “Congo”[Title/Abstract] OR “Costa Rica”[Title/Abstract] OR “Croatia”[Title/Abstract] OR “Cuba”[Title/Abstract] OR “Cyprus”[Title/Abstract] OR “Côte d’Ivoire”[Title/Abstract] OR “Democratic Republic of the Congo”[Title/Abstract] OR “Djibouti”[Title/Abstract] OR “Dominica”[Title/Abstract] OR “Dominican Republic”[Title/Abstract] OR “Ecuador”[Title/Abstract] OR “Egypt”[Title/Abstract] OR “El Salvador”[Title/Abstract] OR “Equatorial Guinea”[Title/Abstract] OR “Eritrea”[Title/Abstract] OR “Ethiopia”[Title/Abstract] OR “Federated States of Micronesia”[Title/Abstract] OR “Fiji”[Title/Abstract] OR “Gabon”[Title/Abstract] OR “Georgia”[Title/Abstract] OR “Ghana”[Title/Abstract] OR “Grenada”[Title/Abstract] OR “Guatemala”[Title/Abstract] OR “Guinea”[Title/Abstract] OR “Guinea-Bissau”[Title/Abstract] OR “Guyana”[Title/Abstract] OR “Haiti”[Title/Abstract] OR “Honduras”[Title/Abstract] OR “India”[Title/Abstract] OR “Indonesia”[Title/Abstract] OR “Iran”[Title/Abstract] OR “Iraq”[Title/Abstract] OR “Jamaica”[Title/Abstract] OR “Jordan”[Title/Abstract] OR “Kazakhstan”[Title/Abstract] OR “Kenya”[Title/Abstract] OR “Kiribati”[Title/Abstract] OR “Kuwait”[Title/Abstract] OR “Kyrgyzstan”[Title/Abstract] OR “Laos”[Title/Abstract] OR “Latvia”[Title/Abstract] OR “Lebanon”[Title/Abstract] OR “Lesotho”[Title/Abstract] OR “Liberia”[Title/Abstract] OR “Libya”[Title/Abstract] OR “Lithuania”[Title/Abstract] OR “Macedonia”[Title/Abstract] OR “Madagascar”[Title/Abstract] OR “Malawi”[Title/Abstract] OR “Malaysia”[Title/Abstract] OR “Maldives”[Title/Abstract] OR “Mali”[Title/Abstract] OR “Malta”[Title/Abstract] OR “Marshall Islands”[Title/Abstract] OR “Mauritania”[Title/Abstract] OR “Mauritius”[Title/Abstract] OR “Moldova”[Title/Abstract] OR “Mongolia”[Title/Abstract] OR “Montenegro”[Title/Abstract] OR “Morocco”[Title/Abstract] OR “Mozambique”[Title/Abstract] OR “Myanmar”[Title/Abstract] OR “Namibia”[Title/Abstract] OR “Nepal”[Title/Abstract] OR “Nicaragua”[Title/Abstract] OR “Niger”[Title/Abstract] OR “Nigeria”[Title/Abstract] OR “North Korea”[Title/Abstract] OR “Oman”[Title/Abstract] OR “Pakistan”[Title/Abstract] OR “Palestine”[Title/Abstract] OR “Panama”[Title/Abstract] OR “Papua New Guinea”[Title/Abstract] OR “Paraguay”[Title/Abstract] OR “Peru”[Title/Abstract] OR “Philippines”[Title/Abstract] OR “Qatar”[Title/Abstract] OR “Romania”[Title/Abstract] OR “Russia”[Title/Abstract] OR “Rwanda”[Title/Abstract] OR “Saint Lucia”[Title/Abstract] OR “Saint Vincent and the Grenadines”[Title/Abstract] OR “Samoa”[Title/Abstract] OR “Saudi Arabia”[Title/Abstract] OR “Senegal”[Title/Abstract] OR “Serbia”[Title/Abstract] OR “Seychelles”[Title/Abstract] OR “Sierra Leone”[Title/Abstract] OR “Singapore”[Title/Abstract] OR “Solomon Islands”[Title/Abstract] OR “Somalia”[Title/Abstract] OR

“South Africa”[Title/Abstract] OR "South Sudan"[Title/Abstract] OR “Sri Lanka”[Title/Abstract] OR “Sudan”[Title/Abstract] OR “Suriname”[Title/Abstract] OR “Swaziland”[Title/Abstract] OR “Syria”[Title/Abstract] OR “São Tomé and Príncipe”[Title/Abstract] OR “Taiwan”[Title/Abstract] OR “Tajikistan”[Title/Abstract] OR “Tanzania”[Title/Abstract] OR “Thailand”[Title/Abstract] OR “The Bahamas”[Title/Abstract] OR “The Gambia”[Title/Abstract] OR “Timor-Leste”[Title/Abstract] OR “Togo”[Title/Abstract] OR “Tonga”[Title/Abstract] OR “Trinidad and Tobago”[Title/Abstract] OR “Tunisia”[Title/Abstract] OR “Turkmenistan”[Title/Abstract] OR “Uganda”[Title/Abstract] OR “Ukraine”[Title/Abstract] OR “United Arab Emirates”[Title/Abstract] OR “Uruguay”[Title/Abstract] OR “Uzbekistan”[Title/Abstract] OR “Vanuatu”[Title/Abstract] OR “Venezuela”[Title/Abstract] OR “Vietnam”[Title/Abstract] OR “Yemen”[Title/Abstract] OR “Zambia”[Title/Abstract] OR “Zimbabwe”[Title/Abstract]) NOT (animals[MeSH] NOT humans[MeSH]) NOT (“demographic and health survey*”[Title/Abstract] OR DHS[Title/Abstract] OR “reproductive health survey*”[Title/Abstract] OR RHS[Title/Abstract]))) AND (2016/08/15[PDat] : 2017/12/31[PDat]))))) OR ((HIV[Title/Abstract] OR "Acquired Immunodeficiency Syndrome"[Title/Abstract] OR AIDS[Title/Abstract]) AND (“pregnant”[Title/Abstract] OR “pregnancy”[Title/Abstract] OR “postpartum”[Title/Abstract] OR "post partum"[Title/Abstract]) AND (“mortality”[Title/Abstract] OR “death”[Title/Abstract]) NOT "case report" NOT (animals[MeSH] NOT humans[MeSH]) AND (2016/08/15[PDat] : 2017/12/31[PDat]))

All data from all geographies were reviewed in CODEm models. Outliers were identified as those data where age patterns or temporal patterns were inconsistent with neighbouring age groups or locations or where sparse data were predicting implausible overall temporal or age patterns for a given location.

We used scientific literature data identified through the search string above to inform DisMod-MR 2.1 aetiology proportion models as well as data from the COD database.

A total of 6,554 literature sources were reviewed for their title and abstract. Of those selected for full text review, 41 of them were extracted to inform maternal disorder models (fatal and non-fatal). All cause-specific maternal mortality data were prepped as “proportion” of total maternal deaths due to that cause. Because many sources do not include the entire cause list, a series of study covariates were used to facilitate crosswalking back to the reference definition. The reference definition ***includes*** “other” direct obstetric complications, indirect maternal deaths, and late maternal death. All aetiology-specific COD data were processed to be “proportion” data by calculating the cause-specific deaths divided by the total maternal deaths for the matching data source, year, age, and location. Late maternal death data were only included for the subset of locations where they were reliably coded in raw VR. All data were uploaded to the non-fatal database.

Modelling strategy

Overall maternal mortality was estimated with CODEm. Covariates included in this model and their level and directionality are show in the table below:

Level	Covariate	Direction
Level 1	Age-specific fertility rate	+
	Total fertility rate (log-transformed)	+
	Maternal education (years per capita)	–
	In-facility delivery (proportion)	–
	Skilled birth attendance (proportion)	–
	Neonatal mortality ratio (log-transformed)	+

	Age-specific HIV mortality in females 10-54 (log-transformed)	+
Level 2	Antenatal care 1-visit coverage (proportion)<br>Antenatal care 4-visits coverage (proportion)<br>Age-standardised wasting (weight-for-height) summary exposure value<br>Age-standardised stunting (height-for-age) summary exposure value<br>Healthcare access and quality index<br>Prevalence of obesity	–<br>–<br>+<br>+<br>-<br>+
Level 3	Socio-demographic Index<br>Mortality shock (cumulative rate in last 10 years)<br>LDI (log-transformed)<br>Hospital beds (per 1,000 population)	+<br>–<br>+<br>–<br>–

We used STGPR to estimate MMRs for each of the eight maternal subcauses. This modeling strategy requires data to be in standard GBD age groups. To achieve this, we used the age pattern of the COD data for each cause and applied it to the literature data that were not in the standard GBD age groups. STGPR also requires variance for each data point. In order to compute variance we ran a Lowess regression on the data by year and used the variance of the residuals resulting from the difference between the data and the predicted values.

Country covariates were specific for each model and included abortion legality (for abortion and miscarriage as well as ectopic pregnancy), log-transformed lag-distributed income (other maternal deaths, and indirect maternal deaths), unsafe sanitation summary exposure value (for maternal sepsis and other maternal infections), Socio-demographic Index (for late maternal deaths), logit-transformed in-facility delivery proportion (for haemorrhage), mean systolic blood pressure (for hypertensive disorders of pregnancy), and age-specific underweight women (for obstructed labour).

Aetiology-specific estimates were derived by scaling the results from the STGPR subcause-specific models scaled in relation to each other to equal one and then multiplying them by the total maternal deaths, corrected for late maternal deaths, for that age group, location, and year. HIV-related maternal deaths were estimated for all locations using the PAF approach described above for mortality data processing. Incidental HIV deaths during pregnancy were by definition excluded.

3.1.2, 3.8.1 Skilled Birth Attendance SDG Capstone Appendix

Flowchart

3.1.2 Skilled Birth Attendance Coverage Estimation Flowchart

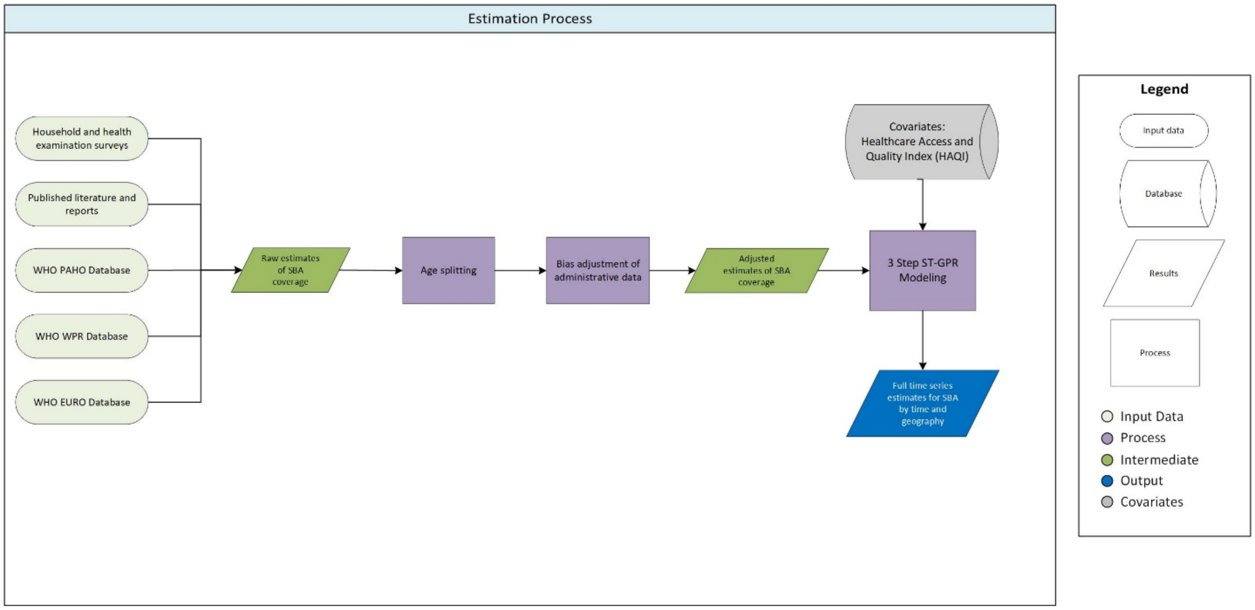

Input data & Methodological summary

Indicator definition

This modeling strategy pertains to the indicator associated with skilled birth attendance (SBA) (SDG indicator 3.1.2), which is also included in the universal health coverage (UHC) index (SDG indicator 3.8.1d).

Indicator 3.1.2

As a component of SDG Goal 3. Ensure healthy lives and promote well-being for all at all ages, SDG Target 3.1, by 2030, reduce the global maternal mortality ratio to less than 70 per 100,000 live births, is measured by SDG indicator 3.1.2, proportion of births attended by skilled health personnel (doctors, nurses or midwives). Note that SBA is included in the UHC index (SDG indicator 3.8.1d).

Input data

For the present analysis, we used individual-level microdata from population health surveys and tabulated survey report data on skilled birth attendance (SBA). As defined by the World Health Organization (WHO), SBA reflects the proportion of births in a given year where a doctor, nurse, or midwife was present.[1]

Survey data which provided individual-level data, and specifically among female respondents, were identified and extracted. Major multi-country survey programs included in the analysis include the Demographic and Health Surveys (DHS),[1] Multiple Indicator Cluster Surveys (MICS),[2] Reproductive Health

Surveys (RHS),[3] Living Standards Measurement Study (LSMS) surveys,[4] and World Health Surveys (WHS).[5] We also conducted a comprehensive search of the Global Health Data Exchange (GHDx),[6] as well as targeted internet searches and review of Ministry of Health websites, to identify national surveys and other multi-country survey programs. In addition, we utilized tabulated report data from regional WHO databases, when available, including the PAHO[7], WHO WPR[8], and the WHO European Health for All databases[9].

We excluded all data sources that were not nationally representative or had high levels of missingness. We applied survey weights based on survey sampling frames whenever they were available to generate weighted national estimates of SBA coverage accompanied by estimates of standard error (SE). Estimates of SE, as well as sample sizes, were used to calculate uncertainty, as described below. Any point estimates with sample sizes less than 50 were reviewed to ensure that they were not substantive outliers and would otherwise have an undue influence on our analysis.

Due to potential bias in recall, we limited our analysis to women who gave birth up to five years prior to the time of survey; due to data limitations, we used a limit of up to two years for some surveys. We also had to standardize the definition of "skilled health professional" across countries, which varied by differences in quality of training or health professional roles. For this analysis, doctors, nurses, and midwives were included as our foundational definition for SBA, and we extended this to include country-specific medical staff based on the number of years of training they received and/or their comparable ability to intervene in an emergency situation (eg, clinical officers). Care received during delivery by traditional health personnel was not considered a birth overseen by a skilled attendant.

Modeling strategy

Data processing

Age splitting

Most household surveys collect information on maternal and child health (MCH) indicators for children under 5 and/or mothers who gave birth within five years prior to the time of survey. To maximize data use for our model, we included SBA information for children aged 12 to 59 months at the time of survey. Children younger than 12 months of age were excluded to minimize the influence of potentially censored observations. SBA coverage estimates were assigned to birth-cohort years based on a child's age prior to the time of survey: we used responses recorded for children aged 12 to 23 months for SBA coverage for one year prior to the time of survey, children aged 24 to 35 months for coverage two years prior to the time of survey, and so forth.

Age-specific estimates are easily computed from individual-level microdata, but many published reports and survey summaries present data in broader age aggregates (eg, SBA coverage for children aged 12 to 35 months). To standardize these age groups, we applied an age-splitting model used in the GBD study,[10] as well as analyses that generated smoking and obesity prevalence by age group.[11,12]

Using surveys with microdata as the reference, we used the following model to generate standardized age group-specific estimates for SBA:

$$\tilde{P}_{a,c,t,k} = P^{a+x}_{a,c,t,k} \frac{P_{a,c,t,j}}{P^{a+x}_{a,c,t,j}}$$

where $\tilde{P}_{a,c,k}$ is the adjusted estimate of coverage for target age group a in country c and year t of survey k; and $P^{a+x}_{a,c,k}$ is coverage reported from survey k, for country c in year t for the age group spanning age a to age $(a + x)$. The ratio of coverage between the target age group and broader age group from a survey j with microdata from the same country-year was used to split data from survey k. Surveys to be split were ideally matched with DHS or MICS surveys. If microdata were not available for the same year, ratios within five years of the survey that required age-splitting were applied.

Bias adjustments

Intervention coverage estimates based on administrative sources can be biased, yet the direction and magnitude of such biases are not universal. Some studies show that coverage estimates from administrative data source are systematically higher than those of survey-based estimates,[13] while other studies show that bias directionality is more heterogeneous.[14] Such biases may arise for a number of reasons, including discrepancies in the accurate reporting of services or interventions provided (eg, number of skilled attendants) and target population (eg, number of children born), as well as capturing these data in a timely manner from both public and private sector facilities and healthcare providers.

For SBA, we view individual-level data collected through population health surveys as the most accurate and least biased source of information, particularly for geographies with incomplete health information systems. We thus used SBA coverage estimates from household surveys to calculate country-specific adjustment factors:

$$logit(P_{s,\,c,t}) = \beta_0 + \beta_1 logit(\tilde{P}_{a,\,c,t}) + \sum_{k=2}^{2+B} \beta_k S_k + \varepsilon_{c,t}$$

where $P_{s,\,c,t}$ is the survey-based estimate for SBA coverage (s) in country c for year t; $\tilde{P}_{a,\,c,t}$ is the administrative estimate for coverage in country c in year t; S_k is a spline basis used to capture the secular trend in coverage; β_1 is the estimated adjustment factor used to correct for the administrative bias; and ε is the error term for country c in year t.

To quantify uncertainty for bias-adjusted estimates from the mixed-effects models described above, we calculated prediction error, $\widehat{PE}$, as follows:

$$\widehat{PE} = X^2 var(\hat{\beta})$$

where $var(\hat{\beta})$ is the variance for the estimated fixed-effects coefficient of the adjustment factor and X is the independent variable. Proper estimation of prediction errors is crucial as the data synthesis procedure, Gaussian process regression (GPR) (as described in the subsequent section), accounts for uncertainty from point estimates and bias adjustments when generating fitted values. More weight is given to data with less uncertainty. Prediction errors estimated from the bias adjustment were incorporated into the data variance and propagated through the GPR step to obtain estimates of SBA coverage and uncertainty intervals (UIs).

To assess the accuracy of our estimates in the bias adjustment, we performed cross-validation analyses by randomly holding out 20% of the sample and, if available, the corresponding administrative estimates for the given indicator of the same country and year, 10 separate times. We computed the average root mean squared errors (RMSE) across each country. Error in the bias adjustments was calculated as the mean difference between the adjusted administrative estimate for a given country, year, and corresponding survey-level estimates (which were considered the "gold-standard").

Trend estimation

We used a spatiotemporal Gaussian process regression (ST-GPR) to synthesize point estimates from multiple data sources and derive a complete time series for SBA coverage. This method has been used extensively in GBD and related studies, and accounts for uncertainty pertaining to each point estimate while borrowing strength across geographic space and time.[10, 11,15,16] Briefly, we assumed the Gaussian process was defined by a mean function *m* n o ri n e un tion *Cov* .

We estimated the mean function using a two-step approach. Specifically, $m_c(t)$ can be expressed as:

$$m_c(t) = X\beta + h(r_{c,t})$$

where $X\beta$ is a linear model and $h(r_{c,t})$ is a smoothing function for the residuals; and $r_{c,t}$ is derived from the linear model. The following linear model was used for estimating SBA:

$$logit(P_{c,t}) = \beta_0 + \beta_1 HAQ_{\mathrm{c,t}} + \alpha_c + \gamma_{R[c]} + \omega_{\mathrm{SR[c]}} + \varepsilon_{c,t}$$

where $P_{c,t}$ is SBA coverage for country c year t; $HAQ_{c,t}$ is value of the Healthcare Access and Quality Index[16] for country c and year t; α_c, $\gamma_{R[c]}$, and $\omega_{\mathrm{SR[c]}}$ are country, region, and super-region random intercepts, respectively. These estimates were then modeled through ST-GPR.

Random draws of 1,000 samples were obtained from the distributions above for every country for a given vaccine. Ninety-five percent uncertainty intervals were calculated by taking the ordinal 25th and 975th draws from the sample distribution.

To assess the accuracy of our modeled estimates, we performed cross-validation analyses using a knockout structure as previously described[17]. ST-GPR hyperparameters were selected on models that minimized the overall root-mean squared error (RMSE) of the model across a set of 10 knockouts.

References

1 Measure DHS: Demographic and Health Surveys. http://www.measuredhs.com (accessed Aug 11, 2015).

2 UNICEF Stat. Monit. Multiple Indicator Cluster Survey (MICS). http://www.unicef.org/statistics/index_24302.html (accessed Aug 11, 2015).

3 Cent. Dis. Control Prev. Reproductive Health Surveys (RHS). http://www.cdc.gov/reproductivehealth/Global/surveys.htm (accessed Aug 11, 2015).

4 World Bank. Living Standard Measurement Studies (LSMS). http://go.worldbank.org/UK1ETMHBN0 (accessed Aug 11, 2015).

5 WHO Multi-Ctry. Stud. Data Arch. World Health Survey (WHS). http://apps.who.int/healthinfo/systems/surveydata/index.php/catalog/whs/about (accessed Aug 11, 2015).

6 IHME GHDx. Global Helath Data Exchange. http://ghdx.healthdata.org/ (accessed Aug 11, 2015).

7 PAHO/WHO Data - Home. http://www.paho.org/data/index.php/en/ (accessed June 12, 2017).

8 WPR - Health Information & Intelligence Platform > Data & analytics. http://hiip.wpro.who.int/portal/Dataanalytics.aspx (accessed June 12, 2017).

9 European Health for All Family of Databases (HFA-DB). 2017; published online June 12. http://www.euro.who.int/en/data-and-evidence/databases/european-health-for-all-family-of-databases-hfa-db (accessed June 12, 2017).

10 Prospective Studies Collaboration. Age-specific relevance of usual blood pressure to vascular mortality: a meta-analysis of individual data for one million adults in 61 prospective studies. *The Lancet* 2002; **360**: 1903–13.

11 Ng M, Freeman MK, Fleming TD, *et al.* Smoking Prevalence and Cigarette Consumption in 187 Countries, 1980-2012. *JAMA* 2014; **311**: 183.

12 Ng M, Fleming T, Robinson M, *et al.* Global, regional, and national prevalence of overweight and obesity in children and adults during 1980–2013: a systematic analysis for the Global Burden of Disease Study 2013. *The Lancet* 2014; **384**: 766–81.

13 Murray CJ, Shengelia B, Gupta N, Moussavi S, Tandon A, Thieren M. Validity of reported vaccination coverage in 45 countries. *The Lancet* 2003; **362**: 1022–7.

14 Haddad S, Bicaba A, Feletto M, Fournier P, Zunzunegui MV. Heterogeneity in the validity of administrative-based estimates of immunization coverage across health districts in Burkina Faso: implications for measurement, monitoring and planning. *Health Policy Plan* 2010; **25**: 393–405.

15 Wang H, Liddell CA, Coates MM, *et al.* Global, regional, and national levels of neonatal, infant, and under-5 mortality during 1990–2013: a systematic analysis for the Global Burden of Disease Study 2013. *The Lancet* 13; **384**: 957–79.

16 Barber RM, Fullman N, Sorensen RJD, *et al.* Healthcare Access and Quality Index based on mortality from causes amenable to personal health care in 195 countries and territories, 1990?2015: a novel analysis from the Global Burden of Disease Study 2015. *The Lancet* 2017; published online May. DOI:10.1016/S0140-6736(17)30818-8.

17 Foreman KJ, Lozano R, Lopez AD, Murray CJ. Modeling causes of death: an integrated approach using CODEm. *Popul Health Metr* 2012; **10**: 1.

3.2.1 and 3.2.2 Under-5 Mortality and Neonatal Mortality SDG Capstone Appendix

Flowchart

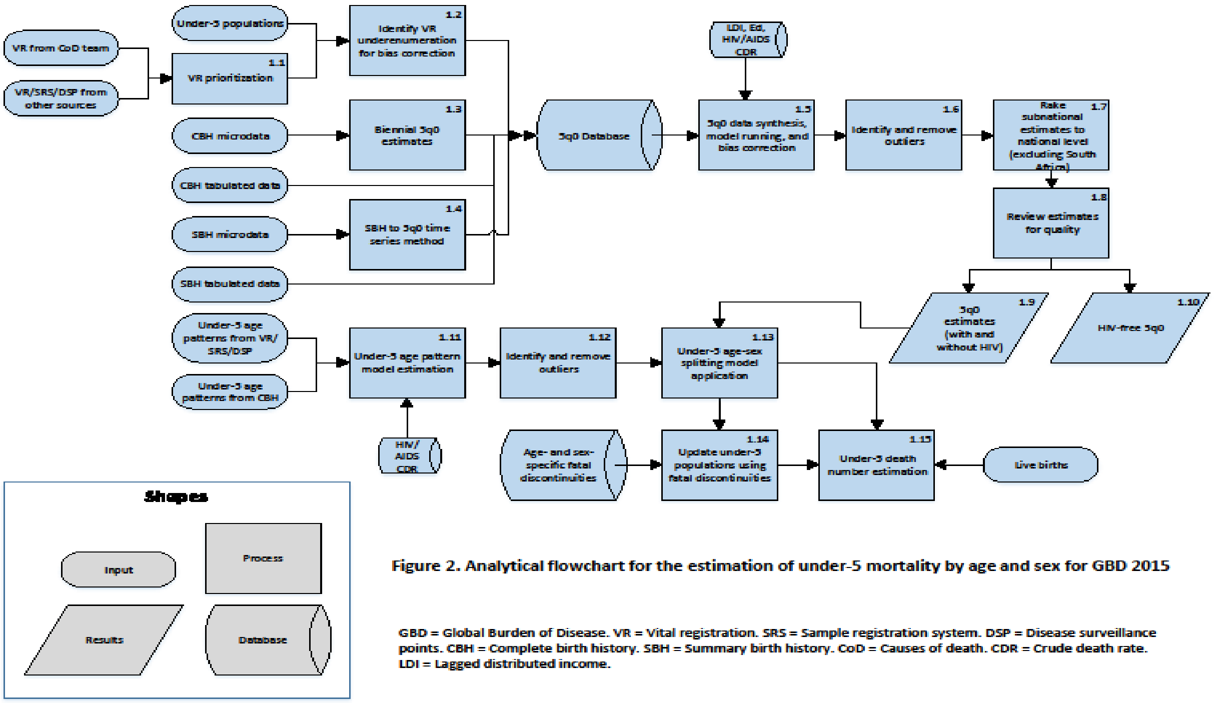

Figure 2. Analytical flowchart for the estimation of under-5 mortality by age and sex for GBD 2015

GBD = Global Burden of Disease. VR = Vital registration. SRS = Sample registration system. DSP = Disease surveillance points. CBH = Complete birth history. SBH = Summary birth history. CoD = Causes of death. CDR = Crude death rate. LDI = Lagged distributed income.

Input Data & Methodological Summary

Indicator definition

This modeling strategy encompassed the indicator associated with under-5 mortality (3.2.1) and neonatal mortality (3.2.2)

Indicator 3.2.1

As a component of SDG Goal 3. Ensure healthy lives and promote well-being for all at all ages, SDG Target 3.2, by 2030, end preventable deaths of newborns and children under 5 years of age, with all countries aiming to reduce neonatal mortality to at least as low as 12 per 1000 live births and under-5 mortality to at least as low as 25 per 1,000 live births, is measured using SDG Indicator 3.2.1, under-5 mortality rate (probability of dying before the age of 5 per 1,000 live births).

Indicator 3.2.2

As a component of SDG Goal 3. Ensure healthy lives and promote well-being for all at all ages, SDG Target 3.2, by 2030, end preventable deaths of newborns and children under 5 years of age, with all countries aiming to reduce neonatal mortality to at least as low as 12 per 1000 live births and under-5 mortality to at least as low as 25 per 1,000 live births, is measured using SDG Indicator 3.2.2, neonatal mortality rate (probability of dying during the first 28 days of life per 1,000 live births).

Input data

Vital registration from Causes of Death team

Data were provided by the GBD causes of death (CoD) research team and were aggregated into total age-sex-specific all-cause mortality for each location-year. This aggregation occurred after the data were adjusted and mapped to the GBD cause list.

Data intended for use in causes of death modeling were assessed for quality with respect to consistency of cause fractions, diagnostic accuracy, and missing data, whereas for all-cause mortality modeling it was more important that data were fully representative of the given estimation area and were consistent with other all-cause mortality data sources. Thus, there were cases in which VR data prepared for cause-specific modeling could be used in all-cause modeling or had to be adjusted based on degree of completeness before being used.

In our vetting of CoD VR data, we dropped points with a more than 1% difference from corresponding points in the WHO database. There were instances where VR data used in cause-specific mortality analysis had been collapsed to Basic Tabulation List (BTL) format rather than in full cause classification list format (e.g., ICD9). In some of these cases, we elected to use WHO data instead.

Vital registration, sample registration systems, and Disease Surveillance Points from other sources

We endeavored to include all available data from VR systems as inputs in our all-cause mortality estimation process. To achieve this, we utilized a number of multi-country VR sources, including the WHO Mortality Database, the Human Mortality Database, United Nations Demographic Yearbooks, and OECD (Organisation for Economic Co-operation and Development) databases. These multi-country sources were regularly updated in our systems when new data were added. Beyond multi-country sources, for all ongoing national VR systems (for example, the USA National Vital Statistics System) we cataloged all data sources from each system where possible.

Some countries that do not have well-performing VR systems implement sample registration systems that are incomplete by design. We made use of these data, paying close attention to the proper weighting of sampled data and consistency with other representative sources. We have systematically extracted data from the Sample Registration System Statistical Report series published by the Registrar General of India. For the Disease Surveillance Points (DSP) system of China, we obtained both national and provincial level DSP data through a data usage agreement with the Chinese Center for Disease Control and Prevention. Census data were systematically extracted from Demographic Yearbook series, Integrated Public Use Microdata Series (IPUMS), and statistical reports from the national statistical bureaus.

Under-5 populations and live births

For GBD 2017, live births were produced as part of the population and fertility estimation.

Complete birth history microdata

Complete birth histories (CBHs), the preferred method for data collection on child mortality in the absence of VR, rely on administering surveys to mothers. The questionnaires ask about all living and deceased children, including date of birth, survival status, and date of death. These modules were included in many routine survey series, including the World Fertility Surveys (WFS), Demographic and Health Surveys (DHS), Multiple Indicator Cluster Surveys (MICS), and many national survey programs. When available, we downloaded and used microdata that included individual-level survey responses as opposed to tabulated results.

Complete birth history tabulated data

In some instances, tabulated records from reports became available before survey microdata, and we incorporated those data points into our database of 5q0 data as well. However, as microdata became available, we updated with point estimates from our processed microdata rather than the tabulated report estimates.

Summary birth history microdata

Summary birth history (SBH) questionnaires are a shorter alternative to complete birth histories. Instead of asking in detail about each child, summary birth histories simply ask mothers how many children they have given birth to and how many of the children have died. The questionnaires are shorter and can be more easily attached to other surveys. Often, censuses and MICS surveys contain summary birth histories. For GBD, we compiled all available SBH data with microdata, which enabled us to apply the updated SBH method to produce a more accurate and timely assessment of U5MR.[1]

Summary birth history tabulated data

In cases where we did not have access to the microdata on SBH modules from surveys and censuses, we utilized the reported estimates of U5MR from survey or census reports and outliered the first two data points based on mothers aged 15-19 and 20-24.

Under-5 age-sex patterns from VR/SRS/DSP

VR systems were the primary source of data for the under-5 age pattern of mortality in high-income countries. Often, these data were classified into several age groups: early neonatal (0-6 days), late neonatal (7-27 days), post-neonatal (28-364 days), and 1 to 4 years. Some country-years of data had other age groupings with less specificity, with the early and late neonatal age groups combined, or all of the under-1 age groups combined. Sample Registration Systems (SRS) also provided data for the age-sex patterns of under-5 mortality in several countries (notably India and Bangladesh). The DSP system in China provided data on age-sex under-5 mortality as well.

Under-5 age-sex patterns from complete birth history

In many countries without VR systems, CBH surveys were used to obtain age-sex patterns of mortality in under-5 age groups. These sources are described above in the “CBH microdata” section. For all CBH microdata sources, we applied direct estimation methods to obtain probabilities of death for each of the under-5 age groups. Within each survey, where each observation is a child recalled by a mother,

observations were grouped into 5-year groups in time to provide a data point of probability of death for each of the under-5 age-sex groups. Recall was cut off 15 years before the survey, limiting data points estimated from the survey to the 15 years prior. All of these estimates were then put in the database of estimates for the age-sex pattern of under-5 mortality.

Modeling Strategy

VR prioritization

Our continual evaluation of VR data sources led us to develop a general hierarchy of preferred VR sources. When considering which of multiple sources to use for a given location-year, we preferred to first use WHO data from GBD cause-specific mortality estimation, then unadjusted WHO data, then Human Mortality Database (HMD) data, then UN Demographic Yearbook data. There were exceptions to this hierarchy where we had reason to believe that there were quality issues with a certain source. For instance, where available we preferred to use HMD VR over WHO data for Germany, Taiwan, and Spain due to WHO data producing mortality rates that were inconsistent with previously established trends. Single-country VR sources were evaluated based on consistency with other data sources and also VR system documentation.

Identify VR under-enumeration for bias correction

The approach to estimating the completeness of VR systems for deaths under age 5 was the same as that of the previous three GBD studies. However, the VR-specific correction in previous GBD rounds has been removed for GBD 2017. Most biased VR is corrected in the mixed effects non-linear model outline in section 2.2.6.

Similar to previous GBD rounds, there were countries for which only VR data were available and the VR systems were considered biased. This was a problem particularly in English-speaking Caribbean countries, so for these countries we adjusted 5q0 estimates from VR using the regional average VR bias in a given year for those countries with both VR and survey 5q0 estimates. The countries for which VR systems were adjusted using this method include Antigua and Barbuda, Bahamas, Barbados, Bermuda, Dominica, Grenada, Saint Lucia, and Saint Vincent and the Grenadines. While there was no direct evidence on the level of VR bias in these countries, assuming they were complete when similar countries in the region exhibited under-registration seemed unwarranted.

Biennial 5q0 estimates

Complete birth history 5q0 computation

Microdata (individual-level survey data) from CBH yielded direct calculation of death numbers and probabilities of death in the under-5 age group. Observations were grouped into two-year intervals such that biennial estimates of 5q0 were obtained from these survey data. In GBD 2017, we unpooled surveys for our analysis, whereas surveys were pooled by series in GBD 2013.3 Instead of grouping observations from all DHS complete birth history questionnaires from a country into one full set of observations and all MICS observations from multiple survey years into another full set of observations, we analyzed each survey separately by location (e.g., DHS 2012, DHS 1996, MICS 2002). This allowed for a greater ability to address known data quality issues in specific surveys. To compensate for the decreased sample size and to generate greater stability in the unpooled data points, we created two-year estimates of under-5 mortality, pooling observations over two-year periods instead of single years.

Tabular complete birth history processing

In some instances, microdata from surveys were not available. If survey reports could be obtained but the microdata were not available for us to do our own calculations to obtain 5q0, we used report data point estimates. These estimates were added directly to the under-5 mortality database.

Summary birth history time series method

Summary birth history method from microdata

Rajaratnam and colleagues have developed an updated summary birth history method that is able to provide more accurate and timely estimates of U5MR from micro data on SBH from surveys and censuses.[1]

Summary birth history analysis from tabular data

When only tabular data are available for the numbers of children ever born and number of children that have died by mother's age, we apply the Maternal Age Cohort model from the method developed by Rajaratnam and colleagues.[1]

5q0 data synthesis, model running, and bias correction

Data synthesis using ST-GPR and bias correction

We applied the child mortality estimation methodology as reported by Wang and colleagues.3 Based on the under-5 mortality data synthesis model for the Global Burden of Disease Study 2010,4,5 2013,3 2015,6 and 20167 we incorporated data bias adjustment into the modeling process. Specifically, we included a fixed effect for source type across all locations to detect systematic differences in the level of child mortality, controlling for covariates for one source type versus another. The groups of sources used to make this adjustment are listed below. In addition, we included a random effect for each country-source. By choosing a reference source (country-by-country or using the mean of a set of sources, we adjusted on a country-by-country basis for the problem of compositional bias created by substantial source-specific non-sampling error. Reference sources were not adjusted, even if multiple sources were used as reference. Once the systematic difference in sources were removed, we were able to avoid estimating false trends due to partial overlap of sources with different levels of non-sampling variance. We then applied the combination of non-linear mixed effects model, spatiotemporal regression, and GPR to synthesize raw child mortality data after data bias adjustment to obtain consistent time series estimates of mortality with 95% uncertainty intervals for every country.

We apply the child mortality estimation methodology as reported by Wang et al.[4] Based on the under-five mortality data synthesis model for the Global Burden of Disease Study 2010, 2013, 2015, and 2016 we have incorporated data bias adjustment into the modeling process. Specifically, we have included a fixed effect for source type across all locations to detect systematic differences in the level of child mortality, controlling for covariates for one source type versus another. The groups of sources to make this adjustment are listed in the table below. In addition, we include a random effect for each country-source. By choosing a reference source country-by-country or using the mean of a set of sources, we can adjust on a country-by-country basis for the problem of compositional bias created by substantial source-specific non-sampling error. Once the systematic difference in sources is removed, we are able to avoid estimating false trends due to partial overlap of sources with different levels of non-sampling variance. We then apply the combination of non-linear mixed effects model, spatial-temporal regression and Gaussian process regression to synthesize raw child mortality data after data bias adjustment to obtain consistent time series estimates of mortality with 95% uncertainty intervals for every country.

Table: Source types used in child mortality bias correction

Data Source Type
Complete Birth History-Demographic and Health Survey
Complete Birth History-AIDS Indicator Survey and Malaria Indicators Survey
Complete Birth History-World Fertility Survey
Complete Birth History-Multiple Indicator Cluster Survey
Complete Birth History-Census
Complete Birth History-Other survey Series
Summary Birth History-Demographic and Health Survey
Summary Birth History-Multiple Indicator Cluster Survey
Summary Birth History-Other survey series
Summary Birth History-AIDS Indicator Survey and Malaria Indicators Survey
Summary Birth History-Census
Summary Birth History-World Fertility Survey
Vital Registration/Sample Registration/Surveillance- complete
Vital Registration/Sample Registration/Surveillance- incomplete
Household Death Recall-Other survey series
Household Death Recall-Census
Household Death Recall – incomplete Vital Registration/Sample Registration/Surveillance

Mixed effect non-linear model and the bias adjustment for raw U5MR sources

In this stage, we used a nonlinear mixed effects regression to estimate data bias and provide first stage predictions.

The nonlinear mixed effects regression model is

$$ {}_5m_{0cys} = \exp[(\beta_1 + \gamma_{1c}) * \log\left(LDI_{cy}\right) + (\beta_2 + \gamma_{2c}) * education_{cy} + \gamma_c + \gamma_{cs} + \alpha_t] + \beta_3 * HIV_{cy} + \varepsilon_{cys} $$

where *c* is country, *y* is year, *s* is source, and *t* is source type; each source was categorized into one of 17 source types across all countries, as listed in the table above.
Additionally,

$_{5}m_{0}$ is under five mortality rate

LDI is lagged distributed income per capita

education is mean years of education for women of reproductive age (15-49 years)

HIV is death rate due to HIV in age groups 0-4

is r n o e e t

is i e e e t on sour e t e ross ountries

$_{i}$ is a fixed covariate coefficient

is the resi u

For each country, we rely on expert opinion to choose a source, or combination of sources, which are believed to be the least biased. If a country has vital registration which we deem to be complete (described in detail in an earlier section), this is the reference source. If a country does not have complete vital registration, but has DHS estimates from complete birth histories, these were chosen as the reference source. If a country has neither of these types of data or DHS surveys are deemed unreliable, we assigned the surveys conducted after 1980, in combination, as the reference (incomplete vital registration data were not included). Additionally, in many countries we chose other surveys as the reference. For accurate estimation, it is important to have local knowledge on specific data sources' accuracy. All-cause mortality experts draw from their familiarity with data quality to help us to choose the reference category.
Each data source has an associated random effect as well as a source type fixed effect. The values of these random and fixed effects for the reference sources are deemed to be the true deviation from unbiased mortality level. In countries with multiple high-quality sources, the mean of the random and fixed effects from these sources is taken as this true deviation. We adjusted all other sources by including these reference values for the random and fixed effects values instead of those estimated for each individual source, as shown below.

$$adjusted_5 m_{0,cys} = \exp[(\beta_1 + \gamma_{1c}) * \log(LDI_{cy}) + (\beta_2 + \gamma_{2c}) * education_{cy} + \gamma_c + \gamma_{ref,c} + \alpha_{ref,c}] + (\beta_3 + \gamma_{3c}) * HIV_{cy} + \varepsilon_{cys}$$

Spatio-temporal smoothing

The spatio-temporal stage smooths the residuals between the predicted time series of $_{5}q_{0}$ and the adjusted raw data over time and across countries in the same GBD region. The predicted time series for this smoother was obtained from the equation below; no random effects or survey type fixed effects are included.

$$predicted_5 m_{0,cy} = \exp[\beta_1 * \log(LDI_{cy}) + \beta_2 * education_{cy} + \alpha_{intercept}] + \beta_3 * HIV_{cy}$$

We first found the residuals between the predicted time series, above, and the adjusted points. We then applied a combination of smoothing functions to these residuals. For each country year, we weighted all the data points in this region based on their proximity to this country-year in space and time. We gave 99% of the weight to in-country residuals, and 1% of the weight to out-of-country residuals. Additionally,

we used a modified tricubic window, as specified below, to give more weight to points closer in time, and less weight to points further in time.

$$w_t = \left(1 - \left(\frac{|r_t - r_{est}|}{1 + \text{argmax}_t |r_t - r_{est}|}\right)^{\lambda}\right)^3$$

The r_t and r_{est} terms are, respectively, the year of interest and the year of the residual being weighted. The $\text{argmax}_t|r_t - r_{est}|$ term is the maximum distance between the year of interest and a residual within the region. The parameter in this weighting function dictates how quickly the weights fall off as the distance in time increases: a larger implies that the assigned weights will diminish slowly with time, while a smaller allows the weights to diminish more rapidly with time.

ues ere hosen usin the r eter se ection process described below. We then created one estimate of the smoothed residuals using a linear fit to this weighted data; this is similar to a Loess fit. Additionally, we created a second estimate of the smoothed residuals by calculating the weighted average of this data.

We then combined these two estimates for a final estimate of the smoothed residuals. In data-dense countries, more weight was given to the local linear fit; in data sparse countries, more weight was given to the weighted average. The equation for this is as follows.

$$final\ smoothed\ residual = k * linear\ estimate + (1 - k) * weighted\ average$$

where $k = \frac{number\ of\ in\ country\ data\ points}{number\ of\ in\ country\ data\ points + number\ of\ country\ years\ with\ no\ data}$

Finally, the smoothed residuals were added back to the predictions from above; this smoothed approximation to the adjusted data was used as the prior for the Gaussian process regression, described below.

Third stage: Gaussian process regression (GPR)

The output of the space-time local smoother was used as a prior for the Gaussian process regression, which produced a final time series of point estimates, as well as confidence bounds. Parameters for the GPR were chosen through cross-validation described in section 1.5.E.

The model for the Gaussian process regression is shown below, where μ_t is the true $\log_{10}({}_5q_0)$ at time t, f(t) is the baseline mortality risk, and S_t captures excess mortality due to war and disasters. S_t is estimated independently of f(t). M and C describe the Gaussian process, giving the mean and covariance, respectively.

$$\mu_t = f(t) + S_t$$
$$f(t) \sim GP(M, C)$$

We specified a prior distribution for f(t) from the spatio-temporal regression, and a likelihood function which describes the data generation process; the specified prior distributions and likelihood function are described below. We then used Markov Chain Monte Carlo (MCMC)[5] to approximate the posterior distribution of f(t) which also incorporates information from the observed empirical estimates of adult mortality. An MCMC chain of length 5000 was produced; the first 3000 samples were discarded and the remaining 2000 were thinned by a factor of 2 for a total of 1000 simulations retained. The reported best estimates and confidence intervals were generated from the mean and the 2.5th and 97.5th percentiles of the 1000 samples, respectively.

The prior distribution of f(t) can be described in terms of the mean prior—the prior for M—and the covariance prior—the prior for C. We utilized the second stage predictions as the mean prior and used a Matern covariance function to describe the covariance prior.

Likelihood

The likelihood describes the probability of observing the data given a particular set of parameters. As shown in the equation below, we used a normal model for describing the probability of observing a particular value of log($_5q_0$) where the mean is given by f(t) and the variance by V_t, the data variance.

$$\log_{10}({}_5q_{0_t}) \sim \mathrm{Normal}(f(t), V_t)$$

Data variance was calculated for each empirical observation of $_5q_0$ and incorporated both sampling and non-sampling variation. The method for calculating the data variance depended on the type of data:

1. For estimates derived from complete vital registration data we assumed that there was no non-sampling variance and included only sampling variance as computed from a binomial model. We set N equal to the national population aged 0 to 5 years and p equal to the mortality rate, $_5m_0$. We calculated the variance of $_5m_0$ from p(1-p)/N and then transformed this to the variance of $\log_{10}({}_5q_0)$ using the delta method.[6]
2. For estimates derived from incomplete vital registration data, we wanted to include not only sampling variance but also the non-sampling variance that arises from uncertainty in the completeness estimate. For these data, the total data variance was given by the sum of the sampling variance (calculated as for complete vital registration data) and the variance of the completeness estimate;
3. For estimates derived from complete birth histories we generate 1000 simulations of $_5q_0$, convert these estimates into $\log_{10}$ space and calculate the sampling variance from these 1,000 simulations;
4. For estimates derived from summary birth histories we use the standard error from the mean residuals;
5. For estimates not covered under the above 4 calculations the missing data variance is determined as the maximum standard error from non-VR points in the country, if the data variance is still missing it is calculated as the maximum standard error from non-VR data in the GBD region.
6. Finally, for each source type, we calculate the within-source-type variance of the source-specific random effect. This additional non-sampling variance is then converted to $\log_{10}$ space and added to the variance as calculated above for all data points not classified as complete vital registration.

Hyper-parameter selection for under-5 mortality rate ST-GPR

For GBD 2017, hyperparameters were selected based on a newly-created data density score for a given location. The data density score was calculated for each location based on the number of deaths from VR sources as well as the number of unique CBH and SBH available. The data density score was computed using the following steps:

1. Calculate complete VR score: this component of data density was computed based on the number of deaths from an unbiased VR sources in the location. Using the death counts, we capped the number of deaths at 500 for each year and then divided that number 500. The result was a score for each year between 0 and 1 where 1 represents a complete VR system with at least 500 registered deaths. To get the final complete VR score for a location, we added up the score for each year across the full time series. The result was a complete VR score between 0 and 68 (the range of our full 1950-2017 estimation time series).
2. Calculate incomplete VR score: this component of data density is computed in the same manner as the complete VR score using biased VR instead of unbiased VR.
3. Total CBH sources: this is simply a count of the unique complete birth histories for a location
4. Total SBH sources this is simply a count of the unique summary birth histories for a location
5. Once the intermediate calculations were completed, the following formula was used to compute the final data density

$$data\_density = complete\_vr\_deaths' + (0.5 \times incomplete\_vr\_deaths) + (2 \times cbh\_sources) + (0.25 * sbh\_sources)$$

Once the data density for a location was calculated, we assigned hyperpameters:

Table A

Data Density	Zeta	Lambda	Scale
0 to 9	0.7	0.7	15
10 to 19	0.7	0.5	15
20 to 29	0.8	0.4	15
30 to 49	0.9	0.3	10
50 plus	0.99	0.2	5

Identify and remove outliers

There are several important quality-control steps in reviewing child mortality data and estimates. First, data points from years in which fatal discontinuities occurred are outliered, unless they are VR data points with sufficient information that the fatal discontinuities can simply be subtracted out of the VR data. The intent is to capture the underlying mortality risk rather than large stochastic variations. These fatal discontinuities are then added on in a later step (see section 5). Secondly, we outlier data sources with quality concerns such as the Afghanistan DHS from 2010. Our extensive collaborator network allows for review of sources, and collaborators can raise concerns over known issues with data sources about which they have expert knowledge.

Rake subnational estimates to national level (excluding South Africa)

The estimation process for 5q0 does not enforce consistency between subnational estimates and national estimates. To ensure consistency throughout the GBD hierarchy, we rescaled the subnational estimates to the national level by population-weighting to get an implied national estimate from the subnational estimates, creating a scalar of the national-level estimate from GPR to the aggregated subnational estimates, and then multiplying all of the subnational estimates by this scalar to obtain the scaled estimates. In most cases, we considered national-level estimates to be more reliable, so we chose this strategy of subnational scaling. In locations with high-quality vital registration data, this scaling has a minimal effect, but the effect can be greater in locations with more subnational units and variable-quality data. In South Africa, it was essential that the state-specific mortality patterns be consistent with HIV

models is essential, since such a large part of the trend is driven by deaths due to HIV/AIDS. In this case, instead of scaling provincial-level estimates to national-level GPR estimates, we aggregated province-level GPR estimates to generate the national-level estimates.

Review estimates for quality

Estimates of 5q0 from the ST-GPR process were reviewed in comparison to UNICEF estimates from their 2015 revision and GBD 2015 results. Any differences were traced to either changes in available data or changes induced by changes in hyperparameters or input covariates. Revisions were made through this review process and through expert consultation with the GBD mortality collaborator network.

Under-5 mortality rates with HIV

The U5MR ST-GPR process generates U5MR for all GBD 2017 locations that is inclusive of the impact of all causes of death excluding fatal discontinuities, which are added in a separate step (see section 5).

HIV-free 5q0

As a result of the Non-linear mixed effects model, we are able to generate HIV free 5q0 counterfactuals where the crude death rate due to HIV in age group 0-4 is set to zero. This is a crucial input to the GBD model life table system as described in section 3.

Under-5 age pattern model estimation

The process used to break down under-5 mortality into age- and sex- specific groups has been previously described.[7] The current process is largely similar but has been modified to improve the accuracy of predictions for countries affected by HIV/AIDS. As pointed out by Bradshaw et al., neonatal mortality tends to be overestimated if the all-cause child mortality rate is used as the only predictor.[8] We use a two-stage modeling process to generate sex-specific estimates of early neonatal (days 0 to 6), late neonatal (days 7 to 27), post-neonatal (the remainder of the first year), and childhood (ages 1 to 4) mortality. First, the ratio of male to female under-5 probability of death is estimated, then age- and sex-specific mortality estimates are generated using this ratio. To fit models to obtain estimates, data from vital registration, sample vital registration, and complete birth histories are converted to mortality risks for specific age groups. Sources have differing levels of age specificity and at least include infant (composed of early neonatal, late neonatal, and post-neonatal) and child mortality, but can include all 4 smaller age groups. The two models – first the sex model, then the age-specific and sex specific model – are fit on the data.

The sex model predicts the ratio of male probability of death under age 5 ($_5q_0$) to female $_5q_0$ for each country *i* in region *j* in year *t*. The data are ordered by observed $_5q_0$, and categorized into 20 evenly sized bins. Then, the model is fit to the data as described in the equation below.

$$\left(\frac{\text{Male }_5q_0}{\text{Female }_5q_0}\right)_{\text{jit}} = \beta + \gamma_{_5q_0\text{ bin}} + \gamma_{\text{j}} + \gamma_{\text{i}} + \varepsilon_{\text{jit}}$$

The ratio is predicted by nested location and region random effects $_i$ n $_j$, a random effect on the $_5q_0$ bin, and an intercept term, . A oess re ression is then use to s ooth the esti te $\gamma_{_5q_0\text{ bin}}$ on $_5q_0$, creating a continuous $\gamma'_{_5q_0\text{ bin}}$. Then, the equation below is used to predict the ratio of male to female $_5q_0$:

$$\left(\frac{\text{Male }_5q_0}{\text{Female }_5q_0}\right)_{\text{jit}} = \hat{\beta} + \gamma'_{_5q_0\text{ bin}}\left({_5q_0}_{\text{jit}}\right) + \hat{\gamma}_{\text{j}} + \hat{\gamma}_{\text{i}}$$

The male and female $_5q_0$ values are found using the system of equations that includes the model above and equation below, where r_{birth} is the sex-ratio at birth.

$$_5q_0 = \left(\frac{1}{1+r_{birth}}\right) * (female_5q_0) + \left(\frac{r_{birth}}{1+r_{birth}}\right) * (male_5q_0)$$

Age-specific models are then fit for each age group on sex-specific data. A separate model is fit for each age group yielding five models for each sex: early neonatal, late neonatal, postneonatal, infant, and child. The log of the probability that an under-5 death occurs in a given age group is modeled instead of the mortality risk, simplifying the scaling process and restricting risks to be between 0 and 1. Because evidence suggests HIV has differential effects on different under-5 age groups,[8,9] the crude death rates from HIV/AIDS in the under-5 age group were included in the model. We used crude death rate due to HIV from the GBD 2015 model (see section 3). The inclusion of this covariate improves both the fit and prediction of the model in countries with HIV. In addition, in this version of GBD, we added two new covariates to improve model fit. First, we included the maternal education covariate that is also used in the 5q0 first-stage model. Second, we used the completeness of the source-specific 5q0 estimate for the data-point used in the regression. This completeness was calculated by taking the source-specific 5q0 point estimate and dividing by the final 5q0 estimate from GPR. The functional form of the model is below.

$$\log(\Pr(\text{death at age y}|\text{u5 death})_{jit}) = \beta_1 + \beta_2 * HIV_{it} + \beta_3 * Mat.Ed._{it} + \beta_4 * Completeness_{sit} + \gamma_{_5q_0\,\text{bin}} + \gamma_j + \gamma_i + \varepsilon_{jit}$$

Similar to the sex model, the sex-specific age prediction uses $_5q_0$ bins and smooths the random effect on the bin using $_5q_0$. The prediction equation for age *y* in country in region *j* at time *t* is seen below, with nested random effects on country ($\hat{\gamma}_i$) and region ($\hat{\gamma}_j$), an intercept term ($\hat{\beta}_1$), a smoothed random effect on $_5q_0$ bin ($\hat{\gamma}'_{_5q_0\,\text{bin}}(_5q_{0_{jit}})$), a coefficient on the under-5 crude death rate from HIV ($\hat{\beta}_2$), a coefficient on maternal education ($\hat{\beta}_3$), and a coefficient on completeness ($\hat{\beta}_4$):

$$\log(\Pr(\text{death at age y}|\text{u5 death})_{jit}) = \hat{\beta}_1 + \hat{\beta}_2 * HIV_{it} + \hat{\beta}_3 * Mat.Ed._{it} + \hat{\beta}_4 * 1 + \hat{\gamma}'_{_5q_0\,\text{bin}}(_5q_{0_{jit}}) + \hat{\gamma}_j + \hat{\gamma}_i \quad 13$$

Note that for prediction, the completeness coefficient gets multiplied by 1 instead of a source-specific completeness, as we seek to predict based on a hypothetically complete source.
Once each of these predictions is made by age group, they are rescaled such that the probabilities of death in the Early Neonatal, Late Neonatal, Post Neonatal, and 1-4 year age groups aggregate to the 5q0 estimates from the under-5 model.

Identify and remove outliers

There are several criteria for removing outliers for the under-5 age-sex pattern model. First, sources may be marked as outliers if they contain low population numbers or very few deaths. If data come from vital registration and the under-5 population of the country is less than 20,000 person-years, then the data are outliered. If the total number of deaths in a VR system among both sexes under-5 is less than 200, the data are also outliered. VR data that are considered incomplete are marked as outliers. To be considered incomplete, the 9-year rolling average of the VR data 5q0 value is compared to the 9-year rolling average of the 5q0 estimates. Then, for a given data-year, the value of 5q0 in the raw data are compared to our final 5q0 estimate. A value of 90% would be considered incomplete and outliered, unless the ratio of the 9-year rolling average above is above 90% complete. Any data that are chosen as outliers as part of the 5q0 analysis are also marked as outliers in the age-sex pattern analysis. If the female-to-male ratio of 5q0 in the raw data are less than .5 or greater than 2, the data are outliered because of an implausible sex ratio. If a country has both VR and CBH data, they are typically both used, unless the two conflict, in which case the VR data are used. CBH data points more than 15 years before the survey are outliered. Lastly, some data points are manually outliered. For example, the definition of live birth changed in some Eastern European countries in the 1990s, leading to inconsistencies. For these examples, age group data in ages that would include childbirth deaths (early neonatal, neonatal, and ages 1-4) are outliered if the definition of live birth contains a minimum weight, as it did in some of these locations.

Under-5 age-sex splitting model application

The prediction method from the age-specific model is described above in 1.11. First, the results of the sex model are applied, yielding sex-specific 5q0 estimates. Once age-sex-specific predictions of the log conditional probability of death are made, these are exponentiated and rescaled so that they some to 1. First, the under-1 and 1-4 conditional probabilities are scaled to add to 1. Then, the early neonatal, late neonatal, and post neonatal conditional probabilities are scaled to the under-1 conditional probability. Then, the probabilities of death can be calculated so that they properly aggregate to the final 5q0 prediction. For example, to calculate the probability of death in the early neonatal age group, the rescaled conditional probability of early neonatal death given under-5 death is multiplied by the probability of under-5 death. Then, to obtain the probability of death in the late neonatal age group, the rescaled conditional probability of death in the late neonatal age group given under-5 death is multiplied by the probability of under-5 death and then divided by the probability of survival to the beginning of the age group, and so on. Equations below represent this process, where enn represents early neonatal and lnn represents late neonatal.

$$q_{enn} = \Pr(death\ in\ enn\,|\,u5\ death) * 5q0$$
$$q_{lnn} = \Pr(death\ in\ lnn\,|\,u5\ death) * 5q0/(1 - q_{enn})$$

The rest of the older age groups are also calculated in this manner, yielding probabilities of death in each of the under-5 age-sex groups.

Update under-5 populations using fatal discontinuities

To obtain denominators for vital registration death numbers and to estimate death numbers for age groups under-5, we need to obtain age-specific populations for the under-5 age groups. Taking final probability of death estimates including impacts of fatal discontinuities from the first run of the all-cause mortality process as the mortality risks, we take our input birth numbers and create person-year estimates of population as described in section 1.15. These person-year estimates are then the input as populations for the final run of the estimation process.

Under-5 death number estimation

Assigning under-5 deaths to GBD age-sex groups

To estimate the number of under-5 deaths, we run an estimation process that ages birth cohorts through our estimated probabilities of death. This process separates our yearly birth numbers for each location into week-sized cohorts and ages each of these cohorts through our mortality estimates in week-long steps to estimate the number of person-years and deaths in each of the early neonatal, late neonatal, post neonatal, and 1-4 years age groups.

3.3.1 HIV SDG Capstone Appendix

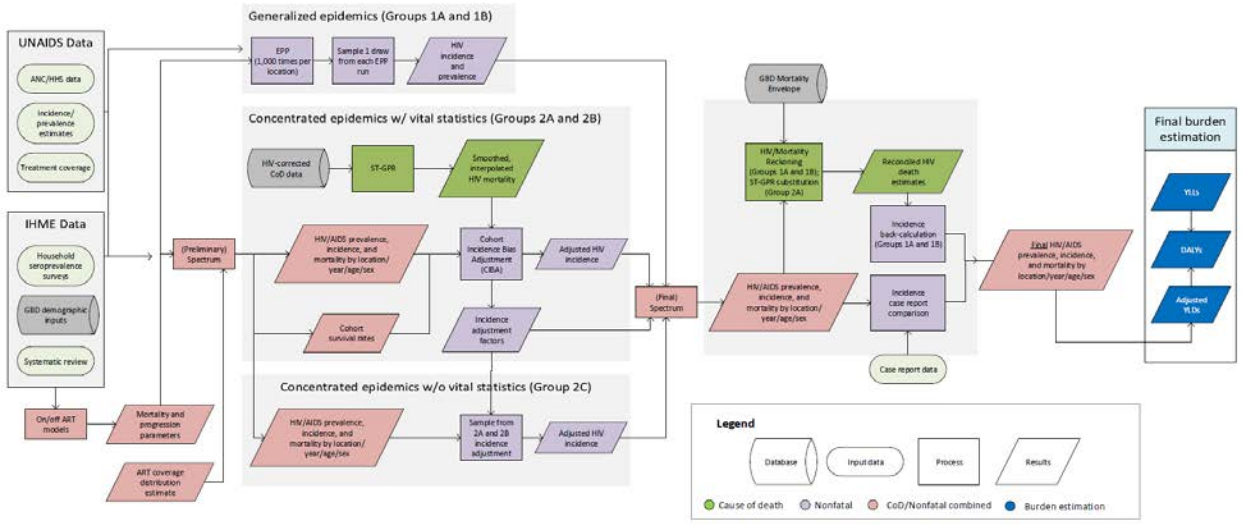

Input data and Methodology

Indicator definition

This modeling strategy encompassed the indicator associated with HIV incidence (3.3.1).

Indicator 3.3.1

As a component of SDG Goal 3. Ensure healthy lives and promote well-being for all at all ages, SDG Target 3.3, by 2030, end the epidemics of AIDS, tuberculosis, malaria and neglected tropical diseases and combat hepatitis, water-borne diseases and other communicable diseases, is measured using SDG Indicator 3.3.1, number of new HIV infections per 1,000.

Case definition

Infection with the human immunodeficiency virus (HIV) causes influenza-like symptoms during the acute period following infection, and can lead to acquired immunodeficiency syndrome (AIDS) if untreated. HIV attacks the immune system of its host, leaving infected individuals more susceptible to opportunistic infections like tuberculosis. Although there are two different subtypes of HIV, HIV-1 and HIV-2, no distinction is made in our estimation process or presentation of results. For HIV, ICD 10 codes are B20-B24, C46-C469, D84.9; ICD 9 codes are 042-044, 112-118 (after 1980), 130 (after 1980), 136.3-136.8 (after 1980), 176.0-176.9 (after 1980), 279 (after 1980); and ICD9 BTL codes are B184-B185.

Input data

Household seroprevalence surveys

Geographically representative HIV seroprevalence survey results were used as inputs to the model for countries with generalised HIV epidemics where available.

GBD demographic inputs

Location-specific population, fertility, and HIV-free survival rates from GBD 2017 and migration data from UNAIDS were used as inputs in modeling all locations.

UNAIDS data

The files compiled by UNAIDS for their HIV/AIDS estimation process were our main source of data for producing estimates of HIV burden. These files are typically country-specific and contain both demographic data (population, fertility, migration, and HIV-free survival rates) and HIV-specific information. In all cases except migration, we substituted in our own, internally consistent demographic estimates. The HIV-specific information includes what is needed to run both the Spectrum and Estimation and Projection Package (EPP) models. Spectrum requires the following input data: AIDS mortality among people living with HIV with and without ART, CD4 progression among people living with HIV not on-ART, ART coverage among adults and children, Cotrimoxazole coverage among children, coverage of breastfeeding among women living with HIV, prevention of mother-to-child transmission coverage, and CD4 thresholds for treatment eligibility. EPP uses many of the same assumptions as Spectrum but fits a simpler model to HIV prevalence data from surveillance sites and representative surveys. Antenatal care, incidence, prevalence, and treatment coverage data from UNAIDS were used in modeling for all locations. We extracted all of these data from the proprietary format used by UNAIDS.

For GBD 2017, we received updated national-level files for 97 countries and updated subnational-level files for eight countries. For many of the GBD locations not covered by these files, we had UNAIDS files from an earlier year of estimation, which we used again. After combining, we were left with a set of 35 countries for which we have never received a UNAIDS file, many of them countries with small populations and/or low HIV prevalence. In those places, we generated regional averages of all needed inputs. This enabled us to run Spectrum for every GBD location.

In several cases, we have modified the structure or data in the UNAIDS files. In South Africa, Russia, Iran, New Zealand, Great Britain, Kenya, Japan, Indonesia, Mexico, United States, Norway, Brazil, Ukraine, and China, which we estimate at the subnational level, we split the national-level UNAIDS file into subnational datasets using assumptions from GBD 2017 demographics and GBD 2016 HIV prevalence. We also estimate at the subnational level in Ethiopia, Kenya, and India, but have subnational-level UNAIDS files for these locations; however, in Kenya and India, we must split larger subnationals to more granular locations. The subnational locations in Ethiopia, Kenya, India, and South Africa are fit as separate subpopulations in EPP, so we extracted the prevalence data for the individual subnationals. In Benin, Cote d'Ivoire, Haiti, Moldova, Mozambique, Nigeria, Togo, Zambia, and Zimbabwe, the country files that we received from UNAIDS contained only subnational data without national-level aggregates. In these cases, we aggregated the UNAIDS files and ran EPP and Spectrum at the national level.

Vital registration data

We used all available sources of vital registration and sample registration data from the GBD Causes of Death database after garbage code redistribution and HIV/AIDS mis-coding correction, except in Group 1A countries as described below.[1, 2] There are two different cause of death data sources for HIV/AIDS in China: the Disease Surveillance Point (DSP) system and the Notifiable Infectious Disease Reporting (NIDR) system. Both systems are administered by the Chinese Center for Disease Control and Prevention, but the reported number of deaths due to HIV is significantly lower in DSP. Therefore, we have used the provincial-level ratio of deaths due to HIV/AIDS from NIDR to those from DSP, choosing the larger ratio between years 2013 and 2014, and scaled the reported deaths in the DSP system, which is in turn used in the Space-Time Gaussian Process Regression (ST-GPR).

On-ART literature data

Data were identified by using search terms "HIV," "mortality," and "antiretroviral therapy" in PubMed searches across the literature. To be included, studies must include only HIV-positive people who receive antiretroviral therapy (ART) but who were ART-naïve prior to the study. In addition, studies must report either a duration-specific mortality proportion or a hazard ratio across age or sex, and must not include children.

For duration-specific survival data, studies must report uncertainty on mortality estimates or provide stratum-specific sample sizes and must include duration-specific data to allow for calculation of 0-6, 7-12, or 13-24 month conditional mortality. In addition, studies must either report separate mortality and loss-to-follow-up (LTFU) curves, be corrected for LTFU using vital registration data or double sampling, or be conducted in a high-income setting. Finally, studies must report the percent of participants who are male and the median age of participants.
Hazard ratio data for ages or sexes can only be used if the hazard ratios are controlled for other variables of interest (age, sex, and CD4 category). In GBD 2013, we identified 102 papers for extraction. For GBD 2015, we included 13 additional studies informing the duration-specific mortality estimation process and 26 studies informing the age and sex hazard ratio estimation process (some studies were used and counted in both). We also added one study to our LTFU analysis. For GBD 2016, we included 12 additional studies informing the duration-specific mortality estimation process and 11 studies informing the age and sex hazard ratio estimation process (some studies were used and counted in both). For GBD 2017, we included 17 additional studies informing the duration-specific mortality estimation process and 13 studies informing the age and sex hazard ratio estimation process (some studies were used and counted in both). We also included two new studies in our LTFU analysis.

Off-ART literature data

In GBD 2013, to characterize uncertainty in the progression and death rates, we systematically reviewed the literature on mortality without ART. We searched terms related to pre-ART or ART-naive survival since seroconversion.[3] After screening, we identified 13 cohort studies that included the cohorts used by UNAIDS from which we extracted survival at each one-year point after infection. Screening for additional, recently published studies in GBD 2015, GBD 2016 and GBD 2017 identified no new cohort studies for inclusion in this analysis.

Severity splits and disability weights

The basis of the GBD disability weight survey assessments are lay descriptions of sequelae highlighting major functional consequences and symptoms. The lay descriptions and disability weights for HIV/AIDS severity levels are shown below.

Severity level	Lay description	DW (95% CI)
Symptomatic HIV	has weight loss, fatigue, and frequent infections.	0.274 (0.184-0.377)

AIDS with antiretroviral treatment	has occasional fevers and infections. The person takes daily medication that sometimes causes diarrhea.	0.078 (0.052-0.111)
AIDS without antiretroviral treatment	has severe weight loss, weakness, fatigue, cough and fever, and frequent infections, skin rashes, and diarrhea.	0.582 (0.406-0.743)

The proportion of people living with HIV/AIDS who are being treated with antiretroviral therapy is an output of Spectrum, the compartmental model used to make consistent incidence, prevalence, and mortality estimates described below.

Modelling strategy

In GBD 2017, our general modelling strategy for estimating HIV incidence, prevalence, and mortality is very similar to the strategy used in GBD 2016. We continue to use the Spectrum program rewritten in Python for GBD 2013 to facilitate faster and more flexible execution necessary for our more intensive computational needs. We made several changes to the modeling strategy in Spectrum comparing to the Spectrum software used by UNAIDS. We also, again, ran EPP using an open-source computer program in R written by Jeffrey Eaton.[4] We ran EPP for all Group 1 countries in order to produce incidence and prevalence estimates that were consistent with the demographic and epidemiological assumptions used in GBD 2017.

On-ART

First, we corrected reported probabilities of death for loss to follow-up using an update of the approach developed by Verguet and colleagues.[5] Verguet and colleagues used tracing and follow-up studies to empirically estimate the relationship between death in LTFU and the rate of LTFU.
To create estimates of age-specific hazard ratios, we synthesized hazard ratio data in five broad age groups: 15-25, 25-35, 35-45, 45-55, 55-100, and modeled the data using DisMod-MR 2.1.

To create estimates of sex-specific hazard ratios, we use the *metan* function in Stata to create estimates of relative risks separately by region, using female age groups as the reference group.
The age and sex hazard ratios were applied to the study level mortality rates, accounting for the distribution of ages and sexes in the mortality data. We then subtracted HIV-free mortality from the model life table process to calculate study level age-sex HIV-specific mortality.

We used DisMod-MR 2.1 to synthesize the age-sex split study level data into estimates of conditional probability of death over initial CD4 count.[3] We modeled the data separately by duration, age, sex and region and added a fixed effect on whether the study was conducted prior to 2002. Finally we replaced our on-ART mortality rates with those estimated off treatment if they were higher.

Off-ART

Following UNAIDS assumptions, no-ART mortality is modeled as shown in the figure below.[3]

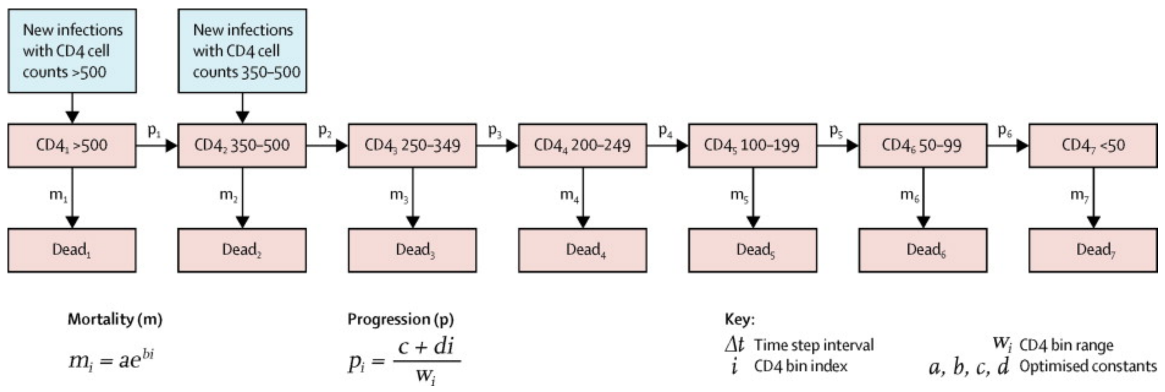

The death and progression rates between CD4 categories vary by age according to four age groups: 15–24 years, 25–34 years, 35–44 years, and 45 years or older. We modeled the logit of the conditional probability of death between years in these studies using the following formula:

$$logit\,(m_{ijk}) = \beta_0 + \sum_{i=1}^{4} \beta_{1i}\, a_i + \sum_{j=1}^{12} \beta_{2j} t_j + u_k + \varepsilon_{ijk}$$

In the formula, m is conditional probability of death from year t_j to t_{j+1}, a_i is an indicator variable for age group at seroconversion (15–24 years, 25–34 years, 35–44 years, and 45 years or older), t_j is an indicator variable of year since seroconversion, and u is a study-level random effect.

By sampling the variance-covariance matrix of the regression coefficients and the study-level random effect, we generated 1,000 survival curves for each age group that capture the systematic variation in survival across the available studies. For each of the 1,000 survival curves, we used a framework modeled after the UNAIDS optimization framework in which we find a set of progression and death rates that minimizes the sum of the squared errors for the fit to the survival curve.[8, 9]

Changes for GBD 2017

In GBD 2017, we chose to estimate mortality for each region in its own DisMod model, whereas previous GBD iterations estimated all regions together with fixed effects. This change was driven by new results from the IeDEA cohort collaboration which provided enough data to estimate mortality trends by CD4 in each region separately.[6] We also added a year covariate to our LTFU model reflecting evidence from a large meta analysis by Zurcher and colleagues, which showed that mortality among the LTFU has declined in recent years.[7] Finally we replaced our estimated on-ART mortality rates by rates off art, accounting for progression to lower CD4 categories, if the on-ART rates were higher. This was done to ensure individuals would not experience higher mortality when they entered treatment in spectrum.

Burden estimation overview

UNAIDS uses two key analytical components in their epidemiological estimation. EPP is used to estimate incidence and prevalence trajectories that are consistent with prevalence surveys and other prevalence measurements such as antenatal clinic sero-surveillance. Spectrum is a compartmental HIV progression model used to generate age-specific incidence, prevalence, and death rates from the EPP incidence and prevalence curves and assumptions about intervention scale-up and local variation in epidemiology.

Due to the substantial differences in the quality and types of data available across different countries, we used three different methodologies to produce year-, age-, and sex-specific estimates of HIV incidence, prevalence, and mortality.

Spectrum

For GBD 2013, we created an exact replica of Spectrum in Python. This enabled us to run thousands of iterations of the model at once on our computing cluster and allowed for more flexible input data structures. Additionally, in order to generate estimates with more realistic ranges of uncertainty than those in UNAIDS 2012, we adjusted all input data by uniformly sampled factors between 0.9 and 1.1. These changes, along with our new estimation of with- and without-ART mortality and CD4 progression parameters, persist into GBD 2017.

Changes for GBD 2017

For GBD 2017, we implemented a new approach to address selection bias resulting from temporal and geographic variation in ANC reporting, which has the potential to skew unadjusted estimates, especially early in the epidemic when there are no nationally representative prevalence surveys to anchor estimates.[14] To address this issue, the specification of the likelihood of observed ANC clinic data within EPP includes random intercepts for each clinic. While this approach largely accounts for differences in level between clinics, it does not impact the estimated shape of the epidemic. In order to leverage available information from nearby geographies, we developed a model for data imputation which establishes an epidemic peak from a first-stage model fit to ANC clinic data from a location and its nearest neighbors. The model included random effects for country, clinic, and time. The year of the largest random effect was used as t_{max} the location of a single knot in an imputation model which predicted the logit of prevalence in each year for a clinic as a linear spline. We can write this method mathematically in the following way:

$$logit(\rho_i(t)) = \beta_0 + S(t) + \beta_3 X_i + \epsilon$$

$$S(t) = \beta_1 S_1(t) + \beta_2 S_2(t)$$

$$S_1(t) = \begin{cases} t_{max} - t & t \leq t_{max} \\ 0 & t > t_{max} \end{cases}$$

$$S_2(t) = \begin{cases} t - t_{max} & t \geq t_{max} \\ 0 & t < t_{max} \end{cases}$$

$\rho_i(t)$ is prevalence among ANC attending pregnant women in clinic *i*, with location-level intercept β_0, linear spline $S(t)$ with a knot at t_{max}, and site-specific fixed effects X_i.

One thousand draws of imputed clinic prevalence, accounting for covariance between predictors, were generated for clinic-years where at least one clinic had an observation in a given year. These draws were used for each of the one thousand EPP runs we ran for each location.

Additionally, for GBD 2017 we improved our sex-specific modeling strategy in Spectrum by sex-splitting incidence based on a model fit to the sex ratio of prevalence observed in countries with representative surveys. We also updated the Spectrum pediatric module to reflect changes made by UNAIDS.[10] Our child module was revised to include CD4 progression and CD4-specific mortality rates taken from a model fit to survival data from IeDEA. We also updated child initiation of ART to include data on-ART distribution from IeDEA.

ART Coverage Distribution

In UNAIDS' implementation of Spectrum, initiation of ART is constrained by eligibility criteria and distributed across CD4 count groups according to both the expected number of deaths and the number of people in each untreated CD4 count group - groups with a large proportion of PLHIV and high numbers

of expected deaths initiated the most individuals into treatment. Three surveys were available at the time of publication that contained questions which can help inform the CD4 count distribution of ART coverage, Uganda AIS 2011 and Kenya AIS 2007 and 2012. Both of these surveys conducted CD4 count measurements and include a question regarding the amount of time that an individual receiving ART had been enrolled in treatment. Survey data provides cross-sectional CD4 count information; however, the Spectrum modeling framework tracks individuals by categorical CD4 count at the initiation of treatment. In order to cross-walk the cross-sectional survey data into estimates of CD4 count at treatment initiation, we built a model using relevant cohort data which tracked changes in CD4 count after initiation of treatment to translate an individual's current CD4 count and duration on treatment into CD4 count at initiation of treatment. The functional form for changes in CD4 count as a function of duration on treatment was a natural spline on duration with knots at 3, 12, 24 and 36 months, and an interaction between initial CD4 count and duration.

After cross-walking, we predicted the probability of being on treatment as a function individual income (measured through an asset-based index), stratified by CD4 count, age, and sex. The results of this prediction were translated into country-specific age-sex-year-CD4 count probabilities of coverage using a conversion factor between individual income and LDI. We used stochastic frontier analysis to constrain the maximum possible coverage for a given degree of income and CD4 count. Predicted probabilities of coverage were input to Spectrum to inform the distribution, and not the overall level, of ART treatment by CD4 count. Spectrum converted counts of expected individuals on treatment in each CD4 count group and scaled the distribution across CD4 count groups to match the input data on the number of people on-ART coming from UNAIDS country files. In cases where the predicted number of individuals initiating treatment exceeds the total number of untreated individuals in a CD4 count group, we reallocate treatment evenly to other CD4 count groups.

Countries with seroprevalence surveys and antenatal clinic data (Groups 1A and 1B)

We identified 50 countries – as well as subnational locations in India, Kenya, Ethiopia, and South Africa – with at least 0.5% adult HIV prevalence and at least one geographically representative HIV seroprevalence survey or available antenatal care clinic (ANC) data. In order to ensure that our estimates of incidence and prevalence in these places were consistent with our estimates of HIV progression, we used a version of EPP written in R and C++ by Jeffrey Eaton to create new fits to the available prevalence data. The version of EPP used in GBD 2017 was updated in 2017 by Jeffrey Eaton. In this new version, an ANC prevalence adjustment was included and incorporated with the 2016 lookup database for the relative risk between pregnant women and the whole adult population and an additional parameter to estimate ANC variance inflation was included as well.

For adjusting ANC data to align with the national 15 to 49 both sexes population, we extracted data on HIV prevalence among pregnant women who gave birth within the last year and attended an ANC clinic from available DHS surveys. A simple model with regional random effects was run to generate location-specific prior distributions for the ANC bias adjustment where surveys were available, and regional priors for locations without a survey. The adjustment using a time-series of relative risk between pregnant women and the adult population was removed, and the ANC bias parameter was changed to account for all of the biases observed between these two populations.

In the new version of EPP, in addition to the equilibrium prior assumption of the force of infection in projection, a random walk approach is available as an alternative method. For locations with two or more prevalence surveys and a declining trend between the mean of the most recent two surveys, the random walk approach was chosen to project the force of infection. We assumed the change of the log scaled force of infection was following a normal distribution with mean equal to the median of the change of the

modeled force of infection among the years having ART implemented or prevalence data, and the SD was equal to the default setting as the mean SD of the change of the modeled force of infections among the years having prevalence data. The projection year was chosen from the most recent year between the year with the lowest model force of infection and the year of the second latest survey data.

In the new EPP code, an optimization step was added into IMIS function to speed up the parameter sampling step based on Raftery and Bao.[10] Two optimization methods have been introduced. The main algorithm is Broyden–Fletcher–Goldfarb–Shanno (BFGS) optimization. If BFGS fails, Nelder-Mead optimum is used instead. In our 2016 EPP model, by substituting in our own assumptions about HIV progression rates and on/off ART mortality, we were able to ensure that the implied relationship between incidence and mortality/prevalence in EPP is similar to that in Spectrum.

To incorporate uncertainty in our mortality and progression parameters, we run EPP with separate draws of each of these parameters. This process produced 1,000 sets of EPP output for each of the locations that make up the 48 countries in the group. Every set of EPP outputs contains 500 consistent draws of HIV incidence and prevalence in adults aged 15-49.

For every location in the group, we sampled one of the 500 incidence/prevalence draws from each of the sets of EPP results. By sampling one draw from each set, we ensured that the distribution of progression parameters dictating the relationship between incidence and prevalence was exactly the same as the distribution of the sorted parameters generated in the previous step. At the end of this process, for every location in the set of 48 countries, we were left with 1,000 linked draws of adult incidence and prevalence and the exact mortality and progression parameters that generated those draws. We then ran these results, along with the previously described demographic and HIV-specific inputs, through Spectrum to produce location-, year-, age-, and sex-specific estimates of HIV incidence, prevalence, and mortality.

The HIV/mortality reckoning process is intended as a method of reconciling separate estimates of HIV mortality (and its resulting effect on estimates of HIV-free and all-cause mortality) in Group 1 countries by averaging estimates of HIV mortality from the model life table process and EPP-Spectrum. Additional details on the reckoning can be found in the GBD 2017 mortality manuscript.[11]

Since Spectrum produces HIV incidence, prevalence, and deaths that are consistent with one another over time, the reckoning process results in death numbers that are no longer consistent with the incidence and prevalence produced in Spectrum. In order to recreate this consistency, we recalculated incidence for all Group 1 locations using reckoned deaths and prevalence produced by Spectrum. The updated incidence is calculated by aggregating counts of new infections, HIV deaths from Spectrum, and HIV deaths after reckoning at the year-sex level. The difference between reckoned HIV deaths and HIV deaths from Spectrum is added to Spectrum incidence, and we calculate the ratio between updated incidence and Spectrum incidence. Age-specific counts of new infections are then scaled by their corresponding sex-year ratios.

Countries with vital registration data (Group 2A and 2B)

Vital registration is one of the highest-quality sources of data on HIV burden in many countries, so generating estimates that are consistent with these data with necessary adjustment to account for any potential underreporting is critical. We identified 108 countries – as well as 574 subnational locations from Brazil, China, Japan, Indonesia, India, Mexico, Sweden, the United Kingdom, Ukraine, Russia, New Zealand, Iran, Norway and the United States – with usable points of vital registration data, verbal autopsy (VA) data, or sample registration system (SRS) data. In India, Vietnam and Indonesia, we used SRS and VA

data, respectively, as input mortality for CIBA. For India we extracted the resulting age-sex distribution of incidence, but scaled the level to match the adult incidence rate estimated from EPP for each state.

We imputed missing years of data to generate a complete time series for HIV from the estimated start year of the epidemic using ST-GPR. We analyzed mortality trends using ST-GPR starting in 1981, the year that HIV was first identified in the United States.[12] For ST-GPR, we adjusted the lambda (time weight) and GPR scale according to the completeness of vital registration data, with 4- and 5-star quality VR using parameters designed to follow the data more closely. We produced separate splines by country/age group, up to the peak year of death rate. We then ran a linear regression with fixed effects on region, age, and sex. Following this, we ran space-time residual smoothing, in which time, age, and space weights are used to inform smoothing of the residuals between data points and the linear regression estimate. From this process, we generated space-time estimates with the applied weights, along with the median absolute deviation (MAD) of the space-time estimates from the data. The MAD was calculated at various levels of the geographic hierarchy (e.g., subnational and national), and was added into the data variance term. The data variance and space-time estimates were then analyzed using Gaussian Process Regression to return a final estimate of mortality along with uncertainty.

Although Spectrum produces HIV mortality estimates that are within the realm of possibility in most countries using the incidence curves provided in the UNAIDS country files, it is a deterministic model that has not yet been integrated into an optimizable framework. Therefore, in order to “fit” it to vital registration data, we need to adjust input incidence.

To improve the fit of this process, in GBD 2015, we restructured Spectrum to track cohorts by year of HIV infection. With this version of Spectrum we can output, among many other metrics, HIV deaths by year, age, sex, and infection cohort. This enables us to adjust incidence to fit to death much more precisely and without making any rigid assumptions about the time from HIV infection to HIV death.

We have incorporated these improvements into a cohort incidence bias adjustment (CIBA) process. First, we ran Spectrum normally to produce 1,000 draws of incidence, prevalence and mortality. Then, by year, age, and sex, we took the ratio of VR deaths to Spectrum deaths to quantify the amount of bias in Spectrum. Using draw-level duration data from the new version of Spectrum, for every year-, age-, and sex-specific infection cohort, we calculated the share of all HIV deaths observed over the course of the projection period in that cohort that would occur in each year after the year of infection. For example, projecting from 1970 through 2016, we identified the cohort of men infected in 1992 at the age of 16, calculated the total number of HIV deaths in that cohort in all subsequent years through the end of 2016, and divided the annual number of deaths by that total. This showed us the distribution of deaths among that cohort over the projection period. In the most extreme case (infections in 2015), we could only produce one point of that distribution (2016), so that single value is exactly 1·0; 100% of the deaths observed in that cohort occurred in 2016.

We then used these distributions of death to weigh the ratio of VR deaths to Spectrum deaths, meaning that ratios in the years where we expect the largest share of deaths were weighed most heavily. We then multiplied the initial size of that cohort from the normal run of Spectrum by the sum of the combined ratios to get a new estimate of new cases in that year/age/sex combination.

We can write this method mathematically in the following way:

$$r_t = \frac{VR_t}{D_t}$$

$$\rho_t^{t-i} = \frac{d_t^{t-i}}{\sum_{k=t-i+1}^{n} d_k^{t-i}}$$
$$\alpha^{t-i} = \sum_{k=t-i+1}^{n} r_k * \rho_k^{t-i}$$
$$n_{\text{adjusted}}^{t-i} = \alpha^{t-i} * n^{t-i}$$

VR_t is the number of HIV/AIDS deaths in year t from ST-GPR, and D_t is the number of HIV/AIDS deaths from the first run of Spectrum. In the second equation, d_t^{t-i} is the number of HIV/AIDS deaths among members of infection cohort $t-i$ in year t, with $i \geq 1$, from the new, duration-tracking version of Spectrum, and n is final year of the projection. Therefore, ρ_t^{t-i} is the share of observed deaths in cohort $t-i$ that we expect to occur in year t. It follows that α^{t-i} is the weighted adjustment ratio described above, which we multiply by the estimated initial size of infection cohort $t-i$ as calculated in the first-stage Spectrum run to get the adjusted number of new cases, $n_{\text{adjusted}}^{t-i}$. This process is run separately for every sex, single-age, and draw.

CIBA allows ratios in each year after a given infection year to influence the final adjustment to incidence. The size of that influence is determined by the relative importance of that year in the cohort-year's distribution of deaths over time. The result is a new set of 1,000 draws of incidence and a set of 1,000 ratios of post-adjustment incidence to pre-adjustment incidence. We perform this adjustment using mean durations from the new version of Spectrum in order to try to shift the mean of the regular distribution of deaths.

Finally, to produce location-, year-, age-, and sex-specific estimates of HIV incidence, prevalence, and mortality, we ran the new estimates of incidence and all previously input data through Spectrum.

Countries without survey data and vital registration data (Group 2C)
The remaining 30 countries – as well as Macao Special Administrative Region of China – had neither geographically representative seroprevalence surveys nor reliable vital registration systems. To produce estimates of HIV burden in these countries, we assumed that Spectrum is similarly biased as in other Group 2 countries within the same super-region. This involved running Spectrum, adjusting incidence using 1,000 adjustment ratios randomly sampled from CIBA results from the same super-region, and rerunning Spectrum using the new draws of adjusted incidence. As above, the estimates of incidence, prevalence, and mortality were incorporated into the rest of the machinery via the reckoning process.

HIV/AIDS resulting in other diseases

There are two Level 4 causes under the HIV/AIDS Level 3 cause in the GBD 2015 cause hierarchy. The modeling process for HIV/AIDS-tuberculosis is detailed in a separate part of this appendix. We computed deaths for HIV resulting in other diseases by subtracting HIV/AIDS-tuberculosis deaths from all HIV deaths at the 1,000 draw level.

Source Counts

HIV on/off treatment	**Other**
**Site-years (total)**	17045
**Number of countries with data**	148

Number of GBD regions with data (out of 21 regions)	21
Number of GBD super-regions with data (out of 7 super-regions)	7

HIV Prevalence	Other
Site-years (total)	2037
Number of countries with data	51
Number of GBD regions with data (out of 21 regions)	9
Number of GBD super-regions with data (out of 7 super-regions)	5

HIV Incidence case reports	Other
Site-years (total)	666
Number of countries with data	54
Number of GBD regions with data (out of 21 regions)	7
Number of GBD super-regions with data (out of 7 super-regions)	3

References

1. Global, regional, and national age–sex specific all-cause and cause-specific mortality for 240 causes of death, 1990–2013: a systematic analysis for the Global Burden of Disease Study 2013. *The Lancet* 2015; **385**: 117–71.

2. Birnbaum JK, Murray CJ, Lozano R. Exposing misclassified HIV/AIDS deaths in South Africa. *Bull World Health Organ* 2011; **89**: 278–85.

3. Murray CJL, Ortblad KF, Guinovart C, *et al.* Global, regional, and national incidence and mortality for HIV, tuberculosis, and malaria during 1990–2013: a systematic analysis for the Global Burden of Disease Study 2013. *The Lancet* 2014; **384**: 1005–70.

4. jeffeaton/epp. GitHub. https://github.com/jeffeaton/epp (accessed April 21, 2016).

5. Verguet S, Lim SS, Murray CJL, Gakidou E, Salomon JA. Incorporating Loss to Follow-up in Estimates of Survival Among HIV-Infected Individuals in Sub-Saharan Africa Enrolled in Antiretroviral Therapy Programs. *J Infect Dis* 2013; **207**: 72–9.

6. Anderegg N, Johnson LF, Zaniewski E, *et al*. All-cause mortality in HIV-positive adults starting combination antiretroviral therapy: correcting for loss to follow-up. *AIDS* 2017; **31 Suppl 1:** S31-40.

7. Zürcher K, Mooser A, Anderegg N, *et* al. Outcomes of HIV-positive patients lost to follow-up in African treatment programmes. *Trop Med Int Health* 2017; **22**: 375-387.

8. Ghys PD, Zaba B, Prins M. Survival and mortality of people infected with HIV in low and middle income countries: results from the extended ALPHA network. *AIDS Lond Engl* 2007; **21 Suppl 6**: S1–4.

9. Hallett TB, Zaba B, Todd J, *et al.*, ALPHA Network. Estimating incidence from prevalence in generalised HIV epidemics: methods and validation. *PLoS Med* 2008; **5**: e80.

10. Raftery AE, Bao L. Estimating and Projecting Trends in HIV/AIDS Generalized Epidemics Using Incremental Mixture Importance Sampling. *Biometrics* 2010; **66**: 1162–73.

11. Wang H, Murray CJ, Carter A, He F. Global, regional, and national under-5 mortality, adult mortality, age-specific mortality, and life expectancy, 1970-2016: a systematic analysis for the Global Burden of Disease Study 2016. *The Lancet* 2017; **390**: 1151-1210.

12. CDC. Pneumocystis Pneumonia --- Los Angeles. MMWR Wkly. 1981; published online June 5. http://www.cdc.gov/mmwr/preview/mmwrhtml/june_5.htm (accessed April 21, 2016).

13. CDC. Pneumocystis Pneumonia --- Los Angeles. MMWR Wkly. 1981; published online June 5. http://www.cdc.gov/mmwr/preview/mmwrhtml/june_5.htm (accessed April 21, 2016).

14. Ng M, Gakidou E, Murray CJL, Lim S. A comparison of missing data procedures for addressing selection bias in HIV sentinel surveillance data. *Population Health Metrics* 2013; **11:** 12.

3.3.2 Tuberculosis SDG Capstone Appendix

Flowchart

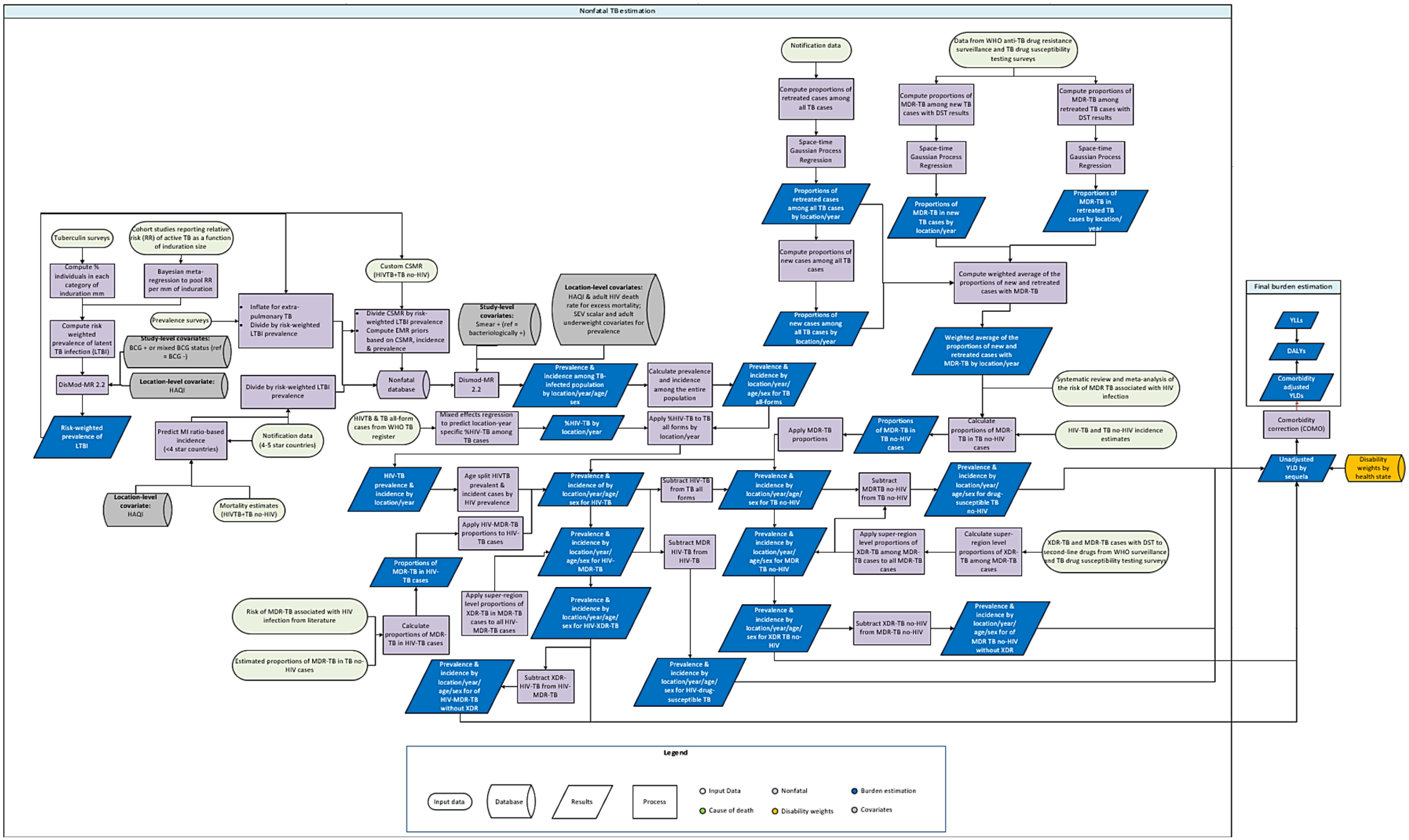

Input Data & Methodological Summary

Indicator definition

This modeling strategy encompassed the indicator associated with tuberculosis incidence (3.3.2).

Indicator 3.3.2

As a component of SDG Goal 3. Ensure healthy lives and promote well-being for all at all ages, SDG Target 3.3, by 2030, end the epidemics of AIDS, tuberculosis, malaria and neglected tropical diseases and combat hepatitis, water-borne diseases and other communicable diseases, is measured using SDG Indicator 3.3.2, number of new and relapsed TB cases per 100,000.

Case Definition

Tuberculosis (TB) is an infectious disease caused by Mycobacterium tuberculosis. The case definition includes all forms of TB including pulmonary TB and extrapulmonary TB which are bacteriologically confirmed or clinically diagnosed. For TB, the ICD 10 codes are A10-A19.9, B90-B90.9, K67.3, K93.0, M49.0, P37.0, and ICD 9 codes are 010-019.9, 137-137.9, 138.0, 138.9, 139.9, 320.4, 730.4-730.6. For HIV-TB, the ICD 10 code is B20.0.

Latent TB infection is defined as an infection with Mycobacterium tuberculosis, without any symptoms or signs of active TB disease.

We separately estimated the incidence and prevalence of multidrug-resistant tuberculosis and extensively drug-resistant tuberculosis by HIV status. The case definitions are shown below.

(1) Multidrug-resistant TB without extensive drug resistance: a form of TB (among HIV-negative individuals) that is resistant to the two most effective first-line anti-tuberculosis drugs (isoniazid and rifampicin), but is not resistant to any fluoroquinolone and any second-line injectable drugs (amikacin, kanamycin, or capreomycin).

(2) Extensively drug-resistant TB: a form of TB (among HIV-negative individuals) that is resistant to isoniazid and rifampicin, plus any fluoroquinolone and any second-line injectable drugs.

(3) Drug-susceptible TB: TB (among HIV-negative individuals) that is susceptible to isoniazid and rifampicin

(4) HIV/AIDS - Multidrug-resistant TB without extensive drug resistance: a form of TB (among HIV-positive individuals) that is resistant to the two most effective first-line anti-tuberculosis drugs (isoniazid and rifampicin), but is not resistant to any fluoroquinolone and any second-line injectable drugs (amikacin, kanamycin, or capreomycin).

(5) HIV/AIDS - Extensively drug-resistant TB: a form of TB (among HIV-positive individuals) that is resistant to isoniazid and rifampicin, plus any fluoroquinolone and any second-line injectable drugs

(6) HIV/AIDS - Drug-susceptible TB: TB (among HIV-positive individuals) that is susceptible to isoniazid and rifampicin

Input data

Model Inputs

Input data for TB include annual case notifications, data from prevalence surveys, and estimated cause-specific mortality rates (CSMR) of TB among HIV-positive and HIV-negative individuals. From these inputs, we calculated 'priors' (expected values) on excess mortality to give more guidance to the model. Input data for latent TB infection (LTBI) include: (1) population-based tuberculin surveys, and (2) cohort studies examining the risk of developing active TB disease as a function of induration size. An updated systematic review was done for GBD 2017. The search terms, number of studies identified, and number of studies included are shown in the table below.

Outcome	Search Terms	Total number of studies identified	Number of studies included
Tuberculosis	Pubmed: ("tuberculosis"[MeSH] OR tuberculosis[Title/Abstract]) OR TB[Title/Abstract] OR Mycobacterium tuberculosis[Title/Abstract] AND prevalence[Title/Abstract] AND ("2016/08/01"[PDAT] : "2017/09/15[PDAT]) NOT (animals[MESH] NOT humans[MESH])	732	13
LTBI (tuberculin surveys)	Pubmed: ("tuberculin survey"[tiab] OR (("risk"[MeSH Terms] OR "risk"[tiab] OR "risk of"[tiab]) AND ("tuberculosis"[MeSH Terms] OR "tuberculosis"[tiab] OR "tuberculous"[tiab]) AND ("infection"[MeSH Terms] OR "infection"[tiab])) OR (("risk"[MeSH Terms] OR "risk"[tiab] OR "risk of"[tiab]) AND TB[tiab] AND ("infection"[MeSH Terms] OR "infection"[tiab])) OR "latent tuberculosis infection"[tiab] OR "latent TB infection"[tiab] OR "latent tuberculosis"[MESH]) AND ("survey"[tiab] OR "surveys"[tiab]) NOT (animals[MESH] NOT humans[MESH]) ("2016/08/01"[PDAT] : "2017/09/07"[PDAT])<br><br>Google Scholar: ("tuberculin survey" OR "risk of tuberculous infection" OR "risk of tuberculosis infection" OR "risk of TB infection" OR "latent tuberculosis infection" OR "latent TB infection") AND "survey". (01-01-2016 to 09-08-2017).	54;<br>1326	3;<br>5
LTBI (cohort studies)	Pubmed: ("tuberculin"[tiab] OR ("tuberculin"[tiab] AND "positive"[tiab]) OR "Mantoux"[tiab] OR ("Mantoux"[tiab] AND "positive"[tiab]) OR "induration"[tiab]) AND (active[tiab] AND ("tuberculosis"[MeSH] OR "tuberculosis"[tiab])) AND ("risk"[MeSH] OR "risk"[tiab]) AND ("prospective"[tiab] OR "follow up"[tiab] OR "longitudinal"[tiab]) NOT (animals[MESH] NOT	20	0

	humans[MESH]) ("2016/08/01"[PDAT] : "2017/09/21"[PDAT])		

Input data for multidrug-resistant TB (MDR-TB) and extensively drug-resistant TB (XDR-TB) include: (i) the number of MDR-TB cases, XDR-TB cases, new and retreated TB cases with a drug sensitivity testing (DST) result for isoniazid and rifampicin, and MDR-TB cases with DST for second-line drugs from routine surveillance and surveys reported to the World Health Organization, and (ii) the risk of MDR-TB associated with HIV infection from the literature.[1]

Modelling Strategy

Overview

Our TB Modelling strategy has not changed substantially from GBD 2016, but we made a refinement in the MI ratio regression approach: we used the Health Care Access and Quality index (HAQI) instead of using SDI in the MI ratio regression (as HAQI is a better health-related index than SDI for TB). First, we estimated risk-weighted prevalence of LTBI by location, year, age and sex using data from population-based tuberculin surveys and cohort studies reporting the risk of developing active TB disease as a function of induration size. Next, we divided the inputs on prevalence (from surveys in low and middle income countries), incidence (notification data from countries with a four or five-star rating, and estimated incidence for countries with a less than four-star rating), and cause specific mortality rate (CSMR) by the risk-weighted LTBI prevalence in order to model TB among those at risk in each country. We first ran a mixed effects regression (with region random effects) using MI ratios (logit transformed) from locations with a 4 or 5-star rating on causes of death with HAQI as a covariate anchoring the lower end of the HAQI scale with a data point from the Bangalore study[2] reporting that 49.2% of 126 untreated new pulmonary TB cases were dead at the end of the 5-year follow up period, to predict age-sex specific MI ratios for all locations and years. We then estimated age-sex specific incidence using the predicted MI ratios and CSMR estimates. We used DisMod-MR 2.1, the GBD Bayesian meta-regression tool to generate consistent trends in all parameters. We then multiplied the DisMod-MR 2.1 outputs by the risk-weighted prevalence of LTBI to get population-level estimates of incidence and prevalence. Because the output from DisMod-MR 2.1 are for all forms of TB, we split them into MDR-TB and XDR-TB by HIV status. To do so, we estimated the proportions of TB cases with MDR-TB for all locations and years, using data from notifications and survey data. We then estimated the proportions of MDR-TB among HIV-negative individuals and MDR-TB among HIV-positive individuals based on the risk of MDR-TB associated with HIV infection from a meta-analysis[1]. To split MDR-TB into MDR-TB with and without extensive drug resistance, we pooled the limited notification and survey data on the proportion of MDR-TB cases with extensive drug resistance by super-region, and applied these proportions to MDR-TB cases among HIV-negative and HIV-positive individuals respectively.

Modelling risk-weighted latent TB infection prevalence

Input data for Modelling risk-weighted LTBI prevalence were from two sources: (i) population based tuberculin skin test (TST) surveys, and (ii) cohort studies examining the risk of developing active TB

disease as a function of induration size. First, we extracted the prevalence of tuberculin skin testing results by induration size using the most detailed induration categories reported by studies. Second, from cohort studies reporting on the relative risk of developing active TB disease as a function of induration size, we pooled the risk of developing active TB by induration size in millimeters using the DisMod Ode computational engine. Third, we multiplied the LTBI prevalence by induration in millimeters ranging from 0-20+ with the relative risk of developing active TB at each induration size, and summed them up to derive risk-weighted LTBI prevalence for each age group.

Available evidence[3] suggests that people with very advanced HIV infection (CD4 counts <200 cells/mm^3) may have a false-negative TST (0mm induration) due to profound immune suppression, but still have very high risk for TB. For those who are HIV-positive, but with higher CD4 counts, the risk for active TB increases with greater induration size as in HIV-negative individuals (i.e., the shape of the tuberculin response curve is similar to that for the general population). To take into account the false-negative TST response in HIV cases with profound immune suppression, we first computed the proportion of HIV-positive individuals with CD4 counts <200 cells/mm for the 0mm induration group using our HIV prevalence estimates for that particular category. We then multiplied that proportion by the relative risk of developing active TB disease in the 0mm induration group compared with the 20+ mm induration group among HIV positive individuals. The relative risk was computed using data from a prospective, multicenter cohort study of HIV-positive people in the United States.[3]

Using the risk-weighted LTBI prevalence (adjusting for a false-negative TST among people with advanced HIV infection) as input data, we ran a DisMod MR 2.1 model with the HAQI covariate to help inform variation over year and geography, with priors that at higher HAQI values, LTBI prevalence decreases. We included two study covariates (BCG positive, and mixed BCG status) where the reference category is BCG negative. We found no statistically significant difference between studies using different dosages of tuberculin purified protein derivative (PPD). We therefore did not include different PPD dosages as study covariates but added more uncertainty to data points from studies that used dosages larger or smaller than the standard dose of 5 tuberculin units per test dose of 0.1 ml, by entering them as z-covariates in DisMod.

Modelling TB incidence

Incidence inputs were from two different sources: (1) incidence from notification data for countries with a four or five-star rating on their cause of death data[4] as a proxy for the quality of health-related administrative data systems, and (2) estimated incidence for countries with a less than four-star rating. We used the age and sex-specific notifications (all new and relapse cases combined) in our analysis. Prior to 2013, notification data were available by case type (new pulmonary smear-positive, new pulmonary smear-negative, and new extra-pulmonary) and there were missing age data especially for younger age-groups in some countries. We imputed the missing age-groups for the three forms of TB notifications. Smear-positive age-specific notifications were inflated with the proportion smear-unknown and relapsed cases only reported at the country-year level. Some countries reported only pulmonary smear-positive cases for selected years. Missing smear-negative and extra-pulmonary cases were predicted from the adjusted smear-positive cases using a seemingly unrelated regression. All three types of notifications were added together to represent TB-all form incidence for countries with a four or five-star rating.

To generate incidence estimates for locations with a less than four-star rating, we ran a mixed effects regression (with region random effects), using MI ratios (logit transformed) from locations with a 4 or 5-star rating on causes of death as input data with HAQI as a covariate anchoring the lower end of the HAQI scale with a data point from a cohort study in the 1960s[2] reporting that 49.2% of 126 untreated new pulmonary TB cases were dead at the end of the 5-year follow up period, in order to predict age-sex specific MI ratios for all locations and years. We then used the MI ratios and cause specific mortality estimates to compute the incidence input for DisMod-MR 2.1 for locations with a less than four-star rating. In locations where estimated MI ratios were greater than notification-based MI ratios, we used the latter to compute the incidence input. Notification-based MI ratios were computed using notification data and estimated CSMR for 2010. For other years, we assumed a similar proportional difference between predicted MI ratios and notifications-based MI ratios as in 2010 and adjusted the predicted MI ratios accordingly, which were then used to compute the incidence input. We computed the age-sex specific incidence of TB among the latent TB-infected population, using TB incidence as the numerator and our estimated risk-weighted latent TB infection prevalence as the denominator.

Modelling TB prevalence

Data from prevalence surveys reporting on pulmonary smear-positive TB and bacteriologically positive TB were included. Because incidence data are for all forms of TB, we adjusted prevalence surveys to account for extra-pulmonary cases. We ran a spatiotemporal Gaussian process regression to predict location-year-age-sex specific proportions of extra-pulmonary TB among all TB cases using data on the three forms of TB from the incidence data above. We then computed the extra-pulmonary inflation factor as *1+(proportion of extrapulmonary TB /(1- proportion of extrapulmonary TB))*, and applied it to data from prevalence surveys. We then computed the prevalence of TB among the TB-infected population, using TB prevalence as the numerator and our estimated risk-weighted LTBI prevalence as the denominator. We included a study covariate indicating whether it was bacteriologically positive TB (reference category) or smear-positive TB. We found no systematic bias between studies that used both symptoms and chest X-ray as screening methods and studies that used only one of the methods. We therefore did not adjust them for systematic bias but added more uncertainty to data points from studies that used only one of the screening methods (by using it as a z-covariate in DisMod). We also added more uncertainty to data points from sub-national surveys. We included two location-level covariates, namely, age-standardized adult underweight prevalence and log-transformed age-standardized Summary Exposure Variable (SEV) scalar for TB (a summary variable of the exposure levels of TB risk factors weighted by relative risk) to help inform variation of TB prevalence over year and geography.

Modelling TB excess mortality

We matched each prevalence data point and TB CSMR (TB and HIV-TB combined) by location, year, age, and sex to calculate excess mortality rate (EMR) as *EMR=CSMR/prevalence*. We also matched each incidence data point and TB CSMR by location, year, age, and sex to calculate EMR for countries with a four or five-star rating on their cause of death data. To reflect a gradient in EMR, we added the HAQI and adult HIV death rates as country-level covariates.

DisMod-MR 2.1

For each location, we included the following as input in the DisMod model: case notifications for locations with a four or five-star rating, predicted MI-ratio-based incidence for locations with a less than four-star rating, prevalence survey data where available, excess mortality estimates, and CSMR (TB and HIV-TB combined) by age and sex.

The output from the DisMod model was for all forms of TB in TB-infected population including both HIV-negative and HIV-positive individuals. We computed the incidence and prevalence of TB among the entire population, by multiplying the prevalence of LTBI with the DisMod model estimates.

Betas and exponentiated values from the DisMod model are shown in the table below.

Covariate	Parameter	Beta (95% CI)	Exponentiated beta (95% CI)
Smear positive TB	Prevalence	-0.75	0.47 (0.47 — 0.47)
Sex (male)	Prevalence	0.17	1.18 (1.10 — 1.27)
Sex (male)	Incidence	0.32	1.38 (1.38 — 1.38)
Age-standardized proportion adult underweight	Prevalence	2.65	14.16 (8.81 — 19.47)
Age-standardized SEV scalar (log-transformed)	Prevalence	0.76	2.13 (2.12 — 2.17)
HAQI (log-transformed)	Excess mortality	-1.50	0.22 (0.21 — 0.23)
Adult HIV death rate	Excess mortality	0.64	1.89 (1.03 — 6.23)

HIV-TB incidence and prevalence

To distinguish HIV-TB from all forms of TB, we first estimated the proportions of HIV-TB cases among all TB cases using data on the number of TB cases recorded as HIV-positive and the number of TB cases with an HIV test result recorded in the WHO TB notifications register. We ran a mixed effects regression using the adult HIV death rate as a covariate to predict location-year specific HIV-TB proportions, which were then applied to TB incident and prevalent cases from DisMod, to generate HIV-TB incident and prevalent cases by location and year. These cases were then age-sex split based on the age-sex pattern of estimated HIV prevalence by location-year to generate location-year-age-sex specific HIV-TB incident and prevalent cases.

Multidrug-resistant TB, extensively drug-resistant TB and drug-susceptible TB

We ran spatiotemporal Gaussian process regressions to predict the proportions of new TB cases with MDR-TB, proportions of retreated TB cases with MDR-TB, and proportions of retreated cases among all TB cases for all locations and years. We calculated the proportions of new TB cases among all TB cases as *1- estimated proportions of retreated cases*. Next, we computed the weighted average of the proportions of new and retreated cases with MDR-TB at the 1000 draw level. We then used the weighted average proportions of MDR-TB, along with the HIV-TB and TB no-HIV incidence estimates, and the relative risk of

MDR-TB associated with HIV infection from the literature[1] to compute the proportions of MDR-TB cases among HIV negative TB cases $\left(PnoHIV_{c,y,a,s}\right)$ by location, year, age, and sex using the following formula:

$$PnoHIV_{c,y,a,s} = \frac{MDR_{c,y}}{\left(1 + \left(RR\,\frac{HIVTB_{c,y,a,s}}{TBnoHIV_{c,y,a,s}}\right)\right) TBnoHIV_{c,y,a,s}}$$

where $MDR_{c,y}$ is the number of all MDR-TB cases among HIV-positive and HIV-negative individuals by location and year, *RR* is the relative risk of MDR-TB associated with HIV infection, $HIVTB_{c,y,a,s}$ is the number of HIV-TB incident cases by location, year, age, and sex, and $TBnoHIV_{c,y,a,s}$ is the number of TB no-HIV incident cases by location, year, age, and sex.

We then applied the predicted proportions of MDR-TB cases among HIV negative TB cases to our predicted HIV-negative TB incident and prevalent cases to generate MDR-TB incident and prevalent cases by location, year, age, and sex. Next, we subtracted MDR-TB cases from all HIV-negative TB cases to generate drug-susceptible TB cases by location, year, age, and sex. To distinguish XDR-TB from MDR-TB, we aggregated the XDR-TB cases and MDR-TB cases (with drug sensitivity testing for second-line drugs) up to the super-region level and calculated the super-region level proportions of XDR-TB among MDR-TB cases, which were then applied to MDR-TB cases in corresponding countries within the super-regions to produce XDR-TB cases by location, year, age, and sex. We linearly extrapolated XDR-TB prevalence and incidence back assuming the rates were zero in 1992, one year before 1993 when XDR-TB was first recorded in USA surveillance data.[5] Finally, we subtracted XDR-TB cases from MDR-TB cases to generate MDR-TB (without XDR) cases by location, year, age, and sex.

HIV/AIDS - Multidrug-resistant TB, HIV/AIDS - extensively drug-resistant TB, and HIV/AIDS - drug-susceptible TB

To split HIV-TB into HIV-MDR-TB and HIV-drug-susceptible-TB, we first calculated the proportions of HIV-MDR-TB among all HIV-TB cases ($PHIV_{c,y,a,s}$) for each location, year, age, and sex using the following formula:

$$PHIV_{c,y,a,s} = PnoHIV_{c,y,a,s}RR$$

where $PnoHIV_{c,y,a,s}$ is the proportions of MDR-TB among all HIV-negative TB cases for each location, year, age, and sex and RR is the relative risk of MDR-TB associated with HIV infection. We then applied the predicted proportions of MDR-TB cases among HIV-TB cases to our estimated HIV-TB incident and prevalent cases to generate HIV-MDR-TB incident and prevalent cases by location, year, age, and sex. Next, we subtracted HIV-MDR-TB cases from all HIV-TB cases to generate HIV-drug-susceptible-TB cases by location, year, age, and sex. To separate out HIV-XDR-TB from HIV-MDR-TB, we applied the super-region level proportions of XDR-TB among MDR-TB cases, to HIV-MDR-TB cases in corresponding countries within the super-regions to produce HIV-XDR-TB cases by location, year, age, and sex. We linearly extrapolated HIV-XDR-TB prevalence and incidence back assuming the rates were zero in 1992, one year before 1993 when XDR-TB was first recorded in USA surveillance data.[5] Finally, we subtracted HIV-XDR-TB cases from HIV-MDR-TB cases to generate HIV-MDR-TB (without extensive drug resistance) cases by location, year, age, and sex.

New MDR-TB and XDR-TB cases among retreated cases by HIV status

Because we split TB incidence (new and relapse cases combined) by drug-resistance type, the above estimation did not capture new MDR-TB and XDR-TB cases arising from retreated TB cases other than relapse cases. We therefore separately estimated new MDR-TB and XDR-TB cases arising from retreated TB cases and added them to the incident cases estimated above. To do so, we first ran a spatiotemporal Gaussian process regression using notification data and HAQI as a covariate to predict the proportion of retreated cases (excluding relapse cases) among all TB patients for all locations and years. Next, we computed retreated cases as *(retreated proportion*estimated incident cases)/(1-retreated proportion)*. We then computed the total number of TB cases by summing estimated incident cases and retreated cases. Similar to our estimation for MDR-TB and XDR-TB among TB incident cases by HIV status, we estimated MDR-TB and XDR-TB cases among all TB cases (incident cases and retreated cases combined) by HIV- status. Finally, the number of retreated cases with MDR-TB was computed by subtracting MDR-TB among TB incident cases from MDR-TB among all TB cases (incident cases and retreated cases combined), separately for HIV positive and HIV negative individuals. Similarly, the number of retreated cases with XDR-TB was computed by subtracting XDR-TB among TB incident cases from XDR-TB among all TB cases, separately for HIV positive and HIV negative individuals. All computations were done at the 1000 draw level.

Disability weights

The lay descriptions and disability weights for severity levels derived from the GBD Disability Weights study are shown below.

Health state Name	Lay description	Disability Weights (95% CI)
Tuberculosis, not HIV infected	has a persistent cough and fever, is short of breath, feels weak, and has lost a lot of weight	0.333 (0.224-0.454)
Tuberculosis, HIV infected	has a persistent cough and fever, shortness of breath, night sweats, weakness and fatigue and severe weight loss	0.408 (0.274-0.549)

For drug-susceptible TB, MDR-TB without extensive drug resistance, and XDR-TB, we used the same disability weight [0.333 (0.224-0.454)] as in non-HIV-infected TB. For HIV-drug-susceptible-TB, HIV-MDR-TB without extensive drug resistance, and HIV-XDR-TB, we used the same disability weight [0.408 (0.274-0.549))] as in HIV-infected TB.

Source Counts

All forms Tuberculosis	Incidence	Prevalence
**Site-years (total)**	1751	166
**Number of countries with data**	76	28
**Number of GBD regions with data (out of 21 regions)**	15	10
**Number of GBD super-regions with data (out of 7 super-regions)**	5	7

Latent Tuberculosis	Prevalence
**Site-years (total)**	228
**Number of countries with data**	48
**Number of GBD regions with data (out of 21 regions)**	15
**Number of GBD super-regions with data (out of 7 super-regions)**	7

Proportion of HIV-TB among all TB cases	Other
**Site-years (total)**	1533
**Number of countries with data**	167
**Number of GBD regions with data (out of 21 regions)**	21
**Number of GBD super-regions with data (out of 7 super-regions)**	7

MDR-TB and MDR-HIV-TB Proportions	Proportion
**Site-years (total)**	853
**Number of countries with data**	139
**Number of GBD regions with data (out of 21 regions)**	21
**Number of GBD super-regions with data (out of 7 super-regions)**	7

XDR-TB and XDR-HIV-TB Proportions	Other
**Site-years (total)**	85
**Number of countries with data**	84
**Number of GBD regions with data (out of 21 regions)**	19
**Number of GBD super-regions with data (out of 7 super-regions)**	7

Risk of MDR-TB associated with HIV infection	Other
**Site-years (total)**	1
**Number of countries with data**	0
**Number of GBD regions with data (out of 21 regions)**	0
**Number of GBD super-regions with data (out of 7 super-regions)**	0

Latent Tuberculosis Relative Risk	Other
**Site-years (total)**	27
**Number of countries with data**	21
**Number of GBD regions with data (out of 21 regions)**	11
**Number of GBD super-regions with data (out of 7 super-regions)**	6

References

1. Mesfin YM, Hailemariam D, Biadglign S, Kibret KT. Association between HIV/AIDS and multi-drug resistance tuberculosis: a systematic review and meta-analysis. *PLoS One*. 2014 Jan 8;9(1):e82235.
2. Institute NT. Tuberculosis in a rural population of South India: a five-year epidemiological study. *Bulletin of the World Health Organization*. 1974;51(5):473.
3. Markowitz N, Hansen NI, Hopewell PC, Glassroth J, Kvale PA, Mangura BT, Wilcosky TC, Wallace JM, Rosen MJ, Reichman LB. Incidence of tuberculosis in the United States among HIV-infected persons. *Annals of internal medicine*. 1997 Jan 15;126(2):123-32.
4. GBD 2016 Mortality and Causes of Death Collaborators. Global, regional, and national age-sex specific mortality for 264 causes of death, 1980–2016: a systematic analysis for the Global Burden of Disease Study 2016. *The Lancet* (under review)
5. Centers for Disease Control and Prevention (CDC). Extensively Drug-Resistant Tuberculosis --- United States, 1993—2006. *MMWR*. 2007; 56(11);250-253.

3.3.3 Malaria SDG Capstone Appendix

Flowchart

Malaria non-fatal outcomes (parasite rate and case incidence) in Sub-Saharan Africa

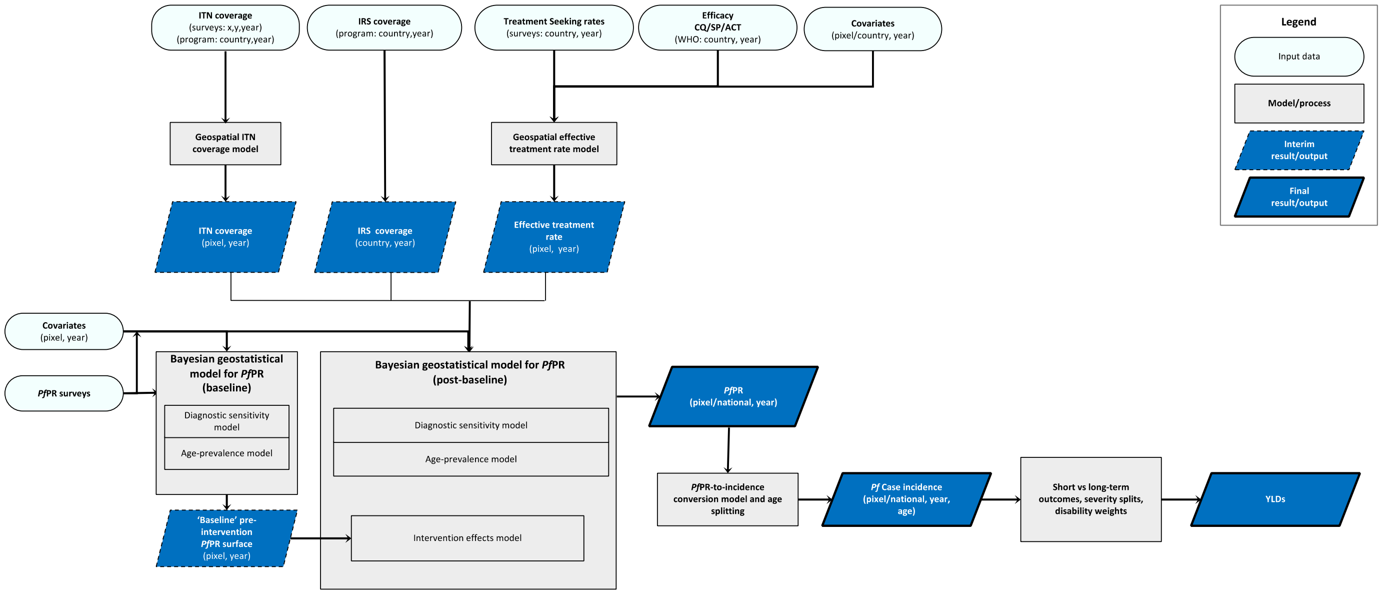

Malaria non-fatal outcomes (parasite rate and case incidence) outside Sub-Saharan Africa

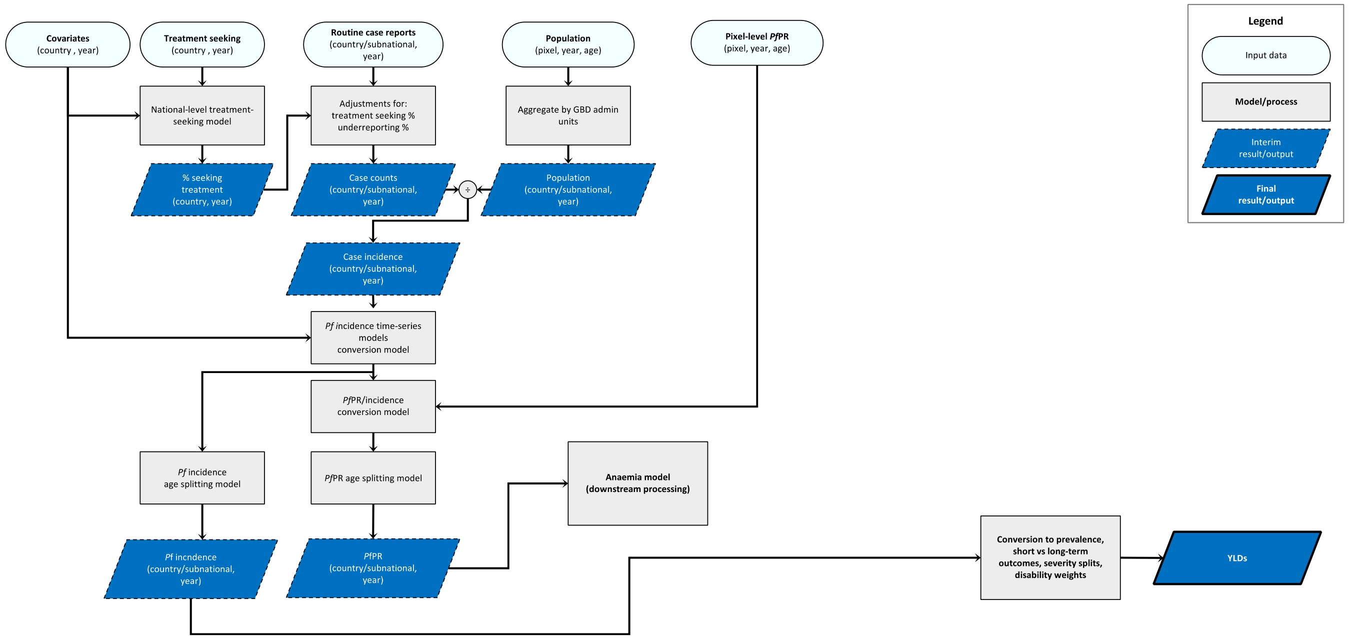

Input Data & Methodological Summary

Indicator definition

This modeling strategy encompassed the indicator associated with malaria incidence (3.3.3).

Indicator 3.3.3

As a component of SDG Goal 3. Ensure healthy lives and promote well-being for all at all ages, SDG Target 3.3, by 2030, end the epidemics of AIDS, tuberculosis, malaria and neglected tropical diseases and combat hepatitis, water-borne diseases and other communicable diseases, is measured using SDG Indicator 3.3.3, malaria cases per 1,000.

Case definition

Malaria is an acute parasitic mosquito-borne disease. An individual with uncomplicated malaria experiences one to two weeks of persistent fever, chills/shivering, sweating, joint pains, and headache. The individual will likely be lethargic and feverish, causing loss of daily function during the attack. Individuals with an untreated *P. falciparum* infection may develop severe malaria, which includes the symptoms of uncomplicated malaria but may also involve swelling, difficulty breathing, unconsciousness, and death. Microscopy is considered the gold-standard diagnostic approach for the purposes of GBD. The relevant ICD-10 codes are B50-B54.

Data input

Primary data inputs were:

(i) Routine malaria case reports from national routine surveillance systems. These were obtained at the national level from the WHO World Malaria Report and at the subnational administrative level, wherever possible, via an exhaustive search of published and grey literature sources along with online data portals hosted by national ministries of health. Each retained record consisted of an annual count of malaria cases along with a distinction between confirmed and unconfirmed diagnoses, and differentiation by malaria parasite species.

(ii) Cross-sectional, geolocated, and community-representative observations of infection prevalence for *Plasmodium falciparum* (referred to hereafter as *P. falciparum* parasite rate, *Pf*PR).

These malaria epidemiological metrics were augmented in the modelling by:

(iii) Malaria Atlas Project (MAP) modelled estimates of malaria control intervention population coverage (ITNs, IRS, and effective treatment with an antimalarial drug) resolved to 5 km x 5 km pixel-year level (for sub-Saharan Africa) and country-year level (outside sub-Saharan Africa).

(iv) A large suite of environmental, sociodemographic, and economic covariates resolved to 5 km x 5 km pixel-year level (for sub-Saharan Africa) and country-year level (outside sub-Saharan Africa).

Modelling strategy

The suitability, availability, and quality of *Pf*PR and routine case reporting data, as well as detailed intervention coverage information, differ markedly inside versus outside sub-Saharan Africa. As such, we developed separate modelling strategies for countries inside sub-Saharan Africa versus those outside. The exceptions were Algeria, Egypt, Morocco, Comoros, Mauritius, Cape Verde, Sao Tome and Principe, Rwanda, Botswana, Namibia, Eritrea, Djibouti, and South Africa. Despite being part of Africa, these countries exhibit epidemiological trends and have data availability/quality more akin to non-African settings.

*Pf*PR and case incidence modelling: Africa

Modelling was conducted in the following steps:

(i) The large assembly of geolocated *Pf*PR surveys maintained by MAP was used in a Bayesian spatiotemporal geostatistical model to predict *Pf*PR for every pixel-year in sub-Saharan Africa, representing an update to earlier work (Bhatt et al Nature, Gething et al NEJM). The model took into account (i) *Pf*PR survey participant age ranges and diagnostic type; (ii) coverage of ITNs, IRS, and effective antimalarial drug coverage, and how these metrics changed through time at each date and prediction location; (iii) environmental conditions at each date and prediction location (including density of vegetation, temperature, humidity, rainfall, elevation, and proximity to populated areas). The outcome was a predicted space-time "cube" of *Pf*PR, standardized to the 2-10 age range, for each year 1980–2017.

(ii) The *Pf*PR cube was then converted into an equivalent cube of the predicted incidence rate of clinical malaria. This conversion was achieved using an established model (Cameron et al Nature Communications) and provided estimates stratified first into three broad age bins (0-5; 5-15; <15) and then into the final 23 GBD 2017 age bins.

*Pf*PR and case incidence modelling: Outside Africa

Malaria endemic countries outside Africa tend to have less *Pf*PR data than those inside, in part because prevalence is generally lower. Thus, *Pf*PR becomes an inefficient way to measure malaria risk. Routine surveillance systems outside Africa are generally stronger, meaning that reports of malaria cases from health systems are more reliable and provide some insight into the total malaria burden in the community. Modelling outside Africa was carried out in the following steps:

(i) National and subnational case reports were first subject to adjustments to identify and minimise bias. Bias in reported case numbers arises from various sources. First, a fraction of cases in the community will fail to seek treatment or will attend a private or informal health care provider who will not provide a record of that case to the routine surveillance system. We adjusted for these factors by modelling the fraction of cases seeking care from different provider categories based on data from nationally representative cross-sectional household surveys (primarily from the Demographic and Health Survey (DHS) program and the Multiple Indicator Cluster Survey program). Another factor for which we must adjust is cases reaching formal clinics may not be subject to a confirmatory diagnostic test. We adjusted for this by assuming the fraction of unconfirmed cases that were truly malaria would equal the fraction of positives among all those tested. A final factor we adjust for is incomplete data as many routine surveillance systems fail to capture all case reports, with facilities/regions missing

from the national totals in a given year. We adjusted for this based on reporting completeness statistics published nationally by WHO.

(ii) These adjusted routine case reports were georeferenced using digitised administrative boundary data using a spatial database of such boundaries collated and maintained by MAP.

(iii) Each case report was converted to an estimate of clinical incidence rate by dividing it by the estimated population in each unit, with the latter quantity derived by combing high-resolution gridded population data and the aforementioned administrative boundaries.

(iv) Bayesian time-series models were then applied to the case reports for each country to impute incidence rates for years with missing data. The results from this analysis, in conjunction with the adjusted case reports, constitute the incidence values delivered for GBD 2017.

(v) The incidence rate for each country-year was then converted to an inferred *Pf*PR value using the same model described earlier (Cameron et al). This allowed us to utilise these polygon-level surveillance data and the *Pf*PR point-level data (where present) within the same modelling framework.

(vi) The combined *Pf*PR survey point data and (pseudo) *Pf*PR administrative unit data were then used in a Bayesian spatiotemporal geostatistical model to predict *Pf*PR at pixel-year level across all countries. As for the Africa model, *Pf*PR was standardised by age and diagnostic type and informed by a wide suite of covariates. An additional mechanism was developed to allow polygon (ie, administrative unit) and point (ie, survey) data to be used jointly to infer the predicted space-time surfaces.

(vii) The predicted *Pf*PR cube was then adjusted to ensure that, after conversion to pixel-level incidence, the incidence counts per country-year would precisely match the incidence results from step (iv). The summarised *Pf*PR values (ie, population-weighted and tallied for each country-year) from the adjusted *Pf*PR cube constitute the *Pf*PR values delivered for GBD 2017.

Total malaria cases by country, year, sex

The pixel-level predictions of clinical incidence rate (both inside and outside Africa) were combined with high-resolution gridded population data to estimate total cases per pixel-year. These were then aggregated to GBD national/subnational geographies. Inside sub-Saharan Africa, for countries endemic for *P. vivax* and *P. falciparum*, we calculated the number of cases due to *P. vivax* by applying the fraction of *P. vivax* and *P. falciparum* obtained from WHO and a literature review. Outside sub-Saharan Africa we followed the identical procedure for *P. vivax* and *P. falciparum.* Final age-splitting was accomplished using age-versus-incidence rate relationships gleaned from the paper by Cameron and colleagues (2014).

Determining YLDs for malaria

As in GBD 2016, we use a two-step process for determining malaria severity. For acute cases, severity splits for mild, moderate, and severe malaria were produced by analysis of MEPS data. These sequelae and their associated disability weights are presented below.

Table 1. Severity level, lay description, and DW

Severity level	Lay description	DW (95% CI)
Mild	Has a low fever and mild discomfort but no difficulty with daily activities.	0.006<br>(0.002–0.012)

Moderate	Has a fever and aches and feels weak, which causes some difficulty with daily activities.	0.051<br>(0.032–0.074)
Severe	Has a high fever and pain and feels very weak, which causes great difficulty with daily activities.	0.133<br>(0.088–0.19)

To determine long-term neurological burden due to malaria, we use the work by Roca-Felter and colleagues (2008) that examined the number of uncomplicated cases that led to longer-term impairment. Analytically, this means multiplying incidence estimates (described in the section above for persons under 20 by 0.00029 (0.000077–0.00057). This adjusted case estimate is then combined with excess mortality rates derived from all-cause mortality and standardised mortality ratios for neonatal encephalopathy (NE) in a DisMod model to produce prevalence estimates of long-term sequelae for all estimation years. Implicit in this process is an assumption that the disability and trend of impairment due to severe malaria follow NE. The subsequent severity splitting follows NE as well.

To determine the burden of acute (short-term) malaria, the incidence estimation results are combined and converted to prevalence by matching each draw with a draw of duration of clinical illness. Consistent with GBD 2016, we use a uniform distribution between 14 and 28 days for duration.

References

Bhatt S, Weiss DJ, Cameron E, et al. The effect of malaria control on Plasmodium falciparum in Africa between 2000 and 2015. *Nature* 2015; 526: 207–11.

Cameron, E., K.E. Battle, S. Bhatt, D.J. Weiss, D. Bisanzio, B. Mappin, U. Dalrymple, S.I. Hay, D.L. Smith, J.T. Griffin, E.A. Wenger, P.A. Eckhoff, T.A. Smith, M.A. Penny, and P.W. Gething. 2015. Defining the relationship between infection prevalence and clinical incidence of Plasmodium falciparum malaria. *Nature Communications* 6:8170.

Gething, P.W., D.C. Casey, D.J. Weiss, D. Bisanzio, S. Bhatt, E. Cameron, K.E. Battle, U. Dalrymple, J. Rozier, P.C. Rao, M.J. Kutz, R.M. Barber, C. Huynh, K.A. Shackelford, M.M. Coates, G. Nguyen, M.S. Fraser, R. Kulikoff, H. Wang, M. Naghavi, D.L. Smith, C.J.L. Murray, S.I. Hay, and S.S. Lim. 2016. "Mapping Plasmodium falciparum Mortality in Africa between 1990 and 2015." New England Journal of Medicine 375 (25):2435-2445.

3.3.4 Hepatitis SDG Capstone Appendix

Acute hepatitis A

Flowchart

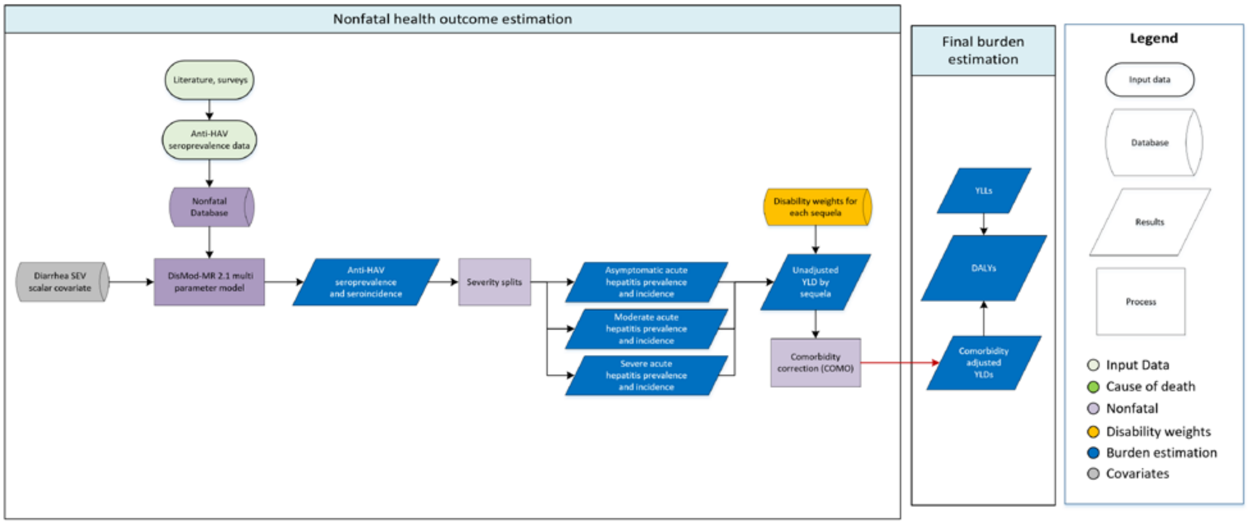

Case definition

We define acute hepatitis A as an infection with the hepatitis A virus resulting in anti-HAV IgG seroconversion, regardless of symptoms. It includes all ICD-10 codes under the heading B15 (Acute hepatitis A).

Input data

Model inputs

Our case definition is infection with hepatitis A, irrespective of symptomology. We use anti-hepatitis A virus (HAV) seroprevalence data from population-based studies and surveys to inform these estimates. We present a summary of the data sources in Table 1 below. Updates to systematic reviews are performed on an ongoing schedule across all GBD causes; an update for hepatitis A will be performed in the next one to two iterations. The last systematic review was performed as part of GBD 2013.

Table 1: Data inputs for acute hepatitis A morbidity modelling by parameter

	Prevalence
Site-years (total)	562
Number of countries/subnational locations with data	119
Number of GBD regions with data (out of 21 regions)	21
Number of GBD super-regions with data (out of 7 super-regions)	7

Severity splits & disability weights

The table below illustrates the sequelae associated with acute hepatitis A, as well as the lay descriptions and associated disability weights.

Sequela	Description	Disability Weight
Moderate	Has a fever and aches, and feels weak, which causes some difficulty with daily activities.	0.051 (0.032–0.074)
Severe	Has a high fever and pain, and feels very weak, which causes great difficulty with daily activities.	0.133 (0.088–0.19)
Asymptomatic	Infection with no apparent illness of	NA

We calculate acute symptomatic infections by multiplying incidence of acute infection by the probability of acute symptomatic infection. The probability of symptomatic infection comes from Armstrong and Bell and is shown in the figure below (where probability of symptomatic infection is represented as "probability of jaundice") [1]. The probability increases with age from ~1% in the first year of life to ~85% in adulthood. The probability function is:

$$Prob\ (symptomatic) = 0.852 * (1 - e^{-0.01244 * age^{1.903}})$$

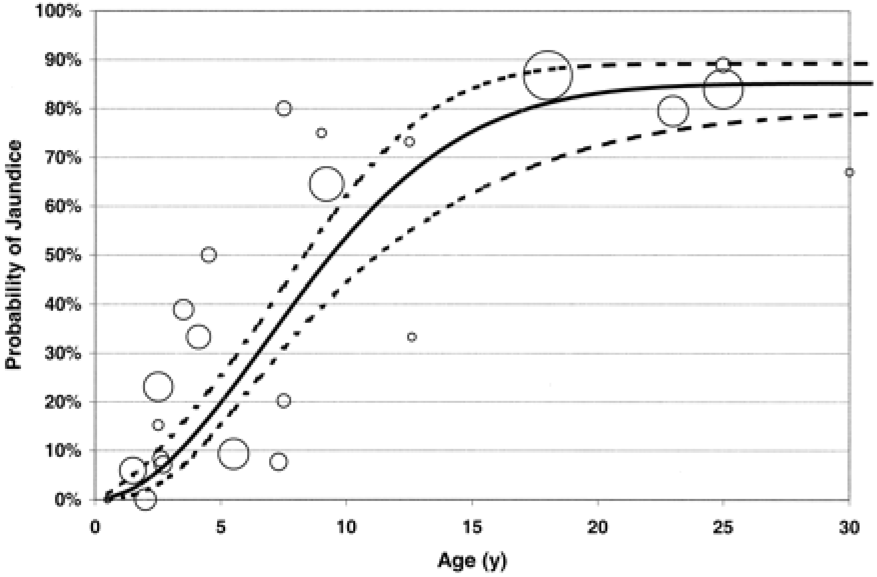

The remainder of acute infections are assumed to be asymptomatic.

We then base severity splits for moderate and severe on expert opinion that the probability of severe infection follows a beta distribution with mean 0.6% (table 2 reports percentiles of this distribution.) We assume the rest of symptomatic infections are moderate.

Table 2. Percentiles of the probability distribution of severe acute hepatitis A

0 percentile	25 percentile	50 percentile	75 percentile	100 percentile
0.0024	0.0054	0.006	0.007	0.01

Modelling strategy

We model the seroprevalence of anti-hepatitis A virus IgG using a DisMod-MR 2.1 model of anti-HAV seroprevalence. Remission and excess mortality value priors of zero were used, and an incidence value prior range between 0 and 0.5 was used. Given its reasonably stable force of infection among susceptible people across age groups, we derive incidence from the prevalence estimates using the following formula:

$$incid = \frac{-\ln(1 - prev)}{\text{age}_{\text{mid}}} * (1 - prev)$$

Changes from GBD 2016 to GBD 2017

This approach is a modification from GBD 2016, where instead of utilising DisMod-MR 2.1, we estimated anti-HAV seroprevalence via a catalytic binomial generalised linear model with a complementary log-log link, and an offset term for log-age. That previous model used a predictive covariate derived from principal components analysis of lag-distributed income (LDI) and the proportion of the population with access to improved water.

Acute hepatitis B and C

Flowchart

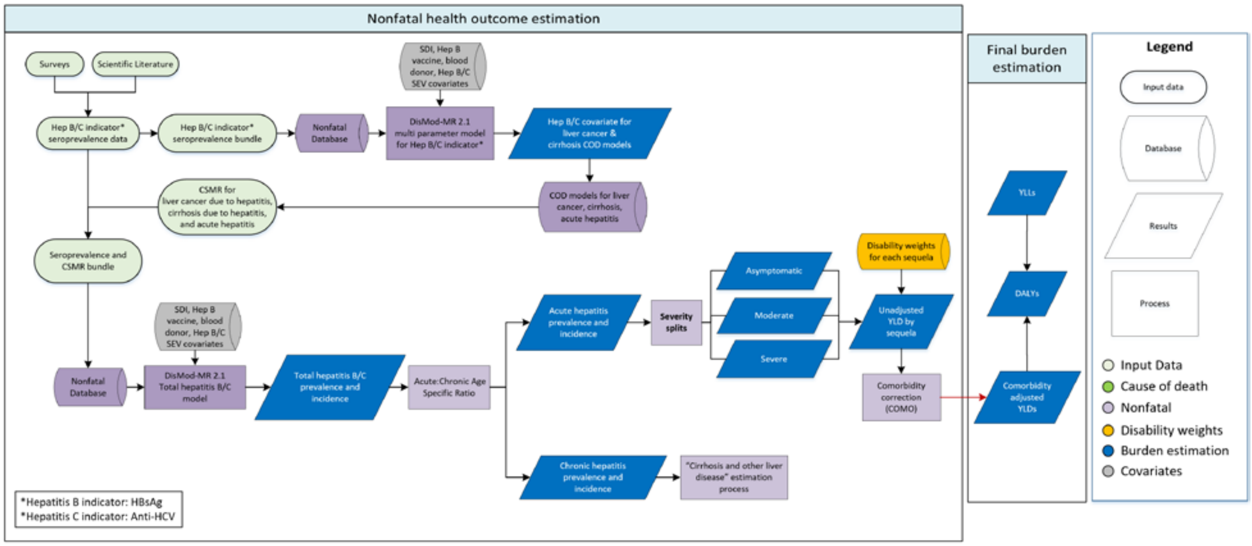

Acute hepatitis B

Case definition

We define acute hepatitis B as the period corresponding to initial infection with the hepatitis B virus, regardless of symptoms. It includes all ICD-10 codes under the heading B16 (Acute hepatitis B).

Input data

Model inputs

We use hepatitis B surface antigen (HBsAg) seroprevalence data from population-based studies and surveys. We present a summary of the seroprevalence data sources in Table 3 below. The last systematic review was performed as part of GBD 2013. Updates to systematic reviews are performed on an ongoing schedule across all GBD causes; an update for hepatitis B will be performed in the next one to two iterations.

Table 3: Data Inputs for acute hepatitis B morbidity modelling by parameter.

	Prevalence
Site-years (total)	420
Number of countries/subnational locations with data	74
Number of GBD regions with data (out of 21 regions)	19
Number of GBD super-regions with data (out of 7 super-regions)	7

We also use cause-specific mortality rate (CSMR) data, which is obtained through the hepatitis mortality modelling process. The generation and incorporation of the CSMR data is described further in the modelling strategy section below.

Modelling strategy

We model the incidence of chronic HBsAg carriage using a full DisMod-MR 2.1 model of HBsAg seroprevalence. We then convert incidence of chronic carriage to total incidence of hepatitis B infection by dividing age-specific estimates of the incidence of chronic carriage by age-specific estimates of the probability of infection resulting in carriage based on Edmunds and colleagues [2]:

$$P(carrier \mid age \leq 6\ months) = 0.885$$

$$P(carrier \mid 6\ months \leq age < 25\ years) = e^{-0.645 \times age^{0.455}}$$

$$P(carrier \mid age \geq 25\ years) = e^{-0.645 \times 25^{0.455}} = 0.061$$

Starting this round, we introduced a second modelling step after the initial estimation of prevalence and incidence. The initial prevalence and incidence estimates are used as covariates for hepatitis B mortality due to acute hepatitis, cirrhosis, and liver cancer, as detailed in the causes of death paper. Following completion of CoDCorrect, we summed the cause-specific mortality rate (CSMR) due to all three and combined the aggregated data with the same dataset of HBsAg seroprevalence. This step ensured internal consistency between all hepatitis prevalence and acute hepatitis incidence estimates. We convert the incidence of chronic HBsAg carriage to incidence of acute hepatitis B through the same calculation described above.

We then split symptomatic cases into moderate (73%) and severe (27%) severities based on data from McMahon and colleagues [3].

Sequela	Description	Disability Weight
Moderate	Has a fever and aches, and feels weak, which causes some difficulty with daily activities.	0.051 (0.032–0.074)
Severe	Has a high fever and pain, and feels very weak, which causes great difficulty with daily activities.	0.133 (0.088–0.19)
Asymptomatic	Infection with no apparent illness.	NA

A limitation of this model is that it does not account for lifetime immunity after initial HBsAg infection, which may cause incidence estimates to be inflated. In future iterations of GBD, we plan to update the model so that individuals can only be considered infected once.

Changes from GBD 2016 to GBD 2017

We have introduced a second round of modelling incidence and prevalence that combines the seroprevalence data with cause-specific mortality rate data from the hepatitis mortality estimation process. A new covariate for hepatitis B childhood vaccination was also added to the DisMod-MR 2.1 model.

Acute hepatitis C

Case definition

We define acute hepatitis C as the period corresponding to initial infection with the hepatitis C virus, resulting in anti-HCV IgG seroconversion, regardless of symptoms. It includes all ICD-10 codes under the heading B17.1 (Acute hepatitis C).

Input data

Model inputs

To estimate morbidity for hepatitis C, we use anti-HCV seroprevalence data from population-based studies and surveys to estimate incidence and prevalence of hepatitis C infection. The last systematic review was performed as part of GBD 2013. Updates to systematic reviews are performed on an ongoing schedule across all GBD causes; an update for hepatitis C will be performed in the next one to two iterations. We present a summary of the data sources in Table 4 below.

Table 4: Data Inputs for acute hepatitis C morbidity modelling by parameter.

	Prevalence
Site-years (total)	333

**Number of countries/subnational locations with data**	75
**Number of GBD regions with data (out of 21 regions)**	20
**Number of GBD super-regions with data (out of 7 super-regions)**	7

We also use cause-specific mortality rate (CSMR) data, which is obtained through the hepatitis mortality modelling process. The generation and incorporation of the CSMR data is described further in the modelling strategy section below.

Modelling strategy

We model the incidence and prevalence of hepatitis C infection using a full DisMod-MR 2.1 model of anti-HCV seroprevalence data. Starting this round, we introduce a new modelling step after the initial estimation of prevalence and incidence. The initial prevalence and incidence estimates are used to estimate hepatitis C mortality, as detailed in the causes of death paper. We then run a second DisMod-MR 2.1 model using anti-HCV seroprevalence data, with the addition of cause-specific mortality rate data derived from the mortality estimates for acute hepatitis C, liver cancer due to hepatitis C, and cirrhosis due to hepatitis C.

We estimate chronic infections from total incident infections by multiplying by the probability an incident infection will be chronic. We estimate this probability using cases reported in Guadagnino and colleagues 1997, sampling from a beta distribution (table 6) [4].

Table 5. Percentiles of the probability distribution of chronic hepatitis C

0 percentile	25 percentile	50 percentile	75 percentile	100 percentile
0.65	0.73	0.75	0.76	0.83

Of the remaining acute infections, we divide incident infections into asymptomatic (75%), moderate (24%), and severe (1%) states based on expert opinion.

Sequela	Description	Disability Weight
Moderate	Has a fever and aches, and feels weak, which causes some difficulty with daily activities.	0.051 (0.032–0.074)
Severe	Has a high fever and pain, and feels very weak, which causes great difficulty with daily activities.	0.133 (0.088–0.19)
Asymptomatic	Infection with no apparent illness.	NA

Changes from GBD 2016 to GBD 2017

We have introduced a second round of modelling incidence and prevalence that combines the seroprevalence data with cause-specific mortality rate data from the hepatitis mortality estimation process.

Acute hepatitis E

Flowchart

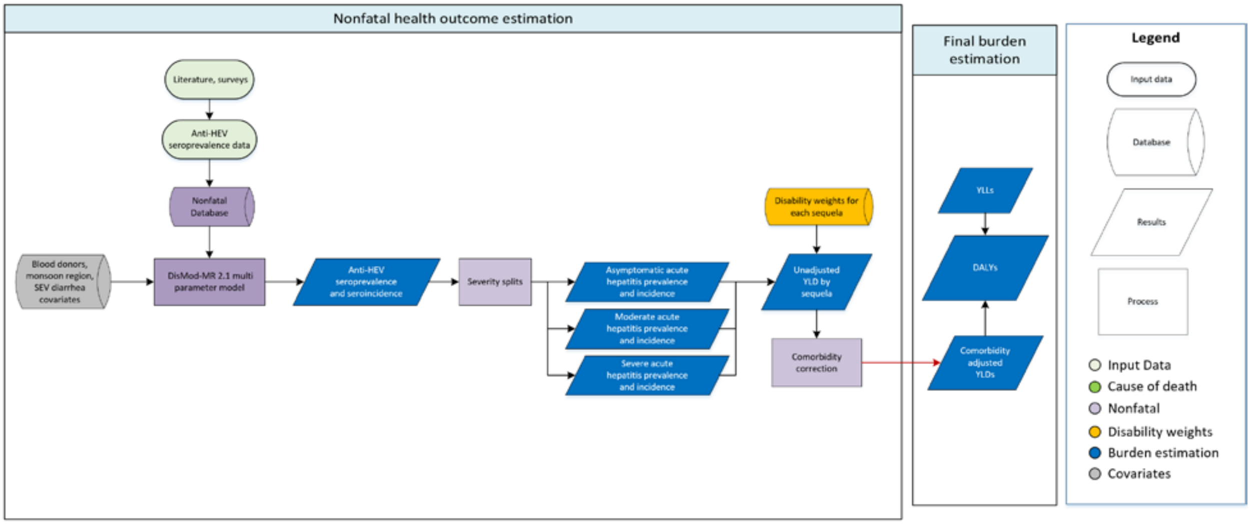

Case definition

We define acute hepatitis E as an infection with the hepatitis E virus resulting in anti-HEV IgG seroconversion, regardless of symptoms. It includes all ICD-10 codes under the heading B17.2 (Acute hepatitis E).

Input data

Model inputs

We use anti-HEV seroprevalence data from population-based studies and surveys to estimate incidence of infection. The last systematic review was performed as part of GBD 2013. Updates to systematic reviews are performed on an ongoing schedule across all GBD causes; an update for hepatitis E will be performed in the next one to two iterations. We present a summary of the data sources in Table 5 below.

Table 5: Data Inputs for acute hepatitis E morbidity modelling by parameter.

	Prevalence
Site-years (total)	91
Number of countries/subnational locations with data	43
Number of GBD regions with data (out of 21 regions)	19
Number of GBD super-regions with data (out of 7 super-regions)	7

Modelling Strategy

We model the incidence of hepatitis E using a full DisMod-MR 2.1 model of anti-HEV seroprevalence, assuming no remission. Based on information published by Rein and colleagues [5], we assume that the probability of symptomatic infection increases with age from ~1% in the first year of life to ~60% in adulthood.

The table below illustrates the sequelae associated with acute hepatitis E, along with their descriptions and disability weights.

Sequela	Description	Disability Weight
Moderate	Has a fever and aches, and feels weak, which causes some difficulty with daily activities.	0.051 (0.032–0.074)
Severe	Has a high fever and pain, and feels very weak, which causes great difficulty with daily activities.	0.133 (0.088–0.19)
Asymptomatic	Infection with no apparent illness.	NA

Changes from GBD 2016 to GBD 2017

We have made no substantive changes in the modelling strategy from GBD 2016.

References

1. Armstrong GL, Bell BP. Hepatitis A Virus Infections in the United States: Model-Based Estimates and Implications for Childhood Immunization. Pediatrics. 2002 May 1;109(5):839–45.
2. Edmunds WJ, Medley GF, Nokes DJ, Hall AJ, Whittle HC. The influence of age on the development of the hepatitis B carrier state. Proc Biol Sci. 1993 Aug 23;253(1337):197–201.
3. McMahon BJ, Alward WL, Hall DB, Heyward WL, Bender TR, Francis DP, et al. Acute hepatitis B virus infection: relation of age to the clinical expression of disease and subsequent development of the carrier state. J Infect Dis. 1985 Apr;151(4):599–603).
4. Guadagnino, Vincenzo, et al. "Prevalence, risk factors, and genotype distribution of hepatitis C virus infection in the general population: a community-based survey in southern Italy." Hepatology 26.4 (1997): 1006-1011.
5. Rein DB, Stevens GA, Theaker J, Wittenborn JS, Wiersma ST. The global burden of hepatitis E virus genotypes 1 and 2 in 2005. Hepatology. 2012 Apr 1;55(4):988–97.

Neglected Tropical Diseases (NTDs) SDG Capstone Appendix

African trypanosomiasis, Chagas disease, cystic echinococcosis, cysticerosis, dengue, food-borne trematodiases, intestinal nematode infections, leishmaniosis, leprosy, lymphatic filariasis, onchocerciasis, rabies, schistosomiasis, and trachoma

Indicator definition

This modeling strategy encompassed the indicator associated with neglected tropical disease prevalence (3.3.5).

Indicator 3.3.5

As a component of SDG Goal 3. Ensure healthy lives and promote well-being for all at all ages, SDG Target 3.3, by 2030, end the epidemics of AIDS, tuberculosis, malaria and neglected tropical diseases and combat hepatitis, water-borne diseases and other communicable diseases, is measured using SDG Indicator 3.3.3, prevalence of neglected tropical diseases.

3.3.5 Human African Trypanosomiasis (HAT) SDG Capstone Appendix

Flowchart

African trypanosomiasis

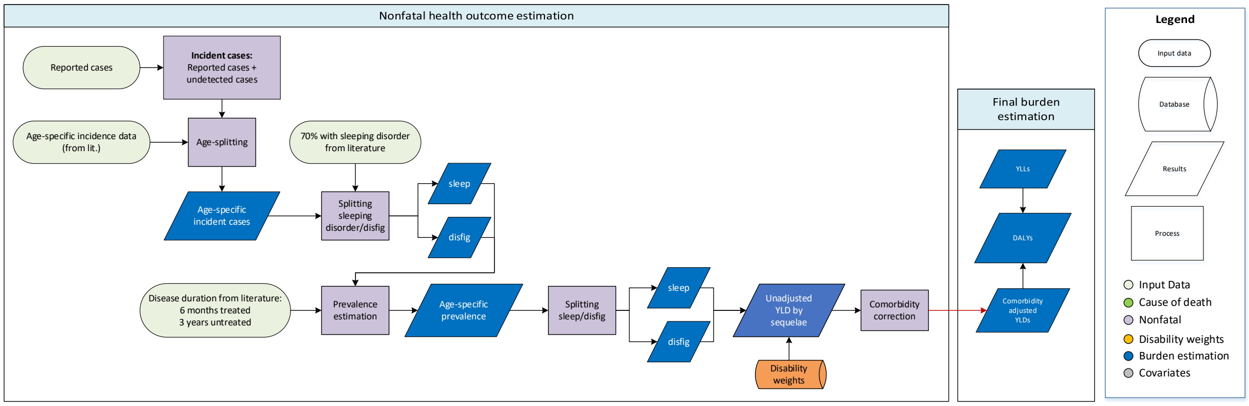

Input Data & Methodological Summary

Case Definition

Human African trypanosomiasis (HAT), also known as sleeping sickness, is a vector-borne disease which is transmitted by the bite of the tsetse fly. It is caused by the parasite *Trypanosoma brucei* with two subspecies, namely *T.b. rhodesience* (makes up less than 5% of total HAT cases) and *T.b. gambiense*. Cases are diagnosed through laboratory methods which rest on finding the parasite in body fluid or tissue by microscopy. In highly endemic or epidemic areas where the likelihood of false positives in serological tests is deemed lower, a seropositive individual is considered affected even in the absence of parasitological confirmation. The ICD-10 codes for HAT are B56.0, B56.1 and B56.9.

Input data

Model inputs

Data sources for GBD 2017:

1) Annual case totals 1980–2016: National-level annual case totals from 1990–2016 were obtained from the publicly available data via WHO, available here: http://apps.who.int/gho/data/node.main.A1635?lang=en

Table of case data counts

	Incidence
**Site-years (total)**	611
**Number of countries with data**	25
**Number of GBD regions with data (out of 21 regions)**	4
**Number of GBD super-regions with data (out of 7 super-regions)**	1

Subnational data:

Kenya: Kenyan subnational estimates are attributed to Busia County. Identification of subnational locations for Kenyan case data were obtained via studies published in the peer-reviewed literature[1] and review of maps published from via the WHO HAT Atlas[2]: http://www.who.int/entity/trypanosomiasis_african/country/Kenya_whole_0014.jpg?ua=1.

2) Age/sex data: Data on the age and sex distribution of HAT cases were extracted from the peer-reviewed literature via a systematic review of sources identified in PubMed using the following search string:

((African trypanosomiasis[Title/Abstract] AND (incidence[Title/Abstract] OR burden[Title/Abstract] OR prevalence[Title/Abstract] OR community[Title/Abstract])) AND (“1990”[Date – Publication] : “2017”[Date – Publication]))

This yielded 219 studies, of which only three met the inclusion criteria and were extracted[3-5]. The inclusion criteria were:

1. Studies representative of the national population
2. Population-based studies
3. Studies with primary data on incidence
4. Studies of human African trypanosomiasis (excluded studies on animal African trypanosomiasis)

Table data counts for age/sex-specific prevalence and incidence

	Incidence	Prevalence
**Site-years (total)**	2	1
**Number of countries with data**	2	1
**Number of GBD regions with data (out of 21 regions)**	2	1
**Number of GBD super-regions with data (out of 7 super-regions)**	1	1

3) Population at risk estimates 1980–2015: population at risk estimates from GBD 2010 ArcGIS analysis using geocoded case notifications for 2000 to 2009[2] and population Count Grid estimates from Gridded Population of the World.

Table of data counts for population at risk estimates

	Other
**Site-years (total)**	751
**Number of countries with data**	24
**Number of GBD regions with data (out of 21 regions)**	4
**Number of GBD super-regions with data (out of 7 super-regions)**	1

4) Screening coverage: Data on active versus passive screening coverage were obtained from a Weekly Epidemiological Report[6] identifying the population screened from 1997 to 2004 at the national level.

Table of data counts for screening coverage data

	Other
**Site-years (total)**	109
**Number of countries with data**	29
**Number of GBD regions with data (out of 21 regions)**	4
**Number of GBD super-regions with data (out of 7 super-regions)**	1

5) Geographic restrictions: Data file of all GBD locations, defining location as either endemic or non-endemic for HAT. Estimates are not produced for non-endemic countries, nor are they generated for countries with a history of HAT transmission but no data reported by WHO from 1990 to 2016.

Modelling strategy

Geographic restrictions

For countries historically considered endemic for HAT, but which have no reported case data or estimate of the population at risk, estimates are not produced. These countries include Botswana, Ethiopia, Guinea-Bissau, and Rwanda.

Among countries where population at-risk data are available, if no cases were reported to WHO, we assume the incidence of HAT is zero for those years and generate model estimates accordingly.

Modelling steps

Non-fatal estimates for HAT were generated as follows:

1. The incidence of reported HAT cases among the population at-risk was calculated as the total number of reported cases divided by the population at-risk estimates generated by the GBD working group for the period 1980–2015. Population at-risk estimates for 2016–2017 were generated by assuming an annual 2% rate of population growth.

2. To estimate the number of cases that were likely undetected by country and year, a multi-level mixed-effects linear regression of log-transformed incidence rate (ratio of reported HAT cases to population at risk) on log-transformed screening coverage (ratio of number screened for HAT to population at risk), with country random effects, was performed. Gaps were then filled using interpolation between years and extrapolation from 2016 to 2017 for reported cases. This model generates a beta-coefficient which is used to estimate the case detection rate (see step 4).

   For country-years in which no screening coverage data were reported:
   - Among countries with data reported, 1997–2004, the proportion of the at-risk population screened from 1997 was used retrospectively for the period 1980–1996 and the screening coverage from 2004 was carried forward from 2005–2017.
   - For countries with no screening data reported, the mean screening coverage for the region was used to impute a value over time.

3. Assuming the same proportion in treated (reported) and untreated (undetected) cases, the incidence estimates were then split into the two sequelae, skin disfigurement and sleeping disorder. This was done by generating 1,000 draws of the splitting proportion for the sequelae (70%–74% with sleeping disorder) based on a study that reported presence of symptoms at admission of patients in treatment centers[7]. Draws were generated from a beta distribution with alpha parameter = 1884 and beta parameter = 649.

4. To compute prevalence of HAT, 1,000 draws of total duration of symptoms in untreated cases were generated from a normal distribution with mean = [ln(3) – 0.5 * sigma^2], and standard deviation = sigma, where sigma = [ln(4.39)-ln(1.92))/(invnormal(0.975)*2)]: these parameters were based on a study of *T.b. gambiense*[7] which estimated an average duration of three years to untreated cases. An estimated duration of six months was applied to cases that received treatment, based on findings from a paper about *T.b. rhodesiense* in Uganda[8].

5. Prevalence was then estimated from the incident cases before applying age pattern. Prevalence of treated and untreated cases were summed up, assuming that untreated cases have been prevalent up to their death for a certain duration[9]. For untreated cases, it was assumed that half the duration is spent with sleeping disorder (severe motor and cognitive impairment) and disfigurement[7]. Treated (ie, reported) cases are assumed to have been prevalent for 0.5 years, and for the fraction of treated cases that present with sleeping disorder, it was assumed that this is present for half the total duration and that the rest of the duration is spent suffering from disfiguring skin disease. Among reported cases assumed to be detected prior to stage 2 infection, we do not attribute any of the duration of morbidity to sleeping disorder.

6. Finally, an age-pattern was applied to the prevalence estimates using the incidence studies from Sudan[5], DRC[3], and Uganda[4]. The age-pattern in GBD 2017 employed a cubic spline to account for the higher risk of infection among working-age adults.

Severity splits/sequelae

The basis of the GBD disability weight (DW) survey assessments are lay descriptions of sequelae highlighting major functional consequences and symptoms. The lay descriptions and disability weights for HAT sequelae due to HAT are shown below.

Sequela	Lay description	DW (95% CI)
Skin disfigurement, level 1	has a slight, visible physical deformity that is sometimes sore or itchy. Others notice the deformity, which causes some worry and discomfort.	0.027 (0.015–0.042)
Motor plus cognitive impairments, severe	cannot move around without help, and cannot lift or hold objects, get dressed or sit upright. The person also has very low intelligence, speaks few words, and needs constant supervision and help with all daily activities	0.542 (0.37–0.702)

References

1. Rutto JJ, Osano O, Thuranira EG, Kurgat RK, Odenyo VA. Socio-economic and cultural determinants of human african trypanosomiasis at the Kenya - Uganda transboundary. *PLoS Negl Trop Dis* 2013; **7**(4): e2186.

2. Simarro PP, Cecchi G, Paone M, et al. The Atlas of human African trypanosomiasis: a contribution to global mapping of neglected tropical diseases. *Int J Health Geogr* 2010; **9**: 57.

3. Lutumba P, Makieya E, Shaw A, Meheus F, Boelaert M. Human African trypanosomiasis in a rural community, Democratic Republic of Congo. *Emerg Infect Dis* 2007; **13**(2): 248-54.

4. Fevre EM, Odiit M, Coleman PG, Woolhouse ME, Welburn SC. Estimating the burden of rhodesiense sleeping sickness during an outbreak in Serere, eastern Uganda. *BMC Public Health* 2008; **8**: 96.

5. Moore A, Richer M, Enrile M, Losio E, Roberts J, Levy D. Resurgence of sleeping sickness in Tambura County, Sudan. *Am J Trop Med Hyg* 1999; **61**(2): 315-8.

6. World Health Organization. Human African trypanosomiasis (sleeping sickness): epidemiological update. *Weekly epidemiological record* 2006; **February 24**(8): 69-80.

7. Blum J, Schmid C, Burri C. Clinical aspects of 2541 patients with second stage human African trypanosomiasis. *Acta Trop* 2006; **97**(1): 55-64.

8. Odiit M, Kansiime F, Enyaru JC. Duration of symptoms and case fatality of sleeping sickness caused by Trypanosoma brucei rhodesiense in Tororo, Uganda. *East Afr Med J* 1997; **74**(12): 792-5.

9. Checchi F, Filipe JA, Haydon DT, Chandramohan D, Chappuis F. Estimates of the duration of the early and late stage of gambiense sleeping sickness. *BMC Infect Dis* 2008; **8**: 16.

3.3.5 Chagas disease SDG Capstone Appendix

Flowchart

Chagas disease

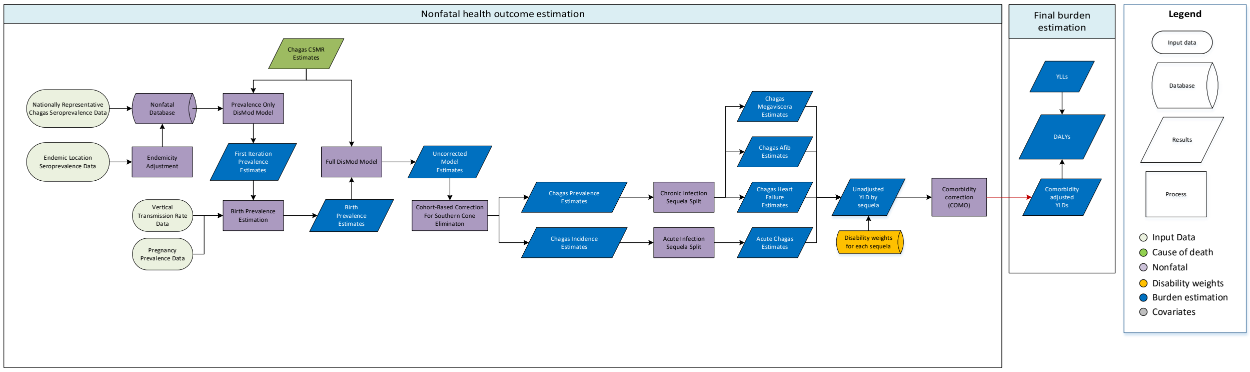

Case definition

Chagas disease is defined by infection with the protozoa *Trypanosoma cruzi,* which is transmitted by *Triatominae* insect vectors (most common), blood transfusion, organ transplant, and congenital transmission. It includes an acute phase corresponding with the time of infection, and is typically asymptomatic. Chronic infection may be latent (ie, asymptomatic), or result in cardiovascular or digestive sequelae. It includes all ICD-10 codes under the heading B57 (Chagas disease), with codes B57.0-B75.1 corresponding to the acute phase, B57.2 corresponding to chronic cardiovascular sequelae, and B57.3 corresponding to chronic digestive sequelae.

Input data

Model inputs

For GBD 2017 estimation, we used seroprevalence data to model Chagas. The table below illustrates the geographic distribution of model input data for the estimation process.

Table 1. Data Coverage

	Prevalence
Site-years (total)	78
Number of countries with data	13
Number of GBD regions with data (out of 21 regions)	4
Number of GBD super-regions with data (out of 7 super-regions)	2

We also use CSMR estimates in the modelling process, which will be addressed in further detail below.

Modelling strategy

We modelled Chagas disease using a full DisMod-MR 2.1 Bayesian meta-regression model incorporating seroprevalence data, as above, and CSMR estimates. We assume no remission. We eliminate all new infections, except those via vertical transmission, in Chile and Uruguay for years after the interruption of vector-based transmission (Abad-Franch F, Diotaiuti L, Gurgel-Gonçalves R, Gürtler RE. Certifying the interruption of Chagas disease transmission by native vectors: cui bono? Mem Inst Oswaldo Cruz 2013;108:251–4.; Coura JR. Chagas disease: control, elimination and eradication. Is it possible? Mem Inst Oswaldo Cruz 2013;108:962–7.). For non-endemic countries, we estimate the prevalence of imported chronic infections based on migration. For each non-endemic country, we estimate the total number of people infected with Chagas as the sum of the number of immigrants from each endemic country multiplied by the corresponding prevalence of Chagas in that endemic country.

We estimate five sequelae: symptomatic acute infection from incidence; and megaviscera, heart failure, atrial fibrillation, and chronic asymptomatic infection from prevalence. We assume that 5% of acute infections will be symptomatic (Teixeira AR, Nitz N, Guimaro MC, Gomes C, Santos-Buch CA. Chagas disease. Postgrad Med J 2006;82:788–98.). The proportion of chronic infections resulting in a given sequela varies by sex and age: the prevalence of megaviscera among those infected with Chagas ranges from 0% in children to nearly 10% among older adults (Coura JR, Naranjo MA, Willcox HP. Chagas' disease in the Brazilian Amazon: II. A serological survey. Rev Inst Med Trop São Paulo 1995; 37:103–7.); the prevalence of atrial fibrillation attributable to Chagas ranges from 0% among children to approximately 10% in men over 80 years of age (Ribeiro AL, Marcolino MS, Prineas RJ, Lima-Costa MF. Electrocardiographic abnormalities in elderly Chagas disease patients: 10-year follow-up of the Bambuí Cohort Study of Aging. J Am Heart Assoc 2014;3:e000632.); and the prevalence of heart failure attributable to Chagas among those who are infected ranges from 0% among young children, to a maximum of 23% among men over 80 years of age (Sabino EC, Ribeiro AL, Salemi VM, et al., for the National Heart, Lung, and Blood Institute Retrovirus Epidemiology Donor Study-II (REDS-II), International Component. Ten-year incidence of Chagas cardiomyopathy among asymptomatic Trypanosoma cruzi-seropositive former blood donors. Circulation 2013;127:1105–15.).

Severity splits and disability weights

The table below illustrates the sequelae, lay descriptions, and DWs for Chagas disease.

Table 2. Sequelae, lay description and DWs

Sequelae	Description	Disability Weight
Atrial fibrillation and flutter due to Chagas disease	Has periods of rapid and irregular heartbeats and occasional fainting.	0.224 (0.151–0.312)

Mild heart failure due to Chagas disease	Is short of breath and easily tires with moderate physical activity, such as walking uphill or more than a quarter-mile on level ground. The person feels comfortable at rest or during activities requiring less effort.	0.041 (0.026–0.062)
Moderate heart failure due to Chagas disease	Is short of breath and easily tires with minimal physical activity, such as walking only a short distance. The person feels comfortable at rest but avoids moderate activity.	0.072 (0.047–0.103)
Severe heart failure due to Chagas disease	Is short of breath and feels tired when at rest. The person avoids any physical activity, for fear of worsening the breathing problems.	0.179 (0.122–0.251)
Mild chronic digestive disease due to Chagas disease	Has some pain in the belly that causes nausea but does not interfere with daily activities.	0.011 (0.005–0.021)
Moderate chronic digestive disease due to Chagas disease	Has pain in the belly and feels nauseated. The person has difficulties with daily activities.	0.114 (0.078–0.159)
Acute Chagas disease	Has a fever and aches, and feels weak, which causes some difficulty with daily activities.	0.051 (0.032–0.074)
Asymptomatic Chagas disease	Latent Chagas infection (ie, chronic infection with no apparent symptoms)	NA

Changes from GBD 2016 to GBD 2017

We have made no substantive changes in the modelling strategy for endemic countries from GBD 2016 to GBD 2017.

3.3.5 Cystic Echinococcosis SDG Capstone Appendix

Flowchart

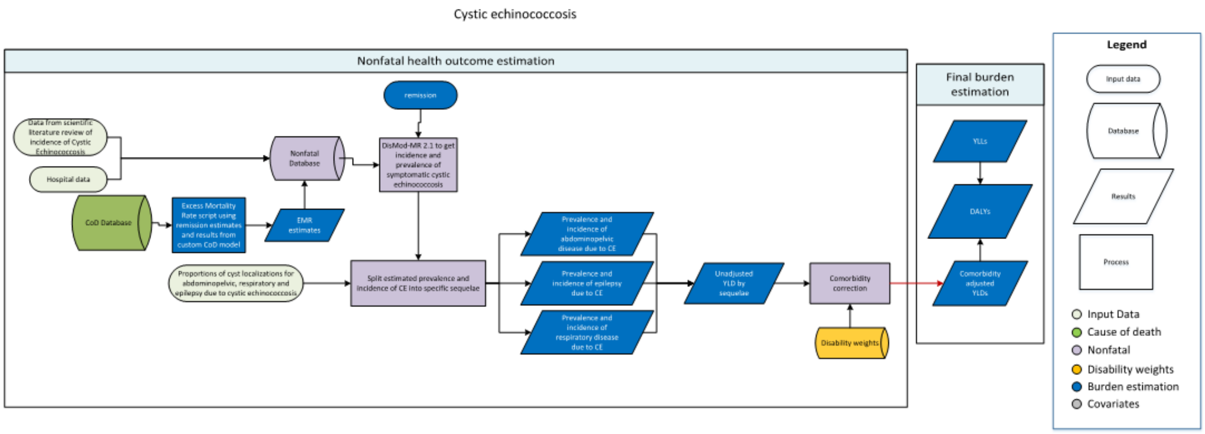

Input Data & Methodological Summary

Case definition

Cystic echinococcosis is a parasitic disease caused by infection with the *Echinococcus granulosis* tapeworm. It is a natural parasite of canines, with sheep being the most common intermediate host in the two-stage lifecycle, but can be spread to humans through ingestion of soil, water, or food contaminated with the fecal matter of an infected dog containing infective eggs. Diagnosis is made by clinical findings, imaging, serology, and tissue pathology. The ICD-9 and ICD-10 codes for echinococcosis are 122.0-122.9 and B67-B67.9, respectively.

Input data

Systematic Literature Review

The non-fatal estimation for cystic echinococcosis (CE) focused on estimating incidence and prevalence of CE and its sequelae. A systematic review of literature was conducted in PubMed for GBD 2015 using the following search string:

> ("echinococcosis"[Title/Abstract] OR "hydatid disease"[Title/Abstract] OR "hydatidosis"[Title/Abstract] OR "echinococcal disease"[Title/Abstract] OR "Echinococcus granulosus infection"[Title/Abstract]) AND ("1990"[Date – Publication] : "2015"[Date – Publication]) AND (epidemiology OR incidence OR prevalence).

This yielded 1,619 studies of which 279 were included during the title/abstract screening. Following the full-text screening, 77 studies (32 incidence, 43 prevalence, and 2 both) were included and extracted – studies were excluded because of one or more of the following reasons:

1. study not population-based
2. study does not have primary data on prevalence and/or incidence
3. study not in humans
4. study on sub-populations
5. review study

Since we were interested in modelling symptomatic CE cases, we only used data on incidence of patients diagnosed by imaging techniques (mainly ultrasonography). Therefore, we excluded prevalence data, which were mostly from serological studies. Data from these extracted studies were combined with data from studies extracted during GBD 2013.

Hospital data

Hospital data prepared by the GBD team were used as additional input into our models. These data were adjusted to account for multiple hospital episodes of a single case and non-primary diagnoses. The table below displays the site-years by geography for both the systematic review and hospital data combined.

Table 1a. Site-years from systematic review and hospital data for GBD 2017

	Incidence
Site-years (total)	1,338
Number of countries with data	49
Number of GBD regions with data (out of 21 regions)	17
Number of GBD super-regions with data (out of 7 super-regions)	7

Geographic restrictions

We conducted a literature review to determine the geographic extent of the disease and classify locations based on whether the disease is absent or present in each year. Locations that were geographically restricted in any given year did not have estimates made. Of note, we did not attempt a complete systematic review, since a single high-quality source could offer sufficient evidence of presence. Evidence of absence or presence was not available for every location for each year, and so assumptions were made for missing years by taking into consideration the epidemiological characteristics of the disease.

If evidence indicated disease presence for two non-consecutive years, we assumed presence for all years between the two. If evidence indicated disease absence for two non-consecutive years, we assumed absence for all years between the two. If evidence indicated a change in status (ie, from absent to present, or present to absent) between two non-consecutive years, then we conducted targeted searches to ascertain the relevant year of introduction or elimination for that location. In the cases where presence or absence information was missing for the start or end years of our study interval (1990–2017) without evidence of any introduction or elimination events within the interval, we applied the status of the first and last presence/absence observations respectively to all years between the interval bound and the observation year. For cystic echinococcosis, we performed targeted searches to classify location-years in PubMed and Google Scholar. Geographic restrictions were populated by reviewing sources referenced by Deplazes and colleagues along with ad hoc searches in PubMed for evidence of active transmission of cystic echinococcosis in respective countries [1].

Sequelae due to cystic echinococcosis

The table below shows the sequelae due to echinococcosis and their associated disability weights.

Table 2. Sequelae, lay descriptions, and disability weights (DWs)

Sequela	Lay description	DW (95% CI)
Chronic respiratory disease	"has cough and shortness of breath after heavy physical activity, but is able to walk long distances and climb stairs."	0.019 (0.011–0.033)
Abdominal problems	"has pain in the belly and feels nauseated. The person has difficulties with daily activities."	0.114 (0.078–0.159)
Epilepsy	(Combined DW)	NA

Modelling strategy

The morbidity model for cystic echinococcosis involved a multi-step process. First, DisMod-MR was used to model incidence and prevalence of symptomatic cystic echinococcosis using incidence data from systematic reviews in GBD 2013 and 2015 and hospital data, excess mortality rate estimates, and an assumed remission of 0.15–0.25 per case per year (duration 2–6.7 years, average 5 years). Estimates of excess mortality rate were obtained by pulling death estimates from our CoD model. The following steps were followed to estimate excess mortality rate: 1) create custom age groups for CE deaths with uncertainty; 2) calculate CSMR as CSMR=deaths/population at the 1,000 draw level – calculate mean CSMR, uncertainty interval, and standard error; and 3) calculate EMR as EMR=CSMR/(prevalence), where prevalence = (incidence*5) – standard error of EMR was calculated taking into consideration the standard errors of both prevalence and CSMR. Geographic restrictions were applied to set incidence and prevalence to zero in location-years where the disease was not endemic. These computations provided 655 site-years of EMR data.

Table 3. DisMod model covariates

Covariate	Type	Parameter	Exponentiated beta
Sex	Study-level	Incidence	0.66 (0.63–0.70)
Urbanicity	Country-level	Incidence	1.00 (0.98–1.00)
Echinococcosis endemicity	Country-level	Incidence	6.03 (5.75–6.37)
Proportion of population involved in agricultural activities	Country-level	Incidence	1.00 (1.00–1.00)
Sex	Study-level	Excess mortality rate	1.63 (1.56–1.70)

After producing all-case prevalence draws, 1,000 draws of proportions for abdominal, respiratory, and epileptic symptoms among echinococcosis cases adding up to 1 were generated. Uncertainty in the splitting proportions was captured by drawing them from a Dirichlet distribution, informed by published data on cysts localization [2]. On average, the proportions of abdominal, respiratory, and epileptic symptoms due to echinococcosis were 0.5, 0.47, and 0.03, respectively. These proportions were used to split the prevalence and incidence from DisMod into the three sequelae.

Model evaluation was done by separately assessing the fit of the DisMod MR model and checking the estimates produced after estimating incidence and prevalence of sequelae due to cystic echinococcosis. Plots of time trends of incidence and prevalence across locations and age were used to evaluate the

results. In addition, maps of the global distribution of incidence and prevalence were assessed across time.

References

1. Deplazes P, Rinaldi L, Alvarez Rojas CA, Torgerson PR, Harandi MF, Romig T, Antolova D, Schrurer JM, Lahmar S, Cringoli G, Magambo J, Thompson RC, Jenkins EJ. Global Distribution of Alveolar and Cystic Echinococcosis. Advanced Parasitology. 2017. 95: 315-493.
2. Raether W, Hänel H. Epidemiology, clinical manifestations and diagnosis of zoonotic cestode infections: an update. Parasitology Research. 2003. 91:412-438.

3.3.5 Cysticercosis SDG Capstone Appendix

Flowchart

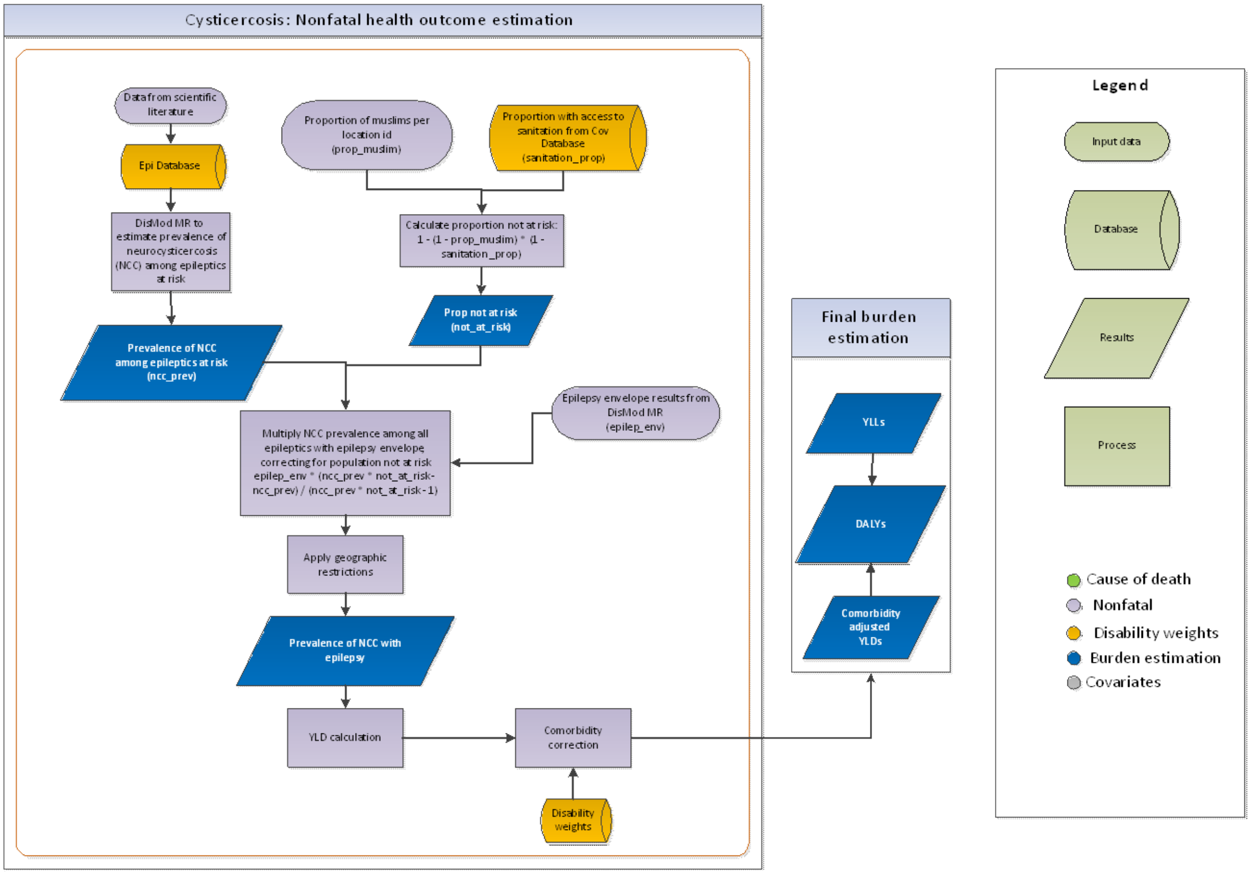

Input Data & Methodological Summary

Case Definition

Cysticercosis, or neurocysticercosis (NCC), is a parasitic disease caused by the pig tapeworm *Taenia solium*. It is transmitted via ingestion of eggs or gravid proglottids shed by a human or non-human host with an intestinal infection of the same helminth known as Taeniasis. In rare cases, auto-infection is also possible among people with intestinal infections. Diagnosis is made by magnetic resonance imaging (MRI) or computerized tomography (CT) brain scans to identify cysts. The ICD-10 codes for cysticercosis are B69-B69.9.

Input data

Systematic literature review

The nonfatal estimation for cysticercosis focused on estimating prevalence of NCC among epileptics at risk as well as the prevalence of NCC with epilepsy. A systematic review of literature was conducted in PubMed for GBD 2015 using the following search string:

("cysticercosis"[Title/Abstract] OR "neurocysticercosis"[Title/Abstract] OR "cysticerciasis"[Title/Abstract] OR "Taenia solium"[Title/Abstract]) AND (“1990”[Date – Publication] : “2015”[Date – Publication]) AND (epidemiology OR prevalence)).

This yielded 1,038 studies, of which 166 were included during the title/abstract screening. Following the full-text screening, 17 studies were included and extracted – studies were excluded because of one or more of the following reasons:

1. study not in epileptics
2. study not population-based
3. study does not have primary data on prevalence of NCC among epileptics at risk
4. study not in humans (some studies were on cysticercosis in pigs)
5. study on comorbidities with NCC (other than epilepsy)
6. study on sub-population, eg, patients with neurological disorders
7. review study

The table below displays the number of site-years by geography:

Table 1. Site-years for GBD 2017

	Prevalence
Site-years (total)	31
Number of countries with data	14
Number of GBD regions with data (out of 21 regions)	8
Number of GBD super-regions with data (out of 7 super-regions)	4

A study-level covariate was also created in GBD 2015 to indicate the type of diagnosis for each study, ie, definitive or probable. Of the 77 rows of country-year-age-sex data, there were 15 rows with definitive diagnosis and 62 rows with probable diagnosis.

Covariates

Data were ascertained from the PEW Research Center [1] on the proportion of the population that is Muslim and incorporated as a continuous covariate with a range between 0 and 1.

Epilepsy envelope

The modelling process incorporates 1,000 draws of epilepsy envelope prevalence from the GBD 2017 epilepsy DisMod-MR model – details on this modelling process can be found elsewhere.

Geographic restrictions

We conducted a literature review to determine the geographic extent of the disease and classify locations based on whether the disease is absent or present in each year. Locations that were geographically restricted in any given year did not have estimates made for them. Of note, we did not attempt a complete systematic review, since a single high-quality source could offer sufficient evidence of presence. Evidence of absence or presence was not available for every location for each year, and so assumptions

were made for missing years by taking into consideration the epidemiological characteristics of the disease.

If evidence indicated disease presence for two non-consecutive years, we assumed presence for all years between the two. If evidence indicated disease absence for two non-consecutive years, we assumed absence for all years between the two. If evidence indicated a change in status (ie, from absent to present, or present to absent) between two non-consecutive years, then we conducted targeted searches to ascertain the relevant year of introduction or elimination for that location. In the cases where presence or absence information was missing for the start or end years of our study interval (1990–2017) without evidence of any introduction or elimination events within the interval, we applied the status of the first and last presence/absence observations, respectively, to all years between the interval bound and the observation year. For cysticercosis, we performed targeted searches to classify location-years in PubMed and Google Scholar. In our searches, we compiled 21 peer-reviewed articles, meta-analyses, and WHO reports.

Modelling strategy

DisMod-MR was used to model the prevalence of NCC among epileptics at risk. In the model, pigs raised in extensive agricultural systems per capita, SDI, and religion (binary, >50% Muslim) were used as country-level covariates. In addition, the prevalence of "definitive diagnosis" was transformed to that of "probable and definitive diagnosis" so as to not underestimate overall prevalence.

Table 2. DisMod model covariates

Covariate	Type	Parameter	Exponentiated beta
Sex	Study-level	Prevalence	0.76 (0.31–1.73)
Definitive diagnosis	Study-level	Prevalence	0.56 (0.37–0.87)
Religion (binary, > 50% Muslim)	Country-level	Prevalence	0.48 (0.17–0.98)
Socio-demographic Index	Country-level	Prevalence	0.35 (0.14–0.95)
Pigs raised in extensive agricultural systems per capita	Country-level	Prevalence	2.31 (1.02–6.79)

After running DisMod, we adjusted the fraction of people with epilepsy attributable to cysticercosis in endemic countries for the population at risk based on the proportion of the population without access to sanitation and the proportion of the population that is Muslim. The following is the computation for estimating NCC prevalence among epileptics at risk:

$$Prevalence_{NCC\ prevalence} = Prevalence_{epilepsy} * \frac{NM - N}{NM - 1}$$

Where prevalence = prevalence of all-cause epilepsy in total population, N = proportion of NCC among epileptics at risk (non-Muslims without access to sanitation), and M = proportion of population not at risk of contracting NCC. It was assumed that the prevalence of epilepsy due to causes other than NCC is the same regardless of whether a population is at risk or not. It was also assumed that Muslims and non-

Muslims have equal access to sanitation. Geographic restrictions were applied to set prevalence to zero in non-endemic locations.

Model evaluation was done by separately assessing the fit of the DisMod-MR model and checking the estimates produced after estimating prevalence of NCC with epilepsy. Plots of time trends of prevalence across locations and age were used to evaluate the results. In addition, maps of the global distribution of prevalence of NCC among epileptics at risk and prevalence of NCC with epilepsy were also assessed across time.

Several changes were made compared to the GBD 2016 modelling strategy. First, we made slight changes to model parameters in DisMod-MR to improve model fit. Second, we incorporated two new covariates (ie, pigs raised in extensive agricultural systems per capita, SDI) to better inform the model. Lastly, we updated geographic restrictions and updated proportion of population with Muslim data by imputing subnational locations with national proportions due to a lack of data at the subnational level.

References:

1. "Table: Muslim Population by Country Pew Research Center, Washington, D.C." (July 7, 2017). http://www.pewforum.org/2011/01/27/table-muslim-population-by-country/

3.3.5 Dengue SDG Capstone Appendix

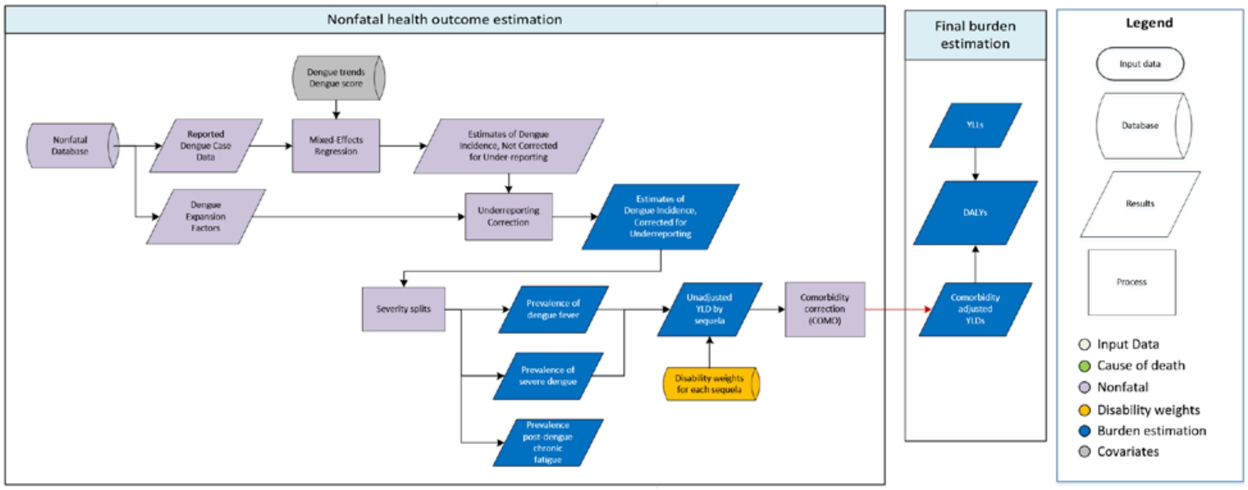

Case definition

Dengue is mosquito-borne viral infection that causes febrile illness and, in severe cases, jaundice, hemorrhage, and death. It includes all ICD-10 codes under the heading A90 (Dengue fever [classical dengue]) and A91 (Dengue hemorrhagic fever).

Input data

Model inputs

For GBD 2016, we modelled dengue incidence based on officially reported cases. The table below illustrates the geographic distribution of data points used in our analysis.

Table 1. Geographies

Level	Incidence
Data points	2,920
Studies	70
Locations	201
Regions	15

While no systematic update was conducted, we did incorporate new expansion factor data that were provided by collaborators and have updated to the latest available case reports for GBD 2017.

Modelling strategy

The methods used to model dengue incidence remain unchanged from GBD 2016, and are an improved variant of the methods used for GBD 2013 that were described by Stanaway et al. Briefly, we derive two dengue-specific covariates: first a variable to define the expected spatial distribution of the disease based

on principal components analysis of dengue CSMR estimates and dengue transmission probability and, second, a variable to define the country-specific trends, based on a mixed-effects model of reported cases. We then estimate a mixed-effects negative binomial model with number of reported cases as the dependent variable, fixed effects on the aforementioned spatial and temporal covariates, and random effects on location. These random effects are assumed to correspond to deviations in reporting completeness and, calibrating against published expansion factor data (ie, estimates of the degree of underreporting), they are inflated to adjust for underreporting estimates. The resulting incidence estimates are split into moderate (94.5%) and severe (5.5%) sequelae, based on the proportion of reported cases that were severe. We assume that 8.4% of symptomatic infections will produce post-acute chronic fatigue lasting an average of six months (Teixeira L de AS, Lopes JSM, Martins AG da C, Campos FAB, Miranzi S de SC, Nascentes GAN. Persistence of dengue symptoms in patients in Uberaba, Minas Gerais State, Brazil. *Cad Saúde Pública* 2010; **26**: 624–30.).

Severity splits and disability weights

Table 2. Sequelae, lay descriptions, and DWs

Sequela	Lay description	Disability Weight (DW)
Moderate	Has a fever and aches, and feels weak, which causes some difficulty with daily activities.	0.051 (0.032–0.074)
Severe	Has a high fever and pain, and feels very weak, which causes great difficulty with daily activities.	0.133 (0.088–0.19)
Asymptomatic	Infection with no apparent illness.	NA

Changes from GBD 2016 to GBD 2017

We have made no substantive changes in the modelling strategy from GBD 2016 to GBD 2017.

References

1. Stanaway JD, Shepard DS, Undurraga EA, Halasa YA, Coffeng LE, Brady OJ, et al. The global burden of dengue: an analysis from the Global Burden of Disease Study 2013. *The Lancet Infectious Diseases* [Internet]. 2016 Feb [cited 2016 May 23].

2. Bhatt S, Gething PW, Brady OJ, Messina JP, Farlow AW, Moyes CL, et al. The global distribution and burden of dengue. *Nature*. 2013 Apr 25;496(7446):504–7.

3.3.5 Foodborne Trematodiases SDG Capstone Appendix

Clonorchiasis

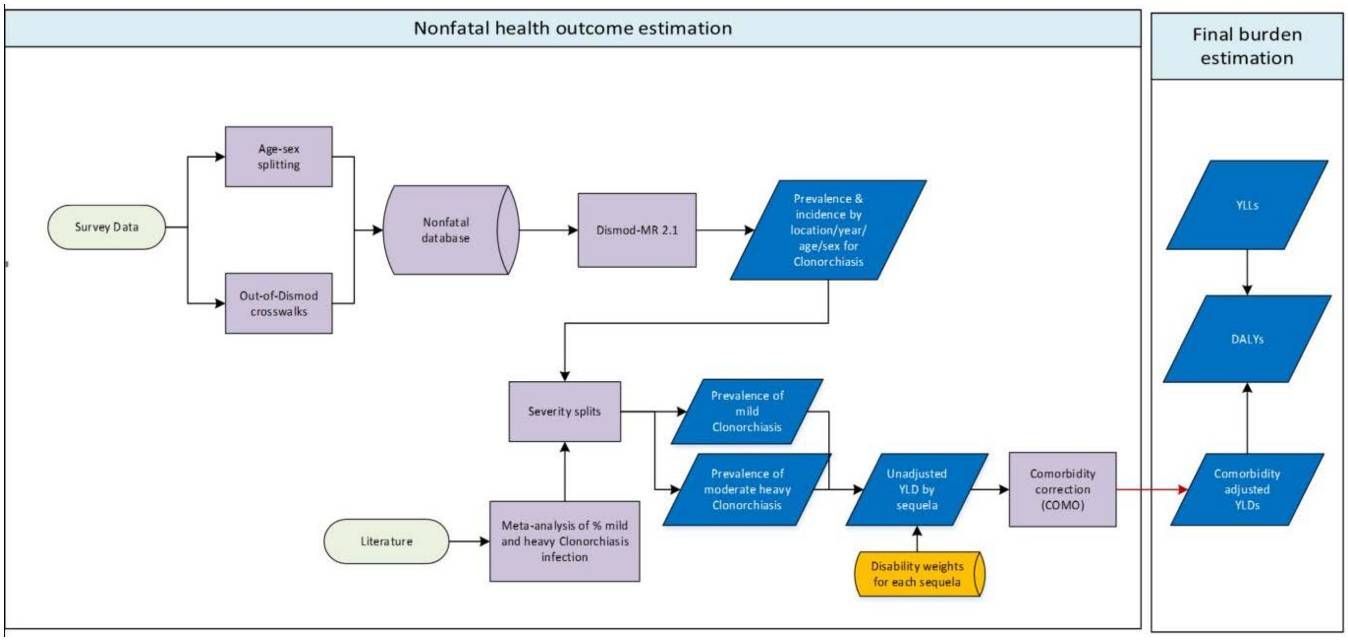

Fascioliasis

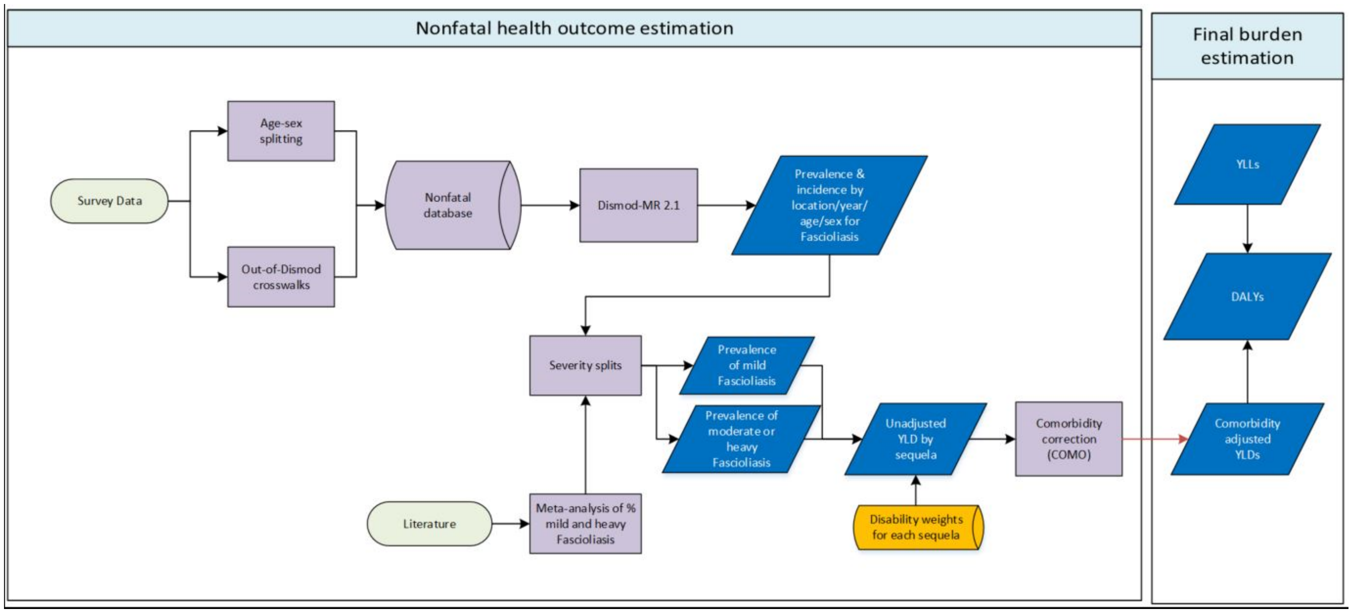

Intestinal Fluke

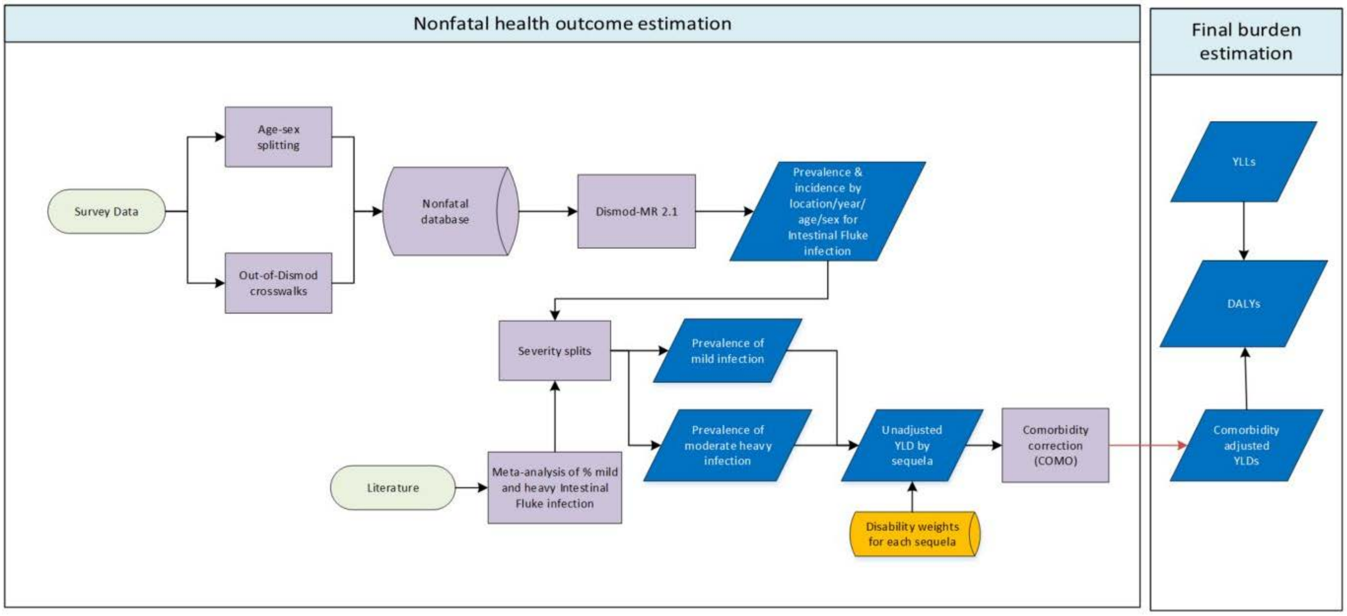

Opisthorchiasis

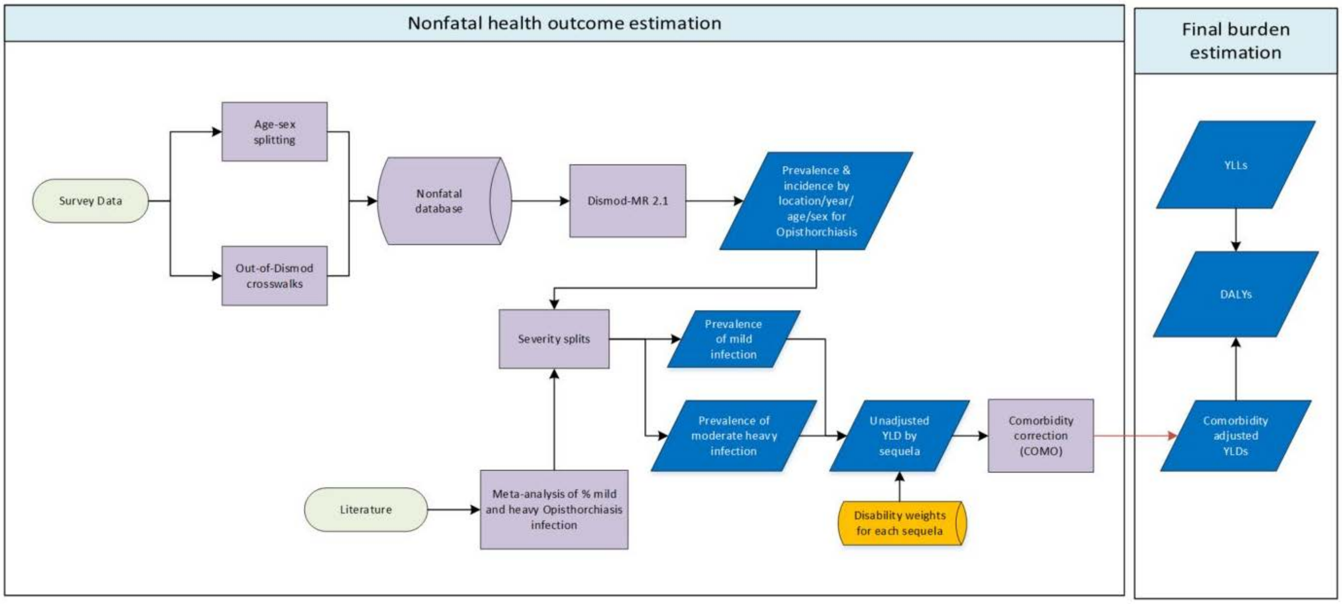

Paragonimiasis

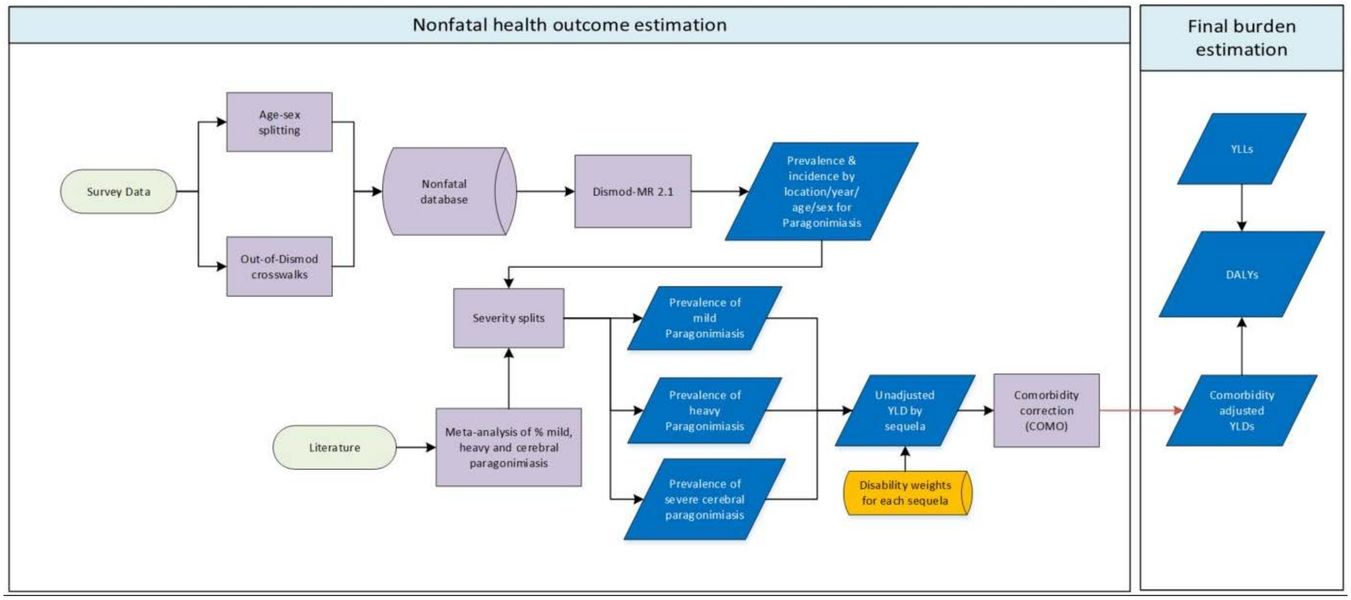

Input Data & Methodological Summary

Case definition

Human foodborne trematodiases (FBT) is defined as the infection with parasitic worms of the class trematoda, which are also known as flukes. Trematodes are transmitted via contaminated food, and infection is highly related to food habits. Definitive hosts, including humans, become infected when ingesting viable metacercariae by consuming contaminated aquatic products (eg, watercress). In the ICD-10, FBT are listed under code B66 [1].

FBT is subdivided into six types of FBT (see Table 1):

- Clonorchiasis
- Fascioliasis
- Intestinal fluke
- Opisthorchiasis
- Paragonimiasis (normal and cerebral infections)

Table 1. Subtypes of FBT

	Species of FBT	Also known as:	Carcinogen
1	Chlonorchiasis	(Chinese) Liver fluke	Associated with choliangiocarcinoma
2	Opisthorchiasis<br><br>(*O viverrini & O felineus*)	Liver fluke	Associated with choliangiocarcinoma<br><br>(*O viverrini*)
3	Fascioliasis	Liver fluke	No available evidence

4	Intenstinal fluke	Liver fluke	No available evidence
5	Paragonimiasis	Lung fluke	

Thresholds for heavy infection and duration by species of FBT

The majority of people infected with FBTs are asymptomatic. When symptoms do occur, they are often non-specific. Among the clinical symptomatic group, severity is associated with worm burden, typically measured by fecal egg counts, and the duration of infection. The thresholds for heavy infection and duration by species of FBT are shown in Table 2. The clinical presentation of FBT depends on the target organs (liver, lung, or intestines). Clonorchiasis and opisthorchiasis patients may suffer from loss of appetite, fullness, indigestion, diarrhoea, pain in the right upper quadrant, lassitude, weight loss, ascites, and oedema.[2, 3] Cholangitis, obstructive jaundice, intra-abdominal mass, cholecystitis, and gallbladder or intrahepatic stones may occur as complications.[3, 4]

Table 2. Thresholds for heavy infection and duration by species of FBT

	Species of FBT	Case thresholds for heavy infection	Duration
1	Chlonorchiasis	10,000 eggs per g of feces	lifelong
2	Opisthorchiasis	10,000 eggs per g of feces	lifelong
3	Fascioliasis	1,000 eggs per g of faces	lifelong
4	Intenstinal fluke	1,000 eggs per g of faces	lifelong
5	Paragonimiasis	100 eggs per 5 ml sputum	lifelong
6	Cerebral paragonimiasis	Any infection of the brain with flukes and/or eggs of *Paragonimus* spp.	lifelong

Input data

Model inputs

For GBD 2010, the data came from the expert group and is the result of their analysis. The expert group analysis used the results of a systematic literature review performed by Furst and colleagues as a starting point for the analysis.[5] Furst and colleagues searched PubMed, WHOLIS, FAOBIB, Embase, CAB Abstracts, Literatura Latino Americana e do Caribe em Ciências de Saùde (LILACS), ISI Web of Science, BIOSIS preview, Science Direct, African Journals OnLine (AJOL), and the System for Information on Grey Literature in Europe (SIGLE), period Jan 1, 1980, to Dec 31, 2008. The initial number of studies identified through the literature review was ~34,000 references. The literature review included extracted data from 181 studies. For GBD 2013 and GBD 2015, the search strategy was replicated to capture epidemiological studies published between 2008 and 2015.

Input data for the assessment of the total national number of infected people

Only studies that used countrywide surveys to estimate the national prevalence rates were included (or for China, province-wide surveys). Reason for choosing only national studies is that FBT shows a highly focal spatial distribution and local cross-sectional surveys would profoundly under- or overestimate true national prevalences. We decided not to model national and subnational together and get a coefficient on subnational, because there is not a one-fits-all relationship across the world. Infection is highly related to food habits, and there are highly varying differences between national and subnational prevalence rates. The final GBD 2016 dataset contained 29 prevalence studies from 17 countries. We used raw data from the selected studies as input for DisMod.

Prevalence of intestinal fluke infection

Intestinal fluke is different from the other types of FBT, because there are several pathogens that fall under intestinal fluke infection. It can be caused by pathogens, such as *Metagonimus* spp., *Echinostoma* spp., and *Neodiplostomatidae*.[6] When assessing the prevalence of intestinal fluke infection, we added the identified prevalence for each parasite species in order to obtain the overall prevalence of intestinal fluke infections. This approach may lead to a certain overestimation of the true prevalence, because people may be co-infected with more than one intestinal fluke species. There is no sufficient evidence about the proportion of co-infections, but the resulting overestimation of the true prevalence may be more than offset by the assumptions made in our previous modelling approach and the many challenges in generating the underlying epidemiological parameters (eg, diagnostic inaccuracy in the detection of infections with the more than 50 intestinal fluke species). Also of note: the transmission source of intestinal fluke infections are species-specific and therefore vary. For instance, *Fasciolopsis buski* is usually transmitted by eating raw water plants with the infective parasite stage attached to the water plants, whereas *Neodiplostomatidae* are transmitted by eating undercooked and infested frogs, snakes, and tadpoles. Because of these different transmission pathways, the rate of co-infection might in fact be smaller than expected.

Input data to differentiate between asymptomatic and heavy infections

We estimated the proportion of heavily infected among all infected in all available national and regional cross-sectional surveys. It is expected that heavy infection increases with age and there are data available on heavy infection by age group. We therefore decided to include age-dependent rates of heavy infection for clonorchiasis, opisthorchiasis, and intenstinal fluke infection. For (cerebral) paragonimiasis and fascioliasis there were not sufficient age-dependent data on high intensity FBT infection.

Total data inputs – Chlonorchiasis

	Prevalence
Site-years (total)	121
Number of countries with data	4
Number of GBD regions with data (out of 21 regions)	4
Number of GBD super-regions with data (out of 7 super-regions)	3

Total data inputs – Fascioliasis

	Prevalence
Site-years (total)	65
Number of countries with data	8
Number of GBD regions with data (out of 21 regions)	4
Number of GBD super-regions with data (out of 7 super-regions)	4

Total data inputs – Intestinal flukes

	Prevalence
Site-years (total)	101
Number of countries with data	7
Number of GBD regions with data (out of 21 regions)	5
Number of GBD super-regions with data (out of 7 super-regions)	4

Total data inputs – Opisthorchiasis

	Prevalence
Site-years (total)	10
Number of countries with data	5
Number of GBD regions with data (out of 21 regions)	3
Number of GBD super-regions with data (out of 7 super-regions)	2

Total data inputs – Paragonimiasis

	Prevalence
Site-years (total)	74
Number of countries with data	5
Number of GBD regions with data (out of 21 regions)	4
Number of GBD super-regions with data (out of 7 super-regions)	3

Total data inputs – Cerebral paragonimiasis

	Prevalence
Site-years (total)	4
Number of countries with data	2
Number of GBD regions with data (out of 21 regions)	2
Number of GBD super-regions with data (out of 7 super-regions)	2

Modelling strategy

We used a three-step process for the disease modelling of FBT. In the first step we used DisMod-MR 2.0 to estimate the prevalence of FBT by age, sex, year, and country. In the second we differentiated between asymptomatic and heavy infections. MetaXL (a meta-analysis add-in for Microsoft Excel) was used to estimate the proportion of heavily infected among all infected by age group for clonorchiasis, opisthorchiasis, and intenstinal fluke infection (see Table 3 and 4). These proportions were used to estimate the prevalence of heavy FBT infection.

The third step consisted of deselecting countries that have no autochtonous case reports of FBT (input 34,000 references from literature review).

Table 3. Percentage of high-intensity infection by age group and type of FBT (based on eight FBT prevalence studies)

Age category	Clonorchiasis			Opisthorchiasis			Intestinal fluke infection		
	Mean	Low	High	Mean	Low	High	Mean	Low	High
0-9	30%	17%	44%	10%	0%	29%	8%	3%	14%
10-19	15%	0%	43%	15%	0%	69%	11%	8%	14%
20-29	18%	10%	29%	16%	0%	52%	18%	15%	21%
30-39	17%	5%	34%	21%	0%	56%	22%	17%	28%
40-49	22%	13%	32%	28%	1%	68%	22%	13%	32%
50-59	18%	0%	49%	29%	0%	75%	17%	9%	28%
60+	32%	18%	47%	25%	0%	64%	15%	8%	23%

Table 4. Percentage of high-intensity infection by type of FBT (based on four FBT prevalence studies)

Type of FBT	Mean	Low	High
Paragonimiasis	23%	0%	59%
Fascioliasis	19%	3%	41%

Cerebral paragonimiasis

It was assumed that 0.8% of paragonimiasis cases have cerebral involvement. This proportion was used to estimate the prevalence of cerebral paragonimiasis. This proportion is based on one study. The data are from Oh SJ. The rate of cerebral involvement in paragonimiasis: an epidemiologic study. *Jpn J Parasitol* 1969;18:211-14. The study was performed in Paju, South Korea. This is an area with 6,738 inhabitants, and according to the survey, it was estimated that 29.6% of all individuals would react to intradermal test (= an immunological reaction indicating previous or current contact with the parasite). 25% of all "positive reactors" may have eggs in their sputum (= active infection with the parasite currently present in the human host). If these rates are applied to the community as a whole, the number of patients with active paragonimiasis would be at least 498 (=6,738*0.296*0.250). Furthermore, four cases of cerebral paragonimiasis were found in this community. Therefore, four out of 498 individuals with active paragonimus infection suffered from cerebral infection (=0.80%; 95% confidence interval 0.019%–1.587%).

Severity splits and disability weights

For GBD 2016, FBT was not split into health states with different severities. The table below shows the GBD 2016 disability weights that were used to calculate the burden of FBT in YLDs.

Table 5. Disability weights that were used to calculate FBT YLDs

Sequelae	Severity description	Health state name	Disability weight
Asymptomatic clonorchiasis	Clonorchiasis, currently without symptoms	N/A	0.000 (0.000–0.000)

Heavy clonorchiasis	Abdominal pain and nausea reported as moderate	Abdominopelvic problem, moderate	0.114 (0.078–0.159)
Asymptomatic opisthorchiasis	Opisthorchiasis, currently without symptoms	N/A	0.000 (0.000–0.000)
Heavy opisthorchiasis	Abdominal pain and nausea reported as moderate	Abdominopelvic problem, moderate	0.114 (0.078–0.159)
Asymptomatic fascioliasis	Fascioliasis, currently without symptoms	N/A	0.000 (0.000–0.000)
Heavy fascioliasis	Abdominal pain and nausea reported as moderate	Abdominopelvic problem, moderate	0.114 (0.078–0.159)
Asymptomatic intestinal fluke infection	Intestinal fluke infection, currently without symptoms	N/A	0.000 (0.000–0.000)
Heavy intestinal fluke infection	Abdominal pain and nausea reported as moderate	Abdominopelvic problem, moderate	0.114 (0.078–0.159)
Asymptomatic paragonimiasis	Paragonimiasis, currently without symptoms	N/A	0.000 (0.000–0.000)
Heavy paragonimiasis	Cough, fever, and weight loss	Tuberculosis, not HIV-infected	0.333 (0.224–0.454)
Cerebral paragonimiasis	Epilepsy due to cerebral paragonimiasis	Epilepsy, less severe (seizures < once per month)	0.263 (0.173–0.367)
		Epilepsy, severe (seizures >= once per month	0.552 (0.375–0.710)

Note. N/A: not applicable

Changes from GBD 2016 to GBD 2017

We conducted an abbreviated literature search based on citations included in published reviews (5) and recommendations by Dr. Thomas Furst. We completed extractions for all but five records during the allotted timeline. Sources were unable to be extracted due to language barriers and lack of detailed citation information.

References

1. WHO. *International Statistical Classification of Diseases and Related Health Problems. 10th Revision. Version for 2007.* 2007 [cited 2009 October 14, 2009]; Available from: http://apps.who.int/classifications/apps/icd/icd10online/.
2. Rim, H.J., *Clonorchiasis: an update.* J Helminthol, 2005. **79**(3): p. 269-81.
3. Pungpak, S., et al., *Clinical features in severe opisthorchiasis viverrini.* Southeast Asian J Trop Med Public Health, 1985. **16**(3): p. 405-9.
4. Rim, H.J., *The current pathobiology and chemotherapy of clonorchiasis.* Korean J Parasitol, 1986. **24**(Suppl.): p. 1-141.
5. Furst, T., J. Keiser, and J. Utzinger, *Global burden of human food-borne trematodiasis: a systematic review and meta-analysis.* Lancet Infect Dis, 2012. **12**(3): p. 210-21.
6. Furst, T., et al., *Manifestation, diagnosis, and management of foodborne trematodiasis.* BMJ, 2012. **344**: p. e4093.

3.3.5 Dracunculiasis (Guinea worm) SDG Capstone Appendix

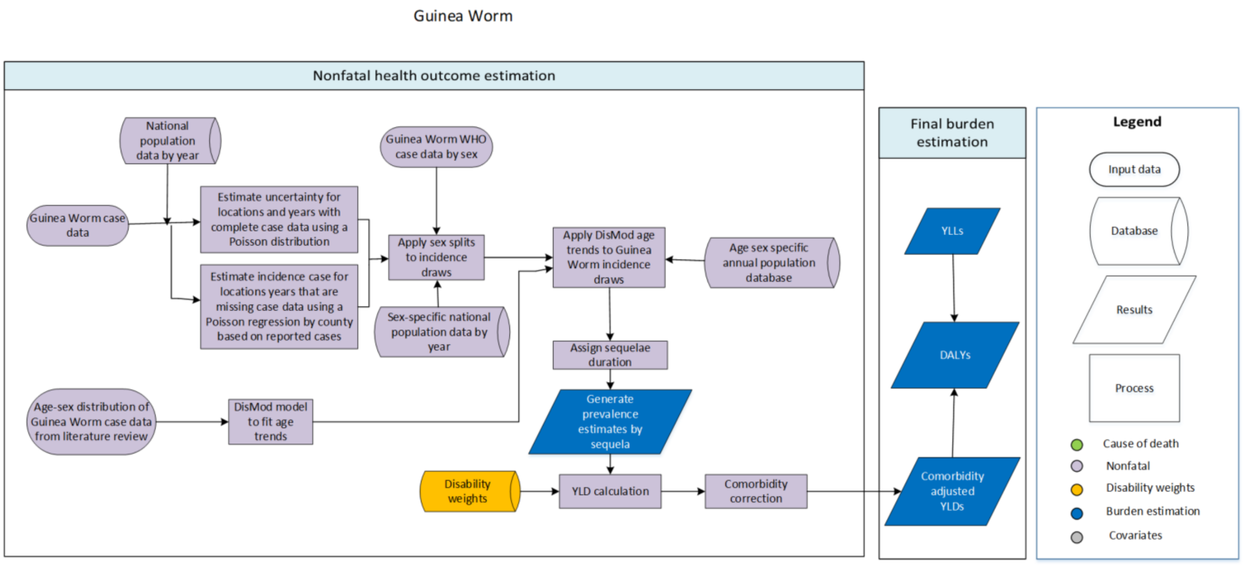

Background

Guinea-worm disease is caused by the parasitic worm *Dracunculus medinensis*. The transmission cycle begins when Guinea worm larvae are released in stagnant water (e.g., ponds, lakes, open wells) where they are ingested by freshwater copepods (small crustaceans sometimes called water fleas) of the genus *Cyclops* [1]. When a person consumes water containing *Cyclops*, the copepods are dissolved by gastric acids and intestinal enzymes and the larvae are released. Larvae then migrate through the intestinal wall and travel to the connective tissues. The larvae mature and mate 60–90 days after infection; shortly thereafter, the male dies and the pregnant female worm continues to move through the victim's connective tissues. Approximately 10–14 months post-infection, the adult worm creates a painful burning blister on the skin that develops and enlarges over several days, usually from the feet or lower limbs. Blister formation may be preceded by a slight fever, itchy rash, nausea, vomiting, and diarrhoea. To relieve the pain associated with the worm's emergence, infected persons immerse the infected part of their body in local stagnant water sources, such as ponds. Upon entering the water, the female worm will expel her larvae and the cycle can begin again [1-4].

The global campaign to eradicate Guinea worm began in 1980, when the US Centers for Disease Control and Prevention (CDC) suggested that Guinea worm eradication would be an ideal indicator of the success of the International Drinking Water Supply and Sanitation Decade of 1981–1990; in 1981, Guinea worm eradication was adopted as a sub-goal of this United Nations advocacy effort [1, 5]. In 1986, the World Health Assembly adopted a resolution to eliminate Guinea worm disease, and since then, the Carter Center has led a coalition that includes ministries of health of endemic countries, CDC, the World Health Organization (WHO), the United Nations Children's Fund (UNICEF), thousands of village volunteers, and supervisory staff supported by numerous donors [5].

To break the cycle of transmission, ministries of health in endemic countries implement a suite of interventions: case detection and containment, provision of safe water sources, distribution of filter cloths and pipe filters, water source treatment with Abate® (a larvacide), and health education.

By design, the Guinea worm eradication programmatic infrastructure covers the entire at-risk population in endemic countries. Since case containment[6] is a key intervention designed to not only interrupt transmission but also monitor progress toward eradication, incident cases of Guinea worm disease are nationally representative. To implement case containment as an intervention, all cases of Guinea worm disease are identified. Containment is defined as detection within 24 hours of the worm's emergence; the patient did not contaminate any water source; the patient received proper wound care and health education on not entering any water source; a supervisor verified the case as dracunculiasis within seven days; and Abate® is used if there is any uncertainty about contamination of water sources or known contamination of water sources [7]. Case reporting occurs at the village level on a monthly basis; case data are then aggregated within the national Guinea Worm Eradication Program and reported to WHO. In settings where annual case reports are low (suggesting no transmission) or transmission has been interrupted, cash rewards are promoted to enhance surveillance activities.

Input Data & Methodological Summary

Case Definition

A Guinea worm case is defined as an individual with Guinea worm disease. A person is counted as a case only once in a calendar year, ie, when the first Guinea worm emerged from that person, although an individual may have more than one worm emerge at a time and/or more than one worm emerge during the year. These cases are confirmed through the Guinea worm eradication program infrastructure by clinical exam and verification by local supervisors. All specimens from case-patients are sent to the CDC for laboratory evaluation and confirmation [7].

Input data

Model inputs

Geographic restrictions

Only the following countries were identified as guinea-worm endemic as of 1990[8]: Benin, Burkina Faso, Cameroon, Central African Republic, Chad, Cote d'Ivoire, Ethiopia, Ghana, India, Kenya, Mali, Mauritania, Niger, Nigeria, Pakistan, Senegal, Sudan, South Sudan, Togo, Uganda, and Yemen[8]. Any country not reporting Guinea worm as of 1990 is not included in the GBD model.

Geographic restrictions by year were also implemented to account for the period post-transmission to reflect the accomplishments of the Guinea worm eradication campaign. Geographic restriction for countries that were endemic in 1990 was defined based on data reported post-interruption of transmission. In the GBD analysis, Guinea worm disease was no longer modelled for the year that followed the last reported case (imported or indigenous) provided that the subsequent years through 2017 also had no case reports. To ensure that cases were attributed to burden in the country in which the case was detected, both indigenous and imported cases were included. For example, Kenya reported its last (imported) case in 2005, and as no other cases were reported through 2017, incidence from 2006

onward is zero. For Chad, a country that had years during which no cases were reported, the model covers the entire period 1990–2017.

Data sources

1) Case data by geography, by year
2) Literature review of age/sex distribution
3) Literature review for sequelae (type, duration, and proportion)

Case data: Annual case data were reported by WHO in the Weekly Epidemiological Record for the period 1990–2017. For years or geographies for which WER reports were not published, the following sources were also used to extract case counts:

1) CDC's MMWR reports
2) 1990–1999 total country reports from Hopkins *et al*[8]
3) India subnational estimates: India MOH report (1984–1999)
4) The Carter Center's Guinea worm wrap-up: disaggregation of case totals for Sudan and South Sudan pre-2011 (independence) to ensure case totals from 1990–2010 are consistent with current national boundaries; 2016 provisional case data.

The number of cases annually was compared to official total numbers published in WER 2016 to ensure accuracy of data entry.

Table of incident case data counts

	**Incidence**
**Site-years (total)**	816
**Number of countries with data**	21
**Number of GBD regions with data (out of 21 regions)**	5
**Number of GBD super-regions with data (out of 7 super-regions)**	3

Subnational data

India: Subnational data for India were obtained from the Ministry of Health for the period 1984–1999; cases were reported by year and state: http://www.ncdc.gov.in/index2.asp?slid=329&sublinkid=216.

Kenya: Subnational data from Kenya were requested from the MOH but not obtained. To split cases by subnational unit, the Carter Center Guinea Worm Wrap-Up was reviewed to identify districts with endemic villages. A national survey conducted 1993/1994 found cases in Turkana and West Pokot counties, but case totals were not reported by county. Indigenous transmission was interrupted in 1995, with imported cases reported until 2005. WER reports from 1999 to 2006 document that all imported cases from 1998 to 2005 occurred in Turkana County. All cases in Kenya are currently analysed in GBD as occurring in Turkana County as we are unable to disaggregate the data.

Accounting for possible under-reporting

Once national eradication programs were initiated, national case searches were conducted to improve the accuracy of national case estimates. These searches were designed to enumerate prevalent Guinea worm disease cases and identify endemic villages to direct intervention and surveillance activities. For the majority of years included in the GBD analysis, the total number of Guinea worm cases reported is equivalent to a national census, as all cases are identified and reported. Nevertheless, not all endemic countries were able to initiate full national surveillance as of 1990.

The model does not account for the possibility that cases occurred in communities that were not included in routine surveillance or did not achieve 100% reporting coverage over time. However, any cases that may have been undetected would likely not have been a significant increase over annual totals given the comprehensive nature of Guinea worm disease surveillance activities. Nevertheless, there are years for which the annual case data is inconsistent with preceding/following annual case totals and could not be accounted for in our model. For example, Niger reported 500 cases in 1992, despite reporting 32,829 cases in 1991 and 25,346 cases in 1993. In those instances, the following data points were identified as outliers and excluded from analysis as follows:

Table 1. List of reported case data outliered in the analysis to account for possible under-reporting

Country	Year	Reported Cases
Central African Republic	1996	9
Central African Republic	1997	5
Ethiopia	1992	303
Kenya (Turkana County)	1990	6
Uganda	1990	4,704
Uganda*	1992	126,369
Benin	1991	4,006
Benin	1992	4,315
Chad	1992	156
Cote d'Ivoire	1990	1,360
Mali	1990	884
Mauritania	1992	1,557
Niger	1992	500
Senegal	1990	38
Togo	1990	3,042
Togo	1991	5,118
South Sudan*	1996	116,844
Sudan	1994	132

*For these two data points, we do not dispute that over 100,000 cases of Guinea worm likely occurred. However, given the amount of missing data in the early time series for these two countries, inclusion of these resulted in implausibly high case predictions (over 1 million cases in Uganda in 1990 and over 1.5 million for South Sudan from 1990 to 1995).

Age/sex distribution

Generally, the risk of Guinea worm infection varies according to sex- or age-specific differences in access to safe drinking water. A study in Ethiopia found women were more likely to experience Guinea worm disease than men; in India, men experienced greater risk of infection [1]. Exposure to unsafe water sources varies largely on mobility patterns and type of water sources: communities in which infected water is carried in for consumption are more likely to see more Guinea worm disease in children and older adults [9]. Once interventions to control the spread of Guinea worm infection are implemented, the age and sex distribution likely changes to reflect variation in coverage and uptake of eradication interventions, such as larvacide of water sources and case-containment rates; age/sex case data are currently not available.

Table of age-specific prevalence data inputs

	**Prevalence**
**Site-years (total)**	7
**Number of countries with data**	4
**Number of GBD regions with data (out of 21 regions)**	3
**Number of GBD super-regions with data (out of 7 super-regions)**	2

The evidence base available to describe risk of infection by age is as follows:

1) Studies from Nigeria:
    a. Adeyeba *et al* [10]: Guinea worm disease not common among children <1 year of age; increase in risk by age
    b. Kale *et al* [11]: More boys ages 5-9 years than girls were infected (11.9% v. 6.8%); Women ages 20-29 years had higher prevalence of infection than men (13.4% v. 4.7%); Overall, the prevalence in both men and women was highest in ages 10-14 years and 30 years or older.
    c. Greenwood *et al* [12]: The mean age of male cases was 25.8 years (95% CI: 23.9, 27.7) and 26.9 years for females (95% CI: 23.7, 30.1).
2) Other countries:
    a. Sudan [13]: No significant age trend among lower-endemicity villages; higher-endemicity villages (n=4) had higher prevalence in children and older adults. This study attributes the difference in age trends to community-level water source.
    b. Ghana [14]: The trend in age of first infection reported was similar for males and females, with more females experiencing first infection between 15 and 19 years and males between 20 and 24 years of age. The proportion of men with Guinea worm disease was much higher than among women 25-54 years of age. Adults >15 years of age were more likely to be infected than children.

The evidence base available to describe the risk of infection by gender is as follows:

1) Studies from Nigeria:
    a. Adeyeba *et al* [10]: No difference among males and females.
    b. Kale *et al* [11]: No overall gender difference comparing total males infected to total females infected, although gender differences for certain age groups (see notes above).
    c. Greenwood *et al* [12]: Two-thirds of cases reported among 47 villages from 1971 to 1974 were male.

WHO Weekly Epidemiological Record (WER) age reports: Age and sex data were reported by country for 2009 onward; these data capture the age distribution for Chad, Ethiopia, Ghana, Mali, and South Sudan. We excluded these data as the age/sex distribution is only described for children <15 years or adults, which does not permit fitting an age trend across multiple categories.

WER sex-specific data: Sex-specific differences in the burden of Guinea worm disease could reflect differing levels of access to eradication program interventions, in addition to risk factors associated with local transmission dynamics. Since the data reported from 2009 to 2015 are the only available nationally representative data, we used the overall sex difference to generate sex-specific incidence and prevalence, with females experiencing a slightly higher risk (53%) compared to males (47%):

Table 2. WHO Weekly Epidemiological Record total worm burden by gender, by year

Year	Female	Male	Total	% Fem	% Male
**2009**	1699	1490	3189	53%	47%
**2010**	976	821	1797	54%	46%
**2011**	524	534	1058	50%	50%
**2012**	273	269	542	50%	50%
**2013**	79	69	148	53%	47%
**2014**	63	63	126	50%	50%
**2015**	9	13	22	41%	59%
**Total**	3623	3259	6882	53%	47%

There is limited evidence to suggest that risk varies jointly by sex and age; however, evidence for this modification also suggests that such age- and sex-specific risks may vary by endemic community within a given geography (in some settings, women at higher risk, in others men, but not for all age strata). Without additional data sources in which cases are disaggregated by age and sex, this joint relationship is not modelled.

To model age-specific variation, we used data from seven studies with age-specific case data to generate an age-trend in a DisMod model. We further assumed no Guinea worm disease occurred in infants less than 1 year of age.

Severity splits/sequelae

Sequelae associated with Guinea worm relate to the wound at the site of the worm's emergence, which can include abscesses and chronic ulcerations. Joint and tissue damage can occur, as well as secondary

infection in connective tissues [15]. During the worm's emergence, which takes approximately one month to exit the body, the ulcer is painful and itchy [1]. The wound is subject to secondary infection and scarring. Possible long-term consequences of Guinea worm infection include arthritis or other permanent damage to connective tissues; however, data on this are limited. In the Greenwood study, 41.7% of all cases experienced infection at the site of emergence, and the annual proportion of cases with definite arthritis ranged from 1.6% to 7.3% of all cases.

While an individual experiences Guinea worm disease, they are generally unable to work and have limited mobility at the time prior and during emergence and in the subsequent period in which they are healing. Although most worms emerge in the feet and lower legs, there are reports of worms exiting at other sites [15], which could cause other disability not accounted for here. A study in Nigeria found that 98% of worms emerged in the lower limbs[16]. The Greenwood study also observed that 88.4% emerged in the lower limbs. Therefore, for the purposes of estimating the burden of Guinea worm disease in GBD, all disability associated with Guinea worm disease is attributed to lower limb conditions, pain, and lack of mobility. Due to limited data, we cannot account for differential disability based on number of worms emerging at the same time.

The following evidence base was reviewed to determine the proportion of cases attributed to each sequela, as well as duration of sequelae.

Duration of disability and type of disability:

Studies from Nigeria:

1) Adeyeba *et al* [10]: 93.4% incapacitated for an average of 26 days.
2) Smith *et al* [17]: Average disability duration 12.7 weeks; 58% unable to leave the home for a mean duration of 4.2 weeks; duration of disability greater among those older than 50 years compared to those younger than 50 years.
3) Okoye *et al* [16]: 21% of cases were totally incapacitated due to their infection (not permanently disabled).
4) Kate *et al* [11]: A survey of 17 villages from 1971 to 1975 found that duration of disability was approximately 100 days.
5) Greenwood *et al* [12]: Weekly visits to 47 villages from 1971 to 1974 reported mean duration of illness ranging from 4.2 weeks to 7.2 weeks. 17.4% of cases had an active infection which persisted for 10 weeks or more.

Other countries:

6) Benin [18]: From two villages in highly endemic areas, estimated 39-59 days of disability experienced after worm emergence.
7) Ghana [19]: 28.2% experienced pain 12-18 months post-emergence; 5% unable to carry out at least one daily activity, 0.5% permanently impaired (ligament damage to thumb).
8) Ghana [14]: Complete disability experienced among males with Guinea worm disease lasted approximately 5 weeks among those untreated. Among cases provided supportive care (wound management), the duration of disability was 2.5 weeks.

For all cases, we assume each experiences pain and disfigurement (level 2), and musculoskeletal problems, lower limb (moderate) for a period of one month, followed by two months of pain and

disfigurement (mild). We then assume that 30% of all cases will then experience disfigurement level 1 with itch/pain for an additional nine months (approximately a year of disability) to account for longer-term disability associated with recovery.

Table 3. Sequela associated with Guinea worm disease in the Global Burden of Disease study

Sequela	Lay description	DW (95% CI)
Disfigurement, level 2, with itch/pain	Has a visible physical deformity that is sore and itchy. Other people stare and comment, which causes the person to worry. The person has trouble sleeping and concentrating.	0.188 (0.125–0.267)
Disfigurement, level 1, with itch/pain	Has a slight, visible physical deformity that is sometimes sore or itchy. Others notice the deformity, which causes some worry and discomfort.	0.027 (0.015–0.042)
Musculoskeletal problems, lower limbs, moderate	Has moderate pain in the leg, which makes the person limp, and causes some difficulty walking, standing, lifting and carrying heavy things, getting up and down and sleeping.	0.079 (0.054–0.11)

Modelling strategy

Total incidence

The incidence of Guinea worm disease is modelled in GBD using two approaches: for years and locations for which case data were reported, 1,000 draws of incidence were estimated using a beta distribution of cases and total population minus cases. For years and locations for which case data were missing (largely the early 1990s) a Poisson regression of all case data was implemented per country, using the total population as the offset. The predicted incidence and standard error were used to generate a random distribution of 1,000 incidence draws. Incidence is multiplied by duration of sequelae to calculate prevalence. Country-level incidence predictions are shown in the following figures.

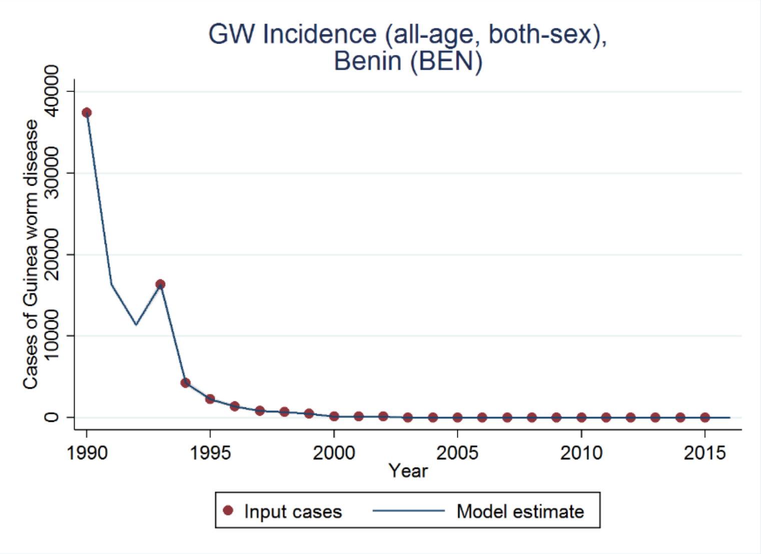

GW Incidence (all-age, both-sex),
Benin (BEN)
Cases of Guinea worm disease
0
10000
20000
30000
40000
1990
1995
2000
2005
2010
2015
Year
Input cases
Model estimate

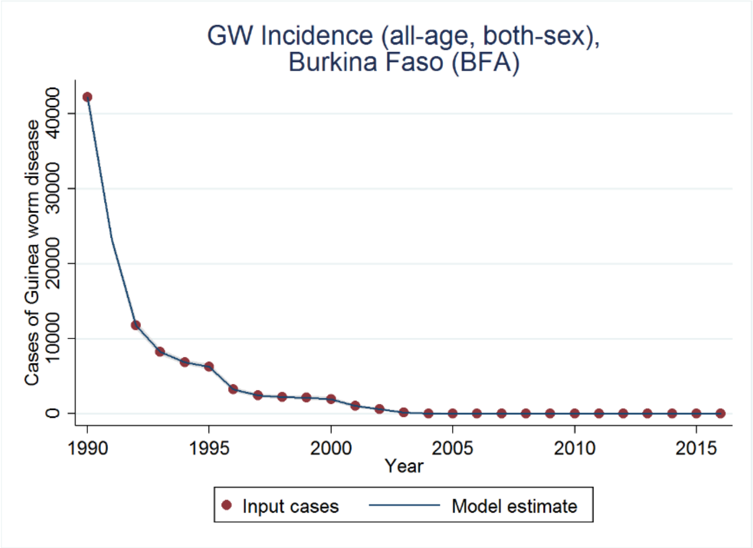

GW Incidence (all-age, both-sex),
Burkina Faso (BFA)
Cases of Guinea worm disease
0
10000
20000
30000
40000
1990
1995
2000
2005
2010
2015
Year
Input cases
Model estimate

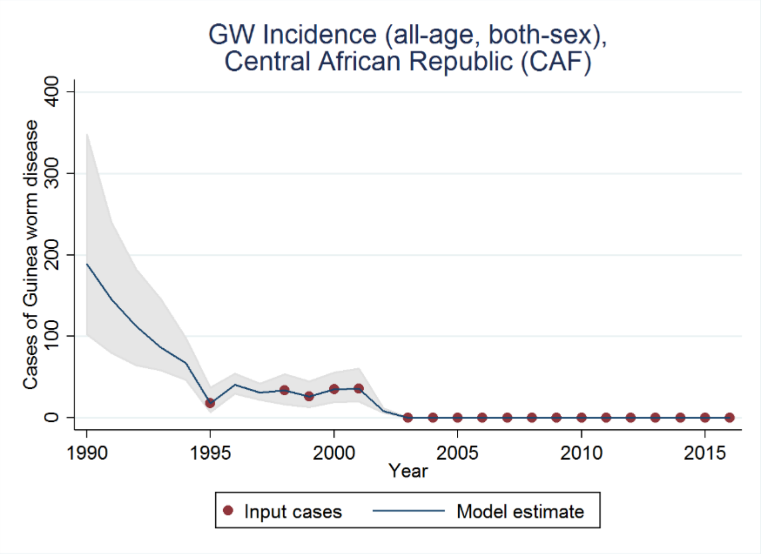

GW Incidence (all-age, both-sex),
Central African Republic (CAF)
Cases of Guinea worm disease
0
100
200
300
400
1990
1995
2000
2005
2010
2015
Year
Input cases
Model estimate

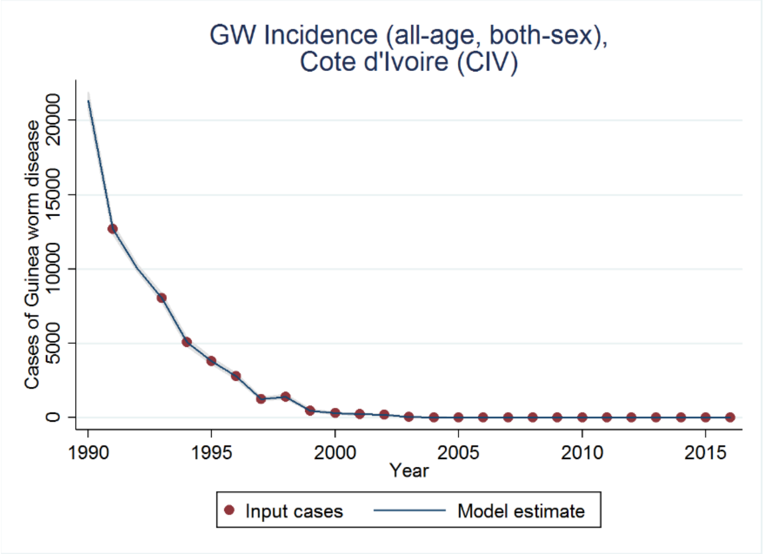

GW Incidence (all-age, both-sex),
Cote d'Ivoire (CIV)
Cases of Guinea worm disease
0
5000
10000
15000
20000
1990
1995
2000
2005
2010
2015
Year
Input cases
Model estimate

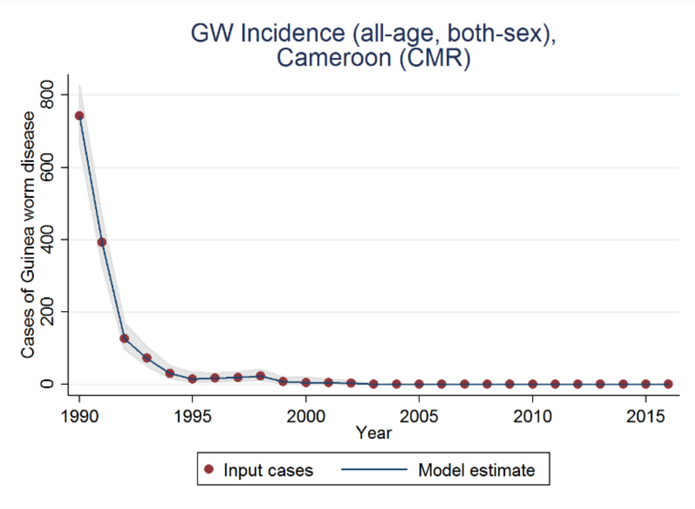
GW Incidence (all-age, both-sex),
Cameroon (CMR)
Cases of Guinea worm disease
0
200
400
600
800
1990
1995
2000
2005
2010
2015
Year
Input cases
Model estimate

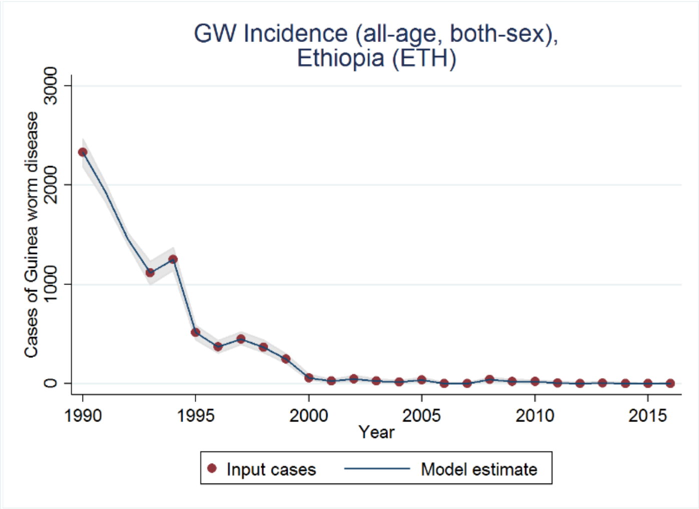
GW Incidence (all-age, both-sex),
Ethiopia (ETH)
Cases of Guinea worm disease
0
1000
2000
3000
1990
1995
2000
2005
2010
2015
Year
Input cases
Model estimate

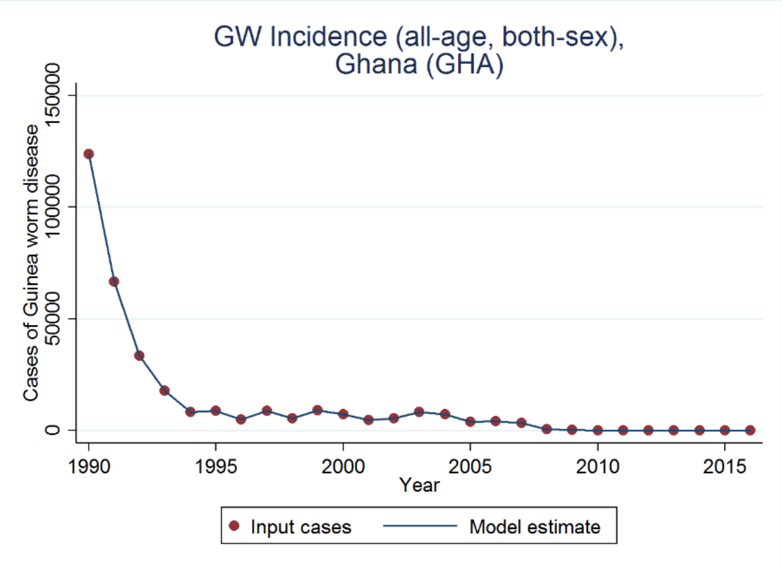
GW Incidence (all-age, both-sex),
Ghana (GHA)
Cases of Guinea worm disease
0
50000
100000
150000
1990
1995
2000
2005
2010
2015
Year
Input cases
Model estimate

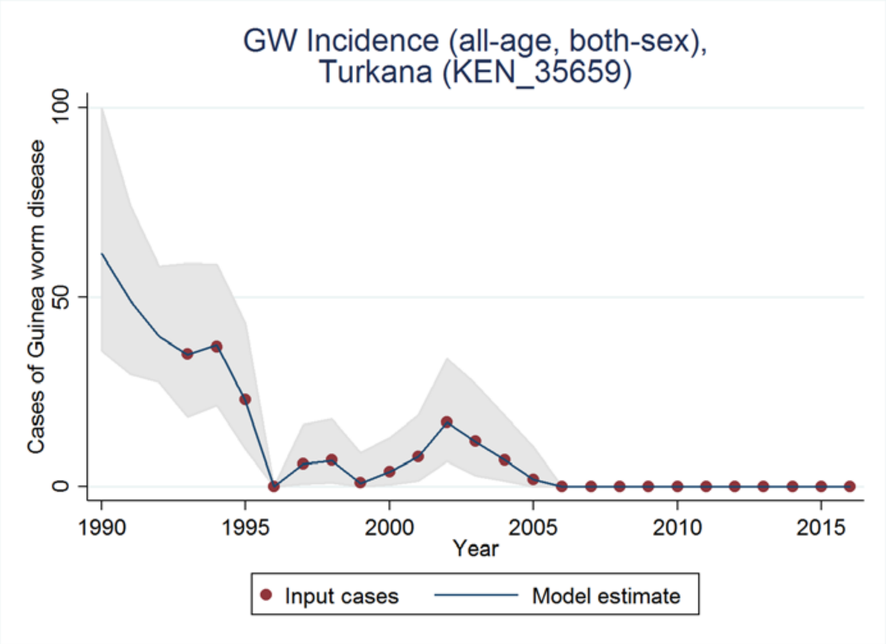
GW Incidence (all-age, both-sex),
Turkana (KEN_35659)
Cases of Guinea worm disease
0
50
100
1990
1995
2000
2005
2010
2015
Year
Input cases
Model estimate

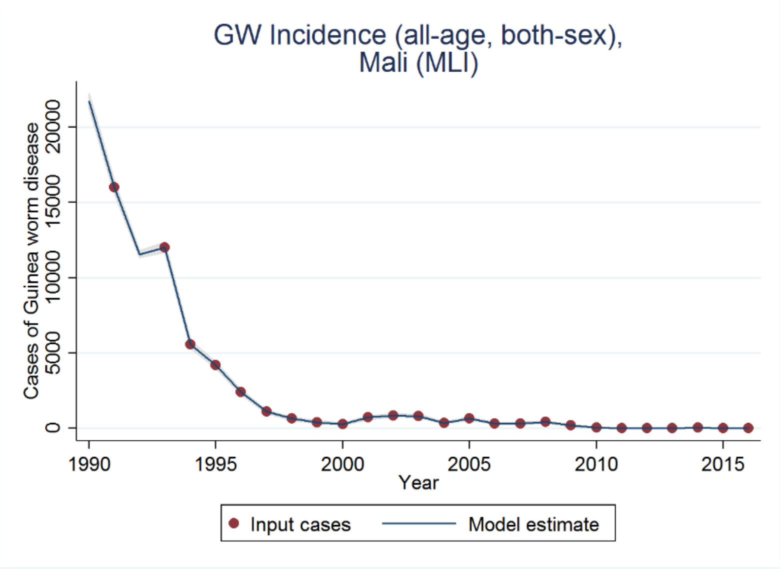
GW Incidence (all-age, both-sex),
Mali (MLI)
Cases of Guinea worm disease
0
5000
10000
15000
20000
1990
1995
2000
2005
2010
2015
Year
Input cases
Model estimate

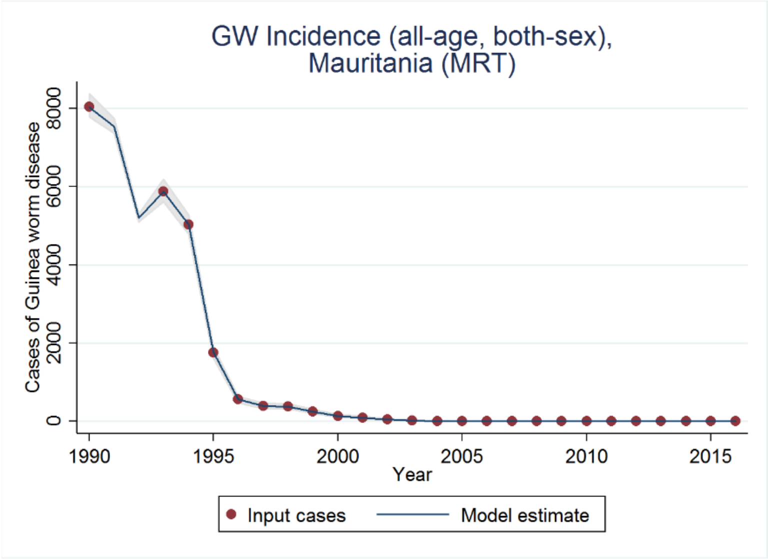
GW Incidence (all-age, both-sex),
Mauritania (MRT)
Cases of Guinea worm disease
0
2000
4000
6000
8000
1990
1995
2000
2005
2010
2015
Year
Input cases
Model estimate

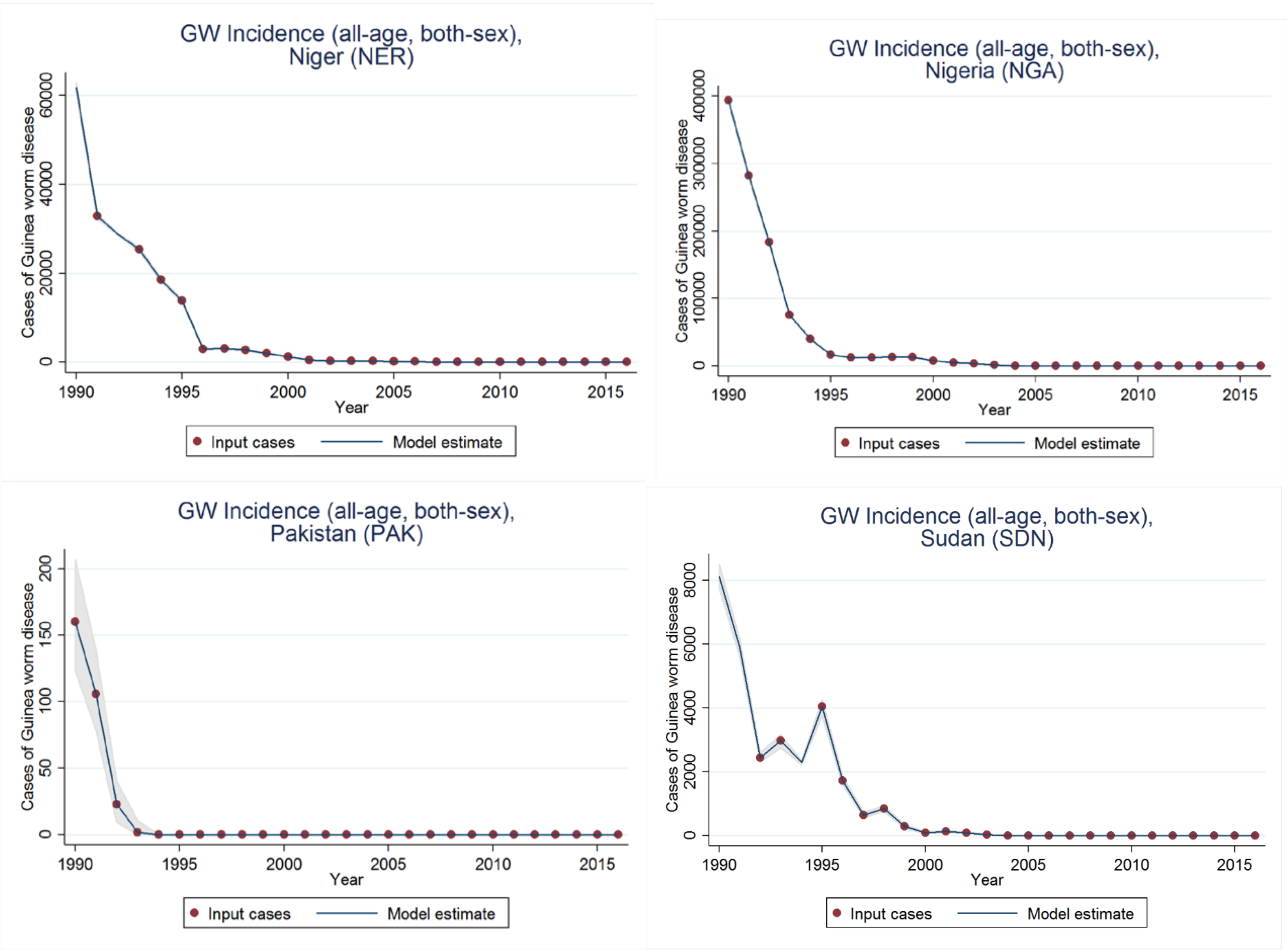

GW Incidence (all-age, both-sex), Niger (NER)
Cases of Guinea worm disease
0
20000
40000
60000
1990
1995
2000
2005
2010
2015
Year
Input cases
Model estimate
GW Incidence (all-age, both-sex), Nigeria (NGA)
Cases of Guinea worm disease
0
100000
200000
300000
400000
1990
1995
2000
2005
2010
2015
Year
Input cases
Model estimate
GW Incidence (all-age, both-sex), Pakistan (PAK)
Cases of Guinea worm disease
0
50
100
150
200
1990
1995
2000
2005
2010
2015
Year
Input cases
Model estimate
GW Incidence (all-age, both-sex), Sudan (SDN)
Cases of Guinea worm disease
0
2000
4000
6000
8000
1990
1995
2000
2005
2010
2015
Year
Input cases
Model estimate

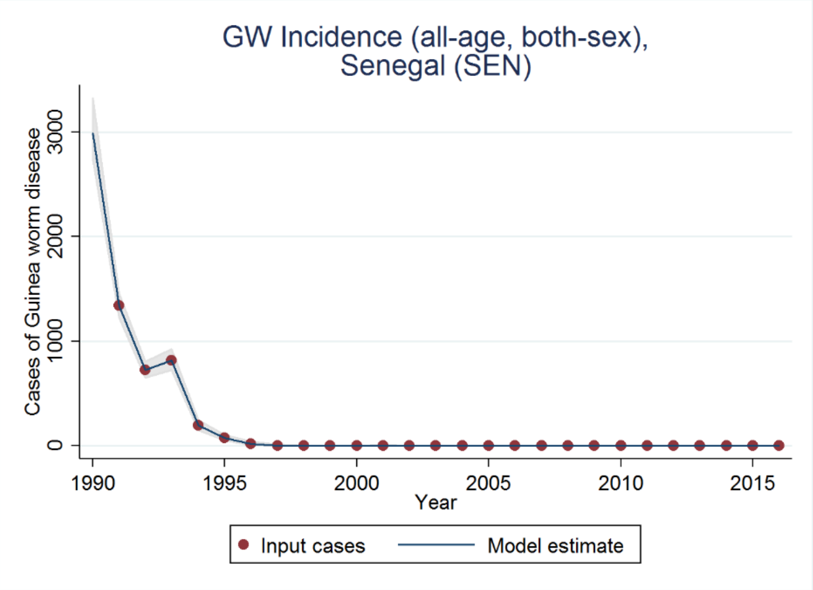
GW Incidence (all-age, both-sex),
Senegal (SEN)
Cases of Guinea worm disease
0
1000
2000
3000
1990
1995
2000
2005
2010
2015
Year
Input cases
Model estimate

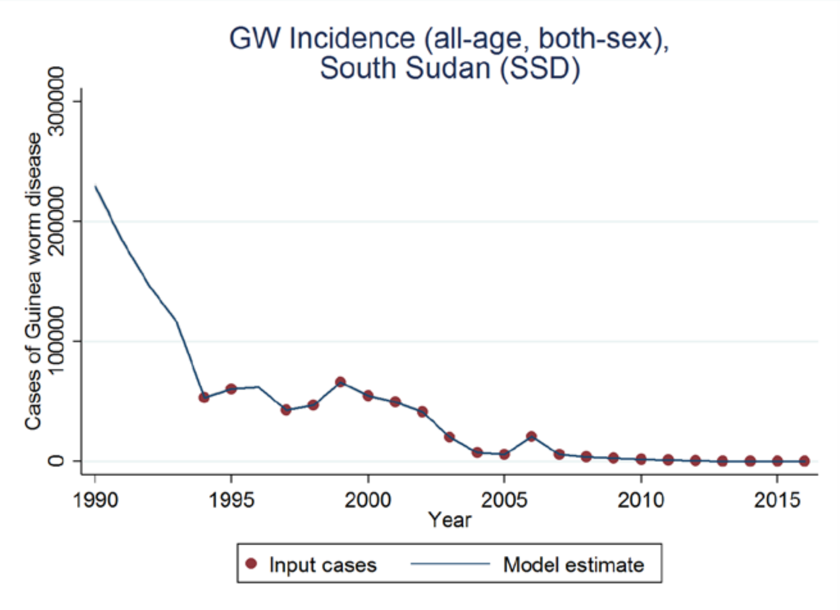
GW Incidence (all-age, both-sex),
South Sudan (SSD)
Cases of Guinea worm disease
0
100000
200000
300000
1990
1995
2000
2005
2010
2015
Year
Input cases
Model estimate

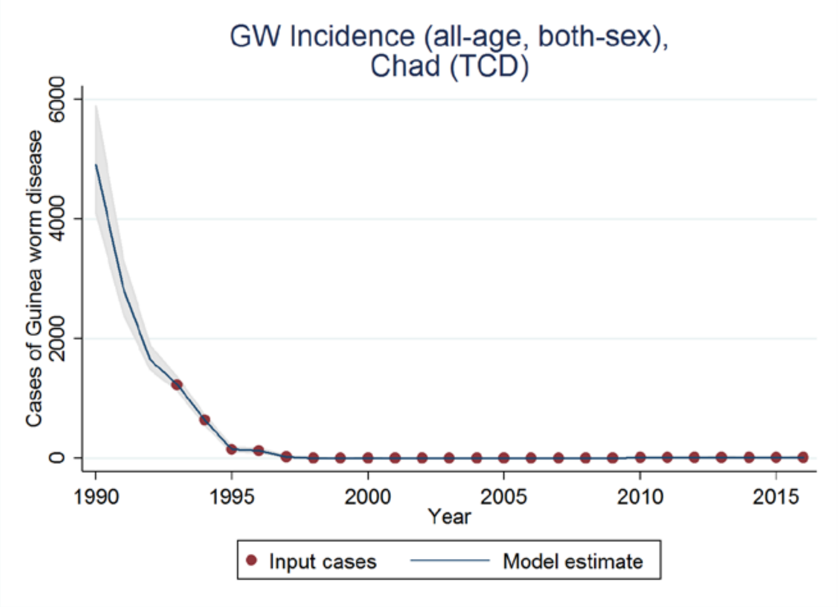
GW Incidence (all-age, both-sex),
Chad (TCD)
Cases of Guinea worm disease
0
2000
4000
6000
1990
1995
2000
2005
2010
2015
Year
Input cases
Model estimate

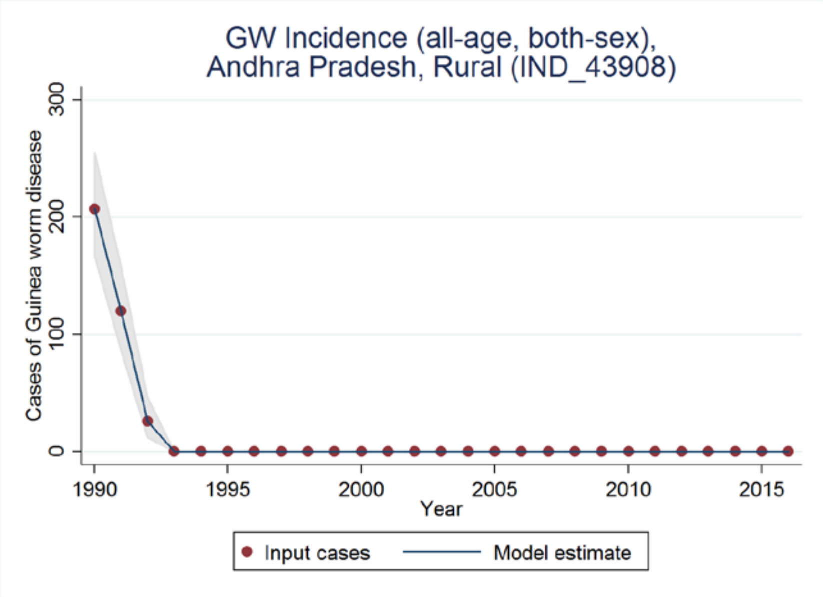
GW Incidence (all-age, both-sex),
Andhra Pradesh, Rural (IND_43908)
Cases of Guinea worm disease
0
100
200
300
1990
1995
2000
2005
2010
2015
Year
Input cases
Model estimate

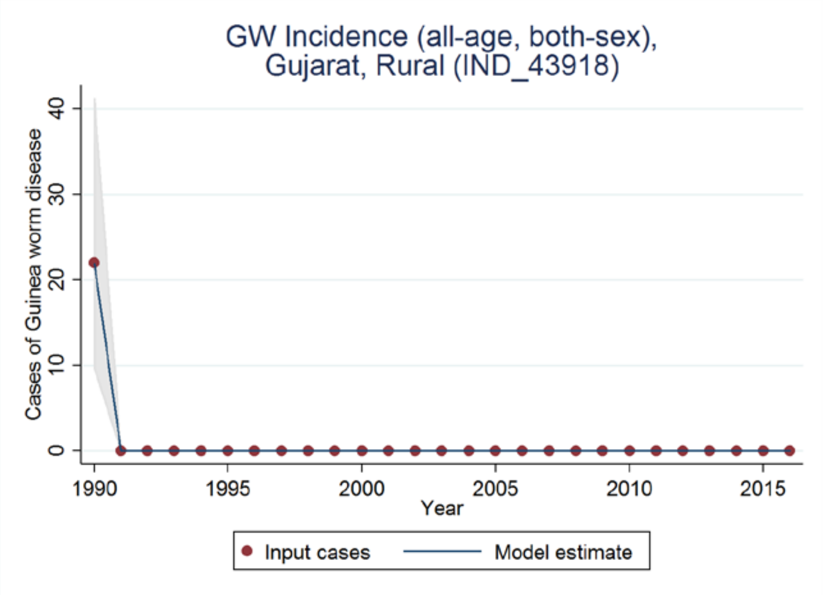

GW Incidence (all-age, both-sex),
Gujarat, Rural (IND_43918)
Cases of Guinea worm disease
0
10
20
30
40
1990
1995
2000
2005
2010
2015
Year
Input cases
Model estimate

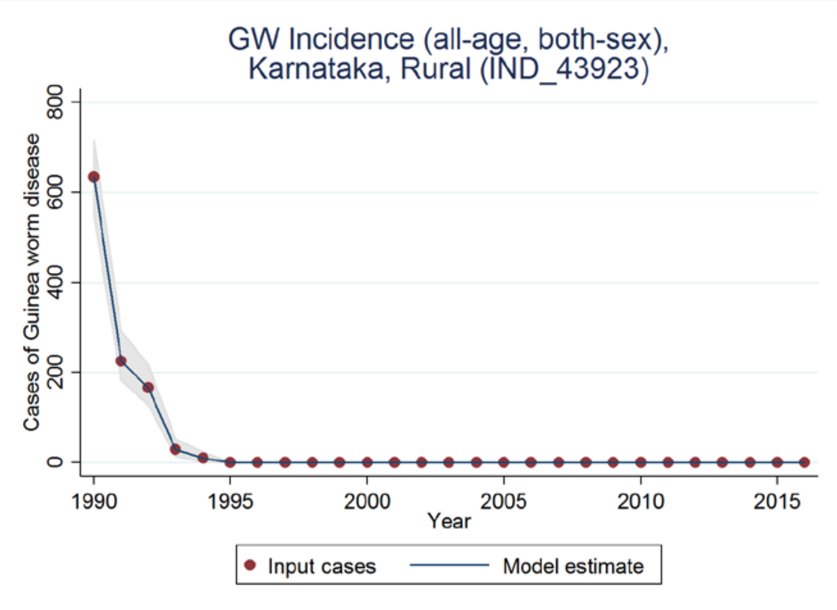

GW Incidence (all-age, both-sex),
Karnataka, Rural (IND_43923)
Cases of Guinea worm disease
0
200
400
600
800
1990
1995
2000
2005
2010
2015
Year
Input cases
Model estimate

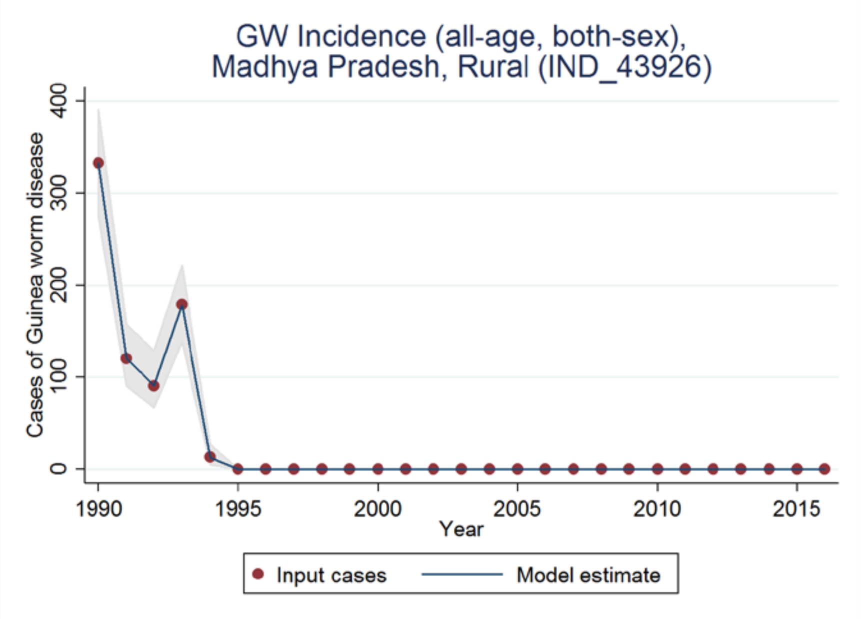

GW Incidence (all-age, both-sex),
Madhya Pradesh, Rural (IND_43926)
Cases of Guinea worm disease
0
100
200
300
400
1990
1995
2000
2005
2010
2015
Year
Input cases
Model estimate

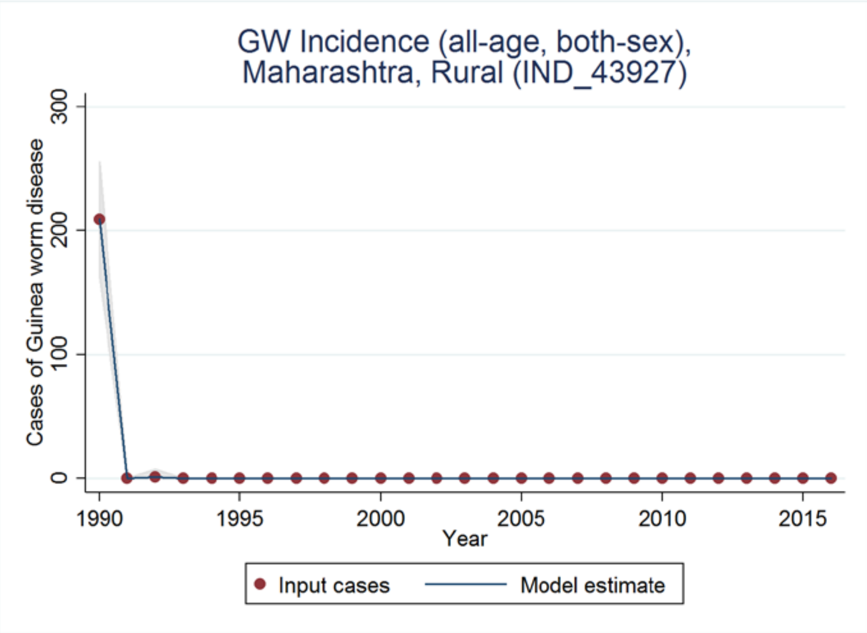
GW Incidence (all-age, both-sex),
Maharashtra, Rural (IND_43927)
Cases of Guinea worm disease
0
100
200
300
1990
1995
2000
2005
2010
2015
Year
Input cases
Model estimate

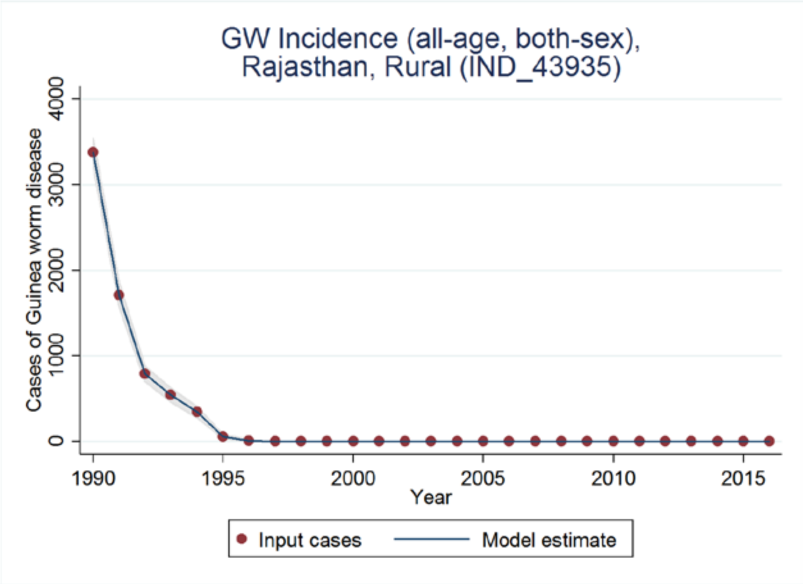
GW Incidence (all-age, both-sex),
Rajasthan, Rural (IND_43935)
Cases of Guinea worm disease
0
1000
2000
3000
4000
1990
1995
2000
2005
2010
2015
Year
Input cases
Model estimate

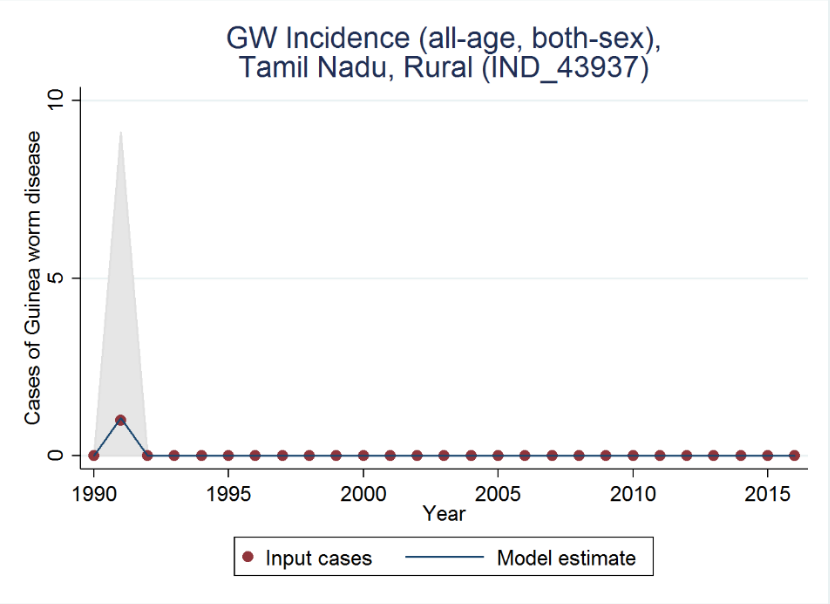
GW Incidence (all-age, both-sex),
Tamil Nadu, Rural (IND_43937)
Cases of Guinea worm disease
0
5
10
1990
1995
2000
2005
2010
2015
Year
Input cases
Model estimate

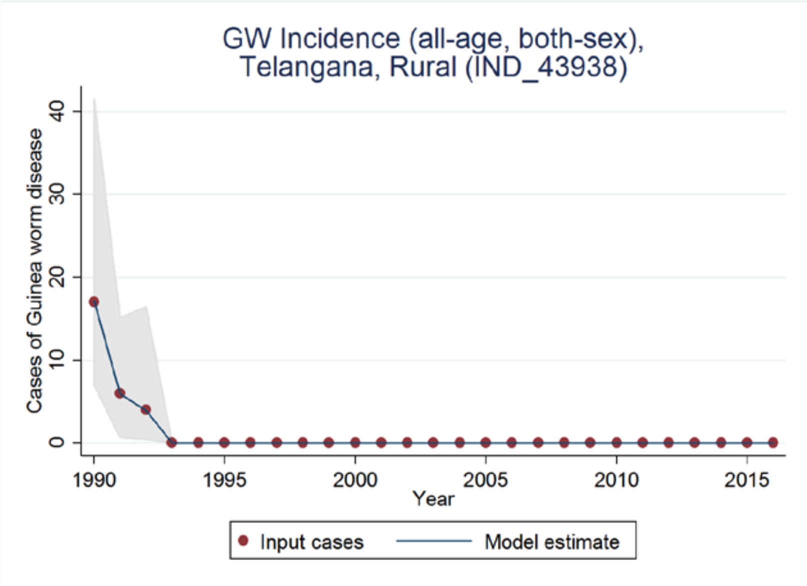
GW Incidence (all-age, both-sex),
Telangana, Rural (IND_43938)
Cases of Guinea worm disease
0
10
20
30
40
1990
1995
2000
2005
2010
2015
Year
Input cases
Model estimate

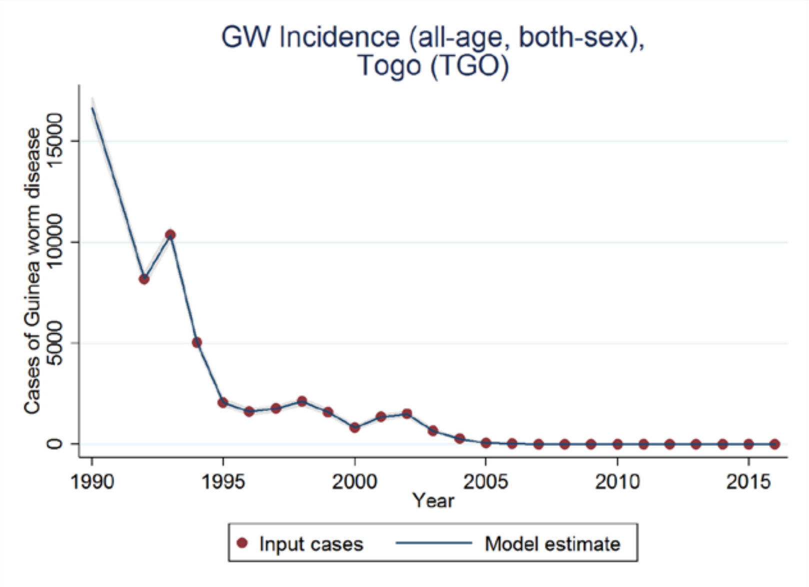

GW Incidence (all-age, both-sex),
Togo (TGO)
Cases of Guinea worm disease
0
5000
10000
15000
1990
1995
2000
2005
2010
2015
Year
Input cases
Model estimate

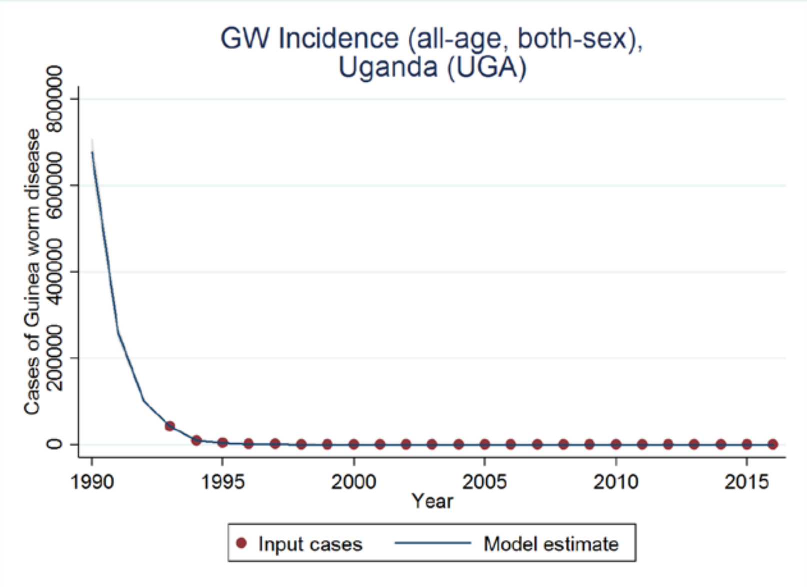

GW Incidence (all-age, both-sex),
Uganda (UGA)
Cases of Guinea worm disease
0
200000
400000
600000
800000
1990
1995
2000
2005
2010
2015
Year
Input cases
Model estimate

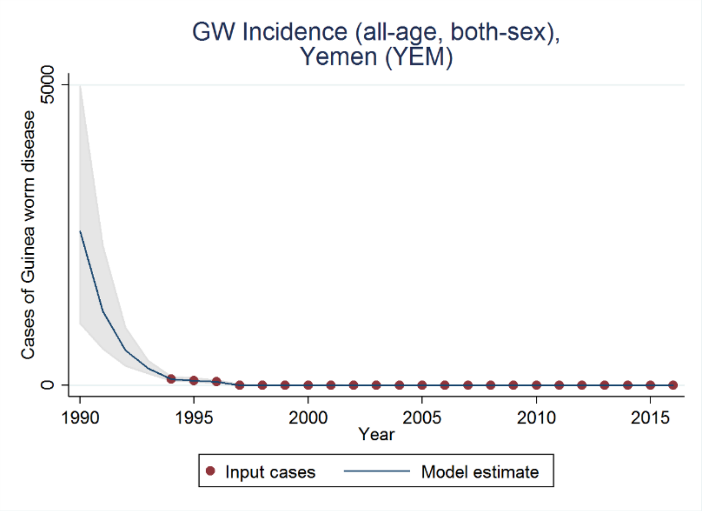

GW Incidence (all-age, both-sex),
Yemen (YEM)
Cases of Guinea worm disease
0
5000
1990
1995
2000
2005
2010
2015
Year
Input cases
Model estimate

Figure 1. Overall comparison of model versus reported cases (excluding outliers)

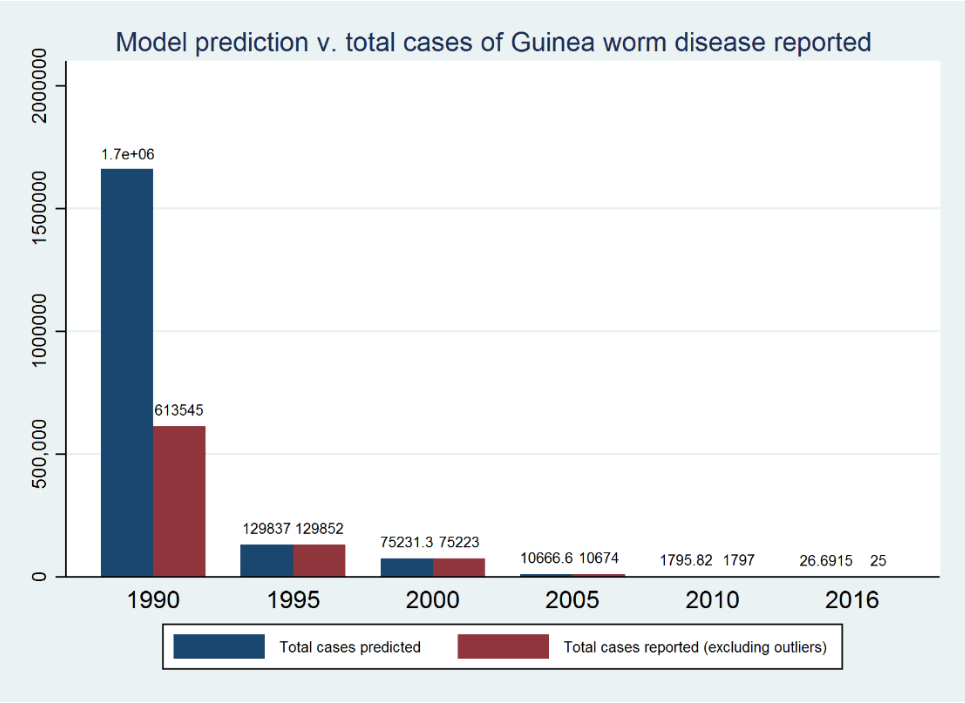

Sex-specific incidence

To account for the proportion of cases in females compared to males (53% to 47%), the incidence draws were multiplied by the sex proportion and the total population (to estimate number of cases by sex), then divided by the sex-specific total population for that year to calculate sex-specific incidence.

Age-specific incidence

In order to generate age-specific incidence, a literature search was conducted to identify national and subnational data sources in which age-specific prevalence was reported. The only nationally representative data available were WER reports from 2009 onward; however, age was only reported as less than 15 years of age or older than 15 years of age. In order to generate a trend over the life course, eight subnational data sources were identified. The prevalence of Guinea worm disease was extracted by age category reported in the original paper. An age trend was then fit using DisMod 2.0, with the following model settings:

Age mesh points: 0 0.01 5 10 15 20 25 30 35 40 45 50 55 60 65 70 75 80 85 90 1000

Drill year: 2000; Drill location: Global; no birth prevalence; 30 year time window

The age data were used to generate one single-age trend that we assumed applied to all geographies and all estimation periods from 1990 to 2017.

Figure 2. Age-specific prevalence model generated by DisMod

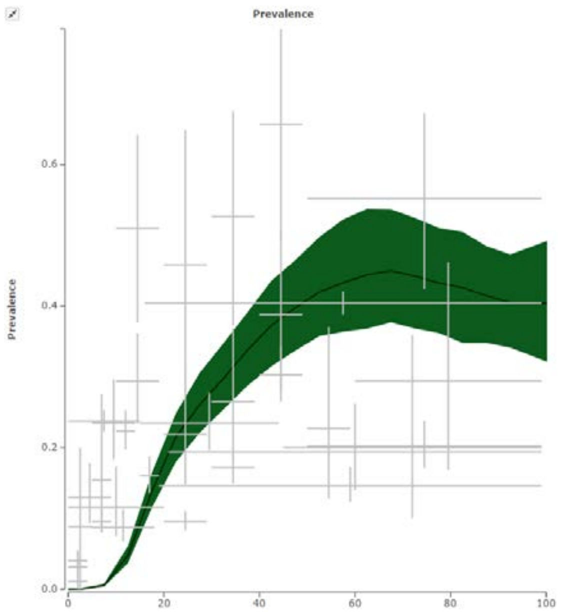

To apply this age prevalence curve to the sex-split incidence draws, 1,000 draws of output were downloaded from DisMod and applied to the incidence data as follows:

j indexes the age strata

i indexes the draw (1 to 1,000)

sex cases draw is the total number of cases for the sex stratum (all ages)

$$age\ cases_j = \ DisMod\ Draw_{i,j} * age\ population_j$$

$$age\ incidence\ draw_i = \frac{age\ cases_j \left(\frac{sex\ cases\ draw_i}{total\ cases}\right)}{age\ population_j}$$

Under the assumption that Guinea worm disease occurs approximately one year post-infection, incidence among children aged less than 1 year was set to zero.

Sequelae splits

Prevalence of the sequelae listed in Table 3 was calculated by multiplying the age- and sex-specific incidence draw by the duration of the health state (in years).

1) Guinea worm pain associated with worm emergence (Level 2): all cases, 1 month
2) Guinea worm pain associated with worm emergence (Level 1): all cases, 2 months plus 30% of cases for an additional 9 months
3) Lower limb musculoskeletal problems: all cases, 1 month

References

1. Cairncross S, Muller R, Zagaria N. Dracunculiasis (Guinea worm disease) and the eradication initiative. Clin Microbiol Rev. 2002;15(2):223-46.
2. Biswas G, Sankara DP, Agua-Agum J, Maiga A. Dracunculiasis (guinea worm disease): eradication without a drug or a vaccine. Philos Trans R Soc Lond B Biol Sci. 2013;368(1623):20120146.
3. Ruiz-Tiben E, Hopkins DR. Dracunculiasis (Guinea worm disease) eradication. Adv Parasitol. 2006;61:275-309.
4. Greenaway C. Dracunculiasis (guinea worm disease). CMAJ. 2004;170(4):495-500.
5. Hopkins DR, Ruiz-Tiben E, Downs P, Withers PC, Jr., Roy S. Dracunculiasis eradication: neglected no longer. Am J Trop Med Hyg. 2008;79(4):474-9.
6. Kappus KD, Hopkins DR, Ruiz-Tiben E, Imtiaz R, Andersen J, Azam M, et al. A strategy to speed the eradication of dracunculiasis. World Health Forum. 1991;12(2):220-5.
7. Prevention CfDCa. Guinea worm wrap-up Atlanta, GA: WHO Collborating center for Research, Training and Eradication of Dracunculiasis, CDC; 2015.
8. Hopkins DR, Ruiz-Tiben E, Diallo N, Withers PC, Jr., Maguire JH. Dracunculiasis eradication: and now, Sudan. Am J Trop Med Hyg. 2002;67(4):415-22.
9. Watts SJ, Brieger WR, Yacoob M. Guinea worm: an in-depth study of what happens to mothers, families and communities. Soc Sci Med. 1989;29(9):1043-9.
10. Adeyeba OA, Kale OO. Epidemiology of dracunculiasis and its socio-economic impact in a village in south-west Nigeria. West Afr J Med. 1991;10(3-4):208-15.
11. Kale OO. The clinico-epidemiological profile of guinea worm in the Ibadan district of Nigeria. Am J Trop Med Hyg. 1977;26(2):208-14.
12. Greenwood B, Greenwood A, Bradley A. Guinea worm infection in northern Nigeria: reflections on a disease approaching eradication. Trop Med Int Health. 2017.
13. Tayeh A, Cairncross S. The impact of dracunculiasis on the nutritional status of children in South Kordofan, Sudan. Ann Trop Paediatr. 1996;16(3):221-6.
14. Belcher DW, Wurapa FK, Ward WB, Lourie IM. Guinea worm in southern Ghana: its epidemiology and impact on agricultural productivity. Am J Trop Med Hyg. 1975;24(2):243-9.
15. Muller R. Guinea worm disease: epidemiology, control, and treatment. Bull World Health Organ. 1979;57(5):683-9.
16. Okoye SN, Onwuliri CO, Anosike JC. A survey of predilection sites and degree of disability associated with guineaworm (Dracunculus medinensis). Int J Parasitol. 1995;25(9):1127-9.
17. Smith GS, Blum D, Huttly SR, Okeke N, Kirkwood BR, Feachem RG. Disability from dracunculiasis: effect on mobility. Ann Trop Med Parasitol. 1989;83(2):151-8.
18. Chippaux JP, Banzou A, Agbede K. [Social and economic impact of dracunculosis: a longitudinal study carried out in 2 villages in Benin]. Bull World Health Organ. 1992;70(1):73-8.
19. Hours M, Cairncross S. Long-term disability due to guinea worm disease. Trans R Soc Trop Med Hyg. 1994;88(5):559-60.

3.3.5 Ascariasis SDG Appendix

Flowchart

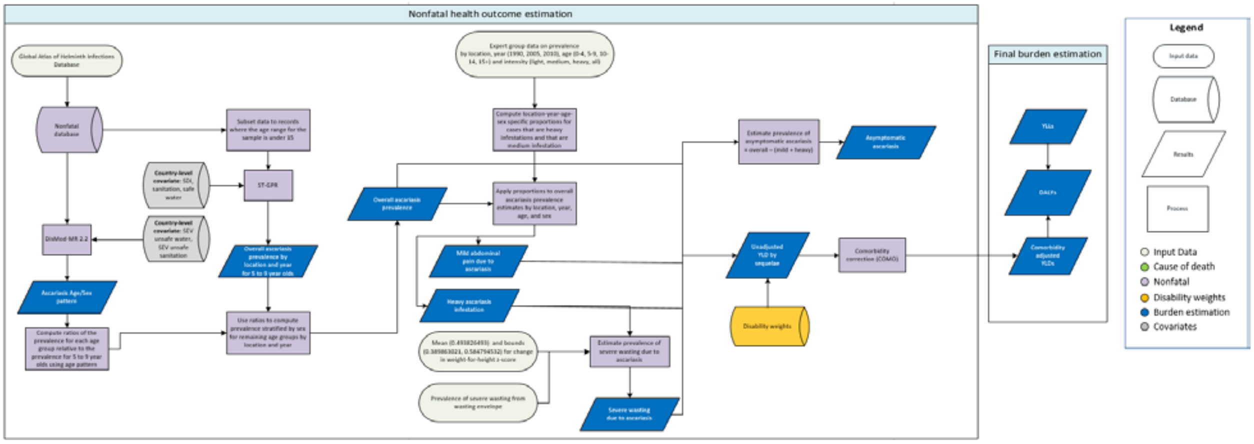

Input data and methodological summary

Case definition

Ascariasis is a helminthic disease caused by the parasitic roundworm *Ascaris lumbricoides*. It is one of the three intestinal nematode infections (INI), or soil-transmitted helminthiasis (STH), that are modelled in GBD. Diagnosis is made by examination of stool by microscope or PCR, with or without concentration procedures. The ICD-10 codes for ascariasis are B77-B77.9.

Input data

Global Atlas of Helminth Infections Data

Input data for this model were primarily compiled from the Global Atlas of Helminth Infections (GAHI) database. The GAHI database collates an exhaustive catalog of surveys and studies conducted by scientists that attempt to estimate the burden of STH [1]. Each record in the database contained metadata (ie, location, year, age range, sex) of each study sample and the prevalence of ascariasis in that sample. We excluded data points where the age range of the sample was unknown and retained only those surveys where Kato-Katz diagnostics were used. The table below displays the number of site-years by geography:

Table 1a. Site-years for ascariasis from the Global Atlas of Helminth Infections data used in GBD 2017

	Prevalence
Site-years (total)	353
Number of countries with data	62
Number of GBD regions with data (out of 21 regions)	13
Number of GBD super-regions with data (out of 7 super-regions)	6

Expert Group Data

Since GBD 2010, we have used prevalence data prepared by the GBD expert group (EG) containing mean prevalence with confidence intervals, stratified by location, year (1990, 2005, 2010), age group (0-4, 5-9, 10-14, 15+ years) and intensity of infection (light, medium, heavy, all). In order to move toward updating inputs and methods, we altered our use of these data. For some stages of our processes, we retain information from previous GBD cycles and the expert group, detailed below.

Geographic restrictions

We conducted a literature review to determine the geographic extent of the disease and classify locations based on whether the disease is absent or present in each year. Locations that were geographically restricted in any given year did not have estimates made for them. Of note, we did not attempt a complete systematic review, since a single high-quality source could offer sufficient evidence of presence. Evidence of absence or presence was not available for every location for each year, and so assumptions were made for missing years by taking into consideration the epidemiological characteristics of the disease.

If evidence indicated disease presence for two non-consecutive years, we assumed presence for all years between the two. If evidence indicated disease absence for two non-consecutive years, we assumed absence for all years between the two. If evidence indicated a change in status (ie, from absent to present, or present to absent) between two non-consecutive years, than we conducted targeted searches to ascertain the relevant year of introduction or elimination for that location. In the cases where presence or absence information was missing for the start or end years of our study interval (1990–2017) without evidence of any introduction or elimination events within the interval, we applied the status of the first and last presence/absence observations, respectively, to all years between the interval bound and the observation year. Our search was done in conjunction with the title/abstract screening portion of a systematic literature review for prevalence data. The search strings and yield can be viewed in the table below for each of the databases queried.

Table 1b. Geographic restriction search strings

Database	Search String	Yield
PubMed	(Ascariasis[Title/Abstract] OR Ascaris[Title/Abstract] OR "A. lumbricoides"[Title/Abstract] OR Ascaris[MeSH] OR Trichuris[Title/Abstract] OR Trichuriasis[Title/Abstract] OR "Whip Worm"[Title/Abstract] OR "T. trichura"[Title/Abstract] OR Trichuris[MeSH] OR Hookworm[Title/Abstract] OR "A. duodenale"[Title/Abstract] OR "Ancylostoma duodenale"[Title/Abstract] OR ancylostomiasis[Title/Abstract] OR "N. americanus"[Title/Abstract] OR "Necator americanus"[Title/Abstract] OR necatoriasis[Title/Abstract] OR Ancylostoma [MeSH] OR Necator[MeSH]) AND (prevalence[Title/Abstract] OR incidence[Title/Abstract] OR epidemiology[Title/Abstract] OR surveillance[Title/Abstract]) NOT(Animals[MeSH] NOT Humans[MeSH])	2,376
Web of Science	(Ascariasis OR Ascaris OR A. lumbricoides OR Trichuris OR Trichuriasis OR Whip Worm OR T. trichura OR Hookworm OR A. duodenale OR Ancylostoma duodenale OR anclyostomiasis OR N. americanus OR Necator americanus OR necatoriasis) AND TOPIC:(prevalence OR incidence OR epidemiology OR surveillance) NOTTOPIC: ((Animals NOT Humans))<br>Timespan: 1980-2016. Indexes: SCI-EXPANDED, SSCI, A&HCI, ESCI.	2,266

SCOPUS	TITLE-ABS_KEY (ascariasis OR ascaris OR a. lumbricoides OR trichuris OR trichuriasis OR whip worm OR t. trichura OR hookworm OR a. duodenale OR ancylostoma duodenale OR anclyostomiasis OR n. americanus OR necator americanus OR necatoriasis) AND PUBYEAR>1979	29

These papers were used to classify location-years for all locations and years present in the literature. We only utilised papers that are explicitly concerned with ascariasis. Additionally, systematic literature reviews, meta-analyses, national health statistics publications, and collaborator input were used to classify location-years not present in the literature review wherever possible.

Health states/sequelae

The table below shows the list of sequelae due to ascariasis and the associated disability weights (DW). Prevalence of medium infection and heavy infection were mapped to *mild abdominopelvic problems* and *heavy infestation of ascariasiss,* respectively. Light infection or asymptomatic was not attributed any disability. To inform the wasting model, 1,000 draws of severe wasting prevalence among children under 5 years were ascertained from GBD 2017 estimates – the methods used to generate estimates of wasting prevalence are detailed elsewhere (part of risk factors documentation) [2].

Table 2. Sequelae, lay descriptions, and disability weights (DWs)

Sequela	Lay description	DW
Mild abdominopelvic problems	"has some pain in the belly that causes nausea but does not interfere with daily activities"	0.011 (0.005–0.021)
Heavy infestation	"has cramping pain and a bloated feeling in the belly"	0.027 (0.015–0.043)
Severe wasting	"is extremely skinny and has no energy"	0.128 (0.082–0.183)
Asymptomatic ascariasis	N/A	N/A

Modelling strategy

DisMod-MR 2.2

In the estimation of overall morbidity due to ascariasis, we implemented a three-stage modelling framework. The first stage of the modelling process was using DisMod-MR to generate a global age-sex curve to disaggregate all-age, both-sex prevalence data. DisMod is an integrated meta-regression framework that allows for multiple datasets to be used within a singular analysis regardless of age-binning, sources, and geographies. As a result, a variety of differently aggregated information can be evaluated to generate a consensus output. Our final model contained all processed GAHI data as input and was informed by two country-level covariates (ie, all risk factors SEV for unsafe water, and all risk factors SEV for unsafe sanitation). From this model, the global fits were used.

Table 3a. DisMod model covariates

**Covariate**	**Type**	**Parameter**	**Exponentiated beta**
SEV unsafe water	Country-level	Proportion	4.41 (4.22–4.48)

SEV unsafe sanitation	Country-level	Proportion	4.45 (4.35–4.48)

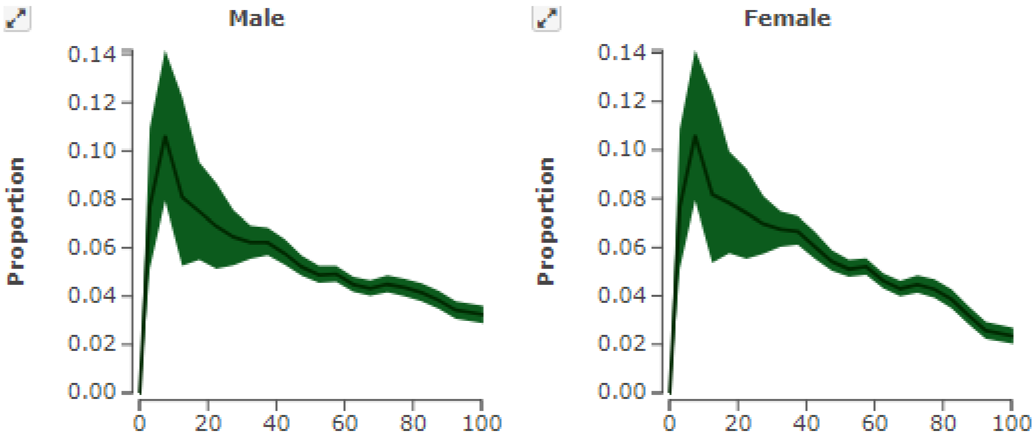

Figure 1: Global age-specific prevalence estimates for males (left) and females (right) for the year 2017. Proportion (prevalence) is on the Y-axis, and age in years on the X-axis. Screenshot from EpiViz tool.

Figure 1 shows the age-specific variation in prevalence rates, differentiated by sex. When considered as a global aggregate, we see that reported male and female prevalence are very similar. This is mostly a function of data used for modelling mainly being reported for both sexes. The highest prevalence rates are among adolescents and then decline among adults.

ST-GPR

After obtaining a global age-sex pattern from DisMod, we utilise a spatiotemporal Gaussian process regression (ST-GPR) to generate a complete time series of estimates for each location where there are no geographic restrictions. ST-GPR attempts to model non-linear trends utilising a Gaussian process to fit a trend. The following model specifications were used:

$$Prevalence = Proportion\ Sanitation + Proportion\ Safe\ Water + Sociodemographic\ Index + (1|level\ 2) + (1|level\ 3)$$

Where Levels 2 and 3 refer to GBD location hierarchies, or random effects for region and location. Notably, the covariates for the model were sanitation or proportion of population with access to improved toilet types, and safe water or proportion of population with access to improved water sources. Improved toilet types and improved water sources are defined by the Joint Monitoring Program. The following hyperparameters were used: st-lambda = 0.25, st-omega =2, st-zeta = 0.01, gpr-scale = 15. We selected these hyperparameters as they provided more weight to country-level data rather than region-level data when estimating the prevalence for a given location-year. In other words, these hyperparameters ensure that the Gaussian process regressions follow country-specific data rather than region-specific data when estimating a time series for a location.

It is important to note that we did not use all processed GAHI data for the ST-GPR model. We opted to run a child-only model because the bulk of our data is among adolescents and there is more granular age information that we can leverage during modelling processes. More specifically, any data points that had

age bins between 0 and 15 years were assigned to the 5 to 9 age group. We selected all data with age bins between 0 and 15 because they fall within the peak in prevalence across all age groups; this is where a majority of data are, and this provides sufficient statistical power for our model. This left us with 210 site-years of global input data for ST-GPR.

Table 3b. ST-GPR model covariates

Covariate	Exponentiated beta	Standard error
Socio-demographic Index	-9.99	2.09
Safe water	-2.56	1.06
Sanitation	3.95	0.79

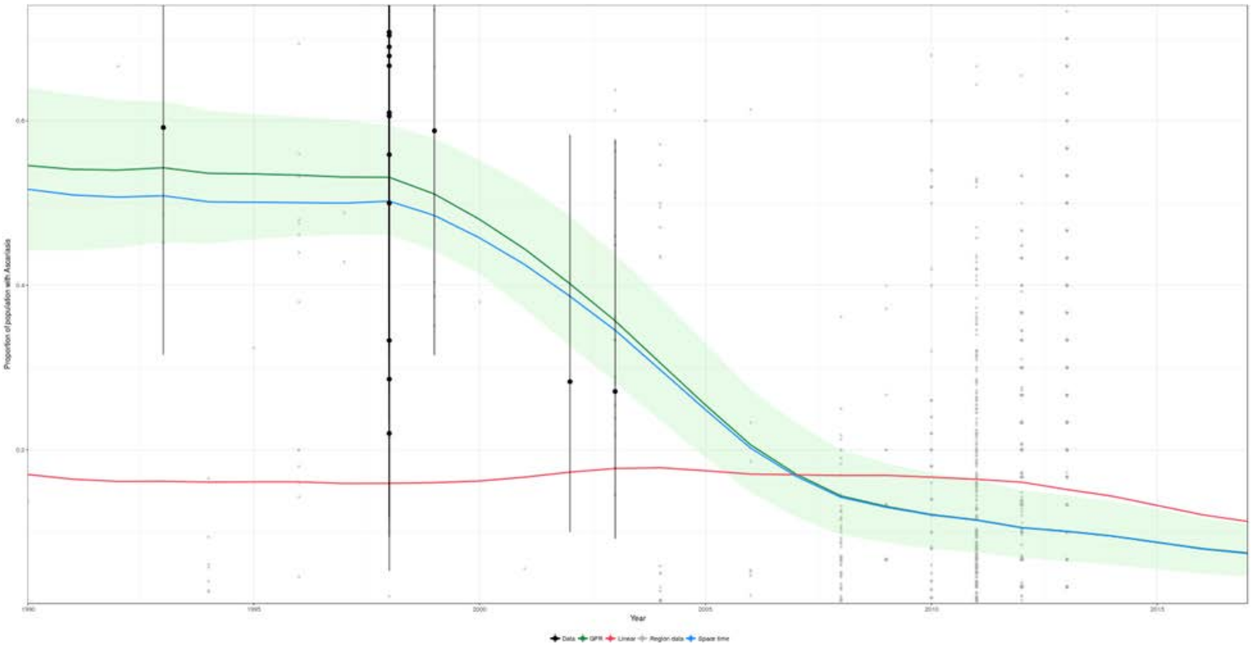

Figure 2: ST-GPR estimates for Cameroon (0- to 15-year-olds, both sex) for years 1990–2017. Black dots represent input data points, with the black lines indicating variance. The green line represents the mean GPR estimated values, with uncertainty shown by the green polygon. The blue line indicates the space-time component of the ST-GPR; the red line indicates the linear regression component derived from global data. Transparent black dots represent data from other locations in the GBD region (Western sub-Saharan Africa).

Figures 2 displays the time trends as computed by ST-GPR. For the most part, locations looked similar to Cameroon, where we see consistent declines in prevalence throughout time.

Imputations

The final stage of the overall prevalence modelling process is to impute the remaining age groups by borrowing information from the ST-GPR time series for 5- to 9-year-olds and the DisMod global age-sex pattern. First, we assign each age group a ratio of how much larger or smaller the prevalence is compared

to the prevalence for 5- to 9-year-olds using the DisMod global age-sex pattern. More specifically, the following is the computation for each age group:

$$Ratio = \frac{prevalence_{[age\ start] to\ [age\ end]}}{prevalence_{5\ to\ 9}}$$

We opted not to use the age-sex curves by location or region, because DisMod performed better at disaggregating our heterogeneous data at the global level. With a ratio for every age group by sex, we multiplied the ratio by the ST-GPR location-year estimates to impute estimates for the remaining age groups.

Health states/sequelae

Following computations of location-year-age-sex-specific prevalence of ascariasis, we leverage information from the 2010 EG data to conduct sequelae splits. The 2010 EG data provided estimates for heavy infestation, mild abdominopelvic problems, and asymptomatic ascariasis by location and for 1990, 2005, and 2010. These three values add up to all cases of ascariasis. Thus, for heavy infestation and mild abdominopelvic problems, we computed the proportion of cases that belong to our sequelae of interest over all cases of ascariasis. More specifically, the following is the computation by heavy infestation and mild abdominopelvic problems:

$$Proportion_{sequelae} = \frac{prevalence_{sequelae}}{prevalence_{all\ cases}}$$

This calculation was done for every location, year, and age group available. Because the EG data only had four age groups (0-4, 5-9, 10-14, 15+ years), we applied the 15+ age group proportion for all remaining age groups. In addition, for 1995 and 2000 we applied the 1990 proportions, and for 2017 we applied the 2010 proportions. Using these location-year-age-specific proportions, we multiplied the total ascariasis estimates to compute heavy infestation and mild abdominopelvic prevalence. To estimate the prevalence of asymptomatic ascariasis, prevalence of mild and heavy infestation was subtracted from the overall ascariasis prevalence.

The final step in the modelling process was to estimate the prevalence of severe wasting due to ascariasis in age groups 28–364 days and 1–4 years. This was done separately using 1,000 draws of prevalence of heavy infestation due to ascariasis and the wasting envelope prevalence. The initial step in determining prevalence of severe wasting due to ascariasis was generating 1,000 draws of change in weight-for-height z-score per heavy prevalent case from a random normal distribution with mean = 0.493826493 and standard deviation = 0.04972834 (calculated from upper and lower bounds of the mean estimate). The mean, upper, and lower bounds were based on a published article [2]. The prevalence of severe wasting due to ascariasis was then obtained as a function of change in weight-for-height z-score. The following are the computations:

$$Prevalence_{wasting\ due\ to\ ascariasis} = wasting - \Phi(\Phi^{-1}(\text{wasting}) - \text{z score} * \text{heavy infestation})$$

Where Φ is the standard normal cumulative distribution function and Φ^{-1} is the inverse standard normal cumulative distribution function.

Changes from GBD 2016

Significant changes have been made compared to prior GBD cycles in an effort to build a database of ascariasis data points and to continuously update methodologies. The following are changes since prior versions of GBD:

Data – In prior years we used estimates from the 2010 EG data. Here, we transition to utilising a comprehensive database of ascariasis data points so we may annually update our inputs to reflect up-to-date data.

Age-sex pattern – Given a substantial amount of heterogeneity in our input data, age-sex curves were generated from a DisMod model.

Gaussian process regressions – To obtain location-year prevalence estimates, we implemented ST-GPR methodology consistently across the globe.

Limitations

As we attempt to improve the modelling processes for ascariasis, we recognise that there are several limitations. A substantial limitation is with regard to our data. While the GAHI database represents a comprehensive synthesis of ascariasis data points, numerous data points were excluded due to our specific case definition. We opted to only include studies where Kato-Katz was used to identify infected individuals, forcing us to drop a large proportion of the GAHI database. Inclusion of these studies may provide substantially more information with regard to our age patterns and time trends. Upcoming GBD cycles will explore methods for combining data with idiosyncratic diagnostic tools.

A secondary limitation to our data is that several included studies are not considered to be nationally representative, and therefore at a location level, the data are highly heterogeneous (Figure 3). Numerous studies within the database were conducted in districts or townships, and in some cases the studies were done in known areas where prevalence is high. Upcoming GBD cycles will continue to rigorously vet these data points and update the database with literature searches. In addition, exploring the use of methodologies, such as model-based geostatistics, that implicitly model the spatial heterogeneity with this focal condition, are likely necessary.

Furthermore, we made a large assumption that the global age-sex distributions were applicable to all locations. While we believe that prevalence should peak among adolescents and slowly decline afterward, there is likely variation across regions and locations. Given that our data are either among children or all-age, it is very difficult to build an age trend at granular location levels. Thus, we allowed DisMod to disaggregate our heterogeneous data in an effort to provide sensible age-sex curves.

We believe that more work needs to be done to improve our sequelae split methods. Since the EG data do not provide all estimation years and age groups, several assumptions had to be made. Thus, we will explore conducting literature searches to provide novel data points for sequelae estimations. Lastly, we ought to explore covariate effects in our ST-GPR model. Surprisingly, the sanitation covariate had a positive relationship with ascariasis prevalence, suggesting that our covariates may need to be reevaluated for future GBD cycles.

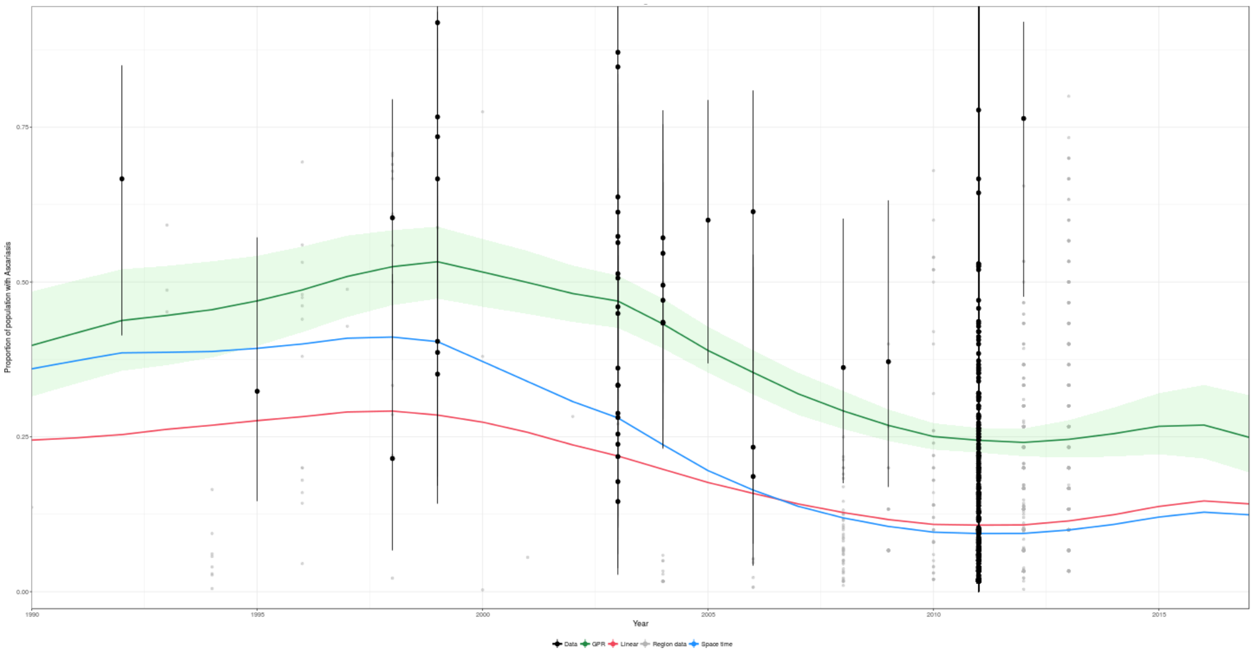

Figure 3: ST-GPR estimates for Nigeria (0 to 15 year olds, both sex) for years 1990–2017. Coloration and symbols are as stated in caption for Figure 2.

Figure 3 show the time trend for Nigeria as computed by ST-GPR. For some locations, we estimate this fluctuating time trend which is a function of the heterogeneity in our input data. As explained above, some of these data points are representative of townships in Nigeria, causing a great deal of heterogeneity throughout time.

References

1. London School of Hygiene and Tropical Medicine. Global Atlas of Helminth Infections – Soil Transmitted Helminths. London, United Kingdom: London School of Hygiene and Tropical Medicine.
2. Hall A, Hewitt G, Tuffrey V, de Silva N. A review and meta-analysis of the impact of intestinal worms on child growth and nutrition. *Maternal and Child Nutrition*. 2008. 4. 118-236.

3.3.5 Hookworm Disease SDG Capstone Appendix

Flowchart

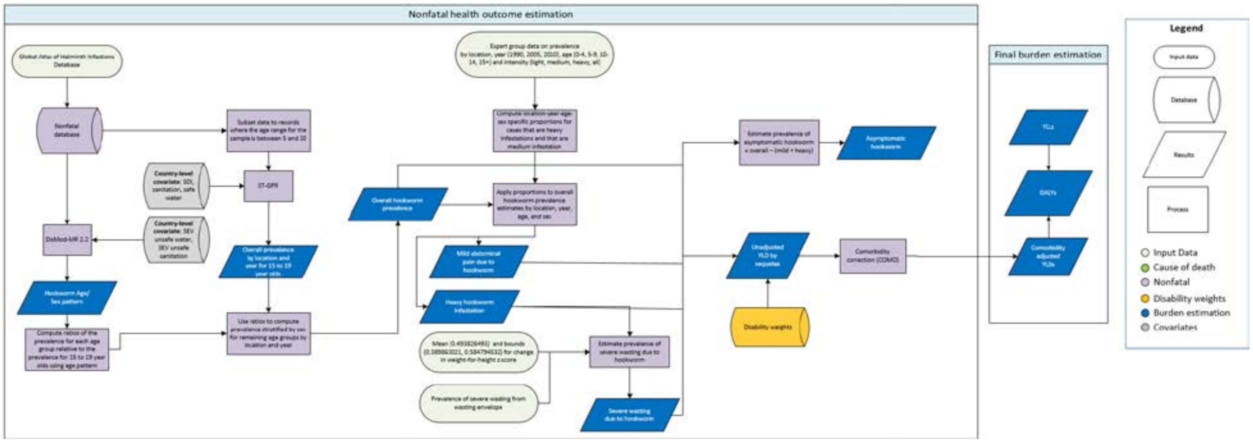

Input data and methodological summary

Case Definition

Hookworm disease is a helminthic disease caused by intestinal parasites in the roundworm group, *Ancylostoma duodenale* and *Necator americanus*. It is one of the three intestinal nematode infections (INI), or soil-transmitted helminthiasis (STH), that we model in GBD. Diagnosis is made by examination of stool by microscope or PCR, with or without concentration procedures. The ICD-10 codes for hookworm disease are B76-B76.9.

Input data

Global Atlas of Helminth Infections Data

Input data for this model were primarily compiled from the Global Atlas of Helminth Infections (GAHI) database. The GAHI database collates an exhaustive catalog of surveys and studies conducted by scientists that attempt to estimate the burden of STH [1]. Each record in the database contained metadata (ie, location, year, age range, sex) of each study sample and the prevalence of hookworm in that sample. We excluded data points where the age range of the sample was unknown and retained only those surveys where Kato-Katz diagnostics were used. The table below displays the number of site-years by geography:

Table 1a. Site-years for hookworm from the Global Atlas of Helminth Infections data used in GBD 2017

	Prevalence
Site-years (total)	312
Number of countries with data	60

**Number of GBD regions with data (out of 21 regions)**	10
**Number of GBD super-regions with data (out of 7 super-regions)**	5

Expert Group Data

Since GBD 2010, we have used prevalence data prepared by the GBD expert group (EG) containing mean prevalence with confidence intervals, stratified by location, year (1990, 2005, 2010), age group (0-4, 5-9, 10-14, 15+ years) and intensity of infection (light, medium, heavy, all). In order to move toward updating inputs and methods, we altered our use of these data. For some stages of our processes, we retain information from previous GBD cycles and the expert group, detailed below.

Geographic Restrictions

We conducted a literature review to determine the geographic extent of the disease and classify locations based on whether the disease is absent or present in each year. Locations that were geographically restricted in any given year did not have estimates made for them. Of note, we did not attempt a complete systematic review, since a single high-quality source could offer sufficient evidence of presence. Evidence of absence or presence was not available for every location for each year, and so assumptions were made for missing years by taking into consideration the epidemiological characteristics of the disease.

If evidence indicated disease presence for two non-consecutive years, we assumed presence for all years between the two. If evidence indicated disease absence for two non-consecutive years, we assumed absence for all years between the two. If evidence indicated a change in status (ie, from absent to present, or present to absent) between two non-consecutive years, then we conducted targeted searches to ascertain the relevant year of introduction or elimination for that location. In the cases where presence or absence information was missing for the start or end years of our study interval (1990–2017) without evidence of any introduction or elimination events within the interval, we applied the status of the first and last presence/absence observations, respectively, to all years between the interval bound and the observation year. Our search was done in conjunction with the title/abstract screening portion of a systematic literature review for prevalence data. The search strings and yield can be viewed in the table below for each of the databases queried.

Table 1b. Geographic Restriction Search Strings

Database	Search String	Yield
PubMed	(Ascariasis[Title/Abstract] OR Ascaris[Title/Abstract] OR "A. lumbricoides"[Title/Abstract] OR Ascaris[MeSH] OR Trichuris[Title/Abstract] OR Trichuriasis[Title/Abstract] OR "Whip Worm"[Title/Abstract] OR "T. trichura"[Title/Abstract] OR Trichuris[MeSH] OR Hookworm[Title/Abstract] OR "A. duodenale"[Title/Abstract] OR "Ancylostoma duodenale"[Title/Abstract] OR ancylostomiasis[Title/Abstract] OR "N. americanus"[Title/Abstract] OR "Necator americanus"[Title/Abstract] OR necatoriasis[Title/Abstract] OR Ancylostoma [MeSH] OR Necator[MeSH]) AND (prevalence[Title/Abstract] OR incidence[Title/Abstract] OR epidemiology[Title/Abstract] OR surveillance[Title/Abstract]) NOT(Animals[MeSH] NOT Humans[MeSH])	2,376
Web of Science	(Ascariasis OR Ascaris OR A. lumbricoides OR Trichuris OR Trichuriasis OR Whip Worm OR T. trichura OR Hookworm OR A. duodenale OR Ancylostoma	2,266

	duodenale OR anclyostomiasis OR N. americanus OR Necator americanus OR necatoriasis) AND TOPIC:(prevalence OR incidence OR epidemiology OR surveillance) NOTTOPIC: ((Animals NOT Humans))<br>Timespan: 1980-2016. Indexes: SCI-EXPANDED, SSCI, A&HCI, ESCI.	
SCOPUS	TITLE-ABS_KEY (ascariasis OR ascaris OR a. lumbricoides OR trichuris OR trichuriasis OR whip worm OR t. trichura OR hookworm OR a. duodenale OR ancylostoma duodenale OR anclyostomiasis OR n. americanus OR necator americanus OR necatoriasis) AND PUBYEAR>1979	29

These papers were used to classify location-years for all locations and years present in the literature. We only utilised papers that are explicitly concerned with hookworm. Additionally, systematic literature reviews, meta-analyses, national health statistics publications and collaborator input were used to classify location-years not present in the literature review wherever possible.

Health states/sequelae

The table below shows the list of sequelae due to hookworm and the associated disability weights (DW). Prevalence of medium infection and heavy infection were mapped to *mild abdominopelvic problems* and *heavy infestation of hookworm,* respectively. Light infection was not attributed any disability. To inform the wasting model, 1,000 draws of severe wasting prevalence among children under 5 years were ascertained from GBD 2017 estimates – the methods used to generate estimates of wasting prevalence are detailed elsewhere (part of risk factors documentation) [2].

Table 2. Sequelae, lay descriptions, and disability weights (DWs)

Sequela	Lay description	DW
Mild abdominopelvic problems	"has some pain in the belly that causes nausea but does not interfere with daily activities"	0.011 (0.005–0.021)
Heavy infestation	"has cramping pain and a bloated feeling in the belly"	0.027 (0.015–0.044)
Severe wasting	"is extremely skinny and has no energy"	0.128 (0.082–0.183)
Asymptomatic hookworm disease	NA	NA
Mild anaemia	"feels slightly tired and weak at times, but this does not interfere with normal daily activities"	0.004 (0.001–0.008)
Moderate anaemia	"feels moderate fatigue, weakness, and shortness of breath after exercise, making daily activities more difficult"	0.052 (0.034–0.076)
Severe anaemia	"feels very weak, tired and short of breath, and has problems with activities that require physical effort or deep concentration"	0.149 (0.101–0.210)

Modelling strategy

DisMod-MR 2.2

In the estimation of overall morbidity due to hookworm, we implemented a three-stage modelling framework. The first stage of the modelling process was using DisMod-MR to generate a global age-sex curve to disaggregate all-age, both-sex prevalence data. DisMod is an integrated meta-regression framework that allows for multiple datasets to be used within a singular analysis regardless of age-binning, sources, and geographies. As a result, a variety of differently aggregated information can be evaluated to generate a consensus output. Our final model contained all processed GAHI data as input and was informed by two country-level covariates (ie, all risk factors SEV for unsafe water, and all risk factors SEV for unsafe sanitation). From this model, the global fits were used.

Table 3a. DisMod model covariates

Covariate	Type	Parameter	Exponentiated beta
SEV unsafe water	Country-level	Proportion	4.38 (4.14–4.48)
SEV unsafe sanitation	Country-level	Proportion	4.44 (4.25–4.48)

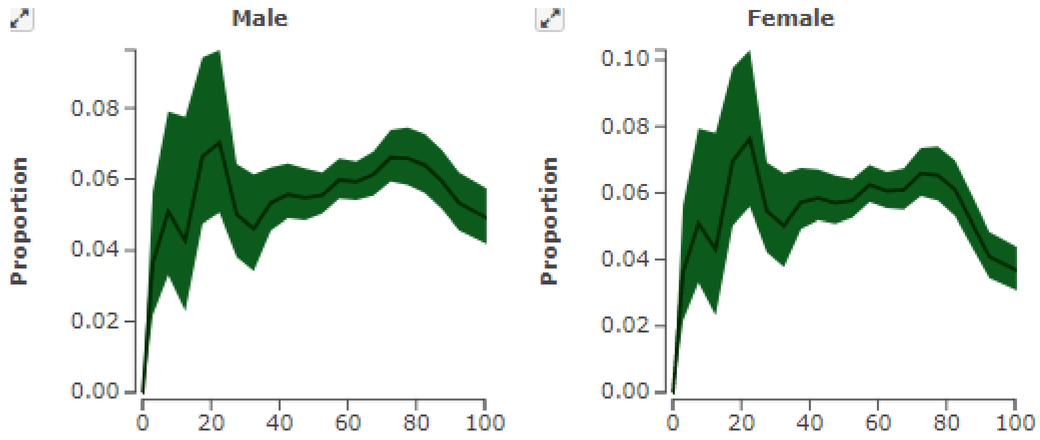

Figure 1: Global age-specific prevalence estimates for males (left) and females (right) for the year 2017. Proportion (prevalence) is on the Y-axis, and age in years on the X-axis. Screenshot from EpiViz tool.

Figure 1 shows the age-specific variation in prevalence rates, differentiated by sex. When considered as a global aggregate, we see that reported male and female prevalence are very similar. This is mostly a function of data used for modelling mainly being reported for both sexes. Prevalence peaks among young adults, followed by a decline and then stabilising during adulthood. These age-sex curves are similar to what has been reported in the literature [3, 4].

ST-GPR

After obtaining a global age-sex pattern from DisMod, we utilise a spatiotemporal Gaussian process regression (ST-GPR) to generate a complete time series of estimates for each location where there are no geographic restrictions. ST-GPR attempts to model non-linear trends utilising a Gaussian process to fit a trend. The following model specifications were used:

$$Prevalence = Proportion\ Sanitation + Proportion\ Safe\ Water + Sociodemographic\ Index + (1|level\ 2) + (1|level\ 3)$$

Where levels 2 and 3 refer to GBD location hierarchies, or random effects for region and location. Notably, the covariates for the model were sanitation or proportion of population with access to improved toilet types, and safe water or proportion of population with access to improved water sources. Improved toilet types and improved water sources are defined by the Joint Monitoring Programme. The following hyperparameters were used: st-lambda = 0.25, st-omega =2, st-zeta = 0.01, gpr-scale = 15. We selected these hyperparameters as they provided more weight to country-level data rather than region-level data when estimating the prevalence for a given location-year. In other words, these hyperparameters ensure that the Gaussian process regressions follow country-specific data rather than region-specific data when estimating a time series for a location.

It is important to note that we did not use all processed GAHI data for the ST-GPR model. We opted to run an adolescent-only model because the bulk of our data are among children and there is more granular age information that we can leverage during modelling processes. More specifically, any data points that had age bins between 5 and 20 years were assigned to the 15 to 19 age group. We selected all data with age bins between 5 and 20 because this falls right below the peak in prevalence across all age groups, this is where a majority of data are, and it provides sufficient statistical power for our model. This left us with 199 site-years of global input data for ST-GPR.

Table 3b. ST-GPR model covariates

Covariate	Exponentiated beta	Standard error
Socio-demographic Index	-0.12	1.69
Safe water	-2.89	0.66
Sanitation	-2.40	0.82

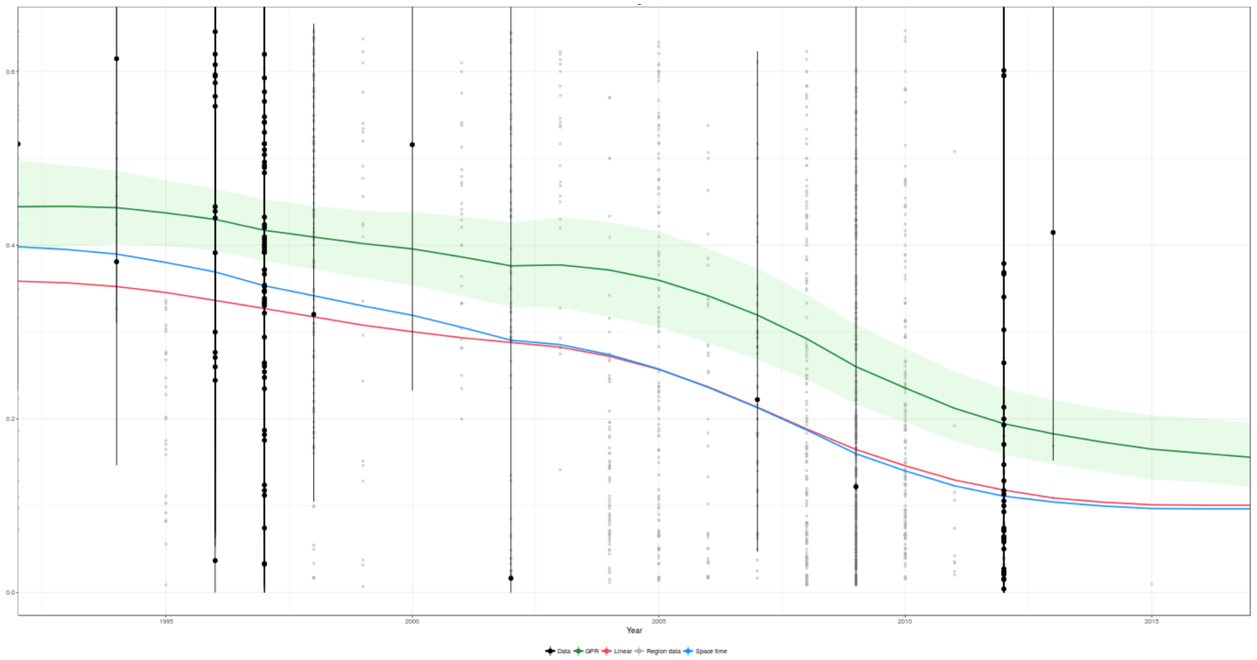

Figure 2: ST-GPR estimates for Tanzania (5- to 20-year-olds, both sexes) for years 1990–2017. Black dots represent input data points, with the black lines indicating variance. The green line represents the mean GPR estimated values, with uncertainty shown by the green polygon. The blue line indicates the space-time component of the ST-GPR; the red line indicates the linear regression component derived from global data. Transparent black dots represent data from other locations in the GBD region (Western sub-Saharan Africa).

Figures 2 displays the time trends as computed by ST-GPR. For the most part, locations looked similar to Tanzania, where we see steady declines in prevalence throughout time.

Imputation

The final stage of the overall prevalence modelling process is to impute the remaining age groups by borrowing information from the ST-GPR time series for 15- to 19-year-olds and the DisMod global age-sex pattern. First, we assign each age group a ratio of how much larger or smaller the prevalence is compared to the prevalence for 15- to 19-year-olds using the DisMod global age-sex pattern. More specifically, the following is the computation for each age group:

$$Ratio = \frac{prevalence_{[age\ start] to\ [age\ end]}}{prevalence_{15\ to\ 19}}$$

We opted not to use the age-sex curves by location or region, because DisMod performed better at disaggregating our heterogeneous data at the global level. With a ratio for every age group by sex, we multiplied the ratio by the ST-GPR location-year estimates to impute estimates for the remaining age groups.

Health states/sequelae

Following computations of location-year-age-sex-specific prevalence of hookworm, we leverage information from the 2010 EG data to conduct sequelae splits. The 2010 EG data provided estimates for

heavy infestation, mild abdominopelvic problems, and asymptomatic hookworm by location and for 1990, 2005, and 2010. These three values add up to all cases of hookworm. Thus, for heavy infestation and mild abdominopelvic problems, we computed the proportion of cases that belong to our sequelae of interest over all cases of hookworm. More specifically, the following is the computation by heavy infestation and mild abdominopelvic problems:

$$Proportion_{sequelae} = \frac{prevalence_{sequelae}}{prevalence_{all\ cases}}$$

This calculation was done for every location, year, and age group available. Because the EG data only had four age groups (0-4, 5-9, 10-14, 15+ years), we applied the 15+ age group proportion for all remaining age groups. In addition, for 1995 and 2000 we applied the 1990 proportions, and for 2017 we applied the 2010 proportions. Using these location-year-age specific proportions, we multiplied the total hookworm estimates to compute heavy infestation and mild abdominopelvic prevalence. To estimate the prevalence of asymptomatic hookworm, prevalence of mild and heavy infestation was subtracted from the overall hookworm prevalence.

The final step in the modelling process was to estimate the prevalence of severe wasting due to hookworm in age groups 28–364 days and 1–4 years. This was done separately using 1,000 draws of prevalence of heavy infestation due to hookworm and the wasting envelope prevalence. The initial step in determining prevalence of severe wasting due to hookworm was generating 1,000 draws of change in weight-for-height z-score per heavy prevalent case from a random normal distribution with mean = 0.493826493 and standard deviation = 0.04972834 (calculated from upper and lower bounds of the mean estimate). The mean, upper, and lower bounds were based on a published article [2]. The prevalence of severe wasting due to hookworm was then obtained as a function of change in weight-for-height z-score. The following are the computations:

$$Prevalence_{wasting\ due\ to\ hookworm} = wasting - \Phi(\Phi^{-1}(\text{wasting}) - \text{z score} * \text{heavy infestation})$$

Where Φ is the standard normal cumulative distribution function and Φ^{-1} is the inverse standard normal cumulative distribution function. Finally, the burden of anaemia due to hookworm disease was estimated separately (see anaemia documentation for details).

Changes from GBD 2016

Significant changes have been made compared to prior GBD cycles in an effort to build a database of hookworm data points and to continuously update methodologies. The following changes have been made since prior versions of GBD:

Data – In prior years we used estimates from the 2010 EG data. Here, we transition to utilising a comprehensive database of hookworm data points so we may annually update our inputs to reflect up-to-date data.

Age-sex pattern – Given a substantial amount of heterogeneity in our input data, age-sex curves were generated from a DisMod model.

Gaussian process regression – To obtain location-year prevalence estimates, we implemented ST-GPR methodology consistently across the globe.

Limitations

As we attempt to improve the modelling processes for hookworm, we recognise that there are several limitations. A substantial limitation is with regard to our data. While the GAHI database represents a comprehensive synthesis of hookworm data points, numerous data points were excluded due to our specific case definition. We opted to only include studies where Kato-Katz was used to identify infected individuals, forcing us to drop a large proportion of the GAHI database. Inclusion of these studies may provide substantially more information with regard to our age patterns and time trends. Upcoming GBD cycles will explore methods for combining data with idiosyncratic diagnostic tools.

A secondary limitation to our data is that several included studies are not considered to be nationally representative, and therefore, at a location level, the data are highly heterogeneous (Figure 3). Numerous studies within the database were conducted in districts or townships, and in some cases the studies were done in known areas where prevalence is high. Upcoming GBD cycles will continue to rigorously vet these data points and update the database with literature searches. In addition, exploring the use of methodologies, such as model-based geostatistics, that implicitly model the spatial heterogeneity with this focal condition, are likely necessary.

Furthermore, we made a large assumption that the global age-sex distributions were applicable to all locations. While we believe that prevalence should peak among young adults and slowly decline afterward, there is likely variation across regions and locations. Given that our data are either among adolescents or all-age, it is very difficult to build an age trend at granular location levels. Thus, we allowed DisMod to disaggregate our heterogeneous data in an effort to provide sensible age-sex curves.

Lastly, we believe that more work needs to be done to improve our sequelae split methods. Since the EG data do not provide all estimation years and age groups, several assumptions had to be made. Thus, we will explore conducting literature searches to provide novel data points for sequelae estimations.

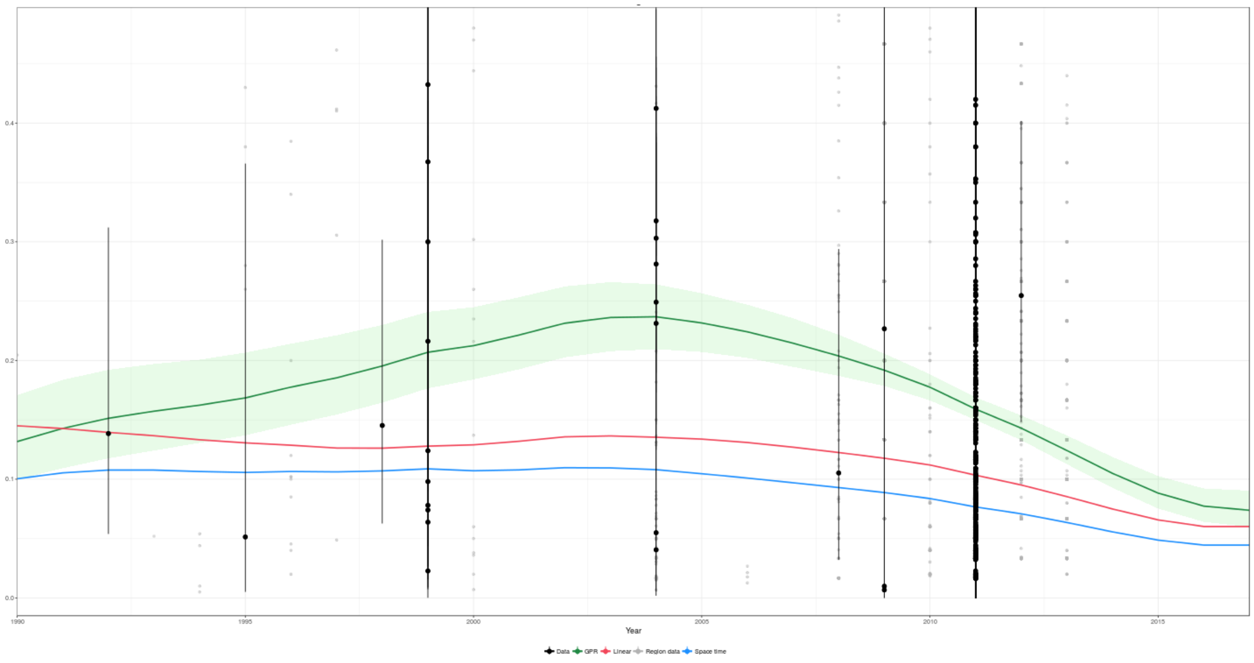

Figure 3: ST-GPR estimates for Nigeria (5- to 20-year-olds, both sexes) for years 1990–2017. Colouration and symbols are as stated in caption for Figure 2.

Figure 3 show the time trend for Nigeria as computed by ST-GPR. For some locations, we estimate this fluctuating time trend, which is a function of the heterogeneity in our input data. As explained above, some of these data points are representative of townships in Nigeria causing a great deal of heterogeneity throughout time.

References

1. London School of Hygiene and Tropical Medicine. Global Atlas of Helminth Infections – Soil Transmitted Helminths. London, United Kingdom: London School of Hygiene and Tropical Medicine.
2. Hall A, Hewitt G, Tuffrey V, de Silva N. A review and meta-analysis of the impact of intestinal worms on child growth and nutrition. *Maternal and Child Nutrition*. 2008. 4. 118-236.
3. Riess H, Clowes P, Kroidl, Kowuor D, Nsojo A, Mangu C, Schule S, Mansmann U, Geldmacher C, Mhina S, Maboko L, Hoelscher M, Saathoff E. Hookworm Infection and Environmental Factors in Mbeya Region, Tanzania: A Cross-Sectional, Population-Based Study. *PLoS Neglected Tropical Diseases*. 2013. 7. e2408.
4. Pullan R, Kabatereine N, Quinnell R, Brooker S. Spatial and Genetic Epidemiology of Hookworm in a Rural Community in Uganda. *PLoS Neglected Tropical Diseases*. 2010. 4. e713.

3.3.5 Cutaneous & Mucocutaneous Leishmaniasis SDG Capstone Appendix

Cutaneous & Mucocutaneous Leishmaniasis

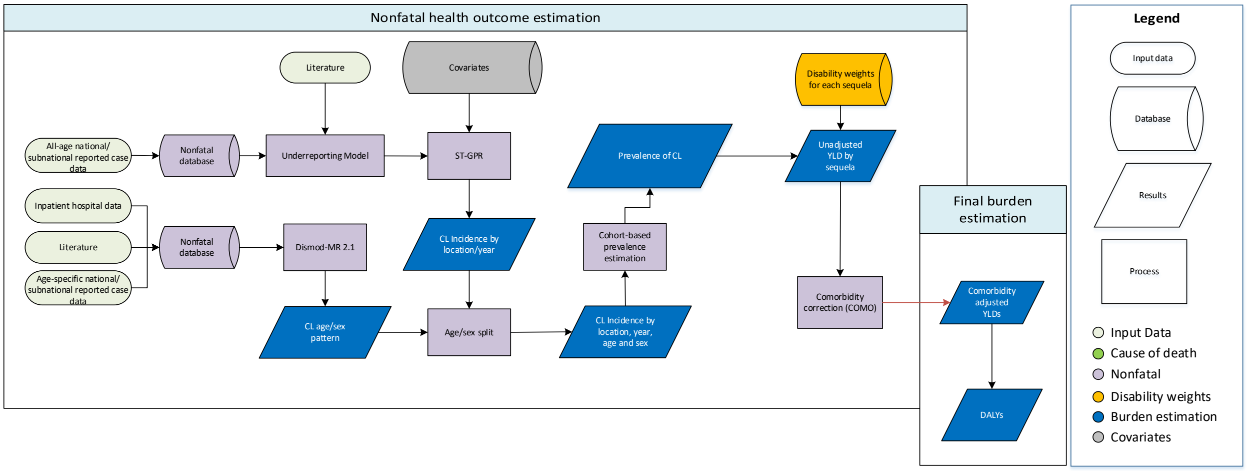

Description of general methodology

The non-fatal estimation process for cutaneous leishmaniasis is built from incident case notification data representative of the GBD geographic location, which are adjusted for underreporting. The upscaled all-age, both sex, case counts are modelled using spatiotemporal Gaussian process regression (ST-GPR) in order to impute for missing location-year combinations as well as to account for further biases and inaccuracies in reporting. Datasets that disaggregate CL cases by age and sex are modelled using DisMod to produce a global age-sex split which is applied to the all-age, both-sex, envelope estimates resulting from ST-GPR. These incidence estimates are used to derive prevalence measures, as well as compute the resulting years lived with disability values.

Input Data – Case Notification time series

Current estimation for the all-age, both-sex, incidence envelope is based upon location-representative information rather than site-specific epidemiological measures due to the absence of global foci maps allowing for upscaling of geographically precise information. The primary data resource therefore is the case notification time-series reported by National Control Programs and Ministries of Health to the World Health Organization. This is supplemented by systematic literature review (last updated for GBD 2015) to identify alternate sources of data for years missing information. For countries with subnational estimates, in-country collaborators have compiled information for respective programs, or identified key resources, again supplemented by literature reviews. Where possible, information disaggregating location-level statistics by age and sex were extracted

	Incidence
Site-years (total)	848
Number of countries with data	56
Number of GBD regions with data (out of 21 regions)	13

Number of GBD super-regions with data (out of 7 super-regions)	7

Table 1: Summary statistics for data used in cutaneous leishmaniasis estimation.

Method – Geographic restrictions

There are strong climatic and biogeographic constraints on the geographic distribution of CL resulting in a focal, rather than cosmopolitan global distribution. As a result, it is necessary to identify locations burdened by the disease through space and time as distinct from countries where CL is absent. Tags were assigned to each location-year based upon the outcome of a search of IHME databases, as well as location-specific searches of PubMed. Each location-year is tagged as follows:

- Present – where a specific citation of either an autochthonous laboratory-confirmed case (ie, a case with PCR, serological, or parasitological diagnosis), reported case (ie, a case noted as CL, but with no supporting diagnostic), or supporting evidence (ie, confirmed infection in animal reservoirs or sandfly vectors)
- Protocol Present – for a given location-year, where no specific citation is used, but is present for another year in the same location, it is assumed that CL is present given that eradication of the pathogen has not been achieved
- Absent – where PubMed location-specific searches returned zero relevant results, in locations scoring -25 or lower as evaluated by Pigott and colleagues (2014) [the threshold for “absence” in that study], locations were tagged as Absent
- Protocol Absent – as with Absent, locations with zero relevant PubMed results, but with greater than -25 as evaluated by Pigott and colleagues (2014), were tagged as Protocol Absent

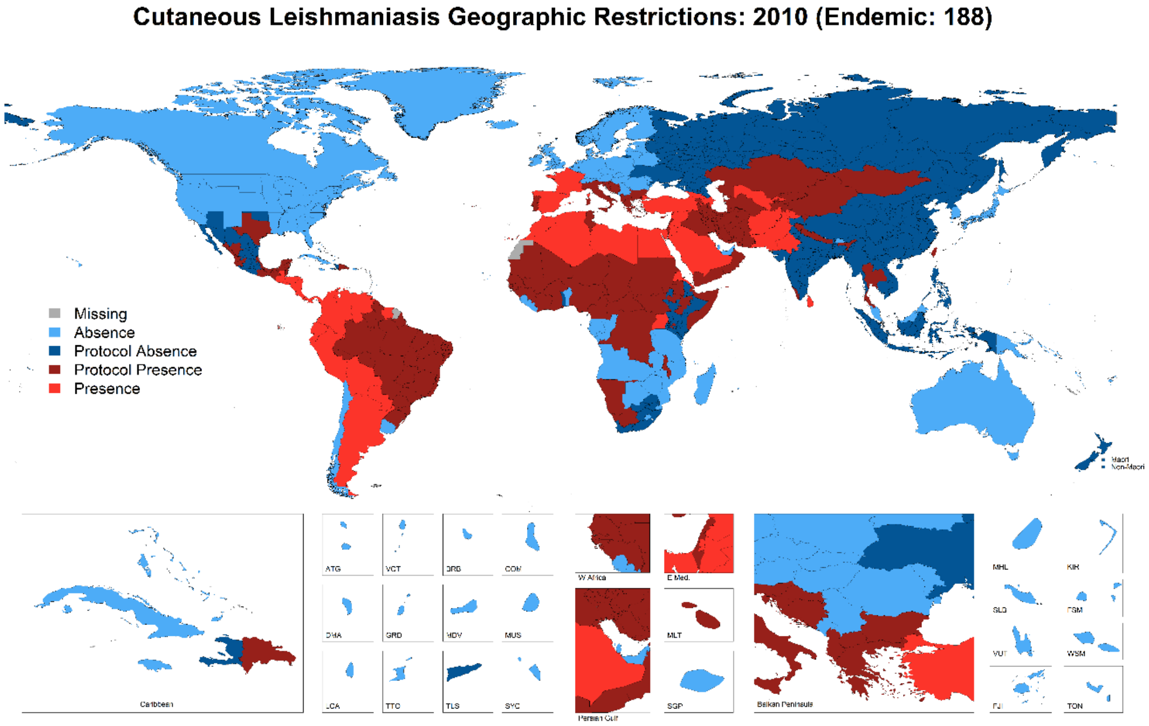

Figure 1: Cutaneous Leishmaniasis geographic restrictions for the year 2010. GBD locations tagged as present are coloured in red, dark red represents protocol presence, dark blue represents protocol absence, and absence is represented by light blue. Locations missing tags are presented in grey.

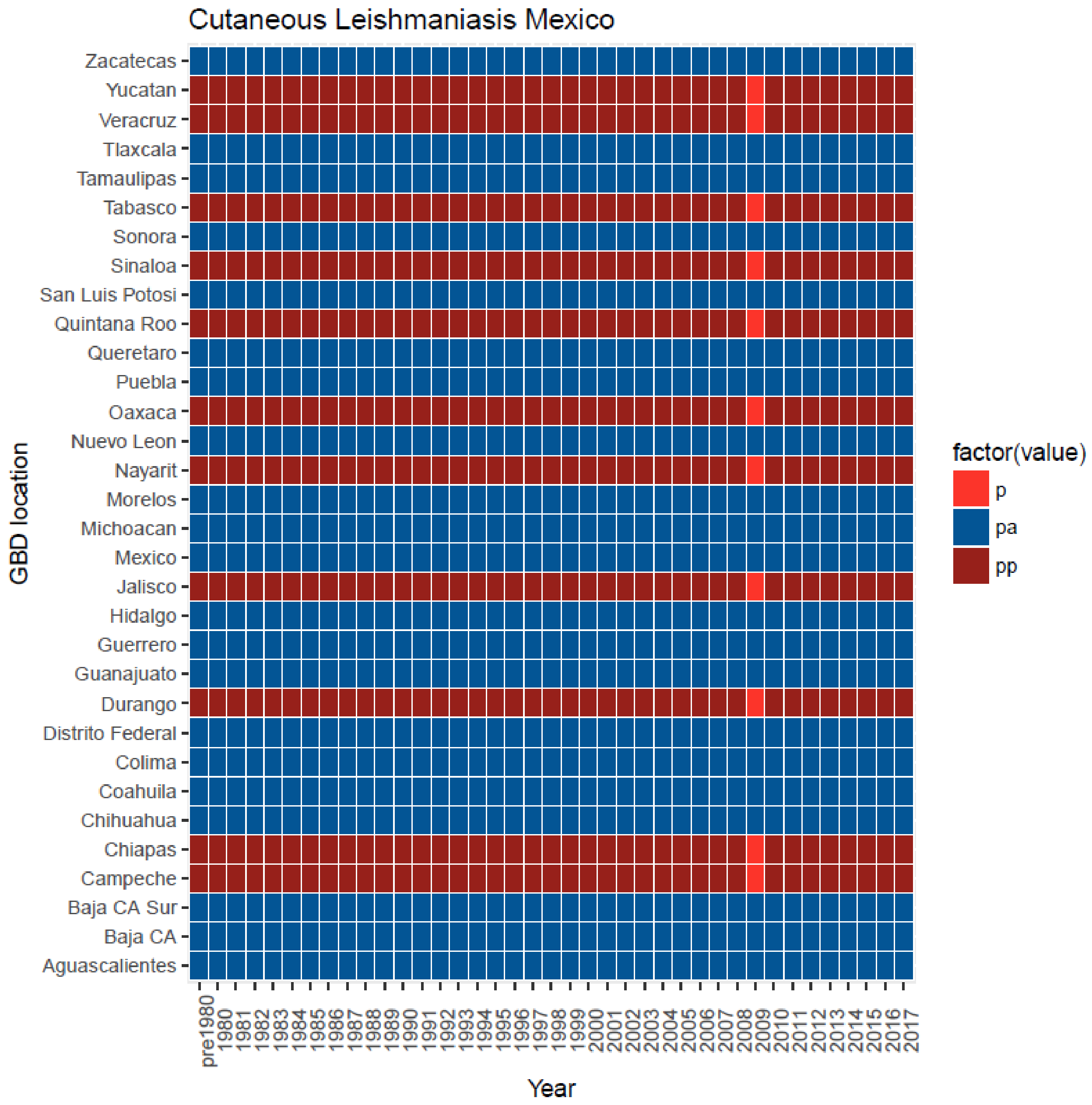

Figure 2: Cutaneous Leishmaniasis geographic restrictions for Mexican subnationals. Locations tagged as present are coloured in red, dark red represents protocol presence, and dark blue represents protocol absence.

Full time series of maps and tables, with relevant GHDx NIDs are available upon request from gbdsec@uw.edu.

Method – ST-GPR

Using existing IHME tools, the summarised values were modelled using ST-GPR to produce a complete time series of estimates for each location-year tagged “Present” or “Protocol Present”. In short, ST-GPR attempts to model non-linear trends utilizing a Gaussian process to fit a trend, rather than a definitive

functional form. Case count data were translated into estimates of true case counts by using underreporting scalars as identified by Alvar et al. (2012).

Method – DisMod

DisMod was used to generate an age-sex curve to disaggregate all-age, both-sex, incidence data. DisMod is an integrated meta-regression framework that allows for multiple datasets to be integrated into a singular analysis regardless of age-binning, sources, and geographies. As a consequence, a variety of differently aggregated information can be evaluated to generate a consensus output. From this model, the global fit was used.

Method – YLD estimation (incorporating duration and disability weighting) / COMO

Following standard GBD estimation protocols, incidence estimates were used to calculate disease prevalence (by multiplication with duration), disaggregated by disease sequelae. One health state is assigned to Cutaneous Leishmaniasis, [Table 2]. Duration value of initial acute infection was set to six months (Reithinger et al. 2007). Prevalence of long-term sequelae was based upon the proportion of cases that would result in facial scarring. The average proportion of sores that occurred on the face was calculated based upon a sample-weighted average of the proportion from four studies conducted in North Africa/Middle East. This proportion was 0.476. Of these people, only those who did not have appropriate access to health care were assigned long-term sequelae, estimated via the Healthcare Access and Quality Index. CL incidence, multiplied by proportion of people with facial sores, times the proportion of people without adequate health care access in each location-year, was used to obtain incidence of people with long-term sequelae, with cohorts streamed through time.

Sequela	Health state lay description	Disability weight	Duration
Cutaneous and mucocutaneous leishmaniasis	"has a slight, visible physical deformity that others notice, which causes some worry and discomfort"	0.011 (0.005–0.021)	6 months (46.7% * HAQ Index) Lifelong

Table 2: Sequelae and associated metadata. For the sequelae used in GBD 2017, the lay descriptor health state, disability weight, and duration are listed.

Central processing is used to generate the final estimates, including co-morbidity simulations.

Changes from GBD 2016

There were no substantive changes from the GBD 2016 methodology

Limitations

As with any modelling process, a number of limitations are known, which will be the focus of additional effort in upcoming GBD cycles and engagement with collaborators. Given the focus on location-representative estimates, the existing model is focused on national case counts. This excludes a large resource of published literature and grey literature focused on site-specific surveillance or surveys. While some pathogens have integrated subnational approaches as a building block for national estimates (eg, schistosomiasis) this has yet to be implemented for cutaneous leishmaniasis. Regardless of contribution

to the global incidence model, these data can be used to inform age-sex splits, as well as a variety of other key parameters, particularly duration parameters, which are currently lacking uncertainty.

References

Alvar et al. (2012) Leishmaniasis Worldwide and Global Estimates of Its Incidence. PLoS One 7(5):e35671

Pigott et al. (2014) Global Distribution Maps of the Leishmaniases. eLife 3:e02851

Reithinger et al. (2007) Cutaneous Leishmaniasis. Lancet Infect Dis 7(9):581-96

3.3.5 Visceral Leishmaniasis SDG Capstone Appendix

Visceral leishmaniasis – GBD2017

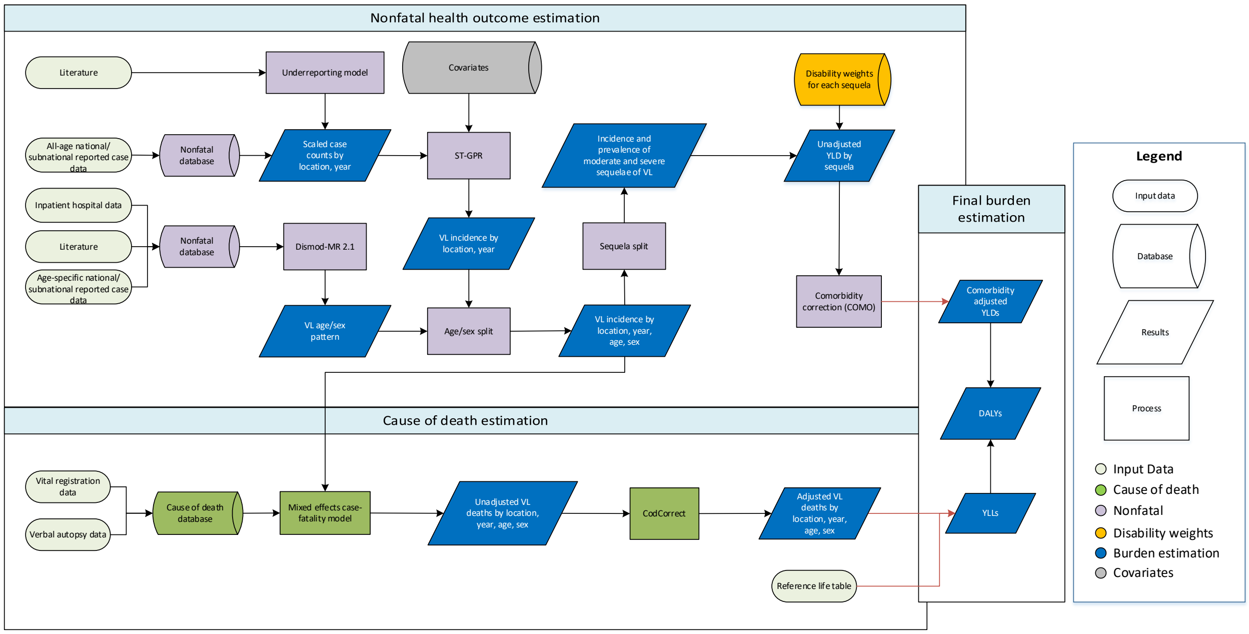

Visceral leishmaniasis (VL) is the most serious manifestation of disease caused by the *Leishmania* parasite, transmitted through the bite of phlebotomine sandflies. Those infected typically present with fever, weight loss, anaemia, leukopenia, thrombocytopenia, and enlargement of the spleen and liver. If left untreated, it can be fatal. Transmission varies by geographic region, with a variety of reservoir hosts implicated, and different vector species associated, maintaining both zoonotic and anthroponotic transmission cycles. The ICD9 code related to visceral leishmaniasis is 085.0, and the ICD10 code is B55.0.

Description of general methodology

The fatal estimation process for visceral leishmaniasis is built from incident case notification data representative of the GBD geographic location, which is adjusted for underreporting. The upscaled all-age, both-sex case counts are modelled using spatiotemporal Gaussian process regression (ST-GPR) in order to impute for missing location-year combinations as well as to account for further biases and inaccuracies in reporting. Datasets that disaggregate VL cases by age and sex are modelled using DisMod to produce a global age-sex split which is applied to the all-age, both-sex envelope estimates resulting from ST-GPR. The mean incidence estimates are compared with estimated death counts to generate a case-fatality rate model that is subsequently used to estimate deaths for each age, sex, location, year.

Input Data – Case Notification time series

Current estimation for the all-age, both-sex incidence envelope is based upon location-representative information rather than site-specific epidemiological measures due to the absence of global foci maps allowing for upscaling of geographically precise information. The primary data resource therefore is the case notification time-series reported by National Control Programs and Ministries of Health to the World Health Organization. This is supplemented by systematic literature review (last updated for GBD 2015) to

identify alternate sources of data for years missing information. For countries with subnational estimates, in-country collaborators have compiled information for respective programs, or identified key resources. Notifications from 1,151 location-years were available.

Input Data – Underreporting assessments

It is recognised that case notification series record only a subset of the true cases present. A review was undertaken to identify articles that compared reported cases with alternate measures to estimate the degree of underreporting. The following search strings were used: 'leish* AND under*'; 'active passive leish*'. Inclusion criteria were broad to maximise spatiotemporal coverage in potential estimates – any report that compared reported statistics with some notion of "truth" (whether capture-recapture, active surveillance, etc.) were extracted. Values for both cutaneous and visceral leishmaniasis were included. For GBD 2017, 12 articles were included, summarised in Table 1.

Citation	GBD location	Time period	Pathogen	Method synopsis	Proportion of "true" cases reported
Copeland *et al.*, 1990 "Comparison of active and passive case detection of cutaneous leishmaniasis in Guatemala" (Copeland, Arana, and Navin 1990)	Guatemala	1990	CL	Comparison of Ministry of Health data with cross-sectional population-based survey to inform estimated number of cases	64/2574
Yadon *et al.* 2001 "Assessment of Leishmaniasis notification system in Santiago del Estero, Argentina, 1990-1993" (Yadón et al. 2001)	Argentina	1990–1993	CL	Capture-recapture methods were used to evaluate four reporting sources.	94/210
Sesma *et al.* 1997 "Leishmaniasis in Navarra: a review of activities" (Sesma and Barricarte 1997)	Spain	1990–1997	CL, VL	Comparison of active searching within the region with reporting via Epidemiological Surveillance System	8/21
Maia-Elkhoury *et al.* 2007 "Analysis of visceral leishmaniasis reports by the capture-recapture method" (Maia-Elkhoury et al. 2007)	Brazil	2002–2003	VL	Comparison of three notification systems for completeness	5896/10691
Singh *et al.* 2006 "Serious underreporting of visceral leishmaniasis through passive case	Bihar, India	2003	VL	Comparison of actively detected cases (identified via household surveys) and governmental health	8/65

reporting in Bihar, India” (S. P. Singh et al. 2006)				system records. Estimate is among study population	
Singh *et al.* 2006 “Serious underreporting of visceral leishmaniasis through passive case reporting in Bihar, India” (S. P. Singh et al. 2006)	Bihar, India	2003	VL	Comparison of actively detected cases (identified via household surveys) and governmental health system records.	109/876
Gkolfinopoulou *et al.* 2013 “Epidemiology of human leishmaniasis in Greece, 1981-2011” (Gkolfinopoulou et al. 2013)	Greece	2004–2009	VL	Comparing number of cases identified at national reference laboratory with mandatory notification system.	260/361
Singh *et al.* 2010 “Estimation of under-reporting of Visceral Leishmaniasis cases in Bihar India” (V. P. Singh et al. 2010)	Bihar, India	2006	VL	Comparison of actual reported number of cases with estimates age-sex stratified incidence proportions for a cohort of 31,324 persons	34/177
Hirve *et al.* 2010 “Effectiveness and feasibility of active and passive case detection in the Visceral Leishmaniasis Elimination Initiative in India, Bangladesh, and Nepal” (Hirve et al. 2010)	Bihar, India<br>Nepal<br>Bangladesh	2008	VL	Comparing active case detection evaluations (conducting via house-to-house screening) with passive case detection systems	111/130<br>119/127<br>18/25<br>20/32
Faraj *et al.* 2016 “Effectiveness and cost of insecticide-treated bed nets and indoor residual spraying for the control of cutaneous leishmaniasis: A cluster-randomized control trial in Morocco” (Faraj et al. 2016)	Morocco	2008–2013	CL	Comparison of incidence of new CL cases by both active and passive case detection	409/670
Das *et al.* 2014 “Active and passive case detection strategies for the control of leishmaniasis in	Bangladesh	2010–2011	VL	Comparing two districts’ estimates [identified in the paper as being directly comparable] of cases, one via active case	756/1087

Bangladesh" (Das et al. 2014)				detection, the other via passive case detection. Active case detection was via community education and outreach workers targeting households	
Rahman *et al.* 2015 "Performance of Kala-azar surveillance in Gaffargaon subdistrict of Mymensingh, Bangladesh" (Rahman et al. 2015)	Bangladesh	2010–2011	VL	Comparison of cases reported to the local health complex versus active search for kala-azar cases	29/58
Eid *et al.* 2017 "Assessment of a Leishmaniasis reporting system in tropical Bolivia using the capture-recapture method" (Eid et al. 2017)	Bolivia	2013–2014	CL	Active surveillance during medical campaigns were compared to registered cases reported by the National Program of Leishmaniasis Control	23/86.4

Table 1: Metadata for underreporting scalars used in GBD 2017. For each record, a citation, GBD location of relevance, year, pathogen, brief summary of methods, and output values used in modelling are listed.

Input data – age/sex-split data

Where possible, information disaggregating location-level statistics by age and sex were extracted.

	Incidence
Site-years (total)	1519
Number of countries with data	70
Number of GBD regions with data (out of 21 regions)*	14
Number of GBD super-regions with data (out of 7 super-regions)	7

Table 2: Summary statistics for data used to inform age/sex split modelling. *It should be noted that not all GBD regions have leishmaniasis-endemic countries within them.

Method – geographic restrictions

There are strong climatic and biogeographic constraints on the geographic distribution of VL resulting in a focal rather than cosmopolitan global distribution. As a result, it is necessary to identify locations burdened by the disease through space and time as distinct from countries were VL is absent. Tags were assigned to each location-year based upon the outcome of a search of IHME databases, as well as location-specific searches of PubMed. Each location-year is tagged as follows:

- Present – where a specific citation of either an autochthonous laboratory-confirmed case (ie, a case with PCR, serological, or parasitological diagnosis), reported case (ie, a case noted as VL, but with no supporting diagnostic), or supporting evidence (ie, confirmed infection in animal reservoirs or sandfly vectors)

- Protocol Present – for a given location-year, where no specific citation is used, but is present for another year in the same location, it is assumed that VL is present given that eradication of the pathogen has not been achieved
- Absent – where PubMed location-specific searches returned zero relevant results, in locations scoring -25 or lower as evaluated by Pigott *et al.* (2014) [the threshold for "absence" in that study (Pigott et al. 2014)], locations were tagged as Absent
- Protocol Absent – as with Absent, locations with zero relevant PubMed results, but with greater than -25 as evaluated by Pigott *et al.* (2014), were tagged as Protocol Absent (Pigott et al. 2014)

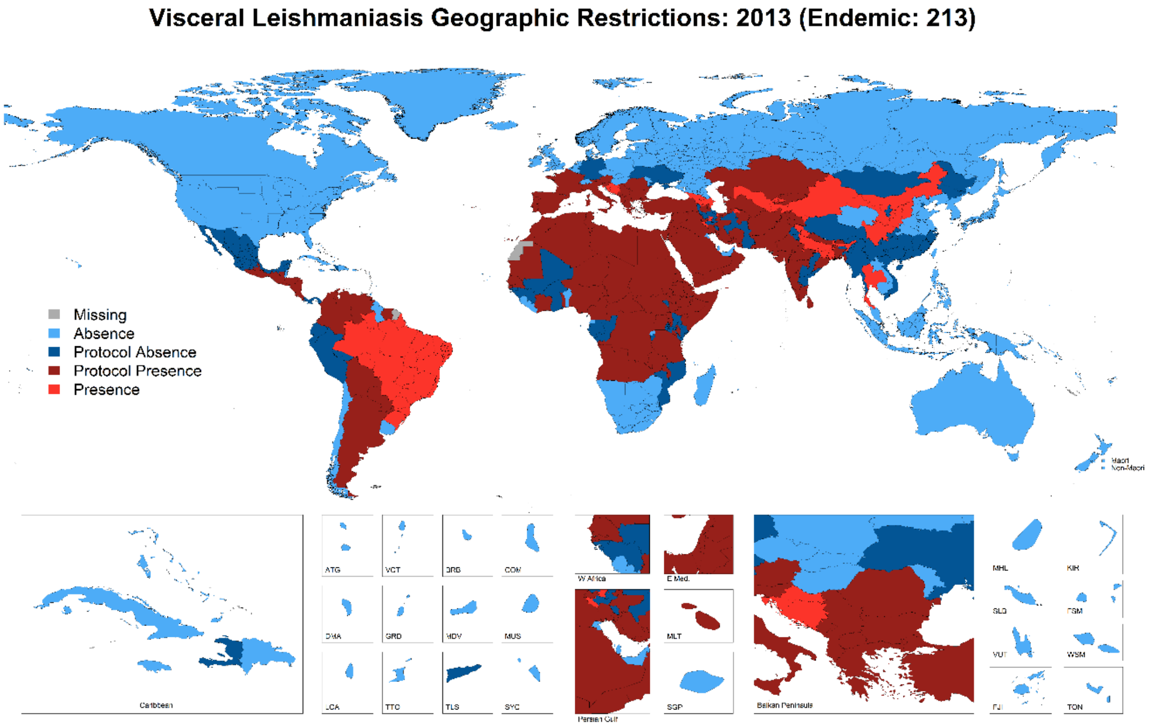

Figure 1: Visceral Leishmaniasis geographic restrictions for the year 2013. GBD locations tagged as present are coloured in red, yellow represents protocol presence, dark blue represents protocol absence, and absence is represented by light blue. Locations missing tags are presented in grey.

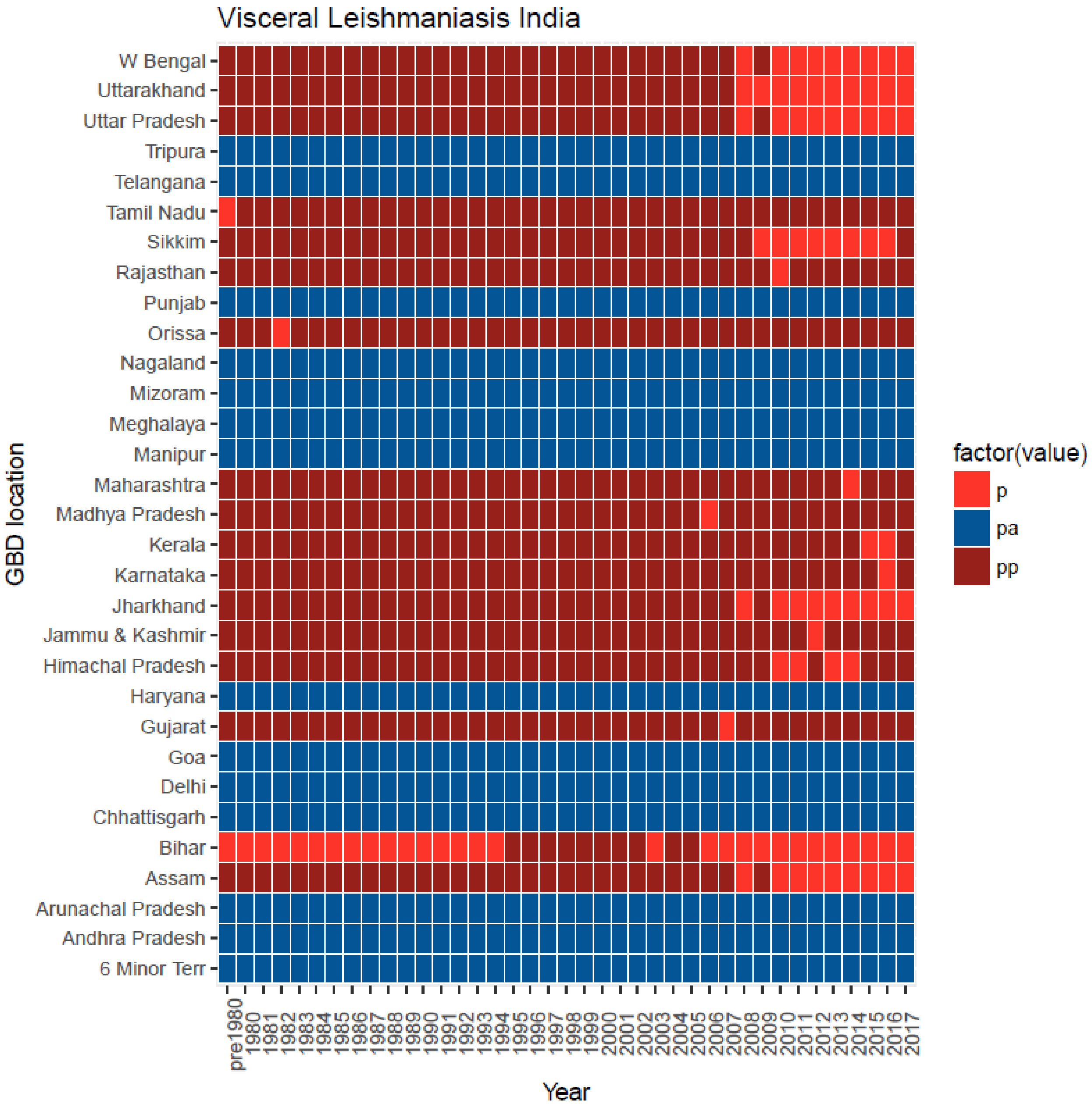

Figure 2: Visceral Leishmaniasis geographic restrictions for Indian subnationals. Locations tagged as present are coloured in red, yellow represents protocol presence, and dark blue represents protocol absence.

Full time series of maps and tables, with relevant GHDx NIDs, are available upon request from gbdsec@uw.edu.

Method – underreporting modelling and scaled case counts

Underreporting scalars were modelled as a generalised linear model estimating the proportion of true cases captured by reporting systems: a value of 1 therefore represents all actual cases of leishmaniasis being reported through notification systems. The specific models is as follows:

$$\frac{reported\ cases}{\text{"true" cases}} = Pathogen + Year + Sociodemographic\ Index$$

To account for potential biases inherently present based upon differing survey methods or location-specific confounders, 1,000 models were run, with each model randomly dropping all data from a specific location, and then one additional data point from the remaining dataset. Similarly, for estimates that spanned multiple years, for each model one of the years within the range of possible years was randomly assigned.
To generate scaled case counts, for each of the 1,000 models a random number was generated, using a normal distribution with mean being that of the mean estimated scalar bounded by the upper and lower confidence interval. With these 1,000 scalars, 1,000 scaled case counts were calculated and summarised for modelling within ST-GPR.

Method – ST-GPR

Using existing IHME tools, the summarised values were modelled using ST-GPR to produce a complete time series of estimates for each location-year tagged "Present" or "Protocol Present". In short, ST-GPR attempts to model non-linear trends utilising a Gaussian process to fit a trend, rather than a definitive functional form. The following model specifications were used:

$$Incidence = Healthcare\ Access\ and\ Quality\ Index + Sociodemographic\ Index + (1|level\ 1) + (1|level\ 2) + (1|level\ 3)$$

where levels 1, 2, and 3, referring to GBD location hierarchies, treated as random effects. The following hyperparameters were used: st-lambda = 0.4, st-omega = 1, st-zeta = 0.01, gpr-scale = 10.

Method – DisMod

DisMod was used to generate an age-sex curve to disaggregate all-age, both-sex incidence data. DisMod is an integrated meta-regression framework that allows for multiple datasets to be integrated into a singular analysis regardless of age-binning, sources, and geographies. As a consequence, a variety of differently aggregated information can be evaluated to generate a consensus output. From this model, the global fit was used.

Method – YLD estimation (incorporating duration and disability weighting) / COMO

Following standard GBD estimation protocols, incidence estimates were used to calculate disease prevalence (by multiplication with duration), disaggregated by disease sequelae. In total, two health states are assigned to visceral leishmaniasis, "moderate visceral leishmaniasis" and "severe visceral leishmaniasis" [Table 3]. Duration values were taken from Murray et *al.* (2005).

Sequela	Health state lay description	Disability weight	Duration
Moderate visceral leishmaniasis	Infectious disease, acute episode, moderate "has a fever and aches, and feels weak, which causes some difficulty in daily activities"	0.051 (0.032–0.074)	2.5 months

Severe visceral leishmaniasis	Infectious disease, acute episode, severe<br>"has a high fever and pain, and feels very weak, which causes great difficulty with daily activities"	0.133 (0.088–0.19)	15 days

Table 3: Sequelae and associated metadata. For the sequelae used in GBD 2017, the lay descriptor health state, disability weight, and duration are listed.

Central processing is used to generate the final estimates, including co-morbidity simulations.

Changes from GBD 2016

A number of changes to the methodology were implemented for GBD 2017:

Geographic restrictions – to improve transparency and tractability of geographic restrictions, maps of restricted locations and years are available, with clear designation of data (or assumptions) used to inform a GBD location-year's status. As a result of updating, the status of some GBD locations has changed in the light of new evidence (eg, Angola). While we explore how best to host this information, it is currently available upon request to gbdsec@uw.edu.

All-age, both-sex incidence envelope – new data were acquired and an ST-GPR methodology implemented consistently across the globe. Relevant covariates were updated from GBD 2016. One important change was the removal of the "High endemicity" covariate, which constrained predictions, particularly in low SDI countries in Africa, since its construction and subsequent use in models are not independent of each other.

Age-sex breakdown – age-sex curves were taken from a DisMod model using an updated dataset of age-sex specific information

Underreporting model – considerable changes were undertaken from GBD 2016 for underreporting. Rather than using a single scalar, taken from expert opinion (Alvar et al. 2012), applied across the entire time series, a model was developed, parameterised by real data, allowing for spatiotemporal variation in estimates. These variable scalars were then applied to their relevant location-year case count values.

Results specific to visceral leishmaniasis model

The aim here is to provide insights in some of the sub-models that are involved in the VL estimation process that are not published as part of the GBD capstones or readily available via the supplemental materials. For further questions, please direct toward gbdsec@uw.edu.

Underreporting

Coefficients
Pathogen: 0.6371 (-0.0456 to 1.5868) (where pathogen order is CL, VL)
Year: 0.1350 (0.0714–0.2058)
SDI: 4.6230 (2.0290–9.3287)

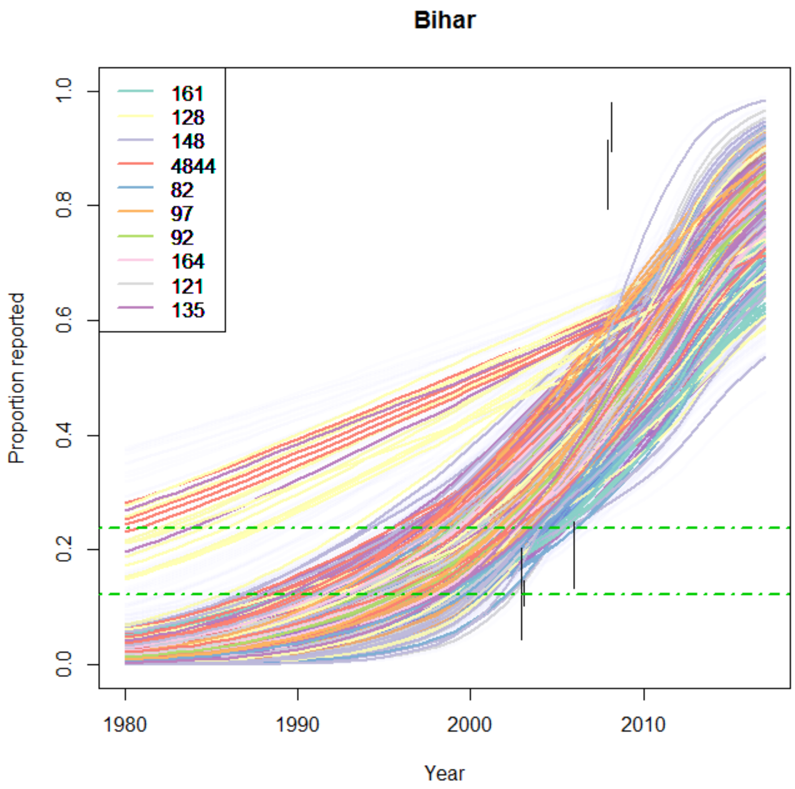

Figure 3: Example of VL underreporting model for Bihar, India. Plot showing each of the 1,000 iterations of the underreporting model run, coloured by the location that has been held out [colours coded by their GBD location id: 161 = Bangladesh, 128 = Guatemala, 148 = Morocco, 4844 = Bihar, India, 82 = Greece, 97 = Argentina, 92 = Spain, 164 = Nepal, 121 = Bolivia, 135 = Brazil]. The black vertical lines represent data points (with standard errors) for Bihar as listed in Table 1, and the green dashed line is the upper and lower bound of the underreporting factor recorded by Alvar *et al.* (2012), which was applied across all time in GBD 2016.

Age- and sex-specific trends in incidence rate

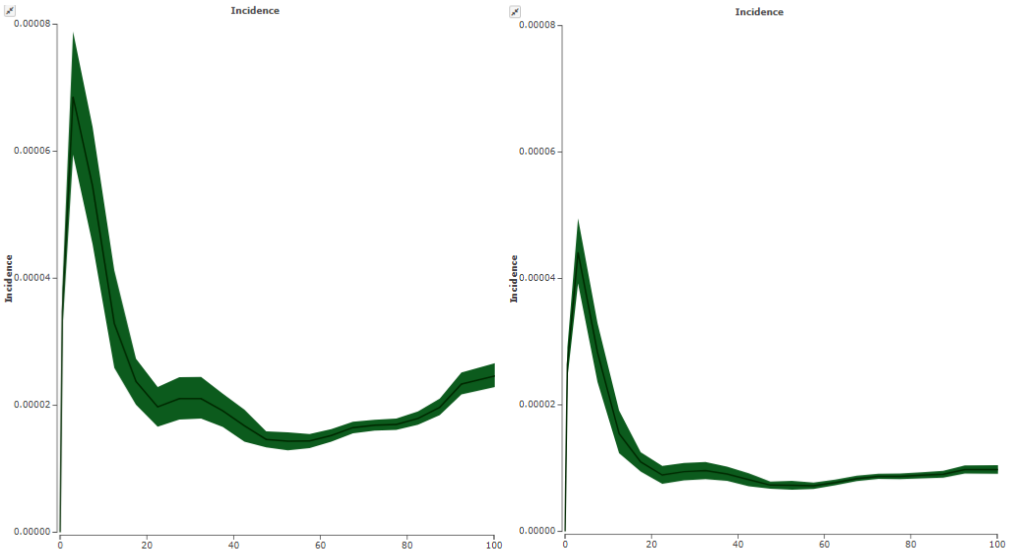

Figure 4: Global age-specific incidence estimates for males (left) and females (right) for the year 2010. Incidence is on the Y-axis (rate per total population), and age in years on the X-axis. Screenshot from EpiViz.

Figure 4 shows the age-specific variation in incidence rates, differentiated by sex. When considered as a global aggregate, we see that reported male incident rates are approximately double those of females, with highest rates observed in younger age groupings. In adults, levels are comparatively flat, but there is an uptick in older age groups.

ST-GPR

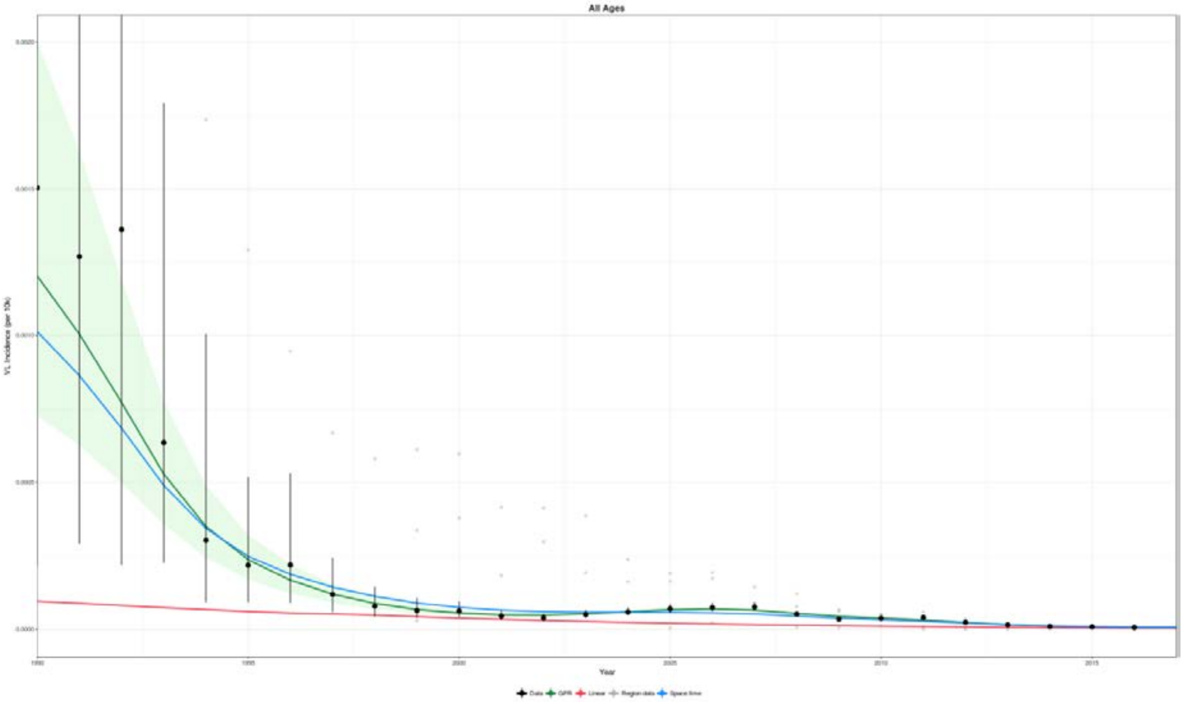

Figure 5: ST-GPR estimates for India (all-age, both sex) for years 1990–2017. Black dots represent input data points (post processing for underreporting) with the black lines indicating variance. The green line represents the mean GPR estimated value, with uncertainty shown by the green polygon. The blue line indicates the space-time component of the ST-GPR; the red indicates the linear regression component derived off of global data. Transparent black dots represent data from other locations in the GBD region CFR.

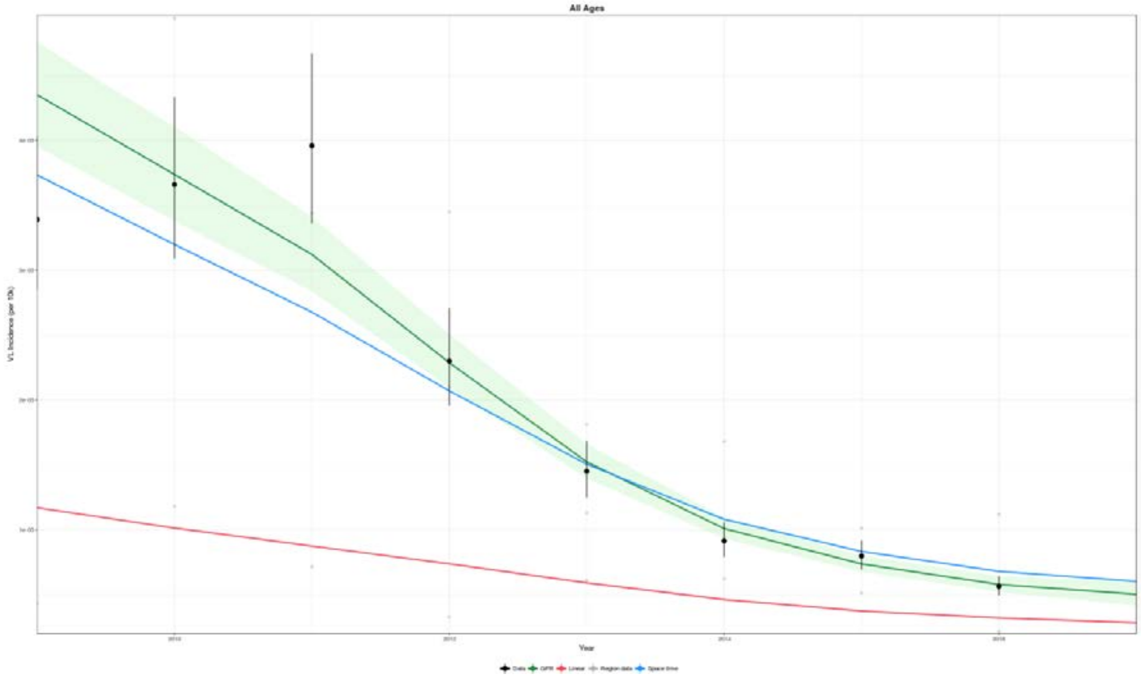

Figure 6: ST-GPR estimates for India (all-age, both sex) for years 2009–2017. Colouration and symbols are as stated in caption for Figure 5.

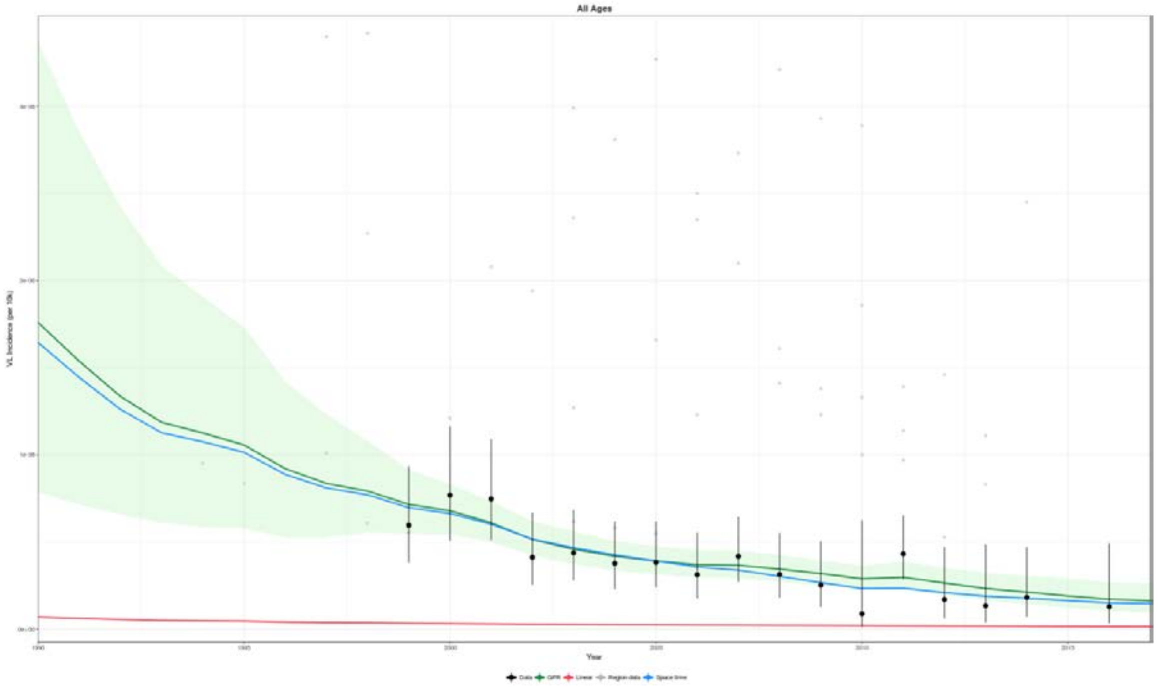

Figure 7: ST-GPR estimates for France (all-age, both sex) for years 1990–2017. Colouration and symbols are as stated in caption for Figure 5.

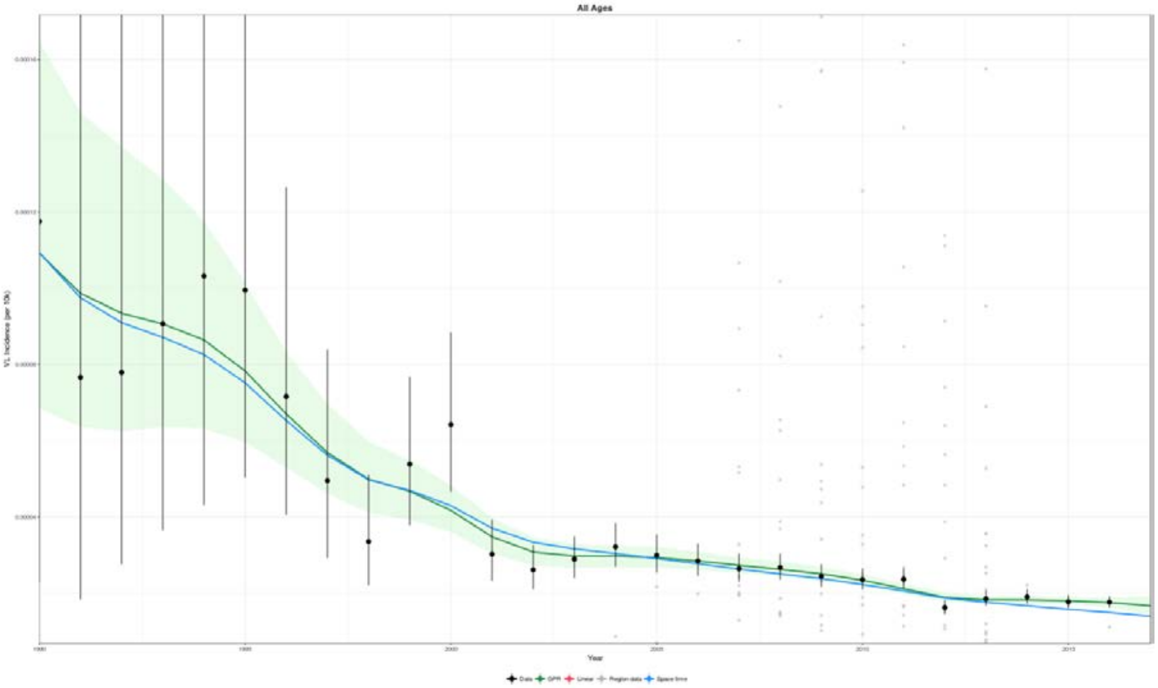

Figure 8: ST-GPR estimates for Brazil (all-age, both sex) for years 1990–2017. Colouration and symbols are as stated in caption for Figure 5.

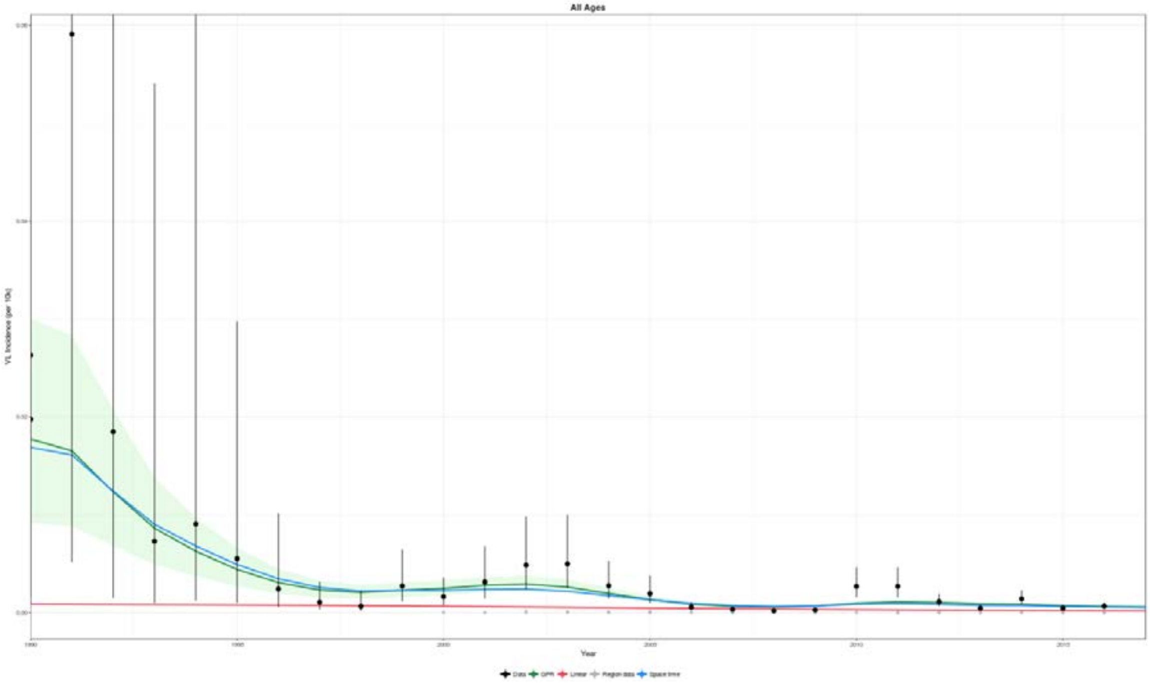

Figure 9: ST-GPR estimates for South Sudan (all-age, both sex) for years 1990–2017. Colouration and symbols are as stated in caption for Figure 5.

Limitations

As with any modelling process, a number of limitations are known, which will be the focus of additional effort in upcoming GBD cycles and engagement with collaborators. Given the focus on location-representative estimates, the existing model is based upon national case counts. This excludes a large

resource of published literature and grey literature focused on site-specific surveillance or surveys. While some pathogens have integrated subnational approaches as a building block for national estimates (eg, schistosomiasis), this is yet to be implemented for visceral leishmaniasis. Regardless of contribution to the global incidence model, these data can be used to inform age-sex splits, as well as a variety of other key parameters, particularly duration parameters, which are currently lacking uncertainty and support from a full literature review.

The removal of the "high endemicity" dataset in the ST-GPR framework led to some sub-Saharan African nations having considerably higher burden estimates than prior cycles. For many nations, this is reflective of the sporadic reporting of cases in these countries (eg, in Angola and the Democratic Republic of Congo), and a consensus on pathogen presence is highly uncertain. It was important to remove this covariate, however, as it was a prior imposed on the model, a model designed to evaluate this status that leveraged the same data that were in the model itself. This high degree of dependence we aimed to eliminate. In the next cycle of GBD, there is a need to identify an independent resource to aid in quantifying the population-at-risk, which the high endemicity layer was designed to approximate, as well as engaging with self-identified pathogen-specific and country-specific collaborators to re-evaluate the presence/protocol presence status assigned to these nations.

Similarly, existing death data are limited in geographic distribution (due to primarily coming from countries with robust vital registration systems), and could lack in external validity when extrapolated to other nations. While region-level random effects help account for some of this (for instance, mitigating some of the higher case fatality rates in immunocompromised individuals in the high-income region in GBD), this doesn't eliminate all possible confounding, and furthermore, does not negate the fact that most of the high-incidence countries do not report a full time series of deaths. Further cycles should explicitly consider the reported case fatality rates in the literature, many of which come from those VR data-poor regions.

Age-sex patterns are highly reflective of the countries from which data are obtained. Importantly, there is a large skew in information coming from Brazil. This information has potential biases due to the nature of the data inputs (notification and hospital data) and the corresponding age-sex variation in health-seeking behaviours. Yet again, consulting some of the detailed household surveys that do exist will increase geographic coverage of these estimates, and provide an important independent comparator to determine whether these disparities are genuine, or an artefact of the reporting systems consulted in this current model.

References

Alvar, Jorge, Iván D Vélez, Caryn Bern, Mercé Herrero, Philippe Desjeux, Jorge Cano, Jean Jannin, and Margriet den Boer. 2012. "Leishmaniasis Worldwide and Global Estimates of Its Incidence." *PLoS One* 7 (5): e35671.

Copeland, H W, B A Arana, and T R Navin. 1990. "Comparison of Active and Passive Case Detection of Cutaneous Leishmaniasis in Guatemala." *Am. J. Trop. Med. Hyg.* 43 (3): 257–259.

Das, A K, A D Harries, S G Hinderaker, R Zachariah, B Ahmed, G N Shah, M A Khogali, G I Das, E M Ahmed, and K Ritmeijer. 2014. "Active and Passive Case Detection Strategies for the Control of Leishmaniasis in Bangladesh." *Public Health Action* 4 (1): 15–21.

Eid, Daniel, Miguel Guzman-Rivero, Ernesto Rojas, Isabel Goicolea, Anna-Karin Hurtig, Daniel Illanes, and Miguel San Sebastian. 2017. “Assessment of a Leishmaniasis Reporting System in Tropical Bolivia Using the Capture-Recapture Method,” October, tpmd170308.

Faraj, Chafika, Joshua Yukich, El Bachir Adlaoui, Rachid Wahabi, Abraham Peter Mnzava, Mustapha Kaddaf, Abderrahmane Laamrani El Idrissi, Btissam Ameur, and Immo Kleinschmidt. 2016. “Effectiveness and Cost of Insecticide-Treated Bed Nets and Indoor Residual Spraying for the Control of Cutaneous Leishmaniasis: A Cluster-Randomized Control Trial in Morocco.” *Am. J. Trop. Med. Hyg.* 94 (3): 679–685.

Gkolfinopoulou, K, N Bitsolas, S Patrinos, L Veneti, A Marka, G Dougas, D Pervanidou, et al. 2013. “Epidemiology of Human Leishmaniasis in Greece, 1981-2011.” *Euro Surveill.* 18 (29): 20532.

Hirve, S, S P Singh, N Kumar, M R Banjara, P Das, S Sundar, S Rijal, et al. 2010. “Effectiveness and Feasibility of Active and Passive Case Detection in the Visceral Leishmaniasis Elimination Initiative in India, Bangladesh, and Nepal.” *Am. J. Trop. Med. Hyg.* 83 (3): 507–511.

Maia-Elkhoury, Ana Nilce Silveira, Eduardo Hage Carmo, Marcia Leite Sousa-Gomes, and Eduardo Mota. 2007. “[Analysis of visceral leishmaniasis reports by the capture-recapture method].” *Rev. Saude Publica* 41 (6): 931–937.

Pigott, David M, Samir Bhatt, Nick Golding, Kirsten A Duda, Katherine E Battle, Oliver J Brady, Jane P Messina, et al. 2014. “Global Distribution Maps of the Leishmaniases.” *Elife* 3 (January): e02851.

Rahman, Kazi Mizanur, Indira V M Samarawickrema, David Harley, Anna Olsen, Colin D Butler, Shariful Amin Sumon, Subrata Kumar Biswas, Stephen P Luby, and Adrian C Sleigh. 2015. “Performance of Kala-Azar Surveillance in Gaffargaon Subdistrict of Mymensingh, Bangladesh.” Edited by Carlos Franco-Paredes. *PLoS Negl. Trop. Dis.* 9 (4): e0003531.

Sesma, B, and A Barricarte. 1997. “[Leishmaniasis in Navarra: review of activities].” *An. Sist. Sanit. Navar.* 20 (2): 209–216.

Singh, S P, D C S Reddy, M Rai, and S Sundar. 2006. “Serious Underreporting of Visceral Leishmaniasis through Passive Case Reporting in Bihar, India.” *Trop. Med. Int. Health* 11 (6): 899–905.

Singh, V P, A Ranjan, R K Topno, R B Verma, N A Siddique, V N Ravidas, N Kumar, K Pandey, and P Das. 2010. “Estimation of Under-Reporting of Visceral Leishmaniasis Cases in Bihar, India.” *Am. J. Trop. Med. Hyg.* 82 (1): 9–11.

Yadón, Z E, M A Quigley, C R Davies, L C Rodrigues, and E L Segura. 2001. “Assessment of Leishmaniasis Notification System in Santiago Del Estero, Argentina, 1990-1993.” *Am. J. Trop. Med. Hyg.* 65 (1): 27–30.

3.3.5 Lymphatic Filariasis SDG Appendix

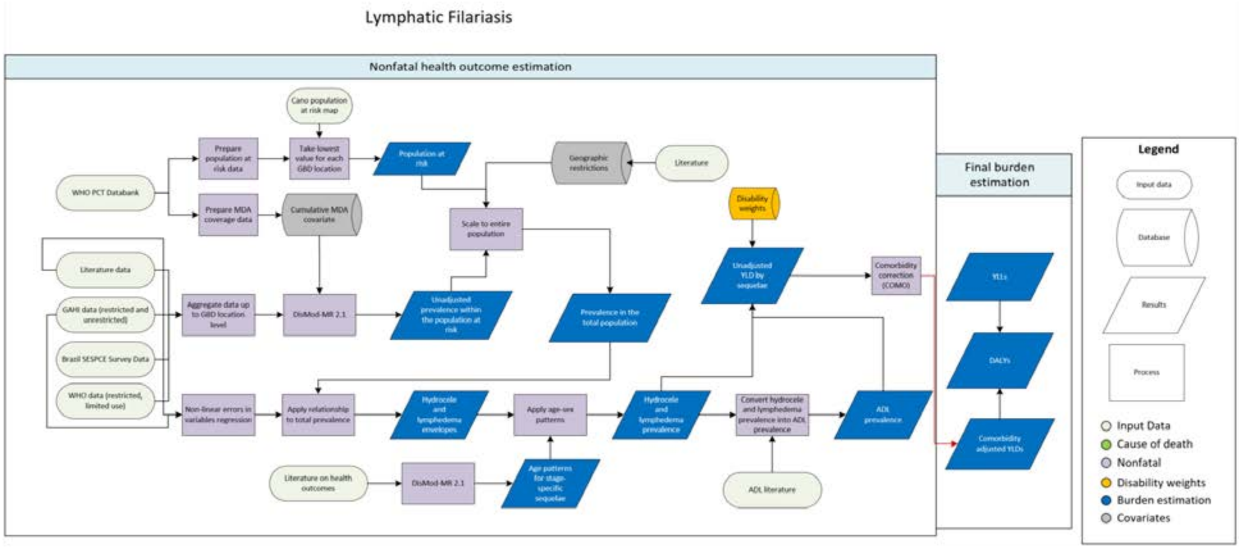

Input Data and Methodological Summary

Case Definition

Lymphatic filariasis (LF) is a neglected tropical disease spread in which threadlike nematodes invade the lymphatic system. The worms responsible – *Wuchereria bancrofti*, *Brugia malayi,* and *Brugia timori* – are spread from human to human via mosquitoes. The most prominent clinical manifestations of LF are lymphedema (a swelling of the legs, also known in its more extreme manifestation as elephantiasis) and hydrocele (a collection of fluid in the sac around the testicles).

Input data

A systematic review of literature for GBD 2016 in the PubMed database was done on October 14, 2016, for prevalence and incidence data using the search (Lymphatic filariasis AND prevalence) OR (Lymphatic filariasis AND (prevalence OR incidence OR "mass drug administration" OR MDA OR coverage)) OR (Lymphedema, hydrocele) OR (Transmission Assessment Survey (TAS)) OR (Lymphatic filariasis AND mapping).

Population at risk and MDA coverage data come from the WHO PCT Databank [1].

Modelling strategy

Data on prevalence of microfilaria is modelled using Dismod-MR 2.1. Due to the focal nature of lymphatic filariasis, we make the assumption that data collected are from endemic locations unless specifically specified in literature or survey methods. If the data are nationally representative, we adjust

the data points by multiplying by the inverse of the proportion of the population at risk. Due to the fact that data is collected in endemic locations or we adjust it so that it is within the population at risk, we then scaled the DisMod-MR 2.1 estimates according to at-risk population in order to attain nationally representative values. We developed a new MDA location-level covariate that is used in the DisMod model based off WHO PCT Databank data, informing prevalence estimates.

For lymphedema and hydrocele, we incorporate survey data from the Global LF Atlas in a non-linear error-in-variables regression that determines the prevalence of lymphedema and hydrocele as functions of microfilaria prevalence, which is then applied to the total microfilaria DisMod model in order to attain an envelope of cases by location-year. Separately, all available prevalence data for these conditions is modeled in DisMod in order to determine an age-sex pattern.

In the estimation of lymphedema and hydrocele prevalence, we perform the same population at-risk correction that is done on microfilaria prevalence. For hydrocele prevalence after treatment, we take the value before MDA rollout in 2000 and reduce that by the same treatment efficacy function described for microfilaria prevalence, using dosage-reduction data specific to hydrocele along with the location-year specific MDA coverage. For lymphedema, we assume no new cases appear among treated individuals. As such, we reduce lymphedema prevalence in post-treatment years in accordance with MDA coverage.

Sequela	Data points	Regions	Countries	Subnational units
Prevalence of detectable microfilaria	1,552	10	40	28
Lymphedema due to lymphatic filariasis	511	10	25	15
Hydrocele due to lymphatic filariasis	265	8	22	12

Changes from GBD 2016 to GBD 2017

We conducted a new literature review, and utilized data from recent years and the MDA covariate to predict the time trend rather than last year's non-linear regression to estimate the reduction of microfilaria as a function of treatments per person. Additionally, we used age-specific data extracted from communities that were pre-MDA and post-MDA to develop age-trends specific to MDA status. We then split out all-age data according to MDA status to provide more granular data to the unadjusted prevalence model.

3.3.5 Leprosy SDG Capstone Appendix

Leprosy

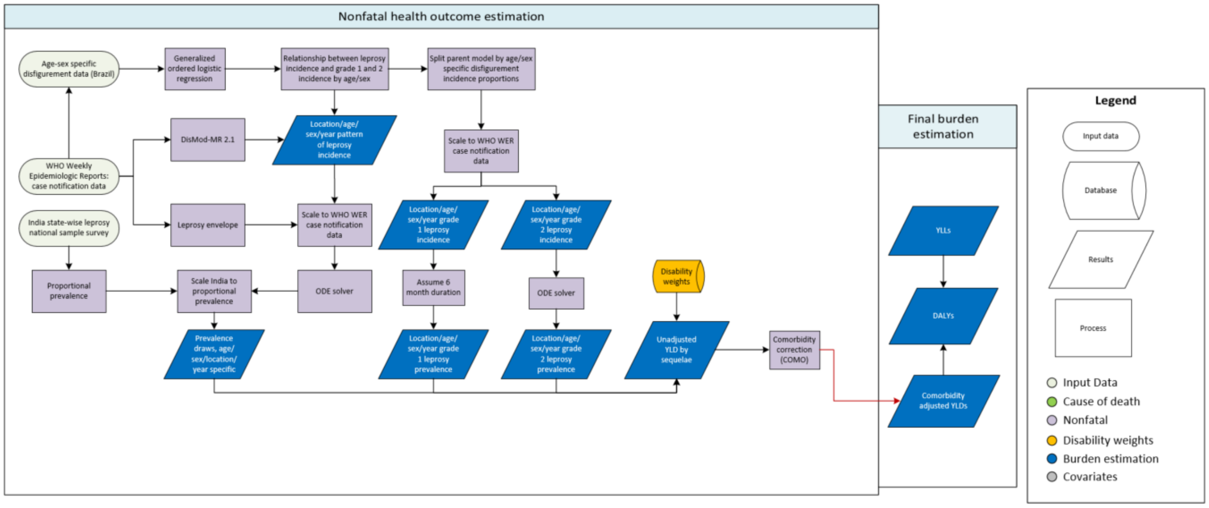

Input Data and Methodological Summary

Case definition

Leprosy is a chronic bacterial infection caused by *Mycobacterium leprae*, primarily affecting the nervous system, skin, respiratory tract, and eyes. Transmission is facilitated through contact with fluid from the nose and mouth of an infected individual. The ICD-10 code for leprosy is A30.9.

Input data

To model non-fatal outcomes due to leprosy, WHO Weekly Epidemiological Record (WER) case notification data were used from 1987 to 2016 to capture incident cases of leprosy. Stage-specific incidence data for grade 1 and grade 2 leprosy that are used to define age-sex patterns came from Brazil case notification data.

Table of data counts for leprosy incidence data

	**Incidence**
**Site-years (total)**	1,374
**Number of countries with data**	147
**Number of GBD regions with data (out of 21 regions)**	21
**Number of GBD super-regions with data (out of 7 super-regions)**	7

Table of data counts for leprosy grade inputs – grade 1

	Prevalence
**Site-years (total)**	12
**Number of countries with data**	1
**Number of GBD regions with data (out of 21 regions)**	1
**Number of GBD super-regions with data (out of 7 super-regions)**	1

Table of data counts for leprosy grade inputs – grade 2

	Prevalence
**Site-years (total)**	710
**Number of countries with data**	121
**Number of GBD regions with data (out of 21 regions)**	17
**Number of GBD super-regions with data (out of 7 super-regions)**	6

Modelling strategy

We used a multi-step process for the disease modelling of leprosy. In the first step, we ran a single-parameter model using DisMod-MR 2.1 to estimate the leprosy incidence age pattern by age, sex, year, and country. Then, we scaled the incidence outputs to the WHO WER cases, and used the ordinary differential equations (ODE) solver to calculate prevalence from the scaled DisMod-MR 2.0 incidence outputs.

Severity data were prepared by running a generalised ordered logistic regression using Brazil case notification data to get the relationship between leprosy incidence and grade 1 and grade 2 incidence by age and sex. We then used this relationship to split the parent DisMod-MR 2.1 model, and again scaled to WHO WER severity-specific cases. For disfigurement grade 1, we apply a duration of six months to get prevalence estimates. For disfigurement grade 2, we again use the ODE solver to get prevalence estimates.

Model evaluation was done by separately assessing the fit of the parent DisMod model and checking the final estimates produced after age-sex splits. Plots of time trends of prevalence across locations and age were used to evaluate the results. In addition, maps of the global distribution of leprosy prevalence and prevalence of sequelae due to leprosy were also assessed across time.

Changes from GBD 2016 to GBD 2017

We extracted WHO WER data from 2013 to 2016 to update prevalence and incidence estimates.

3.3.5 Onchocerciasis SDG Capstone Appendix

Flowchart

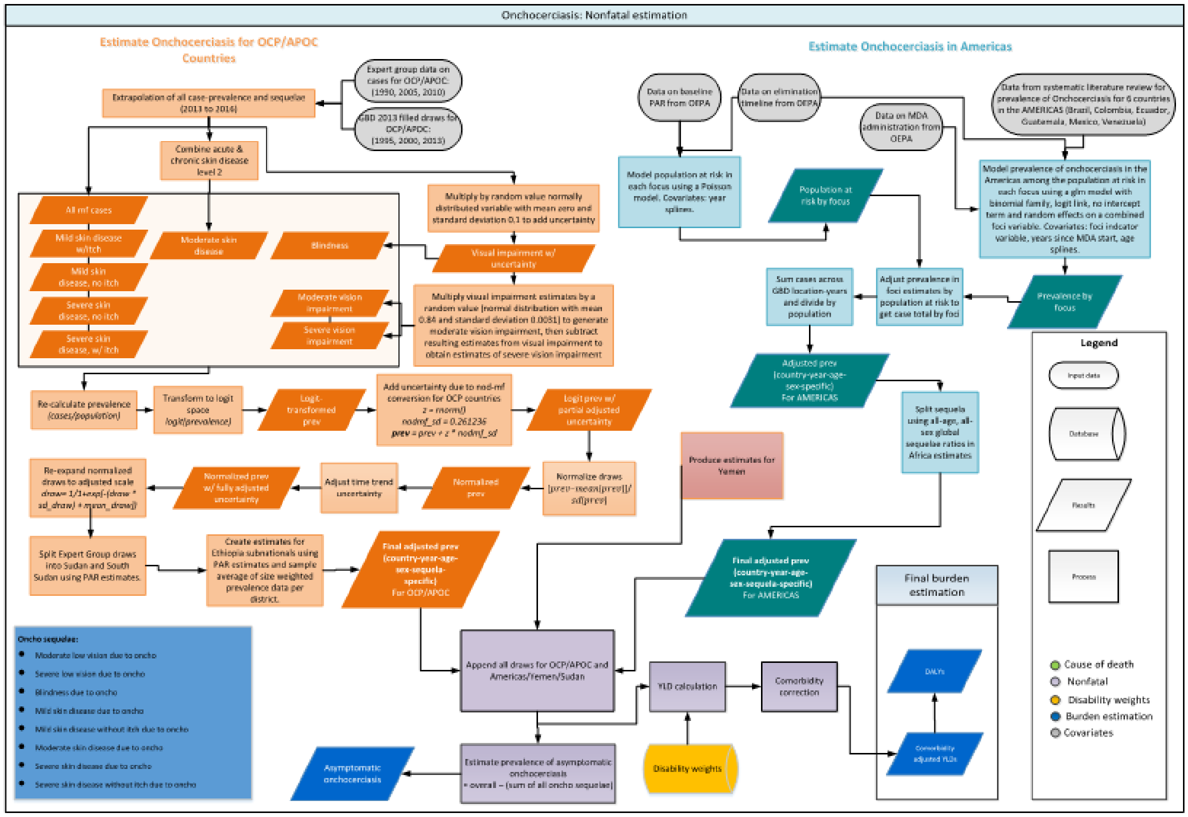

Input data & methodological summary

Case definition

Onchocerciasis, also known as river blindness, is a parasitic disease caused by *Onchocerca volvulus*. It is transmitted via the bite of one of several species of *Similium* blackflies that have historically bred in fast-moving freshwater rivers and tributaries throughout sub-Saharan Africa, Central America, and South America. Diagnosis can be made by skin snip biopsy to identify larvae, surgical removal of nodules and exam for adult worms, slit lamp exam of anterior part of the eye where larvae or lesions caused by them are visible, and antibody tests (mostly useful to visitors to areas with parasites). The ICD-10 code for onchocerciasis is B73.

Input data

Model inputs

Prevalence data prepared by the GBD 2010 expert group (EG) was used for modelling the nonfatal outcomes resulting from onchocerciasis in Africa. This included 1,000 draws of infection and morbidity (visual impairment, blindness, and skin conditions) cases with confidence intervals categorised by

country, age, and sex for years 1990, 1995, 2000, 2005, and 2010. Details about the materials and methods used by the EG to generate these draws can be found elsewhere [1-5]. These data represented all African countries included in the African Programme for Onchocerciasis Control (APOC) and the Onchocerciasis Control Programme (OCP) for which initial Rapid Epidemiological Mapping of Onchocerciasis (REMO) assessments demonstrated a need for Community-Directed Treatment with Ivermectin (CDTI) (defined as having a prevalence of skin nodules greater than 20%). Four countries – Rwanda, Mozambique, Kenya, and Gabon – were designated as hypo-endemic countries after initial REMO assessments and not included due to sparsity of cases and paucity of data. Estimates for Sudan from GBD 2010 were reassigned to South Sudan in GBD 2013 after its independence in 2011 since REMO assessments indicated that the vast majority of cases occurred in that area of the former Sudan. The tables below show the countries included in each program and the number of corresponding GBD locations they represent.

	**APOC Countries**	**OCP Countries**
Countries included	Angola, Burundi, Cameroon, Central African Republic, Chad, Congo, Democratic Republic of Congo, Ethiopia, Equatorial Guinea, Liberia, Malawi, Nigeria, South Sudan, Tanzania, and Uganda	Benin, Burkina Faso, Côte d'Ivoire, Ghana, Guinea Bissau, Guinea, Mali, Niger, Senegal, Sierra Leone, and Togo
Hypo-endemic countries not included	Rwanda, Mozambique, Kenya, Gabon, Sudan	
GBD countries & subnationals provided by EG	15	11
GBD world regions	3	1

Prevalence data for modelling non-fatal outcomes resulting from onchocerciasis in the Americas was extracted via a systematic literature review. Web of Science, Scopus, and PubMed were searched with the following search strings:

**Database**	**Search string**	**Yield**
PubMed	(oncho*[Title/Abstract] OR "river blindness"[Title/Abstract] OR "O. volvulus"[Title/Abstract] OR "robles disease"[Title/Abstract] OR "blinding filariasis"[Title/Abstract] OR "coast erysipelas"[Title/Abstract] OR “sowda” [Title/Abstract] OR “nodding syndrome”[Title/Abstract]) AND (“1980”[Date – Publication] : “2016”[Date – Publication]) AND (epidemiology[Title/Abstract] OR prevalence[Title/Abstract] OR incidence[Title/Abstract] OR surveillance[Title/Abstract] OR”MDA”[Title/Abstract] OR “Mass Drug Administration”[Title/Abstract] OR “Community-directed treatment with ivermectin”[Title/Abstract] OR “CDTI”[Title/Abstract] OR “mass treatment”[Title/Abstract] OR “multiple ivermectin treatments”[Title/Abstract] OR “monthly doses of ivermectin”[Title/Abstract] OR “large scale treatment”[Title/Abstract] OR REMO[Title/Abstract] OR “Rapid epidemiological mapping of onchocerciasis”[Title/Abstract] OR APOC[Title/Abstract] OR “African Programme for Onchocerciasis Control”[Title/Abstract] OR OCP[Title/Abstract] OR “Onchocerciasis Control Programme”[Title/Abstract]) NOT(Animals[MeSH] NOT Humans[MeSH])	986
Web of Science	TS=(oncho* OR "river blindness" OR "O. volvulus" OR "robles disease" OR "blinding filariasis" OR "coast erysipelas" OR sowda OR “nodding syndrome”) AND TS=(epidemiology	1,144

	OR prevalence OR incidence OR surveillance OR MDA OR "Mass Drug Administration" OR "Community-directed treatment with ivermectin" OR CDTI OR "mass treatment" OR "multiple ivermectin treatments" OR "monthly doses of ivermectin" OR "large scale treatment" OR REMO OR "Rapid epidemiological mapping of onchocerciasis" OR APOC OR "African Programme for Onchocerciasis Control" OR OCP OR "Onchocerciasis Control Programme") NOT TS=((Animals NOT Humans))	
SCOPUS	(TITLE-ABS-KEY(oncho* OR "river blindness" OR "O. volvulus" OR "robles disease" OR "blinding filariasis" OR "coast erysipelas")) AND TITLE-ABS-KEY(epidemiology OR prevalence OR incidence OR surveillance OR MDA OR "Mass Drug Administration" OR "Community-directed treatment with ivermectin" OR CDTI OR "mass treatment" OR "multiple ivermectin treatments" OR "monthly doses of ivermectin" OR "large scale treatment" OR REMO OR "Rapid epidemiological mapping of onchocerciasis" OR APOC OR "African Programme for Onchocerciasis Control" OR OCP OR "Onchocerciasis Control Programme") AND NOT KEY(Animals NOT Humans) AND PUBYEAR > 1979	2,000

This yielded 4,130 results in total, which was reduced to 2,502 after removing duplicates. The title and abstracts were screened for inclusion or exclusion with the following criteria:

Exclusion criteria:

- Pre-1980
- Non-original source
- Non-representative population
  - Vulnerable populations (eg, slum-dwellers, prisoners, orphans, high-risk jobs, etc.)
  - Hospital-based samples (including saved stool samples)
  - Non-native peoples (eg, migrants, expats, nomads, etc.)
  - Immunosuppression/illness (eg, HIV, TB, CA, RA, asthma, malaria, handicap, etc.)
- Non-human population
- Does not meet case definition
- Case-control study

Sixty-one articles were identified for full text screening and extraction from the historically endemic American countries: Guatemala, Brazil, Ecuador, Venezuela, Mexico, and Colombia.

Severity splits/sequelae

The table below shows the list of common clinical manifestations of onchocerciasis and the sequelae to which they have been mapped along with the lay description and the associated disability weight (DW) of each sequela.

Clinical manifestation	Sequela name	Lay description	DW
Uveitis; Punctate keratitis; Optic neuritis; Torpid Iritis; Onchochorioretinitis	Moderate vision impairment	"has vision problems that make it difficult to recognize faces or objects across a room"	0.031 (0.019–0.049)
Sclerosing keratitis; Optic neuropathy; Optic atrophy;	Severe vision impairment	"has severe vision loss, which causes difficulty in daily activities, some emotional impact (for example worry), and some	0.184 (0.125–0.258)

Choroidoretinopathy; Cataracts		difficulty going outside the home without assistance”	
Blindness	Blindness	“is completely blind, which causes great difficulty in some daily activities, worry and anxiety, and great difficulty going outside the home without assistance”	0.187 (0.124–0.260)
Acute papular onchodermatitis; Onchocercomata (subcutaneous nodules)	Mild skin disease	“has a slight, visible physical deformity that is sometimes sore or itchy. Others notice the deformity, which causes some worry and discomfort”	0.027 (0.015–0.042)
Chronic papular onchodermatitis; Lichenified onchodermatitis (“sowda”); Lymphadenopathy	Mild skin disease without itch	“has a slight, visible physical deformity that others notice, which causes some worry and discomfort”	0.011 (0.005–0.021)
Skin atrophy; Depigmentation (“leopard skin”)	Moderate skin disease	“has a visible physical deformity that is sore and itchy. Other people stare and comment, which causes the person to worry. The person has trouble sleeping and concentrating”	0.188 (0.124–0.267)
Hanging groin; Lymphoedema	Severe skin disease without itch	“has an obvious physical deformity that makes others uncomfortable, which causes the person to avoid social contact, feel worried, sleep poorly, and think about suicide”	0.405 (0.275–0.546)
	Asymptomatic onchocerciasis	NA	NA

Modelling strategy

The nonfatal modelling for onchocerciasis included six major steps. In the first step, GBD 2010 prevalence was exponentially extrapolated to obtain GBD 2017 estimates. Acute skin disease level 2 and chronic skin disease level 2 were summed to create the moderate skin disease sequela. Uncertainty was quantified and provided by the EG for all estimates except those of visual impairment and blindness. In these cases, for each of the OCP draws the number of cases were multiplied by a random value (the exponent of a normally distributed variable with mean zero and standard deviation 0.1) in order to add uncertainty. Within each draw, the same randomly drawn value was applied to all country-year-age-sex estimates. Visual impairment was then split into moderate and severe vision impairment by first multiplying the visual impairment estimates by a random value (from a normal distribution with mean 0.84 and standard deviation 0.0031) to generate moderate vision impairment, and then subtracting the resulting estimates from visual impairment to obtain estimates of severe vision impairment. Prevalence of sequelae was calculated by dividing the cases by the population.

The second step in modelling morbidity due to onchocerciasis was the adjustment of uncertainty in the conversion of nodule prevalence to microfilaria (mf) prevalence and in the effects of mass drug

administration (MDA). To adjust for uncertainty in translation of nodule prevalence to mf prevalence, the final OCP draws from the first step were logit transformed and uncertainty was added from a random value drawn from a normal distribution to the transformed estimates. The resulting estimates were then normalised and scaled using estimates published elsewhere [1]. To adjust for uncertainty due to MDA, the year when MDA with ivermectin started was set according to the table below.

Country	MDA start year
Angola, Burundi, South Sudan	2005
Congo, Ethiopia, DRC	2001
Cameroon, Central African Republic, Equatorial Guinea, Liberia, Nigeria, Uganda	1999
Chad, Niger, Tanzania	1998
Malawi	1997
All others	1990

The uncertainty in the time trend was then multiplied by the normalised prevalence estimates and the final prevalence was obtained by re-expanding the scaled normalised draws and adjusting the scale back from logit scale.

Third, since EG draws were provided before the independence of South Sudan in 2011, Sudan estimates from the EG were partitioned between Sudan and South Sudan. Population at risk (PAR) estimates pre- and post-Abu Hamed foci elimination in 2015 in Sudan were used to proportionally split cases between the two countries [2]. REMO maps showing definite needs for community-directed treatment with ivermectin (CTDI) were digitised and overlaid with population per pixel rasters to produce estimates of PAR pre-Abu Hamed elimination. Post-Abu Hamed elimination in 2015, REMO maps were edited to remove the foci as a definite CDTI areas and estimates were reproduced.

In the fourth step, prevalence in the Ethiopia subnationals was estimated separately and appended to the Africa model. Subnational draws were split proportionally based on sample size weighted prevalence from prevalence data, using population at risk estimates derived from digitising a map of onchocerciasis endemic districts in 2015 from Meribo and colleagues to convert into case space [3]. A proportion of cases falling into each subnational was then used to split national case numbers provided by EG draws into each subnational.

In the fifth step, prevalence of onchocerciasis in Yemen was modelled separately and combined with the Africa model. Due to limited data, this was done utilising one data point from the Ministry of Health published in 1991 only accounting for population change [22]. Furthermore, the global age-sex trend was imposed to produce age-sex-specific estimates. The clinical manifestation of Yemeni onchocerciasis is different from other regions, notably the atypical and most severe cutaneous manifestation known as sowda [23]. Therefore, all cases of onchocerciasis are being mapped to mild skin disease due to onchocerciasis without itch.

In the sixth step, prevalence of onchocerciasis in the Americas was modelled separately and combined with the Africa and Yemen models. For the GBD estimation period, onchocerciasis is known to have occurred in six countries of Central and Southern America: Mexico, Guatemala, Colombia, Ecuador, Brazil and Venezuela. The epidemiology of onchocerciasis is very different in these countries than in Africa because it has only occurred in relatively small, well defined foci. These foci have been mapped

and thoroughly monitored since the early 1990s with the formation of the Onchocerciasis Elimination Program of the Americas (OEPA) and all of the prevalence surveys conducted are only representative of these areas. Additionally, certain foci are geographically continuous across national boundaries. Therefore, we modelled onchocerciasis in these countries at the focus level among the population at risk in each focus instead of at the national level.

Population at risk for each focus was modelled using data from OEPA on baseline population at risk [6] and data from OEPA and peer-reviewed studies on dates of elimination in each focus [6-19]. This was done with a Poisson model using year splines as a covariate, and 1,000 draws of the population at risk were drawn from the predicted mean and standard error. The prevalence of disease among the population at risk was subsequently modelled using a generalised linear model with a binomial family, logit link, no intercept term, and random effects on a combined-foci variable created by grouping foci by geographic contiguity and nearness when data were sparse. Covariates included an indicator term on the foci, the number of years since MDA began, and splines on age. One thousand draws of prevalence were calculated from 1,000 draws of beta values from the variance-covariance matrix and adjusted by the estimated population at risk in each focus-year to determine the number of cases. The cases were then summed by GBD geography and year and divided by national population to find the national prevalence. While the model predicted case values very close to zero in the countries where elimination has occurred, these were overwritten to zero values for all years after certified elimination. The ratio of global all-age, all-sex prevalence of each sequela to the all-cases prevalence from the Africa estimates was applied to all-cases prevalence from the Americas to calculate prevalence of each sequelae.

Lastly, to estimate the prevalence of asymptomatic onchocerciasis, the prevalence of morbidity (vision loss, blindness and skin conditions) was subtracted from the overall onchocerciasis prevalence. Moderate vision impairment, severe vision impairment, and blindness estimates were each multiplied by a factor of 8/33 before subtraction to account for cases that have concurring symptoms.

References

1. Zouré HG, Noma M, Tekle AH, Amazigo UV, Diggle PJ, Giorgi E, Remme JH. The geographic distribution of onchocerciasis in the 20 participating countries of the African Programme for Onchocerciasis Control: (2) pre-control endemicity levels and estimated number infected. Parasites & Vectors. 2014. 7-326.
2. Zarroung IM, Hashim K, ElMubark WA, Shumo ZA, Salih KA, ElNojomi NA, Awad HA, Aziz N, Katabarwa M, Hassan HK, Unnasch TR, Machenzie CD, Richards F, Higazi TB. The First Confirmed Elimination of an Onchocerciasis Focus in Africa: Abu Hamed, Sudan. The American Journal of Tropical Medicine and Hygeine. 2016. 95(5):1037-1040.
3. Meribo K, Kebede B, Mekasha Feleke S, Mengistu B, Mulugeta A, Sileshi M, Samuel A, Deribe K, Tadesse Z. Review of Ethiopian Onchocerciasis Elimination Program. Ethiopian Medical Journa. 2017. 55(Suppl 1): 55-63.
4. Coffeng L, Stolk W, Hoerauf A, Habbema D, Bakker R, Hopkins A, de Vlas S. Elimination of African onchocerciasis: modeling the impact of increasing the frequency of ivermectin mass treatment. PLoS One. 2014. 9(12):e115886.
5. Coffeng LE, Stolk WA, Zouré HG, Veerman JL, Agblewonu KB, Murdoch ME, Noma M, Fobi G, Richardus JH, Bundy DA, Habbema D, de Vlas SJ, Amazigo UV. African Programme For

Onchocerciasis Control 1995-2015: model-estimated health impact and cost. PLoS Negl Trop Dis. 2013; 7(1): e2032.
6. Murdoch ME, Asuzu MC, Hagan M, Makunde WH, Ngoumou P, Ogbuagu KF, Okello D, Ozoh G, Remme J. Onchocerciasis: the clinical and epidemiological burden of skin disease in Africa. Ann Trop Med Parasitol. 2002; 96(3): 283-296.
7. Brieger WR, Awedoba AK, Eneanya CI, Hagan M, Ogbuagu KF, Okello DO, Ososanya OO, Ovuga EB, Noma M, Kale OO, Burnham GM, Remme JH. The effects of ivermectin on onchocercal skin disease and severe itching: results of a multicentre trial. Trop Med Int Health. 1998; 3(12): 951-61.
8. México. http://www.oepa.net/Mexico.htm (accessed July 7, 2017).
9. Guatemala. http://www.oepa.net/guatemala.html (accessed July 7, 2017).
10. Venezuela. http://www.oepa.net/venezuela.html (accessed July 7, 2017).
11. Colombia. http://www.oepa.net/colombia.html (accessed July 7, 2017).
12. Ecuador. http://www.oepa.net/ecuador.html (accessed July 7, 2017).
13. Rodríguez-Pérez MA, Unnasch TR, Domínguez-Vázquez A, *et al.* Lack of Active Onchocerca volvulus Transmission in the Northern Chiapas Focus of Mexico. *The American Journal of Tropical Medicine and Hygiene* 2010; **83**: 15–20.
14. Rodríguez-Pérez MA, Domínguez-Vázquez A, Unnasch TR, *et al.* Interruption of Transmission of Onchocerca volvulus in the Southern Chiapas Focus, México. *PLOS Neglected Tropical Diseases* 2013; **7**: e2133.
15. Rodríguez-Pérez MA, Unnasch TR, Domínguez-Vázquez A, *et al.* Interruption of Transmission of Onchocerca volvulus in the Oaxaca Focus, Mexico. *The American Journal of Tropical Medicine and Hygiene* 2010; **83**: 21–7.
16. Cruz-Ortiz N, Gonzalez RJ, Lindblade KA, *et al.* Elimination of Onchocerca volvulus Transmission in the Huehuetenango Focus of Guatemala. Journal of Parasitology Research. 2012. https://www.hindawi.com/journals/jpr/2012/638429/abs/ (accessed July 7, 2017).
17. Jr FR, Rizzo N, Espinoza CED, *et al.* One Hundred Years After Its Discovery in Guatemala by Rodolfo Robles, Onchocerca volvulus Transmission Has Been Eliminated from the Central Endemic Zone. *The American Journal of Tropical Medicine and Hygiene* 2015; **93**: 1295–304.
18. Gonzalez RJ, Cruz-Ortiz N, Rizzo N, *et al.* Successful interruption of transmission of Onchocerca volvulus in the Escuintla-Guatemala focus, Guatemala. *PLoS Negl Trop Dis* 2009; **3**: e404.
19. Lindblade KA, Arana B, Zea-Flores G, *et al.* Elimination of Onchocercia volvulus transmission in the Santa Rosa focus of Guatemala. *Am J Trop Med Hyg* 2007; **77**: 334–41.
20. Convit J, Schuler H, Borges R, *et al.* Interruption of Onchocerca volvulus transmission in Northern Venezuela. *Parasites & Vectors* 2013; **6**: 289.
21. WHO | WHO declares Ecuador free of onchocerciasis (river blindness). WHO. http://www.who.int/neglected_diseases/ecuador_free_from_onchocerciasis/en/ (accessed July 7, 2017).
22. Onchocerciasis and its control: report of a WHO Expert Committee on Onchocerciasis Control. WHO. http://apps.who.int/iris/bitstream/handle/10665/37346/WHO_TRS_852.pdf;jsessionid=023018C4198968F3E918A2EA8334432C?sequence=1
23. Al-Kubati A, Mackenzie CD, Boakye D, Al-Qubati Y, Al-Samie A, Awad IE, Thylefors B, Hopkins A. Onchocerciasis in Yemen: moving forward towards an elimination program. *International Health*. March 2018; 10(1): i89–i96.

3.3.5 Rabies SDG Capstone Appendix

Flowchart

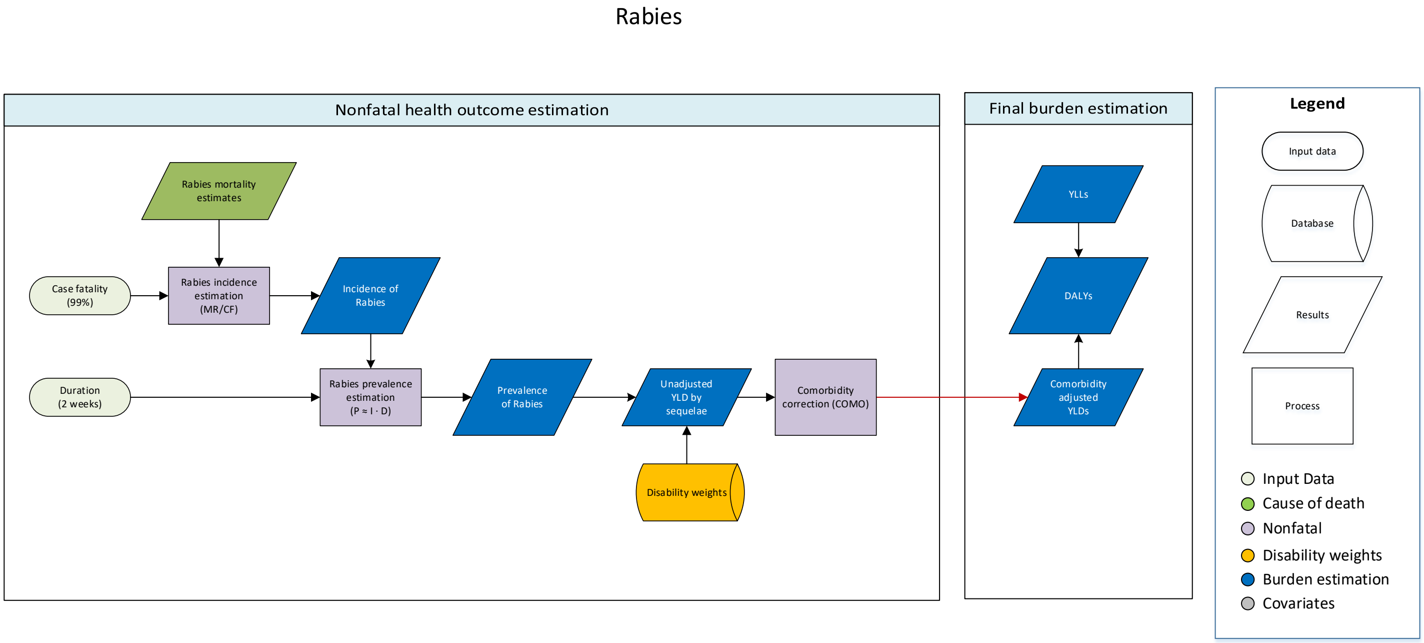

Input data and methodological summary

Case definition

Rabies is a fatal viral infection transmitted by animal bites. Without prophylactic vaccination the disease is almost universally fatal. The disease has a long incubation period (1-3 months), and early intervention with prophylactic vaccination is nearly 100% effective in preventing symptomatic disease. It is considered a neglected tropical disease (NTD). We model symptomatic infections, not including those infections in which intervention prevented the onset of symptomatic disease, corresponding to the ICD10 code A82.

Input data

Model inputs

As we derive our estimate of cases from our estimate of deaths, no incidence data are used in the model. For GBD 2017, we modelled rabies mortality using all available data in the cause of death database. Data points were outliered if they reported an improbable number of rabies deaths (eg, zero rabies deaths in a hyperendemic country) or if their inclusion in the model yielded distorted trends. In some cases, multiple data sources for the same location differed dramatically both in their quality and reported rabies mortality (eg, a verbal autopsy and vital registration source). In these cases, the lower-quality data source was outliered.

Modelling strategy

We derive estimates of the number of symptomatic rabies infections (ie, those not averted through prophylactic vaccination) based on rabies mortality estimates, assuming 99% case fatality. All cases are assumed to be severe.

We modelled rabies mortality using a two-model hybrid approach 1) a global CODEm model of all locations, using all data in the CoD database; and 2) a CODEm model restricted to data-rich countries.

Sequela description and DW

There is only one sequela and associated disability weight for rabies, which is severe. The lay description is included in the table below.

Table 2. Sequela, description, and DW

Sequela	Description	Disability Weight (95% CI)
Severe	Has a high fever and pain, and feels very weak, which causes great difficulty with daily activities.	0.133 (0.088–0.19)

Changes from GBD 2016 to GBD 2017

We have made no substantive changes in the modelling strategy for rabies from GBD 2016.

3.3.5 Schistosomiasis Capstone Appendix

Flowchart

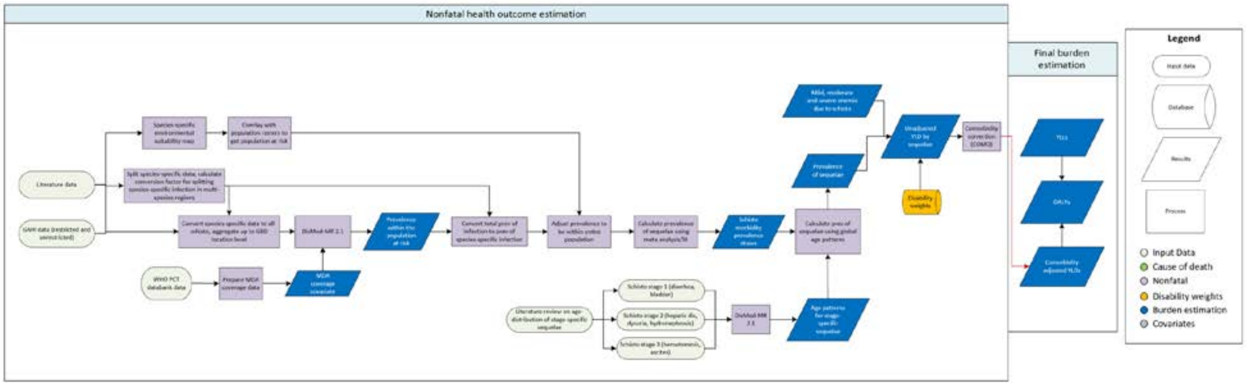

Case definition

Schistosomiasis, also known as bilharzia or "snail fever," is a helminth disease caused by infection with five species of the parasite *Schistosoma,* namely, *S. mansoni, S. japonicum, S. haematobium, S. mekongi, and S. intercalatuma*. It is considered a neglected tropical disease (NTD). The first three species cause the most infection and the last two rarely cause disease. Diagnosis is made by microscopic exam of stool or urine for parasite eggs. For less advanced infections, serologic techniques are used. The ICD-10 codes for schistosomiasis are B65-B65.9.

Input data

Model inputs

To model nonfatal outcomes due to schistosomiasis, we conducted a systematic literature review, extracting prevalence data from 1980 to 2016 for the five species of schistosomiasis listed above. The search string used in the systematic review is (schistosom*[Title/Abstract] OR bilharzia*[Title/Abstract] OR "snail fever"[Title/Abstract]) AND ("1990"[Date - Publication] : "3000"[Date - Publication]) AND (epidemiolog* OR inciden* OR prevalen* OR seroprevalen*) NOT (animals[mesh] NOT humans[mesh]). Additionally, we used data compiled by the Global Atlas of Helminth Infections (GAHI), which includes grey literature and unpublished data.

Mass drug administration data

Mass drug administration data were extracted from the WHO PCT Databank [1].

Severity splits/sequelae

The table below shows the list of clinical sequelae (including mild, moderate, and severe anaemia) due to schistosomiasis, their lay descriptions, and the associated disease stages and disability weights. Using literature [1], a list of eight possible clinical sequelae and anaemia sequelae were defined (mild infection, mild diarrhoea, haematemesis (vomiting blood), hepatomegaly, ascites (buildup of fluid in the peritoneal

cavity), dysuria (painful urination), bladder pathology, hydronephrosis (swelling of kidney due to buildup of urine in the kidney), mild anaemia, moderate anaemia, and severe anaemia).

Table 2. Clinical sequela, lay descriptions, disease stages, and DWs

Clinical sequela	Lay description	Disease stage	Disability weights (DWs)
Mild infection	has a low fever and mild discomfort , but no difficulty with daily activities	1	0.006 (0.002–0.012)
Mild diarrhoea		1	0.056
Hepatomegaly	has some pain in the belly that causes nausea but does not interfere with daily activities	2	0.011 (0.005–0.021)
Dysuria	has some pain in the belly that causes nausea but does not interfere with daily activities	2	0.011 (0.005–0.021)
Hydronephrosis	has some pain in the belly that causes nausea but does not interfere with daily activities	2	0.011 (0.005–0.021)
Haematemesis	vomits blood and feels nauseated	3	0.325 (0.209–0.463)
Ascites	has pain in the belly and feels nauseated. The person has difficulties with daily activities	3	0.114 (0.078–0.159)
Bladder pathology	has some pain in the belly that causes nausea but does not interfere with daily activities	3	0.011 (0.005–0.021)
Mild anaemia	feels slightly tired and weak at times, but this does not interfere with normal daily activities	NA	0.004 (0.001–0.008)
Moderate anaemia	feels moderate fatigue, weakness, and shortness of breath after exercise, making daily activities more difficult	NA	0.052 (0.034–0.076)
Severe anaemia	feels very weak, tired, and short of breath, and has problems with activities that require physical effort or deep concentration	NA	0.149 (0.101–0.210)

Modelling strategy

The morbidity model for schistosomiasis involved a multi-step process. First, we ran a single-parameter prevalence model in DisMod-MR 2.1 using the prevalence data extracted in the systematic review and from the GAHI database. We make the assumption that all of our data are measured within a population at risk – therefore, the estimates from the DisMod model represent prevalence estimates among the population at risk for schistosomiasis. Additionally, we included the MDA treatment data from the WHO as a country-level covariate in the DisMod model. Second, we ran three separate ecological niche maps for the three major species of schistosomiasis (*S. mansoni, S. haematobium,* and *S. japonicum*) using a boosted regression tree and all geolocated data that were extracted from both the literature review and the GAHI database. The output was 1000 maps (representing 1000 draws) for each of the three species representing the suitability for schistosomiasis to exist in each 5x5 km square. Then, we extracted population at risk by optimizing the area under the curve for each of the 1000 maps for each of the three species, overlaid the three species maps over one another, and extracted 1000 draws of proportion of the population at risk for schistosomiasis at the GBD location level.

The BRT was overestimating in Brazil and China. In Brazil, we masked out urban areas from the population at risk rasters, and in China we used year-specific masks based off of published literature on county-specific elimination of schistosomiasis, allowing the geographic restrictions to be implemented at a more detailed level where information is available (5).

We then scaled the prevalence estimates to the population at risk estimates from the ecological niche map to get age/sex/location/year all-schistosomiasis prevalence envelopes. 4) We ran a generalized linear model to get species-specific proportional prevalence on data from literature that reported both *S. haematobium* and *S. mansoni* infection, and 5) literature-informed parameters (a, b, c) for translating infection (x) to morbidity (y): y = (a + bx^c)/(1 + bx^c) – a [2-4]. We used the species-specific conversion factors calculated in step (4) to split the all-schistosomiasis envelope into species-specific schistosomiasis. We then used the parameters determined in step (5) to translate infection into morbidity to get age/sex/year/location-specific prevalence of sequelae. The burden of anemia due to schistosomiasis was estimated (see anaemia documentation for details).

Model evaluation was done by separately assessing the fit of the single-parameter DisMod models and checking the final estimates produced after age-sex splits. Plots of time trends of prevalence across locations and age were used to evaluate the results. In addition, maps of the global distribution of total schistosomiasis prevalence and prevalence of sequelae due to schistosomiasis were also assessed across time.

Changes from GBD 2016 to GBD 2017

The boosted-regression tree environmental suitability maps were further developed in 2017, and were selected based off of improved area under the curve (AUC) statistics. The urban mask for Brazil was also newly implemented this year.

References

1. World Health Organization (WHO). WHO PCT Databank - Schistosomiasis. Geneva, Switzerland: World Health Organization (WHO).
2. van der Werf MJ, de Vlas SJ, Brooker S, et al. Quantification of clinical morbidity associated with schistosome infection in sub-Saharan Africa. Acta Trop. 2003;86(2-3):125-39
3. van der Werf MJ, de Vlas SJ, Looman CW, Nagelkerke NJ, Habbema JD, Engels D. Associating community prevalence of Schistosoma mansoni infection with prevalence of signs and symptoms. Acta Trop. 2002;82(2):127-37
4. van der Werf MJ, de Vlas SJ. Diagnosis of urinary schistosomiasis: A novel approach to compare bladder pathology measured by ultrasound and three methods for hematuria detection. Am. J. Trop. Med. Hyg. 2004;82:98-106
5. Zhou, Xiao-Nong & Bergquist, Robert & Leonardo, Lydia & Olveda, Remigio. (2018). Schistosomiasis: The Disease and its Control.

3.3.5 Vision Impairment due to Trachoma SDG Capstone Appendix

Flowcharts

Vision impairment

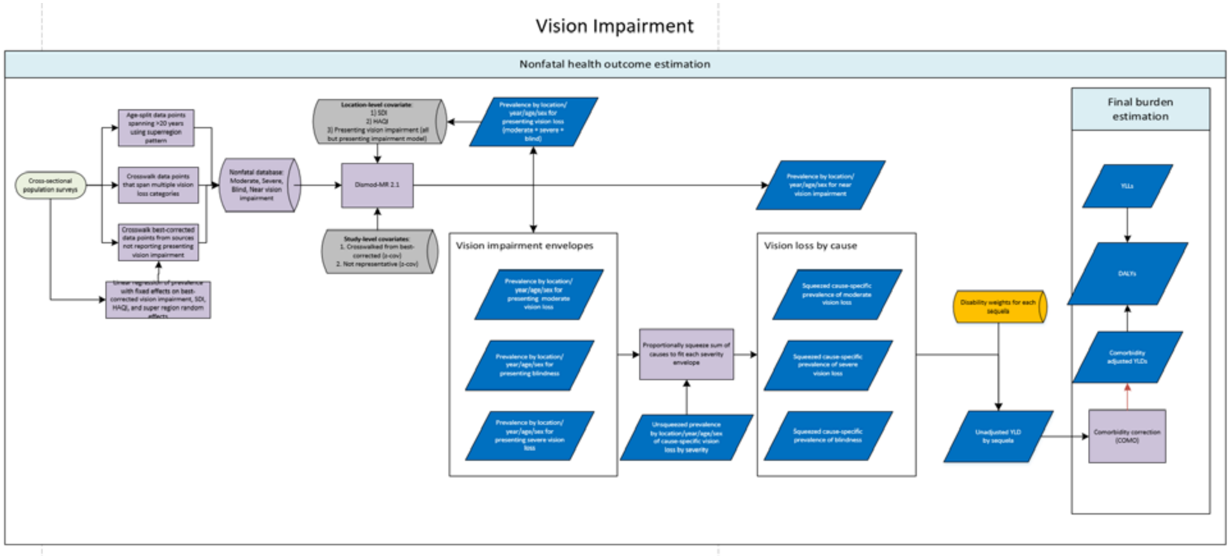

Diabetic retinopathy

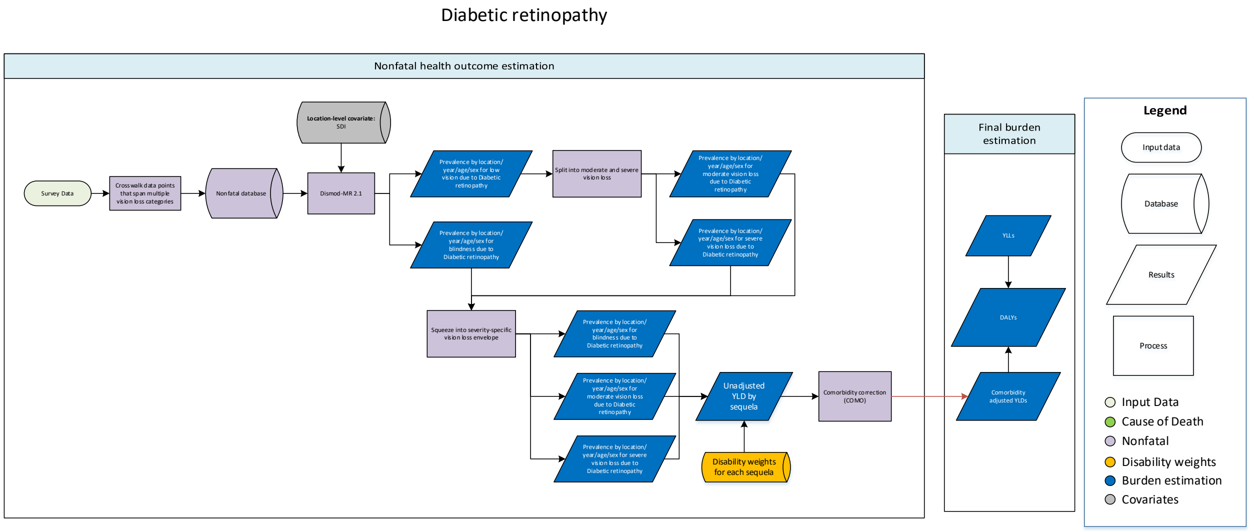

Macular degeneration

Macular degeneration

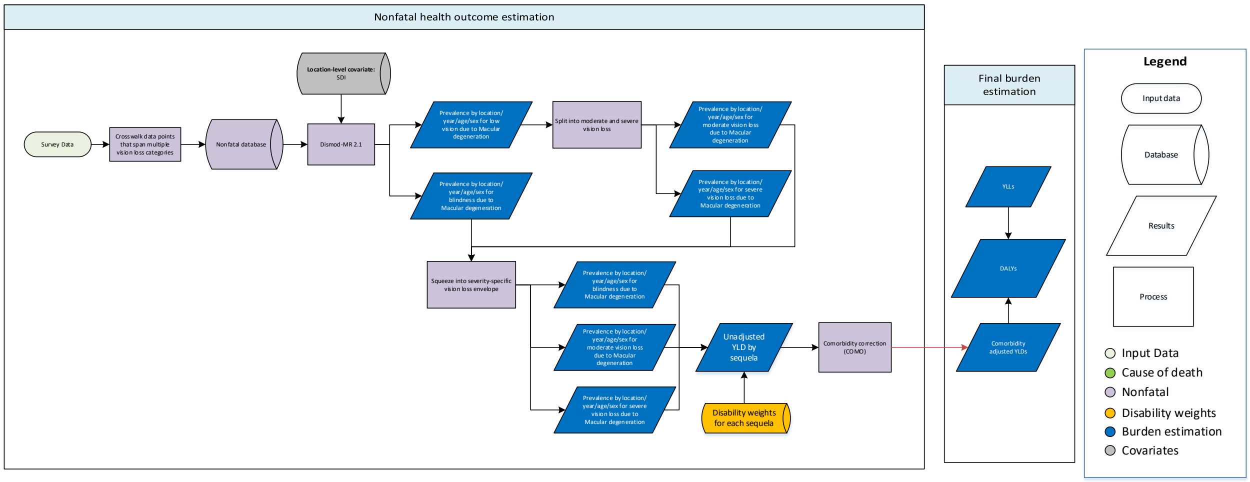

Cataract

Cataract

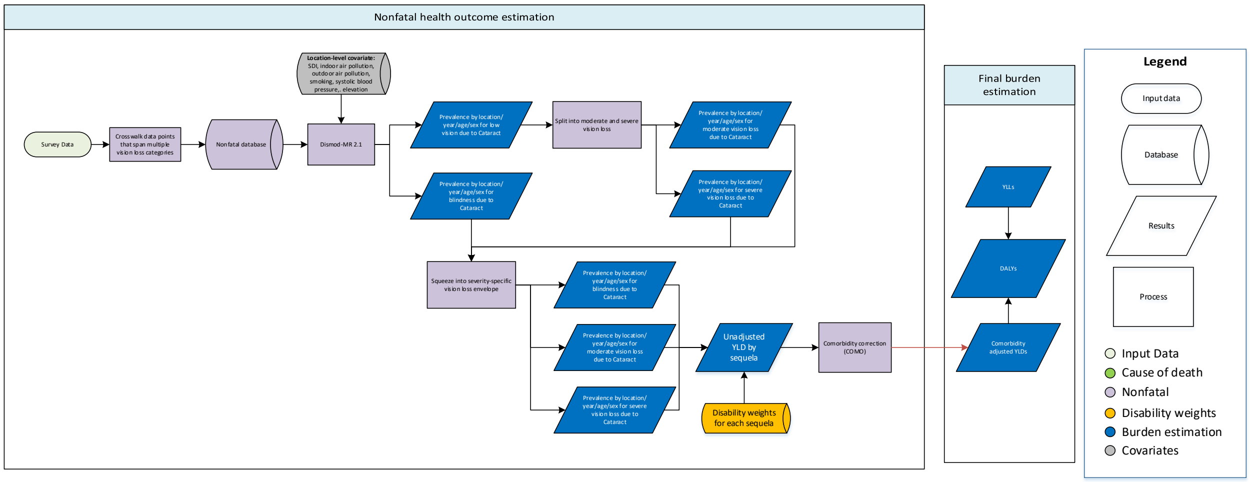

Glaucoma

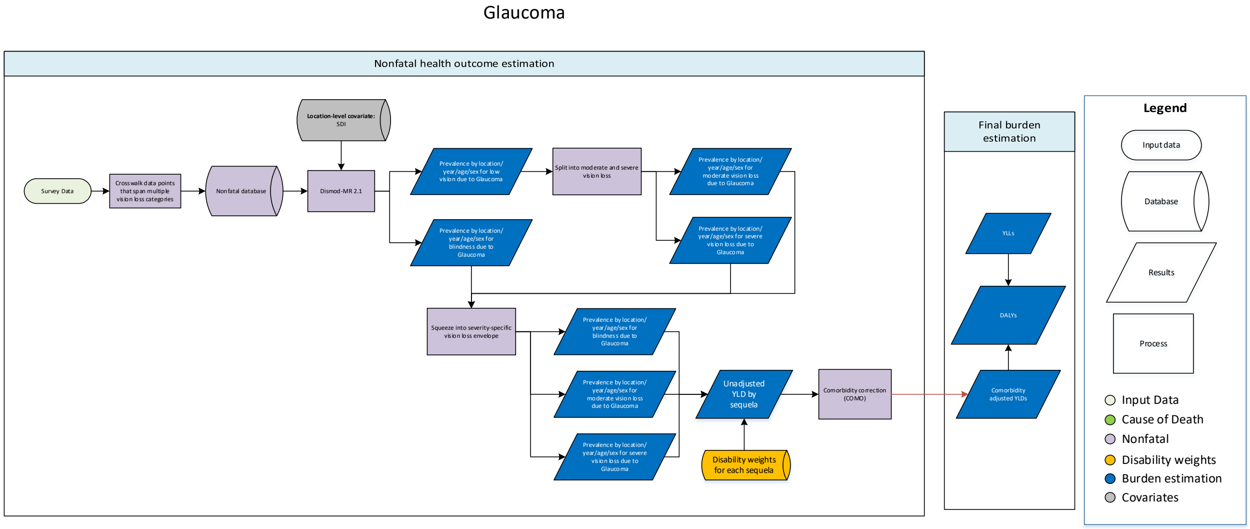

Trachoma

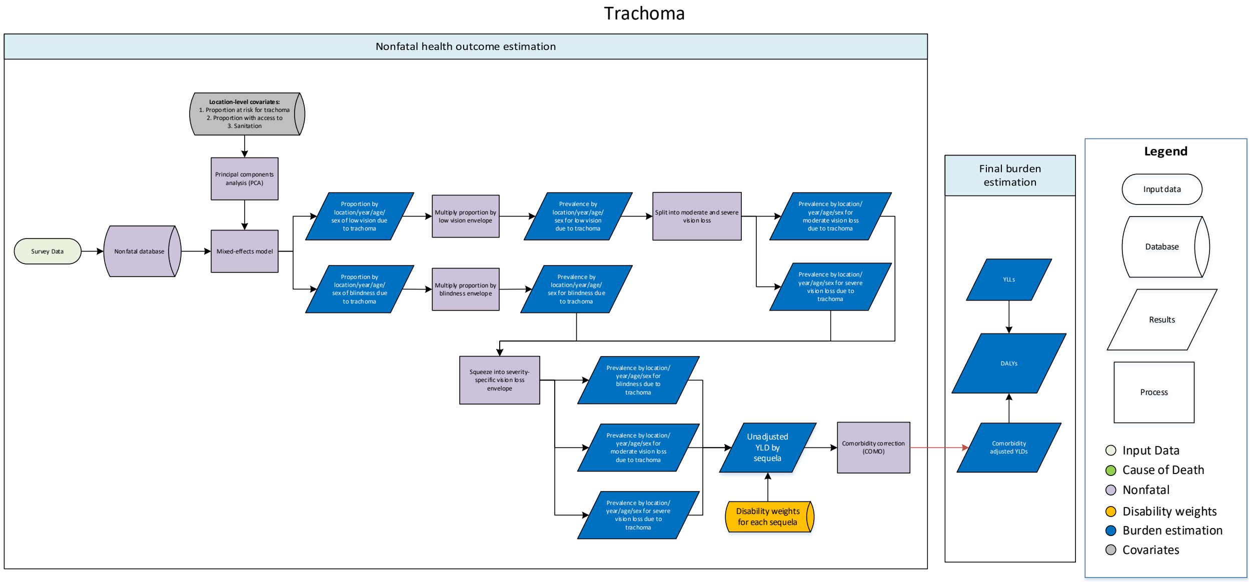

Other vision loss

Other vision loss

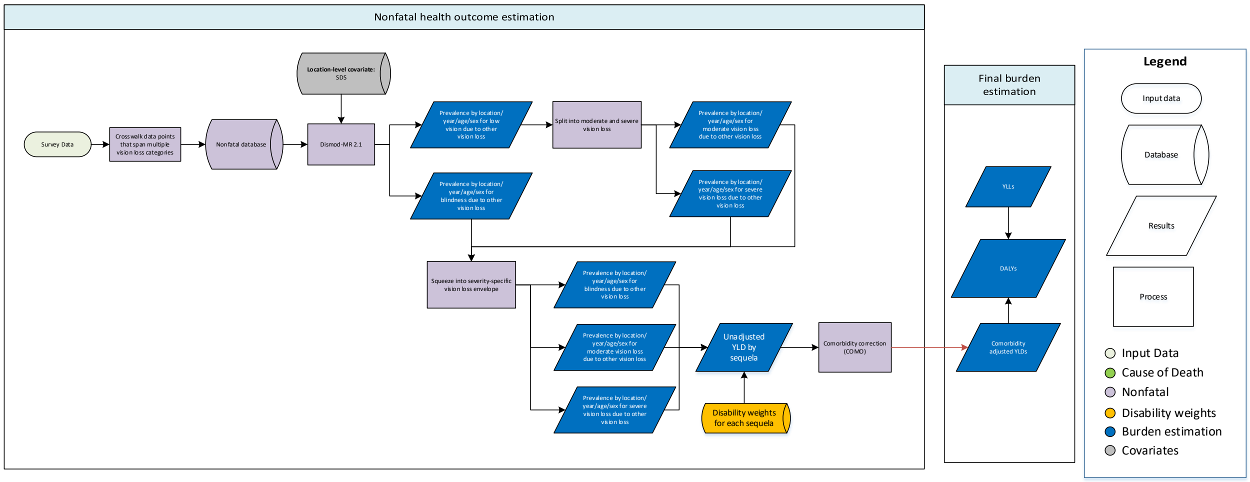

Case definition

We model vision impairment as visual acuity <6/18 according to the Snellen chart. The following impairments are modeled:

Condition	Case definition
Blindness	Visual acuity of <3/60 or <10% visual field around central fixation
Severe vision impairment	3/60 and <6/60
Moderate vision impairment	6/60 and <6/18
Near vision impairment envelope	Near visual acuity of <6/18 distance equivalent

Near vision impairment describes the progressive inability to focus on near objects as individuals age. This impairs the ability to read. The majority of presbyopia can be corrected by the use of reading glasses, contact lenses, or refractive surgery.

We model vision impairment due to the following causes: uncorrected refractive error, cataract, glaucoma, macular degeneration, diabetic retinopathy, trachoma, vitamin A deficiency, retinopathy of prematurity, meningitis, encephalitis, onchocerciasis, and other vision loss. Vision loss due to vitamin A deficiency, retinopathy of prematurity, meningitis, encephalitis, and onchocerciasis are modelled as part of their underlying cause as described in their respective sections.

Refractive error is blurry vision due to the lens's inability to focus. The blurriness caused by refractive error can be addressed through the use of contact lenses, glasses, or refractive surgery. Cataract is

clouding of the lens of the eye due to protein buildup that impairs vision. Glaucoma is a condition with increased intraocular pressure which can lead to damage of the optic nerve. Macular degeneration is a deterioration of the macula, leading to central vision loss. Diabetic retinopathy is damage to the retina caused by damaged blood vessels that can leak blood into the retina and cause scarring of the retina. Trachoma results from a conjunctival bacterial infection (*Chlamydia trachomatis*) that produces inflammation and scarring which leads to an inversion of the eyelids and eyelashes scratching the cornea, which eventually leads to scarring of the cornea and vision impairment or blindness.

Input data

Model inputs

Data on overall vision impairment come from surveys measuring visual acuity in representative population-based studies, either from publications in peer-reviewed and grey literature or surveys for which we had the unit record data. Data were excluded if no test was used of visual acuity that can be converted to the Snellen scale, and if a study did not assess “presenting” or “best-corrected” vision. A subset of these studies that reported vision loss by cause were used to estimate the prevalence of vision loss due to cataract, glaucoma, macular degeneration, diabetic retinopathy, and other causes.

For GBD 2015, we conducted a systematic review for new sources since GBD 2013 (covering 1/1/2013 – 5/20/2015), using the following search string:

((((glaucoma[Title/Abstract] OR cataract[Title/Abstract] OR macular[Title/Abstract] OR 'refractive error'[Title/Abstract] OR presbyopia[Title/Abstract]) OR (('blindness'[MeSH Terms] OR 'blindness'[All Fields]) OR 'vision, low'[MeSH Terms])) AND ('2013'[PDAT] : '3000'[PDAT])) AND 'humans'[MeSH Terms]) AND (prevalence[Title/Abstract] OR incidence[Title/Abstract] OR epidemiology[Title/Abstract])

This yielded 1,169 results, of which we extracted 20 sources. Furthermore, we extracted from the following nationally representative surveys measuring visual acuity: the WHO Studies on Global Ageing and Adult Health (SAGE) and the United States National Health and Examination Surveys (NHANES).

For GBD 2016 and GBD 2017, we did a comprehensive extraction of the Rapid Assessment of Avoidable Blindness (RAAB) repository (http://raabdata.info/), a database of vision impairment studies in developing settings across the world. There are 266 site-years of data, the majority of which have publicly available reports or publications of the data. A standardised methodology was used by all sources in the repository. This allowed us to use all 185 available reports, 70 of which were newly included for GBD 2017. In addition, we extracted two state-level national surveys from India.

Due to the sparse literature reporting measured near-vision visual acuity, we also extracted data from the following nationally representative studies measuring self-reported near vision loss: SAGE; NHANES; the Surveys of Health, Ageing, and Retirement in Europe (SHARE); the Multi-Country Survey Study on Health and Responsiveness (MCSS); and the World Health Surveys (WHS).

Several adjustments were made to raw data.

1) Where studies reported visual acuity spanning multiple thresholds (eg, <6/60, rather than separate severe and blind estimates), we crosswalked using ratios predicted by a linear regression on age, using data from studies reporting vision loss by each severity.
2) Some studies reported best-corrected vision impairment, but not presenting vision impairment (PVI). We crosswalked these data points using a linear regression of logit-transformed PVI prevalence with fixed effects on best-corrected VI, age, and per capita lag-distributed income (LDI) and super-region random effects. This gave us a predicted PVI data points for these studies not explicitly reporting PVI. These crosswalked data points were flagged with a study-level covariate that increased standard error in DisMod.
3) Where data points spanned more than 20 years of age, we age-split using an algorithm that applies the age-pattern of the super-region to split the data to five-year age groups.

Whereas other vision impairment aetiologies are modelled based on prevalence data, vision impairment due to trachoma is modelled as a proportion of the overall best-corrected vision impairment envelope, a strategy that was chosen based on the nature of available data.

The table below shows the number of site-years of data included in envelope models for GBD 2017, as well as the number of countries, regions, and super-regions represented.

**Blindness impairment envelope**	
	**Prevalence**
**Site-years (total)**	415
**Number of countries with data**	100
**Number of GBD regions with data (out of 21 regions)**	20
**Number of GBD super-regions with data (out of 7 super-regions)**	7

**Severe vision impairment envelope**	
	**Prevalence**
**Site-years (total)**	402
**Number of countries with data**	97
**Number of GBD regions with data (out of 21 regions)**	19
**Number of GBD super-regions with data (out of 7 super-regions)**	7

**Moderate vision impairment envelope**	
	**Prevalence**
**Site-years (total)**	378
**Number of countries with data**	94
**Number of GBD regions with data (out of 21 regions)**	19
**Number of GBD super-regions with data (out of 7 super-regions)**	7

Health states and disability weights

Health state name	Health state description	Disability weight
Distance vision, severe impairment	This person has severe vision loss, which causes difficulty in daily activities, some emotional impact (for example, worry), and some difficulty going outside the home without assistance.	0.184 (0.125–0.259)
Distance vision, moderate impairment	This person has vision problems that make it difficult to recognise faces or objects across a room.	0.031 (0.019–0.049)
Distance vision blindness	This person is completely blind, which causes great difficulty in some daily activities, worry and anxiety, and great difficulty going outside the home without assistance.	0.187 (0.124–0.26)
Near vision loss	This person has difficulty seeing things that are nearer than 3 feet, but has no difficulty with seeing things at a distance.	0.011 (0.005–0.02)

Modelling strategy

We modelled the prevalence of vision loss in two steps. In the first step, we estimated the total prevalence estimates of presenting vision loss: moderate vision impairment, severe vision impairment, blindness, and near vision impairment (presbyopia). We directly derived prevalence of near vision impairment from this step, whereas the remaining three models that reflect different severity levels of distance vision loss continued to the next step.

1) Estimate severity-specific vision impairment (the "envelopes")

First, we ran five DisMod-MR 2.1 models to estimate the total prevalence estimates of presenting vision loss: moderate vision impairment, severe vision impairment, blindness, near vision impairment (presbyopia), and presenting vision impairment (moderate + severe + blindness). The presenting vision impairment model was used as a covariate in the severity-specific models to improve consistency across severities.

Betas and exponentiated values, which can be interpreted as an odds ratio, are shown in the table below for each covariate. The best-corrected covariate indicates whether the test measures visual acuity with the level of correction the patient presents with (best_corrected = 0) or the ophthalmologist provides additional correction via pinhole (best_corrected = 1). Rapid-assessment corrects for potential biases in cause-specific vision loss from studies using expedited visual acuity measurement. Socio-demographic Index (SDI) and Healthcare Access and Quality (HAQ) index are used as location covariates as a proxy measure of access to eye care such as cataract surgery. Data points that were crosswalked from best-corrected visual acuity are flagged with a z-cov to adjust uncertainty in the crosswalk process. For near vision impairment, non-standard severity definition is used to crosswalk between the self-report questionnaire of SHARE (nonstandard) and the other surveys. All self-reported data are crosswalked to examination data based on whether the survey question asked about reading at a comfortable distance or recognising an object at arm's length.

Model	Covariate name	Type	Measure	Beta value	Exponentiated value
Vision impairment due to glaucoma unsqueezed	Socio-demographic Index	Country covariate	Prevalence	-0.45 (-0.94 to -0.039)	0.64 (0.39–0.96)

Blindness due to glaucoma unsqueezed	Socio-demographic Index	Country covariate	Prevalence	-1.04 (-1.59 to -0.39)	0.35 (0.20–0.67)
Vision impairment due to cataract unsqueezed	Socio-demographic Index	Country covariate	Prevalence	-0.36 (-0.67 to -0.033)	0.70 (0.51–0.97)
Blindness due to cataract unsqueezed	Socio-demographic Index	Country covariate	Prevalence	-2.9 (-3 to -2.64)	0.055 (0.050–0.072)
Vision impairment due to macular degeneration unsqueezed	Socio-demographic Index	Country covariate	Prevalence	0.50 (-0.21 to 0.97)	1.65 (0.81–2.65)
Blindness due to macular degeneration unsqueezed	Socio-demographic Index	Country covariate	Prevalence	0.027 (-0.85 to 0.91)	1.03 (0.43–2.48)
Near vision loss	Socio-demographic Index	Country covariate	Prevalence	-0.8 (-1.37 to -0.2)	0.45 (0.25–0.82)
Near vision loss	Non-standard severity definition	Study-level x-covariate	Prevalence	-0.19 (-0.2 to -0.17)	0.83 (0.82–0.85)
Near vision loss	Comfortable reading distance	Study-level x-covariate	Prevalence	-0.49 (-0.5 to -0.46)	0.61 (0.61–0.63)
Near vision loss	Recognise object at arm's length	Study-level x-covariate	Prevalence	0.035 (-0.021 to 0.094)	1.04 (0.98–1.10)
Vision impairment due to other vision loss unsqueezed	Socio-demographic Index	Country covariate	Prevalence	-0.064 (-0.23 to -0.0031)	0.94 (0.79–1.00)
Blindness due to other vision loss unsqueezed	Socio-demographic Index	Country covariate	Prevalence	-0.81 (-0.99 to -0.47)	0.45 (0.37–0.63)
Vision impairment envelope	Socio-demographic Index	Country covariate	Prevalence	-0.29 (-0.52 to -0.065)	0.75 (0.59–0.94)
Blindness impairment envelope	Healthcare Access and Quality index	Country covariate	Prevalence	-0.018 (-0.022 to -0.016)	0.98 (0.98–0.98)
Blindness impairment envelope	Presenting vision impairment	Country covariate	Prevalence	0.33 (0.17–0.48)	1.40 (1.18–1.62)
Blindness impairment envelope	Socio-demographic Index	Country covariate	Prevalence	-0.077 (-0.25 to -0.0038)	0.93 (0.78–1.00)
Blindness impairment envelope	Best-corrected crosswalk	Study-level z-covariate	Prevalence	0.0025 (0.00020–0.0070)	1.00 (1.00–1.01)

Moderate vision impairment envelope	Presenting vision impairment	Country covariate	Prevalence	0.74 (0.65–0.83)	2.09 (1.92–2.29)
Moderate vision impairment envelope	Socio-demographic Index	Country covariate	Prevalence	-0.021 (-0.082 to -0.000018)	0.98 (0.92–1.00)
Moderate vision impairment envelope	Best-corrected crosswalk	Study-level z-covariate	Prevalence	0.10 (0.070–0.13)	1.11 (1.07–1.14)
Severe vision impairment envelope	Presenting vision impairment	Country covariate	Prevalence	0.35 (0.24–0.45)	1.42 (1.28–1.58)
Severe vision impairment envelope	Socio-demographic Index	Country covariate	Prevalence	-0.018 (-0.072 to -0.0014)	0.98 (0.93–1.00)
Severe vision impairment envelope	Best-corrected crosswalk	Study-level z-covariate	Prevalence	0.032 (0.0073–0.062)	1.03 (1.01–1.06)
Vision impairment due to diabetes mellitus	Diabetes age-standardised prevalence (proportion)	Country covariate	Prevalence	1.25 (0.85–1.66)	3.50 (2.35–5.24)
Vision impairment due to diabetes mellitus	Socio-demographic Index	Country covariate	Prevalence	-0.066 (-0.24 to -0.00064)	0.94 (0.79–1.00)
Blindness due to diabetes mellitus unsqueezed	Diabetes age-standardised prevalence (proportion)	Country covariate	Prevalence	3.79 (3.19–4.00)	44.40 (24.41–54.33)
Blindness due to diabetes mellitus unsqueezed	Socio-demographic Index	Country covariate	Prevalence	-1.58 (-1.99 to -0.6)	0.20 (0.14–0.55)
Moderate vision impairment due to uncorrected refractive error unsqueezed	Socio-demographic Index	Country covariate	Prevalence	-0.95 (-1 to -0.82)	0.39 (0.37–0.44)
Severe vision impairment due to uncorrected refractive error unsqueezed	Socio-demographic Index	Country covariate	Prevalence	-0.95 (-1 to -0.83)	0.39 (0.37–0.44)
Blindness due to uncorrected refractive error unsqueezed	Socio-demographic Index	Country covariate	Prevalence	-0.98 (-1 to -0.93)	0.37 (0.37–0.40)

2) Estimate cause-specific vision impairment

In the second step, we estimated the prevalence of vision loss due to multiple causes: refractive error, cataract, glaucoma, macular degeneration, diabetic retinopathy, retinopathy due to prematurity, trachoma, vitamin A deficiency, onchocerciasis, meningitis, and other causes not classified elsewhere. The vision loss due to retinopathy of prematurity, vitamin A deficiency, onchocerciasis, meningitis, tetanus, and neonatal conditions was modelled as part of these underlying causes. Vision loss due to trachoma is

modelled as a proportion of the envelope, with separate proportion models for vision impairment and blindness. For each of cataract, glaucoma, macular degeneration, diabetic retinopathy, and other vision loss, we ran two DisMod-MR 2.1 models: one for the combined category of moderate and severe vision loss due to the cause, and one for blindness due to the cause. Moderate and severe vision loss were modelled together because input data were mostly available for the aggregate. Refractive error was modelled in three models, one for each severity. We used the following age restrictions:

Cause	Minimum age
Cataracts	20
Glaucoma	45
Macular degeneration	45
Diabetic retinopathy	20
Trachoma	15
Other vision loss	0

We estimated the proportions of low vision and blindness due to trachoma using custom mixed-effects models. For consistency, the two models (blindness and low vision) were parameterised identically and differ only in their input data. Our model included fixed effects on age (using cubic splines with knots at 0, 40, and 100 years of age), sex, and a covariate derived from a principal components analysis of the proportion of the population at risk for trachoma and the proportion of the population with access to sanitation. We included nested random effects on super-region, region, and country. Finally, we applied geographic and age restrictions to ensure that we estimate zero proportions in non-endemic locations and among those younger than 15 year of age (as scarring of the cornea due to trachoma takes decades to develop). The prevalence of trachoma at each severity level was calculated by multiplying the proportion of vision loss (vision impairment or blindness) due to trachoma by the corresponding best-corrected vision loss envelope.

We split the moderate plus severe vision loss estimates for each cause into moderate and severe using the ratio of presenting moderate and severe vision loss envelopes. As exceptions, onchocerciasis and retinopathy of prematurity were modelled for moderate and severe vision loss as part of the estimation process of these causes.

We scaled the cause-specific vision loss prevalence to the total prevalence of the best-corrected vision loss envelopes for each of the three severity levels. The final result is prevalence of vision loss due to each cause by severity.

The following changes have been implemented since GBD 2016:

- We added an additional 70 RAAB sources
- Several data points from previous rounds have been reevaluated and then re-extracted or determined to not meet our criteria and were taken out of the model
- We elevated presbyopia and refractive error from the sequela-level to the cause-level within the GBD hierarchy
- We crosswalk self-report near vision data by survey question

3.4.1 Non-communicable Disease (NCD) Mortality SDG Capstone Appendix

Cardiovascular diseases, cancers, diabetes mellitus, and chronic respiratory diseases

Indicator definition

This modeling strategy encompasses the indicator associated with non-communicable disease mortality (3.4.1).

Indicator 3.4.1

As a component of SDG Goal 3. Ensure healthy lives and promote well-being for all at all ages, SDG Target 3.4, by 2030, reduce by one third premature mortality from NCDs through prevention and treatment and promote mental health and well-being, is measured using SDG Indicator 3.4.1, deaths due to cardiovascular disease, cancer, diabetes, and chronic respiratory disease among populations aged 30 to 70 per 100,000.

3.4.1: Cardiovascular Diseases

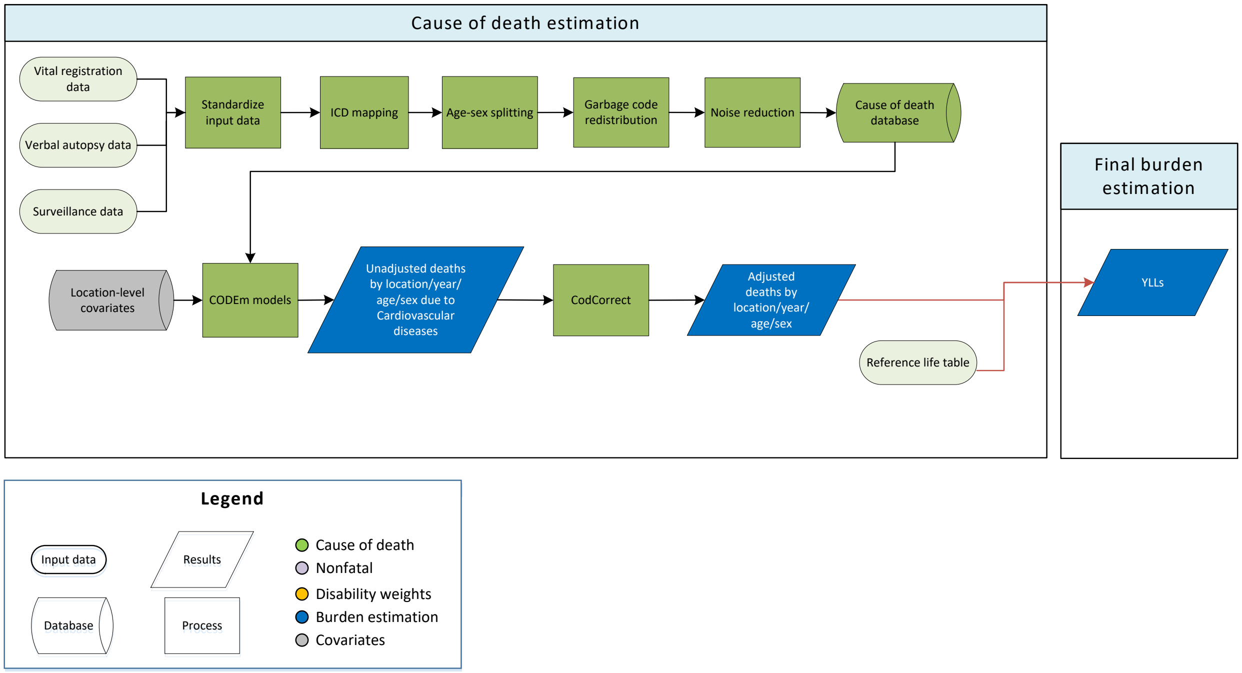

Input data

Vital registration, verbal autopsy, and surveillance data were used to model the parent cardiovascular envelope. We outliered non-representative subnational verbal autopsies from a number of Indian states. We also outliered verbal autopsy data sources that were implausibly low in all age groups and ICD8 and ICD9 BTL data points that were inconsistent with the rest of the data and created implausible time trends.

Modelling strategy

We used a standard CODEm approach to model deaths from cardiovascular diseases. The covariates included in the ensemble modelling process are listed in the table below. There have been no substantive changes from the approach used in GBD 2016.

Table: Selected covariates for CODEm models, cardiovascular diseases

Covariate	Transformation	Level	Direction
Summary exposure variable, cardiovascular disease	None	1	1
Cholesterol (total, mean per capita)	None	1	1
Smoking prevalence	None	1	1
Systolic blood pressure (mmHg)	None	1	1
Trans fatty acid	None	1	1
Mean BMI	None	2	1

Elevation over 1500m (proportion)	None	2	-1
Fasting plasma glucose (mmol/L)	None	2	1
Outdoor pollution ($PM_{2.5}$)	None	2	1
Indoor air pollution (all fuel types)	None	2	1
Healthcare access and quality index	None	2	-1
Lag distributed income per capita (I$)	Log	3	-1
Socio-demographic Index	None	3	0
Omega-3 (kcal/capita, adjusted)	Log	3	-1
Fruits (kcal/capita, adjusted)	None	3	-1
Vegetables (kcal/capita, adjusted)	None	3	-1
Nuts and seeds (kcal/capita, adjusted)	None	3	-1
Whole grains (kcal/capita, adjusted)	None	3	-1
Pulses/legumes (kcal/capita, adjusted)	None	3	-1
PUFA adjusted (percent)	None	3	-1
Alcohol (litres per capita)	None	3	0

3.4.1 Cancers

Input data and methodological summary for all cancers except for non-melanoma skin cancer

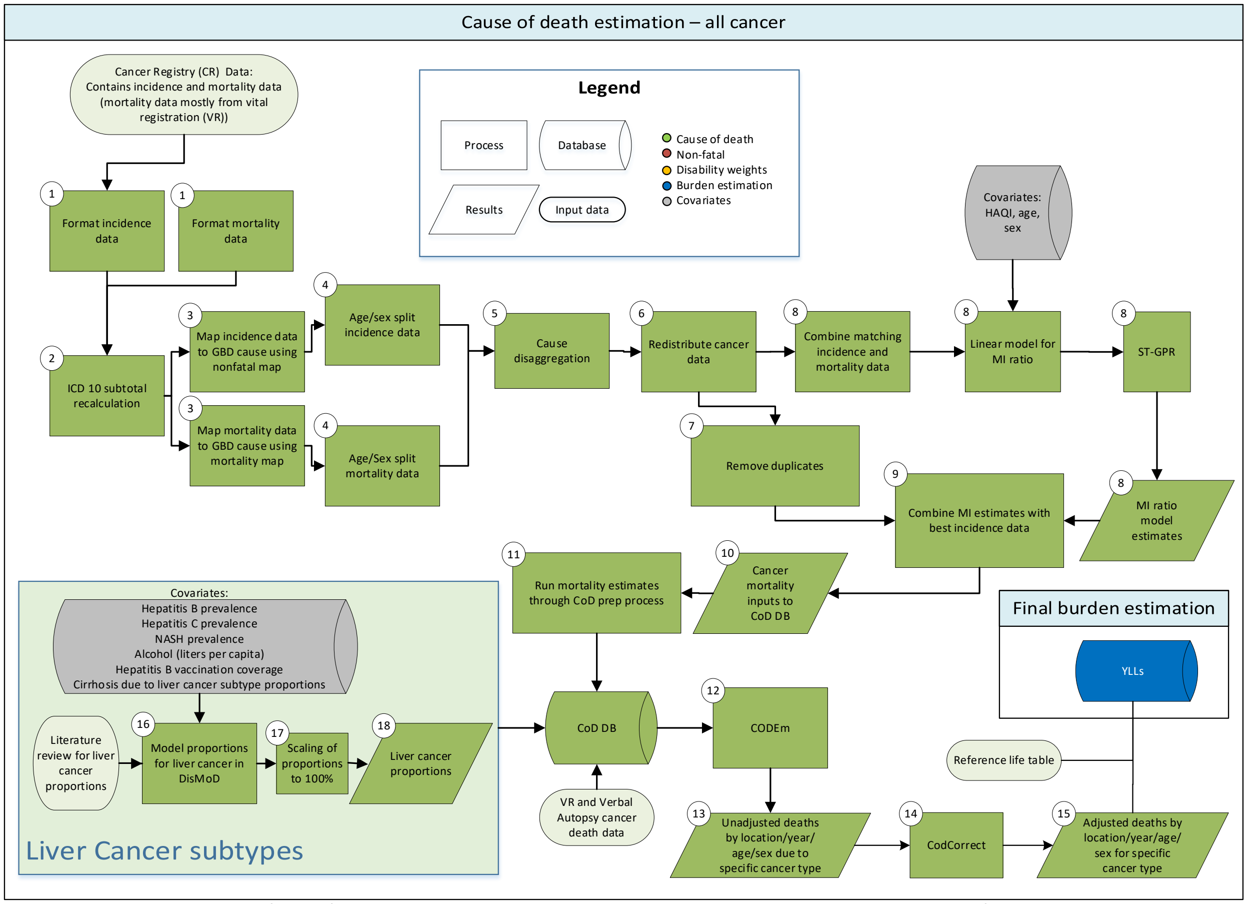

Abbreviations: ICD: International classification of diseases; DB: database, ST-GPR: Space-time smoothing, Gaussian process regression, COD: Causes of death

Data

The cause of death (COD) database contains multiple sources of cancer mortality data. These sources include vital registration, verbal autopsy, and cancer registry data. The cancer registry mortality estimates that are uploaded into the COD database stem from cancer registry incidence data that have been transformed to mortality estimates through the use of mortality-to-incidence ratios (MIR).

Data-seeking processes

Cancer mortality data in the cause of death database other than cancer registry data

Sources for cancer mortality data other than cancer registry data are described in the COD database description (Appendix Section 2 in the Cause of Death Capstone Appendix).

Cancer registry data

Cancer registry data were used from publicly available sources or provided by collaborators. We used all data from GBD 2016 and added registry data from Russia, Iran, Ethiopia, Norway, as well as the newly released CI5 XI (Cancer Registry in Five Continents).

Inclusion and exclusion criteria

Only population-based cancer registries were included, and only those that included all cancers (no specialty registries), data for all age groups, and data for both sexes. Pathology-based cancer registries were included if they had a defined population. Hospital-based cancer registries were excluded.
Cancer registry data were excluded from either the final incidence data input or the MI model input if a more detailed source (eg, providing more detailed age or diagnostic groups) was available for the same population. Preference was given to registries with national coverage over those with only local coverage, except those from countries where the GBD study provides subnational estimates.
Data were excluded if the coverage population was unknown.

Bias of categories of input data

Cancer registry data can be biased in multiple ways. A high proportion of ill-defined cancer cases in the registry data requires redistribution of these cases to other cancers, which introduces a potential for bias. Changes between coding systems can lead to artificial differences in disease estimates; however, we adjust for this bias by mapping the different coding systems to the GBD causes. Underreporting of cancers that require advanced diagnostic techniques (eg, leukaemia and brain, pancreatic, and liver cancer) can be an issue in cancer registries from low-income countries. On the other hand, misclassification of metastatic sites as primary cancer can lead to overestimation of cancer sites that are common sites for metastases, like brain or liver. Since many cancer registries are located in urban areas, the representativeness of the registry for the general population can also be problematic. The accuracy of mortality data reported in cancer registries usually depends on the quality of the vital registration system. If the vital registration system is incomplete or of poor quality, the mortality-to-incidence ratio can be biased to lower ratios.

Data for liver cancer aetiology splits

For GBD 2017, the aetiologies for liver cancer were expanded to include a separate aetiology of liver cancer due to non-alcoholic steatohepatitis (NASH). To find the proportion of liver cancer cases due to the five aetiology groups included in GBD (1. Liver cancer due to hepatitis B, 2. Liver cancer due to hepatitis C, 3. Liver cancer due to alcohol, 4. Liver cancer due to NASH, 5. Liver cancer due to other causes), a systematic literature search was performed in PubMed on 10/24/2016 using the following search string: “("liver neoplasms"[All Fields] OR "HCC"[All Fields] OR "liver cancer"[All Fields] OR "Carcinoma, Hepatocellular"[Mesh]) AND (("hepatitis B"[All Fields] OR "Hepatitis B"[Mesh] OR "Hepatitis B virus"[Mesh] OR "Hepatitis B Antibodies"[Mesh] OR "Hepatitis B Antigens"[Mesh]) OR ("hepatitis C"[All Fields] OR "Hepatitis C"[Mesh] OR "hepatitis C antibodies"[MESH] OR "Hepatitis C Antigens"[Mesh] OR "Hepacivirus"[Mesh]) OR ("alcohol"[All Fields] OR "Alcohol Drinking"[Mesh] OR "Alcohol-Related Disorders"[Mesh] OR "Alcoholism"[Mesh] OR "Alcohol-Induced Disorders"[Mesh])) NOT (animals[MeSH] NOT humans[MeSH])”. Also, studies not found through this search but included in the meta-analysis by de Martel and colleagues were included.[10] We also included the study by Hong and colleagues after the authors provided us with additional data on the overlap in risk factors.[11]

Studies were included if the study population was representative of liver cancer population for the respective location. For each study, the proportions of liver cancer due to the five specific risk factors were calculated. Cases were considered to be due to NASH when the manuscript explicitly listed the aetiology to be NASH or non-alcoholic fatty liver disease (NAFLD). Cases where the aetiology was listed as "cryptogenic", "idiopathic", or "unknown" were included within the "other causes" category. In manuscripts where the aetiology for a case was not known but major categories could not be ruled out (for example, the study tested for hepatitis B and C but did not assess alcohol use), these cases were excluded from the numerator of the study (in other words, did not contribute a proportion to any aetiology). Remaining risk factors were included under a combined "other" group (for example, haemochromatosis, autoimmune hepatitis, Wilson's disease, etc.). If multiple risk factors were reported for an individual patient, these were apportioned proportionally to the individual risk factors.

Methods

Steps of analysis and data transformation processes

Cancer registry data went through multiple processing steps before integration with the COD database. First, the original data were transformed into standardised files, which included standardisation of format, categorisation, and registry names (#1 in flowchart).

Second, some cancer registries report individual codes as well as aggregated totals (eg, C18, C19, and C20 are reported individually but the aggregated group of C18-C20 [colorectal cancer] is also reported in the registry data). The data processing step "subtotal recalculation" (#2 in flowchart) verifies these totals and subtracts the values of any individual codes from the aggregates.

In the third step (#3 in the flowchart), cancer registry incidence data and cancer registry mortality data are mapped to GBD causes. A different map is used for incidence and for mortality data because of the assumption that there are no deaths for certain cancers. One example is basal cell carcinoma of the skin. In the cancer registry incidence data, basal cell carcinoma is mapped to non-melanoma skin cancer (basal cell carcinoma). However, if basal cell skin cancer is recorded in the cancer registry mortality data, the deaths are instead mapped to non-melanoma skin cancer (squamous cell carcinoma) under the assumption that they were indeed misclassified squamous cell skin cancers. Other examples are benign or in situ neoplasms. Benign or in situ neoplasms found in the cancer registry incidence dataset were simply dropped from that dataset. The same neoplasms reported in a cancer registry mortality dataset were mapped to the respective invasive cancer (eg, melanoma in situ in the cancer registry incidence dataset was dropped from the dataset; melanoma in situ in the cancer registry mortality dataset was mapped to melanoma).

In the fourth data processing step (#4 in the flowchart) cancer registry data were standardised to the GBD age groups. Age-specific incidence rates were generated using all datasets that include microdata, and datasets that report age groups up to 95+ years of age, while age-specific mortality rates were generated from the CoD data. Age-specific proportions were then generated by applying the age-specific rates to a given registry population that required age-splitting to produce the expected number of cases/deaths for that registry by age. The expected number of cases/deaths for each sex, age, and cancer were then normalised to 1, creating final, age-specific proportions. These proportions were then applied to the total number of cases/deaths by sex and cancer to get the age-specific number of cases/deaths.

In the rare case that the cancer registry only contained data for both sexes combined, the now-age-specific cases/deaths were split and reassigned to separate sexes using the same weights that are used for the age-splitting process. Starting from the expected number of deaths, proportions were generated by sex for each age (eg, if for ages 15 to 19 years old there are six expected deaths for males and four expected deaths for females, then 60% of the combined-sex deaths for ages 15-19 years would be assigned to males and the remaining 40% would be assigned to females).

In the fifth step (#5 in the flowchart) data for cause entries that are aggregates of GBD causes were redistributed. Examples of these aggregated causes include some registries reporting ICD10 codes C00-C14 together as "lip, oral cavity, and pharyngeal cancer." These groups were broken down into sub-causes that could be mapped to single GBD causes. In this example, those include lip and oral cavity cancer (C00-C08), nasopharyngeal cancer (C11), cancer of other parts of the pharynx (C09-C10, C12-C13), and "Malignant neoplasm of other and ill-defined sites in the lip, oral cavity, and pharynx" (C14). To redistribute the data, weights were created using the same "rate-applied-to-population" method employed in age-sex splitting (see step four above). For the undefined code (C14 in the example) an "average all cancer" weight was used, which was generated by adding all cases from SEER/NORDCAN/CI5 and dividing the total by the combined population. Then, proportions were generated by sub-cause for each aggregate cause as in the sex-splitting example above (see step four). The total number of cases from the aggregated group (C00-C14) was then recalculated for each subgroup and the undefined code (C14). C14 was then redistributed as a "garbage code" in step six. Distinct proportions were used for C44 (non-melanoma skin cancer) and C46 (Kaposi's sarcoma). Non-melanoma skin cancer processing is described under section "Input data and methodological summary for non-melanoma skin cancer (squamous-cell carcinoma)." C46 entries were redistributed as "other cancer," and HIV using proportions described in Appendix Section 2 of the Cause of Death Capstone Appendix.
In the sixth step (#6 in the flowchart) unspecified codes ("garbage codes") were redistributed. Redistribution of cancer registry incidence and mortality data mirrored the process of the redistribution used in the cause of death database.

In the seventh step (#7 in the flowchart) duplicate or redundant sources were removed from the processed cancer registry dataset. Duplicate sources were present if, for example, the cancer registry was part of the CI5 database but we also had data from the registry directly. Redundancies occurred and were removed as described in "Inclusion and Exclusion Criteria," where more detailed data were available, or when national registry data could replace regionally representative data. From here, two parallel selection processes were run to generate input data for the MI models and to generate incidence for final mortality estimation. Higher priority was given to registry data from the most standardised source when creating the final incidence input, whereas for the MI model input only sources that reported incidence and mortality were used.

In the eighth step (#8 in the flowchart) the processed incidence and mortality data from cancer registries were matched by cancer, age, sex, year, and location to generate MI ratios. These MI ratios were used as input for a three-step modelling approach using the updated GBD 2017 ST-GPR approach. with the Healthcare Access and Quality (HAQ) Index as a covariate in the linear step mixed effects model using a logit link function. This is different from GBD 2016, where we used Socio-demographic Index (SDI) as a predictive covariate. Predictions were made without the random effects. The ST-GPR model has three

main hyper-parameters that control for smoothing across time, age, and geography. The time adjustment parameter (λ) was set to 2, which aims to borrow strength from neighbouring time points (ie, the exposure in this year is highly correlated with exposure in the previous year but less so further back in ti e . he e ust ent r eter s set to . hi h borro s stren th ro t in nei hbouring age groups. The space adjustment parameter ξ was set to 0.95 in locations with data and to 0.5 in locations without data (the higher ξ was applied when at least one age-sex group in the country of estimation had at least five unique data points. The lower ξ was applied when estimating data-scarce countries). Zeta aims to borrow strength across the hierarchy of geographical locations.[12] For the amplitude parameter in the Gaussian process regression we used 2, and for the scale we used a value of 15.

For GBD 2017 we slightly changed the data cleaning process and used HAQ rather than SDI to exclude data. For each cancer, MI ratios from locations in HAQ quintiles 1-4 were dropped if they were below the median of MI ratios from locations in HAQ quintile 5. We also dropped MI ratios from locations in HAQ quintiles 1-4 if the MI ratios were above the third quartile + 1.5 * IQR (inter-quartile range). We dropped all MIR that were based on less than 25 cases to avoid noise due to small numbers except for mesothelioma and acute lymphoid leukaemia, where we dropped MIR that were based on fewer than ten cases because of lower data availability for these two cancers. We also aggregated incidence and mortality to the youngest five-year age bin where we had at least 50 data points to avoid MIR predictions in young age groups that were based on few data points. The MIR in the age-bin that was used to aggregate MIR was used to backfill the MIR for younger age groups.

Since MI ratios can be above 1, especially in older age groups and cancers with low cure rates, we used the 95th percentile of the cleaned dataset that only included MIR that were based on 50 or more cases, to cap the MIR input data. This "upper cap" was used to allow MIR over 1 but to constrain the MIR to a maximum level. To run the logit model, the input data were divided by the upper caps and model predictions after ST-GPR was rescaled by multiplying them by the upper caps. To constrain the model at the lower end, we used the fifth percentile of the cancer-specific cleaned MIR input data to replace all model predictions with this lower cap.

Final MI ratios were matched with the cancer registry incidence dataset in the ninth step (#9 in the flowchart) to generate mortality estimates (Incidence * Mortality/Incidence = Mortality) (#10 in the flowchart). The final mortality estimates were then uploaded into the COD database (#11 in the flowchart). Cancer-specific mortality modelling then followed the general CODEm process.

Liver cancer aetiology split models

The proportion data found through the systematic literature review were used as input for five separate DisMod-MR 2.1 models to determine the proportion of liver cancers due to the five subgroups for all locations, both sexes, and all age groups (step #16 in the flowchart). A study covariate was used for publications that only assessed liver cancer in a cirrhotic population. The reference, or "gold standard", that was used for crosswalking was the compilation of all studies that assessed the aetiology of liver cancer in a general population. For liver cancer due to hepatitis C and hepatitis B, a prior value of 0 was set between age 0 and 0.01. For liver cancer due to alcohol, a prior value of 0 was set for ages 0 to 5 years. For liver cancer due to hepatitis C, hepatitis C (IgG) seroprevalence was used as a covariate as well as a covariate for alcohol (litres per capita), hepatitis B prevalence (HBsAg seroprevalence), and

NASH/NAFLD prevalence, forcing a negative relationship between the alcohol, hepatitis B, hepatitis C, and NASH/NAFLD covariates and the outcome of liver cancer due to alcohol proportion. For liver cancer due to hepatitis B, seroprevalence of HBsAg was used as a covariate as well as a covariate for alcohol, hepatitis C IgG seroprevalence, NASH/NAFLD prevalence, and the population coverage of three-dose hepatitis B vaccination, forcing a negative relationship between these covariates and the outcome of liver cancer due to hepatitis B proportion. For liver cancer due to alcohol, alcohol (litres per capita) was used as a covariate as well as a covariate for proportion of alcohol abstainers, hepatitis B and hepatitis C seroprevalence, and NASH/NAFLD prevalence, forcing a negative relationship between the proportion of alcohol abstainers, NASH/NAFLD, and hepatitis B and hepatitis C covariates and the outcome of liver cancer due to alcohol proportion. For liver cancer due to NASH, NASH/NAFLD prevalence was used as a covariate as well as a covariate for obesity prevalence and mean body-mass index (BMI), forcing a positive relationship between these covariates and the outcome of liver cancer due to NASH proportion. All covariates used were modelled independently. To ensure consistency between cirrhosis and liver cancer estimates and to take advantage of the data for the respective other related cause (eg, liver cancer due to hepatitis C and the related cause cirrhosis due to hepatitis C), we generated covariates from the liver cancer proportion models that we used in the cirrhosis aetiology proportion models. We then created covariates from the cirrhosis aetiology proportion models and used those in the liver cancer aetiology models.

Since the proportion models are run independently of each other, the final proportion models were scaled to sum to 100% within each age, sex, year, and location, by dividing each proportion by the sum of the five (step # 17). For the liver cancer subtype mortality estimates, we multiplied the parent cause "liver cancer" by the corresponding scaled proportions (step # 18). Single-cause estimates were adjusted to fit into the separately modelled all-cause mortality in the process CoDCorrect.

Results

Interpretation of results

Cancer mortality estimates for GBD 2017 can differ from the GBD 2016 results for multiple reasons. Updated cancer mortality data were added from vital registration system data, verbal autopsy studies, as well as cancer registry incidence data. A new cause "Myelodysplastic, myeloproliferative, and other haematopoietic neoplasms" was added. In GBD 2016 all deaths due to this new cause were counted in the category "other neoplasms". The mortality-to-incidence ratio estimation has been updated compared to GBD 2016, using HAQ rather than SDI in the data cleaning and modelling process, and the ST-GPR approach was also updated. Covariate inputs for the CODEm models were changed based on recommendations from collaborators. Covariates used in CODEm models were updated for GBD 2017.

The other group producing country-level cancer mortality estimates is the International Agency for Research on Cancer (IARC) with their GLOBOCAN database. Significantly different methods between the GBD study and GLOBOCAN can lead to differences in results. Whereas estimates in GLOBOCAN are based on the assumption that there are "In theory, […] as many methods as countries,"[13] the cancer estimation process for the GBD study follows a coherent, well-documented method for all cancers, which allows cross-validation of models as well as determination of uncertainty. Another major difference is the ability in the GBD study to adjust single-cause estimates to the all-cause mortality, which is being determined independently. This also allows us to adjust individual causes of death to the all-cause mortality envelope,

which permits us to correct for the underdiagnosis of cancer in countries with inadequate diagnostic resources. Redistribution of a fraction of undefined causes of death to certain cancers is another methodological advantage the GBD study has over GLOBOCAN, and estimates for cancer mortality can therefore differ substantially in countries with a large proportion of undefined causes of deaths in their vital registration data or a large proportion of undefined cancer cases in their cancer registry data.

Limitations

There are certain limitations to consider when interpreting the GBD cancer mortality estimates. First, even though every effort is made to include the most recently available data for each country, data-seeking resources are not limitless and new data cannot always be accessed as soon as they are made available. It is therefore possible that the GBD study does not include all available data sources for cancer incidence or cancer mortality. Second, different redistribution methods can potentially change the cancer estimates substantially if the data sources used for the estimated location contain a large number of undefined causes; however, neglecting to account for these undefined deaths would likely introduce an even greater bias in the disease estimates. Third, using mortality-to-incidence ratios to transform cancer registry incidence data to mortality estimates requires accurate MIR. For GBD 2017 we have made further changes to the MIR estimation, but the method remains sensitive to underdiagnosis of cancer cases or underascertainment of cancer deaths. However, given that the majority of data used for the cancer mortality estimation come from vital registration data and not cancer registry data, this is not a major limitation.

Non-melanoma skin cancer (squamous cell carcinoma)

Data

Data seeking processes

Since squamous cell carcinomas are only very infrequently recorded by cancer registries, only vital registration system data were used as input for the squamous cell carcinoma mortality modelling.

Inclusion and exclusion criteria

Inclusion and exclusion criteria followed the same methods as described for the vital registration data sources (Appendix Section 2 of the Cause of Death Capstone Appendix).

Bias of categories of input data

The potential biases of the input data are the same as for other cancers (see above).

Methods

Overall methodological process

Vital registration system data were used as input to model deaths due to squamous cell skin cancer.

Steps of analysis and data transformation processes

Since mortality estimates for non-melanoma skin cancer are only produced for squamous cell carcinoma under the assumption that basal cell carcinoma causes almost no deaths, all mortalities reported as “C44” or “173” were mapped to the “squamous cell carcinoma” GBD cause.

Model selection

The modelling strategy for non-melanoma skin cancer (squamous cell carcinoma) followed the general CODEm process.

Model performance and sensitivity

The modelling performance and sensitivity for non-melanoma skin cancer (squamous cell carcinoma) mirrored that of the general CODEm process.

Uncertainty intervals

Uncertainty was determined using standard CODEm methodology.

Results

Interpretation of results

Non-melanoma skin cancer mortality estimates are not available from other sources. GLOBOCAN, for example, does not report deaths due to non-melanoma skin cancer. Even though the data availability for non-melanoma skin cancer is poor, the fact that it is the most common incident cancer, with rates expected to rise, makes it a necessity to include the disease in the GBD framework.

Limitations

Cancer registry data for non-melanoma skin cancer incidence have to be interpreted with caution due to a substantial amount of underreporting or rules that only the first non-melanoma skin cancer has to be registered. Many cancer registries therefore do not include non-melanoma skin cancers at all. However, information on whether registries capture NMSC or not is not consistently available. Therefore, no cancer registry data were used to estimate deaths due to squamous cell carcinoma of the skin. For vital registration data, we make the assumption that there are no deaths due to basal cell non-melanoma skin cancer; therefore, all deaths attributed to basal cell carcinoma were included instead as squamous cell carcinoma.

Covariates by cancer:

Lip and oral cavity cancer

Level	*Covariate*	*Direction*
1	*Alcohol (litres per capita)*	+
	Cumulative cigarettes (5 years)	+
	Cumulative cigarettes (10 years)	+
	Cumulative cigarettes (15 years)	+
	Cumulative cigarettes (20 years)	+
	Smoking prevalence	+
	Tobacco (cigarettes per capita)	+
	Log-transformed SEV scalar: Mouth C	+
	Vegetables adjusted (g)	–
2	*Red meats adjusted (g)*	+
	Fruits adjusted (g)	–
	*Health system access 2 (unitless)**	–
	Healthcare access and quality index	–
3	*Education (years per capita)*	–
	LDI (I$ per capita)	*0*
	Socio-demographic Index	*0*

Nasopharynx cancer

Level	*Covariate*	*Direction*
1	*Alcohol (litres per capita)*	+
	Cumulative cigarettes (5 years)	+
	Cumulative cigarettes (10 years)	+
	Cumulative cigarettes (15 years)	+
	Cumulative cigarettes (20 years)	+
	Smoking prevalence	+
	Tobacco (cigarettes per capita)	+
	Log-transformed SEV scalar: Nasoph C	+
	Vegetables adjusted (g)	–
2	*Red meats adjusted (g)*	+
	Fruits adjusted (g)	–
	*Health system access 2 (unitless)**	–
	Healthcare access and quality index	–
3	*Education (years per capita)*	–
	LDI (I$ per capita)	*0*
	Socio-demographic Index	*0*

Oesophageal cancer

Level	*Covariate*	*Direction*
1	*Alcohol (litres per capita)*	+
	Log-transformed SEV scalar: Esophag C	+
	Log-transformed age-standardized SEV scalar: Esophag C	+
	Mean BMI	+
	Smoking prevalence	+
	Tobacco (cigarettes per capita)	+
	Fruits adjusted (g)	-
2	*Indoor air pollution (all cooking fuels)*	+
	Sanitation (proportion with access)	–
	Vegetables adjusted (g)	–
	Improved water source (proportion with access)	–
	Healthcare access and quality index	–
3	*Education (years per capita)*	–
	LDI (I$ per capita)	*0*
	Socio-demographic Index	*0*

Other pharynx cancer

Level	*Covariate*	*Direction*
1	*Alcohol (litres per capita)*	+
	Smoking prevalence	+
	Log-transformed SEV scalar: Oth Phar C	+
	Vegetables adjusted (g)	–
2	*Cumulative cigarettes (5 years)*	+
	Fruits adjusted (g)	–
	Vegetables adjusted (g)	–
	Population density (over 1000 ppl/sqkm, proportion)	+
	Population density (under 150 ppl/sqkm, proportion)	+
	Healthcare access and quality index	–
3	*Education (years per capita)*	–
	LDI (I$ per capita)	*0*
	Socio-demographic Index	*0*

Stomach cancer

Level	*Covariate*	*Direction*
1	*Diet high in sodium*	+
	Cumulative cigarettes (5 years)	+
	Cumulative cigarettes (10 years)	+
	Cumulative cigarettes (15 years)	+
	Cumulative cigarettes (20 years)	+
	Smoking prevalence	+
	Tobacco (cigarettes per capita)	+
	Log-transformed SEV scalar: Stomach C	+
	SEV unsafe water	+
	SEV unsafe sanitation	+
2	*Vegetables adjusted (g)*	–
	Fruits adjusted (g)	–
	Mean BMI	+
	Sanitation (proportion with access)	–
	Improved water source (proportion with access)	–
	Healthcare access and quality index	–
3	*Education (years per capita)*	–
	LDI (I$ per capita)	*0*
	Socio-demographic Index	*0*

Colon and rectum cancer

Level	*Covariate*	*Direction*
1	*Alcohol (litres per capita)*	+
	Mean BMI	+
	Smoking prevalence	+
	Tobacco (cigarettes per capita)	+
	Log-transformed SEV scalar: Colorect C	+
	Red meats adjusted (g)	+
2	*Milk adjusted (g)*	–
	Fruits adjusted (g)	–
	Nuts seeds adjusted (g)	–
	PUFA adjusted (percent)	–
	Vegetables adjusted (g)	–
	Cumulative cigarettes (5 years)	+
	Cumulative cigarettes (10 years)	+
	Cumulative cigarettes (15 years)	+
	Cumulative cigarettes (20 years)	+
	Diabetes age-specific prevalence (proportion)	+
	Healthcare access and quality index	–
3	*Education (years per capita)*	–
	LDI (I$ per capita)	*0*
	Socio-demographic Index	*0*

Liver cancer

Level	*Covariate*	*Direction*
1	*Alcohol (litres per capita)*	+
	HIV age-standardised prevalence	+
	Hepatitis B (HBsAg) seroprevalence	+
	Hepatitis C (IgG) seroprevalence	+
	Log-transformed SEV scalar: Liver C	+
2	*Hepatitis B 3-dose coverage (proportion)*	–
	Hepatitis B 3-dose coverage (proportion), lagged 5 years	–
	Hepatitis B 3-dose coverage (proportion), lagged 10 years	–
	Hepatitis B vaccine coverage (proportion), aged through time	–
	Intravenous drug use (age-standardised proportion)	+
	Cumulative cigarettes (5 years)	+
	Cumulative cigarettes (10 years)	+
	Cumulative cigarettes (15 years)	+
	Cumulative cigarettes (20 years)	+
	Diabetes age-specific prevalence (proportion)	+
	Diabetes fasting plasma glucose (mmol/L)	+

Liver cancer, continued

Level	*Covariate*	*Direction*
2	*Mean BMI*	+
	Tobacco (cigarettes per capita)	+
	Red meats adjusted (g)	+
	Healthcare access and quality index	–
3	*Education (years per capita)*	–
	LDI (I$ per capita)	0
	Socio-demographic Index	0

Gallbladder and biliary tract cancer

Level	*Covariate*	*Direction*
1	*Log-transformed SEV scalar: Gallblad C*	+
	Mean BMI	+
2	*Alcohol (litres per capita)*	+
	Cumulative cigarettes (5 years)	+
	Cumulative cigarettes (10 years)	+
	Smoking prevalence	+
	Tobacco (cigarettes per capita)	+
	Fruits adjusted (g)	–
	Vegetables adjusted (g)	–
	Diabetes age-standardised prevalence (proportion)	+
	Healthcare access and quality index	–
3	*Education (years per capita)*	–
	LDI (I$ per capita)	0
	Socio-demographic Index	0

Pancreatic cancer

Level	*Covariate*	*Direction*
1	*Alcohol (litres per capita)*	+
	Cumulative cigarettes (5 years)	+
	Cumulative cigarettes (10 years)	+
	Cumulative cigarettes (15 years)	+
	Cumulative cigarettes (20 years)	+
	Smoking prevalence	+
	Tobacco (cigarettes per capita)	+
	Log-transformed SEV scalar: Pancreas C	+
	Mean BMI	+
2	*Red meats adjusted (g)*	+
	Fruits adjusted (g)	–
	Vegetables adjusted (g)	–
	Energy unadjusted (kcal)	+
	Diabetes fasting plasma glucose (mmol/L)	+
	Diabetes age-standardised prevalence (proportion)	+
	Healthcare access and quality index	–
3	*Education (years per capita)*	–
	LDI (I$ per capita)	*0*
	Socio-demographic Index	*0*

Larynx cancer

Level	*Covariate*	*Direction*
1	*Alcohol (litres per capita)*	+
	Log-transformed SEV scalar: Larynx C	+
2	*Smoking prevalence*	+
	Tobacco (cigarettes per capita)	+
	Asbestos consumption (metric tons per year per capita)	+
	Fruits adjusted (g)	–
	Vegetables adjusted (g)	–
	Cumulative cigarettes (5 years)	+
	Cumulative cigarettes (10 years)	+
	Cumulative cigarettes (15 years)	+
	Cumulative cigarettes (20 years)	+
	Population density (over 1000 ppl/sqkm, proportion)	+
	Population density (under 150 ppl/sqkm, proportion)	+
	Healthcare access and quality index	–
3	*Education (years per capita)*	–
	LDI (I$ per capita)	*0*
	Socio-demographic Index	*0*

Tracheal, bronchus, and lung cancer

Level	*Covariate*	*Direction*
1	*Alcohol (litres per capita)*	+
	Cumulative cigarettes (5 yYears)	+
	Cumulative cigarettes (10 years)	+
	Cumulative cigarettes (15 years)	+
	Cumulative cigarettes (20 years)	+
	Smoking prevalence	+
	Tobacco (cigarettes per capita)	+
	Secondhand smoke	+
	Log-transformed SEV scalar: Lung C	+
	Log-transformed age-standardised SEV scalar: Lung C	+
2	*Indoor air pollution (all cooking fuels)*	+
	Outdoor air pollution ($PM_{2.5}$)	+
	Residential radon	+
	Diabetes fasting plasma glucose (mmol/L)	+
	Healthcare access and quality index	–
3	*Education (years per capita)*	–
	LDI (I$ per capita)	*0*
	Socio-demographic Index	*0*

Malignant skin melanoma

Level	*Covariate*	*Direction*
1	*Alcohol (litres per capita)*	*+*
2	*Fruits adjusted (g)*	*–*
	Vegetables adjusted (g)	*–*
	Latitude under 15 (proportion)	*–*
	Latitude 15 to 30 (proportion)	*0*
	Latitude 30 to 45 (proportion)	*–*
	Latitude over 45 (proportion)	*–*
	Healthcare access and quality index	*–*
3	*Education (years per capita)*	*–*
	LDI (I$ per capita)	*0*
	Socio-demographic Index	*0*

Non-melanoma skin cancer

Level	*Covariate*	*Direction*
1	*Cumulative cigarettes (5 years)*	*+*
	Cumulative cigarettes (10 years)	*+*
	Cumulative cigarettes (15 years)	*+*
	Smoking prevalence	*+*
2	*Average latitude*	*0*
	Healthcare access and quality index	*–*
3	*Education (years per capita)*	*0*
	LDI (I$ per capita)	*0*
	Socio-demographic Index	*0*

Breast cancer

Level	*Covariate*	*Direction*
1	*Alcohol (litres per capita)*	*+*
	Mean BMI	*+*
	Log-transformed SEV scalar: Breast C	*+*
2	*Age-specific fertility rate*	*–*
	Total fertility rate	*–*
	Fruits adjusted (g)	*–*
	Vegetables adjusted (g)	*–*
	Cumulative cigarettes (5 years)	*+*
	Cumulative cigarettes (10 years)	*+*
	Cumulative cigarettes (15 years)	*+*
	Cumulative cigarettes (20 years)	*+*
	Smoking prevalence	*+*
	Secondhand smoke	*+*
	Diabetes fasting plasma glucose (mmol/L)	*+*
	Healthcare access and quality index	*–*
3	*Education (years per capita)*	*–*
	LDI (I$ per capita)	*0*
	Socio-demographic Index	*0*

Cervical cancer

Level	*Covariate*	*Direction*
1	*Cumulative cigarettes (5 years)*	+
	Cumulative cigarettes (10 years)	+
	Cumulative cigarettes (15 years)	+
	HIV age-standardised prevalence	+
2	*Age-specific fertility rate*	+
	Total fertility rate	+
	Smoking prevalence	+
	Fruits adjusted (g)	–
	Vegetables adjusted (g)	–
	Healthcare access and quality index	–
3	*Education (years per capita)*	–
	LDI (I$ per capita)	*0*
	Socio-demographic Index	*0*

Uterine cancer

Level	*Covariate*	*Direction*
1	*Log-transformed SEV scalar: Uterus C*	+
	Mean BMI	+
2	*Cumulative cigarettes (5 years)*	+
	Cumulative cigarettes (10 years)	+
	Smoking prevalence	+
	Tobacco (cigarettes per capita)	+
	Diabetes age-standardized prevalence (proportion)	+
	Total fertility rate	*0*
	Fruits adjusted (g)	–
	Vegetables adjusted (g)	–
	Healthcare access and quality index	–
3	*Education (years per capita)*	–
	LDI (I$ per capita)	*0*
	Socio-demographic Index	*0*

Prostate cancer

Level	*Covariate*	*Direction*
1	*Log-transformed SEV scalar: Prostate C*	+
2	*Smoking prevalence*	*0*
	Healthcare access and quality index	–
3	*Education (years per capita)*	*0*
	LDI (I$ per capita)	*0*
	Socio-demographic Index	*0*

Ovarian cancer

Level	Covariate	Direction
1	Alcohol (litres per capita)	+
	Cumulative cigarettes (5 years)	+
	Cumulative cigarettes (10 years)	+
	Cumulative cigarettes (15 years)	+
	Cumulative cigarettes (20 years)	+
	Tobacco (cigarettes per capita)	+
	Contraception (modern) prevalence (proportion)	–
	Log-transformed SEV scalar: Ovary C	+
2	Asbestos consumption (metric tons per year per capita)	+
	Smoking prevalence	+
	Total fertility rate	0
	Energy unadjusted (kcal)	+
	Fruits adjusted (g)	–
	Vegetables adjusted (g)	–
	Mean BMI	+
	Diabetes age-standardised prevalence (proportion)	+
	Diabetes fasting plasma glucose (mmol/L)	+
	Healthcare access and quality index	–

Ovarian cancer, continued

Level	Covariate	Direction
3	Education (years per capita)	–
	LDI (I$ per capita)	0
	Socio-demographic Index	0

Testicular cancer

Level	Covariate	Direction
2	Cumulative cigarettes (5 years)	+
	Cumulative cigarettes (10 years)	+
	Cumulative cigarettes (15 years)	+
	Cumulative cigarettes (20 years)	+
	Tobacco (cigarettes per capita)	+
	Smoking prevalence	+
	Fruits adjusted (g)	–
	Vegetables adjusted (g)	–
	Healthcare access and quality index	–
3	Education (years per capita)	–
	LDI (I$ per capita)	0
	Socio-demographic Index	0

Kidney cancer

Level	Covariate	Direction
1	Cumulative cigarettes (5 years)	+
	Cumulative cigarettes (10 years)	+
	Cumulative cigarettes (15 years)	+
	Mean BMI	+
	Log-transformed SEV scalar: Kidney C	+
2	Alcohol (litres per capita)	+
	Diabetes age-standardised prevalence (proportion)	+
	Systolic blood pressure (mmHg)	+
	Smoking prevalence	+
	Healthcare access and quality index	–
3	Education (years per capita)	–
	LDI (I$ per capita)	0
	Socio-demographic Index	0

Bladder cancer

Level	Covariate	Direction
1	Schistosomiasis prevalence (proportion)	+
	Cumulative cigarettes (5 years)	+
	Cumulative cigarettes (10 years)	+
	Cumulative cigarettes (15 years)	+
	Smoking prevalence	+
	Log-transformed SEV scalar: Bladder C	+
2	Alcohol (litres per capita)	+
	Diabetes gasting plasma glucose (mmol/L)	+
	Vegetables adjusted (g)	–
	Fruits adjusted (g)	–
	Healthcare access and quality index	–
3	Education (years per capita)	–
	LDI (I$ per capita)	0
	Socio-demographic Index	0

Brain and nervous system cancer

Level	Covariate	Direction
1	Alcohol (litres per capita)	+
	Cumulative cigarettes (10 years)	+
	Cumulative cigarettes (15 years)	+
	Smoking prevalence	+
2	Cholesterol (total, mean per capita)	+
	Systolic blood pressure (mmHg)	+
	Red meats adjusted (g)	+
	Vegetables adjusted (g)	–
	Fruits adjusted (g)	–
	Healthcare access and quality index	–
3	Education (years per capita)	–
	LDI (I$ per capita)	0
	Socio-demographic Index	0

Thyroid cancer

Level	*Covariate*	*Direction*
1	*Alcohol (litres per capita)*	*+*
	Smoking prevalence	*+*
	Log-transformed SEV scalar: Thyroid C	*+*
2	*Vegetables adjusted (g)*	*–*
	Fruits adjusted (g)	*–*
	Red meats adjusted (g)	*+*
	Tobacco (cigarettes per capita)	*+*
	Mean BMI	*+*
	Smoking prevalence	*+*
	Sanitation (proportion with access)	*–*
	Improved water source (proportion with access)	*–*
	Healthcare access and quality index	*–*
3	*Education (years per capita)*	*–*
	LDI (I$ per capita)	*0*
	Socio-demographic Index	*0*

Mesothelioma

Level	*Covariate*	*Direction*
1	*Asbestos consumption (metric tons per year per capita)*	*+*
	Cumulative cigarettes (5 years)	*+*
	Asbestos production (binary)	*+*
	Smoking prevalence	*+*
	Indoor air pollution (all cooking fuels)	*+*
	Log-transformed SEV scalar: Mesothel	*+*
	Log-transformed age-standardised SEV scalar: Mesothel	*+*
2	*Asbestos production (kg) per capita*	*+*
	Gold production (binary)	*+*
	Gold production (kg) per capita	*+*
	Population density (over 1000 ppl/sqkm, proportion)	*+*
	Healthcare access and quality index	*–*
3	*Education (years per capita)*	*–*
	LDI (I$ per capita)	*0*
	Socio-demographic Index	*0*

Hodgkin's lymphoma

Level	*Covariate*	*Direction*
2	*Healthcare access and quality index*	*–*
3	*Education (years per capita)*	*–*
	LDI (I$ per capita)	*0*
	Socio-demographic Index	*0*

Non-Hodgkin lymphoma

Level	*Covariate*	*Direction*
2	*Cumulative cigarettes (5 years)*	+
	Cumulative cigarettes (10 years)	+
	Cumulative cigarettes (15 years)	+
	Cumulative cigarettes (20 years)	+
	Alcohol (litres per capita)	+
	Smoking prevalence	+
	Mean BMI	+
	Healthcare access and quality index	–
3	*Total fertility rate*	*0*
	LDI (I$ per capita)	*0*
	Socio-demographic Index	*0*

Multiple myeloma

Level	*Covariate*	*Direction*
1	*Alcohol (litres per capita)*	+
	Smoking prevalence	+
	Tobacco (cigarettes per capita)	+
2	*Vegetables adjusted (g)*	–
	Fruits adjusted (g)	–
	Red meats adjusted (g)	+
	Tobacco (cigarettes per capita)	+
	Mean BMI	+
	Sanitation (proportion with access)	–
	Improved water source (proportion with access)	–
	Healthcare access and quality index	–
3	*Education (years per capita)*	–
	LDI (I$ per capita)	*0*
	Socio-demographic Index	*0*

Leukaemia

Level	*Covariate*	*Direction*
1	*Log-transformed age-standardised SEV scalar: Leukaemia*	+
	Log-transformed SEV scalar: Leukaemia	+
2	*Alcohol (litres per capita)*	+
	Mean BMI	+
	Cumulative cigarettes (5 years)	+
	Cumulative cigarettes (10 years)	+
	Cumulative cigarettes (15 years)	+
	Cumulative cigarettes (20 years)	+
	Smoking prevalence	+
	Tobacco (cigarettes per capita)	+
	Healthcare access and quality index	–
3	*Education (years per capita)*	–
	LDI (I$ per capita)	*0*
	Socio-demographic Index	*0*

Myelodysplastic, myeloproliferative, other haematopoietic neoplasms

Level	*Covariate*	*Direction*
1	*Log-transformed age-standardised SEV scalar: Leukaemia*	+
	Log-transformed SEV scalar: Leukaemia	+
2	*Alcohol (litres per capita)*	+
	Mean BMI	+
	Cumulative cigarettes (5 years)	+
	Cumulative cigarettes (10 years)	+
	Cumulative cigarettes (15 years)	+
	Cumulative cigarettes (20 years)	+
	Smoking prevalence	+
	Tobacco (cigarettes per capita)	+
	Healthcare access and quality index	–
3	*Education (years per capita)*	–
	LDI (I$ per capita)	*0*
	Socio-demographic Index	*0*

Other malignant cancers

Level	*Covariate*	*Direction*
1	*Smoking prevalence*	+
	Tobacco (cigarettes per capita)	+
2	*Vegetables adjusted (g)*	–
	Fruits adjusted (g)	–
	Nuts seeds adjusted (g)	–
	PUFA adjusted (percent)	–
	Mean BMI	–
	Healthcare access and quality index	–
3	*Education (years per capita)*	–
	LDI (I$ per capita)	*0*
	Socio-demographic Index	*0*

Other neoplasms

Level	*Covariate*	*Direction*
2	*Healthcare access and quality index*	–
3	*Education (years per capita)*	-
	LDI (I$ per capita)	*0*
	Socio-demographic Index	*0*

References

1 Waterhouse J, Muir C, Shanmugaratnam K, Powell J. Cancer Incidence in Five Continents IV. Lyon: IARC, 1982.

2 Curado M, Edwards B, Shin H, *et al.* Cancer Incidence in Five Continents IX. Lyon: IARC, 2007 http://www.iarc.fr/en/publications/pdfs-online/epi/sp160/CI5vol9-A.pdf.

3 Muir C, Mack T, Powell J, Whelan S. Cancer Incidence in Five Continents V. Lyon: IARC, 1987.

4 Parkin D, Muir C, Whelan S, Gao Y, Ferlay J, Powell J. Cancer Incidence in Five Continents VI. Lyon: IARC, 1992.

5 Parkin D, Whelan S, Ferlay J, Raymond L, Young J. Cancer Incidence in Five Continents VII. Lyon: IARC, 1997.

6 Parkin D, Whelan S, Ferlay J, Teppo L, Thomas D. Cancer Incidence in Five Continents VIII. Lyon: IARC, 2002.

7 Forman D, Bray F, Brewster D, *et al.* Cancer Incidence in Five Continents X. 2013. http://ci5.iarc.fr.

8 Engholm G, Ferlay J, Christensen N, *et al.* NORDCAN: Cancer Incidence, Mortality, Prevalence and Survival in the Nordic Countries, Version 7.3 Association of the Nordic Cancer Registries. Danish Cancer Society. 2016; published online Aug 7. http://www.ancr.nu.

9 Steliarova-Foucher E, O'Callaghan M, Ferlay J, Masuyer E, Forman D, Comber H, Bray F. European Cancer Observatory: Cancer Incidence, Mortality, Prevalence and Survival in Europe. Version 1.0 European Network of Cancer Registries, International Agency for Research on Cancer. 2012; published online Sept. http://eco.iarc.fr.

10 de Martel C, Maucort-Boulch D, Plummer M, Franceschi S. World-wide relative contribution of hepatitis B and C viruses in hepatocellular carcinoma. *Hepatology* 2015; **62**: 1190–200.

11 Hong TP, Gow P, Fink M, *et al.* Novel population-based study finding higher than reported hepatocellular carcinoma incidence suggests an updated approach is needed. *Hepatology* 2016; **63**: 1205–12.

12 GBD 2015 Risk Factors Collaborators. Global, regional, and national comparative risk assessment of 79 behavioural, environmental and occupational, and metabolic risks or clusters of risks, 1990–2015: a systematic analysis for the Global Burden of Disease Study 2015. *The Lancet* 2016; **388**: 1659–724.

13 International Agency for Research on Cancer, World Health Organization. GLOBOCAN estimated cancer incidence, mortality, and prevalence worldwide in 2012. Lyon, France: IARC, 2014 http://globocan.iarc.fr/Default.aspx (accessed April 19, 2016).

14 Karagas MR, Greenberg ER, Spencer SK, Stukel TA, Mott LA. Increase in incidence rates of basal cell and squamous cell skin cancer in New Hampshire, USA. New Hampshire Skin Cancer Study Group. *Int J Cancer* 1999; **81**: 555–9.

3.4.1: Diabetes Mellitus

Diabetes mellitus mortality was estimated for overall diabetes mellitus, diabetes mellitus type 1, and diabetes mellitus type 2 in GBD 2017. We included neonatal diabetes with type 1 diabetes and gestational diabetes with type 2 diabetes.

Overall Diabetes Mellitus

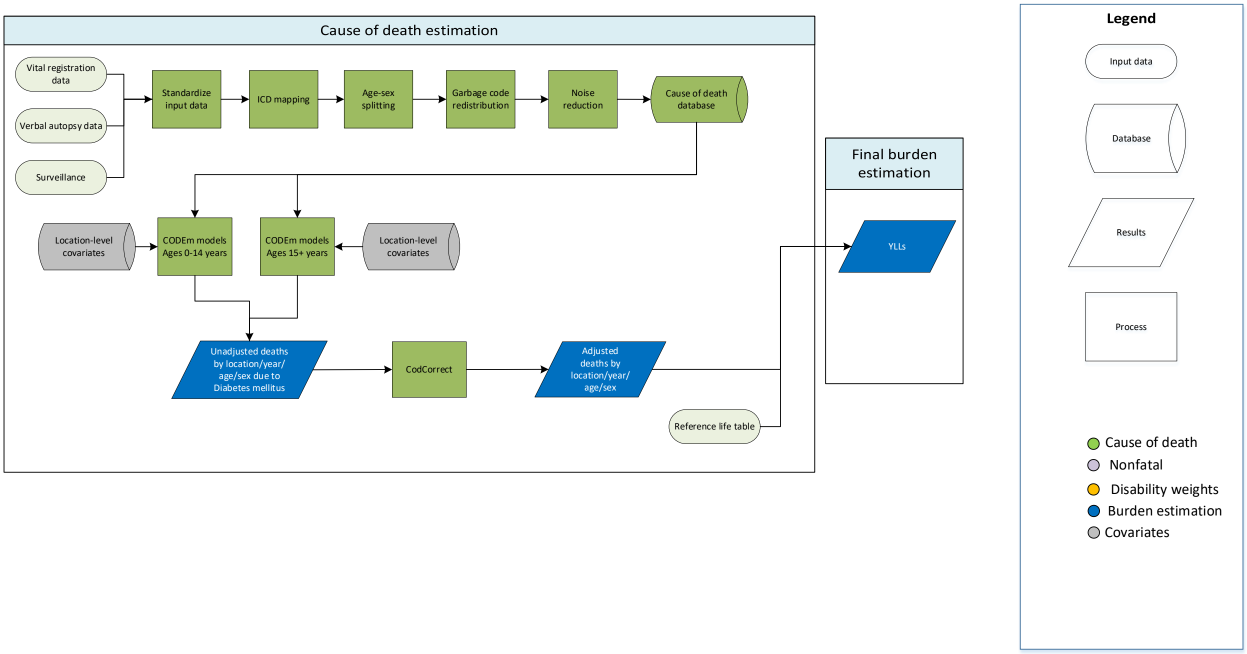

Input data

Overall diabetes mellitus mortality was estimated using deaths directly attributed to diabetes mellitus. We used verbal autopsy and vital registration data as inputs into the model.

Verbal autopsy data: We outliered data points from sources where there were zero deaths estimated in an age group as this was not realistic for deaths due to diabetes and we determined that these data sources were unreliable.

Vital registration data: We outliered all data from the India Medical Certification of Cause of Death report since the source of the data was unreliable according to expert opinion. We also outliered ICD9BTL data points that were inconsistent with the rest of the data series and created unlikely time trends.

Modelling strategy

The Cause of Death Ensemble model (CODEm) was used for deaths due to diabetes mellitus estimation.

In the overall diabetes mellitus model, we used two models to estimate overall diabetes deaths with different age restrictions. This is because deaths in younger age groups are almost exclusively due to type 1 diabetes, while deaths in older ages are primarily due to type 2 diabetes. This allowed us to select

predictive covariates that are specific to the pathophysiology of diabetes type 1 and type 2. We set the younger age model from 0-14 years and the older age model from 15-95+ years. We determined the age threshold based on evidence that the onset age of diabetes type 2 is occurring at younger ages.

Covariate selection

The following are the covariates included in the model. We were able to set an expected direction on each covariate. This requires that the covariate selected for the model must have the directional relationship with diabetes mellitus deaths.

Model	Level	Covariate	Direction
0-14 years	1	Healthcare access and quality index	-
	3	Education years per capita	-
	2	Age-standardised fertility rate	+
	2	Latitude	+
	2	Age-standardised underweight (weight-for-age) summary exposure variable	-
	2	Percentage of births occurring in women >35 years old	+
	2	Percentage of births occurring in women >40 years old	+
	3	Socio-demographic Index	-
	2	Age-standardised stunting (height-for-age) summary exposure variable	-
	2	Mean birth weight	-
15 + model	1	Age-standardised mean fasting plasma glucose (mmol/L)	+
	1	Age-standardised prevalence of diabetes	+
	3	Education years per capita	+/-
	3	Lag-distributed income per capita	+/-
	1	Mean BMI	+
	2	Mean cholesterol	+/-
	2	Mean systolic blood pressure	+/-
	1	Prevalence of obesity	+
	2	Energy-adjusted grams of fruits	+/-
	2	Energy-adjusted grams of sugar	+
	2	Energy-adjusted grams of vegetables	+/-
	3	Healthcare access and quality index	+/-

Diabetes mellitus Type 1 and Type 2

Diabetes mellitus Type 1

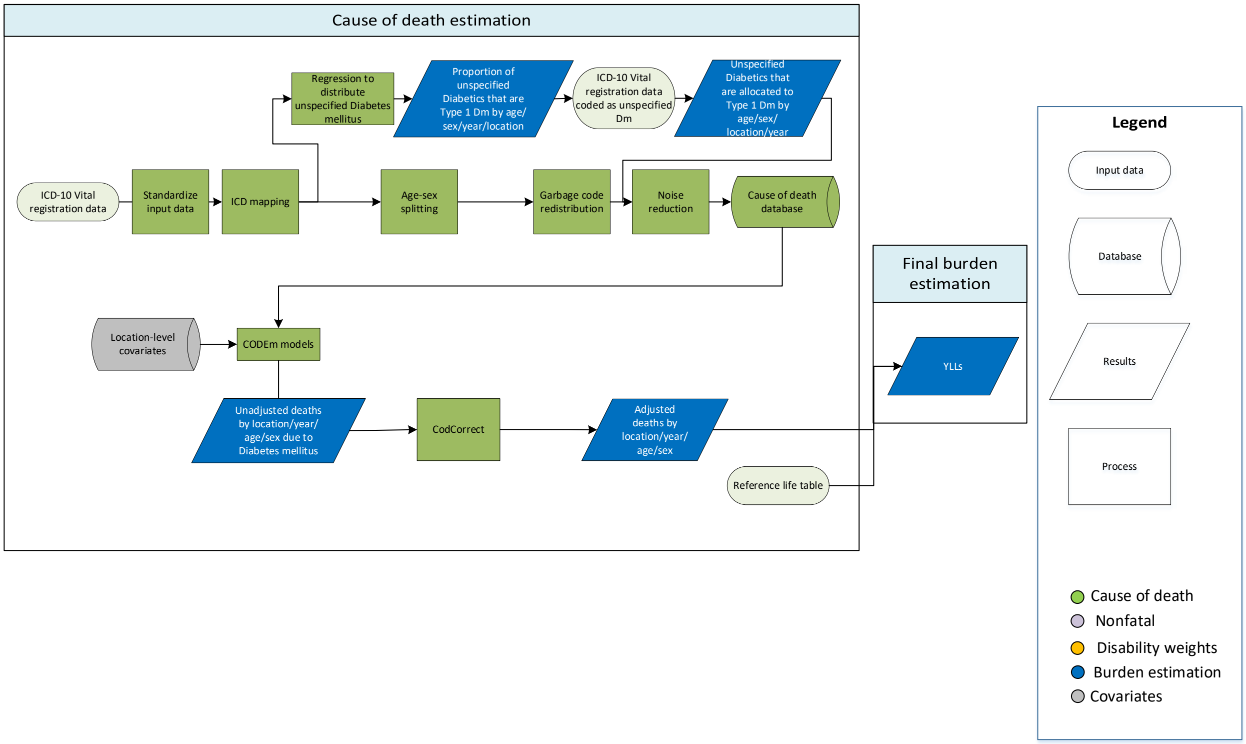

Diabetes mellitus Type 2

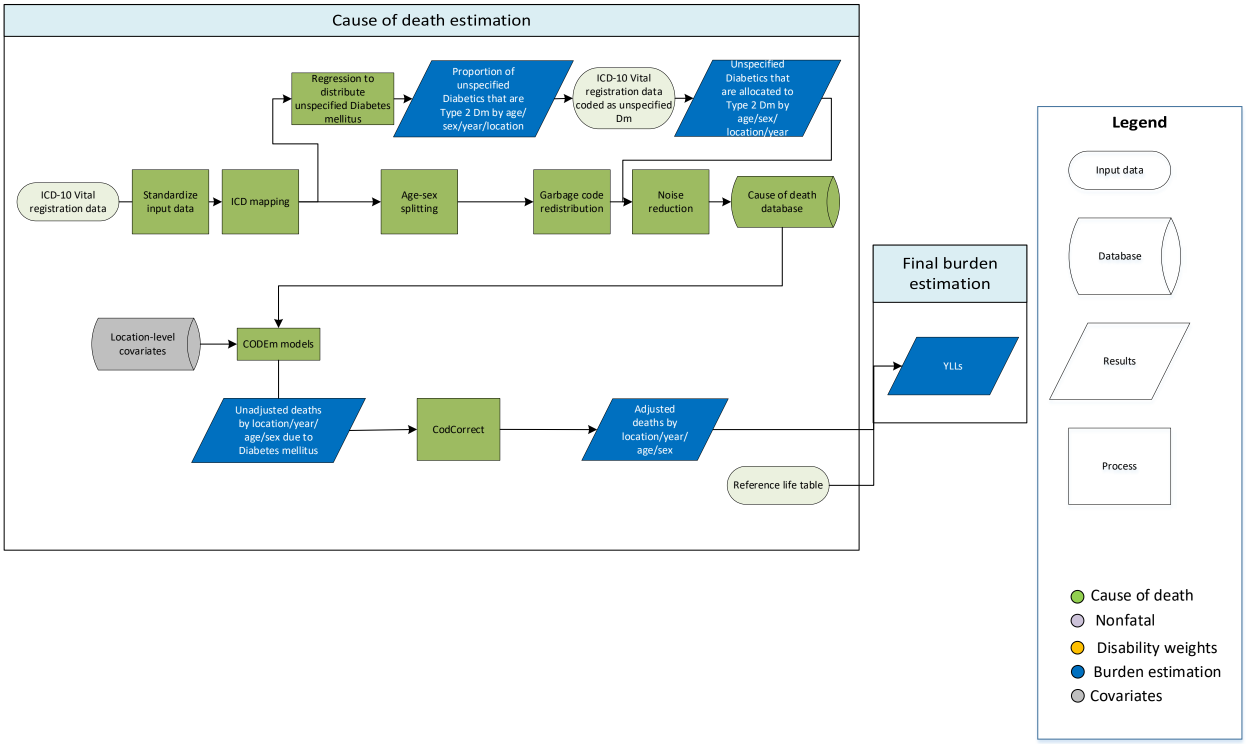

Input data

Type-specific diabetes mellitus mortality was estimated using deaths from vital registration sources in ICD-10 codes only. Diabetes type-specific information was not available in ICD-9 codes or deaths determined by verbal autopsy.

Modelling strategy

The Cause of Death Ensemble model (CODEm) was used for deaths due to diabetes mellitus estimation.

Deaths in younger age groups are almost exclusively due to type 1 diabetes, while deaths in older ages are primarily due to type 2 diabetes. To account for this age pattern, we set the age range of the diabetes type 1 model to 0-95+ years and the age range of the diabetes type 2 model to 15-95+ years. We used the same covariates in the diabetes type 1 model and diabetes type 2 model as the 0-14 year and 15-95+ year in the overall diabetes models, respectively.

There were two unique data manipulation steps that occurred in order to prepare the data as part of the modelling process.

1. We assumed that all deaths <15 years were due to type 1 regardless of the ICD-10 code assigned to the death. We imposed 100% attribution of diabetes mellitus deaths in <15 years to type 1 diabetes mellitus.

2. ICD-10 diabetes data were reported as type 1, type 2, or unspecified. We developed a regression to estimate the fraction of unspecified diabetes mellitus that was type 1 and type 2. Since there is a separate regression to estimate the proportion of type 1 diabetes mellitus and type 2 diabetes mellitus, we scaled the predicted proportions to one. These scaled proportions were then applied to number of deaths coded to unspecified diabetes in each location, year, sex where ICD-10 data was reported.

Regression equation

Type 1:

$$\text{logit}\left(\frac{\text{number type 1 DM}}{\text{number total DM}}\right) \sim \text{logit}\left(\frac{\text{number unspecified DM}}{\text{number total DM}}\right) + \beta_1 \text{age group} + \beta_2 \text{age-st prev obesity*age group} + \text{age-st prev obesity}$$

Type 2:

$$\text{logit}\left(\frac{\text{number type 2 DM}}{\text{number total DM}}\right) \sim \text{logit}\left(\frac{\text{number unspecified DM}}{\text{number total DM}}\right) + \beta_1 \text{age group} + \beta_2 \text{age-st prev obesity*age group} + \text{age-st prev obesit}$$

Covariate selection

The following are the covariates included in the model. We were able to set an expected direction on each covariate. This requires that the covariate selected for the model must have the directional relationship with diabetes mellitus deaths.

Model	Level	Covariate	Direction
Type 1	1	Healthcare access and quality index	-
	3	Education years per capita	-
	2	Age-standardised fertility rate	+
	2	Latitude	+
	2	Age-standardised underweight (weight-for-age) summary exposure variable	-
	2	Percentage of births occurring in women >35 years old	+
	2	Percentage of births occurring in women >40 years old	+
	3	Socio-demographic Index	-
	2	Age-standardised stunting (height-for-age) summary exposure variable	-
	2	Mean birth weight	-

Type 2	1	Age-standardised mean fasting plasma glucose (mmol/L)	+
	1	Age-standardised prevalence of diabetes	+
	3	Education years per capita	+/-
	3	Lag-distributed income per capita	+/-
	1	Mean BMI	+
	2	Mean cholesterol	+/-
	2	Mean systolic blood pressure	+/-
	1	Prevalence of obesity	+
	2	Energy-adjusted grams of fruits	+/-
	2	Energy-adjusted grams of sugar	+
	2	Energy-adjusted grams of vegetables	+/-
	3	Healthcare access and quality index	+/-

Chronic Kidney Disease

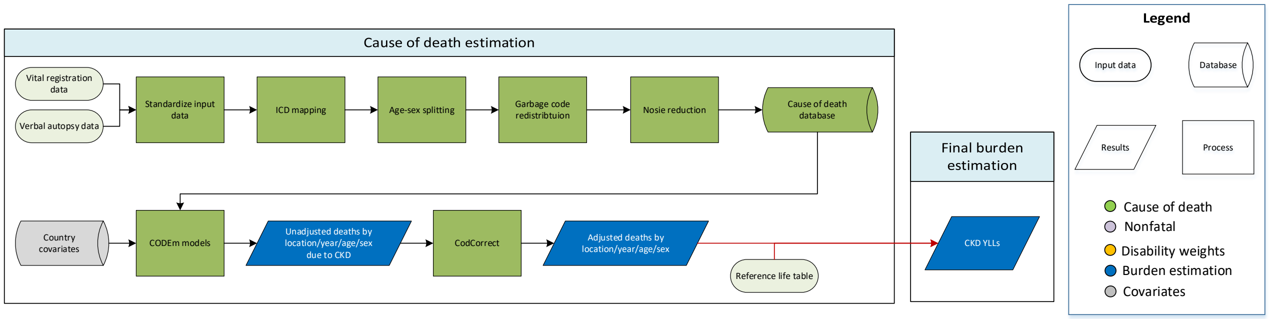

Input data

Vital registration and verbal autopsy data were used to model mortality due to chronic kidney disease. Outliers were identified by systematic examination of data points for all location-years. Data were standardised and mapped according to the GBD causes of death ICD mapping method. These data were then age-sex split, and appropriate redistribution of garbage code data was performed. Data points that violated well-established age or time trends or that resulted in extremely high or low cause fractions were determined to be outliers. Deaths due to congenital kidney anomalies (cystic kidney disease and reflux hydronephrosis) were attributed to chronic kidney disease, marking a change from GBD 2015, when these deaths were assigned to congenital anomalies.

Modelling strategy

The estimation strategy used for fatal chronic kidney disease is largely similar to methods used in GBD 2016. A standard CODEm model with location-level covariates was used to model deaths due to chronic kidney disease. Iterations of models were assessed at the location/year/age group/sex level to determine whether data points merited exclusion via outliering. Unadjusted death estimates were adjusted using CoDCorrect to produce final estimates of YLLs. The covariates used are displayed below.

Level	Covariate	Direction
1	Diabetes fasting plasma glucose (mmol/L)	+
	Diabetes age-standardised prevalence (proportion)	+
	Mean systolic blood pressure (mmHg)	+
	Mean BMI	+
	Healthcare access and quality index	
2	Mean cholesterol	+
	Total calories (kcal per capita)	
	Red meat (kcal per capita)	0
	Whole grains (kcal per capita)	0
	Animal fat (kcal per capita)	0
3	Socio-demographic Index	0
	Education (years per capita)	
	Log LDI ($I per capita)	

3.4.1: Chronic Respiratory Diseases

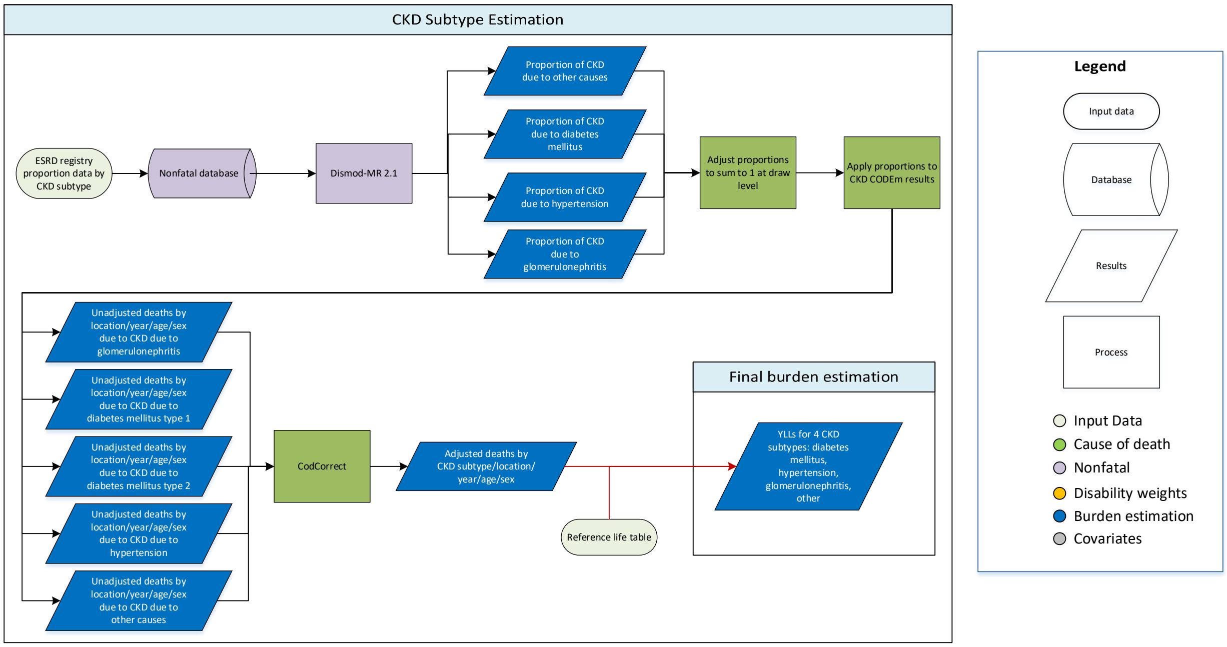

Input data

We estimated deaths due to five subtypes of chronic kidney disease: diabetes mellitus (DM) type 1, diabetes mellitus (DM) type 2, hypertension, glomerulonephritis, and other causes. Data from end-stage renal disease registries were used to inform estimates of proportion of CKD mortality attributable to each CKD subtype. Age-specific data on the proportion of ESRD by subtype was available from the United States, Australia, New Zealand, Nigeria, and Russia. Given the geographic spread in availability of age-specific proportion data, input data were not age-split, marking a change from GBD 2016.

Vital registration (VR) data were excluded from estimates, as aetiology coding in VR sources was considered highly variable and inconsistent between countries.

Modelling strategy

We ran DisMod-MR 2.1 models including diabetes prevalence and mean systolic blood pressure as country-level covariates to obtain estimates of proportions for each subtype by location, year, age, and sex. Data for CKD due to overall DM were more widely available than data by type of DM. In order to make use of all available data, we modelled the proportion of CKD due to overall DM, DM type 1, and DM type 2. Proportion of CKD due to DM type 1 and DM type 2 were then scaled to sum to the proportion of overall DM at the gender, age, and country-matched level. The results from all subtype-specific models were adjusted so that estimates across the subtypes equaled 1 at each of 1,000 draws. These adjusted proportions were applied to the parent CKD CODEm model.

Model	Covariate	Value	Exponentiated
CKD proportion due to diabetes mellitus	Diabetes age-standardised prevalence	0.49 (0.36–0.61)	1.63 (1.44–1.84)
CKD proportion due to hypertension	Mean systolic blood pressure	0.30 (0.010–1.05)	1.35 (1.01–2.86)

3.4.2, 3.6.1, 3.9.3, 16.1.1 Injuries SDG Capstone Appendix

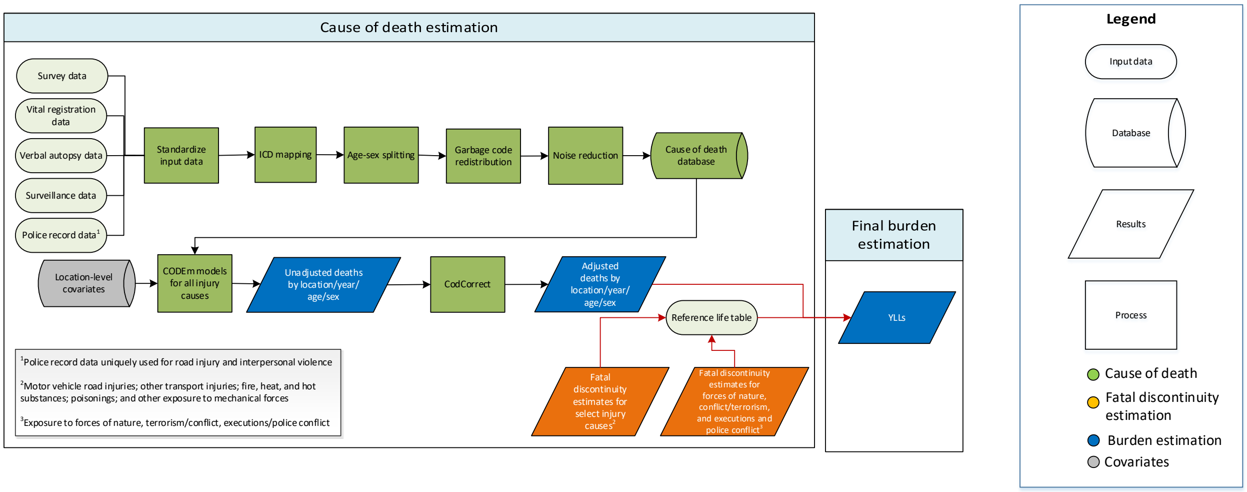

Indicator definition

This modeling strategy encompassed the indicator associated with mortality due to self-harm (3.4.2), road injuries (3.6.1), unintentional poisonings (3.9.3), and interpersonal violence (16.1.1).

Indicator 3.4.2

As a component of SDG Goal 3. Ensure healthy lives and promote well-being for all at all ages, SDG Target 3.4, reduce by one third premature mortality from NCDs through prevention and treatment and promote mental health and well-being, is measured using SDG Indicator 3.4.2, deaths due to self-harm per 100,000.

Indicator 3.6.1

As a component of SDG Goal 3. Ensure healthy lives and promote well-being for all at all ages, SDG Target 3.6, by 2030, halve the number of global deaths and injuries from road traffic accidents, is measured using SDG Indicator 3.6.1, deaths due to road injuries per 100,000.

Indicator 3.9.3

As a component of SDG Goal 3. Ensure healthy lives and promote well-being for all at all ages, SDG Target 3.9, by 2030, substantially reduce the number of deaths and illnesses from hazardous chemicals and air, water and soil pollution and contamination, is measured using SDG Indicator 3.9.3, deaths due to unintentional poisoning per 100,000.

Indicator 16.1.1

As a component of SDG Goal 16. Promote peaceful and inclusive societies for sustainable development, provide access to justice for all and build effective, accountable and inclusive institutions at all levels, SDG Target 16.1, by 2030, significantly reduce all forms of violence and related death rates everywhere, is measured using SDG Indicator 16.1.1, deaths due to interpersonal violence per 100,000.

Input data

In GBD 2017, we estimated injury mortality from vital registration, verbal autopsy, mortality surveillance, censuses, surveys, and police record data. Police and crime reports were data sources uniquely used for the estimation of deaths from road traffic injury and interpersonal violence. The police data were collected from published studies, national agencies, and institutional surveys such as the United Nations Crime Trends Survey and the WHO Global Status Report on Road Safety Survey. For countries with vital registration data we did not use police records, except if the recorded number of road injury and interpersonal violence deaths from police records exceeded that in the vital registration.

Infrequently, data points were marked as outliers. Outlier criteria excluded data points that (1) were implausibly high or low relative to global or regional patterns, (2) substantially conflicted with established age or temporal patterns, or (3) significantly conflicted with other data sources conducted from the same locations or locations with similar characteristics (ie, Socio-demographic Index).

Modelling strategy

Overview

In GBD 2017, the standard CODEm modelling approach was applied to estimate deaths due to all causes of injury, excluding "Exposure to forces of nature," and "Conflict and terrorism". These causes were modelled solely outside of the CODEm process as fatal discontinuities estimation.

Fatal discontinuity was estimated for ten injury causes also modelled in CODEm. These causes included "Road injuries", "Motor vehicle road injuries", "Other transport injuries", "Fire, heat, and hot substances", "Poisonings", "Environmental exposure to heat and cold ", "Other unintentional injuries", "Interpersonal violence", "Other exposure to mechanical forces", and "Executions and police conflict". Final fatal discontinuity estimations for these causes were merged with CODEm results post-CoDCorrect to produce final cause of death results.

Refer to the table at the end of this section for a complete list of the cause-of-injury categories, modelling strategies, and covariate changes from GBD 2016.

GBD injury codes and categories

The International Classification of Diseases (ICD) was used to classify injuries. In GBD, injury incidence and death are defined as ICD-9 codes E000-E999 and ICD-10 chapters V to Y. There is one exception: deaths and cases of alcohol poisoning and drug overdoses are classified under drug and alcohol use disorders. In GBD 2017, injury causes were organized into 28 mutually exclusive and collectively exhaustive external cause-of-injury categories. For GBD 2017, "Poisoning" was differentiated into "Poisoning by carbon monoxide," and "Poisoning by other", and "Unintentional suffocation" was removed as its ICD codes were added to the "Pulmonary aspiration and foreign body in airway" cause.

Preparation of data

The preparation of cause of death data includes age splitting, age-sex splitting, smoothing, and outlier detection. These steps are described in detail by Naghavi and colleagues and Lozano and colleagues.[1,2,3] The concept of "garbage codes" and redistribution of these codes was proposed in GBD 1990.[4] Garbage codes are causes of death that should not be identified as specific underlying causes of death but have been entered as the underlying cause of death on death certificates. A classic example of these types of codes in injuries chapters are "Exposure to unspecified factor" (X59 in ICD-10 and E887 in ICD-9) and all

undetermined intent codes (Y10-Y34 in ICD-10 and E980-E988 in ICD-9). Other examples of garbage codes in injuries are the coding of an injury death to intermediate codes like septicaemia or peritonitis or as an ill-defined and unknown cause of mortality (R99). Approximately 2% of total deaths in countries with vital registration data are assigned to these three injury garbage code categories.

Splitting into sublevel causes

In countries with non-detail ICD code data, cause-of-injury categories were proportionally split into sublevel cause-of-injury categories. The sublevel cause-of-injury causes were created in the CoDCorrect process. One of the countries with non-detail ICD code data is South Africa, and in GBD 2013 the proportions of sublevel cause-of-injury were based on vital registration data. For GBD iterations of 2015, 2016, and 2017, the proportions were based on post-mortem investigation of injury deaths as described in the paper by Matzopoulos and colleagues 2015.[5]

Limitations and model assumptions

We added police data for road injuries and interpersonal violence to help predict level and age patterns in countries with sparse or absent cause of death data even though we know from countries with near-complete vital registration data that police records tend to underestimate the true level of deaths. However, we applied police data estimates in instances where reported deaths were higher than vital registration numbers.

Covariates

The following covariates were included.

**Transport injuries**		
Level	Covariate	Direction
1	Alcohol (litres per capita)	+
1	Vehicles – 2 wheels fraction (proportion)	+
1	Vehicles – 2+4 wheels (per capita)	+
2	Healthcare access and quality index	-
2	LDI (I$ per capita)	0
2	Population density (300-500 ppl/sqkm, proportion)	0
2	Population density (500-1000 ppl/sqkm, proportion)	0
3	Education (years per capita)	-
3	Rainfall quintile 5 (proportion)	+
3	Socio-demographic Index	-
**Road injuries**		
Level	Covariate	Direction
1	Alcohol (litres per capita)	+
1	Log-transformed SEV scalar: Road Inj	+
1	Vehicles – 2 wheels (per capita)	+
1	Vehicles – 2 wheels fraction (proportion)	+
1	Vehicles – 2+4 wheels (per capita)	+

1	Vehicles – 4 wheels (per capita)	+
2	Healthcare access and quality index	-
2	Population 15 to 30 (proportion)	+
2	Population density (300-500 ppl/sqkm, proportion)	0
2	Population density (500-1000 ppl/sqkm, proportion)	0
3	Education (years per capita)	-
3	LDI (I$ per capita)	0
3	Socio-demographic Index	-
Pedestrian road injuries		
Level	Covariate	Direction
1	Alcohol (litres per capita)	+
1	Log-transformed SEV scalar: Pedest	+
1	Vehicles – 2 wheels fraction (proportion)	+
1	Vehicles – 2+4 wheels (per capita)	+
2	Healthcare access and quality index	-
2	LDI (I$ per capita)	0
2	Population density (300-500 ppl/sqkm, proportion)	0
2	Population density (500-1000 ppl/sqkm, proportion)	0
3	Education (years per capita)	-
3	Rainfall quintile 5 (proportion)	+
3	Socio-demographic Index	-
Cyclist road injuries		
Level	Covariate	Direction
1	Alcohol (litres per capita)	+
1	Log-transformed SEV scalar: Cyclist	+
1	Vehicles – 2 wheels fraction (proportion)	+
1	Vehicles – 2+4 wheels (per capita)	+
2	Healthcare access and quality index	-
2	LDI (I$ per capita)	0
2	Population density (300-500 ppl/sqkm, proportion)	0
2	Population density (500-1000 ppl/sqkm, proportion)	0
3	Education (years per capita)	-
3	Socio-demographic Index	0
Motorcyclist road injuries		
Level	Covariate	Direction
1	Alcohol (litres per capita)	+

1	Log-transformed SEV scalar: Mot Cyc	+
1	Vehicles – 2 wheels (per capita)	+
2	Healthcare access and quality index	-
2	LDI (I$ per capita)	0
2	Population density (300-500 ppl/sqkm, proportion)	0
2	Population density (500-1000 ppl/sqkm, proportion)	0
3	Education (years per capita)	-
3	Rainfall quintile 5 (proportion)	+
3	Socio-demographic Index	0
Motor vehicle road injuries		
Level	Covariate	Direction
1	Alcohol (litres per capita)	+
1	Log-transformed SEV scalar: Mot Veh	+
1	Vehicles – 4 wheels (per capita)	+
2	Healthcare access and quality index	-
2	Population density (300-500 ppl/sqkm, proportion)	0
2	Population density (500-1000 ppl/sqkm, proportion)	0
3	Education (years per capita)	0
3	LDI (I$ per capita)	0
3	Rainfall quintile 5 (proportion)	+
3	Socio-demographic Index	0
Other road injuries		
Level	Covariate	Direction
1	Alcohol (litres per capita)	+
1	Log-transformed SEV scalar: Oth Road	+
1	Vehicles – 2 wheels fraction (proportion)	+
1	Vehicles – 2+4 wheels (per capita)	+
2	Healthcare access and quality index	-
2	LDI (I$ per capita)	0
3	Rainfall quintile 5 (proportion)	+
3	Socio-demographic Index	-
Other transport injuries		
Level	Covariate	Direction
1	Alcohol (litres per capita)	+
1	Log-transformed SEV scalar: Oth Trans	+
1	Vehicles – 2 wheels fraction (proportion)	+

1	Vehicles – 2+4 wheels (per capita)	+
2	Healthcare access and quality index	-
2	Population density (300-500 ppl/sqkm, proportion)	0
2	Population density (500-1000 ppl/sqkm, proportion)	0
3	Education (years per capita)	0
3	LDI (I$ per capita)	0
3	LDI (I$ per capita)	+
3	Rainfall quintile 5 (proportion)	+
3	Socio-demographic Index	0
Unintentional injuries		
Level	Covariate	Direction
1	Cumulative cigarettes (5 Years)	+
1	Diabetes fasting plasma glucose (mmol/L)	+
1	Health system access 2 (unitless)	-
1	Indoor air pollution (all cooking fuels)	+
1	Smoking prevalence	+
1	Underweight (proportion <2SD weight for age, <5 years)	+
2	Alcohol (litres per capita)	+
2	Population density (500-1000 ppl/sqkm, proportion)	+
2	Population density (over 1000 ppl/sqkm, proportion)	+
3	Education (years per capita)	-
3	LDI (I$ per capita)	-
Falls		
Level	Covariate	Direction
1	Alcohol (litres per capita)	+
1	Log-transformed SEV scalar: Falls	+
2	Healthcare access and quality index	-
2	Milk adjusted (g)	-
3	Elevation over 1500m (proportion)	+
3	LDI (I$ per capita)	0
3	Socio-demographic Index	0
Drowning		
Level	Covariate	Direction
1	Alcohol (litres per capita)	+
1	Coastal population within 10km (proportion)	+
1	Landlocked nation (binary)	-

1	Log-transformed SEV scalar: Drown	+
1	Rainfall quintile 1 (proportion)	-
1	Rainfall quintile 5 (proportion)	+
2	Elevation under 100m (proportion)	+
3	Education (years per capita)	-
3	LDI (I$ per capita)	0
3	Socio-demographic Index	-
**Fire, heat, and hot substances**		
Level	Covariate	Direction
1	Log-transformed SEV scalar: Fire	+
2	Alcohol (litres per capita)	+
2	Healthcare access and quality index	-
2	Indoor air pollution (all cooking fuels)	+
2	Population density (over 1000 ppl/sqkm, proportion)	0
2	Tobacco (cigarettes per capita)	+
3	Education (years per capita)	-
3	LDI (I$ per capita)	0
3	Socio-demographic Index	-
**Poisonings**		
Level	Covariate	Direction
1	Log-transformed SEV scalar: Poison	+
1	Opium cultivation (binary)	+
2	Healthcare access and quality index	-
2	Population density (over 1000 ppl/sqkm, proportion)	0
2	Population density (under 150 ppl/sqkm, proportion)	0
3	Education (years per capita)	-
3	LDI (I$ per capita)	0
3	Socio-demographic Index	-
**Poisoning by carbon monoxide**		
Level	Covariate	Direction
3	Education (years per capita)	-
3	Healthcare access and quality index	-
3	LDI (I$ per capita)	0
3	Socio-demographic Index	0
**Poisoning by other means**		

Level	Covariate	Direction
3	Education (years per capita)	-
3	Healthcare access and quality index	-
3	LDI (I$ per capita)	0
3	Socio-demographic Index	0
Exposure to mechanical forces		
Level	Covariate	Direction
2	Alcohol (litres per capita)	+
2	Healthcare access and quality index	-
2	Population density (over 1000 ppl/sqkm, proportion)	0
2	Population density (under 150 ppl/sqkm, proportion)	0
3	Education (years per capita)	-
3	LDI (I$ per capita)	0
3	Socio-demographic Index	-
Unintentional firearm injuries		
Level	Covariate	Direction
1	Log-transformed SEV scalar: Mech Gun	+
2	Alcohol (litres per capita)	+
2	Health system access (unitless)	-
2	Healthcare access and quality index	-
3	Education (years per capita)	-
3	LDI (I$ per capita)	0
3	Population density (over 1000 ppl/sqkm, proportion)	0
3	Population density (under 150 ppl/sqkm, proportion)	0
3	Socio-demographic Index	-
Other exposure to mechanical forces		
Level	Covariate	Direction
1	Log-transformed SEV scalar: Oth Mech	+
2	Alcohol (litres per capita)	+
2	Health system access (unitless)	-
2	Healthcare access and quality index	-
2	Population density (over 1000 ppl/sqkm, proportion)	0
2	Population density (under 150 ppl/sqkm, proportion)	0
3	Education (years per capita)	-
3	LDI (I$ per capita)	0
3	Socio-demographic Index	-

Adverse effects of medical treatment		
Level	Covariate	Direction
2	Healthcare access and quality index	0
3	LDI (I$ per capita)	0
3	Socio-demographic Index	0
Animal contact		
Level	Covariate	Direction
1	Alcohol (litres per capita)	+
1	Log-transformed SEV scalar: Animal	+
2	Healthcare access and quality index	-
2	Population 15 to 30 (proportion)	+
3	Education (years per capita)	-
3	Elevation over 1500m (proportion)	0
3	Elevation under 100m (proportion)	0
3	LDI (I$ per capita)	0
3	Population density (over 1000 ppl/sqkm, proportion)	0
3	Population density (under 150 ppl/sqkm, proportion)	0
3	Socio-demographic Index	-
Venomous animal contact		
Level	Covariate	Direction
1	Alcohol (litres per capita)	+
1	Log-transformed SEV scalar: Venom	+
2	Healthcare access and quality index	-
3	Education (years per capita)	-
3	Elevation over 1500m (proportion)	0
3	Elevation under 100m (proportion)	0
3	LDI (I$ per capita)	0
3	Population density (over 1000 ppl/sqkm, proportion)	0
3	Population density (under 150 ppl/sqkm, proportion)	0
3	Socio-demographic Index	-
Non-venomous animal contact		
Level	Covariate	Direction
1	Alcohol (litres per capita)	+
1	Log-transformed SEV scalar: Non Ven	+
2	Healthcare access and quality index	-

3	Alcohol (litres per capita)	+
3	Education (years per capita)	-
3	Elevation over 1500m (proportion)	0
3	Elevation under 100m (proportion)	0
3	Healthcare access and quality index	-
3	LDI (I$ per capita)	0
3	Population density (over 1000 ppl/sqkm, proportion)	0
3	Population density (under 150 ppl/sqkm, proportion)	0
3	Socio-demographic Index	-
**Foreign body**		
Level	Covariate	Direction
1	Education (years per capita)	+
1	Indoor air pollution (all cooking fuels)	+
1	LDI (I$ per capita)	+
1	Population density (over 1000 ppl/sqkm, proportion)	+
1	Population over 65 (proportion)	+
2	Healthcare access and quality index	-
3	Socio-demographic Index	0
**Pulmonary aspiration and foreign body in airway**		
Level	Covariate	Direction
1	Log-transformed SEV scalar: F Body Asp	+
2	Alcohol (litres per capita)	+
2	Alcohol binge drinker proportion, age-standardised	+
2	Healthcare access and quality index	-
2	Mean BMI	+
3	Education (years per capita)	-
3	LDI (I$ per capita)	0
3	Socio-demographic Index	0
**Foreign body in other body part**		
Level	Covariate	Direction
1	Alcohol (litres per capita)	+
1	Log-transformed SEV scalar: Oth F Body	+
2	Healthcare access and quality index	-
3	Education (years per capita)	-
3	Elevation Over 1500m (proportion)	0
3	Elevation Under 100m (proportion)	0

3	LDI (I$ per capita)	0
3	Population density (over 1000 ppl/sqkm, proportion)	0
3	Population density (under 150 ppl/sqkm, proportion)	0
3	Socio-demographic Index	-
**Other unintentional injuries**		
Level	Covariate	Direction
1	Alcohol (liters per capita)	+
1	Log-transformed SEV scalar: Oth Unint	+
1	Vehicles – 2 wheels (per capita)	+
1	Vehicles – 4 wheels (per capita)	0
2	Healthcare access and quality index	-
3	Education (years per capita)	-
3	Elevation over 1500m (proportion)	0
3	Elevation under 100m (proportion)	0
3	LDI (I$ per capita)	0
3	Population density (over 1000 ppl/sqkm, proportion)	0
3	Population density (under 150 ppl/sqkm, proportion)	0
3	Socio-demographic Index	0
**Self-harm and interpersonal violence**		
Level	Covariate	Direction
1	Alcohol (litres per capita)	+
1	Healthcare access and quality index	+
1	Log-transformed SEV scalar: Oth Unint	+
3	Education (years per capita)	-
3	Elevation over 1500m (proportion)	0
3	Elevation under 100m (proportion)	0
3	LDI (I$ per capita)	0
3	Population density (over 1000 ppl/sqkm, proportion)	0
3	Population density (under 150 ppl/sqkm, proportion)	0
**Self-harm**		
Level	Covariate	Direction
1	Alcohol (litres per capita)	+
1	Log-transformed SEV scalar: Self Harm	+
1	Major depressive disorder	+
1	Non-partner lifetime prevalence of sexual violence (female-only)	+
1	Risk of self-harm due to major depressive disorder	+

2	Healthcare access and quality index	-
2	Muslim religion (proportion of population)	-
2	Population density (150-300 ppl/sqkm, proportion)	0
2	Population density (300-500 ppl/sqkm, proportion)	0
2	Population density (500-1000 ppl/sqkm, proportion)	0
2	Population density (over 1000 ppl/sqkm, proportion)	0
2	Population density (under 150 ppl/sqkm, proportion)	0
2	Religion (binary, >50% Muslim)	-
3	Education (years per capita)	0
3	LDI (I$ per capita)	0
3	Socio-demographic Index	0
Self-harm by firearm		
Level	Covariate	Direction
1	Alcohol (litres per capita)	+
1	Log-transformed SEV scalar: Self Harm	+
1	Major depressive disorder	+
2	Healthcare access and quality index	-
2	Population density (150-300 ppl/sqkm, proportion)	0
2	Population density (300-500 ppl/sqkm, proportion)	0
2	Population density (500-1000 ppl/sqkm, proportion)	0
2	Population density (over 1000 ppl/sqkm, proportion)	0
2	Population density (under 150 ppl/sqkm, proportion)	0
2	Religion (binary, >50% Muslim)	-
3	Education (years per capita)	0
3	LDI (I$ per capita)	0
3	Socio-demographic Index	0
Self-harm by other specified means		
Level	Covariate	Direction
1	Alcohol (litres per capita)	+
1	Log-transformed SEV scalar: Self Harm	+
1	Major depressive disorder	+
2	Healthcare access and quality index	-
2	Population density (150-300 ppl/sqkm, proportion)	0
2	Population density (300-500 ppl/sqkm, proportion)	0
2	Population density (500-1000 ppl/sqkm, proportion)	0
2	Population density (over 1000 ppl/sqkm, proportion)	0
2	Population density (under 150 ppl/sqkm, proportion)	0

2	Religion (binary, >50% Muslim)	-
3	Education (years per capita)	0
3	LDI (I$ per capita)	0
3	Socio-demographic Index	0
**Interpersonal violence**		
Level	Covariate	Direction
1	Alcohol (liters per capita)	+
1	Log-transformed SEV scalar: Violence	+
2	Healthcare access and quality index	-
2	Opium cultivation (binary)	+
2	Population density (over 1000 ppl/sqkm, proportion)	+
3	Education (years per capita)	0
3	LDI (I$ per capita)	0
3	Socio-demographic Index	0
**Assault by firearm**		
Level	Covariate	Direction
1	Alcohol (liters per capita)	+
1	Log-transformed SEV scalar: Viol Gun	+
2	Healthcare access and quality index	-
2	Opium cultivation (binary)	+
2	Population density (over 1000 ppl/sqkm, proportion)	+
3	Education (years per capita)	0
3	LDI (I$ per capita)	0
3	Socio-demographic Index	0
**Assault by sharp object**		
Level	Covariate	Direction
1	Alcohol (litres per capita)	+
1	Log-transformed SEV scalar: Viol Knife	+
2	Healthcare access and quality index	-
2	Opium cultivation (binary)	+
2	Population density (over 1000 ppl/sqkm, proportion)	+
3	Education (years per capita)	0
3	LDI (I$ per capita)	0
3	Socio-demographic Index	0
**Assault by other means**		

Level	Covariate	Direction
1	Alcohol (litres per capita)	+
1	Log-transformed SEV scalar: Oth Viol	+
2	Healthcare access and quality index	-
2	Opium cultivation (binary)	+
2	Population density (over 1000 ppl/sqkm, proportion)	+
3	Education (years per capita)	0
3	LDI (I$ per capita)	0
3	Socio-demographic Index	0
**Environmental heat and cold exposure**		
Level	Covariate	Direction
2	Healthcare access and quality index	-
3	90th percentile climatic temperature in the given country-year	0
3	Education (years per capita)	-
3	Elevation 500 to 1500m (proportion)	0
3	Elevation over 1500m (proportion)	0
3	LDI (I$ per capita)	0
3	Population fensity (150-300 ppl/sqkm, proportion)	0
3	Population-weighted mean temperature	0
3	Rainfall (quintiles 4-5)	0
3	Sanitation (proportion with access)	0
3	Socio-demographic Index	-

Table – Injury cause list

ID	Cause	Modelling strategy	Covariate changes from GBD 2016
1	Transport injuries	CODEm	
1.1	Road injuries	CODEm and fatal discontinuity estimation	
1.1.1	Pedestrian road injuries	CODEm	
1.1.2	Cyclist road injuries	CODEm	
1.1.3	Motorcyclist road injuries	CODEm	
1.1.4	Motor vehicle road injuries	CODEm and fatal discontinuity estimation	
1.1.5	Other road injuries	CODEm	
1.2	Other transport injuries	CODEm and fatal discontinuity estimation	

Table – Injury cause list			
ID	Cause	Modelling strategy	Covariate changes from GBD 2016
2	Unintentional injuries	CODEm	
2.1	Falls	CODEm	
2.2	Drowning	CODEm	
2.3	Fire, heat, and hot substances	CODEm and fatal discontinuity estimation	
2.4	Poisonings	CODEm and fatal discontinuity estimation	
2.4.1	Poisoning by carbon monoxide	CODEm	Same covariates used as Poisoning in GBD 2016
2.4.2	Poisoning by other means	CODEm	Same covariates used as Poisoning in GBD 2016
2.5	Exposure to mechanical forces	CODEm	
2.5.1	Unintentional firearm injuries	CODEm	
2.5.2	Other exposure to mechanical forces	CODEm and fatal discontinuity estimation	
2.6	Adverse effects of medical treatment	CODEm	
2.7	Animal contact	CODEm	
2.7.1	Venomous animal contact	CODEm	
2.7.2	Non-venomous animal contact	CODEm	
2.8	Foreign body	CODEm	
2.8.1	Pulmonary aspiration and foreign body in airway	CODEm	
2.8.2	Foreign body in other body part	CODEm	
2.9	Environmental exposure to heat and cold	CODEm and fatal discontinuity estimation	
2.10	Exposure to forces of nature	Fatal discontinuity estimation	
2.11	Other unintentional injuries	CODEm and fatal discontinuity estimation	
3	Self-harm and interpersonal violence	CODEm	
3.1	Self-harm	CODEm	
3.1.1	Self-harm by firearm	CODEm	
3.1.2	Self-harm by other specified means	CODEm	
3.2	Interpersonal violence	CODEm and fatal discontinuity estimation	

Table – Injury cause list			
ID	Cause	Modelling strategy	Covariate changes from GBD 2016
3.2.1	Physical violence by firearm	CODEm	
3.2.2	Physical violence by sharp object	CODEm	
3.2.3	Physical violence by other means	CODEm	
3.3	Conflict and terrorism	Fatal discontinuity estimation	
3.4	Executions and police conflict	CODEm and fatal discontinuity estimation	

References

1 Lozano R, Naghavi M, Foreman K, *et al.* Global and regional mortality from 235 causes of death for 20 age groups in 1990 and 2010: a systematic analysis for the Global Burden of Disease Study 2010. *The Lancet* 2012; **380**: 2095–128.

2 Global, regional, and national age–sex specific all-cause and cause-specific mortality for 240 causes of death, 1990–2013: a systematic analysis for the Global Burden of Disease Study 2013. *The Lancet* 2015; **385**: 117–71.

3 Global, regional, and national life expectancy, all-cause mortality, and cause-specific mortality for 249 causes of death, 1980-2015: a systematic analysis for the Global Burden of Disease Study 2015. *The Lancet* 2016; **388**: 1459-1544.

4 Murray CJL, Lopez AD, Harvard School of Public Health, World Health Organization, World Bank. The global burden of disease: a comprehensive assessment of mortality and disability from diseases, injuries, and risk factors in 1990 and projected to 2020. Cambridge, MA: Published by the Harvard School of Public Health on behalf of the Wor e th r ni tion n the or n istribute b Harvard University Press, 1996.

5 Matzopoulos R, Prinsloo M, Wyk VP, Gwebushe N, Mathews S, et al. Injury-related mortality in South Africa: a retrospective descriptive study of postmortem investigations. *Bull World Health Organ* 2015; **93**: 303–13.

3.5.2 Alcohol Use SDG Capstone Appendix

Flowchart

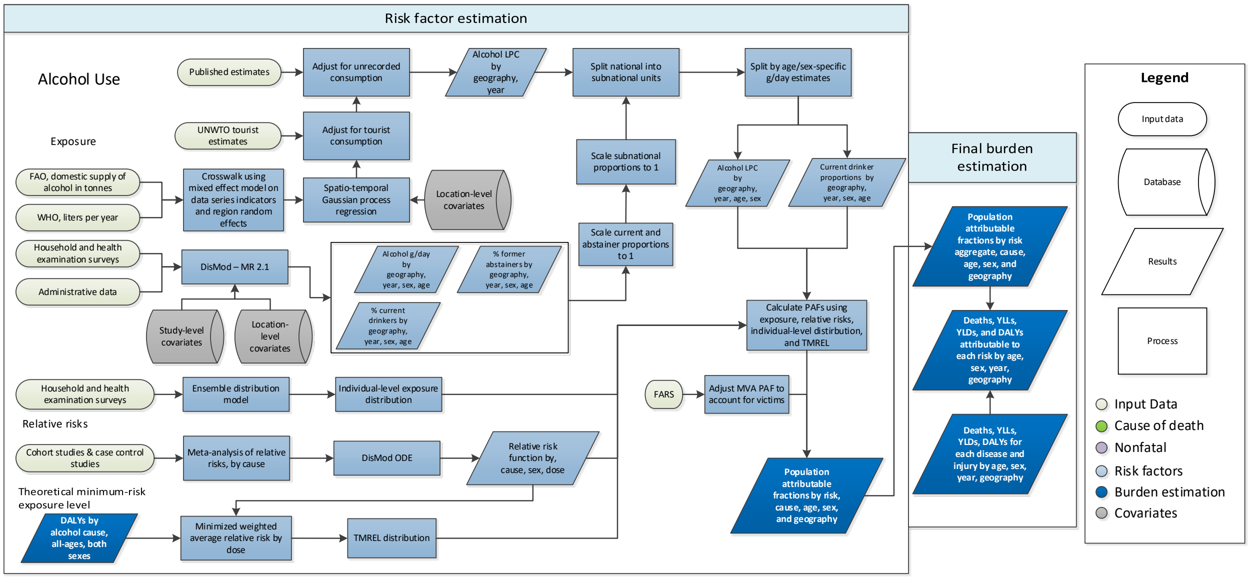

Input data & methodological summary

Exposure

Case definition

We defined exposure as the grams per day of pure alcohol consumed amongst drinkers. We constructed this exposure using the indicators outlined below:

1. Current drinkers, defined as the proportion of individuals who have consumed at least one alcoholic beverage (or some approximation) in a 12-month period.
2. Lifetime abstainers, defined as the proportion of individuals who have never consumed an alcoholic beverage.
3. Alcohol consumption (in grams per day), defined as grams of alcohol consumed by current drinkers, per day, over a 12-month period.
4. Alcohol liters per capita stock, defined in liters per capita of pure alcohol, over a 12-month period.

We also used three additional indicators to adjust alcohol exposure estimates to account for different types of bias:

1. Number of tourists within a location, defined as the total amount of visitors to a location within a 12 month period.
2. Tourists’ duration of stay, defined as the number of days resided in a hosting country.
3. Unrecorded alcohol stock, defined as a percentage of the total alcohol stock produced outside established markets.

Input data

A systematic review of the literature was performed to extract data on our primary indicators. The Global Health Exchange (GHDx), IHME's online database of health-related data, was searched for population survey data containing participant-level information from which we could formulate the required alcohol use indicators on current drinkers, lifetime abstainers, alcohol consumption, and binge drinkers. Data-sources were included if they captured a sample representative of the geographic location under study. We documented relevant survey variables from each data-source in a spreadsheet and extracted using STATA 13.1 and R 3.3 . A total of 2,821 potential data-sources were available in the GHDx across countries with subnational locations, out of which 191 data-sources (corresponding 88,734 tabulated data-points by location/year/sex/age) were included across the four indicators mentioned above.

Within the grams per day, current drinkers, and abstainers model, we had a large amount of data on male drinking but not female drinking. To ensure a balanced dataset between sexes for use within DisMod MR 2.1, we imputed for missing sex observations within locations where data existed on male drinking but not female drinking. We used the following models to do so:

For grams per day:

$$y_i \sim Gamma(\mu, \phi)$$
$$\mu = log^{-1}(1 + SDI + sex + age + sex * age + (1 + sex|super\ region) + (1 + sex|region)$$

Where y is average amount of grams per day within a demographic, μ a parameter for the mean of the average amounts, and ϕ is a dispersion parameter

For current drinking and abstention:

$$y_i \sim Binomial(\pi_i, n)$$
$$\pi_i \sim Beta(\mu_i, \theta_i)$$
$$\mu_i = cloglog^{-1}(1 + SDI + sex + age + sex * age + (1 + sex|super\ region) + (1 + sex|region)$$

We then sampled 1000 draws from the above estimator for both sex = male & sex = female, with all other variables fixed by demographic unit. For sampling draws, we assumed the parameters were Gaussian multivariately distributed. For each demographic unit with only male observations, we multiplied male data by the ratio between the draws with sex = male & sex = female to impute for female observations.

To generate estimates of alcohol consumption in liters per capita (LPC), we obtained data from FAOSTAT, and WHO GISAH database.[1,2] To provide more stable time trends in the model, we transformed FAO sales data (which calculates stock based on primary inputs) to a lagged five-year average. Given WHO uses FAO data in locations where WHO could not find data using their own methods, we removed FAO data in the locations where WHO used FAO data in place of their own. To correct for bias in the underlying data sources, we adjusted the input data (crosswalked), by running a mixed effect model on the log average of the data with dummy variables for the data series, as well as random effects on super region, region, country, and time. We adjusted the data points using the following equation:

$$Log\ Average\ Data = D + (Super\ Region\ |D, Region\ |\ D, Country\ |D, Year|\ D)$$

$$Transformed\ data = \ data\ * \ e^{\widehat{\beta_1} + \widehat{\beta_3}}$$

where:

$D\ is\ a\ dummy\ variable\ for\ a\ data\ source$

None of the data sources on liters per capita provided estimates of uncertainty, which is a component required for our eventual modelling strategy. To generate uncertainty, we ran a Loess model on the adjusted data points and the standard deviation between the difference of the Loess smoothed model and the adjusted data points across a five-year span was used as the standard deviation of the data. (i.e., if the total stock changes more variably in a narrow time frame, we believe the data to be more uncertain).

We obtained data on the number of tourists and their duration of stay from the UNWTO.[3] We applied a crosswalk across different tourist categories, similar to the one used for the liters per capita data, to arrive at a consistent definition (i.e. visitors to a country).

We obtained estimates on unrecorded alcohol stock from six published papers,[4-9] consisting of 166 locations.

Modelling strategy

While population-based surveys provide accurate estimates of the prevalence of lifetime abstainers and current drinkers, they typically underestimate real alcohol consumption levels.[10-12] As a result, we considered the liter per capita input to be a better estimate of overall volume of consumption. Per capita consumption, however, does not provide age- and sex-specific consumption estimates needed to compute alcohol-attributable burden of disease. Therefore, we use the age-sex pattern of consumption among drinkers modelled from the population survey data and the overall volume of consumption from FAO and GISAH to determine the total amount of alcohol consumed within a location. In the paragraphs we outline how we estimated each primary input in the alcohol exposure model, as well as how we combined these inputs to arrive at our final estimate of grams per day of pure alcohol. We estimated all models below using 1,000 draws.

For data obtained through surveys, we used DisMod-MR 2.1 to construct estimates for each country/year/age/sex. We chose to use DisMod due to its ability to leverage information across the heterogeneous age groups reported in the surveys, through age-integration, as well as the model's ability to leverage information available from data in nearby locations or time-periods.[13]

We modelled the alcohol liters per capita data, as well as the total number of tourists, using a spatio-temporal Gaussian process regression (ST-GPR). We chose parameters, as well as our final model, using out-of-sample 10-fold cross validation.

Given the heterogeneous nature of the estimates on unrecorded consumption, as well as the wide variation across countries and time-periods, we took 1,000 draws from the uniform distribution of the lowest and highest estimates available for a given country. We did this to incorporate the diffuse uncertainty within the unrecorded estimates reported. We used these 1,000 draws in the above equation. We adjusted LPC only for countries where estimates were available.

We adjusted the alcohol LPC for unrecorded consumption using the following equation:

$$Alcohol\ LPC = \frac{Alcohol\ LPC}{(1 - \%\ Unrecorded)}$$

We then adjusted the estimates for alcohol LPC for tourist consumption by adding in the per capita rate of consumption abroad and subtracting the per capita rate of tourist consumption domestically.

$$Alcohol\ LPC_d = Unadjusted\ Alcohol\ LPC_d + Alcohol\ LPC_{Domestic\ consumption\ abroad} - Alcohol\ LPC_{Tourist\ consumption\ domestically}$$

$$Alcohol\ LPC_i = \frac{\sum_l Tourist\ Population_l * Proportion\ of\ tourists_{i,l} * Unadjusted\ Alcohol\ LPC_l * \frac{Average\ length\ of\ stay_{i,l}}{365} *}{Population_d}$$

where:

l *is the set of all locations*, i *is either* Domestic consumption abroad *or* Tourist consumption domestically,

and d *is a domestic location*

After adjusting alcohol LPC by tourist consumption and unrecorded consumption for all location/years reported, sex-specific and age-specific estimates were generated by incorporating estimates modelled in DisMod for percentage of current drinkers within a location/year/sex/age, as well as consumption trends modelled in the DisMod g/day model. We do this by first making sure the sum of percent current drinkers and percent abstainers sum to one for a given location/year/age/sex. We then calculate the proportion of total consumption for a given location/year by age and sex, using the estimates of alcohol consumed per day, the population size, and the percentage of current drinkers. Lastly, we then multiply this proportion of total stock for a given location/year/sex/age by the total stock for a given location/year to calculate the consumption in terms of liter per capita for a given location/year/sex/age. We then convert these estimates to be in terms of grams/per day. The following equations describe these calculations:

$$\%\ Current\ drinkers_{l,y,s,a} = \frac{\%\ Current\ drinkers_{l,y,s,a}}{\%\ Current\ drinkers_{l,y,s,a} + \%\ Abstainers_{l,y,s,a}}$$

$$Proportion\ of\ total\ consumption_{l,y,s,a} = \frac{Alcohol\ g/day_{l,y,s,a} * Population_{l,y,s,a} * \%\ Current\ drinkers_{l,y,s,a}}{\sum_{s,a} Alcohol\ g/day_{l,y,s,a} * Population_{l,y,s,a} * \%\ Current\ drinkers_{l,y,s,a}}$$

$$Alcohol\ LPC_{l,y,s,a} = \frac{Alcohol\ LPC_{l,y} * Population_{l,y} * Proportion\ of\ total\ consumption_{l,y,s,a}}{\%\ Current\ drinkers_{l,y,s,a} * Population_{l,y,s,a}}$$

$$Alcohol\ g/day_{l,y,s,a} = Alcohol\ LPC_{l,y,s,a} * \frac{1000}{365}$$

where:

$$l \text{ is a location}, y \text{ is a year}, s \text{ is a sex}, \text{and } a \text{ is an age group}.$$

We then used the gamma distribution to estimate individual level variation within location, year, sex, age drinking populations, following the recommendations of other published alcohol studies.[7,8] We chose parameters of the gamma distribution based on the mean and standard deviation of the 1,000 draws of alcohol g/day exposure for a given population.

Theoretical minimum-risk exposure level

We calculated TMREL by first calculating the overall risk attributable to alcohol. We did this by weighting each relative risk curve by the share of overall DALYs for a given cause. We then took the minimum of this overall-risk curve as the TMREL of alcohol-use. More formally,

$$TMREL = argmin\ average\ overall\ risk_{\omega}(g/day)$$

$$Average\ overall\ risk_{\omega}(g/day) = \sum_{i}^{\omega} RR_i(g/day) * \frac{DALY_i}{\sum_i^{\omega} DALY_i}$$

Where:

ω *is the set of causes associated with alcohol*, i *is a given cause from that set*, $DALY$ *is the global DALY rate in* 2010, *and* RR *is the dose response curve for a given cause and exposure level in grams per day.*

In other words, we chose TMREL as being the exposure that minimises your risk of suffering burden from any given cause related to alcohol. We weight the risk for a particular cause in our aggregation by the proportion of DALYs due to that cause. (e.g. since more observed people die from IHD, we weight the risk for IHD more in the above calculation of average risk compared to, say, diabetes, even if both have the same relative risk for a given level of consumption)

Relative risks

For GBD 2016, we performed a systematic literature review of all cohort and case-control studies reporting a relative risk, hazard ratio, or odds ratio for any risk-outcome pairs studied in GBD 2016. Studies were included if they reported a categorical or continuous dose for alcohol consumption, as well as uncertainty measures for their outcomes, and the population under study was representative.

We then used these studies to calculate a dose-response, modelled using DisMod ODE. We chose DisMod ODE rather than a conventional mixed effect meta-regression because of its ability to estimate nonparametric splines over doses (i.e. for most alcohol causes, there is a non-linear relationship with different doses) and incorporate heterogeneous doses through dose-integration (i.e. most studies report doses categorically in wide ranges. DisMod ODE estimates specific doses when categories overlap across studies, through an integration step.) We used the results of the meta-regression to estimate a non-parametric curve for all doses between 0-150 g/day and their corresponding relative risks. For all

causes, we assumed the relative risk was the same for all-ages and sexes, with the exception of ischemic heart disease, ischemic stroke, hemorrhagic stroke, and diabetes, which we estimated by sex.

Regarding injuries outcomes, we constructed relative risks based on chronic exposure rather than acute, which has a weaker relationship to the outcome, though still significant.[15,16,18-21] We decided to use chronic exposure given the lack of available data on acute exposure, as well as, the lack of cohort studies using acute exposure as a metric. Further, using chronic exposure allowed us to construct relative risks curves for unintentional injuries, interpersonal violence, motor vehicle accidents, and self-harm using the same method as reported above.

In the case of motor vehicle accidents, we adjusted the PAF to account for victims of drunk drivers that are involved in accidents. Using data from the Fatality Analysis Reporting System in the US,[17] we calculated the average number of fatalities in a car crash involving alcohol, as well as the percentage of those fatalities distributed by age and sex (figures 1 and 2). We aggregated FARS data across the years 1985-2015, given there was little variation in the data temporally and the number of cases in old age groups had too much variance when constructing estimates by year. To adjust PAFs, we multiplied attributable deaths by the average number of fatalities from FARS and redistributed the PAF amongst each population, based on the probability of being a victim to a certain drunk driver by age and sex, based on the FARS data. The following equation describes this process:

$$Adjusted\ PAF_i = \frac{\sum_d PAF_d * DALY_d * Avg\ Fatalities_d *\ P(i\ is\ a\ victim)_d}{DALY_i}$$

where:

$i\ is\ a\ population\ by\ location, year, age, sex\ and$
$d\ is\ the\ set\ of\ all\ age\ and\ sex\ exposed\ groups\ within\ that\ location\ and\ year.$

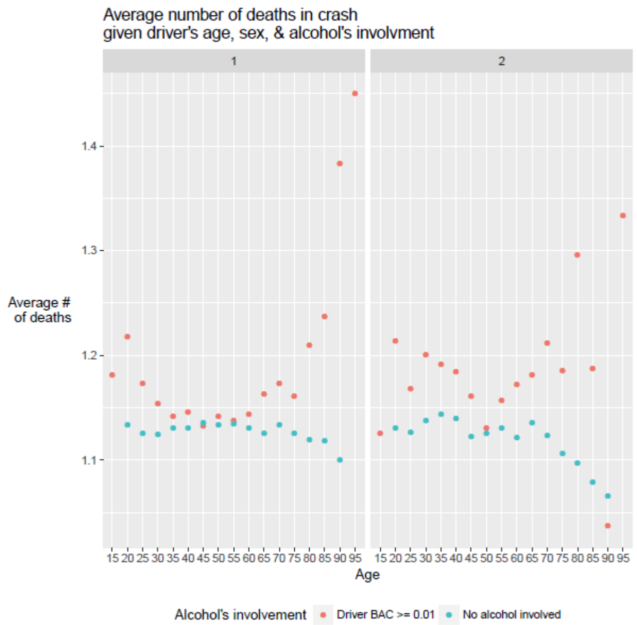

Figure 1

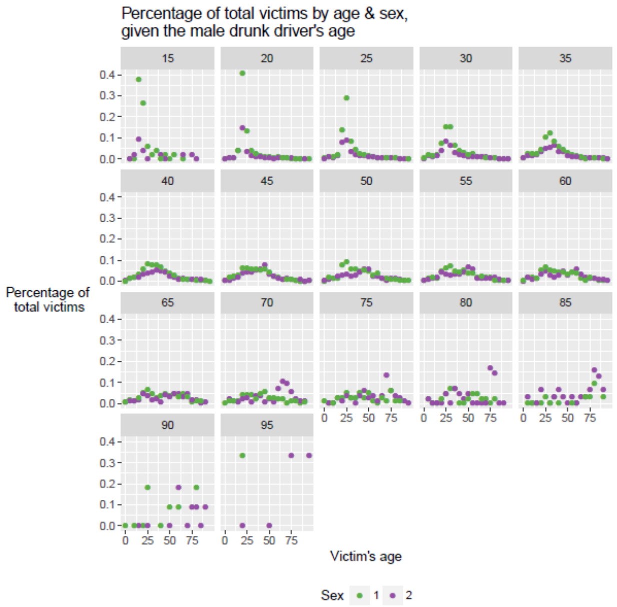

Figure 2

Population attributable fraction

For all causes, we defined PAF as:

$$PAF(x) = \frac{P_A + \int_0^{150} P(x) * RR_C(x)\, dx - 1}{P_A + \int_0^{150} P(x) * RR_C(x)\, dx} \qquad P(x) = P_C * \Gamma(\boldsymbol{p})$$

where:

P_C *is the prevalence of current drinkers,* P_a *is the prevalence of abstainers,* $RR_c(x)$ *is the relative risk function for current drinkers, and* $\boldsymbol{p}$ *are parameters determined by the mean and sd of exposure*

We performed the above equation for 1,000 draws of the exposure and relative risk models. We then used the estimated PAF draws to calculate YLL, YLDs, and DALYs, as per the other risk factors.

References

1. Food and Agriculture Organization of the United Nations (FAO). FAOSTAT Food Balance Sheets, October 2014. Rome, Italy: Food and Agriculture Organization of the United Nations (FAO).
2. World Health Organization (WHO). WHO Global Health Observatory - Recorded adult per capita alcohol consumption, Total per country. Geneva, Switzerland: World Health Organization (WHO).
3. UN World Tourism Organization (UNWTO). UN World Tourism Organization Compendium of Tourism Statistics 2015 [Electronic]. Madrid, Spain: UN World Tourism Organization (UNWTO), 2016.
4. Norstrom, Thor. "Estimating changes in unrecorded alcohol consumption in Norway using indicators of harm." *Addiction* 93.10 (1998): 1531-1538.
5. Macdonald, Scott. "Unrecorded alcohol consumption in Ontario, Canada: estimation procedures and research implications." *Drug and Alcohol Review* 18.1 (1999): 21-29.
6. Meier, Petra Sylvia, et al. "Adjusting for unrecorded consumption in survey and per capita sales data: quantification of impact on gender-and age-specific alcohol-attributable fractions for oral and pharyngeal cancers in Great Britain." *Alcohol and Alcoholism* 48.2 (2013): 241-249.
7. Hao, Wei, Hanhui Chen, and Zhonghua Su. "China: alcohol today." *Addiction* 100.6 (2005): 737-741.
8. Rehm, Jürgen, and Vladimir Poznyak. "On monitoring unrecorded alcohol consumption." *Alcoholism and Drug Addiction* 28.2 (2015): 79-89.
9. Probst et al. “Unrecorded Alcohol Use: A global modeling study based on Delphi assessments and survey data”. Toronto, Canada: CAMH.
10. Ramstedt, Mats. "How much alcohol do you buy? A comparison of self-reported alcohol purchases with actual sales." *Addiction* 105.4 (2010): 649-654.
11. Stockwell, Tim, et al. "Under-reporting of alcohol consumption in household surveys: a comparison of quantity–frequency, graduated–frequency and recent recall." *Addiction* 99.8 (2004): 1024-1033.
12. Kerr, William C., and Thomas K. Greenfield. "Distribution of alcohol consumption and expenditures and the impact of improved measurement on coverage of alcohol sales in the 2000 National Alcohol Survey." *Alcoholism: Clinical and Experimental Research* 31.10 (2007): 1714-1722.
13. An Integrative Metaregression Framework for Descriptive Epidemiology. Abraham D. Flaxman, Theo Vos, Christopher J. L. Murray. Seattle: University of Washington Press, [2015]
14. Taylor, Bruce, et al. "The more you drink, the harder you fall: a systematic review and meta-analysis of how acute alcohol consumption and injury or collision risk increase together." *Drug and alcohol dependence* 110.1 (2010): 108-116.
15. Vinson, Daniel C., Guilherme Borges, and Cheryl J. Cherpitel. "The risk of intentional injury with acute and chronic alcohol exposures: a case-control and case-crossover study." *Journal of studies on alcohol* 64.3 (2003): 350-357.
16. Vinson, Daniel C., et al. "A population-based case-crossover and case-control study of alcohol and the risk of injury." *Journal of studies on alcohol*64.3 (2003): 358-366.
17. Fatal Accident Reporting System (FARS). National Highway Traffic Safety Administration, National Center for Statistics and Analysis Data Reporting and Information Division (NVS-424); 1985, 1990, 1995, 2000, 2005, 2010, 2015

18. Chen, Li-Hui, Susan P. Baker, and Guohua Li. "Drinking history and risk of fatal injury: comparison among specific injury causes." *Accident Analysis & Prevention* 37.2 (2005): 245-251.
19. Bell, Nicole S., et al. "Self-reported risk-taking behaviors and hospitalization for motor vehicle injury among active duty army personnel." *American journal of preventive medicine* 18.3 (2000): 85-95.
20. Margolis, Karen L., et al. "Risk factors for motor vehicle crashes in older women." *The Journals of Gerontology Series A: Biological Sciences and Medical Sciences* 57.3 (2002): M186-M191.
21. Sorock, Gary S., et al. "Alcohol-drinking history and fatal injury in older adults." *Alcohol* 40.3 (2006): 193-199.

3.7.1 Met Need for Family Planning with Modern Methods SDG Capstone Appendix

Flowcharts

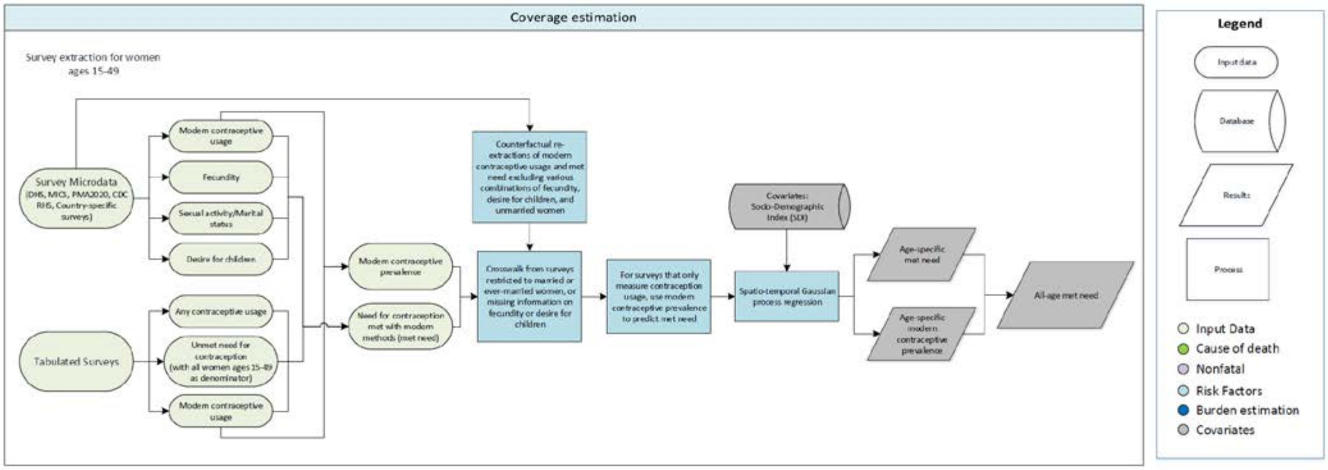

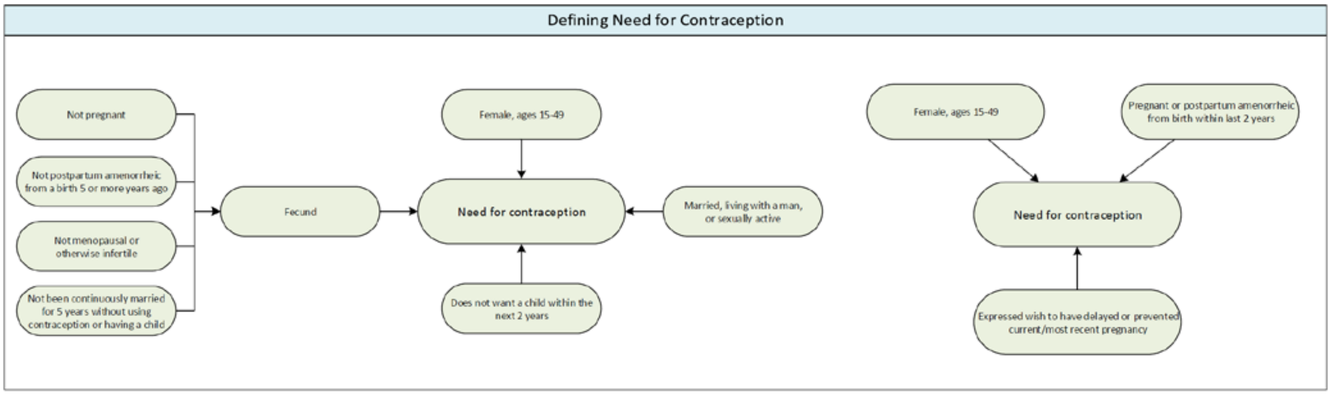

Input Data & Methodological Summary

Indicator definition

This modeling strategy encompassed the indicator associated with the proportion of women aged 15 to 49 years with their family planning needs met with modern contraception methods (3.7.1). This indicator also is an individual component of Indicator 3.8.1, which is the composite indicator for universal health coverage (UHC) tracer interventions.

Indicator 3.7.1

As a component of SDG Goal 3, SDG Target 3.7 is measured using SDG Indicator 3.7.1:

> SDG Goal 3: *Ensure healthy lives and promote well-being for all at all ages*
>
> SDG Target 3.7: *By 2030, ensure universal access to sexual and reproductive health-care services, including for family planning, information and education, and the integration of reproductive health into national strategies and programmes*

SDG Indicator 3.7.1: *The proportion of women of reproductive age (15 to 49 years) who are sexually active and have their need for family planning satisfied with modern methods*

We defined modern contraceptive methods as the current use of male or female sterilization, male or female condoms, diaphragms, spermicide foam/jelly, oral hormonal pills, implants, injections, intra-uterine devices (IUDs), or emergency contraceptives. Traditional contraceptive methods were defined as the current use of alternative methods including but not limited to withdrawal, periodic abstinence, the rhythm method, and the lactational amenorrhea method (LAM).

Women between the ages of 15 and 49 were defined as having a need for family planning if they were using any method of contraception, or if they were fecund, sexually active, and did not wish to become pregnant within the next two years. Sexual activity was assumed for all currently married or in-union women. We defined met need with modern methods as the proportion of women with a need for contraception that are actually using modern methods. It is important to note that with respect to most family planning literature, the GBD met need indicator corresponds to the proportion of demand satisfied with modern methods, not the indicator for unmet need (for which the denominator is all women, not just women with a need for contraception). Women were assumed to be fecund unless they met one or more of the following criteria:

(1) they were pregnant

(2) they were postpartum amenorrheic from a birth that occurred 5 or more years ago

(3) they had not menstruated within the last 6 months (unless postpartum amenorrheic for less than 5 years)

(4) they had been continuously married/in a union for 5 or more years without having a child and without ever having used any method of contraceptive (modern or traditional)

(5) they otherwise indicated that they were infertile (ex. mentioned having had a hysterectomy).

Women who were pregnant or postpartum amenorrheic from a birth within the last 2 years were considered separately, and were determined to have a need for contraception if they indicated a desire to have delayed or avoided their current or most recent pregnancy.

Input data

The present study used two primary types of input data in order to ultimately generate a time series of met need for family planning with modern methods:

(1) individual-level microdata from which met need for family planning with modern methods could be directly estimated

(2) tabulated data from survey reports for which met need with modern methods could be directly extracted (as demand satisfied with modern methods) or indirectly calculated based on reported estimates of modern contraception coverage, any contraception coverage, and unmet need for family planning.

Our primary data sources for met need with modern methods included multi-country survey series such as Demographic and Health Surveys (DHS), Multiple Indicator Cluster Surveys (MICS), and Centers for Disease Control and Prevention Reproductive Health Surveys (CDC RHS). In addition, we extracted data

from the Performance Monitoring and Accountability 2020 (PMA2020) surveys, to which we were granted access. We originally sought a wider universe of population surveys, but our search was somewhat restricted to the survey series for which information on contraception use by method and marital status was readily available for all women of reproductive age. Notably, relatively few microdata sources were available for higher-income countries; subsequently, we heavily relied on tabulated data for these geographies.

The below table shows the number of studies included in the 2017 SDG Capstone paper.

Survey Series	Number of Included Surveys
DHS	310
MICS	200
CDC RHS	71
PMA2020	50
Country-specific	356

Among the surveys for which we had access to microdata, we applied survey weights based on survey sampling frames to generate weighted national estimates of met need accompanied by estimates of standard error (SE). In the absence of microdata or survey sampling information, we used survey sample sizes as a mechanism for informing uncertainty estimation.

A number of the tabulated report data sources did not include estimates of met need with modern methods; instead, such sources provided information on prevalence of modern contraception use, prevalence of any contraception use, and prevalence of unmet need for family planning among women of reproductive age. Following the recommended analytic approach from DHS and the Inter-agency Expert Group on the SDG Indicators (IAEG-SDGs)[1,2], we estimated met need with modern methods based on the following formula:

$$Prev_{MetMod} = \frac{Prev_{Mod}}{Prev_{Any} + Prev_{Unmet}}$$

where $Prev_{MetMod}$ is the prevalence of met need with modern methods (the GBD indicator); $Prev_{Mod}$ is the modern contraceptive prevalence among women aged 15 to 49 years; $Prev_{Any}$ is the total contraceptive prevalence among women aged 15 to 49 years; and $Prev_{Unmet}$ is the prevalence of women who have need for family planning but are not currently using any method of contraception, where the denominator is all women aged 15 to 49 years. In future iterations of this analysis, we will prioritize gaining access to microdata for these surveys, so that we can directly estimate met need with modern methods from individual-level data.

For a subset of surveys, contraceptive use and met need was only collected for women who were currently or had ever been married. To estimate contraceptive use and met need for all women using such restricted data, we performed counterfactual re-extractions on surveys for which microdata was available for all women, this time subsetting our re-analysis to only currently married or ever-married women. We used the average difference between the original unrestricted estimates and the counterfactual re-extractions to crosswalk modern contraceptive use and met need for those surveys which only sampled current or ever-married women. These crosswalks were performed separately within

each 5-year age group to account for the differential effect by age of restricting data collection based on marital status. Additionally, some surveys did not ask questions related to fecundity or the desire for children within the next two years, creating further sources of potential bias in our met need estimates. For these issues we used the same crosswalk methodology, accounting for missing variables by re-extracting available microdata under counterfactual scenarios to inform age-specific crosswalks. This allowed us to predict met need for women in countries where contraceptive information was gathered, but information on fecundity and desire for children were unavailable. When multiple issues applied to a survey (for example, when a survey was restricted to married women and also lacked questions related to fecundity), we crosswalked only once, using a counterfactual re-extraction that matched the data issues of the original survey in order to account for potential interactions between missing and restricted variables.

After accounting for differences in survey sampling frames and missing variables, we leveraged the relationship between modern contraception use and met need with modern contraception to predict met need where only modern contraceptive prevalence data was available. To do this, we ran a regression of met need observations against modern contraceptive prevalence by age group. To account for geographical differences in the relationship between modern contraceptive prevalence and met need, the regression included an interaction term between modern contraceptive use and GBD super-region.

Modelling strategy

For the present analysis, we used Spatiotemporal Gaussian process regression (ST-GPR), a three-stage model used widely within the GBD study to synthesize coherent trends and uncertainty from multiple sources of data. The first stage included fitting a linear model with fixed effects on age and the Socio-Demographic Index. The second stage involved smoothing over space-time based on the residuals from the first stage linear model. The third stage used Gaussian Process Regression (GPR) to generate a cohesive time series of met need with modern contraception and uncertainty for all GBD locations and from 1990 to 2017.

After generating age-specific estimates of met need and modern contraceptive prevalence, we could calculate age-specific estimates of need for contraception using the formula:

$$Prev_{Need} = \frac{Prev_{Mod}}{Prev_{MetMod}}$$

where $Prev_{Need}$ is the age-specific prevalence of need for contraception among women aged 15 to 49 (referred to as demand for contraception in most family planning literature). Using $Prev_{Need}$ and GBD population estimates we could calculate the weights needed to aggregate age-specific met need for contraception into total met need among women aged 15 to 49, which is the SDG indicator reported in this paper.

References

1. Bradley, S. E. K., Croft, T. N. & Fishel, J. D. Revising Unmet Need for Family Planning: DHS Analytical Studies No. 25. 63 (2012).

2. United Nations Department of Economics and Social Affairs. *Goal 3: Ensure healthy lives and promote well-being for all at all ages*. (2015). at <https://sustainabledevelopment.un.org/sdg3>

3.7.2 Adolescent Fertility SDG Capstone Appendix

Flowcharts

Appendix Figure 1b: Analytical Flowchart for the GBD 2017 Fertility Estimation Process

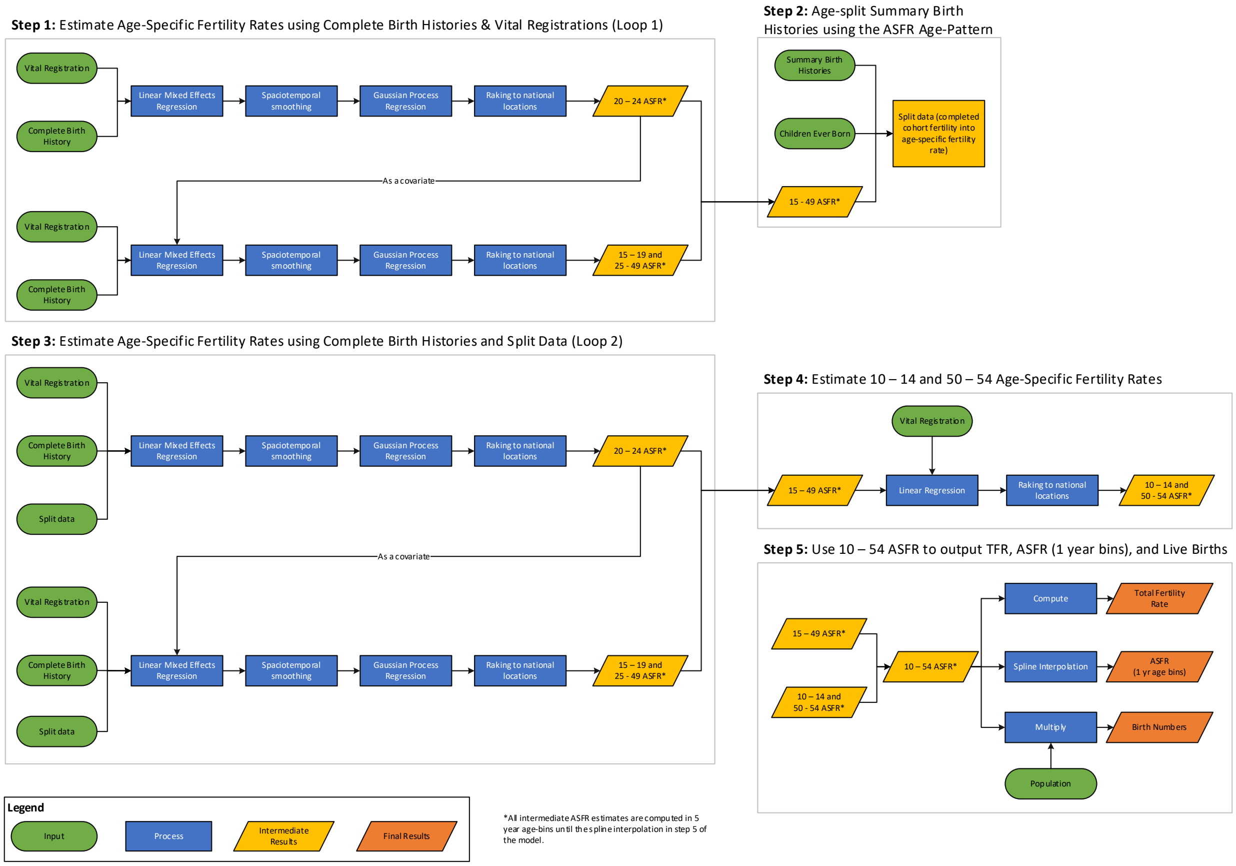

Input Data & Methodological Summary

Indicator definition

This modeling strategy encompassed the indicator associated with the number of births per 1,000 women aged 10-14 and aged 15-19 years (3.7.2).

Indicator 3.7.1

As a component of SDG Goal 3, SDG Target 3.7 is measured using SDG Indicator 3.7.1:

SDG Goal 3: *Ensure healthy lives and promote well-being for all at all ages*

SDG Target 3.7: *By 2030, ensure universal access to sexual and reproductive health-care services, including for family planning, information and education, and the integration of reproductive health into national strategies and programmes*

SDG Indicator 3.7.2: *Number of births per 1,000 women aged 10-14 years and women aged 15 to 19 years*

Input data

We used three primary source types for the fertility analysis: (1) the number of live births by age of mother reported through vital registration (VR) systems; (2) complete birth histories (CBH); and (3) summary birth histories (SBH). In total, we compiled 9548 location-years of data for women aged 10 to 54 for the 1950 to 2017 period. Below we present a brief description of each of these source types and the results of data synthesis.

Fertility Data Source Types

Accurate and complete registration data of live births by age of the mother are typically regarded as the gold standard source of information on fertility; in theory, these regular (usually annual) reports should capture all births in a given country or subnational unit within a given year. High-income countries tended to have high-quality VR systems that contained the date and location of the birth, as well as detailed demographic characteristics of the mother and the date and location of birth. In lower-income countries, however, birth registration systems tended to suffer from interrupted and/or delayed reporting and incomplete coverage. Birth registries provided almost all of the fertility information pertaining to women aged 10-14 and 50-54, as the overwhelming majority of household surveys only collected birth histories from women aged 15-49 at the time of survey.

In cases where the completeness and quality of birth registration data were poor, we relied heavily on other types of data sources (namely household surveys and censuses) to triangulate the level and age-pattern of fertility. Fertility information in household surveys and censuses was predominantly in two forms— complete birth histories (CBH) and summary birth histories (SBH). CBHs, which grew to prominence with the World Fertility Surveys (WFS) administered in the 1970s and 80s, collected information about a surveyed mother's date of birth, as well as the dates of birth and death of all children she can recall bearing in her lifetime. Since each birth could be linked to the time of birth and the mother's concurrent age, this permitted the calculation of period and age-specific fertility rates in the years prior to the survey under assumptions of no survivor, migrant, or recall bias. Many major survey programs contain CBH modules, including the Demographic and Health Surveys (DHS), Multiple Indicator Cluster Surveys (MICS), and the Reproductive Health Surveys (RHS).

SBHs, on the other hand, collected no information about the dates of birth of children, but instead only recorded the total number of children ever born (CEB) to a woman over her lifetime. Also collected is the mother's date of birth or age at the time of interview. These data provided valuable information about the overall level of fertility experienced by cohorts over time but could not be used by themselves to estimate period and age-specific fertility. Using assumptions similar to CBH (no survivor or migrant bias), cohort age patterns of fertility derived from other data sources could be used to split CEB information into period age-specific fertility rates. Availability of SBHs far surpassed that of CBHs, largely due to their relative simplicity but also their precedence. CEB questions have been featured in censuses since the turn of the 20th century and thus comprise a large share of information about fertility in low-income settings prior to the mid 1970s. In addition to censuses, a number of other survey families featured SBH, including a subset of MICS, the Living Standards and Measurement Surveys (LSMS), and a variety of country-specific surveys.

Fertility Data Identification and Synthesis

Registry data were identified through the UN Demographic Yearbook (DYB; UN Statistical Division [UNSTAT]),[6] the Human Fertility Collection (HFC; Max Planck Institute for Demographic Research [MPIDR]),[7] official publications, online data portals of national statistical offices, and international collaborators. The DYB and HFC compile registry-based fertility data as reported by national statistical offices and country research institutes. DYB reports of live births by age of mother were extracted for every year available from 1948, and the complete set of age-specific HFC data were downloaded in October 2017. Estimates provided to HFC by individual researchers were excluded from our analyses (i.e. we incorporated only empirical data, per MPIDR's source categorizations); country-year-ages already covered by the DYB were also excluded. We also extracted data from sample registration systems (SRS) typical of South Asian countries, including India, Pakistan, and Bangladesh. In total, we compiled 7,817 country years of VR data, with 2,421 of them coming prior to 1970 and 1,755 of them coming after 2000. We included 31 location-years of data from SRS, the majority of which cover India.

Fertility data from household surveys and censuses were initially identified using the Global Health Data Exchange (GHDx). Records classified as "survey" or "census" and that contained any of the keywords "complete birth history," "summary birth history," or "fertility" were then compiled and reviewed by research team members to verify that they contained sufficient detail for inclusion in GBD analysis. Additional seeking was conducted for identified gaps in data, primarily through country statistical office websites as well as major survey families such as DHS, MICS, WFS, and RHS. In cases where sufficiently-detailed data were not publicly available, in-country collaborators assisted in its procurement. Fertility data from the 1950s and 60s in low-income settings (particularly sub-Saharan Africa) was specifically sought in colonial censuses containing SBH information. Where sources provided microdata, we standardized and processed CBHs to compute period age-specific fertility rates (ASFR) every three years over a fifteen-year recall and collapsed SBHs to tabulations of average children ever born by mother's age to be later split by cohort age patterns from the first modeling stage (see Section 2.2.2 for further details). Where microdata were unavailable, we extracted period ASFRs or average CEB by mother's age as documented in reports or other publications. In total, we extracted and processed 429 CBHs and 643 SBHs, out of 4259 identified surveys and censuses. Occasionally, the recall type of a survey for which tabulated period ASFRs were available was unable to be identified from the report or available documentation. These accounted for only 59 location-years.

Modelling strategy

Age-specific fertility rate estimation

Using all the data described above, we estimated age-specific fertility rates by 5-year age groups from ages 10 to 54 years in two broad steps. First, we estimated age-specific fertility rates for 15 to 49 years of age using spatio-temporal Gaussian process regression (ST-GPR). Next, we estimated fertility rates for 10 to 14 year olds as a function of estimated fertility in 15 to 19 year olds. The sections below provide further estimation process details.

Age-specific fertility rate estimation for 15 to 49 years

ASFR for age groups 15-19, 20-24, 25-29, 30-34, 35-39, 40-44, and 45-49 were estimated using ST-GPR, which has been covered in detail elsewhere.[1,2] The estimation of ASFR involved the following sequential

steps: (1) Estimation of ASFR 20-24 using age-specific data from CBH and VR and using mean years of education in 20-24 year olds as a predictor; (2) Estimation of ASFR for the remaining age groups using age-specific data from CBH and VR and using age-specific mean years of education and estimated ASFR 20-24; (3) Split SBH data by age and period using the estimated location, time and age-specific estimates of ASFR; (iv) Re-estimate ASFR 20-24 using CBH, VR and the period-age-split SBH data; and (v) Re-estimate ASFR using CBH, VR and the period-age-split SBH data.

The ST-GPR models for ASFR were implemented as follows. The first stage mixed effect regression was fit in bounded logit space:

$$Logit\left(\frac{ASFR\ data - lower\ bound_{age}}{upper\ bound_{age} - lower\ bound_{age}}\right)$$

The lower bound was the minimum fertility by age across time and geography, and the upper bound was the 99.3 percentile of fertility by age across time and geography, after dropping implausibly high ASFRs above 0.5. This upper bound on ASFR data produced an implied maximum TFR of 10.5.

The specifications of the mixed effects regression are below.

$$logit_{bound}(ASFR_{20-24}) = \beta_1 + \beta_2 * female\ education_{c.y} + \gamma_{locsource}$$

$$logit_{bound}(ASFR_{n-n+5}) = \beta_1 + \beta_2 * female\ education_{c,y} + spline(ASFR_{20-24,c,y}) + \gamma_{locsource}$$

Where n is between 25 and 45, β_1 is the intercept, β_2 is the coefficient on female education, female education and the ASFR 20-24 estimates are specific by country and year, and $\gamma_{locsource}$ is a location-source random intercept.

Data Source Adjustment

After the mixed-effects model was computed, the random intercept on the concatenation of location and source was used to adjust data to a reference or standard source. The adjustment factor was the sum of the difference between the reference source fixed and random effects, and the fixed and random effects on the data point for the specific source, as below, and was then added to the data to derive an adjusted value.

$$Adjustment\ Factor = (Location\ Source\ RE_{ref} - Location\ Source\ RE_{data\ point})$$

Where RE represents a random intercept of either a reference source or a data-point specific location-source. When more than one reference source was selected in a single location, the values of the location source random effects for each reference source were averaged to produce the first term of the equation.

Reference sources were initially chosen as: (1) complete VR for locations with complete VR, (2) an average of complete birth history sources for locations with 1 or more complete birth history, (3) and agnostically (as an average of all the sources for each location) for locations with neither complete VR nor complete birth histories. VR was designated as complete for a country if the median of child death registration completeness for the location over all available years was over 95%.[5] For some locations, reference sources were chosen based on expert judgement. For example, data from past censuses (1950s and 1960s) in Sub-Saharan Africa were often chosen as reference sources to accurately capture the depressed fertility during that time period.

Hyper-parameter Selection

The residual smoothing and GPR stages of ST-GPR were implemented using the output of the mixed effects regression and data source adjustments. Hyper-parameters for the residual smoothing and GPR stages were chosen based on a location and age-specific data density score. For locations with VR, the VR component of the score was calculated as the sum of the years for which VR data were available, and then down-weighted if the number of births in the age group was less than 100. Incomplete VR was down-weighted by 0.5. For non-VR sources, the number of sources was counted instead of the number of years. For example, one DHS survey would count as one source, even if it contributed more than 1 data point.

The data density score was calculated as follows:

$$\begin{aligned} DD\ Score_{loc,age} &= Complete\ VR\ years_{loc,age} + \left(2 * Number\ CBH\ Sources_{loc,age}\right) \\ &+ \left(0.25 * Number\ SBH\ sources_{loc,age}\right) + \left(0.5 * Incomplete\ VR\ years_{loc,age}\right) \\ &+ Number\ Other\ Sources_{loc,age} \end{aligned}$$

Where DD stands for data density, CBH is complete birth history, SBH is summary birth history, and all elements of the equation incorporate year and age.

ST-GPR hyper-parameters λ, ξ, and scale were designated by categories of data density, as shown in Table B below.

Table B: Hyper-parameter Values by Data Density

Data Density	Lambda	Zeta	Scale
Over 50	0.2	0.99	5
Between 30 and 50	0.4	0.9	10
Between 20 and 30	0.6	0.8	15
Between 10 and 20	0.8	0.7	15
Under 10	1	0.6	15

For non-complete VR sources, data variance was calculated as the variance between the spatio-temporal prediction and the unadjusted data. For location-ages with fewer than 5 data points, the maximum data variance in the associated GBD region was used. For complete VR sources, we assumed that non-sampling variance was 0 and calculated sampling variance using the binomial equation shown below:

$$Sampling\ Variance = \frac{ASFR * (1 - ASFR)}{Births}$$

To calculate amplitude, we computed the mean of the location-specific standard deviation of the difference between the first stage mixed-effect regression and the second stage spatio-temporal smoothing, restricted to national locations with a data density score of over 50 in the years between 1990 to 2017. This amplitude was applied to all locations.

SBH Methods

SBHs that collected CEB data were more frequently available at early time points than CBHs. For example, questions about CEB were often included in early colonial censuses in Africa. Multiple techniques exist to

compute period-and-age-specific fertility from SBH information, the most widely used of which is the Brass Parity/Fertility ratio method. This method assumes, however, that age-specific fertility remains constant over time. To relax this assumption, we used the first set of estimates of age-specific fertility that were based on CBH and registry data, an estimate of dynamic cohort age patterns over time, to split SBH into period-and-age-specific ASFR. Using the CBH and registry data-based estimates, we calculated the implied cohort fertility as the weighted average of the upper and lower bounding age groups, taking into account a cohort aging into the next 5-year age interval and adopting the fertility associated with that age interval. The cumulative sum of the implied cohort fertility yielded CEB implied by the CBH and registry based model for birth cohorts between 1940 (who began to experience AFSR 10-14 in 1950 at the beginning of our estimation period) and 2007 (who began to experience ASFR 10-14 in 2017 at the end of our estimation period).

To split SBH data into period and age specific ASFR, we applied the ratio of implied CEB from the CBH and registry based model and the empirical children from SBH to the cohort age pattern from the CBH and registry based model. This approach provided additional information about the all-age level of fertility over time, and represented a large increase in the availability of past data. Once the data was split, we reran the estimation process, incorporating all registry data, CBH, and period-and-age split SBH data.

Age-specific fertility rate estimation for 10 to 14 year olds

ASFR for 10-14 was estimated separately, given the scarcity of the data for those age groups in any locations without a vital registration system. In both models, we leverage the relationship between ASFR in one age group and the neighboring age group. In age 10-14, we ran a mixed effects regression on the log of the ratio of ASFR 10-15 over ASFR 15-19, and used ASFR 15-19 as a predictor along with nested random intercepts by super-region, region, and location, as follows:

$$\log\left(\frac{ASFR\ 10-14}{ASFR\ 15-19}\right) = \beta_1 + \beta_2 \log(\mathrm{ASFR}\ 15-19) + \gamma_{\mathrm{sr}} + \gamma_r + \gamma_{\mathrm{loc}}$$

Where β_1 is the intercept and γ_{sr}, γ_r, and γ_{loc} are nested super-region, region, and location random intercepts.

References

1 Wang H, Naghavi M, Allen C, *et al.* Global, regional, and national life expectancy, all-cause mortality, and cause-specific mortality for 249 causes of death, 1980–2015: a systematic analysis for the Global Burden of Disease Study 2015. *The Lancet* 2016; **388**: 1459–544.

2 Wang H, Abajobir AA, Abate KH, *et al.* Global, regional, and national under-5 mortality, adult mortality, age-specific mortality, and life expectancy, 1970–2016: a systematic analysis for the Global Burden of Disease Study 2016. *The Lancet* 2017; **390**: 1084–150.

3 United Nations Population Division | Department of Economic and Social Affairs. http://www.un.org/en/development/desa/population/

4 Human Mortality Database. http://www.mortality.org

5 Gakidou E. Global, regional, and national under-5 mortality, adult mortality, age-specific mortality, and life expectancy, 1950-2017: a systematic analysis for the Global Burden of Disease Study 2017. Under Review.

3.8.1 Universal Health Coverage (UHC) Service Coverage Index SDG Capstone Appendix

Input data & Methodological summary

Indicator definition

This modeling strategy involves the construction of a composite measure for the universal health coverage (UHC) service coverage index (SDG Indicator 3.8.1). The UHC service coverage index includes nine measures of coverage for a subset of interventions for communicable diseases and maternal and child health and the 32 causes that comprise the Healthcare Access and Quality (HAQ) Index, a summary measure of personal healthcare access and quality based on risk-standardised death rates and mortality-to-incidence ratios (MIRs) from causes amenable to healthcare.[1,2] For GBD 2017, using MIRs instead of risk-standardised death rates for cancers was a key improvement for better approximating access to quality cancer care.[2] Each component of the UHC service coverage index was scaled on a scale of 0 to 100, with 0 being the worst observed from 1990 to 2017 and 100 being the best observed during this time, and then the arithmetic mean was taken of each component. We then projected the UHC service coverage index, based on past trends, as a composite indicator from 2018 to 2030.

The measures of intervention coverage are as follows: three doses of diphtheria-pertussis-tetanus (DPT3), measles vaccine, three doses of the oral polio vaccine or inactivated polio vaccine; met need for family planning with modern methods; antenatal care (ANC) coverage (one ANC visit [ANC1] and four ANC visits [ANC4]); skilled birth attendance (SBA); in-facility delivery rates; and coverage of antiretroviral therapy (ART) among people living with HIV.

The causes that comprise the HAQ Index are as follows: tuberculosis, diarrheal diseases, lower respiratory infections, upper respiratory infections, diphtheria, whooping cough, tetanus, measles, maternal disorders, neonatal disorders, colon and rectum cancer, non-melanoma cancer, breast cancer, cervical cancer, uterine cancer, testicular cancer, Hodgkin's lymphoma, leukemia, rheumatic heart disease, ischemic heart disease, cerebrovascular disease, hypertensive heart disease, peptic ulcer disease, appendicitis, hernia, gallbladder and biliary diseases, epilepsy, diabetes, chronic kidney disease, congenital heart anomalies, and adverse effects of medical treatment.

Indicator 3.8.1

As a component of SDG Goal 3. Ensure healthy lives and promote well-being for all at all ages, SDG Target 3.8, achieve universal health coverage, including financial risk protection, access to quality essential health-care services and access to safe, effective, quality and affordable essential medicines and vaccines for all, is measured using SDG Indicator 3.8.1.

The UN definition of Indicator 3.8.1 is “Coverage of essential health services (defined as the average coverage of essential services based on tracer interventions that include reproductive, maternal, newborn and child health, infectious diseases, non-communicable diseases and service capacity and access),” with which we sought to align by combining measures of RMNCH, ART intervention coverage, and component parts of the HAQ Index.

UHC service coverage index indicator input data

Individual UHC service coverage index components serve as the input data for the overall UHC service coverage index, and unless otherwise specified, their write-ups are included in this portion of the appendix. Exceptions were interventions or causes covered by other indicator write-ups, such as skilled birth attendance (SDG indicator 3.1.2); maternal disorders (SDG indicator 3.1.1); and cardiovascular diseases, cancers, diabetes, and chronic respiratory diseases considered amenable to healthcare (SDG indicator 3.4.1).

UHC service coverage index component	Appendix content
Vaccine coverage (diphtheria-pertussis-tetanus vaccination, three doses [DPT3], measles, and polio vaccination, 3 doses)	Under 3.8.1
Antenatal care (ANC), 1 visit (ANC1) and 4 visits (ANC4)	Under 3.8.1
Skilled birth attendance (SBA)	Indicator 3.1.2
In-facility delivery rate (IFD)	Under 3.8.1
Met need for family planning with modern contraception methods	Indicator 3.7.1
Antiretroviral therapy (ART) coverage among people living with HIV	Under 3.3.1
Causes of death included in the Healthcare Access and Quality (HAQ) Index: tuberculosis, diarrheal diseases, lower respiratory infections, upper respiratory infections, diphtheria, whooping cough, tetanus, measles, maternal disorders, neonatal disorders, colon and rectum cancer, non-melanoma cancer, breast cancer, cervical cancer, uterine cancer, testicular cancer, Hodgkin's lymphoma, leukemia, rheumatic heart disease, ischemic heart disease, cerebrovascular disease, hypertensive heart disease, chronic respiratory diseases, peptic ulcer disease, appendicitis, hernia, gallbladder and biliary diseases, epilepsy, diabetes, chronic kidney disease, congenital heart anomalies, and adverse effects of medical treatment.	Maternal disorders covered by Indicator 3.1.1; cancers, cardiovascular diseases, diabetes, and chronic respiratory diseases are covered under Indicator 3.4.1 Adverse effects of medical treatment are covered in the injuries estimation process.

In sum, each component is estimated within the broader GBD study, with many of the measures of intervention coverage used as covariates to inform cause-specific models. Most of the individual interventions use population health survey microdata, or tabulated report data when microdata are not publicly available, as their primary input data sources. For vaccination, administrative data sources are also used to supplement survey-based estimates.

Each cause of death considered amenable to healthcare is estimated as part of the GBD cause of death analysis. Risk-standardisation of non-cancer death rates is based on the joint population attributable fraction (PAF) of environmental or occupational and behavioral risks as quantified by the GBD comparative risk assessment.[3] The construction of MIRs came from incidence and linked mortality data recorded in cancer registries. Additional information on risk-standardization and MIRs is provided in the next section.

UHC service coverage index modeling strategy

Summary

To construct the composite UHC service coverage index, we risk-standardised non-cancer death rates, constructed MIRs, and computed intervention coverage with draw-level estimates from GBD 2017. For each input, 1,000 draws were used in order to estimate uncertainty. We then scaled each UHC service coverage index component on a scale of 0 to 100 from 1990 to 2017, and took the arithmetic mean across components.

Risk standardisation of non-cancers

For non-cancers, location-year-age-sex-specific cause of death estimates from GBD 2017 are used directly. We estimated a joint-risk exposure (ie, population attributable fraction, or PAF) for each cause, using all risks except three metabolic risks (high blood pressure, high total cholesterol, and high fasting plasma glucose), which are inextricably linked to personal healthcare, and thus would not be appropriate for risk-standardisation steps explained later. Assumptions about how one risk factor is mediated through other risk factors are needed in order to estimate the joint-risk factor burden for combinations of behavioural or environmental risks. To accomplish this, for every two risk factors for an outcome, we used published studies to estimate the fraction of risk that was mediated through the other risk. This resulted in a matrix of parameters containing each possible pairing of risk factors included in the GBD 2017.[3] Using this matrix, we computed the aggregated burden of disease at each level of the GBD 2017 hierarchy and for all risk factors using the following formula:

$$PAF_{J,o,a,s,l,t} = 1 - \prod_{j=1}^{J}\left(1 - PAF_{j,o,a,s,l,t}\prod_{i=1}^{J}(1 - MF_{j,i,o})\right)$$

where J is a set of risk factors for the aggregation; $PAF_{j,o,a,s,l,t}$ is the PAF for risk j for cause o, age group a, sex s, location l, and year t; and $MF_{j,i,o}$ is the mediation factor for risk j mediated through i for cause o.

The aim of the risk-standardisation process is to eliminate the residual effects of localised risk exposure that would otherwise act as a confounding element in our ability to draw inferences about healthcare from mortality due to amenable causes. By imposing a global level of exposure on all locations, we de-contextualise them and create a measure of mortality that is a more appropriate proxy for healthcare access and quality. For non-cancer causes, death rates are risk-standardised using the formula:

$$RSD_{l,y,a,s} = D_{l,y,a,s} \times \left(1 - PAF_{l,y,a,s}\right) \times \frac{1}{1 - GPAF_{a,s}}$$

where $RSD_{l,y,a,s}$ is the risk-standardised deaths in location l, year y, age group a, and sex s; $D_{l,y,a,s}$ is the deaths for those specifications; $PAF_{l,y,a,s}$ is the PAF for those specifications; and $GPAF_{a,s}$ is the global PAF over all six estimation years for age group a, and sex s. If for a given cause no risk attribution is present or all deaths are attributed to risks (ie, PAF of 0 or 1), the observed deaths are used. If any cause

has a maximum observed mean joint-risk PAF greater than 0.9 but less than 1 for a given age and sex, PAFs across all location years are scaled such that the maximum is scaled down to 0.9.

Mortality-to-incidence ratios (MIRs) for cancers

Due to the expansion of cancer registry data quantity and quality in GBD 2016, we tested the use of MIRs instead of risk-standardised death rates for the HAQ Index.[4] MIRs were not produced for non-melanoma skin cancer (squamous-cell carcinoma) for GBD 2017 due to under-ascertainment of this cancer in registry data, and since MIRs calculated within the overall cancer cause-of-death estimation process are recalibrated to final mortality outputs, we constructed MIRs for each cancer based on post-CoDCorrect deaths (ie, a process that ensures internal consistently between all-cause mortality and cause-specific deaths) by age, sex, and location-year and final incidence estimates for the same dimensions.[5] We used the GBD 2017 final incidence and mortality estimates by age, sex, cancer, and location-year to generate the MIRs and limited the age groups to the bounds defined by the Nolte and McKee cause list. We age-standardised mortality and incidence rates per the age standardisation process described below, and then took the ratio of mortality to incidence to calculate MIRs.

Age-standardisation

Deaths outside of the defined age groups were eliminated,[1,2] as only deaths in those ages were deemed highly amenable to healthcare. We then aggregated cause-specific mortality rates by sex to both sexes. Using the GBD population age standard, we compiled risk-standardised mortality rates for non-cancers and the initial components of cancer MIRs (ie, mortality and incidence) for both sexes by location, year, and amenable cause:

$$RSASD_{l,y} = \sum_{a=1}^{n} RSD_{l,y,a} \times PAS_a$$

here $RSASD_{l,y,d}$ is the age-standardised risk-standardised death rate or component parts for MIRs for location l, and year y; $RSD_{l,y,a}$ is the risk-standardised death rate or component parts for MIRs in location l, year y, and age group a; and $PAS_a$is the population age standard for age group a.

Creating the UHC service coverage index and projections based on past trends

Using the above methodology, we created 1,000 draws of location-year index values for 32 causes of death amenable to healthcare. In combination with the 1,000 draws for each of the nine intervention coverage indicators, we created a composite measure – the UHC service coverage index – based on these 41 components.

To do this, we first log-transformed each of the amenable causes and then scaled them on a scale of 0 to 100, with 0 being the worst observed from 1990 to 2017 and 100 being the best observed during this time. No transformation was needed for the intervention coverage measures, so we applied the same scaling approach to them as well.

We then took the arithmetic mean of each of the 41 components to construct the UHC service coverage index, by country, from 1990 to 2017. We then projected the UHC service coverage index from 2018 to 2030 on the basis of past trends; additional detail on the projection methodologies used in the present study can be found in this appendix.

References

1 Barber RM, Fullman N, Sorensen RJD, *et al.* Healthcare Access and Quality Index based on mortality from causes amenable to personal health care in 195 countries and territories, 1990–2015: a novel analysis from the Global Burden of Disease Study 2015. *The Lancet* 2017; **0**. DOI:10.1016/S0140-6736(17)30818-8.

2 GBD 2016 Healthcare Access and Quality Collaborators. Measuring performance on the Healthcare Access and Quality Index for 195 countries and territories and selected subnational locations: a systematic analysis from the Global Burden of Disease Study 2016. *The Lancet* Under review.

3 Gakidou E, Afshin A, Abajobir AA, *et al.* Global, regional, and national comparative risk assessment of 84 behavioural, environmental and occupational, and metabolic risks or clusters of risks, 1990–2016: a systematic analysis for the Global Burden of Disease Study 2016. *The Lancet* 2017; **390**: 1345–422.

4 Naghavi M, Abajobir AA, Abbafati C, *et al.* Global, regional, and national age-sex specific mortality for 264 causes of death, 1980–2016: a systematic analysis for the Global Burden of Disease Study 2016. *The Lancet* 2017; **390**: 1151–210.

5 Vos T, Abajobir AA, Abate KH, *et al.* Global, regional, and national incidence, prevalence, and years lived with disability for 328 diseases and injuries for 195 countries, 1990–2016: a systematic analysis for the Global Burden of Disease Study 2016. *The Lancet* 2017; **390**: 1211–59.

3.8.1 UHC Service Coverage Index – Immunizations SDG Capstone Appendix

Flowchart

3.8.1 UHC – Immunization Coverage Estimation Flowchart

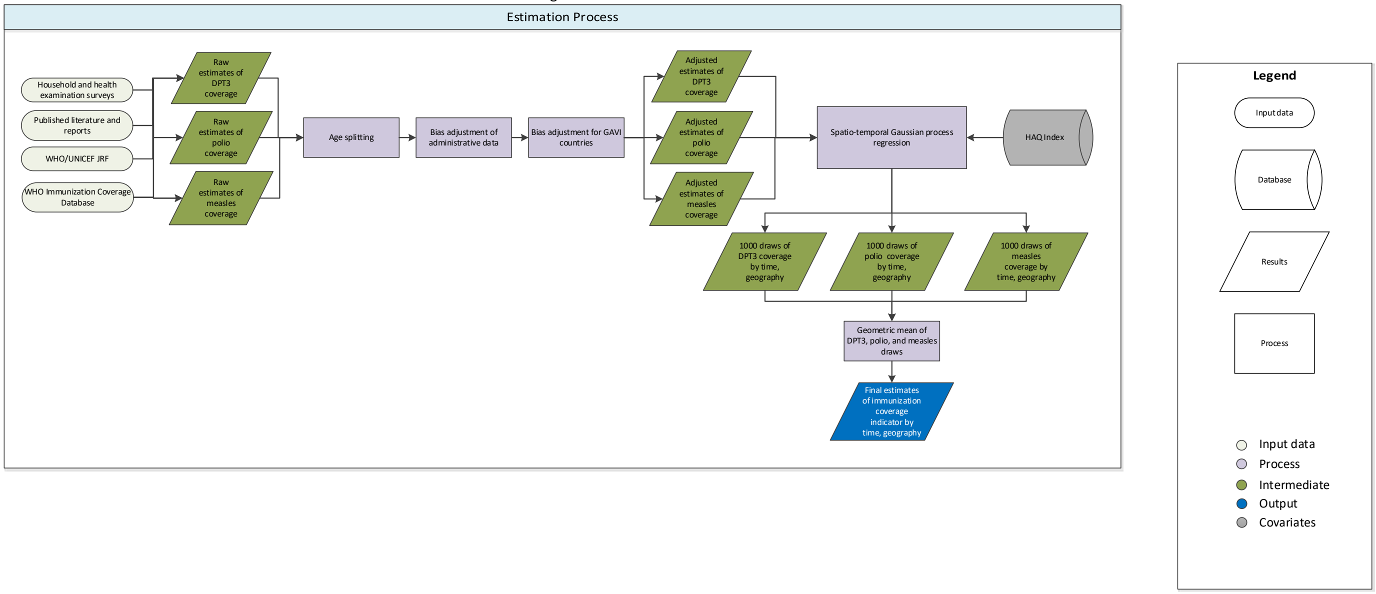

Input data & Methodological summary

Indicator definition

This modeling strategy pertains to the composite universal health coverage (UHC) service coverage index (Indicator 3.8.1) and specifically the estimation of immunization coverage for diphtheria-pertussis-tetanus (DTP3), measles vaccine, and three doses of the oral polio vaccine or inactivated polio vaccine (OPV3 or IPV3).

Indicator 3.8.1

As a component of SDG Goal 3. Ensure healthy lives and promote well-being for all at all ages, SDG Target 3.8, achieve universal health coverage, including financial risk protection, access to quality essential health-care services and access to safe, effective, quality and affordable essential medicines and vaccines for all, is measured using SDG Indicator 3.8.1, three measures of immunization coverage: DTP3, measles, and polio (OPV3 and/or IPV3) among children aged 12 to 23 months.

Input data

The present study used data from household-level surveys as well as administrative reports of immunization coverage. Survey data which provided person-level information on immunization were identified and extracted. Major multi-country survey programs included in the analysis include the Demographic and Health Surveys (DHS),[1] Multiple Indicator Cluster Surveys (MICS),[2] Reproductive Health Surveys (RHS),[3] Living Standards Measurement Study (LSMS) surveys,[4] and World Health Surveys (WHS).[5]

We also conducted a comprehensive search of the Global Health Data Exchange (GHDx),[6] as well as targeted internet searches and review of Ministry of Health websites, to identify national surveys and other multi-country survey programs.

Administrative estimates of immunization coverage were obtained from the Joint Reporting Process,[7] through which the World Health Organization (WHO) and UNICEF collates annual estimates of immunization coverage reported UN member states. These immunization coverage estimates are separate from those synthesized by WHO, and are calculated by dividing the number of doses of a given vaccine delivered to the target population (i.e., children aged 12 to 23) by the number of individuals in that target population.

We excluded all data sources that were not nationally representative or had high levels of missingness. We applied survey weights based on survey sampling frames whenever they were available to generate weighted national estimates of vaccination coverage accompanied by estimates of standard error (SE). Estimates of SE, as well as sample sizes, were used to calculate uncertainty, as described below. Any point estimates with sample sizes less than 50 were reviewed to ensure that they were not substantive outliers and would otherwise have an undue influence on our analysis.

Modeling strategy

Data processing

Age splitting

Most household surveys collect information on maternal and child health (MCH) indicators for children under 5 and/or mothers who gave birth within five years prior to the time of survey. To maximize data use for our model, we included immunization data for children aged 12 to 59 at the time of survey. Children younger than 12 months of age were excluded to minimize the influence of potentially censored observations. For each vaccine, coverage estimates were assigned to birth-cohort years based on a child's age prior to the time of survey: we used responses recorded for children aged 12 to 23 months for immunization coverage for one year prior to the time of survey, children aged 24 to 35 months for coverage two years prior to the time of survey, and so forth.

Age-specific estimates are easily computed from individual-level microdata, but many published reports and survey summaries present data in broader age aggregates (e.g., DTP3 coverage for children aged 12 to 35 months). To standardize these age groups, we applied an age-splitting model used in the GBD study,[8] as well as analyses that generated smoking and obesity prevalence by age group.[9,10]

Using surveys with microdata as the reference, we used the following model to generate standardized age group-specific estimates of immunization coverage:

$$\tilde{P}_{a,c,t,k} = P_{a,c,t,k}^{a+x} \frac{P_{a,c,t,j}}{P_{a,c,t,j}^{a+x}}$$

where $\tilde{P}_{a,c,k}$ is the adjusted estimate of coverage for target age group a in country c and year t of survey k; and $P_{a,c,k}^{a+x}$ is coverage reported from survey k, for country c in year t for the age group spanning age a to age $(a + x)$. The ratio of coverage between the target age group and broader age group from a survey j with microdata from the same country-year was used to split data from survey k. Surveys to be split

were ideally matched with DHS or MICS surveys. If microdata were not available for the same year, ratios within five years of the survey that required age-splitting were applied.

Administrative bias adjustment

Intervention coverage estimates based on administrative sources can be biased. Such biases may arise for a number of reasons, including discrepancies in the accurate reporting of services or interventions provided (e.g., number of vaccine doses administered) and target population (e.g., number of children in need of vaccines), as well as capturing these data in a timely manner from both public and private-sector facilities and health care providers. We implemented a vaccine-specific bias adjustment process to account for bias in administrative reports of immunization coverage in the JRF. Given that the magnitude, direction, and cause of such biases are heterogeneous across space, time, and antigen,[11,12] a vaccine-specific, time-varying, all-location bias correction factor was used for all three antigens.

For immunization coverage, we view individual-level data collected through population health surveys as the most accurate and least biased source of information of vaccination coverage, particularly for geographies with incomplete health information systems. We thus compute administrative bias as the ratio between estimates of coverage from surveys (where available) and matched administrative coverage. We model this bias in a spatiotemporal Gaussian process regression (ST-GPR) framework, described further in the subsequent section of this appendix, using the Socio-demographic Index (SDI) as a predictor. This method allows us to estimate antigen-specific administrative bias factors for all geographies and years since 1980, even in places without survey data, by borrowing strength in data across space and time. The GPR framework properly estimates prediction errors in the data synthesis procedure by for uncertainty in bias ratios when generating fitted values. In this framework, more weight is given to survey data with less uncertainty.

Antigen-specific modeled estimates of administrative bias are then used to adjust administrative coverage data for over- or under-reporting to reflect observed survey coverage. Adjusted administrative data are used as inputs into the trend estimation process.

Trend estimation

We used a spatiotemporal Gaussian process regression (ST-GPR) to synthesize point estimates from multiple data sources and derive a complete time series for each vaccine. This method has been used extensively in GBD and related studies, and accounts for uncertainty pertaining to each point estimate while borrowing strength across geographic space and time.[10, 11,15,16] Briefly, we assumed the Gaussian process was defined by a mean function *m* n o ri n e un tion *Cov* .

We estimated the mean function using a two-step approach. Specifically, $m_c(t)$ can be expressed as:

$$m_c(t) = X\beta + h(r_{c,t})$$

where $X\beta$ is a linear model and $h(r_{c,t})$ is a smoothing function for the residuals; and $r_{c,t}$ is derived from the linear model. The following linear model was used to model DTP3, measles, and polio coverage:

$$logit(P_{c,t}) = \beta_0 + \beta_1 \mathrm{HAQ_{c,t}} + \alpha_c + \gamma_{R[c]} + \omega_{\mathrm{SR[c]}} + \varepsilon_{c,t}$$

where $P_{c,t}$ is vaccination coverage for country c year t; $HAQ_{c,t}$ is value of the Healthcare Access and Quality Index[15] for country c and year t; α_c, $\gamma_{R[c]}$, and $\omega_{\mathrm{SR[c]}}$ are country, region, and super-region random intercepts, respectively. These estimates were then modeled through ST-GPR.

Random draws of 1,000 samples were obtained from the distributions above for every country for a given vaccine. Ninety-five percent uncertainty intervals were calculated by taking the ordinal 25th and 975th draws from the sample distribution.

To assess the accuracy of our modeled estimates, we performed cross-validation analyses using a knockout structure as previously described[16]. ST-GPR hyperparameters were selected on models that minimized the overall root-mean squared error (RMSE) of the model across a set of 10 knockouts.

References

1 Measure DHS: Demographic and Health Surveys. http://www.measuredhs.com (accessed Aug 11, 2015).

2 UNICEF Stat. Monit. Multiple Indicator Cluster Survey (MICS). http://www.unicef.org/statistics/index_24302.html (accessed Aug 11, 2015).

3 Cent. Dis. Control Prev. Reproductive Health Surveys (RHS). http://www.cdc.gov/reproductivehealth/Global/surveys.htm (accessed Aug 11, 2015).

4 World Bank. Living Standard Measruement Studies (LSMS). http://go.worldbank.org/UK1ETMHBN0 (accessed Aug 11, 2015).

5 WHO Multi-Ctry. Stud. Data Arch. World Health Survey (WHS). http://apps.who.int/healthinfo/systems/surveydata/index.php/catalog/whs/about (accessed Aug 11, 2015).

6 IHME GHDx. Global Helath Data Exchange. http://ghdx.healthdata.org/ (accessed Aug 11, 2015).

7 WHO | WHO/UNICEF Joint Reporting Process. WHO. http://www.who.int/immunization/monitoring_surveillance/routine/reporting/reporting/en/ (accessed Aug 17, 2015).

8 Prospective Studies Collaboration. Age-specific relevance of usual blood pressure to vascular mortality: a meta-analysis of individual data for one million adults in 61 prospective studies. *The Lancet* 2002; **360**: 1903–13.

9 Ng M, Freeman MK, Fleming TD, *et al.* Smoking Prevalence and Cigarette Consumption in 187 Countries, 1980-2012. *JAMA* 2014; **311**: 183.

10 Ng M, Fleming T, Robinson M, *et al.* Global, regional, and national prevalence of overweight and obesity in children and adults during 1980–2013: a systematic analysis for the Global Burden of Disease Study 2013. *The Lancet* 2014; **384**: 766–81.

11 Murray CJ, Shengelia B, Gupta N, Moussavi S, Tandon A, Thieren M. Validity of reported vaccination coverage in 45 countries. *The Lancet* 2003; **362**: 1022–7.

12 Haddad S, Bicaba A, Feletto M, Fournier P, Zunzunegui MV. Heterogeneity in the validity of administrative-based estimates of immunization coverage across health districts in Burkina Faso: implications for measurement, monitoring and planning. *Health Policy Plan* 2010; **25**: 393–405.

13 Lim SS, Stein DB, Charrow A, Murray CJ. Tracking progress towards universal childhood immunisation and the impact of global initiatives: a systematic analysis of three-dose diphtheria, tetanus, and pertussis immunisation coverage. *The Lancet* 2008; **372**: 2031–46.

14 Wang H, Liddell CA, Coates MM, *et al.* Global, regional, and national levels of neonatal, infant, and under-5 mortality during 1990–2013: a systematic analysis for the Global Burden of Disease Study 2013. *The Lancet* 13; **384**: 957–79.

15 Barber RM, Fullman N, Sorensen RJD, *et al.* Healthcare Access and Quality Index based on mortality from causes amenable to personal health care in 195 countries and territories, 1990?2015: a novel analysis from the Global Burden of Disease Study 2015. *The Lancet* 2017; published online May. DOI:10.1016/S0140-6736(17)30818-8.

16 Foreman KJ, Lozano R, Lopez AD, Murray CJ. Modeling causes of death: an integrated approach using CODEm. *Popul Health Metr* 2012; **10**: 1.

3.8.1 UHC Service Coverage Index: Antenatal Care (ANC) SDG Capstone Appendix

Flowchart

3.8.1 UHC – ANC1/ANC4 Coverage Estimation Flowchart

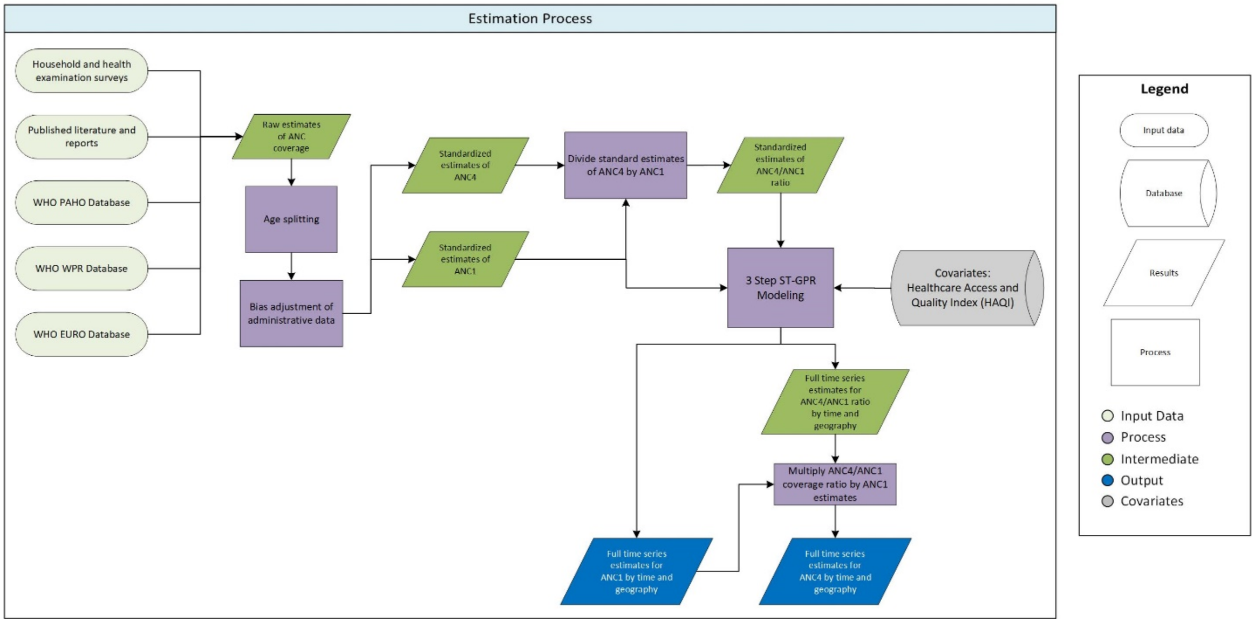

Input data & Methodological summary

Indicator definition

This modeling strategy pertains to the composite universal health coverage (UHC) service coverage index (Indicator 3.8.1) and specifically the estimation of antenatal care (ANC), as defined by the proportion of women who attended at least one ANC visit (ANC1) and/or at least four ANC visits (ANC4) for previous births, as provided by a skilled attendant.

Indicator 3.8.1b

As a component of SDG Goal 3. Ensure healthy lives and promote well-being for all at all ages, SDG Target 3.8, achieve universal health coverage, including financial risk protection, access to quality essential health-care services and access to safe, effective, quality and affordable essential medicines and vaccines for all, is measured using SDG Indicator 3.8.1b, ANC1 and ANC4 coverage.

Input data

For the present analysis, we used individual-level microdata from population health surveys and tabulated survey report data on skilled ANC1 and ANC4. As defined by the World Health Organization (WHO), ANC is considered attended by a skilled health professional when a doctor, nurse, or trained midwife are in attendance.[1]

We identified and extracted survey data which provided individual-level data, and specifically among female respondents. Major multi-country survey programs included in the analysis include the Demographic and Health Surveys (DHS),[1] Multiple Indicator Cluster Surveys (MICS),[2] Reproductive Health Surveys (RHS),[3] Living Standards Measurement Study (LSMS) surveys,[4] and World Health Surveys (WHS).[5] We also conducted a comprehensive search of the Global Health Data Exchange (GHDx),[6] as well as targeted internet searches and reviews of Ministry of Health websites, to identify national surveys and other multi-country survey programs. In addition, we utilized tabulated report data from regional WHO databases when available, including the PAHO[7], WHO WPR[8], and the WHO European Health for All databases[9].

We excluded all data sources that were not nationally representative or had high levels of missingness. We applied survey weights based on survey sampling frames whenever they were available to generate weighted national estimates of ANC1 and ANC4 coverage accompanied by estimates of standard error (SE). Estimates of SE, as well as sample sizes, were used to calculate uncertainty, as described below. Any point estimates with sample sizes less than 50 were reviewed to ensure that were not substantive outliers and would otherwise have an undue influence on our analysis.

Due to potential bias in recall, we limited our analysis to women who gave birth up to five years prior to the time of survey; due to data limitations, we used a limit of up to two years for some surveys. We also had to standardize the definition of “skilled health professional” across countries, which varied by differences in quality of training or health professional roles. For this analysis, doctors, nurses, and midwives were included as our foundational definition for skilled ANC, and we extended this to include country-specific medical staff based on the number of years of training they received and/or their comparable ability to intervene in an emergency situation (e.g., clinical officers). Care received during an ANC visit by traditional health personnel was not considered a skilled ANC visit.

Modeling strategy

Data processing

Age splitting

Most household surveys collection information on maternal and child health (MCH) indicators for children under 5 and/or mothers who gave birth within five years prior to the time of survey. To maximize data use for our model, we included ANC information for children aged 12 to 59 months at the time of survey. Children younger than 12 months of age were excluded to minimize the influence of potentially censored observations. ANC coverage estimates were assigned to birth-cohort years based on a child’s age prior to the time of survey: we used responses recorded for children aged 12 to 23 months for ANC coverage for one year prior to the time of survey, children aged 24 to 35 months for coverage two years prior to the time of survey, and so forth.

Age-specific estimates are easily computed from individual-level microdata, but many published reports and survey summaries present data in broader age aggregates (eg, ANC coverage for children aged 12 to 35 months). To standardize these age groups, we applied an age-splitting model used in the GBD study,[10] as well as analyses that generated smoking and obesity prevalence by age group.[11,12]

Using surveys with microdata as the reference, we used the following model to generate standardized age group-specific estimates for ANC:

$$\tilde{P}_{a,c,t,k} = P^{a+x}_{a,c,t,k} \frac{P_{a,c,t,j}}{P^{a+x}_{a,c,t,j}}$$

where $\tilde{P}_{a,c,k}$ is the adjusted estimate of coverage for target age group a in country c and year t of survey k; and $P^{a+x}_{a,c,k}$ is coverage reported from survey k, for country c in year t for the age group spanning age a to age $(a + x)$. The ratio of coverage between the target age group and broader age group from a survey j with microdata from the same country-year was used to split data from survey k. Surveys to be split were ideally matched with DHS or MICS surveys. If microdata were not available for the same year, ratios within five years of the survey that required age-splitting were applied.

Bias adjustments

Intervention coverage estimates based on administrative sources can be biased, yet the direction and magnitude of such biases are not universal. Some studies show that coverage estimates from administrative data source are systematically higher than those of survey-based estimates,[13] while other studies show that bias directionality is more heterogeneous.[14] Such biases may arise for a number of reasons, including discrepancies in the accurate reporting of services or interventions provided (eg, number of ANC visits) and target population (eg, number of children born), as well as capturing these data in a timely manner from both public and private sector facilities and healthcare providers.

For ANC, we view individual-level data collected through population health surveys as the most accurate and least biased source of information, particularly for geographies with incomplete health information systems. We thus used ANC coverage estimates from household surveys to calculate country-specific adjustment factors:

$$logit(P_{s,\,c,t}) = \beta_0 + \beta_1 logit(\tilde{P}_{a,\,c,t}) + \sum_{k=2}^{2+B} \beta_k S_k + \varepsilon_{c,t}$$

where $P_{s,\,c,t}$ is the survey-based estimate for ANC coverage (s) in country c for year t; $\tilde{P}_{a,\,c,t}$ is the administrative estimate for coverage in country c in year t; S_k is a spline basis used to capture the secular trend in coverage; β_1 is the estimated adjustment factor used to correct for the administrative bias; and ε is the error term for country c in year t.

To quantify uncertainty for bias-adjusted estimates from the mixed-effects models described above, we calculated prediction error, $\widehat{PE}$, as follows:

$$\widehat{PE} = X^2 var(\hat{\beta})$$

where $var(\hat{\beta})$ is the variance for the estimated fixed-effects coefficient of the adjustment factor and X is the independent variable. Proper estimation of prediction errors is crucial as the data synthesis procedure, Gaussian process regression (GPR) (as described in the subsequent section), accounts for uncertainty from point estimates and bias adjustments when generating fitted values. More weight is given to data with less uncertainty. Prediction errors estimated from the bias adjustment were

incorporated into the data variance and propagated through the GPR step to obtain estimates of ANC coverage and uncertainty intervals (UIs).

To assess the accuracy of our estimates in the bias adjustment, we performed cross-validation analyses by randomly holding out 20% of the sample and, if available, the corresponding administrative estimates for the given indicator of the same country and year, 10 separate times. We computed the average root mean squared errors (RMSE) across each country. Error in the bias adjustments was calculated as the mean difference between the adjusted administrative estimate for a given country, year, and corresponding survey-level estimates (which were considered the “gold-standard”).

Trend estimation

We used a spatiotemporal Gaussian process regression (ST-GPR) to synthesize point estimates from multiple data sources and derive a complete time series for ANC coverage. This method has been used extensively in GBD and related studies, and accounts for uncertainty pertaining to each point estimate while borrowing strength across geographic space and time.[10, 11,15,16] Briefly, we assumed the Gaussian process was defined by a mean function *m* n o ri n e un tion *Cov* .

We estimated the mean function using a two-step approach. Specifically, $m_c(t)$ can be expressed as:

$$m_c(t) = X\beta + h(r_{c,t})$$

where $X\beta$ is a linear model and $h(r_{c,t})$ is a smoothing function for the residuals; and $r_{c,t}$ is derived from the linear model. The following linear model was used for the estimation of ANC indicators:

$$logit(P_{c,t}) = \beta_0 + \beta_1 \mathrm{HAQ_{c,t}} + \alpha_c + \gamma_{R[c]} + \omega_{\mathrm{SR[c]}} + \varepsilon_{c,t}$$

where $P_{c,t}$ is ANC coverage for country c year t; $HAQ_{c,t}$ is value of the Healthcare Access and Quality Index[16] for country c and year t; α_c, $\gamma_{R[c]}$, and $\omega_{\mathrm{SR[c]}}$ are country, region, and super-region random intercepts, respectively. These estimates were then modeled through ST-GPR.

By definition, point estimates from a given survey-year for ANC4 cannot exceed ANC1. To ensure definitional consistency for levels of ANC1 and ANC4 coverage, we estimated the coverage of ANC4 by first calculating the ratio of ANC4/ANC1 by survey-year, modeling the ratio of ANC4/ANC1 through ST-GPR, and subsequently multiplying out by the final estimated coverage of ANC4.

Random draws of 1,000 samples were obtained from the distributions above for every country for a given vaccine. Ninety-five percent uncertainty intervals were calculated by taking the ordinal 25th and 975th draws from the sample distribution.

To assess the accuracy of our modeled estimates, we performed cross-validation analyses using a knockout structure as previously described[17]. ST-GPR hyper-parameters were selected on models that minimized the overall root-mean squared error (RMSE) of the model across a set of 10 knockouts.

References

1 Measure DHS: Demographic and Health Surveys. http://www.measuredhs.com (accessed Aug 11, 2015).

2 UNICEF Stat. Monit. Multiple Indicator Cluster Survey (MICS). http://www.unicef.org/statistics/index_24302.html (accessed Aug 11, 2015).

3 Cent. Dis. Control Prev. Reproductive Health Surveys (RHS). http://www.cdc.gov/reproductivehealth/Global/surveys.htm (accessed Aug 11, 2015).

4 World Bank. Living Standard Measruement Studies (LSMS). http://go.worldbank.org/UK1ETMHBN0 (accessed Aug 11, 2015).

5 WHO Multi-Ctry. Stud. Data Arch. World Health Survey (WHS). http://apps.who.int/healthinfo/systems/surveydata/index.php/catalog/whs/about (accessed Aug 11, 2015).

6 IHME GHDx. Global Health Data Exchange. http://ghdx.healthdata.org/ (accessed Aug 11, 2015).

7 PAHO/WHO Data - Home. http://www.paho.org/data/index.php/en/ (accessed June 12, 2017).

8 WPR - Health Information & Intelligence Platform > Data & analytics. http://hiip.wpro.who.int/portal/Dataanalytics.aspx (accessed June 12, 2017).

9 European Health for All Family of Databases (HFA-DB). 2017; published online June 12. http://www.euro.who.int/en/data-and-evidence/databases/european-health-for-all-family-of-databases-hfa-db (accessed June 12, 2017).

10 Prospective Studies Collaboration. Age-specific relevance of usual blood pressure to vascular mortality: a meta-analysis of individual data for one million adults in 61 prospective studies. *The Lancet* 2002; **360**: 1903–13.

11 Ng M, Freeman MK, Fleming TD, *et al.* Smoking Prevalence and Cigarette Consumption in 187 Countries, 1980-2012. *JAMA* 2014; **311**: 183.

12 Ng M, Fleming T, Robinson M, *et al.* Global, regional, and national prevalence of overweight and obesity in children and adults during 1980–2013: a systematic analysis for the Global Burden of Disease Study 2013. *The Lancet* 2014; **384**: 766–81.

13 Murray CJ, Shengelia B, Gupta N, Moussavi S, Tandon A, Thieren M. Validity of reported vaccination coverage in 45 countries. *The Lancet* 2003; **362**: 1022–7.

14 Haddad S, Bicaba A, Feletto M, Fournier P, Zunzunegui MV. Heterogeneity in the validity of administrative-based estimates of immunization coverage across health districts in Burkina Faso: implications for measurement, monitoring and planning. *Health Policy Plan* 2010; **25**: 393–405.

15 Wang H, Liddell CA, Coates MM, *et al.* Global, regional, and national levels of neonatal, infant, and under-5 mortality during 1990–2013: a systematic analysis for the Global Burden of Disease Study 2013. *The Lancet* 13; **384**: 957–79.

16 Barber RM, Fullman N, Sorensen RJD, *et al.* Healthcare Access and Quality Index based on mortality from causes amenable to personal health care in 195 countries and territories, 1990?2015: a novel analysis from the Global Burden of Disease Study 2015. *The Lancet* 2017; published online May. DOI:10.1016/S0140-6736(17)30818-8.

17 Foreman KJ, Lozano R, Lopez AD, Murray CJ. Modeling causes of death: an integrated approach using CODEm. *Popul Health Metr* 2012; **10**: 1.

3.8.1 UHC Service Coverage Index: In-Facility Birth Rate (IFD) SDG Capstone Appendix

Input data & Methodological summary

3.8.1 UHC – In-Facility Delivery Coverage Estimation Flowchart

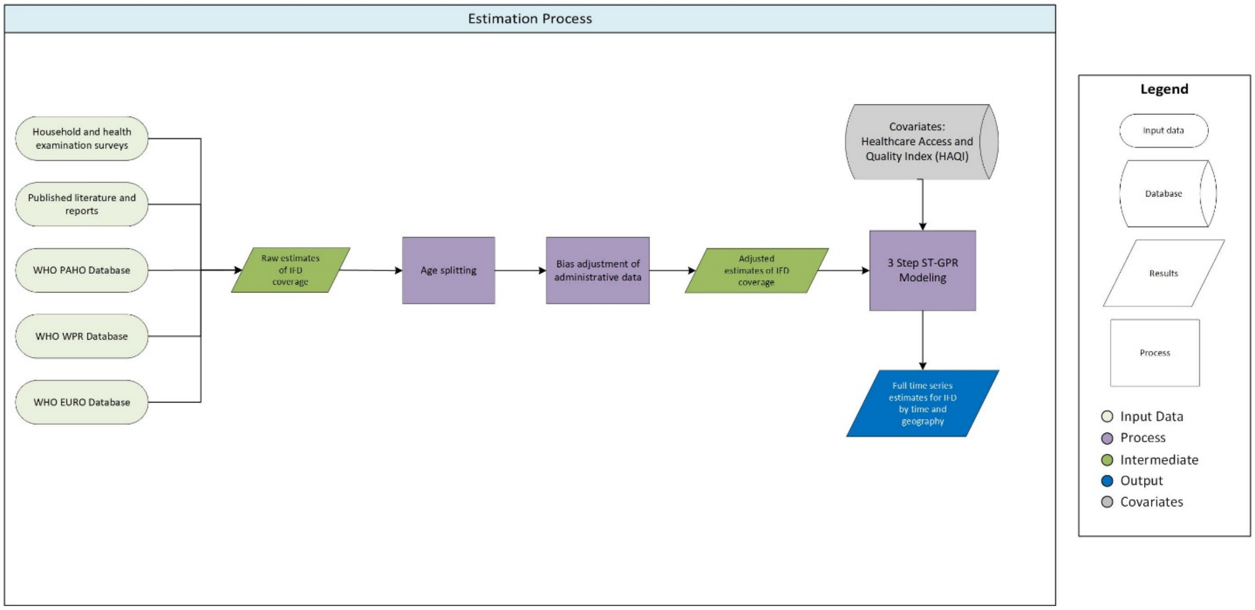

Indicator definition

This modeling strategy pertains to the composite universal health coverage (UHC) service coverage index (Indicator 3.8.1) and specifically the estimation of in-facility delivery (IFD), as defined by the proportion of births that were delivered in a health facility.

Indicator 3.8.1

As a component of SDG Goal 3. Ensure healthy lives and promote well-being for all at all ages, SDG Target 3.8, achieve universal health coverage, including financial risk protection, access to quality essential health-care services and access to safe, effective, quality and affordable essential medicines and vaccines for all, is measured using SDG Indicator 3.8.1c, in-facility birth rate (IFD).

Input data

Our study included data from household-level surveys as well as administrative reports of in-facility delivery (IFD), defined by WHO as the proportion of births in a given year delivered in a health facility.[1] Survey data which provided person-level information were identified and extracted. Major multi-country survey programs included in the analysis include the Demographic and Health Surveys (DHS)[1], the Multiple Indicator Cluster Surveys (MICS)[2], the Reproductive Health Surveys (RHS)[3], the Living Standards Measurement Studies[4], and the World Health Surveys (WHS)[5]. In additional, a comprehensive search was performed on the Global Health Data Exchange (GHDx)[6], as well as a targeted Google search and a search

on the websites of national ministries of health, to identify national surveys and smaller multi-country surveys. In addition we utilized administrative estimates from regional WHO databases, when available, including the PAHO[7], WHO WPR[8], and the WHO European Health for All databases[9].

We excluded all data sources that were not nationally representative or had high levels of missingness. We applied survey weights based on survey sampling frames whenever they were available to generate weighted national estimates of IFD coverage accompanied by estimates of standard error (SE). Estimates of SE, as well as sample sizes, were used to calculate uncertainty, as described below. Any point estimates with sample sizes less than 50 were reviewed to ensure that there were no substantive outliers and would otherwise have an undue influence on our analysis.

Modeling strategy

Data processing

Age Splitting

Household-level surveys typically collect information about MCH indicators for children under 5 years of age or mothers who have given birth at most 5 years prior to the time of survey. For the sake of utilizing as much data as available, we incorporated estimates for births 0–59 months prior to the survey for analysis. For each indicator, estimates were assigned to a given birth cohort year based on the birth age prior to the time of interview – we used the responses recorded for children aged 12–23 months to estimate coverage 1 year prior to the survey, 24–35 months to estimate coverage 2 years prior to the survey, and so forth.

While information aggregated to these specific age ranges was easily extracted from surveys with person-level data, many published reports and summaries of surveys presented data in broader age groups. We disaggregated these data into the age grouping of interest in this study by applying a splitting model previously used in the Global Burden of Diseases, Injuries, and Risk Factors Study (GBD)[10], as well as in a studies estimating global smoking[11] and obesity prevalence[12].

Using surveys that provided person-level data as references, the following model was applied on estimates with the broader age groups. Specifically, let $\tilde{P}_{a,c,k}$ be the adjusted estimate of coverage for a given indicator for the target age group a in country c and year t of survey k. To disaggregate data that were reported in a broader age group, the following formula was used:

$$\tilde{P}_{a,c,t,k} = P_{a,c,t,k}^{a+x} \frac{P_{a,c,t,j}}{P_{a,c,t,j}^{a+x}}$$

Where $P_{a,c,k}^{a+x}$ denotes the coverage reported from survey k, for country c in year t, but of the age group spanning age a to age $(a + x)$. The ratio of coverage between the age group of interest and the broader age group from a survey j with person-level data from the same country and year was used to split data

from survey k. Surveys to be split were ideally matched with DHS or MICS surveys. If person-level data were not available for the same year, data within five years to be split were used.

Bias adjustments

Administrative estimates of IFD are most typically produced using data gathered from supply-side registries. The quality and accuracy of the data therefore depends on the completeness of the nation's health information system.[13] Previous studies have reported that administrative reports of MCH coverage indicators tend to be biased.[8,12,13]

To reduce the impact of these biases on the final results, we performed adjustments on administrative data to account for overall systematic error. Using mixed effects models, we compared administrative data and survey data to derive appropriate adjustment ratios:

$$logit(P_{s,\,c,t}) = \beta_0 + \beta_1 logit(\tilde{P}_{a,\,c,t}) + \varepsilon_{c,t}$$

where $P_{s,\,c,t}$ is the survey-based coverage for a specific indicator for country c in year t, $\tilde{P}_{a,\,c,t}$ is the administrative coverage for country *c* in year *t*, β_1 is the estimated adjustment factor used to correct for the administrative bias.

To assess the accuracy of our estimates in the bias adjustment, we performed cross-validation analyses by randomly holding out 20% of the sample and, if available, the corresponding administrative estimates for the given indicator of the same country and year, 10 separate times. We computed the average root mean squared errors (RMSE) across each country. Error in the bias adjustments was calculated as the mean difference between the adjusted administrative estimate for a given country, year, and corresponding survey-level estimates (which were considered the "gold-standard").

Trend estimation

We used a spatiotemporal Gaussian process regression (ST-GPR) to synthesize information from the various data sources in order to derive a complete time series for each indicator for all countries. This method has been used extensively in other studies to combine information from different sources, taking into account uncertainty for each data point as well as to interpolate nonlinear trends by borrowing strength across geographic space and time.[9-11] Briefly, we assumed the Gaussian process was defined by a mean function *m* n o ri n e un tion *Cov* .

We estimated the mean function using a two-step approach. Specifically, $m_c(t)$ can be expressed as:

$$m_c(t) = X\beta + h(r_{c,t})$$

where $X\beta$ is a linear model and $h(r_{c,t})$ is a smoothing function for the residuals; and $r_{c,t}$ is derived from the linear model. The following linear model was used for the estimation of ANC indicators:

$$logit(P_{c,t}) = \beta_0 + \beta_1 \mathrm{HAQ_{c,t}} + \alpha_c + \gamma_{R[c]} + \omega_{\mathrm{SR[c]}} + \varepsilon_{c,t}$$

where $P_{c,t}$ is IFD coverage for country c year t; $HAQ_{c,t}$ is value of the Healthcare Access and Quality Index[15] for country c and year t; α_c, $\gamma_{R[c]}$, and $\omega_{\mathrm{SR[c]}}$ are country, region, and super-region random intercepts, respectively. These estimates were then modeled through ST-GPR.

Random draws of 1,000 samples were obtained from the distributions above for every country for a given vaccine. Ninety-five percent uncertainty intervals were calculated by taking the ordinal 25th and 975th draws from the sample distribution.

To assess the accuracy of our modeled estimates, we performed cross-validation analyses using a knockout structure as previously described[16]. ST-GPR hyperparameters were selected on models that minimized the overall root-mean squared error (RMSE) of the model across a set of 10 knockouts.

References

1 Measure DHS: Demographic and Health Surveys. http://www.measuredhs.com (accessed Aug 11, 2015).

2 UNICEF Stat. Monit. Multiple Indicator Cluster Survey (MICS). http://www.unicef.org/statistics/index_24302.html (accessed Aug 11, 2015).

3 Cent. Dis. Control Prev. Reproductive Health Surveys (RHS). http://www.cdc.gov/reproductivehealth/Global/surveys.htm (accessed Aug 11, 2015).

4 World Bank. Living Standard Measruement Studies (LSMS). http://go.worldbank.org/UK1ETMHBN0 (accessed Aug 11, 2015).

5 WHO Multi-Ctry. Stud. Data Arch. World Health Survey (WHS). http://apps.who.int/healthinfo/systems/surveydata/index.php/catalog/whs/about (accessed Aug 11, 2015).

6 IHME GHDx. Global Helath Data Exchange. http://ghdx.healthdata.org/ (accessed Aug 11, 2015).

7 PAHO/WHO Data - Home. http://www.paho.org/data/index.php/en/ (accessed June 12, 2017).

8 WPR - Health Information & Intelligence Platform > Data & analytics. http://hiip.wpro.who.int/portal/Dataanalytics.aspx (accessed June 12, 2017).

9 European Health for All Family of Databases (HFA-DB). 2017; published online June 12. http://www.euro.who.int/en/data-and-evidence/databases/european-health-for-all-family-of-databases-hfa-db (accessed June 12, 2017).

10 Prospective Studies Collaboration. Age-specific relevance of usual blood pressure to vascular mortality: a meta-analysis of individual data for one million adults in 61 prospective studies. *The Lancet* 2002; **360**: 1903–13.

11 Ng M, Freeman MK, Fleming TD, *et al.* Smoking Prevalence and Cigarette Consumption in 187 Countries, 1980-2012. *JAMA* 2014; **311**: 183.

12 Ng M, Fleming T, Robinson M, *et al.* Global, regional, and national prevalence of overweight and obesity in children and adults during 1980–2013: a systematic analysis for the Global Burden of Disease Study 2013. *The Lancet* 2014; **384**: 766–81.

13 Murray CJ, Shengelia B, Gupta N, Moussavi S, Tandon A, Thieren M. Validity of reported vaccination coverage in 45 countries. *The Lancet* 2003; **362**: 1022–1027.

14 Haddad S, Bicaba A, Feletto M, Fournier P, Zunzunegui MV. Heterogeneity in the validity of administrative-based estimates of immunization coverage across health districts in Burkina Faso: implications for measurement, monitoring and planning. *Health Policy Plan* 2010; **25**: 393–405.

15 Barber RM, Fullman N, Sorensen RJD, *et al.* Healthcare Access and Quality Index based on mortality from causes amenable to personal health care in 195 countries and territories, 1990?2015: a novel analysis from the Global Burden of Disease Study 2015. *The Lancet* 2017; published online May. DOI:10.1016/S0140-6736(17)30818-8.

16 Foreman KJ, Lozano R, Lopez AD, Murray CJ. Modeling causes of death: an integrated approach using CODEm. *Popul Health Metr* 2012; **10**: 1.

3.8.1 UHC Index: Tuberculosis SDG Capstone Appendix

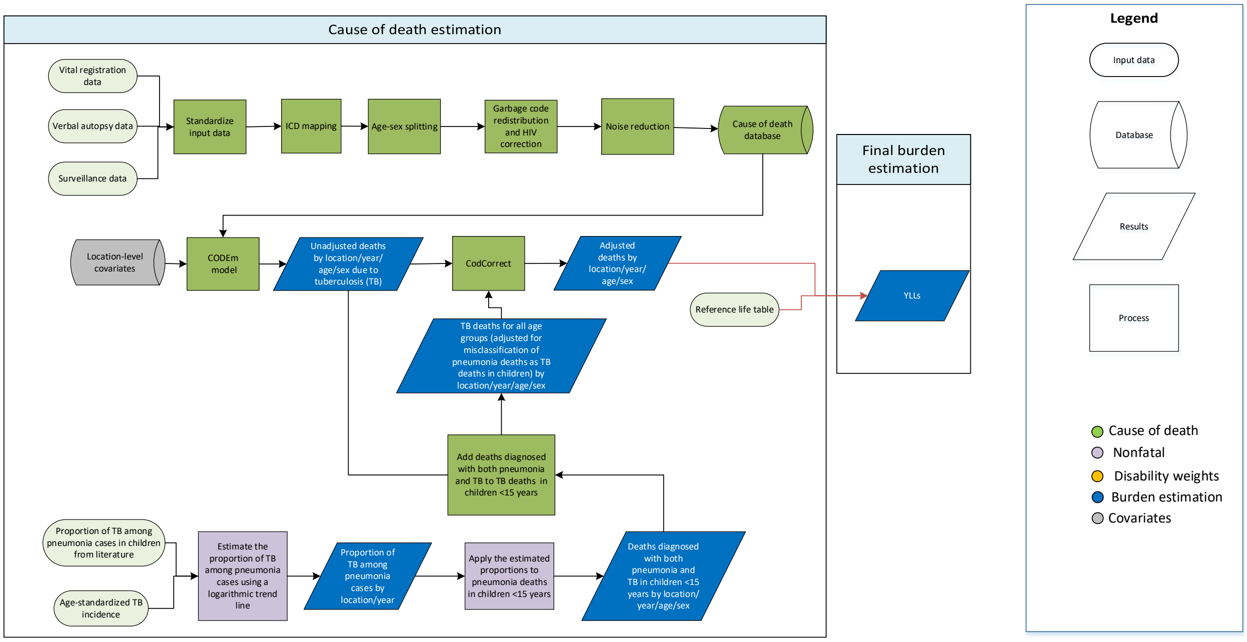

Input data

Input data for modelling tuberculosis (TB) mortality among HIV-negative individuals include vital registration, verbal autopsy, and surveillance data. Vital registration data were adjusted for garbage coding (including ill-defined codes and the use of intermediate causes) following GBD algorithms and misclassified HIV deaths (ie, HIV deaths being assigned to other underlying causes of death such as tuberculosis or diarrhea because of stigma or misdiagnosis).

Verbal autopsy data in countries with age-standardised HIV prevalence greater than 5% were removed because of a high probability of misclassification, as verbal autopsy studies have poor validity in distinguishing HIV deaths from HIV-TB deaths.

Modelling strategy

A general CODEm modelling strategy was used. We added a new covariate, namely the TB strain prevalence-weighted transmission risk. We also included the cigarettes per capita covariate. Other location-level covariates included in the CODEm model were the same as in GBD 2016: adult underweight proportion, alcohol (litres per capita), diabetes (fasting plasma glucose mmol/L), education (years per capita), Healthcare Access and Quality Index, lag-distributed income, indoor air pollution, outdoor air pollution, population density, prevalence of active tuberculosis, prevalence of latent tuberculosis infection, smoking prevalence, Socio-demographic Index, and a summary exposure variable reflecting the average exposure to all of the risk factors.

Covariate table

	Covariate	Direction
Level 1	TB prevalence<br>Latent TB infection prevalence<br>SEV scalar<br>Alcohol per capita<br>Smoking prevalence<br>Cigarettes per capita<br>Fasting plasma glucose<br>TB strain prevalence-weighted transmission risk	+<br>+<br>+<br>+<br>+<br>+<br>+<br>+
Level 2	HAQ Index<br>Adult underweight proportion<br>Indoor air pollution<br>Outdoor air pollution<br>Population density	-<br>+<br>+<br>+<br>+
Level 3	Log LDI<br>Education (years per capita)<br>Socio-demographic Index (SDI)	-<br>-<br>-

Correcting for a potential misclassification of tuberculosis deaths as pneumonia deaths in children

In locations with high TB burden, TB deaths may be misclassified as pneumonia deaths in children,[1] and we addressed this potential misclassification in GBD 2017. First, we estimated the proportion of tuberculosis among pneumonia cases as a function of age-standardised TB incidence using data from eight clinical studies[2,3,4,5,6,7,8,9] reporting the proportion of pneumonia cases that had tuberculosis (or the data to calculate them) and the age-standardised TB incidence estimates. We used a logarithmic trend line to fit these data. Next, we applied the estimated proportions to pneumonia deaths (estimated for GBD 2017) among children younger than 15 years to compute the number of deaths diagnosed with both pneumonia and TB, which were then added to child TB deaths from the CODEm model. Finally, these estimates were adjusted using CoDCorrect, which ensures that the number of deaths from each cause add up to all-cause mortality deaths for a given year.

References

1. Graham SM, Sismanidis C, Menzies HJ, Marais BJ, Detjen AK, Black RE. Importance of tuberculosis control to address child survival. *Lancet* 2014; **383**(9928): 1605-7.
2. Adegbola RA, Falade AG, Sam BE, et al. The etiology of pneumonia in malnourished and well-nourished Gambian children. Pediatr Infect Dis J 1994; 13: 975–82.

3. Chisti MJ, Graham SM, Duke T, et al. A prospective study of the prevalence of tuberculosis and bacteraemia in Bangladeshi children with severe malnutrition and pneumonia including an evaluation of Xpert MTB/RIF assay. PloS One 2014; 9: e93776.
4. Madhi SA, Petersen K, Madhi A, Khoosal M, Klugman KP. Increased disease burden and antibiotic resistance of bacteria causing severe community-acquired lower respiratory tract infections in hu n i uno e ien irus t e -infected children. Clin Infect Dis 2000; 31: 170–76.
5. een ee et . e t o e o i robi ise se n tern status on treatment response and cause of severe pneumonia in South African children: a prospective descriptive study. Lancet 2007; 369: 1440–51.
6. Moore DP, Klugman KP, Madhi SA. Role of Streptococcus pneumoniae in hospitalisation for acute community-acquired pneumonia associated with culture- on r e ob teriu tuber u osis in children: a pneumococcal conjugate vaccine probe study. Pediatr Infect Dis J 2010; 29: 1099–104.
7. Nantongo JM, Wobudeya E, Mupere E, et al. High incidence of pulmonary tuberculosis in children admitted with severe pneumonia in Uganda. BMC Pediatr 2013; 13: 16.
8. Zar HJ, Hanslo D, Tannenbaum E, et al. Aetiology and outcome of pneumonia in human i uno e ien irus-infected children hospitalized in South Africa. Acta Paediatr 2001; 90: 119–25.
9. Moore DP, Higdon MM, Hammitt LL, Prosperi C, DeLuca AN, Da Silva P, Baillie VL, Adrian PV, Mudau A, Deloria Knoll M, Feikin DR. The incremental value of repeated induced sputum and gastric aspirate samples for the diagnosis of pulmonary tuberculosis in young children with acute community-acquired pneumonia. Clinical Infectious Diseases. 2017 May 27;64(suppl_3):S309-16.

TB strain prevalence-weighted transmission risk covariate

We added a new TB covariate added in this round of GBD that incorporated data on the global distribution of TB strains and the relative risk of transmission associated with those strains. For this covariate, we defined TB strains according to the seven phylogenetic lineages of the *Mycobacterium tuberculosis* complex (MTBC) identified by S. Gagneaux and colleagues.[1] We determined the global distribution of these strains using a systematic review of human TB molecular epidemiology studies from 1990 to 2017 in PubMed and Scopus, as described in greater detail elsewhere.[2] All studies that used population-based sampling methods or collected isolates from all culture-positive TB cases in a given location and time period were included. All genotypes that could be converted to phylogenetic lineages were extracted, including genotypes determined by spoligotyping, MIRU-VNTR typing, and PCR or whole-genome sequencing. Studies of sub-populations, such as prison populations or drug-resistant cases only, were excluded. In total, 206 studies representing 85 countries and over 200,000 bacterial isolates were included. A map of these strains highlighted the widespread global distribution of Euro-American Lineage 4 strains and East Asian Lineage 2 strains, and the geographic restriction of Lineage 5 and 6 strains to West Africa. Thirty of these studies also reported transmission chains associated with bacterial genotypes, as defined by genetic clustering.[3]

We used spatiotemporal Gaussian process regression (ST-GPR) to model the distribution of each strain in each GBD location across all ages and sexes, as described in greater detail elsewhere.[4] The covariates tested in each model included HIV age-standardised prevalence, population density, and a custom-made human movement covariate. The human movement covariate took into account (1) immigration and emigration patterns[5] and (2) airplane passenger flow[6] to and from each country. In the

ST-GPR models we assumed strong correlation and smoothing over both space and time. We then used a random-effects meta-analysis to determine the relative risk (RR) of transmission associated with each strain, as defined by genetic clustering. We used the most widespread strains, Euro-American Lineage 4 strains, as the reference group. We found that East Asian Lineage 2 strains were associated with increased risk of transmission overall (Relative Risk [95% CI] = 1.24 [1.07, 1.45]), while West African Lineage 5 and 6 strains were associated with reduced transmission (Relative Risk [95% CI] = 0.61 [0.43, 0.86]). We used the following formula to calculate a TB strain prevalence-weighted risk of transmission based on these estimates:

$$\sum_{i=1}^{n} Pr_i RR_i$$ i=TB strain; Pr=proportion; RR=relative risk

References

1. Comas I, Coscolla M, Luo T, *et al.* Out-of-Africa migration and Neolithic coexpansion of *Mycobacterium tuberculosis* with modern humans. *Nat Genet* 2013; **45**: 1176–82.
2. Wiens KE, Woyczynski LP, Ledesma JR, *et al*. Global variation in bacterial strains that cause tuberculosis disease: a systematic review and meta-analysis. *BMC Medicine*; In Review.
3. Dheda K, Gumbo T, Maartens G, *et al.* The epidemiology, pathogenesis, transmission, diagnosis, and management of multidrug-resistant, extensively drug-resistant, and incurable tuberculosis. *Lancet Respir Med* 2017; **5**: 291–360.
4. Manuscript in preparation.
5. United Nations Population Division. United Nations Trends in International Migrant Stock: The 2015 Revision. New York City, United States: United Nations Population Division, 2015.
6. Huang Z, Wu X, Garcia AJ, *et al*. An open-access modeled passenger flow matrix for the global air network in 2010. *PLoS ONE* **8(5)**: e64317.

3.8.1 UHC Index: Diarrheal Diseases SDG Capstone Appendix

Diarrheal diseases

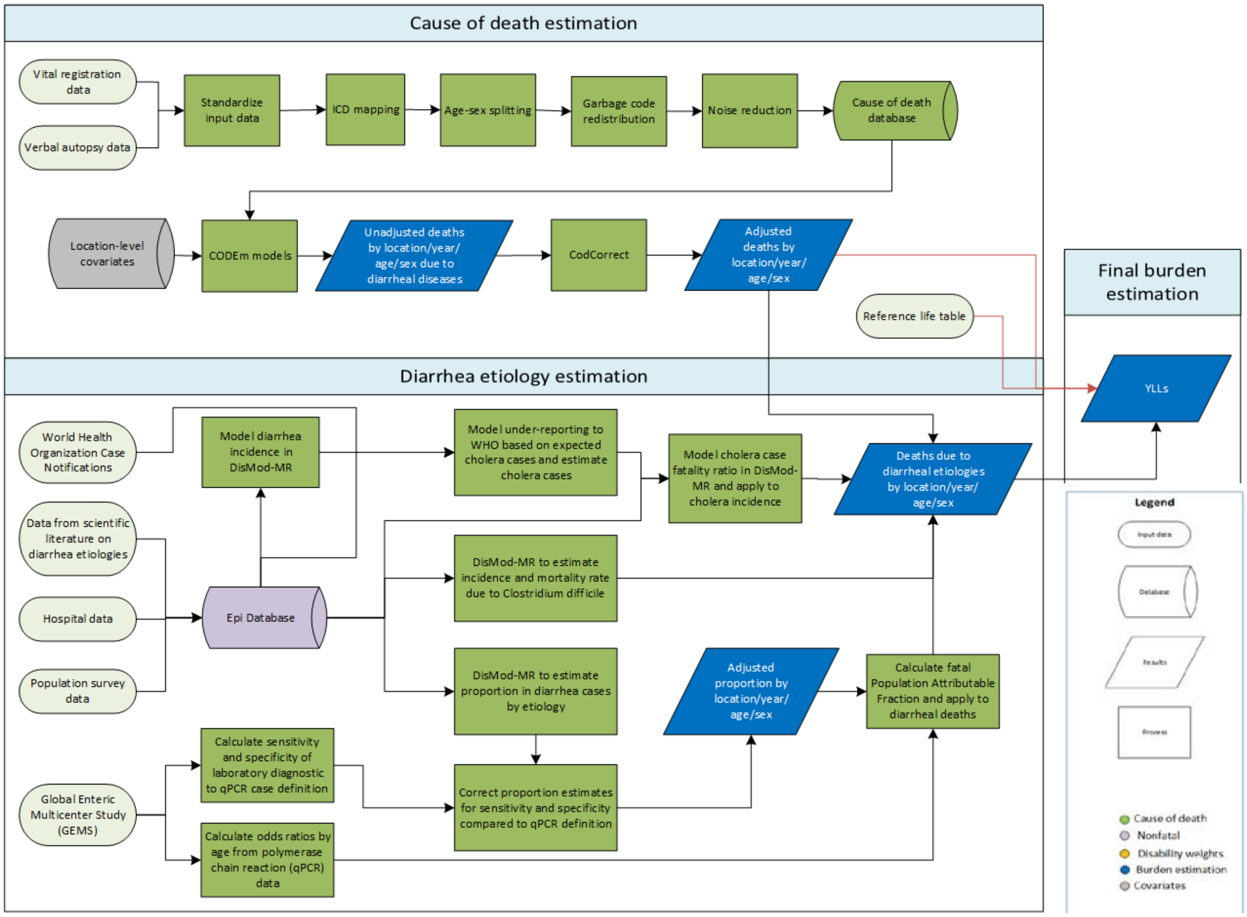

Input data

Cause of death. We used all available data from vital registration systems, surveillance systems and verbal autopsy (**Table 1 and Figure 1**). We checked for and excluded outliers from our data by country or region. We also excluded early neonatal mortality data in the Philippines (1994–1998) and India Civil Registration System data and medically certified cause of death (MCCD) data in all states (1986–2013).

Aetiologies. We conducted a systematic literature review for the proportion of diarrhoea cases that tested positive for each aetiology. We updated our review of literature to include studies published between January 2016 and May 2017. The search was performed in PubMed using the following search string:

(diarrhoea[title/abstract] OR diarrhea[title/abstract]) AND (2016/01/01:2017/12/31[PDat]) AND Humans[MeSH Terms] AND (incidence[title/abstract] OR prevalence[title/abstract] OR epidemiology[title/abstract] OR salmonella[title/abstract] OR aeromonad*[title/abstract] OR shigell*[title/abstract] OR enteropathogenic[title/abstract] OR enterotoxigenic[title/abstract] OR campylobacter[title/abstract] OR amoebiasis[title/abstract] OR entamoeb*[title/abstract] OR cryptosporid*[title/abstract] OR rotavirus[title/abstract] OR norovirus[title/abstract] OR adenovirus[title/abstract] OR etiology[title/abstract]) NOT (appendicitis[title/abstract] OR esophag*[title/abstract] OR surger*[title/abstract] OR gastritis[title/abstract] OR liver[title/abstract] OR case report[title] OR case-report[title] OR therapy[title] OR treatment[title] Crohn[title/abstract] OR "inflammatory bowel"[title/abstract] OR irritable[title/abstract] OR travel*[title] OR Outbreak[title] OR Review[ptyp] OR vomiting[title/abstract)

Inclusion criteria included diarrhoea as the case definition, studies with a sample size of at least 100, and studies with at least one year of follow-up. We excluded studies that reported on diarrhoeal outbreaks exclusively and those that used acute gastroenteritis with or without diarrhoea. We identified 225 studies, of which 51 met our criteria of inclusion and were included. We extracted data points for location, sex, year, and age. We assigned an age range based on the prevalence-weighted mean age of diarrhoea in the appropriate year/sex/location if the age of the study participants was not reported.

We used the Global Enteric Multicenter Study (GEMS), a seven-site, case-control study of moderate-to-severe diarrhoea in children under 5 years,[1] to calculate odds ratios for the diarrhoeal pathogens based on a molecular diagnostic case definition.

For GBD 2017, we added an additional 40,000 stool samples analyzed using quantitative polymerase chain reaction (qPCR) from The Etiology, Risk Factors, and Interactions of Enteric Infections and Malnutrition and the Consequences for Child Health and Development Project (MAL-ED) study to the roughly 10,000 samples from the GEMS reanalysis.[2–5]

Modelling strategy

Cause of death. Diarrhoeal disease mortality was estimated in the Cause of Death Ensemble modelling platform (CODEm). We estimated diarrhoea mortality separately for males and females and for children under 5 years and older than 5 years. We used country-level covariates to inform our CODEm models (Table 2). We evaluated our diarrhoeal disease cause of death models using in and out of sample predictive performance.

Aetiologies. We estimated diarrhoeal disease aetiologies independently from overall diarrhoea mortality using a counterfactual strategy for enteric adenovirus, *Aeromonas*, *Entamoeba histolytica* (amoebiasis), *Campylobacter*, *Cryptosporidium*, typical enteropathogenic *Escherichia coli* (t-EPEC), enterotoxigenic *Escherichia coli* (ETEC), norovirus, non-typhoidal salmonella

infections, rotavirus, and *Shigella*. *Vibrio cholerae* and *Clostridium difficile* were modelled separately.

Diarrhoeal aetiologies are attributed to diarrhoeal deaths using a counterfactual approach. We calculated a population attributable fraction (PAF) from the proportion of severe diarrhoea cases that are positive for each aetiology. The PAF represents the relative reduction in diarrhoea mortality if there was no exposure to a given aetiology. As diarrhoea can be caused by multiple pathogens and the pathogens may co-infect, PAFs can overlap and add up to more than 100%. We calculated the PAF from the proportion of severe diarrhoea cases that are positive for each aetiology. We assumed that hospitalised diarrhoea cases are a proxy of severe and fatal cases. We used the following formula to estimate PAF:[6]

$$PAF = Proportion * (1 - \frac{1}{OR})$$

Where *Proportion* is the proportion of diarrhoea cases positive for an aetiology and *OR* is the odds ratio of diarrhoea given the presence of the pathogen.

We dichotomised the continuous qPCR test result using the value of the cycle threshold (Ct) that most accurately discriminated between cases and controls. The Ct values range from 0 to 35 cycles representing the relative concentration of the target gene in the stool sample. A low value indicates a higher concentration of the pathogen while a value of 35 indicates the absence of the target in the sample. We used the lower Ct value when we had multiple Ct values for the cutpoint. The case definition for each pathogen is a Ct value that is below the established cutoff point.

We used a mixed effects conditional logistic regression model to calculate the odds ratio for under 1 year and 1-4 years old for each of our pathogens. The stool samples from cases and controls in GEMS were used exclusively to calculate these odds ratios as we assumed that the association between pathogens and moderate-to-severe diarrhoea is a proxy for fatal outcomes. The odds ratio for 1-4 years was applied to all GBD age groups over 5 years. There were three pathogen-age odds ratios that were not statistically significant: Aeromonas and Amoebiasis in under 1 year and Campylobacter in 1-4 years. The mean value of the odds ratio was above 1 in all three cases so we transformed the odds ratios for these three exceptions only in log-space such that exponentiated values could not be below 1. The transformation was:

$$\text{Odds ratio} = exp(log(OR) - 1)) + 1$$

We modelled the proportion data using the meta-regression tool DisMod-MR to estimate the proportion of positive diarrhoea cases for each separate aetiology by location/year/age/sex and to adjust for the covariates.

We used the estimated sensitivity and specificity of the original laboratory diagnostic test results from the pooled GEMS and MALED qPCR stool samples compared to the qPCR test result to adjust our proportion before we computed the PAF:[7]

$$Proportion_{True} = \frac{(Proportion_{Observed} + Specificity - 1)}{(Sensitivity + Specificity - 1)}$$

We used this correction to account for the fact that the proportions we used are based on a new test that is not consistent with the laboratory-based case definition (qPCR versus GEMS conventional laboratory testing for pathogens).[8] Because differences in the type of PCR used in the original (non-reference qPCR diagnostic) between GEMS and MALED in detecting norovirus, we combined the sensitivity and specificity results for norovirus such that 50% of the draws were coming from GEMS test results exclusively and 50% of the draws were coming from MALED test results exclusively. Additionally, because the original laboratory diagnostic technique used for *Campylobacter* in MALED was one not commonly used, we only used GEMS to determine the sensitivity and specificity of bacterial culture compared to qPCR in detecting *Campylobacter.*[9]

Our literature review extracted the proportion of any enteropathogenic *Escherichia coli* (EPEC) without differentiating between typical (tEPEC) and atypical (aEPEC). In order to be consistent with the odds ratios that we obtained, we adjusted our proportion estimates of any EPEC to typical EPEC only. This adjustment was informed by a subset of our literature review that reported both atypical and typical EPEC. We estimated a ratio by super-region of tEPEC to any EPEC and adjusted our proportion estimates accordingly. We found that the majority of EPEC diarrhoea cases were positive for atypical EPEC, consistent with other published work.[10]

For *Vibrio cholerae* (cholera), we used the literature review to estimate expected number of cholera cases for each country-year using the incidence of diarrhoea, estimated using DisMod-MR, and the proportion of diarrhoea cases that are positive for cholera. We assigned cholera PAF using odds ratios from the qPCR results to estimate a number of cholera-attributable cases. We compared this expected number of cholera cases to the number reported to the World Health Organization at the country-year level.[11] We modelled the underreporting fraction to correct the cholera case notification data for all countries using health system access and the diarrhoea SEV scalar to predict total cholera cases. We used the age-specific proportion of positive cholera samples in DisMod and our incidence estimates to predict the number of cholera cases for each age/sex/year/location. Finally, we modelled the case fatality ratio of cholera using DisMod-MR and to estimate the number of cholera deaths.

For *C. difficile*, we modelled incidence and mortality in DisMod-MR for each age, sex, year, location. DisMod-MR is a Bayesian meta-regression tool that uses spatiotemporal information as priors to estimate prevalence, incidence, remission, and mortality for *C. difficile* infection. DisMod-MR uses a compartmental model to relate prevalence, incidence, remission, and mortality. We set remission in our model to 1 month.

Table 1. Cause-specific mortality input data.

Input data	GBD 2017
**Total data sources**	**19,665 geography-years**
Vital registration data	17,734 geography-years
Sample registration data	740 geography-years
Verbal autopsy data	1,042 geography-years
Surveillance data	509 geography-years

Figure 1. Number of geography-years of mortality data used in diarrhoea mortality modelling

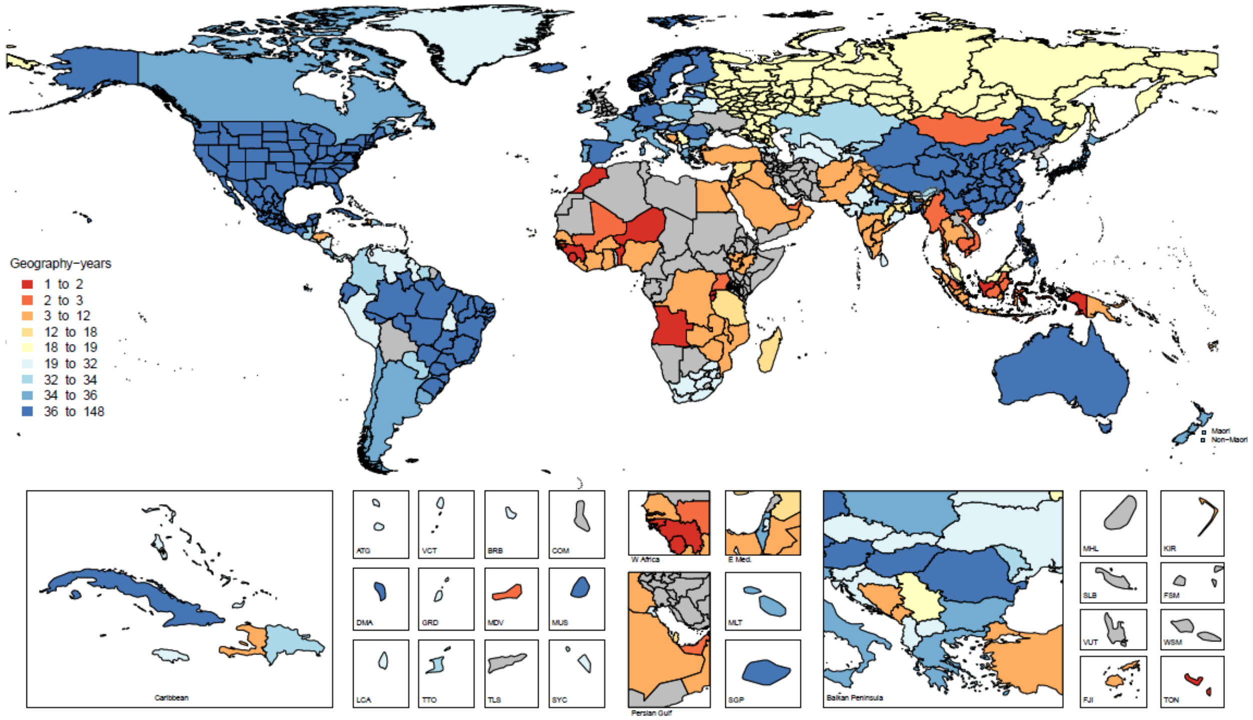

Table 2. The covariates used in diarrhoea mortality modelling. Table 2A shows the covariates used in the 0-4 years model and Table 2B shows the covariates used in the 5-95+ years model. The *Level* represents the strength of the association between the covariate and diarrhoea mortality from 1 (proximally related) to 3 (distally related). The *Direction* indicates the positive or negative association between the covariate and diarrhoea mortality.

Table 2A. The covariates used in the 0-4 years model

Level	Covariate	Direction
1	Diarrhoea SEV	+
	Childhood stunting SEV	+
	Sanitation SEV	+
	Water SEV	+
	Childhood underweight SEV	+
	Childhood wasting SEV	+
	Short gestation SEV	+
	Low weight gestation SEV	+
	Oral rehydration solution treatment	-
	Safe sanitation	-
	Safe water	-
2	Vitamin A deficiency	+
	Zinc deficiency	+
	Healthcare access and quality index	-
	Rotavirus vaccine	-
	Zinc treatment for diarrhoea	-
3	Breastfeeding SEV	+
	Handwashing	-
	LDI per capita	-
	Maternal education years	-
	Socio-demographic Index	-
	Population density < 150/km2	0
	Population density > 1000/km2	0

Table 2B. The covariates used in the 5-95+ years model.

Level	Covariate	Direction
1	Diarrhoea summary exposure variable	+
	Safe sanitation summary exposure variable	+
	Safe water summary exposure variable	+
	Improved sanitation	-
	Improved water	-
2	Healthcare access and quality index	-
	Rotavirus vaccine coverage	-
3	Education years per capita	-
	LDI per capita	-
	Mean BMI	-
	Socio-demographic Index	-
	Population density greater than 1000/km^2	0

References

1 Kotloff KL, Nataro JP, Blackwelder WC, *et al.* Burden and aetiology of diarrhoeal disease in infants and young children in developing countries (the Global Enteric Multicenter Study, GEMS): a prospective, case-control study. *Lancet Lond Engl* 2013; **382**: 209–22.

2 Platts-Mills JA, Babji S, Bodhidatta L, *et al.* Pathogen-specific burdens of community diarrhoea in developing countries: a multisite birth cohort study (MAL-ED). *Lancet Glob Health* 2015; **3**: e564-575.

3 MAL-ED Network Investigators. The MAL-ED study: a multinational and multidisciplinary approach to understand the relationship between enteric pathogens, malnutrition, gut physiology, physical growth, cognitive development, and immune responses in infants and children up to 2 years of age in resource-poor environments. *Clin Infect Dis Off Publ Infect Dis Soc Am* 2014; **59 Suppl 4**: S193-206.

4 Kotloff KL, Nataro JP, Blackwelder WC, *et al.* Burden and aetiology of diarrhoeal disease in infants and young children in developing countries (the Global Enteric Multicenter Study, GEMS): a prospective, case-control study. *Lancet Lond Engl* 2013; **382**: 209–22.

5 Liu J, Platts-Mills JA, Juma J, *et al.* Use of quantitative molecular diagnostic methods to identify causes of diarrhoea in children: a reanalysis of the GEMS case-control study. *Lancet Lond Engl* 2016; **388**: 1291–301.

6 Miettinen OS. Proportion of disease caused or prevented by a given exposure, trait or intervention. *Am J Epidemiol* 1974; **99**: 325–32.

7 Reiczigel J, Földi J, Ozsvári L. Exact confidence limits for prevalence of a disease with an imperfect diagnostic test. *Epidemiol Infect* 2010; **138**: 1674–8.

8 Platts-Mills JA, Operario DJ, Houpt ER. Molecular diagnosis of diarrhea: current status and future potential. *Curr Infect Dis Rep* 2012; **14**: 41–6.

9 Platts-Mills JA, Liu J, Gratz J, *et al.* Detection of Campylobacter in stool and determination of significance by culture, enzyme immunoassay, and PCR in developing countries. *J Clin Microbiol* 2014; **52**: 1074–80.

10 Ochoa TJ, Barletta F, Contreras C, Mercado E. New insights into the epidemiology of enteropathogenic Escherichia coli infection. *Trans R Soc Trop Med Hyg* 2008; **102**: 852–6.

11 World Health Organization. Global Health Observatory data repository: Cholera. 2016. http://apps.who.int/gho/data/node.main.174?lang=en (accessed Aug 25, 2016).

3.8.1 UHC Index: Lower Respiratory Infections SDG Capstone Appendix

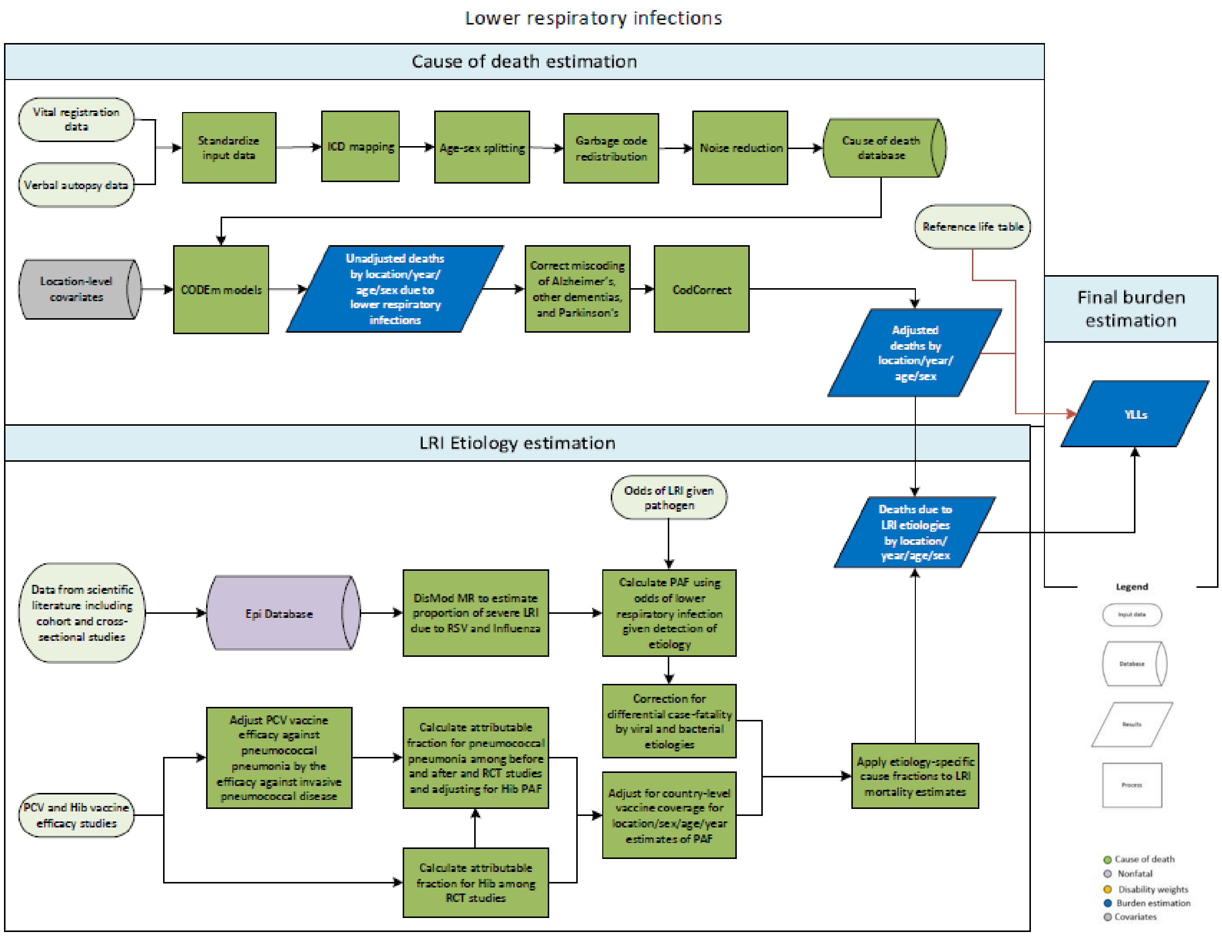

Input data

Cause of death

Lower respiratory infection (LRI) mortality was estimated in CODEm. We estimated LRI mortality separately for males and females and for children under 5 years and older than 5 years. We used all available data from vital registration systems, surveillance systems, and verbal autopsy (**Table 1**). We checked for and excluded outliers from our data by country or region. We also excluded ICD9-coded mortality data in Sri Lanka (1982, 1987–1992), ICD9-coded neonatal mortality data in Guatemala (1980, 1981, 1984, 2000–2004), and medically coded cause of death data (MCCD) and Civil Registration System data in many Indian states (1986–2013).

Table 1. Summary of cause-specific mortality modelling input data.

Input data	GBD 2017
**Total data sources**	**19,827 geography-years**
Vital registration data	17,374 geography-years
Sample registration data	740 geography-years
Verbal autopsy data	1,153 geography-years
Surveillance	560 geography-years

Figure 1. The number of geography-years of LRI mortality data by GBD geography is shown

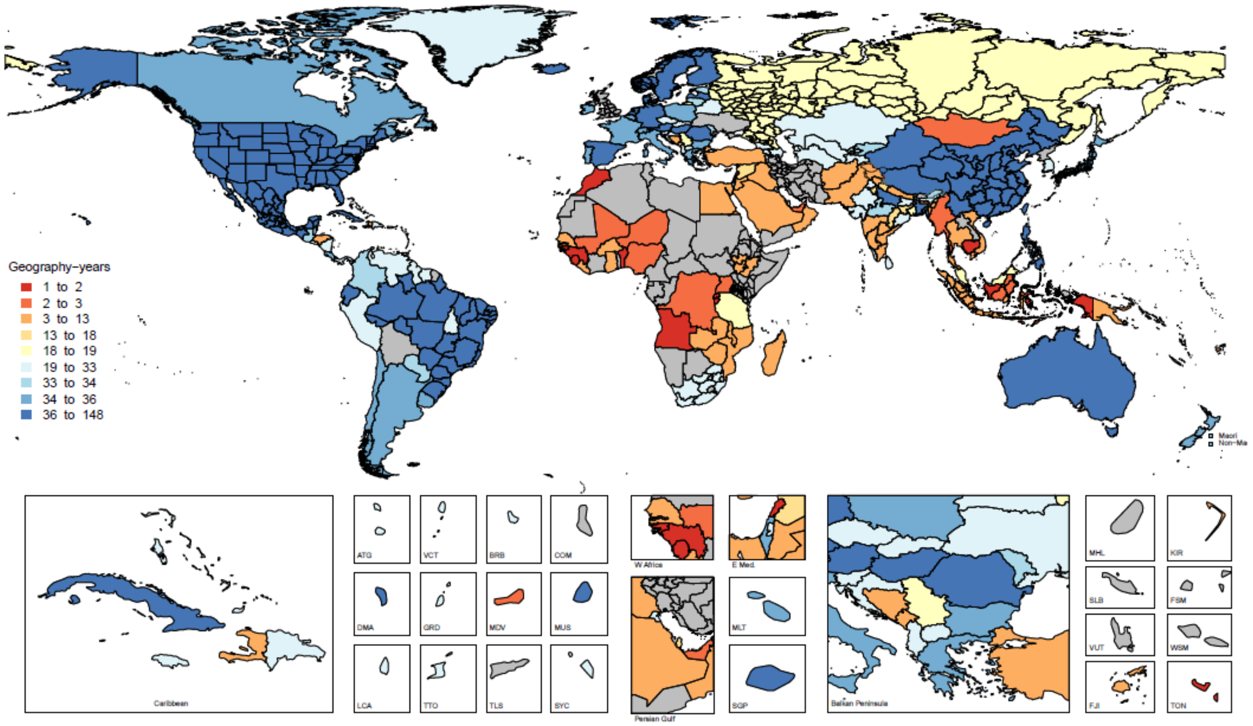

Aetiologies

We updated our systematic review of scientific literature for the proportion of LRI that tested positive for influenza and respiratory syncytial virus (RSV) to include all data from GBD 2016 and from studies published between January 1, 2016 and May 26, 2017. We performed the search using PubMed and the following search string:

("lower respiratory"[title/abstract] OR pneumonia[title/abstract]) AND (2016/01/01[PDat] : 2017/12/31[PDat]) AND (incidence OR prevalence OR epidemiology OR etiolog*[title/abstract] OR influenza[title/abstract] OR "respiratory syncytial virus"[title/abstract]) AND Humans[MeSH Terms] NOT(autoimmune[title/abstract] OR COPD [title/abstract] OR "cystic fibrosis"[title/abstract] OR Review[ptyp])

Inclusion criteria were studies that had a sample size of at least 100, studies that were at least one year in duration, and studies describing lower respiratory infections, pneumonia, or bronchiolitis as the case definition. During our literature review we identified 595 studies, of which 75 met our inclusion criteria and were extracted. We excluded studies that described pandemic H1N1 influenza solely and studies that used influenza-like illness as the case definition. We assigned an age range based on the prevalence-weighted mean age of LRI in the appropriate year/sex/location if the ages of the study participants were not reported.

We also conducted a systematic literature review of studies on the Hib vaccine and PCV effectiveness studies against X-ray-confirmed pneumonia and against pneumococcal and Hib disease until May 2017. For PCV studies, we extracted, if available, the distribution of pneumococcal pneumonia serotypes and the serotypes included in the PCV used in the study. No new studies were identified for GBD 2017. We excluded observational and case-control studies due to implausibly high vaccine efficacy estimates. Hib trial data were exclusively from children <5 years, so we did not include the effect of Hib on ages over 5 years of age. PCV trial data are also frequently limited to younger populations. To understand the contribution of pneumococcal pneumonia in older populations, we also included PCV efficacy studies that used before-after approaches.

Modelling strategy

Cause of death. We used country-level covariates to inform our CODEm models (**Table 2**). We evaluated our LRI cause of death models using in and out of sample predictive performance.

Like all models of mortality in GBD, LRI mortality models are single-cause, requiring in effect that the sum of all mortality models must be equal to the all-cause mortality envelope. We correct LRI mortality estimates, and other causes of mortality, by rescaling them according to the uncertainty around the cause-specific mortality rate. This process is called CoDCorrect and is essential to ensure internal consistency among causes of death. Before CoDCorrect, we also adjust LRI mortality for unreliable estimates due to improper death certification and ICD coding among elderly adults where the underlying cause of death should be Alzheimer's or Parkinson's disease. This process occurs in a step new to GBD 2016 called OldCorrect and scales LRI mortality among adult age groups 70+ years into a new envelope without Alzheimer's and Parkinson's. Further details can be found in the main text and appendix for the OldCorrect process.

Table 2. Covariates used in LRI mortality modelling. Table 2A is for children under 5 and Table 2B shows the covariates used for ages 5-95+. The *Level* is the associated strength of relationship between the covariate and LRI mortality, ranked from 1 (proximally related) to 3 (distally related). The direction is the forced direction of the association between the covariate and LRI mortality.

Table 2A. Covariates used in under 5 years model

Level	Covariate	Direction
1	Childhood stunting SEV	+
	Childhood underweight SEV	+
	Childhood wasted SEV	+
	Indoor air pollution	+
	Short gestation SEV	+
	Low weight gestation	+
	LRI summary exposure variable	+
	Second-hand smoking prevalence	+
	Antibiotics for LRI	-
	Hib vaccine coverage	-
	Pneumococcal conjugate vaccine coverage	-
2	Discontinued breastfeeding SEV	+
	Vitamin A deficiency	+
	Zinc deficiency	+
	DTP3 vaccine coverage	-
	Healthcare access and quality index	-
3	Outdoor air pollution ($PM_{2.5}$)	+
	Population density > 1000/km^2	+
	Sanitation SEV	+
	Handwashing	-
	LDI per capita	-
	Maternal education	-
	Socio-demographic Index	-

Table 2B. Covariates used in 5-95+ years model

Level	Covariate	Direction
1	Indoor air pollution	+
	LRI summary exposure variable	+
	Outdoor air pollution	+
	Secondhand smoking prevalence	+
	Smoking prevalence	+
2	DTP3 vaccine coverage	-
	Healthcare access and quality index	-
	Mean BMI	-
	Pneumococcal conjugate vaccine coverage	-
	Handwashing	+
3	Education years per capita	-
	LDI per capita	-

	Socio-demographic Index	-
	Alcohol consumption	+
	Sanitation summary exposure variable	+

Aetiologies

We estimated LRI aetiologies separately from overall LRI mortality using two distinct counterfactual modelling strategies to estimate population attributable fractions (PAFs), described in detail below. The PAF represents the relative reduction in LRI mortality if there was no exposure to a given aetiology. As LRIs can be caused by multiple pathogens and the pathogens may co-infect, PAFs can overlap and add up to more than 100%. Separate strategies were used for viral – influenza and respiratory syncytial virus (RSV) – and bacterial – *Streptococcus pneumoniae* and *Haemophilus influenzae* type B – aetiologies. We did not attribute aetiologies to neonatal pneumonia deaths due to a dearth of reliable data in this age group. We calculated uncertainty of our PAF estimates from 1,000 draws of each parameter using normal distributions in log space.

Influenza and RSV. We calculated the population attributable fraction (PAF) from the proportion of severe LRI cases positive for influenza and RSV. We assumed that hospitalised LRI cases are a proxy of severe cases. We used the following formula to estimate PAF:[1]

$$PAF = Proportion * (1-1/OR)$$

Where *Proportion* is the proportion of LRI cases that test positive for influenza or RSV and *OR* is the odds ratio of LRI given the presence of the pathogen. We used an odds ratio of 5.1 (3.19–8.14) for influenza and 9.79 (4.98–19.27) for RSV from a recently published meta-analysis.[2]

We modelled the proportion data using the meta-regression tool DisMod-MR to estimate the proportion of LRI cases that are positive for influenza and RSV, separately, by location/year/age/sex. We accounted for study-level covariates in our models such as PCR as the diagnostic technique, studies that investigated RSV or influenza exclusively, and studies from inpatient populations.

As the case-fatality of viral causes of pneumonia is lower than for bacterial causes, we adjusted for differential case-fatality by determining the aetiological fractions for mortality attributable to RSV and influenza (**Table 3**). We measured the aetiologic fractions by applying a relative case-fatality adjustment based on in-hospital case-fatality, which we coded to specific pneumonia aetiologies. Hospital admissions data of this type were limited to data from Austria, Brazil, Chile, China, Ecuador, Italy, Kenya, Mexico, New Zealand, the Philippines, Portugal, and the United States. We generated the pooled estimate of the case-fatality differential between bacterial (pneumococcus, Hib) and viral aetiologies (RSV, influenza) using DisMod-MR to determine an age pattern for this ratio.

Pneumococcal pneumonia and Hib. For *Streptococcus pneumoniae* (pneumococcal pneumonia) and *Haemophilus influenzae* type B (Hib), we calculated the population attributable fraction using a vaccine probe design.[3,4] The ratio of vaccine effectiveness against nonspecific pneumonia to pathogen-specific disease represents the fraction of pneumonia cases attributable to each pathogen.

To estimate the PAF for Hib and pneumococcal pneumonia, we calculated the ratio of vaccine effectiveness against nonspecific pneumonia to pathogen-specific pneumonia (Equations 1 and 3). We

estimated a study-level estimate of PAF from a meta-analysis of these ratios. To estimate the PAF for Hib, we only used randomised controlled trials because of implausibly high values of vaccine efficacy in case-control studies. To estimate the PAF for pneumococcal pneumonia, we included RCTs and before and after vaccine introduction longitudinal studies.

We adjusted the study-level PAF estimate by vaccine coverage and expected vaccine performance to estimate country- and year-specific PAF values. For pneumococcal pneumonia, we adjusted the PAF by the final Hib PAF estimate and by vaccine serotype coverage. Finally, we used an age distribution of PAF modelled in DisMod to determine the PAF by age. Because of an absence of data describing vaccine efficacy against Hib in children older than 2 years, we did not attribute Hib to episodes of LRI in ages 5 years and older.

We used a vaccine probe design to estimate the PAF for pneumococcal pneumonia and (Hib) by first calculating the ratio of vaccine effectiveness against nonspecific pneumonia to pathogen-specific pneumonia at the study level (Equations 1 and 2).[3–5] We then adjusted this estimate by vaccine coverage and expected vaccine performance to estimate country- and year-specific PAF values (Equations 3 and 4).

$$1)\quad HibPAF_{Base} = 1 - \frac{VE_{Pneumonia}}{VE_{Hib}}$$

$$2)\quad PneumoPAF_{Base} = 1 - \frac{VE_{Pneumonia} * (1 - PAF_{Hib} * VE_{Hib\ Optimal})}{VE_{Streptococcus} * Cov_{Serotype}}$$

$$3)\quad PAF_{Hib} = PAF_{Base} * \frac{(1 - Cov_{Hib} * VE_{Hib\ Optimal})}{(1 - PAF_{Base} * Cov_{Hib} * VE_{Hib\ Optimal})}$$

$$4)\quad PAF_{Pneumo} = \frac{PAF_{Base} * (1 - Cov_{PCV} * VE_{PCV\ Optimal})}{(1 - PAF_{Hib} * Cov_{Hib} * VE_{Hib\ Optimal}) * \left(1 - \frac{PAF_{Base} Cov_{PCV} * VE_{PCV\ Optimal}}{(1 - PAF_{Hib} * Cov_{Hib} * VE_{Hib\ Optimal})}\right)}$$

Where $VE_{Pneumonia}$ is the vaccine efficacy against nonspecific pneumonia, VE_{Hib} is the vaccine efficacy against invasive Hib disease, $VE_{Streptococcus}$ is the vaccine efficacy against serotype-specific pneumococcal pneumonia, $Cov_{serotype}$ is the serotype-specific vaccine coverage for PCV,[6] $VE_{Hib\ Optimal}$ is the Hib effectiveness in the community (0.8)[7], PAF_{Hib} is the final PAF for Hib, Cov_{PCV} is the PCV coverage, Cov_{Hib} is the Hib coverage by country, and $VE_{PCV\ Optimal}$ is the vaccine effectiveness in the community (0.8).[8]

For Hib, we assumed that the vaccine efficacy against invasive Hib disease is the same against Hib pneumonia. For pneumococcal pneumonia, a recent study in adults[9] found that the vaccine efficacy against invasive pneumococcal disease may be significantly higher than against pneumococcal pneumonia. We used this ratio to adjust estimates of vaccine efficacy against invasive pneumococcal disease from other studies. However, recognising that the study is unique in that it uses a urine antigen test among adults, we added uncertainty around our adjustment using a wide uniform distribution (median 0.65, 0.3–1.0). This has increased the estimates of pneumococcal pneumonia mortality in a meaningful way.

The only substantive changes to the cause of death estimation for LRI in GBD 2017 were the addition of new cause of death data and inclusion of several additional covariates. The ratio of mortality in bacterial to viral aaetiologies was updated for GBD 2017 and the new results substantively increase the attribution of influenza and RSV to LRI deaths.

Table 3: **The median values for the ratio of viral to bacterial pneumonia case fatality ratio by age is shown.** These estimates are modelled using hospital-based, ICD-coded admissions and mortality for aetiology-specified pneumonia. Values in parentheses represent 95% uncertainty interval.

Age Group	Ratio
Early Neonatal	0.59 (0.36–0.84)
Late Neonatal	0.58 (0.37–0.84)
Post Neonatal	0.58 (0.41–0.77)
1 to 4	0.69 (0.64–0.74)
5 to 9	0.85 (0.77–0.93)
10 to 14	0.84 (0.79–0.89)
15 to 19	0.83 (0.78–0.87)
20 to 24	0.82 (0.77–0.87)
25 to 29	0.82 (0.78–0.86)
30 to 34	0.82 (0.79–0.85)
35 to 39	0.82 (0.8–0.85)
40 to 44	0.82 (0.8-0.85)
45 to 49	0.82 (0.8–0.85)
50 to 54	0.82 (0.79–0.85)
55 to 59	0.82 (0.79–0.86)
60 to 64	0.82 (0.79–0.86)
65 to 69	0.82 (0.8–0.85)
70 to 74	0.82 (0.79–0.85)
75 to 79	0.82 (0.78–0.85)
80 to 84	0.83 (0.8–0.87)
85 to 89	0.86 (0.83–0.89)
90 to 94	0.89 (0.85–0.93)
95 to 99	0.92 (0.86–0.97)

References

1 Miettinen OS. Proportion of disease caused or prevented by a given exposure, trait or intervention. *Am J Epidemiol* 1974; **99**: 325–32.

2 Shi T, McLean K, Campbell H, Nair H. Aetiological role of common respiratory viruses in acute lower respiratory infections in children under five years: A systematic review and meta-analysis. *J Glob Health* 2015; **5**: 10408.

3 Feikin DR, Scott JAG, Gessner BD. Use of vaccines as probes to define disease burden. *Lancet Lond Engl* 2014; **383**: 1762–70.

4 O'Brien KL, Wolfson LJ, Watt JP, *et al.* Burden of disease caused by Streptococcus pneumoniae in children younger than 5 years: global estimates. *Lancet Lond Engl* 2009; **374**: 893–902.

5 Watt JP, Wolfson LJ, O'Brien KL, *et al.* Burden of disease caused by Haemophilus influenzae type b in children younger than 5 years: global estimates. *Lancet Lond Engl* 2009; **374**: 903–11.

6 Johnson HL, Deloria-Knoll M, Levine OS, *et al.* Systematic evaluation of serotypes causing invasive pneumococcal disease among children under five: the pneumococcal global serotype project. *PLoS Med* 2010; **7**. DOI:10.1371/journal.pmed.1000348.

7 Swingler G, Fransman D, Hussey G. Conjugate vaccines for preventing Haemophilus influenzae type B infections. *Cochrane Database Syst Rev* 2007; : CD001729.

8 Lucero MG, Dulalia VE, Nillos LT, *et al.* Pneumococcal conjugate vaccines for preventing vaccine-type invasive pneumococcal disease and X-ray defined pneumonia in children less than two years of age. *Cochrane Database Syst Rev* 2009; : CD004977.

9 Bonten MJM, Huijts SM, Bolkenbaas M, *et al.* Polysaccharide conjugate vaccine against pneumococcal pneumonia in adults. *N Engl J Med* 2015; **372**: 1114–25.

3.8.1 UHC Index: Upper Respiratory Infections SDG Capstone Appendix

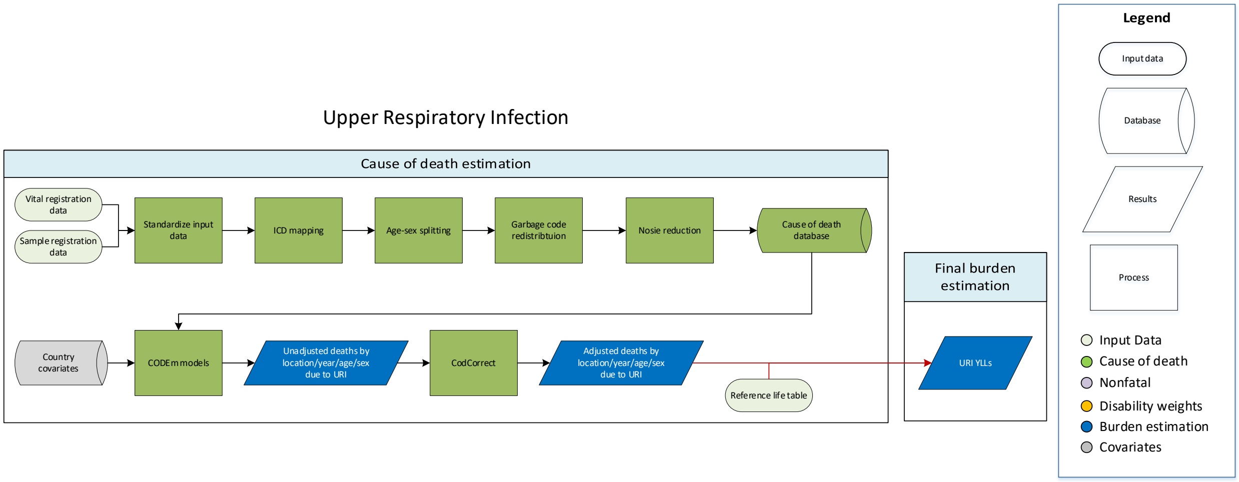

Input data

Vital registration and surveillance data from the cause of death database were used. Outliers were identified by systematic examination of data points. Data points that violated well-established age or time trends, were inconsistent with other country- or region-specific points, or that resulted in extremely high or low mortality rates were determined to be outliers.

Modelling strategy

A generic CODEm approach was used to estimate mortality due to upper respiratory infections (URI) in GBD 2017. In GBD 2016, mortality from URI was modelled using a negative binomial regression. It was determined that a negative binomial regression was an appropriate approach for estimating URI due to a small number of deaths due to URI in the cause of death database. However, due to changes in how we redistribute cause of death codes, more deaths were attributed to URI in the COD database and thus it was determined that a generic CODEm approach is feasible for estimating URI mortality in GBD 2017. The covariates used are displayed below.

Level	Covariate	Direction
1	Smoking prevalence	+
2	Indoor pollution	+
	Outdoor pollution ($PM_{2.5}$)	+
	Healthcare Access and Quality Index	+
3	SDI	-
	LDI	-
	Education (years per capita)	-

3.8.1 UHC Index: Diphtheria SDG Capstone Appendix

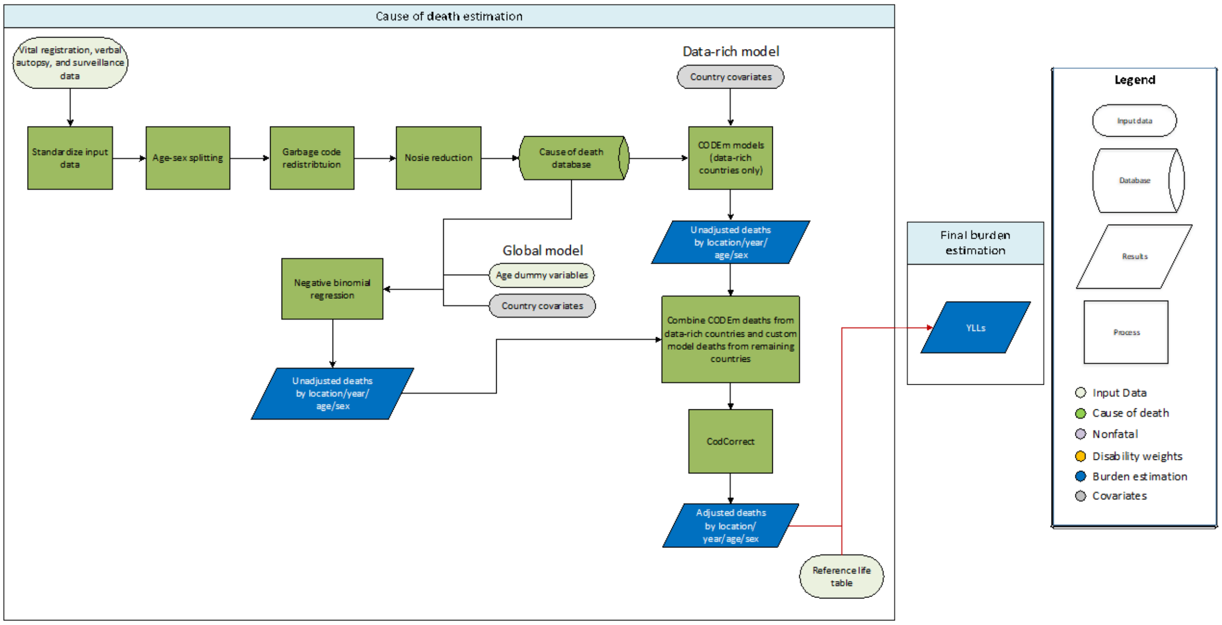

Modelling strategy overview

For this round of the GBD, we implemented two separate methods for modelling diphtheria mortality based on the quality of available vital registration data. For countries with well-defined vital registration (ie, “data-rich” countries), we used a cause of death ensemble model (CODEm) approach. For the remaining countries, we used a custom count model approach.

Data-rich countries

For data-rich countries, we used a CODEm strategy in count space to model vital registration (VR) data through time using the following country covariates:

Level	Covariate	Direction
1	Diphtheria-tetanus-pertussis third-dose vaccination coverage (DTP3)	-
	Health systems access (capped)	-
	Healthcare access and quality (HAQ) index	-
	Wasting (proportion under 2SD)	+
3	Lagged-distributed income (LDI)	-
	Socio-demographic Index (SDI)	-
	Mean years of education per capita	-

Models in count space had lower out-of-sample root mean squared error (RMSE) than rate-space models and were thus chosen as final models for these data-rich countries.

Custom count model

To inform the custom model of diphtheria mortality, vital registration and surveillance data from the cause of death database were used. Data with very high cause fractions (those greater than the 99th percentile values) were excluded.

Due to the small number of recorded deaths, diphtheria mortality was modelled using a negative binomial regression. Cause fractions representing number of deaths as a proportion of the all-cause mortality envelope were regressed on the diphtheria-pertussis-tetanus third-dose (DPT3) vaccine coverage covariate with dummy variables for each GBD age group, with death counts as the dependent variable and the offset as the total number of deaths:

$$Y_{ij} = \beta_0 + \beta_1\ DTP3_{ij} + age_{a\ ij} + e_{ij}\ ,$$

where Y_{ij} is the log-transformed cause fraction, β_0 is the fixed-effect intercept, β_1 is the fixed-effects slope on vaccine coverage, $age_{a\ ij}$ is the dummy variable for each GBD age group in the estimation, e_{ij} is the residual, *i* is the year, and *j* is the location. The negative binomial model was used here over a Poisson count model because it more appropriately accounts for large variance (over-dispersion) in the data. Uncertainty was estimated by taking 1,000 iterations of the predictions based on the variance-covariance matrix and a random sample of the dispersion parameter from a gamma distribution.

3.8.1 UHC Index: Whooping cough (Pertussis) SDG Capstone Appendix

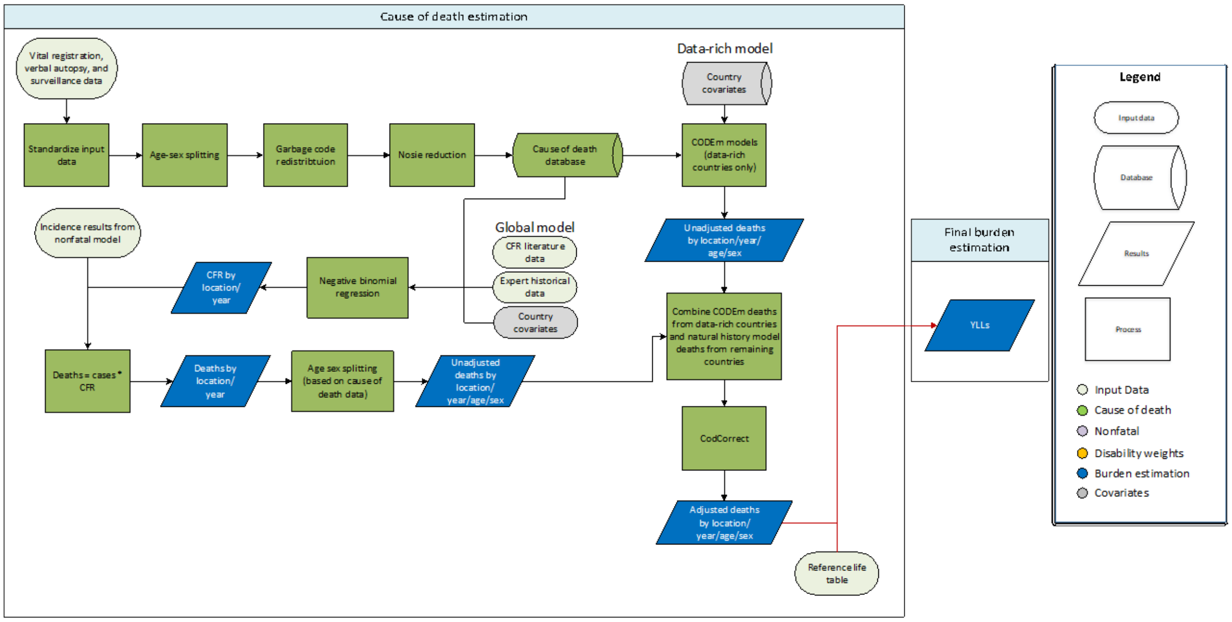

Modelling strategy overview

We implemented two separate methods for modeling pertussis mortality based on the quality of available vital registration data. For countries with well-defined vital registration (ie, "data-rich" countries), we used a cause of death ensemble model (CODEm) approach. For the remaining countries, we used a natural history model approach. For all countries, we estimated for the age range post-neonatal to 59 years.

Data-rich countries

For data-rich countries (ie, countries with vital registration more than 65% complete for all GBD estimation years, 1980–2017), we used a strategy in count space to model vital registration (VR) data through time using the following country covariates:

Level	Covariate	Direction
1	Diphtheria-tetanus-pertussis third-dose vaccination coverage (DTP3)	-
	Health system access (capped)	-
	Malnutrition	+
	Healthcare access and quality (HAQ) index	-
3	Lagged-distributed income (LDI)	-
	Socio-demographic Index (SDI)	-
	Mean years of education per capita	-

Models in count space (as opposed to rate space) had lower out-of-sample root mean squared error (RMSE) and were thus chosen as final models for these countries.

Natural history model

To inform the natural history model that was used as the modelling approach for countries without well-defined vital registration, we used data from the following sources: World Health Organization (WHO) case notifications; historical case notifications for the United Kingdom back to 1940; vital registration (VR) data in countries defined as "data-rich"; case fatality data identified by collaborators; and case fatality data identified through systematic literature reviews. The PubMed search query was: (whooping cough [Title/Abstract]) OR (pertussis [Title/Abstract]) AND (case fatality [Title/Abstract]). Studies were included if they reported case fatality rate, number of deaths, and number of cases. Studies were excluded if they included non-representative samples only.

Pertussis mortality in these remaining countries was modelled using a natural history-based model because CODEm does not predict well for countries without data.

First, we modelled log-transformed incidence from whooping cough case notifications reported to WHO (1985–2017) with diphtheria-tetanus-pertussis dose three (DTP3) vaccination coverage using the following equation:

$$Y_{ij} = \beta_0 + \beta_1\ DTP3_{ij} + u_j + e_{ij}\ ,$$

where Y_{ij} is the log-transformed incidence rate (in cases per 100,000 persons using WHO case notifications and GBD populations), β_0 is the fixed effect intercept, β_1 is the fixed effects slope on the log-transformed proportion of unvaccinated individuals, u_j is the country random effect, e_{ij} is the residual, i is the year, and j is the location. Historical data of UK pertussis cases and UK DTP3 coverage rates (both back to 1940) were also used to inform the incidence model. The random effect by country allowed for registration completeness to vary by country.

The results of this model were then used to predict incidence as a function of vaccine coverage. To correct for underreporting in case notifications, we used a value of the random effect that matched the highest random effect in a high-income region – Switzerland (which has a pertussis monitoring system which captures a high percentage of cases) – to get an implied attack rate assumed to be the same for all unvaccinated populations. Uncertainty was estimated by taking 1,000 iterations of the predictions based on the variance-covariance matrix.

Second, we modelled the pertussis case fatality rate using a negative binomial model with the healthcare access and quality (HAQ) index as a covariate:

$$Y_{ij} = \beta_0 + \beta_1 HAQ_{ij} + u_j + e_{ij}\ ,$$

where Y_{ij} is the number of deaths (using pertussis cases as the offset term); β_0 is the fixed-effect intercept; β_1, *is* the fixed-effects slopes on the healthcare access and quality (HAQ) index; u_j is country-level random effects; e_{ij} is the residual; i is the year; and j is the location. Uncertainty was estimated by taking 1,000 iterations of the predictions based on the variance-covariance matrix and a random sample from a gamma distribution of the dispersion parameter.

Finally, whooping cough deaths were calculated at the 1,000-draw level as

$$deaths = incidence * CFR\ .$$

We estimated overall number of deaths and then assigned an age-sex distribution based on the age- and sex-specific patterns found in the cause of death data.

3.8.1 UHC Index: Tetanus SDG Capstone Appendix

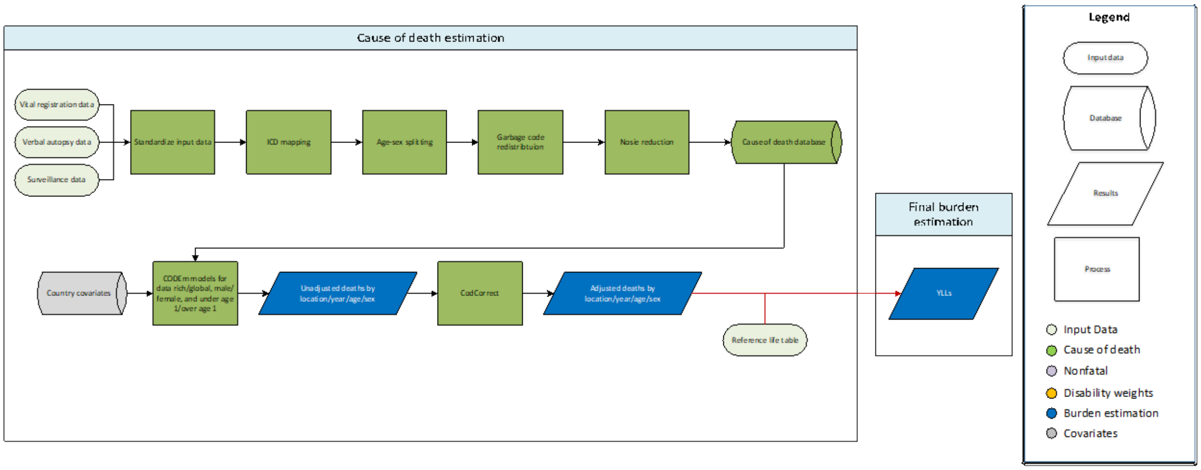

Input data

Mortality data from vital registration, verbal autopsy, and surveillance sources were used in tetanus cause of death models. Data were excluded if they largely conflicted with the majority of data from other studies conducted either in the same or different countries in the same region with similar sociodemographic characteristics.

Modelling strategy

A count-space cause of death ensemble modelling strategy (CODEm) was used. We ran separate models by age (under 1 year and 1 to 95+ years of age), sex (male and female), and data quality (data-rich and global). We used the following covariates for the under-1 models:

Level	Covariate	Direction
1	Diphtheria-tetanus-pertussis third-dose vaccination coverage (DTP3)	-
	Tetanus toxoid coverage	-
2	In-facility deliveries (proportion)	-
	Skilled birth attendance (proportion	-
	Health systems access (capped)	-
	Healthcare access and quality index (HAQ)	-
3	Lagged-distributed income (LDI)	-
	Socio-demographic Index (SDI)	-
	Mean years of education per capita	-

and the following covariates for the 1+ models:

Level	Covariate	Direction
1	Diphtheria-tetanus-pertussis third-dose vaccination coverage (DTP3)	-
2	Health systems access (capped)	-

	Healthcare access and quality index (HAQ)	-
3	Sanitation (proportion)	-
	Lagged-distributed income (LDI)	-
	Socio-demographic Index (SDI)	-
	Mean years of education per capita	-

Models in count space had lower out-of-sample root mean squared error (RMSE) than rate-space models and were thus chosen as final models for these data-rich countries.

3.8.1 UHC Index: Measles SDG Capstone Appendix

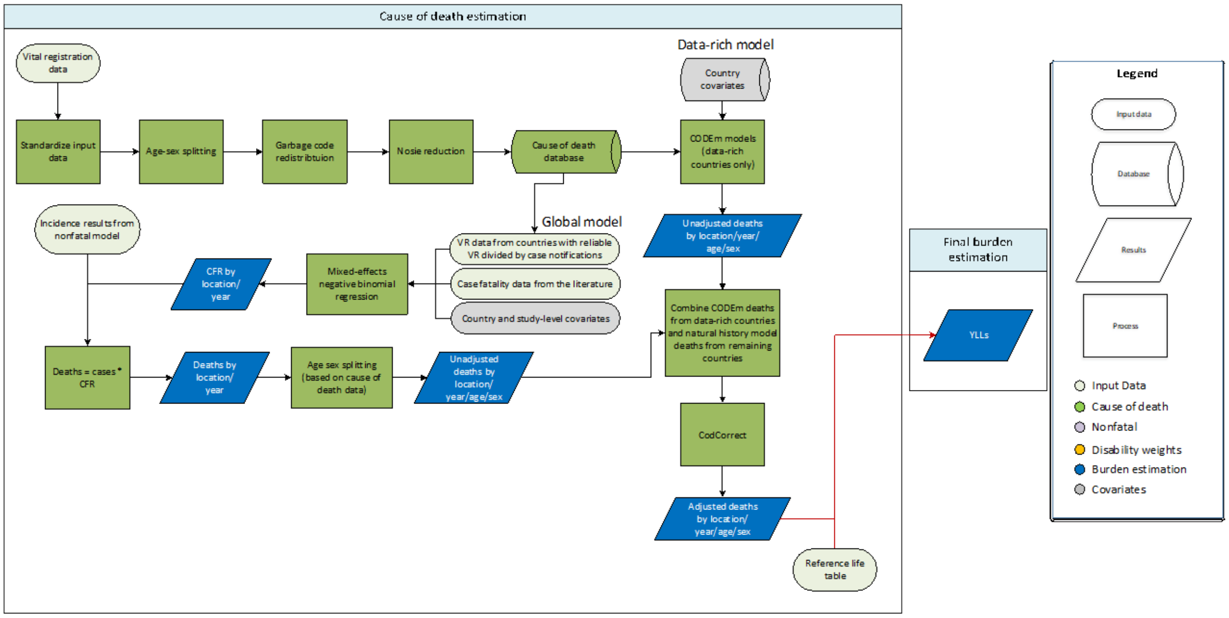

Modelling strategy overview

We implemented two separate methods for modelling measles mortality based on the quality of available vital registration data. For countries with well-defined vital registration (ie, "data-rich" countries), we used a cause of death ensemble model (CODEm) approach. For the remaining countries, we used a natural history model approach. For all countries, we estimated for the age range post-neonatal to 59 years.

Data-rich countries

For data-rich countries, we used a CODEm strategy in count space to model vital registration (VR) data through time using the following country covariates:

Level	Covariate	Direction
1	Measles-containing vaccination dose one (MCV1)	-
2	Healthcare access and quality (HAQ) index	-
3	Health systems access (capped)	-
	Socio-demographic Index (SDI)	-
	Mean years of education per capita	-

Covariates including measles-containing vaccination dose two (MCV2) coverage were excluded due to their collinearity with MCV1. Models in count space (as opposed to rate space) had lower out-of-sample root mean squared error (RMSE) and were thus chosen as final models for these countries.

Natural history model

To inform the natural history model, we used data from the following sources: World Health Organization (WHO) case notifications from 1995 to 2017 (most recently released in June 2018); additional case notification sources identified by collaborators (eg, Japan and USA subnational surveillance); vital registration (VR) data in countries with well-defined vital registration data; and case fatality data identified through systematic literature reviews. Studies were included in the literature review if they reported case fatality rate, number of deaths, and number of cases. Studies were excluded if they were conducted on non-representative samples.

Measles mortality in the non-data-rich countries was modelled using a natural-history-based model.

First, we modelled measles incidence with a mixed-effects linear regression of case notifications from WHO (1995–2017) on both doses of routine measles vaccination rates (MCV1 and MCV2) and supplementary immunization activity (SIA) coverage using the following equation:

$$Y_{ij} = \beta_0 + \beta_1 MCV1_{ij} + \beta_2 MCV2_{ij} + \beta_{a3} SIA_{aij} + u_j + e_{ij} \,,$$

where Y_{ij} is the log-transformed incidence rate (in cases per 100,000 persons using WHO case notifications and GBD populations); β_0 is the fixed-effect intercept; β_1, β_2, and β_{a3} are the fixed-effects slopes on the log-transformed proportion of population without the MCV1 vaccine, log-transformed proportion of population without the MCV2 vaccine, and supplementary vaccination coverage (administered doses over the target population of all under-15s) lagged by a=1-5 years, respectively; u_j is the super-region, region, and country-level random effects; e_{ij} is the residual; i is the year; and j is the location.

The results of this mixed effects regression model were then used to predict location-year-specific incidence as a function of routine vaccine coverage and SIAs. To correct for underreporting in case notifications, we added the effect of a 95% attack rate, assumed to be the same across all unvaccinated populations. Uncertainty was estimated by taking 1,000 iterations of the predictions based on the variance-covariance matrix. For locations in three super-regions – high-income, Central Europe/Eastern Europe/Central Asia and Latin America and Caribbean – we used reported measles cases as incident cases. More information on this part of the natural history model can be found in the non-fatal methods appendix for this round of the GBD.

Second, measles case fatality ratio was modelled using a mixed effects negative binomial regression using the Socio-demographic Index (SDI) as a country covariate and three indicators (hospital-based or not; outbreak or not; and rural or urban/mixed) as study-level covariates, with country random effects:

$$Y_{ij} = \beta_0 + \beta_1 SDI_{ij} + \beta_2 hospital_{ij} + \beta_3 outbreak_{ij} + \beta_3 rural_{ij} + u_j + e_{ij} \,,$$

where Y_{ij} is the number of deaths (using measles cases as the offset term); β_0 is the fixed-effect intercept; β_1, β_2, β_3, and β_4 are the fixed-effects slopes on the Socio-demographic Index (SDI) and hospital, outbreak, and rurality study-level covariates; u_j is country-level random effects; e_{ij} is the residual; i is the year; and j is the location. Uncertainty was estimated by taking 1,000 iterations of the predictions based on the variance-covariance matrix and uncertainty in country random effects. The fit of the model was evaluated using diagnostic plots of predicted versus observed values.

Finally, estimated deaths were calculated at the 1,000-draw level from the two sets of custom model predictions as:

$$deaths = incident\ cases * CFR\ .$$

We estimated overall number of deaths and then assigned an age-sex distribution based on the global-level age- and sex-specific patterns found in the cause of death data.

3.8.1 UHC Index: Neonatal disorders SDG Capstone Appendix

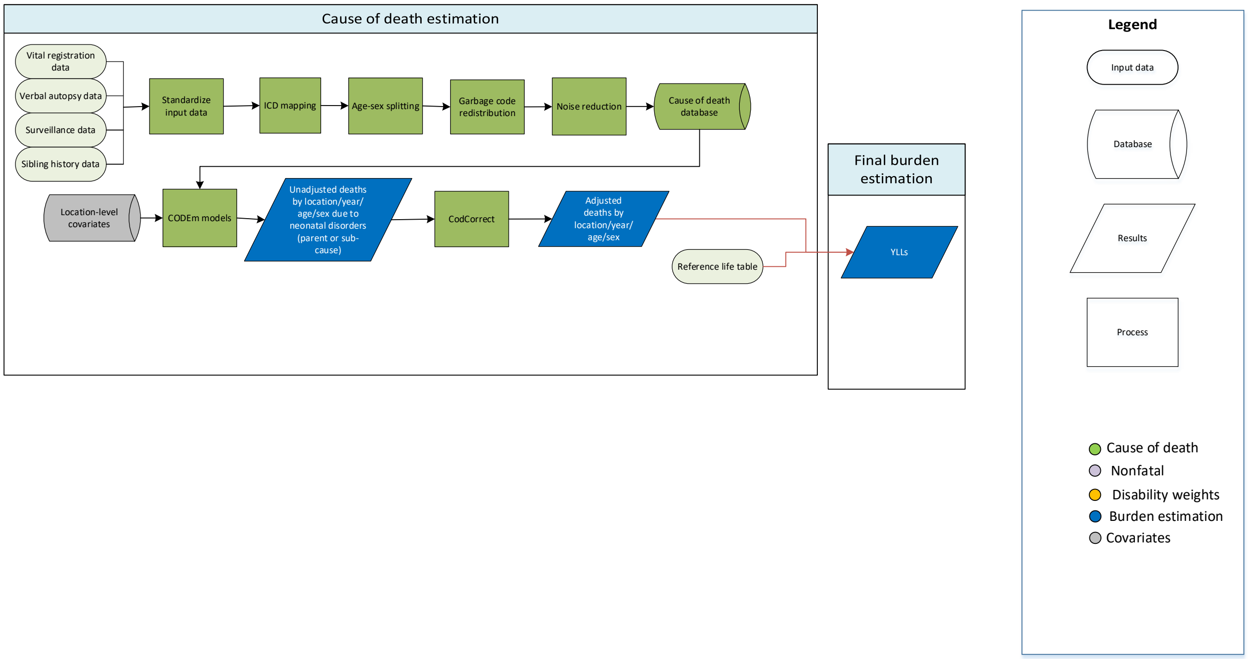

Input data

Mortality for five causes are modeled within "neonatal disorders": preterm birth complications, neonatal encephalopathy and birth trauma, neonatal sepsis and other infections, hemolytic disease and neonatal jaundice, and other neonatal disorders. An overall neonatal disorders "parent" envelope is also estimated, to which all neonatal causes are squeezed.

For the neonatal disorders envelope, preterm birth complications, neonatal encephalopathy and birth trauma, neonatal sepsis and other infections, hemolytic disease and neonatal jaundice, and other neonatal disorders, vital registration and surveillance were the majority of data sources used for GBD 2017 to estimate number of deaths from each condition. In Indian states, only verbal autopsy was used to inform estimates. Only deaths among males and females under age 5 were modelled, in four separate age groups: early neonatal period, late neonatal period, post-neonatal period, and 1-4 years. Data points were selected as outliers if they were implausibly high, low, or significantly conflicted with established age or temporal patterns.

Modelling strategy

For GBD 2017, the standard CODEm modelling approach was used to model each of the neonatal conditions. This same method was employed in GBD 2013, 2015, and 2016.

Varying levels of data quality and coding issues may still have affected our results. Validation studies suggest that verbal autopsy methods tend to be less accurate for cause of death ascertainment in the neonatal age groups.[1–4] Thus, for GBD 2017, except for the Indian states, the majority of verbal autopsy data were excluded.

All neonatal causes used the following pool of covariates in covariate selection:

Level	Covariate	Direction
1	Indoor air pollution (all cooking fuels)	+
	Smoking prevalence (reproductive age-standardized)	+
2	Antenatal care (4 visits) coverage (proportion)	-
	In-facility delivery (proportion)	-
	Live births 35+ (proportion)	+
	Skilled birth attendance (proportion)	-
	Health system access (capped)	-
	Healthcare access and quality index	-
	Age-standardised underweight (weight-for-age) SEV	+
3	Education (years per capita)	-
	Lag distributed income per capita (I$)	-
	Total fertility rate	+
	Socio-demographic Index	-

References

1 Anker M, Black RE, Coldham C, *et al.* A Standard Verbal Autopsy Method for Investigating Causes of Death in Infants and Children. Geneva, Switzerland: World Health Organization Department of Communicable Disease Surveillance and Response; The Johns Hopkins School of Hygiene and Public Health; The London School of Hygiene and Tropical Medicine, 1999.

2 Kalter HD, Gray RH, Black RE, Gultiano SA. Validation of postmortem interviews to ascertain selected causes of death in children. *Int J Epidemiol* 1990; **19**: 380–6.

3 Quigley MA, Armstrong Schellenberg JR, Snow RW. Algorithms for verbal autopsies: a validation study in Kenyan children. *Bull World Health Organ* 1996; **74**: 147–54.

4 Snow RW, Armstrong JR, Forster D, *et al.* Childhood deaths in Africa: uses and limitations of verbal autopsies. *The Lancet* 1992; **340**: 351–5.

3.8.1 UHC Index: Peptic ulcer disease SDG Capstone Appendix

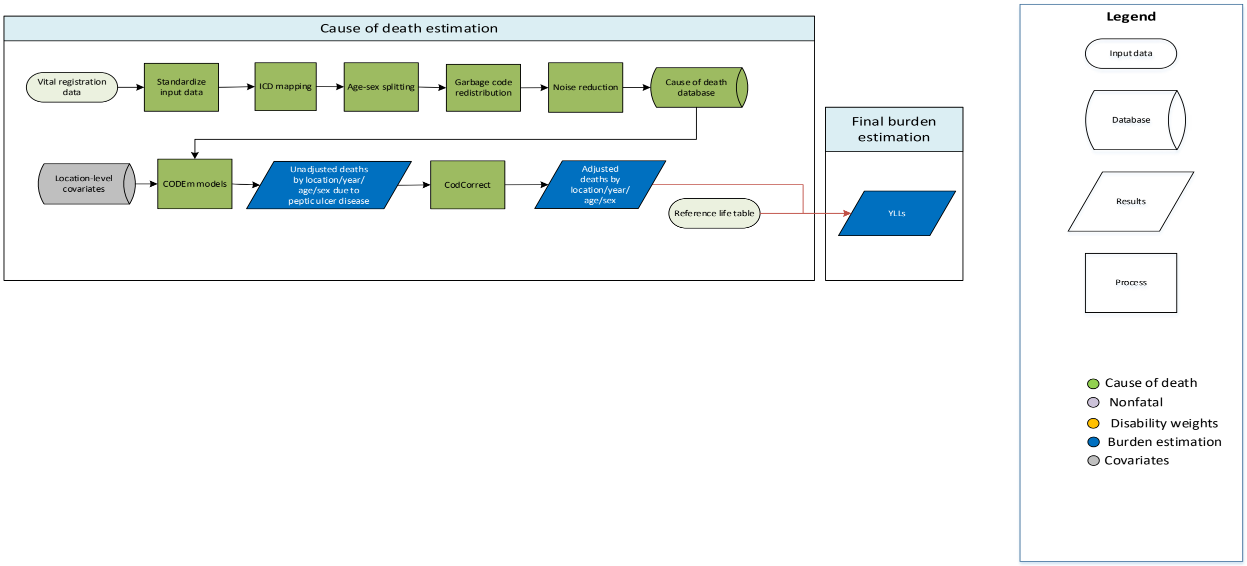

Input data

Data used to estimate mortality of peptic ulcer disease consisted of vital registration data from the cause of death (COD) database. We marked data as outliers in instances where garbage code redistribution and noise reduction, in combination with small sample sizes, resulted in unreasonable cause fractions, and data that violated well-established time or age trends.

Modelling strategy

We modelled deaths due to peptic ulcer disease with a standard CODEm model using the cause of death database and location-level covariates as inputs. The model followed standard parameters, with the exception that the start age of the model was 1 year instead of 0 and the linear floor rate was lowered to 0.0001 in order to better capture low data. We hybridised separate global and data-rich models to acquire unadjusted results, which we finalised and adjusted using CoDCorrect to reach final years of life lost (YLLs) due to peptic ulcer disease. The covariates tried and their expected strengths and directions are unchanged in GBD 2017 compared to GBD 2016.

Covariate	Level	Direction
Alcohol (litres per capita)	1	1
Cumulative cigarettes (10 years)	1	1
Cumulative cigarettes (5 years)	1	1
Lag distributed income (per capita)	3	-1
Sanitation (proportion with access)	2	-1
Smoking (prevalence)	1	1
Maternal education (years per capita)	3	-1
Improved water source (proportion with access)	2	1
Socio-demographic Index	3	-1
Vegetables (grams adjusted)	2	0
Healthcare access and quality index	2	-1

3.8.1 UHC Index: Stroke (Cerebrovascular Disease) SDG Capstone Appendix

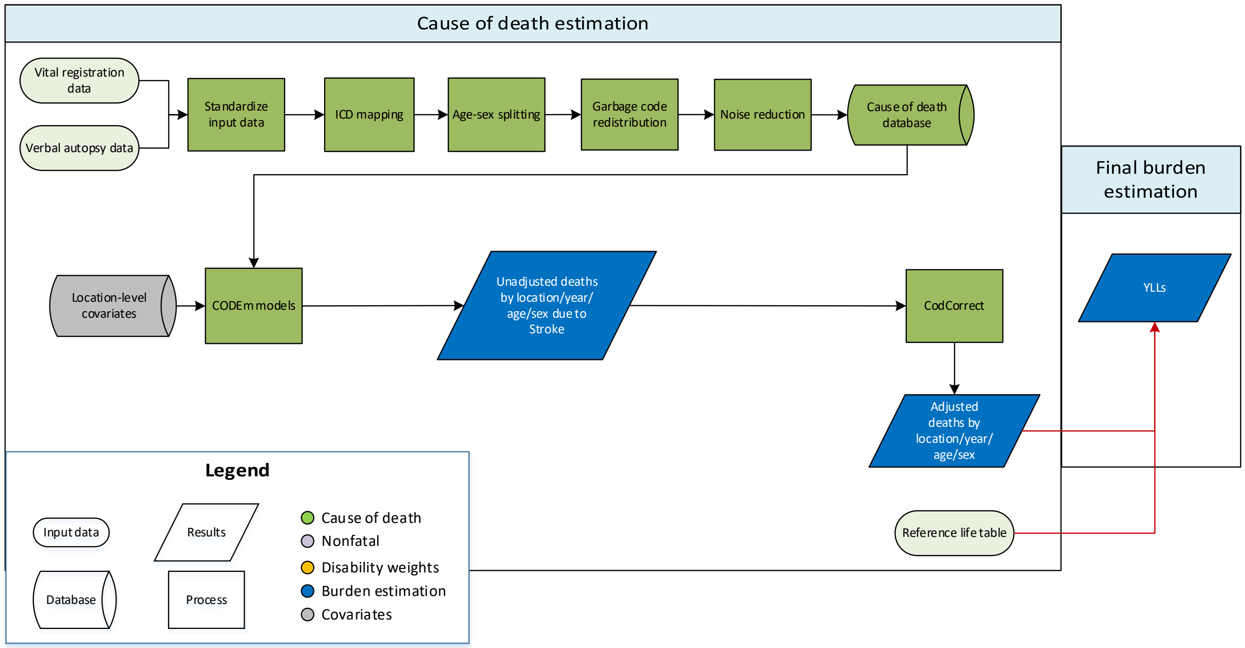

Input data

Verbal autopsy and vital registration data were used to model cerebrovascular disease. We reassigned deaths from verbal autopsy reports for cerebrovascular disease to the parent cardiovascular disease for both sexes for those under 20 years of age. We outliered non-representative subnational verbal autopsy data points. We also outliered ICD8, ICD9 BTL, and ICD10 Tabulated data points which were inconsistent with the rest of the data and created implausible time trends. Data points from sources which were implausibly low in all age groups and data points that were causing the regional estimates to be improbably high were outliered.

Modelling strategy

We used a standard CODEm approach to model deaths from stroke. The covariates included in the ensemble modelling process are listed in the table below. Apart from the updated strategy to reassign mis-coded deaths which should have been attributed to Alzheimer's and other dementias, Parkinson's disease, or atrial fibrillation and flutter, there have been no substantive changes from the approach used in GBD 2016.

Table: Selected covariates for CODEm models, stroke

Covariate	Transformation	Level	Direction
Summary exposure variable, stroke	None	1	1
Cholesterol (total, mean per capita)	None	1	1
Smoking prevalence	None	1	1
Systolic blood pressure (mmHg)	None	1	1
Trans fatty acid	None	1	1
Mean BMI	None	2	1
Elevation over 1500m (proportion)	None	2	-1
Fasting plasma glucose	None	2	1
Outdoor pollution ($PM_{2.5}$)	None	2	1
Indoor air pollution	None	2	1
Healthcare access and quality index	None	2	-1
Lag distributed income per capita (I$)	Log	3	-1
Socio-demographic Index	None	3	0
Omega-3 (kcal/capita, adjusted)	Log	3	-1
Fruits (kcal/capita, adjusted)	None	3	-1
Vegetables (kcal/capita, adjusted)	None	3	-1
Nuts and seeds (kcal/capita, adjusted)	None	3	-1
Whole grains (kcal/capita, adjusted)	None	3	-1
Pulses/legumes (kcal/capita, adjusted)	None	3	-1
PUFA adjusted (percent)	None	3	-1
Alcohol (litres per capita)	None	3	0

3.8.1 UHC Index: Appendicitis SDG Capstone Appendix

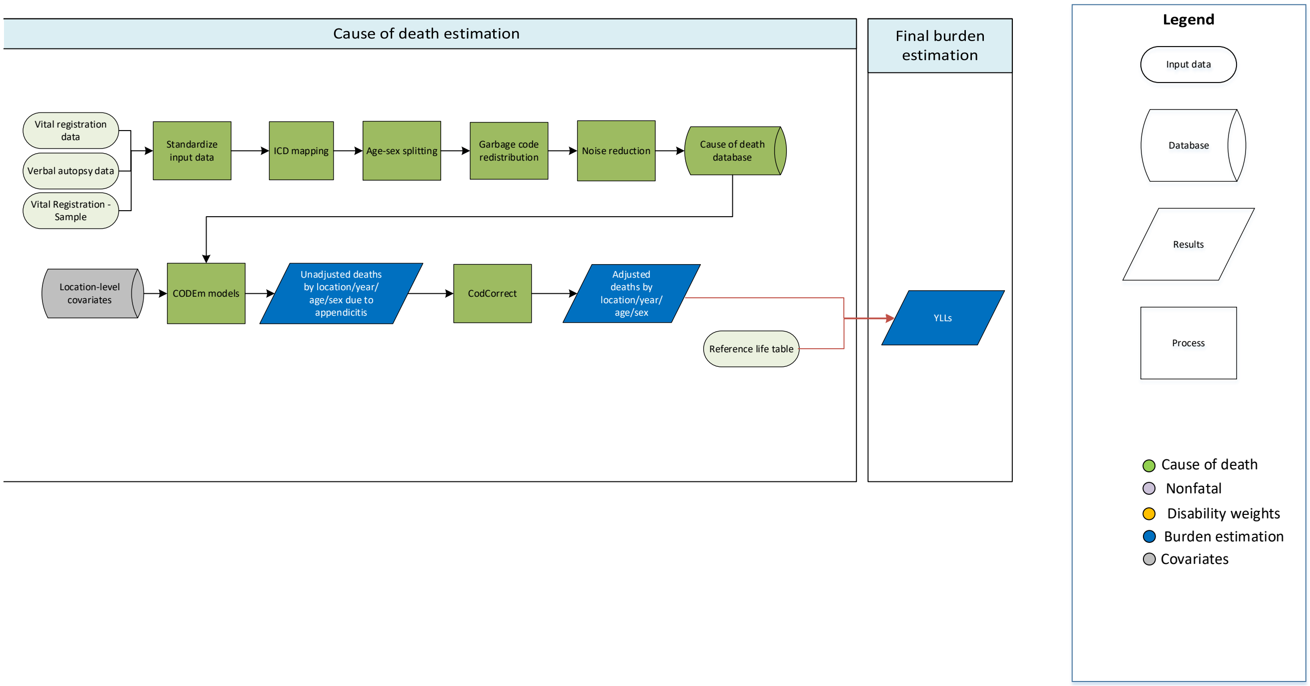

Input data

Data used to estimate appendicitis mortality consisted of vital registration and verbal autopsy data from the cause of death (COD) database. We outliered data in instances where garbage code redistribution and noise reduction, in combination with small sample sizes, resulted in unreasonable cause fractions; and data that violated well-established time or age trends.

Modelling strategy

We modelled deaths due to appendicitis with a standard CODEm model using the cause of death database and location-level covariates as inputs. The model followed standard parameters, with the exception that the start age of the model was 1 year old instead of 0 and the linear floor rate was lowered to 0.0001 in order to better capture low data. We hybridized separate global and data-rich models to acquire unadjusted results, which we finalised and adjusted using CodCorrect to reach final YLLs due to appendicitis. There were no significant changes in the modelling process between GBD 2016 and GBD 2017.

Level	Covariate	Direction
2	Healthcare access and quality index	-
	Fruits adjusted (g)	-
	Vegetables adjusted (g)	-
3	Education (years per capita)	-
	Log LDI (I$ per capita)	-
	Socio-demographic Index	-
	Health system access (capped)	-

3.8.1 UHC Index: Inguinal, Femoral, and Abdominal Hernias SDG Capstone Appendix

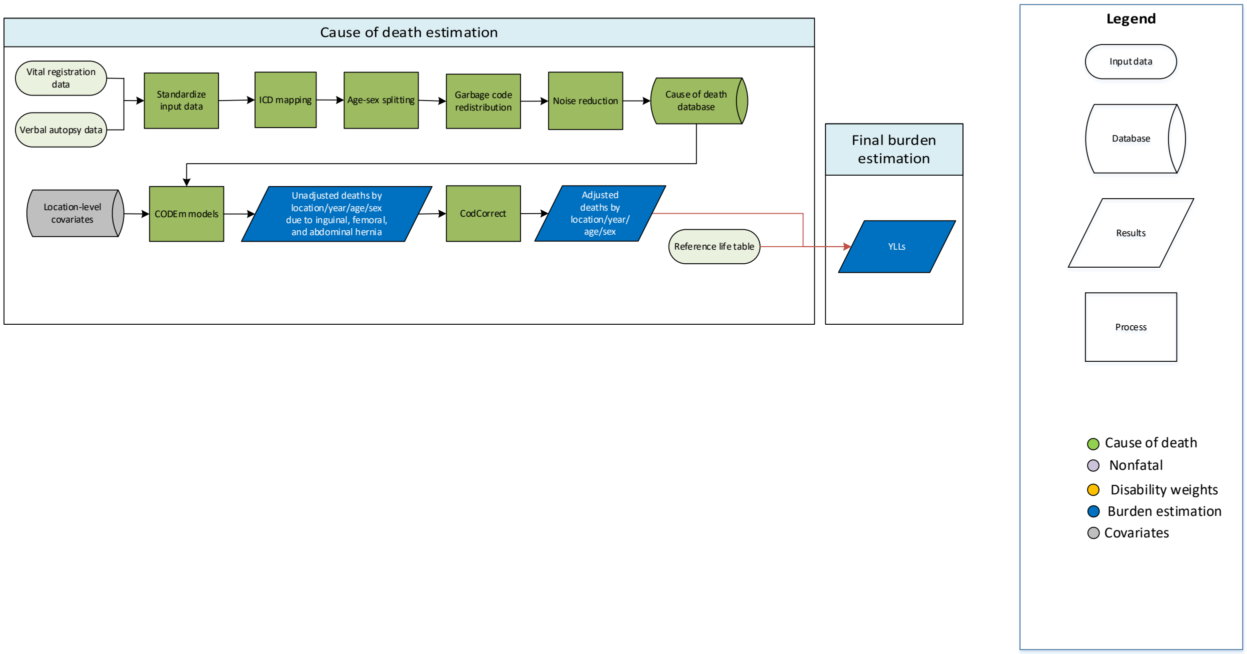

Input data

Vital registration and verbal autopsy data were used to model this cause. We marked data as outliers in instances where garbage code redistribution and noise reduction, in combination with small sample sizes, resulted in unreasonable cause fractions. We also marked as outliers those data that violated well-established time or age trends. Methods for assigning outlier status were consistent across both vital registration and verbal autopsy data.

Modelling strategy

We modelled deaths due to inguinal, femoral, and abdominal hernias with a standard CODEm model using the cause of death database and location-level covariates as inputs. The model followed standard parameters, with the exception that the start age of the model was 1 year instead of 0 and the linear floor rate was lowered to 0.0001 in order to better capture low data. We hybridised separate global and data-rich models to acquire unadjusted results, which we finalised and adjusted using CoDCorrect to reach final years of life lost (YLLs) due to inguinal, femoral, and abdominal hernias. In contrast to GBD 2016, covariates for smoking and BMI were included for possible selection during CODEm modelling in GBD 2017.

Covariate	Level	Direction
Education (years per capita)	3	-1
Lag distributed income (per capita)	3	-1
Socio-demographic Index	3	0
Healthcare access and quality index	2	-1
Cumulative cigarettes (10 years)	1	1
Cumulative cigarettes (5 years)	1	1
Smoking prevalence	1	1
Body mass index (mean)	1	-1

3.8.1 UHC Index: Gallbladder and Biliary Diseases SDG Capstone Appendix

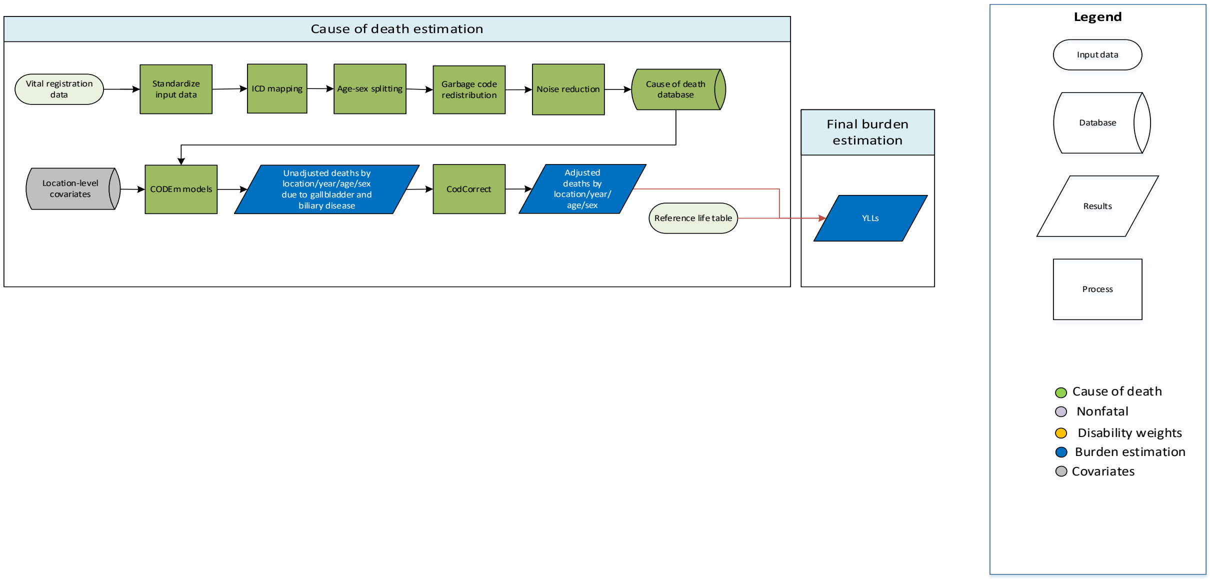

Input data

Data used to estimate mortality of gallbladder and biliary diseases consisted of vital registration data from the cause of death (COD) database. We outliered data in instances where garbage code redistribution and noise reduction, in combination with small sample sizes, resulted in unreasonable cause fractions; and data that violated well-established time or age trends.

Modelling strategy

We modelled deaths due to gallbladder and biliary diseases with a standard CODEm model using the cause of death database and location-level covariates as inputs. The model followed standard parameters, with the exception that the start age of the model was 1 year old instead of 0 and the linear floor rate was lowered to 0.0001 in order to better capture low data. We hybridised separate global and data-rich models to acquire unadjusted results, which we finalised and adjusted using CodCorrect to reach final years of life lost (YLLs) due to gallbladder and biliary diseases. In GBD 2016 we added the Healthcare Access and Quality (HAQ) index covariate and replaced the animal fats (kcal per capita) covariate with an updated saturated fats covariate (adjusted percent). There were no significant changes in the modelling process between GBD 2016 and GBD 2017.

Level	Covariate	Direction
1	Body-mass index (mean)	+
	Saturated fats (adjusted percent)	+
2	Alcohol (litres per capita)	+
	Healthcare access and quality index	-
	Red meats (grams adjusted)	+
	Population over 65 (proportion)	+
3	Socio-demographic Index	0

	Lag distributed income (per capita)	0
	Education (years per capita)	0

3.8.1 UHC Index: Epilepsy SDG Capstone Appendix

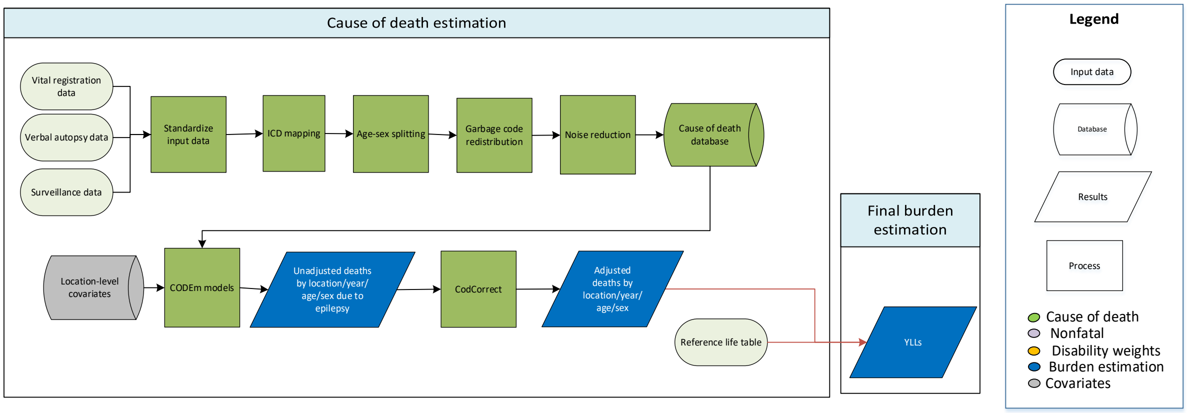

Input data

Data used to estimate epilepsy mortality included vital registration (VR), verbal autopsy, and China mortality surveillance data from the cause of death (COD) database. Our outlier criteria were to exclude data points that (1) were implausibly high or low relative to global or regional patterns, (2) substantially conflicted with established age or temporal patterns, or (3) significantly conflicted with other data sources based from the same locations or locations with similar characteristics (ie, Socio-demographic Index).

Based on these criteria, we excluded ICD-9 BTL data for Sri Lanka, Fiji, and Kiribati as the estimates varied from year to year between zero and high values. We also excluded the Survey of Causes of Death Data and Medical Certification of Cause of Death Data for India, as these data types were not consistent with the Sample Registration System Data and would have led to discontinuities in our estimates over time.

Modelling strategy

The standard CODEm modelling approach was applied to estimate deaths due to epilepsy. Separate models were conducted for male and female mortality, and the age range for both models was 28 days–95+ years. There were no substantial changes for GBD 2017. The covariates used are displayed below.

Level	Covariate	Direction
1	pig meat consumption (kcal per capita)	+
	pigs (per capita)	+
	SEV scalar: epilepsy	+
	mean systolic blood pressure (mmHg)	+
2	healthcare access and quality index	-
	mean body-mass index	+

	mean serum total cholesterol (mmol/L)	+
3	cumulative cigarettes (10 years)	+
	cumulative cigarettes (5 years)	+
	education (years per capita)	-
	log LDI (per capita)	-
	Socio-demographic Index	-

3.8.1 UHC Index: Chronic Kidney Disease SDG Capstone Appendix

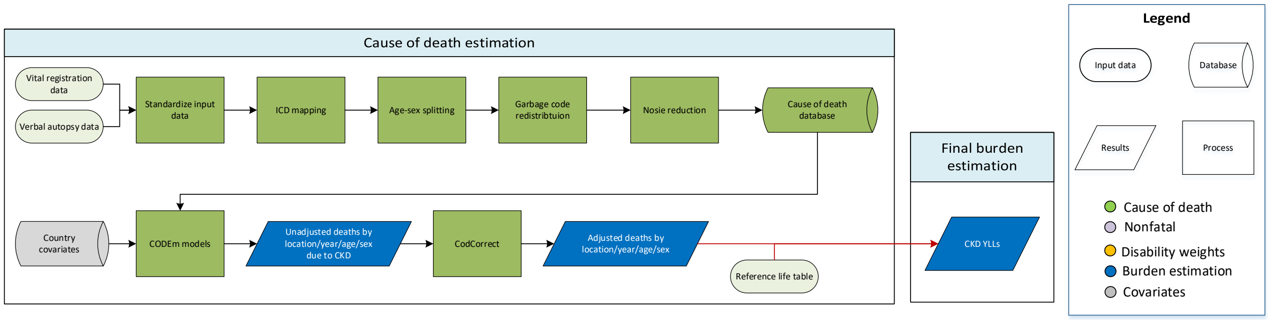

Input data

Vital registration and verbal autopsy data were used to model mortality due to chronic kidney disease. Outliers were identified by systematic examination of data points for all location-years. Data were standardised and mapped according to the GBD causes of death ICD mapping method. These data were then age-sex split, and appropriate redistribution of garbage code data was performed. Data points that violated well-established age or time trends or that resulted in extremely high or low cause fractions were determined to be outliers. Deaths due to congenital kidney anomalies (cystic kidney disease and reflux hydronephrosis) were attributed to chronic kidney disease, marking a change from GBD 2015, when these deaths were assigned to congenital anomalies.

Modelling strategy

The estimation strategy used for fatal chronic kidney disease is largely similar to methods used in GBD 2016. A standard CODEm model with location-level covariates was used to model deaths due to chronic kidney disease. Iterations of models were assessed at the location/year/age group/sex level to determine whether data points merited exclusion via outliering. Unadjusted death estimates were adjusted using CoDCorrect to produce final estimates of YLLs. The covariates used are displayed below.

Level	Covariate	Direction
1	Diabetes fasting plasma glucose (mmol/L)	+
	Diabetes age-standardised prevalence (proportion)	+
	Mean systolic blood pressure (mmHg)	+
	Mean BMI	+
	Healthcare access and quality index	
2	Mean cholesterol	+
	Total calories (kcal per capita)	
	Red meat (kcal per capita)	0
	Whole grains (kcal per capita)	0
	Animal fat (kcal per capita)	0
3	Socio-demographic Index	0
	Education (years per capita)	
	Log LDI ($I per capita)	

Chronic Kidney Disease subtypes

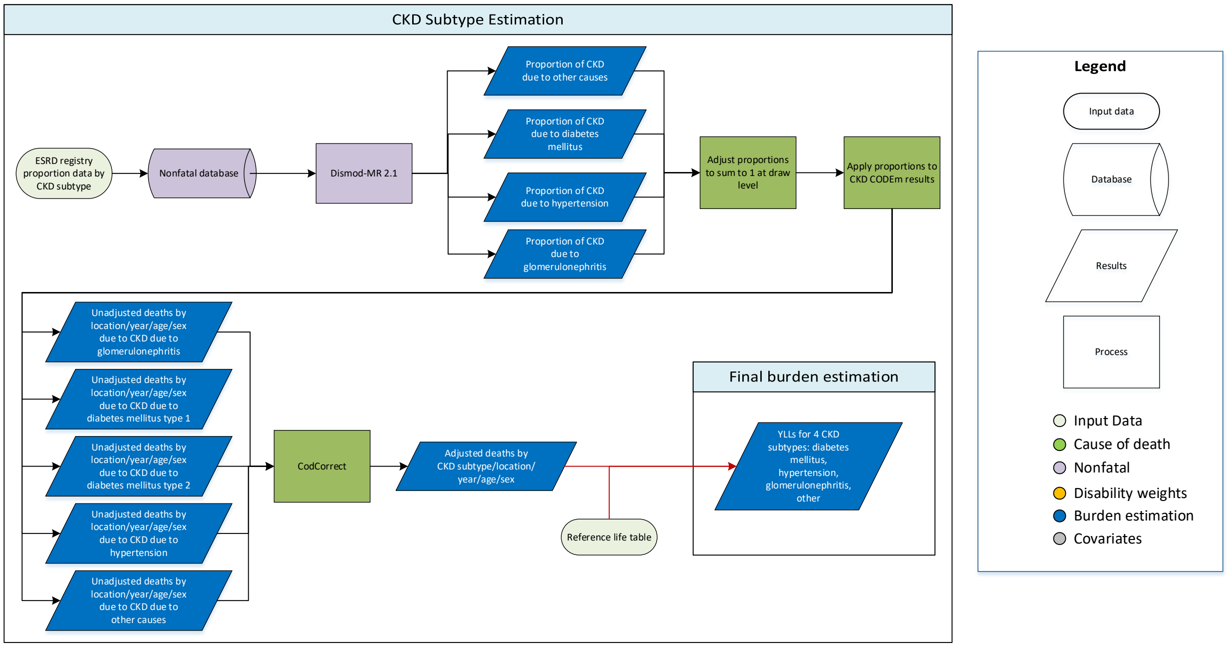

Input data

We estimated deaths due to five subtypes of chronic kidney disease: diabetes mellitus (DM) type 1, diabetes mellitus (DM) type 2, hypertension, glomerulonephritis, and other causes. Data from end-stage renal disease registries were used to inform estimates of proportion of CKD mortality attributable to each CKD subtype. Age-specific data on the proportion of ESRD by subtype was available from the United States, Australia, New Zealand, Nigeria, and Russia. Given the geographic spread in availability of age-specific proportion data, input data were not age-split, marking a change from GBD 2016.

Vital registration (VR) data were excluded from estimates, as aetiology coding in VR sources was considered highly variable and inconsistent between countries.

Modelling strategy

We ran DisMod-MR 2.1 models including diabetes prevalence and mean systolic blood pressure as country-level covariates to obtain estimates of proportions for each subtype by location, year, age, and sex. Data for CKD due to overall DM were more widely available than data by type of DM. In order to make use of all available data, we modelled the proportion of CKD due to overall DM, DM type 1, and DM type 2. Proportion of CKD due to DM type 1 and DM type 2 were then scaled to sum to the proportion of overall DM at the gender, age, and country-matched level. The results from all subtype-specific models were adjusted so that estimates across the subtypes equaled 1 at each of 1,000 draws. These adjusted proportions were applied to the parent CKD CODEm model.

Model	Covariate	Value	Exponentiated
CKD proportion due to diabetes mellitus	Diabetes age-standardised prevalence	0.49 (0.36–0.61)	1.63 (1.44–1.84)
CKD proportion due to hypertension	Mean systolic blood pressure	0.30 (0.010–1.05)	1.35 (1.01–2.86)

3.8.1 UHC Index: Congenital Birth Defects SDG Capstone Appendix

Neural tube defects, congenital heart anomalies, orofacial clefts, Down syndrome, Turner syndrome, Klinefelter syndrome, other chromosomal disorders, congenital musculoskeletal anomalies, urogenital congenital anomalies, digestive congenital anomalies, and other congenital birth defects.

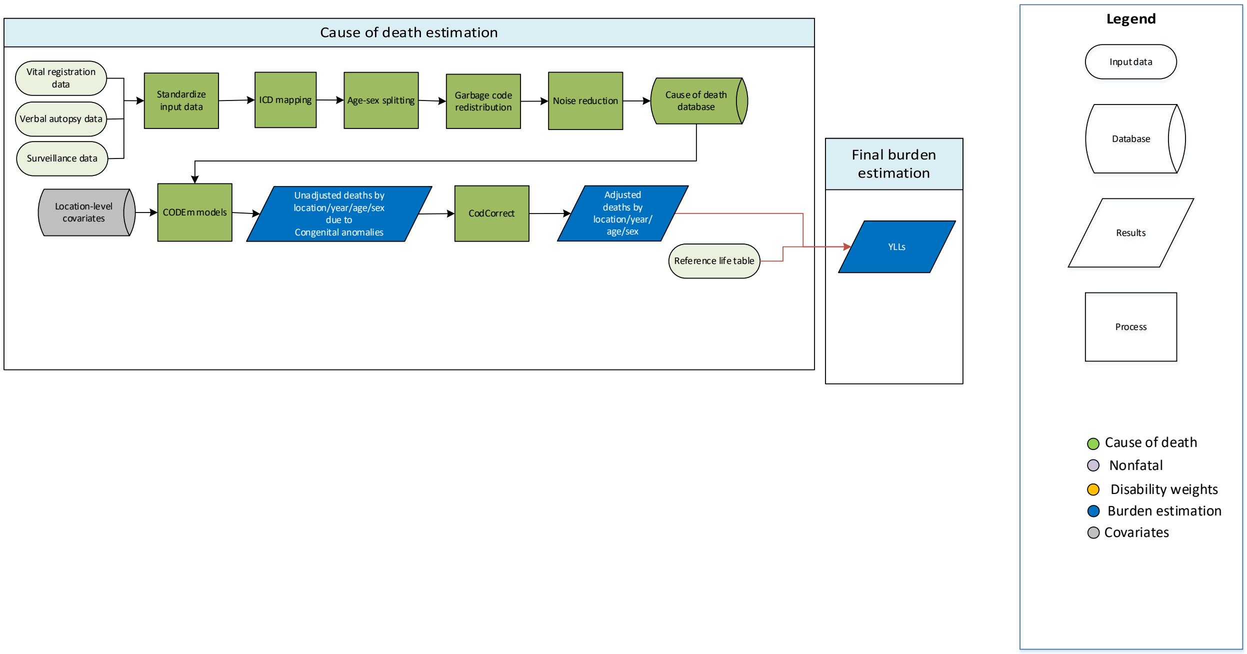

Input data

For GBD 2017, input data for estimating mortality due to congenital anomalies was centrally extracted, processed, and stored in the causes of death (CoD) database. Vital registration (VR) was the dominant data type, followed by verbal autopsy (VA) and surveillance. Those CoD data sources that specified the sub-cause of birth defect were included in estimation of both the parent congenital anomalies model as well as in sub-type-specific models.

For GBD 2017, data exclusions were limited. The majority of VA data were outliered in those over 5 years old as the age patterns were unreliable and led to poor model performance in the under-5 age groups. We also excluded some data sources from the parent model where only a subset of sub-causes were specified (eg, congenital heart disease, neural tube defects, and other congenital anomalies) and the sum of the sub-causes clearly represented systematic underreporting of one of the sub-causes. Systematic underreporting was suspected when sex- and age-specific rates were more than an order of magnitude lower than neighboring or comparable locations. Data sources for those locations were still included by default for sub-cause-specific models because under-reporting of the total was not assumed to necessarily be associated with under-reporting of all of the component conditions.

Modelling strategy

All types of congenital anomalies were estimated using cause of death ensemble modeling (CODEm) for GBD 2017, as was done for previous iterations of the GBD study. Specific causes included neural tube defects, congenital heart anomalies, orofacial clefts, Down syndrome, other chromosomal anomalies, congenital musculoskeletal anomalies, urogenital congenital anomalies, digestive congenital anomalies, and other congenital birth defects. We assumed no mortality from either Klinefelter syndrome or Turner syndrome, for which we model non-fatal outcomes only. For GBD 2017, we modelled congenital anomalies as a cause of death for ages 0-69 years only, assuming that all mortality from congenital conditions occurs before age 70 years of age.

For GBD 2016, we added three new causes to the congenital anomalies: congenital musculoskeletal and limb anomalies; urogenital congenital anomalies; and digestive congenital anomalies. We made no additions to the causes of congenital anomalies for GBD 2017.

Covariates selected for CODEm model of overall congenital birth defects

Covariate	Transformation	Level	Direction
Maternal alcohol consumption during pregnancy (proportion)	None	1	Positive
In-facility delivery (proportion)	None	1	Negative
Live births 35+ (proportion)	None	1	Positive
Folic acid unadjusted (ug)	None	1	Negative
Legality of abortion	None	2	Negative
Antenatal care (1 visit) coverage (proportion)	None	2	Not specified
Smoking prevalence (reproductive-age-standardised)	None	2	Positive
Antenatal care (4 visits) coverage (proportion)	None	2	Negative
Healthcare access and quality index	None	2	Negative
Education (years per capita)	None	2	Negative
Alcohol (litres per capita)	None	3	Positive
Fruits unadjusted (g)	None	3	Positive
Outdoor air pollution ($PM_{2.5}$)	None	3	Positive
Indoor air pollution (all cooking fuels)	None	3	Positive
Socio-demographic Index	None	3	Negative
Vegetables unadjusted (g)	None	3	Positive

Covariates selected for CODEm model of neural tube defects

Covariate	Transformation	Level	Direction
Health system access (capped)	None	1	Negative
Fruits adjusted (g)	None	2	Negative
Vegetables adjusted (g)	None	2	Negative
Healthcare access and quality index	None	2	Negative
Education (years per capita)	None	3	Negative
LDI (I$ per capita)	Log	3	Negative

Socio-demographic Index	None	3	Negative

Covariates selected for CODEm model of congenital heart anomalies

Covariate	Transformation	Level	Direction
Maternal alcohol consumption during pregnancy (proportion)	None	1	Positive
Socio-demographic Index	Log	2	Negative
Smoking prevalence (reproductive-age-standardised)	None	2	Positive
Diabetes age-standardised prevalence (proportion)	None	2	Positive
Healthcare access and quality index	None	2	Negative
Legality of abortion	None	2	Negative
Antenatal care (1 visit) coverage (proportion)	None	2	Negative
In-facility delivery (proportion)	None	2	Negative
Education (years per capita)	None	2	Negative
Alcohol (litres per capita)	None	3	Positive
Antenatal care (4 visits) coverage (proportion)	None	3	Negative
Skilled birth attendance (proportion)	None	3	Negative
Live births 35+ (proportion)	None	3	Positive

Covariates selected for CODEm model of cleft lip and cleft palate

Covariate	Transformation	Level	Direction
Indoor air pollution (all cooking fuels)	None	1	Positive
Diabetes age-standardised prevalence (proportion)	None	2	Positive
Maternal alcohol consumption during pregnancy (proportion)	None	2	Positive
Healthcare access and quality index	None	2	Negative
Outdoor air pollution ($PM_{2.5}$)	None	2	Positive
Legality of abortion	None	2	Negative
Skilled birth attendance (proportion)	None	2	Negative
Smoking prevalence (reproductive-age-standardised)	None	2	Positive
Vegetables unadjusted (g)	None	3	Not specified
Alcohol (litres per capita)	None	3	Positive
Antenatal care (4 visits) coverage (proportion)	None	3	Negative
Education (years per capita)	None	3	Negative
Fruits unadjusted (g)	None	3	Not specified
Antenatal care (1 visit) coverage (proportion)	None	3	Negative

Covariates selected for CODEm model of Down syndrome

Covariate	Transformation	Level	Direction
Live births 35+ (proportion)	None	1	Positive
Legality of abortion	None	1	Negative
Live births 40+ (proportion)	None	1	Positive
Socio-demographic Index	None	2	Negative
LDI (I$ per capita)	Log	2	Negative
In-facility delivery (proportion)	None	2	Negative
Healthcare access and quality index	None	2	Negative
Maternal alcohol consumption during pregnancy (proportion)	None	3	Positive
Antenatal care (1 visit) coverage (proportion)	None	3	Negative
Education (years per capita)	None	3	Negative
Indoor air pollution (all cooking fuels)	None	3	Positive
Antenatal care (4 visits) coverage (proportion)	None	3	Negative
Vegetables unadjusted (g)	None	3	Negative
Smoking prevalence (reproductive age-standardised)	None	3	Positive

Covariates selected for CODEm model of other chromosomal abnormalities

Covariate	Transformation	Level	Direction
Live births 35+ (proportion)	None	1	Positive
Live births 40+ (proportion)	None	1	Positive
Legality of abortion	None	1	Negative
LDI (I$ per capita)	Log	2	Negative
Healthcare access and quality index	None	2	Negative
Antenatal care (4 visits) coverage (proportion)	None	2	Negative
Antenatal care (1 visit) coverage (proportion)	None	2	Negative
In-facility delivery (proportion)	None	2	Negative
Maternal alcohol consumption during pregnancy (proportion)	None	2	Positive
Socio-demographic Index	None	3	Not specified
Alcohol (litres per capita)	None	3	Positive
Smoking prevalence (reproductive age-standardised)	None	3	Positive
Education (years per capita)	None	3	Negative
Skilled birth attendance (proportion)	None	3	Negative

Covariates selected for CODEm model of congenital musculoskeletal and limb anomalies

Covariate	Transformation	Level	Direction
Maternal alcohol consumption during pregnancy (proportion)	None	1	Positive
Legality of abortion	None	1	Negative
In-facility delivery (proportion)	None	2	Negative
Diabetes age-standardised prevalence (proportion)	None	2	Positive

Socio-demographic Index	None	2	Negative
Healthcare access and quality index	None	2	Negative
Indoor air pollution (all cooking fuels)	None	2	Positive
Smoking prevalence (reproductive age standardised)	None	2	Positive
Antenatal care (4 visits) coverage (proportion)	None	3	Negative
Alcohol (litres per capita)	None	3	Positive
Vegetables unadjusted (g)	None	3	Not specified
Fruits unadjusted (g)	None	3	Not specified
Education (years per capita)	None	3	Negative
Antenatal care (1 visit) coverage (proportion)	None	3	Negative

Covariates selected for CODEm model of urogenital congenital anomalies

Covariate	Transformation	Level	Direction
Smoking prevalence (reproductive age-standardised)	None	1	Positive
Maternal alcohol consumption during pregnancy (proportion)	None	1	Positive
Healthcare access and quality index	None	2	Negative
Diabetes age-standardised prevalence (proportion)	None	2	Positive
Socio-demographic Index	None	2	Negative
Outdoor air pollution ($PM_{2.5}$)	None	2	Positive
In-facility delivery (proportion)	None	2	Negative
Indoor air pollution (all cooking fuels)	None	2	Positive
Antenatal care (1 visit) coverage (proportion)	None	3	Negative
Alcohol (litres per capita)	None	3	Positive
Education (years per capita)	None	3	Negative
LDI (I$ per capita)	Log	3	Negative
Antenatal care (4 visits) coverage (proportion)	None	3	Negative

Covariates selected for CODEm model of digestive congenital anomalies

Covariate	Transformation	Level	Direction
Maternal alcohol consumption during pregnancy (proportion)	None	1	Positive
Smoking prevalence (reproductive age-standardised)	None	1	Positive
Indoor air pollution (all cooking fuels)	None	2	Positive
Diabetes age-standardised prevalence (proportion)	None	2	Positive
Socio-demographic Index	None	2	Negative
Prevalence of obesity (age-standardised)	None	2	Positive
In-facility delivery (proportion)	None	2	Negative
Healthcare access and quality index	None	2	Negative
Alcohol (liters per capita)	None	3	Positive
Health system access (capped)	None	3	Negative
Education (years per capita)	None	3	Negative
Vegetables unadjusted (g)	None	3	Not specified
Antenatal care (1 visit) coverage (proportion)	None	3	Negative
Antenatal care (4 visits) coverage (proportion)	None	3	Negative

Fruits unadjusted (g)	None	3	Not specified
LDI (I$ per capita)	Log	3	Negative

Covariates selected for CODEm model of other congenital birth defects

Covariate	Transformation	Level	Direction
Maternal alcohol consumption during pregnancy (proportion)	None	1	Positive
Live births 35+ (proportion)	None	1	Positive
Education (years per capita)	None	2	Negative
Smoking prevalence (reproductive age-standardised)	None	2	Positive
Legality of abortion	None	2	Negative
In-facility delivery (proportion)	None	2	Negative
Indoor air pollution (all cooking fuels)	None	2	Positive
Healthcare access and quality index	None	2	Negative
Antenatal care (1 visit) coverage (proportion)	None	3	Negative
Diabetes age-standardised prevalence (proportion)	None	3	Positive
LDI (I$ per capita)	Log	3	Negative
Socio-demographic Index	None	3	Negative
Antenatal care (4 visits) coverage (proportion)	None	3	Negative
Alcohol (litres per capita)	None	3	Positive

3.9.1, 7.1.2, and 11.6.2 Household Air Pollution SDG Capstone Appendix

Flowchart

Household Air Pollution from Solid Fuels

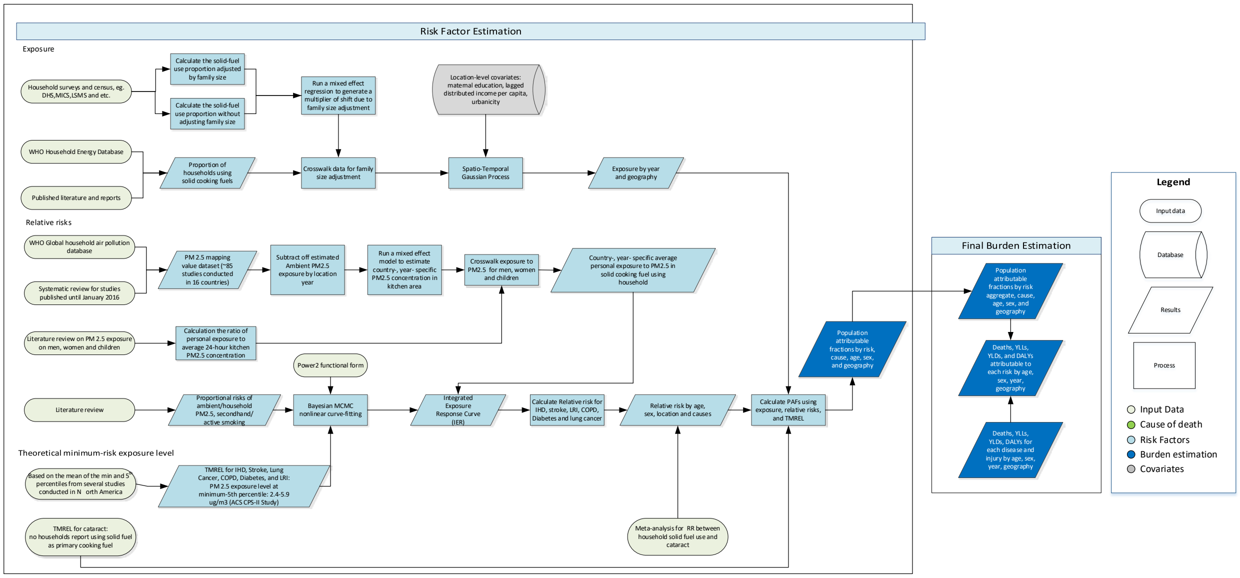

Indicator definition

This modeling strategy encompassed the indicator associated with deaths attributable to household air pollution (3.9.1).

Indicator 3.9.1

As a component of SDG Goal 3. Ensure healthy lives and promote well-being for all at all ages, SDG Target 3.9, by 2030, substantially reduce the number of deaths and illnesses from hazardous chemicals and air, water and soil pollution and contamination, is measured using SDG Indicator 3.9.1, deaths attributable to household air pollution and ambient air pollution per 100,000.

Indicator 7.1.2

As a component of SDG Goal 7. Ensure access to affordable, reliable, sustainable, and modern energy for all, SDG Target 7.1, by 2030, ensure universal access to affordable, reliable and modern energy services, is measured using SDG Indicator 7.1.2, risk weighted prevalence of population using unsafe cooking fuel, which comes from household air pollution (HAP).

Input Data & Methodological Summary

Exposure

Case definition

Exposure to household air pollution from solid fuels (HAP) is defined as the proportion of households using solid cooking fuels. The definition of solid fuel in our analysis includes coal, wood, charcoal, dung, and agricultural residues.

Input data

Data were extracted from the standard multi-country survey series such as Demographic and Health Surveys (DHS), Living Standards Measurement Surveys (LSMS), Multiple Indicator Cluster Surveys (MICS), and World Health Surveys (WHS), as well as country-specific survey series such as Kenya Welfare Monitoring Survey and South Africa General Household Survey. To fill the gaps of data in surveys and censuses, we also downloaded and updated HAP estimates from WHO Energy Database and extracted from literature through systematic review. Each nationally or sub-nationally representative data point provided an estimate for the percentage of households using solid cooking fuels. Estimates for the usage of solid fuels for non-cooking purpose were excluded, i.e. primary fuels for lighting. The database, with estimates from 1980 to 2017, contained about 680 studies from 150 countries. As updates to systematic reviews are performed on an ongoing schedule across all GBD causes and risk factors, an update for household air pollution will be performed in the next 1-2 iterations.

Modelling strategy

Household air pollution was modelled at household level using a three-step modelling strategy that uses linear regression, spatiotemporal regression and Gaussian Process Regression (GPR). The first step is a mixed-effect linear regression of logit-transformed proportion of households using solid cooking fuels. The linear model contains maternal education, proportion of population living in urban areas, and lagged-distributed income as covariates and has nested random effect by GBD region, and GBD super region respectively. The full ST-GPR process is specified elsewhere this appendix. No substantial modelling changes were made in this round compared to GBD 2016.

Theoretical minimum-risk exposure level

For cataract, the TMREL is defined as no households using solid cooking fuel. For outcomes that utilise evidence based on the Integrated Exposure Response (IER), the TMREL is defined as uniform distribution between 2.4 and 5.9 ug/m^3.

Relative risks

In addition to the previously included outcomes of lower respiratory infections (LRI), stroke, Ischemic Heart Disease (IHD), Chronic Obstructive Pulmonary Disease (COPD), lung cancer, and cataract, in GBD 2017 we added Type II Diabetes as a new outcome of household air pollution. The relative risk for cataracts was extracted from a meta-analysis and is 2.47 with 95% (1.61, 3.73).[1] GBD currently only estimates cataracts as an outcome for females.

In GBD 2017, we adopted a new approach for risk attribution using the Integrated Exposure-Response Function (IER). Updates to the IER and the new joint-estimation PAF approach is described in the Ambient Particulate Matter appendix.

$PM_{2.5}$ mapping value

In order to use the IER curve, we must estimate the exposure to particulate matter with diameter of less than 2.5 micrometers ($PM_{2.5}$). Since GBD 2015 we have been using a mapping model relying on a database of now almost 90 studies which measures $PM_{2.5}$ exposure in households using solid cooking fuel. Using socio-demographic index and study-level factors as covariates, we predict exposure for all location-years.

In GBD 2017, we updated the model to estimate the individual exposure to $PM_{2.5}$ over and above ambient levels due to the use of solid cooking fuel. We did this by subtracting off the estimated ambient

level $PM_{2.5}$ for the location-year of each study in the database before inputting them into the model. By doing this we have independent estimates for $PM_{2.5}$ exposure due to ambient and household solid fuel use.

These exposures are cross-walked to values for men, women, and children by generating the ratio of each group's mean exposure to the overall mean personal exposure. The resulting location, year, sex, and age specific $PM_{2.5}$ exposure values are used as inputs in the IER and attributable burden calculation process.

References

1. Smith KR, Bruce N, Balakrishnan K, Adair-Rohani H, Balmes J, Chafe Z, et al. Millions Dead: How Do We Know and What Does It Mean? Methods Used in the Comparative Risk Assessment of Household Air Pollution. Annu Rev Public Health. 2014;35(1):185–206.

3.9.2, 6.1.1, 6.2.1 WaSH SDG Capstone Appendix

Input data & Methodological Summary

Indicator definition

This modeling strategy encompassed the indicator associated with deaths attributable to unsafe water, sanitation, and hygiene (WaSH) (3.9.2).

For GBD 2016, the WaSH category is an aggregate of the risk estimates for water (6.1.1), hygiene (6.2.1b) and sanitation (6.2.1a). These are modeled independently and then aggregated together to generate the overall risk estimates for deaths attributable to WaSH.

Indicator 3.9.2

As a component of SDG Goal 3. Ensure healthy lives and promote well-being for all at all ages, SDG Target 3.9 by 2030, substantially reduce the number of deaths and illnesses from hazardous chemicals and air, water and soil pollution and contamination, is measured using SDG Indicator 3.9.2, deaths attributable to unsafe WaSH per 100,000.

Indicator 6.1.1

As a component of SDG Goal 6. Ensure availability and sustainable management of water and sanitation for all, SDG Target 6.1, by 2030, achieve universal and equitable access to safe and affordable drinking water for all, is measured using SDG Indicator 6.1.1, risk-weighted prevalence of population using unsafe/unimproved water sources.

Indicator 6.2.1a

As a component of SDG Goal 6. Ensure availability and sustainable management of water and sanitation for all, SDG Target 6.2, by 2030, achieve access to adequate and equitable sanitation and hygiene for all and end open defecation, paying special attention to the needs of women and girls and those in vulnerable situations, is measured using SDG Indicator 6.2.1a, risk-weighted prevalence of population using unsafe sanitation practices.

Indicator 6.2.1b

As a component of SDG Goal 6. Ensure availability and sustainable management of water and sanitation for all, SDG Target 6.2, by 2030, achieve access to adequate and equitable sanitation and hygiene for all and end open defecation, paying special attention to the needs of women and girls and those in vulnerable situations, is measured using SDG Indicator 6.2.1b, risk-weighted prevalence of population with no access to a handwashing facility.

3.9.2 and 6.1.1 Unsafe Water SDG Capstone Appendix

Flowchart

Unsafe Drinking Water

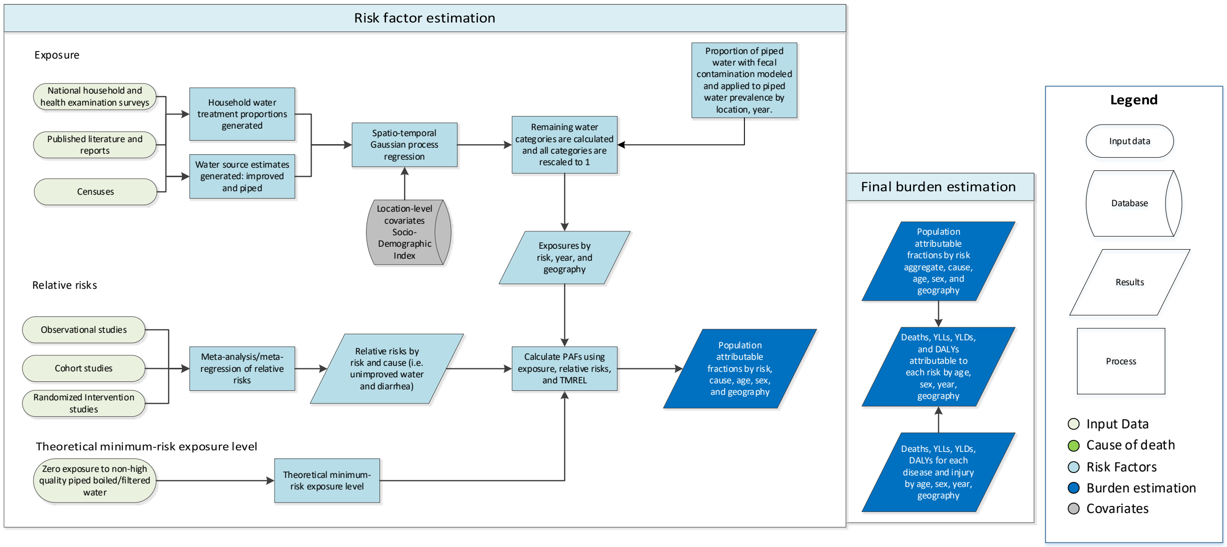

Input data & methodological summary

Exposure

Case definition

For GBD 2017, exposure to unsafe water was defined based on reported primary water source used by the household and use of household water treatment (HWT) to improve the quality of drinking water before consumption. Water sources were defined as "improved" based on the JMP designation,[1] which includes piped water as improved water, and households with access to piped water connection to the house, yard, or plot were defined as having access to piped water supply. Solar treatment, chlorine treatment, boiling, or the use of filters were all established as effective point-of-use household water treatments based on effect sizes calculated from network meta-analysis.

Input data

The search for usable household surveys and censuses was conducted using the Global Health Data Exchange (GHDx) database. HWT input data is primarily limited to two large survey series (DHS and MICS) due to data availability. Water source data includes censuses and nationally representative surveys such as DHS, MICS, AIS, and WHS. For each survey, household sample weights were multiplied by the number of household members to produce a weighting scheme that estimates proportion of individuals, not proportion of households, exposed to a given indicator. Surveys and censuses were then tabulated to the two water source and two water treatment categories of interest for each location.

Modelling

Water source data is modelled using an ordinal framework, with two distinct models: prevalence of piped water and proportion of improved water (excluding piped) within the non-piped population. Both models produce results for each unique location, year combination. This ordinal framework allows us to estimate the category with the most data (piped water prevalence) and leverage that estimate to

anchor the estimates for improved and unimproved water categories. The results of the improved proportion model are multiplied by the piped water prevalence to calculate improved water prevalence. The sum of improved and piped water prevalence are subtracted from 1 to yield unimproved water prevalence.

HWT categories are estimated in a similar ordinal framework, by modelling prevalence of individuals using no water treatment methods and proportions of households that boil/filter water within the population of households that engage in treatment methods. The prevalence of individuals that boil/filter drinking water is calculated by multiplying the proportion that boil/filter modelled previously times prevalence of any water treatment (estimated by subtracting prevalence of no treatment from 1). The prevalence of individuals that treat their water using solar/chlorine methods was estimated by subtracting the sum of prevalence of no treatment estimates and prevalence of filter/boil treatment from 1. By year and location, each of the above categories are modelled using a 3-step modelling scheme of mixed effect linear regression followed by spatio-temporal Gaussian process regression (ST-GPR), which produces full time series estimates for each GBD 2017 location. Socio-demographic index (SDI), a composite metric combining education per capita, income per capita, and fertility, was set as a fixed effect in the linear regression since it proved to be a significant predictor. Random effects were set at GBD 2017 region and super-region levels to fit the models but were not used in the predictions.

The process of vetting and validating models was accomplished primarily through an examination of ST-GPR scatter plots by GBD 2017 location from 1990-2017. Any unfitting data points were re-inspected for error at the level of extraction and survey implementation, and subsequently excluded from analysis if deemed appropriate. In addition to SDI, a number of different potential fixed effects were considered, including lag-distributed income and urbanicity, but SDI proved to be the strongest predictor of the unsafe water categories. Uncertainty in the estimates was initially formed based on standard deviation by survey, then propagated through ST-GPR modelling by means of confidence intervals around each data point that reflect the point-estimate specific variance.

Once models are vetted, full time series outputs from ST-GPR modelling are then converted from proportion to prevalence by year and geography and then rescaled to form 9 mutually exclusive categories that sum up to 1. The table below provides the final result of this rescaling.

Category	Definition
Unimproved, no HWT	Proportion of individuals that primarily use unimproved source, and *do not* use any HWT to purify their drinking water.
Unimproved, chlorine/solar	Proportion of individuals that primarily use unimproved source, and solar or chlorine treatment to purify their drinking water.
Unimproved, boil/filter	Proportion of individuals that primarily use unimproved source, and boil or filter to purify their drinking water.
Improved water except piped, no HWT	Proportion of individuals that primarily use improved sources other than piped water supply, and *do not* use any HWT to purify their drinking water.

Improved water except piped, chlorine/solar	Proportion of individuals that primarily use improved sources other than piped water supply, and use solar or chlorine treatment to purify their drinking water.
Improved water except piped, boil/filter	Proportion of individuals that primarily use improved sources other than piped water supply, and boil/filter their drinking water.
Basic piped water, no HWT	Proportion of individuals that primarily use basic piped water supply, and *do not* use any HWT to purify their drinking water
Basic piped water, chlorine/solar	Proportion of individuals that primarily use basic piped water supply, and *use* solar or chlorine water treatment to purify their drinking water.
Basic piped water, boil/filter	Proportion of individuals that primarily use basic piped water supply, and boil or filter to purify their drinking water
High-quality (HQ) piped water, boil/filter	Proportion of individuals that primarily use basic piped water supply, and boil or filter to purify their drinking water

We modelled the microbiological quality of piped water sources primarily using data a review by Bain et al.[1] that measured proportion of piped water sources contaminated with fecal indicators. We use the value generated from this model to split the prevalence of piped water into basic piped water and high quality piped water by location, year, age, and sex.

A substantial limitation in our analysis is the paucity of data on HWT and piped water quality. The inclusion of more location-specific data on water treatment utilisation at the household level can greatly improve our estimates in future iterations.

Theoretical minimum-risk exposure level

The theoretical minimum-risk exposure level for unsafe water is defined as all households have access to high quality piped water that has been boiled or filtered before drinking.

Relative risks

For GBD 2017, unsafe water was paired with one outcome-diarrheal diseases-given evidence provided by relative risk studies. A meta-analysis by Wolf et al.[3] provided the bulk of the relative risk evidence for the relationship between unsafe water and diarrheal diseases. This meta-analysis was updated through a literature review that searched for related intervention studies post-2014 conducted in PubMed. Search terms used were identical to those provided by Wolf et al.[3] Relative risk values for water-source interventions and point-of-use treatment interventions were calculated using network meta-analysis approach so as to include studies that differ in control groups within the same analysis. This analysis produced distinct relative risks for each water source and water treatment category. The combined effect of a source intervention and point-of-use intervention was assumed to be multiplicative in order to match GBD 2017 exposure definitions.

References

1. "Improved and Unimproved Water Sources and Sanitation Facilities."*WHO / UNICEF Joint Monitoring Programme: Wat/san Categories*. The WHO/UNICEF, n.d. Web. 08 June 2016
2. Bain, R., Cronk, R., Wright, J., Yang, H., Slaymaker, T., & Bartram, J. (2014). Fecal Contamination of Drinking-Water in Low- and Middle-Income Countries: A Systematic Review and Meta-Analysis. PLoS Medicine, 11(5). doi:10.1371/journal.pmed.1001644
3. Wolf, Jennyfer, Annette Prüss-Ustün, Oliver Cumming, Jamie Bartram, Sophie Bonjour, Sandy Cairncross, Thomas Clasen, John M. Colford, Valerie Curtis, Jennifer De France, Lorna Fewtrell, Matthew C. Freeman, Bruce Gordon, Paul R. Hunter, Aurelie Jeandron, Richard B. Johnston, Daniel Mäusezahl, Colin Mathers, Maria Neira, and Julian P. T. Higgins. "Systematic Review: Assessing the Impact of Drinking Water and Sanitation on Diarrhoeal Disease in Low- and Middle-income Settings: Systematic Review and Meta-regression." Trop Med Int Health Tropical Medicine & International Health 19.8 (2014): 928-42. Web.

6.1.2a Unsafe Sanitation Capstone Appendix

Flowchart

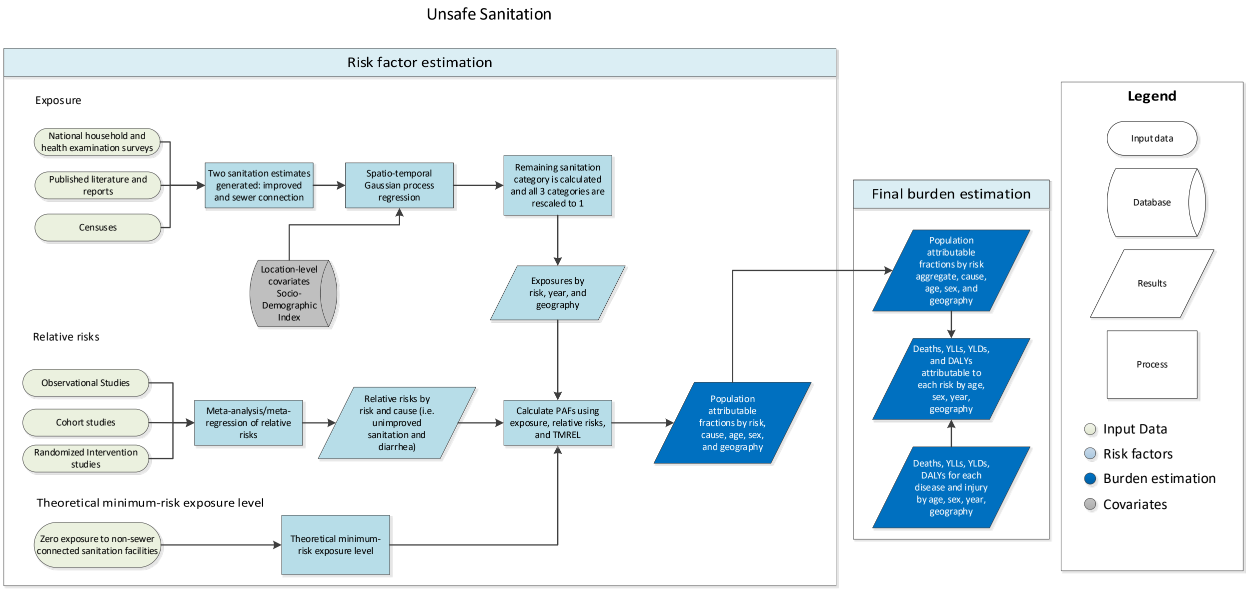

Input data & methodological summary

Exposure

Case definition

Exposure to unsafe sanitation is defined based on the primary toilet type used by households. Improved facilities are defined as such based on JMP designation (WHO). Sewer connection toilets included flush toilets or any toilet with connection to the sewer or septic tank.

Input data

The search for usable household surveys and censuses was conducted using the Global Health Data Exchange (GHDx) database. For each survey, household sample weights were multiplied by the number of household members to produce a weighting scheme that estimates proportion of individuals, not

proportion of households, exposed to a given indicator. Surveys and censuses were then tabulated to two sanitation categories, sewer connection and improved sanitation, for each location. Data in tabulated form was lower priority to add to models and was only updated when time permitted.

Modeling

A change made for GBD 2017 was to model sanitation categories in an ordinal framework instead of independent models. Two distinct indicators were estimated: the prevalence of individuals using sewer connection or septic tank facilities and the proportion of individuals with improved sanitation within the population not connected to sewer or septic tank. This ordinal framework allows us to estimate the category with the most data (sewer connection/septic tank prevalence) and leverage that estimate to anchor the estimates for improved and unimproved sanitation categories. The results of the improved proportion model are multiplied by the sewer connection/septic tank prevalence to calculate improved sanitation prevalence. The sum of improved and sewer connection/septic tank prevalence are subtracted from 1 to yield unimproved sanitation prevalence.

The two indicators were modeled using a 3-step modeling scheme of mixed effect linear regression followed by spatio-temporal Gaussian process regression (ST-GPR), which produced full time series estimates for each GBD 2017 location. Socio-demographic index (SDI), a composite metric combining education per capita, income per capita, and fertility, was set as a fixed effect in the linear regression since it proved to be a significant predictor. Random effects were set at GBD 2017 region and super-region levels to fit the models but were not used in the predictions.

The process of vetting and validating models was accomplished primarily through an examination of ST-GPR scatter plots by GBD 2017 location from 1990-2017. Any unfitting data points were re-inspected for error at the level of extraction and survey implementation, and subsequently excluded from analysis if deemed appropriate. In addition to SDI, a number of different potential fixed effects were considered, including lag-distributed income and urbanicity, but SDI proved to be the strongest predictor of unsafe sanitation in terms of magnitude of the coefficient. Uncertainty in the estimates was initially constructed based on standard deviation around each survey mean, then propagated through ST-GPR modeling by incorporating the variance of each data point in the Gaussian process regression step. A data point with high variance, for example, would contribute relatively less influence to the model than a data point with lower variance.

Once models are vetted, full time series outputs from ST-GPR modeling are then converted from proportion to prevalence by year and geography and then rescaled to form three mutually exclusive categories that sum up to 1. The table below provides the final result of this rescaling.

Category	*Definition*
Unimproved sanitation	Proportion of individuals that use unimproved sanitation facilities.
Improved sanitation	Proportion of individuals with access to improved sanitation facilities, excluding sewer connection or septic tank.
Sanitation facilities with sewer connection or septic tank	Proportion of individuals with access to toilet facilities with sewer connection or septic tank.

Theoretical minimum-risk exposure level

The theoretical minimum-risk exposure level for unsafe sanitation was defined as all individuals have access to a sanitation facility with sewer connection.

Relative risks

For GBD 2017, unsafe sanitation was only paired with one outcome, diarrheal diseases. A meta-analysis by Wolf et al. 2014 provides the bulk of the relative risk evidence for the relationship between unsafe sanitation and diarrheal diseases. This meta-analysis was updated through a literature review that searched for related intervention studies post-2014 conducted in PubMed. Search terms used were identical to those provided by Wolf et al. 2014.

References

1. "Improved and Unimproved Water Sources and Sanitation Facilities."*WHO / UNICEF Joint Monitoring Programme: Wat/san Categories*. The WHO/UNICEF, n.d. Web. 08 June 2016
2. Wolf, Jennyfer, Annette Prüss-Ustün, Oliver Cumming, Jamie Bartram, Sophie Bonjour, Sandy Cairncross, Thomas Clasen, John M. Colford, Valerie Curtis, Jennifer De France, Lorna Fewtrell, Matthew C. Freeman, Bruce Gordon, Paul R. Hunter, Aurelie Jeandron, Richard B. Johnston, Daniel Mäusezahl, Colin Mathers, Maria Neira, and Julian P. T. Higgins. "Systematic Review: Assessing the Impact of Drinking Water and Sanitation on Diarrhoeal Disease in Low- and Middle-income Settings: Systematic Review and Meta-regression." Trop Med Int Health Tropical Medicine & International Health 19.8 (2014): 928-42. Web.

6.2.1a Unsafe Hygiene Capstone Appendix

Flowchart

Unsafe Handwashing

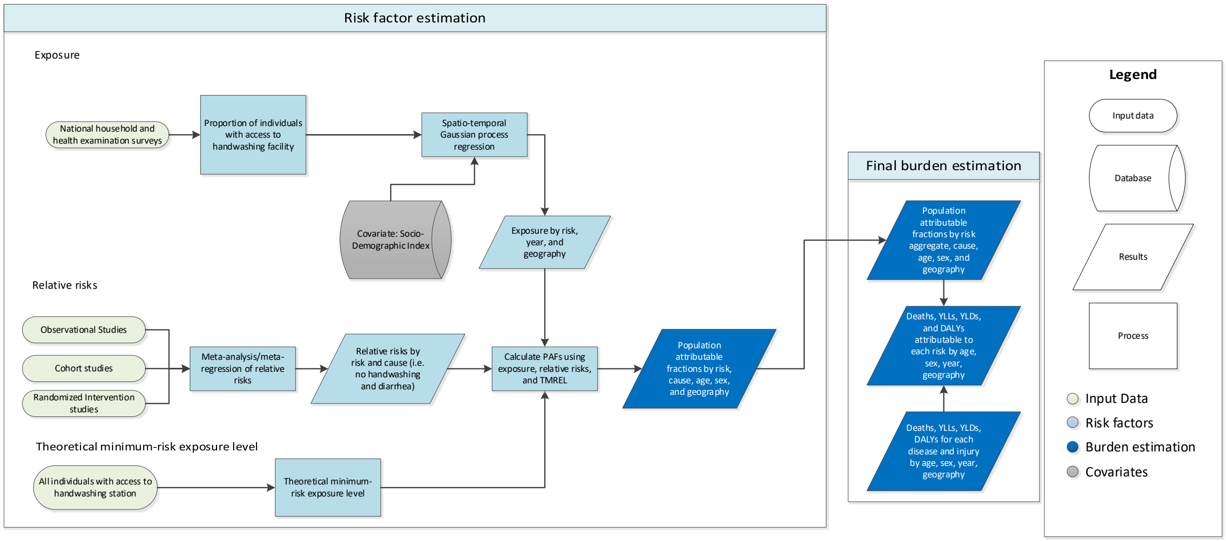

Input data & methodological summary

Exposure

Case definition

Unsafe hygiene is defined as lack of access to a handwashing station with available soap and water. We estimated the burden of unsafe hygiene in both developed and developing settings.

Input data

Since water and soap availability data are very limited, only country-specific Demographic Health Surveys (DHS) and Malaria Indicator Survey Series (MICS) conducted after 2006 were included as input data.

Modelling strategy

By year and location, proportion of households with handwashing facility is modelled using a 3-step modelling scheme of mixed effect linear regression followed by spatio-temporal Gaussian process regression (ST-GPR), which outputs full time series estimates for each GBD 2017 location. Socio-demographic index (SDI), a composite index that include income per capita, education, and fertility, was set as a fixed effect in the linear regression since it proved to have significant coefficient. Random effects were set at GBD 2017 region and super-region levels to fit the model but were not used in the predictions.

The process of vetting and validating models was accomplished primarily through an examination of ST-GPR scatter plots by GBD 2016 location from 1990-2016. Any data points lacking face validity were re-inspected for error at the level of extraction and survey implementation, and subsequently excluded

from analysis if deemed appropriate. In addition to SDI, a number of different potential fixed effects were considered, including lag-distributed income and urbanicity. However, SDI proved to be the strongest predictor.

A considerable limitation for when estimating handwashing practices for over 190 independent locations around the world was data sparseness. Even when data were published on handwashing prevalence, the definition was often altered from the GBD 2017 standard definition or it may only have pertained to certain populations (such as hospital patients) and lacked representativeness at the geographic scale we required. The incorporation of questions about soap and water availability in DHS and MICS added much-needed information but there remains a large data gap to be filled if we are to become more certain in handwashing access estimates.

Theoretical minimum-risk exposure level

The theoretical minimum-risk exposure level for unsafe hygiene is defined as all individuals with access to handwashing facility after any contact with excreta, including children's excreta.

Relative risks

A meta-analysis by Cairncross et al.[1] provide relative risk values describing the relationship between lack of facility access and diarrheal diseases. A meta-analysis by Rabie and Curtis[2] provided relative risk evidence for the relationship between lack of facility access and lower respiratory infection.

References

1. Cairncross, S., Hunt, C., Boisson, S., Bostoen, K., Curtis, V., Fung, I. C., & Schmidt, W. P. (2010). Water, sanitation and hygiene for the prevention of diarrhoea. International Journal of Epidemiology, 39(Supplement 1), I193-I205. doi:10.1093/ije/dyq035
2. Rabie, T., & Curtis, V. (2006). Handwashing and risk of respiratory infections: a quantitative systematic review. Tropical Medicine and International Health, 11(3), 258-267. doi:10.1111/j.1365-3156.2006.01568.x

3.a.1 Smoking Prevalence SDG Capstone Appendix

Flowchart

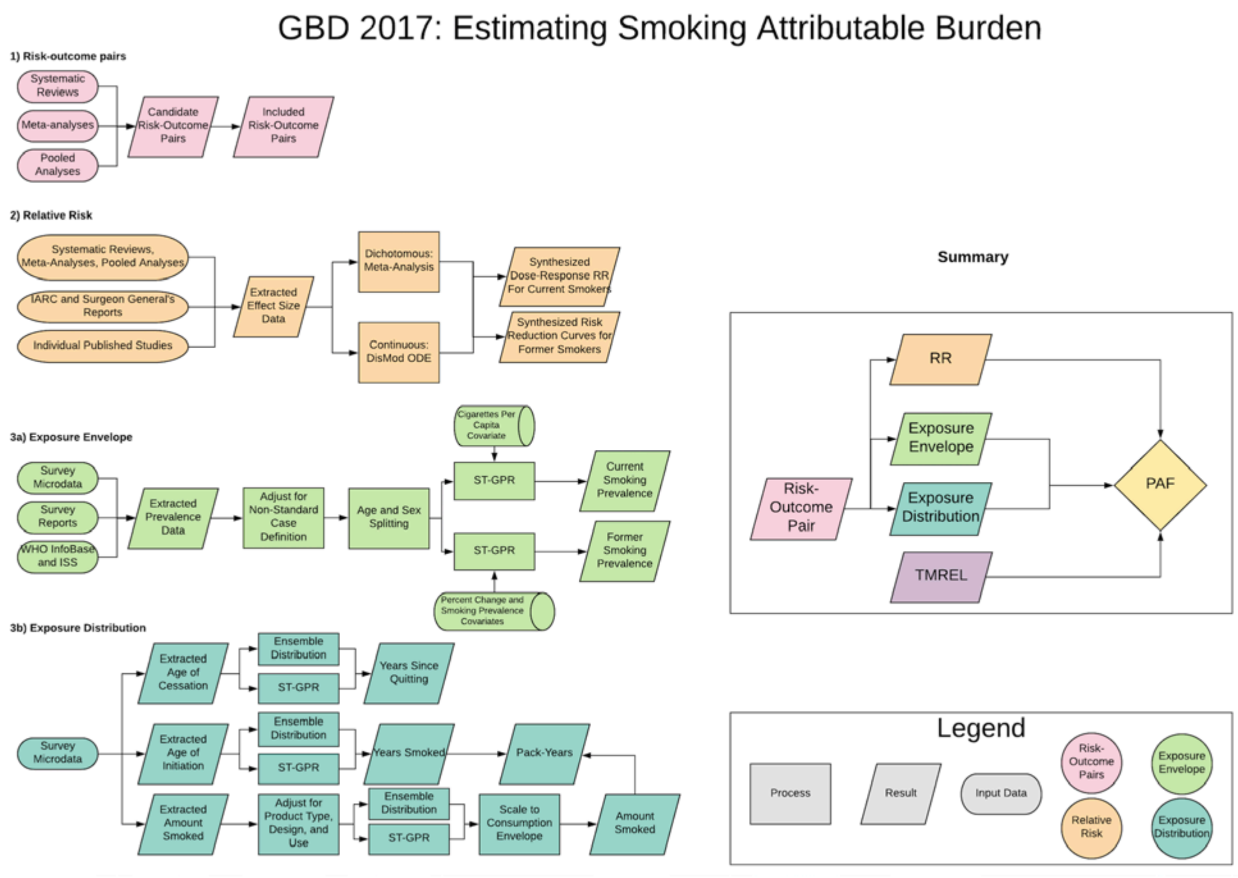

We made significant changes to the methods used to estimate smoking attributable burden in GBD 2017. In previous iterations of the GBD, we have used the Peto-Lopez (Smoking Impact Ratio) method to estimate burden attributable to cancers and chronic respiratory diseases. Although this method provides robust estimates of the burden of cancers and chronic respiratory diseases related to tobacco, it is not fully consistent with the GBD approach of estimating exposure independently of the outcomes affected by exposure. For cardiovascular diseases and all other smoking attributable health outcomes, we used five-year lagged daily smoking prevalence as the exposure. With a growing body of evidence on the association between smoking and several types of cancers and with cardiovascular disease, coupled with good estimates of the distribution of cumulative smoking exposure, direct estimation of attributable burden is possible. In GBD 2017, we have transitioned to using continuous measures of exposure that incorporate dose-response effects among daily, occasional, and former smokers for all health outcomes except fractures.

Current and former smoking prevalence

We estimated the prevalence of current smoking and the prevalence of former smoking using data from cross-sectional nationally representative household surveys. We defined current smokers as individuals

who currently use any smoked tobacco product on a daily or occasional basis. We defined former smokers as individuals who quit using all smoked tobacco products for at least 6 months, where possible, or according to the definition used by the survey. Prior to modelling a complete time series for all demographic groups, we made adjustments for alternative case definitions as well as for data reported in non-standard age or sex groups. We modelled current and former prevalence using spatiotemporal Gaussian process regression.

Data extraction

We extracted primary data from individual-level microdata and survey report tabulations. We extracted data on current, former, and/or ever smoked tobacco use reported as any combination of frequency of use (daily, occasional, and unspecified, which includes both daily and occasional smokers) and type of smoked tobacco used (all smoked tobacco, cigarettes, hookah, and other smoked tobacco products such as cigars or pipes), resulting in 36 possible combinations. Other variants of tobacco products, for example hand-rolled cigarettes, were grouped into the four type categories listed above based on product similarities. Only smoked tobacco products are included, smoked drugs are estimated separately as part of the drug use risk factor.

For microdata, we extracted relevant demographic information, including age, sex, location, and year, as well as survey metadata, including survey weights, primary sampling units, and strata. This information allowed us to tabulate individual-level data in the standard GBD five-year age-sex groups and produce accurate estimates of uncertainty. For survey report tabulations, we extracted data at the most granular age-sex group provided.

Crosswalk

Our GBD smoking case definitions were current smoking of any tobacco product and former smoking of any tobacco product. All other data points were adjusted to be consistent with either of these definitions. Some sources contained information on more than one case definition and these sources were used to develop the adjustment coefficient to transform alternative case definitions to the GBD case definition. The adjustment coefficient was the beta value derived from a linear model with one predictor and no intercept.

We generated separate crosswalk coefficients for the 10-14 age group and the 15-19 age group, as we found the relationships between case definitions differed strongly in the younger age groups compared to the 20+ age groups. To account for this, we attempted to generate a global crosswalk coefficient for both the 10-14 and 15-19 age groups, using the same regression as above. Due to data limitations, none of the crosswalk coefficients met the criteria outlined above, so no data covering youths under 20 years old were crosswalked. In other words, all data from these age groups that appear in the model were asked according to our case definition in the survey.

We propagated uncertainty at the survey level from the crosswalk by incorporating both the variance of the errors and the variance of the adjustment coefficients.

For each source that needed adjusting, we assigned space weights based on GBD region and super region to the sources containing more than one case definition. Data from the same region receiving a full weight of 1, and data from the same super-region received a weight of ½. We explored using a time weight, to control for possible changes in the relationship between smokeless tobacco use behaviours over time. We found incorporating temporal information did not significantly change the estimated

coefficients but did undercut sample sizes, and chose to exclude the time weight. Crosswalk coefficients generated from fewer than 20 data sources were dropped

Age and Sex Splitting

We split data reported in broader age groups than the GBD 5-year age groups or as both sexes combined by adapting the method reported in Ng et al. (http://jamanetwork.com/journals/jama/fullarticle/1812960) to split using a sex- geography- time specific reference age pattern. We separated the data into two sets: a training dataset, with data already falling into GBD sex-specific 5-year age groups, and a split dataset, which reported data in aggregated age or sex groups. We then used spatiotemporal Gaussian Process Regression (ST-GPR) to estimate sex-geography-time specific age patterns using data in the training dataset. The estimated age patterns were used to split each source in the split dataset.

The ST-GPR model used to estimate the age patterns for age-sex splitting used an age weight parameter value that minimises the effect of any age smoothing. This parameter choice allows the estimated age pattern to be driven by data, rather than being enforced by any smoothing parameters of the model. Because these age-sex split data points will be incorporated in the final ST-GPR exposure model, we do not want to doubly enforce a modelled age pattern for a given sex-location-year on a given aggregate data point.

Smoking Prevalence Modelling

We used ST-GPR to model current and former smoking prevalence. Full details on the ST-GPR method are reported elsewhere in the Appendices of the GBD Capstones. Briefly, the mean function input to GPR is a complete time series of estimates generated from a mixed effects hierarchical linear model plus weighted residuals smoothed across time, space and age. The linear model formula for current smoking, fit separately by sex using restricted maximum likelihood in R, is:

$$logit(p_{g,a,t}) = \beta_0 + \beta_1 CPC_{g,t} + \sum_{k=2}^{19} \beta_k I_{A[a]} + \alpha_s + \alpha_r + \alpha_g + \epsilon_{g,a,t}$$

Where $CPC_{g,t}$ is the tobacco consumption covariate by geography g and time t, described above, $I_{A[a]}$ is a dummy variable indicating specific age group A that the prevalence point $p_{g,a,t}$ captures, and α_s, α_r, and α_g are super region, region, and geography random intercepts, respectively. Random effects were used in model fitting but not in prediction.

The linear model formula for former smoking is:

$$logit(p_{g,a,t}) = \beta_0 + \beta_1 PctChange_{A[a],g,t} + \beta_3 CSP_{A[a],g,t} + \sum_{k=3}^{20} \beta_k I_{A[a]} + \alpha_s + \alpha_r + \alpha_g + \epsilon_{g,a,t}$$

Where $PctChange_{A[a],g,t}$ is the percent change in current smoking prevalence from the previous year, and $CSP_{A[a],g,t}$ is the current smoking prevalence by specific age group A, geography g, and time t that point $p_{g,a,t}$ captures, both derived from the current smoking ST-GPR model defined above.

Exposure Among Current and Former Smokers

We estimated exposure among current smokers for two continuous indicators: cigarettes per smoker per day and pack-years. Pack-years incorporates aspects of both duration and amount. One pack-year represents the equivalent of smoking one pack of cigarettes (assuming a 20 cigarette pack) per day for one year. Since the pack-years indicator collapses duration and intensity into a single dimension, one pack-year of exposure can reflect smoking 40 cigarettes per day for six months or smoking 10 cigarettes per day for two years.

To produce these indicators, we simulated individual smoking histories based on distributions of age of initiation and amount smoked. We informed the simulation with cross-sectional survey data capturing these indicators, modelled at the mean level for all locations, years, ages, and sexes using spatiotemporal Gaussian process regression. We rescaled estimates of cigarettes per smoker per day to an envelope of cigarette consumption based on supply-side data. We estimated pack-years of exposure by summing samples from age- and time-specific distributions of cigarettes per smoker for a birth cohort in order to capture both age trends and time trends and avoid the common assumption that the amount someone currently smokes is the amount they have smoked since they began smoking. All distributions were age-, sex-, and region- specific ensemble distributions, which were found to outperform any single distribution.

We estimated exposure among former smokers using years since cessation. We utilised spatiotemporal Gaussian process regression to model mean age of cessation using cross-sectional survey data capturing age of cessation. Using these estimates, we generated ensemble distributions of years since cessation for every location, year, age group, and sex.

Risk-Outcome Pairs

We included the following risk-outcome pairs based on evidence supporting a causal relationship: tuberculosis, lower respiratory tract infections, esophageal cancer, stomach cancer, bladder cancer, liver cancer, laryngeal cancer, lung cancer, breast cancer, cervical cancer, colorectal cancer, lip and oral cancer, nasopharyngeal cancer, other pharyngeal cancer, pancreatic cancer, kidney cancer, leukemia, ischemic heart disease, ischemic stroke, hemorrhagic stroke, subarachnoid hemorrhage, atrial fibrillation and flutter, aortic aneurysm, peripheral arterial disease, chronic obstructive pulmonary disease, other chronic respiratory diseases, asthma, peptic ulcer disease, gallbladder and biliary tract diseases, Alzheimer disease and other dementias, Parkinson disease (protective), multiple sclerosis, type-II diabetes, rheumatoid arthritis, low back pain, cataracts, macular degeneration, and fracture.

Dose-response risk curves

We conducted systematic literature reviews for all risk-outcome pairs identified as being caused by smoking. We extracted effect sizes by cigarettes per smoker per day, pack-years, and years since quitting from cohort and case-control studies. We synthesised these data to produce non-linear dose response curves using a Bayesian meta-regression model. For outcomes with significant differences in effect size by sex or age, we produced sex- or age-specific risk curves.

We estimate risk curves of former smokers compared to never smokers taking into account the rate of risk reduction among former smokers seen in the cohort and case-control studies, and the cumulative exposure among former smokers within each age, sex, location and year group.

PAF Calculation

We estimated population attributable fractions based on the following equation:

$$PAF = \frac{p(n) + p(f) \int \exp(x) * rr(x) + p(c) \int \exp(y) * rr(y) - 1}{p(n) + p(f) \int \exp(x) * rr(x) + p(c) \int \exp(y) * rr(y)}$$

where $p(n)$ is the prevalence of never smokers, $p(f)$ is the prevalence of former smokers, $p(c)$ is the prevalence of current smokers, $\exp(x)$ is a distribution of years since quitting among former smokers, $rr(x)$ is the relative risk for years since quitting, $\exp(y)$ is a distribution of cigarettes per smoker per day or pack-years, and $rr(y)$ is the relative risk for cigarettes per smoker per day or pack-years.

We used pack-years as the exposure definition for cancers and chronic respiratory diseases, and cigarettes per smoker per day for cardiovascular diseases and all other health outcomes.

3.b.1 Vaccine Coverage SDG Capstone Appendix

Flowchart

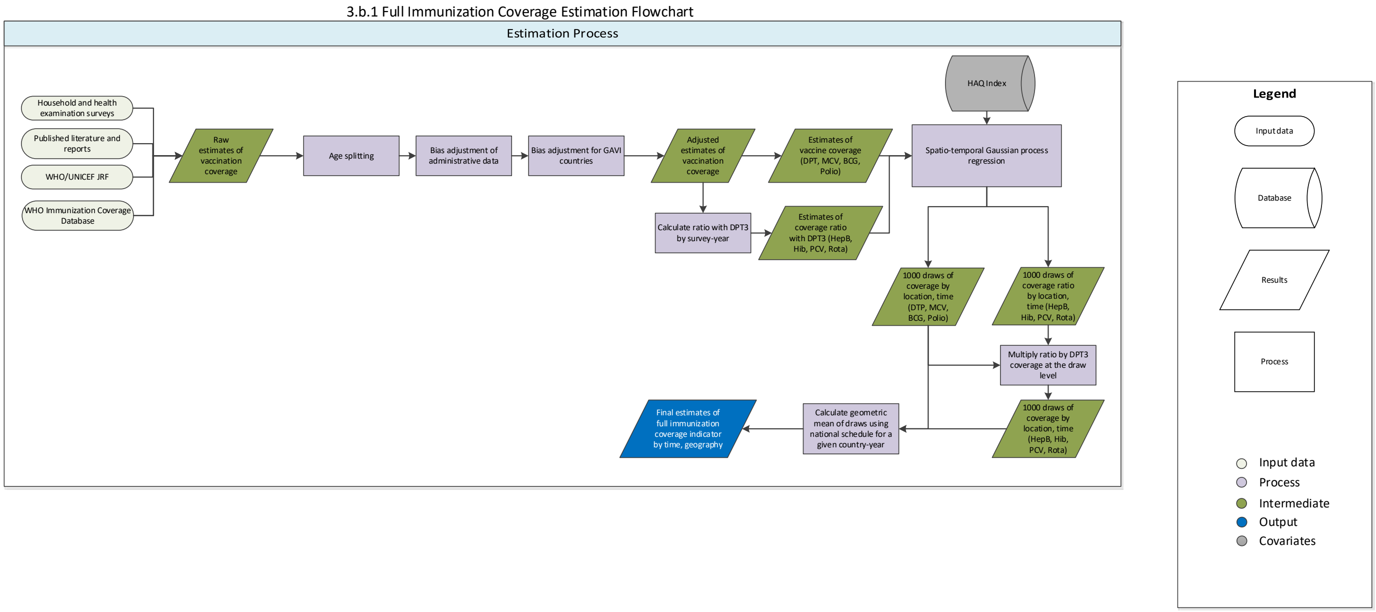

Input data & methodological summary

Indicator definition

This modeling strategy pertains to the vaccine coverage measure (Indicator 3.b.1), the proportion of the target population covered by all vaccines included in the national program, including diphtheria-tetanus-pertussis (DTP, three doses), both doses of measles vaccine (one dose and two doses), polio (three doses), hepatitis B (three doses), *Haemophilus influenzae* type b (Hib, three doses), pneumococcal conjugate vaccine (PCV, three doses), and rotavirus vaccine (two or three doses). We use the arithmetic mean of coverage of these eight vaccines, irrespective of their inclusion in the national vaccine schedule, to compute overall vaccine coverage of target populations.

Indicator 3.b.1

As a component of Goal 3: Ensure healthy lives and promote well-being for all at all ages, Target 3.b: Support the research and development of vaccines and medicines for the communicable and non-communicable diseases that primarily affect developing countries, provide access to affordable essential medicines and vaccines, in accordance with the Doha Declaration on the TRIPS Agreement and Public Health, which affirms the right of developing countries to use to the full the provisions in the Agreement on Trade-Related Aspects of Intellectual Property Rights regarding flexibilities to protect public health, and, in particular, provide access to medicines for all., is measured using SGD Indicator 3.b.1: proportion of the target population covered by all vaccines included in their national programme.

Input data

The present study used data from household-level surveys as well as administrative reports of immunization coverage. Survey data which provided person-level information on immunization were identified and extracted. Major multi-country survey programs included in the analysis include the Demographic and Health Surveys (DHS),[1] Multiple Indicator Cluster Surveys (MICS),[2] Reproductive Health Surveys (RHS),[3] Living Standards Measurement Study (LSMS) surveys,[4] and World Health Surveys (WHS).[5] We also conducted a comprehensive search of the Global Health Data Exchange (GHDx),[6] as well as targeted internet searches and review of Ministry of Health websites, to identify national surveys and other multi-country survey programs.

Administrative estimates of immunization coverage were obtained from the Joint Reporting Process (JRF),[7] through which the World Health Organization (WHO) and UNICEF collate annual estimates of immunization coverage reported by UN member states. These immunization coverage estimates are separate from those synthesized by WHO, and are calculated by dividing the number of doses of a given vaccine delivered to the target population (i.e., children aged 12 to 23) by the number of individuals in that target population.

We excluded all data sources that were not nationally representative or had high levels of missingness. We applied survey weights based on survey sampling frames whenever they were available to generate weighted national estimates of vaccination coverage accompanied by estimates of standard error (SE). Estimates of SE, as well as sample sizes, were used to calculate uncertainty, as described below. Any point estimates with sample sizes less than 50 were reviewed to ensure that were not substantive outliers and would otherwise have an undue influence on our analysis.

Modeling strategy

Data processing

Age splitting

Most household surveys collect information on maternal and child health (MCH) indicators for children under 5 and/or mothers who gave birth within five years prior to the time of survey. To maximize data use for our model, we included immunization data for children aged 12 to 59 at the time of survey. Children younger than 12 months of age were excluded to minimize the influence of potentially censored observations. For each vaccine, coverage estimates were assigned to birth-cohort years based on a child's age prior to the time of survey: we used responses recorded for children aged 12 to 23 months for immunization coverage for one year prior to the time of survey, children aged 24 to 35 months for coverage two years prior to the time of survey, and so forth.

Age-specific estimates are easily computed from individual-level microdata, but many published reports and survey summaries present data in broader age aggregates (e.g., DPT3 coverage for children aged 12 to 35 months). To standardize these age groups, we applied an age-splitting model used in the GBD study,[8] as well as analyses that generated smoking and obesity prevalence by age group.[9,10]

Using surveys with microdata as the reference, we used the following model to generate standardized age group-specific estimates of immunization coverage:

$$\tilde{P}_{a,c,t,k} = P_{a,c,t,k}^{a+x} \frac{P_{a,c,t,j}}{P_{a,c,t,j}^{a+x}}$$

where $\tilde{P}_{a,c,k}$ is the adjusted estimate of coverage for target age group a in country c and year t of survey k; and $P_{a,c,k}^{a+x}$ is coverage reported from survey k, for country c in year t for the age group spanning age a to age $(a + x)$. The ratio of coverage between the target age group and broader age group from a survey j with microdata from the same country-year was used to split data from survey k. Surveys to be split were ideally matched with DHS or MICS surveys. If microdata were not available for the same year, ratios within five years of the survey that required age-splitting were applied.

Administrative bias adjustment

Intervention coverage estimates based on administrative sources can be biased. Such biases may arise for a number of reasons, including discrepancies in the accurate reporting of services or interventions provided (e.g., number of vaccine doses administered) and target population (e.g., number of children in need of vaccines), as well as capturing these data in a timely manner from both public and private-sector facilities and health care providers. We implemented a vaccine-specific bias adjustment process to account for bias in administrative reports of immunization coverage in the JRF. Given that the magnitude, direction, and cause of such biases are heterogeneous across space, time, and antigen,[11,12] a vaccine-specific, time-varying, all-location bias correction factor was used.

For immunization coverage, we view individual-level data collected through population health surveys as the most accurate and least biased source of information of vaccination coverage, particularly for geographies with incomplete health information systems. We thus compute administrative bias as the ratio between estimates of coverage from surveys (where available) and matched administrative coverage. We model this bias in a spatiotemporal Gaussian process regression (ST-GPR) framework, described further in the other appendices of the GBD Capstones, using the Socio-demographic index (SDI) as a predictor. This method allows us to estimate antigen-specific administrative bias factors for all geographies and years since 1980, even in places without survey data, by borrowing strength in data across space and time. The GPR framework properly estimates prediction errors in the data synthesis procedure by for uncertainty in bias ratios when generating fitted values. In this framework, more weight is given to survey data with less uncertainty.

Antigen-specific modeled estimates of administrative bias are then used to adjust administrative coverage data for over- or under-reporting to reflect observed survey coverage. Adjusted administrative data are used as inputs into the trend estimation process.

Trend estimation

We used a spatiotemporal Gaussian process regression (ST-GPR) to synthesize point estimates from multiple data sources and derive a complete time series for each vaccine. This method has been used extensively GBD and related studies, and accounts for uncertainty pertaining to each point estimate while borrowing strength across geographic space and time.[10, 11,15,16] Briefly, we assumed the Gaussian process was defined by a mean function *m* n o ri n e un tion *Cov* .

We estimated the mean function using a two-step approach. Specifically, $m_c(t)$ can be expressed as:

$$m_c(t) = X\beta + h(r_{c,t})$$

where $X\beta$ is a linear model and $h(r_{c,t})$ is a smoothing function for the residuals; and $r_{c,t}$ is derived from the linear model. The following linear model was used to model DPT3, measles, BCG, polio coverage:

$$logit(P_{c,t}) = \beta_0 + \beta_1 \mathrm{HAQ}_{\mathrm{c,t}} + \alpha_c + \gamma_{R[c]} + \omega_{\mathrm{SR[c]}} + \varepsilon_{c,t}$$

where $P_{c,t}$ is vaccination coverage for country c year t; $HAQ_{c,t}$ is value of the Healthcare Access and Quality Index[15] for country c and year t; α_c, $\gamma_{R[c]}$, and $\omega_{\mathrm{SR[c]}}$ are country, region, and super-region random intercepts, respectively. These estimates were then modeled through ST-GPR.

Given their recent introduction, there is limited coverage data for HepB, Hib, PCV, and rotavirus vaccines. To leverage the relatively data-rich DPT3 estimates, we modeled the ramp-up of each vaccine by modeling their ratio with DPT3 coverage. We first calculated the ratio of each particular vaccine with DPT3 by survey-year. We then modeled the full time series of the ratio using ST-GPR and ultimately obtained estimates of coverage by multiplying the modeled ratio by the final estimated DPT3 coverage by location-year. The following linear model was used as the mean function for the HepB, Hib, PCV, and Rota ratio with DPT3:

$$logit(P_{c,i}) = \beta_0 + \beta_1 \mathrm{HAQ}_{\mathrm{c,i}} + \alpha_c + \gamma_{R[c]} + \omega_{\mathrm{SR[c]}} + \varepsilon_{c,i}$$

where $P_{c,i}$ is the coverage ratio for country c time since introduction i; $HAQ_{c,i}$ is value of the Healthcare Access and Quality Index[15] for country c and time since introduction i; α_c, $\gamma_{R[c]}$, and $\omega_{\mathrm{SR[c]}}$ are country, region, and super-region random intercepts, respectively.

Random draws of 1,000 samples were obtained from the distributions above for every country for a given vaccine. Ninety-five percent uncertainty intervals were calculated by taking the ordinal 25th and 975th draws from the sample distribution.

To assess the accuracy of our modeled estimates, we performed cross-validation analyses using a knockout structure as previously described[16]. ST-GPR hyperparameters were selected on models that minimized the overall root-mean squared error (RMSE) of the model across a set of 10 knockouts.

Introduction schedule

National vaccine schedules and vaccine introduction dates were used as reported from WHO[17] or from the country's Ministry of Health website where otherwise unavailable. These data were used to bound estimates of coverage (i.e. x=0 or 0<x<1) based on introduction status. Dates of policy changes for the BCG vaccine were used as reported by the BCG Atlas[18] or directly form the country's ministry of health website.

Full coverage indicator

To synthesize the full vaccination coverage indicator (SDG indicator 3.b.1), we calculated the arithmetic mean of the eight vaccines irrespective of their inclusion in the national vaccine schedule for a given year. In other words, newer-generation vaccinations such as PCV, second-dose measles, and Rota are included in each country-year's calculation as zero percent coverage if the vaccine has not yet been introduced into the national schedule. This means that countries can only achieve "full" (i.e. 100%) vaccine coverage only after all eight vaccines have been introduced and scaled up to cover all 12- to 23-month olds in a given year.

Because of strange trends in coverage of vaccines that are removed from the national schedule (e.g. BCG in several European countries, Australia, and New Zealand), we did not include BCG in the full coverage indicator for this round of the GBD.

References

1 Measure DHS: Demographic and Health Surveys. http://www.measuredhs.com (accessed Aug 11, 2015).

2 UNICEF Stat. Monit. Multiple Indicator Cluster Survey (MICS). http://www.unicef.org/statistics/index_24302.html (accessed Aug 11, 2015).

3 Cent. Dis. Control Prev. Reproductive Health Surveys (RHS). http://www.cdc.gov/reproductivehealth/Global/surveys.htm (accessed Aug 11, 2015).

4 World Bank. Living Standard Measruement Studies (LSMS). http://go.worldbank.org/UK1ETMHBN0 (accessed Aug 11, 2015).

5 WHO Multi-Ctry. Stud. Data Arch. World Health Survey (WHS). http://apps.who.int/healthinfo/systems/surveydata/index.php/catalog/whs/about (accessed Aug 11, 2015).

6 IHME GHDx. Global Helath Data Exchange. http://ghdx.healthdata.org/ (accessed Aug 11, 2015).

7 WHO | WHO/UNICEF Joint Reporting Process. WHO. http://www.who.int/immunization/monitoring_surveillance/routine/reporting/reporting/en/ (accessed Aug 17, 2015).

8 Prospective Studies Collaboration. Age-specific relevance of usual blood pressure to vascular mortality: a meta-analysis of individual data for one million adults in 61 prospective studies. *The Lancet* 2002; **360**: 1903–13.

9 Ng M, Freeman MK, Fleming TD, *et al.* Smoking Prevalence and Cigarette Consumption in 187 Countries, 1980-2012. *JAMA* 2014; **311**: 183.

10 Ng M, Fleming T, Robinson M, *et al.* Global, regional, and national prevalence of overweight and obesity in children and adults during 1980–2013: a systematic analysis for the Global Burden of Disease Study 2013. *The Lancet* 2014; **384**: 766–81.

11 Murray CJ, Shengelia B, Gupta N, Moussavi S, Tandon A, Thieren M. Validity of reported vaccination coverage in 45 countries. *The Lancet* 2003; **362**: 1022–7.

12 Haddad S, Bicaba A, Feletto M, Fournier P, Zunzunegui MV. Heterogeneity in the validity of administrative-based estimates of immunization coverage across health districts in Burkina Faso: implications for measurement, monitoring and planning. *Health Policy Plan* 2010; **25**: 393–405.

13 Lim SS, Stein DB, Charrow A, Murray CJ. Tracking progress towards universal childhood immunisation and the impact of global initiatives: a systematic analysis of three-dose diphtheria, tetanus, and pertussis immunisation coverage. *The Lancet* 2008; **372**: 2031–46.

14 Wang H, Liddell CA, Coates MM, *et al.* Global, regional, and national levels of neonatal, infant, and under-5 mortality during 1990–2013: a systematic analysis for the Global Burden of Disease Study 2013. *The Lancet* 13; **384**: 957–79.

15 Barber RM, Fullman N, Sorensen RJD, *et al.* Healthcare Access and Quality Index based on mortality from causes amenable to personal health care in 195 countries and territories, 1990?2015: a novel analysis from the Global Burden of Disease Study 2015. *The Lancet* 2017; published online May. DOI:10.1016/S0140-6736(17)30818-8.

16 Foreman KJ, Lozano R, Lopez AD, Murray CJ. Modeling causes of death: an integrated approach using CODEm. *Popul Health Metr* 2012; **10**: 1.

17 WHO | Data, statistics and graphics. WHO. http://www.who.int/immunization/monitoring_surveillance/data/en/ (accessed June 12, 2017).

18 The BCG World Atlas: A Database of Global BCG Vaccination Policies and Practices. http://journals.plos.org/plosmedicine/article?id=10.1371/journal.pmed.1001012 (accessed June 12, 2017).

3.c.1 Health Worker Density and Distribution SDG Capstone Appendix

Flowchart

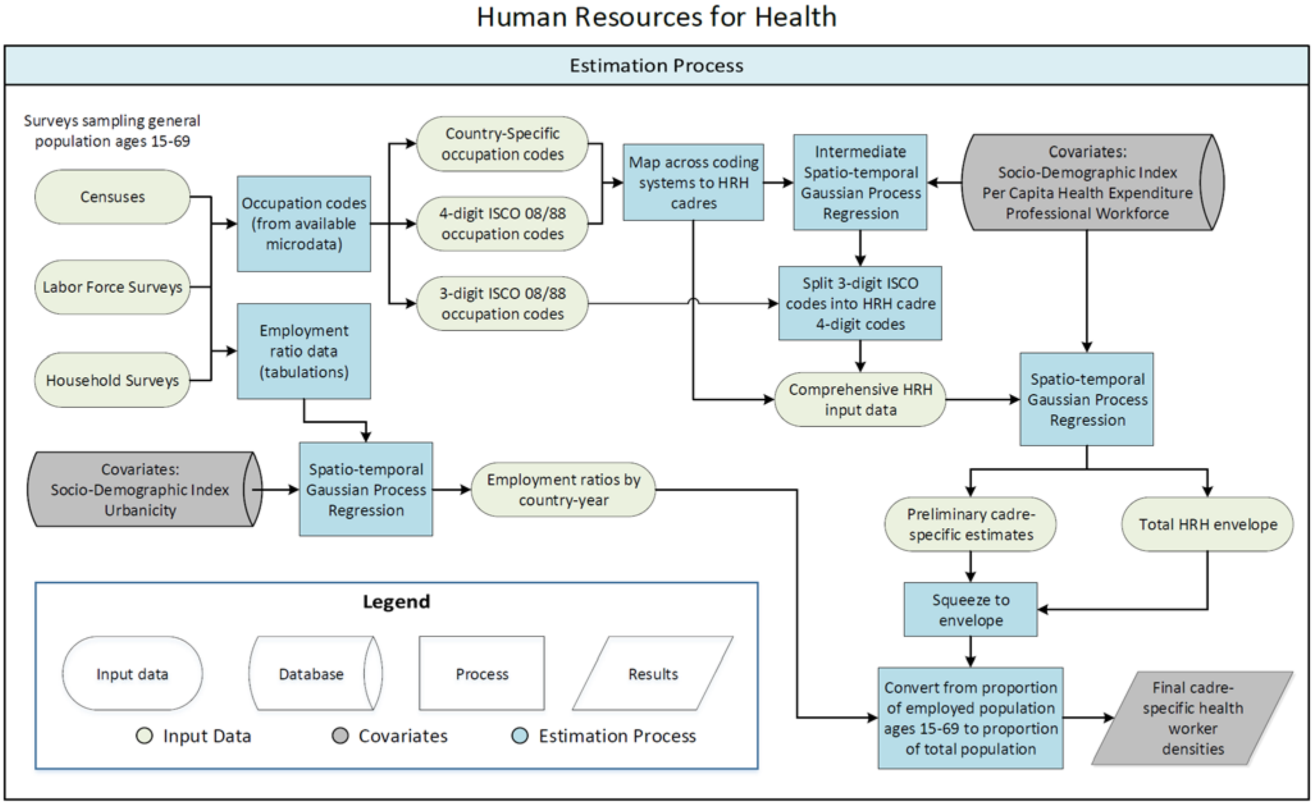

Input Data & Methodological Summary

Indicator definition

This modeling strategy encompasses the indicator associated with health worker densities (3.c.1)

Indicator 3.c.1

As a component of SDG Goal 3, SDG Target 3.c is measured using SDG Indicator 3.c.1[1]:

> SDG Goal 3: *Ensure healthy lives and promote well-being for all at all ages*
>
> SDG Target 3.c: *Substantially increase health financing and the recruitment, development, training, and retention of the health workforce in developing countries, especially in least developed countries and small island developing States*
>
> SDG Indicator 3.c.1: *Health worker density and distribution (health worker prevalence per 10,000 population, by sex and cadre)*

For the purposes of this paper, we constructed indicator 3.c.1 from the densities of physicians, pharmacists, and nurses and midwives. The choice of these cadres and of their corresponding thresholds is discussed in detail below.

Input data

The main data used in this study come from surveys that sampled general working-age populations (ages 15-69) and asked employed respondents about their current main occupation. Such sources included general household surveys, labor force surveys, and censuses. The main indicators extracted from these surveys were employment ratios and the proportion of the employed population ages 15-69 involved in various occupations. Tabulated estimates of employment ratios from these types of sources were acquired from the ILO (International Labour Organization) and used as an envelope in the final estimation of health worker densities. Unfortunately, the vast majority of surveys that inquire about occupations do not code responses to the level of detail required to identify health workers, let alone specific cadres of health workers. In addition, those surveys that do code occupations to the necessary granularity rarely release tabulations of their data at such a detailed level. As a result, data on occupations came exclusively from individual-level microdata that could be obtained from such high-granularity surveys.

There was substantial variability in the occupational coding systems used in the identified surveys. The most common coding systems adhered to the International Standard Classification of Occupations (ISCO), which has established standard codes ranging from 1 to 4 digits in length arranged in a hierarchy of granularity (see figure below for examples). Many surveys used other country-specific coding systems, but sometimes those systems were closely based on ISCO such that relevant occupations could be translated to ISCO codes with little information loss. However, when country-specific codes deviated substantially from ISCO, relevant occupations were only mapped to a standard code when documented descriptions sufficiently matched ISCO categorizations.

Since its original adoption in 1957, ISCO has transitioned through three main versions: ISCO 68, ISCO 88, and ISCO 08. The ISCO 68 system was adopted in 1968 and distinguished occupations by trade, but this version was already phased out for the majority of the time period of interest for this study. ISCO 88 was adopted in 1988 and reframed the coding system to emphasize differences in occupational skill level and specialization. ISCO 08 was adopted in 2008 and updated the ISCO 88 system with additional distinctions relevant to the modern labour market. This update created more detailed distinctions between types of health workers and also consolidated them hierarchically, such that a few general health worker categories could be accurately identified using less granular codes[2].

An example subsection of the ISCO 88 hierarchy:

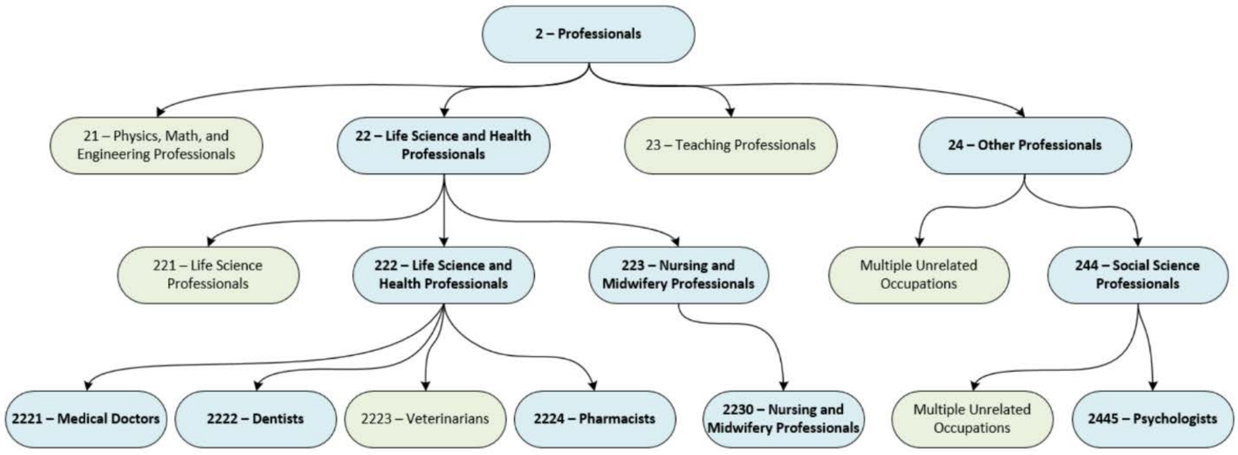

An example subsection of the ISCO 08 hierarchy (only relevant occupations shown):

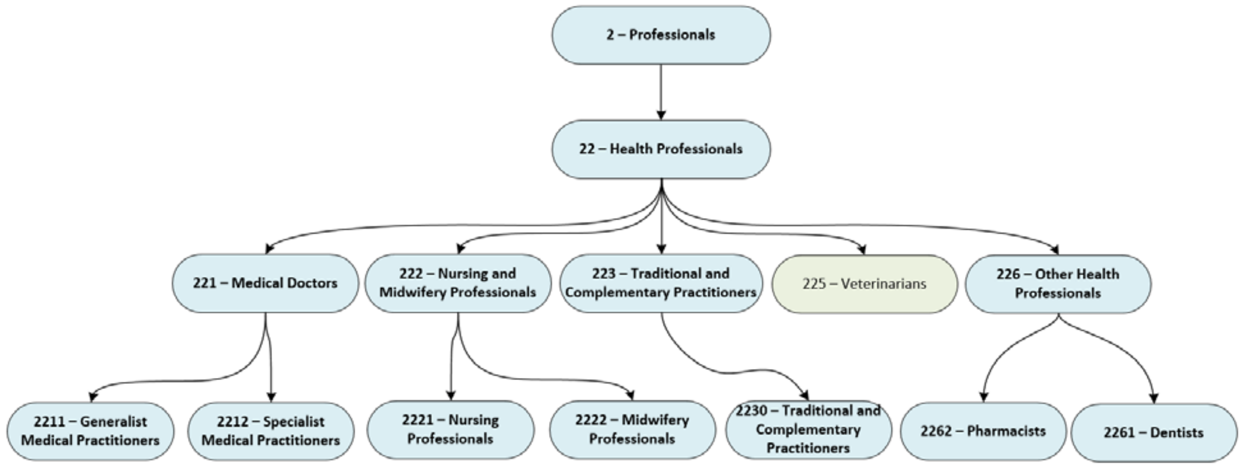

The diagrams above depict ISCO 88 and 08 coding systems at different levels of granularity. The top level is a 1-digit code, which refers to a very large number of occupations (in this case, all professionals). With increased digits (added to the right-hand side of the code at each subsequent level) it becomes possible to differentiate more specialized occupations from one another.

Although ISCO 08 facilitates the identification of health workers with greater ease and detail, surveys have been slow to transition to the newer coding system. Consequently, ISCO 88 makes up the majority of ISCO coded surveys in every year in the study until 2013. Due to the relative paucity of ISCO 08 across the time period of interest and the information loss inherent in mapping from one coding system to another, defining the health worker cadres according to ISCO 08 codes seemed untenable. As a result, we set ISCO 88 as the gold standard coding system for the purposes of this study. This meant that the granularity of health worker cadres estimated in this analysis was constrained to the level of detail provided by ISCO 88 4-digit codes (the most-detailed level in the system). In future analyses—when ISCO 08 surveys cover a greater proportion of the time period of interest—it should be possible to use ISCO 08 as the gold standard coding system, thus increasing the granularity of cadre-specific estimates.

To compile a list of relevant health worker cadres from ISCO codes, we used the WHO Handbook on Monitoring and Evaluation of Human Resources for Health[3].

Most-granular health worker cadres under ISCO 88 coding system:

Health Worker Cadre	4-Digit ISCO 88 Code
Physicians*	2221
Nursing & Midwifery Professionals*	2230
Nursing Associate Professionals*	3231
Midwifery Associate Professionals*	3232
Pharmacists*	2224
Pharmaceutical Assistants	3228
Dentists	2222
Dental Assistants	3225
Physiotherapists & Related Professionals	3226
Medical Equipment Operators	3133
Medical & Life Sciences Laboratory Technicians	3211
Community Health Workers, Clinical Officers, & Medical Assistants	3221
Paramedics, Emergency Medical Technicians, & Healthcare Aides	5132 & 5139
Environmental Health Officers	3222
Optometrists & Opticians	3224
Dieticians & Nutritionists	3223
Audiologists, Speech Therapists, & HIV/Family Planning Counsellors	3229
Psychologists	2445
Home-Based Personal Care Workers	5133
Traditional Medicine Practitioners	3241

***** Included in SDG indicator 3.c.1 in this analysis

Data mapping and splitting

When possible, occupation codes were mapped directly to the corresponding health worker cadres. For less granular surveys, codes had to be split in order to provide cadre-specific estimates.

Among ISCO coded sources, only 3 and 4-digit surveys were used, as it was not feasible to split less granular codes accurately to the cadre-specific level. All 4-digit (and some 3-digit) ISCO codes could be directly mapped to health worker cadres using ISCO concordance documentation to convert ISCO 08 codes to ISCO 88. Not all codes had exact matches between versions; in such cases the closest approximate concordance was used.

Examples of health worker cadre mapping:

Occupation Titles	ISCO 08 Code	ISCO 88 Code	ISCO 88-Defined Health Worker Cadre
Dentists	2261	2222	Dentists
Community Health Workers	3253	3221	CHWs, Clinical Officers, & Medical Assistants
Clinical Officers	2240	3221	CHWs, Clinical Officers, & Medical Assistants
Medical Assistants	3256	3221	CHWs, Clinical Officers, & Medical Assistants
Environmental Health Officers	2263	3222	Environmental Health Officers
Food Inspectors	3257	3222	No Cadre / Environmental Health Officers
Occupational Hygienists	2263	3152	Environmental Health Officers / No Cadre
Quality Controllers, Electrical Product Inspector, etc.	3257	3152	No Cadre

In the table above, occupations and their corresponding codes come from ISCO's concordance documentation[2]. In this example, there is an exact match between codes for dentists in both coding versions (though one codes them as 2261 and the other as 2222). As a result, both codes can be mapped to the same cadre without any information loss. While ISCO 08 separates community health workers, clinical officers, and medical assistants into distinct codes, all three combined form one 4-digit code in ISCO 88. Since the health worker cadres for this analysis are defined by the ISCO 88 hierarchy, the three distinct ISCO 08 codes are all mapped to the same cadre, resulting in a loss of granularity but not a loss of comparability between versions. In the case of environmental health officers, ISCO 08 and 88 codes cannot be aggregated in a way that creates an exact match between versions without incorporating many unrelated occupations as well. In ISCO 88, environmental health officers and food inspectors are grouped together, and are both mapped to the environmental health officer cadre. In ISCO 08, environmental health officers and occupational hygienists are grouped together and are also mapped to the environmental health officer cadre. It would not be appropriate to include occupational hygienists from an ISCO 88 survey in the cadre, nor would it be appropriate to include food inspectors from an ISCO 08 survey, because in both systems these occupations are grouped together with many non-health related positions (such as electrical product inspectors and quality controllers). As a result, those codes and occupations are not mapped to any cadre and are not included in subsequent analyses. This mapping strategy inherently results in some inconsistencies and information loss, but in these cases codes were chosen from ISCO concordance documents so as to minimize inconsistencies to the greatest extent possible.

Most 3-digit ISCO codes were not granular enough to be mapped to particular cadres, but still provided information as an envelope within which certain cadres could be found. Since 3-digit ISCO coded surveys were very common (particularly among identified censuses), relevant 3-digit codes were split out into their underlying cadres and retained in the analysis. In order to perform such splits, we ran preliminary

versions of our final model (more detail on the model below) for each health worker cadre using only 4-digit survey data. We also ran models for the residual categories associated with each 3-digit code of interest. The data for such residual models encompassed all 4-digit codes underlying a 3-digit code of interest that were not associated with a health worker cadre. Each 3-digit code could then be split into cadre-specific data using the preliminary modeled estimates of the code's underlying components for that GBD location and year. This method leveraged available 4-digit data to better inform 3-digit splits, and was therefore preferable to one global split that would have been insensitive to differences across space and time in the proportional makeup of each 3-digit code. However, since the splits were based entirely upon models using only 4-digit data, this meant that all cadre input data obtained from 3-digit surveys were dependent upon the quality and coverage of 4-digit surveys, and could change as more 4-digit surveys were incorporated.

Among non-ISCO country-specific coding systems, levels of granularity varied substantially for different cadres, and was not easy to predict based on a coding system's digit length alone. For example, a 2-digit country-specific coding system might make distinctions between different types of physicians but use one code for all nurses and midwives. Country-specific codes that matched (or could be aggregated up to) a cadre defined by 4-digit ISCO 88 were mapped to those cadres. Other codes that could be directly split into multiple cadres (without any residual groups) were split using the same method applied to 3-digit ISCO codes. All other country-specific codes were excluded from the analysis.

After mapping and splitting all usable surveys and censuses, input data representing proportions of the employed population ages 15-69 in each health worker cadre were ready to enter the final model. We also compiled a dataset of the sum of all cadres from those surveys whose coding systems allowed mapping to every cadre included in the analysis (primarily ISCO 88 and 08 surveys).

Modeling Strategy

All models for this analysis used Spatiotemporal Gaussian process regression (ST-GPR), a three-stage model used widely within the GBD study to synthesize coherent trends and uncertainty from multiple sources of data. The first stage of ST-GPR entails fitting a linear model on the data. The second stage involves smoothing over space-time based on the residuals from the first stage linear model. The third stage uses Gaussian Process Regression (GPR) to generate a cohesive time series of an indicator and uncertainty for all GBD locations from 1990 to 2017. Models were run for each health worker cadre individually, for all health workers together, and for employment ratios.

For health worker cadres and all health workers together, the linear model had fixed effects on Socio-Demographic Index, total national per-capita health expenditure (log-transformed), and professional workforce (the proportion of the employed population working in professional occupations according to ISCO). The Socio-Demographic Index and estimates of total per-capita health expenditure are outputs of other projects within GBD and the Institute for Health Metrics and Evaluation. Estimates of the professional workforce come from the same types of surveys for which health worker cadre data is derived, but because professional occupations can be identified from surveys with only 1 digit of occupation code granularity, many more surveys were available to inform estimates for this covariate. In order to generate a complete time-series for the professional workforce in all GBD locations, it was also modeled in ST-GPR (more details on the professional occupation modeling process can be found in the appendix for Occupational Risk Factors, since that is the primary purpose for the indicator). For health worker cadres, identical model settings were used for the intermediate estimates (used in the splitting process and run on only 4-digit surveys) and the final estimates (run on all available data).

Unlike the health cadres, employment ratios were modeled by age and sex, with fixed effects on the Socio-Demographic Index, urbanicity, and age group, and random effects on location, region, and super-region. Final results were then aggregated using GBD populations to produce an estimate of the employed population ages 15-69 as a proportion of the total population for every location and year.

Because specific health worker cadres make up such a small proportion of the employed population, logit transformations were very unstable for modeling specific cadres. As a result, health worker cadre proportion data was multiplied by 10,000 to represent the number of cadre workers per 10,000 employed population. The data was then modeled in log space, and model results were converted back to proportions. Because all health workers together constituted larger proportions than individual cadres, this indicator did not require such additional transformations and was modeled directly as a proportion in logit space. To further control for unrealistic trends and extrapolations due to stochastic variation in smaller health cadre proportion data, the total estimate of all health workers together was used as an envelope to which all cadre-specific results were squeezed. In the final step, squeezed estimates of health worker cadres as a proportion of the employed population ages 15-69 were converted to proportions of the total population using the output from the employment ratio model.

Indicator Construction

The WHO established a health workforce threshold of 23 physicians, nurses, or midwives per 10,000 population based on the 2006 World Health Report comparison of health worker density and achievement of skilled birth attendance above 80%[4]. This threshold has been widely referenced in global health literature and was extrapolated to represent the threshold necessary for implementing the primary health care interventions enumerated in the Millennium Development Goals. However, evidence for the threshold's utility is rather limited, since it is the product of a single analysis looking at only one measure of healthcare performance, whereas workforce requirements are likely to differ substantially across a variety of services. For this analysis, we aimed to establish new evidence-based thresholds that leveraged workforce associations with a more holistic measure of healthcare performance, the GBD's Healthcare Access and Quality (HAQ) Index.

Following exploratory analyses of health worker cadre associations with HAQ, and after consulting the SDG indicator metadata guidelines, we settled on physicians, pharmacists, and nurses and midwives as components for the SDG indicator used in this analysis. Although dentistry personnel are also mentioned in the SDG indicator metadata, and many other healthcare workers are known to contribute to healthcare access and quality, initial analyses relating additional cadres to the HAQ Index proved challenging, and more research is needed before such cadres can be added to the indicator.

Although ISCO 88 codes allowed the disaggregation of nurses and midwives into nursing and midwifery professionals, nursing associate professionals, and midwifery associate professionals, close inspection of survey data showed that countries and survey series did not exhibit much consistency in their coding of nurses and midwives. Within a particular country and survey series, the aggregate nurses and midwives category tended not to differ much from year to year, whereas the individual cadre subcomponents could differ substantially from one survey to the next. This suggested that the trends in specific cadres of nurses and midwives were not as informative or reliable as the overall trend, so for the purpose of constructing a health workforce indicator the aggregate of all nurses and midwives were treated as one cadre.

Rather than creating an indicator from the simple sum of physicians, pharmacists, and nurses and midwives all together—which masks important distinctions in the cadre composition of a health workforce—we aimed to establish an indicator that distinguishes the importance of individual cadres.

Consequently, each cadre in the analysis was assigned its own minimum threshold value for use in constructing a scaled index (where countries that reached or surpassed the threshold value were all assigned the same maximum value). Establishing such thresholds allowed the scaled workforce indicator to reflect the adequacy of workforce densities on the basis of their association with healthcare access and quality as opposed to just their relative values compared to other countries, many of which may have workforce densities much higher than what is required for good care. However, it was also important to keep observations of cadre workforce densities paired with one another, as a location that is low on one cadre may be able to compensate with higher densities of a different cadre, yielding higher healthcare performance than other locations where workforce densities are low across many cadres.

To establish cadre-specific thresholds, we ran a logistic regression on GBD estimates of HAQ with fixed effects on physicians, pharmacists, and nurses and midwives, as well as random effects on intercept by location. We then used the regression outputs to predict HAQ Index values for every location and year, replacing all location-specific random effects with the fifth percentile of observed random effects across all locations. We then scattered predicted HAQ values against each cadre individually and identified the health worker density at which further increases in workforce began yielding diminishing returns in the predicted HAQ Index. The lowest observed cadre workforce density at which a country's returns in predicted HAQ began diminishing was used as the minimum threshold for the cadre. The threshold values established for each cadre in the construction of SDG indicator 3.c.1 were as follows: 30 physicians per 10,000 population, 100 nurses and midwives per 10,000 population, and 5 pharmacists per 10,000 population.

References

1. United Nations Department of Economics and Social Affairs. *Goal 3: Ensure healthy lives and promote well-being for all at all ages*. (2015). at <https://sustainabledevelopment.un.org/sdg3>

2. International Labour Organization. *International Standard Classification of Occupations: ISCO-08 Structure, index correspondence with ISCO-88*. at < http://www.ilo.org/public/english/bureau/stat/isco/isco08/index.htm>

3. World Health Organization. *Handbook on Monitoring and Evaluation of Human Resources for Health*. (11 Nov. 2014). at <www.who.int/hrh/resources/handbook/en>

4. World Health Organization. *The World Health Report 2006 - Working Together for Health*. (29 July 2013). at <www.who.int/whr/2006/en>

5.2.1, 5.2.2, 16.1.3, 16.2.3, Sexual and Physical Violence SDG Capstone

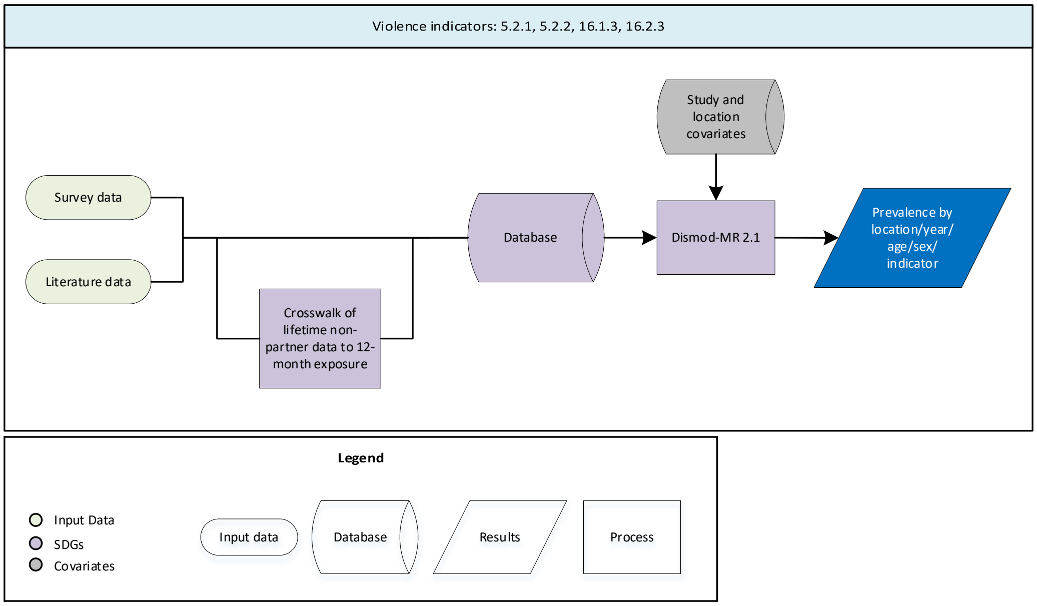

Indicator definition

This modeling strategy relates to all indicators associated with sexual and physical violence as specified below.

Indicator 5.2.1

Indicator 5.2.1 is part of Goal 5: achieve gender equality and empower all women and girls. Target 5.2 is to eliminate all forms of violence against all women and girls in public and private spheres, including trafficking and sexual and other types of exploitation. This indicator measures the proportion of ever-partnered women and girls aged 15 years and older subjected to physical, sexual, or psychological violence by a current or former intimate partner in the previous 12 months, by form of violence and age. Because of the difficulty in measuring psychological violence in surveys, and limited data availability, we have excluded psychological violence from the measurement of this indicator.

Indicator 5.2.2

Indicator 5.2.2 is part of Goal 5: achieve gender equality and empower all women and girls. Target 5.2 is to eliminate all forms of violence against all women and girls in public and private spheres, including trafficking and sexual and other types of exploitation. This indicator measures the proportion of women and girls aged 15 years and older subjected to sexual violence by persons other than an intimate partner

in the previous 12 months, by age and place of occurrence. For this indicator, we do not distinguish the events by place of occurrence.

Indicator 16.1.3

Indicator 16.1.3 is part of Goal 16: promote peaceful and inclusive societies for sustainable development, provide access to justice for all and build effective, accountable and inclusive institutions at all levels. Target 16.1 is to significantly reduce all forms of violence and related death rates everywhere. This indicator measures the proportion of population subjected to physical, psychological, or sexual violence in the previous 12 months. Similarly to Indicator 5.2.1, we have excluded psychological violence from the measurement of this indicator due to data availability issues. New for GBD 2017, we are modeling physical violence and sexual violence separately as indicators 16.1.3.a and 16.1.3.c respectively.

Indicator 16.2.3:

Indicator 16.2.3 is part of Goal 16: promote peaceful and inclusive societies for sustainable development, provide access to justice for all and build effective, accountable and inclusive institutions at all levels. Target 16.2 is to end abuse, exploitations, trafficking and all forms of violence against and torture of children. This indicator measures the proportion of young women and men aged 18-29 years who experienced sexual violence by age 18.

Input data

The main sources of input data for Indicators 5.2.1, 5.2.2, and 16.1.3, and 16.2.3 come from the following:

Demographic and Health Surveys (DHS)

In the GHDx, we identified DHS that had variables related to sexual violence. The vast majority of the DHS only contain questions relating to indicators 5.2.1 and 16.1.3 (the Rwanda 2014-2015 DHS had additional questions relating to indicator 5.2.2, non-partner sexual violence).

The European Union Violence against Women Study

This violence against women study for the European Union provided data for many countries in the European Union. Questions related to sexual violence covered both intimate partner violence, and non-partner sexual violence, contributing data to all three indicators.

The United States Behavioral Risk Factor Surveillance System (BRFSS)

The BRFSS study has U.S. state-level data on all three of the indicators. Not all states choose to expand their BRFSS survey to include sexual and physical violence questions, so the data included are only from a select number of states. The surveys included are from 2005, 2006, and 2007.

China Health and Family Life Survey

The China Health and Family Life Survey from 1999-2000 asks about lifetime prevalence of sexual assault; however, we were able to extract yearly prevalence for indicator 16.1.3.c by pairing a respondent's current age with the reported age of when the sexual assault occurred.

Figures 1 – 4 show the data density of unique source by location for each of the three indicator models.

No data from police reports, Child Protection Services (CPS), or other crime data is used because the reliability and comprehensiveness of these data vary too much geographically to warrant inclusion.

Figure 1. Input data density of unique source by location for Indicator 5.2.1: prevalence of intimate partner sexual or physical violence in the last 12 months among women and girls.*

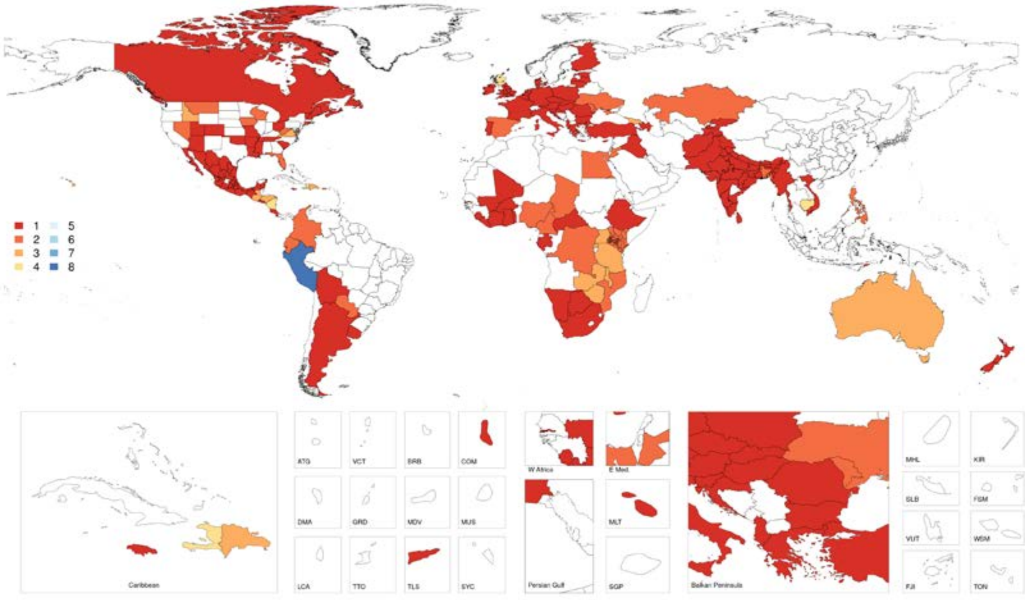

*Country-level sources for China (1), England (1), India (1), Kenya (4), Mexico (2), South Africa (1), Sweden (2), and United States (4) are not included on the map

Figure 2. Input data density of unique source by location for indicator 5.2.2: prevalence of non-partner sexual violence in the last 12 months among women and girls.*

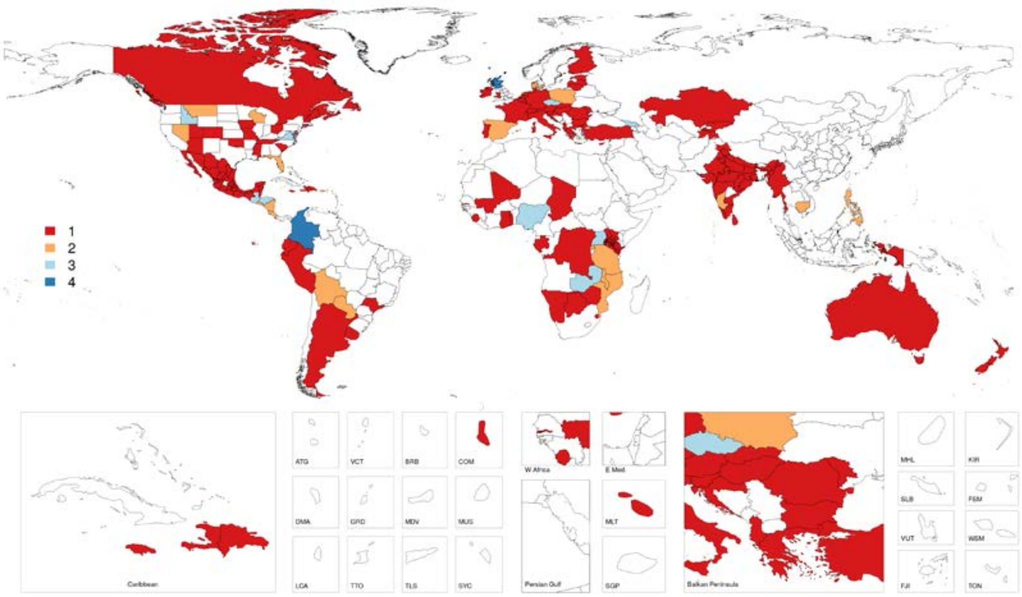

*Country-level sources for China (1), England (1), India (1), Kenya (2), Mexico (1), Sweden (1), and United States (4) are not included on the map

Figure 3. Input data density of unique source by location for indicator 16.1.3.a: prevalence of physical violence in the last 12 months (females).*

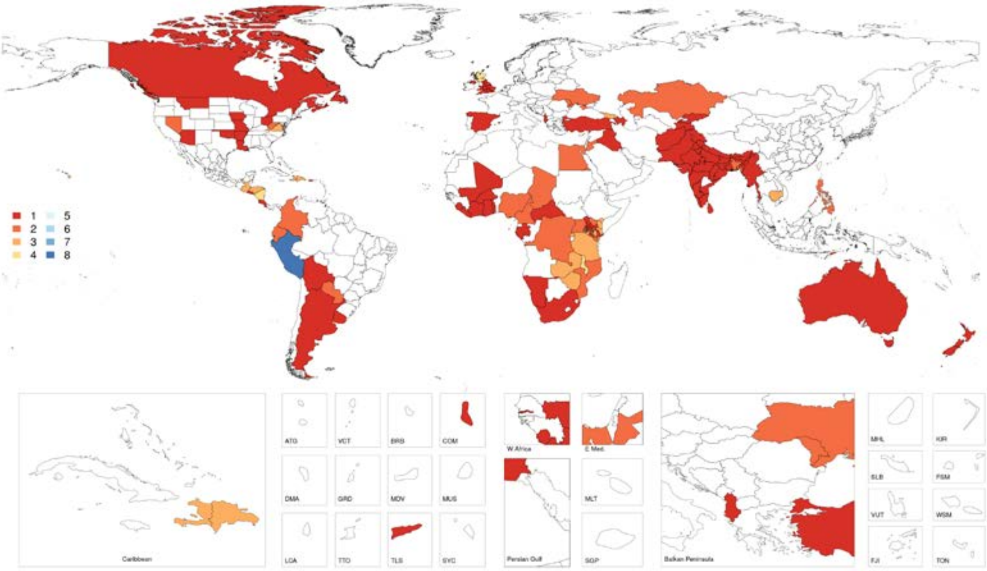

*Country-level sources for China (1), England (1), India (1), Kenya (4), South Africa (1), Sweden (1), and United States (4) are not included on the map

Figure 4. Input data density of unique source by location for indicator 16.1.3.a: prevalence of physical violence in the last 12 months (males).*

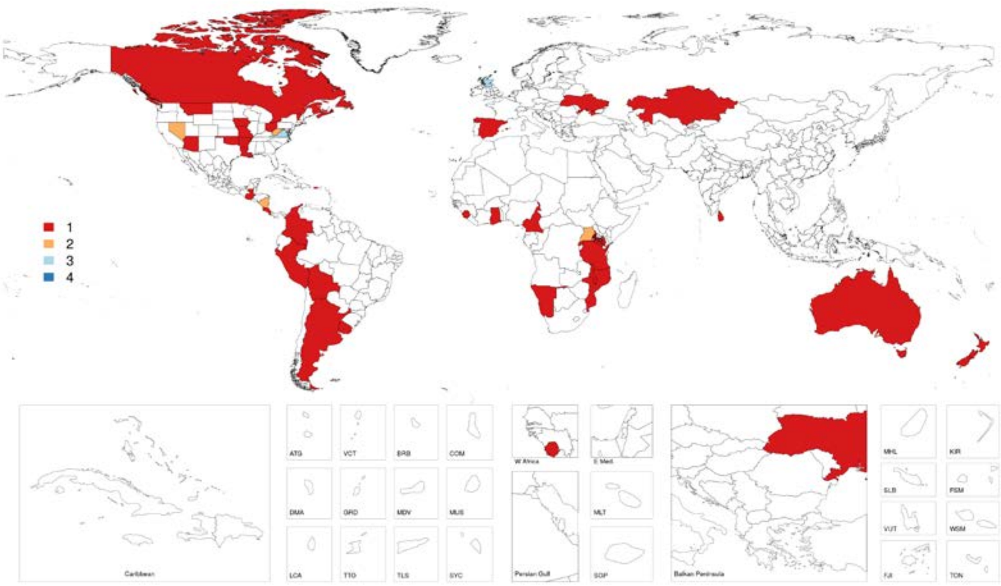

*Country-level sources for China (1), Kenya (2), Sweden (1), and United States (4) are not included on the map

Figure 5. Input data density of unique source by location for indicator 16.1.3.c: prevalence of sexual violence in the last 12 months (females).*

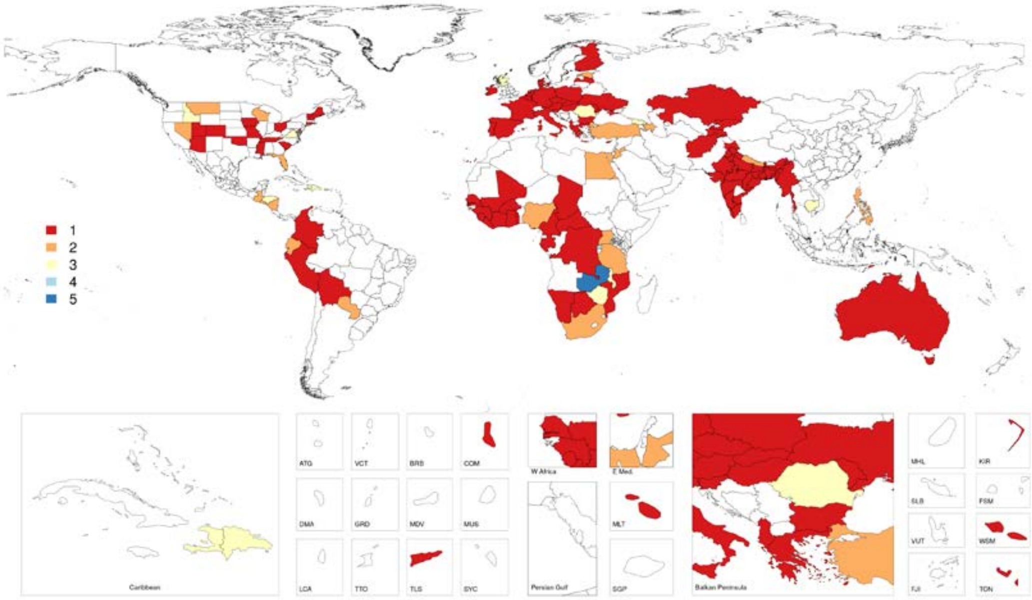

*Country-level sources for China (1), England (1), India (1), Kenya (4), South Africa (2), Sweden (2), and United States (2) are not included on the map

Figure 6. Input data density of unique source by location for indicator 16.1.3.c: prevalence of sexual violence in the last 12 months (males).*

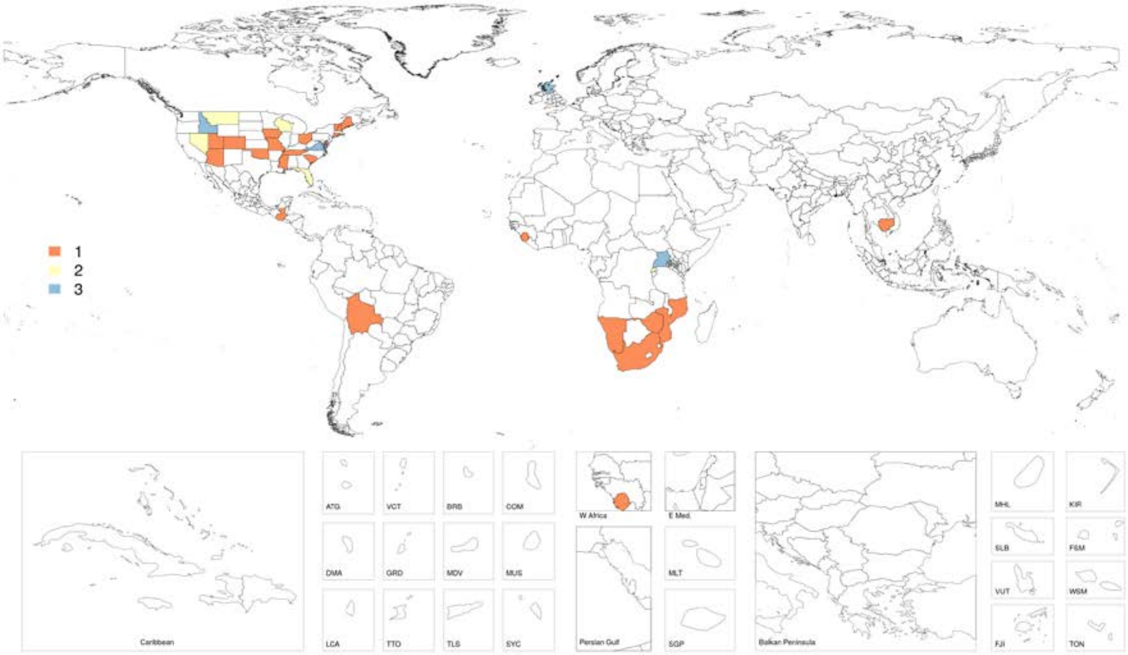

*Country-level sources for China (1), Kenya (1), South Africa (1), Sweden (1), and United States (2) are not included on the map

Modelling Strategy

Overall Modelling Strategy – Indicators 5.2.1, 5.2.2, 16.1.3.a, and 16.1.3.c:

In order to model the prevalence of these four indicators with a 12-month recall, we used DisMod-MR 2.1, a descriptive epidemiological meta-regression tool. For indicators 5.2.1, 5.2.2, and 16.1.3.a, we used a single-parameter modelling approach within DisMod-MR 2.1 to fit the data for prevalence only. To maintain consistency with the modelling of overall sexual violence in the Global Burden of Disease framework, a single-parameter model was not used for indicator 16.1.3.c and instead prevalence, incidence, and remission were estimated simultaneously.

The three main data sources ask questions in different ways, so we have included study-level covariates in order to adjust these data sources to the reference measure of prevalence in the last 12-months. These covariates are described in Table 1 for each of the indicator models. We also present the exponentiated coefficient representing the magnitude of difference between the data points deviating from the reference from each of the indicator models (interpreted as a ratio: larger ratios indicate that the reference data points have lower estimates than those marked with the covariate, and smaller ratios indicate that the gold standard data points have higher estimates that those marked with the covariate). When appropriate, we pre-specified logical bounds for study-level covariates (i.e. studies only asking about penetrative sexual violence must have lower estimates than the reference studies that ask about both penetrative and non-penetrative sexual violence).

To inform estimates in areas where we do not have data, we included country-level covariates in the indicator models. The exponentiated coefficients for these country-level covariates are also included in

Table 1. Where appropriate, we have also included the estimate of the sex covariate (where values < 1 indicate that the indicator is more prevalent in females).

Table 1. Study- and country-level covariates for DisMod-MR 2.1 yearly recall prevalence models for SDG indicators 5.2.1, 5.2.2, and 16.1.3.

Covariate	**Indicator 5.2.1:** intimate partner physical or sexual violence among women	**Indicator 5.2.2:** non-partner sexual violence among women	**Indicator 16.1.3a:** physical violence	**Indicator 16.1.3.c: sexual violence**
**Study-level covariates**				
Does not include sexual violence	0.68 (0.61 — 0.75)			
Does not include physical violence	0.39 (0.27 — 0.61)			
Penetrative sexual violence only		0.66 (0.54 – 0.79)		0.79 (0.66 – 0.93)
Ever-partnered people only (all people for Indicator 5.2.1)	0.88 (0.79 — 0.96)			1.70 (1.18 – 2.52)
Only includes partner violence			0.99 (0.98 – 1.00)	0.85 (0.57 – 1.00)
Physically forced sexual violence only				0.94 (0.82 – 1.00)
Ever-married people only				2.65 (1.85 – 4.67)
Specifies specifically degrading or humiliating sex acts				0.92 (0.75 – 1.00)
Ever had sex				1.38 (1.16 – 1.64)
Ever married or lived with a partner				2.03 (1.49 – 2.87)
Does not include coerced or feared sex in definition				0.92 (0.80 – 1.00)
Does not include non-partner non-penetrative				0.99 (0.96 – 1.00)
Includes attempted sexual violence				1.44 (1.02 – 2.17)
Sex (male)			0.71 (0.67 – 0.75)	0.36 (0.30 – 0.41)
**Country-level covariates**				
Socio-demographic Index	0.23 (0.19 — 0.28)	3.17 (1.70 — 6.11)		
Age-standardized, sex-specific homicide rate	1.13 (1.07 – 1.19)			
Alcohol (liters per capita)				1.11 (1.08 – 1.15)

Lifetime Experience of Non-Partner Sexual Violence

As shown in Figure 2, the model for SDG indicator 5.2.2. Prevalence of non-partner sexual violence in the last 12 months among females is data-sparse for low and middle-income countries. Most of the DHS surveys, which is where the bulk of the sexual violence data for low and middle-income countries come from, do not ask questions about sexual violence committed explicitly by a non-partner in the last year. The result of this is clear in preliminary model results where all of the variation in the low and middle-income countries is driven by the country-level covariates of socio-demographic index and the age-standardized homicide rate in females. Although these covariates may have a relationship with non-partner sexual violence, the regional gradients that they produced in the results for non-partner sexual violence were implausible.

In order to better inform our estimates, we sought to use all of the non-partner sexual violence data available. Although many surveys do not ask about non-partner sexual violence in the last year, they often do have questions about non-partner sexual violence *ever*. Therefore, we have included these sources and performed an age-specific crosswalk of the lifetime recall data to adjust for bias.

Overall Modelling Strategy – indicator 16.2.3:

A single parameter proportion model in DisMod-MR was used to model sexual violence before age 18. Exposure is modeled separately for males and females because we observe little correlation between the prevalence of child abuse among females and males, and modeling both sexes together causes unreasonable estimates in countries where we only have data for one sex. Age mesh points for the female model were set at 0 20 30 40 60 & 100.

Three study-level covariates were used for alternate definitions of the violence.

- Study asked only about intercourse
- Study asked about contact and non-contact abuse
- Study placed restrictions on the relationship between the perpetrator and the victim (e.g. only asked about sexual violence committed by a father)

We also included study-level fixed effects for varying age thresholds across studies.

- Study asked about recall for events before an age below 15 years (versus reference age threshold of 18)
- Study asked about recall for events before an age between 15 and 17 years (versus reference age threshold of 18)

Two study-level covariate fixed effects on variance (z-cov) were also included in both the male and female models, including an indicator that the survey was not nationally representative, as well as whether the survey was administered in schools. These study-level covariates were tested as x-covs first, but we did not find coefficients which would indicate systematic bias. We have not included any country-level covariates to date due to lack of knowledge about a covariate (for which we have a time series for all GBD locations) that predicts childhood sexual abuse prevalence.

8.8.1 DALYs due to Occupational Risk Factors – SDG Appendix

Input Data and Methodological Summary

Indicator definition

This modeling strategy encompassed the indicator associated with DALY rates attributable to occupational risks (8.8.1).

Indicator 8.8.1

As a component of SDG Goal 8. Promote sustained, inclusive and sustainable economic growth, full and productive employment and decent work for all, SDG Target 8.8, protect labour rights and promote safe and secure working environments for all workers, including migrant workers, in particular women migrants, and those in precarious employment, is measured using SDG Indicator 8.8.1, age-standardised all-cause DALY rates (per 100,000) attributable to occupational risks.

Exposure definitions

The following definitions were used for occupational risk factor exposures. All exposures were estimated for ages 15 and older.

Occupational Asbestos	Cumulative lifetime exposure to occupational asbestos, using mesothelioma death rate as an analogue
Occupational Asthmagens	Proportion of the working population exposed to asthmagens, based on population distributions across nine occupational categories
Occupational Carcinogens (arsenic, benzene, beryllium, cadmium, chromium, diesel engine exhaust, formaldehyde, nickel, polycyclic aromatic hydrocarbons, silica, sulfuric acid, and trichloroethylene)	Proportion of the population that was ever occupationally exposed to carcinogens at high or low exposure levels, based on population distributions across seventeen economic activities
Occupational Ergonomic Factors	Proportion of the working population exposed to low back pain-inducing work, based on population distributions across nine occupational categories
Occupational Injuries	Proportion of injuries in the working-age population attributable to occupational work, based on fatal injury rates in seventeen economic activities
Occupational Noise	Proportion of the population occupationally exposed to 85+ decibels of noise, based on population distributions across seventeen economic activities
Occupational Particulates	Proportion of the population occupationally exposed to particulates, based on population distributions across seventeen economic activities

Economic activities and occupations were coded according to the following categories:

Economic Activities	Occupations
Agriculture, hunting, forestry	Legislators, senior officials, and managers
Fishing	Professionals
Mining and Quarrying	Technicians and associate professionals
Manufacturing	Clerks
Electricity, gas, and water	Service workers and shop/market sales workers
Construction	Skilled agricultural and fishery workers
Wholesale and retail trade/repair	Plant and machine operators and assemblers
Hospitality	Craft and related workers
Transport, storage, and communication	Elementary occupations
Financial intermediation	
Real estate/renting	
Public administration/defense; compulsory social security	
Education	
Health and social work	
Other community/social/personal service activities	
Private households	
Extra-territorial organisations/bodies	

Input data

Primary inputs were obtained from the ILO,[1-4] and included raw data on economic activity proportions, occupation proportions, fatal injury rates, and employment to population ratio estimates. A systematic web review was conducted in order to collect the underlying microdata from the ILO's estimates to aid in re-extraction at greater levels of granularity. Where freely available, survey datasets were downloaded from the survey organisations in question. Other datasets were obtained through submission of requests to agencies and through the GBD collaborator network. Microdata was tabulated in order to create survey-weighted estimates of economic activities and occupations for the GBD geographies and years. Various classification systems were crosswalked to ISIC Rev.3 (for economic activities) and ISCO 1988 (for occupations). Subnational estimates for UK and China were added to the datasets for economic activities and occupations.[5,6]

For occupational asbestos, primary inputs were obtained through GBD 2017 cause of death estimates and published studies.[7,13,14]

Uncertainty for inputs where microdata was unavailable was generated by fitting a Loess curve to the data and determining the standard deviation of the data from the fitted curve.

Modelling strategies

A Spatio-temporal Gaussian process regression (ST-GPR) was used to generate estimates for all years and locations for the primary inputs. Study level covariates used in the prior model were education in years per capita, geological covariates (for mining models), the proportion of the population living with access to a coastline (for fishing models), the IHME socio-demographic index (SDI), the mean

temperature/latitude (for agriculture models), and the proportion of the population living in urban areas. Space-time parameters were chosen by maximising out-of-sample cross-validation and minimising RMSE. For economic activity and occupation proportions, estimates from ST-GPR were then re-scaled to sum to 1 across categories by dividing each estimate by the sum of all the estimates.

The following sections describe the modelling approaches for each occupational risk's exposure prevalence.

Occupational carcinogens, occupational noise, and occupational particulates

Prevalence of exposure to these risks was determined using the following equation:

$$Prevalence\ of\ Exposure_{c,y,s,a,r,l} = \sum_{EA} Proportion_{EA,c,y} * EAP_{c,y,s,a} * Exposure\ rate_{EA,r,l,d}$$

where:

EAP = economically active population	c = country	r = risk
EA = economic activity	d = duration	s = sex
a = age	l = level of exposure	y =year

Exposure rate was provided by expert group recommendations and literature[8-11] (see table 1). The CAREX database was used in order to quantify the association between exposure by industry/carcinogen to SDI across all the countries in the database. This effect was used to predict exposure in countries that were not included in CAREX. Duration was considered for occupational carcinogens through application of occupational turnover factors[12] and for occupational noise and particulates by calculating cumulative exposure as the average exposure over the lifetime (the past 50 years) for each age/sex cohort.

Occupational ergonomic factors and occupational asthmagens

Prevalence of exposure to these risks was determined using the following equation:

$$Prevalence\ of\ Exposure_{c,y,s,a,r} = \sum_{EA} Proportion_{OCC,c,y} * EAP_{c,y,s,a}$$

where:

EAP = economically active population	c = country	r = risk
OCC = occupation	a = age	s = sex
		y = year

Occupational injuries

Occupational injury counts were estimated using the following equation:

$$Occupational\ fatal\ injuries_{c,y,a,s} = \sum_{EA} Injury\ rate_{EA,c,y,s} * Population_{c,y,a,s} * EAP_{c,y,s,a} * Proportion_{EA,c,y}$$

where:

EAP = economically active population	c = country	y = year
EA = economic activity	a = age	s = sex

Occupational asbestos

Prevalence of exposure to asbestos was estimated using the asbestos impact ratio (AIR), which is equivalent to the excess deaths due to mesothelioma observed in a population divided by excess deaths due to mesothelioma in a population heavily exposed to asbestos. Formally, this is defined using the following equation:

$$AIR = \frac{Mort_{c,y,s} - N_{c,y,s}}{Mort^{*}_{c,ys,} - N_{c,y,s}}$$

where:

Mort = Mortality rate due to mesothelioma
Mort* = Mortality rate due to mesothelioma in population highly exposed to asbestos
N = Mortality rate due to mesothelioma in population not exposed to asbestos

c = country
y = year
s = sex

Mortality rate due to mesothelioma was estimated from GBD 2017 causes of death.[7] Mortality rate due to mesothelioma in populations not exposed to asbestos was calculated using the model in Lin et al.,[13] while the mortality rate due to high exposure to asbestos was estimated in Goodman et al.[14] Asbestos exposure prevalence created using the AIR was used to estimate PAFs for all asbestos-associated causes except for mesothelioma. Custom PAFs were calculated for mesothelioma by using the ratio of the excess mortality with respect to an unexposed population (Mort – N) divided by the mortality rate in the population in question (Mort). This calculation assumes that all mesothelioma is a product of occupational asbestos exposure and could potentially over-estimate burden due to occupational asbestos exposure in populations with high non-occupational asbestos exposure.

Theoretical minimum-risk exposure level

For all occupational risks, with the exception of occupational asbestos, the theoretical minimum-risk exposure level was assumed to be no exposure to that risk.

Relative risk

Relative risks were obtained for all occupational risks by conducting a systematic review of published meta-analysis. The estimates used, as well as the associated studies, are reported in the Risk Factors Capstone Appendix.

PAFs

For all occupational risks, with the exception of injuries (outlined below) and mesothelioma (outlined above), PAFs were calculated using the prevalences estimated above, using the PAF formula in outlined in the GBD 2017 Risk Factors Capstone methods appendix.

Occupational injuries PAF

The PAFs for occupational injuries were calculated using the following formula:

$$PAF_{c,y,a,s} = \frac{Occupational\ fatal\ injuries_{c,y,a,s} - TMREL}{Fatal\ injuries_{c,y,a,s}}$$

where:

c = country	a = age
y = year	s = sex

Fatal injury totals were obtained from GBD 2017 causes of death.[7]

References

1. International Labour Organization (ILO). International Labour Organization Database (ILOSTAT) - Employment by Sex and Economic Activity. International Labour Organization (ILO).

2. International Labour Organization (ILO). International Labour Organization Database (ILOSTAT) - Employment by Sex and Occupation. International Labour Organization (ILO).

3. International Labour Organization (ILO). International Labour Organization Database (ILOSTAT) - Fatal Injuries by Sex and Economic Activity. International Labour Organization (ILO).

4. International Labour Organization (ILO). International Labour Organization LABORSTA Economically Active Population, Estimates and Projections, October 2011. International Labour Organization (ILO), 2011.

5. Office for National Statistics (United Kingdom). Nomis Official Labor Market Statistics - Annual Population Survey. Newport, United Kingdom: Office for National Statistics (United Kingdom).

6. National Bureau of Statistics of China. China 1% National Population Sample Survey 1995. Ann Arbor, United States: China Data Center, University of Michigan.

7. GBD 2017 Mortality and Causes of Death Collaborators. Global, regional, and national life expectancy, all-cause and cause-specific mortality for 249 causes of death, 1980–2017: a systematic analysis for the Global Burden of Disease Study 2017. *Lancet Rev*.

8. Wilson DH, Walsh PG, Sanchez L, *et al.* The epidemiology of hearing impairment in an Australian adult population. *Int J Epidemiol* 1999; 28: 247–52

9. Kauppinen T, Toikkanen J, Pederson D, Young R, Kogevinas M, Ahrens W, et al. Occupational Exposure to Carcinogens in the European Union in 1990-93. Helsinki, Finland: Finnish Institute of Occupational Health; 1998.

10. Kauppinen T, Toikkanen J, Pedersen D, Young R, Ahrens W, Boffetta P, et al. Occupational exposure to carcinogens in the European Union. *Occup Environ Med* 2000; 57(1): 10–18.

11. Driscoll T, et al. The global burden of non-malignant respiratory disease due to occupational airborne exposures. *American Journal of Industrial Medicine 2005; 48*(6): 432-445.

12. Nelson, D. I., Concha-Barrientos, M., Driscoll, T., Steenland, K., Fingerhut, M., Punnett, L. & Corvalan, C. (2005). The global burden of selected occupational diseases and injury risks: Methodology and summary. *American journal of industrial medicine*, *48*(6), 400-418

13. Lin R-T, Takahashi K, Karjalainen A, *et al.* Ecological association between asbestos-related diseases and historical asbestos consumption: an international analysis. *Lancet* 2007; **369**: 844–9.

14. Goodman M, Morgan RW, Ray R, Malloy CD, Zhao K. Cancer in asbestos-exposed occupational cohorts: a meta-analysis. *Cancer Causes Control* 1999; **10**: 453–65.

17.19.2a Population & Housing Census SDG Appendix

Input data and Methodology

Indicator definition

This strategy encompassed the indicator associated with the indicator associated with housing and population censuses (17.19.2a).

Indicator 17.19.2.a

As a component of SDG Goal 17. To build on existing initiatives to develop measurements of progress on sustainable development that complement gross domestic product, and support statistical capacity-building in developing countries by 2030, is measured using SDG Indicator 17.19.2.a, proportion of countries that have conducted at least one population and housing census in the last 10 years.

Strategy

We searched the Integrated Public Use Microdata Series (IPUMS) questionnaires, the UN Demographic Yearbook, the UN census program census dates, and the International Population Census Biography to identify all censuses conducted between 1980 and 2017.[1–4] In some cases, the same census was reported by different sources in different years. For example, Dominica 1981 incorrectly appears as 1980 on the IPUMS website. We resolved these inconsistencies based on a review of available documentation. A number of countries high income countries do not run censuses directly, preferring to maintain administrative databases that capture population information. We included these population registers in our list. In several cases, the United Nations does not recognize administrative splits in territories, such as Kosovo and Serbia. If a census occurred in either territory, we report the census as occurring in that year in the recognized country.

References

1 Goyer DS. The International population census bibliography, revision and update, 1945-1977. New York: Academic Press, 1980 https://catalog.hathitrust.org/Record/101176113 (accessed March 30, 2018).
2 Ruggles S, Alexander JT, Genadek K, Goeken R, Schroeder MB, Sobek M. Integrated Public Use Microdata Series: Version 5.0 [Machine-readable database]. Minneapolis, MN: Minnesota Population Center [producer and distributor], 2010.
3 United Nations, Department of Economic and Social Affairs, Statistics Division. Population Censuses' Datasets (1995 - Present). https://unstats.un.org/unsd/demographic-social/products/dyb/dybcensusdata.cshtml (accessed March 30, 2018).
4 United Nations, Department of Economic and Social Affairs, Statistics Division. The census program, census dates from 1990 onward. https://unstats.un.org/unsd/demographic/sources/census/censusdates.htm (accessed March 30, 2018).

17.19.2c Well-certified deaths SDG Capstone Appendix

GBD estimates are most accurate when computed with a full time series of complete VR with a low percentage of garbage codes. In GBD 2016, we developed the percent well certified metric to give a picture of the quality of data available in a given country over the full time series used in GBD estimates. Countries improve well-certified death registration as they increase availability, completeness, and detail of their mortality data and reduce the percentage of deaths coded to ill-defined garbage codes or highly aggregated causes. We measure this proportion for each location-year of vital registration (VR) data.

For each year of VR, percent well-certified is:

$$pct_{wellcertified} = completeness \times (1 - pct_{majgarbage})$$

Where:

$$completeness = \frac{registered\ deaths}{GBD\ mortality\ envelope}$$

$$pct_{majgarbage} = \frac{deaths\ coded\ to\ level\ 1\ or\ 2\ garbage\ or\ highly\ aggregated\ cause}{registered\ deaths}$$

Simplifying this equation, "percent well-certified" is the number of deaths that are registered to a well-defined cause (those codes which are not Level 1 or 2 garbage or highly aggregated) divided by the GBD mortality envelope.

Not all locations have VR data from 1990-2017, so ST-GPR is used to interpolate the full time series for all GBD locations where we have VR data. Locations where we do not have any VR data from 1990-2017 are zeroed out. The Wilson interval was used to calculate variance prior to ST-GPR interpolation.[1] The data were modeled as logit percent well-certified, where socio-development index was used as a covariate with 1000 draws.

[1] Wilson, EB. Probable Inference, the Law of Succession, and Statistical Inference. Journal of the American Statistical Association 1927; 158: 209-212.

Part 2. SDG index construction

In this analysis we have constructed an index that represents overall performance on the health-related SDG indicators (referred to as the SDG index). With the exception of health worker density and natural disasters, as described below, we follow the same scaling and index construction approach as we used in GBD 2015 and GBD 2016.[1,2]

For rate-space indicators, we first transformed the values to natural log space; proportion indicators were not transformed. The resultant indicator distributions were then rescaled to a 0 to 100 scale with 0 being the 2.5th percentile and 100 being the 97.5th percentile of the distribution of indicator values over the time period 1990 to 2030. The health-related SDG index was then computed by first determining the geometric mean of each rescaled health-related SDG indicator for a given target and then taking the geometric mean of the resulting values across the targets. This approach weights each of the health-related SDG targets equally and assumes partial substitutability with high values on one target partly compensating for low values on another target.

For health worker density (SDG indicator 3.c.1), we used a modified scaling approach to reflect the important roles of each health worker cadre included in the SDG indicator (ie, physicians, nurses and midwives, and pharmacists). As described in more detail under the write-up for health worker density (appendix section 1), we derived cadre-specific thresholds at which increases in health worker density did not result in additional gains on the Healthcare Access and Quality (HAQ) Index.[3] These thresholds were 30 physicians per 100,000; 100 nurses and midwives per 100,000; and 5 pharmacists per 100,000. For each location-year where estimates met or exceeded these thresholds, a scaled value of 100 was assigned. We selected the 2.5th percentile, by cadre, to set scores of 0, from 1990 to 2030. We then took the geometric mean of the scaled health worker cadres, by location-year, to provide scaled results for overall health worker density.

For natural disasters, given the stochastic nature of the cause, we used the long-term average over the 2018 to 2030 period to generate the estimates for the SDG index.

References

1 Lim SS, Allen K, Bhutta ZA, *et al.* Measuring the health-related Sustainable Development Goals in 188 countries: a baseline analysis from the Global Burden of Disease Study 2015. *The Lancet* 2016; **388**: 1813–50.

2 Fullman N, Barber RM, Abajobir AA, *et al.* Measuring progress and projecting attainment on the basis of past trends of the health-related Sustainable Development Goals in 188 countries: an analysis from the Global Burden of Disease Study 2016. *The Lancet* 2017; **390**: 1423–59.

3 Fullman N, Yearwood J, Abay SM, *et al.* Measuring performance on the Healthcare Access and Quality Index for 195 countries and territories and selected subnational locations: a systematic analysis from the Global Burden of Disease Study 2016. *The Lancet* 2018; **0**. DOI:10.1016/S0140-6736(18)30994-2.

Part 3. Projections for the health-related SDGs

Section 1. Overall projection modeling strategy

We generated projections of the health-related Sustainable Development Goal (SDG) indicators through 2030 by leveraging the forecasting framework developed by Foreman and colleagues.[1] This modelling framework has been designed to account for the relationships between risk factors and other independent drivers of health outcomes (eg, gains in overall sociodemographic development, select interventions such as vaccine coverage and met need for family planning), thus better capturing causal pathways of health change shown in randomised control trials and cohort studies. Here we provide more detail on how these methods were used to produce projections through 2030 for (1) independent drivers, including risk factors, intervention coverage, and some nonfatal health outcomes; (2) cause-specific models; (3) HIV; and (4) universal health coverage (UHC) index that approximates UHC service coverage. The latter uses a modified version of the overarching forecasting framework to account the effects of projected increases in total health spending per capita and potential system efficiencies.[2]

The underlying forecasting framework produces projections through 2040 for most indicators; because we are focused on the SDGs in this analysis, which spans 2015 to 2030, we use these results through 2030 rather than 2040. In addition, many of the inputs into the forecasting framework are based on Global Burden of Disease study (GBD) 2016 results as the 2017 iteration of the GBD study remains in-progress. We use these forecasts, which are produced through the full forecasting estimation pipeline of independent drivers and their causal relationships, and then shift up or down the forecasted series such that the first year of the GBD 2016 forecasts matches last year of the GBD 2017 past estimates, by location, year, and draw. This allows us to incorporate the trends predicted by the forecasting model while preserving consistency with the levels estimated in GBD 2017.

Table 1. Weights selected with out-of-sample validity testing for projecting the health-related SDGs. Air poll mort=Mortality attributable to household air pollution and ambient air pollution. Cert Death Reg=Well-certified death registration. HRH – Nurses/Midwives=Human resources for health – nurses and midwives . HRH – Pharmacists= Human resources for health – pharmacists . HRH – Physicians= Human resources for health – physicians. Int Partner Viol=Intimate partner violence. Malaria incid=Malaria incidence. Mean PM2.5=Mean particulate matter smaller than 2.5 microns in diameter. Met need for Family Planning=Met need for family planning with modern contraception methods. Non-Int Partner Sex Viol=Non-intimate partner sexual violence. Occ burden=Disease burden attributable to occupational risks. Skilled Birth Attend=Skilled birth attendance. Smoking prev=prevalence of current smoking. WaSH mort=Mortality attributable to unsafe water, sanitation, and hygiene. NTD prev=Prevalence of 18 neglected tropical diseases.

Indicator	Selected Weight		Indicator	Selected Weight
Air Poll Mort	1.5		Smoking Prev	1.0
Alcohol Use SEV	4.4		WaSH Mort	1.4
Cert Death Reg	1.0		Water SEV	5.2

Child Overweight	1.7	NTD Prev	
Child Sex Abuse	1.2	African Trypanosomiasis	1.7
Child Stunting	1.7	Ascariasis	1.8
Child Wasting	1.9	Chagas	0.5
Household Air Pollution SEV	6.2	Cutaneous and Mucocutaneous Leishmaniasis	1.7
HRH - Nurses/Midwives	1.0	Cystic Echinococcosis	0.1
HRH - Pharmacists	1.3	Cysticercosis	1.6
HRH - Physicians	1.0	Dengue	0.8
Hygiene SEV	7.0	Food-Borne Trematodiases	1.0
Int Partner Viol	1.5	Guinea Worm	0.6
Malaria Incid	1.0	Hookworm Disease	1.7
Mean PM2.5	0.3	Leprosy	1.8
Met need for family planning	0.6	Lymphatic Filariasis	1.6
Non-Int Partner Sex Viol	1.0	Onchocerciasis	1.0
Occ Burden	1.4	Rabies	1.1
Physical Violence	0.5	Schistosomiasis	1.0
Sanitation SEV	5.7	Trachoma	0.5
Sexual Violence	1.0	Trichuriasis	1.5
Skilled Birth Attend	1.6	Visceral Leishmaniasis	1.8

References

1 Foreman KJ, Marquez N, Dolgert A, *et al.* Forecasting life expectancy, years of life lost, all-cause and cause-specific mortality for 250 causes of death: reference and alternative scenarios 2016–2040 for 195 countries and territories. *The Lancet* Under review.

2 Dieleman JL, Sadat N, Chang AY, *et al.* Trends in future health financing and coverage: future health spending and universal health coverage in 188 countries, 2016–40. *The Lancet* 2018; **391**: 1783–98.

Section 2. Independent drivers

Risks and other SDG indicators

For each risk factor (r), we calculated the annual change in the logit of the SEV for every location (l), age (a), sex (s), and past year (t = 1991, … ,2016). In order to dampen the effect of noisy data we replaced annual changes (first differences) outside the 15th and 85th percentiles with those percentile-values, respectively.

$$d_{l,a,s,t,r} = \text{logit}\left(SEV_{l,a,s,t,r}\right) - \text{logit}\left(SEV_{l,a,s,t-1,r}\right)$$

We then computed the annualized rate of change for each country, age, and sex by calculating the weighted mean of the first difference over time, where the weights w_t are determined by a recency-weighting parameter ω, and scaled to sum to 1.

$$\delta_{l,a,s,r} = \text{mean}\left(d_{l,a,s,r,T}, w_{r,t}\right)$$

$$w_{r,t} = (t - t_{initial} + 1)^{\omega_r}$$

In order to select weighting parameters ω, we used data from just 1990 to 2006 to project each risk factor to 2016 using values of ω ranging, in increments of 0.25, from 0 to 10, and calculated the root-mean-square error (RMSE) to select the weights.

We applied this same model for SDG indicators not expressly included in the independent driver model developed by Foreman and colleagues.[1] Our only adjustment was holding out data from 1990 to 2007 to project each measure from 2008 to 2017.

Vaccines

We forecasted coverage for the following 8 vaccines: diphtheria-tetanus-pertussis (DTP) dose 3, measles (MCV1), polio, rotavirus, pneumococcal (PCV3), Haemophilus influenzae type B (HIB3), hepatitis B dose 3 (HepB3), and measles dose 2 (MCV2). These were divided into two types: simple vaccines and ratio vaccines, based on how they are modeled in the GBD. The simple vaccines, DTP3, measles, and polio, have been introduced in every GBD country, while the ratio vaccines were first introduced more recently and have not yet been added to the routine schedule in all countries. These newer generation vaccines therefore require the additional step of forecasting introduction dates. Due to the typical scheduling of rotavirus, PCV, Hib, and HepB vaccine administration programs, coverage for these vaccines was assumed to converge to DTP3 coverage over time (and thus cannot exceed DTP3 coverage). Similarly, MCV2 is assumed to converge to MCV1 coverage over time. These vaccines are combined into an aggregate indicator of overall vaccine coverage by taking the arithmetic mean of all 8 of them, as is described for past estimates (section 1.3).

Contraceptive met need

We consider the proportion of women of reproductive age (15-49 years) who have their need for family planning satisfied with modern methods.[1]

Reference scenario

We calculated the weighted annual change in logit transformed contraceptive met need (MN) for ages 15 to 49 for every country and past year (1990 to 2016), using the weight selection method described in section 3.1. The ω parameter for met need is 0.6.

Fertility

Fertility scenarios were modeled using age specific fertility rates (ASFR) that were aggregated for the total fertility rate (TFR) scenarios, where a is age group and n_a is the length of an age group (e.g. 6 days or 5 years):

$$\mathrm{TFR} = \sum^{a} n_a \cdot \mathrm{ASFR}_a$$

Reference scenario

We used three separate models for ASFR, corresponding to the following three sets of age groups: 1) the youngest fertile age group (15 to 19 years); 2) fertile age groups (5-year age groups for 20 to 49 years of age); and 3) youngest and oldest age groups (10 to 14 and 50 to 54 years of age).

1) ASFR for the youngest fertile age group was estimated with a two stage model. The first stage was a linear model with maternal education (MEDU) and met need for contraception as independent variables:

$$\mathrm{Logit}(\mathrm{ASFR}_{a,l,t}) = \beta_a \mathrm{MEDU}_{a,l,t} + \gamma_a \mathrm{met\_need}_{a,l,t} + \epsilon_{a,l,t}$$

   where $\epsilon_{a,l,t} \sim \mathcal{N}(0, \sigma_1)$.

   The second stage was used to estimate location specific errors with an autoregressive model of order 1:

$$\epsilon_{a,l,t} = c + \psi \epsilon_{a,l,t-1} + \eta_{a,l,t}$$

   where $\eta_{a,l,t} \sim \mathcal{N}(0, \sigma_2)$

   The forecasts were shifted to match past coverage.

2) ASFR for the fertile age groups were estimated with a similar model, with two changes made to the first stage. First, a cohort lag component was added to the model. Second, a linear spline was applied to the maternal education term (MEDU). The resulting first stage linear model was

$$\mathrm{Logit}\big(\mathrm{ASFR}_{a,l,t}\big) = \beta_a \mathrm{met\_need}_{a,l,t} + \lambda_a \mathrm{ASFR}_{a-N_a,l,t+N_a} + \sum_{x \in X} \gamma_a^{[x]} \mathrm{MEDU}_{a,t}^{[x]} + \epsilon_{a,l,t}$$

   where the knots X were less than 6 years of maternal education, between 6 and 10 years of education, between 10 and 14 years of education, and greater than 14 years of education. There were no changes to the second stage of the model.

3) The youngest and oldest age groups were estimated using LOESS fits on data from the Human Fertility Database used in GBD 2016[3]:

$$\text{ASFR}_{10} \sim \text{LOESS}(\text{ASFR}_{15}),$$

$$\text{ASFR}_{50} \sim \text{LOESS}(\text{ASFR}_{45})$$

Each LOESS was fit using data from all available past years and locations.

References

1 Foreman KJ, Marquez N, Dolgert A, *et al.* Forecasting life expectancy, years of life lost, all-cause and cause-specific mortality for 250 causes of death: reference and alternative scenarios 2016–2040 for 195 countries and territories. *The Lancet* Under review.

2 Fullman N, Barber RM, Abajobir AA, *et al.* Measuring progress and projecting attainment on the basis of past trends of the health-related Sustainable Development Goals in 188 countries: an analysis from the Global Burden of Disease Study 2016. *The Lancet* 2017; **390**: 1423–59.

3 Naghavi M, Abajobir AA, Abbafati C, *et al.* Global, regional, and national age-sex specific mortality for 264 causes of death, 1980–2016: a systematic analysis for the Global Burden of Disease Study 2016. *The Lancet* 2017; **390**: 1151–210.

Section 3. Cause models

Overview

The forecasting model is cause-specific and separate for males and females and also generates estimates of all-cause mortality that are the sum of cause-specific estimates. The logarithm of the cause-specific mortality is modeled as the sum of three components: 1) underlying mortality; 2) a scalar that captures the combined effects of risk factors on the specific cause, accounting for mediation; and 3) and ARIMA model for the unexplained residual. The underlying mortality is modeled with terms for development (SDI) and calendar time. The scalar is a function of all the GBD risk factors relevant to each cause and select interventions tracked in the GBD. The third component is ARIMA forecasting of the residuals of the first two components model (underlying mortality plus combined effect of risk factors).

We develop a forecast or reference scenario which is meant to represent the most likely future trajectory of health given past trends of the independent drivers and the observed past relationships between independent drivers and each cause of death. The forecast is not what will happen only what would most likely happen if past trends and relationships continue into the future.

Cause-Specific Mortality Modeling

Accounting for risk factors

We took advantage of the relationships between drivers and mortality that we will describe in detail in Section 5 to come up with an additive relationship (in log space) between underlying mortality rate m_U, the risk factor scalar $\mathbb{S}$, and total cause-specific mortality m_T:

$$m_T = m_U \times \mathbb{S}$$

Taking the logarithm gives

$$\ln(m_T) = \ln(m_U) + \ln(\mathbb{S}).$$

We thus accounted for risk factors by including $\ln(\mathbb{S})$ as an offset when modeling total cause-specific mortality.

Basic model

We assumed that underlying mortality m_U could be estimated as a function of SDI, location, age, and time. We used a model with location-age-specific intercepts α, a global effect on SDI β, and age-specific effects on the secular trend θ_a.

$$\ln(m_T) \sim \mathcal{N}(\hat{y}, \sigma)$$

$$\hat{y} = \alpha_{la} + \beta_0 SDI_{<0.8} + \beta_1 SDI_{\geq 0.8} + \theta_a t + \ln(\mathbb{S})$$

For some causes, other independent variables with strong known relationships for which data are available (ie, age-specific fertility for maternal causes, HIV mortality for maternal HIV, vehicles per capita

for road injuries) or risk factors which cannot be quantified in terms of RR because they are part of the disease definition (eg, systolic blood pressure for hypertensive heart disease, fasting plasma glucose [FPG] for diabetes, alcohol consumption for alcohol-related cirrhosis; others are listed later in this appendix [pp 14]) were added as additional covariates to the model.

In addition, for a few non-communicable diseases with strong evidence of recent accelerated progress beyond what would be expected by SDI alone, we include an SDI*time interaction effect to capture this. These causes include ischaemic heart disease, diabetes, and all of the child causes of cirrhosis, stroke, and chronic kidney disease. Due to the collinearity of SDI, time, and other covariates in some causes of death, several models with the above formulation did not converge. To address this, all models with one or more coefficients whose standard deviation was more than 1000 times the absolute value of the median coefficient value were rerun without SDI. If the new formulation also did not converge, all covariates besides time were dropped from the underlying mortality formulation and the model was run once more.

Additionally, because SDI is included as an input to the vaccine forecasts, it was excluded as a covariate for all vaccine-dependent causes of death except lower respiratory infections.

Smoothing priors

By including the scalar $\mathbb{S}$ as an offset in the model, we were able to place Girosi-King (2008) type priors on the total cause-specific mortality while modeling underlying mortality.[13] These biased the model towards parameterizations that produce consistent age patterns over time by adding a penalty function based on the dot product of the first derivatives of adjacent age groups over time.

Modeling Latent Trends

The residuals ϵ from the basic model represent latent trends in total cause-specific mortality not captured by risk factors, SDI, and global secular trends.

$$\epsilon = \ln(m_T) - \hat{y}$$

We forecasted the latent trends by using an $ARIMA_{1,0,0}$ model, which combines an autoregressive model to capture overall trends and a differencing step to ensure stationarity and reflect expanding uncertainty in the future. However, running independent ARIMA models on the residuals of every cause, location, and age is not very robust and can lead to extreme forecasts. Therefore, we used a pooled model, which enabled us to share ARIMA parameters within geographic super-regions (s).

$$\epsilon_{lat} \sim \mathcal{N}(\hat{\epsilon}_{lat}, \sigma)$$

$$\hat{\epsilon}_{lat} = \psi_{sa}\hat{\epsilon}_{lat-1} + \delta_{sat}$$

$$\delta_{sat} \sim \mathcal{N}(0, \tau_{sa})$$

Cascading Mortality Models

In addition to using the above framework to model cause-specific latent trends, we also modeled the residuals at higher levels of the cause hierarchy in order to prevent a few unusual cause-specific trends from dominating our all-cause forecasts.

First, we generated all-cause total mortality predictions and residuals by summing up cause-specific forecasts based on risk factors, SDI and global secular trends.

$$\hat{Y} = \ln(\sum_{c}^{C} e^{\hat{y}_c})$$

$$E = \ln(M) - \hat{Y}$$

We then used an equally-weighted blend of arima models to forecast these latent trends in the same way as the cause-specific forecasts. The latent trends in the summed all-cause are more robust than those from the cause-specific models alone, so modeling them at the location-age-sex specific level is more feasible. For the blend, we took an equal number of draws from each of four ARIMA specifications: ARIMA(1, 0, 0), ARIMA(1, 1, 0), ARIMA(1, 0, 0) + constant, and ARIMA(1, 1, 0) + constant. These correspond to

$$E_{lat} \sim \mathcal{N}(\hat{E}_{lat}, \sigma)$$

with ARIMA(1, 0, 0):

$$\hat{E}_{lat} = \psi_{la}\hat{E}_{lat-1} + \delta_{lat}$$
$$\delta_{lat} \sim \mathcal{N}(0, \tau_{la}),$$

ARIMA(1, 1, 0):

$$\hat{E}_{lat} = \hat{E}_{lat-1} + \psi_{la}(\hat{E}_{lat-1} - \hat{E}_{lat-2}) + \delta_{lat}$$
$$\delta_{lat} \sim \mathcal{N}(0, \tau_{la}),$$

ARIMA(1, 0, 0) + constant:

$$\hat{E}_{lat} = \psi_{la}\hat{E}_{lat-1} + \delta_{lat}$$
$$\delta_{lat} \sim \mathcal{N}(\delta_{la}, \tau_{la}),$$

ARIMA(1, 1, 0) + constant:

$$\hat{E}_{lat} = \hat{E}_{lat-1} + \psi_{la}\left(\hat{E}_{lat-1} - \hat{E}_{lat-2}\right) + \delta_{lat}$$
$$\delta_{lat} \sim \mathcal{N}(\delta_{la}, \tau_{la}),$$

By adding our estimated all-cause latent trends to the sum of our cause-specific forecasts (*Note*: not including cause-specific latent trends), we then generated robust all-cause mortality forecasts.

$$\hat{M} = e^{\hat{Y} + \hat{E}}$$

We then repeated this method, though without the blend, for successive levels of the cause hierarchy. For instance, we calculated group I, II, and III mortality by finding residuals at each level and then forecasting them using the pooled AR1 [ARIMA (1,0,0)] method described for the cause-specific models.

Then, we generated robust forecasts of cause-specific mortality at each level by generating a forecasted cause fraction and multiplying it by the forecasted parent cause mortality.

$$\hat{m}_c = e^{\hat{y}_c + \hat{\epsilon}_c}$$

$$CS\hat{M}F_c = \frac{\hat{m}_c}{\sum_i^C \hat{m}_i}$$

$$m_c^* = \hat{M} \times CS\hat{M}F_c$$

We did this successively down the cause hierarchy, so that child causes were constrained to the parent, but the parent causes still reflected the risk factors and underlying trends driving each child cause. Latent trends for group I causes were pooled at the region-age-sex level, and latent trends for groups II, III, and IV were pooled at the super-region-age-sex level. Modelling relationships between drivers and mortality

Modeling overview by cause and sex

We forecasted cause-specific mortality rates m by cause of death c, location l, age group a, and year t. This calculation uses population at mid-year to calculate mortality rate.

$$m_{clat} = \frac{\text{deaths}_{clat}}{\text{pop}_{lat}}$$

The all-cause mortality rate M is the sum of cause-specific mortality rate m_c by location, age group, and year.

$$M_{lat} = \sum_c^C m_{clat}$$

Mediation, Scalars, and Population Attributable Fractions (PAFs)

We generated an estimated risk-specific (r) $P\hat{A}F$ in the future by converting the forecasted $S\hat{E}V$ values to $P\hat{A}F$ as follows (see section 4 for an explanation of $S\hat{E}V$ forecasts):

$$P\hat{A}F_{rclat} = 1 - \frac{1}{S\hat{E}V_{rlat} \times (RR_{rc}^{max} - 1) + 1}$$

$P\hat{A}F$ estimates depend on $S\hat{E}V$, which is not cause-specific; therefore we expect a bias in logit-transformed space, which is the space where exposures are modelled. We try to correct for this bias by forcing our estimated values to agree with the GBD in the year 2016. This is done by first taking a reference PAF directly computed from exposure and cause-specific relative risks available in the GBD:

$$PAF_{rclat} = \frac{\sum_x^X p_{xrlat} \times RR_{xrc} - 1}{\sum_x^X p_{xrlat} \times RR_{xrc}}$$

where x corresponds to the different exposure levels of the risk factor. This is followed by calculating the correction factor CF via comparing (in logit space) the GBD PAF to the $S\hat{E}V$-derived estimated $P\hat{A}F$ in the reference year 2016:

$$CF_{rcla} = \text{logit}(PAF_{rcla2016}) - \text{logit}(P\hat{A}F_{rcla2016})$$

This correction factor is necessary because the $S\hat{E}V$ is summarized across all of the causes of death related to that risk factor. If there are different patterns of relative risk by exposure level for different causes of death for the same risk factor, there is some information loss attributable to this dimensionality reduction. Since that correction factor is relatively stable over time, we can simply add it to each year in the forecast to approximate the cause-risk-specific $P\hat{A}F$ accounting for these different relative risk curves.

We applied the correction factor to the estimated $P\hat{A}F$ to come up with an adjusted estimated PAF^*:

$$PAF^*_{rclat} = \text{expit}(\text{logit}(P\hat{A}F_{rclat}) + CF_{rcla})$$

To properly estimate the joint PAF of all risks, one must take into account of how one risk factor is mediated through other risk factors. The fraction of one risk that is mediated through another is called Mediation Factor (MF).[11] Using risk mediation factors provided in the GBD 2016, we computed the joint (adjusted) PAF of all risks for a cause:

$$PAF_{clat} = 1 - \prod_{j=1}^{J} (1 - PAF_{jclat} \times \prod_{i=1}^{J} (1 - MF_{jic}))$$

Where *J* is a set of risk factors for the aggregation and MF_{jic} is the mediation factor for risk *j* mediated through *i* for cause *c*. Since PAF is the ratio of risk-attributable cause-specific deaths to total cause-specific deaths, we can relate total cause-specific mortality m_T to underlying cause-specific mortality m_U.

$$PAF = \frac{m_A}{m_T}$$

$$\&$$

$$m_T = m_A + m_U$$

$$\therefore$$

$$m_T = m_U \times \frac{1}{1 - PAF}$$

Finally, we generated a risk factor scalar $\mathbb{S}$, corresponding to the ratio of total cause-specific mortality to underlying cause-specific mortality.

$$\mathbb{S}_{clat} = \frac{1}{1 - PAF_{clat}}$$

For some cause-risk factor pairs, the PAF is 1 because exposure to the risk factor is part of the disease definition itself. These are systolic blood pressure for hypertensive heart disease and hypertensive chronic kidney disease, fasting plasma glucose for diabetes mellitus, alcohol consumption for alcohol-related cirrhosis of the liver, underweight for protein energy malnutrition deaths, iron deficiency for aenemia deaths, alcohol consumption for alcohol-related cardiomyopathy, impaired kidney function for chronic kidney disease, low birth-weight for preterm birth complication deaths, and occupational exposure to particulates and silica for certain subtypes of pneumonia deaths. In these cases, the risk factors are excluded from the risk-factor scalar and instead their SEVs are included as additional covariates in the mortality model. In addition, we model rotavirus mortality separate from other diarrhoeal disease mortality since we include rotavirus vaccination coverage forecasts as an independent driver. We then sum up other diarrhoeal and rotavirus diarrhoeal deaths.

Using Causes of Death Forecasts to Inform Nonfatal Outcomes

To preserve the relationships between projected mortality and nonfatal health outcomes, we computed mortality-to-incidence ratios for tuberculosis and hepatitis B to predict incidence through 2030.

Tuberculosis

Incidence of Tuberculosis without HIV (TB) was forecasted using a mixed-effects model with the ratio of mortality to incidence (MI) for TB without HIV as the dependent variable and Healthcare Access and Quality (HAQ) as the independent variable. We used a model with region-age-specific intercepts $\propto_{r,a}$ and a region-age-specific trend on HAQ, $\theta_{r,a}$. The MI ratios for sexes were modeled independently of each other. After fitting the regression, MI ratio predictions were made using forecasted HAQ and then converted to incidence estimates through multiplication of the mortality forecasts.

$$\widehat{logit(MI)}_{a,l,t} = \alpha_{r,a} + \theta_{r,a} HAQ_{l,t}$$

Similarly, incidence of Tuberculosis with HIV (TB/HIV) was forecasted using a mixed-effects model with the same covariate and mixed-effect structure, but with a slightly different dependent variable. Here the incidence to prevalence ratio (IP) being forecasted was generated with the aggregate incidence of Drug-susceptible TB, Multi-drug resistant TB, and Extensively drug resistant TB divided by the prevalence of HIV. The forecasted ratios were then multiplied by HIV prevalence forecasts to generate final TB with HIV incidence estimates. More detail on HIV forecasting methods is described in section 3.4.

$$\mathrm{I}^{TB}_{s,l,a,t} = \sum_{tb}^{TB} i_{h,s,l,a,t}$$

$$\widehat{logit(IP)}_{a,l,t} = \alpha_{r,a} + \theta_{r,a} HAQ_{l,t}$$

Both of these forecasts were then aggregated and intercept shifted to GBD 2017 estimates to generate forecasts of total TB-incidence.

Hepatitis B

Incidence of Hepatitis B was forecasted using the MI ratio of the aggregate mortality rates for acute Hepatitis B, Hepatitis B from liver cancer, and Hepatitis B from cirrhosis divided by the incidence of Acute Hepatitis B.

$$\mathrm{M}_{s,l,a,t}^{Hep\ B} = \sum_{h}^{H} m_{h,s,l,a,t}$$

The MI ratio was modeled using the same specifications as the MI ratio described in the TB without HIV incidence forecasts, and this ratio was then multiplied by the forecasted aggregate mortality in order to generate total Hepatitis B incidence.

$$\widehat{logit(MI)}_{a,l,t} = \alpha_{r,a} + \theta_{r,a} HAQ_{l,t}$$

Causes of Death Forecasted Outside the Main Framework

War, legal interventions, and disasters

Deaths due to stochastic events including wars, terrorism, legal interventions, and natural disasters were forecasted for each year in the future by randomly sampling from past death rates from 1990 to 2016,

$$\widehat{m}_{s,a,f,l} = m_{s,a,r,l},$$

$$\forall a \in A, \forall s \in S, \forall l \in L$$

where $\widehat{m}_{s,a,f,l}$ is the age-sex-location-specific mortality rate for the future year $f \in [2017, 2040]$, and $m_{s,a,r,l}$ is the age-sex-location-specific mortality rate of random past year $r \sim U(1990{,}2016)$, A is all of the ages, S is both of the sexes, and L is all of the countries. To maintain correlation among ages, sexes, and countries, a single past year r was randomly selected for a given future year f. For example, if we randomly selected the year 1997 for the year 2030, then the mortality rate for Canadian 20-24 year-old females in 2030 would be inferred from the mortality Canadian 20-24 year-old females in 1997, while the mortality rate for Japanese 40-44 year-old males in 2030 would be inferred from the mortality Japanese 40-44 year-old males in 1997.

In the next step, we applied an SDI-adjustment factor derived from the degree to which increases in SDI have reduced mortality from those events in the past. To do this, we fit a local regression (LOESS) on past mortality rates, $m_{s,a,t_p,l}$, where $t_p \in [1990, 2016]$, and past and forecasted SDI, $SDI_{t,l}$, where $t \in [1990, 2040]$. We extended the traditional LOESS regression to allow us to extrapolate to SDI forecasts. This regression was performed for each sex and age groups separately, but across all years and countries together. The model is

$$\ln\left(m_{s,a,t,l}^{*}\right) \sim \beta_{s,a} SDI_{t,l}$$

where $m_{s,a,t,l}^{*}$ is the LOESS-predicted sex-age-year-location-specific mortality rate, $SDI_{t,l}$ is the year-location-specific SDI, and $\beta_{s,a}$ is the sex-age-specific effect of SDI on the mortality rate. Here $t \in [2017, 2040]$, so $m_{s,a,t,l}^{*}$ has values for both past and future years.

We used these LOESS-predictions to generate an adjustment factor for the randomly selected past mortality rate. The adjustment factor is the ratio of the LOESS-predicted mortality for the future year divided by the LOESS-predicted mortality of the year that was randomly selected to represent that future year's mortality rate. In log space, this is calculated as

$$\phi_{s,a,f,l} = \ln\left(m^*_{s,a,f,l}\right) - \ln\left(m^*_{s,a,r,l}\right)$$

where $m^*_{s,a,f,l}$ is the LOESS-predicted mortality rate of the future year f and $m^*_{s,a,r,l}$ is the LOESS-predicted mortality rate of the randomly selected past year r.

The final estimate of the future year's mortality rate was the observed value of the randomly selected past year multiplied by the correction factor.

$$\widehat{m}^*{}_{s,a,f,l} = \exp(\phi_{s,a,f,l} + \ln\left(\widehat{m}_{s,a,f,l}\right))$$

Due to the enormous magnitude of the war-related mortality rate during the genocide in Rwanda in 1994, in Rwanda we replace any draws of war-related mortality rates greater than the 95th percentile with the median across draws for each age, sex, year in the forecasts.

References

1 Girosi F, King G. Demographic Forecasting. Princeton: Princeton University Press, 2008.

2 Gakidou E, Afshin A, Abajobir AA, *et al.* Global, regional, and national comparative risk assessment of 84 behavioural, environmental and occupational, and metabolic risks or clusters of risks, 1990–2016: a systematic analysis for the Global Burden of Disease Study 2016. *The Lancet* 2017; **390**: 1345–422.

Section 4. HIV

Antiretroviral therapy for HIV/AIDS coverage

In recent years, we have seen a massive scale up of ART treatment among low-income nations, who through large internal investments and substantial development assistance have been able to increase ART access considerably. For that reason, if past trends in ART coverage for each country are simply scaled up in projections using a logistic curve, all countries would be projected to achieve 100% coverage by 2030. Given limitations on coverage by health system capacity, and due to the cost of treatment, we bound ART projections with a frontier by income level to reflect resource availability.

Cross-walking Cross-Sectional and Spectrum CD4 Definitions

In order to model the relationship between income and ART coverage, we must also consider CD4 count as a major stratifying variable, since individuals who are sicker (with lower CD4 counts) are more likely to have received a diagnosis and treatment. Survey data provides cross-sectional CD4 count information; however, the Spectrum modeling framework tracks individuals by categorical CD4 count at the initiation of treatment. Therefore, in order to model the relationship between CD4-specific ART coverage and income in a format that aligns with Spectrum, we cross- walked cross-sectional CD4 values to CD4 at treatment initiation.

We extracted information on the average CD4 progression over time after the initiation of ART treatment from a number of cohort studies.[1-8] We used a natural spline model to parameterize CD4 count response to treatment over time. Our outcome variable , was the difference in the average CD4 count for a cohort i at time *t* from the value at the beginning of treatment, time *t0*:

$$Yi,=CD4i,t- CD4i,t0$$

We model this change over time using the following model:

$$Y_{i,}=S_1t*CD4_{i,t0}+S_2t$$

Where $S2t$ is a natural spline on the number of months since treatment initiation, and ($S1t*CD4i$,0) is a natural spline on the number of months interacted with the starting average CD4 count of the cohort. Both spline bases use knots at 3, 12, 24, and 36 months. The model was fit, for each of the CD4 cut-points used to define compartmental categories in the Spectrum modeling framework (0-49,50-99,100-199,200-249,250-349, 350-500, and 500+). We then use the progression curves from this model to categorically backcast each individual observed in our cross-sectional survey data sources to one of the aforementioned categories.

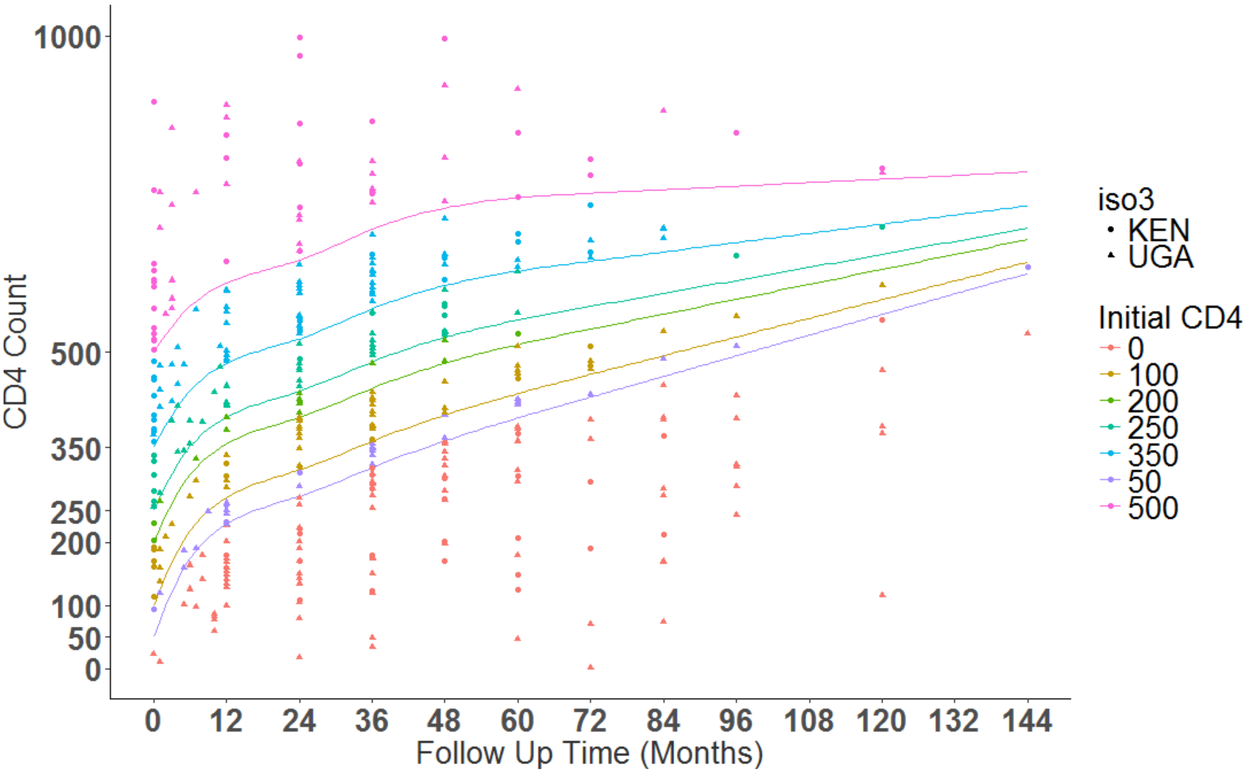

Figure 1. Categorical backcast of survey microdata using modelled progression curves.

Modeling ART Coverage Frontier as a Function of Income and CD4 Count

To obtain realistic forecasts of ART coverage it is important to place bounds on the coverage relative to resources that are expected to be available. We identified two publically available survey datasets, the 2011 Uganda and 2012 Kenya AIDS Indicator Survey, that provide person-level information regarding the distribution of ART coverage by CD4 count. CD4 information for each participant was obtained from laboratory test values, and cross-walked to the Spectrum definition as described in the previous section. As a proxy for income, we used a household asset index based on assets present in the respondent's home, converted to international dollars.[9] A logistic curve describing the relationship between ART coverage and income is then fit, controlling for CD4 count, age and sex, using a logistic regression:

$$(Pcoverage) = \beta 0 + \beta 1 Income + \beta 2 CD4 + \beta 3 Age + \beta 4 Female$$

We used the predicted probabilities from this model to fit a stochastic frontier analysis, which estimates the maximum possible coverage for a given degree of income and CD4 count. Formally, we estimate:

$$\log\left(logit(Pcoverage) + offset\right) = \beta 0 + \beta 1 Income + \beta 2 CD4$$

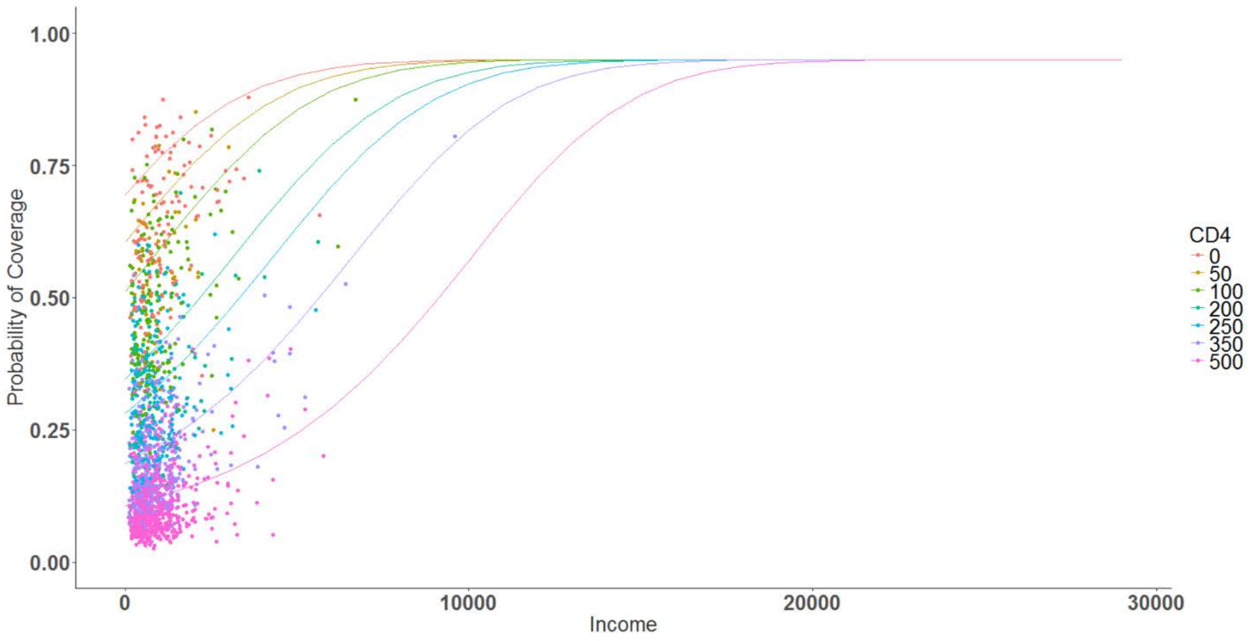

Figure 2. Predicted probabilities of coverage for each individual shown as points. Frontier of coverage as a function of income is shown with lines. Color indicates categorical CD4 count.

ART Price Forecasts

Forecasting ART Prices

In order to forecast ART coverage, an understanding of the cost of ART treatment over time is necessary. We created estimates and projections of the average cost of ART treatment using data from the Global Price Reporting Mechanism (GPRM).28 From the GPRM we obtained 1,175 country-years of data representing the average cost of ART in dollars per person per year, covering 130 countries and spanning 2004-2016. We used a stochastic frontier analysis and Gaussian process regression modelling framework to complete the time series and project the estimates through 2030.

Stochastic Frontier Analysis

In order to bound the future minimum cost plausibly, we use a stochastic frontier analysis to model the minimum ART price possible over time.29 First we create the outcome variable by transforming cost, by rescaling to an inverse zero to one scale, where 0 is the lowest observed cost and 1 is the highest. This is necessary as the stochastic frontier analysis function is used to find a maximum value. Therefore, the outcome must be rescaled to find a minimum cost frontier. We then take the logit of this transformed cost variable, which creates our outcome variable:

$$Yc,=logit(\ (Costi,t-\min\ (Costi,t))\ /\ range(Costi,t)\ +offset)$$

We then fit a stochastic frontier analysis, with time as the independent variable, assuming a truncated normal distribution for the extent to which countries fall short of obtaining the minimum achievable ART price.

Gaussian Process Regression

We used Gaussian process regression (GPR) to complete the time series and make projections through the year 2030. GPR has been used extensively in the Global Burden of Disease estimation framework as a data synthesis tool.[10, 11] The mean function is a linear model which models the log of the difference between the cost frontier and the current cost, as a function of lag-distributed GDP per capita (LDI) and super-region secular trends:[12]

Consistent with prior implementations of GPR, a Matérn covariance function was used to smooth the residuals from the first stage mean function, and produce complete time series with uncertainty.[11]

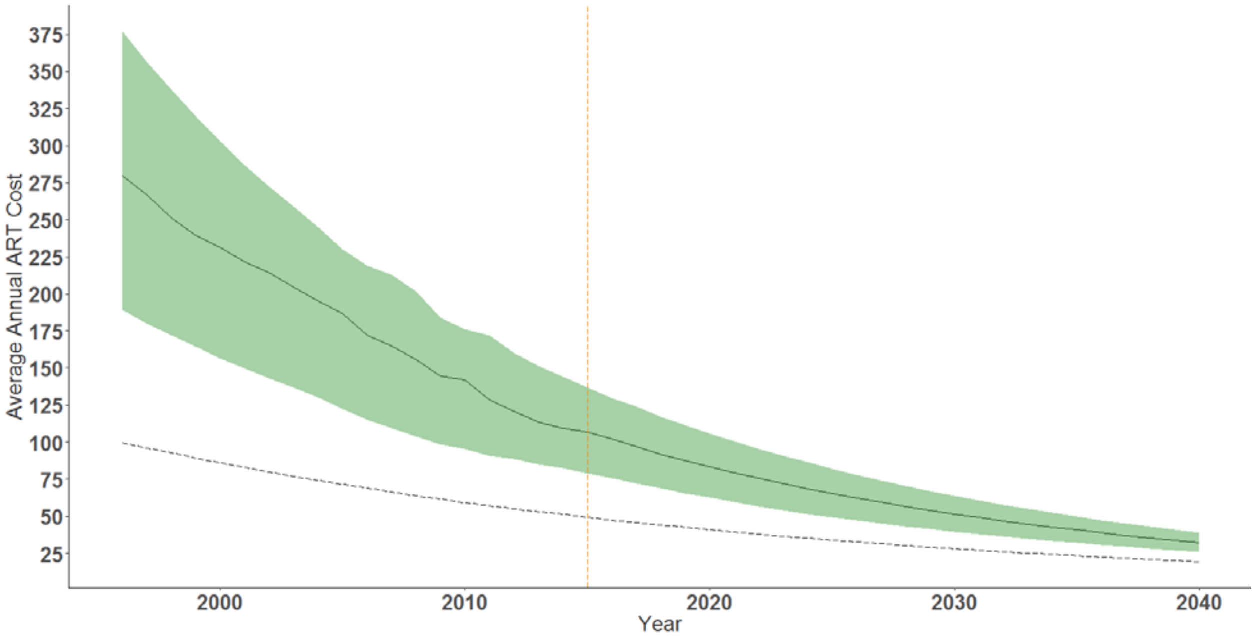

Figure 3. Median and IQR of ART price over time globally, alongside the cost frontier as a dashed line. All series are shown in USD.

Forecasting Spectrum Inputs

A number of inputs to ART forecasting, incidence hazard forecasting, and Spectrum HIV modeling systems are treated as exogenous inputs. Projection for these inputs were created using a rate of change approach, consistent with that used across the forecasting platform. These inputs include:

- ART Price
- Lag Distributed GDP per capita
- Child ART coverage

- Cotrimoxazole coverage among children
- Coverage of medication used to prevent mother-to-child transmission of HIV (PMTCT) in the prenatal and postnatal periods

For each indicator, the distribution of the rate of change across countries was calculated. The time series in each indicator was projected assuming each country grows in the future at the 50th percentile of the past rate of change across countries. Inputs that represent a coverage indicator, including PMTCT, cotrimoxazole, and ART, were forecasted in logit space, while the remaining indicators were modeled in log space.

Forecasting ART Coverage

ART coverage is projected using the ART bounds described above in the ART coverage frontiers section, as well as spending on HIV care and treatment that is forecasted independently.[12] In order to account for the changing costs of ART over time, the HIV spending covariate is rescaled to "dose equivalents" by dividing by ART cost. The relationship between country-year specific ART coverage is then modelled with a slope on dose-equivalents of HIV spending and fixed intercepts for each CD4 group.

$$(ARTc,t)=\beta 1 Spend + (\beta 2 CD40{-}49 * I0{-}49) \ldots (\beta 8 CD4500{+} * I500{+})$$

Projected ART values were bounded using the frontiers estimated as described above, or the largest value observed in the past for the time series in question, whichever is larger. We then forecast ART coverage at the granularity it is used in Spectrum, specific to single-year age and sex groups, as well as draws used in Spectrum to propagate uncertainty:

$$(ART_{c,a,s,t,d})= \beta 1 Spend + (\beta 2 CD40{-}49 * I0{-}49) \ldots (\beta 8 CD4500{+} * I500{+}) + \emptyset c,a,s,t,d$$

where $\emptyset c,a,s,t,d$ is a country-year-age-sex-draw specific intercept shift term, used to ensure no disjunctions in the first year of the forecasts by removing the difference from year 2017 to year 2018 from all forecasted estimates for each time series.

HIV Incidence Hazard

Incidence hazard, the rate of new infections among the susceptible population, is a key input to the Spectrum modeling process. We forecast incidence hazard using ART projections as well as a rate of change approach similar to those described above with respect to the trend in the counterfactual incidence hazard, the expected hazard if ART coverage were zero. A time series of incidence hazard from 1970 through 2017 for each location is taken from GBD 2017 final estimates, then counterfactual incidence hazard is calculated as:

$$Hazard\ Counterfactual_{c,a,s,t,i} = \frac{Hazard_{c,a,s,t,i}}{1-(\mathrm{ART}_{\mathrm{c,a,s,t,i}} * Viral\ Suppression_{\mathrm{c,a,s,t,i}})}$$

$$Viral\ Suppression \sim (.6,.8)$$

Where ART is the proportion of HIV+ individuals receiving ART, hazard is the number of new HIV infections over population at risk, and viral suppression is the proportion of individuals taking ART who achieve viral suppression. We assumed that a mean of 70% of the on-ART population reached viral suppression and created uncertainty by taking draws from a uniform distribution ranging from 60% - 80%, aligning with assumptions in the EPP model developed by UNAIDs.

Consistent with the approach taken to forecast the independent drivers, projections for the secular trend in the counterfactual hazard is created by calculating the rate of change across countries over the previous five years, and assuming each country changes in the future at the 50th percentile of the past rate of change across countries. The final projected hazard rates therefore decreases in response to improvements in ART coverage, as well as change due to the underlying secular trend in the counterfactual hazard.

Projections of HIV incidence

In order to produce age- and sex-specific estimates of HIV incidence, we input the projections of incidence hazard along with the other independent drivers into the Spectrum model. Spectrum is a cohort component model originally developed by UNAIDs that we have modified to incorporate CD4-specific probability of treatment in addition to a number of other methods developments made for GBD.[13, 14] Spectrum ages a population over time using demographic parameters while applying HIV incidence hazard, disease progression, CD4-specific treatment coverage, and mortality. Our final estimates of HIV incidence and ART coverage are age-, sex-, location-specific Spectrum outputs through 2030.

References

[1] Egger S, Petoumenos K, Kamarulzaman A, *et al.* Long-term patterns in CD4 response is determined by an interaction between baseline CD4 cell count, viral load and time: the Asia Pacific HIV Observational Database (APHOD). *J Acquir Immune Defic Syndr 1999* 2009; **50**: 513–20.

[2] Li T, Tubiana R, Katlama C, Calvez V, Mohand HA, Autran B. Long-lasting recovery in CD4 T-cell function and viral-load reduction after highly active antiretroviral therapy in advanced HIV-1 disease. *The Lancet* 1998; **351**: 1682–6.

[3] Mocroft A, Phillips A, Gatell J, *et al.* Normalisation of CD4 counts in patients with HIV-1 infection and maximum virological suppression who are taking combination antiretroviral therapy: an observational cohort study. *The Lancet* 2007; **370**: 407–13.

[4] Montarroyos UR, Miranda-Filho DB, César CC, *et al.* Factors Related to Changes in CD4+ T-Cell Counts over Time in Patients Living with HIV/AIDS: A Multilevel Analysis. *PLOS ONE* 2014; **9**: e84276.

[5] Smith CJ, Sabin CA, Youle MS, *et al.* Factors Influencing Increases in CD4 Cell Counts of HIV-Positive Persons Receiving Long-Term Highly Active Antiretroviral Therapy. *J Infect Dis* 2004; **190**: 1860–8.

[6] Tarwater PM, Margolick JB, Jin J, *et al.* Increase and plateau of CD4 T-cell counts in the 3(1/2) years after initiation of potent antiretroviral therapy. *J Acquir Immune Defic Syndr 1999* 2001; **27**: 168–75. Page 55

[7] Zhou J, Kumarasamy N, Ditangco R, *et al.* The TREAT Asia HIV Observational Database. *J Acquir Immune Defic Syndr 1999* 2005; **38**: 174–9.

[8] The potential for CD4 cell increases in HIV-positive individ... : AIDS. https://journals.lww.com/aidsonline/Fulltext/2003/05020/The_potential_for_CD4_cell_increases_in.4.aspx (accessed April 17, 2018).

[9] Ferguson BD, Tandon A, Gakidou E, Murray CJL. Estimating Permanent Income Using Indicator Variables. ; : 24.

[10] Ng M, Fleming T, Robinson M, *et al.* Global, regional, and national prevalence of overweight and obesity in children and adults during 1980-2013: a systematic analysis for the Global Burden of Disease Study 2013. *Lancet Lond Engl* 2014; **384**: 766–81.

[11] Ng M, Freeman MK, Fleming TD, *et al.* Smoking prevalence and cigarette consumption in 187 countries, 1980-2012. *JAMA* 2014; **311**: 183–92.

[12] Global Burden of Disease Health Financing Collaborator Network. Trends in future health financing and coverage: future health spending and universal health coverage in 188 countries, 2016–40. *The Lancet*. 17 Apr 2018.

[13] Stover J. Updates to the Spectrum Model to Estimate Key HIV Indicators for Adults and Children. *AIDS* 2014; : S427–S434.

[14] Vos T, Allen C, Arora M, *et al.* Global, regional, and national incidence, prevalence, and years lived with disability for 310 diseases and injuries, 1990–2015: a systematic analysis for the Global Burden of Disease Study 2015. *The Lancet* 2016; **388**: 1545–602.

Section 5. UHC index

Universal health coverage (UHC) has emerged as both a global and national health priority, with achieving UHC viewed as a critical path to improved health outcomes and greater equity in health across all populations. This section focuses the method used for forecasting the UHC service coverage index from 2018 through 2040 using our health financing variables, particularly the sum of GHE, DAH and PPP per capita, hereby referred as 'pooled health resources per capita' or 'pooled spending per capita'. Further detail on the construction of the UHC service coverage is found elsewhere in this appendix.

Stochastic Frontier Analysis

We used a stochastic frontier model to forecast the level of UHC index achievable by all countries between 2016 through 2040. Implementing the work of Battese and Coelli,[1,2] our SFA model, with a production function specification, was such:

$$\begin{aligned} \ln(UHC_{i,t}) &= \alpha + \beta \ln(X_{i,t}) - \nu_{i,t} + \epsilon_{i,t} \\ \nu_{i,t} &\sim N^{+}(0, \sigma_{\nu}^{2}) \\ \epsilon_{i,t} &\sim N(0, \sigma_{\epsilon}^{2}) \end{aligned}$$

where our observed outcome was the logged UHC index, with our single covariate X being the country-year specific pooled spending per capita, $\epsilon_{i,t}$ is the noise component and $\nu_{i,t}$ is the estimated technical efficiency that a country would need to achive the optimal, frontier goal. The prior distribution of technical efficiency is a half-normal distribution, describing an unbounded distribution between zero and very high efficiency.

Forecasting Steps

We forecasted the UHC index from 2017 through 2040 in the following steps:

(i) Forecasts of the pooled spending were developed by adding the forecasts of government health expenditure (GHE), development assistance for health (DAH) and prepaid private spending (PPP) per capita, modeled previously using ensembles.[3]

(ii) For each of the error components (efficiency ν and noise ϵ) for a country, we added them together to create a unified residual time series for each country. That series was separately forecasted for each country using a weighted ordinary linear regression (using a linear time trend as a covariate), where recent time periods were weighed higher than the further past.

(iii) Using the draws of reference, better and worse scenarios of $X_{i,t}$ along with forecasts of the summed residuals from (ii), we created reference, better and worse projections of the UHC index from 2017 through 2040.

References

1 BATTESE GE, COELLI TJ. Frontier Production Functions, Technical Efficiency and Panel Data: With Application to Paddy Farmers in India. *J Product Anal* 1992; **3**: 153–69.

2 Battese GE, Coelli TJ. A model for technical inefficiency effects in a stochastic frontier production function for panel data. *Empir Econ* 1995; **20**: 325–32.

3 Dieleman JL, Sadat N, Chang AY, *et al.* Trends in future health financing and coverage: future health spending and universal health coverage in 188 countries, 2016–40. *The Lancet* 2018; **391**: 1783–98.

Part 4. Online tools and abbreviations

Section 1. Online tools

Further results are presented as dynamic visualizations at [link to be added upon acceptance].

SDG indicator data sources are made available in an online data citation tool [link to be added upon acceptance].

Section 2. List of abbreviations

13th General Program of Work (GPW13)

ARCs: Annualised rates of change

GBD: Global Burden of Diseases, Injuries, and Risk Factors Study

GATHER: Guidelines for Accurate and Transparent Health Estimates Reporting

HAQ: Healthcare Access and Quality

IAEG-SDGs: Inter-Agency and Expert Group on Sustainable Development Goal Indicators

ISCO: International Standard Classification of Occupations

IQR: Interquartile range

MDG: Millennium Development Goal

NCDs: non-communicable diseases

NTDs: neglected tropical diseases

SDI: Socio-demographic Index

SDG: Sustainable Development Goal

SDSN: Sustainable Development Solutions Network

SEV: summary exposure value

UN: United Nations

UHC: universal health coverage

UIs: uncertainty intervals

World Health Assembly (WHA)

WHO: World Health Organization

Section 3. List of ISO3 codes and location names

AFG	Afghanistan
AGO	Angola

ALB	Albania
AND	Andorra
ARE	United Arab Emirates
ARG	Argentina
ARM	Armenia
ASM	American Samoa
ATG	Antigua and Barbuda
AUS	Australia
AUT	Austria
AZE	Azerbaijan
BDI	Burundi
BEL	Belgium
BEN	Benin
BFA	Burkina Faso
BGD	Bangladesh
BGR	Bulgaria
BHR	Bahrain
BHS	The Bahamas
BIH	Bosnia and Herzegovina
BLR	Belarus
BLZ	Belize
BMU	Bermuda
BOL	Bolivia
BRA	Brazil
BRB	Barbados
BRN	Brunei
BTN	Bhutan
BWA	Botswana
CAF	Central African Republic
CAN	Canada
CHE	Switzerland
CHL	Chile
CHN	China
CIV	Cote d'Ivoire
CMR	Cameroon
COD	Democratic Republic of the Congo
COG	Congo (Brazzaville)
COL	Colombia
COM	Comoros
CPV	Cape Verde
CRI	Costa Rica
CUB	Cuba
CYP	Cyprus
CZE	Czech Republic

DEU	Germany
DJI	Djibouti
DMA	Dominica
DNK	Denmark
DOM	Dominican Republic
DZA	Algeria
ECU	Ecuador
EGY	Egypt
ERI	Eritrea
ESP	Spain
EST	Estonia
ETH	Ethiopia
FIN	Finland
FJI	Fiji
FRA	France
FSM	Federated States of Micronesia
GAB	Gabon
GBR	United Kingdom
GEO	Georgia
GHA	Ghana
GIN	Guinea
GMB	The Gambia
GNB	Guinea-Bissau
GNQ	Equatorial Guinea
GRC	Greece
GRD	Grenada
GRL	Greenland
GTM	Guatemala
GUM	Guam
GUY	Guyana
HND	Honduras
HRV	Croatia
HTI	Haiti
HUN	Hungary
IDN	Indonesia
IND	India
IRL	Ireland
IRN	Iran
IRQ	Iraq
ISL	Iceland
ISR	Israel
ITA	Italy
JAM	Jamaica
JOR	Jordan

JPN	Japan
KAZ	Kazakhstan
KEN	Kenya
KGZ	Kyrgyzstan
KHM	Cambodia
KIR	Kiribati
KOR	South Korea
KWT	Kuwait
LAO	Laos
LBN	Lebanon
LBR	Liberia
LBY	Libya
LCA	Saint Lucia
LKA	Sri Lanka
LSO	Lesotho
LTU	Lithuania
LUX	Luxembourg
LVA	Latvia
MAR	Morocco
MDA	Moldova
MDG	Madagascar
MDV	Maldives
MEX	Mexico
MHL	Marshall Islands
MKD	Macedonia
MLI	Mali
MLT	Malta
MMR	Myanmar
MNE	Montenegro
MNG	Mongolia
MNP	Northern Mariana Islands
MOZ	Mozambique
MRT	Mauritania
MUS	Mauritius
MWI	Malawi
MYS	Malaysia
NAM	Namibia
NER	Niger
NGA	Nigeria
NIC	Nicaragua
NLD	Netherlands
NOR	Norway
NPL	Nepal
NZL	New Zealand

OMN	Oman
PAK	Pakistan
PAN	Panama
PER	Peru
PHL	Philippines
PNG	Papua New Guinea
POL	Poland
PRI	Puerto Rico
PRK	North Korea
PRT	Portugal
PRY	Paraguay
PSE	Palestine
QAT	Qatar
ROU	Romania
RUS	Russia
RWA	Rwanda
SAU	Saudi Arabia
SDN	Sudan
SEN	Senegal
SGP	Singapore
SLB	Solomon Islands
SLE	Sierra Leone
SLV	El Salvador
SOM	Somalia
SRB	Serbia
SSD	South Sudan
STP	Sao Tome and Principe
SUR	Suriname
SVK	Slovakia
SVN	Slovenia
SWE	Sweden
SWZ	Swaziland
SYC	Seychelles
SYR	Syria
TCD	Chad
TGO	Togo
THA	Thailand
TJK	Tajikistan
TKM	Turkmenistan
TLS	Timor-Leste
TON	Tonga
TTO	Trinidad and Tobago
TUN	Tunisia
TUR	Turkey

TWN	Taiwan (Province of China)
TZA	Tanzania
UGA	Uganda
UKR	Ukraine
URY	Uruguay
USA	United States
UZB	Uzbekistan
VCT	Saint Vincent and the Grenadines
VEN	Venezuela
VIR	Virgin Islands
VNM	Vietnam
VUT	Vanuatu
WSM	Samoa
YEM	Yemen
ZAF	South Africa
ZMB	Zambia
ZWE	Zimbabwe

Appendix Table. Socio-demographic Index groupings by geography, based on 2017 values		
**Geography**	**2017 SDI**	**SDI Quintile**
Global	0.652	
Central Europe, Eastern Europe, and Central Asia	0.766	
Central Asia	0.673	
Armenia	0.702	High-middle SDI
Azerbaijan	0.701	High-middle SDI
Georgia	0.7	High-middle SDI
Kazakhstan	0.735	High-middle SDI
Kyrgyzstan	0.607	Low-middle SDI
Mongolia	0.662	Middle SDI
Tajikistan	0.523	Low-middle SDI
Turkmenistan	0.696	Middle SDI
Uzbekistan	0.63	Middle SDI
Central Europe	0.814	
Albania	0.685	Middle SDI
Bosnia and Herzegovina	0.713	High-middle SDI
Bulgaria	0.792	High-middle SDI
Croatia	0.825	High SDI
Czech Republic	0.851	High SDI
Hungary	0.817	High-middle SDI
Macedonia	0.754	High-middle SDI
Montenegro	0.788	High-middle SDI
Poland	0.844	High SDI
Romania	0.784	High-middle SDI
Serbia	0.752	High-middle SDI
Slovakia	0.842	High SDI
Slovenia	0.86	High SDI
Eastern Europe	0.785	
Belarus	0.773	High-middle SDI
Estonia	0.858	High SDI
Latvia	0.825	High SDI
Lithuania	0.841	High SDI
Moldova	0.676	Middle SDI
Russian Federation	0.792	High-middle SDI
Ukraine	0.74	High-middle SDI
High-income	0.854	
Australasia	0.869	
Australia	0.873	High SDI
New Zealand	0.842	High SDI
High-income Asia-Pacific	0.869	
Brunei	0.856	High SDI
Japan	0.865	High SDI
Aichi	0.875	High SDI
Akita	0.829	High SDI
Aomori	0.825	High SDI

Appendix Table. Socio-demographic Index groupings by geography, based on 2017 values		
**Geography**	**2017 SDI**	**SDI Quintile**
Chiba	0.859	High SDI
Ehime	0.838	High SDI
Fukui	0.852	High SDI
Fukuoka	0.855	High SDI
Fukushima	0.831	High SDI
Gifu	0.849	High SDI
Gunma	0.851	High SDI
Hiroshima	0.863	High SDI
Hokkaidō	0.842	High SDI
Hyōgo	0.86	High SDI
Ibaraki	0.851	High SDI
Ishikawa	0.856	High SDI
Iwate	0.825	High SDI
Kagawa	0.85	High SDI
Kagoshima	0.83	High SDI
Kanagawa	0.875	High SDI
Kōchi	0.825	High SDI
Kumamoto	0.832	High SDI
Kyōto	0.873	High SDI
Mie	0.854	High SDI
Miyagi	0.85	High SDI
Miyazaki	0.823	High SDI
Nagano	0.851	High SDI
Nagasaki	0.826	High SDI
Nara	0.848	High SDI
Niigata	0.843	High SDI
Ōita	0.846	High SDI
Okayama	0.856	High SDI
Okinawa	0.818	High SDI
Ōsaka	0.872	High SDI
Saga	0.834	High SDI
Saitama	0.852	High SDI
Shiga	0.871	High SDI
Shimane	0.831	High SDI
Shizuoka	0.859	High SDI
Tochigi	0.853	High SDI
Tokushima	0.845	High SDI
Tōkyō	0.924	High SDI
Tottori	0.834	High SDI
Toyama	0.86	High SDI
Wakayama	0.84	High SDI
Yamagata	0.832	High SDI
Yamaguchi	0.849	High SDI
Yamanashi	0.854	High SDI
South Korea	0.872	High SDI

Appendix Table. Socio-demographic Index groupings by geography, based on 2017 values		
**Geography**	**2017 SDI**	**SDI Quintile**
Singapore	0.872	High SDI
High-income North America	0.868	
Canada	0.882	High SDI
Greenland	0.76	High-middle SDI
USA	0.867	High SDI
Alabama	0.837	High SDI
Alaska	0.861	High SDI
Arizona	0.845	High SDI
Arkansas	0.826	High SDI
California	0.872	High SDI
Colorado	0.882	High SDI
Connecticut	0.906	High SDI
Delaware	0.874	High SDI
Washington, DC	0.89	High SDI
Florida	0.864	High SDI
Georgia	0.848	High SDI
Hawaii	0.872	High SDI
Idaho	0.841	High SDI
Illinois	0.879	High SDI
Indiana	0.848	High SDI
Iowa	0.87	High SDI
Kansas	0.864	High SDI
Kentucky	0.831	High SDI
Louisiana	0.835	High SDI
Maine	0.872	High SDI
Maryland	0.896	High SDI
Massachusetts	0.913	High SDI
Michigan	0.868	High SDI
Minnesota	0.893	High SDI
Mississippi	0.819	High SDI
Missouri	0.853	High SDI
Montana	0.863	High SDI
Nebraska	0.873	High SDI
Nevada	0.847	High SDI
New Hampshire	0.904	High SDI
New Jersey	0.899	High SDI
New Mexico	0.835	High SDI
New York	0.893	High SDI
North Carolina	0.85	High SDI
North Dakota	0.88	High SDI
Ohio	0.858	High SDI
Oklahoma	0.838	High SDI
Oregon	0.871	High SDI
Pennsylvania	0.879	High SDI
Rhode Island	0.89	High SDI

Appendix Table. Socio-demographic Index groupings by geography, based on 2017 values		
**Geography**	**2017 SDI**	**SDI Quintile**
South Carolina	0.846	High SDI
South Dakota	0.86	High SDI
Tennessee	0.837	High SDI
Texas	0.838	High SDI
Utah	0.856	High SDI
Vermont	0.896	High SDI
Virginia	0.885	High SDI
Washington	0.884	High SDI
West Virginia	0.825	High SDI
Wisconsin	0.878	High SDI
Wyoming	0.869	High SDI
Southern Latin America	0.72	
Argentina	0.71	High-middle SDI
Chile	0.748	High-middle SDI
Uruguay	0.707	High-middle SDI
Western Europe	0.857	
Andorra	0.902	High SDI
Austria	0.866	High SDI
Belgium	0.886	High SDI
Cyprus	0.865	High SDI
Denmark	0.918	High SDI
Finland	0.893	High SDI
France	0.865	High SDI
Germany	0.87	High SDI
Greece	0.817	High SDI
Iceland	0.907	High SDI
Ireland	0.882	High SDI
Israel	0.816	High-middle SDI
Italy	0.843	High SDI
Luxembourg	0.916	High SDI
Malta	0.836	High SDI
Netherlands	0.912	High SDI
Norway	0.911	High SDI
Portugal	0.778	High-middle SDI
Spain	0.825	High SDI
Sweden	0.883	High SDI
Stockholm	0.914	High SDI
Sweden except Stockholm	0.873	High SDI
Switzerland	0.889	High SDI
United Kingdom	0.843	High SDI
England	0.849	High SDI
East Midlands	0.83	High SDI
Derby	0.846	High SDI
Derbyshire	0.817	High SDI
Leicester	0.839	High SDI

Appendix Table. Socio-demographic Index groupings by geography, based on 2017 values		
**Geography**	**2017 SDI**	**SDI Quintile**
Leicestershire	0.846	High SDI
Lincolnshire	0.812	High SDI
Northamptonshire	0.829	High SDI
Nottingham	0.863	High SDI
Nottinghamshire	0.814	High SDI
Rutland	0.833	High SDI
East of England	0.84	High SDI
Bedford	0.838	High SDI
Cambridgeshire	0.871	High SDI
Central Bedfordshire	0.834	High SDI
Essex	0.832	High SDI
Hertfordshire	0.87	High SDI
Luton	0.833	High SDI
Norfolk	0.826	High SDI
Peterborough	0.818	High SDI
Southend-on-Sea	0.811	High SDI
Suffolk	0.821	High SDI
Thurrock	0.807	High SDI
Greater London	0.894	High SDI
Barking and Dagenham	0.802	High SDI
Barnet	0.865	High SDI
Bexley	0.826	High SDI
Brent	0.849	High SDI
Bromley	0.848	High SDI
Camden	0.93	High SDI
Croydon	0.833	High SDI
Ealing	0.865	High SDI
Enfield	0.839	High SDI
Greenwich	0.833	High SDI
Hackney	0.887	High SDI
Hammersmith and Fulham	0.927	High SDI
Haringey	0.854	High SDI
Harrow	0.848	High SDI
Havering	0.824	High SDI
Hillingdon	0.882	High SDI
Hounslow	0.879	High SDI
Islington	0.922	High SDI
Kensington and Chelsea	0.932	High SDI
Kingston upon Thames	0.89	High SDI
Lambeth	0.9	High SDI
Lewisham	0.843	High SDI
Merton	0.873	High SDI
Newham	0.838	High SDI
Redbridge	0.831	High SDI
Richmond upon Thames	0.902	High SDI

Appendix Table. Socio-demographic Index groupings by geography, based on 2017 values		
**Geography**	**2017 SDI**	**SDI Quintile**
Southwark	0.912	High SDI
Sutton	0.843	High SDI
Tower Hamlets	0.905	High SDI
Waltham Forest	0.819	High SDI
Wandsworth	0.911	High SDI
Westminster	0.927	High SDI
North East England	0.821	High SDI
County Durham	0.81	High SDI
Darlington	0.825	High SDI
Gateshead	0.826	High SDI
Hartlepool	0.793	High SDI
Middlesbrough	0.808	High SDI
Newcastle upon Tyne	0.872	High SDI
North Tyneside	0.825	High SDI
Northumberland	0.808	High SDI
Redcar and Cleveland	0.79	High SDI
South Tyneside	0.794	High SDI
Stockton-on-Tees	0.823	High SDI
Sunderland	0.815	High SDI
North West England	0.834	High SDI
Blackburn with Darwen	0.802	High SDI
Blackpool	0.781	High SDI
Bolton	0.805	High SDI
Bury	0.815	High SDI
Cheshire East	0.864	High SDI
Cheshire West and Chester	0.855	High SDI
Cumbria	0.828	High SDI
Halton	0.824	High SDI
Knowsley	0.816	High SDI
Lancashire	0.831	High SDI
Liverpool	0.852	High SDI
Manchester	0.885	High SDI
Oldham	0.79	High SDI
Rochdale	0.795	High SDI
Salford	0.838	High SDI
Sefton	0.812	High SDI
St Helens	0.803	High SDI
Stockport	0.843	High SDI
Tameside	0.797	High SDI
Trafford	0.873	High SDI
Warrington	0.86	High SDI
Wigan	0.798	High SDI
Wirral	0.803	High SDI
South East England	0.856	High SDI
Bracknell Forest	0.869	High SDI

Appendix Table. Socio-demographic Index groupings by geography, based on 2017 values		
**Geography**	**2017 SDI**	**SDI Quintile**
Brighton and Hove	0.885	High SDI
Buckinghamshire	0.865	High SDI
East Sussex	0.814	High SDI
Hampshire	0.85	High SDI
Isle of Wight	0.814	High SDI
Kent	0.828	High SDI
Medway	0.809	High SDI
Milton Keynes	0.86	High SDI
Oxfordshire	0.879	High SDI
Portsmouth	0.86	High SDI
Reading	0.895	High SDI
Slough	0.859	High SDI
Southampton	0.858	High SDI
Surrey	0.883	High SDI
West Berkshire	0.872	High SDI
West Sussex	0.843	High SDI
Windsor and Maidenhead	0.889	High SDI
Wokingham	0.885	High SDI
South West England	0.841	High SDI
Bath and North East Somerset	0.875	High SDI
Bournemouth	0.858	High SDI
Bristol, City of	0.884	High SDI
Cornwall	0.817	High SDI
Devon	0.837	High SDI
Dorset	0.825	High SDI
Gloucestershire	0.85	High SDI
North Somerset	0.832	High SDI
Plymouth	0.836	High SDI
Poole	0.842	High SDI
Somerset	0.816	High SDI
South Gloucestershire	0.867	High SDI
Swindon	0.847	High SDI
Torbay	0.79	High SDI
Wiltshire	0.829	High SDI
West Midlands	0.829	High SDI
Birmingham	0.84	High SDI
Coventry	0.848	High SDI
Dudley	0.799	High SDI
Herefordshire, County of	0.828	High SDI
Sandwell	0.797	High SDI
Shropshire	0.832	High SDI
Solihull	0.855	High SDI
Staffordshire	0.826	High SDI
Stoke-on-Trent	0.804	High SDI
Telford and Wrekin	0.822	High SDI

Appendix Table. Socio-demographic Index groupings by geography, based on 2017 values		
**Geography**	**2017 SDI**	**SDI Quintile**
Walsall	0.791	High SDI
Warwickshire	0.857	High SDI
Wolverhampton	0.811	High SDI
Worcestershire	0.833	High SDI
Yorkshire and the Humber	0.83	High SDI
Barnsley	0.787	High SDI
Bradford	0.807	High SDI
Calderdale	0.827	High SDI
Doncaster	0.791	High SDI
East Riding of Yorkshire	0.822	High SDI
Kingston upon Hull, City of	0.813	High SDI
Kirklees	0.816	High SDI
Leeds	0.868	High SDI
North East Lincolnshire	0.804	High SDI
North Lincolnshire	0.811	High SDI
North Yorkshire	0.839	High SDI
Rotherham	0.796	High SDI
Sheffield	0.853	High SDI
Wakefield	0.806	High SDI
York	0.879	High SDI
Northern Ireland	0.835	High SDI
Scotland	0.805	High SDI
Wales	0.806	High SDI
Latin America and Caribbean	0.64	
Andean Latin America	0.628	
Bolivia	0.587	Low-middle SDI
Ecuador	0.636	Middle SDI
Peru	0.636	Middle SDI
Caribbean	0.638	
Antigua and Barbuda	0.715	High-middle SDI
The Bahamas	0.756	High-middle SDI
Barbados	0.739	High-middle SDI
Belize	0.602	Low-middle SDI
Bermuda	0.805	High-middle SDI
Cuba	0.688	Middle SDI
Dominica	0.687	Middle SDI
Dominican Republic	0.593	Low-middle SDI
Grenada	0.64	Middle SDI
Guyana	0.584	Low-middle SDI
Haiti	0.442	Low SDI
Jamaica	0.679	Middle SDI
Puerto Rico	0.813	High-middle SDI
Saint Lucia	0.653	Middle SDI
Saint Vincent and the Grenadines	0.608	Middle SDI
Suriname	0.641	Middle SDI

Appendix Table. Socio-demographic Index groupings by geography, based on 2017 values		
**Geography**	**2017 SDI**	**SDI Quintile**
Trinidad and Tobago	0.698	Middle SDI
Virgin Islands	0.807	High-middle SDI
Central Latin America	0.623	
Colombia	0.634	Middle SDI
Costa Rica	0.662	Middle SDI
El Salvador	0.593	Low-middle SDI
Guatemala	0.524	Low-middle SDI
Honduras	0.512	Low-middle SDI
Mexico	0.628	Middle SDI
Aguascalientes	0.659	Middle SDI
Baja California	0.657	Middle SDI
Baja California Sur	0.659	Middle SDI
Campeche	0.616	Middle SDI
Chiapas	0.533	Middle SDI
Chihuahua	0.639	Middle SDI
Coahuila	0.645	Middle SDI
Colima	0.654	Middle SDI
Mexico City	0.716	Middle SDI
Durango	0.624	Middle SDI
Guanajuato	0.621	Middle SDI
Guerrero	0.562	Middle SDI
Hidalgo	0.587	Middle SDI
Jalisco	0.649	Middle SDI
México	0.635	Middle SDI
Michoacán de Ocampo	0.586	Middle SDI
Morelos	0.635	Middle SDI
Nayarit	0.62	Middle SDI
Nuevo León	0.677	Middle SDI
Oaxaca	0.561	Middle SDI
Puebla	0.584	Middle SDI
Querétaro	0.639	Middle SDI
Quintana Roo	0.626	Middle SDI
San Luis Potosí	0.621	Middle SDI
Sinaloa	0.649	Middle SDI
Sonora	0.65	Middle SDI
Tabasco	0.611	Middle SDI
Tamaulipas	0.647	Middle SDI
Tlaxcala	0.604	Middle SDI
Veracruz de Ignacio de la Llave	0.592	Middle SDI
Yucatán	0.63	Middle SDI
Zacatecas	0.608	Middle SDI
Nicaragua	0.53	Low-middle SDI
Panama	0.677	Middle SDI
Venezuela	0.655	Middle SDI
Tropical Latin America	0.662	

Appendix Table. Socio-demographic Index groupings by geography, based on 2017 values		
**Geography**	**2017 SDI**	**SDI Quintile**
Brazil	0.663	Middle SDI
Acre	0.602	Low-middle SDI
Alagoas	0.556	Low-middle SDI
Amapá	0.659	Middle SDI
Amazonas	0.629	Middle SDI
Bahia	0.591	Low-middle SDI
Ceará	0.6	Low-middle SDI
Distrito Federal	0.792	High-middle SDI
Espírito Santo	0.677	Middle SDI
Goiás	0.65	Middle SDI
Maranhão	0.507	Low-middle SDI
Mato Grosso	0.662	Middle SDI
Mato Grosso do Sul	0.65	Middle SDI
Minas Gerais	0.661	Middle SDI
Pará	0.579	Low-middle SDI
Paraíba	0.574	Low-middle SDI
Paraná	0.682	Middle SDI
Pernambuco	0.594	Low-middle SDI
Piauí	0.552	Low-middle SDI
Rio de Janeiro	0.709	High-middle SDI
Rio Grande do Norte	0.605	Low-middle SDI
Rio Grande do Sul	0.693	Middle SDI
Rondônia	0.622	Middle SDI
Roraima	0.646	Middle SDI
Santa Catarina	0.702	High-middle SDI
São Paulo	0.72	High-middle SDI
Sergipe	0.616	Middle SDI
Tocantins	0.611	Middle SDI
Paraguay	0.619	Middle SDI
North Africa and Middle East	0.639	
North Africa and Middle East	0.639	
Afghanistan	0.29	Low SDI
Algeria	0.696	Middle SDI
Bahrain	0.712	High-middle SDI
Egypt	0.604	Low-middle SDI
Iran	0.7	High-middle SDI
Iraq	0.585	Low-middle SDI
Jordan	0.697	Middle SDI
Kuwait	0.786	High-middle SDI
Lebanon	0.73	High-middle SDI
Libya	0.761	High-middle SDI
Morocco	0.579	Low-middle SDI
Palestine	0.541	Low-middle SDI
Oman	0.744	High-middle SDI
Qatar	0.766	High-middle SDI

Appendix Table. Socio-demographic Index groupings by geography, based on 2017 values		
**Geography**	**2017 SDI**	**SDI Quintile**
Saudi Arabia	0.779	High-middle SDI
Sudan	0.478	Low-middle SDI
Syria	0.611	Middle SDI
Tunisia	0.675	Middle SDI
Turkey	0.729	High-middle SDI
United Arab Emirates	0.795	High-middle SDI
Yemen	0.43	Low SDI
South Asia	0.534	
South Asia	0.534	
Bangladesh	0.458	Low SDI
Bhutan	0.57	Low-middle SDI
India	0.55	Low-middle SDI
Andhra Pradesh	0.536	Low-middle SDI
Arunachal Pradesh	0.556	Low-middle SDI
Assam	0.53	Low-middle SDI
Bihar	0.433	Low SDI
Chhattisgarh	0.512	Low-middle SDI
Delhi	0.715	High-middle SDI
Goa	0.74	High-middle SDI
Gujarat	0.584	Low-middle SDI
Haryana	0.6	Low-middle SDI
Himachal Pradesh	0.633	Middle SDI
Jammu and Kashmir	0.59	Low-middle SDI
Jharkhand	0.487	Low-middle SDI
Karnataka	0.574	Low-middle SDI
Kerala	0.659	Middle SDI
Madhya Pradesh	0.487	Low-middle SDI
Maharashtra	0.618	Middle SDI
Manipur	0.59	Low-middle SDI
Meghalaya	0.565	Low-middle SDI
Mizoram	0.616	Middle SDI
Nagaland	0.633	Middle SDI
Odisha	0.524	Low-middle SDI
Punjab	0.622	Middle SDI
Rajasthan	0.492	Low-middle SDI
Sikkim	0.628	Middle SDI
Tamil Nadu	0.615	Middle SDI
Telangana	0.575	Low-middle SDI
Tripura	0.543	Low-middle SDI
Uttar Pradesh	0.488	Low-middle SDI
Uttarakhand	0.607	Middle SDI
West Bengal	0.538	Low-middle SDI
Union Territories other than Delhi	0.653	Middle SDI
Nepal	0.429	Low SDI
Pakistan	0.492	Low-middle SDI

Appendix Table. Socio-demographic Index groupings by geography, based on 2017 values		
**Geography**	**2017 SDI**	**SDI Quintile**
Southeast Asia, East Asia, and Oceania	0.685	
East Asia	0.709	
China	0.707	High-middle SDI
North Korea	0.538	Low-middle SDI
Taiwan (Province of China)	0.864	High SDI
Oceania	0.471	
American Samoa	0.702	High-middle SDI
Federated States of Micronesia	0.575	Low-middle SDI
Fiji	0.641	Middle SDI
Guam	0.794	High-middle SDI
Kiribati	0.427	Low SDI
Marshall Islands	0.55	Low-middle SDI
Northern Mariana Islands	0.758	High-middle SDI
Papua New Guinea	0.419	Low SDI
Samoa	0.576	Low-middle SDI
Solomon Islands	0.425	Low SDI
Tonga	0.625	Middle SDI
Vanuatu	0.475	Low-middle SDI
Southeast Asia	0.641	
Cambodia	0.482	Low-middle SDI
Indonesia	0.648	Middle SDI
Laos	0.519	Low-middle SDI
Malaysia	0.759	High-middle SDI
Maldives	0.655	Middle SDI
Mauritius	0.72	High-middle SDI
Myanmar	0.556	Low-middle SDI
Philippines	0.617	Middle SDI
Sri Lanka	0.68	Middle SDI
Seychelles	0.692	Middle SDI
Thailand	0.684	Middle SDI
Timor-Leste	0.505	Low-middle SDI
Vietnam	0.607	Middle SDI
Sub-Saharan Africa	0.446	
Central sub-Saharan Africa	0.457	
Angola	0.461	Low-middle SDI
Central African Republic	0.334	Low SDI
Congo (Brazzaville)	0.574	Low-middle SDI
DR Congo	0.364	Low SDI
Equatorial Guinea	0.625	Middle SDI
Gabon	0.651	Middle SDI
Eastern sub-Saharan Africa	0.387	
Burundi	0.31	Low SDI
Comoros	0.434	Low SDI
Djibouti	0.485	Low-middle SDI
Eritrea	0.409	Low SDI

Appendix Table. Socio-demographic Index groupings by geography, based on 2017 values		
**Geography**	**2017 SDI**	**SDI Quintile**
Ethiopia	0.334	Low SDI
Kenya	0.499	Low-middle SDI
Baringo	0.444	Low-middle SDI
Bomet	0.496	Low-middle SDI
Bungoma	0.463	Low-middle SDI
Busia	0.438	Low-middle SDI
Elgeyo Marakwet	0.496	Low-middle SDI
Embu	0.533	Low-middle SDI
Garissa	0.334	Low-middle SDI
Homa Bay	0.425	Low-middle SDI
Isiolo	0.385	Low-middle SDI
Kajiado	0.534	Low-middle SDI
Kakamega	0.45	Low-middle SDI
Kericho	0.5	Low-middle SDI
Kiambu	0.58	Low-middle SDI
Kilifi	0.456	Low-middle SDI
Kirinyaga	0.533	Low-middle SDI
Kisii	0.522	Low-middle SDI
Kisumu	0.503	Low-middle SDI
Kitui	0.461	Low-middle SDI
Kwale	0.457	Low-middle SDI
Laikipia	0.556	Low-middle SDI
Lamu	0.453	Low-middle SDI
Machakos	0.518	Low-middle SDI
Makueni	0.469	Low-middle SDI
Mandera	0.295	Low-middle SDI
Marsabit	0.34	Low-middle SDI
Meru	0.508	Low-middle SDI
Migori	0.419	Low-middle SDI
Mombasa	0.568	Low-middle SDI
Murang'a	0.528	Low-middle SDI
Nairobi	0.674	Low-middle SDI
Nakuru	0.545	Low-middle SDI
Nandi	0.501	Low-middle SDI
Narok	0.402	Low-middle SDI
Nyamira	0.544	Low-middle SDI
Nyandarua	0.534	Low-middle SDI
Nyeri	0.554	Low-middle SDI
Samburu	0.308	Low-middle SDI
Siaya	0.46	Low-middle SDI
Taita Taveta	0.529	Low-middle SDI
Tana River	0.379	Low-middle SDI
Tharaka Nithi	0.528	Low-middle SDI
Trans Nzoia	0.496	Low-middle SDI
Turkana	0.295	Low-middle SDI

Appendix Table. Socio-demographic Index groupings by geography, based on 2017 values		
**Geography**	**2017 SDI**	**SDI Quintile**
Uasin Gishu	0.545	Low-middle SDI
Vihiga	0.477	Low-middle SDI
Wajir	0.243	Low-middle SDI
West Pokot	0.382	Low-middle SDI
Madagascar	0.331	Low SDI
Malawi	0.349	Low SDI
Mozambique	0.34	Low SDI
Rwanda	0.407	Low SDI
Somalia	0.235	Low SDI
South Sudan	0.275	Low SDI
Tanzania	0.412	Low SDI
Uganda	0.388	Low SDI
Zambia	0.472	Low-middle SDI
Southern sub-Saharan Africa	0.64	
Botswana	0.663	Middle SDI
Lesotho	0.493	Low-middle SDI
Namibia	0.616	Middle SDI
South Africa	0.677	Middle SDI
Swaziland	0.578	Low-middle SDI
Zimbabwe	0.463	Low-middle SDI
Western sub-Saharan Africa	0.441	
Benin	0.373	Low SDI
Burkina Faso	0.284	Low SDI
Cameroon	0.482	Low-middle SDI
Cape Verde	0.549	Low-middle SDI
Chad	0.253	Low SDI
Cote d'Ivoire	0.412	Low SDI
The Gambia	0.405	Low SDI
Ghana	0.537	Low-middle SDI
Guinea	0.325	Low SDI
Guinea-Bissau	0.349	Low SDI
Liberia	0.328	Low SDI
Mali	0.267	Low SDI
Mauritania	0.471	Low-middle SDI
Niger	0.191	Low SDI
Nigeria	0.493	Low-middle SDI
Sao Tome and Principe	0.488	Low-middle SDI
Senegal	0.373	Low SDI
Sierra Leone	0.357	Low SDI
Togo	0.413	Low SDI
