# Supplementary material for: Measuring progress from 1990 to 2017 and projecting attainment to 2030 of the health-related Sustainable Development Goals for 195 countries and territories: a systematic analysis for the Global Burden of Disease Study 2017
Source: Lancet. 2018 Nov 10;392(10159):2091–138. doi: 10.1016/S0140-6736(18)32281-5 (PMC6227911; doi:10.1016/S0140-6736(18)32281-5)
Supplement: Supplementary appendix 2 [file mmc2.pdf]

THE LANCET

Supplementary appendix 2

This appendix formed part of the original submission and has been peer reviewed. We post it as supplied by the authors.

Supplement to: GBD 2017 SDG Collaborators. Measuring progress from 1990 to 2017 and projecting attainment to 2030 of the health-related Sustainable Development Goals for 195 countries and territories: a systematic analysis for the Global Burden of Disease Study 2017. *Lancet* 2018; **392:** 2091–138.

Supplementary results to Measuring progress and projecting attainment on the basis of past trends of the health-related Sustainable Development Goals in 195 countries and territories: an analysis from the Global Burden of Disease Study 2017

The supplementary results appendix offers additional figures and tables.

List of Supplementary Results: Figures and Tables

Supplemental figures and tables providing more detailed results can be found in the supplementary results appendix.

Supplementary figure 1. Census or population registry status and coverage of a population and housing census within the last 10 years, by location, from 1990-2017. Per SDG indicator 17.19.2a, the target is that each country is to have conducted a population and housing census in the last 10 years, or have a population registry from which detailed population and housing statistics are derived. Census years are designated by a dark blue colouring, followed by a lighter blue colour indicating census coverage for the 9 years following a census for a total of 10 years of census coverage. A medium blue colour is used to designate the implementation of a population registry. White indicates that a given location-year is not covered by a population and housing census or population registry. Census status for 1990-2000 was informed by census conducted from 1980-1989, which are not shown here. SDG=Sustainable Development Goal.

Supplementary figure 2. Map of health-related SDG index, by decile, in 1990, 2000, and 2017 for Japan (A), Sweden (B), England (C), USA (D), Mexico (E), Brazil (F), India (G), and Kenya (H). Deciles were based on the distribution of health-related SDG index values for countries and territories in 2017, as found in figure 2 of the main text, and then were applied for subnational locations over time. SDG=Sustainable Development Goal.

Supplementary figure 3. Projected performance, based on past trends, on the health-related SDG index and 40 individual health-related indicators, by country or territory, 2030. All projections were based on past trends and rates of change observed from 1990 to 2017. Countries are ranked by their health-related SDG index from highest to lowest in 2030, which was projected based on past trends. Indices and individual indicators are reported on a scale of 0 to 100, with 0 representing the worst levels from 1990 to 2030 and 100 reflecting the best during that time. SDG indicator 17.19.2a, population census status within the last 10 years, was not included in the health-related SDG index as projections were not generated for this indicator. Definitions of health-related SDG indicators are shown in table 1 in the main manuscript. SDG=Sustainable Development Goal. SDG=Sustainable Development Goal. Mort=mortality. Mat Mort Ratio=maternal mortality ratio; Skill Birth Attend=skilled birth attendance. Incid=incidence. Prev=prevalence. TB=tuberculosis. Hep B=hepatitis B. NTD=neglected tropical diseases. NCD=non-communicable disease. Inj=injury. FP Need Met, Mod=family planning need met with modern contraception methods. Adol=adolescent. UHC Serv Cov Index=universal health coverage, service coverage index. Air Poll=air pollution. WaSH=water, sanitation, and hygiene. Cov=coverage. Int=intimate. Viol=violence. HH=household. Occ=occupational risk. PM2.5= particulate matter smaller than 2.5 microns in diameter. Cert Death Reg=well-certified death registration.

Supplementary figure 4. Comparing attainment of defined health-related SDG indicator targets in 2017 and, based on past trends, projected to be attained in 2030, by country. Countries and territories are ranked by the number of SDG indicator targets they were projected to attain based on the mean estimate for 2030. All projections were based on past trends and rates of change observed from 1990-2017. Of the 41 health-related indicators measured in this study, 25 had defined targets linked to each indicator. SDG target 3.6 aims to reduce road injury mortality by 50% between 2015 and 2020, and thus attainment for

this indicator is based on estimates from 2015 to 2020 rather than 2015 to 2030. Definitions of health-related SDG indicators and targets associated with them, as well as the specific target thresholds applied, are shown in table 1 in the main manuscript. SDG=Sustainable Development Goal. Mort=mortality. Mat Mort Ratio=maternal mortality ratio; Skill Birth Attend=skilled birth attendance. Incid=incidence. Prev=prevalence. TB=tuberculosis. NTD=neglected tropical diseases. NCD=non-communicable disease. Inj=injury. FP Need Met, Mod=family planning need met with modern contraception methods. UHC Serv Cov Index=universal health coverage, service coverage index. Air Poll=air pollution. WaSH=water, sanitation, and hygiene. Cov=coverage. Int=intimate. Viol=violence. HH=household. Cert Death Reg=well-certified death registration.

Supplementary figure 5. Global annualised rate of change required to meet selected SDG targets based on annualised rate of change achieved by countries from 1990–2015 for selected SDG indicators with defined targets: well-certified death registration, child overweight, child sex abuse, child stunting, child wasting, hygiene, intimate partner violence, malaria incidence, neonatal mortality, non-intimate partner sexual violence, NTD prevalence, road injury mortality, sanitation, skilled birth attendance, suicide mortality, and water. For the 25 SDG indicators with defined targets, the global ARC required to meet each target was computed using the global average in 2015 and specific thresholds to be met by 2030 or relative reductions to be achieved by 2030. SDG target 3.6 aims to reduce road injury mortality by 50% between 2015 and 2020, and thus the global ARC is based on the time span from 2015 to 2020 rather than 2015 to 2030. Global ARCs are compared with the ARCs achieved across countries and territories from 1990-2015. The best-performing decile of ARC is shown compared to all other deciles against the global ARC required to meet the defined SDG target. A subset of SDG indicators with defined targets are shown in figure 6 in the main manuscript; the remaining plots are shown here. Definitions of health-related SDG indicators and targets associated with them are shown in table 1 in the main manuscript. ARC=annualised rate of change. SDG=Sustainable Development Goal.

Supplementary figure 6. Comparing 2016 values on the health-related SDG index from GBD 2016 to 2017 values on the health-related SDG index from GBD 2017. Countries are colour-coded by SDI quintile, and are abbreviated according to their ISO3 codes, which are listed in the appendix. The 7 territories included for GBD 2017 are not shown since they were not included in previous GBD SDG analyses. SDG=Sustainable Development Goal. GBD=Global Burden of Disease.

Supplementary figure 7. Comparing 2030 values on the health-related SDG index from GBD 2016 to 2030 values on the health-related SDG index from GBD 2017. Countries are colour-coded by SDI quintile, and are abbreviated according to their ISO3 codes, which are listed in the appendix. The 7 territories included for GBD 2017 are not shown since they were not included in previous GBD SDG analyses. SDG=Sustainable Development Goal. GBD=Global Burden of Disease.

Supplementary figure 8. Comparing 2016 rankings on the health-related SDG index from GBD 2016 to 2017 rankings on the health-related SDG index from GBD 2017. Countries are colour-coded by SDI quintile, and are abbreviated according to their ISO3 codes, which are listed in the appendix. The 7 territories included for GBD 2017 are not shown since they were not included in previous GBD SDG analyses. SDG=Sustainable Development Goal. GBD=Global Burden of Disease.

Supplementary table 1. Health-related SDGs excluded in the present analysis, and measurement needs and strategy for future reporting, by SDG target. Definitions and descriptions of health-related SDG indicators beyond the specific indicators originate from the IAEG-SDGs compilation of metadata for each SDG (as

provided by each indicator's custodial agency). DAH=development assistance for health. DHS=Demographic and Health Survey. GBD=Global Burden of Disease. IAEG-SDGs=Inter-agency and Expert Group on SDG Indicators. IHR=International Health Regulations. ISIC=International Standard Industrial Classification. JEE=Joint External Evaluation. NCD=non-communicable diseases. SDG=Sustainable Development Goal. TRIPS=Agreement on Trade-Related Aspects of Intellectual Property Rights. UHC=universal health coverage. UN=United Nations. WHO=World Health Organization.

Supplementary table 2. Health-related SDG index in 2000, 2015, 2017, and 2030, by country or territory. The health-related SDG index is reported on a scale of 0 to 100, with 0 representing the worst levels from 1990 to 2030 and 100 reflecting the best during that time. SDG=Sustainable Development Goal.

Supplementary table 3. Unscaled values for the 40 individual health-related SDG indicators in 2000, 2015, 2017, and 2030, and projected percent change from 2015 to 2030, by country or territory. Projected estimates for 2030 were produced based on past trends and rates of change observed from 1990 to 2017. Estimates are reported for each country and territory included in this analysis, and ordered by health-related SDG indicator followed by GBD super-region and region. SDG indicator 17.19.2a, population census status within the last 10 years, is not included here as projections were not generated for this indicator. All results from 1990 to 2017 for SDG indicator 17.19.2a can be found in supplementary figure 1. For this table, draws for conflict and disaster mortality were truncated so that any values which fell outside the confidence intervals for any location year were set to the lower or upper bound of that confidence interval. SDG=Sustainable Development Goal. GBD=Global Burden of Disease.

Supplementary table 4. GATHER checklist of information that should be included in reports of global health estimates, with description of compliance and location of information for GBD 2017 SDG Capstone.

Supplementary figure 1 **. Census or population registry status and coverage of a population and housing census within the last 10 years, by location, from 1990-2017**. Per SDG indicator 17.19.2a, the target is that each country is to have conducted a population and housing census in the last 10 years, or have a population registry from which detailed population and housing statistics are derived. Census years are designated by a dark blue colouring, followed by a lighter blue colour indicating census coverage for the 9 years following a census for a total o 10 years of census coverage. A medium blue colour is used to designate the implementation of a population registry. White indicates that a given location-year is not covered by a population and housing census or population registry. Census status for 1990-2000 was informed by censu conducted from 1980-1989, which are not shown here. SDG=Sustainable Development Goal.

Location name	1990	1991	1992	1993	1994	1995	1996	1997	1998	1999	2000	2001	2002	2003	2004	2005	2006	2007	2008	2009	2010	2011	2012	2013	2014	2015	2016	2017
Afghanistan																												
Albania												Census										Census						
Algeria									Census										Census									
American Samoa	Census										Census										Census							
Andorra																												
Angola																									Census			
Antigua and Barbuda		Census										Census										Census						
Argentina		Census										Census									Census							
Armenia												Census										Census						
Australia		Census					Census					Census					Census					Census					Census	
Austria		Census										Census																
Azerbaijan										Census										Census								
Bahrain		Census										Census									Census							
Bangladesh		Census										Census										Census						
Barbados	Census										Census										Census							
Belarus										Census										Census								
Belgium		Census										Census																
Belize		Census									Census										Census							
Benin			Census										Census											Census				
Bermuda		Census									Census										Census							
Bhutan																Census												Census
Bolivia			Census									Census											Census					
Bosnia and Herzegovina		Census																						Census				
Botswana		Census										Census										Census						
Brazil		Census									Census										Census							
Brunei		Census										Census										Census						
Bulgaria			Census																									
Burkina Faso							Census										Census										Census	
Burundi	Census																		Census									
Cambodia									Census										Census									
Cameroon																Census												
Canada		Census					Census					Census					Census					Census					Census	
Cape Verde	Census										Census										Census							
Central African Republic														Census														
Chad				Census																Census								
Chile			Census										Census										Census					Census
China	Census	Census					Census				Census	Census					Census				Census	Census					Census	
Colombia				Census												Census												
Comoros		Census												Census													Census	
Congo							Census											Census										Census
Costa Rica											Census											Census						
Cote d'Ivoire									Census																Census			
Croatia		Census										Census										Census						
Cuba													Census										Census					
Cyprus			Census				Census					Census					Census					Census						
Czech Republic		Census																										
Democratic Republic of the Congo																												
Denmark																												
Djibouti																				Census								
Dominica		Census										Census										Census						

***Supplementary figure 1* . Census or population registry status and coverage of a population and housing census within the last 10 years, by location, from 1990-2017**. Per SDG indicator 17.19.2a, the target is that each country is to have conducted a population and housing census in the last 10 years, or have a population registry from which detailed population and housing statistics are derived. Census years are designated by a dark blue colouring, followed by a lighter blue colour indicating census coverage for the 9 years following a census for a total o 10 years of census coverage. A medium blue colour is used to designate the implementation of a population registry. White indicates that a given location-year is not covered by a population and housing census or population registry. Census status for 1990-2000 was informed by censu conducted from 1980-1989, which are not shown here. SDG=Sustainable Development Goal.

**Location name**	**1990**	**1991**	**1992**	**1993**	**1994**	**1995**	**1996**	**1997**	**1998**	**1999**	**2000**	**2001**	**2002**	**2003**	**2004**	**2005**	**2006**	**2007**	**2008**	**2009**	**2010**	**2011**	**2012**	**2013**	**2014**	**2015**	**2016**	**2017**
Dominican Republic				Census									Census								Census							
Ecuador	Census											Census									Census							
Egypt							Census										Census											Census
El Salvador			Census															Census										
Equatorial Guinea					Census							Census														Census		
Eritrea																												
Estonia																												
Ethiopia					Census													Census										
Federated States of Micronesia					Census						Census										Census							
Fiji							Census											Census										Census
Finland																												
France	Census									Census							Census	Census	Census	Census	Census	Census	Census	Census	Census	Census		
Gabon				Census										Census										Census				
Georgia													Census												Census			
Germany																												
Ghana											Census										Census							
Greece		Census										Census										Census						
Greenland																												
Grenada		Census										Census										Census						
Guam	Census										Census										Census							
Guatemala					Census								Census															
Guinea							Census																		Census			
Guinea-Bissau		Census																		Census								
Guyana		Census											Census										Census					
Haiti														Census														
Honduras												Census												Census				
Hungary	Census											Census										Census	Census	Census	Census	Census	Census	Census
Iceland																												
India		Census										Census										Census						
Indonesia	Census										Census										Census							
Iran							Census										Census					Census					Census	
Iraq								Census																				
Ireland		Census					Census						Census				Census					Census					Census	
Israel						Census													Census									
Italy		Census										Census																
Jamaica		Census										Census										Census						
Japan	Census					Census					Census					Census					Census					Census		
Jordan					Census										Census											Census		
Kazakhstan										Census										Census								
Kenya										Census										Census								
Kiribati	Census					Census					Census					Census					Census					Census		
Kuwait						Census										Census						Census						
Kyrgyzstan										Census										Census								
Laos						Census										Census										Census		
Latvia											Census																	
Lebanon																												
Lesotho							Census										Census										Census	
Liberia																			Census									
Libya						Census											Census											
Lithuania												Census										Census						

Supplementary figure 1 **. Census or population registry status and coverage of a population and housing census within the last 10 years, by location, from 1990-2017**. Per SDG indicator 17.19.2a, the target is that each country is to have conducted a population and housing census in the last 10 years, or have a population registry from which detailed population and housing statistics are derived. Census years are designated by a dark blue colouring, followed by a lighter blue colour indicating census coverage for the 9 years following a census for a total o 10 years of census coverage. A medium blue colour is used to designate the implementation of a population registry. White indicates that a given location-year is not covered by a population and housing census or population registry. Census status for 1990-2000 was informed by censu conducted from 1980-1989, which are not shown here. SDG=Sustainable Development Goal.

Location name	1990	1991	1992	1993	1994	1995	1996	1997	1998	1999	2000	2001	2002	2003	2004	2005	2006	2007	2008	2009	2010	2011	2012	2013	2014	2015	2016	2017
Luxembourg		Census																										
Macedonia					Census								Census															
Madagascar				Census																								
Malawi									Census										Census									
Malaysia	Census	Census							Census		Census										Census							
Maldives	Census					Census					Census						Census								Census			
Mali									Census											Census								
Malta						Census										Census						Census						
Marshall Islands										Census												Census						
Mauritania											Census													Census				
Mauritius	Census										Census											Census						
Mexico	Census					Census					Census					Census					Census							
Moldova															Census										Census	Census		
Mongolia											Census										Census							
Montenegro		Census												Census								Census						
Morocco					Census										Census										Census			
Mozambique								Census										Census										Census
Myanmar																									Census			
Namibia		Census										Census										Census						
Nepal		Census										Census										Census						
Netherlands																												
New Zealand		Census					Census					Census					Census							Census				
Nicaragua						Census										Census												
Niger												Census											Census					
Nigeria		Census															Census								Census			
North Korea				Census															Census									
Northern Mariana Islands	Census					Census					Census										Census							
Norway																												
Oman				Census										Census							Census							
Pakistan									Census																			Census
Palestine								Census										Census										Census
Panama	Census										Census										Census							
Papua New Guinea	Census										Census											Census						
Paraguay			Census										Census															
Peru				Census												Census		Census										
Philippines	Census					Census					Census							Census			Census					Census		
Poland													Census															
Portugal		Census										Census										Census						
Puerto Rico	Census										Census										Census							
Qatar								Census							Census						Census					Census		
Romania			Census										Census									Census						
Russia													Census								Census							
Rwanda		Census											Census										Census					
Saint Lucia		Census										Census									Census							
Saint Vincent and the Grenadines		Census										Census											Census					
Samoa		Census										Census					Census					Census					Census	
Sao Tome and Principe		Census										Census											Census					
Saudi Arabia			Census												Census						Census							
Senegal													Census											Census				
Serbia		Census											Census									Census						

Supplementary figure 1 **. Census or population registry status and coverage of a population and housing census within the last 10 years, by location, from 1990-2017**. Per SDG indicator 17.19.2a, the target is that each country is to have conducted a population and housing census in the last 10 years, or have a population registry from which detailed population and housing statistics are derived. Census years are designated by a dark blue colouring, followed by a lighter blue colour indicating census coverage for the 9 years following a census for a total o 10 years of census coverage. A medium blue colour is used to designate the implementation of a population registry. White indicates that a given location-year is not covered by a population and housing census or population registry. Census status for 1990-2000 was informed by censu conducted from 1980-1989, which are not shown here. SDG=Sustainable Development Goal.

Location name	1990	1991	1992	1993	1994	1995	1996	1997	1998	1999	2000	2001	2002	2003	2004	2005	2006	2007	2008	2009	2010	2011	2012	2013	2014	2015	2016	2017
Seychelles					Census			Census					Census								Census							
Sierra Leone															Census											Census		
Singapore	Census																											
Slovakia																												
Slovenia		Census																										
Solomon Islands										Census										Census								
Somalia																												
South Africa		Census					Census					Census										Census						
South Korea	Census					Census					Census					Census					Census					Census		
South Sudan				Census															Census									
Spain		Census																										
Sri Lanka												Census											Census					
Sudan				Census															Census									
Suriname											Census				Census								Census					
Swaziland								Census										Census										Census
Sweden																												
Switzerland	Census										Census																	
Syria					Census										Census													
Taiwan (Province of China)																												
Tajikistan											Census										Census							
Tanzania													Census										Census					
Thailand	Census										Census										Census							
The Bahamas	Census										Census										Census							
The Gambia				Census										Census										Census				
Timor-Leste	Census														Census						Census					Census		
Togo																					Census							
Tonga							Census										Census					Census					Census	
Trinidad and Tobago	Census										Census											Census						
Tunisia					Census										Census										Census			
Turkey	Census										Census																	
Turkmenistan						Census																	Census			Census		
Uganda		Census											Census												Census			
Ukraine												Census																
United Arab Emirates						Census										Census												
United Kingdom		Census										Census										Census						
United States	Census										Census										Census							
Uruguay							Census								Census							Census						
Uzbekistan																												
Vanuatu										Census										Census							Census	
Venezuela	Census											Census										Census						
Vietnam										Census										Census								
Virgin Islands	Census										Census										Census							
Yemen					Census										Census													
Zambia	Census										Census										Census							
Zimbabwe			Census										Census										Census					

Supplementary figure 2. Map of health-related SDG Index, by decile, in 1990, 2000, and 2017, for Japan (A).

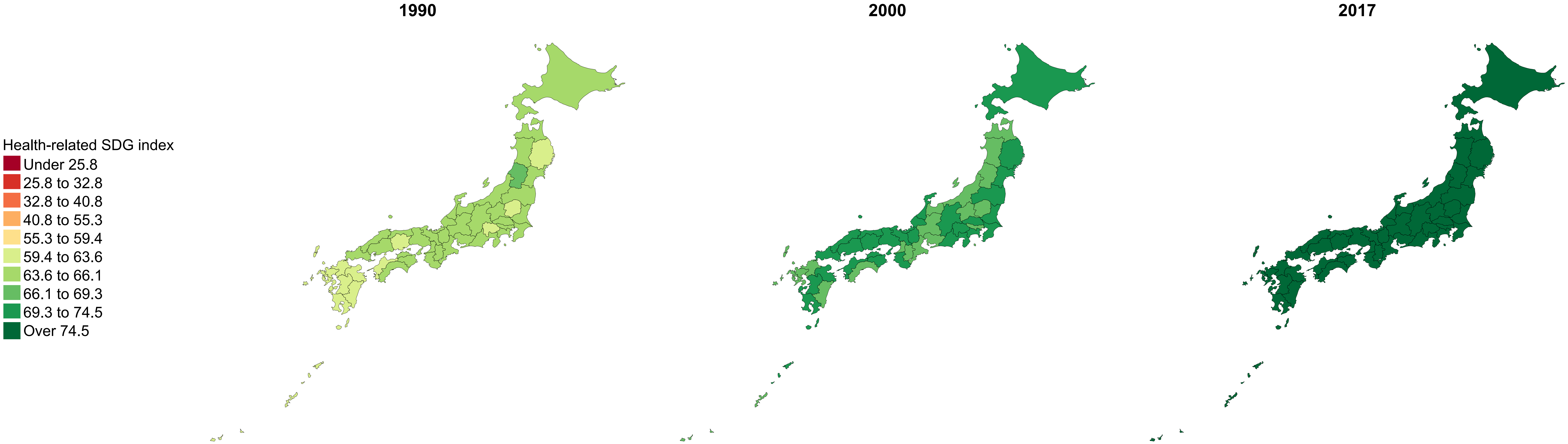

Supplementary figure 2. Map of health-related SDG Index, by decile, in 1990, 2000, and 2017, for Sweden (B).

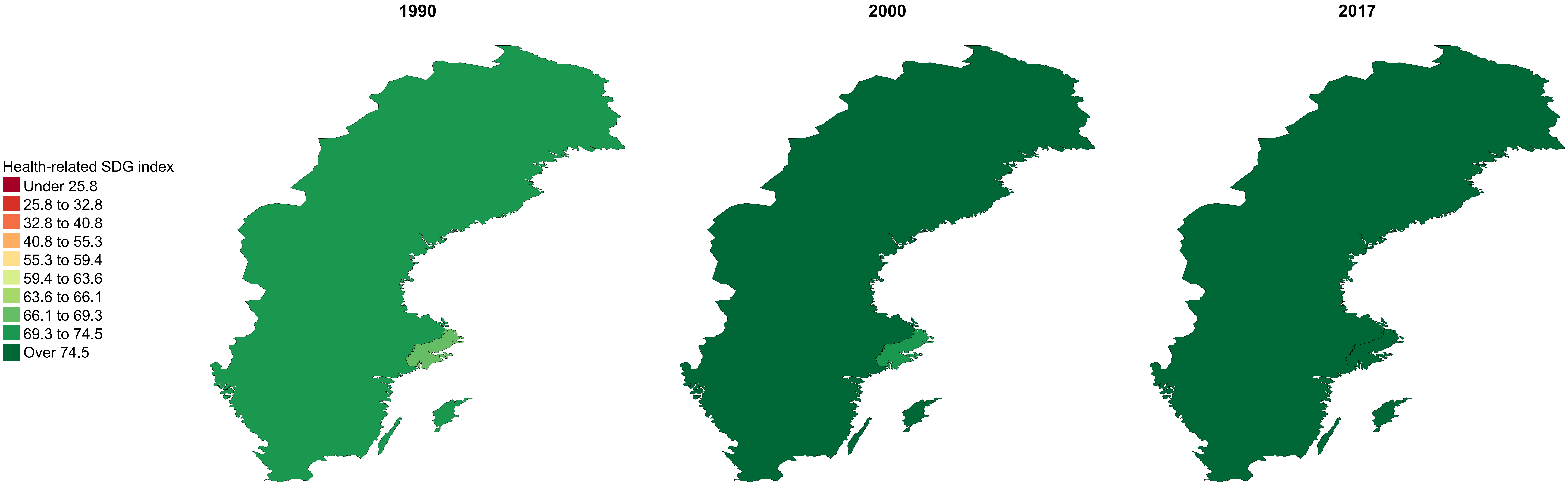

Supplementary figure 2. Map of health-related SDG Index, by decile, in 1990, 2000, and 2017, for England (C).

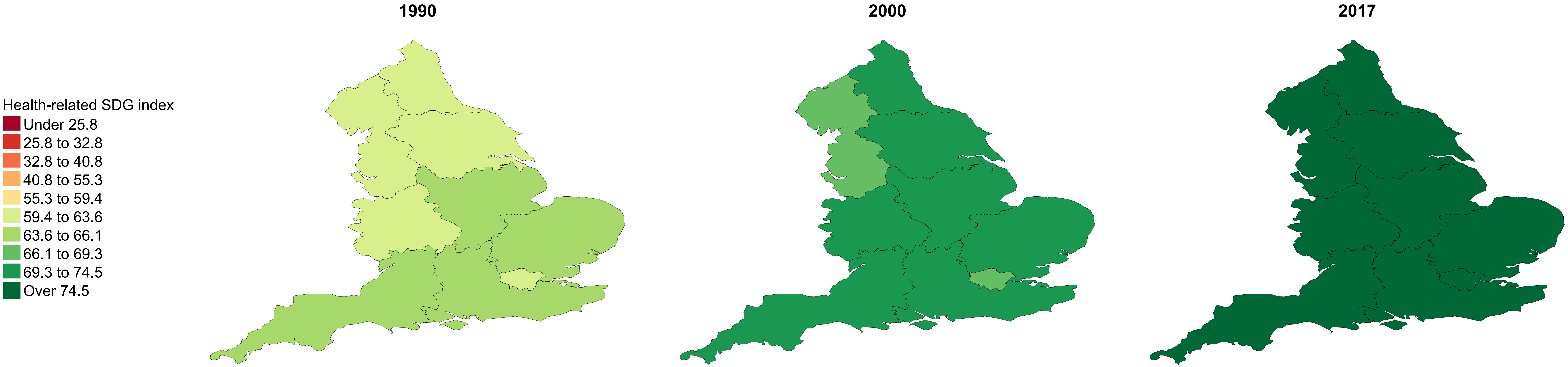

Supplementary figure 2. Map of health-related SDG Index, by decile, in 1990, 2000, and 2017, for the United States (D).

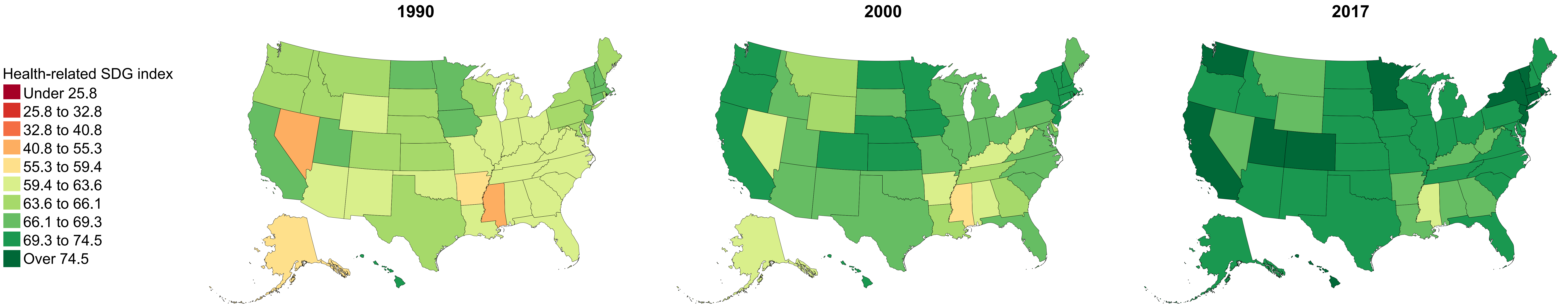

Supplementary figure 2. Map of health-related SDG Index, by decile, in 1990, 2000, and 2017, for Mexico (E).

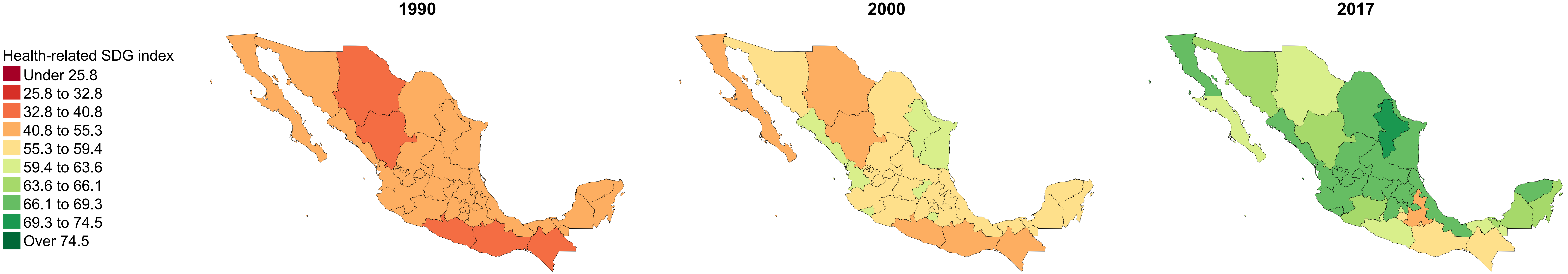

Supplementary figure 2. Map of health-related SDG Index, by decile, in 1990, 2000, and 2017, for Brazil (F).

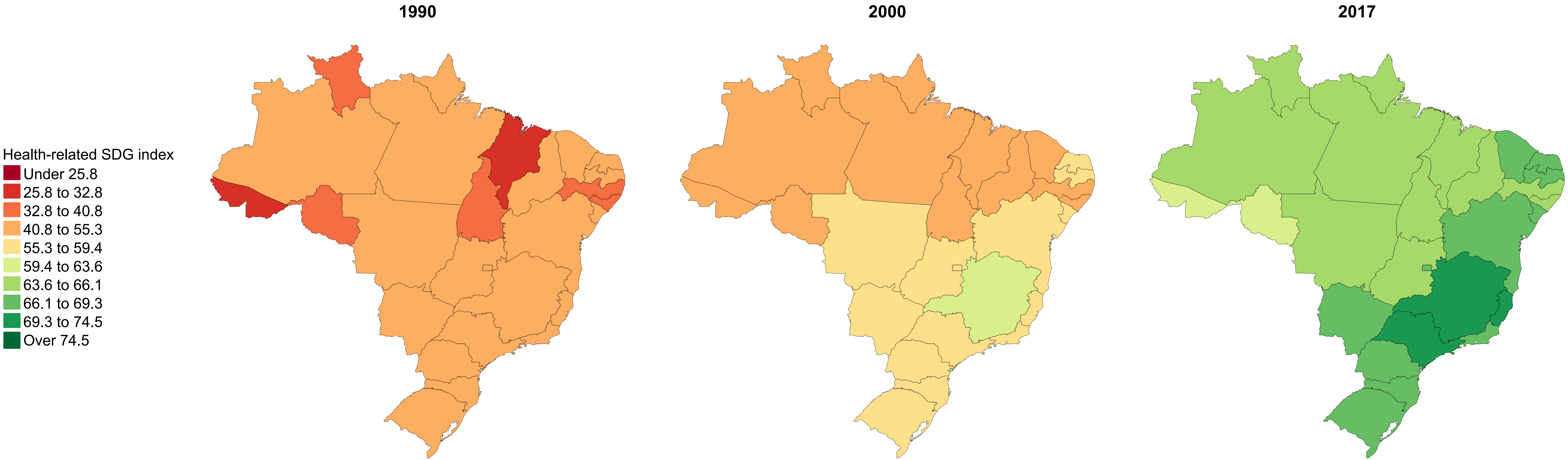

Supplementary figure 2. Map of health-related SDG Index, by decile, in 1990, 2000, and 2017, for India (G).

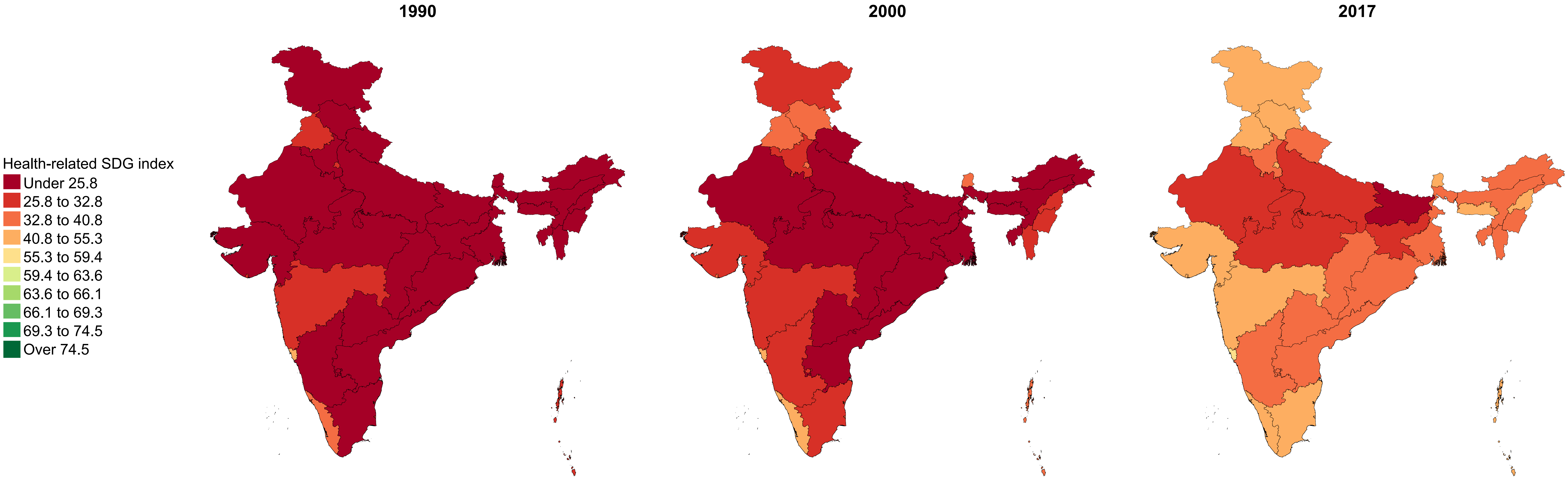

Supplementary figure 2. Map of health-related SDG Index, by decile, in 1990, 2000, and 2017, for Kenya (H).

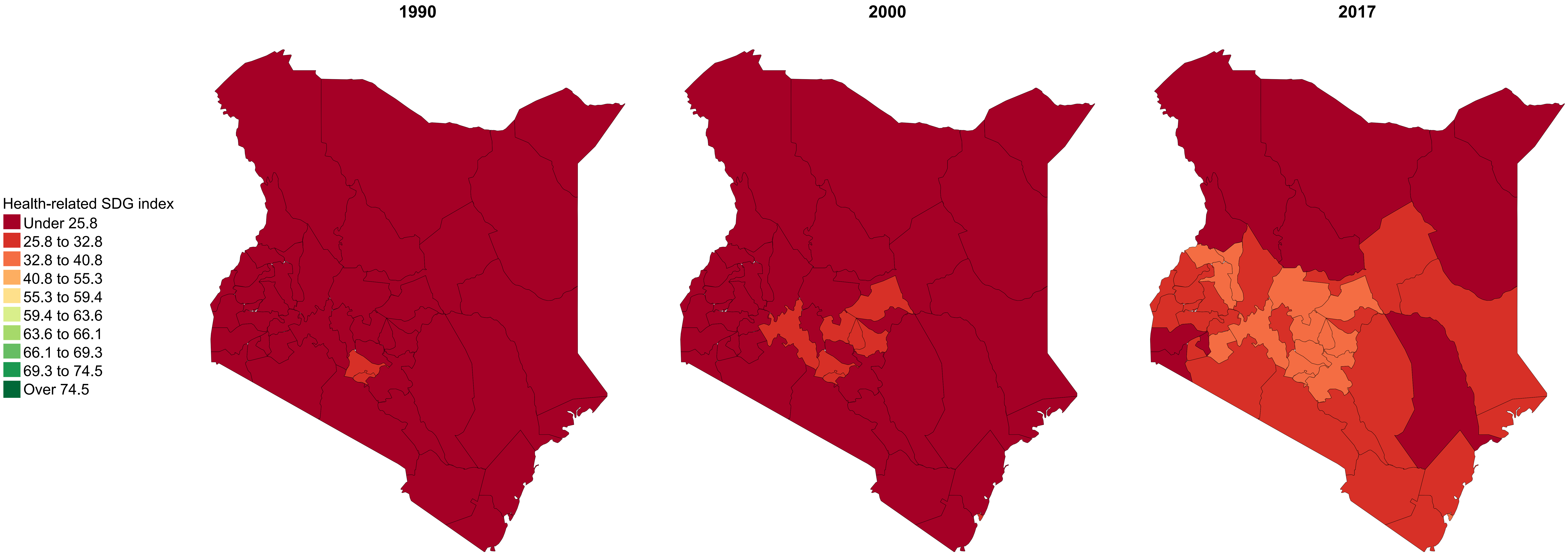

Supplementary figure 3. Projected performance, based on past trends, on the health-related SDG index and 40 individual health-related indicators, by country or territory, 2030

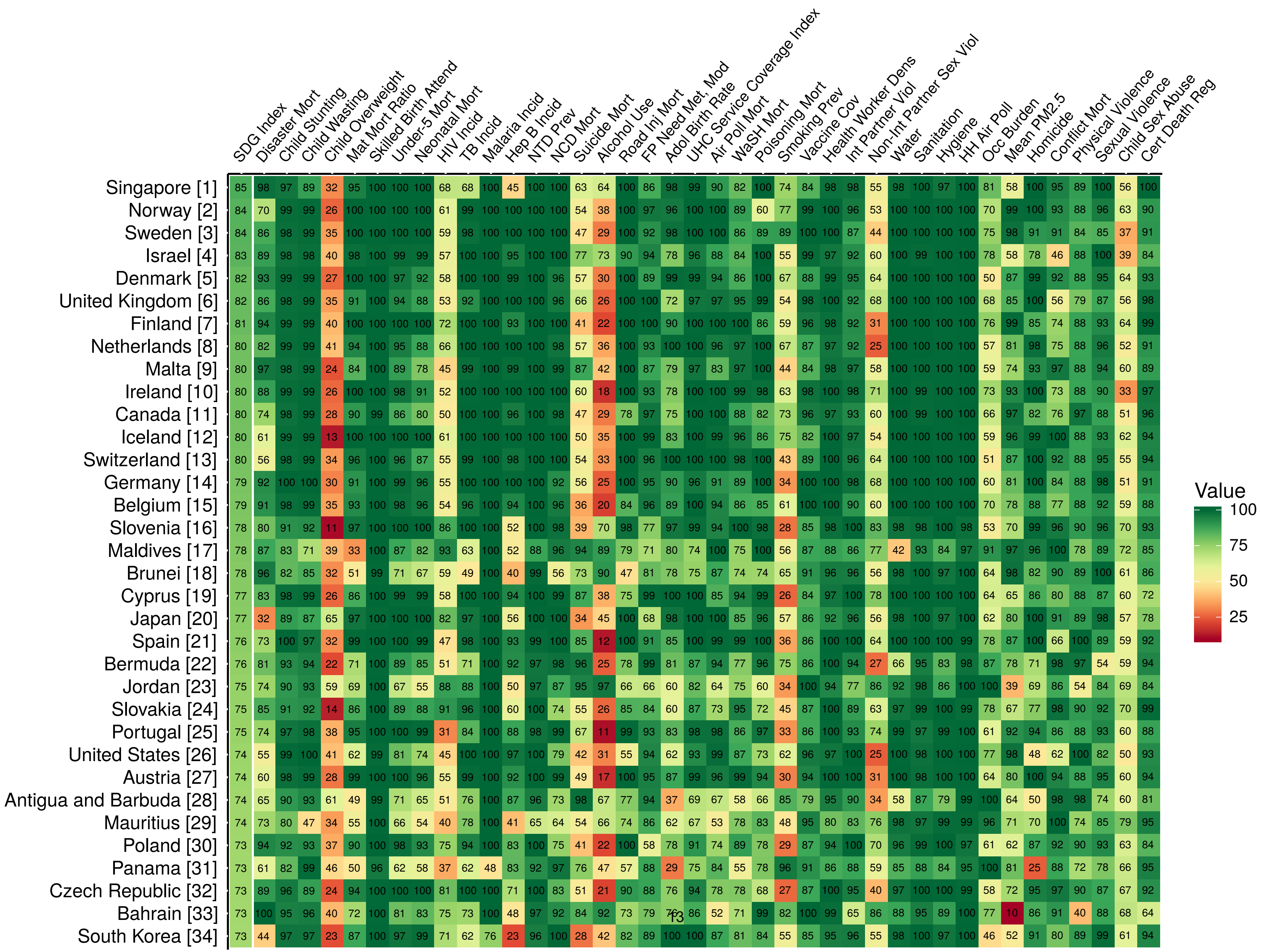

Supplementary figure 3. Projected performance, based on past trends, on the health-related SDG index and 40 individual health-related indicators, by country or territory, 2030

Country or territory	SDG Index	Disaster Mort	Child Stunting	Child Wasting	Child Overweight	Mat Mort Ratio	Skilled Birth Attend	Under-5 Mort	Neonatal Mort	HIV Incid	TB Incid	Malaria Incid	Hep B Incid	NTD Prev	NCD Mort	Suicide Mort	Alcohol Use	Road Inj Mort	FP Need Met, Mod	Adol Birth Rate	UHC Service Coverage Index	Air Poll Mort
Hungary [35]	73	85	90	92	30	88	100	95	92	78	99	100	44	100	59	40	26	90	68	61	89	70
Australia [36]	72	69	95	99	30	99	100	96	91	60	100	100	82	100	100	48	19	89	100	75	100	99
Barbados [37]	72	75	90	93	58	46	99	65	51	45	96	100	91	96	70	87	48	77	78	44	74	64
Kuwait [38]	72	99	98	92	0	81	99	81	73	89	76	100	56	100	98	99	100	57	87	92	89	62
Uruguay [39]	72	92	88	96	43	69	100	77	70	41	75	100	100	100	67	30	46	70	84	36	78	88
Italy [40]	72	49	98	98	23	100	100	100	97	47	100	100	73	100	100	87	40	94	81	96	100	95
Seychelles [41]	71	84	86	77	58	48	99	71	64	58	69	100	45	88	61	58	68	59	91	36	71	58
Malaysia [42]	71	75	77	47	15	50	100	84	80	47	56	64	61	92	74	60	82	54	93	86	72	55
Luxembourg [43]	71	92	99	99	10	95	99	100	100	49	99	100	87	100	99	57	2	98	99	94	100	99
The Bahamas [44]	71	62	91	94	48	37	99	67	60	28	77	100	85	96	53	98	65	49	97	49	65	69
Colombia [45]	71	32	86	100	72	52	99	67	62	46	75	38	68	91	99	77	47	61	100	38	76	90
Mexico [46]	71	46	87	98	50	53	99	65	63	47	77	80	65	87	80	76	59	56	90	32	66	57
Virgin Islands, U.S. [47]	71	79	93	95	19	51	99	81	71	44	84	100	90	100	56	77	71	62	99	39	69	70
New Zealand [48]	70	37	95	98	13	87	100	93	89	62	94	100	67	100	98	50	26	81	94	65	100	100
France [49]	70	74	98	99	39	93	100	97	94	55	100	100	82	100	99	46	24	91	100	88	100	100
Palestine [50]	70	97	94	96	53	76	99	67	69	93	75	100	47	93	63	87	100	68	75	28	72	56
Turkmenistan [51]	70	96	91	96	15	77	100	54	41	65	52	100	36	96	36	51	58	78	90	71	66	50
Paraguay [52]	70	77	97	99	32	43	96	68	71	42	58	100	62	94	70	79	43	40	96	38	69	61
Brazil [53]	70	63	89	98	7	47	100	61	56	37	65	45	55	91	72	68	45	54	100	40	68	77
Argentina [54]	70	71	93	94	66	52	100	72	64	42	73	100	100	95	70	50	37	59	86	34	73	73
Greece [55]	70	62	98	98	29	89	100	96	92	60	100	100	60	100	89	99	27	89	69	85	97	80
Saint Lucia [56]	70	65	97	92	84	43	99	68	54	59	84	100	85	95	61	68	52	60	78	46	65	62
Northern Mariana Islands [57]	70	90	85	81	30	40	99	80	74	76	58	100	22	92	64	40	86	57	91	47	72	64
Uzbekistan [58]	70	96	80	86	35	64	100	56	47	60	58	100	36	96	38	48	53	56	96	62	64	30
Sri Lanka [59]	69	47	86	55	48	70	100	78	68	76	57	100	54	91	84	16	62	68	79	72	82	72
Grenada [60]	69	75	88	92	73	38	99	64	49	55	88	100	85	95	48	80	52	66	91	43	67	52
Oman [61]	69	77	77	66	3	70	100	76	69	52	72	100	50	98	74	97	95	29	58	84	83	53
Tunisia [62]	69	67	90	95	21	68	100	77	69	70	71	100	65	97	82	98	94	27	84	99	80	53
Estonia [63]	69	94	91	94	18	97	100	98	98	44	78	100	71	100	76	39	3	96	85	69	91	99
Croatia [64]	68	94	91	92	17	96	100	97	89	81	95	100	63	100	79	48	39	90	49	84	93	75
Algeria [65]	68	54	89	89	13	51	99	57	47	77	67	94	55	97	84	88	95	48	85	92	68	60
Turkey [66]	68	42	90	99	5	71	99	72	66	88	70	100	57	98	83	99	88	78	78	64	84	57
United Arab Emirates [67]	68	100	84	62	11	75	100	87	82	73	79	100	52	98	48	82	62	16	87	77	67	38

Country or territory	WaSH Mort	Poisoning Mort	Smoking Prev	Vaccine Cov	Health Worker Dens	Int Partner Viol	Non-Int Partner Sex Viol	Water	Sanitation	Hygiene	HH Air Poll	Occ Burden	Mean PM2.5	Homicide	Conflict Mort	Physical Violence	Sexual Violence	Child Sex Abuse	Cert Death Reg
Hungary [35]	76	82	30	88	87	89	70	97	96	100	93	66	70	90	93	90	94	70	98
Australia [36]	93	100	81	99	99	100	0	98	100	97	100	63	90	87	71	89	93	62	96
Barbados [37]	60	92	81	77	97	89	33	78	90	77	100	99	56	34	97	98	69	60	94
Kuwait [38]	72	76	63	87	99	76	84	92	98	91	100	90	16	97	74	38	89	68	89
Uruguay [39]	69	64	56	84	66	83	77	96	100	96	99	46	88	47	97	94	96	78	85
Italy [40]	98	100	45	100	99	89	0	100	99	99	100	71	69	100	71	88	79	60	92
Seychelles [41]	53	65	61	97	82	80	78	69	93	90	98	77	59	51	92	76	82	77	77
Malaysia [42]	54	60	58	82	82	86	75	88	99	96	99	77	68	78	75	75	89	78	63
Luxembourg [43]	94	99	54	100	100	100	44	100	100	100	100	80	86	95	98	88	77	60	92
The Bahamas [44]	61	80	83	90	99	88	32	63	92	80	98	100	67	11	99	97	69	60	95
Colombia [45]	72	97	82	94	83	67	80	84	97	88	96	100	70	13	41	34	85	66	98
Mexico [46]	63	63	74	90	88	97	74	95	91	88	94	80	58	8	86	72	79	71	98
Virgin Islands, U.S. [47]	68	78	98	71	98	97	19	63	95	81	99	68	83	20	91	100	73	59	73
New Zealand [48]	85	100	70	98	100	83	43	98	99	97	100	50	97	87	74	58	87	23	100
France [49]	100	93	8	88	95	92	49	100	100	100	100	42	82	99	60	88	92	96	88
Palestine [50]	66	96	39	100	76	54	88	63	95	80	100	74	39	47	40	37	88	68	85
Turkmenistan [51]	78	64	54	82	97	89	100	84	69	92	100	64	59	57	88	84	99	100	82
Paraguay [52]	62	88	80	100	73	91	76	84	88	79	88	55	79	30	90	85	83	92	90
Brazil [53]	60	98	98	100	97	91	86	92	81	87	97	63	77	10	90	85	81	76	95
Argentina [54]	68	57	51	92	87	73	86	94	98	95	99	45	77	48	87	75	94	76	78
Greece [55]	99	100	6	100	95	89	65	100	99	100	100	71	69	93	74	88	92	60	84
Saint Lucia [56]	61	86	69	76	93	86	38	59	87	84	98	95	57	23	98	98	72	60	94
Northern Mariana Islands [57]	67	81	35	86	87	73	67	82	96	96	92	89	86	54	96	86	41	5	86
Uzbekistan [58]	75	51	81	100	95	88	100	90	50	92	94	62	51	65	90	84	96	100	91
Sri Lanka [59]	61	79	78	87	73	70	86	70	85	90	87	72	86	63	59	87	82	67	83
Grenada [60]	56	68	79	63	87	87	39	62	84	78	98	97	57	50	99	98	72	62	97
Oman [61]	73	65	94	87	99	75	84	86	98	91	100	95	32	100	94	41	89	71	68
Tunisia [62]	69	65	49	84	84	69	86	82	88	87	100	89	34	100	82	37	87	68	63
Estonia [63]	100	70	49	100	95	100	52	96	94	98	97	90	97	64	95	80	68	18	99
Croatia [64]	95	83	4	84	100	100	84	95	98	100	96	58	65	96	77	90	93	70	99
Algeria [65]	76	56	64	84	90	70	86	90	96	90	100	85	37	91	57	37	87	68	79
Turkey [66]	77	70	46	85	79	77	85	81	96	90	100	62	27	77	61	60	87	76	98
United Arab Emirates [67]	58	96	71	96	100	71	84	90	100	90	100	80	31	97	94	41	86	69	85

Value: 100, 75, 50, 25

Supplementary figure 3. Projected performance, based on past trends, on the health-related SDG index and 40 individual health-related indicators, by country or territory, 2030

Country	SDG Index	Disaster Mort	Child Stunting	Child Wasting	Child Overweight	Mat Mort Ratio	Skilled Birth Attend	Under-5 Mort	Neonatal Mort	HIV Incid	TB Incid	Malaria Incid	Hep B Incid	NTD Prev	NCD Mort	Suicide Mort	Alcohol Use	Road Inj Mort	FP Need Met, Mod	Adol Birth Rate	UHC Service Coverage Index	Air Poll Mort	WaSH Mort	Poisoning Mort	Smoking Prev	Vaccine Cov	Health Worker Dens	Int Partner Viol	Non-Int Partner Sex Viol	Water	Sanitation	Hygiene	HH Air Poll	Occ Burden	Mean PM2.5	Homicide	Conflict Mort	Physical Violence	Sexual Violence	Child Sex Abuse	Cert Death Reg
Armenia [68]	68	80	88	79	7	62	100	79	72	60	67	100	43	97	56	61	56	70	45	62	78	50	85	64	43	98	100	90	100	98	82	92	95	52	42	62	73	84	99	100	99
Nicaragua [69]	67	30	84	94	58	50	98	68	59	40	66	42	85	88	96	72	68	63	100	29	70	72	64	79	81	100	62	88	65	70	74	82	82	86	68	42	73	83	87	79	100
Taiwan (Province of China) [70]	67	43	91	96	10	73	100	91	88	80	69	100	2	95	87	36	54	59	100	100	92	70	78	89	62	86	86	83	39	96	98	97	99	85	55	87	90	91	0	80	92
Puerto Rico [71]	67	68	93	95	2	65	100	75	68	58	100	100	82	99	95	73	36	86	100	50	85	92	64	96	82	93	97	98	0	65	99	83	100	100	90	24	75	100	77	60	95
Costa Rica [72]	67	52	90	98	42	63	100	80	70	56	90	100	78	91	90	61	54	56	98	39	74	84	69	86	82	88	77	76	0	95	100	81	96	92	69	37	94	68	86	13	98
Syria [73]	66	94	63	24	22	72	96	60	71	95	75	100	49	88	58	99	97	60	73	58	66	46	78	70	69	53	73	60	87	89	94	88	100	72	31	74	66	37	87	68	69
Saudi Arabia [74]	66	87	90	93	0	67	100	94	91	73	71	73	41	98	86	100	96	30	58	87	76	43	72	64	88	100	99	80	83	92	99	91	100	100	12	99	86	39	89	69	75
Trinidad and Tobago [75]	66	79	95	88	37	47	99	60	48	40	81	100	90	95	55	42	64	63	71	46	59	54	62	91	60	53	76	86	36	64	88	78	100	100	56	16	96	98	79	60	92
Albania [76]	66	76	74	69	37	80	100	70	62	100	81	100	67	100	81	80	50	81	19	75	84	66	95	76	9	88	91	99	88	84	93	98	86	44	65	70	90	90	98	73	81
Cuba [77]	66	67	86	95	31	57	100	90	92	45	92	100	97	96	73	40	49	65	100	42	78	71	63	91	34	84	100	90	36	63	88	86	99	25	63	52	91	98	77	60	99
Jamaica [78]	66	56	91	94	61	43	99	63	47	37	76	100	98	96	51	92	54	72	96	43	65	53	62	84	64	86	91	89	21	56	67	60	95	85	75	8	94	98	77	8	95
Chile [79]	66	42	98	100	29	71	100	81	71	45	82	100	89	96	92	46	30	69	89	44	80	82	75	82	20	82	86	82	82	97	96	96	98	53	59	57	88	86	93	78	97
Libya [80]	65	85	72	70	35	53	99	69	76	76	59	100	43	80	46	59	100	37	66	83	72	37	65	45	74	88	90	61	86	85	92	80	100	86	27	72	82	37	89	68	74
Peru [81]	65	30	93	100	25	47	98	67	65	44	51	32	71	92	100	97	50	60	78	30	74	76	54	64	95	84	87	80	82	84	67	83	89	68	55	63	64	71	91	44	83
China [82]	65	46	89	96	36	85	100	78	82	70	63	100	23	89	76	61	56	56	100	88	88	52	89	39	44	100	99	76	47	84	87	93	94	67	23	93	80	91	0	74	82
Saint Vincent and the Grenadines [83]	65	54	87	91	59	41	99	64	53	42	73	100	89	95	47	59	53	69	95	35	57	54	52	86	78	82	88	83	41	49	79	71	96	99	58	18	100	98	73	60	91
Thailand [84]	65	52	81	58	32	57	100	86	83	40	52	65	26	84	87	44	53	54	100	56	78	72	48	88	57	84	80	81	77	43	100	79	96	54	51	50	60	74	78	92	81
Qatar [85]	65	100	86	88	2	84	100	84	82	91	66	100	38	98	93	79	82	34	84	86	88	52	78	90	81	99	99	70	85	91	97	90	100	78	5	97	96	43	88	72	55
Andorra [86]	65	100	98	99	15	96	100	100	100	66	98	100	98	100	98	62	28	96	99	98	100	96	96	95	40	87	99	96	55	100	100	100	100	59	87	99	100	88	94	61	0
Ecuador [87]	65	39	75	96	46	44	100	62	60	46	65	64	62	90	89	56	53	31	86	34	62	75	63	62	77	85	77	80	77	86	88	81	98	63	72	29	89	62	71	46	78
Latvia [88]	65	95	90	94	39	75	100	96	94	42	67	100	78	100	60	28	10	80	84	63	86	74	96	54	13	100	95	91	56	94	89	97	98	74	78	54	95	80	86	18	100
Kyrgyzstan [89]	65	59	83	94	70	54	100	59	44	50	43	100	35	94	48	48	55	52	71	51	65	52	74	36	52	75	88	70	100	87	23	92	87	67	59	55	86	69	91	100	100
Kazakhstan [90]	64	90	91	93	22	70	100	69	64	50	47	100	44	96	43	10	30	53	93	56	75	63	84	25	43	73	99	96	94	91	68	95	95	62	74	40	88	88	98	96	87
El Salvador [91]	64	35	89	96	62	54	100	72	73	43	70	90	72	92	80	46	58	39	96	37	65	55	51	88	93	98	76	86	54	78	66	83	92	100	55	0	73	78	90	68	80
Georgia [92]	64	77	85	94	11	61	99	76	67	51	52	100	49	97	43	61	39	54	58	43	70	42	80	56	35	94	94	98	100	92	67	95	84	58	57	60	73	95	100	100	62
Bulgaria [93]	64	85	89	90	20	68	100	84	80	73	84	100	62	100	53	54	23	84	63	44	79	58	95	74	10	84	96	88	55	94	99	100	93	59	65	79	95	90	88	70	81
Iraq [94]	64	96	74	73	29	72	96	53	46	91	66	100	60	93	85	91	94	68	67	36	72	73	78	90	55	78	66	24	88	80	88	95	100	76	19	50	32	37	88	68	76
Azerbaijan [95]	64	89	84	78	2	62	99	46	32	69	44	100	32	97	38	89	51	81	32	44	66	43	71	54	40	76	99	90	100	91	71	96	91	56	62	61	77	82	93	100	92
Moldova [96]	63	89	92	96	78	70	100	66	50	45	52	100	34	100	49	31	25	62	65	66	76	65	79	1	51	76	86	83	79	81	51	91	96	87	70	51	96	78	92	18	91
Dominica [97]	63	53	90	93	54	30	99	42	26	51	70	100	87	96	57	83	47	44	96	42	54	60	52	36	86	76	90	88	36	54	87	77	97	95	62	30	93	98	68	60	92
American Samoa [98]	63	63	84	80	26	43	99	78	76	79	76	100	29	98	40	65	83	67	84	42	54	48	56	59	30	83	81	72	67	80	93	95	92	69	76	50	89	86	37	8	79
Cape Verde [99]	63	78	90	91	60	52	96	63	52	32	42	42	24	95	80	29	66	78	80	56	66	52	43	63	97	69	78	67	82	59	72	28	87	70	40	31	100	33	64	32	88
Romania [100]	63	74	87	87	24	76	100	85	80	60	66	100	61	100	64	61	4	84	64	52	83	69	84	50	34	79	90	90	90	93	92	98	95	51	74	85	95	90	89	70	91

Value: 100, 75, 50, 25, 0

Supplementary figure 3. Projected performance, based on past trends, on the health-related SDG index and 40 individual health-related indicators, by country or territory, 2030

Country	SDG Index	Disaster Mort	Child Stunting	Child Wasting	Child Overweight	Mat Mort Ratio	Skilled Birth Attend	Under-5 Mort	Neonatal Mort	HIV Incid	TB Incid	Malaria Incid	Hep B Incid	NTD Prev	NCD Mort	Suicide Mort	Alcohol Use	Road Inj Mort	FP Need Met, Mod	Adol Birth Rate	UHC Service Coverage Index	Air Poll Mort	WaSH Mort	Poisoning Mort	Smoking Prev	Vaccine Cov	Health Worker Dens	Int Partner Viol	Non-Int Partner Sex Viol	Water	Sanitation	Hygiene	HH Air Poll	Occ Burden	Mean PM2.5	Homicide	Conflict Mort	Physical Violence	Sexual Violence	Child Sex Abuse	Cert Death Reg
Macedonia [101]	63	70	94	91	29	88	100	76	64	91	74	100	70	100	59	65	75	82	15	71	76	58	99	82	4	77	91	94	78	94	94	100	92	71	48	80	88	90	100	52	87
Iran [102]	63	14	90	80	31	65	99	69	60	67	79	96	63	97	77	77	100	29	98	59	75	54	70	44	74	88	83	61	86	86	94	89	100	88	30	73	57	37	88	67	92
Guam [103]	63	72	88	84	0	56	98	72	66	61	65	100	23	94	45	20	91	54	94	42	64	55	73	85	37	86	91	77	63	84	97	96	95	81	78	56	82	86	37	2	83
Bosnia and Herzegovina [104]	62	90	92	97	23	88	100	86	72	100	71	100	70	100	62	63	34	88	24	88	74	49	97	64	4	70	83	93	78	93	97	99	85	74	48	74	88	90	92	70	84
Morocco [105]	62	56	83	96	36	36	93	63	51	73	34	100	50	95	57	65	95	41	85	78	54	44	62	50	86	98	77	59	88	76	94	83	99	54	39	89	69	37	88	54	31
Venezuela [106]	62	55	82	72	46	44	98	62	49	42	71	13	77	68	71	54	50	41	92	24	67	66	59	76	70	76	66	84	61	88	93	88	100	68	69	1	70	72	79	66	96
Bolivia [107]	62	50	86	98	25	31	91	52	45	48	50	47	68	89	70	69	67	38	66	34	59	57	53	56	89	96	95	58	68	85	92	83	92	56	59	50	90	1	69	47	88
Tonga [108]	62	62	87	74	39	36	100	62	58	70	55	100	12	93	42	71	88	54	46	70	54	43	56	37	29	82	65	69	70	74	90	94	84	66	82	72	99	86	54	12	72
Russia [109]	62	73	86	88	37	72	100	86	82	24	57	100	64	95	48	11	17	67	68	61	84	68	91	17	33	87	99	85	71	92	82	97	100	60	69	34	70	80	81	13	91
Guatemala [110]	61	45	26	100	66	37	99	54	54	49	72	48	60	89	80	63	68	55	86	30	58	48	36	41	90	86	73	86	81	59	72	76	80	76	55	4	53	86	92	73	88
Serbia [111]	61	90	91	88	52	85	100	92	85	74	73	100	70	100	52	36	8	78	36	71	84	48	88	77	15	84	77	93	78	96	97	100	85	55	53	73	85	90	90	58	74
Tajikistan [112]	61	56	58	44	64	63	95	43	33	59	47	100	40	90	44	73	85	70	59	43	49	34	38	64	90	100	89	74	100	76	34	80	79	62	33	64	76	71	94	100	61
Suriname [113]	61	91	91	84	76	34	98	47	32	39	72	70	77	95	52	10	38	62	72	36	54	53	46	68	50	72	85	86	40	57	89	72	94	99	54	38	95	98	66	59	85
Fiji [114]	60	33	90	70	38	46	99	46	39	60	60	100	42	90	15	45	74	66	85	56	54	32	38	66	41	92	75	67	70	78	82	92	88	83	81	55	98	86	39	5	83
Dominican Republic [115]	60	48	89	92	50	40	99	51	34	40	54	58	74	94	62	52	53	39	98	24	54	61	50	77	89	82	83	74	82	31	88	40	93	62	74	18	88	82	76	61	82
Belize [116]	59	48	84	91	68	39	98	61	50	32	65	89	78	94	62	60	38	42	85	35	58	60	50	61	86	88	87	83	82	21	81	73	91	68	56	12	92	98	64	84	98
Lithuania [117]	59	92	91	94	37	85	100	94	95	61	69	100	74	100	61	4	0	90	85	70	82	73	87	21	22	85	98	96	85	94	89	98	99	75	82	54	97	80	73	18	100
Sao Tome and Principe [118]	58	100	85	83	87	27	92	54	45	83	48	23	2	81	60	94	61	44	70	43	50	27	36	54	100	100	69	53	84	51	70	39	76	67	46	49	100	17	65	32	63
Guyana [119]	58	92	84	80	59	32	95	57	42	30	55	16	78	87	36	10	59	48	64	34	54	47	43	66	69	99	82	83	42	33	86	51	94	89	57	22	92	98	79	58	85
Honduras [120]	58	30	80	98	69	34	98	67	60	60	65	48	68	82	73	84	72	55	93	29	58	54	39	48	79	97	47	83	69	78	78	87	74	65	62	1	76	76	84	63	45
Egypt [121]	57	84	80	76	16	50	99	68	68	88	74	100	3	94	40	77	97	24	98	40	66	24	56	91	56	85	81	74	88	93	82	90	100	74	9	99	67	48	95	68	60
Lebanon [122]	57	78	86	87	36	66	95	79	76	75	74	100	45	98	65	79	81	94	75	52	84	53	71	64	18	82	93	67	85	76	99	90	100	92	40	59	51	37	85	68	0
Montenegro [123]	57	85	86	92	1	90	99	98	93	81	86	100	76	100	61	55	14	76	45	79	83	55	100	65	2	71	89	93	77	88	98	100	85	78	61	67	97	90	93	70	91
Philippines [124]	56	23	51	69	75	42	80	53	47	48	24	61	36	82	46	67	71	60	69	41	51	24	39	86	54	87	40	90	75	50	94	84	74	54	65	23	47	88	75	83	85
Greenland [125]	56	87	96	98	40	74	94	66	62	44	58	100	97	100	54	0	50	96	86	44	77	78	78	43	0	84	93	94	52	100	96	100	100	50	81	43	100	100	87	57	70
Mongolia [126]	55	51	88	99	41	55	99	56	44	74	36	100	16	93	27	26	49	54	75	59	66	45	71	13	29	83	99	85	100	66	36	78	82	71	33	40	91	84	94	100	82
Bhutan [127]	55	73	63	84	60	30	90	56	42	57	42	86	56	93	75	75	92	76	96	60	65	50	38	89	97	72	84	76	96	80	77	84	91	43	36	93	97	64	58	33	0
Belarus [128]	54	94	96	95	34	72	100	92	91	42	69	100	78	100	56	18	0	87	81	76	88	59	100	29	2	87	100	89	72	95	86	98	100	81	64	52	93	80	77	18	94
South Africa [129]	53	72	70	91	17	33	100	48	44	4	7	65	47	87	59	37	36	23	91	39	59	55	29	50	59	88	65	78	60	72	75	45	96	65	49	10	70	62	80	27	82
Ukraine [130]	52	94	80	83	60	54	100	75	72	31	52	100	60	99	32	7	0	53	76	64	76	51	84	21	33	50	98	69	73	86	73	94	98	78	63	40	94	83	82	14	90
Vietnam [131]	50	43	80	82	60	78	99	70	65	43	37	58	33	78	62	59	28	42	85	66	76	50	58	54	58	81	72	84	79	63	86	69	90	50	48	82	70	75	73	81	0
Botswana [132]	47	88	59	76	35	31	99	60	52	9	10	46	37	92	63	45	52	52	96	45	65	40	26	48	66	88	76	68	77	67	72	42	88	70	55	42	97	51	74	0	0
Samoa [133]	47	44	83	87	48	61	81	66	64	62	58	100	30	85	48	47	77	63	46	45	49	36	57	58	26	55	46	64	73	86	97	96	73	82	79	61	100	86	56	5	0

Value
100
75
50
25
0

Supplementary figure 3. Projected performance, based on past trends, on the health-related SDG index and 40 individual health-related indicators, by country or territory, 2030

Country [rank]	SDG Index	Disaster Mort	Child Stunting	Child Wasting	Child Overweight	Mat Mort Ratio	Skilled Birth Attend	Under-5 Mort	Neonatal Mort	HIV Incid	TB Incid	Malaria Incid	Hep B Incid	NTD Prev	NCD Mort	Suicide Mort	Alcohol Use	Road Inj Mort	FP Need Met, Mod	Adol Birth Rate	UHC Service Coverage Index	Air Poll Mort	WaSH Mort	Poisoning Mort	Smoking Prev	Vaccine Cov	Health Worker Dens	Int Partner Viol	Non-Int Partner Sex Viol	Water	Sanitation	Hygiene	HH Air Poll	Occ Burden	Mean PM2.5	Homicide	Conflict Mort	Physical Violence	Sexual Violence	Child Sex Abuse	Cert Death Reg
Cambodia [134]	46	64	50	69	95	32	99	52	42	65	30	37	18	85	62	82	45	31	74	57	62	31	36	65	57	84	78	85	70	59	75	76	54	37	52	70	52	79	77	60	0
Indonesia [135]	46	45	52	42	48	32	95	57	46	58	27	28	41	78	52	95	97	74	90	60	60	46	25	77	16	55	75	80	22	61	90	78	88	40	65	89	59	75	89	78	0
Ghana [136]	46	85	82	76	89	34	85	39	27	30	19	7	19	87	54	48	43	31	57	45	49	27	21	45	100	98	78	69	63	13	47	8	68	56	43	64	87	36	61	31	36
North Korea [137]	45	57	74	81	92	42	91	56	52	75	36	52	12	86	35	40	78	42	88	100	58	24	63	28	56	86	49	55	61	90	68	94	57	26	43	66	94	91	0	81	0
Marshall Islands [138]	45	100	78	75	66	25	96	57	49	62	35	100	19	87	5	13	84	30	94	32	34	38	49	44	58	67	73	63	72	65	81	90	89	70	84	47	100	86	41	3	0
Myanmar [139]	45	58	63	76	66	33	80	45	36	42	29	34	33	79	55	78	85	40	95	66	47	32	34	34	54	69	75	83	96	38	71	66	71	41	38	80	23	82	92	79	0
Laos [140]	44	64	41	72	83	30	80	38	30	63	39	32	37	83	50	55	68	43	80	40	57	32	28	65	50	73	61	80	81	43	47	69	50	44	51	63	62	75	80	79	0
Namibia [141]	44	74	70	62	67	38	94	44	37	12	7	24	55	80	67	39	32	38	99	39	59	48	23	47	69	98	63	51	82	55	59	58	78	65	53	28	58	49	65	32	0
Federated States of Micronesia [142]	44	69	78	75	65	24	99	62	56	43	13	100	21	87	16	24	89	40	74	54	40	36	51	47	24	68	62	61	72	66	82	90	86	69	80	48	100	86	49	3	0
Timor-Leste [143]	43	94	30	37	71	21	82	49	40	56	33	63	43	70	54	61	100	65	56	43	51	31	34	67	12	68	54	56	81	54	43	82	55	58	63	66	53	37	89	67	0
Sudan [144]	42	76	47	55	73	30	89	41	32	44	51	13	25	92	55	82	87	23	35	39	53	37	36	48	99	93	67	51	89	40	26	34	73	65	28	88	43	37	82	68	0
Gabon [145]	41	99	79	91	54	22	96	46	35	38	12	5	39	81	53	36	58	24	60	32	47	43	26	49	86	64	67	30	36	68	68	33	96	78	33	53	96	26	33	36	0
Senegal [146]	41	84	77	65	88	18	52	43	34	40	32	18	18	59	57	39	98	42	67	34	44	24	14	46	93	95	49	61	87	50	34	23	40	54	34	87	77	33	84	32	0
India [147]	40	48	53	26	64	31	95	48	32	57	30	26	73	92	53	27	70	50	84	71	55	24	17	82	88	87	67	50	98	48	81	66	86	56	2	62	70	49	53	52	1
Comoros [148]	40	62	52	64	60	23	90	42	30	83	32	35	46	90	51	56	97	34	35	53	36	34	18	38	90	77	63	72	74	39	30	18	48	30	58	40	97	62	89	44	0
Yemen [149]	39	60	40	6	84	18	57	44	36	73	60	15	38	85	35	74	100	19	60	38	60	36	36	40	59	66	56	42	90	44	73	57	87	63	30	64	56	37	89	68	0
Pakistan [150]	39	34	33	32	98	16	80	39	21	66	35	16	57	89	37	84	100	29	63	58	49	25	23	74	82	90	66	48	97	20	79	63	79	52	20	56	64	46	60	34	0
Mauritania [151]	39	84	56	0	79	11	87	43	30	80	37	14	20	82	69	68	99	30	30	35	53	37	20	55	69	90	54	67	85	33	33	33	72	66	36	56	81	33	86	31	0
Nigeria [152]	38	86	62	51	61	25	27	26	21	25	22	6	16	67	85	63	73	56	50	24	25	38	17	56	100	32	58	84	81	14	66	8	69	52	28	59	73	49	70	20	14
Kiribati [153]	38	90	65	57	72	18	87	40	35	87	26	100	22	71	2	6	85	60	48	55	33	15	18	33	0	66	52	57	77	45	56	84	45	69	82	78	99	86	8	7	44
Cote d'Ivoire [154]	38	91	63	75	70	18	67	29	21	22	28	10	19	73	55	40	79	35	45	23	43	25	16	46	79	86	57	59	86	29	42	16	47	44	51	29	80	28	82	38	0
Nepal [155]	37	28	65	72	93	22	92	56	39	64	39	59	74	72	62	57	88	32	75	44	57	21	21	89	63	83	67	74	97	31	73	68	78	16	6	80	76	67	36	34	0
The Gambia [156]	37	81	72	37	80	8	70	40	31	34	26	27	9	56	44	52	90	35	35	34	34	17	20	44	89	93	50	86	93	31	36	9	35	56	40	89	92	54	91	31	0
Djibouti [157]	37	68	48	23	26	11	97	44	40	32	25	14	41	94	50	56	90	36	62	50	39	40	24	46	72	74	64	50	31	31	39	12	87	60	33	40	80	27	62	44	0
Solomon Islands [158]	37	27	61	78	79	32	96	53	45	62	47	6	24	79	18	27	85	36	65	37	48	9	35	46	35	75	35	52	77	58	39	88	40	73	78	50	93	86	45	2	0
Kenya [159]	36	71	51	80	73	15	84	42	34	12	19	14	46	75	63	41	56	40	91	34	42	34	14	33	91	80	68	34	57	32	36	9	53	37	42	30	67	43	35	3	0
Swaziland [160]	36	86	64	97	48	27	95	40	37	20	5	56	26	91	26	12	78	16	96	29	44	36	17	32	100	94	45	60	0	40	47	45	86	63	64	13	95	51	76	66	0
Haiti [161]	36	19	70	88	90	12	48	37	30	31	52	25	84	79	29	55	59	10	55	43	39	25	28	52	98	61	62	62	0	15	29	22	43	77	72	23	82	85	35	60	74
Zambia [162]	36	91	44	68	5	35	88	35	30	14	11	6	32	78	56	42	74	41	79	21	44	34	15	43	65	100	77	44	57	32	34	13	48	37	52	46	86	25	17	50	0
Equatorial Guinea [163]	36	100	85	95	0	32	99	40	33	21	14	3	36	88	75	51	40	35	45	28	48	46	30	60	94	25	74	61	43	48	83	27	80	91	27	61	93	17	0	39	0
Togo [164]	36	91	69	36	84	20	67	34	29	36	31	0	15	73	55	41	81	32	52	49	42	25	15	49	98	92	59	80	80	11	34	8	43	43	42	46	89	52	74	31	0
Bangladesh [165]	36	13	43	34	97	32	74	53	35	90	37	70	57	54	64	73	100	77	92	39	65	30	31	87	49	83	62	48	97	12	50	29	65	35	16	81	83	45	61	2	0
Vanuatu [166]	36	32	56	75	64	36	85	50	42	61	54	32	16	72	11	24	94	38	57	42	25	19	31	45	57	44	56	61	76	36	40	71	54	29	78	76	100	86	51	10	0

Value: 100, 75, 50, 25, 0

Supplementary figure 3. Projected performance, based on past trends, on the health-related SDG index and 40 individual health-related indicators, by country or territory, 2030

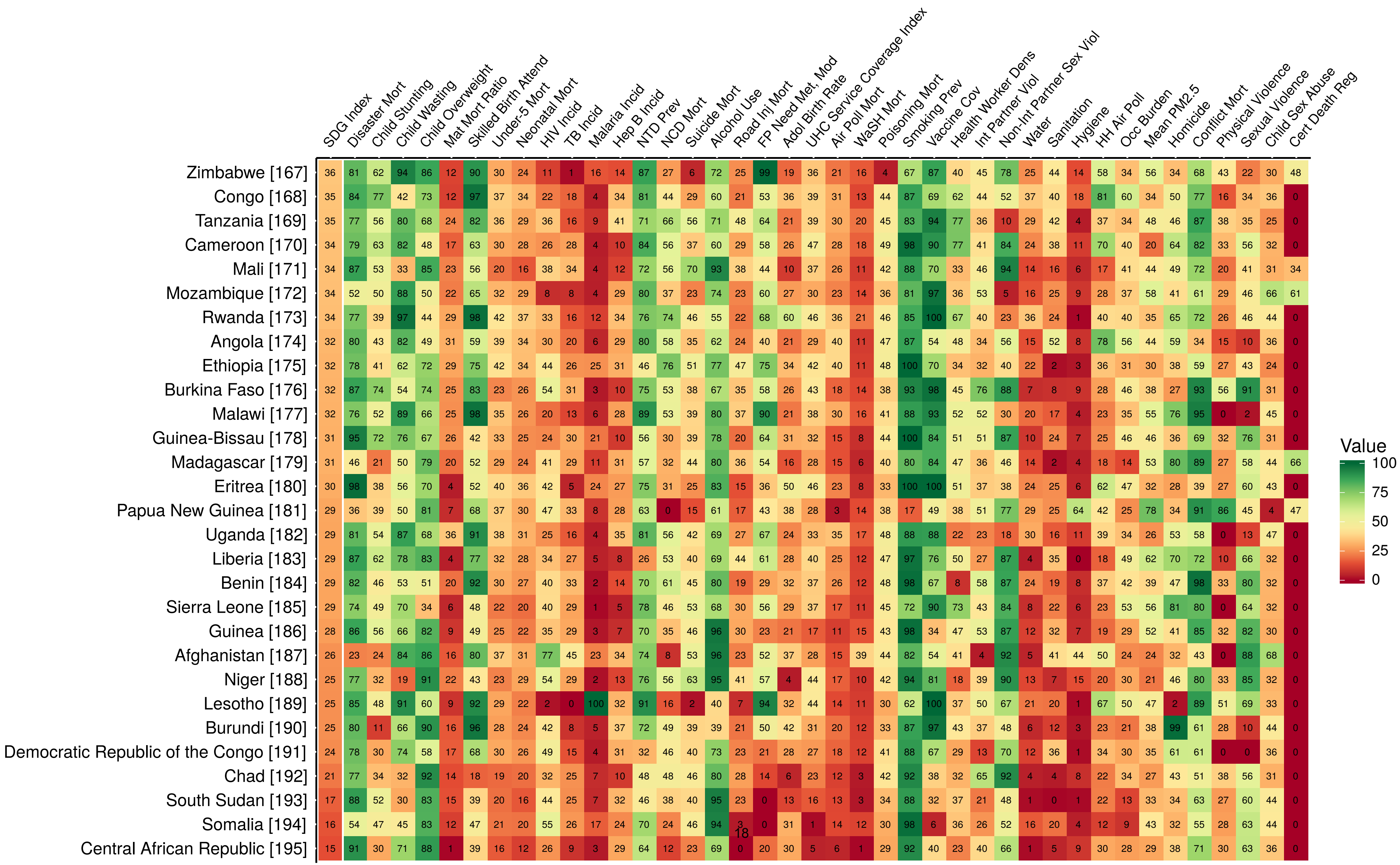

Supplementary Figure 4. Comparing attainment of defined health-related SDG indicator targets in 2017 and, based on past trends, projected to be attained in 2030, by country.

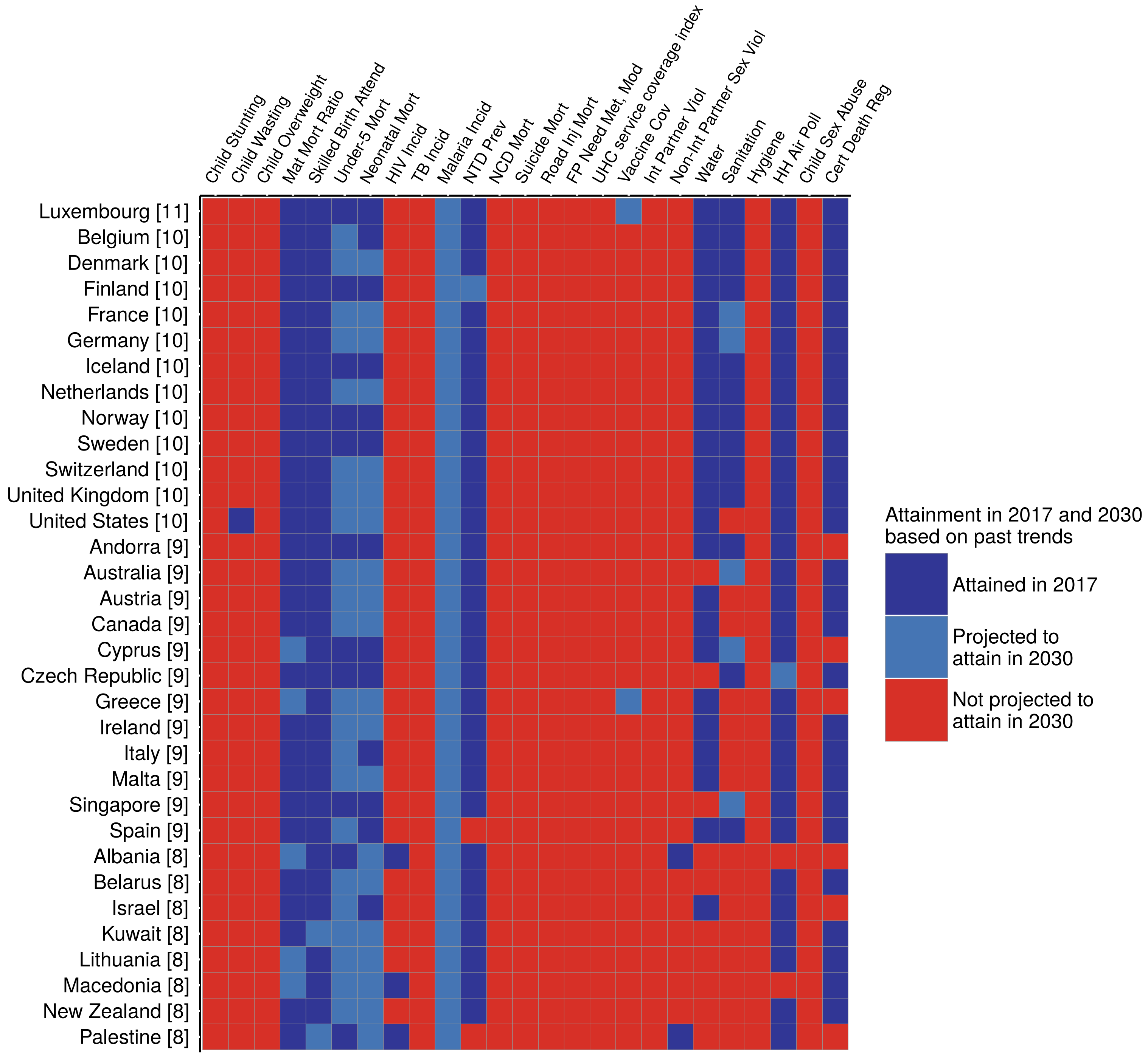

Supplementary Figure 4. Comparing attainment of defined health-related SDG indicator targets in 2017 and, based on past trends, projected to be attained in 2030, by country.

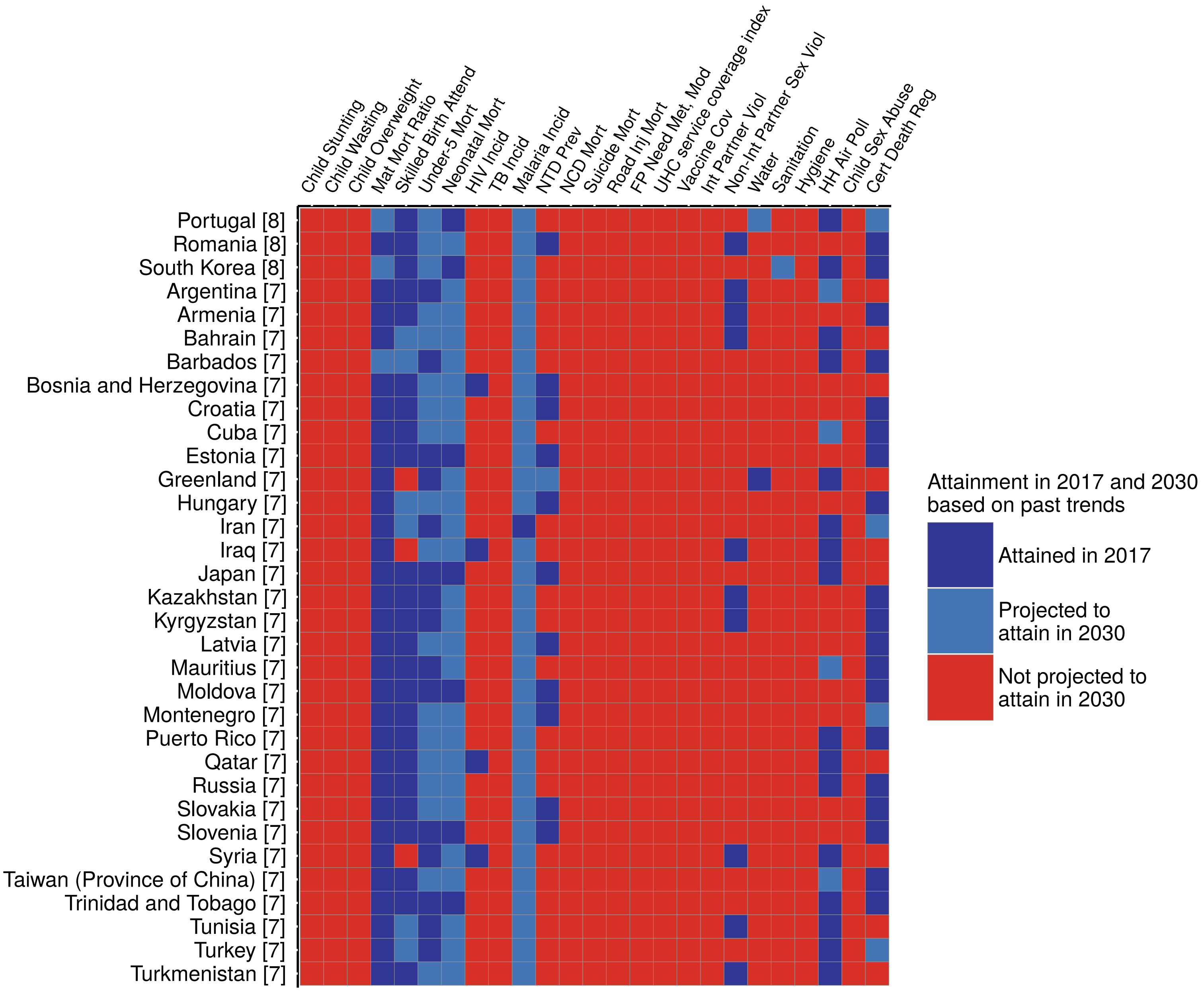

Supplementary Figure 4. Comparing attainment of defined health-related SDG indicator targets in 2017 and, based on past trends, projected to be attained in 2030, by country.

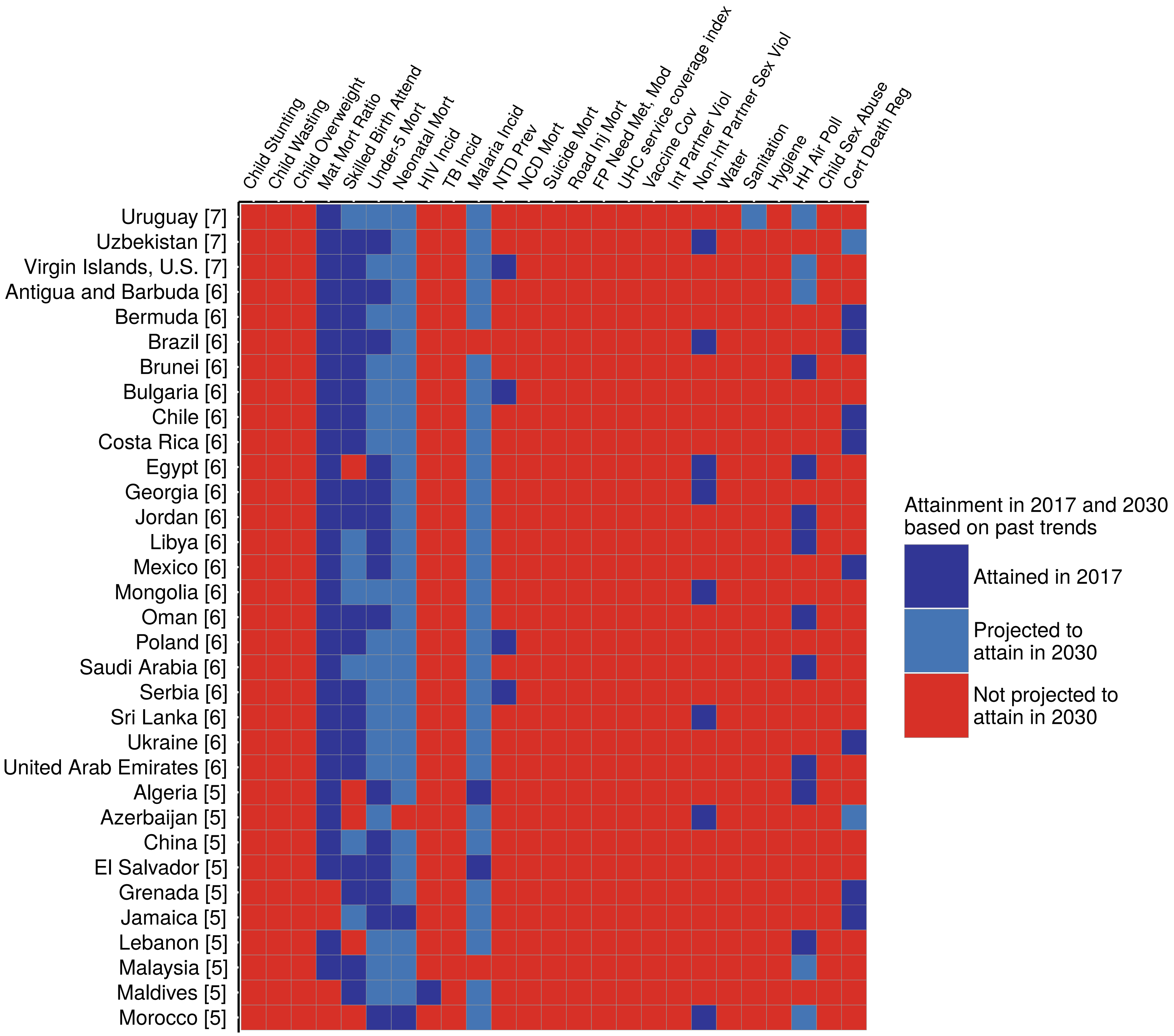

Supplementary Figure 4. Comparing attainment of defined health-related SDG indicator targets in 2017 and, based on past trends, projected to be attained in 2030, by country.

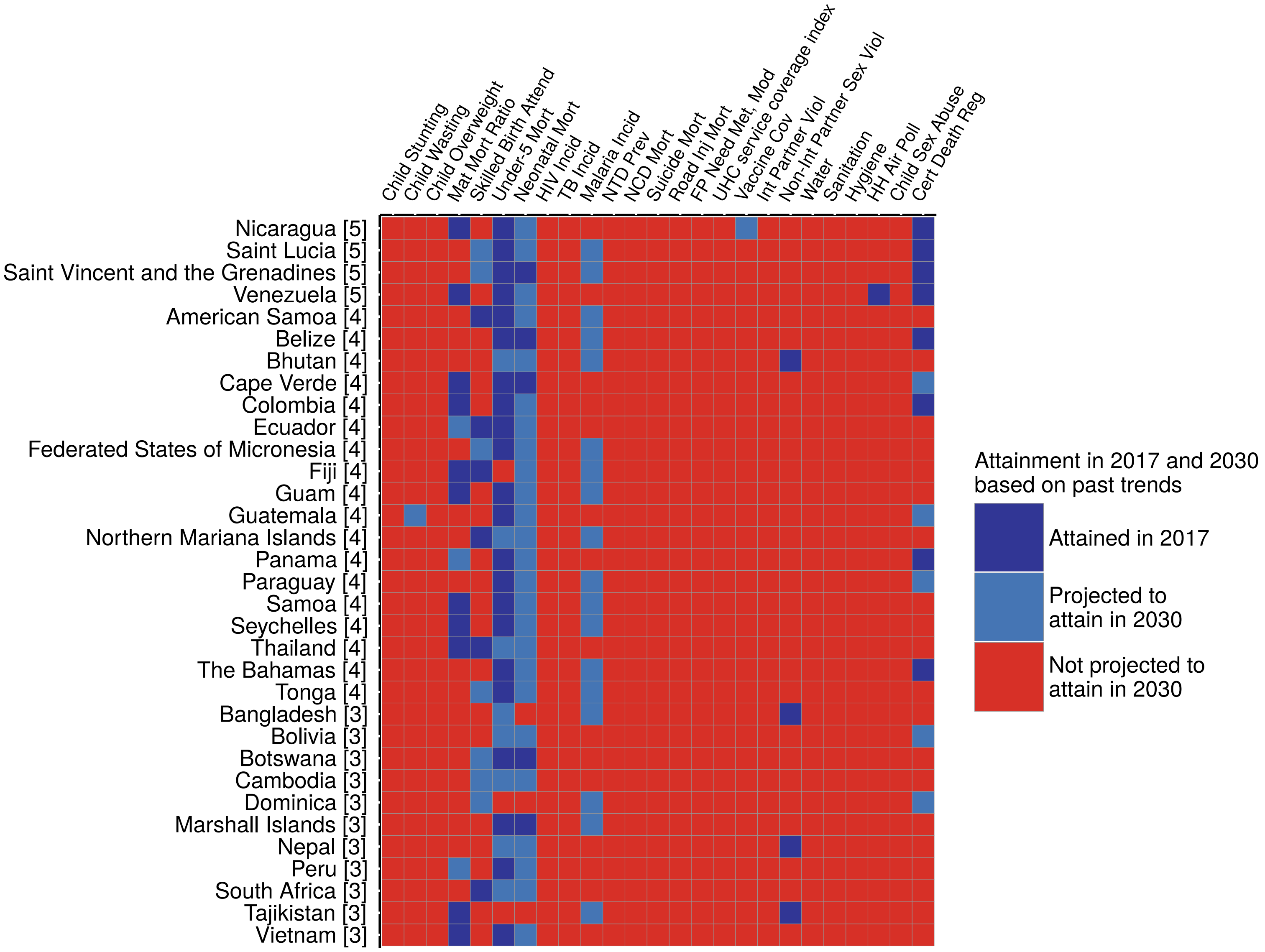

Supplementary Figure 4. Comparing attainment of defined health-related SDG indicator targets in 2017 and, based on past trends, projected to be attained in 2030, by country.

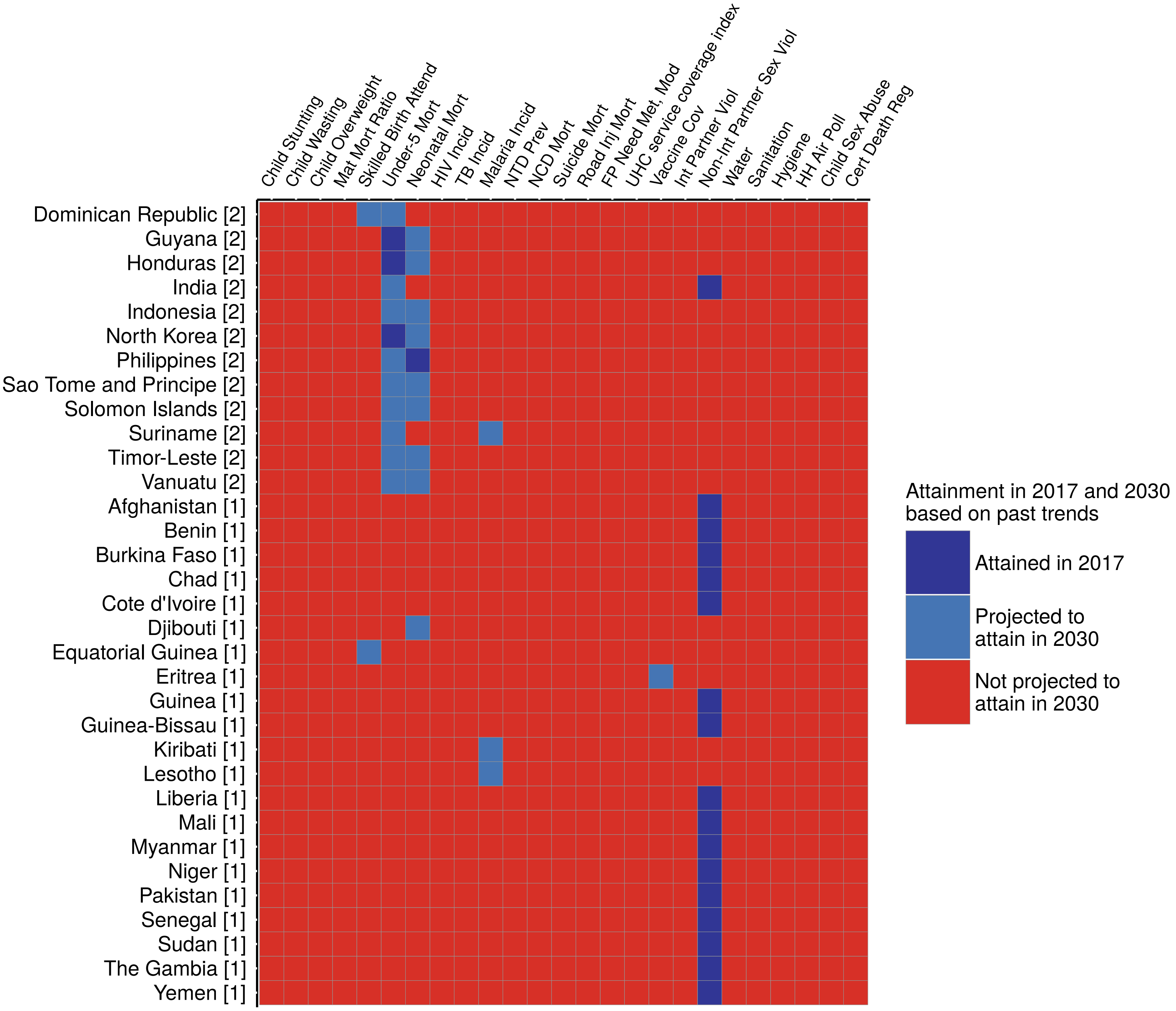

Supplementary Figure 4. Comparing attainment of defined health-related SDG indicator targets in 2017 and, based on past trends, projected to be attained in 2030, by country.

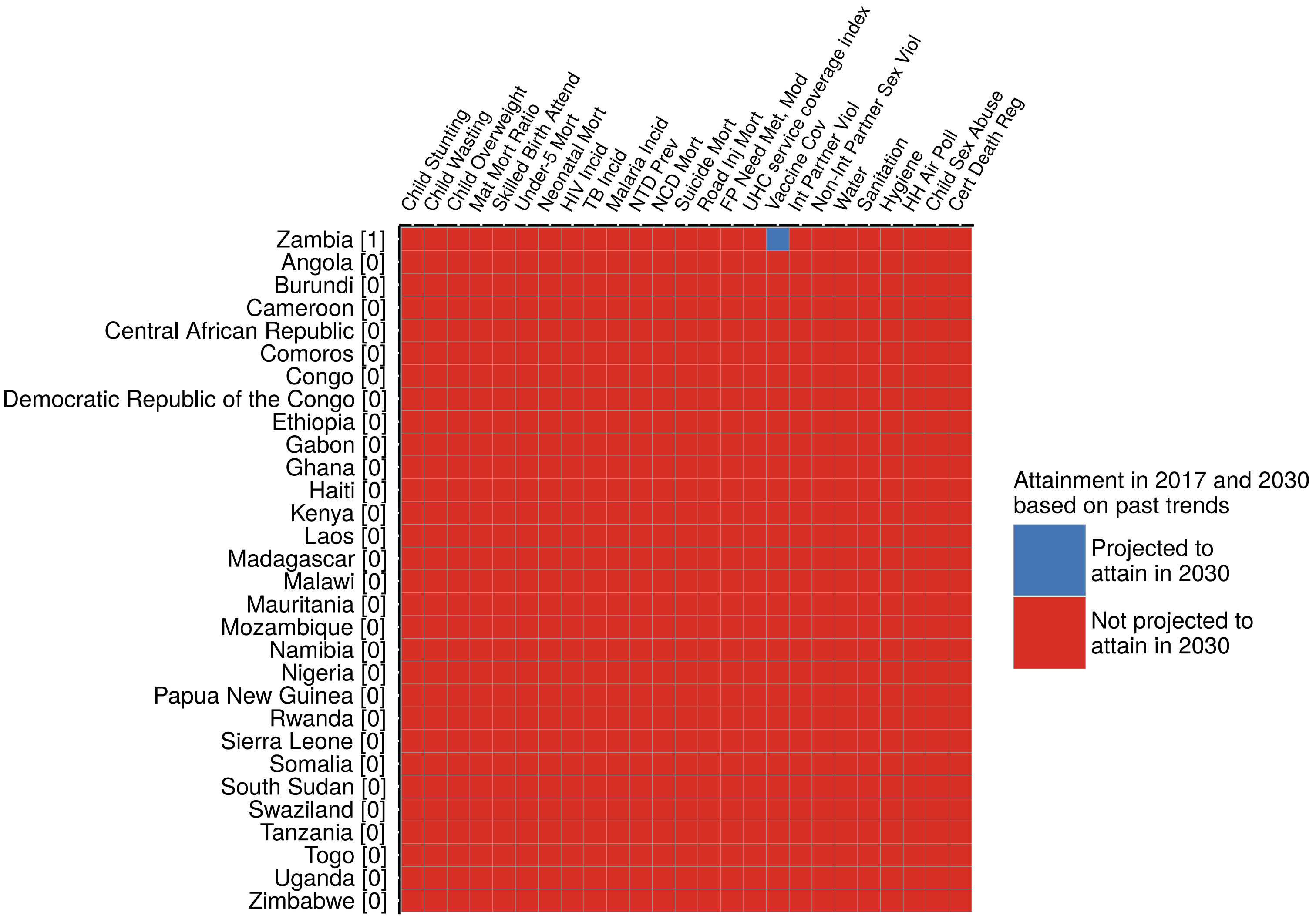

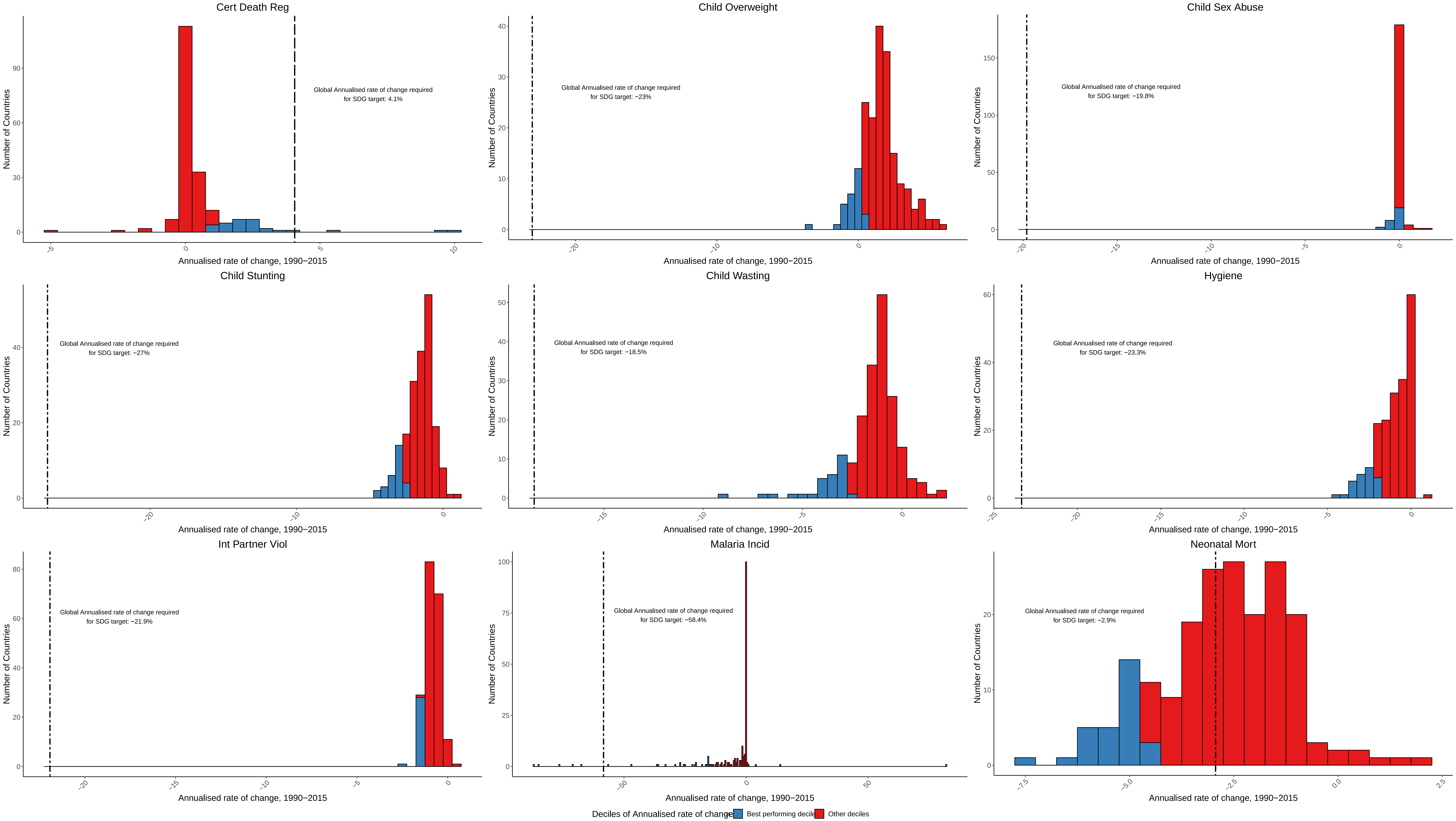

Supplementary Figure 5. Global Annualised rate of change required to meet selected SDG targets based on Annualised rate of change achieved by countries from 1990−2015.

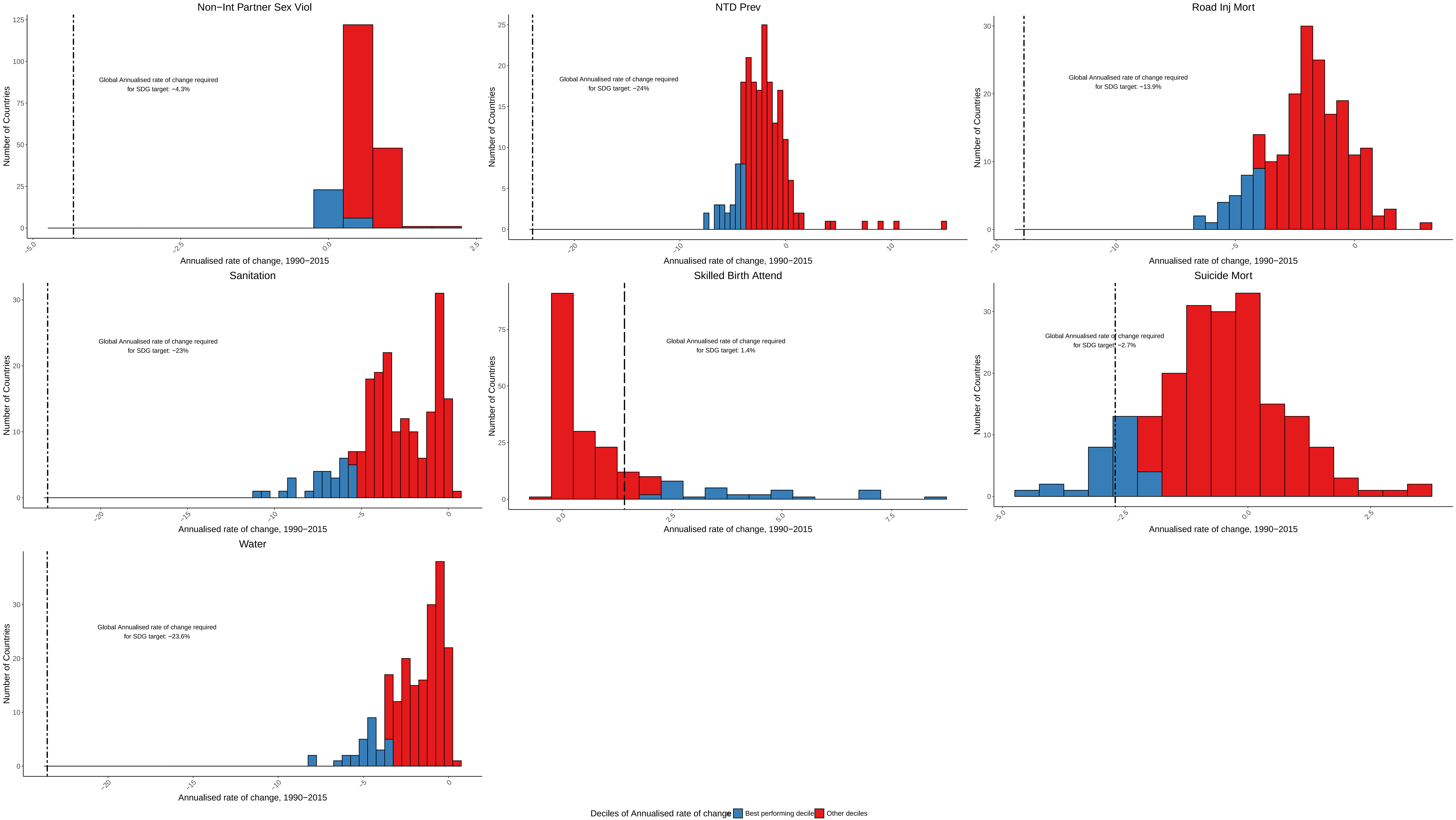

Supplementary Figure 5. Global Annualised rate of change required to meet selected SDG targets based on Annualised rate of change achieved by countries from 1990−2015.

Supplementary figure 6. Comparing 2016 values on the health-related SDG index from GBD 2016 to 2017 values on the health-related SDG index from GBD 2017

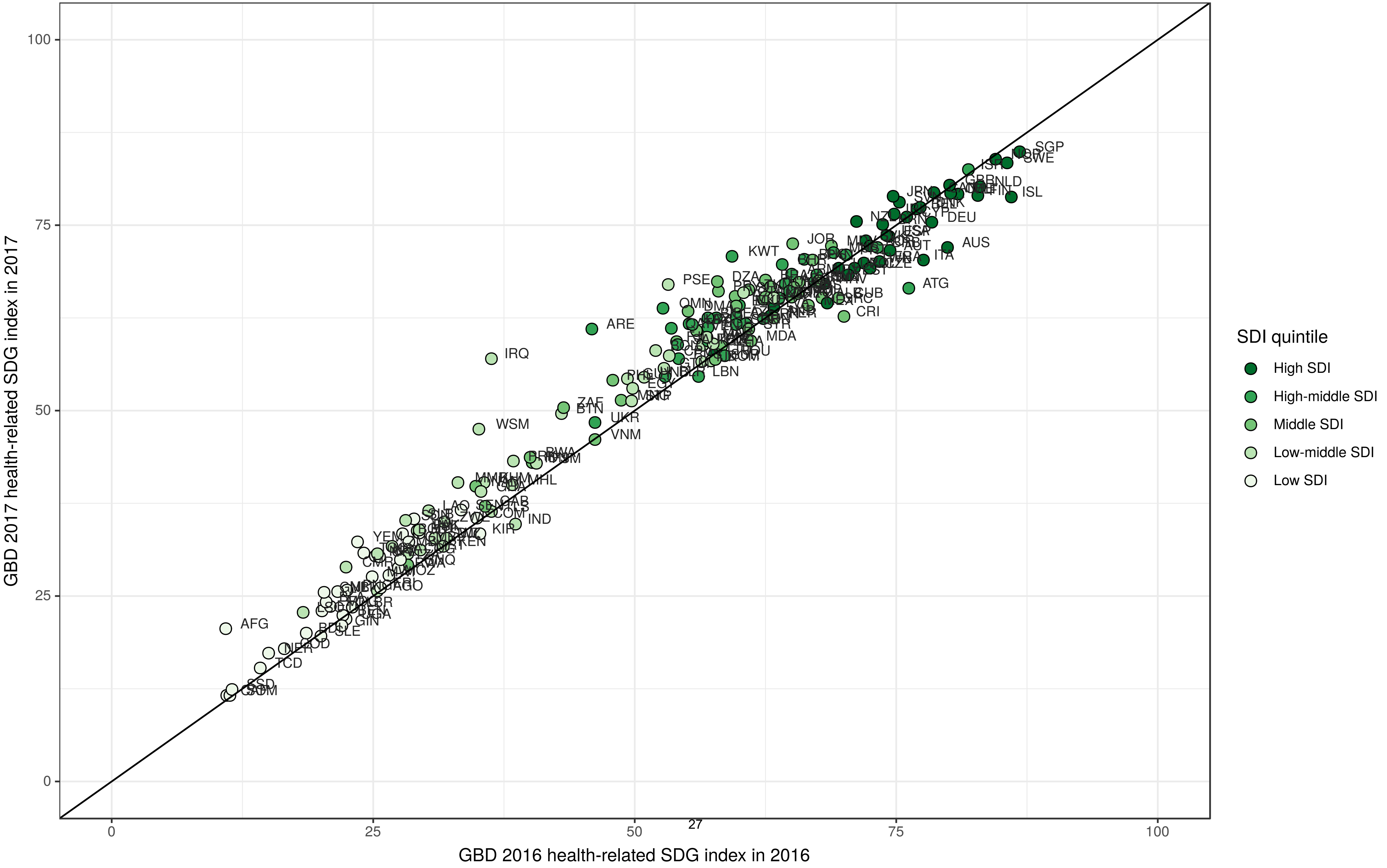

Supplementary figure 7. Comparing 2030 values on the health-related SDG index from GBD 2016 to 2017 values on the health-related SDG index from GBD 2017

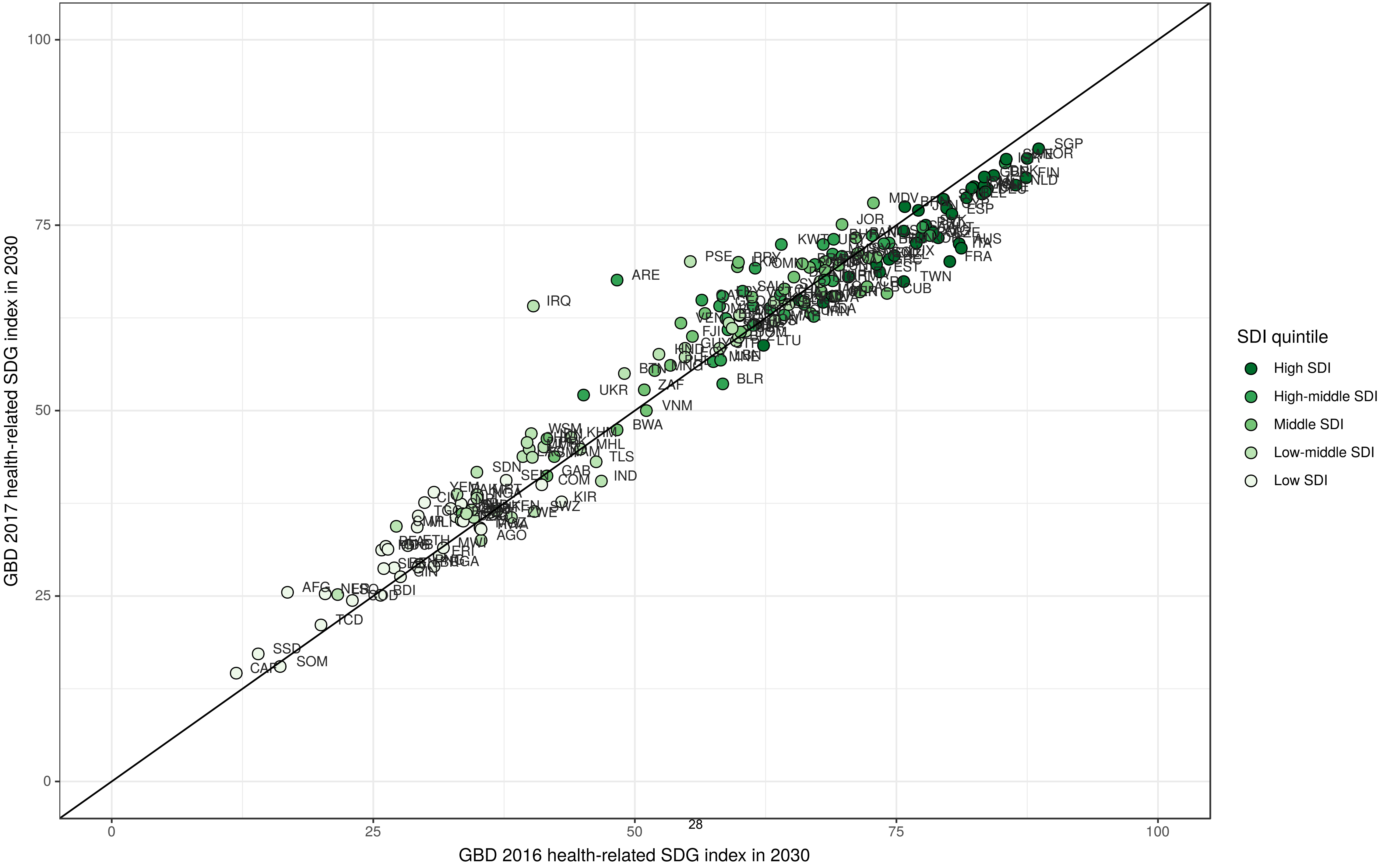

Supplementary figure 8. Comparing 2016 rankings on the health-related SDG index from GBD 2016 to 2017 rankings on the health-related SDG index from GBD 2017.

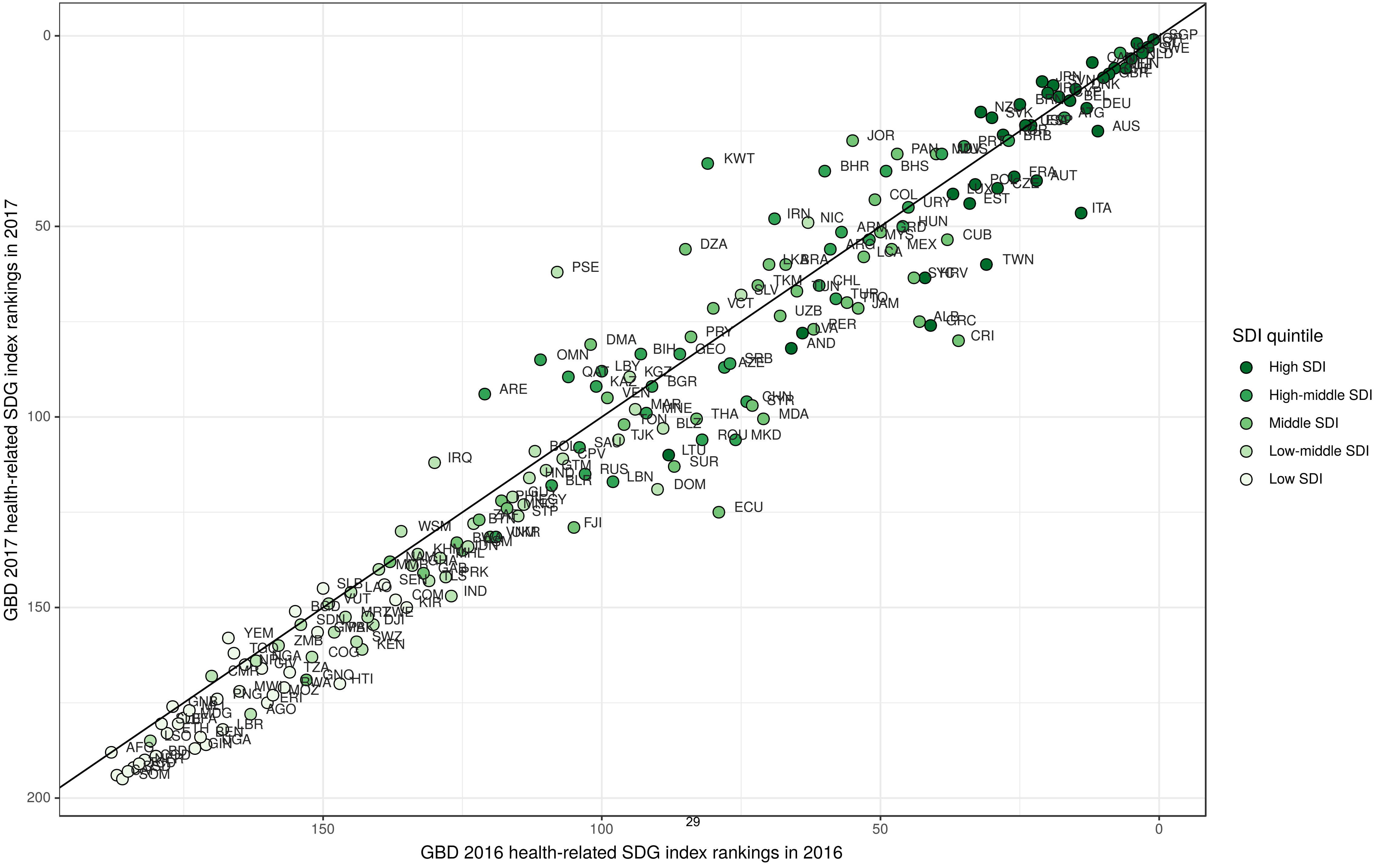

Supplementary Table 1. Health-related SDG index in 2000, 2015, 2017, and 2030, by country or territory.				
	SDG Index in 2000	SDG Index in 2015	SDG Index in 2017	SDG Index in 2030
Central Europe, Eastern Europe, and Central Asia				
Central Asia				
Armenia	57.5 (55.2 to 59.2)	66.8 (63.8 to 68.8)	68.4 (65.4 to 70.6)	67.5 (54.8 to 73.4)
Azerbaijan	43.3 (41.1 to 45.2)	61.1 (58.3 to 63.4)	62.7 (59.5 to 65.2)	63.8 (59.4 to 67.9)
Georgia	57.8 (56.2 to 59.4)	59.1 (56.8 to 60.9)	62.5 (59.8 to 64.6)	64.1 (59.7 to 68.5)
Kazakhstan	47.3 (44.9 to 49.2)	59.5 (56.1 to 62.1)	61.7 (57.9 to 64.6)	64.3 (56.9 to 70.2)
Kyrgyzstan	47.4 (43.6 to 49.5)	56.8 (53.6 to 58.9)	58.8 (56.0 to 60.9)	64.6 (54.9 to 69.0)
Mongolia	37.7 (34.6 to 40.0)	52.7 (48.6 to 56.4)	51.4 (47.7 to 54.8)	55.4 (44.9 to 62.5)
Tajikistan	47.2 (41.3 to 50.1)	56.6 (49.3 to 60.1)	56.6 (49.2 to 60.2)	60.7 (48.7 to 67.3)
Turkmenistan	55.4 (52.0 to 57.8)	65.3 (61.6 to 68.0)	66.3 (63.1 to 69.2)	70.1 (64.2 to 74.8)
Uzbekistan	53.9 (49.0 to 56.5)	63.7 (59.7 to 66.8)	65.3 (61.3 to 68.6)	69.5 (58.8 to 74.4)
Central Europe				
Albania	55.1 (53.5 to 56.4)	64.3 (62.2 to 66.3)	65.2 (62.8 to 67.2)	66.0 (59.3 to 71.0)
Bosnia and Herzegovina	56.7 (54.6 to 58.8)	60.8 (57.4 to 63.7)	62.5 (59.0 to 65.4)	62.4 (56.1 to 68.9)
Bulgaria	50.7 (49.2 to 51.9)	59.0 (55.3 to 62.6)	61.2 (56.5 to 65.0)	64.1 (56.2 to 69.5)
Croatia	61.0 (57.2 to 63.7)	64.5 (60.2 to 68.0)	67.1 (62.1 to 71.0)	68.0 (61.7 to 74.7)
Czech Republic	60.9 (57.8 to 63.4)	68.8 (61.7 to 72.2)	69.2 (61.7 to 73.2)	73.3 (66.4 to 77.5)
Hungary	53.7 (49.1 to 57.7)	66.0 (61.2 to 68.7)	67.4 (59.9 to 70.8)	72.6 (67.4 to 76.4)
Macedonia	57.0 (51.9 to 59.9)	60.5 (56.1 to 63.3)	64.2 (59.3 to 67.3)	62.8 (50.4 to 71.0)
Montenegro	53.8 (48.5 to 58.0)	58.9 (54.0 to 63.5)	59.4 (52.0 to 64.4)	56.6 (46.7 to 65.1)
Poland	61.0 (58.9 to 62.8)	68.6 (65.6 to 70.9)	69.2 (64.7 to 72.0)	73.4 (65.1 to 77.5)
Romania	49.8 (45.1 to 53.0)	58.1 (54.8 to 63.7)	57.4 (54.4 to 63.3)	62.9 (57.2 to 70.3)
Serbia	54.1 (52.1 to 55.8)	60.5 (54.0 to 63.8)	61.6 (54.8 to 65.8)	60.9 (53.8 to 68.0)
Slovakia	63.1 (60.2 to 65.3)	72.5 (69.1 to 74.9)	72.9 (67.5 to 75.8)	75.0 (69.3 to 79.1)
Slovenia	69.7 (67.9 to 71.2)	76.3 (73.6 to 78.2)	78.1 (75.1 to 80.3)	78.5 (74.6 to 82.4)
Eastern Europe				
Belarus	45.7 (41.4 to 48.4)	53.9 (50.7 to 59.7)	54.6 (50.4 to 60.9)	53.6 (48.8 to 59.4)
Estonia	58.2 (56.2 to 59.9)	68.4 (63.6 to 74.5)	68.3 (63.9 to 75.8)	68.7 (63.6 to 77.8)
Latvia	55.6 (53.2 to 57.8)	63.5 (56.8 to 66.9)	63.9 (57.3 to 68.4)	64.6 (56.4 to 71.7)
Lithuania	55.8 (54.0 to 57.3)	56.3 (53.6 to 61.6)	57.5 (54.3 to 63.3)	58.8 (54.5 to 63.7)
Moldova	46.3 (42.3 to 50.9)	58.2 (53.0 to 60.9)	59.4 (52.1 to 62.8)	63.1 (54.7 to 68.5)
Russia	42.4 (40.9 to 43.6)	54.3 (51.7 to 56.5)	57.0 (52.7 to 59.7)	61.5 (53.6 to 67.9)

	SDG Index in 2000	SDG Index in 2015	SDG Index in 2017	SDG Index in 2030
Ukraine	43.4 (38.4 to 47.0)	44.8 (42.6 to 48.3)	48.4 (45.7 to 53.1)	52.1 (46.4 to 58.5)
High-income				
Australasia				
Australia	68.6 (65.4 to 71.8)	71.9 (68.1 to 74.9)	72.0 (65.7 to 75.3)	72.5 (64.7 to 77.0)
New Zealand	67.1 (65.2 to 68.8)	72.5 (69.3 to 74.9)	75.5 (71.2 to 78.2)	70.4 (59.5 to 78.9)
High-income Asia Pacific				
Brunei	69.0 (67.7 to 70.3)	69.4 (68.1 to 70.7)	75.1 (73.8 to 76.3)	77.5 (73.8 to 79.8)
Japan	69.0 (67.4 to 70.1)	78.4 (76.6 to 79.8)	78.9 (76.7 to 80.5)	77.0 (66.6 to 81.0)
South Korea	59.5 (57.6 to 60.9)	71.3 (68.8 to 73.3)	72.2 (69.0 to 74.4)	72.6 (67.1 to 76.6)
Singapore	77.5 (76.0 to 79.1)	84.3 (82.4 to 85.9)	84.9 (83.1 to 86.7)	85.3 (81.7 to 88.0)
High-income North America				
Canada	73.0 (70.7 to 74.6)	79.7 (76.3 to 81.7)	79.4 (75.0 to 81.7)	80.0 (73.6 to 83.6)
United States	68.8 (67.5 to 69.9)	72.7 (70.7 to 74.3)	73.6 (70.9 to 75.4)	74.2 (70.6 to 77.0)
Greenland	48.6 (46.7 to 50.4)	53.7 (51.7 to 55.2)	54.2 (52.3 to 55.8)	55.7 (52.3 to 58.3)
Southern Latin America				
Argentina	57.1 (54.1 to 59.1)	65.5 (62.2 to 67.6)	67.1 (63.1 to 69.6)	69.7 (64.6 to 73.0)
Chile	54.2 (52.4 to 57.1)	60.6 (57.7 to 63.0)	65.2 (61.8 to 68.1)	65.6 (55.0 to 71.9)
Uruguay	54.0 (48.5 to 60.5)	66.3 (63.5 to 68.1)	68.3 (65.6 to 70.6)	72.4 (68.9 to 75.1)
Western Europe				
Andorra	52.3 (50.1 to 62.8)	62.9 (57.7 to 66.0)	62.8 (55.6 to 66.5)	64.9 (60.5 to 68.4)
Austria	58.5 (52.3 to 62.8)	68.5 (61.5 to 72.8)	71.6 (64.4 to 76.9)	74.0 (65.4 to 79.5)
Belgium	68.4 (66.2 to 70.1)	76.8 (70.7 to 79.7)	77.2 (67.9 to 80.8)	78.7 (69.4 to 83.3)
Cyprus	64.5 (59.9 to 67.0)	75.4 (73.1 to 77.4)	76.1 (73.4 to 78.3)	77.3 (70.9 to 81.3)
Denmark	64.5 (58.3 to 69.1)	76.8 (67.7 to 80.7)	77.4 (68.2 to 81.7)	81.7 (75.1 to 85.5)
Finland	70.7 (67.5 to 72.8)	79.6 (70.7 to 83.2)	79.0 (70.2 to 83.4)	81.4 (72.1 to 85.4)
France	57.7 (54.7 to 63.2)	68.6 (61.2 to 73.1)	70.1 (62.2 to 75.1)	70.1 (61.9 to 75.9)
Germany	67.2 (60.3 to 70.4)	74.8 (66.2 to 78.4)	75.4 (66.9 to 79.7)	79.2 (72.9 to 82.9)
Greece	59.5 (57.1 to 61.4)	63.1 (57.2 to 68.1)	64.5 (56.9 to 69.3)	69.7 (61.9 to 76.1)
Iceland	78.3 (75.8 to 79.8)	82.8 (79.2 to 84.9)	78.8 (75.0 to 81.0)	79.9 (70.6 to 85.3)
Ireland	62.7 (58.8 to 67.8)	75.7 (68.6 to 81.1)	76.5 (69.7 to 83.0)	80.2 (70.7 to 85.8)
Israel	73.2 (71.4 to 74.7)	81.9 (78.9 to 83.3)	82.5 (79.4 to 84.0)	83.4 (78.1 to 86.5)
Italy	60.4 (56.7 to 63.0)	69.9 (66.9 to 72.4)	70.3 (66.4 to 73.0)	71.9 (63.2 to 76.2)
Luxembourg	64.7 (59.7 to 67.5)	68.3 (65.3 to 73.1)	69.2 (65.8 to 76.4)	70.9 (66.6 to 79.9)

	SDG Index in 2000	SDG Index in 2015	SDG Index in 2017	SDG Index in 2030
Malta	74.3 (72.7 to 75.7)	79.4 (77.2 to 81.0)	79.2 (76.7 to 81.1)	80.3 (75.7 to 83.6)
Netherlands	71.5 (69.4 to 72.9)	79.9 (77.3 to 81.6)	80.2 (77.0 to 82.3)	80.4 (67.0 to 84.1)
Norway	71.5 (69.4 to 73.1)	80.3 (77.6 to 82.0)	83.9 (81.0 to 85.9)	84.0 (79.1 to 86.9)
Portugal	60.2 (57.2 to 66.3)	70.8 (65.5 to 76.6)	71.0 (66.0 to 77.6)	74.7 (66.5 to 80.4)
Spain	64.2 (59.7 to 68.8)	71.7 (66.2 to 78.0)	73.5 (68.1 to 80.4)	76.5 (68.3 to 83.1)
Sweden	77.0 (74.1 to 78.9)	82.7 (79.1 to 85.0)	83.4 (78.5 to 86.2)	83.9 (77.9 to 87.2)
Switzerland	67.6 (64.0 to 69.8)	75.5 (71.2 to 78.0)	79.3 (73.6 to 82.2)	79.5 (74.4 to 83.1)
United Kingdom	70.6 (68.8 to 72.0)	79.2 (76.7 to 81.0)	80.4 (77.3 to 82.5)	81.5 (76.4 to 84.5)
Latin America and Caribbean				
Andean Latin America				
Bolivia	41.8 (39.7 to 43.7)	55.0 (52.4 to 57.6)	58.1 (55.3 to 61.0)	61.8 (57.0 to 65.8)
Ecuador	50.8 (49.5 to 52.0)	60.7 (59.2 to 62.1)	64.1 (62.3 to 65.9)	64.7 (55.2 to 68.7)
Peru	49.7 (47.3 to 51.6)	62.5 (59.9 to 64.6)	62.5 (59.6 to 64.9)	65.4 (55.4 to 71.5)
Caribbean				
Antigua and Barbuda	69.5 (67.4 to 71.0)	72.6 (70.7 to 74.3)	66.5 (64.4 to 68.4)	73.6 (68.6 to 77.7)
The Bahamas	65.2 (62.7 to 67.1)	56.6 (54.7 to 58.1)	70.4 (68.1 to 72.5)	70.8 (64.9 to 75.3)
Barbados	66.6 (64.4 to 68.5)	71.8 (69.4 to 73.7)	72.0 (69.8 to 74.0)	72.5 (60.4 to 76.8)
Belize	40.3 (37.5 to 41.9)	58.1 (55.3 to 60.4)	59.0 (56.2 to 61.3)	59.4 (49.4 to 66.0)
Cuba	60.7 (58.3 to 62.9)	65.6 (63.3 to 67.9)	65.2 (62.8 to 67.6)	65.8 (54.9 to 70.3)
Dominica	58.4 (56.6 to 59.9)	51.2 (49.7 to 52.5)	63.4 (61.2 to 65.1)	63.1 (53.0 to 68.1)
Dominican Republic	49.5 (47.7 to 51.2)	55.0 (52.9 to 56.8)	56.7 (54.3 to 59.0)	59.9 (51.3 to 64.3)
Grenada	63.2 (60.9 to 64.9)	67.0 (64.8 to 68.7)	67.3 (64.9 to 69.2)	69.3 (57.6 to 73.3)
Guyana	46.1 (43.5 to 47.9)	52.8 (49.9 to 55.2)	54.3 (51.0 to 57.1)	58.4 (52.1 to 62.5)
Haiti	20.4 (18.2 to 23.1)	32.4 (28.7 to 35.5)	33.5 (29.7 to 36.5)	36.3 (30.0 to 42.2)
Jamaica	59.0 (57.5 to 60.8)	64.5 (62.2 to 66.8)	65.5 (63.1 to 68.0)	65.7 (60.0 to 69.4)
Saint Lucia	58.3 (56.4 to 60.0)	66.3 (64.1 to 68.0)	66.6 (64.4 to 68.4)	69.6 (63.0 to 73.8)
Saint Vincent and the Grenadines	59.9 (57.7 to 61.6)	64.7 (62.6 to 66.5)	65.4 (63.0 to 67.3)	65.3 (54.6 to 71.2)
Suriname	49.3 (47.1 to 51.2)	55.9 (53.0 to 58.2)	56.9 (54.0 to 59.3)	60.6 (56.0 to 64.5)
Trinidad and Tobago	59.0 (56.2 to 60.9)	65.2 (62.1 to 67.7)	65.3 (62.1 to 68.0)	66.1 (60.1 to 70.2)
Bermuda	48.8 (44.3 to 53.7)	69.8 (62.8 to 72.7)	70.2 (62.9 to 73.3)	75.7 (69.2 to 80.4)
Puerto Rico	63.1 (59.7 to 66.0)	67.8 (65.0 to 71.1)	54.4 (52.0 to 57.4)	67.3 (56.5 to 72.0)
Virgin Islands, U.S.	65.1 (62.1 to 67.5)	68.6 (65.4 to 71.2)	55.8 (53.1 to 58.1)	70.6 (65.2 to 75.0)
Central Latin America				

	SDG Index in 2000	SDG Index in 2015	SDG Index in 2017	SDG Index in 2030
Colombia	53.6 (51.8 to 55.2)	65.5 (63.4 to 67.3)	65.8 (63.2 to 68.1)	70.7 (60.3 to 75.4)
Costa Rica	58.1 (56.6 to 59.3)	65.9 (64.4 to 67.2)	62.7 (61.0 to 64.1)	66.7 (63.3 to 69.7)
El Salvador	52.4 (50.4 to 53.8)	62.2 (60.2 to 63.8)	65.9 (63.6 to 67.9)	64.3 (54.3 to 70.5)
Guatemala	40.1 (38.5 to 41.4)	55.4 (53.5 to 57.1)	55.7 (53.5 to 57.8)	61.1 (51.2 to 66.4)
Honduras	41.2 (38.5 to 47.4)	54.5 (47.2 to 58.2)	54.5 (47.0 to 57.8)	57.6 (49.0 to 65.0)
Mexico	58.5 (57.5 to 59.5)	66.8 (65.7 to 67.8)	64.2 (62.9 to 65.4)	70.6 (66.4 to 73.2)
Nicaragua	49.8 (43.4 to 51.6)	65.0 (63.2 to 66.6)	65.2 (63.3 to 66.9)	67.4 (57.9 to 74.2)
Panama	63.0 (61.7 to 64.1)	69.2 (67.9 to 70.4)	70.3 (68.7 to 71.7)	73.3 (70.2 to 76.0)
Venezuela	50.6 (48.8 to 52.2)	60.0 (57.9 to 61.8)	60.8 (58.2 to 63.0)	61.8 (51.2 to 66.8)
Tropical Latin America				
Brazil	56.1 (55.1 to 57.1)	66.3 (64.8 to 67.4)	67.6 (65.7 to 68.9)	69.8 (66.9 to 73.3)
Paraguay	51.2 (49.6 to 52.6)	62.5 (60.7 to 64.3)	66.1 (63.6 to 68.5)	70.0 (65.8 to 73.5)
North Africa and Middle East				
North Africa and Middle East				
Algeria	51.7 (45.9 to 54.5)	65.0 (58.1 to 68.1)	67.4 (58.2 to 70.9)	68.0 (57.0 to 75.6)
Bahrain	59.2 (53.5 to 62.2)	69.0 (62.9 to 73.0)	69.7 (63.3 to 73.7)	73.1 (66.1 to 79.3)
Egypt	46.7 (41.4 to 51.1)	51.0 (46.6 to 57.4)	53.0 (48.3 to 59.6)	57.2 (50.8 to 66.0)
Iran	54.0 (50.8 to 55.5)	65.0 (63.9 to 65.9)	62.4 (61.0 to 63.6)	62.7 (55.7 to 70.8)
Iraq	41.8 (36.8 to 45.0)	53.8 (48.0 to 57.8)	57.0 (50.7 to 60.7)	64.1 (55.8 to 70.5)
Jordan	58.7 (56.7 to 60.3)	71.9 (69.0 to 73.8)	72.5 (69.7 to 74.3)	75.1 (64.0 to 79.2)
Kuwait	63.9 (57.1 to 67.3)	68.4 (61.1 to 72.9)	70.8 (63.0 to 74.8)	72.4 (64.2 to 77.6)
Lebanon	47.3 (45.7 to 48.7)	52.5 (50.9 to 54.0)	54.6 (53.0 to 56.1)	56.8 (48.0 to 60.8)
Libya	57.8 (49.9 to 62.0)	63.1 (54.1 to 68.3)	61.6 (53.3 to 66.9)	65.4 (54.8 to 73.6)
Morocco	44.9 (41.4 to 46.9)	58.8 (55.7 to 61.4)	59.9 (56.4 to 62.7)	62.1 (50.9 to 67.6)
Palestine	52.2 (50.2 to 53.8)	63.3 (60.6 to 65.1)	67.0 (64.3 to 68.9)	70.1 (63.9 to 74.4)
Oman	51.1 (47.1 to 53.2)	61.6 (54.6 to 66.9)	63.8 (56.9 to 68.7)	69.2 (59.4 to 75.4)
Qatar	43.7 (39.5 to 48.2)	60.2 (56.5 to 66.7)	61.1 (57.2 to 67.7)	64.9 (59.9 to 72.6)
Saudi Arabia	52.3 (47.0 to 57.7)	57.2 (51.4 to 65.3)	58.9 (52.9 to 66.3)	66.1 (60.0 to 75.3)
Syria	56.5 (51.5 to 58.7)	58.1 (50.9 to 60.8)	61.0 (53.1 to 63.7)	66.4 (56.8 to 71.9)
Tunisia	56.0 (52.7 to 58.4)	65.6 (61.7 to 68.5)	66.7 (62.5 to 69.8)	68.9 (59.9 to 74.4)
Turkey	48.0 (46.0 to 49.8)	64.0 (60.5 to 66.2)	66.1 (62.8 to 68.3)	67.6 (56.8 to 73.8)
United Arab Emirates	52.5 (48.5 to 57.0)	60.0 (54.2 to 65.9)	61.0 (55.2 to 67.0)	67.6 (58.7 to 73.6)
Yemen	18.4 (15.2 to 21.7)	31.6 (26.7 to 35.1)	32.3 (27.4 to 35.8)	39.0 (31.8 to 44.4)

	SDG Index in 2000	SDG Index in 2015	SDG Index in 2017	SDG Index in 2030
Afghanistan	6.8 (5.5 to 8.3)	18.3 (15.6 to 20.8)	20.6 (17.6 to 23.1)	25.5 (20.2 to 30.0)
Sudan	19.1 (16.3 to 21.6)	32.9 (28.2 to 36.1)	35.2 (30.5 to 38.4)	41.7 (35.6 to 46.5)
South Asia				
South Asia				
Bangladesh	18.3 (15.7 to 20.3)	31.6 (28.3 to 34.0)	33.4 (30.0 to 35.6)	35.7 (29.6 to 41.7)
Bhutan	22.6 (20.9 to 24.4)	48.1 (45.5 to 50.0)	49.6 (46.8 to 51.7)	55.0 (45.7 to 59.2)
India	22.3 (20.7 to 24.2)	33.7 (32.2 to 36.3)	34.7 (33.2 to 37.7)	40.5 (38.5 to 44.7)
Nepal	15.4 (13.7 to 17.2)	24.9 (22.1 to 28.1)	30.4 (27.3 to 34.3)	37.4 (31.2 to 42.9)
Pakistan	22.1 (19.4 to 24.1)	30.8 (27.0 to 33.4)	33.9 (30.0 to 36.5)	38.7 (30.5 to 43.6)
Southeast Asia, East Asia, and Oceania				
East Asia				
China	45.9 (44.6 to 47.1)	59.1 (57.7 to 60.5)	61.7 (60.5 to 62.9)	65.4 (55.5 to 68.4)
North Korea	32.7 (30.6 to 34.6)	40.9 (38.9 to 42.7)	43.2 (41.1 to 45.2)	45.1 (41.4 to 48.0)
Taiwan (Province of China)	61.1 (59.5 to 62.5)	69.4 (67.0 to 71.1)	69.9 (67.4 to 71.9)	67.4 (58.5 to 72.1)
Oceania				
Fiji	45.6 (44.0 to 47.3)	58.4 (55.4 to 61.0)	59.3 (55.9 to 62.2)	60.0 (54.7 to 64.5)
Kiribati	26.5 (23.7 to 28.4)	32.5 (29.9 to 34.8)	33.4 (30.7 to 36.0)	37.7 (33.7 to 41.5)
Marshall Islands	33.0 (31.2 to 34.6)	39.1 (37.0 to 41.1)	40.0 (37.7 to 42.7)	44.8 (40.6 to 48.6)
Federated States of Micronesia	36.7 (33.8 to 39.1)	34.5 (31.7 to 37.3)	42.9 (39.4 to 46.5)	43.7 (35.5 to 48.6)
Papua New Guinea	14.9 (13.0 to 17.1)	24.3 (21.1 to 27.1)	25.8 (22.6 to 28.7)	29.2 (23.6 to 33.9)
Samoa	43.5 (41.7 to 45.3)	46.8 (44.9 to 48.7)	47.5 (45.6 to 49.5)	46.9 (39.6 to 52.5)
Solomon Islands	27.0 (23.2 to 29.5)	30.5 (27.7 to 32.9)	35.4 (32.2 to 38.1)	36.6 (31.0 to 42.1)
Tonga	51.2 (49.1 to 53.0)	58.2 (56.2 to 60.0)	59.0 (57.0 to 61.0)	61.6 (56.9 to 65.5)
Vanuatu	27.5 (24.1 to 31.1)	26.8 (24.2 to 29.6)	33.8 (30.5 to 37.3)	35.6 (29.5 to 41.0)
American Samoa	57.3 (55.5 to 59.2)	60.7 (58.8 to 62.6)	61.2 (59.2 to 63.0)	63.0 (52.1 to 69.0)
Guam	59.0 (56.4 to 61.3)	58.3 (56.8 to 60.4)	58.5 (56.9 to 60.6)	62.6 (56.6 to 66.2)
Northern Mariana Islands	63.5 (60.9 to 65.4)	64.6 (62.4 to 66.5)	65.0 (62.8 to 67.0)	69.5 (64.4 to 72.6)
Southeast Asia				
Cambodia	16.0 (14.4 to 17.7)	39.0 (37.0 to 40.6)	40.3 (38.4 to 41.9)	46.4 (42.7 to 49.5)
Indonesia	32.6 (31.6 to 33.6)	42.0 (41.1 to 43.0)	43.0 (42.0 to 44.0)	46.2 (39.1 to 48.8)
Laos	14.8 (12.9 to 16.5)	34.8 (32.9 to 36.8)	36.5 (34.4 to 38.5)	43.8 (39.4 to 47.0)
Malaysia	57.0 (55.7 to 58.4)	64.5 (63.0 to 66.2)	66.3 (64.4 to 68.2)	71.1 (67.2 to 74.8)
Maldives	49.0 (47.4 to 50.4)	70.2 (68.8 to 71.5)	72.2 (70.8 to 73.5)	78.0 (64.6 to 81.2)

	SDG Index in 2000	SDG Index in 2015	SDG Index in 2017	SDG Index in 2030
Myanmar	23.7 (20.8 to 25.7)	36.3 (34.5 to 38.0)	40.3 (38.4 to 42.1)	44.8 (37.2 to 48.5)
Philippines	44.2 (43.0 to 45.5)	52.4 (50.7 to 53.9)	54.1 (52.2 to 56.1)	56.1 (48.0 to 60.1)
Sri Lanka	45.3 (44.0 to 46.6)	62.6 (60.5 to 64.4)	65.0 (62.1 to 67.6)	69.4 (58.1 to 75.5)
Thailand	45.3 (43.2 to 47.2)	59.6 (57.8 to 61.1)	58.7 (57.0 to 60.5)	65.2 (59.7 to 68.7)
Timor-Leste	15.3 (12.8 to 17.5)	34.7 (32.5 to 37.3)	36.4 (34.1 to 38.9)	43.1 (39.4 to 46.4)
Vietnam	35.3 (33.6 to 36.8)	46.0 (43.6 to 48.0)	46.1 (43.5 to 48.3)	50.0 (43.6 to 54.3)
Mauritius	63.5 (62.0 to 64.9)	70.3 (68.4 to 72.0)	71.3 (69.4 to 73.1)	73.6 (68.9 to 76.9)
Seychelles	60.8 (59.2 to 62.3)	66.0 (64.0 to 67.6)	67.5 (65.4 to 69.2)	71.2 (66.1 to 74.6)
Sub-Saharan Africa				
Central Sub-Saharan Africa				
Angola	8.0 (6.7 to 9.6)	23.4 (21.4 to 25.0)	25.7 (23.6 to 27.5)	32.5 (27.9 to 35.5)
Central African Republic	6.8 (5.7 to 7.9)	11.0 (9.1 to 13.2)	11.6 (9.6 to 14.0)	14.6 (11.6 to 17.6)
Congo	15.3 (12.9 to 17.3)	30.2 (26.2 to 32.9)	30.7 (26.4 to 33.4)	35.2 (29.1 to 39.7)
Democratic Republic of the Congo	9.4 (7.9 to 11.2)	17.5 (15.0 to 19.3)	17.9 (15.6 to 19.7)	24.4 (20.8 to 27.3)
Equatorial Guinea	11.9 (9.1 to 14.4)	27.3 (23.0 to 30.6)	29.2 (25.0 to 32.5)	36.1 (30.7 to 39.9)
Gabon	21.8 (18.9 to 23.5)	35.8 (31.0 to 38.3)	37.1 (31.8 to 39.6)	41.2 (34.8 to 45.1)
Eastern Sub-Saharan Africa				
Burundi	8.3 (6.9 to 9.8)	18.5 (16.0 to 20.7)	20.0 (17.3 to 22.2)	25.1 (21.0 to 28.4)
Comoros	21.1 (18.8 to 23.0)	33.6 (31.6 to 35.2)	35.5 (33.4 to 37.1)	40.0 (33.0 to 43.5)
Djibouti	19.0 (15.7 to 22.0)	31.2 (26.6 to 34.5)	32.8 (28.1 to 35.9)	36.6 (29.5 to 42.0)
Eritrea	12.5 (10.4 to 14.7)	24.3 (20.5 to 27.0)	26.1 (22.4 to 29.0)	30.4 (25.1 to 34.5)
Ethiopia	6.4 (5.8 to 7.0)	21.4 (19.2 to 22.8)	23.0 (20.7 to 24.3)	31.8 (28.6 to 33.9)
Kenya	21.9 (20.9 to 23.0)	30.2 (29.1 to 31.3)	31.7 (30.6 to 32.9)	36.5 (34.7 to 38.3)
Madagascar	13.7 (12.3 to 15.4)	22.4 (19.7 to 25.2)	23.6 (20.8 to 26.3)	31.2 (28.1 to 34.3)
Malawi	14.3 (12.5 to 16.3)	23.0 (20.9 to 25.0)	27.6 (25.2 to 29.4)	31.5 (28.4 to 34.0)
Mozambique	8.9 (8.0 to 10.1)	24.3 (21.3 to 26.5)	27.8 (24.8 to 30.1)	34.2 (29.8 to 37.9)
Rwanda	9.2 (8.0 to 10.6)	27.8 (24.6 to 30.2)	28.8 (25.4 to 31.0)	34.0 (28.7 to 37.8)
Somalia	6.3 (5.2 to 7.6)	11.1 (9.2 to 13.4)	11.6 (9.6 to 14.0)	15.5 (12.5 to 18.7)
Tanzania	17.7 (15.9 to 19.4)	27.8 (25.5 to 29.3)	29.9 (27.8 to 31.5)	35.1 (30.3 to 37.8)
Uganda	10.0 (8.8 to 11.3)	20.8 (18.3 to 24.2)	21.9 (19.0 to 25.5)	29.0 (23.2 to 33.5)
Zambia	17.4 (15.7 to 18.7)	30.4 (28.7 to 31.9)	31.7 (29.6 to 33.3)	36.1 (31.5 to 39.0)
South Sudan	6.9 (5.6 to 8.2)	12.4 (10.1 to 14.7)	12.4 (10.2 to 14.6)	17.2 (14.2 to 19.8)
Southern Sub-Saharan Africa				

	SDG Index in 2000	SDG Index in 2015	SDG Index in 2017	SDG Index in 2030
Botswana	31.6 (29.6 to 33.2)	42.0 (40.1 to 43.5)	43.7 (42.0 to 45.3)	47.4 (42.8 to 50.3)
Lesotho	17.1 (14.5 to 19.3)	20.5 (17.3 to 23.8)	22.8 (19.6 to 26.1)	25.2 (20.0 to 30.5)
Namibia	26.0 (24.1 to 27.7)	38.4 (35.8 to 40.4)	39.8 (36.9 to 42.1)	43.8 (37.9 to 47.8)
South Africa	31.9 (31.1 to 32.6)	49.2 (47.7 to 50.5)	50.4 (48.9 to 51.8)	52.8 (44.9 to 57.0)
Swaziland	21.4 (18.4 to 24.1)	30.2 (26.3 to 33.3)	32.8 (29.6 to 35.2)	36.4 (29.7 to 40.9)
Zimbabwe	26.4 (24.4 to 28.6)	32.3 (29.6 to 35.1)	35.0 (31.9 to 37.9)	35.6 (27.1 to 42.3)
Western Sub-Saharan Africa				
Benin	12.9 (11.3 to 15.0)	21.3 (18.3 to 23.5)	22.4 (19.5 to 24.8)	28.8 (23.8 to 34.3)
Burkina Faso	12.3 (10.4 to 13.8)	22.8 (20.0 to 25.4)	24.2 (21.2 to 26.8)	31.7 (27.0 to 34.9)
Cameroon	16.6 (14.5 to 18.4)	27.0 (23.8 to 30.1)	28.9 (25.5 to 32.2)	34.4 (28.6 to 38.9)
Cape Verde	40.0 (37.3 to 42.0)	49.0 (44.1 to 51.9)	57.4 (54.3 to 59.5)	62.9 (54.5 to 67.1)
Chad	7.2 (6.2 to 8.6)	14.0 (11.7 to 16.2)	15.3 (12.8 to 17.7)	21.1 (17.8 to 24.3)
Cote d'Ivoire	21.0 (19.2 to 22.6)	28.4 (26.1 to 30.1)	30.3 (28.2 to 32.1)	37.6 (33.1 to 40.1)
The Gambia	23.3 (20.6 to 25.5)	31.6 (28.8 to 33.7)	32.3 (29.7 to 34.6)	36.8 (32.1 to 39.8)
Ghana	27.6 (24.7 to 29.4)	37.4 (32.9 to 39.8)	39.1 (34.8 to 41.6)	45.7 (38.9 to 49.4)
Guinea	9.2 (8.0 to 10.5)	18.9 (16.6 to 20.9)	21.0 (18.6 to 23.0)	27.6 (24.3 to 30.4)
Guinea-Bissau	11.4 (9.4 to 13.5)	24.2 (21.4 to 26.4)	25.5 (22.9 to 27.7)	31.3 (27.5 to 34.2)
Liberia	10.5 (9.2 to 11.6)	21.9 (19.3 to 24.1)	23.5 (21.1 to 25.7)	28.9 (26.2 to 31.5)
Mali	11.1 (9.5 to 12.8)	24.2 (20.6 to 27.1)	25.6 (22.3 to 28.7)	34.3 (29.2 to 38.2)
Mauritania	20.0 (17.1 to 22.0)	31.9 (27.5 to 34.3)	31.2 (26.9 to 33.7)	38.7 (32.9 to 42.3)
Niger	7.3 (6.1 to 8.7)	16.4 (13.7 to 19.1)	17.3 (14.4 to 20.2)	25.3 (20.5 to 29.7)
Nigeria	15.8 (13.4 to 17.8)	29.7 (25.6 to 33.0)	30.7 (26.5 to 34.0)	38.2 (32.7 to 42.7)
Sao Tome and Principe	31.3 (28.5 to 33.2)	49.4 (47.0 to 51.4)	51.3 (49.0 to 53.3)	58.4 (52.5 to 61.8)
Senegal	25.4 (23.2 to 27.0)	35.4 (31.4 to 37.2)	36.6 (33.6 to 38.5)	40.6 (35.4 to 43.6)
Sierra Leone	10.1 (9.2 to 11.4)	21.8 (19.7 to 23.7)	19.6 (17.8 to 21.2)	28.7 (25.4 to 31.6)
Togo	19.1 (16.9 to 21.0)	29.5 (26.0 to 31.6)	30.8 (28.1 to 32.9)	35.8 (30.4 to 38.5)

Supplementary table 2. Values, by sex, for selected health-related SDG indicators in 2017, by country or territory. Estimates are reported for each country and territory included in this analysis, and ordered by health-related SDG indicator followed by GBD super-region and region. SDG=Sustainable Development Goal. GBD=Global Burden of Disease. UI=uncertainty interval.

	Estimate in 2017, male (95% UIs)	Estimate in 2017, female (95% UIs)
**Indicator 3.3.1: Age-standardised rate of new HIV infections (per 1,000 population)**		
Central Europe, Eastern Europe, and Central Asia		
Central Asia		
Armenia	0.06 (0.04 to 0.09)	0.04 (0.03 to 0.05)
Azerbaijan	0.04 (0.03 to 0.07)	0.02 (0.01 to 0.02)
Georgia	0.15 (0.09 to 0.26)	0.05 (0.03 to 0.08)
Kazakhstan	0.15 (0.1 to 0.19)	0.1 (0.07 to 0.14)
Kyrgyzstan	0.14 (0.09 to 0.22)	0.14 (0.09 to 0.22)
Mongolia	0.03 (0.01 to 0.07)	0.01 (0 to 0.02)
Tajikistan	0.03 (0.02 to 0.04)	0.1 (0.04 to 0.16)
Turkmenistan	0.04 (0.03 to 0.05)	0.02 (0.01 to 0.03)
Uzbekistan	0.06 (0.02 to 0.11)	0.06 (0.02 to 0.11)
Central Europe		
Albania	0 (0 to 0)	0 (0 to 0)
Bosnia and Herzegovina	0 (0 to 0)	0 (0 to 0)
Bulgaria	0.03 (0.02 to 0.03)	0.01 (0.01 to 0.02)
Croatia	0.01 (0.01 to 0.02)	0.01 (0 to 0.01)
Czech Republic	0.02 (0.01 to 0.03)	0 (0 to 0.01)
Hungary	0.02 (0.01 to 0.03)	0 (0 to 0.01)
Macedonia	0 (0 to 0.01)	0 (0 to 0.01)
Montenegro	0.02 (0.01 to 0.02)	0.01 (0.01 to 0.01)
Poland	0.03 (0.01 to 0.05)	0.01 (0.01 to 0.01)
Romania	0.07 (0.05 to 0.09)	0.04 (0.03 to 0.05)
Serbia	0.03 (0.02 to 0.04)	0 (0 to 0.01)
Slovakia	0.01 (0 to 0.01)	0 (0 to 0)
Slovenia	0.01 (0.01 to 0.02)	0 (0 to 0)
Eastern Europe		
Belarus	0.24 (0.18 to 0.33)	0.24 (0.15 to 0.38)
Estonia	0.27 (0.19 to 0.39)	0.08 (0.06 to 0.12)
Latvia	0.27 (0.22 to 0.34)	0.21 (0.15 to 0.24)
Lithuania	0.07 (0.02 to 0.11)	0.03 (0.01 to 0.04)
Moldova	0.18 (0.13 to 0.26)	0.18 (0.13 to 0.27)
Russian Federation	1.24 (1.02 to 1.55)	0.57 (0.48 to 0.72)
Ukraine	0.65 (0.39 to 0.82)	0.46 (0.3 to 0.67)
High-income		
Australasia		
Australia	0.09 (0.05 to 0.13)	0.03 (0.02 to 0.06)
New Zealand	0.07 (0.03 to 0.11)	0.02 (0.01 to 0.03)
High-income Asia Pacific		
Brunei	0.1 (0.06 to 0.17)	0.03 (0.02 to 0.05)
Japan	0.02 (0.01 to 0.02)	0.01 (0 to 0.01)
South Korea	0.04 (0.01 to 0.07)	0.01 (0 to 0.01)

	Estimate in 2017, male (95% UIs)	Estimate in 2017, female (95% UIs)
Singapore	0.03 (0.02 to 0.06)	0.02 (0.01 to 0.02)
High-income North America		
Canada	0.15 (0.06 to 0.25)	0.06 (0.03 to 0.1)
Greenland	0.16 (0.07 to 0.27)	0.24 (0.09 to 0.42)
United States	0.24 (0.09 to 0.38)	0.1 (0.04 to 0.16)
Southern Latin America		
Argentina	0.29 (0.15 to 0.5)	0.26 (0.11 to 0.51)
Chile	0.37 (0.19 to 0.59)	0.07 (0.04 to 0.13)
Uruguay	0.4 (0.32 to 0.5)	0.12 (0.08 to 0.17)
Western Europe		
Andorra	0.1 (0 to 0.48)	0.02 (0 to 0.09)
Austria	0.12 (0.06 to 0.18)	0.04 (0.02 to 0.06)
Belgium	0.13 (0.05 to 0.2)	0.07 (0.03 to 0.1)
Cyprus	0.13 (0.09 to 0.19)	0.03 (0.02 to 0.04)
Denmark	0.09 (0.04 to 0.15)	0.06 (0.02 to 0.09)
Finland	0.04 (0.01 to 0.07)	0.01 (0 to 0.02)
France	0.11 (0.05 to 0.2)	0.05 (0.02 to 0.09)
Germany	0.11 (0.06 to 0.15)	0.04 (0.02 to 0.05)
Greece	0.11 (0.07 to 0.19)	0.01 (0.01 to 0.02)
Iceland	0.12 (0.05 to 0.18)	0 (0 to 0)
Ireland	0.18 (0.07 to 0.28)	0.07 (0.03 to 0.1)
Israel	0.11 (0.06 to 0.16)	0.05 (0.02 to 0.07)
Italy	0.19 (0.11 to 0.28)	0.06 (0.04 to 0.09)
Luxembourg	0.24 (0.11 to 0.35)	0.07 (0.03 to 0.1)
Malta	0.38 (0.2 to 0.63)	0.08 (0.04 to 0.12)
Netherlands	0.04 (0.02 to 0.06)	0.03 (0.01 to 0.04)
Norway	0.08 (0.04 to 0.11)	0.04 (0.02 to 0.06)
Portugal	1.27 (0.65 to 1.92)	0.34 (0.18 to 0.51)
Spain	0.22 (0.09 to 0.39)	0.06 (0.02 to 0.1)
Sweden	0.08 (0.04 to 0.13)	0.06 (0.03 to 0.09)
Switzerland	0.09 (0.03 to 0.14)	0.05 (0.02 to 0.07)
United Kingdom	0.15 (0.09 to 0.22)	0.06 (0.04 to 0.09)
Latin America and Caribbean		
Andean Latin America		
Bolivia	0.27 (0.03 to 0.96)	0.15 (0.01 to 0.64)
Ecuador	0.24 (0.2 to 0.29)	0.12 (0.08 to 0.16)
Peru	0.28 (0.2 to 0.45)	0.12 (0.08 to 0.18)
Caribbean		
Antigua and Barbuda	0.14 (0.11 to 0.18)	0.06 (0.04 to 0.08)
The Bahamas	1.14 (0.95 to 1.41)	0.68 (0.57 to 0.9)
Barbados	0.3 (0.21 to 0.45)	0.2 (0.14 to 0.28)
Belize	0.67 (0.6 to 0.78)	0.53 (0.42 to 0.68)
Bermuda	0.14 (0.12 to 0.17)	0.05 (0.04 to 0.07)
Cuba	0.34 (0.18 to 0.52)	0.11 (0.05 to 0.2)
Dominica	0.13 (0.11 to 0.18)	0.06 (0.04 to 0.1)
Dominican Republic	0.4 (0.22 to 0.65)	0.31 (0.14 to 0.53)
Grenada	0.08 (0.07 to 0.1)	0.03 (0.03 to 0.05)

	Estimate in 2017, male (95% UIs)	Estimate in 2017, female (95% UIs)
Guyana	0.73 (0.51 to 1)	0.86 (0.58 to 1.11)
Haiti	0.59 (0.38 to 0.87)	0.71 (0.46 to 0.97)
Jamaica	0.51 (0.41 to 0.65)	0.34 (0.24 to 0.49)
Puerto Rico	0.08 (0.07 to 0.09)	0.03 (0.03 to 0.04)
Saint Lucia	0.06 (0.05 to 0.08)	0.04 (0.03 to 0.05)
Saint Vincent and the Grenadines	0.23 (0.19 to 0.3)	0.13 (0.11 to 0.18)
Suriname	0.4 (0.32 to 0.53)	0.33 (0.26 to 0.45)
Trinidad and Tobago	0.32 (0.27 to 0.39)	0.29 (0.23 to 0.39)
Virgin Islands, U.S.	0.27 (0.17 to 0.44)	0.08 (0.06 to 0.13)
Central Latin America		
Colombia	0.27 (0.21 to 0.37)	0.09 (0.06 to 0.13)
Costa Rica	0.12 (0.09 to 0.17)	0.04 (0.02 to 0.05)
El Salvador	0.28 (0.16 to 0.38)	0.14 (0.08 to 0.21)
Guatemala	0.17 (0.08 to 0.37)	0.09 (0.04 to 0.19)
Honduras	0.06 (0.04 to 0.08)	0.04 (0.02 to 0.08)
Mexico	0.25 (0.18 to 0.33)	0.06 (0.04 to 0.08)
Nicaragua	0.39 (0.25 to 0.57)	0.22 (0.15 to 0.31)
Panama	0.73 (0.57 to 1.01)	0.22 (0.17 to 0.28)
Venezuela	0.42 (0.34 to 0.51)	0.08 (0.06 to 0.12)
Tropical Latin America		
Brazil	0.49 (0.36 to 0.58)	0.25 (0.18 to 0.3)
Paraguay	0.28 (0.18 to 0.38)	0.18 (0.11 to 0.26)
North Africa and Middle East		
North Africa and Middle East		
Afghanistan	0.04 (0 to 0.2)	0.02 (0 to 0.09)
Algeria	0.03 (0 to 0.1)	0.02 (0 to 0.08)
Bahrain	0.02 (0.01 to 0.02)	0.01 (0.01 to 0.01)
Egypt	0.01 (0.01 to 0.01)	0 (0 to 0)
Iran	0.03 (0.02 to 0.05)	0.04 (0.02 to 0.05)
Iraq	0 (0 to 0.01)	0.01 (0 to 0.01)
Jordan	0.01 (0 to 0.01)	0 (0 to 0.01)
Kuwait	0 (0 to 0.01)	0 (0 to 0)
Lebanon	0.04 (0 to 0.2)	0.02 (0 to 0.09)
Libya	0.03 (0 to 0.14)	0.03 (0 to 0.13)
Morocco	0.03 (0 to 0.16)	0.03 (0 to 0.15)
Palestine	0 (0 to 0.01)	0 (0 to 0.01)
Oman	0.12 (0.05 to 0.19)	0.03 (0.02 to 0.04)
Qatar	0 (0 to 0)	0 (0 to 0)
Saudi Arabia	0.02 (0.01 to 0.03)	0.02 (0.01 to 0.04)
Sudan	0.15 (0.07 to 0.31)	0.19 (0.08 to 0.37)
Syria	0 (0 to 0)	0 (0 to 0)
Tunisia	0.05 (0.01 to 0.17)	0.03 (0 to 0.14)
Turkey	0.01 (0.01 to 0.01)	0 (0 to 0.01)
United Arab Emirates	0.06 (0 to 0.4)	0.02 (0 to 0.07)
Yemen	0.05 (0 to 0.24)	0.03 (0 to 0.11)
South Asia		
South Asia		

	Estimate in 2017, male (95% UIs)	Estimate in 2017, female (95% UIs)
Bangladesh	0.01 (0 to 0.03)	0.01 (0 to 0.02)
Bhutan	0.16 (0 to 0.68)	0.08 (0 to 0.38)
India	0.08 (0.05 to 0.11)	0.06 (0.04 to 0.09)
Nepal	0.1 (0 to 0.47)	0.05 (0 to 0.24)
Pakistan	0.09 (0 to 0.39)	0.05 (0 to 0.2)
Southeast Asia, East Asia, and Oceania		
East Asia		
China	0.03 (0.01 to 0.06)	0.01 (0 to 0.02)
North Korea	0.06 (0 to 0.39)	0.03 (0 to 0.17)
Taiwan (Province of China)	0.02 (0.01 to 0.03)	0 (0 to 0)
Oceania		
American Samoa	0.02 (0.01 to 0.03)	0.01 (0 to 0.01)
Federated States of Micronesia	0.8 (0.01 to 5)	0.73 (0 to 5.02)
Fiji	0.05 (0.03 to 0.07)	0.06 (0.04 to 0.08)
Guam	0.08 (0.04 to 0.13)	0.01 (0.01 to 0.02)
Kiribati	0.01 (0 to 0.01)	0.01 (0 to 0.01)
Marshall Islands	0.13 (0 to 0.91)	0.14 (0 to 0.87)
Northern Mariana Islands	0.02 (0.01 to 0.04)	0.01 (0 to 0.02)
Papua New Guinea	0.28 (0.02 to 1.22)	0.37 (0.03 to 1.57)
Samoa	0.14 (0 to 0.98)	0.14 (0 to 0.96)
Solomon Islands	0.12 (0 to 0.86)	0.14 (0 to 0.87)
Tonga	0.03 (0.02 to 0.05)	0.02 (0.01 to 0.04)
Vanuatu	0.13 (0 to 0.91)	0.14 (0 to 0.96)
Southeast Asia		
Cambodia	0.06 (0.01 to 0.15)	0.06 (0.01 to 0.16)
Indonesia	0.09 (0.07 to 0.12)	0.05 (0.04 to 0.07)
Laos	0.16 (0 to 1.05)	0.06 (0 to 0.37)
Malaysia	0.18 (0.11 to 0.22)	0.08 (0.06 to 0.1)
Maldives	0 (0 to 0)	0.01 (0 to 0.01)
Mauritius	0.29 (0.22 to 0.39)	0.16 (0.11 to 0.2)
Myanmar	0.18 (0.11 to 0.31)	0.17 (0.1 to 0.25)
Philippines	0.19 (0.12 to 0.31)	0.06 (0.03 to 0.09)
Sri Lanka	0.02 (0.01 to 0.03)	0.01 (0.01 to 0.01)
Seychelles	0.06 (0.05 to 0.09)	0.05 (0.04 to 0.07)
Thailand	0.42 (0.34 to 0.58)	0.26 (0.18 to 0.41)
Timor-Leste	0.24 (0 to 1.65)	0.14 (0 to 0.85)
Vietnam	0.31 (0.25 to 0.41)	0.07 (0.04 to 0.12)
Sub-Saharan Africa		
Central Sub-Saharan Africa		
Angola	0.55 (0.21 to 1)	0.97 (0.37 to 1.73)
Central African Republic	0.73 (0.15 to 1.65)	1.53 (0.3 to 3.36)
Congo	1.05 (0.73 to 1.43)	1.75 (1.2 to 2.36)
Democratic Republic of the Congo	0.14 (0.03 to 0.32)	0.25 (0.04 to 0.57)
Equatorial Guinea	1.29 (0.34 to 3.09)	2.45 (0.58 to 5.81)
Gabon	0.28 (0.15 to 0.5)	0.54 (0.3 to 0.96)
Eastern Sub-Saharan Africa		
Burundi	0.24 (0.05 to 0.55)	0.37 (0.08 to 0.9)

	Estimate in 2017, male (95% UIs)	Estimate in 2017, female (95% UIs)
Comoros	0.03 (0 to 0.19)	0.01 (0 to 0.05)
Djibouti	0.57 (0.16 to 1.24)	0.83 (0.23 to 1.79)
Eritrea	0.24 (0.06 to 0.48)	0.36 (0.08 to 0.75)
Ethiopia	0.11 (0.06 to 0.18)	0.18 (0.1 to 0.3)
Kenya	1.63 (1.07 to 2.48)	2.3 (1.55 to 3.36)
Madagascar	0.31 (0.03 to 1.53)	0.43 (0.05 to 2.05)
Malawi	1.39 (0.66 to 2.23)	1.78 (0.79 to 2.96)
Mozambique	4.19 (2.66 to 6.22)	5.14 (3.32 to 7.59)
Rwanda	0.42 (0.26 to 0.67)	0.63 (0.4 to 0.96)
Somalia	0.13 (0 to 0.53)	0.17 (0.01 to 0.67)
South Sudan	0.39 (0.01 to 1.4)	0.6 (0.02 to 2.14)
Tanzania	0.87 (0.03 to 2.83)	1.2 (0.04 to 3.85)
Uganda	0.98 (0.11 to 3.38)	1.28 (0.12 to 4.73)
Zambia	2.41 (1.54 to 3.62)	2.97 (1.95 to 4.39)
Southern Sub-Saharan Africa		
Botswana	3.19 (2.12 to 4.59)	4.11 (2.84 to 5.71)
Lesotho	5.46 (3.77 to 7.42)	6.92 (4.84 to 9.22)
Namibia	3.47 (2.02 to 5.65)	4.36 (2.52 to 7.24)
South Africa	3.96 (2.93 to 5.14)	5.33 (4 to 6.81)
Swaziland	1.78 (0.46 to 3.38)	2.15 (0.48 to 4.09)
Zimbabwe	3.48 (0.11 to 8.08)	4.51 (0.12 to 10.36)
Western Sub-Saharan Africa		
Benin	0.27 (0.15 to 0.43)	0.38 (0.2 to 0.6)
Burkina Faso	0.13 (0.02 to 0.29)	0.18 (0.03 to 0.37)
Cameroon	0.85 (0.26 to 1.43)	1.41 (0.4 to 2.32)
Cape Verde	0.52 (0.34 to 0.76)	0.59 (0.4 to 0.83)
Chad	0.56 (0.19 to 1.02)	0.73 (0.26 to 1.31)
Cote d'Ivoire	1.49 (0.23 to 2.61)	2.31 (0.35 to 4.08)
The Gambia	0.52 (0.16 to 0.97)	0.68 (0.21 to 1.25)
Ghana	0.52 (0.28 to 0.89)	0.99 (0.55 to 1.63)
Guinea	0.41 (0.22 to 0.67)	0.64 (0.34 to 1.05)
Guinea-Bissau	1.1 (0.38 to 2.12)	1.57 (0.54 to 2.94)
Liberia	0.45 (0.19 to 0.83)	0.63 (0.26 to 1.12)
Mali	0.37 (0.24 to 0.52)	0.49 (0.32 to 0.7)
Mauritania	0.03 (0 to 0.2)	0.01 (0 to 0.06)
Niger	0.1 (0.05 to 0.17)	0.13 (0.06 to 0.2)
Nigeria	1.05 (0.71 to 1.48)	1.3 (0.89 to 1.76)
Sao Tome and Principe	0.01 (0 to 0.03)	0 (0 to 0.01)
Senegal	0.16 (0.1 to 0.25)	0.26 (0.16 to 0.4)
Sierra Leone	0.34 (0.04 to 0.78)	0.47 (0.06 to 1.06)
Togo	0.37 (0.07 to 0.81)	0.63 (0.1 to 1.41)
**Indicator 3.3.2: Age-standardised rate of tuberculosis cases (per 100,000 population)**		
Central Europe, Eastern Europe, and Central Asia		
Central Asia		
Armenia	51.21 (46.73 to 55.7)	17.86 (16.01 to 19.76)
Azerbaijan	135.65 (120.31 to 151.99)	74.5 (65.92 to 84.2)

	Estimate in 2017, male (95% UIs)	Estimate in 2017, female (95% UIs)
Georgia	94.24 (85.78 to 102.32)	47.41 (43.2 to 52.06)
Kazakhstan	93.57 (85.24 to 101.97)	58.51 (53.28 to 63.86)
Kyrgyzstan	136.52 (125.17 to 148.29)	94.23 (86.02 to 103.05)
Mongolia	169.95 (152.52 to 189.67)	132.6 (119.53 to 145.96)
Tajikistan	110.47 (98.73 to 122.31)	105.35 (95.12 to 116.74)
Turkmenistan	94.73 (83.91 to 107.58)	62.83 (54.77 to 71.37)
Uzbekistan	74.82 (68.76 to 81.68)	51.56 (47.42 to 56.08)
Central Europe		
Albania	18.87 (16.92 to 21.25)	10 (9.02 to 11.13)
Bosnia and Herzegovina	36.01 (32.36 to 40.12)	21.15 (19.03 to 23.51)
Bulgaria	23.38 (21.57 to 25.23)	14.95 (13.78 to 16.25)
Croatia	11.74 (10.69 to 12.88)	8.49 (7.62 to 9.38)
Czech Republic	6.28 (5.7 to 6.96)	4.18 (3.73 to 4.66)
Hungary	9.06 (8.23 to 9.98)	6.38 (5.79 to 7.03)
Macedonia	26.49 (23.97 to 29.3)	20.02 (17.87 to 22.4)
Montenegro	14.59 (13.09 to 16.29)	13.41 (11.99 to 15.01)
Poland	16.23 (14.87 to 17.78)	7.89 (7.23 to 8.57)
Romania	76.57 (70.28 to 83.2)	41.96 (38.5 to 45.7)
Serbia	26.45 (23.82 to 29.44)	17.35 (15.57 to 19.4)
Slovakia	11.35 (10.17 to 12.69)	7.31 (6.53 to 8.16)
Slovenia	7.08 (6.35 to 7.88)	5.79 (5.12 to 6.5)
Eastern Europe		
Belarus	48.88 (44.47 to 53.48)	19.06 (17.4 to 21)
Estonia	25.94 (23.3 to 28.82)	12.08 (10.7 to 13.57)
Latvia	43.63 (40.05 to 47.34)	21.9 (19.7 to 24.12)
Lithuania	60.83 (55.42 to 66.92)	26.28 (23.71 to 28.98)
Moldova	110.14 (100.19 to 121.43)	44.44 (40.02 to 48.76)
Russia	93.81 (84.22 to 104.32)	44.95 (40.33 to 49.58)
Ukraine	87.22 (76.16 to 100.5)	39.69 (34.31 to 45.64)
High-income		
Australasia		
Australia	4.97 (4.51 to 5.45)	4.88 (4.39 to 5.4)
New Zealand	10.55 (9.51 to 11.66)	11.14 (9.98 to 12.4)
High-income Asia Pacific		
Brunei	103.54 (93.62 to 114.04)	106.82 (96.59 to 118.04)
Japan	10.1 (9.2 to 11.08)	6.22 (5.61 to 6.88)
South Korea	65.45 (59.91 to 71.12)	39.97 (36.5 to 43.71)
Singapore	42.81 (38.87 to 46.68)	29.16 (26.05 to 32.48)
High-income North America		
Canada	4.6 (4.27 to 4.98)	4.2 (3.86 to 4.57)
Greenland	65.33 (60.1 to 70.57)	45.11 (41.2 to 49.28)
United States	3.01 (2.68 to 3.4)	2.06 (1.81 to 2.36)
Southern Latin America		
Argentina	25.57 (23.59 to 27.72)	19.03 (17.48 to 20.55)
Chile	17.9 (16.54 to 19.36)	10.38 (9.54 to 11.27)
Uruguay	28.08 (25.53 to 30.97)	14.26 (12.98 to 15.65)
Western Europe		

	Estimate in 2017, male (95% UIs)	Estimate in 2017, female (95% UIs)
Andorra	7.12 (6.39 to 8.06)	7.24 (6.38 to 8.21)
Austria	9.3 (8.37 to 10.33)	5.57 (4.96 to 6.26)
Belgium	10.49 (9.57 to 11.49)	6.39 (5.76 to 7.07)
Cyprus	8.36 (7.36 to 9.49)	4.97 (4.35 to 5.71)
Denmark	7.46 (6.68 to 8.38)	4.96 (4.39 to 5.58)
Finland	6.1 (5.45 to 6.83)	4.68 (4.15 to 5.24)
France	8.86 (8.02 to 9.69)	5.44 (4.91 to 6.02)
Germany	9.21 (8.25 to 10.28)	4.62 (4.18 to 5.1)
Greece	6.13 (5.53 to 6.8)	3.23 (2.87 to 3.61)
Iceland	5.69 (4.96 to 6.52)	5.34 (4.63 to 6.09)
Ireland	7.68 (6.85 to 8.55)	5.75 (5.12 to 6.39)
Israel	4.85 (4.29 to 5.42)	3.32 (2.92 to 3.75)
Italy	8.09 (6.92 to 9.34)	4.54 (3.92 to 5.19)
Luxembourg	7.58 (6.65 to 8.68)	4.77 (4.15 to 5.49)
Malta	10.02 (8.71 to 11.44)	6.34 (5.46 to 7.29)
Netherlands	6.27 (5.66 to 6.93)	4.81 (4.31 to 5.42)
Norway	7.08 (6.17 to 8.17)	6.15 (5.33 to 7.07)
Portugal	21.02 (18.93 to 23.02)	11.78 (10.64 to 13.03)
Spain	10.34 (8.23 to 12.58)	7.18 (5.79 to 8.84)
Sweden	9.34 (7.94 to 11.1)	6.5 (5.52 to 7.65)
Switzerland	8.23 (7.28 to 9.22)	5.38 (4.81 to 5.96)
United Kingdom	13.83 (12.15 to 15.69)	13.49 (11.78 to 15.34)
Latin America and Caribbean		
Andean Latin America		
Bolivia	104.33 (94.82 to 115.32)	73.14 (65.98 to 80.56)
Ecuador	43.58 (39.92 to 47.17)	24.42 (22.52 to 26.67)
Peru	96.82 (87.78 to 107.2)	74.52 (67.82 to 82.09)
Caribbean		
Antigua and Barbuda	16.44 (14.53 to 18.61)	16.8 (14.72 to 19.17)
The Bahamas	23.02 (20.7 to 25.47)	16.44 (14.74 to 18.09)
Barbados	6.22 (5.41 to 7.14)	7.17 (6.16 to 8.37)
Belize	46.63 (42.37 to 51.22)	26.92 (24.12 to 29.85)
Bermuda	16.51 (14.49 to 18.94)	24.64 (21.72 to 28.15)
Cuba	8.1 (7.33 to 8.95)	3.68 (3.31 to 4.09)
Dominica	32.34 (28.88 to 35.96)	31.44 (28.26 to 34.9)
Dominican Republic	65.51 (59.33 to 72.05)	51.38 (46.1 to 56.85)
Grenada	11.03 (9.79 to 12.44)	12.28 (10.81 to 14.08)
Guyana	85.87 (78.59 to 93.77)	43.72 (39.91 to 47.68)
Haiti	93.35 (84.95 to 102.91)	77.22 (69.65 to 85.08)
Jamaica	17.58 (15.64 to 19.72)	15.64 (13.83 to 17.86)
Puerto Rico	4.43 (3.94 to 4.97)	2.9 (2.52 to 3.28)
Saint Lucia	17.86 (15.85 to 19.98)	11.99 (10.63 to 13.44)
Saint Vincent and the Grenadines	24.68 (22.03 to 27.65)	18.25 (16.15 to 20.48)
Suriname	23.06 (20.6 to 25.67)	17.73 (15.65 to 20.05)
Trinidad and Tobago	20.17 (18.42 to 22.15)	9.93 (8.9 to 10.95)
Virgin Islands, U.S.	14.6 (12.97 to 16.32)	14.58 (12.83 to 16.48)
Central Latin America		

	Estimate in 2017, male (95% UIs)	Estimate in 2017, female (95% UIs)
Colombia	27.73 (25.19 to 30.42)	16.69 (15.13 to 18.37)
Costa Rica	11.4 (10.28 to 12.66)	7.15 (6.33 to 8.04)
El Salvador	34.41 (30.77 to 38.24)	24.74 (22.07 to 27.79)
Guatemala	28.65 (26.25 to 31.24)	19.02 (17.31 to 20.97)
Honduras	45.29 (40.7 to 50.5)	37.39 (33.67 to 41.51)
Mexico	19.86 (17.82 to 22.35)	14.71 (13.02 to 16.71)
Nicaragua	38.86 (35.14 to 43.29)	33.28 (29.82 to 37.12)
Panama	51.4 (46.44 to 56.55)	29.83 (26.92 to 32.96)
Venezuela	29.18 (26.35 to 31.92)	17.98 (16.45 to 19.56)
Tropical Latin America		
Brazil	47.51 (42.64 to 53.49)	24.83 (22.22 to 28.08)
Paraguay	65.26 (57.87 to 73.64)	36.22 (32.3 to 40.46)
North Africa and Middle East		
North Africa and Middle East		
Afghanistan	135.18 (115.4 to 161.38)	167.48 (141.43 to 195.79)
Algeria	38.85 (33.04 to 45.66)	35.59 (30.3 to 41.58)
Bahrain	25.2 (22.96 to 27.69)	17.83 (16.03 to 19.82)
Egypt	21.54 (18.33 to 25.17)	17.85 (15.29 to 20.75)
Iran	16.33 (14.85 to 17.91)	15.22 (13.84 to 16.76)
Iraq	40.27 (36.83 to 43.91)	41.28 (37.87 to 45.09)
Jordan	10.71 (8.86 to 12.99)	10.86 (9 to 13.2)
Kuwait	20.87 (19.24 to 22.69)	18.13 (16.56 to 19.92)
Lebanon	19.77 (17.49 to 22.32)	19.43 (17.03 to 21.97)
Libya	30.83 (27.91 to 33.98)	25.98 (23.52 to 28.63)
Morocco	147.82 (135.07 to 161.4)	142.61 (130.2 to 155.01)
Palestine	16.65 (15.13 to 18.31)	12.65 (11.5 to 13.94)
Oman	20.79 (18.93 to 22.93)	20.27 (18.15 to 22.6)
Qatar	30.99 (27.86 to 34.35)	27.68 (24.85 to 30.8)
Saudi Arabia	37.43 (33.94 to 41.15)	48.79 (44.56 to 53.7)
Sudan	112.11 (101.18 to 124.23)	86.14 (76.22 to 96.97)
Syria	20.86 (18.85 to 23.14)	23.22 (20.83 to 25.86)
Tunisia	32.26 (29.23 to 35.52)	22.42 (20.21 to 24.83)
Turkey	34.42 (30.28 to 38.95)	21.49 (18.92 to 24.61)
United Arab Emirates	18.25 (16.41 to 20.25)	16.35 (14.57 to 18.26)
Yemen	65.09 (59.59 to 71.25)	58.5 (53.49 to 63.92)
South Asia		
South Asia		
Bangladesh	164.4 (148.21 to 181.97)	96.27 (86.73 to 106.52)
Bhutan	112.95 (100.36 to 127.82)	118.87 (105.19 to 134.12)
India	234.3 (214.3 to 256.45)	210.48 (192.94 to 230.4)
Nepal	187.56 (169.68 to 210.01)	167.26 (149.57 to 187.13)
Pakistan	175.46 (161.12 to 191.66)	208.3 (189.93 to 229.08)
Southeast Asia, East Asia, and Oceania		
East Asia		
China	66.73 (60.96 to 72.7)	45.36 (41.11 to 49.82)
North Korea	196.49 (177.44 to 216.51)	151.92 (137.12 to 169.33)
Taiwan	47.45 (43.68 to 51.59)	30.52 (27.84 to 33.32)

	Estimate in 2017, male (95% UIs)	Estimate in 2017, female (95% UIs)
Oceania		
American Samoa	17.84 (16.14 to 19.67)	15.38 (13.68 to 17.16)
Federated States of Micronesia	116.47 (105.72 to 127.68)	133.52 (119.77 to 148.43)
Fiji	35.38 (32.24 to 38.78)	41.51 (37.4 to 46.06)
Guam	41.99 (37.99 to 45.97)	51.63 (46.38 to 56.97)
Kiribati	322.79 (294.02 to 354.83)	275 (246.88 to 306.09)
Marshall Islands	219.5 (199.22 to 240.39)	163.34 (146.17 to 181.94)
Northern Mariana Islands	47.39 (42.96 to 51.93)	57.77 (51.85 to 64.24)
Papua New Guinea	163.95 (149.07 to 179.47)	196.27 (177.35 to 217.81)
Samoa	38.95 (35.64 to 42.64)	45.4 (40.72 to 50.12)
Solomon Islands	75.2 (68.8 to 82.42)	93.59 (84.51 to 103.64)
Tonga	34.58 (31.45 to 38.13)	36.98 (33.2 to 41)
Vanuatu	75.58 (68.82 to 82.65)	74.42 (67.01 to 82.78)
Southeast Asia		
Cambodia	298.21 (269.98 to 330.15)	249.12 (225.03 to 276.2)
Indonesia	251.73 (230.43 to 273.84)	205.2 (186.33 to 225.81)
Laos	184.66 (160 to 211.88)	135.22 (115.86 to 156.2)
Malaysia	70.55 (64.08 to 77.42)	51.83 (46.97 to 57.29)
Maldives	47.62 (43.1 to 52.6)	34.04 (30.7 to 37.66)
Mauritius	20.56 (18.78 to 22.51)	16.43 (14.8 to 18.46)
Myanmar	235.55 (214.97 to 259.7)	188.9 (172.57 to 206.38)
Philippines	328.49 (295.32 to 364.68)	185.12 (167.85 to 204.01)
Sri Lanka	54.2 (46.41 to 63.18)	35.31 (29.84 to 41.09)
Seychelles	31.6 (28.64 to 34.74)	18.06 (16.01 to 20.42)
Thailand	82.53 (74.78 to 90.49)	61.52 (55.58 to 68.15)
Timor-Leste	202.96 (185.01 to 223.02)	170.79 (154.07 to 189.54)
Vietnam	169.64 (145.63 to 193.61)	98.63 (85.17 to 113.96)
Sub-Saharan Africa		
Central Sub-Saharan Africa		
Angola	532.49 (480.47 to 589.15)	407.76 (366.89 to 454.39)
Central African Republic	970.67 (881.82 to 1070.58)	841.17 (763.13 to 925.48)
Congo	455.48 (411.87 to 502.87)	462.8 (419.07 to 511.52)
Democratic Republic of the Congo	601.9 (546.93 to 663.03)	611.17 (553.87 to 674.19)
Equatorial Guinea	421.07 (380.24 to 467.6)	441.66 (396.34 to 494.7)
Gabon	441.02 (398.13 to 486.38)	311.13 (280.85 to 344.72)
Eastern Sub-Saharan Africa		
Burundi	797.17 (729.42 to 871.64)	726.08 (660.63 to 798.73)
Comoros	275.21 (248.27 to 305.2)	232.78 (209.29 to 259.68)
Djibouti	425.55 (377.67 to 478.67)	356.53 (318.06 to 398.95)
Eritrea	689.77 (626.58 to 760.41)	518.13 (470.83 to 566.3)
Ethiopia	314.75 (288 to 344.14)	243.98 (222.84 to 266.77)
Kenya	472.57 (430.76 to 518.8)	389.96 (354.4 to 429.06)
Madagascar	244.11 (203.02 to 294.03)	192.47 (156.55 to 235.3)
Malawi	681.84 (618.48 to 747.59)	535.14 (483.51 to 590.89)
Mozambique	933.71 (845.68 to 1024.46)	783.66 (712.44 to 861.14)
Rwanda	485.24 (445.07 to 532.4)	364.16 (332.65 to 399.53)
Somalia	436.68 (377.42 to 502.67)	357.09 (305.2 to 414.72)

	Estimate in 2017, male (95% UIs)	Estimate in 2017, female (95% UIs)
South Sudan	428.92 (393.19 to 468.24)	369.31 (340.09 to 402.66)
Tanzania	492.11 (445.79 to 543.71)	420 (378.61 to 466.8)
Uganda	710.39 (633.29 to 794.41)	508.18 (454.27 to 569.88)
Zambia	795.16 (709.17 to 884.15)	685.95 (615.52 to 765.9)
Southern Sub-Saharan Africa		
Botswana	734.45 (635.49 to 852.77)	646.43 (556.08 to 751.95)
Lesotho	1484.99 (1347.41 to 1630.21)	1219.09 (1094.73 to 1360.7
Namibia	898.32 (817.2 to 988.65)	771.14 (695.56 to 861.4)
South Africa	869.34 (791.56 to 956.56)	659.15 (594.45 to 734.84)
Swaziland	1078.71 (979.27 to 1195.17)	893.24 (803.57 to 998.63)
Zimbabwe	1070.51 (941.99 to 1202.41)	887.63 (764.47 to 1022.2)
Western Sub-Saharan Africa		
Benin	252.94 (228.86 to 279.25)	225.86 (203.94 to 250.78)
Burkina Faso	354.09 (323.77 to 383.71)	274.67 (252.38 to 301.63)
Cameroon	341.28 (300.75 to 383.47)	288.13 (256.54 to 325.51)
Cape Verde	129.83 (117.82 to 143.07)	139.25 (126.18 to 152.77)
Chad	388.48 (357.56 to 421.95)	351.32 (324.95 to 381.34)
Cote d'Ivoire	337.93 (309.22 to 369.53)	278.42 (253.95 to 307.08)
The Gambia	377.14 (339.36 to 420.75)	320.79 (288.06 to 354.01)
Ghana	381.37 (318.89 to 456.79)	282.24 (235.4 to 336.92)
Guinea	353.74 (324.01 to 387.24)	339.66 (312.99 to 368.65)
Guinea-Bissau	341.94 (311.69 to 375.71)	296.49 (270.05 to 325.35)
Liberia	306.72 (280.28 to 336.57)	297.24 (272.51 to 323.9)
Mali	181.05 (166.18 to 197.12)	181.89 (165.67 to 199.78)
Mauritania	151.41 (137.58 to 165.89)	127.46 (116.14 to 139.99)
Niger	255.69 (235.17 to 279.54)	246.22 (224.25 to 269.82)
Nigeria	412.22 (376.45 to 450.96)	297.29 (272.71 to 322.18)
Sao Tome and Principe	149.35 (136.19 to 163.75)	99.88 (90.15 to 109.27)
Senegal	266.02 (243.2 to 291.85)	248.53 (228.58 to 271.25)
Sierra Leone	396.96 (365.72 to 432.83)	354.65 (325.83 to 387.9)
Togo	323.86 (295.93 to 353.04)	267.28 (244.63 to 293.46)
**Indicator 3.4.1: Age-standardised death rate due to cardiovascular disease, cancer, diabetes, and chronic respiratory disease in populations aged 30-70 (per 100,000 population)**		
Central Europe, Eastern Europe, and Central Asia		
Central Asia		
Armenia	602.31 (578.06 to 626.32)	271.89 (258.26 to 286.93)
Azerbaijan	818 (746.67 to 898.76)	390.94 (353.83 to 430.17)
Georgia	815.28 (787.04 to 846.28)	325.42 (310.88 to 341.26)
Kazakhstan	776.56 (730.06 to 824.57)	347.23 (322.78 to 371.48)
Kyrgyzstan	649.48 (625.64 to 675.23)	337.82 (323.83 to 351.67)
Mongolia	998.25 (887.21 to 1112.12)	483.55 (431.61 to 537.93)
Tajikistan	650.71 (576.15 to 732.15)	435.25 (381.07 to 488.08)
Turkmenistan	859.18 (785.05 to 936.57)	476.03 (428.98 to 525.83)
Uzbekistan	823.39 (715.42 to 945.32)	474.6 (406.68 to 551.39)
Central Europe		

	Estimate in 2017, male (95% UIs)	Estimate in 2017, female (95% UIs)
Albania	383.86 (300.28 to 479.28)	186.2 (138.23 to 242.68)
Bosnia and Herzegovina	498.28 (459.44 to 537.34)	279.28 (255.7 to 304.4)
Bulgaria	725.48 (674.89 to 776.65)	331.08 (307.21 to 357.91)
Croatia	441.54 (409.82 to 472.85)	196.06 (179.01 to 213.92)
Czech Republic	396.83 (368.93 to 427.05)	201.08 (185.64 to 218.37)
Hungary	627.05 (586.31 to 673.03)	292.12 (271.03 to 315.7)
Macedonia	540.07 (500.49 to 583.25)	296.22 (269.57 to 324.43)
Montenegro	551.37 (492.95 to 619.52)	294.65 (267.89 to 323.25)
Poland	511.42 (478.55 to 546.64)	235.66 (219.05 to 254.54)
Romania	635.43 (595.42 to 677.94)	279.39 (258.85 to 298.49)
Serbia	548.83 (511.48 to 587.85)	334.72 (309.74 to 360.11)
Slovakia	494.73 (460.23 to 534.35)	231.32 (211.98 to 251.22)
Slovenia	308.78 (283.52 to 335.74)	149 (134.68 to 165.2)
Eastern Europe		
Belarus	769.87 (722.13 to 823.57)	271.55 (250.34 to 292.74)
Estonia	486 (418.59 to 559.88)	196.97 (166.32 to 231.44)
Latvia	712.47 (622.41 to 810.83)	263.14 (222.08 to 309.58)
Lithuania	683.71 (635.44 to 733.93)	244.32 (222.81 to 265.7)
Moldova	735.48 (708.65 to 763.42)	313.77 (299.27 to 328.3)
Russian Federation	807.75 (798.57 to 817.14)	321.79 (317.54 to 326.19)
Ukraine	1000.12 (941.85 to 1060.48)	348.35 (323.32 to 376.68)
High-income		
Australasia		
Australia	235.65 (203.7 to 270.2)	148.06 (126.67 to 171.33)
New Zealand	261.65 (243.85 to 279.83)	183.93 (171.78 to 197.15)
High-income Asia Pacific		
Brunei	487.46 (441.71 to 535.98)	370.23 (335.59 to 401.99)
Japan	212.78 (205.62 to 220.23)	109.97 (106.35 to 113.88)
South Korea	218.56 (198.79 to 238.98)	99.58 (90.9 to 108.45)
Singapore	218.17 (201.78 to 236.18)	107.02 (97.48 to 117.58)
High-income North America		
Canada	249.42 (232.02 to 267.69)	168.71 (156.97 to 182.25)
Greenland	517.51 (490.13 to 542.8)	334.5 (309.38 to 360.35)
United States	351.37 (340.47 to 362.57)	228.68 (221.17 to 235.97)
Southern Latin America		
Argentina	422.84 (368.79 to 491.4)	254.12 (218.71 to 293.66)
Chile	281.33 (242.49 to 324.69)	195.08 (166.05 to 227.21)
Uruguay	454.65 (395.09 to 520.41)	258.72 (219.33 to 300.3)
Western Europe		
Andorra	248.62 (214.95 to 285.9)	148.52 (119.55 to 180.26)
Austria	263.34 (243.68 to 283.77)	157.96 (145.55 to 171.23)
Belgium	271.26 (251.14 to 291.93)	165.45 (152.09 to 180.43)
Cyprus	294.28 (261.72 to 329.92)	140.1 (124.12 to 158.48)
Denmark	269.57 (250.02 to 292.12)	193.59 (177.86 to 211.31)
Finland	268.32 (249.4 to 290.75)	139.04 (127.09 to 152.44)
France	277.82 (258.66 to 298.36)	143.92 (133.15 to 155.28)
Germany	315.1 (275.36 to 360.63)	180 (153.79 to 208.65)

	Estimate in 2017, male (95% UIs)	Estimate in 2017, female (95% UIs)
Greece	351.75 (326.1 to 376.85)	173.9 (159.63 to 188.44)
Iceland	231.71 (220.12 to 243.08)	130.11 (121.7 to 138.98)
Ireland	233.46 (214.22 to 253.02)	166.71 (152.6 to 183.76)
Israel	215.29 (199.26 to 233.77)	140.31 (128.43 to 152.89)
Italy	226.95 (210.87 to 245.11)	136.25 (124.81 to 148)
Luxembourg	243.12 (213.38 to 275.74)	169.01 (149.11 to 190.56)
Malta	268.66 (251.46 to 283.84)	164.51 (152.54 to 177.64)
Netherlands	242.98 (225.97 to 262.11)	195.13 (179.85 to 211.33)
Norway	215.08 (209.09 to 221.76)	154.75 (150.24 to 159.41)
Portugal	305.19 (281.54 to 328.1)	142.54 (130.86 to 155.18)
Spain	268.16 (249.67 to 286.26)	126.45 (117.34 to 136.22)
Sweden	203.64 (190.05 to 217.76)	151.45 (140.82 to 163.09)
Switzerland	180.41 (165.92 to 195.19)	120.58 (110.24 to 130.92)
United Kingdom	270.08 (265.77 to 274.72)	190.07 (186.92 to 194.25)
Latin America and Caribbean		
Andean Latin America		
Bolivia	357.48 (266.87 to 473.37)	350.6 (266.83 to 438.53)
Ecuador	244.44 (215 to 278.29)	241.71 (212.66 to 274.47)
Peru	187.28 (149.81 to 226.34)	182.15 (146.47 to 222.52)
Caribbean		
Antigua and Barbuda	336.86 (309.75 to 367.03)	292.55 (267.46 to 316.29)
The Bahamas	507.27 (457.3 to 562.4)	366.95 (324.92 to 408.88)
Barbados	349.64 (311.85 to 389.85)	283.59 (253.36 to 313.02)
Belize	410.8 (390.55 to 430.63)	316.89 (301.4 to 332.93)
Bermuda	334.77 (310.89 to 359.82)	126.21 (110.89 to 142.71)
Cuba	385.39 (335.33 to 445.74)	266.49 (228.76 to 307.13)
Dominica	451.22 (415.39 to 490.93)	333.46 (301.41 to 365.87)
Dominican Republic	477.41 (394.61 to 562.6)	329.12 (275.87 to 387.36)
Grenada	515.62 (486.07 to 548.08)	404.97 (379.5 to 431.29)
Guyana	657.1 (570.74 to 753.73)	534.16 (458.44 to 617.44)
Haiti	630.29 (492.25 to 765.86)	716.53 (532.41 to 897.56)
Jamaica	478.45 (395.56 to 574.05)	367.86 (299.41 to 448.57)
Puerto Rico	293.58 (269.64 to 317.1)	180.4 (165.37 to 196.81)
Saint Lucia	431.86 (400.56 to 465.14)	328.65 (300.77 to 358.07)
Saint Vincent and the Grenadines	524.19 (489.18 to 561.12)	406.4 (374.22 to 442.12)
Suriname	533.06 (462.84 to 615.89)	342.17 (300.82 to 387.78)
Trinidad and Tobago	508.48 (399.15 to 643.46)	363.04 (275.19 to 466.49)
Virgin Islands, U.S.	646.67 (509.65 to 746.9)	301.45 (260.73 to 343.11)
Central Latin America		
Colombia	230.63 (200.1 to 264.54)	184.04 (158.71 to 213.89)
Costa Rica	300 (278.38 to 323.31)	183.53 (168.81 to 201.21)
El Salvador	315.56 (252.3 to 386.74)	267.2 (212.27 to 334.31)
Guatemala	291.96 (252.63 to 334.07)	286.82 (247.18 to 331.13)
Honduras	319.32 (244.25 to 410.21)	350.74 (263.9 to 443.86)
Mexico	318.83 (312.14 to 326.17)	239.45 (234.47 to 244.58)
Nicaragua	226.2 (193.83 to 266.72)	207.95 (176.52 to 242.08)
Panama	239.24 (219.55 to 258.41)	191.95 (175.39 to 209.23)

	Estimate in 2017, male (95% UIs)	Estimate in 2017, female (95% UIs)
Venezuela	401.72 (329.82 to 478.56)	261.29 (212.19 to 315.9)
Tropical Latin America		
Brazil	399.62 (394.38 to 405.15)	268.11 (264.49 to 272.45)
Paraguay	380.28 (298.25 to 469.91)	280.72 (219.57 to 349.97)
North Africa and Middle East		
North Africa and Middle East		
Afghanistan	880.32 (729.64 to 1058.01)	1045.95 (860.56 to 1251.53
Algeria	294.06 (265.22 to 319.36)	257.71 (238.6 to 275.88)
Bahrain	256.44 (227.5 to 287.42)	225.04 (201.12 to 251.87)
Egypt	763.59 (668.73 to 870.29)	431.87 (373.07 to 494.46)
Iran	349.5 (345.53 to 356.68)	226.26 (223.44 to 228.87)
Iraq	318.21 (291.92 to 348.04)	228.1 (208.16 to 250.12)
Jordan	302.53 (261.75 to 355.87)	204.41 (176.06 to 232.96)
Kuwait	242.06 (223.5 to 261)	107.86 (99.91 to 115.56)
Lebanon	437.9 (407.86 to 476.14)	288.28 (254.39 to 319.61)
Libya	520.35 (427.09 to 618.02)	424.66 (349.94 to 504.17)
Morocco	472.04 (374.07 to 585.72)	447.92 (358.09 to 546.45)
Palestine	377.26 (345.8 to 415.04)	310.14 (283.38 to 335)
Oman	375.75 (293.38 to 465.19)	261.98 (201.63 to 320.8)
Qatar	233.78 (187 to 284.45)	222.51 (180.12 to 271.49)
Saudi Arabia	343.72 (291.54 to 411.9)	248.71 (212.02 to 291.81)
Sudan	539.21 (409.91 to 706.05)	448.36 (331.97 to 586.27)
Syria	523.23 (414.13 to 650.72)	302.17 (236.64 to 365.8)
Tunisia	355.89 (269.75 to 453.76)	208.49 (155.87 to 273.18)
Turkey	408.31 (366.07 to 454.08)	191.61 (170.17 to 214.81)
United Arab Emirates	609.1 (479.94 to 761.82)	424.05 (338.41 to 521.94)
Yemen	669.2 (512.08 to 854.43)	538.2 (411.35 to 722.78)
South Asia		
South Asia		
Bangladesh	517.13 (447.68 to 596.45)	384.93 (330.97 to 445.28)
Bhutan	391.23 (302.39 to 507.91)	295.47 (233.05 to 370.62)
India	559.81 (533.8 to 580.93)	429.19 (401.61 to 448.17)
Nepal	554.96 (432.01 to 655.53)	359.33 (295.3 to 441.39)
Pakistan	709.64 (555.92 to 879.33)	572.09 (444.37 to 706.21)
Southeast Asia, East Asia, and Oceania		
East Asia		
China	453.13 (431.29 to 474.61)	235.56 (223.91 to 248.43)
North Korea	744.32 (628.93 to 855.27)	423.58 (339.5 to 519.82)
Taiwan	355.25 (332.2 to 380.44)	160.94 (149.17 to 173.67)
Oceania		
American Samoa	660.77 (555.9 to 761.97)	466.55 (422.42 to 512.88)
Federated States of Micronesia	956.52 (733.97 to 1167.93)	660.49 (518.1 to 827.75)
Fiji	1011.89 (865.98 to 1179.23)	719.14 (593.17 to 855.73)
Guam	669.72 (609.73 to 730.02)	399.62 (363.6 to 439.4)
Kiribati	1401.23 (1167.85 to 1662.62)	738.53 (602.39 to 884.77)
Marshall Islands	1207.89 (1022.26 to 1419)	841.88 (703.11 to 1001.3)
Northern Mariana Islands	456.42 (395.88 to 521.48)	273.34 (241.62 to 310.35)

	Estimate in 2017, male (95% UIs)	Estimate in 2017, female (95% UIs)
Papua New Guinea	1521.19 (1256.58 to 1828.01)	1145.9 (937.17 to 1394.85)
Samoa	561.34 (475.36 to 634.4)	424.62 (346.57 to 496.63)
Solomon Islands	883.2 (748.13 to 1028.57)	740.47 (627.32 to 877.31)
Tonga	694.59 (599.34 to 824.66)	390.29 (327.67 to 460.2)
Vanuatu	1099.53 (889.14 to 1365.21)	749.87 (596.72 to 944.33)
Southeast Asia		
Cambodia	530.65 (460.04 to 607.45)	357.21 (296.91 to 438.74)
Indonesia	595.54 (548.23 to 640.04)	419.83 (389.69 to 453)
Laos	646.86 (516.29 to 770.74)	488.86 (395.77 to 611.76)
Malaysia	461.58 (411.21 to 526.84)	271.38 (240.24 to 306.68)
Maldives	265.56 (244.13 to 286.02)	175.82 (159.11 to 192.87)
Mauritius	520.96 (480 to 564.8)	307.76 (279.99 to 337.66)
Myanmar	634.56 (544.04 to 715.79)	409.92 (327.11 to 504.82)
Philippines	691.02 (572.98 to 820.62)	404.97 (332 to 494.77)
Sri Lanka	381.78 (304.61 to 469.08)	206.22 (152.64 to 263.6)
Seychelles	543.75 (512.26 to 576.64)	294.87 (271.3 to 317.92)
Thailand	336.33 (290.9 to 383.92)	176.87 (153.32 to 203.24)
Timor-Leste	534.9 (407.36 to 641.91)	408.03 (327.43 to 504.31)
Vietnam	606.39 (529.27 to 722.46)	260.88 (203.84 to 322.62)
Sub-Saharan Africa		
Central Sub-Saharan Africa		
Angola	521.49 (426.94 to 615.69)	377.31 (295.49 to 478.97)
Central African Republic	834.16 (650.69 to 1024.22)	656.49 (454.36 to 830.37)
Congo	500.79 (404.6 to 598.76)	594.27 (455.7 to 729.59)
Democratic Republic of the Congo	518.77 (415.31 to 623.21)	436.29 (339.07 to 554.84)
Equatorial Guinea	398.48 (288.99 to 524.98)	303.83 (182.7 to 471.76)
Gabon	543.28 (467.74 to 630.4)	314.81 (235.83 to 405.45)
Eastern Sub-Saharan Africa		
Burundi	480.96 (383.55 to 591.24)	443.23 (339.03 to 551.07)
Comoros	457.56 (364.89 to 545.45)	418.82 (332.07 to 528.64)
Djibouti	497.88 (369.62 to 646.39)	398.4 (277.17 to 575.24)
Eritrea	724.17 (569.14 to 884.35)	532.58 (405.65 to 654.9)
Ethiopia	333.15 (296.58 to 368.03)	301.55 (267.19 to 335.23)
Kenya	424.34 (387.21 to 469.07)	301.82 (273.17 to 329.11)
Madagascar	656.88 (508.99 to 811.61)	581 (441.68 to 737.74)
Malawi	525.63 (438.22 to 605.82)	320.11 (252 to 406.3)
Mozambique	745.89 (623.51 to 873.63)	440.64 (334.38 to 549.88)
Rwanda	368.6 (305.27 to 435.22)	295.53 (238.54 to 361.93)
Somalia	646.97 (493.82 to 822.8)	598.63 (449.53 to 780.32)
South Sudan	548.51 (411.61 to 711.76)	451.16 (327.78 to 613.59)
Tanzania	397.64 (331.47 to 471.54)	322.26 (269.04 to 386.5)
Uganda	494.89 (404.91 to 579.17)	308.26 (247.64 to 381.53)
Zambia	490.75 (419.61 to 568.73)	375.85 (296.38 to 459.4)
Southern Sub-Saharan Africa		
Botswana	342.38 (304.36 to 425.81)	355.31 (307.48 to 406.26)
Lesotho	965.39 (813.84 to 1127.68)	589.98 (399.4 to 766.84)
Namibia	511.62 (440.76 to 596.97)	277.74 (217.47 to 367.83)

	Estimate in 2017, male (95% UIs)	Estimate in 2017, female (95% UIs)
South Africa	493.07 (471.81 to 513.43)	307.77 (294.09 to 321.84)
Swaziland	906.99 (738.85 to 1093.47)	475.53 (317.64 to 648.84)
Zimbabwe	627.71 (526.43 to 736.79)	551.59 (436.83 to 668.27)
Western Sub-Saharan Africa		
Benin	415.4 (320.11 to 529.53)	372.28 (284.24 to 485.03)
Burkina Faso	497.86 (415.11 to 592.14)	381.4 (315.67 to 455.32)
Cameroon	479.67 (368.67 to 595.08)	392.84 (291.7 to 526.94)
Cape Verde	354.95 (315.17 to 398.6)	222.25 (199.95 to 236.73)
Chad	451.89 (364.55 to 556.12)	441.01 (348.77 to 555.59)
Cote d'Ivoire	489.17 (391.73 to 601.15)	416.98 (324.62 to 530.59)
The Gambia	564.24 (452.65 to 681.46)	465.12 (361.85 to 584.07)
Ghana	467.63 (402.7 to 531.91)	442.83 (360.47 to 539.22)
Guinea	603.42 (482.46 to 732.04)	555.85 (450.61 to 670.28)
Guinea-Bissau	709.06 (584.51 to 856.32)	608.21 (468.86 to 739.78)
Liberia	399.7 (319.13 to 500.94)	431.66 (333.9 to 539.55)
Mali	417.37 (326.47 to 517.52)	452.16 (368.93 to 555.45)
Mauritania	327.8 (255.74 to 405.74)	400.82 (316.66 to 502.8)
Niger	391.33 (301.82 to 536.43)	392.13 (304.04 to 492.48)
Nigeria	254.12 (192.05 to 359.11)	284.09 (187.94 to 407.98)
Sao Tome and Principe	458.39 (381.02 to 537.89)	424.72 (349.6 to 505.28)
Senegal	434.72 (360.2 to 511.96)	400.71 (323.75 to 485.61)
Sierra Leone	475.4 (382.74 to 582.03)	514.32 (405.67 to 647.75)
Togo	524.17 (413.66 to 635.37)	379.79 (293.71 to 489.29)
**Indicator 3.4.2: Age-standardised death rate due to self-harm (per 100,000 population)**		
Central Europe, Eastern Europe, and Central Asia		
Central Asia		
Armenia	13.81 (12.84 to 14.84)	3.25 (3 to 3.5)
Azerbaijan	6.78 (4.62 to 8.04)	1.58 (1.34 to 1.82)
Georgia	13.35 (12.18 to 14.52)	2.18 (2 to 2.36)
Kazakhstan	39.63 (35.85 to 44.27)	7.67 (7.01 to 8.34)
Kyrgyzstan	16.35 (14.83 to 18.65)	3.91 (3.58 to 4.23)
Mongolia	25.94 (21.36 to 30.64)	4.82 (4.08 to 5.62)
Tajikistan	8.19 (6.51 to 9.55)	3.15 (2.71 to 3.66)
Turkmenistan	13.29 (11.76 to 14.96)	3.52 (3.11 to 3.94)
Uzbekistan	14.15 (11.98 to 16.56)	4.7 (4 to 5.6)
Central Europe		
Albania	7.21 (4.94 to 9.39)	2.88 (2.19 to 3.72)
Bosnia and Herzegovina	11.19 (9.73 to 14.52)	2.59 (2.3 to 2.9)
Bulgaria	15.61 (14.22 to 17)	3.63 (3.28 to 4)
Croatia	18.18 (16.68 to 19.78)	4.41 (3.95 to 4.87)
Czech Republic	17.83 (16.4 to 19.42)	3.69 (3.34 to 4.07)
Hungary	23.51 (21.72 to 25.49)	5.9 (5.37 to 6.46)
Macedonia	10.38 (8.97 to 11.88)	3.31 (2.89 to 3.77)
Montenegro	13.66 (11.32 to 16.01)	5.09 (4.39 to 5.88)
Poland	24.59 (22.47 to 26.89)	3.3 (3 to 3.63)
Romania	15.77 (14.51 to 17.06)	2.51 (2.27 to 2.74)

	Estimate in 2017, male (95% UIs)	Estimate in 2017, female (95% UIs)
Serbia	19.77 (17.58 to 22.95)	5.79 (5.19 to 6.46)
Slovakia	16.35 (13.51 to 19.3)	2.84 (2.5 to 3.23)
Slovenia	24.22 (21.77 to 26.48)	4.59 (4.05 to 5.22)
Eastern Europe		
Belarus	34.43 (30.41 to 39.32)	5.72 (5.13 to 6.35)
Estonia	22.85 (19 to 27.31)	3.42 (2.81 to 4.12)
Latvia	30.98 (26.4 to 35.88)	4.37 (3.62 to 5.23)
Lithuania	50.42 (46.07 to 54.98)	7.41 (6.62 to 8.25)
Moldova	24.63 (22.77 to 26.41)	3.41 (3.14 to 3.71)
Russian Federation	44.03 (42.27 to 45.55)	7.71 (7.48 to 7.95)
Ukraine	46.48 (42.26 to 52.09)	6.53 (5.87 to 7.16)
High-income		
Australasia		
Australia	17.02 (14.6 to 19.7)	4.87 (4.12 to 5.68)
New Zealand	17.27 (15.88 to 18.73)	5.32 (4.88 to 5.81)
High-income Asia Pacific		
Brunei	9.07 (7.47 to 10.67)	3.06 (2.62 to 3.48)
Japan	22.25 (21.06 to 24.08)	8.59 (8.2 to 8.98)
South Korea	30.34 (26.9 to 34.15)	11.83 (10.69 to 12.97)
Singapore	10.06 (9.06 to 11.17)	4.61 (4.16 to 5.13)
High-income North America		
Canada	16.11 (14.7 to 17.62)	5.47 (4.97 to 6.04)
Greenland	73.63 (66.49 to 84.35)	24.7 (22.04 to 27.55)
United States	19.88 (18.72 to 20.72)	5.62 (5.38 to 5.88)
Southern Latin America		
Argentina	17.58 (14.97 to 20.63)	3.9 (3.33 to 4.57)
Chile	16.71 (14.25 to 19.49)	3.63 (3.06 to 4.25)
Uruguay	27.85 (23.83 to 32.18)	6.63 (5.48 to 7.81)
Western Europe		
Andorra	12.34 (9.59 to 15.63)	2.96 (2.41 to 3.58)
Austria	17.46 (15.9 to 19.26)	5.07 (4.58 to 5.63)
Belgium	20.82 (19.05 to 22.8)	7.52 (6.81 to 8.25)
Cyprus	7.5 (6.29 to 8.84)	1.68 (1.44 to 1.95)
Denmark	13.04 (11.82 to 14.32)	4.4 (3.97 to 4.87)
Finland	19.66 (17.91 to 21.71)	5.82 (5.21 to 6.43)
France	19.56 (17.75 to 21.66)	5.48 (4.94 to 6.04)
Germany	15.06 (13.15 to 17.41)	4.67 (3.94 to 5.49)
Greece	5.71 (5.23 to 6.3)	1.27 (1.14 to 1.42)
Iceland	16.37 (15.17 to 17.67)	2.59 (2.36 to 2.83)
Ireland	13.58 (12.07 to 15.28)	2.99 (2.64 to 3.37)
Israel	9.34 (8.44 to 10.4)	2.09 (1.87 to 2.33)
Italy	7.58 (6.86 to 8.43)	1.99 (1.8 to 2.21)
Luxembourg	12.78 (10.85 to 15.14)	4.51 (3.86 to 5.22)
Malta	7.53 (6.9 to 8.19)	1.46 (1.32 to 1.62)
Netherlands	12.61 (11.66 to 13.67)	5.52 (5.03 to 6.07)
Norway	12.91 (12.13 to 14.05)	5.22 (4.99 to 5.45)
Portugal	12.91 (11.43 to 14.43)	2.98 (2.67 to 3.34)

	Estimate in 2017, male (95% UIs)	Estimate in 2017, female (95% UIs)
Spain	8.53 (7.76 to 9.41)	2.53 (2.3 to 2.79)
Sweden	15.45 (14.31 to 16.78)	6.26 (5.73 to 6.84)
Switzerland	13.66 (12.28 to 15.47)	4.88 (4.39 to 5.39)
United Kingdom	11.21 (10.82 to 11.53)	3.29 (3.2 to 3.39)
Latin America and Caribbean		
Andean Latin America		
Bolivia	8.62 (5.81 to 11.35)	4.07 (2.98 to 5.19)
Ecuador	13.84 (11.83 to 16.15)	4.42 (3.82 to 5.13)
Peru	4.71 (3.48 to 5.94)	1.49 (1.18 to 1.85)
Caribbean		
Antigua and Barbuda	4.44 (3.91 to 5.02)	0.91 (0.8 to 1.05)
The Bahamas	5.43 (4.71 to 6.24)	1.03 (0.88 to 1.19)
Barbados	6.8 (5.91 to 7.77)	1.35 (1.18 to 1.54)
Belize	13.63 (12.37 to 15.01)	1.8 (1.65 to 1.97)
Bermuda	6.33 (5.59 to 7.18)	0.83 (0.71 to 0.96)
Cuba	17.85 (15.24 to 20.92)	4.64 (3.98 to 5.41)
Dominica	7.49 (6.69 to 8.47)	1.38 (1.2 to 1.57)
Dominican Republic	14.61 (10.25 to 17.92)	2.98 (2.46 to 3.59)
Grenada	7.55 (6.83 to 8.3)	1.84 (1.66 to 2.05)
Guyana	42.4 (35.66 to 49.46)	9.92 (8.26 to 11.84)
Haiti	13.18 (9.1 to 18.25)	4.17 (3.17 to 6.26)
Jamaica	6.11 (4.18 to 7.71)	1.08 (0.85 to 1.35)
Puerto Rico	10.9 (9.77 to 12.13)	1.57 (1.39 to 1.77)
Saint Lucia	12.28 (10.93 to 13.63)	1.44 (1.28 to 1.63)
Saint Vincent and the Grenadines	12.41 (11.05 to 13.86)	1.55 (1.38 to 1.74)
Suriname	39.38 (33.4 to 46.02)	10.37 (8.92 to 11.97)
Trinidad and Tobago	16.45 (12.56 to 21.16)	3.39 (2.43 to 4.48)
Virgin Islands, U.S.	11.15 (9.24 to 13.02)	2.04 (1.68 to 2.45)
Central Latin America		
Colombia	9.29 (7.84 to 10.9)	1.8 (1.54 to 2.12)
Costa Rica	13.85 (12.12 to 15.36)	2 (1.78 to 2.27)
El Salvador	17.29 (13.32 to 23.19)	3.54 (2.74 to 4.56)
Guatemala	10 (8.47 to 11.69)	2.21 (1.89 to 2.59)
Honduras	7.15 (4.76 to 10.49)	1.73 (1.26 to 2.26)
Mexico	9.92 (9.37 to 10.28)	2.02 (1.95 to 2.09)
Nicaragua	9.13 (7.34 to 11.28)	2.65 (2.17 to 3.21)
Panama	8.31 (7.3 to 9.29)	1.49 (1.31 to 1.68)
Venezuela	15.23 (12.12 to 18.62)	2.02 (1.61 to 2.48)
Tropical Latin America		
Brazil	10.07 (9.39 to 10.42)	2.21 (2.15 to 2.27)
Paraguay	8.46 (5.22 to 11.08)	2.86 (2.2 to 3.62)
North Africa and Middle East		
North Africa and Middle East		
Afghanistan	13.72 (10.33 to 20.63)	4.54 (3.43 to 5.86)
Algeria	5.87 (4.49 to 7.25)	2.19 (1.88 to 2.53)
Bahrain	5.57 (4.39 to 6.99)	1.71 (1.45 to 1.99)
Egypt	7.94 (5.13 to 10.38)	2.66 (2.16 to 3.31)

	Estimate in 2017, male (95% UIs)	Estimate in 2017, female (95% UIs)
Iran	7.55 (6.18 to 8.12)	3.09 (2.83 to 3.31)
Iraq	5.49 (4.7 to 6.53)	2.45 (2.11 to 2.82)
Jordan	4.76 (3.89 to 5.66)	1.26 (1.05 to 1.5)
Kuwait	3.67 (3.16 to 4.34)	0.9 (0.8 to 1.01)
Lebanon	7.33 (5.47 to 8.86)	2.11 (1.7 to 2.56)
Libya	12 (6.73 to 16.87)	3.59 (2.74 to 4.47)
Morocco	6.86 (4.94 to 9.17)	7.15 (5.53 to 9.08)
Palestine	5.35 (3.47 to 6.13)	1.52 (1.35 to 1.71)
Oman	3.78 (2.8 to 5)	1.18 (0.95 to 1.45)
Qatar	5.72 (4.21 to 7.45)	1.11 (0.86 to 1.4)
Saudi Arabia	4.31 (3.13 to 5.65)	0.85 (0.65 to 1.1)
Sudan	7.33 (5.18 to 9.84)	2.5 (1.8 to 3.39)
Syria	4.86 (3.51 to 6.34)	1.15 (0.92 to 1.47)
Tunisia	4.66 (3.27 to 6.3)	1.51 (1.11 to 2)
Turkey	5.25 (4.41 to 6.13)	1.31 (1.13 to 1.5)
United Arab Emirates	6.29 (4.34 to 8.16)	1.77 (1.32 to 2.29)
Yemen	8.22 (5.62 to 11.88)	3.61 (2.63 to 4.89)
South Asia		
South Asia		
Bangladesh	6.12 (4.64 to 7.49)	5.62 (4.56 to 6.79)
Bhutan	8.01 (5.51 to 10.62)	2.96 (2.14 to 3.81)
India	17.62 (12.83 to 19.59)	13.07 (12.08 to 13.89)
Nepal	10.42 (6.55 to 14.19)	6.3 (4.73 to 7.94)
Pakistan	4.41 (3.24 to 5.8)	4.31 (3.16 to 5.49)
Southeast Asia, East Asia, and Oceania		
East Asia		
China	8.65 (7.93 to 9.86)	5.54 (5.19 to 5.91)
North Korea	15.84 (11.13 to 22.02)	6.98 (5.35 to 9.32)
Taiwan	18.31 (16.87 to 19.84)	8.45 (7.72 to 9.26)
Oceania		
American Samoa	9.69 (8.23 to 11.02)	3.6 (3.09 to 4.14)
Federated States of Micronesia	25.32 (15.72 to 34.75)	8.1 (5.44 to 10.91)
Fiji	13.38 (10.93 to 16.29)	5.91 (4.78 to 7.17)
Guam	32.63 (28.16 to 36.78)	6.88 (6 to 7.8)
Kiribati	46.89 (34.59 to 59.44)	6.84 (4.99 to 8.94)
Marshall Islands	32.03 (21.5 to 42.27)	11.58 (8.77 to 15.63)
Northern Mariana Islands	20.2 (16.86 to 23.63)	3.24 (2.74 to 3.84)
Papua New Guinea	34.14 (18.54 to 49.41)	10.6 (7.65 to 14.85)
Samoa	14.53 (10.9 to 18.94)	5.01 (3.6 to 6.38)
Solomon Islands	23.38 (16.39 to 31.37)	8.71 (6.42 to 12.17)
Tonga	7.8 (6.07 to 9.31)	3.85 (3.07 to 4.7)
Vanuatu	27.37 (16.26 to 38.22)	7.41 (5.28 to 10.05)
Southeast Asia		
Cambodia	7.03 (5.49 to 8.65)	2.32 (1.88 to 2.91)
Indonesia	4.63 (3.99 to 5.22)	1.52 (1.4 to 1.66)
Laos	12.61 (7.74 to 17.57)	4.03 (3.09 to 4.99)
Malaysia	11.22 (9.62 to 13.5)	3.96 (3.46 to 4.55)

	Estimate in 2017, male (95% UIs)	Estimate in 2017, female (95% UIs)
Maldives	4.63 (3.72 to 5.57)	1.37 (1.05 to 1.68)
Mauritius	13.42 (11.87 to 14.93)	3.05 (2.72 to 3.44)
Myanmar	8.59 (5.82 to 10.87)	1.78 (1.41 to 2.17)
Philippines	8.92 (7.32 to 10.73)	2.88 (2.38 to 3.49)
Sri Lanka	33.53 (25.57 to 45.87)	7.4 (5.76 to 9.24)
Seychelles	13.27 (11.53 to 17.59)	2.3 (2.04 to 2.59)
Thailand	16.9 (13.69 to 19.75)	3.88 (3.28 to 4.54)
Timor-Leste	10.49 (5.6 to 14.92)	3.97 (2.41 to 5.08)
Vietnam	10.41 (7.79 to 12.5)	4.44 (3.53 to 5.54)
Sub-Saharan Africa		
Central Sub-Saharan Africa		
Angola	20.51 (15.89 to 25.14)	4.49 (3.43 to 5.64)
Central African Republic	27.51 (18.25 to 36.23)	6.59 (4.58 to 9.13)
Congo	20.64 (14.37 to 27.33)	7.45 (5.27 to 9.73)
Democratic Republic of the Congo	19.48 (15.25 to 24.3)	4.5 (3.4 to 5.84)
Equatorial Guinea	15.1 (10.25 to 21.25)	4.39 (2.62 to 6.44)
Gabon	21.73 (14.75 to 28.51)	4.12 (2.99 to 5.28)
Eastern Sub-Saharan Africa		
Burundi	19.35 (14.31 to 25.52)	5.63 (4.42 to 7.33)
Comoros	13.29 (8.08 to 24)	3.74 (2.56 to 5.03)
Djibouti	12.28 (6.8 to 21.85)	3.92 (2.27 to 6.1)
Eritrea	26.79 (18.91 to 35.68)	7.22 (5.18 to 9.54)
Ethiopia	14.19 (11.97 to 16.82)	4.47 (3.79 to 5.21)
Kenya	19.02 (16.28 to 22.98)	3.98 (3.5 to 4.52)
Madagascar	16.92 (12.15 to 22.03)	5.08 (3.95 to 6.43)
Malawi	22.1 (17.58 to 26.62)	3.9 (3.01 to 4.88)
Mozambique	30.83 (23.06 to 38.75)	6 (4.63 to 7.61)
Rwanda	18.78 (14.19 to 24.17)	5.09 (3.85 to 6.55)
Somalia	14.62 (9.45 to 24.86)	5.21 (3.08 to 7.75)
South Sudan	18.35 (12.58 to 25.81)	4.45 (2.68 to 6.72)
Tanzania	14.04 (10.89 to 17.55)	3.9 (3.09 to 4.81)
Uganda	15.47 (12.31 to 19.57)	7.97 (6.37 to 10.02)
Zambia	21 (16.93 to 25.01)	5.36 (4.28 to 6.61)
Southern Sub-Saharan Africa		
Botswana	15.1 (10.14 to 24.19)	5.25 (4.17 to 6.37)
Lesotho	41.1 (29.1 to 54.96)	20.66 (14.14 to 28.22)
Namibia	21.97 (16.24 to 30.15)	2.89 (2.11 to 3.86)
South Africa	18.29 (15.89 to 20.77)	4.41 (3.99 to 4.85)
Swaziland	39.42 (27.78 to 51.81)	6.87 (4.63 to 9.83)
Zimbabwe	40.29 (30.52 to 49.07)	14.21 (10.99 to 17.87)
Western Sub-Saharan Africa		
Benin	16.8 (12.79 to 22.14)	5.76 (4.21 to 7.64)
Burkina Faso	21.49 (17.4 to 26.29)	5.79 (4.71 to 6.93)
Cameroon	20.42 (15.66 to 25.81)	7.55 (5.54 to 9.98)
Cape Verde	27.08 (21.98 to 31.32)	6.01 (5.29 to 6.68)
Chad	14.43 (10.31 to 22.75)	7.08 (4.54 to 9.93)
Cote d'Ivoire	21.15 (16.78 to 26.54)	5.55 (4.26 to 7.08)

	Estimate in 2017, male (95% UIs)	Estimate in 2017, female (95% UIs)
The Gambia	13.83 (8.67 to 23.04)	5.62 (4.45 to 7.1)
Ghana	19.07 (14.99 to 22.59)	1.81 (1.5 to 2.19)
Guinea	15.65 (10.74 to 23.41)	6.51 (4.47 to 8.72)
Guinea-Bissau	20.92 (16.25 to 26.94)	6.88 (4.53 to 9.73)
Liberia	14.88 (10.99 to 20.6)	11.02 (8.53 to 13.89)
Mali	10.08 (5.81 to 22.77)	3.19 (2.16 to 4.31)
Mauritania	10.11 (5.81 to 19.19)	3.01 (2.08 to 4.06)
Niger	10.93 (6.84 to 23.56)	4.21 (2.8 to 5.69)
Nigeria	9.88 (6.79 to 15.23)	5.09 (3.15 to 7.72)
Sao Tome and Principe	5.04 (3.12 to 6.87)	1.95 (1.5 to 2.42)
Senegal	20.97 (16.29 to 26.44)	5.07 (4.18 to 6.11)
Sierra Leone	12.99 (9.24 to 20.17)	6.45 (4.34 to 8.82)
Togo	20.2 (15.75 to 25.26)	5.65 (4.26 to 7.31)
**Indicator 3.5.2: Risk-weighted prevalence of alcohol consumption, as measured by the summary exposure value (SEV) for alcohol use (%)**		
Central Europe, Eastern Europe, and Central Asia		
Central Asia		
Armenia	16.59 (8.71 to 27.09)	7.4 (4.21 to 11.93)
Azerbaijan	23.91 (13.64 to 35.09)	6.43 (3.24 to 10.63)
Georgia	31.91 (19.01 to 46.95)	4.08 (1.68 to 7.9)
Kazakhstan	29.33 (17.74 to 41.89)	15.48 (8.69 to 24.39)
Kyrgyzstan	23.36 (14.11 to 33)	7.73 (4.24 to 12.61)
Mongolia	20.82 (12.26 to 32.67)	8.11 (4.2 to 14.35)
Tajikistan	9.02 (4.37 to 16.5)	2.81 (1.21 to 5.53)
Turkmenistan	20.22 (11.03 to 33)	6.52 (3.21 to 11.67)
Uzbekistan	19.71 (11.3 to 31)	6.09 (3.1 to 10.74)
Central Europe		
Albania	21.7 (12.93 to 30.89)	7.77 (4.47 to 11.86)
Bosnia and Herzegovina	30.23 (23.23 to 37.95)	11.65 (7.88 to 15.52)
Bulgaria	32.15 (19.9 to 46.97)	12.65 (6.66 to 21.82)
Croatia	31.76 (18.79 to 47.04)	10.09 (5.08 to 17.75)
Czech Republic	35.95 (23.74 to 50.41)	17.46 (10.25 to 27.75)
Hungary	37.25 (24.84 to 52.07)	14.81 (8.24 to 24.06)
Macedonia	12.09 (6.02 to 20.8)	4.75 (1.93 to 9.01)
Montenegro	35.89 (22.58 to 49.38)	14.08 (8.11 to 22.23)
Poland	32.68 (20.77 to 47.06)	14.42 (8.16 to 24.09)
Romania	48.17 (36.09 to 59.94)	14.33 (8.12 to 23.04)
Serbia	37.79 (24.95 to 52.07)	15.47 (8.87 to 24.74)
Slovakia	35.13 (21.96 to 49.99)	13.4 (7 to 23.43)
Slovenia	14.76 (3.66 to 28.92)	6.48 (1.44 to 14.15)
Eastern Europe		
Belarus	47.18 (34.2 to 60.94)	21.13 (12.55 to 33.04)
Estonia	43.74 (28.93 to 59.15)	17.84 (9.74 to 28.25)
Latvia	40 (26.07 to 54.87)	15.55 (8.59 to 24.97)
Lithuania	47.02 (34.54 to 59.86)	18.84 (11.23 to 29.36)
Moldova	35.16 (18.86 to 52.21)	13.74 (7.21 to 22.73)

	Estimate in 2017, male (95% UIs)	Estimate in 2017, female (95% UIs)
Russian Federation	35.67 (23.72 to 48.93)	15.12 (8.7 to 23.64)
Ukraine	46.26 (33.99 to 59.41)	27.78 (16.09 to 40.45)
High-income		
Australasia		
Australia	34.44 (21.92 to 49.07)	14.69 (8.1 to 24.29)
New Zealand	38.22 (24.78 to 50.93)	8.76 (4.22 to 16.35)
High-income Asia Pacific		
Brunei	3.65 (1.1 to 8.83)	2.26 (0.7 to 5.58)
Japan	19.12 (10.5 to 30.92)	15.26 (8.32 to 25.23)
South Korea	22.69 (12.49 to 35.34)	16.07 (8.69 to 25.36)
Singapore	14.63 (7.32 to 25.7)	8.94 (4.33 to 16.27)
High-income North America		
Canada	25.52 (15.46 to 38.19)	17.57 (10.2 to 27.71)
Greenland	17.13 (9.2 to 28.53)	12 (5.81 to 21.25)
United States	27.31 (17.65 to 39.25)	15.52 (9.17 to 24.03)
Southern Latin America		
Argentina	27.55 (16.15 to 42.51)	13.68 (7.37 to 24.03)
Chile	28.84 (17.56 to 43.84)	12.97 (7.34 to 22)
Uruguay	25.77 (15.38 to 38.75)	10.48 (5.74 to 17.64)
Western Europe		
Andorra	32.08 (19.81 to 47.27)	18.13 (10.17 to 29.41)
Austria	36.25 (22.67 to 52.44)	19.21 (11.26 to 30.56)
Belgium	31.56 (19.24 to 47.18)	17.37 (9.79 to 27.77)
Cyprus	31.97 (19.74 to 47.87)	8.56 (4.15 to 15.9)
Denmark	30.37 (17.49 to 46.55)	19.22 (10.78 to 30.32)
Finland	35.04 (21.39 to 50.18)	16.7 (9.3 to 27.35)
France	35.65 (21.53 to 51.93)	17.54 (9.96 to 28.31)
Germany	32.51 (19.86 to 48.62)	19.65 (11.38 to 31.32)
Greece	35.17 (21.02 to 51.47)	14.41 (7.84 to 24.14)
Iceland	22.45 (12.98 to 35.62)	13.64 (7.24 to 24.17)
Ireland	35.4 (22.57 to 49.13)	21.27 (12.46 to 32.41)
Israel	9.97 (5.51 to 16.42)	5.13 (2.72 to 8.99)
Italy	29.98 (18.02 to 44.18)	13.61 (7.53 to 22.55)
Luxembourg	48.07 (33.07 to 62.03)	21.29 (12.2 to 33.06)
Malta	21.9 (12.43 to 35.51)	10.9 (5.23 to 20.37)
Netherlands	25.31 (14.93 to 38.73)	15.14 (8.43 to 24.41)
Norway	23.02 (13.82 to 34.77)	15.06 (8.35 to 25.11)
Portugal	46.4 (32.92 to 59.39)	14.26 (8.21 to 22.3)
Spain	41.71 (28.07 to 57.16)	17.98 (10.48 to 28.06)
Sweden	24.61 (14.43 to 38.24)	18.87 (10.91 to 30.7)
Switzerland	24.9 (14.39 to 38.28)	19.78 (11.22 to 30.68)
United Kingdom	27.75 (18.16 to 39.06)	16.29 (9.67 to 25.66)
Latin America and Caribbean		
Andean Latin America		
Bolivia	13 (7.04 to 21.3)	5.79 (2.67 to 10.98)
Ecuador	23.29 (18.15 to 28.44)	7.08 (4.09 to 10.1)
Peru	22.92 (13.65 to 35.03)	11.13 (5.92 to 19.36)

	Estimate in 2017, male (95% UIs)	Estimate in 2017, female (95% UIs)
Caribbean		
Antigua and Barbuda	14.05 (6.87 to 23.44)	5.05 (2.19 to 9.52)
The Bahamas	20.67 (10.1 to 33.21)	6.53 (3.13 to 11.31)
Barbados	24.57 (14.72 to 36.21)	6.92 (3.85 to 11.33)
Belize	30.99 (19.76 to 40.89)	7.13 (4.04 to 11.06)
Bermuda	39.19 (29.48 to 49.46)	12.13 (7.4 to 17.56)
Cuba	21.52 (12.82 to 33.28)	6.67 (3.66 to 11.25)
Dominica	25.14 (14.61 to 36.73)	8.07 (4.46 to 12.87)
Dominican Republic	18.54 (10.21 to 30.01)	8.61 (4.68 to 14.26)
Grenada	24.99 (14.84 to 36.55)	7.46 (4.17 to 12.13)
Guyana	24.24 (14.52 to 35.15)	3.04 (1.38 to 5.75)
Haiti	19.13 (11.46 to 28.5)	5.9 (3.29 to 9.73)
Jamaica	21.31 (11.94 to 32.6)	5.81 (3.22 to 9.73)
Puerto Rico	29.93 (17.25 to 44.53)	8.01 (4.23 to 13.31)
Saint Lucia	26.03 (15.77 to 38.71)	7.9 (4.36 to 12.82)
Saint Vincent and the Grenadines	20.67 (11.61 to 31.71)	6.77 (3.58 to 11.53)
Suriname	28.3 (17.95 to 39.56)	8.62 (4.84 to 13.64)
Trinidad and Tobago	15.1 (8.35 to 23.92)	4.91 (2.45 to 8.87)
Virgin Islands, U.S.	13.15 (0.58 to 28.1)	4.64 (0 to 11.02)
Central Latin America		
Colombia	26.39 (15.69 to 39.26)	6.23 (3.19 to 11.18)
Costa Rica	21.82 (12.72 to 33.82)	7.62 (3.87 to 13.75)
El Salvador	20.89 (12.35 to 31.1)	4.54 (2.4 to 8.18)
Guatemala	16.01 (11.24 to 20.51)	5.17 (3.13 to 7.43)
Honduras	15.72 (11.62 to 19.92)	2.16 (1.29 to 3.37)
Mexico	24.8 (17.2 to 33.44)	2.78 (1.37 to 4.92)
Nicaragua	19.23 (11.5 to 28.56)	2.42 (1.19 to 4.39)
Panama	26.13 (18.18 to 34.6)	4.48 (2.62 to 7.3)
Venezuela	28.08 (19.12 to 36.85)	4.99 (2.85 to 8.05)
Tropical Latin America		
Brazil	24.88 (15.76 to 34.82)	9.64 (5.76 to 14.61)
Paraguay	27.77 (17.09 to 41.33)	7.61 (3.95 to 13.61)
North Africa and Middle East		
North Africa and Middle East		
Afghanistan	2.04 (1.18 to 3.29)	0.86 (0.44 to 1.58)
Algeria	2.93 (1.68 to 4.44)	1.22 (0.66 to 2)
Bahrain	4.5 (2.62 to 6.69)	1.59 (0.86 to 2.57)
Egypt	1.89 (1.38 to 2.4)	0.95 (0.51 to 1.44)
Iran	0.02 (0 to 0.08)	0 (0 to 0.02)
Iraq	2.95 (1.62 to 4.54)	1.21 (0.63 to 2.01)
Jordan	1.82 (0.97 to 3.06)	0.79 (0.38 to 1.44)
Kuwait	0.62 (0.27 to 1.15)	0.25 (0.1 to 0.46)
Lebanon	9.38 (5.94 to 13.1)	2.33 (1.25 to 3.98)
Libya	0.51 (0.21 to 1.01)	0.18 (0.07 to 0.35)
Morocco	2.9 (2.25 to 3.58)	1.15 (0.71 to 1.61)
Palestine	0.39 (0.09 to 0.88)	0.15 (0.03 to 0.35)
Oman	2.54 (1.39 to 4.28)	0.93 (0.46 to 1.63)

	Estimate in 2017, male (95% UIs)	Estimate in 2017, female (95% UIs)
Qatar	7.66 (4.73 to 10.99)	2.15 (1.22 to 3.26)
Saudi Arabia	1.79 (0.9 to 3.1)	0.74 (0.33 to 1.41)
Sudan	5.95 (4.72 to 7.33)	2.96 (1.87 to 3.94)
Syria	2.29 (1.32 to 3.34)	0.55 (0.28 to 0.94)
Tunisia	3.46 (2.01 to 5.38)	0.87 (0.45 to 1.49)
Turkey	6.39 (3.96 to 8.97)	3.46 (1.99 to 5.43)
United Arab Emirates	15.5 (10.23 to 21.88)	8.49 (6.73 to 10.55)
Yemen	0.71 (0.32 to 1.36)	0.28 (0.12 to 0.56)
South Asia		
South Asia		
Bangladesh	0.62 (0.34 to 1.02)	0.08 (0.04 to 0.15)
Bhutan	5.6 (3.24 to 8)	0.78 (0.43 to 1.15)
India	15.76 (12.21 to 19.47)	0.93 (0.59 to 1.32)
Nepal	6.11 (3.51 to 9.22)	1.85 (1.28 to 2.36)
Pakistan	0.07 (0.01 to 0.16)	0.01 (0 to 0.03)
Southeast Asia, East Asia, and Oceania		
East Asia		
China	22.65 (15.09 to 29.63)	1.89 (0.95 to 3.29)
North Korea	12.97 (7.62 to 20.2)	1.58 (0.73 to 2.91)
Taiwan	23.21 (13.29 to 34.55)	2.78 (1.27 to 5)
Oceania		
American Samoa	8.18 (4.25 to 14.22)	2 (0.93 to 3.68)
Federated States of Micronesia	6.78 (3.29 to 12.16)	1.53 (0.66 to 3.11)
Fiji	13.12 (7.25 to 19.9)	1.91 (1.04 to 3.09)
Guam	4.85 (0.71 to 11.62)	1.36 (0.06 to 3.46)
Kiribati	8.71 (4.44 to 14.88)	1.18 (0.52 to 2.22)
Marshall Islands	7.21 (3.35 to 13.33)	1.79 (0.77 to 3.53)
Northern Mariana Islands	6.7 (1.2 to 14.4)	1.61 (0.27 to 3.76)
Papua New Guinea	20.49 (11.2 to 30)	4.94 (2.46 to 8.13)
Samoa	11.73 (6.53 to 19.05)	2.79 (1.49 to 4.87)
Solomon Islands	6.59 (3.2 to 11.72)	1.58 (0.67 to 3.05)
Tonga	6.57 (2.94 to 12.11)	1.56 (0.64 to 3.2)
Vanuatu	4.22 (1.65 to 8.43)	1.01 (0.38 to 2.18)
Southeast Asia		
Cambodia	28.31 (22.46 to 33.93)	6.85 (4.55 to 9.13)
Indonesia	1.37 (0.71 to 2.42)	0.96 (0.53 to 1.56)
Laos	14.28 (8.45 to 23.48)	5.48 (3.11 to 9.07)
Malaysia	8.93 (5.43 to 12.17)	1.94 (1.06 to 2.88)
Maldives	6.36 (3.12 to 11.22)	1.12 (0.48 to 2.18)
Mauritius	19.75 (11.35 to 31.68)	3.12 (1.43 to 6)
Myanmar	8.33 (4.06 to 13.84)	0.64 (0.29 to 1.18)
Philippines	15.23 (8.99 to 24.74)	5.01 (2.59 to 8.64)
Sri Lanka	19.9 (11.48 to 30.68)	1.73 (0.95 to 2.81)
Seychelles	18.83 (11.12 to 27.39)	3.04 (1.67 to 4.86)
Thailand	24.94 (16.37 to 33.51)	4.74 (2.68 to 7.25)
Timor-Leste	0.69 (0.26 to 1.49)	0.11 (0.01 to 0.26)
Vietnam	38.02 (27.73 to 47.97)	5.21 (2.93 to 7.74)

	Estimate in 2017, male (95% UIs)	Estimate in 2017, female (95% UIs)
Sub-Saharan Africa		
Central Sub-Saharan Africa		
Angola	13.95 (8.28 to 21.01)	7.58 (4.43 to 12.12)
Central African Republic	13.97 (7.56 to 21.6)	7.58 (4.09 to 12.31)
Congo	14.55 (8.43 to 21.98)	8.17 (4.67 to 12.91)
Democratic Republic of the Congo	9.82 (5.27 to 16.15)	5.46 (2.76 to 9.35)
Equatorial Guinea	24.09 (16.9 to 30.69)	13.97 (8.69 to 19.67)
Gabon	17.51 (11.08 to 24.45)	9.64 (5.8 to 14.53)
Eastern Sub-Saharan Africa		
Burundi	27.27 (21.22 to 33.12)	13.35 (8.59 to 17.88)
Comoros	1.5 (0.68 to 2.85)	0.84 (0.36 to 1.67)
Djibouti	4.4 (1.98 to 8.61)	1.85 (0.79 to 3.8)
Eritrea	7.86 (3.94 to 13.33)	3.37 (1.5 to 6.24)
Ethiopia	8.93 (5.4 to 14.01)	5.35 (2.88 to 9.51)
Kenya	18.18 (12.24 to 23.88)	8 (5.29 to 10.85)
Madagascar	7.97 (4.13 to 13.7)	3.49 (1.61 to 6.53)
Malawi	11.49 (6.2 to 18.27)	1.73 (0.81 to 3.08)
Mozambique	10.49 (5.33 to 18.15)	5.71 (2.58 to 10.8)
Rwanda	20.9 (12.03 to 32.57)	11.19 (6.01 to 18.37)
Somalia	2.92 (1.28 to 5.9)	1.15 (0.48 to 2.36)
South Sudan	2.66 (1.1 to 5.15)	1.08 (0.43 to 2.17)
Tanzania	15.47 (9.22 to 23.75)	6.39 (3.56 to 10.67)
Uganda	14.85 (8.27 to 23.77)	8.85 (4.66 to 14.97)
Zambia	13.29 (7.41 to 20.88)	3.96 (2.17 to 6.45)
Southern Sub-Saharan Africa		
Botswana	23.12 (16.23 to 29.37)	9.66 (5.65 to 13.6)
Lesotho	27.94 (18.86 to 35.94)	12.76 (7.23 to 18.54)
Namibia	28.16 (18.01 to 36.84)	14.57 (8.61 to 20.69)
South Africa	24.71 (19.88 to 30.08)	14.92 (11.29 to 19.1)
Swaziland	13.22 (8.26 to 18.25)	3.6 (2.04 to 5.72)
Zimbabwe	16.42 (9.96 to 23.25)	2.72 (1.54 to 4.45)
Western Sub-Saharan Africa		
Benin	5.83 (3.18 to 9.59)	6.83 (3.19 to 12.93)
Burkina Faso	14.84 (8.29 to 21.63)	10.06 (5.34 to 16.35)
Cameroon	17.16 (12.77 to 21.45)	9.96 (6.12 to 13.98)
Cape Verde	15.75 (10.48 to 20.62)	7.81 (4.62 to 11.88)
Chad	9.74 (6.15 to 13.61)	4.05 (2.37 to 6.67)
Cote d'Ivoire	11.82 (6.61 to 18.94)	3.69 (1.89 to 6.5)
The Gambia	5.08 (2.78 to 8.41)	2.83 (1.33 to 5.22)
Ghana	24.83 (16.89 to 31.8)	9.87 (5.73 to 14.65)
Guinea	2.04 (0.9 to 3.95)	1.03 (0.42 to 2.03)
Guinea-Bissau	10.3 (6.38 to 14.71)	5.33 (3 to 8.53)
Liberia	12.8 (7.05 to 18.82)	8.29 (4.58 to 12.07)
Mali	2.89 (1.6 to 4.23)	2.41 (1.33 to 3.63)
Mauritania	1.35 (0.66 to 2.45)	0.27 (0.11 to 0.56)
Niger	2.68 (1.1 to 5.18)	1.41 (0.51 to 3.05)
Nigeria	16.8 (9.65 to 25.81)	4.66 (2.09 to 9.24)

	Estimate in 2017, male (95% UIs)	Estimate in 2017, female (95% UIs)
Sao Tome and Principe	16.05 (12.8 to 19.7)	10.46 (7.15 to 13.93)
Senegal	1.3 (0.66 to 2.32)	1.1 (0.56 to 1.99)
Sierra Leone	14.61 (11 to 18.4)	8.92 (5.31 to 12.54)
Togo	8.56 (4.78 to 13.16)	4.49 (2.48 to 7.58)
**Indicator 3.6.1: Age-standardised death rate due to road injuries (per 100,000 population)**		
Central Europe, Eastern Europe, and Central Asia		
Central Asia		
Armenia	11.51 (10.77 to 12.38)	2.69 (2.49 to 2.93)
Azerbaijan	9.95 (8.21 to 11.38)	2.72 (2.3 to 3.21)
Georgia	28.94 (26.09 to 31.24)	5.89 (5.47 to 6.39)
Kazakhstan	22.51 (20.49 to 24.67)	7.48 (6.82 to 8.15)
Kyrgyzstan	22.21 (20.53 to 24.44)	7.11 (6.58 to 7.67)
Mongolia	25.03 (21.5 to 29.2)	7.77 (6.58 to 9.2)
Tajikistan	11.86 (10.44 to 13.52)	3.1 (2.63 to 3.63)
Turkmenistan	10.05 (8.8 to 11.54)	2.42 (2.14 to 2.72)
Uzbekistan	21.08 (17.76 to 24.53)	4.82 (4.15 to 5.6)
Central Europe		
Albania	12.9 (10.04 to 16.16)	2.67 (2.06 to 3.41)
Bosnia and Herzegovina	10.28 (9.18 to 11.39)	2.38 (2.11 to 2.66)
Bulgaria	13.02 (11.79 to 14.49)	4.06 (3.62 to 4.53)
Croatia	11.73 (10.72 to 12.79)	2.74 (2.48 to 3.03)
Czech Republic	9.68 (8.83 to 10.57)	3.35 (3.04 to 3.66)
Hungary	9.85 (9.01 to 10.99)	2.94 (2.67 to 3.25)
Macedonia	9.86 (8.8 to 10.97)	2.89 (2.46 to 3.33)
Montenegro	10.98 (9.69 to 12.56)	4.27 (3.75 to 4.82)
Poland	13.48 (12.36 to 14.61)	3.69 (3.4 to 3.99)
Romania	14.26 (13.09 to 15.44)	4.3 (3.95 to 4.65)
Serbia	10.55 (9.59 to 11.53)	3.35 (3.02 to 3.72)
Slovakia	10.06 (8.97 to 11.41)	3.79 (3.37 to 4.23)
Slovenia	9.22 (8.27 to 10.23)	2.38 (2.11 to 2.67)
Eastern Europe		
Belarus	13.19 (11.87 to 14.87)	4.57 (4.12 to 5.03)
Estonia	8.48 (7.15 to 10.17)	3.12 (2.58 to 3.72)
Latvia	14.45 (12.43 to 16.68)	4.07 (3.41 to 4.82)
Lithuania	14.65 (13.3 to 16.25)	4 (3.58 to 4.42)
Moldova	16.74 (15.69 to 18.01)	4.1 (3.82 to 4.44)
Russian Federation	22.85 (22.13 to 24.65)	7.35 (7.12 to 7.61)
Ukraine	22.19 (18.51 to 24.46)	5.9 (5.26 to 6.47)
High-income		
Australasia		
Australia	8.66 (7.44 to 10.03)	3.29 (2.82 to 3.76)
New Zealand	10.73 (9.92 to 11.57)	4.41 (4.08 to 4.76)
High-income Asia Pacific		
Brunei	22.75 (20.15 to 25.58)	9.17 (8.04 to 10.41)
Japan	5.6 (5.34 to 5.93)	1.93 (1.84 to 2.03)
South Korea	13.75 (12.51 to 15.1)	4.98 (4.52 to 5.41)

	Estimate in 2017, male (95% UIs)	Estimate in 2017, female (95% UIs)
Singapore	4.92 (4.47 to 5.42)	1.3 (1.16 to 1.45)
High-income North America		
Canada	9.05 (8.23 to 9.89)	4.17 (3.78 to 4.58)
Greenland	4.87 (4.21 to 5.67)	3.13 (2.71 to 3.66)
United States	16.89 (15.7 to 17.76)	7.08 (6.75 to 7.4)
Southern Latin America		
Argentina	21.03 (18.23 to 24.4)	6.24 (5.38 to 7.24)
Chile	17.48 (14.83 to 20.28)	4.96 (4.27 to 5.78)
Uruguay	23.16 (19.79 to 26.72)	6.95 (5.98 to 8.05)
Western Europe		
Andorra	5.96 (4.89 to 7.19)	2.06 (1.71 to 2.56)
Austria	6.52 (5.97 to 7.11)	2.22 (2.02 to 2.44)
Belgium	10.42 (9.57 to 11.35)	3.53 (3.23 to 3.88)
Cyprus	15.68 (13.81 to 17.79)	4.73 (4.21 to 5.45)
Denmark	5.52 (5.04 to 6.05)	2.36 (2.09 to 2.65)
Finland	5.99 (5.45 to 6.61)	2.26 (2.03 to 2.49)
France	8.32 (7.64 to 9.12)	2.49 (2.26 to 2.71)
Germany	6.61 (5.72 to 7.64)	2.23 (1.9 to 2.59)
Greece	15.01 (13.84 to 16.3)	4.43 (4.01 to 4.86)
Iceland	5.14 (4.68 to 5.67)	2.08 (1.91 to 2.26)
Ireland	4.94 (4.44 to 5.52)	1.92 (1.73 to 2.14)
Israel	9.03 (8.28 to 9.9)	3.49 (3.19 to 3.83)
Italy	9.29 (8.54 to 10.15)	2.94 (2.69 to 3.2)
Luxembourg	7.54 (6.55 to 8.6)	3.08 (2.7 to 3.53)
Malta	5.19 (4.76 to 5.69)	1.48 (1.33 to 1.64)
Netherlands	5.36 (4.96 to 5.82)	2.11 (1.93 to 2.3)
Norway	4.95 (4.73 to 5.19)	1.77 (1.69 to 1.85)
Portugal	10.35 (9.37 to 11.4)	2.79 (2.5 to 3.07)
Spain	6.34 (5.83 to 6.91)	1.93 (1.76 to 2.11)
Sweden	4.56 (4.16 to 4.98)	1.54 (1.4 to 1.68)
Switzerland	4.47 (4.06 to 5.02)	1.57 (1.41 to 1.73)
United Kingdom	5.15 (5 to 5.34)	1.67 (1.61 to 1.72)
Latin America and Caribbean		
Andean Latin America		
Bolivia	30.07 (21.23 to 39.15)	11.27 (8.37 to 14.35)
Ecuador	42.1 (36.97 to 48.05)	11.85 (10.44 to 13.35)
Peru	20.53 (16.55 to 24.88)	7.1 (5.76 to 8.64)
Caribbean		
Antigua and Barbuda	10.69 (9.37 to 12.16)	3.33 (2.93 to 3.72)
The Bahamas	22.96 (20.06 to 26.23)	7.31 (6.28 to 8.45)
Barbados	13.18 (11.54 to 14.89)	4.7 (4.1 to 5.31)
Belize	31.37 (27.92 to 34.24)	6.46 (5.91 to 7.04)
Bermuda	14.83 (13.04 to 16.77)	1.6 (1.39 to 1.85)
Cuba	12.08 (10.34 to 14.16)	3.84 (3.29 to 4.42)
Dominica	22.71 (19.9 to 25.37)	7.76 (6.78 to 8.76)
Dominican Republic	48.83 (39.93 to 58.23)	10.18 (8.44 to 12.09)
Grenada	13.89 (12.48 to 15.42)	4.59 (4.09 to 5.1)

	Estimate in 2017, male (95% UIs)	Estimate in 2017, female (95% UIs)
Guyana	25.8 (21.36 to 30.46)	5.98 (4.95 to 7.07)
Haiti	63.74 (33.59 to 95.31)	22.74 (13.03 to 31.39)
Jamaica	14.54 (10.74 to 18.14)	3.9 (3.21 to 4.77)
Puerto Rico	16.21 (14.51 to 17.99)	4.1 (3.65 to 4.54)
Saint Lucia	19.51 (16.87 to 22.25)	5.79 (5.05 to 6.58)
Saint Vincent and the Grenadines	13.97 (12.35 to 15.65)	4.43 (3.93 to 4.96)
Suriname	25.89 (21.65 to 30.18)	7.61 (6.59 to 8.79)
Trinidad and Tobago	20.99 (16.37 to 26.08)	6.19 (4.64 to 7.92)
Virgin Islands, U.S.	17.33 (14.79 to 20.37)	5.4 (4.53 to 6.43)
Central Latin America		
Colombia	22.93 (19.76 to 26.45)	5.48 (4.73 to 6.34)
Costa Rica	25.82 (22.36 to 28.98)	5.86 (5.25 to 6.58)
El Salvador	37.14 (29.35 to 45.89)	8.28 (6.59 to 10.36)
Guatemala	29.7 (24.91 to 34.77)	5.95 (5.1 to 6.83)
Honduras	22.14 (14.6 to 29.16)	10.77 (7.94 to 13.73)
Mexico	25.09 (23.88 to 26.15)	6.97 (6.74 to 7.21)
Nicaragua	18.37 (15.42 to 21.86)	3.74 (3.14 to 4.46)
Panama	20.13 (18.02 to 22.23)	5.22 (4.64 to 5.76)
Venezuela	35.47 (28.59 to 43.24)	8.18 (6.7 to 9.99)
Tropical Latin America		
Brazil	32.91 (30.94 to 34.27)	7.92 (7.64 to 8.17)
Paraguay	36 (28.28 to 45.16)	7.72 (5.87 to 9.62)
North Africa and Middle East		
North Africa and Middle East		
Afghanistan	51.71 (39.68 to 66.25)	13.47 (10.17 to 17.69)
Algeria	23.7 (17.68 to 41.82)	10.34 (7.43 to 16.88)
Bahrain	13.34 (11.72 to 15.2)	4.45 (3.92 to 5.19)
Egypt	45.49 (26.91 to 63.29)	16.26 (9.88 to 23.01)
Iran	37.84 (36.97 to 39.04)	13.13 (12.62 to 14.7)
Iraq	12.95 (11.61 to 14.56)	5.46 (4.87 to 6.25)
Jordan	16.1 (14.03 to 18.38)	6.17 (5.3 to 7.15)
Kuwait	21.26 (18.89 to 23.24)	3.84 (3.52 to 4.2)
Lebanon	11.23 (6.95 to 14.15)	2.38 (1.73 to 2.87)
Libya	35.83 (13.31 to 63.06)	13.14 (4.65 to 23.47)
Morocco	32.85 (22.86 to 58.46)	7.57 (5.82 to 9.75)
Palestine	12.12 (10.64 to 13.74)	4.53 (4.01 to 5.15)
Oman	61.41 (47.75 to 75.19)	21.96 (17.99 to 26.42)
Qatar	30.89 (24.78 to 38.22)	7.11 (5.79 to 8.77)
Saudi Arabia	50.02 (33.53 to 61.34)	15.95 (11.51 to 19.85)
Sudan	41.59 (29.18 to 74.36)	17.53 (11.46 to 31.37)
Syria	16.86 (12.78 to 20.85)	5.77 (4.64 to 6.91)
Tunisia	43.17 (33.04 to 54.93)	16.57 (12.02 to 21.65)
Turkey	16.41 (14.52 to 18.42)	4.04 (3.53 to 4.61)
United Arab Emirates	60.96 (46.29 to 77.74)	25.45 (19.33 to 33.09)
Yemen	54.48 (39.87 to 86.42)	21.04 (14.24 to 32.8)
South Asia		
South Asia		

	Estimate in 2017, male (95% UIs)	Estimate in 2017, female (95% UIs)
Bangladesh	13.58 (10.08 to 16.33)	2.47 (1.99 to 2.99)
Bhutan	10.78 (7.7 to 13.76)	4.34 (3.24 to 5.59)
India	25.23 (23.02 to 26.86)	8.32 (7.09 to 8.89)
Nepal	40.49 (18.09 to 65.86)	13.56 (6.11 to 19.29)
Pakistan	39.01 (19.24 to 54.64)	18.9 (9.04 to 26.3)
Southeast Asia, East Asia, and Oceania		
East Asia		
China	22.29 (21 to 23.47)	8.14 (7.74 to 8.64)
North Korea	30.07 (17.89 to 51.49)	9.69 (6.58 to 16.11)
Taiwan	19.19 (17.74 to 20.79)	6.77 (6.18 to 7.36)
Oceania		
American Samoa	9.88 (8.84 to 11.04)	7.39 (6.4 to 8.38)
Federated States of Micronesia	23.56 (14.92 to 31.82)	10.95 (7.41 to 15.11)
Fiji	13.51 (11.36 to 15.93)	6.19 (5.17 to 7.29)
Guam	17.91 (15.63 to 20.23)	8.32 (7.16 to 9.49)
Kiribati	17.8 (13.28 to 22.07)	4.78 (3.66 to 6.03)
Marshall Islands	31.86 (22.55 to 40.01)	15.77 (9.43 to 21.06)
Northern Mariana Islands	14.38 (12.14 to 16.5)	7.13 (6.17 to 8.43)
Papua New Guinea	49.55 (36.94 to 63.28)	17.54 (12.95 to 22.81)
Samoa	13.79 (10.31 to 20.91)	7.49 (5.5 to 10.17)
Solomon Islands	28.68 (20.67 to 39.56)	13.85 (10.02 to 18.31)
Tonga	17.97 (14.36 to 21.18)	5.87 (4.69 to 7.16)
Vanuatu	29.83 (20.26 to 42.25)	11.23 (7.87 to 15.85)
Southeast Asia		
Cambodia	43.4 (32.12 to 57.9)	13.32 (9.86 to 17.87)
Indonesia	21.89 (19.43 to 23.87)	6.78 (6.25 to 7.46)
Laos	38.84 (27.68 to 50.82)	13.99 (10.7 to 17.37)
Malaysia	36.54 (31.4 to 41.58)	8.7 (7.34 to 9.97)
Maldives	11.32 (7.87 to 22.29)	4.24 (3.12 to 7.78)
Mauritius	19.28 (17.35 to 21.38)	3.41 (3.05 to 3.77)
Myanmar	30.58 (24.7 to 37.3)	11.25 (8.83 to 13.62)
Philippines	17.16 (14.16 to 20.38)	5.48 (4.63 to 6.5)
Sri Lanka	20.37 (16.18 to 25.05)	5.03 (3.77 to 6.23)
Seychelles	19.93 (16.83 to 22.59)	5.96 (5.12 to 6.77)
Thailand	39.66 (34.13 to 45.22)	9.31 (7.89 to 10.73)
Timor-Leste	15.32 (8.27 to 29.39)	5.33 (3.12 to 9.74)
Vietnam	34.2 (28.04 to 39.32)	9.18 (7.26 to 11.36)
Sub-Saharan Africa		
Central Sub-Saharan Africa		
Angola	42.34 (33.35 to 54.88)	15.53 (11.9 to 19.76)
Central African Republic	125.93 (62.67 to 172.79)	43.13 (21.91 to 62.41)
Congo	36.84 (27.34 to 49.1)	19.56 (12.99 to 26.22)
Democratic Republic of the Congo	41.34 (30.71 to 53.24)	15.96 (11.33 to 20.89)
Equatorial Guinea	28.57 (20.03 to 40.17)	12.75 (7.67 to 18.92)
Gabon	44.25 (33.76 to 56.1)	13.01 (9.4 to 17.78)
Eastern Sub-Saharan Africa		
Burundi	48.04 (35.37 to 68.99)	21.2 (14.26 to 29.84)

	Estimate in 2017, male (95% UIs)	Estimate in 2017, female (95% UIs)
Comoros	29.14 (23.12 to 36.2)	12.45 (9.57 to 16.32)
Djibouti	31.6 (22.14 to 43.32)	12.68 (8.33 to 18.4)
Eritrea	51.84 (33.37 to 66.32)	17.37 (12.79 to 22.06)
Ethiopia	21.47 (19.18 to 23.81)	8.53 (7.42 to 9.82)
Kenya	27.5 (24.16 to 32.41)	9.46 (8.18 to 11.34)
Madagascar	29.55 (22.77 to 37.82)	11.68 (8.86 to 14.99)
Malawi	31.54 (26.17 to 37.48)	8.98 (7.19 to 10.98)
Mozambique	45.17 (36.23 to 53.97)	12.36 (9.73 to 15.64)
Rwanda	51.9 (29.83 to 80.55)	18.46 (10.98 to 26.65)
Somalia	76.13 (33.61 to 109.55)	25.37 (12.07 to 38.69)
South Sudan	40 (28.63 to 56.73)	14.37 (10.44 to 19.72)
Tanzania	21.91 (18.31 to 26.16)	9.08 (7.5 to 10.97)
Uganda	40.61 (25.64 to 52.82)	13.33 (8.75 to 19.38)
Zambia	28.87 (24.09 to 33.88)	10.74 (8.74 to 12.89)
Southern Sub-Saharan Africa		
Botswana	17.39 (14.99 to 20.54)	12.75 (9.3 to 15.3)
Lesotho	75.49 (57.95 to 95)	16.35 (11.61 to 23.53)
Namibia	36.51 (28.6 to 47)	8.94 (6.75 to 13.15)
South Africa	42.03 (39.18 to 45.34)	14.88 (13.44 to 17.06)
Swaziland	63.98 (45.23 to 82.09)	13.6 (9.64 to 19.58)
Zimbabwe	41.14 (25.6 to 60.18)	11.06 (6.76 to 16.24)
Western Sub-Saharan Africa		
Benin	60.59 (26.93 to 89.32)	23.4 (11.5 to 31.94)
Burkina Faso	35.4 (27.46 to 42.71)	13.86 (11.13 to 16.74)
Cameroon	32.07 (23.56 to 41.37)	13.71 (10.16 to 18.33)
Cape Verde	12.27 (9.83 to 14.3)	4.31 (3.79 to 4.88)
Chad	34.57 (26.87 to 47.39)	15 (11.68 to 19.24)
Cote d'Ivoire	31.2 (26.03 to 37.02)	11.62 (8.71 to 15.65)
The Gambia	29.83 (21.79 to 37.68)	12.43 (10.14 to 15.58)
Ghana	36.91 (30.96 to 43.7)	12.95 (10.66 to 15.41)
Guinea	34.23 (27.91 to 42.23)	14.26 (10.99 to 17.88)
Guinea-Bissau	45.2 (35.59 to 56.15)	20.72 (16.41 to 26.42)
Liberia	21.43 (16.36 to 27.03)	9.76 (7.55 to 12.08)
Mali	26.01 (20.03 to 38.12)	13.38 (10.18 to 17.14)
Mauritania	28.35 (20.87 to 35.18)	17.79 (14.63 to 21.57)
Niger	25.39 (17.93 to 36.51)	10.51 (7.35 to 14.55)
Nigeria	18.63 (13.06 to 24.58)	8.24 (5.81 to 11.14)
Sao Tome and Principe	22.72 (10.84 to 29.84)	8.89 (6.1 to 11.92)
Senegal	26.01 (19.87 to 39.33)	9.43 (7.04 to 13.38)
Sierra Leone	29.08 (23.19 to 36.96)	15.69 (11.86 to 20.55)
Togo	32.21 (24.89 to 40.69)	14.13 (11.02 to 17.31)
**Indicator 3.9.3: Age-standardised death rate due to unintentional poisonings (per 100,000 population)**		
Central Europe, Eastern Europe, and Central Asia		
Central Asia		
Armenia	0.81 (0.72 to 0.89)	0.37 (0.34 to 0.4)
Azerbaijan	1.2 (0.81 to 2.12)	0.51 (0.41 to 0.69)

	Estimate in 2017, male (95% UIs)	Estimate in 2017, female (95% UIs)
Georgia	1.08 (0.96 to 1.2)	0.55 (0.49 to 0.62)
Kazakhstan	3.76 (3.4 to 4.18)	1.38 (1.23 to 1.54)
Kyrgyzstan	2 (1.8 to 2.28)	0.82 (0.75 to 0.9)
Mongolia	3.79 (2.63 to 7.64)	1.69 (1.33 to 2.5)
Tajikistan	1.01 (0.62 to 2.76)	0.31 (0.21 to 0.72)
Turkmenistan	0.8 (0.67 to 0.93)	0.42 (0.37 to 0.49)
Uzbekistan	1.6 (1.36 to 1.89)	0.6 (0.52 to 0.71)
Central Europe		
Albania	0.53 (0.39 to 0.75)	0.22 (0.14 to 0.3)
Bosnia and Herzegovina	0.58 (0.39 to 1.33)	0.33 (0.26 to 0.55)
Bulgaria	0.65 (0.59 to 0.73)	0.28 (0.25 to 0.32)
Croatia	0.39 (0.35 to 0.43)	0.21 (0.19 to 0.23)
Czech Republic	0.84 (0.76 to 0.93)	0.27 (0.24 to 0.3)
Hungary	0.56 (0.51 to 0.62)	0.22 (0.2 to 0.24)
Macedonia	0.46 (0.33 to 0.59)	0.21 (0.14 to 0.25)
Montenegro	0.76 (0.59 to 1)	0.41 (0.34 to 0.52)
Poland	0.68 (0.61 to 0.76)	0.27 (0.25 to 0.3)
Romania	1.72 (1.57 to 1.88)	0.89 (0.82 to 0.98)
Serbia	0.47 (0.38 to 0.71)	0.22 (0.18 to 0.26)
Slovakia	0.68 (0.54 to 1)	0.3 (0.25 to 0.37)
Slovenia	0.28 (0.25 to 0.32)	0.11 (0.1 to 0.12)
Eastern Europe		
Belarus	3.86 (3.35 to 4.44)	1.04 (0.92 to 1.16)
Estonia	0.78 (0.64 to 0.95)	0.19 (0.15 to 0.23)
Latvia	1.49 (1.26 to 1.75)	0.32 (0.26 to 0.38)
Lithuania	4.25 (3.79 to 4.74)	0.91 (0.81 to 1.01)
Moldova	6.29 (5.79 to 6.79)	1.84 (1.69 to 2.02)
Russian Federation	5.65 (5.45 to 5.86)	1.68 (1.61 to 1.75)
Ukraine	4.33 (3.94 to 4.76)	1.04 (0.93 to 1.14)
High-income		
Australasia		
Australia	0.25 (0.22 to 0.29)	0.13 (0.11 to 0.14)
New Zealand	0.29 (0.26 to 0.32)	0.1 (0.09 to 0.11)
High-income Asia Pacific		
Brunei	0.59 (0.47 to 0.92)	0.33 (0.27 to 0.43)
Japan	0.35 (0.33 to 0.38)	0.11 (0.1 to 0.11)
South Korea	0.49 (0.44 to 0.55)	0.16 (0.14 to 0.18)
Singapore	0.07 (0.06 to 0.08)	0.03 (0.03 to 0.04)
High-income North America		
Canada	0.43 (0.38 to 0.48)	0.22 (0.2 to 0.25)
Greenland	1.54 (1.2 to 2.03)	0.71 (0.53 to 0.85)
United States	0.64 (0.61 to 0.67)	0.34 (0.33 to 0.36)
Southern Latin America		
Argentina	0.99 (0.86 to 1.15)	0.53 (0.46 to 0.61)
Chile	0.4 (0.34 to 0.47)	0.18 (0.15 to 0.21)
Uruguay	0.75 (0.64 to 0.88)	0.37 (0.31 to 0.43)
Western Europe		

	Estimate in 2017, male (95% UIs)	Estimate in 2017, female (95% UIs)
Andorra	0.29 (0.21 to 0.43)	0.13 (0.1 to 0.16)
Austria	0.38 (0.34 to 0.43)	0.15 (0.13 to 0.16)
Belgium	0.33 (0.3 to 0.37)	0.21 (0.19 to 0.23)
Cyprus	0.29 (0.2 to 0.37)	0.08 (0.06 to 0.1)
Denmark	0.25 (0.22 to 0.28)	0.08 (0.07 to 0.09)
Finland	0.34 (0.3 to 0.39)	0.12 (0.1 to 0.13)
France	0.31 (0.28 to 0.35)	0.18 (0.16 to 0.2)
Germany	0.15 (0.13 to 0.17)	0.05 (0.05 to 0.06)
Greece	0.23 (0.2 to 0.25)	0.09 (0.08 to 0.1)
Iceland	0.45 (0.41 to 0.49)	0.1 (0.09 to 0.11)
Ireland	0.28 (0.24 to 0.32)	0.1 (0.09 to 0.11)
Israel	0.16 (0.14 to 0.18)	0.08 (0.07 to 0.09)
Italy	0.17 (0.15 to 0.19)	0.09 (0.08 to 0.09)
Luxembourg	0.18 (0.15 to 0.21)	0.12 (0.1 to 0.13)
Malta	0.19 (0.16 to 0.21)	0.04 (0.04 to 0.05)
Netherlands	0.11 (0.1 to 0.12)	0.08 (0.07 to 0.09)
Norway	1.14 (1.02 to 1.27)	0.49 (0.44 to 0.53)
Portugal	0.28 (0.25 to 0.31)	0.14 (0.13 to 0.16)
Spain	0.23 (0.21 to 0.26)	0.12 (0.11 to 0.13)
Sweden	0.4 (0.36 to 0.44)	0.19 (0.17 to 0.21)
Switzerland	0.13 (0.11 to 0.15)	0.07 (0.06 to 0.07)
United Kingdom	0.25 (0.24 to 0.25)	0.13 (0.12 to 0.13)
Latin America and Caribbean		
Andean Latin America		
Bolivia	1.11 (0.74 to 1.65)	0.6 (0.42 to 0.8)
Ecuador	0.83 (0.72 to 0.95)	0.44 (0.38 to 0.5)
Peru	0.89 (0.65 to 1.28)	0.43 (0.33 to 0.59)
Caribbean		
Antigua and Barbuda	0.27 (0.23 to 0.31)	0.68 (0.6 to 0.79)
The Bahamas	0.32 (0.27 to 0.36)	0.28 (0.24 to 0.32)
Barbados	0.2 (0.17 to 0.23)	0.18 (0.16 to 0.2)
Belize	0.68 (0.61 to 0.77)	0.57 (0.51 to 0.63)
Bermuda	0.28 (0.24 to 0.32)	0.09 (0.08 to 0.11)
Cuba	0.13 (0.11 to 0.15)	0.3 (0.26 to 0.34)
Dominica	1.63 (1.41 to 1.88)	1.13 (0.98 to 1.28)
Dominican Republic	0.39 (0.26 to 0.8)	0.33 (0.26 to 0.52)
Grenada	0.33 (0.29 to 0.38)	0.55 (0.48 to 0.62)
Guyana	0.37 (0.31 to 0.44)	0.63 (0.53 to 0.74)
Haiti	1.01 (0.36 to 1.69)	0.84 (0.42 to 1.2)
Jamaica	0.28 (0.2 to 0.36)	0.28 (0.23 to 0.34)
Puerto Rico	0.22 (0.19 to 0.25)	0.17 (0.15 to 0.19)
Saint Lucia	0.26 (0.23 to 0.3)	0.23 (0.2 to 0.26)
Saint Vincent and the Grenadines	0.23 (0.2 to 0.25)	0.24 (0.22 to 0.27)
Suriname	0.35 (0.3 to 0.41)	0.52 (0.45 to 0.58)
Trinidad and Tobago	0.22 (0.17 to 0.28)	0.16 (0.12 to 0.2)
Virgin Islands, U.S.	0.27 (0.23 to 0.32)	0.47 (0.4 to 0.54)
Central Latin America		

	Estimate in 2017, male (95% UIs)	Estimate in 2017, female (95% UIs)
Colombia	0.28 (0.24 to 0.32)	0.08 (0.07 to 0.1)
Costa Rica	0.43 (0.36 to 0.49)	0.14 (0.12 to 0.15)
El Salvador	0.37 (0.25 to 0.49)	0.13 (0.1 to 0.17)
Guatemala	2.22 (1.89 to 2.57)	0.85 (0.72 to 0.99)
Honduras	1.71 (0.99 to 2.64)	0.55 (0.32 to 0.83)
Mexico	0.75 (0.69 to 0.79)	0.4 (0.38 to 0.41)
Nicaragua	0.52 (0.39 to 0.83)	0.24 (0.19 to 0.31)
Panama	0.48 (0.42 to 0.54)	0.23 (0.2 to 0.27)
Venezuela	0.45 (0.37 to 0.55)	0.25 (0.21 to 0.3)
Tropical Latin America		
Brazil	0.22 (0.21 to 0.23)	0.14 (0.13 to 0.14)
Paraguay	0.3 (0.22 to 0.52)	0.18 (0.13 to 0.3)
North Africa and Middle East		
North Africa and Middle East		
Afghanistan	1.05 (0.43 to 1.72)	1.63 (1.04 to 2.2)
Algeria	1.11 (0.33 to 1.82)	0.65 (0.21 to 0.93)
Bahrain	0.18 (0.11 to 0.25)	0.1 (0.08 to 0.15)
Egypt	0.3 (0.17 to 0.47)	0.22 (0.1 to 0.35)
Iran	1.61 (1.13 to 1.86)	0.7 (0.61 to 0.86)
Iraq	0.26 (0.2 to 0.32)	0.31 (0.23 to 0.37)
Jordan	0.67 (0.45 to 0.87)	0.62 (0.49 to 0.78)
Kuwait	0.54 (0.47 to 0.61)	0.2 (0.18 to 0.23)
Lebanon	0.9 (0.25 to 1.53)	0.41 (0.12 to 0.61)
Libya	1.44 (0.42 to 2.16)	0.69 (0.16 to 1.14)
Morocco	1.37 (0.39 to 2.19)	0.85 (0.27 to 1.19)
Palestine	0.18 (0.12 to 0.42)	0.15 (0.13 to 0.21)
Oman	0.91 (0.25 to 1.56)	0.07 (0.05 to 0.09)
Qatar	0.36 (0.2 to 0.52)	0.1 (0.06 to 0.13)
Saudi Arabia	0.89 (0.49 to 1.38)	0.59 (0.35 to 0.84)
Sudan	1.82 (0.51 to 2.82)	1 (0.29 to 1.5)
Syria	0.64 (0.45 to 0.85)	0.39 (0.28 to 0.49)
Tunisia	0.88 (0.23 to 1.59)	0.42 (0.13 to 0.64)
Turkey	0.76 (0.4 to 0.96)	0.31 (0.21 to 0.38)
United Arab Emirates	0.3 (0.13 to 0.46)	0.06 (0.04 to 0.08)
Yemen	2.01 (0.53 to 3.33)	1.1 (0.31 to 1.6)
South Asia		
South Asia		
Bangladesh	0.54 (0.29 to 0.77)	0.15 (0.11 to 0.2)
Bhutan	0.39 (0.23 to 0.72)	0.2 (0.14 to 0.32)
India	0.51 (0.33 to 0.66)	0.27 (0.21 to 0.33)
Nepal	0.39 (0.25 to 0.61)	0.22 (0.15 to 0.36)
Pakistan	0.48 (0.32 to 0.69)	0.57 (0.43 to 0.75)
Southeast Asia, East Asia, and Oceania		
East Asia		
China	1.9 (1.02 to 2.33)	1.4 (0.71 to 1.65)
North Korea	2.52 (1.49 to 3.67)	1.45 (0.94 to 2)
Taiwan	0.37 (0.33 to 0.41)	0.2 (0.18 to 0.22)

	Estimate in 2017, male (95% UIs)	Estimate in 2017, female (95% UIs)
Oceania		
American Samoa	1.34 (1.08 to 1.73)	0.19 (0.15 to 0.25)
Federated States of Micronesia	1.75 (0.97 to 2.66)	0.41 (0.25 to 0.59)
Fiji	0.76 (0.59 to 1.02)	0.23 (0.18 to 0.29)
Guam	0.6 (0.45 to 0.77)	0.13 (0.11 to 0.17)
Kiribati	2.6 (1.62 to 3.64)	1.28 (0.38 to 1.85)
Marshall Islands	2.03 (1.12 to 2.76)	0.49 (0.29 to 0.7)
Northern Mariana Islands	0.56 (0.42 to 0.71)	0.1 (0.08 to 0.14)
Papua New Guinea	2.61 (1 to 4.56)	0.68 (0.28 to 0.98)
Samoa	1.17 (0.73 to 1.95)	0.31 (0.21 to 0.45)
Solomon Islands	1.93 (1.02 to 2.93)	0.54 (0.31 to 0.72)
Tonga	2.22 (1.61 to 2.78)	0.93 (0.72 to 1.2)
Vanuatu	2.04 (0.95 to 3.02)	0.48 (0.27 to 0.67)
Southeast Asia		
Cambodia	0.82 (0.5 to 1.1)	0.46 (0.29 to 0.6)
Indonesia	0.63 (0.26 to 0.79)	0.2 (0.11 to 0.23)
Laos	0.78 (0.43 to 1.08)	0.53 (0.3 to 0.7)
Malaysia	1.04 (0.79 to 1.29)	0.48 (0.35 to 0.59)
Maldives	0.09 (0.07 to 0.12)	0.06 (0.05 to 0.07)
Mauritius	0.36 (0.31 to 0.41)	0.2 (0.17 to 0.22)
Myanmar	2.42 (1 to 3.41)	1.29 (0.67 to 1.67)
Philippines	0.31 (0.26 to 0.36)	0.22 (0.19 to 0.26)
Sri Lanka	0.5 (0.32 to 1)	0.23 (0.17 to 0.35)
Seychelles	0.67 (0.56 to 0.94)	0.48 (0.4 to 0.6)
Thailand	0.34 (0.2 to 0.42)	0.2 (0.13 to 0.24)
Timor-Leste	0.71 (0.37 to 1.04)	0.43 (0.25 to 0.57)
Vietnam	1.7 (0.68 to 2.47)	0.16 (0.1 to 0.2)
Sub-Saharan Africa		
Central Sub-Saharan Africa		
Angola	1.77 (1.23 to 2.35)	0.88 (0.6 to 1.18)
Central African Republic	2.71 (1.44 to 4.13)	1.35 (0.67 to 2.05)
Congo	1.46 (0.93 to 2.17)	0.93 (0.47 to 1.34)
Democratic Republic of the Congo	2.05 (1.14 to 3.99)	0.97 (0.61 to 1.35)
Equatorial Guinea	1.06 (0.64 to 2)	0.58 (0.33 to 0.95)
Gabon	1.39 (0.97 to 2.22)	0.63 (0.4 to 1.03)
Eastern Sub-Saharan Africa		
Burundi	2.3 (1.57 to 3.29)	1.17 (0.8 to 1.55)
Comoros	1.88 (1.2 to 2.95)	1.08 (0.73 to 1.51)
Djibouti	1.57 (1.01 to 2.54)	1.02 (0.61 to 1.66)
Eritrea	2.65 (1.89 to 3.54)	1.42 (0.91 to 1.88)
Ethiopia	1.73 (1.23 to 2.28)	1.1 (0.81 to 1.41)
Kenya	2.99 (1.88 to 4.57)	0.77 (0.6 to 1.04)
Madagascar	1.79 (1.26 to 2.56)	1.04 (0.73 to 1.42)
Malawi	2.02 (1.43 to 2.72)	0.86 (0.65 to 1.17)
Mozambique	2.78 (2.04 to 3.68)	1.22 (0.9 to 1.62)
Rwanda	1.78 (1.24 to 2.59)	0.94 (0.63 to 1.39)
Somalia	2.51 (1.63 to 3.77)	1.35 (0.89 to 1.88)

	Estimate in 2017, male (95% UIs)	Estimate in 2017, female (95% UIs)
South Sudan	2.21 (1.37 to 3.45)	1.42 (0.95 to 1.92)
Tanzania	1.66 (1.23 to 2.41)	1.08 (0.8 to 1.51)
Uganda	1.69 (1.14 to 2.36)	0.8 (0.56 to 1.13)
Zambia	1.9 (1.4 to 2.56)	0.97 (0.75 to 1.23)
Southern Sub-Saharan Africa		
Botswana	1.11 (0.59 to 2.51)	0.96 (0.61 to 1.65)
Lesotho	2.73 (1.76 to 4.5)	1.41 (0.92 to 2.36)
Namibia	1.45 (0.95 to 2.46)	0.92 (0.53 to 1.78)
South Africa	1.01 (0.75 to 1.5)	0.78 (0.61 to 1.1)
Swaziland	2.29 (1.53 to 3.68)	1.28 (0.81 to 2.12)
Zimbabwe	4.42 (2.9 to 5.84)	2.63 (1.63 to 3.55)
Western Sub-Saharan Africa		
Benin	1.65 (1.22 to 2.25)	0.81 (0.45 to 1.1)
Burkina Faso	2.23 (1.62 to 3.08)	1.27 (0.84 to 1.72)
Cameroon	1.59 (1.15 to 2.25)	0.73 (0.33 to 1)
Cape Verde	0.91 (0.68 to 1.16)	0.44 (0.36 to 0.55)
Chad	1.85 (1.3 to 2.71)	1.09 (0.58 to 1.58)
Cote d'Ivoire	1.66 (1.25 to 2.34)	0.91 (0.49 to 1.21)
The Gambia	1.86 (1.31 to 2.54)	0.8 (0.44 to 1.06)
Ghana	1.92 (1.16 to 2.77)	0.71 (0.53 to 0.94)
Guinea	1.99 (1.4 to 2.73)	1.04 (0.55 to 1.36)
Guinea-Bissau	2.01 (1.32 to 2.85)	0.98 (0.54 to 1.29)
Liberia	1.5 (0.93 to 2.69)	0.74 (0.4 to 0.99)
Mali	1.74 (1.21 to 2.43)	1.14 (0.58 to 1.65)
Mauritania	1.19 (0.75 to 1.99)	0.69 (0.37 to 0.94)
Niger	1.89 (1.21 to 2.98)	0.98 (0.54 to 1.58)
Nigeria	1.13 (0.81 to 1.73)	0.65 (0.31 to 0.99)
Sao Tome and Principe	2.02 (1.26 to 3)	0.12 (0.07 to 0.18)
Senegal	1.76 (1.31 to 2.51)	0.84 (0.49 to 1.2)
Sierra Leone	1.7 (1.21 to 2.36)	0.93 (0.44 to 1.31)
Togo	1.65 (1.18 to 2.42)	0.69 (0.37 to 0.92)
**Indicator 3.a.1: Age-standardised prevalence of current smoking in populations aged 10 and older (%)**		
Central Europe, Eastern Europe, and Central Asia		
Central Asia		
Armenia	46.39 (44.07 to 48.59)	2.15 (1.61 to 2.79)
Azerbaijan	43.99 (41.71 to 46.15)	1.69 (1.37 to 2.08)
Georgia	43.49 (40.97 to 45.8)	6.4 (5.02 to 8)
Kazakhstan	40.85 (38.91 to 43)	6.76 (5.42 to 8.36)
Kyrgyzstan	36.32 (34.19 to 38.48)	3.65 (2.84 to 4.74)
Mongolia	44.44 (42.13 to 46.73)	7.18 (5.69 to 8.87)
Tajikistan	18.24 (16.57 to 20.04)	1.15 (0.86 to 1.51)
Turkmenistan	34.4 (31.91 to 37.04)	4.22 (3.29 to 5.28)
Uzbekistan	21.41 (19.67 to 23.29)	1.33 (1.05 to 1.71)
Central Europe		
Albania	41.59 (39.24 to 43.97)	14.97 (12.76 to 17.38)
Bosnia and Herzegovina	38.74 (36.63 to 40.77)	26.94 (23.94 to 29.92)

	Estimate in 2017, male (95% UIs)	Estimate in 2017, female (95% UIs)
Bulgaria	38.57 (36.57 to 40.64)	30.6 (27.98 to 33.21)
Croatia	36.5 (34.49 to 38.66)	32.01 (29.42 to 34.88)
Czech Republic	34.45 (32.4 to 36.61)	23.34 (20.93 to 25.71)
Hungary	32.72 (30.38 to 35.02)	25.79 (23.14 to 28.84)
Macedonia	39.51 (37.12 to 41.89)	27.67 (23.9 to 31.2)
Montenegro	36.92 (34.93 to 39.06)	32.82 (30.12 to 35.57)
Poland	30.68 (28.65 to 32.54)	25.17 (22.84 to 27.5)
Romania	32.95 (30.77 to 35.15)	20.41 (18.06 to 22.94)
Serbia	32.78 (30.42 to 35.15)	28.87 (25.66 to 32.24)
Slovakia	29.75 (27.66 to 31.84)	19.68 (16.97 to 22.09)
Slovenia	28.47 (26.45 to 30.47)	24.13 (21.55 to 27.14)
Eastern Europe		
Belarus	46.32 (44.06 to 48.44)	21.21 (18.46 to 23.99)
Estonia	30.69 (28.57 to 32.66)	16.75 (14.58 to 19.1)
Latvia	42.31 (40.19 to 44.2)	20.38 (18.2 to 22.73)
Lithuania	36.84 (34.82 to 38.85)	19.34 (17.05 to 21.71)
Moldova	34.58 (32.39 to 36.56)	6.18 (4.9 to 7.73)
Russian Federation	41.51 (39.87 to 43.11)	14.57 (13.04 to 16.24)
Ukraine	41.34 (39.09 to 43.51)	15.29 (12.97 to 17.87)
High-income		
Australasia		
Australia	15.94 (14.4 to 17.5)	12.43 (10.33 to 14.72)
New Zealand	19.83 (18.6 to 21.09)	15.45 (14.22 to 16.77)
High-income Asia Pacific		
Brunei	27.6 (25.08 to 30.19)	5.45 (4.34 to 6.81)
Japan	32.36 (30.04 to 34.8)	10.89 (8.72 to 13.25)
South Korea	37.97 (35.67 to 40.36)	4.77 (3.69 to 6.05)
Singapore	19.03 (17.21 to 20.9)	8.39 (6.74 to 10.19)
High-income North America		
Canada	17.36 (15.43 to 19.36)	15.18 (12.06 to 18.82)
Greenland	43.78 (40.88 to 46.8)	46.4 (41.3 to 51.47)
United States	22.2 (20.23 to 24.21)	16.09 (13.87 to 18.43)
Southern Latin America		
Argentina	27.19 (25.01 to 29.19)	17.84 (16.09 to 19.71)
Chile	33.88 (31.56 to 36.29)	29.05 (25.24 to 32.75)
Uruguay	25.69 (23.31 to 27.99)	19.64 (16.8 to 22.61)
Western Europe		
Andorra	28.1 (25.38 to 31.17)	22.39 (17.88 to 26.99)
Austria	33.02 (30.92 to 35.34)	24.43 (21.61 to 27.5)
Belgium	22.51 (20.8 to 24.18)	19.14 (16.71 to 21.82)
Cyprus	39.17 (36.84 to 41.28)	19.77 (17.04 to 22.65)
Denmark	20.26 (18.6 to 21.99)	19.33 (16.92 to 22)
Finland	22.51 (20.69 to 24.35)	16.78 (14.63 to 19.17)
France	33.31 (31.14 to 35.44)	30.37 (27.2 to 33.32)
Germany	29.75 (27.89 to 31.57)	22.88 (20.43 to 25.49)
Greece	38.6 (36.57 to 40.87)	30.43 (27.71 to 33.23)
Iceland	16.12 (14.38 to 18.01)	15 (12.06 to 18.6)

	Estimate in 2017, male (95% UIs)	Estimate in 2017, female (95% UIs)
Ireland	20.67 (18.86 to 22.63)	19.46 (17.2 to 21.98)
Israel	25.54 (23.41 to 28.01)	15.8 (12.98 to 18.88)
Italy	27.5 (25.59 to 29.49)	18.53 (16.03 to 21.13)
Luxembourg	24.97 (22.63 to 27.23)	19.45 (16.31 to 22.64)
Malta	25.53 (23.12 to 28.06)	19.87 (16.9 to 23.02)
Netherlands	21.35 (19.55 to 23.07)	18.04 (15.76 to 20.62)
Norway	17.15 (15.48 to 19.11)	15.75 (12.98 to 18.87)
Portugal	29.32 (27.38 to 31.44)	19.71 (17.3 to 22.4)
Spain	30.19 (27.91 to 32.51)	25.49 (22.95 to 28.42)
Sweden	10.09 (8.96 to 11.27)	12.25 (10.18 to 14.46)
Switzerland	27.02 (24.8 to 29.6)	21.38 (17.89 to 25.5)
United Kingdom	22.77 (20.65 to 24.84)	20.79 (18.02 to 23.57)
Latin America and Caribbean		
Andean Latin America		
Bolivia	10.21 (9.01 to 11.41)	6.93 (5.53 to 8.45)
Ecuador	18.89 (16.96 to 20.67)	5.48 (4.66 to 6.45)
Peru	10.89 (9.58 to 12.29)	4.61 (3.88 to 5.33)
Caribbean		
Antigua and Barbuda	14.26 (12.65 to 16.13)	6.08 (4.63 to 7.9)
The Bahamas	14.55 (12.92 to 16.45)	6 (4.59 to 7.67)
Barbados	16.01 (14.15 to 17.95)	5.96 (4.85 to 7.13)
Belize	18.37 (16.63 to 20.2)	2.99 (2.46 to 3.58)
Bermuda	17.1 (15.33 to 19.09)	8.34 (6.53 to 10.52)
Cuba	32.81 (30.01 to 35.56)	20.69 (17.16 to 24.5)
Dominica	15.06 (13.22 to 16.93)	5.37 (4.12 to 6.76)
Dominican Republic	12.35 (11.21 to 13.63)	7.49 (6.35 to 8.84)
Grenada	18.71 (16.84 to 20.79)	6.04 (4.87 to 7.45)
Guyana	22.85 (20.66 to 25.13)	6.08 (4.94 to 7.37)
Haiti	10.1 (9.09 to 11.23)	4.07 (3.14 to 5.18)
Jamaica	23.99 (21.65 to 26.38)	9.1 (7.43 to 10.99)
Puerto Rico	15.7 (14.28 to 17.21)	8.03 (6.56 to 9.66)
Saint Lucia	20.45 (18.28 to 22.69)	9.11 (7.27 to 11.36)
Saint Vincent and the Grenadines	19.67 (17.68 to 21.91)	6.25 (4.78 to 8.03)
Suriname	31.6 (29.1 to 34.24)	10.03 (7.89 to 12.61)
Trinidad and Tobago	26.39 (24.26 to 28.83)	7.65 (6.14 to 9.32)
Virgin Islands, U.S.	8.21 (7.23 to 9.32)	5.06 (3.81 to 6.5)
Central Latin America		
Colombia	15.71 (14.01 to 17.33)	10.04 (8.71 to 11.54)
Costa Rica	17.71 (16.29 to 19.16)	8.17 (6.71 to 9.8)
El Salvador	12.97 (11.45 to 14.62)	3 (2.25 to 4.06)
Guatemala	17.19 (15.62 to 18.82)	3.31 (2.48 to 4.54)
Honduras	20.88 (18.76 to 22.98)	4.83 (3.9 to 5.92)
Mexico	23.92 (22.47 to 25.44)	8.5 (7.2 to 9.98)
Nicaragua	19.43 (17.4 to 21.64)	4.31 (3.3 to 5.62)
Panama	11.43 (10.16 to 12.92)	3.74 (3.12 to 4.46)
Venezuela	20.69 (18.5 to 22.97)	11.02 (8.64 to 13.79)
Tropical Latin America		

	Estimate in 2017, male (95% UIs)	Estimate in 2017, female (95% UIs)
Brazil	11.66 (10.79 to 12.66)	7.54 (6.46 to 8.75)
Paraguay	20.95 (19.04 to 22.83)	7.29 (6.1 to 8.61)
North Africa and Middle East		
North Africa and Middle East		
Afghanistan	18.79 (16.98 to 20.64)	2.59 (2.13 to 3.15)
Algeria	32.04 (28.97 to 35)	3.74 (2.83 to 4.94)
Bahrain	17.9 (15.79 to 19.91)	4.02 (3.08 to 5.2)
Egypt	36.43 (34.23 to 38.8)	1.23 (0.99 to 1.55)
Iran	21.97 (20.02 to 23.9)	5.02 (3.92 to 6.2)
Iraq	32.88 (30.35 to 35.26)	4.54 (3.54 to 5.77)
Jordan	36.76 (34.23 to 39.4)	11.98 (10.31 to 13.76)
Kuwait	30.05 (27.9 to 32.29)	4.23 (3.6 to 5)
Lebanon	37.36 (35.32 to 39.66)	22.22 (19.7 to 24.77)
Libya	28.32 (26.15 to 30.42)	1.06 (0.78 to 1.47)
Morocco	20.47 (18.55 to 22.55)	1.24 (0.99 to 1.55)
Palestine	36.31 (34.04 to 38.45)	5.87 (5.02 to 6.76)
Oman	13.13 (11.61 to 14.76)	2.35 (1.89 to 2.88)
Qatar	19.37 (17.66 to 21.14)	3.46 (2.66 to 4.54)
Saudi Arabia	17.19 (15.73 to 18.8)	1.91 (1.51 to 2.48)
Sudan	10.96 (9.65 to 12.27)	1.75 (1.28 to 2.32)
Syria	30.2 (27.58 to 32.87)	4.42 (3.42 to 5.72)
Tunisia	40.78 (37.92 to 43.36)	3.12 (2.4 to 3.9)
Turkey	38.11 (36.06 to 40.1)	12.12 (10.65 to 13.71)
United Arab Emirates	23.09 (20.77 to 25.48)	3.83 (2.95 to 4.92)
Yemen	26.03 (24.21 to 27.83)	7.95 (6.59 to 9.41)
South Asia		
South Asia		
Bangladesh	40.28 (38.11 to 42.41)	1.28 (1 to 1.66)
Bhutan	10.83 (9.6 to 12.13)	3.69 (2.98 to 4.53)
India	20.2 (18.93 to 21.52)	2.25 (1.78 to 2.83)
Nepal	29.63 (27.71 to 31.6)	12.75 (11.19 to 14.6)
Pakistan	23.2 (21.59 to 24.93)	3.14 (2.54 to 3.84)
Southeast Asia, East Asia, and Oceania		
East Asia		
China	43.77 (41.53 to 45.75)	2.77 (2.29 to 3.32)
North Korea	39.35 (37.03 to 41.76)	0.37 (0.26 to 0.55)
Taiwan	30.82 (28.44 to 33.1)	5.55 (4.5 to 6.94)
Oceania		
American Samoa	35.13 (32.42 to 38.1)	19.04 (15.37 to 22.96)
Federated States of Micronesia	40.05 (37.06 to 43.27)	17.25 (14.27 to 20.71)
Fiji	38.13 (35.66 to 40.43)	12.12 (10 to 14.59)
Guam	30 (28.11 to 31.99)	21.12 (18.67 to 23.68)
Kiribati	51.91 (49.48 to 54.38)	30.83 (27.06 to 34.58)
Marshall Islands	29.7 (27.18 to 32.2)	7.19 (5.63 to 9.05)
Northern Mariana Islands	34.8 (31.71 to 37.84)	16.69 (12.97 to 20.94)
Papua New Guinea	42.99 (40.21 to 45.82)	20.65 (17.86 to 23.57)
Samoa	40.6 (37.78 to 43.15)	16.07 (13.55 to 18.92)

	Estimate in 2017, male (95% UIs)	Estimate in 2017, female (95% UIs)
Solomon Islands	37.22 (34.62 to 39.77)	14.1 (11.39 to 16.92)
Tonga	42.01 (39.49 to 44.6)	14.13 (11.63 to 16.73)
Vanuatu	35.86 (33.72 to 38.01)	4.9 (3.9 to 6.18)
Southeast Asia		
Cambodia	37.18 (35.3 to 39.07)	4.74 (3.71 to 5.87)
Indonesia	55.75 (53.59 to 57.75)	3.47 (2.67 to 4.39)
Laos	38.79 (36.26 to 41.4)	6.94 (5.6 to 8.5)
Malaysia	37.78 (35.49 to 40.1)	2.84 (2.28 to 3.56)
Maldives	34.33 (32.28 to 36.48)	6.19 (5.2 to 7.35)
Mauritius	37.27 (34.72 to 39.78)	5.17 (4.3 to 6.25)
Myanmar	33.76 (32.08 to 35.58)	8.43 (7.11 to 9.89)
Philippines	37.42 (35.5 to 39.51)	8.1 (6.73 to 9.72)
Sri Lanka	24.94 (23.47 to 26.48)	1.34 (1.03 to 1.72)
Seychelles	31.46 (28.98 to 34.06)	6.46 (5.16 to 8.08)
Thailand	36.41 (34.46 to 38.4)	2.99 (2.33 to 3.79)
Timor-Leste	59.7 (57.65 to 61.87)	5.51 (4.62 to 6.82)
Vietnam	40.14 (38.47 to 41.78)	1.39 (1.2 to 1.63)
Sub-Saharan Africa		
Central Sub-Saharan Africa		
Angola	16.9 (15.43 to 18.51)	3.24 (2.33 to 4.73)
Central African Republic	16.09 (14.32 to 17.91)	2.21 (1.48 to 3.36)
Congo	16.67 (15.16 to 18.31)	3.03 (2.17 to 4.09)
Democratic Republic of the Congo	18 (16.09 to 19.92)	1.57 (1.1 to 2.33)
Equatorial Guinea	14.24 (12.57 to 16.01)	2.23 (1.62 to 3.04)
Gabon	17.75 (15.84 to 19.85)	3.16 (2.38 to 4.3)
Eastern Sub-Saharan Africa		
Burundi	13.5 (11.89 to 15.19)	5.74 (4.5 to 7.03)
Comoros	16.8 (15.21 to 18.55)	2.72 (2.28 to 3.22)
Djibouti	26.23 (24.06 to 28.52)	4.15 (3.35 to 5.1)
Eritrea	7.07 (6.15 to 8.05)	0.36 (0.25 to 0.52)
Ethiopia	6.7 (6.04 to 7.37)	0.92 (0.7 to 1.27)
Kenya	16.95 (15.58 to 18.33)	2.7 (2.15 to 3.49)
Madagascar	22.58 (20.57 to 24.71)	2.18 (1.49 to 3.14)
Malawi	17.39 (15.79 to 19.23)	3.12 (2.45 to 3.96)
Mozambique	18.54 (16.91 to 20.13)	4.97 (4.07 to 5.97)
Rwanda	16.65 (15.36 to 18.09)	5.59 (4.56 to 6.61)
Somalia	12.13 (10.68 to 13.68)	1.62 (1.18 to 2.19)
South Sudan	16.27 (14.44 to 18.2)	2.08 (1.54 to 2.81)
Tanzania	20.04 (18.29 to 21.94)	3.28 (2.61 to 4.08)
Uganda	15.27 (14.09 to 16.54)	4.24 (3.41 to 5.22)
Zambia	23.15 (21.18 to 25.24)	8.1 (6.54 to 9.77)
Southern Sub-Saharan Africa		
Botswana	25.7 (23.41 to 27.98)	8.34 (6.64 to 10.23)
Lesotho	32.5 (30.34 to 34.64)	1.69 (1.13 to 2.58)
Namibia	23.09 (21.21 to 25.12)	10.05 (8.15 to 12.1)
South Africa	29.14 (27.2 to 31.08)	11.12 (9.11 to 13.59)
Swaziland	8.03 (7.04 to 9.03)	1.97 (1.52 to 2.59)

	Estimate in 2017, male (95% UIs)	Estimate in 2017, female (95% UIs)
Zimbabwe	27.75 (25.84 to 29.84)	4.25 (3.39 to 5.27)
Western Sub-Saharan Africa		
Benin	11.9 (10.7 to 13.21)	1.88 (1.43 to 2.5)
Burkina Faso	16.63 (14.97 to 18.33)	0.76 (0.59 to 1)
Cameroon	13.47 (12.16 to 14.96)	1.09 (0.79 to 1.56)
Cape Verde	11.52 (10.22 to 13.04)	2.35 (1.75 to 3.07)
Chad	15.2 (13.82 to 16.6)	2.01 (1.52 to 2.68)
Cote d'Ivoire	20.23 (18.33 to 22.38)	3.35 (2.49 to 4.32)
The Gambia	19.97 (18.36 to 21.72)	1.16 (0.74 to 1.8)
Ghana	7.83 (6.94 to 8.83)	1.4 (1 to 2.04)
Guinea	11.81 (10.25 to 13.43)	1.15 (0.8 to 1.73)
Guinea-Bissau	9.87 (8.61 to 11.32)	0.92 (0.63 to 1.34)
Liberia	11.83 (10.58 to 13.07)	1.6 (1.19 to 2.15)
Mali	17.99 (16.37 to 19.85)	1.24 (0.83 to 1.82)
Mauritania	24.98 (22.54 to 27.48)	7.83 (6.04 to 10.12)
Niger	14.12 (12.52 to 15.82)	0.67 (0.49 to 0.93)
Nigeria	6.75 (5.87 to 7.74)	0.96 (0.67 to 1.39)
Sao Tome and Principe	6.59 (5.79 to 7.46)	1.15 (0.8 to 1.6)
Senegal	17.47 (15.76 to 19.32)	1.48 (1.11 to 1.94)
Sierra Leone	25.92 (24.06 to 27.96)	4.94 (4.11 to 5.99)
Togo	12.26 (10.97 to 13.58)	1.52 (1.09 to 2.07)
**Indicator 16.1.1: Age-standardised death rate due to interpersonal violence (per 100,000 population)**		
Central Europe, Eastern Europe, and Central Asia		
Central Asia		
Armenia	5.27 (4.79 to 5.76)	1.26 (1.15 to 1.38)
Azerbaijan	5.77 (4.02 to 10.21)	1.24 (0.93 to 2.12)
Georgia	5.99 (5.34 to 6.67)	0.97 (0.86 to 1.09)
Kazakhstan	13.14 (11.82 to 14.48)	3.18 (2.87 to 3.51)
Kyrgyzstan	6.98 (6.26 to 7.8)	1.95 (1.75 to 2.23)
Mongolia	12.95 (9.93 to 16.6)	2.45 (1.67 to 3.26)
Tajikistan	4.72 (3.76 to 7.48)	1.31 (1.01 to 2.25)
Turkmenistan	6.09 (5.27 to 6.95)	1.61 (1.39 to 1.86)
Uzbekistan	3.99 (3.39 to 4.7)	1.13 (0.95 to 1.34)
Central Europe		
Albania	3.68 (2.66 to 5.53)	0.87 (0.61 to 1.27)
Bosnia and Herzegovina	3.12 (2.09 to 3.73)	0.85 (0.48 to 1.02)
Bulgaria	2.9 (2.59 to 3.23)	1.04 (0.92 to 1.16)
Croatia	1.07 (0.95 to 1.2)	0.69 (0.6 to 0.82)
Czech Republic	1.15 (1.02 to 1.28)	0.73 (0.65 to 0.81)
Hungary	1.58 (1.43 to 1.75)	0.91 (0.8 to 1.06)
Macedonia	2.21 (1.38 to 2.67)	0.81 (0.5 to 0.99)
Montenegro	3.94 (3.13 to 5.41)	1.87 (1.44 to 2.75)
Poland	2.1 (1.88 to 2.34)	0.63 (0.57 to 0.71)
Romania	2.27 (2.05 to 2.5)	0.95 (0.85 to 1.05)
Serbia	2.77 (1.91 to 3.38)	1.17 (0.88 to 1.64)
Slovakia	2.69 (2.22 to 3.93)	1.2 (0.92 to 1.76)

	Estimate in 2017, male (95% UIs)	Estimate in 2017, female (95% UIs)
Slovenia	0.94 (0.84 to 1.06)	0.61 (0.53 to 0.7)
Eastern Europe		
Belarus	8.14 (7.06 to 9.07)	2.75 (2.44 to 3.12)
Estonia	5.79 (4.79 to 7.05)	1.68 (1.37 to 2.05)
Latvia	8.5 (7.15 to 10)	2.6 (2.12 to 3.16)
Lithuania	7.58 (6.83 to 8.5)	2.29 (2.01 to 2.59)
Moldova	7.42 (6.83 to 8.09)	2.38 (2.17 to 2.64)
Russian Federation	19.8 (19.19 to 20.39)	5.32 (5.1 to 5.69)
Ukraine	13.76 (12.56 to 15.26)	3.56 (3.1 to 3.98)
High-income		
Australasia		
Australia	1.72 (1.46 to 2.01)	0.82 (0.7 to 0.97)
New Zealand	1.83 (1.66 to 2.02)	0.88 (0.8 to 0.97)
High-income Asia Pacific		
Brunei	1.89 (1.22 to 2.29)	0.86 (0.5 to 1.06)
Japan	0.53 (0.49 to 0.56)	0.39 (0.36 to 0.43)
South Korea	1.21 (1.07 to 1.35)	0.87 (0.77 to 0.97)
Singapore	0.43 (0.38 to 0.49)	0.38 (0.34 to 0.43)
High-income North America		
Canada	2.23 (1.94 to 2.52)	0.75 (0.66 to 0.84)
Greenland	7.73 (6.32 to 10.7)	7.48 (5.33 to 9.2)
United States	9.8 (9.32 to 10.41)	2.62 (2.46 to 2.75)
Southern Latin America		
Argentina	10.03 (8.45 to 11.83)	1.78 (1.52 to 2.08)
Chile	7.28 (6.22 to 8.58)	1.01 (0.86 to 1.18)
Uruguay	10.15 (8.55 to 11.87)	1.71 (1.42 to 2.05)
Western Europe		
Andorra	0.83 (0.61 to 1.39)	0.44 (0.33 to 0.8)
Austria	0.72 (0.63 to 0.81)	0.61 (0.54 to 0.69)
Belgium	1.4 (1.25 to 1.56)	0.94 (0.83 to 1.05)
Cyprus	1.98 (1.1 to 2.47)	0.74 (0.44 to 0.93)
Denmark	0.88 (0.77 to 0.99)	0.59 (0.52 to 0.67)
Finland	1.97 (1.74 to 2.22)	0.89 (0.79 to 1.02)
France	1.02 (0.9 to 1.14)	0.5 (0.45 to 0.56)
Germany	0.77 (0.67 to 0.89)	0.6 (0.51 to 0.7)
Greece	1.5 (1.34 to 1.67)	0.5 (0.44 to 0.57)
Iceland	0.85 (0.76 to 0.95)	0.47 (0.42 to 0.53)
Ireland	1 (0.85 to 1.15)	0.29 (0.26 to 0.34)
Israel	3.23 (2.87 to 3.65)	0.9 (0.8 to 1.01)
Italy	0.92 (0.81 to 1.03)	0.37 (0.33 to 0.42)
Luxembourg	0.91 (0.77 to 1.07)	0.89 (0.77 to 1.03)
Malta	1.38 (1.23 to 1.55)	0.76 (0.67 to 0.85)
Netherlands	1 (0.89 to 1.12)	0.57 (0.51 to 0.64)
Norway	0.76 (0.7 to 0.81)	0.56 (0.53 to 0.61)
Portugal	1.44 (1.25 to 1.63)	0.69 (0.61 to 0.8)
Spain	0.76 (0.68 to 0.85)	0.41 (0.36 to 0.46)
Sweden	1.49 (1.35 to 1.65)	0.67 (0.6 to 0.74)

	Estimate in 2017, male (95% UIs)	Estimate in 2017, female (95% UIs)
Switzerland	0.55 (0.47 to 0.62)	0.56 (0.49 to 0.65)
United Kingdom	0.68 (0.65 to 0.7)	0.32 (0.31 to 0.34)
Latin America and Caribbean		
Andean Latin America		
Bolivia	9.35 (5.88 to 13.05)	2.47 (1.52 to 3.44)
Ecuador	23.22 (20.05 to 26.61)	3.91 (3.34 to 4.48)
Peru	5.76 (3.87 to 7.41)	1.26 (0.75 to 1.64)
Caribbean		
Antigua and Barbuda	8.09 (7.1 to 9.23)	1.94 (1.65 to 2.25)
The Bahamas	47.19 (41.67 to 53.34)	6.36 (5.43 to 7.45)
Barbados	16.51 (14.3 to 18.85)	4.59 (3.94 to 5.27)
Belize	46.54 (43.36 to 50.21)	5.19 (4.63 to 5.83)
Bermuda	4.87 (4.11 to 5.77)	0.94 (0.79 to 1.12)
Cuba	8.25 (6.99 to 9.7)	2.59 (2.16 to 3.08)
Dominica	18.28 (16.17 to 20.42)	4.08 (3.44 to 4.8)
Dominican Republic	36.57 (21.02 to 45.83)	5.46 (2.73 to 7.13)
Grenada	7.62 (6.75 to 8.56)	2.3 (2 to 2.63)
Guyana	26.91 (22.58 to 31.62)	6.45 (5.21 to 7.81)
Haiti	28.49 (17.83 to 46.94)	9.12 (5.52 to 12.98)
Jamaica	51.54 (41.31 to 63.38)	7.18 (5.47 to 9.24)
Puerto Rico	40.24 (36.79 to 43.61)	3.4 (2.99 to 3.85)
Saint Lucia	29.85 (26.78 to 32.75)	4.04 (3.53 to 4.6)
Saint Vincent and the Grenadines	31.62 (28.55 to 34.72)	7.38 (6.33 to 8.49)
Suriname	13.31 (11.17 to 15.86)	4.21 (3.51 to 5)
Trinidad and Tobago	36.87 (28.19 to 47.12)	5.23 (3.83 to 6.99)
Virgin Islands, U.S.	48.77 (43.54 to 56.07)	6.28 (4.98 to 7.77)
Central Latin America		
Colombia	51.2 (43.22 to 61.33)	6.03 (5.08 to 7.06)
Costa Rica	17.34 (15.42 to 19.55)	2.05 (1.79 to 2.33)
El Salvador	104.69 (72.79 to 131.5)	11.06 (6.63 to 14.71)
Guatemala	66.06 (57.22 to 75.53)	8.98 (7.66 to 10.37)
Honduras	72.77 (47.84 to 93.73)	22.39 (10.31 to 31.76)
Mexico	57.88 (56.89 to 58.91)	8.56 (8.4 to 8.71)
Nicaragua	12.78 (10.29 to 18.77)	2.14 (1.61 to 3.19)
Panama	28.12 (25.34 to 31.01)	3.43 (2.98 to 3.9)
Venezuela	82.27 (67.42 to 98.49)	6.07 (4.82 to 7.42)
Tropical Latin America		
Brazil	51.18 (50.22 to 52.11)	4.9 (4.73 to 5.08)
Paraguay	20.73 (15.26 to 28.5)	2.93 (2.12 to 4.3)
North Africa and Middle East		
North Africa and Middle East		
Afghanistan	22 (11.29 to 31.09)	1.9 (1.3 to 2.84)
Algeria	1.56 (0.93 to 1.98)	0.44 (0.3 to 0.55)
Bahrain	1.67 (1.1 to 2.12)	0.77 (0.44 to 0.97)
Egypt	1.02 (0.47 to 1.34)	0.3 (0.16 to 0.39)
Iran	3.29 (2.14 to 3.68)	1.03 (0.57 to 1.2)
Iraq	8.45 (4.51 to 10.21)	2.82 (1.39 to 3.56)

	Estimate in 2017, male (95% UIs)	Estimate in 2017, female (95% UIs)
Jordan	3.85 (2.19 to 4.71)	1.08 (0.51 to 1.4)
Kuwait	1.2 (1.05 to 1.37)	0.26 (0.22 to 0.33)
Lebanon	7.07 (3.75 to 9.51)	1 (0.62 to 1.58)
Libya	3.44 (1.66 to 4.87)	0.77 (0.44 to 1.12)
Morocco	1.67 (0.81 to 2.35)	0.51 (0.29 to 0.7)
Palestine	9.15 (3.4 to 11.22)	2.14 (0.65 to 2.98)
Oman	0.56 (0.4 to 0.83)	0.34 (0.22 to 0.46)
Qatar	0.94 (0.64 to 1.25)	0.4 (0.2 to 0.54)
Saudi Arabia	1.03 (0.47 to 1.47)	0.27 (0.18 to 0.38)
Sudan	1.69 (0.37 to 2.45)	0.55 (0.16 to 0.82)
Syria	3.13 (1.53 to 4.2)	0.94 (0.43 to 1.29)
Tunisia	0.88 (0.53 to 1.22)	0.21 (0.15 to 0.3)
Turkey	3.25 (1.85 to 3.98)	0.67 (0.4 to 0.82)
United Arab Emirates	1.27 (0.87 to 1.82)	0.31 (0.22 to 0.46)
Yemen	4.8 (2.87 to 6.8)	1.33 (0.83 to 1.78)
South Asia		
South Asia		
Bangladesh	2.21 (1.69 to 3.77)	0.88 (0.64 to 1.69)
Bhutan	1.39 (0.81 to 1.89)	0.38 (0.24 to 0.54)
India	4.44 (3.34 to 5.63)	1.57 (1.14 to 2.06)
Nepal	2.57 (0.91 to 3.8)	0.78 (0.36 to 1.05)
Pakistan	5.65 (2.83 to 8.28)	2.81 (1.82 to 3.68)
Southeast Asia, East Asia, and Oceania		
East Asia		
China	1.21 (1.04 to 1.84)	0.65 (0.51 to 0.94)
North Korea	3.45 (2.28 to 4.9)	1.69 (1.08 to 2.33)
Taiwan	1.64 (1.48 to 1.81)	0.7 (0.63 to 0.78)
Oceania		
American Samoa	8.96 (7.31 to 13.31)	2.01 (1.32 to 2.57)
Federated States of Micronesia	7.99 (4.26 to 11.93)	3.38 (1.63 to 5.17)
Fiji	5.71 (3.36 to 7.24)	2.09 (1.14 to 2.68)
Guam	6.44 (4.98 to 8.49)	2.87 (1.69 to 3.54)
Kiribati	2.76 (1.55 to 7.94)	0.84 (0.5 to 2.02)
Marshall Islands	9.26 (5.17 to 12.34)	2.98 (1.89 to 4.31)
Northern Mariana Islands	6.76 (5.21 to 9.74)	2.54 (1.72 to 3.35)
Papua New Guinea	16.87 (8.51 to 23.05)	4.43 (2.99 to 6.32)
Samoa	4.55 (2.87 to 7.48)	2.19 (1.16 to 3.31)
Solomon Islands	8.31 (4.17 to 11.35)	2.81 (1.87 to 3.98)
Tonga	3.5 (1.93 to 10.17)	1.12 (0.57 to 3.25)
Vanuatu	2.79 (1.5 to 3.98)	0.77 (0.46 to 1.26)
Southeast Asia		
Cambodia	3.85 (2.52 to 5.4)	0.87 (0.58 to 1.22)
Indonesia	1.37 (1.14 to 2)	0.53 (0.36 to 0.67)
Laos	4.53 (2.75 to 6.78)	1.79 (1 to 2.36)
Malaysia	2.91 (1.86 to 5.33)	0.62 (0.42 to 1.34)
Maldives	1.09 (0.51 to 1.33)	0.34 (0.14 to 0.45)
Mauritius	3.21 (2.82 to 3.64)	1.29 (1.14 to 1.47)

	Estimate in 2017, male (95% UIs)	Estimate in 2017, female (95% UIs)
Myanmar	2.5 (1.56 to 3.44)	0.75 (0.37 to 1.03)
Philippines	27.54 (22.63 to 33.19)	3.68 (3.06 to 4.44)
Sri Lanka	5.68 (4.05 to 8.04)	1.09 (0.7 to 1.48)
Seychelles	7.7 (4.7 to 9.28)	2.79 (1.55 to 3.35)
Thailand	9.41 (5.72 to 11.98)	1.48 (1.06 to 1.93)
Timor-Leste	4.57 (1.88 to 6.41)	1.15 (0.58 to 1.53)
Vietnam	2.46 (1.5 to 3.5)	0.43 (0.26 to 0.57)
Sub-Saharan Africa		
Central Sub-Saharan Africa		
Angola	6.2 (4.1 to 13.13)	1.16 (0.75 to 1.67)
Central African Republic	13.49 (6.85 to 20.12)	2.78 (1.28 to 4.21)
Congo	8.31 (4.44 to 12.46)	2.34 (1.02 to 3.57)
Democratic Republic of the Congo	5.24 (3.37 to 7.12)	1.32 (0.88 to 1.81)
Equatorial Guinea	5.92 (3.07 to 9.43)	1.16 (0.54 to 2.03)
Gabon	8.5 (4.46 to 12.57)	1.32 (0.56 to 2.67)
Eastern Sub-Saharan Africa		
Burundi	0.99 (0.48 to 1.66)	0.28 (0.15 to 0.46)
Comoros	12.93 (4.56 to 18.33)	4.07 (1.36 to 5.83)
Djibouti	12.77 (4.27 to 19.91)	3.6 (1.08 to 5.86)
Eritrea	22.29 (6.64 to 34.88)	5 (1.46 to 7.56)
Ethiopia	13.78 (11.15 to 19.73)	4.92 (3.58 to 6.72)
Kenya	18.35 (13.38 to 25.19)	4.92 (3.4 to 6.89)
Madagascar	2.57 (0.96 to 3.79)	0.68 (0.28 to 0.97)
Malawi	3.21 (1.67 to 10.42)	0.74 (0.33 to 2.32)
Mozambique	13.43 (8.3 to 17.68)	2.46 (1.52 to 3.32)
Rwanda	5.23 (3.51 to 7.25)	1.19 (0.79 to 1.76)
Somalia	17.17 (5.67 to 25.97)	4.77 (1.5 to 7.47)
South Sudan	16.41 (5.27 to 25.37)	4.3 (1.31 to 6.85)
Tanzania	9.91 (7.46 to 14.95)	2.81 (2.08 to 4.95)
Uganda	7.68 (4.41 to 10.57)	1.46 (0.8 to 2.28)
Zambia	10.14 (6 to 13.68)	2.52 (1.52 to 3.89)
Southern Sub-Saharan Africa		
Botswana	12.04 (3.11 to 16.23)	3.22 (0.86 to 4.71)
Lesotho	71.54 (28.88 to 96.37)	8.93 (3.84 to 13.88)
Namibia	26.72 (17.72 to 37.57)	3.81 (2.33 to 5.91)
South Africa	44.54 (32.7 to 50.03)	9.2 (6.45 to 12.12)
Swaziland	43.34 (30.02 to 63.92)	5.2 (3.25 to 9.22)
Zimbabwe	14.77 (10.43 to 20.89)	2.89 (2.04 to 4.33)
Western Sub-Saharan Africa		
Benin	10 (5.08 to 13.9)	2.44 (1.09 to 3.55)
Burkina Faso	20.98 (15.78 to 27.99)	7.15 (5.38 to 10.93)
Cameroon	4.64 (2.63 to 6.48)	1.08 (0.63 to 1.56)
Cape Verde	19.81 (15.57 to 25.94)	4.42 (2.89 to 5.4)
Chad	11.68 (5.84 to 16.17)	2.98 (1.3 to 4.39)
Cote d'Ivoire	20.03 (14.97 to 27.24)	2.85 (1.28 to 4.39)
The Gambia	2.59 (0.66 to 14.24)	0.27 (0.2 to 0.39)
Ghana	4.9 (2.81 to 15.59)	1.58 (0.75 to 5.35)

	Estimate in 2017, male (95% UIs)	Estimate in 2017, female (95% UIs)
Guinea	12.69 (6.25 to 16.85)	3.15 (1.39 to 4.2)
Guinea-Bissau	14.67 (7.77 to 20.33)	5.14 (3.45 to 7.42)
Liberia	3.47 (1.68 to 4.73)	1 (0.57 to 1.34)
Mali	9.16 (4.44 to 16.23)	2.92 (1.3 to 4.32)
Mauritania	7.12 (3.66 to 10.72)	2.02 (0.9 to 2.84)
Niger	10.19 (5.09 to 17.53)	2.61 (1.16 to 4.27)
Nigeria	6.19 (3.33 to 9.1)	1.79 (0.8 to 2.67)
Sao Tome and Principe	7.87 (4.61 to 10.46)	2.54 (1.36 to 3.31)
Senegal	1.76 (1.12 to 2.34)	0.44 (0.29 to 0.61)
Sierra Leone	2.17 (1.13 to 2.99)	0.65 (0.37 to 0.89)
Togo	10.61 (5.29 to 14.44)	2.23 (0.99 to 3.2)

Supplementary table 3. Unscaled values for the 40 individual health-related SDG indicators in 2000, 2015, 2017, and 2030, and annualised rate of change and projected percent change from 2015 to 2030, by country or territory. Projected estimates for 2030 were produced based on past trends and rates of change observed from 1990 to 2017. Estimates are reported for each country and territory included in this analysis, and ordered by health-related SDG indicator followed by GBD super-region and region. SDG indicator 17.19.2a, population census status within the last 10 years, is not included here as projections were not generated for this indicator. All results from 1990 to 2017 for SDG indicator 17.19.2a can be found in Supplementary Figure 1. SDG=Sustainable Development Goal. For this table, forecasted draws (2018 to 2030) for conflict and disaster mortality were truncated so that any values which fell outside the confidence intervals for any location year were set to the lower or upper bound of that confidence interval. ARC=annualised rate of change. GBD=Global Burden of Disease. UIs=uncertainty intervals.

**Indicator 1.5.1: Death rate due to exposure to forces of nature (per 100,000 population)**	**Estimate in 2000 (95% UIs)**	**Estimate in 2015 (95% UIs)**	**Estimate in 2017 (95% UIs)**	**Estimate in 2030 (95% UIs)**	**Annualised rate of change, 2015-2030 (95% UIs)**	**Percent change, 2015-2030 (95% UIs)**
Central Europe, Eastern Europe, and Central Asia						
Central Asia						
Armenia	0.0 (0.0 to 0.0)	0.0 (0.0 to 0.0)	0.0 (0.0 to 0.0)	0.0 (0.0 to 0.1)	12.9 (0.0 to 90.9)	6184077.0 (0.0 to 83038434.9)
Azerbaijan	0.5 (0.5 to 0.5)	0.0 (0.0 to 0.0)	0.0 (0.0 to 0.0)	0.0 (0.0 to 0.3)	12.8 (0.0 to 98.6)	11103613.0 (0.0 to 264422474.3)
Georgia	0.0 (0.0 to 0.0)	0.2 (0.1 to 0.2)	0.0 (0.0 to 0.0)	0.0 (0.0 to 0.3)	-72.0 (-95.6 to 4.2)	-86.1 (-100.0 to 87.2)
Kazakhstan	0.0 (0.0 to 0.0)	0.0 (0.0 to 0.0)	0.0 (0.0 to 0.0)	0.0 (0.0 to 0.0)	-64.5 (-84.1 to 1.1)	-81.1 (-100.0 to 17.5)
Kyrgyzstan	0.2 (0.1 to 0.2)	0.3 (0.3 to 0.3)	0.4 (0.4 to 0.4)	0.1 (0.0 to 1.1)	-67.8 (-99.2 to 9.4)	-59.6 (-100.0 to 307.5)
Mongolia	0.9 (0.9 to 1.0)	0.0 (0.0 to 0.0)	0.5 (0.5 to 0.5)	0.2 (0.0 to 3.2)	25.5 (0.0 to 115.2)	204299326.5 (0.0 to 3214076228.1)
Tajikistan	0.0 (0.0 to 0.0)	0.0 (0.0 to 0.0)	0.1 (0.1 to 0.1)	0.2 (0.0 to 3.9)	-55.9 (-82.4 to 34.2)	697.1 (-100.0 to 16814.1)
Turkmenistan	0.0 (0.0 to 0.0)	0.0 (0.0 to 0.0)	0.0 (0.0 to 0.0)	0.0 (0.0 to 0.0)	19.4 (0.0 to 78.4)	2561547.8 (0.0 to 12755187.1)
Uzbekistan	0.0 (0.0 to 0.0)	0.0 (0.0 to 0.0)	0.0 (0.0 to 0.0)	0.0 (0.0 to 0.0)	9.5 (0.0 to 83.4)	1872259.4 (0.0 to 27239320.3)
Central Europe						
Albania	0.0 (0.0 to 0.0)	0.0 (0.0 to 0.0)	0.0 (0.0 to 0.0)	0.1 (0.0 to 1.0)	-61.1 (-85.4 to 22.0)	58.8 (-100.0 to 2628.4)
Bosnia and Herzegovina	0.1 (0.1 to 0.1)	0.0 (0.0 to 0.0)	0.0 (0.0 to 0.0)	0.0 (0.0 to 0.1)	6.6 (0.0 to 92.6)	6560902.3 (0.0 to 107705166.5)
Bulgaria	0.1 (0.1 to 0.1)	0.1 (0.1 to 0.1)	0.0 (0.0 to 0.0)	0.0 (0.0 to 0.1)	-56.0 (-89.8 to 3.8)	-75.9 (-100.0 to 77.6)
Croatia	0.0 (0.0 to 0.0)	0.0 (0.0 to 0.0)	0.0 (0.0 to 0.0)	0.0 (0.0 to 0.0)	-66.1 (-83.1 to 3.9)	-79.3 (-100.0 to 79.3)
Czech Republic	0.0 (0.0 to 0.0)	0.0 (0.0 to 0.0)	0.0 (0.0 to 0.0)	0.0 (0.0 to 0.1)	21.3 (0.0 to 91.3)	10359792.2 (0.0 to 88323334.9)
Hungary	0.0 (0.0 to 0.0)	0.0 (0.0 to 0.0)	0.0 (0.0 to 0.0)	0.0 (0.0 to 0.1)	15.2 (0.0 to 88.7)	5626253.5 (0.0 to 59698705.2)
Macedonia	0.0 (0.0 to 0.0)	0.3 (0.3 to 0.3)	0.0 (0.0 to 0.0)	0.1 (0.0 to 0.7)	-80.6 (-100.1 to 5.4)	-85.9 (-100.0 to 123.4)
Montenegro	0.0 (0.0 to 0.0)	0.0 (0.0 to 0.0)	0.0 (0.0 to 0.0)	0.0 (0.0 to 0.2)	3.7 (0.0 to 95.7)	5821493.4 (0.0 to 170717220.4)
Poland	0.0 (0.0 to 0.0)	0.0 (0.0 to 0.0)	0.0 (0.0 to 0.0)	0.0 (0.0 to 0.0)	-53.9 (-78.9 to 4.9)	-66.3 (-100.0 to 109.9)
Romania	0.1 (0.1 to 0.1)	0.0 (0.0 to 0.0)	0.1 (0.1 to 0.1)	0.0 (0.0 to 0.3)	-36.7 (-72.3 to 28.2)	434.1 (-100.0 to 6823.6)
Serbia	0.1 (0.1 to 0.1)	0.0 (0.0 to 0.0)	0.0 (0.0 to 0.0)	0.0 (0.0 to 0.1)	-63.7 (-82.6 to 5.9)	-70.6 (-100.0 to 141.1)
Slovakia	0.0 (0.0 to 0.0)	0.0 (0.0 to 0.0)	0.0 (0.0 to 0.0)	0.0 (0.0 to 0.2)	21.7 (0.0 to 96.3)	14350002.8 (0.0 to 188075553.8)
Slovenia	0.0 (0.0 to 0.0)	0.1 (0.0 to 0.1)	0.0 (0.0 to 0.0)	0.0 (0.0 to 0.2)	-58.0 (-87.9 to 7.8)	-47.2 (-100.0 to 224.2)
Eastern Europe						
Belarus	0.0 (0.0 to 0.0)	0.0 (0.0 to 0.0)	0.0 (0.0 to 0.0)	0.0 (0.0 to 0.0)	15.2 (0.0 to 86.0)	5104087.0 (0.0 to 39845863.3)
Estonia	0.1 (0.1 to 0.1)	0.0 (0.0 to 0.0)	0.0 (0.0 to 0.0)	0.0 (0.0 to 0.1)	9.1 (0.0 to 89.9)	6123575.4 (0.0 to 71941218.1)
Latvia	0.0 (0.0 to 0.0)	0.0 (0.0 to 0.0)	0.0 (0.0 to 0.0)	0.0 (0.0 to 0.1)	5.8 (0.0 to 89.1)	3516437.0 (0.0 to 64131484.5)
Lithuania	0.0 (0.0 to 0.0)	0.0 (0.0 to 0.0)	0.0 (0.0 to 0.0)	0.0 (0.0 to 0.1)	12.1 (0.0 to 88.0)	5701093.7 (0.0 to 54249739.7)
Moldova	0.0 (0.0 to 0.0)	0.0 (0.0 to 0.0)	0.0 (0.0 to 0.0)	0.0 (0.0 to 0.1)	10.8 (0.0 to 94.5)	6820378.8 (0.0 to 144192616.0)
Russia	0.1 (0.1 to 0.1)	0.1 (0.1 to 0.1)	0.0 (0.0 to 0.0)	0.0 (0.0 to 0.2)	-49.5 (-89.0 to 9.3)	-43.7 (-100.0 to 301.4)
Ukraine	0.0 (0.0 to 0.0)	0.0 (0.0 to 0.0)	0.0 (0.0 to 0.0)	0.0 (0.0 to 0.1)	-66.9 (-82.2 to 6.7)	-82.2 (-100.0 to 174.0)
High-income						
Australasia						
Australia	0.0 (0.0 to 0.0)	0.0 (0.0 to 0.0)	0.1 (0.1 to 0.1)	0.0 (0.0 to 0.3)	-28.8 (-86.0 to 13.6)	16.2 (-100.0 to 663.4)
New Zealand	0.1 (0.1 to 0.1)	0.1 (0.1 to 0.1)	0.0 (0.0 to 0.0)	1.9 (0.0 to 38.2)	-41.3 (-93.3 to 38.5)	1599.4 (-100.0 to 32196.4)
High-income Asia Pacific						
Brunei	0.0 (0.0 to 0.0)	0.7 (0.5 to 1.0)	0.0 (0.0 to 0.0)	0.0 (0.0 to 0.0)	-105.1 (-107.5 to -102.8)	-100.0 (-100.0 to -100.0)
Japan	0.0 (0.0 to 0.0)	0.0 (0.0 to 0.0)	0.0 (0.0 to 0.0)	0.6 (0.0 to 6.0)	12.3 (-2.2 to 37.0)	2424.7 (-28.2 to 25749.3)
South Korea	0.1 (0.1 to 0.2)	0.0 (0.0 to 0.0)	0.0 (0.0 to 0.0)	0.4 (0.0 to 2.2)	13.0 (-67.4 to 47.0)	20629.8 (-100.0 to 115553.2)
Singapore	0.0 (0.0 to 0.0)	0.0 (0.0 to 0.0)	0.0 (0.0 to 0.0)	0.0 (0.0 to 0.0)	3.5 (0.0 to 79.5)	663495.3 (0.0 to 15018461.6)
High-income North America						
Canada	0.1 (0.0 to 0.1)	0.0 (0.0 to 0.0)	0.0 (0.0 to 0.0)	0.0 (0.0 to 0.2)	50.9 (0.0 to 96.2)	33628572.6 (0.0 to 184970766.7)
Greenland	0.0 (0.0 to 0.0)	0.0 (0.0 to 0.0)	0.0 (0.0 to 0.0)	0.0 (0.0 to 1.1)	4.0 (0.0 to 107.8)	38945077.8 (0.0 to 1052602059.5)
United States	0.1 (0.1 to 0.1)	0.1 (0.1 to 0.1)	0.0 (0.0 to 0.0)	0.1 (0.0 to 0.4)	0.3 (-8.4 to 9.7)	29.1 (-71.7 to 330.8)
Southern Latin America						
Argentina	0.1 (0.1 to 0.1)	0.0 (0.0 to 0.0)	0.0 (0.0 to 0.0)	0.0 (0.0 to 0.3)	-32.6 (-86.0 to 12.8)	3.6 (-100.0 to 583.6)
Chile	0.1 (0.1 to 0.1)	1.3 (1.2 to 1.3)	0.2 (0.1 to 0.2)	0.6 (0.0 to 10.6)	-51.8 (-109.1 to 14.2)	-52.5 (-100.0 to 742.9)
Uruguay	0.0 (0.0 to 0.0)	0.1 (0.1 to 0.1)	0.0 (0.0 to 0.0)	0.0 (0.0 to 0.0)	-73.2 (-89.0 to -2.8)	-91.1 (-100.0 to -34.2)
Western Europe						
Andorra	0.0 (0.0 to 0.0)	0.0 (0.0 to 0.0)	0.0 (0.0 to 0.0)	0.0 (0.0 to 0.0)	0.0 (0.0 to 0.0)	0.0 (0.0 to 0.0)
Austria	0.2 (0.2 to 0.2)	0.2 (0.2 to 0.2)	0.0 (0.0 to 0.0)	0.1 (0.0 to 0.6)	-47.7 (-97.8 to 6.8)	-56.4 (-100.0 to 175.5)
Belgium	0.0 (0.0 to 0.0)	0.0 (0.0 to 0.0)	0.0 (0.0 to 0.0)	0.0 (0.0 to 0.1)	18.1 (0.0 to 93.2)	7832766.1 (0.0 to 118412882.4)
Cyprus	0.0 (0.0 to 0.0)	0.0 (0.0 to 0.0)	0.0 (0.0 to 0.0)	0.0 (0.0 to 0.5)	6.5 (0.0 to 102.3)	18759399.9 (0.0 to 467419236.9)
Denmark	0.0 (0.0 to 0.0)	0.0 (0.0 to 0.0)	0.0 (0.0 to 0.0)	0.0 (0.0 to 0.0)	9.8 (0.0 to 85.6)	3429491.3 (0.0 to 37782905.5)
Finland	0.1 (0.0 to 0.1)	0.0 (0.0 to 0.0)	0.0 (0.0 to 0.0)	0.0 (0.0 to 0.0)	13.9 (0.0 to 84.9)	4204091.3 (0.0 to 34133115.6)
France	0.0 (0.0 to 0.0)	0.0 (0.0 to 0.0)	0.0 (0.0 to 0.0)	0.0 (0.0 to 0.1)	-22.2 (-85.4 to 6.8)	-12.8 (-100.0 to 175.3)
Germany	0.0 (0.0 to 0.0)	0.0 (0.0 to 0.0)	0.0 (0.0 to 0.0)	0.0 (0.0 to 0.1)	-43.6 (-76.7 to 14.1)	-23.7 (-100.0 to 733.4)
Greece	0.0 (0.0 to 0.0)	0.1 (0.1 to 0.1)	0.2 (0.2 to 0.2)	0.1 (0.0 to 0.7)	-38.6 (-90.3 to 15.8)	45.1 (-100.0 to 970.1)
Iceland	0.0 (0.0 to 0.0)	0.0 (0.0 to 0.0)	0.3 (0.2 to 0.4)	0.1 (0.0 to 1.6)	14.2 (0.0 to 110.5)	96401644.0 (0.0 to 1575939293.2)
Ireland	0.0 (0.0 to 0.0)	0.0 (0.0 to 0.0)	0.0 (0.0 to 0.0)	0.0 (0.0 to 0.1)	-64.7 (-82.1 to 8.8)	-59.0 (-100.0 to 272.7)
Israel	0.1 (0.0 to 0.1)	0.0 (0.0 to 0.0)	0.0 (0.0 to 0.0)	0.0 (0.0 to 0.1)	11.5 (0.0 to 91.2)	6700905.3 (0.0 to 87663967.2)
Italy	0.1 (0.1 to 0.1)	0.1 (0.1 to 0.1)	0.1 (0.1 to 0.1)	0.3 (0.0 to 4.0)	-15.0 (-89.3 to 27.6)	325.0 (-100.0 to 6190.7)
Luxembourg	0.5 (0.3 to 0.6)	0.0 (0.0 to 0.0)	0.0 (0.0 to 0.0)	0.0 (0.0 to 0.2)	3.4 (0.0 to 97.7)	8064073.5 (0.0 to 230402861.2)
Malta	0.0 (0.0 to 0.0)	0.0 (0.0 to 0.0)	0.0 (0.0 to 0.0)	0.0 (0.0 to 0.0)	0.0 (0.0 to 0.0)	0.0 (0.0 to 0.0)
Netherlands	0.0 (0.0 to 0.0)	0.0 (0.0 to 0.0)	0.0 (0.0 to 0.0)	0.0 (0.0 to 0.1)	-54.5 (-75.8 to 15.0)	2.3 (-100.0 to 845.3)
Norway	0.0 (0.0 to 0.1)	0.1 (0.1 to 0.1)	0.0 (0.0 to 0.0)	0.1 (0.0 to 0.2)	-52.5 (-94.5 to 3.5)	-63.5 (-100.0 to 69.8)
Portugal	0.0 (0.0 to 0.0)	0.0 (0.0 to 0.0)	0.0 (0.0 to 0.0)	0.0 (0.0 to 0.6)	-44.8 (-76.7 to 27.8)	342.1 (-100.0 to 6405.9)
Spain	0.0 (0.0 to 0.0)	0.0 (0.0 to 0.0)	0.0 (0.0 to 0.0)	0.1 (0.0 to 1.4)	-25.0 (-79.7 to 30.8)	486.3 (-100.0 to 10099.2)
Sweden	0.0 (0.0 to 0.0)	0.0 (0.0 to 0.0)	0.0 (0.0 to 0.0)	0.0 (0.0 to 0.1)	31.8 (0.0 to 93.2)	13836815.6 (0.0 to 118120061.8)
Switzerland	0.4 (0.3 to 0.5)	0.2 (0.2 to 0.3)	0.0 (0.0 to 0.0)	0.1 (0.0 to 1.6)	-52.7 (-98.3 to 12.7)	-36.6 (-100.0 to 574.0)
United Kingdom	0.0 (0.0 to 0.0)	0.0 (0.0 to 0.0)	0.0 (0.0 to 0.0)	0.0 (0.0 to 0.1)	-29.1 (-74.0 to 20.0)	133.7 (-100.0 to 1898.7)
Latin America and Caribbean						

	Estimate in 2000 (95% UIs)	Estimate in 2015 (95% UIs)	Estimate in 2017 (95% UIs)	Estimate in 2030 (95% UIs)	Annualised rate of change, 2015-2030 (95% UIs)	Percent change, 2015-2030 (95% UIs)
Andean Latin America						
Bolivia	0.0 (0.0 to 0.0)	0.2 (0.2 to 0.2)	0.0 (0.0 to 0.0)	0.2 (0.0 to 0.9)	-50.9 (-96.3 to 10.6)	0.8 (-100.0 to 391.5)
Ecuador	0.6 (0.6 to 0.7)	0.2 (0.2 to 0.2)	0.0 (0.0 to 0.0)	0.3 (0.0 to 3.1)	-43.5 (-97.9 to 17.5)	42.0 (-100.0 to 1279.9)
Peru	0.1 (0.1 to 0.2)	0.1 (0.1 to 0.1)	0.3 (0.3 to 0.3)	0.8 (0.0 to 13.4)	-16.3 (-93.1 to 31.9)	615.1 (-100.0 to 11840.2)
Caribbean						
Antigua and Barbuda	0.0 (0.0 to 0.0)	0.0 (0.0 to 0.0)	1.1 (1.0 to 1.3)	0.2 (0.0 to 2.6)	7.8 (0.0 to 113.8)	164870544.3 (0.0 to 2605383142.6)
The Bahamas	0.0 (0.0 to 0.0)	8.9 (7.6 to 10.4)	0.0 (0.0 to 0.0)	0.2 (0.0 to 3.3)	-105.7 (-123.1 to -7.0)	-97.9 (-100.0 to -65.3)
Barbados	0.0 (0.0 to 0.0)	0.0 (0.0 to 0.0)	0.0 (0.0 to 0.0)	0.0 (0.0 to 0.5)	5.6 (0.0 to 103.3)	27189642.7 (0.0 to 539240500.1)
Belize	5.8 (4.9 to 6.9)	0.0 (0.0 to 0.0)	0.0 (0.0 to 0.0)	0.5 (0.0 to 9.3)	14.0 (0.0 to 122.3)	532193983.6 (0.0 to 9259541152.5)
Bermuda	3.2 (1.8 to 5.2)	0.0 (0.0 to 0.0)	0.0 (0.0 to 0.0)	0.1 (0.0 to 2.7)	4.7 (0.0 to 114.0)	108941666.7 (0.0 to 2657244774.6)
Cuba	0.0 (0.0 to 0.0)	0.0 (0.0 to 0.0)	0.1 (0.1 to 0.1)	0.0 (0.0 to 0.3)	-40.7 (-85.9 to 16.3)	46.3 (-100.0 to 1046.1)
Dominica	0.0 (0.0 to 0.0)	8.7 (5.8 to 12.5)	0.0 (0.0 to 0.0)	0.4 (0.0 to 5.3)	-107.8 (-124.3 to -4.1)	-95.3 (-100.0 to -46.0)
Dominican Republic	0.0 (0.0 to 0.1)	0.0 (0.0 to 0.0)	0.0 (0.0 to 0.0)	0.4 (0.0 to 8.2)	-34.2 (-82.0 to 39.9)	2089.7 (-100.0 to 39699.9)
Grenada	0.0 (0.0 to 0.0)	0.0 (0.0 to 0.0)	0.0 (0.0 to 0.0)	0.2 (0.0 to 5.2)	5.4 (0.0 to 118.4)	164770230.4 (0.0 to 5175548899.0)
Guyana	0.0 (0.0 to 0.0)	0.0 (0.0 to 0.0)	0.0 (0.0 to 0.0)	0.0 (0.0 to 0.0)	0.0 (0.0 to 0.0)	0.0 (0.0 to 0.0)
Haiti	0.2 (0.2 to 0.2)	0.1 (0.1 to 0.1)	0.3 (0.2 to 0.3)	3.9 (0.0 to 108.1)	-36.1 (-92.8 to 46.0)	4009.6 (-100.0 to 99611.2)
Jamaica	0.0 (0.0 to 0.1)	0.0 (0.0 to 0.0)	0.0 (0.0 to 0.0)	0.2 (0.0 to 1.9)	33.1 (0.0 to 111.7)	160927323.4 (0.0 to 1902446198.2)
Puerto Rico	0.1 (0.1 to 0.1)	0.0 (0.0 to 0.0)	64.2 (40.2 to 100.6)	0.1 (0.0 to 2.1)	21.8 (0.0 to 112.5)	103657260.4 (0.0 to 2131977504.3)
Saint Lucia	0.0 (0.0 to 0.0)	0.0 (0.0 to 0.0)	0.0 (0.0 to 0.0)	0.3 (0.0 to 6.2)	11.6 (0.0 to 119.6)	287343138.5 (0.0 to 6196988461.1)
Saint Vincent and the Grenadines	0.0 (0.0 to 0.0)	0.0 (0.0 to 0.0)	0.0 (0.0 to 0.0)	0.5 (0.0 to 8.0)	14.5 (0.0 to 121.3)	476303072.1 (0.0 to 7948452573.0)
Suriname	0.0 (0.0 to 0.0)	0.0 (0.0 to 0.0)	0.0 (0.0 to 0.0)	0.0 (0.0 to 0.3)	4.3 (0.0 to 99.6)	12200955.6 (0.0 to 306410276.3)
Trinidad and Tobago	0.0 (0.0 to 0.0)	0.0 (0.0 to 0.0)	0.0 (0.0 to 0.0)	0.0 (0.0 to 0.4)	12.0 (0.0 to 101.6)	28703487.3 (0.0 to 413328323.8)
Virgin Islands, U.S.	0.0 (0.0 to 0.0)	0.0 (0.0 to 0.0)	4.8 (4.1 to 5.5)	0.0 (0.0 to 0.8)	5.3 (0.0 to 106.3)	40901478.4 (0.0 to 846029468.7)
Central Latin America						
Colombia	0.5 (0.5 to 0.6)	0.3 (0.3 to 0.4)	0.7 (0.6 to 0.8)	0.4 (0.0 to 2.7)	-23.7 (-100.9 to 13.3)	18.0 (-100.0 to 639.7)
Costa Rica	0.1 (0.0 to 0.1)	0.0 (0.0 to 0.0)	0.4 (0.4 to 0.4)	0.2 (0.0 to 1.2)	44.5 (0.0 to 108.7)	157745419.5 (0.0 to 1204602929.4)
El Salvador	0.1 (0.1 to 0.2)	0.2 (0.2 to 0.2)	0.0 (0.0 to 0.0)	0.9 (0.0 to 15.8)	-51.9 (-97.7 to 28.2)	325.0 (-100.0 to 6771.3)
Guatemala	0.4 (0.3 to 0.4)	0.0 (0.0 to 0.1)	0.1 (0.1 to 0.1)	0.4 (0.0 to 7.2)	-43.6 (-87.4 to 33.5)	787.9 (-100.0 to 15059.3)
Honduras	0.0 (0.0 to 0.0)	0.0 (0.0 to 0.0)	0.1 (0.1 to 0.1)	6.2 (0.0 to 178.5)	44.4 (0.0 to 142.0)	6254968364.8 (0.0 to 178455454584.2)
Mexico	0.1 (0.1 to 0.1)	0.1 (0.1 to 0.1)	0.4 (0.4 to 0.4)	0.3 (0.0 to 4.6)	-5.9 (-87.7 to 29.8)	446.2 (-100.0 to 8613.5)
Nicaragua	0.2 (0.2 to 0.2)	0.1 (0.0 to 0.1)	0.2 (0.2 to 0.2)	1.5 (0.0 to 31.6)	-42.4 (-88.2 to 42.8)	3045.5 (-100.0 to 61712.7)
Panama	0.1 (0.1 to 0.1)	0.0 (0.0 to 0.0)	0.0 (0.0 to 0.0)	0.1 (0.0 to 0.9)	-44.4 (-85.7 to 23.5)	274.8 (-100.0 to 3279.1)
Venezuela	0.1 (0.1 to 0.1)	0.0 (0.0 to 0.0)	0.0 (0.0 to 0.0)	0.1 (0.0 to 2.1)	41.7 (0.0 to 112.4)	132225287.6 (0.0 to 2106350556.1)
Tropical Latin America						
Brazil	0.1 (0.0 to 0.1)	0.0 (0.0 to 0.1)	0.0 (0.0 to 0.0)	0.1 (0.0 to 0.9)	-15.1 (-86.5 to 21.1)	156.0 (-100.0 to 2275.9)
Paraguay	0.0 (0.0 to 0.0)	0.1 (0.1 to 0.2)	0.0 (0.0 to 0.0)	0.0 (0.0 to 0.4)	-65.0 (-95.2 to 8.3)	-74.5 (-100.0 to 248.0)
North Africa and Middle East						
North Africa and Middle East						
Afghanistan	0.0 (0.0 to 0.0)	0.6 (0.5 to 0.8)	0.5 (0.4 to 0.6)	1.1 (0.0 to 15.2)	-45.3 (-105.2 to 20.5)	75.5 (-100.0 to 2074.0)
Algeria	0.1 (0.1 to 0.2)	0.0 (0.0 to 0.0)	0.0 (0.0 to 0.0)	0.4 (0.0 to 7.6)	-33.4 (-85.0 to 36.2)	1079.2 (-100.0 to 22857.1)
Bahrain	0.0 (0.0 to 0.0)	0.0 (0.0 to 0.0)	0.0 (0.0 to 0.0)	0.0 (0.0 to 0.0)	0.0 (0.0 to 0.0)	0.0 (0.0 to 0.0)
Egypt	0.0 (0.0 to 0.0)	0.0 (0.0 to 0.1)	0.0 (0.0 to 0.0)	0.0 (0.0 to 0.6)	-52.5 (-87.7 to 16.6)	-39.6 (-100.0 to 1103.5)
Iran	0.0 (0.0 to 0.0)	0.1 (0.1 to 0.1)	1.0 (0.9 to 1.2)	4.7 (0.0 to 52.1)	-9.7 (-90.7 to 43.4)	5921.2 (-100.0 to 66696.7)
Iraq	0.0 (0.0 to 0.0)	0.1 (0.1 to 0.2)	0.0 (0.0 to 0.0)	0.0 (0.0 to 0.1)	-83.6 (-95.5 to -6.0)	-97.5 (-100.0 to -59.6)
Jordan	0.2 (0.1 to 0.2)	0.0 (0.0 to 0.0)	0.0 (0.0 to 0.0)	0.1 (0.0 to 1.7)	14.9 (0.0 to 111.1)	71101656.1 (0.0 to 1716127734.5)
Kuwait	0.0 (0.0 to 0.0)	0.0 (0.0 to 0.0)	0.0 (0.0 to 0.0)	0.0 (0.0 to 0.0)	0.0 (0.0 to 0.0)	0.0 (0.0 to 0.0)
Lebanon	0.0 (0.0 to 0.0)	0.1 (0.1 to 0.1)	0.0 (0.0 to 0.0)	0.0 (0.0 to 0.3)	-83.9 (-89.9 to 10.0)	-83.3 (-100.0 to 345.3)
Libya	0.0 (0.0 to 0.0)	0.0 (0.0 to 0.0)	0.0 (0.0 to 0.0)	0.0 (0.0 to 0.2)	3.5 (0.0 to 97.5)	8032945.4 (0.0 to 223137384.1)
Morocco	0.0 (0.0 to 0.0)	0.0 (0.0 to 0.0)	0.0 (0.0 to 0.0)	0.1 (0.0 to 1.3)	35.8 (0.0 to 109.3)	105317226.5 (0.0 to 1313559532.2)
Palestine	0.0 (0.0 to 0.0)	0.1 (0.1 to 0.1)	0.0 (0.0 to 0.0)	0.0 (0.0 to 0.1)	-87.9 (-92.2 to -3.5)	-97.8 (-100.0 to -40.4)
Oman	0.0 (0.0 to 0.0)	0.0 (0.0 to 0.0)	0.0 (0.0 to 0.0)	0.1 (0.0 to 1.2)	11.5 (0.0 to 108.7)	60764565.9 (0.0 to 1212808650.1)
Qatar	0.0 (0.0 to 0.0)	0.0 (0.0 to 0.0)	0.0 (0.0 to 0.0)	0.0 (0.0 to 0.0)	0.0 (0.0 to 0.0)	0.0 (0.0 to 0.0)
Saudi Arabia	0.0 (0.0 to 0.0)	0.1 (0.1 to 0.1)	0.0 (0.0 to 0.0)	0.0 (0.0 to 0.2)	-68.9 (-90.9 to 8.1)	-68.2 (-100.0 to 236.9)
Sudan	0.0 (0.0 to 0.0)	0.1 (0.0 to 0.1)	0.0 (0.0 to 0.0)	0.0 (0.0 to 0.3)	-55.2 (-88.8 to 10.7)	-33.4 (-100.0 to 400.4)
Syria	0.0 (0.0 to 0.0)	0.1 (0.1 to 0.1)	0.0 (0.0 to 0.0)	0.0 (0.0 to 0.3)	-82.2 (-91.0 to 8.7)	-85.3 (-100.0 to 269.5)
Tunisia	0.0 (0.0 to 0.0)	0.0 (0.0 to 0.0)	0.0 (0.0 to 0.0)	0.1 (0.0 to 1.1)	17.8 (0.0 to 108.4)	81227761.2 (0.0 to 1147369144.1)
Turkey	0.0 (0.0 to 0.0)	0.0 (0.0 to 0.0)	0.0 (0.0 to 0.0)	0.4 (0.0 to 5.3)	-18.8 (-83.2 to 35.9)	1628.7 (-100.0 to 21614.7)
United Arab Emirates	0.0 (0.0 to 0.0)	0.0 (0.0 to 0.0)	0.0 (0.0 to 0.0)	0.0 (0.0 to 0.0)	0.0 (0.0 to 0.0)	0.0 (0.0 to 0.0)
Yemen	0.0 (0.0 to 0.0)	0.1 (0.1 to 0.1)	0.2 (0.1 to 0.2)	0.1 (0.0 to 1.5)	-62.0 (-93.8 to 16.4)	-12.7 (-100.0 to 1076.7)
South Asia						
South Asia						
Bangladesh	0.1 (0.1 to 0.1)	0.1 (0.1 to 0.1)	0.1 (0.1 to 0.1)	4.7 (0.0 to 57.2)	-7.5 (-92.4 to 42.1)	4506.1 (-100.0 to 55330.6)
Bhutan	33.2 (31.8 to 34.4)	0.0 (0.0 to 0.0)	0.0 (0.0 to 0.0)	0.2 (0.0 to 4.3)	8.7 (0.0 to 117.2)	216537430.4 (0.0 to 4343377296.7)
India	0.2 (0.2 to 0.2)	0.1 (0.1 to 0.1)	0.1 (0.1 to 0.1)	0.2 (0.0 to 1.0)	-4.9 (-90.9 to 16.7)	90.9 (-100.0 to 1115.4)
Nepal	0.6 (0.6 to 0.6)	30.8 (29.6 to 32.0)	0.5 (0.5 to 0.5)	0.6 (0.0 to 4.2)	-63.2 (-130.5 to -13.3)	-98.1 (-100.0 to -86.4)
Pakistan	0.0 (0.0 to 0.0)	0.3 (0.3 to 0.3)	0.0 (0.0 to 0.0)	0.8 (0.0 to 12.8)	-28.2 (-100.1 to 24.4)	159.6 (-100.0 to 3809.4)
Southeast Asia, East Asia, and Oceania						
East Asia						
China	0.0 (0.0 to 0.0)	0.0 (0.0 to 0.0)	0.0 (0.0 to 0.0)	0.3 (0.0 to 3.7)	-5.4 (-85.2 to 31.0)	735.0 (-100.0 to 10299.7)
North Korea	0.2 (0.2 to 0.2)	0.1 (0.1 to 0.1)	0.0 (0.0 to 0.0)	0.2 (0.0 to 1.9)	-66.1 (-94.4 to 17.8)	35.2 (-100.0 to 1353.7)
Taiwan (Province of China)	0.2 (0.1 to 0.2)	0.0 (0.0 to 0.0)	0.0 (0.0 to 0.0)	0.3 (0.0 to 2.4)	-17.0 (-86.1 to 27.4)	752.9 (-100.0 to 5968.1)
Oceania						
American Samoa	0.0 (0.0 to 0.0)	0.0 (0.0 to 0.0)	0.0 (0.0 to 0.0)	1.6 (0.0 to 45.8)	8.5 (0.0 to 133.0)	1596687769.4 (0.0 to 45836600374.5)
Federated States of Micronesia	0.0 (0.0 to 0.0)	4.8 (4.8 to 4.8)	0.0 (0.0 to 0.0)	1.1 (0.0 to 34.3)	-109.1 (-118.0 to 13.1)	-77.8 (-100.0 to 609.2)
Fiji	0.5 (0.5 to 0.5)	0.0 (0.0 to 0.0)	0.0 (0.0 to 0.0)	0.6 (0.0 to 6.1)	43.0 (0.0 to 119.5)	604066426.6 (0.0 to 6085105276.0)
Guam	0.0 (0.0 to 0.0)	0.0 (0.0 to 0.0)	0.0 (0.0 to 0.0)	0.3 (0.0 to 7.6)	8.8 (0.0 to 120.9)	302875752.3 (0.0 to 7566016204.5)
Kiribati	0.0 (0.0 to 0.0)	0.0 (0.0 to 0.0)	0.0 (0.0 to 0.0)	0.0 (0.0 to 0.0)	0.0 (0.0 to 0.0)	0.0 (0.0 to 0.0)
Marshall Islands	0.0 (0.0 to 0.0)	0.0 (0.0 to 0.0)	0.0 (0.0 to 0.0)	0.0 (0.0 to 0.0)	0.0 (0.0 to 0.0)	0.0 (0.0 to 0.0)
Northern Mariana Islands	0.0 (0.0 to 0.0)	0.0 (0.0 to 0.0)	0.0 (0.0 to 0.0)	0.0 (0.0 to 0.0)	0.0 (0.0 to 0.0)	0.0 (0.0 to 0.0)
Papua New Guinea	0.0 (0.0 to 0.0)	0.0 (0.0 to 0.0)	0.0 (0.0 to 0.0)	1.3 (0.0 to 32.0)	38.4 (0.0 to 130.6)	1264889179.0 (0.0 to 32002799429.2)

	Estimate in 2000 (95% UIs)	Estimate in 2015 (95% UIs)	Estimate in 2017 (95% UIs)	Estimate in 2030 (95% UIs)	Annualised rate of change, 2015-2030 (95% UIs)	Percent change, 2015-2030 (95% UIs)
Samoa	0.0 (0.0 to 0.0)	0.0 (0.0 to 0.0)	0.0 (0.0 to 0.0)	2.6 (0.0 to 62.6)	14.6 (0.0 to 135.0)	2618131035.0 (0.0 to 62629991775.3)
Solomon Islands	0.0 (0.0 to 0.0)	1.5 (1.5 to 1.5)	0.0 (0.0 to 0.0)	1.4 (0.0 to 25.8)	-83.9 (-110.1 to 19.1)	-2.4 (-100.0 to 1646.7)
Tonga	0.0 (0.0 to 0.0)	0.0 (0.0 to 0.0)	0.0 (0.0 to 0.0)	0.2 (0.0 to 5.3)	10.8 (0.0 to 118.6)	252809514.8 (0.0 to 5295559134.3)
Vanuatu	0.0 (0.0 to 0.0)	4.0 (3.9 to 4.0)	0.0 (0.0 to 0.0)	2.3 (0.0 to 44.1)	-96.1 (-116.7 to 16.0)	-42.6 (-100.0 to 1002.7)
Southeast Asia						
Cambodia	2.8 (2.4 to 3.1)	0.0 (0.0 to 0.0)	0.0 (0.0 to 0.0)	0.1 (0.0 to 1.8)	27.0 (0.0 to 111.3)	133816720.9 (0.0 to 1780155360.6)
Indonesia	0.2 (0.2 to 0.2)	0.0 (0.0 to 0.0)	0.0 (0.0 to 0.0)	0.2 (0.0 to 1.8)	-3.4 (-81.9 to 29.4)	847.6 (-100.0 to 8118.0)
Laos	0.3 (0.2 to 0.3)	0.0 (0.0 to 0.0)	0.0 (0.0 to 0.0)	0.1 (0.0 to 0.6)	25.2 (0.0 to 103.9)	63702767.2 (0.0 to 585582072.6)
Malaysia	0.1 (0.1 to 0.1)	0.1 (0.1 to 0.1)	0.0 (0.0 to 0.0)	0.0 (0.0 to 0.4)	-50.8 (-91.6 to 9.8)	-48.2 (-100.0 to 334.4)
Maldives	0.0 (0.0 to 0.0)	0.0 (0.0 to 0.0)	0.0 (0.0 to 0.0)	0.0 (0.0 to 0.0)	0.0 (0.0 to 0.0)	0.0 (0.0 to 0.0)
Mauritius	0.0 (0.0 to 0.0)	0.0 (0.0 to 0.0)	0.0 (0.0 to 0.0)	0.1 (0.0 to 0.9)	16.9 (0.0 to 107.1)	54905831.2 (0.0 to 946715291.0)
Myanmar	0.0 (0.0 to 0.0)	0.4 (0.4 to 0.5)	0.0 (0.0 to 0.0)	0.1 (0.0 to 2.6)	-64.4 (-102.1 to 11.7)	-69.9 (-100.0 to 480.1)
Philippines	0.5 (0.4 to 0.5)	0.1 (0.1 to 0.1)	0.1 (0.1 to 0.1)	0.8 (0.0 to 6.7)	2.7 (-91.6 to 28.0)	783.4 (-100.0 to 6532.6)
Sri Lanka	0.1 (0.1 to 0.1)	0.1 (0.1 to 0.1)	0.2 (0.1 to 0.2)	0.2 (0.0 to 2.9)	-40.0 (-93.0 to 21.7)	96.8 (-100.0 to 2497.0)
Seychelles	0.0 (0.0 to 0.0)	0.0 (0.0 to 0.0)	0.0 (0.0 to 0.0)	0.0 (0.0 to 0.0)	0.0 (0.0 to 0.0)	0.0 (0.0 to 0.0)
Thailand	0.2 (0.1 to 0.2)	0.0 (0.0 to 0.0)	0.1 (0.1 to 0.1)	0.2 (0.0 to 2.0)	-10.8 (-70.5 to 43.1)	6142.1 (-100.0 to 64064.5)
Timor-Leste	0.0 (0.0 to 0.0)	0.0 (0.0 to 0.0)	0.0 (0.0 to 0.0)	0.0 (0.0 to 0.1)	4.6 (0.0 to 90.3)	3610833.9 (0.0 to 75832124.1)
Vietnam	0.7 (0.7 to 0.8)	0.0 (0.0 to 0.1)	0.2 (0.2 to 0.2)	0.3 (0.0 to 3.0)	-21.9 (-87.1 to 27.8)	531.3 (-100.0 to 6374.1)
Sub-Saharan Africa						
Central Sub-Saharan Africa						
Angola	0.4 (0.4 to 0.4)	0.3 (0.3 to 0.3)	0.0 (0.0 to 0.0)	0.0 (0.0 to 0.3)	-78.6 (-99.6 to -0.4)	-89.7 (-100.0 to -5.7)
Central African Republic	0.0 (0.0 to 0.0)	0.0 (0.0 to 0.0)	0.0 (0.0 to 0.0)	0.0 (0.0 to 0.1)	-69.8 (-82.7 to 8.1)	-72.0 (-100.0 to 236.8)
Congo	0.0 (0.0 to 0.0)	0.0 (0.0 to 0.0)	0.0 (0.0 to 0.0)	0.0 (0.0 to 0.2)	7.6 (0.0 to 98.3)	11079255.8 (0.0 to 253766943.3)
Democratic Republic of the Congo	0.0 (0.0 to 0.0)	0.0 (0.0 to 0.1)	0.7 (0.6 to 0.7)	0.0 (0.0 to 0.3)	-61.0 (-87.2 to 13.3)	-39.3 (-100.0 to 639.1)
Equatorial Guinea	0.0 (0.0 to 0.0)	0.0 (0.0 to 0.0)	0.0 (0.0 to 0.0)	0.0 (0.0 to 0.0)	0.0 (0.0 to 0.0)	0.0 (0.0 to 0.0)
Gabon	0.0 (0.0 to 0.0)	0.0 (0.0 to 0.0)	0.0 (0.0 to 0.0)	0.0 (0.0 to 0.0)	0.0 (0.0 to 0.0)	0.0 (0.0 to 0.0)
Eastern Sub-Saharan Africa						
Burundi	0.0 (0.0 to 0.0)	0.4 (0.3 to 0.4)	0.1 (0.1 to 0.1)	0.0 (0.0 to 0.3)	-86.2 (-101.5 to -1.1)	-92.5 (-100.0 to -14.8)
Comoros	0.0 (0.0 to 0.0)	0.0 (0.0 to 0.0)	0.0 (0.0 to 0.0)	0.3 (0.0 to 6.6)	13.5 (0.0 to 120.0)	268091012.4 (0.0 to 6538164269.9)
Djibouti	0.0 (0.0 to 0.0)	0.0 (0.0 to 0.0)	0.0 (0.0 to 0.0)	0.3 (0.0 to 5.5)	6.9 (0.0 to 118.9)	255131942.4 (0.0 to 5533647548.1)
Eritrea	0.0 (0.0 to 0.0)	0.0 (0.0 to 0.0)	0.0 (0.0 to 0.0)	0.0 (0.0 to 0.0)	0.0 (0.0 to 0.0)	0.0 (0.0 to 0.0)
Ethiopia	0.1 (0.1 to 0.1)	0.0 (0.0 to 0.0)	0.1 (0.1 to 0.1)	0.0 (0.0 to 0.3)	31.4 (0.0 to 99.8)	26625288.6 (0.0 to 315699200.0)
Kenya	0.0 (0.0 to 0.0)	0.2 (0.2 to 0.2)	0.1 (0.1 to 0.1)	0.1 (0.0 to 0.4)	-64.5 (-96.1 to 5.1)	-72.0 (-100.0 to 115.7)
Madagascar	1.0 (0.8 to 1.1)	0.1 (0.1 to 0.1)	0.3 (0.3 to 0.3)	0.2 (0.0 to 1.3)	-46.1 (-94.5 to 15.1)	56.4 (-100.0 to 860.4)
Malawi	0.0 (0.0 to 0.0)	1.7 (1.5 to 2.0)	0.0 (0.0 to 0.0)	0.1 (0.0 to 1.3)	-87.2 (-112.0 to -2.3)	-96.9 (-100.0 to -28.7)
Mozambique	4.8 (4.2 to 5.5)	0.6 (0.5 to 0.7)	0.0 (0.0 to 0.0)	0.2 (0.0 to 3.1)	-58.3 (-104.5 to 11.0)	-61.9 (-100.0 to 418.3)
Rwanda	0.0 (0.0 to 0.0)	0.0 (0.0 to 0.0)	0.0 (0.0 to 0.0)	0.1 (0.0 to 0.9)	-63.1 (-81.0 to 25.4)	203.0 (-100.0 to 4430.3)
Somalia	0.0 (0.0 to 0.0)	0.0 (0.0 to 0.0)	0.0 (0.0 to 0.0)	0.1 (0.0 to 2.1)	24.1 (0.0 to 112.6)	151193204.7 (0.0 to 2149119796.1)
South Sudan	0.0 (0.0 to 0.0)	0.0 (0.0 to 0.0)	0.0 (0.0 to 0.0)	0.0 (0.0 to 0.4)	4.5 (0.0 to 101.0)	14047923.2 (0.0 to 382537701.7)
Tanzania	0.1 (0.1 to 0.1)	0.1 (0.1 to 0.1)	0.0 (0.0 to 0.0)	0.0 (0.0 to 0.2)	-62.3 (-93.9 to 3.5)	-78.3 (-100.0 to 68.9)
Uganda	0.0 (0.0 to 0.0)	0.0 (0.0 to 0.0)	0.0 (0.0 to 0.1)	0.0 (0.0 to 0.3)	20.0 (0.0 to 100.3)	19783210.1 (0.0 to 340000592.3)
Zambia	0.0 (0.0 to 0.0)	0.0 (0.0 to 0.0)	0.0 (0.0 to 0.0)	0.0 (0.0 to 0.1)	11.0 (0.0 to 93.3)	7708556.4 (0.0 to 119957715.7)
Southern Sub-Saharan Africa						
Botswana	0.2 (0.2 to 0.2)	0.0 (0.0 to 0.0)	0.0 (0.0 to 0.0)	0.0 (0.0 to 0.3)	6.0 (0.0 to 99.8)	15210700.9 (0.0 to 317872759.6)
Lesotho	0.0 (0.0 to 0.0)	0.0 (0.0 to 0.0)	0.0 (0.0 to 0.0)	0.0 (0.0 to 0.8)	6.4 (0.0 to 106.1)	41959594.3 (0.0 to 821824167.2)
Namibia	0.0 (0.0 to 0.0)	0.0 (0.0 to 0.0)	0.0 (0.0 to 0.0)	0.1 (0.0 to 2.6)	12.6 (0.0 to 114.0)	128926639.7 (0.0 to 2650130492.8)
South Africa	0.2 (0.2 to 0.2)	0.0 (0.0 to 0.0)	0.0 (0.0 to 0.0)	0.0 (0.0 to 0.3)	45.4 (0.0 to 99.0)	39184189.1 (0.0 to 281955498.6)
Swaziland	0.0 (0.0 to 0.0)	0.0 (0.0 to 0.0)	0.0 (0.0 to 0.0)	0.0 (0.0 to 0.7)	4.9 (0.0 to 105.2)	22025251.8 (0.0 to 709810651.2)
Zimbabwe	0.6 (0.5 to 0.7)	0.1 (0.1 to 0.1)	0.0 (0.0 to 0.0)	0.0 (0.0 to 0.5)	-77.9 (-93.3 to 9.3)	-73.8 (-100.0 to 303.5)
Western Sub-Saharan Africa						
Benin	0.0 (0.0 to 0.0)	0.0 (0.0 to 0.0)	0.0 (0.0 to 0.0)	0.0 (0.0 to 0.3)	13.7 (0.0 to 100.2)	20380049.8 (0.0 to 335109556.8)
Burkina Faso	0.0 (0.0 to 0.0)	0.0 (0.0 to 0.1)	0.0 (0.0 to 0.0)	0.0 (0.0 to 0.2)	-70.5 (-86.9 to 9.0)	-66.5 (-100.0 to 285.1)
Cameroon	0.0 (0.0 to 0.0)	0.0 (0.0 to 0.0)	0.0 (0.0 to 0.0)	0.0 (0.0 to 0.2)	-60.2 (-80.3 to 17.7)	-12.7 (-100.0 to 1327.2)
Cape Verde	0.0 (0.0 to 0.0)	1.7 (1.5 to 1.9)	0.0 (0.0 to 0.0)	0.1 (0.0 to 1.0)	-103.8 (-111.7 to -3.5)	-96.9 (-100.0 to -41.1)
Chad	0.0 (0.0 to 0.0)	0.0 (0.0 to 0.0)	0.0 (0.0 to 0.0)	0.0 (0.0 to 0.5)	13.6 (0.0 to 102.4)	35518204.9 (0.0 to 466352340.4)
Cote d'Ivoire	0.0 (0.0 to 0.0)	0.1 (0.1 to 0.1)	0.1 (0.1 to 0.1)	0.0 (0.0 to 0.1)	-79.9 (-90.2 to 3.6)	-88.4 (-100.0 to 71.8)
The Gambia	0.0 (0.0 to 0.0)	0.0 (0.0 to 0.0)	0.0 (0.0 to 0.0)	0.0 (0.0 to 0.2)	9.3 (0.0 to 97.1)	14648398.0 (0.0 to 212251840.7)
Ghana	0.0 (0.0 to 0.0)	0.1 (0.1 to 0.1)	0.1 (0.1 to 0.1)	0.0 (0.0 to 0.2)	-73.6 (-92.0 to 3.8)	-84.3 (-100.0 to 75.6)
Guinea	0.1 (0.0 to 0.1)	0.1 (0.1 to 0.2)	0.0 (0.0 to 0.0)	0.0 (0.0 to 0.1)	-86.9 (-95.2 to -2.8)	-96.5 (-100.0 to -34.0)
Guinea-Bissau	0.0 (0.0 to 0.0)	0.0 (0.0 to 0.0)	0.0 (0.0 to 0.0)	0.0 (0.0 to 0.1)	3.3 (0.0 to 90.1)	2738848.1 (0.0 to 74022923.1)
Liberia	0.0 (0.0 to 0.0)	0.0 (0.0 to 0.0)	0.0 (0.0 to 0.0)	0.0 (0.0 to 0.3)	6.9 (0.0 to 98.8)	9002207.9 (0.0 to 280385488.7)
Mali	0.1 (0.1 to 0.1)	0.0 (0.0 to 0.0)	0.0 (0.0 to 0.0)	0.0 (0.0 to 0.2)	-62.6 (-84.0 to 11.6)	-43.8 (-100.0 to 472.8)
Mauritania	0.0 (0.0 to 0.0)	0.0 (0.0 to 0.0)	0.5 (0.4 to 0.5)	0.0 (0.0 to 0.6)	14.2 (0.0 to 104.0)	29721668.3 (0.0 to 597344607.6)
Niger	0.0 (0.0 to 0.0)	0.1 (0.1 to 0.2)	0.3 (0.3 to 0.4)	0.0 (0.0 to 0.4)	-67.4 (-95.0 to 6.7)	-76.3 (-100.0 to 175.0)
Nigeria	0.0 (0.0 to 0.0)	0.0 (0.0 to 0.0)	0.0 (0.0 to 0.0)	0.0 (0.0 to 0.1)	-56.5 (-84.2 to 9.0)	-53.9 (-100.0 to 284.2)
Sao Tome and Principe	0.0 (0.0 to 0.0)	0.0 (0.0 to 0.0)	0.0 (0.0 to 0.0)	0.0 (0.0 to 0.0)	0.0 (0.0 to 0.0)	0.0 (0.0 to 0.0)
Senegal	0.0 (0.0 to 0.0)	0.0 (0.0 to 0.0)	0.0 (0.0 to 0.0)	0.0 (0.0 to 0.2)	15.8 (0.0 to 96.7)	13387596.6 (0.0 to 198093789.7)
Sierra Leone	0.0 (0.0 to 0.0)	0.1 (0.1 to 0.1)	3.2 (3.1 to 3.3)	0.1 (0.0 to 1.2)	-79.2 (-94.9 to 14.4)	-50.0 (-100.0 to 761.4)
Togo	0.0 (0.0 to 0.0)	0.0 (0.0 to 0.0)	0.0 (0.0 to 0.0)	0.0 (0.0 to 0.2)	5.1 (0.0 to 97.6)	8441673.0 (0.0 to 228712998.3)
**Indicator 2.2.1: Prevalence of stunting in children under 5 (%)**						
Central Europe, Eastern Europe, and Central Asia						
Central Asia						
Armenia	19.6 (17.8 to 21.4)	14.6 (12.8 to 16.4)	12.8 (10.8 to 14.9)	8.7 (6.3 to 11.6)	-3.5 (-4.9 to -2.1)	-40.6 (-52.1 to -27.5)
Azerbaijan	25.4 (23.9 to 27.0)	16.6 (14.2 to 18.9)	15.8 (12.9 to 18.8)	10.9 (7.3 to 15.4)	-2.9 (-4.6 to -1.3)	-34.4 (-49.7 to -18.0)
Georgia	16.4 (14.7 to 18.1)	12.5 (9.8 to 15.4)	12.1 (9.1 to 15.4)	10.0 (5.9 to 15.3)	-1.6 (-3.6 to 0.2)	-21.1 (-41.3 to 2.8)
Kazakhstan	19.2 (17.3 to 21.3)	11.7 (10.3 to 13.2)	10.7 (8.9 to 12.5)	6.9 (4.9 to 9.6)	-3.6 (-5.3 to -2.0)	-41.6 (-54.6 to -25.7)
Kyrgyzstan	28.1 (25.8 to 31.2)	17.7 (16.1 to 19.6)	16.7 (14.5 to 19.0)	11.4 (8.6 to 14.7)	-3.0 (-4.4 to -1.7)	-35.7 (-48.7 to -22.6)
Mongolia	35.0 (32.1 to 39.3)	16.5 (14.6 to 18.4)	15.6 (13.2 to 18.0)	8.6 (6.1 to 11.5)	-4.4 (-6.0 to -2.8)	-47.9 (-59.6 to -34.7)
Tajikistan	40.6 (38.0 to 44.4)	31.4 (27.5 to 38.8)	30.5 (25.5 to 41.1)	24.6 (17.3 to 40.7)	-1.7 (-3.3 to 0.3)	-22.2 (-38.7 to 5.1)
Turkmenistan	26.3 (24.9 to 27.8)	13.4 (11.9 to 15.0)	12.3 (10.5 to 14.2)	6.9 (4.9 to 9.2)	-4.5 (-6.0 to -3.2)	-49.1 (-59.3 to -37.9)

	Estimate in 2000 (95% UIs)	Estimate in 2015 (95% UIs)	Estimate in 2017 (95% UIs)	Estimate in 2030 (95% UIs)	Annualised rate of change, 2015-2030 (95% UIs)	Percent change, 2015-2030 (95% UIs)
Uzbekistan	33.7 (30.5 to 39.5)	20.1 (15.9 to 24.7)	19.3 (14.7 to 24.3)	13.1 (7.9 to 20.1)	-3.0 (-4.9 to -1.2)	-35.6 (-52.3 to -16.7)
Central Europe						
Albania	35.4 (32.3 to 39.8)	22.3 (18.5 to 26.2)	21.8 (17.5 to 26.2)	16.1 (10.6 to 22.7)	-2.3 (-4.1 to -0.8)	-28.8 (-45.9 to -11.3)
Bosnia and Herzegovina	12.6 (10.0 to 15.3)	8.7 (6.6 to 11.4)	8.5 (6.1 to 11.3)	6.6 (3.5 to 10.8)	-2.0 (-4.3 to 0.1)	-25.3 (-47.7 to 0.8)
Bulgaria	12.3 (9.0 to 16.1)	9.6 (6.8 to 13.1)	9.4 (6.4 to 13.2)	8.2 (4.4 to 14.3)	-1.2 (-3.5 to 1.0)	-15.6 (-40.6 to 16.5)
Croatia	10.3 (6.7 to 14.5)	8.1 (5.1 to 11.4)	8.0 (4.9 to 11.5)	7.1 (3.4 to 12.1)	-1.1 (-3.5 to 1.3)	-13.2 (-41.0 to 22.3)
Czech Republic	3.7 (2.5 to 5.3)	3.8 (2.4 to 5.8)	3.8 (2.3 to 5.9)	4.0 (1.9 to 7.5)	0.2 (-2.4 to 2.5)	4.7 (-30.2 to 46.3)
Hungary	10.7 (6.9 to 14.9)	8.4 (5.4 to 11.9)	8.3 (5.2 to 12.1)	7.4 (3.8 to 12.8)	-1.0 (-3.4 to 1.3)	-13.1 (-39.8 to 22.4)
Macedonia	10.0 (7.7 to 12.6)	7.1 (5.2 to 9.3)	6.9 (4.9 to 9.2)	5.6 (3.1 to 9.4)	-1.8 (-4.0 to 0.5)	-22.6 (-45.5 to 8.0)
Montenegro	11.0 (8.2 to 14.2)	9.7 (7.6 to 12.2)	9.7 (7.2 to 12.6)	9.5 (5.6 to 14.4)	-0.3 (-2.6 to 1.7)	-3.4 (-32.3 to 28.6)
Poland	10.5 (6.7 to 15.0)	7.9 (5.0 to 11.7)	7.7 (4.8 to 11.7)	6.4 (3.2 to 11.6)	-1.5 (-4.1 to 0.8)	-19.3 (-45.6 to 13.2)
Romania	17.2 (16.0 to 18.5)	11.8 (8.8 to 15.3)	11.5 (8.3 to 15.3)	9.2 (5.0 to 15.0)	-1.8 (-3.9 to 0.1)	-22.7 (-44.7 to 1.8)
Serbia	11.4 (9.1 to 13.9)	9.0 (7.7 to 10.5)	8.3 (6.7 to 10.3)	6.7 (4.2 to 10.2)	-2.1 (-4.3 to 0.1)	-25.8 (-47.2 to 1.3)
Slovakia	9.7 (6.4 to 13.7)	7.7 (5.1 to 11.2)	7.6 (4.9 to 11.2)	6.9 (3.3 to 11.8)	-1.0 (-3.4 to 1.1)	-12.5 (-40.2 to 18.5)
Slovenia	9.2 (6.0 to 13.3)	7.4 (4.9 to 10.9)	7.3 (4.6 to 10.9)	6.7 (3.3 to 11.8)	-0.9 (-3.3 to 1.5)	-10.7 (-39.0 to 25.0)
Eastern Europe						
Belarus	6.5 (4.9 to 8.5)	4.6 (3.2 to 6.4)	4.6 (3.0 to 6.6)	4.1 (2.0 to 7.6)	-1.1 (-3.6 to 1.5)	-12.9 (-42.0 to 25.1)
Estonia	10.4 (6.8 to 15.0)	7.9 (5.2 to 11.3)	7.8 (5.0 to 11.1)	6.8 (3.3 to 11.6)	-1.3 (-3.8 to 1.1)	-16.0 (-43.2 to 17.9)
Latvia	11.4 (7.3 to 16.1)	8.5 (5.5 to 12.3)	8.4 (5.3 to 12.4)	7.4 (3.8 to 13.0)	-1.1 (-3.6 to 1.2)	-13.7 (-41.9 to 19.8)
Lithuania	10.8 (7.2 to 15.5)	8.1 (5.2 to 12.0)	8.0 (5.0 to 12.0)	7.0 (3.5 to 12.0)	-1.2 (-3.7 to 1.0)	-15.6 (-42.4 to 16.8)
Moldova	13.1 (9.5 to 17.1)	8.4 (6.0 to 11.4)	8.2 (5.6 to 11.3)	6.3 (3.2 to 10.8)	-2.1 (-4.7 to 0.2)	-25.8 (-50.5 to 3.6)
Russia	15.0 (13.6 to 16.6)	11.2 (8.2 to 14.5)	11.1 (7.7 to 14.7)	9.5 (5.2 to 14.8)	-1.3 (-3.3 to 0.5)	-16.8 (-38.9 to 8.0)
Ukraine	21.8 (19.1 to 24.4)	16.0 (11.8 to 20.7)	15.6 (11.3 to 20.6)	12.9 (7.3 to 20.1)	-1.6 (-3.6 to 0.1)	-20.3 (-42.1 to 1.8)
High-income						
Australasia						
Australia	5.3 (3.2 to 8.2)	4.7 (2.9 to 7.1)	4.6 (2.7 to 7.2)	4.5 (2.0 to 8.4)	-0.6 (-3.5 to 2.0)	-6.4 (-41.0 to 35.6)
New Zealand	5.7 (3.5 to 8.6)	5.2 (3.2 to 8.0)	5.1 (3.0 to 7.8)	4.9 (2.3 to 9.2)	-0.6 (-3.1 to 2.0)	-6.7 (-37.4 to 34.4)
High-income Asia Pacific						
Brunei	11.6 (8.2 to 15.3)	12.0 (9.0 to 15.4)	11.6 (8.2 to 15.4)	11.6 (6.3 to 18.0)	-0.4 (-2.7 to 1.7)	-4.4 (-33.6 to 28.8)
Japan	9.2 (8.7 to 9.8)	8.6 (8.2 to 9.0)	8.5 (8.1 to 9.0)	8.0 (7.2 to 8.9)	-0.4 (-1.0 to 0.1)	-6.4 (-13.3 to 0.8)
South Korea	2.9 (2.0 to 3.9)	3.2 (2.6 to 4.1)	3.1 (2.3 to 4.2)	3.4 (1.8 to 5.8)	0.0 (-3.0 to 2.7)	3.1 (-35.9 to 50.1)
Singapore	3.7 (2.8 to 4.9)	3.6 (2.3 to 5.3)	3.5 (2.2 to 5.3)	3.5 (1.6 to 6.6)	-0.3 (-2.9 to 2.0)	-3.3 (-35.3 to 35.5)
High-income North America						
Canada	3.5 (2.1 to 5.5)	3.1 (1.8 to 4.9)	3.0 (1.8 to 4.9)	3.0 (1.4 to 5.8)	-0.3 (-3.2 to 2.3)	-2.7 (-38.3 to 40.8)
Greenland	5.2 (3.1 to 8.0)	4.2 (2.6 to 6.3)	4.2 (2.5 to 6.4)	4.0 (1.7 to 7.3)	-0.7 (-3.5 to 2.1)	-7.3 (-40.4 to 37.2)
United States	3.1 (2.4 to 4.0)	2.4 (1.7 to 3.3)	2.4 (1.6 to 3.4)	2.1 (1.0 to 3.7)	-1.2 (-3.7 to 1.4)	-14.6 (-42.5 to 23.1)
Southern Latin America						
Argentina	13.0 (11.8 to 14.2)	8.1 (5.7 to 10.7)	7.8 (5.3 to 10.7)	5.9 (3.1 to 10.2)	-2.3 (-4.5 to -0.2)	-28.1 (-48.8 to -2.8)
Chile	4.9 (4.2 to 5.7)	4.1 (3.0 to 5.3)	3.9 (2.7 to 5.3)	3.3 (1.6 to 5.7)	-1.7 (-4.2 to 0.8)	-21.3 (-46.4 to 13.0)
Uruguay	11.6 (9.8 to 13.4)	10.2 (7.5 to 13.3)	9.9 (7.0 to 13.3)	8.8 (4.8 to 14.7)	-1.2 (-3.4 to 0.8)	-15.4 (-39.7 to 12.8)
Western Europe						
Andorra	2.6 (1.5 to 4.2)	2.6 (1.5 to 4.2)	2.5 (1.5 to 4.2)	2.7 (1.2 to 5.4)	0.2 (-2.8 to 3.0)	5.1 (-34.3 to 57.7)
Austria	3.0 (1.7 to 4.7)	2.8 (1.7 to 4.5)	2.8 (1.6 to 4.6)	2.9 (1.3 to 5.7)	-0.1 (-2.8 to 2.5)	0.6 (-34.1 to 44.7)
Belgium	2.8 (1.6 to 4.4)	2.7 (1.6 to 4.4)	2.6 (1.6 to 4.4)	2.7 (1.3 to 5.6)	-0.1 (-2.9 to 2.5)	0.3 (-34.9 to 45.8)
Cyprus	3.1 (1.9 to 5.1)	2.8 (1.7 to 4.5)	2.8 (1.6 to 4.5)	2.8 (1.3 to 5.3)	-0.2 (-3.0 to 2.3)	-0.5 (-36.6 to 41.9)
Denmark	2.5 (1.4 to 4.0)	2.4 (1.5 to 3.8)	2.4 (1.4 to 3.8)	2.5 (1.2 to 4.7)	0.0 (-2.7 to 2.6)	2.5 (-33.1 to 47.5)
Finland	2.7 (1.6 to 4.3)	2.6 (1.6 to 4.1)	2.6 (1.6 to 4.1)	2.6 (1.2 to 5.1)	-0.1 (-2.9 to 2.4)	-0.0 (-35.4 to 44.2)
France	3.0 (1.8 to 4.7)	2.9 (1.7 to 4.5)	2.8 (1.6 to 4.6)	2.9 (1.3 to 5.4)	-0.2 (-3.2 to 2.3)	-0.6 (-37.7 to 42.2)
Germany	1.2 (0.7 to 1.9)	1.1 (0.6 to 1.6)	1.1 (0.6 to 1.7)	1.1 (0.5 to 2.2)	-0.2 (-3.0 to 2.7)	0.1 (-36.0 to 49.9)
Greece	3.3 (2.0 to 5.0)	3.1 (1.9 to 5.0)	3.1 (1.9 to 5.1)	3.3 (1.5 to 6.5)	0.1 (-2.4 to 2.7)	4.1 (-30.7 to 49.3)
Iceland	2.7 (1.6 to 4.4)	2.5 (1.5 to 4.0)	2.5 (1.5 to 3.9)	2.5 (1.1 to 4.8)	-0.3 (-2.9 to 2.3)	-1.6 (-35.3 to 41.4)
Ireland	3.0 (1.7 to 4.6)	2.7 (1.6 to 4.2)	2.6 (1.6 to 4.3)	2.6 (1.2 to 5.0)	-0.5 (-3.2 to 2.1)	-4.5 (-38.3 to 36.9)
Israel	3.3 (2.0 to 5.2)	3.2 (2.0 to 5.0)	3.2 (1.9 to 5.0)	3.3 (1.5 to 6.1)	-0.1 (-2.7 to 2.3)	0.9 (-33.0 to 41.2)
Italy	3.0 (1.8 to 4.7)	2.9 (1.8 to 4.6)	2.9 (1.7 to 4.7)	3.1 (1.4 to 6.0)	0.1 (-2.8 to 2.7)	4.2 (-34.3 to 48.8)
Luxembourg	2.5 (1.5 to 4.1)	2.5 (1.5 to 3.8)	2.5 (1.4 to 3.8)	2.5 (1.2 to 4.7)	-0.0 (-2.8 to 2.6)	1.7 (-34.4 to 46.6)
Malta	3.3 (1.9 to 5.3)	3.1 (1.9 to 5.0)	3.0 (1.8 to 5.0)	3.0 (1.4 to 6.0)	-0.3 (-3.1 to 2.3)	-2.5 (-36.8 to 41.9)
Netherlands	2.6 (1.6 to 4.3)	2.5 (1.5 to 4.1)	2.5 (1.4 to 4.1)	2.5 (1.1 to 4.9)	-0.1 (-3.0 to 2.3)	0.1 (-36.3 to 41.8)
Norway	2.6 (1.5 to 4.0)	2.5 (1.5 to 4.0)	2.5 (1.4 to 4.0)	2.5 (1.1 to 4.8)	-0.1 (-3.1 to 2.5)	0.1 (-37.2 to 44.4)
Portugal	4.0 (2.3 to 6.2)	3.6 (2.2 to 5.8)	3.6 (2.1 to 5.7)	3.5 (1.7 to 6.6)	-0.4 (-3.0 to 2.0)	-4.1 (-36.4 to 35.6)
Spain	1.5 (1.0 to 2.3)	1.4 (0.9 to 2.3)	1.4 (0.8 to 2.4)	1.4 (0.6 to 2.9)	-0.2 (-2.8 to 2.5)	-0.4 (-34.5 to 45.0)
Sweden	2.8 (1.6 to 4.6)	2.7 (1.6 to 4.2)	2.6 (1.5 to 4.2)	2.7 (1.2 to 5.2)	-0.2 (-3.2 to 2.4)	-0.3 (-38.1 to 43.4)
Switzerland	2.7 (1.6 to 4.3)	2.6 (1.6 to 4.1)	2.6 (1.5 to 4.1)	2.7 (1.3 to 5.2)	-0.0 (-2.8 to 2.6)	1.8 (-34.2 to 46.9)
United Kingdom	3.2 (1.9 to 5.0)	3.0 (1.8 to 4.6)	2.9 (1.7 to 4.5)	2.9 (1.4 to 5.3)	-0.4 (-3.1 to 2.1)	-4.2 (-37.3 to 36.2)
Latin America and Caribbean						
Andean Latin America						
Bolivia	35.4 (33.5 to 38.2)	19.2 (17.1 to 21.9)	17.6 (14.7 to 21.1)	9.6 (6.5 to 13.8)	-4.8 (-6.5 to -2.8)	-50.5 (-62.4 to -34.7)
Ecuador	31.0 (29.4 to 33.2)	22.7 (20.6 to 25.5)	21.5 (18.5 to 24.8)	15.7 (11.3 to 21.4)	-2.6 (-4.0 to -0.8)	-31.4 (-45.4 to -11.2)
Peru	38.0 (35.4 to 42.4)	18.7 (18.1 to 19.4)	13.6 (12.5 to 14.8)	5.8 (5.2 to 6.5)	-7.8 (-8.4 to -7.2)	-69.0 (-71.8 to -66.1)
Caribbean						
Antigua and Barbuda	9.2 (5.8 to 13.4)	7.8 (4.8 to 11.6)	7.8 (4.7 to 11.5)	7.3 (3.4 to 12.5)	-0.7 (-3.2 to 1.7)	-7.9 (-38.5 to 28.7)
The Bahamas	7.8 (4.8 to 11.7)	6.9 (4.2 to 10.4)	6.9 (4.3 to 10.3)	6.7 (3.5 to 11.6)	-0.4 (-2.8 to 1.8)	-3.7 (-34.3 to 31.9)
Barbados	8.6 (5.5 to 12.4)	7.5 (4.8 to 11.2)	7.5 (4.7 to 11.2)	7.2 (3.6 to 12.3)	-0.5 (-3.2 to 1.8)	-5.8 (-38.5 to 31.0)
Belize	23.7 (20.2 to 27.4)	16.7 (15.6 to 17.7)	15.5 (14.2 to 16.9)	10.8 (8.7 to 13.2)	-2.9 (-4.1 to -1.8)	-35.1 (-45.6 to -23.6)
Bermuda	7.0 (4.2 to 10.5)	6.2 (3.8 to 9.4)	6.2 (3.8 to 9.3)	6.0 (2.9 to 10.5)	-0.4 (-3.0 to 1.8)	-4.2 (-35.9 to 31.6)
Cuba	7.6 (6.4 to 8.9)	8.6 (6.8 to 10.5)	8.6 (6.6 to 10.8)	9.7 (6.1 to 13.9)	0.7 (-1.0 to 2.3)	12.3 (-14.1 to 41.4)
Dominica	11.0 (7.1 to 16.1)	8.7 (5.5 to 12.8)	8.6 (5.2 to 12.6)	7.5 (3.7 to 13.5)	-1.1 (-3.5 to 1.2)	-14.2 (-41.2 to 20.6)
Dominican Republic	13.1 (11.9 to 14.2)	9.9 (8.4 to 11.6)	9.7 (7.9 to 11.9)	7.9 (5.2 to 11.8)	-1.6 (-3.5 to 0.3)	-20.6 (-40.5 to 4.6)
Grenada	11.9 (7.9 to 16.6)	9.7 (6.3 to 13.8)	9.5 (6.0 to 13.8)	8.4 (4.1 to 14.8)	-1.2 (-3.6 to 1.3)	-14.4 (-41.7 to 21.6)
Guyana	16.5 (15.2 to 17.9)	14.5 (12.8 to 16.4)	13.4 (11.2 to 15.6)	10.7 (7.4 to 14.6)	-2.1 (-3.8 to -0.6)	-27.0 (-43.5 to -9.0)
Haiti	30.6 (29.1 to 32.3)	24.0 (22.7 to 25.2)	23.3 (21.3 to 25.1)	18.3 (15.1 to 21.6)	-1.8 (-2.8 to -0.9)	-23.9 (-34.3 to -12.8)

	Estimate in 2000 (95% UIs)	Estimate in 2015 (95% UIs)	Estimate in 2017 (95% UIs)	Estimate in 2030 (95% UIs)	Annualised rate of change, 2015-2030 (95% UIs)	Percent change, 2015-2030 (95% UIs)
Jamaica	8.7 (7.7 to 9.7)	7.7 (6.1 to 9.4)	7.6 (5.7 to 9.6)	7.1 (4.1 to 10.8)	-0.7 (-2.7 to 1.1)	-8.6 (-33.4 to 18.5)
Puerto Rico	7.4 (4.7 to 10.9)	6.3 (3.7 to 9.4)	6.2 (3.6 to 9.4)	5.7 (2.7 to 10.1)	-0.8 (-3.3 to 1.5)	-10.1 (-38.9 to 25.0)
Saint Lucia	6.8 (4.2 to 9.9)	4.4 (2.8 to 6.5)	4.4 (2.8 to 6.4)	3.5 (1.6 to 6.2)	-1.9 (-4.8 to 0.8)	-22.7 (-51.0 to 13.4)
Saint Vincent and the Grenadines	13.1 (9.0 to 17.9)	10.4 (6.8 to 14.4)	10.2 (6.6 to 14.2)	8.8 (4.6 to 14.5)	-1.2 (-3.5 to 0.8)	-15.8 (-41.0 to 12.4)
Suriname	14.2 (12.5 to 16.0)	9.1 (7.1 to 11.4)	8.9 (6.6 to 11.7)	6.8 (3.7 to 11.2)	-2.2 (-4.6 to 0.1)	-26.6 (-49.7 to 1.2)
Trinidad and Tobago	5.3 (3.9 to 7.0)	4.8 (2.9 to 7.1)	4.7 (2.9 to 7.2)	4.7 (2.1 to 8.7)	-0.4 (-3.2 to 2.1)	-4.0 (-37.8 to 36.2)
Virgin Islands, U.S.	7.2 (4.5 to 11.0)	6.1 (3.7 to 9.4)	6.0 (3.6 to 9.4)	5.8 (2.8 to 10.5)	-0.6 (-3.3 to 2.0)	-6.7 (-38.8 to 35.9)
Central Latin America						
Colombia	19.6 (18.2 to 20.9)	13.0 (10.8 to 15.3)	12.7 (9.9 to 15.5)	9.5 (5.9 to 14.5)	-2.2 (-4.2 to -0.3)	-27.4 (-47.0 to -4.5)
Costa Rica	11.5 (7.9 to 15.8)	8.6 (6.0 to 11.6)	8.5 (5.8 to 11.8)	7.6 (3.7 to 12.6)	-1.1 (-3.7 to 1.3)	-13.1 (-42.6 to 20.7)
El Salvador	28.9 (27.8 to 30.5)	14.9 (13.4 to 16.8)	13.9 (11.9 to 16.2)	8.0 (5.7 to 11.0)	-4.3 (-5.9 to -2.6)	-46.8 (-58.5 to -32.5)
Guatemala	51.9 (49.1 to 55.6)	47.8 (42.2 to 55.1)	47.0 (40.4 to 56.0)	42.5 (30.8 to 59.1)	-0.9 (-2.4 to 0.5)	-11.7 (-30.0 to 8.1)
Honduras	42.2 (40.5 to 44.2)	22.7 (20.6 to 24.9)	21.8 (18.8 to 24.7)	13.2 (9.5 to 17.1)	-3.7 (-5.2 to -2.3)	-42.1 (-54.0 to -29.6)
Mexico	20.7 (19.8 to 21.7)	14.4 (13.7 to 15.2)	13.4 (12.6 to 14.3)	9.3 (8.2 to 10.5)	-2.9 (-3.4 to -2.4)	-35.4 (-40.4 to -29.9)
Nicaragua	27.5 (26.3 to 28.9)	16.3 (14.4 to 18.0)	15.5 (13.1 to 18.0)	10.4 (7.3 to 14.1)	-3.0 (-4.6 to -1.6)	-36.1 (-50.1 to -20.9)
Panama	24.4 (22.6 to 26.2)	17.6 (14.9 to 20.2)	16.2 (12.6 to 19.8)	11.7 (7.2 to 17.5)	-2.9 (-4.9 to -1.0)	-34.1 (-52.4 to -13.9)
Venezuela	18.5 (17.4 to 20.1)	14.1 (11.6 to 16.7)	13.9 (10.9 to 17.0)	11.6 (7.2 to 16.7)	-1.4 (-3.3 to 0.2)	-18.8 (-39.4 to 2.5)
Tropical Latin America						
Brazil	12.8 (11.8 to 13.8)	10.1 (9.1 to 11.1)	9.7 (8.7 to 10.8)	7.8 (6.6 to 9.0)	-1.7 (-2.3 to -1.1)	-22.7 (-29.3 to -15.6)
Paraguay	13.5 (10.3 to 16.8)	6.5 (5.2 to 8.1)	6.2 (4.8 to 7.8)	3.7 (2.3 to 5.7)	-4.0 (-5.9 to -1.8)	-44.4 (-58.9 to -23.8)
North Africa and Middle East						
North Africa and Middle East						
Afghanistan	55.0 (53.2 to 57.0)	50.8 (43.5 to 61.1)	49.0 (41.2 to 60.1)	44.1 (30.6 to 64.1)	-1.0 (-2.5 to 0.4)	-13.9 (-31.0 to 6.4)
Algeria	33.3 (31.8 to 35.3)	13.9 (12.0 to 15.8)	13.6 (11.4 to 15.8)	8.1 (5.7 to 10.8)	-3.7 (-5.1 to -2.3)	-42.1 (-53.3 to -29.5)
Bahrain	12.0 (10.2 to 14.0)	6.9 (5.1 to 9.3)	6.7 (4.8 to 9.3)	4.8 (2.6 to 8.1)	-2.7 (-4.9 to -0.6)	-32.2 (-51.7 to -9.1)
Egypt	27.6 (26.5 to 28.7)	22.7 (21.1 to 24.4)	19.2 (17.4 to 21.2)	13.0 (10.9 to 15.5)	-3.7 (-4.5 to -2.9)	-42.6 (-49.0 to -35.4)
Iran	18.9 (17.6 to 20.7)	10.7 (7.9 to 13.7)	10.5 (7.3 to 13.9)	7.4 (3.9 to 11.9)	-2.7 (-5.0 to -0.8)	-32.4 (-52.6 to -10.9)
Iraq	29.0 (28.0 to 30.2)	22.6 (20.9 to 24.4)	21.4 (19.0 to 23.9)	16.1 (12.5 to 20.0)	-2.3 (-3.6 to -1.3)	-28.9 (-41.3 to -17.1)
Jordan	13.9 (12.1 to 15.7)	10.2 (8.4 to 12.3)	9.7 (7.5 to 12.3)	7.4 (4.5 to 11.4)	-2.3 (-4.3 to -0.4)	-28.6 (-47.3 to -5.2)
Kuwait	5.1 (4.3 to 6.2)	3.7 (2.8 to 4.6)	3.6 (2.6 to 4.7)	2.9 (1.6 to 4.7)	-1.8 (-4.3 to 0.6)	-22.2 (-47.2 to 9.2)
Lebanon	17.5 (15.2 to 20.1)	13.1 (10.7 to 15.5)	12.6 (9.7 to 15.4)	9.9 (5.8 to 15.1)	-2.0 (-4.3 to 0.1)	-25.2 (-47.8 to 1.6)
Libya	21.9 (20.2 to 23.9)	18.7 (15.8 to 21.6)	18.7 (15.1 to 22.5)	17.4 (11.1 to 24.4)	-0.6 (-2.4 to 0.9)	-7.6 (-30.7 to 14.6)
Morocco	27.2 (24.7 to 29.9)	16.9 (14.8 to 19.3)	16.3 (13.5 to 19.3)	11.1 (7.4 to 15.5)	-2.9 (-4.9 to -1.2)	-34.9 (-51.8 to -16.8)
Palestine	15.9 (14.8 to 17.1)	10.9 (9.4 to 12.4)	9.0 (7.3 to 11.0)	5.2 (3.7 to 7.3)	-5.0 (-6.4 to -3.4)	-52.3 (-61.8 to -39.6)
Oman	16.7 (15.3 to 18.1)	15.0 (13.7 to 16.4)	15.3 (13.6 to 17.1)	14.6 (11.4 to 18.2)	-0.2 (-1.5 to 0.9)	-2.9 (-20.5 to 14.9)
Qatar	12.3 (9.2 to 16.1)	10.6 (7.0 to 14.9)	10.4 (6.7 to 14.9)	9.6 (4.8 to 16.3)	-0.9 (-3.1 to 1.1)	-10.9 (-37.0 to 18.5)
Saudi Arabia	18.0 (15.3 to 21.0)	10.9 (7.6 to 15.0)	10.5 (7.2 to 14.4)	7.3 (3.9 to 12.1)	-2.8 (-4.8 to -0.8)	-33.6 (-51.4 to -11.7)
Sudan	48.8 (44.0 to 55.1)	39.5 (35.0 to 46.2)	37.6 (32.8 to 45.7)	30.9 (22.6 to 46.3)	-1.7 (-3.3 to 0.1)	-22.2 (-39.5 to 1.6)
Syria	32.0 (30.3 to 33.6)	26.9 (24.6 to 29.6)	26.1 (22.7 to 29.5)	22.2 (16.5 to 28.6)	-1.3 (-2.7 to -0.2)	-17.7 (-33.0 to -2.3)
Tunisia	17.7 (16.4 to 19.0)	10.6 (8.8 to 12.7)	10.4 (8.3 to 12.7)	7.4 (4.9 to 10.9)	-2.5 (-4.3 to -0.8)	-30.6 (-47.3 to -11.0)
Turkey	20.7 (18.9 to 22.6)	12.1 (9.5 to 14.7)	11.5 (8.6 to 14.7)	7.6 (4.3 to 12.0)	-3.3 (-5.3 to -1.3)	-38.0 (-55.0 to -17.2)
United Arab Emirates	16.3 (13.2 to 19.3)	12.8 (8.9 to 17.2)	12.7 (8.7 to 17.2)	10.8 (6.3 to 16.0)	-1.3 (-3.1 to 0.2)	-16.7 (-37.1 to 3.6)
Yemen	57.6 (54.7 to 61.1)	49.1 (44.4 to 54.7)	45.2 (39.0 to 53.5)	35.0 (24.3 to 50.1)	-2.4 (-4.2 to -0.4)	-29.3 (-46.6 to -6.4)
South Asia						
South Asia						
Bangladesh	49.2 (47.3 to 51.5)	39.9 (36.7 to 45.3)	39.1 (34.5 to 47.2)	32.9 (24.3 to 46.7)	-1.4 (-2.8 to 0.4)	-18.1 (-34.4 to 5.8)
Bhutan	52.0 (43.9 to 61.8)	33.2 (29.1 to 40.2)	32.1 (26.9 to 41.2)	22.2 (14.7 to 36.5)	-2.8 (-4.9 to -0.6)	-33.7 (-51.9 to -9.3)
India	55.8 (54.5 to 57.0)	41.9 (41.4 to 42.4)	39.3 (38.7 to 40.1)	27.6 (26.3 to 29.2)	-2.8 (-3.0 to -2.5)	-34.0 (-36.7 to -30.8)
Nepal	57.9 (51.4 to 64.8)	34.0 (30.3 to 41.2)	33.5 (29.7 to 41.6)	21.3 (16.0 to 33.8)	-3.2 (-4.7 to -1.2)	-37.7 (-50.3 to -16.0)
Pakistan	51.4 (46.0 to 57.9)	44.8 (41.3 to 49.5)	44.0 (39.5 to 50.7)	38.8 (29.9 to 51.6)	-1.0 (-2.4 to 0.5)	-13.8 (-29.8 to 7.2)
Southeast Asia, East Asia, and Oceania						
East Asia						
China	17.0 (11.7 to 22.5)	11.1 (7.5 to 15.1)	10.8 (7.3 to 14.7)	8.2 (4.5 to 13.1)	-2.2 (-4.4 to -0.1)	-26.8 (-48.6 to -1.3)
North Korea	49.3 (45.5 to 54.3)	25.6 (23.4 to 28.1)	24.5 (21.7 to 27.8)	16.4 (12.3 to 21.8)	-3.0 (-4.5 to -1.4)	-36.0 (-49.2 to -18.8)
Taiwan (Province of China)	9.6 (6.1 to 13.9)	7.6 (4.7 to 11.3)	7.5 (4.5 to 11.3)	6.9 (3.1 to 12.4)	-0.9 (-3.6 to 1.6)	-10.9 (-42.1 to 27.8)
Oceania						
American Samoa	12.6 (8.0 to 18.0)	11.4 (7.4 to 16.2)	11.1 (7.2 to 15.8)	10.5 (5.6 to 17.1)	-0.7 (-3.0 to 1.2)	-9.1 (-36.4 to 20.5)
Federated States of Micronesia	18.3 (12.7 to 24.6)	15.6 (10.8 to 20.8)	15.3 (10.4 to 20.8)	14.0 (7.8 to 21.4)	-0.9 (-3.0 to 1.0)	-10.9 (-36.4 to 16.3)
Fiji	8.7 (5.4 to 12.5)	7.9 (4.8 to 11.5)	7.7 (4.7 to 11.4)	7.3 (3.4 to 13.7)	-0.8 (-3.6 to 1.9)	-9.3 (-42.1 to 32.8)
Guam	9.9 (6.1 to 14.7)	9.2 (6.0 to 13.1)	8.9 (5.8 to 12.9)	8.6 (4.4 to 14.5)	-0.6 (-3.2 to 1.6)	-7.6 (-38.5 to 27.1)
Kiribati	30.9 (23.8 to 40.0)	25.6 (19.3 to 32.1)	24.7 (18.3 to 31.4)	21.2 (12.6 to 30.9)	-1.3 (-3.2 to 0.2)	-17.5 (-38.1 to 3.5)
Marshall Islands	20.6 (14.6 to 27.7)	16.7 (11.5 to 22.6)	16.1 (11.1 to 22.0)	13.8 (7.9 to 21.6)	-1.4 (-3.2 to 0.5)	-17.9 (-38.5 to 7.9)
Northern Mariana Islands	9.4 (6.0 to 13.7)	9.9 (6.4 to 14.3)	9.8 (6.2 to 14.1)	10.4 (5.2 to 17.6)	0.2 (-2.4 to 2.3)	4.1 (-30.1 to 42.0)
Papua New Guinea	55.6 (48.0 to 63.1)	44.4 (40.2 to 50.4)	43.5 (38.2 to 51.4)	35.4 (26.5 to 49.7)	-1.6 (-3.0 to 0.0)	-20.8 (-36.5 to 0.6)
Samoa	9.9 (7.5 to 13.2)	10.7 (7.2 to 15.1)	10.6 (6.9 to 15.4)	11.3 (5.7 to 19.6)	0.2 (-1.9 to 2.2)	4.1 (-25.3 to 38.7)
Solomon Islands	30.7 (27.8 to 34.4)	27.4 (23.6 to 31.4)	26.4 (21.7 to 31.3)	23.1 (15.4 to 31.9)	-1.3 (-2.9 to 0.3)	-16.6 (-35.4 to 4.1)
Tonga	13.3 (9.7 to 17.0)	10.6 (9.6 to 11.6)	10.2 (8.5 to 11.9)	8.9 (5.7 to 13.0)	-1.3 (-3.7 to 0.9)	-16.2 (-42.4 to 15.0)
Vanuatu	28.1 (25.2 to 31.4)	27.6 (25.1 to 30.2)	27.2 (23.9 to 30.5)	26.0 (19.6 to 32.6)	-0.5 (-1.8 to 0.7)	-6.3 (-23.6 to 10.5)
Southeast Asia						
Cambodia	53.0 (49.4 to 57.8)	41.0 (35.8 to 49.4)	38.8 (32.4 to 49.6)	29.3 (19.4 to 48.0)	-2.4 (-4.3 to 0.0)	-29.4 (-47.3 to 0.3)
Indonesia	41.1 (39.3 to 43.4)	36.0 (35.2 to 37.2)	34.3 (33.3 to 36.0)	28.4 (26.2 to 31.4)	-1.6 (-2.0 to -1.1)	-21.2 (-26.0 to -15.0)
Laos	60.4 (54.7 to 65.2)	46.6 (41.9 to 54.2)	44.7 (39.4 to 53.5)	34.2 (24.8 to 49.4)	-2.2 (-3.7 to -0.4)	-27.0 (-42.6 to -5.6)
Malaysia	25.1 (22.0 to 28.8)	19.1 (17.0 to 21.4)	18.5 (16.4 to 21.0)	14.7 (11.4 to 18.6)	-1.8 (-3.0 to -0.6)	-23.1 (-35.9 to -8.5)
Maldives	35.1 (33.0 to 37.4)	19.5 (16.3 to 23.0)	18.6 (14.8 to 23.0)	11.5 (7.4 to 17.1)	-3.7 (-5.6 to -1.8)	-41.6 (-56.5 to -24.0)
Mauritius	16.6 (13.4 to 20.2)	14.6 (10.4 to 19.6)	14.3 (10.1 to 19.3)	13.0 (6.9 to 20.2)	-0.9 (-2.9 to 0.9)	-11.8 (-34.8 to 13.8)
Myanmar	46.6 (41.3 to 53.9)	33.1 (30.6 to 37.8)	31.4 (28.4 to 38.3)	22.3 (17.3 to 34.1)	-2.7 (-4.0 to -0.6)	-32.8 (-45.2 to -9.1)
Philippines	36.9 (34.5 to 40.0)	33.5 (30.4 to 38.1)	32.4 (28.2 to 39.5)	28.8 (21.6 to 41.0)	-1.1 (-2.4 to 0.5)	-14.4 (-30.7 to 7.2)
Sri Lanka	26.8 (25.7 to 28.0)	15.6 (12.5 to 18.6)	14.9 (11.4 to 18.4)	9.7 (5.8 to 14.4)	-3.3 (-5.4 to -1.6)	-38.5 (-55.2 to -21.2)
Seychelles	10.6 (8.1 to 13.4)	10.0 (8.2 to 12.0)	9.8 (7.7 to 12.2)	9.6 (5.9 to 14.3)	-0.4 (-2.6 to 1.6)	-4.9 (-32.0 to 26.6)
Thailand	17.9 (16.3 to 19.7)	14.7 (13.0 to 16.4)	14.3 (12.1 to 16.7)	12.2 (8.7 to 16.4)	-1.3 (-2.9 to 0.2)	-17.3 (-35.2 to 2.6)

	Estimate in 2000 (95% UIs)	Estimate in 2015 (95% UIs)	Estimate in 2017 (95% UIs)	Estimate in 2030 (95% UIs)	Annualised rate of change, 2015-2030 (95% UIs)	Percent change, 2015-2030 (95% UIs)
Timor-Leste	63.0 (56.3 to 69.2)	51.0 (45.6 to 57.8)	49.6 (42.4 to 58.6)	40.6 (26.7 to 58.0)	-1.7 (-3.8 to 0.2)	-21.0 (-43.6 to 3.7)
Vietnam	40.3 (37.9 to 43.5)	26.3 (24.9 to 27.7)	22.8 (20.1 to 25.4)	12.8 (10.2 to 16.1)	-4.9 (-6.0 to -3.6)	-51.5 (-59.1 to -41.3)
Sub-Saharan Africa						
Central Sub-Saharan Africa						
Angola	55.7 (49.4 to 63.1)	44.8 (38.0 to 54.5)	42.6 (35.9 to 53.2)	33.1 (23.6 to 48.9)	-2.1 (-3.8 to -0.3)	-26.6 (-43.1 to -4.9)
Central African Republic	44.2 (41.7 to 47.8)	43.2 (37.5 to 53.5)	42.7 (35.7 to 54.7)	40.6 (28.8 to 60.6)	-0.5 (-2.0 to 0.9)	-7.0 (-25.6 to 15.1)
Congo	30.2 (26.9 to 34.0)	23.2 (21.8 to 24.8)	21.2 (19.3 to 23.2)	14.8 (11.7 to 17.9)	-3.1 (-4.3 to -2.0)	-36.6 (-47.3 to -26.3)
Democratic Republic of the Congo	45.5 (43.2 to 49.1)	45.4 (41.2 to 52.3)	43.6 (38.9 to 51.4)	40.4 (32.2 to 52.7)	-0.8 (-1.9 to 0.3)	-11.4 (-24.7 to 4.1)
Equatorial Guinea	50.9 (45.0 to 58.6)	22.1 (20.3 to 24.0)	20.9 (18.1 to 23.6)	10.3 (7.3 to 13.8)	-5.1 (-6.8 to -3.6)	-53.4 (-64.1 to -41.4)
Gabon	31.6 (27.1 to 39.7)	20.4 (16.8 to 24.8)	19.4 (15.5 to 23.8)	13.6 (8.9 to 19.5)	-2.8 (-4.5 to -1.3)	-33.5 (-48.8 to -17.2)
Eastern Sub-Saharan Africa						
Burundi	53.8 (51.2 to 57.4)	55.3 (47.4 to 63.4)	54.2 (45.0 to 64.0)	52.2 (36.7 to 68.8)	-0.5 (-2.0 to 0.7)	-6.2 (-25.4 to 11.0)
Comoros	49.1 (43.5 to 58.0)	36.9 (31.1 to 47.5)	35.7 (29.7 to 47.1)	28.1 (19.1 to 45.5)	-2.0 (-3.8 to 0.1)	-24.6 (-43.7 to 0.9)
Djibouti	35.4 (32.6 to 38.7)	33.4 (30.9 to 37.1)	32.7 (29.5 to 36.9)	30.4 (24.5 to 38.4)	-0.7 (-1.7 to 0.3)	-9.2 (-22.9 to 4.6)
Eritrea	48.2 (43.9 to 54.7)	42.4 (34.4 to 53.7)	41.2 (32.7 to 53.6)	35.7 (22.9 to 54.6)	-1.3 (-3.0 to 0.3)	-16.6 (-36.0 to 5.2)
Ethiopia	57.8 (54.0 to 61.9)	45.4 (41.8 to 50.1)	43.8 (40.0 to 49.1)	34.3 (27.9 to 43.5)	-1.9 (-2.9 to -0.8)	-24.7 (-35.8 to -11.2)
Kenya	45.4 (41.4 to 49.8)	36.0 (32.1 to 40.3)	35.1 (31.1 to 39.5)	28.8 (23.3 to 36.0)	-1.5 (-2.4 to -0.6)	-20.0 (-30.7 to -8.4)
Madagascar	54.8 (50.4 to 60.2)	50.6 (43.3 to 60.7)	49.5 (41.4 to 60.7)	45.4 (32.0 to 63.2)	-0.8 (-2.3 to 0.5)	-10.9 (-29.3 to 7.0)
Malawi	53.4 (50.1 to 57.5)	38.8 (35.6 to 43.8)	37.9 (33.9 to 44.7)	28.4 (21.6 to 39.8)	-2.2 (-3.5 to -0.5)	-27.1 (-40.7 to -7.4)
Mozambique	49.7 (46.3 to 54.4)	37.9 (33.4 to 45.5)	37.0 (31.6 to 45.8)	29.1 (20.5 to 43.6)	-1.9 (-3.5 to -0.1)	-23.8 (-40.4 to -1.1)
Rwanda	48.5 (46.0 to 52.4)	41.4 (36.6 to 48.8)	40.4 (34.6 to 50.3)	35.2 (24.6 to 53.2)	-1.2 (-2.9 to 0.7)	-15.8 (-35.7 to 11.3)
Somalia	39.0 (35.5 to 41.9)	34.3 (30.0 to 39.3)	33.8 (28.6 to 40.2)	30.7 (21.4 to 42.7)	-0.8 (-2.4 to 0.7)	-11.0 (-30.0 to 10.4)
South Sudan	41.6 (38.0 to 46.3)	33.3 (30.2 to 38.3)	32.9 (28.9 to 39.4)	28.2 (21.4 to 38.2)	-1.2 (-2.4 to 0.1)	-15.6 (-30.2 to 1.5)
Tanzania	50.6 (45.6 to 57.1)	38.2 (36.0 to 42.1)	35.8 (33.1 to 40.6)	25.7 (20.5 to 35.2)	-2.7 (-4.0 to -1.1)	-32.8 (-45.1 to -14.6)
Uganda	48.8 (43.3 to 57.1)	35.0 (30.8 to 43.2)	34.5 (29.7 to 44.5)	27.3 (19.7 to 42.0)	-1.8 (-3.4 to 0.1)	-22.5 (-40.2 to 1.2)
Zambia	56.3 (52.0 to 61.5)	43.7 (39.5 to 51.2)	41.9 (36.6 to 51.6)	32.2 (23.4 to 48.9)	-2.1 (-3.7 to -0.2)	-26.8 (-42.2 to -2.4)
Southern Sub-Saharan Africa						
Botswana	32.2 (28.4 to 41.8)	29.2 (25.7 to 36.0)	28.1 (23.7 to 36.5)	24.3 (17.1 to 37.5)	-1.3 (-3.0 to 0.4)	-17.3 (-36.6 to 6.2)
Lesotho	49.2 (45.9 to 53.8)	39.4 (33.0 to 51.6)	37.7 (31.0 to 51.6)	30.3 (20.7 to 51.0)	-1.9 (-3.6 to 0.1)	-24.2 (-41.4 to 1.9)
Namibia	31.8 (29.3 to 35.8)	25.9 (23.3 to 29.2)	24.2 (20.8 to 27.7)	18.6 (13.6 to 24.1)	-2.3 (-3.8 to -1.0)	-28.5 (-43.5 to -13.9)
South Africa	34.4 (32.2 to 37.3)	26.2 (24.4 to 28.9)	24.6 (22.5 to 27.7)	18.2 (15.0 to 23.4)	-2.5 (-3.4 to -1.3)	-30.6 (-39.6 to -17.9)
Swaziland	45.0 (37.6 to 55.9)	34.2 (29.6 to 43.3)	30.4 (24.9 to 42.8)	21.8 (14.2 to 40.4)	-3.3 (-5.3 to -0.3)	-37.6 (-54.9 to -4.6)
Zimbabwe	37.1 (33.0 to 44.6)	32.8 (29.8 to 37.6)	29.3 (25.9 to 34.9)	22.4 (17.4 to 32.4)	-2.6 (-3.9 to -0.7)	-31.9 (-44.3 to -9.9)
Western Sub-Saharan Africa						
Benin	44.5 (40.1 to 51.1)	41.9 (38.3 to 47.8)	38.2 (33.2 to 47.0)	31.6 (23.1 to 46.9)	-2.0 (-3.5 to 0.0)	-25.0 (-40.8 to 0.5)
Burkina Faso	49.7 (47.3 to 53.3)	29.2 (28.1 to 30.4)	27.6 (26.1 to 29.2)	16.2 (14.1 to 18.6)	-3.9 (-4.7 to -3.2)	-44.4 (-50.4 to -38.1)
Cameroon	45.8 (41.2 to 54.3)	31.8 (29.5 to 36.3)	30.7 (27.9 to 35.9)	22.3 (17.4 to 31.0)	-2.4 (-3.8 to -0.8)	-30.1 (-43.6 to -11.7)
Cape Verde	22.4 (18.4 to 27.2)	12.3 (8.4 to 17.0)	11.7 (7.8 to 16.4)	7.4 (4.0 to 12.4)	-3.6 (-5.7 to -1.6)	-40.7 (-57.8 to -21.9)
Chad	49.5 (44.8 to 56.0)	43.0 (39.8 to 48.0)	42.4 (39.0 to 48.2)	38.0 (31.2 to 48.1)	-0.9 (-2.0 to 0.2)	-11.7 (-25.4 to 3.6)
Cote d'Ivoire	43.4 (38.5 to 52.1)	32.8 (28.7 to 41.4)	30.4 (25.8 to 40.8)	22.0 (15.2 to 38.0)	-2.8 (-4.6 to -0.2)	-33.4 (-49.9 to -2.9)
The Gambia	31.7 (29.8 to 34.5)	22.6 (20.9 to 24.5)	22.3 (19.9 to 24.7)	17.2 (13.3 to 21.2)	-1.9 (-3.0 to -0.8)	-24.0 (-36.5 to -11.3)
Ghana	35.8 (33.2 to 40.1)	22.2 (20.2 to 24.4)	19.7 (17.0 to 22.4)	11.5 (8.5 to 14.9)	-4.4 (-5.9 to -3.1)	-48.3 (-58.4 to -37.6)
Guinea	41.5 (39.0 to 44.8)	34.7 (31.3 to 41.0)	32.4 (28.3 to 40.8)	25.9 (19.5 to 40.2)	-2.0 (-3.4 to -0.1)	-25.9 (-40.1 to -1.5)
Guinea-Bissau	43.9 (38.9 to 52.3)	28.1 (26.2 to 32.0)	26.5 (24.0 to 30.7)	17.1 (13.1 to 22.2)	-3.4 (-4.8 to -2.0)	-39.3 (-51.2 to -25.8)
Liberia	53.3 (47.6 to 61.1)	35.8 (31.0 to 44.9)	33.7 (28.6 to 44.0)	22.9 (16.0 to 40.0)	-3.1 (-4.7 to -0.7)	-36.8 (-50.8 to -9.6)
Mali	41.5 (39.1 to 46.0)	31.7 (29.4 to 35.4)	31.7 (29.1 to 36.5)	27.7 (23.0 to 35.8)	-0.9 (-1.9 to 0.2)	-12.9 (-25.1 to 2.5)
Mauritania	38.1 (36.3 to 40.9)	26.7 (26.0 to 27.5)	29.2 (27.9 to 30.6)	26.3 (23.9 to 28.6)	-0.1 (-0.6 to 0.3)	-1.7 (-8.8 to 4.7)
Niger	59.1 (53.4 to 65.6)	49.2 (43.0 to 57.3)	47.5 (39.8 to 58.5)	39.0 (25.7 to 59.4)	-1.7 (-3.6 to 0.3)	-21.5 (-41.8 to 4.8)
Nigeria	60.8 (55.8 to 64.3)	43.5 (41.7 to 45.9)	38.1 (36.4 to 40.9)	22.5 (19.9 to 26.5)	-4.4 (-5.1 to -3.6)	-48.2 (-53.2 to -41.8)
Sao Tome and Principe	35.0 (32.5 to 39.0)	19.9 (18.1 to 21.7)	17.9 (15.5 to 20.3)	10.0 (7.5 to 13.0)	-4.7 (-6.0 to -3.4)	-50.2 (-59.6 to -39.7)
Senegal	33.7 (30.8 to 39.1)	22.4 (20.7 to 24.2)	21.1 (18.9 to 23.3)	14.6 (11.6 to 18.0)	-2.9 (-4.1 to -1.8)	-34.9 (-45.6 to -23.2)
Sierra Leone	47.6 (43.0 to 53.5)	38.9 (35.3 to 44.6)	37.1 (32.7 to 45.0)	29.8 (22.1 to 43.8)	-1.9 (-3.3 to -0.1)	-23.8 (-39.4 to -1.1)
Togo	33.6 (31.1 to 36.2)	26.2 (23.7 to 28.6)	24.8 (21.1 to 28.4)	19.1 (13.3 to 25.2)	-2.2 (-3.8 to -0.8)	-27.3 (-43.9 to -10.7)
**Indicator 2.2.2a: Prevalence of wasting in children under 5 (%)**						
Central Europe, Eastern Europe, and Central Asia						
Central Asia						
Armenia	2.7 (2.2 to 3.3)	3.5 (2.9 to 4.3)	3.6 (2.9 to 4.4)	4.3 (2.8 to 5.9)	1.2 (-0.7 to 2.8)	20.5 (-9.3 to 51.1)
Azerbaijan	4.2 (3.7 to 4.7)	4.3 (3.5 to 5.1)	4.3 (3.4 to 5.3)	4.6 (2.9 to 6.6)	0.3 (-1.5 to 1.8)	5.7 (-20.1 to 31.9)
Georgia	2.7 (2.2 to 3.4)	2.0 (1.5 to 2.6)	2.0 (1.4 to 2.6)	1.7 (1.1 to 2.5)	-1.3 (-3.0 to 0.5)	-17.1 (-36.0 to 7.1)
Kazakhstan	3.7 (3.0 to 4.4)	2.7 (2.3 to 3.2)	2.5 (2.1 to 3.0)	1.9 (1.3 to 2.6)	-2.6 (-4.0 to -1.1)	-31.5 (-45.3 to -15.7)
Kyrgyzstan	2.2 (1.8 to 2.6)	1.8 (1.6 to 2.1)	1.8 (1.5 to 2.2)	1.8 (1.2 to 2.5)	-0.3 (-2.2 to 1.4)	-3.8 (-28.1 to 23.1)
Mongolia	4.5 (4.0 to 4.9)	1.4 (1.2 to 1.8)	1.4 (1.1 to 1.7)	0.8 (0.6 to 1.1)	-4.1 (-5.5 to -2.5)	-45.3 (-56.0 to -31.1)
Tajikistan	8.2 (8.0 to 8.5)	10.6 (9.7 to 11.6)	10.4 (9.3 to 11.6)	10.2 (8.4 to 12.5)	-0.2 (-1.0 to 0.6)	-3.4 (-14.0 to 9.9)
Turkmenistan	7.4 (7.2 to 7.6)	3.7 (3.2 to 4.2)	2.9 (2.4 to 3.4)	1.3 (1.0 to 1.7)	-6.9 (-7.7 to -5.9)	-64.3 (-68.3 to -58.6)
Uzbekistan	8.0 (6.6 to 9.6)	4.8 (3.6 to 6.2)	4.5 (3.3 to 6.0)	3.2 (1.8 to 4.9)	-2.9 (-4.7 to -1.1)	-34.4 (-50.5 to -15.7)
Central Europe						
Albania	9.5 (7.9 to 11.2)	7.3 (6.2 to 8.7)	7.1 (5.7 to 8.6)	6.0 (3.9 to 8.7)	-1.4 (-3.2 to 0.2)	-18.4 (-37.8 to 3.3)
Bosnia and Herzegovina	5.1 (4.5 to 5.8)	2.1 (1.6 to 2.6)	2.0 (1.6 to 2.6)	1.2 (0.8 to 1.9)	-3.7 (-5.3 to -2.0)	-42.2 (-55.1 to -26.2)
Bulgaria	3.4 (2.4 to 4.7)	2.8 (2.0 to 3.8)	2.7 (1.9 to 3.8)	2.4 (1.4 to 4.0)	-1.0 (-2.9 to 1.1)	-12.9 (-35.3 to 18.6)
Croatia	2.9 (2.0 to 4.0)	2.4 (1.7 to 3.2)	2.3 (1.6 to 3.2)	2.1 (1.2 to 3.4)	-0.8 (-2.8 to 1.1)	-10.8 (-34.7 to 18.7)
Czech Republic	3.0 (2.4 to 3.6)	2.8 (2.0 to 3.6)	2.7 (2.0 to 3.6)	2.7 (1.6 to 4.1)	-0.3 (-2.1 to 1.4)	-3.8 (-27.3 to 24.2)
Hungary	3.1 (2.2 to 4.3)	2.5 (1.8 to 3.4)	2.4 (1.7 to 3.4)	2.2 (1.3 to 3.5)	-1.0 (-2.9 to 1.0)	-13.2 (-35.6 to 15.5)
Macedonia	2.2 (1.7 to 2.9)	2.5 (1.9 to 3.2)	2.3 (1.8 to 3.1)	2.3 (1.4 to 3.6)	-0.7 (-2.7 to 1.2)	-8.5 (-33.6 to 20.6)
Montenegro	3.7 (2.7 to 5.0)	2.6 (2.0 to 3.4)	2.5 (1.9 to 3.3)	2.0 (1.2 to 3.2)	-1.8 (-3.8 to 0.3)	-22.2 (-43.2 to 4.0)
Poland	3.0 (2.1 to 4.3)	2.4 (1.7 to 3.3)	2.3 (1.6 to 3.2)	2.0 (1.2 to 3.3)	-1.3 (-3.3 to 0.8)	-16.7 (-39.1 to 13.0)
Romania	4.2 (3.8 to 4.6)	3.5 (2.7 to 4.5)	3.4 (2.5 to 4.4)	3.0 (1.7 to 4.8)	-1.3 (-3.2 to 0.6)	-17.2 (-38.3 to 9.6)
Serbia	4.2 (3.1 to 5.2)	3.2 (2.6 to 3.9)	3.1 (2.5 to 4.0)	2.7 (1.7 to 4.2)	-1.3 (-3.5 to 0.9)	-16.2 (-40.4 to 13.7)
Slovakia	2.8 (1.9 to 3.9)	2.3 (1.6 to 3.2)	2.3 (1.6 to 3.1)	2.1 (1.2 to 3.3)	-0.8 (-2.9 to 1.1)	-10.7 (-35.1 to 17.3)
Slovenia	2.7 (1.9 to 3.8)	2.2 (1.6 to 3.1)	2.2 (1.6 to 3.0)	2.0 (1.2 to 3.4)	-0.8 (-2.8 to 1.4)	-10.2 (-34.3 to 23.6)
Eastern Europe						

	Estimate in 2000 (95% UIs)	Estimate in 2015 (95% UIs)	Estimate in 2017 (95% UIs)	Estimate in 2030 (95% UIs)	Annualised rate of change, 2015-2030 (95% UIs)	Percent change, 2015-2030 (95% UIs)
Belarus	2.6 (1.9 to 3.5)	1.8 (1.3 to 2.5)	1.8 (1.3 to 2.5)	1.5 (0.9 to 2.6)	-1.4 (-3.4 to 0.8)	-17.5 (-39.5 to 12.9)
Estonia	2.9 (2.0 to 3.9)	2.1 (1.5 to 2.9)	2.0 (1.4 to 2.9)	1.7 (1.0 to 2.9)	-1.4 (-3.3 to 0.7)	-17.6 (-39.0 to 11.3)
Latvia	3.1 (2.2 to 4.3)	2.2 (1.6 to 3.0)	2.1 (1.5 to 3.0)	1.8 (1.1 to 2.9)	-1.4 (-3.3 to 0.8)	-18.1 (-38.9 to 12.3)
Lithuania	3.0 (2.1 to 4.2)	2.1 (1.5 to 2.9)	2.1 (1.5 to 2.9)	1.7 (1.1 to 2.9)	-1.5 (-3.4 to 0.7)	-19.0 (-40.1 to 10.2)
Moldova	4.6 (3.6 to 5.8)	2.3 (1.7 to 3.0)	2.2 (1.6 to 2.9)	1.4 (0.9 to 2.4)	-3.2 (-5.1 to -1.1)	-37.7 (-53.1 to -15.4)
Russia	4.5 (4.0 to 5.1)	3.4 (2.6 to 4.4)	3.2 (2.4 to 4.3)	2.7 (1.6 to 4.4)	-1.6 (-3.3 to 0.3)	-20.3 (-38.9 to 4.6)
Ukraine	6.7 (5.5 to 8.1)	4.6 (3.4 to 6.0)	4.5 (3.2 to 5.9)	3.6 (2.1 to 5.5)	-1.8 (-3.7 to 0.1)	-22.5 (-42.3 to 1.2)
High-income						
Australasia						
Australia	1.0 (0.7 to 1.4)	1.0 (0.7 to 1.3)	0.9 (0.7 to 1.3)	0.9 (0.6 to 1.4)	-0.3 (-2.3 to 1.5)	-4.1 (-29.0 to 24.4)
New Zealand	1.1 (0.8 to 1.4)	1.0 (0.7 to 1.4)	1.0 (0.7 to 1.4)	1.0 (0.6 to 1.6)	-0.4 (-2.4 to 1.5)	-5.4 (-30.1 to 25.5)
High-income Asia Pacific						
Brunei	3.4 (2.3 to 4.8)	3.4 (2.5 to 4.7)	3.3 (2.3 to 4.6)	3.2 (1.8 to 5.2)	-0.5 (-2.7 to 1.5)	-6.7 (-33.7 to 25.4)
Japan	3.3 (3.1 to 3.5)	3.1 (2.9 to 3.2)	3.0 (2.9 to 3.2)	2.9 (2.7 to 3.2)	-0.3 (-0.7 to 0.0)	-4.6 (-9.4 to 0.7)
South Korea	1.2 (0.8 to 1.7)	1.2 (0.8 to 1.7)	1.2 (0.8 to 1.7)	1.2 (0.7 to 1.9)	-0.2 (-2.3 to 1.8)	-2.5 (-29.4 to 31.6)
Singapore	4.7 (3.9 to 5.6)	3.3 (2.4 to 4.4)	3.2 (2.3 to 4.4)	2.6 (1.6 to 4.2)	-1.6 (-3.4 to 0.3)	-20.0 (-39.5 to 4.8)
High-income North America						
Canada	0.9 (0.7 to 1.3)	0.8 (0.6 to 1.1)	0.8 (0.6 to 1.1)	0.8 (0.5 to 1.3)	-0.4 (-2.5 to 1.6)	-4.8 (-31.0 to 26.5)
Greenland	1.3 (0.9 to 1.8)	1.1 (0.8 to 1.5)	1.1 (0.8 to 1.5)	1.0 (0.6 to 1.6)	-0.6 (-2.6 to 1.5)	-7.7 (-32.0 to 25.8)
United States	0.5 (0.4 to 0.6)	0.4 (0.3 to 0.5)	0.4 (0.3 to 0.5)	0.3 (0.2 to 0.6)	-1.3 (-3.3 to 0.8)	-17.2 (-39.3 to 13.0)
Southern Latin America						
Argentina	3.8 (3.6 to 4.0)	2.5 (2.0 to 3.0)	2.3 (1.8 to 2.8)	1.6 (1.1 to 2.4)	-2.8 (-4.2 to -1.4)	-34.0 (-46.5 to -19.1)
Chile	1.2 (1.1 to 1.4)	1.0 (0.8 to 1.1)	0.8 (0.7 to 1.1)	0.6 (0.4 to 0.9)	-3.4 (-4.9 to -1.6)	-39.1 (-52.3 to -21.6)
Uruguay	1.6 (1.4 to 1.9)	1.5 (1.1 to 1.9)	1.4 (1.1 to 1.9)	1.3 (0.8 to 2.1)	-0.8 (-2.6 to 1.0)	-11.1 (-32.0 to 16.4)
Western Europe						
Andorra	0.9 (0.6 to 1.3)	0.8 (0.6 to 1.1)	0.8 (0.6 to 1.1)	0.8 (0.5 to 1.3)	-0.2 (-2.3 to 1.8)	-1.5 (-29.2 to 31.7)
Austria	1.0 (0.7 to 1.4)	0.9 (0.7 to 1.3)	0.9 (0.7 to 1.3)	0.9 (0.5 to 1.5)	-0.5 (-2.4 to 1.6)	-5.4 (-29.8 to 26.2)
Belgium	1.0 (0.7 to 1.3)	0.9 (0.6 to 1.2)	0.9 (0.6 to 1.2)	0.8 (0.5 to 1.4)	-0.4 (-2.4 to 1.6)	-4.1 (-30.5 to 26.4)
Cyprus	1.1 (0.8 to 1.5)	0.9 (0.7 to 1.3)	0.9 (0.6 to 1.3)	0.9 (0.5 to 1.4)	-0.2 (-2.1 to 1.6)	-2.6 (-27.0 to 27.0)
Denmark	0.9 (0.6 to 1.2)	0.8 (0.6 to 1.1)	0.8 (0.6 to 1.1)	0.8 (0.5 to 1.3)	-0.2 (-2.1 to 1.8)	-2.1 (-27.3 to 31.4)
Finland	1.0 (0.7 to 1.3)	0.9 (0.6 to 1.2)	0.9 (0.6 to 1.2)	0.8 (0.5 to 1.3)	-0.4 (-2.3 to 1.8)	-4.1 (-29.7 to 30.7)
France	1.0 (0.7 to 1.4)	0.9 (0.7 to 1.3)	0.9 (0.6 to 1.2)	0.9 (0.5 to 1.4)	-0.4 (-2.3 to 1.5)	-5.4 (-29.0 to 24.3)
Germany	0.6 (0.4 to 0.8)	0.5 (0.4 to 0.7)	0.5 (0.4 to 0.7)	0.5 (0.3 to 0.8)	-0.3 (-2.3 to 1.9)	-2.5 (-29.6 to 33.9)
Greece	1.1 (0.8 to 1.5)	1.0 (0.7 to 1.4)	1.0 (0.7 to 1.4)	1.0 (0.6 to 1.5)	-0.1 (-2.0 to 1.8)	-0.1 (-26.1 to 30.1)
Iceland	0.9 (0.7 to 1.3)	0.8 (0.6 to 1.1)	0.8 (0.6 to 1.1)	0.8 (0.5 to 1.3)	-0.4 (-2.2 to 1.5)	-5.2 (-28.6 to 26.1)
Ireland	1.0 (0.7 to 1.4)	0.9 (0.6 to 1.2)	0.9 (0.6 to 1.2)	0.8 (0.5 to 1.3)	-0.6 (-2.6 to 1.4)	-7.3 (-31.9 to 23.3)
Israel	1.1 (0.8 to 1.5)	1.0 (0.7 to 1.4)	1.0 (0.7 to 1.4)	1.0 (0.6 to 1.6)	-0.3 (-2.1 to 1.6)	-3.6 (-27.5 to 26.8)
Italy	1.0 (0.7 to 1.4)	0.9 (0.7 to 1.3)	0.9 (0.7 to 1.3)	0.9 (0.6 to 1.6)	-0.1 (-2.0 to 1.7)	-0.7 (-25.6 to 30.0)
Luxembourg	0.9 (0.7 to 1.3)	0.8 (0.6 to 1.1)	0.8 (0.6 to 1.2)	0.8 (0.5 to 1.3)	-0.3 (-2.2 to 1.7)	-2.9 (-28.0 to 28.3)
Malta	1.1 (0.8 to 1.5)	1.0 (0.7 to 1.3)	1.0 (0.7 to 1.3)	0.9 (0.6 to 1.5)	-0.5 (-2.4 to 1.4)	-6.9 (-29.8 to 22.8)
Netherlands	0.9 (0.7 to 1.3)	0.8 (0.6 to 1.1)	0.8 (0.6 to 1.1)	0.8 (0.5 to 1.3)	-0.3 (-2.3 to 1.6)	-3.6 (-28.7 to 27.1)
Norway	0.9 (0.7 to 1.3)	0.9 (0.6 to 1.2)	0.8 (0.6 to 1.1)	0.8 (0.5 to 1.3)	-0.4 (-2.2 to 1.6)	-4.5 (-27.9 to 27.8)
Portugal	1.3 (0.9 to 1.8)	1.1 (0.8 to 1.5)	1.1 (0.8 to 1.5)	1.0 (0.6 to 1.6)	-0.6 (-2.5 to 1.3)	-7.8 (-31.3 to 22.1)
Spain	1.6 (1.1 to 2.1)	1.4 (1.0 to 1.9)	1.4 (0.9 to 1.9)	1.3 (0.8 to 2.1)	-0.6 (-2.7 to 1.3)	-7.5 (-33.6 to 21.6)
Sweden	1.0 (0.7 to 1.4)	0.9 (0.7 to 1.2)	0.9 (0.7 to 1.2)	0.9 (0.5 to 1.4)	-0.4 (-2.3 to 1.3)	-5.0 (-28.8 to 22.0)
Switzerland	0.9 (0.7 to 1.3)	0.9 (0.6 to 1.2)	0.9 (0.6 to 1.2)	0.8 (0.5 to 1.4)	-0.3 (-2.2 to 1.7)	-3.6 (-28.3 to 28.4)
United Kingdom	1.1 (0.8 to 1.5)	0.9 (0.7 to 1.3)	0.9 (0.7 to 1.3)	0.9 (0.5 to 1.4)	-0.5 (-2.4 to 1.5)	-6.0 (-29.8 to 25.4)
Latin America and Caribbean						
Andean Latin America						
Bolivia	2.1 (1.9 to 2.3)	1.4 (1.2 to 1.6)	1.4 (1.1 to 1.6)	1.1 (0.7 to 1.5)	-2.1 (-3.8 to -0.4)	-26.2 (-43.1 to -6.4)
Ecuador	3.0 (2.9 to 3.2)	1.9 (1.6 to 2.3)	1.8 (1.4 to 2.2)	1.3 (0.8 to 2.0)	-2.5 (-4.2 to -0.8)	-30.6 (-46.4 to -12.0)
Peru	2.0 (1.8 to 2.2)	1.0 (0.9 to 1.1)	0.8 (0.7 to 1.0)	0.5 (0.4 to 0.7)	-4.5 (-6.1 to -2.7)	-48.5 (-59.8 to -33.4)
Caribbean						
Antigua and Barbuda	2.3 (1.7 to 3.2)	2.0 (1.4 to 2.6)	2.0 (1.4 to 2.7)	1.9 (1.1 to 3.0)	-0.5 (-2.5 to 1.5)	-6.8 (-31.4 to 25.8)
The Bahamas	2.0 (1.4 to 2.8)	1.8 (1.3 to 2.5)	1.8 (1.3 to 2.5)	1.8 (1.1 to 2.9)	-0.1 (-1.9 to 1.6)	-0.4 (-24.7 to 27.7)
Barbados	2.3 (1.6 to 3.1)	2.0 (1.4 to 2.7)	1.9 (1.4 to 2.7)	1.9 (1.1 to 3.1)	-0.5 (-2.4 to 1.6)	-5.9 (-29.9 to 26.7)
Belize	3.1 (2.4 to 3.9)	2.3 (2.1 to 2.5)	2.3 (2.1 to 2.5)	2.3 (1.9 to 2.6)	-0.2 (-1.3 to 0.8)	-2.3 (-18.2 to 12.5)
Bermuda	1.9 (1.3 to 2.6)	1.7 (1.2 to 2.3)	1.7 (1.2 to 2.3)	1.6 (1.0 to 2.6)	-0.2 (-2.0 to 1.8)	-2.1 (-25.7 to 30.7)
Cuba	2.6 (2.4 to 2.7)	2.0 (1.6 to 2.4)	1.9 (1.5 to 2.3)	1.6 (1.0 to 2.4)	-1.5 (-3.1 to 0.2)	-19.6 (-37.4 to 2.5)
Dominica	2.8 (2.0 to 3.8)	2.2 (1.5 to 3.0)	2.1 (1.5 to 3.0)	1.9 (1.1 to 3.2)	-1.1 (-3.0 to 1.0)	-14.3 (-36.4 to 16.9)
Dominican Republic	2.3 (2.1 to 2.5)	2.1 (1.9 to 2.4)	2.1 (1.8 to 2.5)	2.0 (1.4 to 2.9)	-0.4 (-2.0 to 1.3)	-4.6 (-26.3 to 21.7)
Grenada	3.0 (2.1 to 4.2)	2.4 (1.7 to 3.3)	2.3 (1.6 to 3.2)	2.0 (1.2 to 3.2)	-1.2 (-3.1 to 0.7)	-15.4 (-36.8 to 11.8)
Guyana	11.3 (10.8 to 11.9)	6.3 (5.5 to 7.1)	6.2 (5.3 to 7.1)	4.2 (3.1 to 5.4)	-2.7 (-3.9 to -1.6)	-33.3 (-44.1 to -21.2)
Haiti	7.5 (7.1 to 8.0)	4.5 (4.3 to 4.7)	4.2 (3.9 to 4.5)	2.8 (2.4 to 3.3)	-3.1 (-4.0 to -2.2)	-37.3 (-45.5 to -27.9)
Jamaica	2.9 (2.5 to 3.4)	2.3 (1.8 to 2.9)	2.2 (1.7 to 2.9)	1.8 (1.1 to 2.9)	-1.7 (-3.4 to 0.0)	-21.7 (-39.7 to 0.4)
Puerto Rico	1.9 (1.3 to 2.6)	1.6 (1.2 to 2.2)	1.6 (1.1 to 2.2)	1.5 (0.9 to 2.5)	-0.6 (-2.6 to 1.5)	-6.9 (-32.1 to 26.1)
Saint Lucia	3.3 (2.4 to 4.4)	2.5 (1.8 to 3.3)	2.4 (1.8 to 3.3)	2.1 (1.2 to 3.2)	-1.4 (-3.3 to 0.7)	-17.8 (-38.7 to 10.6)
Saint Vincent and the Grenadines	3.3 (2.2 to 4.6)	2.6 (1.8 to 3.5)	2.5 (1.8 to 3.5)	2.3 (1.4 to 3.4)	-1.0 (-2.9 to 0.8)	-13.2 (-34.9 to 13.3)
Suriname	5.8 (5.0 to 6.6)	4.4 (3.6 to 5.2)	4.2 (3.4 to 5.2)	3.5 (2.2 to 5.2)	-1.5 (-3.4 to 0.3)	-19.7 (-39.6 to 4.8)
Trinidad and Tobago	4.7 (3.8 to 5.7)	3.4 (2.5 to 4.4)	3.3 (2.4 to 4.5)	2.8 (1.7 to 4.5)	-1.3 (-3.1 to 0.8)	-16.4 (-36.7 to 12.4)
Virgin Islands, U.S.	1.9 (1.3 to 2.6)	1.7 (1.2 to 2.3)	1.6 (1.2 to 2.3)	1.6 (0.9 to 2.5)	-0.5 (-2.5 to 1.5)	-6.0 (-31.7 to 25.4)
Central Latin America						
Colombia	1.9 (1.6 to 2.2)	0.9 (0.8 to 1.1)	0.9 (0.7 to 1.1)	0.6 (0.4 to 0.9)	-3.3 (-5.1 to -1.6)	-38.9 (-53.4 to -20.9)
Costa Rica	1.9 (1.4 to 2.6)	1.4 (1.1 to 1.9)	1.4 (1.0 to 1.9)	1.1 (0.7 to 1.8)	-1.7 (-3.6 to 0.3)	-21.7 (-41.4 to 4.0)
El Salvador	1.9 (1.7 to 2.0)	1.5 (1.3 to 1.8)	1.5 (1.3 to 1.8)	1.5 (1.0 to 2.0)	-0.2 (-1.8 to 1.3)	-2.1 (-23.7 to 20.9)
Guatemala	3.7 (3.6 to 4.0)	1.1 (1.0 to 1.3)	1.1 (0.9 to 1.3)	0.5 (0.3 to 0.7)	-5.8 (-7.2 to -4.4)	-58.0 (-66.1 to -48.3)
Honduras	2.0 (1.9 to 2.1)	1.3 (1.1 to 1.6)	1.3 (1.0 to 1.6)	1.0 (0.7 to 1.5)	-1.8 (-3.5 to 0.1)	-23.0 (-40.7 to 0.9)
Mexico	3.3 (3.1 to 3.5)	2.0 (1.9 to 2.1)	1.8 (1.7 to 1.9)	1.1 (0.9 to 1.2)	-4.3 (-4.7 to -3.9)	-47.6 (-50.9 to -44.0)
Nicaragua	2.4 (2.1 to 2.6)	1.8 (1.6 to 2.1)	1.8 (1.5 to 2.2)	1.8 (1.1 to 2.6)	-0.4 (-2.3 to 1.4)	-5.3 (-29.4 to 22.6)
Panama	1.9 (1.6 to 2.2)	1.2 (0.9 to 1.5)	1.1 (0.9 to 1.4)	0.8 (0.5 to 1.3)	-2.7 (-4.7 to -0.6)	-32.1 (-50.6 to -8.8)

	Estimate in 2000 (95% UIs)	Estimate in 2015 (95% UIs)	Estimate in 2017 (95% UIs)	Estimate in 2030 (95% UIs)	Annualised rate of change, 2015-2030 (95% UIs)	Percent change, 2015-2030 (95% UIs)
Venezuela	4.3 (4.2 to 4.5)	5.2 (4.5 to 5.8)	5.1 (4.3 to 6.1)	5.5 (3.8 to 7.7)	0.3 (-1.3 to 1.9)	6.2 (-17.4 to 32.6)
Tropical Latin America						
Brazil	1.6 (1.4 to 1.7)	1.3 (1.2 to 1.4)	1.2 (1.1 to 1.3)	1.0 (0.9 to 1.1)	-1.6 (-2.2 to -1.1)	-21.6 (-27.7 to -15.1)
Paraguay	1.5 (1.3 to 1.7)	1.1 (1.0 to 1.3)	1.1 (0.9 to 1.3)	0.8 (0.6 to 1.2)	-2.2 (-3.9 to -0.1)	-27.1 (-44.4 to -1.4)
North Africa and Middle East						
North Africa and Middle East						
Afghanistan	8.3 (7.8 to 9.2)	5.4 (4.2 to 6.5)	5.1 (3.8 to 6.4)	3.5 (1.9 to 5.4)	-3.1 (-5.4 to -1.1)	-36.3 (-55.2 to -15.3)
Algeria	11.6 (11.4 to 11.8)	4.8 (4.2 to 5.4)	4.4 (3.7 to 5.0)	2.5 (1.9 to 3.4)	-4.3 (-5.5 to -3.0)	-47.3 (-56.3 to -36.1)
Bahrain	5.4 (5.0 to 5.8)	2.5 (2.3 to 2.7)	2.4 (2.2 to 2.6)	1.5 (1.2 to 1.8)	-3.6 (-4.8 to -2.4)	-41.3 (-51.2 to -30.3)
Egypt	6.7 (6.5 to 6.9)	5.8 (5.4 to 6.3)	5.5 (4.9 to 6.1)	4.8 (3.8 to 5.9)	-1.3 (-2.5 to -0.2)	-17.7 (-30.8 to -3.1)
Iran	5.4 (5.3 to 5.6)	4.4 (3.7 to 5.2)	4.4 (3.5 to 5.4)	4.1 (2.7 to 5.8)	-0.6 (-2.1 to 0.9)	-7.8 (-27.5 to 13.9)
Iraq	8.8 (8.7 to 9.0)	6.4 (5.9 to 6.9)	6.3 (5.6 to 7.0)	5.4 (4.1 to 6.7)	-1.2 (-2.4 to -0.1)	-16.7 (-30.3 to -2.1)
Jordan	2.6 (2.2 to 3.0)	2.2 (1.8 to 2.7)	2.1 (1.7 to 2.7)	1.9 (1.2 to 2.8)	-1.2 (-3.1 to 0.5)	-16.3 (-37.1 to 7.6)
Kuwait	2.1 (1.9 to 2.3)	2.1 (1.8 to 2.5)	2.0 (1.7 to 2.5)	2.0 (1.3 to 3.1)	-0.2 (-2.1 to 1.7)	-2.4 (-27.0 to 28.4)
Lebanon	4.9 (3.9 to 5.9)	3.7 (3.0 to 4.6)	3.6 (2.8 to 4.6)	2.9 (1.7 to 4.8)	-1.9 (-4.1 to 0.5)	-24.0 (-45.8 to 7.7)
Libya	4.6 (4.0 to 5.1)	5.3 (4.3 to 6.4)	5.3 (4.2 to 6.7)	5.9 (3.6 to 8.9)	0.5 (-1.5 to 2.3)	9.5 (-19.6 to 42.1)
Morocco	7.6 (7.0 to 8.4)	3.1 (2.6 to 3.8)	2.8 (2.3 to 3.6)	1.5 (1.0 to 2.1)	-5.1 (-6.3 to -3.7)	-53.1 (-61.0 to -42.5)
Palestine	5.2 (5.0 to 5.6)	2.6 (2.2 to 2.9)	2.2 (1.9 to 2.6)	1.4 (1.0 to 1.8)	-4.3 (-5.7 to -2.8)	-47.1 (-57.6 to -34.3)
Oman	9.9 (9.4 to 10.6)	7.8 (7.5 to 8.1)	7.7 (7.2 to 8.2)	6.5 (5.6 to 7.7)	-1.2 (-2.2 to -0.3)	-16.8 (-27.6 to -4.6)
Qatar	2.7 (2.3 to 3.3)	2.7 (2.0 to 3.5)	2.6 (1.9 to 3.5)	2.7 (1.6 to 4.4)	0.0 (-1.8 to 1.8)	1.0 (-23.2 to 31.4)
Saudi Arabia	3.5 (2.6 to 4.5)	2.3 (1.7 to 3.2)	2.3 (1.7 to 3.2)	1.9 (1.1 to 3.0)	-1.6 (-3.5 to 0.3)	-20.8 (-40.9 to 4.4)
Sudan	27.1 (25.4 to 28.9)	15.3 (13.4 to 17.2)	14.2 (12.2 to 16.3)	8.5 (6.1 to 11.2)	-4.0 (-5.4 to -2.6)	-44.5 (-55.3 to -32.4)
Syria	7.0 (6.6 to 7.4)	11.3 (10.1 to 12.5)	11.4 (9.9 to 13.0)	13.8 (10.4 to 17.4)	1.3 (0.2 to 2.3)	21.5 (2.5 to 41.5)
Tunisia	4.0 (3.8 to 4.4)	1.9 (1.6 to 2.3)	2.0 (1.6 to 2.5)	1.5 (1.0 to 2.2)	-1.6 (-3.3 to 0.1)	-20.8 (-39.4 to 0.9)
Turkey	1.5 (1.3 to 1.9)	1.0 (0.7 to 1.3)	1.0 (0.7 to 1.3)	0.8 (0.5 to 1.2)	-1.7 (-3.5 to 0.2)	-21.4 (-40.7 to 3.2)
United Arab Emirates	12.1 (11.0 to 13.3)	8.8 (6.9 to 11.0)	8.6 (6.6 to 10.9)	7.3 (4.9 to 10.1)	-1.3 (-2.6 to -0.1)	-17.2 (-32.2 to -1.4)
Yemen	15.3 (14.7 to 15.9)	14.9 (14.4 to 15.3)	15.7 (14.8 to 16.6)	17.0 (15.2 to 18.7)	0.9 (0.3 to 1.4)	14.4 (4.7 to 23.0)
South Asia						
South Asia						
Bangladesh	13.3 (13.0 to 13.5)	12.6 (12.1 to 13.1)	12.4 (11.6 to 13.2)	12.0 (10.4 to 13.8)	-0.3 (-1.1 to 0.4)	-4.5 (-15.0 to 6.3)
Bhutan	4.1 (3.4 to 4.8)	4.0 (3.4 to 4.7)	3.8 (3.0 to 4.6)	3.4 (2.2 to 5.1)	-1.2 (-3.3 to 0.9)	-15.2 (-38.9 to 14.1)
India	19.2 (18.9 to 19.6)	16.2 (16.1 to 16.3)	15.7 (15.6 to 15.9)	13.4 (13.1 to 13.8)	-1.2 (-1.4 to -1.1)	-16.9 (-19.0 to -14.9)
Nepal	9.6 (9.0 to 10.2)	7.2 (6.7 to 7.8)	7.1 (6.4 to 7.8)	5.5 (4.5 to 6.8)	-1.8 (-2.7 to -0.8)	-23.3 (-33.6 to -11.5)
Pakistan	9.0 (8.2 to 9.8)	12.9 (12.2 to 13.6)	12.2 (11.3 to 13.1)	12.4 (10.7 to 14.3)	-0.3 (-1.1 to 0.5)	-3.7 (-15.1 to 8.3)
Southeast Asia, East Asia, and Oceania						
East Asia						
China	3.0 (2.1 to 4.1)	1.9 (1.4 to 2.7)	1.9 (1.4 to 2.6)	1.4 (0.8 to 2.3)	-2.1 (-4.0 to -0.3)	-26.5 (-45.1 to -3.7)
North Korea	11.8 (11.5 to 12.1)	5.4 (4.6 to 6.1)	5.3 (4.4 to 6.2)	4.0 (2.9 to 5.2)	-2.1 (-3.3 to -0.8)	-26.3 (-39.5 to -11.2)
Taiwan (Province of China)	2.1 (1.5 to 2.9)	1.6 (1.2 to 2.3)	1.6 (1.1 to 2.3)	1.4 (0.8 to 2.4)	-1.0 (-3.1 to 1.1)	-12.4 (-36.8 to 18.4)
Oceania						
American Samoa	4.6 (3.3 to 6.3)	4.3 (3.1 to 5.8)	4.2 (3.0 to 5.8)	4.1 (2.4 to 6.4)	-0.3 (-2.2 to 1.4)	-4.0 (-28.6 to 23.9)
Federated States of Micronesia	6.3 (4.6 to 8.2)	5.4 (3.9 to 7.1)	5.3 (3.8 to 7.0)	4.9 (3.0 to 7.6)	-0.7 (-2.5 to 1.1)	-8.8 (-31.6 to 18.6)
Fiji	7.9 (6.9 to 9.0)	6.8 (5.2 to 8.6)	6.7 (5.0 to 8.6)	5.9 (3.6 to 9.3)	-1.0 (-2.8 to 0.8)	-13.1 (-34.1 to 12.6)
Guam	3.8 (2.8 to 5.2)	3.6 (2.5 to 4.9)	3.5 (2.5 to 4.9)	3.5 (2.1 to 5.6)	-0.3 (-2.1 to 1.6)	-3.8 (-27.0 to 26.6)
Kiribati	10.9 (8.6 to 13.6)	9.4 (7.3 to 11.8)	9.1 (7.0 to 11.6)	8.2 (5.4 to 11.8)	-1.0 (-2.5 to 0.5)	-13.3 (-31.6 to 7.5)
Marshall Islands	7.2 (5.3 to 9.4)	5.8 (4.2 to 7.5)	5.7 (4.1 to 7.4)	5.0 (3.1 to 7.5)	-1.1 (-2.7 to 0.6)	-14.4 (-33.3 to 10.1)
Northern Mariana Islands	3.8 (2.9 to 5.1)	3.8 (2.7 to 5.1)	3.8 (2.7 to 5.2)	4.0 (2.3 to 6.4)	0.2 (-1.7 to 2.1)	4.6 (-22.2 to 36.9)
Papua New Guinea	17.2 (15.9 to 18.6)	12.7 (11.3 to 14.3)	11.8 (10.1 to 13.7)	9.2 (6.5 to 12.5)	-2.2 (-3.7 to -0.7)	-27.9 (-42.2 to -10.3)
Samoa	2.4 (1.9 to 3.0)	2.7 (2.0 to 3.7)	2.7 (1.9 to 3.7)	2.9 (1.8 to 4.6)	0.4 (-1.3 to 2.1)	6.4 (-18.0 to 37.2)
Solomon Islands	6.2 (5.2 to 7.5)	5.2 (4.1 to 6.5)	5.1 (3.9 to 6.5)	4.5 (2.8 to 6.6)	-1.1 (-2.8 to 0.6)	-14.7 (-34.4 to 8.9)
Tonga	7.3 (5.8 to 8.9)	5.9 (5.3 to 6.6)	5.8 (5.0 to 6.6)	5.2 (3.8 to 7.0)	-0.9 (-2.5 to 0.7)	-12.1 (-31.5 to 11.8)
Vanuatu	7.2 (6.0 to 8.5)	5.8 (4.7 to 7.2)	5.7 (4.5 to 7.2)	5.0 (3.3 to 7.5)	-1.0 (-2.7 to 0.7)	-13.8 (-33.4 to 10.8)
Southeast Asia						
Cambodia	15.0 (13.9 to 16.1)	9.2 (8.3 to 10.1)	8.8 (7.6 to 9.9)	6.1 (4.5 to 7.8)	-2.8 (-4.1 to -1.6)	-34.0 (-45.8 to -21.6)
Indonesia	14.9 (14.4 to 15.5)	13.2 (12.9 to 13.6)	12.7 (12.3 to 13.1)	10.7 (10.0 to 11.4)	-1.4 (-1.8 to -1.1)	-19.1 (-23.2 to -15.3)
Laos	6.7 (6.4 to 7.2)	6.1 (5.4 to 6.8)	6.0 (5.2 to 7.0)	5.5 (4.1 to 7.4)	-0.7 (-2.0 to 0.7)	-9.5 (-26.1 to 10.7)
Malaysia	9.3 (8.1 to 10.6)	9.5 (8.7 to 10.2)	9.6 (8.8 to 10.4)	9.9 (8.3 to 11.7)	0.3 (-0.7 to 1.2)	4.2 (-10.1 to 19.8)
Maldives	16.7 (15.7 to 17.7)	9.2 (7.9 to 10.8)	8.9 (7.3 to 10.6)	5.8 (4.0 to 7.9)	-3.2 (-4.7 to -1.7)	-38.0 (-50.5 to -23.1)
Mauritius	13.1 (11.9 to 14.5)	11.2 (8.8 to 13.8)	11.0 (8.6 to 13.8)	9.9 (6.6 to 14.1)	-0.9 (-2.3 to 0.4)	-12.4 (-29.5 to 6.5)
Myanmar	11.4 (10.7 to 12.2)	7.3 (6.7 to 7.8)	6.9 (6.2 to 7.7)	4.8 (3.9 to 6.0)	-2.7 (-3.8 to -1.7)	-33.4 (-43.6 to -22.4)
Philippines	7.6 (7.3 to 7.9)	6.5 (5.8 to 7.3)	6.5 (5.5 to 7.5)	6.1 (4.3 to 8.2)	-0.5 (-2.1 to 0.9)	-7.2 (-26.6 to 14.1)
Sri Lanka	16.3 (15.9 to 16.9)	11.8 (10.4 to 13.2)	11.2 (9.4 to 13.1)	8.5 (6.0 to 11.5)	-2.2 (-3.7 to -0.9)	-28.0 (-42.8 to -12.3)
Seychelles	4.6 (3.6 to 5.9)	4.7 (3.9 to 5.6)	4.6 (3.7 to 5.6)	4.7 (3.2 to 6.6)	-0.1 (-1.8 to 1.6)	-0.1 (-23.4 to 26.9)
Thailand	5.7 (5.4 to 6.2)	6.6 (5.9 to 7.2)	6.7 (5.9 to 7.6)	8.0 (5.9 to 10.0)	1.2 (-0.1 to 2.4)	21.0 (-1.8 to 42.6)
Timor-Leste	22.3 (20.5 to 24.1)	16.4 (15.2 to 17.6)	15.5 (13.9 to 17.2)	11.5 (9.2 to 14.1)	-2.4 (-3.6 to -1.3)	-30.2 (-41.9 to -18.1)
Vietnam	10.3 (10.2 to 10.5)	5.9 (5.6 to 6.2)	5.7 (5.2 to 6.2)	3.9 (3.2 to 4.7)	-2.8 (-3.8 to -1.8)	-34.4 (-43.4 to -24.0)
Sub-Saharan Africa						
Central Sub-Saharan Africa						
Angola	8.6 (7.8 to 9.6)	5.5 (4.8 to 6.2)	5.2 (4.5 to 6.0)	3.9 (2.9 to 5.0)	-2.4 (-3.6 to -1.1)	-29.5 (-41.4 to -15.7)
Central African Republic	9.8 (9.3 to 10.3)	7.2 (6.4 to 8.2)	7.0 (6.1 to 8.1)	5.6 (4.2 to 7.4)	-1.7 (-3.0 to -0.4)	-22.2 (-36.1 to -6.5)
Congo	5.8 (5.2 to 6.4)	8.4 (8.1 to 8.7)	8.5 (8.0 to 8.9)	10.7 (9.2 to 12.3)	1.6 (0.7 to 2.4)	26.6 (11.6 to 42.7)
Democratic Republic of the Congo	11.2 (10.6 to 11.8)	7.8 (7.1 to 8.7)	7.2 (6.3 to 8.2)	5.2 (3.9 to 6.7)	-2.8 (-4.0 to -1.5)	-33.7 (-45.5 to -20.6)
Equatorial Guinea	6.6 (5.8 to 7.5)	3.2 (3.0 to 3.5)	2.9 (2.5 to 3.3)	1.6 (1.2 to 2.2)	-4.7 (-6.4 to -3.0)	-50.5 (-61.6 to -36.4)
Gabon	2.8 (2.3 to 3.5)	2.7 (2.2 to 3.4)	2.6 (2.0 to 3.3)	2.3 (1.6 to 3.6)	-1.1 (-2.7 to 0.7)	-14.7 (-33.2 to 11.3)
Eastern Sub-Saharan Africa						
Burundi	9.1 (8.7 to 9.5)	7.4 (6.5 to 8.4)	7.3 (6.3 to 8.6)	6.5 (4.8 to 8.8)	-0.9 (-2.1 to 0.3)	-12.5 (-27.1 to 5.1)
Comoros	12.1 (10.8 to 13.3)	8.8 (7.6 to 10.1)	8.5 (7.1 to 10.1)	6.8 (4.8 to 9.3)	-1.8 (-3.2 to -0.3)	-22.7 (-38.1 to -4.5)
Djibouti	20.8 (18.9 to 22.8)	17.3 (16.0 to 18.7)	16.6 (14.9 to 18.4)	14.0 (11.0 to 17.3)	-1.5 (-2.6 to -0.4)	-19.6 (-32.0 to -5.6)
Eritrea	14.1 (13.4 to 14.9)	10.5 (8.7 to 12.4)	10.3 (8.2 to 12.4)	8.3 (5.5 to 11.6)	-1.7 (-3.2 to -0.3)	-21.7 (-38.1 to -5.1)
Ethiopia	12.4 (11.7 to 13.1)	9.5 (9.0 to 10.0)	9.2 (8.6 to 9.8)	7.3 (6.4 to 8.3)	-1.7 (-2.3 to -1.1)	-22.8 (-29.3 to -15.4)

	Estimate in 2000 (95% UIs)	Estimate in 2015 (95% UIs)	Estimate in 2017 (95% UIs)	Estimate in 2030 (95% UIs)	Annualised rate of change, 2015-2030 (95% UIs)	Percent change, 2015-2030 (95% UIs)
Kenya	7.2 (6.5 to 8.1)	5.4 (4.8 to 6.0)	5.2 (4.7 to 5.9)	4.2 (3.4 to 5.0)	-1.7 (-2.5 to -0.9)	-22.6 (-31.7 to -13.2)
Madagascar	9.8 (9.2 to 10.4)	9.8 (8.1 to 11.6)	9.6 (7.7 to 11.6)	9.2 (6.0 to 13.0)	-0.5 (-2.2 to 1.0)	-6.6 (-27.9 to 15.5)
Malawi	6.2 (5.8 to 6.6)	3.6 (3.2 to 4.0)	3.6 (3.1 to 4.0)	2.7 (2.0 to 3.4)	-2.0 (-3.4 to -0.8)	-25.9 (-39.6 to -11.7)
Mozambique	7.8 (7.3 to 8.2)	4.3 (3.8 to 4.8)	4.1 (3.5 to 4.9)	2.8 (2.0 to 4.0)	-2.8 (-4.4 to -1.1)	-34.0 (-48.0 to -15.3)
Rwanda	11.1 (10.7 to 11.5)	2.9 (2.5 to 3.3)	2.6 (2.3 to 3.1)	1.1 (0.9 to 1.5)	-6.1 (-7.1 to -5.2)	-60.0 (-65.4 to -53.9)
Somalia	17.9 (17.4 to 18.4)	12.8 (11.3 to 14.4)	12.6 (10.8 to 14.5)	10.1 (7.3 to 13.6)	-1.6 (-3.0 to -0.3)	-21.5 (-36.7 to -3.9)
South Sudan	23.2 (21.4 to 24.9)	18.1 (16.5 to 19.7)	17.0 (15.0 to 19.2)	12.8 (9.9 to 16.2)	-2.3 (-3.5 to -1.2)	-29.3 (-41.0 to -16.5)
Tanzania	5.6 (5.3 to 5.8)	4.3 (4.1 to 4.6)	4.5 (4.1 to 4.9)	4.2 (3.4 to 5.1)	-0.3 (-1.2 to 0.7)	-4.2 (-16.8 to 10.3)
Uganda	5.0 (4.4 to 5.7)	3.7 (3.2 to 4.2)	3.5 (3.0 to 4.2)	2.9 (2.0 to 4.0)	-1.7 (-3.4 to 0.0)	-21.7 (-39.8 to 0.1)
Zambia	5.8 (5.5 to 6.2)	5.9 (5.5 to 6.3)	5.9 (5.3 to 6.6)	6.1 (4.8 to 7.6)	0.2 (-0.9 to 1.3)	3.6 (-12.6 to 21.1)
Southern Sub-Saharan Africa						
Botswana	8.0 (7.2 to 8.9)	6.0 (5.0 to 7.2)	5.9 (4.7 to 7.2)	4.8 (3.1 to 7.2)	-1.5 (-3.2 to 0.1)	-19.9 (-37.9 to 1.8)
Lesotho	3.6 (3.4 to 3.9)	2.2 (1.9 to 2.7)	2.4 (1.9 to 2.9)	2.2 (1.6 to 3.2)	0.0 (-1.4 to 1.3)	0.6 (-19.4 to 21.3)
Namibia	8.8 (8.0 to 9.7)	8.4 (7.6 to 9.2)	8.0 (6.9 to 9.1)	7.2 (5.5 to 9.4)	-1.1 (-2.3 to 0.3)	-14.6 (-29.7 to 4.1)
South Africa	5.2 (4.8 to 5.7)	3.5 (3.2 to 3.8)	3.3 (3.0 to 3.6)	2.2 (1.9 to 2.6)	-3.1 (-3.8 to -2.3)	-37.0 (-43.6 to -29.7)
Swaziland	1.8 (1.5 to 2.2)	1.4 (1.1 to 1.7)	1.4 (1.1 to 1.7)	1.2 (0.8 to 1.7)	-1.2 (-2.8 to 0.5)	-15.5 (-33.9 to 8.5)
Zimbabwe	5.2 (4.6 to 5.8)	2.8 (2.5 to 3.2)	2.6 (2.2 to 3.0)	1.7 (1.2 to 2.3)	-3.4 (-4.7 to -2.0)	-39.3 (-50.6 to -26.2)
Western Sub-Saharan Africa						
Benin	8.7 (8.1 to 9.3)	10.0 (9.3 to 10.7)	9.3 (8.4 to 10.3)	8.8 (7.2 to 10.8)	-0.8 (-1.9 to 0.3)	-11.3 (-24.2 to 4.4)
Burkina Faso	17.9 (17.3 to 18.4)	13.8 (13.2 to 14.3)	12.6 (11.9 to 13.4)	8.7 (7.5 to 9.8)	-3.1 (-3.8 to -2.5)	-37.1 (-43.3 to -31.0)
Cameroon	6.8 (6.2 to 7.4)	5.0 (4.6 to 5.5)	4.8 (4.2 to 5.4)	3.8 (2.9 to 4.8)	-2.0 (-3.3 to -0.8)	-25.2 (-39.2 to -10.8)
Cape Verde	5.7 (4.8 to 6.6)	3.4 (2.5 to 4.4)	3.2 (2.3 to 4.2)	2.2 (1.3 to 3.5)	-3.0 (-4.7 to -1.2)	-35.3 (-50.5 to -17.0)
Chad	14.9 (14.0 to 15.8)	14.1 (13.1 to 15.1)	13.7 (12.5 to 14.9)	12.4 (10.5 to 14.5)	-0.9 (-1.7 to -0.1)	-11.9 (-22.3 to -1.2)
Cote d'Ivoire	7.8 (7.0 to 8.6)	6.2 (5.4 to 7.2)	6.0 (5.0 to 7.1)	5.0 (3.5 to 6.9)	-1.5 (-3.1 to -0.1)	-19.8 (-37.5 to -1.0)
The Gambia	9.2 (8.3 to 10.2)	9.9 (9.1 to 10.8)	10.1 (8.9 to 11.4)	11.6 (9.0 to 14.3)	1.0 (-0.2 to 2.0)	16.1 (-3.2 to 35.0)
Ghana	10.1 (9.5 to 10.6)	7.3 (6.5 to 8.2)	6.7 (5.8 to 7.7)	4.8 (3.6 to 6.3)	-2.9 (-4.2 to -1.6)	-34.6 (-46.6 to -21.6)
Guinea	10.4 (9.9 to 11.0)	7.6 (6.8 to 8.4)	7.6 (6.6 to 8.6)	6.5 (4.8 to 8.2)	-1.1 (-2.5 to 0.0)	-15.3 (-30.9 to 0.7)
Guinea-Bissau	9.1 (8.1 to 10.1)	6.8 (6.3 to 7.3)	6.4 (5.7 to 7.2)	4.8 (3.8 to 6.2)	-2.3 (-3.6 to -1.1)	-28.9 (-41.9 to -14.8)
Liberia	7.0 (5.9 to 8.0)	5.5 (4.7 to 6.4)	5.3 (4.3 to 6.3)	4.4 (3.0 to 6.2)	-1.5 (-3.1 to 0.1)	-19.9 (-37.5 to 1.0)
Mali	14.9 (14.1 to 15.7)	11.4 (10.9 to 12.0)	12.4 (11.6 to 13.2)	12.2 (10.9 to 13.6)	0.4 (-0.0 to 0.9)	7.0 (-0.6 to 14.3)
Mauritania	24.4 (23.1 to 26.0)	24.3 (23.7 to 24.9)	26.6 (26.0 to 27.3)	29.4 (28.3 to 30.6)	1.3 (1.1 to 1.5)	21.1 (17.5 to 24.4)
Niger	17.5 (16.3 to 18.7)	16.5 (15.2 to 17.8)	15.9 (14.1 to 17.6)	14.7 (11.5 to 17.7)	-0.8 (-1.9 to 0.2)	-11.1 (-25.3 to 2.4)
Nigeria	11.7 (10.8 to 12.6)	10.2 (9.7 to 10.8)	10.1 (9.5 to 10.7)	9.2 (8.2 to 10.2)	-0.7 (-1.3 to -0.2)	-10.3 (-17.4 to -3.0)
Sao Tome and Principe	8.2 (7.5 to 9.0)	6.6 (5.8 to 7.4)	5.6 (4.7 to 6.5)	3.7 (2.7 to 4.9)	-3.9 (-5.1 to -2.6)	-44.4 (-53.8 to -32.3)
Senegal	8.8 (8.0 to 9.7)	8.4 (7.8 to 9.0)	7.8 (7.1 to 8.6)	6.7 (5.5 to 7.9)	-1.6 (-2.5 to -0.6)	-20.8 (-31.3 to -9.1)
Sierra Leone	11.1 (10.3 to 12.0)	7.7 (6.9 to 8.5)	7.5 (6.5 to 8.5)	5.9 (4.5 to 7.5)	-1.8 (-3.1 to -0.6)	-23.5 (-37.0 to -8.8)
Togo	8.9 (8.2 to 9.5)	8.9 (8.6 to 9.2)	10.0 (9.5 to 10.5)	11.7 (10.9 to 12.5)	1.8 (1.5 to 2.1)	31.7 (25.8 to 38.0)
**Indicator 2.2.2b: Prevalence of overweight in children aged 2-4 (%)**						
Central Europe, Eastern Europe, and Central Asia						
Central Asia						
Armenia	24.7 (20.4 to 29.0)	32.9 (27.7 to 38.8)	33.5 (27.8 to 40.4)	41.0 (30.3 to 53.9)	1.4 (0.2 to 2.5)	24.4 (3.4 to 44.9)
Azerbaijan	16.1 (12.6 to 20.1)	33.5 (27.1 to 40.5)	34.1 (27.5 to 41.4)	45.6 (33.7 to 58.3)	2.0 (1.0 to 2.9)	36.0 (16.0 to 54.7)
Georgia	26.9 (23.0 to 31.1)	33.8 (27.9 to 40.8)	34.4 (28.3 to 42.1)	38.4 (26.9 to 51.2)	0.8 (-0.4 to 1.9)	13.1 (-6.3 to 32.8)
Kazakhstan	18.4 (15.9 to 20.8)	26.2 (21.9 to 31.2)	27.0 (22.2 to 32.5)	33.2 (24.2 to 44.6)	1.5 (0.3 to 2.6)	26.4 (4.1 to 48.2)
Kyrgyzstan	20.6 (17.4 to 23.8)	16.8 (13.4 to 20.6)	17.2 (13.5 to 21.5)	15.8 (10.1 to 23.2)	-0.5 (-2.3 to 1.1)	-6.7 (-28.9 to 17.7)
Mongolia	22.9 (19.2 to 27.0)	23.5 (19.7 to 27.8)	24.1 (19.7 to 28.8)	26.3 (17.7 to 36.1)	0.7 (-0.9 to 2.0)	11.2 (-12.7 to 36.0)
Tajikistan	13.3 (10.5 to 16.6)	15.5 (12.4 to 18.7)	15.9 (12.6 to 19.7)	18.0 (11.6 to 25.6)	0.9 (-0.9 to 2.5)	14.8 (-13.2 to 44.7)
Turkmenistan	23.7 (17.8 to 30.5)	29.8 (22.9 to 37.0)	30.8 (23.7 to 38.2)	36.5 (24.3 to 48.7)	1.3 (-0.2 to 2.5)	22.0 (-2.6 to 44.9)
Uzbekistan	29.2 (26.1 to 32.2)	27.2 (22.3 to 32.9)	28.0 (22.6 to 34.2)	28.4 (19.1 to 40.2)	0.2 (-1.2 to 1.5)	3.7 (-16.7 to 25.8)
Central Europe						
Albania	48.3 (45.0 to 51.8)	32.6 (27.2 to 38.0)	32.8 (26.7 to 39.3)	27.5 (19.0 to 37.9)	-1.2 (-2.5 to 0.1)	-16.2 (-31.6 to 1.2)
Bosnia and Herzegovina	27.1 (23.9 to 30.6)	30.5 (26.2 to 35.6)	30.6 (25.7 to 36.4)	32.8 (23.6 to 44.0)	0.4 (-1.0 to 1.6)	7.0 (-13.7 to 26.7)
Bulgaria	26.3 (19.9 to 33.0)	29.3 (22.3 to 36.6)	30.1 (22.8 to 37.3)	34.0 (22.5 to 46.7)	0.9 (-0.6 to 2.3)	15.6 (-8.5 to 40.9)
Croatia	24.8 (18.9 to 31.5)	30.2 (23.6 to 38.1)	30.6 (23.9 to 38.0)	35.3 (24.2 to 47.7)	1.0 (-0.4 to 2.3)	16.5 (-6.5 to 41.8)
Czech Republic	27.2 (20.4 to 34.3)	28.6 (22.1 to 35.5)	28.9 (22.2 to 35.9)	32.3 (21.6 to 44.5)	0.8 (-0.8 to 2.2)	12.7 (-11.3 to 40.0)
Hungary	27.0 (20.5 to 34.2)	27.8 (21.4 to 35.0)	28.2 (21.7 to 35.7)	30.2 (19.5 to 42.7)	0.5 (-1.2 to 1.9)	8.5 (-16.2 to 33.1)
Macedonia	22.8 (18.9 to 27.6)	26.5 (22.1 to 31.6)	26.9 (21.8 to 32.2)	30.5 (20.4 to 41.3)	0.9 (-0.7 to 2.1)	14.6 (-10.4 to 37.7)
Montenegro	25.2 (21.3 to 29.8)	35.5 (31.3 to 39.8)	36.2 (31.4 to 41.0)	45.4 (35.1 to 55.4)	1.6 (0.6 to 2.5)	27.7 (8.8 to 45.1)
Poland	20.4 (15.2 to 26.7)	23.6 (17.9 to 30.2)	24.1 (18.1 to 30.6)	27.6 (18.1 to 40.0)	1.0 (-0.6 to 2.5)	16.6 (-8.8 to 45.4)
Romania	24.2 (18.2 to 31.2)	27.1 (20.3 to 34.8)	27.8 (21.0 to 35.5)	32.3 (21.4 to 45.3)	1.1 (-0.3 to 2.5)	19.1 (-4.9 to 46.2)
Serbia	24.1 (20.2 to 28.2)	23.0 (20.1 to 26.1)	23.1 (19.6 to 26.7)	22.0 (15.7 to 29.6)	-0.4 (-1.8 to 1.1)	-4.8 (-23.9 to 17.4)
Slovakia	21.1 (15.8 to 27.3)	29.0 (22.5 to 36.3)	29.5 (22.5 to 36.8)	36.8 (25.0 to 49.5)	1.6 (0.2 to 2.8)	27.0 (3.0 to 52.3)
Slovenia	27.6 (21.1 to 34.9)	32.9 (25.2 to 40.7)	33.3 (25.4 to 41.0)	38.6 (26.3 to 51.1)	1.0 (-0.4 to 2.3)	17.0 (-6.5 to 40.9)
Eastern Europe						
Belarus	14.3 (11.7 to 17.7)	20.9 (16.5 to 26.1)	21.6 (16.8 to 27.2)	28.6 (18.6 to 40.2)	2.0 (0.4 to 3.2)	35.5 (6.8 to 62.0)
Estonia	18.1 (13.1 to 23.8)	26.9 (20.5 to 33.7)	27.3 (20.7 to 34.3)	34.8 (24.1 to 46.9)	1.7 (0.4 to 2.9)	29.3 (5.6 to 55.6)
Latvia	14.8 (10.8 to 19.5)	21.2 (15.7 to 27.4)	21.7 (16.2 to 28.1)	26.9 (17.5 to 38.8)	1.5 (-0.1 to 3.0)	26.8 (-1.4 to 56.4)
Lithuania	11.5 (8.5 to 15.1)	20.1 (15.0 to 25.9)	20.4 (15.3 to 26.4)	27.5 (18.2 to 38.5)	2.0 (0.5 to 3.4)	36.0 (8.1 to 66.9)
Moldova	7.8 (5.7 to 10.2)	9.7 (7.5 to 12.2)	10.1 (7.8 to 12.8)	12.7 (7.9 to 18.9)	1.7 (-0.3 to 3.6)	30.7 (-3.9 to 70.9)
Russia	23.0 (18.6 to 28.0)	25.0 (19.1 to 31.3)	25.3 (19.1 to 32.1)	27.6 (17.7 to 38.9)	0.6 (-1.1 to 1.9)	9.7 (-14.6 to 32.2)
Ukraine	13.0 (9.3 to 17.5)	16.4 (11.9 to 21.5)	16.5 (11.9 to 21.7)	19.3 (11.8 to 29.2)	1.0 (-0.9 to 2.7)	17.4 (-13.0 to 49.9)
High-income						
Australasia						
Australia	18.2 (15.4 to 21.3)	23.9 (19.1 to 29.4)	24.4 (19.1 to 30.2)	30.2 (20.1 to 41.7)	1.5 (0.2 to 2.7)	25.8 (2.3 to 50.0)
New Zealand	26.4 (20.2 to 32.9)	31.4 (25.3 to 38.0)	31.9 (25.5 to 38.5)	37.4 (26.2 to 49.4)	1.1 (-0.4 to 2.4)	18.8 (-5.2 to 42.4)
High-income Asia Pacific						
Brunei	12.6 (9.1 to 16.9)	21.5 (15.8 to 27.8)	21.8 (16.1 to 28.2)	29.4 (19.4 to 41.6)	2.0 (0.5 to 3.5)	36.4 (8.0 to 68.7)
Japan	10.6 (8.4 to 13.0)	13.9 (10.5 to 17.5)	14.2 (10.7 to 18.1)	17.5 (10.5 to 26.0)	1.5 (-0.3 to 3.0)	25.7 (-3.8 to 57.6)
South Korea	16.5 (12.1 to 21.3)	25.8 (19.9 to 32.7)	26.3 (20.2 to 33.2)	32.8 (22.2 to 44.1)	1.6 (0.2 to 2.8)	27.1 (2.4 to 53.3)
Singapore	11.7 (8.3 to 16.0)	20.6 (15.5 to 26.8)	21.0 (15.9 to 27.1)	29.5 (20.0 to 40.1)	2.3 (0.7 to 3.7)	42.7 (11.1 to 75.0)

	Estimate in 2000 (95% UIs)	Estimate in 2015 (95% UIs)	Estimate in 2017 (95% UIs)	Estimate in 2030 (95% UIs)	Annualised rate of change, 2015-2030 (95% UIs)	Percent change, 2015-2030 (95% UIs)
High-income North America						
Canada	20.3 (15.6 to 25.8)	26.1 (19.9 to 32.6)	26.4 (20.2 to 33.0)	31.1 (19.9 to 43.0)	1.1 (-0.5 to 2.5)	18.7 (-7.1 to 45.2)
Greenland	14.5 (10.7 to 19.3)	20.3 (15.2 to 26.5)	20.6 (15.3 to 26.8)	26.4 (16.6 to 37.2)	1.7 (0.1 to 3.2)	29.6 (0.8 to 61.8)
United States	20.8 (18.1 to 23.7)	22.9 (19.1 to 27.2)	23.4 (19.1 to 28.3)	26.2 (18.1 to 36.4)	0.8 (-0.6 to 2.1)	13.8 (-8.7 to 36.3)
Southern Latin America						
Argentina	10.6 (7.6 to 14.5)	13.0 (9.3 to 17.6)	13.4 (9.6 to 18.3)	17.2 (10.3 to 26.0)	1.8 (-0.2 to 3.5)	31.6 (-2.8 to 70.1)
Chile	13.4 (9.8 to 18.3)	21.8 (16.1 to 28.4)	22.3 (16.3 to 28.6)	30.6 (20.3 to 42.2)	2.2 (0.6 to 3.6)	40.3 (9.9 to 70.8)
Uruguay	12.4 (8.7 to 16.9)	18.2 (13.5 to 23.4)	18.8 (14.0 to 24.4)	25.3 (16.4 to 36.2)	2.1 (0.3 to 3.7)	38.8 (5.2 to 74.2)
Western Europe						
Andorra	31.4 (24.4 to 39.7)	33.7 (26.4 to 41.4)	33.8 (26.6 to 41.4)	36.3 (25.5 to 49.1)	0.4 (-1.0 to 1.7)	7.3 (-13.4 to 28.8)
Austria	24.9 (19.1 to 31.6)	27.2 (20.8 to 34.8)	27.5 (20.9 to 35.1)	30.8 (20.1 to 43.8)	0.8 (-0.8 to 2.1)	12.6 (-12.0 to 36.7)
Belgium	24.6 (18.6 to 31.3)	25.6 (19.6 to 32.8)	25.9 (19.6 to 33.1)	28.3 (18.3 to 40.3)	0.6 (-1.1 to 2.0)	9.8 (-14.7 to 35.3)
Cyprus	13.7 (9.9 to 18.1)	24.5 (18.6 to 31.1)	24.6 (18.5 to 31.4)	31.5 (21.2 to 43.0)	1.6 (0.3 to 2.9)	28.4 (3.9 to 54.7)
Denmark	26.4 (19.9 to 33.5)	29.0 (21.9 to 36.5)	29.1 (21.7 to 36.4)	31.4 (20.2 to 42.9)	0.5 (-1.1 to 1.8)	8.1 (-15.5 to 31.4)
Finland	19.3 (14.5 to 24.8)	22.2 (16.9 to 28.1)	22.6 (17.2 to 28.7)	26.6 (17.2 to 37.6)	1.1 (-0.6 to 2.6)	19.6 (-8.5 to 48.0)
France	20.7 (15.3 to 26.8)	23.5 (17.8 to 30.6)	23.8 (18.2 to 30.8)	27.0 (17.0 to 38.7)	0.8 (-0.9 to 2.4)	14.2 (-12.2 to 42.6)
Germany	21.3 (15.8 to 27.6)	25.5 (19.0 to 32.3)	25.9 (19.5 to 32.5)	30.0 (19.0 to 41.6)	1.0 (-0.8 to 2.4)	16.9 (-10.9 to 43.2)
Greece	25.4 (20.2 to 31.8)	28.7 (22.1 to 36.0)	28.6 (21.9 to 35.9)	30.5 (20.1 to 42.7)	0.3 (-1.3 to 1.7)	5.7 (-17.3 to 29.4)
Iceland	26.9 (20.9 to 34.7)	32.3 (24.8 to 40.6)	32.7 (25.1 to 41.2)	37.5 (25.3 to 51.6)	0.9 (-0.5 to 2.2)	15.7 (-6.9 to 38.8)
Ireland	25.7 (20.9 to 31.5)	28.1 (21.6 to 34.8)	28.6 (21.9 to 35.5)	31.5 (20.5 to 44.1)	0.7 (-0.9 to 1.9)	11.6 (-12.8 to 33.7)
Israel	20.2 (15.0 to 26.3)	22.9 (17.0 to 29.0)	23.3 (17.3 to 29.6)	26.4 (16.6 to 37.4)	0.9 (-0.8 to 2.4)	14.6 (-11.3 to 43.3)
Italy	27.4 (21.2 to 34.3)	30.2 (23.2 to 37.8)	30.3 (23.4 to 38.1)	32.8 (22.2 to 45.4)	0.5 (-0.9 to 1.9)	8.2 (-12.4 to 32.1)
Luxembourg	29.0 (22.2 to 36.3)	34.0 (26.6 to 42.4)	34.3 (26.9 to 42.8)	39.0 (27.4 to 52.8)	0.9 (-0.5 to 2.1)	14.6 (-6.8 to 37.3)
Malta	22.7 (17.1 to 30.0)	27.2 (20.6 to 34.7)	27.8 (21.0 to 35.4)	32.6 (21.6 to 44.4)	1.2 (-0.4 to 2.5)	19.6 (-6.3 to 44.9)
Netherlands	19.5 (14.9 to 24.9)	22.1 (17.0 to 28.1)	22.5 (17.1 to 28.5)	26.0 (16.9 to 37.2)	1.0 (-0.6 to 2.5)	17.0 (-9.0 to 45.3)
Norway	23.1 (17.1 to 29.4)	27.1 (20.4 to 34.6)	27.4 (21.0 to 34.8)	31.7 (21.4 to 44.5)	1.0 (-0.5 to 2.4)	16.8 (-7.3 to 42.8)
Portugal	20.4 (15.5 to 26.3)	23.8 (18.0 to 30.4)	24.1 (18.1 to 30.9)	27.2 (17.5 to 39.0)	0.8 (-0.9 to 2.3)	13.9 (-12.1 to 41.6)
Spain	24.3 (18.4 to 30.6)	26.6 (19.8 to 33.9)	26.8 (20.0 to 34.1)	29.4 (18.9 to 42.0)	0.6 (-1.1 to 2.0)	9.8 (-15.1 to 34.7)
Sweden	20.5 (15.0 to 26.5)	24.0 (18.4 to 30.9)	24.4 (18.4 to 31.2)	28.4 (18.1 to 40.2)	1.1 (-0.6 to 2.5)	18.0 (-9.0 to 45.6)
Switzerland	25.0 (19.3 to 31.6)	26.5 (20.4 to 33.5)	26.7 (20.7 to 33.7)	28.6 (18.9 to 39.8)	0.4 (-1.1 to 1.7)	7.5 (-15.0 to 29.6)
United Kingdom	23.3 (20.9 to 25.9)	25.6 (21.6 to 30.2)	25.9 (21.7 to 31.2)	28.3 (20.2 to 38.2)	0.6 (-0.8 to 1.7)	9.7 (-10.9 to 29.6)
Latin America and Caribbean						
Andean Latin America						
Bolivia	25.8 (21.8 to 29.9)	27.1 (21.5 to 33.5)	28.1 (22.1 to 34.7)	32.0 (21.4 to 44.2)	1.0 (-0.3 to 2.2)	17.3 (-4.6 to 39.8)
Ecuador	16.9 (13.8 to 20.4)	19.3 (15.8 to 23.1)	20.0 (16.2 to 24.2)	24.2 (16.1 to 33.3)	1.4 (-0.2 to 2.9)	24.8 (-2.8 to 54.2)
Peru	28.4 (26.3 to 30.3)	27.5 (21.9 to 33.9)	28.6 (22.7 to 35.6)	32.2 (21.7 to 45.0)	1.0 (-0.4 to 2.1)	16.1 (-5.8 to 36.8)
Caribbean						
Antigua and Barbuda	15.9 (11.3 to 21.1)	16.6 (12.0 to 21.8)	17.1 (12.4 to 22.4)	18.8 (11.5 to 27.9)	0.7 (-1.2 to 2.5)	12.5 (-16.0 to 45.9)
The Bahamas	25.7 (19.5 to 33.1)	23.8 (17.5 to 31.2)	23.9 (17.6 to 30.9)	23.6 (15.1 to 35.3)	-0.2 (-1.9 to 1.5)	-1.5 (-24.7 to 24.5)
Barbados	24.1 (18.4 to 30.4)	20.3 (16.4 to 24.7)	20.6 (16.4 to 25.5)	19.9 (13.0 to 28.7)	-0.2 (-2.1 to 1.5)	-2.2 (-26.6 to 26.1)
Belize	18.9 (15.8 to 22.6)	17.2 (13.9 to 20.6)	17.1 (13.6 to 21.0)	16.4 (10.6 to 24.0)	-0.4 (-2.1 to 1.2)	-5.2 (-27.3 to 19.6)
Bermuda	34.6 (26.9 to 42.9)	33.1 (25.8 to 41.5)	33.4 (26.0 to 41.7)	33.1 (22.4 to 45.7)	-0.1 (-1.6 to 1.3)	-0.4 (-20.8 to 21.5)
Cuba	18.7 (14.4 to 23.9)	23.8 (20.3 to 27.9)	24.7 (20.8 to 29.2)	29.8 (21.5 to 39.2)	1.4 (-0.0 to 2.8)	24.8 (-0.3 to 52.7)
Dominica	19.3 (14.1 to 25.8)	19.8 (14.5 to 26.3)	20.1 (14.8 to 26.7)	21.4 (13.0 to 33.0)	0.5 (-1.4 to 2.1)	8.0 (-18.8 to 36.5)
Dominican Republic	14.0 (11.0 to 17.4)	17.8 (13.1 to 23.2)	18.4 (13.7 to 24.6)	22.8 (14.3 to 33.5)	1.6 (0.1 to 3.1)	27.3 (1.0 to 58.5)
Grenada	13.7 (9.8 to 18.2)	13.4 (9.6 to 18.3)	13.8 (9.8 to 18.6)	14.7 (8.4 to 23.5)	0.5 (-1.6 to 2.3)	8.3 (-21.1 to 41.6)
Guyana	11.7 (9.5 to 14.3)	15.4 (12.2 to 19.1)	15.9 (12.2 to 20.1)	19.7 (12.2 to 29.1)	1.5 (-0.3 to 3.0)	26.8 (-3.7 to 58.0)
Haiti	7.4 (5.8 to 9.3)	7.7 (5.8 to 10.4)	7.9 (5.9 to 10.6)	8.5 (5.0 to 13.5)	0.5 (-1.5 to 2.3)	8.8 (-19.9 to 41.5)
Jamaica	17.3 (12.5 to 23.0)	17.6 (12.9 to 23.2)	17.7 (13.1 to 23.0)	18.8 (11.7 to 27.9)	0.4 (-1.5 to 2.0)	6.6 (-19.7 to 35.3)
Puerto Rico	37.7 (30.1 to 45.9)	43.1 (35.3 to 51.2)	43.3 (35.4 to 51.4)	47.2 (34.5 to 60.2)	0.6 (-0.7 to 1.6)	9.4 (-9.5 to 27.2)
Saint Lucia	15.3 (11.4 to 19.8)	11.3 (8.6 to 14.4)	11.6 (8.7 to 14.9)	10.7 (6.6 to 16.5)	-0.5 (-2.5 to 1.5)	-5.6 (-31.1 to 26.1)
Saint Vincent and the Grenadines	12.6 (8.9 to 16.9)	15.9 (11.7 to 21.1)	16.3 (11.9 to 21.6)	19.6 (12.2 to 29.1)	1.3 (-0.5 to 3.0)	22.5 (-6.8 to 56.5)
Suriname	9.9 (7.5 to 13.0)	11.0 (8.3 to 14.1)	11.3 (8.4 to 14.4)	13.7 (8.2 to 20.8)	1.4 (-0.6 to 3.1)	23.8 (-8.3 to 59.3)
Trinidad and Tobago	11.4 (8.8 to 14.6)	20.5 (15.3 to 26.9)	20.7 (15.3 to 27.3)	27.6 (17.3 to 39.5)	1.9 (0.3 to 3.2)	33.9 (4.6 to 62.8)
Virgin Islands, U.S.	33.5 (26.0 to 41.9)	34.5 (26.8 to 43.3)	34.6 (26.8 to 43.2)	34.8 (23.9 to 48.0)	-0.0 (-1.5 to 1.3)	0.2 (-20.0 to 21.5)
Central Latin America						
Colombia	12.2 (9.4 to 15.5)	12.3 (9.1 to 16.0)	12.8 (9.3 to 16.8)	14.9 (8.8 to 22.1)	1.2 (-0.7 to 2.8)	20.1 (-10.3 to 52.4)
Costa Rica	23.9 (18.1 to 31.0)	23.5 (17.6 to 30.4)	24.0 (17.8 to 31.4)	25.9 (16.2 to 37.2)	0.6 (-1.3 to 2.2)	9.9 (-17.4 to 39.0)
El Salvador	12.4 (10.2 to 15.2)	14.8 (11.4 to 18.8)	15.3 (11.7 to 19.7)	18.7 (11.6 to 27.7)	1.4 (-0.2 to 3.0)	25.2 (-2.6 to 57.4)
Guatemala	17.8 (14.9 to 21.0)	16.1 (12.2 to 20.7)	16.4 (12.4 to 21.0)	17.0 (10.4 to 25.1)	0.2 (-1.5 to 1.8)	4.5 (-20.6 to 31.8)
Honduras	11.9 (9.4 to 15.0)	13.4 (10.9 to 16.8)	13.8 (10.8 to 17.6)	16.2 (10.3 to 24.8)	1.1 (-0.6 to 2.8)	19.4 (-9.0 to 51.1)
Mexico	25.4 (19.5 to 31.9)	22.8 (18.2 to 28.2)	23.2 (18.3 to 28.7)	22.9 (15.3 to 32.5)	-0.1 (-1.8 to 1.5)	-0.4 (-23.8 to 24.7)
Nicaragua	15.6 (13.4 to 18.3)	16.8 (13.0 to 21.3)	17.3 (13.3 to 22.2)	20.1 (12.9 to 30.2)	1.1 (-0.6 to 2.6)	18.5 (-8.1 to 47.8)
Panama	14.6 (10.6 to 19.3)	17.9 (13.1 to 23.9)	18.9 (13.9 to 25.0)	24.4 (15.3 to 35.1)	2.0 (0.3 to 3.4)	36.1 (5.4 to 66.7)
Venezuela	21.5 (16.3 to 27.4)	21.6 (15.8 to 27.9)	22.0 (16.1 to 28.5)	24.3 (14.9 to 35.7)	0.7 (-1.0 to 2.2)	12.1 (-13.4 to 39.7)
Tropical Latin America						
Brazil	19.2 (16.0 to 22.7)	29.9 (23.1 to 37.0)	30.7 (23.4 to 37.7)	40.9 (27.1 to 54.2)	2.0 (0.9 to 3.0)	36.2 (14.2 to 57.6)
Paraguay	15.9 (11.8 to 20.7)	21.7 (16.0 to 28.1)	22.5 (16.7 to 29.3)	29.4 (18.9 to 41.8)	2.0 (0.3 to 3.3)	34.9 (5.2 to 63.4)
North Africa and Middle East						
North Africa and Middle East						
Afghanistan	3.2 (2.3 to 4.5)	5.9 (4.1 to 8.2)	6.2 (4.3 to 8.5)	9.9 (5.9 to 15.2)	3.4 (1.4 to 5.1)	68.2 (24.1 to 115.7)
Algeria	18.2 (13.1 to 24.2)	27.2 (20.8 to 34.8)	28.1 (21.5 to 35.5)	37.0 (25.7 to 49.1)	2.0 (0.6 to 3.3)	35.9 (9.3 to 63.0)
Bahrain	23.4 (18.0 to 29.6)	22.4 (17.2 to 28.4)	23.2 (17.7 to 29.2)	26.6 (17.5 to 37.5)	1.1 (-0.5 to 2.4)	17.8 (-6.8 to 44.3)
Egypt	28.2 (24.9 to 31.5)	31.1 (25.5 to 37.1)	31.9 (25.8 to 38.2)	35.8 (24.8 to 47.8)	0.9 (-0.4 to 1.9)	14.6 (-5.5 to 32.9)
Iran	17.3 (12.7 to 22.6)	23.6 (17.9 to 30.5)	24.1 (18.3 to 31.1)	29.9 (19.9 to 42.4)	1.5 (0.0 to 2.9)	26.5 (0.7 to 54.1)
Iraq	15.3 (12.4 to 18.6)	23.5 (18.9 to 28.4)	24.2 (19.3 to 29.4)	30.6 (20.8 to 41.9)	1.7 (0.2 to 2.9)	29.5 (3.6 to 55.6)
Jordan	12.1 (9.5 to 15.0)	15.6 (11.9 to 19.8)	16.2 (12.3 to 20.7)	19.6 (12.7 to 29.8)	1.4 (-0.2 to 3.1)	25.1 (-3.3 to 59.0)
Kuwait	34.5 (27.0 to 42.7)	43.9 (35.7 to 52.4)	44.5 (36.2 to 53.0)	51.9 (38.9 to 64.6)	1.1 (0.0 to 2.1)	18.3 (0.2 to 37.2)
Lebanon	23.1 (17.5 to 29.2)	23.9 (19.1 to 28.7)	24.6 (19.6 to 29.9)	28.0 (18.9 to 38.8)	1.0 (-0.7 to 2.5)	16.9 (-9.7 to 45.0)
Libya	27.0 (20.7 to 34.4)	28.9 (22.4 to 36.3)	28.1 (21.9 to 35.5)	28.5 (18.7 to 40.5)	-0.2 (-1.8 to 1.3)	-1.8 (-23.4 to 21.1)

	Estimate in 2000 (95% UIs)	Estimate in 2015 (95% UIs)	Estimate in 2017 (95% UIs)	Estimate in 2030 (95% UIs)	Annualised rate of change, 2015-2030 (95% UIs)	Percent change, 2015-2030 (95% UIs)
Morocco	19.4 (15.8 to 23.3)	23.1 (18.0 to 29.5)	23.9 (18.5 to 30.5)	27.9 (18.4 to 40.4)	1.2 (-0.4 to 2.5)	19.8 (-6.0 to 45.6)
Palestine	15.9 (12.9 to 19.2)	17.5 (14.1 to 21.8)	18.2 (14.3 to 22.9)	21.7 (14.2 to 31.4)	1.4 (-0.3 to 2.8)	23.4 (-4.5 to 52.6)
Oman	21.2 (15.9 to 27.6)	33.0 (25.9 to 40.7)	33.7 (26.7 to 41.6)	44.5 (31.9 to 57.9)	2.0 (0.7 to 3.1)	34.8 (10.8 to 58.9)
Qatar	37.7 (29.5 to 46.2)	41.1 (32.9 to 49.3)	41.9 (33.7 to 50.1)	45.5 (32.8 to 59.2)	0.6 (-0.6 to 1.8)	10.6 (-9.0 to 30.2)
Saudi Arabia	27.0 (20.4 to 34.5)	39.8 (31.3 to 48.2)	41.4 (32.7 to 49.9)	53.8 (40.2 to 66.6)	2.0 (1.0 to 2.9)	35.5 (16.5 to 54.5)
Sudan	10.3 (8.2 to 12.5)	10.9 (8.4 to 14.2)	11.5 (8.6 to 15.0)	14.8 (9.0 to 21.8)	1.9 (0.2 to 3.4)	33.7 (2.5 to 67.0)
Syria	19.8 (15.6 to 24.5)	27.4 (21.7 to 33.6)	27.8 (21.6 to 34.4)	33.1 (22.1 to 45.0)	1.2 (-0.2 to 2.4)	20.2 (-3.7 to 43.9)
Tunisia	18.8 (13.7 to 24.2)	25.9 (19.4 to 32.9)	26.6 (19.9 to 33.8)	33.6 (22.0 to 45.8)	1.7 (0.2 to 3.1)	29.2 (2.7 to 58.3)
Turkey	16.6 (13.4 to 20.1)	28.6 (22.2 to 36.1)	29.9 (23.1 to 37.5)	42.4 (29.8 to 55.8)	2.6 (1.5 to 3.5)	48.1 (25.7 to 69.7)
United Arab Emirates	23.4 (17.7 to 29.7)	31.5 (24.4 to 39.3)	31.9 (24.9 to 39.3)	38.3 (26.6 to 51.2)	1.3 (-0.2 to 2.4)	21.4 (-2.8 to 44.0)
Yemen	7.8 (5.7 to 10.5)	9.0 (6.5 to 12.2)	9.1 (6.4 to 12.3)	10.8 (6.4 to 16.7)	1.1 (-0.9 to 2.9)	18.8 (-13.1 to 55.2)
South Asia						
South Asia						
Bangladesh	3.3 (2.4 to 4.6)	4.2 (3.0 to 5.8)	4.4 (3.1 to 6.1)	6.0 (3.3 to 9.5)	2.1 (0.0 to 4.2)	39.8 (0.6 to 88.0)
Bhutan	7.4 (5.3 to 10.0)	12.8 (9.5 to 16.9)	13.4 (9.8 to 17.7)	19.3 (11.9 to 27.7)	2.6 (0.8 to 4.3)	49.6 (12.4 to 89.2)
India	6.8 (5.0 to 9.0)	10.8 (7.9 to 14.0)	11.4 (8.5 to 14.9)	17.7 (11.3 to 25.2)	3.3 (1.6 to 4.7)	64.0 (27.8 to 103.5)
Nepal	3.5 (2.5 to 4.9)	5.0 (3.5 to 6.9)	5.3 (3.8 to 7.2)	7.6 (4.5 to 12.0)	2.7 (0.6 to 4.4)	51.1 (10.0 to 94.0)
Pakistan	7.4 (5.5 to 9.6)	5.5 (4.0 to 7.3)	5.7 (4.2 to 7.7)	5.4 (3.3 to 8.4)	-0.2 (-2.1 to 1.5)	-2.5 (-26.8 to 25.5)
Southeast Asia, East Asia, and Oceania						
East Asia						
China	8.4 (7.8 to 9.1)	16.5 (15.4 to 17.5)	17.4 (16.3 to 18.5)	27.8 (25.5 to 30.1)	3.5 (3.1 to 3.8)	68.3 (60.2 to 76.2)
North Korea	7.5 (5.2 to 10.2)	7.5 (5.4 to 10.2)	7.6 (5.6 to 10.3)	8.0 (4.7 to 12.7)	0.3 (-1.8 to 2.5)	5.7 (-23.7 to 44.9)
Taiwan (Province of China)	24.2 (18.3 to 31.2)	32.1 (24.8 to 39.1)	32.6 (25.2 to 39.7)	39.0 (27.4 to 51.1)	1.3 (-0.1 to 2.4)	21.4 (-0.8 to 42.7)
Oceania						
American Samoa	22.6 (17.1 to 29.1)	27.1 (20.4 to 35.0)	27.4 (20.8 to 35.4)	31.7 (21.6 to 44.7)	1.0 (-0.5 to 2.3)	16.6 (-7.8 to 42.0)
Federated States of Micronesia	12.5 (9.1 to 16.8)	14.7 (10.9 to 19.3)	15.0 (11.0 to 19.7)	17.7 (11.0 to 27.0)	1.1 (-0.7 to 3.0)	19.3 (-9.5 to 55.7)
Fiji	17.2 (12.6 to 22.6)	21.9 (16.6 to 28.2)	22.4 (16.8 to 28.8)	27.3 (17.9 to 39.0)	1.4 (-0.2 to 2.9)	24.4 (-2.5 to 53.9)
Guam	36.6 (28.9 to 44.9)	46.4 (37.8 to 55.3)	46.5 (38.1 to 55.2)	54.5 (41.0 to 68.0)	1.1 (0.1 to 2.0)	17.4 (0.9 to 34.1)
Kiribati	9.3 (6.5 to 12.5)	11.6 (8.3 to 15.6)	11.9 (8.6 to 16.1)	15.1 (9.0 to 23.2)	1.6 (-0.4 to 3.5)	29.4 (-5.4 to 69.6)
Marshall Islands	9.8 (7.2 to 13.1)	13.0 (9.6 to 17.2)	13.3 (9.8 to 17.6)	17.4 (10.8 to 26.1)	1.8 (-0.1 to 3.6)	32.8 (-1.9 to 71.6)
Northern Mariana Islands	32.2 (25.0 to 40.3)	31.1 (24.2 to 38.9)	30.9 (23.5 to 38.7)	30.3 (19.6 to 43.1)	-0.3 (-1.9 to 1.2)	-3.0 (-25.0 to 20.5)
Papua New Guinea	8.5 (6.0 to 11.7)	9.6 (6.9 to 13.1)	9.9 (7.0 to 13.5)	11.9 (7.0 to 18.5)	1.3 (-0.7 to 3.2)	23.3 (-9.6 to 62.6)
Samoa	16.0 (11.9 to 21.2)	19.4 (14.2 to 25.1)	19.8 (14.7 to 25.6)	23.4 (14.7 to 34.0)	1.2 (-0.6 to 2.8)	20.4 (-8.4 to 51.5)
Solomon Islands	7.2 (5.2 to 9.8)	9.3 (6.6 to 12.6)	9.6 (6.7 to 12.9)	12.5 (7.1 to 19.9)	1.8 (-0.2 to 3.9)	33.5 (-3.2 to 79.5)
Tonga	30.8 (23.6 to 38.6)	24.9 (18.6 to 31.4)	25.5 (18.7 to 31.9)	26.9 (17.5 to 37.9)	0.4 (-1.3 to 2.0)	7.6 (-17.4 to 34.4)
Vanuatu	10.2 (7.4 to 13.7)	13.7 (10.0 to 18.3)	14.0 (10.1 to 18.5)	17.8 (10.6 to 26.8)	1.6 (-0.2 to 3.3)	29.1 (-3.1 to 62.9)
Southeast Asia						
Cambodia	5.0 (4.3 to 5.9)	5.1 (3.9 to 6.7)	5.4 (4.1 to 7.2)	6.8 (4.0 to 10.8)	1.7 (-0.1 to 3.5)	31.3 (-1.7 to 69.8)
Indonesia	7.8 (6.2 to 9.8)	14.9 (11.2 to 19.3)	15.4 (11.4 to 20.0)	23.8 (15.4 to 34.6)	3.0 (1.5 to 4.4)	58.9 (25.6 to 93.1)
Laos	5.1 (3.8 to 7.0)	6.8 (4.9 to 9.2)	7.3 (5.2 to 9.9)	11.1 (6.9 to 17.4)	3.2 (1.3 to 4.9)	62.1 (21.9 to 107.2)
Malaysia	18.0 (13.5 to 23.3)	26.6 (20.0 to 33.6)	27.4 (20.8 to 34.9)	36.3 (25.2 to 49.8)	2.0 (0.7 to 3.3)	36.0 (10.5 to 64.1)
Maldives	4.5 (3.4 to 5.9)	13.6 (10.9 to 16.7)	14.4 (11.4 to 18.0)	26.7 (18.8 to 35.2)	4.4 (3.2 to 5.5)	95.5 (62.8 to 128.8)
Mauritius	14.4 (10.5 to 19.0)	21.0 (15.5 to 27.2)	21.6 (16.1 to 27.8)	28.5 (18.4 to 41.0)	2.0 (0.4 to 3.4)	35.4 (6.4 to 66.3)
Myanmar	5.0 (3.6 to 6.8)	9.6 (6.7 to 12.9)	10.2 (7.2 to 13.7)	17.1 (10.2 to 25.0)	3.8 (2.0 to 5.4)	77.5 (34.4 to 123.6)
Philippines	5.7 (3.8 to 7.9)	8.9 (6.3 to 12.2)	9.3 (6.6 to 12.6)	14.0 (8.1 to 21.4)	2.9 (0.9 to 4.8)	55.6 (14.4 to 106.3)
Sri Lanka	9.7 (6.9 to 13.2)	15.5 (11.3 to 20.4)	16.3 (11.8 to 21.5)	23.7 (14.3 to 33.8)	2.7 (1.0 to 4.3)	52.2 (15.5 to 90.0)
Seychelles	11.7 (8.4 to 15.8)	14.9 (10.6 to 19.7)	15.5 (11.2 to 20.5)	20.0 (12.2 to 29.1)	1.9 (0.1 to 3.6)	33.7 (1.7 to 70.8)
Thailand	11.3 (8.7 to 14.1)	19.2 (15.9 to 22.5)	19.9 (16.1 to 23.6)	29.2 (20.5 to 38.2)	2.7 (1.3 to 4.0)	51.4 (21.5 to 83.2)
Timor-Leste	7.5 (6.0 to 9.5)	11.6 (8.9 to 14.9)	11.6 (8.8 to 15.0)	15.4 (9.2 to 22.9)	1.8 (-0.1 to 3.4)	31.6 (-2.0 to 66.5)
Vietnam	4.1 (3.0 to 5.5)	10.4 (7.8 to 13.7)	11.0 (8.2 to 14.6)	19.4 (12.4 to 27.8)	4.1 (2.4 to 5.5)	85.1 (43.3 to 128.8)
Sub-Saharan Africa						
Central Sub-Saharan Africa						
Angola	14.0 (10.8 to 17.8)	16.6 (12.1 to 21.9)	17.8 (13.0 to 23.4)	23.2 (14.7 to 34.2)	2.2 (0.5 to 3.6)	39.1 (7.8 to 71.5)
Central African Republic	16.9 (13.9 to 20.2)	10.9 (8.4 to 14.2)	10.9 (8.1 to 14.2)	9.2 (5.7 to 14.1)	-1.3 (-3.0 to 0.5)	-17.0 (-36.3 to 7.2)
Congo	16.0 (12.5 to 19.8)	13.4 (10.1 to 17.0)	14.0 (10.4 to 17.9)	14.8 (9.4 to 21.6)	0.6 (-1.1 to 2.1)	9.5 (-14.9 to 36.2)
Democratic Republic of the Congo	13.5 (10.9 to 16.6)	15.5 (12.0 to 19.6)	16.4 (12.6 to 21.1)	20.1 (13.1 to 29.1)	1.6 (-0.1 to 3.1)	28.7 (-0.7 to 59.8)
Equatorial Guinea	30.3 (25.4 to 34.8)	41.8 (34.1 to 50.0)	42.8 (34.7 to 50.9)	50.8 (37.9 to 63.7)	1.3 (0.3 to 2.1)	21.3 (4.4 to 36.6)
Gabon	15.9 (12.1 to 19.9)	17.9 (14.0 to 22.4)	18.8 (14.4 to 23.9)	21.5 (13.8 to 31.2)	1.1 (-0.5 to 2.7)	19.7 (-6.6 to 49.6)
Eastern Sub-Saharan Africa						
Burundi	8.4 (6.2 to 11.1)	8.2 (6.0 to 11.0)	8.4 (6.0 to 11.3)	8.5 (5.0 to 13.5)	0.1 (-1.9 to 2.0)	2.3 (-24.5 to 35.7)
Comoros	31.6 (27.0 to 36.4)	22.2 (16.9 to 28.4)	22.5 (17.1 to 29.0)	19.5 (12.7 to 29.1)	-1.0 (-2.4 to 0.5)	-13.0 (-30.3 to 8.5)
Djibouti	18.0 (14.3 to 23.0)	24.6 (19.2 to 30.7)	25.6 (19.8 to 31.9)	31.9 (20.1 to 44.1)	1.7 (0.1 to 2.9)	29.1 (1.7 to 55.4)
Eritrea	9.4 (6.9 to 12.4)	12.0 (8.7 to 16.0)	12.5 (9.0 to 16.8)	15.6 (9.3 to 24.3)	1.6 (-0.2 to 3.5)	29.0 (-3.3 to 68.2)
Ethiopia	6.7 (5.0 to 8.8)	9.8 (7.1 to 13.0)	10.6 (7.5 to 14.1)	14.9 (8.9 to 22.7)	2.7 (0.9 to 4.3)	51.4 (13.8 to 91.4)
Kenya	14.3 (11.9 to 17.1)	12.8 (10.2 to 15.7)	13.5 (10.6 to 16.7)	14.6 (9.5 to 21.3)	0.8 (-1.0 to 2.4)	13.2 (-14.0 to 44.2)
Madagascar	10.0 (7.4 to 13.0)	10.7 (7.7 to 14.3)	11.1 (8.1 to 15.0)	12.5 (7.3 to 19.9)	0.9 (-1.0 to 2.7)	16.2 (-14.3 to 50.3)
Malawi	23.8 (20.7 to 27.2)	19.0 (15.5 to 22.7)	19.2 (15.4 to 23.4)	17.1 (11.4 to 24.3)	-0.8 (-2.3 to 0.7)	-10.4 (-29.2 to 10.9)
Mozambique	15.0 (12.4 to 18.0)	17.8 (14.1 to 22.1)	18.7 (14.6 to 23.4)	23.0 (14.8 to 33.0)	1.6 (-0.0 to 3.0)	28.2 (-0.1 to 57.2)
Rwanda	16.4 (13.7 to 19.3)	20.1 (15.9 to 24.7)	20.9 (16.2 to 26.2)	25.1 (15.8 to 36.0)	1.4 (-0.2 to 2.8)	24.0 (-3.2 to 52.9)
Somalia	9.9 (7.2 to 13.0)	10.0 (7.3 to 13.5)	10.3 (7.6 to 14.0)	11.1 (6.7 to 17.4)	0.6 (-1.4 to 2.5)	10.1 (-18.5 to 44.6)
South Sudan	17.9 (15.2 to 21.1)	12.6 (9.2 to 16.7)	12.8 (9.3 to 17.0)	11.0 (6.6 to 17.2)	-1.0 (-2.7 to 0.7)	-13.0 (-33.5 to 10.3)
Tanzania	13.4 (10.5 to 16.4)	13.1 (10.1 to 17.1)	14.0 (10.7 to 18.1)	16.3 (10.2 to 24.0)	1.3 (-0.3 to 2.9)	23.1 (-4.5 to 53.5)
Uganda	15.2 (12.5 to 18.3)	14.8 (11.2 to 18.8)	15.5 (11.4 to 19.8)	16.6 (10.2 to 24.4)	0.6 (-1.1 to 2.2)	10.8 (-14.8 to 38.4)
Zambia	29.3 (26.7 to 31.9)	35.3 (29.9 to 40.9)	36.2 (30.3 to 42.3)	41.7 (30.4 to 53.9)	1.1 (-0.0 to 2.0)	17.7 (-0.7 to 34.2)
Southern Sub-Saharan Africa						
Botswana	15.5 (12.1 to 19.4)	21.0 (15.7 to 27.1)	21.9 (16.5 to 28.3)	28.4 (18.6 to 40.2)	2.0 (0.5 to 3.3)	34.8 (8.2 to 64.0)
Lesotho	14.6 (11.9 to 17.8)	16.2 (12.8 to 20.1)	16.7 (13.0 to 21.1)	19.3 (12.2 to 28.4)	1.0 (-0.7 to 2.6)	17.8 (-10.1 to 48.1)
Namibia	7.9 (5.9 to 10.2)	12.1 (9.2 to 15.8)	12.7 (9.6 to 16.8)	16.8 (10.3 to 25.4)	2.1 (0.1 to 3.8)	37.5 (2.0 to 75.5)
South Africa	26.1 (22.2 to 30.0)	30.4 (26.2 to 35.1)	30.7 (25.9 to 36.2)	35.1 (25.4 to 45.9)	0.9 (-0.4 to 2.0)	14.7 (-6.0 to 35.4)
Swaziland	25.9 (22.2 to 29.8)	22.7 (18.3 to 27.4)	23.1 (18.2 to 28.5)	23.5 (15.1 to 33.9)	0.1 (-1.5 to 1.6)	2.5 (-19.7 to 27.3)

	Estimate in 2000 (95% UIs)	Estimate in 2015 (95% UIs)	Estimate in 2017 (95% UIs)	Estimate in 2030 (95% UIs)	Annualised rate of change, 2015-2030 (95% UIs)	Percent change, 2015-2030 (95% UIs)
Zimbabwe	16.8 (13.9 to 20.0)	11.1 (8.5 to 14.0)	11.5 (8.7 to 14.8)	9.9 (6.2 to 15.1)	-0.8 (-2.4 to 0.9)	-11.2 (-30.6 to 13.9)
Western Sub-Saharan Africa						
Benin	11.5 (9.6 to 13.5)	17.8 (13.9 to 22.1)	18.2 (14.3 to 22.7)	22.5 (14.7 to 31.9)	1.5 (0.1 to 2.8)	25.6 (0.7 to 51.8)
Burkina Faso	11.2 (9.1 to 13.8)	12.5 (9.5 to 16.2)	12.9 (9.7 to 17.2)	14.4 (8.9 to 23.3)	0.8 (-1.0 to 2.6)	14.5 (-14.2 to 48.6)
Cameroon	18.6 (15.7 to 21.9)	19.9 (15.7 to 24.9)	20.9 (16.2 to 26.2)	23.6 (15.4 to 33.9)	1.0 (-0.5 to 2.5)	17.6 (-7.4 to 45.0)
Cape Verde	14.2 (10.2 to 19.0)	16.4 (11.9 to 21.7)	17.1 (12.5 to 22.4)	19.4 (12.1 to 29.3)	1.1 (-0.7 to 2.9)	18.1 (-10.3 to 53.9)
Chad	8.5 (6.8 to 10.3)	7.7 (5.7 to 10.2)	8.0 (5.7 to 10.7)	8.0 (4.7 to 12.8)	0.1 (-1.6 to 1.9)	2.8 (-21.8 to 33.3)
Cote d'Ivoire	13.3 (10.4 to 16.7)	13.7 (10.4 to 17.6)	14.2 (10.7 to 18.3)	15.6 (10.0 to 23.2)	0.7 (-0.9 to 2.4)	12.6 (-13.0 to 44.2)
The Gambia	8.4 (6.5 to 10.5)	9.8 (7.2 to 12.8)	10.2 (7.5 to 13.5)	12.2 (7.2 to 18.7)	1.3 (-0.6 to 3.1)	23.5 (-8.4 to 59.3)
Ghana	11.1 (8.5 to 14.6)	8.9 (6.6 to 11.5)	9.4 (7.0 to 12.3)	9.1 (5.8 to 13.8)	0.1 (-1.6 to 1.7)	1.8 (-20.9 to 28.7)
Guinea	10.0 (7.8 to 12.6)	10.1 (7.4 to 13.3)	10.5 (7.8 to 13.9)	11.3 (6.8 to 17.6)	0.7 (-1.1 to 2.4)	11.7 (-15.4 to 43.6)
Guinea-Bissau	14.8 (12.3 to 17.8)	17.1 (13.4 to 21.2)	16.9 (13.1 to 21.4)	16.7 (11.0 to 23.6)	-0.2 (-1.6 to 1.1)	-3.0 (-21.2 to 17.8)
Liberia	8.9 (6.7 to 11.5)	10.2 (7.6 to 13.5)	10.5 (7.7 to 14.0)	11.2 (6.8 to 17.6)	0.5 (-1.3 to 2.3)	8.5 (-18.3 to 42.0)
Mali	9.1 (7.7 to 10.7)	9.3 (7.0 to 12.1)	9.7 (7.2 to 12.7)	10.4 (6.3 to 16.0)	0.6 (-1.1 to 2.4)	10.4 (-15.2 to 42.7)
Mauritania	10.7 (7.9 to 14.1)	10.4 (7.7 to 13.8)	11.0 (8.1 to 14.4)	12.6 (7.7 to 19.0)	1.1 (-0.8 to 3.0)	19.7 (-10.6 to 56.8)
Niger	5.6 (4.1 to 7.4)	7.0 (5.1 to 9.2)	7.2 (5.2 to 9.5)	8.2 (4.8 to 12.6)	0.9 (-1.1 to 2.8)	16.3 (-14.8 to 51.3)
Nigeria	15.6 (12.4 to 19.5)	16.9 (13.2 to 21.0)	17.2 (13.2 to 21.6)	18.8 (12.0 to 27.3)	0.6 (-1.0 to 2.2)	10.3 (-14.2 to 39.6)
Sao Tome and Principe	13.5 (10.2 to 17.1)	10.2 (7.8 to 13.1)	10.6 (8.0 to 13.8)	9.8 (6.4 to 14.0)	-0.4 (-1.8 to 1.1)	-4.6 (-23.1 to 18.6)
Senegal	8.1 (6.0 to 10.6)	8.1 (5.8 to 10.8)	8.4 (6.1 to 11.2)	9.3 (5.4 to 14.6)	0.9 (-1.2 to 2.8)	15.1 (-16.5 to 51.2)
Sierra Leone	13.9 (11.6 to 16.7)	20.9 (17.2 to 24.7)	21.5 (17.3 to 26.0)	28.5 (19.3 to 39.0)	2.0 (0.5 to 3.2)	35.4 (8.4 to 61.6)
Togo	8.7 (7.1 to 10.6)	8.6 (6.4 to 11.1)	9.2 (6.8 to 12.0)	10.6 (6.6 to 16.3)	1.2 (-0.5 to 3.0)	21.6 (-6.7 to 56.8)
**Indicator 3.1.1: Maternal mortality ratio (maternal deaths per 100,000 livebirths) in women aged 10-54 years**						
Central Europe, Eastern Europe, and Central Asia						
Central Asia						
Armenia	60.3 (55.8 to 65.0)	20.5 (18.9 to 22.2)	23.9 (21.7 to 26.1)	28.7 (18.9 to 40.7)	2.1 (-0.6 to 4.6)	40.0 (-8.1 to 100.5)
Azerbaijan	44.0 (38.3 to 51.0)	25.4 (20.6 to 31.1)	25.8 (20.6 to 32.0)	27.9 (17.5 to 41.1)	0.5 (-2.1 to 3.1)	9.9 (-27.4 to 58.3)
Georgia	22.3 (20.2 to 24.5)	43.2 (40.1 to 47.2)	38.2 (35.0 to 42.0)	28.9 (19.0 to 43.0)	-2.8 (-5.4 to -0.2)	-33.3 (-55.6 to -2.2)
Kazakhstan	73.0 (68.7 to 77.6)	14.2 (13.0 to 15.4)	15.3 (13.5 to 17.0)	19.6 (11.6 to 30.9)	2.0 (-1.2 to 5.2)	38.0 (-16.3 to 117.8)
Kyrgyzstan	62.9 (57.4 to 68.7)	46.0 (42.8 to 49.4)	39.5 (35.5 to 43.6)	41.0 (27.6 to 59.3)	-0.9 (-3.4 to 1.8)	-10.7 (-39.8 to 31.2)
Mongolia	182.8 (162.4 to 204.2)	43.2 (35.1 to 52.8)	42.7 (34.0 to 53.5)	40.8 (24.0 to 65.4)	-0.6 (-3.5 to 2.5)	-5.6 (-41.1 to 45.8)
Tajikistan	49.2 (43.1 to 56.0)	26.6 (21.8 to 32.0)	25.1 (20.1 to 31.0)	26.2 (17.1 to 39.8)	-0.2 (-2.7 to 2.5)	-1.2 (-33.0 to 45.8)
Turkmenistan	12.3 (11.4 to 13.3)	11.2 (10.3 to 12.2)	10.1 (8.7 to 11.6)	13.3 (8.7 to 19.3)	1.0 (-1.6 to 3.5)	19.0 (-21.2 to 69.1)
Uzbekistan	55.5 (51.5 to 59.5)	27.7 (24.7 to 31.1)	26.6 (22.1 to 32.0)	25.6 (16.9 to 36.9)	-0.7 (-3.2 to 1.9)	-7.8 (-38.0 to 32.2)
Central Europe						
Albania	15.2 (12.8 to 18.1)	10.0 (7.2 to 13.4)	9.3 (6.4 to 12.8)	11.4 (7.0 to 17.4)	0.8 (-1.6 to 3.0)	14.6 (-21.2 to 56.5)
Bosnia and Herzegovina	20.5 (16.1 to 25.7)	7.3 (6.1 to 8.5)	7.2 (5.8 to 8.7)	7.9 (6.0 to 10.3)	0.5 (-1.0 to 2.1)	9.1 (-14.0 to 36.6)
Bulgaria	71.2 (66.6 to 76.1)	18.6 (17.3 to 20.1)	19.8 (17.8 to 22.0)	21.1 (15.9 to 27.2)	0.8 (-1.0 to 2.5)	13.6 (-13.6 to 45.6)
Croatia	14.8 (13.7 to 15.9)	5.8 (5.4 to 6.3)	5.9 (5.2 to 6.7)	5.1 (3.9 to 6.7)	-0.9 (-2.6 to 0.9)	-12.1 (-32.0 to 14.0)
Czech Republic	14.6 (13.5 to 15.7)	4.9 (4.5 to 5.3)	5.4 (4.8 to 6.0)	5.7 (4.5 to 7.2)	1.0 (-0.6 to 2.5)	16.4 (-8.4 to 46.5)
Hungary	13.6 (12.7 to 14.7)	15.7 (14.5 to 16.9)	12.2 (10.9 to 13.7)	7.7 (5.6 to 10.0)	-4.8 (-6.7 to -3.0)	-51.1 (-63.6 to -36.3)
Macedonia	18.1 (15.4 to 21.0)	9.3 (7.6 to 11.3)	8.9 (7.3 to 10.9)	7.7 (5.7 to 10.2)	-1.3 (-2.8 to 0.3)	-16.7 (-34.5 to 5.2)
Montenegro	16.4 (13.7 to 19.4)	7.5 (6.0 to 9.2)	7.2 (5.7 to 9.0)	7.1 (5.1 to 9.8)	-0.5 (-2.1 to 1.3)	-5.8 (-26.8 to 22.2)
Poland	13.9 (13.0 to 14.7)	4.3 (4.0 to 4.6)	4.7 (4.2 to 5.2)	6.9 (5.5 to 8.7)	3.2 (1.7 to 4.8)	62.8 (29.5 to 105.4)
Romania	47.1 (44.1 to 50.2)	18.1 (16.9 to 19.4)	17.3 (15.4 to 19.2)	13.8 (10.5 to 17.7)	-1.9 (-3.5 to -0.2)	-24.1 (-40.8 to -3.1)
Serbia	21.2 (18.1 to 24.2)	12.6 (10.7 to 14.9)	12.6 (10.3 to 15.1)	8.9 (6.4 to 12.0)	-2.4 (-4.2 to -0.6)	-29.5 (-46.5 to -9.2)
Slovakia	14.2 (12.2 to 16.1)	7.0 (5.9 to 8.2)	7.7 (6.3 to 9.2)	8.7 (6.4 to 11.4)	1.4 (-0.4 to 3.1)	23.7 (-5.8 to 60.3)
Slovenia	12.6 (11.5 to 13.9)	7.0 (6.4 to 7.6)	6.2 (5.4 to 7.2)	4.8 (3.5 to 6.4)	-2.5 (-4.5 to -0.6)	-30.8 (-49.3 to -8.6)
Eastern Europe						
Belarus	62.7 (57.8 to 67.5)	11.6 (10.2 to 13.3)	11.8 (10.1 to 13.8)	17.3 (11.2 to 25.9)	2.5 (-0.0 to 5.3)	49.5 (-0.0 to 122.1)
Estonia	35.2 (32.5 to 38.1)	2.1 (1.9 to 2.3)	3.4 (2.7 to 4.2)	4.7 (2.7 to 7.8)	5.1 (1.8 to 8.7)	123.7 (30.4 to 268.2)
Latvia	29.9 (27.5 to 32.5)	31.2 (28.2 to 33.9)	14.0 (11.1 to 17.5)	15.2 (8.9 to 23.6)	-5.0 (-8.3 to -1.9)	-51.2 (-71.2 to -25.3)
Lithuania	14.3 (13.1 to 15.5)	12.2 (11.1 to 13.3)	9.4 (8.2 to 10.7)	9.0 (5.5 to 14.5)	-2.2 (-5.3 to 1.1)	-26.1 (-54.8 to 17.9)
Moldova	35.2 (32.7 to 38.1)	27.2 (25.0 to 29.4)	18.5 (16.5 to 20.5)	19.2 (12.1 to 28.3)	-2.5 (-5.4 to 0.3)	-29.3 (-55.7 to 4.5)
Russia	66.0 (64.1 to 68.0)	15.2 (14.8 to 15.6)	15.3 (14.8 to 15.9)	17.9 (10.1 to 30.9)	0.8 (-2.7 to 4.7)	17.6 (-33.0 to 101.7)
Ukraine	103.1 (97.2 to 108.9)	36.5 (32.2 to 39.6)	34.2 (29.8 to 38.6)	42.4 (25.4 to 66.7)	0.8 (-2.4 to 3.9)	16.2 (-30.6 to 79.3)
High-income						
Australasia						
Australia	8.2 (7.6 to 8.8)	4.5 (4.2 to 4.9)	4.7 (3.9 to 5.6)	4.2 (3.2 to 5.5)	-0.6 (-2.3 to 1.2)	-7.4 (-29.0 to 19.9)
New Zealand	13.1 (12.3 to 13.9)	10.6 (9.9 to 11.4)	10.7 (9.6 to 11.8)	8.0 (5.8 to 10.9)	-1.9 (-3.9 to 0.1)	-24.4 (-44.6 to 1.5)
High-income Asia Pacific						
Brunei	39.9 (33.9 to 46.4)	47.3 (38.1 to 58.8)	48.9 (38.3 to 61.7)	48.4 (36.0 to 63.3)	0.1 (-1.2 to 1.5)	2.3 (-16.8 to 25.8)
Japan	10.6 (10.3 to 10.9)	5.3 (5.1 to 5.5)	5.1 (4.8 to 5.4)	4.9 (3.9 to 6.2)	-0.6 (-2.1 to 1.0)	-7.9 (-26.7 to 16.6)
South Korea	18.6 (17.5 to 19.7)	10.3 (9.4 to 11.3)	9.6 (8.4 to 10.9)	8.2 (6.1 to 10.9)	-1.6 (-3.3 to 0.2)	-21.0 (-39.4 to 3.1)
Singapore	13.4 (11.9 to 15.1)	7.3 (6.7 to 7.9)	5.5 (4.9 to 6.2)	5.5 (3.9 to 7.5)	-1.9 (-4.1 to 0.1)	-23.9 (-46.1 to 0.8)
High-income North America						
Canada	7.4 (7.0 to 7.9)	8.3 (7.7 to 9.0)	7.9 (7.0 to 8.9)	6.9 (5.3 to 8.8)	-1.3 (-3.0 to 0.3)	-16.8 (-35.8 to 4.5)
Greenland	17.9 (15.3 to 20.8)	17.3 (14.3 to 20.9)	14.9 (12.1 to 18.4)	15.9 (9.4 to 27.5)	-0.8 (-3.9 to 3.0)	-7.9 (-44.5 to 56.8)
United States	25.1 (24.5 to 25.6)	32.0 (30.9 to 33.0)	30.1 (28.3 to 32.2)	27.2 (20.4 to 35.3)	-1.2 (-3.0 to 0.6)	-15.1 (-36.0 to 9.3)
Southern Latin America						
Argentina	55.5 (52.7 to 58.2)	52.5 (49.8 to 55.5)	50.7 (42.3 to 60.1)	46.1 (34.0 to 60.0)	-0.9 (-2.9 to 0.8)	-12.3 (-35.5 to 13.4)
Chile	29.1 (27.2 to 30.9)	25.4 (23.8 to 27.2)	22.7 (18.9 to 27.0)	18.1 (14.2 to 23.0)	-2.3 (-3.9 to -0.8)	-28.6 (-44.1 to -11.3)
Uruguay	31.6 (29.5 to 33.8)	26.9 (25.0 to 29.2)	25.2 (20.6 to 30.3)	19.6 (14.4 to 25.8)	-2.2 (-4.1 to -0.3)	-27.3 (-46.0 to -4.9)
Western Europe						
Andorra	6.1 (4.6 to 8.2)	4.9 (3.4 to 6.9)	5.0 (3.5 to 7.0)	5.0 (3.1 to 7.8)	0.1 (-1.9 to 2.2)	2.4 (-24.4 to 38.1)
Austria	8.5 (7.9 to 9.2)	5.3 (4.9 to 5.7)	4.3 (3.8 to 4.8)	4.1 (3.1 to 5.4)	-1.7 (-3.6 to 0.2)	-22.2 (-41.5 to 2.8)
Belgium	11.0 (10.2 to 11.8)	7.4 (6.9 to 8.0)	6.6 (6.0 to 7.4)	6.0 (4.5 to 8.0)	-1.4 (-3.3 to 0.5)	-18.3 (-38.8 to 8.4)
Cyprus	26.6 (21.2 to 32.7)	8.4 (6.9 to 10.2)	8.2 (6.6 to 10.1)	8.7 (5.9 to 12.1)	0.1 (-2.0 to 2.2)	3.1 (-26.2 to 39.0)
Denmark	4.2 (3.9 to 4.4)	2.3 (2.1 to 2.5)	3.6 (3.2 to 4.1)	3.1 (2.2 to 4.2)	2.0 (-0.2 to 4.1)	36.1 (-2.3 to 84.2)
Finland	8.6 (8.0 to 9.2)	4.0 (3.7 to 4.3)	3.6 (3.2 to 4.1)	3.5 (2.5 to 4.9)	-0.9 (-3.2 to 1.4)	-11.7 (-37.7 to 23.3)
France	12.0 (11.4 to 12.7)	7.3 (6.8 to 7.9)	7.1 (6.3 to 7.9)	6.1 (4.7 to 7.7)	-1.3 (-3.0 to 0.3)	-16.8 (-35.8 to 5.3)

	Estimate in 2000 (95% UIs)	Estimate in 2015 (95% UIs)	Estimate in 2017 (95% UIs)	Estimate in 2030 (95% UIs)	Annualised rate of change, 2015-2030 (95% UIs)	Percent change, 2015-2030 (95% UIs)
Germany	15.7 (14.9 to 16.7)	7.7 (7.2 to 8.2)	7.9 (6.6 to 9.5)	6.7 (5.0 to 8.9)	-1.0 (-2.8 to 0.8)	-13.2 (-34.6 to 12.7)
Greece	7.4 (6.9 to 8.0)	7.3 (6.8 to 7.8)	9.2 (8.2 to 10.2)	7.5 (5.6 to 9.7)	0.1 (-1.7 to 1.9)	2.2 (-22.1 to 33.8)
Iceland	4.5 (4.1 to 4.8)	2.5 (2.3 to 2.8)	1.2 (1.0 to 1.3)	0.9 (0.6 to 1.4)	-7.1 (-10.0 to -3.8)	-64.3 (-77.6 to -43.5)
Ireland	3.7 (3.4 to 4.0)	4.2 (3.8 to 4.6)	3.9 (3.4 to 4.5)	3.1 (2.3 to 4.1)	-2.2 (-4.1 to -0.2)	-27.0 (-46.2 to -3.5)
Israel	6.3 (5.8 to 6.8)	5.0 (4.7 to 5.5)	5.3 (4.7 to 6.0)	4.5 (3.1 to 6.0)	-0.9 (-3.1 to 1.2)	-11.8 (-37.3 to 19.8)
Italy	7.1 (6.6 to 7.6)	4.4 (4.1 to 4.8)	3.6 (3.2 to 4.1)	3.3 (2.5 to 4.3)	-2.1 (-3.8 to -0.4)	-26.4 (-43.4 to -5.7)
Luxembourg	13.8 (12.7 to 14.9)	8.2 (7.3 to 9.2)	7.2 (6.2 to 8.4)	5.6 (3.8 to 8.4)	-2.7 (-5.1 to 0.1)	-31.5 (-53.1 to 1.5)
Malta	15.8 (14.4 to 17.2)	11.3 (10.2 to 12.4)	11.5 (10.3 to 12.8)	9.6 (5.7 to 15.5)	-1.3 (-4.6 to 2.1)	-14.8 (-49.5 to 36.8)
Netherlands	14.7 (13.7 to 15.7)	5.8 (5.4 to 6.3)	6.4 (5.6 to 7.1)	5.9 (4.3 to 7.9)	0.0 (-2.0 to 2.0)	1.4 (-25.9 to 34.5)
Norway	6.7 (6.5 to 7.0)	2.3 (2.2 to 2.4)	3.1 (3.0 to 3.3)	3.0 (2.2 to 4.0)	1.8 (-0.3 to 3.8)	31.7 (-4.8 to 76.2)
Portugal	12.3 (11.5 to 13.1)	8.2 (7.4 to 9.0)	8.0 (6.9 to 9.2)	5.4 (3.9 to 7.4)	-2.9 (-4.9 to -0.7)	-34.6 (-52.1 to -10.5)
Spain	8.3 (7.8 to 8.9)	5.9 (5.4 to 6.3)	5.1 (4.5 to 5.7)	4.0 (3.0 to 5.3)	-2.6 (-4.3 to -0.6)	-31.2 (-47.3 to -8.8)
Sweden	7.8 (7.4 to 8.2)	2.7 (2.6 to 2.9)	3.2 (3.0 to 3.5)	2.9 (2.2 to 3.7)	0.4 (-1.4 to 2.1)	7.2 (-18.7 to 38.0)
Switzerland	10.6 (9.8 to 11.4)	8.3 (7.6 to 9.0)	6.0 (5.2 to 6.8)	5.2 (3.7 to 7.3)	-3.2 (-5.3 to -1.0)	-37.1 (-54.9 to -13.9)
United Kingdom	13.5 (13.2 to 13.8)	7.0 (6.9 to 7.2)	8.0 (7.7 to 8.2)	6.6 (4.8 to 8.8)	-0.5 (-2.5 to 1.4)	-6.6 (-30.8 to 23.7)
Latin America and Caribbean						
Andean Latin America						
Bolivia	236.8 (191.8 to 275.8)	159.6 (102.5 to 207.6)	153.6 (91.7 to 202.1)	126.7 (72.0 to 183.3)	-1.6 (-3.1 to 0.1)	-20.8 (-37.6 to 1.6)
Ecuador	98.1 (93.3 to 102.5)	82.2 (75.0 to 89.5)	75.7 (64.5 to 87.9)	65.9 (51.1 to 82.7)	-1.5 (-3.1 to 0.0)	-19.7 (-36.8 to 0.5)
Peru	162.4 (143.3 to 183.1)	72.5 (59.8 to 86.1)	71.3 (54.0 to 91.8)	59.3 (37.6 to 89.8)	-1.5 (-4.1 to 1.1)	-18.3 (-45.7 to 18.2)
Caribbean						
Antigua and Barbuda	37.5 (33.3 to 42.4)	67.9 (59.8 to 79.1)	59.3 (50.6 to 71.2)	52.9 (32.1 to 86.4)	-1.9 (-4.9 to 1.4)	-22.2 (-51.9 to 23.4)
The Bahamas	68.0 (61.6 to 75.0)	107.3 (94.9 to 120.9)	104.8 (89.5 to 123.0)	96.2 (63.2 to 142.5)	-0.9 (-3.4 to 1.8)	-10.4 (-39.5 to 30.2)
Barbados	52.7 (48.2 to 58.1)	84.0 (74.3 to 93.8)	79.8 (68.0 to 91.5)	62.0 (41.1 to 91.7)	-2.2 (-4.7 to 0.3)	-26.4 (-50.3 to 5.1)
Belize	42.8 (39.0 to 47.0)	73.6 (67.8 to 80.2)	63.8 (57.6 to 70.9)	87.4 (54.9 to 137.9)	1.0 (-2.0 to 4.1)	18.8 (-25.5 to 86.2)
Bermuda	23.9 (21.4 to 26.6)	21.5 (18.2 to 24.9)	17.9 (14.8 to 21.0)	18.3 (9.1 to 34.2)	-1.4 (-5.5 to 2.8)	-15.0 (-56.4 to 52.9)
Cuba	54.3 (51.2 to 57.6)	53.4 (49.7 to 57.2)	48.4 (40.3 to 57.9)	35.8 (25.3 to 49.7)	-2.8 (-5.0 to -0.5)	-32.9 (-52.9 to -7.7)
Dominica	80.5 (73.0 to 88.3)	179.4 (159.3 to 201.6)	153.2 (133.2 to 175.3)	133.1 (78.7 to 216.1)	-2.2 (-5.4 to 1.1)	-25.9 (-55.7 to 18.6)
Dominican Republic	60.5 (53.1 to 68.7)	117.0 (98.3 to 139.6)	109.3 (86.5 to 137.1)	81.1 (53.6 to 113.8)	-2.5 (-4.9 to -0.2)	-30.6 (-51.9 to -3.2)
Grenada	49.1 (44.7 to 54.3)	99.7 (89.9 to 109.3)	99.2 (88.5 to 110.8)	91.9 (64.7 to 130.0)	-0.6 (-2.9 to 1.7)	-7.8 (-34.8 to 28.7)
Guyana	124.5 (115.2 to 134.3)	128.6 (112.3 to 146.8)	122.4 (99.4 to 149.0)	125.7 (70.1 to 219.3)	-0.4 (-3.9 to 3.4)	-2.4 (-44.3 to 66.6)
Haiti	407.4 (337.6 to 510.8)	330.4 (254.3 to 456.6)	319.8 (241.9 to 441.2)	331.8 (227.9 to 499.1)	-0.0 (-1.6 to 1.6)	0.4 (-21.4 to 27.3)
Jamaica	62.8 (59.0 to 67.2)	76.1 (61.1 to 93.2)	73.6 (55.3 to 97.9)	72.2 (47.8 to 108.7)	-0.4 (-2.4 to 1.6)	-5.4 (-30.0 to 27.7)
Puerto Rico	34.8 (32.5 to 37.4)	36.5 (33.5 to 39.5)	23.8 (20.9 to 27.1)	24.3 (17.0 to 35.0)	-2.8 (-5.1 to -0.4)	-33.4 (-53.8 to -5.6)
Saint Lucia	48.9 (44.7 to 53.4)	85.0 (76.5 to 94.2)	82.1 (72.1 to 93.8)	71.4 (49.8 to 98.9)	-1.3 (-3.5 to 1.0)	-16.0 (-40.5 to 16.4)
Saint Vincent and the Grenadines	69.7 (63.6 to 76.6)	116.5 (105.6 to 128.7)	98.4 (86.5 to 112.3)	77.3 (53.9 to 108.8)	-2.8 (-4.9 to -0.5)	-33.7 (-52.2 to -7.1)
Suriname	82.0 (73.1 to 92.1)	107.7 (93.8 to 123.7)	108.9 (92.6 to 128.0)	109.9 (77.7 to 153.0)	0.0 (-2.0 to 2.1)	2.0 (-25.6 to 37.5)
Trinidad and Tobago	68.5 (62.9 to 74.8)	54.9 (38.8 to 74.7)	55.5 (37.8 to 77.0)	57.9 (34.0 to 89.3)	0.3 (-2.5 to 2.8)	6.1 (-31.0 to 52.2)
Virgin Islands, U.S.	38.3 (31.7 to 45.7)	54.4 (42.8 to 68.6)	52.2 (39.5 to 68.4)	49.4 (28.9 to 79.1)	-0.8 (-3.6 to 1.9)	-9.3 (-41.6 to 33.7)
Central Latin America						
Colombia	75.7 (72.7 to 79.0)	76.7 (73.1 to 80.4)	50.0 (42.5 to 59.5)	44.0 (33.2 to 56.2)	-3.8 (-5.5 to -2.1)	-42.6 (-56.0 to -26.7)
Costa Rica	39.1 (36.5 to 42.1)	28.3 (26.0 to 31.0)	28.3 (24.8 to 32.5)	26.7 (21.2 to 34.2)	-0.4 (-1.9 to 1.0)	-5.8 (-24.3 to 16.4)
El Salvador	38.3 (33.3 to 43.9)	41.9 (33.2 to 51.2)	40.1 (29.1 to 53.8)	40.5 (27.1 to 57.1)	-0.3 (-2.4 to 1.7)	-3.4 (-30.5 to 28.1)
Guatemala	134.9 (129.1 to 141.1)	93.1 (88.1 to 98.2)	110.5 (93.0 to 130.1)	93.1 (68.3 to 122.4)	-0.1 (-2.0 to 1.8)	0.0 (-26.1 to 31.5)
Honduras	170.0 (125.8 to 225.3)	116.2 (81.0 to 166.8)	111.9 (77.6 to 158.1)	108.4 (69.0 to 164.2)	-0.5 (-2.2 to 1.2)	-6.7 (-27.7 to 20.0)
Mexico	56.7 (55.5 to 58.0)	43.5 (42.5 to 44.6)	44.4 (42.9 to 45.9)	42.9 (34.0 to 53.6)	-0.1 (-1.7 to 1.4)	-1.4 (-22.2 to 22.9)
Nicaragua	106.1 (93.8 to 121.9)	59.8 (52.4 to 68.8)	53.9 (41.7 to 66.7)	50.6 (35.2 to 69.2)	-1.2 (-3.3 to 0.8)	-15.4 (-38.9 to 12.1)
Panama	59.8 (55.0 to 64.5)	73.3 (68.5 to 78.7)	71.8 (63.3 to 80.2)	51.2 (36.2 to 69.3)	-2.5 (-4.7 to -0.4)	-30.2 (-50.8 to -5.2)
Venezuela	61.4 (58.5 to 64.2)	68.1 (60.9 to 76.4)	68.5 (53.4 to 85.8)	67.5 (46.6 to 94.6)	-0.2 (-2.3 to 1.9)	-1.0 (-28.8 to 33.9)
Tropical Latin America						
Brazil	68.4 (66.3 to 70.3)	69.1 (67.4 to 70.8)	67.3 (65.2 to 69.8)	58.5 (47.1 to 71.5)	-1.1 (-2.6 to 0.3)	-15.3 (-32.0 to 3.8)
Paraguay	129.9 (116.1 to 144.4)	73.5 (59.5 to 90.3)	74.1 (53.2 to 99.2)	71.4 (47.4 to 104.2)	-0.3 (-2.4 to 1.7)	-3.0 (-30.5 to 29.9)
North Africa and Middle East						
North Africa and Middle East						
Afghanistan	465.7 (316.0 to 626.5)	330.9 (226.0 to 462.9)	299.1 (200.9 to 420.5)	267.7 (155.8 to 410.6)	-1.5 (-3.5 to 0.5)	-19.2 (-41.0 to 8.2)
Algeria	132.0 (113.2 to 150.1)	70.2 (57.9 to 81.8)	65.1 (52.5 to 77.7)	47.7 (36.8 to 58.1)	-2.6 (-3.7 to -1.6)	-32.1 (-42.5 to -21.9)
Bahrain	31.9 (26.6 to 37.7)	19.2 (15.6 to 23.5)	19.1 (15.0 to 23.9)	16.8 (11.4 to 24.1)	-1.0 (-3.0 to 1.2)	-12.7 (-36.0 to 18.9)
Egypt	92.2 (79.6 to 106.5)	61.0 (52.6 to 70.6)	61.8 (48.8 to 77.9)	49.4 (32.4 to 70.8)	-1.5 (-4.0 to 0.9)	-18.9 (-45.4 to 14.6)
Iran	27.6 (24.4 to 30.8)	23.6 (21.9 to 25.8)	23.3 (21.4 to 25.6)	23.8 (18.3 to 29.6)	0.0 (-1.7 to 1.5)	1.0 (-22.0 to 24.4)
Iraq	108.9 (80.8 to 141.3)	24.4 (18.9 to 30.4)	22.0 (17.1 to 27.8)	16.8 (11.6 to 23.5)	-2.5 (-4.4 to -0.6)	-31.0 (-48.2 to -9.1)
Jordan	84.7 (69.7 to 102.5)	21.9 (17.9 to 26.9)	21.2 (16.7 to 27.0)	19.4 (13.3 to 27.5)	-0.9 (-2.9 to 1.0)	-11.6 (-35.3 to 16.7)
Kuwait	13.9 (12.8 to 15.0)	12.0 (10.7 to 13.2)	10.9 (9.5 to 12.4)	11.0 (6.2 to 19.5)	-0.8 (-4.3 to 3.1)	-8.0 (-47.6 to 58.4)
Lebanon	35.5 (27.5 to 44.0)	21.8 (17.2 to 27.0)	21.8 (17.2 to 27.3)	22.8 (15.2 to 31.7)	0.2 (-1.8 to 2.3)	4.6 (-24.1 to 40.3)
Libya	30.1 (23.1 to 37.8)	42.3 (31.9 to 54.6)	41.9 (31.0 to 55.5)	43.4 (30.6 to 59.5)	0.1 (-1.1 to 1.4)	2.6 (-15.7 to 23.3)
Morocco	227.2 (200.2 to 259.5)	138.8 (100.2 to 185.4)	133.5 (92.8 to 186.3)	101.1 (67.7 to 144.8)	-2.1 (-3.5 to -0.7)	-27.1 (-40.7 to -10.2)
Palestine	22.6 (19.0 to 26.7)	14.6 (12.5 to 17.2)	13.2 (11.2 to 15.8)	15.1 (7.7 to 30.5)	-0.2 (-4.2 to 4.9)	3.6 (-46.8 to 110.0)
Oman	26.3 (19.1 to 37.1)	17.8 (13.4 to 23.5)	17.7 (13.2 to 23.2)	19.3 (13.1 to 27.5)	0.5 (-1.2 to 2.2)	8.5 (-16.7 to 38.7)
Qatar	28.1 (21.5 to 35.8)	14.4 (10.7 to 19.1)	11.8 (8.6 to 15.6)	9.6 (6.0 to 14.8)	-2.8 (-5.3 to -0.3)	-33.3 (-54.7 to -4.4)
Saudi Arabia	25.4 (20.9 to 30.6)	25.4 (18.1 to 35.4)	26.1 (17.8 to 36.8)	22.2 (13.8 to 33.6)	-1.0 (-2.8 to 0.9)	-12.4 (-34.5 to 14.3)
Sudan	275.7 (204.3 to 356.5)	189.4 (133.4 to 248.6)	176.5 (125.1 to 229.6)	132.4 (82.2 to 194.4)	-2.5 (-4.4 to -0.6)	-30.2 (-48.1 to -8.1)
Syria	43.8 (36.7 to 52.6)	16.4 (12.4 to 21.9)	16.3 (11.7 to 23.1)	17.3 (12.0 to 25.3)	0.3 (-1.2 to 1.8)	4.9 (-16.2 to 30.8)
Tunisia	47.0 (38.9 to 58.7)	28.6 (19.2 to 40.2)	27.6 (18.3 to 39.2)	20.7 (13.4 to 30.0)	-2.2 (-3.5 to -0.8)	-27.6 (-40.8 to -11.6)
Turkey	78.0 (65.3 to 93.4)	23.6 (20.7 to 27.1)	21.7 (18.1 to 26.4)	17.7 (12.0 to 25.3)	-2.0 (-4.3 to 0.3)	-24.9 (-47.5 to 4.5)
United Arab Emirates	12.8 (9.8 to 16.6)	13.6 (9.0 to 19.9)	15.3 (10.0 to 23.1)	14.6 (8.8 to 22.4)	0.4 (-1.4 to 2.1)	7.3 (-18.6 to 36.9)
Yemen	307.2 (227.5 to 377.8)	208.4 (152.1 to 266.4)	206.1 (150.8 to 263.0)	238.7 (160.9 to 340.7)	0.8 (-0.9 to 2.6)	14.6 (-12.7 to 47.3)
South Asia						
South Asia						
Bangladesh	320.6 (282.6 to 361.2)	176.5 (142.7 to 218.0)	164.1 (128.3 to 212.1)	123.7 (84.5 to 179.0)	-2.5 (-4.5 to -0.4)	-30.0 (-49.1 to -5.8)
Bhutan	310.6 (252.8 to 389.9)	173.8 (106.8 to 237.8)	165.7 (97.3 to 233.8)	134.2 (76.2 to 198.2)	-1.8 (-3.4 to -0.1)	-22.9 (-39.6 to -0.9)
India	379.0 (355.9 to 401.6)	162.6 (152.8 to 173.3)	159.6 (148.4 to 172.4)	129.0 (103.5 to 160.2)	-1.6 (-2.9 to -0.2)	-20.7 (-35.0 to -3.6)

	Estimate in 2000 (95% UIs)	Estimate in 2015 (95% UIs)	Estimate in 2017 (95% UIs)	Estimate in 2030 (95% UIs)	Annualised rate of change, 2015-2030 (95% UIs)	Percent change, 2015-2030 (95% UIs)
Nepal	289.0 (241.7 to 347.8)	234.8 (150.0 to 299.0)	214.0 (139.6 to 274.4)	199.2 (123.1 to 275.0)	-1.1 (-2.4 to 0.2)	-15.2 (-29.8 to 3.1)
Pakistan	475.2 (416.2 to 540.8)	332.0 (250.6 to 418.3)	309.1 (229.4 to 394.7)	268.0 (187.5 to 365.7)	-1.5 (-2.9 to 0.0)	-19.3 (-35.2 to 0.4)
Southeast Asia, East Asia, and Oceania						
East Asia						
China	94.1 (88.3 to 100.8)	14.6 (13.6 to 15.8)	13.7 (12.6 to 15.2)	9.2 (5.8 to 13.5)	-3.2 (-6.1 to -0.6)	-36.7 (-59.9 to -8.0)
North Korea	118.5 (86.5 to 158.2)	86.5 (62.5 to 117.0)	81.6 (57.7 to 113.8)	75.3 (51.8 to 108.8)	-1.0 (-2.0 to 0.1)	-13.0 (-25.5 to 1.2)
Taiwan (Province of China)	13.2 (12.3 to 14.2)	16.7 (15.4 to 17.9)	15.2 (13.5 to 17.1)	16.4 (13.2 to 20.0)	-0.2 (-1.5 to 1.2)	-1.7 (-20.5 to 20.0)
Oceania						
American Samoa	65.7 (55.4 to 77.6)	111.5 (93.5 to 132.7)	105.9 (86.8 to 128.4)	71.5 (51.6 to 96.8)	-3.0 (-4.7 to -1.4)	-35.9 (-50.3 to -18.5)
Federated States of Micronesia	181.0 (122.8 to 249.3)	154.7 (78.6 to 237.3)	151.1 (71.6 to 233.6)	196.1 (71.6 to 381.2)	1.3 (-2.0 to 4.9)	25.2 (-25.6 to 109.9)
Fiji	75.4 (64.4 to 88.2)	62.4 (50.3 to 76.1)	64.1 (48.4 to 82.8)	60.9 (40.7 to 88.0)	-0.2 (-2.3 to 1.8)	-2.5 (-28.7 to 31.6)
Guam	30.7 (25.8 to 36.3)	43.8 (36.2 to 52.5)	43.5 (35.2 to 53.2)	37.0 (26.3 to 50.5)	-1.2 (-3.0 to 0.6)	-15.6 (-36.4 to 8.7)
Kiribati	251.3 (209.5 to 296.7)	235.9 (166.3 to 325.8)	229.8 (161.5 to 319.3)	242.4 (159.2 to 358.1)	0.1 (-1.5 to 1.8)	2.7 (-20.5 to 30.2)
Marshall Islands	268.2 (203.6 to 346.3)	234.1 (160.6 to 324.1)	225.1 (150.6 to 314.8)	171.6 (100.7 to 261.4)	-2.2 (-4.5 to -0.3)	-26.8 (-49.3 to -3.7)
Northern Mariana Islands	33.3 (24.9 to 44.6)	48.9 (37.1 to 62.7)	40.4 (30.8 to 52.1)	83.9 (60.0 to 117.8)	3.5 (2.0 to 5.3)	71.6 (34.6 to 120.6)
Papua New Guinea	581.0 (402.7 to 775.7)	519.2 (360.0 to 708.2)	495.6 (342.6 to 678.8)	412.5 (274.4 to 594.5)	-1.6 (-2.8 to -0.4)	-20.6 (-34.0 to -5.2)
Samoa	51.2 (38.5 to 67.2)	31.4 (19.6 to 42.3)	30.8 (18.5 to 42.8)	29.2 (16.5 to 42.0)	-0.6 (-2.1 to 0.9)	-7.4 (-26.7 to 14.9)
Solomon Islands	164.7 (118.6 to 223.3)	153.2 (105.8 to 208.6)	147.6 (100.3 to 200.6)	123.2 (78.5 to 180.3)	-1.5 (-3.1 to -0.0)	-19.7 (-36.8 to -0.2)
Tonga	131.1 (112.1 to 151.3)	113.8 (82.9 to 150.9)	113.0 (80.5 to 153.5)	100.8 (68.4 to 140.5)	-0.8 (-1.9 to 0.1)	-11.5 (-25.1 to 2.1)
Vanuatu	128.1 (79.8 to 191.8)	114.1 (69.9 to 166.4)	111.1 (66.5 to 166.2)	103.5 (60.7 to 161.4)	-0.7 (-2.1 to 0.8)	-9.2 (-27.1 to 12.0)
Southeast Asia						
Cambodia	345.9 (298.1 to 394.3)	119.5 (93.3 to 155.1)	111.5 (83.9 to 149.3)	123.0 (85.7 to 170.8)	0.1 (-1.5 to 1.6)	2.9 (-20.3 to 27.5)
Indonesia	323.4 (296.4 to 363.1)	170.1 (155.8 to 186.3)	164.4 (149.1 to 181.8)	119.2 (100.4 to 140.0)	-2.4 (-3.3 to -1.5)	-29.9 (-39.3 to -20.4)
Laos	472.8 (384.0 to 574.7)	197.5 (139.8 to 251.3)	177.5 (124.8 to 228.4)	136.1 (89.5 to 192.3)	-2.5 (-4.1 to -0.9)	-31.0 (-46.2 to -13.2)
Malaysia	66.9 (60.2 to 74.4)	56.8 (49.7 to 64.8)	57.0 (45.9 to 68.4)	49.0 (37.1 to 63.1)	-1.0 (-2.4 to 0.3)	-13.8 (-30.6 to 5.0)
Maldives	198.7 (166.4 to 232.7)	77.6 (63.8 to 90.8)	78.8 (64.1 to 94.7)	117.4 (64.3 to 205.7)	2.5 (-1.1 to 6.4)	51.4 (-15.0 to 161.0)
Mauritius	35.2 (32.5 to 37.9)	52.6 (48.4 to 57.1)	55.1 (48.5 to 62.3)	38.3 (29.8 to 48.3)	-2.1 (-3.7 to -0.6)	-27.0 (-42.9 to -8.3)
Myanmar	259.6 (211.9 to 327.3)	223.9 (177.6 to 272.1)	211.1 (164.9 to 262.4)	113.1 (83.3 to 150.5)	-4.6 (-6.0 to -3.2)	-49.5 (-59.5 to -38.5)
Philippines	79.1 (71.3 to 86.6)	91.2 (81.2 to 101.0)	83.3 (66.5 to 101.8)	72.7 (51.3 to 100.4)	-1.6 (-3.6 to 0.4)	-20.3 (-41.3 to 6.4)
Sri Lanka	53.0 (49.0 to 57.0)	31.0 (25.8 to 37.3)	30.3 (23.0 to 39.1)	18.9 (12.5 to 27.2)	-3.4 (-5.6 to -1.3)	-38.9 (-56.5 to -17.6)
Seychelles	61.0 (50.3 to 73.3)	68.3 (57.6 to 80.3)	65.7 (54.1 to 77.3)	55.8 (38.6 to 77.7)	-1.4 (-3.5 to 0.7)	-18.3 (-41.1 to 11.0)
Thailand	84.2 (67.1 to 104.4)	39.1 (31.1 to 46.9)	41.5 (32.5 to 51.5)	36.6 (24.6 to 54.5)	-0.6 (-3.1 to 2.1)	-6.2 (-37.0 to 37.8)
Timor-Leste	444.9 (189.2 to 549.5)	247.3 (83.2 to 326.6)	242.8 (86.2 to 322.5)	211.0 (73.0 to 311.1)	-1.1 (-2.9 to 0.7)	-14.5 (-35.2 to 11.7)
Vietnam	33.0 (25.3 to 42.0)	15.0 (11.2 to 19.8)	14.0 (10.6 to 19.1)	12.5 (8.8 to 17.8)	-1.3 (-2.6 to -0.0)	-16.7 (-32.5 to -0.2)
Sub-Saharan Africa						
Central Sub-Saharan Africa						
Angola	397.4 (314.1 to 491.0)	221.1 (170.0 to 268.4)	200.7 (154.3 to 244.2)	128.8 (92.5 to 178.9)	-3.7 (-5.1 to -1.8)	-41.7 (-53.5 to -23.7)
Central African Republic	763.1 (571.5 to 1026.0)	616.5 (424.9 to 899.2)	584.8 (409.6 to 856.5)	729.3 (427.3 to 1211.4)	1.0 (-1.5 to 3.4)	18.3 (-19.6 to 67.5)
Congo	513.0 (387.2 to 643.0)	403.6 (273.6 to 572.0)	397.1 (263.3 to 570.2)	335.4 (196.1 to 570.1)	-1.4 (-3.4 to 1.4)	-17.0 (-40.0 to 24.1)
Democratic Republic of the Congo	334.0 (269.1 to 403.2)	378.8 (305.9 to 458.0)	346.6 (277.3 to 424.8)	257.2 (187.6 to 352.1)	-2.6 (-4.2 to -1.1)	-32.1 (-46.4 to -14.9)
Equatorial Guinea	476.1 (328.5 to 663.1)	189.5 (103.5 to 289.2)	181.7 (102.0 to 270.6)	127.4 (69.5 to 201.3)	-2.7 (-4.1 to -1.2)	-32.5 (-46.0 to -16.8)
Gabon	300.8 (233.1 to 392.9)	211.0 (155.4 to 280.5)	198.6 (144.6 to 260.1)	204.3 (127.9 to 309.6)	-0.3 (-2.5 to 2.4)	-3.1 (-31.8 to 43.4)
Eastern Sub-Saharan Africa						
Burundi	614.8 (475.8 to 800.1)	267.1 (211.8 to 340.9)	260.1 (208.4 to 329.9)	266.0 (179.3 to 397.9)	-0.1 (-2.2 to 2.1)	-0.4 (-28.3 to 37.9)
Comoros	306.2 (251.3 to 366.1)	179.4 (139.9 to 225.5)	169.7 (131.6 to 214.8)	185.1 (137.5 to 241.4)	0.2 (-0.9 to 1.3)	3.2 (-12.4 to 22.1)
Djibouti	489.9 (363.3 to 625.9)	392.4 (265.7 to 547.5)	375.2 (257.2 to 527.9)	351.5 (215.8 to 525.2)	-0.8 (-2.8 to 1.1)	-10.4 (-34.7 to 17.7)
Eritrea	529.4 (396.5 to 711.2)	455.0 (300.1 to 673.7)	439.2 (286.7 to 649.8)	517.5 (289.5 to 820.0)	0.8 (-1.4 to 2.5)	13.7 (-18.4 to 46.4)
Ethiopia	541.9 (472.7 to 634.9)	226.0 (194.0 to 259.2)	199.5 (168.7 to 231.9)	139.7 (107.6 to 177.0)	-3.2 (-4.3 to -2.0)	-38.2 (-47.8 to -25.6)
Kenya	409.2 (362.9 to 458.1)	301.0 (262.5 to 341.8)	289.3 (249.4 to 331.8)	280.5 (216.7 to 363.0)	-0.5 (-1.8 to 1.2)	-6.8 (-23.8 to 18.9)
Madagascar	395.2 (348.0 to 444.4)	286.0 (223.7 to 372.2)	266.3 (208.3 to 357.9)	219.0 (163.6 to 296.6)	-1.8 (-2.7 to -0.9)	-23.4 (-33.6 to -12.3)
Malawi	303.7 (249.7 to 356.8)	177.3 (133.9 to 226.0)	169.7 (127.0 to 214.5)	172.3 (117.7 to 240.3)	-0.2 (-1.8 to 1.4)	-2.7 (-24.0 to 22.6)
Mozambique	276.4 (228.5 to 325.5)	229.4 (177.9 to 297.9)	210.8 (161.8 to 276.7)	201.2 (130.2 to 315.0)	-1.0 (-3.6 to 1.4)	-12.3 (-42.0 to 24.3)
Rwanda	486.4 (415.4 to 573.0)	186.4 (138.2 to 234.7)	172.7 (128.8 to 221.7)	141.6 (84.5 to 236.8)	-2.0 (-4.7 to 1.3)	-24.1 (-50.2 to 21.3)
Somalia	542.8 (401.2 to 720.0)	356.6 (249.8 to 501.0)	342.8 (241.1 to 488.0)	336.3 (211.2 to 504.5)	-0.5 (-2.1 to 1.3)	-5.7 (-26.8 to 20.9)
South Sudan	418.7 (291.4 to 567.7)	346.3 (247.7 to 451.2)	343.5 (250.1 to 449.3)	282.3 (182.7 to 408.3)	-1.4 (-3.1 to 0.4)	-18.4 (-37.1 to 6.6)
Tanzania	285.1 (232.2 to 344.5)	252.7 (196.5 to 320.0)	245.8 (187.2 to 311.6)	182.5 (132.0 to 241.3)	-2.2 (-3.3 to -1.2)	-27.8 (-38.6 to -16.2)
Uganda	183.6 (151.3 to 216.2)	121.3 (89.8 to 153.9)	112.3 (82.9 to 142.9)	100.7 (67.7 to 143.6)	-1.3 (-2.8 to 0.5)	-16.9 (-34.7 to 7.0)
Zambia	238.5 (199.0 to 286.5)	113.5 (92.2 to 136.8)	112.4 (89.2 to 139.3)	103.8 (69.5 to 144.9)	-0.7 (-3.0 to 1.2)	-8.5 (-36.2 to 19.6)
Southern Sub-Saharan Africa						
Botswana	91.1 (52.8 to 142.3)	91.8 (69.7 to 119.8)	83.0 (62.1 to 110.7)	134.4 (67.3 to 298.1)	2.1 (-1.7 to 7.7)	47.4 (-22.3 to 218.4)
Lesotho	302.6 (208.4 to 415.2)	343.5 (221.6 to 545.0)	295.9 (204.1 to 458.8)	404.6 (208.4 to 771.7)	0.9 (-2.6 to 4.7)	18.9 (-32.3 to 103.7)
Namibia	255.2 (190.0 to 326.7)	100.2 (66.6 to 142.6)	99.8 (67.8 to 145.4)	94.1 (49.4 to 164.1)	-0.6 (-3.7 to 2.8)	-5.8 (-43.0 to 51.3)
South Africa	199.2 (185.2 to 214.8)	97.3 (76.7 to 120.7)	109.0 (90.6 to 129.5)	135.6 (44.7 to 418.6)	1.0 (-5.1 to 9.9)	40.2 (-53.4 to 340.2)
Swaziland	165.5 (109.3 to 246.5)	126.6 (78.0 to 203.4)	111.1 (71.5 to 168.6)	169.6 (67.2 to 388.3)	1.5 (-3.4 to 6.9)	34.8 (-39.8 to 180.8)
Zimbabwe	267.1 (191.9 to 354.1)	263.3 (207.7 to 350.4)	250.3 (198.6 to 330.9)	330.7 (177.4 to 623.6)	1.2 (-2.3 to 5.3)	25.7 (-29.5 to 122.7)
Western Sub-Saharan Africa						
Benin	390.2 (318.9 to 470.4)	286.9 (210.8 to 367.5)	267.5 (196.5 to 341.5)	219.6 (149.8 to 301.8)	-1.8 (-3.3 to -0.3)	-23.5 (-39.3 to -4.7)
Burkina Faso	340.7 (276.3 to 406.2)	228.0 (179.7 to 279.8)	218.3 (171.3 to 270.2)	167.8 (118.3 to 227.2)	-2.1 (-3.7 to -0.7)	-26.4 (-42.9 to -10.4)
Cameroon	470.2 (402.8 to 544.2)	323.8 (244.6 to 402.9)	299.5 (224.8 to 374.2)	250.1 (171.2 to 339.0)	-1.8 (-3.3 to -0.2)	-22.7 (-38.9 to -2.8)
Cape Verde	61.4 (48.4 to 75.5)	50.3 (42.0 to 59.2)	49.8 (40.7 to 60.3)	44.1 (34.8 to 54.3)	-0.9 (-1.7 to -0.1)	-12.3 (-23.0 to -1.6)
Chad	550.6 (455.2 to 646.6)	402.1 (321.2 to 481.8)	381.4 (303.9 to 462.5)	287.8 (201.6 to 387.4)	-2.3 (-3.9 to -0.6)	-28.4 (-44.1 to -8.6)
Cote d'Ivoire	423.7 (344.2 to 503.4)	324.7 (245.8 to 396.2)	303.7 (228.1 to 368.7)	237.2 (173.2 to 318.6)	-2.1 (-3.4 to -0.7)	-26.9 (-39.7 to -10.2)
The Gambia	545.4 (436.6 to 653.8)	462.0 (372.2 to 587.5)	451.6 (359.1 to 585.5)	386.2 (298.7 to 519.9)	-1.2 (-2.1 to -0.1)	-16.4 (-27.5 to -1.4)
Ghana	157.8 (130.3 to 183.1)	130.0 (109.5 to 151.8)	126.0 (102.7 to 149.9)	107.2 (84.3 to 132.4)	-1.3 (-2.4 to -0.3)	-17.6 (-29.8 to -4.9)
Guinea	641.1 (542.1 to 736.1)	482.8 (412.0 to 559.3)	448.7 (373.6 to 525.8)	370.0 (266.6 to 490.1)	-1.8 (-3.5 to -0.3)	-23.4 (-40.8 to -4.1)
Guinea-Bissau	301.3 (243.2 to 371.3)	209.1 (153.2 to 294.1)	198.7 (140.3 to 281.0)	166.6 (117.1 to 239.9)	-1.5 (-2.8 to -0.3)	-20.3 (-33.8 to -5.0)
Liberia	650.1 (548.4 to 753.4)	475.3 (384.1 to 574.5)	451.0 (363.6 to 545.2)	476.4 (350.8 to 635.3)	-0.0 (-1.4 to 1.5)	0.2 (-19.3 to 24.7)
Mali	493.8 (425.2 to 568.2)	304.2 (240.9 to 380.8)	290.4 (224.4 to 367.1)	191.2 (143.9 to 247.7)	-3.1 (-4.0 to -2.1)	-37.1 (-45.3 to -27.3)
Mauritania	714.6 (625.4 to 817.9)	449.9 (357.7 to 552.2)	427.0 (333.2 to 525.1)	340.7 (257.5 to 433.4)	-1.9 (-2.7 to -1.0)	-24.3 (-33.5 to -14.4)
Niger	444.8 (369.5 to 527.8)	306.7 (233.1 to 379.9)	289.9 (217.3 to 358.5)	195.2 (139.5 to 253.6)	-3.0 (-4.0 to -1.9)	-36.3 (-45.0 to -25.2)

	Estimate in 2000 (95% UIs)	Estimate in 2015 (95% UIs)	Estimate in 2017 (95% UIs)	Estimate in 2030 (95% UIs)	Annualised rate of change, 2015-2030 (95% UIs)	Percent change, 2015-2030 (95% UIs)
Nigeria	322.1 (210.9 to 461.0)	237.2 (144.4 to 359.5)	230.0 (147.4 to 346.1)	174.1 (104.2 to 277.2)	-2.1 (-3.5 to -0.6)	-26.4 (-40.5 to -8.9)
Sao Tome and Principe	329.8 (278.3 to 385.6)	184.2 (147.9 to 227.6)	168.8 (131.9 to 210.2)	157.7 (108.1 to 222.6)	-1.1 (-2.9 to 0.9)	-14.4 (-35.4 to 14.2)
Senegal	414.0 (355.0 to 478.5)	298.5 (243.5 to 347.8)	281.9 (225.2 to 332.6)	235.6 (181.6 to 296.3)	-1.6 (-2.7 to -0.5)	-21.1 (-33.3 to -6.6)
Sierra Leone	691.1 (584.4 to 804.3)	483.4 (391.8 to 568.9)	450.6 (362.5 to 537.2)	441.9 (283.4 to 641.7)	-0.7 (-2.9 to 1.7)	-8.6 (-35.7 to 29.8)
Togo	367.8 (290.4 to 447.4)	227.8 (170.1 to 289.3)	221.5 (168.2 to 276.2)	214.4 (154.3 to 282.3)	-0.4 (-1.7 to 0.8)	-5.7 (-22.2 to 12.4)
**Indicator 3.1.2: Proportion of births attended by skilled health personnel (%)**						
Central Europe, Eastern Europe, and Central Asia						
Central Asia						
Armenia	97.7 (96.9 to 98.3)	99.3 (98.9 to 99.6)	99.4 (99.0 to 99.7)	99.7 (99.2 to 99.9)	0.0 (0.0 to 0.0)	0.4 (0.2 to 0.7)
Azerbaijan	88.8 (87.9 to 89.8)	96.6 (93.5 to 98.5)	97.0 (94.3 to 98.7)	98.9 (96.6 to 99.8)	0.2 (0.1 to 0.3)	2.4 (1.2 to 4.2)
Georgia	97.1 (96.4 to 97.7)	99.0 (98.6 to 99.3)	99.0 (98.4 to 99.5)	99.4 (98.5 to 99.8)	0.0 (-0.0 to 0.0)	0.4 (-0.2 to 0.7)
Kazakhstan	99.1 (98.6 to 99.5)	99.4 (99.2 to 99.7)	99.6 (99.3 to 99.8)	99.8 (99.3 to 99.9)	0.0 (0.0 to 0.0)	0.3 (0.1 to 0.5)
Kyrgyzstan	98.3 (97.5 to 99.0)	99.5 (99.2 to 99.7)	99.6 (99.3 to 99.8)	99.8 (99.5 to 100.0)	0.0 (0.0 to 0.0)	0.3 (0.1 to 0.5)
Mongolia	98.2 (97.8 to 98.6)	98.8 (98.2 to 99.3)	99.0 (98.2 to 99.4)	99.2 (98.0 to 99.8)	0.0 (-0.0 to 0.1)	0.4 (-0.5 to 0.8)
Tajikistan	90.5 (87.3 to 93.1)	92.3 (88.7 to 95.1)	93.8 (89.9 to 96.6)	95.9 (89.0 to 98.9)	0.3 (0.0 to 0.4)	3.9 (0.1 to 6.0)
Turkmenistan	97.5 (96.9 to 98.1)	99.2 (98.7 to 99.5)	99.3 (98.8 to 99.6)	99.7 (99.2 to 99.9)	0.0 (0.0 to 0.1)	0.5 (0.2 to 0.8)
Uzbekistan	98.2 (97.4 to 98.8)	99.7 (99.3 to 99.9)	99.7 (99.4 to 99.9)	99.9 (99.7 to 100.0)	0.0 (0.0 to 0.0)	0.2 (0.1 to 0.4)
Central Europe						
Albania	97.3 (96.7 to 97.9)	99.4 (98.7 to 99.7)	99.4 (98.8 to 99.7)	99.7 (99.2 to 100.0)	0.0 (0.0 to 0.0)	0.4 (0.2 to 0.7)
Bosnia and Herzegovina	99.0 (98.3 to 99.5)	99.5 (99.0 to 99.7)	99.5 (99.0 to 99.8)	99.7 (99.0 to 99.9)	0.0 (-0.0 to 0.0)	0.2 (-0.1 to 0.5)
Bulgaria	98.9 (98.1 to 99.4)	99.5 (98.9 to 99.8)	99.5 (99.0 to 99.8)	99.7 (99.1 to 100.0)	0.0 (-0.0 to 0.0)	0.2 (-0.1 to 0.5)
Croatia	98.8 (97.8 to 99.4)	99.4 (98.8 to 99.8)	99.5 (98.9 to 99.8)	99.6 (98.9 to 99.9)	0.0 (-0.0 to 0.0)	0.2 (-0.2 to 0.5)
Czech Republic	98.9 (98.2 to 99.5)	99.4 (98.8 to 99.8)	99.5 (98.9 to 99.8)	99.6 (98.8 to 99.9)	0.0 (-0.0 to 0.0)	0.2 (-0.3 to 0.5)
Hungary	97.0 (95.1 to 98.2)	98.9 (97.9 to 99.5)	99.0 (98.0 to 99.6)	99.4 (98.2 to 99.9)	0.0 (-0.0 to 0.1)	0.5 (-0.1 to 1.1)
Macedonia	96.9 (95.5 to 98.1)	99.0 (98.1 to 99.5)	99.0 (98.1 to 99.5)	99.5 (98.5 to 99.9)	0.0 (0.0 to 0.1)	0.6 (0.1 to 1.0)
Montenegro	98.2 (96.5 to 99.1)	99.0 (98.3 to 99.5)	99.1 (98.3 to 99.5)	99.3 (98.0 to 99.9)	0.0 (-0.0 to 0.1)	0.3 (-0.5 to 0.8)
Poland	99.1 (98.4 to 99.5)	99.7 (99.4 to 99.9)	99.7 (99.4 to 99.9)	99.9 (99.6 to 100.0)	0.0 (0.0 to 0.0)	0.2 (0.0 to 0.3)
Romania	98.4 (97.4 to 99.1)	99.4 (98.9 to 99.8)	99.5 (99.0 to 99.8)	99.8 (99.2 to 100.0)	0.0 (0.0 to 0.0)	0.3 (0.1 to 0.7)
Serbia	99.0 (98.1 to 99.5)	99.5 (99.2 to 99.8)	99.6 (99.2 to 99.8)	99.7 (99.2 to 99.9)	0.0 (-0.0 to 0.0)	0.2 (-0.1 to 0.4)
Slovakia	99.0 (98.2 to 99.4)	99.5 (99.1 to 99.8)	99.6 (99.2 to 99.8)	99.8 (99.2 to 100.0)	0.0 (-0.0 to 0.0)	0.2 (-0.0 to 0.5)
Slovenia	98.9 (98.2 to 99.5)	99.6 (99.2 to 99.8)	99.6 (99.2 to 99.8)	99.8 (99.3 to 100.0)	0.0 (-0.0 to 0.0)	0.2 (-0.1 to 0.4)
Eastern Europe						
Belarus	98.7 (97.7 to 99.3)	99.6 (99.2 to 99.8)	99.6 (99.3 to 99.8)	99.8 (99.5 to 100.0)	0.0 (0.0 to 0.0)	0.3 (0.1 to 0.5)
Estonia	98.9 (98.2 to 99.5)	99.7 (99.4 to 99.9)	99.7 (99.5 to 99.9)	99.9 (99.6 to 100.0)	0.0 (0.0 to 0.0)	0.2 (0.0 to 0.4)
Latvia	99.0 (98.2 to 99.5)	99.6 (99.2 to 99.8)	99.7 (99.3 to 99.9)	99.8 (99.4 to 100.0)	0.0 (-0.0 to 0.0)	0.2 (-0.0 to 0.4)
Lithuania	99.0 (98.4 to 99.5)	99.6 (99.1 to 99.8)	99.6 (99.2 to 99.8)	99.7 (99.2 to 100.0)	0.0 (-0.0 to 0.0)	0.2 (-0.1 to 0.4)
Moldova	99.1 (98.5 to 99.5)	99.3 (98.7 to 99.7)	99.5 (98.9 to 99.8)	99.7 (98.9 to 99.9)	0.0 (-0.0 to 0.0)	0.3 (-0.1 to 0.7)
Russia	98.8 (98.0 to 99.4)	99.6 (99.2 to 99.8)	99.7 (99.3 to 99.9)	99.8 (99.4 to 100.0)	0.0 (0.0 to 0.0)	0.2 (0.0 to 0.4)
Ukraine	99.0 (98.2 to 99.5)	99.3 (98.9 to 99.7)	99.4 (98.9 to 99.7)	99.6 (98.8 to 99.9)	0.0 (-0.0 to 0.0)	0.2 (-0.2 to 0.5)
High-income						
Australasia						
Australia	98.9 (98.0 to 99.5)	99.3 (98.7 to 99.7)	99.4 (98.8 to 99.7)	99.5 (98.5 to 99.9)	0.0 (-0.0 to 0.0)	0.2 (-0.4 to 0.5)
New Zealand	99.0 (98.0 to 99.6)	99.4 (98.8 to 99.8)	99.4 (98.9 to 99.8)	99.6 (98.7 to 99.9)	0.0 (-0.0 to 0.0)	0.2 (-0.3 to 0.5)
High-income Asia Pacific						
Brunei	98.6 (97.5 to 99.3)	99.1 (98.4 to 99.6)	99.2 (98.4 to 99.6)	99.3 (98.0 to 99.9)	0.0 (-0.0 to 0.0)	0.2 (-0.6 to 0.7)
Japan	98.8 (97.7 to 99.5)	99.3 (98.7 to 99.7)	99.3 (98.7 to 99.7)	99.5 (98.4 to 99.9)	0.0 (-0.0 to 0.0)	0.2 (-0.5 to 0.6)
South Korea	98.7 (97.4 to 99.4)	99.6 (99.2 to 99.8)	99.6 (99.2 to 99.8)	99.8 (99.4 to 100.0)	0.0 (-0.0 to 0.0)	0.2 (-0.0 to 0.4)
Singapore	98.9 (97.8 to 99.5)	99.5 (99.0 to 99.8)	99.5 (99.0 to 99.8)	99.7 (99.0 to 99.9)	0.0 (-0.0 to 0.0)	0.2 (-0.2 to 0.4)
High-income North America						
Canada	98.8 (97.8 to 99.4)	99.2 (98.4 to 99.6)	99.2 (98.5 to 99.7)	99.3 (98.1 to 99.9)	0.0 (-0.0 to 0.0)	0.2 (-0.7 to 0.6)
Greenland	82.8 (70.5 to 92.1)	91.1 (83.1 to 96.1)	91.8 (84.1 to 96.3)	95.4 (87.6 to 98.8)	0.3 (0.0 to 0.6)	4.7 (0.5 to 9.9)
United States	99.0 (99.0 to 99.0)	99.2 (99.0 to 99.3)	99.2 (99.1 to 99.3)	99.3 (99.1 to 99.5)	0.0 (0.0 to 0.0)	0.2 (0.0 to 0.3)
Southern Latin America						
Argentina	97.9 (96.7 to 98.7)	99.2 (98.8 to 99.5)	99.2 (98.6 to 99.6)	99.6 (98.8 to 99.9)	0.0 (0.0 to 0.0)	0.4 (0.0 to 0.6)
Chile	98.8 (97.9 to 99.4)	99.4 (98.9 to 99.7)	99.5 (98.9 to 99.8)	99.7 (98.9 to 99.9)	0.0 (-0.0 to 0.0)	0.3 (-0.1 to 0.6)
Uruguay	95.0 (92.8 to 96.8)	98.7 (97.7 to 99.3)	98.9 (97.9 to 99.5)	99.5 (98.5 to 99.9)	0.1 (0.0 to 0.1)	0.8 (0.4 to 1.3)
Western Europe						
Andorra	99.6 (99.2 to 99.8)	99.7 (99.4 to 99.9)	99.7 (99.4 to 99.9)	99.7 (99.2 to 99.9)	0.0 (-0.0 to 0.0)	0.0 (-0.3 to 0.2)
Austria	99.4 (98.8 to 99.8)	99.7 (99.4 to 99.9)	99.7 (99.4 to 99.9)	99.8 (99.4 to 100.0)	0.0 (-0.0 to 0.0)	0.1 (-0.1 to 0.3)
Belgium	99.3 (98.6 to 99.7)	99.6 (99.2 to 99.8)	99.6 (99.3 to 99.8)	99.8 (99.3 to 99.9)	0.0 (-0.0 to 0.0)	0.1 (-0.1 to 0.3)
Cyprus	98.2 (96.5 to 99.2)	99.5 (99.0 to 99.8)	99.5 (99.0 to 99.8)	99.8 (99.3 to 99.9)	0.0 (0.0 to 0.0)	0.3 (0.0 to 0.6)
Denmark	99.2 (98.3 to 99.7)	99.6 (99.2 to 99.8)	99.6 (99.2 to 99.8)	99.7 (99.3 to 99.9)	0.0 (-0.0 to 0.0)	0.1 (-0.1 to 0.4)
Finland	99.3 (98.6 to 99.7)	99.7 (99.4 to 99.9)	99.7 (99.4 to 99.9)	99.8 (99.5 to 100.0)	0.0 (-0.0 to 0.0)	0.1 (-0.1 to 0.3)
France	99.2 (98.5 to 99.7)	99.7 (99.3 to 99.9)	99.7 (99.3 to 99.9)	99.8 (99.3 to 100.0)	0.0 (-0.0 to 0.0)	0.1 (-0.1 to 0.3)
Germany	99.2 (98.5 to 99.7)	99.6 (99.1 to 99.8)	99.6 (99.1 to 99.8)	99.7 (99.2 to 99.9)	0.0 (-0.0 to 0.0)	0.1 (-0.1 to 0.4)
Greece	99.2 (98.3 to 99.7)	99.5 (99.0 to 99.8)	99.5 (99.0 to 99.8)	99.6 (98.9 to 99.9)	0.0 (-0.0 to 0.0)	0.1 (-0.3 to 0.3)
Iceland	99.6 (99.2 to 99.8)	99.8 (99.5 to 99.9)	99.8 (99.5 to 99.9)	99.8 (99.4 to 100.0)	0.0 (-0.0 to 0.0)	0.0 (-0.2 to 0.2)
Ireland	98.5 (97.0 to 99.3)	99.5 (98.9 to 99.8)	99.5 (99.0 to 99.8)	99.7 (99.1 to 99.9)	0.0 (-0.0 to 0.0)	0.2 (-0.1 to 0.6)
Israel	98.3 (96.6 to 99.3)	99.1 (98.2 to 99.7)	99.2 (98.3 to 99.7)	99.5 (98.5 to 99.9)	0.0 (-0.0 to 0.1)	0.3 (-0.2 to 0.8)
Italy	99.4 (98.9 to 99.8)	99.7 (99.4 to 99.9)	99.7 (99.4 to 99.9)	99.8 (99.4 to 100.0)	0.0 (-0.0 to 0.0)	0.1 (-0.1 to 0.2)
Luxembourg	98.8 (97.7 to 99.5)	99.3 (98.6 to 99.7)	99.3 (98.6 to 99.7)	99.4 (98.2 to 99.9)	0.0 (-0.0 to 0.0)	0.2 (-0.6 to 0.5)
Malta	98.6 (97.1 to 99.4)	99.4 (98.8 to 99.7)	99.4 (98.9 to 99.8)	99.7 (98.9 to 99.9)	0.0 (-0.0 to 0.0)	0.3 (-0.0 to 0.6)
Netherlands	99.0 (98.0 to 99.6)	99.5 (99.0 to 99.8)	99.5 (99.0 to 99.8)	99.7 (99.0 to 99.9)	0.0 (-0.0 to 0.0)	0.1 (-0.2 to 0.4)
Norway	99.5 (99.1 to 99.8)	99.7 (99.5 to 99.9)	99.7 (99.5 to 99.9)	99.8 (99.5 to 100.0)	0.0 (-0.0 to 0.0)	0.1 (-0.1 to 0.2)
Portugal	99.0 (98.1 to 99.6)	99.6 (99.2 to 99.8)	99.6 (99.2 to 99.8)	99.8 (99.3 to 100.0)	0.0 (-0.0 to 0.0)	0.2 (-0.0 to 0.4)
Spain	99.1 (98.2 to 99.6)	99.6 (99.2 to 99.8)	99.6 (99.2 to 99.8)	99.8 (99.3 to 99.9)	0.0 (-0.0 to 0.0)	0.2 (-0.1 to 0.4)
Sweden	99.6 (99.2 to 99.8)	99.7 (99.4 to 99.9)	99.7 (99.4 to 99.9)	99.8 (99.3 to 99.9)	0.0 (-0.0 to 0.0)	0.1 (-0.2 to 0.2)
Switzerland	99.6 (99.2 to 99.8)	99.7 (99.4 to 99.9)	99.7 (99.4 to 99.9)	99.8 (99.3 to 100.0)	0.0 (-0.0 to 0.0)	0.0 (-0.2 to 0.2)
United Kingdom	98.9 (98.1 to 99.5)	99.4 (98.8 to 99.8)	99.4 (98.8 to 99.8)	99.6 (98.8 to 99.9)	0.0 (-0.0 to 0.0)	0.2 (-0.3 to 0.5)
Latin America and Caribbean						

	Estimate in 2000 (95% UIs)	Estimate in 2015 (95% UIs)	Estimate in 2017 (95% UIs)	Estimate in 2030 (95% UIs)	Annualised rate of change, 2015-2030 (95% UIs)	Percent change, 2015-2030 (95% UIs)
Andean Latin America						
Bolivia	61.8 (60.2 to 63.4)	83.5 (76.4 to 89.7)	85.3 (75.8 to 91.8)	93.2 (82.1 to 98.2)	0.7 (0.4 to 1.1)	11.8 (5.7 to 17.4)
Ecuador	93.4 (91.1 to 95.4)	99.2 (98.7 to 99.5)	99.2 (98.5 to 99.6)	99.8 (99.3 to 99.9)	0.0 (0.0 to 0.1)	0.6 (0.4 to 0.8)
Peru	63.1 (61.4 to 64.8)	93.4 (90.7 to 95.4)	94.0 (90.4 to 96.6)	98.5 (96.1 to 99.6)	0.4 (0.3 to 0.5)	5.4 (4.0 to 7.0)
Caribbean						
Antigua and Barbuda	98.8 (97.9 to 99.4)	99.2 (98.5 to 99.6)	99.2 (98.5 to 99.7)	99.4 (98.0 to 99.9)	0.0 (-0.0 to 0.0)	0.2 (-0.6 to 0.6)
The Bahamas	98.9 (98.0 to 99.5)	98.9 (98.0 to 99.5)	98.9 (98.0 to 99.5)	98.9 (96.7 to 99.8)	-0.0 (-0.1 to 0.0)	-0.0 (-1.4 to 0.6)
Barbados	96.7 (95.0 to 98.0)	98.8 (97.8 to 99.4)	98.9 (97.8 to 99.5)	99.3 (97.8 to 99.9)	0.0 (-0.0 to 0.1)	0.5 (-0.3 to 1.0)
Belize	92.5 (89.2 to 94.9)	96.6 (94.4 to 98.1)	96.8 (94.3 to 98.4)	98.1 (94.7 to 99.6)	0.1 (-0.0 to 0.2)	1.5 (-0.4 to 2.7)
Bermuda	99.0 (98.2 to 99.5)	99.3 (98.7 to 99.7)	99.4 (98.8 to 99.7)	99.6 (98.7 to 99.9)	0.0 (-0.0 to 0.0)	0.2 (-0.3 to 0.6)
Cuba	98.8 (97.9 to 99.4)	99.2 (98.7 to 99.5)	99.3 (98.8 to 99.6)	99.5 (98.7 to 99.9)	0.0 (-0.0 to 0.0)	0.3 (-0.2 to 0.6)
Dominica	97.9 (96.6 to 98.8)	98.7 (97.5 to 99.4)	98.8 (97.7 to 99.5)	99.2 (97.7 to 99.8)	0.0 (-0.0 to 0.1)	0.6 (-0.3 to 1.2)
Dominican Republic	98.4 (98.2 to 98.7)	98.5 (97.7 to 99.1)	98.7 (97.8 to 99.3)	99.1 (97.7 to 99.8)	0.0 (-0.0 to 0.1)	0.7 (-0.2 to 1.1)
Grenada	98.8 (97.8 to 99.4)	99.1 (98.2 to 99.6)	99.1 (98.2 to 99.6)	99.2 (97.6 to 99.9)	0.0 (-0.0 to 0.1)	0.2 (-0.7 to 0.7)
Guyana	87.7 (85.3 to 89.9)	92.9 (90.4 to 95.0)	93.7 (90.2 to 96.2)	96.1 (90.8 to 98.8)	0.2 (-0.0 to 0.4)	3.5 (-0.5 to 5.4)
Haiti	25.4 (22.5 to 28.5)	41.9 (27.2 to 58.7)	44.7 (28.9 to 62.5)	60.9 (31.6 to 87.0)	2.4 (-0.3 to 4.1)	44.6 (-4.7 to 85.6)
Jamaica	98.0 (97.3 to 98.5)	98.8 (97.7 to 99.4)	98.8 (97.8 to 99.5)	99.2 (97.7 to 99.8)	0.0 (-0.0 to 0.1)	0.5 (-0.4 to 1.0)
Puerto Rico	98.8 (97.8 to 99.4)	99.3 (98.7 to 99.7)	99.3 (98.7 to 99.7)	99.5 (98.4 to 99.9)	0.0 (-0.0 to 0.0)	0.2 (-0.5 to 0.6)
Saint Lucia	98.5 (97.4 to 99.2)	98.9 (97.9 to 99.5)	99.0 (98.0 to 99.5)	99.2 (97.5 to 99.8)	0.0 (-0.0 to 0.1)	0.3 (-0.7 to 0.9)
Saint Vincent and the Grenadines	98.7 (97.7 to 99.3)	98.9 (97.9 to 99.5)	98.9 (97.9 to 99.5)	99.0 (97.1 to 99.8)	0.0 (-0.1 to 0.1)	0.1 (-1.1 to 0.8)
Suriname	87.3 (83.6 to 90.3)	96.3 (93.6 to 98.1)	96.8 (94.1 to 98.5)	98.6 (96.0 to 99.7)	0.2 (0.1 to 0.2)	2.3 (1.1 to 3.6)
Trinidad and Tobago	98.5 (97.4 to 99.2)	99.1 (98.3 to 99.6)	99.2 (98.4 to 99.6)	99.4 (98.2 to 99.9)	0.0 (-0.0 to 0.0)	0.3 (-0.5 to 0.7)
Virgin Islands, U.S.	97.9 (96.2 to 98.9)	99.0 (98.0 to 99.6)	99.0 (98.0 to 99.6)	99.2 (97.8 to 99.8)	0.0 (-0.0 to 0.0)	0.2 (-0.7 to 0.7)
Central Latin America						
Colombia	88.4 (87.5 to 89.3)	96.7 (96.0 to 97.3)	97.2 (96.1 to 98.1)	99.0 (97.9 to 99.6)	0.2 (0.1 to 0.2)	2.3 (1.7 to 2.9)
Costa Rica	98.3 (97.1 to 99.1)	99.1 (98.4 to 99.5)	99.2 (98.5 to 99.7)	99.6 (98.5 to 99.9)	0.0 (-0.0 to 0.1)	0.5 (-0.0 to 0.8)
El Salvador	99.1 (98.8 to 99.3)	99.1 (98.7 to 99.4)	99.3 (98.8 to 99.6)	99.5 (98.6 to 99.8)	0.0 (-0.0 to 0.0)	0.4 (-0.2 to 0.7)
Guatemala	50.4 (48.0 to 52.7)	96.7 (95.7 to 97.5)	96.1 (94.0 to 97.6)	98.9 (97.8 to 99.6)	0.1 (0.1 to 0.2)	2.3 (1.8 to 2.8)
Honduras	60.0 (56.7 to 63.2)	96.8 (94.9 to 98.2)	96.3 (93.5 to 98.0)	98.2 (96.0 to 99.5)	0.1 (0.0 to 0.2)	1.5 (0.6 to 2.4)
Mexico	90.7 (90.0 to 91.4)	98.3 (97.8 to 98.7)	98.3 (97.6 to 99.0)	99.3 (98.5 to 99.8)	0.1 (0.0 to 0.1)	1.0 (0.5 to 1.5)
Nicaragua	91.2 (90.2 to 92.1)	95.8 (92.8 to 97.8)	96.3 (93.4 to 98.2)	98.2 (95.1 to 99.6)	0.2 (0.1 to 0.3)	2.6 (1.2 to 4.2)
Panama	90.4 (88.8 to 91.7)	93.7 (90.9 to 95.9)	94.6 (91.2 to 97.1)	97.1 (92.1 to 99.2)	0.2 (0.0 to 0.3)	3.6 (0.6 to 5.2)
Venezuela	97.0 (95.4 to 98.2)	97.7 (95.8 to 98.9)	97.9 (96.1 to 99.0)	98.5 (95.6 to 99.7)	0.1 (-0.1 to 0.1)	0.8 (-0.9 to 1.9)
Tropical Latin America						
Brazil	96.3 (94.9 to 97.5)	99.0 (98.0 to 99.5)	99.0 (98.1 to 99.6)	99.6 (98.8 to 99.9)	0.0 (0.0 to 0.1)	0.6 (0.3 to 1.1)
Paraguay	79.6 (76.2 to 82.7)	93.4 (91.3 to 95.0)	93.6 (91.1 to 95.7)	97.0 (94.3 to 98.7)	0.3 (0.1 to 0.4)	3.9 (1.8 to 5.8)
North Africa and Middle East						
North Africa and Middle East						
Afghanistan	27.1 (13.9 to 43.1)	57.9 (54.8 to 61.0)	62.1 (54.9 to 69.0)	84.5 (72.8 to 92.2)	2.5 (1.8 to 3.0)	46.0 (30.9 to 57.1)
Algeria	88.0 (79.1 to 94.4)	96.6 (93.3 to 98.6)	97.0 (94.2 to 98.7)	98.9 (97.0 to 99.7)	0.2 (0.1 to 0.3)	2.4 (1.0 to 4.5)
Bahrain	94.6 (89.9 to 97.7)	98.7 (97.2 to 99.5)	98.8 (97.4 to 99.5)	99.5 (98.6 to 99.9)	0.1 (0.0 to 0.1)	0.9 (0.3 to 1.8)
Egypt	67.4 (66.0 to 68.6)	94.0 (92.6 to 95.1)	94.6 (92.3 to 96.4)	98.8 (97.5 to 99.5)	0.3 (0.3 to 0.4)	5.1 (4.3 to 6.0)
Iran	95.6 (91.8 to 97.9)	98.4 (97.0 to 99.3)	98.6 (97.2 to 99.4)	99.3 (97.9 to 99.8)	0.1 (0.0 to 0.1)	0.9 (0.3 to 1.8)
Iraq	72.2 (69.8 to 74.4)	93.0 (89.5 to 95.7)	92.7 (87.7 to 96.0)	96.8 (92.0 to 99.1)	0.3 (0.1 to 0.4)	4.1 (1.9 to 5.9)
Jordan	98.3 (98.0 to 98.6)	99.5 (99.2 to 99.7)	99.6 (99.2 to 99.8)	99.8 (99.5 to 100.0)	0.0 (0.0 to 0.0)	0.3 (0.1 to 0.4)
Kuwait	96.7 (94.5 to 98.3)	98.7 (97.8 to 99.2)	98.8 (97.9 to 99.4)	99.4 (98.4 to 99.9)	0.0 (0.0 to 0.1)	0.7 (0.4 to 1.1)
Lebanon	53.0 (34.8 to 71.3)	87.6 (78.2 to 94.4)	88.5 (79.3 to 94.8)	96.4 (89.9 to 99.3)	0.6 (0.3 to 1.1)	10.2 (4.5 to 18.8)
Libya	93.8 (88.3 to 97.1)	98.6 (97.1 to 99.4)	98.5 (96.9 to 99.4)	99.3 (98.0 to 99.8)	0.0 (0.0 to 0.1)	0.7 (0.1 to 1.5)
Morocco	61.7 (59.6 to 63.8)	84.9 (73.3 to 92.8)	86.6 (76.1 to 94.1)	94.6 (85.0 to 99.0)	0.7 (0.4 to 1.3)	11.6 (5.4 to 21.8)
Palestine	97.3 (97.2 to 97.5)	98.2 (97.2 to 99.0)	98.6 (97.5 to 99.3)	99.3 (98.0 to 99.8)	0.1 (0.0 to 0.1)	1.1 (0.5 to 1.6)
Oman	96.8 (93.9 to 98.5)	99.1 (98.2 to 99.6)	99.2 (98.4 to 99.7)	99.6 (98.9 to 99.9)	0.0 (0.0 to 0.1)	0.6 (0.2 to 1.1)
Qatar	96.8 (94.0 to 98.6)	99.5 (99.0 to 99.8)	99.5 (99.1 to 99.8)	99.8 (99.6 to 100.0)	0.0 (0.0 to 0.0)	0.4 (0.2 to 0.7)
Saudi Arabia	92.4 (89.5 to 94.8)	98.2 (96.7 to 99.2)	98.5 (97.1 to 99.3)	99.5 (98.7 to 99.9)	0.1 (0.0 to 0.2)	1.4 (0.7 to 2.5)
Sudan	67.5 (51.6 to 80.8)	79.6 (75.8 to 82.8)	83.5 (77.1 to 88.8)	91.7 (83.2 to 96.7)	0.9 (0.5 to 1.2)	15.1 (7.9 to 19.7)
Syria	76.5 (74.8 to 78.2)	95.0 (90.7 to 97.8)	94.9 (89.5 to 97.8)	97.2 (91.5 to 99.5)	0.2 (-0.0 to 0.3)	2.3 (-0.2 to 4.5)
Tunisia	92.1 (89.3 to 94.2)	98.8 (97.7 to 99.4)	98.8 (97.7 to 99.5)	99.6 (98.8 to 99.9)	0.1 (0.0 to 0.1)	0.9 (0.5 to 1.5)
Turkey	83.1 (81.4 to 84.8)	96.9 (94.0 to 98.5)	97.4 (95.1 to 98.8)	99.3 (98.0 to 99.9)	0.2 (0.1 to 0.3)	2.6 (1.3 to 4.6)
United Arab Emirates	98.8 (97.5 to 99.5)	99.5 (99.0 to 99.8)	99.5 (99.1 to 99.8)	99.7 (99.2 to 100.0)	0.0 (-0.0 to 0.0)	0.2 (-0.1 to 0.5)
Yemen	32.5 (25.6 to 39.1)	51.6 (44.6 to 58.3)	53.6 (42.1 to 65.3)	67.3 (45.7 to 85.7)	1.7 (-0.0 to 2.8)	30.0 (-0.7 to 52.1)
South Asia						
South Asia						
Bangladesh	11.6 (10.6 to 12.8)	47.9 (42.5 to 53.4)	50.8 (40.8 to 60.8)	80.3 (64.8 to 91.0)	3.4 (2.5 to 4.0)	67.4 (46.3 to 82.5)
Bhutan	27.3 (15.9 to 41.2)	72.2 (62.3 to 80.6)	75.1 (64.1 to 84.6)	92.6 (82.3 to 98.0)	1.7 (1.3 to 2.1)	28.5 (21.0 to 36.8)
India	49.9 (49.3 to 50.5)	85.2 (84.9 to 85.5)	85.4 (84.4 to 86.5)	96.1 (95.4 to 96.8)	0.8 (0.8 to 0.8)	12.8 (12.1 to 13.4)
Nepal	13.2 (12.0 to 14.6)	62.4 (60.2 to 64.6)	67.4 (63.0 to 72.2)	93.6 (90.7 to 96.0)	2.7 (2.5 to 3.0)	50.2 (45.0 to 55.8)
Pakistan	27.9 (22.9 to 33.4)	63.8 (54.4 to 72.4)	66.3 (52.8 to 77.2)	84.7 (65.8 to 94.9)	1.9 (1.1 to 2.4)	32.7 (17.4 to 43.8)
Southeast Asia, East Asia, and Oceania						
East Asia						
China	78.0 (77.5 to 78.5)	98.5 (98.2 to 98.7)	98.6 (98.4 to 98.8)	99.8 (99.7 to 99.8)	0.1 (0.1 to 0.1)	1.3 (1.2 to 1.5)
North Korea	82.4 (68.2 to 91.7)	89.8 (80.8 to 95.6)	90.4 (81.5 to 95.9)	93.1 (81.5 to 98.3)	0.2 (-0.2 to 0.6)	3.7 (-3.5 to 9.9)
Taiwan (Province of China)	98.3 (96.5 to 99.3)	99.6 (99.1 to 99.8)	99.6 (99.2 to 99.8)	99.8 (99.5 to 100.0)	0.0 (0.0 to 0.0)	0.2 (0.1 to 0.5)
Oceania						
American Samoa	98.8 (97.7 to 99.5)	99.2 (98.4 to 99.7)	99.2 (98.4 to 99.7)	99.4 (98.0 to 99.9)	0.0 (-0.0 to 0.0)	0.2 (-0.6 to 0.6)
Federated States of Micronesia	92.8 (89.4 to 95.3)	98.0 (96.2 to 99.2)	98.2 (96.6 to 99.3)	99.2 (97.8 to 99.8)	0.1 (0.0 to 0.2)	1.2 (0.5 to 2.3)
Fiji	98.7 (97.5 to 99.4)	99.1 (98.2 to 99.6)	99.2 (98.4 to 99.6)	99.4 (98.1 to 99.9)	0.0 (-0.0 to 0.1)	0.3 (-0.6 to 0.8)
Guam	99.1 (98.2 to 99.6)	98.9 (97.6 to 99.5)	98.8 (97.6 to 99.5)	98.6 (95.7 to 99.7)	-0.0 (-0.1 to 0.0)	-0.3 (-2.1 to 0.5)
Kiribati	85.2 (79.0 to 90.0)	85.9 (78.5 to 91.4)	87.7 (80.1 to 93.3)	89.8 (76.6 to 97.2)	0.3 (-0.4 to 0.6)	4.5 (-5.5 to 9.4)
Marshall Islands	92.0 (87.7 to 94.8)	93.3 (90.0 to 95.8)	94.3 (90.4 to 97.0)	97.0 (91.8 to 99.2)	0.3 (0.1 to 0.4)	4.0 (1.5 to 5.7)
Northern Mariana Islands	99.1 (98.2 to 99.6)	99.2 (98.5 to 99.7)	99.2 (98.5 to 99.7)	99.2 (97.7 to 99.8)	-0.0 (-0.1 to 0.0)	-0.0 (-1.0 to 0.4)
Papua New Guinea	55.0 (46.0 to 63.8)	64.1 (48.1 to 78.5)	67.0 (49.7 to 81.5)	76.0 (46.5 to 93.8)	1.1 (-0.6 to 2.1)	18.2 (-9.2 to 36.5)

	Estimate in 2000 (95% UIs)	Estimate in 2015 (95% UIs)	Estimate in 2017 (95% UIs)	Estimate in 2030 (95% UIs)	Annualised rate of change, 2015-2030 (95% UIs)	Percent change, 2015-2030 (95% UIs)
Samoa	80.0 (71.1 to 86.6)	82.1 (72.5 to 89.0)	83.4 (73.7 to 90.8)	85.5 (67.0 to 96.2)	0.3 (-0.7 to 0.6)	4.0 (-10.1 to 10.1)
Solomon Islands	86.4 (81.1 to 90.5)	93.0 (87.6 to 96.5)	93.7 (88.5 to 97.2)	96.7 (90.3 to 99.4)	0.3 (0.1 to 0.4)	3.9 (1.3 to 6.8)
Tonga	92.7 (90.1 to 94.7)	98.6 (97.3 to 99.4)	98.7 (97.5 to 99.4)	99.5 (98.5 to 99.9)	0.1 (0.0 to 0.1)	0.9 (0.5 to 1.5)
Vanuatu	86.2 (81.0 to 90.6)	85.0 (74.2 to 92.5)	86.5 (75.1 to 93.4)	88.7 (67.1 to 97.5)	0.3 (-0.8 to 0.7)	4.2 (-11.0 to 11.4)
Southeast Asia						
Cambodia	34.5 (32.6 to 36.6)	93.7 (92.0 to 95.2)	94.3 (91.3 to 96.4)	99.2 (98.1 to 99.7)	0.4 (0.3 to 0.5)	5.8 (4.6 to 7.3)
Indonesia	59.7 (59.1 to 60.2)	88.0 (84.8 to 90.8)	88.6 (82.8 to 93.0)	96.0 (90.7 to 98.7)	0.6 (0.4 to 0.7)	9.1 (6.3 to 11.3)
Laos	30.2 (27.5 to 33.3)	57.6 (45.8 to 69.5)	61.9 (47.2 to 75.9)	85.1 (65.4 to 95.8)	2.6 (1.8 to 3.3)	48.2 (31.7 to 64.3)
Malaysia	94.0 (92.3 to 95.3)	99.3 (98.6 to 99.7)	99.4 (98.8 to 99.7)	99.8 (99.5 to 100.0)	0.0 (0.0 to 0.1)	0.6 (0.3 to 1.1)
Maldives	73.8 (67.5 to 79.7)	99.0 (98.0 to 99.5)	99.0 (98.2 to 99.6)	99.8 (99.5 to 100.0)	0.1 (0.0 to 0.1)	0.9 (0.4 to 1.6)
Mauritius	98.7 (97.6 to 99.3)	99.3 (98.6 to 99.7)	99.4 (98.7 to 99.7)	99.6 (98.7 to 99.9)	0.0 (-0.0 to 0.0)	0.3 (-0.1 to 0.7)
Myanmar	70.4 (65.8 to 74.8)	70.3 (66.4 to 73.8)	76.2 (69.0 to 82.0)	85.1 (72.8 to 92.6)	1.3 (0.5 to 1.7)	20.9 (7.4 to 28.5)
Philippines	60.2 (58.5 to 61.9)	74.1 (60.6 to 85.5)	76.4 (62.1 to 88.1)	84.9 (62.7 to 96.8)	0.9 (-0.1 to 1.5)	14.4 (-1.3 to 25.7)
Sri Lanka	99.1 (98.3 to 99.6)	99.8 (99.7 to 99.9)	99.9 (99.8 to 99.9)	100.0 (99.9 to 100.0)	0.0 (0.0 to 0.0)	0.1 (0.1 to 0.2)
Seychelles	94.8 (89.5 to 97.9)	97.7 (95.4 to 99.0)	98.0 (96.0 to 99.2)	98.9 (96.9 to 99.8)	0.1 (0.0 to 0.2)	1.3 (0.3 to 2.8)
Thailand	94.5 (91.1 to 96.8)	99.2 (98.6 to 99.5)	99.2 (98.6 to 99.6)	99.8 (99.3 to 99.9)	0.0 (0.0 to 0.1)	0.6 (0.4 to 0.9)
Timor-Leste	14.9 (13.6 to 16.3)	58.4 (56.0 to 60.5)	60.0 (55.2 to 64.5)	86.1 (80.6 to 90.6)	2.6 (2.1 to 3.0)	47.6 (37.9 to 56.3)
Vietnam	84.3 (81.6 to 86.8)	95.8 (93.7 to 97.3)	96.5 (94.2 to 98.1)	98.9 (97.2 to 99.7)	0.2 (0.2 to 0.3)	3.3 (2.3 to 4.5)
Sub-Saharan Africa						
Central Sub-Saharan Africa						
Angola	46.9 (43.7 to 50.3)	52.1 (49.7 to 54.5)	57.7 (51.1 to 64.0)	69.2 (56.0 to 80.4)	1.9 (0.7 to 2.7)	32.8 (10.5 to 50.8)
Central African Republic	47.0 (45.4 to 48.7)	51.8 (38.0 to 66.2)	52.2 (36.5 to 68.2)	53.8 (27.2 to 81.6)	0.0 (-2.7 to 1.8)	2.2 (-33.5 to 31.6)
Congo	83.3 (79.1 to 87.0)	94.8 (91.9 to 97.0)	95.2 (91.8 to 97.6)	97.8 (93.8 to 99.5)	0.2 (0.1 to 0.3)	3.1 (1.3 to 4.8)
Democratic Republic of the Congo	47.1 (36.8 to 58.0)	60.5 (46.5 to 73.8)	64.9 (49.0 to 79.3)	75.5 (47.6 to 93.3)	1.4 (-0.1 to 2.4)	24.5 (-2.0 to 42.5)
Equatorial Guinea	65.6 (61.3 to 70.0)	95.1 (90.8 to 97.7)	95.9 (92.1 to 98.2)	99.2 (97.6 to 99.8)	0.3 (0.1 to 0.5)	4.3 (2.1 to 7.8)
Gabon	86.6 (84.8 to 88.5)	93.4 (90.1 to 96.0)	94.5 (90.8 to 97.1)	97.1 (92.4 to 99.3)	0.3 (0.1 to 0.4)	4.0 (1.5 to 5.8)
Eastern Sub-Saharan Africa						
Burundi	27.1 (23.7 to 30.4)	86.6 (85.6 to 87.6)	86.0 (83.6 to 88.2)	97.2 (96.0 to 98.2)	0.8 (0.7 to 0.9)	12.3 (10.6 to 13.8)
Comoros	69.5 (66.1 to 72.8)	86.0 (80.1 to 91.0)	86.9 (78.9 to 92.8)	92.8 (82.0 to 98.2)	0.5 (0.1 to 0.7)	7.8 (0.8 to 11.6)
Djibouti	89.5 (84.0 to 93.4)	95.5 (91.8 to 97.8)	96.1 (92.7 to 98.2)	97.9 (93.6 to 99.6)	0.2 (0.1 to 0.3)	2.5 (1.1 to 4.0)
Eritrea	29.1 (27.1 to 31.2)	44.0 (26.5 to 62.7)	47.5 (29.6 to 67.0)	63.4 (31.6 to 89.4)	2.4 (-0.0 to 4.1)	44.2 (-0.5 to 84.7)
Ethiopia	5.9 (5.0 to 7.0)	30.3 (28.0 to 32.4)	39.7 (34.1 to 45.2)	81.2 (73.3 to 86.6)	6.6 (6.0 to 7.1)	168.8 (144.6 to 191.0)
Kenya	42.4 (40.6 to 44.5)	69.1 (65.5 to 72.2)	72.6 (65.2 to 79.3)	87.8 (78.2 to 94.2)	1.6 (1.1 to 1.9)	26.9 (17.3 to 33.2)
Madagascar	46.2 (43.7 to 48.8)	50.7 (34.8 to 66.8)	55.1 (37.9 to 71.6)	63.8 (32.5 to 88.4)	1.4 (-0.9 to 2.8)	24.9 (-12.2 to 52.9)
Malawi	54.8 (53.3 to 56.4)	91.0 (89.8 to 92.1)	91.4 (88.7 to 93.6)	97.9 (96.3 to 99.0)	0.5 (0.4 to 0.6)	7.6 (6.4 to 8.7)
Mozambique	49.1 (47.5 to 50.8)	59.8 (47.9 to 71.1)	64.0 (49.0 to 77.5)	73.7 (46.9 to 91.8)	1.3 (-0.4 to 2.2)	22.7 (-6.1 to 39.5)
Rwanda	26.0 (24.5 to 27.5)	92.5 (90.7 to 94.1)	92.2 (88.6 to 95.0)	98.2 (96.3 to 99.4)	0.4 (0.3 to 0.5)	6.2 (5.1 to 7.4)
Somalia	32.9 (20.9 to 46.1)	46.7 (34.1 to 59.6)	48.3 (31.6 to 63.6)	59.9 (30.3 to 85.3)	1.5 (-1.1 to 3.0)	26.9 (-15.7 to 58.0)
South Sudan	26.5 (14.6 to 41.9)	43.0 (30.8 to 57.0)	43.3 (28.6 to 59.9)	54.1 (26.9 to 83.2)	1.3 (-1.5 to 3.2)	24.0 (-19.9 to 60.7)
Tanzania	42.8 (40.4 to 45.3)	70.3 (67.8 to 72.8)	71.8 (65.5 to 77.7)	86.1 (77.3 to 92.5)	1.3 (0.8 to 1.7)	22.4 (12.4 to 29.8)
Uganda	38.9 (36.4 to 41.5)	76.8 (75.4 to 78.1)	78.9 (75.6 to 81.8)	93.2 (90.3 to 95.5)	1.3 (1.1 to 1.5)	21.4 (17.4 to 24.8)
Zambia	44.0 (41.9 to 46.0)	74.3 (68.6 to 79.2)	77.1 (67.9 to 84.3)	90.7 (80.0 to 96.5)	1.3 (0.9 to 1.6)	22.1 (14.7 to 27.7)
Southern Sub-Saharan Africa						
Botswana	92.3 (88.5 to 95.2)	97.4 (95.1 to 98.8)	97.8 (95.6 to 99.0)	99.0 (97.0 to 99.8)	0.1 (0.1 to 0.2)	1.6 (0.8 to 2.9)
Lesotho	58.9 (56.6 to 61.3)	83.2 (79.5 to 86.3)	84.4 (78.0 to 89.4)	93.9 (87.2 to 97.7)	0.8 (0.5 to 1.0)	12.8 (7.6 to 16.2)
Namibia	77.5 (75.3 to 79.7)	90.2 (86.8 to 93.0)	91.3 (86.4 to 94.8)	95.6 (89.3 to 98.7)	0.4 (0.1 to 0.5)	6.0 (2.2 to 8.2)
South Africa	87.4 (84.8 to 89.7)	99.0 (98.1 to 99.5)	99.0 (98.0 to 99.6)	99.7 (99.2 to 100.0)	0.1 (0.0 to 0.1)	0.8 (0.4 to 1.4)
Swaziland	75.7 (71.1 to 80.2)	90.0 (87.1 to 92.3)	90.9 (86.8 to 94.3)	96.0 (91.5 to 98.7)	0.4 (0.2 to 0.6)	6.7 (3.7 to 8.7)
Zimbabwe	70.9 (68.3 to 73.3)	82.8 (80.7 to 84.7)	84.3 (79.3 to 88.4)	92.3 (86.1 to 96.3)	0.7 (0.4 to 0.9)	11.6 (5.6 to 15.1)
Western Sub-Saharan Africa						
Benin	77.1 (75.3 to 79.0)	87.5 (84.8 to 89.8)	88.7 (83.8 to 92.2)	93.6 (86.8 to 97.3)	0.4 (0.1 to 0.6)	7.0 (1.7 to 9.9)
Burkina Faso	43.0 (40.7 to 45.2)	76.1 (65.2 to 85.3)	76.1 (63.0 to 87.2)	87.0 (69.6 to 97.0)	0.9 (0.3 to 1.4)	14.2 (3.8 to 22.5)
Cameroon	56.8 (54.7 to 58.9)	63.9 (59.8 to 68.3)	67.4 (58.6 to 75.7)	72.3 (54.9 to 86.2)	0.8 (-0.6 to 1.7)	12.8 (-8.7 to 28.5)
Cape Verde	65.6 (53.0 to 76.8)	89.2 (79.9 to 95.1)	90.8 (82.7 to 95.9)	96.8 (91.0 to 99.3)	0.5 (0.3 to 1.0)	8.6 (4.0 to 15.4)
Chad	14.0 (12.4 to 15.9)	23.6 (20.3 to 27.0)	26.1 (18.9 to 34.1)	38.0 (20.1 to 57.7)	3.0 (-0.3 to 5.3)	59.6 (-4.8 to 120.8)
Cote d'Ivoire	64.6 (62.3 to 66.8)	64.4 (52.7 to 74.7)	67.7 (52.8 to 79.5)	75.0 (48.4 to 91.4)	1.0 (-0.9 to 1.8)	16.0 (-12.5 to 30.7)
The Gambia	57.0 (52.6 to 61.5)	67.1 (60.3 to 73.7)	69.3 (58.4 to 78.8)	77.2 (56.9 to 90.6)	0.9 (-0.4 to 1.6)	14.8 (-6.4 to 27.4)
Ghana	47.5 (45.2 to 49.8)	73.9 (69.1 to 77.9)	75.8 (67.5 to 82.9)	88.3 (77.2 to 95.1)	1.2 (0.6 to 1.5)	19.5 (10.0 to 25.9)
Guinea	33.6 (31.1 to 36.2)	47.5 (37.5 to 58.0)	50.3 (36.9 to 64.2)	61.7 (35.9 to 84.4)	1.6 (-0.6 to 2.9)	28.8 (-9.1 to 55.2)
Guinea-Bissau	37.7 (30.1 to 45.6)	45.4 (40.3 to 50.3)	47.7 (36.8 to 57.7)	56.4 (34.9 to 76.3)	1.3 (-1.1 to 2.9)	23.5 (-15.3 to 55.4)
Liberia	33.2 (25.6 to 41.1)	67.0 (59.8 to 73.5)	67.8 (56.4 to 77.6)	82.9 (66.5 to 93.5)	1.4 (0.5 to 1.9)	23.7 (7.9 to 33.2)
Mali	33.7 (32.5 to 34.9)	50.0 (40.2 to 59.9)	54.0 (40.4 to 67.4)	66.8 (40.7 to 87.0)	1.8 (-0.1 to 3.0)	32.8 (-1.0 to 55.9)
Mauritania	61.9 (58.9 to 65.0)	75.7 (66.9 to 83.8)	79.8 (69.7 to 88.2)	90.0 (76.7 to 97.2)	1.2 (0.7 to 1.5)	18.9 (11.0 to 25.8)
Niger	16.6 (14.4 to 19.1)	36.8 (27.1 to 46.2)	38.2 (25.7 to 52.3)	57.0 (30.6 to 81.9)	2.8 (0.4 to 4.3)	53.3 (5.5 to 89.4)
Nigeria	34.4 (32.0 to 37.0)	35.5 (34.0 to 37.1)	39.8 (35.6 to 43.5)	45.4 (36.0 to 53.9)	1.6 (0.0 to 2.8)	27.8 (0.7 to 51.8)
Sao Tome and Principe	81.3 (73.6 to 87.7)	88.5 (79.8 to 94.5)	90.1 (81.4 to 95.5)	94.0 (82.1 to 98.8)	0.4 (0.0 to 0.7)	6.2 (0.2 to 10.9)
Senegal	59.5 (50.9 to 67.3)	58.1 (56.1 to 60.1)	61.4 (56.2 to 66.3)	63.9 (52.8 to 74.1)	0.6 (-0.6 to 1.6)	9.9 (-9.0 to 26.5)
Sierra Leone	44.3 (40.4 to 48.3)	49.5 (42.8 to 56.8)	52.5 (41.5 to 64.5)	60.7 (38.1 to 81.8)	1.2 (-1.1 to 2.7)	21.8 (-15.4 to 49.0)
Togo	56.0 (52.3 to 59.9)	63.7 (56.7 to 70.5)	67.6 (56.2 to 77.8)	75.3 (53.8 to 90.1)	1.1 (-0.5 to 1.9)	17.9 (-7.4 to 32.3)
**Indicator 3.2.1: Under-5 mortality rate (probability of dying before the age of 5 per 1,000 livebirths)**						
Central Europe, Eastern Europe, and Central Asia						
Central Asia						
Armenia	27.4 (24.6 to 30.5)	10.1 (9.6 to 10.7)	9.6 (8.7 to 10.5)	6.4 (4.4 to 9.0)	-3.2 (-5.5 to -0.9)	-36.9 (-56.4 to -12.9)
Azerbaijan	64.6 (58.4 to 71.3)	37.5 (32.2 to 43.0)	35.2 (30.0 to 41.0)	25.0 (20.1 to 30.5)	-2.7 (-3.6 to -1.8)	-33.3 (-42.0 to -23.2)
Georgia	34.0 (30.9 to 37.6)	12.0 (10.1 to 14.0)	11.1 (9.3 to 13.1)	7.2 (5.4 to 9.6)	-3.4 (-4.7 to -1.9)	-39.7 (-50.9 to -25.0)
Kazakhstan	35.3 (31.6 to 39.7)	14.8 (12.5 to 17.7)	14.1 (11.8 to 17.2)	9.6 (6.8 to 13.4)	-3.0 (-4.8 to -1.0)	-35.1 (-51.6 to -13.4)
Kyrgyzstan	44.4 (40.2 to 49.2)	21.9 (21.3 to 22.5)	20.1 (18.9 to 21.5)	14.3 (13.1 to 15.7)	-2.8 (-3.4 to -2.2)	-34.5 (-40.2 to -28.3)
Mongolia	61.4 (56.5 to 66.8)	27.7 (23.9 to 32.5)	25.7 (21.9 to 30.8)	16.5 (13.0 to 21.5)	-3.5 (-4.7 to -2.1)	-40.4 (-50.3 to -27.3)
Tajikistan	71.9 (65.9 to 78.2)	49.2 (42.7 to 56.6)	46.9 (40.2 to 54.5)	29.0 (23.1 to 36.3)	-3.6 (-4.7 to -2.2)	-41.2 (-50.4 to -27.9)
Turkmenistan	55.9 (50.4 to 62.1)	31.6 (27.4 to 36.1)	29.1 (24.9 to 33.9)	17.8 (14.5 to 21.4)	-3.8 (-4.7 to -3.1)	-43.7 (-50.8 to -37.0)

	Estimate in 2000 (95% UIs)	Estimate in 2015 (95% UIs)	Estimate in 2017 (95% UIs)	Estimate in 2030 (95% UIs)	Annualised rate of change, 2015-2030 (95% UIs)	Percent change, 2015-2030 (95% UIs)
Uzbekistan	45.2 (40.5 to 50.5)	26.1 (22.0 to 30.8)	23.8 (20.1 to 28.8)	16.6 (13.0 to 21.4)	-3.0 (-4.1 to -1.7)	-36.3 (-46.1 to -23.0)
Central Europe						
Albania	28.5 (25.6 to 31.5)	13.5 (11.4 to 16.2)	12.7 (10.5 to 15.2)	9.3 (6.7 to 12.7)	-2.5 (-4.4 to -0.8)	-31.0 (-48.6 to -11.1)
Bosnia and Herzegovina	10.6 (10.0 to 11.3)	6.9 (6.4 to 7.4)	6.4 (5.6 to 7.4)	4.8 (3.6 to 8.8)	-2.6 (-4.2 to 1.5)	-30.4 (-47.0 to 26.0)
Bulgaria	16.9 (16.2 to 17.7)	8.0 (7.5 to 8.5)	7.7 (6.8 to 8.7)	5.1 (3.9 to 6.5)	-3.0 (-4.9 to -1.4)	-36.0 (-51.9 to -18.5)
Croatia	8.2 (7.7 to 8.8)	4.7 (4.3 to 5.2)	4.3 (3.7 to 4.9)	3.0 (2.4 to 3.7)	-3.1 (-4.4 to -1.6)	-36.8 (-48.7 to -20.7)
Czech Republic	5.2 (4.9 to 5.6)	3.1 (2.9 to 3.3)	2.9 (2.5 to 3.2)	2.0 (1.5 to 2.6)	-2.9 (-4.8 to -1.1)	-34.4 (-51.4 to -14.8)
Hungary	10.5 (10.0 to 10.9)	5.6 (5.2 to 5.9)	4.8 (4.2 to 5.4)	3.2 (2.6 to 4.1)	-3.7 (-5.2 to -2.0)	-42.0 (-53.9 to -26.0)
Macedonia	14.9 (14.0 to 15.9)	10.0 (9.3 to 10.8)	9.6 (8.5 to 10.9)	7.1 (5.2 to 10.0)	-2.4 (-4.4 to -0.1)	-29.1 (-48.2 to -0.9)
Montenegro	14.2 (13.1 to 15.5)	4.1 (3.6 to 4.7)	4.0 (3.5 to 4.7)	2.7 (1.9 to 4.1)	-2.8 (-5.0 to -0.1)	-32.8 (-52.4 to -1.5)
Poland	9.3 (9.1 to 9.6)	4.7 (4.5 to 4.8)	4.4 (4.0 to 4.9)	2.8 (2.1 to 3.5)	-3.5 (-5.2 to -1.9)	-40.6 (-53.9 to -24.7)
Romania	22.3 (21.8 to 22.9)	9.3 (8.9 to 9.7)	8.7 (7.9 to 9.6)	4.8 (3.8 to 6.1)	-4.4 (-6.1 to -2.7)	-47.9 (-59.9 to -33.5)
Serbia	16.0 (13.5 to 18.7)	5.2 (4.9 to 5.5)	5.0 (4.5 to 5.6)	3.6 (3.0 to 4.5)	-2.4 (-3.8 to -0.8)	-30.0 (-43.5 to -11.9)
Slovakia	9.7 (9.1 to 10.2)	6.5 (6.1 to 7.0)	6.0 (5.3 to 6.7)	4.1 (3.4 to 5.0)	-3.2 (-4.4 to -1.7)	-37.5 (-48.0 to -22.1)
Slovenia	5.3 (4.9 to 5.8)	2.3 (2.1 to 2.6)	2.2 (1.9 to 2.5)	1.5 (1.2 to 1.9)	-3.0 (-4.4 to -1.5)	-35.6 (-48.5 to -19.8)
Eastern Europe						
Belarus	17.7 (15.5 to 20.0)	7.0 (6.0 to 8.2)	6.5 (5.5 to 7.8)	3.7 (2.8 to 4.9)	-4.3 (-5.9 to -2.5)	-47.1 (-58.5 to -31.7)
Estonia	11.0 (10.2 to 11.9)	3.3 (2.9 to 3.8)	3.0 (2.6 to 3.5)	2.5 (1.4 to 4.0)	-2.2 (-5.8 to 1.1)	-25.7 (-58.1 to 18.6)
Latvia	13.4 (12.6 to 14.2)	5.2 (4.7 to 5.7)	4.9 (4.3 to 5.5)	3.0 (2.0 to 4.5)	-3.7 (-6.2 to -1.1)	-41.8 (-60.6 to -15.5)
Lithuania	10.6 (10.1 to 11.2)	5.1 (4.7 to 5.5)	4.8 (4.3 to 5.4)	3.3 (2.3 to 4.3)	-3.0 (-5.3 to -1.1)	-35.4 (-54.8 to -14.9)
Moldova	29.1 (26.4 to 32.2)	15.8 (13.6 to 18.2)	14.4 (12.3 to 16.8)	11.1 (7.5 to 15.8)	-2.5 (-4.8 to 0.0)	-30.0 (-51.6 to 0.3)
Russia	19.5 (19.2 to 19.7)	8.1 (8.0 to 8.2)	7.4 (7.0 to 7.8)	4.7 (3.7 to 5.8)	-3.7 (-5.2 to -2.2)	-42.3 (-53.9 to -28.0)
Ukraine	14.9 (14.6 to 15.2)	10.4 (10.2 to 10.7)	9.5 (8.9 to 10.1)	7.4 (5.6 to 9.6)	-2.4 (-4.1 to -0.5)	-29.5 (-45.9 to -7.2)
High-income						
Australasia						
Australia	6.3 (6.1 to 6.6)	4.0 (3.8 to 4.2)	3.9 (3.3 to 4.5)	3.1 (2.5 to 3.7)	-1.9 (-3.0 to -0.6)	-24.0 (-36.6 to -9.0)
New Zealand	7.7 (7.2 to 8.2)	5.1 (4.7 to 5.5)	4.5 (3.9 to 5.1)	3.4 (2.6 to 4.4)	-2.7 (-4.5 to -0.9)	-32.2 (-48.7 to -12.0)
High-income Asia Pacific						
Brunei	9.8 (9.0 to 10.5)	9.6 (8.6 to 10.6)	9.0 (7.9 to 10.3)	8.8 (6.3 to 11.7)	-0.6 (-2.7 to 1.2)	-7.9 (-33.4 to 20.5)
Japan	4.4 (4.3 to 4.5)	2.7 (2.6 to 2.8)	2.6 (2.2 to 3.0)	1.9 (1.6 to 2.3)	-2.4 (-3.6 to -1.1)	-29.7 (-42.1 to -15.2)
South Korea	6.6 (6.4 to 6.7)	3.4 (3.3 to 3.5)	3.3 (3.0 to 3.5)	3.1 (1.9 to 15.9)	-1.6 (-4.2 to 10.3)	-9.6 (-46.4 to 368.4)
Singapore	3.3 (3.1 to 3.6)	2.0 (1.9 to 2.2)	1.9 (1.7 to 2.2)	1.5 (1.2 to 1.8)	-2.2 (-3.5 to -0.9)	-27.6 (-40.4 to -13.2)
High-income North America						
Canada	6.0 (5.8 to 6.3)	5.5 (5.4 to 5.8)	5.4 (4.9 to 6.0)	4.7 (3.9 to 5.6)	-1.1 (-2.4 to 0.1)	-14.7 (-29.7 to 1.2)
Greenland	19.4 (17.2 to 21.8)	14.7 (12.8 to 17.0)	13.8 (11.8 to 16.2)	10.6 (8.3 to 13.6)	-2.2 (-3.5 to -0.8)	-27.7 (-40.8 to -10.8)
United States	8.3 (8.2 to 8.4)	6.9 (6.8 to 7.0)	6.7 (6.1 to 7.4)	5.7 (4.8 to 6.7)	-1.3 (-2.4 to -0.2)	-17.2 (-30.7 to -3.3)
Southern Latin America						
Argentina	19.4 (19.0 to 19.7)	11.3 (11.1 to 11.5)	10.9 (9.5 to 12.6)	8.3 (6.9 to 9.9)	-2.1 (-3.3 to -0.9)	-26.6 (-38.9 to -12.8)
Chile	11.4 (11.0 to 11.8)	8.0 (7.7 to 8.3)	7.3 (6.3 to 8.4)	5.8 (4.7 to 7.2)	-2.2 (-3.5 to -0.7)	-27.2 (-40.8 to -10.2)
Uruguay	16.4 (15.6 to 17.2)	9.0 (8.4 to 9.7)	9.1 (7.8 to 10.6)	6.9 (5.3 to 8.8)	-1.8 (-3.4 to -0.1)	-23.2 (-40.3 to -1.3)
Western Europe						
Andorra	3.4 (2.9 to 4.1)	1.9 (1.6 to 2.3)	1.9 (1.6 to 2.3)	1.6 (1.0 to 2.4)	-1.5 (-3.8 to 1.2)	-18.8 (-43.4 to 20.0)
Austria	5.6 (5.3 to 6.0)	3.6 (3.4 to 3.9)	3.4 (3.0 to 3.9)	2.5 (2.1 to 3.1)	-2.4 (-3.8 to -1.0)	-30.1 (-43.1 to -14.0)
Belgium	6.0 (5.7 to 6.3)	3.9 (3.7 to 4.2)	3.6 (3.2 to 4.1)	2.8 (2.4 to 3.3)	-2.2 (-3.4 to -1.1)	-28.3 (-39.8 to -14.6)
Cyprus	7.2 (6.6 to 7.8)	3.1 (2.8 to 3.4)	2.9 (2.6 to 3.3)	2.4 (1.6 to 2.9)	-2.4 (-4.2 to -0.6)	-22.9 (-46.9 to -9.0)
Denmark	5.6 (5.2 to 6.0)	4.1 (3.8 to 4.4)	3.8 (3.3 to 4.3)	3.0 (2.5 to 3.6)	-2.2 (-3.3 to -0.9)	-27.5 (-39.0 to -12.6)
Finland	4.3 (4.0 to 4.6)	2.2 (2.0 to 2.4)	2.2 (1.9 to 2.5)	1.8 (1.4 to 2.2)	-1.5 (-3.0 to -0.0)	-19.7 (-36.2 to -0.4)
France	5.5 (5.3 to 5.6)	4.1 (4.0 to 4.2)	3.9 (3.5 to 4.3)	2.9 (2.5 to 3.5)	-2.2 (-3.3 to -1.0)	-27.9 (-39.1 to -13.6)
Germany	5.3 (5.2 to 5.5)	4.0 (3.9 to 4.2)	3.6 (3.1 to 4.2)	2.7 (2.2 to 3.1)	-2.8 (-3.8 to -1.7)	-34.0 (-43.6 to -23.0)
Greece	6.3 (5.9 to 6.6)	4.7 (4.4 to 5.0)	4.5 (4.0 to 5.0)	3.0 (2.4 to 3.8)	-2.9 (-4.4 to -1.5)	-34.9 (-48.0 to -20.6)
Iceland	4.0 (3.5 to 4.5)	2.3 (2.0 to 2.7)	2.2 (1.9 to 2.6)	1.7 (1.3 to 2.1)	-2.3 (-3.6 to -1.1)	-29.1 (-41.3 to -15.3)
Ireland	7.0 (6.6 to 7.5)	3.8 (3.5 to 4.1)	3.4 (3.0 to 3.9)	2.9 (2.3 to 3.6)	-1.9 (-3.4 to -0.3)	-24.6 (-39.7 to -4.0)
Israel	6.8 (6.5 to 7.2)	3.9 (3.6 to 4.1)	3.6 (3.2 to 4.0)	2.5 (1.9 to 3.6)	-2.9 (-4.8 to -0.5)	-34.4 (-51.1 to -7.5)
Italy	5.4 (5.2 to 5.6)	3.5 (3.3 to 3.6)	3.2 (2.9 to 3.6)	2.4 (2.0 to 2.9)	-2.4 (-3.6 to -1.1)	-30.0 (-41.5 to -15.1)
Luxembourg	4.8 (4.2 to 5.3)	2.3 (2.0 to 2.7)	2.1 (1.8 to 2.5)	1.8 (1.3 to 2.3)	-2.0 (-3.9 to -0.3)	-24.9 (-44.0 to -4.1)
Malta	7.5 (6.8 to 8.3)	6.3 (5.6 to 7.2)	6.0 (5.2 to 6.9)	4.1 (3.3 to 5.1)	-2.9 (-4.1 to -1.7)	-35.2 (-45.7 to -22.9)
Netherlands	6.3 (6.0 to 6.6)	3.9 (3.7 to 4.1)	3.9 (3.5 to 4.4)	3.1 (2.7 to 3.7)	-1.4 (-2.4 to -0.1)	-18.6 (-30.7 to -1.4)
Norway	4.9 (4.5 to 5.2)	2.8 (2.6 to 3.1)	2.6 (2.3 to 3.0)	2.1 (1.7 to 2.5)	-2.0 (-3.2 to -0.8)	-25.7 (-38.1 to -10.9)
Portugal	7.3 (6.9 to 7.7)	3.6 (3.4 to 3.9)	3.4 (3.0 to 3.9)	2.3 (1.7 to 3.0)	-3.2 (-5.0 to -1.3)	-37.1 (-52.6 to -17.4)
Spain	5.5 (5.3 to 5.7)	3.2 (3.1 to 3.4)	3.2 (2.9 to 3.6)	2.3 (1.9 to 2.7)	-2.4 (-3.6 to -1.1)	-30.3 (-42.1 to -15.8)
Sweden	3.9 (3.6 to 4.2)	2.9 (2.7 to 3.1)	2.7 (2.4 to 3.1)	2.1 (1.8 to 2.5)	-2.1 (-3.3 to -1.0)	-26.8 (-38.6 to -13.3)
Switzerland	5.8 (5.5 to 6.2)	4.4 (4.1 to 4.7)	3.8 (3.4 to 4.3)	3.1 (2.5 to 3.7)	-2.4 (-3.8 to -1.1)	-30.3 (-43.8 to -15.5)
United Kingdom	6.5 (6.1 to 7.0)	4.5 (4.3 to 4.6)	4.5 (4.0 to 4.9)	3.4 (2.9 to 3.9)	-1.9 (-2.8 to -0.9)	-24.1 (-33.9 to -12.4)
Latin America and Caribbean						
Andean Latin America						
Bolivia	71.3 (65.4 to 77.1)	32.9 (27.8 to 38.6)	29.6 (24.8 to 35.2)	19.4 (15.9 to 23.3)	-3.5 (-4.2 to -2.8)	-41.1 (-47.1 to -34.2)
Ecuador	34.6 (30.2 to 39.1)	18.6 (15.5 to 21.9)	17.5 (14.6 to 21.0)	12.7 (10.1 to 15.8)	-2.5 (-3.8 to -1.2)	-31.3 (-43.5 to -17.1)
Peru	38.6 (35.6 to 41.9)	16.6 (13.9 to 19.4)	15.5 (12.9 to 18.6)	10.4 (7.8 to 13.5)	-3.2 (-4.6 to -1.6)	-37.1 (-49.9 to -21.4)
Caribbean						
Antigua and Barbuda	14.9 (12.7 to 17.2)	11.4 (9.6 to 13.6)	11.1 (9.3 to 13.3)	8.9 (6.6 to 11.5)	-1.7 (-3.4 to -0.2)	-22.5 (-40.2 to -3.1)
The Bahamas	15.1 (13.1 to 17.2)	13.2 (11.2 to 15.7)	12.5 (10.5 to 15.3)	10.5 (8.4 to 13.2)	-1.6 (-2.6 to -0.4)	-20.7 (-32.4 to -6.1)
Barbados	14.9 (12.8 to 17.2)	13.2 (11.0 to 15.6)	13.0 (10.9 to 15.6)	11.2 (8.8 to 14.0)	-1.2 (-2.2 to -0.1)	-15.7 (-27.7 to -1.2)
Belize	27.9 (25.3 to 30.7)	18.5 (16.0 to 21.5)	17.9 (15.3 to 21.2)	13.2 (10.4 to 16.7)	-2.3 (-3.5 to -1.2)	-28.6 (-40.5 to -16.8)
Bermuda	5.8 (4.9 to 6.8)	5.3 (4.5 to 6.3)	5.1 (4.3 to 6.2)	4.4 (1.7 to 11.2)	-1.9 (-7.6 to 4.6)	-18.2 (-68.1 to 100.2)
Cuba	8.6 (8.2 to 9.0)	5.8 (5.4 to 6.1)	5.2 (4.4 to 6.0)	3.9 (3.2 to 4.8)	-2.6 (-3.9 to -1.3)	-31.6 (-44.1 to -17.6)
Dominica	19.3 (16.7 to 22.2)	30.5 (25.9 to 35.5)	30.3 (25.6 to 36.0)	30.7 (18.4 to 43.9)	-0.1 (-3.3 to 2.2)	0.8 (-39.1 to 39.3)
Dominican Republic	41.8 (38.0 to 45.7)	31.3 (25.8 to 37.5)	29.2 (24.2 to 35.3)	20.6 (15.6 to 26.2)	-2.8 (-4.4 to -1.2)	-33.9 (-48.1 to -17.0)
Grenada	17.1 (14.9 to 19.7)	15.1 (12.9 to 17.7)	14.4 (12.2 to 17.0)	12.0 (8.1 to 16.5)	-1.6 (-3.8 to 0.3)	-20.3 (-43.1 to 4.0)
Guyana	38.1 (35.0 to 41.3)	26.5 (22.8 to 30.5)	24.8 (21.1 to 29.2)	15.8 (10.4 to 20.8)	-3.5 (-5.9 to -1.9)	-40.5 (-59.0 to -25.0)
Haiti	102.9 (95.1 to 111.3)	65.2 (55.9 to 76.6)	59.3 (50.1 to 70.2)	36.8 (27.4 to 46.4)	-3.9 (-5.4 to -2.8)	-43.6 (-55.6 to -34.2)

	Estimate in 2000 (95% UIs)	Estimate in 2015 (95% UIs)	Estimate in 2017 (95% UIs)	Estimate in 2030 (95% UIs)	Annualised rate of change, 2015-2030 (95% UIs)	Percent change, 2015-2030 (95% UIs)
Jamaica	19.9 (17.1 to 22.9)	15.1 (12.7 to 18.0)	14.5 (12.1 to 17.5)	12.3 (9.9 to 15.3)	-1.3 (-2.3 to -0.5)	-18.2 (-28.7 to -7.7)
Puerto Rico	11.0 (10.4 to 11.6)	7.5 (6.9 to 8.1)	10.0 (9.1 to 11.1)	7.5 (6.0 to 9.6)	0.0 (-1.5 to 1.6)	0.8 (-20.1 to 27.7)
Saint Lucia	18.4 (15.9 to 21.2)	14.7 (12.4 to 17.4)	13.8 (11.5 to 16.4)	9.8 (7.1 to 13.1)	-2.8 (-4.5 to -1.0)	-33.3 (-49.2 to -14.0)
Saint Vincent and the Grenadines	24.0 (20.8 to 27.5)	17.5 (14.8 to 20.5)	16.5 (13.9 to 19.6)	11.8 (8.8 to 15.3)	-2.7 (-4.3 to -1.4)	-32.8 (-47.5 to -18.9)
Suriname	42.4 (37.4 to 47.8)	29.5 (25.1 to 34.6)	28.7 (24.3 to 33.9)	23.7 (18.9 to 28.9)	-1.5 (-2.6 to -0.5)	-19.5 (-32.1 to -7.3)
Trinidad and Tobago	25.9 (22.5 to 29.6)	16.8 (13.8 to 20.2)	16.6 (13.8 to 20.1)	13.7 (10.8 to 17.1)	-1.4 (-2.4 to -0.2)	-18.3 (-30.2 to -3.3)
Virgin Islands, U.S.	11.5 (10.3 to 12.9)	8.6 (7.2 to 10.1)	8.6 (7.2 to 10.2)	5.7 (4.5 to 7.2)	-2.8 (-4.1 to -1.5)	-33.6 (-45.9 to -20.5)
Central Latin America						
Colombia	25.6 (23.3 to 28.1)	15.3 (13.0 to 17.8)	14.2 (12.1 to 16.8)	10.2 (8.4 to 12.5)	-2.7 (-3.6 to -1.7)	-33.5 (-42.1 to -22.8)
Costa Rica	12.4 (11.8 to 13.0)	8.8 (7.9 to 9.9)	8.1 (6.8 to 9.6)	6.1 (4.9 to 7.4)	-2.5 (-3.8 to -1.3)	-31.0 (-43.0 to -17.9)
El Salvador	28.4 (25.7 to 31.3)	14.4 (12.3 to 16.6)	12.4 (10.4 to 14.8)	8.4 (6.3 to 12.4)	-3.6 (-5.2 to -1.1)	-41.2 (-54.2 to -15.4)
Guatemala	50.1 (45.5 to 54.9)	27.3 (23.7 to 31.5)	24.6 (20.8 to 29.1)	17.6 (13.5 to 22.5)	-3.0 (-4.3 to -1.4)	-35.4 (-47.5 to -19.4)
Honduras	36.3 (32.4 to 40.3)	16.4 (14.0 to 19.2)	14.6 (12.1 to 17.5)	10.3 (8.2 to 13.4)	-3.2 (-4.2 to -1.4)	-37.5 (-47.0 to -19.0)
Mexico	25.0 (22.3 to 28.1)	16.4 (13.9 to 18.9)	15.7 (13.3 to 18.6)	11.2 (9.2 to 13.4)	-2.6 (-3.4 to -1.8)	-31.7 (-40.0 to -23.3)
Nicaragua	35.7 (32.5 to 39.2)	17.5 (15.0 to 20.6)	15.2 (12.9 to 18.2)	10.3 (7.2 to 16.6)	-3.6 (-5.4 to -0.5)	-40.9 (-55.7 to -7.9)
Panama	23.6 (20.5 to 27.5)	18.0 (15.2 to 21.0)	17.2 (14.5 to 20.4)	13.1 (10.5 to 16.1)	-2.1 (-3.1 to -1.1)	-27.2 (-37.4 to -15.8)
Venezuela	22.6 (22.2 to 23.0)	15.9 (14.4 to 17.4)	15.6 (13.2 to 18.4)	12.8 (10.3 to 15.8)	-1.5 (-2.8 to -0.1)	-19.7 (-33.9 to -1.2)
Tropical Latin America						
Brazil	41.4 (37.8 to 45.1)	19.8 (17.0 to 22.9)	18.4 (15.6 to 21.6)	13.5 (11.4 to 16.0)	-2.6 (-3.2 to -1.9)	-31.8 (-38.4 to -24.7)
Paraguay	28.7 (25.9 to 32.0)	15.1 (13.0 to 17.5)	14.0 (11.9 to 16.4)	10.2 (8.0 to 12.7)	-2.7 (-3.8 to -1.5)	-32.7 (-43.6 to -20.7)
North Africa and Middle East						
North Africa and Middle East						
Afghanistan	120.5 (110.9 to 131.6)	60.5 (52.3 to 69.1)	54.1 (46.4 to 62.8)	36.9 (29.2 to 45.8)	-3.3 (-4.4 to -2.0)	-39.1 (-48.4 to -26.3)
Algeria	33.7 (31.1 to 36.4)	25.9 (23.9 to 27.9)	22.8 (20.6 to 25.1)	16.0 (11.7 to 21.1)	-3.3 (-5.2 to -1.4)	-38.4 (-54.4 to -19.0)
Bahrain	11.7 (11.1 to 12.5)	7.9 (7.1 to 8.7)	7.3 (6.5 to 8.3)	5.9 (3.4 to 9.0)	-2.1 (-5.5 to 0.9)	-24.8 (-56.3 to 15.1)
Egypt	44.4 (40.0 to 49.1)	23.6 (19.8 to 27.6)	19.2 (16.0 to 23.1)	9.9 (7.4 to 13.1)	-5.8 (-7.5 to -4.1)	-57.8 (-67.4 to -45.6)
Iran	39.6 (34.6 to 45.1)	20.0 (19.8 to 20.2)	14.4 (13.8 to 14.9)	9.6 (7.4 to 12.2)	-5.0 (-6.7 to -3.3)	-52.1 (-63.2 to -39.1)
Iraq	41.4 (38.3 to 44.9)	26.6 (23.3 to 30.4)	25.1 (21.9 to 28.7)	18.7 (15.3 to 25.1)	-2.4 (-3.4 to -0.5)	-29.8 (-39.6 to -6.6)
Jordan	27.6 (25.2 to 30.0)	15.3 (13.1 to 18.1)	14.4 (12.2 to 17.3)	10.5 (8.7 to 12.7)	-2.5 (-3.2 to -1.8)	-31.5 (-38.4 to -23.7)
Kuwait	11.7 (11.1 to 12.4)	9.0 (8.4 to 9.6)	7.8 (6.7 to 9.0)	5.9 (3.7 to 9.8)	-3.0 (-5.6 to 0.6)	-34.8 (-57.0 to 8.7)
Lebanon	17.6 (15.2 to 20.6)	8.6 (7.3 to 10.2)	8.1 (6.8 to 9.8)	6.6 (4.5 to 17.8)	-2.2 (-4.0 to 4.7)	-23.5 (-44.7 to 103.7)
Libya	26.4 (22.8 to 30.4)	14.3 (11.9 to 17.1)	14.3 (12.1 to 17.3)	9.7 (5.9 to 14.6)	-2.8 (-5.7 to -0.1)	-31.9 (-57.7 to -2.0)
Morocco	45.7 (40.8 to 50.8)	22.1 (18.2 to 26.5)	19.7 (16.3 to 23.9)	12.4 (9.6 to 15.5)	-3.9 (-5.2 to -2.9)	-43.9 (-53.8 to -35.6)
Palestine	29.6 (27.4 to 31.9)	15.3 (13.3 to 17.4)	13.6 (11.7 to 15.7)	10.9 (6.6 to 30.2)	-2.7 (-5.3 to 4.7)	-28.5 (-54.9 to 101.6)
Oman	16.3 (14.3 to 18.4)	11.2 (10.7 to 11.8)	10.6 (9.9 to 11.3)	7.5 (3.8 to 14.2)	-3.0 (-7.3 to 1.6)	-32.8 (-66.7 to 27.1)
Qatar	14.3 (12.6 to 16.2)	7.9 (6.8 to 9.3)	7.4 (6.2 to 8.7)	5.0 (4.0 to 6.2)	-3.1 (-4.2 to -2.1)	-36.8 (-46.5 to -26.6)
Saudi Arabia	26.5 (21.9 to 31.8)	9.2 (7.6 to 11.2)	7.9 (6.6 to 9.8)	3.4 (1.9 to 5.2)	-6.9 (-10.4 to -3.9)	-63.4 (-79.0 to -44.3)
Sudan	97.3 (89.7 to 105.6)	52.8 (46.1 to 60.9)	47.7 (40.9 to 56.4)	31.2 (24.5 to 39.6)	-3.5 (-4.8 to -2.3)	-40.9 (-51.6 to -29.5)
Syria	20.2 (19.8 to 20.6)	24.4 (22.8 to 26.2)	19.8 (18.2 to 21.6)	14.6 (9.1 to 28.6)	-3.6 (-6.6 to 1.0)	-39.9 (-62.9 to 17.0)
Tunisia	26.8 (23.8 to 29.9)	12.0 (10.0 to 14.3)	10.5 (8.8 to 12.6)	6.9 (5.1 to 9.0)	-3.8 (-5.5 to -2.3)	-42.8 (-56.0 to -28.9)
Turkey	40.1 (36.0 to 44.3)	16.6 (14.1 to 19.7)	14.2 (12.0 to 16.9)	8.5 (6.3 to 11.4)	-4.5 (-6.2 to -2.7)	-48.7 (-60.8 to -33.1)
United Arab Emirates	10.5 (10.0 to 11.1)	8.0 (6.7 to 9.5)	7.1 (6.0 to 8.7)	4.5 (2.8 to 6.7)	-3.9 (-7.2 to -1.4)	-43.2 (-65.9 to -18.7)
Yemen	88.2 (81.4 to 96.0)	46.2 (39.9 to 53.9)	45.9 (39.5 to 54.1)	27.9 (20.9 to 35.1)	-3.4 (-5.1 to -2.1)	-39.5 (-53.2 to -27.4)
South Asia						
South Asia						
Bangladesh	85.8 (80.2 to 91.5)	37.8 (33.2 to 43.2)	33.1 (28.4 to 38.5)	18.6 (14.8 to 24.1)	-4.7 (-5.9 to -3.2)	-50.6 (-58.6 to -38.0)
Bhutan	83.4 (73.5 to 94.0)	33.4 (27.5 to 40.4)	29.3 (24.2 to 36.0)	16.4 (11.8 to 21.1)	-4.8 (-6.6 to -3.5)	-51.1 (-62.9 to -41.1)
India	83.1 (76.7 to 90.1)	48.1 (42.1 to 54.8)	42.4 (36.5 to 50.0)	23.2 (18.6 to 28.6)	-4.9 (-6.0 to -3.9)	-51.8 (-59.2 to -44.3)
Nepal	79.3 (73.9 to 85.0)	36.7 (32.1 to 41.4)	31.4 (27.1 to 36.6)	16.7 (13.1 to 20.7)	-5.3 (-6.6 to -4.1)	-54.4 (-62.7 to -45.6)
Pakistan	90.5 (84.3 to 97.2)	64.5 (55.4 to 75.5)	58.2 (49.5 to 68.6)	34.1 (24.8 to 43.2)	-4.3 (-6.2 to -2.9)	-47.1 (-60.4 to -35.6)
Southeast Asia, East Asia, and Oceania						
East Asia						
China	33.9 (30.6 to 37.3)	13.6 (11.8 to 15.8)	12.0 (10.3 to 14.3)	6.7 (4.8 to 9.2)	-4.8 (-6.9 to -2.9)	-50.9 (-64.3 to -35.4)
North Korea	119.9 (112.3 to 128.3)	25.5 (20.9 to 31.3)	22.7 (18.7 to 28.1)	17.7 (10.0 to 50.3)	-2.9 (-6.2 to 4.8)	-30.5 (-60.7 to 106.9)
Taiwan (Province of China)	7.9 (7.6 to 8.1)	5.0 (4.8 to 5.2)	4.7 (4.2 to 5.2)	3.8 (2.9 to 5.2)	-1.9 (-3.7 to 0.3)	-24.0 (-42.2 to 5.4)
Oceania						
American Samoa	14.6 (12.9 to 16.3)	10.4 (8.9 to 12.1)	10.1 (8.6 to 12.0)	6.5 (4.5 to 8.3)	-3.2 (-5.4 to -1.6)	-37.5 (-55.5 to -21.5)
Federated States of Micronesia	34.4 (29.5 to 40.0)	18.8 (15.6 to 22.7)	17.5 (14.4 to 21.2)	12.6 (9.9 to 15.9)	-2.7 (-3.7 to -1.7)	-32.7 (-42.9 to -22.1)
Fiji	27.3 (23.5 to 31.5)	26.3 (22.0 to 31.3)	25.7 (21.5 to 30.8)	25.6 (16.3 to 36.7)	-0.3 (-2.9 to 2.1)	-2.2 (-35.3 to 38.0)
Guam	11.1 (10.1 to 12.2)	13.5 (12.0 to 15.1)	13.5 (11.7 to 15.5)	8.4 (5.0 to 11.6)	-3.3 (-6.4 to -1.2)	-37.6 (-61.9 to -16.5)
Kiribati	66.5 (58.4 to 75.7)	46.2 (38.8 to 54.9)	43.0 (35.8 to 51.7)	31.7 (24.8 to 39.4)	-2.5 (-3.5 to -1.6)	-31.5 (-40.7 to -21.3)
Marshall Islands	37.0 (33.0 to 41.3)	23.7 (19.5 to 28.3)	22.2 (18.4 to 26.5)	16.1 (12.5 to 20.3)	-2.6 (-3.9 to -1.4)	-32.0 (-44.0 to -18.8)
Northern Mariana Islands	7.3 (6.3 to 8.4)	8.8 (7.4 to 10.2)	8.4 (7.2 to 9.9)	6.2 (4.1 to 9.3)	-2.4 (-4.8 to 0.1)	-29.0 (-51.5 to 1.3)
Papua New Guinea	67.0 (58.0 to 76.7)	55.7 (46.6 to 66.4)	52.6 (43.7 to 63.2)	36.0 (29.2 to 44.0)	-2.9 (-3.6 to -2.1)	-35.4 (-41.8 to -26.5)
Samoa	16.9 (13.9 to 20.5)	14.8 (12.2 to 17.8)	14.1 (11.6 to 17.1)	10.8 (8.2 to 14.2)	-2.2 (-3.2 to -0.5)	-27.2 (-38.0 to -7.3)
Solomon Islands	38.1 (31.6 to 45.4)	27.9 (23.0 to 33.2)	26.0 (21.5 to 31.5)	19.1 (14.7 to 24.3)	-2.5 (-3.6 to -1.3)	-31.4 (-42.0 to -17.6)
Tonga	21.1 (17.6 to 25.1)	17.5 (14.4 to 21.1)	16.6 (13.7 to 20.1)	13.0 (10.3 to 16.3)	-2.0 (-2.9 to -1.0)	-25.7 (-35.2 to -13.9)
Vanuatu	33.8 (28.5 to 39.4)	30.5 (25.1 to 37.4)	29.4 (24.2 to 36.1)	21.3 (16.2 to 28.9)	-2.4 (-3.8 to -0.8)	-30.2 (-43.5 to -10.9)
Southeast Asia						
Cambodia	93.7 (87.3 to 100.7)	35.1 (30.1 to 40.4)	31.5 (26.9 to 36.8)	21.1 (13.9 to 58.3)	-3.8 (-5.9 to 3.2)	-40.0 (-58.9 to 62.1)
Indonesia	53.9 (50.2 to 57.8)	28.5 (24.4 to 33.4)	26.0 (21.7 to 31.1)	15.8 (12.5 to 19.5)	-4.0 (-5.0 to -2.9)	-44.8 (-53.1 to -35.5)
Laos	136.6 (124.6 to 148.6)	64.0 (54.4 to 74.5)	57.5 (48.6 to 67.5)	34.6 (28.4 to 41.5)	-4.1 (-5.0 to -3.3)	-45.9 (-52.4 to -39.1)
Malaysia	9.3 (9.1 to 9.5)	8.4 (8.2 to 8.7)	7.2 (6.3 to 8.4)	5.0 (4.0 to 6.2)	-3.5 (-5.0 to -2.0)	-40.3 (-52.5 to -25.6)
Maldives	29.3 (26.2 to 32.8)	8.9 (7.5 to 10.6)	7.7 (6.5 to 9.3)	4.5 (2.7 to 7.3)	-4.7 (-7.8 to -1.6)	-48.7 (-69.1 to -20.9)
Mauritius	16.8 (15.7 to 18.1)	14.9 (13.7 to 16.1)	13.8 (12.1 to 15.8)	11.0 (9.0 to 13.2)	-2.0 (-3.3 to -0.8)	-25.9 (-39.3 to -11.1)
Myanmar	110.5 (99.9 to 122.7)	49.1 (43.0 to 56.0)	44.3 (38.1 to 51.4)	26.2 (19.9 to 33.6)	-4.2 (-5.7 to -2.8)	-46.5 (-57.7 to -34.5)
Philippines	38.6 (35.4 to 42.1)	29.7 (25.2 to 34.6)	26.6 (22.5 to 31.5)	18.8 (15.7 to 22.4)	-3.0 (-3.7 to -2.3)	-36.6 (-42.9 to -29.2)
Sri Lanka	16.3 (15.9 to 16.7)	9.7 (8.4 to 11.2)	8.5 (7.1 to 10.2)	6.8 (4.3 to 9.8)	-2.5 (-5.3 to 0.1)	-30.2 (-54.9 to 1.7)
Seychelles	13.7 (12.2 to 15.4)	13.2 (11.6 to 15.0)	12.5 (10.8 to 14.5)	8.9 (6.9 to 11.2)	-2.7 (-4.1 to -1.3)	-32.9 (-46.0 to -18.2)
Thailand	17.6 (15.2 to 20.3)	8.9 (8.7 to 9.1)	8.7 (8.2 to 9.2)	4.7 (3.6 to 6.0)	-4.3 (-6.0 to -2.7)	-46.8 (-59.1 to -32.9)

	Estimate in 2000 (95% UIs)	Estimate in 2015 (95% UIs)	Estimate in 2017 (95% UIs)	Estimate in 2030 (95% UIs)	Annualised rate of change, 2015-2030 (95% UIs)	Percent change, 2015-2030 (95% UIs)
Timor-Leste	80.7 (74.2 to 87.7)	37.5 (32.4 to 43.4)	35.7 (30.5 to 41.5)	22.5 (17.2 to 30.3)	-3.4 (-4.9 to -1.6)	-39.8 (-52.2 to -21.2)
Vietnam	27.1 (24.4 to 30.1)	14.4 (12.3 to 17.1)	13.1 (11.0 to 15.9)	9.3 (6.8 to 15.4)	-3.0 (-4.6 to 0.6)	-35.3 (-49.7 to 9.8)
Sub-Saharan Africa						
Central Sub-Saharan Africa						
Angola	181.6 (168.4 to 195.1)	72.4 (63.2 to 82.3)	65.2 (56.3 to 75.2)	33.8 (25.9 to 42.0)	-5.1 (-6.7 to -3.9)	-53.3 (-63.2 to -44.6)
Central African Republic	180.5 (162.7 to 199.5)	131.3 (112.4 to 154.7)	123.8 (104.9 to 148.2)	87.2 (64.6 to 113.5)	-2.8 (-4.3 to -1.3)	-33.6 (-47.5 to -18.2)
Congo	108.5 (98.5 to 118.7)	57.5 (48.9 to 67.9)	55.2 (46.5 to 66.5)	36.2 (27.9 to 48.7)	-3.1 (-4.4 to -1.5)	-37.0 (-48.5 to -19.8)
Democratic Republic of the Congo	151.5 (140.2 to 163.6)	89.5 (77.5 to 103.2)	81.9 (70.1 to 95.2)	50.1 (41.8 to 59.7)	-3.9 (-4.7 to -3.1)	-44.0 (-50.3 to -37.1)
Equatorial Guinea	137.4 (119.4 to 156.4)	57.6 (47.6 to 69.9)	54.1 (44.8 to 66.2)	32.6 (24.7 to 42.5)	-3.8 (-5.3 to -2.3)	-43.4 (-54.6 to -29.1)
Gabon	69.8 (63.2 to 77.3)	38.8 (32.5 to 45.4)	35.2 (29.6 to 41.7)	25.6 (19.7 to 32.0)	-2.8 (-4.0 to -1.7)	-34.0 (-45.1 to -22.3)
Eastern Sub-Saharan Africa						
Burundi	168.9 (157.4 to 181.7)	83.3 (73.9 to 94.5)	80.7 (70.2 to 93.9)	54.5 (44.4 to 67.4)	-2.9 (-3.9 to -1.8)	-34.6 (-44.6 to -23.7)
Comoros	97.7 (88.2 to 107.8)	53.1 (44.8 to 63.0)	48.0 (40.4 to 57.9)	29.9 (24.0 to 37.6)	-3.9 (-4.9 to -2.7)	-43.7 (-52.2 to -33.6)
Djibouti	101.5 (90.4 to 115.5)	51.3 (42.3 to 62.1)	45.1 (37.4 to 55.5)	27.0 (20.8 to 34.5)	-4.3 (-5.6 to -3.2)	-47.4 (-56.6 to -38.3)
Eritrea	91.6 (81.4 to 103.0)	51.6 (42.4 to 62.2)	46.5 (38.5 to 57.1)	32.8 (24.7 to 44.1)	-3.1 (-4.5 to -1.3)	-36.4 (-48.7 to -17.4)
Ethiopia	145.8 (135.8 to 155.8)	64.0 (56.0 to 73.2)	56.1 (48.3 to 66.1)	30.4 (23.4 to 38.9)	-5.0 (-6.4 to -3.6)	-52.5 (-62.0 to -41.7)
Kenya	87.6 (81.4 to 94.1)	48.0 (41.8 to 55.1)	44.0 (37.7 to 51.5)	29.2 (23.4 to 36.7)	-3.3 (-4.5 to -2.2)	-39.2 (-48.8 to -27.6)
Madagascar	109.5 (100.2 to 119.5)	78.2 (66.4 to 93.1)	73.9 (62.2 to 89.0)	50.5 (41.8 to 61.1)	-2.9 (-3.7 to -2.3)	-35.5 (-42.7 to -28.8)
Malawi	156.9 (148.0 to 166.4)	70.7 (62.3 to 80.6)	65.2 (56.4 to 76.4)	39.4 (32.5 to 47.4)	-3.9 (-4.9 to -3.0)	-44.4 (-51.8 to -36.4)
Mozambique	159.2 (148.1 to 170.4)	81.7 (69.9 to 94.3)	73.8 (62.6 to 86.4)	44.7 (37.1 to 53.2)	-4.0 (-4.7 to -3.4)	-45.2 (-50.6 to -40.2)
Rwanda	157.9 (147.8 to 168.1)	51.5 (44.5 to 60.0)	47.0 (40.2 to 55.7)	31.7 (22.2 to 38.6)	-3.8 (-5.2 to -2.3)	-38.3 (-54.3 to -29.0)
Somalia	155.2 (139.9 to 171.3)	107.5 (90.3 to 128.0)	102.5 (86.3 to 123.6)	72.5 (58.8 to 88.8)	-2.6 (-3.5 to -1.9)	-32.5 (-41.0 to -25.4)
South Sudan	144.3 (131.7 to 157.6)	107.6 (91.9 to 125.5)	104.7 (89.0 to 122.4)	75.5 (60.7 to 93.7)	-2.4 (-3.4 to -1.4)	-29.8 (-40.0 to -18.7)
Tanzania	123.6 (115.5 to 131.0)	66.6 (57.9 to 76.3)	61.5 (52.9 to 72.5)	37.8 (32.0 to 45.5)	-3.8 (-4.5 to -3.1)	-43.2 (-48.9 to -36.9)
Uganda	137.0 (129.0 to 145.6)	66.2 (58.4 to 74.3)	60.0 (52.4 to 68.5)	35.5 (29.5 to 42.0)	-4.2 (-5.1 to -3.3)	-46.3 (-53.4 to -38.9)
Zambia	142.4 (131.4 to 153.5)	64.8 (55.7 to 75.8)	62.5 (53.1 to 74.5)	39.5 (32.6 to 48.3)	-3.3 (-4.2 to -2.5)	-39.1 (-46.4 to -31.2)
Southern Sub-Saharan Africa						
Botswana	62.4 (54.9 to 70.5)	24.3 (20.2 to 29.4)	22.7 (18.8 to 27.9)	15.1 (7.6 to 31.2)	-3.6 (-7.6 to 1.4)	-38.0 (-67.9 to 24.1)
Lesotho	96.9 (89.5 to 105.3)	75.2 (64.7 to 86.9)	69.0 (59.0 to 80.7)	51.8 (35.8 to 72.5)	-2.6 (-4.7 to -0.5)	-31.2 (-50.3 to -7.7)
Namibia	64.7 (59.1 to 70.4)	38.2 (32.5 to 44.9)	39.2 (33.1 to 47.0)	28.2 (20.4 to 40.4)	-2.1 (-3.9 to 0.0)	-26.3 (-44.6 to 0.0)
South Africa	77.0 (71.1 to 83.2)	42.2 (36.7 to 48.4)	33.6 (28.6 to 39.2)	23.1 (18.9 to 27.9)	-4.0 (-4.8 to -3.2)	-45.2 (-51.3 to -38.5)
Swaziland	91.3 (84.8 to 99.1)	49.3 (42.5 to 56.3)	45.1 (38.6 to 52.5)	33.6 (21.4 to 49.9)	-2.7 (-5.3 to -0.0)	-31.9 (-55.0 to -0.1)
Zimbabwe	75.9 (70.8 to 81.1)	59.8 (52.5 to 67.3)	56.1 (48.7 to 64.4)	50.5 (32.6 to 80.3)	-1.3 (-3.9 to 1.8)	-15.6 (-44.2 to 31.5)
Western Sub-Saharan Africa						
Benin	140.2 (130.2 to 150.6)	91.3 (80.1 to 104.2)	83.0 (71.6 to 97.5)	48.4 (40.8 to 58.3)	-4.2 (-5.1 to -3.5)	-47.0 (-53.2 to -40.7)
Burkina Faso	179.5 (166.6 to 193.4)	113.6 (97.4 to 132.3)	105.9 (89.9 to 126.5)	65.1 (51.8 to 82.3)	-3.7 (-4.8 to -2.7)	-42.7 (-51.6 to -33.3)
Cameroon	134.2 (125.0 to 144.8)	81.5 (70.7 to 93.8)	73.5 (62.9 to 85.9)	50.0 (38.2 to 64.6)	-3.3 (-4.8 to -1.9)	-38.6 (-51.3 to -24.6)
Cape Verde	50.6 (44.0 to 57.8)	22.4 (19.8 to 25.2)	20.3 (17.5 to 23.8)	12.5 (9.2 to 16.1)	-3.9 (-5.8 to -2.6)	-44.1 (-58.0 to -32.1)
Chad	183.6 (170.0 to 197.7)	123.7 (107.8 to 140.2)	117.0 (101.1 to 134.8)	78.6 (63.3 to 95.2)	-3.0 (-4.2 to -2.2)	-36.4 (-46.4 to -27.7)
Cote d'Ivoire	137.7 (126.8 to 148.5)	90.7 (77.7 to 105.1)	83.1 (70.5 to 97.9)	51.0 (40.5 to 63.1)	-3.9 (-4.9 to -2.7)	-43.7 (-52.0 to -33.6)
The Gambia	86.9 (78.6 to 95.8)	51.3 (43.4 to 60.3)	47.8 (40.3 to 57.5)	32.2 (26.1 to 39.6)	-3.1 (-4.0 to -2.2)	-37.2 (-45.4 to -28.0)
Ghana	100.9 (94.8 to 106.8)	61.7 (54.0 to 70.9)	55.6 (47.9 to 65.5)	34.0 (27.7 to 41.3)	-4.0 (-5.0 to -3.0)	-45.0 (-52.8 to -36.4)
Guinea	171.6 (159.3 to 185.2)	105.3 (90.7 to 122.1)	96.7 (82.4 to 115.0)	59.9 (49.8 to 73.7)	-3.8 (-4.6 to -2.9)	-43.1 (-49.6 to -35.5)
Guinea-Bissau	161.1 (147.4 to 175.9)	80.3 (69.1 to 93.0)	73.2 (62.4 to 85.4)	43.8 (34.4 to 53.3)	-4.1 (-5.3 to -3.2)	-45.4 (-54.6 to -37.8)
Liberia	169.9 (158.5 to 182.2)	82.6 (71.4 to 96.8)	74.8 (63.8 to 88.9)	44.8 (36.4 to 56.0)	-4.1 (-5.1 to -3.0)	-45.8 (-53.5 to -36.5)
Mali	193.0 (181.1 to 205.5)	126.7 (113.0 to 143.0)	119.0 (104.0 to 137.9)	73.8 (61.9 to 89.2)	-3.6 (-4.5 to -2.8)	-41.7 (-49.1 to -34.1)
Mauritania	91.4 (84.7 to 98.9)	49.4 (43.2 to 57.1)	45.0 (38.7 to 53.1)	28.5 (23.2 to 34.9)	-3.7 (-4.7 to -2.8)	-42.3 (-50.4 to -33.9)
Niger	225.7 (208.3 to 244.4)	117.1 (100.3 to 137.6)	108.0 (91.8 to 128.9)	66.7 (55.1 to 80.3)	-3.8 (-4.5 to -3.1)	-43.0 (-49.2 to -37.1)
Nigeria	180.4 (171.2 to 189.9)	110.7 (99.2 to 123.8)	103.2 (90.8 to 117.0)	57.2 (42.2 to 71.2)	-4.5 (-6.4 to -3.2)	-48.3 (-61.5 to -38.1)
Sao Tome and Principe	74.2 (67.6 to 80.9)	32.8 (28.0 to 38.3)	29.3 (24.8 to 34.7)	18.3 (14.5 to 22.3)	-3.9 (-5.0 to -2.9)	-44.1 (-52.8 to -35.1)
Senegal	117.2 (110.1 to 124.8)	52.0 (46.4 to 58.6)	48.0 (41.9 to 55.7)	28.9 (23.7 to 35.1)	-4.0 (-5.1 to -2.9)	-44.5 (-53.3 to -35.3)
Sierra Leone	214.0 (200.8 to 228.9)	120.4 (105.8 to 136.1)	110.7 (95.5 to 127.6)	70.2 (58.3 to 84.1)	-3.6 (-4.4 to -2.7)	-41.7 (-48.5 to -33.0)
Togo	122.8 (112.3 to 133.8)	74.1 (64.1 to 85.7)	66.8 (56.8 to 79.7)	40.8 (33.1 to 49.8)	-4.0 (-4.8 to -3.2)	-44.9 (-51.7 to -38.3)
**Indicator 3.2.2: Neonatal mortality rate (probability of dying during the first 28 days of life per 1,000 livebirths)**						
Central Europe, Eastern Europe, and Central Asia						
Central Asia						
Armenia	14.4 (12.6 to 16.4)	5.7 (5.3 to 6.2)	5.5 (4.9 to 6.1)	3.6 (2.6 to 4.8)	-3.2 (-5.2 to -1.1)	-37.5 (-53.9 to -15.6)
Azerbaijan	30.7 (27.0 to 34.7)	21.3 (18.2 to 24.8)	20.2 (17.2 to 23.7)	15.0 (12.1 to 18.4)	-2.4 (-3.4 to -1.3)	-29.7 (-39.6 to -18.2)
Georgia	20.2 (17.8 to 22.8)	7.1 (5.9 to 8.3)	6.5 (5.4 to 7.7)	4.3 (3.2 to 5.6)	-3.4 (-4.7 to -1.9)	-39.6 (-50.9 to -24.7)
Kazakhstan	17.4 (15.4 to 19.8)	7.3 (6.0 to 8.8)	6.9 (5.5 to 8.5)	4.8 (3.1 to 6.7)	-2.8 (-5.5 to -0.7)	-33.7 (-56.2 to -9.7)
Kyrgyzstan	19.9 (16.9 to 23.1)	14.2 (13.3 to 15.1)	13.0 (12.0 to 14.1)	9.8 (8.8 to 10.8)	-2.5 (-3.2 to -1.8)	-31.2 (-37.7 to -24.0)
Mongolia	22.9 (20.5 to 25.3)	15.1 (13.2 to 17.5)	14.2 (12.3 to 16.4)	9.6 (8.1 to 11.7)	-3.0 (-4.0 to -2.1)	-36.3 (-44.8 to -26.9)
Tajikistan	25.5 (22.7 to 28.5)	21.8 (18.8 to 25.2)	21.0 (18.0 to 24.4)	14.4 (11.7 to 17.4)	-2.8 (-3.9 to -1.7)	-33.9 (-44.0 to -22.9)
Turkmenistan	25.8 (22.7 to 29.4)	16.7 (14.5 to 19.1)	15.7 (13.4 to 18.1)	10.7 (8.3 to 13.2)	-3.0 (-4.5 to -2.0)	-36.3 (-48.8 to -26.0)
Uzbekistan	19.8 (17.5 to 22.3)	13.5 (11.3 to 15.9)	12.4 (10.4 to 15.0)	8.9 (7.0 to 11.3)	-2.8 (-3.9 to -1.6)	-34.1 (-43.9 to -21.4)
Central Europe						
Albania	13.8 (11.5 to 16.3)	7.4 (6.0 to 9.1)	6.8 (5.5 to 8.4)	5.1 (3.4 to 7.0)	-2.5 (-4.9 to -0.8)	-30.3 (-51.7 to -11.6)
Bosnia and Herzegovina	7.6 (7.0 to 8.2)	5.3 (4.9 to 5.8)	5.0 (4.4 to 5.8)	3.6 (2.8 to 4.7)	-2.7 (-4.4 to -0.9)	-33.0 (-48.1 to -12.9)
Bulgaria	7.6 (6.9 to 8.3)	4.1 (3.9 to 4.4)	4.0 (3.6 to 4.5)	2.7 (2.0 to 3.5)	-3.0 (-5.0 to -1.0)	-35.1 (-52.4 to -14.2)
Croatia	5.5 (5.1 to 5.9)	3.0 (2.7 to 3.4)	2.8 (2.4 to 3.1)	1.9 (1.5 to 2.5)	-3.1 (-4.8 to -1.2)	-36.5 (-51.0 to -16.1)
Czech Republic	2.6 (2.5 to 2.9)	1.5 (1.4 to 1.7)	1.4 (1.2 to 1.6)	1.0 (0.7 to 1.4)	-3.1 (-5.6 to -0.5)	-35.7 (-57.0 to -6.9)
Hungary	6.1 (5.6 to 6.5)	3.0 (2.7 to 3.2)	2.6 (2.2 to 3.0)	1.7 (1.2 to 2.4)	-3.7 (-5.8 to -1.5)	-42.0 (-58.2 to -20.3)
Macedonia	8.9 (8.2 to 9.7)	6.5 (5.8 to 7.3)	6.3 (5.3 to 7.3)	4.8 (3.3 to 7.1)	-2.2 (-4.6 to 0.6)	-26.5 (-50.2 to 8.9)
Montenegro	8.7 (7.8 to 9.6)	2.6 (2.3 to 2.9)	2.5 (2.2 to 2.9)	1.7 (1.1 to 2.9)	-2.9 (-5.8 to 0.7)	-33.5 (-57.9 to 11.4)
Poland	5.8 (5.5 to 6.2)	2.9 (2.7 to 3.0)	2.7 (2.4 to 3.0)	1.7 (1.2 to 2.3)	-3.6 (-5.8 to -1.2)	-40.5 (-57.9 to -16.9)
Romania	9.2 (8.4 to 10.2)	4.5 (4.1 to 4.9)	4.2 (3.8 to 4.8)	2.7 (2.0 to 3.7)	-3.4 (-5.6 to -1.2)	-39.0 (-57.0 to -16.8)
Serbia	8.8 (7.3 to 10.3)	3.3 (3.1 to 3.5)	3.1 (2.8 to 3.5)	2.3 (1.8 to 2.8)	-2.5 (-4.1 to -0.9)	-30.6 (-45.7 to -12.2)
Slovakia	5.2 (4.8 to 5.5)	3.2 (2.9 to 3.6)	3.0 (2.5 to 3.5)	2.0 (1.6 to 2.5)	-3.2 (-4.7 to -1.7)	-38.2 (-50.6 to -22.5)
Slovenia	3.2 (3.0 to 3.5)	1.5 (1.4 to 1.7)	1.4 (1.2 to 1.6)	1.0 (0.8 to 1.3)	-2.9 (-4.6 to -1.2)	-35.0 (-50.0 to -16.5)
Eastern Europe						

	Estimate in 2000 (95% UIs)	Estimate in 2015 (95% UIs)	Estimate in 2017 (95% UIs)	Estimate in 2030 (95% UIs)	Annualised rate of change, 2015-2030 (95% UIs)	Percent change, 2015-2030 (95% UIs)
Belarus	9.1 (8.0 to 10.2)	3.8 (3.2 to 4.4)	3.5 (3.0 to 4.2)	1.8 (1.2 to 2.7)	-5.0 (-7.2 to -2.3)	-51.7 (-66.3 to -29.7)
Estonia	5.2 (4.7 to 5.7)	1.4 (1.3 to 1.6)	1.3 (1.2 to 1.4)	1.1 (0.5 to 2.1)	-2.1 (-7.2 to 2.5)	-21.8 (-66.2 to 45.3)
Latvia	6.8 (6.3 to 7.3)	2.7 (2.4 to 3.0)	2.5 (2.1 to 2.9)	1.6 (0.9 to 2.8)	-3.7 (-7.2 to 0.2)	-40.6 (-66.1 to 2.4)
Lithuania	4.7 (4.5 to 5.0)	2.4 (2.2 to 2.6)	2.2 (2.0 to 2.5)	1.6 (0.9 to 2.3)	-3.0 (-6.1 to -0.3)	-35.2 (-60.0 to -4.8)
Moldova	18.7 (16.7 to 20.9)	11.7 (10.1 to 13.4)	10.6 (9.0 to 12.3)	8.0 (5.0 to 11.6)	-2.7 (-5.4 to -0.0)	-31.4 (-55.2 to -0.4)
Russia	10.6 (10.2 to 11.0)	4.4 (4.3 to 4.6)	4.1 (4.0 to 4.2)	2.5 (1.7 to 3.5)	-3.9 (-6.3 to -1.4)	-43.5 (-61.0 to -18.9)
Ukraine	8.4 (7.8 to 8.9)	5.0 (4.6 to 5.4)	4.7 (4.2 to 5.2)	3.6 (2.4 to 5.3)	-2.4 (-5.0 to 0.5)	-29.0 (-53.1 to 7.2)
High-income						
Australasia						
Australia	3.5 (3.3 to 3.8)	2.4 (2.2 to 2.5)	2.2 (1.9 to 2.6)	1.8 (1.5 to 2.3)	-1.7 (-3.1 to -0.2)	-22.5 (-37.3 to -2.9)
New Zealand	3.5 (3.3 to 3.8)	2.8 (2.6 to 3.0)	2.4 (2.1 to 2.8)	1.9 (1.3 to 2.7)	-2.6 (-5.2 to -0.2)	-30.7 (-54.3 to -2.6)
High-income Asia Pacific						
Brunei	5.0 (4.5 to 5.4)	4.5 (4.1 to 5.0)	4.3 (3.8 to 4.9)	4.3 (3.0 to 5.8)	-0.4 (-2.6 to 1.5)	-5.3 (-32.1 to 26.0)
Japan	1.8 (1.7 to 1.9)	1.1 (1.1 to 1.2)	1.1 (0.9 to 1.2)	0.8 (0.6 to 1.0)	-2.8 (-4.5 to -0.9)	-33.6 (-49.3 to -12.4)
South Korea	2.7 (2.4 to 3.0)	1.5 (1.3 to 1.7)	1.5 (1.2 to 1.7)	1.2 (0.8 to 2.4)	-1.7 (-4.3 to 3.3)	-19.7 (-47.2 to 63.7)
Singapore	1.6 (1.5 to 1.7)	0.9 (0.8 to 1.0)	0.9 (0.8 to 1.0)	0.7 (0.5 to 0.8)	-2.2 (-4.0 to -0.7)	-27.8 (-45.3 to -10.1)
High-income North America						
Canada	3.5 (3.3 to 3.7)	3.1 (3.0 to 3.3)	3.1 (2.8 to 3.4)	2.7 (2.2 to 3.3)	-1.1 (-2.6 to 0.4)	-14.1 (-31.8 to 6.4)
Greenland	9.3 (8.2 to 10.5)	7.5 (6.6 to 8.5)	6.9 (6.0 to 7.9)	5.2 (4.0 to 6.7)	-2.5 (-4.0 to -1.0)	-31.0 (-45.3 to -13.8)
United States	4.6 (4.5 to 4.8)	4.0 (3.9 to 4.0)	3.9 (3.5 to 4.2)	3.3 (2.7 to 4.0)	-1.2 (-2.5 to 0.1)	-16.1 (-31.2 to 1.0)
Southern Latin America						
Argentina	10.6 (10.2 to 11.0)	6.5 (6.2 to 6.8)	6.3 (5.5 to 7.3)	4.8 (3.9 to 5.9)	-2.1 (-3.4 to -0.6)	-26.5 (-40.1 to -8.6)
Chile	5.8 (5.4 to 6.1)	5.1 (4.8 to 5.3)	4.6 (3.9 to 5.4)	3.7 (3.0 to 4.6)	-2.1 (-3.5 to -0.6)	-26.4 (-41.2 to -8.3)
Uruguay	8.2 (7.6 to 8.8)	5.0 (4.5 to 5.4)	5.0 (4.1 to 6.1)	3.8 (2.7 to 5.3)	-1.8 (-4.1 to 0.5)	-22.6 (-45.8 to 8.2)
Western Europe						
Andorra	1.4 (1.2 to 1.7)	0.8 (0.7 to 0.9)	0.8 (0.7 to 0.9)	0.6 (0.4 to 1.1)	-1.8 (-5.0 to 2.0)	-21.9 (-52.6 to 34.7)
Austria	3.1 (2.8 to 3.5)	2.2 (1.9 to 2.4)	2.1 (1.8 to 2.3)	1.5 (1.2 to 1.9)	-2.5 (-4.2 to -0.6)	-30.5 (-47.0 to -9.2)
Belgium	3.1 (2.6 to 3.5)	2.1 (1.9 to 2.4)	2.0 (1.7 to 2.2)	1.5 (1.2 to 1.8)	-2.3 (-3.8 to -0.8)	-28.8 (-43.5 to -11.0)
Cyprus	4.2 (3.9 to 4.6)	1.8 (1.6 to 2.0)	1.7 (1.5 to 1.9)	1.2 (0.8 to 1.7)	-3.0 (-5.1 to -0.4)	-34.3 (-53.5 to -5.5)
Denmark	3.2 (2.9 to 3.6)	2.4 (2.2 to 2.6)	2.2 (1.9 to 2.5)	1.8 (1.4 to 2.2)	-2.1 (-3.6 to -0.5)	-26.5 (-41.9 to -7.1)
Finland	2.5 (2.2 to 2.8)	1.2 (1.0 to 1.4)	1.2 (1.0 to 1.4)	1.0 (0.7 to 1.4)	-1.4 (-3.8 to 0.6)	-18.4 (-43.6 to 10.1)
France	2.8 (2.4 to 3.2)	2.2 (2.0 to 2.4)	2.1 (1.9 to 2.3)	1.6 (1.3 to 2.0)	-2.1 (-3.5 to -0.3)	-25.9 (-41.1 to -4.6)
Germany	2.8 (2.4 to 3.1)	2.3 (2.0 to 2.6)	2.0 (1.6 to 2.5)	1.5 (1.2 to 1.9)	-2.9 (-4.2 to -1.5)	-34.7 (-46.9 to -19.7)
Greece	3.9 (3.5 to 4.2)	2.6 (2.3 to 2.9)	2.5 (2.2 to 2.9)	1.7 (1.2 to 2.3)	-2.8 (-5.0 to -0.9)	-33.9 (-52.4 to -12.6)
Iceland	2.0 (1.7 to 2.3)	1.0 (0.9 to 1.1)	0.9 (0.8 to 1.1)	0.7 (0.5 to 0.9)	-2.7 (-4.3 to -1.0)	-33.2 (-47.3 to -14.5)
Ireland	4.0 (3.6 to 4.4)	2.3 (2.1 to 2.6)	2.1 (1.8 to 2.4)	1.8 (1.4 to 2.4)	-1.9 (-3.7 to 0.4)	-23.6 (-42.9 to 6.8)
Israel	3.5 (3.0 to 4.1)	2.1 (1.9 to 2.3)	1.9 (1.7 to 2.1)	1.3 (0.9 to 1.8)	-3.4 (-5.6 to -0.9)	-39.0 (-56.9 to -12.9)
Italy	3.3 (3.0 to 3.7)	2.1 (1.9 to 2.2)	1.9 (1.7 to 2.1)	1.4 (1.1 to 1.8)	-2.6 (-4.2 to -0.9)	-31.6 (-47.0 to -12.3)
Luxembourg	2.6 (2.3 to 2.9)	1.2 (1.0 to 1.5)	1.1 (0.9 to 1.4)	0.9 (0.6 to 1.4)	-1.9 (-4.8 to 0.6)	-22.9 (-51.4 to 9.9)
Malta	5.1 (4.6 to 5.7)	4.4 (3.9 to 5.0)	4.2 (3.6 to 4.8)	2.9 (2.2 to 3.6)	-3.0 (-4.4 to -1.5)	-35.7 (-48.7 to -20.6)
Netherlands	3.8 (3.4 to 4.2)	2.5 (2.2 to 2.7)	2.5 (2.2 to 2.8)	2.0 (1.7 to 2.5)	-1.3 (-2.7 to 0.3)	-17.0 (-33.3 to 4.0)
Norway	2.6 (2.4 to 2.8)	1.5 (1.4 to 1.6)	1.4 (1.2 to 1.6)	1.1 (0.9 to 1.4)	-1.9 (-3.3 to -0.5)	-24.4 (-39.4 to -7.0)
Portugal	3.6 (3.1 to 4.0)	2.0 (1.7 to 2.2)	1.8 (1.6 to 2.1)	1.2 (0.9 to 1.7)	-3.4 (-5.5 to -0.9)	-38.9 (-56.1 to -13.3)
Spain	2.9 (2.5 to 3.3)	1.8 (1.6 to 2.0)	1.8 (1.6 to 2.0)	1.3 (1.0 to 1.6)	-2.4 (-4.1 to -0.7)	-30.1 (-46.0 to -9.9)
Sweden	2.1 (1.9 to 2.4)	1.6 (1.4 to 1.7)	1.5 (1.3 to 1.7)	1.2 (0.9 to 1.4)	-2.0 (-3.5 to -0.6)	-25.9 (-41.2 to -8.4)
Switzerland	3.5 (3.1 to 4.0)	3.0 (2.7 to 3.3)	2.6 (2.3 to 2.9)	2.1 (1.7 to 2.6)	-2.4 (-3.8 to -0.9)	-29.3 (-43.4 to -12.5)
United Kingdom	3.7 (3.5 to 4.0)	2.6 (2.5 to 2.8)	2.6 (2.4 to 2.9)	2.0 (1.7 to 2.3)	-1.9 (-3.0 to -0.9)	-24.8 (-35.9 to -12.5)
Latin America and Caribbean						
Andean Latin America						
Bolivia	25.8 (22.5 to 29.4)	15.3 (12.9 to 18.2)	14.1 (11.8 to 16.7)	9.4 (7.8 to 11.4)	-3.2 (-4.1 to -2.3)	-38.3 (-45.8 to -28.7)
Ecuador	16.3 (13.9 to 18.9)	8.3 (6.3 to 10.6)	7.9 (5.9 to 10.0)	5.6 (3.9 to 7.4)	-2.7 (-4.6 to -0.8)	-32.4 (-50.2 to -11.8)
Peru	15.5 (13.9 to 17.4)	7.6 (6.0 to 9.3)	6.9 (5.8 to 8.3)	4.6 (3.5 to 6.1)	-3.4 (-5.3 to -1.4)	-39.0 (-54.7 to -19.5)
Caribbean						
Antigua and Barbuda	9.4 (8.0 to 11.1)	5.7 (4.8 to 6.7)	5.6 (4.8 to 6.7)	4.5 (3.2 to 6.2)	-1.6 (-3.8 to 0.2)	-20.4 (-43.0 to 2.9)
The Bahamas	7.3 (6.3 to 8.5)	6.7 (5.7 to 7.9)	6.5 (5.4 to 7.9)	5.5 (4.1 to 7.5)	-1.3 (-2.9 to 0.5)	-17.0 (-35.1 to 7.8)
Barbados	9.5 (8.2 to 11.1)	9.2 (7.7 to 10.9)	9.1 (7.6 to 10.8)	7.7 (5.9 to 9.7)	-1.3 (-2.4 to -0.1)	-16.9 (-30.2 to -1.6)
Belize	13.3 (11.6 to 15.3)	10.6 (9.0 to 12.3)	10.3 (8.7 to 12.1)	7.8 (6.2 to 9.6)	-2.1 (-3.2 to -1.0)	-26.5 (-38.2 to -13.6)
Bermuda	2.8 (2.3 to 3.4)	2.7 (2.2 to 3.3)	2.6 (2.1 to 3.2)	2.5 (0.8 to 8.0)	-1.6 (-8.0 to 7.3)	-6.9 (-69.8 to 200.6)
Cuba	4.2 (3.8 to 4.7)	2.6 (2.3 to 2.8)	2.3 (2.0 to 2.7)	1.8 (1.4 to 2.2)	-2.5 (-4.3 to -0.8)	-30.7 (-47.2 to -11.0)
Dominica	11.5 (9.9 to 13.2)	18.1 (15.4 to 21.1)	18.1 (15.2 to 21.4)	18.9 (9.7 to 28.9)	0.0 (-4.0 to 2.9)	4.4 (-45.4 to 54.4)
Dominican Republic	23.3 (20.3 to 26.5)	20.4 (16.7 to 24.9)	19.3 (16.0 to 23.2)	13.9 (10.0 to 18.3)	-2.6 (-4.6 to -0.7)	-31.5 (-49.7 to -10.6)
Grenada	10.2 (8.9 to 11.8)	10.0 (8.6 to 11.5)	9.5 (8.1 to 11.0)	8.3 (4.9 to 11.8)	-1.4 (-4.5 to 1.0)	-16.9 (-48.9 to 16.5)
Guyana	23.3 (20.3 to 26.6)	17.0 (14.4 to 19.7)	16.0 (13.6 to 18.8)	10.6 (6.4 to 14.1)	-3.3 (-6.3 to -1.5)	-37.6 (-61.2 to -20.3)
Haiti	31.9 (27.7 to 36.8)	26.1 (19.8 to 34.0)	24.4 (18.0 to 31.9)	16.2 (11.1 to 22.1)	-3.2 (-4.8 to -1.9)	-38.0 (-51.3 to -24.9)
Jamaica	12.9 (11.0 to 15.1)	10.9 (8.9 to 13.1)	10.4 (8.4 to 12.6)	8.9 (7.0 to 11.3)	-1.4 (-2.4 to -0.5)	-18.4 (-30.2 to -6.7)
Puerto Rico	7.4 (6.8 to 8.0)	5.0 (4.6 to 5.5)	5.4 (4.8 to 6.0)	4.1 (3.0 to 5.7)	-1.4 (-3.4 to 0.9)	-17.6 (-40.1 to 13.9)
Saint Lucia	11.7 (10.2 to 13.5)	10.6 (9.0 to 12.5)	9.9 (8.3 to 11.7)	6.9 (4.5 to 9.6)	-3.0 (-5.5 to -0.8)	-35.2 (-56.0 to -11.2)
Saint Vincent and the Grenadines	14.5 (12.6 to 16.6)	11.3 (9.6 to 13.2)	10.6 (8.9 to 12.5)	7.1 (4.9 to 9.4)	-3.2 (-5.3 to -1.6)	-37.1 (-55.0 to -21.5)
Suriname	24.0 (21.1 to 27.1)	18.2 (15.5 to 21.4)	17.8 (15.0 to 21.0)	15.2 (11.6 to 19.0)	-1.2 (-2.7 to -0.0)	-16.4 (-33.1 to -0.2)
Trinidad and Tobago	15.4 (13.1 to 17.8)	10.5 (8.7 to 12.6)	10.4 (8.6 to 12.5)	8.5 (6.6 to 11.0)	-1.4 (-2.5 to 0.1)	-18.4 (-31.6 to 0.8)
Virgin Islands, U.S.	7.5 (6.7 to 8.5)	5.7 (4.8 to 6.7)	5.7 (4.8 to 6.7)	3.7 (2.8 to 4.8)	-2.9 (-4.6 to -1.5)	-34.9 (-50.0 to -19.6)
Central Latin America						
Colombia	13.8 (12.5 to 15.2)	8.1 (6.8 to 9.4)	7.4 (6.3 to 8.7)	5.2 (4.1 to 6.5)	-3.0 (-4.3 to -1.6)	-35.6 (-47.2 to -21.4)
Costa Rica	7.4 (6.8 to 7.9)	5.8 (5.2 to 6.5)	5.3 (4.5 to 6.3)	3.9 (3.0 to 4.9)	-2.7 (-4.3 to -1.2)	-32.7 (-47.5 to -16.8)
El Salvador	12.1 (10.1 to 14.1)	6.3 (4.9 to 8.0)	5.6 (4.0 to 7.4)	3.5 (2.3 to 5.0)	-4.1 (-6.0 to -2.2)	-45.3 (-59.5 to -28.2)
Guatemala	16.2 (14.2 to 18.5)	10.0 (8.5 to 11.7)	9.2 (7.8 to 10.8)	6.8 (5.5 to 8.2)	-2.6 (-3.8 to -1.5)	-31.9 (-43.9 to -19.7)
Honduras	17.3 (15.1 to 19.6)	8.5 (7.0 to 10.0)	7.6 (6.3 to 9.1)	5.6 (4.5 to 6.8)	-2.8 (-3.8 to -1.7)	-34.0 (-43.6 to -22.7)
Mexico	11.0 (9.7 to 12.4)	7.2 (6.0 to 8.2)	6.8 (5.7 to 8.0)	4.8 (3.9 to 6.0)	-2.6 (-3.7 to -1.5)	-32.3 (-43.0 to -20.7)
Nicaragua	13.0 (11.5 to 14.6)	10.1 (8.4 to 11.9)	8.7 (7.1 to 10.7)	5.7 (4.3 to 7.4)	-3.8 (-5.3 to -2.5)	-43.3 (-54.6 to -31.3)
Panama	10.4 (8.8 to 12.2)	8.0 (6.7 to 9.5)	7.7 (6.5 to 9.1)	5.8 (4.8 to 6.9)	-2.2 (-3.1 to -1.3)	-28.0 (-37.4 to -17.4)

	Estimate in 2000 (95% UIs)	Estimate in 2015 (95% UIs)	Estimate in 2017 (95% UIs)	Estimate in 2030 (95% UIs)	Annualised rate of change, 2015-2030 (95% UIs)	Percent change, 2015-2030 (95% UIs)
Venezuela	11.5 (10.5 to 12.4)	9.6 (8.5 to 10.9)	9.4 (8.0 to 11.1)	8.1 (6.4 to 10.2)	-1.2 (-2.7 to 0.5)	-15.7 (-33.5 to 8.4)
Tropical Latin America						
Brazil	17.5 (15.9 to 19.1)	9.2 (7.9 to 10.6)	8.5 (7.2 to 9.9)	6.4 (5.2 to 7.6)	-2.5 (-3.3 to -1.7)	-30.6 (-39.0 to -22.9)
Paraguay	15.9 (14.5 to 17.5)	5.7 (4.9 to 6.7)	5.3 (4.5 to 6.2)	3.7 (2.9 to 4.7)	-3.0 (-4.3 to -1.8)	-35.5 (-47.2 to -23.5)
North Africa and Middle East						
North Africa and Middle East						
Afghanistan	41.9 (34.9 to 48.7)	24.0 (20.4 to 28.0)	22.5 (19.3 to 26.1)	15.7 (12.8 to 18.7)	-2.9 (-3.9 to -1.8)	-34.6 (-44.0 to -24.2)
Algeria	17.6 (15.2 to 20.0)	15.4 (13.8 to 17.0)	13.7 (11.9 to 15.4)	9.0 (5.6 to 11.7)	-3.7 (-6.8 to -2.0)	-41.8 (-64.2 to -25.9)
Bahrain	5.1 (4.6 to 5.6)	3.2 (2.9 to 3.6)	3.1 (2.7 to 3.5)	2.5 (1.2 to 4.4)	-2.0 (-6.5 to 2.3)	-22.3 (-62.6 to 41.7)
Egypt	21.2 (18.3 to 24.1)	8.6 (6.8 to 10.6)	7.3 (5.8 to 9.0)	4.2 (3.0 to 5.7)	-4.8 (-6.8 to -2.6)	-50.9 (-63.8 to -32.7)
Iran	21.7 (19.1 to 24.7)	11.9 (11.6 to 12.2)	8.3 (7.8 to 8.7)	5.5 (4.2 to 6.8)	-5.2 (-7.0 to -3.8)	-53.7 (-65.1 to -43.4)
Iraq	21.4 (19.4 to 23.6)	13.5 (11.6 to 15.6)	12.4 (10.7 to 14.4)	8.9 (7.4 to 10.7)	-2.8 (-3.6 to -1.9)	-33.8 (-41.7 to -24.8)
Jordan	16.6 (15.1 to 18.2)	9.3 (8.0 to 11.0)	8.7 (7.3 to 10.5)	6.5 (5.3 to 7.8)	-2.4 (-3.2 to -1.6)	-30.5 (-37.8 to -21.4)
Kuwait	6.2 (5.7 to 6.8)	5.3 (4.8 to 5.9)	4.7 (4.0 to 5.5)	3.5 (2.2 to 5.9)	-3.0 (-5.8 to 0.6)	-34.4 (-57.9 to 9.8)
Lebanon	9.0 (7.4 to 10.8)	4.6 (3.8 to 5.5)	4.3 (3.6 to 5.2)	3.1 (2.3 to 4.3)	-2.7 (-4.2 to -0.7)	-32.6 (-46.5 to -10.0)
Libya	12.7 (10.2 to 15.1)	5.3 (4.5 to 6.1)	5.2 (4.5 to 5.9)	3.1 (1.6 to 4.8)	-3.7 (-8.0 to -0.7)	-40.1 (-69.7 to -9.5)
Morocco	24.6 (21.5 to 28.0)	13.3 (10.9 to 15.9)	11.8 (9.8 to 14.3)	7.7 (5.6 to 9.9)	-3.7 (-5.3 to -2.5)	-42.1 (-55.0 to -30.9)
Palestine	11.2 (10.0 to 12.3)	7.2 (6.2 to 8.3)	6.4 (5.5 to 7.4)	4.1 (2.2 to 6.5)	-4.0 (-7.9 to -0.8)	-42.8 (-69.2 to -11.4)
Oman	8.1 (6.8 to 9.4)	6.0 (5.5 to 6.4)	5.7 (5.2 to 6.2)	4.2 (1.9 to 8.0)	-2.8 (-7.8 to 1.9)	-29.9 (-68.8 to 32.1)
Qatar	7.4 (6.2 to 8.7)	4.1 (3.4 to 4.8)	3.8 (3.1 to 4.6)	2.5 (1.9 to 3.3)	-3.2 (-4.7 to -1.8)	-37.5 (-50.3 to -24.0)
Saudi Arabia	13.8 (10.8 to 17.1)	4.8 (4.0 to 5.9)	4.3 (3.5 to 5.3)	1.8 (1.0 to 3.0)	-6.8 (-10.6 to -3.5)	-62.5 (-79.6 to -40.4)
Sudan	33.8 (29.4 to 38.8)	24.3 (19.6 to 29.6)	22.3 (17.2 to 27.6)	15.0 (10.4 to 20.2)	-3.3 (-5.0 to -1.8)	-38.3 (-53.0 to -23.7)
Syria	7.6 (6.6 to 8.6)	6.0 (5.1 to 7.1)	5.3 (4.3 to 6.4)	3.7 (2.1 to 5.5)	-3.4 (-6.9 to -0.9)	-38.1 (-64.3 to -12.5)
Tunisia	15.8 (13.7 to 18.0)	7.0 (5.8 to 8.3)	6.2 (5.2 to 7.4)	4.0 (2.8 to 5.3)	-3.8 (-5.9 to -2.1)	-42.9 (-58.5 to -27.4)
Turkey	18.4 (16.1 to 21.1)	9.1 (7.4 to 11.2)	7.6 (5.8 to 9.5)	4.5 (2.9 to 6.2)	-4.8 (-7.2 to -2.9)	-51.0 (-65.9 to -35.1)
United Arab Emirates	5.5 (4.9 to 6.0)	4.4 (3.7 to 5.3)	4.0 (3.4 to 4.9)	2.5 (1.3 to 4.2)	-4.0 (-7.9 to -0.5)	-42.5 (-69.4 to -7.5)
Yemen	34.4 (30.4 to 38.6)	22.1 (17.3 to 27.2)	21.2 (16.3 to 26.7)	13.1 (8.2 to 18.0)	-3.6 (-6.2 to -1.9)	-40.6 (-60.3 to -25.1)
South Asia						
South Asia						
Bangladesh	42.5 (37.8 to 47.3)	23.5 (19.3 to 27.6)	21.0 (17.2 to 25.1)	13.2 (10.3 to 16.3)	-3.8 (-4.8 to -3.0)	-43.7 (-51.4 to -36.3)
Bhutan	41.4 (36.4 to 46.7)	20.5 (16.9 to 24.9)	18.3 (15.1 to 22.6)	10.7 (6.9 to 14.2)	-4.5 (-7.1 to -3.0)	-48.0 (-65.7 to -36.0)
India	38.0 (34.2 to 41.6)	26.0 (22.5 to 30.0)	23.5 (20.1 to 27.8)	14.7 (12.2 to 17.5)	-3.8 (-4.6 to -3.0)	-43.6 (-50.2 to -36.4)
Nepal	39.4 (35.8 to 43.1)	21.5 (17.8 to 25.5)	19.4 (15.7 to 23.5)	11.6 (8.6 to 14.7)	-4.2 (-5.6 to -3.1)	-46.1 (-56.5 to -36.8)
Pakistan	46.8 (40.2 to 53.2)	39.3 (32.6 to 47.3)	35.9 (29.3 to 44.0)	22.1 (15.4 to 29.0)	-3.9 (-5.9 to -2.5)	-43.6 (-58.8 to -31.0)
Southeast Asia, East Asia, and Oceania						
East Asia						
China	13.0 (11.8 to 14.3)	5.5 (4.8 to 6.4)	4.8 (4.1 to 5.8)	2.5 (1.6 to 3.7)	-5.3 (-8.2 to -2.8)	-53.5 (-70.9 to -34.7)
North Korea	12.7 (10.4 to 15.2)	10.7 (8.8 to 13.2)	9.7 (7.9 to 12.0)	7.3 (5.5 to 10.1)	-2.6 (-4.0 to -0.8)	-31.9 (-45.4 to -10.9)
Taiwan (Province of China)	3.5 (3.2 to 3.8)	2.6 (2.4 to 2.8)	2.4 (2.0 to 2.8)	2.0 (1.4 to 2.9)	-1.8 (-3.9 to 1.0)	-21.9 (-44.4 to 16.4)
Oceania						
American Samoa	7.3 (6.5 to 8.2)	4.7 (4.1 to 5.3)	4.6 (4.0 to 5.2)	3.0 (2.4 to 3.8)	-2.9 (-4.6 to -1.4)	-35.0 (-49.9 to -19.1)
Federated States of Micronesia	14.5 (12.3 to 17.1)	9.3 (7.7 to 11.2)	8.7 (7.2 to 10.6)	6.2 (4.9 to 7.8)	-2.7 (-3.8 to -1.6)	-32.9 (-43.5 to -21.4)
Fiji	13.3 (11.2 to 15.4)	12.5 (10.5 to 14.8)	12.3 (10.3 to 14.7)	11.5 (8.3 to 15.6)	-0.6 (-2.5 to 1.2)	-8.0 (-31.7 to 19.4)
Guam	5.6 (5.1 to 6.2)	7.1 (6.3 to 8.2)	7.2 (6.2 to 8.3)	4.6 (2.5 to 6.8)	-3.1 (-6.8 to -0.4)	-35.5 (-64.2 to -6.0)
Kiribati	23.5 (20.2 to 27.2)	18.6 (15.5 to 22.2)	17.6 (14.7 to 21.2)	13.3 (10.8 to 16.2)	-2.2 (-3.2 to -1.4)	-28.3 (-37.7 to -19.3)
Marshall Islands	16.3 (14.2 to 18.6)	11.9 (9.1 to 14.9)	11.2 (8.5 to 14.2)	8.1 (5.6 to 10.8)	-2.6 (-3.8 to -1.5)	-32.2 (-43.8 to -20.6)
Northern Mariana Islands	3.8 (3.3 to 4.4)	4.5 (3.7 to 5.5)	4.4 (3.5 to 5.4)	3.4 (2.0 to 5.8)	-2.1 (-5.3 to 1.6)	-24.7 (-55.0 to 26.7)
Papua New Guinea	25.5 (22.0 to 29.3)	22.4 (18.7 to 26.6)	21.4 (17.8 to 25.8)	15.8 (13.0 to 19.2)	-2.3 (-3.0 to -1.6)	-29.1 (-36.3 to -21.5)
Samoa	7.9 (6.1 to 9.8)	6.8 (5.6 to 8.1)	6.4 (5.3 to 7.8)	4.7 (3.6 to 6.1)	-2.5 (-3.7 to -1.0)	-30.6 (-42.8 to -14.2)
Solomon Islands	16.9 (14.0 to 20.2)	13.4 (11.0 to 15.9)	12.6 (10.4 to 15.3)	9.4 (7.4 to 11.8)	-2.3 (-3.4 to -1.3)	-29.5 (-40.2 to -17.9)
Tonga	9.7 (8.2 to 11.3)	8.0 (6.5 to 9.7)	7.6 (6.2 to 9.2)	5.8 (4.7 to 7.2)	-2.1 (-3.0 to -1.2)	-26.9 (-35.9 to -16.9)
Vanuatu	15.6 (13.2 to 18.3)	14.5 (11.9 to 17.8)	14.1 (11.7 to 17.4)	10.6 (8.3 to 13.5)	-2.1 (-3.3 to -1.0)	-26.7 (-38.6 to -14.0)
Southeast Asia						
Cambodia	32.5 (29.7 to 35.8)	17.7 (15.2 to 20.5)	16.2 (13.9 to 18.9)	10.6 (8.1 to 14.8)	-3.5 (-4.8 to -1.3)	-40.5 (-51.6 to -17.2)
Indonesia	24.9 (23.1 to 26.7)	14.8 (12.6 to 17.5)	13.7 (11.3 to 16.4)	8.9 (6.9 to 11.3)	-3.4 (-4.7 to -2.2)	-39.6 (-51.0 to -28.6)
Laos	50.4 (46.1 to 54.7)	28.0 (23.6 to 33.0)	25.6 (21.6 to 30.1)	16.2 (12.6 to 20.2)	-3.7 (-4.9 to -2.5)	-42.2 (-52.0 to -30.9)
Malaysia	4.7 (4.0 to 5.3)	4.6 (3.9 to 5.2)	3.9 (3.1 to 4.8)	2.7 (1.9 to 3.6)	-3.6 (-5.8 to -1.6)	-40.6 (-57.8 to -21.3)
Maldives	17.1 (15.1 to 19.2)	5.4 (4.5 to 6.4)	4.7 (4.0 to 5.7)	2.5 (1.6 to 4.4)	-5.3 (-7.8 to -1.5)	-53.2 (-69.0 to -20.2)
Mauritius	11.2 (10.4 to 12.1)	9.7 (8.9 to 10.6)	9.0 (7.9 to 10.3)	6.9 (5.6 to 8.6)	-2.3 (-3.7 to -0.8)	-28.7 (-42.5 to -11.6)
Myanmar	41.9 (36.0 to 49.4)	23.1 (19.9 to 26.9)	21.3 (18.3 to 24.6)	13.2 (9.2 to 17.1)	-3.8 (-5.8 to -2.2)	-42.6 (-58.3 to -28.3)
Philippines	16.4 (14.2 to 18.6)	13.1 (10.2 to 16.4)	12.0 (9.0 to 15.1)	8.8 (6.6 to 11.1)	-2.7 (-3.6 to -1.8)	-32.9 (-41.8 to -23.1)
Sri Lanka	8.7 (7.9 to 9.5)	6.0 (5.1 to 7.0)	5.4 (4.5 to 6.5)	4.2 (2.2 to 6.4)	-2.7 (-6.6 to 0.4)	-30.8 (-62.9 to 6.7)
Seychelles	7.6 (6.8 to 8.5)	7.2 (6.3 to 8.2)	6.9 (5.9 to 8.0)	4.7 (3.5 to 6.0)	-2.8 (-4.7 to -1.6)	-34.2 (-50.5 to -20.9)
Thailand	8.5 (7.1 to 10.0)	4.3 (3.9 to 4.7)	4.3 (3.7 to 4.8)	2.4 (1.8 to 3.4)	-4.1 (-5.8 to -1.6)	-44.9 (-58.3 to -21.7)
Timor-Leste	24.7 (22.2 to 27.2)	16.6 (14.3 to 19.2)	16.0 (13.8 to 18.6)	11.4 (8.9 to 14.6)	-2.5 (-3.9 to -1.1)	-31.3 (-43.9 to -15.7)
Vietnam	13.7 (12.5 to 15.1)	7.7 (6.6 to 9.2)	7.0 (5.8 to 8.4)	4.5 (3.6 to 5.8)	-3.6 (-4.7 to -2.4)	-41.2 (-50.6 to -29.8)
Sub-Saharan Africa						
Central Sub-Saharan Africa						
Angola	44.6 (38.1 to 51.1)	24.5 (20.9 to 28.3)	22.9 (19.7 to 26.5)	13.9 (10.7 to 17.0)	-3.8 (-5.4 to -2.7)	-43.4 (-55.2 to -32.9)
Central African Republic	47.7 (40.1 to 56.0)	41.2 (33.9 to 49.7)	39.4 (32.9 to 47.5)	31.2 (23.1 to 40.2)	-1.9 (-3.5 to -0.3)	-24.1 (-41.3 to -4.8)
Congo	28.9 (24.5 to 33.9)	20.4 (15.9 to 25.5)	19.8 (15.2 to 24.9)	14.2 (10.1 to 19.5)	-2.5 (-4.0 to -0.9)	-30.4 (-45.1 to -12.5)
Democratic Republic of the Congo	35.0 (31.3 to 39.1)	27.1 (21.5 to 33.7)	25.4 (19.5 to 32.6)	18.4 (13.3 to 24.6)	-2.6 (-3.8 to -1.5)	-32.2 (-43.5 to -20.1)
Equatorial Guinea	38.0 (32.7 to 43.8)	21.0 (17.3 to 25.7)	20.1 (16.7 to 24.7)	14.6 (9.4 to 21.9)	-2.6 (-5.0 to 0.0)	-30.6 (-52.7 to 0.2)
Gabon	27.0 (24.3 to 29.9)	18.9 (15.9 to 22.1)	17.4 (14.6 to 20.6)	13.5 (9.7 to 17.4)	-2.3 (-3.9 to -0.9)	-28.4 (-43.9 to -12.2)
Eastern Sub-Saharan Africa						
Burundi	42.0 (38.5 to 45.7)	26.9 (23.6 to 30.7)	26.3 (23.0 to 30.5)	19.9 (16.5 to 24.1)	-2.0 (-3.0 to -1.2)	-25.9 (-36.2 to -16.0)
Comoros	42.6 (38.4 to 47.1)	27.5 (23.0 to 32.8)	25.2 (21.2 to 30.5)	16.4 (12.2 to 20.6)	-3.5 (-5.2 to -2.4)	-40.3 (-54.3 to -29.9)
Djibouti	30.3 (26.9 to 34.7)	20.1 (16.5 to 24.4)	18.4 (15.2 to 22.7)	11.5 (7.8 to 15.2)	-3.8 (-6.2 to -2.5)	-42.8 (-60.8 to -31.4)
Eritrea	24.7 (21.5 to 28.3)	18.3 (14.8 to 22.5)	17.0 (14.1 to 21.0)	12.7 (9.8 to 16.1)	-2.5 (-3.8 to -1.4)	-30.5 (-43.4 to -18.4)
Ethiopia	43.5 (40.3 to 46.6)	25.8 (22.4 to 29.7)	23.2 (19.9 to 27.5)	13.9 (11.5 to 17.0)	-4.1 (-5.1 to -3.1)	-45.9 (-53.2 to -37.6)

	Estimate in 2000 (95% UIs)	Estimate in 2015 (95% UIs)	Estimate in 2017 (95% UIs)	Estimate in 2030 (95% UIs)	Annualised rate of change, 2015-2030 (95% UIs)	Percent change, 2015-2030 (95% UIs)
Kenya	27.0 (25.1 to 29.1)	19.9 (17.2 to 22.9)	18.7 (15.9 to 21.8)	13.7 (10.6 to 17.6)	-2.5 (-3.9 to -1.1)	-31.3 (-44.3 to -15.7)
Madagascar	32.0 (29.3 to 34.9)	27.0 (22.8 to 32.1)	25.9 (21.8 to 31.3)	19.9 (15.6 to 25.2)	-2.1 (-3.4 to -0.9)	-26.4 (-39.7 to -13.2)
Malawi	36.8 (32.9 to 41.0)	26.9 (23.6 to 30.9)	25.7 (22.2 to 30.2)	18.5 (15.0 to 22.3)	-2.5 (-3.6 to -1.6)	-31.4 (-41.7 to -21.6)
Mozambique	41.7 (37.7 to 46.4)	26.3 (22.4 to 30.7)	24.5 (20.7 to 28.9)	16.4 (12.7 to 20.2)	-3.2 (-4.5 to -2.2)	-37.5 (-49.2 to -28.0)
Rwanda	38.2 (30.1 to 46.7)	19.5 (14.8 to 24.5)	18.3 (13.7 to 23.3)	12.5 (9.1 to 16.0)	-3.0 (-4.0 to -1.9)	-36.1 (-45.3 to -25.3)
Somalia	40.2 (36.0 to 45.0)	31.6 (26.3 to 37.4)	30.4 (25.5 to 36.5)	22.7 (18.2 to 27.7)	-2.2 (-3.1 to -1.4)	-28.0 (-37.0 to -18.8)
South Sudan	39.6 (35.8 to 43.9)	35.1 (29.8 to 41.1)	34.3 (28.9 to 40.5)	26.6 (20.2 to 33.1)	-1.9 (-3.2 to -0.9)	-24.2 (-38.4 to -12.2)
Tanzania	33.5 (29.9 to 36.8)	25.5 (22.2 to 29.1)	24.0 (20.6 to 28.4)	16.4 (13.6 to 20.0)	-2.9 (-3.8 to -1.9)	-35.5 (-43.8 to -25.3)
Uganda	34.3 (32.1 to 36.7)	26.1 (22.9 to 29.4)	24.4 (21.2 to 27.9)	15.3 (11.4 to 19.1)	-3.6 (-5.4 to -2.3)	-41.3 (-55.2 to -29.6)
Zambia	32.8 (29.1 to 36.9)	23.3 (19.8 to 27.2)	22.4 (19.0 to 26.7)	16.4 (13.0 to 20.5)	-2.4 (-3.6 to -1.2)	-29.7 (-41.9 to -16.3)
Southern Sub-Saharan Africa						
Botswana	20.0 (16.2 to 23.9)	12.0 (9.9 to 14.8)	11.2 (9.3 to 13.8)	7.5 (4.6 to 11.3)	-3.3 (-6.3 to -0.5)	-37.5 (-61.1 to -7.5)
Lesotho	38.5 (32.6 to 44.5)	30.7 (23.4 to 38.4)	28.8 (21.3 to 36.6)	21.3 (14.7 to 30.0)	-2.5 (-4.1 to -0.7)	-30.5 (-45.6 to -9.9)
Namibia	22.2 (20.5 to 24.0)	16.8 (14.1 to 19.7)	16.6 (14.0 to 20.0)	12.6 (9.5 to 16.7)	-1.9 (-3.5 to -0.3)	-24.7 (-40.7 to -4.3)
South Africa	23.3 (21.5 to 25.2)	14.9 (13.0 to 17.1)	12.9 (11.0 to 15.1)	9.7 (7.3 to 12.4)	-2.9 (-4.4 to -1.8)	-35.0 (-48.7 to -23.2)
Swaziland	20.9 (18.9 to 23.0)	16.0 (14.1 to 18.1)	15.5 (13.5 to 17.5)	12.4 (9.5 to 16.4)	-1.7 (-3.3 to -0.0)	-22.4 (-39.3 to -0.2)
Zimbabwe	23.9 (22.0 to 25.9)	25.3 (22.3 to 28.6)	24.1 (20.9 to 27.7)	20.2 (15.3 to 25.9)	-1.5 (-3.0 to -0.0)	-20.2 (-36.1 to -0.3)
Western Sub-Saharan Africa						
Benin	39.9 (35.5 to 44.4)	31.5 (27.2 to 36.7)	29.3 (25.0 to 33.9)	18.1 (13.3 to 22.3)	-3.7 (-5.5 to -2.5)	-42.3 (-55.9 to -31.6)
Burkina Faso	37.5 (34.4 to 40.9)	27.9 (23.6 to 33.0)	26.4 (22.2 to 31.5)	18.7 (14.6 to 23.3)	-2.7 (-4.0 to -1.6)	-32.9 (-45.3 to -21.0)
Cameroon	32.9 (28.5 to 37.5)	24.9 (19.4 to 30.5)	23.4 (17.7 to 29.5)	17.7 (12.6 to 23.8)	-2.3 (-3.8 to -1.1)	-29.2 (-43.5 to -15.4)
Cape Verde	17.3 (14.9 to 19.9)	13.0 (11.5 to 14.7)	11.8 (10.2 to 13.8)	7.5 (4.7 to 9.9)	-3.8 (-6.8 to -2.2)	-42.6 (-63.9 to -27.7)
Chad	41.5 (37.4 to 45.8)	32.7 (28.0 to 38.0)	31.5 (27.1 to 36.7)	23.3 (18.1 to 29.2)	-2.3 (-3.8 to -1.1)	-28.8 (-43.4 to -15.2)
Cote d'Ivoire	46.0 (41.1 to 50.7)	34.5 (27.1 to 42.2)	32.1 (24.7 to 40.2)	22.1 (15.8 to 29.3)	-3.0 (-4.3 to -1.6)	-35.9 (-47.7 to -21.7)
The Gambia	31.6 (28.3 to 35.4)	23.1 (19.5 to 27.3)	21.8 (18.2 to 26.4)	15.6 (12.6 to 19.1)	-2.6 (-3.5 to -1.6)	-32.3 (-40.7 to -21.9)
Ghana	35.9 (32.9 to 39.0)	29.0 (24.2 to 34.3)	26.6 (21.6 to 32.3)	17.6 (13.5 to 22.1)	-3.3 (-4.7 to -2.2)	-39.1 (-50.9 to -28.0)
Guinea	48.3 (44.2 to 52.7)	33.2 (28.6 to 38.5)	31.2 (26.6 to 37.1)	21.4 (17.7 to 25.6)	-2.9 (-3.9 to -2.2)	-35.5 (-44.0 to -28.4)
Guinea-Bissau	50.8 (45.7 to 56.8)	33.4 (28.7 to 38.9)	31.0 (26.4 to 36.3)	19.5 (15.5 to 23.5)	-3.6 (-4.8 to -2.8)	-41.6 (-51.0 to -34.0)
Liberia	41.2 (37.7 to 45.1)	26.6 (23.4 to 30.1)	25.1 (22.3 to 28.4)	17.2 (14.8 to 20.0)	-2.9 (-3.7 to -2.1)	-35.2 (-42.2 to -26.6)
Mali	52.8 (47.3 to 58.6)	39.9 (35.1 to 45.9)	37.9 (33.1 to 44.0)	26.5 (21.0 to 32.5)	-2.8 (-4.1 to -1.5)	-33.5 (-46.3 to -20.0)
Mauritania	36.8 (32.6 to 41.1)	25.6 (22.1 to 29.6)	23.5 (20.2 to 27.2)	15.8 (13.0 to 18.8)	-3.3 (-4.3 to -2.4)	-38.5 (-47.4 to -30.1)
Niger	40.8 (36.0 to 45.5)	25.4 (21.4 to 30.1)	24.0 (20.4 to 28.6)	16.7 (13.9 to 20.0)	-2.8 (-3.6 to -1.9)	-34.1 (-41.8 to -25.1)
Nigeria	47.5 (42.8 to 52.4)	36.5 (32.2 to 40.8)	34.7 (30.5 to 39.5)	22.1 (14.4 to 28.3)	-3.4 (-6.2 to -1.9)	-39.5 (-60.4 to -24.5)
Sao Tome and Principe	20.6 (18.1 to 23.1)	14.5 (12.3 to 17.0)	13.3 (11.2 to 15.8)	9.3 (7.4 to 11.5)	-3.0 (-4.0 to -2.0)	-35.8 (-45.4 to -25.4)
Senegal	35.3 (31.5 to 39.2)	21.9 (19.4 to 24.7)	20.6 (17.9 to 23.9)	13.7 (10.6 to 16.6)	-3.2 (-4.8 to -2.1)	-37.6 (-51.1 to -27.1)
Sierra Leone	54.8 (50.5 to 59.2)	34.5 (29.7 to 39.7)	32.7 (28.0 to 37.9)	23.4 (19.1 to 28.0)	-2.6 (-3.6 to -1.7)	-32.2 (-42.0 to -22.9)
Togo	36.5 (31.5 to 41.6)	26.7 (22.7 to 31.2)	24.8 (21.1 to 29.7)	16.5 (13.6 to 19.9)	-3.2 (-4.2 to -2.2)	-37.9 (-46.7 to -27.6)
**Indicator 3.3.1: Age-standardised rate of new HIV infections (per 1,000 population)**						
Central Europe, Eastern Europe, and Central Asia						
Central Asia						
Armenia	0.0 (0.0 to 0.0)	0.1 (0.1 to 0.1)	0.1 (0.0 to 0.1)	0.1 (0.1 to 0.1)	0.3 (-0.3 to 0.9)	4.8 (-3.9 to 13.9)
Azerbaijan	0.0 (0.0 to 0.0)	0.0 (0.0 to 0.0)	0.0 (0.0 to 0.0)	0.0 (0.0 to 0.1)	0.9 (0.2 to 3.1)	14.8 (3.6 to 58.5)
Georgia	0.0 (0.0 to 0.0)	0.1 (0.1 to 0.2)	0.1 (0.1 to 0.2)	0.1 (0.1 to 0.2)	2.0 (1.5 to 2.5)	34.3 (24.6 to 45.3)
Kazakhstan	0.0 (0.0 to 0.0)	0.1 (0.1 to 0.1)	0.1 (0.1 to 0.2)	0.1 (0.1 to 0.2)	2.0 (1.2 to 3.2)	34.9 (19.6 to 60.4)
Kyrgyzstan	0.0 (0.0 to 0.1)	0.1 (0.1 to 0.1)	0.1 (0.1 to 0.2)	0.1 (0.1 to 0.2)	2.3 (1.4 to 3.1)	41.0 (22.5 to 58.8)
Mongolia	0.0 (0.0 to 0.0)	0.0 (0.0 to 0.1)	0.0 (0.0 to 0.0)	0.0 (0.0 to 0.1)	-0.0 (-1.0 to 1.5)	-0.2 (-14.1 to 24.5)
Tajikistan	0.0 (0.0 to 0.0)	0.1 (0.0 to 0.1)	0.1 (0.0 to 0.1)	0.1 (0.0 to 0.1)	1.2 (0.3 to 3.3)	20.7 (4.7 to 63.9)
Turkmenistan	0.0 (0.0 to 0.1)	0.0 (0.0 to 0.0)	0.0 (0.0 to 0.0)	0.1 (0.0 to 0.1)	3.9 (3.4 to 4.4)	78.7 (66.5 to 92.1)
Uzbekistan	0.0 (0.0 to 0.1)	0.1 (0.0 to 0.1)	0.1 (0.0 to 0.1)	0.1 (0.0 to 0.1)	1.3 (-0.0 to 3.5)	22.6 (-0.3 to 68.5)
Central Europe						
Albania	0.0 (0.0 to 0.0)	0.0 (0.0 to 0.0)	0.0 (0.0 to 0.0)	0.0 (0.0 to 0.0)	0.4 (0.1 to 1.2)	6.8 (1.1 to 19.9)
Bosnia and Herzegovina	0.0 (0.0 to 0.0)	0.0 (0.0 to 0.0)	0.0 (0.0 to 0.0)	0.0 (0.0 to 0.0)	2.5 (2.0 to 3.0)	46.0 (34.9 to 56.0)
Bulgaria	0.0 (0.0 to 0.0)	0.0 (0.0 to 0.0)	0.0 (0.0 to 0.0)	0.0 (0.0 to 0.0)	1.4 (0.7 to 3.6)	23.7 (10.3 to 72.6)
Croatia	0.0 (0.0 to 0.0)	0.0 (0.0 to 0.0)	0.0 (0.0 to 0.0)	0.0 (0.0 to 0.0)	1.5 (1.0 to 3.2)	25.3 (15.4 to 62.3)
Czech Republic	0.0 (0.0 to 0.0)	0.0 (0.0 to 0.0)	0.0 (0.0 to 0.0)	0.0 (0.0 to 0.0)	0.8 (0.4 to 1.6)	13.2 (5.6 to 26.3)
Hungary	0.0 (0.0 to 0.0)	0.0 (0.0 to 0.0)	0.0 (0.0 to 0.0)	0.0 (0.0 to 0.0)	4.0 (3.3 to 4.8)	82.0 (63.9 to 104.7)
Macedonia	0.0 (0.0 to 0.0)	0.0 (0.0 to 0.0)	0.0 (0.0 to 0.0)	0.0 (0.0 to 0.0)	0.6 (0.2 to 1.1)	9.8 (3.6 to 18.6)
Montenegro	0.0 (0.0 to 0.0)	0.0 (0.0 to 0.0)	0.0 (0.0 to 0.0)	0.0 (0.0 to 0.0)	0.4 (0.1 to 0.8)	6.7 (1.9 to 12.4)
Poland	0.0 (0.0 to 0.0)	0.0 (0.0 to 0.0)	0.0 (0.0 to 0.0)	0.0 (0.0 to 0.0)	1.1 (0.6 to 2.9)	17.6 (8.8 to 55.2)
Romania	0.0 (0.0 to 0.0)	0.1 (0.0 to 0.1)	0.1 (0.0 to 0.1)	0.1 (0.1 to 0.1)	1.1 (0.6 to 2.0)	18.9 (9.6 to 35.4)
Serbia	0.0 (0.0 to 0.1)	0.0 (0.0 to 0.0)	0.0 (0.0 to 0.0)	0.0 (0.0 to 0.0)	2.3 (1.6 to 2.9)	41.5 (28.1 to 54.8)
Slovakia	0.0 (0.0 to 0.0)	0.0 (0.0 to 0.0)	0.0 (0.0 to 0.0)	0.0 (0.0 to 0.0)	0.9 (0.4 to 1.8)	14.5 (5.6 to 30.0)
Slovenia	0.0 (0.0 to 0.0)	0.0 (0.0 to 0.0)	0.0 (0.0 to 0.0)	0.0 (0.0 to 0.0)	2.0 (1.0 to 3.4)	35.9 (15.3 to 65.3)
Eastern Europe						
Belarus	0.1 (0.0 to 0.1)	0.2 (0.1 to 0.3)	0.2 (0.2 to 0.3)	0.3 (0.2 to 0.4)	1.8 (1.2 to 2.4)	30.6 (19.1 to 43.2)
Estonia	0.1 (0.1 to 0.1)	0.2 (0.1 to 0.2)	0.2 (0.1 to 0.3)	0.2 (0.2 to 0.3)	1.2 (0.3 to 2.2)	20.8 (5.0 to 39.8)
Latvia	0.1 (0.1 to 0.1)	0.2 (0.2 to 0.2)	0.2 (0.2 to 0.3)	0.3 (0.2 to 0.3)	1.9 (1.4 to 2.3)	33.8 (23.9 to 41.3)
Lithuania	0.0 (0.0 to 0.1)	0.1 (0.0 to 0.1)	0.1 (0.0 to 0.1)	0.1 (0.0 to 0.1)	1.4 (0.7 to 3.6)	25.3 (11.3 to 71.1)
Moldova	0.1 (0.1 to 0.1)	0.2 (0.1 to 0.2)	0.2 (0.1 to 0.3)	0.2 (0.1 to 0.3)	1.5 (0.9 to 3.2)	26.2 (15.0 to 60.7)
Russia	0.1 (0.1 to 0.2)	0.9 (0.8 to 1.1)	0.9 (0.8 to 1.1)	1.1 (0.9 to 1.3)	0.8 (0.5 to 1.2)	12.8 (7.9 to 19.3)
Ukraine	0.3 (0.2 to 0.5)	0.4 (0.3 to 0.5)	0.6 (0.4 to 0.7)	0.6 (0.5 to 0.8)	3.2 (2.8 to 3.8)	62.5 (51.1 to 77.0)
High-income						
Australasia						
Australia	0.1 (0.0 to 0.1)	0.1 (0.0 to 0.1)	0.1 (0.0 to 0.1)	0.1 (0.0 to 0.1)	1.5 (1.1 to 2.0)	26.0 (18.1 to 35.0)
New Zealand	0.0 (0.0 to 0.1)	0.0 (0.0 to 0.1)	0.0 (0.0 to 0.1)	0.1 (0.0 to 0.1)	2.3 (1.4 to 3.2)	41.5 (23.5 to 62.7)
High-income Asia Pacific						
Brunei	0.1 (0.0 to 0.1)	0.1 (0.0 to 0.1)	0.1 (0.0 to 0.1)	0.1 (0.1 to 0.1)	0.8 (0.5 to 1.8)	13.0 (7.8 to 30.6)
Japan	0.0 (0.0 to 0.0)	0.0 (0.0 to 0.0)	0.0 (0.0 to 0.0)	0.0 (0.0 to 0.0)	0.4 (0.1 to 1.4)	5.5 (1.6 to 23.6)
South Korea	0.1 (0.0 to 0.1)	0.0 (0.0 to 0.0)	0.0 (0.0 to 0.0)	0.0 (0.0 to 0.1)	2.1 (1.4 to 2.9)	38.3 (24.1 to 55.0)
Singapore	0.0 (0.0 to 0.1)	0.0 (0.0 to 0.0)	0.0 (0.0 to 0.0)	0.0 (0.0 to 0.1)	3.3 (2.4 to 4.7)	65.4 (42.8 to 101.6)

	Estimate in 2000 (95% UIs)	Estimate in 2015 (95% UIs)	Estimate in 2017 (95% UIs)	Estimate in 2030 (95% UIs)	Annualised rate of change, 2015-2030 (95% UIs)	Percent change, 2015-2030 (95% UIs)
High-income North America						
Canada	0.1 (0.1 to 0.1)	0.1 (0.1 to 0.2)	0.1 (0.1 to 0.2)	0.1 (0.1 to 0.2)	2.5 (1.6 to 3.5)	45.0 (26.2 to 67.9)
Greenland	0.1 (0.1 to 0.2)	0.2 (0.1 to 0.3)	0.2 (0.1 to 0.3)	0.2 (0.1 to 0.4)	2.3 (1.2 to 3.3)	41.8 (19.3 to 64.1)
United States	0.1 (0.1 to 0.2)	0.2 (0.1 to 0.2)	0.2 (0.1 to 0.3)	0.2 (0.1 to 0.4)	2.4 (1.7 to 3.1)	44.0 (29.7 to 59.4)
Southern Latin America						
Argentina	0.2 (0.1 to 0.4)	0.3 (0.1 to 0.5)	0.3 (0.1 to 0.5)	0.3 (0.1 to 0.5)	0.7 (0.2 to 1.4)	10.5 (2.4 to 23.0)
Chile	0.1 (0.1 to 0.2)	0.2 (0.1 to 0.3)	0.2 (0.1 to 0.4)	0.2 (0.1 to 0.4)	0.8 (0.6 to 1.0)	12.4 (9.0 to 15.8)
Uruguay	0.2 (0.1 to 0.3)	0.2 (0.2 to 0.3)	0.3 (0.2 to 0.3)	0.3 (0.2 to 0.4)	0.9 (0.0 to 2.5)	15.2 (0.4 to 45.7)
Western Europe						
Andorra	0.0 (0.0 to 0.2)	0.1 (0.0 to 0.3)	0.1 (0.0 to 0.3)	0.1 (0.0 to 0.3)	1.1 (-0.1 to 2.0)	18.2 (-0.8 to 35.5)
Austria	0.1 (0.0 to 0.1)	0.1 (0.0 to 0.1)	0.1 (0.1 to 0.1)	0.1 (0.1 to 0.2)	2.4 (1.6 to 4.2)	44.9 (28.0 to 87.9)
Belgium	0.1 (0.0 to 0.1)	0.1 (0.0 to 0.1)	0.1 (0.0 to 0.1)	0.1 (0.1 to 0.2)	1.3 (0.3 to 2.6)	22.3 (4.6 to 48.1)
Cyprus	0.0 (0.0 to 0.0)	0.1 (0.1 to 0.1)	0.1 (0.1 to 0.1)	0.1 (0.1 to 0.1)	0.5 (0.0 to 1.1)	7.8 (0.6 to 18.0)
Denmark	0.1 (0.0 to 0.1)	0.1 (0.0 to 0.1)	0.1 (0.0 to 0.1)	0.1 (0.0 to 0.1)	0.9 (0.1 to 2.1)	15.6 (1.8 to 36.4)
Finland	0.0 (0.0 to 0.0)	0.0 (0.0 to 0.0)	0.0 (0.0 to 0.0)	0.0 (0.0 to 0.1)	1.0 (0.1 to 2.9)	17.5 (0.8 to 55.1)
France	0.1 (0.1 to 0.1)	0.1 (0.0 to 0.1)	0.1 (0.0 to 0.1)	0.1 (0.1 to 0.2)	1.9 (1.3 to 2.5)	33.1 (22.4 to 44.5)
Germany	0.0 (0.0 to 0.1)	0.1 (0.0 to 0.1)	0.1 (0.0 to 0.1)	0.1 (0.1 to 0.1)	2.5 (1.5 to 3.4)	45.0 (25.7 to 66.9)
Greece	0.0 (0.0 to 0.1)	0.1 (0.0 to 0.1)	0.1 (0.0 to 0.1)	0.1 (0.1 to 0.1)	0.8 (0.4 to 1.1)	12.6 (5.7 to 18.3)
Iceland	0.0 (0.0 to 0.1)	0.1 (0.0 to 0.1)	0.1 (0.0 to 0.1)	0.1 (0.0 to 0.1)	0.5 (-0.1 to 2.9)	8.4 (-2.2 to 55.5)
Ireland	0.1 (0.0 to 0.1)	0.1 (0.0 to 0.2)	0.1 (0.1 to 0.2)	0.1 (0.1 to 0.2)	0.7 (0.1 to 1.8)	11.8 (2.0 to 31.5)
Israel	0.1 (0.0 to 0.1)	0.1 (0.0 to 0.1)	0.1 (0.0 to 0.1)	0.1 (0.1 to 0.1)	0.8 (0.3 to 2.0)	13.5 (5.3 to 34.7)
Italy	0.1 (0.1 to 0.1)	0.1 (0.1 to 0.2)	0.1 (0.1 to 0.2)	0.2 (0.1 to 0.3)	3.1 (2.1 to 4.1)	59.0 (36.1 to 84.1)
Luxembourg	0.1 (0.1 to 0.2)	0.1 (0.1 to 0.2)	0.1 (0.1 to 0.2)	0.2 (0.1 to 0.2)	0.8 (0.2 to 2.0)	13.7 (2.6 to 35.2)
Malta	0.1 (0.0 to 0.1)	0.2 (0.1 to 0.3)	0.2 (0.1 to 0.4)	0.2 (0.1 to 0.4)	0.4 (0.2 to 0.5)	5.5 (2.6 to 8.4)
Netherlands	0.1 (0.1 to 0.1)	0.0 (0.0 to 0.1)	0.0 (0.0 to 0.1)	0.0 (0.0 to 0.1)	1.4 (0.6 to 2.1)	22.8 (10.0 to 36.0)
Norway	0.1 (0.0 to 0.1)	0.1 (0.0 to 0.1)	0.1 (0.0 to 0.1)	0.1 (0.0 to 0.1)	0.9 (0.4 to 2.2)	15.5 (5.8 to 38.3)
Portugal	0.6 (0.4 to 0.8)	0.8 (0.4 to 1.2)	0.8 (0.4 to 1.2)	0.6 (0.3 to 0.9)	-1.3 (-2.0 to -0.4)	-17.6 (-26.2 to -5.3)
Spain	0.1 (0.1 to 0.1)	0.1 (0.1 to 0.2)	0.1 (0.1 to 0.2)	0.2 (0.1 to 0.3)	2.4 (1.5 to 3.3)	43.5 (24.7 to 64.2)
Sweden	0.1 (0.0 to 0.1)	0.1 (0.0 to 0.1)	0.1 (0.0 to 0.1)	0.1 (0.0 to 0.1)	0.1 (-0.2 to 0.6)	2.0 (-2.3 to 9.8)
Switzerland	0.1 (0.1 to 0.1)	0.1 (0.0 to 0.1)	0.1 (0.0 to 0.1)	0.1 (0.0 to 0.1)	2.3 (1.5 to 3.2)	42.5 (25.1 to 60.8)
United Kingdom	0.1 (0.1 to 0.2)	0.1 (0.1 to 0.1)	0.1 (0.1 to 0.1)	0.1 (0.1 to 0.2)	0.5 (0.1 to 1.1)	8.0 (2.0 to 17.6)
Latin America and Caribbean						
Andean Latin America						
Bolivia	0.1 (0.0 to 0.4)	0.2 (0.0 to 0.8)	0.2 (0.0 to 0.8)	0.2 (0.0 to 0.9)	1.3 (0.1 to 3.1)	21.8 (2.1 to 58.1)
Ecuador	0.1 (0.1 to 0.2)	0.2 (0.2 to 0.2)	0.2 (0.1 to 0.2)	0.2 (0.2 to 0.2)	0.1 (-0.1 to 0.3)	1.8 (-1.4 to 5.1)
Peru	0.1 (0.1 to 0.1)	0.2 (0.1 to 0.3)	0.2 (0.1 to 0.3)	0.2 (0.2 to 0.3)	0.9 (0.3 to 1.7)	14.9 (4.6 to 28.6)
Caribbean						
Antigua and Barbuda	0.1 (0.1 to 0.1)	0.1 (0.1 to 0.1)	0.1 (0.1 to 0.1)	0.1 (0.1 to 0.2)	2.1 (1.8 to 2.6)	37.9 (30.2 to 46.8)
The Bahamas	0.7 (0.6 to 0.8)	0.9 (0.8 to 1.0)	0.9 (0.8 to 1.1)	0.8 (0.7 to 1.0)	-0.7 (-1.6 to 0.2)	-9.8 (-21.7 to 3.3)
Barbados	0.3 (0.3 to 0.3)	0.2 (0.2 to 0.3)	0.2 (0.2 to 0.4)	0.2 (0.1 to 0.3)	-1.1 (-2.3 to 0.0)	-14.6 (-28.9 to 0.4)
Belize	0.7 (0.1 to 0.9)	0.6 (0.5 to 0.7)	0.6 (0.5 to 0.7)	0.6 (0.5 to 0.7)	-0.2 (-0.7 to 0.3)	-3.4 (-10.6 to 5.3)
Bermuda	0.1 (0.1 to 0.1)	0.1 (0.1 to 0.1)	0.1 (0.1 to 0.1)	0.1 (0.1 to 0.2)	2.6 (2.2 to 3.0)	47.3 (39.3 to 57.7)
Cuba	0.0 (0.0 to 0.1)	0.2 (0.1 to 0.3)	0.2 (0.1 to 0.3)	0.2 (0.1 to 0.3)	0.3 (-0.1 to 0.8)	5.2 (-2.1 to 13.5)
Dominica	0.1 (0.1 to 0.1)	0.1 (0.1 to 0.1)	0.1 (0.1 to 0.1)	0.1 (0.1 to 0.2)	2.1 (1.7 to 2.5)	36.6 (28.1 to 45.7)
Dominican Republic	0.8 (0.6 to 1.1)	0.4 (0.2 to 0.6)	0.4 (0.2 to 0.5)	0.3 (0.2 to 0.5)	-1.5 (-2.2 to -0.7)	-20.0 (-28.2 to -9.5)
Grenada	0.1 (0.1 to 0.1)	0.1 (0.1 to 0.1)	0.1 (0.1 to 0.1)	0.1 (0.1 to 0.1)	3.0 (2.5 to 3.5)	56.6 (45.6 to 69.8)
Guyana	1.1 (0.9 to 1.3)	0.9 (0.6 to 1.1)	0.8 (0.6 to 1.0)	0.7 (0.5 to 0.9)	-1.9 (-2.7 to -1.2)	-24.2 (-32.8 to -16.4)
Haiti	1.1 (0.6 to 1.5)	0.8 (0.6 to 1.1)	0.7 (0.5 to 0.9)	0.7 (0.5 to 0.9)	-1.8 (-2.5 to -1.2)	-24.1 (-31.4 to -16.6)
Jamaica	0.3 (0.3 to 0.4)	0.5 (0.4 to 0.6)	0.4 (0.3 to 0.6)	0.4 (0.3 to 0.5)	-1.2 (-2.0 to -0.5)	-16.8 (-25.6 to -6.8)
Puerto Rico	0.1 (0.1 to 0.1)	0.1 (0.1 to 0.1)	0.1 (0.1 to 0.1)	0.1 (0.1 to 0.1)	2.9 (2.5 to 3.3)	54.8 (45.8 to 64.9)
Saint Lucia	0.1 (0.1 to 0.1)	0.1 (0.0 to 0.1)	0.1 (0.0 to 0.1)	0.1 (0.1 to 0.1)	2.6 (2.2 to 3.1)	48.3 (39.7 to 59.9)
Saint Vincent and the Grenadines	0.2 (0.2 to 0.3)	0.2 (0.2 to 0.2)	0.2 (0.1 to 0.2)	0.3 (0.2 to 0.4)	2.4 (1.9 to 2.9)	43.4 (33.6 to 55.6)
Suriname	0.6 (0.5 to 0.6)	0.4 (0.3 to 0.5)	0.4 (0.3 to 0.5)	0.3 (0.3 to 0.5)	-0.5 (-1.4 to 0.3)	-7.4 (-18.6 to 5.0)
Trinidad and Tobago	0.4 (0.4 to 0.5)	0.3 (0.3 to 0.4)	0.3 (0.3 to 0.4)	0.3 (0.2 to 0.4)	-0.5 (-1.3 to 0.3)	-6.6 (-17.9 to 5.4)
Virgin Islands, U.S.	0.1 (0.1 to 0.1)	0.2 (0.1 to 0.3)	0.2 (0.1 to 0.3)	0.2 (0.2 to 0.4)	2.0 (0.4 to 2.8)	36.1 (6.5 to 51.4)
Central Latin America						
Colombia	0.1 (0.1 to 0.1)	0.2 (0.1 to 0.2)	0.2 (0.1 to 0.2)	0.2 (0.1 to 0.3)	0.5 (-0.0 to 1.6)	8.3 (-0.2 to 27.6)
Costa Rica	0.1 (0.1 to 0.1)	0.1 (0.1 to 0.1)	0.1 (0.1 to 0.1)	0.1 (0.1 to 0.1)	1.2 (0.4 to 2.1)	19.7 (5.5 to 37.3)
El Salvador	0.3 (0.2 to 0.3)	0.2 (0.1 to 0.3)	0.2 (0.1 to 0.3)	0.2 (0.1 to 0.3)	0.0 (-1.1 to 1.3)	1.1 (-15.2 to 21.1)
Guatemala	0.2 (0.2 to 0.4)	0.1 (0.1 to 0.2)	0.1 (0.1 to 0.3)	0.2 (0.1 to 0.3)	2.6 (1.5 to 3.6)	49.1 (25.1 to 72.7)
Honduras	0.1 (0.0 to 0.1)	0.1 (0.0 to 0.1)	0.1 (0.0 to 0.1)	0.1 (0.1 to 0.1)	1.9 (0.8 to 3.3)	33.3 (12.0 to 63.4)
Mexico	0.1 (0.1 to 0.1)	0.1 (0.1 to 0.2)	0.1 (0.1 to 0.2)	0.2 (0.1 to 0.2)	1.5 (0.8 to 2.3)	25.0 (12.5 to 40.6)
Nicaragua	0.1 (0.1 to 0.1)	0.3 (0.2 to 0.4)	0.3 (0.2 to 0.4)	0.3 (0.2 to 0.5)	0.1 (-0.5 to 1.0)	1.4 (-6.9 to 16.0)
Panama	0.3 (0.2 to 0.3)	0.5 (0.4 to 0.6)	0.5 (0.4 to 0.6)	0.4 (0.3 to 0.6)	-1.0 (-1.5 to -0.6)	-14.3 (-20.4 to -8.2)
Venezuela	0.2 (0.1 to 0.2)	0.2 (0.2 to 0.3)	0.2 (0.2 to 0.3)	0.3 (0.2 to 0.3)	0.6 (0.3 to 1.0)	8.7 (4.1 to 15.6)
Tropical Latin America						
Brazil	0.2 (0.2 to 0.3)	0.4 (0.3 to 0.4)	0.4 (0.3 to 0.4)	0.4 (0.3 to 0.5)	0.7 (0.5 to 0.9)	10.8 (8.6 to 14.1)
Paraguay	0.1 (0.1 to 0.2)	0.3 (0.2 to 0.3)	0.2 (0.1 to 0.3)	0.3 (0.2 to 0.4)	0.5 (-0.2 to 1.4)	7.9 (-2.4 to 23.3)
North Africa and Middle East						
North Africa and Middle East						
Afghanistan	0.0 (0.0 to 0.0)	0.0 (0.0 to 0.1)	0.0 (0.0 to 0.1)	0.0 (0.0 to 0.1)	0.8 (-0.3 to 2.8)	14.6 (-4.5 to 52.2)
Algeria	0.0 (0.0 to 0.1)	0.0 (0.0 to 0.1)	0.0 (0.0 to 0.1)	0.0 (0.0 to 0.1)	-2.5 (-36.7 to 1.9)	-2.4 (-99.6 to 33.9)
Bahrain	0.0 (0.0 to 0.0)	0.0 (0.0 to 0.0)	0.0 (0.0 to 0.0)	0.0 (0.0 to 0.0)	2.6 (2.1 to 3.2)	48.0 (37.1 to 61.6)
Egypt	0.0 (0.0 to 0.0)	0.0 (0.0 to 0.0)	0.0 (0.0 to 0.0)	0.0 (0.0 to 0.0)	0.9 (0.2 to 2.5)	15.5 (2.6 to 46.5)
Iran	0.0 (0.0 to 0.0)	0.0 (0.0 to 0.1)	0.0 (0.0 to 0.1)	0.0 (0.0 to 0.1)	1.0 (0.4 to 1.8)	16.1 (5.7 to 30.2)
Iraq	0.0 (0.0 to 0.0)	0.0 (0.0 to 0.0)	0.0 (0.0 to 0.0)	0.0 (0.0 to 0.0)	0.6 (0.1 to 1.8)	9.4 (0.9 to 30.4)
Jordan	0.0 (0.0 to 0.0)	0.0 (0.0 to 0.0)	0.0 (0.0 to 0.0)	0.0 (0.0 to 0.0)	1.3 (0.7 to 2.0)	22.4 (11.6 to 35.4)
Kuwait	0.0 (0.0 to 0.0)	0.0 (0.0 to 0.0)	0.0 (0.0 to 0.0)	0.0 (0.0 to 0.0)	3.4 (2.6 to 4.2)	65.6 (48.8 to 86.9)
Lebanon	0.0 (0.0 to 0.1)	0.0 (0.0 to 0.1)	0.0 (0.0 to 0.1)	0.0 (0.0 to 0.2)	2.0 (0.6 to 3.5)	36.3 (9.0 to 69.7)
Libya	0.0 (0.0 to 0.1)	0.0 (0.0 to 0.1)	0.0 (0.0 to 0.1)	0.0 (0.0 to 0.1)	1.6 (0.6 to 3.2)	28.3 (9.4 to 61.5)

	Estimate in 2000 (95% UIs)	Estimate in 2015 (95% UIs)	Estimate in 2017 (95% UIs)	Estimate in 2030 (95% UIs)	Annualised rate of change, 2015-2030 (95% UIs)	Percent change, 2015-2030 (95% UIs)
Morocco	0.1 (0.0 to 0.2)	0.0 (0.0 to 0.1)	0.0 (0.0 to 0.1)	0.0 (0.0 to 0.2)	2.0 (0.4 to 3.7)	35.6 (5.9 to 73.3)
Palestine	0.0 (0.0 to 0.0)	0.0 (0.0 to 0.0)	0.0 (0.0 to 0.0)	0.0 (0.0 to 0.0)	0.5 (0.2 to 1.1)	7.1 (2.5 to 17.4)
Oman	0.1 (0.1 to 0.1)	0.1 (0.0 to 0.1)	0.1 (0.0 to 0.1)	0.1 (0.1 to 0.2)	4.4 (2.8 to 6.7)	94.3 (53.2 to 171.4)
Qatar	0.0 (0.0 to 0.0)	0.0 (0.0 to 0.0)	0.0 (0.0 to 0.0)	0.0 (0.0 to 0.0)	3.7 (3.1 to 4.5)	75.3 (58.6 to 95.2)
Saudi Arabia	0.0 (0.0 to 0.0)	0.0 (0.0 to 0.0)	0.0 (0.0 to 0.0)	0.0 (0.0 to 0.0)	2.2 (0.9 to 2.9)	38.8 (14.6 to 54.5)
Sudan	0.2 (0.2 to 0.3)	0.2 (0.1 to 0.4)	0.2 (0.1 to 0.3)	0.2 (0.1 to 0.4)	0.2 (-1.0 to 1.1)	4.0 (-13.5 to 18.5)
Syria	0.0 (0.0 to 0.0)	0.0 (0.0 to 0.0)	0.0 (0.0 to 0.0)	0.0 (0.0 to 0.0)	1.6 (0.6 to 2.9)	28.4 (9.9 to 54.1)
Tunisia	0.0 (0.0 to 0.1)	0.0 (0.0 to 0.1)	0.0 (0.0 to 0.1)	0.0 (0.0 to 0.2)	0.8 (0.2 to 2.2)	13.3 (2.8 to 40.0)
Turkey	0.0 (0.0 to 0.0)	0.0 (0.0 to 0.0)	0.0 (0.0 to 0.0)	0.0 (0.0 to 0.0)	0.6 (0.3 to 1.5)	9.3 (4.6 to 24.5)
United Arab Emirates	0.0 (0.0 to 0.1)	0.0 (0.0 to 0.2)	0.0 (0.0 to 0.3)	0.1 (0.0 to 0.3)	2.6 (-3.3 to 8.5)	59.5 (-39.5 to 258.6)
Yemen	0.0 (0.0 to 0.1)	0.0 (0.0 to 0.2)	0.0 (0.0 to 0.2)	0.0 (0.0 to 0.2)	1.5 (0.0 to 3.3)	26.4 (0.7 to 64.4)
South Asia						
South Asia						
Bangladesh	0.0 (0.0 to 0.0)	0.0 (0.0 to 0.0)	0.0 (0.0 to 0.0)	0.0 (0.0 to 0.0)	1.4 (0.1 to 2.8)	23.4 (1.4 to 52.6)
Bhutan	0.1 (0.0 to 0.3)	0.1 (0.0 to 0.6)	0.1 (0.0 to 0.6)	0.2 (0.0 to 0.7)	1.5 (0.5 to 3.2)	26.2 (7.3 to 61.0)
India	0.3 (0.2 to 0.3)	0.1 (0.1 to 0.1)	0.1 (0.0 to 0.1)	0.1 (0.1 to 0.1)	0.6 (-0.3 to 1.5)	9.7 (-3.7 to 26.1)
Nepal	0.3 (0.0 to 1.2)	0.1 (0.0 to 0.4)	0.1 (0.0 to 0.3)	0.1 (0.0 to 0.4)	1.3 (-0.3 to 2.6)	21.8 (-4.6 to 48.6)
Pakistan	0.0 (0.0 to 0.0)	0.1 (0.0 to 0.3)	0.1 (0.0 to 0.3)	0.1 (0.0 to 0.3)	1.0 (0.1 to 2.9)	17.4 (1.1 to 54.6)
Southeast Asia, East Asia, and Oceania						
East Asia						
China	0.0 (0.0 to 0.1)	0.0 (0.0 to 0.0)	0.0 (0.0 to 0.0)	0.0 (0.0 to 0.1)	2.0 (1.4 to 2.8)	35.5 (24.0 to 51.4)
North Korea	0.0 (0.0 to 0.3)	0.0 (0.0 to 0.3)	0.1 (0.0 to 0.3)	0.1 (0.0 to 0.3)	1.0 (-0.2 to 2.1)	16.5 (-3.3 to 36.7)
Taiwan (Province of China)	0.0 (0.0 to 0.0)	0.0 (0.0 to 0.0)	0.0 (0.0 to 0.0)	0.0 (0.0 to 0.0)	1.8 (1.3 to 2.2)	31.5 (22.4 to 39.8)
Oceania						
American Samoa	0.0 (0.0 to 0.0)	0.0 (0.0 to 0.0)	0.0 (0.0 to 0.0)	0.0 (0.0 to 0.0)	2.4 (1.8 to 2.9)	43.5 (30.0 to 53.4)
Federated States of Micronesia	0.1 (0.0 to 0.4)	0.9 (0.0 to 5.4)	0.8 (0.0 to 4.5)	0.8 (0.0 to 4.4)	-0.5 (-8.4 to 5.8)	6.0 (-71.5 to 138.8)
Fiji	0.0 (0.0 to 0.0)	0.1 (0.0 to 0.1)	0.1 (0.0 to 0.1)	0.1 (0.1 to 0.1)	2.3 (1.5 to 4.1)	42.3 (25.7 to 83.6)
Guam	0.0 (0.0 to 0.0)	0.0 (0.0 to 0.1)	0.1 (0.0 to 0.1)	0.1 (0.0 to 0.1)	2.8 (2.4 to 3.2)	53.3 (43.8 to 62.4)
Kiribati	0.0 (0.0 to 0.0)	0.0 (0.0 to 0.0)	0.0 (0.0 to 0.0)	0.0 (0.0 to 0.0)	2.8 (1.7 to 4.8)	53.6 (28.5 to 104.7)
Marshall Islands	0.0 (0.0 to 0.3)	0.1 (0.0 to 0.7)	0.1 (0.0 to 0.9)	0.2 (0.0 to 1.0)	2.3 (0.5 to 3.8)	43.5 (7.7 to 76.2)
Northern Mariana Islands	0.0 (0.0 to 0.0)	0.0 (0.0 to 0.0)	0.0 (0.0 to 0.0)	0.0 (0.0 to 0.0)	1.4 (-0.6 to 2.4)	23.3 (-8.6 to 42.5)
Papua New Guinea	0.9 (0.2 to 2.4)	0.3 (0.0 to 1.1)	0.3 (0.0 to 1.3)	0.3 (0.0 to 1.2)	-0.1 (-3.1 to 2.8)	1.7 (-36.9 to 51.8)
Samoa	0.0 (0.0 to 0.3)	0.1 (0.0 to 0.8)	0.1 (0.0 to 0.9)	0.2 (0.0 to 1.0)	2.0 (0.2 to 3.6)	36.6 (3.7 to 70.4)
Solomon Islands	0.0 (0.0 to 0.3)	0.1 (0.0 to 0.7)	0.1 (0.0 to 0.8)	0.2 (0.0 to 1.0)	2.4 (0.5 to 3.9)	44.0 (7.5 to 78.3)
Tonga	0.0 (0.0 to 0.0)	0.0 (0.0 to 0.0)	0.0 (0.0 to 0.0)	0.0 (0.0 to 0.1)	2.3 (0.6 to 3.3)	42.2 (10.1 to 63.0)
Vanuatu	0.1 (0.0 to 0.3)	0.1 (0.0 to 0.8)	0.1 (0.0 to 0.9)	0.2 (0.0 to 1.0)	2.4 (0.5 to 3.9)	45.0 (8.2 to 79.4)
Southeast Asia						
Cambodia	0.6 (0.2 to 1.2)	0.1 (0.0 to 0.2)	0.1 (0.0 to 0.1)	0.1 (0.0 to 0.1)	-2.2 (-4.0 to -1.2)	-27.4 (-45.0 to -16.9)
Indonesia	0.0 (0.0 to 0.0)	0.1 (0.0 to 0.1)	0.1 (0.1 to 0.1)	0.1 (0.1 to 0.1)	0.5 (0.1 to 1.1)	7.9 (1.9 to 18.1)
Laos	0.2 (0.0 to 1.2)	0.1 (0.0 to 0.7)	0.1 (0.0 to 0.7)	0.1 (0.0 to 0.8)	1.5 (0.2 to 2.7)	26.1 (2.8 to 49.2)
Malaysia	0.1 (0.1 to 0.2)	0.1 (0.1 to 0.1)	0.1 (0.1 to 0.2)	0.2 (0.1 to 0.2)	3.1 (2.3 to 3.9)	60.4 (40.5 to 79.7)
Maldives	0.0 (0.0 to 0.0)	0.0 (0.0 to 0.0)	0.0 (0.0 to 0.0)	0.0 (0.0 to 0.0)	2.2 (1.7 to 2.7)	40.0 (29.7 to 50.6)
Mauritius	0.1 (0.0 to 0.1)	0.3 (0.2 to 0.4)	0.2 (0.2 to 0.3)	0.3 (0.2 to 0.4)	-0.1 (-0.9 to 0.7)	-1.8 (-12.8 to 10.4)
Myanmar	0.7 (0.6 to 0.8)	0.2 (0.1 to 0.3)	0.2 (0.1 to 0.2)	0.3 (0.2 to 0.4)	2.0 (1.4 to 2.6)	35.8 (22.8 to 47.4)
Philippines	0.1 (0.1 to 0.1)	0.1 (0.1 to 0.2)	0.1 (0.1 to 0.2)	0.2 (0.1 to 0.3)	2.5 (1.2 to 3.5)	46.8 (19.2 to 68.9)
Sri Lanka	0.0 (0.0 to 0.0)	0.0 (0.0 to 0.0)	0.0 (0.0 to 0.0)	0.0 (0.0 to 0.0)	3.2 (2.2 to 4.0)	62.9 (38.2 to 81.8)
Seychelles	0.1 (0.1 to 0.1)	0.1 (0.1 to 0.1)	0.1 (0.0 to 0.1)	0.1 (0.1 to 0.1)	1.8 (1.5 to 2.1)	30.3 (25.3 to 36.2)
Thailand	0.3 (0.1 to 0.5)	0.4 (0.3 to 0.5)	0.3 (0.3 to 0.5)	0.3 (0.2 to 0.5)	-0.9 (-1.3 to -0.4)	-12.2 (-17.8 to -6.2)
Timor-Leste	0.3 (0.0 to 2.1)	0.2 (0.0 to 1.2)	0.2 (0.0 to 1.2)	0.2 (0.0 to 1.5)	0.9 (-0.3 to 1.9)	15.0 (-4.7 to 32.6)
Vietnam	0.2 (0.2 to 0.3)	0.2 (0.2 to 0.3)	0.2 (0.1 to 0.2)	0.2 (0.2 to 0.3)	1.0 (0.3 to 1.6)	15.6 (5.1 to 27.2)
Sub-Saharan Africa						
Central Sub-Saharan Africa						
Angola	1.2 (0.9 to 1.6)	1.0 (0.5 to 1.5)	0.8 (0.3 to 1.4)	0.7 (0.3 to 1.2)	-2.2 (-4.9 to -0.8)	-27.2 (-51.8 to -11.2)
Central African Republic	5.3 (3.7 to 7.2)	1.2 (0.4 to 2.2)	1.1 (0.2 to 2.5)	1.1 (0.2 to 2.4)	-1.1 (-4.2 to 1.2)	-13.5 (-46.4 to 19.4)
Congo	2.1 (1.5 to 2.9)	1.5 (1.1 to 2.0)	1.4 (1.0 to 1.9)	1.3 (0.9 to 1.7)	-1.2 (-1.7 to -0.7)	-16.9 (-22.5 to -10.3)
Democratic Republic of the Congo	1.4 (1.1 to 1.8)	0.2 (0.1 to 0.4)	0.2 (0.0 to 0.4)	0.2 (0.0 to 0.4)	-0.5 (-2.5 to 0.8)	-6.0 (-31.1 to 13.0)
Equatorial Guinea	3.3 (2.3 to 4.5)	2.7 (1.1 to 5.1)	1.8 (0.4 to 4.2)	1.5 (0.3 to 3.6)	-4.3 (-9.4 to -1.9)	-45.5 (-75.4 to -24.9)
Gabon	5.1 (3.6 to 7.3)	0.7 (0.4 to 1.1)	0.4 (0.2 to 0.8)	0.4 (0.2 to 0.7)	-4.0 (-5.4 to -2.4)	-44.9 (-55.3 to -30.1)
Eastern Sub-Saharan Africa						
Burundi	2.3 (1.1 to 3.5)	0.3 (0.1 to 0.7)	0.3 (0.1 to 0.8)	0.3 (0.1 to 0.7)	-1.1 (-3.0 to 0.5)	-14.1 (-36.4 to 7.5)
Comoros	0.0 (0.0 to 0.0)	0.0 (0.0 to 0.1)	0.0 (0.0 to 0.1)	0.0 (0.0 to 0.1)	2.5 (1.2 to 3.7)	45.2 (19.3 to 75.3)
Djibouti	3.1 (2.1 to 4.1)	0.7 (0.3 to 1.3)	0.7 (0.2 to 1.5)	0.7 (0.2 to 1.4)	-0.7 (-3.3 to 0.9)	-8.2 (-39.2 to 14.1)
Eritrea	1.4 (0.8 to 2.0)	0.3 (0.1 to 0.6)	0.3 (0.1 to 0.6)	0.3 (0.1 to 0.6)	-0.7 (-3.7 to 0.8)	-9.0 (-43.0 to 12.8)
Ethiopia	1.2 (0.7 to 1.8)	0.2 (0.1 to 0.3)	0.1 (0.1 to 0.2)	0.2 (0.1 to 0.4)	0.8 (-0.3 to 1.9)	13.3 (-3.9 to 32.0)
Kenya	4.7 (3.6 to 6.0)	2.2 (1.6 to 3.2)	2.0 (1.4 to 2.9)	2.6 (1.8 to 3.5)	0.9 (0.3 to 1.5)	14.8 (4.2 to 24.6)
Madagascar	0.4 (0.3 to 0.6)	0.2 (0.0 to 0.6)	0.4 (0.0 to 1.8)	0.4 (0.1 to 1.9)	5.5 (1.2 to 10.4)	141.1 (19.5 to 379.2)
Malawi	9.4 (7.0 to 12.1)	2.2 (1.4 to 3.2)	1.6 (0.7 to 2.6)	1.6 (0.7 to 2.5)	-2.7 (-4.7 to -1.3)	-32.3 (-50.9 to -17.5)
Mozambique	8.6 (7.2 to 10.4)	6.2 (4.6 to 8.2)	4.7 (3.1 to 6.8)	3.8 (2.4 to 5.6)	-3.3 (-4.7 to -2.4)	-38.8 (-50.4 to -29.9)
Rwanda	2.5 (1.6 to 3.4)	0.7 (0.5 to 1.0)	0.5 (0.3 to 0.8)	0.5 (0.3 to 0.8)	-1.8 (-3.1 to -0.7)	-23.9 (-36.8 to -9.5)
Somalia	0.6 (0.1 to 2.2)	0.2 (0.0 to 0.7)	0.1 (0.0 to 0.6)	0.2 (0.0 to 0.8)	0.3 (-4.1 to 2.6)	8.7 (-46.1 to 48.2)
South Sudan	1.5 (0.1 to 4.9)	0.7 (0.0 to 2.2)	0.5 (0.0 to 1.8)	0.4 (0.0 to 1.6)	-3.5 (-6.3 to -2.0)	-40.0 (-60.9 to -25.5)
Tanzania	4.7 (2.7 to 6.5)	1.2 (0.2 to 2.7)	1.0 (0.0 to 3.4)	0.9 (0.0 to 2.9)	-5.7 (-31.8 to 0.7)	-38.3 (-99.2 to 11.8)
Uganda	3.6 (2.6 to 4.7)	1.8 (0.7 to 3.9)	1.1 (0.1 to 4.0)	1.2 (0.2 to 4.1)	-3.5 (-9.9 to 0.6)	-38.0 (-77.4 to 8.8)
Zambia	8.8 (6.8 to 11.0)	3.5 (2.6 to 4.6)	2.7 (1.8 to 4.0)	2.3 (1.4 to 3.4)	-3.0 (-4.2 to -1.8)	-35.8 (-47.0 to -23.2)
Southern Sub-Saharan Africa						
Botswana	14.8 (12.2 to 18.2)	6.0 (4.5 to 7.8)	3.6 (2.6 to 5.0)	3.4 (2.4 to 4.6)	-3.9 (-5.2 to -2.7)	-43.8 (-54.3 to -33.2)
Lesotho	16.2 (13.7 to 19.3)	9.6 (7.6 to 11.9)	6.2 (4.5 to 8.2)	5.7 (4.0 to 7.7)	-3.5 (-4.6 to -2.5)	-40.6 (-50.1 to -31.5)
Namibia	13.0 (10.8 to 15.4)	4.5 (3.0 to 6.5)	3.9 (2.3 to 6.4)	2.7 (1.7 to 4.1)	-3.6 (-4.8 to -2.4)	-41.2 (-51.1 to -30.4)
South Africa	14.3 (12.8 to 16.5)	5.8 (4.7 to 7.1)	4.6 (3.5 to 5.8)	4.8 (3.5 to 6.2)	-1.3 (-2.3 to -0.4)	-17.6 (-28.9 to -6.3)
Swaziland	17.5 (13.9 to 21.9)	4.5 (2.8 to 6.7)	2.0 (0.5 to 3.7)	1.8 (0.2 to 3.5)	-7.3 (-16.6 to -3.9)	-63.2 (-91.7 to -43.9)

	Estimate in 2000 (95% UIs)	Estimate in 2015 (95% UIs)	Estimate in 2017 (95% UIs)	Estimate in 2030 (95% UIs)	Annualised rate of change, 2015-2030 (95% UIs)	Percent change, 2015-2030 (95% UIs)
Zimbabwe	12.0 (6.9 to 16.4)	4.0 (0.7 to 7.8)	4.0 (0.1 to 9.0)	4.1 (0.1 to 8.8)	-1.1 (-13.3 to 1.6)	-6.9 (-86.4 to 27.4)
Western Sub-Saharan Africa						
Benin	0.9 (0.5 to 1.3)	0.4 (0.3 to 0.6)	0.3 (0.2 to 0.5)	0.3 (0.2 to 0.5)	-2.1 (-3.7 to -1.0)	-26.1 (-42.8 to -14.2)
Burkina Faso	0.8 (0.4 to 1.1)	0.2 (0.1 to 0.3)	0.2 (0.0 to 0.3)	0.1 (0.0 to 0.3)	-2.7 (-9.3 to -0.3)	-29.3 (-75.1 to -4.1)
Cameroon	4.9 (4.2 to 5.9)	1.4 (0.7 to 2.0)	1.1 (0.3 to 1.9)	1.0 (0.3 to 1.7)	-2.2 (-5.6 to -0.7)	-27.2 (-57.0 to -10.6)
Cape Verde	0.8 (0.6 to 1.1)	0.6 (0.4 to 0.8)	0.6 (0.4 to 0.8)	0.6 (0.4 to 0.8)	0.0 (-0.4 to 0.5)	0.3 (-5.9 to 7.1)
Chad	1.8 (1.1 to 2.6)	0.7 (0.3 to 1.0)	0.7 (0.2 to 1.1)	0.6 (0.2 to 1.1)	-0.6 (-2.6 to 1.0)	-6.9 (-32.7 to 15.7)
Cote d'Ivoire	2.6 (1.4 to 3.7)	1.7 (0.6 to 2.7)	1.9 (0.3 to 3.3)	1.6 (0.3 to 3.0)	-1.0 (-4.5 to 1.2)	-11.5 (-49.4 to 20.0)
The Gambia	1.6 (1.2 to 2.2)	0.7 (0.3 to 1.2)	0.6 (0.2 to 1.1)	0.6 (0.2 to 1.0)	-2.1 (-5.0 to -0.7)	-26.1 (-52.8 to -9.4)
Ghana	1.7 (1.3 to 2.1)	0.8 (0.5 to 1.1)	0.8 (0.4 to 1.3)	0.7 (0.4 to 1.2)	-0.5 (-1.7 to 0.6)	-6.3 (-22.3 to 9.9)
Guinea	1.4 (1.1 to 1.7)	0.6 (0.4 to 0.9)	0.5 (0.3 to 0.9)	0.5 (0.2 to 0.8)	-2.0 (-3.3 to -0.8)	-26.1 (-39.3 to -11.4)
Guinea-Bissau	2.8 (2.0 to 3.8)	1.4 (0.6 to 2.4)	1.3 (0.5 to 2.5)	1.2 (0.4 to 2.2)	-1.6 (-3.9 to -0.3)	-20.5 (-44.6 to -4.5)
Liberia	1.8 (1.0 to 2.5)	0.5 (0.3 to 0.9)	0.5 (0.2 to 0.9)	0.5 (0.2 to 0.9)	-0.5 (-1.9 to 0.7)	-6.6 (-25.1 to 11.1)
Mali	1.2 (0.8 to 1.5)	0.5 (0.4 to 0.7)	0.4 (0.3 to 0.6)	0.4 (0.2 to 0.5)	-2.6 (-3.2 to -2.0)	-31.9 (-37.8 to -26.3)
Mauritania	0.0 (0.0 to 0.1)	0.0 (0.0 to 0.1)	0.0 (0.0 to 0.1)	0.0 (0.0 to 0.1)	1.4 (0.1 to 2.8)	23.6 (1.1 to 52.6)
Niger	0.9 (0.7 to 1.1)	0.1 (0.1 to 0.2)	0.1 (0.1 to 0.2)	0.1 (0.1 to 0.2)	-0.8 (-1.4 to -0.1)	-10.6 (-18.4 to -1.8)
Nigeria	3.0 (2.3 to 3.7)	1.4 (1.1 to 1.9)	1.2 (0.8 to 1.6)	1.0 (0.7 to 1.3)	-2.5 (-3.1 to -1.9)	-31.6 (-37.6 to -25.3)
Sao Tome and Principe	0.0 (0.0 to 0.0)	0.0 (0.0 to 0.0)	0.0 (0.0 to 0.0)	0.0 (0.0 to 0.0)	1.5 (0.4 to 2.7)	26.2 (5.6 to 50.9)
Senegal	0.7 (0.5 to 0.9)	0.2 (0.1 to 0.3)	0.2 (0.1 to 0.3)	0.3 (0.2 to 0.5)	2.2 (1.4 to 3.2)	38.9 (24.0 to 61.2)
Sierra Leone	1.2 (0.9 to 1.6)	0.4 (0.1 to 0.8)	0.4 (0.1 to 0.9)	0.4 (0.0 to 0.8)	-1.2 (-5.8 to 1.0)	-13.7 (-57.8 to 15.9)
Togo	3.8 (2.8 to 5.0)	0.6 (0.2 to 1.1)	0.5 (0.1 to 1.1)	0.5 (0.1 to 1.0)	-1.8 (-6.4 to 0.1)	-21.9 (-61.4 to 1.6)
**Indicator 3.3.2: Age-standardised rate of tuberculosis cases (per 100,000 population)**						
Central Europe, Eastern Europe, and Central Asia						
Central Asia						
Armenia	44.8 (40.4 to 49.4)	37.7 (34.4 to 41.0)	33.6 (30.7 to 36.5)	28.1 (21.7 to 36.7)	-2.0 (-3.5 to -0.3)	-25.6 (-40.6 to -4.8)
Azerbaijan	148.5 (134.2 to 164.0)	106.0 (95.0 to 118.3)	104.1 (92.7 to 116.7)	95.0 (71.3 to 124.6)	-0.8 (-2.4 to 0.9)	-10.4 (-30.1 to 14.6)
Georgia	79.0 (71.1 to 87.0)	77.6 (71.0 to 84.5)	69.6 (63.9 to 75.5)	59.7 (47.5 to 74.3)	-1.8 (-3.2 to -0.4)	-23.1 (-38.3 to -5.5)
Kazakhstan	159.9 (145.0 to 176.0)	89.9 (82.0 to 98.2)	74.7 (68.4 to 81.0)	80.2 (54.7 to 114.9)	-0.9 (-3.2 to 1.4)	-10.7 (-38.6 to 22.5)
Kyrgyzstan	134.4 (122.3 to 148.4)	110.2 (101.3 to 119.7)	114.0 (105.0 to 123.5)	97.4 (75.3 to 124.3)	-0.9 (-2.5 to 0.7)	-11.6 (-31.5 to 10.3)
Mongolia	179.7 (163.9 to 197.8)	153.2 (138.9 to 168.3)	150.3 (135.7 to 165.1)	140.9 (108.2 to 186.6)	-0.6 (-2.2 to 1.1)	-8.1 (-27.9 to 18.0)
Tajikistan	135.7 (122.4 to 149.2)	110.7 (99.8 to 121.7)	107.7 (97.3 to 118.9)	79.5 (64.9 to 98.7)	-2.2 (-3.4 to -1.0)	-28.1 (-40.3 to -14.3)
Turkmenistan	99.2 (89.5 to 110.3)	76.4 (68.2 to 85.2)	78.9 (69.6 to 89.5)	60.1 (45.6 to 80.1)	-1.6 (-3.2 to 0.1)	-21.3 (-37.8 to 0.8)
Uzbekistan	85.0 (77.2 to 93.3)	64.7 (59.8 to 70.2)	62.5 (57.8 to 68.1)	45.3 (37.0 to 55.9)	-2.4 (-3.7 to -1.1)	-30.0 (-42.4 to -14.7)
Central Europe						
Albania	17.3 (15.6 to 19.1)	14.1 (12.8 to 15.7)	14.3 (12.9 to 16.0)	13.5 (10.4 to 17.0)	-0.3 (-1.9 to 1.2)	-4.3 (-24.7 to 19.1)
Bosnia and Herzegovina	37.8 (34.5 to 41.5)	29.1 (26.4 to 32.1)	28.3 (25.5 to 31.4)	21.9 (18.0 to 27.4)	-1.9 (-3.1 to -0.6)	-24.7 (-37.3 to -7.9)
Bulgaria	30.3 (27.4 to 33.4)	20.6 (18.9 to 22.2)	19.1 (17.6 to 20.5)	11.5 (8.1 to 16.0)	-4.0 (-6.1 to -1.8)	-44.3 (-59.7 to -23.7)
Croatia	19.1 (17.1 to 21.6)	10.8 (9.8 to 11.9)	10.0 (9.1 to 11.0)	6.3 (4.7 to 8.4)	-3.7 (-5.5 to -1.8)	-41.8 (-56.3 to -24.0)
Czech Republic	9.4 (8.5 to 10.4)	5.5 (5.0 to 6.1)	5.2 (4.7 to 5.8)	3.2 (2.5 to 4.1)	-3.7 (-5.0 to -2.1)	-42.2 (-52.9 to -27.6)
Hungary	17.6 (16.1 to 19.4)	8.8 (8.0 to 9.6)	7.6 (6.9 to 8.3)	4.8 (3.7 to 6.6)	-4.1 (-5.8 to -2.0)	-45.2 (-57.8 to -25.5)
Macedonia	39.5 (35.8 to 43.5)	23.2 (21.0 to 25.5)	23.1 (20.9 to 25.5)	19.2 (14.3 to 24.5)	-1.3 (-3.1 to 0.2)	-17.2 (-37.0 to 3.1)
Montenegro	17.7 (15.8 to 19.6)	14.1 (12.8 to 15.6)	13.9 (12.6 to 15.4)	10.3 (7.6 to 14.0)	-2.2 (-4.0 to -0.1)	-27.1 (-45.4 to -2.1)
Poland	19.3 (17.7 to 21.2)	12.4 (11.3 to 13.5)	11.9 (10.9 to 12.9)	6.5 (5.2 to 8.3)	-4.3 (-5.6 to -2.8)	-47.2 (-57.0 to -34.2)
Romania	88.1 (80.0 to 95.6)	62.2 (57.3 to 67.1)	59.1 (54.4 to 63.6)	30.0 (21.2 to 43.4)	-5.0 (-7.1 to -2.5)	-51.8 (-65.7 to -31.0)
Serbia	31.0 (28.0 to 34.2)	22.8 (20.7 to 25.1)	21.8 (19.7 to 24.1)	20.2 (14.9 to 27.2)	-0.9 (-2.7 to 1.1)	-11.3 (-33.0 to 17.8)
Slovakia	14.0 (12.6 to 15.5)	9.5 (8.6 to 10.5)	9.2 (8.3 to 10.2)	5.9 (4.5 to 7.8)	-3.3 (-4.9 to -1.3)	-38.2 (-52.2 to -17.8)
Slovenia	11.7 (10.5 to 13.0)	7.0 (6.2 to 7.8)	6.4 (5.7 to 7.2)	3.7 (2.7 to 4.8)	-4.4 (-6.1 to -2.6)	-47.9 (-59.9 to -32.6)
Eastern Europe						
Belarus	51.0 (44.6 to 57.7)	38.7 (35.3 to 42.3)	33.1 (30.2 to 36.1)	24.4 (19.2 to 30.7)	-3.1 (-4.5 to -1.7)	-36.9 (-49.1 to -22.7)
Estonia	41.2 (36.9 to 45.8)	20.7 (18.6 to 22.9)	18.8 (16.9 to 20.7)	15.7 (11.4 to 21.8)	-1.9 (-3.9 to 0.3)	-24.2 (-44.1 to 4.5)
Latvia	62.6 (57.0 to 68.7)	34.0 (31.1 to 36.9)	32.1 (29.4 to 34.7)	27.5 (20.8 to 37.0)	-1.5 (-3.2 to 0.5)	-19.3 (-37.8 to 7.8)
Lithuania	61.3 (55.1 to 67.8)	44.9 (40.9 to 49.1)	42.5 (38.8 to 46.4)	24.8 (17.6 to 34.6)	-4.0 (-6.1 to -1.8)	-44.7 (-60.1 to -23.9)
Moldova	74.2 (66.7 to 82.1)	81.8 (74.3 to 89.6)	76.0 (69.3 to 83.0)	60.4 (46.3 to 79.1)	-2.1 (-3.8 to -0.3)	-26.2 (-43.1 to -3.9)
Russia	95.2 (84.3 to 108.1)	80.0 (70.8 to 89.9)	67.9 (61.1 to 75.3)	47.5 (34.9 to 64.2)	-3.6 (-5.4 to -1.5)	-40.6 (-55.7 to -19.8)
Ukraine	67.2 (59.1 to 76.5)	64.5 (56.6 to 73.7)	62.0 (54.1 to 71.5)	61.3 (49.1 to 77.5)	-0.4 (-1.6 to 1.0)	-5.1 (-21.6 to 15.5)
High-income						
Australasia						
Australia	5.0 (4.5 to 5.6)	5.1 (4.6 to 5.6)	4.9 (4.5 to 5.4)	3.1 (2.6 to 3.7)	-3.3 (-4.4 to -2.2)	-39.1 (-48.1 to -28.6)
New Zealand	11.0 (10.1 to 11.8)	10.2 (9.3 to 11.3)	10.8 (9.7 to 12.0)	6.8 (5.6 to 8.3)	-2.7 (-3.8 to -1.5)	-33.4 (-43.2 to -20.4)
High-income Asia Pacific						
Brunei	152.4 (138.9 to 167.1)	107.2 (97.7 to 117.3)	104.3 (94.5 to 114.5)	70.6 (55.8 to 99.0)	-2.9 (-4.2 to -0.5)	-34.2 (-46.7 to -7.2)
Japan	15.8 (14.0 to 18.0)	8.2 (7.5 to 9.0)	8.0 (7.3 to 8.8)	5.7 (4.5 to 7.3)	-2.5 (-3.8 to -0.9)	-30.5 (-43.7 to -12.4)
South Korea	64.8 (59.4 to 70.8)	56.3 (51.7 to 61.0)	51.9 (47.7 to 56.1)	34.9 (28.9 to 42.0)	-3.2 (-4.4 to -2.1)	-37.9 (-48.0 to -26.9)
Singapore	36.2 (32.7 to 40.3)	32.5 (29.4 to 35.7)	35.5 (32.3 to 39.0)	26.7 (22.4 to 31.2)	-1.3 (-2.3 to -0.4)	-17.8 (-29.3 to -6.4)
High-income North America						
Canada	5.4 (4.9 to 5.9)	4.4 (4.0 to 4.7)	4.4 (4.0 to 4.7)	3.7 (3.1 to 4.2)	-1.1 (-2.3 to -0.3)	-14.3 (-29.4 to -4.1)
Greenland	73.8 (67.8 to 80.4)	59.2 (54.6 to 64.0)	55.7 (51.4 to 60.3)	44.8 (36.5 to 56.3)	-1.9 (-3.1 to -0.4)	-24.3 (-37.2 to -5.2)
United States	5.7 (5.2 to 6.3)	2.7 (2.5 to 3.0)	2.5 (2.2 to 2.9)	1.9 (1.6 to 2.3)	-2.3 (-3.4 to -1.5)	-29.0 (-39.6 to -20.4)
Southern Latin America						
Argentina	30.2 (27.6 to 33.1)	21.1 (19.5 to 22.9)	22.1 (20.5 to 23.8)	20.3 (17.6 to 23.4)	-0.3 (-1.0 to 0.5)	-4.2 (-14.3 to 8.5)
Chile	21.4 (19.7 to 23.2)	14.7 (13.6 to 16.0)	14.0 (12.9 to 15.1)	12.1 (10.6 to 13.8)	-1.3 (-2.1 to -0.6)	-17.7 (-26.9 to -8.4)
Uruguay	19.7 (17.9 to 21.5)	19.8 (18.1 to 21.6)	20.9 (19.1 to 22.7)	18.4 (16.1 to 20.9)	-0.5 (-1.2 to 0.2)	-7.3 (-16.1 to 2.7)
Western Europe						
Andorra	9.8 (8.7 to 11.1)	7.3 (6.5 to 8.2)	7.2 (6.4 to 8.1)	5.2 (4.2 to 6.4)	-2.2 (-3.5 to -0.9)	-27.7 (-41.1 to -12.5)
Austria	10.8 (9.7 to 12.1)	7.5 (6.7 to 8.3)	7.4 (6.7 to 8.3)	4.9 (4.0 to 6.0)	-2.9 (-4.1 to -1.6)	-34.8 (-45.9 to -21.2)
Belgium	10.2 (9.2 to 11.4)	8.6 (7.8 to 9.4)	8.4 (7.7 to 9.2)	6.0 (4.8 to 7.4)	-2.4 (-3.7 to -1.1)	-30.2 (-42.9 to -14.6)
Cyprus	9.2 (8.2 to 10.4)	6.7 (5.9 to 7.6)	6.7 (5.9 to 7.6)	4.5 (3.5 to 5.9)	-2.6 (-4.2 to -1.0)	-32.3 (-46.6 to -13.8)
Denmark	9.1 (8.1 to 10.2)	6.4 (5.7 to 7.2)	6.2 (5.6 to 7.0)	4.2 (3.3 to 5.3)	-2.9 (-4.2 to -1.5)	-34.6 (-47.1 to -19.7)
Finland	7.7 (6.9 to 8.6)	5.7 (5.0 to 6.3)	5.4 (4.8 to 6.0)	3.2 (2.3 to 4.4)	-3.8 (-5.8 to -1.8)	-43.0 (-58.0 to -24.1)
France	10.9 (9.9 to 12.0)	7.2 (6.6 to 8.0)	7.1 (6.5 to 7.8)	4.5 (3.6 to 5.6)	-3.2 (-4.6 to -1.8)	-38.2 (-49.7 to -23.9)

	Estimate in 2000 (95% UIs)	Estimate in 2015 (95% UIs)	Estimate in 2017 (95% UIs)	Estimate in 2030 (95% UIs)	Annualised rate of change, 2015-2030 (95% UIs)	Percent change, 2015-2030 (95% UIs)
Germany	8.2 (7.2 to 9.5)	6.1 (5.5 to 6.7)	7.0 (6.3 to 7.7)	4.5 (3.6 to 5.6)	-2.0 (-3.2 to -0.7)	-25.6 (-38.1 to -10.5)
Greece	6.0 (5.4 to 6.7)	5.0 (4.5 to 5.6)	4.7 (4.2 to 5.2)	2.9 (2.3 to 3.6)	-3.7 (-5.0 to -2.3)	-42.0 (-52.5 to -29.0)
Iceland	6.8 (6.0 to 7.8)	5.8 (5.1 to 6.6)	5.5 (4.8 to 6.3)	4.1 (2.9 to 5.7)	-2.4 (-4.3 to -0.1)	-29.5 (-47.4 to -1.9)
Ireland	9.1 (8.2 to 10.2)	7.2 (6.4 to 8.0)	6.7 (6.0 to 7.4)	4.6 (3.7 to 5.7)	-3.0 (-4.1 to -1.6)	-35.6 (-46.3 to -21.7)
Israel	7.0 (6.3 to 7.8)	4.3 (3.9 to 4.9)	4.1 (3.6 to 4.6)	3.3 (2.6 to 4.1)	-1.9 (-3.2 to -0.5)	-24.4 (-38.3 to -6.6)
Italy	7.2 (6.3 to 8.3)	6.9 (6.0 to 7.9)	6.3 (5.5 to 7.2)	4.2 (3.4 to 5.2)	-3.4 (-4.5 to -2.1)	-39.3 (-49.4 to -27.1)
Luxembourg	7.4 (6.4 to 8.5)	6.2 (5.5 to 7.1)	6.2 (5.4 to 7.0)	4.9 (3.4 to 7.0)	-1.8 (-3.9 to 0.6)	-22.0 (-44.3 to 9.7)
Malta	7.8 (6.8 to 8.8)	8.0 (7.0 to 9.1)	8.2 (7.2 to 9.4)	5.1 (3.9 to 6.9)	-3.0 (-4.7 to -1.2)	-36.0 (-50.5 to -16.6)
Netherlands	8.4 (7.6 to 9.3)	5.8 (5.2 to 6.5)	5.5 (5.0 to 6.1)	3.8 (3.1 to 4.6)	-2.9 (-4.0 to -1.6)	-35.4 (-45.4 to -21.8)
Norway	6.6 (5.8 to 7.4)	7.0 (6.1 to 8.1)	6.6 (5.8 to 7.6)	4.7 (3.6 to 6.1)	-2.8 (-4.3 to -1.1)	-33.4 (-47.5 to -15.4)
Portugal	34.5 (31.0 to 38.3)	17.5 (15.9 to 19.2)	16.2 (14.7 to 17.7)	11.4 (9.2 to 14.1)	-2.9 (-4.1 to -1.5)	-35.0 (-46.2 to -19.9)
Spain	14.8 (11.9 to 18.0)	9.6 (7.7 to 11.5)	8.7 (7.0 to 10.6)	5.4 (4.0 to 7.5)	-3.8 (-5.3 to -2.1)	-43.4 (-54.8 to -27.2)
Sweden	6.1 (5.3 to 6.9)	7.8 (6.7 to 9.1)	8.0 (6.8 to 9.4)	5.2 (4.0 to 6.9)	-2.8 (-4.2 to -1.1)	-33.8 (-46.4 to -15.0)
Switzerland	7.8 (7.0 to 8.8)	6.7 (6.0 to 7.5)	6.8 (6.1 to 7.6)	4.7 (3.6 to 6.1)	-2.5 (-4.1 to -0.8)	-30.6 (-45.7 to -10.9)
United Kingdom	15.6 (13.7 to 17.9)	15.2 (13.3 to 17.3)	13.6 (12.0 to 15.5)	7.5 (5.9 to 9.5)	-4.8 (-6.0 to -3.4)	-50.9 (-59.4 to -40.2)
Latin America and Caribbean						
Andean Latin America						
Bolivia	138.3 (126.7 to 151.3)	90.7 (83.1 to 99.6)	88.3 (80.5 to 97.2)	67.7 (54.6 to 86.5)	-2.0 (-3.2 to -0.5)	-25.3 (-38.3 to -6.7)
Ecuador	58.2 (52.3 to 65.1)	34.8 (32.1 to 37.6)	33.8 (31.0 to 36.5)	30.0 (26.1 to 34.2)	-1.0 (-1.8 to -0.3)	-13.9 (-23.2 to -3.7)
Peru	136.2 (124.6 to 149.1)	87.0 (79.6 to 95.1)	85.5 (77.7 to 93.8)	62.9 (49.6 to 81.1)	-2.2 (-3.7 to -0.7)	-27.8 (-42.5 to -9.5)
Caribbean						
Antigua and Barbuda	17.8 (15.9 to 19.8)	16.6 (14.7 to 18.8)	16.6 (14.6 to 18.8)	17.1 (13.3 to 22.9)	0.1 (-1.3 to 2.1)	2.8 (-17.8 to 36.4)
The Bahamas	26.3 (23.7 to 29.1)	19.2 (17.4 to 21.2)	19.5 (17.6 to 21.5)	16.7 (13.6 to 20.9)	-1.0 (-2.2 to 0.4)	-13.4 (-27.6 to 5.6)
Barbados	7.9 (7.0 to 9.0)	6.8 (5.9 to 7.8)	6.7 (5.8 to 7.7)	6.0 (4.8 to 7.3)	-0.9 (-1.9 to 0.2)	-12.1 (-25.0 to 3.5)
Belize	54.5 (49.4 to 60.0)	39.4 (35.9 to 43.1)	36.7 (33.4 to 40.3)	30.9 (26.1 to 36.9)	-1.6 (-2.6 to -0.5)	-21.5 (-32.2 to -7.3)
Bermuda	22.3 (19.7 to 25.3)	21.0 (18.6 to 23.9)	20.7 (18.3 to 23.6)	21.9 (17.7 to 27.6)	0.3 (-0.8 to 1.6)	4.4 (-11.4 to 26.3)
Cuba	9.2 (8.3 to 10.1)	6.4 (5.8 to 7.1)	5.9 (5.3 to 6.5)	7.4 (6.3 to 8.5)	1.0 (0.1 to 1.7)	15.7 (1.3 to 29.0)
Dominica	41.9 (37.5 to 46.3)	32.6 (29.4 to 36.0)	31.9 (28.8 to 35.3)	24.0 (19.2 to 30.6)	-2.1 (-3.4 to -0.6)	-26.4 (-39.5 to -8.2)
Dominican Republic	78.3 (71.4 to 85.8)	58.7 (53.6 to 64.5)	58.4 (53.0 to 64.1)	53.3 (44.9 to 63.7)	-0.7 (-1.6 to 0.4)	-9.2 (-21.6 to 6.4)
Grenada	13.6 (12.2 to 15.4)	11.5 (10.3 to 13.1)	11.6 (10.3 to 13.1)	9.3 (7.9 to 11.0)	-1.5 (-2.2 to -0.7)	-19.5 (-28.5 to -10.2)
Guyana	68.0 (62.0 to 74.4)	66.8 (61.4 to 72.5)	64.2 (59.0 to 69.6)	51.8 (42.9 to 64.0)	-1.7 (-2.8 to -0.5)	-22.5 (-34.6 to -6.7)
Haiti	125.0 (114.5 to 136.5)	88.1 (80.6 to 97.0)	84.6 (77.2 to 93.3)	61.9 (53.0 to 72.5)	-2.4 (-3.2 to -1.5)	-29.8 (-38.0 to -20.0)
Jamaica	19.6 (17.5 to 21.8)	16.4 (14.6 to 18.5)	16.6 (14.7 to 18.6)	16.8 (14.3 to 19.7)	0.1 (-0.6 to 0.9)	2.2 (-8.9 to 13.6)
Puerto Rico	6.7 (6.0 to 7.4)	4.0 (3.5 to 4.5)	3.6 (3.2 to 4.0)	3.2 (2.6 to 3.8)	-1.6 (-2.6 to -0.4)	-20.6 (-32.3 to -5.8)
Saint Lucia	20.3 (18.2 to 22.6)	15.0 (13.4 to 16.7)	14.9 (13.2 to 16.5)	11.5 (9.6 to 13.9)	-1.8 (-2.7 to -0.7)	-23.2 (-33.7 to -10.3)
Saint Vincent and the Grenadines	28.7 (25.8 to 31.9)	21.8 (19.5 to 24.3)	21.5 (19.2 to 24.0)	19.7 (16.8 to 23.3)	-0.7 (-1.4 to 0.2)	-9.3 (-19.5 to 3.4)
Suriname	26.5 (23.6 to 29.6)	20.7 (18.5 to 23.1)	20.3 (18.1 to 22.7)	21.0 (17.9 to 24.2)	0.1 (-0.6 to 0.8)	1.6 (-8.8 to 13.2)
Trinidad and Tobago	19.1 (17.3 to 20.9)	15.5 (14.1 to 16.9)	15.0 (13.7 to 16.5)	13.4 (11.6 to 15.5)	-1.0 (-1.8 to -0.2)	-13.1 (-23.3 to -2.3)
Virgin Islands, U.S.	16.0 (14.3 to 17.9)	14.7 (13.1 to 16.5)	14.5 (12.9 to 16.3)	11.0 (8.8 to 13.9)	-1.9 (-3.2 to -0.6)	-24.7 (-38.1 to -8.3)
Central Latin America						
Colombia	27.8 (25.4 to 30.6)	21.6 (19.7 to 23.7)	21.9 (20.0 to 24.0)	17.7 (15.1 to 20.3)	-1.3 (-2.2 to -0.6)	-18.0 (-27.7 to -8.0)
Costa Rica	15.6 (14.1 to 17.2)	9.8 (8.8 to 10.8)	9.2 (8.3 to 10.1)	8.1 (6.9 to 9.4)	-1.3 (-2.0 to -0.5)	-17.5 (-26.1 to -7.0)
El Salvador	32.8 (29.8 to 36.2)	26.8 (24.2 to 29.7)	28.9 (26.0 to 32.1)	24.1 (21.2 to 28.3)	-0.7 (-1.3 to 0.0)	-9.9 (-18.1 to 0.7)
Guatemala	33.8 (30.7 to 37.1)	23.9 (21.9 to 26.1)	23.4 (21.5 to 25.4)	21.2 (18.3 to 24.5)	-0.8 (-1.6 to 0.0)	-11.2 (-21.3 to 0.3)
Honduras	69.0 (62.6 to 76.6)	41.7 (37.9 to 46.2)	41.1 (37.3 to 45.5)	30.8 (26.6 to 35.8)	-2.0 (-2.7 to -1.3)	-26.2 (-33.2 to -18.0)
Mexico	21.9 (19.6 to 24.5)	17.0 (15.4 to 19.0)	17.1 (15.3 to 19.3)	16.0 (13.5 to 19.2)	-0.4 (-1.2 to 0.4)	-6.0 (-16.3 to 6.6)
Nicaragua	60.8 (55.0 to 67.3)	36.3 (32.9 to 40.1)	35.9 (32.4 to 39.7)	28.9 (24.5 to 34.2)	-1.5 (-2.4 to -0.7)	-20.3 (-30.3 to -9.7)
Panama	43.2 (39.2 to 47.7)	40.7 (36.8 to 44.6)	40.6 (36.7 to 44.5)	35.2 (29.1 to 42.8)	-1.0 (-2.0 to 0.3)	-13.5 (-26.3 to 4.3)
Venezuela	25.6 (23.0 to 28.6)	22.4 (20.4 to 24.4)	23.4 (21.4 to 25.4)	22.1 (18.3 to 26.8)	-0.1 (-1.2 to 1.1)	-1.4 (-16.4 to 18.2)
Tropical Latin America						
Brazil	45.0 (40.2 to 50.5)	35.8 (32.4 to 39.7)	35.7 (31.9 to 40.0)	30.6 (25.2 to 36.6)	-1.1 (-2.1 to -0.1)	-14.4 (-27.0 to -1.2)
Paraguay	59.5 (53.2 to 66.7)	52.0 (46.8 to 58.1)	50.7 (45.4 to 56.7)	44.7 (35.4 to 55.6)	-1.0 (-2.4 to 0.3)	-14.1 (-30.1 to 3.9)
North Africa and Middle East						
North Africa and Middle East						
Afghanistan	186.3 (160.3 to 216.8)	160.1 (140.8 to 182.5)	151.1 (129.6 to 177.5)	89.8 (69.1 to 114.4)	-3.9 (-5.2 to -2.6)	-43.9 (-54.4 to -32.2)
Algeria	51.1 (44.0 to 59.8)	38.6 (33.2 to 44.9)	37.2 (32.1 to 43.3)	28.2 (23.6 to 33.9)	-2.1 (-2.8 to -1.4)	-27.0 (-34.7 to -18.9)
Bahrain	37.1 (33.9 to 40.5)	22.9 (21.0 to 25.3)	22.5 (20.5 to 24.8)	19.9 (16.1 to 24.4)	-1.0 (-2.1 to 0.2)	-13.4 (-27.4 to 2.8)
Egypt	22.4 (19.2 to 25.7)	20.3 (17.5 to 23.4)	19.7 (16.9 to 22.8)	19.0 (15.0 to 23.4)	-0.5 (-1.6 to 0.6)	-6.5 (-21.2 to 9.7)
Iran	16.5 (15.1 to 18.1)	15.8 (14.4 to 17.4)	15.8 (14.3 to 17.3)	14.8 (12.5 to 17.4)	-0.5 (-1.4 to 0.4)	-6.7 (-18.4 to 6.6)
Iraq	81.3 (74.4 to 88.8)	44.3 (40.7 to 48.0)	40.8 (37.4 to 44.2)	29.2 (24.1 to 35.3)	-2.8 (-4.0 to -1.7)	-34.1 (-44.9 to -23.0)
Jordan	16.6 (13.8 to 19.8)	10.9 (9.3 to 13.0)	10.8 (9.2 to 13.0)	9.1 (7.2 to 11.6)	-1.2 (-2.3 to -0.1)	-16.7 (-29.6 to -0.9)
Kuwait	31.2 (28.6 to 34.0)	22.4 (20.6 to 24.4)	19.9 (18.3 to 21.6)	17.3 (11.7 to 27.3)	-1.9 (-4.2 to 1.1)	-22.7 (-47.1 to 18.4)
Lebanon	26.1 (23.3 to 29.1)	20.1 (17.8 to 22.6)	19.5 (17.3 to 21.9)	18.7 (15.0 to 23.4)	-0.5 (-1.7 to 0.8)	-6.7 (-22.4 to 12.9)
Libya	31.5 (28.8 to 34.5)	28.3 (25.7 to 31.0)	28.5 (25.9 to 31.2)	41.4 (35.0 to 48.3)	2.5 (1.7 to 3.4)	46.5 (28.4 to 65.7)
Morocco	207.9 (192.1 to 224.7)	151.3 (139.4 to 164.4)	145.0 (133.1 to 157.5)	156.9 (137.7 to 177.2)	0.2 (-0.4 to 0.8)	3.6 (-5.7 to 13.0)
Palestine	19.9 (18.1 to 21.9)	15.2 (13.9 to 16.7)	14.7 (13.3 to 16.1)	18.4 (14.8 to 22.3)	1.2 (0.1 to 2.4)	21.1 (0.8 to 44.4)
Oman	26.5 (24.1 to 29.0)	21.6 (19.6 to 23.8)	20.9 (19.0 to 23.1)	21.5 (17.0 to 26.1)	-0.1 (-1.4 to 1.2)	-0.4 (-19.2 to 18.9)
Qatar	45.9 (42.0 to 50.4)	30.8 (27.8 to 34.0)	30.5 (27.6 to 33.7)	29.3 (22.3 to 39.3)	-0.4 (-2.0 to 1.5)	-5.0 (-25.8 to 24.6)
Saudi Arabia	58.8 (53.9 to 63.6)	44.6 (40.9 to 48.7)	42.4 (38.8 to 46.4)	22.7 (17.8 to 28.2)	-4.5 (-6.0 to -3.2)	-49.2 (-59.2 to -38.2)
Sudan	127.2 (115.5 to 139.4)	101.8 (91.9 to 112.4)	99.3 (89.2 to 110.1)	63.7 (53.7 to 75.8)	-3.1 (-4.0 to -2.2)	-37.5 (-45.3 to -28.3)
Syria	29.2 (26.5 to 32.1)	22.2 (20.1 to 24.6)	22.0 (19.9 to 24.4)	18.2 (15.2 to 22.0)	-1.3 (-2.3 to -0.3)	-18.0 (-29.6 to -3.7)
Tunisia	32.0 (29.2 to 35.0)	27.4 (24.9 to 30.0)	27.2 (24.7 to 29.9)	22.5 (19.5 to 26.1)	-1.3 (-2.1 to -0.6)	-17.8 (-27.0 to -8.7)
Turkey	50.4 (45.1 to 56.3)	28.4 (25.3 to 31.9)	27.9 (24.6 to 31.5)	23.8 (17.7 to 31.8)	-1.2 (-2.9 to 0.5)	-16.2 (-35.2 to 8.3)
United Arab Emirates	21.6 (19.4 to 23.9)	18.4 (16.6 to 20.5)	18.1 (16.4 to 20.1)	14.6 (11.6 to 18.5)	-1.6 (-2.9 to -0.1)	-20.7 (-35.6 to -2.0)
Yemen	81.5 (74.7 to 89.0)	62.6 (57.6 to 68.1)	61.6 (56.6 to 67.2)	40.4 (33.5 to 49.6)	-3.0 (-4.0 to -1.7)	-35.6 (-44.7 to -23.0)
South Asia						
South Asia						
Bangladesh	183.4 (166.4 to 202.4)	127.5 (116.1 to 139.8)	131.0 (118.8 to 144.5)	135.4 (111.3 to 165.7)	0.4 (-0.7 to 1.5)	6.2 (-9.4 to 25.4)
Bhutan	173.2 (155.7 to 192.4)	118.3 (106.0 to 132.5)	115.7 (103.2 to 130.1)	101.1 (85.7 to 119.1)	-1.1 (-1.8 to -0.3)	-14.5 (-23.6 to -4.2)
India	292.7 (268.2 to 319.4)	234.8 (215.4 to 256.5)	221.4 (202.9 to 242.0)	189.0 (162.7 to 218.0)	-1.5 (-2.3 to -0.7)	-19.5 (-28.7 to -9.7)

	Estimate in 2000 (95% UIs)	Estimate in 2015 (95% UIs)	Estimate in 2017 (95% UIs)	Estimate in 2030 (95% UIs)	Annualised rate of change, 2015-2030 (95% UIs)	Percent change, 2015-2030 (95% UIs)
Nepal	251.3 (228.3 to 278.1)	181.6 (164.0 to 201.2)	176.4 (158.7 to 196.0)	117.9 (100.0 to 137.7)	-2.9 (-3.7 to -2.1)	-35.1 (-42.3 to -27.3)
Pakistan	253.0 (232.5 to 276.4)	197.2 (180.8 to 213.7)	191.6 (175.3 to 208.5)	148.7 (127.6 to 173.4)	-1.9 (-2.7 to -1.1)	-24.6 (-33.2 to -15.0)
Southeast Asia, East Asia, and Oceania						
East Asia						
China	124.0 (113.4 to 135.1)	58.2 (53.1 to 63.5)	55.8 (50.8 to 60.9)	34.1 (27.1 to 43.1)	-3.6 (-5.0 to -2.1)	-41.4 (-52.8 to -27.1)
North Korea	166.1 (151.0 to 182.2)	169.0 (153.8 to 186.0)	171.3 (155.2 to 189.0)	139.9 (121.8 to 162.8)	-1.3 (-2.0 to -0.6)	-17.2 (-25.4 to -8.0)
Taiwan (Province of China)	54.5 (50.3 to 58.8)	39.0 (36.5 to 41.6)	38.4 (35.4 to 41.6)	24.6 (20.0 to 30.7)	-3.1 (-4.3 to -1.7)	-36.8 (-47.6 to -22.9)
Oceania						
American Samoa	20.7 (18.7 to 22.9)	17.0 (15.4 to 18.7)	16.5 (14.9 to 18.2)	17.2 (14.8 to 20.1)	0.1 (-0.6 to 0.8)	1.4 (-8.9 to 13.5)
Federated States of Micronesia	152.9 (139.6 to 167.2)	125.4 (114.2 to 137.4)	124.0 (112.4 to 136.4)	596.7 (151.6 to 1481.1)	9.2 (1.2 to 16.2)	375.4 (19.3 to 1042.5)
Fiji	44.2 (40.6 to 48.5)	38.0 (34.7 to 41.8)	38.1 (34.7 to 42.0)	39.9 (32.9 to 50.5)	0.3 (-0.8 to 1.7)	5.0 (-11.3 to 28.7)
Guam	56.0 (50.9 to 61.3)	47.2 (43.0 to 51.7)	46.4 (42.1 to 51.0)	30.4 (23.4 to 39.3)	-3.0 (-4.5 to -1.5)	-35.7 (-49.1 to -19.6)
Kiribati	300.9 (273.5 to 328.6)	283.5 (258.4 to 311.3)	292.5 (266.2 to 321.5)	240.3 (195.9 to 292.4)	-1.1 (-2.3 to 0.1)	-15.2 (-28.9 to 1.7)
Marshall Islands	233.7 (212.4 to 259.3)	195.4 (177.6 to 214.5)	191.4 (173.8 to 210.1)	146.2 (118.7 to 195.4)	-2.0 (-3.2 to -0.2)	-25.2 (-37.9 to -2.6)
Northern Mariana Islands	76.3 (69.3 to 84.1)	53.2 (48.4 to 58.6)	52.3 (47.5 to 57.7)	45.5 (38.5 to 53.8)	-1.1 (-2.0 to -0.2)	-14.4 (-25.8 to -2.4)
Papua New Guinea	191.9 (174.5 to 209.4)	176.9 (161.4 to 193.7)	179.8 (163.3 to 197.6)	169.8 (138.7 to 218.9)	-0.3 (-1.4 to 1.2)	-4.0 (-18.9 to 19.9)
Samoa	52.9 (48.2 to 57.9)	42.1 (38.5 to 46.0)	41.7 (37.9 to 45.7)	44.2 (36.0 to 59.8)	0.3 (-0.9 to 2.2)	5.1 (-12.5 to 38.5)
Solomon Islands	102.1 (93.3 to 111.1)	87.3 (80.0 to 95.3)	84.2 (77.0 to 92.0)	78.9 (64.8 to 98.6)	-0.7 (-1.8 to 0.6)	-9.6 (-23.8 to 9.6)
Tonga	43.4 (39.8 to 47.4)	36.3 (33.0 to 39.8)	35.5 (32.2 to 39.1)	52.0 (40.2 to 77.1)	2.3 (0.9 to 4.9)	43.3 (13.7 to 109.1)
Vanuatu	95.1 (87.0 to 103.6)	76.1 (69.5 to 83.1)	75.3 (68.6 to 82.1)	55.4 (45.0 to 81.0)	-2.2 (-3.3 to 0.3)	-27.3 (-39.3 to 4.3)
Southeast Asia						
Cambodia	398.3 (361.9 to 438.8)	278.3 (252.9 to 304.7)	267.9 (243.0 to 294.8)	196.0 (163.4 to 239.4)	-2.4 (-3.4 to -1.2)	-29.6 (-39.6 to -16.8)
Indonesia	276.1 (253.2 to 301.2)	235.9 (215.7 to 257.7)	226.5 (206.7 to 247.6)	230.7 (198.9 to 269.2)	-0.2 (-0.9 to 0.6)	-2.2 (-13.2 to 9.7)
Laos	224.8 (197.2 to 254.9)	167.8 (147.3 to 190.5)	158.8 (138.1 to 182.0)	119.0 (95.4 to 146.5)	-2.3 (-3.4 to -1.2)	-29.1 (-39.8 to -16.9)
Malaysia	72.5 (66.5 to 79.1)	60.4 (55.3 to 66.1)	61.2 (55.8 to 67.3)	48.6 (39.0 to 60.5)	-1.5 (-2.8 to -0.0)	-19.5 (-34.7 to -0.0)
Maldives	60.5 (55.5 to 66.0)	40.7 (37.0 to 44.6)	41.5 (37.7 to 45.7)	34.9 (25.4 to 48.2)	-1.1 (-3.0 to 0.9)	-14.4 (-36.5 to 13.6)
Mauritius	21.1 (19.0 to 23.4)	19.1 (17.4 to 21.2)	18.3 (16.6 to 20.2)	15.3 (12.8 to 17.8)	-1.5 (-2.5 to -0.5)	-20.3 (-30.8 to -7.9)
Myanmar	317.4 (291.2 to 347.3)	222.2 (203.9 to 241.6)	209.1 (191.3 to 228.2)	206.4 (172.2 to 248.4)	-0.5 (-1.5 to 0.5)	-7.1 (-20.5 to 7.2)
Philippines	216.7 (194.7 to 240.7)	223.0 (201.1 to 245.9)	253.7 (229.3 to 279.1)	271.5 (228.5 to 321.9)	1.3 (0.3 to 2.3)	21.8 (4.8 to 40.4)
Sri Lanka	56.0 (48.3 to 64.7)	44.1 (38.4 to 50.6)	43.9 (37.9 to 50.6)	47.7 (35.7 to 62.6)	0.5 (-1.0 to 2.1)	8.2 (-14.3 to 36.2)
Seychelles	29.8 (27.0 to 32.8)	24.6 (22.3 to 27.1)	24.7 (22.4 to 27.2)	24.6 (19.7 to 30.4)	-0.0 (-1.2 to 1.3)	-0.2 (-16.5 to 21.0)
Thailand	114.6 (104.8 to 125.0)	72.5 (66.0 to 79.3)	71.2 (64.7 to 78.0)	62.0 (49.4 to 79.1)	-1.1 (-2.4 to 0.4)	-14.4 (-29.9 to 6.4)
Timor-Leste	172.3 (158.8 to 188.0)	164.7 (150.3 to 180.4)	186.6 (169.6 to 204.7)	163.8 (133.5 to 201.1)	-0.1 (-1.3 to 1.1)	-0.6 (-17.3 to 17.8)
Vietnam	150.3 (131.0 to 171.7)	133.8 (117.0 to 151.3)	130.8 (114.3 to 148.5)	137.1 (108.2 to 176.7)	0.1 (-1.1 to 1.6)	2.4 (-14.9 to 27.8)
Sub-Saharan Africa						
Central Sub-Saharan Africa						
Angola	598.9 (547.1 to 655.9)	483.1 (439.5 to 532.3)	465.1 (421.6 to 516.4)	330.8 (271.2 to 405.6)	-2.6 (-3.7 to -1.3)	-31.5 (-42.9 to -17.8)
Central African Republic	1183.9 (1086.5 to 1295.2)	953.8 (873.2 to 1039.1)	904.4 (826.5 to 987.4)	599.3 (494.8 to 734.8)	-3.1 (-4.2 to -2.0)	-37.2 (-46.7 to -25.6)
Congo	680.5 (616.5 to 747.8)	472.6 (430.4 to 517.1)	458.9 (416.6 to 505.4)	363.1 (305.8 to 430.4)	-1.8 (-2.7 to -0.6)	-23.2 (-32.9 to -8.1)
Democratic Republic of the Congo	764.5 (697.8 to 837.6)	637.8 (581.9 to 693.5)	605.5 (549.8 to 661.7)	423.9 (342.4 to 524.0)	-2.8 (-4.0 to -1.5)	-33.5 (-45.0 to -19.8)
Equatorial Guinea	595.1 (544.6 to 652.5)	439.6 (399.4 to 484.3)	428.8 (388.4 to 475.2)	449.3 (383.6 to 532.1)	0.1 (-0.8 to 1.1)	2.2 (-10.6 to 17.6)
Gabon	553.0 (503.8 to 604.6)	391.6 (357.1 to 429.8)	373.3 (336.9 to 411.3)	496.7 (430.6 to 573.6)	1.6 (0.9 to 2.3)	26.8 (13.6 to 41.9)
Eastern Sub-Saharan Africa						
Burundi	1025.9 (946.6 to 1110.5)	792.0 (728.3 to 863.8)	762.8 (701.2 to 831.7)	603.6 (472.9 to 771.5)	-1.9 (-3.3 to -0.2)	-23.8 (-38.7 to -3.4)
Comoros	276.2 (252.6 to 302.1)	253.3 (230.7 to 278.5)	252.7 (228.3 to 280.5)	178.3 (149.1 to 216.3)	-2.4 (-3.3 to -1.3)	-29.6 (-39.3 to -17.1)
Djibouti	488.0 (437.2 to 542.4)	407.4 (365.2 to 454.5)	392.7 (351.1 to 439.4)	252.8 (200.3 to 312.9)	-3.2 (-4.5 to -2.1)	-38.0 (-49.4 to -26.6)
Eritrea	641.3 (586.8 to 702.0)	612.9 (560.5 to 669.1)	595.0 (542.9 to 651.7)	714.0 (551.3 to 902.2)	1.0 (-0.6 to 2.4)	16.5 (-8.2 to 44.0)
Ethiopia	893.1 (814.3 to 976.2)	302.0 (276.0 to 329.8)	279.7 (256.0 to 305.9)	245.2 (200.5 to 298.4)	-1.4 (-2.5 to -0.3)	-18.8 (-31.6 to -3.8)
Kenya	442.5 (403.6 to 484.2)	456.0 (415.0 to 500.6)	429.5 (390.9 to 471.7)	353.4 (304.1 to 416.2)	-1.7 (-2.5 to -0.8)	-22.5 (-31.0 to -10.8)
Madagascar	258.8 (215.3 to 305.5)	221.1 (183.7 to 264.4)	217.7 (180.1 to 259.8)	208.4 (164.2 to 261.4)	-0.4 (-1.3 to 0.6)	-5.7 (-18.1 to 8.7)
Malawi	850.3 (771.6 to 932.3)	658.6 (599.8 to 721.3)	602.2 (547.7 to 659.3)	476.4 (416.6 to 546.0)	-2.2 (-2.8 to -1.4)	-27.7 (-34.4 to -19.4)
Mozambique	800.2 (733.9 to 869.9)	910.4 (833.5 to 988.9)	850.5 (775.9 to 923.9)	616.0 (528.3 to 721.0)	-2.6 (-3.4 to -1.8)	-32.3 (-40.2 to -23.4)
Rwanda	824.6 (759.1 to 895.0)	439.0 (405.6 to 475.9)	417.3 (384.0 to 454.5)	416.6 (294.8 to 644.9)	-0.5 (-2.7 to 2.4)	-5.1 (-33.2 to 44.0)
Somalia	432.2 (371.1 to 501.1)	405.1 (351.0 to 464.6)	396.3 (342.9 to 455.9)	235.9 (191.8 to 286.6)	-3.6 (-4.6 to -2.7)	-41.8 (-49.9 to -33.2)
South Sudan	391.7 (359.5 to 425.3)	407.5 (376.2 to 441.4)	400.0 (368.4 to 433.5)	257.3 (214.8 to 313.1)	-3.1 (-4.1 to -1.8)	-36.8 (-46.1 to -23.8)
Tanzania	594.0 (537.8 to 652.6)	478.7 (436.5 to 524.5)	454.0 (412.4 to 500.6)	416.6 (359.5 to 485.8)	-0.9 (-1.7 to -0.1)	-13.0 (-22.5 to -1.9)
Uganda	948.9 (849.5 to 1062.6)	647.6 (586.5 to 719.6)	601.4 (542.4 to 669.5)	399.8 (334.9 to 487.0)	-3.2 (-4.3 to -2.1)	-38.3 (-47.3 to -27.2)
Zambia	1060.9 (951.6 to 1178.2)	791.9 (713.6 to 878.0)	739.2 (665.2 to 821.0)	520.3 (454.3 to 600.0)	-2.8 (-3.5 to -2.1)	-34.3 (-40.6 to -26.6)
Southern Sub-Saharan Africa						
Botswana	981.5 (857.6 to 1120.8)	740.5 (649.2 to 849.7)	684.2 (597.0 to 794.1)	561.8 (440.0 to 775.8)	-1.9 (-3.2 to 0.1)	-24.1 (-37.8 to 1.8)
Lesotho	1363.8 (1233.3 to 1499.4)	1421.1 (1289.9 to 1563.3)	1328.9 (1200.6 to 1463.3)	1089.3 (942.8 to 1275.2)	-1.8 (-2.5 to -1.0)	-23.3 (-31.1 to -14.0)
Namibia	1043.1 (947.0 to 1146.5)	864.2 (787.1 to 953.8)	824.1 (748.6 to 910.1)	661.8 (556.9 to 816.7)	-1.8 (-2.8 to -0.5)	-23.4 (-34.0 to -6.9)
South Africa	1087.9 (988.6 to 1201.2)	1043.9 (949.1 to 1149.8)	754.5 (685.0 to 830.9)	679.9 (512.4 to 1031.2)	-3.0 (-4.6 to -0.2)	-34.9 (-49.7 to -2.7)
Swaziland	1251.7 (1131.1 to 1387.1)	1129.8 (1026.8 to 1244.2)	972.4 (881.6 to 1070.3)	739.6 (612.3 to 915.4)	-2.9 (-3.9 to -1.5)	-34.5 (-43.9 to -20.2)
Zimbabwe	1586.2 (1391.8 to 1827.8)	1143.9 (1002.9 to 1296.1)	964.1 (841.0 to 1091.3)	908.1 (746.1 to 1146.5)	-1.6 (-2.6 to -0.2)	-20.6 (-32.1 to -3.1)
Western Sub-Saharan Africa						
Benin	320.1 (291.6 to 351.0)	244.1 (221.9 to 268.1)	238.4 (215.8 to 261.9)	161.5 (140.2 to 185.1)	-2.8 (-3.4 to -2.1)	-33.8 (-39.6 to -27.1)
Burkina Faso	412.0 (379.9 to 447.0)	332.4 (306.7 to 359.7)	310.8 (286.1 to 336.2)	180.9 (156.6 to 207.5)	-4.1 (-4.8 to -3.3)	-45.6 (-51.6 to -39.2)
Cameroon	395.3 (352.8 to 443.6)	335.1 (299.4 to 374.5)	314.1 (280.3 to 350.7)	214.3 (184.4 to 246.3)	-3.0 (-3.6 to -2.4)	-36.1 (-41.8 to -29.8)
Cape Verde	181.2 (166.6 to 196.7)	139.4 (127.7 to 151.9)	134.8 (123.2 to 147.3)	102.0 (89.9 to 115.3)	-2.1 (-2.7 to -1.5)	-26.8 (-33.0 to -20.1)
Chad	511.6 (473.9 to 554.0)	384.9 (356.7 to 415.1)	370.4 (343.0 to 401.0)	248.3 (216.4 to 281.6)	-2.9 (-3.7 to -2.2)	-35.5 (-42.3 to -28.2)
Cote d'Ivoire	447.5 (413.1 to 484.0)	328.4 (302.1 to 357.5)	309.9 (284.3 to 338.8)	217.5 (194.6 to 244.0)	-2.8 (-3.3 to -2.2)	-33.8 (-38.7 to -28.2)
The Gambia	377.9 (342.6 to 420.0)	353.6 (318.7 to 391.3)	348.0 (314.2 to 383.9)	235.2 (204.6 to 270.1)	-2.7 (-3.3 to -2.1)	-33.5 (-39.3 to -26.8)
Ghana	456.5 (384.4 to 545.9)	351.7 (298.2 to 414.7)	326.1 (274.5 to 388.7)	342.3 (276.3 to 418.6)	-0.2 (-1.1 to 0.7)	-2.7 (-14.9 to 11.3)
Guinea	388.0 (359.2 to 420.2)	365.1 (337.1 to 394.8)	346.4 (319.1 to 375.6)	199.7 (169.8 to 231.2)	-4.0 (-4.9 to -3.2)	-45.3 (-52.1 to -37.7)
Guinea-Bissau	328.7 (302.0 to 356.1)	335.7 (308.5 to 364.6)	316.8 (290.3 to 344.6)	193.2 (163.7 to 228.6)	-3.7 (-4.5 to -2.7)	-42.4 (-49.1 to -33.8)
Liberia	431.4 (398.7 to 467.0)	311.5 (287.6 to 337.9)	302.3 (278.4 to 329.6)	228.1 (186.1 to 279.9)	-2.1 (-3.3 to -0.9)	-26.8 (-38.7 to -12.2)
Mali	246.1 (227.6 to 265.6)	184.8 (170.0 to 201.0)	181.7 (167.4 to 198.1)	160.1 (141.9 to 182.4)	-1.0 (-1.6 to -0.3)	-13.4 (-20.9 to -3.9)
Mauritania	185.2 (169.3 to 202.8)	142.4 (130.5 to 156.2)	138.9 (127.0 to 152.3)	135.5 (115.0 to 163.8)	-0.3 (-1.3 to 0.8)	-4.8 (-17.7 to 12.5)
Niger	338.0 (312.0 to 365.0)	254.3 (233.4 to 276.8)	250.5 (230.0 to 272.5)	206.0 (179.6 to 234.0)	-1.4 (-2.1 to -0.7)	-19.0 (-26.8 to -10.2)

	Estimate in 2000 (95% UIs)	Estimate in 2015 (95% UIs)	Estimate in 2017 (95% UIs)	Estimate in 2030 (95% UIs)	Annualised rate of change, 2015-2030 (95% UIs)	Percent change, 2015-2030 (95% UIs)
Nigeria	549.6 (504.9 to 596.3)	366.1 (336.5 to 396.5)	351.7 (323.0 to 382.1)	290.5 (249.2 to 334.8)	-1.6 (-2.3 to -0.7)	-20.6 (-29.4 to -10.4)
Sao Tome and Principe	152.9 (139.8 to 166.9)	124.8 (114.5 to 136.2)	124.0 (113.0 to 134.9)	73.4 (56.8 to 95.0)	-3.6 (-5.1 to -1.9)	-41.2 (-53.5 to -24.6)
Senegal	349.0 (324.3 to 376.5)	260.1 (240.4 to 282.4)	256.4 (236.4 to 278.8)	170.1 (149.1 to 194.6)	-2.8 (-3.5 to -2.1)	-34.6 (-41.0 to -27.5)
Sierra Leone	474.6 (438.8 to 514.5)	406.1 (374.0 to 439.8)	376.2 (345.8 to 409.1)	208.8 (177.4 to 246.2)	-4.5 (-5.4 to -3.5)	-48.6 (-55.4 to -40.8)
Togo	371.0 (341.8 to 401.4)	308.8 (284.6 to 334.3)	292.0 (267.7 to 318.9)	184.8 (158.3 to 216.7)	-3.4 (-4.2 to -2.6)	-40.2 (-47.0 to -32.3)
**Indicator 3.3.3: Age-standardised rate of malaria cases (per 1,000 population)**						
Central Europe, Eastern Europe, and Central Asia						
Central Asia						
Armenia	0.1 (0.1 to 0.2)	0.0 (0.0 to 0.0)	0.0 (0.0 to 0.0)	0.0 (0.0 to 0.0)	0.0 (0.0 to 0.0)	0.0 (0.0 to 0.0)
Azerbaijan	0.6 (0.4 to 0.8)	0.0 (0.0 to 0.0)	0.0 (0.0 to 0.0)	0.0 (0.0 to 0.0)	-86.3 (-86.5 to -86.3)	-100.0 (-100.0 to -100.0)
Georgia	0.2 (0.1 to 0.2)	0.0 (0.0 to 0.0)	0.0 (0.0 to 0.0)	0.0 (0.0 to 0.0)	0.0 (0.0 to 0.0)	0.0 (0.0 to 0.0)
Kazakhstan	0.0 (0.0 to 0.0)	0.0 (0.0 to 0.0)	0.0 (0.0 to 0.0)	0.0 (0.0 to 0.0)	0.0 (0.0 to 0.0)	0.0 (0.0 to 0.0)
Kyrgyzstan	0.0 (0.0 to 0.0)	0.0 (0.0 to 0.0)	0.0 (0.0 to 0.0)	0.0 (0.0 to 0.0)	0.0 (0.0 to 0.0)	0.0 (0.0 to 0.0)
Mongolia	0.0 (0.0 to 0.0)	0.0 (0.0 to 0.0)	0.0 (0.0 to 0.0)	0.0 (0.0 to 0.0)	0.0 (0.0 to 0.0)	0.0 (0.0 to 0.0)
Tajikistan	13.5 (9.9 to 18.1)	0.0 (0.0 to 0.0)	0.0 (0.0 to 0.0)	0.0 (0.0 to 0.0)	-92.9 (-93.0 to -92.9)	-100.0 (-100.0 to -100.0)
Turkmenistan	0.0 (0.0 to 0.0)	0.0 (0.0 to 0.0)	0.0 (0.0 to 0.0)	0.0 (0.0 to 0.0)	0.0 (0.0 to 0.0)	0.0 (0.0 to 0.0)
Uzbekistan	0.0 (0.0 to 0.0)	0.0 (0.0 to 0.0)	0.0 (0.0 to 0.0)	0.0 (0.0 to 0.0)	0.0 (0.0 to 0.0)	0.0 (0.0 to 0.0)
Central Europe						
Albania	0.0 (0.0 to 0.0)	0.0 (0.0 to 0.0)	0.0 (0.0 to 0.0)	0.0 (0.0 to 0.0)	0.0 (0.0 to 0.0)	0.0 (0.0 to 0.0)
Bosnia and Herzegovina	0.0 (0.0 to 0.0)	0.0 (0.0 to 0.0)	0.0 (0.0 to 0.0)	0.0 (0.0 to 0.0)	0.0 (0.0 to 0.0)	0.0 (0.0 to 0.0)
Bulgaria	0.0 (0.0 to 0.0)	0.0 (0.0 to 0.0)	0.0 (0.0 to 0.0)	0.0 (0.0 to 0.0)	0.0 (0.0 to 0.0)	0.0 (0.0 to 0.0)
Croatia	0.0 (0.0 to 0.0)	0.0 (0.0 to 0.0)	0.0 (0.0 to 0.0)	0.0 (0.0 to 0.0)	0.0 (0.0 to 0.0)	0.0 (0.0 to 0.0)
Czech Republic	0.0 (0.0 to 0.0)	0.0 (0.0 to 0.0)	0.0 (0.0 to 0.0)	0.0 (0.0 to 0.0)	0.0 (0.0 to 0.0)	0.0 (0.0 to 0.0)
Hungary	0.0 (0.0 to 0.0)	0.0 (0.0 to 0.0)	0.0 (0.0 to 0.0)	0.0 (0.0 to 0.0)	0.0 (0.0 to 0.0)	0.0 (0.0 to 0.0)
Macedonia	0.0 (0.0 to 0.0)	0.0 (0.0 to 0.0)	0.0 (0.0 to 0.0)	0.0 (0.0 to 0.0)	0.0 (0.0 to 0.0)	0.0 (0.0 to 0.0)
Montenegro	0.0 (0.0 to 0.0)	0.0 (0.0 to 0.0)	0.0 (0.0 to 0.0)	0.0 (0.0 to 0.0)	0.0 (0.0 to 0.0)	0.0 (0.0 to 0.0)
Poland	0.0 (0.0 to 0.0)	0.0 (0.0 to 0.0)	0.0 (0.0 to 0.0)	0.0 (0.0 to 0.0)	0.0 (0.0 to 0.0)	0.0 (0.0 to 0.0)
Romania	0.0 (0.0 to 0.0)	0.0 (0.0 to 0.0)	0.0 (0.0 to 0.0)	0.0 (0.0 to 0.0)	0.0 (0.0 to 0.0)	0.0 (0.0 to 0.0)
Serbia	0.0 (0.0 to 0.0)	0.0 (0.0 to 0.0)	0.0 (0.0 to 0.0)	0.0 (0.0 to 0.0)	0.0 (0.0 to 0.0)	0.0 (0.0 to 0.0)
Slovakia	0.0 (0.0 to 0.0)	0.0 (0.0 to 0.0)	0.0 (0.0 to 0.0)	0.0 (0.0 to 0.0)	0.0 (0.0 to 0.0)	0.0 (0.0 to 0.0)
Slovenia	0.0 (0.0 to 0.0)	0.0 (0.0 to 0.0)	0.0 (0.0 to 0.0)	0.0 (0.0 to 0.0)	0.0 (0.0 to 0.0)	0.0 (0.0 to 0.0)
Eastern Europe						
Belarus	0.0 (0.0 to 0.0)	0.0 (0.0 to 0.0)	0.0 (0.0 to 0.0)	0.0 (0.0 to 0.0)	0.0 (0.0 to 0.0)	0.0 (0.0 to 0.0)
Estonia	0.0 (0.0 to 0.0)	0.0 (0.0 to 0.0)	0.0 (0.0 to 0.0)	0.0 (0.0 to 0.0)	0.0 (0.0 to 0.0)	0.0 (0.0 to 0.0)
Latvia	0.0 (0.0 to 0.0)	0.0 (0.0 to 0.0)	0.0 (0.0 to 0.0)	0.0 (0.0 to 0.0)	0.0 (0.0 to 0.0)	0.0 (0.0 to 0.0)
Lithuania	0.0 (0.0 to 0.0)	0.0 (0.0 to 0.0)	0.0 (0.0 to 0.0)	0.0 (0.0 to 0.0)	0.0 (0.0 to 0.0)	0.0 (0.0 to 0.0)
Moldova	0.0 (0.0 to 0.0)	0.0 (0.0 to 0.0)	0.0 (0.0 to 0.0)	0.0 (0.0 to 0.0)	0.0 (0.0 to 0.0)	0.0 (0.0 to 0.0)
Russia	0.0 (0.0 to 0.0)	0.0 (0.0 to 0.0)	0.0 (0.0 to 0.0)	0.0 (0.0 to 0.0)	0.0 (0.0 to 0.0)	0.0 (0.0 to 0.0)
Ukraine	0.0 (0.0 to 0.0)	0.0 (0.0 to 0.0)	0.0 (0.0 to 0.0)	0.0 (0.0 to 0.0)	0.0 (0.0 to 0.0)	0.0 (0.0 to 0.0)
High-income						
Australasia						
Australia	0.0 (0.0 to 0.0)	0.0 (0.0 to 0.0)	0.0 (0.0 to 0.0)	0.0 (0.0 to 0.0)	0.0 (0.0 to 0.0)	0.0 (0.0 to 0.0)
New Zealand	0.0 (0.0 to 0.0)	0.0 (0.0 to 0.0)	0.0 (0.0 to 0.0)	0.0 (0.0 to 0.0)	0.0 (0.0 to 0.0)	0.0 (0.0 to 0.0)
High-income Asia Pacific						
Brunei	0.0 (0.0 to 0.0)	0.0 (0.0 to 0.0)	0.0 (0.0 to 0.0)	0.0 (0.0 to 0.0)	0.0 (0.0 to 0.0)	0.0 (0.0 to 0.0)
Japan	0.0 (0.0 to 0.0)	0.0 (0.0 to 0.0)	0.0 (0.0 to 0.0)	0.0 (0.0 to 0.0)	0.0 (0.0 to 0.0)	0.0 (0.0 to 0.0)
South Korea	0.2 (0.2 to 0.3)	0.0 (0.0 to 0.0)	0.0 (0.0 to 0.0)	0.0 (0.0 to 0.0)	-15.6 (-17.8 to -13.6)	-90.3 (-93.1 to -87.0)
Singapore	0.0 (0.0 to 0.0)	0.0 (0.0 to 0.0)	0.0 (0.0 to 0.0)	0.0 (0.0 to 0.0)	0.0 (0.0 to 0.0)	0.0 (0.0 to 0.0)
High-income North America						
Canada	0.0 (0.0 to 0.0)	0.0 (0.0 to 0.0)	0.0 (0.0 to 0.0)	0.0 (0.0 to 0.0)	0.0 (0.0 to 0.0)	0.0 (0.0 to 0.0)
Greenland	0.0 (0.0 to 0.0)	0.0 (0.0 to 0.0)	0.0 (0.0 to 0.0)	0.0 (0.0 to 0.0)	0.0 (0.0 to 0.0)	0.0 (0.0 to 0.0)
United States	0.0 (0.0 to 0.0)	0.0 (0.0 to 0.0)	0.0 (0.0 to 0.0)	0.0 (0.0 to 0.0)	0.0 (0.0 to 0.0)	0.0 (0.0 to 0.0)
Southern Latin America						
Argentina	0.0 (0.0 to 0.0)	0.0 (0.0 to 0.0)	0.0 (0.0 to 0.0)	0.0 (0.0 to 0.0)	0.0 (0.0 to 0.0)	0.0 (0.0 to 0.0)
Chile	0.0 (0.0 to 0.0)	0.0 (0.0 to 0.0)	0.0 (0.0 to 0.0)	0.0 (0.0 to 0.0)	0.0 (0.0 to 0.0)	0.0 (0.0 to 0.0)
Uruguay	0.0 (0.0 to 0.0)	0.0 (0.0 to 0.0)	0.0 (0.0 to 0.0)	0.0 (0.0 to 0.0)	0.0 (0.0 to 0.0)	0.0 (0.0 to 0.0)
Western Europe						
Andorra	0.0 (0.0 to 0.0)	0.0 (0.0 to 0.0)	0.0 (0.0 to 0.0)	0.0 (0.0 to 0.0)	0.0 (0.0 to 0.0)	0.0 (0.0 to 0.0)
Austria	0.0 (0.0 to 0.0)	0.0 (0.0 to 0.0)	0.0 (0.0 to 0.0)	0.0 (0.0 to 0.0)	0.0 (0.0 to 0.0)	0.0 (0.0 to 0.0)
Belgium	0.0 (0.0 to 0.0)	0.0 (0.0 to 0.0)	0.0 (0.0 to 0.0)	0.0 (0.0 to 0.0)	0.0 (0.0 to 0.0)	0.0 (0.0 to 0.0)
Cyprus	0.0 (0.0 to 0.0)	0.0 (0.0 to 0.0)	0.0 (0.0 to 0.0)	0.0 (0.0 to 0.0)	0.0 (0.0 to 0.0)	0.0 (0.0 to 0.0)
Denmark	0.0 (0.0 to 0.0)	0.0 (0.0 to 0.0)	0.0 (0.0 to 0.0)	0.0 (0.0 to 0.0)	0.0 (0.0 to 0.0)	0.0 (0.0 to 0.0)
Finland	0.0 (0.0 to 0.0)	0.0 (0.0 to 0.0)	0.0 (0.0 to 0.0)	0.0 (0.0 to 0.0)	0.0 (0.0 to 0.0)	0.0 (0.0 to 0.0)
France	0.0 (0.0 to 0.0)	0.0 (0.0 to 0.0)	0.0 (0.0 to 0.0)	0.0 (0.0 to 0.0)	0.0 (0.0 to 0.0)	0.0 (0.0 to 0.0)
Germany	0.0 (0.0 to 0.0)	0.0 (0.0 to 0.0)	0.0 (0.0 to 0.0)	0.0 (0.0 to 0.0)	0.0 (0.0 to 0.0)	0.0 (0.0 to 0.0)
Greece	0.0 (0.0 to 0.0)	0.0 (0.0 to 0.0)	0.0 (0.0 to 0.0)	0.0 (0.0 to 0.0)	0.0 (0.0 to 0.0)	0.0 (0.0 to 0.0)
Iceland	0.0 (0.0 to 0.0)	0.0 (0.0 to 0.0)	0.0 (0.0 to 0.0)	0.0 (0.0 to 0.0)	0.0 (0.0 to 0.0)	0.0 (0.0 to 0.0)
Ireland	0.0 (0.0 to 0.0)	0.0 (0.0 to 0.0)	0.0 (0.0 to 0.0)	0.0 (0.0 to 0.0)	0.0 (0.0 to 0.0)	0.0 (0.0 to 0.0)
Israel	0.0 (0.0 to 0.0)	0.0 (0.0 to 0.0)	0.0 (0.0 to 0.0)	0.0 (0.0 to 0.0)	0.0 (0.0 to 0.0)	0.0 (0.0 to 0.0)
Italy	0.0 (0.0 to 0.0)	0.0 (0.0 to 0.0)	0.0 (0.0 to 0.0)	0.0 (0.0 to 0.0)	0.0 (0.0 to 0.0)	0.0 (0.0 to 0.0)
Luxembourg	0.0 (0.0 to 0.0)	0.0 (0.0 to 0.0)	0.0 (0.0 to 0.0)	0.0 (0.0 to 0.0)	0.0 (0.0 to 0.0)	0.0 (0.0 to 0.0)
Malta	0.0 (0.0 to 0.0)	0.0 (0.0 to 0.0)	0.0 (0.0 to 0.0)	0.0 (0.0 to 0.0)	0.0 (0.0 to 0.0)	0.0 (0.0 to 0.0)
Netherlands	0.0 (0.0 to 0.0)	0.0 (0.0 to 0.0)	0.0 (0.0 to 0.0)	0.0 (0.0 to 0.0)	0.0 (0.0 to 0.0)	0.0 (0.0 to 0.0)
Norway	0.0 (0.0 to 0.0)	0.0 (0.0 to 0.0)	0.0 (0.0 to 0.0)	0.0 (0.0 to 0.0)	0.0 (0.0 to 0.0)	0.0 (0.0 to 0.0)
Portugal	0.0 (0.0 to 0.0)	0.0 (0.0 to 0.0)	0.0 (0.0 to 0.0)	0.0 (0.0 to 0.0)	0.0 (0.0 to 0.0)	0.0 (0.0 to 0.0)
Spain	0.0 (0.0 to 0.0)	0.0 (0.0 to 0.0)	0.0 (0.0 to 0.0)	0.0 (0.0 to 0.0)	0.0 (0.0 to 0.0)	0.0 (0.0 to 0.0)
Sweden	0.0 (0.0 to 0.0)	0.0 (0.0 to 0.0)	0.0 (0.0 to 0.0)	0.0 (0.0 to 0.0)	0.0 (0.0 to 0.0)	0.0 (0.0 to 0.0)
Switzerland	0.0 (0.0 to 0.0)	0.0 (0.0 to 0.0)	0.0 (0.0 to 0.0)	0.0 (0.0 to 0.0)	0.0 (0.0 to 0.0)	0.0 (0.0 to 0.0)
United Kingdom	0.0 (0.0 to 0.0)	0.0 (0.0 to 0.0)	0.0 (0.0 to 0.0)	0.0 (0.0 to 0.0)	0.0 (0.0 to 0.0)	0.0 (0.0 to 0.0)
Latin America and Caribbean						

	Estimate in 2000 (95% UIs)	Estimate in 2015 (95% UIs)	Estimate in 2017 (95% UIs)	Estimate in 2030 (95% UIs)	Annualised rate of change, 2015-2030 (95% UIs)	Percent change, 2015-2030 (95% UIs)
Andean Latin America						
Bolivia	10.5 (8.6 to 12.8)	1.3 (1.1 to 1.5)	1.0 (0.8 to 1.2)	0.2 (0.1 to 0.2)	-14.4 (-16.8 to -12.1)	-88.2 (-92.0 to -83.6)
Ecuador	31.2 (26.4 to 36.5)	0.2 (0.2 to 0.2)	0.2 (0.1 to 0.2)	0.0 (0.0 to 0.0)	-20.0 (-21.3 to -18.8)	-95.0 (-95.9 to -94.0)
Peru	6.2 (5.5 to 6.9)	2.4 (2.2 to 2.6)	3.3 (2.9 to 3.6)	1.8 (1.3 to 2.4)	-2.0 (-3.9 to -0.3)	-25.4 (-44.3 to -5.0)
Caribbean						
Antigua and Barbuda	0.0 (0.0 to 0.0)	0.0 (0.0 to 0.0)	0.0 (0.0 to 0.0)	0.0 (0.0 to 0.0)	0.0 (0.0 to 0.0)	0.0 (0.0 to 0.0)
The Bahamas	0.0 (0.0 to 0.0)	0.0 (0.0 to 0.0)	0.0 (0.0 to 0.0)	0.0 (0.0 to 0.0)	0.0 (0.0 to 0.0)	0.0 (0.0 to 0.0)
Barbados	0.0 (0.0 to 0.0)	0.0 (0.0 to 0.0)	0.0 (0.0 to 0.0)	0.0 (0.0 to 0.0)	0.0 (0.0 to 0.0)	0.0 (0.0 to 0.0)
Belize	13.4 (10.7 to 16.6)	0.1 (0.1 to 0.1)	0.0 (0.0 to 0.0)	0.0 (0.0 to 0.0)	-43.0 (-45.5 to -40.7)	-99.8 (-99.9 to -99.8)
Bermuda	0.0 (0.0 to 0.0)	0.0 (0.0 to 0.0)	0.0 (0.0 to 0.0)	0.0 (0.0 to 0.0)	0.0 (0.0 to 0.0)	0.0 (0.0 to 0.0)
Cuba	0.0 (0.0 to 0.0)	0.0 (0.0 to 0.0)	0.0 (0.0 to 0.0)	0.0 (0.0 to 0.0)	0.0 (0.0 to 0.0)	0.0 (0.0 to 0.0)
Dominica	0.0 (0.0 to 0.0)	0.0 (0.0 to 0.0)	0.0 (0.0 to 0.0)	0.0 (0.0 to 0.0)	0.0 (0.0 to 0.0)	0.0 (0.0 to 0.0)
Dominican Republic	0.3 (0.3 to 0.4)	0.2 (0.2 to 0.2)	0.1 (0.1 to 0.1)	0.0 (0.0 to 0.0)	-14.6 (-16.0 to -13.2)	-88.8 (-90.9 to -86.3)
Grenada	0.0 (0.0 to 0.0)	0.0 (0.0 to 0.0)	0.0 (0.0 to 0.0)	0.0 (0.0 to 0.0)	0.0 (0.0 to 0.0)	0.0 (0.0 to 0.0)
Guyana	68.3 (57.1 to 82.5)	45.9 (40.3 to 52.0)	44.7 (38.1 to 52.3)	24.6 (18.2 to 32.7)	-4.2 (-5.5 to -2.9)	-46.7 (-55.9 to -35.4)
Haiti	14.4 (10.1 to 19.5)	28.4 (22.8 to 34.7)	11.4 (8.2 to 15.2)	5.6 (3.0 to 9.6)	-11.1 (-13.9 to -8.3)	-80.6 (-87.5 to -71.1)
Jamaica	0.0 (0.0 to 0.0)	0.0 (0.0 to 0.0)	0.0 (0.0 to 0.0)	0.0 (0.0 to 0.0)	0.0 (0.0 to 0.0)	0.0 (0.0 to 0.0)
Puerto Rico	0.0 (0.0 to 0.0)	0.0 (0.0 to 0.0)	0.0 (0.0 to 0.0)	0.0 (0.0 to 0.0)	0.0 (0.0 to 0.0)	0.0 (0.0 to 0.0)
Saint Lucia	0.0 (0.0 to 0.0)	0.0 (0.0 to 0.0)	0.0 (0.0 to 0.0)	0.0 (0.0 to 0.0)	0.0 (0.0 to 0.0)	0.0 (0.0 to 0.0)
Saint Vincent and the Grenadines	0.0 (0.0 to 0.0)	0.0 (0.0 to 0.0)	0.0 (0.0 to 0.0)	0.0 (0.0 to 0.0)	0.0 (0.0 to 0.0)	0.0 (0.0 to 0.0)
Suriname	93.8 (74.7 to 113.5)	0.9 (0.8 to 1.0)	0.2 (0.2 to 0.3)	0.0 (0.0 to 0.0)	-37.1 (-39.1 to -35.2)	-99.6 (-99.7 to -99.5)
Trinidad and Tobago	0.0 (0.0 to 0.0)	0.0 (0.0 to 0.0)	0.0 (0.0 to 0.0)	0.0 (0.0 to 0.0)	0.0 (0.0 to 0.0)	0.0 (0.0 to 0.0)
Virgin Islands, U.S.	0.0 (0.0 to 0.0)	0.0 (0.0 to 0.0)	0.0 (0.0 to 0.0)	0.0 (0.0 to 0.0)	0.0 (0.0 to 0.0)	0.0 (0.0 to 0.0)
Central Latin America						
Colombia	10.0 (8.4 to 11.9)	2.6 (2.3 to 2.9)	2.1 (1.8 to 2.4)	0.6 (0.5 to 0.8)	-9.7 (-11.0 to -8.4)	-76.4 (-80.9 to -71.8)
Costa Rica	1.1 (0.9 to 1.4)	0.0 (0.0 to 0.0)	0.0 (0.0 to 0.0)	0.0 (0.0 to 0.0)	-97.8 (-98.7 to -96.7)	-100.0 (-100.0 to -100.0)
El Salvador	0.4 (0.3 to 0.5)	0.0 (0.0 to 0.0)	0.0 (0.0 to 0.0)	0.0 (0.0 to 0.0)	-23.2 (-29.7 to -17.9)	-96.6 (-98.8 to -93.1)
Guatemala	21.8 (15.6 to 30.3)	0.9 (0.7 to 1.1)	0.8 (0.6 to 1.1)	0.1 (0.1 to 0.2)	-13.8 (-16.1 to -11.5)	-87.2 (-91.0 to -82.3)
Honduras	15.8 (12.7 to 19.4)	1.1 (1.0 to 1.2)	0.9 (0.8 to 1.0)	0.1 (0.1 to 0.1)	-14.4 (-15.4 to -13.5)	-88.4 (-90.1 to -86.7)
Mexico	0.2 (0.2 to 0.2)	0.0 (0.0 to 0.0)	0.0 (0.0 to 0.0)	0.0 (0.0 to 0.0)	-19.2 (-35.9 to -4.9)	-89.6 (-99.5 to -52.0)
Nicaragua	10.6 (9.1 to 12.2)	0.8 (0.7 to 0.9)	1.7 (1.5 to 2.0)	0.3 (0.2 to 0.4)	-6.3 (-7.1 to -5.5)	-61.1 (-65.6 to -56.0)
Panama	0.8 (0.6 to 0.9)	0.2 (0.2 to 0.2)	0.3 (0.2 to 0.3)	0.1 (0.1 to 0.2)	-3.8 (-5.4 to -2.2)	-42.7 (-55.3 to -27.7)
Venezuela	3.0 (2.5 to 3.7)	11.5 (10.2 to 13.0)	17.9 (15.2 to 20.9)	36.5 (29.2 to 45.3)	7.7 (6.8 to 8.6)	215.9 (176.2 to 261.8)
Tropical Latin America						
Brazil	7.0 (3.6 to 12.6)	2.2 (2.1 to 2.2)	1.0 (0.8 to 1.2)	0.2 (0.1 to 0.3)	-15.7 (-18.1 to -13.1)	-90.3 (-93.4 to -86.0)
Paraguay	3.8 (3.0 to 4.8)	0.0 (0.0 to 0.0)	0.0 (0.0 to 0.0)	0.0 (0.0 to 0.0)	-87.4 (-89.2 to -85.8)	-100.0 (-100.0 to -100.0)
North Africa and Middle East						
North Africa and Middle East						
Afghanistan	36.1 (27.1 to 47.0)	14.2 (12.1 to 16.5)	15.4 (12.3 to 18.9)	7.8 (5.2 to 11.3)	-4.1 (-5.9 to -2.3)	-45.1 (-58.9 to -29.3)
Algeria	0.0 (0.0 to 0.0)	0.0 (0.0 to 0.0)	0.0 (0.0 to 0.0)	0.0 (0.0 to 0.0)	-12.9 (-16.9 to -9.0)	-84.9 (-92.1 to -74.2)
Bahrain	0.0 (0.0 to 0.0)	0.0 (0.0 to 0.0)	0.0 (0.0 to 0.0)	0.0 (0.0 to 0.0)	0.0 (0.0 to 0.0)	0.0 (0.0 to 0.0)
Egypt	0.0 (0.0 to 0.0)	0.0 (0.0 to 0.0)	0.0 (0.0 to 0.0)	0.0 (0.0 to 0.0)	0.0 (0.0 to 0.0)	0.0 (0.0 to 0.0)
Iran	0.6 (0.4 to 0.9)	0.0 (0.0 to 0.0)	0.0 (0.0 to 0.0)	0.0 (0.0 to 0.0)	-39.2 (-42.9 to -34.4)	-99.7 (-99.8 to -99.4)
Iraq	0.4 (0.2 to 0.6)	0.0 (0.0 to 0.0)	0.0 (0.0 to 0.0)	0.0 (0.0 to 0.0)	0.0 (0.0 to 0.0)	0.0 (0.0 to 0.0)
Jordan	0.0 (0.0 to 0.0)	0.0 (0.0 to 0.0)	0.0 (0.0 to 0.0)	0.0 (0.0 to 0.0)	0.0 (0.0 to 0.0)	0.0 (0.0 to 0.0)
Kuwait	0.0 (0.0 to 0.0)	0.0 (0.0 to 0.0)	0.0 (0.0 to 0.0)	0.0 (0.0 to 0.0)	0.0 (0.0 to 0.0)	0.0 (0.0 to 0.0)
Lebanon	0.0 (0.0 to 0.0)	0.0 (0.0 to 0.0)	0.0 (0.0 to 0.0)	0.0 (0.0 to 0.0)	0.0 (0.0 to 0.0)	0.0 (0.0 to 0.0)
Libya	0.0 (0.0 to 0.0)	0.0 (0.0 to 0.0)	0.0 (0.0 to 0.0)	0.0 (0.0 to 0.0)	0.0 (0.0 to 0.0)	0.0 (0.0 to 0.0)
Morocco	0.0 (0.0 to 0.0)	0.0 (0.0 to 0.0)	0.0 (0.0 to 0.0)	0.0 (0.0 to 0.0)	0.0 (0.0 to 0.0)	0.0 (0.0 to 0.0)
Palestine	0.0 (0.0 to 0.0)	0.0 (0.0 to 0.0)	0.0 (0.0 to 0.0)	0.0 (0.0 to 0.0)	0.0 (0.0 to 0.0)	0.0 (0.0 to 0.0)
Oman	0.0 (0.0 to 0.0)	0.0 (0.0 to 0.0)	0.0 (0.0 to 0.0)	0.0 (0.0 to 0.0)	0.0 (0.0 to 0.0)	0.0 (0.0 to 0.0)
Qatar	0.0 (0.0 to 0.0)	0.0 (0.0 to 0.0)	0.0 (0.0 to 0.0)	0.0 (0.0 to 0.0)	0.0 (0.0 to 0.0)	0.0 (0.0 to 0.0)
Saudi Arabia	0.5 (0.5 to 0.5)	0.0 (0.0 to 0.0)	0.0 (0.0 to 0.0)	0.0 (0.0 to 0.0)	-9.6 (-10.1 to -9.0)	-76.1 (-78.1 to -74.0)
Sudan	77.6 (56.3 to 103.0)	37.0 (23.9 to 52.3)	43.9 (22.4 to 68.3)	37.5 (13.6 to 77.5)	-0.4 (-3.9 to 2.9)	-2.3 (-44.2 to 54.0)
Syria	0.0 (0.0 to 0.0)	0.0 (0.0 to 0.0)	0.0 (0.0 to 0.0)	0.0 (0.0 to 0.0)	0.0 (0.0 to 0.0)	0.0 (0.0 to 0.0)
Tunisia	0.0 (0.0 to 0.0)	0.0 (0.0 to 0.0)	0.0 (0.0 to 0.0)	0.0 (0.0 to 0.0)	0.0 (0.0 to 0.0)	0.0 (0.0 to 0.0)
Turkey	0.2 (0.2 to 0.2)	0.0 (0.0 to 0.0)	0.0 (0.0 to 0.0)	0.0 (0.0 to 0.0)	0.0 (0.0 to 0.0)	0.0 (0.0 to 0.0)
United Arab Emirates	0.0 (0.0 to 0.0)	0.0 (0.0 to 0.0)	0.0 (0.0 to 0.0)	0.0 (0.0 to 0.0)	0.0 (0.0 to 0.0)	0.0 (0.0 to 0.0)
Yemen	90.7 (51.4 to 142.1)	45.6 (40.0 to 52.2)	40.2 (34.2 to 47.6)	25.1 (18.6 to 34.3)	-4.0 (-5.4 to -2.5)	-45.2 (-55.7 to -30.9)
South Asia						
South Asia						
Bangladesh	8.1 (6.5 to 10.1)	0.3 (0.3 to 0.4)	0.1 (0.1 to 0.1)	0.0 (0.0 to 0.0)	-30.1 (-32.6 to -27.9)	-98.9 (-99.2 to -98.5)
Bhutan	43.5 (34.8 to 54.3)	0.1 (0.1 to 0.1)	0.0 (0.0 to 0.0)	0.0 (0.0 to 0.0)	-41.8 (-44.0 to -39.7)	-99.8 (-99.9 to -99.7)
India	24.8 (23.8 to 25.9)	12.4 (11.9 to 12.8)	10.2 (9.7 to 10.7)	4.6 (4.1 to 5.2)	-6.6 (-7.2 to -5.9)	-62.6 (-66.0 to -59.0)
Nepal	2.0 (1.7 to 2.5)	0.5 (0.5 to 0.6)	0.2 (0.1 to 0.2)	0.0 (0.0 to 0.0)	-21.8 (-23.1 to -20.5)	-96.2 (-96.9 to -95.4)
Pakistan	29.3 (27.5 to 31.2)	31.3 (30.1 to 32.4)	25.1 (23.8 to 26.4)	22.0 (18.4 to 25.7)	-2.4 (-3.4 to -1.4)	-29.6 (-40.2 to -18.9)
Southeast Asia, East Asia, and Oceania						
East Asia						
China	0.1 (0.0 to 0.3)	0.0 (0.0 to 0.0)	0.0 (0.0 to 0.0)	0.0 (0.0 to 0.0)	-55.6 (-61.7 to -51.3)	-100.0 (-100.0 to -100.0)
North Korea	16.0 (11.0 to 22.8)	0.8 (0.6 to 0.9)	0.4 (0.3 to 0.6)	0.1 (0.0 to 0.1)	-17.1 (-20.1 to -14.5)	-92.1 (-95.1 to -88.7)
Taiwan (Province of China)	0.0 (0.0 to 0.0)	0.0 (0.0 to 0.0)	0.0 (0.0 to 0.0)	0.0 (0.0 to 0.0)	0.0 (0.0 to 0.0)	0.0 (0.0 to 0.0)
Oceania						
American Samoa	0.0 (0.0 to 0.0)	0.0 (0.0 to 0.0)	0.0 (0.0 to 0.0)	0.0 (0.0 to 0.0)	0.0 (0.0 to 0.0)	0.0 (0.0 to 0.0)
Federated States of Micronesia	0.0 (0.0 to 0.0)	0.0 (0.0 to 0.0)	0.0 (0.0 to 0.0)	0.0 (0.0 to 0.0)	0.0 (0.0 to 0.0)	0.0 (0.0 to 0.0)
Fiji	0.0 (0.0 to 0.0)	0.0 (0.0 to 0.0)	0.0 (0.0 to 0.0)	0.0 (0.0 to 0.0)	0.0 (0.0 to 0.0)	0.0 (0.0 to 0.0)
Guam	0.0 (0.0 to 0.0)	0.0 (0.0 to 0.0)	0.0 (0.0 to 0.0)	0.0 (0.0 to 0.0)	0.0 (0.0 to 0.0)	0.0 (0.0 to 0.0)
Kiribati	0.0 (0.0 to 0.0)	0.0 (0.0 to 0.0)	0.0 (0.0 to 0.0)	0.0 (0.0 to 0.0)	0.0 (0.0 to 0.0)	0.0 (0.0 to 0.0)
Marshall Islands	0.0 (0.0 to 0.0)	0.0 (0.0 to 0.0)	0.0 (0.0 to 0.0)	0.0 (0.0 to 0.0)	0.0 (0.0 to 0.0)	0.0 (0.0 to 0.0)
Northern Mariana Islands	0.0 (0.0 to 0.0)	0.0 (0.0 to 0.0)	0.0 (0.0 to 0.0)	0.0 (0.0 to 0.0)	0.0 (0.0 to 0.0)	0.0 (0.0 to 0.0)
Papua New Guinea	311.8 (263.1 to 369.7)	131.2 (116.6 to 147.1)	127.3 (107.9 to 148.4)	79.7 (60.4 to 102.8)	-3.4 (-4.5 to -2.3)	-39.4 (-49.3 to -28.8)

	Estimate in 2000 (95% UIs)	Estimate in 2015 (95% UIs)	Estimate in 2017 (95% UIs)	Estimate in 2030 (95% UIs)	Annualised rate of change, 2015-2030 (95% UIs)	Percent change, 2015-2030 (95% UIs)
Samoa	0.0 (0.0 to 0.0)	0.0 (0.0 to 0.0)	0.0 (0.0 to 0.0)	0.0 (0.0 to 0.0)	0.0 (0.0 to 0.0)	0.0 (0.0 to 0.0)
Solomon Islands	275.6 (158.0 to 455.4)	164.4 (150.0 to 181.3)	157.0 (131.3 to 184.4)	117.5 (84.5 to 158.8)	-2.3 (-4.0 to -0.6)	-28.8 (-45.1 to -9.1)
Tonga	0.0 (0.0 to 0.0)	0.0 (0.0 to 0.0)	0.0 (0.0 to 0.0)	0.0 (0.0 to 0.0)	0.0 (0.0 to 0.0)	0.0 (0.0 to 0.0)
Vanuatu	171.4 (145.6 to 198.8)	23.8 (21.0 to 26.9)	12.3 (10.0 to 15.0)	1.7 (1.1 to 2.5)	-17.7 (-19.5 to -15.8)	-92.9 (-94.6 to -90.7)
Southeast Asia						
Cambodia	46.0 (39.8 to 52.1)	4.5 (4.3 to 4.7)	3.4 (3.2 to 3.6)	0.7 (0.6 to 0.8)	-12.1 (-12.7 to -11.5)	-83.7 (-85.2 to -82.2)
Indonesia	7.0 (6.4 to 7.5)	6.1 (6.0 to 6.4)	4.9 (4.6 to 5.3)	3.2 (2.7 to 3.9)	-4.3 (-5.3 to -3.3)	-47.4 (-55.0 to -39.3)
Laos	77.5 (50.6 to 111.6)	6.6 (5.6 to 7.8)	6.3 (5.1 to 8.0)	1.8 (1.2 to 2.7)	-8.8 (-10.6 to -6.8)	-73.0 (-79.7 to -64.0)
Malaysia	1.8 (1.5 to 2.1)	0.1 (0.1 to 0.1)	0.1 (0.1 to 0.1)	0.0 (0.0 to 0.0)	-16.4 (-17.6 to -15.1)	-91.4 (-92.9 to -89.6)
Maldives	0.0 (0.0 to 0.0)	0.0 (0.0 to 0.0)	0.0 (0.0 to 0.0)	0.0 (0.0 to 0.0)	0.0 (0.0 to 0.0)	0.0 (0.0 to 0.0)
Mauritius	0.0 (0.0 to 0.0)	0.0 (0.0 to 0.0)	0.0 (0.0 to 0.0)	0.0 (0.0 to 0.0)	0.0 (0.0 to 0.0)	0.0 (0.0 to 0.0)
Myanmar	32.3 (24.8 to 41.6)	17.7 (15.3 to 20.4)	5.0 (4.4 to 5.8)	1.1 (0.9 to 1.5)	-18.3 (-19.4 to -17.2)	-93.5 (-94.5 to -92.4)
Philippines	1.6 (1.3 to 1.8)	0.2 (0.2 to 0.2)	0.1 (0.1 to 0.2)	0.0 (0.0 to 0.0)	-17.1 (-18.5 to -15.8)	-92.3 (-93.8 to -90.6)
Sri Lanka	31.7 (26.9 to 37.2)	0.0 (0.0 to 0.0)	0.0 (0.0 to 0.0)	0.0 (0.0 to 0.0)	-106.6 (-107.4 to -105.7)	-100.0 (-100.0 to -100.0)
Seychelles	0.0 (0.0 to 0.0)	0.0 (0.0 to 0.0)	0.0 (0.0 to 0.0)	0.0 (0.0 to 0.0)	0.0 (0.0 to 0.0)	0.0 (0.0 to 0.0)
Thailand	2.9 (2.6 to 3.4)	0.4 (0.4 to 0.5)	0.1 (0.1 to 0.1)	0.0 (0.0 to 0.0)	-26.8 (-28.0 to -25.7)	-98.2 (-98.5 to -97.9)
Timor-Leste	56.5 (45.8 to 69.7)	25.7 (25.0 to 26.3)	0.1 (0.1 to 0.1)	0.0 (0.0 to 0.0)	-51.8 (-53.5 to -50.2)	-100.0 (-100.0 to -99.9)
Vietnam	3.9 (3.2 to 4.7)	0.3 (0.3 to 0.4)	0.2 (0.1 to 0.2)	0.0 (0.0 to 0.0)	-18.0 (-19.7 to -16.6)	-93.2 (-94.8 to -91.7)
Sub-Saharan Africa						
Central Sub-Saharan Africa						
Angola	173.6 (104.9 to 246.1)	115.0 (93.3 to 143.8)	129.8 (104.2 to 162.4)	119.1 (88.0 to 163.0)	0.2 (-1.2 to 1.7)	3.5 (-16.1 to 29.6)
Central African Republic	295.2 (198.0 to 392.3)	259.3 (151.3 to 380.6)	239.3 (126.9 to 365.6)	205.8 (79.2 to 372.5)	-2.0 (-5.7 to 0.4)	-23.7 (-57.3 to 6.3)
Congo	245.9 (153.8 to 332.4)	177.7 (114.5 to 256.0)	180.6 (109.5 to 291.3)	160.6 (74.6 to 317.1)	-1.0 (-3.4 to 1.5)	-11.9 (-39.9 to 24.9)
Democratic Republic of the Congo	303.7 (244.8 to 379.6)	222.6 (179.8 to 277.6)	219.5 (172.8 to 274.3)	175.4 (117.7 to 227.7)	-1.6 (-2.9 to -0.8)	-21.5 (-35.3 to -11.7)
Equatorial Guinea	253.1 (169.1 to 321.5)	232.9 (162.3 to 298.5)	224.0 (117.6 to 301.6)	199.6 (70.2 to 308.7)	-1.4 (-5.6 to 0.8)	-16.1 (-56.8 to 12.5)
Gabon	262.5 (177.8 to 353.4)	157.4 (97.5 to 259.4)	169.0 (95.3 to 297.0)	160.6 (51.9 to 381.2)	-0.5 (-4.9 to 2.7)	-2.4 (-52.3 to 49.1)
Eastern Sub-Saharan Africa						
Burundi	292.3 (177.9 to 384.5)	152.6 (109.0 to 200.3)	169.8 (125.4 to 214.7)	140.2 (96.5 to 187.3)	-0.6 (-1.6 to 0.6)	-8.1 (-21.4 to 9.3)
Comoros	331.4 (22.2 to 1572.8)	123.9 (99.0 to 154.6)	5.4 (4.0 to 7.1)	1.1 (0.5 to 1.8)	-32.0 (-35.9 to -28.9)	-99.2 (-99.5 to -98.7)
Djibouti	8.2 (5.8 to 11.5)	20.1 (15.8 to 24.5)	33.4 (24.6 to 41.5)	36.2 (23.9 to 49.2)	3.8 (2.5 to 5.2)	77.9 (45.1 to 119.1)
Eritrea	46.3 (21.3 to 88.5)	17.2 (14.8 to 20.1)	13.4 (10.5 to 17.4)	6.8 (4.1 to 10.6)	-6.4 (-8.9 to -3.9)	-61.1 (-73.7 to -44.5)
Ethiopia	76.4 (45.6 to 117.4)	12.3 (8.5 to 17.4)	13.9 (8.8 to 23.4)	5.6 (2.4 to 11.5)	-5.8 (-9.7 to -2.2)	-56.0 (-76.5 to -28.3)
Kenya	152.2 (117.7 to 193.3)	52.1 (36.5 to 67.8)	53.1 (37.9 to 72.7)	30.3 (18.4 to 42.8)	-3.7 (-5.4 to -2.2)	-42.4 (-55.8 to -28.0)
Madagascar	109.6 (81.6 to 135.2)	71.1 (53.6 to 87.2)	70.9 (53.1 to 87.3)	52.3 (35.5 to 70.6)	-2.1 (-3.5 to -0.9)	-26.7 (-40.9 to -12.7)
Malawi	349.5 (290.1 to 429.8)	202.1 (146.1 to 264.6)	176.0 (106.5 to 242.8)	118.9 (56.4 to 195.0)	-3.8 (-6.6 to -1.6)	-42.2 (-63.0 to -21.1)
Mozambique	335.5 (266.9 to 426.0)	216.4 (178.8 to 262.6)	207.9 (169.5 to 258.2)	153.6 (118.5 to 200.8)	-2.3 (-3.2 to -1.4)	-29.1 (-38.4 to -18.9)
Rwanda	103.6 (39.6 to 190.2)	44.6 (32.9 to 57.6)	50.4 (36.8 to 68.0)	47.1 (26.1 to 78.5)	0.1 (-2.4 to 4.2)	4.7 (-30.3 to 89.1)
Somalia	69.8 (40.5 to 108.2)	23.9 (17.1 to 33.2)	30.9 (18.9 to 48.6)	21.3 (8.4 to 42.4)	-1.2 (-4.8 to 2.4)	-13.5 (-51.6 to 43.9)
South Sudan	213.3 (144.4 to 291.6)	136.7 (97.2 to 189.1)	136.9 (86.2 to 204.4)	110.0 (55.6 to 195.7)	-1.7 (-4.2 to 0.3)	-20.8 (-46.7 to 5.4)
Tanzania	227.8 (178.2 to 289.1)	108.7 (81.1 to 142.3)	107.4 (81.9 to 141.4)	72.0 (52.4 to 97.7)	-2.8 (-4.0 to -1.6)	-34.0 (-45.1 to -21.9)
Uganda	314.4 (247.1 to 402.3)	212.7 (168.7 to 273.8)	206.1 (160.4 to 263.9)	158.9 (117.3 to 199.0)	-2.0 (-2.7 to -1.3)	-25.4 (-32.9 to -17.3)
Zambia	181.7 (143.2 to 234.9)	123.7 (99.8 to 158.8)	139.1 (110.2 to 179.6)	127.7 (91.6 to 178.2)	0.1 (-1.2 to 1.5)	2.7 (-16.0 to 24.5)
Southern Sub-Saharan Africa						
Botswana	4.1 (3.3 to 5.2)	1.0 (0.9 to 1.2)	0.6 (0.5 to 0.7)	0.2 (0.1 to 0.3)	-11.7 (-13.7 to -9.9)	-82.6 (-87.2 to -77.4)
Lesotho	0.0 (0.0 to 0.0)	0.0 (0.0 to 0.0)	0.0 (0.0 to 0.0)	0.0 (0.0 to 0.0)	0.0 (0.0 to 0.0)	0.0 (0.0 to 0.0)
Namibia	109.6 (10.6 to 464.6)	6.7 (5.9 to 7.6)	16.0 (13.5 to 19.0)	6.4 (3.9 to 10.0)	-0.5 (-3.3 to 2.4)	-4.7 (-38.7 to 43.5)
South Africa	0.1 (0.1 to 0.2)	0.1 (0.1 to 0.2)	0.1 (0.0 to 0.1)	0.0 (0.0 to 0.0)	-19.8 (-22.0 to -17.5)	-94.8 (-96.3 to -92.8)
Swaziland	5.7 (4.4 to 7.3)	0.3 (0.3 to 0.4)	0.3 (0.2 to 0.3)	0.0 (0.0 to 0.1)	-15.4 (-18.3 to -12.6)	-89.8 (-93.5 to -85.0)
Zimbabwe	171.9 (101.8 to 275.5)	53.8 (45.5 to 63.1)	46.9 (36.3 to 59.5)	24.6 (15.8 to 36.5)	-5.3 (-7.3 to -3.5)	-54.6 (-66.4 to -40.6)
Western Sub-Saharan Africa						
Benin	285.4 (210.4 to 385.9)	256.5 (187.2 to 334.9)	253.8 (166.7 to 360.5)	237.4 (124.4 to 371.2)	-0.7 (-3.4 to 1.0)	-8.9 (-40.2 to 16.6)
Burkina Faso	387.9 (307.3 to 468.6)	311.8 (250.2 to 374.0)	257.0 (196.6 to 326.5)	189.7 (128.9 to 278.3)	-3.4 (-5.0 to -1.7)	-39.4 (-52.7 to -22.7)
Cameroon	242.7 (182.4 to 316.9)	193.2 (141.8 to 260.9)	195.4 (125.1 to 269.3)	170.1 (86.6 to 273.7)	-1.1 (-3.6 to 1.0)	-13.4 (-42.1 to 15.7)
Cape Verde	0.6 (0.5 to 0.8)	0.3 (0.2 to 0.3)	0.3 (0.3 to 0.4)	0.3 (0.2 to 0.5)	1.4 (-0.3 to 3.0)	23.6 (-4.7 to 57.1)
Chad	157.2 (97.0 to 248.2)	166.8 (123.5 to 215.2)	129.8 (65.7 to 210.9)	107.9 (30.7 to 218.4)	-3.6 (-9.1 to 0.2)	-37.9 (-74.5 to 3.5)
Cote d'Ivoire	331.6 (258.9 to 408.4)	185.6 (138.5 to 238.7)	127.8 (78.8 to 184.0)	68.5 (32.1 to 112.7)	-7.0 (-10.0 to -4.5)	-63.9 (-77.7 to -48.8)
The Gambia	172.3 (119.7 to 241.5)	27.6 (19.9 to 36.3)	16.8 (9.4 to 28.1)	4.1 (1.4 to 9.7)	-13.4 (-18.0 to -8.6)	-85.8 (-93.2 to -72.4)
Ghana	313.5 (244.7 to 408.6)	214.1 (166.3 to 271.9)	164.3 (120.3 to 209.7)	105.5 (70.2 to 150.4)	-4.8 (-6.0 to -3.4)	-51.0 (-59.1 to -40.0)
Guinea	291.0 (211.4 to 385.7)	268.8 (217.7 to 322.7)	237.8 (169.7 to 296.7)	203.3 (121.2 to 307.8)	-2.0 (-4.1 to 0.3)	-24.9 (-45.9 to 4.0)
Guinea-Bissau	249.6 (123.5 to 380.4)	56.5 (28.6 to 100.9)	38.0 (12.8 to 90.3)	14.7 (2.1 to 49.6)	-11.0 (-18.9 to -3.8)	-77.2 (-94.1 to -43.6)
Liberia	223.3 (121.5 to 336.8)	211.6 (145.1 to 297.3)	183.2 (91.0 to 308.6)	166.9 (37.6 to 383.5)	-2.4 (-8.8 to 2.5)	-25.5 (-73.2 to 46.1)
Mali	276.1 (217.6 to 354.4)	212.0 (164.2 to 253.7)	195.4 (150.8 to 232.5)	149.9 (110.3 to 205.3)	-2.4 (-3.9 to -1.2)	-29.4 (-44.3 to -16.9)
Mauritania	51.0 (23.3 to 108.4)	32.1 (19.3 to 51.3)	36.5 (20.0 to 65.9)	33.7 (11.9 to 88.4)	-0.3 (-5.0 to 4.9)	0.3 (-53.0 to 108.0)
Niger	214.0 (111.6 to 326.5)	259.2 (161.5 to 341.9)	237.2 (139.3 to 341.0)	250.6 (111.2 to 419.3)	-0.5 (-3.3 to 2.1)	-4.9 (-38.7 to 36.1)
Nigeria	283.6 (211.9 to 351.0)	198.6 (159.0 to 245.5)	166.0 (127.1 to 214.5)	110.6 (75.5 to 156.9)	-4.0 (-5.4 to -2.7)	-44.7 (-55.4 to -32.9)
Sao Tome and Principe	360.3 (269.0 to 471.4)	20.8 (17.9 to 23.8)	20.1 (16.3 to 24.0)	6.9 (4.8 to 9.3)	-7.5 (-9.0 to -6.0)	-67.1 (-74.1 to -59.6)
Senegal	158.6 (114.6 to 214.3)	53.7 (41.3 to 67.0)	38.1 (27.8 to 50.8)	16.1 (10.1 to 25.1)	-8.1 (-9.9 to -6.2)	-70.1 (-77.3 to -60.8)
Sierra Leone	287.4 (146.2 to 392.4)	314.8 (264.5 to 391.2)	285.0 (223.1 to 368.9)	275.1 (188.9 to 418.3)	-1.0 (-3.0 to 0.9)	-12.8 (-36.1 to 13.6)
Togo	333.8 (247.8 to 437.3)	282.7 (223.7 to 370.1)	302.4 (225.6 to 393.4)	297.8 (181.0 to 406.1)	0.3 (-1.3 to 2.0)	5.0 (-17.5 to 34.6)
**Indicator 3.3.4: Age-standardised rate of hepatitis B incidence (per 100,000 population)**						
Central Europe, Eastern Europe, and Central Asia						
Central Asia						
Armenia	2207.9 (1849.0 to 2575.1)	2080.2 (1790.5 to 2387.0)	2105.4 (1755.8 to 2476.9)	1691.0 (1299.0 to 2226.9)	-1.4 (-2.8 to 0.1)	-18.8 (-34.1 to 2.3)
Azerbaijan	3093.6 (2594.0 to 3639.9)	2839.1 (2430.8 to 3267.9)	2889.3 (2391.4 to 3421.0)	2346.7 (1835.5 to 2884.8)	-1.3 (-2.4 to -0.3)	-17.4 (-29.9 to -4.5)
Georgia	2085.0 (1713.7 to 2524.6)	1949.5 (1628.9 to 2344.9)	1962.3 (1599.7 to 2434.3)	1414.5 (1075.2 to 1854.6)	-2.2 (-3.5 to -0.8)	-27.5 (-40.9 to -11.5)
Kazakhstan	2051.2 (1673.1 to 2485.8)	2246.4 (1858.5 to 2657.6)	2318.4 (1875.8 to 2803.7)	1655.6 (1174.3 to 2222.0)	-2.1 (-3.9 to -0.4)	-26.4 (-44.0 to -6.5)
Kyrgyzstan	2928.8 (2402.8 to 3590.2)	2915.1 (2436.9 to 3496.8)	2924.3 (2382.6 to 3610.5)	2153.7 (1586.2 to 2887.7)	-2.1 (-3.7 to -0.6)	-26.2 (-42.3 to -8.5)
Mongolia	4764.9 (4059.8 to 5582.6)	5016.2 (4377.0 to 5716.8)	5011.2 (4279.8 to 5930.9)	3778.1 (3076.7 to 4682.8)	-1.9 (-3.0 to -0.9)	-24.7 (-36.4 to -12.0)
Tajikistan	2407.8 (1930.1 to 2904.5)	2288.0 (1909.4 to 2708.7)	2319.8 (1894.5 to 2842.4)	1869.1 (1402.0 to 2423.1)	-1.4 (-2.8 to -0.0)	-18.4 (-34.4 to -0.2)
Turkmenistan	3065.3 (2510.0 to 3817.8)	2922.1 (2403.5 to 3533.2)	2974.7 (2390.5 to 3733.4)	2047.5 (1560.8 to 2713.7)	-2.4 (-3.6 to -1.0)	-30.0 (-41.8 to -13.9)

	Estimate in 2000 (95% UIs)	Estimate in 2015 (95% UIs)	Estimate in 2017 (95% UIs)	Estimate in 2030 (95% UIs)	Annualised rate of change, 2015-2030 (95% UIs)	Percent change, 2015-2030 (95% UIs)
Uzbekistan	2880.4 (2347.7 to 3567.8)	2733.1 (2277.6 to 3288.4)	2781.5 (2265.8 to 3400.0)	2072.1 (1627.1 to 2629.3)	-1.9 (-3.0 to -0.8)	-24.2 (-36.0 to -12.0)
Central Europe						
Albania	899.8 (751.4 to 1053.3)	899.0 (761.8 to 1041.2)	899.8 (753.3 to 1069.8)	822.5 (662.9 to 1018.6)	-0.6 (-1.7 to 0.3)	-8.5 (-22.0 to 4.1)
Bosnia and Herzegovina	903.6 (761.2 to 1061.3)	929.2 (793.0 to 1096.1)	933.8 (775.5 to 1121.1)	750.7 (610.8 to 925.7)	-1.4 (-2.2 to -0.6)	-19.3 (-27.9 to -9.0)
Bulgaria	1173.2 (979.3 to 1408.5)	1216.5 (1032.2 to 1434.8)	1232.5 (1035.4 to 1477.6)	939.8 (739.0 to 1163.9)	-1.7 (-2.7 to -0.8)	-22.8 (-33.7 to -11.1)
Croatia	1027.0 (871.1 to 1214.8)	1047.8 (906.9 to 1211.0)	1062.4 (899.1 to 1253.2)	922.4 (745.4 to 1146.2)	-0.9 (-1.8 to 0.2)	-12.0 (-23.6 to 3.8)
Czech Republic	994.6 (838.3 to 1193.3)	900.8 (779.7 to 1050.4)	902.7 (761.5 to 1074.3)	739.7 (607.9 to 908.5)	-1.3 (-2.3 to -0.5)	-17.9 (-28.7 to -6.7)
Hungary	1942.2 (1631.0 to 2325.4)	1978.2 (1700.1 to 2281.2)	1961.0 (1644.3 to 2311.5)	1650.8 (1209.3 to 2253.3)	-1.3 (-3.1 to 0.7)	-16.6 (-36.8 to 11.0)
Macedonia	893.8 (757.5 to 1072.5)	821.5 (712.1 to 946.1)	824.0 (692.2 to 983.0)	748.8 (598.2 to 940.6)	-0.6 (-1.6 to 0.5)	-8.9 (-21.1 to 7.0)
Montenegro	790.6 (665.2 to 937.4)	723.5 (619.1 to 832.1)	749.8 (618.7 to 878.4)	625.6 (496.4 to 768.4)	-1.0 (-2.0 to -0.1)	-13.6 (-25.7 to -1.1)
Poland	666.4 (573.2 to 782.1)	681.5 (593.8 to 788.9)	680.0 (578.6 to 809.8)	514.8 (424.6 to 629.6)	-1.9 (-2.7 to -1.0)	-24.5 (-33.7 to -14.1)
Romania	1371.4 (1134.5 to 1644.0)	1470.2 (1270.7 to 1694.0)	1492.8 (1252.2 to 1783.4)	995.2 (777.2 to 1275.3)	-2.6 (-3.8 to -1.4)	-32.4 (-43.5 to -19.4)
Serbia	937.0 (773.1 to 1130.8)	849.4 (738.9 to 981.3)	855.8 (727.8 to 1010.5)	745.7 (604.3 to 950.0)	-0.9 (-1.9 to 0.5)	-12.3 (-24.5 to 7.7)
Slovakia	1200.9 (1020.2 to 1410.7)	1254.9 (1083.5 to 1450.5)	1259.3 (1055.5 to 1492.3)	1026.2 (824.8 to 1263.4)	-1.4 (-2.3 to -0.4)	-18.2 (-29.2 to -6.3)
Slovenia	1409.3 (1194.9 to 1683.8)	1465.4 (1280.7 to 1690.2)	1466.4 (1236.7 to 1743.5)	1283.9 (993.4 to 1626.2)	-0.9 (-2.1 to 0.3)	-12.5 (-27.3 to 4.5)
Eastern Europe						
Belarus	848.1 (672.9 to 1046.7)	848.3 (690.4 to 1046.7)	857.8 (684.6 to 1081.0)	596.2 (435.6 to 803.7)	-2.4 (-3.7 to -1.2)	-29.8 (-42.6 to -15.9)
Estonia	917.0 (750.0 to 1144.6)	906.4 (746.0 to 1076.9)	919.1 (738.2 to 1110.4)	735.5 (520.3 to 990.8)	-1.5 (-3.5 to 0.3)	-18.8 (-40.7 to 4.9)
Latvia	805.2 (639.6 to 995.5)	859.5 (685.5 to 1042.9)	863.5 (678.5 to 1070.8)	609.0 (415.1 to 844.4)	-2.4 (-4.7 to -0.4)	-29.1 (-50.3 to -5.7)
Lithuania	914.3 (739.5 to 1112.1)	949.3 (787.0 to 1162.2)	960.8 (774.8 to 1210.0)	672.2 (506.3 to 897.4)	-2.3 (-3.8 to -0.9)	-29.2 (-43.7 to -12.0)
Moldova	2254.6 (1878.5 to 2691.5)	2356.0 (2041.5 to 2726.9)	2388.0 (2018.1 to 2828.8)	2217.3 (1625.8 to 2951.1)	-0.5 (-2.2 to 1.2)	-5.9 (-28.3 to 19.5)
Russia	1175.4 (966.3 to 1405.8)	1259.6 (1051.5 to 1504.8)	1236.2 (1018.6 to 1493.5)	901.7 (624.2 to 1229.9)	-2.3 (-4.2 to -0.6)	-28.4 (-47.1 to -8.3)
Ukraine	1223.4 (983.9 to 1512.4)	1436.0 (1167.2 to 1730.5)	1408.5 (1124.4 to 1740.1)	1029.8 (741.6 to 1383.7)	-2.3 (-3.9 to -0.8)	-28.3 (-44.0 to -10.8)
High-income						
Australasia						
Australia	643.7 (554.6 to 745.1)	647.1 (548.1 to 736.9)	649.2 (532.6 to 758.5)	533.5 (422.2 to 652.8)	-1.3 (-2.2 to -0.3)	-17.6 (-27.8 to -4.3)
New Zealand	868.0 (714.0 to 1031.7)	953.9 (784.3 to 1139.2)	1012.1 (824.6 to 1226.0)	829.9 (629.5 to 1057.5)	-1.0 (-2.0 to 0.0)	-13.1 (-26.1 to 0.5)
High-income Asia Pacific						
Brunei	1740.1 (1471.7 to 2053.5)	1740.1 (1505.1 to 2026.4)	1763.2 (1486.8 to 2096.4)	1863.4 (1470.9 to 2374.4)	0.4 (-0.8 to 1.7)	7.0 (-11.6 to 29.0)
Japan	1269.7 (1064.8 to 1486.0)	1173.3 (1007.8 to 1344.9)	1217.6 (1034.3 to 1419.1)	1144.6 (945.1 to 1369.2)	-0.2 (-0.9 to 0.5)	-2.5 (-12.8 to 8.6)
South Korea	2984.7 (2568.4 to 3447.3)	2988.3 (2636.0 to 3357.2)	2981.2 (2546.1 to 3457.7)	3076.1 (2461.1 to 3859.6)	0.2 (-1.0 to 1.4)	2.9 (-13.8 to 23.7)
Singapore	1632.4 (1401.4 to 1878.3)	1583.4 (1404.1 to 1776.7)	1559.0 (1343.9 to 1807.3)	1566.6 (1239.1 to 1921.1)	-0.1 (-1.3 to 1.0)	-1.1 (-18.3 to 15.8)
High-income North America						
Canada	467.9 (379.9 to 551.7)	430.8 (364.1 to 503.3)	428.3 (360.6 to 504.9)	348.0 (280.4 to 424.0)	-1.4 (-2.2 to -0.7)	-19.3 (-28.0 to -9.8)
Greenland	336.3 (274.1 to 409.6)	348.0 (286.9 to 410.8)	356.0 (282.4 to 433.1)	321.5 (223.8 to 469.8)	-0.6 (-2.4 to 1.7)	-7.7 (-30.3 to 29.4)
United States	313.3 (265.3 to 364.6)	313.9 (270.3 to 361.3)	328.4 (278.4 to 384.6)	334.5 (265.7 to 417.8)	0.4 (-0.9 to 1.5)	6.5 (-12.4 to 25.7)
Southern Latin America						
Argentina	302.8 (262.3 to 343.0)	294.1 (259.9 to 332.6)	296.9 (254.4 to 349.9)	245.4 (204.4 to 293.2)	-1.2 (-1.9 to -0.5)	-16.6 (-25.1 to -6.6)
Chile	501.2 (429.0 to 579.0)	477.1 (419.9 to 541.8)	478.8 (412.1 to 556.0)	427.1 (356.6 to 509.2)	-0.8 (-1.5 to -0.0)	-10.5 (-20.2 to -0.3)
Uruguay	261.3 (225.2 to 300.0)	266.5 (234.2 to 302.3)	264.4 (226.5 to 305.1)	227.6 (188.0 to 273.1)	-1.1 (-1.9 to -0.2)	-14.7 (-24.5 to -2.8)
Western Europe						
Andorra	352.3 (295.9 to 412.7)	341.9 (291.0 to 395.6)	339.6 (284.9 to 400.7)	321.6 (251.6 to 401.2)	-0.4 (-1.5 to 0.7)	-6.0 (-20.5 to 11.0)
Austria	486.8 (410.3 to 573.7)	452.5 (389.9 to 524.1)	450.8 (382.4 to 534.4)	395.7 (320.3 to 487.3)	-0.9 (-1.8 to -0.0)	-12.6 (-24.1 to -0.4)
Belgium	454.7 (386.6 to 529.2)	424.8 (376.5 to 486.1)	426.4 (367.3 to 502.6)	371.4 (311.5 to 455.7)	-0.9 (-1.7 to -0.0)	-12.6 (-22.0 to -0.7)
Cyprus	422.2 (360.5 to 489.2)	418.3 (356.3 to 475.2)	414.5 (346.2 to 486.1)	374.6 (298.2 to 477.6)	-0.8 (-1.9 to 0.5)	-10.5 (-24.9 to 7.9)
Denmark	380.4 (322.0 to 446.9)	378.2 (323.6 to 440.4)	377.1 (314.1 to 447.6)	300.9 (236.0 to 386.0)	-1.6 (-2.7 to -0.4)	-20.5 (-33.4 to -5.4)
Finland	458.6 (382.8 to 540.8)	468.9 (399.2 to 548.1)	470.8 (395.2 to 563.9)	375.8 (296.2 to 476.3)	-1.5 (-2.7 to -0.3)	-19.9 (-33.6 to -5.1)
France	656.0 (548.2 to 786.3)	590.1 (512.0 to 676.2)	583.8 (499.9 to 689.8)	531.1 (442.6 to 643.1)	-0.7 (-1.4 to 0.1)	-10.1 (-19.0 to 1.0)
Germany	370.0 (310.4 to 436.5)	347.1 (302.5 to 393.0)	346.0 (297.0 to 401.2)	297.6 (247.0 to 354.1)	-1.0 (-1.9 to -0.2)	-14.3 (-24.9 to -3.3)
Greece	1126.8 (974.8 to 1289.0)	1108.4 (981.9 to 1258.3)	1114.7 (960.2 to 1293.1)	1021.9 (845.3 to 1220.0)	-0.6 (-1.3 to 0.3)	-7.9 (-18.0 to 3.9)
Iceland	316.6 (264.5 to 377.7)	311.4 (266.5 to 361.4)	305.6 (255.5 to 364.9)	273.8 (207.9 to 364.5)	-0.9 (-2.2 to 0.7)	-12.1 (-28.4 to 10.9)
Ireland	359.9 (306.4 to 420.5)	321.6 (274.3 to 374.8)	324.0 (272.1 to 384.7)	264.6 (215.8 to 330.6)	-1.3 (-2.1 to -0.4)	-17.8 (-27.2 to -5.3)
Israel	415.7 (352.8 to 478.5)	417.9 (362.3 to 480.9)	418.0 (353.9 to 493.1)	353.4 (279.0 to 443.8)	-1.1 (-2.2 to -0.1)	-15.5 (-28.5 to -1.6)
Italy	765.9 (661.8 to 894.3)	781.7 (673.7 to 892.5)	784.0 (657.7 to 927.5)	683.9 (554.6 to 832.1)	-0.9 (-1.7 to -0.1)	-12.6 (-22.2 to -2.1)
Luxembourg	517.7 (435.2 to 605.7)	521.2 (448.3 to 601.0)	522.7 (442.4 to 620.6)	451.6 (331.7 to 618.8)	-1.0 (-2.8 to 0.7)	-13.4 (-33.9 to 11.8)
Malta	398.5 (335.6 to 467.0)	361.0 (310.0 to 419.9)	359.7 (302.1 to 424.2)	305.9 (232.2 to 403.1)	-1.2 (-2.6 to 0.5)	-15.3 (-32.3 to 7.6)
Netherlands	373.4 (315.4 to 435.4)	332.4 (291.9 to 382.0)	306.6 (261.7 to 361.4)	260.6 (216.8 to 313.7)	-1.6 (-2.3 to -0.9)	-21.7 (-29.5 to -12.8)
Norway	373.5 (315.5 to 437.6)	353.1 (300.1 to 408.9)	330.1 (274.9 to 389.1)	289.6 (229.2 to 364.8)	-1.3 (-2.4 to -0.2)	-18.0 (-30.1 to -3.5)
Portugal	550.5 (462.7 to 647.4)	545.0 (470.9 to 632.5)	544.3 (455.9 to 652.3)	444.2 (343.6 to 579.6)	-1.4 (-2.7 to 0.1)	-18.6 (-32.9 to 1.0)
Spain	449.6 (390.0 to 521.3)	447.1 (395.0 to 504.2)	447.6 (384.1 to 519.5)	379.2 (302.1 to 489.5)	-1.1 (-2.3 to 0.3)	-15.2 (-29.7 to 5.1)
Sweden	291.1 (245.6 to 337.3)	311.7 (265.1 to 358.9)	327.9 (277.6 to 385.3)	254.1 (203.6 to 314.0)	-1.4 (-2.2 to -0.4)	-18.5 (-28.4 to -5.4)
Switzerland	471.8 (397.2 to 548.7)	459.3 (397.8 to 526.8)	441.9 (375.0 to 519.7)	322.2 (245.5 to 417.5)	-2.4 (-3.8 to -1.0)	-29.9 (-43.4 to -14.2)
United Kingdom	356.1 (293.2 to 429.9)	378.2 (323.8 to 440.8)	379.8 (321.4 to 450.2)	290.4 (238.9 to 355.8)	-1.8 (-2.6 to -0.9)	-23.2 (-32.6 to -13.2)
Latin America and Caribbean						
Andean Latin America						
Bolivia	1110.9 (950.1 to 1293.3)	1114.4 (969.4 to 1262.0)	1120.8 (964.0 to 1303.1)	809.6 (678.9 to 961.3)	-2.1 (-2.9 to -1.5)	-27.4 (-34.9 to -19.6)
Ecuador	1157.3 (987.1 to 1336.6)	1135.6 (991.1 to 1303.9)	1143.8 (969.7 to 1342.3)	960.1 (801.7 to 1149.8)	-1.1 (-1.7 to -0.5)	-15.5 (-23.0 to -7.7)
Peru	1049.9 (893.6 to 1224.4)	1014.1 (882.4 to 1160.0)	1030.4 (876.5 to 1202.9)	737.7 (568.8 to 946.4)	-2.2 (-3.7 to -0.9)	-27.3 (-42.3 to -12.3)
Caribbean						
Antigua and Barbuda	460.4 (394.7 to 533.5)	470.2 (411.0 to 534.0)	473.1 (403.0 to 552.3)	459.9 (347.4 to 615.2)	-0.2 (-1.6 to 1.6)	-2.2 (-21.8 to 27.4)
The Bahamas	546.9 (459.2 to 650.0)	493.2 (430.8 to 561.7)	493.1 (419.0 to 579.2)	483.0 (376.9 to 628.8)	-0.2 (-1.5 to 1.4)	-2.1 (-20.2 to 23.9)
Barbados	454.0 (379.5 to 533.8)	427.3 (373.8 to 483.8)	424.6 (362.1 to 496.0)	402.1 (330.7 to 490.9)	-0.4 (-1.3 to 0.5)	-6.0 (-17.6 to 7.9)
Belize	619.8 (532.4 to 731.5)	616.8 (544.0 to 704.0)	617.7 (534.8 to 725.9)	594.1 (464.7 to 762.1)	-0.3 (-1.6 to 1.1)	-3.7 (-20.9 to 18.1)
Bermuda	385.2 (324.4 to 448.2)	394.8 (340.6 to 452.5)	398.1 (333.3 to 468.5)	387.9 (243.7 to 667.6)	-0.3 (-3.2 to 3.2)	-1.8 (-37.9 to 61.5)
Cuba	403.0 (340.9 to 467.6)	401.8 (353.0 to 456.3)	402.6 (346.8 to 470.0)	339.9 (284.1 to 414.4)	-1.1 (-2.0 to -0.2)	-15.4 (-25.8 to -2.6)
Dominica	497.9 (423.8 to 579.1)	446.9 (389.7 to 510.4)	450.9 (385.9 to 530.1)	453.9 (354.3 to 592.8)	0.1 (-1.2 to 1.5)	1.5 (-16.3 to 25.4)
Dominican Republic	715.8 (619.4 to 835.5)	704.8 (618.6 to 800.5)	704.9 (602.8 to 816.9)	659.5 (543.6 to 802.6)	-0.5 (-1.3 to 0.5)	-6.5 (-17.3 to 7.3)
Grenada	505.5 (427.9 to 593.8)	508.1 (445.3 to 577.7)	508.2 (438.8 to 588.8)	483.9 (393.9 to 589.8)	-0.3 (-1.3 to 0.6)	-4.8 (-17.9 to 10.0)
Guyana	731.2 (618.5 to 870.2)	658.4 (572.8 to 759.1)	662.5 (565.8 to 784.3)	598.0 (465.4 to 790.4)	-0.7 (-2.0 to 0.9)	-9.2 (-25.4 to 13.9)
Haiti	659.2 (557.5 to 768.2)	635.7 (555.4 to 730.2)	618.7 (527.7 to 728.2)	499.4 (419.7 to 597.0)	-1.6 (-2.2 to -1.0)	-21.5 (-28.6 to -14.2)

	Estimate in 2000 (95% UIs)	Estimate in 2015 (95% UIs)	Estimate in 2017 (95% UIs)	Estimate in 2030 (95% UIs)	Annualised rate of change, 2015-2030 (95% UIs)	Percent change, 2015-2030 (95% UIs)
Jamaica	358.1 (303.4 to 418.5)	321.1 (277.7 to 367.3)	324.0 (275.4 to 378.6)	324.5 (258.9 to 405.1)	0.0 (-0.9 to 1.3)	1.0 (-13.0 to 21.9)
Puerto Rico	578.9 (489.5 to 674.1)	593.7 (513.6 to 685.7)	595.2 (502.2 to 705.3)	530.4 (421.8 to 668.9)	-0.8 (-1.9 to 0.3)	-10.7 (-25.0 to 5.0)
Saint Lucia	543.5 (461.0 to 631.8)	498.7 (435.4 to 569.9)	503.0 (432.2 to 592.8)	486.6 (398.0 to 590.6)	-0.2 (-1.1 to 0.8)	-2.5 (-15.1 to 13.1)
Saint Vincent and the Grenadines	481.1 (400.7 to 569.5)	436.6 (378.5 to 500.1)	436.9 (370.6 to 510.2)	428.4 (341.5 to 535.8)	-0.2 (-1.2 to 1.1)	-1.9 (-15.9 to 17.2)
Suriname	661.1 (563.8 to 771.8)	615.4 (538.0 to 701.7)	618.8 (528.4 to 725.5)	603.1 (503.7 to 722.3)	-0.1 (-0.8 to 0.6)	-2.0 (-11.8 to 9.7)
Trinidad and Tobago	473.1 (400.6 to 556.4)	452.6 (391.5 to 521.9)	450.5 (381.4 to 530.7)	411.2 (333.7 to 514.8)	-0.7 (-1.6 to 0.4)	-9.2 (-21.5 to 6.8)
Virgin Islands, U.S.	502.6 (429.9 to 585.5)	498.1 (437.6 to 564.2)	501.1 (432.0 to 583.6)	409.6 (334.4 to 497.2)	-1.3 (-2.3 to -0.4)	-17.8 (-29.5 to -5.8)
Central Latin America						
Colombia	962.3 (781.7 to 1144.5)	908.6 (752.7 to 1096.2)	902.2 (724.5 to 1095.5)	796.7 (601.6 to 1029.2)	-0.9 (-1.9 to 0.1)	-12.4 (-24.9 to 1.5)
Costa Rica	663.9 (557.0 to 783.9)	653.3 (557.6 to 759.5)	645.3 (537.2 to 773.1)	587.4 (468.8 to 715.1)	-0.7 (-1.5 to -0.0)	-10.2 (-20.1 to -0.4)
El Salvador	770.2 (638.1 to 944.6)	762.0 (656.9 to 909.7)	770.7 (648.0 to 932.6)	707.4 (559.5 to 902.8)	-0.5 (-1.6 to 0.6)	-7.3 (-21.3 to 8.7)
Guatemala	1251.1 (1048.3 to 1481.4)	1131.5 (964.4 to 1312.8)	1129.2 (953.4 to 1334.2)	1028.8 (832.7 to 1290.4)	-0.7 (-1.6 to 0.4)	-9.1 (-20.8 to 6.3)
Honduras	898.9 (748.7 to 1077.4)	894.0 (751.8 to 1042.5)	898.3 (746.9 to 1076.8)	799.4 (634.4 to 980.2)	-0.8 (-1.5 to -0.1)	-10.7 (-19.9 to -0.8)
Mexico	896.6 (752.2 to 1050.2)	819.2 (706.8 to 946.4)	881.0 (751.8 to 1030.0)	863.1 (708.5 to 1048.1)	0.3 (-0.5 to 1.2)	5.3 (-6.9 to 19.6)
Nicaragua	545.6 (465.9 to 647.9)	540.9 (463.8 to 628.0)	541.0 (458.4 to 638.0)	488.1 (395.7 to 614.6)	-0.7 (-1.6 to 0.3)	-9.8 (-21.7 to 5.0)
Panama	533.0 (441.6 to 634.1)	522.7 (437.9 to 609.5)	520.7 (430.2 to 623.7)	514.9 (402.1 to 646.5)	-0.1 (-1.1 to 0.9)	-1.6 (-14.6 to 13.8)
Venezuela	690.6 (573.4 to 813.5)	627.3 (534.9 to 743.0)	621.2 (520.3 to 752.0)	620.3 (488.8 to 800.3)	-0.1 (-1.1 to 1.1)	-1.2 (-15.8 to 17.5)
Tropical Latin America						
Brazil	1319.2 (1108.0 to 1550.5)	1235.5 (1059.2 to 1438.1)	1259.3 (1065.2 to 1486.2)	1161.5 (935.4 to 1411.2)	-0.4 (-1.2 to 0.4)	-6.1 (-16.5 to 5.5)
Paraguay	1069.5 (886.5 to 1286.7)	996.8 (837.0 to 1177.0)	996.3 (833.3 to 1203.2)	962.0 (741.1 to 1236.6)	-0.3 (-1.5 to 0.9)	-3.5 (-19.6 to 14.6)
North Africa and Middle East						
North Africa and Middle East						
Afghanistan	3025.8 (2448.0 to 3701.1)	2735.5 (2310.8 to 3231.6)	2722.6 (2263.8 to 3298.7)	2227.9 (1788.6 to 2750.3)	-1.4 (-2.1 to -0.8)	-18.6 (-26.7 to -10.8)
Algeria	1611.0 (1342.1 to 1942.8)	1421.5 (1214.0 to 1658.3)	1428.5 (1197.5 to 1700.6)	1174.2 (956.9 to 1410.8)	-1.3 (-1.9 to -0.7)	-17.4 (-24.7 to -10.0)
Bahrain	1593.0 (1352.3 to 1899.6)	1687.7 (1433.1 to 1960.4)	1698.4 (1406.8 to 2032.6)	1449.4 (1112.3 to 1871.4)	-1.1 (-2.4 to 0.4)	-14.2 (-29.9 to 6.0)
Egypt	6474.8 (5628.7 to 7468.3)	6413.6 (5741.8 to 7266.2)	6450.1 (5660.0 to 7491.5)	5508.9 (4711.6 to 6470.1)	-1.0 (-1.7 to -0.4)	-14.2 (-22.5 to -5.8)
Iran	938.1 (789.6 to 1096.4)	1040.7 (901.1 to 1188.0)	1049.2 (899.2 to 1222.6)	924.6 (764.1 to 1111.0)	-0.8 (-1.6 to -0.0)	-11.2 (-21.5 to -0.6)
Iraq	1299.0 (1049.6 to 1580.4)	1253.3 (1037.3 to 1458.4)	1264.0 (1038.5 to 1504.9)	1024.9 (828.0 to 1248.0)	-1.4 (-2.0 to -0.7)	-18.3 (-25.8 to -9.6)
Jordan	1585.8 (1312.6 to 1868.9)	1608.2 (1348.2 to 1887.7)	1611.1 (1329.2 to 1939.1)	1382.0 (1098.8 to 1730.2)	-1.0 (-1.8 to -0.2)	-14.2 (-24.0 to -2.9)
Kuwait	1130.9 (951.4 to 1346.4)	1132.3 (954.5 to 1326.5)	1138.5 (948.5 to 1358.0)	1149.3 (780.4 to 1676.1)	0.0 (-2.2 to 2.4)	1.4 (-27.7 to 43.3)
Lebanon	1736.6 (1458.3 to 2059.3)	1791.5 (1534.3 to 2084.5)	1800.9 (1510.6 to 2144.8)	1582.6 (1245.3 to 2001.2)	-0.9 (-2.0 to 0.3)	-11.7 (-25.4 to 4.5)
Libya	1678.9 (1410.0 to 1971.1)	1779.8 (1537.6 to 2056.0)	1801.0 (1529.3 to 2127.6)	1691.6 (1377.9 to 2020.0)	-0.4 (-1.1 to 0.4)	-5.0 (-15.8 to 5.6)
Morocco	1688.7 (1414.9 to 1993.2)	1602.1 (1344.6 to 1861.8)	1610.5 (1331.7 to 1916.1)	1374.6 (1120.9 to 1649.1)	-1.0 (-1.6 to -0.5)	-14.2 (-21.2 to -7.4)
Palestine	1572.0 (1290.4 to 1861.1)	1505.3 (1257.4 to 1733.0)	1490.8 (1213.5 to 1759.8)	1491.9 (1095.0 to 2144.3)	-0.1 (-1.7 to 2.1)	-1.0 (-22.4 to 36.2)
Oman	1482.9 (1200.0 to 1764.2)	1497.4 (1256.7 to 1764.0)	1501.2 (1241.7 to 1809.6)	1348.4 (1040.0 to 1727.4)	-0.7 (-1.9 to 0.5)	-10.0 (-24.4 to 7.7)
Qatar	2056.9 (1700.6 to 2446.9)	2202.6 (1874.9 to 2553.4)	2228.0 (1846.4 to 2658.4)	1946.3 (1432.3 to 2631.6)	-0.9 (-2.4 to 0.7)	-11.7 (-30.0 to 11.2)
Saudi Arabia	2016.8 (1669.0 to 2345.7)	2058.6 (1766.5 to 2386.4)	2067.7 (1742.8 to 2443.0)	1804.2 (1399.5 to 2273.8)	-0.9 (-2.2 to 0.3)	-12.4 (-28.1 to 4.2)
Sudan	3667.6 (2915.6 to 4545.5)	3115.8 (2615.8 to 3651.4)	3112.6 (2578.9 to 3727.8)	2842.2 (2303.0 to 3465.2)	-0.6 (-1.3 to 0.0)	-8.8 (-17.6 to 0.0)
Syria	1507.3 (1263.2 to 1787.1)	1527.5 (1302.8 to 1778.6)	1550.6 (1285.2 to 1846.7)	1395.3 (1116.0 to 1714.8)	-0.6 (-1.5 to 0.3)	-8.7 (-20.6 to 4.9)
Tunisia	1045.0 (855.5 to 1246.5)	1032.5 (877.0 to 1212.0)	1030.5 (868.2 to 1223.4)	879.7 (713.8 to 1080.1)	-1.1 (-1.8 to -0.5)	-14.9 (-23.3 to -6.6)
Turkey	1306.4 (1095.5 to 1545.1)	1264.3 (1087.8 to 1450.1)	1272.8 (1059.2 to 1494.9)	1113.6 (871.6 to 1393.3)	-0.9 (-2.0 to 0.2)	-12.0 (-26.1 to 3.0)
United Arab Emirates	1418.7 (1148.2 to 1712.8)	1433.5 (1212.7 to 1710.1)	1444.5 (1201.1 to 1759.7)	1272.8 (1023.7 to 1607.0)	-0.8 (-1.7 to 0.1)	-11.3 (-22.4 to 1.1)
Yemen	2545.9 (2084.5 to 3077.4)	2216.8 (1883.6 to 2605.7)	2230.8 (1867.7 to 2662.3)	1977.7 (1630.9 to 2408.1)	-0.8 (-1.4 to -0.1)	-10.8 (-19.1 to -2.1)
South Asia						
South Asia						
Bangladesh	1797.3 (1503.9 to 2107.8)	1666.3 (1432.5 to 1918.9)	1667.0 (1408.4 to 1970.6)	1114.1 (911.7 to 1364.7)	-2.7 (-3.5 to -1.8)	-33.2 (-41.2 to -24.0)
Bhutan	1706.5 (1441.0 to 2029.2)	1738.2 (1475.8 to 2009.0)	1737.1 (1450.9 to 2068.5)	1126.8 (928.6 to 1360.8)	-2.9 (-3.5 to -2.2)	-35.2 (-40.9 to -28.3)
India	1077.9 (881.2 to 1295.7)	1151.1 (956.0 to 1370.4)	1141.4 (938.0 to 1376.6)	682.2 (543.0 to 844.5)	-3.5 (-4.3 to -2.8)	-40.8 (-47.4 to -33.9)
Nepal	1043.0 (856.8 to 1225.9)	932.5 (804.0 to 1074.5)	929.9 (785.9 to 1099.6)	676.0 (565.1 to 809.5)	-2.2 (-2.7 to -1.6)	-27.5 (-33.5 to -21.0)
Pakistan	1781.5 (1515.0 to 2081.9)	1604.9 (1399.7 to 1814.7)	1597.3 (1366.3 to 1854.5)	1092.9 (909.0 to 1305.3)	-2.6 (-3.4 to -1.8)	-31.9 (-39.6 to -23.3)
Southeast Asia, East Asia, and Oceania						
East Asia						
China	4087.5 (3459.1 to 4778.7)	3560.2 (3106.6 to 4106.6)	3389.1 (2900.1 to 3986.3)	3030.6 (2450.8 to 3767.8)	-1.1 (-2.1 to -0.0)	-14.9 (-27.1 to -0.4)
North Korea	4713.3 (3935.1 to 5560.9)	4378.5 (3758.6 to 5090.8)	4399.0 (3669.5 to 5230.4)	4272.6 (3492.1 to 5128.8)	-0.2 (-0.8 to 0.4)	-2.5 (-11.8 to 7.0)
Taiwan (Province of China)	5156.9 (4495.3 to 5900.9)	5305.9 (4653.3 to 6042.1)	5265.2 (4458.6 to 6210.3)	5744.8 (4770.3 to 6988.1)	0.5 (-0.4 to 1.5)	8.2 (-5.5 to 24.8)
Oceania						
American Samoa	2901.9 (2444.5 to 3396.2)	2935.3 (2544.7 to 3397.6)	2919.3 (2478.1 to 3454.5)	2559.7 (2128.3 to 3090.4)	-0.9 (-1.6 to -0.3)	-12.8 (-21.8 to -3.7)
Federated States of Micronesia	3257.2 (2701.3 to 3956.7)	3412.1 (2906.6 to 4040.1)	3464.3 (2907.0 to 4171.4)	3273.6 (2578.8 to 4262.7)	-0.3 (-1.5 to 0.9)	-4.2 (-19.7 to 14.2)
Fiji	1929.4 (1589.4 to 2335.0)	1898.7 (1614.5 to 2233.2)	1890.7 (1584.5 to 2247.3)	1733.5 (1339.5 to 2247.3)	-0.6 (-1.8 to 0.8)	-8.7 (-23.8 to 13.1)
Guam	3738.1 (3181.3 to 4426.4)	3669.5 (3197.4 to 4192.6)	3657.9 (3122.5 to 4273.6)	3083.2 (2472.7 to 3831.3)	-1.2 (-2.2 to -0.2)	-16.0 (-28.6 to -2.5)
Kiribati	3588.5 (2948.5 to 4349.5)	3591.6 (3030.8 to 4385.1)	3597.8 (2971.4 to 4409.5)	3170.3 (2560.1 to 3981.0)	-0.8 (-1.5 to -0.2)	-11.8 (-19.6 to -3.1)
Marshall Islands	4108.7 (3432.0 to 4887.6)	4078.7 (3488.8 to 4761.0)	4132.0 (3478.6 to 4930.5)	3408.0 (2705.1 to 4251.1)	-1.2 (-2.2 to -0.4)	-16.5 (-28.4 to -5.8)
Northern Mariana Islands	3776.3 (3227.1 to 4396.0)	3822.1 (3355.5 to 4378.9)	3777.1 (3222.4 to 4428.4)	3125.7 (2585.5 to 3753.4)	-1.4 (-2.1 to -0.6)	-18.3 (-26.8 to -9.1)
Papua New Guinea	3258.0 (2599.4 to 4079.3)	3193.8 (2588.9 to 4009.6)	3211.0 (2514.8 to 4065.6)	2650.8 (2003.9 to 3427.7)	-1.3 (-2.0 to -0.6)	-17.1 (-25.9 to -8.9)
Samoa	2756.6 (2335.3 to 3263.4)	2851.8 (2454.9 to 3369.1)	2834.8 (2385.5 to 3392.5)	2487.2 (2073.7 to 3021.0)	-0.9 (-1.5 to -0.4)	-12.9 (-19.8 to -5.4)
Solomon Islands	3634.2 (2985.1 to 4485.1)	3498.4 (2905.8 to 4324.4)	3497.8 (2825.6 to 4395.3)	2999.9 (2360.3 to 3866.4)	-1.0 (-1.8 to -0.3)	-14.3 (-23.8 to -4.5)
Tonga	4644.5 (3890.2 to 5477.6)	4735.3 (4103.1 to 5520.6)	4745.3 (4010.5 to 5660.7)	4258.2 (3507.3 to 5138.2)	-0.7 (-1.3 to -0.2)	-10.1 (-17.9 to -2.5)
Vanuatu	4268.4 (3477.2 to 5133.1)	4331.6 (3597.9 to 5166.1)	4341.8 (3579.5 to 5306.6)	3734.5 (2976.1 to 4613.6)	-1.0 (-1.6 to -0.4)	-13.9 (-21.9 to -5.4)
Southeast Asia						
Cambodia	5651.2 (4780.4 to 6640.0)	5323.8 (4647.1 to 6076.8)	5360.8 (4587.6 to 6270.8)	3544.7 (2984.9 to 4219.7)	-2.7 (-3.3 to -2.1)	-33.4 (-39.4 to -26.8)
Indonesia	2565.7 (2187.6 to 2988.9)	2501.2 (2173.8 to 2892.4)	2502.1 (2149.1 to 2945.0)	1762.3 (1475.3 to 2104.2)	-2.3 (-3.0 to -1.7)	-29.6 (-36.6 to -22.4)
Laos	3343.8 (2735.9 to 3947.7)	2944.2 (2527.9 to 3438.2)	2963.4 (2491.1 to 3536.5)	2020.6 (1661.8 to 2431.8)	-2.5 (-3.1 to -2.0)	-31.4 (-37.2 to -25.6)
Malaysia	1205.2 (1043.8 to 1388.6)	1215.0 (1071.4 to 1379.3)	1221.2 (1051.1 to 1430.8)	993.8 (827.0 to 1197.9)	-1.4 (-2.1 to -0.5)	-18.3 (-27.4 to -7.1)
Maldives	1359.6 (1144.2 to 1598.3)	1435.9 (1197.9 to 1676.9)	1440.0 (1193.1 to 1714.1)	1294.1 (904.0 to 1794.3)	-0.8 (-2.6 to 1.2)	-9.9 (-32.4 to 19.7)
Mauritius	2048.5 (1712.5 to 2455.7)	2100.9 (1777.6 to 2454.8)	2113.0 (1752.7 to 2541.4)	1805.7 (1427.6 to 2293.5)	-1.0 (-2.1 to -0.1)	-14.1 (-26.6 to -1.4)
Myanmar	3488.3 (2887.4 to 4213.0)	3399.6 (2903.4 to 3950.3)	3462.7 (2882.3 to 4117.6)	2271.8 (1870.0 to 2758.9)	-2.7 (-3.5 to -1.9)	-33.2 (-40.4 to -25.1)
Philippines	2808.0 (2313.9 to 3455.4)	2801.2 (2393.3 to 3279.2)	2809.0 (2343.5 to 3351.7)	2042.9 (1667.1 to 2488.1)	-2.1 (-2.8 to -1.5)	-27.1 (-34.2 to -19.6)
Sri Lanka	1807.4 (1516.5 to 2131.2)	1653.5 (1436.7 to 1889.3)	1658.4 (1400.2 to 1951.9)	1219.3 (971.2 to 1518.8)	-2.1 (-3.2 to -0.9)	-26.3 (-38.1 to -13.2)
Seychelles	2144.8 (1775.8 to 2544.8)	2275.6 (1959.6 to 2636.7)	2303.8 (1940.2 to 2744.3)	1566.2 (1262.6 to 1935.4)	-2.5 (-3.5 to -1.5)	-31.2 (-41.2 to -19.6)
Thailand	2805.2 (2364.8 to 3350.3)	2966.8 (2550.9 to 3422.0)	2933.9 (2471.3 to 3459.6)	2782.4 (2119.9 to 3778.8)	-0.5 (-2.0 to 1.3)	-6.3 (-25.9 to 21.7)

	Estimate in 2000 (95% UIs)	Estimate in 2015 (95% UIs)	Estimate in 2017 (95% UIs)	Estimate in 2030 (95% UIs)	Annualised rate of change, 2015-2030 (95% UIs)	Percent change, 2015-2030 (95% UIs)
Timor-Leste	2537.0 (2118.1 to 2985.9)	2318.9 (1989.3 to 2694.8)	2307.2 (1925.1 to 2737.8)	1690.4 (1365.7 to 2072.7)	-2.1 (-3.0 to -1.2)	-27.1 (-36.0 to -16.3)
Vietnam	3141.7 (2652.9 to 3678.9)	3223.0 (2796.0 to 3675.1)	3203.6 (2705.8 to 3763.0)	2271.6 (1909.5 to 2688.9)	-2.3 (-2.9 to -1.7)	-29.6 (-35.7 to -22.3)
Sub-Saharan Africa						
Central Sub-Saharan Africa						
Angola	3528.2 (2838.0 to 4398.6)	3340.0 (2750.9 to 4098.8)	3386.7 (2766.3 to 4227.9)	2524.3 (1958.5 to 3280.7)	-1.9 (-2.9 to -0.8)	-24.5 (-35.1 to -11.7)
Central African Republic	2976.7 (2254.6 to 3793.2)	2833.5 (2239.2 to 3581.2)	2837.0 (2240.8 to 3649.9)	2546.3 (1852.1 to 3401.9)	-0.8 (-2.1 to 0.6)	-10.2 (-27.5 to 8.7)
Congo	2938.7 (2399.6 to 3586.8)	2761.0 (2262.8 to 3344.6)	2720.0 (2183.6 to 3348.5)	2188.6 (1618.7 to 2920.1)	-1.6 (-3.0 to 0.1)	-20.8 (-35.8 to 1.7)
Democratic Republic of the Congo	3269.4 (2606.0 to 4102.6)	3012.4 (2460.8 to 3654.4)	3017.7 (2433.9 to 3728.1)	2398.6 (1867.8 to 3087.2)	-1.5 (-2.4 to -0.6)	-20.5 (-29.9 to -9.0)
Equatorial Guinea	2651.8 (2074.2 to 3277.0)	2804.8 (2311.1 to 3365.3)	2784.6 (2260.9 to 3408.3)	2083.4 (1610.1 to 2690.6)	-2.0 (-3.1 to -0.8)	-25.7 (-37.5 to -11.0)
Gabon	2805.8 (2271.3 to 3453.9)	2590.1 (2173.1 to 3073.5)	2592.1 (2142.0 to 3114.9)	1906.4 (1501.3 to 2436.5)	-2.1 (-3.1 to -0.9)	-26.4 (-37.6 to -12.7)
Eastern Sub-Saharan Africa						
Burundi	2627.9 (2221.6 to 3096.7)	2401.8 (2054.6 to 2802.8)	2396.7 (2008.2 to 2867.7)	1989.7 (1585.9 to 2528.4)	-1.3 (-2.4 to -0.1)	-17.2 (-30.0 to -1.2)
Comoros	2012.5 (1649.2 to 2422.7)	1879.0 (1590.8 to 2199.2)	1871.1 (1549.8 to 2250.3)	1533.2 (1225.6 to 1913.6)	-1.4 (-2.3 to -0.5)	-18.5 (-28.7 to -6.6)
Djibouti	2201.9 (1817.2 to 2610.1)	2094.3 (1776.7 to 2444.1)	2130.2 (1792.6 to 2543.6)	1762.8 (1401.0 to 2271.7)	-1.2 (-2.3 to -0.0)	-15.9 (-29.7 to -0.2)
Eritrea	3311.1 (2731.7 to 3944.4)	2987.8 (2539.7 to 3509.1)	2993.9 (2498.9 to 3593.2)	2692.1 (2096.3 to 3423.8)	-0.7 (-1.9 to 0.2)	-10.0 (-25.0 to 3.6)
Ethiopia	3569.3 (2991.2 to 4248.1)	3291.6 (2786.4 to 3876.1)	3271.0 (2732.7 to 3907.8)	2390.1 (1944.1 to 2986.6)	-2.2 (-2.9 to -1.3)	-27.4 (-35.6 to -17.6)
Kenya	2127.9 (1782.6 to 2513.0)	2018.2 (1720.9 to 2358.7)	1905.7 (1603.2 to 2279.0)	1549.9 (1186.5 to 2039.8)	-1.8 (-2.9 to -0.3)	-23.3 (-35.7 to -4.2)
Madagascar	3297.6 (2679.2 to 4109.0)	2964.0 (2459.4 to 3537.6)	2990.2 (2436.4 to 3658.4)	2420.5 (1935.1 to 3004.3)	-1.4 (-2.0 to -0.8)	-18.4 (-25.9 to -11.3)
Malawi	3422.3 (2849.9 to 4048.6)	3074.9 (2624.8 to 3600.2)	3088.5 (2583.5 to 3699.8)	2644.8 (2006.3 to 3453.2)	-1.0 (-2.4 to 0.4)	-14.0 (-30.5 to 6.9)
Mozambique	3548.6 (2988.7 to 4247.4)	2972.9 (2565.5 to 3430.2)	2942.8 (2487.1 to 3469.7)	2560.9 (2044.5 to 3240.1)	-1.0 (-2.1 to 0.1)	-13.9 (-27.1 to 1.5)
Rwanda	2896.8 (2358.2 to 3533.2)	2819.4 (2362.0 to 3375.1)	2843.3 (2346.4 to 3459.2)	2190.3 (1527.1 to 3147.5)	-1.8 (-3.8 to 0.5)	-22.4 (-43.5 to 7.8)
Somalia	3374.2 (2767.6 to 4061.1)	3367.1 (2780.0 to 4037.1)	3366.3 (2740.9 to 4100.1)	2951.0 (2282.5 to 3740.3)	-0.9 (-1.9 to 0.0)	-12.4 (-24.7 to 0.2)
South Sudan	2829.1 (2312.9 to 3418.5)	2808.2 (2362.8 to 3289.2)	2731.0 (2248.2 to 3283.2)	2316.6 (1744.7 to 3025.0)	-1.3 (-2.5 to 0.1)	-17.6 (-31.2 to 1.1)
Tanzania	2360.0 (1976.5 to 2841.1)	2196.5 (1893.2 to 2510.8)	2187.4 (1844.0 to 2560.2)	1795.8 (1474.3 to 2176.1)	-1.4 (-2.2 to -0.5)	-18.3 (-27.6 to -7.8)
Uganda	2908.4 (2374.4 to 3539.4)	2516.1 (2136.7 to 2953.8)	2511.3 (2083.5 to 2995.2)	2135.1 (1625.9 to 2716.1)	-1.1 (-2.4 to 0.0)	-15.2 (-29.8 to 0.6)
Zambia	3232.8 (2674.3 to 3864.6)	2959.6 (2550.6 to 3450.0)	2981.5 (2533.0 to 3543.9)	2371.2 (1670.2 to 3205.4)	-1.5 (-3.6 to 0.1)	-19.9 (-41.4 to 2.3)
Southern Sub-Saharan Africa						
Botswana	1980.0 (1644.4 to 2369.8)	1998.4 (1686.2 to 2362.5)	2026.8 (1676.0 to 2442.8)	2030.4 (1461.4 to 3084.3)	0.0 (-1.7 to 2.7)	1.5 (-22.1 to 49.6)
Lesotho	2699.0 (2176.3 to 3235.9)	2314.6 (1922.7 to 2787.2)	2318.6 (1901.7 to 2860.4)	2345.8 (1617.9 to 3424.9)	-0.0 (-2.1 to 2.2)	1.2 (-26.7 to 38.2)
Namibia	1623.0 (1332.4 to 1970.3)	1481.7 (1255.6 to 1754.5)	1485.1 (1234.1 to 1806.1)	1165.5 (886.4 to 1523.8)	-1.6 (-2.8 to -0.3)	-21.4 (-34.6 to -4.3)
South Africa	2047.8 (1702.5 to 2467.4)	1460.7 (1204.0 to 1744.2)	1438.9 (1176.2 to 1746.2)	1554.6 (916.3 to 2708.1)	0.2 (-2.7 to 3.8)	6.3 (-33.2 to 77.7)
Swaziland	2801.6 (2329.7 to 3298.8)	2736.7 (2344.0 to 3225.3)	2799.3 (2344.7 to 3332.2)	2847.2 (1953.2 to 4411.2)	0.2 (-2.0 to 2.9)	4.0 (-25.4 to 54.5)
Zimbabwe	3860.8 (3208.3 to 4629.6)	3567.2 (2995.6 to 4254.2)	3593.4 (2976.7 to 4374.2)	4070.9 (2489.9 to 7168.9)	0.7 (-2.3 to 4.4)	14.1 (-28.8 to 93.0)
Western Sub-Saharan Africa						
Benin	5288.5 (4366.9 to 6338.0)	4907.3 (4122.6 to 5775.5)	4949.5 (4089.2 to 5972.8)	3934.5 (3115.7 to 4926.4)	-1.5 (-2.4 to -0.6)	-19.9 (-29.9 to -8.5)
Burkina Faso	6261.7 (5178.5 to 7400.9)	5553.0 (4725.6 to 6470.3)	5538.2 (4650.3 to 6575.0)	4500.6 (3584.1 to 5505.6)	-1.4 (-2.3 to -0.6)	-19.0 (-29.1 to -9.0)
Cameroon	6025.1 (5040.9 to 7197.9)	5478.3 (4593.8 to 6392.6)	5527.1 (4555.3 to 6661.4)	4542.5 (3557.5 to 5753.7)	-1.3 (-2.2 to -0.1)	-17.1 (-28.6 to -1.9)
Cape Verde	3884.4 (3119.1 to 4753.2)	3599.1 (3068.1 to 4263.4)	3631.1 (3036.4 to 4376.6)	2948.8 (2362.6 to 3709.1)	-1.3 (-2.4 to -0.5)	-18.1 (-30.2 to -6.6)
Chad	5487.2 (4496.6 to 6534.2)	5281.6 (4308.0 to 6411.4)	5243.1 (4122.8 to 6542.1)	4457.9 (3368.1 to 5748.1)	-1.2 (-2.2 to -0.1)	-15.7 (-27.7 to -2.0)
Cote d'Ivoire	4839.6 (3967.1 to 5790.0)	4603.9 (3816.2 to 5386.5)	4529.5 (3698.6 to 5401.0)	3423.1 (2695.8 to 4269.0)	-2.0 (-2.8 to -1.1)	-25.7 (-34.4 to -14.7)
The Gambia	5342.1 (4456.5 to 6246.8)	5330.2 (4509.5 to 6269.9)	5322.2 (4394.0 to 6489.8)	4605.2 (3699.4 to 5744.3)	-1.0 (-1.7 to -0.2)	-13.7 (-23.0 to -2.9)
Ghana	5195.7 (4292.8 to 6121.0)	4731.8 (4087.4 to 5475.8)	4733.8 (4012.3 to 5595.9)	3390.9 (2836.4 to 4023.9)	-2.2 (-2.9 to -1.6)	-28.4 (-35.0 to -21.5)
Guinea	6297.1 (5252.4 to 7483.9)	5924.8 (5014.1 to 6920.9)	5973.1 (4910.1 to 7157.0)	4864.2 (3732.8 to 6180.8)	-1.3 (-2.5 to -0.2)	-17.9 (-30.9 to -3.3)
Guinea-Bissau	6079.8 (5009.8 to 7315.1)	5558.3 (4680.0 to 6503.0)	5585.5 (4581.1 to 6725.5)	4440.2 (3579.0 to 5433.9)	-1.5 (-2.2 to -0.8)	-20.2 (-28.2 to -10.8)
Liberia	6273.7 (5161.8 to 7522.8)	5772.7 (4906.1 to 6814.6)	5757.6 (4815.4 to 6920.8)	4819.9 (3935.1 to 5941.6)	-1.2 (-2.0 to -0.4)	-16.6 (-26.0 to -6.2)
Mali	6032.4 (5030.0 to 7200.2)	5701.4 (4719.2 to 6867.1)	5723.8 (4645.2 to 7088.2)	4214.7 (3340.2 to 5248.5)	-2.0 (-2.7 to -1.3)	-26.1 (-33.3 to -17.8)
Mauritania	4788.3 (3943.6 to 5681.2)	4503.8 (3830.0 to 5225.5)	4477.1 (3738.4 to 5374.0)	3277.4 (2624.3 to 4112.2)	-2.1 (-3.1 to -1.1)	-27.3 (-37.0 to -15.7)
Niger	5985.0 (4891.2 to 7201.6)	5417.9 (4500.4 to 6501.8)	5424.4 (4485.1 to 6617.4)	4134.6 (3354.6 to 5198.7)	-1.8 (-2.5 to -1.1)	-23.7 (-31.5 to -14.9)
Nigeria	4942.8 (4218.2 to 5767.5)	4595.1 (3945.6 to 5317.8)	4575.0 (3866.4 to 5442.8)	3710.4 (3075.9 to 4449.1)	-1.4 (-2.1 to -0.7)	-19.3 (-27.5 to -9.6)
Sao Tome and Principe	7429.7 (6215.2 to 8895.9)	6887.3 (5877.5 to 8122.6)	6887.4 (5789.2 to 8237.5)	5986.6 (4591.0 to 7812.4)	-1.0 (-2.3 to 0.4)	-13.1 (-29.5 to 7.0)
Senegal	4865.0 (3989.1 to 5750.5)	4371.7 (3704.0 to 5144.1)	4379.5 (3597.1 to 5265.9)	3571.3 (2878.1 to 4438.3)	-1.4 (-2.2 to -0.5)	-18.4 (-28.1 to -7.5)
Sierra Leone	6598.4 (5428.1 to 7800.9)	5973.1 (5057.5 to 7071.9)	5994.1 (4939.2 to 7231.5)	5286.9 (4030.5 to 6975.4)	-0.9 (-2.1 to 0.4)	-11.6 (-26.6 to 6.7)
Togo	5062.0 (4163.2 to 6100.5)	4698.3 (3992.9 to 5548.0)	4688.5 (3917.3 to 5644.9)	3854.3 (3039.5 to 4881.1)	-1.3 (-2.3 to -0.2)	-18.0 (-29.3 to -3.3)
**Indicator 3.3.5: Age-standardised prevalence of the sum of 15 neglected tropical diseases (NTDs) (%)**						
Central Europe, Eastern Europe, and Central Asia						
Central Asia						
Armenia	7.8 (6.0 to 10.3)	4.4 (3.5 to 5.7)	4.3 (3.4 to 5.5)	3.3 (2.2 to 5.0)	-2.1 (-3.9 to -0.1)	-25.9 (-44.1 to -1.4)
Azerbaijan	9.2 (7.2 to 11.5)	4.7 (3.7 to 6.0)	4.5 (3.5 to 5.7)	3.3 (2.0 to 5.2)	-2.5 (-4.5 to -0.3)	-30.0 (-49.2 to -3.9)
Georgia	6.8 (5.2 to 9.0)	4.6 (3.5 to 5.9)	4.3 (3.2 to 5.6)	3.3 (1.9 to 5.1)	-2.3 (-4.4 to -0.2)	-28.5 (-48.6 to -2.5)
Kazakhstan	6.1 (4.9 to 7.5)	4.3 (3.4 to 5.3)	4.2 (3.2 to 5.2)	3.5 (2.3 to 5.3)	-1.6 (-3.2 to 0.4)	-20.2 (-38.5 to 5.8)
Kyrgyzstan	10.6 (8.1 to 13.7)	7.5 (5.9 to 9.6)	7.1 (5.5 to 9.1)	5.8 (3.7 to 9.0)	-1.8 (-3.9 to 0.2)	-22.9 (-44.2 to 3.6)
Mongolia	12.3 (9.5 to 15.3)	8.6 (6.9 to 10.6)	8.2 (6.5 to 10.1)	7.0 (4.6 to 10.1)	-1.4 (-3.2 to 0.4)	-18.5 (-37.9 to 5.7)
Tajikistan	25.2 (19.1 to 32.5)	14.2 (10.7 to 18.8)	13.6 (10.0 to 18.0)	9.6 (5.8 to 15.2)	-2.8 (-4.8 to -0.6)	-32.9 (-51.4 to -9.1)
Turkmenistan	10.6 (8.2 to 13.4)	5.6 (4.4 to 7.0)	5.1 (4.0 to 6.4)	3.5 (2.2 to 5.2)	-3.3 (-5.3 to -1.1)	-38.7 (-54.7 to -15.3)
Uzbekistan	11.3 (8.4 to 14.9)	6.6 (5.0 to 8.4)	6.2 (4.7 to 7.9)	4.4 (2.7 to 6.8)	-2.7 (-4.8 to -0.7)	-32.8 (-51.1 to -9.8)
Central Europe						
Albania	0.0 (0.0 to 0.0)	0.0 (0.0 to 0.0)	0.0 (0.0 to 0.0)	0.0 (0.0 to 0.0)	-0.1 (-0.7 to 1.2)	-1.2 (-10.6 to 20.2)
Bosnia and Herzegovina	0.1 (0.1 to 0.1)	0.1 (0.1 to 0.1)	0.1 (0.1 to 0.1)	0.1 (0.1 to 0.1)	-0.6 (-1.1 to -0.1)	-8.3 (-15.5 to -0.9)
Bulgaria	0.0 (0.0 to 0.0)	0.0 (0.0 to 0.0)	0.0 (0.0 to 0.0)	0.0 (0.0 to 0.0)	-0.0 (-0.3 to 0.2)	-0.6 (-4.3 to 3.4)
Croatia	0.1 (0.1 to 0.1)	0.1 (0.1 to 0.1)	0.1 (0.1 to 0.1)	0.1 (0.1 to 0.1)	-0.7 (-1.3 to -0.1)	-9.9 (-17.9 to -1.7)
Czech Republic	0.1 (0.1 to 0.1)	0.1 (0.1 to 0.1)	0.1 (0.1 to 0.1)	0.1 (0.1 to 0.1)	-0.0 (-0.5 to 0.4)	-0.6 (-6.8 to 5.6)
Hungary	0.1 (0.1 to 0.1)	0.1 (0.1 to 0.1)	0.1 (0.1 to 0.1)	0.1 (0.1 to 0.1)	-0.7 (-1.2 to -0.1)	-9.3 (-16.4 to -2.0)
Macedonia	0.1 (0.1 to 0.1)	0.1 (0.1 to 0.1)	0.1 (0.1 to 0.1)	0.1 (0.0 to 0.1)	-0.1 (-0.6 to 0.4)	-1.1 (-8.3 to 5.9)
Montenegro	0.1 (0.1 to 0.1)	0.1 (0.1 to 0.1)	0.1 (0.1 to 0.1)	0.1 (0.1 to 0.1)	0.7 (0.1 to 1.4)	11.1 (2.2 to 22.8)
Poland	0.1 (0.1 to 0.1)	0.1 (0.1 to 0.1)	0.1 (0.1 to 0.1)	0.1 (0.1 to 0.1)	-0.6 (-1.1 to -0.0)	-8.5 (-15.4 to -0.6)
Romania	0.1 (0.1 to 0.1)	0.1 (0.1 to 0.1)	0.1 (0.1 to 0.1)	0.1 (0.1 to 0.1)	-0.2 (-0.7 to 0.3)	-2.8 (-9.3 to 3.9)
Serbia	0.1 (0.1 to 0.1)	0.1 (0.1 to 0.1)	0.1 (0.1 to 0.1)	0.1 (0.1 to 0.1)	-0.3 (-0.8 to 0.3)	-3.9 (-11.3 to 4.8)
Slovakia	0.1 (0.1 to 0.1)	0.1 (0.1 to 0.1)	0.1 (0.1 to 0.1)	0.1 (0.1 to 0.1)	-0.9 (-1.4 to -0.5)	-12.8 (-18.8 to -6.5)
Slovenia	0.1 (0.1 to 0.1)	0.1 (0.1 to 0.1)	0.1 (0.1 to 0.1)	0.1 (0.1 to 0.1)	-0.0 (-0.6 to 0.6)	-0.1 (-8.1 to 8.7)
Eastern Europe						

	Estimate in 2000 (95% UIs)	Estimate in 2015 (95% UIs)	Estimate in 2017 (95% UIs)	Estimate in 2030 (95% UIs)	Annualised rate of change, 2015-2030 (95% UIs)	Percent change, 2015-2030 (95% UIs)
Belarus	0.1 (0.1 to 0.1)	0.1 (0.1 to 0.1)	0.1 (0.1 to 0.1)	0.1 (0.1 to 0.1)	-1.2 (-1.6 to -0.8)	-16.4 (-21.0 to -11.6)
Estonia	0.1 (0.1 to 0.1)	0.1 (0.1 to 0.1)	0.1 (0.1 to 0.1)	0.1 (0.0 to 0.1)	-1.5 (-2.0 to -1.1)	-20.4 (-25.7 to -14.7)
Latvia	0.1 (0.1 to 0.1)	0.1 (0.1 to 0.1)	0.1 (0.1 to 0.1)	0.1 (0.0 to 0.1)	-1.6 (-2.1 to -1.1)	-21.7 (-27.1 to -15.8)
Lithuania	0.1 (0.1 to 0.1)	0.1 (0.1 to 0.1)	0.1 (0.1 to 0.1)	0.1 (0.0 to 0.1)	-1.3 (-1.8 to -0.8)	-17.9 (-23.9 to -11.2)
Moldova	0.1 (0.1 to 0.1)	0.1 (0.1 to 0.1)	0.1 (0.1 to 0.1)	0.1 (0.1 to 0.1)	-1.1 (-1.4 to -0.8)	-15.3 (-19.3 to -11.4)
Russia	4.8 (4.3 to 5.3)	4.7 (4.3 to 5.2)	4.7 (4.3 to 5.2)	4.7 (4.2 to 5.2)	-0.0 (-0.1 to 0.0)	-0.5 (-1.1 to 0.1)
Ukraine	0.6 (0.5 to 0.6)	0.6 (0.5 to 0.6)	0.6 (0.5 to 0.6)	0.6 (0.5 to 0.6)	-0.1 (-0.2 to -0.0)	-1.4 (-2.6 to -0.3)
High-income						
Australasia						
Australia	0.0 (0.0 to 0.0)	0.0 (0.0 to 0.0)	0.0 (0.0 to 0.0)	0.0 (0.0 to 0.1)	0.3 (-2.4 to 4.2)	7.5 (-30.0 to 87.4)
New Zealand	0.1 (0.0 to 0.1)	0.0 (0.0 to 0.1)	0.0 (0.0 to 0.1)	0.0 (0.0 to 0.1)	-1.1 (-1.9 to -0.3)	-14.7 (-24.8 to -4.9)
High-income Asia Pacific						
Brunei	0.1 (0.1 to 0.2)	0.4 (0.3 to 0.5)	0.3 (0.2 to 0.4)	0.7 (0.3 to 2.1)	2.5 (-0.4 to 10.8)	60.2 (-6.5 to 408.0)
Japan	0.1 (0.1 to 0.1)	0.1 (0.1 to 0.1)	0.1 (0.1 to 0.1)	0.1 (0.1 to 0.1)	0.0 (-0.1 to 0.1)	0.2 (-1.0 to 1.3)
South Korea	6.2 (5.4 to 7.2)	4.8 (4.2 to 5.5)	4.7 (4.1 to 5.5)	4.4 (3.6 to 5.6)	-0.6 (-1.4 to 0.5)	-7.8 (-19.2 to 7.4)
Singapore	0.0 (0.0 to 0.1)	0.1 (0.1 to 0.2)	0.1 (0.1 to 0.2)	0.5 (0.2 to 1.5)	7.6 (5.5 to 15.5)	241.7 (126.5 to 917.4)
High-income North America						
Canada	0.0 (0.0 to 0.0)	0.0 (0.0 to 0.0)	0.0 (0.0 to 0.0)	0.0 (0.0 to 0.0)	-0.5 (-0.8 to -0.2)	-6.9 (-11.7 to -2.4)
Greenland	0.0 (0.0 to 0.0)	0.0 (0.0 to 0.0)	0.0 (0.0 to 0.0)	0.0 (0.0 to 0.0)	0.1 (-0.2 to 0.4)	1.0 (-2.5 to 5.9)
United States	0.1 (0.1 to 0.1)	0.1 (0.1 to 0.1)	0.1 (0.1 to 0.1)	0.1 (0.1 to 0.1)	0.6 (0.3 to 0.7)	8.8 (4.9 to 11.7)
Southern Latin America						
Argentina	9.5 (7.9 to 11.2)	6.5 (5.5 to 7.6)	6.0 (5.0 to 7.2)	4.6 (3.3 to 6.4)	-2.4 (-3.8 to -0.7)	-29.7 (-43.6 to -10.0)
Chile	7.6 (6.3 to 9.2)	5.1 (4.3 to 6.2)	4.9 (4.0 to 5.9)	3.8 (2.8 to 5.4)	-2.0 (-3.3 to -0.4)	-25.5 (-39.5 to -6.2)
Uruguay	1.2 (1.0 to 1.4)	0.7 (0.6 to 0.9)	0.7 (0.6 to 0.8)	0.5 (0.4 to 0.6)	-2.4 (-2.5 to -2.2)	-29.8 (-31.6 to -28.1)
Western Europe						
Andorra	0.0 (0.0 to 0.0)	0.0 (0.0 to 0.0)	0.0 (0.0 to 0.0)	0.0 (0.0 to 0.1)	1.5 (-0.8 to 3.9)	27.3 (-11.1 to 80.1)
Austria	0.0 (0.0 to 0.0)	0.0 (0.0 to 0.0)	0.0 (0.0 to 0.0)	0.0 (0.0 to 0.0)	-0.6 (-1.1 to -0.1)	-9.2 (-15.8 to -1.6)
Belgium	0.0 (0.0 to 0.0)	0.0 (0.0 to 0.0)	0.0 (0.0 to 0.0)	0.0 (0.0 to 0.0)	0.7 (0.5 to 1.0)	11.2 (7.0 to 16.1)
Cyprus	0.0 (0.0 to 0.0)	0.0 (0.0 to 0.0)	0.0 (0.0 to 0.0)	0.0 (0.0 to 0.0)	0.3 (0.0 to 0.6)	6.2 (0.7 to 8.9)
Denmark	0.0 (0.0 to 0.0)	0.0 (0.0 to 0.0)	0.0 (0.0 to 0.0)	0.0 (0.0 to 0.0)	1.0 (0.1 to 2.0)	16.5 (1.4 to 34.7)
Finland	0.0 (0.0 to 0.0)	0.0 (0.0 to 0.0)	0.0 (0.0 to 0.0)	0.0 (0.0 to 0.0)	-0.4 (-1.3 to 0.5)	-6.1 (-17.4 to 7.1)
France	0.0 (0.0 to 0.0)	0.0 (0.0 to 0.0)	0.0 (0.0 to 0.0)	0.0 (0.0 to 0.0)	-0.5 (-0.8 to -0.1)	-7.2 (-11.8 to -2.1)
Germany	0.0 (0.0 to 0.0)	0.0 (0.0 to 0.0)	0.0 (0.0 to 0.0)	0.0 (0.0 to 0.0)	-0.4 (-0.8 to -0.0)	-5.8 (-11.7 to -0.1)
Greece	0.0 (0.0 to 0.0)	0.0 (0.0 to 0.0)	0.0 (0.0 to 0.0)	0.0 (0.0 to 0.0)	3.5 (1.5 to 5.9)	71.4 (25.6 to 143.5)
Iceland	0.0 (0.0 to 0.0)	0.0 (0.0 to 0.0)	0.0 (0.0 to 0.0)	0.0 (0.0 to 0.0)	2.9 (0.2 to 6.4)	57.7 (2.4 to 160.9)
Ireland	0.0 (0.0 to 0.0)	0.0 (0.0 to 0.0)	0.0 (0.0 to 0.0)	0.0 (0.0 to 0.0)	6.3 (5.8 to 6.8)	158.2 (140.0 to 176.0)
Israel	0.0 (0.0 to 0.0)	0.0 (0.0 to 0.0)	0.0 (0.0 to 0.0)	0.0 (0.0 to 0.0)	-3.0 (-3.9 to -1.6)	-36.1 (-44.1 to -21.0)
Italy	0.1 (0.1 to 0.1)	0.1 (0.1 to 0.1)	0.1 (0.1 to 0.1)	0.1 (0.1 to 0.1)	0.5 (0.0 to 0.9)	7.4 (0.7 to 14.3)
Luxembourg	0.0 (0.0 to 0.0)	0.0 (0.0 to 0.0)	0.0 (0.0 to 0.0)	0.0 (0.0 to 0.0)	2.7 (1.1 to 4.5)	51.4 (17.5 to 97.6)
Malta	0.0 (0.0 to 0.0)	0.0 (0.0 to 0.0)	0.0 (0.0 to 0.0)	0.0 (0.0 to 0.0)	-5.3 (-14.8 to 3.8)	-39.5 (-89.2 to 77.4)
Netherlands	0.0 (0.0 to 0.0)	0.0 (0.0 to 0.0)	0.0 (0.0 to 0.0)	0.0 (0.0 to 0.0)	1.1 (0.6 to 1.6)	17.7 (9.4 to 26.4)
Norway	0.1 (0.1 to 0.1)	0.1 (0.1 to 0.1)	0.1 (0.1 to 0.1)	0.1 (0.1 to 0.1)	-0.9 (-1.4 to -0.5)	-12.6 (-18.5 to -7.0)
Portugal	2.6 (2.1 to 3.0)	2.3 (1.9 to 2.8)	2.3 (1.9 to 2.8)	2.3 (1.9 to 2.8)	-0.1 (-0.4 to 0.2)	-1.5 (-6.4 to 3.7)
Spain	0.8 (0.7 to 1.0)	0.9 (0.7 to 1.0)	0.8 (0.7 to 1.0)	0.9 (0.7 to 1.0)	0.0 (-0.3 to 0.3)	0.4 (-4.1 to 4.7)
Sweden	0.0 (0.0 to 0.0)	0.0 (0.0 to 0.0)	0.0 (0.0 to 0.0)	0.0 (0.0 to 0.0)	-0.6 (-1.2 to -0.0)	-8.3 (-16.0 to -0.4)
Switzerland	0.0 (0.0 to 0.0)	0.0 (0.0 to 0.0)	0.0 (0.0 to 0.0)	0.0 (0.0 to 0.0)	0.8 (0.4 to 1.1)	12.2 (5.9 to 18.8)
United Kingdom	0.1 (0.1 to 0.1)	0.1 (0.1 to 0.1)	0.1 (0.1 to 0.1)	0.1 (0.0 to 0.1)	-2.0 (-2.6 to -1.5)	-26.2 (-32.6 to -20.6)
Latin America and Caribbean						
Andean Latin America						
Bolivia	24.1 (21.6 to 27.3)	15.6 (13.8 to 17.7)	14.7 (12.9 to 16.8)	11.1 (9.2 to 13.6)	-2.3 (-3.0 to -1.5)	-29.0 (-35.8 to -19.9)
Ecuador	33.3 (27.8 to 39.1)	15.3 (11.6 to 19.5)	14.1 (10.5 to 18.3)	9.3 (5.9 to 14.2)	-3.5 (-5.2 to -1.5)	-39.8 (-54.1 to -20.3)
Peru	18.6 (14.9 to 22.9)	9.7 (8.2 to 11.5)	9.2 (7.8 to 11.0)	7.6 (5.9 to 10.0)	-1.6 (-2.7 to -0.5)	-21.6 (-33.8 to -6.5)
Caribbean						
Antigua and Barbuda	6.5 (5.0 to 8.6)	5.1 (4.1 to 6.5)	5.0 (4.0 to 6.4)	4.4 (3.0 to 6.8)	-1.0 (-2.7 to 1.0)	-13.5 (-32.8 to 15.9)
The Bahamas	5.1 (3.7 to 6.8)	3.8 (3.0 to 4.9)	3.8 (3.0 to 4.8)	3.7 (2.4 to 5.6)	-0.3 (-2.1 to 1.9)	-3.2 (-27.0 to 33.8)
Barbados	5.2 (3.9 to 6.6)	4.0 (3.2 to 5.1)	3.9 (3.1 to 5.0)	3.9 (2.6 to 5.7)	-0.3 (-2.1 to 2.1)	-3.4 (-26.8 to 38.0)
Belize	11.8 (9.0 to 15.3)	8.0 (6.3 to 10.3)	7.8 (5.9 to 10.2)	6.0 (3.8 to 9.1)	-2.0 (-4.0 to -0.1)	-25.3 (-45.1 to -1.1)
Bermuda	4.1 (3.1 to 5.4)	3.0 (2.3 to 3.8)	3.0 (2.2 to 3.8)	2.6 (1.6 to 4.1)	-1.1 (-3.2 to 0.9)	-14.4 (-37.7 to 14.2)
Cuba	9.1 (6.7 to 12.2)	5.2 (4.0 to 6.5)	4.9 (3.7 to 6.3)	3.7 (2.3 to 5.6)	-2.4 (-4.3 to -0.3)	-29.2 (-47.5 to -3.7)
Dominica	6.7 (5.0 to 8.8)	5.0 (3.9 to 6.4)	4.8 (3.7 to 6.3)	4.2 (2.6 to 6.4)	-1.2 (-3.2 to 1.0)	-15.9 (-38.3 to 15.4)
Dominican Republic	18.7 (14.6 to 23.6)	9.9 (7.5 to 13.2)	9.4 (7.2 to 12.5)	6.2 (4.0 to 9.3)	-3.3 (-5.1 to -1.2)	-37.9 (-53.4 to -15.9)
Grenada	8.4 (6.5 to 10.8)	6.2 (4.7 to 7.8)	5.9 (4.4 to 7.7)	4.6 (2.8 to 7.1)	-2.1 (-4.2 to -0.1)	-26.1 (-47.0 to -0.9)
Guyana	32.1 (28.1 to 36.7)	19.6 (16.6 to 23.1)	18.2 (15.3 to 21.9)	12.4 (9.0 to 17.6)	-3.1 (-4.5 to -1.3)	-37.0 (-48.8 to -17.4)
Haiti	52.3 (45.4 to 60.0)	33.4 (28.3 to 39.3)	28.6 (23.6 to 34.5)	20.4 (13.9 to 29.1)	-3.4 (-5.1 to -1.7)	-39.2 (-53.5 to -22.4)
Jamaica	8.0 (6.1 to 10.5)	5.0 (4.0 to 6.4)	4.9 (3.9 to 6.3)	4.4 (2.9 to 6.7)	-1.0 (-2.8 to 0.8)	-13.4 (-34.0 to 12.8)
Puerto Rico	0.5 (0.3 to 0.6)	0.5 (0.4 to 0.7)	0.5 (0.4 to 0.7)	0.7 (0.5 to 1.6)	1.9 (0.6 to 7.5)	38.4 (9.5 to 205.8)
Saint Lucia	7.9 (6.0 to 10.0)	6.5 (5.1 to 8.3)	6.2 (4.8 to 8.1)	5.1 (3.2 to 7.9)	-1.7 (-3.7 to 0.4)	-21.6 (-42.6 to 5.5)
Saint Vincent and the Grenadines	8.8 (6.9 to 11.4)	6.7 (5.2 to 8.7)	6.5 (4.9 to 8.5)	4.9 (3.2 to 7.7)	-2.2 (-4.1 to -0.1)	-27.2 (-45.7 to -1.9)
Suriname	10.6 (8.2 to 13.5)	7.0 (5.4 to 8.8)	6.7 (5.1 to 8.5)	5.0 (3.2 to 7.8)	-2.3 (-4.1 to -0.3)	-28.6 (-45.7 to -4.5)
Trinidad and Tobago	6.4 (5.0 to 8.2)	4.9 (3.8 to 6.4)	4.9 (3.7 to 6.4)	4.7 (3.0 to 7.3)	-0.5 (-2.5 to 1.8)	-5.4 (-30.8 to 30.4)
Virgin Islands, U.S.	0.1 (0.0 to 0.1)	0.1 (0.1 to 0.2)	0.1 (0.1 to 0.2)	0.3 (0.1 to 0.9)	3.9 (1.6 to 12.5)	99.7 (27.6 to 549.3)
Central Latin America						
Colombia	39.3 (30.5 to 47.7)	17.1 (12.7 to 22.4)	15.5 (11.2 to 20.6)	8.8 (5.0 to 14.9)	-4.6 (-7.0 to -2.2)	-49.3 (-64.8 to -27.9)
Costa Rica	34.3 (26.9 to 42.9)	14.4 (10.6 to 18.9)	13.4 (9.6 to 17.8)	8.4 (4.9 to 14.2)	-3.8 (-6.0 to -1.4)	-42.1 (-59.3 to -18.9)
El Salvador	31.7 (24.9 to 39.6)	11.7 (9.3 to 14.6)	11.1 (8.7 to 13.9)	7.5 (5.3 to 10.8)	-3.0 (-4.6 to -1.1)	-35.9 (-49.9 to -15.7)
Guatemala	48.2 (39.9 to 57.8)	19.0 (15.0 to 24.1)	17.9 (13.8 to 22.9)	10.6 (6.7 to 16.2)	-4.0 (-5.9 to -1.9)	-44.6 (-58.8 to -24.7)
Honduras	47.5 (38.9 to 56.5)	25.9 (20.7 to 32.1)	24.7 (19.0 to 31.0)	17.8 (10.6 to 27.8)	-2.7 (-4.8 to -0.4)	-32.2 (-51.4 to -6.2)
Mexico	30.9 (24.2 to 39.1)	17.0 (12.9 to 22.3)	16.3 (12.2 to 21.5)	13.1 (7.6 to 21.6)	-1.9 (-4.4 to 0.5)	-23.8 (-48.3 to 7.5)
Nicaragua	32.6 (25.6 to 40.0)	14.9 (11.8 to 18.6)	14.2 (11.1 to 18.1)	11.3 (7.0 to 18.1)	-2.0 (-4.2 to 0.4)	-24.7 (-46.6 to 6.9)
Panama	22.6 (17.8 to 28.4)	10.1 (7.9 to 13.0)	9.6 (7.3 to 12.7)	7.4 (4.4 to 12.6)	-2.3 (-4.6 to 0.5)	-27.3 (-49.6 to 7.8)

	Estimate in 2000 (95% UIs)	Estimate in 2015 (95% UIs)	Estimate in 2017 (95% UIs)	Estimate in 2030 (95% UIs)	Annualised rate of change, 2015-2030 (95% UIs)	Percent change, 2015-2030 (95% UIs)
Venezuela	54.0 (45.8 to 62.8)	40.6 (32.9 to 49.3)	39.4 (31.3 to 48.7)	31.1 (20.6 to 43.3)	-1.9 (-3.6 to -0.4)	-23.9 (-41.6 to -5.9)
Tropical Latin America						
Brazil	13.9 (11.8 to 16.2)	10.4 (9.1 to 12.0)	10.1 (8.7 to 11.7)	8.5 (7.0 to 11.0)	-1.3 (-2.2 to -0.2)	-18.0 (-27.8 to -3.6)
Paraguay	13.4 (10.6 to 17.3)	8.7 (6.8 to 11.1)	8.2 (6.3 to 10.7)	5.7 (3.7 to 8.7)	-2.9 (-4.8 to -0.9)	-34.3 (-51.1 to -13.1)
North Africa and Middle East						
North Africa and Middle East						
Afghanistan	52.0 (44.4 to 61.2)	36.0 (28.9 to 43.6)	33.9 (26.7 to 41.5)	25.2 (18.1 to 34.9)	-2.4 (-3.6 to -1.1)	-30.2 (-42.0 to -15.7)
Algeria	6.0 (4.4 to 8.3)	3.7 (2.8 to 4.7)	3.5 (2.6 to 4.5)	2.7 (1.7 to 4.5)	-2.1 (-4.2 to 0.2)	-25.8 (-46.4 to 3.6)
Bahrain	3.7 (2.7 to 4.9)	2.9 (2.2 to 3.9)	2.9 (2.1 to 3.9)	2.8 (1.7 to 4.4)	-0.5 (-2.6 to 1.7)	-6.2 (-32.3 to 29.2)
Egypt	46.5 (42.6 to 51.1)	10.3 (8.6 to 12.7)	8.7 (7.2 to 10.8)	5.8 (4.2 to 8.4)	-3.9 (-5.4 to -2.2)	-43.9 (-55.5 to -28.0)
Iran	6.1 (4.5 to 8.3)	3.5 (2.6 to 4.5)	3.4 (2.6 to 4.5)	2.8 (1.8 to 4.3)	-1.5 (-3.4 to 0.4)	-19.2 (-40.3 to 5.6)
Iraq	10.6 (8.4 to 13.7)	8.2 (6.7 to 9.9)	8.0 (6.5 to 9.7)	7.0 (5.3 to 9.5)	-1.0 (-2.1 to 0.3)	-13.9 (-27.5 to 4.0)
Jordan	6.6 (4.7 to 9.2)	3.6 (2.7 to 4.8)	3.4 (2.5 to 4.5)	2.6 (1.6 to 4.0)	-2.3 (-4.3 to -0.1)	-28.7 (-47.5 to -2.1)
Kuwait	0.0 (0.0 to 0.0)	0.0 (0.0 to 0.0)	0.0 (0.0 to 0.0)	0.0 (0.0 to 0.0)	0.3 (-0.1 to 0.9)	4.6 (-2.1 to 13.9)
Lebanon	7.1 (4.9 to 9.8)	3.2 (2.4 to 4.2)	3.0 (2.2 to 4.0)	2.2 (1.3 to 3.5)	-2.8 (-4.9 to -0.3)	-33.2 (-52.3 to -5.0)
Libya	24.5 (22.0 to 27.4)	21.2 (19.0 to 23.8)	21.3 (18.9 to 23.9)	19.9 (17.2 to 22.6)	-0.4 (-0.8 to -0.0)	-6.3 (-11.2 to -0.6)
Morocco	12.7 (9.3 to 16.7)	7.6 (5.6 to 10.0)	7.1 (5.3 to 9.4)	5.2 (3.3 to 7.7)	-2.6 (-4.6 to -0.8)	-32.1 (-49.5 to -11.1)
Palestine	16.5 (11.4 to 22.5)	10.2 (7.3 to 13.6)	9.4 (6.7 to 12.7)	6.5 (4.0 to 10.0)	-3.1 (-5.1 to -1.2)	-36.6 (-53.2 to -16.0)
Oman	15.1 (10.8 to 20.7)	3.8 (3.0 to 4.7)	3.6 (2.8 to 4.5)	2.2 (1.6 to 3.5)	-3.5 (-5.3 to -1.3)	-40.0 (-54.8 to -17.4)
Qatar	3.0 (2.1 to 4.1)	2.3 (1.7 to 2.9)	2.2 (1.6 to 2.9)	2.1 (1.3 to 3.3)	-0.8 (-2.9 to 1.4)	-9.5 (-34.8 to 22.9)
Saudi Arabia	5.1 (3.9 to 6.6)	2.9 (2.4 to 3.7)	2.7 (2.1 to 3.4)	1.9 (1.3 to 2.9)	-2.9 (-4.6 to -1.0)	-34.4 (-49.6 to -14.2)
Sudan	17.3 (14.2 to 21.1)	11.6 (9.7 to 13.7)	10.4 (8.7 to 12.6)	8.2 (5.8 to 11.2)	-2.4 (-3.9 to -0.8)	-29.4 (-43.9 to -11.8)
Syria	11.3 (8.1 to 15.0)	7.7 (5.9 to 9.7)	7.4 (5.7 to 9.4)	11.3 (7.6 to 16.0)	2.5 (0.4 to 5.0)	48.6 (6.4 to 110.2)
Tunisia	8.1 (5.8 to 11.1)	4.3 (3.3 to 5.6)	4.1 (3.0 to 5.3)	3.1 (2.0 to 4.5)	-2.4 (-4.1 to -0.4)	-29.0 (-46.1 to -5.7)
Turkey	7.0 (4.9 to 9.7)	3.2 (2.4 to 4.2)	2.9 (2.1 to 3.8)	1.9 (1.1 to 3.0)	-3.6 (-6.0 to -1.1)	-40.8 (-59.3 to -15.7)
United Arab Emirates	2.6 (1.9 to 3.5)	1.9 (1.5 to 2.5)	2.0 (1.5 to 2.6)	1.9 (1.2 to 3.1)	-0.4 (-2.5 to 1.8)	-4.1 (-30.9 to 31.8)
Yemen	42.0 (36.5 to 47.8)	23.4 (20.2 to 27.1)	19.3 (16.1 to 22.9)	14.6 (10.2 to 20.6)	-3.3 (-4.9 to -1.4)	-38.1 (-52.0 to -19.1)
South Asia						
South Asia						
Bangladesh	56.5 (51.7 to 61.7)	48.7 (40.8 to 56.7)	47.7 (39.1 to 55.8)	44.9 (31.5 to 58.9)	-0.6 (-2.0 to 0.5)	-8.2 (-26.0 to 8.0)
Bhutan	36.5 (29.0 to 45.4)	15.2 (11.7 to 19.4)	13.9 (10.6 to 18.6)	7.1 (4.2 to 11.8)	-5.2 (-7.4 to -2.9)	-53.6 (-66.8 to -35.2)
India	39.7 (32.7 to 48.1)	18.1 (14.2 to 23.0)	15.4 (11.9 to 19.7)	7.5 (5.0 to 11.2)	-6.0 (-7.8 to -4.1)	-58.9 (-68.8 to -45.7)
Nepal	90.7 (82.7 to 98.2)	49.0 (40.4 to 57.8)	44.4 (35.9 to 53.1)	27.4 (17.8 to 38.4)	-4.0 (-5.7 to -2.3)	-44.4 (-57.6 to -29.7)
Pakistan	52.4 (43.3 to 61.9)	22.7 (17.6 to 28.9)	21.0 (15.8 to 27.5)	10.8 (6.6 to 17.1)	-5.1 (-7.1 to -3.0)	-53.0 (-65.3 to -36.4)
Southeast Asia, East Asia, and Oceania						
East Asia						
China	16.4 (13.8 to 20.0)	11.6 (9.7 to 13.9)	11.6 (9.6 to 14.1)	11.0 (8.3 to 14.7)	-0.4 (-1.6 to 0.9)	-5.6 (-21.1 to 14.0)
North Korea	16.1 (12.3 to 20.4)	13.5 (10.4 to 17.1)	13.4 (10.1 to 17.4)	13.8 (9.1 to 20.1)	0.1 (-1.6 to 1.7)	2.1 (-21.5 to 28.2)
Taiwan (Province of China)	6.5 (4.9 to 8.9)	4.9 (3.7 to 6.5)	4.9 (3.6 to 6.5)	5.3 (3.4 to 8.3)	0.5 (-1.4 to 2.4)	8.6 (-18.6 to 43.0)
Oceania						
American Samoa	8.9 (7.9 to 10.2)	2.3 (2.1 to 2.5)	2.3 (2.1 to 2.5)	1.5 (1.2 to 2.6)	-3.0 (-4.1 to 0.8)	-34.9 (-45.8 to 12.6)
Federated States of Micronesia	33.1 (28.3 to 38.9)	18.5 (14.9 to 22.7)	19.1 (15.4 to 23.5)	13.0 (8.8 to 19.0)	-2.5 (-3.9 to -0.8)	-30.4 (-44.2 to -11.0)
Fiji	40.1 (35.8 to 45.7)	13.3 (10.9 to 16.1)	10.4 (7.9 to 13.4)	10.0 (6.0 to 15.8)	-2.0 (-4.2 to 0.2)	-25.4 (-46.9 to 2.8)
Guam	5.6 (4.2 to 7.3)	5.2 (4.1 to 6.7)	5.3 (4.0 to 6.9)	5.5 (3.3 to 8.7)	0.2 (-2.0 to 2.3)	4.2 (-26.0 to 41.1)
Kiribati	25.7 (21.0 to 31.1)	25.4 (20.0 to 31.7)	25.4 (19.7 to 32.3)	28.4 (18.1 to 42.9)	0.6 (-1.3 to 2.3)	10.9 (-17.8 to 41.5)
Marshall Islands	21.1 (17.3 to 25.7)	14.6 (11.8 to 18.1)	15.1 (12.1 to 18.7)	12.8 (8.2 to 19.3)	-1.0 (-3.0 to 1.0)	-13.2 (-36.3 to 15.3)
Northern Mariana Islands	5.4 (4.2 to 6.9)	5.9 (4.6 to 7.6)	6.1 (4.6 to 8.0)	7.5 (4.5 to 11.5)	1.4 (-0.7 to 3.3)	24.9 (-10.2 to 65.1)
Papua New Guinea	61.6 (53.2 to 70.2)	45.8 (39.4 to 52.9)	44.5 (37.9 to 52.0)	36.7 (27.0 to 48.9)	-1.5 (-2.8 to -0.2)	-20.1 (-33.9 to -3.3)
Samoa	21.6 (17.5 to 26.4)	15.5 (12.1 to 19.5)	15.0 (11.2 to 19.4)	14.6 (8.7 to 23.1)	-0.5 (-2.7 to 1.7)	-6.4 (-32.8 to 28.3)
Solomon Islands	38.4 (32.5 to 44.5)	28.5 (23.5 to 34.0)	26.9 (21.7 to 32.5)	20.6 (13.3 to 29.6)	-2.3 (-4.2 to -0.5)	-28.1 (-46.7 to -7.2)
Tonga	16.0 (13.1 to 19.5)	8.7 (7.0 to 10.6)	8.3 (6.6 to 10.3)	6.8 (4.2 to 10.1)	-1.8 (-3.8 to 0.1)	-22.5 (-43.3 to 1.7)
Vanuatu	56.6 (49.5 to 64.1)	37.1 (30.4 to 44.8)	35.1 (27.7 to 43.2)	27.9 (17.8 to 40.9)	-2.0 (-3.7 to -0.3)	-25.3 (-43.0 to -4.3)
Southeast Asia						
Cambodia	43.1 (38.0 to 48.5)	21.1 (16.1 to 26.4)	19.6 (14.3 to 25.6)	14.8 (8.2 to 24.1)	-2.6 (-5.1 to -0.2)	-30.7 (-53.4 to -3.1)
Indonesia	37.5 (31.4 to 43.8)	25.5 (21.0 to 30.6)	24.1 (19.2 to 29.7)	21.7 (14.8 to 31.0)	-1.2 (-2.8 to 0.5)	-15.4 (-34.3 to 7.8)
Laos	33.3 (29.4 to 37.4)	21.0 (17.4 to 25.1)	20.3 (16.5 to 24.4)	16.4 (11.2 to 22.8)	-1.7 (-3.2 to -0.3)	-22.5 (-37.9 to -3.8)
Malaysia	20.7 (15.3 to 26.4)	9.5 (7.1 to 12.5)	9.4 (6.9 to 12.3)	8.3 (5.2 to 12.0)	-1.0 (-2.9 to 0.9)	-12.7 (-34.9 to 15.1)
Maldives	16.9 (12.9 to 21.6)	12.2 (9.4 to 15.8)	12.2 (9.1 to 15.8)	12.0 (7.8 to 17.6)	-0.2 (-2.1 to 1.5)	-2.1 (-27.0 to 25.4)
Mauritius	41.3 (34.1 to 49.4)	34.5 (28.6 to 41.0)	34.5 (28.5 to 41.1)	34.3 (27.6 to 41.8)	-0.1 (-0.6 to 0.6)	-0.9 (-9.1 to 9.6)
Myanmar	77.5 (70.2 to 84.7)	35.1 (28.5 to 42.2)	33.7 (26.7 to 41.4)	20.5 (13.0 to 31.3)	-3.7 (-5.6 to -1.9)	-42.2 (-56.8 to -24.7)
Philippines	70.3 (66.8 to 73.8)	36.8 (34.2 to 39.7)	28.0 (24.2 to 32.2)	17.2 (13.3 to 22.6)	-5.1 (-6.4 to -3.7)	-53.4 (-61.8 to -42.4)
Sri Lanka	22.8 (18.8 to 27.7)	11.2 (8.7 to 14.3)	10.7 (8.1 to 13.9)	8.6 (5.4 to 13.1)	-1.9 (-3.9 to -0.0)	-24.1 (-44.1 to -0.7)
Seychelles	14.5 (10.7 to 19.5)	11.2 (8.5 to 14.6)	11.3 (8.4 to 14.9)	12.1 (7.7 to 18.1)	0.4 (-1.3 to 2.1)	7.4 (-17.9 to 37.5)
Thailand	27.6 (21.2 to 34.9)	18.0 (14.8 to 22.4)	17.6 (14.4 to 22.0)	16.2 (11.8 to 21.9)	-0.8 (-2.1 to 0.6)	-10.2 (-27.5 to 9.6)
Timor-Leste	56.4 (49.0 to 65.2)	34.6 (29.8 to 40.2)	34.4 (29.4 to 40.2)	29.1 (21.7 to 38.5)	-1.2 (-2.5 to 0.1)	-16.2 (-31.0 to 1.4)
Vietnam	62.1 (55.6 to 68.5)	35.8 (29.6 to 43.2)	33.1 (27.3 to 40.3)	22.0 (16.6 to 28.9)	-3.3 (-4.4 to -2.2)	-38.7 (-48.3 to -27.9)
Sub-Saharan Africa						
Central Sub-Saharan Africa						
Angola	69.3 (60.5 to 78.2)	32.8 (28.5 to 37.4)	29.9 (25.9 to 34.4)	19.2 (15.4 to 24.0)	-3.6 (-4.6 to -2.5)	-41.5 (-49.7 to -31.5)
Central African Republic	69.7 (60.9 to 79.1)	47.4 (41.9 to 53.8)	46.2 (40.7 to 52.8)	34.9 (28.2 to 43.2)	-2.1 (-2.9 to -1.1)	-26.5 (-35.7 to -15.1)
Congo	61.0 (56.6 to 66.0)	38.3 (35.6 to 41.4)	30.9 (28.3 to 33.7)	18.5 (16.3 to 21.2)	-4.9 (-5.4 to -4.3)	-51.8 (-55.4 to -47.3)
Democratic Republic of the Congo	113.9 (102.3 to 126.2)	97.5 (88.5 to 109.7)	87.4 (77.5 to 101.4)	66.7 (56.1 to 82.0)	-2.5 (-3.2 to -1.8)	-31.7 (-38.2 to -23.8)
Equatorial Guinea	41.9 (36.2 to 48.7)	19.4 (16.4 to 23.5)	18.6 (15.5 to 22.9)	11.7 (9.3 to 15.6)	-3.4 (-4.1 to -2.6)	-39.8 (-45.7 to -32.0)
Gabon	29.5 (25.4 to 33.9)	22.2 (19.1 to 25.7)	21.5 (18.3 to 24.9)	18.4 (15.3 to 21.7)	-1.3 (-1.7 to -0.8)	-17.4 (-22.8 to -11.0)
Eastern Sub-Saharan Africa						
Burundi	60.9 (54.4 to 67.7)	47.1 (41.7 to 52.7)	40.1 (34.4 to 46.5)	27.0 (20.2 to 36.3)	-3.8 (-5.0 to -2.4)	-43.0 (-53.1 to -30.3)
Comoros	32.1 (28.3 to 36.5)	16.6 (14.4 to 19.2)	15.6 (13.3 to 18.1)	9.7 (7.3 to 12.8)	-3.6 (-4.8 to -2.3)	-41.7 (-51.3 to -29.0)
Djibouti	15.5 (12.0 to 19.8)	8.6 (6.5 to 10.9)	8.0 (6.0 to 10.3)	5.5 (3.4 to 8.7)	-3.1 (-5.0 to -1.0)	-36.6 (-52.5 to -14.2)
Eritrea	23.6 (20.4 to 27.3)	29.1 (24.8 to 34.0)	26.5 (22.2 to 31.2)	24.7 (18.6 to 31.7)	-1.1 (-2.3 to -0.0)	-15.1 (-28.8 to -0.5)
Ethiopia	77.7 (69.3 to 86.8)	58.5 (52.5 to 64.5)	58.0 (51.6 to 64.4)	52.5 (43.4 to 62.6)	-0.7 (-1.5 to 0.1)	-10.4 (-20.6 to 0.8)

	Estimate in 2000 (95% UIs)	Estimate in 2015 (95% UIs)	Estimate in 2017 (95% UIs)	Estimate in 2030 (95% UIs)	Annualised rate of change, 2015-2030 (95% UIs)	Percent change, 2015-2030 (95% UIs)
Kenya	40.5 (35.6 to 45.8)	28.4 (26.0 to 31.1)	27.9 (25.6 to 30.5)	24.3 (21.9 to 27.3)	-1.1 (-1.5 to -0.5)	-14.7 (-20.2 to -7.4)
Madagascar	62.4 (55.6 to 69.4)	52.7 (46.2 to 60.1)	49.3 (42.7 to 56.8)	42.0 (31.7 to 55.0)	-1.6 (-2.9 to -0.3)	-20.6 (-35.5 to -4.1)
Malawi	52.1 (48.5 to 56.3)	24.8 (22.2 to 27.9)	18.1 (15.8 to 21.0)	10.4 (7.5 to 14.4)	-5.9 (-7.4 to -4.1)	-58.2 (-67.0 to -45.9)
Mozambique	71.0 (61.8 to 80.9)	38.9 (33.2 to 45.3)	34.8 (29.2 to 41.1)	19.3 (14.1 to 26.0)	-4.7 (-6.0 to -3.4)	-50.5 (-59.6 to -40.1)
Rwanda	81.4 (74.2 to 88.8)	46.4 (41.0 to 52.5)	40.3 (34.5 to 46.9)	23.7 (17.3 to 32.5)	-4.5 (-5.9 to -2.9)	-49.0 (-59.0 to -35.6)
Somalia	65.4 (56.6 to 74.6)	40.8 (35.0 to 47.6)	39.6 (33.6 to 46.7)	29.5 (22.5 to 39.1)	-2.2 (-3.3 to -0.9)	-27.9 (-38.7 to -13.1)
South Sudan	68.0 (58.4 to 79.0)	61.6 (54.0 to 71.9)	59.4 (50.5 to 71.8)	53.3 (42.4 to 68.1)	-1.0 (-1.8 to -0.2)	-13.6 (-23.3 to -2.8)
Tanzania	120.4 (114.8 to 126.5)	62.5 (57.5 to 68.0)	52.1 (46.6 to 58.6)	28.5 (21.0 to 38.0)	-5.3 (-6.9 to -3.8)	-54.5 (-64.3 to -43.1)
Uganda	133.6 (129.7 to 137.5)	61.4 (58.3 to 64.7)	51.0 (47.3 to 54.8)	18.7 (15.5 to 22.4)	-8.0 (-9.0 to -7.0)	-69.7 (-73.9 to -64.8)
Zambia	63.9 (59.1 to 68.9)	37.8 (34.0 to 42.0)	33.9 (30.1 to 38.2)	22.1 (18.5 to 26.9)	-3.6 (-4.3 to -2.8)	-41.7 (-47.2 to -34.4)
Southern Sub-Saharan Africa						
Botswana	20.2 (16.7 to 24.6)	10.9 (9.4 to 12.6)	10.6 (9.1 to 12.5)	8.2 (6.6 to 10.3)	-1.9 (-2.8 to -0.9)	-25.0 (-34.6 to -12.2)
Lesotho	27.1 (22.0 to 33.2)	13.8 (11.1 to 17.1)	13.3 (10.4 to 16.5)	8.8 (5.8 to 12.7)	-3.1 (-4.8 to -1.4)	-36.5 (-51.4 to -19.5)
Namibia	26.8 (22.8 to 31.6)	25.1 (22.3 to 28.1)	23.2 (20.3 to 26.6)	19.6 (15.6 to 24.5)	-1.7 (-2.7 to -0.6)	-22.0 (-33.7 to -9.1)
South Africa	42.9 (38.9 to 47.4)	16.5 (14.8 to 18.5)	16.2 (14.5 to 18.2)	12.4 (10.9 to 14.4)	-1.9 (-2.6 to -1.2)	-25.0 (-31.9 to -16.2)
Swaziland	28.9 (23.4 to 35.1)	15.9 (12.9 to 19.3)	14.6 (11.6 to 18.1)	9.1 (6.2 to 12.9)	-3.8 (-5.4 to -2.2)	-42.9 (-55.3 to -28.0)
Zimbabwe	31.7 (28.3 to 35.4)	23.9 (21.0 to 27.5)	18.1 (15.4 to 21.3)	12.9 (9.2 to 18.4)	-4.2 (-5.9 to -2.2)	-46.4 (-58.6 to -28.1)
Western Sub-Saharan Africa						
Benin	92.9 (85.4 to 101.1)	59.5 (54.5 to 64.6)	50.3 (45.7 to 55.5)	29.2 (23.4 to 36.4)	-4.8 (-5.9 to -3.6)	-51.0 (-58.5 to -41.8)
Burkina Faso	108.7 (101.5 to 117.5)	34.2 (29.2 to 39.9)	28.9 (23.7 to 35.1)	24.3 (16.0 to 35.9)	-2.4 (-4.4 to -0.3)	-29.3 (-48.4 to -5.0)
Cameroon	118.7 (110.3 to 128.3)	44.4 (40.3 to 49.6)	37.0 (33.0 to 42.6)	15.9 (13.2 to 20.1)	-6.9 (-7.7 to -5.9)	-64.2 (-68.4 to -58.6)
Cape Verde	21.4 (17.2 to 26.1)	7.8 (6.2 to 9.6)	7.4 (5.8 to 9.3)	4.5 (3.0 to 6.9)	-3.8 (-5.6 to -1.7)	-42.4 (-56.6 to -22.3)
Chad	65.5 (57.3 to 74.4)	60.4 (52.9 to 69.4)	56.6 (48.8 to 65.5)	50.8 (39.3 to 63.9)	-1.2 (-2.3 to -0.2)	-16.0 (-28.8 to -2.8)
Cote d'Ivoire	58.1 (53.4 to 63.4)	44.8 (41.0 to 49.0)	38.9 (35.3 to 42.6)	26.5 (22.8 to 31.0)	-3.5 (-4.1 to -2.8)	-40.9 (-46.2 to -34.8)
The Gambia	66.8 (61.4 to 72.2)	52.6 (49.2 to 56.4)	48.4 (44.9 to 52.0)	43.2 (38.3 to 49.1)	-1.3 (-1.9 to -0.7)	-17.9 (-25.0 to -10.0)
Ghana	34.8 (30.8 to 38.8)	22.0 (19.6 to 24.7)	18.4 (15.7 to 21.5)	12.3 (8.8 to 17.0)	-3.9 (-5.5 to -2.3)	-44.2 (-56.5 to -28.8)
Guinea	74.8 (67.1 to 82.9)	47.3 (41.6 to 54.2)	43.1 (37.5 to 50.1)	29.2 (23.4 to 37.6)	-3.3 (-4.1 to -2.2)	-38.5 (-45.7 to -28.4)
Guinea-Bissau	94.8 (84.6 to 105.1)	58.9 (52.1 to 66.5)	56.2 (49.3 to 63.5)	42.7 (33.9 to 53.0)	-2.2 (-3.2 to -1.2)	-27.7 (-38.2 to -16.1)
Liberia	159.4 (147.7 to 172.8)	116.6 (105.5 to 133.6)	103.2 (90.9 to 121.8)	72.5 (59.5 to 90.2)	-3.2 (-4.0 to -2.4)	-38.0 (-45.0 to -30.2)
Mali	129.6 (119.5 to 140.9)	49.2 (42.9 to 57.4)	45.2 (38.4 to 53.2)	27.9 (18.0 to 40.0)	-3.9 (-6.0 to -2.1)	-43.6 (-59.4 to -27.0)
Mauritania	28.9 (23.9 to 34.1)	24.7 (20.5 to 29.0)	22.7 (18.4 to 27.0)	17.6 (12.5 to 23.3)	-2.3 (-3.7 to -1.0)	-28.9 (-42.2 to -13.8)
Niger	43.6 (40.1 to 47.2)	38.8 (33.5 to 44.4)	30.9 (25.9 to 36.3)	23.8 (17.3 to 31.4)	-3.3 (-4.7 to -1.9)	-38.9 (-50.3 to -25.2)
Nigeria	112.0 (106.7 to 117.5)	58.4 (55.1 to 61.7)	52.6 (48.1 to 57.2)	32.2 (26.8 to 39.0)	-4.0 (-4.9 to -2.9)	-44.9 (-52.0 to -35.1)
Sao Tome and Principe	53.6 (45.1 to 62.8)	16.9 (13.3 to 21.6)	18.7 (14.3 to 24.6)	18.9 (12.5 to 27.9)	0.7 (-0.8 to 2.1)	11.0 (-11.1 to 37.2)
Senegal	78.5 (70.6 to 87.5)	60.3 (54.5 to 66.7)	50.0 (44.1 to 56.5)	40.5 (31.3 to 51.2)	-2.7 (-4.0 to -1.5)	-33.0 (-45.1 to -19.7)
Sierra Leone	78.3 (71.0 to 85.6)	41.0 (35.9 to 46.4)	37.0 (31.1 to 43.9)	21.6 (15.2 to 29.4)	-4.4 (-5.9 to -2.9)	-47.7 (-58.9 to -34.8)
Togo	105.3 (98.4 to 112.4)	62.4 (58.5 to 66.6)	39.6 (35.0 to 45.1)	26.9 (19.2 to 36.5)	-5.7 (-7.6 to -3.9)	-57.1 (-67.9 to -43.9)
**Indicator 3.4.1: Age-standardised death rate due to cardiovascular disease, cancer, diabetes, and chronic respiratory disease in populations aged 30-70 (per 100,000 population)**						
Central Europe, Eastern Europe, and Central Asia						
Central Asia						
Armenia	538.1 (532.1 to 543.6)	424.8 (419.6 to 429.9)	420.3 (406.9 to 433.4)	369.8 (318.9 to 414.2)	-0.9 (-1.9 to -0.2)	-12.9 (-25.1 to -2.7)
Azerbaijan	695.4 (670.4 to 720.9)	594.1 (568.4 to 620.0)	587.5 (550.1 to 631.5)	502.1 (411.5 to 584.9)	-1.1 (-2.5 to -0.2)	-15.5 (-31.5 to -3.2)
Georgia	510.0 (490.9 to 529.3)	523.7 (517.5 to 530.3)	545.5 (529.8 to 561.7)	461.2 (394.8 to 521.6)	-0.9 (-1.9 to -0.0)	-11.9 (-24.4 to -0.4)
Kazakhstan	860.0 (854.3 to 865.4)	547.8 (538.5 to 560.6)	535.7 (511.8 to 561.6)	467.2 (345.9 to 577.0)	-1.1 (-3.1 to 0.3)	-14.7 (-36.8 to 5.4)
Kyrgyzstan	725.0 (695.1 to 758.8)	516.9 (510.9 to 523.9)	478.4 (464.6 to 493.5)	432.2 (309.1 to 557.3)	-1.3 (-3.5 to 0.5)	-16.4 (-40.6 to 7.7)
Mongolia	1031.6 (997.0 to 1064.5)	729.3 (684.8 to 771.0)	716.3 (660.2 to 774.2)	614.4 (474.7 to 760.9)	-1.2 (-2.8 to 0.2)	-15.8 (-34.3 to 3.0)
Tajikistan	540.5 (518.0 to 565.9)	535.4 (502.2 to 568.4)	539.3 (490.7 to 587.9)	455.8 (357.1 to 556.4)	-1.1 (-2.7 to 0.2)	-14.9 (-33.0 to 2.8)
Turkmenistan	748.1 (740.8 to 755.7)	659.2 (643.4 to 674.2)	653.4 (612.1 to 696.2)	518.5 (428.5 to 596.4)	-1.6 (-2.9 to -0.7)	-21.3 (-34.8 to -10.2)
Uzbekistan	706.0 (685.2 to 727.8)	659.3 (618.8 to 700.8)	636.1 (573.0 to 704.9)	505.2 (402.6 to 606.4)	-1.8 (-3.2 to -0.6)	-23.4 (-38.0 to -9.2)
Central Europe						
Albania	326.0 (310.1 to 341.1)	287.2 (240.5 to 337.2)	283.9 (236.7 to 338.3)	244.2 (195.3 to 300.2)	-1.1 (-2.0 to -0.2)	-15.0 (-25.8 to -2.9)
Bosnia and Herzegovina	448.3 (408.6 to 487.1)	392.0 (376.5 to 399.7)	383.5 (360.1 to 406.9)	334.5 (300.2 to 369.8)	-1.1 (-1.8 to -0.4)	-14.7 (-23.3 to -5.7)
Bulgaria	615.8 (610.8 to 620.5)	512.8 (507.3 to 518.1)	517.4 (489.4 to 544.9)	390.4 (334.6 to 460.4)	-1.8 (-2.9 to -0.7)	-23.9 (-35.0 to -10.3)
Croatia	455.8 (450.4 to 461.5)	344.7 (339.8 to 349.5)	313.4 (296.4 to 331.4)	252.5 (224.2 to 287.6)	-2.1 (-2.9 to -1.2)	-26.7 (-35.1 to -16.6)
Czech Republic	460.3 (456.4 to 463.9)	302.5 (298.8 to 306.3)	295.5 (278.9 to 312.9)	235.1 (210.7 to 261.6)	-1.7 (-2.4 to -1.0)	-22.3 (-30.5 to -13.5)
Hungary	627.2 (622.4 to 632.7)	498.8 (494.2 to 503.8)	445.4 (424.8 to 468.5)	353.0 (316.4 to 400.2)	-2.3 (-3.0 to -1.5)	-29.2 (-36.6 to -19.7)
Macedonia	515.7 (506.9 to 524.0)	419.7 (412.1 to 427.3)	417.4 (394.4 to 443.7)	351.3 (309.4 to 391.8)	-1.2 (-2.0 to -0.4)	-16.3 (-26.1 to -6.4)
Montenegro	531.0 (518.3 to 544.1)	422.4 (401.1 to 446.9)	418.0 (385.3 to 454.0)	345.0 (293.4 to 396.0)	-1.4 (-2.4 to -0.5)	-18.3 (-30.1 to -7.3)
Poland	513.8 (510.8 to 516.7)	378.2 (375.1 to 381.3)	365.4 (347.2 to 384.2)	268.7 (245.4 to 296.8)	-2.3 (-2.9 to -1.6)	-29.0 (-35.1 to -21.4)
Romania	562.2 (558.2 to 566.2)	445.0 (440.6 to 449.0)	448.0 (426.4 to 469.3)	324.9 (281.4 to 384.2)	-2.1 (-3.1 to -0.9)	-27.0 (-36.9 to -13.2)
Serbia	603.2 (596.2 to 608.3)	468.2 (462.6 to 473.4)	438.1 (415.6 to 459.7)	400.6 (336.9 to 461.8)	-1.1 (-2.2 to -0.1)	-14.4 (-27.9 to -0.9)
Slovakia	526.1 (520.1 to 532.8)	380.0 (372.1 to 395.6)	355.2 (335.3 to 377.4)	276.1 (244.0 to 315.8)	-2.1 (-3.0 to -1.3)	-27.3 (-36.0 to -17.2)
Slovenia	374.7 (368.9 to 381.3)	237.1 (232.2 to 242.1)	228.2 (213.6 to 244.2)	181.7 (162.2 to 200.8)	-1.8 (-2.5 to -1.1)	-23.4 (-31.6 to -14.9)
Eastern Europe						
Belarus	725.9 (718.1 to 731.5)	499.5 (491.4 to 506.4)	486.4 (462.2 to 512.0)	375.4 (296.2 to 467.8)	-1.9 (-3.5 to -0.5)	-24.8 (-40.7 to -6.6)
Estonia	576.4 (567.3 to 586.8)	338.1 (330.3 to 346.5)	325.1 (286.1 to 364.9)	264.9 (206.4 to 333.1)	-1.7 (-3.3 to -0.2)	-21.7 (-38.7 to -2.3)
Latvia	604.9 (597.0 to 613.3)	465.9 (457.4 to 474.2)	459.1 (413.1 to 508.8)	352.8 (265.6 to 476.5)	-1.9 (-3.7 to 0.1)	-24.3 (-43.0 to 2.1)
Lithuania	500.9 (494.4 to 506.7)	443.8 (437.3 to 450.5)	435.8 (410.9 to 461.5)	346.5 (267.3 to 465.9)	-1.7 (-3.4 to 0.3)	-21.9 (-40.2 to 4.9)
Moldova	621.1 (614.5 to 628.0)	546.2 (539.8 to 554.2)	499.0 (484.7 to 514.3)	423.8 (325.1 to 600.3)	-1.8 (-3.5 to 0.6)	-22.4 (-40.6 to 9.7)
Russia	839.3 (837.6 to 843.9)	566.0 (563.6 to 567.9)	531.9 (527.0 to 536.5)	428.7 (285.9 to 607.4)	-2.0 (-4.6 to 0.5)	-24.3 (-49.7 to 7.3)
Ukraine	755.4 (749.8 to 760.6)	694.8 (687.6 to 702.0)	627.5 (596.8 to 658.4)	561.9 (432.3 to 721.2)	-1.5 (-3.2 to 0.2)	-19.1 (-37.8 to 3.7)
High-income						
Australasia						
Australia	263.4 (261.2 to 265.4)	189.2 (187.3 to 191.5)	190.8 (171.9 to 211.4)	166.1 (147.2 to 186.9)	-0.9 (-1.7 to -0.1)	-12.2 (-22.0 to -1.3)
New Zealand	321.1 (317.4 to 325.1)	217.0 (213.9 to 220.2)	221.4 (211.5 to 233.0)	183.7 (163.7 to 207.2)	-1.1 (-1.9 to -0.3)	-15.4 (-24.8 to -4.7)
High-income Asia Pacific						
Brunei	499.9 (479.8 to 519.1)	435.8 (413.0 to 461.1)	428.8 (399.6 to 460.2)	372.3 (314.1 to 440.4)	-1.1 (-2.1 to -0.0)	-14.6 (-26.8 to -0.7)
Japan	222.6 (221.9 to 223.3)	162.5 (160.7 to 163.6)	160.6 (156.6 to 164.9)	133.7 (119.3 to 150.3)	-1.3 (-2.1 to -0.5)	-17.7 (-26.8 to -7.7)
South Korea	335.5 (333.4 to 337.8)	164.6 (158.6 to 170.5)	158.4 (148.0 to 169.1)	133.4 (111.3 to 158.0)	-1.4 (-2.6 to -0.3)	-18.9 (-32.3 to -4.1)
Singapore	313.8 (292.4 to 335.8)	168.8 (165.9 to 171.6)	161.6 (151.8 to 171.6)	128.9 (110.1 to 148.2)	-1.8 (-2.8 to -0.9)	-23.6 (-34.7 to -12.1)

	Estimate in 2000 (95% UIs)	Estimate in 2015 (95% UIs)	Estimate in 2017 (95% UIs)	Estimate in 2030 (95% UIs)	Annualised rate of change, 2015-2030 (95% UIs)	Percent change, 2015-2030 (95% UIs)
High-income North America						
Canada	286.4 (284.2 to 288.4)	213.5 (211.6 to 215.6)	208.0 (197.2 to 219.6)	180.6 (167.0 to 194.1)	-1.1 (-1.6 to -0.6)	-15.4 (-21.8 to -8.8)
Greenland	627.5 (610.1 to 646.8)	450.2 (436.0 to 465.6)	438.3 (417.5 to 456.9)	387.1 (306.9 to 494.0)	-1.1 (-2.6 to 0.6)	-14.0 (-31.9 to 9.2)
United States	369.3 (368.2 to 370.5)	281.3 (279.9 to 284.2)	287.9 (281.4 to 294.4)	253.9 (212.2 to 293.5)	-0.7 (-1.9 to 0.3)	-9.7 (-24.5 to 4.5)
Southern Latin America						
Argentina	432.7 (429.3 to 435.9)	333.1 (329.3 to 336.8)	333.4 (301.1 to 371.7)	293.8 (262.5 to 329.6)	-0.8 (-1.6 to -0.1)	-11.8 (-21.0 to -0.9)
Chile	288.0 (284.9 to 291.5)	235.8 (232.9 to 239.3)	235.8 (212.4 to 261.6)	201.5 (170.1 to 235.0)	-1.1 (-2.2 to -0.0)	-14.6 (-28.0 to -0.6)
Uruguay	445.4 (439.4 to 450.9)	349.9 (343.9 to 355.6)	349.4 (314.7 to 386.8)	311.5 (275.7 to 347.2)	-0.8 (-1.6 to -0.1)	-11.0 (-20.9 to -1.0)
Western Europe						
Andorra	241.0 (218.1 to 266.5)	203.1 (183.3 to 224.8)	200.9 (180.0 to 225.1)	179.1 (155.4 to 205.1)	-0.8 (-1.5 to -0.2)	-11.8 (-19.8 to -3.4)
Austria	299.4 (296.2 to 303.1)	216.9 (213.3 to 222.2)	208.9 (196.8 to 221.3)	178.5 (160.6 to 197.1)	-1.3 (-2.0 to -0.6)	-17.7 (-26.0 to -8.9)
Belgium	310.0 (306.9 to 313.0)	228.5 (225.5 to 231.8)	217.4 (205.5 to 229.8)	189.4 (174.6 to 206.9)	-1.3 (-1.8 to -0.7)	-17.1 (-23.8 to -9.5)
Cyprus	325.7 (308.6 to 343.9)	222.9 (208.4 to 239.3)	216.8 (198.6 to 236.6)	175.0 (154.5 to 196.7)	-1.6 (-2.2 to -1.0)	-21.5 (-28.4 to -14.4)
Denmark	364.8 (360.1 to 369.6)	227.4 (223.7 to 231.7)	231.1 (217.7 to 244.4)	189.8 (174.1 to 207.8)	-1.2 (-1.8 to -0.6)	-16.5 (-23.9 to -8.4)
Finland	290.8 (287.2 to 294.8)	199.3 (195.4 to 204.0)	202.4 (191.1 to 215.4)	169.7 (151.3 to 190.0)	-1.1 (-1.9 to -0.3)	-14.8 (-24.4 to -4.8)
France	284.1 (282.2 to 285.9)	216.0 (213.7 to 218.3)	208.3 (197.3 to 220.0)	178.0 (164.4 to 192.8)	-1.3 (-1.8 to -0.8)	-17.6 (-23.7 to -10.8)
Germany	316.4 (314.4 to 318.3)	244.8 (242.9 to 247.1)	245.9 (221.0 to 271.9)	201.6 (178.2 to 227.9)	-1.3 (-2.1 to -0.5)	-17.6 (-26.9 to -6.9)
Greece	283.1 (280.4 to 285.7)	260.8 (257.8 to 264.0)	260.1 (245.2 to 275.0)	212.2 (191.3 to 234.7)	-1.4 (-2.1 to -0.7)	-18.6 (-26.6 to -9.8)
Iceland	246.0 (240.3 to 251.9)	181.1 (176.0 to 186.4)	181.3 (174.0 to 188.7)	154.1 (138.4 to 174.1)	-1.1 (-1.8 to -0.3)	-14.9 (-23.8 to -3.7)
Ireland	352.3 (347.6 to 357.0)	205.7 (202.2 to 209.3)	199.8 (187.6 to 212.7)	164.3 (148.5 to 183.9)	-1.5 (-2.2 to -0.8)	-20.1 (-28.4 to -10.7)
Israel	287.9 (284.3 to 291.6)	180.3 (177.2 to 183.7)	176.1 (165.9 to 186.8)	145.4 (118.6 to 176.7)	-1.5 (-2.8 to -0.1)	-19.3 (-34.4 to -2.2)
Italy	270.0 (268.5 to 271.6)	192.5 (190.9 to 194.7)	179.9 (169.6 to 190.3)	148.4 (136.7 to 159.3)	-1.7 (-2.3 to -1.3)	-22.9 (-29.2 to -17.2)
Luxembourg	314.1 (308.0 to 320.8)	210.6 (197.9 to 223.3)	206.6 (188.8 to 225.4)	171.5 (141.2 to 202.9)	-1.4 (-2.6 to -0.4)	-18.6 (-31.8 to -5.2)
Malta	282.5 (276.1 to 288.7)	209.4 (203.0 to 215.7)	216.5 (206.4 to 226.8)	171.4 (140.1 to 208.5)	-1.4 (-2.6 to 0.0)	-18.1 (-32.2 to 0.3)
Netherlands	328.9 (326.0 to 331.7)	219.5 (217.1 to 221.7)	218.9 (207.5 to 232.0)	184.1 (169.9 to 199.9)	-1.2 (-1.7 to -0.6)	-16.1 (-22.7 to -8.6)
Norway	286.4 (284.3 to 288.5)	185.2 (183.1 to 187.9)	185.2 (181.3 to 189.2)	157.4 (146.2 to 168.2)	-1.1 (-1.6 to -0.7)	-15.1 (-21.1 to -9.5)
Portugal	314.3 (311.0 to 317.3)	213.9 (210.7 to 217.3)	219.1 (205.8 to 232.1)	171.1 (146.6 to 203.3)	-1.5 (-2.5 to -0.3)	-20.0 (-31.8 to -4.9)
Spain	270.2 (268.2 to 272.1)	202.9 (201.3 to 204.4)	195.2 (184.9 to 204.9)	156.4 (140.0 to 174.2)	-1.7 (-2.5 to -1.0)	-22.9 (-31.0 to -14.4)
Sweden	253.2 (250.6 to 256.0)	183.5 (181.2 to 185.9)	177.5 (168.5 to 186.4)	147.7 (135.8 to 160.0)	-1.5 (-2.0 to -0.9)	-19.5 (-26.1 to -12.6)
Switzerland	239.8 (237.0 to 242.5)	159.9 (157.4 to 162.6)	150.2 (141.5 to 159.2)	128.4 (114.6 to 142.8)	-1.5 (-2.2 to -0.8)	-19.7 (-28.6 to -10.7)
United Kingdom	325.8 (324.3 to 327.3)	225.1 (224.1 to 227.5)	229.3 (226.6 to 232.8)	188.2 (170.6 to 205.4)	-1.2 (-1.8 to -0.6)	-16.4 (-24.1 to -8.7)
Latin America and Caribbean						
Andean Latin America						
Bolivia	468.6 (424.8 to 518.1)	361.6 (298.1 to 427.4)	353.3 (286.5 to 420.5)	292.5 (231.5 to 355.4)	-1.4 (-2.1 to -0.7)	-19.1 (-27.5 to -10.3)
Ecuador	269.1 (266.0 to 272.4)	239.7 (228.7 to 251.0)	243.1 (221.8 to 266.1)	214.8 (189.7 to 243.3)	-0.7 (-1.6 to 0.1)	-10.3 (-21.2 to 2.2)
Peru	245.3 (228.2 to 261.4)	187.8 (171.2 to 203.1)	184.7 (158.9 to 211.3)	152.6 (116.2 to 190.1)	-1.4 (-3.1 to 0.0)	-18.7 (-37.3 to 0.5)
Caribbean						
Antigua and Barbuda	396.8 (381.4 to 412.2)	315.7 (301.3 to 330.4)	313.9 (295.6 to 332.4)	278.1 (230.9 to 326.3)	-0.9 (-2.0 to 0.2)	-11.9 (-26.2 to 3.3)
The Bahamas	477.2 (466.2 to 488.2)	435.7 (413.3 to 457.6)	432.7 (400.0 to 465.2)	393.0 (328.3 to 472.2)	-0.7 (-1.8 to 0.4)	-9.8 (-23.7 to 6.6)
Barbados	373.1 (363.0 to 384.0)	316.7 (298.3 to 333.5)	314.3 (289.7 to 338.8)	295.1 (258.4 to 329.5)	-0.5 (-1.3 to 0.2)	-6.8 (-17.1 to 2.5)
Belize	477.2 (466.0 to 488.8)	364.0 (355.3 to 373.0)	364.9 (352.6 to 378.6)	333.9 (279.4 to 399.0)	-0.6 (-1.7 to 0.6)	-8.3 (-23.0 to 10.2)
Bermuda	332.0 (320.5 to 344.3)	226.5 (215.3 to 237.6)	223.9 (209.5 to 237.9)	172.1 (129.3 to 223.5)	-1.9 (-3.6 to -0.2)	-24.0 (-41.8 to -3.1)
Cuba	359.8 (356.7 to 362.9)	327.7 (323.9 to 331.4)	323.9 (292.5 to 358.4)	280.3 (246.7 to 316.3)	-1.1 (-1.9 to -0.3)	-14.5 (-24.5 to -3.9)
Dominica	382.4 (369.4 to 394.8)	397.4 (378.0 to 415.5)	395.6 (369.9 to 420.6)	367.1 (312.6 to 431.5)	-0.5 (-1.5 to 0.5)	-7.7 (-20.7 to 7.4)
Dominican Republic	327.9 (300.5 to 354.5)	404.3 (369.2 to 438.7)	402.1 (349.0 to 452.0)	338.0 (274.9 to 411.2)	-1.2 (-2.4 to -0.1)	-16.4 (-30.3 to -1.1)
Grenada	441.1 (428.5 to 453.2)	462.6 (447.9 to 478.2)	462.4 (443.1 to 483.6)	422.0 (383.4 to 462.1)	-0.6 (-1.2 to -0.1)	-8.8 (-16.1 to -0.8)
Guyana	716.0 (705.1 to 727.4)	614.8 (579.3 to 653.0)	593.3 (532.0 to 657.2)	520.0 (439.6 to 602.1)	-1.1 (-2.1 to -0.3)	-15.4 (-27.4 to -4.4)
Haiti	811.1 (710.9 to 917.4)	688.2 (568.0 to 805.8)	676.2 (558.6 to 790.6)	592.6 (490.6 to 703.2)	-1.0 (-1.5 to -0.6)	-13.9 (-20.1 to -7.9)
Jamaica	424.7 (419.0 to 430.4)	418.4 (377.3 to 463.5)	422.7 (369.5 to 479.3)	407.1 (342.6 to 487.4)	-0.2 (-1.0 to 0.8)	-2.7 (-14.0 to 12.1)
Puerto Rico	312.4 (307.7 to 317.5)	218.5 (214.2 to 223.8)	232.5 (219.2 to 246.6)	193.7 (171.3 to 216.7)	-0.8 (-1.6 to -0.0)	-11.3 (-21.6 to -0.5)
Saint Lucia	426.2 (416.2 to 436.6)	377.3 (361.8 to 392.9)	379.6 (358.8 to 402.3)	343.2 (296.5 to 391.6)	-0.6 (-1.5 to 0.2)	-9.0 (-20.3 to 2.4)
Saint Vincent and the Grenadines	476.5 (464.2 to 489.5)	469.8 (452.4 to 487.3)	467.9 (443.9 to 493.9)	434.4 (392.6 to 478.6)	-0.5 (-1.1 to 0.1)	-7.5 (-15.6 to 0.9)
Suriname	510.8 (484.2 to 541.2)	435.6 (404.3 to 469.4)	433.3 (390.3 to 477.4)	402.1 (341.1 to 462.3)	-0.5 (-1.4 to 0.2)	-7.7 (-19.0 to 3.1)
Trinidad and Tobago	603.4 (593.1 to 612.7)	427.0 (364.2 to 500.5)	435.4 (361.6 to 520.9)	378.2 (301.1 to 461.4)	-0.8 (-1.9 to 0.3)	-11.3 (-25.1 to 5.4)
Virgin Islands, U.S.	476.4 (445.3 to 507.2)	472.0 (413.5 to 516.4)	463.5 (393.8 to 514.6)	372.1 (310.3 to 430.5)	-1.6 (-2.3 to -0.9)	-21.2 (-29.6 to -12.4)
Central Latin America						
Colombia	310.5 (308.1 to 312.8)	206.2 (203.2 to 208.6)	205.7 (185.8 to 227.9)	168.1 (138.5 to 202.9)	-1.4 (-2.7 to -0.1)	-18.5 (-32.9 to -1.7)
Costa Rica	269.3 (265.1 to 273.8)	224.1 (219.9 to 228.4)	238.2 (224.7 to 252.1)	211.6 (186.6 to 235.7)	-0.4 (-1.2 to 0.3)	-5.6 (-17.0 to 5.1)
El Salvador	296.8 (284.0 to 316.9)	292.4 (263.3 to 320.8)	288.0 (244.2 to 339.0)	249.4 (203.3 to 306.2)	-1.1 (-2.2 to 0.0)	-14.8 (-27.9 to 0.6)
Guatemala	307.7 (303.4 to 311.8)	280.3 (276.3 to 284.5)	289.3 (261.7 to 320.4)	250.3 (216.8 to 284.9)	-0.8 (-1.7 to 0.1)	-10.7 (-22.4 to 1.7)
Honduras	388.9 (323.7 to 456.8)	341.1 (286.4 to 409.0)	335.9 (280.1 to 397.6)	282.2 (233.1 to 339.4)	-1.3 (-1.9 to -0.7)	-17.2 (-24.6 to -10.2)
Mexico	304.7 (303.3 to 306.3)	270.2 (268.6 to 272.0)	276.9 (272.9 to 281.3)	248.7 (221.9 to 277.0)	-0.6 (-1.3 to 0.1)	-8.0 (-18.0 to 1.9)
Nicaragua	277.9 (260.5 to 295.9)	228.3 (221.6 to 243.7)	216.3 (193.1 to 244.7)	185.8 (151.6 to 222.9)	-1.4 (-2.7 to -0.2)	-18.6 (-33.2 to -2.5)
Panama	237.8 (233.8 to 241.5)	224.2 (220.4 to 228.4)	215.5 (202.4 to 227.9)	184.3 (165.5 to 203.1)	-1.3 (-2.0 to -0.7)	-17.8 (-26.4 to -9.3)
Venezuela	377.0 (374.1 to 379.9)	327.1 (310.3 to 342.9)	328.5 (286.1 to 378.7)	290.4 (238.9 to 345.7)	-0.8 (-1.9 to 0.2)	-11.3 (-24.7 to 3.3)
Tropical Latin America						
Brazil	444.9 (442.2 to 447.5)	323.3 (321.4 to 326.4)	329.6 (326.2 to 333.8)	282.8 (261.1 to 306.4)	-0.9 (-1.4 to -0.4)	-12.5 (-19.1 to -5.2)
Paraguay	362.6 (343.7 to 384.2)	336.4 (303.8 to 375.1)	330.1 (281.7 to 388.6)	294.1 (231.0 to 363.3)	-0.9 (-2.4 to 0.3)	-12.6 (-29.7 to 5.1)
North Africa and Middle East						
North Africa and Middle East						
Afghanistan	1224.2 (1045.3 to 1406.0)	982.1 (861.3 to 1115.0)	965.2 (844.2 to 1096.8)	833.9 (706.2 to 980.8)	-1.1 (-1.8 to -0.4)	-15.1 (-23.3 to -6.2)
Algeria	359.4 (334.2 to 384.4)	283.4 (267.4 to 295.8)	276.5 (258.9 to 292.4)	231.4 (204.8 to 258.0)	-1.4 (-2.0 to -0.7)	-18.3 (-26.2 to -9.8)
Bahrain	501.0 (483.6 to 520.9)	247.1 (230.9 to 263.2)	245.3 (224.7 to 267.4)	200.6 (156.9 to 263.1)	-1.4 (-3.0 to 0.4)	-18.8 (-36.4 to 6.4)
Egypt	576.5 (557.8 to 598.0)	617.4 (594.0 to 641.9)	608.5 (552.7 to 674.0)	492.8 (419.8 to 574.8)	-1.5 (-2.5 to -0.5)	-20.2 (-31.4 to -7.3)
Iran	345.3 (338.9 to 350.9)	300.4 (298.5 to 304.7)	287.2 (284.7 to 291.2)	260.8 (226.3 to 293.7)	-1.0 (-1.9 to -0.1)	-13.2 (-24.8 to -2.1)
Iraq	768.9 (692.9 to 852.1)	291.8 (276.6 to 309.1)	274.0 (257.1 to 293.1)	227.8 (205.1 to 253.0)	-1.7 (-2.2 to -1.0)	-21.9 (-28.6 to -14.5)
Jordan	475.2 (438.9 to 515.6)	258.1 (238.4 to 280.9)	256.2 (230.3 to 287.4)	222.2 (186.0 to 263.0)	-1.0 (-2.0 to -0.1)	-14.0 (-26.2 to -2.0)
Kuwait	302.7 (298.2 to 306.9)	188.2 (183.9 to 192.3)	187.5 (176.3 to 199.2)	166.1 (117.6 to 234.4)	-0.9 (-3.1 to 1.4)	-11.7 (-37.1 to 23.8)
Lebanon	429.8 (390.9 to 468.9)	360.6 (339.8 to 382.8)	357.7 (333.8 to 382.9)	322.1 (257.0 to 404.5)	-0.8 (-2.3 to 0.8)	-10.7 (-28.8 to 12.7)
Libya	465.3 (426.9 to 509.7)	460.2 (407.8 to 517.7)	472.8 (413.2 to 536.1)	445.2 (376.2 to 514.9)	-0.2 (-0.8 to 0.3)	-3.3 (-11.4 to 4.7)

	Estimate in 2000 (95% UIs)	Estimate in 2015 (95% UIs)	Estimate in 2017 (95% UIs)	Estimate in 2030 (95% UIs)	Annualised rate of change, 2015-2030 (95% UIs)	Percent change, 2015-2030 (95% UIs)
Morocco	589.3 (549.8 to 635.2)	473.5 (408.3 to 542.9)	460.6 (392.4 to 534.3)	366.2 (308.2 to 429.0)	-1.7 (-2.2 to -1.2)	-22.7 (-28.0 to -16.8)
Palestine	495.3 (466.9 to 525.2)	355.2 (339.4 to 371.2)	344.2 (323.2 to 367.1)	330.1 (269.4 to 404.2)	-0.5 (-1.7 to 0.8)	-7.1 (-22.6 to 12.8)
Oman	473.8 (402.4 to 558.6)	343.6 (286.3 to 402.2)	326.5 (271.8 to 384.5)	278.9 (211.6 to 353.3)	-1.4 (-2.8 to -0.2)	-18.9 (-34.0 to -2.2)
Qatar	434.0 (383.8 to 489.2)	237.3 (201.0 to 275.3)	230.4 (193.8 to 270.5)	198.2 (144.9 to 269.4)	-1.3 (-2.9 to 0.5)	-16.5 (-35.3 to 7.0)
Saudi Arabia	307.9 (275.5 to 349.0)	323.8 (287.4 to 367.7)	306.2 (272.4 to 352.8)	224.8 (165.8 to 297.6)	-2.5 (-4.3 to -0.7)	-30.6 (-47.5 to -10.3)
Sudan	660.0 (558.5 to 772.8)	512.6 (410.6 to 617.2)	498.7 (400.1 to 605.3)	379.0 (301.6 to 470.6)	-2.0 (-2.6 to -1.3)	-26.0 (-32.7 to -17.8)
Syria	564.4 (518.2 to 611.5)	422.1 (362.6 to 487.8)	418.7 (354.2 to 491.1)	359.0 (287.0 to 444.0)	-1.1 (-2.0 to -0.1)	-15.0 (-26.3 to -1.1)
Tunisia	372.8 (343.2 to 403.1)	288.2 (237.0 to 349.9)	281.8 (229.1 to 344.4)	239.7 (190.5 to 292.4)	-1.2 (-1.9 to -0.6)	-16.8 (-24.4 to -8.7)
Turkey	460.3 (438.4 to 482.5)	312.9 (295.6 to 330.4)	297.5 (272.9 to 323.3)	235.3 (199.8 to 272.0)	-1.9 (-2.9 to -1.0)	-24.8 (-35.4 to -14.0)
United Arab Emirates	621.5 (555.4 to 698.6)	564.5 (471.4 to 674.8)	562.8 (460.3 to 680.6)	430.0 (353.8 to 517.4)	-1.8 (-2.4 to -1.1)	-23.8 (-30.7 to -15.7)
Yemen	757.8 (581.9 to 974.4)	598.1 (456.4 to 782.2)	603.2 (459.4 to 790.6)	533.3 (398.6 to 702.7)	-0.8 (-1.4 to -0.1)	-10.8 (-19.2 to -1.6)
South Asia						
South Asia						
Bangladesh	513.8 (484.6 to 544.1)	463.4 (423.9 to 505.0)	453.7 (407.6 to 502.7)	326.0 (273.7 to 379.2)	-2.4 (-3.2 to -1.6)	-29.7 (-38.3 to -21.2)
Bhutan	458.2 (403.5 to 516.9)	355.0 (291.9 to 415.8)	345.5 (284.5 to 411.9)	269.9 (213.0 to 329.0)	-1.8 (-2.6 to -1.1)	-24.0 (-32.0 to -15.3)
India	546.1 (519.2 to 564.7)	496.3 (477.0 to 508.8)	494.0 (473.9 to 508.1)	393.3 (329.3 to 464.2)	-1.6 (-2.7 to -0.5)	-20.8 (-33.3 to -7.1)
Nepal	471.2 (426.4 to 524.2)	458.9 (392.3 to 518.5)	453.5 (384.4 to 513.4)	340.0 (281.1 to 394.3)	-2.0 (-2.6 to -1.4)	-25.9 (-32.1 to -18.9)
Pakistan	735.8 (689.7 to 782.1)	657.5 (558.6 to 764.9)	644.1 (539.6 to 754.3)	517.1 (419.8 to 622.5)	-1.6 (-2.5 to -1.0)	-21.4 (-31.5 to -13.3)
Southeast Asia, East Asia, and Oceania						
East Asia						
China	442.0 (434.4 to 449.8)	364.1 (352.7 to 375.7)	344.4 (332.3 to 357.2)	266.6 (234.0 to 294.9)	-2.1 (-2.9 to -1.4)	-26.8 (-35.2 to -19.0)
North Korea	588.1 (508.6 to 662.8)	585.2 (508.4 to 653.7)	570.9 (497.6 to 639.3)	530.6 (448.8 to 608.0)	-0.7 (-1.3 to -0.1)	-9.3 (-18.2 to -1.1)
Taiwan (Province of China)	333.7 (331.2 to 336.6)	252.3 (249.4 to 254.7)	255.6 (242.9 to 269.5)	222.0 (195.8 to 248.5)	-0.9 (-1.7 to -0.1)	-12.0 (-22.4 to -1.6)
Oceania						
American Samoa	593.7 (563.5 to 628.6)	565.6 (517.9 to 612.0)	561.9 (506.2 to 617.4)	484.2 (427.5 to 545.9)	-1.0 (-1.7 to -0.5)	-14.4 (-22.2 to -6.7)
Federated States of Micronesia	865.0 (773.2 to 967.2)	814.2 (675.7 to 943.7)	807.5 (654.4 to 942.4)	731.3 (585.0 to 886.0)	-0.7 (-1.6 to 0.0)	-10.2 (-21.5 to 0.5)
Fiji	1004.6 (989.3 to 1019.3)	877.0 (806.1 to 947.5)	864.2 (762.8 to 965.9)	749.6 (627.3 to 896.5)	-1.1 (-2.0 to -0.0)	-14.6 (-25.4 to -0.7)
Guam	516.6 (499.4 to 536.1)	539.1 (511.7 to 568.3)	538.0 (503.7 to 575.1)	449.2 (382.2 to 516.6)	-1.2 (-2.2 to -0.4)	-16.7 (-28.2 to -5.8)
Kiribati	1137.9 (1079.1 to 1202.4)	1062.2 (940.3 to 1203.3)	1041.2 (917.2 to 1183.8)	935.4 (810.9 to 1085.7)	-0.9 (-1.4 to -0.3)	-11.9 (-18.9 to -4.0)
Marshall Islands	1178.2 (1111.3 to 1247.1)	1054.3 (940.4 to 1181.2)	1027.3 (907.7 to 1159.5)	880.8 (752.5 to 1024.4)	-1.2 (-1.9 to -0.7)	-16.5 (-24.3 to -9.7)
Northern Mariana Islands	389.3 (354.2 to 427.3)	372.1 (335.4 to 410.4)	368.5 (330.3 to 405.9)	324.4 (280.0 to 369.0)	-0.9 (-1.6 to -0.3)	-12.8 (-21.7 to -4.2)
Papua New Guinea	1486.3 (1304.0 to 1684.4)	1375.4 (1207.8 to 1588.0)	1347.1 (1181.3 to 1556.9)	1122.5 (969.6 to 1300.3)	-1.4 (-1.8 to -1.0)	-18.4 (-23.6 to -13.4)
Samoa	554.6 (496.6 to 620.6)	497.3 (440.0 to 547.0)	492.9 (435.0 to 548.1)	422.6 (366.5 to 475.3)	-1.1 (-1.6 to -0.6)	-15.0 (-20.9 to -8.9)
Solomon Islands	891.8 (793.0 to 1005.3)	824.5 (732.9 to 925.2)	814.0 (721.6 to 917.8)	703.7 (608.5 to 815.0)	-1.1 (-1.5 to -0.6)	-14.7 (-20.6 to -8.5)
Tonga	555.1 (535.1 to 575.0)	543.1 (492.7 to 605.5)	536.5 (482.2 to 605.2)	471.1 (419.1 to 538.9)	-1.0 (-1.3 to -0.6)	-13.3 (-17.3 to -9.1)
Vanuatu	1002.3 (795.6 to 1235.2)	939.3 (750.3 to 1174.7)	931.7 (751.1 to 1163.2)	800.2 (631.0 to 1002.1)	-1.1 (-1.6 to -0.6)	-14.8 (-20.8 to -8.7)
Southeast Asia						
Cambodia	594.4 (545.7 to 642.0)	437.1 (391.5 to 490.5)	429.0 (381.5 to 482.5)	338.5 (297.7 to 385.9)	-1.7 (-2.1 to -1.3)	-22.6 (-27.4 to -17.4)
Indonesia	535.4 (513.9 to 563.7)	520.9 (493.5 to 548.5)	506.7 (478.5 to 536.2)	400.4 (352.4 to 457.5)	-1.8 (-2.5 to -1.0)	-23.1 (-31.5 to -14.0)
Laos	826.5 (742.6 to 923.7)	587.8 (497.3 to 682.3)	566.7 (478.1 to 660.1)	415.9 (344.4 to 484.6)	-2.3 (-2.7 to -1.9)	-29.2 (-33.6 to -24.5)
Malaysia	452.9 (444.4 to 470.7)	364.5 (349.6 to 395.0)	367.4 (335.2 to 405.3)	272.9 (236.7 to 312.7)	-1.9 (-2.8 to -1.2)	-25.2 (-34.2 to -16.3)
Maldives	498.3 (482.5 to 511.8)	227.0 (216.3 to 237.1)	224.3 (210.3 to 237.7)	187.7 (152.2 to 233.9)	-1.3 (-2.5 to 0.1)	-17.3 (-31.6 to 1.4)
Mauritius	580.3 (570.5 to 590.7)	421.0 (412.6 to 429.4)	410.9 (385.9 to 437.5)	327.0 (281.8 to 372.1)	-1.7 (-2.7 to -0.8)	-22.3 (-33.5 to -11.5)
Myanmar	755.9 (666.6 to 855.5)	528.4 (468.2 to 591.5)	510.2 (448.6 to 572.7)	379.5 (326.1 to 435.0)	-2.2 (-2.8 to -1.7)	-28.2 (-34.2 to -22.0)
Philippines	481.6 (456.4 to 507.8)	556.1 (517.6 to 598.2)	543.1 (473.3 to 614.0)	439.6 (371.1 to 517.0)	-1.6 (-2.5 to -0.7)	-21.0 (-31.7 to -9.5)
Sri Lanka	443.3 (435.4 to 463.4)	301.7 (265.5 to 337.5)	287.9 (240.9 to 335.9)	232.0 (182.9 to 289.4)	-1.8 (-3.2 to -0.4)	-23.1 (-37.9 to -6.4)
Seychelles	536.0 (517.9 to 553.5)	423.1 (408.1 to 437.6)	422.0 (402.4 to 441.9)	341.6 (296.0 to 394.8)	-1.4 (-2.3 to -0.5)	-19.3 (-29.4 to -7.3)
Thailand	385.4 (366.0 to 405.9)	250.7 (235.6 to 270.5)	252.8 (226.7 to 281.8)	220.7 (194.0 to 251.6)	-0.9 (-1.7 to -0.1)	-11.9 (-22.7 to -1.1)
Timor-Leste	509.7 (386.9 to 580.7)	460.3 (369.4 to 528.5)	470.5 (382.0 to 542.3)	388.1 (304.4 to 473.6)	-1.2 (-1.9 to -0.3)	-15.7 (-25.2 to -4.6)
Vietnam	482.8 (443.5 to 522.4)	425.2 (380.1 to 483.4)	419.5 (372.0 to 482.9)	337.3 (293.8 to 393.4)	-1.6 (-2.0 to -1.0)	-20.7 (-26.2 to -14.2)
Sub-Saharan Africa						
Central Sub-Saharan Africa						
Angola	646.1 (563.0 to 736.3)	454.5 (390.6 to 517.1)	444.0 (377.4 to 512.9)	361.4 (298.5 to 444.3)	-1.5 (-2.3 to -0.5)	-20.5 (-29.1 to -7.8)
Central African Republic	849.6 (712.0 to 977.6)	761.3 (615.1 to 885.9)	743.9 (595.1 to 868.6)	784.8 (607.5 to 996.3)	0.2 (-0.8 to 1.3)	3.1 (-11.0 to 20.7)
Congo	775.2 (693.2 to 857.8)	552.8 (469.7 to 635.5)	546.8 (461.7 to 632.1)	456.7 (349.4 to 592.8)	-1.3 (-2.7 to 0.2)	-17.4 (-33.5 to 3.3)
Democratic Republic of the Congo	531.1 (455.4 to 597.4)	481.9 (405.0 to 559.4)	474.6 (397.7 to 550.8)	440.3 (359.3 to 529.4)	-0.6 (-1.4 to 0.2)	-8.6 (-18.6 to 3.4)
Equatorial Guinea	556.3 (407.8 to 728.0)	346.5 (227.1 to 510.3)	343.4 (227.5 to 484.7)	273.6 (181.0 to 413.7)	-1.6 (-2.6 to -0.6)	-21.0 (-32.2 to -8.1)
Gabon	571.8 (503.5 to 649.6)	445.4 (388.7 to 514.5)	432.7 (377.3 to 495.1)	391.4 (315.4 to 478.4)	-0.9 (-2.0 to 0.1)	-12.1 (-26.1 to 1.9)
Eastern Sub-Saharan Africa						
Burundi	707.4 (612.6 to 807.0)	470.5 (394.8 to 548.4)	463.2 (385.9 to 543.4)	423.1 (318.9 to 548.5)	-0.7 (-2.2 to 0.7)	-10.1 (-28.0 to 11.7)
Comoros	577.4 (505.3 to 655.3)	439.8 (378.6 to 511.1)	434.7 (371.6 to 505.7)	404.4 (337.9 to 479.2)	-0.6 (-1.1 to 0.1)	-8.0 (-15.6 to 1.3)
Djibouti	565.8 (419.6 to 749.3)	462.9 (333.4 to 629.6)	454.1 (330.7 to 614.6)	418.8 (294.9 to 575.5)	-0.7 (-1.6 to 0.2)	-9.4 (-21.0 to 2.3)
Eritrea	840.4 (731.1 to 946.6)	649.0 (543.8 to 758.3)	612.0 (513.8 to 718.2)	569.2 (460.3 to 677.8)	-0.9 (-1.7 to -0.3)	-12.3 (-22.0 to -3.9)
Ethiopia	547.3 (504.5 to 594.1)	327.2 (302.1 to 350.9)	317.9 (291.8 to 343.1)	263.8 (225.9 to 300.4)	-1.4 (-2.3 to -0.7)	-19.4 (-28.9 to -9.4)
Kenya	425.1 (390.4 to 464.6)	372.9 (345.1 to 398.4)	362.1 (335.0 to 388.9)	331.9 (282.4 to 403.1)	-0.8 (-1.7 to 0.5)	-11.0 (-22.7 to 7.2)
Madagascar	729.8 (660.3 to 800.3)	631.1 (528.3 to 735.9)	618.4 (516.1 to 725.6)	564.4 (466.0 to 669.3)	-0.7 (-1.2 to -0.4)	-10.6 (-16.6 to -5.3)
Malawi	595.8 (525.8 to 667.7)	421.6 (369.8 to 472.7)	412.5 (357.6 to 469.9)	393.7 (306.3 to 502.0)	-0.5 (-1.9 to 1.0)	-6.6 (-24.5 to 16.5)
Mozambique	601.1 (534.7 to 666.6)	602.4 (522.4 to 685.6)	581.3 (500.2 to 664.5)	517.7 (419.1 to 629.5)	-1.0 (-2.0 to 0.1)	-14.0 (-26.1 to 0.9)
Rwanda	624.8 (561.5 to 689.7)	333.1 (291.6 to 379.6)	326.4 (283.3 to 377.1)	281.3 (195.2 to 399.6)	-1.2 (-3.3 to 1.1)	-15.6 (-39.0 to 18.4)
Somalia	781.8 (604.1 to 971.5)	632.2 (482.8 to 803.4)	621.5 (471.8 to 790.7)	649.5 (488.6 to 837.2)	0.2 (-0.3 to 0.7)	2.8 (-4.7 to 11.2)
South Sudan	595.7 (441.2 to 766.7)	499.1 (375.4 to 662.3)	503.9 (379.0 to 671.9)	509.4 (363.8 to 692.9)	0.1 (-0.8 to 1.2)	2.1 (-10.9 to 19.0)
Tanzania	415.7 (369.6 to 466.1)	360.6 (319.2 to 404.9)	359.8 (315.4 to 406.9)	314.7 (269.2 to 363.2)	-0.9 (-1.5 to -0.2)	-12.7 (-20.4 to -3.5)
Uganda	556.5 (487.0 to 632.0)	404.6 (352.8 to 459.9)	392.9 (340.4 to 449.3)	370.1 (303.7 to 443.6)	-0.6 (-1.4 to 0.2)	-8.5 (-19.4 to 3.5)
Zambia	640.5 (576.9 to 703.0)	435.8 (381.0 to 490.1)	430.7 (375.7 to 488.1)	375.5 (249.6 to 497.1)	-1.1 (-3.7 to 0.7)	-13.8 (-42.8 to 10.4)
Southern Sub-Saharan Africa						
Botswana	391.7 (313.1 to 474.8)	369.5 (334.5 to 416.9)	348.3 (316.2 to 392.2)	334.9 (243.8 to 496.0)	-0.8 (-2.7 to 1.9)	-9.3 (-32.9 to 33.3)
Lesotho	739.4 (643.6 to 829.0)	815.2 (666.0 to 945.4)	748.8 (614.6 to 872.2)	745.7 (526.7 to 1042.3)	-0.7 (-2.6 to 1.4)	-8.5 (-32.4 to 23.5)
Namibia	703.2 (638.9 to 767.8)	385.8 (334.5 to 447.0)	379.7 (327.8 to 443.6)	309.1 (235.3 to 407.4)	-1.5 (-3.0 to 0.1)	-19.9 (-36.0 to 1.1)
South Africa	677.4 (661.4 to 693.0)	415.6 (401.9 to 429.4)	389.3 (375.6 to 403.4)	355.1 (259.2 to 486.8)	-1.1 (-3.1 to 1.1)	-14.5 (-37.1 to 17.8)
Swaziland	808.3 (701.6 to 908.8)	731.9 (593.5 to 869.6)	664.8 (547.1 to 791.6)	628.5 (418.2 to 925.1)	-1.1 (-3.6 to 1.5)	-14.0 (-41.3 to 24.5)

	Estimate in 2000 (95% UIs)	Estimate in 2015 (95% UIs)	Estimate in 2017 (95% UIs)	Estimate in 2030 (95% UIs)	Annualised rate of change, 2015-2030 (95% UIs)	Percent change, 2015-2030 (95% UIs)
Zimbabwe	673.7 (601.0 to 741.4)	609.4 (528.5 to 687.2)	583.2 (498.2 to 664.3)	629.5 (383.1 to 1078.3)	0.0 (-3.0 to 3.7)	3.3 (-36.2 to 75.0)
Western Sub-Saharan Africa						
Benin	455.1 (403.0 to 512.0)	402.7 (334.7 to 485.4)	393.1 (323.8 to 475.7)	345.3 (278.9 to 425.2)	-1.0 (-1.7 to -0.4)	-14.3 (-22.7 to -5.2)
Burkina Faso	456.0 (399.1 to 522.3)	438.8 (384.8 to 499.0)	435.1 (378.7 to 495.8)	394.0 (335.5 to 458.0)	-0.7 (-1.3 to -0.2)	-10.2 (-17.8 to -3.1)
Cameroon	548.0 (486.0 to 613.5)	453.8 (370.8 to 541.0)	436.3 (355.2 to 524.8)	370.9 (298.8 to 460.7)	-1.4 (-2.0 to -0.6)	-18.2 (-25.8 to -8.4)
Cape Verde	308.9 (282.5 to 338.0)	284.3 (268.7 to 300.3)	283.9 (262.2 to 306.1)	248.6 (214.5 to 280.9)	-0.9 (-1.8 to -0.2)	-12.6 (-23.3 to -3.5)
Chad	468.0 (412.8 to 526.6)	453.5 (390.4 to 521.8)	447.1 (382.8 to 522.7)	424.1 (350.4 to 505.5)	-0.5 (-1.2 to 0.2)	-6.5 (-16.1 to 3.5)
Cote d'Ivoire	565.4 (495.7 to 639.9)	476.1 (404.4 to 553.3)	455.7 (384.4 to 532.9)	377.6 (314.0 to 452.1)	-1.6 (-2.2 to -1.0)	-20.7 (-27.8 to -13.8)
The Gambia	510.4 (435.9 to 594.2)	525.2 (447.9 to 606.1)	515.3 (439.2 to 595.1)	457.1 (376.5 to 540.1)	-0.9 (-1.5 to -0.3)	-13.0 (-20.2 to -4.4)
Ghana	484.9 (425.1 to 550.2)	467.3 (411.5 to 528.0)	454.0 (396.3 to 516.4)	382.2 (324.6 to 445.5)	-1.3 (-2.0 to -0.7)	-18.2 (-25.5 to -9.7)
Guinea	525.9 (471.9 to 583.4)	593.3 (509.7 to 675.1)	579.8 (492.0 to 669.6)	536.3 (428.8 to 649.7)	-0.7 (-1.6 to 0.2)	-9.6 (-21.7 to 2.8)
Guinea-Bissau	742.1 (648.7 to 836.8)	677.6 (579.0 to 775.1)	656.2 (563.1 to 756.2)	573.2 (485.5 to 672.2)	-1.1 (-1.5 to -0.6)	-15.4 (-20.5 to -9.3)
Liberia	446.4 (395.6 to 498.2)	422.5 (357.9 to 490.5)	414.9 (350.5 to 484.7)	394.9 (318.0 to 486.9)	-0.5 (-1.3 to 0.4)	-6.5 (-17.7 to 5.6)
Mali	518.3 (465.5 to 570.1)	435.6 (374.1 to 504.4)	434.6 (371.7 to 506.9)	376.8 (315.5 to 452.7)	-1.0 (-1.7 to -0.3)	-13.5 (-23.0 to -4.6)
Mauritania	460.2 (406.9 to 520.7)	372.1 (312.8 to 435.7)	364.8 (304.4 to 429.1)	300.8 (246.7 to 360.6)	-1.4 (-2.1 to -0.7)	-19.2 (-27.3 to -10.4)
Niger	439.8 (390.2 to 495.9)	394.5 (331.9 to 483.5)	391.8 (328.7 to 474.2)	373.5 (308.7 to 452.5)	-0.4 (-0.8 to 0.2)	-5.3 (-11.8 to 2.3)
Nigeria	313.1 (210.2 to 428.4)	267.6 (188.3 to 380.3)	270.3 (193.2 to 379.1)	229.1 (163.6 to 324.7)	-1.0 (-1.8 to -0.3)	-14.2 (-24.2 to -4.1)
Sao Tome and Principe	452.9 (405.4 to 507.5)	447.1 (392.6 to 503.8)	440.8 (384.7 to 500.9)	352.4 (284.9 to 432.2)	-1.6 (-2.6 to -0.6)	-21.2 (-32.4 to -7.9)
Senegal	431.5 (392.9 to 476.2)	418.7 (365.9 to 478.1)	417.5 (361.8 to 479.0)	365.9 (307.9 to 432.2)	-0.9 (-1.6 to -0.1)	-12.6 (-21.4 to -1.8)
Sierra Leone	524.2 (467.3 to 582.0)	508.7 (432.3 to 597.3)	494.4 (416.8 to 582.6)	446.4 (356.4 to 554.3)	-0.9 (-1.9 to 0.1)	-12.3 (-25.2 to 1.7)
Togo	499.3 (427.5 to 568.9)	451.4 (374.9 to 537.7)	445.1 (370.0 to 529.3)	382.2 (302.3 to 466.8)	-1.1 (-2.0 to -0.2)	-15.3 (-26.0 to -3.2)
**Indicator 3.4.2: Age-standardised death rate due to self-harm (per 100,000 population)**						
Central Europe, Eastern Europe, and Central Asia						
Central Asia						
Armenia	3.6 (3.5 to 3.8)	8.4 (8.0 to 8.8)	8.1 (7.6 to 8.6)	7.3 (5.6 to 9.7)	-1.0 (-2.5 to 1.0)	-13.2 (-31.5 to 17.0)
Azerbaijan	3.1 (2.8 to 3.8)	4.1 (3.1 to 4.7)	4.0 (3.0 to 4.6)	3.8 (2.6 to 5.0)	-0.7 (-2.3 to 1.0)	-8.7 (-29.0 to 15.9)
Georgia	5.3 (5.0 to 5.7)	7.3 (6.9 to 7.7)	7.3 (6.8 to 7.9)	7.2 (5.4 to 9.6)	-0.1 (-2.0 to 1.7)	-1.2 (-25.7 to 29.6)
Kazakhstan	32.8 (31.8 to 33.9)	22.4 (21.4 to 23.7)	22.5 (20.7 to 24.7)	24.1 (16.1 to 33.9)	0.4 (-2.0 to 2.8)	7.3 (-26.2 to 51.6)
Kyrgyzstan	16.8 (15.8 to 18.0)	10.6 (10.0 to 11.2)	9.8 (9.1 to 10.9)	9.7 (7.0 to 13.0)	-0.6 (-2.7 to 1.3)	-7.8 (-33.3 to 22.0)
Mongolia	20.3 (17.9 to 22.4)	15.5 (13.3 to 17.3)	15.1 (12.7 to 17.4)	16.5 (12.7 to 21.1)	0.4 (-1.0 to 1.8)	6.3 (-14.0 to 31.4)
Tajikistan	4.5 (4.1 to 5.6)	5.5 (4.7 to 6.1)	5.6 (4.7 to 6.3)	5.5 (4.1 to 7.0)	-0.1 (-1.7 to 1.4)	-1.1 (-22.6 to 23.4)
Turkmenistan	11.2 (10.7 to 11.7)	8.3 (7.8 to 8.9)	8.5 (7.7 to 9.4)	9.2 (6.9 to 12.7)	0.5 (-1.2 to 2.7)	9.8 (-16.6 to 50.6)
Uzbekistan	10.7 (10.2 to 11.2)	9.3 (8.6 to 10.2)	9.2 (8.1 to 10.4)	9.9 (7.6 to 12.9)	0.3 (-1.3 to 2.0)	6.1 (-17.4 to 34.8)
Central Europe						
Albania	4.8 (4.3 to 5.3)	5.1 (3.9 to 6.2)	5.0 (3.8 to 6.2)	4.7 (3.3 to 6.5)	-0.6 (-2.3 to 1.1)	-7.2 (-29.2 to 18.5)
Bosnia and Herzegovina	8.5 (7.4 to 9.9)	6.9 (6.3 to 8.3)	6.7 (6.0 to 8.3)	6.9 (5.7 to 8.5)	0.0 (-1.0 to 1.1)	0.4 (-14.4 to 17.2)
Bulgaria	15.2 (14.8 to 15.7)	9.1 (8.6 to 9.5)	9.3 (8.6 to 10.0)	8.6 (6.8 to 10.8)	-0.4 (-1.8 to 1.1)	-5.0 (-24.1 to 17.6)
Croatia	16.4 (15.9 to 16.9)	11.8 (11.4 to 12.3)	10.8 (10.1 to 11.6)	9.8 (8.4 to 11.4)	-1.3 (-2.2 to -0.3)	-17.1 (-28.4 to -4.2)
Czech Republic	13.7 (13.3 to 14.1)	11.0 (10.6 to 11.4)	10.6 (9.8 to 11.4)	9.1 (7.7 to 10.8)	-1.2 (-2.3 to -0.2)	-16.7 (-28.9 to -2.2)
Hungary	23.2 (22.6 to 23.9)	15.9 (15.4 to 16.4)	14.0 (13.1 to 15.0)	11.9 (9.3 to 15.2)	-2.0 (-3.5 to -0.3)	-25.1 (-41.2 to -4.3)
Macedonia	7.7 (7.2 to 9.0)	6.6 (6.0 to 7.3)	6.7 (6.0 to 7.5)	6.5 (5.3 to 8.0)	-0.1 (-1.2 to 1.1)	-0.7 (-15.9 to 17.6)
Montenegro	11.0 (10.2 to 12.6)	9.5 (8.3 to 10.5)	9.2 (8.1 to 10.4)	8.2 (6.6 to 10.1)	-0.9 (-2.1 to 0.2)	-12.9 (-26.9 to 2.7)
Poland	16.2 (15.7 to 16.7)	14.2 (13.7 to 14.8)	13.7 (12.6 to 14.8)	11.5 (9.7 to 13.3)	-1.5 (-2.5 to -0.5)	-19.5 (-30.9 to -7.1)
Romania	10.3 (10.0 to 10.7)	9.1 (8.8 to 9.4)	9.0 (8.3 to 9.6)	7.2 (5.8 to 9.1)	-1.6 (-3.1 to -0.1)	-21.0 (-36.8 to -0.9)
Serbia	18.8 (17.4 to 19.9)	13.2 (12.5 to 14.8)	12.4 (11.3 to 14.2)	13.1 (10.6 to 17.3)	-0.1 (-1.3 to 1.3)	-1.2 (-18.1 to 20.7)
Slovakia	12.8 (11.0 to 13.5)	10.2 (8.7 to 11.0)	9.3 (7.9 to 10.7)	8.3 (6.8 to 10.1)	-1.4 (-2.5 to -0.3)	-18.3 (-31.3 to -4.1)
Slovenia	24.8 (24.0 to 25.5)	14.3 (13.7 to 15.0)	14.1 (12.8 to 15.3)	12.0 (9.5 to 14.9)	-1.2 (-2.7 to 0.3)	-16.0 (-33.1 to 4.7)
Eastern Europe						
Belarus	33.1 (32.0 to 34.3)	19.2 (17.9 to 20.9)	18.7 (16.8 to 20.8)	19.6 (13.8 to 26.1)	0.1 (-2.0 to 1.9)	2.1 (-25.4 to 32.7)
Estonia	27.5 (26.4 to 28.5)	12.8 (12.0 to 13.8)	12.5 (10.6 to 14.7)	12.4 (7.4 to 19.3)	-0.4 (-3.7 to 2.6)	-3.1 (-42.6 to 47.6)
Latvia	27.6 (26.6 to 28.6)	16.8 (16.0 to 17.8)	16.7 (14.5 to 19.0)	16.1 (9.6 to 25.5)	-0.5 (-3.8 to 2.8)	-4.0 (-43.2 to 51.7)
Lithuania	39.6 (38.5 to 41.1)	28.9 (27.8 to 30.0)	27.4 (25.3 to 29.6)	28.3 (20.3 to 39.0)	-0.2 (-2.4 to 1.9)	-2.0 (-30.1 to 33.7)
Moldova	16.6 (16.0 to 17.2)	14.3 (13.7 to 14.9)	13.4 (12.4 to 14.2)	14.8 (10.0 to 21.0)	0.1 (-2.4 to 2.6)	3.5 (-30.2 to 46.7)
Russia	43.3 (42.5 to 43.9)	26.1 (25.5 to 26.7)	24.6 (23.8 to 25.3)	24.4 (15.0 to 38.8)	-0.6 (-3.7 to 2.6)	-6.7 (-42.5 to 48.6)
Ukraine	29.0 (28.1 to 30.0)	27.9 (26.5 to 30.1)	25.1 (23.1 to 27.9)	26.8 (16.1 to 40.4)	-0.5 (-3.6 to 2.5)	-4.1 (-41.9 to 46.2)
High-income						
Australasia						
Australia	12.2 (11.9 to 12.5)	11.1 (10.7 to 11.4)	10.8 (9.6 to 12.2)	9.8 (8.0 to 12.1)	-0.8 (-2.2 to 0.6)	-11.2 (-28.1 to 9.1)
New Zealand	12.9 (12.5 to 13.4)	10.7 (10.3 to 11.2)	11.1 (10.3 to 11.9)	9.3 (8.1 to 10.8)	-0.9 (-1.8 to 0.1)	-12.9 (-24.0 to 0.8)
High-income Asia Pacific						
Brunei	3.9 (3.5 to 5.5)	6.0 (5.2 to 6.7)	6.1 (5.3 to 6.9)	5.5 (4.5 to 6.6)	-0.6 (-1.5 to 0.3)	-8.3 (-20.0 to 4.7)
Japan	17.7 (17.6 to 17.9)	15.4 (15.0 to 16.1)	15.3 (14.7 to 16.2)	13.6 (11.8 to 15.5)	-0.8 (-1.8 to 0.0)	-11.7 (-23.2 to 0.3)
South Korea	18.1 (17.7 to 18.5)	22.0 (20.9 to 23.0)	20.4 (18.7 to 22.1)	15.6 (12.6 to 18.9)	-2.3 (-3.6 to -1.0)	-29.1 (-41.6 to -14.5)
Singapore	11.5 (10.6 to 12.4)	7.5 (7.2 to 7.8)	7.2 (6.6 to 7.8)	6.9 (5.8 to 8.1)	-0.5 (-1.7 to 0.5)	-7.4 (-22.7 to 8.4)
High-income North America						
Canada	12.0 (11.7 to 12.3)	11.1 (10.7 to 11.5)	10.7 (9.9 to 11.5)	9.9 (8.6 to 11.3)	-0.7 (-1.7 to 0.1)	-10.3 (-22.1 to 1.1)
Greenland	77.1 (73.0 to 84.0)	51.7 (48.0 to 57.7)	50.1 (45.8 to 56.5)	50.6 (36.8 to 72.4)	-0.2 (-2.3 to 2.2)	-2.1 (-29.7 to 38.7)
United States	10.7 (10.6 to 10.8)	12.3 (12.0 to 12.4)	12.6 (12.0 to 13.0)	11.2 (8.5 to 14.0)	-0.6 (-2.4 to 0.9)	-8.5 (-30.1 to 13.9)
Southern Latin America						
Argentina	12.1 (11.7 to 12.5)	10.4 (9.9 to 10.8)	10.4 (9.1 to 11.9)	9.3 (7.9 to 11.2)	-0.7 (-1.8 to 0.5)	-9.9 (-23.9 to 7.7)
Chile	12.6 (12.2 to 13.0)	9.9 (9.6 to 10.3)	9.9 (8.7 to 11.3)	10.2 (8.3 to 12.3)	0.2 (-1.1 to 1.3)	3.4 (-15.4 to 22.3)
Uruguay	16.2 (15.7 to 16.8)	16.8 (16.2 to 17.4)	16.5 (14.6 to 18.7)	14.8 (12.1 to 18.0)	-0.9 (-2.2 to 0.4)	-11.5 (-27.7 to 6.4)
Western Europe						
Andorra	9.3 (7.9 to 11.2)	7.8 (6.5 to 9.4)	7.8 (6.4 to 9.4)	7.0 (5.6 to 8.7)	-0.8 (-1.4 to -0.2)	-10.7 (-18.8 to -2.6)
Austria	15.6 (15.2 to 16.0)	11.5 (11.2 to 11.9)	11.0 (10.2 to 11.9)	9.5 (8.2 to 10.9)	-1.3 (-2.3 to -0.4)	-17.4 (-28.8 to -5.5)
Belgium	18.1 (17.6 to 18.6)	14.6 (14.2 to 15.1)	14.0 (13.0 to 15.0)	12.8 (11.0 to 14.8)	-0.9 (-1.9 to 0.1)	-12.5 (-24.7 to 0.9)
Cyprus	4.8 (4.2 to 6.5)	4.6 (4.0 to 5.2)	4.5 (3.9 to 5.2)	4.0 (3.2 to 4.9)	-1.0 (-2.1 to 0.1)	-14.1 (-27.0 to 2.3)
Denmark	12.8 (12.4 to 13.2)	8.3 (7.9 to 8.7)	8.6 (8.0 to 9.3)	8.0 (6.5 to 9.7)	-0.3 (-1.6 to 1.0)	-4.1 (-20.8 to 15.9)
Finland	20.7 (20.2 to 21.3)	12.4 (11.9 to 12.9)	12.7 (11.8 to 13.8)	11.5 (9.3 to 14.0)	-0.5 (-1.9 to 0.8)	-7.2 (-24.7 to 12.2)
France	17.2 (16.8 to 17.6)	12.7 (12.2 to 13.3)	12.2 (11.2 to 13.2)	10.3 (9.0 to 11.7)	-1.5 (-2.3 to -0.6)	-19.5 (-28.9 to -8.7)

	Estimate in 2000 (95% UIs)	Estimate in 2015 (95% UIs)	Estimate in 2017 (95% UIs)	Estimate in 2030 (95% UIs)	Annualised rate of change, 2015-2030 (95% UIs)	Percent change, 2015-2030 (95% UIs)
Germany	11.9 (11.6 to 12.2)	9.5 (9.2 to 9.8)	9.7 (8.6 to 11.0)	8.2 (6.9 to 9.6)	-1.0 (-2.0 to 0.1)	-14.1 (-26.5 to 0.8)
Greece	3.0 (2.8 to 3.1)	3.6 (3.4 to 3.8)	3.4 (3.2 to 3.7)	2.8 (2.4 to 3.3)	-1.6 (-2.6 to -0.7)	-21.0 (-31.8 to -10.0)
Iceland	11.5 (10.9 to 12.1)	9.7 (9.1 to 10.2)	9.4 (8.8 to 10.1)	9.4 (7.5 to 12.2)	-0.2 (-1.7 to 1.5)	-2.3 (-22.1 to 25.8)
Ireland	12.5 (12.1 to 12.9)	8.6 (8.1 to 9.1)	8.2 (7.4 to 9.0)	7.3 (6.2 to 8.8)	-1.1 (-2.1 to 0.1)	-14.7 (-27.2 to 0.9)
Israel	8.7 (8.4 to 9.0)	5.8 (5.5 to 6.0)	5.6 (5.2 to 6.1)	5.0 (4.1 to 6.1)	-1.0 (-2.3 to 0.3)	-13.4 (-28.8 to 4.7)
Italy	6.2 (6.0 to 6.4)	5.0 (4.9 to 5.2)	4.7 (4.3 to 5.1)	3.9 (3.5 to 4.4)	-1.7 (-2.4 to -1.0)	-22.4 (-30.7 to -13.9)
Luxembourg	12.2 (11.7 to 12.8)	8.8 (8.0 to 9.6)	8.5 (7.5 to 9.6)	8.0 (5.9 to 10.6)	-0.7 (-2.4 to 1.0)	-8.8 (-30.4 to 16.0)
Malta	4.7 (4.4 to 4.9)	4.3 (4.0 to 4.5)	4.4 (4.1 to 4.8)	3.9 (3.2 to 4.9)	-0.6 (-1.9 to 0.8)	-8.6 (-25.1 to 12.5)
Netherlands	9.4 (9.1 to 9.6)	9.0 (8.8 to 9.3)	9.0 (8.4 to 9.7)	8.0 (7.0 to 9.1)	-0.9 (-1.7 to 0.0)	-11.8 (-22.3 to 0.1)
Norway	12.3 (12.0 to 12.6)	9.2 (8.8 to 9.6)	9.1 (8.7 to 9.6)	8.4 (7.2 to 9.9)	-0.6 (-1.5 to 0.5)	-7.9 (-20.3 to 8.0)
Portugal	9.8 (9.4 to 10.1)	7.6 (7.2 to 8.1)	7.5 (6.8 to 8.2)	6.2 (5.2 to 7.6)	-1.4 (-2.5 to -0.2)	-18.5 (-31.6 to -3.4)
Spain	6.6 (6.4 to 6.8)	5.7 (5.5 to 5.9)	5.4 (5.0 to 5.8)	4.2 (3.4 to 5.2)	-2.2 (-3.4 to -0.7)	-27.5 (-40.4 to -10.4)
Sweden	12.7 (12.3 to 13.0)	11.4 (11.1 to 11.7)	10.9 (10.2 to 11.6)	9.9 (8.6 to 11.5)	-1.0 (-1.8 to 0.0)	-13.2 (-24.2 to 0.4)
Switzerland	16.0 (15.6 to 16.4)	9.8 (9.4 to 10.1)	9.1 (8.4 to 10.0)	8.4 (6.8 to 10.4)	-1.0 (-2.4 to 0.4)	-13.8 (-30.3 to 6.2)
United Kingdom	8.3 (8.2 to 8.4)	7.1 (7.0 to 7.3)	7.2 (7.0 to 7.4)	6.4 (5.6 to 7.3)	-0.7 (-1.6 to 0.1)	-10.4 (-21.3 to 1.8)
Latin America and Caribbean						
Andean Latin America						
Bolivia	8.0 (6.6 to 9.2)	6.4 (4.7 to 7.8)	6.3 (4.7 to 7.7)	6.1 (4.3 to 7.8)	-0.3 (-1.2 to 0.5)	-4.8 (-15.9 to 8.3)
Ecuador	7.5 (7.2 to 7.8)	9.3 (8.6 to 10.0)	9.0 (8.0 to 10.2)	8.1 (6.9 to 9.3)	-0.9 (-1.9 to 0.0)	-12.8 (-24.3 to 0.1)
Peru	4.5 (3.8 to 5.0)	3.1 (2.6 to 3.5)	3.1 (2.5 to 3.7)	3.0 (2.2 to 3.8)	-0.4 (-1.9 to 1.2)	-4.6 (-25.2 to 18.8)
Caribbean						
Antigua and Barbuda	2.6 (2.4 to 2.8)	2.6 (2.4 to 2.9)	2.6 (2.3 to 2.9)	2.8 (2.1 to 3.9)	0.3 (-1.5 to 2.5)	5.5 (-20.4 to 45.1)
The Bahamas	3.1 (2.9 to 3.4)	3.1 (2.8 to 3.4)	3.1 (2.8 to 3.5)	2.9 (2.3 to 3.8)	-0.5 (-1.8 to 1.2)	-6.0 (-23.7 to 20.0)
Barbados	4.5 (4.1 to 4.8)	3.9 (3.5 to 4.2)	3.9 (3.5 to 4.4)	3.9 (3.3 to 4.8)	0.1 (-0.8 to 1.1)	1.3 (-11.9 to 18.1)
Belize	8.9 (8.3 to 9.5)	7.4 (6.9 to 7.9)	7.7 (7.0 to 8.4)	7.4 (5.9 to 9.2)	-0.1 (-1.4 to 1.5)	-0.3 (-19.2 to 25.5)
Bermuda	4.0 (3.7 to 4.3)	3.4 (3.1 to 3.8)	3.4 (3.0 to 3.8)	3.0 (2.0 to 4.8)	-1.0 (-3.4 to 2.4)	-11.9 (-40.0 to 43.2)
Cuba	15.0 (14.6 to 15.4)	10.8 (10.4 to 11.3)	11.1 (9.8 to 12.6)	11.9 (9.7 to 14.3)	0.6 (-0.7 to 1.9)	9.7 (-10.0 to 32.3)
Dominica	4.3 (4.0 to 4.6)	4.4 (4.0 to 4.8)	4.5 (4.0 to 5.0)	4.3 (3.5 to 5.4)	-0.2 (-1.3 to 1.2)	-2.5 (-18.2 to 19.4)
Dominican Republic	7.2 (5.7 to 8.1)	8.7 (6.8 to 10.0)	8.8 (6.6 to 10.4)	8.9 (6.4 to 11.2)	0.1 (-1.1 to 1.3)	2.4 (-15.0 to 22.4)
Grenada	4.9 (4.5 to 5.2)	4.7 (4.4 to 5.1)	4.8 (4.4 to 5.2)	4.6 (4.0 to 5.4)	-0.1 (-1.0 to 0.8)	-1.7 (-13.3 to 12.3)
Guyana	22.8 (21.8 to 23.8)	26.8 (24.4 to 29.0)	25.6 (22.1 to 29.1)	24.0 (18.5 to 31.8)	-0.8 (-2.2 to 1.0)	-10.4 (-28.2 to 16.3)
Haiti	9.3 (7.6 to 11.9)	8.5 (6.7 to 10.9)	8.4 (6.5 to 10.8)	8.4 (6.3 to 10.8)	-0.1 (-0.8 to 0.6)	-1.6 (-10.9 to 9.3)
Jamaica	2.3 (2.0 to 2.5)	3.7 (2.7 to 4.4)	3.5 (2.6 to 4.3)	3.5 (2.4 to 4.8)	-0.4 (-1.9 to 1.4)	-5.7 (-24.4 to 22.8)
Puerto Rico	7.3 (7.0 to 7.7)	5.5 (5.2 to 5.9)	6.0 (5.4 to 6.6)	5.5 (4.4 to 6.7)	-0.1 (-1.4 to 1.2)	-0.7 (-18.5 to 19.5)
Saint Lucia	7.0 (6.5 to 7.4)	6.5 (5.9 to 7.0)	6.8 (6.1 to 7.5)	6.2 (5.2 to 7.4)	-0.3 (-1.3 to 0.8)	-4.2 (-17.3 to 12.0)
Saint Vincent and the Grenadines	7.1 (6.6 to 7.6)	6.9 (6.3 to 7.4)	7.1 (6.4 to 7.8)	7.5 (6.2 to 9.5)	0.6 (-0.7 to 2.0)	9.3 (-9.9 to 34.4)
Suriname	24.3 (22.6 to 26.1)	24.5 (22.1 to 27.2)	24.3 (21.2 to 27.5)	24.0 (19.7 to 28.7)	-0.2 (-1.1 to 0.8)	-2.2 (-15.8 to 12.8)
Trinidad and Tobago	12.6 (12.0 to 13.3)	9.8 (7.9 to 12.0)	9.9 (7.9 to 12.3)	11.3 (8.3 to 15.3)	0.9 (-0.8 to 2.8)	15.7 (-10.9 to 51.7)
Virgin Islands, U.S.	6.2 (5.7 to 6.7)	6.2 (5.4 to 6.9)	6.2 (5.3 to 7.1)	5.0 (4.0 to 6.1)	-1.4 (-2.5 to -0.4)	-19.3 (-31.3 to -6.1)
Central Latin America						
Colombia	6.4 (6.2 to 6.7)	5.4 (5.0 to 5.7)	5.4 (4.7 to 6.2)	5.0 (3.9 to 6.2)	-0.6 (-2.1 to 0.9)	-7.4 (-27.1 to 15.1)
Costa Rica	7.1 (6.8 to 7.4)	7.3 (6.8 to 7.7)	7.7 (6.8 to 8.4)	7.2 (6.2 to 8.4)	-0.1 (-1.0 to 0.8)	-0.5 (-13.5 to 12.9)
El Salvador	10.2 (9.3 to 12.0)	9.9 (8.4 to 12.4)	9.6 (7.7 to 12.4)	10.4 (8.0 to 13.9)	0.3 (-1.0 to 1.7)	5.2 (-13.8 to 28.4)
Guatemala	13.9 (13.2 to 14.6)	5.2 (4.9 to 5.4)	5.8 (5.1 to 6.6)	6.8 (5.5 to 8.5)	1.8 (0.7 to 3.2)	32.5 (10.4 to 62.0)
Honduras	5.1 (4.0 to 6.9)	4.4 (3.1 to 6.0)	4.3 (3.1 to 6.0)	4.3 (3.0 to 6.0)	-0.1 (-0.9 to 0.7)	-1.8 (-13.1 to 10.4)
Mexico	4.8 (4.7 to 4.9)	5.6 (5.5 to 5.7)	5.8 (5.5 to 6.0)	5.0 (4.2 to 6.3)	-0.8 (-1.9 to 0.8)	-10.4 (-25.4 to 12.3)
Nicaragua	9.3 (7.5 to 10.3)	6.1 (5.6 to 7.0)	5.8 (4.9 to 6.8)	5.7 (4.6 to 7.0)	-0.5 (-1.7 to 0.7)	-7.5 (-22.8 to 11.4)
Panama	6.1 (5.8 to 6.4)	4.9 (4.6 to 5.2)	4.9 (4.4 to 5.4)	5.0 (4.2 to 6.0)	0.2 (-0.9 to 1.4)	3.0 (-12.5 to 22.5)
Venezuela	9.9 (9.4 to 10.4)	8.4 (7.6 to 9.2)	8.5 (6.9 to 10.2)	8.6 (6.3 to 11.7)	0.1 (-1.6 to 2.1)	2.8 (-21.5 to 36.2)
Tropical Latin America						
Brazil	6.2 (6.1 to 6.3)	5.9 (5.7 to 6.0)	6.0 (5.7 to 6.2)	6.0 (5.3 to 6.8)	0.2 (-0.6 to 0.9)	2.9 (-8.4 to 15.3)
Paraguay	5.3 (4.2 to 5.8)	5.7 (4.1 to 6.7)	5.6 (4.0 to 7.0)	4.8 (3.3 to 6.3)	-1.2 (-2.8 to 0.2)	-16.1 (-34.0 to 3.4)
North Africa and Middle East						
North Africa and Middle East						
Afghanistan	10.9 (8.3 to 14.4)	9.1 (7.4 to 12.4)	9.0 (7.3 to 12.3)	8.8 (7.0 to 11.7)	-0.3 (-0.9 to 0.4)	-3.9 (-12.6 to 5.7)
Algeria	5.2 (4.3 to 6.0)	4.2 (3.5 to 4.9)	4.0 (3.3 to 4.8)	3.8 (3.1 to 4.6)	-0.7 (-1.3 to -0.0)	-9.3 (-17.3 to -0.0)
Bahrain	7.0 (5.8 to 7.7)	4.2 (3.5 to 5.0)	4.2 (3.4 to 5.0)	4.2 (3.1 to 5.6)	-0.0 (-1.4 to 1.6)	-0.0 (-18.7 to 27.1)
Egypt	4.8 (3.9 to 5.7)	5.3 (3.9 to 6.3)	5.4 (4.0 to 6.6)	5.0 (3.6 to 6.3)	-0.4 (-1.4 to 0.5)	-6.1 (-19.2 to 7.8)
Iran	6.4 (5.8 to 7.0)	5.5 (4.8 to 5.9)	5.3 (4.7 to 5.7)	5.0 (4.1 to 5.8)	-0.8 (-1.8 to 0.2)	-10.5 (-23.2 to 2.4)
Iraq	9.2 (7.2 to 11.5)	4.3 (3.9 to 4.9)	4.0 (3.6 to 4.6)	3.6 (3.1 to 4.2)	-1.2 (-1.9 to -0.4)	-15.9 (-24.8 to -6.3)
Jordan	5.1 (4.5 to 5.8)	3.2 (2.8 to 3.6)	3.2 (2.7 to 3.7)	3.3 (2.6 to 4.0)	0.2 (-0.7 to 1.1)	3.0 (-9.4 to 18.6)
Kuwait	3.3 (3.1 to 3.5)	2.4 (2.2 to 2.7)	2.4 (2.2 to 2.8)	2.4 (1.6 to 3.5)	-0.2 (-2.5 to 2.2)	-2.2 (-31.3 to 38.9)
Lebanon	5.0 (4.2 to 5.7)	4.7 (3.8 to 5.5)	4.7 (3.7 to 5.5)	4.8 (3.6 to 6.1)	0.1 (-1.1 to 1.3)	1.6 (-14.6 to 22.1)
Libya	6.8 (5.0 to 8.3)	8.2 (5.4 to 10.6)	7.9 (5.2 to 10.3)	7.7 (5.0 to 10.1)	-0.4 (-1.1 to 0.2)	-6.2 (-15.2 to 3.0)
Morocco	9.0 (7.9 to 10.3)	7.2 (6.0 to 8.7)	7.0 (5.7 to 8.4)	6.6 (5.3 to 8.0)	-0.7 (-1.2 to -0.2)	-9.4 (-16.2 to -2.6)
Palestine	4.8 (3.7 to 5.5)	3.6 (2.7 to 4.0)	3.5 (2.5 to 3.9)	4.0 (2.7 to 5.5)	0.7 (-0.7 to 2.6)	11.5 (-10.0 to 48.6)
Oman	3.4 (2.7 to 4.5)	3.0 (2.4 to 3.8)	2.9 (2.2 to 3.7)	3.0 (2.3 to 4.0)	-0.0 (-1.0 to 0.9)	-0.1 (-13.8 to 14.3)
Qatar	6.3 (5.2 to 7.5)	4.5 (3.4 to 5.7)	4.5 (3.4 to 5.8)	4.7 (3.3 to 6.6)	0.2 (-1.2 to 1.9)	4.4 (-16.1 to 33.5)
Saudi Arabia	2.5 (2.1 to 3.0)	2.9 (2.2 to 3.7)	2.9 (2.2 to 3.7)	2.5 (1.8 to 3.3)	-1.2 (-2.4 to -0.1)	-15.8 (-29.7 to -0.8)
Sudan	5.8 (4.3 to 8.0)	5.0 (3.9 to 6.5)	4.9 (3.8 to 6.3)	4.4 (3.4 to 5.8)	-0.8 (-1.4 to -0.2)	-11.1 (-18.8 to -3.1)
Syria	3.2 (2.8 to 3.6)	3.0 (2.4 to 3.7)	3.0 (2.4 to 3.8)	2.8 (2.1 to 3.6)	-0.5 (-1.4 to 0.4)	-7.1 (-19.0 to 6.0)
Tunisia	3.6 (3.1 to 4.2)	3.1 (2.4 to 3.9)	3.0 (2.3 to 3.9)	2.9 (2.2 to 3.7)	-0.4 (-1.1 to 0.2)	-6.1 (-15.4 to 3.4)
Turkey	3.4 (3.0 to 4.0)	3.3 (2.9 to 3.6)	3.2 (2.8 to 3.7)	2.8 (2.2 to 3.5)	-1.1 (-2.3 to 0.3)	-14.1 (-29.4 to 5.1)
United Arab Emirates	4.7 (3.5 to 5.7)	5.0 (3.6 to 6.4)	5.0 (3.5 to 6.3)	4.4 (3.1 to 5.7)	-0.9 (-1.7 to -0.2)	-12.5 (-22.3 to -2.7)
Yemen	6.7 (4.7 to 9.6)	5.8 (4.3 to 8.0)	5.9 (4.4 to 8.1)	5.4 (4.0 to 7.4)	-0.5 (-1.1 to 0.1)	-6.9 (-15.2 to 1.3)
South Asia						
South Asia						
Bangladesh	7.7 (6.9 to 8.8)	6.1 (5.2 to 6.9)	5.9 (5.0 to 6.8)	5.4 (4.3 to 6.5)	-0.8 (-1.5 to -0.0)	-10.9 (-20.5 to -0.7)
Bhutan	7.0 (6.0 to 8.4)	5.7 (4.2 to 7.1)	5.6 (4.1 to 7.0)	5.3 (3.8 to 6.7)	-0.6 (-1.1 to -0.1)	-8.0 (-15.1 to -1.2)
India	19.3 (16.3 to 20.8)	15.4 (12.7 to 16.4)	15.3 (12.8 to 16.4)	15.9 (12.6 to 18.4)	0.2 (-0.5 to 1.0)	3.2 (-7.8 to 15.6)

	Estimate in 2000 (95% UIs)	Estimate in 2015 (95% UIs)	Estimate in 2017 (95% UIs)	Estimate in 2030 (95% UIs)	Annualised rate of change, 2015-2030 (95% UIs)	Percent change, 2015-2030 (95% UIs)
Nepal	9.2 (7.4 to 11.1)	8.4 (6.2 to 10.2)	8.2 (6.2 to 9.9)	8.0 (5.8 to 9.9)	-0.4 (-0.9 to 0.2)	-5.2 (-13.1 to 3.6)
Pakistan	5.5 (4.8 to 6.2)	4.5 (3.7 to 5.4)	4.4 (3.6 to 5.3)	4.2 (3.3 to 5.2)	-0.5 (-1.3 to 0.5)	-6.6 (-18.1 to 7.3)
Southeast Asia, East Asia, and Oceania						
East Asia						
China	14.0 (12.1 to 14.6)	7.3 (6.9 to 8.0)	7.1 (6.7 to 7.7)	7.1 (6.1 to 8.4)	-0.2 (-1.0 to 0.8)	-2.1 (-14.5 to 12.3)
North Korea	12.4 (10.2 to 15.2)	11.0 (8.6 to 14.0)	10.8 (8.5 to 14.1)	11.8 (9.1 to 15.6)	0.5 (-0.3 to 1.3)	8.0 (-3.8 to 22.2)
Taiwan (Province of China)	13.6 (13.3 to 14.0)	12.9 (12.6 to 13.3)	13.3 (12.5 to 14.1)	12.9 (10.9 to 15.4)	-0.0 (-1.2 to 1.1)	-0.0 (-16.7 to 18.2)
Oceania						
American Samoa	6.5 (5.8 to 7.1)	6.7 (5.9 to 7.3)	6.6 (5.8 to 7.3)	6.5 (5.6 to 7.5)	-0.1 (-0.8 to 0.6)	-1.9 (-11.2 to 8.7)
Federated States of Micronesia	18.9 (14.7 to 23.3)	16.8 (11.5 to 21.6)	16.7 (11.1 to 21.8)	17.2 (11.5 to 23.2)	0.1 (-0.7 to 1.0)	2.4 (-10.1 to 16.0)
Fiji	10.8 (10.0 to 12.0)	9.8 (8.6 to 11.1)	9.6 (8.2 to 11.2)	10.5 (8.3 to 13.6)	0.5 (-0.7 to 2.0)	7.7 (-9.7 to 34.4)
Guam	17.0 (15.7 to 18.5)	20.3 (18.2 to 22.3)	20.2 (17.8 to 22.4)	18.6 (14.9 to 22.9)	-0.6 (-1.8 to 0.7)	-8.7 (-23.6 to 10.8)
Kiribati	26.9 (22.8 to 30.4)	26.2 (20.2 to 32.1)	25.9 (19.9 to 31.8)	26.1 (19.8 to 32.4)	-0.0 (-0.5 to 0.5)	-0.4 (-7.4 to 8.0)
Marshall Islands	24.6 (19.6 to 29.8)	22.5 (17.3 to 28.2)	22.0 (16.8 to 27.9)	22.3 (16.1 to 28.6)	-0.1 (-0.9 to 0.7)	-1.0 (-13.3 to 11.7)
Northern Mariana Islands	9.9 (8.6 to 11.2)	11.9 (10.2 to 13.6)	11.9 (10.2 to 13.8)	11.7 (9.6 to 14.2)	-0.1 (-1.0 to 0.8)	-1.4 (-13.6 to 13.0)
Papua New Guinea	24.8 (17.2 to 34.0)	23.0 (15.4 to 31.3)	22.6 (15.1 to 30.6)	21.6 (14.4 to 29.4)	-0.4 (-1.0 to 0.1)	-6.2 (-13.9 to 2.0)
Samoa	12.5 (10.1 to 14.7)	9.8 (7.9 to 12.3)	9.8 (7.7 to 12.3)	10.0 (7.9 to 12.6)	0.1 (-0.4 to 0.7)	2.1 (-5.5 to 10.5)
Solomon Islands	18.8 (14.4 to 24.2)	16.3 (12.7 to 20.5)	16.1 (12.4 to 20.2)	15.9 (12.0 to 20.4)	-0.2 (-0.7 to 0.5)	-2.2 (-10.5 to 7.0)
Tonga	5.7 (5.1 to 6.3)	5.9 (5.0 to 6.8)	5.8 (4.9 to 6.8)	5.7 (4.7 to 6.7)	-0.3 (-0.8 to 0.3)	-3.9 (-11.0 to 4.2)
Vanuatu	19.0 (13.2 to 25.1)	17.4 (11.6 to 23.9)	17.4 (11.4 to 23.9)	17.3 (11.4 to 23.8)	-0.1 (-0.6 to 0.5)	-0.8 (-8.5 to 7.4)
Southeast Asia						
Cambodia	5.6 (4.8 to 6.6)	4.5 (3.8 to 5.3)	4.5 (3.7 to 5.3)	4.4 (3.6 to 5.4)	-0.2 (-0.9 to 0.5)	-2.7 (-13.2 to 8.5)
Indonesia	3.7 (3.2 to 4.0)	3.1 (2.8 to 3.4)	3.1 (2.7 to 3.4)	3.3 (2.7 to 3.8)	0.3 (-0.4 to 1.0)	5.1 (-6.0 to 15.7)
Laos	13.9 (10.6 to 18.1)	8.6 (5.9 to 11.1)	8.3 (5.7 to 10.7)	8.3 (5.6 to 11.1)	-0.2 (-0.9 to 0.5)	-2.4 (-12.2 to 7.8)
Malaysia	8.8 (8.1 to 9.6)	7.5 (6.9 to 8.9)	7.7 (6.9 to 9.0)	7.3 (6.1 to 8.9)	-0.2 (-1.2 to 0.8)	-2.8 (-16.6 to 13.0)
Maldives	6.0 (4.9 to 7.1)	3.3 (2.8 to 3.8)	3.3 (2.7 to 3.8)	3.3 (2.4 to 4.5)	-0.0 (-1.6 to 2.0)	0.8 (-21.5 to 34.4)
Mauritius	10.9 (10.5 to 11.5)	8.6 (8.1 to 9.1)	8.2 (7.4 to 8.9)	8.5 (7.0 to 10.2)	-0.1 (-1.3 to 1.1)	-1.0 (-17.4 to 18.6)
Myanmar	6.9 (5.0 to 8.8)	5.1 (3.8 to 6.2)	5.0 (3.7 to 6.0)	4.8 (3.4 to 6.1)	-0.4 (-1.3 to 0.6)	-5.1 (-17.3 to 8.6)
Philippines	4.8 (4.5 to 5.1)	6.0 (5.5 to 6.5)	5.9 (5.0 to 6.8)	6.2 (5.0 to 7.6)	0.3 (-0.9 to 1.5)	4.3 (-12.9 to 24.8)
Sri Lanka	38.8 (36.4 to 41.4)	19.6 (16.8 to 25.1)	19.4 (15.6 to 25.3)	21.2 (14.9 to 30.3)	0.4 (-1.5 to 2.3)	7.7 (-19.8 to 41.9)
Seychelles	9.1 (8.2 to 11.4)	7.7 (6.9 to 10.0)	7.9 (6.9 to 10.2)	7.8 (6.3 to 10.0)	0.0 (-1.1 to 1.2)	1.0 (-14.8 to 19.4)
Thailand	16.5 (14.5 to 18.4)	9.8 (8.7 to 10.8)	10.2 (8.6 to 11.7)	10.7 (8.0 to 14.7)	0.5 (-1.3 to 2.5)	8.9 (-17.4 to 45.1)
Timor-Leste	8.8 (4.5 to 12.1)	7.2 (4.0 to 9.6)	7.2 (4.1 to 9.6)	7.4 (4.3 to 9.9)	0.2 (-0.6 to 1.1)	3.0 (-8.4 to 18.0)
Vietnam	8.4 (7.1 to 9.6)	7.3 (6.1 to 8.4)	7.3 (6.0 to 8.4)	7.6 (5.9 to 9.2)	0.2 (-0.7 to 1.1)	3.6 (-9.7 to 18.5)
Sub-Saharan Africa						
Central Sub-Saharan Africa						
Angola	15.5 (12.9 to 18.4)	11.9 (9.8 to 14.1)	11.7 (9.5 to 14.1)	13.2 (10.4 to 16.3)	0.6 (-0.2 to 1.6)	10.5 (-2.4 to 27.4)
Central African Republic	17.5 (13.4 to 21.8)	16.8 (12.5 to 21.2)	16.6 (12.4 to 21.0)	17.7 (12.8 to 23.3)	0.3 (-0.6 to 1.3)	5.2 (-8.7 to 20.7)
Congo	16.9 (13.7 to 19.9)	13.7 (10.9 to 17.0)	13.8 (10.7 to 17.3)	15.5 (11.0 to 21.0)	0.8 (-0.6 to 2.2)	12.8 (-9.2 to 39.8)
Democratic Republic of the Congo	11.8 (9.8 to 14.1)	11.3 (9.3 to 13.8)	11.4 (9.3 to 13.8)	11.9 (9.4 to 15.0)	0.3 (-0.5 to 1.2)	5.1 (-6.7 to 19.0)
Equatorial Guinea	12.4 (9.4 to 15.8)	9.0 (6.0 to 12.5)	9.2 (6.2 to 12.7)	9.2 (6.2 to 13.2)	0.2 (-0.9 to 1.2)	2.6 (-12.1 to 19.4)
Gabon	13.6 (11.0 to 16.4)	12.4 (9.1 to 15.7)	12.3 (8.8 to 15.6)	12.9 (8.9 to 17.0)	0.2 (-0.7 to 1.1)	3.7 (-10.2 to 18.1)
Eastern Sub-Saharan Africa						
Burundi	17.5 (14.1 to 21.7)	12.6 (9.8 to 15.8)	12.4 (9.7 to 15.6)	12.2 (9.1 to 16.2)	-0.2 (-1.3 to 0.9)	-3.1 (-17.4 to 14.1)
Comoros	8.7 (6.0 to 13.8)	8.2 (5.8 to 13.7)	8.2 (5.8 to 13.4)	8.2 (5.6 to 13.6)	0.0 (-0.6 to 0.7)	0.7 (-8.2 to 11.5)
Djibouti	9.5 (5.9 to 16.1)	8.3 (5.2 to 13.9)	8.3 (5.2 to 13.8)	8.3 (5.0 to 14.1)	0.0 (-0.8 to 0.8)	0.4 (-11.4 to 12.4)
Eritrea	19.6 (14.0 to 23.5)	15.9 (12.3 to 19.8)	15.4 (12.0 to 19.3)	16.8 (12.7 to 21.5)	0.3 (-0.6 to 1.1)	5.0 (-8.4 to 18.7)
Ethiopia	15.1 (13.2 to 17.0)	9.7 (8.5 to 11.1)	9.4 (8.2 to 11.0)	9.2 (7.6 to 11.3)	-0.4 (-1.1 to 0.5)	-5.2 (-15.7 to 8.3)
Kenya	13.1 (10.9 to 16.5)	11.1 (9.7 to 12.9)	10.8 (9.5 to 12.7)	11.6 (9.2 to 15.1)	0.3 (-0.7 to 1.6)	4.7 (-10.5 to 26.7)
Madagascar	12.8 (10.5 to 15.5)	10.9 (8.6 to 13.6)	10.8 (8.5 to 13.4)	10.7 (8.3 to 13.5)	-0.1 (-0.7 to 0.4)	-2.1 (-9.4 to 5.4)
Malawi	14.8 (12.4 to 17.1)	12.1 (10.2 to 14.1)	11.9 (9.9 to 14.1)	12.1 (9.2 to 15.9)	-0.1 (-1.3 to 1.4)	-0.2 (-18.3 to 23.4)
Mozambique	15.5 (13.2 to 18.0)	17.3 (13.9 to 20.8)	16.9 (13.4 to 20.3)	17.6 (13.3 to 22.2)	0.1 (-0.8 to 1.0)	1.6 (-11.6 to 16.1)
Rwanda	19.8 (16.5 to 23.7)	11.0 (8.8 to 13.8)	10.7 (8.6 to 13.5)	10.4 (7.2 to 14.7)	-0.4 (-2.2 to 1.5)	-5.3 (-27.9 to 26.1)
Somalia	11.7 (7.7 to 18.7)	9.9 (6.5 to 15.6)	9.8 (6.4 to 15.5)	10.5 (6.9 to 16.7)	0.4 (-0.2 to 1.0)	6.2 (-2.7 to 15.4)
South Sudan	13.0 (8.9 to 18.5)	11.8 (8.3 to 16.6)	11.8 (8.3 to 16.5)	12.1 (8.2 to 17.2)	0.1 (-0.7 to 1.1)	2.4 (-9.9 to 18.1)
Tanzania	9.9 (8.5 to 11.5)	8.6 (7.2 to 10.3)	8.7 (7.1 to 10.5)	8.1 (6.6 to 10.0)	-0.4 (-1.0 to 0.3)	-5.4 (-14.3 to 4.8)
Uganda	17.8 (15.0 to 21.6)	11.7 (10.1 to 13.8)	11.3 (9.7 to 13.4)	11.3 (9.3 to 14.0)	-0.2 (-1.0 to 0.6)	-3.0 (-14.3 to 9.5)
Zambia	16.6 (14.1 to 19.2)	12.8 (10.8 to 14.7)	12.8 (10.6 to 14.9)	11.4 (7.9 to 15.3)	-0.8 (-3.0 to 0.9)	-10.7 (-36.3 to 15.1)
Southern Sub-Saharan Africa						
Botswana	12.8 (8.7 to 20.2)	10.1 (7.8 to 14.7)	9.8 (7.5 to 14.3)	10.6 (7.2 to 16.6)	0.3 (-1.3 to 2.3)	4.9 (-17.3 to 41.6)
Lesotho	27.6 (22.6 to 34.4)	33.6 (25.5 to 41.4)	31.1 (23.9 to 38.5)	32.3 (21.5 to 46.7)	-0.4 (-2.4 to 1.8)	-3.9 (-29.9 to 30.1)
Namibia	20.7 (17.4 to 25.8)	11.7 (9.2 to 15.6)	11.6 (8.9 to 15.4)	12.2 (8.6 to 17.9)	0.2 (-1.3 to 1.8)	3.6 (-17.7 to 30.4)
South Africa	23.8 (20.4 to 25.3)	10.6 (9.6 to 11.8)	10.9 (9.8 to 12.1)	12.8 (8.9 to 19.9)	1.2 (-1.0 to 4.1)	21.0 (-13.4 to 85.5)
Swaziland	25.4 (19.2 to 31.8)	24.2 (17.6 to 30.8)	21.9 (16.4 to 28.1)	22.9 (14.9 to 33.8)	-0.5 (-2.4 to 1.8)	-5.4 (-30.1 to 30.1)
Zimbabwe	29.4 (24.1 to 34.0)	26.7 (22.1 to 30.8)	25.9 (21.3 to 30.1)	28.8 (17.3 to 50.0)	0.3 (-2.7 to 4.0)	8.0 (-33.5 to 81.9)
Western Sub-Saharan Africa						
Benin	12.0 (9.7 to 14.8)	11.2 (9.0 to 13.9)	10.9 (8.8 to 13.6)	10.6 (8.4 to 13.5)	-0.4 (-1.0 to 0.3)	-5.3 (-13.8 to 4.3)
Burkina Faso	13.1 (11.2 to 15.3)	13.0 (11.0 to 15.1)	13.0 (10.9 to 15.2)	12.4 (10.2 to 15.0)	-0.3 (-0.9 to 0.2)	-4.6 (-12.1 to 2.9)
Cameroon	16.0 (13.6 to 18.9)	14.2 (11.4 to 17.1)	13.8 (11.1 to 16.8)	12.8 (9.9 to 16.1)	-0.7 (-1.4 to 0.1)	-9.8 (-18.8 to 2.2)
Cape Verde	15.4 (12.3 to 18.0)	16.6 (13.6 to 18.4)	16.4 (13.4 to 18.6)	15.2 (12.1 to 17.8)	-0.6 (-1.4 to 0.1)	-8.1 (-18.8 to 1.6)
Chad	11.4 (8.4 to 16.0)	10.9 (8.1 to 15.5)	10.8 (8.0 to 15.5)	10.4 (7.5 to 15.0)	-0.3 (-1.0 to 0.4)	-4.3 (-13.6 to 6.1)
Cote d'Ivoire	15.6 (13.4 to 18.1)	14.0 (11.5 to 16.8)	13.6 (11.1 to 16.4)	11.9 (9.6 to 14.4)	-1.1 (-1.6 to -0.5)	-14.9 (-21.6 to -7.2)
The Gambia	9.3 (7.0 to 13.9)	9.6 (7.0 to 14.1)	9.5 (6.9 to 14.0)	9.1 (6.4 to 13.6)	-0.3 (-0.9 to 0.3)	-5.0 (-12.7 to 3.8)
Ghana	8.1 (6.9 to 9.6)	9.7 (7.8 to 11.2)	9.5 (7.7 to 11.2)	9.8 (7.4 to 12.0)	0.1 (-0.8 to 0.9)	1.8 (-11.1 to 15.1)
Guinea	9.5 (6.9 to 14.6)	11.1 (8.1 to 15.5)	10.9 (7.9 to 15.1)	10.4 (7.3 to 15.1)	-0.5 (-1.3 to 0.4)	-6.4 (-17.9 to 6.7)
Guinea-Bissau	14.4 (11.4 to 17.4)	13.6 (10.8 to 16.6)	13.3 (10.6 to 16.4)	12.1 (9.5 to 15.0)	-0.8 (-1.3 to -0.2)	-10.9 (-18.0 to -3.6)
Liberia	11.9 (9.5 to 15.7)	13.1 (10.7 to 16.4)	13.0 (10.6 to 16.3)	11.9 (9.3 to 15.5)	-0.7 (-1.5 to 0.2)	-9.2 (-19.7 to 3.1)
Mali	6.9 (4.7 to 12.8)	6.6 (4.2 to 13.3)	6.7 (4.2 to 13.2)	6.2 (3.8 to 12.5)	-0.5 (-1.1 to 0.1)	-7.2 (-15.0 to 0.8)
Mauritania	7.1 (5.2 to 11.9)	6.5 (4.5 to 11.0)	6.5 (4.4 to 10.9)	6.4 (4.2 to 11.2)	-0.2 (-1.0 to 0.8)	-2.5 (-14.1 to 13.2)
Niger	8.2 (5.6 to 14.8)	7.5 (5.1 to 13.8)	7.5 (5.1 to 13.6)	7.1 (4.7 to 13.1)	-0.4 (-0.9 to 0.2)	-5.1 (-12.6 to 2.5)

	Estimate in 2000 (95% UIs)	Estimate in 2015 (95% UIs)	Estimate in 2017 (95% UIs)	Estimate in 2030 (95% UIs)	Annualised rate of change, 2015-2030 (95% UIs)	Percent change, 2015-2030 (95% UIs)
Nigeria	8.9 (6.1 to 12.7)	7.4 (5.3 to 10.6)	7.4 (5.3 to 10.5)	6.9 (5.0 to 9.7)	-0.5 (-1.0 to 0.2)	-6.5 (-14.3 to 2.4)
Sao Tome and Principe	3.0 (2.5 to 3.5)	3.5 (2.6 to 4.4)	3.5 (2.5 to 4.4)	3.3 (2.3 to 4.5)	-0.4 (-1.5 to 0.8)	-5.2 (-20.2 to 12.3)
Senegal	12.6 (10.9 to 14.7)	12.7 (10.5 to 15.4)	12.7 (10.4 to 15.4)	12.2 (9.8 to 15.0)	-0.3 (-0.9 to 0.3)	-4.7 (-12.6 to 5.2)
Sierra Leone	9.2 (7.0 to 12.6)	9.8 (7.2 to 14.0)	9.7 (7.1 to 13.8)	8.8 (6.2 to 12.9)	-0.8 (-1.6 to 0.0)	-10.7 (-20.8 to 0.3)
Togo	12.9 (10.5 to 15.6)	12.2 (9.9 to 14.7)	12.0 (9.8 to 14.6)	11.5 (8.9 to 14.4)	-0.4 (-1.3 to 0.6)	-5.7 (-17.4 to 8.7)
**Indicator 3.5.2: Risk-weighted prevalence of alcohol consumption, as measured by the summary exposure value (SEV) for alcohol use (%)**						
Central Europe, Eastern Europe, and Central Asia						
Central Asia						
Armenia	9.8 (5.6 to 16.1)	11.4 (6.3 to 18.0)	11.8 (6.5 to 18.9)	14.0 (7.7 to 22.0)	1.4 (0.9 to 1.9)	22.9 (14.8 to 32.3)
Azerbaijan	13.7 (8.7 to 19.1)	15.1 (8.9 to 21.8)	15.0 (8.4 to 22.4)	15.4 (8.7 to 23.1)	0.1 (-0.6 to 0.9)	1.7 (-8.6 to 14.0)
Georgia	11.7 (7.1 to 17.9)	17.2 (10.3 to 25.4)	17.7 (10.4 to 26.7)	19.2 (11.4 to 28.6)	0.7 (0.3 to 1.3)	11.9 (4.2 to 21.3)
Kazakhstan	19.9 (12.7 to 28.3)	21.9 (13.2 to 31.3)	22.1 (13.0 to 32.4)	22.1 (12.8 to 32.3)	0.0 (-0.5 to 0.6)	0.5 (-7.5 to 8.8)
Kyrgyzstan	15.2 (10.0 to 20.8)	15.3 (9.7 to 21.4)	15.3 (9.2 to 21.7)	14.1 (8.4 to 20.4)	-0.6 (-1.2 to -0.1)	-7.9 (-16.0 to -0.9)
Mongolia	10.1 (5.9 to 16.2)	14.2 (8.3 to 22.2)	14.2 (8.2 to 22.3)	16.1 (9.5 to 25.0)	0.9 (0.4 to 1.4)	13.9 (6.0 to 22.6)
Tajikistan	7.0 (3.8 to 12.4)	5.9 (2.9 to 10.9)	5.9 (2.8 to 10.9)	5.1 (2.4 to 9.5)	-1.0 (-1.5 to -0.6)	-14.2 (-20.0 to -8.7)
Turkmenistan	12.3 (7.3 to 19.2)	13.0 (7.3 to 21.0)	13.4 (7.3 to 22.1)	13.3 (7.2 to 22.1)	0.1 (-0.4 to 0.9)	1.7 (-5.9 to 14.7)
Uzbekistan	10.6 (6.2 to 16.8)	12.6 (7.4 to 19.6)	12.7 (7.3 to 20.3)	15.0 (8.8 to 23.6)	1.2 (0.7 to 1.7)	19.5 (11.2 to 28.7)
Central Europe						
Albania	10.1 (6.3 to 15.1)	14.4 (9.0 to 20.4)	14.8 (8.8 to 21.3)	15.8 (9.5 to 23.2)	0.6 (-0.1 to 1.4)	9.5 (-1.1 to 23.5)
Bosnia and Herzegovina	19.0 (15.8 to 22.7)	20.6 (16.2 to 25.3)	20.9 (16.2 to 26.0)	20.4 (15.8 to 25.3)	-0.1 (-0.3 to 0.2)	-1.0 (-4.5 to 2.9)
Bulgaria	18.7 (11.8 to 27.0)	21.6 (13.2 to 31.3)	22.4 (13.4 to 33.2)	24.2 (14.4 to 35.9)	0.7 (0.0 to 1.2)	11.8 (0.2 to 18.9)
Croatia	23.4 (16.2 to 31.1)	20.3 (12.1 to 29.8)	20.8 (12.2 to 31.1)	19.3 (11.1 to 29.2)	-0.4 (-0.9 to 0.1)	-5.4 (-12.6 to 1.5)
Czech Republic	26.3 (18.3 to 35.3)	26.6 (17.9 to 37.4)	26.7 (17.3 to 38.0)	24.8 (15.8 to 35.8)	-0.5 (-1.0 to 0.1)	-7.0 (-14.2 to 1.6)
Hungary	27.7 (20.5 to 35.9)	25.3 (16.9 to 35.5)	25.7 (16.6 to 36.8)	23.4 (14.9 to 34.3)	-0.6 (-1.0 to -0.1)	-8.1 (-14.0 to -2.1)
Macedonia	10.1 (5.8 to 17.0)	8.1 (3.9 to 14.2)	8.5 (4.0 to 15.0)	8.1 (3.9 to 14.5)	0.0 (-0.8 to 0.7)	0.8 (-11.2 to 10.5)
Montenegro	23.2 (14.3 to 32.9)	24.4 (15.4 to 34.1)	24.9 (15.5 to 35.4)	27.1 (17.2 to 38.3)	0.7 (0.4 to 1.1)	11.3 (5.5 to 18.1)
Poland	18.8 (12.0 to 27.6)	22.5 (14.2 to 33.2)	23.4 (14.6 to 34.6)	24.6 (15.2 to 36.7)	0.6 (0.2 to 1.0)	9.4 (2.9 to 16.5)
Romania	28.4 (22.1 to 35.0)	30.7 (22.8 to 38.8)	31.1 (22.7 to 40.4)	31.0 (22.0 to 40.6)	0.1 (-0.3 to 0.4)	0.9 (-4.8 to 5.9)
Serbia	18.2 (11.2 to 27.0)	25.9 (17.2 to 35.9)	26.6 (17.2 to 37.9)	29.2 (19.0 to 40.6)	0.8 (0.4 to 1.3)	12.6 (5.9 to 21.1)
Slovakia	24.9 (17.5 to 33.6)	23.5 (15.0 to 33.4)	24.1 (14.9 to 34.9)	23.1 (14.4 to 34.2)	-0.1 (-0.6 to 0.3)	-1.9 (-9.2 to 5.2)
Slovenia	12.7 (6.6 to 21.7)	10.0 (3.0 to 19.3)	10.7 (2.5 to 21.2)	9.6 (2.3 to 19.4)	-0.6 (-2.7 to 1.1)	-5.2 (-33.2 to 17.7)
Eastern Europe						
Belarus	27.3 (19.4 to 35.4)	32.7 (23.3 to 42.7)	33.5 (23.2 to 44.8)	35.1 (24.5 to 46.8)	0.5 (0.1 to 0.8)	7.3 (1.2 to 13.3)
Estonia	19.1 (12.7 to 28.1)	29.5 (19.9 to 39.7)	30.5 (19.6 to 41.9)	32.5 (20.6 to 44.5)	0.6 (0.0 to 1.3)	9.7 (0.4 to 20.9)
Latvia	20.0 (13.0 to 28.7)	26.7 (17.8 to 36.7)	27.4 (17.5 to 37.8)	28.6 (18.3 to 39.4)	0.4 (-0.1 to 1.0)	6.7 (-1.1 to 15.6)
Lithuania	22.1 (15.0 to 30.1)	31.4 (22.4 to 40.7)	32.3 (22.6 to 42.4)	34.6 (24.3 to 45.8)	0.6 (0.2 to 1.1)	10.0 (3.0 to 17.6)
Moldova	29.1 (19.3 to 38.8)	23.5 (13.3 to 34.5)	24.2 (13.2 to 36.0)	23.7 (12.9 to 35.8)	0.0 (-0.6 to 0.6)	0.8 (-9.1 to 9.6)
Russia	19.5 (13.0 to 27.4)	24.5 (16.6 to 33.9)	24.9 (16.1 to 35.2)	25.9 (16.5 to 36.7)	0.3 (-0.2 to 1.1)	5.5 (-3.6 to 17.4)
Ukraine	27.6 (18.7 to 37.2)	36.2 (25.5 to 47.7)	36.5 (25.1 to 49.2)	36.8 (25.1 to 49.6)	0.1 (-0.4 to 0.7)	1.6 (-6.1 to 10.3)
High-income						
Australasia						
Australia	22.1 (14.8 to 31.5)	24.4 (15.7 to 34.6)	24.5 (15.4 to 35.1)	25.4 (16.1 to 36.6)	0.3 (-0.2 to 0.7)	4.1 (-3.1 to 10.8)
New Zealand	21.0 (14.7 to 28.5)	23.3 (15.0 to 31.6)	23.1 (14.4 to 31.8)	23.3 (14.5 to 32.6)	-0.0 (-0.5 to 0.4)	-0.2 (-7.5 to 5.7)
High-income Asia Pacific						
Brunei	2.3 (0.6 to 6.1)	2.9 (0.9 to 7.0)	3.0 (0.9 to 7.1)	3.4 (1.0 to 8.0)	1.0 (-0.2 to 1.7)	15.8 (-2.7 to 28.5)
Japan	15.0 (8.9 to 24.3)	16.3 (9.2 to 26.5)	17.2 (9.5 to 28.5)	17.6 (9.7 to 28.5)	0.5 (0.0 to 0.9)	7.4 (0.1 to 14.0)
South Korea	16.4 (10.0 to 25.7)	18.0 (10.3 to 28.2)	19.4 (10.7 to 30.4)	18.4 (9.9 to 29.1)	0.1 (-0.5 to 0.8)	1.6 (-6.6 to 12.1)
Singapore	10.1 (5.4 to 17.8)	11.3 (5.7 to 20.1)	11.7 (5.9 to 20.9)	11.4 (5.7 to 20.4)	0.1 (-0.3 to 0.6)	1.2 (-4.8 to 10.2)
High-income North America						
Canada	18.9 (11.5 to 28.4)	20.9 (12.9 to 31.0)	21.5 (13.0 to 32.1)	22.2 (13.2 to 32.9)	0.4 (-0.0 to 0.9)	6.4 (-0.6 to 14.6)
Greenland	13.4 (6.3 to 23.3)	14.4 (7.4 to 24.2)	14.7 (7.5 to 24.8)	15.8 (8.1 to 26.4)	0.6 (0.2 to 1.0)	9.6 (2.3 to 16.8)
United States	18.5 (12.4 to 26.4)	21.1 (13.6 to 30.3)	21.4 (13.5 to 31.7)	21.8 (13.7 to 31.9)	0.2 (-0.2 to 0.6)	3.2 (-2.8 to 9.6)
Southern Latin America						
Argentina	21.1 (13.8 to 30.4)	19.9 (11.9 to 30.6)	20.5 (11.8 to 32.0)	20.0 (11.4 to 31.7)	0.0 (-0.6 to 0.5)	0.3 (-9.0 to 7.5)
Chile	16.1 (9.7 to 24.9)	20.2 (12.7 to 30.6)	20.8 (12.7 to 32.0)	22.0 (13.2 to 33.4)	0.5 (0.1 to 1.0)	8.4 (1.4 to 15.6)
Uruguay	19.1 (12.7 to 26.4)	17.4 (10.6 to 26.1)	17.9 (10.6 to 27.5)	17.1 (9.9 to 26.6)	-0.2 (-0.6 to 0.3)	-2.2 (-9.2 to 4.7)
Western Europe						
Andorra	36.2 (6.6 to 46.7)	24.8 (15.7 to 36.0)	25.3 (15.4 to 37.4)	22.6 (13.4 to 34.4)	-0.7 (-1.1 to -0.2)	-9.3 (-15.7 to -2.6)
Austria	28.0 (18.9 to 38.8)	27.4 (17.6 to 39.2)	27.7 (17.4 to 40.2)	26.2 (16.3 to 38.6)	-0.3 (-0.8 to 0.1)	-4.3 (-11.1 to 1.9)
Belgium	17.9 (10.8 to 27.8)	24.2 (15.1 to 35.7)	24.5 (14.9 to 36.9)	25.1 (15.1 to 37.9)	0.2 (-0.2 to 0.8)	3.9 (-3.6 to 11.9)
Cyprus	19.5 (12.8 to 27.5)	19.8 (12.4 to 29.3)	20.3 (12.4 to 30.9)	19.6 (11.8 to 30.0)	-0.1 (-0.5 to 0.3)	-1.4 (-7.8 to 5.4)
Denmark	28.0 (18.2 to 39.6)	25.0 (15.2 to 37.6)	24.9 (14.4 to 38.1)	22.1 (12.4 to 35.2)	-0.8 (-1.5 to -0.1)	-11.8 (-20.0 to -1.8)
Finland	24.5 (16.3 to 33.7)	25.8 (16.1 to 37.3)	26.0 (15.9 to 38.0)	24.5 (14.8 to 36.4)	-0.4 (-0.9 to 0.2)	-5.2 (-13.0 to 3.7)
France	31.4 (21.6 to 42.3)	26.8 (17.0 to 38.3)	26.5 (16.0 to 38.6)	23.8 (14.1 to 35.3)	-0.8 (-1.4 to -0.3)	-11.6 (-19.1 to -5.1)
Germany	26.6 (17.4 to 37.4)	25.8 (16.1 to 37.7)	26.1 (15.8 to 39.5)	23.6 (14.0 to 36.3)	-0.6 (-1.1 to -0.2)	-8.6 (-15.1 to -2.4)
Greece	23.6 (15.5 to 33.0)	24.5 (15.1 to 35.7)	24.8 (14.8 to 37.3)	23.1 (13.4 to 35.3)	-0.4 (-0.9 to 0.0)	-6.2 (-12.9 to 0.7)
Iceland	13.2 (7.5 to 22.4)	17.6 (10.2 to 28.2)	18.1 (10.3 to 28.9)	20.5 (11.8 to 32.5)	1.0 (0.6 to 1.5)	17.0 (10.0 to 25.3)
Ireland	29.9 (22.2 to 38.0)	27.8 (18.3 to 38.1)	28.3 (17.6 to 40.3)	26.0 (15.9 to 37.7)	-0.5 (-1.2 to 0.4)	-6.9 (-16.1 to 6.4)
Israel	6.0 (3.3 to 10.9)	7.3 (4.0 to 12.2)	7.5 (4.1 to 12.4)	8.8 (4.9 to 14.6)	1.2 (0.8 to 1.7)	20.5 (13.3 to 28.9)
Italy	26.6 (18.4 to 36.2)	21.3 (13.0 to 31.4)	21.8 (12.9 to 32.7)	19.1 (10.9 to 29.5)	-0.8 (-1.3 to -0.3)	-10.8 (-17.6 to -3.7)
Luxembourg	26.2 (17.4 to 35.3)	35.6 (24.8 to 46.4)	34.8 (23.5 to 46.6)	34.0 (22.2 to 45.9)	-0.3 (-1.1 to 0.4)	-4.8 (-14.7 to 6.4)
Malta	12.4 (7.2 to 20.8)	15.8 (9.0 to 25.7)	16.5 (9.1 to 27.5)	18.4 (10.5 to 29.8)	1.0 (0.6 to 1.5)	16.4 (8.7 to 24.4)
Netherlands	18.2 (11.6 to 27.8)	19.4 (11.7 to 29.4)	20.2 (12.0 to 31.0)	20.1 (12.0 to 30.9)	0.2 (-0.2 to 0.7)	3.7 (-3.3 to 11.0)
Norway	16.5 (10.1 to 26.2)	18.9 (11.4 to 29.0)	19.1 (11.2 to 29.7)	19.5 (11.4 to 30.1)	0.2 (-0.3 to 0.8)	3.1 (-4.1 to 12.5)
Portugal	30.9 (25.4 to 36.5)	29.3 (21.0 to 37.5)	29.9 (20.5 to 39.4)	27.8 (18.7 to 37.3)	-0.4 (-0.8 to 0.0)	-5.4 (-11.5 to 0.7)
Spain	29.6 (21.3 to 37.6)	29.5 (20.0 to 40.5)	29.8 (19.6 to 41.5)	27.7 (17.8 to 39.3)	-0.4 (-1.0 to 0.0)	-6.4 (-13.5 to 0.3)
Sweden	21.9 (13.7 to 32.7)	21.4 (13.0 to 33.4)	21.8 (12.7 to 34.1)	22.2 (13.1 to 34.7)	0.2 (-0.2 to 0.6)	3.7 (-2.7 to 10.2)
Switzerland	22.1 (13.9 to 33.5)	21.9 (12.9 to 33.5)	22.4 (13.0 to 34.4)	21.1 (12.0 to 33.1)	-0.3 (-0.8 to 0.1)	-4.2 (-11.6 to 2.1)
United Kingdom	21.3 (14.5 to 29.7)	21.5 (13.9 to 31.0)	22.0 (14.1 to 32.4)	23.3 (14.9 to 33.8)	0.5 (0.2 to 0.9)	8.4 (2.6 to 14.1)
Latin America and Caribbean						

	Estimate in 2000 (95% UIs)	Estimate in 2015 (95% UIs)	Estimate in 2017 (95% UIs)	Estimate in 2030 (95% UIs)	Annualised rate of change, 2015-2030 (95% UIs)	Percent change, 2015-2030 (95% UIs)
Andean Latin America						
Bolivia	8.7 (4.8 to 14.5)	9.3 (5.0 to 15.7)	9.4 (4.9 to 15.9)	10.7 (5.7 to 18.3)	0.9 (0.2 to 1.4)	15.3 (3.3 to 23.8)
Ecuador	14.5 (11.5 to 17.7)	15.1 (11.5 to 18.8)	15.1 (11.2 to 18.9)	14.7 (10.8 to 18.6)	-0.2 (-0.5 to 0.2)	-2.9 (-7.5 to 3.8)
Peru	18.3 (11.6 to 27.1)	16.8 (10.1 to 25.7)	17.0 (9.9 to 26.3)	15.7 (9.1 to 24.6)	-0.5 (-1.0 to -0.0)	-6.9 (-13.5 to -0.1)
Caribbean						
Antigua and Barbuda	7.0 (3.5 to 12.4)	9.4 (4.8 to 15.8)	9.4 (4.5 to 16.1)	10.7 (5.2 to 18.0)	0.8 (-0.0 to 1.7)	12.6 (-0.5 to 28.3)
The Bahamas	14.4 (8.8 to 21.6)	13.1 (7.0 to 20.9)	13.4 (6.5 to 21.4)	11.3 (5.4 to 18.4)	-1.1 (-1.8 to -0.4)	-14.8 (-23.8 to -5.7)
Barbados	12.9 (8.4 to 19.0)	15.2 (9.5 to 22.1)	15.5 (9.4 to 22.9)	16.5 (10.0 to 24.2)	0.5 (0.0 to 1.0)	7.9 (0.3 to 16.9)
Belize	12.8 (8.2 to 18.1)	18.9 (12.5 to 25.0)	19.0 (11.8 to 25.5)	19.4 (12.2 to 26.3)	0.1 (-0.5 to 0.7)	2.3 (-7.0 to 11.2)
Bermuda	28.9 (23.8 to 33.8)	25.6 (19.5 to 32.0)	25.3 (18.4 to 32.3)	23.2 (16.7 to 30.0)	-0.7 (-1.1 to -0.3)	-9.3 (-15.4 to -4.8)
Cuba	10.5 (6.3 to 16.4)	13.5 (8.3 to 20.6)	14.2 (8.5 to 21.6)	16.1 (9.7 to 24.4)	1.2 (0.7 to 1.7)	19.5 (11.1 to 28.8)
Dominica	16.0 (10.6 to 22.2)	16.5 (10.2 to 23.4)	16.8 (9.8 to 24.6)	16.7 (9.6 to 24.4)	0.0 (-0.6 to 0.6)	0.7 (-8.2 to 9.5)
Dominican Republic	12.4 (7.5 to 19.4)	13.3 (7.6 to 21.2)	13.6 (7.5 to 22.2)	14.8 (8.3 to 23.9)	0.7 (0.3 to 1.1)	10.8 (4.1 to 18.5)
Grenada	16.1 (11.1 to 21.9)	16.1 (10.1 to 23.2)	16.5 (9.9 to 24.1)	15.3 (9.2 to 22.6)	-0.3 (-0.9 to 0.2)	-5.0 (-13.1 to 3.7)
Guyana	17.4 (12.5 to 22.4)	13.2 (8.2 to 18.8)	13.4 (8.1 to 19.6)	13.1 (7.7 to 19.2)	-0.1 (-0.8 to 0.5)	-1.3 (-11.3 to 8.4)
Haiti	10.8 (7.0 to 15.9)	12.0 (7.4 to 17.6)	12.1 (7.2 to 18.3)	13.0 (7.8 to 19.4)	0.5 (0.1 to 1.0)	8.3 (0.9 to 16.4)
Jamaica	10.1 (6.0 to 15.7)	13.2 (7.7 to 19.6)	13.4 (7.6 to 20.6)	14.7 (8.4 to 22.4)	0.7 (0.1 to 1.3)	11.2 (2.2 to 21.0)
Puerto Rico	14.0 (7.3 to 22.4)	17.9 (10.2 to 26.7)	18.7 (10.7 to 28.3)	20.3 (11.7 to 30.3)	0.8 (0.4 to 1.3)	13.4 (6.2 to 21.3)
Saint Lucia	19.7 (13.9 to 25.6)	16.7 (10.4 to 24.3)	16.9 (10.3 to 25.4)	15.3 (9.1 to 23.2)	-0.6 (-1.2 to -0.1)	-8.7 (-16.3 to -0.7)
Saint Vincent and the Grenadines	10.6 (6.6 to 16.6)	13.6 (8.0 to 20.7)	13.9 (7.9 to 21.6)	14.8 (8.6 to 23.0)	0.6 (0.1 to 1.1)	9.0 (1.6 to 18.2)
Suriname	13.3 (8.6 to 19.6)	18.2 (11.6 to 25.3)	18.3 (11.2 to 25.8)	19.5 (12.1 to 28.2)	0.5 (-0.1 to 1.1)	7.3 (-1.9 to 17.4)
Trinidad and Tobago	7.7 (4.4 to 12.8)	9.8 (5.5 to 15.5)	10.0 (5.5 to 16.0)	11.5 (6.4 to 18.1)	1.1 (0.3 to 1.6)	17.8 (5.4 to 26.5)
Virgin Islands, U.S.	7.0 (0.2 to 15.7)	8.1 (1.3 to 17.0)	8.8 (0.3 to 19.1)	9.4 (0.3 to 20.5)	-2.6 (-15.4 to 3.4)	12.7 (-90.1 to 66.6)
Central Latin America						
Colombia	15.1 (9.7 to 22.0)	15.6 (9.4 to 23.2)	16.0 (9.3 to 24.0)	16.6 (9.8 to 25.0)	0.4 (-0.1 to 0.9)	6.3 (-2.1 to 14.1)
Costa Rica	15.3 (9.8 to 22.1)	14.3 (8.6 to 22.1)	14.5 (8.3 to 22.6)	14.6 (8.5 to 23.1)	0.1 (-0.5 to 0.6)	2.0 (-7.1 to 9.9)
El Salvador	9.8 (6.1 to 14.8)	11.6 (7.1 to 17.2)	11.9 (7.0 to 18.0)	13.2 (7.8 to 19.8)	0.8 (0.3 to 1.4)	13.6 (5.1 to 23.3)
Guatemala	10.0 (7.3 to 12.5)	10.2 (7.0 to 13.1)	10.3 (6.9 to 13.4)	10.1 (6.8 to 13.2)	-0.1 (-0.4 to 0.2)	-1.0 (-6.4 to 3.7)
Honduras	8.0 (5.8 to 10.0)	8.5 (6.4 to 10.8)	8.6 (6.2 to 11.0)	8.9 (6.4 to 11.5)	0.3 (-0.1 to 0.8)	4.9 (-1.9 to 13.1)
Mexico	15.4 (11.7 to 19.1)	13.3 (9.4 to 18.0)	13.4 (9.1 to 18.4)	12.9 (8.7 to 18.0)	-0.2 (-0.7 to 0.2)	-3.2 (-9.4 to 2.8)
Nicaragua	10.6 (6.8 to 15.1)	10.3 (6.4 to 15.3)	10.5 (6.2 to 15.9)	10.3 (6.1 to 15.7)	-0.0 (-0.5 to 0.4)	-0.5 (-7.3 to 6.3)
Panama	12.4 (8.8 to 16.3)	14.8 (10.6 to 19.4)	15.3 (10.6 to 20.4)	16.5 (11.5 to 21.8)	0.7 (0.3 to 1.1)	11.3 (5.3 to 17.7)
Venezuela	16.1 (12.3 to 19.9)	16.4 (11.8 to 21.3)	16.4 (11.0 to 21.7)	15.9 (10.7 to 21.2)	-0.2 (-0.7 to 0.1)	-3.4 (-10.0 to 2.0)
Tropical Latin America						
Brazil	15.7 (10.5 to 21.9)	16.3 (10.6 to 22.9)	17.1 (10.7 to 24.3)	17.4 (11.1 to 24.9)	0.4 (0.0 to 0.9)	6.8 (0.5 to 14.6)
Paraguay	21.1 (14.3 to 28.3)	17.1 (10.8 to 25.6)	17.8 (10.8 to 26.6)	18.1 (10.9 to 27.1)	0.4 (-0.3 to 0.9)	5.6 (-4.6 to 15.2)
North Africa and Middle East						
North Africa and Middle East						
Afghanistan	0.4 (0.2 to 0.8)	1.4 (0.8 to 2.4)	1.4 (0.8 to 2.4)	1.7 (1.0 to 2.9)	1.3 (0.7 to 2.0)	21.9 (11.1 to 35.4)
Algeria	1.9 (1.1 to 2.9)	2.1 (1.2 to 3.1)	2.1 (1.2 to 3.2)	2.0 (1.1 to 3.1)	-0.4 (-1.1 to 0.5)	-5.3 (-14.6 to 8.3)
Bahrain	5.0 (3.9 to 6.2)	3.4 (2.2 to 4.9)	3.4 (2.0 to 5.0)	2.9 (1.7 to 4.3)	-1.0 (-1.8 to -0.5)	-14.3 (-23.9 to -6.6)
Egypt	1.4 (1.1 to 1.7)	1.4 (1.0 to 1.9)	1.4 (1.0 to 1.9)	1.3 (0.9 to 1.7)	-0.6 (-1.0 to -0.1)	-7.9 (-14.0 to -1.2)
Iran	0.2 (0.1 to 0.4)	0.0 (0.0 to 0.0)	0.0 (0.0 to 0.0)	0.0 (0.0 to 0.0)	-7.1 (-103.6 to 2.5)	-6.7 (-100.0 to 45.4)
Iraq	2.5 (1.5 to 3.6)	2.0 (1.1 to 3.1)	2.1 (1.1 to 3.3)	2.2 (1.2 to 3.5)	0.5 (-0.6 to 1.1)	8.3 (-8.6 to 18.6)
Jordan	1.1 (0.6 to 2.0)	1.4 (0.7 to 2.2)	1.4 (0.7 to 2.3)	1.3 (0.7 to 2.3)	-0.1 (-0.7 to 0.8)	-0.9 (-9.9 to 12.2)
Kuwait	0.3 (0.1 to 0.6)	0.4 (0.2 to 0.8)	0.5 (0.2 to 0.8)	0.5 (0.2 to 1.0)	1.3 (0.4 to 1.8)	20.9 (6.6 to 31.1)
Lebanon	5.6 (3.9 to 7.5)	5.7 (3.7 to 7.9)	5.8 (3.7 to 8.3)	6.1 (3.9 to 8.8)	0.5 (-0.4 to 1.0)	7.8 (-5.8 to 16.0)
Libya	0.2 (0.1 to 0.3)	0.3 (0.1 to 0.6)	0.3 (0.1 to 0.7)	0.4 (0.1 to 0.7)	0.8 (0.1 to 1.5)	12.6 (1.5 to 25.6)
Morocco	2.3 (1.8 to 2.8)	2.0 (1.6 to 2.5)	2.0 (1.5 to 2.5)	1.8 (1.4 to 2.3)	-0.7 (-1.1 to -0.4)	-10.3 (-15.2 to -6.5)
Palestine	0.3 (0.1 to 0.6)	0.3 (0.1 to 0.6)	0.3 (0.1 to 0.6)	0.2 (0.1 to 0.6)	-1.7 (-3.3 to 0.8)	-12.5 (-38.6 to 13.3)
Oman	1.6 (1.0 to 2.5)	2.0 (1.1 to 3.1)	1.9 (1.1 to 3.2)	2.0 (1.1 to 3.3)	-0.0 (-0.8 to 0.7)	-0.3 (-11.1 to 11.5)
Qatar	3.0 (1.8 to 4.5)	5.8 (3.8 to 7.9)	5.8 (3.7 to 8.0)	6.0 (3.8 to 8.4)	0.2 (-0.4 to 0.8)	3.8 (-5.3 to 12.9)
Saudi Arabia	1.0 (0.5 to 1.8)	1.2 (0.6 to 2.2)	1.4 (0.7 to 2.4)	1.5 (0.7 to 2.6)	1.1 (0.2 to 1.6)	18.0 (3.7 to 27.9)
Sudan	4.8 (3.9 to 5.8)	4.5 (3.5 to 5.6)	4.5 (3.4 to 5.5)	4.4 (3.3 to 5.5)	-0.2 (-0.5 to 0.1)	-3.0 (-7.8 to 2.2)
Syria	1.6 (1.0 to 2.1)	1.5 (0.9 to 2.2)	1.4 (0.8 to 2.1)	1.2 (0.7 to 1.9)	-1.3 (-2.1 to -0.3)	-18.1 (-27.2 to -4.3)
Tunisia	1.9 (1.2 to 2.9)	2.1 (1.3 to 3.2)	2.1 (1.2 to 3.3)	2.3 (1.3 to 3.5)	0.5 (-0.0 to 1.0)	7.9 (-0.1 to 16.5)
Turkey	7.4 (5.7 to 9.2)	4.9 (3.2 to 7.0)	4.9 (3.0 to 7.1)	4.1 (2.5 to 6.0)	-1.2 (-1.8 to -0.4)	-16.9 (-23.3 to -5.3)
United Arab Emirates	11.2 (8.8 to 13.7)	12.8 (9.4 to 16.7)	12.6 (9.2 to 16.7)	11.9 (8.7 to 15.9)	-0.5 (-0.8 to -0.1)	-6.5 (-11.1 to -1.5)
Yemen	0.9 (0.5 to 1.7)	0.5 (0.2 to 0.9)	0.5 (0.2 to 1.0)	0.5 (0.2 to 1.0)	0.3 (-0.7 to 0.8)	4.0 (-10.6 to 13.4)
South Asia						
South Asia						
Bangladesh	0.3 (0.1 to 0.5)	0.3 (0.2 to 0.6)	0.3 (0.2 to 0.6)	0.4 (0.2 to 0.6)	0.7 (0.1 to 1.4)	10.7 (1.8 to 23.5)
Bhutan	5.0 (3.8 to 6.4)	3.4 (2.0 to 4.7)	3.4 (1.9 to 4.8)	2.9 (1.6 to 4.1)	-1.1 (-1.6 to -0.7)	-15.4 (-21.5 to -10.2)
India	6.8 (5.3 to 8.5)	8.2 (6.4 to 10.1)	8.5 (6.5 to 10.5)	9.6 (7.4 to 12.0)	1.0 (0.8 to 1.4)	17.1 (12.3 to 22.9)
Nepal	2.7 (1.8 to 4.0)	3.8 (2.4 to 5.3)	3.8 (2.4 to 5.4)	4.1 (2.5 to 6.0)	0.6 (-0.1 to 1.6)	9.4 (-2.1 to 27.0)
Pakistan	0.2 (0.1 to 0.5)	0.0 (0.0 to 0.1)	0.0 (0.0 to 0.1)	0.0 (0.0 to 0.1)	-1.6 (-3.1 to 0.5)	-5.7 (-37.6 to 7.9)
Southeast Asia, East Asia, and Oceania						
East Asia						
China	9.2 (6.5 to 12.1)	12.1 (8.3 to 15.8)	12.5 (8.3 to 16.6)	13.9 (9.3 to 18.4)	0.9 (0.6 to 1.2)	15.1 (8.8 to 20.5)
North Korea	8.0 (5.2 to 11.4)	7.1 (4.2 to 10.8)	7.2 (4.3 to 11.2)	7.2 (4.2 to 11.1)	0.1 (-0.6 to 0.7)	1.3 (-8.1 to 10.8)
Taiwan (Province of China)	9.7 (5.4 to 14.4)	12.4 (7.0 to 18.4)	13.1 (7.4 to 19.7)	14.4 (8.3 to 21.4)	1.0 (0.6 to 1.5)	16.9 (9.1 to 25.9)
Oceania						
American Samoa	4.5 (2.2 to 8.3)	5.0 (2.5 to 8.7)	5.1 (2.6 to 8.8)	5.6 (2.8 to 9.7)	0.7 (0.2 to 1.2)	11.3 (3.8 to 19.3)
Federated States of Micronesia	4.9 (2.7 to 8.4)	4.1 (2.1 to 7.5)	4.2 (2.0 to 7.7)	3.7 (1.8 to 6.9)	-0.7 (-1.2 to -0.3)	-10.0 (-16.7 to -3.7)
Fiji	6.0 (3.5 to 9.2)	7.5 (4.4 to 11.3)	7.6 (4.3 to 11.5)	8.4 (4.8 to 12.7)	0.7 (0.1 to 1.4)	11.9 (1.7 to 22.8)
Guam	2.6 (0.2 to 6.6)	3.1 (0.6 to 7.3)	3.2 (0.4 to 7.6)	3.3 (0.4 to 7.8)	-1.3 (-2.3 to 1.9)	3.7 (-29.2 to 32.2)
Kiribati	4.6 (2.6 to 8.0)	4.7 (2.4 to 8.0)	4.7 (2.4 to 8.2)	4.9 (2.5 to 8.5)	0.3 (-0.1 to 0.7)	4.6 (-2.2 to 11.6)
Marshall Islands	4.2 (1.9 to 7.7)	4.5 (2.2 to 8.4)	4.6 (2.1 to 8.5)	5.2 (2.5 to 9.8)	1.0 (0.5 to 1.5)	17.0 (7.9 to 25.9)
Northern Mariana Islands	3.6 (0.7 to 7.6)	4.1 (0.9 to 8.9)	4.2 (0.7 to 9.2)	4.8 (0.8 to 10.3)	-0.3 (-1.4 to 2.4)	15.5 (-19.4 to 42.9)
Papua New Guinea	14.8 (9.0 to 20.6)	12.8 (7.0 to 19.0)	12.9 (7.1 to 19.3)	12.3 (6.7 to 18.7)	-0.3 (-0.9 to 0.3)	-4.0 (-12.1 to 4.0)

	Estimate in 2000 (95% UIs)	Estimate in 2015 (95% UIs)	Estimate in 2017 (95% UIs)	Estimate in 2030 (95% UIs)	Annualised rate of change, 2015-2030 (95% UIs)	Percent change, 2015-2030 (95% UIs)
Samoa	6.7 (4.0 to 10.8)	7.3 (4.2 to 11.8)	7.4 (4.1 to 11.9)	7.5 (4.1 to 12.3)	0.2 (-0.4 to 0.7)	2.8 (-5.3 to 11.5)
Solomon Islands	2.7 (1.3 to 5.4)	4.1 (2.0 to 7.3)	4.1 (2.0 to 7.4)	5.0 (2.4 to 8.9)	1.3 (0.8 to 1.9)	22.0 (13.2 to 32.1)
Tonga	3.8 (1.8 to 7.1)	4.0 (1.8 to 7.5)	4.1 (1.8 to 7.7)	4.2 (1.8 to 8.1)	0.4 (-0.4 to 1.5)	6.2 (-5.3 to 25.6)
Vanuatu	4.1 (2.1 to 7.2)	2.6 (1.1 to 5.2)	2.6 (1.0 to 5.3)	2.3 (0.9 to 4.6)	-1.0 (-1.8 to -0.4)	-14.0 (-23.4 to -6.2)
Southeast Asia						
Cambodia	13.4 (9.7 to 16.6)	17.0 (13.5 to 20.6)	17.1 (13.5 to 20.9)	17.0 (13.3 to 21.3)	0.0 (-0.4 to 0.8)	0.1 (-5.5 to 13.2)
Indonesia	1.0 (0.5 to 1.7)	1.1 (0.6 to 1.9)	1.2 (0.6 to 2.0)	1.3 (0.7 to 2.1)	0.8 (0.4 to 1.2)	13.2 (5.5 to 20.4)
Laos	9.8 (6.0 to 15.2)	9.7 (5.7 to 15.6)	9.9 (5.8 to 16.0)	10.4 (6.2 to 17.0)	0.5 (-0.0 to 1.0)	7.4 (-0.6 to 15.6)
Malaysia	4.8 (3.0 to 6.5)	5.4 (3.4 to 7.5)	5.6 (3.3 to 7.7)	5.7 (3.5 to 8.1)	0.3 (-0.3 to 0.9)	5.4 (-5.1 to 14.4)
Maldives	4.1 (2.3 to 6.8)	4.1 (2.1 to 7.1)	4.2 (2.0 to 7.5)	3.7 (1.8 to 6.8)	-0.8 (-1.7 to 0.7)	-10.6 (-22.8 to 10.7)
Mauritius	11.1 (7.0 to 16.4)	10.9 (6.4 to 17.3)	11.4 (6.5 to 18.2)	10.8 (6.1 to 17.4)	-0.1 (-0.6 to 0.3)	-1.8 (-8.3 to 5.4)
Myanmar	3.1 (1.6 to 5.0)	4.1 (2.0 to 6.8)	4.3 (2.1 to 7.3)	5.1 (2.5 to 8.7)	1.5 (0.9 to 2.2)	24.5 (14.4 to 38.5)
Philippines	10.5 (6.5 to 16.4)	10.0 (5.8 to 16.0)	10.2 (5.9 to 16.3)	9.4 (5.4 to 15.4)	-0.4 (-0.9 to 0.1)	-6.3 (-13.1 to 1.6)
Sri Lanka	4.2 (2.2 to 7.2)	9.9 (5.9 to 14.9)	10.5 (6.1 to 16.2)	12.0 (7.3 to 18.2)	1.3 (0.6 to 2.0)	21.3 (10.0 to 34.4)
Seychelles	12.4 (8.9 to 16.2)	11.3 (7.1 to 16.1)	11.4 (6.8 to 16.6)	10.1 (5.9 to 15.0)	-0.8 (-1.5 to 0.1)	-11.5 (-20.2 to 2.2)
Thailand	12.3 (8.6 to 16.3)	14.2 (9.6 to 19.0)	14.7 (9.6 to 19.9)	14.7 (9.6 to 20.2)	0.2 (-0.3 to 0.7)	3.2 (-3.8 to 11.4)
Timor-Leste	0.9 (0.4 to 1.9)	0.4 (0.1 to 0.9)	0.4 (0.1 to 0.9)	0.4 (0.1 to 0.9)	0.2 (-0.7 to 1.1)	3.8 (-9.7 to 18.5)
Vietnam	10.2 (6.0 to 15.4)	21.3 (15.6 to 26.9)	21.4 (15.3 to 27.1)	22.5 (15.9 to 28.9)	0.4 (-0.2 to 1.1)	5.7 (-2.6 to 18.6)
Sub-Saharan Africa						
Central Sub-Saharan Africa						
Angola	5.2 (2.8 to 9.2)	10.5 (6.4 to 15.7)	10.6 (6.2 to 16.2)	12.2 (7.1 to 18.5)	0.9 (0.2 to 1.7)	15.4 (3.2 to 29.6)
Central African Republic	11.2 (6.7 to 16.8)	10.8 (6.1 to 16.6)	10.7 (5.8 to 17.0)	10.0 (5.5 to 15.9)	-0.5 (-1.1 to -0.0)	-7.3 (-14.6 to -0.3)
Congo	9.8 (5.8 to 15.1)	11.0 (6.7 to 16.6)	11.3 (6.7 to 17.4)	12.6 (7.5 to 19.2)	0.9 (0.3 to 1.5)	14.9 (4.9 to 24.3)
Democratic Republic of the Congo	6.3 (3.4 to 10.6)	7.5 (4.1 to 12.3)	7.6 (4.0 to 12.6)	8.7 (4.6 to 14.3)	1.0 (0.5 to 1.6)	16.9 (8.4 to 26.8)
Equatorial Guinea	10.1 (6.4 to 14.8)	18.7 (13.1 to 24.2)	18.9 (12.8 to 24.7)	18.9 (12.6 to 25.3)	0.0 (-0.7 to 0.9)	0.9 (-10.1 to 14.4)
Gabon	12.6 (8.3 to 17.6)	13.2 (8.5 to 18.4)	13.4 (8.3 to 19.2)	13.3 (8.3 to 19.1)	0.0 (-0.5 to 0.5)	0.7 (-7.2 to 8.5)
Eastern Sub-Saharan Africa						
Burundi	22.0 (17.9 to 26.3)	20.8 (16.0 to 25.7)	20.4 (15.3 to 25.4)	18.8 (13.9 to 23.5)	-0.7 (-1.1 to -0.4)	-9.9 (-15.1 to -5.3)
Comoros	0.9 (0.4 to 1.8)	1.1 (0.5 to 2.2)	1.2 (0.5 to 2.3)	1.3 (0.6 to 2.5)	0.9 (0.3 to 1.5)	14.2 (5.1 to 24.5)
Djibouti	3.3 (1.6 to 6.2)	3.2 (1.4 to 6.2)	3.2 (1.4 to 6.3)	3.3 (1.5 to 6.5)	0.2 (-0.3 to 0.7)	3.7 (-4.2 to 11.1)
Eritrea	6.5 (3.6 to 10.7)	5.3 (2.6 to 9.2)	5.5 (2.6 to 9.5)	5.6 (2.7 to 9.8)	0.4 (-0.1 to 0.9)	6.1 (-1.0 to 13.9)
Ethiopia	6.4 (3.8 to 10.5)	7.0 (4.2 to 11.4)	7.1 (4.2 to 11.6)	7.4 (4.3 to 12.0)	0.3 (-0.1 to 0.9)	5.2 (-1.8 to 14.3)
Kenya	13.5 (9.6 to 17.1)	13.0 (8.8 to 17.1)	13.0 (8.7 to 17.2)	13.7 (9.2 to 18.1)	0.4 (0.0 to 0.7)	5.6 (0.6 to 10.3)
Madagascar	6.2 (3.4 to 10.3)	5.6 (2.9 to 9.9)	5.7 (2.9 to 10.1)	6.5 (3.3 to 11.1)	0.9 (-0.3 to 1.5)	15.0 (-3.9 to 25.6)
Malawi	5.7 (3.3 to 8.9)	6.4 (3.5 to 10.2)	6.4 (3.4 to 10.1)	6.5 (3.4 to 10.4)	0.0 (-0.7 to 0.7)	0.6 (-9.5 to 11.5)
Mozambique	6.5 (3.2 to 12.0)	7.8 (4.0 to 13.7)	7.9 (3.9 to 14.2)	8.5 (4.2 to 15.0)	0.6 (0.0 to 1.4)	9.0 (0.4 to 23.0)
Rwanda	19.6 (12.5 to 28.3)	16.1 (9.5 to 25.0)	15.8 (9.0 to 25.1)	14.2 (8.0 to 22.8)	-0.9 (-1.4 to -0.4)	-12.3 (-18.8 to -5.8)
Somalia	1.7 (0.7 to 3.7)	2.0 (0.9 to 4.1)	2.0 (0.9 to 4.2)	2.2 (0.9 to 4.5)	0.6 (0.0 to 1.1)	9.5 (0.5 to 18.3)
South Sudan	1.9 (0.8 to 3.8)	1.9 (0.8 to 3.7)	1.9 (0.8 to 3.6)	1.9 (0.8 to 3.8)	0.2 (-0.3 to 0.7)	3.4 (-4.7 to 11.2)
Tanzania	15.7 (10.6 to 20.4)	11.3 (7.0 to 17.1)	10.7 (6.2 to 16.8)	9.2 (5.3 to 14.6)	-1.4 (-2.1 to -0.9)	-19.2 (-26.9 to -12.2)
Uganda	16.4 (10.4 to 23.2)	12.4 (7.2 to 19.6)	11.7 (6.4 to 18.9)	9.9 (5.3 to 16.4)	-1.5 (-2.2 to -0.8)	-20.5 (-28.2 to -11.6)
Zambia	10.1 (6.2 to 14.7)	8.6 (4.9 to 13.4)	8.6 (4.8 to 13.6)	8.3 (4.6 to 13.1)	-0.3 (-0.8 to 0.2)	-4.2 (-10.9 to 3.1)
Southern Sub-Saharan Africa						
Botswana	14.5 (10.7 to 18.0)	16.4 (11.6 to 21.0)	16.2 (10.9 to 20.9)	14.9 (10.0 to 19.9)	-0.7 (-1.4 to 0.2)	-9.8 (-18.6 to 3.8)
Lesotho	19.1 (13.2 to 24.5)	20.1 (13.1 to 26.3)	20.0 (12.5 to 26.5)	18.8 (11.7 to 25.8)	-0.4 (-1.0 to 0.1)	-6.3 (-13.7 to 2.1)
Namibia	10.8 (6.5 to 17.1)	21.5 (13.9 to 28.2)	21.0 (12.9 to 28.4)	21.2 (13.0 to 29.2)	-0.1 (-0.9 to 0.9)	-1.4 (-12.8 to 15.0)
South Africa	17.8 (14.5 to 21.2)	19.4 (15.7 to 23.5)	19.7 (15.7 to 24.3)	19.9 (15.8 to 24.6)	0.2 (-0.1 to 0.4)	2.4 (-1.1 to 6.0)
Swaziland	11.4 (9.2 to 13.8)	8.2 (5.4 to 11.1)	8.1 (5.0 to 11.3)	7.0 (4.3 to 9.8)	-1.0 (-1.6 to -0.6)	-14.2 (-21.3 to -8.4)
Zimbabwe	8.8 (5.8 to 12.1)	8.9 (5.7 to 12.7)	9.1 (5.5 to 13.1)	9.0 (5.5 to 13.0)	0.0 (-0.4 to 0.5)	0.5 (-6.0 to 8.3)
Western Sub-Saharan Africa						
Benin	6.0 (3.1 to 10.3)	6.3 (3.2 to 11.0)	6.3 (3.2 to 11.2)	6.6 (3.4 to 11.5)	0.4 (-0.1 to 0.9)	5.6 (-1.9 to 14.7)
Burkina Faso	15.2 (9.6 to 20.7)	12.7 (7.3 to 18.6)	12.3 (6.8 to 18.7)	10.6 (5.7 to 16.5)	-1.2 (-1.8 to -0.5)	-16.5 (-24.2 to -7.0)
Cameroon	12.4 (9.5 to 15.6)	13.8 (10.0 to 17.3)	13.5 (9.6 to 17.2)	12.4 (8.6 to 16.3)	-0.7 (-1.3 to -0.0)	-9.9 (-17.7 to -0.0)
Cape Verde	11.0 (8.5 to 13.7)	11.9 (8.1 to 15.7)	11.8 (7.7 to 16.0)	10.8 (6.9 to 14.9)	-0.7 (-1.4 to 0.2)	-9.6 (-18.4 to 2.8)
Chad	6.0 (4.0 to 8.3)	6.8 (4.4 to 9.4)	6.8 (4.3 to 9.7)	6.7 (4.0 to 9.9)	-0.1 (-0.9 to 0.9)	-1.9 (-13.1 to 14.3)
Cote d'Ivoire	10.5 (6.3 to 15.0)	8.2 (4.7 to 13.0)	7.9 (4.4 to 12.8)	6.8 (3.6 to 11.2)	-1.3 (-2.0 to -0.3)	-17.7 (-26.3 to -4.9)
The Gambia	5.3 (3.3 to 8.1)	4.2 (2.4 to 6.9)	3.9 (2.1 to 6.7)	3.5 (1.8 to 6.2)	-1.4 (-2.3 to -0.1)	-18.1 (-29.0 to -1.7)
Ghana	13.9 (8.5 to 18.9)	16.9 (11.3 to 22.2)	16.9 (11.0 to 22.4)	17.8 (11.5 to 23.8)	0.3 (-0.2 to 0.7)	5.2 (-2.7 to 11.8)
Guinea	1.5 (0.7 to 2.8)	1.5 (0.6 to 2.8)	1.5 (0.6 to 2.9)	1.5 (0.6 to 2.9)	0.2 (-0.3 to 0.6)	2.6 (-4.8 to 9.5)
Guinea-Bissau	8.2 (5.5 to 11.0)	7.8 (5.0 to 11.3)	7.7 (4.7 to 11.3)	7.1 (4.3 to 10.6)	-0.6 (-1.2 to -0.1)	-9.0 (-16.1 to -2.2)
Liberia	12.6 (8.7 to 16.5)	10.3 (6.0 to 14.7)	10.5 (5.8 to 15.3)	9.9 (5.4 to 14.5)	-0.3 (-1.1 to 0.4)	-4.3 (-14.7 to 5.5)
Mali	2.7 (1.6 to 3.8)	2.6 (1.5 to 3.9)	2.6 (1.5 to 3.9)	2.5 (1.4 to 3.8)	-0.3 (-0.7 to 0.2)	-3.9 (-10.6 to 3.2)
Mauritania	0.7 (0.3 to 1.2)	0.8 (0.4 to 1.4)	0.8 (0.4 to 1.5)	0.8 (0.4 to 1.5)	0.3 (-0.6 to 0.8)	4.1 (-8.5 to 13.2)
Niger	1.8 (0.8 to 3.5)	2.0 (0.8 to 3.9)	2.0 (0.8 to 4.1)	2.0 (0.8 to 3.9)	0.0 (-0.6 to 0.7)	0.3 (-8.3 to 11.7)
Nigeria	18.7 (13.7 to 23.7)	10.9 (6.5 to 16.8)	10.4 (5.8 to 16.5)	8.8 (4.8 to 14.0)	-1.5 (-2.2 to -0.7)	-20.0 (-27.9 to -9.6)
Sao Tome and Principe	13.2 (10.6 to 16.0)	13.2 (10.5 to 15.9)	13.2 (10.2 to 16.2)	12.1 (9.2 to 15.2)	-0.6 (-1.0 to 0.3)	-8.2 (-14.0 to 4.9)
Senegal	1.3 (0.7 to 2.2)	1.2 (0.6 to 2.1)	1.2 (0.6 to 2.1)	1.1 (0.6 to 2.1)	-0.2 (-0.9 to 0.4)	-2.1 (-12.6 to 5.8)
Sierra Leone	13.7 (11.0 to 16.6)	12.1 (8.8 to 15.4)	11.7 (8.3 to 15.1)	10.2 (7.1 to 13.4)	-1.2 (-1.7 to -0.2)	-15.7 (-22.8 to -2.9)
Togo	6.2 (3.7 to 9.4)	6.3 (3.6 to 9.6)	6.4 (3.6 to 9.9)	6.3 (3.6 to 9.9)	0.1 (-0.5 to 0.5)	0.9 (-7.4 to 8.4)
**Indicator 3.6.1: Age-standardised death rate due to road injuries (per 100,000 population)**						
Central Europe, Eastern Europe, and Central Asia						
Central Asia						
Armenia	9.2 (8.9 to 9.6)	7.3 (7.0 to 7.7)	6.9 (6.5 to 7.3)	8.1 (6.3 to 10.9)	0.6 (-1.0 to 2.7)	11.0 (-14.0 to 49.5)
Azerbaijan	7.8 (7.4 to 8.4)	6.2 (5.5 to 6.9)	6.1 (5.3 to 6.8)	6.0 (4.8 to 7.8)	-0.2 (-1.6 to 1.2)	-2.6 (-20.9 to 18.8)
Georgia	7.9 (7.5 to 8.5)	18.0 (17.2 to 18.8)	17.1 (15.8 to 18.3)	12.6 (9.5 to 17.1)	-2.4 (-4.2 to -0.4)	-29.7 (-46.8 to -6.4)
Kazakhstan	15.0 (14.4 to 15.5)	15.4 (14.7 to 16.3)	14.6 (13.5 to 15.8)	12.9 (9.4 to 17.6)	-1.2 (-3.2 to 0.9)	-16.1 (-38.2 to 14.5)
Kyrgyzstan	14.7 (13.9 to 15.7)	16.4 (15.7 to 17.2)	14.4 (13.5 to 15.5)	13.2 (10.2 to 17.1)	-1.5 (-3.3 to 0.3)	-19.8 (-38.6 to 4.0)
Mongolia	16.1 (14.6 to 18.1)	16.6 (15.1 to 18.5)	16.1 (14.2 to 18.3)	12.7 (10.2 to 15.4)	-1.8 (-3.0 to -0.6)	-23.8 (-36.7 to -8.8)
Tajikistan	6.6 (6.1 to 7.2)	7.4 (6.7 to 8.2)	7.5 (6.7 to 8.3)	8.0 (6.2 to 9.9)	0.5 (-1.1 to 1.9)	7.8 (-15.1 to 32.4)
Turkmenistan	10.4 (10.0 to 10.9)	6.5 (6.0 to 6.9)	6.3 (5.7 to 7.1)	6.5 (5.0 to 8.9)	-0.0 (-1.5 to 2.0)	0.6 (-20.6 to 35.0)

	Estimate in 2000 (95% UIs)	Estimate in 2015 (95% UIs)	Estimate in 2017 (95% UIs)	Estimate in 2030 (95% UIs)	Annualised rate of change, 2015-2030 (95% UIs)	Percent change, 2015-2030 (95% UIs)
Uzbekistan	13.0 (12.5 to 13.7)	13.2 (12.1 to 14.4)	12.6 (11.0 to 14.2)	12.0 (9.5 to 14.8)	-0.7 (-2.0 to 0.7)	-9.3 (-26.1 to 10.7)
Central Europe						
Albania	8.1 (7.5 to 8.9)	8.1 (6.7 to 9.7)	7.8 (6.3 to 9.5)	6.0 (4.3 to 8.2)	-2.0 (-3.7 to -0.4)	-25.6 (-42.9 to -5.7)
Bosnia and Herzegovina	5.2 (4.6 to 5.9)	6.5 (6.0 to 6.9)	6.3 (5.7 to 6.8)	4.9 (4.2 to 5.8)	-1.9 (-2.9 to -0.8)	-24.4 (-34.9 to -11.5)
Bulgaria	12.0 (11.6 to 12.3)	8.6 (8.1 to 9.1)	8.5 (7.9 to 9.3)	5.5 (4.4 to 6.8)	-3.0 (-4.4 to -1.6)	-35.7 (-48.4 to -21.8)
Croatia	13.7 (13.3 to 14.1)	7.8 (7.5 to 8.1)	7.2 (6.6 to 7.7)	4.7 (4.0 to 5.5)	-3.5 (-4.5 to -2.3)	-40.5 (-49.4 to -29.7)
Czech Republic	13.3 (12.9 to 13.6)	6.7 (6.5 to 7.0)	6.5 (6.0 to 7.0)	4.7 (4.0 to 5.5)	-2.4 (-3.5 to -1.4)	-30.3 (-40.5 to -18.6)
Hungary	13.1 (12.8 to 13.4)	6.9 (6.6 to 7.1)	6.2 (5.8 to 6.8)	4.7 (3.8 to 5.9)	-2.6 (-3.9 to -0.9)	-32.1 (-44.4 to -13.1)
Macedonia	7.0 (6.6 to 7.4)	6.4 (6.0 to 6.8)	6.3 (5.7 to 6.9)	5.8 (4.8 to 7.0)	-0.7 (-1.7 to 0.6)	-9.2 (-22.4 to 8.9)
Montenegro	10.3 (9.7 to 11.0)	7.7 (7.1 to 8.3)	7.6 (6.8 to 8.4)	6.9 (5.7 to 8.2)	-0.7 (-1.9 to 0.4)	-10.1 (-24.9 to 6.0)
Poland	17.4 (17.0 to 17.8)	8.8 (8.5 to 9.1)	8.5 (7.9 to 9.1)	3.4 (2.9 to 4.0)	-6.3 (-7.3 to -5.3)	-61.0 (-66.5 to -54.6)
Romania	13.3 (12.9 to 13.7)	9.3 (9.0 to 9.6)	9.2 (8.6 to 9.8)	5.5 (4.4 to 6.8)	-3.5 (-5.0 to -2.0)	-41.0 (-52.5 to -26.2)
Serbia	12.0 (11.5 to 12.6)	7.4 (7.0 to 7.7)	6.9 (6.4 to 7.4)	6.5 (5.2 to 8.7)	-0.9 (-2.2 to 0.9)	-11.6 (-28.6 to 14.4)
Slovakia	14.7 (14.2 to 15.3)	7.3 (6.9 to 8.1)	6.8 (6.3 to 7.6)	5.4 (4.6 to 6.3)	-2.1 (-3.1 to -1.0)	-26.3 (-37.3 to -14.1)
Slovenia	14.6 (14.2 to 15.1)	5.8 (5.5 to 6.1)	5.8 (5.3 to 6.4)	3.6 (2.8 to 4.7)	-3.2 (-4.9 to -1.3)	-37.1 (-52.3 to -18.2)
Eastern Europe						
Belarus	19.4 (18.7 to 20.1)	9.1 (8.6 to 9.7)	8.6 (7.9 to 9.5)	5.1 (3.7 to 6.8)	-3.9 (-6.0 to -1.9)	-43.7 (-59.2 to -24.6)
Estonia	17.2 (16.6 to 18.0)	5.9 (5.6 to 6.4)	5.7 (4.9 to 6.5)	3.9 (2.4 to 5.9)	-3.0 (-6.1 to -0.1)	-34.6 (-59.8 to -1.2)
Latvia	24.1 (23.3 to 25.0)	9.1 (8.7 to 9.6)	9.0 (7.9 to 10.2)	6.4 (3.9 to 10.1)	-2.6 (-5.6 to 0.6)	-30.1 (-56.9 to 9.8)
Lithuania	21.1 (20.4 to 21.8)	9.7 (9.2 to 10.1)	9.1 (8.4 to 9.9)	4.7 (3.4 to 6.3)	-5.0 (-7.0 to -2.9)	-51.9 (-64.9 to -35.1)
Moldova	15.6 (15.1 to 16.2)	11.2 (10.8 to 11.7)	10.2 (9.6 to 10.8)	10.0 (6.9 to 14.3)	-0.9 (-3.3 to 1.7)	-10.6 (-39.0 to 28.4)
Russia	26.2 (26.0 to 26.5)	16.0 (15.7 to 16.5)	14.8 (14.4 to 15.7)	9.0 (5.6 to 13.1)	-4.0 (-6.9 to -1.4)	-44.1 (-64.7 to -18.8)
Ukraine	17.3 (16.8 to 17.9)	15.1 (12.6 to 16.1)	13.7 (12.0 to 14.9)	13.2 (8.1 to 20.3)	-1.1 (-4.1 to 1.9)	-12.8 (-46.3 to 33.7)
High-income						
Australasia						
Australia	10.1 (9.8 to 10.4)	5.9 (5.7 to 6.1)	5.9 (5.3 to 6.6)	4.9 (4.0 to 6.1)	-1.3 (-2.7 to 0.2)	-17.5 (-33.0 to 2.4)
New Zealand	13.2 (12.8 to 13.6)	7.3 (7.0 to 7.5)	7.5 (7.0 to 8.0)	6.0 (5.1 to 7.0)	-1.3 (-2.4 to -0.2)	-17.4 (-30.3 to -3.5)
High-income Asia Pacific						
Brunei	21.9 (20.5 to 23.4)	16.0 (14.7 to 17.3)	16.1 (14.5 to 17.7)	15.1 (12.8 to 17.9)	-0.4 (-1.3 to 0.6)	-5.5 (-17.9 to 9.8)
Japan	7.8 (7.7 to 7.8)	3.7 (3.6 to 3.8)	3.7 (3.6 to 3.9)	3.3 (2.8 to 4.0)	-0.8 (-2.0 to 0.3)	-11.4 (-25.7 to 4.9)
South Korea	24.5 (24.0 to 25.1)	9.4 (9.0 to 9.9)	9.1 (8.4 to 9.8)	5.8 (4.8 to 7.2)	-3.2 (-4.5 to -1.9)	-38.1 (-49.3 to -24.3)
Singapore	5.9 (5.4 to 6.3)	3.2 (3.1 to 3.4)	3.0 (2.8 to 3.2)	2.9 (2.3 to 3.6)	-0.7 (-2.3 to 0.6)	-9.1 (-28.9 to 10.2)
High-income North America						
Canada	10.0 (9.7 to 10.2)	6.7 (6.4 to 7.0)	6.6 (6.1 to 7.0)	6.4 (5.5 to 7.3)	-0.4 (-1.3 to 0.5)	-5.0 (-17.7 to 7.7)
Greenland	8.1 (7.3 to 9.0)	4.1 (3.7 to 4.6)	4.0 (3.6 to 4.5)	3.8 (2.8 to 5.3)	-0.5 (-2.4 to 1.7)	-6.2 (-30.3 to 29.4)
United States	15.9 (15.8 to 16.1)	11.4 (11.2 to 11.6)	11.9 (11.4 to 12.4)	12.2 (9.7 to 15.3)	0.4 (-1.1 to 1.9)	7.2 (-14.8 to 33.3)
Southern Latin America						
Argentina	14.3 (13.9 to 14.7)	13.9 (13.4 to 14.4)	13.4 (11.9 to 15.1)	10.8 (8.9 to 13.0)	-1.7 (-2.9 to -0.5)	-22.2 (-35.2 to -6.8)
Chile	14.9 (14.5 to 15.3)	11.3 (10.9 to 11.7)	11.0 (9.6 to 12.4)	8.2 (6.8 to 10.0)	-2.1 (-3.4 to -0.9)	-26.9 (-39.7 to -12.6)
Uruguay	15.3 (14.8 to 15.8)	15.4 (14.8 to 16.1)	14.7 (12.9 to 16.5)	8.0 (6.3 to 10.2)	-4.4 (-5.8 to -2.9)	-48.1 (-58.0 to -35.0)
Western Europe						
Andorra	6.3 (5.4 to 7.3)	4.2 (3.6 to 4.8)	4.1 (3.5 to 4.8)	4.0 (3.2 to 4.8)	-0.2 (-1.1 to 0.7)	-3.2 (-15.5 to 10.8)
Austria	10.8 (10.4 to 11.1)	4.6 (4.4 to 4.8)	4.3 (4.0 to 4.7)	3.1 (2.5 to 3.9)	-2.6 (-4.0 to -1.1)	-32.0 (-45.4 to -14.9)
Belgium	15.6 (15.2 to 16.0)	7.2 (6.9 to 7.4)	6.9 (6.4 to 7.4)	5.5 (4.4 to 6.8)	-1.7 (-3.2 to -0.4)	-22.6 (-38.3 to -5.6)
Cyprus	23.5 (21.8 to 25.6)	10.1 (9.4 to 11.0)	10.0 (9.1 to 11.1)	7.0 (5.4 to 9.2)	-2.5 (-4.0 to -0.7)	-30.7 (-44.9 to -10.1)
Denmark	9.3 (9.0 to 9.6)	3.7 (3.5 to 3.9)	3.9 (3.6 to 4.2)	3.2 (2.3 to 4.2)	-1.1 (-2.9 to 0.8)	-13.8 (-35.2 to 13.4)
Finland	8.0 (7.8 to 8.3)	4.0 (3.8 to 4.2)	4.1 (3.8 to 4.4)	2.9 (2.2 to 3.6)	-2.2 (-3.8 to -0.8)	-28.2 (-43.7 to -11.1)
France	13.3 (12.8 to 13.6)	5.6 (5.4 to 5.8)	5.3 (5.0 to 5.8)	4.6 (3.8 to 5.6)	-1.4 (-2.7 to -0.0)	-18.4 (-32.8 to -0.7)
Germany	9.6 (9.4 to 9.8)	4.3 (4.2 to 4.5)	4.4 (4.0 to 4.9)	3.1 (2.4 to 3.8)	-2.3 (-3.8 to -0.7)	-28.2 (-43.7 to -10.4)
Greece	16.6 (16.3 to 17.0)	9.8 (9.5 to 10.1)	9.7 (9.1 to 10.3)	4.8 (4.0 to 5.8)	-4.7 (-6.0 to -3.5)	-50.7 (-59.5 to -41.2)
Iceland	7.0 (6.6 to 7.5)	3.5 (3.3 to 3.7)	3.6 (3.4 to 3.9)	2.8 (2.1 to 4.0)	-1.5 (-3.3 to 0.8)	-18.9 (-38.7 to 13.5)
Ireland	10.4 (10.1 to 10.8)	3.5 (3.3 to 3.7)	3.4 (3.1 to 3.7)	2.5 (2.0 to 3.1)	-2.3 (-3.6 to -0.7)	-28.3 (-41.4 to -10.6)
Israel	11.9 (11.5 to 12.2)	6.4 (6.2 to 6.7)	6.2 (5.8 to 6.6)	4.7 (3.9 to 5.8)	-2.1 (-3.3 to -0.8)	-26.5 (-39.3 to -10.9)
Italy	13.8 (13.6 to 14.1)	6.3 (6.2 to 6.5)	6.0 (5.6 to 6.4)	4.2 (3.5 to 5.0)	-2.8 (-3.9 to -1.7)	-33.6 (-43.9 to -22.1)
Luxembourg	11.8 (11.3 to 12.3)	5.4 (5.0 to 5.8)	5.3 (4.8 to 5.9)	3.6 (2.4 to 5.3)	-2.8 (-5.2 to -0.3)	-33.7 (-53.9 to -4.8)
Malta	5.7 (5.4 to 6.0)	3.1 (2.9 to 3.4)	3.3 (3.1 to 3.6)	2.3 (1.7 to 3.0)	-2.1 (-4.0 to -0.3)	-26.9 (-45.2 to -4.6)
Netherlands	8.0 (7.8 to 8.2)	3.6 (3.5 to 3.8)	3.7 (3.5 to 3.9)	3.0 (2.5 to 3.5)	-1.4 (-2.5 to -0.2)	-18.9 (-31.7 to -2.4)
Norway	7.7 (7.5 to 7.9)	3.3 (3.2 to 3.4)	3.4 (3.2 to 3.5)	2.5 (2.1 to 3.1)	-1.8 (-3.2 to -0.3)	-23.3 (-37.9 to -5.1)
Portugal	19.6 (19.1 to 20.1)	6.5 (6.2 to 6.8)	6.4 (5.9 to 6.9)	3.3 (2.6 to 4.3)	-4.6 (-6.2 to -2.7)	-49.2 (-60.3 to -32.9)
Spain	13.9 (13.6 to 14.2)	4.2 (4.0 to 4.3)	4.1 (3.8 to 4.4)	2.5 (1.9 to 3.6)	-3.4 (-5.3 to -0.9)	-39.3 (-55.1 to -12.9)
Sweden	6.2 (6.0 to 6.4)	3.2 (3.0 to 3.3)	3.0 (2.8 to 3.3)	2.7 (2.2 to 3.2)	-1.2 (-2.4 to 0.0)	-15.5 (-29.8 to 0.7)
Switzerland	7.3 (7.0 to 7.5)	3.2 (3.0 to 3.3)	3.0 (2.8 to 3.3)	2.6 (1.9 to 3.4)	-1.5 (-3.3 to 0.4)	-19.7 (-39.3 to 6.0)
United Kingdom	6.6 (6.5 to 6.7)	3.3 (3.3 to 3.4)	3.4 (3.3 to 3.5)	3.0 (2.4 to 3.6)	-0.9 (-2.2 to 0.5)	-11.7 (-27.8 to 7.8)
Latin America and Caribbean						
Andean Latin America						
Bolivia	31.2 (26.2 to 35.6)	21.2 (16.3 to 25.5)	20.4 (15.5 to 24.9)	19.4 (14.4 to 24.2)	-0.6 (-1.3 to 0.1)	-8.6 (-17.6 to 1.9)
Ecuador	25.7 (25.0 to 26.5)	28.2 (26.5 to 29.9)	26.7 (24.1 to 29.7)	23.5 (20.6 to 26.8)	-1.2 (-2.1 to -0.3)	-16.6 (-27.0 to -4.6)
Peru	17.4 (16.1 to 18.7)	14.4 (12.9 to 16.0)	13.7 (11.8 to 16.0)	10.6 (8.2 to 13.3)	-2.1 (-3.5 to -0.7)	-26.2 (-40.9 to -10.0)
Caribbean						
Antigua and Barbuda	9.4 (8.8 to 10.0)	6.9 (6.4 to 7.6)	6.9 (6.2 to 7.6)	6.7 (5.0 to 9.2)	-0.3 (-2.0 to 1.9)	-3.6 (-26.4 to 33.8)
The Bahamas	19.6 (18.3 to 21.1)	15.1 (13.9 to 16.4)	14.8 (13.4 to 16.5)	14.4 (11.1 to 19.5)	-0.4 (-1.9 to 1.7)	-4.6 (-24.4 to 28.3)
Barbados	12.0 (11.3 to 12.8)	8.8 (8.2 to 9.5)	8.7 (7.9 to 9.7)	6.7 (5.5 to 8.2)	-1.8 (-2.9 to -0.7)	-23.9 (-35.5 to -9.5)
Belize	28.5 (27.1 to 29.9)	18.8 (17.6 to 20.0)	18.8 (17.1 to 20.2)	17.3 (13.3 to 22.9)	-0.6 (-2.1 to 1.2)	-8.2 (-26.5 to 20.6)
Bermuda	12.4 (11.5 to 13.3)	7.9 (7.1 to 8.7)	7.8 (7.0 to 8.8)	6.7 (4.1 to 12.0)	-1.4 (-4.2 to 2.7)	-15.2 (-46.7 to 50.6)
Cuba	14.0 (13.6 to 14.4)	8.0 (7.7 to 8.3)	7.9 (7.1 to 9.0)	9.2 (7.4 to 11.7)	0.9 (-0.5 to 2.6)	16.0 (-6.7 to 47.5)
Dominica	17.4 (16.4 to 18.4)	15.4 (14.2 to 16.6)	15.4 (14.0 to 16.9)	16.3 (12.8 to 21.2)	0.3 (-1.1 to 2.0)	5.4 (-15.0 to 36.0)
Dominican Republic	25.9 (23.6 to 28.3)	30.1 (26.9 to 33.8)	29.4 (24.7 to 34.2)	18.9 (14.9 to 23.4)	-3.2 (-4.4 to -1.8)	-37.4 (-48.3 to -24.0)
Grenada	10.9 (10.2 to 11.7)	9.3 (8.7 to 10.0)	9.4 (8.6 to 10.2)	8.9 (7.5 to 10.6)	-0.4 (-1.3 to 0.8)	-4.9 (-18.2 to 12.2)
Guyana	16.7 (15.9 to 17.5)	16.7 (15.2 to 18.5)	15.6 (13.4 to 18.1)	14.8 (11.3 to 19.4)	-0.9 (-2.2 to 0.9)	-11.7 (-28.5 to 14.8)
Haiti	54.6 (38.4 to 74.3)	43.6 (28.9 to 59.9)	42.0 (27.7 to 57.6)	42.2 (27.3 to 59.2)	-0.2 (-0.9 to 0.4)	-3.4 (-12.3 to 6.5)

	Estimate in 2000 (95% UIs)	Estimate in 2015 (95% UIs)	Estimate in 2017 (95% UIs)	Estimate in 2030 (95% UIs)	Annualised rate of change, 2015-2030 (95% UIs)	Percent change, 2015-2030 (95% UIs)
Jamaica	5.7 (5.3 to 6.0)	9.8 (7.8 to 11.3)	9.1 (7.3 to 10.9)	7.6 (5.8 to 10.4)	-1.7 (-3.0 to 0.1)	-21.9 (-36.7 to 1.2)
Puerto Rico	15.7 (15.2 to 16.3)	9.2 (8.8 to 9.7)	9.9 (9.1 to 10.8)	5.2 (4.2 to 6.6)	-3.8 (-5.2 to -2.2)	-43.4 (-53.9 to -28.4)
Saint Lucia	16.8 (16.0 to 17.9)	12.5 (11.5 to 13.6)	12.6 (11.2 to 14.1)	10.5 (8.6 to 12.7)	-1.2 (-2.3 to 0.0)	-16.5 (-29.4 to 0.2)
Saint Vincent and the Grenadines	11.5 (10.8 to 12.2)	9.3 (8.6 to 10.1)	9.3 (8.5 to 10.2)	8.3 (6.8 to 10.5)	-0.8 (-2.0 to 0.6)	-10.8 (-25.8 to 9.9)
Suriname	19.7 (18.4 to 21.2)	16.6 (15.0 to 18.4)	16.5 (14.4 to 18.6)	10.0 (8.3 to 12.0)	-3.5 (-4.5 to -2.4)	-40.2 (-49.0 to -30.2)
Trinidad and Tobago	15.4 (14.8 to 16.1)	13.6 (11.4 to 16.2)	13.6 (11.1 to 16.4)	9.7 (7.4 to 12.8)	-2.3 (-3.8 to -0.5)	-28.4 (-43.7 to -6.7)
Virgin Islands, U.S.	13.2 (12.4 to 14.0)	10.9 (9.7 to 12.2)	10.9 (9.6 to 12.4)	10.0 (8.2 to 12.0)	-0.6 (-1.6 to 0.3)	-7.9 (-21.9 to 5.1)
Central Latin America						
Colombia	20.0 (19.4 to 20.6)	14.4 (13.7 to 15.0)	13.9 (12.3 to 15.7)	10.3 (8.1 to 12.9)	-2.3 (-3.7 to -0.7)	-28.5 (-42.9 to -10.4)
Costa Rica	19.1 (18.6 to 19.8)	15.0 (14.1 to 15.8)	15.4 (13.8 to 16.9)	12.0 (10.3 to 13.9)	-1.5 (-2.4 to -0.6)	-20.3 (-30.0 to -8.7)
El Salvador	33.9 (31.9 to 37.7)	21.4 (18.5 to 24.4)	21.0 (17.4 to 25.4)	18.8 (14.6 to 24.1)	-0.9 (-2.0 to 0.3)	-12.3 (-26.1 to 4.0)
Guatemala	15.6 (15.0 to 16.3)	17.0 (16.2 to 18.0)	17.0 (14.8 to 19.4)	12.3 (10.0 to 15.4)	-2.2 (-3.4 to -0.8)	-27.7 (-40.3 to -11.2)
Honduras	21.9 (17.5 to 26.2)	16.7 (12.6 to 20.3)	16.2 (12.1 to 19.8)	12.2 (8.9 to 15.5)	-2.1 (-2.7 to -1.3)	-26.5 (-33.6 to -18.3)
Mexico	19.6 (19.4 to 19.9)	15.4 (15.1 to 15.7)	15.7 (15.1 to 16.3)	11.7 (9.8 to 14.4)	-1.9 (-3.0 to -0.5)	-24.1 (-36.2 to -7.2)
Nicaragua	16.4 (15.1 to 18.3)	11.5 (10.8 to 12.9)	10.8 (9.3 to 12.6)	9.9 (8.1 to 12.0)	-1.0 (-2.1 to 0.2)	-14.1 (-27.5 to 2.6)
Panama	18.6 (17.9 to 19.4)	13.3 (12.7 to 13.9)	12.7 (11.6 to 13.8)	11.4 (9.7 to 13.6)	-1.0 (-2.1 to 0.1)	-14.1 (-26.6 to 2.0)
Venezuela	25.2 (24.5 to 25.8)	21.7 (20.1 to 23.5)	21.6 (18.2 to 25.8)	18.1 (13.5 to 24.4)	-1.3 (-3.0 to 0.6)	-17.0 (-36.5 to 8.6)
Tropical Latin America						
Brazil	25.0 (24.4 to 25.5)	20.6 (20.2 to 21.0)	20.0 (19.1 to 20.7)	12.5 (10.6 to 14.9)	-3.3 (-4.4 to -2.2)	-39.0 (-48.6 to -27.7)
Paraguay	18.2 (16.8 to 19.8)	22.9 (19.7 to 26.2)	21.9 (18.0 to 26.6)	18.4 (13.4 to 25.0)	-1.5 (-3.3 to 0.3)	-19.5 (-39.3 to 4.2)
North Africa and Middle East						
North Africa and Middle East						
Afghanistan	53.8 (39.0 to 66.5)	33.7 (27.1 to 41.3)	32.6 (26.3 to 40.0)	29.5 (23.5 to 36.5)	-0.9 (-1.4 to -0.4)	-12.5 (-18.7 to -6.0)
Algeria	29.8 (24.2 to 45.3)	18.4 (14.6 to 30.2)	17.1 (13.5 to 27.9)	15.0 (11.7 to 24.0)	-1.4 (-1.8 to -0.9)	-18.6 (-24.2 to -13.0)
Bahrain	23.3 (22.0 to 24.6)	9.8 (9.0 to 10.8)	9.6 (8.6 to 10.8)	7.5 (6.1 to 9.3)	-1.8 (-3.0 to -0.6)	-23.7 (-36.2 to -8.0)
Egypt	38.5 (30.5 to 48.1)	32.6 (23.2 to 41.2)	31.2 (21.3 to 40.2)	28.5 (19.1 to 37.1)	-0.9 (-1.8 to -0.1)	-12.6 (-23.5 to -0.8)
Iran	44.6 (42.5 to 49.2)	27.3 (26.7 to 28.4)	25.6 (25.1 to 26.8)	24.9 (21.8 to 28.2)	-0.6 (-1.5 to 0.2)	-8.6 (-19.9 to 2.7)
Iraq	26.0 (19.1 to 30.1)	9.9 (9.2 to 10.9)	9.3 (8.6 to 10.3)	8.4 (7.5 to 9.5)	-1.1 (-1.7 to -0.6)	-15.5 (-22.3 to -8.1)
Jordan	19.9 (17.8 to 22.1)	11.8 (10.8 to 13.0)	11.5 (10.2 to 12.9)	8.9 (7.7 to 10.4)	-1.9 (-2.6 to -1.2)	-24.4 (-32.0 to -16.1)
Kuwait	23.3 (22.2 to 24.1)	14.1 (13.3 to 14.7)	13.6 (12.2 to 14.8)	11.6 (8.8 to 15.1)	-1.4 (-3.2 to 0.3)	-17.9 (-38.0 to 4.9)
Lebanon	9.4 (8.2 to 10.8)	6.9 (4.6 to 8.3)	6.7 (4.5 to 8.2)	4.1 (2.5 to 5.4)	-3.5 (-4.7 to -2.3)	-40.7 (-50.4 to -29.3)
Libya	29.8 (19.9 to 42.8)	25.8 (13.3 to 39.5)	24.8 (12.8 to 38.5)	20.6 (10.5 to 32.0)	-1.5 (-2.0 to -0.9)	-20.3 (-26.5 to -13.2)
Morocco	30.0 (24.6 to 47.2)	21.0 (15.9 to 34.3)	20.2 (14.9 to 33.2)	17.9 (13.2 to 29.5)	-1.1 (-1.5 to -0.7)	-14.8 (-19.6 to -9.6)
Palestine	13.0 (11.8 to 14.1)	8.9 (8.0 to 9.8)	8.4 (7.5 to 9.3)	8.6 (7.0 to 10.8)	-0.2 (-1.3 to 1.1)	-3.1 (-18.1 to 18.7)
Oman	73.7 (62.0 to 87.8)	49.5 (40.7 to 58.6)	46.2 (37.7 to 55.0)	24.7 (19.5 to 30.4)	-4.7 (-5.5 to -3.8)	-50.2 (-56.4 to -43.4)
Qatar	39.4 (34.2 to 45.4)	24.6 (20.2 to 29.4)	24.3 (19.7 to 29.9)	21.7 (16.1 to 29.7)	-0.9 (-2.2 to 0.9)	-11.7 (-28.5 to 15.2)
Saudi Arabia	41.5 (37.1 to 46.6)	37.9 (26.9 to 44.8)	36.0 (25.4 to 43.2)	24.5 (17.1 to 31.0)	-2.9 (-4.1 to -1.9)	-35.4 (-46.0 to -24.4)
Sudan	50.2 (38.1 to 74.4)	31.6 (23.7 to 48.7)	29.8 (22.5 to 46.4)	29.4 (21.9 to 45.5)	-0.5 (-0.9 to 0.0)	-6.8 (-12.7 to 0.3)
Syria	15.7 (14.2 to 17.3)	11.6 (9.6 to 13.5)	11.3 (9.2 to 13.5)	10.7 (8.6 to 13.2)	-0.5 (-1.3 to 0.3)	-7.3 (-17.5 to 4.1)
Tunisia	43.7 (38.3 to 48.3)	30.8 (24.6 to 38.0)	29.6 (23.6 to 36.4)	26.1 (20.9 to 32.2)	-1.1 (-1.7 to -0.5)	-15.2 (-22.5 to -7.5)
Turkey	11.8 (10.6 to 13.1)	10.6 (9.8 to 11.3)	10.1 (9.1 to 11.2)	6.5 (5.3 to 8.0)	-3.3 (-4.5 to -1.9)	-38.5 (-49.0 to -25.2)
United Arab Emirates	51.6 (43.9 to 60.8)	50.1 (40.2 to 61.1)	49.0 (38.7 to 59.9)	34.9 (26.8 to 43.9)	-2.4 (-3.2 to -1.6)	-30.3 (-38.4 to -21.2)
Yemen	57.8 (41.8 to 82.8)	38.3 (28.7 to 55.8)	37.7 (28.2 to 54.9)	33.0 (24.4 to 47.7)	-1.0 (-1.4 to -0.6)	-14.0 (-19.5 to -8.6)
South Asia						
South Asia						
Bangladesh	7.7 (6.7 to 8.8)	8.7 (7.1 to 10.1)	8.1 (6.3 to 9.4)	6.6 (5.2 to 7.9)	-1.8 (-2.4 to -1.2)	-23.5 (-30.6 to -16.8)
Bhutan	13.4 (11.4 to 15.6)	8.1 (6.2 to 9.8)	7.7 (5.8 to 9.4)	6.9 (5.2 to 8.4)	-1.1 (-1.6 to -0.5)	-14.8 (-21.1 to -7.6)
India	18.9 (17.9 to 19.8)	17.4 (16.5 to 18.2)	16.8 (15.4 to 17.8)	14.1 (12.1 to 16.2)	-1.4 (-2.3 to -0.6)	-19.2 (-29.1 to -8.8)
Nepal	28.4 (16.4 to 46.4)	27.0 (15.6 to 40.1)	26.1 (14.9 to 38.5)	23.4 (13.2 to 34.9)	-0.9 (-1.4 to -0.3)	-13.0 (-19.2 to -5.0)
Pakistan	30.0 (20.1 to 37.1)	30.2 (18.2 to 39.0)	29.1 (17.6 to 38.0)	25.0 (14.8 to 33.2)	-1.3 (-2.0 to -0.6)	-17.4 (-25.9 to -8.5)
Southeast Asia, East Asia, and Oceania						
East Asia						
China	22.2 (21.2 to 23.0)	16.6 (15.9 to 17.1)	15.3 (14.5 to 15.9)	11.7 (10.3 to 13.3)	-2.3 (-3.1 to -1.5)	-29.2 (-37.0 to -20.4)
North Korea	18.9 (13.9 to 27.7)	20.0 (13.6 to 29.9)	19.8 (13.2 to 29.9)	17.6 (11.7 to 27.1)	-0.9 (-1.6 to -0.1)	-11.9 (-21.1 to -2.1)
Taiwan (Province of China)	24.3 (23.9 to 24.8)	12.7 (12.4 to 13.0)	12.9 (12.1 to 13.7)	10.9 (9.5 to 12.6)	-1.0 (-1.9 to -0.1)	-14.1 (-24.8 to -1.0)
Oceania						
American Samoa	10.6 (9.8 to 11.4)	8.7 (8.0 to 9.4)	8.7 (7.9 to 9.4)	8.7 (7.8 to 9.8)	0.1 (-0.5 to 0.6)	0.9 (-6.5 to 9.2)
Federated States of Micronesia	19.8 (15.9 to 25.2)	17.5 (12.5 to 22.1)	17.3 (12.0 to 21.7)	18.3 (12.6 to 24.0)	0.3 (-0.5 to 1.0)	4.5 (-6.7 to 16.9)
Fiji	12.9 (12.0 to 13.7)	10.0 (9.0 to 11.0)	9.8 (8.6 to 11.2)	8.9 (7.3 to 11.2)	-0.8 (-1.8 to 0.5)	-10.8 (-24.0 to 7.0)
Guam	14.8 (13.9 to 15.8)	13.1 (11.9 to 14.3)	13.3 (12.0 to 14.7)	12.5 (10.5 to 14.9)	-0.3 (-1.3 to 0.7)	-4.1 (-17.2 to 11.7)
Kiribati	13.4 (11.9 to 14.9)	11.3 (9.0 to 13.7)	11.0 (8.6 to 13.2)	10.6 (8.3 to 13.0)	-0.5 (-1.0 to 0.0)	-6.9 (-13.3 to 0.4)
Marshall Islands	27.2 (23.5 to 30.9)	24.8 (19.9 to 29.3)	24.0 (19.0 to 28.9)	23.8 (18.4 to 29.4)	-0.3 (-1.0 to 0.3)	-4.0 (-13.7 to 5.2)
Northern Mariana Islands	13.8 (11.6 to 15.9)	10.7 (9.6 to 11.9)	10.9 (9.5 to 12.2)	11.4 (9.8 to 13.2)	0.4 (-0.2 to 1.1)	6.7 (-3.0 to 17.1)
Papua New Guinea	39.8 (32.4 to 47.6)	35.0 (28.1 to 42.8)	34.0 (27.4 to 41.4)	34.4 (27.5 to 41.9)	-0.1 (-0.5 to 0.3)	-1.7 (-7.0 to 4.2)
Samoa	12.2 (9.9 to 16.8)	10.8 (8.7 to 15.3)	10.7 (8.5 to 14.9)	9.9 (7.9 to 14.2)	-0.6 (-1.0 to -0.2)	-8.4 (-14.2 to -2.6)
Solomon Islands	24.1 (18.9 to 31.7)	21.8 (17.1 to 28.1)	21.3 (16.5 to 27.3)	20.8 (16.1 to 26.9)	-0.3 (-0.8 to 0.1)	-4.9 (-10.7 to 1.4)
Tonga	12.7 (11.6 to 13.8)	12.2 (10.2 to 13.9)	11.9 (9.9 to 13.7)	12.5 (10.3 to 14.5)	0.1 (-0.3 to 0.6)	2.1 (-3.8 to 9.2)
Vanuatu	22.0 (15.9 to 30.0)	20.7 (14.6 to 28.6)	20.6 (14.5 to 28.1)	19.8 (13.8 to 27.1)	-0.3 (-0.8 to 0.2)	-4.6 (-11.3 to 2.7)
Southeast Asia						
Cambodia	40.2 (32.0 to 50.6)	28.2 (22.6 to 36.3)	27.1 (21.7 to 34.5)	23.6 (18.4 to 30.1)	-1.2 (-1.7 to -0.7)	-16.3 (-22.6 to -9.4)
Indonesia	25.1 (23.7 to 26.7)	14.7 (13.4 to 15.8)	14.2 (12.9 to 15.4)	7.3 (6.3 to 8.3)	-4.7 (-5.4 to -4.0)	-50.5 (-55.3 to -45.5)
Laos	40.8 (35.4 to 47.3)	27.9 (21.4 to 34.8)	26.3 (20.1 to 32.9)	17.1 (12.8 to 21.6)	-3.3 (-3.8 to -2.7)	-38.7 (-43.2 to -33.5)
Malaysia	26.2 (25.2 to 27.2)	23.2 (21.2 to 24.5)	22.9 (20.2 to 25.6)	12.6 (10.6 to 15.0)	-4.1 (-5.0 to -3.0)	-45.5 (-52.9 to -36.4)
Maldives	16.4 (13.8 to 24.1)	8.6 (6.5 to 14.9)	8.3 (6.2 to 14.4)	6.5 (4.5 to 11.6)	-1.8 (-3.2 to -0.4)	-23.7 (-37.7 to -6.5)
Mauritius	14.4 (13.7 to 15.0)	11.8 (11.2 to 12.4)	11.2 (10.2 to 12.3)	7.3 (6.2 to 8.6)	-3.2 (-4.2 to -2.2)	-38.1 (-46.8 to -28.2)
Myanmar	32.9 (28.3 to 38.0)	21.4 (18.2 to 25.2)	20.3 (17.3 to 24.1)	18.3 (15.2 to 21.9)	-1.1 (-1.7 to -0.5)	-14.7 (-22.5 to -6.7)
Philippines	10.8 (10.2 to 11.4)	12.0 (10.9 to 13.1)	11.3 (9.9 to 13.0)	10.6 (8.9 to 12.6)	-0.8 (-1.8 to 0.1)	-11.5 (-23.8 to 1.6)
Sri Lanka	17.0 (16.1 to 17.9)	12.8 (11.1 to 14.6)	12.1 (10.1 to 14.3)	8.5 (6.5 to 11.1)	-2.8 (-4.2 to -1.3)	-33.8 (-46.8 to -17.3)
Seychelles	15.6 (14.2 to 16.8)	13.2 (11.9 to 14.6)	13.1 (11.4 to 14.7)	11.0 (9.2 to 13.1)	-1.2 (-2.0 to -0.3)	-16.9 (-26.1 to -4.8)
Thailand	44.6 (41.2 to 48.4)	23.7 (22.2 to 25.4)	24.2 (21.3 to 27.0)	12.4 (9.7 to 16.7)	-4.4 (-6.0 to -2.4)	-47.6 (-59.1 to -30.7)

	Estimate in 2000 (95% UIs)	Estimate in 2015 (95% UIs)	Estimate in 2017 (95% UIs)	Estimate in 2030 (95% UIs)	Annualised rate of change, 2015-2030 (95% UIs)	Percent change, 2015-2030 (95% UIs)
Timor-Leste	13.1 (7.4 to 25.5)	10.3 (6.0 to 18.0)	10.3 (6.1 to 17.9)	9.6 (5.6 to 16.8)	-0.5 (-1.2 to 0.2)	-7.2 (-16.0 to 3.8)
Vietnam	23.7 (21.0 to 26.7)	21.9 (18.8 to 24.4)	21.0 (17.8 to 23.7)	17.5 (14.6 to 20.9)	-1.5 (-2.2 to -0.6)	-19.9 (-28.6 to -9.1)
Sub-Saharan Africa						
Central Sub-Saharan Africa						
Angola	50.3 (42.1 to 59.3)	30.0 (24.8 to 36.5)	28.1 (23.1 to 34.2)	28.4 (22.2 to 37.1)	-0.4 (-1.4 to 0.8)	-5.1 (-19.0 to 13.0)
Central African Republic	86.2 (57.7 to 109.2)	86.5 (50.1 to 112.7)	83.8 (49.7 to 109.1)	92.7 (54.5 to 127.3)	0.4 (-0.8 to 1.6)	7.3 (-10.7 to 26.7)
Congo	43.5 (35.0 to 50.5)	28.7 (22.8 to 35.6)	28.0 (22.0 to 34.8)	30.8 (21.9 to 42.2)	0.4 (-1.1 to 2.1)	7.3 (-15.6 to 36.9)
Democratic Republic of the Congo	34.8 (27.9 to 41.5)	29.6 (23.7 to 36.4)	28.3 (22.6 to 35.1)	29.1 (22.1 to 37.9)	-0.1 (-1.0 to 1.0)	-1.6 (-14.3 to 16.8)
Equatorial Guinea	38.6 (28.6 to 50.1)	20.7 (13.9 to 29.7)	20.5 (14.0 to 29.6)	21.3 (14.0 to 31.6)	0.2 (-1.3 to 1.7)	3.1 (-17.2 to 29.6)
Gabon	36.2 (30.2 to 44.9)	29.5 (23.7 to 36.8)	27.9 (22.2 to 35.0)	28.6 (21.6 to 36.9)	-0.2 (-1.4 to 0.9)	-3.0 (-19.0 to 14.8)
Eastern Sub-Saharan Africa						
Burundi	45.4 (37.2 to 54.5)	35.4 (28.0 to 47.1)	34.4 (27.1 to 45.7)	31.2 (23.7 to 42.2)	-0.8 (-1.9 to 0.3)	-11.6 (-24.7 to 4.0)
Comoros	25.8 (21.3 to 32.4)	20.8 (17.5 to 25.0)	20.3 (16.9 to 24.3)	21.9 (17.8 to 26.7)	0.3 (-0.3 to 1.0)	4.9 (-3.8 to 16.4)
Djibouti	31.8 (23.4 to 43.5)	23.4 (16.6 to 32.1)	22.6 (16.3 to 31.4)	20.8 (14.8 to 29.4)	-0.8 (-1.6 to 0.0)	-10.8 (-20.8 to 0.4)
Eritrea	47.9 (29.4 to 59.1)	34.2 (25.1 to 41.8)	32.4 (24.1 to 39.8)	36.4 (26.1 to 45.5)	0.4 (-0.4 to 1.1)	6.5 (-5.5 to 18.7)
Ethiopia	27.2 (24.1 to 29.9)	15.8 (14.4 to 17.2)	15.1 (13.8 to 16.6)	15.1 (12.9 to 17.5)	-0.4 (-1.2 to 0.5)	-5.0 (-16.0 to 8.4)
Kenya	21.3 (18.9 to 26.3)	18.5 (16.8 to 20.8)	17.8 (16.2 to 20.2)	18.3 (15.2 to 22.6)	-0.1 (-1.0 to 1.2)	-0.9 (-14.0 to 19.3)
Madagascar	26.6 (23.2 to 30.4)	21.0 (17.3 to 25.5)	20.4 (16.7 to 24.6)	20.3 (16.5 to 24.5)	-0.2 (-0.7 to 0.2)	-3.3 (-9.8 to 3.8)
Malawi	28.3 (24.4 to 31.8)	19.8 (17.2 to 22.6)	19.1 (16.4 to 22.0)	19.8 (15.6 to 25.1)	-0.1 (-1.3 to 1.4)	-0.2 (-17.6 to 22.9)
Mozambique	35.2 (30.4 to 40.4)	28.5 (24.1 to 32.9)	27.0 (22.9 to 31.3)	29.0 (23.1 to 35.7)	0.1 (-0.8 to 1.0)	1.8 (-11.9 to 16.4)
Rwanda	56.4 (43.5 to 67.6)	34.2 (23.3 to 45.8)	32.6 (22.3 to 43.6)	30.7 (19.3 to 46.8)	-0.8 (-2.6 to 1.2)	-10.1 (-32.0 to 19.4)
Somalia	58.9 (35.2 to 80.8)	52.0 (28.1 to 73.6)	50.1 (27.3 to 70.6)	55.0 (28.9 to 77.9)	0.4 (-0.2 to 0.9)	5.8 (-2.8 to 15.0)
South Sudan	31.0 (21.7 to 42.6)	28.4 (20.9 to 39.3)	27.7 (20.5 to 38.3)	29.1 (20.8 to 41.8)	0.2 (-0.6 to 1.2)	2.8 (-9.2 to 18.9)
Tanzania	18.8 (16.4 to 21.6)	15.5 (13.6 to 17.6)	15.1 (13.1 to 17.4)	14.8 (12.5 to 17.4)	-0.3 (-1.0 to 0.4)	-4.5 (-13.6 to 5.6)
Uganda	33.8 (27.1 to 41.8)	26.8 (19.5 to 34.6)	25.6 (18.5 to 32.7)	26.2 (18.3 to 34.7)	-0.2 (-1.1 to 0.7)	-2.3 (-15.7 to 11.1)
Zambia	30.7 (25.9 to 34.5)	20.0 (17.5 to 22.6)	19.4 (16.9 to 22.2)	18.0 (12.9 to 23.8)	-0.8 (-2.7 to 0.9)	-10.1 (-33.8 to 13.9)
Southern Sub-Saharan Africa						
Botswana	16.5 (12.8 to 21.1)	15.7 (13.1 to 17.8)	14.8 (12.4 to 16.7)	13.4 (10.0 to 18.6)	-1.1 (-2.6 to 1.3)	-14.3 (-32.7 to 21.0)
Lesotho	42.3 (35.1 to 50.8)	48.0 (37.6 to 58.3)	44.1 (35.0 to 53.4)	46.1 (30.6 to 66.8)	-0.4 (-2.3 to 1.8)	-3.9 (-29.7 to 30.7)
Namibia	38.4 (33.0 to 44.2)	22.3 (18.1 to 27.6)	21.6 (17.4 to 27.0)	19.6 (14.5 to 26.4)	-0.9 (-2.3 to 0.7)	-11.8 (-29.3 to 10.3)
South Africa	60.5 (57.6 to 64.3)	27.8 (26.1 to 30.2)	27.7 (26.0 to 29.8)	29.4 (20.9 to 46.5)	0.2 (-1.8 to 3.4)	5.5 (-23.7 to 65.6)
Swaziland	47.2 (38.9 to 55.2)	41.1 (30.4 to 50.5)	37.0 (28.3 to 45.3)	36.0 (24.5 to 49.8)	-0.9 (-2.9 to 1.3)	-12.1 (-35.3 to 21.2)
Zimbabwe	33.7 (25.8 to 40.3)	26.2 (18.9 to 34.9)	24.9 (18.0 to 33.6)	28.9 (16.1 to 51.3)	0.4 (-2.5 to 4.4)	10.6 (-31.4 to 92.5)
Western Sub-Saharan Africa						
Benin	55.1 (31.9 to 74.2)	43.0 (25.7 to 58.6)	40.8 (24.5 to 54.9)	33.0 (19.4 to 45.6)	-1.8 (-2.4 to -1.1)	-23.3 (-29.7 to -15.8)
Burkina Faso	26.0 (22.5 to 29.9)	24.4 (20.6 to 27.9)	23.8 (19.9 to 27.6)	20.8 (17.2 to 24.6)	-1.1 (-1.6 to -0.5)	-14.9 (-21.8 to -7.7)
Cameroon	30.9 (26.1 to 35.3)	23.9 (19.1 to 29.0)	22.6 (17.9 to 27.9)	24.7 (19.0 to 31.4)	0.2 (-0.5 to 1.0)	3.4 (-6.7 to 16.7)
Cape Verde	11.5 (9.5 to 13.3)	8.4 (7.4 to 9.3)	8.2 (7.0 to 9.3)	7.5 (6.2 to 8.7)	-0.8 (-1.6 to -0.1)	-11.0 (-20.9 to -1.7)
Chad	26.2 (20.6 to 35.2)	25.7 (21.0 to 33.2)	25.0 (20.3 to 32.0)	25.5 (20.5 to 33.0)	-0.1 (-0.7 to 0.7)	-0.6 (-10.4 to 10.9)
Cote d'Ivoire	28.6 (24.5 to 32.9)	23.0 (19.7 to 26.6)	21.7 (18.5 to 25.3)	21.1 (17.8 to 25.0)	-0.6 (-1.1 to 0.0)	-8.0 (-15.5 to 0.2)
The Gambia	22.3 (18.4 to 26.6)	21.2 (16.9 to 25.4)	20.7 (16.5 to 24.8)	21.3 (16.9 to 26.2)	0.0 (-0.5 to 0.6)	0.2 (-7.8 to 9.3)
Ghana	25.1 (22.0 to 28.9)	24.7 (21.4 to 28.2)	23.9 (20.7 to 27.4)	23.6 (19.6 to 28.4)	-0.3 (-1.1 to 0.5)	-4.7 (-14.6 to 7.1)
Guinea	26.6 (22.4 to 33.4)	24.9 (21.2 to 29.1)	23.9 (20.3 to 28.1)	24.4 (19.6 to 29.8)	-0.1 (-1.0 to 0.7)	-1.8 (-13.5 to 11.4)
Guinea-Bissau	45.8 (38.9 to 53.8)	33.4 (27.9 to 39.9)	32.0 (26.6 to 38.5)	32.0 (26.1 to 38.6)	-0.3 (-0.8 to 0.2)	-4.3 (-10.9 to 2.7)
Liberia	21.4 (18.3 to 24.5)	16.2 (13.5 to 19.3)	15.6 (12.8 to 18.9)	16.7 (13.3 to 21.0)	0.2 (-0.6 to 1.0)	2.7 (-8.6 to 15.4)
Mali	24.3 (20.0 to 32.1)	19.7 (16.1 to 26.3)	19.7 (16.2 to 26.2)	19.3 (15.5 to 26.2)	-0.2 (-0.7 to 0.4)	-2.3 (-10.6 to 6.7)
Mauritania	32.8 (27.6 to 38.0)	23.8 (20.0 to 27.5)	22.9 (19.0 to 26.8)	23.8 (19.1 to 28.9)	0.0 (-0.8 to 0.9)	0.4 (-11.4 to 14.8)
Niger	23.5 (18.5 to 31.0)	18.0 (13.1 to 23.9)	17.8 (13.1 to 23.8)	18.1 (13.2 to 24.2)	0.0 (-0.5 to 0.5)	0.4 (-7.1 to 8.5)
Nigeria	16.6 (11.6 to 22.4)	13.4 (9.7 to 17.9)	13.3 (9.7 to 17.6)	11.9 (8.7 to 16.2)	-0.8 (-1.4 to -0.1)	-10.8 (-18.6 to -2.2)
Sao Tome and Principe	15.3 (11.9 to 17.9)	16.2 (10.2 to 20.2)	15.6 (9.7 to 19.8)	16.4 (9.9 to 21.7)	0.1 (-0.9 to 1.1)	1.1 (-12.2 to 18.8)
Senegal	19.9 (16.3 to 27.9)	17.6 (14.4 to 24.9)	17.4 (14.2 to 24.2)	17.2 (13.7 to 24.1)	-0.2 (-0.8 to 0.5)	-2.1 (-10.8 to 8.0)
Sierra Leone	27.2 (22.4 to 34.3)	23.4 (19.8 to 28.1)	22.3 (18.8 to 27.0)	24.1 (19.6 to 30.1)	0.2 (-0.6 to 0.9)	2.8 (-8.4 to 15.2)
Togo	25.9 (21.3 to 33.3)	23.1 (19.1 to 27.4)	22.1 (18.1 to 26.6)	22.7 (17.9 to 28.4)	-0.1 (-0.9 to 0.8)	-1.7 (-12.8 to 12.3)
**Indicator 3.7.1: Proportion of women of reproductive age (15-49 years) who have their need for family planning satisfied with modern methods (%)**						
Central Europe, Eastern Europe, and Central Asia						
Central Asia						
Armenia	27.2 (25.0 to 29.7)	40.2 (37.5 to 42.8)	41.3 (37.8 to 44.6)	47.0 (43.5 to 50.4)	1.0 (0.8 to 1.4)	17.0 (11.9 to 22.6)
Azerbaijan	20.2 (17.9 to 22.5)	30.5 (25.0 to 36.2)	32.0 (25.7 to 38.1)	37.3 (30.9 to 44.1)	1.4 (0.9 to 1.9)	22.6 (13.6 to 32.4)
Georgia	39.2 (34.5 to 44.2)	51.2 (45.1 to 57.5)	52.7 (46.0 to 59.2)	57.5 (50.6 to 64.1)	0.8 (0.4 to 1.1)	12.3 (6.7 to 17.9)
Kazakhstan	65.6 (63.2 to 67.9)	77.3 (75.0 to 79.3)	78.8 (76.2 to 81.2)	84.7 (82.6 to 86.7)	0.6 (0.5 to 0.7)	9.6 (8.2 to 11.0)
Kyrgyzstan	66.4 (62.7 to 70.1)	63.7 (60.6 to 66.8)	64.8 (61.2 to 68.8)	67.7 (64.1 to 71.3)	0.4 (0.3 to 0.6)	6.3 (3.9 to 8.7)
Mongolia	72.5 (68.8 to 75.9)	67.8 (64.1 to 71.2)	68.4 (64.2 to 72.5)	70.6 (66.3 to 74.6)	0.3 (0.1 to 0.4)	4.2 (1.4 to 6.9)
Tajikistan	48.6 (42.9 to 54.3)	52.3 (49.9 to 54.4)	52.5 (50.0 to 55.0)	58.0 (55.5 to 60.5)	0.7 (0.5 to 0.9)	11.1 (8.3 to 13.9)
Turkmenistan	56.9 (52.6 to 61.1)	73.6 (71.3 to 75.8)	75.0 (72.4 to 77.4)	81.9 (79.8 to 83.8)	0.7 (0.6 to 0.8)	11.3 (9.8 to 12.8)
Uzbekistan	75.6 (72.9 to 78.2)	82.0 (76.9 to 85.9)	82.8 (77.8 to 86.8)	87.4 (83.4 to 90.5)	0.4 (0.3 to 0.6)	6.7 (4.7 to 9.0)
Central Europe						
Albania	17.1 (14.3 to 20.4)	21.1 (17.1 to 25.8)	22.2 (17.7 to 27.6)	26.7 (21.3 to 32.9)	1.6 (1.0 to 2.1)	26.6 (16.2 to 36.9)
Bosnia and Herzegovina	24.3 (19.8 to 29.5)	25.0 (21.2 to 29.1)	25.7 (21.4 to 30.1)	30.6 (26.0 to 35.5)	1.3 (1.0 to 1.7)	22.5 (16.2 to 29.2)
Bulgaria	52.8 (45.6 to 59.2)	58.0 (50.0 to 66.2)	58.6 (50.6 to 66.7)	61.5 (53.3 to 69.3)	0.4 (0.1 to 0.7)	6.1 (1.6 to 10.4)
Croatia	48.9 (40.6 to 57.7)	47.7 (39.1 to 56.0)	48.2 (39.8 to 56.6)	50.4 (41.9 to 58.6)	0.4 (0.1 to 0.7)	5.7 (1.8 to 10.2)
Czech Republic	66.9 (61.4 to 72.2)	75.4 (69.0 to 81.2)	75.6 (69.3 to 81.6)	80.6 (75.2 to 85.6)	0.5 (0.3 to 0.6)	7.0 (4.4 to 10.1)
Hungary	67.4 (60.5 to 73.8)	63.3 (55.2 to 71.1)	63.3 (55.1 to 71.3)	65.5 (57.5 to 73.2)	0.2 (0.1 to 0.4)	3.5 (0.8 to 6.3)
Macedonia	16.0 (12.7 to 20.1)	19.1 (16.1 to 22.3)	19.8 (16.4 to 23.8)	23.8 (19.8 to 28.3)	1.5 (1.1 to 1.8)	24.8 (18.1 to 31.1)
Montenegro	42.7 (36.7 to 48.9)	43.6 (39.4 to 48.1)	44.4 (39.4 to 49.4)	47.5 (42.4 to 52.5)	0.6 (0.3 to 0.8)	8.9 (5.2 to 12.4)
Poland	41.2 (34.0 to 49.1)	50.1 (41.4 to 58.5)	50.9 (42.1 to 59.2)	57.4 (48.6 to 65.7)	0.9 (0.5 to 1.4)	14.8 (7.5 to 22.8)
Romania	43.2 (36.9 to 49.8)	52.9 (45.0 to 60.3)	53.4 (45.5 to 60.9)	61.6 (54.2 to 68.6)	1.0 (0.7 to 1.4)	16.7 (11.8 to 22.6)
Serbia	42.0 (36.3 to 48.2)	36.5 (31.4 to 41.8)	36.7 (31.1 to 42.2)	40.5 (34.6 to 46.3)	0.7 (0.4 to 0.9)	10.7 (6.3 to 15.2)
Slovakia	75.2 (69.1 to 80.3)	75.6 (69.3 to 81.3)	75.9 (69.7 to 81.5)	77.9 (71.8 to 83.5)	0.2 (0.0 to 0.4)	3.1 (0.4 to 5.9)
Slovenia	70.1 (63.3 to 76.0)	69.7 (62.3 to 76.6)	70.2 (63.0 to 77.1)	71.9 (64.7 to 78.7)	0.2 (0.0 to 0.4)	3.2 (0.7 to 6.2)
Eastern Europe						

	Estimate in 2000 (95% UIs)	Estimate in 2015 (95% UIs)	Estimate in 2017 (95% UIs)	Estimate in 2030 (95% UIs)	Annualised rate of change, 2015-2030 (95% UIs)	Percent change, 2015-2030 (95% UIs)
Belarus	64.8 (58.7 to 70.2)	71.7 (68.5 to 74.7)	72.5 (68.7 to 76.0)	75.3 (71.3 to 78.8)	0.3 (0.2 to 0.5)	5.0 (2.5 to 7.4)
Estonia	67.3 (60.6 to 73.7)	74.8 (68.3 to 80.5)	75.1 (68.6 to 80.8)	78.6 (73.0 to 83.9)	0.3 (0.2 to 0.5)	5.2 (2.5 to 8.4)
Latvia	59.7 (52.9 to 66.3)	71.3 (65.3 to 77.3)	71.9 (65.8 to 77.8)	78.0 (72.8 to 83.2)	0.6 (0.4 to 0.8)	9.5 (5.9 to 13.5)
Lithuania	56.7 (49.5 to 63.2)	70.2 (63.6 to 76.2)	70.5 (63.8 to 76.6)	78.6 (72.8 to 83.4)	0.8 (0.5 to 1.0)	12.0 (8.5 to 16.0)
Moldova	55.1 (51.7 to 58.3)	59.0 (55.7 to 62.7)	60.1 (55.8 to 64.3)	62.6 (58.2 to 66.8)	0.4 (0.1 to 0.6)	6.0 (2.1 to 9.9)
Russia	49.6 (46.6 to 52.8)	61.9 (55.7 to 67.8)	62.2 (55.4 to 68.5)	64.9 (58.4 to 71.8)	0.3 (0.0 to 0.6)	4.9 (0.3 to 9.8)
Ukraine	60.0 (55.3 to 64.5)	66.0 (61.0 to 70.7)	66.8 (61.2 to 72.0)	71.6 (66.2 to 76.4)	0.5 (0.3 to 0.8)	8.4 (4.8 to 12.4)
High-income						
Australasia						
Australia	85.7 (81.2 to 89.0)	88.1 (84.4 to 91.2)	88.3 (85.0 to 91.4)	90.7 (87.8 to 93.3)	0.2 (0.1 to 0.3)	3.0 (1.8 to 4.5)
New Zealand	79.9 (74.6 to 84.2)	82.7 (77.8 to 87.0)	83.0 (78.2 to 87.2)	85.5 (81.2 to 89.3)	0.2 (0.1 to 0.3)	3.4 (2.1 to 5.0)
High-income Asia Pacific						
Brunei	67.2 (60.0 to 74.0)	71.8 (64.6 to 78.1)	72.3 (65.7 to 78.4)	75.6 (69.4 to 81.1)	0.3 (0.2 to 0.5)	5.4 (3.1 to 8.0)
Japan	61.9 (57.3 to 66.3)	62.9 (55.5 to 70.2)	63.5 (56.2 to 71.0)	65.5 (58.3 to 72.5)	0.3 (0.1 to 0.5)	4.2 (1.4 to 7.4)
South Korea	72.0 (67.6 to 75.9)	75.1 (70.5 to 79.4)	76.3 (71.5 to 80.8)	81.3 (77.4 to 85.1)	0.5 (0.4 to 0.7)	8.4 (6.6 to 10.3)
Singapore	66.6 (60.2 to 72.4)	74.1 (66.9 to 80.0)	74.6 (67.6 to 80.5)	79.2 (73.0 to 84.3)	0.4 (0.3 to 0.6)	7.0 (4.7 to 9.6)
High-income North America						
Canada	84.9 (81.7 to 87.9)	86.1 (82.1 to 89.7)	86.3 (81.9 to 89.8)	88.1 (84.2 to 91.2)	0.1 (0.1 to 0.2)	2.3 (1.2 to 3.5)
Greenland	72.7 (65.5 to 79.2)	76.0 (69.5 to 81.4)	75.9 (69.4 to 81.2)	79.3 (73.5 to 84.2)	0.3 (0.1 to 0.5)	4.5 (1.9 to 7.3)
United States	80.5 (76.9 to 83.8)	82.6 (78.2 to 86.0)	82.8 (78.3 to 86.4)	85.3 (81.3 to 88.5)	0.2 (0.1 to 0.3)	3.3 (2.2 to 4.5)
Southern Latin America						
Argentina	67.1 (61.1 to 73.0)	73.6 (68.0 to 78.4)	74.3 (68.8 to 79.0)	78.8 (73.9 to 82.9)	0.5 (0.3 to 0.6)	7.1 (5.3 to 9.3)
Chile	68.4 (61.8 to 74.3)	75.5 (69.6 to 81.0)	76.0 (70.0 to 81.3)	81.3 (76.3 to 85.8)	0.5 (0.3 to 0.7)	7.7 (5.4 to 10.4)
Uruguay	65.8 (58.2 to 72.8)	72.7 (65.8 to 78.4)	73.6 (66.6 to 79.2)	78.0 (72.0 to 82.8)	0.5 (0.3 to 0.6)	7.4 (5.2 to 10.1)
Western Europe						
Andorra	86.1 (81.4 to 89.9)	87.9 (83.8 to 91.2)	88.1 (83.9 to 91.4)	89.7 (85.9 to 92.7)	0.1 (0.1 to 0.2)	2.1 (1.1 to 3.2)
Austria	72.5 (66.5 to 77.8)	81.4 (76.3 to 85.9)	81.6 (76.4 to 86.1)	86.4 (82.2 to 89.9)	0.4 (0.3 to 0.5)	6.1 (4.2 to 8.4)
Belgium	81.4 (75.9 to 86.1)	84.9 (80.0 to 88.8)	85.0 (79.9 to 89.0)	87.4 (82.9 to 90.8)	0.2 (0.1 to 0.3)	3.0 (1.8 to 4.4)
Cyprus	81.6 (76.0 to 86.3)	85.6 (80.6 to 89.5)	85.7 (80.7 to 89.6)	90.1 (86.4 to 92.8)	0.3 (0.2 to 0.5)	5.2 (3.4 to 7.5)
Denmark	78.2 (72.3 to 83.4)	79.9 (74.3 to 84.7)	80.0 (74.6 to 84.9)	81.3 (75.9 to 85.9)	0.1 (0.0 to 0.2)	1.8 (0.1 to 3.5)
Finland	88.2 (84.4 to 91.1)	89.5 (86.2 to 92.3)	89.5 (86.3 to 92.3)	90.9 (88.1 to 93.3)	0.1 (0.1 to 0.2)	1.7 (0.9 to 2.6)
France	89.0 (87.0 to 91.0)	91.5 (88.8 to 93.6)	91.6 (88.8 to 93.7)	93.3 (91.0 to 95.0)	0.1 (0.1 to 0.2)	2.0 (1.3 to 2.9)
Germany	79.1 (74.0 to 83.6)	82.6 (77.1 to 86.8)	82.8 (77.3 to 87.1)	86.2 (81.7 to 89.9)	0.3 (0.2 to 0.4)	4.5 (2.8 to 6.5)
Greece	51.2 (44.0 to 58.5)	60.3 (52.5 to 67.9)	60.4 (52.3 to 68.3)	66.0 (58.6 to 73.4)	0.6 (0.4 to 0.8)	9.6 (6.2 to 13.4)
Iceland	85.3 (80.8 to 89.2)	87.9 (84.2 to 91.3)	88.1 (84.3 to 91.4)	90.4 (87.2 to 93.1)	0.2 (0.1 to 0.3)	2.8 (1.7 to 4.2)
Ireland	75.5 (70.7 to 79.9)	78.7 (73.4 to 83.4)	79.2 (73.5 to 84.2)	84.8 (80.4 to 88.4)	0.5 (0.3 to 0.7)	7.9 (5.2 to 10.9)
Israel	80.5 (74.7 to 85.5)	82.4 (76.9 to 87.2)	82.8 (77.3 to 87.2)	85.6 (80.8 to 89.5)	0.3 (0.2 to 0.4)	3.9 (2.5 to 5.7)
Italy	62.5 (55.9 to 68.8)	70.0 (63.1 to 76.1)	70.3 (63.6 to 76.6)	75.4 (69.1 to 80.7)	0.5 (0.3 to 0.7)	7.6 (5.1 to 10.5)
Luxembourg	86.7 (82.4 to 90.5)	88.5 (84.2 to 91.6)	88.6 (84.3 to 91.8)	90.3 (86.7 to 93.1)	0.1 (0.1 to 0.2)	2.1 (1.2 to 3.3)
Malta	67.7 (60.8 to 73.9)	74.4 (67.7 to 80.2)	75.0 (68.5 to 80.5)	80.2 (74.2 to 85.3)	0.5 (0.3 to 0.7)	7.9 (5.0 to 11.1)
Netherlands	77.4 (72.3 to 81.6)	82.1 (77.7 to 86.1)	82.4 (77.7 to 86.4)	84.4 (80.2 to 88.2)	0.2 (0.1 to 0.3)	2.8 (1.2 to 4.5)
Norway	85.5 (81.8 to 88.6)	85.6 (81.3 to 89.1)	85.8 (81.2 to 89.2)	87.6 (83.7 to 90.7)	0.2 (0.1 to 0.2)	2.3 (1.3 to 3.5)
Portugal	74.9 (69.1 to 80.1)	80.0 (74.5 to 84.9)	80.4 (75.0 to 85.4)	84.7 (80.1 to 88.8)	0.4 (0.2 to 0.5)	5.8 (3.8 to 8.4)
Spain	77.4 (72.6 to 81.2)	79.4 (73.8 to 84.3)	79.7 (74.3 to 84.7)	83.2 (78.5 to 87.3)	0.3 (0.2 to 0.5)	4.8 (2.5 to 7.3)
Sweden	79.8 (75.3 to 83.9)	81.5 (76.4 to 86.0)	81.8 (76.7 to 86.3)	83.5 (78.9 to 87.7)	0.2 (0.1 to 0.3)	2.5 (0.9 to 4.1)
Switzerland	83.0 (78.3 to 87.4)	85.2 (80.7 to 89.0)	85.3 (80.8 to 89.1)	86.8 (82.6 to 90.2)	0.1 (0.1 to 0.2)	1.9 (0.8 to 3.1)
United Kingdom	88.3 (87.3 to 89.3)	89.3 (86.6 to 91.6)	89.5 (86.7 to 91.9)	91.3 (88.9 to 93.3)	0.1 (0.1 to 0.2)	2.2 (1.4 to 3.0)
Latin America and Caribbean						
Andean Latin America						
Bolivia	36.3 (34.5 to 38.1)	48.8 (46.2 to 51.3)	51.0 (48.1 to 53.7)	63.3 (60.6 to 65.8)	1.7 (1.6 to 1.9)	29.6 (26.6 to 32.9)
Ecuador	62.0 (57.5 to 66.5)	71.4 (65.1 to 77.2)	72.1 (65.7 to 77.6)	79.1 (73.9 to 83.6)	0.7 (0.5 to 0.9)	10.8 (7.9 to 13.9)
Peru	57.7 (56.1 to 59.1)	63.5 (61.7 to 65.4)	64.5 (61.8 to 67.3)	73.1 (70.8 to 75.5)	0.9 (0.9 to 1.0)	15.1 (13.7 to 16.5)
Caribbean						
Antigua and Barbuda	78.5 (72.3 to 83.6)	81.7 (76.4 to 85.7)	82.0 (77.0 to 86.2)	85.8 (81.1 to 89.2)	0.3 (0.2 to 0.5)	5.0 (3.3 to 7.0)
The Bahamas	84.1 (79.5 to 87.9)	85.1 (80.9 to 89.1)	85.1 (81.1 to 89.2)	88.2 (84.8 to 91.4)	0.2 (0.1 to 0.4)	3.7 (2.3 to 5.4)
Barbados	68.3 (61.7 to 74.0)	65.4 (61.2 to 69.3)	65.9 (61.3 to 70.5)	73.1 (69.0 to 77.1)	0.7 (0.6 to 0.9)	11.8 (9.7 to 14.0)
Belize	67.5 (62.0 to 72.6)	69.0 (64.8 to 72.8)	69.7 (65.2 to 73.9)	78.3 (74.6 to 81.5)	0.8 (0.7 to 1.0)	13.4 (11.1 to 15.9)
Bermuda	86.0 (81.6 to 89.5)	87.0 (83.2 to 90.4)	87.0 (83.2 to 90.3)	89.5 (86.3 to 92.3)	0.2 (0.1 to 0.3)	2.9 (1.8 to 4.1)
Cuba	84.7 (81.2 to 87.8)	87.8 (86.0 to 89.4)	88.4 (86.3 to 90.1)	90.5 (88.8 to 92.0)	0.2 (0.2 to 0.2)	3.1 (2.4 to 3.8)
Dominica	79.5 (73.8 to 84.6)	81.8 (77.2 to 86.3)	82.1 (77.3 to 86.4)	86.9 (83.4 to 90.2)	0.4 (0.3 to 0.5)	6.3 (4.4 to 8.5)
Dominican Republic	77.8 (76.3 to 79.3)	82.9 (81.3 to 84.5)	83.5 (81.2 to 85.4)	88.8 (87.2 to 90.2)	0.5 (0.4 to 0.5)	7.1 (6.4 to 7.9)
Grenada	75.4 (69.3 to 81.1)	78.0 (71.6 to 83.2)	78.7 (72.3 to 83.8)	83.4 (77.8 to 87.8)	0.4 (0.3 to 0.7)	7.0 (3.8 to 10.9)
Guyana	55.7 (49.9 to 61.6)	55.3 (50.9 to 59.6)	55.9 (51.0 to 60.6)	62.4 (56.9 to 67.6)	0.8 (0.5 to 1.1)	12.8 (7.7 to 17.6)
Haiti	31.3 (29.1 to 33.8)	44.2 (42.3 to 46.3)	44.5 (42.1 to 47.2)	55.3 (52.9 to 57.8)	1.5 (1.3 to 1.6)	24.9 (21.8 to 27.8)
Jamaica	76.5 (73.8 to 79.1)	83.7 (80.2 to 86.9)	83.8 (80.0 to 87.1)	87.3 (84.3 to 90.3)	0.3 (0.2 to 0.4)	4.4 (3.1 to 5.9)
Puerto Rico	81.7 (78.0 to 85.2)	86.7 (82.7 to 90.0)	86.8 (82.7 to 90.2)	90.7 (87.6 to 93.2)	0.3 (0.2 to 0.4)	4.7 (3.2 to 6.3)
Saint Lucia	64.4 (57.6 to 70.9)	66.3 (61.3 to 70.9)	66.9 (61.7 to 71.8)	73.3 (68.6 to 77.7)	0.7 (0.5 to 0.8)	10.6 (8.4 to 13.1)
Saint Vincent and the Grenadines	83.6 (78.5 to 87.8)	81.6 (76.4 to 86.3)	82.0 (76.9 to 86.4)	86.2 (81.8 to 89.9)	0.4 (0.2 to 0.5)	5.7 (3.7 to 7.9)
Suriname	62.9 (58.0 to 67.5)	64.7 (60.5 to 68.5)	65.5 (60.6 to 70.0)	68.7 (64.0 to 73.0)	0.4 (0.3 to 0.5)	6.2 (4.3 to 8.3)
Trinidad and Tobago	55.0 (49.8 to 60.4)	61.0 (56.1 to 65.6)	61.3 (55.9 to 66.2)	67.6 (62.6 to 72.2)	0.7 (0.5 to 0.9)	10.9 (8.1 to 14.1)
Virgin Islands, U.S.	84.9 (80.2 to 88.7)	87.0 (83.0 to 90.6)	87.0 (83.0 to 90.5)	90.3 (86.9 to 93.0)	0.2 (0.2 to 0.3)	3.8 (2.4 to 5.4)
Central Latin America						
Colombia	74.3 (72.7 to 75.8)	85.4 (83.6 to 87.1)	86.3 (84.3 to 88.3)	91.6 (90.3 to 92.8)	0.5 (0.4 to 0.5)	7.2 (6.4 to 8.2)
Costa Rica	81.4 (77.8 to 84.7)	85.4 (82.6 to 87.9)	86.0 (83.1 to 88.7)	88.7 (86.3 to 91.0)	0.3 (0.2 to 0.3)	3.9 (2.9 to 4.9)
El Salvador	74.8 (72.2 to 77.3)	81.2 (78.2 to 84.0)	81.3 (77.9 to 84.3)	86.7 (84.1 to 89.0)	0.4 (0.4 to 0.5)	6.8 (5.6 to 8.2)
Guatemala	59.9 (56.7 to 63.2)	69.8 (66.5 to 73.2)	70.0 (66.1 to 73.7)	78.8 (75.5 to 81.7)	0.8 (0.7 to 0.9)	12.9 (11.0 to 15.0)
Honduras	64.8 (62.6 to 66.9)	77.3 (74.3 to 79.9)	78.0 (74.4 to 81.0)	84.4 (81.7 to 86.7)	0.6 (0.5 to 0.7)	9.2 (8.0 to 10.4)
Mexico	69.8 (66.0 to 73.5)	75.0 (70.9 to 78.3)	75.8 (71.4 to 79.4)	82.5 (78.9 to 85.3)	0.6 (0.5 to 0.8)	10.0 (8.4 to 11.9)
Nicaragua	77.0 (75.2 to 78.7)	86.3 (82.7 to 89.6)	86.8 (83.2 to 90.1)	91.6 (89.3 to 93.8)	0.4 (0.3 to 0.5)	6.2 (4.6 to 8.1)
Panama	74.8 (69.3 to 79.3)	73.4 (70.2 to 76.3)	74.5 (71.0 to 77.7)	80.4 (77.4 to 83.1)	0.6 (0.5 to 0.7)	9.6 (8.0 to 11.4)

	Estimate in 2000 (95% UIs)	Estimate in 2015 (95% UIs)	Estimate in 2017 (95% UIs)	Estimate in 2030 (95% UIs)	Annualised rate of change, 2015-2030 (95% UIs)	Percent change, 2015-2030 (95% UIs)
Venezuela	72.8 (67.3 to 78.0)	78.5 (72.9 to 83.4)	79.0 (73.2 to 83.9)	84.2 (79.5 to 88.2)	0.5 (0.3 to 0.6)	7.3 (5.1 to 9.9)
Tropical Latin America						
Brazil	81.4 (78.8 to 83.7)	87.6 (84.4 to 90.5)	88.0 (84.5 to 90.9)	92.0 (89.5 to 94.0)	0.3 (0.2 to 0.4)	5.0 (3.7 to 6.4)
Paraguay	65.5 (62.0 to 69.0)	78.9 (75.6 to 82.0)	79.9 (76.6 to 83.1)	86.8 (84.3 to 89.0)	0.6 (0.5 to 0.8)	10.1 (8.1 to 12.2)
North Africa and Middle East						
North Africa and Middle East						
Afghanistan	18.4 (13.8 to 23.5)	40.6 (37.4 to 43.6)	41.8 (38.1 to 45.3)	52.9 (49.2 to 56.5)	1.8 (1.5 to 2.0)	30.3 (25.8 to 34.6)
Algeria	69.4 (66.0 to 72.5)	73.2 (69.7 to 76.5)	74.2 (70.3 to 77.8)	78.7 (75.2 to 82.0)	0.5 (0.4 to 0.6)	7.5 (5.5 to 9.5)
Bahrain	55.9 (48.5 to 63.2)	65.5 (58.1 to 72.7)	66.4 (59.4 to 73.6)	74.0 (67.4 to 80.1)	0.8 (0.6 to 1.1)	13.0 (9.4 to 17.5)
Egypt	77.0 (75.7 to 78.3)	82.5 (80.1 to 84.5)	83.5 (80.9 to 85.8)	88.7 (86.9 to 90.3)	0.5 (0.4 to 0.6)	7.6 (6.5 to 8.9)
Iran	81.1 (76.0 to 85.5)	84.2 (79.2 to 88.2)	84.8 (80.3 to 88.6)	88.5 (84.5 to 91.5)	0.3 (0.1 to 0.5)	5.0 (2.2 to 8.4)
Iraq	48.8 (42.6 to 54.4)	57.1 (52.9 to 61.4)	58.6 (53.5 to 63.7)	64.2 (59.4 to 68.9)	0.8 (0.7 to 0.9)	12.4 (10.3 to 14.5)
Jordan	52.9 (50.6 to 55.3)	56.1 (53.6 to 58.7)	55.3 (52.6 to 58.2)	63.9 (61.4 to 66.6)	0.9 (0.7 to 1.0)	13.8 (11.8 to 16.0)
Kuwait	59.7 (53.3 to 66.3)	71.9 (64.5 to 78.1)	72.9 (65.8 to 78.9)	80.0 (74.3 to 84.8)	0.7 (0.5 to 1.0)	11.3 (7.3 to 16.2)
Lebanon	49.0 (44.8 to 53.3)	60.6 (54.0 to 66.7)	61.9 (54.9 to 67.9)	70.5 (64.0 to 76.1)	1.0 (0.7 to 1.3)	16.4 (11.0 to 22.2)
Libya	42.2 (38.1 to 46.5)	57.7 (52.1 to 62.8)	57.4 (51.4 to 62.7)	63.6 (58.5 to 67.9)	0.6 (0.5 to 0.9)	10.2 (7.4 to 13.8)
Morocco	68.0 (65.4 to 70.6)	69.6 (64.3 to 74.1)	70.6 (64.6 to 75.5)	78.6 (73.7 to 82.5)	0.8 (0.6 to 1.0)	12.9 (10.1 to 15.9)
Palestine	53.4 (49.8 to 56.8)	62.3 (59.2 to 65.4)	63.8 (60.1 to 67.4)	70.6 (67.2 to 73.8)	0.8 (0.7 to 1.0)	13.3 (11.3 to 15.3)
Oman	38.8 (32.8 to 45.5)	40.8 (38.0 to 43.4)	42.4 (38.9 to 45.9)	57.7 (54.0 to 61.3)	2.3 (2.1 to 2.6)	41.5 (36.2 to 47.3)
Qatar	57.4 (51.2 to 63.4)	68.7 (65.4 to 71.7)	69.7 (65.9 to 73.2)	77.9 (74.9 to 80.8)	0.8 (0.7 to 1.0)	13.5 (11.6 to 15.5)
Saudi Arabia	47.5 (40.4 to 54.4)	42.7 (35.7 to 49.4)	43.1 (36.1 to 49.9)	57.4 (50.1 to 64.0)	2.0 (1.6 to 2.4)	34.6 (27.7 to 42.9)
Sudan	18.5 (15.6 to 21.9)	27.7 (24.7 to 30.5)	29.9 (26.4 to 33.4)	39.7 (35.6 to 43.7)	2.4 (2.2 to 2.7)	43.3 (38.2 to 48.9)
Syria	50.1 (45.5 to 54.4)	56.0 (49.5 to 62.2)	57.0 (50.5 to 63.4)	68.8 (62.9 to 74.3)	1.4 (1.1 to 1.6)	23.0 (18.7 to 28.1)
Tunisia	69.3 (65.6 to 73.1)	70.7 (66.2 to 74.9)	71.8 (66.6 to 76.2)	77.8 (73.5 to 81.7)	0.6 (0.5 to 0.8)	10.2 (7.9 to 12.7)
Turkey	53.3 (51.1 to 55.6)	59.6 (56.6 to 62.5)	61.2 (57.5 to 64.8)	72.6 (69.4 to 75.6)	1.3 (1.2 to 1.5)	21.9 (19.5 to 24.3)
United Arab Emirates	55.6 (48.4 to 62.5)	70.4 (62.5 to 77.6)	70.7 (62.7 to 77.6)	79.8 (73.6 to 84.8)	0.8 (0.5 to 1.3)	13.5 (7.9 to 20.8)
Yemen	21.2 (18.6 to 23.9)	43.3 (40.3 to 46.4)	43.8 (40.1 to 47.7)	58.8 (55.1 to 62.3)	2.0 (1.8 to 2.2)	35.7 (32.0 to 39.9)
South Asia						
South Asia						
Bangladesh	65.2 (63.2 to 67.2)	75.9 (73.7 to 78.2)	77.3 (74.5 to 79.9)	83.6 (81.5 to 85.6)	0.6 (0.6 to 0.7)	10.1 (8.9 to 11.4)
Bhutan	64.7 (59.5 to 69.4)	78.9 (75.7 to 81.7)	79.9 (76.2 to 83.1)	86.8 (84.5 to 89.1)	0.6 (0.5 to 0.8)	10.0 (8.3 to 12.0)
India	65.0 (62.9 to 67.0)	70.4 (67.7 to 73.0)	71.4 (68.5 to 74.2)	77.2 (74.7 to 79.6)	0.6 (0.5 to 0.7)	9.7 (7.8 to 11.8)
Nepal	54.0 (51.4 to 56.8)	59.8 (57.5 to 62.0)	59.8 (57.1 to 62.6)	70.4 (68.0 to 72.8)	1.1 (1.0 to 1.2)	17.8 (15.9 to 19.9)
Pakistan	34.2 (31.3 to 37.3)	45.4 (42.7 to 48.2)	47.1 (43.5 to 50.6)	61.1 (57.7 to 64.4)	2.0 (1.8 to 2.1)	34.5 (31.3 to 38.0)
Southeast Asia, East Asia, and Oceania						
East Asia						
China	92.3 (90.2 to 94.0)	95.6 (94.1 to 96.9)	95.9 (94.5 to 97.1)	97.4 (96.4 to 98.2)	0.1 (0.1 to 0.2)	1.9 (1.3 to 2.7)
North Korea	66.9 (62.0 to 71.6)	77.0 (74.3 to 79.6)	79.0 (76.4 to 81.5)	81.0 (78.4 to 83.4)	0.3 (0.2 to 0.4)	5.2 (3.7 to 6.7)
Taiwan (Province of China)	95.2 (93.3 to 96.7)	95.8 (94.2 to 97.1)	95.9 (94.3 to 97.2)	97.2 (96.1 to 98.0)	0.1 (0.1 to 0.2)	1.5 (0.9 to 2.3)
Oceania						
American Samoa	73.7 (66.6 to 79.7)	73.3 (66.6 to 79.4)	73.6 (66.8 to 79.6)	77.6 (69.0 to 84.5)	0.4 (-0.1 to 0.8)	5.9 (-0.8 to 12.4)
Federated States of Micronesia	60.0 (51.8 to 68.0)	60.3 (52.4 to 67.7)	61.0 (52.8 to 68.2)	69.5 (61.7 to 76.2)	0.9 (0.6 to 1.3)	15.3 (9.3 to 21.7)
Fiji	70.9 (64.3 to 77.3)	70.8 (64.2 to 77.0)	71.8 (65.2 to 77.8)	78.7 (73.0 to 83.6)	0.7 (0.5 to 0.9)	11.1 (8.0 to 14.9)
Guam	82.2 (77.1 to 86.8)	81.5 (76.2 to 86.2)	81.5 (76.2 to 86.3)	85.8 (81.6 to 89.7)	0.3 (0.2 to 0.5)	5.3 (3.6 to 7.6)
Kiribati	51.3 (45.4 to 57.8)	41.9 (36.3 to 48.3)	43.3 (37.0 to 49.8)	49.9 (43.3 to 56.6)	1.2 (0.9 to 1.4)	19.0 (14.4 to 23.8)
Marshall Islands	73.5 (69.1 to 77.8)	80.8 (77.2 to 84.4)	81.5 (77.5 to 85.1)	85.6 (82.2 to 88.7)	0.4 (0.3 to 0.5)	6.0 (4.7 to 7.3)
Northern Mariana Islands	82.3 (76.0 to 87.1)	79.4 (73.7 to 84.2)	78.6 (72.4 to 83.9)	82.8 (75.0 to 88.7)	0.3 (-0.2 to 0.7)	4.3 (-3.0 to 10.5)
Papua New Guinea	35.4 (31.4 to 39.8)	39.5 (33.0 to 46.1)	40.7 (33.7 to 47.3)	45.6 (38.2 to 52.4)	1.0 (0.7 to 1.2)	15.4 (11.5 to 19.5)
Samoa	47.7 (41.2 to 54.2)	44.1 (37.9 to 51.2)	45.1 (38.7 to 52.4)	47.9 (41.2 to 55.1)	0.5 (0.3 to 0.8)	8.6 (4.2 to 13.1)
Solomon Islands	50.3 (42.8 to 58.3)	57.1 (49.5 to 64.5)	58.3 (50.8 to 65.4)	63.0 (55.3 to 69.9)	0.7 (0.5 to 0.9)	10.4 (7.0 to 14.0)
Tonga	39.4 (33.0 to 46.1)	39.0 (34.6 to 43.9)	40.0 (34.7 to 45.6)	47.6 (42.3 to 53.3)	1.3 (1.0 to 1.6)	22.2 (17.0 to 27.4)
Vanuatu	50.5 (43.9 to 56.9)	49.6 (45.1 to 54.6)	50.3 (45.2 to 55.6)	56.7 (51.8 to 61.8)	0.9 (0.7 to 1.1)	14.3 (10.6 to 18.5)
Southeast Asia						
Cambodia	32.2 (29.8 to 34.5)	57.2 (54.4 to 60.0)	58.7 (55.0 to 62.2)	70.1 (67.0 to 73.0)	1.4 (1.2 to 1.5)	22.5 (19.9 to 25.3)
Indonesia	76.3 (72.9 to 79.6)	77.2 (74.7 to 79.5)	77.7 (74.8 to 80.2)	82.0 (79.5 to 84.2)	0.4 (0.3 to 0.5)	6.2 (4.9 to 7.6)
Laos	49.8 (44.0 to 55.4)	62.4 (58.2 to 66.9)	64.3 (59.5 to 69.3)	74.8 (70.8 to 78.9)	1.2 (1.0 to 1.4)	19.9 (16.9 to 23.1)
Malaysia	62.6 (55.6 to 69.3)	75.8 (69.3 to 81.9)	76.8 (70.4 to 82.7)	84.5 (79.8 to 88.8)	0.7 (0.5 to 1.0)	11.5 (8.0 to 15.7)
Maldives	46.1 (40.2 to 52.1)	52.1 (46.1 to 58.6)	54.4 (48.0 to 61.5)	67.6 (62.0 to 73.6)	1.7 (1.4 to 2.1)	29.8 (23.3 to 36.6)
Mauritius	62.4 (55.7 to 68.7)	72.8 (66.4 to 78.6)	73.5 (67.0 to 79.5)	79.0 (73.3 to 84.2)	0.6 (0.4 to 0.7)	8.6 (6.2 to 11.3)
Myanmar	53.9 (48.1 to 59.5)	76.0 (73.7 to 78.4)	77.0 (74.4 to 79.5)	86.0 (84.2 to 87.6)	0.8 (0.7 to 0.9)	13.2 (11.4 to 15.0)
Philippines	44.5 (42.2 to 46.8)	55.5 (53.2 to 57.9)	57.3 (54.5 to 60.1)	66.2 (63.7 to 68.7)	1.2 (1.0 to 1.3)	19.3 (16.8 to 21.6)
Sri Lanka	63.8 (58.6 to 68.7)	63.5 (60.6 to 66.0)	63.8 (61.0 to 66.5)	73.9 (71.6 to 76.0)	1.0 (0.9 to 1.2)	16.4 (13.9 to 19.2)
Seychelles	71.9 (64.9 to 78.6)	77.4 (71.0 to 83.3)	78.1 (71.6 to 83.9)	83.1 (77.8 to 87.7)	0.5 (0.3 to 0.7)	7.5 (4.8 to 10.6)
Thailand	84.4 (81.3 to 87.0)	89.0 (87.5 to 90.2)	89.5 (87.8 to 90.9)	92.0 (90.7 to 93.1)	0.2 (0.2 to 0.3)	3.5 (2.9 to 4.1)
Timor-Leste	37.0 (32.6 to 41.4)	46.2 (41.6 to 51.2)	45.9 (41.1 to 51.1)	55.6 (50.6 to 60.6)	1.2 (1.0 to 1.5)	20.4 (16.5 to 24.4)
Vietnam	68.1 (65.9 to 70.5)	72.9 (70.4 to 75.6)	74.4 (71.2 to 77.5)	78.7 (75.8 to 81.5)	0.5 (0.4 to 0.6)	8.0 (6.4 to 9.5)
Sub-Saharan Africa						
Central Sub-Saharan Africa						
Angola	10.8 (8.3 to 13.6)	28.5 (25.3 to 31.6)	30.4 (27.1 to 34.2)	43.0 (39.0 to 47.1)	2.8 (2.5 to 3.1)	51.3 (44.4 to 58.5)
Central African Republic	14.4 (12.3 to 16.8)	21.0 (17.7 to 24.3)	21.5 (17.7 to 25.4)	27.4 (23.0 to 31.8)	1.8 (1.5 to 2.0)	30.1 (24.9 to 35.5)
Congo	22.9 (19.0 to 26.9)	40.2 (36.7 to 43.6)	41.8 (37.5 to 45.7)	53.5 (49.0 to 57.5)	1.9 (1.7 to 2.1)	32.9 (28.9 to 36.9)
Democratic Republic of the Congo	11.3 (9.2 to 13.8)	20.0 (17.7 to 22.6)	21.6 (18.5 to 25.0)	28.3 (24.6 to 32.3)	2.3 (2.0 to 2.6)	41.2 (35.1 to 47.6)
Equatorial Guinea	12.9 (9.8 to 16.5)	31.5 (25.1 to 38.1)	33.0 (26.4 to 39.9)	47.1 (39.3 to 54.7)	2.7 (2.2 to 3.1)	50.1 (39.9 to 60.3)
Gabon	25.7 (23.5 to 28.1)	46.0 (42.2 to 49.7)	47.8 (43.0 to 52.2)	58.9 (54.5 to 63.3)	1.7 (1.5 to 1.8)	28.3 (24.5 to 32.0)
Eastern Sub-Saharan Africa						
Burundi	20.4 (16.4 to 24.4)	37.2 (33.3 to 41.0)	38.1 (33.8 to 42.2)	51.0 (46.7 to 55.1)	2.1 (1.8 to 2.4)	37.1 (31.1 to 43.2)
Comoros	25.9 (22.4 to 29.2)	30.8 (27.0 to 34.8)	32.5 (28.1 to 37.3)	39.1 (34.3 to 44.1)	1.6 (1.3 to 1.8)	27.0 (22.3 to 31.8)
Djibouti	25.6 (20.8 to 31.2)	49.3 (43.8 to 54.9)	50.9 (44.9 to 56.8)	60.2 (54.4 to 65.6)	1.3 (1.0 to 1.6)	22.1 (16.8 to 27.8)
Eritrea	14.5 (12.8 to 16.4)	27.4 (22.1 to 34.1)	29.2 (23.2 to 35.9)	40.2 (33.6 to 47.7)	2.6 (2.0 to 3.1)	47.1 (35.7 to 58.9)
Ethiopia	21.0 (17.9 to 24.3)	57.0 (54.8 to 59.0)	59.9 (57.4 to 62.3)	70.7 (68.5 to 72.8)	1.4 (1.3 to 1.6)	24.1 (21.5 to 26.7)

	**Estimate in 2000 (95% UIs)**	**Estimate in 2015 (95% UIs)**	**Estimate in 2017 (95% UIs)**	**Estimate in 2030 (95% UIs)**	**Annualised rate of change, 2015-2030 (95% UIs)**	**Percent change, 2015-2030 (95% UIs)**
Kenya	46.1 (43.1 to 49.3)	71.0 (68.9 to 73.1)	72.8 (70.3 to 75.2)	82.8 (81.1 to 84.6)	1.0 (0.9 to 1.1)	16.7 (15.0 to 18.5)
Madagascar	27.6 (24.0 to 31.7)	41.4 (35.5 to 47.7)	42.0 (35.5 to 48.7)	54.0 (47.2 to 60.6)	1.8 (1.5 to 2.1)	30.7 (25.5 to 36.1)
Malawi	40.1 (36.5 to 43.9)	70.0 (66.6 to 73.3)	71.1 (67.6 to 74.7)	82.0 (79.5 to 84.5)	1.1 (0.9 to 1.2)	17.1 (14.7 to 19.7)
Mozambique	24.8 (21.3 to 28.2)	44.9 (40.7 to 49.4)	47.2 (42.1 to 52.3)	58.9 (54.0 to 63.8)	1.8 (1.6 to 2.0)	31.0 (26.6 to 35.5)
Rwanda	21.1 (17.5 to 25.2)	54.5 (48.6 to 60.1)	55.8 (49.5 to 61.8)	65.3 (59.8 to 70.5)	1.2 (0.9 to 1.5)	19.9 (14.4 to 25.8)
Somalia	2.1 (1.5 to 2.8)	3.7 (2.6 to 5.0)	3.9 (2.8 to 5.3)	5.7 (4.0 to 7.8)	2.9 (2.4 to 3.4)	54.7 (43.6 to 66.6)
South Sudan	3.8 (2.7 to 5.4)	5.5 (3.9 to 7.4)	5.8 (4.1 to 7.8)	7.1 (5.1 to 9.4)	1.7 (1.1 to 2.3)	29.0 (18.5 to 42.1)
Tanzania	37.0 (34.1 to 40.0)	46.7 (43.4 to 50.1)	47.7 (43.9 to 51.6)	62.2 (58.6 to 65.8)	1.9 (1.7 to 2.1)	33.2 (29.6 to 37.2)
Uganda	26.8 (24.3 to 29.5)	46.6 (45.1 to 48.3)	48.8 (46.9 to 50.8)	64.3 (62.6 to 66.1)	2.1 (2.0 to 2.3)	38.0 (34.8 to 41.0)
Zambia	34.9 (31.9 to 38.0)	60.1 (55.4 to 64.6)	61.7 (56.7 to 66.4)	74.0 (69.8 to 77.8)	1.4 (1.2 to 1.6)	23.3 (19.7 to 27.0)
Southern Sub-Saharan Africa						
Botswana	71.1 (65.2 to 76.1)	79.8 (74.2 to 84.5)	80.4 (75.0 to 85.2)	86.8 (82.8 to 90.3)	0.6 (0.4 to 0.8)	8.9 (5.8 to 12.5)
Lesotho	54.3 (49.6 to 58.6)	76.3 (72.8 to 79.5)	77.4 (73.5 to 80.9)	85.0 (82.2 to 87.5)	0.7 (0.6 to 0.9)	11.5 (9.3 to 13.9)
Namibia	69.0 (66.5 to 71.2)	81.5 (79.1 to 83.8)	82.2 (79.3 to 84.8)	89.1 (87.2 to 90.8)	0.6 (0.5 to 0.7)	9.3 (8.1 to 10.7)
South Africa	79.3 (77.6 to 81.2)	77.5 (75.4 to 79.7)	77.7 (75.4 to 79.7)	83.1 (81.3 to 84.7)	0.5 (0.4 to 0.6)	7.2 (5.6 to 8.8)
Swaziland	59.8 (54.7 to 64.5)	81.1 (78.1 to 84.1)	81.8 (78.5 to 85.0)	87.4 (84.8 to 89.6)	0.5 (0.4 to 0.6)	7.7 (5.8 to 9.9)
Zimbabwe	72.1 (69.6 to 74.3)	83.4 (81.3 to 85.2)	84.2 (81.9 to 86.3)	89.7 (88.1 to 91.0)	0.5 (0.4 to 0.6)	7.6 (6.4 to 9.0)
Western Sub-Saharan Africa						
Benin	13.9 (12.5 to 15.4)	24.1 (21.2 to 27.2)	25.3 (21.9 to 29.4)	34.7 (30.4 to 39.5)	2.4 (2.2 to 2.7)	44.1 (38.1 to 49.8)
Burkina Faso	21.9 (19.2 to 25.1)	41.2 (38.6 to 43.7)	42.9 (39.9 to 45.8)	57.7 (54.6 to 60.4)	2.2 (2.1 to 2.5)	40.1 (36.2 to 44.7)
Cameroon	23.7 (21.4 to 25.9)	43.1 (40.0 to 46.3)	44.7 (40.6 to 48.7)	57.0 (53.0 to 60.8)	1.9 (1.6 to 2.1)	32.1 (28.0 to 36.6)
Cape Verde	54.9 (49.2 to 60.5)	69.6 (62.8 to 75.7)	70.3 (63.2 to 76.4)	74.5 (68.0 to 80.3)	0.5 (0.2 to 0.7)	7.1 (3.6 to 11.2)
Chad	6.9 (5.6 to 8.6)	14.7 (12.7 to 17.0)	15.8 (13.3 to 18.4)	22.7 (19.4 to 26.1)	2.9 (2.5 to 3.3)	54.6 (45.9 to 62.9)
Cote d'Ivoire	21.8 (19.5 to 24.4)	33.7 (31.8 to 35.7)	35.5 (33.3 to 37.7)	47.1 (44.7 to 49.6)	2.2 (2.0 to 2.5)	40.0 (35.5 to 44.6)
The Gambia	21.9 (17.9 to 26.4)	29.8 (26.7 to 33.2)	30.7 (26.8 to 35.0)	39.4 (35.0 to 44.1)	1.9 (1.6 to 2.1)	32.2 (27.5 to 36.8)
Ghana	30.0 (25.3 to 35.1)	40.5 (36.2 to 44.6)	42.6 (37.8 to 47.2)	56.4 (51.6 to 60.9)	2.2 (2.0 to 2.5)	39.2 (34.0 to 44.6)
Guinea	15.7 (13.6 to 17.7)	20.8 (18.4 to 23.3)	21.3 (18.6 to 24.1)	29.9 (26.4 to 33.4)	2.4 (2.1 to 2.8)	44.1 (37.0 to 52.3)
Guinea-Bissau	19.3 (14.9 to 24.3)	48.1 (41.2 to 54.9)	49.0 (41.7 to 55.7)	61.8 (55.0 to 67.7)	1.7 (1.3 to 2.0)	28.7 (22.2 to 35.7)
Liberia	17.9 (14.8 to 21.0)	43.7 (40.5 to 47.1)	45.8 (42.0 to 50.2)	60.0 (56.2 to 64.3)	2.1 (1.9 to 2.3)	37.5 (33.7 to 41.4)
Mali	15.8 (14.0 to 17.6)	30.9 (28.6 to 33.0)	32.5 (29.4 to 35.3)	46.3 (42.7 to 49.5)	2.7 (2.5 to 2.9)	50.0 (45.1 to 54.6)
Mauritania	13.1 (11.0 to 15.5)	27.8 (25.0 to 30.8)	29.5 (26.2 to 32.8)	35.9 (32.2 to 39.7)	1.7 (1.4 to 2.0)	29.3 (24.0 to 34.5)
Niger	19.3 (16.4 to 22.3)	37.4 (33.9 to 41.0)	41.6 (37.7 to 45.6)	56.5 (52.2 to 60.7)	2.8 (2.5 to 3.0)	51.4 (45.2 to 57.4)
Nigeria	23.4 (20.1 to 26.9)	37.2 (34.4 to 40.0)	39.2 (35.7 to 42.6)	51.0 (47.4 to 54.4)	2.1 (1.9 to 2.3)	37.0 (32.5 to 41.8)
Sao Tome and Principe	47.5 (41.6 to 53.6)	55.7 (50.5 to 60.9)	57.1 (51.3 to 62.6)	66.5 (61.1 to 71.4)	1.2 (1.0 to 1.4)	19.4 (15.8 to 23.1)
Senegal	17.9 (15.9 to 19.8)	44.6 (43.0 to 46.2)	48.5 (46.5 to 50.8)	64.4 (62.5 to 66.5)	2.5 (2.3 to 2.6)	44.6 (41.5 to 47.8)
Sierra Leone	16.6 (13.6 to 20.3)	41.3 (36.9 to 46.1)	42.8 (37.9 to 48.2)	55.5 (50.2 to 60.7)	2.0 (1.7 to 2.2)	34.5 (30.0 to 39.3)
Togo	23.6 (20.5 to 26.8)	37.5 (34.2 to 41.0)	39.4 (35.3 to 44.0)	52.8 (48.5 to 57.6)	2.3 (2.1 to 2.5)	41.1 (36.1 to 45.9)
**Indicator 3.7.2: Number of livebirths per 1,000 women aged 10-19 years old**						
Central Europe, Eastern Europe, and Central Asia						
Central Asia						
Armenia	14.9 (13.7 to 16.2)	12.7 (11.0 to 14.5)	12.1 (10.4 to 14.1)	8.6 (6.1 to 11.9)	-2.7 (-4.6 to -0.6)	-32.5 (-50.1 to -8.0)
Azerbaijan	15.5 (13.5 to 17.8)	24.2 (20.7 to 28.4)	21.8 (18.5 to 25.7)	16.6 (12.2 to 21.6)	-2.6 (-4.5 to -0.8)	-31.5 (-48.9 to -11.0)
Georgia	17.7 (15.2 to 20.4)	24.6 (20.9 to 28.6)	23.1 (19.6 to 27.2)	17.3 (12.4 to 23.1)	-2.4 (-4.3 to -0.5)	-29.5 (-47.9 to -7.2)
Kazakhstan	13.2 (12.5 to 14.0)	16.0 (14.3 to 17.8)	14.2 (12.4 to 16.1)	10.2 (7.4 to 13.8)	-3.0 (-5.0 to -1.0)	-35.9 (-52.8 to -14.1)
Kyrgyzstan	17.1 (15.3 to 19.0)	20.4 (17.7 to 23.3)	18.8 (16.0 to 22.0)	13.2 (9.2 to 18.1)	-3.0 (-5.1 to -1.0)	-35.5 (-53.5 to -13.9)
Mongolia	13.4 (12.6 to 14.4)	13.5 (12.2 to 15.1)	13.2 (11.6 to 15.0)	9.4 (6.8 to 13.0)	-2.5 (-4.5 to -0.4)	-30.2 (-48.9 to -6.5)
Tajikistan	27.6 (24.4 to 31.0)	27.7 (23.6 to 32.4)	27.5 (23.3 to 32.3)	17.6 (12.7 to 23.8)	-3.1 (-5.0 to -1.1)	-36.2 (-52.5 to -15.1)
Turkmenistan	7.8 (7.1 to 8.7)	9.3 (8.1 to 10.8)	9.3 (8.0 to 10.9)	6.2 (4.4 to 8.7)	-2.8 (-4.7 to -0.6)	-33.1 (-50.4 to -9.2)
Uzbekistan	15.8 (14.1 to 17.8)	16.5 (14.2 to 19.2)	16.1 (13.7 to 19.0)	9.0 (6.4 to 12.2)	-4.1 (-6.3 to -2.2)	-45.5 (-60.9 to -27.8)
Central Europe						
Albania	12.1 (10.8 to 13.4)	11.0 (9.3 to 12.9)	10.6 (8.9 to 12.7)	6.2 (4.0 to 9.4)	-3.9 (-6.7 to -1.1)	-43.1 (-63.3 to -15.5)
Bosnia and Herzegovina	10.0 (9.2 to 10.8)	5.6 (4.9 to 6.5)	5.4 (4.7 to 6.3)	3.7 (2.4 to 5.7)	-2.9 (-5.8 to 0.1)	-34.1 (-57.9 to 1.6)
Bulgaria	23.3 (22.3 to 24.5)	20.4 (18.5 to 22.4)	19.7 (17.1 to 22.6)	16.8 (10.8 to 24.6)	-1.4 (-4.2 to 1.2)	-17.8 (-47.0 to 20.5)
Croatia	7.3 (6.7 to 8.1)	5.3 (4.7 to 6.1)	5.2 (4.5 to 6.1)	4.2 (2.7 to 6.2)	-1.6 (-4.4 to 1.0)	-20.0 (-48.2 to 16.2)
Czech Republic	6.6 (5.9 to 7.2)	6.0 (5.2 to 6.9)	5.9 (5.0 to 7.0)	5.0 (3.1 to 7.9)	-1.4 (-4.3 to 1.6)	-16.6 (-47.8 to 26.9)
Hungary	11.6 (10.8 to 12.4)	11.8 (10.6 to 13.0)	11.0 (9.5 to 12.8)	9.4 (5.9 to 14.6)	-1.6 (-4.5 to 1.3)	-19.7 (-49.5 to 21.2)
Macedonia	14.1 (13.1 to 15.3)	8.7 (7.6 to 10.0)	8.6 (7.3 to 9.9)	6.9 (4.3 to 10.3)	-1.8 (-4.6 to 1.0)	-21.2 (-49.5 to 16.8)
Montenegro	11.4 (10.1 to 13.0)	5.9 (5.0 to 6.9)	5.9 (5.0 to 7.0)	5.0 (3.1 to 8.0)	-1.2 (-3.9 to 1.8)	-14.0 (-44.7 to 31.0)
Poland	8.6 (8.2 to 9.1)	6.7 (6.0 to 7.4)	6.5 (5.5 to 7.6)	5.1 (3.2 to 7.7)	-2.0 (-4.9 to 1.0)	-23.5 (-51.7 to 15.7)
Romania	18.0 (17.4 to 18.7)	18.0 (16.8 to 19.4)	17.5 (15.3 to 20.3)	13.0 (8.3 to 19.7)	-2.3 (-5.2 to 0.6)	-27.8 (-53.9 to 10.1)
Serbia	13.9 (13.1 to 14.8)	7.5 (6.7 to 8.4)	7.8 (6.7 to 9.1)	6.5 (4.1 to 9.6)	-1.1 (-4.1 to 1.7)	-13.7 (-45.8 to 29.0)
Slovakia	11.1 (10.3 to 12.0)	12.4 (11.2 to 13.6)	11.8 (10.3 to 13.5)	9.8 (6.2 to 14.7)	-1.7 (-4.6 to 1.2)	-20.7 (-50.0 to 19.1)
Slovenia	3.4 (2.9 to 4.0)	2.5 (2.1 to 3.0)	2.5 (2.1 to 3.0)	2.4 (1.6 to 3.7)	-0.5 (-3.2 to 2.4)	-5.0 (-38.3 to 44.0)
Eastern Europe						
Belarus	11.5 (10.6 to 12.6)	9.8 (8.6 to 11.1)	9.3 (8.1 to 10.9)	5.2 (4.1 to 6.6)	-4.3 (-5.7 to -2.8)	-47.0 (-57.4 to -33.9)
Estonia	11.1 (9.8 to 12.4)	6.7 (5.6 to 7.8)	6.7 (5.6 to 8.0)	6.5 (4.9 to 8.3)	-0.2 (-1.8 to 1.4)	-2.4 (-23.4 to 23.8)
Latvia	8.2 (7.3 to 9.2)	9.2 (8.0 to 10.6)	8.9 (7.6 to 10.5)	8.0 (6.1 to 10.3)	-1.0 (-2.6 to 0.6)	-13.3 (-32.1 to 9.3)
Lithuania	9.5 (8.7 to 10.4)	8.2 (7.2 to 9.3)	8.6 (7.4 to 10.0)	7.0 (5.5 to 8.8)	-1.1 (-2.6 to 0.6)	-14.0 (-32.3 to 8.6)
Moldova	18.6 (17.1 to 20.2)	12.2 (10.7 to 13.9)	11.7 (10.1 to 13.5)	7.4 (5.6 to 9.5)	-3.4 (-4.9 to -2.0)	-39.4 (-51.8 to -25.5)
Russia	13.6 (13.3 to 14.0)	12.6 (11.5 to 13.7)	12.1 (10.4 to 14.1)	8.5 (6.6 to 10.9)	-2.6 (-4.2 to -1.1)	-32.3 (-46.9 to -14.7)
Ukraine	15.5 (14.4 to 16.6)	14.0 (12.2 to 15.8)	13.1 (11.4 to 15.1)	8.1 (6.2 to 10.1)	-3.7 (-5.2 to -2.2)	-42.3 (-54.5 to -28.4)
High-income						
Australasia						
Australia	8.8 (8.1 to 9.5)	6.7 (6.0 to 7.5)	6.7 (5.7 to 7.8)	5.4 (4.3 to 6.7)	-1.4 (-2.7 to -0.2)	-18.8 (-33.1 to -2.4)
New Zealand	13.7 (12.2 to 15.3)	10.2 (9.0 to 11.6)	10.4 (8.9 to 12.2)	7.8 (6.2 to 9.7)	-1.8 (-3.1 to -0.5)	-23.3 (-37.5 to -7.3)
High-income Asia Pacific						
Brunei	12.2 (10.4 to 14.3)	6.5 (5.4 to 7.7)	6.5 (5.4 to 7.8)	5.5 (2.0 to 11.5)	-1.7 (-7.6 to 3.7)	-15.5 (-67.9 to 75.4)
Japan	2.8 (2.4 to 3.2)	2.1 (1.7 to 2.5)	2.1 (1.8 to 2.6)	2.1 (0.8 to 4.6)	-0.7 (-6.0 to 5.2)	-0.3 (-59.5 to 119.7)
South Korea	2.0 (1.7 to 2.3)	0.9 (0.8 to 1.1)	1.0 (0.8 to 1.2)	0.8 (0.4 to 1.5)	-1.6 (-5.4 to 2.9)	-16.7 (-55.3 to 55.0)
Singapore	3.9 (3.3 to 4.7)	2.6 (2.2 to 3.1)	2.7 (2.2 to 3.3)	2.2 (1.0 to 4.7)	-1.6 (-6.5 to 3.8)	-14.8 (-62.5 to 77.0)

	Estimate in 2000 (95% UIs)	Estimate in 2015 (95% UIs)	Estimate in 2017 (95% UIs)	Estimate in 2030 (95% UIs)	Annualised rate of change, 2015-2030 (95% UIs)	Percent change, 2015-2030 (95% UIs)
High-income North America						
Canada	8.4 (7.9 to 8.9)	6.5 (5.6 to 7.6)	6.5 (5.5 to 7.8)	5.5 (4.3 to 6.8)	-1.2 (-2.8 to 0.1)	-16.1 (-34.5 to 1.7)
Greenland	26.2 (22.3 to 30.6)	21.3 (18.1 to 24.9)	21.3 (18.0 to 25.4)	16.6 (12.8 to 20.9)	-1.7 (-3.2 to -0.2)	-22.1 (-37.8 to -3.6)
United States	23.5 (23.1 to 24.0)	10.9 (10.6 to 11.2)	11.0 (10.0 to 12.0)	8.7 (7.1 to 10.5)	-1.6 (-2.8 to -0.3)	-20.3 (-34.3 to -3.7)
Southern Latin America						
Argentina	31.6 (30.5 to 32.9)	31.3 (30.2 to 32.7)	30.6 (27.3 to 34.4)	24.7 (20.1 to 29.7)	-1.6 (-2.9 to -0.5)	-21.2 (-35.6 to -6.6)
Chile	29.9 (29.0 to 31.0)	21.8 (20.4 to 23.2)	21.8 (19.2 to 24.8)	17.3 (14.0 to 20.9)	-1.6 (-2.9 to -0.4)	-20.7 (-35.1 to -5.3)
Uruguay	31.3 (29.5 to 33.1)	29.5 (27.3 to 31.9)	28.3 (24.7 to 32.3)	22.8 (18.7 to 27.7)	-1.7 (-2.9 to -0.6)	-22.6 (-35.7 to -8.3)
Western Europe						
Andorra	3.8 (3.2 to 4.4)	2.5 (2.2 to 2.9)	2.6 (2.3 to 3.0)	2.3 (1.6 to 3.2)	-0.7 (-2.7 to 1.6)	-9.3 (-33.6 to 27.4)
Austria	7.0 (6.2 to 7.9)	4.6 (4.0 to 5.2)	4.7 (4.0 to 5.5)	3.6 (2.4 to 5.2)	-1.7 (-4.2 to 0.8)	-20.9 (-47.1 to 12.4)
Belgium	5.5 (4.9 to 6.2)	3.7 (3.1 to 4.4)	3.8 (3.2 to 4.6)	3.4 (2.2 to 4.9)	-0.8 (-3.2 to 1.8)	-9.5 (-38.1 to 31.1)
Cyprus	5.1 (4.2 to 6.1)	2.0 (1.6 to 2.4)	2.1 (1.7 to 2.5)	1.7 (1.2 to 2.5)	-1.1 (-3.5 to 1.3)	-13.8 (-41.2 to 21.7)
Denmark	3.5 (3.0 to 4.2)	2.3 (1.9 to 2.7)	2.4 (2.0 to 2.9)	2.0 (1.4 to 2.9)	-0.9 (-3.2 to 1.6)	-11.0 (-38.2 to 27.6)
Finland	4.2 (3.7 to 4.8)	3.5 (3.0 to 4.2)	3.5 (2.9 to 4.1)	3.1 (2.1 to 4.6)	-1.0 (-3.5 to 1.8)	-11.6 (-40.8 to 31.3)
France	4.9 (4.6 to 5.2)	3.8 (3.4 to 4.2)	3.8 (3.3 to 4.4)	3.4 (2.3 to 5.1)	-0.9 (-3.2 to 1.9)	-10.5 (-38.2 to 32.0)
Germany	7.2 (6.8 to 7.6)	4.0 (3.6 to 4.4)	4.2 (3.6 to 4.8)	3.3 (2.2 to 4.8)	-1.4 (-4.0 to 1.2)	-18.0 (-44.8 to 19.5)
Greece	5.6 (5.0 to 6.2)	4.5 (3.9 to 5.0)	4.6 (4.0 to 5.3)	3.8 (2.5 to 5.4)	-1.2 (-3.7 to 1.3)	-15.1 (-42.7 to 21.6)
Iceland	10.4 (8.8 to 12.3)	4.7 (4.0 to 5.5)	4.9 (4.1 to 5.7)	4.2 (2.8 to 6.1)	-0.9 (-3.4 to 1.8)	-11.3 (-40.1 to 30.3)
Ireland	9.9 (9.0 to 11.0)	5.0 (4.4 to 5.6)	5.2 (4.5 to 6.1)	4.9 (3.2 to 7.1)	-0.2 (-2.8 to 2.3)	-1.1 (-34.5 to 41.8)
Israel	8.2 (7.5 to 9.0)	5.3 (4.6 to 6.0)	5.5 (4.6 to 6.5)	4.9 (3.2 to 7.2)	-0.7 (-3.4 to 2.0)	-7.6 (-39.8 to 35.4)
Italy	3.6 (3.2 to 3.9)	2.8 (2.4 to 3.1)	2.8 (2.4 to 3.3)	2.5 (1.7 to 3.6)	-0.8 (-3.3 to 1.6)	-9.0 (-38.6 to 27.5)
Luxembourg	4.8 (4.0 to 5.7)	3.1 (2.5 to 3.7)	3.2 (2.7 to 3.9)	2.8 (1.8 to 4.1)	-0.9 (-3.5 to 1.6)	-11.1 (-41.2 to 26.8)
Malta	8.2 (7.4 to 9.1)	6.8 (6.2 to 7.5)	6.7 (5.8 to 7.7)	5.0 (3.3 to 7.2)	-2.2 (-4.8 to 0.3)	-27.0 (-51.7 to 5.4)
Netherlands	3.1 (2.7 to 3.5)	2.0 (1.7 to 2.4)	2.1 (1.8 to 2.5)	1.9 (1.3 to 2.8)	-0.3 (-2.7 to 2.1)	-2.0 (-33.1 to 36.6)
Norway	5.2 (4.5 to 6.0)	2.8 (2.3 to 3.3)	2.9 (2.4 to 3.5)	2.5 (1.6 to 3.5)	-0.9 (-3.5 to 1.6)	-10.5 (-41.3 to 27.8)
Portugal	11.0 (10.2 to 11.9)	5.0 (4.3 to 5.7)	5.4 (4.6 to 6.3)	4.2 (2.9 to 6.2)	-1.2 (-3.7 to 1.3)	-15.3 (-42.7 to 21.6)
Spain	4.9 (4.5 to 5.4)	3.9 (3.4 to 4.5)	4.0 (3.4 to 4.7)	3.7 (2.5 to 5.4)	-0.5 (-3.0 to 2.0)	-5.3 (-36.3 to 35.3)
Sweden	3.0 (2.5 to 3.5)	2.5 (2.1 to 3.0)	2.5 (2.1 to 3.0)	2.1 (1.4 to 3.0)	-1.2 (-3.7 to 1.2)	-15.2 (-42.5 to 19.5)
Switzerland	2.6 (2.2 to 3.1)	1.7 (1.4 to 2.1)	1.8 (1.4 to 2.1)	1.4 (1.0 to 2.1)	-1.3 (-3.6 to 1.2)	-16.3 (-41.5 to 19.1)
United Kingdom	13.5 (13.0 to 14.0)	7.5 (7.1 to 8.1)	7.7 (6.8 to 8.7)	6.1 (4.0 to 8.7)	-1.5 (-4.2 to 1.0)	-18.8 (-46.5 to 15.9)
Latin America and Caribbean						
Andean Latin America						
Bolivia	41.6 (36.8 to 46.7)	39.5 (34.7 to 45.0)	37.6 (32.4 to 43.5)	24.4 (19.0 to 31.0)	-3.2 (-4.7 to -1.8)	-38.0 (-50.6 to -24.0)
Ecuador	38.9 (34.9 to 43.4)	32.0 (27.4 to 37.0)	31.6 (26.9 to 36.9)	25.0 (20.4 to 29.9)	-1.6 (-2.9 to -0.4)	-21.5 (-35.7 to -5.4)
Peru	40.0 (35.5 to 44.9)	41.3 (35.6 to 47.7)	39.4 (33.9 to 46.2)	27.8 (22.1 to 34.1)	-2.7 (-3.9 to -1.5)	-32.6 (-44.1 to -20.6)
Caribbean						
Antigua and Barbuda	32.3 (29.0 to 36.2)	27.7 (24.2 to 31.7)	27.4 (23.6 to 31.6)	22.7 (17.8 to 28.2)	-1.4 (-2.7 to -0.0)	-18.1 (-33.8 to -0.5)
The Bahamas	23.1 (20.5 to 25.7)	16.5 (13.8 to 19.2)	16.5 (13.9 to 19.6)	13.6 (10.2 to 17.6)	-1.3 (-3.0 to 0.4)	-16.9 (-36.3 to 6.6)
Barbados	29.1 (27.1 to 31.2)	20.6 (18.1 to 23.1)	21.1 (18.1 to 24.7)	17.1 (13.1 to 21.8)	-1.3 (-2.8 to 0.4)	-17.0 (-34.5 to 6.7)
Belize	42.9 (39.1 to 46.9)	28.7 (24.9 to 32.7)	29.6 (25.6 to 34.0)	23.0 (18.0 to 29.4)	-1.5 (-2.9 to -0.1)	-19.7 (-35.5 to -1.6)
Bermuda	15.0 (13.2 to 16.9)	4.1 (3.6 to 4.7)	4.4 (3.8 to 5.1)	4.3 (3.3 to 5.5)	0.4 (-1.2 to 1.9)	6.2 (-16.8 to 33.7)
Cuba	24.0 (22.9 to 25.3)	26.0 (24.8 to 27.4)	25.3 (22.8 to 28.2)	19.6 (15.4 to 24.6)	-1.9 (-3.5 to -0.4)	-24.8 (-40.8 to -6.0)
Dominica	35.0 (29.8 to 40.5)	22.0 (19.4 to 25.1)	22.9 (19.5 to 27.0)	18.2 (14.1 to 23.7)	-1.3 (-2.9 to 0.4)	-16.9 (-35.3 to 5.5)
Dominican Republic	64.3 (57.4 to 71.7)	47.1 (41.0 to 54.0)	47.3 (40.7 to 54.5)	35.9 (28.8 to 43.3)	-1.8 (-3.2 to -0.5)	-23.7 (-38.6 to -7.7)
Grenada	27.4 (25.1 to 29.7)	25.9 (22.8 to 29.3)	25.1 (21.8 to 28.9)	18.2 (14.5 to 22.6)	-2.4 (-3.9 to -1.0)	-29.5 (-44.6 to -14.0)
Guyana	44.8 (41.8 to 48.0)	36.1 (31.1 to 41.3)	36.5 (31.6 to 42.1)	25.6 (20.2 to 31.6)	-2.3 (-3.7 to -0.9)	-29.1 (-42.9 to -12.9)
Haiti	32.0 (29.0 to 35.1)	24.8 (21.3 to 28.7)	24.8 (21.2 to 28.9)	17.0 (12.9 to 22.1)	-2.6 (-4.1 to -1.1)	-31.6 (-46.1 to -15.7)
Jamaica	34.6 (31.1 to 38.1)	21.9 (19.4 to 24.6)	22.3 (19.5 to 25.4)	18.9 (14.9 to 23.7)	-1.0 (-2.6 to 0.4)	-13.8 (-32.0 to 6.6)
Puerto Rico	34.5 (32.7 to 36.3)	16.5 (15.0 to 18.2)	16.7 (14.5 to 19.1)	14.6 (11.3 to 18.7)	-0.9 (-2.5 to 0.7)	-11.5 (-31.3 to 11.4)
Saint Lucia	28.6 (25.2 to 32.3)	23.8 (20.3 to 27.9)	23.5 (19.9 to 27.4)	16.3 (12.9 to 20.3)	-2.6 (-4.0 to -1.1)	-31.3 (-45.4 to -14.9)
Saint Vincent and the Grenadines	39.0 (35.5 to 42.7)	31.6 (27.3 to 36.1)	30.9 (26.4 to 36.0)	23.3 (18.4 to 29.3)	-2.1 (-3.7 to -0.5)	-26.0 (-42.4 to -7.4)
Suriname	35.9 (32.9 to 39.5)	30.7 (27.4 to 34.2)	29.6 (25.6 to 34.2)	22.8 (17.9 to 28.4)	-2.0 (-3.5 to -0.5)	-25.6 (-40.6 to -7.1)
Trinidad and Tobago	17.2 (15.9 to 18.6)	19.8 (17.5 to 22.3)	19.5 (17.0 to 22.4)	15.4 (12.1 to 19.7)	-1.7 (-3.3 to -0.2)	-21.8 (-38.8 to -2.7)
Virgin Islands, U.S.	35.1 (30.6 to 40.4)	26.9 (22.9 to 31.2)	26.4 (22.6 to 30.9)	19.4 (15.2 to 24.1)	-2.2 (-3.8 to -0.8)	-27.5 (-43.8 to -11.0)
Central Latin America						
Colombia	46.2 (42.3 to 50.2)	33.6 (29.1 to 38.6)	32.6 (28.1 to 37.7)	21.3 (16.5 to 26.7)	-3.1 (-4.6 to -1.4)	-36.4 (-50.1 to -19.1)
Costa Rica	35.0 (32.9 to 37.3)	29.9 (28.4 to 31.5)	28.7 (25.8 to 32.0)	21.2 (16.2 to 26.9)	-2.4 (-4.0 to -0.8)	-29.3 (-45.1 to -10.9)
El Salvador	45.9 (43.0 to 48.8)	34.7 (30.8 to 38.7)	34.5 (30.3 to 39.1)	23.6 (18.4 to 29.7)	-2.6 (-4.2 to -1.1)	-31.9 (-46.5 to -15.5)
Guatemala	51.7 (48.9 to 54.5)	39.3 (34.6 to 44.0)	39.0 (33.8 to 44.9)	28.2 (22.0 to 35.2)	-2.2 (-3.8 to -0.7)	-28.1 (-43.6 to -9.8)
Honduras	57.2 (53.7 to 61.1)	45.4 (40.2 to 51.6)	45.1 (39.2 to 51.9)	29.8 (24.0 to 36.5)	-2.8 (-4.3 to -1.5)	-34.3 (-47.6 to -19.6)
Mexico	40.7 (35.8 to 45.8)	35.9 (30.9 to 41.5)	35.8 (30.7 to 42.0)	25.9 (19.5 to 34.0)	-2.2 (-3.8 to -0.8)	-27.7 (-43.7 to -11.0)
Nicaragua	53.8 (50.1 to 57.5)	42.4 (37.0 to 48.6)	41.5 (35.8 to 47.7)	28.7 (22.6 to 35.3)	-2.6 (-4.1 to -1.2)	-32.2 (-45.9 to -16.6)
Panama	41.7 (36.8 to 46.8)	40.7 (34.9 to 46.9)	39.6 (34.1 to 45.9)	29.1 (23.0 to 36.1)	-2.3 (-3.7 to -0.7)	-28.3 (-43.0 to -10.4)
Venezuela	46.6 (45.5 to 48.2)	51.5 (49.8 to 53.5)	46.4 (41.8 to 51.4)	34.0 (27.6 to 41.4)	-2.8 (-4.1 to -1.5)	-33.9 (-46.1 to -19.8)
Tropical Latin America						
Brazil	38.4 (35.1 to 41.5)	28.3 (24.7 to 32.8)	27.0 (23.4 to 31.4)	19.8 (15.5 to 25.0)	-2.4 (-3.7 to -1.0)	-30.1 (-42.7 to -14.5)
Paraguay	35.5 (31.4 to 39.9)	34.2 (29.4 to 39.6)	32.7 (27.9 to 38.0)	21.0 (16.6 to 26.2)	-3.3 (-4.7 to -1.9)	-38.5 (-50.9 to -24.9)
North Africa and Middle East						
North Africa and Middle East						
Afghanistan	59.9 (53.9 to 66.0)	47.0 (40.8 to 54.0)	46.2 (40.1 to 53.8)	20.7 (14.7 to 27.7)	-5.5 (-7.3 to -3.9)	-56.0 (-66.5 to -43.9)
Algeria	9.5 (8.1 to 10.9)	5.3 (4.5 to 6.3)	4.8 (4.1 to 5.8)	2.9 (2.1 to 4.0)	-4.1 (-6.2 to -2.1)	-45.6 (-60.4 to -26.5)
Bahrain	10.6 (9.1 to 12.3)	7.4 (6.2 to 8.8)	7.5 (6.3 to 9.0)	5.2 (3.6 to 7.3)	-2.5 (-4.4 to -0.4)	-29.9 (-48.5 to -5.1)
Egypt	29.3 (25.8 to 33.2)	34.1 (29.0 to 39.3)	29.9 (25.5 to 35.0)	18.7 (13.7 to 24.7)	-4.1 (-5.9 to -2.1)	-45.2 (-58.9 to -26.7)
Iran	27.2 (23.4 to 31.1)	14.0 (11.9 to 16.4)	12.9 (10.9 to 15.6)	9.5 (6.5 to 13.6)	-2.7 (-4.9 to -0.4)	-32.0 (-52.0 to -5.9)
Iraq	40.0 (35.8 to 44.3)	29.5 (25.2 to 34.7)	28.9 (24.7 to 34.4)	21.6 (15.9 to 28.6)	-2.1 (-4.0 to -0.2)	-26.6 (-45.0 to -3.5)
Jordan	19.1 (16.4 to 22.2)	12.6 (10.6 to 14.8)	12.4 (10.4 to 14.8)	9.0 (6.1 to 12.4)	-2.3 (-4.6 to -0.2)	-28.3 (-49.5 to -2.9)
Kuwait	9.1 (7.9 to 10.4)	4.0 (3.3 to 4.7)	3.6 (3.0 to 4.5)	2.6 (1.8 to 3.6)	-2.9 (-5.1 to -0.9)	-34.4 (-53.4 to -13.1)
Lebanon	46.5 (40.6 to 52.7)	29.3 (25.1 to 35.0)	28.4 (24.1 to 33.9)	12.3 (9.1 to 15.9)	-5.8 (-7.7 to -3.9)	-57.9 (-68.5 to -44.6)
Libya	9.6 (8.2 to 11.2)	6.9 (5.7 to 8.3)	6.6 (5.5 to 8.1)	4.0 (2.9 to 5.6)	-3.6 (-5.6 to -1.5)	-40.8 (-56.8 to -19.8)

	Estimate in 2000 (95% UIs)	Estimate in 2015 (95% UIs)	Estimate in 2017 (95% UIs)	Estimate in 2030 (95% UIs)	Annualised rate of change, 2015-2030 (95% UIs)	Percent change, 2015-2030 (95% UIs)
Morocco	19.9 (17.1 to 22.8)	10.5 (8.7 to 12.6)	9.9 (8.2 to 11.8)	4.8 (3.4 to 6.6)	-5.3 (-7.4 to -3.3)	-54.6 (-67.3 to -39.3)
Palestine	54.8 (50.1 to 59.8)	38.5 (33.5 to 44.5)	36.7 (31.8 to 42.9)	28.9 (20.7 to 38.2)	-2.0 (-3.8 to -0.3)	-25.0 (-43.5 to -4.8)
Oman	11.6 (9.8 to 13.4)	6.3 (5.5 to 7.2)	6.0 (5.2 to 6.9)	3.8 (2.7 to 5.1)	-3.4 (-5.5 to -1.5)	-39.3 (-56.5 to -19.6)
Qatar	9.0 (7.9 to 10.1)	4.9 (4.2 to 5.7)	4.8 (4.1 to 5.7)	3.1 (2.2 to 4.3)	-3.0 (-5.2 to -1.0)	-35.7 (-53.8 to -13.8)
Saudi Arabia	17.1 (14.3 to 20.3)	5.4 (4.6 to 6.3)	5.1 (4.3 to 6.0)	3.6 (2.5 to 5.0)	-2.7 (-4.8 to -0.5)	-32.3 (-51.2 to -7.4)
Sudan	69.9 (63.8 to 76.1)	42.0 (36.5 to 48.3)	39.6 (34.3 to 46.0)	19.0 (13.9 to 25.2)	-5.3 (-7.3 to -3.4)	-54.6 (-66.4 to -40.3)
Syria	32.5 (31.5 to 33.5)	16.6 (14.4 to 19.4)	15.6 (13.3 to 18.6)	8.9 (6.2 to 12.6)	-4.3 (-6.3 to -2.1)	-46.5 (-61.2 to -27.4)
Tunisia	8.4 (7.1 to 9.8)	3.4 (2.8 to 4.1)	3.1 (2.6 to 3.8)	2.1 (1.5 to 2.9)	-3.2 (-5.0 to -1.3)	-37.7 (-52.7 to -17.7)
Turkey	28.7 (25.3 to 32.4)	14.2 (12.1 to 16.7)	13.4 (11.3 to 15.8)	8.5 (5.9 to 11.8)	-3.5 (-5.4 to -1.5)	-40.2 (-55.5 to -19.8)
United Arab Emirates	16.7 (14.2 to 19.5)	6.3 (5.4 to 7.5)	6.6 (5.5 to 7.9)	5.4 (3.7 to 7.7)	-1.2 (-3.2 to 1.0)	-14.8 (-38.2 to 15.8)
Yemen	67.4 (61.1 to 73.7)	37.6 (32.6 to 43.4)	36.9 (31.9 to 43.0)	18.7 (13.6 to 24.9)	-4.7 (-6.7 to -2.9)	-50.1 (-63.1 to -35.5)
South Asia						
South Asia						
Bangladesh	52.6 (47.6 to 57.7)	37.0 (31.9 to 42.5)	35.6 (30.5 to 41.3)	20.3 (16.2 to 24.8)	-4.0 (-5.2 to -2.9)	-45.1 (-54.1 to -35.4)
Bhutan	31.2 (26.5 to 36.3)	18.4 (15.5 to 22.0)	17.6 (14.8 to 21.2)	9.4 (7.6 to 11.6)	-4.5 (-5.5 to -3.3)	-48.7 (-56.4 to -39.4)
India	26.8 (23.7 to 30.1)	12.7 (10.9 to 15.0)	13.0 (10.9 to 15.7)	6.5 (5.2 to 8.0)	-4.5 (-5.8 to -3.3)	-49.0 (-58.4 to -39.1)
Nepal	53.3 (48.6 to 58.2)	30.8 (26.6 to 35.6)	30.4 (26.1 to 35.2)	17.1 (13.5 to 21.5)	-4.0 (-5.1 to -2.8)	-44.7 (-53.4 to -34.4)
Pakistan	38.0 (33.6 to 42.7)	21.6 (18.3 to 25.4)	21.5 (18.2 to 25.8)	10.0 (8.2 to 12.1)	-5.1 (-6.2 to -4.0)	-53.4 (-60.6 to -45.0)
Southeast Asia, East Asia, and Oceania						
East Asia						
China	2.6 (2.3 to 3.0)	4.7 (4.5 to 5.0)	4.7 (4.3 to 5.1)	3.6 (2.0 to 6.0)	-2.1 (-5.6 to 1.6)	-23.6 (-57.1 to 27.2)
North Korea	2.7 (2.2 to 3.3)	1.1 (0.9 to 1.3)	1.0 (0.8 to 1.2)	0.9 (0.6 to 1.3)	-1.5 (-3.6 to 1.1)	-18.4 (-41.5 to 17.1)
Taiwan (Province of China)	7.5 (6.9 to 8.0)	2.2 (1.9 to 2.6)	2.3 (1.9 to 2.8)	1.2 (0.7 to 1.9)	-4.4 (-7.6 to -1.1)	-46.4 (-68.2 to -14.6)
Oceania						
American Samoa	22.8 (19.4 to 26.3)	18.1 (15.2 to 21.2)	18.0 (15.2 to 21.3)	18.2 (14.6 to 22.6)	0.0 (-1.3 to 1.2)	1.0 (-17.2 to 20.3)
Federated States of Micronesia	22.2 (20.4 to 24.1)	18.8 (16.0 to 21.9)	18.6 (15.7 to 21.8)	11.7 (9.1 to 14.6)	-3.2 (-4.5 to -1.9)	-37.5 (-48.9 to -25.0)
Fiji	20.0 (17.6 to 22.6)	17.4 (14.8 to 20.1)	16.6 (14.1 to 19.5)	10.4 (8.4 to 12.8)	-3.4 (-4.8 to -2.2)	-40.1 (-51.5 to -28.0)
Guam	29.4 (26.1 to 32.8)	22.4 (19.4 to 25.7)	22.7 (19.3 to 26.6)	17.9 (14.5 to 21.9)	-1.5 (-2.8 to -0.3)	-20.3 (-34.5 to -4.1)
Kiribati	21.0 (18.8 to 23.3)	19.2 (16.3 to 22.5)	18.6 (15.8 to 21.9)	10.8 (8.7 to 13.4)	-3.8 (-5.3 to -2.5)	-43.4 (-54.5 to -31.6)
Marshall Islands	40.7 (38.3 to 43.1)	32.4 (28.8 to 36.5)	31.4 (27.6 to 36.2)	24.6 (19.8 to 30.5)	-1.8 (-3.0 to -0.7)	-23.9 (-36.5 to -10.2)
Northern Mariana Islands	23.3 (19.7 to 26.9)	20.9 (18.3 to 23.7)	23.2 (20.3 to 26.5)	17.9 (14.0 to 22.0)	-1.1 (-2.4 to 0.1)	-14.6 (-29.8 to 1.7)
Papua New Guinea	33.0 (28.5 to 37.9)	30.1 (25.7 to 35.3)	29.4 (24.9 to 34.5)	20.6 (16.5 to 25.5)	-2.5 (-3.7 to -1.4)	-31.4 (-42.6 to -19.4)
Samoa	15.9 (13.4 to 18.7)	20.7 (17.8 to 24.4)	20.1 (17.1 to 24.0)	15.4 (12.4 to 18.7)	-2.0 (-3.3 to -0.8)	-25.8 (-39.1 to -10.8)
Solomon Islands	37.7 (32.3 to 43.3)	30.0 (25.8 to 35.0)	29.4 (25.0 to 34.4)	20.6 (15.7 to 26.0)	-2.5 (-3.9 to -1.3)	-31.4 (-44.2 to -18.2)
Tonga	10.9 (9.4 to 12.6)	8.3 (7.0 to 9.9)	8.2 (6.8 to 9.8)	6.0 (4.8 to 7.5)	-2.1 (-3.4 to -0.7)	-27.0 (-40.1 to -10.3)
Vanuatu	31.4 (27.6 to 35.8)	25.1 (21.4 to 29.6)	24.2 (20.6 to 28.8)	17.1 (13.7 to 21.1)	-2.6 (-3.8 to -1.4)	-31.6 (-43.3 to -18.4)
Southeast Asia						
Cambodia	31.3 (28.3 to 34.4)	22.5 (19.2 to 26.5)	21.6 (18.4 to 25.8)	10.4 (7.9 to 13.8)	-5.2 (-6.8 to -3.5)	-53.4 (-63.8 to -40.4)
Indonesia	21.4 (19.3 to 23.6)	13.7 (11.6 to 16.1)	13.6 (11.5 to 15.9)	9.2 (6.8 to 12.2)	-2.7 (-4.4 to -1.1)	-33.1 (-48.5 to -14.6)
Laos	43.5 (39.6 to 47.4)	32.7 (28.2 to 38.1)	31.7 (27.2 to 37.4)	19.4 (15.2 to 24.7)	-3.5 (-5.0 to -2.0)	-40.5 (-52.8 to -25.8)
Malaysia	6.6 (6.1 to 7.0)	6.2 (5.6 to 6.8)	5.8 (5.0 to 6.8)	3.7 (2.8 to 5.0)	-3.4 (-5.0 to -1.5)	-39.3 (-53.1 to -20.4)
Maldives	15.8 (13.7 to 18.1)	9.1 (7.8 to 10.8)	8.6 (7.2 to 10.3)	4.5 (3.2 to 6.2)	-4.8 (-6.5 to -3.1)	-51.0 (-62.1 to -36.8)
Mauritius	21.4 (19.2 to 23.8)	11.9 (10.3 to 13.6)	12.3 (10.5 to 14.4)	9.2 (6.9 to 12.3)	-1.8 (-3.6 to -0.0)	-22.8 (-41.5 to -0.1)
Myanmar	21.1 (18.1 to 24.2)	12.6 (10.8 to 15.0)	12.5 (10.5 to 15.0)	7.8 (5.8 to 10.2)	-3.3 (-5.0 to -1.7)	-38.5 (-52.9 to -22.5)
Philippines	26.2 (22.8 to 29.7)	28.1 (24.0 to 32.9)	27.3 (23.2 to 32.5)	18.9 (14.6 to 23.7)	-2.7 (-4.3 to -1.1)	-32.5 (-47.4 to -15.1)
Sri Lanka	17.3 (14.6 to 20.5)	9.5 (8.0 to 11.2)	9.1 (7.5 to 10.8)	6.1 (4.5 to 8.0)	-3.0 (-4.8 to -1.2)	-35.7 (-51.0 to -16.9)
Seychelles	28.1 (25.2 to 31.2)	30.8 (27.1 to 34.8)	29.1 (25.4 to 33.3)	22.2 (17.6 to 28.0)	-2.2 (-3.5 to -0.8)	-27.9 (-41.0 to -10.8)
Thailand	27.1 (23.3 to 31.3)	18.3 (15.6 to 21.6)	17.5 (14.7 to 21.2)	11.9 (8.9 to 15.6)	-2.9 (-4.6 to -1.2)	-35.1 (-50.0 to -17.1)
Timor-Leste	63.6 (56.9 to 70.8)	29.3 (24.7 to 34.4)	29.9 (25.3 to 35.0)	16.9 (12.8 to 21.6)	-3.7 (-5.3 to -2.2)	-42.1 (-55.0 to -27.7)
Vietnam	17.2 (15.5 to 19.1)	13.3 (11.4 to 15.8)	12.4 (10.6 to 14.9)	7.7 (5.8 to 10.1)	-3.7 (-5.5 to -2.1)	-42.5 (-55.9 to -27.0)
Sub-Saharan Africa						
Central Sub-Saharan Africa						
Angola	77.5 (70.1 to 84.7)	58.1 (51.3 to 65.7)	53.9 (47.2 to 61.1)	34.8 (30.8 to 38.9)	-3.4 (-4.0 to -2.8)	-40.1 (-45.2 to -34.6)
Central African Republic	66.7 (59.5 to 74.0)	45.6 (39.5 to 52.7)	42.8 (36.8 to 49.4)	27.4 (22.1 to 34.1)	-3.4 (-4.3 to -2.5)	-40.0 (-47.5 to -31.8)
Congo	59.8 (56.1 to 63.9)	35.4 (31.1 to 40.5)	32.6 (28.3 to 37.9)	21.0 (18.2 to 24.4)	-3.5 (-4.2 to -2.8)	-40.6 (-46.8 to -34.3)
Democratic Republic of the Congo	59.3 (55.3 to 63.4)	42.4 (38.0 to 47.0)	40.7 (36.0 to 45.7)	28.0 (24.8 to 31.4)	-2.8 (-3.4 to -2.2)	-34.0 (-40.3 to -28.1)
Equatorial Guinea	82.0 (73.6 to 90.2)	53.5 (46.8 to 61.3)	51.7 (44.7 to 59.1)	29.0 (25.6 to 32.5)	-4.1 (-4.7 to -3.4)	-45.7 (-50.9 to -40.3)
Gabon	53.5 (46.3 to 60.3)	33.0 (27.9 to 39.0)	31.3 (26.7 to 37.2)	25.1 (22.0 to 29.1)	-1.8 (-2.5 to -1.1)	-23.7 (-31.1 to -15.8)
Eastern Sub-Saharan Africa						
Burundi	28.5 (25.2 to 31.9)	24.7 (21.4 to 28.8)	24.7 (21.2 to 28.9)	16.9 (14.3 to 19.9)	-2.5 (-3.4 to -1.7)	-31.6 (-39.6 to -22.6)
Comoros	42.9 (37.8 to 48.1)	22.6 (19.3 to 26.5)	21.5 (18.3 to 25.2)	11.5 (9.7 to 13.5)	-4.5 (-5.4 to -3.6)	-49.0 (-55.3 to -41.6)
Djibouti	60.6 (52.7 to 68.6)	26.0 (21.8 to 30.8)	24.8 (20.9 to 29.2)	13.5 (11.2 to 15.9)	-4.4 (-5.3 to -3.5)	-48.1 (-55.1 to -40.6)
Eritrea	44.8 (38.6 to 51.3)	24.6 (20.8 to 29.3)	23.7 (20.0 to 28.2)	13.2 (11.0 to 15.6)	-4.2 (-5.0 to -3.3)	-46.3 (-53.1 to -38.9)
Ethiopia	74.1 (68.4 to 79.5)	43.8 (38.5 to 50.0)	42.6 (37.0 to 49.2)	23.0 (19.8 to 26.4)	-4.3 (-5.1 to -3.6)	-47.5 (-53.3 to -41.7)
Kenya	52.6 (47.8 to 57.8)	34.5 (29.7 to 39.9)	34.3 (29.4 to 40.3)	23.0 (18.9 to 27.9)	-2.7 (-3.8 to -1.6)	-33.3 (-43.5 to -21.5)
Madagascar	73.5 (68.1 to 78.8)	62.4 (55.5 to 69.8)	61.4 (54.2 to 69.2)	45.0 (40.4 to 50.2)	-2.2 (-2.8 to -1.6)	-27.8 (-34.0 to -20.9)
Malawi	83.5 (79.7 to 87.5)	54.4 (49.2 to 60.0)	53.3 (47.6 to 59.7)	36.8 (32.4 to 41.9)	-2.6 (-3.3 to -2.0)	-32.4 (-38.7 to -25.9)
Mozambique	82.5 (79.2 to 85.7)	48.0 (43.6 to 53.1)	46.0 (41.1 to 51.7)	28.7 (23.9 to 34.3)	-3.4 (-4.3 to -2.6)	-40.2 (-47.7 to -32.1)
Rwanda	22.3 (20.1 to 24.9)	14.9 (12.7 to 17.5)	14.6 (12.4 to 17.4)	8.8 (7.4 to 10.5)	-3.6 (-4.4 to -2.7)	-41.2 (-48.7 to -33.3)
Somalia	68.8 (60.5 to 77.0)	44.9 (38.9 to 51.7)	44.4 (38.3 to 51.6)	24.3 (20.8 to 28.2)	-4.1 (-4.8 to -3.4)	-45.7 (-51.4 to -39.8)
South Sudan	72.8 (64.7 to 80.8)	61.2 (53.6 to 69.9)	60.0 (52.6 to 68.6)	48.0 (42.6 to 54.5)	-1.6 (-2.2 to -1.1)	-21.4 (-27.6 to -15.1)
Tanzania	60.9 (57.3 to 64.5)	48.9 (43.6 to 55.2)	47.4 (41.7 to 54.0)	36.5 (32.3 to 41.3)	-2.0 (-2.6 to -1.3)	-25.4 (-32.3 to -17.6)
Uganda	84.2 (80.3 to 88.0)	52.4 (47.6 to 57.7)	51.4 (46.2 to 57.5)	33.1 (29.2 to 37.4)	-3.1 (-3.7 to -2.4)	-36.9 (-42.9 to -30.3)
Zambia	71.4 (64.7 to 78.0)	52.3 (46.0 to 60.3)	50.1 (43.6 to 57.8)	37.3 (32.9 to 42.2)	-2.3 (-3.0 to -1.6)	-28.6 (-36.0 to -21.0)
Southern Sub-Saharan Africa						
Botswana	36.1 (32.2 to 40.1)	23.4 (20.3 to 27.2)	22.5 (19.4 to 26.4)	15.8 (13.1 to 19.1)	-2.6 (-3.6 to -1.7)	-32.2 (-41.5 to -23.0)
Lesotho	41.5 (38.8 to 44.6)	35.8 (31.8 to 40.5)	34.9 (30.5 to 40.3)	26.0 (22.6 to 30.0)	-2.1 (-2.9 to -1.3)	-27.2 (-35.6 to -17.4)
Namibia	36.4 (33.9 to 39.4)	29.3 (26.1 to 33.2)	27.8 (24.4 to 32.0)	19.8 (16.5 to 23.3)	-2.6 (-3.5 to -1.8)	-32.4 (-41.0 to -23.9)
South Africa	36.6 (31.6 to 42.2)	31.1 (26.6 to 36.9)	29.3 (24.9 to 34.9)	20.0 (16.8 to 23.6)	-2.9 (-3.9 to -2.0)	-35.6 (-44.4 to -25.8)
Swaziland	47.6 (44.3 to 51.0)	37.8 (33.2 to 43.1)	36.8 (32.1 to 42.7)	29.1 (25.3 to 33.8)	-1.7 (-2.6 to -1.0)	-22.8 (-32.1 to -13.3)

	Estimate in 2000 (95% UIs)	Estimate in 2015 (95% UIs)	Estimate in 2017 (95% UIs)	Estimate in 2030 (95% UIs)	Annualised rate of change, 2015-2030 (95% UIs)	Percent change, 2015-2030 (95% UIs)
Zimbabwe	53.5 (50.0 to 56.9)	50.1 (44.9 to 55.9)	48.5 (43.2 to 54.8)	40.7 (34.6 to 47.4)	-1.4 (-2.1 to -0.7)	-18.8 (-27.5 to -9.7)
Western Sub-Saharan Africa						
Benin	56.5 (51.9 to 62.2)	40.9 (35.9 to 46.8)	39.9 (34.9 to 46.0)	24.0 (20.3 to 28.6)	-3.6 (-4.3 to -2.9)	-41.3 (-47.4 to -35.1)
Burkina Faso	59.7 (56.2 to 63.3)	46.3 (41.6 to 51.8)	46.1 (40.8 to 52.0)	29.6 (25.9 to 33.4)	-3.0 (-3.6 to -2.4)	-36.1 (-42.0 to -29.7)
Cameroon	68.7 (63.8 to 73.5)	45.4 (40.0 to 51.7)	42.8 (37.2 to 49.2)	29.0 (25.3 to 33.0)	-3.0 (-3.7 to -2.3)	-36.1 (-42.9 to -28.8)
Cape Verde	36.0 (32.1 to 40.1)	19.6 (16.6 to 23.3)	18.5 (15.6 to 21.9)	11.1 (9.2 to 13.2)	-3.8 (-4.7 to -2.9)	-43.6 (-50.6 to -35.7)
Chad	92.9 (87.3 to 98.4)	79.2 (72.0 to 87.1)	78.0 (70.4 to 86.3)	60.7 (54.9 to 68.0)	-1.8 (-2.1 to -1.5)	-23.4 (-27.2 to -19.7)
Cote d'Ivoire	65.8 (59.9 to 71.9)	48.9 (43.4 to 55.2)	47.6 (41.8 to 54.2)	33.5 (29.6 to 38.1)	-2.5 (-3.2 to -1.8)	-31.4 (-38.3 to -24.0)
The Gambia	56.6 (49.7 to 63.8)	36.2 (30.7 to 42.3)	35.0 (29.9 to 41.1)	23.3 (19.8 to 27.1)	-2.9 (-3.7 to -2.2)	-35.4 (-42.6 to -27.7)
Ghana	39.5 (34.8 to 44.6)	26.1 (22.2 to 30.5)	24.6 (20.8 to 28.9)	15.6 (13.3 to 18.5)	-3.4 (-4.4 to -2.5)	-40.1 (-48.6 to -31.1)
Guinea	79.5 (74.7 to 84.5)	58.2 (52.8 to 64.4)	55.9 (50.4 to 62.4)	36.6 (30.7 to 43.3)	-3.1 (-3.9 to -2.4)	-37.2 (-44.7 to -30.1)
Guinea-Bissau	66.4 (58.8 to 74.2)	45.3 (39.2 to 52.3)	43.3 (37.4 to 50.0)	25.2 (21.5 to 29.2)	-3.9 (-4.7 to -3.2)	-44.2 (-50.4 to -37.9)
Liberia	75.0 (69.8 to 79.9)	49.5 (43.9 to 56.1)	47.4 (41.7 to 54.3)	28.4 (25.0 to 32.3)	-3.7 (-4.5 to -3.0)	-42.5 (-48.8 to -36.5)
Mali	86.2 (82.1 to 90.3)	69.7 (64.0 to 76.2)	68.7 (62.3 to 75.7)	52.7 (47.8 to 58.4)	-1.9 (-2.3 to -1.4)	-24.3 (-29.5 to -18.9)
Mauritania	49.0 (44.3 to 53.8)	34.1 (29.8 to 39.4)	33.0 (28.5 to 38.4)	21.5 (18.2 to 24.9)	-3.1 (-3.8 to -2.4)	-37.0 (-43.7 to -29.9)
Niger	100.6 (95.4 to 106.0)	81.0 (73.5 to 89.0)	79.4 (71.7 to 87.7)	65.8 (59.8 to 73.3)	-1.4 (-1.7 to -1.1)	-18.7 (-22.4 to -14.8)
Nigeria	56.8 (52.2 to 61.8)	44.0 (38.9 to 50.1)	42.8 (37.4 to 49.0)	32.3 (26.7 to 38.6)	-2.1 (-2.9 to -1.3)	-26.6 (-35.3 to -18.0)
Sao Tome and Principe	54.3 (50.1 to 58.2)	28.5 (25.0 to 32.3)	26.9 (23.3 to 30.8)	16.2 (13.9 to 18.8)	-3.8 (-4.6 to -2.9)	-43.1 (-49.9 to -35.4)
Senegal	50.5 (45.1 to 56.5)	37.1 (32.0 to 42.9)	36.2 (31.1 to 42.3)	23.3 (19.9 to 27.2)	-3.1 (-3.9 to -2.3)	-37.0 (-44.2 to -28.8)
Sierra Leone	71.0 (65.1 to 76.5)	50.2 (44.3 to 57.1)	48.4 (42.3 to 55.5)	28.6 (23.3 to 35.3)	-3.8 (-4.7 to -2.8)	-43.0 (-50.5 to -34.0)
Togo	44.1 (41.3 to 47.3)	25.8 (22.8 to 29.2)	24.6 (21.4 to 28.2)	12.7 (10.7 to 15.1)	-4.7 (-5.5 to -3.9)	-50.8 (-56.5 to -44.5)
**Indicator 3.8.1: Coverage of essential health services, as defined by the UHC service coverage index comprised of the coverage of 9 tracer interventions and 32 causes amenable to healthcare (scale of 0 to 100)**						
Central Europe, Eastern Europe, and Central Asia						
Central Asia						
Armenia	65.0 (63.9 to 66.0)	75.3 (74.2 to 76.6)	75.8 (74.6 to 77.2)	79.6 (76.4 to 82.7)	0.4 (0.1 to 0.6)	5.7 (1.4 to 9.3)
Azerbaijan	52.4 (51.0 to 54.0)	68.0 (65.9 to 70.0)	68.7 (66.4 to 70.8)	72.2 (67.1 to 76.6)	0.4 (-0.1 to 0.8)	6.1 (-1.4 to 12.9)
Georgia	68.7 (67.3 to 69.8)	70.6 (69.5 to 71.5)	71.2 (69.9 to 72.3)	74.4 (68.5 to 79.0)	0.3 (-0.2 to 0.8)	5.4 (-3.0 to 12.0)
Kazakhstan	62.5 (61.3 to 63.6)	72.6 (71.2 to 73.8)	73.1 (71.6 to 74.5)	78.0 (74.7 to 80.9)	0.5 (0.2 to 0.8)	7.5 (2.3 to 12.3)
Kyrgyzstan	56.7 (55.5 to 57.8)	67.6 (66.4 to 68.6)	68.6 (67.3 to 69.8)	71.3 (66.2 to 75.1)	0.3 (-0.2 to 0.7)	5.4 (-2.3 to 11.5)
Mongolia	50.5 (48.7 to 52.3)	63.7 (61.5 to 65.6)	65.7 (63.4 to 67.8)	71.8 (67.1 to 75.6)	0.8 (0.3 to 1.2)	12.7 (4.1 to 20.2)
Tajikistan	55.9 (54.4 to 57.6)	60.4 (58.6 to 62.2)	60.6 (58.6 to 62.6)	60.6 (54.5 to 66.4)	0.0 (-0.7 to 0.6)	0.3 (-9.5 to 9.4)
Turkmenistan	60.3 (59.2 to 61.4)	67.1 (65.4 to 68.6)	68.2 (66.2 to 70.0)	72.0 (66.5 to 77.2)	0.5 (-0.1 to 0.9)	7.4 (-1.1 to 14.9)
Uzbekistan	61.0 (59.3 to 62.2)	67.0 (65.5 to 68.5)	67.8 (66.0 to 69.7)	71.1 (66.9 to 75.0)	0.4 (-0.0 to 0.7)	6.1 (-0.6 to 11.8)
Central Europe						
Albania	70.9 (69.3 to 72.5)	79.7 (77.4 to 82.0)	80.3 (77.8 to 82.6)	84.2 (81.0 to 86.7)	0.4 (0.1 to 0.7)	5.7 (1.4 to 10.3)
Bosnia and Herzegovina	72.2 (70.5 to 73.8)	75.7 (74.2 to 76.9)	76.6 (74.9 to 78.0)	77.4 (73.5 to 79.9)	0.1 (-0.2 to 0.4)	2.3 (-3.3 to 6.9)
Bulgaria	73.6 (72.9 to 74.2)	77.3 (76.5 to 78.1)	77.7 (76.5 to 78.8)	80.3 (77.7 to 82.7)	0.3 (0.0 to 0.5)	3.9 (0.3 to 7.0)
Croatia	80.5 (79.8 to 81.2)	85.7 (85.0 to 86.3)	87.2 (86.2 to 88.1)	90.0 (89.0 to 91.2)	0.3 (0.2 to 0.4)	5.0 (3.5 to 6.7)
Czech Republic	83.8 (83.2 to 84.3)	88.2 (87.6 to 88.8)	88.6 (87.7 to 89.6)	90.8 (89.9 to 91.8)	0.2 (0.1 to 0.3)	3.0 (1.9 to 4.1)
Hungary	77.8 (76.9 to 78.8)	82.2 (81.6 to 83.1)	84.2 (83.0 to 85.4)	87.3 (85.9 to 88.6)	0.4 (0.3 to 0.5)	6.2 (4.3 to 8.1)
Macedonia	72.8 (71.7 to 74.0)	77.6 (76.6 to 78.6)	77.7 (76.4 to 78.9)	78.6 (77.5 to 79.8)	0.1 (-0.0 to 0.2)	1.3 (-0.6 to 3.1)
Montenegro	74.4 (73.3 to 75.7)	80.8 (79.6 to 81.9)	80.7 (79.3 to 82.0)	83.3 (81.9 to 84.7)	0.2 (0.1 to 0.3)	3.2 (1.0 to 5.3)
Poland	77.5 (76.9 to 78.1)	84.0 (83.1 to 84.9)	84.5 (83.4 to 85.6)	88.3 (87.4 to 89.3)	0.3 (0.2 to 0.4)	5.2 (3.6 to 6.8)
Romania	70.6 (69.6 to 71.6)	79.5 (78.5 to 80.3)	79.6 (78.4 to 80.7)	83.0 (80.9 to 84.7)	0.3 (0.1 to 0.5)	4.5 (1.5 to 7.3)
Serbia	69.7 (68.6 to 70.7)	79.5 (78.7 to 80.5)	80.5 (79.3 to 81.7)	84.2 (82.9 to 85.5)	0.4 (0.3 to 0.5)	5.8 (3.9 to 7.8)
Slovakia	77.2 (76.2 to 78.0)	82.1 (81.3 to 82.9)	83.2 (81.9 to 84.3)	86.0 (84.3 to 87.6)	0.3 (0.2 to 0.4)	4.7 (2.4 to 6.8)
Slovenia	82.0 (81.3 to 82.7)	90.4 (89.7 to 91.0)	90.7 (89.7 to 91.7)	93.5 (92.6 to 94.5)	0.2 (0.2 to 0.3)	3.5 (2.3 to 4.8)
Eastern Europe						
Belarus	71.5 (70.3 to 72.5)	83.1 (81.7 to 84.2)	83.6 (82.0 to 84.9)	86.3 (84.4 to 88.4)	0.3 (0.1 to 0.4)	3.9 (1.6 to 6.3)
Estonia	75.4 (74.7 to 76.0)	84.8 (83.9 to 85.6)	85.5 (84.0 to 87.2)	88.3 (86.5 to 90.1)	0.3 (0.1 to 0.4)	4.1 (1.7 to 6.3)
Latvia	74.8 (74.0 to 75.6)	80.8 (79.7 to 82.0)	81.9 (80.0 to 83.8)	85.4 (83.4 to 87.7)	0.4 (0.2 to 0.5)	5.8 (3.2 to 8.3)
Lithuania	75.1 (74.5 to 75.7)	80.4 (79.8 to 81.4)	81.2 (80.0 to 82.4)	82.3 (79.9 to 84.7)	0.1 (-0.0 to 0.3)	2.3 (-0.7 to 4.6)
Moldova	65.9 (64.7 to 67.2)	71.1 (70.0 to 72.3)	72.6 (71.3 to 73.9)	78.4 (75.4 to 81.4)	0.7 (0.4 to 0.9)	10.3 (5.9 to 14.3)
Russia	67.3 (66.7 to 68.0)	79.8 (79.1 to 80.6)	81.1 (80.2 to 82.1)	84.2 (81.8 to 86.3)	0.4 (0.2 to 0.5)	5.6 (2.3 to 8.6)
Ukraine	72.0 (71.1 to 72.9)	73.2 (72.2 to 74.0)	75.3 (74.0 to 76.5)	78.8 (76.7 to 80.7)	0.5 (0.3 to 0.7)	7.7 (4.7 to 10.8)
High-income						
Australasia						
Australia	91.1 (90.7 to 91.6)	95.0 (94.5 to 95.5)	95.2 (94.1 to 96.2)	96.3 (95.4 to 97.2)	0.1 (0.0 to 0.2)	1.4 (0.4 to 2.3)
New Zealand	87.2 (86.7 to 87.7)	92.6 (92.2 to 93.0)	92.7 (92.0 to 93.3)	94.3 (93.7 to 94.8)	0.1 (0.1 to 0.2)	1.8 (1.1 to 2.6)
High-income Asia Pacific						
Brunei	73.5 (72.2 to 74.8)	76.4 (75.0 to 77.8)	76.5 (74.9 to 78.0)	78.0 (74.0 to 81.2)	0.1 (-0.2 to 0.4)	2.0 (-3.2 to 6.6)
Japan	88.4 (87.9 to 88.9)	93.5 (93.1 to 93.9)	93.7 (93.2 to 94.1)	94.6 (94.1 to 95.1)	0.1 (0.0 to 0.1)	1.2 (0.5 to 1.9)
South Korea	81.8 (81.1 to 82.3)	92.0 (91.1 to 92.8)	92.6 (91.5 to 93.4)	94.7 (93.7 to 95.5)	0.2 (0.1 to 0.3)	3.0 (1.7 to 4.3)
Singapore	85.2 (84.3 to 86.2)	91.8 (91.2 to 92.6)	92.4 (91.7 to 93.3)	94.1 (93.1 to 95.3)	0.2 (0.1 to 0.2)	2.5 (1.4 to 3.6)
High-income North America						
Canada	89.7 (89.2 to 90.1)	92.4 (91.9 to 92.9)	92.8 (91.9 to 93.8)	94.3 (93.2 to 95.1)	0.1 (0.1 to 0.2)	2.0 (0.9 to 3.1)
Greenland	71.6 (70.2 to 73.0)	75.8 (74.2 to 77.6)	77.0 (75.2 to 78.8)	79.4 (77.7 to 81.5)	0.3 (0.1 to 0.5)	4.8 (1.8 to 8.0)
United States	86.2 (85.8 to 86.6)	87.9 (87.6 to 88.3)	87.8 (87.3 to 88.2)	89.7 (88.2 to 90.6)	0.1 (0.0 to 0.2)	2.0 (0.3 to 3.0)
Southern Latin America						
Argentina	70.5 (69.8 to 71.1)	74.4 (73.8 to 75.0)	74.7 (72.8 to 76.3)	76.7 (73.7 to 79.5)	0.2 (-0.1 to 0.4)	3.1 (-1.0 to 6.8)
Chile	74.2 (73.6 to 74.9)	80.7 (80.0 to 81.6)	81.0 (79.2 to 83.1)	81.5 (79.0 to 84.1)	0.1 (-0.1 to 0.3)	1.0 (-2.1 to 4.1)
Uruguay	72.8 (72.0 to 73.4)	76.6 (75.7 to 77.5)	77.1 (75.3 to 78.9)	80.3 (78.1 to 82.8)	0.3 (0.1 to 0.5)	4.9 (2.0 to 8.0)
Western Europe						
Andorra	90.5 (88.4 to 92.3)	93.8 (92.2 to 95.1)	93.9 (92.2 to 95.3)	94.3 (92.7 to 95.8)	0.0 (-0.1 to 0.2)	0.6 (-1.4 to 2.8)
Austria	88.4 (87.9 to 89.0)	92.0 (91.5 to 92.4)	92.2 (91.3 to 93.0)	93.6 (92.8 to 94.3)	0.1 (0.1 to 0.2)	1.8 (0.9 to 2.7)
Belgium	87.8 (87.2 to 88.4)	92.9 (92.3 to 93.5)	93.5 (92.5 to 94.4)	94.8 (94.0 to 95.6)	0.1 (0.1 to 0.2)	2.1 (1.0 to 3.1)
Cyprus	84.3 (83.2 to 85.4)	93.3 (92.1 to 94.3)	93.6 (92.4 to 94.7)	95.7 (94.8 to 96.5)	0.2 (0.1 to 0.3)	2.7 (1.3 to 4.2)
Denmark	86.1 (85.5 to 86.7)	91.9 (91.2 to 92.6)	91.8 (90.8 to 92.9)	93.7 (92.7 to 94.6)	0.1 (0.1 to 0.2)	1.9 (0.8 to 3.1)
Finland	87.7 (87.0 to 88.4)	92.8 (92.1 to 93.3)	92.5 (91.6 to 93.2)	94.2 (93.4 to 95.0)	0.1 (0.0 to 0.2)	1.5 (0.6 to 2.5)
France	89.0 (88.5 to 89.5)	93.5 (92.8 to 94.0)	93.6 (92.7 to 94.4)	94.9 (94.1 to 95.5)	0.1 (0.0 to 0.2)	1.5 (0.5 to 2.6)

	Estimate in 2000 (95% UIs)	Estimate in 2015 (95% UIs)	Estimate in 2017 (95% UIs)	Estimate in 2030 (95% UIs)	Annualised rate of change, 2015-2030 (95% UIs)	Percent change, 2015-2030 (95% UIs)
Germany	87.7 (87.2 to 88.1)	90.8 (90.4 to 91.2)	90.8 (89.3 to 92.1)	92.1 (90.8 to 93.4)	0.1 (0.0 to 0.2)	1.5 (0.0 to 2.9)
Greece	88.2 (87.5 to 89.0)	90.9 (90.3 to 91.6)	90.9 (90.0 to 91.8)	92.7 (91.8 to 93.5)	0.1 (0.1 to 0.2)	1.9 (0.9 to 3.0)
Iceland	92.8 (92.1 to 93.5)	95.3 (94.8 to 95.9)	95.0 (94.3 to 95.7)	96.8 (96.2 to 97.2)	0.1 (0.0 to 0.1)	1.5 (0.7 to 2.3)
Ireland	86.4 (85.8 to 87.0)	94.0 (93.2 to 94.5)	94.5 (93.5 to 95.3)	96.5 (95.8 to 97.2)	0.2 (0.1 to 0.2)	2.8 (1.8 to 3.7)
Israel	83.7 (82.8 to 84.5)	89.3 (88.6 to 89.9)	89.6 (88.8 to 90.5)	91.6 (90.8 to 92.3)	0.2 (0.1 to 0.2)	2.6 (1.5 to 3.7)
Italy	90.0 (89.6 to 90.5)	94.0 (93.5 to 94.4)	94.7 (93.9 to 95.4)	96.1 (95.5 to 96.7)	0.2 (0.1 to 0.2)	2.3 (1.5 to 3.1)
Luxembourg	90.2 (89.4 to 91.0)	94.1 (93.2 to 94.8)	94.2 (93.1 to 95.2)	95.9 (95.0 to 96.6)	0.1 (0.0 to 0.2)	2.0 (0.7 to 3.2)
Malta	85.5 (84.7 to 86.3)	89.7 (88.8 to 90.4)	89.6 (88.5 to 90.5)	92.5 (91.6 to 93.2)	0.2 (0.1 to 0.3)	3.1 (1.9 to 4.4)
Netherlands	89.5 (88.9 to 90.1)	94.7 (94.2 to 95.1)	94.5 (93.7 to 95.1)	95.9 (95.2 to 96.4)	0.1 (0.0 to 0.1)	1.3 (0.5 to 2.0)
Norway	88.9 (88.1 to 89.4)	95.3 (94.7 to 95.7)	95.1 (94.5 to 95.6)	96.4 (95.8 to 96.9)	0.1 (0.0 to 0.1)	1.2 (0.4 to 2.0)
Portugal	82.1 (81.5 to 82.7)	90.0 (89.3 to 90.6)	90.2 (89.1 to 91.1)	93.2 (92.3 to 94.0)	0.2 (0.2 to 0.3)	3.6 (2.3 to 4.7)
Spain	88.1 (87.7 to 88.6)	93.4 (92.9 to 93.8)	93.6 (92.9 to 94.3)	95.3 (94.7 to 95.8)	0.1 (0.1 to 0.2)	2.0 (1.2 to 2.7)
Sweden	91.7 (91.2 to 92.1)	94.2 (93.8 to 94.8)	94.6 (93.9 to 95.4)	95.3 (94.5 to 96.1)	0.1 (0.0 to 0.1)	1.2 (0.2 to 2.1)
Switzerland	91.7 (91.1 to 92.2)	95.2 (94.6 to 95.7)	95.7 (94.8 to 96.5)	96.2 (95.3 to 97.0)	0.1 (0.0 to 0.1)	1.1 (0.1 to 2.0)
United Kingdom	86.7 (86.3 to 87.2)	91.0 (90.5 to 91.5)	91.0 (90.5 to 91.6)	92.5 (91.7 to 93.4)	0.1 (0.1 to 0.2)	1.7 (0.7 to 2.5)
Latin America and Caribbean						
Andean Latin America						
Bolivia	45.5 (43.7 to 47.4)	60.0 (56.8 to 63.7)	61.0 (57.8 to 65.0)	67.5 (62.6 to 72.0)	0.8 (0.2 to 1.4)	12.7 (3.0 to 22.8)
Ecuador	61.7 (60.6 to 62.7)	67.8 (66.4 to 69.0)	67.9 (66.0 to 69.7)	69.4 (65.5 to 72.7)	0.2 (-0.2 to 0.5)	2.5 (-3.2 to 8.2)
Peru	58.4 (57.0 to 59.9)	71.4 (69.6 to 73.2)	71.9 (69.5 to 74.4)	77.6 (74.5 to 80.6)	0.6 (0.2 to 0.9)	8.8 (3.5 to 14.1)
Caribbean						
Antigua and Barbuda	67.0 (65.9 to 68.3)	71.2 (69.7 to 72.5)	71.5 (70.0 to 73.1)	74.0 (68.6 to 78.2)	0.3 (-0.3 to 0.7)	3.9 (-3.7 to 10.9)
The Bahamas	66.3 (65.3 to 67.4)	69.4 (68.1 to 70.8)	69.5 (67.9 to 71.2)	71.5 (68.7 to 74.2)	0.2 (-0.1 to 0.5)	3.0 (-1.3 to 7.6)
Barbados	70.7 (69.7 to 71.8)	74.4 (73.0 to 75.7)	74.6 (72.9 to 76.2)	77.6 (74.6 to 79.9)	0.3 (-0.0 to 0.5)	4.3 (-0.0 to 8.4)
Belize	59.2 (58.0 to 60.4)	66.0 (64.7 to 67.3)	66.1 (64.6 to 67.5)	66.9 (62.9 to 70.5)	0.1 (-0.4 to 0.5)	1.4 (-5.2 to 7.0)
Bermuda	78.7 (77.3 to 80.0)	84.8 (83.4 to 86.3)	85.3 (83.7 to 86.8)	85.7 (82.2 to 88.4)	0.1 (-0.2 to 0.3)	1.0 (-3.4 to 4.8)
Cuba	73.6 (72.6 to 74.5)	77.4 (76.6 to 78.2)	78.1 (76.2 to 79.8)	80.0 (78.0 to 82.0)	0.2 (0.0 to 0.4)	3.4 (0.5 to 6.1)
Dominica	65.0 (63.7 to 66.4)	63.9 (62.3 to 65.4)	64.0 (62.3 to 65.8)	64.1 (59.7 to 68.3)	0.0 (-0.5 to 0.5)	0.4 (-6.6 to 7.0)
Dominican Republic	59.7 (57.9 to 61.3)	63.6 (61.4 to 65.8)	64.3 (61.4 to 66.8)	64.5 (59.6 to 68.3)	0.1 (-0.5 to 0.6)	1.4 (-6.9 to 9.0)
Grenada	64.3 (63.0 to 65.5)	67.1 (65.5 to 68.6)	67.7 (66.0 to 69.3)	72.8 (68.8 to 75.9)	0.5 (0.1 to 0.9)	8.5 (2.1 to 14.4)
Guyana	56.3 (55.0 to 57.4)	60.2 (58.7 to 61.6)	61.0 (59.0 to 62.9)	63.9 (58.1 to 68.6)	0.4 (-0.3 to 0.9)	6.2 (-4.2 to 14.7)
Haiti	36.1 (33.6 to 39.0)	46.2 (43.1 to 49.2)	47.2 (44.1 to 50.4)	54.4 (49.1 to 60.0)	1.1 (0.3 to 1.9)	17.8 (4.1 to 32.7)
Jamaica	68.0 (66.4 to 69.2)	70.2 (68.1 to 72.1)	70.7 (68.1 to 73.0)	71.2 (67.3 to 74.5)	0.1 (-0.3 to 0.5)	1.5 (-4.7 to 7.2)
Puerto Rico	74.9 (73.8 to 76.0)	82.6 (81.8 to 83.4)	81.7 (80.5 to 82.8)	84.5 (83.3 to 85.7)	0.2 (0.0 to 0.3)	2.3 (0.6 to 4.0)
Saint Lucia	64.1 (62.9 to 65.4)	68.5 (67.1 to 69.9)	68.6 (67.0 to 70.1)	71.6 (67.3 to 75.5)	0.3 (-0.1 to 0.7)	4.5 (-1.9 to 10.8)
Saint Vincent and the Grenadines	61.5 (60.3 to 62.5)	63.7 (62.4 to 64.9)	64.2 (62.9 to 65.7)	66.2 (62.6 to 69.8)	0.3 (-0.1 to 0.7)	4.0 (-1.9 to 10.2)
Suriname	56.2 (54.6 to 57.6)	62.3 (60.7 to 64.0)	62.5 (60.6 to 64.4)	64.3 (58.3 to 69.5)	0.2 (-0.5 to 0.7)	3.2 (-6.9 to 11.6)
Trinidad and Tobago	60.1 (58.8 to 61.2)	67.4 (64.6 to 70.1)	67.4 (64.5 to 70.3)	67.7 (62.1 to 72.6)	0.0 (-0.6 to 0.5)	0.4 (-8.5 to 8.4)
Virgin Islands, U.S.	68.4 (66.4 to 70.2)	72.6 (70.4 to 74.8)	72.9 (70.6 to 75.5)	74.2 (71.5 to 77.1)	0.1 (-0.1 to 0.5)	2.3 (-2.2 to 7.3)
Central Latin America						
Colombia	66.9 (66.0 to 67.9)	74.3 (73.2 to 75.5)	74.8 (73.0 to 76.7)	78.8 (76.5 to 81.1)	0.4 (0.2 to 0.6)	6.1 (2.6 to 9.5)
Costa Rica	72.0 (71.3 to 72.7)	77.0 (76.3 to 77.7)	76.7 (75.5 to 77.8)	77.2 (75.0 to 79.3)	0.0 (-0.2 to 0.2)	0.3 (-2.8 to 3.3)
El Salvador	62.2 (60.7 to 63.6)	69.3 (66.9 to 71.4)	70.1 (67.1 to 73.0)	71.5 (68.0 to 74.4)	0.2 (-0.2 to 0.6)	3.3 (-2.5 to 8.9)
Guatemala	53.4 (51.3 to 54.7)	61.8 (59.8 to 63.3)	61.8 (59.3 to 64.1)	66.5 (63.3 to 69.2)	0.5 (0.1 to 0.8)	7.7 (1.6 to 13.5)
Honduras	52.4 (49.8 to 55.1)	61.8 (59.0 to 64.6)	62.7 (60.1 to 65.7)	66.6 (62.2 to 70.4)	0.5 (-0.0 to 1.0)	7.8 (-0.4 to 15.7)
Mexico	65.5 (64.7 to 66.2)	68.1 (67.3 to 68.7)	70.5 (69.7 to 71.2)	72.4 (70.8 to 73.8)	0.4 (0.3 to 0.6)	6.3 (3.9 to 9.0)
Nicaragua	57.8 (56.4 to 59.4)	70.1 (68.7 to 71.3)	71.2 (69.1 to 73.0)	74.8 (71.8 to 77.7)	0.4 (0.1 to 0.7)	6.8 (1.9 to 11.8)
Panama	69.8 (68.8 to 70.7)	72.7 (71.4 to 74.0)	73.8 (72.0 to 75.6)	77.9 (75.1 to 80.5)	0.5 (0.2 to 0.7)	7.1 (3.0 to 11.3)
Venezuela	64.5 (63.5 to 65.7)	70.8 (69.6 to 72.0)	70.9 (68.5 to 73.1)	72.8 (66.7 to 77.2)	0.2 (-0.4 to 0.6)	2.9 (-6.0 to 9.6)
Tropical Latin America						
Brazil	63.1 (62.4 to 63.8)	69.9 (69.3 to 70.5)	70.0 (69.4 to 70.7)	73.2 (69.4 to 75.4)	0.3 (-0.0 to 0.5)	4.8 (-0.6 to 7.9)
Paraguay	59.1 (57.5 to 60.5)	68.7 (66.6 to 70.8)	69.6 (66.7 to 72.4)	73.8 (69.9 to 77.2)	0.5 (0.1 to 0.9)	7.5 (0.9 to 14.0)
North Africa and Middle East						
North Africa and Middle East						
Afghanistan	25.6 (21.8 to 30.1)	43.9 (40.7 to 47.3)	46.0 (42.6 to 49.5)	47.0 (42.6 to 51.9)	0.4 (-0.4 to 1.3)	7.0 (-5.5 to 21.2)
Algeria	62.5 (60.8 to 64.4)	71.1 (69.5 to 72.9)	72.3 (70.7 to 74.3)	73.2 (69.2 to 77.6)	0.2 (-0.2 to 0.5)	3.0 (-2.8 to 8.2)
Bahrain	69.4 (68.3 to 70.5)	80.8 (79.5 to 82.2)	81.5 (79.9 to 83.0)	85.3 (82.2 to 87.7)	0.4 (0.1 to 0.6)	5.5 (1.6 to 9.2)
Egypt	54.9 (52.8 to 56.6)	63.4 (61.3 to 65.4)	65.2 (62.6 to 67.5)	72.1 (68.0 to 75.9)	0.9 (0.4 to 1.2)	13.7 (6.9 to 19.9)
Iran	70.1 (69.0 to 71.4)	74.5 (73.1 to 75.6)	76.7 (75.3 to 77.9)	78.1 (73.6 to 82.0)	0.3 (-0.1 to 0.6)	4.9 (-1.3 to 9.9)
Iraq	56.9 (55.0 to 58.8)	69.5 (68.1 to 70.9)	71.1 (69.4 to 72.6)	75.9 (70.2 to 80.4)	0.6 (0.0 to 1.0)	9.2 (0.7 to 15.9)
Jordan	64.0 (62.4 to 65.8)	77.0 (75.2 to 78.6)	77.3 (75.4 to 79.2)	82.3 (79.6 to 84.9)	0.4 (0.2 to 0.7)	7.0 (2.7 to 11.1)
Kuwait	75.0 (74.3 to 75.7)	84.1 (83.2 to 84.8)	84.8 (83.5 to 85.9)	87.3 (82.8 to 89.6)	0.2 (-0.1 to 0.5)	3.8 (-1.6 to 7.1)
Lebanon	72.2 (70.4 to 74.3)	80.9 (78.8 to 82.8)	81.2 (79.2 to 83.2)	83.7 (79.2 to 87.2)	0.2 (-0.1 to 0.5)	3.5 (-2.1 to 8.4)
Libya	65.7 (63.4 to 67.7)	70.8 (68.5 to 73.3)	70.6 (68.1 to 73.1)	76.1 (72.6 to 79.2)	0.5 (0.1 to 0.8)	7.4 (2.1 to 12.6)
Morocco	49.8 (48.0 to 51.6)	60.1 (57.5 to 63.1)	61.1 (58.4 to 64.1)	64.4 (60.5 to 69.0)	0.5 (-0.0 to 0.9)	7.1 (-0.7 to 15.2)
Palestine	63.2 (61.5 to 64.5)	70.7 (69.0 to 72.2)	71.8 (70.0 to 73.4)	75.7 (73.9 to 77.4)	0.5 (0.2 to 0.7)	7.1 (3.7 to 11.1)
Oman	73.4 (70.9 to 75.9)	78.6 (76.5 to 80.8)	79.3 (77.3 to 81.5)	83.1 (79.8 to 86.3)	0.4 (0.1 to 0.7)	5.7 (0.9 to 10.3)
Qatar	71.7 (69.8 to 73.6)	82.5 (80.5 to 84.7)	83.7 (81.4 to 85.9)	86.2 (81.9 to 89.5)	0.3 (-0.1 to 0.6)	4.5 (-1.2 to 9.5)
Saudi Arabia	68.8 (66.8 to 70.9)	77.1 (74.8 to 79.5)	77.8 (75.4 to 80.1)	78.8 (73.8 to 83.0)	0.1 (-0.3 to 0.5)	2.2 (-4.3 to 8.2)
Sudan	46.7 (42.7 to 51.1)	58.8 (55.3 to 62.3)	60.5 (57.0 to 63.9)	63.7 (57.1 to 70.3)	0.5 (-0.3 to 1.2)	8.5 (-4.0 to 20.2)
Syria	60.3 (59.0 to 61.7)	68.5 (66.2 to 70.7)	69.9 (67.5 to 72.3)	71.9 (66.1 to 76.8)	0.3 (-0.2 to 0.8)	5.0 (-3.2 to 12.5)
Tunisia	69.2 (67.6 to 70.9)	78.4 (75.6 to 81.3)	79.4 (76.5 to 82.2)	81.6 (78.6 to 85.0)	0.3 (-0.1 to 0.6)	4.1 (-0.9 to 9.5)
Turkey	61.0 (59.5 to 62.5)	76.3 (75.0 to 77.7)	77.8 (76.2 to 79.3)	84.2 (81.4 to 86.0)	0.7 (0.4 to 0.9)	10.3 (6.3 to 13.9)
United Arab Emirates	65.0 (62.5 to 67.3)	67.8 (65.0 to 70.7)	69.0 (66.1 to 72.2)	72.6 (67.3 to 78.1)	0.5 (-0.1 to 0.9)	7.1 (-2.1 to 15.3)
Yemen	45.5 (41.1 to 50.0)	55.8 (51.0 to 60.1)	56.2 (51.5 to 60.8)	68.0 (63.3 to 72.5)	1.3 (0.6 to 2.0)	22.1 (9.3 to 35.6)
South Asia						
South Asia						
Bangladesh	40.8 (39.3 to 42.4)	58.5 (56.6 to 60.5)	59.8 (57.6 to 62.0)	71.4 (68.3 to 74.6)	1.3 (1.0 to 1.7)	22.1 (15.7 to 28.2)
Bhutan	46.5 (44.1 to 49.2)	62.7 (59.7 to 66.1)	64.1 (61.0 to 67.7)	71.3 (65.1 to 76.6)	0.9 (0.2 to 1.4)	13.9 (2.5 to 23.1)
India	42.1 (40.8 to 43.3)	56.0 (54.8 to 57.2)	56.8 (55.5 to 58.0)	64.5 (62.2 to 66.5)	0.9 (0.6 to 1.2)	15.3 (9.9 to 20.5)

	Estimate in 2000 (95% UIs)	Estimate in 2015 (95% UIs)	Estimate in 2017 (95% UIs)	Estimate in 2030 (95% UIs)	Annualised rate of change, 2015-2030 (95% UIs)	Percent change, 2015-2030 (95% UIs)
Nepal	43.0 (40.7 to 45.2)	57.9 (55.4 to 61.1)	59.0 (56.5 to 62.2)	66.4 (62.1 to 70.8)	0.9 (0.4 to 1.4)	14.7 (5.9 to 24.1)
Pakistan	37.5 (36.0 to 39.2)	49.8 (47.0 to 52.7)	51.6 (48.8 to 54.7)	61.0 (55.3 to 66.8)	1.4 (0.7 to 2.0)	22.7 (11.0 to 34.3)
Southeast Asia, East Asia, and Oceania						
East Asia						
China	61.9 (60.6 to 63.1)	78.3 (77.4 to 79.3)	80.4 (79.4 to 81.3)	86.2 (84.6 to 87.8)	0.6 (0.5 to 0.8)	10.0 (7.5 to 12.6)
North Korea	57.2 (54.6 to 60.0)	63.8 (61.3 to 66.5)	64.7 (62.0 to 67.3)	66.7 (64.1 to 69.3)	0.3 (-0.1 to 0.7)	4.6 (-1.0 to 10.5)
Taiwan (Province of China)	79.6 (79.0 to 80.2)	86.4 (85.9 to 87.0)	86.5 (85.6 to 87.3)	88.8 (87.6 to 90.1)	0.2 (0.1 to 0.3)	2.8 (1.3 to 4.2)
Oceania						
American Samoa	61.9 (60.5 to 63.2)	63.5 (61.7 to 65.2)	63.7 (61.6 to 65.6)	64.5 (62.4 to 66.5)	0.1 (-0.2 to 0.4)	1.7 (-2.7 to 5.9)
Federated States of Micronesia	48.6 (46.3 to 50.9)	55.1 (51.8 to 60.4)	55.1 (51.5 to 61.4)	55.1 (50.6 to 61.9)	0.0 (-0.8 to 0.9)	0.3 (-11.6 to 14.1)
Fiji	54.4 (52.7 to 55.8)	59.4 (56.8 to 62.0)	59.9 (57.1 to 63.0)	63.9 (59.2 to 69.3)	0.5 (-0.0 to 1.0)	7.6 (-0.7 to 16.2)
Guam	71.6 (70.2 to 72.9)	70.6 (69.2 to 72.0)	70.6 (68.9 to 72.2)	70.5 (68.9 to 72.1)	-0.0 (-0.2 to 0.2)	-0.1 (-3.0 to 2.9)
Kiribati	40.0 (38.1 to 42.1)	43.1 (40.5 to 45.9)	43.7 (41.0 to 46.6)	50.6 (47.0 to 54.5)	1.1 (0.5 to 1.6)	17.4 (7.6 to 26.9)
Marshall Islands	43.1 (41.1 to 45.1)	48.5 (46.1 to 51.0)	49.3 (46.8 to 51.9)	51.0 (43.7 to 56.5)	0.3 (-0.8 to 1.1)	5.2 (-10.9 to 18.6)
Northern Mariana Islands	73.5 (71.8 to 75.2)	73.9 (71.9 to 75.9)	74.2 (72.3 to 76.1)	76.1 (74.2 to 78.0)	0.2 (-0.0 to 0.4)	3.0 (-0.6 to 6.8)
Papua New Guinea	42.1 (39.5 to 44.7)	46.3 (43.3 to 49.2)	46.6 (43.6 to 49.4)	46.8 (42.4 to 51.3)	0.1 (-0.7 to 0.8)	1.2 (-10.1 to 12.0)
Samoa	57.6 (55.2 to 60.0)	59.6 (57.2 to 62.2)	59.9 (57.5 to 62.8)	61.1 (54.4 to 67.8)	0.2 (-0.7 to 0.9)	2.6 (-9.4 to 14.1)
Solomon Islands	50.4 (47.9 to 52.9)	54.9 (52.3 to 57.5)	55.6 (53.0 to 58.1)	60.6 (54.7 to 66.1)	0.7 (-0.1 to 1.3)	10.5 (-1.4 to 21.2)
Tonga	58.0 (56.4 to 59.6)	60.2 (58.0 to 62.7)	60.7 (58.3 to 63.3)	64.3 (59.0 to 69.6)	0.4 (-0.1 to 1.0)	6.9 (-1.9 to 15.4)
Vanuatu	47.0 (43.2 to 51.2)	45.7 (41.4 to 50.6)	46.8 (42.2 to 52.0)	45.3 (39.1 to 52.9)	-0.1 (-1.1 to 1.0)	-0.7 (-15.6 to 15.9)
Southeast Asia						
Cambodia	41.1 (39.3 to 43.2)	61.1 (58.8 to 63.2)	61.9 (59.6 to 64.0)	69.7 (65.1 to 73.7)	0.9 (0.4 to 1.3)	14.1 (5.6 to 21.9)
Indonesia	50.5 (49.5 to 51.6)	60.8 (59.6 to 62.0)	61.8 (60.5 to 63.1)	68.1 (65.7 to 70.9)	0.8 (0.5 to 1.0)	12.0 (7.6 to 16.7)
Laos	32.0 (29.4 to 34.7)	51.9 (49.0 to 55.1)	53.0 (50.1 to 56.3)	65.9 (61.9 to 70.4)	1.6 (1.0 to 2.1)	27.1 (16.9 to 36.7)
Malaysia	64.0 (63.1 to 65.3)	71.6 (70.4 to 72.8)	72.9 (71.1 to 74.7)	76.2 (73.7 to 78.5)	0.4 (0.2 to 0.6)	6.4 (2.8 to 9.9)
Maldives	61.0 (59.6 to 62.6)	72.6 (71.4 to 73.9)	78.2 (76.8 to 79.5)	77.5 (74.8 to 79.9)	0.4 (0.2 to 0.7)	6.7 (2.5 to 10.3)
Mauritius	68.3 (67.4 to 69.5)	71.1 (70.3 to 71.9)	71.7 (70.3 to 72.9)	72.7 (70.1 to 75.4)	0.1 (-0.1 to 0.4)	2.2 (-1.6 to 5.8)
Myanmar	45.2 (42.6 to 48.0)	56.3 (54.0 to 58.6)	58.4 (56.0 to 60.7)	59.3 (55.8 to 62.6)	0.3 (-0.1 to 0.8)	5.3 (-2.1 to 12.6)
Philippines	52.8 (51.7 to 53.9)	56.7 (55.3 to 58.1)	57.9 (55.8 to 60.0)	62.0 (58.6 to 65.3)	0.6 (0.2 to 1.0)	9.3 (2.7 to 16.4)
Sri Lanka	65.5 (64.5 to 66.5)	75.9 (73.7 to 78.0)	77.1 (74.3 to 79.8)	82.9 (79.8 to 86.0)	0.6 (0.3 to 0.9)	9.2 (4.2 to 14.1)
Seychelles	63.7 (62.5 to 64.9)	68.5 (67.1 to 69.9)	69.0 (67.6 to 70.5)	75.6 (69.8 to 79.6)	0.7 (0.1 to 1.0)	10.5 (2.1 to 16.2)
Thailand	66.8 (65.3 to 68.2)	76.3 (75.0 to 77.9)	76.4 (74.6 to 78.4)	80.1 (77.6 to 82.8)	0.3 (0.1 to 0.5)	5.0 (1.3 to 8.4)
Timor-Leste	35.4 (32.0 to 41.9)	53.5 (49.9 to 60.6)	54.5 (50.9 to 61.4)	62.2 (58.0 to 69.1)	1.0 (0.1 to 1.8)	16.5 (2.1 to 30.1)
Vietnam	58.8 (57.0 to 60.8)	70.8 (68.8 to 73.2)	71.7 (69.5 to 74.1)	78.5 (75.3 to 81.5)	0.7 (0.4 to 1.0)	10.9 (5.7 to 16.1)
Sub-Saharan Africa						
Central Sub-Saharan Africa						
Angola	29.1 (25.1 to 33.7)	41.7 (37.8 to 45.5)	43.2 (39.0 to 47.1)	48.0 (40.4 to 56.7)	0.9 (-0.2 to 1.9)	15.3 (-3.3 to 33.5)
Central African Republic	25.2 (21.8 to 29.4)	30.3 (26.4 to 34.5)	30.3 (26.3 to 34.5)	31.7 (26.0 to 38.3)	0.3 (-1.2 to 1.9)	5.3 (-16.5 to 32.5)
Congo	32.3 (29.0 to 35.8)	48.0 (44.1 to 51.6)	48.5 (44.6 to 52.4)	54.0 (45.9 to 62.1)	0.8 (-0.3 to 1.7)	12.7 (-3.9 to 29.1)
Democratic Republic of the Congo	30.9 (27.5 to 34.5)	42.6 (39.4 to 45.7)	43.9 (40.5 to 47.1)	46.2 (39.5 to 54.0)	0.5 (-0.5 to 1.5)	8.5 (-7.8 to 25.1)
Equatorial Guinea	32.5 (27.1 to 38.2)	50.8 (43.2 to 58.6)	52.2 (45.0 to 60.1)	60.4 (50.0 to 69.6)	1.2 (-0.3 to 2.5)	19.7 (-4.8 to 46.5)
Gabon	41.6 (38.1 to 45.6)	55.0 (51.4 to 59.3)	56.0 (52.3 to 60.3)	59.7 (53.1 to 66.6)	0.5 (-0.2 to 1.2)	8.5 (-3.2 to 19.9)
Eastern Sub-Saharan Africa						
Burundi	31.2 (28.1 to 34.8)	44.6 (40.7 to 48.5)	45.1 (41.3 to 49.2)	48.9 (43.2 to 55.3)	0.6 (-0.2 to 1.5)	9.7 (-3.4 to 25.1)
Comoros	35.2 (31.9 to 38.5)	46.0 (42.1 to 49.5)	47.4 (43.4 to 51.0)	52.6 (44.5 to 59.7)	0.9 (-0.2 to 1.7)	14.5 (-2.7 to 29.7)
Djibouti	33.2 (27.8 to 38.5)	47.0 (40.4 to 53.4)	48.9 (42.4 to 55.4)	54.5 (45.3 to 62.6)	1.0 (-0.4 to 2.2)	16.5 (-6.1 to 40.1)
Eritrea	27.0 (24.2 to 30.1)	38.7 (35.2 to 42.6)	40.4 (37.0 to 44.3)	58.7 (53.4 to 64.4)	2.8 (2.0 to 3.5)	52.0 (34.6 to 69.9)
Ethiopia	19.1 (16.8 to 21.8)	41.6 (38.9 to 44.5)	44.2 (41.4 to 47.1)	56.3 (48.9 to 65.2)	2.0 (1.1 to 2.8)	35.4 (18.4 to 51.7)
Kenya	44.7 (42.8 to 46.9)	52.4 (50.5 to 54.6)	53.3 (51.3 to 55.8)	56.5 (52.9 to 61.1)	0.5 (0.1 to 0.9)	7.8 (1.5 to 14.2)
Madagascar	33.9 (31.0 to 37.7)	40.5 (37.0 to 44.0)	41.6 (38.2 to 45.0)	46.8 (40.6 to 53.5)	1.0 (-0.1 to 1.9)	15.9 (-1.2 to 33.5)
Malawi	42.5 (39.9 to 45.2)	52.1 (48.6 to 55.9)	52.4 (48.5 to 56.4)	53.3 (46.7 to 60.6)	0.1 (-0.7 to 0.9)	2.4 (-9.5 to 15.0)
Mozambique	34.9 (32.6 to 37.6)	44.2 (41.3 to 47.4)	45.9 (42.7 to 49.2)	48.3 (42.3 to 55.1)	0.6 (-0.3 to 1.5)	9.3 (-4.3 to 25.7)
Rwanda	27.9 (25.1 to 30.7)	49.2 (45.7 to 53.2)	50.1 (46.5 to 54.1)	58.6 (52.8 to 65.3)	1.2 (0.4 to 1.8)	19.1 (6.7 to 31.1)
Somalia	17.3 (13.4 to 21.8)	24.7 (19.4 to 30.7)	25.8 (20.2 to 31.8)	26.8 (20.2 to 34.7)	0.5 (-1.7 to 2.7)	9.7 (-22.8 to 49.4)
South Sudan	19.9 (14.8 to 25.6)	31.3 (25.9 to 36.4)	30.7 (25.5 to 35.9)	39.3 (33.2 to 45.7)	1.5 (-0.0 to 2.9)	26.5 (-0.4 to 55.6)
Tanzania	42.4 (40.4 to 44.7)	49.1 (46.1 to 52.2)	50.2 (46.9 to 53.4)	54.1 (47.8 to 60.7)	0.6 (-0.1 to 1.4)	10.2 (-2.1 to 22.9)
Uganda	32.5 (29.6 to 35.4)	45.4 (42.3 to 48.6)	46.9 (43.8 to 50.3)	50.6 (44.6 to 57.9)	0.7 (-0.1 to 1.5)	11.6 (-1.1 to 25.4)
Zambia	37.7 (35.6 to 39.8)	52.5 (49.9 to 55.4)	54.0 (51.1 to 57.1)	58.0 (52.3 to 64.2)	0.7 (-0.1 to 1.3)	10.4 (-0.8 to 22.0)
Southern Sub-Saharan Africa						
Botswana	59.3 (55.2 to 63.8)	65.0 (62.3 to 67.4)	66.6 (63.7 to 69.1)	71.2 (67.0 to 75.0)	0.6 (0.1 to 1.1)	9.6 (2.1 to 17.6)
Lesotho	41.8 (39.0 to 44.8)	48.6 (44.9 to 52.5)	52.6 (48.8 to 56.5)	57.8 (53.1 to 62.2)	1.1 (0.4 to 1.9)	18.9 (6.9 to 32.5)
Namibia	44.8 (42.4 to 47.1)	59.8 (56.7 to 63.0)	61.6 (58.3 to 64.9)	67.2 (62.9 to 71.3)	0.8 (0.3 to 1.3)	12.4 (4.2 to 21.4)
South Africa	50.4 (49.0 to 51.7)	64.7 (63.5 to 65.9)	65.2 (64.1 to 66.5)	67.5 (65.2 to 70.2)	0.3 (0.1 to 0.5)	4.4 (0.8 to 8.5)
Swaziland	44.6 (41.3 to 47.9)	52.5 (48.8 to 56.4)	54.7 (51.0 to 58.5)	57.9 (52.2 to 63.4)	0.6 (-0.2 to 1.4)	10.4 (-3.1 to 24.2)
Zimbabwe	43.9 (41.5 to 46.5)	49.0 (46.4 to 51.8)	50.2 (47.5 to 53.1)	52.1 (45.5 to 58.5)	0.4 (-0.6 to 1.3)	6.5 (-8.3 to 21.7)
Western Sub-Saharan Africa						
Benin	42.8 (40.3 to 45.3)	49.9 (46.9 to 53.2)	50.6 (47.2 to 53.9)	53.0 (46.9 to 59.5)	0.4 (-0.5 to 1.2)	6.2 (-6.8 to 20.1)
Burkina Faso	39.6 (37.2 to 42.0)	50.7 (47.4 to 53.8)	51.1 (47.6 to 54.2)	57.1 (51.8 to 62.8)	0.8 (0.1 to 1.5)	12.6 (1.2 to 25.6)
Cameroon	39.6 (37.3 to 42.0)	51.0 (47.6 to 54.6)	53.0 (49.6 to 56.7)	59.3 (54.0 to 65.0)	1.0 (0.3 to 1.7)	16.3 (5.2 to 28.7)
Cape Verde	55.2 (53.1 to 57.3)	67.8 (66.2 to 69.4)	68.2 (66.4 to 70.1)	71.9 (66.8 to 76.2)	0.4 (-0.1 to 0.8)	6.0 (-1.4 to 13.1)
Chad	25.4 (22.8 to 28.1)	38.8 (35.8 to 41.8)	39.6 (36.4 to 42.8)	43.5 (37.3 to 50.1)	0.8 (-0.3 to 1.8)	12.3 (-4.7 to 30.5)
Cote d'Ivoire	43.3 (40.7 to 45.6)	48.9 (46.2 to 51.6)	50.3 (47.5 to 53.3)	56.8 (53.0 to 60.2)	1.0 (0.5 to 1.5)	16.2 (7.0 to 25.1)
The Gambia	45.2 (42.4 to 48.0)	50.5 (47.5 to 53.3)	51.3 (48.2 to 54.3)	50.7 (44.5 to 56.7)	0.0 (-0.9 to 0.8)	0.5 (-12.2 to 13.5)
Ghana	48.4 (46.2 to 50.7)	56.2 (53.5 to 59.3)	57.1 (54.2 to 60.0)	60.9 (54.5 to 66.0)	0.5 (-0.2 to 1.2)	8.4 (-3.0 to 19.4)
Guinea	28.2 (25.7 to 30.9)	34.7 (31.9 to 37.8)	35.8 (33.0 to 38.9)	39.8 (34.5 to 45.3)	0.9 (-0.1 to 1.8)	14.7 (-2.2 to 31.0)
Guinea-Bissau	32.1 (29.1 to 35.1)	44.4 (41.5 to 47.6)	45.0 (42.1 to 48.2)	49.8 (44.0 to 57.0)	0.7 (-0.1 to 1.8)	12.2 (-2.1 to 30.5)
Liberia	38.7 (35.1 to 42.6)	50.1 (46.6 to 53.4)	51.4 (47.9 to 54.7)	54.6 (48.5 to 62.0)	0.6 (-0.3 to 1.4)	9.2 (-4.0 to 23.1)
Mali	36.4 (33.4 to 39.3)	46.5 (43.5 to 49.7)	46.6 (43.5 to 50.0)	53.1 (46.5 to 59.8)	0.9 (-0.1 to 1.7)	14.1 (-1.5 to 29.8)
Mauritania	40.6 (38.2 to 43.2)	55.0 (52.1 to 57.8)	56.1 (53.1 to 59.0)	63.8 (57.1 to 70.4)	1.0 (0.2 to 1.6)	16.1 (3.5 to 26.9)
Niger	28.2 (25.2 to 31.3)	47.0 (43.3 to 50.8)	46.8 (43.1 to 50.6)	57.5 (52.0 to 62.8)	1.3 (0.5 to 2.1)	22.6 (8.5 to 36.0)

	Estimate in 2000 (95% UIs)	Estimate in 2015 (95% UIs)	Estimate in 2017 (95% UIs)	Estimate in 2030 (95% UIs)	Annualised rate of change, 2015-2030 (95% UIs)	Percent change, 2015-2030 (95% UIs)
Nigeria	37.6 (32.5 to 42.5)	46.7 (40.9 to 52.1)	46.4 (40.8 to 51.6)	44.9 (35.2 to 57.0)	-0.3 (-1.9 to 1.2)	-3.6 (-25.0 to 19.6)
Sao Tome and Principe	47.6 (45.5 to 49.6)	57.1 (54.6 to 59.5)	58.6 (56.0 to 61.2)	61.3 (53.4 to 68.7)	0.5 (-0.4 to 1.2)	7.4 (-6.2 to 20.2)
Senegal	41.8 (39.8 to 44.0)	51.6 (49.2 to 54.1)	52.6 (49.9 to 55.3)	57.7 (53.1 to 62.4)	0.7 (0.1 to 1.3)	11.7 (2.1 to 21.2)
Sierra Leone	35.3 (31.6 to 39.1)	47.7 (44.4 to 51.1)	49.5 (46.0 to 52.7)	53.2 (47.4 to 58.5)	0.7 (-0.1 to 1.5)	11.7 (-1.7 to 25.4)
Togo	41.5 (38.9 to 44.1)	51.9 (48.8 to 55.4)	53.3 (50.2 to 56.8)	56.6 (50.8 to 62.6)	0.6 (-0.2 to 1.4)	9.1 (-3.4 to 22.7)
**Indicator 3.9.1: Age-standardised death rate attributable to household air pollution and ambient air pollution (per 100,000 population)**						
Central Europe, Eastern Europe, and Central Asia						
Central Asia						
Armenia	77.6 (64.6 to 91.2)	54.5 (42.9 to 66.5)	52.3 (40.4 to 64.9)	39.1 (24.9 to 55.8)	-2.3 (-4.8 to 0.0)	-27.9 (-51.6 to 0.2)
Azerbaijan	92.9 (71.9 to 115.4)	67.4 (47.7 to 88.0)	64.5 (44.7 to 85.6)	51.3 (26.6 to 83.0)	-2.0 (-6.2 to 2.1)	-22.4 (-60.6 to 36.1)
Georgia	73.2 (61.9 to 84.3)	57.5 (46.1 to 68.8)	58.0 (47.4 to 69.4)	51.5 (36.0 to 69.8)	-0.8 (-2.9 to 1.3)	-10.2 (-35.5 to 21.4)
Kazakhstan	72.3 (33.8 to 106.4)	42.6 (12.9 to 64.5)	39.9 (11.5 to 63.8)	29.0 (5.2 to 57.8)	-3.1 (-13.9 to 6.3)	-20.7 (-87.5 to 156.7)
Kyrgyzstan	96.2 (74.3 to 124.6)	60.6 (44.5 to 80.3)	55.3 (40.4 to 73.6)	38.1 (21.5 to 63.1)	-3.3 (-6.6 to 0.2)	-36.9 (-63.1 to 2.8)
Mongolia	142.3 (114.5 to 168.8)	72.7 (53.1 to 88.9)	69.8 (50.2 to 85.4)	46.5 (26.2 to 64.8)	-3.1 (-6.3 to -0.4)	-35.6 (-61.4 to -6.4)
Tajikistan	105.7 (89.1 to 123.2)	82.8 (64.9 to 102.0)	81.1 (62.4 to 102.5)	68.2 (40.9 to 102.2)	-1.4 (-4.6 to 1.6)	-16.9 (-50.1 to 27.6)
Turkmenistan	78.5 (44.0 to 112.8)	56.9 (30.4 to 81.0)	54.6 (30.6 to 76.0)	41.5 (17.4 to 74.0)	-2.4 (-8.1 to 2.9)	-20.8 (-70.4 to 55.1)
Uzbekistan	99.0 (67.5 to 129.1)	91.8 (57.6 to 123.2)	86.5 (54.1 to 116.8)	78.8 (36.4 to 132.4)	-1.3 (-6.4 to 3.1)	-11.4 (-61.5 to 58.7)
Central Europe						
Albania	55.4 (47.6 to 63.2)	34.7 (28.1 to 42.4)	33.0 (26.1 to 40.8)	24.2 (15.7 to 35.5)	-2.5 (-4.7 to -0.3)	-30.4 (-50.9 to -4.0)
Bosnia and Herzegovina	59.4 (49.8 to 69.9)	51.0 (41.6 to 60.2)	48.4 (38.9 to 57.1)	41.4 (28.6 to 54.1)	-1.4 (-3.7 to 0.4)	-18.6 (-42.2 to 6.4)
Bulgaria	64.5 (53.7 to 75.5)	43.0 (35.0 to 51.3)	41.0 (33.0 to 49.6)	30.6 (20.6 to 42.2)	-2.4 (-5.1 to 0.3)	-28.5 (-53.4 to 5.4)
Croatia	42.1 (33.8 to 50.9)	29.1 (22.7 to 36.1)	25.9 (20.0 to 31.9)	18.0 (10.9 to 27.1)	-3.3 (-6.4 to -0.5)	-37.8 (-61.7 to -7.5)
Czech Republic	37.5 (29.4 to 48.1)	25.1 (19.7 to 32.8)	23.3 (18.2 to 30.6)	16.3 (10.3 to 25.7)	-3.0 (-5.9 to -0.1)	-34.6 (-58.7 to -1.7)
Hungary	46.1 (39.2 to 53.5)	33.8 (27.7 to 40.0)	29.5 (23.8 to 35.5)	20.5 (14.6 to 27.2)	-3.4 (-5.4 to -1.4)	-39.1 (-55.7 to -18.4)
Macedonia	64.4 (54.5 to 75.6)	43.4 (35.9 to 52.1)	42.0 (34.6 to 50.6)	31.0 (22.8 to 40.4)	-2.3 (-3.9 to -0.7)	-28.6 (-44.3 to -9.8)
Montenegro	52.2 (44.5 to 60.5)	41.1 (34.8 to 48.1)	39.8 (33.0 to 47.2)	33.9 (24.4 to 44.6)	-1.3 (-3.1 to 0.5)	-17.4 (-37.4 to 7.0)
Poland	45.5 (35.5 to 54.5)	29.8 (21.7 to 37.5)	27.3 (20.1 to 34.2)	18.6 (10.9 to 27.2)	-3.2 (-6.6 to -0.3)	-36.9 (-62.7 to -4.3)
Romania	57.5 (47.3 to 68.5)	34.0 (26.6 to 41.4)	32.4 (25.1 to 39.7)	21.8 (13.5 to 29.8)	-3.1 (-5.9 to -0.8)	-35.8 (-58.7 to -10.9)
Serbia	72.0 (61.0 to 81.8)	56.5 (47.8 to 66.1)	51.6 (42.7 to 60.5)	41.7 (30.2 to 54.6)	-2.1 (-4.1 to -0.3)	-25.9 (-45.9 to -3.8)
Slovakia	46.5 (39.4 to 54.1)	30.5 (25.3 to 35.9)	27.9 (23.2 to 33.2)	18.9 (13.6 to 25.4)	-3.2 (-5.3 to -1.1)	-37.7 (-54.7 to -15.4)
Slovenia	31.2 (25.5 to 37.0)	16.2 (13.0 to 19.7)	15.3 (12.1 to 18.7)	9.3 (6.3 to 12.5)	-3.8 (-6.1 to -1.7)	-42.4 (-59.8 to -21.9)
Eastern Europe						
Belarus	61.5 (48.7 to 76.8)	41.7 (32.5 to 51.6)	40.0 (31.1 to 50.9)	29.5 (18.1 to 44.1)	-2.4 (-5.3 to 0.3)	-29.0 (-55.1 to 4.7)
Estonia	33.8 (26.1 to 41.4)	12.4 (7.4 to 17.4)	11.7 (6.6 to 16.7)	5.8 (2.4 to 10.5)	-5.3 (-10.5 to -0.9)	-51.9 (-79.3 to -13.1)
Latvia	44.6 (34.8 to 56.0)	28.6 (21.6 to 37.1)	27.4 (20.0 to 36.6)	18.4 (10.7 to 28.3)	-3.1 (-6.0 to -0.7)	-35.7 (-59.6 to -9.9)
Lithuania	34.5 (28.4 to 41.5)	26.9 (21.6 to 33.1)	25.6 (19.7 to 31.8)	19.1 (12.1 to 26.8)	-2.4 (-4.7 to -0.1)	-29.0 (-50.5 to -1.5)
Moldova	78.6 (66.8 to 91.3)	44.6 (35.4 to 53.9)	39.6 (31.1 to 48.9)	24.8 (15.3 to 36.0)	-4.0 (-7.3 to -1.4)	-44.0 (-66.5 to -18.4)
Russia	59.9 (50.2 to 69.6)	36.5 (30.0 to 43.1)	33.8 (27.6 to 39.4)	21.7 (16.3 to 26.6)	-3.5 (-4.8 to -2.3)	-40.6 (-51.3 to -29.0)
Ukraine	70.9 (49.8 to 92.4)	56.5 (39.5 to 75.7)	50.6 (35.1 to 68.2)	39.8 (19.8 to 70.6)	-2.6 (-7.4 to 1.9)	-27.8 (-66.9 to 33.2)
High-income						
Australasia						
Australia	15.2 (6.7 to 25.1)	8.6 (1.8 to 15.8)	8.1 (1.7 to 15.3)	5.5 (0.7 to 12.5)	-3.6 (-11.8 to 2.2)	-34.3 (-82.8 to 39.0)
New Zealand	13.2 (5.0 to 20.2)	7.0 (2.0 to 11.9)	6.3 (1.4 to 11.3)	4.1 (0.6 to 9.3)	-4.3 (-14.7 to 3.7)	-34.2 (-89.0 to 74.0)
High-income Asia Pacific						
Brunei	25.8 (8.9 to 39.1)	18.8 (5.5 to 31.2)	15.8 (3.5 to 28.1)	11.8 (1.9 to 25.4)	-3.8 (-12.1 to 3.8)	-33.4 (-83.8 to 77.8)
Japan	13.9 (11.6 to 16.6)	9.1 (7.5 to 11.1)	8.6 (7.0 to 10.6)	5.8 (4.5 to 7.5)	-3.0 (-3.7 to -2.3)	-36.1 (-43.0 to -29.1)
South Korea	33.7 (28.4 to 38.9)	19.3 (15.5 to 23.0)	17.6 (14.1 to 21.1)	11.9 (8.1 to 16.2)	-3.3 (-5.4 to -1.3)	-38.2 (-55.6 to -17.4)
Singapore	32.0 (24.1 to 40.3)	18.3 (13.0 to 23.1)	16.7 (11.6 to 21.2)	10.9 (5.9 to 15.9)	-3.6 (-6.7 to -0.7)	-40.0 (-63.3 to -10.0)
High-income North America						
Canada	14.5 (6.9 to 20.4)	8.3 (2.2 to 12.8)	7.1 (1.6 to 11.4)	4.6 (0.8 to 9.3)	-4.5 (-14.4 to 4.2)	17.0 (-88.4 to 88.5)
Greenland	40.2 (20.3 to 62.2)	24.9 (8.3 to 41.0)	23.9 (8.0 to 39.8)	17.6 (4.0 to 38.7)	-2.9 (-12.8 to 5.9)	-17.7 (-85.4 to 142.7)
United States	20.3 (16.1 to 24.4)	12.8 (9.5 to 16.1)	11.8 (8.5 to 15.3)	7.8 (5.0 to 10.8)	-3.4 (-5.0 to -2.3)	-39.6 (-52.7 to -29.6)
Southern Latin America						
Argentina	35.3 (21.6 to 52.6)	26.9 (14.1 to 46.0)	25.0 (11.6 to 41.0)	20.4 (6.3 to 44.3)	-2.3 (-10.4 to 5.0)	-13.6 (-79.0 to 112.1)
Chile	32.8 (23.0 to 39.7)	20.7 (13.5 to 25.9)	20.1 (13.1 to 25.7)	14.3 (6.7 to 21.4)	-2.6 (-6.8 to 0.9)	-29.8 (-63.8 to 14.1)
Uruguay	24.1 (15.5 to 34.4)	16.0 (7.8 to 24.8)	15.5 (8.4 to 23.7)	11.8 (4.5 to 23.7)	-2.3 (-8.9 to 4.4)	-20.4 (-73.8 to 93.5)
Western Europe						
Andorra	15.1 (11.3 to 20.8)	11.6 (8.3 to 16.5)	10.6 (7.3 to 15.3)	8.1 (4.3 to 13.8)	-2.6 (-5.9 to -0.1)	-30.6 (-58.7 to -1.0)
Austria	21.9 (16.4 to 26.5)	13.8 (10.0 to 17.1)	12.7 (9.0 to 16.2)	8.6 (4.8 to 12.7)	-3.3 (-6.5 to -0.5)	-37.3 (-62.3 to -6.8)
Belgium	24.5 (18.5 to 30.0)	15.1 (10.8 to 19.3)	14.0 (10.0 to 18.0)	9.3 (5.3 to 13.3)	-3.3 (-6.3 to -0.7)	-37.8 (-61.0 to -9.8)
Cyprus	38.0 (31.1 to 45.2)	21.2 (16.9 to 25.9)	19.7 (15.4 to 24.1)	12.8 (8.8 to 17.4)	-3.4 (-5.3 to -1.4)	-39.3 (-54.7 to -19.5)
Denmark	23.0 (17.6 to 28.8)	14.1 (9.9 to 18.3)	13.6 (9.4 to 18.1)	9.3 (4.8 to 14.3)	-3.0 (-6.7 to -0.0)	-33.9 (-63.3 to -0.5)
Finland	13.9 (3.4 to 19.5)	6.0 (1.4 to 9.0)	5.8 (1.3 to 9.0)	3.5 (0.6 to 6.6)	-4.1 (-12.7 to 3.9)	-27.6 (-85.0 to 79.8)
France	15.5 (11.5 to 19.7)	9.8 (6.8 to 12.7)	8.9 (6.2 to 11.7)	5.9 (3.2 to 8.8)	-3.5 (-7.0 to -0.6)	-38.8 (-65.0 to -9.2)
Germany	22.4 (18.1 to 27.8)	15.0 (11.1 to 18.9)	14.4 (10.7 to 18.6)	10.5 (6.3 to 15.7)	-2.5 (-5.4 to 0.3)	-29.4 (-55.2 to 4.5)
Greece	27.2 (21.1 to 34.5)	20.4 (14.8 to 27.3)	19.3 (13.9 to 25.4)	15.2 (8.7 to 23.9)	-2.1 (-5.4 to 1.4)	-24.8 (-55.5 to 22.8)
Iceland	12.6 (6.2 to 22.1)	7.2 (2.5 to 14.0)	6.4 (2.2 to 13.4)	4.5 (0.9 to 13.2)	-3.8 (-11.5 to 4.0)	-30.6 (-82.2 to 82.7)
Ireland	25.8 (19.5 to 32.8)	11.4 (7.7 to 14.9)	10.5 (7.3 to 14.0)	5.9 (3.1 to 9.0)	-4.5 (-8.1 to -1.4)	-47.5 (-70.3 to -19.1)
Israel	30.5 (23.7 to 37.0)	18.3 (13.7 to 24.5)	16.9 (12.6 to 22.6)	11.7 (7.1 to 19.6)	-3.1 (-6.2 to 0.3)	-35.8 (-60.4 to 4.0)
Italy	20.6 (14.4 to 27.2)	14.1 (9.4 to 18.9)	12.6 (8.1 to 17.1)	9.0 (4.4 to 14.7)	-3.2 (-6.9 to 0.8)	-35.0 (-64.7 to 12.6)
Luxembourg	20.2 (15.7 to 24.5)	12.1 (8.9 to 15.2)	11.2 (8.2 to 14.2)	7.2 (4.3 to 10.4)	-3.6 (-6.2 to -1.2)	-40.4 (-60.5 to -16.3)
Malta	28.3 (22.6 to 33.7)	18.3 (14.4 to 22.1)	18.2 (14.2 to 22.4)	13.6 (9.5 to 18.3)	-2.0 (-3.7 to -0.5)	-25.8 (-42.2 to -7.2)
Netherlands	26.1 (21.0 to 31.4)	14.1 (11.0 to 17.6)	13.5 (10.3 to 16.9)	8.7 (5.6 to 12.2)	-3.3 (-5.9 to -1.1)	-38.4 (-58.6 to -15.3)
Norway	15.7 (12.4 to 19.1)	7.7 (5.5 to 10.2)	7.3 (5.0 to 9.7)	4.1 (2.4 to 6.0)	-4.3 (-6.1 to -2.9)	-47.5 (-60.2 to -35.4)
Portugal	23.9 (14.7 to 32.9)	11.8 (5.3 to 17.5)	11.1 (4.6 to 16.8)	6.8 (1.9 to 12.1)	-4.1 (-11.0 to 1.0)	-41.1 (-80.9 to 16.0)
Spain	17.8 (12.5 to 23.1)	10.8 (6.7 to 14.4)	9.6 (5.8 to 13.4)	6.3 (2.8 to 10.3)	-3.8 (-8.5 to 0.2)	-40.2 (-72.1 to 3.4)
Sweden	12.8 (6.6 to 18.0)	6.7 (2.5 to 11.2)	6.4 (2.2 to 10.5)	4.3 (1.1 to 8.3)	-3.4 (-10.9 to 3.4)	-31.4 (-80.6 to 67.5)
Switzerland	16.6 (12.4 to 20.7)	9.1 (6.6 to 11.8)	8.2 (5.8 to 10.7)	4.9 (2.9 to 7.4)	-4.3 (-7.4 to -1.3)	-46.1 (-67.0 to -17.6)
United Kingdom	24.2 (20.4 to 28.4)	13.4 (10.8 to 16.6)	13.0 (10.4 to 16.1)	8.4 (6.3 to 10.9)	-3.1 (-3.8 to -2.6)	-37.5 (-43.8 to -32.3)
Latin America and Caribbean						

	Estimate in 2000 (95% UIs)	Estimate in 2015 (95% UIs)	Estimate in 2017 (95% UIs)	Estimate in 2030 (95% UIs)	Annualised rate of change, 2015-2030 (95% UIs)	Percent change, 2015-2030 (95% UIs)
Andean Latin America						
Bolivia	81.8 (65.3 to 100.2)	48.7 (33.1 to 64.4)	46.1 (30.6 to 64.3)	32.5 (15.0 to 57.1)	-3.0 (-7.2 to 1.2)	-32.7 (-66.2 to 20.4)
Ecuador	31.7 (23.6 to 40.2)	24.0 (15.9 to 31.2)	23.1 (14.8 to 30.5)	17.9 (8.6 to 28.9)	-2.2 (-6.1 to 1.8)	-24.5 (-59.8 to 31.8)
Peru	49.5 (39.0 to 59.8)	28.9 (20.5 to 37.3)	27.0 (18.1 to 36.4)	17.5 (8.6 to 28.4)	-3.6 (-7.6 to -0.2)	-39.1 (-68.2 to -2.6)
Caribbean						
Antigua and Barbuda	39.6 (28.9 to 50.5)	29.9 (20.7 to 38.5)	28.8 (18.9 to 37.5)	23.4 (11.9 to 36.9)	-1.8 (-5.7 to 1.5)	-21.0 (-57.5 to 26.0)
The Bahamas	35.5 (23.3 to 47.3)	28.7 (18.2 to 39.2)	27.4 (16.6 to 37.5)	22.6 (9.3 to 39.1)	-1.9 (-7.3 to 2.8)	-19.2 (-66.4 to 53.2)
Barbados	41.9 (30.6 to 51.3)	32.4 (22.8 to 41.4)	31.5 (22.4 to 40.3)	26.0 (14.4 to 40.2)	-1.6 (-5.1 to 1.9)	-18.8 (-53.7 to 33.9)
Belize	61.0 (42.1 to 79.3)	40.0 (26.9 to 52.6)	38.4 (25.5 to 50.7)	29.7 (13.5 to 48.9)	-2.2 (-7.2 to 2.3)	-23.5 (-66.1 to 41.3)
Bermuda	24.3 (17.9 to 30.7)	14.6 (10.2 to 18.9)	13.9 (9.4 to 18.3)	9.4 (5.8 to 13.1)	-3.0 (-3.8 to -2.3)	-35.7 (-43.5 to -28.9)
Cuba	36.2 (21.1 to 50.0)	27.5 (14.6 to 39.1)	26.3 (14.1 to 37.8)	21.3 (7.5 to 39.2)	-2.1 (-8.8 to 4.0)	-18.4 (-73.3 to 83.4)
Dominica	41.0 (32.2 to 49.4)	34.5 (25.5 to 43.0)	33.4 (23.9 to 42.0)	29.2 (15.7 to 44.5)	-1.3 (-4.6 to 1.8)	-15.2 (-50.0 to 30.1)
Dominican Republic	29.8 (20.6 to 38.6)	32.2 (19.3 to 43.7)	30.3 (17.0 to 42.4)	29.1 (11.2 to 51.4)	-1.0 (-7.2 to 4.1)	-6.6 (-66.0 to 84.5)
Grenada	51.7 (39.4 to 64.6)	45.2 (33.0 to 57.4)	43.4 (30.3 to 55.6)	37.6 (21.0 to 58.6)	-1.4 (-5.1 to 1.8)	-16.2 (-53.2 to 31.9)
Guyana	73.9 (51.9 to 94.6)	58.7 (38.7 to 78.2)	55.3 (35.9 to 74.7)	45.8 (21.6 to 76.2)	-1.9 (-6.7 to 2.7)	-19.8 (-63.1 to 50.3)
Haiti	147.4 (127.3 to 168.8)	112.9 (92.8 to 133.7)	108.5 (89.8 to 129.1)	87.8 (65.2 to 113.9)	-1.7 (-3.1 to -0.3)	-22.2 (-37.5 to -4.4)
Jamaica	40.5 (31.3 to 49.3)	36.1 (25.5 to 45.8)	36.1 (24.1 to 46.6)	36.6 (19.4 to 54.4)	-0.1 (-3.0 to 2.3)	1.0 (-36.2 to 40.7)
Puerto Rico	22.7 (11.3 to 31.9)	13.4 (4.2 to 20.1)	13.5 (3.4 to 20.9)	9.5 (1.6 to 16.1)	-2.7 (-6.7 to -1.2)	-32.2 (-63.4 to -15.9)
Saint Lucia	49.3 (38.0 to 58.9)	35.5 (26.3 to 43.8)	35.0 (25.8 to 43.6)	26.9 (15.5 to 39.8)	-2.0 (-5.1 to 1.0)	-23.3 (-53.4 to 15.9)
Saint Vincent and the Grenadines	61.0 (48.9 to 71.9)	44.8 (33.4 to 56.1)	43.3 (32.6 to 54.8)	35.6 (20.9 to 52.6)	-1.7 (-5.0 to 1.2)	-20.0 (-52.9 to 19.7)
Suriname	53.7 (39.9 to 67.9)	44.3 (29.4 to 57.0)	42.5 (28.7 to 55.4)	36.6 (18.0 to 60.2)	-1.5 (-6.0 to 2.5)	-16.0 (-59.3 to 46.3)
Trinidad and Tobago	65.7 (46.6 to 81.8)	44.8 (28.5 to 59.1)	45.9 (29.2 to 62.2)	36.5 (17.0 to 61.6)	-1.6 (-6.2 to 2.5)	-17.5 (-60.4 to 46.5)
Virgin Islands, U.S.	30.5 (19.8 to 40.9)	25.6 (14.4 to 35.7)	24.4 (12.2 to 35.0)	21.1 (8.5 to 31.9)	-1.4 (-3.3 to -0.3)	-18.8 (-39.1 to -4.5)
Central Latin America						
Colombia	33.6 (24.6 to 43.2)	18.5 (11.7 to 25.1)	17.5 (10.1 to 25.2)	11.1 (4.4 to 19.9)	-3.8 (-9.1 to 1.3)	-38.5 (-74.3 to 21.8)
Costa Rica	25.6 (20.3 to 30.4)	16.3 (12.2 to 19.8)	16.7 (12.3 to 20.3)	13.3 (8.1 to 18.3)	-1.5 (-4.0 to 0.6)	-18.4 (-45.0 to 9.9)
El Salvador	49.0 (42.1 to 56.7)	42.4 (34.0 to 51.1)	39.6 (30.7 to 50.0)	33.8 (20.5 to 51.7)	-1.7 (-4.5 to 1.1)	-20.1 (-49.0 to 18.0)
Guatemala	107.3 (92.6 to 121.3)	61.1 (51.5 to 70.9)	60.1 (49.6 to 69.9)	42.2 (29.8 to 55.5)	-2.5 (-4.4 to -0.8)	-30.8 (-48.0 to -11.5)
Honduras	61.7 (49.7 to 73.5)	45.6 (36.3 to 55.6)	43.1 (34.0 to 52.5)	34.5 (22.8 to 47.8)	-1.9 (-4.0 to -0.1)	-24.5 (-44.9 to -1.6)
Mexico	42.4 (34.7 to 48.3)	36.6 (28.9 to 42.6)	36.0 (28.5 to 41.6)	31.6 (23.6 to 38.0)	-1.0 (-1.8 to -0.3)	-13.8 (-24.2 to -3.7)
Nicaragua	46.2 (40.0 to 52.0)	31.9 (25.8 to 37.4)	28.8 (22.6 to 34.5)	19.7 (12.8 to 26.7)	-3.3 (-5.7 to -1.3)	-38.3 (-57.7 to -18.0)
Panama	25.1 (19.4 to 30.7)	19.3 (13.5 to 24.9)	17.9 (10.9 to 23.7)	13.5 (5.9 to 21.1)	-2.6 (-6.7 to 0.4)	-29.8 (-63.6 to 6.8)
Venezuela	33.2 (20.2 to 45.2)	30.7 (18.4 to 42.6)	28.4 (14.9 to 40.3)	24.7 (8.5 to 45.1)	-1.8 (-8.5 to 3.5)	-15.3 (-72.2 to 67.9)
Tropical Latin America						
Brazil	41.6 (35.0 to 48.1)	25.4 (20.5 to 30.7)	24.2 (19.4 to 29.2)	16.3 (11.6 to 21.3)	-3.0 (-4.6 to -1.6)	-35.9 (-49.5 to -21.2)
Paraguay	48.8 (40.6 to 56.4)	38.8 (29.5 to 47.5)	36.0 (26.6 to 45.6)	27.9 (16.0 to 43.0)	-2.4 (-5.6 to 0.5)	-27.9 (-56.9 to 8.5)
North Africa and Middle East						
North Africa and Middle East						
Afghanistan	259.1 (217.8 to 300.6)	171.3 (147.5 to 197.2)	163.7 (140.5 to 189.3)	120.8 (93.1 to 152.9)	-2.4 (-3.9 to -0.9)	-29.4 (-43.9 to -12.6)
Algeria	46.4 (32.0 to 62.7)	36.2 (23.9 to 49.5)	35.8 (22.9 to 49.1)	29.9 (14.1 to 53.0)	-1.5 (-6.6 to 3.3)	-15.1 (-63.0 to 64.4)
Bahrain	95.7 (81.8 to 108.0)	57.4 (45.0 to 65.4)	55.0 (43.4 to 63.1)	37.4 (26.1 to 46.9)	-2.9 (-4.3 to -1.5)	-35.0 (-47.5 to -20.1)
Egypt	98.8 (75.9 to 122.3)	97.6 (70.7 to 120.4)	95.8 (69.3 to 121.9)	94.5 (51.3 to 145.7)	-0.4 (-4.4 to 3.0)	-1.8 (-48.0 to 56.8)
Iran	45.4 (40.8 to 49.8)	38.5 (34.4 to 42.7)	37.5 (33.5 to 41.7)	34.2 (29.2 to 39.5)	-0.8 (-1.5 to -0.1)	-11.1 (-19.6 to -2.1)
Iraq	100.4 (76.6 to 124.3)	39.6 (30.4 to 48.8)	36.4 (28.5 to 44.7)	18.8 (11.5 to 27.9)	-5.1 (-8.4 to -2.0)	-51.9 (-71.5 to -25.4)
Jordan	58.5 (46.2 to 68.9)	33.8 (26.5 to 41.2)	33.0 (25.8 to 40.5)	25.6 (17.3 to 36.5)	-1.9 (-4.1 to 0.1)	-24.1 (-46.2 to 2.3)
Kuwait	41.6 (35.2 to 48.5)	31.4 (26.1 to 36.9)	30.4 (25.1 to 36.3)	26.5 (18.8 to 35.4)	-1.2 (-3.4 to 0.9)	-15.5 (-40.0 to 13.9)
Lebanon	45.6 (37.9 to 54.0)	40.8 (34.0 to 48.2)	40.0 (32.9 to 46.9)	36.2 (25.4 to 48.4)	-0.8 (-3.0 to 1.3)	-10.7 (-35.9 to 21.5)
Libya	55.7 (37.8 to 76.0)	58.5 (38.1 to 82.7)	60.5 (38.3 to 84.3)	63.9 (28.2 to 115.3)	0.3 (-5.0 to 5.2)	11.9 (-52.8 to 117.1)
Morocco	68.8 (53.6 to 85.8)	56.0 (43.0 to 71.3)	55.1 (41.2 to 71.2)	48.5 (27.9 to 77.7)	-1.1 (-4.6 to 2.3)	-12.8 (-49.9 to 41.6)
Palestine	65.7 (53.1 to 80.9)	46.5 (35.5 to 58.2)	44.2 (33.8 to 55.8)	33.0 (20.4 to 48.3)	-2.4 (-4.8 to 0.1)	-28.9 (-51.5 to 1.0)
Oman	63.8 (48.2 to 82.6)	49.4 (37.8 to 63.9)	45.8 (34.0 to 59.0)	36.9 (20.3 to 58.1)	-2.1 (-5.7 to 1.2)	-24.5 (-57.3 to 19.6)
Qatar	101.2 (84.4 to 118.8)	57.2 (45.3 to 67.8)	54.5 (43.0 to 65.5)	37.3 (25.8 to 50.9)	-2.9 (-4.6 to -1.1)	-34.8 (-50.2 to -15.3)
Saudi Arabia	57.7 (41.8 to 74.9)	59.6 (40.0 to 80.0)	54.9 (38.7 to 72.4)	51.5 (26.9 to 83.6)	-1.2 (-5.5 to 3.1)	-11.6 (-56.2 to 58.3)
Sudan	137.0 (113.9 to 161.7)	87.5 (67.3 to 111.2)	82.9 (64.9 to 106.3)	60.1 (37.9 to 90.6)	-2.6 (-5.4 to 0.1)	-30.9 (-55.5 to 1.5)
Syria	64.5 (50.9 to 82.2)	52.6 (40.3 to 69.5)	51.8 (39.0 to 67.6)	45.8 (26.8 to 73.7)	-1.1 (-4.6 to 2.2)	-12.4 (-49.9 to 38.5)
Tunisia	54.0 (42.9 to 67.2)	42.0 (31.3 to 55.2)	41.8 (30.9 to 54.1)	36.6 (20.8 to 60.9)	-1.1 (-4.5 to 2.3)	-11.9 (-48.7 to 40.5)
Turkey	53.9 (44.6 to 63.8)	39.6 (31.7 to 48.0)	37.2 (29.1 to 45.4)	32.0 (19.4 to 46.1)	-1.5 (-4.6 to 1.2)	-18.8 (-50.1 to 19.3)
United Arab Emirates	78.0 (60.9 to 95.2)	66.3 (49.8 to 84.4)	64.8 (47.8 to 82.9)	60.2 (34.0 to 93.0)	-0.8 (-4.0 to 2.2)	-8.6 (-45.2 to 38.4)
Yemen	128.1 (98.0 to 165.5)	84.6 (58.6 to 115.7)	81.6 (56.4 to 113.4)	63.3 (33.4 to 111.1)	-2.1 (-6.0 to 1.8)	-24.2 (-59.5 to 30.8)
South Asia						
South Asia						
Bangladesh	132.7 (115.3 to 149.9)	98.3 (86.1 to 110.6)	93.5 (80.0 to 106.1)	73.8 (57.3 to 92.0)	-1.9 (-3.1 to -0.7)	-24.9 (-37.4 to -9.7)
Bhutan	104.1 (85.8 to 122.3)	60.3 (47.0 to 76.0)	56.4 (43.2 to 72.2)	39.3 (25.1 to 58.8)	-3.0 (-5.0 to -1.0)	-35.1 (-52.7 to -14.0)
India	149.0 (128.4 to 169.2)	113.0 (97.5 to 127.3)	108.6 (93.3 to 123.3)	89.5 (75.1 to 104.8)	-1.6 (-2.1 to -1.1)	-20.9 (-26.9 to -15.3)
Nepal	132.1 (110.8 to 154.2)	113.4 (94.0 to 133.6)	110.2 (89.3 to 130.8)	101.9 (72.5 to 135.4)	-0.8 (-2.5 to 1.0)	-10.0 (-31.7 to 16.3)
Pakistan	131.7 (113.5 to 150.1)	107.8 (89.6 to 128.2)	103.2 (83.9 to 123.8)	89.1 (61.5 to 123.3)	-1.3 (-3.3 to 0.6)	-17.4 (-38.8 to 9.4)
Southeast Asia, East Asia, and Oceania						
East Asia						
China	104.9 (88.2 to 118.9)	61.7 (53.1 to 70.1)	56.5 (48.0 to 64.9)	36.7 (30.6 to 43.3)	-3.5 (-4.1 to -2.9)	-40.5 (-45.8 to -35.3)
North Korea	142.0 (113.6 to 173.5)	114.5 (91.0 to 138.6)	108.4 (84.8 to 131.8)	91.9 (66.2 to 119.0)	-1.5 (-2.8 to -0.2)	-19.7 (-34.2 to -2.6)
Taiwan (Province of China)	37.1 (29.0 to 43.6)	26.8 (20.1 to 32.3)	26.1 (19.8 to 32.0)	20.9 (13.0 to 29.0)	-1.7 (-4.3 to 0.3)	-21.9 (-47.2 to 4.8)
Oceania						
American Samoa	66.1 (48.8 to 81.0)	52.9 (35.1 to 68.0)	51.3 (32.7 to 67.1)	42.6 (23.3 to 60.0)	-1.5 (-3.0 to -0.5)	-20.2 (-36.7 to -6.5)
Federated States of Micronesia	104.1 (79.2 to 128.1)	81.1 (46.4 to 107.5)	78.5 (46.4 to 105.8)	64.8 (28.6 to 105.9)	-1.7 (-7.1 to 2.8)	-17.7 (-65.6 to 52.7)
Fiji	126.1 (91.2 to 153.2)	97.3 (56.7 to 126.7)	90.9 (45.9 to 124.3)	75.8 (27.4 to 127.8)	-2.0 (-8.1 to 3.0)	-20.1 (-70.3 to 56.3)
Guam	41.4 (30.7 to 51.3)	37.1 (26.6 to 46.9)	36.1 (25.6 to 46.3)	34.1 (22.1 to 45.9)	-0.6 (-1.5 to 0.2)	-8.7 (-20.6 to 2.3)
Kiribati	168.4 (143.6 to 188.4)	145.4 (118.5 to 169.8)	139.7 (115.5 to 163.0)	123.2 (90.4 to 156.5)	-1.1 (-2.6 to 0.1)	-15.3 (-32.3 to 1.6)
Marshall Islands	116.7 (83.3 to 147.2)	86.3 (52.0 to 115.3)	80.0 (44.8 to 108.5)	61.7 (25.6 to 104.0)	-2.5 (-7.8 to 2.1)	-26.6 (-69.2 to 37.1)
Northern Mariana Islands	38.3 (28.3 to 48.1)	30.5 (20.3 to 40.1)	29.0 (19.0 to 38.1)	25.1 (14.1 to 36.3)	-1.4 (-2.9 to -0.2)	-18.2 (-35.2 to -2.5)
Papua New Guinea	262.3 (217.7 to 310.0)	227.6 (184.7 to 276.4)	218.5 (175.6 to 265.3)	188.3 (133.3 to 249.9)	-1.3 (-3.0 to 0.5)	-17.2 (-36.4 to 7.3)

	Estimate in 2000 (95% UIs)	Estimate in 2015 (95% UIs)	Estimate in 2017 (95% UIs)	Estimate in 2030 (95% UIs)	Annualised rate of change, 2015-2030 (95% UIs)	Percent change, 2015-2030 (95% UIs)
Samoa	77.5 (62.5 to 92.9)	71.3 (55.7 to 85.9)	68.0 (52.6 to 84.2)	62.0 (38.9 to 88.1)	-1.0 (-3.9 to 1.4)	-12.8 (-44.1 to 24.2)
Solomon Islands	205.2 (174.9 to 239.8)	176.7 (148.7 to 204.7)	171.8 (143.1 to 198.6)	149.3 (111.6 to 192.2)	-1.2 (-2.7 to 0.3)	-15.4 (-33.1 to 4.0)
Tonga	86.3 (68.0 to 101.5)	66.7 (45.4 to 86.2)	63.2 (39.8 to 82.6)	50.8 (23.1 to 79.7)	-2.0 (-6.6 to 1.5)	-23.0 (-62.7 to 25.6)
Vanuatu	150.0 (116.2 to 185.5)	126.5 (95.4 to 158.8)	123.5 (93.4 to 157.6)	106.9 (70.3 to 151.6)	-1.2 (-3.3 to 0.6)	-15.4 (-38.9 to 9.8)
Southeast Asia						
Cambodia	170.4 (146.8 to 191.9)	102.6 (88.2 to 116.5)	98.7 (85.2 to 114.0)	73.0 (57.0 to 92.3)	-2.3 (-3.5 to -1.0)	-28.8 (-41.1 to -14.3)
Indonesia	75.3 (66.1 to 84.8)	58.7 (50.6 to 67.3)	55.7 (47.6 to 64.3)	45.1 (35.9 to 54.4)	-1.8 (-2.7 to -0.9)	-23.3 (-33.7 to -13.3)
Laos	194.4 (168.7 to 222.7)	113.2 (96.7 to 131.6)	105.8 (88.5 to 124.1)	70.1 (51.6 to 90.9)	-3.2 (-4.8 to -1.9)	-38.1 (-51.1 to -24.2)
Malaysia	55.6 (32.4 to 72.5)	44.3 (23.6 to 61.0)	42.1 (22.1 to 59.3)	35.0 (12.8 to 59.5)	-1.9 (-7.4 to 2.8)	-19.1 (-66.9 to 51.6)
Maldives	64.8 (54.6 to 76.0)	18.0 (13.4 to 22.6)	15.2 (10.3 to 20.0)	5.3 (2.8 to 7.8)	-8.4 (-10.8 to -6.9)	-71.2 (-80.1 to -64.4)
Mauritius	50.1 (36.3 to 63.2)	43.5 (26.0 to 57.5)	42.5 (24.4 to 56.5)	36.9 (15.8 to 60.9)	-1.3 (-5.8 to 2.5)	-13.5 (-57.8 to 46.1)
Myanmar	173.6 (144.5 to 202.5)	105.2 (86.0 to 123.3)	99.7 (81.3 to 117.1)	70.7 (52.7 to 89.1)	-2.7 (-3.9 to -1.4)	-32.7 (-44.1 to -18.8)
Philippines	90.6 (73.6 to 105.6)	102.2 (84.9 to 119.2)	95.2 (76.1 to 113.5)	93.5 (63.2 to 128.1)	-0.7 (-3.0 to 1.4)	-8.5 (-35.9 to 23.2)
Sri Lanka	69.3 (61.0 to 79.4)	46.2 (38.0 to 54.6)	33.3 (19.4 to 47.1)	20.9 (9.1 to 36.3)	-5.7 (-10.4 to -1.9)	-54.8 (-78.9 to -24.9)
Seychelles	44.9 (33.4 to 56.7)	37.5 (25.5 to 51.1)	36.7 (25.2 to 49.9)	31.6 (17.1 to 51.1)	-1.3 (-3.1 to 0.5)	-16.6 (-37.5 to 7.8)
Thailand	50.4 (41.2 to 59.6)	29.1 (22.3 to 35.2)	28.5 (20.7 to 36.2)	19.9 (10.8 to 30.9)	-2.7 (-6.6 to 0.5)	-31.2 (-63.1 to 7.7)
Timor-Leste	130.8 (110.1 to 150.7)	87.9 (71.1 to 104.5)	86.7 (70.5 to 103.6)	72.6 (51.5 to 96.5)	-1.3 (-2.9 to 0.3)	-17.5 (-35.6 to 4.3)
Vietnam	77.1 (64.7 to 88.9)	54.2 (41.8 to 66.2)	51.5 (37.9 to 63.0)	39.3 (22.1 to 56.8)	-2.3 (-5.6 to 0.4)	-27.1 (-57.0 to 6.8)
Sub-Saharan Africa						
Central Sub-Saharan Africa						
Angola	174.9 (146.2 to 202.6)	89.0 (72.9 to 107.2)	84.3 (68.3 to 103.3)	55.6 (37.8 to 79.4)	-3.2 (-5.6 to -0.9)	-37.4 (-57.0 to -13.1)
Central African Republic	213.8 (179.7 to 251.0)	189.6 (154.3 to 223.8)	183.8 (149.6 to 219.7)	166.4 (123.2 to 218.7)	-0.9 (-2.3 to 0.5)	-12.2 (-29.5 to 8.5)
Congo	167.0 (143.8 to 193.9)	105.4 (82.8 to 130.1)	100.7 (79.4 to 126.2)	75.0 (47.3 to 112.3)	-2.4 (-5.3 to 0.4)	-28.5 (-54.5 to 6.3)
Democratic Republic of the Congo	165.6 (140.0 to 191.4)	135.2 (113.0 to 159.7)	129.5 (107.8 to 152.1)	110.5 (84.2 to 142.3)	-1.4 (-2.8 to 0.1)	-18.2 (-33.9 to 1.4)
Equatorial Guinea	146.8 (113.6 to 187.6)	73.4 (51.4 to 101.8)	71.4 (49.8 to 99.8)	46.7 (27.0 to 76.4)	-3.1 (-5.6 to -0.6)	-36.3 (-56.9 to -8.7)
Gabon	92.8 (71.1 to 118.0)	66.0 (45.6 to 88.3)	64.1 (43.2 to 87.2)	50.8 (26.2 to 87.5)	-2.0 (-6.4 to 2.3)	-21.3 (-61.6 to 42.0)
Eastern Sub-Saharan Africa						
Burundi	197.7 (166.8 to 228.6)	135.9 (112.8 to 160.2)	131.9 (109.5 to 155.0)	103.2 (77.6 to 131.0)	-1.9 (-3.2 to -0.6)	-24.1 (-38.1 to -8.2)
Comoros	135.8 (114.8 to 156.3)	89.2 (74.0 to 104.8)	85.8 (71.2 to 101.5)	65.4 (48.1 to 85.5)	-2.1 (-3.6 to -0.7)	-26.7 (-41.3 to -10.4)
Djibouti	117.3 (87.7 to 152.4)	74.6 (49.4 to 105.0)	71.8 (49.8 to 100.1)	55.6 (29.3 to 96.8)	-2.1 (-6.2 to 1.7)	-24.1 (-60.5 to 30.0)
Eritrea	171.9 (143.6 to 201.2)	129.8 (106.0 to 156.1)	121.8 (99.7 to 147.3)	94.1 (67.0 to 124.6)	-2.2 (-3.9 to -0.6)	-27.5 (-44.1 to -9.0)
Ethiopia	155.3 (137.8 to 172.5)	86.8 (74.4 to 99.6)	82.1 (70.1 to 95.6)	55.0 (43.6 to 69.4)	-3.1 (-3.9 to -2.1)	-36.8 (-44.6 to -27.1)
Kenya	113.4 (99.9 to 129.5)	85.9 (75.4 to 98.5)	82.6 (72.2 to 95.1)	65.8 (55.4 to 77.8)	-1.8 (-2.2 to -1.4)	-23.4 (-28.1 to -18.9)
Madagascar	184.8 (160.4 to 210.4)	148.9 (122.9 to 175.8)	143.6 (119.3 to 170.7)	123.0 (91.4 to 162.5)	-1.3 (-2.8 to 0.0)	-17.5 (-34.0 to 0.5)
Malawi	161.0 (136.1 to 185.1)	100.4 (85.4 to 115.8)	96.5 (81.5 to 112.3)	74.0 (56.5 to 93.5)	-2.1 (-3.3 to -0.8)	-26.3 (-39.1 to -11.9)
Mozambique	160.6 (137.9 to 183.2)	123.4 (104.9 to 143.9)	116.4 (97.4 to 136.3)	94.7 (70.2 to 123.3)	-1.8 (-3.2 to -0.4)	-23.3 (-38.3 to -6.3)
Rwanda	190.3 (161.9 to 216.3)	93.1 (77.6 to 111.0)	89.7 (74.2 to 106.8)	61.2 (46.0 to 79.2)	-2.8 (-4.1 to -1.5)	-34.3 (-46.1 to -20.6)
Somalia	218.1 (171.6 to 259.5)	160.3 (124.6 to 202.0)	154.8 (119.9 to 197.1)	129.1 (89.8 to 176.9)	-1.5 (-3.1 to 0.1)	-19.5 (-36.9 to 0.9)
South Sudan	178.9 (141.0 to 218.4)	150.3 (118.1 to 186.3)	145.6 (116.7 to 181.9)	129.6 (95.4 to 178.6)	-1.0 (-2.5 to 0.5)	-13.8 (-31.4 to 7.2)
Tanzania	123.6 (103.9 to 142.8)	91.8 (76.8 to 106.8)	89.2 (75.1 to 104.9)	74.6 (56.8 to 95.9)	-1.4 (-2.7 to -0.1)	-18.7 (-33.6 to -2.0)
Uganda	143.3 (120.9 to 167.0)	90.2 (76.5 to 106.1)	86.2 (72.6 to 100.1)	63.8 (47.9 to 81.0)	-2.4 (-3.7 to -1.1)	-29.4 (-42.2 to -15.0)
Zambia	150.1 (128.0 to 172.7)	92.6 (77.7 to 108.5)	89.1 (74.1 to 104.7)	66.5 (49.5 to 86.8)	-2.2 (-3.7 to -0.7)	-28.1 (-42.4 to -10.4)
Southern Sub-Saharan Africa						
Botswana	66.2 (51.6 to 84.6)	64.9 (51.8 to 79.3)	60.1 (48.0 to 74.2)	55.7 (36.0 to 81.6)	-1.1 (-3.8 to 1.6)	-13.8 (-43.8 to 26.5)
Lesotho	139.2 (115.6 to 164.8)	149.4 (119.8 to 178.8)	135.0 (107.0 to 161.9)	127.6 (87.2 to 174.5)	-1.1 (-2.9 to 0.5)	-14.9 (-35.1 to 7.1)
Namibia	128.9 (108.7 to 150.4)	68.6 (54.8 to 85.2)	65.1 (51.2 to 81.9)	42.9 (27.8 to 63.1)	-3.2 (-6.0 to -0.6)	-37.3 (-59.5 to -8.3)
South Africa	91.4 (80.5 to 102.0)	56.7 (47.8 to 64.6)	49.7 (41.5 to 57.0)	33.8 (26.2 to 41.6)	-3.5 (-4.6 to -2.4)	-40.4 (-50.2 to -29.9)
Swaziland	145.6 (121.3 to 173.2)	103.8 (80.8 to 131.0)	92.8 (70.3 to 116.8)	63.6 (40.0 to 93.4)	-3.4 (-5.5 to -1.4)	-38.8 (-55.9 to -18.7)
Zimbabwe	124.3 (102.8 to 147.6)	119.8 (97.3 to 143.0)	112.6 (91.3 to 135.0)	101.7 (72.8 to 136.5)	-1.1 (-2.9 to 0.5)	-15.1 (-35.3 to 8.3)
Western Sub-Saharan Africa						
Benin	142.3 (120.5 to 164.0)	110.5 (91.3 to 132.8)	104.5 (86.1 to 125.0)	85.7 (63.5 to 114.5)	-1.7 (-3.3 to -0.3)	-22.4 (-38.7 to -4.3)
Burkina Faso	160.0 (137.2 to 183.6)	129.2 (111.0 to 147.9)	124.5 (105.6 to 144.0)	111.0 (84.5 to 141.0)	-1.1 (-2.5 to 0.3)	-14.1 (-31.1 to 4.9)
Cameroon	152.1 (127.5 to 176.7)	112.7 (87.3 to 139.9)	104.5 (81.2 to 130.2)	80.8 (52.2 to 116.3)	-2.3 (-4.7 to 0.1)	-28.1 (-50.4 to 1.7)
Cape Verde	62.1 (52.5 to 72.2)	47.2 (37.7 to 56.7)	45.0 (36.0 to 54.2)	37.6 (24.3 to 52.6)	-1.6 (-4.3 to 0.8)	-20.0 (-47.7 to 13.1)
Chad	179.1 (152.7 to 205.7)	153.1 (128.5 to 175.5)	148.8 (124.0 to 171.6)	134.6 (102.0 to 170.2)	-0.9 (-2.4 to 0.5)	-12.1 (-29.8 to 7.7)
Cote d'Ivoire	150.9 (127.0 to 176.3)	117.7 (97.9 to 137.8)	109.3 (91.2 to 128.9)	87.7 (65.4 to 115.2)	-2.0 (-3.5 to -0.6)	-25.4 (-41.2 to -8.1)
The Gambia	139.5 (117.3 to 163.3)	127.0 (106.7 to 148.1)	123.2 (102.9 to 145.2)	113.6 (86.1 to 147.8)	-0.8 (-2.1 to 0.5)	-10.6 (-27.0 to 8.1)
Ghana	120.7 (101.2 to 140.2)	102.1 (85.1 to 122.4)	96.6 (78.6 to 117.2)	84.3 (58.2 to 120.6)	-1.4 (-3.5 to 0.8)	-17.2 (-40.9 to 12.8)
Guinea	174.9 (149.8 to 200.1)	155.6 (131.2 to 184.6)	148.7 (123.7 to 176.5)	138.1 (102.7 to 177.7)	-0.8 (-2.3 to 0.6)	-11.2 (-29.3 to 8.8)
Guinea-Bissau	205.1 (173.3 to 237.8)	156.7 (131.7 to 183.2)	149.3 (125.6 to 175.5)	122.3 (92.3 to 155.8)	-1.7 (-3.0 to -0.3)	-22.0 (-36.6 to -4.9)
Liberia	147.8 (125.7 to 170.0)	108.5 (91.6 to 127.3)	104.7 (88.1 to 124.4)	87.9 (66.2 to 116.3)	-1.4 (-2.8 to -0.2)	-19.0 (-34.1 to -2.3)
Mali	127.6 (109.2 to 146.6)	99.0 (83.7 to 115.7)	98.0 (82.3 to 113.9)	84.8 (62.7 to 108.6)	-1.1 (-2.7 to 0.4)	-14.4 (-33.2 to 5.9)
Mauritania	110.3 (90.0 to 134.4)	79.7 (62.7 to 100.6)	76.1 (58.8 to 97.7)	60.7 (37.0 to 92.9)	-2.0 (-5.1 to 1.2)	-23.3 (-53.6 to 19.0)
Niger	175.8 (149.3 to 201.4)	132.5 (107.4 to 155.8)	130.0 (107.1 to 154.7)	113.6 (85.2 to 147.2)	-1.1 (-2.6 to 0.4)	-14.2 (-31.9 to 6.6)
Nigeria	118.6 (91.5 to 149.6)	77.2 (57.3 to 106.8)	74.8 (54.7 to 101.2)	59.1 (36.2 to 96.3)	-1.9 (-4.5 to 1.1)	-23.0 (-49.1 to 17.5)
Sao Tome and Principe	139.4 (118.3 to 160.8)	100.7 (82.0 to 120.3)	97.8 (80.2 to 118.2)	82.3 (57.5 to 113.4)	-1.4 (-3.4 to 0.4)	-18.2 (-40.0 to 6.4)
Senegal	112.6 (95.8 to 129.9)	99.9 (84.2 to 117.8)	97.3 (82.4 to 114.7)	92.1 (70.2 to 117.0)	-0.6 (-2.0 to 0.7)	-7.8 (-26.5 to 10.5)
Sierra Leone	184.5 (154.0 to 214.9)	145.5 (122.8 to 170.1)	138.4 (115.7 to 161.1)	115.2 (86.6 to 147.6)	-1.6 (-2.9 to -0.3)	-20.9 (-35.5 to -4.7)
Togo	142.1 (118.9 to 165.1)	111.3 (90.8 to 132.3)	105.6 (87.4 to 126.3)	87.4 (64.6 to 115.5)	-1.7 (-3.1 to -0.3)	-21.5 (-37.1 to -4.1)
**Indicator 3.9.2: Age-standardised death rate attributable to unsafe water, sanitation, and hygiene (WaSH) (per 100,000 population)**						
Central Europe, Eastern Europe, and Central Asia						
Central Asia						
Armenia	3.9 (2.9 to 5.1)	0.6 (0.5 to 0.8)	0.6 (0.4 to 0.8)	0.3 (0.2 to 0.5)	-4.8 (-6.0 to -3.6)	-50.8 (-59.3 to -41.7)
Azerbaijan	12.7 (9.8 to 15.6)	2.5 (1.7 to 3.5)	2.4 (1.6 to 3.4)	0.9 (0.5 to 1.6)	-6.9 (-8.8 to -4.9)	-64.3 (-73.3 to -52.2)
Georgia	2.9 (2.2 to 3.8)	0.7 (0.5 to 0.9)	0.6 (0.5 to 0.8)	0.5 (0.3 to 0.6)	-2.8 (-3.7 to -1.8)	-33.8 (-42.9 to -23.8)
Kazakhstan	4.5 (3.5 to 5.6)	0.8 (0.6 to 1.1)	0.8 (0.6 to 1.0)	0.3 (0.2 to 0.5)	-6.1 (-7.3 to -4.9)	-59.5 (-66.7 to -51.7)
Kyrgyzstan	10.6 (8.6 to 12.5)	2.1 (1.7 to 2.5)	2.0 (1.6 to 2.4)	0.8 (0.5 to 1.1)	-7.0 (-8.5 to -5.2)	-64.5 (-72.1 to -54.5)
Mongolia	7.1 (5.1 to 9.5)	2.1 (1.5 to 2.8)	1.9 (1.4 to 2.6)	0.9 (0.6 to 1.4)	-5.8 (-7.2 to -4.1)	-57.6 (-65.9 to -45.7)
Tajikistan	32.2 (25.9 to 38.9)	16.0 (11.8 to 21.6)	15.3 (11.0 to 20.4)	10.8 (6.7 to 16.8)	-2.7 (-4.4 to -1.0)	-33.2 (-48.4 to -13.3)
Turkmenistan	17.7 (14.2 to 21.3)	2.1 (1.5 to 2.9)	1.9 (1.3 to 2.6)	0.5 (0.3 to 0.8)	-9.3 (-10.4 to -8.0)	-75.1 (-79.1 to -69.9)

	Estimate in 2000 (95% UIs)	Estimate in 2015 (95% UIs)	Estimate in 2017 (95% UIs)	Estimate in 2030 (95% UIs)	Annualised rate of change, 2015-2030 (95% UIs)	Percent change, 2015-2030 (95% UIs)
Uzbekistan	5.2 (3.9 to 6.5)	1.7 (1.2 to 2.2)	1.5 (1.1 to 2.0)	0.7 (0.4 to 1.0)	-6.1 (-7.3 to -4.8)	-59.6 (-66.3 to -51.4)
Central Europe						
Albania	1.0 (0.7 to 1.4)	0.3 (0.2 to 0.5)	0.3 (0.2 to 0.4)	0.2 (0.1 to 0.3)	-5.1 (-6.8 to -3.3)	-52.7 (-63.7 to -38.8)
Bosnia and Herzegovina	0.3 (0.2 to 0.5)	0.2 (0.1 to 0.3)	0.2 (0.1 to 0.3)	0.1 (0.1 to 0.2)	-3.1 (-4.8 to -1.2)	-36.8 (-51.6 to -16.5)
Bulgaria	0.4 (0.3 to 0.6)	0.2 (0.1 to 0.4)	0.2 (0.1 to 0.4)	0.2 (0.1 to 0.3)	-3.3 (-5.0 to -1.4)	-38.1 (-52.5 to -18.3)
Croatia	0.2 (0.1 to 0.3)	0.2 (0.1 to 0.3)	0.2 (0.1 to 0.3)	0.2 (0.1 to 0.3)	-1.6 (-3.4 to 0.1)	-21.0 (-40.0 to 2.0)
Czech Republic	0.1 (0.1 to 0.2)	0.4 (0.2 to 0.8)	0.4 (0.2 to 0.7)	0.6 (0.2 to 1.1)	1.6 (0.1 to 3.3)	28.9 (1.3 to 65.2)
Hungary	0.1 (0.1 to 0.1)	0.6 (0.3 to 1.0)	0.5 (0.2 to 0.8)	0.7 (0.3 to 1.2)	0.3 (-1.2 to 1.8)	6.1 (-16.2 to 31.1)
Macedonia	1.6 (0.9 to 2.3)	0.2 (0.1 to 0.3)	0.2 (0.1 to 0.3)	0.1 (0.0 to 0.2)	-5.7 (-7.9 to -3.4)	-57.1 (-69.5 to -40.3)
Montenegro	0.3 (0.2 to 0.4)	0.1 (0.1 to 0.2)	0.1 (0.1 to 0.2)	0.1 (0.1 to 0.1)	-2.3 (-3.7 to -0.9)	-28.6 (-42.6 to -12.5)
Poland	0.2 (0.1 to 0.3)	0.2 (0.1 to 0.4)	0.2 (0.1 to 0.3)	0.2 (0.1 to 0.4)	-0.6 (-1.9 to 0.7)	-7.5 (-24.8 to 11.9)
Romania	1.9 (1.5 to 2.4)	0.6 (0.4 to 0.8)	0.5 (0.4 to 0.7)	0.3 (0.2 to 0.5)	-4.1 (-5.4 to -2.8)	-45.8 (-55.6 to -34.6)
Serbia	0.3 (0.2 to 0.4)	0.3 (0.1 to 0.5)	0.3 (0.1 to 0.5)	0.3 (0.1 to 0.8)	-0.1 (-3.8 to 3.6)	4.4 (-43.8 to 70.5)
Slovakia	0.3 (0.2 to 0.4)	0.2 (0.1 to 0.3)	0.2 (0.1 to 0.3)	0.1 (0.1 to 0.3)	-2.4 (-3.9 to -0.7)	-29.1 (-44.5 to -9.4)
Slovenia	0.2 (0.1 to 0.3)	0.1 (0.1 to 0.1)	0.1 (0.1 to 0.1)	0.1 (0.0 to 0.1)	-3.1 (-4.5 to -1.7)	-36.9 (-49.4 to -22.4)
Eastern Europe						
Belarus	0.9 (0.7 to 1.1)	0.1 (0.1 to 0.2)	0.1 (0.1 to 0.2)	0.1 (0.1 to 0.1)	-4.8 (-6.1 to -3.5)	-51.1 (-59.8 to -40.5)
Estonia	0.4 (0.2 to 0.5)	0.1 (0.1 to 0.2)	0.1 (0.1 to 0.2)	0.1 (0.1 to 0.1)	-3.2 (-4.9 to -1.7)	-37.7 (-52.3 to -22.0)
Latvia	0.5 (0.3 to 0.6)	0.2 (0.2 to 0.3)	0.2 (0.1 to 0.3)	0.1 (0.1 to 0.2)	-3.1 (-4.6 to -1.6)	-36.5 (-50.2 to -20.8)
Lithuania	0.3 (0.2 to 0.4)	0.3 (0.2 to 0.4)	0.3 (0.2 to 0.4)	0.3 (0.2 to 0.4)	-0.8 (-2.0 to 0.5)	-10.2 (-26.2 to 8.1)
Moldova	3.2 (2.4 to 4.1)	1.0 (0.7 to 1.4)	0.9 (0.7 to 1.2)	0.5 (0.3 to 0.7)	-4.8 (-6.0 to -3.7)	-51.5 (-59.6 to -42.3)
Russia	1.2 (1.0 to 1.5)	0.4 (0.3 to 0.6)	0.4 (0.3 to 0.5)	0.2 (0.1 to 0.3)	-4.7 (-5.8 to -3.6)	-50.4 (-57.9 to -41.9)
Ukraine	0.8 (0.6 to 1.0)	0.5 (0.3 to 0.6)	0.4 (0.3 to 0.6)	0.3 (0.2 to 0.5)	-2.3 (-3.3 to -1.2)	-28.5 (-38.8 to -17.0)
High-income						
Australasia						
Australia	0.2 (0.1 to 0.3)	0.2 (0.1 to 0.3)	0.2 (0.1 to 0.3)	0.2 (0.1 to 0.3)	-0.5 (-2.1 to 1.0)	-6.0 (-26.6 to 17.0)
New Zealand	0.2 (0.1 to 0.2)	0.3 (0.2 to 0.5)	0.3 (0.2 to 0.5)	0.3 (0.2 to 0.6)	-0.1 (-1.6 to 1.4)	-1.2 (-21.9 to 23.8)
High-income Asia Pacific						
Brunei	0.7 (0.4 to 1.1)	0.7 (0.4 to 1.1)	0.7 (0.4 to 1.0)	0.7 (0.3 to 1.2)	0.0 (-2.1 to 1.8)	1.5 (-27.2 to 31.0)
Japan	0.5 (0.3 to 0.7)	0.4 (0.3 to 0.6)	0.4 (0.2 to 0.6)	0.3 (0.2 to 0.5)	-1.3 (-2.5 to -0.1)	-17.8 (-31.4 to -1.7)
South Korea	0.5 (0.3 to 0.7)	0.5 (0.3 to 0.8)	0.5 (0.3 to 0.7)	0.4 (0.2 to 0.7)	-1.0 (-2.4 to 0.5)	-13.4 (-29.7 to 7.4)
Singapore	0.7 (0.4 to 1.0)	0.5 (0.3 to 0.8)	0.5 (0.3 to 0.7)	0.4 (0.2 to 0.6)	-1.6 (-3.1 to -0.1)	-21.0 (-37.6 to -2.1)
High-income North America						
Canada	0.2 (0.1 to 0.3)	0.3 (0.2 to 0.4)	0.2 (0.2 to 0.4)	0.3 (0.1 to 0.4)	-0.4 (-2.0 to 1.2)	-5.2 (-26.5 to 19.7)
Greenland	0.8 (0.5 to 1.6)	0.7 (0.4 to 1.1)	0.7 (0.3 to 1.0)	0.6 (0.2 to 1.1)	-1.6 (-4.2 to 0.9)	-20.0 (-46.9 to 14.8)
United States	0.3 (0.2 to 0.4)	0.3 (0.2 to 0.4)	0.3 (0.2 to 0.4)	0.3 (0.2 to 0.4)	-0.5 (-1.9 to 0.9)	-7.0 (-24.3 to 13.8)
Southern Latin America						
Argentina	2.5 (1.9 to 3.1)	1.6 (1.1 to 2.2)	1.5 (1.0 to 2.1)	1.2 (0.7 to 1.8)	-2.3 (-3.8 to -0.8)	-28.5 (-43.4 to -10.8)
Chile	1.8 (1.3 to 2.3)	1.0 (0.7 to 1.4)	0.9 (0.6 to 1.4)	0.7 (0.4 to 1.1)	-2.8 (-4.4 to -1.0)	-33.4 (-48.5 to -13.6)
Uruguay	1.9 (1.3 to 2.5)	1.4 (0.8 to 2.2)	1.3 (0.8 to 2.0)	1.1 (0.5 to 2.0)	-1.9 (-3.6 to -0.0)	-23.6 (-42.0 to -0.3)
Western Europe						
Andorra	0.2 (0.1 to 0.3)	0.1 (0.1 to 0.2)	0.1 (0.1 to 0.2)	0.1 (0.1 to 0.2)	-0.5 (-2.2 to 1.2)	-6.4 (-28.0 to 19.4)
Austria	0.1 (0.0 to 0.1)	0.1 (0.1 to 0.1)	0.1 (0.1 to 0.1)	0.1 (0.1 to 0.1)	0.6 (-1.2 to 2.4)	9.9 (-16.6 to 44.1)
Belgium	0.2 (0.2 to 0.3)	0.3 (0.2 to 0.5)	0.3 (0.2 to 0.4)	0.3 (0.2 to 0.5)	-0.4 (-1.9 to 1.1)	-4.8 (-24.5 to 18.4)
Cyprus	0.2 (0.1 to 0.4)	0.2 (0.1 to 0.3)	0.2 (0.1 to 0.3)	0.2 (0.1 to 0.4)	-1.2 (-3.9 to 2.1)	-13.0 (-44.7 to 36.0)
Denmark	0.2 (0.1 to 0.2)	0.3 (0.2 to 0.4)	0.3 (0.2 to 0.4)	0.3 (0.2 to 0.5)	0.5 (-0.9 to 2.1)	8.5 (-12.9 to 37.5)
Finland	0.2 (0.1 to 0.3)	0.1 (0.0 to 0.1)	0.1 (0.0 to 0.1)	0.0 (0.0 to 0.0)	-4.6 (-5.8 to -3.2)	-49.2 (-58.4 to -38.3)
France	0.3 (0.2 to 0.4)	0.1 (0.1 to 0.2)	0.1 (0.1 to 0.2)	0.1 (0.1 to 0.1)	-3.4 (-4.8 to -2.0)	-39.9 (-51.4 to -25.8)
Germany	0.1 (0.1 to 0.1)	0.2 (0.1 to 0.3)	0.2 (0.1 to 0.3)	0.2 (0.1 to 0.4)	0.7 (-1.1 to 2.4)	11.5 (-14.6 to 42.7)
Greece	0.1 (0.1 to 0.1)	0.1 (0.1 to 0.1)	0.1 (0.1 to 0.1)	0.1 (0.1 to 0.2)	0.2 (-1.2 to 1.6)	3.3 (-16.7 to 27.2)
Iceland	0.2 (0.1 to 0.2)	0.1 (0.1 to 0.2)	0.1 (0.1 to 0.2)	0.1 (0.1 to 0.2)	0.2 (-1.3 to 1.6)	3.0 (-17.9 to 27.8)
Ireland	0.3 (0.2 to 0.4)	0.1 (0.1 to 0.2)	0.1 (0.1 to 0.2)	0.1 (0.1 to 0.2)	-2.7 (-4.2 to -1.1)	-32.5 (-46.4 to -15.7)
Israel	0.3 (0.2 to 0.4)	0.3 (0.2 to 0.5)	0.3 (0.2 to 0.5)	0.3 (0.2 to 0.6)	-0.1 (-1.7 to 1.5)	-0.2 (-22.0 to 26.0)
Italy	0.1 (0.0 to 0.1)	0.1 (0.1 to 0.2)	0.1 (0.1 to 0.1)	0.1 (0.1 to 0.2)	0.6 (-0.8 to 2.2)	10.9 (-11.4 to 38.9)
Luxembourg	0.2 (0.1 to 0.2)	0.2 (0.1 to 0.3)	0.2 (0.1 to 0.3)	0.2 (0.1 to 0.3)	-0.4 (-2.0 to 1.2)	-5.6 (-26.4 to 20.2)
Malta	0.2 (0.1 to 0.2)	0.1 (0.1 to 0.2)	0.1 (0.1 to 0.2)	0.1 (0.1 to 0.2)	-0.8 (-2.2 to 0.5)	-10.5 (-28.0 to 8.5)
Netherlands	0.2 (0.1 to 0.3)	0.1 (0.1 to 0.2)	0.1 (0.1 to 0.2)	0.1 (0.1 to 0.2)	-1.3 (-2.8 to 0.1)	-17.6 (-34.1 to 2.0)
Norway	0.2 (0.2 to 0.3)	0.3 (0.2 to 0.4)	0.2 (0.2 to 0.4)	0.2 (0.1 to 0.4)	-0.5 (-1.9 to 0.7)	-7.2 (-24.4 to 11.3)
Portugal	0.3 (0.2 to 0.4)	0.3 (0.2 to 0.4)	0.3 (0.2 to 0.4)	0.3 (0.2 to 0.5)	-0.4 (-1.7 to 1.0)	-5.0 (-23.0 to 16.6)
Spain	0.1 (0.1 to 0.2)	0.1 (0.1 to 0.2)	0.1 (0.1 to 0.2)	0.1 (0.1 to 0.2)	-1.0 (-2.5 to 0.5)	-13.7 (-31.5 to 7.0)
Sweden	0.1 (0.1 to 0.2)	0.2 (0.2 to 0.4)	0.2 (0.1 to 0.4)	0.3 (0.2 to 0.5)	0.9 (-0.7 to 2.6)	16.1 (-10.5 to 47.1)
Switzerland	0.1 (0.1 to 0.1)	0.1 (0.1 to 0.2)	0.1 (0.1 to 0.2)	0.1 (0.1 to 0.2)	-0.7 (-2.3 to 0.8)	-9.9 (-29.2 to 13.3)
United Kingdom	0.3 (0.2 to 0.4)	0.2 (0.1 to 0.3)	0.2 (0.1 to 0.3)	0.1 (0.1 to 0.2)	-1.9 (-3.2 to -0.7)	-24.7 (-38.2 to -10.0)
Latin America and Caribbean						
Andean Latin America						
Bolivia	25.2 (18.8 to 33.0)	7.5 (5.2 to 11.2)	6.9 (4.7 to 10.6)	3.6 (2.1 to 6.0)	-5.0 (-6.4 to -3.6)	-52.7 (-61.8 to -41.7)
Ecuador	9.8 (7.7 to 11.8)	3.2 (2.3 to 4.1)	3.3 (2.3 to 4.2)	1.7 (1.0 to 2.4)	-4.5 (-5.9 to -3.2)	-49.1 (-58.5 to -37.8)
Peru	11.9 (9.2 to 15.6)	5.3 (3.8 to 8.1)	5.2 (3.5 to 7.9)	3.2 (2.0 to 5.6)	-3.4 (-5.0 to -1.8)	-39.3 (-53.0 to -23.9)
Caribbean						
Antigua and Barbuda	4.2 (3.3 to 5.2)	3.1 (2.3 to 3.9)	3.0 (2.2 to 3.8)	2.5 (1.7 to 3.4)	-1.5 (-2.5 to -0.5)	-19.7 (-31.4 to -7.3)
The Bahamas	2.8 (2.2 to 3.6)	2.2 (1.6 to 2.9)	2.2 (1.6 to 2.9)	1.9 (1.3 to 2.7)	-1.0 (-1.9 to -0.0)	-13.4 (-25.3 to -0.2)
Barbados	3.2 (2.3 to 4.0)	2.5 (1.8 to 3.2)	2.5 (1.8 to 3.2)	2.1 (1.4 to 3.0)	-1.1 (-2.1 to -0.1)	-14.7 (-27.5 to -1.0)
Belize	9.3 (7.8 to 10.8)	5.9 (4.7 to 7.2)	5.8 (4.5 to 7.0)	4.4 (3.2 to 5.8)	-2.1 (-2.9 to -0.9)	-26.4 (-35.5 to -13.3)
Bermuda	1.2 (0.9 to 1.6)	0.8 (0.6 to 1.0)	0.8 (0.5 to 1.0)	0.6 (0.4 to 0.8)	-2.1 (-3.1 to -1.0)	-26.6 (-36.7 to -14.4)
Cuba	4.4 (3.6 to 5.2)	2.5 (1.9 to 3.0)	2.4 (1.8 to 2.9)	1.6 (1.1 to 2.2)	-2.9 (-4.2 to -1.5)	-35.0 (-46.7 to -19.8)
Dominica	4.6 (3.6 to 5.5)	4.0 (3.1 to 5.0)	3.9 (3.0 to 4.9)	3.9 (2.5 to 5.7)	-0.4 (-1.7 to 1.2)	-4.8 (-22.0 to 20.0)
Dominican Republic	13.1 (10.5 to 17.4)	6.9 (5.2 to 9.6)	6.5 (4.8 to 9.1)	4.4 (2.8 to 6.8)	-3.0 (-4.6 to -1.6)	-36.3 (-50.0 to -21.4)
Grenada	5.0 (3.7 to 6.3)	3.6 (2.6 to 4.8)	3.4 (2.4 to 4.5)	2.7 (1.8 to 3.9)	-2.0 (-3.0 to -1.0)	-25.5 (-35.8 to -13.5)
Guyana	21.1 (18.2 to 23.9)	11.6 (9.4 to 13.8)	10.9 (8.7 to 13.0)	7.6 (5.6 to 10.0)	-2.9 (-3.9 to -1.8)	-35.0 (-44.6 to -23.9)
Haiti	66.5 (53.5 to 80.6)	43.0 (32.2 to 55.5)	38.5 (28.5 to 51.6)	22.1 (14.3 to 33.4)	-4.5 (-6.0 to -3.1)	-48.9 (-59.3 to -37.0)

	Estimate in 2000 (95% UIs)	Estimate in 2015 (95% UIs)	Estimate in 2017 (95% UIs)	Estimate in 2030 (95% UIs)	Annualised rate of change, 2015-2030 (95% UIs)	Percent change, 2015-2030 (95% UIs)
Jamaica	3.9 (3.1 to 4.7)	2.3 (1.8 to 3.0)	2.3 (1.7 to 3.0)	1.8 (1.2 to 2.6)	-1.9 (-3.0 to -0.7)	-24.0 (-36.1 to -9.8)
Puerto Rico	1.7 (1.2 to 2.2)	1.5 (1.1 to 1.9)	1.6 (1.2 to 2.0)	1.5 (1.0 to 2.0)	-0.1 (-1.2 to 0.9)	-1.8 (-16.7 to 15.2)
Saint Lucia	4.7 (3.9 to 5.5)	2.7 (2.2 to 3.3)	2.7 (2.1 to 3.2)	1.9 (1.4 to 2.5)	-2.4 (-3.4 to -1.6)	-30.5 (-40.1 to -21.1)
Saint Vincent and the Grenadines	7.8 (6.5 to 9.2)	5.1 (4.1 to 6.2)	4.9 (3.9 to 6.0)	3.8 (2.8 to 5.0)	-2.0 (-2.8 to -1.1)	-25.3 (-34.1 to -14.8)
Suriname	17.0 (14.2 to 19.7)	8.9 (7.1 to 10.4)	8.6 (6.8 to 10.2)	6.0 (4.4 to 7.7)	-2.7 (-3.6 to -1.7)	-32.6 (-42.1 to -23.0)
Trinidad and Tobago	4.5 (3.6 to 5.4)	2.4 (1.8 to 3.0)	2.4 (1.8 to 3.1)	1.8 (1.1 to 2.5)	-2.1 (-3.5 to -0.6)	-26.1 (-40.9 to -8.9)
Virgin Islands, U.S.	1.8 (1.4 to 2.2)	1.4 (1.0 to 1.8)	1.4 (1.0 to 1.8)	1.2 (0.8 to 1.7)	-1.2 (-2.4 to -0.1)	-16.7 (-29.9 to -1.7)
Central Latin America						
Colombia	5.4 (4.2 to 6.4)	1.8 (1.2 to 2.3)	1.7 (1.2 to 2.2)	0.8 (0.5 to 1.2)	-5.1 (-6.4 to -3.7)	-53.1 (-61.8 to -42.3)
Costa Rica	3.0 (2.1 to 3.9)	1.5 (1.0 to 2.1)	1.5 (1.0 to 2.1)	1.0 (0.6 to 1.6)	-2.7 (-3.9 to -1.4)	-32.7 (-44.4 to -19.2)
El Salvador	16.0 (12.3 to 21.4)	6.7 (4.3 to 11.6)	6.4 (4.1 to 11.3)	4.2 (2.1 to 8.4)	-3.3 (-5.2 to -1.4)	-38.1 (-54.4 to -19.0)
Guatemala	57.8 (50.8 to 64.0)	23.6 (19.9 to 27.4)	23.2 (19.0 to 27.6)	12.7 (9.4 to 16.9)	-4.2 (-5.3 to -3.0)	-46.5 (-55.1 to -36.5)
Honduras	32.7 (20.9 to 47.1)	16.0 (9.3 to 25.0)	15.1 (8.6 to 24.1)	10.6 (5.0 to 18.3)	-2.9 (-4.7 to -0.9)	-34.3 (-50.8 to -12.5)
Mexico	6.8 (5.3 to 8.2)	3.0 (2.1 to 3.8)	2.9 (2.0 to 3.7)	1.6 (1.1 to 2.3)	-3.9 (-4.8 to -3.2)	-44.4 (-51.1 to -38.2)
Nicaragua	17.0 (13.9 to 21.6)	3.9 (2.6 to 6.7)	3.6 (2.5 to 6.3)	1.6 (0.8 to 3.3)	-6.3 (-8.2 to -4.1)	-60.6 (-70.9 to -45.9)
Panama	5.9 (4.8 to 7.1)	4.7 (3.5 to 5.8)	4.2 (3.1 to 5.4)	3.0 (1.9 to 4.4)	-3.0 (-4.4 to -1.4)	-35.5 (-48.2 to -19.5)
Venezuela	8.4 (5.9 to 10.4)	3.8 (2.5 to 4.9)	3.6 (2.4 to 4.9)	2.3 (1.4 to 3.4)	-3.4 (-4.8 to -1.9)	-39.6 (-51.4 to -24.9)
Tropical Latin America						
Brazil	11.6 (9.8 to 13.3)	3.7 (2.9 to 4.5)	3.6 (2.8 to 4.4)	2.1 (1.5 to 2.7)	-3.9 (-4.7 to -3.2)	-44.4 (-50.8 to -38.2)
Paraguay	12.3 (9.4 to 16.2)	3.8 (2.6 to 5.6)	3.6 (2.5 to 5.3)	1.9 (1.1 to 3.0)	-4.8 (-6.5 to -3.3)	-51.2 (-62.1 to -39.0)
North Africa and Middle East						
North Africa and Middle East						
Afghanistan	46.0 (33.4 to 59.6)	18.9 (14.1 to 24.5)	17.8 (13.0 to 23.0)	10.3 (6.8 to 15.4)	-4.1 (-5.7 to -2.5)	-45.6 (-57.2 to -30.8)
Algeria	4.1 (2.8 to 5.8)	1.3 (0.8 to 1.8)	1.1 (0.7 to 1.6)	0.6 (0.4 to 1.0)	-4.8 (-6.2 to -3.4)	-51.2 (-60.5 to -40.2)
Bahrain	2.9 (1.5 to 4.4)	1.5 (0.8 to 2.3)	1.4 (0.8 to 2.2)	0.9 (0.5 to 1.5)	-3.4 (-4.7 to -2.1)	-39.5 (-50.9 to -27.0)
Egypt	20.8 (16.0 to 26.6)	6.8 (4.4 to 9.8)	5.9 (3.8 to 8.6)	2.8 (1.4 to 4.7)	-6.2 (-8.3 to -3.8)	-59.9 (-71.2 to -43.8)
Iran	2.3 (1.7 to 2.9)	1.4 (0.9 to 2.1)	1.2 (0.8 to 1.9)	1.0 (0.6 to 1.7)	-2.4 (-3.4 to -1.4)	-30.3 (-40.3 to -19.4)
Iraq	3.3 (2.2 to 4.5)	1.5 (1.0 to 2.1)	1.2 (0.8 to 1.8)	0.6 (0.3 to 1.0)	-6.6 (-8.8 to -4.3)	-62.1 (-73.1 to -47.7)
Jordan	2.3 (1.6 to 3.0)	1.1 (0.7 to 1.5)	1.0 (0.7 to 1.5)	0.7 (0.4 to 1.1)	-3.3 (-4.6 to -1.9)	-38.6 (-49.7 to -24.3)
Kuwait	0.8 (0.5 to 1.0)	0.8 (0.5 to 1.2)	0.8 (0.5 to 1.1)	0.9 (0.5 to 1.4)	0.1 (-1.4 to 1.7)	2.0 (-19.2 to 29.7)
Lebanon	2.5 (1.6 to 3.4)	1.4 (0.9 to 2.0)	1.3 (0.8 to 1.9)	0.9 (0.5 to 1.5)	-2.8 (-4.5 to -1.1)	-33.4 (-49.2 to -14.9)
Libya	3.8 (2.5 to 5.6)	1.9 (1.2 to 2.6)	1.9 (1.2 to 2.6)	1.4 (0.8 to 2.0)	-2.1 (-3.4 to -0.8)	-27.2 (-40.1 to -11.4)
Morocco	14.1 (10.1 to 18.3)	4.3 (2.9 to 5.9)	3.8 (2.6 to 5.3)	1.8 (1.1 to 2.8)	-5.9 (-7.4 to -4.2)	-58.4 (-67.1 to -47.0)
Palestine	3.3 (2.3 to 4.3)	2.1 (1.4 to 3.1)	1.9 (1.3 to 2.9)	1.3 (0.8 to 2.2)	-3.2 (-4.5 to -1.7)	-37.6 (-48.7 to -22.3)
Oman	3.9 (2.5 to 5.6)	1.7 (1.0 to 2.5)	1.5 (0.9 to 2.3)	0.8 (0.4 to 1.5)	-4.8 (-6.6 to -2.7)	-51.1 (-63.0 to -33.7)
Qatar	1.0 (0.6 to 1.4)	0.7 (0.4 to 1.0)	0.7 (0.4 to 0.9)	0.6 (0.3 to 0.8)	-1.7 (-3.6 to 0.1)	-21.5 (-41.4 to 1.1)
Saudi Arabia	5.6 (3.8 to 7.8)	1.9 (1.2 to 2.9)	1.8 (1.0 to 2.7)	0.9 (0.5 to 1.5)	-5.6 (-7.2 to -3.6)	-56.3 (-65.9 to -42.1)
Sudan	46.5 (30.6 to 63.2)	22.3 (15.2 to 30.9)	20.5 (13.7 to 28.8)	13.2 (7.5 to 22.1)	-3.7 (-5.4 to -1.5)	-41.6 (-55.4 to -20.1)
Syria	1.7 (1.2 to 2.2)	0.8 (0.6 to 1.2)	0.8 (0.5 to 1.1)	0.6 (0.3 to 0.9)	-2.8 (-4.3 to -1.1)	-33.7 (-47.4 to -15.5)
Tunisia	2.3 (1.6 to 3.1)	1.5 (1.0 to 2.2)	1.4 (0.9 to 2.0)	1.1 (0.6 to 1.8)	-2.3 (-3.9 to -0.6)	-28.8 (-44.4 to -9.0)
Turkey	6.7 (5.0 to 8.8)	1.0 (0.7 to 1.4)	0.9 (0.6 to 1.2)	0.6 (0.3 to 0.9)	-3.9 (-6.7 to -1.6)	-43.5 (-63.2 to -21.4)
United Arab Emirates	4.1 (1.5 to 7.1)	3.1 (1.1 to 5.8)	3.0 (1.1 to 5.6)	2.6 (0.8 to 5.9)	-1.3 (-3.1 to 0.8)	-17.3 (-36.8 to 12.9)
Yemen	60.0 (41.9 to 78.3)	20.2 (13.1 to 29.6)	22.0 (15.3 to 30.9)	12.2 (7.4 to 19.9)	-3.4 (-5.0 to -1.6)	-39.5 (-52.7 to -21.2)
South Asia						
South Asia						
Bangladesh	85.9 (61.8 to 130.6)	32.1 (21.8 to 60.3)	32.1 (21.4 to 58.7)	19.6 (10.8 to 40.6)	-3.4 (-5.2 to -1.5)	-39.6 (-54.1 to -20.6)
Bhutan	76.7 (38.9 to 115.2)	23.8 (11.1 to 38.8)	22.4 (10.3 to 36.6)	11.2 (4.3 to 22.2)	-5.3 (-7.0 to -2.8)	-53.9 (-64.9 to -34.7)
India	149.7 (107.7 to 221.0)	85.1 (58.6 to 128.7)	80.5 (54.0 to 122.5)	53.2 (32.0 to 87.0)	-3.2 (-4.2 to -2.0)	-37.9 (-46.5 to -26.4)
Nepal	131.5 (82.5 to 181.5)	64.8 (36.2 to 93.7)	61.6 (34.2 to 89.7)	40.3 (19.2 to 66.0)	-3.3 (-4.8 to -1.6)	-38.6 (-51.1 to -21.2)
Pakistan	77.2 (54.1 to 115.9)	48.4 (31.3 to 82.5)	45.3 (28.8 to 78.2)	33.8 (17.4 to 65.8)	-2.6 (-4.3 to -0.5)	-31.3 (-47.2 to -7.6)
Southeast Asia, East Asia, and Oceania						
East Asia						
China	5.6 (4.7 to 6.5)	0.9 (0.7 to 1.2)	0.7 (0.5 to 1.0)	0.2 (0.1 to 0.4)	-9.3 (-10.4 to -7.6)	-75.2 (-79.1 to -68.2)
North Korea	4.2 (2.6 to 9.3)	2.5 (1.5 to 6.0)	2.3 (1.4 to 5.7)	1.8 (0.9 to 4.1)	-2.3 (-4.2 to -0.0)	-28.6 (-46.6 to -0.6)
Taiwan (Province of China)	0.4 (0.3 to 0.6)	0.5 (0.3 to 0.7)	0.5 (0.3 to 0.7)	0.5 (0.3 to 0.8)	0.3 (-0.9 to 1.6)	4.9 (-13.1 to 27.7)
Oceania						
American Samoa	4.3 (2.6 to 7.0)	3.4 (1.9 to 5.5)	3.4 (1.9 to 5.4)	2.9 (1.4 to 5.0)	-1.3 (-2.8 to 0.4)	-17.2 (-34.3 to 6.6)
Federated States of Micronesia	27.0 (21.7 to 38.0)	6.3 (3.8 to 12.3)	6.1 (3.6 to 11.9)	4.2 (2.2 to 8.0)	-2.7 (-4.0 to -1.1)	-33.4 (-45.3 to -14.9)
Fiji	15.5 (10.6 to 26.8)	13.5 (8.0 to 21.0)	12.9 (7.5 to 20.3)	11.6 (5.5 to 19.8)	-1.1 (-2.9 to 1.0)	-14.8 (-35.0 to 15.6)
Guam	1.1 (0.7 to 1.8)	0.9 (0.5 to 1.5)	0.9 (0.5 to 1.5)	0.8 (0.4 to 1.4)	-1.0 (-2.2 to 0.3)	-13.0 (-27.9 to 4.4)
Kiribati	97.7 (60.6 to 142.2)	63.6 (32.2 to 99.2)	62.6 (31.5 to 98.6)	50.4 (20.8 to 93.5)	-1.8 (-3.4 to 0.4)	-22.5 (-39.9 to 6.1)
Marshall Islands	26.5 (20.2 to 39.3)	8.4 (5.2 to 16.2)	8.0 (4.9 to 15.5)	5.0 (2.8 to 10.0)	-3.6 (-4.8 to -2.2)	-41.3 (-51.2 to -28.6)
Northern Mariana Islands	1.4 (0.8 to 2.3)	1.3 (0.7 to 2.1)	1.3 (0.7 to 2.0)	1.2 (0.6 to 2.1)	-0.4 (-1.6 to 1.3)	-4.7 (-21.8 to 21.4)
Papua New Guinea	103.8 (73.9 to 160.8)	80.3 (55.7 to 125.3)	77.8 (53.9 to 122.6)	65.0 (42.5 to 103.2)	-1.4 (-2.4 to -0.1)	-19.0 (-30.2 to -1.7)
Samoa	5.5 (3.1 to 11.1)	3.7 (1.8 to 7.9)	3.6 (1.7 to 7.6)	2.8 (1.2 to 6.1)	-1.9 (-3.5 to 0.1)	-24.2 (-40.8 to 2.0)
Solomon Islands	32.1 (21.6 to 52.8)	20.5 (13.8 to 34.6)	19.6 (12.9 to 33.9)	13.9 (8.7 to 23.7)	-2.6 (-3.6 to -1.5)	-32.1 (-42.0 to -20.5)
Tonga	8.0 (5.2 to 12.6)	4.3 (2.6 to 7.2)	4.2 (2.5 to 6.6)	2.8 (1.4 to 4.9)	-3.1 (-4.6 to -1.0)	-36.5 (-49.7 to -13.7)
Vanuatu	39.4 (23.7 to 73.6)	25.4 (14.8 to 50.0)	24.9 (14.7 to 48.7)	19.1 (10.4 to 37.1)	-1.9 (-3.2 to -0.5)	-25.0 (-37.7 to -7.3)
Southeast Asia						
Cambodia	63.6 (45.2 to 86.0)	22.8 (14.9 to 34.0)	21.9 (14.2 to 32.4)	12.7 (6.8 to 22.0)	-4.1 (-5.7 to -1.9)	-45.2 (-57.6 to -24.9)
Indonesia	60.3 (34.4 to 83.7)	40.6 (19.9 to 56.7)	39.3 (19.1 to 55.5)	29.9 (12.0 to 49.5)	-2.2 (-3.8 to -0.4)	-27.7 (-43.4 to -6.4)
Laos	65.9 (43.5 to 90.1)	35.7 (22.4 to 50.0)	33.2 (20.6 to 46.5)	23.3 (12.1 to 38.1)	-3.0 (-4.7 to -1.0)	-35.6 (-50.3 to -13.9)
Malaysia	3.2 (1.9 to 5.5)	3.4 (1.9 to 5.2)	3.2 (1.8 to 5.0)	3.2 (1.6 to 5.2)	-0.4 (-2.1 to 1.3)	-4.5 (-27.4 to 22.2)
Maldives	5.9 (4.2 to 8.8)	1.6 (1.1 to 2.5)	1.5 (1.1 to 2.4)	0.7 (0.4 to 1.1)	-5.8 (-7.1 to -4.2)	-57.9 (-65.7 to -46.5)
Mauritius	1.1 (0.8 to 1.7)	0.8 (0.5 to 1.3)	0.8 (0.5 to 1.3)	0.6 (0.3 to 1.0)	-2.7 (-4.2 to -1.2)	-33.0 (-46.6 to -16.6)
Myanmar	56.0 (39.4 to 78.8)	26.0 (18.0 to 37.6)	24.1 (16.4 to 35.2)	14.5 (8.5 to 22.9)	-4.0 (-5.5 to -2.3)	-44.6 (-56.2 to -29.4)
Philippines	15.9 (13.0 to 18.7)	13.5 (10.4 to 16.8)	12.4 (9.4 to 15.5)	10.2 (7.1 to 13.7)	-2.0 (-3.2 to -0.7)	-25.0 (-38.2 to -9.8)
Sri Lanka	11.3 (7.3 to 17.9)	4.1 (2.6 to 7.7)	3.9 (2.4 to 7.1)	2.0 (1.0 to 4.1)	-5.1 (-7.1 to -3.0)	-52.9 (-65.7 to -36.4)
Seychelles	4.6 (3.2 to 7.0)	4.2 (3.0 to 6.1)	4.0 (2.8 to 5.9)	3.6 (2.2 to 5.3)	-1.2 (-2.4 to -0.2)	-16.5 (-29.8 to -2.3)
Thailand	8.7 (5.8 to 13.6)	6.1 (4.0 to 10.0)	6.1 (4.0 to 9.8)	5.0 (2.9 to 8.5)	-1.4 (-3.1 to -0.0)	-18.5 (-37.1 to -0.5)

	Estimate in 2000 (95% UIs)	Estimate in 2015 (95% UIs)	Estimate in 2017 (95% UIs)	Estimate in 2030 (95% UIs)	Annualised rate of change, 2015-2030 (95% UIs)	Percent change, 2015-2030 (95% UIs)
Timor-Leste	39.9 (22.8 to 62.2)	21.6 (12.4 to 33.0)	20.9 (12.2 to 32.0)	15.4 (7.8 to 26.0)	-2.4 (-4.1 to -0.2)	-29.1 (-45.7 to -2.4)
Vietnam	9.2 (5.3 to 13.2)	4.1 (2.5 to 5.8)	3.9 (2.4 to 5.7)	2.5 (1.3 to 4.8)	-3.6 (-5.4 to -0.7)	-40.7 (-55.8 to -10.4)
Sub-Saharan Africa						
Central Sub-Saharan Africa						
Angola	186.5 (144.3 to 246.2)	114.0 (81.6 to 164.8)	106.2 (75.7 to 154.2)	84.4 (51.3 to 128.8)	-2.1 (-3.4 to -0.5)	-26.4 (-40.0 to -7.2)
Central African Republic	196.7 (136.1 to 265.5)	189.2 (127.4 to 253.0)	189.2 (125.8 to 251.5)	203.7 (118.8 to 303.0)	0.4 (-1.0 to 2.1)	7.0 (-14.3 to 36.2)
Congo	129.5 (88.1 to 179.2)	93.7 (59.9 to 134.2)	88.0 (55.6 to 125.5)	70.4 (38.9 to 109.3)	-2.0 (-3.3 to -0.6)	-25.5 (-38.7 to -8.1)
Democratic Republic of the Congo	126.4 (94.0 to 180.3)	90.7 (63.3 to 140.5)	87.0 (60.5 to 136.2)	74.3 (44.2 to 129.3)	-1.4 (-2.8 to 0.0)	-18.8 (-33.8 to 0.3)
Equatorial Guinea	123.2 (82.4 to 173.9)	39.0 (22.9 to 60.5)	37.8 (21.9 to 57.9)	20.6 (9.5 to 39.1)	-4.5 (-6.4 to -2.1)	-48.3 (-61.9 to -26.8)
Gabon	72.9 (47.4 to 102.5)	41.1 (25.9 to 57.8)	38.9 (24.6 to 54.8)	26.7 (14.8 to 42.5)	-3.0 (-4.2 to -1.5)	-35.7 (-46.4 to -19.7)
Eastern Sub-Saharan Africa						
Burundi	166.0 (115.8 to 227.2)	100.3 (68.2 to 144.9)	97.6 (65.7 to 142.4)	77.3 (46.6 to 120.4)	-1.8 (-2.9 to -0.5)	-23.5 (-35.4 to -7.4)
Comoros	144.8 (110.9 to 191.0)	68.5 (48.2 to 99.8)	66.7 (46.5 to 98.7)	48.0 (30.2 to 73.6)	-2.4 (-3.6 to -1.0)	-30.3 (-41.5 to -13.8)
Djibouti	103.1 (69.6 to 147.4)	48.0 (27.0 to 79.3)	46.8 (27.1 to 78.1)	32.1 (15.9 to 60.7)	-2.8 (-4.4 to -1.2)	-34.1 (-48.5 to -16.2)
Eritrea	211.2 (151.3 to 284.0)	140.7 (93.3 to 199.2)	133.3 (87.7 to 189.0)	101.7 (58.2 to 152.8)	-2.2 (-3.5 to -0.8)	-28.3 (-40.4 to -10.8)
Ethiopia	146.4 (106.2 to 197.7)	97.4 (71.0 to 138.4)	96.2 (69.1 to 137.6)	80.0 (51.7 to 118.3)	-1.4 (-2.6 to -0.3)	-18.2 (-32.1 to -4.9)
Kenya	130.1 (95.3 to 177.7)	88.0 (63.8 to 122.8)	86.0 (62.4 to 119.3)	65.5 (46.7 to 93.0)	-2.0 (-2.5 to -1.4)	-25.7 (-31.7 to -18.6)
Madagascar	146.0 (114.7 to 201.6)	129.0 (95.9 to 186.5)	126.2 (93.6 to 183.9)	121.5 (79.6 to 184.3)	-0.5 (-1.6 to 0.7)	-6.3 (-21.2 to 11.1)
Malawi	172.9 (121.1 to 244.4)	81.7 (56.4 to 120.2)	80.0 (54.5 to 118.9)	55.5 (32.2 to 90.2)	-2.7 (-4.2 to -1.1)	-32.8 (-47.1 to -15.7)
Mozambique	123.2 (82.6 to 158.1)	82.3 (47.0 to 112.5)	79.5 (45.3 to 110.0)	65.9 (30.2 to 112.5)	-1.7 (-3.3 to 0.2)	-21.6 (-38.7 to 3.3)
Rwanda	186.2 (144.2 to 242.3)	63.3 (42.4 to 98.5)	61.4 (41.1 to 94.6)	38.6 (20.8 to 66.4)	-3.4 (-5.1 to -1.7)	-39.9 (-53.6 to -22.3)
Somalia	194.8 (144.5 to 264.3)	106.9 (73.9 to 159.1)	103.4 (70.8 to 153.8)	74.9 (45.7 to 119.0)	-2.5 (-3.9 to -0.8)	-30.4 (-44.0 to -11.4)
South Sudan	165.0 (115.8 to 225.4)	156.4 (112.2 to 216.0)	153.4 (108.9 to 212.6)	152.4 (100.7 to 217.1)	-0.2 (-1.4 to 1.1)	-2.7 (-18.8 to 17.9)
Tanzania	122.6 (87.7 to 174.5)	62.5 (41.6 to 99.0)	61.2 (40.6 to 96.9)	41.9 (23.3 to 77.9)	-2.9 (-4.5 to -0.9)	-34.2 (-49.1 to -12.4)
Uganda	108.9 (74.5 to 154.2)	67.7 (46.1 to 102.2)	65.8 (44.4 to 99.5)	52.4 (31.3 to 82.3)	-1.8 (-3.0 to -0.4)	-23.1 (-36.3 to -5.8)
Zambia	183.3 (132.1 to 264.9)	88.5 (64.2 to 136.1)	86.5 (62.5 to 132.5)	61.9 (39.9 to 94.2)	-2.4 (-3.6 to -0.9)	-30.3 (-42.0 to -13.2)
Southern Sub-Saharan Africa						
Botswana	74.0 (45.8 to 111.4)	43.0 (28.9 to 60.4)	40.3 (27.2 to 57.1)	27.2 (16.4 to 41.8)	-3.1 (-4.8 to -1.3)	-36.8 (-51.4 to -17.6)
Lesotho	157.9 (107.8 to 224.5)	110.4 (75.0 to 157.3)	103.6 (70.6 to 147.4)	84.5 (51.8 to 133.3)	-1.9 (-3.1 to -0.2)	-23.9 (-37.6 to -2.5)
Namibia	90.6 (62.8 to 126.6)	51.3 (34.4 to 72.7)	48.4 (32.0 to 68.3)	33.0 (19.3 to 50.1)	-3.0 (-4.4 to -1.5)	-36.0 (-48.4 to -19.7)
South Africa	67.4 (52.6 to 95.4)	37.3 (27.7 to 57.0)	33.6 (24.6 to 51.7)	22.1 (15.2 to 37.0)	-3.6 (-4.2 to -2.7)	-41.3 (-47.0 to -33.0)
Swaziland	137.8 (94.8 to 189.4)	83.6 (55.7 to 118.4)	76.6 (51.1 to 107.7)	53.3 (31.7 to 83.2)	-3.1 (-4.4 to -1.4)	-36.6 (-48.5 to -19.3)
Zimbabwe	67.4 (45.3 to 88.6)	63.6 (43.1 to 84.5)	60.5 (41.1 to 80.9)	55.4 (34.5 to 81.5)	-1.0 (-2.1 to 0.2)	-13.5 (-27.0 to 3.7)
Western Sub-Saharan Africa						
Benin	130.7 (94.2 to 181.9)	95.4 (70.2 to 140.9)	90.6 (65.7 to 134.5)	74.4 (48.2 to 114.4)	-1.7 (-2.9 to -0.5)	-22.4 (-34.9 to -7.9)
Burkina Faso	180.0 (137.7 to 241.8)	94.5 (66.5 to 133.2)	93.3 (64.8 to 131.4)	67.4 (40.0 to 102.8)	-2.3 (-3.7 to -0.9)	-29.3 (-42.9 to -12.5)
Cameroon	153.0 (113.9 to 203.4)	78.5 (54.9 to 114.3)	75.2 (53.0 to 109.3)	50.9 (31.7 to 79.5)	-3.0 (-4.3 to -1.6)	-35.7 (-47.5 to -21.2)
Cape Verde	31.1 (23.5 to 43.3)	12.6 (9.3 to 17.4)	12.2 (9.0 to 17.1)	7.6 (5.2 to 10.6)	-3.4 (-4.4 to -2.2)	-39.3 (-48.6 to -28.2)
Chad	220.8 (167.9 to 292.7)	170.6 (131.4 to 230.0)	166.9 (126.5 to 223.4)	146.1 (100.1 to 207.9)	-1.1 (-2.1 to 0.1)	-14.6 (-27.0 to 0.9)
Cote d'Ivoire	107.0 (78.8 to 151.0)	75.3 (56.6 to 107.8)	70.8 (52.2 to 103.2)	55.8 (37.6 to 82.0)	-2.0 (-3.1 to -0.8)	-26.2 (-37.5 to -11.5)
The Gambia	106.6 (78.8 to 156.6)	59.3 (43.2 to 89.1)	57.9 (41.6 to 87.8)	42.0 (26.9 to 65.3)	-2.4 (-3.6 to -0.9)	-29.4 (-41.5 to -13.3)
Ghana	78.2 (56.2 to 102.5)	50.0 (36.9 to 73.2)	49.0 (36.0 to 72.5)	39.5 (26.0 to 63.8)	-1.7 (-2.9 to -0.2)	-21.6 (-34.9 to -3.7)
Guinea	140.3 (106.7 to 194.4)	82.3 (60.9 to 118.4)	79.8 (58.6 to 116.1)	59.3 (39.4 to 90.0)	-2.2 (-3.4 to -1.0)	-28.3 (-40.4 to -14.4)
Guinea-Bissau	172.4 (129.0 to 238.3)	128.7 (90.3 to 188.0)	124.0 (86.4 to 181.3)	103.8 (65.5 to 155.3)	-1.5 (-2.6 to -0.2)	-19.7 (-32.1 to -3.1)
Liberia	180.4 (134.8 to 250.7)	98.4 (66.7 to 150.1)	95.4 (63.9 to 146.0)	76.2 (45.1 to 125.5)	-1.8 (-3.1 to -0.3)	-23.3 (-36.8 to -4.1)
Mali	214.1 (157.2 to 294.3)	111.5 (73.6 to 175.0)	113.0 (75.1 to 175.8)	84.2 (47.9 to 139.8)	-2.0 (-3.4 to -0.4)	-25.1 (-40.1 to -6.2)
Mauritania	149.7 (105.9 to 201.8)	64.5 (44.8 to 97.2)	62.4 (43.2 to 94.4)	40.8 (25.4 to 65.3)	-3.1 (-4.4 to -1.8)	-37.2 (-48.2 to -23.4)
Niger	243.4 (184.1 to 313.2)	126.9 (94.1 to 180.8)	122.1 (89.9 to 175.0)	88.0 (57.3 to 134.1)	-2.5 (-3.6 to -1.2)	-30.9 (-42.0 to -17.0)
Nigeria	153.4 (113.6 to 204.2)	88.5 (64.3 to 123.3)	82.3 (58.8 to 114.5)	54.2 (33.3 to 86.6)	-3.4 (-4.9 to -1.4)	-39.2 (-52.0 to -18.7)
Sao Tome and Principe	72.2 (57.1 to 95.0)	24.2 (16.4 to 35.5)	23.2 (15.6 to 34.6)	13.3 (7.1 to 24.5)	-4.2 (-6.0 to -2.1)	-45.8 (-59.4 to -26.8)
Senegal	136.2 (101.2 to 184.9)	78.8 (54.7 to 117.1)	77.4 (53.5 to 116.3)	63.4 (40.1 to 99.5)	-1.5 (-2.6 to -0.3)	-20.0 (-32.2 to -4.1)
Sierra Leone	181.9 (126.9 to 235.4)	109.8 (71.0 to 155.5)	106.1 (68.0 to 150.9)	83.2 (47.9 to 134.4)	-2.0 (-3.4 to -0.5)	-25.0 (-39.7 to -7.6)
Togo	124.4 (91.3 to 168.1)	80.3 (57.3 to 118.9)	76.5 (53.9 to 113.8)	59.7 (37.5 to 94.7)	-2.0 (-3.2 to -0.9)	-26.2 (-38.0 to -12.5)
**Indicator 3.9.3: Age-standardised death rate due to unintentional poisonings (per 100,000 population)**						
Central Europe, Eastern Europe, and Central Asia						
Central Asia						
Armenia	1.0 (0.9 to 1.1)	0.6 (0.6 to 0.6)	0.6 (0.5 to 0.6)	0.4 (0.3 to 0.6)	-2.6 (-4.3 to -0.7)	-31.7 (-47.3 to -9.4)
Azerbaijan	1.1 (1.0 to 1.7)	0.9 (0.7 to 1.3)	0.8 (0.7 to 1.3)	0.6 (0.4 to 1.0)	-2.4 (-3.9 to -0.8)	-29.7 (-44.1 to -11.9)
Georgia	0.6 (0.6 to 0.6)	0.8 (0.7 to 0.8)	0.8 (0.7 to 0.9)	0.5 (0.4 to 0.7)	-2.4 (-4.1 to -0.7)	-29.5 (-45.7 to -10.3)
Kazakhstan	4.7 (4.4 to 5.0)	2.6 (2.5 to 2.8)	2.5 (2.3 to 2.7)	1.6 (1.1 to 2.2)	-3.4 (-5.6 to -1.2)	-38.7 (-56.8 to -16.5)
Kyrgyzstan	3.3 (3.1 to 3.5)	1.5 (1.4 to 1.6)	1.4 (1.3 to 1.5)	1.1 (0.8 to 1.4)	-2.3 (-4.3 to -0.3)	-28.1 (-47.5 to -4.6)
Mongolia	8.8 (5.2 to 10.5)	2.8 (2.1 to 4.8)	2.7 (2.1 to 4.7)	2.6 (1.7 to 4.7)	-0.6 (-2.1 to 1.0)	-7.3 (-27.0 to 16.9)
Tajikistan	1.8 (1.2 to 2.1)	0.7 (0.5 to 1.6)	0.7 (0.4 to 1.6)	0.5 (0.3 to 1.1)	-3.3 (-5.0 to -1.6)	-38.7 (-52.6 to -20.8)
Turkmenistan	1.2 (1.1 to 1.3)	0.6 (0.5 to 0.6)	0.6 (0.5 to 0.7)	0.4 (0.3 to 0.6)	-2.3 (-4.1 to -0.3)	-28.8 (-46.0 to -5.0)
Uzbekistan	1.5 (1.4 to 1.6)	1.1 (1.0 to 1.2)	1.1 (1.0 to 1.2)	0.7 (0.5 to 0.8)	-3.8 (-5.3 to -2.1)	-42.9 (-54.8 to -27.1)
Central Europe						
Albania	0.7 (0.5 to 0.8)	0.4 (0.3 to 0.5)	0.4 (0.3 to 0.5)	0.3 (0.2 to 0.4)	-2.3 (-4.0 to -0.7)	-28.6 (-44.8 to -10.6)
Bosnia and Herzegovina	0.9 (0.8 to 1.2)	0.5 (0.3 to 0.9)	0.5 (0.3 to 0.9)	0.4 (0.3 to 0.8)	-0.4 (-1.6 to 0.9)	-6.0 (-21.4 to 13.8)
Bulgaria	1.2 (1.1 to 1.3)	0.5 (0.4 to 0.5)	0.5 (0.4 to 0.5)	0.3 (0.2 to 0.4)	-3.1 (-5.0 to -1.0)	-36.9 (-52.9 to -13.9)
Croatia	0.6 (0.5 to 0.6)	0.3 (0.3 to 0.3)	0.3 (0.3 to 0.3)	0.2 (0.2 to 0.3)	-2.9 (-4.3 to -1.4)	-35.0 (-47.4 to -19.3)
Czech Republic	1.0 (0.9 to 1.0)	0.6 (0.5 to 0.6)	0.6 (0.5 to 0.6)	0.4 (0.3 to 0.5)	-3.1 (-4.4 to -1.6)	-36.4 (-48.7 to -21.0)
Hungary	0.6 (0.6 to 0.6)	0.4 (0.4 to 0.5)	0.4 (0.3 to 0.4)	0.2 (0.2 to 0.3)	-4.4 (-6.3 to -2.3)	-47.7 (-61.2 to -29.5)
Macedonia	0.4 (0.3 to 0.5)	0.3 (0.2 to 0.4)	0.3 (0.2 to 0.4)	0.2 (0.1 to 0.3)	-2.9 (-4.3 to -1.5)	-34.5 (-47.3 to -20.3)
Montenegro	0.9 (0.7 to 1.0)	0.6 (0.5 to 0.7)	0.6 (0.5 to 0.7)	0.4 (0.3 to 0.5)	-2.5 (-4.0 to -0.9)	-30.9 (-45.0 to -12.2)
Poland	1.1 (1.0 to 1.1)	0.5 (0.5 to 0.5)	0.5 (0.4 to 0.5)	0.2 (0.2 to 0.3)	-4.2 (-5.5 to -3.0)	-46.7 (-56.2 to -35.8)
Romania	3.4 (3.2 to 3.5)	1.3 (1.2 to 1.4)	1.3 (1.2 to 1.4)	0.7 (0.5 to 0.9)	-4.4 (-6.4 to -2.2)	-48.0 (-61.8 to -28.0)
Serbia	0.6 (0.5 to 0.7)	0.3 (0.3 to 0.5)	0.3 (0.3 to 0.5)	0.3 (0.2 to 0.4)	-2.0 (-3.7 to -0.1)	-25.7 (-42.9 to -2.1)
Slovakia	0.7 (0.6 to 0.9)	0.5 (0.5 to 0.7)	0.5 (0.4 to 0.6)	0.3 (0.2 to 0.5)	-3.2 (-4.7 to -1.6)	-38.0 (-50.6 to -21.9)
Slovenia	0.5 (0.5 to 0.6)	0.2 (0.2 to 0.2)	0.2 (0.2 to 0.2)	0.1 (0.1 to 0.1)	-3.5 (-5.0 to -1.9)	-40.0 (-52.6 to -24.6)
Eastern Europe						

	Estimate in 2000 (95% UIs)	Estimate in 2015 (95% UIs)	Estimate in 2017 (95% UIs)	Estimate in 2030 (95% UIs)	Annualised rate of change, 2015-2030 (95% UIs)	Percent change, 2015-2030 (95% UIs)
Belarus	6.6 (6.3 to 7.0)	2.5 (2.2 to 2.7)	2.3 (2.1 to 2.6)	1.4 (1.0 to 1.9)	-3.8 (-5.7 to -1.9)	-42.9 (-57.4 to -24.4)
Estonia	1.7 (1.5 to 1.8)	0.5 (0.4 to 0.5)	0.5 (0.4 to 0.6)	0.3 (0.2 to 0.5)	-2.4 (-5.3 to 0.5)	-28.9 (-54.7 to 7.8)
Latvia	2.7 (2.5 to 2.9)	0.8 (0.8 to 0.9)	0.9 (0.8 to 1.0)	0.6 (0.4 to 0.9)	-2.4 (-5.3 to 0.7)	-28.2 (-54.6 to 11.7)
Lithuania	3.8 (3.6 to 4.0)	2.5 (2.4 to 2.7)	2.4 (2.2 to 2.7)	1.9 (1.3 to 2.6)	-2.1 (-4.2 to 0.3)	-25.4 (-46.6 to 4.0)
Moldova	5.5 (5.3 to 5.9)	4.3 (4.1 to 4.5)	3.9 (3.6 to 4.1)	4.2 (3.1 to 5.7)	-0.2 (-2.1 to 1.8)	-2.3 (-26.6 to 31.3)
Russia	7.7 (7.5 to 7.8)	3.8 (3.7 to 3.9)	3.5 (3.4 to 3.6)	2.1 (1.4 to 3.1)	-3.9 (-6.6 to -1.3)	-43.4 (-62.6 to -17.7)
Ukraine	6.3 (6.1 to 6.6)	2.8 (2.6 to 3.0)	2.5 (2.4 to 2.8)	1.9 (1.2 to 2.7)	-2.9 (-5.6 to -0.3)	-33.8 (-56.6 to -4.5)
High-income						
Australasia						
Australia	0.3 (0.3 to 0.3)	0.2 (0.2 to 0.2)	0.2 (0.2 to 0.2)	0.1 (0.1 to 0.1)	-3.6 (-4.9 to -2.4)	-41.3 (-51.8 to -29.8)
New Zealand	0.2 (0.2 to 0.2)	0.2 (0.2 to 0.2)	0.2 (0.2 to 0.2)	0.1 (0.1 to 0.1)	-4.4 (-5.4 to -3.4)	-48.3 (-55.8 to -39.9)
High-income Asia Pacific						
Brunei	0.6 (0.5 to 0.7)	0.5 (0.4 to 0.7)	0.5 (0.4 to 0.7)	0.3 (0.2 to 0.4)	-2.9 (-4.2 to -1.3)	-35.1 (-46.4 to -18.1)
Japan	0.3 (0.3 to 0.3)	0.2 (0.2 to 0.2)	0.2 (0.2 to 0.2)	0.1 (0.1 to 0.2)	-3.6 (-4.7 to -2.3)	-41.4 (-50.9 to -29.0)
South Korea	0.9 (0.9 to 0.9)	0.3 (0.3 to 0.3)	0.3 (0.3 to 0.3)	0.2 (0.2 to 0.2)	-3.0 (-4.1 to -1.8)	-35.6 (-45.8 to -23.3)
Singapore	0.1 (0.1 to 0.1)	0.1 (0.1 to 0.1)	0.1 (0.1 to 0.1)	0.0 (0.0 to 0.0)	-2.9 (-4.2 to -1.7)	-35.3 (-46.7 to -23.0)
High-income North America						
Canada	0.4 (0.4 to 0.4)	0.3 (0.3 to 0.4)	0.3 (0.3 to 0.3)	0.2 (0.2 to 0.3)	-3.0 (-4.0 to -1.8)	-36.1 (-45.5 to -23.5)
Greenland	1.7 (1.4 to 2.0)	1.2 (1.0 to 1.4)	1.1 (0.9 to 1.4)	0.8 (0.6 to 1.2)	-2.1 (-3.8 to -0.2)	-26.6 (-43.5 to -2.8)
United States	0.4 (0.4 to 0.4)	0.5 (0.5 to 0.5)	0.5 (0.5 to 0.5)	0.3 (0.2 to 0.4)	-3.3 (-4.8 to -1.9)	-39.1 (-51.0 to -25.1)
Southern Latin America						
Argentina	1.0 (0.9 to 1.0)	0.8 (0.7 to 0.8)	0.8 (0.7 to 0.8)	0.5 (0.4 to 0.6)	-2.6 (-3.6 to -1.5)	-32.2 (-42.0 to -20.5)
Chile	0.5 (0.5 to 0.6)	0.3 (0.3 to 0.3)	0.3 (0.2 to 0.3)	0.2 (0.2 to 0.3)	-1.9 (-3.1 to -0.6)	-24.5 (-37.1 to -8.4)
Uruguay	0.8 (0.7 to 0.8)	0.6 (0.5 to 0.6)	0.6 (0.5 to 0.6)	0.4 (0.3 to 0.5)	-2.1 (-3.3 to -0.8)	-26.4 (-39.1 to -11.3)
Western Europe						
Andorra	0.3 (0.2 to 0.3)	0.2 (0.2 to 0.3)	0.2 (0.2 to 0.3)	0.1 (0.1 to 0.2)	-2.8 (-3.7 to -1.8)	-33.7 (-42.5 to -23.4)
Austria	0.2 (0.2 to 0.2)	0.3 (0.3 to 0.3)	0.3 (0.2 to 0.3)	0.1 (0.1 to 0.2)	-4.3 (-5.5 to -3.2)	-47.7 (-56.2 to -37.8)
Belgium	0.5 (0.5 to 0.5)	0.3 (0.3 to 0.3)	0.3 (0.2 to 0.3)	0.2 (0.2 to 0.2)	-2.6 (-3.6 to -1.4)	-31.6 (-41.6 to -19.2)
Cyprus	0.3 (0.2 to 0.3)	0.2 (0.1 to 0.2)	0.2 (0.1 to 0.2)	0.1 (0.1 to 0.1)	-3.3 (-4.7 to -1.8)	-38.8 (-50.3 to -23.8)
Denmark	0.3 (0.3 to 0.3)	0.2 (0.1 to 0.2)	0.2 (0.1 to 0.2)	0.1 (0.1 to 0.1)	-2.8 (-4.3 to -1.1)	-33.9 (-47.7 to -15.2)
Finland	0.5 (0.5 to 0.5)	0.2 (0.2 to 0.2)	0.2 (0.2 to 0.2)	0.2 (0.1 to 0.2)	-1.2 (-2.7 to 0.2)	-15.9 (-32.9 to 3.7)
France	0.3 (0.3 to 0.4)	0.3 (0.2 to 0.3)	0.2 (0.2 to 0.3)	0.1 (0.1 to 0.2)	-3.7 (-4.6 to -2.6)	-42.0 (-50.2 to -32.2)
Germany	0.1 (0.1 to 0.1)	0.1 (0.1 to 0.1)	0.1 (0.1 to 0.1)	0.1 (0.1 to 0.1)	-3.9 (-5.1 to -2.6)	-44.1 (-53.5 to -31.9)
Greece	0.3 (0.3 to 0.3)	0.1 (0.1 to 0.2)	0.2 (0.1 to 0.2)	0.1 (0.1 to 0.1)	-2.4 (-3.4 to -1.2)	-29.6 (-40.3 to -17.0)
Iceland	0.3 (0.3 to 0.3)	0.3 (0.2 to 0.3)	0.3 (0.2 to 0.3)	0.2 (0.1 to 0.2)	-2.0 (-3.4 to -0.4)	-24.9 (-40.2 to -5.3)
Ireland	0.3 (0.3 to 0.4)	0.2 (0.2 to 0.2)	0.2 (0.2 to 0.2)	0.1 (0.1 to 0.1)	-3.2 (-4.4 to -2.0)	-37.9 (-47.9 to -25.4)
Israel	0.2 (0.2 to 0.2)	0.1 (0.1 to 0.1)	0.1 (0.1 to 0.1)	0.1 (0.1 to 0.1)	-4.1 (-5.4 to -2.6)	-45.7 (-55.7 to -32.8)
Italy	0.2 (0.2 to 0.3)	0.1 (0.1 to 0.1)	0.1 (0.1 to 0.1)	0.1 (0.1 to 0.1)	-2.4 (-3.3 to -1.5)	-30.2 (-39.0 to -20.2)
Luxembourg	0.2 (0.2 to 0.3)	0.1 (0.1 to 0.2)	0.1 (0.1 to 0.2)	0.1 (0.1 to 0.1)	-2.1 (-3.7 to -0.5)	-26.2 (-42.2 to -6.7)
Malta	0.2 (0.1 to 0.2)	0.1 (0.1 to 0.1)	0.1 (0.1 to 0.1)	0.1 (0.1 to 0.1)	-3.6 (-4.8 to -2.1)	-41.0 (-51.6 to -26.7)
Netherlands	0.1 (0.1 to 0.1)	0.1 (0.1 to 0.1)	0.1 (0.1 to 0.1)	0.1 (0.1 to 0.1)	-3.2 (-4.1 to -2.3)	-38.1 (-46.2 to -28.9)
Norway	0.5 (0.5 to 0.6)	0.9 (0.8 to 1.0)	0.8 (0.8 to 0.9)	0.5 (0.4 to 0.5)	-4.4 (-5.4 to -3.4)	-47.9 (-55.3 to -39.9)
Portugal	0.4 (0.4 to 0.4)	0.2 (0.2 to 0.2)	0.2 (0.2 to 0.2)	0.1 (0.1 to 0.1)	-3.4 (-4.7 to -2.2)	-39.7 (-50.4 to -27.6)
Spain	0.3 (0.3 to 0.3)	0.2 (0.2 to 0.2)	0.2 (0.2 to 0.2)	0.1 (0.1 to 0.1)	-3.6 (-4.8 to -2.1)	-41.3 (-51.0 to -27.1)
Sweden	0.3 (0.3 to 0.3)	0.3 (0.3 to 0.3)	0.3 (0.3 to 0.3)	0.2 (0.1 to 0.2)	-4.1 (-5.2 to -2.9)	-45.9 (-54.4 to -35.1)
Switzerland	0.1 (0.1 to 0.2)	0.1 (0.1 to 0.1)	0.1 (0.1 to 0.1)	0.1 (0.1 to 0.1)	-3.9 (-5.2 to -2.4)	-43.8 (-54.3 to -30.4)
United Kingdom	0.4 (0.4 to 0.4)	0.2 (0.2 to 0.2)	0.2 (0.2 to 0.2)	0.1 (0.1 to 0.1)	-3.2 (-4.2 to -2.2)	-38.1 (-46.6 to -28.0)
Latin America and Caribbean						
Andean Latin America						
Bolivia	1.5 (0.9 to 1.9)	0.9 (0.7 to 1.2)	0.8 (0.6 to 1.1)	0.6 (0.4 to 0.8)	-3.3 (-4.1 to -2.3)	-38.5 (-45.8 to -29.6)
Ecuador	0.9 (0.9 to 1.0)	0.7 (0.6 to 0.7)	0.6 (0.6 to 0.7)	0.4 (0.4 to 0.5)	-2.6 (-3.5 to -1.8)	-32.6 (-41.2 to -23.1)
Peru	1.2 (0.9 to 1.4)	0.7 (0.6 to 0.9)	0.7 (0.5 to 0.9)	0.4 (0.3 to 0.6)	-3.2 (-4.6 to -1.8)	-38.2 (-50.2 to -24.2)
Caribbean						
Antigua and Barbuda	0.6 (0.6 to 0.7)	0.5 (0.4 to 0.6)	0.5 (0.4 to 0.6)	0.4 (0.3 to 0.5)	-1.7 (-2.9 to -0.4)	-22.1 (-34.9 to -6.1)
The Bahamas	0.4 (0.4 to 0.4)	0.3 (0.3 to 0.3)	0.3 (0.3 to 0.3)	0.2 (0.2 to 0.3)	-1.7 (-2.9 to -0.4)	-22.3 (-34.9 to -5.3)
Barbados	0.3 (0.2 to 0.3)	0.2 (0.2 to 0.2)	0.2 (0.2 to 0.2)	0.1 (0.1 to 0.2)	-1.6 (-2.6 to -0.7)	-21.5 (-31.9 to -9.6)
Belize	1.1 (1.0 to 1.2)	0.6 (0.6 to 0.7)	0.6 (0.6 to 0.7)	0.5 (0.4 to 0.6)	-2.0 (-3.1 to -0.7)	-26.0 (-37.7 to -10.6)
Bermuda	0.4 (0.3 to 0.4)	0.2 (0.2 to 0.2)	0.2 (0.2 to 0.2)	0.1 (0.1 to 0.2)	-2.2 (-4.9 to 1.1)	-26.2 (-52.1 to 17.8)
Cuba	0.4 (0.3 to 0.4)	0.2 (0.2 to 0.2)	0.2 (0.2 to 0.2)	0.2 (0.1 to 0.2)	-2.5 (-3.5 to -1.4)	-30.6 (-40.4 to -18.9)
Dominica	1.7 (1.6 to 1.9)	1.4 (1.2 to 1.5)	1.4 (1.2 to 1.6)	1.1 (0.9 to 1.4)	-1.6 (-2.7 to -0.3)	-20.6 (-33.2 to -4.9)
Dominican Republic	0.4 (0.4 to 0.7)	0.4 (0.3 to 0.6)	0.4 (0.3 to 0.6)	0.3 (0.2 to 0.5)	-2.2 (-3.3 to -1.1)	-28.1 (-39.4 to -15.4)
Grenada	0.7 (0.7 to 0.8)	0.5 (0.4 to 0.5)	0.5 (0.4 to 0.5)	0.3 (0.3 to 0.4)	-2.0 (-2.9 to -1.0)	-25.3 (-34.9 to -14.5)
Guyana	0.8 (0.7 to 0.8)	0.5 (0.5 to 0.6)	0.5 (0.5 to 0.6)	0.4 (0.3 to 0.5)	-2.4 (-3.8 to -1.0)	-30.1 (-43.5 to -13.8)
Haiti	1.5 (0.5 to 2.2)	1.0 (0.4 to 1.4)	0.9 (0.4 to 1.3)	0.6 (0.3 to 0.9)	-2.8 (-3.8 to -1.8)	-33.9 (-43.4 to -23.6)
Jamaica	0.3 (0.3 to 0.3)	0.3 (0.2 to 0.3)	0.3 (0.2 to 0.3)	0.2 (0.2 to 0.3)	-2.3 (-3.4 to -1.2)	-29.3 (-40.3 to -16.6)
Puerto Rico	0.3 (0.3 to 0.3)	0.2 (0.2 to 0.2)	0.2 (0.2 to 0.2)	0.1 (0.1 to 0.2)	-1.9 (-3.3 to -0.4)	-24.8 (-38.8 to -6.3)
Saint Lucia	0.4 (0.3 to 0.4)	0.2 (0.2 to 0.3)	0.2 (0.2 to 0.3)	0.2 (0.2 to 0.2)	-1.9 (-2.8 to -1.0)	-25.1 (-34.7 to -13.4)
Saint Vincent and the Grenadines	0.3 (0.3 to 0.4)	0.2 (0.2 to 0.3)	0.2 (0.2 to 0.3)	0.2 (0.2 to 0.2)	-1.6 (-2.6 to -0.4)	-20.7 (-31.8 to -5.9)
Suriname	0.6 (0.6 to 0.7)	0.5 (0.4 to 0.5)	0.5 (0.4 to 0.5)	0.3 (0.3 to 0.4)	-1.7 (-2.5 to -0.8)	-22.5 (-31.4 to -11.6)
Trinidad and Tobago	0.3 (0.3 to 0.3)	0.2 (0.2 to 0.2)	0.2 (0.2 to 0.2)	0.2 (0.1 to 0.2)	-1.1 (-2.3 to 0.1)	-15.3 (-28.9 to 1.3)
Virgin Islands, U.S.	0.5 (0.5 to 0.6)	0.4 (0.3 to 0.4)	0.4 (0.3 to 0.4)	0.2 (0.2 to 0.3)	-2.9 (-4.1 to -1.6)	-35.3 (-45.7 to -21.1)
Central Latin America						
Colombia	0.3 (0.3 to 0.3)	0.2 (0.2 to 0.2)	0.2 (0.2 to 0.2)	0.1 (0.1 to 0.2)	-2.4 (-3.7 to -1.0)	-29.7 (-42.4 to -14.0)
Costa Rica	0.3 (0.3 to 0.3)	0.3 (0.2 to 0.3)	0.3 (0.2 to 0.3)	0.2 (0.2 to 0.2)	-2.5 (-3.5 to -1.4)	-30.7 (-40.7 to -19.2)
El Salvador	0.4 (0.2 to 0.5)	0.2 (0.2 to 0.3)	0.2 (0.2 to 0.3)	0.2 (0.1 to 0.2)	-2.1 (-3.2 to -1.0)	-27.0 (-38.5 to -13.6)
Guatemala	1.3 (1.2 to 1.4)	1.5 (1.4 to 1.6)	1.5 (1.3 to 1.7)	0.9 (0.8 to 1.1)	-3.4 (-4.6 to -2.2)	-39.4 (-49.5 to -27.6)
Honduras	1.6 (1.0 to 2.2)	1.1 (0.7 to 1.6)	1.1 (0.7 to 1.6)	0.7 (0.4 to 1.1)	-3.0 (-3.8 to -2.1)	-36.5 (-43.8 to -27.5)
Mexico	0.8 (0.8 to 0.8)	0.6 (0.6 to 0.6)	0.6 (0.5 to 0.6)	0.4 (0.4 to 0.5)	-1.9 (-2.8 to -1.0)	-25.0 (-34.0 to -13.3)
Nicaragua	0.7 (0.5 to 0.8)	0.4 (0.3 to 0.6)	0.4 (0.3 to 0.5)	0.2 (0.2 to 0.3)	-3.2 (-4.3 to -2.0)	-37.8 (-47.2 to -25.7)
Panama	0.6 (0.5 to 0.6)	0.3 (0.3 to 0.4)	0.3 (0.3 to 0.4)	0.2 (0.2 to 0.3)	-2.3 (-3.2 to -1.3)	-28.8 (-38.5 to -17.8)

	Estimate in 2000 (95% UIs)	Estimate in 2015 (95% UIs)	Estimate in 2017 (95% UIs)	Estimate in 2030 (95% UIs)	Annualised rate of change, 2015-2030 (95% UIs)	Percent change, 2015-2030 (95% UIs)
Venezuela	0.5 (0.5 to 0.5)	0.4 (0.3 to 0.4)	0.3 (0.3 to 0.4)	0.3 (0.2 to 0.3)	-1.9 (-3.3 to -0.4)	-24.2 (-39.3 to -5.5)
Tropical Latin America						
Brazil	0.3 (0.3 to 0.3)	0.2 (0.2 to 0.2)	0.2 (0.2 to 0.2)	0.1 (0.1 to 0.1)	-2.7 (-3.6 to -1.8)	-32.9 (-41.9 to -24.0)
Paraguay	0.4 (0.3 to 0.5)	0.2 (0.2 to 0.4)	0.2 (0.2 to 0.4)	0.2 (0.1 to 0.3)	-2.1 (-3.4 to -0.8)	-26.2 (-39.6 to -10.7)
North Africa and Middle East						
North Africa and Middle East						
Afghanistan	1.8 (1.1 to 2.4)	1.4 (0.8 to 1.9)	1.3 (0.8 to 1.8)	0.8 (0.5 to 1.2)	-3.4 (-4.2 to -2.5)	-39.7 (-46.7 to -31.3)
Algeria	1.3 (0.5 to 1.6)	0.9 (0.3 to 1.3)	0.9 (0.3 to 1.3)	0.6 (0.2 to 0.8)	-3.4 (-4.1 to -2.6)	-39.5 (-45.6 to -32.5)
Bahrain	0.2 (0.2 to 0.3)	0.2 (0.1 to 0.2)	0.1 (0.1 to 0.2)	0.1 (0.1 to 0.2)	-2.3 (-3.6 to -0.9)	-28.6 (-41.4 to -12.9)
Egypt	0.3 (0.2 to 0.4)	0.3 (0.2 to 0.4)	0.3 (0.1 to 0.4)	0.2 (0.1 to 0.2)	-3.6 (-4.8 to -2.5)	-41.6 (-51.0 to -31.5)
Iran	1.9 (1.4 to 2.2)	1.2 (1.0 to 1.4)	1.2 (0.9 to 1.3)	0.8 (0.6 to 1.0)	-2.6 (-3.6 to -1.6)	-31.6 (-42.0 to -21.1)
Iraq	0.6 (0.4 to 0.8)	0.3 (0.2 to 0.3)	0.3 (0.2 to 0.3)	0.2 (0.1 to 0.2)	-4.0 (-4.7 to -3.2)	-44.9 (-50.9 to -37.7)
Jordan	1.4 (0.9 to 1.6)	0.7 (0.5 to 0.8)	0.7 (0.5 to 0.8)	0.5 (0.4 to 0.6)	-2.3 (-3.2 to -1.4)	-29.2 (-37.8 to -18.8)
Kuwait	0.7 (0.6 to 0.7)	0.4 (0.4 to 0.4)	0.4 (0.3 to 0.4)	0.3 (0.2 to 0.4)	-2.8 (-5.0 to -0.3)	-33.4 (-52.9 to -4.2)
Lebanon	0.9 (0.3 to 1.3)	0.7 (0.2 to 1.1)	0.7 (0.2 to 1.0)	0.4 (0.1 to 0.7)	-2.7 (-3.7 to -1.6)	-33.3 (-43.0 to -21.8)
Libya	1.2 (0.4 to 1.7)	1.1 (0.4 to 1.5)	1.1 (0.4 to 1.5)	0.8 (0.3 to 1.2)	-1.8 (-2.6 to -0.6)	-23.1 (-31.9 to -9.2)
Morocco	1.5 (0.5 to 2.0)	1.2 (0.4 to 1.6)	1.1 (0.4 to 1.6)	0.7 (0.2 to 1.0)	-3.4 (-4.0 to -2.7)	-39.6 (-45.1 to -33.1)
Palestine	0.3 (0.2 to 0.5)	0.2 (0.1 to 0.3)	0.2 (0.1 to 0.3)	0.1 (0.1 to 0.2)	-2.1 (-3.6 to -0.2)	-26.9 (-41.7 to -3.1)
Oman	0.8 (0.2 to 1.2)	0.6 (0.2 to 1.0)	0.6 (0.2 to 1.0)	0.4 (0.1 to 0.8)	-2.5 (-3.9 to -1.0)	-30.5 (-44.6 to -13.8)
Qatar	0.3 (0.2 to 0.4)	0.3 (0.2 to 0.4)	0.3 (0.2 to 0.4)	0.2 (0.1 to 0.3)	-3.6 (-5.1 to -1.9)	-41.4 (-53.7 to -24.6)
Saudi Arabia	1.0 (0.7 to 1.2)	0.8 (0.5 to 1.1)	0.8 (0.5 to 1.1)	0.4 (0.2 to 0.6)	-4.3 (-5.7 to -3.1)	-47.7 (-57.5 to -37.2)
Sudan	2.1 (0.7 to 3.5)	1.5 (0.5 to 2.1)	1.4 (0.5 to 2.0)	0.8 (0.3 to 1.1)	-4.4 (-5.3 to -3.4)	-48.0 (-55.1 to -40.1)
Syria	0.7 (0.6 to 0.8)	0.5 (0.4 to 0.6)	0.5 (0.4 to 0.6)	0.3 (0.2 to 0.4)	-3.0 (-3.9 to -2.2)	-36.4 (-44.6 to -27.7)
Tunisia	1.0 (0.3 to 1.4)	0.7 (0.2 to 1.1)	0.7 (0.2 to 1.1)	0.4 (0.1 to 0.7)	-3.0 (-3.8 to -2.2)	-36.1 (-43.4 to -28.3)
Turkey	0.5 (0.4 to 0.8)	0.6 (0.3 to 0.7)	0.5 (0.3 to 0.6)	0.3 (0.2 to 0.5)	-3.4 (-4.7 to -2.0)	-39.5 (-50.8 to -25.8)
United Arab Emirates	0.2 (0.1 to 0.3)	0.2 (0.1 to 0.3)	0.2 (0.1 to 0.3)	0.1 (0.1 to 0.2)	-4.4 (-5.4 to -3.3)	-48.3 (-55.6 to -39.3)
Yemen	2.1 (0.7 to 3.4)	1.6 (0.5 to 2.4)	1.6 (0.5 to 2.5)	1.0 (0.3 to 1.6)	-2.9 (-3.7 to -2.1)	-35.3 (-42.5 to -27.4)
South Asia						
South Asia						
Bangladesh	0.3 (0.3 to 0.6)	0.4 (0.2 to 0.5)	0.3 (0.2 to 0.5)	0.2 (0.1 to 0.2)	-4.9 (-5.8 to -3.9)	-51.7 (-57.9 to -44.6)
Bhutan	0.6 (0.4 to 0.7)	0.3 (0.2 to 0.5)	0.3 (0.2 to 0.5)	0.2 (0.1 to 0.3)	-4.1 (-4.9 to -3.1)	-45.7 (-52.1 to -37.3)
India	0.6 (0.4 to 0.8)	0.4 (0.3 to 0.5)	0.4 (0.3 to 0.5)	0.2 (0.2 to 0.3)	-4.4 (-5.2 to -3.6)	-48.4 (-54.3 to -41.8)
Nepal	0.4 (0.3 to 0.6)	0.3 (0.2 to 0.5)	0.3 (0.2 to 0.5)	0.2 (0.1 to 0.3)	-4.0 (-4.7 to -3.2)	-45.0 (-50.8 to -37.9)
Pakistan	0.9 (0.7 to 1.1)	0.6 (0.4 to 0.7)	0.5 (0.4 to 0.7)	0.3 (0.2 to 0.4)	-4.3 (-5.5 to -3.1)	-47.1 (-56.2 to -36.9)
Southeast Asia, East Asia, and Oceania						
East Asia						
China	1.9 (1.6 to 2.4)	1.8 (1.0 to 2.0)	1.6 (0.9 to 1.9)	1.0 (0.5 to 1.2)	-4.0 (-4.8 to -3.1)	-44.6 (-51.6 to -37.1)
North Korea	1.8 (1.2 to 2.4)	2.0 (1.3 to 2.4)	1.9 (1.3 to 2.4)	1.4 (1.0 to 1.9)	-2.0 (-2.7 to -1.3)	-26.0 (-33.2 to -17.5)
Taiwan (Province of China)	1.1 (1.1 to 1.2)	0.3 (0.3 to 0.3)	0.3 (0.3 to 0.3)	0.2 (0.1 to 0.2)	-3.1 (-4.1 to -2.2)	-37.4 (-46.2 to -27.6)
Oceania						
American Samoa	0.8 (0.7 to 1.1)	0.8 (0.6 to 1.0)	0.8 (0.6 to 1.0)	0.5 (0.4 to 0.6)	-2.9 (-3.6 to -2.2)	-35.3 (-41.8 to -28.5)
Federated States of Micronesia	1.2 (0.8 to 1.6)	1.1 (0.7 to 1.5)	1.1 (0.7 to 1.5)	0.8 (0.5 to 1.1)	-2.5 (-3.3 to -1.6)	-30.8 (-39.1 to -21.3)
Fiji	0.7 (0.5 to 0.8)	0.5 (0.4 to 0.6)	0.5 (0.4 to 0.6)	0.4 (0.3 to 0.5)	-1.8 (-3.1 to -0.2)	-23.1 (-37.5 to -3.2)
Guam	0.3 (0.3 to 0.4)	0.4 (0.3 to 0.5)	0.4 (0.3 to 0.5)	0.2 (0.1 to 0.3)	-4.1 (-5.5 to -2.7)	-45.3 (-56.3 to -33.1)
Kiribati	2.0 (1.3 to 2.4)	2.0 (1.0 to 2.5)	1.9 (1.0 to 2.6)	1.2 (0.6 to 1.7)	-3.1 (-3.9 to -2.2)	-37.0 (-44.5 to -28.3)
Marshall Islands	1.5 (0.9 to 1.9)	1.3 (0.8 to 1.7)	1.3 (0.8 to 1.6)	0.8 (0.5 to 1.1)	-3.1 (-3.9 to -2.3)	-36.9 (-44.2 to -28.7)
Northern Mariana Islands	0.3 (0.2 to 0.4)	0.3 (0.3 to 0.4)	0.3 (0.3 to 0.4)	0.2 (0.2 to 0.3)	-2.5 (-3.4 to -1.6)	-30.8 (-40.2 to -21.5)
Papua New Guinea	1.8 (0.8 to 3.0)	1.7 (0.8 to 2.8)	1.7 (0.8 to 2.8)	1.1 (0.5 to 1.8)	-3.0 (-3.7 to -2.3)	-36.6 (-43.0 to -28.9)
Samoa	0.9 (0.6 to 1.3)	0.8 (0.5 to 1.1)	0.7 (0.5 to 1.1)	0.5 (0.3 to 0.8)	-2.7 (-3.4 to -2.0)	-32.8 (-39.8 to -26.0)
Solomon Islands	1.4 (0.8 to 1.9)	1.3 (0.8 to 1.8)	1.2 (0.7 to 1.8)	0.8 (0.5 to 1.1)	-3.2 (-3.9 to -2.5)	-37.9 (-44.1 to -31.2)
Tonga	1.7 (1.4 to 2.0)	1.6 (1.2 to 1.9)	1.6 (1.2 to 1.9)	1.1 (0.8 to 1.3)	-2.8 (-3.4 to -2.2)	-33.9 (-40.0 to -27.6)
Vanuatu	1.4 (0.7 to 1.9)	1.3 (0.7 to 1.8)	1.3 (0.7 to 1.8)	0.8 (0.4 to 1.2)	-2.9 (-3.6 to -2.2)	-34.8 (-41.4 to -28.0)
Southeast Asia						
Cambodia	0.8 (0.4 to 1.0)	0.7 (0.4 to 0.8)	0.6 (0.4 to 0.8)	0.4 (0.3 to 0.5)	-3.3 (-4.0 to -2.6)	-38.7 (-44.9 to -31.9)
Indonesia	0.4 (0.3 to 0.5)	0.4 (0.2 to 0.5)	0.4 (0.2 to 0.5)	0.3 (0.1 to 0.3)	-3.1 (-3.9 to -2.5)	-37.6 (-44.0 to -31.0)
Laos	1.1 (0.8 to 1.5)	0.7 (0.5 to 0.9)	0.7 (0.4 to 0.9)	0.4 (0.3 to 0.5)	-3.7 (-4.5 to -2.9)	-42.3 (-48.7 to -35.3)
Malaysia	1.1 (0.8 to 1.2)	0.8 (0.6 to 0.9)	0.8 (0.6 to 0.9)	0.5 (0.4 to 0.6)	-3.2 (-4.4 to -2.1)	-38.3 (-48.5 to -27.3)
Maldives	0.1 (0.1 to 0.2)	0.1 (0.1 to 0.1)	0.1 (0.1 to 0.1)	0.1 (0.0 to 0.1)	-2.4 (-3.9 to -0.7)	-29.5 (-44.3 to -10.6)
Mauritius	0.1 (0.1 to 0.1)	0.3 (0.3 to 0.3)	0.3 (0.2 to 0.3)	0.2 (0.2 to 0.2)	-2.3 (-3.3 to -1.4)	-29.3 (-38.6 to -18.8)
Myanmar	2.9 (1.6 to 3.8)	1.9 (1.0 to 2.5)	1.9 (0.9 to 2.4)	1.2 (0.6 to 1.6)	-3.3 (-4.0 to -2.6)	-38.6 (-44.9 to -31.8)
Philippines	0.4 (0.4 to 0.4)	0.3 (0.3 to 0.3)	0.3 (0.2 to 0.3)	0.2 (0.2 to 0.2)	-2.7 (-3.6 to -1.9)	-33.1 (-41.4 to -24.3)
Sri Lanka	0.7 (0.6 to 1.0)	0.4 (0.3 to 0.6)	0.3 (0.3 to 0.6)	0.2 (0.2 to 0.4)	-2.6 (-3.9 to -1.2)	-32.2 (-44.6 to -16.5)
Seychelles	0.8 (0.7 to 0.9)	0.6 (0.5 to 0.8)	0.6 (0.5 to 0.8)	0.4 (0.3 to 0.5)	-2.8 (-3.7 to -1.8)	-34.0 (-42.2 to -24.0)
Thailand	0.5 (0.3 to 0.6)	0.3 (0.2 to 0.3)	0.3 (0.2 to 0.3)	0.2 (0.1 to 0.2)	-2.8 (-4.3 to -1.1)	-33.4 (-47.3 to -14.6)
Timor-Leste	0.7 (0.4 to 0.9)	0.6 (0.3 to 0.7)	0.6 (0.3 to 0.8)	0.4 (0.2 to 0.5)	-2.8 (-3.6 to -1.8)	-34.1 (-41.5 to -24.1)
Vietnam	1.3 (0.6 to 1.8)	0.9 (0.4 to 1.3)	0.9 (0.4 to 1.3)	0.6 (0.3 to 0.9)	-2.9 (-3.8 to -1.9)	-35.3 (-43.5 to -24.6)
Sub-Saharan Africa						
Central Sub-Saharan Africa						
Angola	2.3 (1.6 to 3.0)	1.4 (1.1 to 1.7)	1.3 (1.0 to 1.6)	0.7 (0.5 to 1.0)	-4.2 (-5.2 to -3.2)	-46.4 (-53.9 to -37.9)
Central African Republic	2.2 (1.4 to 2.9)	2.0 (1.3 to 2.6)	2.0 (1.3 to 2.6)	1.4 (0.9 to 1.9)	-2.6 (-3.7 to -1.5)	-31.9 (-42.5 to -19.6)
Congo	1.7 (1.3 to 2.2)	1.2 (0.9 to 1.6)	1.2 (0.8 to 1.6)	0.8 (0.6 to 1.1)	-2.7 (-3.8 to -1.4)	-32.7 (-43.7 to -18.3)
Democratic Republic of the Congo	1.7 (1.2 to 2.4)	1.5 (1.1 to 2.5)	1.5 (1.0 to 2.4)	0.9 (0.6 to 1.5)	-3.4 (-4.2 to -2.5)	-39.5 (-47.1 to -31.1)
Equatorial Guinea	1.5 (1.1 to 2.0)	0.8 (0.5 to 1.3)	0.8 (0.5 to 1.4)	0.5 (0.3 to 0.8)	-3.4 (-4.6 to -2.1)	-39.6 (-49.8 to -27.2)
Gabon	1.2 (0.9 to 1.5)	1.0 (0.8 to 1.5)	1.0 (0.7 to 1.5)	0.7 (0.5 to 1.1)	-2.7 (-3.6 to -1.6)	-32.6 (-41.6 to -21.7)
Eastern Sub-Saharan Africa						
Burundi	2.2 (1.5 to 2.9)	1.8 (1.4 to 2.3)	1.7 (1.3 to 2.2)	1.2 (0.9 to 1.6)	-2.7 (-3.6 to -1.6)	-32.7 (-41.7 to -21.7)
Comoros	1.8 (1.5 to 2.2)	1.5 (1.1 to 2.1)	1.5 (1.0 to 2.1)	1.0 (0.7 to 1.5)	-2.5 (-3.2 to -1.8)	-31.1 (-37.7 to -23.9)
Djibouti	1.9 (1.4 to 2.5)	1.4 (0.9 to 2.1)	1.3 (0.9 to 2.1)	0.8 (0.5 to 1.3)	-3.6 (-4.5 to -2.7)	-41.8 (-48.7 to -33.4)
Eritrea	2.5 (1.6 to 3.5)	2.0 (1.6 to 2.5)	1.9 (1.5 to 2.4)	1.2 (0.9 to 1.5)	-3.4 (-4.2 to -2.6)	-39.6 (-46.9 to -31.8)
Ethiopia	2.2 (1.7 to 2.7)	1.5 (1.1 to 1.8)	1.4 (1.1 to 1.8)	0.7 (0.5 to 0.9)	-4.8 (-5.7 to -3.8)	-51.0 (-57.3 to -43.7)

	Estimate in 2000 (95% UIs)	Estimate in 2015 (95% UIs)	Estimate in 2017 (95% UIs)	Estimate in 2030 (95% UIs)	Annualised rate of change, 2015-2030 (95% UIs)	Percent change, 2015-2030 (95% UIs)
Kenya	2.0 (1.6 to 2.8)	1.9 (1.3 to 2.6)	1.8 (1.3 to 2.6)	1.2 (0.8 to 1.9)	-2.9 (-3.8 to -1.5)	-34.5 (-43.7 to -20.5)
Madagascar	1.7 (1.4 to 2.1)	1.4 (1.1 to 1.8)	1.4 (1.1 to 1.8)	0.9 (0.7 to 1.2)	-2.9 (-3.4 to -2.3)	-35.2 (-40.3 to -29.5)
Malawi	1.9 (1.4 to 2.4)	1.4 (1.1 to 1.8)	1.4 (1.1 to 1.8)	0.9 (0.7 to 1.2)	-3.0 (-4.1 to -1.8)	-35.6 (-46.2 to -23.3)
Mozambique	2.4 (2.0 to 2.9)	2.0 (1.6 to 2.4)	1.9 (1.5 to 2.4)	1.1 (0.9 to 1.4)	-3.9 (-4.8 to -3.0)	-44.4 (-51.4 to -36.5)
Rwanda	2.3 (1.5 to 3.0)	1.4 (1.0 to 1.8)	1.3 (0.9 to 1.8)	0.8 (0.5 to 1.2)	-3.7 (-5.2 to -2.0)	-42.1 (-54.5 to -25.4)
Somalia	2.5 (1.6 to 3.4)	2.0 (1.4 to 2.8)	1.9 (1.3 to 2.7)	1.3 (0.9 to 1.9)	-2.6 (-3.3 to -1.9)	-32.1 (-39.2 to -25.1)
South Sudan	2.0 (1.3 to 3.0)	1.9 (1.3 to 2.6)	1.8 (1.3 to 2.6)	1.2 (0.8 to 1.7)	-3.0 (-4.0 to -1.9)	-36.3 (-45.4 to -25.2)
Tanzania	1.6 (1.3 to 1.8)	1.4 (1.1 to 1.9)	1.4 (1.1 to 1.8)	0.8 (0.6 to 1.1)	-3.8 (-4.5 to -3.0)	-43.0 (-49.0 to -35.9)
Uganda	1.6 (1.3 to 1.9)	1.3 (0.9 to 1.6)	1.2 (0.9 to 1.6)	0.7 (0.5 to 0.9)	-3.9 (-4.7 to -3.0)	-43.9 (-50.9 to -35.8)
Zambia	2.1 (1.6 to 2.6)	1.5 (1.2 to 1.8)	1.4 (1.1 to 1.8)	0.9 (0.6 to 1.2)	-3.5 (-5.3 to -2.1)	-40.7 (-54.7 to -27.4)
Southern Sub-Saharan Africa						
Botswana	1.3 (0.8 to 2.2)	1.1 (0.7 to 1.9)	1.0 (0.6 to 1.9)	0.8 (0.4 to 1.4)	-2.4 (-4.2 to 0.1)	-29.4 (-46.5 to 1.6)
Lesotho	2.2 (1.4 to 3.5)	2.2 (1.5 to 3.4)	2.0 (1.4 to 3.2)	1.4 (0.9 to 2.4)	-3.1 (-5.0 to -1.0)	-36.1 (-52.5 to -13.3)
Namibia	2.1 (1.6 to 3.0)	1.2 (0.8 to 1.9)	1.2 (0.8 to 1.9)	0.8 (0.5 to 1.2)	-3.0 (-4.4 to -1.5)	-36.0 (-48.1 to -20.3)
South Africa	2.2 (1.5 to 2.4)	0.9 (0.7 to 1.2)	0.9 (0.7 to 1.2)	0.7 (0.4 to 1.2)	-2.0 (-4.1 to 1.7)	-24.0 (-46.2 to 28.3)
Swaziland	2.3 (1.6 to 3.4)	1.9 (1.4 to 2.8)	1.8 (1.3 to 2.6)	1.3 (0.8 to 2.1)	-2.7 (-4.8 to -0.2)	-32.1 (-51.2 to -3.0)
Zimbabwe	3.8 (3.0 to 4.8)	3.6 (2.8 to 4.3)	3.5 (2.6 to 4.2)	3.6 (2.2 to 5.4)	-0.2 (-2.6 to 2.7)	-0.6 (-32.5 to 50.4)
Western Sub-Saharan Africa						
Benin	1.6 (1.2 to 2.0)	1.3 (1.0 to 1.6)	1.2 (0.9 to 1.5)	0.7 (0.5 to 0.9)	-4.0 (-4.8 to -3.2)	-44.8 (-51.3 to -37.7)
Burkina Faso	2.0 (1.6 to 2.4)	1.7 (1.3 to 2.2)	1.7 (1.3 to 2.3)	1.0 (0.8 to 1.4)	-3.5 (-4.3 to -2.6)	-40.5 (-47.8 to -32.1)
Cameroon	1.6 (1.2 to 2.0)	1.2 (0.9 to 1.5)	1.1 (0.9 to 1.4)	0.7 (0.5 to 0.9)	-3.7 (-4.6 to -2.7)	-42.7 (-49.8 to -33.4)
Cape Verde	0.9 (0.7 to 1.0)	0.7 (0.6 to 0.8)	0.7 (0.6 to 0.8)	0.4 (0.3 to 0.5)	-3.2 (-4.1 to -2.3)	-37.8 (-45.9 to -29.7)
Chad	1.8 (1.2 to 2.4)	1.5 (1.1 to 2.0)	1.5 (1.1 to 1.9)	0.9 (0.6 to 1.2)	-3.7 (-4.5 to -2.8)	-42.1 (-49.0 to -34.8)
Cote d'Ivoire	1.6 (1.1 to 2.1)	1.4 (1.1 to 1.7)	1.3 (1.0 to 1.6)	0.8 (0.6 to 0.9)	-4.0 (-4.8 to -3.1)	-45.0 (-51.6 to -37.5)
The Gambia	1.5 (1.1 to 1.8)	1.4 (1.0 to 1.6)	1.3 (0.9 to 1.6)	0.8 (0.6 to 1.1)	-3.3 (-4.0 to -2.6)	-38.8 (-44.8 to -32.3)
Ghana	1.4 (1.1 to 1.7)	1.3 (0.9 to 1.7)	1.3 (0.9 to 1.7)	0.8 (0.5 to 1.1)	-3.1 (-3.9 to -2.2)	-37.3 (-44.5 to -28.6)
Guinea	1.8 (1.2 to 2.3)	1.6 (1.1 to 1.9)	1.5 (1.1 to 1.9)	0.9 (0.6 to 1.1)	-3.9 (-4.9 to -3.0)	-44.4 (-52.1 to -36.0)
Guinea-Bissau	2.0 (1.3 to 2.8)	1.5 (1.1 to 1.9)	1.5 (1.1 to 1.8)	0.8 (0.6 to 1.1)	-4.0 (-4.7 to -3.3)	-45.0 (-50.6 to -38.7)
Liberia	1.6 (1.1 to 2.3)	1.2 (0.8 to 1.8)	1.1 (0.8 to 1.8)	0.8 (0.5 to 1.2)	-3.0 (-3.9 to -2.1)	-35.6 (-44.3 to -26.6)
Mali	1.8 (1.1 to 2.4)	1.4 (1.0 to 1.9)	1.4 (1.0 to 1.9)	0.9 (0.6 to 1.2)	-3.1 (-4.0 to -2.2)	-37.4 (-45.2 to -28.4)
Mauritania	1.3 (1.0 to 1.6)	1.0 (0.7 to 1.4)	0.9 (0.7 to 1.4)	0.6 (0.4 to 0.8)	-3.6 (-4.4 to -2.7)	-41.4 (-48.2 to -33.0)
Niger	1.9 (1.2 to 2.6)	1.4 (1.0 to 2.0)	1.4 (1.0 to 2.0)	0.9 (0.6 to 1.3)	-3.1 (-3.9 to -2.4)	-37.2 (-44.1 to -30.1)
Nigeria	1.2 (0.8 to 1.6)	0.9 (0.6 to 1.2)	0.9 (0.6 to 1.2)	0.5 (0.4 to 0.8)	-3.3 (-4.4 to -2.3)	-38.5 (-48.6 to -29.0)
Sao Tome and Principe	1.2 (0.9 to 1.6)	1.1 (0.7 to 1.6)	1.0 (0.7 to 1.5)	0.6 (0.4 to 0.9)	-3.8 (-4.9 to -2.7)	-43.6 (-51.7 to -32.9)
Senegal	1.6 (1.2 to 1.9)	1.3 (1.1 to 1.7)	1.3 (1.0 to 1.6)	0.8 (0.6 to 1.0)	-3.6 (-4.3 to -2.9)	-41.8 (-47.4 to -35.2)
Sierra Leone	1.7 (1.1 to 2.3)	1.4 (1.0 to 1.8)	1.3 (1.0 to 1.7)	0.8 (0.6 to 1.1)	-3.6 (-4.5 to -2.7)	-41.8 (-49.2 to -33.5)
Togo	1.5 (1.2 to 1.9)	1.2 (0.9 to 1.5)	1.1 (0.8 to 1.5)	0.7 (0.5 to 0.9)	-3.6 (-4.5 to -2.7)	-41.8 (-48.9 to -33.7)
**Indicator 3.a.1: Age-standardised prevalence of current smoking in populations aged 10 and older (%)**						
Central Europe, Eastern Europe, and Central Asia						
Central Asia						
Armenia	53.6 (51.5 to 55.6)	49.3 (47.2 to 51.4)	48.5 (46.2 to 50.9)	45.7 (41.9 to 49.9)	-0.5 (-0.9 to -0.1)	-7.4 (-12.3 to -2.2)
Azerbaijan	43.2 (40.7 to 45.3)	46.4 (44.3 to 48.4)	45.7 (43.4 to 47.9)	47.2 (43.4 to 51.4)	0.1 (-0.2 to 0.5)	1.8 (-3.4 to 7.5)
Georgia	52.9 (50.4 to 55.7)	50.8 (48.0 to 53.4)	49.9 (46.9 to 52.8)	50.2 (44.7 to 56.0)	-0.1 (-0.5 to 0.4)	-1.0 (-7.6 to 5.5)
Kazakhstan	50.6 (48.4 to 53.1)	48.8 (46.7 to 51.1)	47.6 (45.2 to 50.1)	45.8 (41.6 to 50.3)	-0.4 (-0.8 to -0.0)	-6.2 (-11.5 to -0.4)
Kyrgyzstan	41.0 (39.4 to 42.6)	40.8 (38.9 to 42.9)	40.0 (37.8 to 42.1)	40.1 (36.2 to 44.4)	-0.1 (-0.5 to 0.3)	-1.9 (-7.7 to 4.7)
Mongolia	49.0 (46.7 to 51.1)	52.3 (49.9 to 54.8)	51.6 (48.9 to 54.4)	54.2 (49.1 to 59.6)	0.2 (-0.2 to 0.6)	3.6 (-2.4 to 10.1)
Tajikistan	23.5 (21.6 to 25.5)	19.6 (17.9 to 21.4)	19.4 (17.7 to 21.3)	17.0 (14.6 to 19.9)	-1.0 (-1.5 to -0.3)	-13.4 (-20.2 to -4.8)
Turkmenistan	39.0 (36.0 to 41.9)	38.9 (36.2 to 41.6)	38.6 (35.8 to 41.4)	39.0 (34.7 to 43.4)	0.0 (-0.4 to 0.5)	0.1 (-6.1 to 7.2)
Uzbekistan	24.1 (22.4 to 25.9)	23.3 (21.5 to 25.0)	22.7 (21.0 to 24.7)	22.6 (19.6 to 25.9)	-0.2 (-0.8 to 0.3)	-3.1 (-10.9 to 5.3)
Central Europe						
Albania	48.4 (45.9 to 50.9)	56.8 (53.6 to 59.8)	56.6 (53.2 to 60.0)	66.3 (58.8 to 74.7)	1.0 (0.5 to 1.6)	16.7 (7.9 to 26.8)
Bosnia and Herzegovina	60.5 (58.6 to 62.3)	67.3 (64.2 to 70.3)	65.7 (62.0 to 69.3)	69.8 (62.4 to 77.6)	0.2 (-0.2 to 0.7)	3.8 (-3.6 to 11.0)
Bulgaria	77.9 (73.4 to 82.2)	70.0 (67.2 to 72.8)	69.2 (66.0 to 72.4)	65.9 (59.8 to 72.8)	-0.4 (-0.8 to 0.1)	-5.8 (-11.6 to 1.2)
Croatia	66.9 (63.4 to 70.8)	69.4 (66.5 to 72.4)	68.5 (65.3 to 72.0)	69.6 (63.2 to 76.0)	0.0 (-0.4 to 0.4)	0.3 (-6.1 to 6.6)
Czech Republic	63.6 (61.1 to 65.9)	58.7 (56.0 to 61.5)	57.8 (54.7 to 61.0)	55.5 (50.1 to 61.4)	-0.4 (-0.8 to 0.1)	-5.4 (-11.4 to 0.8)
Hungary	69.7 (66.4 to 72.9)	60.3 (57.0 to 63.6)	58.5 (54.9 to 62.4)	53.6 (47.8 to 60.3)	-0.8 (-1.3 to -0.3)	-11.0 (-17.4 to -4.1)
Macedonia	66.2 (61.4 to 71.3)	67.7 (63.7 to 71.4)	67.2 (62.9 to 71.4)	70.6 (61.5 to 80.4)	0.3 (-0.4 to 0.9)	4.3 (-5.3 to 14.3)
Montenegro	68.9 (64.1 to 73.8)	70.5 (67.7 to 73.2)	69.7 (66.4 to 73.1)	72.9 (65.5 to 81.2)	0.2 (-0.3 to 0.7)	3.4 (-4.2 to 11.8)
Poland	62.1 (58.6 to 65.7)	55.3 (52.8 to 57.9)	55.9 (52.8 to 58.8)	54.3 (49.2 to 59.8)	-0.1 (-0.6 to 0.4)	-1.8 (-8.5 to 5.4)
Romania	62.3 (58.7 to 65.8)	53.7 (51.1 to 56.8)	53.4 (50.3 to 56.7)	50.9 (45.4 to 57.6)	-0.4 (-0.9 to 0.2)	-5.4 (-12.4 to 2.4)
Serbia	64.3 (61.8 to 66.9)	62.6 (59.0 to 66.1)	61.6 (57.7 to 65.7)	62.9 (55.5 to 70.9)	0.0 (-0.5 to 0.5)	0.4 (-7.1 to 8.4)
Slovakia	60.7 (57.2 to 64.3)	49.8 (47.0 to 52.8)	49.4 (46.1 to 52.9)	44.5 (38.9 to 50.3)	-0.8 (-1.3 to -0.3)	-10.8 (-17.9 to -3.8)
Slovenia	50.7 (47.2 to 54.3)	52.8 (49.8 to 55.9)	52.6 (49.1 to 56.1)	54.5 (48.1 to 61.5)	0.2 (-0.3 to 0.8)	3.2 (-5.1 to 12.2)
Eastern Europe						
Belarus	67.6 (64.8 to 70.5)	67.9 (65.1 to 70.8)	67.5 (64.1 to 71.2)	72.3 (64.7 to 80.3)	0.4 (-0.1 to 0.9)	6.4 (-1.4 to 14.9)
Estonia	56.1 (54.4 to 57.9)	49.2 (46.8 to 52.0)	47.4 (44.4 to 50.5)	41.7 (37.3 to 46.3)	-1.1 (-1.6 to -0.6)	-15.4 (-21.2 to -9.2)
Latvia	62.5 (60.4 to 64.5)	63.8 (61.2 to 66.5)	62.7 (59.6 to 65.9)	63.7 (58.0 to 70.3)	-0.0 (-0.4 to 0.4)	-0.2 (-6.3 to 6.6)
Lithuania	55.0 (53.1 to 57.0)	56.9 (54.2 to 59.6)	56.2 (53.1 to 59.5)	58.2 (52.1 to 65.1)	0.1 (-0.3 to 0.7)	2.2 (-5.0 to 10.2)
Moldova	42.8 (40.2 to 45.6)	41.8 (39.6 to 43.9)	40.8 (38.2 to 43.1)	40.9 (36.4 to 45.7)	-0.2 (-0.7 to 0.3)	-2.2 (-9.3 to 5.2)
Russia	67.0 (65.8 to 68.2)	56.9 (55.5 to 58.3)	56.1 (53.9 to 58.4)	51.7 (47.9 to 56.1)	-0.6 (-1.1 to -0.2)	-9.1 (-14.6 to -3.1)
Ukraine	65.9 (63.9 to 68.0)	58.7 (55.8 to 61.5)	56.6 (53.2 to 60.0)	51.4 (45.7 to 57.5)	-0.9 (-1.4 to -0.4)	-12.5 (-18.7 to -5.4)
High-income						
Australasia						
Australia	43.1 (41.7 to 44.3)	29.0 (27.0 to 31.1)	28.4 (25.7 to 31.2)	22.1 (18.7 to 26.0)	-1.8 (-2.5 to -1.1)	-23.7 (-31.6 to -14.6)
New Zealand	51.7 (49.9 to 53.7)	35.6 (34.2 to 37.0)	35.3 (33.5 to 37.2)	29.0 (26.4 to 32.0)	-1.4 (-1.9 to -0.8)	-18.4 (-24.4 to -11.9)
High-income Asia Pacific						
Brunei	35.4 (32.9 to 38.0)	33.3 (30.4 to 36.0)	33.0 (30.2 to 35.8)	31.9 (27.6 to 36.3)	-0.3 (-0.8 to 0.2)	-4.4 (-11.3 to 3.1)
Japan	58.9 (57.2 to 60.6)	42.8 (40.2 to 45.7)	43.2 (39.9 to 46.7)	37.2 (32.5 to 42.7)	-1.0 (-1.5 to -0.4)	-13.2 (-20.2 to -5.3)
South Korea	53.9 (51.4 to 56.5)	43.3 (41.1 to 45.6)	42.8 (40.3 to 45.3)	38.1 (34.0 to 42.4)	-0.9 (-1.3 to -0.4)	-12.0 (-17.8 to -5.9)
Singapore	30.2 (27.8 to 32.7)	28.1 (25.8 to 30.7)	27.4 (24.8 to 30.2)	26.5 (22.1 to 31.7)	-0.4 (-1.1 to 0.4)	-5.6 (-15.0 to 5.5)

	Estimate in 2000 (95% UIs)	Estimate in 2015 (95% UIs)	Estimate in 2017 (95% UIs)	Estimate in 2030 (95% UIs)	Annualised rate of change, 2015-2030 (95% UIs)	Percent change, 2015-2030 (95% UIs)
High-income North America						
Canada	47.1 (44.8 to 49.7)	32.4 (28.7 to 36.1)	32.5 (28.7 to 36.5)	27.2 (22.1 to 32.9)	-1.2 (-1.9 to -0.4)	-16.1 (-25.3 to -5.4)
Greenland	96.0 (90.7 to 101.0)	91.5 (85.7 to 97.5)	90.2 (84.0 to 96.7)	88.4 (78.8 to 98.9)	-0.2 (-0.7 to 0.2)	-3.4 (-9.4 to 2.9)
United States	50.2 (48.7 to 51.8)	38.1 (35.8 to 40.5)	38.3 (35.2 to 41.4)	34.1 (29.7 to 38.9)	-0.8 (-1.4 to -0.2)	-10.7 (-18.5 to -2.8)
Southern Latin America						
Argentina	57.9 (54.1 to 61.5)	45.6 (43.2 to 47.9)	45.0 (42.2 to 47.8)	40.7 (35.9 to 45.6)	-0.8 (-1.3 to -0.2)	-10.8 (-17.9 to -3.2)
Chile	73.3 (69.6 to 77.1)	63.4 (59.4 to 67.3)	62.9 (58.5 to 67.5)	59.2 (51.5 to 67.4)	-0.5 (-1.0 to 0.1)	-6.6 (-14.1 to 0.8)
Uruguay	66.5 (63.3 to 69.5)	45.9 (42.5 to 49.3)	45.3 (41.5 to 49.2)	37.8 (31.9 to 44.1)	-1.3 (-2.0 to -0.7)	-17.7 (-25.6 to -9.6)
Western Europe						
Andorra	58.0 (53.8 to 62.3)	51.0 (45.9 to 56.2)	50.5 (45.4 to 55.7)	47.5 (40.5 to 55.2)	-0.5 (-1.0 to 0.1)	-6.9 (-14.0 to 1.2)
Austria	68.3 (65.6 to 70.9)	59.0 (55.8 to 62.3)	57.5 (53.9 to 61.2)	53.3 (47.7 to 59.5)	-0.7 (-1.1 to -0.2)	-9.7 (-15.5 to -3.0)
Belgium	59.9 (57.7 to 62.3)	42.8 (40.4 to 45.3)	41.7 (38.7 to 44.7)	34.7 (30.6 to 39.4)	-1.4 (-2.0 to -0.8)	-19.0 (-26.1 to -11.8)
Cyprus	67.2 (63.6 to 71.1)	60.2 (56.6 to 63.6)	58.9 (55.3 to 62.6)	55.8 (49.5 to 62.5)	-0.5 (-1.0 to -0.0)	-7.5 (-13.9 to -0.4)
Denmark	65.3 (63.5 to 67.2)	40.2 (37.8 to 42.7)	39.6 (36.7 to 42.7)	30.7 (26.9 to 35.0)	-1.8 (-2.4 to -1.2)	-23.7 (-30.6 to -16.0)
Finland	48.1 (46.7 to 49.5)	39.6 (37.1 to 42.2)	39.3 (36.5 to 42.5)	35.6 (31.5 to 40.5)	-0.7 (-1.3 to -0.1)	-10.0 (-17.2 to -1.4)
France	65.3 (63.1 to 67.4)	61.6 (58.4 to 64.8)	63.7 (59.8 to 67.4)	66.8 (60.0 to 73.3)	0.5 (0.1 to 1.0)	8.6 (1.0 to 15.6)
Germany	61.0 (59.6 to 62.4)	52.7 (50.0 to 55.4)	52.6 (49.5 to 55.9)	51.3 (45.8 to 57.1)	-0.2 (-0.7 to 0.3)	-2.7 (-9.7 to 4.5)
Greece	74.4 (71.9 to 76.8)	71.1 (68.2 to 73.8)	69.0 (65.5 to 72.5)	68.2 (62.3 to 74.6)	-0.3 (-0.7 to 0.1)	-4.0 (-10.1 to 2.0)
Iceland	46.2 (43.5 to 49.5)	30.7 (27.5 to 34.2)	31.1 (27.6 to 35.0)	25.9 (21.1 to 31.6)	-1.2 (-1.9 to -0.3)	-15.7 (-25.2 to -4.4)
Ireland	57.6 (55.8 to 59.4)	42.1 (39.5 to 44.9)	40.1 (37.2 to 43.2)	33.4 (28.8 to 38.1)	-1.6 (-2.2 to -0.9)	-20.9 (-28.2 to -13.1)
Israel	49.4 (46.6 to 52.2)	41.4 (38.1 to 44.8)	41.3 (37.7 to 45.3)	38.4 (32.5 to 45.0)	-0.5 (-1.1 to 0.2)	-7.4 (-15.7 to 2.5)
Italy	49.7 (48.7 to 50.8)	46.5 (43.9 to 49.3)	46.0 (42.9 to 49.5)	44.1 (38.9 to 49.7)	-0.4 (-0.9 to 0.2)	-5.4 (-13.3 to 2.7)
Luxembourg	58.2 (55.4 to 61.5)	45.4 (41.8 to 49.0)	44.4 (40.6 to 48.1)	38.7 (33.1 to 44.8)	-1.1 (-1.7 to -0.5)	-14.9 (-22.3 to -6.7)
Malta	47.9 (45.1 to 50.9)	45.7 (42.1 to 49.3)	45.4 (41.7 to 49.3)	44.6 (38.1 to 52.3)	-0.2 (-0.8 to 0.5)	-2.4 (-11.1 to 7.7)
Netherlands	59.9 (59.1 to 60.6)	40.2 (37.8 to 42.6)	39.4 (36.5 to 42.4)	31.0 (27.1 to 35.7)	-1.7 (-2.3 to -1.1)	-22.9 (-29.5 to -15.2)
Norway	63.6 (61.2 to 66.2)	32.8 (29.9 to 35.9)	32.9 (29.7 to 36.4)	25.0 (20.9 to 29.9)	-1.8 (-2.6 to -1.0)	-23.8 (-31.8 to -14.2)
Portugal	51.9 (49.7 to 54.3)	48.5 (45.8 to 51.3)	49.0 (46.1 to 52.3)	51.7 (45.8 to 57.9)	0.4 (-0.2 to 1.0)	6.5 (-2.6 to 15.7)
Spain	63.5 (61.6 to 65.3)	57.8 (54.9 to 61.0)	55.7 (52.3 to 59.3)	49.8 (44.6 to 55.6)	-1.0 (-1.5 to -0.5)	-13.9 (-20.1 to -7.2)
Sweden	38.5 (36.8 to 40.2)	23.0 (21.1 to 25.1)	22.4 (20.0 to 24.9)	17.3 (14.4 to 20.9)	-1.9 (-2.7 to -1.0)	-24.7 (-33.3 to -14.2)
Switzerland	57.6 (55.4 to 60.0)	48.9 (44.9 to 53.2)	48.4 (44.4 to 53.2)	45.5 (39.0 to 54.0)	-0.5 (-1.1 to 0.2)	-6.9 (-15.1 to 2.5)
United Kingdom	53.9 (52.8 to 55.0)	45.6 (42.7 to 48.5)	43.6 (40.1 to 47.0)	39.1 (34.0 to 44.7)	-1.0 (-1.6 to -0.4)	-14.3 (-21.8 to -6.4)
Latin America and Caribbean						
Andean Latin America						
Bolivia	18.0 (16.3 to 19.8)	17.6 (15.9 to 19.6)	17.1 (15.3 to 19.1)	17.5 (14.0 to 21.8)	-0.1 (-1.0 to 0.9)	-1.2 (-13.5 to 14.2)
Ecuador	25.7 (23.9 to 27.4)	25.3 (23.4 to 27.1)	24.4 (22.3 to 26.4)	24.6 (21.0 to 28.3)	-0.2 (-0.8 to 0.4)	-2.8 (-11.3 to 6.1)
Peru	20.4 (18.6 to 22.4)	15.9 (14.5 to 17.6)	15.5 (14.0 to 17.2)	14.0 (11.9 to 16.6)	-0.9 (-1.6 to -0.2)	-12.3 (-20.9 to -2.7)
Caribbean						
Antigua and Barbuda	22.1 (19.6 to 24.6)	20.6 (18.3 to 23.1)	20.3 (18.1 to 22.9)	20.1 (16.9 to 23.9)	-0.2 (-0.8 to 0.5)	-2.4 (-11.7 to 8.0)
The Bahamas	21.9 (19.5 to 24.3)	20.8 (18.6 to 23.4)	20.6 (18.4 to 23.1)	21.0 (17.5 to 25.0)	0.1 (-0.7 to 0.8)	0.9 (-9.3 to 12.2)
Barbados	23.6 (21.2 to 26.1)	22.2 (20.1 to 24.4)	22.0 (19.8 to 24.2)	22.1 (18.4 to 26.0)	-0.1 (-0.7 to 0.7)	-0.7 (-10.4 to 10.5)
Belize	25.3 (23.3 to 27.2)	21.8 (20.0 to 23.6)	21.4 (19.5 to 23.4)	19.6 (16.9 to 22.7)	-0.7 (-1.3 to -0.1)	-10.3 (-17.7 to -2.2)
Bermuda	27.0 (24.2 to 30.1)	25.8 (23.3 to 28.7)	25.4 (23.0 to 28.4)	25.7 (21.9 to 30.4)	-0.0 (-0.6 to 0.6)	-0.3 (-9.2 to 9.7)
Cuba	60.8 (56.8 to 65.2)	54.5 (50.2 to 58.9)	53.5 (49.0 to 57.8)	50.7 (43.6 to 58.1)	-0.5 (-1.1 to 0.1)	-7.2 (-14.7 to 0.8)
Dominica	23.2 (21.0 to 25.5)	21.0 (18.7 to 23.2)	20.4 (18.1 to 22.7)	19.5 (16.0 to 23.5)	-0.5 (-1.2 to 0.4)	-7.3 (-16.8 to 5.5)
Dominican Republic	26.8 (25.2 to 28.5)	20.3 (18.6 to 22.0)	19.8 (18.2 to 21.7)	17.5 (15.0 to 20.9)	-1.0 (-1.6 to -0.4)	-13.8 (-21.0 to -5.2)
Grenada	27.6 (25.2 to 30.3)	25.2 (23.0 to 27.7)	24.8 (22.6 to 27.2)	23.8 (20.5 to 27.9)	-0.4 (-1.0 to 0.2)	-5.7 (-13.9 to 3.1)
Guyana	30.1 (27.6 to 32.8)	29.4 (27.1 to 32.1)	28.9 (26.4 to 31.6)	29.4 (25.1 to 34.4)	-0.0 (-0.7 to 0.6)	-0.3 (-9.4 to 10.0)
Haiti	19.0 (17.3 to 20.9)	14.6 (13.1 to 16.1)	14.2 (12.7 to 15.7)	12.1 (10.2 to 14.5)	-1.3 (-2.0 to -0.5)	-17.4 (-25.4 to -7.5)
Jamaica	37.0 (34.6 to 39.7)	33.9 (31.2 to 36.8)	33.1 (30.3 to 36.0)	32.5 (27.7 to 37.8)	-0.3 (-0.9 to 0.4)	-4.2 (-12.6 to 5.6)
Puerto Rico	30.0 (28.3 to 31.9)	24.1 (22.3 to 26.0)	23.7 (21.7 to 25.9)	21.5 (18.3 to 24.9)	-0.8 (-1.5 to -0.1)	-10.9 (-20.0 to -1.0)
Saint Lucia	32.0 (28.8 to 35.4)	30.1 (27.2 to 33.4)	29.6 (26.6 to 32.8)	29.9 (25.1 to 35.6)	-0.1 (-0.7 to 0.7)	-1.1 (-10.6 to 11.4)
Saint Vincent and the Grenadines	29.8 (27.2 to 32.5)	26.3 (23.9 to 29.0)	25.9 (23.5 to 28.5)	24.5 (20.8 to 28.8)	-0.5 (-1.0 to 0.2)	-7.0 (-14.5 to 2.6)
Suriname	44.1 (40.5 to 48.0)	42.7 (39.3 to 46.4)	41.6 (38.2 to 45.2)	41.2 (35.9 to 46.9)	-0.3 (-0.7 to 0.3)	-3.6 (-10.6 to 4.1)
Trinidad and Tobago	34.4 (32.1 to 36.9)	34.3 (31.9 to 36.7)	34.0 (31.5 to 36.8)	34.9 (30.0 to 40.6)	0.1 (-0.5 to 0.7)	1.6 (-7.2 to 11.6)
Virgin Islands, U.S.	18.2 (16.6 to 19.8)	13.6 (11.9 to 15.2)	13.3 (11.6 to 15.0)	12.0 (9.5 to 15.2)	-0.8 (-1.7 to 0.2)	-11.4 (-22.0 to 2.5)
Central Latin America						
Colombia	39.1 (36.5 to 41.8)	26.2 (24.2 to 28.1)	25.7 (23.6 to 27.9)	21.9 (18.7 to 25.8)	-1.2 (-1.9 to -0.4)	-16.4 (-24.6 to -5.9)
Costa Rica	36.2 (34.4 to 38.1)	26.2 (24.4 to 28.0)	25.9 (23.8 to 28.0)	21.7 (18.5 to 25.3)	-1.3 (-1.9 to -0.6)	-17.2 (-24.4 to -9.2)
El Salvador	19.0 (17.1 to 21.1)	16.4 (14.7 to 18.3)	16.0 (14.2 to 17.9)	14.8 (12.1 to 17.7)	-0.7 (-1.5 to 0.0)	-10.2 (-19.9 to 0.7)
Guatemala	28.8 (26.5 to 31.2)	21.0 (19.2 to 22.9)	20.5 (18.6 to 22.5)	16.8 (14.3 to 19.9)	-1.5 (-2.1 to -0.8)	-20.1 (-26.6 to -11.5)
Honduras	31.4 (29.1 to 33.8)	26.3 (24.0 to 28.5)	25.7 (23.4 to 28.0)	23.4 (19.9 to 26.9)	-0.8 (-1.4 to -0.2)	-11.2 (-19.0 to -3.3)
Mexico	45.7 (43.9 to 47.6)	32.8 (31.0 to 34.6)	32.4 (30.4 to 34.4)	26.8 (23.9 to 30.0)	-1.4 (-1.8 to -0.9)	-18.3 (-24.0 to -12.3)
Nicaragua	26.6 (24.0 to 29.0)	24.4 (21.9 to 27.0)	23.8 (21.3 to 26.2)	22.6 (19.1 to 26.5)	-0.5 (-1.1 to 0.1)	-7.4 (-15.3 to 1.5)
Panama	20.7 (18.9 to 22.8)	15.5 (14.1 to 17.1)	15.2 (13.7 to 16.8)	13.0 (10.9 to 15.5)	-1.2 (-1.8 to -0.5)	-16.2 (-23.8 to -7.6)
Venezuela	37.5 (34.4 to 40.5)	32.4 (29.1 to 35.8)	31.7 (28.4 to 35.3)	29.0 (23.6 to 34.8)	-0.8 (-1.5 to -0.0)	-10.7 (-19.7 to -0.5)
Tropical Latin America						
Brazil	43.0 (40.4 to 45.8)	20.7 (19.4 to 22.0)	19.2 (17.7 to 20.7)	11.7 (10.2 to 13.5)	-3.8 (-4.4 to -3.2)	-43.6 (-48.0 to -38.0)
Paraguay	41.5 (39.2 to 43.9)	29.0 (26.7 to 31.2)	28.2 (25.8 to 30.6)	23.0 (19.9 to 26.4)	-1.6 (-2.1 to -1.0)	-20.7 (-26.8 to -14.1)
North Africa and Middle East						
North Africa and Middle East						
Afghanistan	21.3 (19.1 to 23.5)	22.2 (20.3 to 24.1)	21.4 (19.5 to 23.3)	22.0 (19.0 to 25.3)	-0.1 (-0.6 to 0.5)	-0.9 (-9.0 to 7.8)
Algeria	40.8 (37.7 to 44.0)	36.8 (33.6 to 39.9)	35.8 (32.6 to 39.0)	32.9 (28.4 to 37.5)	-0.8 (-1.2 to -0.3)	-10.8 (-16.9 to -4.2)
Bahrain	22.6 (21.1 to 24.2)	22.1 (19.9 to 24.3)	21.9 (19.7 to 24.2)	21.9 (18.4 to 25.7)	-0.1 (-0.6 to 0.5)	-1.1 (-9.1 to 7.9)
Egypt	38.3 (35.8 to 40.9)	38.3 (36.3 to 40.5)	37.7 (35.4 to 40.0)	37.6 (33.7 to 41.7)	-0.1 (-0.6 to 0.3)	-2.0 (-8.4 to 4.6)
Iran	28.4 (26.5 to 30.6)	27.7 (25.5 to 29.9)	27.0 (24.6 to 29.2)	26.7 (22.7 to 31.2)	-0.3 (-0.9 to 0.4)	-3.9 (-12.1 to 5.6)
Iraq	35.7 (33.5 to 37.9)	38.4 (35.9 to 41.0)	37.4 (34.9 to 40.1)	38.1 (33.9 to 42.6)	-0.1 (-0.5 to 0.4)	-0.8 (-7.1 to 5.9)
Jordan	48.5 (46.0 to 50.9)	49.5 (46.8 to 52.3)	48.7 (45.7 to 51.8)	51.0 (45.5 to 57.3)	0.2 (-0.3 to 0.7)	3.0 (-4.5 to 10.4)
Kuwait	36.2 (33.9 to 38.5)	35.7 (33.5 to 37.9)	34.3 (32.0 to 36.5)	33.3 (29.5 to 37.3)	-0.5 (-0.9 to 0.0)	-6.7 (-13.2 to 0.0)
Lebanon	58.3 (55.9 to 61.0)	61.4 (58.4 to 64.3)	59.6 (56.4 to 62.8)	60.9 (54.7 to 67.4)	-0.1 (-0.5 to 0.4)	-0.9 (-7.4 to 5.8)
Libya	34.2 (32.3 to 36.3)	30.4 (28.4 to 32.4)	29.4 (27.2 to 31.5)	26.7 (23.6 to 30.1)	-0.9 (-1.3 to -0.4)	-12.1 (-18.2 to -6.0)

	Estimate in 2000 (95% UIs)	Estimate in 2015 (95% UIs)	Estimate in 2017 (95% UIs)	Estimate in 2030 (95% UIs)	Annualised rate of change, 2015-2030 (95% UIs)	Percent change, 2015-2030 (95% UIs)
Morocco	27.3 (26.0 to 28.6)	22.0 (20.1 to 24.1)	21.7 (19.8 to 23.9)	19.0 (16.2 to 22.2)	-1.0 (-1.5 to -0.4)	-13.9 (-20.4 to -6.2)
Palestine	43.6 (42.7 to 44.5)	42.8 (40.7 to 44.9)	42.2 (39.8 to 44.6)	47.8 (42.8 to 53.2)	0.7 (0.2 to 1.3)	11.7 (3.3 to 21.0)
Oman	17.7 (16.2 to 19.5)	15.9 (14.3 to 17.6)	15.5 (13.9 to 17.1)	14.4 (12.2 to 16.9)	-0.7 (-1.3 to -0.0)	-9.3 (-17.5 to -0.3)
Qatar	24.8 (22.7 to 26.9)	23.5 (21.7 to 25.4)	22.8 (21.0 to 24.9)	22.4 (19.0 to 26.2)	-0.3 (-1.0 to 0.4)	-4.9 (-13.9 to 6.3)
Saudi Arabia	21.7 (20.1 to 23.2)	20.2 (18.6 to 21.8)	19.1 (17.6 to 20.8)	18.0 (15.5 to 20.7)	-0.8 (-1.3 to -0.2)	-10.9 (-18.2 to -3.4)
Sudan	17.0 (15.3 to 18.7)	13.3 (11.9 to 14.7)	12.7 (11.3 to 14.1)	10.8 (9.0 to 12.8)	-1.4 (-2.0 to -0.7)	-18.7 (-26.0 to -10.2)
Syria	44.7 (41.8 to 47.5)	35.7 (33.0 to 38.6)	34.6 (31.9 to 37.5)	29.6 (26.0 to 33.5)	-1.3 (-1.7 to -0.8)	-17.2 (-22.4 to -11.7)
Tunisia	47.5 (45.4 to 49.5)	45.1 (42.2 to 47.8)	43.9 (41.0 to 46.8)	41.8 (37.1 to 46.5)	-0.5 (-0.9 to -0.1)	-7.4 (-12.9 to -1.8)
Turkey	64.7 (62.5 to 66.9)	51.4 (49.2 to 53.5)	50.2 (47.8 to 52.8)	43.4 (39.6 to 47.6)	-1.1 (-1.5 to -0.7)	-15.5 (-20.6 to -10.0)
United Arab Emirates	26.0 (24.0 to 28.0)	27.4 (25.1 to 29.8)	26.9 (24.6 to 29.4)	28.5 (24.4 to 33.0)	0.3 (-0.3 to 0.8)	4.0 (-4.6 to 13.1)
Yemen	33.4 (31.4 to 35.3)	34.5 (32.4 to 36.7)	34.0 (31.6 to 36.3)	35.9 (31.5 to 40.9)	0.3 (-0.3 to 0.8)	3.9 (-4.2 to 13.3)
South Asia						
South Asia						
Bangladesh	40.8 (39.5 to 42.1)	42.0 (40.0 to 44.0)	41.6 (39.3 to 43.8)	41.7 (37.6 to 45.6)	-0.1 (-0.4 to 0.3)	-0.9 (-6.0 to 4.3)
Bhutan	19.0 (17.2 to 21.0)	14.9 (13.5 to 16.3)	14.5 (13.1 to 16.0)	12.7 (10.6 to 15.1)	-1.1 (-1.7 to -0.4)	-14.8 (-22.9 to -5.5)
India	31.0 (29.6 to 32.6)	23.8 (22.5 to 25.1)	22.4 (21.0 to 23.9)	18.0 (16.1 to 20.1)	-1.9 (-2.3 to -1.3)	-24.3 (-29.6 to -18.2)
Nepal	66.2 (64.0 to 68.6)	43.8 (41.4 to 46.2)	42.4 (39.7 to 45.1)	33.4 (29.9 to 37.3)	-1.8 (-2.2 to -1.4)	-23.8 (-28.4 to -18.7)
Pakistan	37.2 (35.0 to 39.5)	27.1 (25.4 to 28.8)	26.3 (24.6 to 28.1)	21.6 (19.2 to 24.4)	-1.5 (-2.0 to -1.0)	-20.2 (-25.9 to -14.4)
Southeast Asia, East Asia, and Oceania						
East Asia						
China	48.7 (47.7 to 49.7)	47.3 (45.6 to 49.1)	46.5 (44.4 to 48.7)	44.9 (41.2 to 48.6)	-0.4 (-0.7 to -0.0)	-5.3 (-10.5 to -0.1)
North Korea	44.5 (42.1 to 46.8)	40.4 (38.2 to 42.8)	39.7 (37.3 to 42.2)	37.3 (33.7 to 41.1)	-0.5 (-0.9 to -0.2)	-7.7 (-12.8 to -2.5)
Taiwan (Province of China)	42.5 (40.0 to 45.2)	37.0 (34.5 to 39.5)	36.4 (33.8 to 39.0)	33.9 (29.9 to 38.5)	-0.6 (-1.1 to -0.1)	-8.3 (-15.0 to -1.0)
Oceania						
American Samoa	58.3 (54.1 to 62.9)	54.8 (50.3 to 59.8)	54.2 (49.6 to 59.2)	53.2 (46.5 to 61.2)	-0.2 (-0.7 to 0.3)	-3.0 (-9.9 to 4.8)
Federated States of Micronesia	60.4 (55.6 to 65.4)	57.9 (53.9 to 62.3)	57.3 (53.2 to 62.0)	57.3 (50.8 to 64.9)	-0.1 (-0.5 to 0.5)	-1.1 (-7.8 to 7.4)
Fiji	58.3 (55.5 to 60.8)	50.8 (47.6 to 53.8)	50.2 (46.9 to 53.4)	47.0 (41.6 to 52.5)	-0.5 (-1.0 to -0.1)	-7.7 (-13.6 to -0.9)
Guam	58.3 (54.9 to 61.9)	51.6 (48.9 to 54.4)	51.1 (48.0 to 54.5)	49.4 (43.4 to 56.0)	-0.3 (-0.9 to 0.3)	-4.4 (-12.3 to 4.2)
Kiribati	90.6 (87.9 to 93.6)	84.5 (80.2 to 88.5)	82.7 (78.1 to 87.2)	81.5 (73.3 to 90.0)	-0.3 (-0.6 to 0.2)	-3.7 (-9.2 to 2.6)
Marshall Islands	38.8 (36.5 to 41.1)	37.2 (34.4 to 40.2)	36.9 (34.0 to 40.1)	36.6 (31.9 to 42.3)	-0.1 (-0.6 to 0.4)	-1.8 (-8.7 to 6.8)
Northern Mariana Islands	56.0 (51.2 to 61.4)	51.9 (47.0 to 56.9)	51.5 (46.6 to 56.7)	50.6 (43.7 to 58.8)	-0.2 (-0.7 to 0.4)	-2.4 (-9.7 to 6.0)
Papua New Guinea	70.4 (66.2 to 74.6)	64.3 (60.7 to 68.1)	63.6 (59.8 to 67.8)	61.3 (55.1 to 68.7)	-0.3 (-0.8 to 0.1)	-4.8 (-10.9 to 2.2)
Samoa	60.4 (57.1 to 64.0)	57.5 (53.7 to 61.4)	56.7 (52.9 to 60.7)	55.7 (49.6 to 63.0)	-0.2 (-0.6 to 0.2)	-3.1 (-9.2 to 3.6)
Solomon Islands	54.2 (51.0 to 57.8)	52.4 (49.1 to 56.0)	51.3 (47.9 to 55.1)	50.3 (44.3 to 57.0)	-0.3 (-0.8 to 0.2)	-4.2 (-10.9 to 3.3)
Tonga	62.2 (58.9 to 65.5)	56.8 (53.2 to 60.3)	56.1 (52.4 to 59.9)	53.9 (47.4 to 61.0)	-0.4 (-0.9 to 0.2)	-5.1 (-12.2 to 2.7)
Vanuatu	49.2 (46.7 to 51.6)	41.6 (39.6 to 43.9)	40.8 (38.5 to 43.2)	37.0 (33.2 to 41.1)	-0.8 (-1.2 to -0.3)	-11.1 (-17.0 to -4.6)
Southeast Asia						
Cambodia	53.3 (51.8 to 54.9)	42.6 (40.7 to 44.7)	41.9 (39.7 to 44.1)	36.9 (33.4 to 40.5)	-1.0 (-1.3 to -0.6)	-13.4 (-18.2 to -8.3)
Indonesia	57.2 (55.8 to 58.6)	59.9 (57.8 to 62.0)	59.2 (56.8 to 61.5)	61.9 (57.6 to 65.8)	0.2 (-0.1 to 0.5)	3.2 (-1.0 to 7.1)
Laos	56.7 (53.7 to 59.2)	46.7 (43.9 to 49.5)	45.7 (42.8 to 48.8)	41.1 (36.7 to 45.8)	-0.9 (-1.3 to -0.4)	-12.0 (-17.2 to -6.2)
Malaysia	49.0 (46.7 to 51.3)	41.6 (39.5 to 43.8)	40.6 (38.3 to 43.0)	36.2 (32.5 to 40.2)	-0.9 (-1.4 to -0.5)	-13.0 (-18.7 to -7.2)
Maldives	47.1 (44.4 to 50.1)	41.5 (39.5 to 43.7)	40.5 (38.3 to 42.9)	37.6 (33.6 to 41.9)	-0.7 (-1.2 to -0.2)	-9.4 (-15.9 to -2.4)
Mauritius	43.0 (40.9 to 45.2)	43.0 (40.5 to 45.6)	42.4 (39.7 to 45.3)	42.4 (37.5 to 47.3)	-0.1 (-0.6 to 0.4)	-1.6 (-8.1 to 5.5)
Myanmar	51.5 (49.3 to 53.7)	42.5 (40.5 to 44.5)	42.2 (40.0 to 44.5)	38.9 (35.2 to 43.0)	-0.6 (-1.0 to -0.2)	-8.5 (-13.9 to -2.4)
Philippines	61.2 (58.7 to 63.6)	46.4 (44.4 to 48.7)	45.5 (43.2 to 48.0)	38.5 (34.8 to 42.4)	-1.3 (-1.7 to -0.8)	-17.1 (-22.3 to -11.4)
Sri Lanka	30.9 (29.5 to 32.4)	26.7 (25.3 to 28.1)	26.3 (24.8 to 27.8)	23.9 (21.3 to 26.4)	-0.7 (-1.2 to -0.3)	-10.5 (-16.5 to -4.4)
Seychelles	46.2 (43.3 to 49.4)	38.8 (35.9 to 42.0)	37.9 (35.0 to 41.0)	34.7 (30.4 to 39.6)	-0.7 (-1.2 to -0.3)	-10.6 (-16.5 to -3.8)
Thailand	45.4 (43.1 to 47.7)	40.1 (38.2 to 41.9)	39.4 (37.3 to 41.4)	36.9 (33.4 to 40.6)	-0.5 (-1.0 to -0.1)	-7.8 (-13.6 to -1.8)
Timor-Leste	67.2 (64.8 to 69.7)	65.8 (63.5 to 68.1)	65.2 (62.9 to 67.7)	64.8 (61.1 to 68.9)	-0.1 (-0.3 to 0.1)	-1.6 (-4.9 to 2.0)
Vietnam	52.6 (51.3 to 53.9)	42.2 (40.7 to 43.7)	41.5 (39.8 to 43.1)	36.4 (33.8 to 39.1)	-1.0 (-1.3 to -0.7)	-13.8 (-17.6 to -10.0)
Sub-Saharan Africa						
Central Sub-Saharan Africa						
Angola	23.3 (21.0 to 25.6)	20.9 (19.0 to 23.0)	20.1 (18.4 to 22.2)	18.8 (16.2 to 21.9)	-0.7 (-1.3 to -0.1)	-10.0 (-17.3 to -0.7)
Central African Republic	24.8 (22.3 to 27.4)	18.9 (16.8 to 21.1)	18.3 (16.3 to 20.5)	15.5 (13.1 to 18.4)	-1.3 (-1.9 to -0.7)	-17.9 (-25.1 to -9.5)
Congo	21.4 (19.5 to 23.6)	20.5 (18.8 to 22.4)	19.7 (17.9 to 21.6)	18.8 (16.1 to 22.3)	-0.6 (-1.2 to 0.1)	-8.2 (-16.6 to 2.2)
Democratic Republic of the Congo	22.6 (20.5 to 25.1)	20.0 (18.0 to 22.1)	19.6 (17.5 to 21.7)	17.8 (15.0 to 20.7)	-0.8 (-1.3 to -0.2)	-11.2 (-18.1 to -2.7)
Equatorial Guinea	20.6 (18.3 to 23.1)	17.1 (15.3 to 19.1)	16.5 (14.7 to 18.4)	14.4 (12.1 to 17.1)	-1.2 (-1.8 to -0.5)	-16.0 (-23.7 to -7.7)
Gabon	25.1 (22.7 to 27.7)	21.7 (19.6 to 24.1)	20.9 (18.8 to 23.2)	19.2 (16.4 to 22.6)	-0.8 (-1.4 to -0.1)	-11.4 (-18.7 to -1.8)
Eastern Sub-Saharan Africa						
Burundi	21.4 (19.2 to 23.8)	19.6 (17.7 to 21.5)	19.2 (17.2 to 21.1)	18.7 (15.5 to 22.0)	-0.3 (-1.0 to 0.4)	-4.7 (-14.4 to 5.6)
Comoros	24.8 (22.6 to 27.1)	20.2 (18.5 to 22.0)	19.5 (17.9 to 21.3)	16.9 (14.7 to 19.5)	-1.2 (-1.8 to -0.6)	-16.6 (-23.7 to -9.2)
Djibouti	36.0 (33.9 to 38.1)	31.4 (29.1 to 33.8)	30.4 (28.1 to 32.9)	27.6 (24.1 to 31.7)	-0.9 (-1.4 to -0.3)	-12.1 (-18.5 to -4.8)
Eritrea	9.2 (8.2 to 10.4)	7.7 (6.8 to 8.7)	7.4 (6.5 to 8.4)	6.5 (5.3 to 7.8)	-1.2 (-1.9 to -0.5)	-16.2 (-24.3 to -6.9)
Ethiopia	9.4 (8.4 to 10.4)	7.8 (7.2 to 8.6)	7.6 (6.9 to 8.4)	6.8 (5.7 to 7.9)	-1.0 (-1.6 to -0.3)	-13.8 (-21.8 to -4.6)
Kenya	28.6 (27.0 to 30.2)	20.3 (18.9 to 21.9)	19.6 (18.1 to 21.3)	16.1 (13.9 to 18.5)	-1.6 (-2.1 to -1.0)	-21.0 (-26.9 to -14.5)
Madagascar	28.6 (26.2 to 31.0)	25.2 (23.1 to 27.5)	24.8 (22.6 to 27.1)	22.8 (19.6 to 26.5)	-0.7 (-1.2 to -0.1)	-9.8 (-16.8 to -2.0)
Malawi	25.5 (23.7 to 27.4)	21.2 (19.5 to 23.1)	20.5 (18.7 to 22.5)	17.9 (15.3 to 21.1)	-1.1 (-1.7 to -0.5)	-15.7 (-22.8 to -7.3)
Mozambique	27.2 (25.3 to 29.2)	24.6 (22.9 to 26.4)	23.5 (21.7 to 25.4)	22.2 (19.1 to 25.7)	-0.7 (-1.3 to -0.1)	-9.5 (-17.4 to -1.2)
Rwanda	26.3 (24.3 to 28.5)	23.1 (21.5 to 24.7)	22.2 (20.6 to 23.9)	20.0 (17.3 to 22.9)	-1.0 (-1.6 to -0.4)	-13.3 (-21.2 to -5.4)
Somalia	18.0 (16.1 to 20.0)	14.1 (12.6 to 15.9)	13.8 (12.3 to 15.4)	11.7 (9.8 to 13.7)	-1.3 (-1.9 to -0.7)	-17.4 (-24.5 to -9.7)
South Sudan	20.2 (18.1 to 22.3)	18.7 (16.7 to 20.9)	18.4 (16.4 to 20.4)	17.7 (15.1 to 20.6)	-0.4 (-0.9 to 0.2)	-5.3 (-12.9 to 2.5)
Tanzania	29.0 (27.0 to 31.2)	24.2 (22.3 to 26.2)	23.3 (21.4 to 25.4)	20.9 (18.1 to 24.1)	-1.0 (-1.5 to -0.4)	-14.0 (-20.3 to -6.5)
Uganda	24.8 (23.0 to 26.6)	20.4 (19.1 to 22.0)	19.5 (18.1 to 21.2)	17.9 (15.2 to 21.4)	-0.9 (-1.7 to 0.1)	-12.5 (-22.0 to 0.9)
Zambia	32.0 (30.1 to 34.1)	32.5 (30.1 to 34.9)	31.2 (28.8 to 33.9)	32.1 (27.3 to 37.9)	-0.1 (-0.7 to 0.7)	-1.2 (-10.6 to 10.7)
Southern Sub-Saharan Africa						
Botswana	39.9 (37.1 to 42.7)	34.8 (32.3 to 38.0)	34.0 (31.3 to 37.3)	31.5 (27.2 to 36.7)	-0.7 (-1.2 to -0.1)	-9.6 (-16.8 to -1.3)
Lesotho	35.2 (32.8 to 37.9)	34.9 (32.9 to 37.0)	34.2 (32.1 to 36.4)	34.0 (30.2 to 37.9)	-0.2 (-0.7 to 0.3)	-2.4 (-9.7 to 4.4)
Namibia	40.8 (38.6 to 43.2)	34.0 (31.5 to 36.6)	33.1 (30.6 to 36.0)	29.6 (25.6 to 34.2)	-0.9 (-1.5 to -0.4)	-12.8 (-19.8 to -5.2)
South Africa	51.1 (49.1 to 53.4)	41.0 (38.4 to 43.7)	40.3 (37.5 to 43.3)	35.5 (31.1 to 40.8)	-1.0 (-1.5 to -0.4)	-13.5 (-20.1 to -5.7)
Swaziland	17.4 (16.0 to 18.9)	10.5 (9.3 to 11.7)	10.0 (8.8 to 11.2)	7.5 (6.0 to 9.1)	-2.3 (-3.0 to -1.5)	-28.5 (-35.9 to -19.6)

	Estimate in 2000 (95% UIs)	Estimate in 2015 (95% UIs)	Estimate in 2017 (95% UIs)	Estimate in 2030 (95% UIs)	Annualised rate of change, 2015-2030 (95% UIs)	Percent change, 2015-2030 (95% UIs)
Zimbabwe	35.6 (33.3 to 38.0)	32.6 (30.5 to 34.7)	32.0 (29.9 to 34.3)	31.1 (27.5 to 35.2)	-0.3 (-0.8 to 0.3)	-4.7 (-11.8 to 3.8)
Western Sub-Saharan Africa						
Benin	18.6 (16.9 to 20.4)	14.2 (12.9 to 15.7)	13.8 (12.5 to 15.3)	11.6 (9.9 to 13.7)	-1.4 (-1.9 to -0.7)	-18.2 (-25.2 to -10.5)
Burkina Faso	22.9 (20.8 to 25.0)	17.9 (16.3 to 19.6)	17.4 (15.7 to 19.1)	14.8 (12.6 to 17.3)	-1.3 (-1.9 to -0.6)	-17.2 (-25.2 to -8.6)
Cameroon	20.3 (18.4 to 22.2)	15.1 (13.7 to 16.8)	14.6 (13.2 to 16.1)	11.8 (10.1 to 13.9)	-1.7 (-2.3 to -1.0)	-22.2 (-29.4 to -14.1)
Cape Verde	17.0 (15.2 to 18.9)	14.3 (12.8 to 15.8)	13.9 (12.4 to 15.4)	12.3 (10.3 to 14.6)	-1.0 (-1.6 to -0.4)	-14.0 (-21.8 to -5.7)
Chad	21.2 (19.5 to 22.8)	17.8 (16.3 to 19.1)	17.2 (15.8 to 18.7)	15.3 (13.2 to 17.6)	-1.0 (-1.5 to -0.4)	-13.8 (-20.7 to -6.3)
Cote d'Ivoire	24.3 (22.2 to 26.6)	24.3 (22.1 to 26.6)	23.6 (21.4 to 25.8)	23.4 (20.1 to 27.0)	-0.3 (-0.8 to 0.3)	-3.7 (-11.9 to 4.4)
The Gambia	29.0 (26.7 to 31.5)	21.7 (20.1 to 23.4)	21.1 (19.5 to 23.0)	17.4 (15.1 to 20.0)	-1.5 (-2.1 to -0.9)	-19.7 (-26.5 to -12.6)
Ghana	11.8 (10.6 to 13.1)	9.7 (8.7 to 11.0)	9.2 (8.2 to 10.5)	8.0 (6.5 to 9.9)	-1.3 (-2.0 to -0.5)	-17.6 (-26.4 to -6.8)
Guinea	15.7 (13.9 to 17.7)	13.3 (11.8 to 15.0)	13.0 (11.4 to 14.6)	11.7 (9.5 to 13.9)	-0.9 (-1.6 to -0.2)	-12.8 (-21.4 to -3.6)
Guinea-Bissau	12.7 (11.2 to 14.3)	11.1 (9.7 to 12.6)	10.8 (9.4 to 12.3)	9.6 (7.9 to 11.6)	-1.0 (-1.7 to -0.4)	-14.1 (-22.2 to -5.8)
Liberia	15.7 (14.1 to 17.4)	13.8 (12.5 to 15.3)	13.4 (12.1 to 14.9)	12.3 (10.4 to 14.4)	-0.8 (-1.4 to -0.2)	-11.4 (-18.8 to -3.2)
Mali	21.6 (19.7 to 23.6)	19.8 (18.1 to 21.8)	19.2 (17.5 to 21.2)	18.1 (15.6 to 21.2)	-0.6 (-1.1 to -0.0)	-8.6 (-15.8 to -0.0)
Mauritania	40.6 (38.0 to 43.3)	34.1 (31.1 to 37.2)	32.8 (29.8 to 36.1)	29.6 (24.9 to 35.0)	-1.0 (-1.6 to -0.3)	-13.2 (-21.5 to -4.1)
Niger	16.4 (14.7 to 18.1)	15.3 (13.8 to 16.9)	14.8 (13.2 to 16.4)	14.3 (11.9 to 17.0)	-0.4 (-1.1 to 0.2)	-6.2 (-15.8 to 3.8)
Nigeria	10.8 (9.6 to 12.0)	7.9 (7.0 to 9.0)	7.7 (6.7 to 8.8)	6.4 (5.2 to 8.0)	-1.4 (-2.2 to -0.6)	-19.2 (-28.6 to -8.6)
Sao Tome and Principe	10.0 (8.9 to 11.2)	8.0 (7.1 to 9.0)	7.7 (6.8 to 8.7)	6.7 (5.5 to 8.0)	-1.2 (-1.9 to -0.5)	-16.7 (-25.1 to -7.3)
Senegal	26.0 (24.0 to 28.1)	19.5 (17.9 to 21.4)	18.9 (17.2 to 20.9)	15.1 (12.8 to 17.9)	-1.7 (-2.4 to -1.0)	-22.8 (-30.0 to -14.5)
Sierra Leone	38.8 (36.0 to 41.7)	31.8 (29.9 to 34.0)	30.9 (28.8 to 33.1)	27.5 (24.2 to 31.1)	-1.0 (-1.5 to -0.5)	-13.6 (-20.4 to -6.9)
Togo	18.1 (16.4 to 19.9)	14.3 (13.0 to 15.8)	13.8 (12.4 to 15.3)	11.9 (10.0 to 14.2)	-1.3 (-1.9 to -0.5)	-17.0 (-25.0 to -7.6)
**Indicator 3.b.1: Coverage of eight vaccines in target populations (%)**						
Central Europe, Eastern Europe, and Central Asia						
Central Asia						
Armenia	51.2 (49.4 to 53.0)	90.6 (87.6 to 93.0)	93.9 (90.4 to 96.3)	95.7 (95.6 to 95.7)	0.4 (0.2 to 0.6)	5.6 (2.9 to 9.1)
Azerbaijan	17.5 (16.1 to 18.9)	73.3 (69.8 to 76.2)	74.5 (70.1 to 78.1)	77.5 (77.4 to 77.5)	0.4 (0.1 to 0.7)	5.8 (1.7 to 11.0)
Georgia	35.8 (33.8 to 37.6)	77.8 (74.2 to 81.0)	84.6 (79.8 to 88.3)	92.2 (92.1 to 92.2)	1.1 (0.9 to 1.4)	18.5 (13.7 to 24.2)
Kazakhstan	57.3 (55.9 to 58.4)	68.7 (64.7 to 72.0)	70.1 (64.8 to 74.4)	74.8 (74.7 to 74.8)	0.6 (0.2 to 1.0)	8.9 (3.8 to 15.6)
Kyrgyzstan	45.3 (43.2 to 47.3)	65.8 (63.1 to 68.0)	74.9 (70.5 to 78.6)	77.0 (77.0 to 77.0)	1.0 (0.8 to 1.3)	17.0 (13.2 to 22.0)
Mongolia	54.6 (53.0 to 56.0)	69.4 (66.9 to 71.2)	81.4 (78.5 to 83.4)	83.5 (83.5 to 83.6)	1.2 (1.1 to 1.5)	20.4 (17.4 to 24.9)
Tajikistan	38.7 (37.2 to 40.1)	83.0 (80.8 to 84.6)	83.7 (81.3 to 85.3)	97.0 (97.0 to 97.0)	1.0 (0.9 to 1.2)	16.9 (14.7 to 20.0)
Turkmenistan	46.0 (44.9 to 47.0)	67.8 (65.4 to 69.6)	68.4 (65.7 to 70.3)	82.4 (82.4 to 82.4)	1.3 (1.1 to 1.5)	21.6 (18.4 to 26.0)
Uzbekistan	51.7 (49.5 to 54.0)	94.9 (92.0 to 96.8)	95.3 (92.1 to 97.2)	97.0 (97.0 to 97.0)	0.2 (0.0 to 0.4)	2.3 (0.3 to 5.5)
Central Europe						
Albania	48.5 (47.5 to 49.2)	87.3 (87.1 to 87.4)	87.3 (87.1 to 87.4)	87.4 (87.4 to 87.4)	0.0 (-0.0 to 0.0)	0.1 (-0.0 to 0.3)
Bosnia and Herzegovina	55.2 (53.5 to 56.6)	62.5 (59.1 to 65.2)	57.8 (53.2 to 61.7)	72.5 (72.5 to 72.5)	1.0 (0.7 to 1.4)	16.2 (11.2 to 22.7)
Bulgaria	58.2 (56.6 to 59.4)	81.9 (79.1 to 83.9)	82.2 (79.0 to 84.3)	83.8 (83.8 to 83.8)	0.2 (-0.0 to 0.4)	2.4 (-0.1 to 5.9)
Croatia	59.4 (58.1 to 60.4)	72.5 (70.9 to 73.5)	71.4 (69.0 to 72.9)	84.1 (84.1 to 84.1)	1.0 (0.9 to 1.1)	16.0 (14.4 to 18.6)
Czech Republic	48.8 (48.0 to 49.3)	73.7 (73.1 to 74.2)	73.3 (72.5 to 73.9)	86.6 (86.6 to 86.6)	1.1 (1.0 to 1.1)	17.5 (16.8 to 18.5)
Hungary	62.0 (61.5 to 62.2)	74.1 (73.6 to 74.4)	74.5 (74.1 to 74.7)	87.2 (87.2 to 87.2)	1.1 (1.1 to 1.1)	17.7 (17.1 to 18.4)
Macedonia	46.5 (45.4 to 47.4)	67.1 (64.7 to 69.2)	65.3 (61.9 to 68.1)	78.8 (78.8 to 78.8)	1.1 (0.9 to 1.3)	17.4 (13.9 to 21.7)
Montenegro	34.2 (32.5 to 35.4)	61.8 (58.4 to 64.8)	59.2 (54.5 to 63.3)	73.5 (73.5 to 73.6)	1.2 (0.8 to 1.5)	19.0 (13.6 to 26.0)
Poland	61.4 (60.8 to 61.7)	73.2 (72.2 to 73.8)	73.2 (72.1 to 73.9)	86.6 (86.6 to 86.6)	1.1 (1.1 to 1.2)	18.3 (17.3 to 20.0)
Romania	61.6 (61.2 to 61.9)	67.4 (64.3 to 69.6)	65.2 (61.5 to 68.0)	80.0 (80.0 to 80.1)	1.1 (0.9 to 1.5)	18.8 (15.0 to 24.5)
Serbia	45.9 (44.8 to 46.8)	71.3 (69.3 to 72.8)	71.0 (68.7 to 72.7)	84.2 (84.2 to 84.2)	1.1 (1.0 to 1.3)	18.0 (15.7 to 21.4)
Slovakia	70.3 (69.0 to 71.4)	86.5 (85.8 to 87.0)	86.3 (85.2 to 86.9)	86.5 (86.5 to 86.5)	0.0 (-0.0 to 0.1)	0.0 (-0.5 to 0.9)
Slovenia	71.0 (69.2 to 72.4)	77.1 (75.0 to 79.3)	80.0 (77.5 to 82.3)	85.3 (85.3 to 85.3)	0.7 (0.5 to 0.9)	10.6 (7.5 to 13.7)
Eastern Europe						
Belarus	58.9 (57.5 to 60.1)	86.6 (85.8 to 87.0)	86.7 (85.8 to 87.1)	87.0 (87.0 to 87.0)	0.0 (0.0 to 0.1)	0.5 (0.1 to 1.4)
Estonia	49.2 (46.4 to 52.9)	80.5 (78.5 to 82.1)	83.2 (81.3 to 84.5)	97.0 (97.0 to 97.0)	1.2 (1.1 to 1.4)	20.6 (18.2 to 23.6)
Latvia	67.7 (65.5 to 69.3)	90.3 (88.2 to 92.3)	94.3 (92.5 to 95.7)	98.4 (98.3 to 98.4)	0.6 (0.4 to 0.7)	8.9 (6.6 to 11.5)
Lithuania	58.7 (57.4 to 59.8)	81.5 (79.3 to 83.3)	83.3 (81.6 to 84.7)	85.3 (85.3 to 85.3)	0.3 (0.2 to 0.5)	4.8 (2.4 to 7.6)
Moldova	46.1 (45.1 to 46.9)	68.8 (64.1 to 73.2)	71.0 (65.0 to 76.8)	77.9 (77.8 to 77.9)	0.8 (0.4 to 1.3)	13.4 (6.4 to 21.5)
Russia	48.5 (47.2 to 49.6)	77.1 (75.0 to 78.7)	81.3 (79.5 to 82.9)	86.8 (86.8 to 86.8)	0.8 (0.6 to 1.0)	12.6 (10.2 to 15.6)
Ukraine	49.6 (49.1 to 49.8)	41.1 (36.7 to 46.0)	45.2 (40.6 to 50.1)	56.2 (56.2 to 56.2)	2.1 (1.3 to 2.8)	37.2 (22.3 to 53.3)
High-income						
Australasia						
Australia	67.8 (65.3 to 69.6)	94.2 (91.5 to 96.0)	95.4 (92.6 to 97.2)	96.4 (96.4 to 96.4)	0.2 (0.0 to 0.4)	2.4 (0.4 to 5.4)
New Zealand	62.4 (59.7 to 64.9)	88.9 (85.7 to 91.6)	92.7 (89.4 to 95.0)	95.3 (95.3 to 95.3)	0.5 (0.3 to 0.7)	7.2 (4.1 to 11.2)
High-income Asia Pacific						
Brunei	59.9 (58.5 to 60.8)	73.7 (72.7 to 74.2)	74.1 (73.4 to 74.5)	90.0 (90.0 to 90.0)	1.3 (1.3 to 1.4)	22.2 (21.2 to 23.9)
Japan	34.8 (33.6 to 35.6)	72.8 (71.4 to 73.7)	84.6 (82.3 to 86.0)	86.0 (86.0 to 86.0)	1.1 (1.0 to 1.2)	18.1 (16.6 to 20.5)
South Korea	48.6 (45.7 to 51.0)	84.7 (83.4 to 85.6)	84.7 (83.1 to 85.7)	85.4 (85.4 to 85.4)	0.1 (-0.0 to 0.2)	0.8 (-0.3 to 2.4)
Singapore	59.7 (58.4 to 60.7)	80.4 (78.1 to 82.0)	81.9 (79.3 to 83.7)	84.4 (84.4 to 84.4)	0.3 (0.2 to 0.5)	5.0 (2.8 to 8.0)
High-income North America						
Canada	54.3 (52.3 to 56.3)	78.8 (75.1 to 82.0)	82.7 (77.9 to 86.7)	93.8 (93.8 to 93.8)	1.2 (0.9 to 1.5)	19.0 (14.4 to 24.9)
Greenland	58.6 (57.3 to 59.5)	69.2 (66.9 to 71.0)	70.8 (68.8 to 72.2)	84.3 (84.3 to 84.3)	1.3 (1.1 to 1.5)	21.8 (18.7 to 26.1)
United States	72.7 (71.4 to 73.8)	90.2 (87.7 to 92.2)	91.1 (87.9 to 93.5)	94.3 (94.3 to 94.3)	0.3 (0.1 to 0.5)	4.6 (2.3 to 7.5)
Southern Latin America						
Argentina	57.9 (54.7 to 61.1)	84.7 (81.1 to 87.8)	86.2 (81.3 to 89.9)	90.9 (90.9 to 90.9)	0.5 (0.2 to 0.8)	7.3 (3.5 to 12.1)
Chile	58.5 (57.1 to 59.6)	79.8 (77.2 to 81.9)	80.2 (76.7 to 82.6)	82.2 (82.2 to 82.2)	0.2 (0.0 to 0.4)	3.0 (0.4 to 6.5)
Uruguay	67.6 (65.6 to 69.4)	81.8 (78.9 to 83.8)	82.1 (79.1 to 84.2)	83.8 (83.8 to 83.8)	0.2 (0.0 to 0.4)	2.6 (0.1 to 6.2)
Western Europe						
Andorra	65.7 (63.3 to 67.6)	83.7 (82.1 to 84.8)	85.0 (83.8 to 85.8)	86.5 (86.5 to 86.5)	0.2 (0.1 to 0.3)	3.3 (2.0 to 5.3)
Austria	47.3 (44.4 to 50.1)	81.2 (78.2 to 83.1)	79.0 (74.8 to 81.9)	92.7 (92.7 to 92.7)	0.9 (0.7 to 1.1)	14.3 (11.6 to 18.6)
Belgium	55.7 (52.5 to 58.6)	95.3 (94.0 to 96.3)	96.0 (94.6 to 97.0)	98.7 (98.7 to 98.7)	0.2 (0.2 to 0.3)	3.6 (2.5 to 5.0)
Cyprus	57.4 (55.5 to 59.1)	81.2 (79.1 to 82.8)	82.4 (80.3 to 84.0)	84.5 (84.5 to 84.5)	0.3 (0.1 to 0.4)	4.1 (2.0 to 6.8)
Denmark	58.7 (57.4 to 59.7)	69.4 (67.2 to 71.1)	71.2 (69.5 to 72.4)	87.9 (87.9 to 87.9)	1.6 (1.4 to 1.8)	26.6 (23.6 to 30.7)
Finland	58.3 (56.8 to 59.5)	81.4 (79.1 to 83.2)	80.1 (75.9 to 82.8)	94.0 (94.0 to 94.0)	1.0 (0.8 to 1.2)	15.5 (13.0 to 18.8)
France	55.1 (52.7 to 57.6)	80.2 (78.2 to 81.9)	81.0 (78.4 to 82.8)	87.6 (87.6 to 87.6)	0.6 (0.5 to 0.8)	9.2 (7.0 to 12.0)

	Estimate in 2000 (95% UIs)	Estimate in 2015 (95% UIs)	Estimate in 2017 (95% UIs)	Estimate in 2030 (95% UIs)	Annualised rate of change, 2015-2030 (95% UIs)	Percent change, 2015-2030 (95% UIs)
Germany	52.4 (49.3 to 54.9)	83.4 (80.6 to 86.0)	87.2 (84.0 to 90.1)	97.1 (97.1 to 97.1)	1.0 (0.8 to 1.2)	16.4 (12.8 to 20.5)
Greece	63.2 (59.7 to 66.2)	85.0 (83.4 to 86.4)	88.1 (86.1 to 89.9)	99.1 (99.1 to 99.1)	1.0 (0.9 to 1.1)	16.6 (14.7 to 18.8)
Iceland	59.3 (57.5 to 60.6)	68.3 (65.7 to 70.4)	68.0 (65.0 to 70.2)	82.9 (82.9 to 82.9)	1.3 (1.1 to 1.6)	21.4 (17.8 to 26.2)
Ireland	49.6 (47.2 to 51.9)	82.3 (80.2 to 84.0)	83.0 (80.3 to 85.0)	95.6 (95.6 to 95.6)	1.0 (0.9 to 1.2)	16.1 (13.9 to 19.2)
Israel	71.3 (69.7 to 72.4)	94.3 (91.6 to 96.2)	95.2 (92.2 to 97.2)	96.5 (96.5 to 96.5)	0.2 (0.0 to 0.3)	2.3 (0.3 to 5.3)
Italy	53.1 (51.5 to 54.5)	82.7 (80.7 to 84.2)	85.1 (82.8 to 87.0)	97.1 (97.1 to 97.1)	1.1 (0.9 to 1.2)	17.5 (15.3 to 20.3)
Luxembourg	65.2 (62.4 to 67.6)	94.9 (93.7 to 95.9)	93.8 (92.2 to 95.2)	99.5 (99.5 to 99.5)	0.3 (0.2 to 0.4)	4.9 (3.8 to 6.2)
Malta	39.6 (37.6 to 41.5)	70.7 (69.0 to 72.0)	70.0 (67.8 to 71.5)	84.5 (84.4 to 84.5)	1.2 (1.1 to 1.3)	19.5 (17.3 to 22.3)
Netherlands	60.3 (59.0 to 61.2)	82.2 (79.8 to 84.0)	82.2 (79.2 to 84.2)	86.6 (86.6 to 86.6)	0.3 (0.2 to 0.5)	5.4 (3.1 to 8.5)
Norway	56.2 (53.9 to 58.0)	72.1 (70.1 to 73.8)	84.5 (80.4 to 87.4)	96.6 (96.6 to 96.6)	2.0 (1.8 to 2.1)	34.0 (30.9 to 37.9)
Portugal	62.0 (59.4 to 64.2)	82.1 (79.0 to 84.3)	84.5 (82.7 to 85.8)	86.2 (86.2 to 86.2)	0.3 (0.1 to 0.6)	5.1 (2.3 to 9.1)
Spain	67.7 (65.8 to 69.2)	72.2 (70.4 to 73.3)	83.1 (80.4 to 84.8)	85.5 (85.5 to 85.5)	1.1 (1.0 to 1.3)	18.5 (16.7 to 21.4)
Sweden	59.7 (58.6 to 60.6)	80.9 (79.3 to 82.3)	84.7 (82.5 to 86.9)	98.1 (98.1 to 98.1)	1.3 (1.2 to 1.4)	21.3 (19.2 to 23.8)
Switzerland	52.0 (49.7 to 53.9)	72.1 (70.1 to 74.0)	75.8 (73.2 to 78.2)	88.6 (88.6 to 88.6)	1.4 (1.2 to 1.6)	22.9 (19.6 to 26.4)
United Kingdom	55.0 (53.1 to 56.5)	81.7 (79.3 to 83.3)	92.4 (87.6 to 95.4)	95.9 (95.9 to 95.9)	1.1 (0.9 to 1.3)	17.5 (15.1 to 20.9)
Latin America and Caribbean						
Andean Latin America						
Bolivia	48.1 (46.3 to 49.8)	79.7 (76.3 to 82.2)	79.6 (75.6 to 82.7)	93.8 (93.8 to 93.9)	1.1 (0.9 to 1.4)	17.8 (14.2 to 22.9)
Ecuador	37.3 (35.3 to 39.3)	77.8 (71.5 to 83.1)	77.8 (70.4 to 83.5)	85.0 (85.0 to 85.1)	0.6 (0.2 to 1.2)	9.4 (2.3 to 18.9)
Peru	35.3 (34.2 to 36.3)	76.0 (71.8 to 79.8)	75.5 (69.6 to 80.4)	83.8 (83.7 to 83.8)	0.6 (0.3 to 1.0)	10.2 (5.0 to 16.6)
Caribbean						
Antigua and Barbuda	63.2 (60.5 to 65.6)	67.2 (65.1 to 69.1)	66.3 (63.6 to 68.6)	80.4 (80.4 to 80.5)	1.2 (1.0 to 1.4)	19.7 (16.4 to 23.5)
The Bahamas	43.1 (41.5 to 44.4)	76.5 (73.3 to 79.4)	82.1 (77.4 to 86.1)	89.3 (89.3 to 89.3)	1.0 (0.8 to 1.3)	16.7 (12.4 to 21.8)
Barbados	45.5 (42.5 to 48.5)	76.1 (72.8 to 78.9)	75.2 (70.8 to 78.5)	78.1 (78.1 to 78.1)	0.2 (-0.1 to 0.5)	2.6 (-0.9 to 7.2)
Belize	38.5 (36.7 to 40.1)	65.0 (62.2 to 67.6)	67.5 (63.4 to 71.6)	87.3 (87.3 to 87.4)	2.0 (1.7 to 2.3)	34.4 (29.3 to 40.4)
Bermuda	36.5 (35.6 to 37.1)	36.9 (36.1 to 37.2)	36.8 (36.1 to 37.2)	85.9 (85.9 to 85.9)	5.6 (5.6 to 5.8)	133.1 (130.6 to 137.6)
Cuba	53.6 (51.4 to 55.5)	70.9 (69.2 to 72.3)	71.2 (69.3 to 72.6)	84.2 (84.2 to 84.2)	1.1 (1.0 to 1.3)	18.7 (16.4 to 21.7)
Dominica	34.9 (34.0 to 35.6)	66.5 (63.6 to 68.7)	64.2 (60.1 to 67.3)	77.4 (77.4 to 77.4)	1.0 (0.8 to 1.3)	16.5 (12.7 to 21.6)
Dominican Republic	31.0 (29.7 to 32.1)	56.8 (53.1 to 60.3)	59.0 (53.4 to 63.9)	82.8 (82.7 to 82.9)	2.5 (2.1 to 3.0)	45.8 (37.3 to 56.1)
Grenada	58.5 (56.0 to 60.9)	65.0 (62.4 to 67.3)	62.7 (59.0 to 65.9)	66.6 (66.6 to 66.7)	0.2 (-0.1 to 0.4)	2.6 (-0.9 to 6.8)
Guyana	33.6 (32.7 to 34.4)	93.4 (90.8 to 95.5)	93.9 (90.9 to 96.1)	96.4 (96.4 to 96.4)	0.2 (0.1 to 0.4)	3.2 (0.9 to 6.1)
Haiti	22.1 (20.9 to 23.4)	47.1 (42.8 to 51.3)	52.8 (46.5 to 58.8)	65.5 (65.5 to 65.6)	2.2 (1.6 to 2.8)	39.4 (27.7 to 53.0)
Jamaica	33.6 (32.7 to 34.3)	76.4 (74.8 to 77.8)	78.8 (77.2 to 80.1)	86.1 (86.0 to 86.1)	0.8 (0.7 to 0.9)	12.6 (10.6 to 15.0)
Puerto Rico	52.0 (44.3 to 57.5)	78.5 (72.0 to 82.5)	78.9 (71.8 to 83.2)	91.9 (91.9 to 91.9)	1.1 (0.7 to 1.6)	17.3 (11.3 to 27.7)
Saint Lucia	42.2 (40.3 to 43.6)	65.8 (63.4 to 67.7)	62.6 (59.2 to 65.5)	77.4 (77.4 to 77.5)	1.1 (0.9 to 1.3)	17.7 (14.4 to 22.2)
Saint Vincent and the Grenadines	46.6 (45.5 to 47.5)	68.9 (66.6 to 70.6)	69.2 (66.5 to 71.1)	82.1 (82.1 to 82.2)	1.2 (1.0 to 1.4)	19.3 (16.4 to 23.3)
Suriname	31.4 (30.4 to 32.3)	56.0 (53.1 to 58.6)	62.6 (57.4 to 67.3)	74.7 (74.7 to 74.7)	1.9 (1.6 to 2.3)	33.5 (27.4 to 40.7)
Trinidad and Tobago	32.5 (30.8 to 34.3)	54.6 (49.9 to 58.9)	54.7 (48.6 to 60.1)	59.0 (59.0 to 59.1)	0.5 (0.0 to 1.1)	8.4 (0.2 to 18.4)
Virgin Islands, U.S.	35.9 (27.3 to 44.4)	60.9 (50.3 to 69.7)	60.9 (48.9 to 71.2)	73.4 (73.4 to 73.4)	1.3 (0.3 to 2.5)	21.4 (5.4 to 45.8)
Central Latin America						
Colombia	56.8 (54.8 to 58.5)	87.4 (82.8 to 90.7)	88.5 (83.7 to 92.1)	92.7 (92.6 to 92.7)	0.4 (0.1 to 0.8)	6.1 (2.1 to 11.9)
Costa Rica	63.2 (60.0 to 65.8)	85.7 (84.8 to 86.3)	86.6 (86.1 to 86.9)	87.3 (87.3 to 87.3)	0.1 (0.1 to 0.2)	1.9 (1.1 to 2.8)
El Salvador	56.4 (54.2 to 58.2)	90.8 (86.6 to 93.7)	93.1 (89.2 to 95.8)	95.7 (95.7 to 95.7)	0.4 (0.1 to 0.7)	5.5 (2.2 to 10.5)
Guatemala	34.9 (34.2 to 35.5)	63.3 (56.2 to 69.7)	78.1 (70.9 to 84.1)	85.4 (85.3 to 85.5)	2.0 (1.4 to 2.8)	35.3 (22.6 to 52.0)
Honduras	56.1 (54.1 to 57.7)	78.1 (74.3 to 81.2)	80.8 (77.3 to 83.3)	94.7 (94.6 to 94.7)	1.3 (1.0 to 1.6)	21.3 (16.6 to 27.4)
Mexico	64.0 (61.9 to 65.8)	82.1 (77.2 to 86.6)	84.2 (78.5 to 88.9)	89.0 (88.9 to 89.0)	0.5 (0.2 to 0.9)	8.5 (2.7 to 15.3)
Nicaragua	52.9 (50.7 to 54.6)	87.3 (87.1 to 87.4)	98.2 (97.4 to 98.9)	99.9 (99.9 to 99.9)	0.9 (0.9 to 0.9)	14.5 (14.3 to 14.7)
Panama	63.3 (61.2 to 65.4)	82.4 (77.3 to 86.7)	85.0 (79.2 to 89.8)	90.3 (90.2 to 90.3)	0.6 (0.3 to 1.0)	9.7 (4.1 to 16.8)
Venezuela	25.9 (24.1 to 27.8)	60.7 (56.0 to 65.2)	63.4 (57.0 to 69.2)	77.6 (77.5 to 77.6)	1.6 (1.2 to 2.2)	28.0 (19.1 to 38.5)
Tropical Latin America						
Brazil	58.9 (57.6 to 59.9)	94.8 (93.6 to 95.7)	92.3 (90.5 to 93.8)	97.2 (97.2 to 97.2)	0.2 (0.1 to 0.2)	2.6 (1.5 to 3.8)
Paraguay	30.9 (29.6 to 32.0)	91.1 (88.6 to 93.2)	93.8 (91.7 to 95.4)	97.7 (97.7 to 97.7)	0.5 (0.3 to 0.6)	7.3 (4.8 to 10.2)
North Africa and Middle East						
North Africa and Middle East						
Afghanistan	10.2 (8.8 to 11.8)	45.1 (41.0 to 49.3)	53.2 (47.6 to 58.6)	59.5 (59.4 to 59.5)	1.9 (1.3 to 2.5)	32.2 (20.6 to 45.0)
Algeria	45.6 (44.2 to 46.8)	70.9 (68.4 to 72.5)	83.0 (80.1 to 85.0)	84.5 (84.5 to 84.5)	1.2 (1.0 to 1.4)	19.2 (16.5 to 23.5)
Bahrain	71.8 (70.5 to 72.8)	98.6 (97.5 to 99.3)	98.5 (97.1 to 99.3)	99.0 (98.9 to 99.0)	0.0 (-0.0 to 0.1)	0.3 (-0.4 to 1.5)
Egypt	56.4 (54.9 to 57.6)	71.7 (70.0 to 72.9)	72.2 (70.4 to 73.4)	85.3 (85.3 to 85.3)	1.2 (1.1 to 1.3)	19.1 (17.1 to 21.9)
Iran	61.8 (61.4 to 62.1)	74.4 (73.6 to 74.7)	74.4 (73.7 to 74.7)	87.1 (87.1 to 87.1)	1.1 (1.0 to 1.1)	17.1 (16.6 to 18.4)
Iraq	38.1 (36.4 to 39.6)	55.5 (50.6 to 60.3)	64.0 (56.4 to 69.9)	79.5 (79.4 to 79.6)	2.4 (1.8 to 3.0)	43.6 (31.8 to 57.2)
Jordan	54.1 (53.3 to 54.7)	83.9 (81.8 to 85.2)	84.6 (82.5 to 85.9)	97.9 (97.9 to 97.9)	1.0 (0.9 to 1.2)	16.7 (14.9 to 19.7)
Kuwait	68.9 (66.7 to 70.5)	86.2 (84.8 to 86.9)	86.6 (85.4 to 87.1)	86.8 (86.8 to 86.8)	0.1 (-0.0 to 0.2)	0.8 (-0.1 to 2.4)
Lebanon	53.1 (51.0 to 54.9)	74.0 (69.8 to 77.6)	77.0 (73.0 to 80.2)	82.2 (82.2 to 82.2)	0.7 (0.4 to 1.1)	11.2 (5.9 to 17.7)
Libya	55.6 (53.2 to 57.7)	91.2 (87.4 to 94.2)	87.5 (81.7 to 91.7)	87.7 (87.7 to 87.7)	-0.3 (-0.5 to 0.0)	-3.8 (-6.8 to 0.4)
Morocco	39.4 (37.8 to 41.1)	92.5 (89.6 to 94.8)	93.5 (90.0 to 95.9)	95.8 (95.7 to 95.8)	0.2 (0.1 to 0.4)	3.5 (1.0 to 6.9)
Palestine	45.0 (43.7 to 46.0)	84.3 (83.1 to 85.3)	95.5 (93.8 to 96.7)	99.0 (99.0 to 99.0)	1.1 (1.0 to 1.2)	17.4 (16.1 to 19.2)
Oman	62.1 (61.7 to 62.2)	86.6 (85.6 to 87.1)	86.6 (85.5 to 87.1)	86.9 (86.9 to 86.9)	0.0 (-0.0 to 0.1)	0.4 (-0.2 to 1.5)
Qatar	64.8 (62.6 to 66.7)	94.8 (91.8 to 96.7)	95.0 (92.1 to 97.0)	96.9 (96.9 to 96.9)	0.1 (0.0 to 0.4)	2.2 (0.2 to 5.6)
Saudi Arabia	59.1 (57.5 to 60.3)	97.3 (95.2 to 98.4)	97.3 (95.0 to 98.6)	98.0 (98.0 to 98.0)	0.1 (-0.0 to 0.2)	0.8 (-0.4 to 3.0)
Sudan	22.8 (21.2 to 24.3)	80.5 (76.1 to 84.5)	83.0 (77.6 to 87.4)	91.7 (91.7 to 91.8)	0.9 (0.6 to 1.2)	14.0 (8.6 to 20.6)
Syria	51.6 (49.6 to 53.3)	40.4 (36.4 to 44.8)	44.3 (39.1 to 49.5)	59.0 (58.9 to 59.0)	2.5 (1.8 to 3.2)	46.5 (31.7 to 61.9)
Tunisia	54.1 (51.3 to 56.4)	71.3 (69.3 to 72.8)	71.5 (69.3 to 73.0)	84.5 (84.5 to 84.6)	1.1 (1.0 to 1.3)	18.7 (16.1 to 22.1)
Turkey	45.4 (43.1 to 47.4)	82.4 (80.1 to 83.8)	82.7 (80.5 to 84.1)	85.0 (85.0 to 85.0)	0.2 (0.1 to 0.4)	3.2 (1.5 to 6.2)
United Arab Emirates	64.9 (62.3 to 67.2)	92.7 (89.0 to 95.3)	92.4 (88.2 to 95.5)	94.2 (94.2 to 94.2)	0.1 (-0.1 to 0.4)	1.7 (-1.1 to 5.8)
Yemen	26.9 (25.5 to 28.3)	57.6 (51.9 to 62.9)	58.5 (51.0 to 65.5)	69.1 (69.0 to 69.2)	1.2 (0.6 to 1.9)	20.2 (9.9 to 33.1)
South Asia						
South Asia						
Bangladesh	30.0 (29.0 to 30.9)	75.5 (72.3 to 78.0)	78.5 (75.4 to 81.0)	83.4 (83.4 to 83.4)	0.7 (0.4 to 1.0)	10.6 (6.9 to 15.4)
Bhutan	47.6 (46.6 to 48.3)	72.9 (71.7 to 73.8)	73.4 (72.1 to 74.2)	74.2 (74.2 to 74.2)	0.1 (0.0 to 0.2)	1.7 (0.5 to 3.5)
India	22.6 (22.2 to 23.2)	50.8 (48.5 to 52.6)	63.0 (60.9 to 64.9)	87.0 (86.9 to 87.1)	3.6 (3.4 to 3.9)	71.5 (65.5 to 79.2)

	Estimate in 2000 (95% UIs)	Estimate in 2015 (95% UIs)	Estimate in 2017 (95% UIs)	Estimate in 2030 (95% UIs)	Annualised rate of change, 2015-2030 (95% UIs)	Percent change, 2015-2030 (95% UIs)
Nepal	32.0 (31.1 to 32.8)	65.6 (62.0 to 68.6)	71.4 (67.7 to 74.7)	83.3 (83.2 to 83.3)	1.6 (1.3 to 2.0)	27.0 (21.3 to 34.3)
Pakistan	27.5 (26.3 to 28.5)	62.3 (58.7 to 65.7)	70.9 (65.5 to 75.5)	88.8 (88.7 to 88.9)	2.4 (2.0 to 2.8)	42.6 (35.2 to 51.2)
Southeast Asia, East Asia, and Oceania						
East Asia						
China	46.0 (40.0 to 49.6)	81.0 (76.3 to 85.3)	87.6 (82.0 to 91.8)	98.5 (98.5 to 98.5)	1.3 (1.0 to 1.7)	21.8 (15.5 to 29.1)
North Korea	32.3 (30.6 to 33.8)	72.9 (71.1 to 73.9)	73.3 (71.7 to 74.2)	85.8 (85.8 to 85.8)	1.1 (1.0 to 1.2)	17.7 (16.1 to 20.6)
Taiwan (Province of China)	57.3 (51.1 to 60.5)	84.8 (82.0 to 86.4)	85.2 (82.6 to 86.6)	85.7 (85.7 to 85.7)	0.1 (-0.1 to 0.3)	1.0 (-0.8 to 4.5)
Oceania						
American Samoa	35.9 (34.4 to 36.8)	35.5 (33.6 to 36.6)	35.4 (33.6 to 36.5)	83.2 (83.2 to 83.2)	5.7 (5.5 to 6.0)	134.7 (127.6 to 147.6)
Federated States of Micronesia	48.8 (45.6 to 51.9)	59.3 (54.3 to 63.7)	56.9 (50.4 to 62.8)	71.0 (71.0 to 71.1)	1.2 (0.7 to 1.8)	20.0 (11.5 to 30.8)
Fiji	50.6 (48.1 to 52.8)	85.6 (82.2 to 88.7)	86.6 (82.3 to 90.0)	90.8 (90.8 to 90.9)	0.4 (0.2 to 0.7)	6.1 (2.4 to 10.5)
Guam	37.1 (36.6 to 37.3)	36.6 (35.5 to 37.1)	36.5 (35.4 to 37.1)	85.4 (85.4 to 85.4)	5.7 (5.6 to 5.8)	133.5 (130.2 to 140.4)
Kiribati	29.4 (27.1 to 31.4)	59.6 (53.7 to 65.2)	61.7 (54.2 to 68.8)	69.3 (69.2 to 69.3)	1.0 (0.4 to 1.7)	16.4 (6.2 to 29.0)
Marshall Islands	36.2 (33.5 to 39.3)	54.3 (49.2 to 59.4)	56.0 (49.6 to 62.5)	70.1 (70.0 to 70.2)	1.7 (1.1 to 2.4)	29.4 (18.1 to 42.6)
Northern Mariana Islands	37.1 (36.6 to 37.3)	36.9 (36.2 to 37.2)	36.9 (36.2 to 37.2)	86.0 (86.0 to 86.0)	5.6 (5.6 to 5.8)	133.0 (130.8 to 137.2)
Papua New Guinea	28.3 (26.1 to 30.3)	40.6 (37.1 to 44.1)	46.9 (41.3 to 52.6)	55.7 (55.6 to 55.8)	2.1 (1.6 to 2.7)	37.5 (26.2 to 50.3)
Samoa	36.6 (34.8 to 38.3)	45.7 (41.9 to 49.0)	46.2 (41.3 to 50.8)	60.6 (60.5 to 60.6)	1.9 (1.4 to 2.5)	32.7 (23.6 to 44.7)
Solomon Islands	37.0 (34.9 to 39.0)	57.3 (53.6 to 60.5)	61.5 (57.2 to 64.9)	76.4 (76.4 to 76.5)	1.9 (1.6 to 2.4)	33.6 (26.3 to 42.6)
Tonga	44.6 (43.0 to 46.0)	67.7 (65.2 to 69.8)	68.4 (65.5 to 70.7)	82.2 (82.2 to 82.2)	1.3 (1.1 to 1.5)	21.5 (17.7 to 26.0)
Vanuatu	43.5 (41.9 to 44.7)	37.9 (34.6 to 41.2)	38.7 (34.5 to 42.9)	51.2 (51.2 to 51.3)	2.0 (1.5 to 2.6)	35.7 (24.4 to 48.1)
Southeast Asia						
Cambodia	24.0 (22.5 to 25.4)	76.0 (72.7 to 78.6)	79.3 (75.7 to 82.0)	84.0 (84.0 to 84.0)	0.7 (0.4 to 1.0)	10.5 (6.9 to 15.5)
Indonesia	30.3 (28.6 to 32.0)	49.2 (45.7 to 52.6)	50.7 (46.2 to 54.7)	60.4 (60.4 to 60.5)	1.4 (0.9 to 1.9)	23.0 (14.8 to 32.2)
Laos	12.2 (11.0 to 13.6)	53.7 (49.6 to 57.3)	55.4 (50.0 to 59.8)	75.0 (75.0 to 75.1)	2.2 (1.8 to 2.8)	39.9 (30.9 to 51.4)
Malaysia	41.2 (39.2 to 42.9)	68.8 (66.0 to 70.8)	69.0 (66.1 to 71.1)	82.5 (82.4 to 82.5)	1.2 (1.0 to 1.5)	20.0 (16.5 to 25.0)
Maldives	45.0 (43.6 to 46.3)	74.2 (73.4 to 74.6)	74.3 (73.6 to 74.7)	86.9 (86.9 to 86.9)	1.1 (1.0 to 1.1)	17.1 (16.4 to 18.3)
Mauritius	40.5 (38.4 to 42.4)	76.8 (73.5 to 79.7)	84.3 (79.1 to 89.0)	93.0 (92.9 to 93.0)	1.3 (1.0 to 1.6)	21.1 (16.7 to 26.5)
Myanmar	32.5 (31.5 to 33.4)	53.0 (49.6 to 56.2)	61.8 (56.4 to 66.7)	71.7 (71.7 to 71.8)	2.0 (1.6 to 2.5)	35.4 (27.7 to 44.6)
Philippines	34.2 (32.9 to 35.5)	71.8 (67.1 to 75.8)	77.1 (71.2 to 81.9)	86.4 (86.3 to 86.4)	1.2 (0.9 to 1.7)	20.3 (14.0 to 28.8)
Sri Lanka	35.8 (34.9 to 36.5)	73.4 (72.0 to 74.2)	73.5 (72.2 to 74.3)	86.4 (86.4 to 86.4)	1.1 (1.0 to 1.2)	17.6 (16.4 to 19.9)
Seychelles	56.5 (54.6 to 58.0)	69.7 (67.3 to 71.6)	87.9 (82.7 to 92.3)	94.8 (94.7 to 94.8)	2.0 (1.9 to 2.3)	36.0 (32.4 to 40.7)
Thailand	58.7 (57.5 to 59.8)	59.0 (57.5 to 60.1)	59.1 (57.4 to 60.3)	84.1 (84.1 to 84.1)	2.4 (2.2 to 2.5)	42.6 (39.9 to 46.4)
Timor-Leste	12.0 (10.2 to 13.9)	54.9 (52.6 to 56.8)	63.8 (61.2 to 66.1)	70.8 (70.8 to 70.8)	1.7 (1.5 to 2.0)	29.0 (24.6 to 34.5)
Vietnam	38.7 (37.2 to 40.0)	65.7 (62.9 to 68.0)	66.6 (63.6 to 69.1)	81.5 (81.5 to 81.5)	1.4 (1.2 to 1.7)	24.1 (19.9 to 29.7)
Sub-Saharan Africa						
Central Sub-Saharan Africa						
Angola	18.9 (17.4 to 20.3)	36.5 (31.7 to 41.8)	37.5 (31.0 to 44.3)	59.5 (59.4 to 59.7)	3.3 (2.4 to 4.2)	63.8 (42.6 to 87.8)
Central African Republic	16.0 (14.6 to 17.5)	33.5 (29.4 to 38.0)	35.5 (29.2 to 41.9)	47.9 (47.8 to 48.0)	2.4 (1.5 to 3.3)	43.8 (25.9 to 63.2)
Congo	14.2 (12.4 to 15.9)	53.4 (47.3 to 58.8)	53.4 (45.9 to 60.1)	71.6 (71.5 to 71.7)	2.0 (1.3 to 2.8)	34.4 (21.7 to 51.2)
Democratic Republic of the Congo	14.8 (13.3 to 16.4)	53.2 (49.3 to 56.8)	54.9 (50.2 to 59.2)	70.4 (70.3 to 70.5)	1.9 (1.4 to 2.4)	32.5 (24.0 to 42.7)
Equatorial Guinea	11.2 (9.9 to 12.6)	17.2 (14.1 to 20.6)	20.0 (15.8 to 24.3)	35.3 (35.2 to 35.4)	4.8 (3.6 to 6.1)	106.5 (71.6 to 151.2)
Gabon	13.7 (12.2 to 15.3)	45.1 (42.2 to 47.8)	45.4 (41.5 to 48.5)	68.1 (68.0 to 68.1)	2.8 (2.4 to 3.2)	51.2 (42.3 to 61.5)
Eastern Sub-Saharan Africa						
Burundi	29.2 (28.0 to 30.1)	88.5 (85.5 to 90.8)	89.9 (85.9 to 92.6)	94.9 (94.9 to 94.9)	0.5 (0.3 to 0.7)	7.2 (4.6 to 11.0)
Comoros	24.8 (23.3 to 26.3)	50.7 (47.9 to 53.1)	59.9 (56.2 to 63.0)	78.4 (78.3 to 78.4)	2.9 (2.6 to 3.3)	54.7 (47.7 to 63.7)
Djibouti	19.5 (18.0 to 21.0)	60.8 (55.4 to 66.3)	62.6 (55.9 to 69.3)	76.1 (76.0 to 76.2)	1.5 (0.9 to 2.1)	25.5 (14.8 to 37.6)
Eritrea	31.6 (30.3 to 32.6)	97.9 (96.9 to 98.6)	98.5 (97.8 to 99.1)	99.8 (99.8 to 99.8)	0.1 (0.1 to 0.2)	2.0 (1.3 to 3.0)
Ethiopia	10.7 (9.5 to 11.8)	46.2 (41.5 to 51.0)	48.7 (42.2 to 55.2)	72.4 (72.2 to 72.5)	3.0 (2.3 to 3.7)	57.1 (41.5 to 74.2)
Kenya	26.9 (25.5 to 28.2)	72.0 (68.1 to 75.7)	71.8 (66.0 to 76.7)	81.2 (81.2 to 81.2)	0.8 (0.5 to 1.2)	12.9 (7.2 to 19.2)
Madagascar	23.1 (21.8 to 24.3)	68.0 (63.3 to 72.0)	70.5 (64.9 to 75.2)	84.1 (84.1 to 84.2)	1.4 (1.0 to 1.9)	23.8 (16.8 to 33.0)
Malawi	30.9 (29.8 to 31.8)	80.4 (76.9 to 83.2)	83.2 (78.6 to 86.9)	91.6 (91.6 to 91.7)	0.9 (0.6 to 1.2)	14.1 (10.1 to 19.2)
Mozambique	25.5 (24.1 to 26.9)	75.8 (71.7 to 79.5)	85.3 (81.9 to 87.9)	95.1 (95.1 to 95.1)	1.5 (1.2 to 1.9)	25.5 (19.6 to 32.6)
Rwanda	30.7 (29.5 to 31.8)	94.9 (92.8 to 96.4)	95.7 (93.4 to 97.2)	97.9 (97.9 to 97.9)	0.2 (0.1 to 0.4)	3.2 (1.6 to 5.5)
Somalia	12.4 (11.0 to 13.9)	12.8 (10.4 to 15.6)	12.8 (10.0 to 16.2)	19.7 (19.6 to 19.7)	2.9 (1.6 to 4.3)	55.2 (26.3 to 89.2)
South Sudan	7.2 (4.2 to 11.2)	32.3 (28.9 to 35.8)	27.7 (23.2 to 32.2)	41.4 (41.4 to 41.5)	1.7 (1.0 to 2.4)	28.6 (15.7 to 43.6)
Tanzania	30.9 (29.8 to 31.8)	82.0 (78.1 to 85.2)	85.0 (80.3 to 88.7)	92.8 (92.8 to 92.8)	0.8 (0.6 to 1.2)	13.2 (8.9 to 18.9)
Uganda	22.3 (21.1 to 23.6)	57.5 (54.1 to 60.7)	65.6 (60.9 to 70.2)	87.3 (87.3 to 87.4)	2.8 (2.4 to 3.2)	52.0 (43.9 to 61.3)
Zambia	30.9 (30.0 to 31.7)	89.4 (87.2 to 91.1)	94.0 (92.8 to 95.1)	99.3 (99.3 to 99.3)	0.7 (0.6 to 0.9)	11.1 (9.1 to 13.9)
Southern Sub-Saharan Africa						
Botswana	38.1 (36.0 to 40.1)	83.2 (76.7 to 88.4)	81.2 (72.7 to 87.8)	87.5 (87.5 to 87.6)	0.3 (-0.1 to 0.9)	5.4 (-0.9 to 14.1)
Lesotho	37.9 (35.9 to 39.7)	79.0 (76.2 to 81.2)	93.5 (91.6 to 95.1)	98.2 (98.2 to 98.2)	1.5 (1.3 to 1.7)	24.4 (21.0 to 28.9)
Namibia	26.8 (25.5 to 28.0)	77.0 (73.3 to 79.9)	85.0 (81.4 to 87.9)	96.1 (96.1 to 96.1)	1.5 (1.2 to 1.8)	25.0 (20.3 to 31.1)
South Africa	57.8 (55.3 to 60.0)	84.5 (79.8 to 88.1)	82.6 (76.3 to 87.3)	87.2 (87.1 to 87.2)	0.2 (-0.1 to 0.6)	3.2 (-1.1 to 9.2)
Swaziland	47.7 (45.3 to 49.7)	86.1 (81.7 to 89.2)	86.7 (80.8 to 90.7)	92.2 (92.1 to 92.2)	0.5 (0.2 to 0.8)	7.1 (3.3 to 12.8)
Zimbabwe	29.4 (27.6 to 31.2)	81.4 (77.4 to 85.1)	81.1 (76.0 to 85.7)	86.5 (86.5 to 86.5)	0.4 (0.1 to 0.7)	6.3 (1.7 to 11.7)
Western Sub-Saharan Africa						
Benin	26.1 (25.0 to 27.3)	54.5 (50.3 to 57.8)	51.8 (46.0 to 56.5)	70.1 (70.0 to 70.2)	1.7 (1.3 to 2.2)	28.9 (21.2 to 39.6)
Burkina Faso	23.8 (22.5 to 25.0)	85.7 (82.3 to 88.5)	87.0 (83.3 to 90.0)	95.4 (95.3 to 95.4)	0.7 (0.5 to 1.0)	11.3 (7.8 to 15.8)
Cameroon	23.9 (22.6 to 25.1)	68.1 (63.6 to 71.9)	72.1 (66.8 to 76.1)	89.2 (89.1 to 89.2)	1.8 (1.4 to 2.3)	31.0 (24.0 to 40.3)
Cape Verde	28.2 (26.8 to 29.6)	70.6 (68.4 to 72.2)	70.5 (67.6 to 72.4)	72.1 (72.1 to 72.1)	0.1 (-0.0 to 0.4)	2.2 (-0.1 to 5.4)
Chad	10.9 (9.7 to 12.0)	31.0 (27.9 to 34.2)	30.6 (26.5 to 35.1)	46.2 (46.2 to 46.3)	2.7 (2.0 to 3.4)	49.5 (35.6 to 65.8)
Cote d'Ivoire	28.6 (26.5 to 30.8)	53.8 (49.0 to 57.6)	63.9 (58.4 to 68.5)	85.7 (85.7 to 85.8)	3.1 (2.7 to 3.7)	59.7 (48.9 to 75.2)
The Gambia	50.7 (48.4 to 52.7)	85.8 (81.6 to 89.1)	85.0 (80.0 to 89.0)	91.5 (91.5 to 91.6)	0.4 (0.2 to 0.8)	6.7 (2.8 to 12.2)
Ghana	29.7 (28.6 to 30.7)	86.8 (82.1 to 90.2)	91.2 (87.0 to 94.0)	95.6 (95.6 to 95.6)	0.6 (0.4 to 1.0)	10.2 (5.9 to 16.5)
Guinea	16.7 (15.3 to 18.1)	28.7 (24.9 to 32.5)	28.6 (23.9 to 33.5)	43.2 (43.1 to 43.4)	2.7 (1.9 to 3.7)	51.3 (33.0 to 73.7)
Guinea-Bissau	20.2 (18.6 to 21.8)	68.5 (64.0 to 72.1)	66.2 (59.8 to 71.3)	84.5 (84.4 to 84.5)	1.4 (1.1 to 1.8)	23.4 (17.1 to 31.9)
Liberia	21.3 (19.9 to 22.7)	47.4 (42.1 to 52.1)	61.1 (54.1 to 67.0)	77.7 (77.6 to 77.7)	3.3 (2.7 to 4.1)	64.2 (49.0 to 84.5)
Mali	19.2 (17.6 to 20.9)	52.9 (48.7 to 56.8)	52.2 (45.8 to 58.2)	73.1 (73.0 to 73.2)	2.2 (1.7 to 2.7)	38.6 (28.8 to 50.1)
Mauritania	21.0 (19.4 to 22.6)	65.0 (59.6 to 69.7)	70.7 (64.5 to 75.8)	88.8 (88.8 to 88.9)	2.1 (1.6 to 2.7)	36.8 (27.5 to 49.2)
Niger	14.2 (12.7 to 15.7)	60.8 (55.4 to 65.7)	68.6 (61.7 to 74.7)	81.4 (81.3 to 81.4)	1.9 (1.4 to 2.6)	34.0 (23.8 to 46.8)

	Estimate in 2000 (95% UIs)	Estimate in 2015 (95% UIs)	Estimate in 2017 (95% UIs)	Estimate in 2030 (95% UIs)	Annualised rate of change, 2015-2030 (95% UIs)	Percent change, 2015-2030 (95% UIs)
Nigeria	13.0 (11.7 to 14.5)	30.0 (26.3 to 33.9)	28.1 (23.1 to 33.5)	41.2 (41.2 to 41.3)	2.1 (1.3 to 3.0)	38.0 (21.8 to 56.6)
Sao Tome and Principe	31.4 (29.9 to 32.6)	81.6 (79.2 to 83.3)	93.2 (90.0 to 95.3)	97.7 (97.7 to 97.8)	1.2 (1.1 to 1.4)	19.9 (17.3 to 23.4)
Senegal	25.0 (23.4 to 26.5)	80.9 (78.0 to 83.7)	85.3 (81.7 to 88.4)	93.5 (93.5 to 93.5)	1.0 (0.7 to 1.2)	15.6 (11.7 to 19.8)
Sierra Leone	18.2 (16.8 to 19.6)	79.5 (74.7 to 83.6)	80.6 (75.3 to 85.1)	89.5 (89.4 to 89.5)	0.8 (0.5 to 1.2)	12.6 (7.1 to 19.9)
Togo	23.0 (21.6 to 24.4)	70.8 (66.3 to 74.3)	74.5 (69.3 to 78.2)	90.6 (90.6 to 90.7)	1.6 (1.3 to 2.1)	28.1 (22.0 to 36.6)
**Indicator 3.c.1: Physicians, nurses and midwives, and pharmacist per 1,000 population**						
Central Europe, Eastern Europe, and Central Asia						
Central Asia						
Armenia	5.5 (4.0 to 7.2)	11.6 (9.0 to 14.8)	12.1 (9.1 to 15.7)	21.6 (13.1 to 32.9)	4.0 (2.0 to 5.8)	84.5 (34.5 to 139.4)
Azerbaijan	6.0 (4.2 to 8.4)	12.4 (8.2 to 17.4)	12.8 (8.7 to 18.1)	20.6 (11.6 to 33.9)	3.2 (1.3 to 5.1)	63.4 (21.2 to 114.4)
Georgia	7.9 (5.4 to 11.1)	12.3 (9.0 to 16.3)	12.9 (9.4 to 17.2)	18.9 (10.7 to 29.7)	2.7 (0.5 to 4.8)	52.7 (8.3 to 106.6)
Kazakhstan	7.9 (5.5 to 11.2)	13.2 (8.8 to 18.5)	13.8 (9.1 to 19.3)	20.7 (10.9 to 34.7)	2.8 (0.6 to 4.8)	54.8 (9.7 to 106.9)
Kyrgyzstan	4.2 (2.9 to 5.8)	5.9 (4.1 to 8.3)	6.1 (4.2 to 8.5)	8.0 (4.4 to 12.9)	1.8 (-0.3 to 3.8)	33.1 (-5.0 to 77.8)
Mongolia	6.0 (4.7 to 7.5)	12.1 (8.7 to 16.6)	12.6 (8.8 to 17.6)	22.6 (12.4 to 36.3)	4.0 (2.1 to 5.8)	84.6 (36.6 to 139.8)
Tajikistan	3.5 (2.6 to 4.7)	7.2 (5.4 to 9.4)	7.6 (5.5 to 10.2)	13.4 (7.7 to 21.6)	4.0 (2.0 to 5.8)	83.1 (34.7 to 139.9)
Turkmenistan	5.6 (3.7 to 7.7)	9.6 (6.4 to 13.5)	10.0 (6.9 to 13.9)	15.7 (8.7 to 25.6)	3.2 (1.1 to 5.2)	63.2 (18.4 to 117.7)
Uzbekistan	3.8 (2.6 to 5.3)	7.2 (4.9 to 9.9)	7.7 (5.2 to 10.5)	13.3 (7.1 to 21.6)	4.0 (1.8 to 6.0)	83.5 (31.6 to 145.0)
Central Europe						
Albania	4.7 (3.8 to 5.7)	7.0 (5.8 to 8.3)	7.3 (5.9 to 8.9)	10.5 (6.8 to 15.1)	2.6 (0.8 to 4.2)	48.2 (13.4 to 88.8)
Bosnia and Herzegovina	2.8 (1.9 to 4.0)	4.1 (2.8 to 5.7)	4.3 (2.9 to 6.1)	6.5 (3.5 to 10.9)	2.9 (0.8 to 5.1)	57.2 (13.1 to 113.9)
Bulgaria	6.8 (5.2 to 8.4)	10.2 (7.3 to 13.7)	10.4 (7.4 to 14.2)	14.5 (7.7 to 23.8)	2.2 (0.1 to 4.2)	40.1 (1.6 to 88.2)
Croatia	8.7 (7.4 to 10.1)	12.0 (10.9 to 13.3)	12.3 (10.8 to 13.9)	16.3 (12.4 to 20.6)	2.0 (0.7 to 3.1)	34.9 (11.7 to 60.3)
Czech Republic	13.2 (12.0 to 14.4)	16.7 (14.9 to 18.4)	17.1 (14.8 to 19.6)	20.9 (16.2 to 26.7)	1.5 (0.3 to 2.6)	25.1 (4.8 to 47.2)
Hungary	7.8 (7.3 to 8.4)	7.1 (6.2 to 8.0)	7.0 (6.0 to 8.2)	6.7 (5.1 to 8.8)	-0.4 (-1.6 to 0.7)	-6.0 (-21.2 to 11.8)
Macedonia	3.3 (2.3 to 4.7)	5.8 (4.0 to 8.3)	6.1 (4.1 to 8.6)	9.4 (5.2 to 15.5)	3.0 (1.0 to 5.2)	58.5 (16.8 to 116.8)
Montenegro	4.7 (3.2 to 6.6)	6.3 (4.4 to 8.7)	6.5 (4.6 to 9.2)	8.6 (5.0 to 14.5)	2.0 (-0.2 to 4.0)	36.2 (-2.4 to 83.5)
Poland	6.9 (5.5 to 8.5)	9.1 (6.7 to 11.9)	9.2 (6.7 to 12.2)	11.9 (6.9 to 19.1)	1.7 (-0.2 to 3.5)	29.8 (-2.5 to 69.2)
Romania	4.5 (3.8 to 5.4)	6.3 (5.7 to 7.0)	6.5 (5.6 to 7.4)	8.4 (6.2 to 11.1)	1.9 (0.5 to 3.4)	33.2 (7.5 to 65.7)
Serbia	3.1 (2.2 to 4.2)	4.5 (3.1 to 6.3)	4.7 (3.2 to 6.7)	6.5 (3.5 to 11.2)	2.3 (0.2 to 4.4)	42.7 (3.8 to 92.1)
Slovakia	11.0 (10.0 to 12.1)	14.4 (12.9 to 16.1)	14.7 (12.7 to 16.9)	19.1 (14.7 to 24.4)	1.8 (0.7 to 3.0)	32.3 (11.8 to 56.9)
Slovenia	8.4 (6.9 to 10.2)	12.1 (9.9 to 14.7)	12.3 (9.8 to 15.1)	16.6 (11.1 to 23.9)	2.0 (0.5 to 3.6)	36.6 (7.1 to 70.4)
Eastern Europe						
Belarus	7.6 (5.6 to 10.3)	14.3 (10.6 to 18.3)	14.5 (10.4 to 18.8)	23.1 (13.1 to 37.7)	3.0 (0.8 to 5.3)	59.5 (13.0 to 121.2)
Estonia	9.4 (8.3 to 10.6)	9.8 (8.4 to 11.4)	9.8 (8.0 to 11.7)	10.3 (7.2 to 14.4)	0.2 (-1.3 to 1.8)	4.2 (-17.8 to 31.1)
Latvia	10.3 (9.3 to 11.3)	10.2 (9.1 to 11.4)	10.2 (8.8 to 11.7)	10.3 (7.8 to 13.3)	0.1 (-1.1 to 1.2)	1.2 (-15.6 to 19.6)
Lithuania	8.6 (7.7 to 9.7)	10.7 (9.5 to 12.0)	10.9 (9.4 to 12.6)	13.6 (10.3 to 17.5)	1.5 (0.3 to 2.7)	26.6 (4.7 to 50.5)
Moldova	2.5 (1.7 to 3.5)	4.5 (3.0 to 6.1)	4.7 (3.2 to 6.4)	6.9 (3.9 to 11.0)	2.8 (0.8 to 4.9)	53.5 (13.1 to 107.6)
Russia	10.3 (8.6 to 12.1)	14.5 (11.5 to 17.8)	14.6 (11.2 to 18.4)	18.5 (11.6 to 28.2)	1.5 (-0.3 to 3.2)	25.6 (-4.1 to 61.0)
Ukraine	8.4 (6.0 to 11.3)	12.7 (9.2 to 16.7)	12.9 (9.2 to 17.5)	17.4 (9.7 to 28.2)	1.9 (-0.3 to 4.0)	35.3 (-3.7 to 83.1)
High-income						
Australasia						
Australia	18.6 (15.6 to 21.8)	16.4 (12.9 to 20.8)	16.8 (12.9 to 21.8)	16.9 (11.0 to 26.0)	0.1 (-1.5 to 1.8)	1.9 (-19.9 to 30.3)
New Zealand	16.4 (13.5 to 19.5)	24.3 (18.8 to 30.6)	25.1 (19.0 to 32.2)	34.1 (21.2 to 51.0)	2.1 (0.4 to 3.8)	38.6 (6.1 to 75.9)
High-income Asia Pacific						
Brunei	6.9 (4.6 to 9.6)	10.0 (6.7 to 13.9)	10.4 (7.0 to 14.6)	14.9 (8.0 to 25.3)	2.5 (0.4 to 4.6)	47.3 (6.8 to 98.0)
Japan	11.6 (8.8 to 15.1)	13.6 (10.8 to 16.8)	13.6 (10.6 to 17.2)	16.1 (10.2 to 24.3)	1.0 (-0.8 to 2.9)	17.4 (-11.8 to 55.1)
South Korea	4.3 (3.3 to 5.5)	8.3 (6.7 to 10.2)	8.8 (6.9 to 11.1)	15.2 (9.3 to 23.3)	3.9 (1.9 to 5.7)	80.5 (32.5 to 135.8)
Singapore	7.2 (4.9 to 10.3)	12.9 (9.0 to 18.4)	13.3 (9.1 to 18.8)	21.1 (11.6 to 34.4)	3.1 (1.1 to 5.0)	61.2 (17.7 to 112.0)
High-income North America						
Canada	15.9 (13.0 to 19.2)	19.0 (14.0 to 24.9)	19.6 (14.2 to 26.2)	24.0 (13.9 to 38.4)	1.4 (-0.4 to 3.3)	24.7 (-5.9 to 64.8)
Greenland	6.7 (4.4 to 9.8)	8.7 (5.8 to 12.9)	8.9 (5.9 to 13.1)	11.4 (5.9 to 19.9)	1.6 (-0.5 to 3.8)	29.2 (-7.7 to 77.0)
United States	12.5 (10.3 to 15.1)	13.2 (10.8 to 16.1)	13.0 (10.4 to 16.2)	13.8 (9.2 to 20.1)	0.2 (-1.4 to 1.9)	3.8 (-19.2 to 32.2)
Southern Latin America						
Argentina	4.1 (2.8 to 5.5)	5.4 (3.7 to 7.4)	5.5 (3.7 to 7.5)	7.4 (3.8 to 12.3)	1.8 (-0.7 to 4.1)	33.7 (-9.6 to 85.3)
Chile	3.3 (2.9 to 3.8)	6.3 (5.4 to 7.3)	6.6 (5.5 to 8.0)	11.8 (8.4 to 16.6)	4.1 (2.7 to 5.6)	86.8 (49.7 to 132.4)
Uruguay	5.5 (4.6 to 6.4)	9.6 (7.7 to 11.7)	10.2 (7.9 to 12.8)	16.6 (10.6 to 24.4)	3.5 (1.8 to 5.1)	71.0 (31.2 to 116.4)
Western Europe						
Andorra	16.3 (10.5 to 23.7)	18.4 (11.5 to 26.3)	18.7 (11.8 to 27.1)	21.8 (10.6 to 38.8)	0.9 (-1.4 to 3.2)	16.7 (-19.1 to 61.0)
Austria	13.0 (11.5 to 14.4)	15.2 (12.9 to 17.6)	15.6 (12.6 to 18.5)	18.8 (12.7 to 26.1)	1.3 (-0.3 to 2.8)	22.4 (-4.3 to 51.5)
Belgium	17.1 (16.0 to 18.1)	17.5 (16.0 to 19.2)	17.3 (15.4 to 19.4)	17.8 (14.3 to 22.0)	0.1 (-0.8 to 1.0)	1.2 (-11.9 to 16.6)
Cyprus	8.0 (7.1 to 9.0)	10.5 (9.4 to 11.8)	11.1 (9.6 to 12.8)	14.7 (11.1 to 19.3)	2.2 (0.9 to 3.4)	39.0 (15.0 to 65.5)
Denmark	14.6 (12.7 to 16.6)	15.3 (12.5 to 18.4)	15.0 (11.7 to 18.8)	15.5 (9.9 to 23.0)	-0.0 (-1.7 to 1.6)	0.5 (-22.7 to 27.3)
Finland	21.1 (19.2 to 22.9)	17.5 (15.4 to 19.8)	17.6 (14.9 to 20.5)	15.9 (11.7 to 21.2)	-0.7 (-2.0 to 0.7)	-9.2 (-26.4 to 10.4)
France	13.1 (12.1 to 14.1)	12.4 (11.0 to 14.0)	11.9 (9.9 to 13.9)	10.9 (8.2 to 14.3)	-0.9 (-2.1 to 0.3)	-12.8 (-26.8 to 4.6)
Germany	14.5 (13.6 to 15.5)	23.5 (21.6 to 25.5)	24.6 (22.0 to 27.5)	36.0 (29.1 to 44.3)	2.8 (1.8 to 3.8)	52.9 (30.3 to 76.8)
Greece	8.0 (7.3 to 8.7)	9.4 (8.6 to 10.3)	9.4 (8.2 to 10.5)	10.8 (8.3 to 13.5)	0.9 (-0.3 to 2.0)	14.2 (-4.2 to 34.0)
Iceland	19.9 (17.7 to 22.2)	20.2 (17.7 to 23.0)	20.4 (17.2 to 23.9)	21.2 (15.5 to 28.7)	0.2 (-1.1 to 1.6)	4.3 (-14.7 to 27.8)
Ireland	12.8 (11.5 to 14.2)	16.8 (14.6 to 19.2)	16.8 (14.1 to 19.8)	21.3 (15.0 to 28.3)	1.5 (0.2 to 2.8)	26.1 (2.4 to 52.0)
Israel	7.5 (6.0 to 9.2)	9.7 (7.7 to 12.0)	10.0 (7.7 to 12.8)	12.8 (7.8 to 19.1)	1.7 (-0.1 to 3.4)	30.9 (-0.8 to 67.7)
Italy	9.2 (8.2 to 10.2)	12.0 (10.6 to 13.7)	12.3 (10.5 to 14.4)	15.8 (11.5 to 21.0)	1.8 (0.4 to 3.1)	30.7 (6.6 to 58.2)
Luxembourg	9.9 (8.8 to 11.1)	13.6 (11.7 to 15.7)	13.6 (11.4 to 16.3)	17.8 (12.9 to 24.7)	1.7 (0.3 to 3.2)	30.6 (5.2 to 61.4)
Malta	7.3 (5.0 to 10.4)	10.8 (7.0 to 16.1)	11.2 (7.2 to 16.8)	15.8 (8.1 to 27.5)	2.3 (0.1 to 4.4)	44.1 (1.3 to 94.8)
Netherlands	23.1 (21.0 to 25.6)	26.0 (22.6 to 30.0)	26.1 (22.0 to 30.9)	29.8 (22.1 to 40.4)	0.8 (-0.4 to 2.0)	13.8 (-5.5 to 36.0)
Norway	18.9 (17.0 to 21.2)	27.1 (23.2 to 31.3)	27.4 (22.7 to 32.8)	35.9 (25.2 to 49.4)	1.8 (0.3 to 3.3)	31.7 (4.9 to 63.2)
Portugal	6.6 (5.8 to 7.6)	11.1 (9.5 to 12.8)	11.8 (9.8 to 14.2)	18.6 (13.2 to 25.6)	3.4 (2.0 to 4.8)	67.1 (34.5 to 104.4)
Spain	9.4 (8.6 to 10.3)	13.6 (12.2 to 15.2)	13.6 (11.8 to 15.8)	18.1 (14.0 to 23.8)	1.8 (0.6 to 3.0)	32.0 (10.2 to 57.6)
Sweden	12.3 (11.1 to 13.7)	19.5 (17.1 to 22.2)	20.2 (17.0 to 23.8)	27.4 (19.6 to 37.1)	2.2 (0.9 to 3.6)	40.0 (14.0 to 71.0)
Switzerland	20.5 (19.1 to 22.0)	22.6 (20.5 to 24.8)	22.5 (19.8 to 25.6)	25.8 (20.1 to 32.4)	0.9 (-0.3 to 1.9)	14.1 (-3.7 to 32.5)
United Kingdom	16.2 (15.2 to 17.3)	18.7 (16.7 to 20.7)	18.7 (16.0 to 21.4)	21.0 (15.7 to 26.6)	0.7 (-0.5 to 1.8)	11.8 (-7.0 to 31.5)
Latin America and Caribbean						

Andean Latin America	Estimate in 2000 (95% UIs)	Estimate in 2015 (95% UIs)	Estimate in 2017 (95% UIs)	Estimate in 2030 (95% UIs)	Annualised rate of change, 2015-2030 (95% UIs)	Percent change, 2015-2030 (95% UIs)
Bolivia	3.9 (3.2 to 4.6)	6.8 (4.8 to 9.3)	7.2 (5.1 to 9.9)	12.3 (6.8 to 20.0)	3.7 (1.7 to 5.6)	76.7 (29.8 to 131.3)
Ecuador	3.1 (2.6 to 3.6)	5.2 (3.9 to 6.8)	5.4 (4.0 to 7.2)	8.1 (4.6 to 13.3)	2.8 (0.9 to 4.8)	53.6 (14.0 to 105.9)
Peru	4.2 (2.8 to 6.2)	5.7 (3.7 to 8.0)	5.8 (3.8 to 8.2)	7.8 (4.1 to 13.7)	2.0 (-0.2 to 4.1)	36.0 (-2.9 to 86.0)
Caribbean						
Antigua and Barbuda	6.9 (4.5 to 9.9)	9.4 (6.0 to 13.6)	9.8 (6.4 to 14.1)	13.4 (6.6 to 22.4)	2.2 (-0.0 to 4.3)	41.1 (-0.4 to 90.5)
The Bahamas	13.4 (9.1 to 19.3)	18.9 (12.7 to 26.9)	19.3 (12.9 to 27.0)	27.4 (14.5 to 47.6)	2.3 (0.2 to 4.3)	43.0 (2.7 to 91.9)
Barbados	9.3 (6.1 to 13.3)	11.8 (7.7 to 16.7)	12.1 (7.8 to 17.0)	15.5 (8.0 to 26.3)	1.6 (-0.4 to 3.8)	29.7 (-6.3 to 75.7)
Belize	2.6 (1.8 to 3.8)	4.8 (3.1 to 6.9)	5.0 (3.2 to 7.2)	8.3 (4.2 to 14.2)	3.5 (1.3 to 5.7)	72.3 (21.6 to 133.6)
Bermuda	18.1 (11.8 to 25.8)	27.9 (17.7 to 40.3)	27.6 (17.3 to 40.2)	37.3 (18.0 to 65.1)	1.8 (-0.4 to 3.9)	32.0 (-5.9 to 79.8)
Cuba	12.7 (11.1 to 14.4)	23.5 (17.8 to 30.6)	24.5 (18.2 to 32.7)	39.0 (23.5 to 61.9)	3.2 (1.5 to 5.0)	63.3 (24.4 to 111.2)
Dominica	4.0 (2.7 to 5.8)	6.0 (4.0 to 8.5)	6.3 (4.2 to 8.9)	9.2 (4.8 to 16.0)	2.7 (0.6 to 4.9)	51.1 (9.0 to 109.1)
Dominican Republic	4.7 (3.6 to 6.1)	5.8 (3.9 to 8.3)	6.0 (3.9 to 8.8)	7.7 (3.5 to 14.9)	1.6 (-1.0 to 4.1)	28.6 (-13.9 to 86.2)
Grenada	4.1 (2.7 to 5.9)	5.7 (3.6 to 8.2)	5.9 (3.7 to 8.4)	8.2 (4.2 to 13.8)	2.2 (0.1 to 4.3)	41.6 (1.1 to 89.2)
Guyana	2.6 (1.8 to 3.7)	4.3 (2.8 to 6.1)	4.5 (3.0 to 6.4)	6.7 (3.5 to 11.6)	2.8 (0.8 to 5.0)	54.5 (12.1 to 112.1)
Haiti	1.2 (0.8 to 1.7)	1.9 (1.3 to 2.8)	2.0 (1.4 to 2.9)	3.0 (1.6 to 5.1)	2.8 (0.7 to 4.7)	53.2 (11.1 to 103.3)
Jamaica	5.1 (3.4 to 7.5)	6.9 (4.5 to 10.1)	7.2 (4.8 to 10.6)	9.9 (5.2 to 17.3)	2.2 (-0.1 to 4.2)	41.0 (-1.5 to 86.5)
Puerto Rico	9.3 (5.9 to 13.6)	12.7 (8.5 to 18.6)	12.9 (8.6 to 18.7)	17.2 (9.0 to 30.2)	1.9 (-0.4 to 4.2)	34.7 (-6.5 to 87.0)
Saint Lucia	4.7 (3.1 to 6.8)	7.3 (4.8 to 10.3)	7.7 (5.0 to 10.9)	11.7 (5.8 to 19.7)	2.9 (1.0 to 5.0)	56.9 (15.7 to 110.7)
Saint Vincent and the Grenadines	3.5 (2.4 to 5.1)	5.6 (3.5 to 8.0)	5.8 (3.7 to 8.4)	8.6 (4.2 to 15.5)	2.7 (0.6 to 5.0)	52.3 (10.1 to 110.4)
Suriname	5.2 (3.4 to 7.4)	6.8 (4.6 to 9.2)	6.7 (4.6 to 9.1)	8.1 (4.7 to 13.4)	1.1 (-1.0 to 3.2)	19.0 (-13.4 to 62.7)
Trinidad and Tobago	4.0 (3.1 to 5.1)	6.0 (3.8 to 8.9)	6.0 (3.8 to 8.9)	8.2 (4.0 to 15.4)	1.9 (-0.4 to 4.0)	35.1 (-5.6 to 82.4)
Virgin Islands, U.S.	11.8 (7.5 to 16.8)	15.5 (9.9 to 22.8)	15.6 (9.9 to 23.0)	19.7 (10.3 to 35.1)	1.4 (-0.7 to 3.6)	25.3 (-9.7 to 72.1)
Central Latin America						
Colombia	3.1 (2.1 to 4.4)	4.9 (3.3 to 7.1)	5.2 (3.5 to 7.4)	7.7 (4.1 to 12.8)	2.8 (0.7 to 4.8)	54.8 (10.8 to 106.5)
Costa Rica	3.4 (2.7 to 4.2)	3.8 (2.8 to 5.1)	3.9 (2.8 to 5.4)	4.8 (2.7 to 8.3)	1.4 (-0.8 to 3.4)	24.2 (-10.9 to 66.5)
El Salvador	2.4 (1.8 to 3.1)	4.0 (3.2 to 5.0)	4.3 (3.3 to 5.4)	6.9 (4.4 to 10.3)	3.5 (1.7 to 5.3)	69.9 (28.1 to 122.3)
Guatemala	1.8 (1.3 to 2.5)	3.1 (2.1 to 4.4)	3.2 (2.2 to 4.6)	5.3 (2.9 to 8.8)	3.3 (1.2 to 5.5)	65.4 (19.7 to 127.5)
Honduras	1.7 (1.1 to 2.4)	2.9 (2.0 to 3.9)	3.0 (2.1 to 4.2)	4.9 (2.7 to 7.8)	3.4 (1.4 to 5.2)	67.5 (23.1 to 119.1)
Mexico	5.1 (3.9 to 6.4)	7.7 (6.0 to 9.8)	7.8 (5.8 to 10.2)	11.2 (6.6 to 17.8)	2.4 (0.4 to 4.3)	44.0 (6.2 to 89.4)
Nicaragua	1.1 (0.8 to 1.6)	2.1 (1.4 to 3.0)	2.2 (1.5 to 3.3)	3.8 (1.9 to 6.6)	3.7 (1.5 to 5.9)	77.3 (25.4 to 141.2)
Panama	4.5 (3.5 to 5.6)	6.6 (4.5 to 9.0)	7.0 (4.8 to 9.6)	10.2 (5.4 to 16.7)	2.8 (0.8 to 4.6)	53.5 (13.3 to 100.2)
Venezuela	4.2 (3.3 to 5.1)	5.1 (3.8 to 6.8)	5.0 (3.5 to 6.7)	5.8 (3.3 to 9.1)	0.6 (-1.1 to 2.4)	11.0 (-14.8 to 43.5)
Tropical Latin America						
Brazil	4.4 (3.4 to 5.7)	7.8 (6.5 to 9.2)	8.1 (6.4 to 9.9)	12.9 (8.1 to 19.7)	3.2 (1.3 to 5.3)	64.4 (20.7 to 121.0)
Paraguay	2.6 (2.1 to 3.1)	3.5 (2.7 to 4.6)	3.8 (2.8 to 5.0)	5.3 (3.1 to 8.4)	2.6 (0.7 to 4.4)	48.5 (10.7 to 94.1)
North Africa and Middle East						
North Africa and Middle East						
Afghanistan	0.4 (0.3 to 0.6)	0.7 (0.5 to 1.0)	0.7 (0.5 to 1.0)	1.1 (0.6 to 1.8)	2.9 (0.9 to 4.9)	55.5 (15.2 to 109.6)
Algeria	2.0 (1.4 to 2.9)	4.6 (3.2 to 6.3)	4.8 (3.4 to 6.7)	9.3 (5.2 to 15.0)	4.6 (2.5 to 6.5)	102.0 (45.6 to 165.6)
Bahrain	7.5 (5.3 to 10.7)	12.6 (8.8 to 17.3)	12.9 (9.0 to 17.7)	19.7 (11.5 to 32.7)	2.8 (0.9 to 4.7)	54.9 (14.2 to 103.7)
Egypt	3.4 (2.6 to 4.3)	4.0 (3.6 to 4.2)	4.1 (3.7 to 4.5)	5.0 (3.8 to 6.3)	1.5 (0.1 to 2.9)	26.1 (1.6 to 53.9)
Iran	1.8 (1.5 to 2.1)	3.4 (2.6 to 4.3)	3.7 (2.8 to 4.8)	6.3 (3.7 to 10.0)	4.0 (2.1 to 5.9)	85.4 (36.5 to 142.9)
Iraq	1.3 (0.9 to 1.8)	1.8 (1.4 to 2.2)	1.9 (1.5 to 2.4)	2.7 (1.6 to 4.1)	2.7 (0.5 to 4.9)	52.1 (7.3 to 108.7)
Jordan	5.6 (4.9 to 6.3)	7.7 (5.9 to 9.9)	8.0 (6.0 to 10.4)	11.3 (6.5 to 17.7)	2.4 (0.6 to 4.1)	44.4 (9.3 to 84.5)
Kuwait	9.2 (6.3 to 13.2)	14.7 (10.2 to 20.4)	15.3 (10.7 to 21.3)	23.2 (13.0 to 37.0)	2.9 (0.9 to 4.9)	56.6 (14.6 to 108.5)
Lebanon	3.8 (2.6 to 5.4)	6.5 (4.6 to 8.8)	6.8 (4.8 to 9.2)	11.1 (6.5 to 17.9)	3.5 (1.4 to 5.4)	70.9 (24.3 to 125.7)
Libya	5.8 (4.1 to 8.2)	6.7 (4.6 to 9.2)	7.0 (4.8 to 9.6)	8.6 (5.1 to 14.2)	1.6 (0.0 to 3.4)	27.6 (0.1 to 65.8)
Morocco	1.3 (0.9 to 1.8)	2.5 (1.7 to 3.5)	2.7 (1.9 to 3.7)	4.8 (2.7 to 7.8)	4.1 (2.1 to 6.2)	88.3 (37.9 to 155.0)
Palestine	1.3 (0.9 to 1.9)	2.3 (1.6 to 3.2)	2.5 (1.7 to 3.4)	4.1 (2.4 to 6.4)	3.8 (1.9 to 5.7)	79.4 (32.0 to 135.9)
Oman	3.8 (2.6 to 5.4)	9.6 (6.5 to 13.7)	10.1 (6.9 to 14.5)	20.8 (12.1 to 33.5)	5.1 (3.4 to 6.7)	115.5 (66.0 to 173.7)
Qatar	11.9 (8.0 to 16.9)	18.2 (12.6 to 25.2)	18.7 (12.9 to 26.3)	26.8 (15.6 to 44.3)	2.4 (0.5 to 4.6)	45.4 (7.2 to 98.0)
Saudi Arabia	4.6 (3.1 to 6.3)	10.4 (7.0 to 14.5)	11.4 (7.7 to 16.0)	25.1 (13.8 to 40.8)	5.7 (3.7 to 7.4)	138.4 (74.8 to 205.0)
Sudan	0.7 (0.5 to 1.0)	1.4 (1.0 to 2.0)	1.6 (1.1 to 2.2)	2.9 (1.6 to 4.7)	4.5 (2.6 to 6.4)	98.5 (47.1 to 159.6)
Syria	1.6 (1.1 to 2.2)	2.4 (1.6 to 3.3)	2.5 (1.8 to 3.6)	3.8 (2.2 to 6.1)	2.9 (1.1 to 4.8)	56.6 (17.4 to 105.8)
Tunisia	2.2 (1.6 to 3.1)	3.9 (2.7 to 5.5)	4.1 (2.8 to 5.8)	6.7 (3.7 to 11.3)	3.5 (1.6 to 5.5)	70.1 (26.8 to 126.5)
Turkey	2.2 (1.6 to 3.0)	3.1 (2.4 to 3.9)	3.3 (2.5 to 4.3)	4.9 (3.0 to 7.6)	3.0 (0.8 to 5.1)	59.7 (12.4 to 114.5)
United Arab Emirates	11.4 (8.0 to 16.0)	18.4 (12.7 to 26.1)	18.5 (12.8 to 26.1)	27.8 (15.5 to 46.4)	2.6 (0.8 to 4.5)	49.7 (13.2 to 95.9)
Yemen	0.9 (0.6 to 1.3)	1.3 (0.9 to 1.9)	1.3 (0.9 to 1.8)	1.8 (1.1 to 3.0)	1.9 (0.1 to 3.8)	35.0 (1.8 to 76.9)
South Asia						
South Asia						
Bangladesh	0.7 (0.5 to 1.0)	1.2 (0.9 to 1.8)	1.4 (0.9 to 2.0)	2.3 (1.2 to 4.0)	3.8 (1.6 to 5.9)	79.8 (26.8 to 143.2)
Bhutan	1.4 (1.0 to 2.1)	2.9 (2.0 to 4.3)	3.2 (2.1 to 4.6)	6.0 (3.1 to 10.1)	4.6 (2.3 to 6.5)	100.9 (41.6 to 165.4)
India	0.7 (0.6 to 1.0)	1.4 (1.0 to 1.8)	1.5 (1.1 to 2.0)	2.8 (1.5 to 4.7)	4.4 (2.1 to 6.5)	95.0 (36.3 to 163.2)
Nepal	0.7 (0.5 to 1.1)	1.4 (1.0 to 2.0)	1.5 (1.0 to 2.2)	2.8 (1.5 to 4.8)	4.2 (1.9 to 6.3)	91.7 (33.5 to 156.7)
Pakistan	1.0 (0.7 to 1.4)	1.6 (1.2 to 2.1)	1.7 (1.3 to 2.2)	2.8 (1.7 to 4.2)	3.4 (1.5 to 5.6)	68.2 (24.6 to 132.4)
Southeast Asia, East Asia, and Oceania						
East Asia						
China	3.4 (2.5 to 4.5)	7.8 (6.0 to 10.1)	8.3 (6.2 to 10.8)	17.5 (10.6 to 25.8)	5.2 (3.5 to 7.1)	121.7 (67.9 to 189.1)
North Korea	1.2 (0.8 to 1.8)	1.4 (0.9 to 2.1)	1.4 (1.0 to 2.1)	1.7 (0.9 to 2.9)	1.1 (-1.1 to 3.2)	18.8 (-15.7 to 61.2)
Taiwan (Province of China)	3.5 (2.7 to 4.5)	5.3 (4.3 to 6.7)	5.5 (4.3 to 7.1)	8.1 (5.1 to 12.7)	2.7 (0.7 to 4.7)	50.8 (11.7 to 102.0)
Oceania						
American Samoa	4.3 (2.9 to 6.1)	5.0 (3.3 to 7.0)	5.1 (3.5 to 7.2)	6.1 (3.3 to 9.8)	1.2 (-0.9 to 3.3)	21.1 (-12.0 to 63.1)
Federated States of Micronesia	1.2 (0.8 to 1.8)	2.1 (1.4 to 3.1)	1.9 (1.3 to 2.7)	2.6 (1.6 to 4.2)	1.3 (0.1 to 2.4)	21.8 (1.5 to 43.6)
Fiji	2.8 (2.0 to 4.1)	3.5 (2.3 to 5.1)	3.6 (2.4 to 5.3)	4.6 (2.4 to 7.9)	1.6 (-0.5 to 3.6)	29.0 (-7.6 to 71.4)
Guam	9.4 (6.4 to 13.6)	10.0 (6.7 to 14.2)	10.1 (6.6 to 14.4)	11.0 (6.0 to 18.5)	0.5 (-1.5 to 2.5)	8.7 (-19.9 to 46.5)
Kiribati	1.4 (0.9 to 2.0)	2.0 (1.3 to 2.9)	2.1 (1.4 to 3.1)	2.9 (1.7 to 4.7)	2.4 (0.7 to 4.2)	44.3 (11.0 to 87.9)
Marshall Islands	2.3 (1.6 to 3.3)	3.6 (2.4 to 5.3)	3.6 (2.4 to 5.4)	5.3 (3.0 to 8.5)	2.4 (1.0 to 3.8)	43.4 (15.5 to 75.9)
Northern Mariana Islands	7.1 (4.8 to 10.2)	7.0 (4.7 to 10.1)	7.3 (4.8 to 10.4)	8.1 (4.5 to 13.9)	0.8 (-1.2 to 3.0)	14.6 (-16.2 to 56.2)
Papua New Guinea	0.8 (0.5 to 1.2)	0.9 (0.6 to 1.4)	0.9 (0.6 to 1.4)	1.2 (0.6 to 2.0)	1.3 (-0.7 to 3.5)	23.3 (-9.7 to 68.7)

	**Estimate in 2000 (95% UIs)**	**Estimate in 2015 (95% UIs)**	**Estimate in 2017 (95% UIs)**	**Estimate in 2030 (95% UIs)**	**Annualised rate of change, 2015-2030 (95% UIs)**	**Percent change, 2015-2030 (95% UIs)**
Samoa	1.4 (0.9 to 2.0)	1.4 (0.9 to 2.0)	1.4 (0.9 to 2.0)	1.6 (0.9 to 2.7)	1.0 (-0.7 to 2.8)	17.5 (-9.8 to 52.2)
Solomon Islands	0.6 (0.4 to 0.8)	0.8 (0.5 to 1.1)	0.8 (0.5 to 1.1)	1.1 (0.6 to 1.7)	2.2 (0.3 to 4.0)	39.9 (4.5 to 83.5)
Tonga	1.6 (1.1 to 2.3)	2.0 (1.4 to 3.0)	2.2 (1.5 to 3.2)	3.0 (1.7 to 4.9)	2.4 (0.8 to 4.0)	44.8 (12.0 to 81.1)
Vanuatu	1.0 (0.7 to 1.3)	1.5 (1.0 to 2.2)	1.6 (1.1 to 2.3)	2.1 (1.2 to 3.4)	2.1 (0.6 to 3.6)	37.8 (10.2 to 70.5)
Southeast Asia						
Cambodia	1.1 (0.8 to 1.4)	2.9 (2.4 to 3.6)	3.0 (2.4 to 3.8)	6.0 (3.7 to 8.8)	4.6 (2.7 to 6.3)	102.6 (50.8 to 158.9)
Indonesia	1.6 (1.1 to 2.2)	3.1 (2.1 to 4.4)	3.3 (2.3 to 4.7)	5.8 (3.2 to 9.6)	4.0 (2.0 to 6.0)	84.3 (34.7 to 144.3)
Laos	0.9 (0.6 to 1.3)	1.4 (1.0 to 2.1)	1.5 (1.0 to 2.2)	2.6 (1.4 to 4.4)	3.7 (1.7 to 5.7)	75.8 (29.5 to 135.9)
Malaysia	3.4 (2.6 to 4.3)	5.9 (3.9 to 8.4)	6.3 (4.2 to 9.1)	10.6 (5.2 to 18.4)	3.6 (1.3 to 5.9)	74.1 (22.1 to 142.8)
Maldives	2.2 (1.4 to 3.3)	5.0 (3.1 to 7.5)	5.5 (3.4 to 8.1)	11.2 (5.7 to 19.2)	5.2 (3.1 to 7.2)	122.5 (58.5 to 194.5)
Mauritius	2.2 (1.4 to 3.1)	3.9 (2.6 to 5.5)	4.0 (2.7 to 5.9)	6.8 (3.6 to 11.6)	3.6 (1.4 to 5.8)	73.7 (23.0 to 138.6)
Myanmar	0.6 (0.4 to 0.8)	2.0 (1.3 to 2.9)	2.2 (1.4 to 3.1)	5.0 (2.7 to 8.0)	6.0 (4.2 to 7.5)	146.7 (87.3 to 207.8)
Philippines	2.5 (2.2 to 2.8)	1.9 (1.6 to 2.4)	2.0 (1.6 to 2.5)	2.0 (1.3 to 3.0)	0.2 (-1.4 to 1.9)	4.1 (-19.5 to 32.6)
Sri Lanka	1.6 (1.0 to 2.3)	2.6 (1.7 to 3.8)	2.7 (1.8 to 4.0)	4.3 (2.2 to 7.7)	3.3 (1.3 to 5.5)	66.3 (20.9 to 129.3)
Seychelles	3.3 (2.2 to 4.8)	4.5 (2.9 to 6.7)	4.8 (3.1 to 7.0)	6.8 (3.5 to 12.1)	2.5 (0.4 to 4.7)	48.2 (5.6 to 103.6)
Thailand	4.4 (3.1 to 6.0)	7.0 (4.8 to 9.8)	7.4 (5.0 to 10.3)	11.0 (6.0 to 18.5)	2.9 (0.9 to 4.8)	55.6 (15.3 to 106.8)
Timor-Leste	0.5 (0.3 to 0.7)	1.1 (0.7 to 1.5)	1.2 (0.8 to 1.6)	1.9 (1.1 to 3.0)	3.7 (2.1 to 5.3)	76.6 (37.2 to 121.6)
Vietnam	0.9 (0.6 to 1.4)	2.0 (1.3 to 3.0)	2.2 (1.4 to 3.3)	4.2 (2.1 to 7.5)	4.7 (2.2 to 6.9)	104.4 (39.7 to 181.2)
Sub-Saharan Africa						
Central Sub-Saharan Africa						
Angola	0.5 (0.3 to 0.7)	0.8 (0.5 to 1.2)	0.9 (0.6 to 1.3)	1.5 (0.8 to 2.4)	3.7 (1.3 to 6.0)	76.6 (22.4 to 144.9)
Central African Republic	0.4 (0.2 to 0.5)	0.4 (0.3 to 0.6)	0.5 (0.3 to 0.7)	0.6 (0.3 to 1.0)	2.1 (0.5 to 3.9)	38.5 (7.6 to 79.2)
Congo	0.8 (0.5 to 1.1)	1.4 (1.0 to 2.0)	1.5 (1.0 to 2.1)	2.6 (1.4 to 4.3)	3.8 (1.6 to 5.8)	79.1 (27.4 to 137.1)
Democratic Republic of the Congo	0.3 (0.2 to 0.4)	0.5 (0.3 to 0.7)	0.5 (0.3 to 0.8)	0.8 (0.4 to 1.3)	2.8 (1.0 to 4.8)	53.5 (15.9 to 105.2)
Equatorial Guinea	0.7 (0.4 to 1.0)	1.8 (1.1 to 2.6)	1.9 (1.2 to 2.9)	4.5 (2.2 to 7.7)	6.0 (3.8 to 8.1)	150.0 (77.6 to 239.5)
Gabon	1.2 (0.8 to 1.8)	1.8 (1.2 to 2.6)	1.9 (1.3 to 2.8)	3.0 (1.6 to 5.2)	3.3 (1.1 to 5.5)	66.3 (17.5 to 126.6)
Eastern Sub-Saharan Africa						
Burundi	0.9 (0.6 to 1.3)	1.4 (0.9 to 1.9)	1.4 (0.9 to 2.0)	2.0 (1.1 to 3.3)	2.3 (0.2 to 4.2)	42.1 (3.7 to 88.7)
Comoros	1.5 (0.9 to 2.1)	2.4 (1.7 to 3.4)	2.6 (1.8 to 3.6)	4.1 (2.3 to 6.6)	3.3 (1.1 to 5.4)	65.6 (18.8 to 124.2)
Djibouti	0.9 (0.6 to 1.3)	2.1 (1.5 to 3.0)	2.3 (1.6 to 3.2)	4.3 (2.4 to 7.0)	4.5 (2.4 to 6.5)	100.0 (44.1 to 165.4)
Eritrea	1.2 (0.8 to 1.8)	1.6 (1.1 to 2.2)	1.7 (1.2 to 2.4)	2.5 (1.5 to 4.0)	3.0 (1.4 to 4.7)	58.2 (23.5 to 102.3)
Ethiopia	0.2 (0.1 to 0.3)	0.5 (0.3 to 0.7)	0.5 (0.3 to 0.8)	1.0 (0.5 to 1.7)	4.5 (2.5 to 6.3)	98.6 (45.9 to 158.0)
Kenya	1.9 (1.2 to 2.6)	3.0 (2.0 to 4.3)	3.2 (2.2 to 4.6)	5.0 (2.7 to 8.4)	3.3 (1.2 to 5.5)	66.4 (19.3 to 127.2)
Madagascar	1.4 (0.9 to 1.9)	1.7 (1.1 to 2.3)	1.8 (1.2 to 2.5)	2.2 (1.2 to 3.7)	1.8 (-0.2 to 3.8)	32.3 (-3.2 to 76.1)
Malawi	0.9 (0.6 to 1.3)	1.7 (1.1 to 2.4)	1.8 (1.2 to 2.5)	3.0 (1.6 to 5.0)	3.7 (1.7 to 5.4)	75.7 (28.7 to 125.9)
Mozambique	0.4 (0.3 to 0.5)	0.8 (0.6 to 1.0)	0.8 (0.6 to 1.1)	1.6 (0.8 to 2.6)	4.7 (2.5 to 6.8)	103.7 (46.4 to 176.8)
Rwanda	0.8 (0.6 to 1.0)	1.7 (1.4 to 2.0)	1.8 (1.5 to 2.2)	3.1 (2.1 to 4.5)	3.9 (2.2 to 5.7)	82.1 (40.0 to 134.1)
Somalia	0.6 (0.4 to 0.8)	1.0 (0.6 to 1.4)	1.1 (0.7 to 1.5)	1.6 (0.8 to 2.8)	3.4 (1.5 to 5.3)	69.1 (24.9 to 119.9)
South Sudan	0.8 (0.5 to 1.1)	1.1 (0.7 to 1.6)	1.2 (0.8 to 1.7)	1.7 (0.9 to 2.8)	2.6 (0.5 to 4.8)	50.5 (8.1 to 105.8)
Tanzania	1.3 (1.0 to 1.5)	3.2 (2.6 to 4.0)	3.6 (2.8 to 4.6)	7.6 (4.8 to 11.3)	5.6 (3.9 to 7.3)	133.0 (78.5 to 199.1)
Uganda	1.1 (0.9 to 1.2)	1.8 (1.4 to 2.2)	1.9 (1.4 to 2.4)	2.9 (1.7 to 4.4)	3.1 (1.4 to 4.7)	60.6 (24.1 to 103.7)
Zambia	1.6 (1.3 to 1.8)	2.4 (2.0 to 2.9)	2.6 (2.0 to 3.2)	4.3 (2.6 to 6.3)	3.7 (1.9 to 5.3)	75.6 (32.8 to 123.0)
Southern Sub-Saharan Africa						
Botswana	1.5 (1.0 to 2.1)	3.0 (2.0 to 4.3)	3.2 (2.1 to 4.5)	6.0 (3.0 to 10.1)	4.3 (2.0 to 6.4)	93.1 (34.1 to 162.6)
Lesotho	0.4 (0.3 to 0.7)	0.7 (0.4 to 1.0)	0.7 (0.4 to 1.1)	1.1 (0.5 to 2.0)	3.2 (0.9 to 5.5)	64.9 (13.8 to 128.1)
Namibia	1.1 (0.7 to 1.9)	1.6 (1.0 to 2.4)	1.7 (1.0 to 2.5)	2.6 (1.2 to 4.9)	3.1 (0.1 to 5.9)	63.1 (2.0 to 143.8)
South Africa	2.6 (2.0 to 3.3)	3.0 (2.3 to 3.8)	2.9 (2.2 to 3.8)	3.5 (2.1 to 5.6)	0.9 (-0.9 to 2.9)	15.7 (-13.2 to 54.7)
Swaziland	0.7 (0.4 to 1.2)	1.0 (0.6 to 1.6)	1.1 (0.6 to 1.6)	1.6 (0.7 to 2.9)	2.6 (0.1 to 5.1)	51.3 (1.3 to 116.0)
Zimbabwe	1.1 (0.6 to 1.7)	1.0 (0.6 to 1.5)	1.0 (0.6 to 1.6)	1.1 (0.6 to 2.0)	0.9 (-1.2 to 3.1)	15.9 (-16.5 to 59.7)
Western Sub-Saharan Africa						
Benin	0.7 (0.4 to 1.0)	0.9 (0.6 to 1.3)	0.9 (0.6 to 1.4)	1.3 (0.7 to 2.2)	2.4 (0.1 to 4.7)	45.3 (0.8 to 103.2)
Burkina Faso	0.7 (0.4 to 1.0)	1.1 (0.7 to 1.6)	1.1 (0.7 to 1.7)	1.7 (0.9 to 2.9)	2.8 (0.5 to 5.0)	53.6 (7.5 to 113.1)
Cameroon	1.6 (1.1 to 2.3)	2.5 (1.7 to 3.6)	2.7 (1.9 to 3.9)	4.4 (2.5 to 7.8)	3.6 (1.5 to 5.9)	74.8 (24.5 to 141.7)
Cape Verde	1.3 (0.8 to 1.9)	2.6 (1.7 to 3.9)	2.8 (1.8 to 4.0)	4.9 (2.5 to 8.1)	4.0 (1.8 to 6.0)	85.8 (31.4 to 147.8)
Chad	0.6 (0.3 to 0.8)	0.7 (0.5 to 1.1)	0.8 (0.5 to 1.2)	1.1 (0.5 to 1.9)	2.4 (0.1 to 4.7)	44.7 (2.0 to 102.1)
Cote d'Ivoire	1.2 (0.8 to 1.7)	1.5 (1.0 to 2.3)	1.6 (1.1 to 2.4)	2.3 (1.2 to 3.9)	2.5 (0.3 to 4.6)	46.9 (5.2 to 100.0)
The Gambia	1.0 (0.7 to 1.4)	1.3 (0.8 to 1.9)	1.4 (0.9 to 1.9)	1.7 (0.9 to 2.9)	1.8 (-0.3 to 4.0)	33.2 (-4.1 to 81.0)
Ghana	1.7 (1.2 to 2.3)	3.0 (2.3 to 3.9)	3.0 (2.2 to 4.0)	4.5 (2.6 to 7.1)	2.5 (0.5 to 4.4)	47.3 (8.0 to 92.8)
Guinea	0.5 (0.4 to 0.7)	0.8 (0.6 to 1.1)	0.8 (0.6 to 1.1)	1.3 (0.7 to 2.1)	2.7 (0.8 to 4.5)	50.9 (13.0 to 95.7)
Guinea-Bissau	0.8 (0.5 to 1.2)	1.2 (0.8 to 1.8)	1.2 (0.8 to 1.8)	1.9 (1.0 to 3.0)	3.0 (1.2 to 4.6)	57.2 (18.9 to 100.5)
Liberia	0.7 (0.5 to 1.0)	1.7 (1.0 to 2.7)	1.5 (1.0 to 2.3)	2.7 (1.4 to 4.5)	3.0 (1.3 to 4.6)	58.5 (22.0 to 98.3)
Mali	0.6 (0.4 to 0.9)	0.8 (0.5 to 1.2)	0.9 (0.5 to 1.3)	1.2 (0.6 to 2.1)	2.3 (0.1 to 4.7)	44.4 (0.8 to 103.6)
Mauritania	1.1 (0.7 to 1.6)	1.3 (0.9 to 1.9)	1.4 (0.9 to 2.0)	1.9 (1.0 to 3.3)	2.3 (0.3 to 4.3)	42.5 (4.1 to 90.8)
Niger	0.5 (0.3 to 0.8)	0.6 (0.4 to 0.9)	0.6 (0.4 to 1.0)	0.8 (0.4 to 1.4)	1.5 (-0.8 to 3.9)	27.0 (-11.1 to 80.6)
Nigeria	1.2 (0.9 to 1.6)	2.0 (1.6 to 2.4)	2.1 (1.6 to 2.6)	3.0 (1.8 to 4.6)	2.4 (0.5 to 4.5)	45.4 (7.7 to 95.6)
Sao Tome and Principe	1.1 (0.7 to 1.7)	1.9 (1.2 to 2.6)	2.0 (1.3 to 2.9)	3.3 (1.9 to 5.4)	3.7 (1.8 to 5.4)	74.8 (31.6 to 123.6)
Senegal	1.1 (0.9 to 1.3)	1.2 (0.8 to 1.7)	1.2 (0.8 to 1.8)	1.5 (0.8 to 2.6)	1.3 (-0.9 to 3.3)	22.5 (-12.3 to 63.4)
Sierra Leone	1.5 (1.0 to 2.1)	2.8 (2.1 to 3.6)	2.8 (2.1 to 3.6)	4.5 (2.9 to 6.7)	3.1 (1.2 to 4.9)	60.2 (20.4 to 107.8)
Togo	0.9 (0.6 to 1.4)	1.4 (0.9 to 2.1)	1.5 (1.0 to 2.3)	2.4 (1.3 to 4.1)	3.3 (1.2 to 5.4)	65.8 (19.0 to 124.5)
**Indicator 3.c.1b: Nurses and midwives per 1,000 population**						
Central Europe, Eastern Europe, and Central Asia						
Central Asia						
Armenia	3.3 (2.3 to 4.6)	7.0 (5.3 to 9.2)	7.3 (5.3 to 9.9)	12.9 (7.1 to 21.5)	3.8 (1.6 to 6.1)	80.8 (28.0 to 150.7)
Azerbaijan	3.2 (1.9 to 4.7)	6.7 (3.9 to 10.1)	6.9 (4.1 to 10.6)	10.9 (5.2 to 20.0)	3.1 (0.9 to 5.1)	61.1 (14.0 to 115.9)
Georgia	2.2 (1.4 to 3.4)	3.5 (2.3 to 5.1)	3.7 (2.4 to 5.4)	5.3 (2.7 to 9.1)	2.5 (0.1 to 4.9)	49.2 (0.8 to 109.4)
Kazakhstan	4.3 (2.6 to 6.4)	7.0 (4.2 to 10.9)	7.3 (4.3 to 11.5)	10.7 (4.7 to 20.6)	2.6 (0.1 to 5.0)	49.6 (1.0 to 112.3)
Kyrgyzstan	2.3 (1.4 to 3.5)	3.2 (2.0 to 4.8)	3.2 (2.1 to 4.9)	4.1 (2.1 to 7.3)	1.6 (-0.7 to 3.8)	28.6 (-10.4 to 76.1)
Mongolia	3.6 (2.7 to 4.5)	7.0 (4.7 to 10.3)	7.3 (4.9 to 10.8)	12.7 (6.3 to 23.7)	3.8 (1.6 to 6.0)	78.1 (26.2 to 145.1)
Tajikistan	2.2 (1.5 to 3.0)	4.5 (3.1 to 6.2)	4.8 (3.2 to 6.7)	8.3 (4.2 to 14.4)	3.8 (1.4 to 6.3)	80.6 (22.9 to 156.1)
Turkmenistan	3.1 (1.9 to 4.7)	5.1 (3.0 to 8.0)	5.4 (3.2 to 8.4)	8.2 (3.7 to 16.0)	2.9 (0.4 to 5.3)	57.6 (7.0 to 120.5)

	Estimate in 2000 (95% UIs)	Estimate in 2015 (95% UIs)	Estimate in 2017 (95% UIs)	Estimate in 2030 (95% UIs)	Annualised rate of change, 2015-2030 (95% UIs)	Percent change, 2015-2030 (95% UIs)
Uzbekistan	2.0 (1.2 to 3.1)	3.8 (2.3 to 5.8)	4.0 (2.5 to 6.1)	6.8 (3.3 to 12.6)	3.7 (1.3 to 6.2)	77.8 (20.7 to 154.7)
Central Europe						
Albania	3.7 (3.0 to 4.6)	5.6 (4.6 to 6.7)	5.8 (4.5 to 7.1)	8.2 (5.1 to 12.2)	2.5 (0.6 to 4.2)	46.1 (9.5 to 87.9)
Bosnia and Herzegovina	2.0 (1.2 to 3.1)	2.9 (1.8 to 4.4)	3.1 (1.9 to 4.7)	4.6 (2.2 to 8.5)	2.8 (0.4 to 5.2)	55.5 (6.4 to 119.4)
Bulgaria	5.2 (3.9 to 6.6)	7.7 (5.2 to 10.4)	7.9 (5.3 to 11.1)	10.8 (5.5 to 18.8)	2.1 (-0.2 to 4.4)	38.2 (-3.1 to 93.8)
Croatia	6.3 (5.2 to 7.5)	8.6 (7.7 to 9.7)	8.8 (7.6 to 10.1)	11.2 (8.2 to 14.9)	1.7 (0.3 to 3.0)	29.9 (4.3 to 57.4)
Czech Republic	10.7 (9.5 to 11.9)	12.9 (11.4 to 14.6)	13.2 (11.2 to 15.4)	15.6 (11.5 to 20.9)	1.2 (-0.2 to 2.5)	19.9 (-2.3 to 45.4)
Hungary	5.9 (5.4 to 6.4)	5.0 (4.3 to 5.7)	5.0 (4.1 to 5.9)	4.5 (3.3 to 6.1)	-0.7 (-2.0 to 0.6)	-9.7 (-25.9 to 9.3)
Macedonia	2.4 (1.5 to 3.6)	4.2 (2.6 to 6.3)	4.4 (2.7 to 6.6)	6.7 (3.4 to 11.8)	2.9 (0.7 to 5.3)	56.9 (11.2 to 120.6)
Montenegro	3.4 (2.1 to 5.0)	4.5 (2.9 to 6.6)	4.7 (3.1 to 6.9)	6.1 (3.1 to 10.6)	1.9 (-0.5 to 4.1)	34.0 (-7.4 to 85.8)
Poland	5.1 (4.0 to 6.4)	6.5 (4.7 to 8.6)	6.6 (4.7 to 8.9)	8.2 (4.5 to 13.6)	1.4 (-0.8 to 3.4)	24.7 (-10.7 to 66.7)
Romania	2.6 (2.0 to 3.4)	4.2 (3.7 to 4.7)	4.3 (3.6 to 5.0)	6.0 (4.1 to 8.5)	2.4 (0.5 to 4.2)	44.2 (7.1 to 88.0)
Serbia	2.5 (1.8 to 3.5)	3.8 (2.5 to 5.4)	3.9 (2.6 to 5.7)	5.4 (2.7 to 9.5)	2.2 (0.0 to 4.4)	41.4 (0.1 to 93.6)
Slovakia	8.8 (7.9 to 9.8)	10.6 (9.3 to 12.0)	10.9 (9.3 to 12.8)	13.4 (9.7 to 18.0)	1.5 (0.1 to 2.9)	25.4 (2.1 to 53.4)
Slovenia	6.7 (5.2 to 8.3)	9.2 (7.2 to 11.5)	9.3 (7.2 to 11.9)	12.2 (7.7 to 18.6)	1.8 (0.0 to 3.6)	32.0 (0.5 to 72.0)
Eastern Europe						
Belarus	5.5 (3.8 to 7.5)	10.4 (7.6 to 13.8)	10.5 (7.4 to 14.4)	16.7 (9.0 to 29.2)	2.9 (0.6 to 5.4)	57.4 (9.8 to 124.0)
Estonia	6.3 (5.5 to 7.2)	6.2 (5.2 to 7.4)	6.2 (4.9 to 7.6)	6.1 (4.0 to 9.2)	-0.2 (-1.9 to 1.6)	-2.2 (-24.4 to 26.4)
Latvia	7.2 (6.3 to 8.0)	6.5 (5.5 to 7.6)	6.5 (5.3 to 7.7)	6.0 (4.2 to 8.3)	-0.7 (-2.0 to 0.8)	-8.9 (-26.3 to 12.2)
Lithuania	5.8 (5.0 to 6.7)	7.0 (5.9 to 8.1)	7.1 (5.9 to 8.6)	8.7 (6.1 to 12.1)	1.4 (-0.1 to 2.9)	24.4 (-0.9 to 55.4)
Moldova	1.3 (0.8 to 1.9)	2.4 (1.5 to 3.6)	2.5 (1.6 to 3.7)	3.6 (1.8 to 6.5)	2.7 (0.4 to 5.0)	52.0 (6.4 to 111.7)
Russia	6.3 (5.0 to 7.6)	8.7 (6.6 to 11.2)	8.6 (6.3 to 11.2)	10.6 (6.1 to 16.8)	1.2 (-0.8 to 3.1)	20.5 (-10.8 to 59.7)
Ukraine	6.6 (4.6 to 9.1)	9.8 (7.0 to 13.4)	10.0 (7.0 to 14.0)	13.2 (6.8 to 22.3)	1.7 (-0.9 to 4.0)	31.8 (-13.0 to 83.1)
High-income						
Australasia						
Australia	15.5 (12.7 to 18.4)	12.6 (9.4 to 16.4)	12.8 (9.3 to 17.0)	12.0 (7.1 to 19.4)	-0.5 (-2.1 to 1.4)	-6.1 (-27.4 to 23.6)
New Zealand	12.8 (10.3 to 15.5)	19.0 (14.3 to 25.0)	19.6 (14.5 to 26.0)	26.2 (15.6 to 41.8)	2.0 (0.2 to 3.8)	36.2 (3.4 to 78.1)
High-income Asia Pacific						
Brunei	5.3 (3.4 to 7.8)	7.4 (4.7 to 11.1)	7.8 (4.9 to 11.5)	10.7 (5.3 to 19.2)	2.2 (-0.0 to 4.5)	41.9 (-0.7 to 97.8)
Japan	9.5 (7.1 to 12.5)	11.2 (8.6 to 14.2)	11.3 (8.4 to 14.6)	13.4 (8.1 to 20.6)	1.0 (-1.0 to 3.1)	17.5 (-13.8 to 59.2)
South Korea	3.3 (2.5 to 4.3)	6.5 (5.1 to 8.2)	6.8 (5.2 to 8.9)	11.9 (7.0 to 18.8)	3.9 (1.6 to 6.0)	81.0 (27.4 to 144.1)
Singapore	5.4 (3.5 to 8.0)	9.8 (6.4 to 14.0)	10.0 (6.5 to 14.7)	15.9 (8.1 to 27.9)	3.1 (0.8 to 5.2)	60.9 (12.4 to 119.1)
High-income North America						
Canada	12.7 (10.1 to 15.8)	15.8 (11.2 to 21.0)	16.4 (11.6 to 22.4)	20.5 (11.2 to 34.7)	1.5 (-0.4 to 3.7)	27.7 (-5.9 to 73.1)
Greenland	5.0 (3.1 to 7.6)	6.5 (4.0 to 10.1)	6.7 (4.0 to 10.4)	8.5 (4.1 to 15.8)	1.5 (-0.9 to 4.0)	28.2 (-12.7 to 82.7)
United States	9.9 (8.1 to 12.1)	10.2 (8.2 to 12.5)	10.0 (7.9 to 12.6)	10.3 (6.5 to 15.8)	-0.1 (-1.8 to 1.7)	0.1 (-24.0 to 29.8)
Southern Latin America						
Argentina	1.9 (1.1 to 2.8)	2.5 (1.6 to 3.6)	2.5 (1.5 to 3.8)	3.2 (1.4 to 5.9)	1.5 (-1.5 to 4.1)	28.1 (-20.3 to 84.4)
Chile	2.2 (1.8 to 2.7)	4.9 (4.1 to 5.9)	5.2 (4.2 to 6.4)	9.9 (6.7 to 14.3)	4.6 (3.0 to 6.2)	100.8 (57.7 to 153.4)
Uruguay	3.1 (2.4 to 3.9)	5.3 (3.9 to 6.9)	5.6 (4.0 to 7.5)	8.9 (5.1 to 14.4)	3.3 (1.3 to 5.3)	66.1 (22.3 to 121.5)
Western Europe						
Andorra	11.1 (6.5 to 17.0)	12.6 (7.3 to 19.4)	12.8 (7.5 to 20.0)	14.8 (6.7 to 28.3)	0.8 (-1.9 to 3.2)	15.4 (-24.6 to 62.4)
Austria	9.5 (8.4 to 10.7)	10.2 (8.4 to 12.1)	10.4 (8.3 to 12.8)	11.8 (7.6 to 16.9)	0.8 (-0.9 to 2.4)	14.0 (-12.1 to 43.1)
Belgium	13.0 (11.9 to 14.1)	13.5 (11.9 to 15.2)	13.3 (11.4 to 15.4)	13.7 (10.3 to 17.7)	0.0 (-1.1 to 1.1)	0.6 (-15.2 to 18.7)
Cyprus	4.2 (3.5 to 4.9)	5.5 (4.7 to 6.6)	5.8 (4.8 to 7.2)	7.6 (5.2 to 11.3)	2.0 (0.4 to 3.7)	36.4 (6.4 to 74.7)
Denmark	10.6 (9.2 to 12.2)	10.2 (8.0 to 12.6)	10.0 (7.5 to 12.9)	9.8 (5.8 to 15.5)	-0.4 (-2.3 to 1.4)	-5.4 (-29.1 to 22.6)
Finland	16.8 (15.2 to 18.5)	13.7 (11.9 to 15.6)	13.7 (11.4 to 16.1)	12.2 (8.7 to 16.7)	-0.9 (-2.3 to 0.5)	-11.6 (-29.5 to 8.5)
France	9.2 (8.3 to 10.1)	8.9 (7.7 to 10.2)	8.5 (7.0 to 10.2)	7.9 (5.8 to 10.8)	-0.9 (-2.1 to 0.6)	-11.6 (-27.1 to 9.2)
Germany	10.2 (9.4 to 11.0)	17.6 (16.0 to 19.2)	18.4 (16.3 to 20.9)	28.0 (22.0 to 35.1)	3.1 (1.9 to 4.1)	58.8 (33.7 to 85.8)
Greece	3.4 (3.1 to 3.8)	4.8 (4.3 to 5.4)	4.8 (4.1 to 5.5)	6.1 (4.5 to 8.0)	1.5 (0.2 to 2.8)	26.5 (3.8 to 51.8)
Iceland	15.2 (13.3 to 17.2)	14.8 (12.8 to 17.0)	15.0 (12.4 to 17.7)	15.1 (10.6 to 20.5)	0.1 (-1.5 to 1.4)	1.4 (-19.7 to 24.2)
Ireland	10.7 (9.5 to 11.9)	13.6 (11.8 to 15.6)	13.6 (11.3 to 16.3)	16.7 (11.9 to 22.8)	1.3 (-0.1 to 2.7)	22.1 (-2.1 to 48.9)
Israel	5.5 (4.2 to 7.0)	6.4 (4.8 to 8.3)	6.5 (4.8 to 8.6)	7.6 (4.3 to 12.2)	1.0 (-1.2 to 3.0)	17.4 (-16.0 to 56.4)
Italy	5.7 (4.9 to 6.4)	7.2 (6.1 to 8.3)	7.3 (6.1 to 8.8)	9.2 (6.4 to 13.1)	1.6 (0.0 to 3.1)	27.6 (0.5 to 59.5)
Luxembourg	6.8 (5.9 to 7.7)	8.9 (7.5 to 10.5)	8.8 (7.0 to 10.8)	10.7 (7.2 to 15.2)	1.1 (-0.5 to 2.7)	19.5 (-7.6 to 49.7)
Malta	4.9 (3.0 to 7.5)	7.2 (4.3 to 11.1)	7.5 (4.4 to 11.6)	10.6 (5.0 to 19.6)	2.3 (-0.2 to 4.6)	43.3 (-2.9 to 100.7)
Netherlands	20.4 (18.3 to 22.9)	21.9 (18.7 to 25.7)	21.8 (17.9 to 26.2)	23.4 (16.4 to 32.5)	0.4 (-1.0 to 1.7)	6.2 (-13.6 to 30.0)
Norway	15.9 (14.2 to 17.8)	22.2 (18.7 to 26.1)	22.6 (18.3 to 27.4)	29.2 (19.4 to 41.6)	1.7 (0.1 to 3.3)	30.5 (1.9 to 63.4)
Portugal	4.6 (4.0 to 5.3)	7.1 (6.0 to 8.4)	7.5 (6.1 to 9.2)	11.0 (7.5 to 15.6)	2.8 (1.2 to 4.4)	53.5 (19.6 to 92.6)
Spain	6.4 (5.7 to 7.2)	9.3 (8.1 to 10.7)	9.2 (7.7 to 11.1)	12.2 (8.6 to 16.9)	1.7 (0.3 to 3.2)	30.8 (4.9 to 62.2)
Sweden	9.7 (8.6 to 10.9)	14.2 (12.1 to 16.7)	14.7 (11.9 to 18.0)	18.7 (12.6 to 27.2)	1.7 (0.1 to 3.4)	30.3 (1.7 to 65.8)
Switzerland	15.9 (14.7 to 17.3)	15.2 (13.5 to 17.1)	14.9 (12.8 to 17.3)	14.7 (10.9 to 19.0)	-0.3 (-1.5 to 1.0)	-3.8 (-20.4 to 16.4)
United Kingdom	13.8 (12.8 to 14.8)	15.3 (13.5 to 17.2)	15.3 (12.9 to 17.8)	16.8 (12.2 to 22.0)	0.5 (-0.7 to 1.8)	8.9 (-10.3 to 30.1)
Latin America and Caribbean						
Andean Latin America						
Bolivia	2.1 (1.7 to 2.6)	3.8 (2.6 to 5.4)	4.0 (2.7 to 5.7)	6.8 (3.3 to 11.8)	3.6 (1.2 to 5.7)	74.0 (20.5 to 136.7)
Ecuador	1.2 (0.9 to 1.5)	2.5 (1.7 to 3.3)	2.5 (1.7 to 3.5)	4.1 (2.1 to 7.1)	3.2 (0.9 to 5.6)	64.7 (13.6 to 130.7)
Peru	2.0 (1.2 to 3.3)	2.8 (1.7 to 4.4)	2.9 (1.7 to 4.5)	3.9 (1.8 to 7.7)	1.9 (-0.7 to 4.6)	36.0 (-10.4 to 98.6)
Caribbean						
Antigua and Barbuda	4.9 (2.9 to 7.4)	6.6 (4.0 to 10.2)	6.9 (4.2 to 10.6)	9.4 (4.5 to 17.0)	2.1 (-0.3 to 4.5)	40.0 (-4.5 to 95.6)
The Bahamas	9.8 (6.0 to 14.6)	13.8 (8.5 to 20.8)	14.1 (8.8 to 21.0)	20.0 (9.7 to 34.9)	2.3 (-0.1 to 4.7)	43.1 (-1.0 to 102.4)
Barbados	6.6 (4.1 to 9.7)	8.3 (5.0 to 12.5)	8.5 (5.1 to 12.7)	10.7 (5.0 to 19.6)	1.5 (-0.9 to 4.0)	27.1 (-12.3 to 81.7)
Belize	1.9 (1.2 to 2.8)	3.4 (2.1 to 5.0)	3.5 (2.1 to 5.2)	5.7 (2.7 to 10.1)	3.4 (1.0 to 5.7)	68.4 (15.8 to 135.7)
Bermuda	13.4 (8.1 to 19.9)	21.1 (12.6 to 32.2)	20.9 (12.5 to 32.2)	28.5 (13.3 to 50.9)	1.8 (-0.5 to 4.0)	33.2 (-7.1 to 82.3)
Cuba	7.2 (5.9 to 8.7)	14.0 (9.5 to 19.2)	14.6 (9.7 to 20.1)	23.4 (11.8 to 39.8)	3.2 (1.1 to 5.2)	63.7 (17.3 to 117.5)
Dominica	2.8 (1.7 to 4.1)	4.1 (2.5 to 6.3)	4.3 (2.6 to 6.5)	6.1 (3.0 to 11.1)	2.5 (0.2 to 4.7)	47.5 (3.4 to 103.0)
Dominican Republic	3.5 (2.5 to 4.6)	4.2 (2.7 to 6.4)	4.3 (2.7 to 6.6)	5.5 (2.3 to 11.4)	1.4 (-1.4 to 4.2)	26.6 (-18.5 to 88.0)
Grenada	3.0 (1.8 to 4.5)	4.0 (2.4 to 6.2)	4.1 (2.4 to 6.3)	5.5 (2.5 to 9.8)	1.9 (-0.6 to 4.2)	35.4 (-8.8 to 86.7)
Guyana	1.8 (1.2 to 2.7)	2.9 (1.8 to 4.5)	3.1 (1.9 to 4.7)	4.5 (2.1 to 8.4)	2.6 (0.3 to 5.2)	50.9 (4.7 to 116.9)
Haiti	0.8 (0.5 to 1.3)	1.4 (0.8 to 2.1)	1.4 (0.9 to 2.2)	2.0 (1.0 to 3.8)	2.5 (0.1 to 4.9)	48.3 (2.2 to 107.9)

	Estimate in 2000 (95% UIs)	Estimate in 2015 (95% UIs)	Estimate in 2017 (95% UIs)	Estimate in 2030 (95% UIs)	Annualised rate of change, 2015-2030 (95% UIs)	Percent change, 2015-2030 (95% UIs)
Jamaica	3.5 (2.1 to 5.5)	4.7 (2.9 to 7.4)	4.9 (2.9 to 7.7)	6.6 (3.2 to 12.3)	2.0 (-0.4 to 4.3)	37.2 (-6.1 to 90.2)
Puerto Rico	6.7 (4.0 to 10.6)	9.1 (5.6 to 13.8)	9.2 (5.6 to 14.1)	12.2 (5.8 to 23.0)	1.7 (-0.6 to 4.2)	32.1 (-9.0 to 89.1)
Saint Lucia	3.3 (2.0 to 5.1)	5.2 (3.1 to 7.8)	5.4 (3.3 to 8.1)	8.0 (3.7 to 14.2)	2.7 (0.5 to 5.0)	52.3 (7.2 to 112.5)
Saint Vincent and the Grenadines	2.5 (1.6 to 3.8)	3.9 (2.4 to 6.0)	4.0 (2.4 to 6.3)	5.9 (2.7 to 11.1)	2.5 (0.0 to 4.9)	47.6 (0.7 to 109.9)
Suriname	4.5 (2.8 to 6.5)	5.7 (3.7 to 7.9)	5.5 (3.7 to 7.8)	6.5 (3.5 to 10.7)	0.7 (-1.5 to 3.0)	12.2 (-19.6 to 56.3)
Trinidad and Tobago	3.4 (2.5 to 4.5)	5.1 (3.2 to 7.9)	5.2 (3.2 to 7.9)	7.1 (3.3 to 13.6)	1.9 (-0.5 to 4.2)	35.5 (-7.1 to 86.4)
Virgin Islands, U.S.	8.6 (5.0 to 12.6)	11.3 (6.5 to 17.6)	11.4 (6.5 to 17.9)	14.2 (6.2 to 27.6)	1.3 (-1.1 to 3.8)	23.2 (-15.5 to 76.3)
Central Latin America						
Colombia	1.8 (1.1 to 2.7)	2.8 (1.7 to 4.2)	2.9 (1.8 to 4.4)	4.3 (1.9 to 7.8)	2.7 (0.2 to 5.1)	52.2 (2.5 to 114.6)
Costa Rica	0.8 (0.5 to 1.3)	1.1 (0.6 to 1.8)	1.1 (0.6 to 1.9)	1.6 (0.6 to 3.2)	1.9 (-0.9 to 4.7)	37.1 (-13.2 to 102.3)
El Salvador	1.3 (0.9 to 1.7)	2.2 (1.6 to 3.0)	2.4 (1.7 to 3.2)	4.0 (2.3 to 6.6)	3.7 (1.6 to 5.9)	76.2 (27.0 to 143.4)
Guatemala	1.0 (0.6 to 1.6)	1.8 (1.1 to 2.6)	1.8 (1.1 to 2.7)	2.9 (1.4 to 5.4)	3.1 (0.6 to 5.7)	61.4 (9.3 to 134.6)
Honduras	1.1 (0.7 to 1.7)	2.0 (1.3 to 3.1)	2.1 (1.3 to 3.2)	3.5 (1.7 to 6.2)	3.4 (1.1 to 5.6)	70.3 (17.3 to 131.9)
Mexico	3.3 (2.5 to 4.2)	5.2 (3.9 to 6.6)	5.3 (3.8 to 6.9)	7.7 (4.3 to 12.7)	2.5 (0.3 to 4.6)	47.7 (4.7 to 100.4)
Nicaragua	0.3 (0.2 to 0.5)	0.6 (0.3 to 1.0)	0.6 (0.4 to 1.1)	1.1 (0.4 to 2.1)	3.4 (0.6 to 6.1)	70.6 (9.4 to 149.1)
Panama	2.9 (2.2 to 3.7)	4.3 (2.8 to 6.1)	4.5 (2.9 to 6.6)	6.6 (3.2 to 11.8)	2.7 (0.5 to 4.8)	51.7 (7.1 to 106.9)
Venezuela	3.0 (2.3 to 3.8)	3.8 (2.6 to 5.3)	3.6 (2.4 to 5.2)	4.3 (2.1 to 7.3)	0.6 (-1.5 to 2.7)	11.1 (-19.9 to 50.9)
Tropical Latin America						
Brazil	3.3 (2.4 to 4.4)	5.6 (4.5 to 6.8)	5.8 (4.4 to 7.3)	9.2 (5.3 to 14.4)	3.1 (0.8 to 5.2)	60.9 (13.0 to 117.2)
Paraguay	1.7 (1.3 to 2.1)	2.3 (1.6 to 3.1)	2.4 (1.7 to 3.4)	3.5 (1.8 to 5.8)	2.5 (0.2 to 4.5)	47.4 (3.7 to 97.0)
North Africa and Middle East						
North Africa and Middle East						
Afghanistan	0.3 (0.2 to 0.4)	0.4 (0.2 to 0.6)	0.4 (0.3 to 0.7)	0.6 (0.3 to 1.1)	2.4 (0.1 to 4.9)	45.7 (1.5 to 108.3)
Algeria	1.3 (0.8 to 2.0)	2.9 (1.8 to 4.4)	3.1 (1.9 to 4.7)	5.9 (2.7 to 10.4)	4.6 (1.9 to 7.0)	102.1 (33.8 to 184.0)
Bahrain	4.8 (2.9 to 7.3)	8.1 (5.0 to 12.1)	8.3 (5.2 to 12.4)	12.6 (6.3 to 22.6)	2.8 (0.4 to 5.0)	54.3 (6.2 to 112.9)
Egypt	2.0 (1.5 to 2.7)	2.4 (2.2 to 2.6)	2.5 (2.2 to 2.8)	3.1 (2.3 to 4.1)	1.6 (-0.1 to 3.3)	27.5 (-2.2 to 63.4)
Iran	1.3 (1.0 to 1.6)	2.4 (1.8 to 3.1)	2.6 (1.9 to 3.5)	4.4 (2.3 to 7.4)	3.9 (1.7 to 6.0)	82.1 (28.1 to 145.6)
Iraq	0.9 (0.6 to 1.3)	1.2 (0.9 to 1.5)	1.3 (0.9 to 1.7)	1.8 (1.0 to 2.8)	2.4 (-0.0 to 4.8)	46.4 (-0.1 to 106.2)
Jordan	2.9 (2.4 to 3.4)	4.0 (2.8 to 5.4)	4.2 (2.8 to 5.8)	6.0 (2.9 to 10.6)	2.4 (-0.1 to 4.6)	45.4 (-1.3 to 99.8)
Kuwait	5.9 (3.5 to 8.9)	9.4 (5.9 to 14.2)	9.8 (6.1 to 14.6)	14.6 (7.1 to 26.1)	2.7 (0.2 to 5.1)	53.5 (3.7 to 113.5)
Lebanon	2.4 (1.5 to 3.7)	4.0 (2.5 to 6.0)	4.3 (2.7 to 6.2)	6.8 (3.4 to 12.2)	3.3 (0.9 to 5.7)	66.8 (14.4 to 134.6)
Libya	3.6 (2.2 to 5.5)	4.2 (2.5 to 6.2)	4.3 (2.6 to 6.6)	5.3 (2.7 to 9.7)	1.4 (-0.5 to 3.5)	24.5 (-7.3 to 69.4)
Morocco	0.8 (0.5 to 1.2)	1.6 (0.9 to 2.4)	1.7 (1.0 to 2.6)	3.1 (1.4 to 5.6)	4.2 (1.8 to 6.7)	91.4 (30.4 to 171.7)
Palestine	0.8 (0.5 to 1.2)	1.4 (0.8 to 2.0)	1.5 (0.9 to 2.2)	2.4 (1.1 to 4.2)	3.5 (1.2 to 5.9)	73.1 (20.2 to 141.1)
Oman	2.4 (1.4 to 3.7)	6.2 (3.8 to 9.7)	6.6 (4.0 to 10.4)	13.6 (6.5 to 24.4)	5.0 (2.8 to 7.1)	114.8 (52.5 to 188.0)
Qatar	7.6 (4.5 to 11.6)	12.0 (7.3 to 18.1)	12.3 (7.5 to 19.0)	17.7 (8.4 to 32.6)	2.4 (-0.0 to 4.8)	45.6 (-0.6 to 104.6)
Saudi Arabia	2.9 (1.9 to 4.3)	6.8 (4.0 to 10.1)	7.5 (4.3 to 11.0)	16.5 (7.6 to 29.3)	5.7 (3.5 to 7.8)	139.0 (67.9 to 222.6)
Sudan	0.4 (0.2 to 0.6)	0.9 (0.5 to 1.3)	0.9 (0.6 to 1.4)	1.7 (0.8 to 3.0)	4.4 (2.3 to 6.7)	96.2 (40.5 to 173.2)
Syria	0.9 (0.6 to 1.4)	1.4 (0.9 to 2.2)	1.6 (1.0 to 2.4)	2.3 (1.1 to 4.2)	2.9 (0.6 to 5.2)	56.9 (9.8 to 117.6)
Tunisia	1.4 (0.9 to 2.1)	2.5 (1.5 to 3.8)	2.6 (1.6 to 4.1)	4.3 (1.9 to 8.1)	3.4 (0.9 to 5.7)	70.3 (15.2 to 133.5)
Turkey	1.7 (1.1 to 2.4)	2.2 (1.6 to 2.9)	2.4 (1.7 to 3.2)	3.3 (1.8 to 5.5)	2.5 (0.0 to 4.9)	49.0 (0.6 to 109.8)
United Arab Emirates	7.4 (4.7 to 11.3)	11.8 (7.1 to 17.9)	11.8 (7.2 to 18.0)	17.6 (8.4 to 32.8)	2.5 (0.1 to 4.9)	47.2 (1.8 to 109.6)
Yemen	0.6 (0.4 to 0.9)	0.8 (0.5 to 1.2)	0.8 (0.5 to 1.1)	1.0 (0.5 to 1.8)	1.5 (-0.7 to 3.8)	26.6 (-10.6 to 76.6)
South Asia						
South Asia						
Bangladesh	0.4 (0.2 to 0.6)	0.7 (0.4 to 1.1)	0.7 (0.4 to 1.1)	1.2 (0.6 to 2.3)	3.6 (0.9 to 6.0)	75.1 (15.3 to 146.3)
Bhutan	0.8 (0.5 to 1.3)	1.6 (1.0 to 2.5)	1.7 (1.0 to 2.7)	3.0 (1.4 to 5.5)	4.1 (1.6 to 6.6)	88.9 (27.1 to 169.1)
India	0.4 (0.3 to 0.5)	0.7 (0.5 to 1.0)	0.8 (0.6 to 1.1)	1.5 (0.8 to 2.7)	4.5 (2.0 to 7.0)	100.7 (35.3 to 186.0)
Nepal	0.4 (0.2 to 0.6)	0.8 (0.5 to 1.2)	0.8 (0.5 to 1.3)	1.4 (0.6 to 2.7)	3.8 (1.1 to 6.2)	80.3 (18.0 to 153.1)
Pakistan	0.6 (0.4 to 0.9)	0.9 (0.6 to 1.2)	0.9 (0.7 to 1.2)	1.3 (0.8 to 2.1)	2.6 (0.3 to 5.2)	50.2 (4.0 to 117.4)
Southeast Asia, East Asia, and Oceania						
East Asia						
China	1.4 (1.0 to 1.9)	4.0 (2.9 to 5.5)	4.4 (3.1 to 6.1)	10.3 (5.6 to 17.0)	6.0 (3.9 to 8.1)	149.5 (79.4 to 238.0)
North Korea	0.9 (0.6 to 1.4)	1.1 (0.6 to 1.6)	1.1 (0.7 to 1.6)	1.2 (0.6 to 2.3)	0.8 (-1.6 to 3.3)	14.0 (-21.7 to 64.6)
Taiwan (Province of China)	2.7 (1.9 to 3.6)	4.0 (3.1 to 5.1)	4.2 (3.1 to 5.6)	6.1 (3.6 to 10.2)	2.6 (0.3 to 4.9)	50.2 (5.2 to 108.2)
Oceania						
American Samoa	3.4 (2.2 to 5.0)	3.8 (2.4 to 5.5)	3.9 (2.4 to 5.7)	4.5 (2.2 to 7.7)	0.9 (-1.3 to 3.2)	16.3 (-17.9 to 60.6)
Federated States of Micronesia	0.9 (0.6 to 1.4)	1.6 (1.0 to 2.4)	1.4 (0.9 to 2.0)	1.9 (1.0 to 3.0)	1.0 (-0.3 to 2.2)	16.9 (-4.2 to 38.4)
Fiji	2.1 (1.4 to 3.3)	2.6 (1.6 to 4.0)	2.7 (1.6 to 4.2)	3.3 (1.7 to 6.1)	1.4 (-0.9 to 3.6)	25.2 (-12.1 to 71.6)
Guam	7.5 (4.7 to 11.3)	7.9 (4.9 to 11.7)	8.0 (5.0 to 12.0)	8.5 (4.2 to 15.0)	0.3 (-1.9 to 2.6)	5.6 (-24.9 to 48.4)
Kiribati	1.2 (0.8 to 1.8)	1.7 (1.1 to 2.6)	1.9 (1.2 to 2.9)	2.4 (1.3 to 4.2)	2.2 (0.4 to 4.0)	39.8 (6.4 to 83.1)
Marshall Islands	2.0 (1.3 to 3.0)	3.1 (1.9 to 4.7)	3.1 (1.9 to 4.6)	4.3 (2.4 to 7.0)	2.0 (0.5 to 3.5)	36.7 (8.6 to 69.9)
Northern Mariana Islands	5.3 (3.3 to 8.0)	5.3 (3.2 to 8.1)	5.5 (3.3 to 8.5)	6.0 (3.0 to 11.3)	0.7 (-1.5 to 3.0)	13.1 (-19.8 to 57.4)
Papua New Guinea	0.6 (0.4 to 1.0)	0.7 (0.5 to 1.1)	0.7 (0.4 to 1.1)	0.9 (0.4 to 1.6)	1.0 (-1.4 to 3.4)	18.1 (-18.9 to 67.3)
Samoa	1.0 (0.7 to 1.5)	1.0 (0.6 to 1.6)	1.1 (0.6 to 1.6)	1.2 (0.6 to 2.2)	1.1 (-0.8 to 3.0)	19.6 (-11.4 to 56.5)
Solomon Islands	0.5 (0.3 to 0.7)	0.6 (0.4 to 0.9)	0.6 (0.4 to 0.9)	0.8 (0.4 to 1.4)	1.9 (-0.2 to 3.8)	33.9 (-2.9 to 77.2)
Tonga	1.2 (0.7 to 1.7)	1.5 (0.9 to 2.2)	1.6 (1.0 to 2.5)	2.1 (1.1 to 3.6)	2.4 (0.7 to 4.0)	44.1 (11.3 to 83.3)
Vanuatu	0.7 (0.5 to 1.0)	1.1 (0.7 to 1.7)	1.2 (0.7 to 1.8)	1.6 (0.8 to 2.5)	1.9 (0.4 to 3.3)	33.8 (6.3 to 65.2)
Southeast Asia						
Cambodia	0.8 (0.6 to 1.1)	2.3 (1.8 to 2.9)	2.4 (1.8 to 3.1)	4.7 (2.9 to 7.2)	4.5 (2.5 to 6.4)	99.2 (46.3 to 161.2)
Indonesia	1.3 (0.9 to 1.8)	2.6 (1.7 to 3.6)	2.7 (1.8 to 3.8)	4.8 (2.5 to 8.1)	3.9 (1.8 to 6.0)	83.3 (30.4 to 147.6)
Laos	0.7 (0.4 to 1.1)	1.1 (0.7 to 1.6)	1.2 (0.7 to 1.8)	1.8 (0.9 to 3.3)	3.2 (1.0 to 5.6)	64.1 (15.8 to 132.6)
Malaysia	3.0 (2.3 to 3.9)	5.2 (3.4 to 7.5)	5.6 (3.6 to 8.2)	9.2 (4.4 to 16.2)	3.5 (1.2 to 5.9)	72.1 (19.3 to 141.2)
Maldives	1.9 (1.1 to 2.8)	4.1 (2.5 to 6.4)	4.5 (2.7 to 6.9)	8.9 (4.1 to 15.9)	5.0 (2.6 to 7.1)	113.9 (48.4 to 188.7)
Mauritius	1.6 (1.1 to 2.4)	2.9 (1.8 to 4.4)	3.1 (1.9 to 4.7)	5.1 (2.4 to 9.3)	3.5 (1.0 to 5.9)	72.7 (15.9 to 140.7)
Myanmar	0.4 (0.3 to 0.7)	1.5 (0.9 to 2.3)	1.7 (1.0 to 2.5)	3.8 (1.9 to 6.4)	5.8 (3.9 to 7.5)	142.5 (80.4 to 208.5)
Philippines	1.9 (1.6 to 2.2)	1.5 (1.2 to 1.8)	1.6 (1.2 to 2.0)	1.5 (0.9 to 2.3)	-0.1 (-1.8 to 1.7)	0.2 (-24.1 to 29.5)
Sri Lanka	1.2 (0.8 to 1.8)	1.9 (1.2 to 2.9)	2.0 (1.2 to 3.1)	3.1 (1.5 to 5.9)	3.0 (0.7 to 5.5)	60.5 (11.6 to 128.0)
Seychelles	2.6 (1.6 to 4.0)	3.5 (2.1 to 5.3)	3.7 (2.2 to 5.6)	5.1 (2.4 to 9.6)	2.3 (0.0 to 4.7)	42.8 (0.0 to 101.5)
Thailand	4.0 (2.8 to 5.5)	6.3 (4.2 to 9.0)	6.7 (4.5 to 9.4)	9.9 (5.3 to 16.8)	2.8 (0.8 to 4.8)	54.4 (12.4 to 105.6)

	Estimate in 2000 (95% UIs)	Estimate in 2015 (95% UIs)	Estimate in 2017 (95% UIs)	Estimate in 2030 (95% UIs)	Annualised rate of change, 2015-2030 (95% UIs)	Percent change, 2015-2030 (95% UIs)
Timor-Leste	0.4 (0.2 to 0.6)	0.8 (0.5 to 1.2)	0.9 (0.5 to 1.3)	1.4 (0.8 to 2.3)	3.5 (1.7 to 5.2)	70.3 (29.6 to 118.2)
Vietnam	0.7 (0.4 to 1.0)	1.4 (0.8 to 2.3)	1.6 (0.9 to 2.5)	3.0 (1.4 to 5.9)	4.6 (1.9 to 7.2)	104.7 (32.5 to 196.6)
Sub-Saharan Africa						
Central Sub-Saharan Africa						
Angola	0.4 (0.2 to 0.6)	0.6 (0.4 to 0.9)	0.7 (0.4 to 1.0)	1.0 (0.5 to 1.8)	2.9 (0.2 to 5.5)	57.5 (2.5 to 129.7)
Central African Republic	0.3 (0.2 to 0.4)	0.3 (0.2 to 0.5)	0.3 (0.2 to 0.5)	0.4 (0.2 to 0.7)	1.8 (0.1 to 3.7)	33.0 (2.1 to 74.4)
Congo	0.6 (0.3 to 0.8)	1.0 (0.6 to 1.5)	1.1 (0.7 to 1.6)	1.8 (0.9 to 3.1)	3.5 (1.2 to 5.7)	73.0 (19.8 to 135.0)
Democratic Republic of the Congo	0.2 (0.1 to 0.3)	0.4 (0.2 to 0.6)	0.4 (0.2 to 0.6)	0.6 (0.3 to 1.0)	2.5 (0.5 to 4.7)	47.8 (8.0 to 103.3)
Equatorial Guinea	0.6 (0.3 to 0.8)	1.4 (0.8 to 2.1)	1.5 (0.9 to 2.4)	3.3 (1.6 to 6.0)	5.6 (3.1 to 8.0)	136.5 (58.2 to 230.0)
Gabon	0.9 (0.6 to 1.4)	1.3 (0.8 to 2.0)	1.4 (0.9 to 2.1)	2.0 (1.0 to 3.6)	2.7 (0.2 to 5.1)	52.7 (2.9 to 115.5)
Eastern Sub-Saharan Africa						
Burundi	0.8 (0.5 to 1.1)	1.1 (0.7 to 1.7)	1.2 (0.8 to 1.7)	1.6 (0.8 to 2.8)	2.2 (0.1 to 4.3)	41.3 (1.5 to 90.8)
Comoros	1.2 (0.8 to 1.8)	2.0 (1.4 to 3.0)	2.2 (1.4 to 3.1)	3.3 (1.7 to 5.8)	3.1 (0.8 to 5.4)	62.0 (12.4 to 123.6)
Djibouti	0.7 (0.5 to 1.1)	1.8 (1.2 to 2.6)	1.9 (1.3 to 2.8)	3.6 (2.0 to 6.2)	4.5 (2.3 to 6.5)	99.2 (41.7 to 165.9)
Eritrea	1.0 (0.7 to 1.5)	1.3 (0.9 to 1.9)	1.4 (0.9 to 2.0)	2.0 (1.1 to 3.5)	2.9 (1.2 to 4.7)	56.6 (19.8 to 102.6)
Ethiopia	0.2 (0.1 to 0.3)	0.4 (0.2 to 0.6)	0.4 (0.2 to 0.7)	0.8 (0.3 to 1.4)	4.1 (1.9 to 6.1)	86.2 (32.6 to 150.8)
Kenya	1.6 (1.0 to 2.3)	2.5 (1.6 to 3.8)	2.7 (1.7 to 4.0)	4.2 (2.2 to 7.4)	3.3 (1.1 to 5.5)	65.6 (17.4 to 126.7)
Madagascar	1.1 (0.7 to 1.6)	1.4 (0.9 to 2.1)	1.5 (1.0 to 2.1)	1.9 (1.0 to 3.2)	1.7 (-0.5 to 3.8)	30.9 (-6.8 to 77.7)
Malawi	0.8 (0.5 to 1.1)	1.4 (0.9 to 2.1)	1.5 (1.0 to 2.2)	2.5 (1.3 to 4.3)	3.6 (1.5 to 5.6)	75.1 (25.7 to 131.4)
Mozambique	0.3 (0.2 to 0.4)	0.6 (0.5 to 0.8)	0.7 (0.5 to 1.0)	1.3 (0.7 to 2.2)	4.7 (2.4 to 6.9)	104.6 (43.5 to 180.1)
Rwanda	0.6 (0.4 to 0.7)	1.1 (0.9 to 1.3)	1.1 (0.9 to 1.4)	1.8 (1.1 to 2.7)	3.2 (1.3 to 5.2)	62.7 (20.9 to 118.4)
Somalia	0.5 (0.3 to 0.7)	0.8 (0.5 to 1.2)	0.9 (0.6 to 1.3)	1.4 (0.7 to 2.4)	3.5 (1.4 to 5.4)	70.3 (22.6 to 124.9)
South Sudan	0.7 (0.4 to 1.0)	0.9 (0.6 to 1.4)	1.0 (0.6 to 1.4)	1.4 (0.7 to 2.4)	2.5 (0.3 to 4.9)	48.7 (4.7 to 109.2)
Tanzania	1.0 (0.8 to 1.2)	2.7 (2.1 to 3.4)	3.0 (2.4 to 3.9)	6.5 (4.0 to 9.8)	5.7 (3.9 to 7.5)	138.2 (79.0 to 208.5)
Uganda	1.0 (0.9 to 1.1)	1.6 (1.3 to 2.1)	1.7 (1.3 to 2.3)	2.7 (1.6 to 4.2)	3.1 (1.4 to 4.8)	61.4 (23.2 to 104.4)
Zambia	1.3 (1.1 to 1.6)	1.8 (1.4 to 2.1)	1.9 (1.5 to 2.4)	2.7 (1.6 to 4.0)	2.7 (0.7 to 4.4)	50.7 (11.9 to 93.0)
Southern Sub-Saharan Africa						
Botswana	1.2 (0.8 to 1.7)	2.5 (1.6 to 3.5)	2.6 (1.6 to 3.8)	4.8 (2.3 to 8.4)	4.2 (1.6 to 6.5)	91.4 (27.1 to 164.2)
Lesotho	0.3 (0.2 to 0.6)	0.5 (0.3 to 0.8)	0.5 (0.3 to 0.9)	0.8 (0.3 to 1.6)	3.1 (0.2 to 5.8)	62.6 (2.8 to 137.2)
Namibia	0.8 (0.4 to 1.5)	1.0 (0.6 to 1.7)	1.1 (0.6 to 1.8)	1.5 (0.6 to 3.0)	2.0 (-1.3 to 5.4)	39.2 (-18.1 to 125.9)
South Africa	2.1 (1.6 to 2.7)	2.4 (1.8 to 3.1)	2.3 (1.7 to 3.1)	2.8 (1.6 to 4.5)	0.8 (-1.3 to 2.9)	13.4 (-17.1 to 54.4)
Swaziland	0.6 (0.3 to 0.9)	0.8 (0.4 to 1.2)	0.8 (0.4 to 1.3)	1.1 (0.5 to 2.3)	2.5 (-0.3 to 5.4)	49.1 (-4.6 to 123.2)
Zimbabwe	0.8 (0.4 to 1.4)	0.7 (0.4 to 1.3)	0.8 (0.4 to 1.3)	0.8 (0.3 to 1.6)	0.3 (-2.0 to 3.0)	6.7 (-26.3 to 57.1)
Western Sub-Saharan Africa						
Benin	0.6 (0.3 to 1.0)	0.8 (0.4 to 1.2)	0.8 (0.5 to 1.2)	1.0 (0.5 to 1.9)	1.8 (-0.8 to 4.4)	34.3 (-11.0 to 94.8)
Burkina Faso	0.6 (0.3 to 0.9)	0.9 (0.6 to 1.4)	0.9 (0.6 to 1.4)	1.4 (0.6 to 2.5)	2.5 (0.0 to 5.1)	48.1 (0.7 to 114.3)
Cameroon	1.1 (0.7 to 1.6)	1.6 (1.1 to 2.4)	1.8 (1.2 to 2.6)	2.6 (1.3 to 4.9)	2.8 (0.5 to 5.2)	54.8 (7.0 to 119.3)
Cape Verde	1.0 (0.6 to 1.6)	1.9 (1.2 to 3.0)	2.0 (1.2 to 3.1)	3.3 (1.5 to 5.8)	3.3 (0.9 to 5.7)	68.2 (14.0 to 135.8)
Chad	0.5 (0.3 to 0.8)	0.6 (0.4 to 1.0)	0.7 (0.4 to 1.0)	0.9 (0.4 to 1.6)	1.9 (-0.8 to 4.6)	35.7 (-10.9 to 98.6)
Cote d'Ivoire	0.9 (0.6 to 1.4)	1.2 (0.7 to 1.9)	1.3 (0.8 to 2.0)	1.7 (0.8 to 3.0)	2.1 (-0.4 to 4.5)	38.9 (-6.0 to 96.0)
The Gambia	0.8 (0.5 to 1.2)	1.0 (0.6 to 1.5)	1.0 (0.7 to 1.6)	1.3 (0.6 to 2.3)	1.4 (-0.9 to 3.7)	25.2 (-12.9 to 74.9)
Ghana	1.1 (0.7 to 1.5)	1.9 (1.4 to 2.6)	1.9 (1.3 to 2.6)	2.7 (1.5 to 4.4)	2.1 (-0.1 to 4.2)	39.2 (-0.8 to 88.9)
Guinea	0.3 (0.2 to 0.4)	0.5 (0.3 to 0.7)	0.5 (0.3 to 0.7)	0.7 (0.3 to 1.2)	2.1 (-0.0 to 4.3)	38.9 (-0.0 to 90.3)
Guinea-Bissau	0.7 (0.4 to 1.1)	1.0 (0.6 to 1.5)	1.0 (0.6 to 1.5)	1.4 (0.7 to 2.5)	2.5 (0.6 to 4.2)	46.5 (9.2 to 87.0)
Liberia	0.6 (0.3 to 0.9)	1.5 (0.8 to 2.5)	1.3 (0.8 to 2.1)	2.4 (1.2 to 4.0)	2.8 (0.9 to 4.5)	53.4 (15.3 to 95.0)
Mali	0.5 (0.3 to 0.8)	0.7 (0.4 to 1.1)	0.7 (0.4 to 1.1)	1.0 (0.4 to 1.8)	2.0 (-0.5 to 4.6)	38.2 (-6.8 to 99.8)
Mauritania	0.8 (0.5 to 1.3)	1.0 (0.6 to 1.6)	1.1 (0.6 to 1.6)	1.4 (0.6 to 2.5)	1.8 (-0.5 to 4.0)	32.5 (-7.1 to 82.9)
Niger	0.5 (0.2 to 0.7)	0.5 (0.3 to 0.8)	0.6 (0.3 to 0.9)	0.7 (0.3 to 1.3)	1.3 (-1.2 to 4.0)	23.5 (-17.1 to 81.9)
Nigeria	1.0 (0.7 to 1.3)	1.6 (1.2 to 1.9)	1.6 (1.2 to 2.1)	2.3 (1.3 to 3.8)	2.4 (0.3 to 4.7)	45.0 (4.1 to 102.6)
Sao Tome and Principe	0.9 (0.6 to 1.5)	1.4 (0.9 to 2.1)	1.5 (0.9 to 2.3)	2.2 (1.1 to 3.8)	2.7 (0.6 to 4.6)	51.7 (8.6 to 99.6)
Senegal	0.8 (0.7 to 1.0)	0.8 (0.6 to 1.2)	0.9 (0.6 to 1.3)	1.0 (0.5 to 1.8)	0.8 (-1.4 to 3.0)	13.6 (-18.9 to 57.3)
Sierra Leone	1.3 (0.8 to 1.8)	2.3 (1.7 to 3.0)	2.2 (1.6 to 3.0)	3.4 (1.9 to 5.3)	2.4 (0.3 to 4.6)	45.9 (4.8 to 98.6)
Togo	0.7 (0.4 to 1.1)	1.1 (0.7 to 1.7)	1.1 (0.7 to 1.8)	1.7 (0.9 to 3.2)	3.0 (0.5 to 5.3)	59.1 (7.3 to 121.4)
**Indicator 3.c.1c: Pharmacists per 1,000 population**						
Central Europe, Eastern Europe, and Central Asia						
Central Asia						
Armenia	0.2 (0.1 to 0.4)	0.6 (0.4 to 0.8)	0.6 (0.4 to 0.8)	1.1 (0.5 to 2.2)	4.2 (0.8 to 7.5)	94.4 (12.7 to 209.9)
Azerbaijan	0.3 (0.2 to 0.6)	0.8 (0.5 to 1.5)	0.9 (0.5 to 1.5)	1.6 (0.6 to 3.4)	4.0 (0.4 to 7.0)	87.4 (6.9 to 186.8)
Georgia	0.6 (0.3 to 0.9)	0.9 (0.5 to 1.4)	0.9 (0.5 to 1.5)	1.5 (0.5 to 3.1)	2.8 (-0.9 to 6.2)	58.8 (-12.9 to 153.8)
Kazakhstan	0.5 (0.3 to 0.9)	0.9 (0.5 to 1.6)	1.0 (0.5 to 1.7)	1.5 (0.5 to 3.4)	3.0 (-0.4 to 6.5)	62.2 (-6.0 to 166.3)
Kyrgyzstan	0.3 (0.1 to 0.5)	0.4 (0.2 to 0.7)	0.4 (0.2 to 0.8)	0.6 (0.2 to 1.5)	2.2 (-1.4 to 6.0)	44.7 (-18.4 to 144.4)
Mongolia	0.3 (0.2 to 0.4)	0.6 (0.3 to 1.0)	0.6 (0.3 to 1.1)	1.2 (0.4 to 2.5)	3.9 (0.7 to 7.0)	84.7 (11.3 to 185.0)
Tajikistan	0.1 (0.0 to 0.1)	0.1 (0.1 to 0.2)	0.1 (0.1 to 0.2)	0.2 (0.1 to 0.6)	4.0 (0.3 to 7.4)	88.8 (5.0 to 202.9)
Turkmenistan	0.4 (0.2 to 0.6)	0.7 (0.4 to 1.1)	0.7 (0.4 to 1.2)	1.2 (0.5 to 2.7)	3.4 (0.1 to 6.8)	71.6 (1.9 to 175.6)
Uzbekistan	0.2 (0.1 to 0.4)	0.5 (0.3 to 0.8)	0.5 (0.3 to 0.9)	0.9 (0.3 to 2.0)	4.1 (0.8 to 7.6)	92.8 (11.9 to 212.8)
Central Europe						
Albania	0.2 (0.1 to 0.3)	0.3 (0.2 to 0.4)	0.3 (0.2 to 0.5)	0.5 (0.2 to 0.9)	2.6 (-0.5 to 5.6)	51.3 (-7.7 to 132.9)
Bosnia and Herzegovina	0.2 (0.1 to 0.3)	0.3 (0.2 to 0.5)	0.3 (0.2 to 0.6)	0.5 (0.2 to 1.1)	2.9 (-0.4 to 6.4)	60.4 (-6.1 to 161.3)
Bulgaria	0.4 (0.3 to 0.7)	0.7 (0.4 to 1.1)	0.7 (0.4 to 1.1)	1.0 (0.4 to 2.3)	2.2 (-1.0 to 5.3)	44.2 (-14.2 to 122.5)
Croatia	0.6 (0.4 to 0.8)	0.8 (0.6 to 1.0)	0.8 (0.6 to 1.1)	1.1 (0.6 to 2.1)	2.1 (-0.6 to 4.8)	39.9 (-9.1 to 105.8)
Czech Republic	0.5 (0.4 to 0.6)	0.7 (0.5 to 0.9)	0.7 (0.5 to 1.0)	0.9 (0.5 to 1.6)	1.6 (-0.9 to 4.2)	30.6 (-13.0 to 86.7)
Hungary	0.6 (0.5 to 0.6)	0.6 (0.4 to 0.8)	0.6 (0.4 to 0.8)	0.6 (0.3 to 1.1)	0.3 (-1.9 to 2.8)	6.9 (-25.2 to 51.4)
Macedonia	0.2 (0.1 to 0.4)	0.4 (0.2 to 0.7)	0.5 (0.2 to 0.8)	0.7 (0.3 to 1.6)	3.1 (-0.2 to 6.3)	65.6 (-2.5 to 157.9)
Montenegro	0.3 (0.2 to 0.6)	0.5 (0.3 to 0.8)	0.5 (0.3 to 0.8)	0.7 (0.3 to 1.5)	2.1 (-1.3 to 5.5)	41.2 (-17.5 to 126.7)
Poland	0.5 (0.3 to 0.8)	0.9 (0.6 to 1.4)	0.9 (0.6 to 1.4)	1.5 (0.6 to 3.0)	2.9 (-0.2 to 6.1)	59.0 (-3.2 to 149.3)
Romania	0.4 (0.4 to 0.5)	0.6 (0.5 to 0.7)	0.6 (0.5 to 0.8)	0.8 (0.5 to 1.4)	1.8 (-0.6 to 4.2)	33.6 (-8.6 to 89.2)
Serbia	0.2 (0.1 to 0.4)	0.3 (0.2 to 0.6)	0.4 (0.2 to 0.6)	0.5 (0.2 to 1.2)	2.5 (-0.9 to 6.0)	50.3 (-12.9 to 144.8)
Slovakia	0.5 (0.4 to 0.6)	0.8 (0.6 to 0.9)	0.8 (0.6 to 1.0)	1.2 (0.6 to 2.0)	2.7 (0.3 to 5.0)	51.7 (4.7 to 112.8)
Slovenia	0.6 (0.4 to 0.9)	1.0 (0.7 to 1.5)	1.0 (0.6 to 1.5)	1.5 (0.7 to 2.8)	2.2 (-0.7 to 5.3)	43.5 (-10.4 to 120.4)
Eastern Europe						

	Estimate in 2000 (95% UIs)	Estimate in 2015 (95% UIs)	Estimate in 2017 (95% UIs)	Estimate in 2030 (95% UIs)	Annualised rate of change, 2015-2030 (95% UIs)	Percent change, 2015-2030 (95% UIs)
Belarus	0.5 (0.3 to 0.7)	0.8 (0.5 to 1.2)	0.8 (0.5 to 1.3)	1.4 (0.5 to 2.8)	2.9 (-0.5 to 6.4)	59.6 (-7.6 to 160.2)
Estonia	0.6 (0.4 to 0.7)	0.7 (0.5 to 0.9)	0.7 (0.5 to 1.0)	0.9 (0.4 to 1.7)	1.3 (-1.4 to 4.0)	23.6 (-19.2 to 81.9)
Latvia	0.6 (0.5 to 0.8)	0.8 (0.6 to 1.1)	0.8 (0.6 to 1.2)	1.1 (0.5 to 1.9)	1.6 (-1.0 to 4.3)	29.7 (-13.9 to 89.6)
Lithuania	0.6 (0.5 to 0.7)	0.8 (0.6 to 1.0)	0.8 (0.6 to 1.1)	1.2 (0.6 to 2.0)	2.1 (-0.4 to 4.5)	39.5 (-5.7 to 96.8)
Moldova	0.2 (0.1 to 0.4)	0.4 (0.2 to 0.7)	0.4 (0.2 to 0.7)	0.7 (0.2 to 1.4)	2.9 (-0.5 to 6.2)	60.1 (-6.7 to 154.3)
Russia	0.7 (0.5 to 1.0)	1.1 (0.7 to 1.7)	1.1 (0.7 to 1.8)	1.7 (0.7 to 3.5)	2.3 (-0.8 to 5.5)	45.7 (-10.8 to 129.8)
Ukraine	0.4 (0.2 to 0.7)	0.6 (0.3 to 0.9)	0.6 (0.3 to 1.0)	0.8 (0.3 to 1.7)	1.7 (-1.6 to 5.1)	33.9 (-21.4 to 114.8)
High-income						
Australasia						
Australia	0.7 (0.4 to 1.0)	0.9 (0.5 to 1.3)	0.9 (0.5 to 1.4)	1.1 (0.5 to 2.3)	1.4 (-1.6 to 4.5)	26.5 (-21.1 to 95.0)
New Zealand	0.7 (0.4 to 1.1)	0.9 (0.5 to 1.4)	0.9 (0.5 to 1.5)	1.2 (0.5 to 2.5)	1.2 (-1.7 to 4.5)	24.0 (-22.7 to 96.0)
High-income Asia Pacific						
Brunei	0.6 (0.3 to 1.0)	0.9 (0.5 to 1.6)	1.0 (0.6 to 1.7)	1.5 (0.6 to 3.3)	2.8 (-0.9 to 6.2)	57.0 (-12.6 to 153.1)
Japan	1.0 (0.6 to 1.5)	1.2 (0.8 to 1.7)	1.2 (0.7 to 1.8)	1.4 (0.6 to 2.8)	1.0 (-1.9 to 4.3)	18.8 (-25.2 to 91.6)
South Korea	0.4 (0.3 to 0.7)	0.8 (0.5 to 1.1)	0.8 (0.5 to 1.2)	1.3 (0.5 to 2.5)	3.1 (-0.1 to 6.5)	64.8 (-1.3 to 163.7)
Singapore	0.7 (0.4 to 1.2)	1.2 (0.7 to 2.1)	1.3 (0.7 to 2.2)	2.0 (0.7 to 4.4)	2.7 (-0.6 to 6.1)	56.1 (-8.0 to 151.0)
High-income North America						
Canada	0.7 (0.5 to 1.1)	0.8 (0.5 to 1.4)	0.8 (0.4 to 1.4)	0.9 (0.4 to 2.0)	0.4 (-2.4 to 3.7)	9.3 (-30.2 to 74.2)
Greenland	0.4 (0.2 to 0.7)	0.5 (0.3 to 0.9)	0.5 (0.3 to 0.9)	0.7 (0.3 to 1.6)	1.6 (-1.7 to 5.3)	31.9 (-23.1 to 122.1)
United States	0.6 (0.4 to 0.8)	0.7 (0.5 to 1.0)	0.7 (0.5 to 1.1)	0.9 (0.4 to 1.8)	0.9 (-1.7 to 4.1)	17.9 (-22.8 to 85.9)
Southern Latin America						
Argentina	0.4 (0.2 to 0.6)	0.5 (0.3 to 0.9)	0.6 (0.3 to 0.9)	0.8 (0.3 to 1.6)	2.0 (-1.2 to 5.3)	39.8 (-17.0 to 122.7)
Chile	0.1 (0.1 to 0.1)	0.1 (0.1 to 0.2)	0.1 (0.1 to 0.2)	0.2 (0.1 to 0.5)	3.4 (0.7 to 6.3)	70.4 (10.3 to 156.3)
Uruguay	0.0 (0.0 to 0.0)	0.0 (0.0 to 0.0)	0.0 (0.0 to 0.0)	0.1 (0.0 to 0.1)	3.9 (0.8 to 7.3)	83.8 (12.7 to 199.0)
Western Europe						
Andorra	1.1 (0.6 to 1.9)	1.2 (0.6 to 2.1)	1.2 (0.6 to 2.2)	1.4 (0.5 to 3.5)	0.7 (-2.6 to 4.4)	15.2 (-32.0 to 92.8)
Austria	0.6 (0.5 to 0.7)	0.7 (0.5 to 1.0)	0.8 (0.5 to 1.0)	1.0 (0.5 to 1.7)	1.5 (-1.1 to 4.0)	26.7 (-14.9 to 81.0)
Belgium	0.9 (0.8 to 1.0)	0.9 (0.7 to 1.1)	0.9 (0.6 to 1.2)	0.9 (0.5 to 1.6)	-0.0 (-2.4 to 2.3)	1.6 (-30.3 to 41.7)
Cyprus	1.3 (1.0 to 1.6)	1.7 (1.3 to 2.2)	1.8 (1.3 to 2.4)	2.3 (1.2 to 4.1)	1.8 (-0.8 to 4.3)	32.6 (-10.7 to 90.6)
Denmark	0.7 (0.6 to 0.8)	0.9 (0.7 to 1.2)	0.9 (0.6 to 1.3)	1.1 (0.5 to 2.0)	0.8 (-1.7 to 3.5)	15.7 (-22.1 to 68.2)
Finland	0.6 (0.5 to 0.7)	0.6 (0.4 to 0.8)	0.6 (0.4 to 0.8)	0.6 (0.3 to 1.1)	-0.0 (-2.5 to 2.7)	1.6 (-31.4 to 50.6)
France	0.7 (0.7 to 0.8)	0.7 (0.5 to 0.9)	0.7 (0.5 to 0.9)	0.7 (0.4 to 1.1)	-0.6 (-2.6 to 1.8)	-6.7 (-32.3 to 31.4)
Germany	0.8 (0.8 to 0.9)	1.2 (0.9 to 1.5)	1.3 (0.9 to 1.7)	1.8 (0.9 to 2.9)	2.1 (-0.3 to 4.3)	39.0 (-4.8 to 90.6)
Greece	0.6 (0.6 to 0.7)	0.6 (0.4 to 0.7)	0.6 (0.4 to 0.8)	0.6 (0.3 to 1.0)	-0.2 (-2.3 to 2.1)	-2.1 (-29.5 to 36.5)
Iceland	1.1 (0.9 to 1.4)	1.5 (1.1 to 2.0)	1.5 (1.1 to 2.1)	1.9 (1.0 to 3.5)	1.4 (-1.3 to 4.0)	25.0 (-17.5 to 81.3)
Ireland	0.6 (0.5 to 0.7)	0.7 (0.6 to 1.0)	0.8 (0.5 to 1.0)	1.0 (0.5 to 1.8)	1.8 (-0.8 to 4.3)	33.2 (-10.7 to 89.3)
Israel	0.4 (0.3 to 0.6)	0.7 (0.4 to 0.9)	0.7 (0.4 to 1.0)	1.1 (0.5 to 2.0)	3.0 (-0.3 to 6.0)	60.8 (-3.7 to 145.9)
Italy	0.6 (0.5 to 0.6)	0.8 (0.6 to 1.0)	0.8 (0.6 to 1.1)	1.2 (0.6 to 1.9)	2.3 (-0.2 to 4.7)	43.5 (-2.9 to 102.3)
Luxembourg	1.0 (0.8 to 1.2)	0.9 (0.7 to 1.3)	0.9 (0.7 to 1.4)	1.0 (0.5 to 2.0)	0.3 (-1.9 to 3.0)	7.4 (-25.4 to 56.1)
Malta	0.6 (0.3 to 0.9)	0.8 (0.4 to 1.4)	0.8 (0.4 to 1.4)	1.1 (0.4 to 2.5)	2.1 (-1.5 to 5.5)	41.2 (-20.0 to 129.8)
Netherlands	0.4 (0.4 to 0.5)	0.4 (0.3 to 0.5)	0.4 (0.3 to 0.5)	0.4 (0.2 to 0.7)	-0.0 (-2.4 to 2.5)	1.5 (-30.0 to 45.2)
Norway	0.5 (0.4 to 0.5)	0.7 (0.5 to 0.9)	0.7 (0.5 to 0.9)	0.9 (0.4 to 1.6)	1.7 (-0.9 to 4.4)	31.7 (-12.9 to 93.9)
Portugal	0.6 (0.5 to 0.7)	0.7 (0.5 to 0.9)	0.7 (0.5 to 1.0)	0.9 (0.4 to 1.6)	1.3 (-1.3 to 4.0)	25.1 (-18.2 to 83.5)
Spain	0.6 (0.5 to 0.7)	0.7 (0.5 to 0.9)	0.7 (0.5 to 0.9)	0.8 (0.4 to 1.3)	0.5 (-1.6 to 3.0)	10.2 (-20.9 to 56.2)
Sweden	0.4 (0.3 to 0.5)	0.6 (0.5 to 0.8)	0.7 (0.5 to 0.9)	0.9 (0.5 to 1.6)	2.3 (-0.2 to 4.9)	43.2 (-2.8 to 108.1)
Switzerland	0.7 (0.7 to 0.8)	1.1 (0.8 to 1.4)	1.1 (0.8 to 1.5)	1.5 (0.9 to 2.6)	2.1 (-0.1 to 4.5)	39.9 (-1.3 to 95.3)
United Kingdom	0.5 (0.4 to 0.5)	0.6 (0.5 to 0.8)	0.6 (0.5 to 0.8)	0.8 (0.4 to 1.2)	1.0 (-1.2 to 3.2)	18.4 (-16.4 to 62.1)
Latin America and Caribbean						
Andean Latin America						
Bolivia	0.6 (0.5 to 0.8)	1.1 (0.7 to 1.8)	1.2 (0.7 to 2.0)	2.1 (0.8 to 4.5)	3.7 (0.8 to 6.7)	77.6 (12.6 to 173.3)
Ecuador	0.1 (0.0 to 0.1)	0.1 (0.1 to 0.1)	0.1 (0.1 to 0.1)	0.1 (0.1 to 0.3)	3.2 (0.1 to 6.4)	66.8 (1.2 to 159.7)
Peru	0.2 (0.1 to 0.4)	0.3 (0.2 to 0.6)	0.4 (0.2 to 0.6)	0.5 (0.2 to 1.1)	2.5 (-1.1 to 5.8)	51.4 (-15.3 to 137.1)
Caribbean						
Antigua and Barbuda	0.3 (0.2 to 0.6)	0.4 (0.2 to 0.8)	0.5 (0.2 to 0.8)	0.6 (0.2 to 1.4)	2.1 (-1.3 to 5.6)	42.1 (-17.8 to 131.7)
The Bahamas	0.4 (0.2 to 0.7)	0.6 (0.3 to 1.0)	0.6 (0.3 to 1.0)	0.8 (0.3 to 1.6)	1.8 (-1.3 to 5.1)	35.3 (-18.3 to 113.9)
Barbados	0.4 (0.2 to 0.7)	0.5 (0.3 to 0.9)	0.5 (0.3 to 1.0)	0.7 (0.2 to 1.7)	1.7 (-1.8 to 5.4)	33.1 (-23.7 to 124.5)
Belize	0.1 (0.1 to 0.2)	0.2 (0.1 to 0.4)	0.2 (0.1 to 0.4)	0.5 (0.1 to 1.0)	4.0 (0.5 to 7.8)	88.4 (7.2 to 223.5)
Bermuda	0.6 (0.3 to 1.1)	0.8 (0.4 to 1.4)	0.8 (0.4 to 1.4)	1.0 (0.3 to 2.2)	1.0 (-2.3 to 4.9)	21.4 (-29.5 to 107.3)
Cuba	0.5 (0.4 to 0.7)	0.9 (0.5 to 1.5)	0.9 (0.5 to 1.6)	1.5 (0.5 to 3.5)	2.7 (-0.5 to 5.7)	53.3 (-7.6 to 136.1)
Dominica	0.2 (0.1 to 0.3)	0.3 (0.2 to 0.6)	0.3 (0.2 to 0.6)	0.6 (0.2 to 1.2)	3.0 (-0.3 to 6.5)	63.8 (-4.8 to 165.9)
Dominican Republic	0.1 (0.1 to 0.2)	0.2 (0.1 to 0.3)	0.2 (0.1 to 0.4)	0.3 (0.1 to 0.7)	2.0 (-1.3 to 5.5)	39.9 (-18.0 to 129.9)
Grenada	0.2 (0.1 to 0.3)	0.3 (0.1 to 0.5)	0.3 (0.1 to 0.5)	0.4 (0.1 to 1.0)	2.8 (-0.7 to 6.3)	57.8 (-10.1 to 158.1)
Guyana	0.1 (0.1 to 0.2)	0.2 (0.1 to 0.3)	0.2 (0.1 to 0.4)	0.3 (0.1 to 0.8)	3.1 (-0.3 to 6.7)	66.2 (-5.1 to 172.8)
Haiti	0.1 (0.0 to 0.1)	0.1 (0.0 to 0.1)	0.1 (0.1 to 0.1)	0.2 (0.1 to 0.3)	3.6 (0.3 to 6.9)	76.8 (3.9 to 183.0)
Jamaica	0.2 (0.1 to 0.4)	0.3 (0.2 to 0.6)	0.3 (0.2 to 0.6)	0.5 (0.2 to 1.1)	2.4 (-1.0 to 5.9)	49.1 (-13.5 to 141.3)
Puerto Rico	0.3 (0.2 to 0.6)	0.5 (0.2 to 0.8)	0.5 (0.2 to 0.8)	0.7 (0.2 to 1.5)	1.9 (-1.8 to 5.5)	37.1 (-23.3 to 129.4)
Saint Lucia	0.2 (0.1 to 0.3)	0.3 (0.2 to 0.6)	0.3 (0.2 to 0.6)	0.6 (0.2 to 1.2)	3.1 (-0.5 to 6.7)	66.2 (-6.7 to 173.5)
Saint Vincent and the Grenadines	0.1 (0.1 to 0.2)	0.2 (0.1 to 0.4)	0.3 (0.1 to 0.5)	0.4 (0.1 to 0.9)	3.1 (-0.2 to 7.0)	66.3 (-3.4 to 184.0)
Suriname	0.2 (0.1 to 0.3)	0.3 (0.1 to 0.5)	0.3 (0.2 to 0.5)	0.5 (0.2 to 1.0)	3.1 (-0.5 to 6.4)	64.0 (-7.2 to 162.9)
Trinidad and Tobago	0.1 (0.1 to 0.1)	0.1 (0.1 to 0.3)	0.1 (0.1 to 0.3)	0.2 (0.1 to 0.5)	1.5 (-1.4 to 4.8)	28.3 (-19.4 to 105.2)
Virgin Islands, U.S.	0.5 (0.2 to 0.8)	0.6 (0.3 to 1.0)	0.6 (0.3 to 1.0)	0.8 (0.3 to 1.8)	1.5 (-1.7 to 5.0)	29.2 (-22.6 to 111.5)
Central Latin America						
Colombia	0.1 (0.0 to 0.1)	0.1 (0.1 to 0.3)	0.2 (0.1 to 0.3)	0.3 (0.1 to 0.6)	3.6 (0.2 to 7.0)	78.5 (2.5 to 187.3)
Costa Rica	0.5 (0.4 to 0.6)	0.6 (0.4 to 0.8)	0.6 (0.4 to 0.9)	0.7 (0.3 to 1.5)	1.4 (-1.4 to 4.1)	25.7 (-19.1 to 85.1)
El Salvador	0.0 (0.0 to 0.1)	0.1 (0.0 to 0.1)	0.1 (0.1 to 0.1)	0.1 (0.1 to 0.3)	4.0 (0.7 to 7.2)	88.6 (11.3 to 194.3)
Guatemala	0.0 (0.0 to 0.1)	0.1 (0.1 to 0.1)	0.1 (0.1 to 0.1)	0.2 (0.1 to 0.4)	4.0 (0.3 to 7.5)	89.1 (5.3 to 208.6)
Honduras	0.0 (0.0 to 0.0)	0.0 (0.0 to 0.0)	0.0 (0.0 to 0.0)	0.0 (0.0 to 0.1)	3.4 (-0.2 to 7.2)	74.3 (-3.0 to 196.7)
Mexico	0.1 (0.0 to 0.1)	0.1 (0.1 to 0.2)	0.1 (0.1 to 0.2)	0.2 (0.1 to 0.5)	3.9 (0.7 to 6.8)	85.3 (11.6 to 178.4)
Nicaragua	0.1 (0.0 to 0.1)	0.1 (0.1 to 0.1)	0.1 (0.1 to 0.2)	0.2 (0.1 to 0.4)	4.2 (1.1 to 7.3)	92.9 (17.2 to 198.1)
Panama	0.1 (0.1 to 0.1)	0.1 (0.1 to 0.2)	0.1 (0.1 to 0.2)	0.2 (0.1 to 0.5)	3.1 (-0.2 to 6.3)	64.1 (-3.1 to 159.0)

	Estimate in 2000 (95% UIs)	Estimate in 2015 (95% UIs)	Estimate in 2017 (95% UIs)	Estimate in 2030 (95% UIs)	Annualised rate of change, 2015-2030 (95% UIs)	Percent change, 2015-2030 (95% UIs)
Venezuela	0.1 (0.0 to 0.1)	0.1 (0.0 to 0.1)	0.1 (0.0 to 0.1)	0.1 (0.0 to 0.2)	0.5 (-2.5 to 3.9)	10.3 (-31.0 to 80.1)
Tropical Latin America						
Brazil	0.2 (0.1 to 0.3)	0.5 (0.4 to 0.6)	0.5 (0.3 to 0.7)	0.8 (0.4 to 1.6)	3.6 (0.5 to 6.8)	75.6 (7.0 to 178.6)
Paraguay	0.1 (0.1 to 0.1)	0.1 (0.1 to 0.2)	0.1 (0.1 to 0.2)	0.2 (0.1 to 0.3)	2.4 (-0.8 to 5.7)	48.4 (-10.7 to 134.1)
North Africa and Middle East						
North Africa and Middle East						
Afghanistan	0.1 (0.0 to 0.1)	0.1 (0.1 to 0.1)	0.1 (0.1 to 0.2)	0.2 (0.1 to 0.4)	4.0 (0.4 to 7.3)	87.4 (6.8 to 198.1)
Algeria	0.3 (0.2 to 0.6)	0.7 (0.4 to 1.1)	0.7 (0.4 to 1.2)	1.3 (0.5 to 2.9)	4.2 (0.6 to 7.4)	94.4 (9.9 to 201.3)
Bahrain	1.1 (0.6 to 1.8)	1.8 (0.9 to 3.0)	1.8 (1.0 to 3.1)	2.8 (1.0 to 6.2)	2.6 (-0.7 to 6.1)	52.6 (-9.4 to 149.5)
Egypt	0.4 (0.2 to 0.6)	0.5 (0.5 to 0.6)	0.6 (0.5 to 0.7)	0.8 (0.5 to 1.3)	2.7 (0.1 to 5.5)	53.3 (0.9 to 128.7)
Iran	0.2 (0.1 to 0.3)	0.4 (0.3 to 0.6)	0.5 (0.3 to 0.7)	0.8 (0.3 to 1.7)	4.1 (0.9 to 6.9)	90.4 (14.9 to 183.2)
Iraq	0.1 (0.1 to 0.2)	0.2 (0.1 to 0.3)	0.2 (0.2 to 0.4)	0.4 (0.2 to 0.9)	3.5 (0.1 to 7.2)	75.7 (1.7 to 192.9)
Jordan	0.8 (0.6 to 0.9)	1.1 (0.7 to 1.7)	1.1 (0.7 to 1.8)	1.7 (0.7 to 3.7)	2.3 (-0.7 to 5.4)	45.3 (-9.9 to 123.7)
Kuwait	1.4 (0.7 to 2.3)	2.2 (1.1 to 3.7)	2.3 (1.2 to 3.9)	3.5 (1.3 to 7.2)	2.8 (-0.5 to 6.1)	58.0 (-7.8 to 150.1)
Lebanon	0.5 (0.3 to 0.9)	0.9 (0.5 to 1.6)	1.0 (0.5 to 1.7)	1.6 (0.6 to 3.4)	3.4 (0.1 to 6.7)	72.4 (1.6 to 174.0)
Libya	0.8 (0.5 to 1.4)	0.9 (0.5 to 1.6)	1.0 (0.5 to 1.6)	1.2 (0.5 to 2.5)	1.3 (-1.3 to 4.4)	24.4 (-17.8 to 92.3)
Morocco	0.2 (0.1 to 0.4)	0.4 (0.2 to 0.7)	0.4 (0.2 to 0.7)	0.8 (0.3 to 1.7)	4.1 (0.5 to 7.3)	89.9 (8.5 to 199.6)
Palestine	0.2 (0.1 to 0.3)	0.3 (0.1 to 0.5)	0.3 (0.2 to 0.6)	0.6 (0.2 to 1.2)	4.2 (0.4 to 7.6)	94.1 (6.9 to 211.3)
Oman	0.6 (0.3 to 0.9)	1.4 (0.8 to 2.4)	1.5 (0.8 to 2.6)	3.2 (1.3 to 6.8)	5.1 (2.0 to 7.9)	121.9 (34.0 to 227.2)
Qatar	1.7 (0.9 to 2.9)	2.7 (1.4 to 4.6)	2.8 (1.4 to 4.7)	4.2 (1.4 to 8.7)	2.4 (-1.0 to 5.8)	49.3 (-14.0 to 139.6)
Saudi Arabia	0.6 (0.3 to 1.0)	1.5 (0.8 to 2.6)	1.7 (0.9 to 2.9)	3.9 (1.5 to 8.0)	6.0 (2.5 to 8.9)	151.5 (45.7 to 277.9)
Sudan	0.1 (0.1 to 0.1)	0.2 (0.1 to 0.4)	0.2 (0.1 to 0.4)	0.5 (0.2 to 1.0)	5.0 (1.7 to 8.2)	117.6 (29.3 to 242.5)
Syria	0.2 (0.1 to 0.4)	0.3 (0.2 to 0.6)	0.4 (0.2 to 0.6)	0.6 (0.2 to 1.2)	2.9 (-0.4 to 6.2)	60.5 (-5.4 to 153.9)
Tunisia	0.3 (0.2 to 0.5)	0.6 (0.3 to 1.0)	0.6 (0.3 to 1.1)	1.1 (0.4 to 2.5)	3.4 (0.1 to 6.9)	72.6 (1.2 to 180.3)
Turkey	0.1 (0.1 to 0.2)	0.2 (0.1 to 0.3)	0.2 (0.1 to 0.4)	0.4 (0.2 to 0.9)	4.3 (1.0 to 7.5)	96.1 (16.3 to 210.1)
United Arab Emirates	1.7 (0.9 to 3.0)	2.7 (1.4 to 4.5)	2.7 (1.5 to 4.6)	4.0 (1.6 to 8.3)	2.3 (-0.8 to 5.6)	45.7 (-11.4 to 130.2)
Yemen	0.1 (0.1 to 0.1)	0.2 (0.1 to 0.3)	0.2 (0.1 to 0.3)	0.3 (0.1 to 0.6)	3.0 (-0.3 to 6.2)	61.9 (-4.0 to 154.0)
South Asia						
South Asia						
Bangladesh	0.1 (0.1 to 0.1)	0.2 (0.1 to 0.3)	0.2 (0.1 to 0.3)	0.3 (0.1 to 0.8)	4.5 (1.0 to 7.7)	102.0 (15.3 to 219.7)
Bhutan	0.2 (0.1 to 0.3)	0.4 (0.2 to 0.6)	0.4 (0.2 to 0.7)	0.8 (0.3 to 1.7)	5.0 (1.8 to 8.3)	119.6 (30.1 to 244.9)
India	0.1 (0.1 to 0.1)	0.2 (0.1 to 0.3)	0.2 (0.1 to 0.3)	0.4 (0.1 to 0.8)	4.9 (1.6 to 7.9)	115.5 (27.7 to 227.3)
Nepal	0.1 (0.1 to 0.1)	0.2 (0.1 to 0.3)	0.2 (0.1 to 0.3)	0.4 (0.1 to 0.9)	5.0 (1.7 to 8.2)	120.3 (28.2 to 243.5)
Pakistan	0.1 (0.0 to 0.1)	0.2 (0.1 to 0.3)	0.2 (0.1 to 0.3)	0.3 (0.1 to 0.7)	4.5 (1.1 to 7.8)	103.9 (17.9 to 220.1)
Southeast Asia, East Asia, and Oceania						
East Asia						
China	0.3 (0.2 to 0.4)	0.6 (0.3 to 0.9)	0.6 (0.3 to 0.9)	1.1 (0.4 to 2.3)	4.2 (1.2 to 7.4)	93.3 (18.9 to 204.6)
North Korea	0.1 (0.1 to 0.2)	0.1 (0.1 to 0.2)	0.1 (0.1 to 0.2)	0.2 (0.1 to 0.4)	1.7 (-1.6 to 5.4)	34.0 (-21.9 to 125.0)
Taiwan (Province of China)	0.4 (0.2 to 0.7)	0.7 (0.4 to 1.0)	0.7 (0.4 to 1.0)	1.0 (0.4 to 2.0)	2.3 (-0.8 to 5.6)	45.7 (-11.9 to 133.1)
Oceania						
American Samoa	0.3 (0.2 to 0.6)	0.4 (0.2 to 0.7)	0.4 (0.2 to 0.7)	0.5 (0.2 to 1.1)	1.7 (-1.6 to 5.0)	33.3 (-21.6 to 111.6)
Federated States of Micronesia	0.1 (0.1 to 0.2)	0.2 (0.1 to 0.3)	0.2 (0.1 to 0.3)	0.2 (0.1 to 0.5)	2.0 (-0.3 to 4.2)	37.1 (-4.6 to 88.9)
Fiji	0.2 (0.1 to 0.4)	0.3 (0.1 to 0.5)	0.3 (0.2 to 0.5)	0.4 (0.1 to 0.9)	1.8 (-1.5 to 5.3)	36.1 (-19.7 to 120.3)
Guam	0.6 (0.3 to 1.1)	0.7 (0.4 to 1.2)	0.7 (0.4 to 1.3)	0.9 (0.3 to 2.0)	0.9 (-2.3 to 4.2)	18.5 (-29.2 to 87.2)
Kiribati	0.0 (0.0 to 0.1)	0.1 (0.0 to 0.1)	0.1 (0.0 to 0.1)	0.1 (0.0 to 0.2)	3.3 (-0.3 to 7.0)	70.3 (-4.3 to 184.2)
Marshall Islands	0.1 (0.1 to 0.2)	0.1 (0.1 to 0.3)	0.2 (0.1 to 0.3)	0.3 (0.1 to 0.6)	3.4 (0.1 to 6.9)	73.9 (0.9 to 182.9)
Northern Mariana Islands	0.7 (0.4 to 1.2)	0.6 (0.3 to 1.1)	0.7 (0.3 to 1.1)	0.7 (0.3 to 1.5)	0.5 (-2.6 to 3.9)	10.6 (-32.4 to 79.2)
Papua New Guinea	0.1 (0.0 to 0.1)	0.1 (0.0 to 0.1)	0.1 (0.0 to 0.1)	0.1 (0.0 to 0.2)	2.2 (-1.1 to 5.8)	44.8 (-14.9 to 137.1)
Samoa	0.1 (0.1 to 0.2)	0.1 (0.1 to 0.2)	0.1 (0.1 to 0.2)	0.1 (0.1 to 0.3)	0.2 (-2.5 to 3.3)	5.6 (-31.5 to 64.6)
Solomon Islands	0.0 (0.0 to 0.1)	0.1 (0.0 to 0.1)	0.1 (0.0 to 0.1)	0.1 (0.0 to 0.2)	3.1 (-0.4 to 6.5)	63.9 (-6.4 to 163.7)
Tonga	0.2 (0.1 to 0.3)	0.2 (0.1 to 0.4)	0.2 (0.1 to 0.4)	0.3 (0.1 to 0.7)	2.3 (-1.0 to 5.6)	46.1 (-13.3 to 130.6)
Vanuatu	0.1 (0.1 to 0.1)	0.1 (0.1 to 0.2)	0.1 (0.1 to 0.2)	0.2 (0.1 to 0.5)	2.5 (-0.8 to 5.8)	51.2 (-10.7 to 137.5)
Southeast Asia						
Cambodia	0.1 (0.0 to 0.1)	0.1 (0.1 to 0.2)	0.2 (0.1 to 0.3)	0.3 (0.1 to 0.7)	5.0 (1.7 to 8.2)	117.4 (28.8 to 242.3)
Indonesia	0.1 (0.0 to 0.1)	0.1 (0.1 to 0.2)	0.2 (0.1 to 0.3)	0.3 (0.1 to 0.7)	4.1 (0.8 to 7.6)	90.4 (13.0 to 212.1)
Laos	0.1 (0.0 to 0.1)	0.2 (0.1 to 0.3)	0.2 (0.1 to 0.3)	0.4 (0.1 to 0.8)	5.2 (1.8 to 8.3)	124.1 (31.3 to 247.5)
Malaysia	0.2 (0.1 to 0.2)	0.3 (0.2 to 0.6)	0.4 (0.2 to 0.6)	0.7 (0.2 to 1.6)	4.1 (0.6 to 7.3)	89.7 (9.6 to 199.5)
Maldives	0.1 (0.1 to 0.3)	0.4 (0.2 to 0.7)	0.5 (0.2 to 0.8)	1.2 (0.4 to 2.5)	6.6 (2.9 to 9.5)	176.3 (55.1 to 312.7)
Mauritius	0.3 (0.1 to 0.5)	0.5 (0.3 to 0.8)	0.5 (0.3 to 0.9)	0.9 (0.3 to 1.9)	3.5 (0.1 to 6.9)	75.4 (1.5 to 181.5)
Myanmar	0.1 (0.0 to 0.1)	0.2 (0.1 to 0.4)	0.2 (0.1 to 0.4)	0.6 (0.2 to 1.2)	6.6 (3.6 to 9.1)	173.4 (70.6 to 289.6)
Philippines	0.4 (0.3 to 0.5)	0.4 (0.2 to 0.5)	0.4 (0.2 to 0.6)	0.4 (0.2 to 0.9)	1.0 (-1.7 to 3.9)	19.6 (-22.1 to 78.8)
Sri Lanka	0.2 (0.1 to 0.3)	0.3 (0.2 to 0.6)	0.3 (0.2 to 0.6)	0.6 (0.2 to 1.3)	3.9 (0.3 to 7.2)	86.0 (5.4 to 196.6)
Seychelles	0.3 (0.2 to 0.6)	0.5 (0.3 to 0.9)	0.5 (0.3 to 0.9)	0.8 (0.3 to 1.9)	3.0 (-0.5 to 6.5)	61.4 (-7.0 to 165.2)
Thailand	0.1 (0.1 to 0.2)	0.2 (0.1 to 0.3)	0.2 (0.1 to 0.3)	0.3 (0.1 to 0.7)	3.3 (-0.2 to 6.8)	71.1 (-2.4 to 177.0)
Timor-Leste	0.1 (0.0 to 0.1)	0.1 (0.1 to 0.2)	0.1 (0.1 to 0.2)	0.3 (0.1 to 0.6)	4.5 (1.4 to 7.5)	102.8 (23.6 to 210.0)
Vietnam	0.1 (0.1 to 0.2)	0.2 (0.1 to 0.4)	0.3 (0.1 to 0.5)	0.5 (0.2 to 1.2)	4.6 (1.3 to 8.0)	107.4 (21.2 to 234.4)
Sub-Saharan Africa						
Central Sub-Saharan Africa						
Angola	0.0 (0.0 to 0.0)	0.1 (0.0 to 0.1)	0.1 (0.0 to 0.1)	0.2 (0.1 to 0.4)	6.4 (3.2 to 9.2)	167.2 (62.6 to 298.4)
Central African Republic	0.0 (0.0 to 0.0)	0.0 (0.0 to 0.1)	0.0 (0.0 to 0.1)	0.1 (0.0 to 0.1)	2.9 (-0.5 to 6.3)	60.2 (-7.0 to 155.7)
Congo	0.1 (0.0 to 0.1)	0.1 (0.1 to 0.2)	0.1 (0.1 to 0.3)	0.3 (0.1 to 0.7)	4.7 (1.3 to 7.9)	109.3 (21.2 to 226.3)
Democratic Republic of the Congo	0.0 (0.0 to 0.0)	0.0 (0.0 to 0.1)	0.0 (0.0 to 0.1)	0.1 (0.0 to 0.1)	4.1 (0.9 to 6.8)	90.2 (13.7 to 179.2)
Equatorial Guinea	0.0 (0.0 to 0.1)	0.1 (0.1 to 0.2)	0.1 (0.1 to 0.3)	0.5 (0.2 to 0.9)	7.7 (4.5 to 10.2)	224.1 (95.9 to 360.5)
Gabon	0.1 (0.0 to 0.2)	0.2 (0.1 to 0.3)	0.2 (0.1 to 0.3)	0.4 (0.1 to 0.8)	4.8 (1.3 to 8.2)	114.5 (21.3 to 242.9)
Eastern Sub-Saharan Africa						
Burundi	0.0 (0.0 to 0.1)	0.0 (0.0 to 0.1)	0.0 (0.0 to 0.1)	0.1 (0.0 to 0.1)	2.3 (-1.2 to 6.0)	45.4 (-16.4 to 144.6)
Comoros	0.0 (0.0 to 0.1)	0.1 (0.0 to 0.1)	0.1 (0.0 to 0.1)	0.2 (0.1 to 0.3)	4.0 (0.4 to 7.5)	88.5 (6.8 to 207.6)
Djibouti	0.0 (0.0 to 0.1)	0.1 (0.0 to 0.1)	0.1 (0.0 to 0.1)	0.2 (0.1 to 0.4)	5.0 (1.4 to 8.2)	118.7 (23.0 to 243.8)
Eritrea	0.0 (0.0 to 0.1)	0.1 (0.0 to 0.1)	0.1 (0.0 to 0.1)	0.1 (0.0 to 0.2)	3.4 (0.2 to 6.6)	71.9 (3.8 to 167.8)
Ethiopia	0.0 (0.0 to 0.0)	0.0 (0.0 to 0.0)	0.0 (0.0 to 0.1)	0.1 (0.0 to 0.1)	6.4 (3.3 to 8.9)	166.2 (63.8 to 277.9)

	Estimate in 2000 (95% UIs)	Estimate in 2015 (95% UIs)	Estimate in 2017 (95% UIs)	Estimate in 2030 (95% UIs)	Annualised rate of change, 2015-2030 (95% UIs)	Percent change, 2015-2030 (95% UIs)
Kenya	0.1 (0.0 to 0.1)	0.1 (0.1 to 0.2)	0.1 (0.1 to 0.2)	0.2 (0.1 to 0.5)	3.5 (-0.0 to 7.0)	76.0 (-0.4 to 185.4)
Madagascar	0.0 (0.0 to 0.1)	0.1 (0.0 to 0.1)	0.1 (0.0 to 0.1)	0.1 (0.0 to 0.2)	2.2 (-1.1 to 5.5)	44.1 (-15.3 to 129.0)
Malawi	0.0 (0.0 to 0.0)	0.1 (0.0 to 0.1)	0.1 (0.0 to 0.1)	0.1 (0.0 to 0.2)	4.0 (0.6 to 7.2)	88.6 (10.0 to 195.0)
Mozambique	0.0 (0.0 to 0.0)	0.0 (0.0 to 0.0)	0.0 (0.0 to 0.0)	0.1 (0.0 to 0.1)	5.0 (1.7 to 8.2)	118.3 (29.5 to 240.9)
Rwanda	0.0 (0.0 to 0.0)	0.1 (0.1 to 0.1)	0.1 (0.1 to 0.1)	0.2 (0.1 to 0.4)	5.1 (2.2 to 8.0)	119.7 (38.2 to 233.4)
Somalia	0.0 (0.0 to 0.0)	0.0 (0.0 to 0.1)	0.0 (0.0 to 0.1)	0.1 (0.0 to 0.1)	3.2 (-0.5 to 6.7)	67.0 (-6.9 to 172.2)
South Sudan	0.0 (0.0 to 0.0)	0.0 (0.0 to 0.1)	0.0 (0.0 to 0.1)	0.1 (0.0 to 0.1)	3.0 (-0.6 to 6.3)	63.9 (-9.0 to 158.8)
Tanzania	0.1 (0.0 to 0.1)	0.1 (0.1 to 0.1)	0.1 (0.1 to 0.2)	0.2 (0.1 to 0.4)	4.4 (1.0 to 7.6)	101.0 (16.5 to 211.0)
Uganda	0.0 (0.0 to 0.0)	0.0 (0.0 to 0.0)	0.0 (0.0 to 0.0)	0.0 (0.0 to 0.1)	3.8 (0.6 to 7.1)	82.7 (9.5 to 189.7)
Zambia	0.1 (0.1 to 0.1)	0.3 (0.2 to 0.4)	0.3 (0.2 to 0.4)	0.8 (0.4 to 1.4)	5.9 (2.9 to 8.8)	149.0 (55.1 to 274.7)
Southern Sub-Saharan Africa						
Botswana	0.1 (0.1 to 0.2)	0.2 (0.1 to 0.3)	0.2 (0.1 to 0.4)	0.5 (0.2 to 1.1)	4.5 (0.8 to 8.1)	103.6 (12.7 to 238.2)
Lesotho	0.1 (0.0 to 0.1)	0.1 (0.0 to 0.1)	0.1 (0.0 to 0.1)	0.1 (0.1 to 0.3)	3.6 (0.7 to 6.9)	77.9 (11.3 to 181.7)
Namibia	0.2 (0.1 to 0.3)	0.3 (0.2 to 0.5)	0.3 (0.2 to 0.6)	0.7 (0.3 to 1.5)	4.7 (1.0 to 8.3)	110.6 (15.4 to 247.7)
South Africa	0.1 (0.1 to 0.2)	0.2 (0.1 to 0.2)	0.2 (0.1 to 0.3)	0.2 (0.1 to 0.5)	1.5 (-1.5 to 4.7)	28.2 (-19.7 to 102.5)
Swaziland	0.1 (0.0 to 0.1)	0.1 (0.1 to 0.2)	0.1 (0.1 to 0.2)	0.2 (0.1 to 0.4)	3.0 (-0.4 to 6.2)	62.7 (-6.2 to 154.3)
Zimbabwe	0.1 (0.1 to 0.2)	0.1 (0.1 to 0.2)	0.1 (0.1 to 0.2)	0.2 (0.1 to 0.3)	2.5 (-0.4 to 5.4)	49.5 (-5.5 to 125.4)
Western Sub-Saharan Africa						
Benin	0.1 (0.0 to 0.1)	0.1 (0.1 to 0.2)	0.1 (0.1 to 0.2)	0.2 (0.1 to 0.5)	4.6 (0.9 to 7.9)	108.2 (13.6 to 228.9)
Burkina Faso	0.0 (0.0 to 0.1)	0.1 (0.0 to 0.1)	0.1 (0.0 to 0.1)	0.1 (0.1 to 0.3)	4.0 (0.3 to 7.6)	88.2 (3.9 to 214.5)
Cameroon	0.2 (0.1 to 0.3)	0.4 (0.2 to 0.6)	0.4 (0.2 to 0.6)	0.8 (0.3 to 1.8)	5.0 (1.7 to 8.2)	120.0 (29.2 to 242.0)
Cape Verde	0.1 (0.1 to 0.2)	0.3 (0.1 to 0.5)	0.3 (0.2 to 0.6)	0.7 (0.3 to 1.5)	5.7 (2.3 to 9.0)	143.6 (40.9 to 284.3)
Chad	0.0 (0.0 to 0.0)	0.0 (0.0 to 0.1)	0.0 (0.0 to 0.1)	0.1 (0.0 to 0.2)	4.5 (0.9 to 7.9)	103.9 (14.5 to 228.8)
Cote d'Ivoire	0.1 (0.0 to 0.2)	0.1 (0.1 to 0.2)	0.1 (0.1 to 0.3)	0.2 (0.1 to 0.6)	3.6 (-0.1 to 7.3)	77.5 (-0.8 to 198.6)
The Gambia	0.1 (0.0 to 0.1)	0.1 (0.1 to 0.2)	0.1 (0.1 to 0.2)	0.2 (0.1 to 0.5)	3.3 (-0.2 to 6.8)	70.2 (-3.2 to 178.5)
Ghana	0.2 (0.1 to 0.3)	0.4 (0.2 to 0.6)	0.4 (0.3 to 0.7)	0.7 (0.3 to 1.5)	3.7 (0.1 to 7.1)	79.1 (2.0 to 188.4)
Guinea	0.1 (0.1 to 0.1)	0.1 (0.1 to 0.2)	0.1 (0.1 to 0.2)	0.2 (0.1 to 0.5)	3.6 (0.5 to 6.7)	76.5 (7.3 to 174.1)
Guinea-Bissau	0.0 (0.0 to 0.1)	0.1 (0.0 to 0.1)	0.1 (0.1 to 0.2)	0.2 (0.1 to 0.4)	4.6 (1.1 to 7.9)	106.3 (18.0 to 226.9)
Liberia	0.0 (0.0 to 0.1)	0.1 (0.0 to 0.1)	0.1 (0.0 to 0.1)	0.1 (0.1 to 0.3)	5.0 (2.4 to 7.7)	114.8 (42.9 to 215.8)
Mali	0.0 (0.0 to 0.0)	0.0 (0.0 to 0.1)	0.1 (0.0 to 0.1)	0.1 (0.0 to 0.2)	4.0 (0.2 to 7.6)	87.9 (2.8 to 212.7)
Mauritania	0.1 (0.0 to 0.1)	0.1 (0.1 to 0.2)	0.1 (0.1 to 0.2)	0.2 (0.1 to 0.5)	3.6 (0.3 to 6.8)	78.1 (4.2 to 178.7)
Niger	0.0 (0.0 to 0.0)	0.0 (0.0 to 0.1)	0.0 (0.0 to 0.1)	0.0 (0.0 to 0.1)	2.7 (-0.9 to 6.2)	56.0 (-12.2 to 154.1)
Nigeria	0.1 (0.0 to 0.1)	0.1 (0.1 to 0.1)	0.1 (0.1 to 0.1)	0.1 (0.1 to 0.2)	2.3 (-1.0 to 5.7)	46.3 (-13.7 to 135.3)
Sao Tome and Principe	0.1 (0.0 to 0.1)	0.2 (0.1 to 0.3)	0.2 (0.1 to 0.3)	0.4 (0.2 to 0.9)	6.4 (3.2 to 9.2)	170.1 (60.7 to 296.2)
Senegal	0.1 (0.1 to 0.1)	0.1 (0.1 to 0.2)	0.1 (0.1 to 0.2)	0.2 (0.1 to 0.5)	2.5 (-0.8 to 5.7)	49.6 (-10.9 to 135.3)
Sierra Leone	0.1 (0.0 to 0.1)	0.1 (0.1 to 0.2)	0.1 (0.1 to 0.2)	0.3 (0.1 to 0.6)	5.6 (1.9 to 8.6)	138.8 (34.0 to 260.6)
Togo	0.1 (0.0 to 0.1)	0.1 (0.1 to 0.2)	0.1 (0.1 to 0.2)	0.3 (0.1 to 0.6)	4.1 (0.8 to 7.7)	90.4 (12.7 to 215.3)
**Indicator 3.c.1a: Physicians per 1,000 population**						
Central Europe, Eastern Europe, and Central Asia						
Central Asia						
Armenia	1.9 (1.2 to 2.9)	4.0 (2.9 to 5.5)	4.2 (2.9 to 6.0)	7.6 (3.5 to 13.7)	3.9 (0.8 to 6.8)	85.4 (13.4 to 176.4)
Azerbaijan	2.5 (1.4 to 4.0)	4.9 (2.8 to 8.1)	5.1 (2.8 to 8.3)	8.1 (3.1 to 17.0)	2.9 (-0.0 to 5.9)	59.2 (-0.4 to 140.9)
Georgia	5.1 (3.1 to 8.0)	7.8 (4.7 to 11.4)	8.2 (4.9 to 12.2)	12.1 (5.3 to 22.6)	2.6 (-0.4 to 5.4)	51.7 (-5.6 to 124.3)
Kazakhstan	3.0 (1.7 to 5.0)	5.3 (2.8 to 8.3)	5.5 (2.9 to 8.7)	8.4 (3.1 to 17.3)	2.8 (-0.4 to 5.8)	56.5 (-6.2 to 137.4)
Kyrgyzstan	1.6 (0.9 to 2.6)	2.4 (1.3 to 3.8)	2.4 (1.3 to 3.9)	3.2 (1.2 to 6.7)	1.7 (-1.5 to 5.0)	34.0 (-19.6 to 110.2)
Mongolia	2.2 (1.6 to 2.9)	4.5 (2.6 to 7.3)	4.7 (2.7 to 7.6)	8.8 (3.2 to 17.8)	4.0 (1.2 to 6.7)	87.3 (19.0 to 173.1)
Tajikistan	1.3 (0.8 to 2.0)	2.6 (1.6 to 4.0)	2.8 (1.7 to 4.3)	4.9 (2.0 to 9.7)	3.8 (0.7 to 6.7)	82.0 (11.0 to 174.0)
Turkmenistan	2.1 (1.2 to 3.5)	3.7 (2.0 to 6.1)	3.9 (2.1 to 6.2)	6.3 (2.4 to 12.4)	3.2 (-0.0 to 6.1)	65.5 (-0.1 to 150.2)
Uzbekistan	1.5 (0.8 to 2.5)	2.9 (1.6 to 4.7)	3.1 (1.7 to 5.0)	5.5 (2.0 to 10.8)	3.9 (0.8 to 6.9)	85.2 (12.3 to 183.0)
Central Europe						
Albania	0.7 (0.5 to 1.1)	1.1 (0.8 to 1.6)	1.2 (0.8 to 1.8)	1.8 (0.8 to 3.5)	2.6 (-0.6 to 5.7)	52.2 (-8.2 to 134.2)
Bosnia and Herzegovina	0.6 (0.3 to 1.1)	0.8 (0.4 to 1.4)	0.9 (0.5 to 1.6)	1.4 (0.5 to 3.0)	2.8 (-0.5 to 6.5)	57.1 (-7.2 to 163.9)
Bulgaria	1.2 (0.7 to 1.9)	1.8 (1.0 to 3.0)	1.9 (1.1 to 3.0)	2.6 (1.0 to 5.7)	2.1 (-1.0 to 5.3)	40.3 (-14.3 to 121.0)
Croatia	1.8 (1.3 to 2.2)	2.6 (2.2 to 3.1)	2.7 (2.2 to 3.4)	3.9 (2.5 to 5.9)	2.5 (0.5 to 4.6)	47.7 (8.2 to 100.3)
Czech Republic	2.0 (1.7 to 2.4)	3.1 (2.5 to 3.7)	3.2 (2.5 to 4.0)	4.5 (2.8 to 6.7)	2.3 (0.4 to 4.2)	43.8 (6.4 to 88.8)
Hungary	1.4 (1.2 to 1.6)	1.5 (1.2 to 1.8)	1.4 (1.1 to 1.8)	1.5 (1.0 to 2.2)	-0.1 (-1.7 to 1.8)	0.1 (-22.1 to 31.4)
Macedonia	0.7 (0.4 to 1.2)	1.2 (0.6 to 2.1)	1.3 (0.7 to 2.2)	2.0 (0.7 to 4.3)	2.8 (-0.5 to 6.3)	57.2 (-7.6 to 158.5)
Montenegro	1.0 (0.5 to 1.7)	1.3 (0.7 to 2.3)	1.4 (0.7 to 2.4)	1.9 (0.7 to 4.2)	1.9 (-1.6 to 5.4)	36.6 (-21.3 to 125.5)
Poland	1.3 (0.8 to 1.9)	1.6 (1.1 to 2.5)	1.7 (1.0 to 2.6)	2.2 (0.9 to 4.3)	1.4 (-1.4 to 4.3)	27.5 (-19.4 to 90.0)
Romania	1.4 (1.2 to 1.8)	1.5 (1.3 to 1.8)	1.5 (1.2 to 1.9)	1.6 (1.1 to 2.3)	0.1 (-1.8 to 2.0)	1.8 (-23.3 to 34.0)
Serbia	0.3 (0.1 to 0.5)	0.4 (0.2 to 0.7)	0.4 (0.2 to 0.8)	0.6 (0.2 to 1.4)	2.0 (-1.5 to 5.6)	40.9 (-19.8 to 130.6)
Slovakia	1.7 (1.4 to 2.1)	3.0 (2.5 to 3.5)	3.0 (2.4 to 3.8)	4.5 (2.9 to 6.7)	2.6 (0.5 to 4.6)	50.1 (8.2 to 98.9)
Slovenia	1.1 (0.8 to 1.7)	1.9 (1.3 to 2.7)	1.9 (1.2 to 2.8)	3.0 (1.4 to 5.6)	2.5 (-0.2 to 5.5)	50.1 (-3.5 to 128.2)
Eastern Europe						
Belarus	1.7 (1.0 to 2.6)	3.0 (1.8 to 4.6)	3.1 (1.8 to 4.8)	5.0 (1.8 to 10.7)	2.9 (-0.8 to 6.4)	60.1 (-10.9 to 160.6)
Estonia	2.5 (2.0 to 3.0)	2.9 (2.4 to 3.6)	2.9 (2.2 to 3.8)	3.3 (2.0 to 5.4)	0.7 (-1.4 to 2.9)	11.8 (-19.5 to 54.1)
Latvia	2.5 (2.1 to 3.0)	2.9 (2.3 to 3.4)	3.0 (2.3 to 3.7)	3.3 (2.0 to 5.0)	0.8 (-1.3 to 2.7)	14.0 (-17.3 to 49.7)
Lithuania	2.3 (1.9 to 2.7)	2.9 (2.5 to 3.4)	2.9 (2.3 to 3.6)	3.7 (2.4 to 5.4)	1.5 (-0.5 to 3.5)	26.1 (-6.7 to 67.9)
Moldova	1.0 (0.5 to 1.6)	1.7 (0.9 to 2.7)	1.8 (0.9 to 2.8)	2.6 (1.0 to 5.4)	2.5 (-0.5 to 5.6)	50.7 (-6.8 to 131.3)
Russia	3.4 (2.5 to 4.5)	4.8 (3.3 to 6.5)	4.8 (3.2 to 6.8)	6.2 (3.0 to 11.1)	1.4 (-1.3 to 4.0)	26.7 (-17.1 to 83.3)
Ukraine	1.4 (0.8 to 2.4)	2.2 (1.3 to 3.7)	2.3 (1.3 to 3.9)	3.4 (1.3 to 7.2)	2.3 (-1.1 to 5.7)	45.4 (-15.8 to 136.3)
High-income						
Australasia						
Australia	2.5 (1.7 to 3.5)	3.0 (2.0 to 4.5)	3.0 (1.9 to 4.7)	3.8 (1.6 to 7.8)	1.2 (-1.6 to 4.0)	23.4 (-21.8 to 83.5)
New Zealand	2.8 (1.9 to 4.1)	4.5 (2.8 to 6.9)	4.6 (2.8 to 7.2)	6.8 (2.6 to 14.0)	2.3 (-0.8 to 5.4)	45.7 (-10.8 to 123.8)
High-income Asia Pacific						
Brunei	1.0 (0.5 to 1.7)	1.6 (0.8 to 2.9)	1.7 (0.9 to 3.0)	2.6 (0.9 to 6.2)	2.9 (-0.5 to 6.2)	59.4 (-7.3 to 155.1)
Japan	1.1 (0.6 to 1.8)	1.1 (0.7 to 1.8)	1.1 (0.7 to 1.9)	1.3 (0.5 to 2.8)	0.3 (-2.8 to 3.6)	8.3 (-34.6 to 72.5)
South Korea	0.6 (0.3 to 1.0)	1.1 (0.7 to 1.7)	1.1 (0.7 to 1.8)	2.0 (0.8 to 4.4)	3.7 (0.1 to 7.1)	79.8 (1.4 to 188.9)
Singapore	1.1 (0.6 to 2.0)	2.0 (1.0 to 3.4)	2.0 (1.0 to 3.5)	3.2 (1.1 to 7.1)	2.9 (-0.3 to 6.3)	58.6 (-4.8 to 158.6)

High-income North America	Estimate in 2000 (95% UIs)	Estimate in 2015 (95% UIs)	Estimate in 2017 (95% UIs)	Estimate in 2030 (95% UIs)	Annualised rate of change, 2015-2030 (95% UIs)	Percent change, 2015-2030 (95% UIs)
Canada	2.5 (1.6 to 3.7)	2.4 (1.3 to 4.0)	2.4 (1.3 to 4.1)	2.6 (1.0 to 5.7)	0.2 (-2.7 to 3.2)	5.2 (-33.3 to 60.7)
Greenland	1.3 (0.7 to 2.3)	1.7 (0.9 to 3.0)	1.7 (0.9 to 3.1)	2.2 (0.8 to 5.2)	1.4 (-1.9 to 4.7)	27.4 (-24.9 to 103.4)
United States	2.0 (1.5 to 2.7)	2.3 (1.7 to 3.1)	2.3 (1.6 to 3.3)	2.6 (1.3 to 5.0)	0.6 (-2.0 to 3.3)	12.4 (-26.3 to 63.8)
Southern Latin America						
Argentina	1.8 (1.0 to 2.9)	2.5 (1.4 to 3.8)	2.5 (1.4 to 4.0)	3.4 (1.3 to 7.3)	1.8 (-1.6 to 5.1)	34.8 (-20.9 to 115.3)
Chile	1.0 (0.8 to 1.3)	1.2 (0.9 to 1.7)	1.3 (0.9 to 1.9)	1.7 (0.8 to 3.0)	1.7 (-0.8 to 4.0)	30.4 (-11.0 to 83.5)
Uruguay	2.4 (1.8 to 3.0)	4.3 (3.0 to 5.9)	4.5 (3.2 to 6.4)	7.6 (3.9 to 13.4)	3.6 (1.1 to 6.0)	73.8 (17.3 to 146.2)
Western Europe						
Andorra	4.1 (2.1 to 7.2)	4.6 (2.3 to 7.9)	4.7 (2.4 to 8.0)	5.5 (1.9 to 12.7)	0.8 (-2.4 to 4.4)	17.3 (-30.3 to 93.0)
Austria	2.9 (2.3 to 3.6)	4.3 (3.3 to 5.4)	4.4 (3.2 to 5.9)	6.0 (3.3 to 10.4)	2.1 (-0.2 to 4.5)	39.4 (-3.4 to 96.2)
Belgium	3.1 (2.8 to 3.5)	3.1 (2.6 to 3.6)	3.1 (2.5 to 3.8)	3.2 (2.2 to 4.6)	0.1 (-1.5 to 1.8)	1.8 (-20.4 to 31.7)
Cyprus	2.4 (2.0 to 2.9)	3.3 (2.7 to 3.9)	3.5 (2.8 to 4.3)	4.8 (3.0 to 7.2)	2.3 (0.3 to 4.3)	43.6 (5.2 to 90.6)
Denmark	3.3 (2.8 to 3.8)	4.2 (3.4 to 5.2)	4.1 (3.1 to 5.2)	4.7 (2.9 to 7.2)	0.6 (-1.2 to 2.5)	10.1 (-17.0 to 46.5)
Finland	3.7 (3.2 to 4.2)	3.2 (2.6 to 4.0)	3.3 (2.5 to 4.2)	3.2 (1.9 to 5.2)	-0.2 (-2.2 to 1.9)	-2.4 (-27.8 to 33.1)
France	3.1 (2.8 to 3.5)	2.9 (2.4 to 3.4)	2.7 (2.1 to 3.3)	2.3 (1.7 to 3.4)	-1.4 (-2.7 to 0.1)	-18.8 (-33.4 to 2.1)
Germany	3.5 (3.2 to 3.8)	4.7 (4.3 to 5.2)	4.9 (4.3 to 5.6)	6.3 (4.8 to 8.2)	1.9 (0.6 to 3.3)	33.8 (9.6 to 64.7)
Greece	3.9 (3.6 to 4.3)	4.0 (3.6 to 4.5)	4.0 (3.4 to 4.6)	4.1 (3.0 to 5.4)	0.1 (-1.3 to 1.4)	1.3 (-17.8 to 24.2)
Iceland	3.5 (2.8 to 4.4)	4.0 (3.1 to 5.0)	3.9 (2.9 to 5.2)	4.2 (2.5 to 7.1)	0.2 (-1.7 to 2.5)	5.2 (-22.8 to 45.7)
Ireland	1.5 (1.3 to 1.8)	2.4 (2.0 to 2.9)	2.4 (1.9 to 3.0)	3.5 (2.2 to 5.3)	2.4 (0.4 to 4.2)	44.4 (5.5 to 88.7)
Israel	1.6 (1.1 to 2.2)	2.6 (1.9 to 3.7)	2.8 (1.9 to 4.0)	4.1 (2.0 to 7.7)	2.7 (0.1 to 5.5)	52.7 (1.3 to 129.7)
Italy	2.9 (2.6 to 3.3)	4.0 (3.5 to 4.6)	4.2 (3.5 to 5.0)	5.4 (3.8 to 7.4)	1.8 (0.3 to 3.3)	32.6 (5.3 to 65.1)
Luxembourg	2.2 (1.9 to 2.6)	3.7 (3.0 to 4.6)	3.9 (3.0 to 5.0)	6.1 (3.7 to 9.6)	3.1 (1.1 to 5.1)	61.2 (17.6 to 115.5)
Malta	1.9 (1.0 to 3.3)	2.8 (1.4 to 4.9)	2.9 (1.5 to 5.0)	4.1 (1.4 to 9.2)	2.1 (-1.2 to 5.4)	42.5 (-15.9 to 125.3)
Netherlands	2.3 (1.9 to 2.6)	3.8 (3.2 to 4.4)	4.0 (3.2 to 4.9)	6.0 (3.9 to 8.9)	3.0 (1.1 to 4.9)	58.0 (17.7 to 107.5)
Norway	2.6 (2.2 to 3.0)	4.2 (3.5 to 5.0)	4.2 (3.3 to 5.3)	5.8 (3.6 to 8.7)	2.0 (0.1 to 3.9)	36.1 (1.6 to 79.8)
Portugal	1.5 (1.2 to 1.7)	3.2 (2.7 to 3.9)	3.5 (2.8 to 4.4)	6.7 (4.3 to 9.8)	4.7 (2.9 to 6.5)	104.7 (55.2 to 165.9)
Spain	2.4 (2.1 to 2.7)	3.7 (3.3 to 4.2)	3.7 (3.1 to 4.4)	5.1 (3.7 to 7.1)	2.1 (0.6 to 3.7)	37.9 (9.0 to 73.3)
Sweden	2.2 (1.8 to 2.7)	4.6 (4.0 to 5.3)	4.9 (4.0 to 5.9)	7.8 (5.3 to 11.1)	3.4 (1.5 to 5.1)	67.3 (25.1 to 116.0)
Switzerland	3.9 (3.4 to 4.4)	6.3 (5.5 to 7.2)	6.5 (5.4 to 7.8)	9.6 (6.8 to 13.4)	2.7 (1.1 to 4.3)	51.4 (18.4 to 90.4)
United Kingdom	2.0 (1.8 to 2.2)	2.7 (2.4 to 3.1)	2.7 (2.2 to 3.3)	3.4 (2.4 to 4.9)	1.5 (-0.2 to 3.1)	25.8 (-2.6 to 59.3)
Latin America and Caribbean						
Andean Latin America						
Bolivia	1.1 (0.8 to 1.3)	1.9 (1.2 to 2.8)	2.0 (1.2 to 3.1)	3.4 (1.4 to 6.7)	3.5 (0.6 to 6.3)	73.8 (9.5 to 158.1)
Ecuador	1.9 (1.6 to 2.2)	2.6 (1.8 to 3.8)	2.8 (1.8 to 4.1)	3.8 (1.7 to 7.0)	2.1 (-0.6 to 4.8)	39.7 (-8.3 to 105.3)
Peru	2.0 (1.0 to 3.2)	2.5 (1.3 to 4.3)	2.6 (1.3 to 4.4)	3.4 (1.3 to 7.2)	1.6 (-1.2 to 4.6)	31.2 (-17.0 to 99.2)
Caribbean						
Antigua and Barbuda	1.7 (0.9 to 3.0)	2.3 (1.2 to 3.9)	2.4 (1.2 to 4.1)	3.4 (1.2 to 7.3)	2.0 (-1.5 to 5.3)	39.9 (-20.6 to 120.9)
The Bahamas	3.2 (1.6 to 5.6)	4.5 (2.3 to 8.2)	4.5 (2.3 to 8.1)	6.5 (2.2 to 15.5)	2.0 (-1.3 to 5.5)	39.7 (-17.8 to 128.8)
Barbados	2.3 (1.2 to 4.1)	3.0 (1.6 to 5.0)	3.1 (1.6 to 5.2)	4.1 (1.5 to 8.8)	1.6 (-1.6 to 5.1)	32.4 (-21.9 to 115.7)
Belize	0.7 (0.3 to 1.2)	1.2 (0.6 to 2.1)	1.2 (0.6 to 2.2)	2.1 (0.8 to 5.2)	3.5 (-0.0 to 6.9)	74.9 (-0.3 to 182.1)
Bermuda	4.1 (2.1 to 7.2)	5.9 (3.0 to 10.3)	5.8 (2.9 to 10.2)	7.7 (2.7 to 17.6)	1.3 (-1.8 to 4.9)	25.7 (-23.5 to 108.4)
Cuba	4.9 (4.1 to 5.9)	8.6 (5.3 to 13.2)	9.0 (5.4 to 14.2)	14.1 (5.8 to 29.4)	2.9 (0.0 to 5.6)	57.5 (0.2 to 130.3)
Dominica	1.0 (0.5 to 1.8)	1.6 (0.8 to 2.7)	1.6 (0.9 to 2.9)	2.5 (0.9 to 5.5)	2.6 (-0.7 to 6.0)	53.3 (-9.6 to 146.7)
Dominican Republic	1.1 (0.7 to 1.6)	1.4 (0.8 to 2.2)	1.4 (0.8 to 2.4)	1.9 (0.6 to 4.2)	1.4 (-1.9 to 4.9)	28.0 (-24.5 to 107.6)
Grenada	1.0 (0.5 to 1.7)	1.4 (0.7 to 2.4)	1.5 (0.7 to 2.5)	2.2 (0.8 to 5.0)	2.5 (-0.8 to 6.0)	51.5 (-10.7 to 145.1)
Guyana	0.7 (0.4 to 1.2)	1.2 (0.6 to 2.0)	1.2 (0.6 to 2.1)	1.9 (0.6 to 4.0)	2.8 (-0.6 to 6.1)	56.5 (-8.0 to 151.0)
Haiti	0.3 (0.2 to 0.5)	0.5 (0.3 to 0.9)	0.5 (0.3 to 0.9)	0.8 (0.3 to 1.8)	2.8 (-0.5 to 6.0)	57.9 (-6.7 to 144.1)
Jamaica	1.4 (0.7 to 2.4)	1.9 (1.0 to 3.2)	2.0 (1.0 to 3.4)	2.9 (1.0 to 6.3)	2.2 (-1.0 to 5.6)	44.7 (-14.0 to 132.7)
Puerto Rico	2.2 (1.1 to 3.9)	3.1 (1.5 to 5.5)	3.2 (1.6 to 5.6)	4.4 (1.5 to 10.0)	1.9 (-1.5 to 5.2)	37.6 (-19.6 to 117.8)
Saint Lucia	1.2 (0.6 to 2.0)	1.9 (0.9 to 3.2)	1.9 (1.0 to 3.3)	3.1 (1.1 to 6.7)	3.0 (-0.5 to 6.4)	63.3 (-7.4 to 160.8)
Saint Vincent and the Grenadines	0.9 (0.5 to 1.5)	1.4 (0.7 to 2.5)	1.5 (0.7 to 2.6)	2.3 (0.8 to 5.3)	2.9 (-0.4 to 6.2)	58.4 (-5.3 to 153.7)
Suriname	0.6 (0.3 to 1.0)	0.8 (0.4 to 1.4)	0.8 (0.4 to 1.4)	1.2 (0.4 to 2.6)	2.2 (-1.4 to 5.8)	45.3 (-18.5 to 138.5)
Trinidad and Tobago	0.5 (0.3 to 0.8)	0.7 (0.3 to 1.2)	0.7 (0.3 to 1.2)	0.9 (0.3 to 2.1)	1.4 (-1.7 to 4.7)	28.0 (-23.1 to 101.2)
Virgin Islands, U.S.	2.8 (1.4 to 4.7)	3.6 (1.8 to 6.3)	3.6 (1.8 to 6.4)	4.7 (1.6 to 10.5)	1.4 (-1.8 to 4.7)	27.5 (-24.1 to 102.7)
Central Latin America						
Colombia	1.3 (0.7 to 2.2)	2.0 (1.0 to 3.3)	2.1 (1.1 to 3.5)	3.2 (1.1 to 6.6)	2.6 (-0.6 to 5.7)	52.9 (-9.0 to 134.7)
Costa Rica	2.1 (1.6 to 2.7)	2.1 (1.4 to 3.1)	2.2 (1.4 to 3.3)	2.5 (1.1 to 5.0)	0.7 (-1.9 to 3.5)	14.1 (-25.2 to 70.1)
El Salvador	1.1 (0.7 to 1.5)	1.7 (1.2 to 2.4)	1.8 (1.2 to 2.6)	2.8 (1.4 to 5.1)	2.9 (0.3 to 5.6)	57.9 (4.7 to 130.4)
Guatemala	0.7 (0.4 to 1.2)	1.3 (0.7 to 2.2)	1.3 (0.7 to 2.3)	2.2 (0.8 to 4.7)	3.2 (-0.0 to 6.2)	66.3 (-0.4 to 152.9)
Honduras	0.5 (0.3 to 0.9)	0.8 (0.4 to 1.4)	0.9 (0.4 to 1.5)	1.4 (0.5 to 3.0)	2.7 (-0.4 to 6.1)	55.2 (-6.5 to 148.3)
Mexico	1.8 (1.3 to 2.4)	2.4 (1.6 to 3.5)	2.4 (1.6 to 3.6)	3.3 (1.4 to 6.7)	1.6 (-1.2 to 4.6)	30.6 (-16.3 to 98.3)
Nicaragua	0.8 (0.5 to 1.1)	1.4 (0.9 to 2.2)	1.5 (0.9 to 2.4)	2.6 (1.1 to 5.0)	3.6 (0.7 to 6.3)	76.2 (11.2 to 156.4)
Panama	1.5 (1.0 to 2.1)	2.2 (1.2 to 3.5)	2.3 (1.3 to 3.7)	3.4 (1.3 to 6.8)	2.6 (-0.3 to 5.3)	51.5 (-4.1 to 123.0)
Venezuela	1.1 (0.7 to 1.6)	1.3 (0.7 to 2.0)	1.2 (0.7 to 1.9)	1.4 (0.5 to 2.9)	0.2 (-2.6 to 3.1)	6.2 (-32.4 to 60.3)
Tropical Latin America						
Brazil	0.9 (0.6 to 1.3)	1.7 (1.3 to 2.1)	1.7 (1.3 to 2.3)	2.9 (1.5 to 5.0)	3.4 (0.7 to 6.2)	70.5 (10.3 to 152.9)
Paraguay	0.8 (0.6 to 1.1)	1.1 (0.8 to 1.7)	1.2 (0.8 to 1.9)	1.7 (0.7 to 3.5)	2.4 (-0.5 to 5.2)	46.6 (-7.5 to 119.6)
North Africa and Middle East						
North Africa and Middle East						
Afghanistan	0.1 (0.1 to 0.2)	0.2 (0.1 to 0.3)	0.2 (0.1 to 0.3)	0.3 (0.1 to 0.6)	2.8 (-0.4 to 6.1)	56.1 (-6.1 to 149.8)
Algeria	0.5 (0.2 to 0.8)	1.0 (0.5 to 1.8)	1.1 (0.5 to 1.9)	2.1 (0.7 to 4.5)	4.3 (1.1 to 7.4)	97.6 (17.4 to 204.1)
Bahrain	1.7 (0.9 to 2.9)	2.7 (1.4 to 4.6)	2.8 (1.5 to 4.8)	4.3 (1.5 to 9.7)	2.5 (-0.8 to 5.9)	51.6 (-11.0 to 140.5)
Egypt	1.0 (0.6 to 1.4)	1.0 (0.9 to 1.1)	1.0 (0.8 to 1.1)	1.1 (0.7 to 1.6)	0.4 (-1.6 to 2.5)	7.2 (-21.5 to 46.0)
Iran	0.3 (0.2 to 0.4)	0.6 (0.3 to 0.8)	0.6 (0.4 to 0.9)	1.1 (0.4 to 2.3)	3.9 (0.6 to 6.9)	83.5 (9.4 to 181.7)
Iraq	0.3 (0.2 to 0.5)	0.3 (0.2 to 0.5)	0.4 (0.2 to 0.6)	0.5 (0.2 to 1.1)	2.5 (-1.1 to 6.0)	50.3 (-14.7 to 145.2)
Jordan	2.0 (1.6 to 2.4)	2.6 (1.6 to 3.8)	2.6 (1.6 to 4.0)	3.6 (1.4 to 7.2)	1.8 (-1.0 to 4.6)	34.7 (-14.1 to 98.0)
Kuwait	2.0 (1.0 to 3.4)	3.1 (1.6 to 5.3)	3.3 (1.6 to 5.6)	5.0 (1.8 to 11.1)	2.8 (-0.5 to 6.3)	57.5 (-7.2 to 155.5)
Lebanon	0.9 (0.5 to 1.5)	1.5 (0.8 to 2.5)	1.6 (0.8 to 2.7)	2.6 (0.9 to 5.5)	3.4 (-0.0 to 6.7)	73.1 (-0.7 to 174.7)
Libya	1.3 (0.7 to 2.3)	1.6 (0.8 to 2.8)	1.7 (0.9 to 2.9)	2.1 (0.8 to 4.7)	1.7 (-0.9 to 4.8)	33.0 (-12.9 to 106.2)

	Estimate in 2000 (95% UIs)	Estimate in 2015 (95% UIs)	Estimate in 2017 (95% UIs)	Estimate in 2030 (95% UIs)	Annualised rate of change, 2015-2030 (95% UIs)	Percent change, 2015-2030 (95% UIs)
Morocco	0.3 (0.1 to 0.5)	0.5 (0.2 to 0.9)	0.5 (0.3 to 0.9)	0.9 (0.3 to 1.9)	3.2 (-0.1 to 6.4)	68.2 (-2.1 to 162.7)
Palestine	0.4 (0.2 to 0.6)	0.6 (0.3 to 1.0)	0.7 (0.3 to 1.1)	1.1 (0.4 to 2.5)	3.7 (0.3 to 6.7)	78.7 (4.9 to 175.2)
Oman	0.8 (0.4 to 1.4)	1.9 (0.9 to 3.3)	2.0 (1.0 to 3.5)	3.9 (1.4 to 8.4)	4.5 (1.2 to 7.3)	103.0 (19.8 to 196.8)
Qatar	2.6 (1.3 to 4.4)	3.6 (1.8 to 6.2)	3.6 (1.9 to 6.2)	5.0 (1.7 to 11.2)	1.7 (-1.5 to 5.2)	34.3 (-20.7 to 117.6)
Saudi Arabia	1.1 (0.6 to 1.9)	2.1 (1.1 to 3.4)	2.3 (1.2 to 3.8)	4.6 (1.7 to 9.5)	5.0 (1.8 to 8.0)	116.5 (31.9 to 229.5)
Sudan	0.2 (0.1 to 0.3)	0.4 (0.2 to 0.6)	0.4 (0.2 to 0.7)	0.7 (0.2 to 1.5)	3.9 (0.5 to 7.0)	84.1 (8.1 to 187.7)
Syria	0.4 (0.2 to 0.7)	0.6 (0.3 to 1.0)	0.6 (0.3 to 1.1)	0.9 (0.3 to 2.0)	2.4 (-0.9 to 5.6)	47.2 (-12.5 to 130.2)
Tunisia	0.5 (0.3 to 0.8)	0.8 (0.4 to 1.4)	0.8 (0.4 to 1.4)	1.3 (0.4 to 3.0)	2.8 (-0.7 to 6.3)	58.1 (-9.5 to 156.8)
Turkey	0.4 (0.2 to 0.7)	0.7 (0.4 to 1.0)	0.7 (0.5 to 1.1)	1.2 (0.5 to 2.4)	3.6 (0.0 to 6.7)	77.2 (0.7 to 175.1)
United Arab Emirates	2.2 (1.2 to 4.0)	3.9 (2.0 to 6.9)	3.9 (2.0 to 6.9)	6.2 (2.2 to 13.4)	2.7 (-0.8 to 5.7)	53.6 (-10.9 to 134.7)
Yemen	0.3 (0.1 to 0.4)	0.4 (0.2 to 0.6)	0.4 (0.2 to 0.6)	0.5 (0.2 to 1.1)	1.8 (-1.1 to 5.0)	34.4 (-15.2 to 111.9)
South Asia						
South Asia						
Bangladesh	0.3 (0.1 to 0.4)	0.4 (0.2 to 0.7)	0.5 (0.2 to 0.7)	0.8 (0.3 to 1.6)	3.5 (0.2 to 6.8)	74.2 (3.0 to 175.6)
Bhutan	0.5 (0.2 to 0.8)	1.0 (0.5 to 1.7)	1.1 (0.6 to 1.9)	2.1 (0.8 to 4.4)	4.7 (1.4 to 7.8)	107.9 (23.9 to 220.5)
India	0.3 (0.2 to 0.4)	0.5 (0.3 to 0.7)	0.5 (0.3 to 0.8)	0.9 (0.4 to 1.8)	3.5 (0.5 to 6.4)	72.9 (8.5 to 161.5)
Nepal	0.2 (0.1 to 0.4)	0.5 (0.3 to 0.8)	0.5 (0.3 to 0.8)	0.9 (0.3 to 2.0)	4.2 (0.8 to 7.5)	93.7 (13.4 to 209.1)
Pakistan	0.3 (0.2 to 0.6)	0.6 (0.4 to 0.9)	0.7 (0.4 to 0.9)	1.1 (0.5 to 2.1)	3.8 (0.8 to 6.8)	81.5 (12.1 to 178.1)
Southeast Asia, East Asia, and Oceania						
East Asia						
China	1.7 (1.2 to 2.3)	3.2 (2.0 to 4.8)	3.4 (2.0 to 5.0)	6.1 (2.6 to 11.9)	4.0 (1.1 to 6.6)	85.0 (18.8 to 168.4)
North Korea	0.2 (0.1 to 0.3)	0.2 (0.1 to 0.4)	0.2 (0.1 to 0.4)	0.3 (0.1 to 0.6)	1.4 (-2.1 to 5.0)	28.7 (-26.9 to 110.4)
Taiwan (Province of China)	0.4 (0.2 to 0.7)	0.7 (0.4 to 1.0)	0.7 (0.4 to 1.1)	1.1 (0.4 to 2.1)	2.5 (-0.7 to 5.9)	51.4 (-9.6 to 141.4)
Oceania						
American Samoa	0.6 (0.3 to 1.1)	0.8 (0.4 to 1.4)	0.8 (0.4 to 1.4)	1.1 (0.3 to 2.4)	1.6 (-1.8 to 5.2)	32.8 (-23.3 to 118.4)
Federated States of Micronesia	0.2 (0.1 to 0.4)	0.4 (0.2 to 0.6)	0.3 (0.2 to 0.6)	0.5 (0.2 to 1.0)	1.8 (-0.5 to 4.1)	32.5 (-6.8 to 85.9)
Fiji	0.5 (0.2 to 0.8)	0.6 (0.3 to 1.1)	0.6 (0.3 to 1.1)	0.8 (0.3 to 2.0)	1.8 (-1.6 to 5.2)	35.7 (-21.2 to 117.3)
Guam	1.2 (0.6 to 2.2)	1.4 (0.7 to 2.4)	1.4 (0.7 to 2.5)	1.6 (0.5 to 3.8)	0.7 (-2.9 to 4.1)	15.5 (-35.2 to 86.1)
Kiribati	0.1 (0.1 to 0.2)	0.2 (0.1 to 0.4)	0.2 (0.1 to 0.4)	0.3 (0.1 to 0.8)	3.2 (-0.4 to 6.7)	68.4 (-5.9 to 173.3)
Marshall Islands	0.2 (0.1 to 0.4)	0.4 (0.2 to 0.7)	0.4 (0.2 to 0.8)	0.7 (0.2 to 1.6)	3.6 (0.1 to 7.0)	77.3 (1.9 to 184.8)
Northern Mariana Islands	1.1 (0.6 to 1.9)	1.1 (0.6 to 2.0)	1.2 (0.6 to 2.1)	1.4 (0.5 to 3.0)	0.9 (-2.3 to 4.3)	18.7 (-29.4 to 89.8)
Papua New Guinea	0.1 (0.1 to 0.2)	0.1 (0.1 to 0.2)	0.1 (0.1 to 0.2)	0.2 (0.1 to 0.4)	1.7 (-1.6 to 5.2)	34.5 (-21.7 to 117.9)
Samoa	0.2 (0.1 to 0.4)	0.2 (0.1 to 0.4)	0.2 (0.1 to 0.4)	0.2 (0.1 to 0.5)	0.5 (-2.4 to 3.8)	10.7 (-29.9 to 76.2)
Solomon Islands	0.1 (0.0 to 0.1)	0.1 (0.1 to 0.2)	0.1 (0.1 to 0.2)	0.2 (0.1 to 0.4)	2.7 (-1.0 to 6.2)	54.6 (-13.9 to 154.2)
Tonga	0.3 (0.1 to 0.5)	0.4 (0.2 to 0.7)	0.4 (0.2 to 0.7)	0.5 (0.2 to 1.2)	2.1 (-1.0 to 5.4)	42.1 (-14.4 to 125.6)
Vanuatu	0.2 (0.1 to 0.3)	0.2 (0.1 to 0.4)	0.2 (0.1 to 0.4)	0.4 (0.1 to 0.8)	2.2 (-1.1 to 5.7)	43.8 (-15.8 to 133.6)
Southeast Asia						
Cambodia	0.2 (0.1 to 0.3)	0.4 (0.3 to 0.6)	0.5 (0.3 to 0.7)	0.9 (0.4 to 1.7)	4.8 (2.0 to 7.5)	109.7 (34.2 to 209.5)
Indonesia	0.2 (0.1 to 0.4)	0.4 (0.2 to 0.7)	0.4 (0.2 to 0.8)	0.8 (0.3 to 1.8)	3.7 (0.2 to 6.7)	78.6 (3.1 to 172.5)
Laos	0.1 (0.1 to 0.2)	0.2 (0.1 to 0.3)	0.2 (0.1 to 0.3)	0.4 (0.1 to 0.8)	4.1 (0.7 to 7.6)	92.9 (11.6 to 213.6)
Malaysia	0.2 (0.1 to 0.3)	0.4 (0.2 to 0.7)	0.4 (0.2 to 0.7)	0.7 (0.2 to 1.6)	3.4 (-0.1 to 6.8)	73.3 (-1.3 to 175.5)
Maldives	0.2 (0.1 to 0.4)	0.5 (0.2 to 0.8)	0.5 (0.2 to 0.9)	1.1 (0.3 to 2.6)	5.6 (1.9 to 8.8)	138.2 (33.9 to 271.5)
Mauritius	0.2 (0.1 to 0.5)	0.4 (0.2 to 0.8)	0.5 (0.2 to 0.8)	0.8 (0.3 to 1.8)	3.2 (-0.4 to 6.9)	68.3 (-6.0 to 179.7)
Myanmar	0.1 (0.0 to 0.1)	0.2 (0.1 to 0.4)	0.2 (0.1 to 0.5)	0.6 (0.2 to 1.2)	5.6 (2.2 to 8.4)	138.4 (39.4 to 254.5)
Philippines	0.2 (0.1 to 0.2)	0.1 (0.1 to 0.1)	0.1 (0.1 to 0.1)	0.1 (0.0 to 0.2)	-0.6 (-3.2 to 2.2)	-6.7 (-37.9 to 40.0)
Sri Lanka	0.2 (0.1 to 0.3)	0.3 (0.2 to 0.6)	0.3 (0.2 to 0.7)	0.6 (0.2 to 1.4)	3.3 (-0.1 to 6.7)	71.3 (-2.1 to 174.8)
Seychelles	0.3 (0.2 to 0.6)	0.5 (0.3 to 1.0)	0.6 (0.3 to 1.0)	0.9 (0.3 to 2.1)	3.0 (-0.8 to 6.5)	63.0 (-11.7 to 166.9)
Thailand	0.3 (0.2 to 0.6)	0.5 (0.2 to 0.9)	0.5 (0.3 to 0.9)	0.8 (0.3 to 2.0)	2.8 (-0.7 to 6.2)	57.5 (-10.6 to 153.7)
Timor-Leste	0.1 (0.0 to 0.1)	0.1 (0.1 to 0.2)	0.2 (0.1 to 0.3)	0.3 (0.1 to 0.6)	3.9 (0.9 to 6.8)	83.2 (14.1 to 179.2)
Vietnam	0.2 (0.1 to 0.3)	0.3 (0.2 to 0.6)	0.3 (0.2 to 0.6)	0.6 (0.2 to 1.4)	4.0 (0.5 to 7.3)	88.6 (7.3 to 199.1)
Sub-Saharan Africa						
Central Sub-Saharan Africa						
Angola	0.1 (0.0 to 0.1)	0.1 (0.1 to 0.2)	0.1 (0.1 to 0.3)	0.3 (0.1 to 0.7)	5.0 (1.7 to 7.9)	116.7 (28.9 to 227.8)
Central African Republic	0.1 (0.0 to 0.1)	0.1 (0.1 to 0.1)	0.1 (0.1 to 0.2)	0.1 (0.1 to 0.3)	2.3 (-1.0 to 5.5)	44.8 (-14.3 to 128.4)
Congo	0.2 (0.1 to 0.3)	0.3 (0.1 to 0.5)	0.3 (0.1 to 0.5)	0.5 (0.2 to 1.1)	3.7 (0.4 to 7.0)	79.5 (6.2 to 186.5)
Democratic Republic of the Congo	0.1 (0.0 to 0.1)	0.1 (0.1 to 0.2)	0.1 (0.1 to 0.2)	0.1 (0.1 to 0.3)	2.8 (-0.3 to 5.7)	56.6 (-4.8 to 136.5)
Equatorial Guinea	0.1 (0.0 to 0.1)	0.3 (0.1 to 0.5)	0.3 (0.1 to 0.5)	0.7 (0.2 to 1.6)	6.5 (3.0 to 9.5)	173.4 (57.5 to 315.5)
Gabon	0.2 (0.1 to 0.3)	0.3 (0.2 to 0.6)	0.4 (0.2 to 0.7)	0.6 (0.2 to 1.5)	4.0 (0.4 to 7.3)	88.0 (5.5 to 197.2)
Eastern Sub-Saharan Africa						
Burundi	0.1 (0.1 to 0.2)	0.2 (0.1 to 0.3)	0.2 (0.1 to 0.3)	0.3 (0.1 to 0.6)	2.1 (-1.4 to 5.3)	41.9 (-19.0 to 122.0)
Comoros	0.2 (0.1 to 0.3)	0.3 (0.1 to 0.6)	0.3 (0.2 to 0.6)	0.6 (0.2 to 1.3)	3.5 (-0.3 to 7.1)	75.9 (-4.1 to 188.2)
Djibouti	0.1 (0.1 to 0.2)	0.3 (0.1 to 0.5)	0.3 (0.1 to 0.5)	0.5 (0.2 to 1.2)	4.1 (0.4 to 7.4)	92.1 (6.1 to 205.2)
Eritrea	0.1 (0.1 to 0.3)	0.2 (0.1 to 0.4)	0.2 (0.1 to 0.4)	0.3 (0.1 to 0.7)	2.9 (-0.1 to 6.1)	59.4 (-1.0 to 149.2)
Ethiopia	0.0 (0.0 to 0.1)	0.1 (0.0 to 0.1)	0.1 (0.1 to 0.1)	0.2 (0.1 to 0.4)	5.3 (2.2 to 8.0)	128.5 (38.5 to 231.2)
Kenya	0.2 (0.1 to 0.4)	0.4 (0.2 to 0.6)	0.4 (0.2 to 0.7)	0.6 (0.2 to 1.4)	3.0 (-0.8 to 6.7)	62.6 (-10.6 to 173.6)
Madagascar	0.2 (0.1 to 0.3)	0.2 (0.1 to 0.4)	0.2 (0.1 to 0.4)	0.3 (0.1 to 0.7)	1.7 (-2.0 to 5.1)	33.6 (-25.6 to 116.1)
Malawi	0.1 (0.1 to 0.2)	0.2 (0.1 to 0.4)	0.2 (0.1 to 0.4)	0.4 (0.1 to 0.9)	3.3 (-0.2 to 6.6)	70.3 (-3.6 to 171.0)
Mozambique	0.1 (0.0 to 0.1)	0.1 (0.1 to 0.2)	0.1 (0.1 to 0.2)	0.2 (0.1 to 0.4)	3.9 (0.5 to 7.2)	84.9 (8.0 to 193.3)
Rwanda	0.2 (0.1 to 0.3)	0.6 (0.4 to 0.7)	0.6 (0.4 to 0.8)	1.2 (0.6 to 2.0)	4.9 (2.3 to 7.3)	111.4 (41.8 to 198.2)
Somalia	0.1 (0.0 to 0.1)	0.1 (0.1 to 0.2)	0.1 (0.1 to 0.2)	0.2 (0.1 to 0.5)	2.7 (-1.0 to 6.3)	56.8 (-13.6 to 156.0)
South Sudan	0.1 (0.0 to 0.2)	0.1 (0.1 to 0.3)	0.1 (0.1 to 0.3)	0.2 (0.1 to 0.5)	2.6 (-1.0 to 6.2)	54.1 (-14.3 to 152.7)
Tanzania	0.2 (0.1 to 0.3)	0.4 (0.3 to 0.6)	0.5 (0.3 to 0.7)	0.9 (0.4 to 1.8)	4.5 (1.4 to 7.5)	101.4 (23.6 to 209.8)
Uganda	0.1 (0.1 to 0.1)	0.1 (0.1 to 0.2)	0.1 (0.1 to 0.2)	0.2 (0.1 to 0.4)	2.2 (-0.9 to 5.2)	42.5 (-12.1 to 119.1)
Zambia	0.1 (0.1 to 0.2)	0.4 (0.3 to 0.5)	0.4 (0.3 to 0.6)	0.9 (0.4 to 1.7)	5.2 (2.1 to 7.9)	124.2 (37.9 to 229.0)
Southern Sub-Saharan Africa						
Botswana	0.2 (0.1 to 0.3)	0.3 (0.2 to 0.6)	0.4 (0.2 to 0.7)	0.7 (0.2 to 1.5)	3.9 (0.4 to 7.4)	87.0 (5.9 to 204.9)
Lesotho	0.1 (0.0 to 0.1)	0.1 (0.0 to 0.2)	0.1 (0.1 to 0.2)	0.1 (0.1 to 0.4)	2.8 (-0.6 to 6.0)	56.2 (-9.3 to 145.8)
Namibia	0.1 (0.1 to 0.3)	0.3 (0.1 to 0.4)	0.3 (0.1 to 0.5)	0.5 (0.2 to 1.1)	4.0 (0.1 to 7.6)	90.5 (0.9 to 211.5)
South Africa	0.3 (0.2 to 0.5)	0.4 (0.3 to 0.7)	0.4 (0.2 to 0.6)	0.5 (0.2 to 1.1)	1.0 (-1.5 to 4.1)	19.2 (-19.9 to 85.6)
Swaziland	0.1 (0.1 to 0.2)	0.1 (0.1 to 0.2)	0.1 (0.1 to 0.2)	0.2 (0.1 to 0.5)	2.3 (-1.0 to 5.6)	45.8 (-13.6 to 131.9)

	Estimate in 2000 (95% UIs)	Estimate in 2015 (95% UIs)	Estimate in 2017 (95% UIs)	Estimate in 2030 (95% UIs)	Annualised rate of change, 2015-2030 (95% UIs)	Percent change, 2015-2030 (95% UIs)
Zimbabwe	0.1 (0.1 to 0.3)	0.1 (0.1 to 0.2)	0.1 (0.1 to 0.3)	0.2 (0.1 to 0.4)	1.7 (-1.5 to 5.1)	33.5 (-20.1 to 114.6)
Western Sub-Saharan Africa						
Benin	0.0 (0.0 to 0.0)	0.0 (0.0 to 0.0)	0.0 (0.0 to 0.0)	0.0 (0.0 to 0.1)	3.9 (0.3 to 7.6)	87.8 (4.2 to 214.2)
Burkina Faso	0.1 (0.0 to 0.1)	0.1 (0.1 to 0.2)	0.1 (0.1 to 0.2)	0.2 (0.1 to 0.5)	3.2 (-0.6 to 6.9)	67.9 (-8.4 to 180.4)
Cameroon	0.3 (0.2 to 0.4)	0.5 (0.3 to 0.8)	0.6 (0.3 to 0.9)	1.0 (0.4 to 2.2)	4.2 (0.9 to 7.4)	94.6 (15.3 to 203.3)
Cape Verde	0.2 (0.1 to 0.3)	0.4 (0.2 to 0.7)	0.4 (0.2 to 0.8)	0.9 (0.3 to 2.0)	5.0 (1.2 to 8.3)	119.4 (18.9 to 249.8)
Chad	0.0 (0.0 to 0.1)	0.1 (0.0 to 0.1)	0.1 (0.0 to 0.1)	0.1 (0.0 to 0.3)	3.8 (0.1 to 7.1)	83.4 (1.5 to 191.2)
Cote d'Ivoire	0.1 (0.1 to 0.3)	0.2 (0.1 to 0.4)	0.2 (0.1 to 0.4)	0.4 (0.1 to 0.9)	3.0 (-0.5 to 6.5)	61.7 (-6.6 to 164.1)
The Gambia	0.1 (0.1 to 0.2)	0.2 (0.1 to 0.3)	0.2 (0.1 to 0.3)	0.3 (0.1 to 0.6)	2.3 (-1.1 to 5.8)	46.9 (-15.3 to 139.8)
Ghana	0.4 (0.2 to 0.7)	0.7 (0.4 to 1.1)	0.7 (0.4 to 1.1)	1.1 (0.4 to 2.1)	2.4 (-0.6 to 5.4)	46.4 (-9.2 to 124.2)
Guinea	0.1 (0.1 to 0.2)	0.2 (0.1 to 0.4)	0.2 (0.1 to 0.4)	0.3 (0.1 to 0.7)	2.7 (-0.2 to 5.5)	54.4 (-3.4 to 127.7)
Guinea-Bissau	0.1 (0.0 to 0.1)	0.1 (0.1 to 0.2)	0.2 (0.1 to 0.3)	0.3 (0.1 to 0.6)	4.0 (0.8 to 7.6)	89.5 (12.1 to 211.4)
Liberia	0.1 (0.0 to 0.1)	0.1 (0.1 to 0.2)	0.1 (0.1 to 0.3)	0.2 (0.1 to 0.5)	4.1 (1.4 to 7.0)	89.9 (24.2 to 185.7)
Mali	0.1 (0.0 to 0.1)	0.1 (0.0 to 0.1)	0.1 (0.0 to 0.1)	0.1 (0.0 to 0.3)	3.1 (-0.8 to 6.7)	65.4 (-11.0 to 173.1)
Mauritania	0.1 (0.1 to 0.2)	0.2 (0.1 to 0.3)	0.2 (0.1 to 0.4)	0.3 (0.1 to 0.8)	3.0 (-0.3 to 6.4)	63.6 (-4.2 to 162.5)
Niger	0.0 (0.0 to 0.1)	0.1 (0.0 to 0.1)	0.1 (0.0 to 0.1)	0.1 (0.0 to 0.2)	2.1 (-1.5 to 5.5)	41.2 (-19.6 to 126.8)
Nigeria	0.2 (0.1 to 0.3)	0.4 (0.3 to 0.5)	0.4 (0.2 to 0.5)	0.6 (0.2 to 1.1)	2.1 (-1.0 to 5.5)	41.9 (-14.3 to 129.9)
Sao Tome and Principe	0.1 (0.1 to 0.2)	0.3 (0.1 to 0.5)	0.3 (0.1 to 0.5)	0.6 (0.2 to 1.4)	5.4 (1.9 to 8.1)	132.0 (32.3 to 239.4)
Senegal	0.2 (0.1 to 0.2)	0.2 (0.1 to 0.3)	0.2 (0.1 to 0.4)	0.3 (0.1 to 0.7)	1.6 (-1.7 to 4.8)	31.9 (-22.1 to 106.5)
Sierra Leone	0.2 (0.1 to 0.4)	0.4 (0.2 to 0.6)	0.4 (0.3 to 0.7)	0.8 (0.3 to 1.8)	4.9 (1.2 to 8.0)	114.4 (19.6 to 231.1)
Togo	0.1 (0.1 to 0.2)	0.2 (0.1 to 0.4)	0.2 (0.1 to 0.4)	0.4 (0.1 to 0.9)	3.5 (0.1 to 7.0)	76.0 (1.9 to 186.8)
**Indicator 5.2.1: Age-standardised prevalence of ever-partnered women aged 15 years and older who experienced physical or sexual violence by a current or former intimate partner in the last 12 months (%)**						
Central Europe, Eastern Europe, and Central Asia						
Central Asia						
Armenia	9.2 (7.8 to 10.7)	7.6 (6.5 to 8.9)	7.5 (6.3 to 8.8)	6.6 (5.5 to 7.8)	-1.0 (-1.3 to -0.7)	-13.8 (-18.2 to -9.5)
Azerbaijan	9.2 (8.2 to 10.2)	7.6 (6.8 to 8.3)	7.5 (6.7 to 8.3)	6.5 (5.7 to 7.3)	-1.0 (-1.4 to -0.7)	-14.4 (-19.2 to -9.6)
Georgia	4.7 (4.2 to 5.3)	4.4 (3.9 to 4.8)	4.4 (3.9 to 4.9)	4.2 (3.7 to 4.8)	-0.2 (-0.6 to 0.2)	-3.0 (-8.1 to 2.8)
Kazakhstan	6.7 (6.0 to 7.5)	5.7 (5.2 to 6.3)	5.6 (5.0 to 6.2)	4.9 (4.2 to 5.5)	-1.1 (-1.5 to -0.7)	-15.0 (-19.8 to -9.7)
Kyrgyzstan	14.6 (13.0 to 16.3)	13.6 (12.5 to 14.8)	13.4 (12.1 to 14.6)	12.4 (11.1 to 13.8)	-0.6 (-1.0 to -0.3)	-9.2 (-13.7 to -4.3)
Mongolia	10.5 (8.9 to 12.4)	9.1 (7.7 to 10.6)	8.9 (7.6 to 10.4)	8.0 (6.7 to 9.4)	-0.8 (-1.2 to -0.5)	-11.8 (-15.9 to -7.6)
Tajikistan	14.5 (13.0 to 16.2)	12.3 (11.3 to 13.4)	12.2 (11.1 to 13.4)	11.1 (9.9 to 12.3)	-0.7 (-1.0 to -0.4)	-10.2 (-14.4 to -5.2)
Turkmenistan	9.2 (7.8 to 10.8)	8.0 (6.8 to 9.3)	7.7 (6.6 to 9.1)	6.8 (5.6 to 8.1)	-1.0 (-1.4 to -0.7)	-14.5 (-18.8 to -10.2)
Uzbekistan	9.8 (8.3 to 11.6)	8.2 (7.0 to 9.7)	8.1 (6.9 to 9.6)	7.1 (5.9 to 8.4)	-1.0 (-1.3 to -0.7)	-14.1 (-17.8 to -9.8)
Central Europe						
Albania	5.6 (5.1 to 6.2)	4.5 (4.0 to 5.0)	4.4 (4.0 to 5.0)	3.9 (3.4 to 4.4)	-1.0 (-1.4 to -0.7)	-13.9 (-18.4 to -9.9)
Bosnia and Herzegovina	8.1 (6.9 to 9.4)	6.8 (5.8 to 7.9)	6.7 (5.7 to 7.8)	5.8 (4.9 to 6.8)	-1.1 (-1.5 to -0.7)	-15.0 (-19.6 to -10.2)
Bulgaria	9.6 (8.6 to 10.7)	8.2 (7.4 to 9.1)	8.1 (7.3 to 9.0)	7.2 (6.4 to 8.2)	-0.8 (-1.2 to -0.5)	-11.9 (-16.1 to -7.6)
Croatia	4.3 (3.8 to 4.8)	3.6 (3.3 to 4.1)	3.6 (3.2 to 4.0)	3.2 (2.8 to 3.7)	-0.8 (-1.1 to -0.5)	-11.5 (-15.7 to -7.2)
Czech Republic	6.2 (5.5 to 6.9)	5.5 (5.0 to 6.1)	5.5 (4.9 to 6.1)	5.0 (4.4 to 5.6)	-0.7 (-1.0 to -0.3)	-9.5 (-13.8 to -4.9)
Hungary	8.9 (8.0 to 10.0)	7.7 (7.0 to 8.5)	7.7 (7.0 to 8.5)	6.9 (6.2 to 7.8)	-0.7 (-1.1 to -0.4)	-10.5 (-14.6 to -6.2)
Macedonia	7.4 (6.3 to 8.7)	6.3 (5.4 to 7.3)	6.2 (5.3 to 7.2)	5.5 (4.7 to 6.5)	-0.9 (-1.2 to -0.5)	-12.0 (-16.3 to -7.6)
Montenegro	7.3 (6.3 to 8.6)	6.3 (5.4 to 7.4)	6.3 (5.3 to 7.3)	5.7 (4.8 to 6.7)	-0.7 (-1.1 to -0.4)	-10.6 (-14.6 to -6.1)
Poland	4.1 (3.7 to 4.6)	3.4 (3.0 to 3.8)	3.4 (3.0 to 3.7)	2.9 (2.6 to 3.2)	-1.1 (-1.4 to -0.7)	-14.8 (-18.4 to -10.6)
Romania	9.0 (8.1 to 10.0)	7.5 (6.8 to 8.3)	7.4 (6.7 to 8.2)	6.5 (5.8 to 7.3)	-1.0 (-1.3 to -0.6)	-13.5 (-17.9 to -9.2)
Serbia	7.8 (6.7 to 9.1)	6.5 (5.6 to 7.6)	6.5 (5.5 to 7.6)	5.8 (4.9 to 6.9)	-0.8 (-1.2 to -0.5)	-11.1 (-16.2 to -7.2)
Slovakia	8.8 (7.9 to 9.8)	7.7 (6.9 to 8.6)	7.6 (6.9 to 8.5)	6.8 (6.1 to 7.8)	-0.8 (-1.1 to -0.5)	-11.1 (-15.3 to -6.7)
Slovenia	3.6 (3.3 to 4.0)	3.1 (2.8 to 3.5)	3.1 (2.8 to 3.5)	2.8 (2.5 to 3.2)	-0.8 (-1.1 to -0.4)	-10.7 (-14.7 to -6.1)
Eastern Europe						
Belarus	8.9 (7.9 to 9.9)	7.8 (6.8 to 8.8)	7.6 (6.6 to 8.7)	6.8 (5.7 to 8.0)	-0.9 (-1.3 to -0.5)	-12.4 (-18.0 to -6.7)
Estonia	5.2 (4.6 to 5.7)	4.2 (3.8 to 4.7)	4.2 (3.8 to 4.7)	3.7 (3.3 to 4.2)	-1.0 (-1.3 to -0.7)	-13.6 (-17.7 to -9.4)
Latvia	8.5 (7.5 to 9.4)	7.1 (6.4 to 7.8)	7.0 (6.3 to 7.8)	6.2 (5.5 to 7.0)	-0.9 (-1.2 to -0.6)	-12.4 (-16.8 to -8.1)
Lithuania	6.5 (5.9 to 7.3)	5.5 (5.0 to 6.1)	5.5 (4.9 to 6.1)	4.8 (4.3 to 5.4)	-1.0 (-1.3 to -0.7)	-13.3 (-17.3 to -9.3)
Moldova	11.8 (10.9 to 12.9)	10.2 (9.2 to 11.2)	9.9 (8.9 to 10.9)	8.5 (7.4 to 9.7)	-1.2 (-1.7 to -0.8)	-16.7 (-22.1 to -10.9)
Russia	9.7 (8.7 to 10.8)	8.7 (7.8 to 9.7)	8.7 (7.8 to 9.7)	8.0 (7.1 to 8.9)	-0.6 (-0.7 to -0.5)	-8.3 (-9.7 to -6.6)
Ukraine	12.6 (11.3 to 14.0)	12.2 (10.9 to 13.6)	12.4 (11.1 to 13.9)	12.6 (11.1 to 14.3)	0.2 (-0.1 to 0.5)	3.5 (-1.1 to 7.7)
High-income						
Australasia						
Australia	3.5 (3.1 to 3.9)	3.3 (2.9 to 3.7)	3.2 (2.9 to 3.7)	3.1 (2.7 to 3.6)	-0.4 (-0.7 to -0.0)	-5.5 (-10.6 to -0.6)
New Zealand	10.0 (8.9 to 11.4)	9.4 (8.4 to 10.6)	9.2 (8.2 to 10.4)	8.4 (7.4 to 9.6)	-0.8 (-1.0 to -0.5)	-10.7 (-14.4 to -6.9)
High-income Asia Pacific						
Brunei	5.9 (5.2 to 6.7)	5.3 (4.7 to 6.1)	5.3 (4.7 to 6.0)	4.9 (4.2 to 5.7)	-0.6 (-1.0 to -0.2)	-8.4 (-13.4 to -3.3)
Japan	5.8 (5.2 to 6.6)	5.3 (4.8 to 6.0)	5.3 (4.7 to 6.0)	4.9 (4.4 to 5.6)	-0.5 (-0.6 to -0.4)	-7.7 (-8.9 to -6.5)
South Korea	6.3 (5.6 to 7.3)	5.5 (4.8 to 6.3)	5.5 (4.8 to 6.2)	4.9 (4.2 to 5.6)	-0.8 (-1.2 to -0.5)	-11.5 (-15.9 to -6.6)
Singapore	5.9 (5.2 to 6.8)	5.0 (4.4 to 5.7)	4.9 (4.3 to 5.7)	4.3 (3.7 to 5.0)	-0.9 (-1.3 to -0.5)	-12.8 (-17.7 to -7.2)
High-income North America						
Canada	6.1 (5.4 to 7.0)	5.9 (5.2 to 6.8)	5.9 (5.1 to 6.7)	5.6 (4.8 to 6.6)	-0.3 (-0.7 to 0.1)	-4.4 (-10.3 to 1.4)
Greenland	6.7 (5.4 to 8.4)	6.0 (4.8 to 7.6)	6.0 (4.8 to 7.4)	5.5 (4.4 to 6.8)	-0.6 (-1.0 to -0.2)	-8.6 (-14.1 to -2.2)
United States	2.8 (2.4 to 3.3)	2.5 (2.2 to 3.0)	2.6 (2.3 to 3.1)	2.6 (2.3 to 3.1)	0.3 (0.1 to 0.4)	3.9 (1.7 to 6.4)
Southern Latin America						
Argentina	14.1 (12.0 to 16.5)	12.7 (10.7 to 14.8)	12.6 (10.6 to 14.7)	11.5 (9.5 to 13.6)	-0.7 (-1.0 to -0.2)	-9.4 (-14.1 to -3.5)
Chile	11.7 (9.7 to 14.0)	10.1 (8.4 to 12.1)	9.9 (8.2 to 11.9)	8.7 (7.1 to 10.6)	-1.0 (-1.4 to -0.6)	-13.7 (-18.6 to -8.7)
Uruguay	10.4 (8.7 to 12.6)	9.3 (7.8 to 11.4)	9.3 (7.7 to 11.3)	8.5 (6.9 to 10.6)	-0.6 (-1.1 to -0.2)	-8.8 (-14.7 to -3.2)
Western Europe						
Andorra	5.3 (4.6 to 6.2)	5.1 (4.3 to 6.0)	5.1 (4.3 to 5.9)	4.9 (4.1 to 5.8)	-0.3 (-0.6 to 0.1)	-3.9 (-8.6 to 1.5)
Austria	4.6 (4.0 to 5.3)	4.1 (3.6 to 4.7)	4.0 (3.5 to 4.6)	3.7 (3.2 to 4.2)	-0.7 (-1.1 to -0.3)	-9.7 (-14.8 to -4.0)
Belgium	7.4 (6.6 to 8.4)	6.9 (6.1 to 7.7)	6.8 (6.0 to 7.6)	6.4 (5.5 to 7.3)	-0.5 (-0.9 to -0.1)	-7.3 (-12.3 to -2.1)
Cyprus	5.7 (5.1 to 6.5)	3.9 (3.4 to 4.4)	3.9 (3.4 to 4.4)	3.2 (2.8 to 3.7)	-1.3 (-1.7 to -0.9)	-17.4 (-22.4 to -12.9)
Denmark	6.2 (5.5 to 6.9)	5.6 (5.0 to 6.3)	5.6 (4.9 to 6.3)	5.2 (4.5 to 6.0)	-0.6 (-0.9 to -0.2)	-8.1 (-12.8 to -3.2)
Finland	7.0 (6.2 to 7.9)	6.5 (5.8 to 7.3)	6.3 (5.6 to 7.1)	5.8 (5.1 to 6.7)	-0.7 (-1.0 to -0.3)	-10.0 (-14.5 to -5.1)
France	7.2 (6.4 to 8.2)	6.7 (6.0 to 7.5)	6.5 (5.9 to 7.4)	6.1 (5.3 to 6.9)	-0.6 (-0.9 to -0.2)	-8.6 (-13.2 to -2.8)

	Estimate in 2000 (95% UIs)	Estimate in 2015 (95% UIs)	Estimate in 2017 (95% UIs)	Estimate in 2030 (95% UIs)	Annualised rate of change, 2015-2030 (95% UIs)	Percent change, 2015-2030 (95% UIs)
Germany	5.3 (4.7 to 6.1)	4.8 (4.2 to 5.4)	4.7 (4.1 to 5.3)	4.3 (3.7 to 5.0)	-0.7 (-1.1 to -0.3)	-9.7 (-14.6 to -4.9)
Greece	7.8 (7.0 to 8.8)	7.2 (6.5 to 8.1)	7.2 (6.4 to 8.1)	6.8 (6.0 to 7.7)	-0.4 (-0.7 to -0.1)	-5.6 (-10.0 to -1.0)
Iceland	5.6 (4.8 to 6.5)	5.0 (4.3 to 5.8)	5.0 (4.3 to 5.8)	4.6 (3.9 to 5.5)	-0.6 (-0.9 to -0.3)	-8.1 (-13.1 to -3.7)
Ireland	5.4 (4.7 to 6.1)	4.8 (4.3 to 5.4)	4.7 (4.2 to 5.3)	4.3 (3.7 to 4.9)	-0.8 (-1.1 to -0.4)	-10.9 (-15.6 to -6.2)
Israel	6.8 (5.9 to 8.0)	6.3 (5.4 to 7.3)	6.3 (5.4 to 7.3)	5.9 (5.0 to 6.9)	-0.5 (-0.8 to -0.2)	-6.9 (-11.2 to -2.3)
Italy	8.0 (7.2 to 9.1)	7.4 (6.6 to 8.2)	7.3 (6.6 to 8.2)	6.9 (6.1 to 7.8)	-0.5 (-0.8 to -0.1)	-6.6 (-11.3 to -2.0)
Luxembourg	3.9 (3.5 to 4.4)	3.6 (3.2 to 4.0)	3.6 (3.2 to 4.0)	3.4 (3.0 to 3.8)	-0.5 (-0.8 to -0.2)	-6.8 (-11.5 to -2.4)
Malta	5.8 (5.1 to 6.6)	5.2 (4.6 to 5.9)	5.1 (4.5 to 5.8)	4.6 (4.1 to 5.3)	-0.8 (-1.1 to -0.4)	-10.7 (-15.7 to -5.7)
Netherlands	7.2 (6.4 to 8.1)	6.6 (5.9 to 7.4)	6.5 (5.8 to 7.3)	6.0 (5.3 to 6.9)	-0.6 (-0.9 to -0.3)	-8.5 (-12.8 to -3.8)
Norway	5.8 (5.2 to 6.5)	5.3 (4.7 to 5.9)	5.2 (4.6 to 5.8)	4.8 (4.3 to 5.4)	-0.7 (-0.8 to -0.5)	-9.4 (-10.9 to -7.8)
Portugal	7.7 (6.8 to 8.7)	6.6 (5.9 to 7.5)	6.5 (5.8 to 7.4)	5.8 (5.1 to 6.6)	-0.9 (-1.2 to -0.5)	-12.1 (-16.5 to -7.7)
Spain	4.0 (3.5 to 4.5)	3.6 (3.2 to 4.0)	3.5 (3.1 to 4.0)	3.3 (2.9 to 3.7)	-0.5 (-0.9 to -0.2)	-7.8 (-12.3 to -3.1)
Sweden	7.0 (6.2 to 7.8)	7.1 (6.3 to 8.0)	7.1 (6.4 to 8.0)	7.3 (6.4 to 8.3)	0.1 (-0.1 to 0.4)	2.0 (-1.8 to 6.5)
Switzerland	5.6 (4.8 to 6.6)	5.2 (4.5 to 6.0)	5.2 (4.5 to 6.0)	4.9 (4.2 to 5.7)	-0.4 (-0.8 to -0.1)	-6.3 (-11.3 to -1.3)
United Kingdom	6.4 (5.7 to 7.2)	6.2 (5.5 to 7.0)	6.2 (5.5 to 6.9)	6.0 (5.4 to 6.8)	-0.1 (-0.2 to -0.1)	-2.2 (-3.0 to -1.4)
Latin America and Caribbean						
Andean Latin America						
Bolivia	21.5 (19.2 to 24.1)	18.1 (16.3 to 20.2)	17.9 (16.0 to 20.2)	15.8 (13.8 to 18.2)	-0.9 (-1.3 to -0.5)	-12.9 (-17.7 to -7.1)
Ecuador	11.1 (10.3 to 12.2)	10.8 (9.6 to 12.2)	10.4 (9.3 to 11.8)	9.5 (8.2 to 11.0)	-0.8 (-1.2 to -0.4)	-11.6 (-16.9 to -6.2)
Peru	13.6 (12.5 to 14.7)	11.4 (10.9 to 11.8)	11.0 (10.5 to 11.6)	9.3 (8.6 to 10.0)	-1.4 (-1.7 to -1.0)	-18.5 (-22.3 to -14.3)
Caribbean						
Antigua and Barbuda	7.5 (6.4 to 8.9)	7.1 (6.1 to 8.3)	7.0 (6.0 to 8.3)	6.6 (5.6 to 7.9)	-0.4 (-0.8 to -0.0)	-6.4 (-11.5 to -0.3)
The Bahamas	7.7 (6.5 to 9.0)	7.3 (6.2 to 8.5)	7.3 (6.2 to 8.6)	7.0 (5.8 to 8.4)	-0.3 (-0.7 to 0.1)	-4.3 (-10.2 to 0.9)
Barbados	7.4 (6.3 to 8.8)	7.1 (6.1 to 8.3)	7.1 (6.0 to 8.3)	6.8 (5.7 to 8.2)	-0.3 (-0.6 to 0.1)	-4.2 (-9.0 to 1.7)
Belize	10.4 (8.8 to 12.3)	9.3 (8.0 to 11.0)	9.2 (7.9 to 10.9)	8.5 (7.1 to 10.1)	-0.7 (-1.0 to -0.3)	-9.5 (-14.2 to -4.7)
Bermuda	6.2 (5.2 to 7.3)	5.8 (4.9 to 6.8)	5.7 (4.8 to 6.7)	5.3 (4.4 to 6.3)	-0.5 (-0.9 to -0.2)	-7.6 (-12.8 to -2.5)
Cuba	8.2 (7.0 to 9.7)	7.4 (6.3 to 8.6)	7.2 (6.2 to 8.5)	6.6 (5.6 to 7.8)	-0.8 (-1.1 to -0.4)	-10.6 (-15.1 to -6.3)
Dominica	8.2 (6.9 to 9.5)	7.7 (6.6 to 9.1)	7.6 (6.5 to 9.0)	7.2 (6.0 to 8.6)	-0.5 (-0.9 to -0.2)	-7.5 (-13.2 to -2.6)
Dominican Republic	12.5 (11.2 to 14.2)	11.8 (10.7 to 12.8)	11.7 (10.6 to 12.9)	11.2 (9.8 to 12.7)	-0.3 (-0.8 to 0.2)	-4.4 (-10.9 to 3.1)
Grenada	8.8 (7.5 to 10.3)	8.0 (6.8 to 9.4)	7.9 (6.8 to 9.4)	7.3 (6.2 to 8.8)	-0.6 (-1.0 to -0.2)	-8.6 (-14.4 to -3.1)
Guyana	10.3 (8.8 to 12.1)	9.4 (8.1 to 11.1)	9.3 (7.9 to 11.0)	8.4 (7.0 to 10.1)	-0.8 (-1.2 to -0.4)	-10.7 (-15.9 to -6.2)
Haiti	17.6 (16.2 to 19.2)	15.5 (14.3 to 17.0)	15.5 (14.2 to 17.2)	14.4 (12.8 to 16.4)	-0.5 (-0.9 to -0.1)	-7.3 (-13.1 to -1.9)
Jamaica	7.4 (6.5 to 8.3)	7.0 (6.2 to 7.8)	7.0 (6.2 to 7.9)	6.8 (5.8 to 7.8)	-0.2 (-0.6 to 0.2)	-3.2 (-9.0 to 3.8)
Puerto Rico	5.2 (4.6 to 5.8)	4.7 (4.1 to 5.3)	4.6 (4.0 to 5.2)	4.2 (3.6 to 4.9)	-0.7 (-1.1 to -0.3)	-9.7 (-15.0 to -4.1)
Saint Lucia	9.1 (7.8 to 10.7)	8.4 (7.2 to 9.8)	8.3 (7.1 to 9.8)	7.7 (6.5 to 9.2)	-0.6 (-1.0 to -0.2)	-8.4 (-14.3 to -3.0)
Saint Vincent and the Grenadines	9.9 (8.5 to 11.7)	9.2 (7.8 to 10.8)	9.1 (7.7 to 10.7)	8.5 (7.1 to 10.2)	-0.5 (-0.9 to -0.1)	-7.2 (-12.5 to -1.7)
Suriname	9.0 (7.6 to 10.5)	8.2 (7.0 to 9.7)	8.2 (6.9 to 9.6)	7.6 (6.4 to 9.1)	-0.5 (-0.9 to -0.2)	-7.6 (-12.1 to -3.0)
Trinidad and Tobago	8.4 (7.1 to 9.9)	8.1 (6.9 to 9.5)	8.0 (6.8 to 9.4)	7.7 (6.4 to 9.1)	-0.3 (-0.7 to 0.0)	-4.7 (-9.7 to 0.2)
Virgin Islands, U.S.	5.3 (4.7 to 6.0)	4.8 (4.3 to 5.4)	4.9 (4.3 to 5.5)	4.6 (4.1 to 5.3)	-0.3 (-0.7 to 0.1)	-4.3 (-9.3 to 1.1)
Central Latin America						
Colombia	17.0 (15.2 to 19.1)	15.1 (14.0 to 16.0)	14.7 (13.3 to 15.8)	13.1 (11.3 to 14.5)	-1.0 (-1.5 to -0.5)	-13.3 (-19.7 to -7.4)
Costa Rica	12.8 (11.5 to 14.3)	11.6 (10.4 to 12.9)	11.4 (10.2 to 12.7)	10.4 (9.2 to 11.7)	-0.8 (-1.1 to -0.4)	-10.7 (-14.8 to -6.3)
El Salvador	9.7 (8.7 to 10.8)	8.3 (7.5 to 9.2)	8.3 (7.5 to 9.2)	7.6 (6.7 to 8.5)	-0.6 (-1.0 to -0.1)	-8.7 (-14.2 to -1.7)
Guatemala	9.4 (8.4 to 10.4)	8.3 (7.7 to 9.0)	8.3 (7.5 to 9.2)	7.6 (6.6 to 8.6)	-0.6 (-1.1 to -0.2)	-8.9 (-14.9 to -2.5)
Honduras	9.4 (8.7 to 10.3)	9.0 (8.3 to 9.8)	8.9 (8.2 to 9.8)	8.4 (7.5 to 9.5)	-0.4 (-0.9 to -0.0)	-6.0 (-12.2 to -0.4)
Mexico	4.5 (3.8 to 5.2)	4.5 (3.9 to 5.2)	4.5 (3.9 to 5.2)	4.5 (3.8 to 5.2)	0.0 (-0.1 to 0.2)	0.2 (-1.9 to 2.4)
Nicaragua	10.0 (9.2 to 10.8)	7.8 (7.1 to 8.6)	7.9 (7.1 to 8.9)	7.0 (6.0 to 8.2)	-0.7 (-1.3 to -0.2)	-10.0 (-17.5 to -3.0)
Panama	8.8 (7.5 to 10.3)	8.3 (7.1 to 9.7)	8.0 (6.8 to 9.3)	7.2 (6.1 to 8.5)	-0.9 (-1.2 to -0.6)	-12.8 (-17.1 to -7.9)
Venezuela	9.4 (8.0 to 11.1)	8.8 (7.5 to 10.4)	8.7 (7.4 to 10.2)	8.1 (6.8 to 9.7)	-0.6 (-1.0 to -0.2)	-8.2 (-13.3 to -2.7)
Tropical Latin America						
Brazil	8.6 (7.7 to 9.7)	7.3 (6.5 to 8.2)	7.2 (6.4 to 8.0)	6.2 (5.6 to 7.0)	-1.1 (-1.2 to -1.0)	-14.9 (-16.0 to -13.6)
Paraguay	7.9 (7.1 to 8.9)	7.1 (6.4 to 8.0)	7.0 (6.2 to 7.9)	6.4 (5.5 to 7.5)	-0.7 (-1.2 to -0.2)	-10.3 (-16.5 to -3.3)
North Africa and Middle East						
North Africa and Middle East						
Afghanistan	53.7 (48.3 to 59.6)	39.8 (36.9 to 44.0)	39.1 (36.0 to 43.6)	31.9 (28.4 to 37.0)	-1.5 (-2.0 to -1.0)	-19.8 (-25.6 to -13.3)
Algeria	18.2 (15.7 to 21.1)	14.8 (12.7 to 17.0)	14.5 (12.5 to 16.7)	12.3 (10.5 to 14.3)	-1.2 (-1.6 to -0.8)	-16.5 (-21.0 to -11.9)
Bahrain	16.6 (14.4 to 19.3)	14.9 (12.8 to 17.2)	14.8 (12.7 to 17.1)	13.7 (11.6 to 16.0)	-0.6 (-0.9 to -0.2)	-8.4 (-13.1 to -3.5)
Egypt	15.7 (14.4 to 17.0)	13.4 (12.3 to 14.8)	13.0 (11.8 to 14.6)	11.2 (9.7 to 13.2)	-1.2 (-1.8 to -0.7)	-16.6 (-23.3 to -9.4)
Iran	20.7 (18.5 to 23.1)	17.2 (15.3 to 19.2)	17.1 (15.2 to 19.0)	14.8 (13.2 to 16.5)	-1.0 (-1.1 to -0.9)	-14.0 (-15.2 to -12.8)
Iraq	32.7 (29.4 to 36.6)	28.2 (25.7 to 30.9)	28.1 (25.1 to 31.3)	25.5 (22.0 to 29.4)	-0.7 (-1.2 to -0.2)	-9.5 (-16.7 to -2.6)
Jordan	13.8 (12.3 to 15.4)	11.6 (10.8 to 12.6)	11.5 (10.5 to 12.7)	10.1 (9.1 to 11.4)	-0.9 (-1.3 to -0.6)	-13.0 (-17.9 to -8.2)
Kuwait	15.4 (13.3 to 17.9)	12.7 (11.0 to 14.6)	12.5 (10.8 to 14.4)	10.6 (9.1 to 12.3)	-1.2 (-1.5 to -0.8)	-16.4 (-20.6 to -11.8)
Lebanon	20.1 (17.2 to 23.3)	16.1 (13.9 to 18.5)	15.8 (13.7 to 18.2)	13.2 (11.3 to 15.4)	-1.3 (-1.7 to -1.0)	-18.1 (-22.0 to -14.1)
Libya	16.9 (14.5 to 19.7)	15.4 (13.2 to 17.7)	15.5 (13.3 to 18.0)	14.8 (12.6 to 17.5)	-0.3 (-0.6 to 0.1)	-3.7 (-8.5 to 1.1)
Morocco	21.1 (18.2 to 24.1)	18.1 (15.5 to 20.8)	17.7 (15.1 to 20.5)	15.5 (13.1 to 18.2)	-1.0 (-1.4 to -0.7)	-14.3 (-19.1 to -9.3)
Palestine	22.9 (19.8 to 26.5)	20.0 (17.1 to 23.1)	19.5 (16.6 to 22.5)	16.8 (14.0 to 19.6)	-1.2 (-1.7 to -0.8)	-16.2 (-21.9 to -11.5)
Oman	16.0 (13.7 to 18.4)	13.1 (11.3 to 15.2)	12.9 (11.1 to 14.9)	10.9 (9.3 to 12.8)	-1.2 (-1.6 to -0.9)	-16.5 (-21.1 to -12.5)
Qatar	14.5 (12.5 to 16.8)	13.4 (11.5 to 15.4)	13.2 (11.4 to 15.2)	12.1 (10.3 to 14.1)	-0.7 (-1.0 to -0.3)	-9.4 (-14.0 to -4.3)
Saudi Arabia	14.8 (12.6 to 17.1)	11.7 (10.1 to 13.4)	11.3 (9.8 to 13.1)	9.3 (7.9 to 10.8)	-1.6 (-1.9 to -1.2)	-20.7 (-24.4 to -16.7)
Sudan	25.0 (21.5 to 29.1)	21.8 (18.8 to 25.2)	20.9 (18.0 to 24.2)	17.6 (14.9 to 20.4)	-1.4 (-1.9 to -1.0)	-19.5 (-24.4 to -14.5)
Syria	22.8 (19.6 to 26.4)	18.1 (15.6 to 20.9)	17.9 (15.3 to 20.6)	15.1 (12.8 to 17.7)	-1.2 (-1.6 to -0.9)	-17.1 (-21.1 to -13.0)
Tunisia	17.7 (15.3 to 20.4)	14.8 (12.8 to 17.2)	14.6 (12.6 to 16.9)	12.7 (10.8 to 14.9)	-1.1 (-1.4 to -0.7)	-14.7 (-19.1 to -10.0)
Turkey	15.9 (14.2 to 17.9)	12.3 (11.2 to 13.6)	12.2 (10.9 to 13.6)	10.4 (8.9 to 12.0)	-1.2 (-1.7 to -0.6)	-15.9 (-22.0 to -8.8)
United Arab Emirates	14.6 (12.6 to 16.8)	13.2 (11.3 to 15.3)	13.1 (11.1 to 15.3)	12.0 (10.0 to 14.1)	-0.6 (-1.0 to -0.3)	-9.1 (-13.9 to -4.7)
Yemen	30.4 (26.2 to 35.0)	24.4 (20.9 to 28.2)	24.1 (20.6 to 27.8)	20.3 (17.3 to 23.8)	-1.2 (-1.6 to -0.8)	-16.6 (-21.4 to -11.7)
South Asia						
South Asia						
Bangladesh	27.1 (24.2 to 30.4)	21.8 (19.7 to 24.1)	21.5 (19.3 to 24.1)	18.5 (16.1 to 21.1)	-1.1 (-1.6 to -0.7)	-15.2 (-21.0 to -9.3)
Bhutan	16.0 (14.4 to 17.9)	12.4 (11.2 to 13.8)	12.3 (11.0 to 13.7)	10.5 (9.1 to 12.2)	-1.1 (-1.6 to -0.6)	-15.1 (-21.2 to -8.3)
India	18.8 (17.0 to 20.9)	18.0 (16.1 to 20.1)	18.1 (16.2 to 20.4)	17.8 (15.7 to 20.2)	-0.1 (-0.3 to 0.2)	-1.0 (-4.1 to 2.5)

	Estimate in 2000 (95% UIs)	Estimate in 2015 (95% UIs)	Estimate in 2017 (95% UIs)	Estimate in 2030 (95% UIs)	Annualised rate of change, 2015-2030 (95% UIs)	Percent change, 2015-2030 (95% UIs)
Nepal	15.7 (13.9 to 17.6)	12.7 (11.5 to 14.2)	12.6 (11.4 to 14.2)	11.0 (9.6 to 12.5)	-1.0 (-1.4 to -0.6)	-13.7 (-18.4 to -8.4)
Pakistan	28.9 (25.8 to 32.6)	22.7 (20.8 to 25.1)	22.3 (20.4 to 24.7)	18.5 (16.6 to 20.8)	-1.4 (-1.8 to -1.0)	-18.6 (-23.5 to -13.5)
Southeast Asia, East Asia, and Oceania						
East Asia						
China	14.5 (13.0 to 16.2)	12.3 (11.0 to 13.7)	12.0 (10.7 to 13.4)	10.5 (9.4 to 11.7)	-1.1 (-1.1 to -1.0)	-14.9 (-15.8 to -14.0)
North Korea	14.8 (13.1 to 16.8)	15.9 (14.0 to 17.8)	15.8 (13.9 to 17.8)	16.5 (14.3 to 18.8)	0.3 (-0.1 to 0.6)	3.9 (-1.7 to 9.8)
Taiwan (Province of China)	10.2 (9.0 to 11.6)	9.1 (8.1 to 10.3)	9.1 (8.0 to 10.3)	8.6 (7.4 to 9.8)	-0.4 (-0.8 to -0.1)	-6.1 (-10.9 to -1.3)
Oceania						
American Samoa	13.0 (11.5 to 14.8)	12.1 (10.7 to 13.8)	12.1 (10.6 to 13.7)	11.6 (10.1 to 13.4)	-0.3 (-0.7 to 0.0)	-4.6 (-9.5 to 0.7)
Federated States of Micronesia	15.7 (13.9 to 17.8)	15.3 (13.5 to 17.3)	15.2 (13.4 to 17.2)	14.8 (12.9 to 17.0)	-0.2 (-0.6 to 0.1)	-3.2 (-8.4 to 1.9)
Fiji	14.9 (13.1 to 16.8)	13.9 (12.3 to 15.6)	13.8 (12.2 to 15.6)	13.1 (11.5 to 15.0)	-0.4 (-0.8 to -0.0)	-5.7 (-10.7 to -0.5)
Guam	10.6 (9.3 to 12.0)	10.5 (9.3 to 11.9)	10.4 (9.2 to 11.8)	10.3 (8.9 to 11.8)	-0.2 (-0.5 to 0.2)	-2.3 (-7.1 to 2.5)
Kiribati	17.2 (15.2 to 19.4)	16.8 (14.8 to 19.1)	16.5 (14.5 to 18.8)	15.9 (13.8 to 18.3)	-0.4 (-0.7 to -0.1)	-5.6 (-10.6 to -0.8)
Marshall Islands	16.7 (14.8 to 18.9)	15.6 (13.8 to 17.6)	15.4 (13.5 to 17.5)	14.4 (12.5 to 16.6)	-0.6 (-0.9 to -0.2)	-7.9 (-12.3 to -2.7)
Northern Mariana Islands	10.8 (9.6 to 12.3)	11.1 (9.8 to 12.7)	11.2 (9.8 to 12.8)	11.5 (9.9 to 13.2)	0.2 (-0.1 to 0.6)	3.4 (-2.0 to 8.9)
Papua New Guinea	19.2 (16.9 to 22.0)	18.8 (16.6 to 21.3)	18.6 (16.4 to 20.9)	17.8 (15.6 to 20.4)	-0.4 (-0.7 to -0.1)	-5.4 (-10.3 to -0.8)
Samoa	14.8 (13.0 to 16.8)	14.4 (12.7 to 16.2)	14.3 (12.6 to 16.2)	13.9 (12.1 to 16.1)	-0.2 (-0.6 to 0.1)	-3.0 (-8.6 to 1.9)
Solomon Islands	19.0 (16.8 to 21.5)	18.7 (16.6 to 21.1)	18.4 (16.2 to 20.7)	17.5 (15.3 to 20.0)	-0.4 (-0.8 to -0.1)	-6.4 (-11.1 to -2.1)
Tonga	14.1 (12.5 to 16.1)	13.3 (11.8 to 15.1)	13.2 (11.6 to 15.0)	12.5 (10.9 to 14.4)	-0.4 (-0.8 to -0.1)	-6.4 (-11.2 to -1.3)
Vanuatu	16.1 (14.2 to 18.2)	15.5 (13.7 to 17.6)	15.4 (13.5 to 17.4)	14.7 (12.8 to 17.0)	-0.3 (-0.7 to 0.0)	-5.0 (-10.0 to 0.2)
Southeast Asia						
Cambodia	11.8 (10.8 to 13.1)	9.4 (8.6 to 10.5)	9.4 (8.6 to 10.5)	8.1 (7.2 to 9.2)	-1.0 (-1.4 to -0.7)	-14.5 (-18.6 to -10.1)
Indonesia	13.1 (11.5 to 14.8)	11.2 (9.9 to 12.8)	11.0 (9.7 to 12.4)	9.5 (8.4 to 10.8)	-1.1 (-1.2 to -1.0)	-15.2 (-16.8 to -13.3)
Laos	14.5 (12.3 to 17.1)	11.8 (9.9 to 13.9)	11.4 (9.6 to 13.6)	9.5 (7.8 to 11.4)	-1.5 (-1.8 to -1.1)	-19.6 (-23.8 to -14.9)
Malaysia	10.5 (8.8 to 12.3)	8.9 (7.5 to 10.5)	8.7 (7.4 to 10.3)	7.5 (6.3 to 8.9)	-1.1 (-1.5 to -0.8)	-15.4 (-20.1 to -10.9)
Maldives	15.0 (12.6 to 17.7)	10.2 (8.6 to 12.0)	9.9 (8.4 to 11.7)	7.6 (6.3 to 9.0)	-2.0 (-2.3 to -1.6)	-25.9 (-29.5 to -21.6)
Mauritius	11.1 (9.3 to 13.1)	9.9 (8.3 to 11.6)	9.7 (8.1 to 11.4)	8.6 (7.2 to 10.2)	-0.9 (-1.2 to -0.5)	-12.8 (-17.1 to -7.7)
Myanmar	14.5 (12.9 to 16.3)	10.8 (9.8 to 12.1)	10.6 (9.6 to 11.9)	8.5 (7.5 to 9.7)	-1.6 (-2.0 to -1.2)	-21.3 (-25.7 to -16.6)
Philippines	8.9 (7.9 to 10.0)	7.5 (6.8 to 8.3)	7.4 (6.7 to 8.3)	6.5 (5.7 to 7.5)	-0.9 (-1.4 to -0.5)	-12.8 (-18.4 to -7.3)
Sri Lanka	18.8 (16.7 to 21.2)	15.1 (13.5 to 17.0)	14.7 (13.1 to 16.6)	12.2 (10.7 to 13.9)	-1.5 (-1.9 to -1.1)	-19.7 (-24.3 to -15.5)
Seychelles	12.6 (10.6 to 14.8)	10.8 (9.1 to 12.8)	10.7 (8.9 to 12.6)	9.4 (7.8 to 11.3)	-0.9 (-1.3 to -0.6)	-13.2 (-18.3 to -8.4)
Thailand	13.8 (11.6 to 16.3)	11.1 (9.3 to 12.9)	10.9 (9.2 to 12.8)	9.2 (7.7 to 10.9)	-1.2 (-1.6 to -0.8)	-16.6 (-21.1 to -11.6)
Timor-Leste	26.9 (24.0 to 30.2)	20.6 (18.6 to 22.8)	20.1 (17.9 to 22.6)	16.1 (13.9 to 18.6)	-1.6 (-2.2 to -1.2)	-21.8 (-27.6 to -16.0)
Vietnam	11.6 (10.4 to 13.0)	9.6 (8.6 to 10.7)	9.5 (8.5 to 10.7)	8.3 (7.2 to 9.6)	-0.9 (-1.4 to -0.4)	-13.0 (-19.2 to -6.1)
Sub-Saharan Africa						
Central Sub-Saharan Africa						
Angola	36.0 (31.7 to 40.7)	29.6 (26.2 to 33.4)	28.3 (25.1 to 32.2)	22.7 (19.8 to 26.1)	-1.8 (-2.1 to -1.4)	-23.4 (-27.2 to -19.0)
Central African Republic	24.0 (21.6 to 27.0)	21.6 (19.5 to 24.1)	21.9 (19.5 to 24.7)	20.9 (18.2 to 24.3)	-0.2 (-0.6 to 0.3)	-3.1 (-8.9 to 4.0)
Congo	29.9 (26.5 to 33.9)	24.5 (21.6 to 27.7)	23.8 (21.0 to 27.0)	19.7 (17.2 to 22.8)	-1.4 (-1.8 to -1.1)	-19.5 (-23.8 to -14.9)
Democratic Republic of the Congo	42.8 (38.5 to 47.8)	36.9 (33.2 to 41.2)	35.1 (31.2 to 39.6)	28.7 (23.9 to 34.0)	-1.7 (-2.4 to -1.1)	-22.4 (-29.9 to -14.7)
Equatorial Guinea	31.1 (27.2 to 35.5)	20.8 (18.3 to 23.5)	20.2 (17.8 to 23.0)	14.9 (13.0 to 17.4)	-2.2 (-2.6 to -1.8)	-28.1 (-32.6 to -23.6)
Gabon	33.0 (29.6 to 37.0)	27.9 (25.8 to 30.5)	27.4 (25.2 to 30.0)	23.6 (21.4 to 26.4)	-1.1 (-1.5 to -0.7)	-15.4 (-20.4 to -10.0)
Eastern Sub-Saharan Africa						
Burundi	24.2 (21.7 to 27.0)	23.0 (20.7 to 25.8)	22.8 (20.5 to 25.5)	21.7 (19.2 to 24.8)	-0.4 (-0.7 to -0.0)	-5.9 (-10.6 to -0.7)
Comoros	16.3 (14.7 to 18.2)	13.5 (12.2 to 15.0)	13.3 (11.9 to 14.8)	11.6 (10.3 to 13.1)	-1.0 (-1.3 to -0.7)	-14.1 (-18.1 to -9.4)
Djibouti	23.8 (21.2 to 26.6)	20.7 (18.5 to 23.0)	20.2 (18.1 to 22.5)	17.8 (15.7 to 20.3)	-1.0 (-1.3 to -0.6)	-13.9 (-18.1 to -9.3)
Eritrea	26.7 (23.9 to 29.8)	24.3 (21.8 to 27.1)	23.9 (21.3 to 26.7)	21.7 (19.1 to 24.7)	-0.8 (-1.2 to -0.4)	-10.8 (-16.0 to -6.0)
Ethiopia	38.5 (34.6 to 42.6)	30.4 (27.4 to 33.8)	29.3 (26.4 to 32.6)	23.2 (20.8 to 25.9)	-1.8 (-2.0 to -1.6)	-23.7 (-26.1 to -21.2)
Kenya	23.1 (20.1 to 26.6)	22.7 (20.1 to 25.8)	22.7 (20.1 to 25.7)	22.4 (19.9 to 25.2)	-0.1 (-0.2 to 0.1)	-1.3 (-3.3 to 1.1)
Madagascar	25.9 (23.2 to 29.0)	24.0 (21.6 to 26.7)	23.5 (21.1 to 26.2)	22.0 (19.5 to 24.9)	-0.6 (-0.9 to -0.2)	-8.1 (-12.6 to -3.2)
Malawi	19.8 (17.9 to 22.2)	18.1 (16.7 to 19.5)	18.2 (16.5 to 19.8)	17.3 (14.9 to 19.4)	-0.3 (-0.9 to 0.2)	-4.9 (-12.7 to 2.7)
Mozambique	24.8 (22.3 to 27.5)	21.1 (19.3 to 23.0)	20.2 (18.1 to 22.5)	17.1 (14.9 to 19.6)	-1.4 (-1.9 to -0.9)	-19.0 (-24.5 to -12.6)
Rwanda	30.1 (27.0 to 33.8)	25.3 (22.9 to 28.1)	24.8 (22.2 to 27.8)	20.9 (18.1 to 24.2)	-1.3 (-1.8 to -0.8)	-17.3 (-23.6 to -11.4)
Somalia	30.8 (27.4 to 34.4)	27.4 (24.6 to 30.7)	27.2 (24.3 to 30.5)	24.9 (22.0 to 28.6)	-0.7 (-1.0 to -0.3)	-9.4 (-13.9 to -4.1)
South Sudan	30.7 (27.6 to 34.5)	28.4 (25.6 to 31.7)	28.1 (25.2 to 31.4)	26.2 (23.2 to 29.7)	-0.6 (-0.9 to -0.2)	-7.9 (-12.1 to -3.1)
Tanzania	30.2 (27.1 to 33.7)	25.7 (23.9 to 27.8)	25.1 (23.3 to 27.7)	21.9 (19.7 to 25.1)	-1.1 (-1.6 to -0.6)	-14.9 (-20.9 to -8.7)
Uganda	39.1 (35.2 to 43.6)	30.4 (27.8 to 33.1)	30.2 (27.0 to 33.7)	25.6 (21.8 to 29.7)	-1.1 (-1.7 to -0.6)	-15.6 (-22.8 to -8.3)
Zambia	31.1 (28.2 to 34.5)	25.4 (23.3 to 28.1)	24.4 (22.1 to 27.3)	19.7 (17.0 to 23.0)	-1.7 (-2.3 to -1.2)	-22.7 (-29.0 to -16.2)
Southern Sub-Saharan Africa						
Botswana	17.2 (15.1 to 19.7)	14.8 (12.9 to 16.8)	14.5 (12.7 to 16.4)	12.9 (11.1 to 14.8)	-0.9 (-1.3 to -0.5)	-12.8 (-17.3 to -7.4)
Lesotho	23.6 (20.7 to 27.0)	20.5 (18.0 to 23.6)	20.2 (17.7 to 23.1)	17.9 (15.5 to 20.7)	-0.9 (-1.3 to -0.6)	-13.0 (-17.6 to -8.1)
Namibia	25.3 (22.5 to 28.4)	21.1 (19.0 to 23.6)	20.7 (18.5 to 23.1)	17.7 (15.6 to 20.1)	-1.2 (-1.6 to -0.8)	-16.3 (-21.4 to -11.0)
South Africa	10.0 (8.8 to 11.6)	9.9 (8.7 to 11.4)	9.9 (8.7 to 11.4)	10.0 (8.8 to 11.4)	0.1 (-0.1 to 0.2)	1.2 (-1.4 to 3.8)
Swaziland	19.7 (17.3 to 22.3)	17.2 (15.2 to 19.6)	16.9 (14.8 to 19.3)	15.0 (12.9 to 17.3)	-0.9 (-1.3 to -0.6)	-12.9 (-17.5 to -8.5)
Zimbabwe	22.1 (20.4 to 24.1)	21.2 (19.4 to 23.1)	20.7 (18.5 to 23.4)	19.4 (16.1 to 23.2)	-0.6 (-1.4 to 0.1)	-8.5 (-18.8 to 1.5)
Western Sub-Saharan Africa						
Benin	20.8 (18.0 to 23.8)	18.2 (15.8 to 20.8)	17.8 (15.4 to 20.5)	15.7 (13.5 to 18.1)	-1.0 (-1.3 to -0.7)	-13.8 (-17.9 to -9.4)
Burkina Faso	13.0 (11.7 to 14.6)	11.2 (10.1 to 12.4)	11.2 (10.1 to 12.6)	10.5 (9.2 to 12.0)	-0.4 (-0.8 to 0.0)	-6.2 (-11.7 to 0.7)
Cameroon	26.6 (24.5 to 29.4)	24.4 (22.3 to 26.9)	23.2 (20.9 to 26.1)	20.5 (17.9 to 23.6)	-1.2 (-1.6 to -0.6)	-16.1 (-21.5 to -9.1)
Cape Verde	19.5 (16.9 to 22.5)	15.9 (13.8 to 18.2)	15.6 (13.5 to 17.8)	13.2 (11.3 to 15.3)	-1.2 (-1.6 to -0.9)	-17.0 (-21.5 to -12.2)
Chad	19.8 (17.7 to 22.1)	16.2 (15.1 to 17.8)	15.9 (14.5 to 17.8)	13.6 (11.8 to 15.8)	-1.2 (-1.7 to -0.7)	-16.6 (-23.0 to -9.5)
Cote d'Ivoire	20.2 (18.1 to 22.7)	17.8 (16.5 to 19.4)	17.4 (16.0 to 19.0)	15.5 (14.0 to 17.0)	-0.9 (-1.3 to -0.5)	-13.0 (-17.9 to -7.9)
The Gambia	10.9 (9.8 to 12.2)	9.0 (8.1 to 10.1)	8.9 (8.0 to 10.1)	7.8 (6.8 to 8.9)	-1.0 (-1.4 to -0.7)	-14.2 (-18.8 to -9.6)
Ghana	17.8 (16.0 to 19.9)	14.9 (13.5 to 16.6)	14.6 (13.0 to 16.3)	12.6 (10.9 to 14.3)	-1.2 (-1.5 to -0.8)	-15.9 (-20.6 to -11.0)
Guinea	22.6 (19.7 to 26.0)	19.9 (17.2 to 22.8)	19.4 (16.8 to 22.2)	17.0 (14.6 to 19.9)	-1.0 (-1.4 to -0.7)	-14.5 (-19.4 to -9.9)
Guinea-Bissau	23.6 (20.5 to 27.1)	20.6 (18.1 to 23.6)	20.1 (17.5 to 23.1)	17.7 (15.3 to 20.4)	-1.0 (-1.3 to -0.7)	-14.0 (-18.3 to -9.5)
Liberia	34.0 (30.6 to 38.0)	28.2 (25.7 to 30.9)	27.7 (24.9 to 30.9)	24.5 (21.1 to 28.2)	-0.9 (-1.5 to -0.4)	-13.0 (-19.6 to -6.1)
Mali	25.3 (22.7 to 28.3)	21.9 (20.4 to 23.8)	21.5 (19.9 to 23.5)	19.0 (17.1 to 21.0)	-0.9 (-1.3 to -0.6)	-13.2 (-17.8 to -8.2)
Mauritania	17.8 (15.5 to 20.4)	15.4 (13.4 to 17.7)	15.1 (13.1 to 17.4)	13.2 (11.2 to 15.4)	-1.0 (-1.4 to -0.6)	-14.4 (-18.9 to -9.2)
Niger	25.3 (21.9 to 28.9)	23.3 (20.2 to 26.7)	22.9 (19.9 to 26.3)	21.0 (17.9 to 24.6)	-0.7 (-1.1 to -0.3)	-9.6 (-14.9 to -4.5)

	Estimate in 2000 (95% UIs)	Estimate in 2015 (95% UIs)	Estimate in 2017 (95% UIs)	Estimate in 2030 (95% UIs)	Annualised rate of change, 2015-2030 (95% UIs)	Percent change, 2015-2030 (95% UIs)
Nigeria	12.7 (11.4 to 14.2)	10.0 (9.2 to 11.0)	9.8 (9.0 to 11.1)	8.2 (7.1 to 9.8)	-1.3 (-1.9 to -0.8)	-18.0 (-25.1 to -10.7)
Sao Tome and Principe	25.0 (22.3 to 28.1)	20.6 (18.6 to 22.7)	20.1 (18.0 to 22.4)	17.0 (14.8 to 19.6)	-1.3 (-1.7 to -0.8)	-17.2 (-22.9 to -11.4)
Senegal	18.2 (15.8 to 20.8)	16.6 (14.4 to 18.9)	16.3 (14.1 to 18.6)	14.9 (12.6 to 17.2)	-0.7 (-1.1 to -0.4)	-10.2 (-14.9 to -5.9)
Sierra Leone	29.8 (26.6 to 33.3)	24.4 (22.7 to 26.5)	23.8 (21.9 to 26.0)	20.0 (18.1 to 22.3)	-1.3 (-1.7 to -0.9)	-17.9 (-22.9 to -12.7)
Togo	12.5 (11.1 to 13.9)	10.9 (10.0 to 11.9)	10.6 (9.7 to 11.8)	9.4 (8.4 to 10.4)	-1.0 (-1.4 to -0.7)	-14.2 (-18.8 to -9.6)
**Indicator 5.2.2: Age-standardised prevalence of women aged 15 years and older who experienced physical or sexual violence by non-intimate partner in the last 12 months (%)**						
Central Europe, Eastern Europe, and Central Asia						
Central Asia						
Armenia	0.1 (0.1 to 0.1)	0.1 (0.1 to 0.1)	0.1 (0.1 to 0.1)	0.1 (0.1 to 0.1)	0.6 (0.2 to 1.0)	9.2 (2.8 to 16.1)
Azerbaijan	0.1 (0.1 to 0.1)	0.1 (0.1 to 0.1)	0.1 (0.1 to 0.1)	0.1 (0.1 to 0.1)	0.5 (0.1 to 1.0)	8.1 (1.3 to 16.0)
Georgia	0.1 (0.1 to 0.1)	0.1 (0.1 to 0.1)	0.1 (0.1 to 0.1)	0.1 (0.1 to 0.1)	0.6 (0.0 to 1.2)	9.5 (0.1 to 18.9)
Kazakhstan	0.2 (0.2 to 0.3)	0.2 (0.2 to 0.3)	0.2 (0.2 to 0.3)	0.2 (0.2 to 0.3)	0.5 (0.1 to 0.9)	7.7 (0.8 to 14.9)
Kyrgyzstan	0.0 (0.0 to 0.0)	0.0 (0.0 to 0.0)	0.0 (0.0 to 0.0)	0.0 (0.0 to 0.1)	0.3 (-0.4 to 0.8)	4.8 (-5.3 to 13.0)
Mongolia	0.1 (0.1 to 0.1)	0.1 (0.1 to 0.1)	0.1 (0.1 to 0.1)	0.1 (0.1 to 0.1)	0.5 (0.1 to 1.0)	7.9 (1.6 to 16.3)
Tajikistan	0.0 (0.0 to 0.1)	0.0 (0.0 to 0.1)	0.0 (0.0 to 0.1)	0.0 (0.0 to 0.1)	0.4 (-0.1 to 0.9)	6.9 (-1.1 to 14.8)
Turkmenistan	0.1 (0.1 to 0.1)	0.1 (0.1 to 0.1)	0.1 (0.1 to 0.1)	0.1 (0.1 to 0.1)	0.6 (0.2 to 1.1)	9.5 (2.4 to 17.3)
Uzbekistan	0.1 (0.1 to 0.1)	0.1 (0.1 to 0.1)	0.1 (0.1 to 0.1)	0.1 (0.1 to 0.1)	0.7 (0.2 to 1.1)	10.4 (2.8 to 17.4)
Central Europe						
Albania	0.4 (0.3 to 0.5)	0.4 (0.3 to 0.6)	0.4 (0.3 to 0.6)	0.4 (0.3 to 0.6)	0.4 (-0.2 to 0.8)	5.5 (-2.5 to 13.3)
Bosnia and Herzegovina	0.6 (0.5 to 0.7)	0.7 (0.5 to 0.8)	0.7 (0.5 to 0.8)	0.7 (0.6 to 0.9)	0.7 (0.2 to 1.2)	11.4 (3.8 to 19.1)
Bulgaria	1.1 (0.7 to 1.6)	1.3 (0.9 to 1.9)	1.3 (0.9 to 1.9)	1.4 (1.0 to 2.1)	0.7 (0.2 to 1.2)	10.8 (2.8 to 20.3)
Croatia	0.5 (0.3 to 0.7)	0.5 (0.4 to 0.7)	0.5 (0.4 to 0.7)	0.6 (0.4 to 0.8)	0.7 (0.3 to 1.2)	11.1 (3.9 to 19.8)
Czech Republic	1.9 (1.5 to 2.3)	1.8 (1.5 to 2.3)	1.9 (1.5 to 2.3)	1.9 (1.5 to 2.4)	0.3 (-0.2 to 0.7)	4.0 (-2.4 to 11.4)
Hungary	0.9 (0.7 to 1.1)	0.9 (0.8 to 1.2)	0.9 (0.8 to 1.2)	1.0 (0.8 to 1.3)	0.5 (0.0 to 1.0)	7.3 (0.1 to 15.9)
Macedonia	0.6 (0.5 to 0.8)	0.7 (0.6 to 0.9)	0.7 (0.6 to 0.9)	0.8 (0.6 to 0.9)	0.5 (0.2 to 0.9)	8.5 (2.4 to 14.6)
Montenegro	0.7 (0.5 to 0.8)	0.7 (0.6 to 0.9)	0.7 (0.6 to 0.9)	0.8 (0.6 to 1.0)	0.5 (0.0 to 0.9)	7.5 (0.4 to 14.7)
Poland	0.8 (0.7 to 1.1)	0.9 (0.7 to 1.1)	0.9 (0.7 to 1.1)	1.0 (0.8 to 1.2)	0.6 (0.1 to 1.1)	9.4 (1.1 to 18.3)
Romania	0.3 (0.2 to 0.5)	0.4 (0.2 to 0.5)	0.3 (0.2 to 0.5)	0.4 (0.3 to 0.5)	0.4 (-0.2 to 0.8)	5.8 (-2.3 to 13.1)
Serbia	0.6 (0.5 to 0.8)	0.7 (0.6 to 0.8)	0.7 (0.6 to 0.9)	0.7 (0.6 to 0.9)	0.5 (0.1 to 1.0)	8.2 (1.2 to 15.5)
Slovakia	1.1 (0.8 to 1.3)	1.1 (0.9 to 1.4)	1.1 (0.9 to 1.4)	1.2 (1.0 to 1.5)	0.5 (0.1 to 0.9)	7.6 (0.8 to 14.5)
Slovenia	0.5 (0.4 to 0.7)	0.6 (0.4 to 0.7)	0.6 (0.4 to 0.7)	0.6 (0.5 to 0.8)	0.4 (-0.0 to 0.9)	6.0 (-0.3 to 14.4)
Eastern Europe						
Belarus	0.7 (0.6 to 0.9)	0.8 (0.7 to 1.0)	0.8 (0.7 to 1.0)	0.9 (0.7 to 1.1)	0.7 (0.3 to 1.1)	10.8 (5.1 to 17.8)
Estonia	1.3 (1.0 to 1.6)	1.4 (1.1 to 1.7)	1.4 (1.1 to 1.8)	1.5 (1.2 to 1.9)	0.6 (0.2 to 1.1)	9.7 (3.4 to 17.1)
Latvia	1.3 (1.0 to 1.6)	1.4 (1.1 to 1.7)	1.4 (1.1 to 1.7)	1.4 (1.1 to 1.7)	0.4 (0.0 to 0.7)	5.8 (0.6 to 11.3)
Lithuania	0.4 (0.3 to 0.6)	0.5 (0.4 to 0.6)	0.5 (0.4 to 0.6)	0.5 (0.4 to 0.7)	0.5 (0.1 to 0.9)	8.1 (1.5 to 14.9)
Moldova	0.6 (0.5 to 0.8)	0.6 (0.5 to 0.9)	0.7 (0.5 to 0.9)	0.7 (0.5 to 0.9)	0.6 (0.1 to 1.0)	9.2 (1.8 to 16.4)
Russia	0.8 (0.7 to 1.0)	0.9 (0.7 to 1.1)	0.9 (0.7 to 1.1)	0.9 (0.8 to 1.2)	0.4 (0.4 to 0.5)	6.5 (5.4 to 7.8)
Ukraine	0.8 (0.6 to 1.0)	0.8 (0.7 to 1.0)	0.8 (0.7 to 1.0)	0.9 (0.7 to 1.1)	0.4 (-0.0 to 0.7)	5.8 (-0.2 to 11.8)
High-income						
Australasia						
Australia	3.1 (2.4 to 3.9)	3.2 (2.5 to 4.0)	3.2 (2.5 to 4.1)	3.3 (2.5 to 4.3)	0.3 (-0.2 to 0.7)	4.4 (-2.2 to 11.7)
New Zealand	1.9 (1.4 to 2.4)	1.8 (1.4 to 2.3)	1.8 (1.4 to 2.3)	1.8 (1.4 to 2.3)	-0.1 (-0.4 to 0.3)	-1.1 (-6.4 to 5.4)
High-income Asia Pacific						
Brunei	1.2 (1.0 to 1.6)	1.3 (1.0 to 1.7)	1.3 (1.0 to 1.7)	1.4 (1.1 to 1.8)	0.4 (0.0 to 1.0)	7.0 (0.3 to 16.7)
Japan	1.3 (1.1 to 1.7)	1.4 (1.1 to 1.7)	1.4 (1.1 to 1.7)	1.4 (1.1 to 1.8)	0.2 (0.1 to 0.3)	3.6 (1.8 to 5.3)
South Korea	1.3 (1.0 to 1.6)	1.3 (1.1 to 1.7)	1.4 (1.1 to 1.7)	1.4 (1.1 to 1.9)	0.5 (0.1 to 0.8)	7.5 (1.3 to 13.4)
Singapore	1.3 (1.0 to 1.6)	1.4 (1.1 to 1.7)	1.4 (1.1 to 1.7)	1.4 (1.1 to 1.9)	0.4 (0.0 to 0.9)	6.6 (0.7 to 13.7)
High-income North America						
Canada	1.1 (0.9 to 1.4)	1.2 (0.9 to 1.6)	1.2 (0.9 to 1.6)	1.3 (1.0 to 1.7)	0.5 (-0.1 to 1.0)	7.4 (-0.8 to 15.9)
Greenland	1.4 (1.1 to 1.8)	1.5 (1.1 to 1.9)	1.5 (1.1 to 1.9)	1.6 (1.2 to 2.0)	0.4 (-0.0 to 0.7)	5.6 (-0.3 to 11.0)
United States	2.2 (1.7 to 2.9)	2.3 (1.8 to 3.0)	2.3 (1.8 to 3.0)	2.4 (1.8 to 3.1)	0.2 (-0.1 to 0.4)	2.6 (-1.4 to 6.7)
Southern Latin America						
Argentina	0.4 (0.3 to 0.6)	0.4 (0.3 to 0.7)	0.4 (0.3 to 0.7)	0.5 (0.3 to 0.8)	0.6 (0.0 to 1.2)	9.4 (0.5 to 19.8)
Chile	0.5 (0.4 to 0.6)	0.5 (0.4 to 0.7)	0.6 (0.4 to 0.7)	0.6 (0.5 to 0.8)	0.7 (0.3 to 1.1)	11.3 (4.0 to 18.3)
Uruguay	0.7 (0.5 to 0.9)	0.7 (0.5 to 1.0)	0.7 (0.5 to 1.0)	0.8 (0.6 to 1.1)	0.8 (0.3 to 1.3)	13.1 (4.4 to 21.8)
Western Europe						
Andorra	1.4 (1.1 to 1.7)	1.4 (1.1 to 1.7)	1.4 (1.1 to 1.8)	1.4 (1.1 to 1.9)	0.2 (-0.3 to 0.6)	2.4 (-4.2 to 8.9)
Austria	2.0 (1.6 to 2.4)	2.1 (1.6 to 2.6)	2.1 (1.6 to 2.6)	2.2 (1.7 to 2.8)	0.3 (-0.1 to 0.7)	5.1 (-0.8 to 11.4)
Belgium	1.2 (0.9 to 1.5)	1.2 (1.0 to 1.5)	1.2 (1.0 to 1.5)	1.3 (1.0 to 1.6)	0.4 (-0.0 to 0.8)	5.9 (-0.5 to 12.9)
Cyprus	0.7 (0.5 to 0.8)	0.7 (0.6 to 0.9)	0.7 (0.6 to 0.9)	0.8 (0.6 to 0.9)	0.3 (-0.1 to 0.8)	5.4 (-1.1 to 12.8)
Denmark	1.1 (0.9 to 1.4)	1.1 (0.9 to 1.4)	1.1 (0.9 to 1.4)	1.2 (0.9 to 1.5)	0.2 (-0.2 to 0.6)	3.1 (-2.4 to 9.1)
Finland	2.0 (1.6 to 2.5)	2.1 (1.7 to 2.5)	2.1 (1.7 to 2.6)	2.2 (1.8 to 2.7)	0.4 (-0.1 to 0.9)	5.7 (-1.2 to 13.7)
France	1.5 (1.2 to 1.9)	1.6 (1.2 to 1.9)	1.6 (1.3 to 1.9)	1.6 (1.3 to 2.1)	0.4 (-0.1 to 0.8)	5.5 (-0.8 to 12.8)
Germany	1.1 (0.8 to 1.6)	1.0 (0.7 to 1.5)	1.0 (0.7 to 1.5)	1.1 (0.7 to 1.5)	0.1 (-0.6 to 0.7)	1.5 (-7.9 to 10.8)
Greece	1.1 (0.9 to 1.4)	1.1 (0.9 to 1.4)	1.1 (0.9 to 1.4)	1.1 (0.9 to 1.4)	0.2 (-0.1 to 0.6)	3.0 (-2.0 to 8.9)
Iceland	1.3 (1.1 to 1.7)	1.4 (1.1 to 1.8)	1.4 (1.1 to 1.8)	1.5 (1.2 to 1.9)	0.3 (-0.0 to 0.8)	5.3 (-0.7 to 13.1)
Ireland	0.8 (0.7 to 1.1)	0.9 (0.7 to 1.1)	0.9 (0.7 to 1.1)	0.9 (0.8 to 1.2)	0.6 (0.1 to 1.0)	8.9 (1.8 to 16.9)
Israel	1.2 (1.0 to 1.6)	1.3 (1.0 to 1.6)	1.3 (1.0 to 1.6)	1.3 (1.0 to 1.6)	0.2 (-0.2 to 0.6)	3.8 (-2.5 to 10.0)
Italy	3.5 (2.8 to 4.4)	3.5 (2.8 to 4.3)	3.5 (2.8 to 4.4)	3.5 (2.8 to 4.5)	0.1 (-0.3 to 0.6)	1.7 (-4.6 to 8.9)
Luxembourg	1.7 (1.3 to 2.1)	1.7 (1.4 to 2.1)	1.7 (1.4 to 2.1)	1.8 (1.4 to 2.2)	0.2 (-0.2 to 0.6)	3.5 (-2.6 to 9.7)
Malta	1.2 (0.9 to 1.6)	1.3 (1.0 to 1.6)	1.3 (1.0 to 1.7)	1.4 (1.0 to 1.8)	0.5 (-0.0 to 1.0)	7.9 (-0.4 to 15.9)
Netherlands	2.1 (1.7 to 2.6)	2.2 (1.8 to 2.7)	2.2 (1.8 to 2.8)	2.4 (1.9 to 3.0)	0.4 (-0.1 to 0.9)	6.1 (-1.4 to 14.1)
Norway	1.4 (1.1 to 1.7)	1.4 (1.2 to 1.8)	1.4 (1.2 to 1.8)	1.5 (1.2 to 1.9)	0.3 (0.2 to 0.5)	5.2 (3.4 to 7.2)
Portugal	0.7 (0.5 to 0.9)	0.7 (0.6 to 1.0)	0.8 (0.6 to 1.0)	0.9 (0.7 to 1.1)	1.1 (0.5 to 1.5)	17.4 (7.4 to 25.8)
Spain	1.0 (0.8 to 1.3)	1.1 (0.9 to 1.4)	1.1 (0.8 to 1.4)	1.1 (0.9 to 1.5)	0.4 (-0.1 to 0.8)	5.6 (-0.9 to 12.8)
Sweden	1.9 (1.5 to 2.3)	1.8 (1.5 to 2.2)	1.8 (1.5 to 2.2)	1.8 (1.4 to 2.2)	-0.1 (-0.4 to 0.2)	-1.3 (-6.0 to 3.7)
Switzerland	1.1 (0.9 to 1.3)	1.1 (0.9 to 1.4)	1.1 (0.9 to 1.4)	1.2 (0.9 to 1.5)	0.2 (-0.2 to 0.7)	3.7 (-2.6 to 10.7)
United Kingdom	1.1 (0.8 to 1.3)	1.1 (0.8 to 1.3)	1.1 (0.8 to 1.3)	1.1 (0.8 to 1.3)	0.0 (-0.1 to 0.1)	0.1 (-1.3 to 1.5)
Latin America and Caribbean						

	Estimate in 2000 (95% UIs)	Estimate in 2015 (95% UIs)	Estimate in 2017 (95% UIs)	Estimate in 2030 (95% UIs)	Annualised rate of change, 2015-2030 (95% UIs)	Percent change, 2015-2030 (95% UIs)
Andean Latin America						
Bolivia	0.9 (0.7 to 1.1)	0.9 (0.7 to 1.2)	1.0 (0.7 to 1.2)	1.1 (0.8 to 1.4)	0.9 (0.3 to 1.4)	14.2 (4.4 to 24.2)
Ecuador	0.6 (0.5 to 0.8)	0.7 (0.5 to 0.9)	0.7 (0.6 to 0.9)	0.8 (0.6 to 1.0)	0.7 (0.2 to 1.1)	10.6 (2.8 to 18.2)
Peru	0.5 (0.4 to 0.7)	0.6 (0.4 to 0.8)	0.6 (0.4 to 0.8)	0.6 (0.4 to 0.9)	0.6 (-0.0 to 1.2)	9.4 (-0.5 to 20.4)
Caribbean						
Antigua and Barbuda	1.8 (1.4 to 2.4)	2.0 (1.5 to 2.5)	2.0 (1.5 to 2.5)	2.1 (1.6 to 2.7)	0.4 (-0.0 to 0.9)	5.8 (-0.5 to 14.4)
The Bahamas	1.9 (1.5 to 2.5)	2.1 (1.6 to 2.7)	2.1 (1.6 to 2.7)	2.2 (1.7 to 2.8)	0.3 (-0.2 to 0.7)	4.1 (-2.7 to 10.3)
Barbados	1.9 (1.5 to 2.4)	2.0 (1.6 to 2.6)	2.0 (1.6 to 2.6)	2.1 (1.6 to 2.8)	0.3 (-0.1 to 0.7)	5.4 (-1.9 to 11.1)
Belize	0.5 (0.4 to 0.7)	0.6 (0.4 to 0.8)	0.6 (0.4 to 0.8)	0.6 (0.5 to 0.9)	0.7 (0.1 to 1.2)	10.7 (1.5 to 19.8)
Bermuda	2.0 (1.6 to 2.6)	2.2 (1.7 to 2.8)	2.2 (1.7 to 2.9)	2.3 (1.8 to 3.0)	0.3 (-0.2 to 0.8)	4.9 (-2.3 to 13.3)
Cuba	1.7 (1.3 to 2.2)	1.9 (1.5 to 2.4)	1.9 (1.5 to 2.5)	2.0 (1.5 to 2.6)	0.5 (0.1 to 0.9)	7.8 (2.0 to 13.8)
Dominica	1.7 (1.3 to 2.2)	1.9 (1.5 to 2.4)	1.9 (1.5 to 2.4)	2.0 (1.6 to 2.6)	0.5 (0.1 to 0.9)	8.0 (2.2 to 14.4)
Dominican Republic	0.5 (0.4 to 0.7)	0.6 (0.5 to 0.7)	0.6 (0.5 to 0.7)	0.6 (0.5 to 0.8)	0.5 (0.0 to 1.0)	8.2 (0.2 to 15.9)
Grenada	1.7 (1.3 to 2.1)	1.8 (1.4 to 2.3)	1.8 (1.4 to 2.3)	1.9 (1.5 to 2.5)	0.5 (0.1 to 1.1)	8.3 (1.0 to 17.1)
Guyana	1.5 (1.2 to 2.0)	1.7 (1.3 to 2.1)	1.7 (1.3 to 2.2)	1.8 (1.4 to 2.4)	0.6 (0.2 to 1.0)	9.0 (2.3 to 16.0)
Haiti	6.3 (4.9 to 8.2)	7.0 (5.7 to 8.4)	7.0 (5.8 to 8.5)	7.6 (6.2 to 9.3)	0.6 (-0.0 to 1.2)	9.5 (-0.1 to 19.2)
Jamaica	2.1 (1.6 to 2.7)	2.3 (1.8 to 2.9)	2.3 (1.8 to 3.0)	2.5 (1.9 to 3.2)	0.6 (0.1 to 0.9)	9.0 (1.9 to 15.1)
Puerto Rico	2.9 (2.3 to 3.8)	3.1 (2.4 to 3.9)	3.1 (2.4 to 4.0)	3.3 (2.5 to 4.3)	0.5 (0.0 to 1.1)	7.7 (0.1 to 17.5)
Saint Lucia	1.7 (1.3 to 2.2)	1.8 (1.4 to 2.3)	1.8 (1.4 to 2.4)	2.0 (1.5 to 2.5)	0.5 (0.0 to 1.0)	7.7 (0.7 to 15.7)
Saint Vincent and the Grenadines	1.6 (1.2 to 2.0)	1.7 (1.3 to 2.2)	1.7 (1.3 to 2.2)	1.9 (1.4 to 2.5)	0.5 (0.1 to 0.9)	8.3 (1.2 to 15.2)
Suriname	1.6 (1.3 to 2.1)	1.8 (1.4 to 2.3)	1.8 (1.4 to 2.3)	1.9 (1.5 to 2.5)	0.4 (-0.0 to 0.9)	6.8 (-0.2 to 13.9)
Trinidad and Tobago	1.8 (1.4 to 2.3)	1.9 (1.5 to 2.5)	1.9 (1.5 to 2.5)	2.0 (1.6 to 2.6)	0.3 (-0.1 to 0.7)	4.4 (-1.8 to 11.7)
Virgin Islands, U.S.	2.2 (1.7 to 2.9)	2.5 (1.9 to 3.1)	2.4 (1.9 to 3.1)	2.5 (2.0 to 3.3)	0.3 (-0.2 to 0.8)	3.9 (-3.5 to 12.9)
Central Latin America						
Colombia	0.6 (0.5 to 0.8)	0.6 (0.5 to 0.8)	0.7 (0.5 to 0.8)	0.7 (0.5 to 0.9)	0.8 (-0.1 to 1.4)	12.2 (-1.2 to 23.5)
Costa Rica	4.8 (3.8 to 6.0)	5.2 (4.1 to 6.7)	5.3 (4.1 to 6.8)	5.7 (4.4 to 7.4)	0.6 (0.1 to 0.9)	9.2 (2.2 to 15.2)
El Salvador	1.2 (0.9 to 1.5)	1.3 (1.0 to 1.7)	1.3 (1.1 to 1.7)	1.5 (1.1 to 1.9)	0.8 (0.2 to 1.4)	12.1 (2.9 to 22.6)
Guatemala	0.5 (0.4 to 0.7)	0.6 (0.5 to 0.7)	0.6 (0.5 to 0.8)	0.6 (0.5 to 0.8)	0.5 (0.0 to 1.1)	8.5 (0.7 to 18.0)
Honduras	0.9 (0.7 to 1.1)	0.9 (0.7 to 1.2)	0.9 (0.8 to 1.2)	1.0 (0.8 to 1.3)	0.7 (-0.0 to 1.3)	10.6 (-0.4 to 21.8)
Mexico	0.8 (0.7 to 1.1)	0.8 (0.7 to 1.1)	0.8 (0.7 to 1.1)	0.8 (0.7 to 1.1)	0.1 (-0.0 to 0.3)	1.5 (-0.4 to 4.0)
Nicaragua	0.9 (0.7 to 1.2)	1.0 (0.8 to 1.3)	1.0 (0.8 to 1.3)	1.1 (0.9 to 1.5)	0.7 (0.3 to 1.2)	10.8 (4.8 to 19.0)
Panama	1.1 (0.9 to 1.4)	1.2 (0.9 to 1.5)	1.2 (1.0 to 1.6)	1.3 (1.0 to 1.8)	0.7 (0.2 to 1.1)	10.8 (3.1 to 18.2)
Venezuela	1.1 (0.9 to 1.4)	1.2 (0.9 to 1.5)	1.2 (0.9 to 1.5)	1.3 (1.0 to 1.6)	0.4 (-0.1 to 1.0)	6.8 (-1.1 to 15.7)
Tropical Latin America						
Brazil	0.4 (0.3 to 0.5)	0.4 (0.3 to 0.5)	0.4 (0.3 to 0.6)	0.5 (0.4 to 0.6)	1.0 (0.8 to 1.1)	15.6 (12.9 to 18.2)
Paraguay	0.7 (0.5 to 0.8)	0.7 (0.6 to 0.9)	0.7 (0.6 to 0.9)	0.8 (0.6 to 1.1)	0.6 (0.1 to 1.1)	9.4 (1.0 to 17.1)
North Africa and Middle East						
North Africa and Middle East						
Afghanistan	0.2 (0.2 to 0.3)	0.3 (0.2 to 0.4)	0.3 (0.2 to 0.4)	0.3 (0.2 to 0.4)	0.9 (0.5 to 1.3)	13.9 (7.1 to 21.4)
Algeria	0.4 (0.3 to 0.5)	0.5 (0.3 to 0.6)	0.5 (0.3 to 0.6)	0.5 (0.4 to 0.7)	0.8 (0.3 to 1.2)	12.3 (4.4 to 20.0)
Bahrain	0.4 (0.3 to 0.6)	0.5 (0.4 to 0.6)	0.5 (0.4 to 0.6)	0.5 (0.4 to 0.6)	0.4 (-0.1 to 0.8)	5.7 (-1.6 to 13.4)
Egypt	0.4 (0.3 to 0.5)	0.4 (0.3 to 0.5)	0.4 (0.3 to 0.5)	0.4 (0.3 to 0.6)	0.7 (0.3 to 1.2)	10.9 (4.1 to 19.8)
Iran	0.4 (0.3 to 0.5)	0.5 (0.4 to 0.6)	0.5 (0.4 to 0.6)	0.5 (0.4 to 0.7)	0.7 (0.5 to 0.8)	10.3 (8.3 to 12.4)
Iraq	0.3 (0.3 to 0.5)	0.4 (0.3 to 0.5)	0.4 (0.3 to 0.5)	0.4 (0.3 to 0.6)	0.8 (0.3 to 1.3)	12.2 (4.7 to 21.8)
Jordan	0.4 (0.3 to 0.5)	0.5 (0.3 to 0.6)	0.5 (0.3 to 0.6)	0.5 (0.4 to 0.7)	0.7 (0.2 to 1.1)	11.6 (3.2 to 18.6)
Kuwait	0.4 (0.3 to 0.6)	0.5 (0.4 to 0.6)	0.5 (0.4 to 0.7)	0.6 (0.4 to 0.7)	0.8 (0.3 to 1.2)	12.0 (4.5 to 19.8)
Lebanon	0.4 (0.3 to 0.5)	0.5 (0.4 to 0.6)	0.5 (0.4 to 0.6)	0.5 (0.4 to 0.7)	0.8 (0.3 to 1.3)	13.2 (5.2 to 22.1)
Libya	0.5 (0.4 to 0.6)	0.5 (0.4 to 0.6)	0.5 (0.4 to 0.6)	0.5 (0.4 to 0.7)	0.1 (-0.4 to 0.5)	1.2 (-5.6 to 8.4)
Morocco	0.3 (0.3 to 0.5)	0.4 (0.3 to 0.5)	0.4 (0.3 to 0.5)	0.4 (0.3 to 0.6)	0.8 (0.4 to 1.3)	13.0 (5.7 to 21.3)
Palestine	0.3 (0.2 to 0.4)	0.4 (0.3 to 0.5)	0.4 (0.3 to 0.5)	0.4 (0.3 to 0.5)	0.9 (0.5 to 1.3)	14.0 (7.5 to 21.6)
Oman	0.4 (0.3 to 0.5)	0.5 (0.4 to 0.6)	0.5 (0.4 to 0.6)	0.6 (0.4 to 0.7)	1.0 (0.5 to 1.5)	16.6 (8.1 to 25.3)
Qatar	0.5 (0.3 to 0.6)	0.5 (0.4 to 0.6)	0.5 (0.4 to 0.6)	0.5 (0.4 to 0.7)	0.6 (0.1 to 1.0)	8.8 (2.2 to 16.5)
Saudi Arabia	0.4 (0.3 to 0.5)	0.5 (0.4 to 0.6)	0.5 (0.4 to 0.7)	0.6 (0.5 to 0.8)	1.3 (0.8 to 1.7)	21.0 (12.5 to 29.6)
Sudan	0.3 (0.2 to 0.4)	0.3 (0.3 to 0.5)	0.3 (0.3 to 0.5)	0.4 (0.3 to 0.5)	1.2 (0.8 to 1.6)	19.6 (12.0 to 27.6)
Syria	0.3 (0.3 to 0.5)	0.4 (0.3 to 0.5)	0.4 (0.3 to 0.5)	0.5 (0.3 to 0.6)	0.9 (0.3 to 1.3)	14.5 (5.2 to 21.8)
Tunisia	0.4 (0.3 to 0.5)	0.4 (0.3 to 0.6)	0.4 (0.3 to 0.6)	0.5 (0.4 to 0.6)	0.8 (0.4 to 1.3)	12.8 (6.2 to 21.7)
Turkey	0.4 (0.3 to 0.5)	0.5 (0.4 to 0.6)	0.5 (0.4 to 0.6)	0.5 (0.4 to 0.7)	0.8 (0.3 to 1.3)	12.6 (4.8 to 20.8)
United Arab Emirates	0.5 (0.4 to 0.6)	0.5 (0.4 to 0.7)	0.5 (0.4 to 0.7)	0.5 (0.4 to 0.7)	0.4 (-0.0 to 0.9)	6.6 (-0.7 to 14.8)
Yemen	0.3 (0.2 to 0.4)	0.3 (0.3 to 0.4)	0.3 (0.3 to 0.4)	0.4 (0.3 to 0.5)	0.9 (0.4 to 1.4)	14.4 (6.8 to 23.5)
South Asia						
South Asia						
Bangladesh	0.1 (0.1 to 0.2)	0.1 (0.1 to 0.2)	0.1 (0.1 to 0.2)	0.2 (0.1 to 0.2)	0.9 (0.2 to 1.4)	14.2 (2.5 to 24.3)
Bhutan	0.1 (0.1 to 0.2)	0.2 (0.1 to 0.2)	0.2 (0.1 to 0.2)	0.2 (0.1 to 0.2)	1.0 (0.3 to 1.6)	15.5 (5.3 to 27.1)
India	0.1 (0.1 to 0.2)	0.1 (0.1 to 0.2)	0.1 (0.1 to 0.2)	0.1 (0.1 to 0.2)	0.1 (-0.3 to 0.4)	1.0 (-3.8 to 6.1)
Nepal	0.1 (0.1 to 0.1)	0.1 (0.1 to 0.2)	0.1 (0.1 to 0.2)	0.1 (0.1 to 0.2)	0.9 (0.4 to 1.5)	15.3 (6.0 to 24.8)
Pakistan	0.1 (0.1 to 0.2)	0.1 (0.1 to 0.2)	0.1 (0.1 to 0.2)	0.2 (0.1 to 0.2)	0.8 (0.3 to 1.4)	13.4 (3.9 to 23.8)
Southeast Asia, East Asia, and Oceania						
East Asia						
China	1.3 (1.0 to 1.7)	1.5 (1.2 to 1.9)	1.5 (1.2 to 1.9)	1.7 (1.3 to 2.1)	0.7 (0.6 to 0.8)	11.3 (9.6 to 13.1)
North Korea	1.2 (0.9 to 1.5)	1.2 (0.9 to 1.6)	1.2 (0.9 to 1.6)	1.2 (0.9 to 1.6)	0.2 (-0.2 to 0.6)	2.8 (-3.1 to 10.1)
Taiwan (Province of China)	1.6 (1.2 to 2.1)	1.8 (1.4 to 2.3)	1.8 (1.4 to 2.3)	1.9 (1.5 to 2.5)	0.5 (0.1 to 1.0)	8.6 (1.6 to 16.9)
Oceania						
American Samoa	0.9 (0.7 to 1.2)	1.0 (0.8 to 1.3)	1.0 (0.8 to 1.3)	1.1 (0.8 to 1.4)	0.5 (0.0 to 0.9)	7.5 (0.4 to 14.6)
Federated States of Micronesia	0.8 (0.6 to 1.0)	0.9 (0.7 to 1.1)	0.9 (0.7 to 1.1)	0.9 (0.7 to 1.2)	0.5 (-0.0 to 1.0)	7.8 (-0.1 to 15.7)
Fiji	0.9 (0.7 to 1.1)	0.9 (0.7 to 1.2)	0.9 (0.7 to 1.2)	1.0 (0.8 to 1.3)	0.5 (0.0 to 0.9)	8.0 (0.1 to 14.9)
Guam	1.1 (0.8 to 1.4)	1.1 (0.9 to 1.4)	1.1 (0.9 to 1.5)	1.2 (0.9 to 1.6)	0.5 (-0.0 to 0.9)	7.4 (-0.7 to 14.6)
Kiribati	0.7 (0.5 to 0.9)	0.7 (0.6 to 0.9)	0.7 (0.6 to 0.9)	0.8 (0.6 to 1.0)	0.5 (-0.0 to 0.9)	7.5 (-0.2 to 14.5)
Marshall Islands	0.8 (0.6 to 1.0)	0.8 (0.7 to 1.1)	0.8 (0.7 to 1.1)	0.9 (0.7 to 1.2)	0.7 (0.1 to 1.1)	11.1 (2.1 to 18.1)
Northern Mariana Islands	1.1 (0.8 to 1.4)	1.1 (0.8 to 1.4)	1.1 (0.8 to 1.4)	1.1 (0.8 to 1.4)	0.1 (-0.3 to 0.6)	1.6 (-4.7 to 8.7)
Papua New Guinea	0.7 (0.5 to 0.9)	0.7 (0.6 to 0.9)	0.7 (0.6 to 0.9)	0.8 (0.6 to 1.0)	0.6 (0.1 to 1.1)	9.0 (1.9 to 18.4)

	Estimate in 2000 (95% UIs)	Estimate in 2015 (95% UIs)	Estimate in 2017 (95% UIs)	Estimate in 2030 (95% UIs)	Annualised rate of change, 2015-2030 (95% UIs)	Percent change, 2015-2030 (95% UIs)
Samoa	0.8 (0.6 to 1.1)	0.9 (0.7 to 1.1)	0.9 (0.7 to 1.1)	0.9 (0.7 to 1.2)	0.3 (-0.3 to 0.8)	4.8 (-4.1 to 13.0)
Solomon Islands	0.7 (0.5 to 0.9)	0.7 (0.6 to 0.9)	0.7 (0.6 to 0.9)	0.8 (0.6 to 1.0)	0.6 (0.2 to 1.1)	9.1 (2.4 to 17.4)
Tonga	0.9 (0.7 to 1.1)	0.9 (0.7 to 1.2)	0.9 (0.7 to 1.2)	1.0 (0.8 to 1.3)	0.5 (0.1 to 0.9)	8.4 (1.2 to 15.0)
Vanuatu	0.7 (0.6 to 0.9)	0.8 (0.6 to 1.0)	0.8 (0.6 to 1.0)	0.8 (0.6 to 1.1)	0.5 (0.0 to 1.0)	7.5 (0.3 to 15.5)
Southeast Asia						
Cambodia	0.7 (0.5 to 0.9)	0.8 (0.7 to 1.1)	0.8 (0.7 to 1.1)	1.0 (0.8 to 1.3)	1.0 (0.4 to 1.6)	16.8 (6.5 to 26.9)
Indonesia	2.0 (1.6 to 2.6)	2.2 (1.7 to 2.8)	2.3 (1.8 to 2.9)	2.5 (1.9 to 3.1)	0.7 (0.5 to 0.8)	10.3 (8.4 to 12.5)
Laos	0.5 (0.4 to 0.6)	0.6 (0.4 to 0.7)	0.6 (0.4 to 0.7)	0.7 (0.5 to 0.8)	1.1 (0.7 to 1.6)	17.9 (11.0 to 26.4)
Malaysia	0.7 (0.5 to 0.9)	0.7 (0.6 to 0.9)	0.8 (0.6 to 1.0)	0.8 (0.6 to 1.1)	0.8 (0.3 to 1.3)	13.2 (5.1 to 21.9)
Maldives	0.6 (0.4 to 0.7)	0.7 (0.5 to 0.8)	0.7 (0.5 to 0.8)	0.8 (0.6 to 1.0)	1.1 (0.7 to 1.5)	17.6 (10.3 to 25.0)
Mauritius	0.6 (0.5 to 0.8)	0.7 (0.6 to 0.9)	0.7 (0.6 to 0.9)	0.8 (0.6 to 1.0)	0.8 (0.3 to 1.2)	12.5 (4.8 to 20.5)
Myanmar	0.1 (0.1 to 0.2)	0.1 (0.1 to 0.2)	0.2 (0.1 to 0.2)	0.2 (0.1 to 0.2)	1.1 (0.5 to 1.7)	17.5 (8.6 to 28.3)
Philippines	0.8 (0.6 to 1.0)	0.8 (0.6 to 1.0)	0.8 (0.6 to 1.1)	0.8 (0.6 to 1.1)	0.3 (-0.5 to 0.8)	4.1 (-6.8 to 13.0)
Sri Lanka	0.3 (0.2 to 0.5)	0.4 (0.3 to 0.6)	0.4 (0.3 to 0.6)	0.5 (0.3 to 0.7)	1.1 (0.5 to 1.7)	18.8 (7.3 to 29.1)
Seychelles	0.6 (0.5 to 0.8)	0.7 (0.5 to 0.9)	0.7 (0.5 to 0.9)	0.8 (0.6 to 1.0)	0.6 (0.2 to 1.1)	10.0 (2.4 to 18.1)
Thailand	0.6 (0.5 to 0.8)	0.7 (0.5 to 0.9)	0.7 (0.5 to 0.9)	0.8 (0.6 to 1.0)	0.8 (0.4 to 1.2)	13.2 (6.1 to 19.1)
Timor-Leste	0.5 (0.3 to 0.6)	0.6 (0.4 to 0.7)	0.6 (0.4 to 0.7)	0.7 (0.5 to 0.8)	1.1 (0.6 to 1.5)	18.1 (9.3 to 25.0)
Vietnam	0.5 (0.4 to 0.7)	0.6 (0.5 to 0.8)	0.6 (0.5 to 0.8)	0.7 (0.5 to 0.9)	1.0 (0.5 to 1.5)	15.9 (7.8 to 24.8)
Sub-Saharan Africa						
Central Sub-Saharan Africa						
Angola	1.0 (0.8 to 1.2)	1.2 (0.9 to 1.5)	1.2 (0.9 to 1.5)	1.4 (1.1 to 1.8)	1.2 (0.8 to 1.7)	20.0 (12.4 to 29.9)
Central African Republic	0.9 (0.7 to 1.2)	1.0 (0.8 to 1.3)	1.0 (0.8 to 1.3)	1.1 (0.8 to 1.5)	0.5 (0.1 to 1.0)	8.3 (1.6 to 15.6)
Congo	1.2 (0.9 to 1.5)	1.4 (1.0 to 1.7)	1.4 (1.1 to 1.8)	1.6 (1.2 to 2.0)	0.9 (0.4 to 1.3)	14.9 (7.0 to 22.4)
Democratic Republic of the Congo	0.8 (0.6 to 1.1)	0.9 (0.7 to 1.2)	0.9 (0.7 to 1.2)	1.0 (0.7 to 1.3)	0.8 (0.3 to 1.2)	13.0 (3.8 to 20.1)
Equatorial Guinea	1.1 (0.8 to 1.4)	1.4 (1.1 to 1.8)	1.5 (1.1 to 1.9)	1.8 (1.4 to 2.4)	1.6 (1.1 to 2.1)	27.5 (18.2 to 36.8)
Gabon	1.5 (1.2 to 1.9)	1.7 (1.4 to 2.2)	1.8 (1.4 to 2.3)	2.0 (1.6 to 2.6)	1.0 (0.5 to 1.5)	15.9 (7.7 to 25.6)
Eastern Sub-Saharan Africa						
Burundi	1.5 (1.2 to 2.0)	1.6 (1.2 to 2.0)	1.6 (1.2 to 2.1)	1.7 (1.2 to 2.1)	0.3 (-0.2 to 0.7)	4.3 (-2.7 to 11.8)
Comoros	0.7 (0.5 to 1.0)	0.8 (0.6 to 1.0)	0.8 (0.6 to 1.0)	0.9 (0.7 to 1.1)	0.6 (-0.1 to 1.3)	10.2 (-1.5 to 21.9)
Djibouti	1.7 (1.3 to 2.2)	1.9 (1.5 to 2.5)	2.0 (1.5 to 2.5)	2.2 (1.7 to 2.8)	0.8 (0.3 to 1.3)	13.0 (4.0 to 22.0)
Eritrea	1.6 (1.2 to 2.1)	1.8 (1.4 to 2.3)	1.8 (1.4 to 2.3)	2.0 (1.5 to 2.6)	0.7 (0.2 to 1.2)	10.6 (2.3 to 19.7)
Ethiopia	1.3 (1.1 to 1.7)	1.6 (1.2 to 2.0)	1.6 (1.3 to 2.1)	1.9 (1.5 to 2.4)	1.1 (0.9 to 1.4)	18.5 (14.8 to 22.5)
Kenya	1.4 (1.0 to 1.8)	1.4 (1.1 to 1.8)	1.4 (1.1 to 1.7)	1.4 (1.1 to 1.8)	-0.1 (-0.3 to 0.1)	-1.6 (-4.7 to 0.9)
Madagascar	1.6 (1.2 to 2.0)	1.6 (1.3 to 2.1)	1.6 (1.3 to 2.1)	1.7 (1.3 to 2.2)	0.4 (-0.1 to 1.0)	5.7 (-1.4 to 15.6)
Malawi	1.7 (1.3 to 2.1)	2.0 (1.5 to 2.6)	2.0 (1.5 to 2.6)	2.2 (1.7 to 3.0)	0.8 (0.1 to 1.4)	13.3 (1.0 to 23.9)
Mozambique	2.3 (1.8 to 3.0)	2.6 (2.0 to 3.3)	2.6 (2.0 to 3.4)	3.0 (2.2 to 3.9)	1.1 (0.5 to 1.6)	17.9 (8.6 to 27.8)
Rwanda	2.0 (1.5 to 2.5)	2.1 (1.7 to 2.6)	2.2 (1.7 to 2.8)	2.4 (1.9 to 3.1)	0.8 (0.3 to 1.4)	13.0 (4.1 to 22.8)
Somalia	1.4 (1.1 to 1.7)	1.4 (1.1 to 1.8)	1.5 (1.1 to 1.9)	1.6 (1.2 to 2.0)	0.4 (-0.0 to 1.0)	6.9 (-0.5 to 15.6)
South Sudan	1.4 (1.1 to 1.8)	1.5 (1.2 to 1.9)	1.5 (1.2 to 2.0)	1.6 (1.2 to 2.1)	0.5 (0.1 to 0.9)	8.0 (1.9 to 14.5)
Tanzania	2.5 (2.0 to 3.3)	2.9 (2.3 to 3.6)	2.8 (2.1 to 3.5)	2.8 (2.1 to 3.7)	-0.1 (-0.9 to 0.5)	-1.8 (-12.0 to 8.1)
Uganda	1.9 (1.4 to 2.4)	2.2 (1.8 to 2.8)	2.3 (1.8 to 2.9)	2.6 (1.9 to 3.4)	1.0 (0.4 to 1.6)	17.0 (5.7 to 27.3)
Zambia	1.2 (0.9 to 1.5)	1.2 (0.9 to 1.5)	1.3 (1.0 to 1.6)	1.4 (1.0 to 1.8)	0.9 (0.2 to 1.5)	14.4 (3.0 to 25.5)
Southern Sub-Saharan Africa						
Botswana	0.6 (0.5 to 0.8)	0.7 (0.5 to 0.9)	0.7 (0.5 to 0.9)	0.8 (0.6 to 1.0)	0.8 (0.1 to 1.4)	12.9 (1.1 to 23.5)
Lesotho	0.9 (0.7 to 1.1)	1.0 (0.8 to 1.2)	1.0 (0.8 to 1.3)	1.1 (0.8 to 1.4)	0.7 (0.2 to 1.2)	11.4 (3.6 to 19.8)
Namibia	0.5 (0.3 to 0.7)	0.6 (0.4 to 0.7)	0.6 (0.4 to 0.8)	0.6 (0.5 to 0.9)	0.8 (0.2 to 1.4)	13.5 (3.0 to 23.7)
South Africa	1.1 (0.9 to 1.4)	1.2 (0.9 to 1.6)	1.2 (0.9 to 1.6)	1.3 (1.0 to 1.7)	0.5 (0.3 to 0.7)	7.6 (5.0 to 10.3)
Swaziland	4.7 (3.6 to 6.1)	5.2 (4.0 to 6.7)	5.2 (4.1 to 6.7)	5.6 (4.3 to 7.2)	0.5 (0.0 to 1.0)	8.2 (0.6 to 16.6)
Zimbabwe	0.7 (0.6 to 0.9)	0.7 (0.5 to 0.9)	0.7 (0.5 to 0.9)	0.7 (0.6 to 1.0)	0.5 (0.0 to 1.1)	8.3 (0.3 to 18.3)
Western Sub-Saharan Africa						
Benin	0.4 (0.3 to 0.5)	0.4 (0.3 to 0.6)	0.4 (0.3 to 0.6)	0.5 (0.4 to 0.6)	0.7 (0.2 to 1.1)	11.0 (2.4 to 18.7)
Burkina Faso	0.3 (0.3 to 0.5)	0.4 (0.3 to 0.5)	0.4 (0.3 to 0.5)	0.4 (0.3 to 0.6)	0.6 (0.3 to 1.0)	9.8 (4.5 to 15.6)
Cameroon	0.4 (0.3 to 0.6)	0.5 (0.4 to 0.6)	0.5 (0.4 to 0.6)	0.6 (0.4 to 0.7)	0.8 (0.4 to 1.4)	13.6 (5.6 to 22.5)
Cape Verde	0.5 (0.3 to 0.6)	0.5 (0.4 to 0.7)	0.5 (0.4 to 0.7)	0.6 (0.5 to 0.8)	0.9 (0.4 to 1.4)	15.3 (6.9 to 23.9)
Chad	0.3 (0.2 to 0.3)	0.3 (0.2 to 0.4)	0.3 (0.2 to 0.4)	0.3 (0.2 to 0.4)	0.6 (0.0 to 1.2)	9.8 (0.4 to 19.5)
Cote d'Ivoire	0.4 (0.3 to 0.5)	0.5 (0.3 to 0.6)	0.5 (0.3 to 0.6)	0.5 (0.4 to 0.7)	0.7 (0.2 to 1.2)	10.3 (2.3 to 20.5)
The Gambia	0.2 (0.1 to 0.3)	0.2 (0.2 to 0.3)	0.2 (0.2 to 0.3)	0.3 (0.2 to 0.4)	0.7 (0.1 to 1.2)	11.6 (1.8 to 20.0)
Ghana	1.0 (0.7 to 1.2)	1.1 (0.8 to 1.4)	1.1 (0.8 to 1.4)	1.2 (0.9 to 1.6)	0.7 (0.2 to 1.3)	10.9 (2.6 to 21.5)
Guinea	0.4 (0.3 to 0.5)	0.4 (0.3 to 0.5)	0.4 (0.3 to 0.5)	0.5 (0.3 to 0.6)	0.8 (0.3 to 1.2)	12.1 (4.4 to 19.1)
Guinea-Bissau	0.4 (0.3 to 0.5)	0.4 (0.3 to 0.5)	0.4 (0.3 to 0.6)	0.5 (0.3 to 0.6)	0.7 (0.2 to 1.3)	12.0 (2.9 to 20.7)
Liberia	0.3 (0.3 to 0.5)	0.4 (0.3 to 0.5)	0.4 (0.3 to 0.5)	0.5 (0.3 to 0.6)	0.8 (0.4 to 1.3)	12.7 (5.5 to 21.1)
Mali	0.2 (0.2 to 0.3)	0.2 (0.2 to 0.3)	0.2 (0.2 to 0.3)	0.2 (0.2 to 0.3)	0.6 (-0.0 to 1.2)	8.9 (-0.1 to 19.5)
Mauritania	0.4 (0.3 to 0.6)	0.5 (0.4 to 0.6)	0.5 (0.4 to 0.6)	0.5 (0.4 to 0.7)	0.7 (0.2 to 1.2)	11.5 (3.8 to 20.6)
Niger	0.3 (0.2 to 0.4)	0.3 (0.3 to 0.5)	0.3 (0.3 to 0.5)	0.4 (0.3 to 0.5)	0.4 (0.0 to 0.8)	6.9 (0.0 to 12.9)
Nigeria	0.5 (0.4 to 0.7)	0.6 (0.5 to 0.7)	0.6 (0.5 to 0.8)	0.7 (0.5 to 0.8)	0.7 (0.1 to 1.4)	11.3 (1.2 to 22.8)
Sao Tome and Principe	0.4 (0.3 to 0.6)	0.5 (0.4 to 0.6)	0.5 (0.4 to 0.7)	0.6 (0.4 to 0.8)	1.0 (0.4 to 1.5)	15.9 (6.1 to 25.7)
Senegal	0.4 (0.3 to 0.5)	0.4 (0.3 to 0.6)	0.4 (0.3 to 0.6)	0.5 (0.3 to 0.6)	0.5 (0.1 to 1.1)	8.4 (1.1 to 18.2)
Sierra Leone	0.4 (0.3 to 0.6)	0.5 (0.4 to 0.7)	0.5 (0.4 to 0.7)	0.6 (0.4 to 0.7)	0.8 (0.1 to 1.4)	13.3 (2.2 to 22.4)
Togo	0.6 (0.4 to 0.7)	0.6 (0.5 to 0.8)	0.6 (0.5 to 0.8)	0.7 (0.5 to 0.9)	0.7 (0.1 to 1.2)	10.8 (2.2 to 20.1)
**Indicator 6.1.1: Risk-weighted prevalence of populations using unsafe or unimproved water sources, as measured by the summary exposure value (SEV) for unsafe water (%)**						
Central Europe, Eastern Europe, and Central Asia						
Central Asia						
Armenia	8.0 (4.5 to 10.3)	3.4 (1.9 to 4.8)	3.1 (1.7 to 4.5)	2.2 (1.2 to 3.1)	-3.1 (-3.7 to -2.4)	-36.7 (-42.3 to -30.4)
Azerbaijan	25.0 (18.9 to 29.0)	10.8 (7.2 to 14.2)	10.3 (6.6 to 13.8)	7.6 (5.0 to 10.3)	-2.3 (-2.9 to -1.7)	-29.2 (-35.1 to -22.7)
Georgia	12.7 (7.6 to 16.6)	10.6 (6.0 to 13.9)	9.8 (5.4 to 13.2)	7.2 (3.9 to 9.8)	-2.6 (-3.1 to -2.0)	-31.9 (-37.3 to -26.3)
Kazakhstan	16.4 (9.0 to 20.5)	11.0 (6.3 to 14.1)	10.0 (5.4 to 13.1)	7.7 (4.2 to 10.2)	-2.4 (-3.0 to -1.7)	-30.2 (-36.1 to -22.1)
Kyrgyzstan	21.7 (13.9 to 26.2)	16.3 (10.7 to 20.0)	15.7 (10.1 to 19.6)	11.6 (7.3 to 14.6)	-2.2 (-2.7 to -1.8)	-28.6 (-33.7 to -23.7)
Mongolia	31.6 (18.7 to 38.7)	31.0 (19.3 to 37.4)	30.1 (18.6 to 36.3)	29.0 (17.6 to 35.0)	-0.4 (-0.7 to -0.2)	-6.4 (-9.3 to -3.5)
Tajikistan	27.7 (17.6 to 33.3)	24.2 (15.3 to 29.6)	23.6 (14.7 to 29.2)	20.9 (13.2 to 26.0)	-1.0 (-1.4 to -0.5)	-13.3 (-18.7 to -6.7)
Turkmenistan	25.4 (19.0 to 31.0)	20.5 (15.2 to 24.2)	19.2 (13.8 to 23.1)	14.0 (9.8 to 16.9)	-2.6 (-3.0 to -2.2)	-31.8 (-36.5 to -27.6)

	Estimate in 2000 (95% UIs)	Estimate in 2015 (95% UIs)	Estimate in 2017 (95% UIs)	Estimate in 2030 (95% UIs)	Annualised rate of change, 2015-2030 (95% UIs)	Percent change, 2015-2030 (95% UIs)
Uzbekistan	15.2 (6.3 to 19.8)	12.4 (4.9 to 17.4)	11.6 (4.7 to 16.6)	9.1 (4.6 to 13.1)	-2.0 (-2.6 to -0.3)	-25.4 (-32.1 to -4.6)
Central Europe						
Albania	27.7 (23.8 to 31.9)	19.3 (14.4 to 24.4)	18.3 (13.2 to 23.8)	14.0 (10.0 to 18.5)	-2.2 (-2.8 to -1.6)	-27.6 (-34.0 to -21.9)
Bosnia and Herzegovina	13.0 (10.3 to 15.8)	7.7 (5.6 to 10.4)	7.2 (5.1 to 9.9)	6.4 (4.4 to 8.8)	-1.3 (-2.0 to -0.6)	-17.7 (-25.8 to -9.1)
Bulgaria	9.5 (6.5 to 13.6)	6.3 (4.3 to 9.1)	6.0 (4.1 to 8.7)	5.4 (3.6 to 7.9)	-1.1 (-1.7 to -0.4)	-14.6 (-22.9 to -5.9)
Croatia	7.2 (5.0 to 10.2)	4.7 (3.0 to 6.8)	4.6 (2.9 to 6.8)	4.3 (2.8 to 6.4)	-0.6 (-1.3 to 0.1)	-8.2 (-17.1 to 1.1)
Czech Republic	3.5 (2.3 to 5.0)	2.8 (1.8 to 4.1)	2.7 (1.8 to 4.0)	2.6 (1.7 to 3.8)	-0.5 (-1.2 to 0.2)	-7.2 (-16.8 to 2.7)
Hungary	4.9 (3.4 to 6.7)	3.5 (2.3 to 5.0)	3.3 (2.2 to 4.9)	3.1 (2.1 to 4.6)	-0.7 (-1.4 to -0.0)	-10.3 (-19.0 to -0.1)
Macedonia	9.5 (6.6 to 12.9)	6.2 (4.4 to 8.3)	5.9 (4.2 to 8.2)	5.4 (3.8 to 7.5)	-0.9 (-1.5 to -0.3)	-12.7 (-20.6 to -4.5)
Montenegro	18.7 (13.3 to 25.5)	13.6 (9.9 to 18.6)	13.0 (9.2 to 18.2)	10.5 (7.3 to 14.8)	-1.7 (-2.4 to -1.1)	-22.8 (-30.2 to -15.0)
Poland	7.0 (4.8 to 10.1)	4.2 (2.8 to 6.0)	4.0 (2.6 to 5.7)	3.6 (2.4 to 5.1)	-1.0 (-1.7 to -0.4)	-14.3 (-22.2 to -5.5)
Romania	18.3 (13.8 to 23.5)	8.9 (6.0 to 12.6)	8.4 (5.7 to 12.1)	6.5 (4.4 to 9.4)	-2.1 (-2.8 to -1.5)	-27.0 (-34.7 to -19.6)
Serbia	10.7 (6.0 to 14.4)	5.5 (2.9 to 7.5)	5.3 (2.7 to 7.3)	4.0 (2.0 to 5.6)	-2.2 (-2.8 to -1.5)	-27.7 (-34.4 to -20.5)
Slovakia	4.6 (3.1 to 6.5)	3.1 (2.1 to 4.6)	3.1 (2.0 to 4.5)	2.9 (1.9 to 4.2)	-0.7 (-1.3 to -0.0)	-9.3 (-18.0 to -0.0)
Slovenia	3.4 (2.3 to 4.7)	2.5 (1.8 to 3.6)	2.4 (1.7 to 3.4)	2.3 (1.6 to 3.2)	-0.6 (-1.3 to 0.1)	-8.5 (-17.3 to 1.4)
Eastern Europe						
Belarus	16.7 (13.7 to 19.6)	6.0 (4.4 to 8.1)	5.7 (4.0 to 7.8)	4.9 (3.5 to 6.7)	-1.4 (-2.2 to -0.7)	-19.2 (-28.6 to -9.6)
Estonia	7.8 (5.5 to 10.7)	4.7 (3.0 to 6.6)	4.6 (2.9 to 6.6)	4.1 (2.6 to 6.0)	-0.9 (-1.6 to -0.2)	-11.9 (-20.8 to -2.3)
Latvia	9.7 (6.8 to 13.1)	6.0 (4.0 to 8.5)	5.8 (3.8 to 8.2)	5.3 (3.4 to 7.4)	-0.9 (-1.5 to -0.2)	-12.2 (-20.6 to -3.0)
Lithuania	10.2 (6.7 to 14.2)	5.8 (3.8 to 8.5)	5.7 (3.7 to 8.2)	5.0 (3.2 to 7.3)	-0.9 (-1.6 to -0.3)	-12.9 (-21.1 to -3.9)
Moldova	36.0 (31.4 to 40.3)	23.2 (19.4 to 27.2)	22.3 (17.9 to 26.6)	16.6 (13.2 to 20.1)	-2.2 (-2.8 to -1.7)	-28.6 (-33.8 to -22.9)
Russia	12.2 (9.1 to 15.6)	8.3 (5.8 to 11.9)	8.0 (5.5 to 11.2)	7.2 (4.9 to 10.2)	-1.0 (-1.6 to -0.4)	-13.3 (-20.9 to -5.6)
Ukraine	15.5 (12.4 to 19.1)	14.1 (10.8 to 17.4)	13.7 (10.2 to 17.2)	12.1 (8.9 to 15.3)	-1.0 (-1.6 to -0.5)	-14.2 (-21.1 to -7.1)
High-income						
Australasia						
Australia	1.7 (1.1 to 2.5)	1.8 (1.2 to 2.7)	1.7 (1.2 to 2.6)	1.7 (1.1 to 2.5)	-0.4 (-1.1 to 0.3)	-5.4 (-15.3 to 4.4)
New Zealand	2.2 (1.4 to 3.1)	2.3 (1.5 to 3.4)	2.3 (1.5 to 3.3)	2.2 (1.4 to 3.1)	-0.4 (-1.1 to 0.3)	-5.7 (-14.9 to 4.8)
High-income Asia Pacific						
Brunei	2.3 (1.5 to 3.4)	2.0 (1.4 to 2.9)	2.0 (1.3 to 2.8)	1.9 (1.2 to 2.7)	-0.5 (-1.2 to 0.2)	-7.2 (-16.7 to 2.7)
Japan	1.6 (1.0 to 2.4)	1.9 (1.2 to 2.7)	1.9 (1.2 to 2.7)	1.8 (1.2 to 2.6)	-0.3 (-1.0 to 0.4)	-4.6 (-13.7 to 5.5)
South Korea	2.1 (1.3 to 3.0)	1.8 (1.2 to 2.8)	1.8 (1.2 to 2.6)	1.7 (1.1 to 2.5)	-0.5 (-1.2 to 0.2)	-7.2 (-16.8 to 2.9)
Singapore	2.1 (1.4 to 3.0)	1.8 (1.2 to 2.6)	1.7 (1.1 to 2.5)	1.7 (1.1 to 2.4)	-0.5 (-1.2 to 0.2)	-6.7 (-15.9 to 3.2)
High-income North America						
Canada	0.9 (0.6 to 1.4)	0.3 (0.2 to 0.5)	0.3 (0.2 to 0.5)	0.3 (0.2 to 0.4)	-0.3 (-1.0 to 0.2)	-5.0 (-13.8 to 3.7)
Greenland	2.5 (1.7 to 3.7)	0.7 (0.4 to 1.0)	0.7 (0.4 to 1.0)	0.5 (0.3 to 0.8)	-1.4 (-2.3 to -0.6)	-18.5 (-29.7 to -8.0)
United States	1.3 (0.9 to 1.9)	0.3 (0.2 to 0.5)	0.3 (0.2 to 0.5)	0.3 (0.2 to 0.4)	-0.9 (-1.8 to -0.1)	-12.0 (-23.5 to -1.2)
Southern Latin America						
Argentina	18.8 (14.1 to 23.7)	8.7 (6.2 to 11.9)	8.2 (5.8 to 11.3)	5.8 (4.1 to 8.1)	-2.7 (-3.4 to -2.0)	-32.8 (-39.6 to -26.2)
Chile	6.5 (4.7 to 8.7)	3.8 (2.6 to 5.4)	3.7 (2.5 to 5.1)	2.5 (1.7 to 3.5)	-2.7 (-3.3 to -2.2)	-33.5 (-39.1 to -27.8)
Uruguay	8.9 (6.9 to 11.4)	6.1 (4.5 to 8.2)	5.7 (4.2 to 7.9)	4.0 (2.9 to 5.5)	-2.8 (-3.4 to -2.3)	-34.7 (-40.2 to -28.9)
Western Europe						
Andorra	0.3 (0.2 to 0.4)	0.2 (0.2 to 0.3)	0.2 (0.2 to 0.3)	0.2 (0.2 to 0.3)	-0.3 (-0.8 to 0.3)	-4.1 (-11.4 to 4.2)
Austria	0.3 (0.2 to 0.5)	0.2 (0.2 to 0.3)	0.2 (0.2 to 0.3)	0.2 (0.1 to 0.3)	-1.7 (-2.3 to -1.2)	-22.4 (-28.8 to -16.1)
Belgium	0.4 (0.3 to 0.5)	0.3 (0.2 to 0.4)	0.3 (0.2 to 0.4)	0.3 (0.2 to 0.4)	-0.4 (-0.9 to 0.1)	-5.1 (-12.4 to 2.3)
Cyprus	0.6 (0.5 to 0.8)	0.5 (0.4 to 0.7)	0.5 (0.4 to 0.6)	0.5 (0.3 to 0.6)	-0.8 (-1.8 to -0.2)	-11.8 (-23.4 to -3.3)
Denmark	0.3 (0.2 to 0.4)	0.3 (0.2 to 0.3)	0.3 (0.2 to 0.3)	0.2 (0.2 to 0.3)	-0.3 (-0.7 to 0.2)	-4.1 (-10.6 to 2.9)
Finland	0.3 (0.2 to 0.4)	0.2 (0.2 to 0.3)	0.2 (0.2 to 0.3)	0.2 (0.2 to 0.3)	-0.4 (-1.0 to 0.1)	-5.9 (-14.3 to 2.2)
France	0.7 (0.5 to 1.0)	0.5 (0.4 to 0.7)	0.5 (0.4 to 0.7)	0.5 (0.3 to 0.7)	-0.4 (-1.1 to 0.2)	-6.1 (-14.7 to 3.1)
Germany	0.4 (0.3 to 0.6)	0.3 (0.2 to 0.4)	0.3 (0.2 to 0.4)	0.2 (0.2 to 0.3)	-1.8 (-2.4 to -1.3)	-24.1 (-30.3 to -18.1)
Greece	0.3 (0.2 to 0.4)	0.2 (0.2 to 0.3)	0.2 (0.2 to 0.3)	0.2 (0.2 to 0.3)	-0.2 (-0.7 to 0.2)	-2.7 (-9.4 to 3.8)
Iceland	0.3 (0.2 to 0.4)	0.2 (0.2 to 0.3)	0.2 (0.2 to 0.3)	0.2 (0.2 to 0.3)	-0.6 (-1.2 to -0.0)	-8.3 (-15.9 to -0.2)
Ireland	0.4 (0.3 to 0.5)	0.3 (0.2 to 0.4)	0.3 (0.2 to 0.4)	0.2 (0.2 to 0.3)	-0.8 (-1.5 to -0.2)	-11.0 (-20.4 to -2.8)
Israel	0.5 (0.3 to 0.7)	0.4 (0.3 to 0.6)	0.4 (0.3 to 0.6)	0.3 (0.2 to 0.5)	-1.4 (-2.1 to -0.7)	-19.2 (-27.3 to -10.3)
Italy	0.6 (0.4 to 0.7)	0.5 (0.4 to 0.7)	0.5 (0.4 to 0.7)	0.4 (0.3 to 0.6)	-1.3 (-1.9 to -0.8)	-17.7 (-24.9 to -11.3)
Luxembourg	0.3 (0.2 to 0.4)	0.3 (0.2 to 0.3)	0.3 (0.2 to 0.3)	0.3 (0.2 to 0.3)	-0.3 (-0.7 to 0.2)	-4.2 (-10.5 to 2.6)
Malta	0.5 (0.3 to 0.7)	0.3 (0.2 to 0.5)	0.3 (0.2 to 0.5)	0.3 (0.2 to 0.4)	-1.5 (-2.2 to -0.8)	-19.7 (-28.4 to -11.0)
Netherlands	0.3 (0.2 to 0.4)	0.2 (0.2 to 0.3)	0.2 (0.2 to 0.3)	0.2 (0.2 to 0.3)	-0.3 (-0.9 to 0.2)	-5.0 (-12.6 to 2.7)
Norway	0.3 (0.2 to 0.4)	0.2 (0.2 to 0.3)	0.2 (0.2 to 0.3)	0.2 (0.2 to 0.3)	-0.4 (-0.9 to 0.2)	-5.3 (-13.1 to 2.7)
Portugal	2.0 (1.3 to 2.8)	1.1 (0.8 to 1.6)	1.1 (0.8 to 1.6)	0.8 (0.5 to 1.1)	-2.3 (-3.0 to -1.6)	-28.7 (-35.9 to -21.3)
Spain	0.6 (0.4 to 0.8)	0.5 (0.3 to 0.6)	0.4 (0.3 to 0.6)	0.4 (0.3 to 0.6)	-0.3 (-0.8 to 0.2)	-4.3 (-11.5 to 3.5)
Sweden	0.3 (0.2 to 0.5)	0.3 (0.2 to 0.4)	0.3 (0.2 to 0.4)	0.3 (0.2 to 0.4)	-0.5 (-1.1 to 0.0)	-7.8 (-15.7 to 0.2)
Switzerland	0.3 (0.2 to 0.4)	0.3 (0.2 to 0.4)	0.3 (0.2 to 0.4)	0.2 (0.2 to 0.3)	-0.4 (-0.9 to 0.2)	-5.4 (-12.3 to 3.3)
United Kingdom	0.5 (0.3 to 0.7)	0.3 (0.2 to 0.5)	0.3 (0.2 to 0.5)	0.3 (0.2 to 0.4)	-1.8 (-2.5 to -1.2)	-24.1 (-31.4 to -16.5)
Latin America and Caribbean						
Andean Latin America						
Bolivia	33.7 (29.3 to 37.8)	19.3 (14.9 to 23.1)	18.1 (13.9 to 22.1)	13.1 (10.2 to 16.1)	-2.6 (-3.0 to -2.2)	-32.0 (-36.1 to -28.1)
Ecuador	21.7 (17.7 to 25.6)	16.7 (12.5 to 20.6)	16.4 (12.1 to 20.2)	12.0 (8.9 to 15.0)	-2.2 (-2.6 to -1.9)	-28.4 (-32.4 to -24.9)
Peru	30.0 (22.0 to 34.3)	19.0 (13.3 to 22.2)	18.6 (12.9 to 21.9)	13.6 (9.9 to 16.1)	-2.2 (-2.5 to -1.9)	-28.4 (-31.7 to -25.1)
Caribbean						
Antigua and Barbuda	42.3 (37.0 to 47.5)	40.0 (34.3 to 45.5)	39.4 (33.8 to 44.6)	36.1 (30.8 to 41.2)	-0.7 (-0.9 to -0.5)	-9.8 (-12.2 to -7.5)
The Bahamas	31.9 (26.5 to 37.4)	33.4 (28.0 to 38.5)	33.3 (28.0 to 38.4)	31.8 (26.5 to 36.8)	-0.3 (-0.5 to -0.1)	-4.9 (-7.8 to -2.2)
Barbados	22.9 (18.0 to 28.2)	21.7 (16.6 to 27.0)	21.7 (16.7 to 26.8)	19.6 (15.0 to 24.4)	-0.7 (-1.0 to -0.4)	-9.7 (-14.2 to -5.4)
Belize	69.4 (61.3 to 78.5)	70.9 (62.6 to 80.2)	70.3 (62.2 to 79.7)	68.4 (59.9 to 78.0)	-0.2 (-0.3 to -0.1)	-3.5 (-4.8 to -2.2)
Bermuda	31.4 (26.0 to 36.9)	31.9 (27.1 to 37.1)	31.8 (27.1 to 36.9)	29.9 (25.5 to 34.8)	-0.5 (-0.6 to -0.3)	-6.5 (-9.1 to -3.7)
Cuba	51.9 (46.3 to 57.1)	41.5 (35.8 to 46.8)	40.2 (34.6 to 45.1)	31.8 (26.8 to 36.5)	-1.8 (-2.1 to -1.6)	-23.5 (-26.5 to -20.9)
Dominica	50.0 (44.1 to 55.4)	44.2 (38.6 to 49.3)	43.5 (38.0 to 48.7)	39.4 (34.1 to 44.5)	-0.8 (-0.9 to -0.6)	-10.7 (-13.1 to -8.5)
Dominican Republic	65.2 (58.9 to 70.3)	63.3 (57.1 to 68.3)	62.4 (56.3 to 67.4)	59.9 (53.7 to 65.0)	-0.4 (-0.5 to -0.3)	-5.4 (-6.8 to -3.9)
Grenada	43.4 (37.3 to 49.5)	40.4 (34.3 to 45.9)	39.7 (33.7 to 45.1)	33.4 (27.8 to 38.6)	-1.3 (-1.6 to -1.0)	-17.2 (-21.2 to -13.9)
Guyana	57.7 (51.1 to 63.3)	62.6 (56.0 to 67.9)	61.9 (55.4 to 67.0)	58.1 (51.6 to 63.6)	-0.5 (-0.6 to -0.3)	-7.2 (-9.2 to -4.9)
Haiti	77.5 (72.2 to 82.6)	74.8 (69.7 to 79.4)	74.4 (68.9 to 79.0)	73.4 (67.8 to 78.2)	-0.1 (-0.2 to -0.1)	-1.9 (-2.7 to -1.3)

	Estimate in 2000 (95% UIs)	Estimate in 2015 (95% UIs)	Estimate in 2017 (95% UIs)	Estimate in 2030 (95% UIs)	Annualised rate of change, 2015-2030 (95% UIs)	Percent change, 2015-2030 (95% UIs)
Jamaica	43.9 (38.4 to 48.6)	41.2 (36.3 to 46.1)	40.8 (35.9 to 45.5)	37.6 (32.7 to 42.3)	-0.6 (-0.8 to -0.4)	-8.7 (-11.3 to -6.4)
Puerto Rico	32.9 (28.0 to 38.0)	31.4 (26.3 to 36.8)	31.2 (26.2 to 36.6)	30.0 (25.1 to 35.3)	-0.3 (-0.5 to -0.1)	-4.2 (-7.6 to -1.2)
Saint Lucia	42.2 (35.9 to 48.5)	40.8 (34.8 to 46.1)	40.1 (34.1 to 45.7)	35.7 (30.2 to 41.1)	-0.9 (-1.1 to -0.7)	-12.4 (-15.5 to -9.8)
Saint Vincent and the Grenadines	50.6 (44.5 to 57.0)	48.5 (42.4 to 54.6)	47.7 (41.5 to 53.9)	44.0 (38.0 to 50.1)	-0.6 (-0.8 to -0.5)	-9.2 (-11.7 to -7.0)
Suriname	48.4 (42.6 to 54.1)	44.7 (38.8 to 50.0)	44.4 (38.5 to 49.8)	37.4 (31.7 to 42.6)	-1.2 (-1.4 to -1.0)	-16.4 (-19.0 to -13.8)
Trinidad and Tobago	42.2 (37.8 to 46.1)	35.1 (29.8 to 40.0)	34.5 (29.3 to 39.6)	31.3 (25.8 to 36.2)	-0.8 (-1.2 to -0.5)	-11.0 (-16.4 to -6.7)
Virgin Islands, U.S.	33.9 (28.5 to 39.3)	33.4 (28.2 to 38.5)	33.3 (28.1 to 38.5)	32.1 (27.0 to 37.3)	-0.3 (-0.5 to -0.1)	-3.9 (-7.1 to -0.9)
Central Latin America						
Colombia	24.8 (20.3 to 29.8)	20.5 (17.0 to 24.3)	19.5 (16.1 to 23.0)	14.3 (11.7 to 17.1)	-2.4 (-2.7 to -2.1)	-30.2 (-33.5 to -26.6)
Costa Rica	10.1 (7.7 to 12.8)	7.2 (5.4 to 9.3)	6.5 (4.9 to 8.5)	4.5 (3.4 to 5.9)	-3.1 (-3.5 to -2.7)	-37.1 (-41.1 to -33.2)
El Salvador	32.4 (27.3 to 37.9)	24.8 (19.9 to 30.1)	23.6 (18.6 to 28.7)	19.0 (14.8 to 23.4)	-1.8 (-2.2 to -1.4)	-23.4 (-27.8 to -18.9)
Guatemala	53.2 (47.0 to 59.1)	45.5 (40.7 to 50.2)	43.0 (38.2 to 47.9)	35.9 (31.3 to 40.9)	-1.6 (-1.8 to -1.3)	-21.2 (-24.2 to -18.3)
Honduras	30.6 (25.8 to 35.6)	25.1 (20.0 to 30.7)	23.6 (18.4 to 29.0)	19.4 (15.1 to 24.3)	-1.8 (-2.1 to -1.4)	-23.1 (-27.5 to -18.7)
Mexico	10.4 (8.6 to 12.4)	7.0 (5.4 to 9.1)	6.8 (5.1 to 8.9)	4.8 (3.6 to 6.4)	-2.5 (-3.0 to -2.0)	-31.2 (-36.4 to -25.6)
Nicaragua	51.4 (46.8 to 56.0)	31.9 (26.4 to 37.6)	30.1 (24.6 to 35.8)	25.6 (20.8 to 31.0)	-1.5 (-1.8 to -1.1)	-19.9 (-24.2 to -15.3)
Panama	28.7 (24.1 to 33.6)	18.9 (15.0 to 23.2)	17.2 (13.5 to 21.2)	13.5 (10.5 to 17.0)	-2.2 (-2.7 to -1.8)	-28.5 (-33.0 to -23.8)
Venezuela	20.5 (17.0 to 24.4)	15.5 (12.6 to 18.8)	14.7 (11.9 to 18.1)	10.7 (8.6 to 13.3)	-2.5 (-2.8 to -2.1)	-30.8 (-34.7 to -27.1)
Tropical Latin America						
Brazil	18.0 (14.3 to 22.4)	10.3 (7.7 to 13.1)	9.8 (7.4 to 12.8)	7.5 (5.7 to 10.0)	-2.1 (-2.6 to -1.5)	-26.5 (-32.0 to -20.5)
Paraguay	38.5 (34.0 to 43.2)	19.9 (15.6 to 25.0)	18.7 (14.4 to 23.7)	13.7 (10.4 to 17.6)	-2.5 (-3.0 to -2.0)	-31.1 (-36.6 to -25.9)
North Africa and Middle East						
North Africa and Middle East						
Afghanistan	89.3 (84.8 to 94.7)	83.4 (76.6 to 91.8)	82.6 (75.7 to 91.5)	81.9 (74.7 to 91.0)	-0.1 (-0.2 to -0.1)	-1.8 (-2.9 to -0.9)
Algeria	20.6 (16.9 to 24.6)	13.1 (10.4 to 16.2)	12.3 (9.5 to 15.7)	8.7 (6.7 to 11.2)	-2.7 (-3.2 to -2.2)	-33.5 (-38.3 to -28.1)
Bahrain	14.6 (10.3 to 19.9)	14.0 (10.1 to 19.1)	13.5 (9.8 to 18.2)	10.9 (7.9 to 15.0)	-1.7 (-2.2 to -1.1)	-21.9 (-28.5 to -14.8)
Egypt	17.5 (13.1 to 22.3)	9.9 (6.9 to 13.4)	8.9 (6.2 to 12.2)	6.2 (4.3 to 8.7)	-3.1 (-3.7 to -2.5)	-37.3 (-42.8 to -31.2)
Iran	19.0 (14.0 to 25.0)	15.0 (11.4 to 19.2)	14.4 (11.0 to 18.3)	12.0 (9.1 to 15.4)	-1.5 (-2.0 to -1.0)	-19.7 (-26.3 to -13.5)
Iraq	22.5 (19.0 to 26.4)	21.7 (16.9 to 27.2)	21.4 (16.3 to 27.4)	17.5 (13.2 to 22.7)	-1.4 (-2.0 to -0.8)	-19.2 (-26.3 to -11.8)
Jordan	14.2 (10.2 to 18.9)	9.8 (6.8 to 13.7)	9.4 (6.5 to 13.2)	6.7 (4.5 to 9.4)	-2.6 (-3.2 to -1.9)	-31.8 (-38.3 to -25.0)
Kuwait	12.9 (9.1 to 17.9)	9.7 (7.0 to 13.3)	9.3 (6.8 to 12.6)	7.1 (5.1 to 9.8)	-2.1 (-2.8 to -1.6)	-27.5 (-34.4 to -20.8)
Lebanon	36.5 (31.0 to 42.3)	29.6 (24.5 to 35.1)	27.3 (22.3 to 33.0)	20.8 (16.5 to 25.4)	-2.4 (-2.8 to -1.9)	-29.7 (-34.0 to -24.6)
Libya	15.5 (11.7 to 19.8)	15.6 (11.7 to 20.1)	15.8 (12.0 to 20.5)	13.4 (10.0 to 17.9)	-1.0 (-1.8 to -0.4)	-13.9 (-23.6 to -5.5)
Morocco	44.8 (39.6 to 49.4)	29.9 (23.2 to 36.7)	28.2 (21.7 to 35.3)	21.0 (15.8 to 26.9)	-2.4 (-2.9 to -1.9)	-29.8 (-35.1 to -24.3)
Palestine	38.1 (32.0 to 44.8)	38.9 (35.0 to 42.8)	37.3 (32.3 to 42.3)	32.4 (27.8 to 37.2)	-1.2 (-1.7 to -0.8)	-16.8 (-23.1 to -10.8)
Oman	44.5 (36.6 to 53.7)	18.6 (13.4 to 24.4)	17.6 (12.8 to 23.1)	12.7 (9.0 to 17.0)	-2.6 (-3.1 to -2.1)	-32.0 (-37.6 to -26.5)
Qatar	12.0 (8.5 to 16.6)	10.8 (7.9 to 14.2)	10.4 (7.6 to 13.9)	8.3 (6.1 to 11.3)	-1.7 (-2.3 to -1.2)	-22.5 (-29.3 to -16.1)
Saudi Arabia	23.4 (17.5 to 30.6)	10.4 (7.2 to 14.6)	9.5 (6.6 to 13.5)	6.7 (4.5 to 9.6)	-2.9 (-3.6 to -2.3)	-35.6 (-41.7 to -29.7)
Sudan	65.2 (58.8 to 71.5)	63.1 (56.6 to 70.4)	59.8 (52.6 to 67.5)	52.2 (44.7 to 60.7)	-1.3 (-1.7 to -0.8)	-17.3 (-22.0 to -12.0)
Syria	21.3 (15.3 to 28.9)	14.9 (10.6 to 20.3)	14.1 (10.0 to 19.4)	10.0 (7.0 to 14.0)	-2.6 (-3.2 to -2.1)	-32.6 (-38.5 to -26.7)
Tunisia	25.9 (20.4 to 32.2)	21.4 (16.3 to 27.2)	20.2 (15.3 to 26.0)	15.9 (12.0 to 20.9)	-2.0 (-2.6 to -1.4)	-25.7 (-32.0 to -18.6)
Turkey	36.0 (31.9 to 40.6)	25.2 (19.5 to 31.6)	22.7 (17.4 to 28.9)	16.8 (12.6 to 21.8)	-2.7 (-3.2 to -2.2)	-33.4 (-38.0 to -28.4)
United Arab Emirates	15.7 (11.9 to 20.4)	11.9 (8.8 to 15.8)	11.7 (8.6 to 15.6)	9.1 (6.6 to 12.3)	-1.8 (-2.2 to -1.3)	-23.2 (-28.6 to -17.1)
Yemen	70.3 (63.4 to 77.5)	55.1 (47.4 to 64.1)	54.0 (46.2 to 63.1)	48.4 (40.9 to 58.1)	-0.9 (-1.2 to -0.5)	-12.2 (-16.6 to -7.5)
South Asia						
South Asia						
Bangladesh	80.9 (71.0 to 94.2)	77.8 (67.9 to 90.9)	77.3 (67.5 to 90.4)	76.2 (66.1 to 89.8)	-0.1 (-0.2 to -0.1)	-2.1 (-2.8 to -1.2)
Bhutan	46.0 (38.2 to 53.9)	23.6 (18.4 to 29.7)	22.2 (17.2 to 28.4)	17.9 (13.4 to 23.6)	-1.9 (-2.6 to -1.2)	-24.4 (-32.0 to -16.5)
India	57.2 (53.5 to 59.7)	52.3 (47.6 to 56.1)	51.0 (46.3 to 54.6)	45.2 (40.6 to 49.1)	-1.0 (-1.2 to -0.7)	-13.7 (-15.9 to -10.5)
Nepal	78.7 (71.0 to 87.7)	68.3 (60.1 to 77.8)	67.0 (59.0 to 76.5)	60.1 (51.5 to 70.3)	-0.9 (-1.1 to -0.6)	-12.1 (-15.4 to -8.6)
Pakistan	69.8 (62.3 to 78.7)	71.9 (63.1 to 83.2)	71.7 (62.7 to 83.0)	68.8 (59.4 to 80.7)	-0.3 (-0.4 to -0.2)	-4.4 (-6.3 to -2.5)
Southeast Asia, East Asia, and Oceania						
East Asia						
China	37.2 (33.8 to 40.4)	19.8 (15.4 to 24.6)	18.1 (13.5 to 23.0)	13.8 (10.1 to 17.6)	-2.4 (-3.0 to -1.9)	-30.5 (-35.8 to -24.9)
North Korea	13.4 (9.9 to 17.1)	9.7 (6.7 to 13.3)	9.3 (6.3 to 12.8)	8.5 (5.7 to 11.6)	-0.9 (-1.5 to -0.2)	-12.4 (-20.7 to -3.6)
Taiwan (Province of China)	8.5 (5.7 to 12.0)	4.3 (2.7 to 6.4)	4.1 (2.6 to 6.1)	3.7 (2.4 to 5.5)	-1.0 (-1.6 to -0.3)	-13.5 (-21.7 to -4.6)
Oceania						
American Samoa	30.0 (24.7 to 35.0)	20.4 (16.1 to 24.7)	20.0 (15.8 to 24.3)	17.2 (13.4 to 21.0)	-1.1 (-1.5 to -0.8)	-15.7 (-20.6 to -10.9)
Federated States of Micronesia	47.0 (41.4 to 52.0)	34.5 (29.0 to 40.0)	33.8 (28.2 to 39.2)	29.4 (24.1 to 34.6)	-1.1 (-1.5 to -0.7)	-14.8 (-19.7 to -10.5)
Fiji	40.0 (35.0 to 44.5)	24.3 (20.0 to 29.1)	22.9 (18.6 to 27.7)	19.2 (15.5 to 23.4)	-1.5 (-1.9 to -1.2)	-20.7 (-24.7 to -16.7)
Guam	19.6 (15.1 to 24.0)	14.9 (10.9 to 18.7)	14.8 (11.1 to 18.9)	14.0 (10.4 to 17.8)	-0.4 (-0.7 to -0.1)	-6.1 (-10.4 to -1.4)
Kiribati	64.0 (59.0 to 68.4)	53.5 (47.2 to 59.0)	52.1 (46.1 to 57.8)	47.8 (41.9 to 53.4)	-0.7 (-0.9 to -0.6)	-10.6 (-13.2 to -8.2)
Marshall Islands	54.0 (48.7 to 58.9)	38.1 (32.4 to 43.3)	37.0 (31.3 to 42.1)	30.3 (25.3 to 35.1)	-1.5 (-1.8 to -1.2)	-20.5 (-24.0 to -17.0)
Northern Mariana Islands	19.1 (14.9 to 23.6)	16.4 (12.6 to 20.7)	16.4 (12.5 to 20.7)	15.6 (11.8 to 19.7)	-0.3 (-0.7 to -0.0)	-5.1 (-9.5 to -0.5)
Papua New Guinea	72.9 (69.1 to 76.1)	65.5 (60.7 to 69.5)	64.8 (59.9 to 68.8)	61.7 (56.8 to 66.0)	-0.4 (-0.5 to -0.3)	-5.8 (-7.3 to -4.4)
Samoa	24.7 (19.9 to 29.4)	15.6 (12.0 to 19.3)	15.2 (11.6 to 18.7)	12.3 (9.5 to 15.4)	-1.6 (-2.0 to -1.2)	-20.9 (-25.7 to -16.5)
Solomon Islands	53.5 (47.3 to 59.3)	43.2 (37.2 to 49.1)	41.9 (35.6 to 48.0)	36.6 (30.6 to 42.3)	-1.1 (-1.4 to -0.8)	-15.2 (-18.6 to -11.5)
Tonga	40.4 (35.1 to 46.1)	28.6 (23.5 to 33.9)	27.7 (22.7 to 33.2)	22.9 (18.5 to 27.8)	-1.5 (-1.8 to -1.2)	-19.9 (-23.6 to -16.5)
Vanuatu	67.3 (62.7 to 70.9)	60.3 (55.6 to 64.4)	59.7 (54.9 to 63.7)	55.6 (50.8 to 59.9)	-0.5 (-0.6 to -0.4)	-7.8 (-9.2 to -6.5)
Southeast Asia						
Cambodia	54.9 (47.5 to 59.9)	41.0 (33.5 to 45.9)	40.5 (32.6 to 45.5)	35.4 (28.5 to 40.1)	-1.0 (-1.2 to -0.8)	-13.7 (-16.1 to -11.6)
Indonesia	36.2 (26.2 to 41.7)	36.4 (26.1 to 42.4)	35.8 (25.4 to 41.9)	33.9 (23.6 to 39.8)	-0.5 (-0.7 to -0.3)	-6.9 (-9.5 to -4.3)
Laos	66.4 (62.7 to 70.0)	55.1 (50.5 to 59.3)	53.7 (49.1 to 58.0)	49.5 (45.2 to 53.8)	-0.7 (-0.9 to -0.5)	-10.2 (-12.4 to -7.8)
Malaysia	13.6 (10.2 to 17.6)	12.5 (8.8 to 16.2)	12.0 (8.3 to 15.6)	10.7 (7.4 to 13.9)	-1.0 (-1.5 to -0.6)	-14.5 (-19.9 to -7.9)
Maldives	69.0 (61.9 to 76.3)	57.9 (50.8 to 64.1)	56.1 (49.3 to 62.5)	49.9 (43.1 to 56.4)	-1.0 (-1.2 to -0.7)	-13.9 (-16.9 to -10.5)
Mauritius	3.1 (2.2 to 4.3)	2.3 (1.6 to 3.2)	2.3 (1.6 to 3.1)	2.1 (1.5 to 2.8)	-0.7 (-1.2 to -0.3)	-10.3 (-16.3 to -3.9)
Myanmar	70.3 (66.1 to 73.6)	59.9 (54.5 to 64.0)	58.8 (53.4 to 63.0)	53.6 (48.7 to 58.0)	-0.7 (-0.9 to -0.6)	-10.4 (-12.3 to -8.3)
Philippines	53.5 (48.6 to 57.5)	50.5 (44.9 to 55.5)	49.0 (43.4 to 54.1)	43.1 (37.5 to 48.5)	-1.1 (-1.4 to -0.7)	-14.8 (-18.7 to -10.1)
Sri Lanka	49.8 (45.0 to 54.1)	34.9 (28.9 to 39.9)	33.3 (27.1 to 38.5)	26.0 (21.4 to 30.5)	-1.9 (-2.3 to -1.6)	-25.2 (-29.0 to -21.5)
Seychelles	33.2 (27.7 to 38.9)	31.4 (25.4 to 37.0)	29.9 (24.3 to 35.3)	27.1 (21.9 to 32.1)	-1.0 (-1.3 to -0.6)	-13.7 (-18.1 to -8.0)
Thailand	64.6 (60.4 to 68.3)	54.1 (49.2 to 58.2)	52.7 (47.8 to 56.9)	49.5 (44.5 to 53.8)	-0.6 (-0.8 to -0.4)	-8.6 (-11.0 to -6.4)

	Estimate in 2000 (95% UIs)	Estimate in 2015 (95% UIs)	Estimate in 2017 (95% UIs)	Estimate in 2030 (95% UIs)	Annualised rate of change, 2015-2030 (95% UIs)	Percent change, 2015-2030 (95% UIs)
Timor-Leste	54.4 (49.6 to 58.9)	46.4 (40.7 to 51.2)	46.3 (40.4 to 51.3)	39.8 (34.1 to 44.9)	-1.0 (-1.4 to -0.0)	-14.2 (-18.4 to -0.6)
Vietnam	44.8 (37.8 to 49.9)	35.4 (26.7 to 40.6)	34.3 (25.7 to 39.4)	32.0 (24.9 to 36.9)	-0.7 (-0.8 to -0.3)	-9.6 (-11.8 to -4.3)
Sub-Saharan Africa						
Central Sub-Saharan Africa						
Angola	86.1 (81.4 to 91.4)	78.9 (72.6 to 86.4)	77.2 (70.0 to 84.8)	73.6 (65.7 to 82.0)	-0.5 (-0.7 to -0.3)	-6.8 (-9.4 to -4.3)
Central African Republic	88.6 (82.1 to 97.1)	86.3 (78.5 to 96.3)	86.2 (78.4 to 96.1)	85.8 (78.0 to 96.0)	-0.0 (-0.1 to -0.0)	-0.6 (-1.2 to -0.1)
Congo	73.3 (65.5 to 81.9)	61.6 (52.9 to 71.4)	59.6 (50.6 to 69.2)	54.9 (45.9 to 64.8)	-0.8 (-1.1 to -0.5)	-11.0 (-15.2 to -7.2)
Democratic Republic of the Congo	80.5 (72.2 to 91.2)	78.4 (69.6 to 90.8)	77.1 (68.0 to 89.4)	75.9 (66.5 to 88.7)	-0.2 (-0.3 to -0.1)	-3.2 (-4.9 to -1.8)
Equatorial Guinea	82.2 (74.8 to 90.8)	55.4 (46.0 to 65.2)	53.1 (43.9 to 63.0)	45.3 (36.4 to 55.5)	-1.4 (-1.7 to -1.0)	-18.3 (-23.0 to -14.0)
Gabon	56.6 (48.1 to 66.3)	36.6 (29.2 to 45.4)	34.5 (27.1 to 43.3)	28.1 (21.7 to 36.2)	-1.8 (-2.3 to -1.3)	-23.3 (-29.1 to -18.0)
Eastern Sub-Saharan Africa						
Burundi	84.7 (78.5 to 91.3)	81.6 (74.4 to 89.7)	81.5 (74.4 to 89.7)	80.9 (73.7 to 89.3)	-0.1 (-0.1 to -0.0)	-0.8 (-1.6 to -0.1)
Comoros	71.1 (64.9 to 76.7)	59.4 (53.0 to 65.2)	58.6 (52.2 to 64.3)	52.6 (45.9 to 58.8)	-0.8 (-1.1 to -0.6)	-11.5 (-14.7 to -8.7)
Djibouti	73.5 (66.3 to 81.7)	63.8 (56.0 to 73.7)	62.4 (54.5 to 72.3)	59.4 (51.0 to 69.8)	-0.5 (-0.7 to -0.3)	-6.9 (-10.6 to -4.1)
Eritrea	74.9 (70.1 to 79.5)	70.1 (64.5 to 75.2)	69.1 (63.3 to 74.1)	65.9 (59.9 to 71.3)	-0.4 (-0.5 to -0.3)	-6.1 (-7.9 to -4.6)
Ethiopia	83.1 (78.6 to 87.8)	71.4 (65.0 to 78.4)	69.5 (63.1 to 76.9)	67.7 (60.9 to 75.5)	-0.3 (-0.5 to -0.2)	-5.1 (-7.4 to -2.9)
Kenya	69.0 (64.9 to 73.0)	62.7 (58.2 to 66.8)	61.6 (56.8 to 65.8)	58.5 (53.6 to 62.9)	-0.5 (-0.6 to -0.4)	-6.7 (-8.1 to -5.5)
Madagascar	76.4 (73.1 to 79.2)	75.4 (71.7 to 78.4)	74.8 (71.0 to 78.0)	74.3 (70.5 to 77.5)	-0.1 (-0.2 to -0.0)	-1.4 (-2.3 to -0.6)
Malawi	80.0 (75.0 to 85.0)	73.0 (66.1 to 79.6)	72.3 (65.4 to 79.0)	69.5 (62.2 to 76.7)	-0.3 (-0.4 to -0.2)	-4.7 (-6.2 to -3.4)
Mozambique	85.6 (81.4 to 90.0)	76.1 (70.7 to 81.5)	74.8 (68.7 to 80.4)	72.7 (66.6 to 78.6)	-0.3 (-0.4 to -0.2)	-4.5 (-6.3 to -3.0)
Rwanda	71.9 (67.5 to 75.2)	62.5 (57.8 to 66.4)	61.6 (56.8 to 65.5)	55.4 (50.5 to 59.4)	-0.8 (-0.9 to -0.7)	-11.3 (-13.1 to -9.8)
Somalia	83.5 (78.9 to 87.8)	75.3 (69.7 to 81.3)	75.2 (69.3 to 81.4)	72.3 (65.8 to 79.1)	-0.3 (-0.4 to -0.1)	-4.0 (-6.1 to -2.0)
South Sudan	88.5 (84.8 to 92.2)	86.2 (81.4 to 91.0)	85.7 (81.0 to 90.6)	85.3 (80.5 to 90.3)	-0.1 (-0.1 to -0.0)	-1.0 (-1.7 to -0.4)
Tanzania	72.1 (68.3 to 75.7)	64.4 (59.8 to 68.6)	63.2 (58.4 to 67.5)	61.5 (56.6 to 66.0)	-0.3 (-0.5 to -0.2)	-4.5 (-7.0 to -2.4)
Uganda	74.5 (70.9 to 77.7)	63.9 (59.3 to 67.6)	63.1 (58.5 to 66.8)	60.4 (55.9 to 64.3)	-0.4 (-0.5 to -0.3)	-5.4 (-6.7 to -4.2)
Zambia	66.8 (62.6 to 70.2)	63.2 (58.4 to 67.0)	61.4 (56.6 to 65.5)	58.6 (53.7 to 62.8)	-0.5 (-0.7 to -0.3)	-7.3 (-9.5 to -5.0)
Southern Sub-Saharan Africa						
Botswana	63.7 (56.0 to 71.8)	35.5 (29.2 to 42.1)	34.1 (27.6 to 41.1)	28.6 (22.7 to 35.1)	-1.5 (-1.8 to -1.1)	-19.5 (-23.6 to -15.4)
Lesotho	79.7 (72.2 to 88.9)	73.5 (65.4 to 82.9)	72.5 (64.2 to 81.8)	68.2 (59.3 to 78.4)	-0.5 (-0.7 to -0.3)	-7.3 (-9.7 to -5.0)
Namibia	62.8 (54.5 to 71.2)	48.2 (40.5 to 56.7)	46.1 (38.4 to 54.5)	38.8 (31.3 to 46.9)	-1.5 (-1.9 to -1.1)	-19.6 (-24.2 to -15.4)
South Africa	32.0 (27.6 to 37.0)	28.8 (24.5 to 33.2)	28.0 (23.5 to 32.8)	24.0 (20.0 to 28.5)	-1.2 (-1.6 to -0.8)	-16.6 (-21.3 to -11.7)
Swaziland	72.7 (65.4 to 81.0)	60.5 (52.4 to 69.1)	58.9 (51.1 to 67.8)	51.9 (43.8 to 61.3)	-1.0 (-1.3 to -0.7)	-14.3 (-17.7 to -10.3)
Zimbabwe	62.4 (55.2 to 69.9)	68.1 (60.3 to 75.9)	66.6 (58.5 to 74.5)	65.0 (56.9 to 73.0)	-0.3 (-0.5 to -0.2)	-4.6 (-7.0 to -2.6)
Western Sub-Saharan Africa						
Benin	77.5 (70.7 to 85.7)	68.3 (60.0 to 77.7)	67.0 (58.8 to 76.7)	65.8 (57.3 to 75.7)	-0.2 (-0.4 to -0.1)	-3.6 (-6.4 to -1.2)
Burkina Faso	87.4 (80.9 to 95.9)	82.3 (74.6 to 92.6)	81.8 (74.0 to 92.1)	80.1 (71.8 to 91.2)	-0.2 (-0.3 to -0.1)	-2.8 (-4.3 to -1.3)
Cameroon	79.5 (73.6 to 86.5)	70.5 (62.5 to 79.9)	68.5 (60.4 to 77.7)	65.8 (57.4 to 75.5)	-0.5 (-0.6 to -0.3)	-6.7 (-9.3 to -4.4)
Cape Verde	66.3 (58.1 to 74.6)	45.0 (36.4 to 53.8)	43.8 (35.1 to 52.3)	35.9 (27.8 to 44.3)	-1.5 (-2.0 to -1.1)	-20.4 (-25.9 to -15.3)
Chad	88.6 (83.5 to 94.6)	84.0 (78.0 to 91.6)	83.4 (77.2 to 91.2)	82.6 (76.1 to 90.7)	-0.1 (-0.2 to -0.1)	-1.7 (-2.8 to -0.8)
Cote d'Ivoire	69.4 (62.3 to 77.6)	67.1 (58.5 to 76.8)	65.6 (57.0 to 75.5)	61.2 (52.4 to 71.7)	-0.6 (-0.9 to -0.4)	-8.9 (-12.5 to -5.9)
The Gambia	75.6 (67.7 to 85.6)	65.4 (57.0 to 74.8)	64.6 (56.1 to 74.2)	59.6 (50.7 to 69.8)	-0.6 (-0.9 to -0.4)	-8.8 (-12.2 to -5.5)
Ghana	78.8 (72.2 to 87.4)	76.3 (68.0 to 86.8)	75.5 (67.4 to 85.7)	75.0 (66.8 to 85.3)	-0.1 (-0.2 to -0.0)	-1.8 (-3.1 to -0.7)
Guinea	87.1 (82.9 to 91.7)	79.8 (73.3 to 87.4)	78.7 (72.1 to 86.5)	76.2 (68.9 to 84.8)	-0.3 (-0.5 to -0.2)	-4.5 (-6.6 to -2.6)
Guinea-Bissau	83.8 (78.3 to 90.1)	80.3 (73.3 to 88.5)	79.5 (72.5 to 87.7)	78.2 (71.0 to 86.8)	-0.2 (-0.3 to -0.1)	-2.7 (-4.3 to -1.5)
Liberia	87.3 (82.0 to 94.0)	83.8 (77.1 to 92.7)	83.3 (76.3 to 92.2)	82.9 (75.8 to 91.9)	-0.1 (-0.1 to -0.0)	-1.0 (-1.9 to -0.3)
Mali	84.6 (79.5 to 90.6)	76.6 (70.1 to 83.7)	75.7 (69.2 to 82.8)	74.6 (67.9 to 82.2)	-0.2 (-0.3 to -0.1)	-2.6 (-4.4 to -1.2)
Mauritania	73.7 (67.4 to 80.3)	65.6 (57.4 to 73.2)	64.0 (56.0 to 72.1)	57.6 (49.4 to 66.2)	-0.9 (-1.2 to -0.6)	-12.3 (-16.0 to -8.6)
Niger	86.9 (81.6 to 93.1)	80.7 (73.7 to 88.5)	80.0 (72.9 to 88.0)	74.9 (66.7 to 84.3)	-0.5 (-0.7 to -0.3)	-7.2 (-10.5 to -3.9)
Nigeria	81.5 (77.0 to 85.8)	76.0 (69.3 to 83.1)	75.6 (68.8 to 82.6)	74.2 (67.3 to 81.5)	-0.2 (-0.2 to -0.1)	-2.3 (-3.5 to -1.2)
Sao Tome and Principe	73.4 (65.7 to 82.8)	47.3 (39.5 to 56.1)	45.4 (37.1 to 54.0)	42.5 (34.4 to 51.2)	-0.7 (-1.1 to -0.4)	-10.3 (-15.0 to -5.9)
Senegal	60.0 (54.7 to 65.5)	49.3 (42.9 to 55.9)	48.9 (41.7 to 56.5)	43.4 (36.5 to 51.2)	-0.9 (-1.3 to -0.5)	-12.1 (-17.2 to -7.1)
Sierra Leone	87.0 (82.4 to 92.9)	81.6 (75.1 to 89.3)	80.8 (74.2 to 88.6)	79.8 (73.1 to 87.9)	-0.1 (-0.2 to -0.1)	-2.2 (-3.5 to -1.2)
Togo	82.9 (76.4 to 90.0)	78.9 (71.4 to 87.8)	77.8 (70.2 to 87.1)	76.9 (69.1 to 86.4)	-0.2 (-0.3 to -0.1)	-2.5 (-4.0 to -1.3)
**Indicator 6.2.1a: Risk-weighted prevalence of populations using unsafe or unimproved sanitation, as measured by the summary exposure value (SEV) for unsafe sanitation (%)**						
Central Europe, Eastern Europe, and Central Asia						
Central Asia						
Armenia	33.3 (26.6 to 42.5)	22.6 (17.1 to 30.2)	22.6 (16.7 to 30.9)	17.1 (12.4 to 23.8)	-1.9 (-2.5 to -1.3)	-24.7 (-31.7 to -17.2)
Azerbaijan	59.9 (46.1 to 79.3)	39.2 (28.0 to 53.0)	37.6 (26.4 to 51.1)	26.9 (17.3 to 38.9)	-2.6 (-3.6 to -1.7)	-31.6 (-42.1 to -22.3)
Georgia	50.3 (38.2 to 64.9)	37.0 (25.9 to 50.0)	34.5 (23.5 to 48.0)	30.5 (20.2 to 43.3)	-1.3 (-2.0 to -0.7)	-17.8 (-25.4 to -10.4)
Kazakhstan	44.0 (37.3 to 53.2)	42.5 (37.6 to 48.3)	37.6 (32.3 to 43.9)	29.3 (24.6 to 35.2)	-2.5 (-3.1 to -2.0)	-31.1 (-37.2 to -25.5)
Kyrgyzstan	64.1 (49.2 to 86.0)	75.6 (66.6 to 86.9)	74.2 (64.9 to 85.7)	70.1 (59.6 to 83.1)	-0.5 (-0.8 to -0.2)	-7.3 (-11.6 to -3.4)
Mongolia	76.1 (66.7 to 88.5)	69.4 (60.4 to 80.3)	68.0 (58.5 to 78.8)	59.1 (48.9 to 71.7)	-1.1 (-1.5 to -0.7)	-15.0 (-20.7 to -9.3)
Tajikistan	63.2 (43.9 to 91.6)	63.4 (41.9 to 96.7)	63.0 (42.1 to 96.7)	60.7 (39.1 to 96.2)	-0.3 (-0.6 to -0.0)	-4.6 (-8.4 to -0.2)
Turkmenistan	58.8 (40.6 to 84.7)	46.0 (28.0 to 73.9)	44.0 (26.4 to 71.0)	29.0 (15.2 to 55.8)	-3.2 (-4.2 to -1.9)	-38.3 (-46.7 to -25.2)
Uzbekistan	69.5 (55.4 to 91.3)	60.9 (45.4 to 81.8)	58.3 (43.0 to 79.3)	46.0 (30.7 to 69.6)	-1.9 (-2.7 to -1.1)	-25.1 (-33.0 to -15.0)
Central Europe						
Albania	28.2 (21.6 to 36.9)	15.3 (10.3 to 22.1)	13.7 (9.0 to 20.4)	7.4 (4.7 to 11.4)	-4.9 (-5.7 to -4.0)	-51.7 (-57.4 to -45.3)
Bosnia and Herzegovina	17.1 (13.1 to 23.0)	6.6 (4.2 to 10.3)	6.2 (3.8 to 9.7)	3.4 (2.1 to 5.5)	-4.5 (-5.6 to -3.4)	-48.6 (-56.8 to -40.3)
Bulgaria	2.1 (1.1 to 3.5)	1.4 (0.8 to 2.4)	1.4 (0.7 to 2.3)	1.3 (0.7 to 2.1)	-1.0 (-1.8 to -0.2)	-13.4 (-23.5 to -2.2)
Croatia	4.7 (2.7 to 7.7)	2.5 (1.4 to 4.5)	2.4 (1.4 to 4.2)	2.2 (1.3 to 3.9)	-0.8 (-1.7 to 0.0)	-11.4 (-22.4 to 0.4)
Czech Republic	1.0 (0.5 to 1.6)	0.7 (0.4 to 1.2)	0.7 (0.4 to 1.2)	0.6 (0.3 to 1.1)	-0.7 (-1.5 to 0.1)	-9.5 (-20.1 to 2.2)
Hungary	11.5 (6.6 to 18.2)	6.0 (3.3 to 10.2)	5.7 (3.1 to 9.6)	4.5 (2.4 to 7.7)	-2.0 (-3.1 to -1.0)	-26.1 (-37.5 to -14.4)
Macedonia	13.3 (7.8 to 20.2)	7.4 (4.4 to 11.8)	6.9 (4.0 to 11.1)	5.8 (3.3 to 9.5)	-1.6 (-2.6 to -0.8)	-21.7 (-32.4 to -11.0)
Montenegro	8.1 (4.5 to 13.7)	3.7 (2.1 to 6.5)	3.5 (1.9 to 6.1)	2.2 (1.2 to 4.0)	-3.5 (-4.7 to -2.4)	-40.8 (-50.9 to -29.9)
Poland	5.5 (3.1 to 8.6)	2.4 (1.3 to 4.0)	2.2 (1.2 to 3.7)	1.7 (1.0 to 3.0)	-2.0 (-2.9 to -1.2)	-26.0 (-34.9 to -15.9)
Romania	36.4 (24.9 to 50.8)	17.9 (10.6 to 28.8)	16.8 (9.7 to 27.2)	8.1 (4.5 to 14.0)	-5.3 (-6.2 to -4.4)	-55.0 (-60.4 to -48.4)
Serbia	12.4 (8.0 to 19.0)	5.4 (3.2 to 8.4)	5.2 (3.0 to 8.3)	3.7 (2.1 to 6.0)	-2.6 (-4.0 to -1.4)	-31.9 (-45.5 to -18.7)
Slovakia	3.3 (1.9 to 5.5)	1.8 (1.0 to 3.0)	1.7 (0.9 to 2.8)	1.6 (0.8 to 2.6)	-0.8 (-1.6 to 0.0)	-10.9 (-21.5 to 0.6)
Slovenia	4.4 (2.5 to 7.0)	2.5 (1.4 to 4.1)	2.4 (1.3 to 3.9)	2.2 (1.2 to 3.6)	-0.8 (-1.6 to 0.1)	-11.2 (-21.8 to 1.6)
Eastern Europe						

	Estimate in 2000 (95% UIs)	Estimate in 2015 (95% UIs)	Estimate in 2017 (95% UIs)	Estimate in 2030 (95% UIs)	Annualised rate of change, 2015-2030 (95% UIs)	Percent change, 2015-2030 (95% UIs)
Belarus	30.4 (24.3 to 38.7)	17.4 (13.2 to 23.0)	16.4 (11.8 to 22.7)	13.8 (9.7 to 19.6)	-1.6 (-2.6 to -0.7)	-20.6 (-32.0 to -9.8)
Estonia	13.6 (8.1 to 20.9)	7.1 (4.1 to 11.2)	6.9 (4.0 to 10.7)	6.4 (3.7 to 10.0)	-0.7 (-1.5 to 0.1)	-9.8 (-19.6 to 1.0)
Latvia	21.3 (13.8 to 30.7)	12.0 (7.5 to 18.1)	11.4 (6.8 to 17.2)	10.6 (6.3 to 16.1)	-0.9 (-1.6 to -0.1)	-12.3 (-21.4 to -1.4)
Lithuania	21.6 (13.7 to 31.9)	12.3 (7.6 to 18.7)	12.0 (7.3 to 18.9)	10.7 (6.5 to 17.0)	-1.0 (-1.7 to -0.2)	-13.1 (-23.0 to -3.1)
Moldova	66.6 (58.2 to 76.5)	58.3 (52.2 to 65.6)	55.9 (48.1 to 64.0)	45.0 (37.6 to 53.3)	-1.8 (-2.3 to -1.3)	-23.1 (-29.5 to -17.3)
Russia	35.9 (27.7 to 46.6)	21.8 (15.1 to 30.4)	20.8 (14.2 to 29.8)	16.6 (11.2 to 24.4)	-1.8 (-2.6 to -1.2)	-23.9 (-31.8 to -16.1)
Ukraine	31.0 (22.4 to 42.8)	31.6 (23.0 to 43.0)	30.7 (21.7 to 43.2)	25.5 (17.6 to 36.7)	-1.5 (-2.1 to -0.8)	-19.7 (-27.5 to -11.8)
High-income						
Australasia						
Australia	2.4 (1.4 to 3.9)	1.5 (0.8 to 2.4)	1.4 (0.8 to 2.3)	0.9 (0.5 to 1.4)	-3.5 (-4.3 to -2.7)	-41.0 (-47.8 to -32.9)
New Zealand	3.2 (1.9 to 4.9)	2.1 (1.2 to 3.3)	2.0 (1.1 to 3.2)	1.3 (0.7 to 2.0)	-3.2 (-4.1 to -2.3)	-38.3 (-45.8 to -29.5)
High-income Asia Pacific						
Brunei	3.5 (2.1 to 5.6)	1.8 (1.0 to 2.9)	1.7 (0.9 to 2.7)	1.1 (0.6 to 1.8)	-3.3 (-4.5 to -2.2)	-39.0 (-49.4 to -28.3)
Japan	2.1 (1.3 to 3.4)	1.6 (0.9 to 2.5)	1.5 (0.9 to 2.5)	1.1 (0.7 to 1.9)	-2.2 (-3.1 to -1.3)	-27.8 (-37.5 to -17.4)
South Korea	3.1 (1.8 to 4.9)	1.5 (0.9 to 2.4)	1.4 (0.8 to 2.3)	0.8 (0.5 to 1.4)	-3.9 (-4.9 to -3.0)	-44.3 (-52.1 to -36.3)
Singapore	3.2 (1.8 to 4.9)	1.5 (0.9 to 2.4)	1.4 (0.8 to 2.3)	0.8 (0.5 to 1.4)	-4.0 (-5.2 to -2.9)	-44.8 (-53.9 to -35.5)
High-income North America						
Canada	3.3 (1.9 to 5.2)	1.9 (1.1 to 3.2)	1.9 (1.1 to 3.2)	1.6 (0.9 to 2.6)	-1.6 (-2.4 to -0.7)	-20.7 (-30.3 to -9.4)
Greenland	12.7 (7.6 to 18.5)	6.5 (3.8 to 10.3)	6.3 (3.6 to 10.1)	4.3 (2.5 to 7.2)	-2.7 (-3.6 to -1.8)	-32.8 (-42.0 to -23.1)
United States	5.2 (3.2 to 8.2)	3.1 (1.7 to 5.0)	3.0 (1.6 to 5.0)	2.5 (1.4 to 4.2)	-1.5 (-2.4 to -0.7)	-20.4 (-29.9 to -9.5)
Southern Latin America						
Argentina	19.2 (15.5 to 23.3)	5.8 (3.6 to 8.7)	5.3 (3.3 to 8.1)	2.5 (1.5 to 3.9)	-5.7 (-6.8 to -4.7)	-57.1 (-64.0 to -50.4)
Chile	17.2 (13.4 to 21.4)	8.0 (4.9 to 11.8)	7.6 (4.7 to 11.7)	3.9 (2.3 to 6.0)	-4.8 (-5.7 to -3.9)	-51.1 (-57.3 to -44.1)
Uruguay	10.2 (7.9 to 12.9)	2.5 (1.5 to 3.7)	2.1 (1.3 to 3.2)	0.9 (0.5 to 1.4)	-6.9 (-8.0 to -6.0)	-64.5 (-69.7 to -59.0)
Western Europe						
Andorra	1.1 (0.7 to 1.9)	0.8 (0.5 to 1.2)	0.8 (0.5 to 1.2)	0.6 (0.3 to 0.9)	-1.9 (-2.8 to -1.0)	-24.6 (-34.1 to -14.3)
Austria	4.9 (3.0 to 7.6)	3.2 (1.9 to 5.2)	3.1 (1.8 to 5.0)	2.3 (1.3 to 3.7)	-2.3 (-3.1 to -1.4)	-28.8 (-37.4 to -19.5)
Belgium	1.6 (1.0 to 2.4)	0.9 (0.6 to 1.5)	0.9 (0.5 to 1.4)	0.7 (0.4 to 1.1)	-2.0 (-2.9 to -1.0)	-25.3 (-35.6 to -14.3)
Cyprus	2.5 (1.4 to 3.8)	1.1 (0.7 to 1.8)	1.1 (0.7 to 1.8)	0.8 (0.5 to 1.3)	-2.1 (-3.4 to -1.0)	-26.5 (-39.7 to -13.4)
Denmark	1.0 (0.6 to 1.6)	0.7 (0.4 to 1.1)	0.7 (0.4 to 1.0)	0.5 (0.3 to 0.8)	-1.8 (-2.8 to -0.8)	-23.8 (-34.5 to -11.3)
Finland	1.5 (0.9 to 2.4)	0.9 (0.5 to 1.4)	0.8 (0.5 to 1.4)	0.6 (0.4 to 1.0)	-2.2 (-3.3 to -1.2)	-28.4 (-39.2 to -17.1)
France	2.0 (1.2 to 3.2)	1.2 (0.7 to 1.9)	1.1 (0.7 to 1.8)	0.9 (0.5 to 1.4)	-1.9 (-3.0 to -0.9)	-24.9 (-35.8 to -13.2)
Germany	1.9 (1.2 to 3.1)	1.1 (0.7 to 1.7)	1.1 (0.6 to 1.7)	0.8 (0.5 to 1.2)	-2.5 (-3.4 to -1.6)	-31.0 (-40.1 to -21.9)
Greece	2.8 (1.7 to 4.4)	1.8 (1.1 to 2.8)	1.8 (1.1 to 2.9)	1.5 (0.9 to 2.4)	-1.2 (-2.7 to -0.1)	-16.3 (-33.6 to -1.2)
Iceland	1.4 (0.8 to 2.2)	0.8 (0.4 to 1.2)	0.7 (0.4 to 1.1)	0.5 (0.3 to 0.7)	-3.2 (-4.1 to -2.2)	-37.9 (-46.2 to -28.4)
Ireland	4.1 (2.4 to 6.6)	2.1 (1.2 to 3.4)	2.0 (1.2 to 3.1)	1.3 (0.8 to 2.1)	-3.2 (-4.1 to -2.3)	-37.7 (-46.3 to -28.9)
Israel	2.8 (1.6 to 4.5)	1.9 (1.1 to 3.0)	1.8 (1.0 to 2.9)	1.3 (0.8 to 2.1)	-2.2 (-3.0 to -1.3)	-27.5 (-36.5 to -18.0)
Italy	2.2 (1.4 to 3.5)	1.6 (1.0 to 2.5)	1.6 (1.0 to 2.5)	1.3 (0.8 to 2.0)	-1.6 (-2.5 to -0.7)	-21.2 (-31.2 to -9.6)
Luxembourg	1.1 (0.6 to 1.7)	0.7 (0.4 to 1.1)	0.7 (0.4 to 1.1)	0.5 (0.3 to 0.8)	-1.9 (-2.9 to -0.9)	-24.5 (-35.4 to -13.3)
Malta	2.9 (1.8 to 4.6)	1.6 (0.9 to 2.5)	1.5 (0.9 to 2.4)	1.0 (0.6 to 1.6)	-3.1 (-4.0 to -2.2)	-36.6 (-44.8 to -27.9)
Netherlands	1.2 (0.7 to 1.9)	0.7 (0.4 to 1.2)	0.7 (0.4 to 1.1)	0.5 (0.3 to 0.8)	-2.1 (-3.2 to -1.1)	-26.4 (-37.8 to -14.6)
Norway	1.3 (0.8 to 2.0)	0.7 (0.4 to 1.1)	0.7 (0.4 to 1.1)	0.5 (0.3 to 0.8)	-2.2 (-3.3 to -1.3)	-28.4 (-39.5 to -17.3)
Portugal	10.6 (7.4 to 14.4)	5.8 (3.5 to 8.8)	5.6 (3.4 to 8.6)	3.8 (2.2 to 5.9)	-2.9 (-3.8 to -2.0)	-35.3 (-43.5 to -26.5)
Spain	1.5 (0.9 to 2.4)	0.6 (0.3 to 0.9)	0.5 (0.3 to 0.9)	0.3 (0.2 to 0.5)	-4.1 (-5.2 to -3.0)	-45.4 (-54.0 to -36.2)
Sweden	1.6 (0.9 to 2.4)	1.0 (0.6 to 1.6)	0.9 (0.6 to 1.5)	0.7 (0.5 to 1.2)	-1.9 (-2.7 to -1.0)	-24.3 (-33.6 to -13.4)
Switzerland	1.3 (0.8 to 2.1)	0.9 (0.5 to 1.4)	0.9 (0.5 to 1.4)	0.6 (0.4 to 1.0)	-2.5 (-3.4 to -1.6)	-30.7 (-39.9 to -21.2)
United Kingdom	0.4 (0.2 to 0.6)	0.2 (0.1 to 0.4)	0.2 (0.1 to 0.3)	0.2 (0.1 to 0.2)	-2.6 (-3.5 to -1.7)	-31.7 (-40.5 to -22.3)
Latin America and Caribbean						
Andean Latin America						
Bolivia	38.3 (31.1 to 47.9)	19.2 (12.8 to 27.6)	16.6 (10.8 to 24.5)	8.2 (5.1 to 12.6)	-5.8 (-6.6 to -4.9)	-57.8 (-63.0 to -52.1)
Ecuador	27.1 (22.4 to 33.2)	12.9 (9.2 to 18.1)	12.0 (8.1 to 17.2)	11.1 (7.5 to 16.0)	-1.0 (-1.8 to -0.3)	-14.3 (-23.8 to -4.6)
Peru	42.0 (35.7 to 51.0)	35.0 (26.9 to 46.3)	32.7 (24.1 to 44.4)	30.4 (22.0 to 42.0)	-1.0 (-1.6 to -0.4)	-13.2 (-21.0 to -5.8)
Caribbean						
Antigua and Barbuda	25.4 (19.3 to 34.0)	14.8 (8.7 to 23.5)	14.1 (8.3 to 22.4)	12.2 (7.2 to 19.6)	-1.3 (-2.2 to -0.6)	-17.8 (-28.3 to -8.0)
The Bahamas	12.1 (8.5 to 16.6)	8.1 (4.6 to 13.6)	8.1 (4.6 to 13.7)	7.5 (4.2 to 12.6)	-0.5 (-1.2 to 0.3)	-6.9 (-17.0 to 3.9)
Barbados	20.7 (13.0 to 31.3)	11.8 (6.9 to 19.2)	11.5 (6.8 to 18.9)	9.6 (5.6 to 16.1)	-1.4 (-2.1 to -0.6)	-18.7 (-27.2 to -8.7)
Belize	42.3 (32.0 to 56.5)	25.8 (16.3 to 39.4)	25.0 (15.2 to 38.5)	18.3 (10.7 to 29.4)	-2.4 (-3.3 to -1.4)	-29.6 (-39.0 to -19.2)
Bermuda	10.5 (6.1 to 16.6)	5.6 (3.0 to 9.2)	5.5 (3.0 to 9.2)	4.9 (2.6 to 8.1)	-0.9 (-1.8 to -0.2)	-13.0 (-23.3 to -2.4)
Cuba	31.9 (22.1 to 45.5)	20.3 (13.8 to 29.4)	18.5 (11.9 to 27.4)	11.3 (7.0 to 17.7)	-4.0 (-5.0 to -2.9)	-44.7 (-52.4 to -35.7)
Dominica	38.6 (26.9 to 52.2)	19.2 (11.4 to 30.0)	18.6 (10.8 to 29.0)	12.9 (7.2 to 20.9)	-2.7 (-3.6 to -1.9)	-33.3 (-42.1 to -24.8)
Dominican Republic	45.7 (39.7 to 53.4)	22.1 (17.4 to 27.7)	20.0 (15.1 to 25.7)	11.1 (8.0 to 14.9)	-4.6 (-5.6 to -3.6)	-49.7 (-57.0 to -42.2)
Grenada	41.4 (33.1 to 52.3)	25.0 (16.6 to 37.3)	23.4 (14.9 to 34.7)	15.6 (9.6 to 24.3)	-3.2 (-4.2 to -2.3)	-38.1 (-47.1 to -29.5)
Guyana	50.9 (38.5 to 68.9)	26.6 (18.3 to 38.8)	25.1 (16.9 to 37.0)	13.2 (8.5 to 21.2)	-4.7 (-5.6 to -3.8)	-50.6 (-56.9 to -43.8)
Haiti	83.6 (71.6 to 100.0)	76.3 (62.8 to 94.8)	75.3 (61.6 to 93.6)	65.1 (49.0 to 89.9)	-1.1 (-1.7 to -0.3)	-15.2 (-22.3 to -4.8)
Jamaica	43.5 (34.7 to 55.9)	35.2 (24.3 to 51.0)	33.9 (22.4 to 49.6)	30.4 (19.8 to 45.4)	-1.0 (-1.6 to -0.5)	-13.9 (-21.5 to -7.4)
Puerto Rico	3.0 (1.7 to 4.8)	2.2 (1.3 to 3.6)	2.1 (1.2 to 3.5)	2.0 (1.1 to 3.2)	-0.6 (-1.4 to 0.2)	-9.0 (-19.3 to 3.1)
Saint Lucia	41.0 (32.6 to 51.4)	20.3 (14.5 to 29.2)	19.3 (13.5 to 29.1)	12.4 (8.0 to 19.7)	-3.4 (-4.9 to -2.2)	-39.5 (-51.9 to -28.2)
Saint Vincent and the Grenadines	44.1 (35.2 to 55.1)	27.7 (17.5 to 40.1)	26.4 (16.5 to 38.5)	20.0 (12.2 to 30.2)	-2.2 (-3.1 to -1.4)	-28.1 (-37.3 to -19.3)
Suriname	28.3 (22.1 to 36.5)	17.0 (11.4 to 24.8)	16.6 (10.7 to 24.4)	10.6 (6.6 to 15.8)	-3.2 (-4.1 to -2.3)	-38.1 (-46.2 to -28.8)
Trinidad and Tobago	27.2 (21.1 to 35.4)	13.5 (8.3 to 21.4)	13.7 (8.0 to 22.4)	11.6 (6.7 to 18.8)	-1.0 (-2.4 to 0.0)	-13.7 (-30.5 to 0.0)
Virgin Islands, U.S.	11.9 (6.7 to 19.3)	5.5 (3.1 to 9.1)	5.5 (3.0 to 9.1)	5.1 (2.8 to 8.5)	-0.5 (-1.4 to 0.3)	-7.5 (-18.4 to 4.3)
Central Latin America						
Colombia	22.9 (19.7 to 26.7)	7.6 (5.4 to 10.7)	7.0 (4.8 to 10.3)	3.4 (2.3 to 5.1)	-5.4 (-6.2 to -4.6)	-55.4 (-60.7 to -49.8)
Costa Rica	7.9 (5.3 to 11.1)	2.1 (1.2 to 3.5)	1.9 (1.1 to 3.2)	1.1 (0.6 to 1.9)	-4.3 (-5.5 to -3.2)	-47.2 (-56.4 to -38.2)
El Salvador	50.9 (42.4 to 61.3)	39.5 (29.5 to 53.9)	36.8 (27.2 to 50.4)	31.6 (22.7 to 44.3)	-1.5 (-2.1 to -1.0)	-20.1 (-26.7 to -13.9)
Guatemala	48.8 (40.6 to 59.1)	38.9 (31.5 to 48.3)	36.8 (29.0 to 47.0)	26.5 (20.1 to 35.3)	-2.6 (-3.3 to -1.9)	-31.9 (-38.9 to -25.2)
Honduras	54.2 (44.4 to 67.0)	35.2 (26.5 to 45.7)	33.0 (24.2 to 44.6)	21.0 (14.6 to 30.2)	-3.5 (-4.2 to -2.7)	-40.6 (-47.0 to -33.5)
Mexico	22.4 (18.8 to 27.0)	11.8 (8.6 to 16.2)	11.5 (8.1 to 16.3)	8.8 (6.1 to 12.6)	-2.0 (-3.2 to -1.2)	-26.1 (-37.7 to -15.9)
Nicaragua	65.7 (53.8 to 82.0)	44.9 (33.8 to 59.8)	41.8 (30.5 to 55.4)	24.5 (16.1 to 35.6)	-4.1 (-5.0 to -3.2)	-45.8 (-52.9 to -38.2)
Panama	41.7 (34.9 to 51.4)	25.7 (19.9 to 33.0)	22.3 (16.4 to 29.8)	11.3 (8.0 to 15.7)	-5.5 (-6.3 to -4.7)	-56.3 (-61.4 to -50.4)

	Estimate in 2000 (95% UIs)	Estimate in 2015 (95% UIs)	Estimate in 2017 (95% UIs)	Estimate in 2030 (95% UIs)	Annualised rate of change, 2015-2030 (95% UIs)	Percent change, 2015-2030 (95% UIs)
Venezuela	13.7 (10.8 to 16.9)	9.6 (5.9 to 14.6)	9.2 (5.7 to 14.3)	7.1 (4.3 to 11.0)	-2.1 (-2.9 to -1.3)	-26.4 (-35.2 to -17.4)
Tropical Latin America						
Brazil	36.4 (30.2 to 44.4)	24.8 (18.3 to 33.9)	23.3 (16.3 to 32.8)	18.1 (12.4 to 25.9)	-2.1 (-2.8 to -1.4)	-27.3 (-34.7 to -19.3)
Paraguay	41.5 (35.0 to 49.3)	18.4 (12.4 to 26.6)	17.0 (10.7 to 24.9)	11.4 (6.9 to 17.9)	-3.3 (-4.6 to -2.1)	-38.3 (-50.1 to -27.4)
North Africa and Middle East						
North Africa and Middle East						
Afghanistan	89.7 (80.6 to 100.0)	73.9 (60.3 to 92.7)	72.3 (58.2 to 91.7)	54.6 (38.1 to 83.2)	-2.1 (-3.0 to -0.8)	-26.9 (-36.7 to -10.7)
Algeria	21.8 (17.7 to 26.7)	9.3 (5.8 to 13.9)	8.4 (5.1 to 12.9)	4.2 (2.5 to 6.6)	-5.3 (-6.4 to -4.3)	-54.8 (-61.6 to -47.2)
Bahrain	12.0 (7.4 to 18.0)	7.3 (4.5 to 11.3)	6.9 (4.3 to 10.7)	4.9 (3.0 to 7.7)	-2.7 (-3.5 to -1.9)	-33.0 (-40.7 to -25.0)
Egypt	65.9 (61.4 to 71.0)	27.7 (21.5 to 35.1)	23.9 (17.6 to 31.9)	16.8 (12.1 to 23.1)	-3.4 (-4.2 to -2.7)	-39.7 (-46.5 to -32.9)
Iran	21.9 (14.3 to 31.9)	8.5 (5.2 to 13.1)	7.8 (4.7 to 12.2)	5.8 (3.6 to 9.4)	-2.5 (-3.5 to -1.6)	-31.1 (-40.9 to -21.4)
Iraq	26.0 (20.6 to 33.1)	22.5 (16.6 to 30.5)	20.3 (14.4 to 28.8)	11.7 (7.8 to 17.3)	-4.4 (-5.4 to -3.5)	-48.4 (-55.7 to -40.7)
Jordan	15.9 (11.8 to 20.7)	7.6 (4.6 to 11.4)	5.8 (3.4 to 9.1)	2.5 (1.5 to 4.1)	-7.3 (-8.1 to -6.5)	-66.5 (-70.4 to -62.4)
Kuwait	10.2 (6.2 to 15.1)	3.8 (2.2 to 5.9)	3.4 (2.0 to 5.5)	2.8 (1.6 to 4.5)	-2.0 (-3.1 to -0.9)	-25.3 (-37.6 to -12.9)
Lebanon	16.7 (12.6 to 21.4)	2.6 (1.5 to 4.2)	2.4 (1.3 to 3.8)	1.9 (1.1 to 3.1)	-1.9 (-3.1 to -1.0)	-25.0 (-37.6 to -14.1)
Libya	39.6 (31.5 to 48.1)	12.1 (7.4 to 18.1)	13.2 (8.1 to 19.7)	8.0 (4.7 to 12.7)	-2.8 (-4.3 to -1.5)	-33.7 (-47.3 to -20.4)
Morocco	34.9 (30.8 to 39.5)	14.2 (9.8 to 19.6)	12.8 (8.5 to 18.2)	5.9 (3.8 to 8.8)	-5.9 (-6.7 to -5.0)	-58.6 (-63.5 to -52.9)
Palestine	25.9 (19.1 to 34.2)	9.5 (7.2 to 12.2)	8.5 (6.1 to 11.4)	5.0 (3.4 to 7.0)	-4.3 (-5.4 to -3.3)	-47.6 (-55.2 to -39.2)
Oman	7.2 (4.5 to 10.6)	2.7 (1.6 to 4.3)	2.5 (1.4 to 3.9)	2.1 (1.2 to 3.4)	-1.6 (-2.9 to -0.6)	-21.1 (-35.6 to -8.3)
Qatar	9.0 (5.4 to 13.6)	4.5 (2.6 to 6.9)	4.1 (2.4 to 6.5)	3.3 (1.9 to 5.3)	-1.9 (-3.0 to -0.9)	-24.6 (-35.9 to -12.7)
Saudi Arabia	20.3 (13.4 to 29.7)	4.1 (2.5 to 6.4)	3.6 (2.1 to 5.6)	2.0 (1.1 to 3.2)	-4.9 (-6.5 to -3.6)	-51.5 (-62.6 to -41.4)
Sudan	88.1 (81.2 to 97.3)	82.5 (73.0 to 95.5)	80.6 (70.6 to 94.0)	68.2 (55.1 to 88.8)	-1.3 (-1.9 to -0.5)	-17.6 (-25.1 to -7.2)
Syria	24.1 (19.3 to 30.5)	10.7 (6.7 to 16.3)	9.7 (5.9 to 15.1)	6.5 (3.7 to 10.7)	-3.3 (-4.8 to -2.2)	-38.8 (-51.1 to -28.1)
Tunisia	18.8 (13.5 to 25.6)	15.8 (11.0 to 21.7)	14.2 (9.5 to 19.8)	11.9 (8.0 to 16.8)	-1.9 (-2.7 to -1.2)	-25.0 (-32.8 to -16.1)
Turkey	30.4 (26.6 to 34.6)	10.4 (7.1 to 14.9)	8.9 (5.8 to 13.1)	4.8 (3.0 to 7.1)	-5.2 (-6.2 to -4.3)	-54.3 (-60.4 to -47.3)
United Arab Emirates	2.2 (1.3 to 3.5)	1.4 (0.8 to 2.2)	1.4 (0.8 to 2.2)	1.2 (0.7 to 1.9)	-1.1 (-2.0 to -0.1)	-14.4 (-25.5 to -1.3)
Yemen	86.0 (79.4 to 94.5)	44.9 (38.4 to 52.8)	44.2 (36.3 to 53.1)	25.3 (19.3 to 33.0)	-3.9 (-4.7 to -3.0)	-43.9 (-50.9 to -36.0)
South Asia						
South Asia						
Bangladesh	78.5 (67.7 to 94.2)	66.8 (54.1 to 84.2)	65.4 (52.4 to 83.5)	46.4 (32.9 to 69.4)	-2.5 (-3.4 to -1.4)	-31.0 (-39.8 to -18.7)
Bhutan	69.1 (59.6 to 81.1)	43.3 (33.0 to 54.5)	40.1 (29.6 to 52.2)	21.5 (14.3 to 30.9)	-4.7 (-5.9 to -3.7)	-50.8 (-58.7 to -42.9)
India	73.1 (68.2 to 79.2)	39.5 (33.2 to 46.5)	34.9 (27.9 to 43.3)	18.0 (13.4 to 23.5)	-5.3 (-6.2 to -4.3)	-54.6 (-60.5 to -47.9)
Nepal	78.3 (74.4 to 83.0)	48.0 (41.8 to 54.0)	45.0 (38.0 to 52.3)	25.1 (19.8 to 31.0)	-4.3 (-5.2 to -3.6)	-47.8 (-54.1 to -41.3)
Pakistan	54.8 (50.3 to 59.9)	36.1 (31.8 to 41.9)	34.9 (29.3 to 41.4)	19.5 (15.7 to 24.2)	-4.1 (-4.8 to -3.4)	-46.1 (-51.2 to -40.3)
Southeast Asia, East Asia, and Oceania						
East Asia						
China	54.8 (48.4 to 63.5)	26.7 (18.2 to 36.9)	24.7 (16.4 to 34.2)	12.6 (7.9 to 18.6)	-5.1 (-6.0 to -4.1)	-53.2 (-59.2 to -46.2)
North Korea	43.8 (33.2 to 54.3)	35.0 (24.2 to 46.8)	33.6 (23.1 to 45.2)	30.1 (20.3 to 41.4)	-1.0 (-1.6 to -0.5)	-14.2 (-21.5 to -7.2)
Taiwan (Province of China)	12.4 (7.2 to 18.6)	4.7 (2.8 to 7.4)	4.4 (2.6 to 6.9)	2.8 (1.6 to 4.4)	-3.4 (-4.4 to -2.5)	-39.8 (-48.5 to -31.3)
Oceania						
American Samoa	16.6 (10.7 to 24.8)	8.5 (5.0 to 13.2)	8.2 (4.9 to 12.3)	7.3 (4.3 to 11.2)	-1.0 (-1.9 to -0.2)	-13.7 (-24.5 to -2.5)
Federated States of Micronesia	41.5 (29.2 to 54.3)	24.6 (16.2 to 34.3)	23.5 (15.6 to 33.0)	17.3 (11.1 to 25.5)	-2.4 (-3.5 to -1.4)	-29.7 (-41.1 to -19.4)
Fiji	30.3 (21.6 to 40.0)	19.8 (13.1 to 28.5)	18.4 (11.8 to 26.4)	16.8 (10.8 to 24.4)	-1.1 (-2.0 to -0.4)	-15.3 (-25.5 to -5.5)
Guam	6.6 (4.0 to 9.9)	3.4 (2.0 to 5.3)	3.4 (2.0 to 5.3)	3.1 (1.9 to 5.0)	-0.5 (-1.3 to 0.3)	-7.0 (-17.2 to 4.4)
Kiribati	71.8 (61.3 to 81.9)	58.7 (46.7 to 70.0)	56.8 (44.7 to 68.0)	40.3 (29.0 to 52.5)	-2.6 (-3.4 to -1.8)	-31.7 (-40.0 to -24.0)
Marshall Islands	52.4 (40.1 to 64.5)	30.3 (21.1 to 41.2)	28.7 (19.5 to 39.6)	17.9 (11.6 to 25.9)	-3.5 (-4.5 to -2.8)	-41.2 (-49.0 to -33.8)
Northern Mariana Islands	5.9 (3.5 to 9.2)	4.8 (2.8 to 7.5)	4.7 (2.8 to 7.4)	4.4 (2.6 to 6.9)	-0.6 (-1.4 to 0.2)	-8.4 (-18.6 to 3.6)
Papua New Guinea	87.4 (80.6 to 95.5)	81.1 (72.2 to 89.9)	79.8 (71.0 to 89.1)	68.4 (57.0 to 81.7)	-1.1 (-1.7 to -0.6)	-15.8 (-22.2 to -8.7)
Samoa	10.8 (6.7 to 16.1)	4.4 (2.7 to 6.7)	4.0 (2.4 to 6.1)	3.5 (2.1 to 5.5)	-1.4 (-2.3 to -0.6)	-18.6 (-28.8 to -8.0)
Solomon Islands	81.0 (73.3 to 88.6)	73.0 (63.3 to 82.2)	71.3 (61.0 to 81.5)	55.7 (44.1 to 68.7)	-1.8 (-2.5 to -1.2)	-23.9 (-31.6 to -15.9)
Tonga	30.6 (21.5 to 41.2)	17.0 (11.0 to 24.9)	16.0 (10.3 to 23.6)	9.7 (6.0 to 15.1)	-3.7 (-4.6 to -2.9)	-42.9 (-50.1 to -35.0)
Vanuatu	78.6 (70.4 to 87.4)	70.2 (60.0 to 80.3)	69.0 (58.3 to 78.9)	54.8 (42.8 to 67.0)	-1.7 (-2.3 to -1.1)	-22.1 (-29.6 to -15.5)
Southeast Asia						
Cambodia	83.0 (77.4 to 87.8)	46.5 (38.4 to 54.7)	43.1 (34.3 to 52.0)	23.6 (17.4 to 30.7)	-4.6 (-5.6 to -3.6)	-49.4 (-56.6 to -42.0)
Indonesia	48.1 (41.6 to 56.4)	22.7 (16.4 to 30.4)	20.7 (14.4 to 28.0)	9.9 (6.6 to 14.0)	-5.6 (-6.5 to -4.8)	-56.7 (-62.3 to -51.1)
Laos	74.2 (68.6 to 80.6)	66.5 (59.1 to 74.5)	62.9 (54.0 to 71.9)	48.6 (39.3 to 59.0)	-2.1 (-2.7 to -1.5)	-27.0 (-33.4 to -20.6)
Malaysia	7.1 (4.5 to 10.7)	3.2 (1.9 to 5.1)	2.9 (1.7 to 4.6)	1.9 (1.1 to 3.2)	-3.4 (-4.5 to -2.4)	-39.7 (-49.3 to -30.4)
Maldives	45.7 (35.1 to 58.3)	17.7 (11.7 to 25.8)	16.1 (10.2 to 23.7)	7.2 (4.4 to 11.2)	-6.0 (-7.1 to -5.0)	-59.4 (-65.7 to -52.9)
Mauritius	12.5 (9.3 to 16.5)	4.8 (3.0 to 7.4)	4.6 (2.9 to 7.1)	3.4 (2.0 to 5.2)	-2.4 (-3.6 to -1.4)	-30.4 (-42.1 to -19.3)
Myanmar	86.3 (77.7 to 97.5)	52.4 (43.2 to 64.6)	47.8 (38.7 to 60.0)	27.1 (20.2 to 37.9)	-4.4 (-5.3 to -3.5)	-48.5 (-55.0 to -40.8)
Philippines	31.3 (28.4 to 34.6)	12.6 (9.8 to 15.9)	11.8 (8.7 to 15.6)	5.9 (4.2 to 8.1)	-5.1 (-6.1 to -4.2)	-53.5 (-59.7 to -46.4)
Sri Lanka	66.1 (55.4 to 76.4)	31.6 (21.8 to 43.2)	28.8 (19.6 to 39.8)	14.2 (8.6 to 21.4)	-5.4 (-6.4 to -4.4)	-55.4 (-61.6 to -48.3)
Seychelles	23.6 (15.6 to 32.0)	13.3 (8.6 to 19.7)	11.9 (7.5 to 17.7)	6.7 (4.2 to 10.5)	-4.6 (-5.6 to -3.5)	-49.4 (-57.1 to -40.9)
Thailand	9.2 (5.5 to 14.2)	2.4 (1.4 to 3.8)	2.1 (1.2 to 3.5)	1.1 (0.7 to 1.8)	-5.0 (-6.1 to -4.1)	-52.9 (-59.8 to -46.0)
Timor-Leste	91.5 (84.0 to 100.0)	72.5 (61.3 to 83.7)	72.1 (61.0 to 83.1)	52.5 (39.6 to 67.5)	-2.2 (-3.1 to -1.4)	-27.9 (-37.1 to -18.4)
Vietnam	70.3 (60.6 to 83.9)	29.3 (23.6 to 36.6)	26.7 (20.4 to 34.4)	13.6 (9.8 to 18.4)	-5.2 (-6.2 to -4.2)	-53.9 (-60.8 to -46.9)
Sub-Saharan Africa						
Central Sub-Saharan Africa						
Angola	71.4 (60.4 to 85.0)	60.1 (49.2 to 72.8)	56.4 (44.9 to 70.1)	44.1 (32.4 to 58.2)	-2.1 (-3.0 to -1.3)	-27.0 (-35.9 to -18.3)
Central African Republic	88.5 (76.9 to 100.0)	88.0 (76.0 to 100.0)	87.9 (75.8 to 100.0)	86.3 (73.0 to 100.0)	-0.1 (-0.3 to -0.0)	-2.0 (-4.7 to -0.0)
Congo	80.4 (67.8 to 98.2)	71.5 (57.6 to 89.0)	69.6 (55.6 to 87.0)	54.7 (38.9 to 78.1)	-1.8 (-2.8 to -0.8)	-24.0 (-34.3 to -11.5)
Democratic Republic of the Congo	85.2 (73.3 to 100.0)	76.7 (65.2 to 92.3)	74.9 (63.0 to 90.9)	58.6 (44.3 to 82.4)	-1.9 (-2.7 to -0.8)	-24.1 (-33.1 to -11.1)
Equatorial Guinea	75.1 (63.5 to 90.9)	30.9 (20.3 to 44.6)	28.2 (17.8 to 42.1)	16.3 (9.7 to 26.5)	-4.3 (-5.6 to -3.2)	-47.7 (-56.6 to -38.6)
Gabon	65.7 (51.3 to 86.5)	49.8 (37.1 to 66.9)	46.1 (33.9 to 62.3)	29.7 (19.8 to 45.0)	-3.5 (-4.4 to -2.5)	-40.8 (-48.3 to -31.6)
Eastern Sub-Saharan Africa						
Burundi	87.8 (76.0 to 100.0)	85.1 (71.2 to 100.0)	85.1 (71.2 to 100.0)	81.0 (64.5 to 100.0)	-0.4 (-0.8 to -0.0)	-5.1 (-11.8 to -0.0)
Comoros	84.8 (72.0 to 100.0)	79.1 (66.8 to 96.6)	78.2 (65.8 to 95.5)	64.5 (47.7 to 91.7)	-1.4 (-2.3 to -0.3)	-19.0 (-28.8 to -5.1)
Djibouti	81.4 (67.5 to 98.8)	70.6 (55.6 to 89.7)	68.5 (53.7 to 87.5)	56.2 (40.1 to 80.6)	-1.6 (-2.5 to -0.7)	-20.8 (-30.9 to -10.2)
Eritrea	86.6 (78.3 to 96.7)	82.1 (72.0 to 96.0)	80.9 (71.0 to 95.2)	68.8 (55.3 to 90.8)	-1.2 (-1.9 to -0.4)	-16.4 (-24.4 to -5.4)
Ethiopia	95.6 (91.8 to 99.8)	91.7 (86.3 to 98.4)	90.9 (85.3 to 97.9)	89.8 (83.5 to 97.6)	-0.1 (-0.3 to -0.0)	-2.1 (-4.1 to -0.6)

	Estimate in 2000 (95% UIs)	Estimate in 2015 (95% UIs)	Estimate in 2017 (95% UIs)	Estimate in 2030 (95% UIs)	Annualised rate of change, 2015-2030 (95% UIs)	Percent change, 2015-2030 (95% UIs)
Kenya	78.8 (66.8 to 94.5)	71.5 (60.0 to 86.3)	70.3 (58.5 to 85.4)	58.5 (45.1 to 77.7)	-1.4 (-1.9 to -0.7)	-18.6 (-24.8 to -10.0)
Madagascar	90.5 (83.5 to 99.5)	92.9 (87.5 to 99.3)	92.5 (86.8 to 98.9)	90.0 (82.4 to 98.5)	-0.2 (-0.4 to -0.1)	-3.2 (-5.7 to -0.7)
Malawi	87.1 (76.1 to 100.0)	82.2 (68.0 to 100.0)	81.9 (67.4 to 100.0)	76.3 (58.8 to 100.0)	-0.5 (-1.2 to -0.0)	-7.6 (-16.7 to -0.0)
Mozambique	90.8 (82.8 to 100.0)	83.0 (73.0 to 96.0)	82.0 (71.8 to 95.3)	68.6 (54.7 to 90.5)	-1.3 (-2.0 to -0.4)	-17.7 (-26.5 to -5.4)
Rwanda	78.7 (57.3 to 100.0)	77.6 (56.1 to 100.0)	77.5 (56.1 to 100.0)	69.7 (44.9 to 100.0)	-0.8 (-1.7 to -0.0)	-11.0 (-22.3 to -0.0)
Somalia	87.9 (77.0 to 100.0)	82.7 (70.3 to 100.0)	82.3 (69.4 to 99.3)	73.2 (56.7 to 98.8)	-0.8 (-1.6 to -0.1)	-11.8 (-21.1 to -0.9)
South Sudan	91.5 (82.6 to 100.0)	92.0 (83.7 to 100.0)	91.8 (83.6 to 100.0)	91.2 (82.5 to 100.0)	-0.1 (-0.1 to -0.0)	-0.9 (-2.1 to -0.0)
Tanzania	81.7 (66.3 to 100.0)	72.9 (60.8 to 91.7)	71.9 (59.3 to 91.3)	53.9 (38.6 to 82.6)	-2.1 (-3.1 to -0.8)	-26.7 (-37.5 to -10.9)
Uganda	83.8 (68.1 to 100.0)	81.2 (63.6 to 100.0)	80.9 (63.4 to 100.0)	76.5 (56.0 to 100.0)	-0.4 (-1.1 to -0.0)	-6.1 (-14.8 to -0.0)
Zambia	78.9 (72.8 to 88.3)	73.1 (63.8 to 85.9)	71.0 (61.1 to 84.1)	60.5 (49.5 to 76.7)	-1.3 (-1.8 to -0.7)	-17.5 (-23.6 to -9.9)
Southern Sub-Saharan Africa						
Botswana	69.1 (55.2 to 88.5)	47.0 (34.5 to 65.1)	44.2 (31.6 to 61.8)	25.9 (16.7 to 41.6)	-4.1 (-5.2 to -2.9)	-45.6 (-53.8 to -35.0)
Lesotho	86.7 (74.4 to 100.0)	80.9 (64.6 to 100.0)	80.1 (64.0 to 100.0)	72.9 (53.4 to 100.0)	-0.7 (-1.4 to -0.0)	-10.3 (-18.8 to -0.0)
Namibia	65.1 (58.9 to 72.5)	54.5 (46.9 to 64.0)	51.4 (42.7 to 61.8)	37.7 (29.0 to 48.5)	-2.5 (-3.4 to -1.5)	-31.0 (-39.9 to -20.7)
South Africa	39.0 (31.9 to 48.9)	32.6 (23.7 to 46.7)	31.2 (22.4 to 44.7)	23.6 (16.2 to 36.0)	-2.2 (-2.8 to -1.6)	-27.8 (-34.3 to -20.8)
Swaziland	76.0 (63.9 to 93.0)	67.3 (52.9 to 86.5)	65.2 (51.0 to 84.1)	48.8 (33.8 to 71.9)	-2.2 (-3.1 to -1.1)	-28.1 (-37.4 to -15.8)
Zimbabwe	60.7 (51.4 to 72.6)	61.4 (50.3 to 76.7)	60.0 (48.2 to 75.9)	52.0 (39.9 to 69.4)	-1.1 (-1.7 to -0.6)	-15.7 (-22.2 to -9.2)
Western Sub-Saharan Africa						
Benin	89.7 (79.7 to 100.0)	83.6 (72.6 to 99.4)	82.5 (71.1 to 98.4)	74.4 (59.5 to 97.2)	-0.8 (-1.4 to -0.1)	-11.3 (-19.1 to -1.6)
Burkina Faso	91.7 (83.5 to 100.0)	89.1 (80.6 to 100.0)	88.7 (79.9 to 100.0)	83.8 (72.0 to 100.0)	-0.4 (-0.8 to -0.0)	-6.1 (-10.7 to -0.0)
Cameroon	80.2 (64.2 to 100.0)	72.3 (56.8 to 94.0)	70.0 (54.4 to 91.3)	57.2 (39.9 to 85.7)	-1.6 (-2.4 to -0.6)	-21.5 (-30.1 to -8.9)
Cape Verde	75.9 (62.6 to 93.1)	46.2 (33.8 to 62.0)	44.0 (32.0 to 59.5)	25.9 (17.1 to 39.2)	-3.9 (-5.0 to -2.9)	-44.5 (-52.9 to -35.3)
Chad	92.5 (84.4 to 100.0)	89.9 (80.5 to 100.0)	89.5 (80.3 to 100.0)	88.0 (77.8 to 100.0)	-0.1 (-0.3 to -0.0)	-2.2 (-4.4 to -0.0)
Cote d'Ivoire	75.3 (65.2 to 89.2)	67.7 (55.8 to 83.1)	66.2 (53.5 to 81.7)	53.0 (39.6 to 71.8)	-1.7 (-2.3 to -0.9)	-22.1 (-29.4 to -12.7)
The Gambia	79.3 (64.7 to 100.0)	68.6 (54.3 to 88.4)	68.0 (53.3 to 88.0)	59.3 (43.3 to 83.0)	-1.0 (-2.0 to -0.3)	-13.9 (-25.5 to -4.9)
Ghana	81.6 (69.3 to 100.0)	68.9 (55.8 to 88.8)	67.1 (54.2 to 87.4)	48.9 (34.9 to 76.1)	-2.4 (-3.2 to -1.1)	-29.7 (-38.2 to -15.0)
Guinea	88.1 (77.5 to 100.0)	78.5 (65.9 to 93.8)	76.9 (64.0 to 92.5)	62.8 (46.7 to 86.1)	-1.5 (-2.4 to -0.6)	-20.5 (-30.6 to -8.1)
Guinea-Bissau	85.3 (74.2 to 100.0)	79.4 (66.4 to 96.6)	78.1 (65.2 to 95.6)	69.6 (53.3 to 92.8)	-0.9 (-1.6 to -0.2)	-12.7 (-20.8 to -3.6)
Liberia	87.2 (77.3 to 99.3)	72.7 (62.6 to 87.0)	72.1 (61.2 to 86.4)	59.5 (46.3 to 78.1)	-1.4 (-2.1 to -0.6)	-18.5 (-27.3 to -9.2)
Mali	85.1 (70.7 to 100.0)	83.0 (69.7 to 100.0)	82.6 (69.1 to 100.0)	77.2 (60.3 to 100.0)	-0.5 (-1.1 to -0.0)	-7.4 (-15.8 to -0.0)
Mauritania	87.4 (74.3 to 100.0)	77.9 (63.9 to 97.7)	76.6 (62.5 to 96.3)	61.3 (43.7 to 92.5)	-1.7 (-2.7 to -0.4)	-22.0 (-32.8 to -5.5)
Niger	93.3 (86.1 to 100.0)	90.9 (82.3 to 100.0)	90.6 (81.7 to 100.0)	84.8 (71.7 to 100.0)	-0.5 (-0.9 to -0.0)	-6.9 (-12.9 to -0.0)
Nigeria	77.7 (64.7 to 97.6)	55.0 (46.1 to 67.8)	50.4 (41.6 to 62.5)	31.5 (23.9 to 42.8)	-3.8 (-4.5 to -2.9)	-43.0 (-49.1 to -35.1)
Sao Tome and Principe	78.2 (66.0 to 93.3)	50.5 (37.4 to 67.4)	47.3 (34.3 to 63.9)	28.0 (18.1 to 42.8)	-4.0 (-5.0 to -2.9)	-45.2 (-52.9 to -35.3)
Senegal	72.2 (61.5 to 88.4)	63.7 (48.9 to 86.2)	64.1 (49.1 to 86.7)	61.1 (45.8 to 85.1)	-0.3 (-0.8 to 0.0)	-4.4 (-11.4 to 0.7)
Sierra Leone	88.1 (76.6 to 100.0)	82.2 (69.4 to 100.0)	81.2 (68.4 to 100.0)	71.1 (54.3 to 100.0)	-1.0 (-1.7 to -0.0)	-14.1 (-22.1 to -0.0)
Togo	84.2 (73.4 to 99.5)	75.4 (62.5 to 93.0)	73.8 (60.6 to 91.7)	60.2 (44.2 to 85.6)	-1.6 (-2.3 to -0.6)	-20.6 (-29.7 to -8.2)
**Indicator 6.2.1b: Risk-weighted prevalence of populations without access to a handwashing facility, as measured by the summary exposure value (SEV) for unsafe hygiene (%)**						
Central Europe, Eastern Europe, and Central Asia						
Central Asia						
Armenia	18.1 (15.3 to 21.4)	12.5 (10.3 to 14.9)	12.2 (9.8 to 14.5)	10.1 (8.1 to 12.1)	-1.5 (-1.7 to -1.2)	-19.6 (-22.9 to -16.4)
Azerbaijan	24.3 (20.2 to 28.9)	9.4 (7.6 to 11.6)	9.2 (7.4 to 11.3)	6.5 (5.2 to 8.0)	-2.5 (-2.8 to -2.2)	-31.7 (-34.6 to -28.6)
Georgia	15.1 (12.5 to 18.4)	10.8 (8.7 to 13.2)	10.2 (8.3 to 12.6)	7.7 (6.2 to 9.6)	-2.2 (-2.5 to -2.0)	-28.5 (-31.5 to -25.5)
Kazakhstan	19.2 (15.9 to 23.2)	11.1 (9.0 to 13.5)	10.4 (8.4 to 12.7)	7.7 (6.2 to 9.4)	-2.5 (-2.8 to -2.2)	-30.8 (-33.8 to -27.7)
Kyrgyzstan	22.9 (19.2 to 27.2)	13.7 (11.4 to 16.1)	13.3 (11.0 to 15.8)	9.7 (7.9 to 11.6)	-2.3 (-2.6 to -2.0)	-29.3 (-32.2 to -26.3)
Mongolia	34.5 (29.8 to 39.4)	31.0 (27.4 to 34.8)	29.4 (25.6 to 33.1)	22.8 (19.6 to 26.1)	-2.1 (-2.4 to -1.8)	-26.7 (-29.9 to -23.5)
Tajikistan	30.4 (25.9 to 35.2)	26.1 (22.8 to 30.0)	25.0 (21.7 to 28.9)	21.4 (18.4 to 24.8)	-1.4 (-1.6 to -1.1)	-18.4 (-21.3 to -15.4)
Turkmenistan	23.0 (19.1 to 27.2)	16.6 (13.5 to 20.2)	15.4 (12.5 to 18.8)	10.6 (8.4 to 13.0)	-3.0 (-3.3 to -2.7)	-36.4 (-39.1 to -33.7)
Uzbekistan	23.8 (19.9 to 28.2)	16.1 (13.2 to 19.3)	15.0 (12.2 to 18.1)	10.2 (8.3 to 12.5)	-3.0 (-3.3 to -2.7)	-36.2 (-39.0 to -33.5)
Central Europe						
Albania	8.3 (6.6 to 10.5)	6.0 (4.8 to 7.5)	5.8 (4.5 to 7.1)	4.5 (3.6 to 5.6)	-1.9 (-2.2 to -1.6)	-25.2 (-28.6 to -21.7)
Bosnia and Herzegovina	5.3 (4.2 to 6.5)	4.2 (3.3 to 5.2)	4.0 (3.2 to 5.1)	3.6 (2.9 to 4.5)	-1.0 (-1.3 to -0.7)	-14.2 (-17.9 to -10.2)
Bulgaria	3.9 (3.1 to 4.8)	3.3 (2.6 to 4.1)	3.2 (2.5 to 4.1)	2.8 (2.2 to 3.5)	-1.1 (-1.4 to -0.8)	-15.3 (-18.8 to -11.6)
Croatia	3.4 (2.7 to 4.2)	3.0 (2.4 to 3.7)	3.0 (2.4 to 3.7)	2.8 (2.2 to 3.4)	-0.6 (-0.9 to -0.3)	-8.2 (-12.3 to -3.9)
Czech Republic	2.8 (2.2 to 3.5)	2.7 (2.1 to 3.4)	2.7 (2.1 to 3.4)	2.5 (2.0 to 3.2)	-0.5 (-0.8 to -0.2)	-6.6 (-10.9 to -2.4)
Hungary	3.0 (2.3 to 3.6)	2.8 (2.2 to 3.5)	2.8 (2.2 to 3.5)	2.6 (2.0 to 3.3)	-0.4 (-0.7 to -0.1)	-6.2 (-10.4 to -1.7)
Macedonia	4.0 (3.1 to 5.0)	3.3 (2.6 to 4.1)	3.3 (2.6 to 4.1)	3.0 (2.4 to 3.7)	-0.7 (-1.0 to -0.4)	-10.1 (-14.1 to -6.3)
Montenegro	4.7 (3.8 to 5.8)	3.9 (3.0 to 4.8)	3.8 (3.0 to 4.7)	3.1 (2.4 to 3.9)	-1.4 (-1.7 to -1.1)	-19.2 (-22.8 to -15.5)
Poland	3.4 (2.7 to 4.3)	2.9 (2.3 to 3.7)	2.9 (2.3 to 3.6)	2.5 (2.0 to 3.1)	-1.0 (-1.3 to -0.7)	-13.7 (-17.3 to -9.7)
Romania	8.8 (7.0 to 10.6)	5.5 (4.3 to 6.8)	5.2 (4.1 to 6.3)	4.0 (3.2 to 5.0)	-2.1 (-2.4 to -1.8)	-26.8 (-30.2 to -23.2)
Serbia	6.3 (4.9 to 7.8)	3.4 (2.7 to 4.2)	3.3 (2.6 to 4.1)	2.8 (2.2 to 3.5)	-1.3 (-1.7 to -0.9)	-17.2 (-22.3 to -12.2)
Slovakia	3.0 (2.3 to 3.7)	2.7 (2.2 to 3.4)	2.7 (2.1 to 3.4)	2.5 (2.0 to 3.1)	-0.6 (-0.9 to -0.3)	-9.0 (-12.9 to -4.9)
Slovenia	3.4 (2.7 to 4.2)	2.8 (2.3 to 3.5)	2.8 (2.2 to 3.5)	2.7 (2.1 to 3.3)	-0.4 (-0.7 to -0.2)	-6.5 (-10.6 to -2.4)
Eastern Europe						
Belarus	11.8 (9.3 to 14.4)	6.1 (4.8 to 7.5)	6.0 (4.7 to 7.5)	5.0 (4.0 to 6.3)	-1.3 (-1.7 to -0.9)	-17.4 (-22.0 to -12.9)
Estonia	7.0 (5.6 to 8.7)	5.5 (4.3 to 6.8)	5.4 (4.3 to 6.8)	4.8 (3.7 to 6.0)	-0.9 (-1.2 to -0.6)	-13.2 (-17.1 to -9.2)
Latvia	7.8 (6.2 to 9.7)	6.0 (4.8 to 7.5)	5.9 (4.7 to 7.4)	5.5 (4.4 to 6.8)	-0.6 (-0.9 to -0.3)	-8.5 (-12.9 to -4.1)
Lithuania	8.0 (6.5 to 9.8)	5.8 (4.7 to 7.2)	5.8 (4.6 to 7.2)	5.0 (3.9 to 6.2)	-1.1 (-1.4 to -0.8)	-15.5 (-19.2 to -11.8)
Moldova	28.6 (24.1 to 33.1)	16.3 (13.6 to 19.5)	15.6 (12.8 to 18.7)	10.8 (8.8 to 13.0)	-2.8 (-3.1 to -2.5)	-34.2 (-36.9 to -31.3)
Russia	8.7 (7.1 to 10.7)	6.8 (5.4 to 8.5)	6.7 (5.3 to 8.3)	5.9 (4.6 to 7.3)	-1.0 (-1.3 to -0.7)	-14.3 (-18.0 to -10.3)
Ukraine	10.9 (8.7 to 13.4)	10.2 (8.2 to 12.6)	10.0 (8.1 to 12.2)	8.7 (7.0 to 10.7)	-1.1 (-1.4 to -0.8)	-15.0 (-18.7 to -11.5)
High-income						
Australasia						
Australia	6.6 (5.2 to 8.2)	5.8 (4.5 to 7.1)	5.8 (4.6 to 7.2)	5.5 (4.3 to 6.8)	-0.3 (-0.6 to -0.1)	-5.1 (-9.2 to -0.8)
New Zealand	6.6 (5.3 to 8.1)	6.0 (4.7 to 7.4)	5.9 (4.7 to 7.4)	5.7 (4.5 to 7.0)	-0.4 (-0.6 to -0.1)	-5.1 (-9.0 to -1.1)
High-income Asia Pacific						
Brunei	6.7 (5.3 to 8.4)	5.8 (4.6 to 7.2)	5.8 (4.6 to 7.2)	5.5 (4.4 to 6.8)	-0.4 (-0.7 to -0.1)	-5.2 (-9.4 to -0.9)
Japan	6.5 (5.2 to 8.1)	5.9 (4.7 to 7.3)	5.8 (4.7 to 7.2)	5.7 (4.6 to 7.0)	-0.2 (-0.5 to 0.1)	-3.1 (-7.2 to 1.2)
South Korea	6.8 (5.4 to 8.4)	5.9 (4.6 to 7.3)	5.8 (4.6 to 7.2)	5.5 (4.3 to 6.8)	-0.4 (-0.7 to -0.1)	-6.3 (-10.6 to -2.1)
Singapore	6.8 (5.5 to 8.5)	5.8 (4.6 to 7.2)	5.8 (4.6 to 7.2)	5.5 (4.3 to 6.7)	-0.4 (-0.7 to -0.1)	-6.1 (-10.2 to -1.9)

	Estimate in 2000 (95% UIs)	Estimate in 2015 (95% UIs)	Estimate in 2017 (95% UIs)	Estimate in 2030 (95% UIs)	Annualised rate of change, 2015-2030 (95% UIs)	Percent change, 2015-2030 (95% UIs)
High-income North America						
Canada	3.2 (2.5 to 4.0)	3.0 (2.4 to 3.7)	3.0 (2.4 to 3.7)	2.9 (2.3 to 3.6)	-0.2 (-0.5 to 0.1)	-2.7 (-7.3 to 1.7)
Greenland	3.7 (2.9 to 4.6)	3.2 (2.5 to 4.1)	3.2 (2.5 to 4.0)	3.1 (2.4 to 3.9)	-0.3 (-0.7 to 0.0)	-4.6 (-9.4 to 0.2)
United States	3.5 (2.8 to 4.4)	3.0 (2.4 to 3.8)	3.0 (2.4 to 3.8)	2.9 (2.3 to 3.7)	-0.2 (-0.5 to 0.0)	-3.5 (-7.6 to 0.5)
Southern Latin America						
Argentina	13.5 (11.0 to 16.4)	8.7 (6.8 to 10.6)	8.5 (6.8 to 10.3)	7.0 (5.5 to 8.5)	-1.5 (-1.8 to -1.1)	-19.6 (-23.8 to -15.6)
Chile	9.7 (7.8 to 11.9)	7.3 (5.9 to 9.0)	7.2 (5.8 to 8.9)	6.3 (5.0 to 7.7)	-1.0 (-1.3 to -0.7)	-13.8 (-17.7 to -10.0)
Uruguay	9.1 (7.3 to 11.2)	7.3 (5.7 to 9.1)	7.1 (5.6 to 8.9)	6.0 (4.7 to 7.5)	-1.2 (-1.5 to -0.9)	-17.0 (-20.4 to -13.3)
Western Europe						
Andorra	3.1 (2.5 to 3.9)	3.0 (2.4 to 3.8)	3.0 (2.4 to 3.7)	2.9 (2.3 to 3.6)	-0.2 (-0.5 to 0.1)	-2.6 (-6.8 to 1.7)
Austria	3.4 (2.7 to 4.2)	3.2 (2.6 to 4.0)	3.2 (2.5 to 4.0)	3.1 (2.5 to 3.9)	-0.2 (-0.6 to 0.1)	-3.5 (-8.0 to 1.3)
Belgium	3.1 (2.5 to 3.9)	3.0 (2.4 to 3.8)	3.0 (2.4 to 3.7)	2.9 (2.4 to 3.6)	-0.2 (-0.5 to 0.1)	-3.2 (-7.5 to 1.6)
Cyprus	3.5 (2.8 to 4.4)	3.1 (2.4 to 3.8)	3.1 (2.4 to 3.8)	3.0 (2.4 to 3.7)	-0.2 (-0.5 to 0.1)	-3.1 (-7.1 to 1.2)
Denmark	3.1 (2.4 to 3.9)	3.0 (2.4 to 3.7)	3.0 (2.3 to 3.7)	2.9 (2.3 to 3.6)	-0.2 (-0.5 to 0.1)	-3.2 (-7.5 to 1.4)
Finland	3.1 (2.5 to 3.9)	3.0 (2.4 to 3.8)	3.0 (2.4 to 3.8)	2.9 (2.3 to 3.7)	-0.2 (-0.6 to 0.1)	-3.6 (-8.2 to 0.8)
France	3.2 (2.5 to 4.1)	3.1 (2.5 to 3.9)	3.1 (2.5 to 4.0)	3.1 (2.4 to 3.9)	-0.2 (-0.5 to 0.1)	-3.0 (-7.1 to 1.3)
Germany	3.1 (2.5 to 4.0)	3.1 (2.5 to 3.8)	3.0 (2.5 to 3.8)	3.0 (2.4 to 3.7)	-0.2 (-0.5 to 0.1)	-3.2 (-7.6 to 0.9)
Greece	3.1 (2.5 to 3.9)	3.0 (2.4 to 3.8)	3.0 (2.4 to 3.8)	3.0 (2.4 to 3.7)	-0.2 (-0.5 to 0.1)	-2.5 (-6.9 to 2.1)
Iceland	3.1 (2.5 to 3.9)	3.0 (2.4 to 3.8)	3.0 (2.4 to 3.8)	2.9 (2.3 to 3.6)	-0.3 (-0.6 to 0.0)	-4.2 (-8.4 to 0.4)
Ireland	3.2 (2.5 to 4.0)	3.0 (2.4 to 3.8)	3.0 (2.4 to 3.8)	2.9 (2.3 to 3.6)	-0.3 (-0.6 to -0.0)	-4.4 (-9.0 to -0.1)
Israel	3.2 (2.6 to 4.0)	3.2 (2.5 to 3.9)	3.1 (2.5 to 3.9)	3.1 (2.4 to 3.8)	-0.2 (-0.5 to 0.1)	-3.1 (-7.5 to 1.2)
Italy	3.6 (2.9 to 4.4)	3.4 (2.7 to 4.3)	3.4 (2.7 to 4.3)	3.3 (2.6 to 4.2)	-0.2 (-0.5 to 0.1)	-3.2 (-7.6 to 1.0)
Luxembourg	3.1 (2.4 to 3.8)	3.0 (2.4 to 3.8)	3.0 (2.4 to 3.7)	2.9 (2.3 to 3.6)	-0.2 (-0.5 to 0.1)	-2.9 (-7.0 to 1.6)
Malta	3.2 (2.6 to 4.0)	3.1 (2.5 to 3.9)	3.1 (2.5 to 3.9)	3.0 (2.4 to 3.8)	-0.3 (-0.6 to 0.0)	-4.3 (-8.7 to 0.1)
Netherlands	3.1 (2.4 to 3.8)	3.0 (2.3 to 3.7)	3.0 (2.3 to 3.7)	2.9 (2.2 to 3.6)	-0.2 (-0.5 to 0.1)	-3.3 (-7.3 to 1.3)
Norway	3.1 (2.5 to 3.9)	3.0 (2.4 to 3.8)	3.0 (2.3 to 3.8)	2.9 (2.3 to 3.6)	-0.2 (-0.5 to 0.1)	-3.5 (-7.6 to 0.8)
Portugal	3.7 (2.9 to 4.5)	3.4 (2.7 to 4.2)	3.4 (2.7 to 4.2)	3.2 (2.6 to 4.0)	-0.4 (-0.7 to -0.1)	-5.3 (-9.3 to -1.0)
Spain	3.2 (2.5 to 4.0)	3.1 (2.5 to 3.9)	3.1 (2.5 to 3.9)	3.0 (2.4 to 3.8)	-0.2 (-0.5 to 0.1)	-2.8 (-7.1 to 2.0)
Sweden	3.1 (2.5 to 3.9)	3.1 (2.4 to 3.8)	3.1 (2.4 to 3.8)	3.0 (2.3 to 3.7)	-0.2 (-0.5 to 0.1)	-2.9 (-7.1 to 1.6)
Switzerland	3.1 (2.5 to 3.8)	3.0 (2.4 to 3.8)	3.0 (2.4 to 3.8)	2.9 (2.3 to 3.6)	-0.2 (-0.5 to 0.1)	-3.1 (-7.2 to 1.2)
United Kingdom	3.3 (2.6 to 4.1)	3.1 (2.4 to 3.9)	3.1 (2.4 to 3.9)	3.0 (2.3 to 3.8)	-0.3 (-0.6 to 0.0)	-3.9 (-8.2 to 0.3)
Latin America and Caribbean						
Andean Latin America						
Bolivia	38.1 (33.0 to 44.1)	24.9 (20.8 to 29.4)	23.9 (19.8 to 28.3)	18.6 (15.2 to 22.3)	-2.0 (-2.2 to -1.7)	-25.5 (-28.4 to -22.4)
Ecuador	27.4 (22.9 to 32.3)	23.7 (20.0 to 28.1)	23.3 (19.5 to 27.9)	20.1 (16.6 to 24.2)	-1.1 (-1.4 to -0.8)	-15.5 (-18.9 to -11.8)
Peru	33.0 (27.7 to 38.5)	25.2 (20.9 to 29.9)	24.6 (20.3 to 29.4)	18.8 (15.3 to 22.6)	-2.0 (-2.2 to -1.7)	-25.6 (-28.5 to -22.6)
Caribbean						
Antigua and Barbuda	28.0 (23.4 to 32.9)	26.1 (21.9 to 30.7)	25.6 (21.6 to 30.1)	22.5 (18.8 to 26.7)	-1.0 (-1.2 to -0.8)	-13.8 (-16.9 to -10.6)
The Bahamas	21.3 (17.4 to 25.4)	21.3 (17.6 to 25.3)	21.3 (17.6 to 25.2)	20.7 (17.1 to 24.6)	-0.2 (-0.4 to 0.1)	-2.9 (-6.2 to 0.7)
Barbados	26.7 (22.6 to 31.2)	25.1 (21.6 to 28.9)	24.9 (21.4 to 28.9)	24.4 (20.9 to 28.4)	-0.2 (-0.4 to 0.0)	-2.8 (-6.4 to 0.7)
Belize	26.3 (22.3 to 30.6)	33.0 (28.5 to 37.6)	31.8 (27.4 to 36.3)	27.5 (23.4 to 31.9)	-1.2 (-1.5 to -0.9)	-16.7 (-19.9 to -13.3)
Bermuda	20.0 (16.3 to 23.7)	19.4 (16.0 to 23.3)	19.3 (16.1 to 23.3)	18.4 (15.2 to 22.3)	-0.4 (-0.6 to -0.1)	-5.2 (-8.6 to -1.8)
Cuba	28.3 (23.7 to 33.1)	20.7 (17.4 to 24.3)	19.9 (16.7 to 23.4)	15.7 (13.1 to 18.6)	-1.9 (-2.1 to -1.6)	-24.2 (-27.6 to -21.2)
Dominica	34.8 (29.1 to 40.1)	29.3 (24.4 to 34.3)	28.8 (23.9 to 33.5)	24.3 (19.9 to 28.5)	-1.3 (-1.5 to -1.0)	-17.3 (-20.3 to -14.4)
Dominican Republic	67.1 (61.8 to 72.0)	64.2 (59.7 to 68.5)	63.2 (58.6 to 67.6)	58.5 (53.6 to 63.1)	-0.6 (-0.8 to -0.4)	-8.9 (-11.6 to -6.3)
Grenada	31.1 (26.3 to 36.1)	27.8 (23.4 to 32.6)	27.1 (22.8 to 31.9)	23.1 (19.2 to 27.5)	-1.2 (-1.5 to -1.0)	-16.9 (-20.1 to -13.5)
Guyana	43.1 (37.8 to 48.1)	57.3 (53.8 to 60.8)	55.3 (51.6 to 59.1)	47.8 (43.8 to 52.1)	-1.2 (-1.5 to -1.0)	-16.6 (-19.6 to -13.4)
Haiti	78.4 (74.9 to 81.9)	77.3 (74.1 to 80.6)	76.7 (73.1 to 80.4)	75.0 (71.3 to 78.9)	-0.2 (-0.3 to -0.1)	-3.0 (-4.3 to -1.7)
Jamaica	46.8 (41.2 to 52.0)	44.3 (40.1 to 48.4)	43.5 (39.1 to 48.0)	39.5 (35.2 to 44.0)	-0.8 (-1.0 to -0.6)	-11.0 (-13.6 to -8.4)
Puerto Rico	20.6 (16.9 to 24.5)	18.9 (15.5 to 22.5)	18.8 (15.3 to 22.4)	18.4 (15.0 to 21.9)	-0.2 (-0.4 to 0.1)	-2.7 (-6.3 to 1.0)
Saint Lucia	20.8 (17.2 to 24.4)	18.9 (16.1 to 22.1)	18.6 (15.8 to 21.9)	17.1 (14.4 to 20.1)	-0.7 (-0.9 to -0.4)	-9.6 (-13.2 to -6.2)
Saint Vincent and the Grenadines	36.9 (31.6 to 42.9)	34.3 (29.2 to 39.9)	33.6 (28.5 to 38.9)	29.5 (24.8 to 34.5)	-1.0 (-1.2 to -0.8)	-14.1 (-17.1 to -11.3)
Suriname	39.3 (34.1 to 44.1)	34.6 (30.9 to 38.5)	34.3 (30.4 to 38.4)	28.6 (25.1 to 32.4)	-1.3 (-1.5 to -1.1)	-17.5 (-20.4 to -14.7)
Trinidad and Tobago	26.7 (22.2 to 31.7)	24.5 (20.5 to 28.7)	24.5 (20.5 to 28.9)	23.3 (19.4 to 27.6)	-0.3 (-0.6 to -0.1)	-4.9 (-8.8 to -1.1)
Virgin Islands, U.S.	21.9 (18.3 to 25.8)	20.8 (17.0 to 24.8)	20.8 (17.1 to 24.7)	20.3 (16.7 to 24.1)	-0.2 (-0.4 to 0.1)	-2.6 (-6.1 to 1.0)
Central Latin America						
Colombia	24.3 (20.3 to 29.0)	18.7 (15.3 to 22.5)	18.1 (14.9 to 21.7)	13.9 (11.2 to 16.8)	-2.0 (-2.3 to -1.7)	-26.0 (-28.9 to -23.0)
Costa Rica	28.1 (24.3 to 32.1)	24.2 (21.1 to 27.6)	23.3 (20.2 to 26.6)	20.6 (17.8 to 23.7)	-1.1 (-1.3 to -0.8)	-14.7 (-18.0 to -11.6)
El Salvador	30.2 (25.8 to 35.4)	24.6 (20.3 to 29.1)	23.8 (19.6 to 28.4)	18.8 (15.3 to 22.6)	-1.8 (-2.1 to -1.5)	-23.8 (-26.7 to -20.6)
Guatemala	37.9 (32.4 to 43.0)	33.0 (28.1 to 38.3)	31.8 (27.2 to 36.8)	25.2 (21.3 to 29.7)	-1.8 (-2.1 to -1.6)	-23.6 (-26.7 to -20.8)
Honduras	21.7 (18.2 to 25.4)	19.3 (16.5 to 22.4)	18.4 (15.4 to 21.6)	14.8 (12.3 to 17.5)	-1.8 (-2.0 to -1.5)	-23.2 (-26.5 to -19.9)
Mexico	17.7 (14.6 to 21.5)	15.8 (12.8 to 18.9)	15.7 (12.8 to 18.8)	14.0 (11.3 to 16.9)	-0.8 (-1.1 to -0.5)	-11.4 (-14.9 to -7.8)
Nicaragua	36.9 (31.5 to 42.6)	27.4 (22.8 to 32.2)	26.1 (21.6 to 30.8)	18.9 (15.5 to 22.8)	-2.5 (-2.7 to -2.2)	-30.8 (-33.6 to -27.8)
Panama	30.4 (25.8 to 35.5)	21.9 (18.1 to 26.3)	20.5 (16.8 to 24.6)	17.1 (13.9 to 20.8)	-1.6 (-1.9 to -1.4)	-21.8 (-24.9 to -18.9)
Venezuela	19.5 (15.9 to 23.6)	16.8 (13.8 to 20.2)	16.6 (13.5 to 19.9)	14.3 (11.6 to 17.3)	-1.1 (-1.3 to -0.8)	-14.8 (-18.3 to -11.0)
Tropical Latin America						
Brazil	25.4 (21.4 to 30.3)	19.0 (15.6 to 22.6)	18.6 (15.3 to 22.2)	15.1 (12.2 to 18.1)	-1.5 (-1.8 to -1.2)	-20.3 (-23.7 to -17.1)
Paraguay	42.4 (36.7 to 47.7)	28.2 (23.6 to 33.3)	27.4 (22.8 to 32.0)	22.3 (18.3 to 26.6)	-1.6 (-1.9 to -1.3)	-21.0 (-24.7 to -17.2)
North Africa and Middle East						
North Africa and Middle East						
Afghanistan	62.7 (58.0 to 67.5)	58.1 (53.9 to 62.1)	57.4 (52.9 to 61.7)	55.0 (50.6 to 59.5)	-0.4 (-0.5 to -0.2)	-5.2 (-7.2 to -3.3)
Algeria	22.1 (18.6 to 25.9)	16.9 (14.4 to 19.7)	16.4 (13.9 to 19.2)	12.1 (10.2 to 14.3)	-2.2 (-2.5 to -1.9)	-28.1 (-31.2 to -24.9)
Bahrain	14.9 (12.1 to 17.8)	14.9 (12.0 to 17.9)	14.7 (11.8 to 17.7)	13.1 (10.3 to 15.8)	-0.9 (-1.2 to -0.6)	-12.4 (-16.2 to -8.2)
Egypt	18.7 (15.4 to 22.5)	13.7 (11.3 to 16.2)	13.3 (11.0 to 15.8)	12.0 (9.9 to 14.3)	-0.9 (-1.2 to -0.6)	-12.3 (-16.0 to -8.5)
Iran	16.2 (13.4 to 19.5)	14.3 (11.8 to 17.7)	14.1 (11.6 to 17.5)	13.3 (10.9 to 16.6)	-0.5 (-0.7 to -0.2)	-6.9 (-10.5 to -3.3)
Iraq	11.0 (9.0 to 13.5)	9.9 (7.9 to 12.0)	9.5 (7.6 to 11.6)	7.2 (5.7 to 8.9)	-2.1 (-2.5 to -1.6)	-26.8 (-31.5 to -21.5)
Jordan	20.6 (16.6 to 24.6)	18.0 (14.8 to 21.5)	17.3 (14.2 to 20.6)	15.2 (12.5 to 18.2)	-1.1 (-1.4 to -0.9)	-15.4 (-18.7 to -12.0)
Kuwait	14.1 (11.5 to 17.1)	12.7 (10.2 to 15.4)	12.4 (10.1 to 15.3)	10.9 (8.8 to 13.4)	-1.0 (-1.3 to -0.7)	-14.0 (-17.7 to -10.1)
Lebanon	22.3 (18.4 to 26.5)	17.6 (14.4 to 21.3)	16.8 (13.8 to 20.4)	11.8 (9.6 to 14.5)	-2.6 (-2.9 to -2.3)	-32.7 (-35.5 to -29.7)
Libya	24.3 (20.3 to 28.8)	23.1 (19.2 to 27.5)	23.1 (19.2 to 27.5)	21.5 (17.7 to 25.7)	-0.5 (-0.8 to -0.2)	-6.8 (-10.7 to -2.7)

	Estimate in 2000 (95% UIs)	Estimate in 2015 (95% UIs)	Estimate in 2017 (95% UIs)	Estimate in 2030 (95% UIs)	Annualised rate of change, 2015-2030 (95% UIs)	Percent change, 2015-2030 (95% UIs)
Morocco	38.2 (32.8 to 44.1)	27.0 (22.7 to 32.0)	25.7 (21.6 to 30.5)	18.7 (15.4 to 22.5)	-2.5 (-2.7 to -2.2)	-30.9 (-33.5 to -27.9)
Palestine	33.2 (28.1 to 38.7)	28.1 (23.3 to 32.9)	26.6 (22.1 to 31.5)	21.7 (17.9 to 26.0)	-1.7 (-2.1 to -1.3)	-22.6 (-26.7 to -17.7)
Oman	29.6 (24.8 to 34.6)	16.2 (13.2 to 19.5)	15.8 (12.8 to 19.1)	11.2 (9.0 to 13.7)	-2.5 (-2.8 to -2.2)	-30.9 (-34.0 to -27.8)
Qatar	13.5 (10.9 to 16.4)	13.5 (10.9 to 16.4)	13.3 (10.7 to 16.0)	12.2 (9.8 to 14.8)	-0.7 (-0.9 to -0.4)	-9.7 (-13.1 to -6.1)
Saudi Arabia	25.1 (21.0 to 29.8)	16.7 (13.7 to 20.1)	15.8 (12.9 to 19.0)	10.9 (8.8 to 13.2)	-2.9 (-3.1 to -2.6)	-34.9 (-37.6 to -32.3)
Sudan	77.9 (73.9 to 81.4)	75.0 (71.6 to 78.4)	72.6 (69.0 to 76.3)	64.1 (60.0 to 68.5)	-1.1 (-1.2 to -0.9)	-14.7 (-16.9 to -12.3)
Syria	19.7 (16.2 to 23.4)	16.8 (13.8 to 20.1)	16.4 (13.4 to 19.6)	13.6 (11.1 to 16.4)	-1.4 (-1.7 to -1.1)	-18.9 (-22.0 to -15.7)
Tunisia	24.2 (20.5 to 28.1)	20.2 (17.4 to 23.3)	19.6 (16.8 to 22.6)	14.9 (12.5 to 17.6)	-2.1 (-2.5 to -1.6)	-26.6 (-30.9 to -21.8)
Turkey	26.9 (22.5 to 32.2)	18.2 (15.0 to 22.1)	17.2 (14.0 to 20.9)	12.0 (9.7 to 14.8)	-2.8 (-3.1 to -2.5)	-34.2 (-37.2 to -31.4)
United Arab Emirates	14.9 (12.2 to 18.3)	13.8 (11.3 to 16.8)	13.8 (11.2 to 16.6)	11.8 (9.6 to 14.3)	-1.1 (-1.4 to -0.8)	-15.1 (-18.4 to -11.5)
Yemen	63.1 (58.2 to 68.1)	51.5 (47.9 to 55.3)	50.4 (46.6 to 54.3)	43.0 (39.0 to 46.9)	-1.2 (-1.5 to -1.0)	-16.6 (-19.7 to -13.7)
South Asia						
South Asia						
Bangladesh	76.0 (71.8 to 79.7)	72.7 (69.2 to 75.9)	72.0 (68.3 to 75.6)	68.2 (64.3 to 72.2)	-0.4 (-0.5 to -0.3)	-6.1 (-7.8 to -4.4)
Bhutan	35.6 (30.9 to 40.5)	19.6 (16.7 to 22.8)	18.6 (15.7 to 21.9)	17.6 (14.8 to 20.6)	-0.7 (-1.0 to -0.5)	-10.3 (-13.6 to -6.9)
India	55.7 (49.8 to 61.6)	45.6 (39.6 to 51.2)	44.1 (38.3 to 49.8)	34.3 (29.0 to 39.5)	-1.9 (-2.2 to -1.6)	-24.9 (-27.9 to -21.9)
Nepal	57.9 (52.8 to 62.8)	43.2 (40.0 to 46.3)	41.5 (38.1 to 45.0)	32.1 (29.0 to 35.3)	-2.0 (-2.3 to -1.7)	-25.8 (-28.8 to -22.8)
Pakistan	51.4 (46.2 to 56.4)	44.7 (40.7 to 48.9)	43.1 (38.9 to 47.4)	37.1 (33.2 to 41.3)	-1.2 (-1.5 to -1.0)	-16.9 (-20.3 to -13.5)
Southeast Asia, East Asia, and Oceania						
East Asia						
China	27.1 (26.1 to 28.1)	15.1 (14.4 to 15.8)	13.8 (13.2 to 14.4)	9.4 (9.0 to 9.9)	-3.1 (-3.2 to -3.1)	-37.5 (-38.1 to -36.9)
North Korea	10.2 (8.2 to 12.5)	9.3 (7.5 to 11.3)	9.2 (7.3 to 11.1)	8.3 (6.7 to 10.1)	-0.8 (-1.0 to -0.5)	-10.7 (-14.4 to -6.6)
Taiwan (Province of China)	7.5 (6.0 to 9.4)	5.9 (4.7 to 7.3)	5.8 (4.6 to 7.2)	5.3 (4.2 to 6.7)	-0.6 (-0.9 to -0.4)	-9.2 (-12.9 to -5.2)
Oceania						
American Samoa	11.6 (9.3 to 14.3)	8.3 (6.6 to 10.3)	8.2 (6.5 to 10.1)	7.1 (5.6 to 8.8)	-1.1 (-1.4 to -0.8)	-15.0 (-18.8 to -11.2)
Federated States of Micronesia	20.2 (16.7 to 24.0)	14.3 (11.5 to 17.5)	13.9 (11.2 to 16.9)	11.7 (9.4 to 14.4)	-1.3 (-1.6 to -1.0)	-17.9 (-21.6 to -14.0)
Fiji	17.6 (14.3 to 21.2)	11.6 (9.1 to 14.1)	11.2 (8.8 to 13.5)	9.6 (7.6 to 11.7)	-1.2 (-1.5 to -1.0)	-17.0 (-20.5 to -13.4)
Guam	7.3 (5.9 to 9.1)	6.2 (5.0 to 7.7)	6.2 (5.0 to 7.7)	6.0 (4.8 to 7.5)	-0.2 (-0.5 to 0.0)	-3.7 (-7.9 to 0.6)
Kiribati	32.2 (27.2 to 37.2)	24.2 (20.2 to 28.8)	23.2 (19.2 to 27.5)	17.6 (14.4 to 21.2)	-2.1 (-2.4 to -1.9)	-27.3 (-30.2 to -24.7)
Marshall Islands	25.8 (21.8 to 30.7)	16.2 (13.2 to 19.7)	15.6 (12.7 to 19.1)	11.8 (9.4 to 14.5)	-2.1 (-2.4 to -1.8)	-27.3 (-30.4 to -24.2)
Northern Mariana Islands	7.3 (5.7 to 9.1)	6.9 (5.5 to 8.6)	6.8 (5.5 to 8.6)	6.6 (5.3 to 8.3)	-0.3 (-0.5 to 0.0)	-3.8 (-7.9 to 0.4)
Papua New Guinea	50.3 (44.9 to 56.3)	43.4 (37.6 to 49.2)	42.5 (36.7 to 48.3)	35.8 (30.4 to 41.4)	-1.3 (-1.5 to -1.0)	-17.5 (-20.4 to -14.5)
Samoa	10.5 (8.4 to 12.9)	7.9 (6.2 to 9.8)	7.8 (6.1 to 9.6)	6.5 (5.1 to 8.1)	-1.3 (-1.6 to -1.0)	-17.5 (-21.0 to -14.0)
Solomon Islands	25.4 (21.2 to 29.8)	20.4 (16.6 to 24.5)	19.7 (16.0 to 23.7)	14.3 (11.4 to 17.4)	-2.4 (-2.7 to -2.1)	-30.1 (-32.9 to -27.2)
Tonga	16.6 (13.4 to 20.1)	11.7 (9.4 to 14.2)	11.3 (9.1 to 13.7)	8.3 (6.7 to 10.1)	-2.3 (-2.5 to -2.0)	-28.7 (-31.7 to -25.6)
Vanuatu	42.9 (37.1 to 48.9)	37.8 (32.8 to 43.8)	37.0 (31.8 to 42.7)	29.8 (25.2 to 34.9)	-1.6 (-1.9 to -1.4)	-21.4 (-24.4 to -18.6)
Southeast Asia						
Cambodia	50.7 (45.0 to 56.3)	32.3 (28.4 to 36.5)	32.5 (28.3 to 36.8)	25.4 (21.8 to 29.1)	-1.6 (-1.9 to -1.4)	-21.5 (-24.5 to -18.6)
Indonesia	25.4 (21.4 to 29.6)	26.9 (23.2 to 30.9)	25.9 (22.2 to 30.0)	22.8 (19.5 to 26.6)	-1.1 (-1.4 to -0.8)	-15.4 (-18.9 to -11.6)
Laos	41.8 (36.0 to 47.7)	39.1 (33.5 to 45.0)	37.5 (32.1 to 43.0)	31.5 (26.5 to 36.8)	-1.4 (-1.7 to -1.1)	-19.5 (-23.1 to -15.8)
Malaysia	6.6 (5.3 to 8.2)	7.0 (5.5 to 8.7)	6.8 (5.4 to 8.4)	6.2 (5.0 to 7.8)	-0.7 (-1.0 to -0.4)	-10.3 (-13.9 to -6.3)
Maldives	41.6 (35.8 to 47.4)	25.9 (21.6 to 30.2)	24.0 (20.0 to 27.9)	17.2 (14.1 to 20.3)	-2.8 (-3.0 to -2.5)	-33.8 (-36.6 to -31.1)
Mauritius	4.2 (3.3 to 5.3)	3.9 (3.1 to 4.9)	3.8 (3.0 to 4.8)	3.7 (2.9 to 4.7)	-0.3 (-0.6 to 0.0)	-4.1 (-8.4 to 0.0)
Myanmar	46.1 (40.5 to 52.2)	42.0 (36.1 to 48.2)	40.9 (34.9 to 46.8)	34.5 (28.9 to 40.1)	-1.3 (-1.6 to -1.1)	-18.0 (-21.2 to -14.9)
Philippines	25.5 (21.0 to 30.3)	25.2 (21.0 to 29.9)	23.9 (19.9 to 28.7)	18.0 (14.8 to 21.9)	-2.2 (-2.6 to -1.9)	-28.5 (-32.3 to -24.6)
Sri Lanka	27.5 (22.8 to 32.6)	18.0 (14.5 to 21.9)	16.9 (13.7 to 20.6)	11.7 (9.4 to 14.5)	-2.9 (-3.2 to -2.6)	-34.9 (-37.7 to -31.9)
Seychelles	16.8 (13.5 to 20.2)	15.2 (12.3 to 18.3)	14.4 (11.6 to 17.4)	12.4 (10.0 to 15.1)	-1.3 (-1.6 to -1.0)	-18.2 (-21.8 to -14.2)
Thailand	31.8 (26.9 to 37.3)	28.2 (23.6 to 33.3)	26.6 (22.1 to 31.5)	22.3 (18.3 to 26.6)	-1.6 (-1.9 to -1.3)	-21.1 (-24.7 to -17.6)
Timor-Leste	25.7 (21.3 to 30.4)	26.6 (22.2 to 31.2)	26.6 (22.1 to 31.2)	19.8 (16.2 to 23.8)	-2.0 (-2.3 to -1.6)	-25.6 (-29.3 to -21.5)
Vietnam	42.6 (37.6 to 47.6)	38.9 (35.0 to 43.3)	36.9 (32.9 to 41.4)	31.5 (27.7 to 35.8)	-1.4 (-1.7 to -1.1)	-19.0 (-22.1 to -15.6)
Sub-Saharan Africa						
Central Sub-Saharan Africa						
Angola	93.3 (91.7 to 94.7)	91.7 (89.9 to 93.3)	91.1 (89.2 to 92.9)	87.5 (84.9 to 89.9)	-0.3 (-0.4 to -0.2)	-4.6 (-5.7 to -3.6)
Central African Republic	87.5 (84.9 to 89.8)	87.1 (84.7 to 89.3)	87.1 (84.6 to 89.3)	86.7 (84.2 to 89.1)	-0.0 (-0.1 to 0.0)	-0.4 (-1.0 to 0.2)
Congo	89.2 (86.8 to 91.2)	84.8 (81.7 to 87.8)	83.8 (80.4 to 87.0)	78.9 (74.6 to 82.9)	-0.5 (-0.6 to -0.4)	-7.0 (-8.9 to -5.4)
Democratic Republic of the Congo	96.0 (95.0 to 96.8)	95.8 (94.9 to 96.7)	95.6 (94.6 to 96.5)	94.2 (92.9 to 95.5)	-0.1 (-0.1 to -0.1)	-1.7 (-2.2 to -1.2)
Equatorial Guinea	92.7 (90.9 to 94.1)	80.1 (76.4 to 83.5)	78.7 (74.7 to 82.4)	70.6 (65.7 to 75.2)	-0.8 (-1.0 to -0.7)	-11.9 (-14.1 to -9.9)
Gabon	82.0 (78.0 to 85.3)	73.2 (68.8 to 77.6)	71.7 (67.2 to 75.9)	64.6 (59.4 to 69.5)	-0.8 (-1.0 to -0.7)	-11.9 (-14.2 to -9.8)
Eastern Sub-Saharan Africa						
Burundi	95.2 (94.0 to 96.2)	94.8 (93.5 to 95.9)	94.7 (93.5 to 95.9)	93.1 (91.5 to 94.6)	-0.1 (-0.2 to -0.1)	-1.8 (-2.3 to -1.3)
Comoros	90.5 (88.5 to 92.3)	85.6 (82.9 to 88.1)	85.2 (82.4 to 87.8)	79.0 (75.4 to 82.4)	-0.5 (-0.7 to -0.4)	-7.7 (-9.3 to -6.2)
Djibouti	91.5 (89.6 to 93.2)	88.6 (86.0 to 90.8)	88.0 (85.2 to 90.2)	83.8 (80.4 to 86.8)	-0.4 (-0.5 to -0.3)	-5.4 (-6.8 to -4.2)
Eritrea	94.0 (92.7 to 95.2)	93.0 (91.4 to 94.3)	92.7 (91.1 to 94.1)	90.2 (88.1 to 92.0)	-0.2 (-0.3 to -0.2)	-3.0 (-3.8 to -2.3)
Ethiopia	97.2 (96.5 to 97.8)	95.6 (94.7 to 96.5)	95.3 (94.3 to 96.2)	93.0 (91.6 to 94.3)	-0.2 (-0.2 to -0.1)	-2.7 (-3.3 to -2.1)
Kenya	94.0 (92.7 to 95.1)	91.3 (89.8 to 92.5)	90.9 (89.2 to 92.3)	87.0 (84.6 to 88.9)	-0.3 (-0.4 to -0.3)	-4.7 (-5.9 to -3.8)
Madagascar	90.5 (88.5 to 92.2)	91.8 (90.1 to 93.3)	91.8 (90.0 to 93.3)	91.4 (89.5 to 93.0)	-0.0 (-0.1 to -0.0)	-0.5 (-0.9 to -0.1)
Malawi	90.8 (89.1 to 92.3)	92.8 (91.3 to 94.2)	92.7 (91.2 to 94.2)	91.9 (90.2 to 93.5)	-0.1 (-0.1 to -0.0)	-1.0 (-1.5 to -0.5)
Mozambique	92.9 (91.3 to 94.3)	91.1 (89.3 to 92.7)	90.8 (88.9 to 92.5)	86.8 (84.1 to 89.1)	-0.3 (-0.4 to -0.3)	-4.8 (-6.1 to -3.8)
Rwanda	97.8 (97.3 to 98.3)	96.7 (96.0 to 97.4)	96.6 (95.9 to 97.3)	94.9 (93.8 to 95.9)	-0.1 (-0.2 to -0.1)	-1.9 (-2.4 to -1.5)
Somalia	95.0 (93.9 to 96.0)	93.6 (92.1 to 94.9)	93.5 (91.9 to 94.9)	91.5 (89.4 to 93.2)	-0.2 (-0.2 to -0.1)	-2.3 (-3.1 to -1.7)
South Sudan	95.5 (94.4 to 96.4)	95.2 (94.0 to 96.2)	95.1 (93.9 to 96.1)	94.6 (93.3 to 95.7)	-0.0 (-0.1 to -0.0)	-0.6 (-0.9 to -0.4)
Tanzania	94.1 (92.7 to 95.3)	93.0 (91.4 to 94.5)	92.8 (91.1 to 94.2)	92.0 (90.1 to 93.6)	-0.1 (-0.1 to -0.0)	-1.1 (-1.5 to -0.7)
Uganda	92.7 (91.0 to 94.0)	89.5 (87.7 to 91.1)	89.2 (87.3 to 90.9)	85.2 (82.7 to 87.5)	-0.3 (-0.4 to -0.3)	-4.8 (-5.9 to -3.8)
Zambia	90.2 (88.1 to 92.1)	87.4 (84.9 to 89.5)	86.6 (84.1 to 89.0)	83.7 (80.9 to 86.5)	-0.3 (-0.4 to -0.2)	-4.2 (-5.4 to -3.1)
Southern Sub-Saharan Africa						
Botswana	77.3 (73.0 to 81.2)	66.8 (61.4 to 71.8)	65.0 (59.5 to 70.1)	56.4 (50.4 to 62.0)	-1.1 (-1.3 to -0.9)	-15.7 (-18.3 to -13.2)
Lesotho	97.4 (96.7 to 97.9)	96.3 (95.4 to 97.1)	96.1 (95.1 to 96.9)	94.5 (93.1 to 95.6)	-0.1 (-0.2 to -0.1)	-2.0 (-2.5 to -1.5)
Namibia	67.1 (61.9 to 71.7)	53.1 (49.3 to 57.0)	51.4 (47.3 to 55.5)	41.8 (37.9 to 45.9)	-1.6 (-1.8 to -1.4)	-21.2 (-23.8 to -18.6)
South Africa	60.6 (55.1 to 66.2)	55.0 (49.7 to 60.8)	54.2 (48.9 to 60.2)	53.5 (48.1 to 59.4)	-0.2 (-0.3 to -0.1)	-2.8 (-4.8 to -0.8)
Swaziland	74.3 (70.3 to 78.0)	63.5 (59.4 to 67.3)	62.4 (58.0 to 66.4)	53.9 (49.2 to 58.1)	-1.1 (-1.3 to -0.9)	-15.2 (-17.6 to -12.8)

	Estimate in 2000 (95% UIs)	Estimate in 2015 (95% UIs)	Estimate in 2017 (95% UIs)	Estimate in 2030 (95% UIs)	Annualised rate of change, 2015-2030 (95% UIs)	Percent change, 2015-2030 (95% UIs)
Zimbabwe	78.0 (74.2 to 81.4)	83.1 (80.2 to 85.4)	82.6 (79.5 to 85.0)	82.2 (79.1 to 84.7)	-0.1 (-0.1 to -0.0)	-1.1 (-2.0 to -0.2)
Western Sub-Saharan Africa						
Benin	94.7 (93.5 to 95.8)	91.4 (89.6 to 93.0)	91.0 (89.2 to 92.8)	88.3 (85.9 to 90.6)	-0.2 (-0.3 to -0.2)	-3.3 (-4.3 to -2.4)
Burkina Faso	92.3 (90.7 to 93.7)	91.0 (89.1 to 92.6)	90.8 (89.0 to 92.5)	87.5 (85.0 to 89.8)	-0.3 (-0.3 to -0.2)	-3.8 (-4.8 to -3.0)
Cameroon	90.5 (88.3 to 92.4)	88.3 (85.6 to 90.7)	87.6 (84.8 to 90.1)	85.4 (82.2 to 88.2)	-0.2 (-0.3 to -0.2)	-3.3 (-4.3 to -2.5)
Cape Verde	86.3 (83.2 to 89.0)	77.0 (72.5 to 81.2)	76.1 (71.2 to 80.3)	69.7 (64.3 to 74.5)	-0.7 (-0.8 to -0.5)	-9.5 (-11.8 to -7.5)
Chad	89.4 (87.2 to 91.1)	88.3 (86.3 to 90.1)	88.5 (86.4 to 90.3)	88.2 (86.1 to 90.1)	-0.0 (-0.0 to 0.0)	-0.1 (-0.6 to 0.4)
Cote d'Ivoire	85.6 (82.6 to 88.3)	86.0 (83.5 to 88.3)	85.5 (82.7 to 88.0)	80.2 (76.9 to 83.6)	-0.5 (-0.6 to -0.3)	-6.7 (-8.4 to -5.1)
The Gambia	92.6 (90.9 to 94.1)	91.1 (89.3 to 92.7)	90.8 (88.9 to 92.5)	87.2 (84.5 to 89.4)	-0.3 (-0.4 to -0.2)	-4.4 (-5.4 to -3.5)
Ghana	90.0 (88.1 to 91.7)	88.4 (86.7 to 90.1)	88.2 (86.4 to 89.9)	88.0 (86.1 to 89.6)	-0.0 (-0.1 to 0.0)	-0.5 (-1.1 to 0.0)
Guinea	93.0 (91.4 to 94.4)	92.1 (90.2 to 93.6)	91.8 (89.9 to 93.3)	88.8 (86.2 to 90.9)	-0.2 (-0.3 to -0.2)	-3.5 (-4.5 to -2.7)
Guinea-Bissau	91.9 (89.8 to 93.5)	91.3 (89.4 to 93.0)	91.0 (89.2 to 92.8)	89.2 (87.1 to 91.4)	-0.2 (-0.2 to -0.1)	-2.2 (-3.0 to -1.6)
Liberia	97.0 (96.3 to 97.6)	97.0 (96.3 to 97.6)	97.0 (96.3 to 97.6)	96.6 (95.8 to 97.3)	-0.0 (-0.0 to -0.0)	-0.5 (-0.7 to -0.3)
Mali	91.2 (89.4 to 92.8)	90.1 (88.1 to 91.8)	89.9 (87.9 to 91.6)	89.4 (87.4 to 91.3)	-0.1 (-0.1 to -0.0)	-0.8 (-1.3 to -0.3)
Mauritania	80.9 (77.5 to 84.0)	74.5 (71.3 to 77.6)	74.0 (70.5 to 77.2)	65.1 (60.9 to 68.8)	-0.9 (-1.1 to -0.8)	-12.7 (-14.9 to -10.8)
Niger	89.7 (87.5 to 91.6)	87.3 (84.9 to 89.5)	87.1 (84.6 to 89.3)	81.8 (78.5 to 84.8)	-0.4 (-0.5 to -0.3)	-6.3 (-7.7 to -5.1)
Nigeria	90.2 (88.2 to 92.0)	88.8 (86.8 to 90.5)	88.6 (86.6 to 90.4)	87.6 (85.4 to 89.5)	-0.1 (-0.1 to -0.1)	-1.4 (-2.1 to -0.8)
Sao Tome and Principe	83.3 (80.0 to 86.1)	67.3 (63.5 to 70.6)	65.8 (61.9 to 69.1)	58.9 (54.8 to 62.5)	-0.9 (-1.1 to -0.7)	-12.5 (-14.6 to -10.6)
Senegal	69.8 (66.4 to 73.4)	74.8 (71.7 to 77.6)	75.2 (71.8 to 78.1)	73.7 (70.4 to 76.8)	-0.1 (-0.2 to -0.0)	-1.4 (-2.8 to -0.1)
Sierra Leone	91.2 (89.3 to 92.8)	90.2 (88.4 to 91.8)	90.0 (88.0 to 91.7)	89.7 (87.7 to 91.5)	-0.0 (-0.1 to -0.0)	-0.5 (-1.0 to -0.1)
Togo	90.6 (88.6 to 92.5)	89.9 (87.9 to 91.8)	89.5 (87.3 to 91.5)	88.2 (85.8 to 90.4)	-0.1 (-0.2 to -0.1)	-1.9 (-2.6 to -1.3)
**Indicator 7.1.2: Risk-weighted prevalence of household air pollution, as measured by the summary exposure value (SEV) for household air pollution (%)**						
Central Europe, Eastern Europe, and Central Asia						
Central Asia						
Armenia	20.9 (14.2 to 29.1)	9.0 (4.5 to 14.9)	8.5 (4.3 to 14.1)	3.9 (1.9 to 6.7)	-5.6 (-9.0 to -2.1)	-55.5 (-74.2 to -27.4)
Azerbaijan	18.6 (12.8 to 25.2)	10.5 (5.2 to 16.0)	10.1 (4.9 to 15.9)	6.7 (3.0 to 10.9)	-3.0 (-6.7 to 0.3)	-34.1 (-63.4 to 5.0)
Georgia	25.9 (18.5 to 35.9)	20.1 (13.7 to 28.3)	19.3 (13.1 to 27.1)	11.9 (7.7 to 17.5)	-3.5 (-5.4 to -1.5)	-40.0 (-55.7 to -20.7)
Kazakhstan	15.5 (8.9 to 21.4)	8.9 (4.0 to 14.5)	8.0 (3.5 to 13.7)	3.8 (1.6 to 6.8)	-5.7 (-10.6 to -1.5)	-55.3 (-79.5 to -20.4)
Kyrgyzstan	25.2 (16.3 to 35.5)	18.6 (11.5 to 26.1)	18.1 (11.2 to 24.6)	10.0 (5.9 to 14.1)	-4.2 (-6.9 to -1.6)	-45.9 (-64.4 to -21.1)
Mongolia	31.9 (21.6 to 47.0)	21.7 (13.7 to 32.2)	21.2 (13.1 to 30.9)	13.8 (8.2 to 20.8)	-3.1 (-5.7 to -0.5)	-35.5 (-57.3 to -6.7)
Tajikistan	35.2 (22.6 to 52.5)	22.2 (13.2 to 32.2)	22.1 (13.1 to 31.3)	16.4 (9.3 to 24.2)	-2.1 (-5.2 to 1.1)	-24.7 (-54.3 to 17.3)
Turkmenistan	0.8 (0.3 to 1.6)	0.2 (0.1 to 0.5)	0.2 (0.1 to 0.5)	0.2 (0.1 to 0.4)	-2.1 (-8.7 to 4.4)	-15.8 (-73.0 to 93.4)
Uzbekistan	18.2 (10.3 to 26.0)	10.1 (4.3 to 16.8)	9.4 (4.3 to 15.9)	4.6 (2.0 to 8.0)	-5.3 (-9.8 to -0.8)	-52.0 (-76.9 to -11.2)
Central Europe						
Albania	29.6 (21.5 to 41.2)	19.1 (13.6 to 26.8)	18.5 (12.6 to 25.9)	10.4 (6.7 to 15.0)	-4.1 (-5.9 to -2.4)	-45.4 (-58.5 to -30.1)
Bosnia and Herzegovina	27.1 (19.1 to 37.5)	17.7 (11.4 to 25.3)	17.3 (10.7 to 25.0)	11.2 (6.7 to 16.6)	-3.0 (-5.4 to -0.8)	-35.7 (-55.8 to -11.2)
Bulgaria	11.8 (6.2 to 18.1)	8.2 (4.0 to 13.7)	7.8 (3.6 to 13.4)	5.1 (2.3 to 9.0)	-3.2 (-6.8 to 0.8)	-35.2 (-64.0 to 13.2)
Croatia	8.9 (4.5 to 14.3)	5.5 (2.5 to 10.4)	5.2 (2.5 to 9.9)	3.1 (1.5 to 6.1)	-3.7 (-8.0 to 0.6)	-39.5 (-69.9 to 9.2)
Czech Republic	1.5 (0.7 to 3.1)	1.1 (0.5 to 2.3)	1.1 (0.4 to 2.3)	0.7 (0.3 to 1.4)	-3.4 (-7.8 to 1.4)	-36.4 (-69.0 to 22.6)
Hungary	11.7 (6.2 to 17.6)	8.1 (4.0 to 13.3)	7.8 (3.9 to 13.1)	5.3 (2.6 to 9.1)	-2.9 (-6.4 to 0.6)	-32.7 (-61.5 to 8.8)
Macedonia	19.8 (13.3 to 27.9)	12.2 (6.7 to 18.3)	11.7 (6.1 to 18.2)	6.5 (3.2 to 10.3)	-4.3 (-7.5 to -1.2)	-46.4 (-67.6 to -16.8)
Montenegro	18.1 (12.1 to 24.7)	15.2 (9.5 to 21.7)	15.0 (9.5 to 21.3)	11.8 (7.2 to 16.9)	-1.7 (-4.3 to 0.9)	-21.3 (-47.6 to 14.4)
Poland	7.4 (3.6 to 13.5)	3.9 (1.7 to 7.9)	3.8 (1.6 to 7.4)	2.1 (0.9 to 4.3)	-4.0 (-8.3 to 0.8)	-41.4 (-71.4 to 13.1)
Romania	15.7 (10.6 to 21.2)	8.8 (4.7 to 13.6)	8.3 (4.5 to 13.2)	4.2 (2.2 to 6.8)	-5.0 (-8.2 to -1.8)	-51.3 (-70.8 to -24.0)
Serbia	22.3 (15.8 to 31.4)	16.3 (10.1 to 23.1)	15.9 (9.9 to 22.9)	11.3 (6.8 to 16.6)	-2.4 (-5.1 to -0.0)	-29.4 (-53.6 to -0.2)
Slovakia	2.4 (1.1 to 4.5)	1.6 (0.7 to 3.2)	1.6 (0.7 to 3.1)	1.1 (0.5 to 2.1)	-2.9 (-7.0 to 1.3)	-31.4 (-65.2 to 22.2)
Slovenia	6.1 (3.0 to 11.0)	3.0 (1.3 to 6.0)	2.8 (1.2 to 5.8)	1.5 (0.6 to 3.1)	-4.7 (-8.9 to -0.4)	-47.7 (-73.5 to -5.7)
Eastern Europe						
Belarus	2.1 (1.0 to 4.0)	0.5 (0.2 to 1.0)	0.5 (0.2 to 0.9)	0.2 (0.1 to 0.7)	-5.7 (-10.9 to 1.0)	-52.1 (-80.5 to 16.1)
Estonia	10.4 (5.6 to 15.2)	5.7 (2.3 to 10.7)	5.1 (1.9 to 9.9)	2.4 (0.9 to 5.0)	-5.7 (-10.5 to -1.4)	-55.1 (-79.3 to -18.6)
Latvia	7.1 (3.5 to 12.2)	4.0 (1.6 to 7.6)	3.6 (1.6 to 7.0)	1.8 (0.8 to 3.6)	-5.2 (-8.6 to -1.5)	-52.4 (-72.5 to -20.6)
Lithuania	0.9 (0.4 to 1.9)	0.8 (0.3 to 1.7)	0.8 (0.3 to 1.7)	0.6 (0.2 to 1.3)	-2.1 (-6.7 to 2.4)	-23.0 (-63.5 to 43.8)
Moldova	22.0 (16.6 to 29.6)	7.5 (4.0 to 12.6)	6.7 (3.4 to 11.5)	3.0 (1.5 to 5.3)	-6.2 (-9.1 to -3.4)	-59.4 (-74.5 to -40.2)
Russia	0.1 (0.1 to 0.2)	0.1 (0.1 to 0.2)	0.1 (0.1 to 0.2)	0.1 (0.0 to 0.2)	-3.6 (-8.7 to 0.6)	-36.1 (-72.7 to 9.1)
Ukraine	5.2 (2.3 to 9.5)	2.8 (1.2 to 5.6)	2.7 (1.1 to 5.4)	1.3 (0.5 to 2.6)	-5.3 (-10.2 to -0.6)	-52.1 (-78.5 to -8.8)
High-income						
Australasia						
Australia	0.3 (0.1 to 0.7)	0.2 (0.0 to 0.4)	0.2 (0.0 to 0.4)	0.1 (0.0 to 0.4)	-1.4 (-8.2 to 4.8)	-6.8 (-71.0 to 105.2)
New Zealand	0.3 (0.1 to 0.7)	0.2 (0.0 to 0.5)	0.2 (0.0 to 0.5)	0.1 (0.0 to 0.3)	-3.2 (-12.2 to 3.3)	-27.6 (-84.0 to 64.5)
High-income Asia Pacific						
Brunei	0.7 (0.2 to 1.5)	0.1 (0.0 to 0.3)	0.1 (0.0 to 0.3)	0.1 (0.0 to 0.3)	-2.0 (-10.6 to 3.9)	-16.3 (-79.6 to 78.8)
Japan	0.1 (0.1 to 0.2)	0.1 (0.0 to 0.1)	0.1 (0.0 to 0.1)	0.1 (0.0 to 0.1)	-0.3 (-1.4 to 0.7)	-4.8 (-18.8 to 11.1)
South Korea	0.1 (0.1 to 0.2)	0.0 (0.0 to 0.1)	0.0 (0.0 to 0.1)	0.0 (0.0 to 0.1)	-0.3 (-5.0 to 4.8)	3.2 (-53.0 to 107.0)
Singapore	0.3 (0.1 to 0.6)	0.1 (0.0 to 0.1)	0.1 (0.0 to 0.1)	0.1 (0.0 to 0.1)	-0.6 (-5.2 to 4.2)	-3.1 (-54.3 to 88.4)
High-income North America						
Canada	0.1 (0.0 to 0.2)	0.0 (0.0 to 0.1)	0.0 (0.0 to 0.1)	0.0 (0.0 to 0.1)	-0.7 (-10.6 to 6.5)	19.2 (-79.6 to 164.0)
Greenland	1.0 (0.4 to 2.0)	0.6 (0.2 to 1.2)	0.5 (0.2 to 1.1)	0.3 (0.1 to 0.6)	-4.2 (-9.3 to 1.2)	-39.9 (-75.3 to 19.8)
United States	0.2 (0.1 to 0.3)	0.1 (0.0 to 0.2)	0.1 (0.0 to 0.2)	0.1 (0.0 to 0.2)	-0.5 (-2.0 to 0.8)	-6.3 (-25.4 to 13.5)
Southern Latin America						
Argentina	7.9 (4.0 to 12.3)	2.4 (1.0 to 4.5)	2.2 (0.8 to 4.2)	0.9 (0.4 to 1.9)	-6.5 (-11.5 to -1.6)	-59.4 (-82.2 to -21.8)
Chile	10.2 (5.9 to 15.2)	4.8 (2.3 to 9.0)	4.6 (2.0 to 8.5)	1.8 (0.8 to 3.4)	-6.7 (-10.6 to -2.9)	-61.7 (-79.5 to -35.1)
Uruguay	3.5 (1.6 to 6.8)	1.7 (0.7 to 3.3)	1.5 (0.6 to 3.2)	0.7 (0.3 to 1.5)	-5.9 (-9.8 to -1.9)	-56.7 (-77.0 to -25.3)
Western Europe						
Andorra	0.6 (0.2 to 1.3)	0.3 (0.1 to 0.7)	0.3 (0.1 to 0.7)	0.2 (0.1 to 0.4)	-4.0 (-8.6 to 0.7)	-41.1 (-72.4 to 11.5)
Austria	1.1 (0.5 to 2.2)	0.5 (0.2 to 1.0)	0.5 (0.2 to 1.0)	0.2 (0.1 to 0.6)	-4.5 (-9.0 to 0.1)	-46.0 (-74.0 to 1.0)
Belgium	0.4 (0.1 to 0.8)	0.2 (0.1 to 0.4)	0.2 (0.1 to 0.4)	0.1 (0.1 to 0.3)	-2.0 (-6.5 to 3.1)	-20.4 (-62.4 to 59.3)
Cyprus	0.8 (0.3 to 1.6)	0.3 (0.1 to 0.6)	0.3 (0.1 to 0.6)	0.1 (0.1 to 0.3)	-4.3 (-9.1 to 0.2)	-44.4 (-74.3 to 3.8)
Denmark	0.3 (0.1 to 0.6)	0.2 (0.1 to 0.3)	0.1 (0.1 to 0.3)	0.1 (0.1 to 0.3)	-1.1 (-6.2 to 3.8)	-9.2 (-60.3 to 75.5)
Finland	0.6 (0.1 to 1.4)	0.2 (0.0 to 0.6)	0.2 (0.0 to 0.6)	0.1 (0.0 to 0.4)	-3.6 (-14.2 to 5.4)	-26.3 (-88.1 to 125.6)
France	0.6 (0.2 to 1.2)	0.2 (0.1 to 0.5)	0.2 (0.1 to 0.5)	0.1 (0.1 to 0.3)	-3.2 (-7.9 to 1.5)	-34.3 (-69.4 to 25.3)

	Estimate in 2000 (95% UIs)	Estimate in 2015 (95% UIs)	Estimate in 2017 (95% UIs)	Estimate in 2030 (95% UIs)	Annualised rate of change, 2015-2030 (95% UIs)	Percent change, 2015-2030 (95% UIs)
Germany	0.9 (0.4 to 1.7)	0.5 (0.2 to 0.9)	0.4 (0.2 to 0.9)	0.2 (0.1 to 0.5)	-3.9 (-8.4 to 0.4)	-41.3 (-71.5 to 6.9)
Greece	0.8 (0.3 to 1.6)	0.4 (0.1 to 0.8)	0.4 (0.2 to 0.8)	0.2 (0.1 to 0.5)	-3.9 (-8.6 to 1.0)	-40.6 (-72.4 to 16.0)
Iceland	0.6 (0.2 to 1.4)	0.3 (0.1 to 0.7)	0.2 (0.0 to 0.6)	0.2 (0.0 to 0.5)	-3.4 (-11.4 to 3.2)	-30.2 (-81.9 to 61.8)
Ireland	0.9 (0.4 to 1.9)	0.4 (0.1 to 0.8)	0.4 (0.1 to 0.8)	0.2 (0.1 to 0.5)	-4.4 (-9.2 to 0.4)	-45.0 (-74.9 to 5.7)
Israel	0.5 (0.2 to 1.1)	0.3 (0.1 to 0.7)	0.3 (0.1 to 0.6)	0.2 (0.1 to 0.4)	-3.6 (-8.3 to 0.9)	-38.3 (-71.3 to 14.4)
Italy	0.8 (0.3 to 1.7)	0.4 (0.2 to 0.9)	0.4 (0.2 to 0.8)	0.2 (0.1 to 0.5)	-4.1 (-8.9 to 0.9)	-41.8 (-73.9 to 13.8)
Luxembourg	0.5 (0.2 to 1.1)	0.2 (0.1 to 0.5)	0.2 (0.1 to 0.5)	0.1 (0.1 to 0.3)	-3.3 (-8.3 to 1.7)	-34.3 (-71.2 to 30.0)
Malta	0.9 (0.4 to 1.9)	0.4 (0.2 to 0.9)	0.4 (0.2 to 0.8)	0.2 (0.1 to 0.4)	-5.3 (-9.5 to -1.4)	-52.7 (-75.9 to -18.8)
Netherlands	0.3 (0.1 to 0.7)	0.1 (0.1 to 0.3)	0.1 (0.1 to 0.3)	0.1 (0.0 to 0.3)	-1.3 (-6.4 to 3.8)	-10.2 (-61.8 to 76.6)
Norway	0.8 (0.4 to 1.6)	0.3 (0.1 to 0.7)	0.3 (0.1 to 0.6)	0.2 (0.1 to 0.3)	-4.4 (-6.1 to -2.5)	-47.5 (-60.0 to -30.9)
Portugal	3.9 (1.7 to 7.7)	1.1 (0.4 to 2.4)	0.9 (0.3 to 2.1)	0.3 (0.1 to 0.8)	-7.7 (-12.4 to -3.4)	-66.4 (-84.5 to -40.2)
Spain	2.1 (1.0 to 4.0)	0.9 (0.4 to 1.9)	0.9 (0.4 to 1.9)	0.5 (0.2 to 1.0)	-4.4 (-8.6 to -0.3)	-45.8 (-72.4 to -5.0)
Sweden	0.8 (0.3 to 1.7)	0.3 (0.1 to 0.8)	0.3 (0.1 to 0.7)	0.2 (0.0 to 0.5)	-2.7 (-8.9 to 3.7)	-25.6 (-73.7 to 74.6)
Switzerland	0.9 (0.4 to 2.0)	0.5 (0.2 to 1.1)	0.5 (0.2 to 1.0)	0.2 (0.1 to 0.6)	-4.7 (-9.5 to -0.1)	-47.2 (-76.1 to -0.8)
United Kingdom	1.0 (0.5 to 1.9)	0.4 (0.2 to 0.7)	0.3 (0.2 to 0.7)	0.2 (0.1 to 0.4)	-4.6 (-5.8 to -3.2)	-49.9 (-58.3 to -38.2)
Latin America and Caribbean						
Andean Latin America						
Bolivia	28.6 (20.8 to 39.3)	16.0 (9.8 to 22.0)	14.4 (8.6 to 20.1)	5.8 (3.4 to 8.3)	-6.8 (-9.7 to -4.5)	-63.1 (-76.7 to -49.4)
Ecuador	10.1 (5.5 to 15.1)	4.1 (1.9 to 7.5)	3.6 (1.6 to 6.5)	1.3 (0.6 to 2.4)	-7.9 (-10.8 to -4.9)	-68.5 (-80.2 to -52.3)
Peru	27.5 (19.6 to 39.1)	17.4 (11.2 to 24.1)	16.4 (10.3 to 22.7)	8.4 (5.0 to 12.0)	-4.9 (-7.3 to -2.8)	-51.2 (-66.7 to -34.0)
Caribbean						
Antigua and Barbuda	3.1 (1.4 to 5.9)	1.5 (0.7 to 3.0)	1.4 (0.6 to 2.8)	0.6 (0.3 to 1.3)	-5.9 (-9.9 to -1.9)	-56.7 (-77.3 to -24.4)
The Bahamas	3.9 (1.6 to 7.5)	2.1 (0.8 to 4.2)	2.0 (0.8 to 4.1)	1.3 (0.5 to 2.5)	-3.4 (-8.3 to 1.4)	-36.2 (-71.2 to 23.1)
Barbados	0.1 (0.1 to 0.2)	0.0 (0.0 to 0.1)	0.0 (0.0 to 0.1)	0.0 (0.0 to 0.1)	-0.3 (-4.8 to 3.9)	0.6 (-51.1 to 78.3)
Belize	18.6 (11.5 to 25.8)	11.7 (6.0 to 17.2)	11.2 (5.6 to 16.6)	7.2 (3.5 to 10.9)	-3.3 (-7.4 to 0.6)	-36.6 (-67.0 to 8.8)
Bermuda	3.3 (1.5 to 6.4)	2.6 (1.1 to 5.0)	2.5 (1.1 to 5.0)	1.7 (0.7 to 3.4)	-3.0 (-6.6 to 0.7)	-33.4 (-62.7 to 10.6)
Cuba	4.6 (2.0 to 9.3)	2.1 (0.9 to 4.5)	1.9 (0.8 to 3.8)	0.8 (0.3 to 1.7)	-6.1 (-10.8 to -1.6)	-57.3 (-80.1 to -20.9)
Dominica	15.6 (10.1 to 21.8)	7.0 (3.4 to 11.8)	6.4 (3.2 to 11.2)	2.6 (1.2 to 4.7)	-6.7 (-10.1 to -3.1)	-62.0 (-78.0 to -36.9)
Dominican Republic	19.1 (13.6 to 25.5)	10.9 (5.8 to 15.7)	9.8 (5.2 to 14.5)	5.3 (2.7 to 8.1)	-4.9 (-8.3 to -2.1)	-50.7 (-71.0 to -26.6)
Grenada	10.2 (5.5 to 15.7)	3.6 (1.7 to 6.7)	3.2 (1.4 to 6.2)	1.2 (0.6 to 2.4)	-7.1 (-10.8 to -3.5)	-63.9 (-80.2 to -40.8)
Guyana	15.2 (8.4 to 21.5)	9.8 (4.5 to 15.3)	9.1 (4.2 to 14.7)	4.3 (1.9 to 7.2)	-5.6 (-9.6 to -1.3)	-54.4 (-76.3 to -17.3)
Haiti	63.6 (45.0 to 80.9)	53.8 (39.1 to 74.7)	52.4 (37.8 to 73.1)	43.7 (29.3 to 67.7)	-1.5 (-3.0 to -0.0)	-19.0 (-36.6 to -0.5)
Jamaica	14.3 (8.9 to 19.8)	7.5 (3.8 to 12.5)	7.1 (3.7 to 11.9)	3.6 (1.8 to 6.3)	-5.0 (-7.4 to -2.4)	-51.6 (-67.0 to -30.6)
Puerto Rico	0.2 (0.1 to 0.4)	0.2 (0.1 to 0.4)	0.2 (0.0 to 0.4)	0.2 (0.0 to 0.4)	-0.5 (-4.8 to 3.4)	-2.5 (-51.3 to 67.5)
Saint Lucia	15.2 (9.1 to 21.6)	5.6 (2.6 to 10.1)	4.9 (2.3 to 9.5)	1.7 (0.8 to 3.5)	-7.8 (-11.5 to -4.2)	-67.7 (-82.1 to -46.5)
Saint Vincent and the Grenadines	23.8 (16.9 to 32.8)	9.1 (4.4 to 14.8)	8.2 (4.2 to 13.1)	3.3 (1.6 to 5.4)	-6.8 (-10.2 to -3.6)	-63.1 (-78.3 to -42.1)
Suriname	16.1 (9.9 to 22.9)	10.2 (5.2 to 15.3)	9.5 (4.9 to 14.9)	5.0 (2.5 to 8.1)	-4.8 (-8.6 to -1.1)	-49.3 (-72.5 to -15.1)
Trinidad and Tobago	1.3 (0.5 to 2.8)	0.6 (0.3 to 1.3)	0.6 (0.2 to 1.3)	0.4 (0.2 to 0.9)	-2.8 (-8.4 to 2.8)	-27.9 (-71.8 to 52.2)
Virgin Islands, U.S.	1.9 (0.8 to 3.9)	1.1 (0.4 to 2.2)	1.0 (0.4 to 2.1)	0.6 (0.2 to 1.3)	-3.7 (-7.4 to 0.0)	-40.3 (-67.1 to 0.6)
Central Latin America						
Colombia	17.8 (12.6 to 23.8)	7.9 (4.1 to 12.8)	6.9 (3.2 to 11.6)	2.9 (1.3 to 5.1)	-6.9 (-11.1 to -3.6)	-63.3 (-81.0 to -41.3)
Costa Rica	13.4 (8.2 to 18.4)	6.8 (3.4 to 11.6)	6.2 (3.0 to 11.1)	2.9 (1.4 to 5.4)	-5.7 (-8.2 to -3.0)	-56.4 (-70.6 to -36.6)
El Salvador	28.4 (20.4 to 39.3)	15.7 (10.3 to 20.9)	14.7 (9.6 to 19.7)	5.8 (3.6 to 8.0)	-6.7 (-8.9 to -4.7)	-62.7 (-73.6 to -50.5)
Guatemala	43.5 (31.9 to 59.9)	30.8 (22.5 to 42.3)	29.2 (21.3 to 41.0)	15.6 (10.6 to 23.8)	-4.6 (-6.1 to -3.1)	-49.6 (-59.9 to -37.4)
Honduras	44.2 (32.1 to 61.7)	32.5 (23.9 to 44.4)	31.0 (22.8 to 42.5)	20.0 (14.0 to 29.0)	-3.3 (-4.6 to -1.9)	-38.6 (-49.9 to -24.3)
Mexico	14.3 (9.7 to 20.1)	10.6 (7.2 to 14.9)	10.2 (6.7 to 14.4)	4.4 (2.8 to 6.5)	-5.9 (-6.8 to -4.7)	-58.4 (-64.2 to -50.7)
Nicaragua	43.4 (32.0 to 61.5)	28.8 (20.8 to 40.0)	26.8 (19.6 to 36.8)	13.8 (9.5 to 20.2)	-4.9 (-6.3 to -3.6)	-52.1 (-61.2 to -41.3)
Panama	17.4 (12.1 to 23.5)	9.6 (5.4 to 14.4)	8.6 (4.6 to 13.1)	3.6 (1.8 to 5.7)	-6.7 (-9.6 to -4.3)	-62.8 (-76.1 to -47.3)
Venezuela	1.4 (0.6 to 2.8)	0.5 (0.2 to 1.0)	0.4 (0.2 to 0.9)	0.2 (0.1 to 0.4)	-6.8 (-12.0 to -0.9)	-60.2 (-83.5 to -12.9)
Tropical Latin America						
Brazil	15.1 (10.9 to 20.8)	6.4 (3.8 to 9.8)	5.6 (3.2 to 8.8)	2.0 (1.1 to 3.2)	-7.8 (-8.8 to -6.8)	-68.8 (-73.4 to -64.0)
Paraguay	34.0 (25.5 to 45.6)	23.8 (17.4 to 32.9)	22.1 (15.8 to 30.1)	9.5 (6.5 to 13.7)	-6.2 (-7.6 to -4.8)	-60.2 (-67.8 to -51.3)
North Africa and Middle East						
North Africa and Middle East						
Afghanistan	70.6 (50.4 to 86.9)	53.9 (37.3 to 75.8)	52.3 (35.6 to 76.3)	38.6 (23.2 to 67.8)	-2.4 (-5.1 to 0.4)	-28.2 (-53.6 to 5.8)
Algeria	1.5 (0.6 to 3.2)	0.2 (0.1 to 0.5)	0.2 (0.1 to 0.4)	0.1 (0.0 to 0.3)	-5.0 (-12.5 to 2.6)	-44.4 (-84.7 to 47.6)
Bahrain	0.6 (0.3 to 1.2)	0.3 (0.1 to 0.5)	0.2 (0.1 to 0.4)	0.1 (0.0 to 0.2)	-7.2 (-10.9 to -3.4)	-64.7 (-80.4 to -40.3)
Egypt	4.7 (2.3 to 8.5)	0.5 (0.2 to 1.1)	0.4 (0.1 to 0.8)	0.3 (0.1 to 0.7)	-3.8 (-10.5 to 2.4)	-36.8 (-79.3 to 43.4)
Iran	2.2 (1.2 to 3.8)	0.4 (0.2 to 0.6)	0.3 (0.1 to 0.5)	0.2 (0.1 to 0.3)	-5.6 (-8.9 to -1.9)	-55.1 (-73.8 to -24.7)
Iraq	1.3 (0.5 to 2.5)	0.3 (0.1 to 0.6)	0.2 (0.1 to 0.5)	0.1 (0.0 to 0.2)	-8.0 (-13.1 to -3.2)	-67.8 (-85.9 to -37.7)
Jordan	0.1 (0.1 to 0.3)	0.0 (0.0 to 0.1)	0.0 (0.0 to 0.0)	0.0 (0.0 to 0.0)	-0.9 (-4.4 to 2.9)	-8.7 (-48.6 to 55.0)
Kuwait	0.6 (0.3 to 1.2)	0.1 (0.0 to 0.2)	0.1 (0.0 to 0.2)	0.1 (0.0 to 0.1)	-2.3 (-7.1 to 2.7)	-24.3 (-65.7 to 49.0)
Lebanon	0.0 (0.0 to 0.1)	0.0 (0.0 to 0.0)	0.0 (0.0 to 0.0)	0.0 (0.0 to 0.0)	-1.1 (-4.9 to 2.5)	-12.0 (-51.7 to 45.9)
Libya	0.3 (0.1 to 0.7)	0.0 (0.0 to 0.1)	0.0 (0.0 to 0.1)	0.0 (0.0 to 0.1)	-2.3 (-10.2 to 6.0)	-14.5 (-78.3 to 146.7)
Morocco	5.1 (2.4 to 9.6)	1.9 (0.9 to 3.6)	1.4 (0.6 to 2.7)	0.5 (0.2 to 0.9)	-9.1 (-13.3 to -4.8)	-72.9 (-86.5 to -51.7)
Palestine	1.8 (0.8 to 3.3)	0.6 (0.2 to 1.1)	0.4 (0.2 to 0.9)	0.2 (0.1 to 0.3)	-8.2 (-12.1 to -4.2)	-69.7 (-83.7 to -46.9)
Oman	7.4 (3.4 to 13.7)	0.6 (0.2 to 1.2)	0.4 (0.2 to 0.9)	0.1 (0.1 to 0.3)	-9.1 (-14.4 to -3.3)	-72.2 (-88.4 to -39.0)
Qatar	0.0 (0.0 to 0.0)	0.0 (0.0 to 0.0)	0.0 (0.0 to 0.0)	0.0 (0.0 to 0.0)	-1.1 (-5.3 to 3.4)	-10.2 (-54.9 to 66.7)
Saudi Arabia	3.3 (1.2 to 6.7)	0.2 (0.1 to 0.5)	0.1 (0.0 to 0.4)	0.1 (0.0 to 0.2)	-7.1 (-14.8 to 0.4)	-59.1 (-89.1 to 6.8)
Sudan	60.6 (39.0 to 81.0)	34.6 (20.5 to 53.0)	32.5 (20.4 to 47.8)	20.7 (11.8 to 33.4)	-3.5 (-7.2 to 0.5)	-38.3 (-65.9 to 7.6)
Syria	0.4 (0.2 to 0.8)	0.1 (0.0 to 0.2)	0.1 (0.0 to 0.1)	0.1 (0.0 to 0.1)	-2.0 (-6.2 to 2.5)	-21.7 (-60.8 to 46.6)
Tunisia	3.2 (1.4 to 5.9)	0.2 (0.1 to 0.5)	0.2 (0.1 to 0.3)	0.1 (0.0 to 0.3)	-4.5 (-9.9 to 1.0)	-43.9 (-77.4 to 15.9)
Turkey	6.0 (2.9 to 10.5)	0.6 (0.2 to 1.1)	0.4 (0.2 to 0.9)	0.1 (0.1 to 0.3)	-8.9 (-13.4 to -4.5)	-72.3 (-86.5 to -48.7)
United Arab Emirates	0.9 (0.4 to 1.7)	0.2 (0.1 to 0.4)	0.2 (0.1 to 0.4)	0.1 (0.0 to 0.2)	-4.7 (-10.8 to 1.8)	-44.6 (-80.2 to 31.6)
Yemen	43.7 (28.4 to 62.7)	21.3 (11.8 to 31.1)	19.4 (10.2 to 29.2)	10.2 (5.0 to 16.2)	-5.0 (-9.3 to -1.3)	-50.4 (-75.3 to -17.6)
South Asia						
South Asia						
Bangladesh	55.4 (39.4 to 77.4)	40.1 (28.1 to 56.2)	38.9 (27.6 to 55.9)	26.8 (17.8 to 43.0)	-2.8 (-4.6 to -0.9)	-33.1 (-49.5 to -12.1)
Bhutan	42.0 (29.8 to 61.5)	20.4 (12.8 to 27.9)	18.5 (11.1 to 25.9)	7.3 (4.1 to 10.7)	-6.9 (-9.1 to -5.4)	-64.4 (-74.5 to -55.2)
India	43.3 (32.5 to 57.3)	26.1 (19.6 to 35.1)	23.7 (18.1 to 32.6)	10.8 (7.6 to 15.6)	-5.9 (-6.8 to -4.8)	-58.9 (-64.2 to -51.4)

	Estimate in 2000 (95% UIs)	Estimate in 2015 (95% UIs)	Estimate in 2017 (95% UIs)	Estimate in 2030 (95% UIs)	Annualised rate of change, 2015-2030 (95% UIs)	Percent change, 2015-2030 (95% UIs)
Nepal	51.2 (33.5 to 74.4)	34.1 (22.4 to 50.3)	31.2 (20.4 to 45.5)	17.0 (10.0 to 27.0)	-4.7 (-8.0 to -1.7)	-49.6 (-69.8 to -22.3)
Pakistan	46.8 (32.5 to 69.5)	30.1 (20.9 to 43.3)	27.9 (18.5 to 39.2)	15.9 (10.1 to 24.1)	-4.3 (-6.9 to -1.9)	-46.6 (-64.4 to -24.3)
Southeast Asia, East Asia, and Oceania						
East Asia						
China	29.6 (22.5 to 39.5)	13.4 (9.6 to 18.1)	12.2 (8.7 to 16.7)	4.8 (3.3 to 6.7)	-6.9 (-7.6 to -6.2)	-64.4 (-68.0 to -60.3)
North Korea	45.1 (32.2 to 64.6)	40.1 (28.8 to 56.7)	40.3 (28.9 to 56.7)	32.7 (22.4 to 49.2)	-1.4 (-3.1 to 0.3)	-18.3 (-36.8 to 4.3)
Taiwan (Province of China)	7.2 (3.5 to 12.5)	2.3 (0.9 to 4.7)	2.1 (0.9 to 4.2)	0.7 (0.3 to 1.5)	-7.6 (-12.1 to -3.0)	-66.1 (-83.7 to -36.2)
Oceania						
American Samoa	16.8 (11.0 to 23.6)	11.4 (6.2 to 17.1)	10.8 (5.5 to 16.4)	5.8 (2.9 to 9.0)	-4.6 (-6.8 to -2.3)	-49.2 (-63.7 to -29.4)
Federated States of Micronesia	31.8 (23.3 to 44.8)	21.5 (15.0 to 30.5)	20.6 (13.9 to 29.5)	10.8 (7.0 to 16.0)	-4.6 (-6.2 to -3.2)	-49.8 (-60.3 to -38.1)
Fiji	27.1 (19.8 to 37.7)	18.9 (13.1 to 26.3)	17.6 (11.4 to 24.5)	8.9 (5.5 to 12.7)	-5.0 (-7.0 to -3.6)	-52.6 (-65.2 to -41.4)
Guam	5.4 (2.5 to 9.7)	5.1 (2.3 to 9.6)	5.0 (2.3 to 9.7)	3.8 (1.7 to 7.4)	-1.9 (-5.2 to 1.3)	-22.6 (-54.1 to 22.1)
Kiribati	62.9 (45.4 to 79.8)	54.5 (38.7 to 75.0)	52.6 (37.5 to 73.3)	42.5 (27.6 to 66.3)	-1.7 (-3.0 to -0.3)	-22.3 (-36.2 to -4.4)
Marshall Islands	26.4 (19.5 to 36.0)	18.6 (12.7 to 25.3)	17.3 (11.3 to 23.9)	8.6 (5.4 to 12.6)	-5.2 (-7.3 to -3.4)	-53.4 (-66.6 to -40.2)
Northern Mariana Islands	11.9 (6.7 to 17.1)	9.8 (4.9 to 15.4)	9.3 (4.8 to 14.6)	5.8 (2.9 to 9.4)	-3.5 (-6.0 to -1.0)	-39.6 (-59.5 to -13.9)
Papua New Guinea	63.9 (45.4 to 80.9)	56.2 (40.2 to 76.1)	54.6 (38.9 to 75.2)	44.4 (28.8 to 69.0)	-1.6 (-3.3 to 0.0)	-21.2 (-39.4 to 0.5)
Samoa	38.4 (27.8 to 53.7)	34.5 (25.2 to 47.5)	32.9 (24.1 to 45.5)	20.6 (14.2 to 30.7)	-3.5 (-5.0 to -2.0)	-40.4 (-53.1 to -25.8)
Solomon Islands	64.8 (46.0 to 81.5)	57.5 (39.8 to 77.2)	56.2 (40.3 to 76.1)	46.3 (30.2 to 70.0)	-1.5 (-3.0 to 0.1)	-19.7 (-36.6 to 0.9)
Tonga	34.4 (25.0 to 48.2)	22.6 (16.4 to 31.0)	21.4 (15.6 to 29.4)	11.9 (8.4 to 17.1)	-4.3 (-5.6 to -3.0)	-47.3 (-56.7 to -36.7)
Vanuatu	56.9 (40.6 to 76.0)	47.3 (34.4 to 67.1)	46.0 (32.7 to 65.9)	35.6 (23.7 to 56.8)	-2.0 (-3.4 to -0.5)	-25.0 (-40.0 to -7.8)
Southeast Asia						
Cambodia	65.3 (46.9 to 82.1)	51.1 (36.4 to 72.6)	49.5 (35.2 to 69.8)	35.1 (22.4 to 59.1)	-2.6 (-4.6 to -0.7)	-31.7 (-49.7 to -10.0)
Indonesia	33.4 (25.5 to 45.1)	23.0 (17.6 to 30.6)	21.3 (16.1 to 28.5)	9.4 (6.6 to 13.2)	-6.0 (-6.9 to -5.0)	-59.2 (-64.4 to -52.6)
Laos	62.5 (44.3 to 80.5)	48.1 (34.0 to 68.7)	46.5 (32.8 to 67.0)	38.1 (25.2 to 60.3)	-1.6 (-3.5 to 0.3)	-20.7 (-40.7 to 5.0)
Malaysia	4.9 (2.3 to 9.1)	1.7 (0.7 to 3.6)	1.4 (0.6 to 3.0)	0.8 (0.2 to 2.1)	-5.0 (-11.0 to 1.5)	-46.7 (-80.8 to 25.9)
Maldives	29.9 (22.4 to 41.4)	9.2 (5.0 to 13.8)	7.4 (3.5 to 11.8)	2.7 (1.2 to 4.5)	-8.4 (-10.7 to -6.2)	-71.0 (-80.0 to -60.4)
Mauritius	6.6 (3.1 to 11.2)	2.4 (1.1 to 4.6)	2.1 (0.9 to 4.1)	0.8 (0.3 to 1.5)	-7.6 (-11.5 to -3.5)	-66.4 (-82.3 to -40.6)
Myanmar	55.8 (39.8 to 76.1)	38.3 (26.9 to 54.6)	37.5 (26.9 to 53.1)	22.4 (14.7 to 35.7)	-3.6 (-5.6 to -1.6)	-41.4 (-56.8 to -20.9)
Philippines	29.6 (21.9 to 40.8)	29.9 (22.1 to 41.8)	28.5 (20.9 to 40.1)	20.1 (14.1 to 29.4)	-2.7 (-4.1 to -1.2)	-32.5 (-45.9 to -16.2)
Sri Lanka	36.9 (27.1 to 51.2)	22.8 (15.7 to 32.6)	22.3 (15.3 to 32.1)	10.2 (6.5 to 15.6)	-5.4 (-7.4 to -3.4)	-55.3 (-66.9 to -39.7)
Seychelles	6.1 (2.9 to 10.8)	3.7 (1.6 to 7.2)	3.1 (1.4 to 6.4)	1.3 (0.5 to 3.2)	-7.2 (-10.7 to -2.0)	-63.9 (-80.0 to -25.4)
Thailand	24.8 (18.0 to 34.0)	10.5 (5.7 to 16.4)	8.9 (4.6 to 14.3)	3.3 (1.6 to 5.5)	-7.8 (-11.2 to -4.7)	-68.1 (-81.5 to -50.5)
Timor-Leste	68.4 (49.3 to 83.8)	48.6 (34.4 to 69.4)	48.0 (34.6 to 67.5)	34.7 (22.6 to 56.8)	-2.3 (-4.1 to -0.4)	-28.7 (-45.9 to -5.8)
Vietnam	41.2 (28.7 to 60.0)	21.6 (15.1 to 29.6)	19.9 (13.7 to 27.8)	8.0 (5.2 to 11.7)	-6.7 (-9.0 to -4.8)	-62.8 (-74.2 to -51.3)
Sub-Saharan Africa						
Central Sub-Saharan Africa						
Angola	62.6 (42.0 to 81.0)	37.6 (25.5 to 52.8)	35.9 (25.2 to 50.1)	16.9 (10.6 to 26.8)	-5.4 (-7.8 to -2.8)	-55.0 (-68.9 to -34.2)
Central African Republic	69.0 (46.6 to 86.0)	60.1 (36.5 to 82.2)	60.0 (37.7 to 81.9)	54.0 (31.5 to 78.3)	-0.8 (-4.2 to 2.6)	-8.1 (-47.0 to 48.3)
Congo	48.7 (32.1 to 73.1)	30.6 (19.8 to 44.8)	28.2 (16.9 to 41.8)	14.4 (7.8 to 23.4)	-5.1 (-8.7 to -1.7)	-52.1 (-72.9 to -22.9)
Democratic Republic of the Congo	68.7 (46.5 to 85.5)	60.7 (39.1 to 80.5)	58.6 (38.2 to 80.1)	51.0 (30.7 to 75.6)	-1.2 (-4.1 to 1.3)	-15.0 (-46.3 to 22.0)
Equatorial Guinea	57.1 (38.0 to 78.3)	29.9 (18.5 to 44.2)	27.9 (17.0 to 41.4)	15.6 (8.9 to 25.2)	-4.4 (-7.9 to -1.1)	-46.6 (-69.3 to -14.9)
Gabon	22.9 (13.5 to 32.5)	8.7 (3.6 to 15.2)	7.3 (3.1 to 13.8)	2.8 (1.1 to 5.5)	-7.8 (-13.6 to -2.0)	-65.4 (-86.9 to -25.8)
Eastern Sub-Saharan Africa						
Burundi	69.8 (48.7 to 85.6)	67.0 (45.8 to 84.3)	66.1 (46.6 to 83.4)	59.0 (38.2 to 79.2)	-0.9 (-3.2 to 1.5)	-11.2 (-38.0 to 24.7)
Comoros	66.4 (47.5 to 82.6)	51.1 (36.6 to 73.1)	49.2 (35.1 to 69.9)	40.0 (26.6 to 63.0)	-1.7 (-3.2 to 0.0)	-21.7 (-38.3 to 0.3)
Djibouti	42.3 (28.4 to 62.0)	21.8 (12.5 to 32.4)	20.4 (10.6 to 31.2)	10.0 (4.8 to 15.9)	-5.3 (-9.6 to -1.9)	-53.2 (-76.3 to -25.0)
Eritrea	57.4 (38.6 to 78.9)	48.5 (31.4 to 70.5)	47.0 (30.7 to 69.4)	29.5 (16.6 to 55.5)	-3.5 (-6.6 to -0.3)	-38.7 (-63.1 to -3.8)
Ethiopia	69.2 (53.6 to 82.7)	62.9 (46.4 to 78.4)	60.4 (44.2 to 77.0)	49.7 (32.8 to 69.6)	-1.6 (-3.0 to -0.5)	-21.5 (-36.3 to -7.1)
Kenya	54.3 (41.6 to 69.3)	46.4 (35.2 to 60.9)	44.9 (34.1 to 59.2)	35.9 (25.8 to 50.8)	-1.7 (-2.2 to -1.2)	-22.9 (-28.6 to -16.0)
Madagascar	73.6 (55.4 to 86.3)	69.5 (49.6 to 84.1)	68.5 (49.2 to 83.0)	63.0 (42.5 to 79.8)	-0.7 (-2.4 to 0.8)	-9.1 (-30.0 to 13.1)
Malawi	75.2 (54.9 to 88.2)	66.1 (47.4 to 82.5)	65.2 (45.7 to 81.5)	59.4 (38.6 to 78.1)	-0.8 (-2.6 to 0.9)	-10.1 (-32.0 to 14.4)
Mozambique	79.8 (60.1 to 90.4)	68.4 (49.4 to 83.5)	66.7 (47.4 to 82.4)	55.8 (34.6 to 75.8)	-1.5 (-3.6 to 0.3)	-18.7 (-41.6 to 3.8)
Rwanda	67.9 (47.4 to 84.2)	57.0 (38.3 to 77.7)	55.2 (35.6 to 77.1)	46.0 (26.8 to 71.5)	-1.5 (-4.3 to 0.9)	-18.8 (-47.2 to 14.2)
Somalia	79.5 (59.9 to 90.2)	74.9 (54.2 to 87.9)	74.2 (54.5 to 87.6)	67.6 (45.4 to 83.9)	-0.7 (-2.8 to 1.0)	-9.4 (-34.3 to 17.0)
South Sudan	75.4 (53.3 to 88.9)	69.0 (47.2 to 85.2)	68.3 (45.9 to 84.9)	59.7 (35.5 to 79.8)	-1.0 (-3.7 to 1.6)	-12.7 (-42.3 to 26.3)
Tanzania	68.0 (48.3 to 83.8)	59.4 (41.0 to 78.4)	57.5 (39.1 to 77.7)	48.3 (29.9 to 72.0)	-1.5 (-3.7 to 0.5)	-18.6 (-42.8 to 7.5)
Uganda	72.7 (51.7 to 87.6)	56.7 (38.2 to 77.8)	54.5 (36.7 to 76.4)	46.6 (29.1 to 71.3)	-1.4 (-3.8 to 0.9)	-17.2 (-43.4 to 15.3)
Zambia	58.4 (40.9 to 78.5)	48.9 (33.4 to 70.9)	47.5 (33.1 to 67.0)	40.0 (26.5 to 60.4)	-1.4 (-3.2 to 0.6)	-17.7 (-38.5 to 9.8)
Southern Sub-Saharan Africa						
Botswana	31.9 (23.5 to 43.6)	23.0 (16.4 to 31.9)	21.9 (15.6 to 29.9)	9.2 (6.2 to 13.1)	-6.2 (-8.3 to -4.1)	-59.8 (-71.2 to -46.2)
Lesotho	44.2 (32.4 to 62.8)	36.8 (27.0 to 51.4)	35.5 (25.8 to 49.6)	25.3 (17.4 to 38.4)	-2.6 (-4.2 to -1.0)	-31.4 (-47.0 to -14.5)
Namibia	38.3 (27.2 to 54.8)	28.0 (20.0 to 38.8)	26.4 (18.2 to 37.5)	16.6 (11.0 to 25.2)	-3.5 (-5.9 to -1.5)	-40.2 (-58.9 to -20.6)
South Africa	20.1 (15.2 to 27.2)	8.5 (6.0 to 11.5)	7.3 (5.1 to 10.1)	2.9 (1.9 to 4.0)	-7.3 (-8.4 to -6.2)	-66.2 (-71.7 to -60.5)
Swaziland	44.9 (32.8 to 62.2)	25.4 (18.9 to 34.9)	24.0 (18.1 to 33.2)	10.7 (7.7 to 15.6)	-5.8 (-7.1 to -4.4)	-57.7 (-65.3 to -48.3)
Zimbabwe	43.4 (31.4 to 60.8)	44.6 (32.7 to 63.3)	42.6 (31.1 to 59.8)	31.9 (21.8 to 48.3)	-2.3 (-4.0 to -0.6)	-28.3 (-45.5 to -8.2)
Western Sub-Saharan Africa						
Benin	69.0 (46.4 to 84.9)	58.0 (37.4 to 78.7)	56.5 (35.5 to 78.0)	48.2 (27.7 to 72.9)	-1.3 (-4.2 to 1.2)	-16.1 (-46.9 to 19.3)
Burkina Faso	75.5 (53.2 to 88.9)	66.6 (44.5 to 84.8)	66.0 (44.6 to 84.0)	55.4 (32.7 to 77.8)	-1.3 (-4.3 to 1.5)	-15.9 (-47.7 to 25.0)
Cameroon	48.6 (29.3 to 73.3)	33.8 (21.0 to 51.0)	31.1 (18.8 to 46.2)	23.1 (13.1 to 36.5)	-2.6 (-6.3 to 1.0)	-29.7 (-61.1 to 15.6)
Cape Verde	41.4 (28.5 to 58.9)	24.6 (16.5 to 35.9)	23.4 (15.6 to 33.2)	10.1 (6.3 to 15.3)	-6.0 (-8.5 to -4.0)	-58.8 (-72.0 to -44.7)
Chad	74.8 (50.7 to 89.9)	66.1 (40.6 to 85.2)	65.6 (39.4 to 85.5)	59.9 (33.2 to 82.5)	-0.7 (-4.5 to 2.6)	-7.4 (-49.2 to 46.8)
Cote d'Ivoire	62.0 (43.6 to 80.5)	53.7 (35.7 to 75.7)	52.3 (36.2 to 74.0)	40.9 (25.5 to 67.3)	-1.9 (-4.1 to 0.3)	-23.6 (-45.9 to 5.0)
The Gambia	67.0 (45.5 to 83.3)	56.9 (38.4 to 77.4)	56.1 (38.7 to 76.8)	49.8 (32.1 to 72.9)	-0.9 (-3.0 to 1.2)	-11.9 (-36.1 to 19.0)
Ghana	53.3 (36.5 to 74.8)	38.5 (26.4 to 55.3)	36.1 (24.2 to 52.2)	24.4 (15.2 to 38.6)	-3.1 (-5.7 to -0.5)	-36.1 (-57.5 to -7.5)
Guinea	77.4 (58.9 to 88.8)	69.0 (47.8 to 84.7)	67.6 (47.3 to 83.3)	62.0 (40.4 to 79.9)	-0.7 (-2.8 to 1.1)	-9.6 (-34.2 to 17.8)
Guinea-Bissau	74.1 (54.6 to 87.4)	65.0 (44.9 to 81.9)	64.1 (45.0 to 81.7)	58.0 (37.7 to 78.0)	-0.8 (-2.7 to 1.1)	-10.5 (-33.5 to 18.7)
Liberia	79.5 (60.9 to 90.0)	70.3 (51.9 to 84.2)	69.3 (51.1 to 83.4)	63.2 (43.2 to 79.5)	-0.7 (-2.4 to 0.7)	-10.0 (-30.0 to 10.9)
Mali	76.9 (54.7 to 90.3)	70.1 (45.9 to 86.3)	69.8 (46.9 to 85.8)	63.9 (39.2 to 82.3)	-0.7 (-3.6 to 1.8)	-7.9 (-41.4 to 31.1)
Mauritania	43.0 (27.2 to 64.0)	33.4 (20.3 to 47.4)	31.6 (19.8 to 46.4)	21.4 (12.6 to 34.1)	-3.0 (-6.5 to 0.3)	-34.2 (-62.0 to 5.3)
Niger	74.9 (47.2 to 90.8)	70.1 (39.4 to 88.7)	68.1 (39.7 to 88.0)	61.4 (31.7 to 84.6)	-1.0 (-5.1 to 2.8)	-9.5 (-53.4 to 52.2)

	Estimate in 2000 (95% UIs)	Estimate in 2015 (95% UIs)	Estimate in 2017 (95% UIs)	Estimate in 2030 (95% UIs)	Annualised rate of change, 2015-2030 (95% UIs)	Percent change, 2015-2030 (95% UIs)
Nigeria	48.3 (26.3 to 73.4)	34.5 (16.4 to 52.4)	33.7 (15.7 to 52.3)	23.6 (10.2 to 39.9)	-2.6 (-7.7 to 2.4)	-28.3 (-68.5 to 42.3)
Sao Tome and Principe	60.2 (42.3 to 79.2)	38.6 (27.4 to 54.3)	36.1 (25.4 to 51.7)	18.7 (12.0 to 30.7)	-4.9 (-6.8 to -2.8)	-51.8 (-64.0 to -34.2)
Senegal	54.0 (37.8 to 74.7)	53.2 (36.6 to 75.5)	53.4 (37.1 to 75.2)	45.9 (29.8 to 70.6)	-1.0 (-3.2 to 1.3)	-13.0 (-38.4 to 21.4)
Sierra Leone	76.1 (56.1 to 88.4)	66.6 (47.6 to 82.5)	65.5 (47.7 to 81.9)	59.0 (39.9 to 77.9)	-0.8 (-2.6 to 0.7)	-11.2 (-32.2 to 11.1)
Togo	65.9 (45.8 to 83.0)	55.7 (38.0 to 76.8)	54.1 (36.2 to 75.7)	43.8 (26.3 to 69.2)	-1.7 (-4.3 to 0.7)	-21.1 (-47.9 to 10.6)
**Indicator 8.8.1: Age-standardised all-cause disability-adjusted life year (DALY) rates attributable to occupational risks (per 100,000 population)**						
Central Europe, Eastern Europe, and Central Asia						
Central Asia						
Armenia	1003.6 (821.2 to 1192.2)	850.2 (686.4 to 1025.5)	840.8 (678.0 to 1018.1)	745.5 (584.4 to 920.7)	-0.9 (-1.2 to -0.6)	-12.4 (-16.6 to -8.3)
Azerbaijan	910.1 (741.4 to 1090.1)	752.9 (599.7 to 928.0)	743.9 (588.4 to 920.7)	696.8 (533.4 to 886.1)	-0.5 (-0.9 to -0.1)	-7.6 (-13.2 to -1.7)
Georgia	807.2 (642.8 to 978.4)	752.3 (610.7 to 905.5)	741.9 (603.3 to 893.4)	679.1 (552.2 to 827.0)	-0.7 (-1.0 to -0.4)	-9.7 (-13.8 to -5.6)
Kazakhstan	1149.9 (980.4 to 1344.5)	842.1 (707.6 to 989.0)	806.1 (672.6 to 950.5)	635.1 (518.2 to 773.5)	-1.9 (-2.2 to -1.6)	-24.7 (-28.5 to -21.0)
Kyrgyzstan	1024.1 (847.1 to 1209.6)	747.5 (601.3 to 893.6)	716.7 (574.6 to 860.2)	589.6 (462.7 to 722.6)	-1.6 (-2.0 to -1.2)	-21.2 (-25.5 to -16.8)
Mongolia	975.9 (802.6 to 1158.7)	707.4 (572.2 to 858.1)	681.5 (550.8 to 829.0)	554.2 (432.7 to 687.8)	-1.6 (-2.0 to -1.2)	-21.7 (-26.4 to -17.0)
Tajikistan	1080.5 (869.1 to 1319.4)	808.2 (640.8 to 1001.4)	773.9 (612.2 to 961.5)	634.7 (484.4 to 801.0)	-1.6 (-2.1 to -1.1)	-21.6 (-27.5 to -15.7)
Turkmenistan	865.8 (692.0 to 1061.3)	703.7 (557.3 to 879.1)	691.5 (546.3 to 870.1)	619.0 (467.3 to 801.7)	-0.9 (-1.4 to -0.4)	-12.2 (-18.5 to -5.9)
Uzbekistan	1003.1 (814.3 to 1210.5)	759.7 (607.5 to 921.6)	735.9 (587.0 to 900.1)	639.9 (494.3 to 806.4)	-1.2 (-1.6 to -0.7)	-15.9 (-21.6 to -10.3)
Central Europe						
Albania	997.5 (784.0 to 1246.4)	904.4 (702.0 to 1154.0)	889.0 (686.1 to 1133.3)	847.0 (635.4 to 1116.4)	-0.5 (-0.9 to 0.0)	-6.5 (-12.3 to 0.1)
Bosnia and Herzegovina	851.8 (682.5 to 1039.9)	647.1 (500.5 to 801.9)	625.1 (481.8 to 776.4)	525.1 (396.7 to 670.5)	-1.4 (-1.9 to -1.0)	-19.0 (-24.4 to -13.7)
Bulgaria	521.4 (416.8 to 631.1)	574.6 (460.9 to 700.8)	587.8 (469.4 to 716.9)	664.1 (525.6 to 820.2)	1.0 (0.6 to 1.3)	15.5 (10.0 to 20.9)
Croatia	661.8 (533.0 to 798.3)	663.3 (538.0 to 801.5)	663.8 (541.4 to 802.9)	679.4 (548.7 to 824.2)	0.2 (-0.2 to 0.5)	2.4 (-2.3 to 7.1)
Czech Republic	946.4 (775.4 to 1128.5)	774.0 (618.2 to 957.3)	761.5 (602.8 to 949.6)	680.8 (519.9 to 885.6)	-0.9 (-1.3 to -0.4)	-12.2 (-17.9 to -6.3)
Hungary	722.9 (593.7 to 861.5)	647.6 (516.0 to 782.7)	642.0 (508.9 to 776.2)	600.4 (465.3 to 745.3)	-0.5 (-0.8 to -0.2)	-7.4 (-11.7 to -3.6)
Macedonia	579.4 (461.7 to 715.4)	553.5 (427.5 to 693.9)	555.7 (426.4 to 698.7)	550.9 (407.7 to 706.7)	-0.1 (-0.5 to 0.4)	-0.7 (-7.5 to 6.7)
Montenegro	551.9 (439.8 to 674.4)	513.3 (401.0 to 632.4)	511.8 (398.3 to 633.4)	493.7 (374.4 to 623.5)	-0.3 (-0.7 to 0.1)	-3.9 (-9.8 to 1.7)
Poland	894.0 (731.2 to 1061.1)	732.7 (598.4 to 884.3)	721.0 (587.1 to 873.7)	646.9 (514.9 to 794.2)	-0.8 (-1.2 to -0.5)	-11.8 (-16.1 to -7.7)
Romania	1018.9 (826.8 to 1229.2)	837.3 (666.4 to 1026.2)	824.5 (653.3 to 1014.0)	752.5 (582.5 to 940.1)	-0.7 (-1.0 to -0.4)	-10.2 (-14.1 to -6.4)
Serbia	770.1 (613.1 to 942.3)	735.4 (579.0 to 907.1)	730.4 (574.1 to 902.6)	709.9 (543.4 to 893.8)	-0.2 (-0.6 to 0.1)	-3.5 (-8.7 to 1.4)
Slovakia	636.3 (519.1 to 764.4)	545.5 (433.3 to 665.6)	541.5 (428.0 to 664.8)	496.9 (381.0 to 623.9)	-0.6 (-1.0 to -0.3)	-9.1 (-13.6 to -4.4)
Slovenia	778.1 (631.3 to 932.1)	742.1 (588.3 to 904.0)	746.4 (588.3 to 916.8)	732.0 (564.3 to 924.0)	-0.1 (-0.5 to 0.3)	-1.5 (-7.1 to 3.9)
Eastern Europe						
Belarus	708.5 (588.6 to 839.8)	554.5 (449.0 to 675.7)	543.9 (436.7 to 665.6)	470.4 (363.2 to 591.5)	-1.1 (-1.5 to -0.8)	-15.3 (-20.0 to -10.7)
Estonia	698.9 (574.4 to 836.1)	531.8 (420.4 to 655.2)	509.1 (401.1 to 634.0)	411.2 (311.3 to 523.8)	-1.7 (-2.1 to -1.4)	-22.8 (-27.0 to -18.8)
Latvia	693.7 (569.1 to 827.7)	600.0 (482.0 to 725.7)	587.4 (470.8 to 712.6)	526.3 (406.3 to 659.6)	-0.9 (-1.3 to -0.5)	-12.4 (-17.2 to -7.1)
Lithuania	706.6 (580.5 to 839.0)	601.5 (485.6 to 729.5)	586.2 (468.6 to 712.4)	518.6 (404.0 to 648.3)	-1.0 (-1.4 to -0.6)	-13.9 (-19.2 to -8.8)
Moldova	691.6 (553.5 to 842.8)	521.8 (416.8 to 641.7)	505.3 (402.7 to 623.7)	426.2 (329.8 to 542.0)	-1.4 (-1.8 to -1.0)	-18.4 (-23.1 to -13.4)
Russia	902.4 (769.0 to 1054.0)	738.7 (615.1 to 878.6)	732.2 (604.6 to 876.9)	653.0 (522.1 to 805.1)	-0.8 (-1.1 to -0.5)	-11.7 (-15.8 to -7.2)
Ukraine	806.6 (681.4 to 942.8)	616.2 (506.9 to 737.8)	599.6 (491.6 to 717.6)	497.2 (396.9 to 615.0)	-1.4 (-1.8 to -1.1)	-19.4 (-23.2 to -15.1)
High-income						
Australasia						
Australia	795.8 (666.1 to 928.7)	698.4 (572.0 to 833.9)	686.2 (559.5 to 822.4)	629.0 (498.5 to 771.7)	-0.7 (-1.1 to -0.4)	-10.1 (-14.6 to -5.5)
New Zealand	903.4 (737.4 to 1067.9)	809.3 (652.7 to 988.7)	805.3 (645.7 to 987.7)	768.9 (603.6 to 974.1)	-0.4 (-0.7 to 0.0)	-5.1 (-10.3 to 0.4)
High-income Asia Pacific						
Brunei	1125.0 (957.9 to 1313.1)	820.2 (675.8 to 979.8)	790.2 (647.6 to 949.5)	620.4 (477.7 to 782.7)	-1.9 (-2.5 to -1.3)	-24.5 (-30.8 to -18.1)
Japan	809.4 (651.2 to 979.2)	707.3 (555.7 to 879.2)	697.5 (544.4 to 874.9)	637.3 (480.8 to 826.9)	-0.7 (-1.1 to -0.3)	-10.1 (-15.2 to -4.2)
South Korea	1159.7 (974.1 to 1365.6)	966.1 (768.7 to 1188.6)	940.6 (738.2 to 1164.2)	820.0 (601.1 to 1072.4)	-1.1 (-1.7 to -0.6)	-15.4 (-23.0 to -8.0)
Singapore	778.5 (637.4 to 930.1)	578.8 (452.6 to 724.0)	563.2 (438.3 to 712.1)	469.9 (348.8 to 620.0)	-1.4 (-2.0 to -0.8)	-19.1 (-25.9 to -11.8)
High-income North America						
Canada	887.2 (756.3 to 1033.0)	712.9 (584.8 to 847.3)	696.3 (568.2 to 830.9)	598.5 (471.1 to 734.6)	-1.2 (-1.6 to -0.8)	-16.2 (-21.1 to -11.9)
Greenland	1279.0 (1091.2 to 1450.2)	986.6 (840.1 to 1136.8)	947.5 (802.1 to 1092.8)	759.9 (630.9 to 902.0)	-1.8 (-2.1 to -1.4)	-23.0 (-27.4 to -18.5)
United States	703.3 (598.4 to 811.0)	583.4 (492.8 to 678.4)	570.7 (481.0 to 664.2)	501.0 (416.7 to 588.6)	-1.0 (-1.2 to -0.8)	-14.2 (-17.0 to -11.4)
Southern Latin America						
Argentina	1003.8 (836.5 to 1179.0)	904.7 (741.9 to 1068.5)	893.2 (729.1 to 1056.9)	833.3 (653.0 to 1014.0)	-0.6 (-1.0 to -0.2)	-8.0 (-14.0 to -2.4)
Chile	631.1 (514.5 to 751.6)	665.3 (535.7 to 808.1)	672.4 (539.3 to 819.7)	733.9 (572.1 to 924.0)	0.6 (0.3 to 1.0)	10.2 (4.4 to 15.5)
Uruguay	689.7 (580.8 to 807.8)	741.0 (620.7 to 874.5)	747.1 (623.5 to 884.5)	811.3 (660.0 to 978.0)	0.6 (0.2 to 0.9)	9.4 (3.8 to 14.8)
Western Europe						
Andorra	730.0 (596.6 to 865.6)	707.1 (571.0 to 847.0)	686.2 (554.8 to 826.5)	664.5 (523.1 to 824.2)	-0.4 (-0.8 to -0.1)	-6.1 (-10.7 to -1.6)
Austria	729.9 (599.0 to 864.4)	666.5 (539.2 to 804.9)	657.6 (530.1 to 796.7)	616.0 (478.1 to 765.9)	-0.5 (-0.9 to -0.2)	-7.7 (-12.6 to -2.9)
Belgium	759.2 (642.8 to 879.7)	638.7 (528.7 to 759.6)	624.0 (513.5 to 745.5)	561.7 (446.9 to 690.1)	-0.9 (-1.2 to -0.5)	-12.2 (-16.9 to -7.5)
Cyprus	778.4 (636.1 to 931.8)	679.8 (546.4 to 827.5)	671.9 (539.4 to 818.5)	615.3 (479.3 to 767.5)	-0.7 (-1.1 to -0.3)	-9.6 (-14.6 to -5.0)
Denmark	817.7 (688.3 to 952.7)	784.2 (642.8 to 940.3)	775.5 (631.8 to 935.2)	765.0 (599.9 to 948.9)	-0.2 (-0.5 to 0.2)	-2.6 (-7.8 to 2.7)
Finland	641.2 (522.9 to 769.5)	566.0 (451.0 to 688.2)	557.9 (442.5 to 678.3)	513.6 (399.4 to 644.0)	-0.7 (-1.0 to -0.3)	-9.4 (-14.2 to -4.5)
France	853.4 (713.0 to 996.8)	846.7 (695.1 to 1015.9)	850.6 (696.1 to 1024.4)	872.6 (693.9 to 1084.6)	0.2 (-0.2 to 0.6)	2.9 (-2.9 to 9.3)
Germany	767.7 (629.4 to 908.3)	713.9 (576.1 to 860.5)	701.6 (566.5 to 845.1)	653.1 (514.8 to 801.0)	-0.6 (-0.9 to -0.3)	-8.6 (-12.1 to -5.1)
Greece	609.0 (484.7 to 741.5)	566.8 (445.8 to 695.3)	568.7 (446.3 to 700.4)	553.9 (427.1 to 693.1)	-0.2 (-0.4 to 0.1)	-2.4 (-6.2 to 1.3)
Iceland	836.2 (665.8 to 1013.8)	744.2 (594.0 to 911.7)	729.0 (580.5 to 892.8)	669.6 (523.9 to 825.3)	-0.7 (-1.0 to -0.5)	-10.1 (-13.4 to -6.9)
Ireland	704.6 (579.0 to 834.6)	581.1 (466.4 to 708.7)	577.9 (458.6 to 708.9)	533.9 (407.7 to 674.9)	-0.6 (-1.0 to -0.2)	-8.3 (-14.0 to -2.3)
Israel	586.6 (471.8 to 707.0)	527.6 (411.7 to 650.7)	522.8 (407.1 to 648.0)	492.0 (373.2 to 625.0)	-0.5 (-0.8 to -0.1)	-6.9 (-11.9 to -1.9)
Italy	800.7 (674.0 to 938.0)	639.7 (526.4 to 772.2)	627.4 (515.1 to 758.9)	549.0 (432.1 to 683.9)	-1.0 (-1.4 to -0.6)	-14.3 (-19.0 to -9.2)
Luxembourg	716.3 (591.8 to 851.6)	591.8 (481.8 to 716.7)	567.4 (463.0 to 687.7)	480.7 (378.6 to 597.5)	-1.4 (-1.7 to -1.0)	-18.9 (-23.1 to -14.2)
Malta	701.8 (577.2 to 835.4)	665.2 (536.5 to 803.9)	666.5 (536.4 to 807.7)	668.4 (520.7 to 834.9)	0.0 (-0.3 to 0.4)	0.3 (-4.6 to 5.7)
Netherlands	797.4 (675.1 to 927.4)	720.8 (597.3 to 850.9)	711.9 (587.4 to 843.5)	683.6 (551.7 to 830.9)	-0.4 (-0.7 to -0.0)	-5.3 (-9.9 to -0.5)
Norway	691.3 (557.2 to 827.8)	620.9 (495.6 to 749.0)	609.7 (485.0 to 737.0)	563.5 (441.1 to 693.0)	-0.7 (-0.9 to -0.4)	-9.3 (-12.6 to -5.8)
Portugal	777.0 (632.2 to 932.5)	687.2 (545.3 to 851.7)	681.7 (536.6 to 849.4)	644.0 (495.9 to 824.9)	-0.4 (-0.8 to -0.1)	-6.5 (-11.2 to -1.1)
Spain	589.1 (490.9 to 695.1)	509.3 (413.8 to 616.7)	514.9 (415.9 to 628.0)	493.0 (383.4 to 620.6)	-0.2 (-0.6 to 0.1)	-3.4 (-8.1 to 1.5)
Sweden	617.9 (496.4 to 747.7)	564.3 (443.1 to 692.4)	555.5 (435.3 to 682.4)	518.1 (399.2 to 653.5)	-0.6 (-0.8 to -0.3)	-8.3 (-11.9 to -4.6)
Switzerland	873.4 (714.1 to 1033.6)	803.1 (638.0 to 976.5)	794.5 (627.0 to 972.4)	756.9 (578.6 to 963.0)	-0.4 (-0.8 to 0.0)	-6.0 (-11.5 to 0.0)
United Kingdom	790.0 (672.5 to 912.2)	663.4 (555.0 to 777.5)	648.8 (540.0 to 763.4)	576.0 (466.4 to 692.4)	-1.0 (-1.2 to -0.7)	-13.3 (-17.0 to -9.7)
Latin America and Caribbean						

Andean Latin America	Estimate in 2000 (95% UIs)	Estimate in 2015 (95% UIs)	Estimate in 2017 (95% UIs)	Estimate in 2030 (95% UIs)	Annualised rate of change, 2015-2030 (95% UIs)	Percent change, 2015-2030 (95% UIs)
Bolivia	835.0 (677.0 to 999.9)	756.2 (601.9 to 921.7)	746.8 (593.7 to 911.0)	701.2 (544.3 to 869.1)	-0.5 (-0.9 to -0.2)	-7.4 (-12.0 to -2.4)
Ecuador	786.3 (657.9 to 920.4)	697.2 (570.0 to 826.8)	686.0 (557.4 to 820.1)	626.5 (487.8 to 770.3)	-0.7 (-1.1 to -0.3)	-10.3 (-15.6 to -4.5)
Peru	704.7 (564.3 to 856.9)	635.6 (498.9 to 780.6)	626.6 (490.1 to 769.8)	579.8 (442.0 to 729.0)	-0.6 (-0.9 to -0.3)	-8.9 (-13.2 to -4.7)
Caribbean						
Antigua and Barbuda	348.5 (284.6 to 422.3)	324.7 (255.6 to 399.2)	324.4 (254.2 to 400.4)	316.8 (241.5 to 403.2)	-0.2 (-0.5 to 0.2)	-2.6 (-7.8 to 2.9)
The Bahamas	360.7 (296.6 to 433.2)	328.2 (262.8 to 400.2)	325.3 (259.9 to 396.9)	308.7 (242.4 to 381.1)	-0.4 (-0.7 to -0.1)	-6.0 (-10.4 to -1.8)
Barbados	386.3 (315.1 to 469.1)	356.8 (285.4 to 435.2)	355.6 (283.3 to 435.8)	345.0 (268.4 to 430.8)	-0.2 (-0.5 to 0.0)	-3.4 (-7.8 to 0.6)
Belize	780.2 (670.0 to 899.2)	664.2 (566.5 to 772.2)	646.5 (548.0 to 755.0)	574.1 (474.2 to 691.2)	-1.0 (-1.4 to -0.6)	-13.6 (-19.1 to -8.0)
Bermuda	519.8 (434.4 to 609.6)	456.5 (370.8 to 555.0)	453.7 (367.6 to 556.4)	428.2 (331.5 to 550.9)	-0.5 (-0.9 to 0.1)	-6.5 (-13.2 to 1.4)
Cuba	748.5 (646.0 to 867.8)	944.5 (802.3 to 1122.3)	976.6 (824.1 to 1163.8)	1136.0 (926.4 to 1379.4)	1.2 (0.8 to 1.6)	20.1 (13.1 to 26.2)
Dominica	453.2 (368.2 to 543.8)	414.4 (333.7 to 498.6)	409.6 (329.1 to 494.0)	379.6 (299.1 to 468.5)	-0.6 (-0.9 to -0.3)	-8.5 (-12.6 to -4.4)
Dominican Republic	684.4 (579.2 to 794.9)	658.3 (556.4 to 770.1)	655.8 (551.4 to 773.1)	639.5 (517.3 to 781.6)	-0.2 (-0.6 to 0.2)	-2.9 (-9.1 to 3.1)
Grenada	450.4 (373.1 to 533.4)	389.5 (315.1 to 472.0)	390.9 (314.9 to 475.4)	364.9 (283.1 to 453.6)	-0.4 (-0.9 to -0.1)	-6.5 (-12.0 to -1.4)
Guyana	505.2 (426.2 to 595.3)	452.7 (375.7 to 539.3)	444.4 (367.1 to 531.3)	412.9 (334.5 to 503.5)	-0.6 (-1.0 to -0.3)	-8.9 (-14.2 to -3.8)
Haiti	654.2 (549.4 to 764.8)	565.4 (470.8 to 667.4)	549.2 (454.3 to 651.1)	499.4 (402.2 to 607.0)	-0.8 (-1.3 to -0.4)	-11.8 (-17.6 to -6.0)
Jamaica	556.1 (458.5 to 663.3)	493.1 (403.5 to 591.4)	486.0 (398.4 to 584.1)	442.9 (356.6 to 539.7)	-0.7 (-1.1 to -0.4)	-10.2 (-14.8 to -5.3)
Puerto Rico	278.0 (236.3 to 328.1)	255.6 (209.8 to 310.9)	254.2 (207.0 to 310.1)	242.9 (188.5 to 308.5)	-0.4 (-0.9 to 0.1)	-5.2 (-12.4 to 2.1)
Saint Lucia	491.2 (403.3 to 583.5)	421.8 (341.1 to 513.8)	416.8 (336.5 to 510.5)	376.4 (294.5 to 467.8)	-0.8 (-1.1 to -0.4)	-10.9 (-15.2 to -6.3)
Saint Vincent and the Grenadines	365.3 (291.5 to 450.0)	350.7 (279.1 to 433.9)	351.1 (279.1 to 435.8)	348.6 (273.3 to 442.2)	-0.0 (-0.4 to 0.3)	-0.6 (-5.6 to 4.5)
Suriname	413.6 (342.9 to 489.8)	362.9 (297.6 to 434.9)	362.8 (296.7 to 435.4)	347.9 (276.1 to 428.0)	-0.3 (-0.6 to 0.1)	-4.2 (-9.1 to 1.0)
Trinidad and Tobago	329.0 (266.0 to 397.5)	310.5 (246.5 to 380.0)	312.9 (248.0 to 383.4)	312.7 (241.4 to 388.5)	0.0 (-0.2 to 0.3)	0.6 (-3.2 to 4.1)
Virgin Islands, U.S.	610.2 (515.1 to 715.1)	585.2 (489.1 to 691.5)	585.3 (489.6 to 693.0)	574.4 (460.4 to 697.1)	-0.1 (-0.6 to 0.3)	-2.0 (-8.1 to 4.2)
Central Latin America						
Colombia	528.2 (442.4 to 615.7)	413.7 (333.7 to 495.2)	402.1 (323.2 to 484.1)	337.4 (260.3 to 420.6)	-1.4 (-1.8 to -1.0)	-18.6 (-23.7 to -13.6)
Costa Rica	524.6 (446.0 to 615.0)	443.2 (370.3 to 525.5)	436.8 (364.0 to 519.3)	395.8 (322.6 to 480.6)	-0.8 (-1.1 to -0.5)	-10.8 (-15.3 to -6.6)
El Salvador	502.5 (419.2 to 591.0)	359.0 (281.9 to 442.7)	341.9 (266.7 to 424.5)	282.0 (210.9 to 359.6)	-1.6 (-2.1 to -1.2)	-21.6 (-26.5 to -17.1)
Guatemala	1043.5 (903.7 to 1197.3)	706.3 (594.2 to 827.6)	675.0 (562.9 to 796.4)	504.7 (401.3 to 621.5)	-2.3 (-2.8 to -1.8)	-28.7 (-34.1 to -23.5)
Honduras	747.8 (639.7 to 867.5)	665.6 (566.6 to 781.4)	651.7 (550.7 to 768.2)	604.9 (493.9 to 737.6)	-0.6 (-1.1 to -0.2)	-9.2 (-14.6 to -3.5)
Mexico	674.2 (584.2 to 773.7)	563.2 (479.8 to 657.0)	551.7 (468.8 to 647.4)	481.0 (398.1 to 574.9)	-1.1 (-1.4 to -0.8)	-14.7 (-18.7 to -10.9)
Nicaragua	574.1 (478.2 to 687.2)	495.0 (402.9 to 597.8)	485.4 (393.0 to 587.7)	434.6 (341.9 to 539.4)	-0.9 (-1.3 to -0.5)	-12.3 (-17.5 to -7.2)
Panama	489.6 (408.0 to 575.1)	391.6 (314.9 to 474.6)	381.2 (304.7 to 463.7)	326.8 (249.4 to 410.1)	-1.2 (-1.7 to -0.8)	-16.7 (-22.1 to -11.9)
Venezuela	1089.4 (960.0 to 1233.3)	776.6 (659.1 to 900.0)	749.8 (630.8 to 876.8)	573.4 (447.8 to 705.3)	-2.0 (-2.7 to -1.5)	-26.3 (-33.0 to -19.8)
Tropical Latin America						
Brazil	846.9 (718.9 to 978.5)	713.8 (587.1 to 843.6)	703.6 (574.7 to 836.0)	624.2 (495.1 to 756.5)	-0.9 (-1.3 to -0.6)	-12.7 (-17.3 to -8.3)
Paraguay	895.3 (742.9 to 1060.4)	789.9 (646.4 to 949.3)	776.3 (632.6 to 934.4)	706.1 (552.7 to 866.9)	-0.8 (-1.2 to -0.3)	-10.7 (-16.7 to -4.9)
North Africa and Middle East						
North Africa and Middle East						
Afghanistan	1846.3 (1578.9 to 2122.1)	1333.2 (1129.8 to 1545.3)	1262.7 (1062.3 to 1470.5)	1150.6 (934.4 to 1393.7)	-1.0 (-1.4 to -0.5)	-13.8 (-19.3 to -7.5)
Algeria	672.7 (559.9 to 791.2)	534.2 (436.6 to 645.5)	523.6 (426.7 to 633.7)	441.5 (344.2 to 559.6)	-1.3 (-1.8 to -0.7)	-17.5 (-24.2 to -9.5)
Bahrain	1145.1 (952.6 to 1345.4)	670.3 (534.8 to 805.6)	653.8 (519.9 to 791.2)	502.1 (386.1 to 629.5)	-1.9 (-2.4 to -1.6)	-25.2 (-29.9 to -20.8)
Egypt	595.9 (502.1 to 704.6)	557.8 (458.7 to 665.1)	550.2 (450.8 to 658.7)	526.2 (417.3 to 646.7)	-0.4 (-0.8 to -0.0)	-5.8 (-11.4 to -0.7)
Iran	642.4 (540.5 to 749.8)	505.7 (423.6 to 595.5)	497.7 (415.6 to 586.1)	424.0 (345.4 to 513.2)	-1.2 (-1.6 to -0.7)	-16.2 (-21.7 to -10.6)
Iraq	782.3 (663.6 to 920.8)	633.5 (513.8 to 767.6)	612.3 (492.5 to 747.5)	514.4 (382.7 to 662.5)	-1.4 (-2.2 to -0.7)	-19.0 (-27.6 to -10.2)
Jordan	623.1 (516.5 to 735.6)	420.8 (341.2 to 503.8)	391.8 (315.7 to 471.3)	278.6 (217.2 to 345.3)	-2.8 (-3.2 to -2.3)	-33.9 (-38.2 to -29.4)
Kuwait	866.3 (699.5 to 1043.1)	572.9 (454.3 to 691.5)	536.6 (423.9 to 651.6)	412.1 (316.4 to 514.3)	-2.2 (-2.6 to -1.8)	-28.2 (-32.8 to -23.4)
Lebanon	660.0 (562.0 to 772.5)	503.8 (415.9 to 604.3)	490.3 (402.0 to 592.7)	395.1 (307.2 to 497.2)	-1.6 (-2.2 to -1.1)	-21.7 (-28.1 to -15.6)
Libya	610.5 (510.1 to 715.6)	499.5 (414.7 to 589.0)	487.6 (404.7 to 576.5)	431.7 (345.2 to 530.2)	-1.0 (-1.5 to -0.4)	-13.7 (-20.7 to -6.4)
Morocco	841.4 (701.9 to 981.1)	768.9 (627.0 to 917.4)	760.8 (617.4 to 912.2)	720.1 (557.1 to 904.5)	-0.5 (-1.0 to 0.0)	-6.5 (-13.9 to 0.2)
Palestine	831.2 (703.3 to 971.4)	658.9 (537.0 to 796.0)	638.8 (518.7 to 777.4)	526.2 (406.5 to 674.3)	-1.5 (-2.2 to -0.9)	-20.3 (-27.7 to -12.5)
Oman	935.6 (785.0 to 1098.5)	550.6 (455.8 to 655.0)	511.6 (420.1 to 610.4)	375.2 (296.8 to 458.8)	-2.6 (-3.0 to -2.2)	-31.9 (-36.0 to -28.3)
Qatar	1836.1 (1546.4 to 2187.4)	799.5 (650.9 to 959.6)	742.5 (600.1 to 896.3)	495.8 (386.0 to 619.1)	-3.2 (-3.6 to -2.8)	-38.1 (-41.8 to -34.5)
Saudi Arabia	711.7 (593.2 to 840.9)	472.6 (386.5 to 564.4)	442.7 (360.4 to 531.4)	307.4 (237.4 to 379.3)	-2.9 (-3.5 to -2.3)	-35.1 (-40.4 to -29.7)
Sudan	1110.0 (943.9 to 1277.9)	820.3 (674.5 to 965.1)	780.1 (640.5 to 923.5)	606.9 (477.7 to 749.1)	-2.0 (-2.6 to -1.5)	-26.1 (-32.6 to -19.8)
Syria	760.6 (639.3 to 903.3)	634.6 (524.7 to 766.7)	623.9 (513.0 to 757.2)	540.1 (422.0 to 691.8)	-1.1 (-1.6 to -0.5)	-15.1 (-21.9 to -7.4)
Tunisia	584.8 (492.9 to 680.1)	479.9 (398.5 to 568.5)	471.7 (389.9 to 561.5)	414.3 (330.3 to 513.4)	-1.0 (-1.5 to -0.5)	-13.8 (-19.6 to -7.6)
Turkey	1260.5 (1082.8 to 1446.8)	876.4 (742.7 to 1025.2)	835.2 (703.1 to 982.5)	632.6 (513.6 to 774.6)	-2.2 (-2.7 to -1.8)	-27.9 (-33.0 to -23.1)
United Arab Emirates	1331.8 (1118.5 to 1569.5)	711.9 (591.0 to 827.0)	668.2 (552.6 to 780.2)	481.6 (388.9 to 574.9)	-2.6 (-3.0 to -2.2)	-32.4 (-36.4 to -28.1)
Yemen	1253.0 (1078.5 to 1443.2)	866.1 (732.8 to 1006.0)	825.6 (692.8 to 964.5)	625.4 (497.6 to 766.3)	-2.2 (-2.8 to -1.6)	-27.9 (-34.6 to -20.8)
South Asia						
South Asia						
Bangladesh	1689.3 (1443.8 to 1930.4)	1234.8 (1032.9 to 1447.1)	1191.1 (993.2 to 1403.6)	970.2 (773.3 to 1187.6)	-1.6 (-2.1 to -1.1)	-21.6 (-27.5 to -15.5)
Bhutan	1818.3 (1550.0 to 2132.9)	1229.0 (1015.4 to 1461.4)	1172.2 (962.5 to 1399.8)	853.9 (679.7 to 1048.8)	-2.4 (-2.8 to -2.0)	-30.6 (-34.5 to -26.4)
India	1328.7 (1166.3 to 1503.8)	951.0 (825.7 to 1086.4)	912.9 (791.2 to 1048.1)	699.1 (592.1 to 821.0)	-2.1 (-2.4 to -1.7)	-26.5 (-30.3 to -22.5)
Nepal	1976.8 (1701.0 to 2258.3)	1573.5 (1325.1 to 1845.5)	1529.3 (1282.4 to 1803.3)	1317.9 (1062.8 to 1605.9)	-1.2 (-1.6 to -0.8)	-16.4 (-21.4 to -11.5)
Pakistan	1110.4 (954.4 to 1284.3)	904.6 (766.0 to 1063.8)	870.8 (733.6 to 1027.3)	738.1 (604.1 to 911.0)	-1.4 (-1.8 to -0.9)	-18.5 (-24.0 to -12.8)
Southeast Asia, East Asia, and Oceania						
East Asia						
China	1280.8 (1121.9 to 1440.9)	835.6 (708.9 to 959.1)	794.3 (672.4 to 915.5)	582.4 (474.4 to 696.6)	-2.4 (-2.8 to -2.0)	-30.4 (-34.5 to -26.0)
North Korea	1375.6 (1192.8 to 1603.3)	1227.7 (1062.9 to 1415.2)	1213.4 (1046.7 to 1398.0)	1123.1 (939.3 to 1315.7)	-0.6 (-1.0 to -0.2)	-8.6 (-14.1 to -3.3)
Taiwan (Province of China)	569.5 (488.7 to 655.5)	477.9 (401.3 to 562.6)	473.4 (396.4 to 558.4)	438.1 (357.2 to 529.2)	-0.6 (-0.9 to -0.3)	-8.4 (-12.8 to -4.0)
Oceania						
American Samoa	664.0 (576.2 to 761.9)	621.6 (524.6 to 726.1)	608.2 (510.9 to 713.8)	571.6 (466.1 to 690.9)	-0.6 (-1.0 to -0.2)	-8.2 (-13.5 to -2.7)
Federated States of Micronesia	895.7 (775.2 to 1018.8)	714.7 (604.1 to 831.0)	681.9 (572.6 to 797.3)	570.4 (455.0 to 686.0)	-1.5 (-2.0 to -1.0)	-20.3 (-26.1 to -14.2)
Fiji	662.1 (570.5 to 760.5)	535.2 (454.1 to 625.0)	526.0 (443.0 to 616.9)	452.6 (367.1 to 547.3)	-1.1 (-1.6 to -0.7)	-15.5 (-20.9 to -10.3)
Guam	560.8 (482.3 to 650.2)	510.1 (431.6 to 596.8)	504.6 (425.2 to 589.8)	467.7 (385.2 to 560.1)	-0.6 (-0.9 to -0.3)	-8.4 (-12.7 to -4.0)
Kiribati	665.5 (575.9 to 769.5)	592.7 (499.1 to 692.4)	589.4 (492.9 to 690.1)	567.5 (454.0 to 693.8)	-0.3 (-0.8 to 0.2)	-4.4 (-11.6 to 2.8)
Marshall Islands	946.2 (824.5 to 1080.8)	719.2 (613.8 to 835.5)	689.5 (585.2 to 801.2)	560.7 (461.3 to 671.5)	-1.7 (-2.1 to -1.3)	-22.1 (-27.2 to -17.2)
Northern Mariana Islands	534.5 (450.7 to 624.6)	492.8 (408.6 to 585.5)	479.4 (398.0 to 571.4)	412.8 (333.9 to 499.4)	-1.2 (-1.4 to -1.0)	-16.3 (-19.2 to -13.4)
Papua New Guinea	1843.4 (1607.3 to 2107.1)	1428.8 (1238.8 to 1634.9)	1369.8 (1180.8 to 1573.6)	1142.1 (948.7 to 1345.4)	-1.5 (-1.9 to -1.1)	-20.1 (-25.3 to -15.0)

	Estimate in 2000 (95% UIs)	Estimate in 2015 (95% UIs)	Estimate in 2017 (95% UIs)	Estimate in 2030 (95% UIs)	Annualised rate of change, 2015-2030 (95% UIs)	Percent change, 2015-2030 (95% UIs)
Samoa	709.5 (586.3 to 832.5)	558.0 (450.4 to 668.7)	542.7 (435.9 to 652.3)	464.1 (361.9 to 574.4)	-1.2 (-1.6 to -0.8)	-17.0 (-21.8 to -11.6)
Solomon Islands	744.9 (645.5 to 859.6)	622.3 (528.3 to 725.5)	604.6 (510.9 to 706.3)	530.4 (430.8 to 639.6)	-1.1 (-1.5 to -0.6)	-14.9 (-20.7 to -8.8)
Tonga	713.4 (605.6 to 823.4)	640.9 (536.4 to 758.7)	634.9 (526.2 to 754.3)	600.8 (484.4 to 737.6)	-0.4 (-0.9 to -0.1)	-6.4 (-12.1 to -0.8)
Vanuatu	1533.7 (1312.0 to 1775.4)	1254.0 (1047.7 to 1484.4)	1227.1 (1021.9 to 1460.5)	1061.6 (848.6 to 1299.9)	-1.1 (-1.5 to -0.7)	-15.5 (-20.6 to -10.5)
Southeast Asia						
Cambodia	1632.2 (1382.9 to 1897.2)	1225.8 (1012.0 to 1452.1)	1183.6 (972.1 to 1412.1)	943.2 (751.6 to 1152.6)	-1.8 (-2.1 to -1.4)	-23.2 (-27.4 to -19.3)
Indonesia	1409.4 (1214.4 to 1626.5)	1113.0 (940.0 to 1311.5)	1077.8 (905.6 to 1275.0)	896.5 (718.1 to 1090.5)	-1.5 (-1.9 to -1.0)	-19.6 (-24.7 to -14.3)
Laos	1382.3 (1171.7 to 1613.6)	1084.6 (894.0 to 1284.4)	1043.9 (854.2 to 1242.8)	849.6 (672.2 to 1042.3)	-1.6 (-2.0 to -1.2)	-21.8 (-26.4 to -16.8)
Malaysia	732.5 (630.8 to 841.3)	600.8 (504.7 to 697.2)	588.5 (492.0 to 685.0)	498.8 (403.5 to 597.2)	-1.3 (-1.7 to -0.9)	-17.1 (-22.3 to -12.1)
Maldives	1084.1 (919.6 to 1262.9)	662.3 (542.4 to 791.6)	609.5 (495.6 to 728.6)	401.2 (313.5 to 502.2)	-3.4 (-3.8 to -2.9)	-39.5 (-43.4 to -35.7)
Mauritius	492.6 (404.4 to 584.0)	412.8 (329.4 to 495.9)	407.2 (323.1 to 490.5)	369.2 (283.5 to 458.1)	-0.8 (-1.0 to -0.5)	-10.7 (-14.5 to -6.9)
Myanmar	1177.6 (999.8 to 1371.8)	1008.9 (854.6 to 1171.6)	984.8 (828.5 to 1150.8)	882.9 (720.8 to 1064.7)	-0.9 (-1.4 to -0.5)	-12.6 (-18.6 to -6.8)
Philippines	908.0 (760.9 to 1067.4)	801.3 (667.0 to 948.3)	791.9 (657.8 to 939.9)	724.9 (598.0 to 870.6)	-0.7 (-1.0 to -0.4)	-9.6 (-13.5 to -5.6)
Sri Lanka	692.4 (579.7 to 816.1)	600.9 (492.5 to 722.5)	592.2 (482.9 to 715.5)	538.1 (422.3 to 667.6)	-0.7 (-1.1 to -0.4)	-10.6 (-15.4 to -5.9)
Seychelles	744.2 (615.0 to 886.6)	600.2 (491.2 to 710.1)	586.5 (478.6 to 696.9)	497.7 (392.4 to 601.6)	-1.3 (-1.6 to -0.9)	-17.1 (-21.2 to -13.2)
Thailand	1193.0 (1013.0 to 1375.2)	904.9 (745.5 to 1065.6)	881.5 (719.0 to 1045.0)	720.0 (562.2 to 877.1)	-1.5 (-2.0 to -1.1)	-20.6 (-25.4 to -15.8)
Timor-Leste	1215.8 (1029.4 to 1396.9)	887.4 (732.4 to 1035.7)	849.1 (695.5 to 995.0)	678.5 (539.6 to 817.7)	-1.8 (-2.2 to -1.4)	-23.6 (-28.5 to -19.0)
Vietnam	1040.0 (883.2 to 1205.2)	866.1 (721.1 to 1018.2)	847.0 (703.4 to 998.6)	760.9 (614.4 to 915.5)	-0.9 (-1.2 to -0.5)	-12.2 (-16.9 to -7.8)
Sub-Saharan Africa						
Central Sub-Saharan Africa						
Angola	1464.5 (1253.1 to 1689.1)	992.7 (828.1 to 1175.4)	938.0 (774.1 to 1118.9)	694.5 (554.0 to 858.7)	-2.4 (-2.9 to -1.9)	-30.2 (-35.4 to -24.9)
Central African Republic	1504.7 (1310.7 to 1731.2)	1297.1 (1118.1 to 1505.6)	1253.9 (1078.1 to 1464.9)	1089.7 (895.0 to 1318.0)	-1.2 (-1.7 to -0.7)	-16.1 (-22.0 to -9.9)
Congo	1131.4 (963.1 to 1317.6)	854.2 (717.6 to 1010.9)	820.4 (685.7 to 977.0)	653.5 (526.6 to 800.0)	-1.8 (-2.3 to -1.3)	-23.6 (-29.2 to -18.0)
Democratic Republic of the Congo	1465.6 (1245.1 to 1697.9)	1251.9 (1049.2 to 1489.7)	1211.8 (1013.9 to 1451.7)	1058.5 (848.6 to 1302.5)	-1.1 (-1.7 to -0.6)	-15.6 (-22.1 to -8.5)
Equatorial Guinea	1312.1 (1119.9 to 1519.9)	724.9 (597.8 to 868.2)	665.5 (542.8 to 799.7)	403.1 (309.4 to 507.4)	-3.9 (-4.5 to -3.3)	-44.5 (-49.0 to -39.4)
Gabon	841.1 (716.0 to 979.8)	638.0 (529.0 to 753.7)	608.7 (501.9 to 722.4)	494.9 (394.0 to 610.0)	-1.7 (-2.1 to -1.3)	-22.5 (-27.4 to -17.5)
Eastern Sub-Saharan Africa						
Burundi	1821.1 (1556.9 to 2108.1)	1486.6 (1231.0 to 1747.3)	1432.7 (1183.2 to 1691.1)	1215.5 (969.6 to 1486.5)	-1.4 (-1.8 to -0.9)	-18.4 (-23.3 to -13.3)
Comoros	1366.5 (1138.1 to 1613.8)	1197.6 (966.4 to 1441.3)	1171.7 (940.7 to 1415.4)	1054.5 (815.5 to 1303.5)	-0.9 (-1.3 to -0.5)	-12.1 (-17.3 to -7.0)
Djibouti	1055.4 (868.7 to 1256.6)	824.1 (669.4 to 986.2)	793.9 (641.8 to 956.5)	652.4 (509.8 to 812.1)	-1.6 (-2.0 to -1.2)	-20.9 (-25.5 to -16.0)
Eritrea	1409.2 (1220.9 to 1628.7)	1026.8 (868.9 to 1216.0)	987.4 (832.8 to 1176.4)	806.3 (653.5 to 981.4)	-1.6 (-2.1 to -1.2)	-21.6 (-26.6 to -16.8)
Ethiopia	1715.6 (1442.3 to 2018.1)	1349.4 (1106.8 to 1619.1)	1286.3 (1049.6 to 1544.5)	1032.0 (813.9 to 1290.3)	-1.8 (-2.3 to -1.3)	-23.7 (-29.3 to -18.2)
Kenya	1354.7 (1109.0 to 1612.1)	1141.3 (927.3 to 1366.7)	1100.1 (890.5 to 1320.7)	937.3 (745.8 to 1147.3)	-1.3 (-1.6 to -1.0)	-17.9 (-21.8 to -13.9)
Madagascar	2026.4 (1713.4 to 2369.3)	1675.1 (1384.4 to 1971.8)	1612.0 (1331.4 to 1910.0)	1361.9 (1101.0 to 1661.7)	-1.4 (-1.8 to -1.0)	-18.8 (-23.1 to -13.8)
Malawi	1454.2 (1207.2 to 1722.2)	1202.2 (992.7 to 1437.0)	1147.8 (946.3 to 1370.5)	972.1 (773.8 to 1191.7)	-1.4 (-1.9 to -1.0)	-19.2 (-24.7 to -13.5)
Mozambique	1597.8 (1339.2 to 1862.8)	1243.9 (1031.2 to 1477.2)	1175.9 (971.2 to 1403.8)	942.7 (753.6 to 1151.6)	-1.9 (-2.2 to -1.5)	-24.3 (-28.6 to -20.1)
Rwanda	1938.3 (1628.4 to 2251.6)	1317.6 (1082.3 to 1576.4)	1240.4 (1012.3 to 1489.3)	902.2 (705.1 to 1115.9)	-2.5 (-2.9 to -2.1)	-31.6 (-35.7 to -27.5)
Somalia	1882.1 (1602.9 to 2187.0)	1631.8 (1381.9 to 1919.8)	1577.4 (1330.7 to 1860.5)	1470.6 (1199.3 to 1768.5)	-0.7 (-1.1 to -0.3)	-10.0 (-14.6 to -5.0)
South Sudan	1640.4 (1373.5 to 1951.6)	1474.5 (1223.3 to 1748.0)	1462.9 (1209.1 to 1735.5)	1380.0 (1124.8 to 1669.3)	-0.5 (-0.9 to -0.1)	-6.5 (-12.5 to -1.1)
Tanzania	1372.7 (1130.4 to 1637.6)	1171.2 (954.2 to 1420.8)	1132.1 (918.0 to 1379.0)	981.7 (770.8 to 1234.3)	-1.2 (-1.6 to -0.8)	-16.3 (-21.4 to -11.0)
Uganda	1476.0 (1212.0 to 1749.2)	1212.4 (989.2 to 1448.8)	1162.5 (946.8 to 1396.9)	979.2 (777.2 to 1211.1)	-1.4 (-1.8 to -1.0)	-19.3 (-24.1 to -13.7)
Zambia	1295.8 (1081.2 to 1518.7)	1122.0 (911.3 to 1343.7)	1073.2 (868.0 to 1290.1)	937.8 (741.0 to 1164.8)	-1.2 (-1.6 to -0.8)	-16.5 (-21.0 to -11.8)
Southern Sub-Saharan Africa						
Botswana	926.7 (788.0 to 1076.6)	733.3 (608.9 to 871.8)	700.8 (578.7 to 836.1)	557.8 (445.8 to 676.9)	-1.8 (-2.2 to -1.5)	-24.0 (-28.4 to -19.8)
Lesotho	1046.7 (903.6 to 1192.2)	899.1 (771.2 to 1030.4)	866.1 (740.5 to 997.3)	763.2 (627.3 to 905.7)	-1.1 (-1.6 to -0.6)	-15.2 (-20.9 to -9.2)
Namibia	838.9 (697.1 to 993.5)	727.8 (593.8 to 870.8)	701.9 (570.1 to 843.1)	605.7 (477.9 to 738.0)	-1.2 (-1.6 to -0.9)	-16.9 (-21.0 to -12.9)
South Africa	861.6 (752.8 to 982.6)	727.6 (627.9 to 841.3)	705.2 (607.1 to 817.4)	607.4 (511.8 to 719.2)	-1.2 (-1.6 to -0.9)	-16.6 (-20.9 to -12.5)
Swaziland	954.0 (815.2 to 1100.8)	798.0 (674.2 to 929.5)	760.0 (634.8 to 887.8)	620.9 (502.8 to 745.6)	-1.7 (-2.2 to -1.2)	-22.3 (-27.6 to -17.0)
Zimbabwe	1139.6 (953.3 to 1328.3)	1103.4 (914.0 to 1307.0)	1059.5 (877.1 to 1261.1)	984.0 (795.9 to 1198.1)	-0.8 (-1.1 to -0.5)	-10.9 (-15.2 to -7.0)
Western Sub-Saharan Africa						
Benin	1230.2 (1018.6 to 1456.1)	1049.2 (858.1 to 1259.8)	1012.2 (827.0 to 1216.9)	873.4 (689.5 to 1074.4)	-1.2 (-1.6 to -0.9)	-16.8 (-20.9 to -12.8)
Burkina Faso	1109.3 (896.9 to 1337.5)	957.7 (769.7 to 1164.7)	926.7 (744.6 to 1127.6)	820.9 (641.4 to 1012.5)	-1.0 (-1.3 to -0.8)	-14.4 (-18.1 to -10.9)
Cameroon	1282.2 (1064.8 to 1511.4)	1082.1 (879.4 to 1295.7)	1042.6 (843.1 to 1252.9)	892.7 (701.2 to 1096.6)	-1.3 (-1.6 to -1.0)	-17.6 (-21.4 to -13.9)
Cape Verde	888.9 (710.0 to 1082.8)	688.7 (540.0 to 852.2)	665.5 (516.3 to 826.4)	560.8 (414.4 to 719.7)	-1.4 (-1.8 to -1.0)	-18.7 (-23.6 to -13.9)
Chad	1275.5 (1060.2 to 1511.3)	1141.4 (935.1 to 1366.3)	1104.5 (903.9 to 1326.0)	983.3 (792.6 to 1209.4)	-1.0 (-1.3 to -0.7)	-13.9 (-18.2 to -9.3)
Cote d'Ivoire	971.5 (797.8 to 1148.8)	917.1 (737.9 to 1098.8)	898.4 (719.9 to 1079.0)	838.9 (647.1 to 1038.0)	-0.6 (-1.0 to -0.3)	-8.6 (-13.5 to -3.8)
The Gambia	903.7 (744.5 to 1073.4)	800.7 (639.9 to 958.6)	780.0 (619.7 to 936.1)	701.6 (546.2 to 864.0)	-0.9 (-1.3 to -0.6)	-12.5 (-17.2 to -7.9)
Ghana	916.8 (747.7 to 1093.8)	801.4 (648.9 to 960.6)	778.0 (628.1 to 934.3)	693.9 (552.7 to 849.5)	-1.0 (-1.2 to -0.7)	-13.5 (-16.9 to -10.1)
Guinea	1296.8 (1078.7 to 1510.1)	1186.2 (969.4 to 1401.6)	1156.2 (938.3 to 1373.8)	1071.0 (855.9 to 1307.3)	-0.7 (-1.1 to -0.3)	-9.8 (-15.0 to -5.0)
Guinea-Bissau	1120.8 (940.4 to 1317.0)	962.0 (782.9 to 1146.1)	929.5 (753.0 to 1111.6)	811.7 (638.2 to 989.4)	-1.1 (-1.5 to -0.8)	-15.7 (-20.7 to -11.2)
Liberia	1021.5 (839.9 to 1223.9)	899.2 (729.5 to 1090.3)	872.4 (705.2 to 1062.9)	776.5 (617.7 to 958.6)	-1.0 (-1.3 to -0.6)	-13.7 (-17.9 to -9.2)
Mali	1282.8 (1087.1 to 1493.2)	1072.7 (882.2 to 1274.8)	1033.2 (846.9 to 1234.7)	888.1 (707.5 to 1097.5)	-1.3 (-1.7 to -0.9)	-17.3 (-22.2 to -12.8)
Mauritania	774.1 (628.2 to 931.0)	675.2 (542.3 to 821.9)	656.9 (524.8 to 803.0)	593.9 (454.1 to 743.8)	-0.9 (-1.3 to -0.5)	-12.2 (-17.5 to -7.0)
Niger	1427.7 (1193.3 to 1672.5)	1234.2 (1005.7 to 1479.9)	1194.0 (973.2 to 1439.2)	1058.3 (838.7 to 1305.3)	-1.0 (-1.5 to -0.6)	-14.4 (-19.7 to -9.1)
Nigeria	938.6 (749.3 to 1140.5)	837.7 (653.5 to 1029.3)	813.8 (635.1 to 1002.6)	745.6 (567.1 to 933.4)	-0.8 (-1.1 to -0.5)	-11.1 (-15.8 to -6.9)
Sao Tome and Principe	822.3 (688.5 to 963.8)	694.1 (576.6 to 819.0)	669.1 (555.0 to 790.0)	587.4 (477.9 to 700.4)	-1.1 (-1.5 to -0.8)	-15.4 (-20.2 to -10.7)
Senegal	907.8 (756.0 to 1075.3)	805.9 (657.4 to 967.6)	789.0 (643.6 to 950.3)	718.6 (567.8 to 894.3)	-0.8 (-1.1 to -0.4)	-10.9 (-15.6 to -6.1)
Sierra Leone	1161.6 (966.7 to 1361.1)	916.7 (745.8 to 1089.8)	880.1 (714.8 to 1049.8)	730.4 (579.4 to 888.4)	-1.5 (-1.9 to -1.2)	-20.4 (-24.5 to -16.6)
Togo	1149.9 (952.0 to 1358.5)	994.0 (811.5 to 1196.2)	965.5 (785.0 to 1166.4)	853.0 (673.9 to 1060.5)	-1.0 (-1.4 to -0.6)	-14.3 (-19.6 to -9.1)
**Indicator 11.6.2: Population-weighted mean levels of fine particulate matter smaller than 2.5 microns in diameter (PM2.5)**						
Central Europe, Eastern Europe, and Central Asia						
Central Asia						
Armenia	36.8 (18.7 to 65.1)	34.9 (17.7 to 62.0)	32.5 (16.2 to 59.5)	31.8 (11.8 to 69.7)	-1.0 (-6.8 to 4.9)	-3.9 (-64.1 to 107.2)
Azerbaijan	22.9 (9.9 to 44.7)	21.4 (8.7 to 41.6)	19.9 (8.1 to 41.1)	20.2 (4.7 to 57.0)	-1.1 (-10.2 to 8.4)	9.6 (-78.5 to 254.4)
Georgia	25.3 (13.2 to 41.8)	24.2 (11.7 to 40.9)	22.2 (11.3 to 37.0)	21.3 (7.5 to 43.1)	-1.2 (-7.5 to 5.1)	-5.9 (-67.6 to 115.4)
Kazakhstan	15.9 (3.5 to 44.2)	14.4 (3.3 to 36.5)	13.8 (3.1 to 37.7)	15.1 (1.6 to 50.9)	-1.0 (-15.1 to 13.6)	52.6 (-89.6 to 666.5)
Kyrgyzstan	27.1 (9.6 to 70.3)	24.3 (8.7 to 61.2)	22.7 (7.8 to 54.6)	25.5 (4.4 to 95.6)	-1.0 (-11.4 to 9.4)	15.1 (-82.0 to 311.0)
Mongolia	39.0 (7.8 to 81.0)	42.5 (9.1 to 79.0)	40.1 (9.1 to 73.0)	45.8 (5.4 to 107.3)	-0.1 (-12.0 to 10.5)	35.6 (-83.4 to 382.7)
Tajikistan	51.2 (16.7 to 114.4)	47.8 (16.4 to 106.6)	46.1 (15.0 to 105.6)	49.6 (8.2 to 157.1)	-0.8 (-11.4 to 10.2)	24.0 (-82.0 to 364.8)
Turkmenistan	25.5 (8.3 to 59.1)	23.0 (7.1 to 51.6)	21.8 (7.1 to 45.4)	22.7 (4.4 to 64.7)	-0.9 (-11.6 to 9.4)	21.5 (-82.3 to 308.0)

	Estimate in 2000 (95% UIs)	Estimate in 2015 (95% UIs)	Estimate in 2017 (95% UIs)	Estimate in 2030 (95% UIs)	Annualised rate of change, 2015-2030 (95% UIs)	Percent change, 2015-2030 (95% UIs)
Uzbekistan	31.9 (8.4 to 78.0)	30.4 (8.0 to 68.9)	28.5 (7.6 to 61.6)	29.6 (4.4 to 87.7)	-1.1 (-12.5 to 10.1)	22.9 (-84.6 to 353.0)
Central Europe						
Albania	22.0 (15.0 to 31.6)	19.5 (13.2 to 28.4)	18.2 (12.1 to 27.4)	16.8 (8.4 to 31.9)	-1.3 (-6.2 to 4.1)	-11.7 (-60.5 to 86.1)
Bosnia and Herzegovina	33.2 (16.0 to 53.4)	29.6 (14.9 to 52.0)	27.8 (13.9 to 45.7)	27.9 (9.1 to 59.3)	-0.9 (-7.5 to 4.3)	-3.5 (-67.5 to 92.0)
Bulgaria	24.1 (15.7 to 36.1)	20.8 (13.0 to 31.8)	19.1 (12.0 to 30.6)	16.7 (8.0 to 32.5)	-1.8 (-7.6 to 4.1)	-15.3 (-68.2 to 86.0)
Croatia	21.5 (13.0 to 34.6)	19.2 (11.3 to 30.7)	17.9 (10.8 to 28.1)	16.9 (7.0 to 35.1)	-1.2 (-7.2 to 4.4)	-8.3 (-66.1 to 94.6)
Czech Republic	20.4 (13.2 to 36.4)	17.4 (11.4 to 30.3)	16.1 (10.7 to 27.8)	13.6 (7.0 to 28.9)	-1.9 (-6.7 to 3.3)	-19.3 (-63.3 to 63.8)
Hungary	19.0 (13.8 to 25.6)	17.2 (12.3 to 23.5)	15.9 (11.5 to 21.9)	13.9 (8.4 to 21.3)	-1.5 (-4.8 to 2.0)	-17.9 (-51.3 to 35.4)
Macedonia	36.4 (22.9 to 63.5)	32.0 (19.5 to 54.5)	29.7 (17.9 to 51.0)	25.9 (12.7 to 49.4)	-1.6 (-5.6 to 2.3)	-17.5 (-56.7 to 41.3)
Montenegro	26.0 (17.8 to 38.1)	22.6 (15.7 to 32.5)	20.8 (14.4 to 29.7)	18.1 (9.8 to 31.7)	-1.7 (-5.6 to 2.7)	-18.0 (-57.1 to 49.5)
Poland	25.8 (14.6 to 41.2)	22.7 (11.9 to 36.4)	20.9 (11.2 to 33.3)	18.2 (7.3 to 35.1)	-1.7 (-8.0 to 4.1)	-15.4 (-70.1 to 85.0)
Romania	17.7 (11.2 to 27.2)	15.4 (9.8 to 23.0)	14.6 (9.1 to 21.6)	12.9 (6.3 to 22.1)	-1.4 (-6.3 to 3.3)	-13.5 (-61.0 to 64.3)
Serbia	29.2 (17.5 to 42.1)	26.9 (16.1 to 39.4)	24.7 (14.3 to 35.5)	22.8 (9.9 to 39.9)	-1.4 (-6.6 to 4.1)	-12.0 (-62.6 to 85.6)
Slovakia	21.7 (16.0 to 29.0)	18.8 (13.7 to 24.6)	17.6 (13.1 to 23.1)	15.0 (9.3 to 23.7)	-1.6 (-5.0 to 2.1)	-18.5 (-52.7 to 36.6)
Slovenia	19.1 (13.4 to 25.8)	17.1 (11.6 to 23.5)	16.0 (11.3 to 21.6)	13.8 (8.4 to 21.3)	-1.5 (-4.7 to 1.8)	-17.8 (-50.5 to 30.5)
Eastern Europe						
Belarus	22.6 (13.8 to 37.2)	20.1 (12.4 to 31.4)	18.8 (11.3 to 29.8)	17.2 (7.9 to 34.3)	-1.4 (-6.6 to 4.1)	-12.0 (-62.8 to 85.6)
Estonia	8.2 (6.0 to 11.1)	7.0 (5.0 to 9.5)	6.7 (4.8 to 9.1)	5.9 (3.4 to 9.4)	-1.3 (-5.1 to 2.7)	-14.0 (-53.7 to 50.7)
Latvia	16.7 (10.0 to 29.6)	14.0 (8.3 to 24.9)	13.4 (7.8 to 23.9)	11.7 (5.4 to 25.3)	-1.4 (-5.7 to 2.1)	-15.9 (-57.3 to 37.6)
Lithuania	14.4 (10.7 to 20.4)	12.5 (9.1 to 17.9)	11.8 (8.6 to 16.3)	10.3 (6.2 to 16.4)	-1.4 (-4.5 to 1.8)	-16.6 (-49.3 to 30.9)
Moldova	19.6 (11.8 to 29.8)	17.1 (10.5 to 26.1)	16.2 (9.9 to 26.0)	14.5 (6.3 to 28.6)	-1.4 (-7.3 to 4.1)	-11.2 (-66.7 to 84.3)
Russia	19.1 (14.9 to 24.3)	17.0 (13.0 to 21.6)	16.2 (12.7 to 20.2)	14.2 (10.5 to 19.0)	-1.2 (-3.2 to 0.8)	-16.0 (-37.9 to 12.9)
Ukraine	24.2 (10.6 to 50.6)	21.2 (9.7 to 42.7)	20.3 (9.2 to 44.0)	19.5 (5.2 to 54.2)	-1.4 (-10.4 to 7.9)	2.9 (-78.9 to 228.1)
High-income						
Australasia						
Australia	10.8 (5.2 to 24.7)	9.3 (4.2 to 20.1)	8.6 (4.0 to 18.3)	8.3 (2.7 to 23.3)	-1.4 (-5.8 to 3.7)	-13.4 (-58.1 to 75.1)
New Zealand	7.2 (4.7 to 11.2)	6.3 (4.2 to 9.9)	6.0 (3.9 to 9.2)	5.5 (2.7 to 10.6)	-1.1 (-5.3 to 3.3)	-11.3 (-54.6 to 63.1)
High-income Asia Pacific						
Brunei	6.9 (4.7 to 9.4)	6.5 (4.5 to 8.9)	5.9 (4.1 to 8.1)	5.5 (3.2 to 8.9)	-1.2 (-4.6 to 2.3)	-14.1 (-50.1 to 41.9)
Japan	14.2 (12.5 to 15.7)	12.7 (11.2 to 14.0)	11.7 (10.3 to 13.0)	10.4 (8.9 to 11.8)	-1.3 (-2.0 to -0.7)	-18.1 (-25.7 to -9.3)
South Korea	29.8 (22.4 to 39.1)	28.2 (20.8 to 37.6)	25.0 (18.8 to 32.5)	23.0 (14.0 to 35.5)	-1.5 (-5.0 to 1.8)	-16.9 (-52.5 to 31.3)
Singapore	19.3 (12.6 to 27.1)	20.7 (13.4 to 28.9)	19.1 (12.4 to 26.6)	19.7 (9.9 to 33.5)	-0.5 (-4.7 to 3.2)	-4.1 (-50.5 to 60.9)
High-income North America						
Canada	8.3 (5.1 to 11.9)	7.2 (4.2 to 10.3)	6.4 (3.9 to 8.9)	5.7 (2.7 to 9.5)	-1.7 (-6.1 to 3.0)	-16.9 (-59.9 to 57.7)
Greenland	14.0 (6.0 to 29.9)	11.8 (4.8 to 25.1)	11.6 (4.9 to 24.8)	11.3 (2.9 to 32.9)	-1.0 (-11.1 to 8.6)	12.8 (-81.0 to 262.2)
United States	9.5 (8.0 to 10.9)	8.1 (6.7 to 9.2)	7.4 (6.1 to 8.5)	6.3 (5.1 to 7.5)	-1.6 (-2.5 to -0.5)	-21.1 (-31.3 to -6.6)
Southern Latin America						
Argentina	16.8 (6.6 to 38.8)	14.6 (6.2 to 38.3)	13.3 (5.4 to 31.1)	13.6 (3.2 to 44.4)	-1.3 (-11.3 to 9.4)	10.1 (-81.8 to 311.2)
Chile	26.2 (11.4 to 39.3)	22.6 (9.3 to 34.3)	21.0 (9.1 to 32.7)	20.0 (5.7 to 38.9)	-1.2 (-8.3 to 5.4)	-5.9 (-71.2 to 125.0)
Uruguay	11.4 (6.5 to 20.9)	9.7 (5.5 to 20.2)	9.3 (5.4 to 17.3)	9.0 (3.5 to 21.6)	-0.9 (-7.5 to 6.4)	-0.0 (-67.6 to 162.9)
Western Europe						
Andorra	12.6 (9.5 to 19.7)	11.5 (8.4 to 17.7)	10.3 (7.5 to 15.7)	9.1 (5.1 to 17.9)	-1.8 (-5.1 to 1.2)	-21.6 (-53.6 to 20.6)
Austria	15.4 (9.8 to 21.8)	13.5 (8.8 to 19.3)	12.5 (8.0 to 18.3)	10.9 (5.8 to 18.5)	-1.6 (-5.7 to 2.6)	-17.5 (-57.4 to 47.9)
Belgium	15.7 (10.6 to 21.1)	13.6 (9.3 to 18.2)	12.9 (8.6 to 17.2)	11.2 (6.1 to 16.6)	-1.4 (-4.6 to 1.8)	-16.3 (-49.8 to 30.6)
Cyprus	20.2 (15.3 to 28.2)	19.1 (14.1 to 26.6)	17.3 (12.7 to 23.9)	15.8 (9.8 to 23.2)	-1.3 (-4.1 to 1.8)	-16.3 (-46.3 to 30.0)
Denmark	12.4 (8.8 to 16.4)	10.7 (7.8 to 14.0)	10.0 (7.3 to 13.1)	8.8 (5.3 to 13.7)	-1.4 (-4.9 to 2.1)	-15.8 (-52.4 to 37.4)
Finland	7.1 (4.0 to 9.7)	6.1 (3.8 to 8.0)	5.9 (3.8 to 7.8)	5.2 (2.8 to 7.7)	-1.1 (-4.7 to 2.3)	-11.8 (-50.5 to 41.7)
France	14.7 (9.9 to 20.6)	12.8 (8.8 to 17.6)	11.8 (8.1 to 16.5)	10.3 (6.0 to 15.7)	-1.5 (-5.2 to 1.8)	-17.8 (-54.2 to 31.6)
Germany	14.9 (10.5 to 21.1)	12.8 (9.2 to 17.7)	12.0 (8.6 to 16.7)	10.3 (6.1 to 16.6)	-1.6 (-5.1 to 2.1)	-18.0 (-53.2 to 36.6)
Greece	19.9 (12.5 to 31.9)	17.7 (10.8 to 31.4)	16.2 (10.1 to 26.3)	14.9 (7.1 to 33.4)	-1.4 (-6.3 to 4.5)	-12.9 (-60.9 to 96.0)
Iceland	8.0 (5.1 to 16.6)	6.9 (4.5 to 14.6)	6.5 (4.2 to 13.8)	6.1 (2.9 to 18.2)	-1.3 (-6.5 to 5.1)	-9.7 (-62.6 to 115.8)
Ireland	10.1 (7.5 to 13.0)	8.7 (6.4 to 11.0)	8.2 (6.1 to 10.5)	7.3 (4.6 to 10.8)	-1.2 (-4.3 to 1.8)	-14.9 (-47.2 to 31.1)
Israel	24.5 (14.5 to 39.4)	22.8 (13.6 to 45.9)	21.4 (12.8 to 43.6)	20.8 (9.1 to 59.1)	-1.0 (-6.7 to 4.8)	-7.0 (-63.5 to 107.0)
Italy	19.8 (10.3 to 32.5)	17.9 (9.0 to 29.8)	16.8 (8.6 to 27.8)	15.7 (6.0 to 32.5)	-1.2 (-7.4 to 5.3)	-7.1 (-66.9 to 120.2)
Luxembourg	12.6 (9.4 to 15.9)	11.0 (8.4 to 13.5)	10.4 (7.7 to 12.7)	9.1 (5.9 to 12.6)	-1.4 (-4.0 to 1.0)	-17.0 (-45.0 to 16.5)
Malta	16.5 (12.3 to 21.9)	14.7 (11.1 to 19.4)	13.9 (10.4 to 18.2)	12.7 (7.9 to 19.3)	-1.1 (-3.2 to 1.4)	-13.8 (-38.3 to 23.8)
Netherlands	14.9 (11.6 to 18.5)	12.9 (10.3 to 15.9)	12.0 (9.6 to 14.8)	10.3 (7.2 to 14.3)	-1.6 (-4.3 to 1.0)	-19.2 (-47.4 to 17.0)
Norway	8.7 (7.5 to 9.7)	7.2 (6.2 to 8.0)	7.0 (6.0 to 7.7)	6.0 (5.1 to 6.9)	-1.2 (-2.0 to -0.4)	-16.3 (-25.4 to -5.5)
Portugal	10.1 (6.0 to 16.0)	8.8 (5.3 to 13.7)	8.2 (4.9 to 12.6)	7.4 (3.5 to 14.1)	-1.4 (-6.5 to 3.5)	-13.6 (-62.0 to 69.3)
Spain	11.8 (7.6 to 16.9)	10.4 (6.7 to 15.2)	9.7 (6.1 to 14.2)	8.8 (4.2 to 15.3)	-1.4 (-6.1 to 3.1)	-13.5 (-60.1 to 59.3)
Sweden	7.6 (5.1 to 11.9)	6.3 (4.4 to 10.1)	6.2 (4.3 to 9.7)	5.5 (3.3 to 10.1)	-1.0 (-4.1 to 2.5)	-11.1 (-46.0 to 45.8)
Switzerland	12.7 (8.3 to 17.8)	11.2 (7.6 to 15.4)	10.3 (7.4 to 14.0)	8.9 (5.5 to 13.9)	-1.6 (-4.7 to 1.9)	-18.8 (-50.6 to 33.3)
United Kingdom	12.6 (11.7 to 13.4)	10.8 (10.0 to 11.4)	10.5 (9.7 to 11.2)	9.2 (8.4 to 9.8)	-1.1 (-1.4 to -0.8)	-14.8 (-18.6 to -10.8)
Latin America and Caribbean						
Andean Latin America						
Bolivia	26.2 (10.1 to 53.6)	22.5 (8.8 to 47.4)	21.6 (8.0 to 44.5)	22.1 (4.8 to 63.4)	-0.8 (-10.2 to 9.1)	14.5 (-78.2 to 293.6)
Ecuador	18.7 (9.9 to 30.9)	15.7 (8.4 to 25.9)	14.9 (8.3 to 24.7)	13.7 (5.5 to 28.5)	-1.2 (-6.7 to 5.1)	-7.8 (-63.2 to 116.0)
Peru	30.3 (11.3 to 57.1)	26.0 (9.6 to 49.3)	24.8 (8.9 to 51.3)	24.4 (5.2 to 60.7)	-1.1 (-9.9 to 7.2)	4.1 (-77.5 to 195.9)
Caribbean						
Antigua and Barbuda	22.0 (11.9 to 38.6)	19.7 (10.8 to 34.0)	18.6 (9.9 to 32.0)	18.0 (6.4 to 41.2)	-1.1 (-8.4 to 6.3)	-2.1 (-71.5 to 155.6)
The Bahamas	21.3 (9.2 to 42.9)	18.9 (8.3 to 36.9)	17.4 (7.4 to 33.8)	17.1 (4.6 to 44.6)	-1.4 (-10.2 to 7.3)	2.2 (-78.2 to 197.5)
Barbados	28.5 (15.3 to 48.7)	24.7 (13.6 to 41.7)	23.1 (12.4 to 38.6)	22.0 (8.4 to 48.5)	-1.2 (-8.2 to 5.9)	-4.1 (-70.6 to 142.0)
Belize	27.8 (8.3 to 68.5)	25.2 (9.0 to 54.9)	23.0 (9.1 to 49.6)	25.1 (5.3 to 73.5)	-0.9 (-12.0 to 10.7)	26.5 (-83.5 to 397.7)
Bermuda	14.8 (8.9 to 23.5)	12.4 (7.5 to 19.2)	11.7 (7.0 to 18.0)	11.2 (6.5 to 17.5)	-0.7 (-1.3 to -0.1)	-10.1 (-17.5 to -2.0)
Cuba	24.1 (8.5 to 47.4)	20.9 (7.5 to 40.1)	19.7 (7.1 to 37.9)	19.7 (4.1 to 49.2)	-1.0 (-11.2 to 8.8)	12.6 (-81.4 to 276.2)
Dominica	23.0 (11.7 to 38.8)	20.7 (10.8 to 35.2)	19.5 (10.4 to 33.0)	18.7 (7.0 to 42.6)	-1.1 (-7.8 to 5.8)	-5.0 (-68.9 to 138.2)
Dominican Republic	16.4 (6.6 to 34.5)	14.9 (5.9 to 30.8)	13.7 (5.6 to 28.3)	14.2 (3.2 to 38.3)	-1.1 (-10.6 to 8.2)	11.6 (-79.6 to 243.5)
Grenada	27.5 (13.4 to 50.6)	24.2 (12.2 to 43.4)	22.7 (11.4 to 42.5)	22.3 (7.1 to 53.9)	-1.1 (-8.5 to 6.3)	-1.8 (-72.1 to 156.8)
Guyana	27.4 (9.7 to 62.3)	23.9 (8.6 to 54.4)	22.4 (8.4 to 50.2)	24.1 (5.3 to 73.5)	-0.8 (-10.6 to 9.7)	25.1 (-79.5 to 330.5)
Haiti	17.8 (7.1 to 35.7)	15.5 (6.5 to 31.3)	15.0 (6.3 to 33.0)	15.6 (3.7 to 49.1)	-0.8 (-10.6 to 9.6)	19.9 (-79.7 to 321.5)

	Estimate in 2000 (95% UIs)	Estimate in 2015 (95% UIs)	Estimate in 2017 (95% UIs)	Estimate in 2030 (95% UIs)	Annualised rate of change, 2015-2030 (95% UIs)	Percent change, 2015-2030 (95% UIs)
Jamaica	15.9 (10.8 to 26.4)	14.2 (9.4 to 22.7)	13.4 (8.7 to 21.9)	12.4 (6.5 to 23.4)	-1.1 (-5.1 to 3.1)	-11.0 (-53.3 to 60.2)
Puerto Rico	10.4 (5.7 to 16.9)	8.9 (4.9 to 14.3)	8.3 (4.6 to 13.4)	8.1 (4.3 to 14.0)	-0.7 (-1.3 to 0.1)	-9.3 (-18.2 to 0.8)
Saint Lucia	27.1 (13.5 to 47.2)	24.3 (13.1 to 42.8)	22.4 (12.5 to 38.8)	21.8 (7.9 to 51.3)	-1.2 (-8.3 to 5.9)	-2.0 (-71.4 to 142.5)
Saint Vincent and the Grenadines	27.0 (12.9 to 54.0)	23.5 (12.1 to 44.3)	22.2 (11.4 to 41.7)	21.5 (7.0 to 50.6)	-1.1 (-8.8 to 7.0)	0.7 (-73.3 to 184.2)
Suriname	29.0 (10.8 to 61.6)	26.0 (9.5 to 52.6)	24.8 (9.2 to 48.3)	25.1 (5.5 to 66.0)	-1.0 (-10.2 to 8.9)	11.8 (-78.5 to 277.4)
Trinidad and Tobago	28.8 (11.8 to 56.8)	24.9 (10.8 to 50.8)	24.1 (10.7 to 48.0)	24.3 (6.1 to 66.7)	-0.9 (-10.6 to 9.7)	15.5 (-79.5 to 326.1)
Virgin Islands, U.S.	12.8 (7.0 to 23.6)	11.0 (6.1 to 20.0)	10.3 (5.7 to 18.6)	9.9 (5.3 to 18.4)	-0.7 (-1.3 to -0.1)	-10.4 (-18.2 to -1.6)
Central Latin America						
Colombia	20.1 (8.2 to 40.2)	17.3 (7.4 to 34.4)	16.5 (6.6 to 35.5)	16.5 (4.2 to 46.0)	-1.2 (-9.5 to 9.0)	8.2 (-75.9 to 285.7)
Costa Rica	19.4 (11.6 to 26.6)	16.9 (10.2 to 23.1)	15.7 (9.5 to 21.3)	14.4 (7.0 to 22.1)	-1.2 (-4.7 to 2.1)	-12.8 (-50.9 to 38.0)
El Salvador	31.9 (18.0 to 52.7)	26.5 (15.2 to 44.3)	24.5 (13.3 to 40.7)	22.3 (8.4 to 46.8)	-1.5 (-7.9 to 4.6)	-10.3 (-69.3 to 99.4)
Guatemala	31.1 (17.6 to 46.5)	25.1 (13.7 to 38.5)	24.1 (13.9 to 36.1)	22.1 (9.2 to 41.0)	-1.1 (-6.6 to 4.9)	-6.5 (-62.8 to 108.3)
Honduras	27.6 (13.8 to 48.6)	22.4 (11.8 to 36.4)	20.6 (11.5 to 34.8)	18.6 (7.3 to 40.5)	-1.6 (-7.8 to 5.2)	-11.0 (-68.7 to 117.2)
Mexico	26.8 (18.3 to 34.9)	22.1 (15.2 to 28.8)	20.9 (14.5 to 26.8)	19.1 (12.1 to 26.3)	-1.0 (-2.8 to 0.8)	-13.3 (-34.5 to 12.6)
Nicaragua	23.1 (11.1 to 38.7)	18.6 (8.8 to 32.3)	17.6 (8.4 to 30.4)	16.2 (5.4 to 34.3)	-1.3 (-8.1 to 5.5)	-6.1 (-70.2 to 128.8)
Panama	14.4 (7.9 to 23.7)	12.5 (6.8 to 20.6)	11.4 (6.2 to 18.2)	10.8 (4.0 to 21.9)	-1.3 (-6.6 to 4.1)	-10.1 (-63.1 to 84.3)
Venezuela	21.6 (8.1 to 44.1)	18.3 (7.3 to 38.4)	17.0 (6.6 to 35.0)	16.5 (3.7 to 47.2)	-1.4 (-10.8 to 8.2)	5.8 (-80.1 to 239.7)
Tropical Latin America						
Brazil	15.8 (12.6 to 19.2)	13.6 (11.0 to 16.5)	12.7 (10.2 to 15.3)	11.4 (8.2 to 15.0)	-1.2 (-3.2 to 1.0)	-15.6 (-38.1 to 15.4)
Paraguay	14.2 (7.0 to 24.7)	13.0 (6.1 to 23.5)	11.9 (5.6 to 21.1)	11.5 (3.8 to 25.9)	-1.3 (-8.6 to 6.4)	-2.1 (-72.3 to 159.9)
North Africa and Middle East						
North Africa and Middle East						
Afghanistan	64.9 (28.4 to 115.7)	61.2 (26.9 to 105.0)	56.9 (25.9 to 103.2)	56.7 (16.1 to 132.9)	-1.1 (-8.6 to 7.6)	3.2 (-72.5 to 212.4)
Algeria	37.5 (15.0 to 80.4)	36.1 (12.9 to 83.0)	38.9 (12.8 to 89.4)	43.5 (8.9 to 137.7)	0.3 (-10.9 to 11.7)	48.2 (-80.5 to 474.2)
Bahrain	68.0 (48.5 to 92.8)	72.6 (50.5 to 101.5)	70.8 (49.1 to 99.4)	74.5 (40.5 to 127.9)	-0.0 (-4.4 to 4.4)	5.1 (-48.2 to 93.0)
Egypt	73.3 (32.3 to 132.0)	88.2 (30.9 to 166.8)	87.0 (33.7 to 164.2)	101.4 (22.9 to 261.5)	0.3 (-9.8 to 9.0)	31.4 (-77.0 to 287.7)
Iran	38.4 (33.8 to 43.4)	39.6 (34.7 to 44.9)	39.0 (34.4 to 44.8)	39.7 (32.3 to 49.2)	-0.0 (-1.6 to 1.6)	0.6 (-20.9 to 27.8)
Iraq	62.5 (28.4 to 116.7)	66.3 (30.3 to 129.1)	61.6 (29.0 to 109.7)	66.1 (19.8 to 160.4)	-0.5 (-8.8 to 7.4)	12.6 (-73.3 to 204.3)
Jordan	33.5 (19.7 to 52.2)	34.3 (20.3 to 59.6)	33.0 (19.4 to 53.9)	33.4 (15.2 to 67.4)	-0.4 (-5.5 to 4.0)	0.0 (-56.2 to 81.3)
Kuwait	61.5 (38.1 to 94.7)	65.4 (41.0 to 101.0)	60.8 (39.2 to 90.8)	62.5 (32.1 to 112.5)	-0.5 (-4.8 to 4.0)	-1.4 (-51.6 to 82.3)
Lebanon	30.6 (21.7 to 42.2)	31.2 (20.8 to 44.1)	30.6 (20.6 to 43.4)	31.7 (16.5 to 54.1)	-0.1 (-4.8 to 4.8)	5.1 (-51.6 to 105.7)
Libya	46.3 (16.8 to 105.6)	51.5 (16.1 to 134.0)	54.2 (16.2 to 135.5)	65.8 (10.2 to 233.9)	0.5 (-11.6 to 13.2)	67.6 (-82.4 to 627.1)
Morocco	31.0 (16.5 to 54.3)	31.4 (16.3 to 55.8)	32.6 (17.1 to 55.2)	35.6 (12.3 to 79.2)	0.3 (-6.8 to 7.9)	24.8 (-63.7 to 227.8)
Palestine	33.9 (20.4 to 57.3)	33.9 (18.3 to 63.1)	33.2 (18.0 to 60.3)	34.5 (13.0 to 74.3)	-0.3 (-5.9 to 5.6)	6.1 (-58.7 to 133.0)
Oman	39.9 (19.9 to 77.6)	42.6 (20.8 to 85.5)	41.1 (20.0 to 76.6)	44.5 (14.1 to 107.9)	-0.3 (-8.5 to 7.5)	16.9 (-72.0 to 206.5)
Qatar	83.5 (53.3 to 125.7)	94.4 (57.9 to 144.1)	91.2 (55.5 to 140.7)	96.0 (42.1 to 192.3)	-0.2 (-5.6 to 5.0)	5.6 (-56.8 to 110.2)
Saudi Arabia	79.1 (29.2 to 173.5)	97.4 (29.4 to 239.8)	88.0 (29.6 to 197.9)	109.2 (17.5 to 346.6)	-0.1 (-10.8 to 10.8)	39.2 (-80.3 to 407.1)
Sudan	49.4 (16.2 to 121.2)	58.9 (14.8 to 168.4)	55.4 (15.4 to 142.5)	66.5 (9.0 to 235.8)	-0.4 (-14.9 to 13.0)	59.9 (-89.2 to 603.1)
Syria	41.6 (23.4 to 78.5)	44.3 (23.1 to 89.3)	43.8 (22.7 to 87.8)	46.9 (14.7 to 120.2)	-0.3 (-7.9 to 7.5)	12.7 (-69.4 to 209.7)
Tunisia	35.6 (20.4 to 58.9)	36.3 (19.8 to 65.2)	37.7 (19.5 to 69.0)	40.9 (14.8 to 97.3)	0.3 (-7.1 to 7.2)	24.0 (-65.6 to 192.9)
Turkey	42.7 (26.7 to 62.8)	45.4 (27.0 to 71.1)	44.3 (26.5 to 67.5)	47.2 (19.3 to 88.3)	-0.0 (-6.4 to 5.6)	10.3 (-61.9 to 131.3)
United Arab Emirates	39.7 (22.5 to 64.4)	42.3 (23.3 to 72.0)	40.9 (23.0 to 71.8)	43.7 (16.5 to 93.7)	-0.2 (-7.0 to 6.4)	11.3 (-65.2 to 160.9)
Yemen	50.9 (17.8 to 123.2)	54.3 (16.5 to 127.6)	50.5 (15.8 to 126.0)	58.4 (9.8 to 197.1)	-0.7 (-12.5 to 12.1)	42.9 (-84.6 to 512.2)
South Asia						
South Asia						
Bangladesh	63.0 (40.2 to 91.6)	67.0 (42.8 to 95.9)	60.9 (38.0 to 86.3)	61.4 (28.9 to 105.6)	-0.8 (-5.6 to 4.1)	-4.8 (-56.7 to 84.2)
Bhutan	39.8 (22.7 to 65.2)	39.8 (23.2 to 67.5)	37.9 (21.6 to 66.0)	38.8 (15.2 to 93.9)	-0.7 (-5.9 to 4.3)	-2.7 (-58.6 to 90.2)
India	84.2 (65.2 to 102.5)	89.3 (68.9 to 109.9)	90.9 (67.5 to 113.6)	96.1 (63.8 to 131.9)	0.4 (-1.7 to 2.6)	8.0 (-22.4 to 48.5)
Nepal	88.9 (41.6 to 171.0)	96.2 (44.2 to 170.8)	99.7 (44.6 to 179.5)	113.9 (33.1 to 267.3)	0.6 (-7.2 to 8.4)	30.1 (-66.0 to 255.1)
Pakistan	61.1 (30.4 to 107.3)	60.1 (31.2 to 115.0)	58.3 (31.1 to 106.0)	61.0 (19.6 to 145.1)	-0.3 (-7.7 to 7.5)	11.6 (-68.4 to 207.6)
Southeast Asia, East Asia, and Oceania						
East Asia						
China	60.7 (48.2 to 73.1)	59.1 (46.4 to 70.8)	52.7 (41.0 to 62.8)	48.9 (37.1 to 60.2)	-1.3 (-2.3 to -0.2)	-17.1 (-29.5 to -2.9)
North Korea	38.2 (22.7 to 57.3)	37.4 (21.8 to 57.9)	32.0 (19.2 to 48.4)	30.2 (12.8 to 59.3)	-1.8 (-7.8 to 4.6)	-14.4 (-69.1 to 99.3)
Taiwan (Province of China)	28.8 (17.7 to 41.1)	25.2 (15.8 to 34.8)	23.2 (14.6 to 31.8)	21.6 (10.3 to 35.9)	-1.2 (-5.1 to 2.2)	-13.7 (-53.7 to 39.2)
Oceania						
American Samoa	15.2 (8.1 to 25.4)	13.0 (7.0 to 21.4)	12.5 (6.7 to 20.6)	12.1 (6.3 to 20.9)	-0.5 (-0.9 to 0.0)	-7.4 (-13.1 to 0.4)
Federated States of Micronesia	14.1 (4.8 to 31.1)	11.7 (3.7 to 24.7)	11.3 (4.1 to 22.9)	11.5 (2.3 to 32.4)	-0.9 (-12.3 to 9.7)	21.3 (-84.2 to 326.0)
Fiji	13.1 (4.8 to 27.2)	11.6 (4.5 to 23.0)	10.8 (4.0 to 22.4)	11.1 (2.5 to 32.7)	-1.3 (-11.5 to 9.0)	12.5 (-82.1 to 283.3)
Guam	14.3 (8.7 to 23.1)	12.5 (7.6 to 20.2)	11.6 (7.1 to 18.8)	11.2 (6.5 to 18.6)	-0.8 (-1.4 to -0.1)	-10.7 (-18.9 to -1.4)
Kiribati	13.5 (4.9 to 27.9)	11.5 (4.9 to 23.9)	10.6 (4.2 to 21.8)	10.5 (2.5 to 26.8)	-1.3 (-11.3 to 8.1)	7.9 (-81.7 to 234.6)
Marshall Islands	13.0 (5.1 to 25.3)	10.8 (4.7 to 20.8)	10.2 (4.4 to 20.3)	10.1 (2.6 to 26.1)	-1.2 (-10.5 to 8.0)	7.1 (-79.2 to 231.9)
Northern Mariana Islands	12.2 (7.2 to 19.3)	10.4 (6.1 to 16.7)	9.9 (5.9 to 15.7)	9.1 (5.2 to 15.0)	-1.0 (-1.4 to -0.3)	-13.3 (-19.3 to -3.9)
Papua New Guinea	14.8 (3.9 to 37.5)	13.3 (3.6 to 33.3)	12.3 (3.4 to 35.4)	13.3 (2.0 to 49.2)	-1.3 (-14.5 to 11.9)	38.2 (-88.7 to 499.1)
Samoa	14.3 (4.6 to 31.2)	12.3 (3.9 to 27.1)	11.6 (3.5 to 26.0)	12.2 (1.9 to 39.4)	-1.3 (-12.7 to 10.4)	24.0 (-85.2 to 375.6)
Solomon Islands	13.9 (4.2 to 34.6)	12.7 (4.0 to 33.8)	11.9 (3.8 to 29.5)	13.0 (2.1 to 44.9)	-0.9 (-12.9 to 11.2)	35.8 (-85.7 to 434.4)
Tonga	12.8 (5.1 to 25.0)	11.3 (4.5 to 21.6)	10.8 (4.2 to 21.7)	10.8 (2.6 to 28.9)	-1.1 (-10.3 to 8.2)	10.5 (-78.7 to 244.5)
Vanuatu	14.1 (4.5 to 33.1)	12.2 (3.8 to 28.6)	11.7 (3.8 to 25.8)	12.8 (2.1 to 43.6)	-0.6 (-12.8 to 12.6)	44.1 (-85.2 to 561.3)
Southeast Asia						
Cambodia	30.0 (15.9 to 52.8)	26.8 (14.1 to 49.5)	25.6 (12.7 to 48.5)	25.2 (8.0 to 61.3)	-1.0 (-8.7 to 7.2)	3.4 (-72.8 to 196.5)
Indonesia	17.9 (13.9 to 24.0)	16.6 (13.0 to 22.1)	16.5 (12.9 to 21.7)	15.7 (11.0 to 21.5)	-0.4 (-2.2 to 1.5)	-5.2 (-28.5 to 24.4)
Laos	27.6 (12.1 to 48.2)	26.1 (11.4 to 47.9)	25.1 (9.9 to 45.2)	26.5 (6.9 to 63.9)	-0.6 (-10.0 to 7.8)	14.8 (-77.7 to 224.3)
Malaysia	18.1 (7.3 to 28.9)	17.4 (7.6 to 27.4)	16.0 (6.7 to 25.9)	15.8 (4.5 to 30.5)	-1.0 (-7.6 to 5.3)	-3.6 (-68.0 to 122.8)
Maldives	11.1 (8.0 to 14.9)	9.4 (6.7 to 12.6)	7.8 (5.6 to 10.6)	6.2 (4.5 to 8.4)	-2.8 (-2.9 to -2.6)	-33.8 (-35.3 to -32.3)
Mauritius	15.4 (8.1 to 26.3)	14.4 (7.2 to 27.0)	14.5 (7.2 to 26.8)	14.9 (4.7 to 36.0)	-0.3 (-7.8 to 6.9)	10.1 (-68.8 to 180.9)
Myanmar	39.4 (22.8 to 61.5)	38.5 (21.6 to 60.9)	35.6 (21.2 to 58.5)	34.8 (15.1 to 74.3)	-1.0 (-7.2 to 5.6)	-3.3 (-66.2 to 131.4)
Philippines	22.2 (9.3 to 36.6)	19.6 (8.6 to 31.5)	18.1 (7.8 to 28.8)	17.1 (4.8 to 33.5)	-1.3 (-9.2 to 6.2)	-4.4 (-74.9 to 151.6)
Sri Lanka	30.9 (17.3 to 47.0)	25.3 (15.2 to 38.0)	11.1 (2.4 to 27.3)	8.5 (1.1 to 26.6)	-9.9 (-20.6 to 1.5)	-63.8 (-95.4 to 25.2)
Seychelles	21.6 (12.4 to 35.5)	20.1 (10.4 to 37.1)	20.2 (10.6 to 37.0)	20.5 (7.2 to 51.3)	-0.3 (-3.2 to 2.7)	-1.5 (-38.4 to 51.0)
Thailand	31.0 (16.4 to 49.9)	27.3 (15.8 to 43.5)	26.3 (13.5 to 40.8)	25.2 (8.6 to 50.3)	-0.9 (-8.4 to 5.6)	-1.6 (-71.5 to 131.4)

	Estimate in 2000 (95% UIs)	Estimate in 2015 (95% UIs)	Estimate in 2017 (95% UIs)	Estimate in 2030 (95% UIs)	Annualised rate of change, 2015-2030 (95% UIs)	Percent change, 2015-2030 (95% UIs)
Timor-Leste	23.5 (8.7 to 49.9)	20.6 (7.5 to 42.5)	19.3 (7.4 to 40.3)	19.8 (4.2 to 58.6)	-1.1 (-12.4 to 9.9)	19.3 (-84.4 to 342.5)
Vietnam	36.2 (13.7 to 62.6)	32.5 (13.0 to 57.3)	29.6 (11.2 to 50.5)	28.9 (6.9 to 69.0)	-1.3 (-11.1 to 7.9)	2.2 (-81.0 to 225.6)
Sub-Saharan Africa						
Central Sub-Saharan Africa						
Angola	36.2 (11.5 to 84.8)	32.7 (12.7 to 73.0)	32.4 (11.9 to 70.8)	35.1 (6.6 to 101.5)	-0.6 (-12.3 to 9.7)	31.1 (-84.2 to 330.1)
Central African Republic	51.6 (15.6 to 129.9)	58.3 (15.3 to 154.5)	56.8 (14.3 to 154.5)	66.6 (8.3 to 255.6)	-0.5 (-14.2 to 13.6)	60.6 (-88.0 to 663.6)
Congo	43.6 (13.1 to 118.5)	44.1 (13.5 to 109.0)	46.6 (12.9 to 121.8)	53.3 (7.8 to 194.1)	-0.1 (-13.3 to 12.5)	58.2 (-86.3 to 554.0)
Democratic Republic of the Congo	44.8 (12.3 to 109.9)	44.9 (12.4 to 113.4)	44.9 (11.7 to 105.9)	51.9 (7.1 to 185.5)	-0.2 (-14.3 to 13.4)	59.9 (-88.3 to 641.6)
Equatorial Guinea	51.7 (19.1 to 118.0)	52.6 (19.3 to 117.3)	53.2 (19.3 to 120.8)	59.5 (11.8 to 174.8)	-0.1 (-11.0 to 10.6)	40.0 (-80.7 to 387.8)
Gabon	42.6 (14.3 to 106.7)	44.1 (15.2 to 105.3)	44.4 (15.2 to 102.7)	49.7 (10.7 to 161.8)	-0.1 (-11.1 to 10.8)	41.2 (-80.9 to 405.2)
Eastern Sub-Saharan Africa						
Burundi	41.0 (13.3 to 92.7)	38.4 (14.8 to 86.8)	38.9 (14.5 to 85.2)	42.6 (8.4 to 122.2)	-0.3 (-11.2 to 10.4)	36.2 (-81.4 to 372.7)
Comoros	21.5 (10.9 to 38.5)	20.7 (10.3 to 36.0)	20.6 (9.7 to 37.8)	21.8 (6.1 to 54.2)	-0.3 (-8.8 to 7.7)	16.3 (-73.3 to 218.1)
Djibouti	41.0 (13.7 to 96.1)	46.0 (14.7 to 107.0)	45.5 (14.3 to 114.0)	52.6 (9.0 to 180.9)	-0.2 (-12.6 to 12.3)	52.4 (-84.8 to 532.3)
Eritrea	46.4 (15.4 to 105.5)	49.8 (15.3 to 116.7)	48.0 (15.7 to 114.7)	54.7 (9.4 to 189.0)	-0.5 (-12.6 to 11.1)	38.6 (-84.9 to 430.0)
Ethiopia	35.5 (23.7 to 52.8)	38.9 (25.3 to 58.0)	39.0 (25.5 to 58.2)	42.4 (20.5 to 80.2)	0.3 (-4.9 to 5.6)	14.2 (-52.2 to 130.8)
Kenya	28.4 (25.4 to 31.9)	28.5 (25.3 to 32.1)	28.6 (25.5 to 32.4)	28.8 (23.8 to 35.6)	0.0 (-1.4 to 1.5)	1.1 (-19.4 to 25.2)
Madagascar	21.9 (11.6 to 38.3)	22.9 (11.6 to 41.4)	22.5 (11.2 to 38.2)	24.0 (8.1 to 54.3)	-0.2 (-7.6 to 7.1)	14.8 (-68.1 to 191.2)
Malawi	26.1 (9.8 to 60.5)	24.5 (9.7 to 52.9)	23.6 (9.7 to 47.5)	24.8 (6.0 to 68.0)	-0.7 (-10.3 to 9.0)	19.4 (-78.8 to 287.5)
Mozambique	22.7 (9.4 to 48.8)	21.9 (9.5 to 47.5)	21.3 (9.5 to 43.5)	22.6 (5.7 to 58.7)	-0.5 (-9.8 to 8.7)	18.9 (-77.0 to 271.2)
Rwanda	43.5 (15.3 to 92.9)	42.6 (14.5 to 98.5)	43.2 (14.9 to 99.7)	46.6 (8.6 to 148.7)	-0.3 (-12.0 to 10.7)	37.3 (-83.6 to 395.2)
Somalia	31.4 (13.1 to 65.0)	32.0 (12.8 to 66.9)	32.0 (12.8 to 72.1)	36.1 (7.6 to 108.2)	-0.2 (-10.7 to 10.2)	32.2 (-80.0 to 358.9)
South Sudan	44.1 (14.2 to 104.9)	47.8 (15.1 to 108.9)	45.6 (14.7 to 107.0)	51.5 (8.4 to 182.5)	-0.5 (-12.8 to 11.7)	41.1 (-85.3 to 482.6)
Tanzania	29.0 (12.1 to 62.7)	28.2 (12.7 to 63.8)	29.1 (12.3 to 67.1)	31.5 (7.5 to 101.1)	-0.2 (-10.4 to 10.1)	31.9 (-79.0 to 353.4)
Uganda	46.0 (18.8 to 85.8)	49.0 (20.0 to 94.2)	50.5 (21.1 to 97.2)	56.6 (13.9 to 146.3)	0.3 (-9.0 to 9.6)	35.5 (-74.0 to 319.7)
Zambia	30.9 (10.4 to 76.5)	28.4 (10.1 to 61.6)	27.4 (10.4 to 62.2)	28.9 (5.6 to 88.7)	-0.9 (-12.1 to 10.5)	27.0 (-83.6 to 380.9)
Southern Sub-Saharan Africa						
Botswana	25.3 (11.2 to 51.7)	24.3 (11.3 to 47.1)	23.1 (10.3 to 43.9)	24.4 (6.4 to 63.0)	-0.7 (-9.7 to 7.9)	14.3 (-76.6 to 227.6)
Lesotho	31.5 (18.4 to 51.9)	30.9 (17.5 to 49.6)	28.0 (16.0 to 45.8)	27.9 (11.3 to 56.9)	-1.1 (-7.6 to 5.8)	-2.6 (-67.9 to 138.2)
Namibia	27.4 (11.0 to 58.2)	26.9 (10.9 to 57.8)	25.4 (9.6 to 53.2)	27.0 (5.8 to 82.6)	-0.9 (-11.1 to 9.5)	19.2 (-81.0 to 318.6)
South Africa	26.7 (21.9 to 32.5)	26.1 (21.3 to 32.7)	25.1 (20.5 to 31.1)	24.6 (17.4 to 34.8)	-0.5 (-2.8 to 2.0)	-5.0 (-34.4 to 34.6)
Swaziland	18.7 (10.3 to 30.5)	17.9 (10.9 to 27.8)	17.2 (10.2 to 26.2)	17.1 (7.0 to 33.0)	-0.6 (-6.7 to 5.5)	1.7 (-63.2 to 129.9)
Zimbabwe	23.1 (9.1 to 50.3)	21.7 (9.0 to 45.1)	22.2 (9.0 to 47.3)	24.5 (5.5 to 72.2)	-0.1 (-10.3 to 9.9)	34.9 (-78.6 to 341.0)
Western Sub-Saharan Africa						
Benin	39.8 (14.7 to 104.7)	40.5 (14.2 to 115.1)	39.0 (13.4 to 112.5)	43.1 (8.8 to 162.5)	-0.6 (-12.2 to 10.9)	33.6 (-83.9 to 415.4)
Burkina Faso	44.9 (13.5 to 119.5)	46.6 (11.7 to 122.6)	42.9 (12.2 to 110.0)	46.4 (7.0 to 162.4)	-1.1 (-14.2 to 12.1)	40.8 (-88.2 to 514.3)
Cameroon	70.8 (23.4 to 166.7)	77.0 (21.8 to 180.4)	72.8 (22.2 to 167.7)	79.4 (13.1 to 255.1)	-0.7 (-12.8 to 10.5)	30.2 (-85.4 to 384.7)
Cape Verde	36.6 (14.9 to 75.2)	38.6 (16.1 to 75.5)	34.8 (15.7 to 68.3)	36.6 (9.8 to 100.2)	-1.0 (-10.1 to 7.6)	10.0 (-78.0 to 213.3)
Chad	66.0 (16.4 to 192.6)	68.7 (16.8 to 194.5)	66.0 (14.9 to 194.0)	80.3 (7.4 to 319.6)	-0.8 (-16.4 to 14.5)	72.6 (-91.5 to 774.5)
Cote d'Ivoire	25.3 (8.8 to 60.6)	27.7 (9.0 to 68.3)	25.9 (8.8 to 57.9)	28.8 (5.6 to 84.8)	-0.5 (-12.6 to 11.3)	37.8 (-85.0 to 440.8)
The Gambia	34.6 (15.5 to 66.1)	36.3 (15.7 to 73.5)	34.0 (15.4 to 67.3)	36.5 (10.3 to 99.3)	-0.6 (-9.4 to 8.5)	17.7 (-75.5 to 259.1)
Ghana	35.0 (12.0 to 85.3)	34.7 (12.2 to 87.1)	34.7 (11.6 to 85.8)	37.1 (6.9 to 122.4)	-0.5 (-12.3 to 10.2)	31.4 (-84.3 to 358.7)
Guinea	24.0 (8.5 to 56.2)	26.9 (9.2 to 65.5)	26.1 (8.6 to 68.9)	29.7 (5.3 to 93.9)	-0.4 (-12.1 to 11.1)	42.0 (-83.7 to 428.1)
Guinea-Bissau	30.5 (12.7 to 59.6)	31.8 (12.0 to 67.0)	29.8 (12.4 to 59.4)	32.0 (7.8 to 88.2)	-0.6 (-11.0 to 9.2)	21.9 (-80.8 to 298.8)
Liberia	17.2 (8.5 to 30.4)	18.2 (8.8 to 34.4)	18.0 (8.9 to 34.1)	19.5 (6.2 to 47.1)	-0.1 (-8.5 to 8.5)	20.7 (-72.0 to 256.8)
Mali	39.8 (10.1 to 117.1)	41.9 (9.8 to 122.5)	38.5 (9.7 to 116.4)	44.1 (5.3 to 163.6)	-1.1 (-15.7 to 13.3)	61.6 (-90.5 to 637.8)
Mauritania	48.9 (12.5 to 138.2)	49.5 (12.3 to 137.6)	47.4 (11.7 to 135.4)	52.7 (6.7 to 199.5)	-0.8 (-14.1 to 13.8)	55.9 (-88.0 to 687.5)
Niger	87.5 (19.0 to 264.3)	87.3 (18.7 to 273.4)	94.0 (18.6 to 306.8)	109.2 (8.8 to 463.6)	-0.2 (-15.9 to 14.9)	86.1 (-90.8 to 835.7)
Nigeria	67.5 (20.0 to 213.4)	75.4 (18.6 to 262.4)	71.8 (18.8 to 270.6)	83.0 (10.1 to 400.8)	-0.8 (-15.2 to 13.9)	61.5 (-89.8 to 710.5)
Sao Tome and Principe	30.3 (14.2 to 56.6)	28.4 (14.0 to 54.2)	28.5 (13.1 to 52.2)	30.2 (8.9 to 79.8)	-0.3 (-8.9 to 8.1)	17.9 (-73.6 to 236.1)
Senegal	41.4 (15.9 to 82.0)	44.3 (14.0 to 94.2)	40.7 (15.2 to 79.1)	44.1 (9.9 to 111.5)	-0.6 (-10.9 to 9.3)	23.2 (-80.6 to 302.5)
Sierra Leone	20.5 (9.6 to 38.4)	22.5 (8.9 to 46.1)	21.6 (9.2 to 44.2)	23.8 (5.7 to 64.5)	-0.3 (-10.3 to 9.2)	25.9 (-78.5 to 299.3)
Togo	37.7 (13.1 to 91.1)	38.4 (13.0 to 91.2)	35.7 (12.9 to 79.5)	38.1 (8.1 to 122.6)	-0.9 (-10.9 to 10.3)	23.7 (-80.4 to 369.0)
**Indicator 16.1.1: Age-standardised death rate due to interpersonal violence (per 100,000 population)**						
Central Europe, Eastern Europe, and Central Asia						
Central Asia						
Armenia	4.0 (3.7 to 4.2)	3.2 (3.0 to 3.4)	3.2 (2.9 to 3.4)	2.7 (2.0 to 3.8)	-1.2 (-3.0 to 1.1)	-15.4 (-35.8 to 17.1)
Azerbaijan	5.7 (4.5 to 6.8)	3.4 (2.5 to 5.8)	3.4 (2.5 to 5.8)	2.9 (2.0 to 5.1)	-1.0 (-2.6 to 0.6)	-13.7 (-32.0 to 10.1)
Georgia	3.7 (3.5 to 3.9)	3.2 (3.0 to 3.5)	3.4 (3.1 to 3.8)	2.9 (2.2 to 3.9)	-0.7 (-2.6 to 1.3)	-9.3 (-31.9 to 21.5)
Kazakhstan	17.5 (17.0 to 18.1)	7.7 (7.2 to 8.2)	7.9 (7.3 to 8.6)	7.0 (4.9 to 9.6)	-0.7 (-3.0 to 1.4)	-8.8 (-36.6 to 23.9)
Kyrgyzstan	9.4 (8.8 to 10.0)	4.8 (4.5 to 5.1)	4.4 (4.0 to 4.8)	3.7 (2.7 to 4.8)	-1.8 (-3.7 to 0.0)	-23.0 (-42.8 to 0.4)
Mongolia	11.9 (8.6 to 14.5)	7.8 (6.2 to 9.6)	7.6 (6.0 to 9.3)	6.9 (4.9 to 9.3)	-0.8 (-2.2 to 0.6)	-10.6 (-28.6 to 9.6)
Tajikistan	6.3 (3.5 to 7.2)	3.0 (2.5 to 4.8)	3.0 (2.5 to 4.7)	2.6 (1.8 to 4.0)	-1.0 (-2.7 to 0.6)	-13.7 (-33.4 to 9.1)
Turkmenistan	6.0 (5.7 to 6.2)	3.9 (3.6 to 4.2)	3.9 (3.5 to 4.4)	3.5 (2.6 to 4.6)	-0.8 (-2.5 to 1.2)	-10.6 (-31.8 to 20.1)
Uzbekistan	3.7 (3.6 to 3.9)	2.5 (2.3 to 2.7)	2.5 (2.2 to 2.9)	2.4 (1.8 to 3.0)	-0.4 (-1.9 to 1.0)	-5.0 (-24.9 to 16.5)
Central Europe						
Albania	8.8 (2.8 to 10.7)	2.3 (1.8 to 3.2)	2.3 (1.8 to 3.2)	1.9 (1.3 to 2.8)	-1.3 (-2.9 to 0.1)	-17.2 (-35.4 to 1.2)
Bosnia and Herzegovina	2.9 (1.8 to 3.6)	2.0 (1.4 to 2.3)	2.0 (1.4 to 2.3)	1.7 (1.1 to 2.1)	-1.2 (-2.3 to 0.1)	-16.1 (-29.6 to 1.1)
Bulgaria	3.9 (3.7 to 4.0)	1.9 (1.8 to 2.1)	2.0 (1.8 to 2.1)	1.4 (1.1 to 1.7)	-2.4 (-4.0 to -0.8)	-29.9 (-45.1 to -11.9)
Croatia	2.2 (2.1 to 2.3)	0.9 (0.9 to 1.0)	0.9 (0.8 to 1.0)	0.7 (0.5 to 0.8)	-2.6 (-3.7 to -1.3)	-31.5 (-42.5 to -17.7)
Czech Republic	1.8 (1.7 to 1.9)	1.0 (0.9 to 1.0)	0.9 (0.9 to 1.0)	0.7 (0.6 to 0.8)	-2.5 (-3.6 to -1.4)	-31.4 (-41.5 to -19.4)
Hungary	2.5 (2.5 to 2.7)	1.4 (1.3 to 1.5)	1.2 (1.1 to 1.3)	0.8 (0.7 to 1.1)	-3.3 (-4.5 to -1.7)	-38.5 (-49.0 to -22.7)
Macedonia	2.9 (1.4 to 3.3)	1.5 (1.1 to 1.7)	1.5 (1.1 to 1.8)	1.3 (0.9 to 1.6)	-1.0 (-2.2 to 0.3)	-13.6 (-28.5 to 5.1)
Montenegro	5.0 (3.9 to 5.8)	2.9 (2.5 to 3.8)	2.9 (2.4 to 3.8)	2.2 (1.7 to 3.0)	-2.0 (-3.2 to -0.8)	-25.1 (-37.8 to -10.7)
Poland	2.8 (2.6 to 2.9)	1.4 (1.3 to 1.4)	1.4 (1.2 to 1.5)	0.9 (0.8 to 1.1)	-2.5 (-3.6 to -1.3)	-30.8 (-42.1 to -18.2)
Romania	3.1 (3.0 to 3.2)	1.6 (1.5 to 1.7)	1.6 (1.5 to 1.7)	1.1 (0.8 to 1.4)	-2.8 (-4.4 to -1.1)	-33.6 (-48.5 to -14.7)
Serbia	4.1 (2.4 to 4.5)	2.1 (1.6 to 2.5)	2.0 (1.5 to 2.4)	1.7 (1.4 to 2.4)	-1.3 (-2.7 to 0.3)	-17.7 (-33.2 to 4.3)
Slovakia	3.0 (2.5 to 3.7)	2.0 (1.8 to 2.8)	1.9 (1.6 to 2.6)	1.4 (1.1 to 2.0)	-2.3 (-3.6 to -1.1)	-29.1 (-41.3 to -15.3)
Slovenia	1.6 (1.5 to 1.7)	0.8 (0.7 to 0.8)	0.8 (0.7 to 0.9)	0.5 (0.4 to 0.7)	-2.6 (-4.1 to -1.1)	-32.4 (-45.5 to -15.3)
Eastern Europe						

	Estimate in 2000 (95% UIs)	Estimate in 2015 (95% UIs)	Estimate in 2017 (95% UIs)	Estimate in 2030 (95% UIs)	Annualised rate of change, 2015-2030 (95% UIs)	Percent change, 2015-2030 (95% UIs)
Belarus	11.5 (11.1 to 12.0)	5.4 (5.0 to 5.8)	5.2 (4.7 to 5.7)	4.2 (3.2 to 5.5)	-1.7 (-3.4 to 0.0)	-22.2 (-40.2 to 0.6)
Estonia	13.4 (12.7 to 14.1)	3.6 (3.3 to 4.0)	3.7 (3.1 to 4.3)	2.5 (1.6 to 3.8)	-2.5 (-5.5 to 0.2)	-30.1 (-56.1 to 3.3)
Latvia	11.6 (11.1 to 12.1)	5.4 (5.1 to 5.8)	5.4 (4.7 to 6.2)	3.9 (2.4 to 6.0)	-2.4 (-5.4 to 0.7)	-27.9 (-55.2 to 10.2)
Lithuania	9.1 (8.7 to 9.6)	5.0 (4.7 to 5.3)	4.8 (4.4 to 5.3)	3.9 (2.9 to 5.2)	-1.8 (-3.7 to 0.2)	-22.7 (-42.5 to 2.5)
Moldova	10.9 (10.4 to 11.4)	5.1 (4.8 to 5.4)	4.8 (4.5 to 5.1)	4.4 (3.1 to 6.1)	-1.1 (-3.2 to 1.1)	-13.6 (-38.6 to 18.8)
Russia	29.7 (29.2 to 30.1)	12.8 (12.6 to 13.1)	12.2 (11.9 to 12.5)	9.3 (5.8 to 14.2)	-2.3 (-5.4 to 0.7)	-27.4 (-55.3 to 11.4)
Ukraine	13.9 (13.3 to 14.5)	9.2 (8.7 to 9.8)	8.4 (7.7 to 9.2)	7.0 (4.5 to 10.3)	-2.0 (-4.8 to 0.8)	-24.2 (-51.5 to 12.5)
High-income						
Australasia						
Australia	1.7 (1.6 to 1.8)	1.3 (1.2 to 1.4)	1.3 (1.1 to 1.4)	1.0 (0.8 to 1.1)	-2.0 (-3.0 to -1.0)	-25.4 (-36.3 to -13.5)
New Zealand	1.7 (1.6 to 1.8)	1.3 (1.2 to 1.4)	1.3 (1.2 to 1.4)	0.9 (0.8 to 1.1)	-2.0 (-2.8 to -1.2)	-26.1 (-34.6 to -15.9)
High-income Asia Pacific						
Brunei	1.4 (1.0 to 1.6)	1.4 (1.0 to 1.6)	1.4 (1.0 to 1.6)	1.2 (0.8 to 1.4)	-1.2 (-2.2 to -0.3)	-16.7 (-28.3 to -3.7)
Japan	0.8 (0.8 to 0.8)	0.5 (0.5 to 0.5)	0.5 (0.4 to 0.5)	0.3 (0.3 to 0.4)	-2.0 (-2.7 to -1.3)	-25.8 (-33.5 to -17.5)
South Korea	2.2 (2.1 to 2.3)	1.1 (1.0 to 1.1)	1.0 (0.9 to 1.1)	0.8 (0.7 to 0.9)	-2.1 (-3.1 to -1.1)	-27.1 (-37.3 to -14.7)
Singapore	1.3 (1.2 to 1.4)	0.4 (0.4 to 0.4)	0.4 (0.4 to 0.4)	0.3 (0.3 to 0.4)	-2.0 (-3.1 to -0.9)	-25.4 (-37.6 to -12.9)
High-income North America						
Canada	1.3 (1.3 to 1.4)	1.6 (1.4 to 1.7)	1.5 (1.3 to 1.6)	1.1 (1.0 to 1.3)	-2.2 (-2.9 to -1.4)	-27.5 (-34.8 to -19.5)
Greenland	13.8 (10.6 to 15.8)	7.9 (6.6 to 9.5)	7.6 (6.4 to 9.3)	6.2 (4.5 to 8.5)	-1.7 (-3.4 to 0.4)	-22.0 (-40.1 to 5.5)
United States	6.8 (6.7 to 6.9)	5.9 (5.8 to 6.0)	6.2 (6.0 to 6.5)	4.9 (3.9 to 5.8)	-1.3 (-2.7 to -0.1)	-17.3 (-33.1 to -1.7)
Southern Latin America						
Argentina	6.3 (6.1 to 6.5)	6.0 (5.6 to 6.3)	5.8 (5.0 to 6.7)	4.8 (4.1 to 5.8)	-1.4 (-2.4 to -0.3)	-18.8 (-30.6 to -3.9)
Chile	4.8 (4.6 to 5.0)	4.2 (4.0 to 4.5)	4.1 (3.6 to 4.8)	3.3 (2.8 to 4.0)	-1.6 (-2.7 to -0.5)	-21.5 (-33.7 to -7.5)
Uruguay	5.0 (4.7 to 5.2)	6.2 (5.8 to 6.6)	5.8 (5.0 to 6.7)	5.2 (4.2 to 6.5)	-1.2 (-2.5 to 0.3)	-15.7 (-31.3 to 5.0)
Western Europe						
Andorra	0.8 (0.7 to 1.3)	0.7 (0.5 to 1.0)	0.6 (0.5 to 1.0)	0.5 (0.4 to 0.8)	-1.6 (-2.4 to -0.8)	-21.4 (-29.7 to -11.8)
Austria	0.9 (0.8 to 0.9)	0.7 (0.7 to 0.8)	0.7 (0.6 to 0.7)	0.5 (0.4 to 0.6)	-2.6 (-3.4 to -1.8)	-31.9 (-39.6 to -23.6)
Belgium	2.1 (2.0 to 2.2)	1.2 (1.1 to 1.3)	1.2 (1.1 to 1.3)	0.9 (0.8 to 1.0)	-2.2 (-3.0 to -1.4)	-27.7 (-35.8 to -18.6)
Cyprus	2.1 (1.3 to 2.5)	1.4 (0.9 to 1.6)	1.4 (0.8 to 1.6)	1.0 (0.6 to 1.3)	-2.1 (-3.1 to -1.0)	-27.1 (-37.5 to -13.9)
Denmark	1.4 (1.4 to 1.5)	0.7 (0.7 to 0.7)	0.7 (0.7 to 0.8)	0.5 (0.4 to 0.6)	-1.9 (-3.1 to -0.7)	-24.1 (-36.8 to -9.4)
Finland	2.7 (2.5 to 2.8)	1.4 (1.3 to 1.5)	1.4 (1.3 to 1.6)	1.0 (0.9 to 1.2)	-2.0 (-3.1 to -0.8)	-25.2 (-37.6 to -11.2)
France	1.2 (1.1 to 1.2)	0.8 (0.8 to 0.8)	0.8 (0.7 to 0.8)	0.5 (0.5 to 0.6)	-2.7 (-3.4 to -1.9)	-32.7 (-39.9 to -24.8)
Germany	1.1 (1.1 to 1.2)	0.7 (0.7 to 0.7)	0.7 (0.6 to 0.8)	0.5 (0.4 to 0.6)	-2.3 (-3.2 to -1.4)	-28.9 (-38.1 to -18.4)
Greece	1.0 (0.9 to 1.1)	1.0 (1.0 to 1.1)	1.0 (0.9 to 1.1)	0.7 (0.6 to 0.8)	-2.1 (-3.0 to -1.2)	-27.4 (-36.3 to -16.7)
Iceland	0.9 (0.9 to 1.0)	0.7 (0.6 to 0.7)	0.7 (0.6 to 0.7)	0.6 (0.4 to 0.7)	-1.3 (-2.6 to 0.2)	-17.2 (-31.9 to 3.1)
Ireland	1.0 (1.0 to 1.1)	0.7 (0.6 to 0.7)	0.6 (0.6 to 0.7)	0.5 (0.4 to 0.6)	-2.1 (-3.0 to -1.1)	-27.1 (-36.6 to -15.7)
Israel	3.0 (2.8 to 3.2)	2.1 (2.0 to 2.3)	2.1 (1.9 to 2.3)	1.4 (1.1 to 1.6)	-3.0 (-4.1 to -1.9)	-36.4 (-46.2 to -24.3)
Italy	1.2 (1.1 to 1.2)	0.7 (0.7 to 0.7)	0.7 (0.6 to 0.7)	0.5 (0.4 to 0.5)	-2.9 (-3.6 to -2.1)	-34.8 (-41.5 to -27.4)
Luxembourg	1.5 (1.4 to 1.6)	0.9 (0.9 to 1.0)	0.9 (0.8 to 1.0)	0.7 (0.5 to 0.9)	-2.2 (-3.9 to -0.5)	-27.5 (-44.6 to -7.8)
Malta	1.4 (1.2 to 1.4)	1.1 (1.0 to 1.1)	1.1 (1.0 to 1.2)	0.7 (0.6 to 0.9)	-2.4 (-3.8 to -0.9)	-30.1 (-43.1 to -13.0)
Netherlands	1.4 (1.4 to 1.5)	0.8 (0.7 to 0.8)	0.8 (0.7 to 0.9)	0.6 (0.5 to 0.7)	-2.0 (-2.8 to -1.3)	-26.0 (-34.3 to -17.5)
Norway	1.1 (1.1 to 1.2)	0.7 (0.6 to 0.7)	0.7 (0.6 to 0.7)	0.5 (0.5 to 0.6)	-1.4 (-2.3 to -0.5)	-19.0 (-28.8 to -6.8)
Portugal	2.2 (2.1 to 2.4)	1.1 (1.0 to 1.2)	1.1 (0.9 to 1.1)	0.7 (0.6 to 0.8)	-2.9 (-4.1 to -1.7)	-35.5 (-45.8 to -22.9)
Spain	1.0 (0.9 to 1.0)	0.6 (0.6 to 0.6)	0.6 (0.5 to 0.6)	0.4 (0.3 to 0.5)	-3.4 (-4.6 to -2.0)	-39.9 (-49.6 to -25.9)
Sweden	1.3 (1.2 to 1.4)	1.1 (1.1 to 1.2)	1.1 (1.0 to 1.2)	0.8 (0.7 to 0.9)	-2.4 (-3.2 to -1.5)	-30.0 (-38.3 to -20.3)
Switzerland	1.1 (1.1 to 1.2)	0.6 (0.6 to 0.6)	0.6 (0.5 to 0.6)	0.4 (0.3 to 0.5)	-2.7 (-4.0 to -1.5)	-33.2 (-44.8 to -20.2)
United Kingdom	0.8 (0.7 to 0.8)	0.5 (0.5 to 0.5)	0.5 (0.5 to 0.5)	0.4 (0.3 to 0.4)	-2.0 (-2.7 to -1.3)	-25.8 (-33.4 to -18.0)
Latin America and Caribbean						
Andean Latin America						
Bolivia	9.0 (7.2 to 11.3)	6.1 (4.2 to 8.0)	5.9 (3.9 to 7.8)	4.7 (3.1 to 6.4)	-1.7 (-2.4 to -1.0)	-22.8 (-30.6 to -14.4)
Ecuador	20.2 (19.5 to 20.9)	13.0 (12.1 to 14.1)	13.4 (11.8 to 15.2)	11.2 (9.7 to 12.9)	-1.0 (-2.0 to -0.1)	-14.1 (-25.5 to -1.5)
Peru	4.8 (3.7 to 5.6)	3.6 (2.6 to 4.2)	3.5 (2.5 to 4.3)	2.6 (1.8 to 3.4)	-2.1 (-3.7 to -0.7)	-27.0 (-42.7 to -9.4)
Caribbean						
Antigua and Barbuda	4.4 (4.0 to 4.8)	4.9 (4.5 to 5.4)	4.9 (4.4 to 5.5)	4.5 (3.3 to 6.5)	-0.7 (-2.7 to 1.9)	-8.4 (-33.2 to 32.2)
The Bahamas	19.1 (18.0 to 20.0)	27.5 (25.4 to 29.7)	26.2 (23.5 to 29.2)	23.5 (17.8 to 32.6)	-1.1 (-2.8 to 1.1)	-14.6 (-33.8 to 17.5)
Barbados	9.7 (9.0 to 10.4)	10.5 (9.5 to 11.4)	10.4 (9.3 to 11.5)	9.0 (7.6 to 10.8)	-1.0 (-1.9 to 0.0)	-13.8 (-24.9 to 0.6)
Belize	20.8 (19.7 to 21.9)	25.6 (24.2 to 27.0)	25.6 (23.9 to 27.4)	22.3 (17.8 to 29.1)	-1.0 (-2.4 to 0.8)	-13.1 (-30.3 to 13.0)
Bermuda	2.8 (2.5 to 3.1)	2.9 (2.5 to 3.2)	2.8 (2.5 to 3.3)	1.9 (1.1 to 3.8)	-3.0 (-6.2 to 1.9)	-32.4 (-60.7 to 32.1)
Cuba	5.2 (5.0 to 5.4)	5.4 (5.2 to 5.7)	5.4 (4.8 to 6.2)	4.2 (3.4 to 5.1)	-1.8 (-3.0 to -0.4)	-22.9 (-36.7 to -6.5)
Dominica	6.2 (5.7 to 6.7)	11.4 (10.4 to 12.4)	11.4 (10.2 to 12.5)	10.5 (8.4 to 13.3)	-0.6 (-1.9 to 0.8)	-8.0 (-24.7 to 13.5)
Dominican Republic	13.5 (11.4 to 16.2)	21.8 (12.4 to 25.9)	21.1 (12.4 to 26.0)	18.0 (10.2 to 22.8)	-1.3 (-2.4 to -0.2)	-17.3 (-30.2 to -3.0)
Grenada	4.9 (4.5 to 5.3)	4.9 (4.4 to 5.3)	5.0 (4.6 to 5.6)	4.5 (3.7 to 5.4)	-0.6 (-1.7 to 0.5)	-8.6 (-22.1 to 7.2)
Guyana	16.2 (15.2 to 17.1)	17.3 (15.7 to 18.9)	16.4 (14.1 to 18.9)	14.8 (11.4 to 19.7)	-1.1 (-2.6 to 0.8)	-14.7 (-32.3 to 13.3)
Haiti	21.2 (15.2 to 30.8)	18.7 (12.5 to 27.6)	18.2 (12.2 to 27.5)	14.5 (9.6 to 21.6)	-1.7 (-2.3 to -1.1)	-22.5 (-29.5 to -14.7)
Jamaica	16.7 (16.1 to 17.4)	32.7 (28.1 to 37.7)	29.0 (24.0 to 34.8)	26.9 (20.5 to 35.7)	-1.3 (-2.6 to 0.3)	-17.7 (-32.1 to 4.3)
Puerto Rico	19.3 (18.7 to 19.9)	20.2 (19.5 to 20.9)	21.5 (19.8 to 23.1)	13.9 (11.0 to 17.5)	-2.5 (-4.0 to -1.0)	-31.2 (-45.4 to -14.3)
Saint Lucia	14.5 (13.7 to 15.3)	17.1 (15.8 to 18.3)	16.9 (15.4 to 18.4)	14.4 (12.2 to 17.7)	-1.1 (-2.1 to 0.1)	-15.5 (-27.3 to 1.6)
Saint Vincent and the Grenadines	14.3 (13.4 to 15.2)	19.8 (18.4 to 21.2)	19.7 (18.1 to 21.4)	17.9 (14.6 to 22.3)	-0.7 (-1.8 to 0.7)	-9.9 (-23.9 to 11.6)
Suriname	8.8 (7.9 to 9.7)	8.7 (7.8 to 9.7)	8.7 (7.6 to 10.0)	7.5 (6.2 to 8.9)	-1.0 (-1.9 to -0.1)	-13.7 (-24.5 to -1.3)
Trinidad and Tobago	11.0 (10.5 to 11.6)	22.0 (17.9 to 26.6)	21.2 (16.6 to 26.4)	19.2 (14.3 to 26.4)	-0.9 (-2.6 to 1.0)	-12.3 (-31.8 to 15.7)
Virgin Islands, U.S.	20.3 (19.2 to 21.5)	26.2 (23.7 to 29.8)	26.3 (23.6 to 30.0)	16.0 (13.0 to 19.7)	-3.3 (-4.5 to -2.1)	-38.8 (-49.2 to -26.9)
Central Latin America						
Colombia	81.3 (80.5 to 82.1)	27.5 (26.3 to 29.8)	28.3 (24.3 to 33.1)	21.9 (17.1 to 27.7)	-1.6 (-3.1 to -0.0)	-20.5 (-37.2 to -0.5)
Costa Rica	6.0 (5.8 to 6.3)	9.3 (8.8 to 9.9)	9.4 (8.5 to 10.4)	7.9 (6.8 to 9.0)	-1.1 (-1.9 to -0.3)	-15.2 (-25.2 to -5.1)
El Salvador	56.2 (44.4 to 60.9)	105.8 (90.5 to 114.2)	53.5 (38.3 to 65.4)	46.7 (31.4 to 60.2)	-5.5 (-7.1 to -4.0)	-56.1 (-65.4 to -44.9)
Guatemala	37.8 (36.8 to 39.1)	33.2 (32.0 to 34.4)	35.8 (31.6 to 40.3)	31.3 (26.2 to 37.4)	-0.4 (-1.5 to 0.8)	-5.7 (-20.4 to 12.9)
Honduras	54.9 (40.5 to 66.2)	102.6 (84.9 to 115.2)	46.2 (30.0 to 58.2)	39.1 (25.4 to 49.2)	-6.5 (-8.1 to -5.6)	-62.1 (-70.4 to -56.5)
Mexico	13.4 (13.2 to 13.6)	21.8 (21.5 to 22.0)	32.4 (31.9 to 32.9)	27.4 (22.9 to 33.9)	1.5 (0.3 to 2.9)	25.7 (5.1 to 55.6)
Nicaragua	12.3 (9.6 to 14.0)	7.7 (6.7 to 10.6)	7.3 (6.0 to 10.4)	6.3 (5.0 to 8.8)	-1.3 (-2.4 to -0.1)	-18.0 (-30.3 to -1.2)
Panama	10.2 (9.8 to 10.7)	16.9 (16.1 to 17.6)	15.8 (14.4 to 17.3)	13.0 (10.8 to 15.5)	-1.8 (-2.9 to -0.6)	-22.8 (-35.3 to -8.0)

	Estimate in 2000 (95% UIs)	Estimate in 2015 (95% UIs)	Estimate in 2017 (95% UIs)	Estimate in 2030 (95% UIs)	Annualised rate of change, 2015-2030 (95% UIs)	Percent change, 2015-2030 (95% UIs)
Venezuela	33.2 (32.5 to 34.0)	44.3 (41.1 to 47.5)	44.1 (36.5 to 52.3)	42.7 (29.8 to 60.6)	-0.4 (-2.4 to 1.9)	-3.8 (-30.7 to 32.4)
Tropical Latin America						
Brazil	29.8 (29.3 to 30.3)	27.8 (27.4 to 28.1)	27.7 (27.2 to 28.1)	24.3 (21.4 to 27.0)	-0.9 (-1.8 to -0.2)	-12.5 (-23.2 to -3.0)
Paraguay	18.2 (13.2 to 20.2)	11.8 (9.6 to 15.6)	11.9 (9.1 to 15.9)	10.6 (7.7 to 14.4)	-0.7 (-2.3 to 0.8)	-9.6 (-28.9 to 12.2)
North Africa and Middle East						
North Africa and Middle East						
Afghanistan	16.2 (8.8 to 21.9)	14.3 (8.8 to 19.0)	12.1 (6.9 to 16.6)	9.7 (5.5 to 13.5)	-2.6 (-3.4 to -1.9)	-32.6 (-40.2 to -25.1)
Algeria	1.7 (1.0 to 2.1)	1.1 (0.7 to 1.3)	1.0 (0.7 to 1.2)	0.8 (0.5 to 1.0)	-1.9 (-2.6 to -1.2)	-24.3 (-32.1 to -16.3)
Bahrain	2.0 (1.6 to 2.4)	1.4 (0.9 to 1.6)	1.3 (0.9 to 1.6)	1.0 (0.7 to 1.4)	-2.1 (-3.3 to -0.7)	-26.2 (-39.3 to -9.9)
Egypt	0.4 (0.4 to 0.6)	0.7 (0.4 to 0.8)	0.7 (0.4 to 0.8)	0.5 (0.3 to 0.7)	-1.7 (-2.8 to -0.7)	-22.6 (-34.7 to -10.1)
Iran	2.5 (2.0 to 3.1)	2.3 (1.5 to 2.5)	2.2 (1.4 to 2.4)	1.7 (1.2 to 2.0)	-2.1 (-3.1 to -1.2)	-26.9 (-37.4 to -16.1)
Iraq	7.5 (6.0 to 12.1)	6.3 (3.4 to 7.4)	5.7 (3.2 to 6.8)	4.7 (2.6 to 5.7)	-2.0 (-2.6 to -1.4)	-25.9 (-32.5 to -18.9)
Jordan	4.0 (2.6 to 4.7)	2.6 (1.5 to 3.1)	2.6 (1.5 to 3.1)	2.1 (1.1 to 2.6)	-1.6 (-2.5 to -0.8)	-21.7 (-30.9 to -11.5)
Kuwait	1.8 (1.7 to 1.9)	0.8 (0.7 to 0.8)	0.8 (0.7 to 0.9)	0.6 (0.4 to 0.9)	-1.6 (-4.0 to 0.9)	-20.3 (-44.9 to 14.7)
Lebanon	5.9 (4.2 to 7.0)	6.0 (4.2 to 7.3)	4.1 (2.3 to 5.4)	3.2 (1.7 to 4.4)	-4.3 (-6.0 to -2.8)	-47.2 (-59.6 to -34.5)
Libya	2.2 (1.5 to 2.7)	11.7 (10.6 to 12.5)	2.1 (1.1 to 2.9)	1.8 (1.0 to 2.5)	-12.6 (-16.0 to -10.7)	-84.5 (-91.0 to -79.8)
Morocco	1.3 (0.8 to 1.6)	1.1 (0.6 to 1.5)	1.1 (0.6 to 1.4)	0.9 (0.5 to 1.2)	-1.5 (-2.3 to -0.8)	-20.3 (-28.7 to -11.5)
Palestine	2.8 (2.3 to 5.0)	6.6 (2.3 to 8.2)	5.7 (2.2 to 7.1)	5.4 (1.9 to 7.7)	-1.3 (-2.6 to 0.5)	-17.3 (-32.7 to 7.3)
Oman	0.7 (0.5 to 0.9)	0.5 (0.4 to 0.7)	0.5 (0.4 to 0.7)	0.4 (0.3 to 0.6)	-1.4 (-2.5 to -0.3)	-18.1 (-31.6 to -4.2)
Qatar	0.9 (0.7 to 1.5)	0.8 (0.6 to 1.1)	0.8 (0.6 to 1.0)	0.6 (0.4 to 0.8)	-1.9 (-3.2 to -0.5)	-24.5 (-37.9 to -7.6)
Saudi Arabia	0.8 (0.5 to 0.9)	0.8 (0.4 to 1.0)	0.7 (0.4 to 1.0)	0.5 (0.2 to 0.7)	-2.9 (-4.5 to -1.5)	-35.3 (-49.2 to -20.6)
Sudan	2.2 (2.1 to 2.4)	3.5 (2.6 to 3.9)	1.1 (0.3 to 1.5)	0.9 (0.2 to 1.3)	-9.4 (-15.8 to -7.5)	-74.4 (-90.7 to -67.3)
Syria	2.5 (1.5 to 3.0)	6.3 (5.3 to 6.9)	2.0 (1.1 to 2.6)	1.7 (0.9 to 2.3)	-8.9 (-12.0 to -7.3)	-73.2 (-83.5 to -66.4)
Tunisia	0.8 (0.5 to 0.9)	0.6 (0.4 to 0.7)	0.5 (0.4 to 0.7)	0.5 (0.3 to 0.6)	-1.6 (-2.3 to -0.8)	-20.7 (-29.7 to -11.2)
Turkey	2.6 (2.0 to 3.2)	2.0 (1.2 to 2.3)	2.0 (1.2 to 2.4)	1.4 (0.8 to 1.9)	-2.3 (-3.7 to -0.9)	-28.4 (-42.5 to -12.6)
United Arab Emirates	1.1 (0.9 to 1.5)	1.0 (0.7 to 1.4)	1.0 (0.7 to 1.4)	0.6 (0.4 to 0.9)	-3.3 (-4.3 to -2.3)	-39.2 (-47.5 to -29.2)
Yemen	3.7 (2.5 to 5.0)	4.3 (3.2 to 5.5)	3.1 (2.0 to 4.2)	2.5 (1.6 to 3.5)	-3.6 (-4.7 to -2.7)	-41.8 (-50.5 to -33.4)
South Asia						
South Asia						
Bangladesh	2.5 (2.0 to 4.3)	1.6 (1.3 to 2.6)	1.5 (1.2 to 2.5)	1.2 (0.9 to 2.0)	-1.8 (-2.4 to -1.1)	-23.2 (-30.6 to -15.8)
Bhutan	1.8 (1.0 to 2.1)	1.0 (0.6 to 1.2)	0.9 (0.6 to 1.2)	0.7 (0.5 to 0.9)	-1.9 (-2.4 to -1.3)	-24.3 (-30.7 to -17.3)
India	4.5 (3.4 to 5.1)	3.1 (2.5 to 3.8)	3.0 (2.5 to 3.8)	2.8 (2.2 to 3.5)	-0.8 (-1.5 to -0.1)	-10.6 (-19.9 to -1.3)
Nepal	1.9 (0.9 to 2.4)	1.7 (0.7 to 2.3)	1.6 (0.7 to 2.2)	1.4 (0.6 to 1.9)	-1.4 (-2.0 to -0.8)	-19.2 (-25.9 to -11.7)
Pakistan	5.6 (3.7 to 6.5)	4.5 (2.8 to 5.9)	4.2 (2.7 to 5.6)	3.5 (2.1 to 4.8)	-1.7 (-2.5 to -0.8)	-22.1 (-31.7 to -10.9)
Southeast Asia, East Asia, and Oceania						
East Asia						
China	2.6 (1.8 to 2.8)	1.0 (0.9 to 1.4)	0.9 (0.8 to 1.3)	0.7 (0.6 to 1.1)	-2.1 (-2.9 to -1.2)	-26.8 (-35.2 to -16.6)
North Korea	3.1 (2.2 to 4.0)	2.6 (1.9 to 3.4)	2.6 (1.9 to 3.4)	2.3 (1.6 to 3.1)	-0.8 (-1.6 to -0.0)	-11.3 (-21.5 to -0.0)
Taiwan (Province of China)	3.1 (3.0 to 3.2)	1.1 (1.1 to 1.2)	1.2 (1.1 to 1.3)	0.9 (0.8 to 1.1)	-1.2 (-2.3 to -0.1)	-16.3 (-28.9 to -2.1)
Oceania						
American Samoa	6.5 (5.4 to 8.4)	5.5 (4.7 to 7.7)	5.4 (4.6 to 7.6)	4.6 (3.8 to 6.3)	-1.2 (-1.8 to -0.6)	-16.2 (-24.0 to -8.0)
Federated States of Micronesia	6.6 (4.4 to 9.2)	5.8 (3.4 to 8.1)	5.7 (3.3 to 8.2)	5.1 (2.9 to 7.6)	-0.9 (-2.1 to 0.2)	-12.1 (-26.5 to 3.2)
Fiji	4.7 (3.0 to 5.3)	4.0 (2.5 to 4.7)	3.9 (2.4 to 4.7)	3.7 (2.2 to 4.9)	-0.6 (-1.7 to 0.8)	-7.7 (-22.7 to 12.5)
Guam	3.7 (3.2 to 4.8)	4.7 (3.7 to 5.7)	4.7 (3.7 to 5.7)	3.5 (2.6 to 4.5)	-1.9 (-3.1 to -0.8)	-24.7 (-37.3 to -10.8)
Kiribati	2.2 (1.6 to 5.2)	1.8 (1.1 to 4.8)	1.8 (1.1 to 4.7)	1.5 (0.9 to 4.1)	-1.1 (-1.7 to -0.5)	-15.6 (-22.9 to -7.1)
Marshall Islands	7.1 (5.1 to 8.7)	6.4 (4.3 to 8.0)	6.2 (4.2 to 7.8)	5.3 (3.4 to 7.0)	-1.3 (-2.1 to -0.5)	-17.0 (-27.0 to -6.6)
Northern Mariana Islands	5.1 (4.0 to 6.2)	4.7 (3.8 to 6.1)	4.7 (3.8 to 6.3)	3.9 (3.1 to 5.3)	-1.2 (-1.9 to -0.5)	-16.6 (-25.0 to -7.2)
Papua New Guinea	12.6 (7.7 to 16.4)	11.1 (6.6 to 14.5)	10.8 (6.5 to 14.1)	8.9 (5.2 to 11.9)	-1.5 (-2.1 to -0.9)	-19.8 (-26.9 to -12.4)
Samoa	4.1 (2.8 to 5.7)	3.4 (2.2 to 5.2)	3.4 (2.2 to 5.2)	2.9 (1.9 to 4.4)	-1.2 (-1.9 to -0.6)	-16.8 (-24.3 to -9.2)
Solomon Islands	6.5 (4.2 to 8.3)	5.7 (3.5 to 7.4)	5.6 (3.4 to 7.2)	4.7 (2.8 to 6.2)	-1.3 (-2.0 to -0.7)	-17.9 (-25.7 to -9.5)
Tonga	2.8 (2.0 to 6.4)	2.3 (1.4 to 6.5)	2.3 (1.3 to 6.4)	2.0 (1.2 to 5.8)	-0.9 (-1.6 to -0.3)	-13.1 (-21.0 to -4.5)
Vanuatu	2.0 (1.3 to 2.8)	1.8 (1.1 to 2.5)	1.8 (1.1 to 2.5)	1.5 (1.0 to 2.1)	-1.1 (-1.8 to -0.3)	-14.5 (-23.9 to -4.0)
Southeast Asia						
Cambodia	3.5 (2.2 to 4.0)	2.4 (1.6 to 3.1)	2.3 (1.6 to 3.1)	2.0 (1.4 to 2.6)	-1.3 (-2.0 to -0.7)	-17.9 (-25.8 to -9.6)
Indonesia	1.7 (1.3 to 1.9)	1.0 (0.8 to 1.3)	0.9 (0.8 to 1.3)	0.9 (0.7 to 1.2)	-0.7 (-1.5 to -0.0)	-10.1 (-19.6 to -0.3)
Laos	6.5 (4.0 to 9.3)	3.4 (2.2 to 4.6)	3.2 (2.1 to 4.3)	2.6 (1.7 to 3.7)	-1.6 (-2.3 to -0.9)	-21.3 (-29.7 to -12.9)
Malaysia	2.2 (1.9 to 3.7)	1.9 (1.3 to 3.3)	1.8 (1.2 to 3.3)	1.4 (0.9 to 2.6)	-2.0 (-3.1 to -0.7)	-25.2 (-37.5 to -10.3)
Maldives	1.2 (0.7 to 1.4)	0.8 (0.4 to 1.0)	0.8 (0.4 to 0.9)	0.6 (0.3 to 0.9)	-1.6 (-3.2 to 0.5)	-20.0 (-38.3 to 8.1)
Mauritius	2.0 (1.9 to 2.1)	2.4 (2.2 to 2.6)	2.2 (2.0 to 2.5)	2.0 (1.7 to 2.3)	-1.4 (-2.4 to -0.4)	-18.9 (-30.4 to -6.1)
Myanmar	2.3 (1.7 to 2.8)	1.6 (1.0 to 2.1)	1.6 (1.0 to 2.1)	1.3 (0.8 to 1.7)	-1.5 (-2.3 to -0.8)	-20.5 (-29.4 to -10.9)
Philippines	20.0 (18.5 to 21.6)	15.9 (14.4 to 17.6)	15.7 (13.2 to 18.6)	14.1 (11.7 to 17.0)	-0.8 (-2.0 to 0.2)	-11.7 (-26.0 to 3.3)
Sri Lanka	6.3 (4.8 to 7.6)	3.4 (2.7 to 4.4)	3.3 (2.5 to 4.4)	2.6 (1.8 to 3.8)	-1.7 (-3.4 to 0.2)	-21.8 (-40.3 to 2.6)
Seychelles	7.6 (4.3 to 8.8)	5.4 (3.7 to 6.2)	5.5 (3.6 to 6.4)	4.4 (2.8 to 5.4)	-1.5 (-2.5 to -0.4)	-19.3 (-31.0 to -5.2)
Thailand	13.1 (7.1 to 15.5)	5.2 (3.6 to 6.3)	5.4 (3.6 to 6.7)	4.6 (3.0 to 6.7)	-1.0 (-2.7 to 1.0)	-12.8 (-33.6 to 17.0)
Timor-Leste	3.3 (1.4 to 4.4)	2.9 (1.2 to 3.8)	2.9 (1.3 to 3.9)	2.4 (1.1 to 3.3)	-1.2 (-2.1 to -0.2)	-16.7 (-27.3 to -3.1)
Vietnam	1.8 (1.4 to 2.3)	1.5 (1.0 to 1.9)	1.4 (0.9 to 1.9)	1.2 (0.8 to 1.7)	-1.4 (-2.2 to -0.5)	-18.4 (-28.6 to -6.7)
Sub-Saharan Africa						
Central Sub-Saharan Africa						
Angola	5.4 (4.0 to 10.0)	3.7 (2.7 to 7.0)	3.5 (2.5 to 6.7)	3.2 (2.2 to 6.0)	-1.1 (-2.0 to -0.1)	-15.1 (-26.0 to -1.6)
Central African Republic	8.7 (5.1 to 11.9)	14.5 (10.8 to 18.0)	8.1 (4.4 to 11.4)	7.7 (4.0 to 11.3)	-4.4 (-6.8 to -2.7)	-47.6 (-63.7 to -33.8)
Congo	7.4 (4.4 to 9.6)	5.4 (3.2 to 7.6)	5.3 (3.0 to 7.5)	4.7 (2.5 to 7.0)	-1.0 (-2.4 to 0.6)	-12.8 (-30.3 to 10.2)
Democratic Republic of the Congo	5.8 (4.9 to 6.8)	3.4 (2.4 to 4.3)	3.3 (2.3 to 4.2)	2.9 (1.9 to 3.8)	-1.1 (-2.0 to -0.1)	-15.2 (-25.7 to -1.6)
Equatorial Guinea	5.6 (3.4 to 7.7)	3.5 (1.9 to 5.7)	3.6 (1.9 to 5.6)	2.9 (1.5 to 4.8)	-1.3 (-2.6 to 0.1)	-17.5 (-31.9 to 1.9)
Gabon	6.1 (3.5 to 9.4)	5.0 (2.9 to 7.5)	4.8 (2.7 to 7.0)	4.0 (2.2 to 6.2)	-1.5 (-2.5 to -0.5)	-19.6 (-30.8 to -6.9)
Eastern Sub-Saharan Africa						
Burundi	0.9 (0.5 to 1.1)	0.7 (0.3 to 1.0)	0.6 (0.3 to 1.0)	0.5 (0.3 to 0.8)	-1.8 (-2.8 to -0.8)	-23.6 (-34.2 to -11.2)
Comoros	9.8 (4.2 to 13.9)	8.6 (3.2 to 12.1)	8.4 (3.1 to 11.6)	7.2 (2.5 to 10.2)	-1.2 (-2.0 to -0.3)	-16.4 (-26.4 to -5.1)
Djibouti	10.6 (4.2 to 16.3)	8.7 (3.0 to 13.7)	8.4 (2.9 to 13.2)	7.4 (2.4 to 11.6)	-1.1 (-2.2 to -0.2)	-15.6 (-28.2 to -3.5)
Eritrea	16.6 (5.9 to 23.9)	13.9 (4.6 to 20.5)	13.3 (4.4 to 19.6)	12.2 (3.9 to 18.0)	-0.9 (-1.9 to -0.1)	-12.9 (-24.9 to -1.6)
Ethiopia	16.6 (13.5 to 20.1)	10.2 (8.7 to 13.4)	9.4 (8.0 to 12.3)	7.6 (6.2 to 10.1)	-2.0 (-2.8 to -1.1)	-25.6 (-34.3 to -15.8)

	Estimate in 2000 (95% UIs)	Estimate in 2015 (95% UIs)	Estimate in 2017 (95% UIs)	Estimate in 2030 (95% UIs)	Annualised rate of change, 2015-2030 (95% UIs)	Percent change, 2015-2030 (95% UIs)
Kenya	15.0 (12.2 to 20.5)	12.2 (9.7 to 16.2)	11.5 (9.1 to 15.4)	10.8 (7.9 to 15.9)	-0.9 (-2.1 to 0.6)	-12.2 (-26.6 to 9.0)
Madagascar	3.5 (0.8 to 4.5)	1.7 (0.7 to 2.4)	1.6 (0.7 to 2.3)	1.4 (0.6 to 2.0)	-1.5 (-2.2 to -0.8)	-19.8 (-27.7 to -11.7)
Malawi	2.8 (1.6 to 8.2)	2.0 (1.2 to 5.9)	1.9 (1.1 to 5.9)	1.8 (0.9 to 5.5)	-1.0 (-2.7 to 0.9)	-13.3 (-33.6 to 14.5)
Mozambique	5.8 (4.8 to 8.5)	7.9 (5.1 to 10.0)	7.5 (4.9 to 9.4)	6.8 (4.3 to 8.8)	-1.1 (-2.1 to -0.1)	-14.5 (-27.2 to -1.5)
Rwanda	7.0 (5.3 to 8.3)	3.3 (2.4 to 4.3)	3.1 (2.2 to 4.2)	2.4 (1.5 to 3.6)	-2.2 (-4.2 to 0.1)	-26.8 (-46.7 to 1.7)
Somalia	15.3 (8.1 to 20.2)	11.5 (4.1 to 17.1)	11.1 (3.8 to 16.6)	10.2 (3.3 to 15.7)	-0.8 (-1.7 to -0.0)	-11.3 (-22.2 to -0.5)
South Sudan	9.9 (3.8 to 15.1)	14.1 (7.1 to 19.9)	10.4 (3.5 to 15.9)	9.4 (3.0 to 15.3)	-2.9 (-5.9 to -1.2)	-34.8 (-58.4 to -16.7)
Tanzania	7.4 (5.9 to 10.4)	6.3 (5.0 to 9.1)	6.2 (4.9 to 8.8)	5.3 (4.0 to 7.9)	-1.2 (-2.1 to -0.3)	-16.2 (-27.0 to -4.9)
Uganda	9.1 (6.9 to 10.5)	4.6 (2.8 to 6.4)	4.4 (2.7 to 6.0)	4.1 (2.4 to 5.9)	-0.9 (-2.3 to 0.4)	-12.1 (-28.9 to 6.5)
Zambia	9.3 (6.0 to 11.3)	6.5 (4.1 to 8.5)	6.3 (4.0 to 8.3)	5.5 (3.1 to 8.0)	-1.2 (-3.4 to 0.8)	-15.1 (-39.6 to 12.1)
Southern Sub-Saharan Africa						
Botswana	8.0 (2.8 to 11.9)	7.8 (2.2 to 10.3)	7.5 (2.2 to 9.9)	6.9 (1.9 to 10.7)	-0.9 (-2.4 to 1.3)	-12.2 (-30.7 to 20.7)
Lesotho	43.0 (17.8 to 56.5)	42.8 (18.0 to 55.8)	39.5 (16.8 to 52.1)	40.6 (16.1 to 62.3)	-0.5 (-2.6 to 1.8)	-5.2 (-32.5 to 30.9)
Namibia	27.5 (19.1 to 33.1)	15.2 (10.7 to 20.6)	14.8 (10.2 to 20.5)	11.8 (7.6 to 17.3)	-1.7 (-3.0 to -0.4)	-22.5 (-36.5 to -5.3)
South Africa	59.7 (42.3 to 64.4)	25.9 (19.9 to 30.0)	26.4 (20.3 to 30.2)	24.8 (16.5 to 39.1)	-0.4 (-2.5 to 2.4)	-4.4 (-30.8 to 43.0)
Swaziland	29.9 (23.1 to 41.5)	25.8 (18.4 to 37.3)	23.3 (16.9 to 33.5)	22.7 (14.4 to 35.7)	-0.9 (-3.0 to 1.4)	-12.1 (-36.1 to 24.0)
Zimbabwe	11.2 (8.2 to 13.6)	8.8 (6.8 to 11.9)	8.5 (6.4 to 11.4)	9.4 (4.9 to 18.1)	0.1 (-3.3 to 4.6)	6.5 (-38.9 to 99.2)
Western Sub-Saharan Africa						
Benin	7.1 (4.1 to 9.0)	6.3 (3.3 to 8.5)	6.0 (3.1 to 8.1)	5.2 (2.5 to 7.2)	-1.4 (-2.1 to -0.6)	-18.3 (-27.2 to -8.1)
Burkina Faso	14.5 (11.9 to 20.3)	13.7 (11.0 to 17.5)	13.5 (10.8 to 17.5)	12.2 (9.6 to 15.5)	-0.8 (-1.4 to -0.1)	-11.0 (-19.4 to -1.9)
Cameroon	4.2 (2.5 to 5.2)	3.0 (1.8 to 4.0)	2.8 (1.7 to 3.9)	2.6 (1.5 to 3.6)	-1.0 (-1.9 to 0.2)	-13.1 (-25.3 to 3.5)
Cape Verde	13.7 (11.6 to 17.9)	12.7 (10.3 to 15.8)	12.2 (9.7 to 15.3)	10.3 (8.1 to 12.9)	-1.4 (-2.2 to -0.7)	-19.3 (-28.1 to -10.6)
Chad	8.6 (5.5 to 11.0)	7.4 (3.8 to 10.1)	7.3 (3.7 to 9.8)	6.2 (3.1 to 8.7)	-1.2 (-2.0 to -0.2)	-15.7 (-25.4 to -2.9)
Cote d'Ivoire	15.1 (11.4 to 19.1)	12.4 (9.3 to 16.0)	11.8 (8.9 to 15.4)	11.0 (8.0 to 14.5)	-0.8 (-1.5 to -0.1)	-11.6 (-20.5 to -1.2)
The Gambia	1.5 (0.5 to 7.5)	1.4 (0.5 to 7.1)	1.4 (0.5 to 7.1)	1.4 (0.4 to 6.9)	-0.4 (-1.2 to 0.3)	-6.1 (-16.9 to 4.9)
Ghana	3.9 (2.4 to 10.6)	3.3 (1.9 to 9.7)	3.1 (1.8 to 9.4)	2.9 (1.6 to 8.4)	-1.1 (-1.8 to -0.4)	-14.6 (-23.4 to -5.3)
Guinea	7.8 (4.8 to 10.0)	7.9 (4.1 to 10.1)	7.6 (3.9 to 9.9)	6.8 (3.3 to 9.2)	-1.0 (-2.1 to 0.1)	-14.0 (-26.8 to 1.3)
Guinea-Bissau	11.5 (7.7 to 14.5)	9.9 (6.1 to 12.9)	9.6 (5.9 to 12.7)	8.2 (5.1 to 10.9)	-1.3 (-1.9 to -0.6)	-17.5 (-24.5 to -9.3)
Liberia	2.8 (1.6 to 3.6)	2.3 (1.2 to 3.0)	2.2 (1.2 to 2.9)	2.0 (1.1 to 2.6)	-1.0 (-1.8 to -0.2)	-13.6 (-23.6 to -3.1)
Mali	6.7 (4.2 to 9.7)	6.7 (3.8 to 10.6)	6.0 (3.1 to 9.9)	4.8 (2.5 to 8.1)	-2.3 (-3.1 to -1.5)	-28.6 (-37.3 to -20.5)
Mauritania	5.5 (3.3 to 7.3)	4.6 (2.5 to 6.6)	4.5 (2.4 to 6.5)	3.6 (1.8 to 5.3)	-1.7 (-2.9 to -0.5)	-22.3 (-35.2 to -6.9)
Niger	7.4 (4.4 to 10.8)	6.5 (3.5 to 10.3)	6.3 (3.3 to 10.2)	5.4 (2.8 to 8.9)	-1.2 (-1.9 to -0.5)	-16.2 (-24.5 to -7.0)
Nigeria	6.4 (4.5 to 8.2)	4.5 (2.7 to 6.3)	3.9 (2.1 to 5.7)	3.2 (1.7 to 4.7)	-2.4 (-3.3 to -1.6)	-30.2 (-39.4 to -21.5)
Sao Tome and Principe	6.2 (4.0 to 7.5)	5.5 (3.4 to 6.8)	5.2 (3.3 to 6.6)	4.8 (2.9 to 6.6)	-0.9 (-2.0 to 0.6)	-12.0 (-26.1 to 9.3)
Senegal	1.2 (0.9 to 1.5)	1.1 (0.8 to 1.4)	1.1 (0.7 to 1.4)	0.9 (0.6 to 1.3)	-1.0 (-1.9 to -0.1)	-14.1 (-24.6 to -1.0)
Sierra Leone	1.6 (1.0 to 2.0)	1.4 (0.8 to 1.9)	1.4 (0.8 to 1.8)	1.2 (0.7 to 1.7)	-1.1 (-2.0 to -0.1)	-14.8 (-25.5 to -1.1)
Togo	7.1 (4.2 to 8.9)	6.4 (3.3 to 8.4)	6.1 (3.2 to 8.1)	5.5 (2.6 to 7.7)	-1.1 (-2.1 to 0.1)	-14.8 (-26.7 to 1.4)
**Indicator 16.1.2: Death rate due to conflict and terrorism (per 100,000 population)**						
Central Europe, Eastern Europe, and Central Asia						
Central Asia						
Armenia	0.1 (0.1 to 0.1)	1.7 (1.6 to 1.8)	0.0 (0.0 to 0.0)	0.1 (0.0 to 0.8)	-62.5 (-111.3 to -4.6)	-94.1 (-100.0 to -49.8)
Azerbaijan	0.2 (0.2 to 0.2)	0.6 (0.6 to 0.7)	0.0 (0.0 to 0.0)	0.1 (0.0 to 1.3)	-64.0 (-104.7 to 4.6)	-83.1 (-100.0 to 98.3)
Georgia	0.5 (0.5 to 0.5)	0.1 (0.1 to 0.1)	0.0 (0.0 to 0.0)	0.5 (0.0 to 7.6)	-44.6 (-89.4 to 31.7)	637.9 (-100.0 to 11493.0)
Kazakhstan	0.0 (0.0 to 0.0)	0.1 (0.1 to 0.1)	0.0 (0.0 to 0.0)	0.0 (0.0 to 0.1)	-65.5 (-89.7 to 4.8)	-75.5 (-100.0 to 104.4)
Kyrgyzstan	3.5 (3.4 to 3.6)	0.1 (0.1 to 0.1)	0.0 (0.0 to 0.0)	0.1 (0.0 to 2.2)	-65.1 (-91.2 to 21.6)	2.6 (-100.0 to 2453.5)
Mongolia	0.0 (0.0 to 0.0)	0.1 (0.1 to 0.1)	0.0 (0.0 to 0.0)	0.0 (0.0 to 0.1)	-70.7 (-89.8 to 3.4)	-87.6 (-100.0 to 67.3)
Tajikistan	0.9 (0.9 to 0.9)	0.2 (0.2 to 0.2)	0.0 (0.0 to 0.0)	1.7 (0.0 to 31.9)	-57.3 (-97.1 to 33.6)	733.6 (-100.0 to 15245.7)
Turkmenistan	0.1 (0.1 to 0.1)	0.1 (0.1 to 0.1)	0.0 (0.0 to 0.0)	0.0 (0.0 to 0.1)	-65.2 (-89.7 to 2.4)	-80.6 (-100.0 to 43.8)
Uzbekistan	1.2 (1.2 to 1.2)	0.1 (0.1 to 0.1)	0.0 (0.0 to 0.0)	0.0 (0.0 to 0.2)	-69.8 (-89.7 to 6.8)	-78.2 (-100.0 to 177.8)
Central Europe						
Albania	0.0 (0.0 to 0.0)	0.0 (0.0 to 0.0)	0.0 (0.0 to 0.0)	0.0 (0.0 to 0.5)	18.7 (0.0 to 102.1)	29917301.7 (0.0 to 446243277.6)
Bosnia and Herzegovina	0.0 (0.0 to 0.0)	0.1 (0.1 to 0.1)	0.0 (0.0 to 0.0)	3.7 (0.0 to 71.5)	-72.1 (-93.1 to 42.9)	3129.3 (-100.0 to 62414.7)
Bulgaria	0.1 (0.1 to 0.1)	0.0 (0.0 to 0.0)	0.0 (0.0 to 0.0)	0.0 (0.0 to 0.1)	10.3 (0.0 to 87.9)	2798987.5 (0.0 to 52877555.7)
Croatia	0.2 (0.2 to 0.2)	0.0 (0.0 to 0.0)	0.0 (0.0 to 0.0)	1.1 (0.0 to 24.9)	-43.8 (-83.1 to 45.7)	4456.8 (-100.0 to 95267.9)
Czech Republic	0.0 (0.0 to 0.0)	0.0 (0.0 to 0.0)	0.0 (0.0 to 0.0)	0.0 (0.0 to 0.0)	8.2 (0.0 to 79.3)	1102064.8 (0.0 to 14704265.3)
Hungary	0.0 (0.0 to 0.0)	0.0 (0.0 to 0.0)	0.0 (0.0 to 0.0)	0.0 (0.0 to 0.1)	14.0 (0.0 to 89.3)	3370402.0 (0.0 to 65543738.3)
Macedonia	0.3 (0.3 to 0.3)	0.0 (0.0 to 0.0)	0.0 (0.0 to 0.0)	0.1 (0.0 to 0.6)	20.1 (0.0 to 104.1)	55580556.1 (0.0 to 606798000.0)
Montenegro	0.2 (0.1 to 0.2)	0.0 (0.0 to 0.0)	0.0 (0.0 to 0.0)	0.0 (0.0 to 0.1)	4.6 (0.0 to 91.7)	4736120.7 (0.0 to 94744343.2)
Poland	0.0 (0.0 to 0.0)	0.0 (0.0 to 0.0)	0.0 (0.0 to 0.0)	0.0 (0.0 to 0.1)	19.0 (0.0 to 90.9)	6699049.4 (0.0 to 83190446.2)
Romania	0.0 (0.0 to 0.0)	0.0 (0.0 to 0.0)	0.0 (0.0 to 0.0)	0.0 (0.0 to 0.0)	-58.7 (-72.9 to -1.5)	-90.2 (-100.0 to -20.6)
Serbia	0.3 (0.3 to 0.3)	0.0 (0.0 to 0.0)	0.0 (0.0 to 0.0)	0.3 (0.0 to 9.1)	28.3 (0.0 to 121.7)	257953234.5 (0.0 to 8923090053.7)
Slovakia	0.0 (0.0 to 0.0)	0.0 (0.0 to 0.0)	0.0 (0.0 to 0.0)	0.0 (0.0 to 0.0)	4.2 (0.0 to 76.7)	543256.5 (0.0 to 9899415.8)
Slovenia	0.0 (0.0 to 0.0)	0.0 (0.0 to 0.0)	0.0 (0.0 to 0.0)	0.0 (0.0 to 0.1)	6.8 (0.0 to 88.8)	3220352.1 (0.0 to 60791694.6)
Eastern Europe						
Belarus	0.0 (0.0 to 0.0)	0.0 (0.0 to 0.0)	0.0 (0.0 to 0.0)	0.0 (0.0 to 0.1)	14.5 (0.0 to 87.0)	5852500.2 (0.0 to 46222143.6)
Estonia	0.0 (0.0 to 0.0)	0.0 (0.0 to 0.0)	0.0 (0.0 to 0.0)	0.0 (0.0 to 0.1)	9.6 (0.0 to 89.1)	5287315.2 (0.0 to 63525975.5)
Latvia	0.0 (0.0 to 0.0)	0.0 (0.0 to 0.0)	0.0 (0.0 to 0.0)	0.0 (0.0 to 0.0)	9.9 (0.0 to 84.4)	2867312.4 (0.0 to 31416063.9)
Lithuania	0.0 (0.0 to 0.0)	0.0 (0.0 to 0.0)	0.0 (0.0 to 0.0)	0.0 (0.0 to 0.1)	5.7 (0.0 to 87.2)	2225515.4 (0.0 to 47737780.2)
Moldova	0.0 (0.0 to 0.0)	0.0 (0.0 to 0.0)	0.0 (0.0 to 0.0)	0.0 (0.0 to 0.0)	8.4 (0.0 to 82.3)	1483907.1 (0.0 to 22944547.6)
Russia	4.5 (4.3 to 4.6)	0.2 (0.2 to 0.2)	0.1 (0.1 to 0.1)	0.3 (0.0 to 3.3)	-43.4 (-96.9 to 18.5)	41.5 (-100.0 to 1506.1)
Ukraine	0.0 (0.0 to 0.0)	5.1 (5.0 to 5.2)	1.1 (1.0 to 1.1)	0.1 (0.0 to 3.2)	-105.1 (-118.4 to -3.2)	-98.0 (-100.0 to -37.7)
High-income						
Australasia						
Australia	0.0 (0.0 to 0.0)	0.0 (0.0 to 0.0)	0.0 (0.0 to 0.0)	0.1 (0.0 to 0.4)	-22.1 (-78.6 to 22.4)	436.2 (-100.0 to 2784.8)
New Zealand	0.1 (0.1 to 0.1)	0.0 (0.0 to 0.0)	0.0 (0.0 to 0.0)	0.4 (0.0 to 5.3)	-38.3 (-82.8 to 36.3)	1694.5 (-100.0 to 23124.5)
High-income Asia Pacific						
Brunei	0.0 (0.0 to 0.0)	0.0 (0.0 to 0.0)	0.0 (0.0 to 0.0)	0.0 (0.0 to 0.0)	20.7 (0.0 to 80.6)	2824152.4 (0.0 to 17894550.7)
Japan	0.0 (0.0 to 0.0)	0.0 (0.0 to 0.0)	0.0 (0.0 to 0.0)	0.0 (0.0 to 0.0)	-57.5 (-81.5 to -1.1)	-86.4 (-100.0 to -15.6)
South Korea	0.0 (0.0 to 0.0)	0.0 (0.0 to 0.0)	0.0 (0.0 to 0.0)	11.6 (0.0 to 363.0)	40.2 (0.0 to 146.8)	11560070008.9 (0.0 to 362997387032.7)
Singapore	0.0 (0.0 to 0.0)	0.0 (0.0 to 0.0)	0.0 (0.0 to 0.0)	0.0 (0.0 to 0.1)	9.4 (0.0 to 93.4)	8266703.3 (0.0 to 120791927.1)

	Estimate in 2000 (95% UIs)	Estimate in 2015 (95% UIs)	Estimate in 2017 (95% UIs)	Estimate in 2030 (95% UIs)	Annualised rate of change, 2015-2030 (95% UIs)	Percent change, 2015-2030 (95% UIs)
High-income North America						
Canada	0.0 (0.0 to 0.0)	0.0 (0.0 to 0.0)	0.0 (0.0 to 0.0)	0.0 (0.0 to 0.2)	48.6 (0.0 to 98.0)	37256043.5 (0.0 to 241486657.6)
Greenland	0.0 (0.0 to 0.0)	0.0 (0.0 to 0.0)	0.0 (0.0 to 0.0)	0.0 (0.0 to 0.0)	0.0 (0.0 to 0.0)	0.0 (0.0 to 0.0)
United States	0.0 (0.0 to 0.0)	0.0 (0.0 to 0.0)	0.0 (0.0 to 0.0)	0.4 (0.0 to 3.3)	-8.3 (-81.8 to 33.6)	1752.5 (-100.0 to 15416.7)
Southern Latin America						
Argentina	0.0 (0.0 to 0.0)	0.0 (0.0 to 0.0)	0.0 (0.0 to 0.0)	0.0 (0.0 to 0.3)	30.4 (0.0 to 98.6)	17772338.6 (0.0 to 265416443.5)
Chile	0.0 (0.0 to 0.0)	0.0 (0.0 to 0.0)	0.0 (0.0 to 0.0)	0.0 (0.0 to 0.1)	24.1 (0.0 to 94.1)	14676838.8 (0.0 to 135458030.0)
Uruguay	0.0 (0.0 to 0.0)	0.0 (0.0 to 0.0)	0.0 (0.0 to 0.0)	0.0 (0.0 to 0.0)	5.3 (0.0 to 80.9)	1143862.1 (0.0 to 18657050.5)
Western Europe						
Andorra	0.0 (0.0 to 0.0)	0.0 (0.0 to 0.0)	0.0 (0.0 to 0.0)	0.0 (0.0 to 0.0)	0.0 (0.0 to 0.0)	0.0 (0.0 to 0.0)
Austria	0.0 (0.0 to 0.0)	0.0 (0.0 to 0.0)	0.0 (0.0 to 0.0)	0.0 (0.0 to 0.0)	12.1 (0.0 to 85.0)	3418163.4 (0.0 to 34508147.9)
Belgium	0.0 (0.0 to 0.0)	0.0 (0.0 to 0.0)	0.0 (0.0 to 0.0)	0.0 (0.0 to 0.2)	45.7 (0.0 to 97.7)	32552882.0 (0.0 to 232917363.4)
Cyprus	0.0 (0.0 to 0.0)	0.0 (0.0 to 0.0)	0.0 (0.0 to 0.0)	0.9 (0.0 to 11.4)	31.8 (0.0 to 123.7)	915380749.7 (0.0 to 11363853616.6)
Denmark	0.0 (0.0 to 0.0)	0.0 (0.0 to 0.0)	0.0 (0.0 to 0.0)	0.0 (0.0 to 0.0)	-69.6 (-85.7 to 1.0)	-85.1 (-100.0 to 16.9)
Finland	0.1 (0.0 to 0.1)	0.0 (0.0 to 0.0)	0.0 (0.0 to 0.0)	0.0 (0.0 to 0.3)	48.3 (0.0 to 98.7)	39559904.5 (0.0 to 267629837.7)
France	0.0 (0.0 to 0.0)	0.5 (0.4 to 0.5)	0.0 (0.0 to 0.0)	0.1 (0.0 to 1.6)	-21.8 (-102.2 to 8.4)	-76.6 (-100.0 to 253.3)
Germany	0.0 (0.0 to 0.0)	0.0 (0.0 to 0.0)	0.0 (0.0 to 0.0)	0.0 (0.0 to 0.0)	-24.6 (-71.0 to 13.1)	42.6 (-100.0 to 610.9)
Greece	0.0 (0.0 to 0.0)	0.0 (0.0 to 0.0)	0.0 (0.0 to 0.0)	0.1 (0.0 to 0.5)	-25.0 (-76.8 to 26.0)	506.2 (-100.0 to 4862.2)
Iceland	0.0 (0.0 to 0.0)	0.0 (0.0 to 0.0)	0.0 (0.0 to 0.0)	0.0 (0.0 to 0.0)	0.0 (0.0 to 0.0)	0.0 (0.0 to 0.0)
Ireland	0.0 (0.0 to 0.0)	0.0 (0.0 to 0.0)	0.0 (0.0 to 0.0)	0.0 (0.0 to 0.2)	51.3 (0.0 to 96.9)	42139594.8 (0.0 to 205688716.5)
Israel	1.9 (1.8 to 1.9)	0.4 (0.4 to 0.4)	0.2 (0.2 to 0.3)	2.6 (0.0 to 29.1)	-20.6 (-101.7 to 28.3)	553.3 (-100.0 to 6887.6)
Italy	0.0 (0.0 to 0.0)	0.0 (0.0 to 0.0)	0.0 (0.0 to 0.0)	0.1 (0.0 to 0.5)	63.2 (0.0 to 102.5)	50833970.8 (0.0 to 472214508.4)
Luxembourg	0.0 (0.0 to 0.0)	0.0 (0.0 to 0.0)	0.0 (0.0 to 0.0)	0.0 (0.0 to 0.3)	3.0 (0.0 to 99.6)	9189473.1 (0.0 to 306318969.6)
Malta	0.0 (0.0 to 0.0)	0.0 (0.0 to 0.0)	0.0 (0.0 to 0.0)	0.0 (0.0 to 0.1)	5.5 (0.0 to 94.9)	8456509.2 (0.0 to 152424880.8)
Netherlands	0.0 (0.0 to 0.0)	0.0 (0.0 to 0.0)	0.0 (0.0 to 0.0)	0.0 (0.0 to 0.6)	52.1 (0.0 to 104.2)	41910254.8 (0.0 to 612690994.2)
Norway	0.0 (0.0 to 0.0)	0.0 (0.0 to 0.0)	0.0 (0.0 to 0.0)	0.0 (0.0 to 0.1)	14.9 (0.0 to 89.0)	4080815.8 (0.0 to 62904756.5)
Portugal	0.0 (0.0 to 0.0)	0.0 (0.0 to 0.0)	0.0 (0.0 to 0.0)	0.0 (0.0 to 0.1)	31.0 (0.0 to 87.4)	7066826.3 (0.0 to 49092843.7)
Spain	0.1 (0.1 to 0.1)	0.0 (0.0 to 0.0)	0.0 (0.0 to 0.0)	0.1 (0.0 to 0.8)	65.3 (0.0 to 106.1)	108955679.1 (0.0 to 811920838.8)
Sweden	0.0 (0.0 to 0.0)	0.0 (0.0 to 0.0)	0.0 (0.0 to 0.0)	0.0 (0.0 to 0.0)	-65.0 (-86.7 to -4.2)	-92.8 (-100.0 to -46.8)
Switzerland	0.0 (0.0 to 0.0)	0.0 (0.0 to 0.0)	0.0 (0.0 to 0.0)	0.0 (0.0 to 0.1)	17.0 (0.0 to 91.0)	6218112.0 (0.0 to 84968569.8)
United Kingdom	0.0 (0.0 to 0.0)	0.0 (0.0 to 0.0)	0.0 (0.0 to 0.0)	0.1 (0.0 to 0.6)	12.8 (-71.4 to 32.9)	2608.0 (-100.0 to 13898.5)
Latin America and Caribbean						
Andean Latin America						
Bolivia	0.0 (0.0 to 0.0)	0.0 (0.0 to 0.0)	0.0 (0.0 to 0.0)	0.0 (0.0 to 0.9)	18.9 (0.0 to 106.9)	36779350.1 (0.0 to 921245863.8)
Ecuador	0.0 (0.0 to 0.0)	0.0 (0.0 to 0.0)	0.0 (0.0 to 0.0)	0.0 (0.0 to 0.1)	25.3 (0.0 to 90.4)	6982881.3 (0.0 to 76977740.7)
Peru	0.0 (0.0 to 0.0)	0.1 (0.0 to 0.1)	0.0 (0.0 to 0.0)	1.0 (0.0 to 9.5)	-27.2 (-87.3 to 35.0)	1972.6 (-100.0 to 19033.6)
Caribbean						
Antigua and Barbuda	0.0 (0.0 to 0.0)	0.0 (0.0 to 0.0)	0.0 (0.0 to 0.0)	0.0 (0.0 to 0.9)	2.9 (0.0 to 106.7)	24170927.7 (0.0 to 895219542.5)
The Bahamas	0.0 (0.0 to 0.0)	0.0 (0.0 to 0.0)	0.0 (0.0 to 0.0)	0.0 (0.0 to 0.0)	0.0 (0.0 to 0.0)	0.0 (0.0 to 0.0)
Barbados	0.0 (0.0 to 0.0)	0.0 (0.0 to 0.0)	0.0 (0.0 to 0.0)	0.0 (0.0 to 0.8)	3.4 (0.0 to 105.6)	24310100.1 (0.0 to 759690827.3)
Belize	5.4 (4.4 to 6.8)	0.0 (0.0 to 0.0)	0.0 (0.0 to 0.0)	0.2 (0.0 to 3.2)	11.0 (0.0 to 115.3)	161310918.4 (0.0 to 3226259177.1)
Bermuda	0.0 (0.0 to 0.0)	0.0 (0.0 to 0.0)	0.0 (0.0 to 0.0)	0.0 (0.0 to 0.0)	0.0 (0.0 to 0.0)	0.0 (0.0 to 0.0)
Cuba	0.0 (0.0 to 0.0)	0.0 (0.0 to 0.0)	0.0 (0.0 to 0.0)	0.5 (0.0 to 8.8)	14.6 (0.0 to 121.9)	457181041.2 (0.0 to 8781061119.4)
Dominica	0.0 (0.0 to 0.0)	0.0 (0.0 to 0.0)	0.0 (0.0 to 0.0)	0.1 (0.0 to 2.4)	9.8 (0.0 to 113.4)	152129112.0 (0.0 to 2429463968.1)
Dominican Republic	0.0 (0.0 to 0.0)	0.0 (0.0 to 0.0)	0.0 (0.0 to 0.0)	1.6 (0.0 to 57.7)	24.9 (0.0 to 134.5)	1621191397.9 (0.0 to 57698683236.8)
Grenada	0.0 (0.0 to 0.0)	0.0 (0.0 to 0.0)	0.0 (0.0 to 0.0)	0.0 (0.0 to 0.0)	0.0 (0.0 to 0.0)	0.0 (0.0 to 0.0)
Guyana	0.0 (0.0 to 0.0)	0.0 (0.0 to 0.0)	0.0 (0.0 to 0.0)	0.0 (0.0 to 0.5)	13.3 (0.0 to 102.1)	34950934.9 (0.0 to 447057871.4)
Haiti	0.1 (0.1 to 0.1)	0.0 (0.0 to 0.0)	0.0 (0.0 to 0.0)	0.1 (0.0 to 1.0)	32.0 (0.0 to 107.7)	82865956.8 (0.0 to 1030651110.7)
Jamaica	0.0 (0.0 to 0.0)	0.0 (0.0 to 0.0)	0.0 (0.0 to 0.0)	0.0 (0.0 to 0.1)	11.4 (0.0 to 94.9)	12676154.6 (0.0 to 152781570.7)
Puerto Rico	0.0 (0.0 to 0.0)	0.0 (0.0 to 0.0)	0.0 (0.0 to 0.0)	0.1 (0.0 to 1.6)	46.4 (0.0 to 110.6)	128072952.7 (0.0 to 1593971796.9)
Saint Lucia	1.3 (1.1 to 1.6)	0.0 (0.0 to 0.0)	0.0 (0.0 to 0.0)	0.0 (0.0 to 0.0)	0.0 (0.0 to 0.0)	0.0 (0.0 to 0.0)
Saint Vincent and the Grenadines	0.0 (0.0 to 0.0)	0.0 (0.0 to 0.0)	0.0 (0.0 to 0.0)	0.0 (0.0 to 0.0)	0.0 (0.0 to 0.0)	0.0 (0.0 to 0.0)
Suriname	0.0 (0.0 to 0.0)	0.0 (0.0 to 0.0)	0.0 (0.0 to 0.0)	0.1 (0.0 to 0.9)	7.0 (0.0 to 107.1)	47865722.4 (0.0 to 952340505.6)
Trinidad and Tobago	0.0 (0.0 to 0.0)	0.0 (0.0 to 0.0)	0.0 (0.0 to 0.0)	0.0 (0.0 to 0.2)	6.4 (0.0 to 97.9)	10917646.4 (0.0 to 238226040.7)
Virgin Islands, U.S.	0.0 (0.0 to 0.0)	0.0 (0.0 to 0.0)	0.0 (0.0 to 0.0)	0.2 (0.0 to 3.2)	13.1 (0.0 to 115.2)	217875696.1 (0.0 to 3180467281.8)
Central Latin America						
Colombia	5.4 (5.2 to 5.6)	0.5 (0.5 to 0.5)	0.0 (0.0 to 0.0)	0.9 (0.0 to 3.5)	-13.0 (-103.0 to 12.7)	78.6 (-100.0 to 576.5)
Costa Rica	0.0 (0.0 to 0.0)	0.0 (0.0 to 0.0)	0.0 (0.0 to 0.0)	0.0 (0.0 to 0.1)	12.3 (0.0 to 91.2)	6707069.1 (0.0 to 87546679.5)
El Salvador	0.0 (0.0 to 0.0)	0.0 (0.0 to 0.0)	0.0 (0.0 to 0.0)	7.7 (0.0 to 75.3)	39.2 (0.0 to 136.3)	7704669305.6 (0.0 to 75335545663.4)
Guatemala	0.1 (0.1 to 0.1)	0.0 (0.0 to 0.0)	0.0 (0.0 to 0.0)	3.5 (0.0 to 16.2)	71.0 (0.0 to 126.0)	3551260720.4 (0.0 to 16165806432.0)
Honduras	0.0 (0.0 to 0.0)	0.0 (0.0 to 0.0)	0.0 (0.0 to 0.0)	1.3 (0.0 to 23.4)	39.9 (0.0 to 128.5)	1313431472.4 (0.0 to 23348774747.0)
Mexico	0.0 (0.0 to 0.0)	0.0 (0.0 to 0.0)	0.0 (0.0 to 0.0)	0.0 (0.0 to 0.1)	30.5 (0.0 to 94.1)	12440511.3 (0.0 to 135796038.5)
Nicaragua	0.0 (0.0 to 0.1)	0.0 (0.0 to 0.0)	0.0 (0.0 to 0.0)	12.1 (0.0 to 139.7)	41.4 (0.0 to 140.4)	12061502000.8 (0.0 to 139659317278.6)
Panama	0.0 (0.0 to 0.0)	0.0 (0.0 to 0.0)	0.0 (0.0 to 0.0)	0.0 (0.0 to 0.7)	22.3 (0.0 to 105.3)	39076078.0 (0.0 to 722774882.6)
Venezuela	0.1 (0.1 to 0.1)	0.0 (0.0 to 0.0)	0.0 (0.0 to 0.0)	0.1 (0.0 to 0.7)	-10.6 (-69.9 to 35.7)	2094.0 (-100.0 to 21048.7)
Tropical Latin America						
Brazil	0.0 (0.0 to 0.0)	0.0 (0.0 to 0.0)	0.0 (0.0 to 0.0)	0.0 (0.0 to 0.0)	-31.2 (-65.1 to 14.3)	38.4 (-100.0 to 758.3)
Paraguay	0.0 (0.0 to 0.0)	0.2 (0.1 to 0.3)	0.0 (0.0 to 0.0)	0.0 (0.0 to 0.2)	-77.5 (-99.4 to -0.8)	-93.0 (-100.0 to -11.6)
North Africa and Middle East						
North Africa and Middle East						
Afghanistan	30.3 (29.2 to 31.4)	76.4 (72.0 to 81.2)	5.1 (4.0 to 6.4)	23.7 (0.0 to 193.9)	-59.9 (-136.7 to 6.2)	-69.0 (-100.0 to 151.8)
Algeria	8.0 (7.3 to 8.9)	0.3 (0.3 to 0.3)	0.3 (0.3 to 0.4)	10.4 (0.0 to 87.6)	-35.6 (-99.3 to 38.0)	3500.8 (-100.0 to 29782.0)
Bahrain	0.0 (0.0 to 0.0)	0.3 (0.3 to 0.5)	0.8 (0.6 to 1.0)	0.1 (0.0 to 0.8)	-85.0 (-102.1 to 5.0)	-85.6 (-100.0 to 112.5)
Egypt	0.0 (0.0 to 0.0)	0.2 (0.2 to 0.3)	1.2 (1.0 to 1.5)	0.5 (0.0 to 9.5)	-32.4 (-98.8 to 23.4)	116.9 (-100.0 to 3262.5)
Iran	0.2 (0.2 to 0.2)	0.1 (0.1 to 0.1)	0.1 (0.1 to 0.1)	3.8 (0.0 to 42.7)	-22.2 (-92.3 to 40.5)	3845.1 (-100.0 to 43296.0)
Iraq	1.3 (1.0 to 1.6)	64.3 (52.3 to 80.8)	76.8 (61.6 to 95.9)	21.3 (0.0 to 186.9)	-41.4 (-136.1 to 6.7)	-66.2 (-100.0 to 173.5)
Jordan	0.0 (0.0 to 0.0)	0.1 (0.1 to 0.1)	0.3 (0.3 to 0.4)	0.1 (0.0 to 0.9)	-65.0 (-91.9 to 16.0)	-37.9 (-100.0 to 1008.9)
Kuwait	0.3 (0.2 to 0.6)	1.4 (1.2 to 1.5)	0.0 (0.0 to 0.0)	0.5 (0.0 to 12.2)	-63.9 (-110.2 to 14.1)	-67.5 (-100.0 to 725.7)
Lebanon	0.7 (0.7 to 0.8)	2.3 (1.9 to 2.7)	4.5 (3.6 to 5.7)	16.5 (0.0 to 373.7)	-39.4 (-113.8 to 33.0)	624.5 (-100.0 to 13998.0)
Libya	0.0 (0.0 to 0.1)	0.8 (0.6 to 0.9)	22.1 (17.7 to 27.2)	3.7 (0.0 to 56.2)	-79.0 (-107.0 to 28.3)	401.3 (-100.0 to 6909.3)

	Estimate in 2000 (95% UIs)	Estimate in 2015 (95% UIs)	Estimate in 2017 (95% UIs)	Estimate in 2030 (95% UIs)	Annualised rate of change, 2015-2030 (95% UIs)	Percent change, 2015-2030 (95% UIs)
Morocco	0.0 (0.0 to 0.0)	0.0 (0.0 to 0.0)	0.0 (0.0 to 0.0)	0.5 (0.0 to 3.1)	-19.9 (-74.2 to 41.9)	8815.7 (-100.0 to 53417.9)
Palestine	76.7 (61.4 to 96.3)	5.9 (4.7 to 7.6)	2.6 (2.1 to 3.3)	43.1 (0.0 to 704.6)	-34.9 (-120.4 to 31.4)	640.6 (-100.0 to 11089.0)
Oman	0.0 (0.0 to 0.0)	0.0 (0.0 to 0.0)	0.0 (0.0 to 0.0)	0.6 (0.0 to 10.4)	9.4 (0.0 to 123.1)	550786849.6 (0.0 to 10377948663.3)
Qatar	0.0 (0.0 to 0.0)	0.0 (0.0 to 0.0)	0.0 (0.0 to 0.0)	0.0 (0.0 to 0.1)	6.0 (0.0 to 94.5)	7868017.3 (0.0 to 142503669.4)
Saudi Arabia	0.0 (0.0 to 0.0)	1.0 (0.8 to 1.1)	7.5 (6.0 to 9.6)	0.0 (0.0 to 0.3)	-76.8 (-108.3 to -8.0)	-97.4 (-100.0 to -69.7)
Sudan	12.3 (12.2 to 12.4)	0.0 (0.0 to 0.0)	3.4 (2.7 to 4.2)	3.0 (0.0 to 9.4)	79.9 (0.0 to 122.4)	2972557772.8 (0.0 to 9399411067.0)
Syria	0.0 (0.0 to 0.1)	304.5 (249.8 to 375.7)	228.2 (185.8 to 284.5)	11.2 (0.0 to 133.1)	-91.9 (-146.8 to -5.5)	-96.3 (-100.0 to -56.5)
Tunisia	0.1 (0.0 to 0.1)	0.9 (0.8 to 0.9)	0.2 (0.2 to 0.3)	0.3 (0.0 to 4.8)	-76.4 (-107.0 to 11.2)	-62.8 (-100.0 to 433.8)
Turkey	0.2 (0.2 to 0.2)	2.0 (1.9 to 2.1)	3.7 (3.0 to 4.6)	0.3 (0.0 to 1.8)	-43.6 (-112.2 to -0.4)	-84.8 (-100.0 to -5.9)
United Arab Emirates	0.0 (0.0 to 0.0)	0.0 (0.0 to 0.0)	0.0 (0.0 to 0.0)	0.0 (0.0 to 0.3)	11.3 (0.0 to 99.1)	14948049.9 (0.0 to 286638147.3)
Yemen	0.1 (0.1 to 0.1)	31.5 (29.9 to 33.1)	55.2 (44.2 to 68.3)	7.1 (0.0 to 41.3)	-62.8 (-130.7 to 1.8)	-77.4 (-100.0 to 31.1)
South Asia						
South Asia						
Bangladesh	0.0 (0.0 to 0.0)	0.1 (0.1 to 0.1)	0.1 (0.1 to 0.1)	0.0 (0.0 to 0.1)	-48.2 (-89.6 to 1.7)	-85.8 (-100.0 to 28.3)
Bhutan	0.0 (0.0 to 0.0)	0.0 (0.0 to 0.0)	0.0 (0.0 to 0.0)	0.0 (0.0 to 0.2)	4.7 (0.0 to 97.8)	10590752.5 (0.0 to 235162619.2)
India	0.5 (0.5 to 0.5)	0.1 (0.1 to 0.1)	0.1 (0.1 to 0.1)	0.1 (0.0 to 0.2)	-33.2 (-92.5 to 5.9)	-51.5 (-100.0 to 142.0)
Nepal	1.9 (1.9 to 1.9)	0.0 (0.0 to 0.0)	0.0 (0.0 to 0.0)	0.4 (0.0 to 6.2)	-27.2 (-69.9 to 49.7)	10343.8 (-100.0 to 173079.8)
Pakistan	0.1 (0.1 to 0.1)	1.9 (1.8 to 1.9)	0.2 (0.2 to 0.2)	0.2 (0.0 to 1.6)	-47.5 (-111.7 to -1.3)	-86.7 (-100.0 to -18.2)
Southeast Asia, East Asia, and Oceania						
East Asia						
China	0.0 (0.0 to 0.0)	0.0 (0.0 to 0.0)	0.0 (0.0 to 0.0)	0.1 (0.0 to 1.6)	-30.9 (-83.1 to 27.4)	376.5 (-100.0 to 6018.3)
North Korea	0.0 (0.0 to 0.0)	0.0 (0.0 to 0.0)	0.0 (0.0 to 0.0)	42.8 (0.0 to 1354.6)	10.0 (0.0 to 155.5)	42792279176.3 (0.0 to 1354604211020.1)
Taiwan (Province of China)	0.0 (0.0 to 0.0)	0.0 (0.0 to 0.0)	0.0 (0.0 to 0.0)	0.0 (0.0 to 0.1)	18.8 (0.0 to 91.6)	10033426.2 (0.0 to 93347192.1)
Oceania						
American Samoa	0.0 (0.0 to 0.0)	0.0 (0.0 to 0.0)	0.0 (0.0 to 0.0)	0.3 (0.0 to 2.4)	15.4 (0.0 to 113.4)	258801963.2 (0.0 to 2442762084.8)
Federated States of Micronesia	0.0 (0.0 to 0.0)	0.0 (0.0 to 0.0)	0.0 (0.0 to 0.0)	0.0 (0.0 to 0.0)	0.0 (0.0 to 0.0)	0.0 (0.0 to 0.0)
Fiji	0.9 (0.8 to 0.9)	0.0 (0.0 to 0.0)	0.0 (0.0 to 0.0)	0.0 (0.0 to 0.1)	3.2 (0.0 to 90.0)	2629269.6 (0.0 to 73035265.8)
Guam	0.0 (0.0 to 0.0)	0.0 (0.0 to 0.0)	0.0 (0.0 to 0.0)	0.7 (0.0 to 9.8)	25.7 (0.0 to 122.7)	715482672.3 (0.0 to 9826322599.7)
Kiribati	0.0 (0.0 to 0.0)	0.0 (0.0 to 0.0)	0.0 (0.0 to 0.0)	0.0 (0.0 to 0.0)	0.0 (0.0 to 0.0)	0.0 (0.0 to 0.0)
Marshall Islands	0.0 (0.0 to 0.0)	0.0 (0.0 to 0.0)	0.0 (0.0 to 0.0)	0.0 (0.0 to 0.0)	0.0 (0.0 to 0.0)	0.0 (0.0 to 0.0)
Northern Mariana Islands	0.0 (0.0 to 0.0)	0.0 (0.0 to 0.0)	0.0 (0.0 to 0.0)	0.1 (0.0 to 2.2)	5.8 (0.0 to 112.8)	102654379.1 (0.0 to 2213815816.7)
Papua New Guinea	0.0 (0.0 to 0.0)	0.0 (0.0 to 0.0)	0.0 (0.0 to 0.0)	0.1 (0.0 to 0.9)	14.7 (0.0 to 106.9)	77651235.6 (0.0 to 915744206.3)
Samoa	0.0 (0.0 to 0.0)	0.0 (0.0 to 0.0)	0.0 (0.0 to 0.0)	0.0 (0.0 to 0.0)	0.0 (0.0 to 0.0)	0.0 (0.0 to 0.0)
Solomon Islands	6.1 (5.9 to 6.2)	0.0 (0.0 to 0.0)	0.0 (0.0 to 0.0)	0.1 (0.0 to 1.7)	11.6 (0.0 to 111.1)	66074791.4 (0.0 to 1715211952.6)
Tonga	0.0 (0.0 to 0.0)	0.0 (0.0 to 0.0)	0.0 (0.0 to 0.0)	0.0 (0.0 to 0.0)	0.0 (0.0 to 0.0)	0.0 (0.0 to 0.0)
Vanuatu	0.0 (0.0 to 0.0)	0.0 (0.0 to 0.0)	0.0 (0.0 to 0.0)	0.0 (0.0 to 0.0)	0.0 (0.0 to 0.0)	0.0 (0.0 to 0.0)
Southeast Asia						
Cambodia	0.1 (0.1 to 0.1)	0.0 (0.0 to 0.0)	0.0 (0.0 to 0.0)	82.2 (0.0 to 960.3)	-8.5 (-85.2 to 68.9)	257797.9 (-100.0 to 3060396.7)
Indonesia	0.3 (0.3 to 0.3)	0.2 (0.2 to 0.2)	0.0 (0.0 to 0.0)	0.4 (0.0 to 2.4)	-23.5 (-96.4 to 17.2)	139.8 (-100.0 to 1222.5)
Laos	0.1 (0.1 to 0.1)	0.1 (0.1 to 0.1)	0.0 (0.0 to 0.0)	5.8 (0.0 to 30.5)	-30.3 (-89.4 to 42.0)	9760.1 (-100.0 to 54247.0)
Malaysia	0.0 (0.0 to 0.0)	0.0 (0.0 to 0.0)	0.0 (0.0 to 0.0)	1.0 (0.0 to 11.3)	-29.0 (-70.4 to 54.0)	29358.1 (-100.0 to 328178.3)
Maldives	0.0 (0.0 to 0.0)	0.0 (0.0 to 0.0)	0.0 (0.0 to 0.0)	0.0 (0.0 to 0.0)	0.0 (0.0 to 0.0)	0.0 (0.0 to 0.0)
Mauritius	0.0 (0.0 to 0.0)	0.0 (0.0 to 0.0)	0.0 (0.0 to 0.0)	0.0 (0.0 to 0.0)	0.0 (0.0 to 0.0)	0.0 (0.0 to 0.0)
Myanmar	1.4 (1.4 to 1.5)	1.5 (1.3 to 1.7)	0.1 (0.1 to 0.1)	5.1 (0.1 to 21.5)	1.1 (-17.7 to 17.6)	248.4 (-92.9 to 1306.0)
Philippines	2.6 (2.5 to 2.6)	1.0 (1.0 to 1.1)	1.1 (0.9 to 1.3)	0.5 (0.0 to 2.5)	-22.9 (-108.0 to 5.9)	-51.3 (-100.0 to 143.0)
Sri Lanka	16.1 (15.3 to 16.8)	0.0 (0.0 to 0.0)	0.0 (0.0 to 0.0)	3.1 (0.0 to 17.5)	-18.3 (-79.8 to 47.4)	21948.2 (-100.0 to 122286.7)
Seychelles	0.0 (0.0 to 0.0)	0.0 (0.0 to 0.0)	0.0 (0.0 to 0.0)	0.0 (0.0 to 0.0)	18.2 (0.0 to 82.6)	3382767.7 (0.0 to 23902972.5)
Thailand	0.0 (0.0 to 0.0)	0.2 (0.2 to 0.2)	0.1 (0.0 to 0.1)	0.2 (0.0 to 1.1)	-22.6 (-95.6 to 12.7)	25.9 (-100.0 to 570.2)
Timor-Leste	8.9 (7.7 to 10.2)	0.0 (0.0 to 0.0)	0.0 (0.0 to 0.0)	15.9 (0.0 to 122.5)	63.9 (0.0 to 139.5)	15943336797.2 (0.0 to 122540254017.3)
Vietnam	0.0 (0.0 to 0.0)	0.0 (0.0 to 0.0)	0.0 (0.0 to 0.0)	21.2 (0.0 to 161.8)	46.5 (0.0 to 141.4)	21222442394.8 (0.0 to 161665347909.9)
Sub-Saharan Africa						
Central Sub-Saharan Africa						
Angola	23.0 (21.1 to 25.1)	0.0 (0.0 to 0.0)	0.2 (0.2 to 0.2)	8.8 (0.0 to 22.0)	23.7 (-71.0 to 58.3)	233310.4 (-100.0 to 628946.3)
Central African Republic	0.4 (0.4 to 0.4)	10.2 (9.5 to 10.9)	40.6 (36.3 to 45.8)	1.8 (0.0 to 41.1)	-78.7 (-123.3 to 9.1)	-82.0 (-100.0 to 291.9)
Congo	15.8 (14.1 to 17.8)	0.0 (0.0 to 0.0)	0.5 (0.5 to 0.6)	1.8 (0.0 to 48.1)	36.6 (0.0 to 133.3)	1772919606.2 (0.0 to 48068766576.4)
Democratic Republic of the Congo	5.6 (5.2 to 6.0)	1.5 (1.4 to 1.6)	3.5 (3.1 to 3.9)	1.5 (0.0 to 22.0)	-50.3 (-110.5 to 17.7)	-1.9 (-100.0 to 1319.1)
Equatorial Guinea	0.0 (0.0 to 0.0)	0.0 (0.0 to 0.0)	0.0 (0.0 to 0.0)	0.0 (0.0 to 0.4)	11.1 (0.0 to 102.0)	21692000.6 (0.0 to 440111811.8)
Gabon	0.0 (0.0 to 0.0)	0.1 (0.1 to 0.1)	0.0 (0.0 to 0.0)	0.0 (0.0 to 0.3)	-82.1 (-89.5 to 10.3)	-80.4 (-100.0 to 367.0)
Eastern Sub-Saharan Africa						
Burundi	225.7 (193.8 to 264.2)	6.4 (5.7 to 7.2)	2.2 (1.9 to 2.6)	11.1 (0.0 to 248.8)	-65.0 (-120.5 to 24.0)	72.7 (-100.0 to 3533.6)
Comoros	0.0 (0.0 to 0.0)	0.0 (0.0 to 0.0)	0.0 (0.0 to 0.0)	0.1 (0.0 to 2.9)	4.5 (0.0 to 114.5)	79320484.2 (0.0 to 2886451156.0)
Djibouti	0.0 (0.0 to 0.0)	2.6 (2.2 to 3.0)	0.1 (0.1 to 0.1)	0.4 (0.0 to 4.6)	-83.2 (-114.8 to 3.6)	-83.2 (-100.0 to 72.5)
Eritrea	51.8 (51.2 to 52.3)	0.2 (0.1 to 0.2)	0.0 (0.0 to 0.0)	61.3 (0.0 to 251.0)	-5.1 (-96.6 to 47.9)	34496.0 (-100.0 to 131436.4)
Ethiopia	74.8 (74.3 to 75.3)	0.4 (0.4 to 0.4)	1.0 (0.9 to 1.2)	0.8 (0.0 to 9.2)	-33.2 (-101.7 to 20.6)	99.3 (-100.0 to 2098.4)
Kenya	1.4 (1.3 to 1.5)	1.4 (1.3 to 1.5)	0.8 (0.8 to 0.9)	1.5 (0.0 to 19.9)	-55.7 (-110.0 to 17.6)	7.2 (-100.0 to 1306.8)
Madagascar	0.0 (0.0 to 0.0)	0.7 (0.6 to 0.8)	0.7 (0.6 to 0.8)	0.0 (0.0 to 0.3)	-82.2 (-105.9 to -5.2)	-96.4 (-100.0 to -54.1)
Malawi	0.0 (0.0 to 0.0)	0.1 (0.1 to 0.1)	0.1 (0.1 to 0.1)	0.0 (0.0 to 0.1)	-77.5 (-91.0 to -3.4)	-96.6 (-100.0 to -40.1)
Mozambique	0.0 (0.0 to 0.0)	0.0 (0.0 to 0.0)	0.6 (0.5 to 0.7)	3.8 (0.0 to 37.5)	-18.9 (-79.3 to 52.5)	26699.1 (-100.0 to 262725.4)
Rwanda	24.8 (21.4 to 29.3)	0.0 (0.0 to 0.0)	0.0 (0.0 to 0.0)	2.1 (0.0 to 26.3)	-38.7 (-83.8 to 46.0)	8253.1 (-100.0 to 99759.6)
Somalia	0.3 (0.3 to 0.4)	10.6 (9.5 to 11.7)	32.3 (27.2 to 37.9)	2.2 (0.0 to 32.6)	-57.8 (-123.7 to 6.9)	-79.1 (-100.0 to 186.1)
South Sudan	27.9 (23.7 to 32.9)	1.1 (0.9 to 1.2)	42.5 (35.9 to 50.3)	2.2 (0.0 to 19.5)	-56.1 (-108.7 to 19.0)	113.4 (-100.0 to 1629.1)
Tanzania	0.1 (0.1 to 0.1)	0.1 (0.1 to 0.1)	0.1 (0.1 to 0.1)	0.0 (0.0 to 0.2)	-57.8 (-89.5 to 7.0)	-76.7 (-100.0 to 187.6)
Uganda	6.2 (5.6 to 6.9)	0.1 (0.1 to 0.1)	0.1 (0.1 to 0.1)	3.3 (0.0 to 29.7)	-24.9 (-89.2 to 41.8)	5800.3 (-100.0 to 52540.7)
Zambia	0.0 (0.0 to 0.0)	0.0 (0.0 to 0.1)	0.0 (0.0 to 0.0)	0.0 (0.0 to 0.2)	-56.2 (-87.4 to 10.6)	-67.5 (-100.0 to 386.9)
Southern Sub-Saharan Africa						
Botswana	0.0 (0.0 to 0.0)	0.0 (0.0 to 0.0)	0.0 (0.0 to 0.0)	0.0 (0.0 to 0.1)	5.7 (0.0 to 93.0)	5442827.7 (0.0 to 114892660.3)
Lesotho	0.0 (0.0 to 0.0)	0.2 (0.1 to 0.2)	0.2 (0.2 to 0.2)	0.0 (0.0 to 0.3)	-75.3 (-95.9 to 5.0)	-80.5 (-100.0 to 113.0)
Namibia	8.5 (7.9 to 9.1)	0.1 (0.1 to 0.1)	0.0 (0.0 to 0.1)	10.9 (0.0 to 42.8)	-34.5 (-94.6 to 38.6)	8227.1 (-100.0 to 32440.2)
South Africa	0.1 (0.1 to 0.1)	0.3 (0.2 to 0.3)	0.1 (0.1 to 0.1)	0.1 (0.0 to 0.7)	-42.4 (-98.9 to 6.0)	-69.0 (-100.0 to 146.7)
Swaziland	0.0 (0.0 to 0.0)	0.0 (0.0 to 0.0)	0.0 (0.0 to 0.0)	0.0 (0.0 to 0.1)	8.0 (0.0 to 94.5)	9561031.5 (0.0 to 143990675.4)

	Estimate in 2000 (95% UIs)	Estimate in 2015 (95% UIs)	Estimate in 2017 (95% UIs)	Estimate in 2030 (95% UIs)	Annualised rate of change, 2015-2030 (95% UIs)	Percent change, 2015-2030 (95% UIs)
Zimbabwe	0.2 (0.2 to 0.3)	0.1 (0.1 to 0.1)	0.0 (0.0 to 0.0)	3.6 (0.0 to 76.1)	-35.2 (-91.7 to 45.1)	4242.3 (-100.0 to 87115.5)
Western Sub-Saharan Africa						
Benin	0.0 (0.0 to 0.0)	0.0 (0.0 to 0.0)	0.1 (0.1 to 0.1)	0.0 (0.0 to 0.0)	-77.9 (-84.5 to -4.2)	-97.2 (-100.0 to -46.8)
Burkina Faso	0.0 (0.0 to 0.0)	0.1 (0.1 to 0.1)	0.5 (0.4 to 0.6)	0.0 (0.0 to 0.1)	-78.6 (-94.6 to -1.7)	-95.3 (-100.0 to -22.8)
Cameroon	0.1 (0.1 to 0.1)	6.0 (5.8 to 6.4)	1.6 (1.4 to 1.9)	0.1 (0.0 to 1.8)	-87.2 (-119.8 to -8.3)	-98.2 (-100.0 to -71.1)
Cape Verde	0.0 (0.0 to 0.0)	0.0 (0.0 to 0.0)	0.0 (0.0 to 0.0)	0.0 (0.0 to 0.0)	0.0 (0.0 to 0.0)	0.0 (0.0 to 0.0)
Chad	12.2 (12.2 to 12.2)	3.7 (3.6 to 3.9)	1.9 (1.7 to 2.3)	1.9 (0.0 to 7.2)	-42.4 (-116.4 to 4.3)	-49.3 (-100.0 to 90.9)
Cote d'Ivoire	0.7 (0.6 to 0.7)	0.1 (0.1 to 0.1)	0.1 (0.1 to 0.1)	0.2 (0.0 to 2.5)	-51.6 (-90.0 to 23.8)	170.2 (-100.0 to 3430.3)
The Gambia	0.1 (0.1 to 0.2)	0.0 (0.0 to 0.0)	0.1 (0.1 to 0.2)	0.0 (0.0 to 0.5)	13.4 (0.0 to 102.8)	24351148.9 (0.0 to 494990161.9)
Ghana	0.2 (0.1 to 0.2)	0.1 (0.1 to 0.1)	0.2 (0.1 to 0.2)	0.0 (0.0 to 0.1)	-62.1 (-92.2 to -1.0)	-89.1 (-100.0 to -14.4)
Guinea	14.2 (12.6 to 16.4)	0.1 (0.1 to 0.1)	0.2 (0.2 to 0.3)	0.1 (0.0 to 1.6)	-60.8 (-90.4 to 20.4)	4.6 (-100.0 to 2037.1)
Guinea-Bissau	1.1 (1.0 to 1.3)	0.0 (0.0 to 0.0)	0.0 (0.0 to 0.0)	11.5 (0.0 to 69.8)	46.0 (0.0 to 135.8)	11481458547.3 (0.0 to 69784133539.3)
Liberia	3.5 (3.5 to 3.5)	0.0 (0.0 to 0.0)	0.1 (0.1 to 0.1)	5.3 (0.0 to 60.7)	-40.0 (-82.9 to 52.7)	24316.2 (-100.0 to 270879.7)
Mali	18.2 (15.6 to 21.1)	1.3 (1.2 to 1.4)	4.3 (3.7 to 5.0)	0.8 (0.0 to 11.2)	-65.8 (-109.7 to 14.0)	-40.6 (-100.0 to 711.5)
Mauritania	0.0 (0.0 to 0.0)	0.0 (0.0 to 0.0)	0.1 (0.0 to 0.1)	1.0 (0.0 to 14.2)	-54.0 (-84.3 to 41.8)	3807.4 (-100.0 to 52672.1)
Niger	0.1 (0.1 to 0.1)	3.1 (2.9 to 3.4)	1.4 (1.2 to 1.7)	0.1 (0.0 to 0.6)	-76.3 (-115.5 to -11.1)	-98.0 (-100.0 to -81.1)
Nigeria	0.2 (0.1 to 0.2)	0.0 (0.0 to 0.0)	2.0 (1.7 to 2.3)	0.7 (0.0 to 11.1)	46.6 (0.0 to 123.5)	662677985.1 (0.0 to 11077326568.4)
Sao Tome and Principe	0.0 (0.0 to 0.0)	0.0 (0.0 to 0.0)	0.0 (0.0 to 0.0)	0.0 (0.0 to 0.0)	0.0 (0.0 to 0.0)	0.0 (0.0 to 0.0)
Senegal	0.8 (0.7 to 0.8)	0.0 (0.0 to 0.0)	0.0 (0.0 to 0.0)	0.1 (0.0 to 1.6)	-32.3 (-75.4 to 36.2)	1960.1 (-100.0 to 22578.1)
Sierra Leone	21.2 (20.8 to 21.7)	0.0 (0.0 to 0.0)	0.0 (0.0 to 0.0)	2.2 (0.0 to 34.8)	31.2 (0.0 to 131.1)	2206968530.2 (0.0 to 34556586372.9)
Togo	0.0 (0.0 to 0.0)	0.0 (0.0 to 0.0)	0.3 (0.2 to 0.3)	0.0 (0.0 to 0.4)	20.0 (0.0 to 101.4)	32379458.6 (0.0 to 404845972.6)
**Indicator 16.1.3a: Age-standardised prevalence of physical violence experienced by populations in the past 12 months (%)**						
Central Europe, Eastern Europe, and Central Asia						
Central Asia						
Armenia	4.6 (4.0 to 5.2)	4.6 (4.0 to 5.2)	4.6 (4.0 to 5.2)	4.6 (4.0 to 5.2)	0.0 (-0.0 to 0.0)	0.0 (-0.4 to 0.4)
Azerbaijan	4.9 (4.6 to 5.2)	4.9 (4.6 to 5.1)	4.9 (4.6 to 5.1)	4.8 (4.5 to 5.1)	-0.0 (-0.1 to 0.1)	-0.5 (-1.8 to 1.0)
Georgia	2.6 (2.5 to 2.8)	2.7 (2.5 to 2.9)	2.7 (2.5 to 2.9)	2.7 (2.5 to 2.9)	0.0 (-0.0 to 0.1)	0.5 (-0.1 to 1.0)
Kazakhstan	3.9 (3.7 to 4.2)	4.0 (3.7 to 4.2)	4.0 (3.7 to 4.2)	4.0 (3.7 to 4.2)	-0.0 (-0.1 to 0.1)	-0.0 (-1.2 to 1.2)
Kyrgyzstan	7.1 (6.7 to 7.5)	7.1 (6.7 to 7.4)	7.1 (6.7 to 7.4)	7.1 (6.7 to 7.4)	-0.0 (-0.1 to 0.1)	-0.3 (-1.7 to 1.1)
Mongolia	4.6 (4.0 to 5.2)	4.6 (4.0 to 5.2)	4.6 (4.0 to 5.2)	4.6 (4.0 to 5.2)	0.0 (-0.0 to 0.0)	0.0 (-0.0 to 0.1)
Tajikistan	6.7 (6.3 to 7.1)	6.7 (6.4 to 7.0)	6.7 (6.4 to 7.0)	6.7 (6.4 to 7.0)	-0.0 (-0.1 to 0.1)	-0.1 (-1.5 to 1.5)
Turkmenistan	4.6 (4.0 to 5.2)	4.6 (4.0 to 5.2)	4.5 (4.0 to 5.1)	4.5 (4.0 to 5.1)	-0.0 (-0.0 to -0.0)	-0.2 (-0.3 to -0.0)
Uzbekistan	4.6 (4.0 to 5.2)	4.6 (4.0 to 5.2)	4.6 (4.0 to 5.2)	4.6 (4.0 to 5.2)	-0.0 (-0.0 to -0.0)	-0.2 (-0.4 to -0.0)
Central Europe						
Albania	3.6 (3.4 to 3.9)	3.7 (3.5 to 3.9)	3.7 (3.5 to 3.9)	3.7 (3.5 to 3.9)	-0.0 (-0.0 to 0.0)	-0.0 (-0.3 to 0.3)
Bosnia and Herzegovina	3.7 (3.4 to 3.9)	3.7 (3.4 to 3.9)	3.7 (3.4 to 3.9)	3.7 (3.4 to 3.9)	0.0 (0.0 to 0.0)	0.0 (0.0 to 0.0)
Bulgaria	3.7 (3.4 to 3.9)	3.7 (3.4 to 3.9)	3.7 (3.4 to 3.9)	3.7 (3.4 to 3.9)	-0.0 (-0.0 to -0.0)	-0.2 (-0.2 to -0.1)
Croatia	3.7 (3.4 to 3.9)	3.7 (3.4 to 3.9)	3.7 (3.4 to 3.9)	3.7 (3.4 to 3.9)	-0.0 (-0.0 to -0.0)	-0.0 (-0.0 to -0.0)
Czech Republic	3.7 (3.4 to 3.9)	3.7 (3.4 to 3.9)	3.7 (3.4 to 3.9)	3.7 (3.4 to 3.9)	-0.0 (-0.0 to 0.0)	-0.0 (-0.0 to 0.0)
Hungary	3.7 (3.4 to 3.9)	3.7 (3.4 to 3.9)	3.7 (3.4 to 3.9)	3.7 (3.4 to 3.9)	-0.0 (-0.0 to -0.0)	-0.1 (-0.1 to -0.1)
Macedonia	3.7 (3.4 to 3.9)	3.7 (3.4 to 3.9)	3.7 (3.4 to 3.9)	3.7 (3.4 to 3.9)	0.0 (0.0 to 0.0)	0.1 (0.1 to 0.1)
Montenegro	3.7 (3.4 to 3.9)	3.7 (3.5 to 3.9)	3.7 (3.5 to 3.9)	3.7 (3.5 to 3.9)	-0.0 (-0.0 to -0.0)	-0.1 (-0.1 to -0.0)
Poland	3.7 (3.4 to 3.9)	3.7 (3.4 to 3.9)	3.7 (3.4 to 3.9)	3.7 (3.4 to 3.9)	0.0 (0.0 to 0.0)	0.0 (0.0 to 0.0)
Romania	3.7 (3.4 to 3.9)	3.7 (3.4 to 3.9)	3.7 (3.4 to 3.9)	3.7 (3.4 to 3.9)	-0.0 (-0.0 to -0.0)	-0.1 (-0.1 to -0.0)
Serbia	3.7 (3.4 to 3.9)	3.7 (3.5 to 3.9)	3.7 (3.5 to 3.9)	3.7 (3.5 to 3.9)	-0.0 (-0.0 to 0.0)	-0.0 (-0.0 to 0.0)
Slovakia	3.7 (3.4 to 3.9)	3.7 (3.4 to 3.9)	3.7 (3.4 to 3.9)	3.7 (3.4 to 3.9)	-0.0 (-0.0 to -0.0)	-0.0 (-0.1 to -0.0)
Slovenia	3.7 (3.4 to 3.9)	3.7 (3.4 to 3.9)	3.7 (3.4 to 3.9)	3.7 (3.4 to 3.9)	-0.0 (-0.0 to -0.0)	-0.0 (-0.1 to -0.0)
Eastern Europe						
Belarus	5.3 (5.0 to 5.7)	5.3 (5.0 to 5.7)	5.3 (5.0 to 5.7)	5.3 (5.0 to 5.6)	-0.0 (-0.0 to -0.0)	-0.5 (-0.6 to -0.5)
Estonia	5.3 (5.0 to 5.7)	5.3 (5.0 to 5.6)	5.3 (5.0 to 5.6)	5.3 (5.0 to 5.6)	-0.0 (-0.0 to -0.0)	-0.4 (-0.4 to -0.3)
Latvia	5.3 (5.0 to 5.7)	5.3 (5.0 to 5.6)	5.3 (5.0 to 5.6)	5.3 (5.0 to 5.6)	-0.0 (-0.0 to -0.0)	-0.7 (-0.7 to -0.6)
Lithuania	5.3 (5.0 to 5.7)	5.3 (5.0 to 5.7)	5.3 (5.0 to 5.7)	5.3 (5.0 to 5.6)	-0.0 (-0.0 to -0.0)	-0.6 (-0.7 to -0.6)
Moldova	5.6 (5.3 to 5.9)	5.7 (5.4 to 6.0)	5.6 (5.3 to 5.9)	5.5 (5.2 to 5.8)	-0.2 (-0.3 to -0.1)	-2.7 (-4.6 to -0.8)
Russia	5.3 (5.1 to 5.6)	5.3 (5.1 to 5.6)	5.3 (5.1 to 5.6)	5.3 (5.1 to 5.6)	-0.0 (-0.0 to -0.0)	-0.3 (-0.3 to -0.3)
Ukraine	4.8 (4.6 to 5.0)	4.8 (4.6 to 5.1)	4.8 (4.5 to 5.0)	4.8 (4.5 to 5.0)	-0.1 (-0.2 to 0.0)	-1.3 (-3.0 to 0.4)
High-income						
Australasia						
Australia	3.7 (3.5 to 4.0)	3.7 (3.5 to 4.0)	3.7 (3.4 to 4.0)	3.7 (3.4 to 4.0)	-0.0 (-0.2 to 0.2)	-0.3 (-3.3 to 2.8)
New Zealand	8.9 (8.4 to 9.5)	9.0 (8.5 to 9.5)	8.9 (8.4 to 9.5)	8.9 (8.4 to 9.5)	-0.0 (-0.2 to 0.1)	-0.6 (-2.4 to 1.1)
High-income Asia Pacific						
Brunei	3.7 (3.4 to 4.1)	3.7 (3.4 to 4.0)	3.7 (3.4 to 4.0)	3.7 (3.4 to 4.0)	-0.0 (-0.0 to 0.0)	-0.0 (-0.1 to 0.0)
Japan	3.7 (3.5 to 4.0)	3.7 (3.5 to 4.0)	3.7 (3.5 to 4.0)	3.7 (3.5 to 4.0)	-0.0 (-0.0 to -0.0)	-0.0 (-0.0 to -0.0)
South Korea	3.7 (3.4 to 4.0)	3.7 (3.4 to 4.0)	3.7 (3.4 to 4.0)	3.7 (3.4 to 4.0)	0.0 (0.0 to 0.0)	0.1 (0.0 to 0.2)
Singapore	3.7 (3.3 to 4.0)	3.8 (3.4 to 4.1)	3.8 (3.4 to 4.1)	3.8 (3.4 to 4.1)	-0.0 (-0.0 to -0.0)	-0.1 (-0.2 to -0.0)
High-income North America						
Canada	2.5 (2.3 to 2.7)	2.4 (2.2 to 2.6)	2.4 (2.2 to 2.6)	2.4 (2.2 to 2.6)	-0.0 (-0.1 to 0.0)	-0.4 (-0.8 to 0.1)
Greenland	1.9 (1.7 to 2.2)	1.9 (1.7 to 2.2)	1.9 (1.7 to 2.2)	1.9 (1.7 to 2.2)	-0.0 (-0.0 to 0.0)	-0.0 (-0.1 to 0.1)
United States	1.3 (1.2 to 1.4)	1.3 (1.2 to 1.4)	1.3 (1.3 to 1.4)	1.4 (1.3 to 1.4)	0.4 (0.3 to 0.5)	6.2 (4.5 to 7.8)
Southern Latin America						
Argentina	6.2 (5.5 to 6.8)	6.1 (5.4 to 6.8)	6.1 (5.4 to 6.8)	6.1 (5.4 to 6.8)	0.0 (-0.0 to 0.0)	0.1 (-0.4 to 0.5)
Chile	4.3 (3.6 to 5.2)	4.3 (3.6 to 5.2)	4.3 (3.6 to 5.2)	4.3 (3.6 to 5.2)	-0.0 (-0.0 to -0.0)	-0.1 (-0.2 to -0.1)
Uruguay	3.0 (2.7 to 3.4)	3.0 (2.6 to 3.3)	3.0 (2.6 to 3.3)	3.0 (2.6 to 3.3)	-0.0 (-0.1 to 0.0)	-0.3 (-1.0 to 0.3)
Western Europe						
Andorra	3.9 (3.3 to 4.7)	3.9 (3.3 to 4.7)	3.9 (3.3 to 4.7)	3.9 (3.3 to 4.7)	-0.0 (-0.0 to -0.0)	-0.3 (-0.4 to -0.2)
Austria	4.0 (3.3 to 4.7)	4.0 (3.3 to 4.7)	4.0 (3.3 to 4.7)	3.9 (3.3 to 4.7)	-0.0 (-0.0 to -0.0)	-0.4 (-0.5 to -0.3)
Belgium	4.0 (3.3 to 4.7)	4.0 (3.3 to 4.7)	4.0 (3.3 to 4.7)	4.0 (3.3 to 4.7)	-0.0 (-0.0 to -0.0)	-0.3 (-0.4 to -0.3)
Cyprus	3.9 (3.3 to 4.7)	4.0 (3.3 to 4.7)	4.0 (3.3 to 4.7)	3.9 (3.3 to 4.6)	-0.1 (-0.1 to -0.1)	-1.0 (-1.4 to -0.8)
Denmark	4.0 (3.3 to 4.7)	4.0 (3.3 to 4.7)	4.0 (3.3 to 4.7)	4.0 (3.3 to 4.7)	-0.0 (-0.0 to -0.0)	-0.0 (-0.0 to -0.0)
Finland	4.0 (3.3 to 4.7)	4.0 (3.3 to 4.7)	4.0 (3.3 to 4.7)	4.0 (3.3 to 4.7)	-0.0 (-0.0 to -0.0)	-0.2 (-0.2 to -0.1)
France	4.0 (3.3 to 4.7)	4.0 (3.3 to 4.7)	4.0 (3.3 to 4.7)	4.0 (3.3 to 4.7)	-0.0 (-0.0 to -0.0)	-0.3 (-0.4 to -0.3)

	Estimate in 2000 (95% UIs)	Estimate in 2015 (95% UIs)	Estimate in 2017 (95% UIs)	Estimate in 2030 (95% UIs)	Annualised rate of change, 2015-2030 (95% UIs)	Percent change, 2015-2030 (95% UIs)
Germany	4.0 (3.3 to 4.7)	4.0 (3.3 to 4.7)	3.9 (3.3 to 4.7)	3.9 (3.3 to 4.7)	-0.0 (-0.0 to -0.0)	-0.3 (-0.3 to -0.2)
Greece	3.9 (3.3 to 4.7)	4.0 (3.3 to 4.7)	4.0 (3.3 to 4.7)	4.0 (3.3 to 4.7)	-0.0 (-0.0 to 0.0)	-0.0 (-0.1 to 0.0)
Iceland	4.0 (3.3 to 4.7)	4.0 (3.3 to 4.7)	4.0 (3.3 to 4.7)	4.0 (3.3 to 4.7)	-0.0 (-0.0 to -0.0)	-0.2 (-0.3 to -0.2)
Ireland	4.0 (3.3 to 4.7)	4.0 (3.3 to 4.7)	4.0 (3.3 to 4.7)	4.0 (3.3 to 4.7)	-0.0 (-0.1 to -0.0)	-0.6 (-0.8 to -0.4)
Israel	4.0 (3.3 to 4.7)	4.0 (3.3 to 4.7)	4.0 (3.3 to 4.7)	4.0 (3.3 to 4.7)	-0.0 (-0.0 to -0.0)	-0.3 (-0.3 to -0.2)
Italy	4.0 (3.3 to 4.7)	4.0 (3.3 to 4.7)	4.0 (3.3 to 4.7)	4.0 (3.3 to 4.7)	-0.0 (-0.0 to -0.0)	-0.3 (-0.3 to -0.2)
Luxembourg	4.0 (3.3 to 4.7)	4.0 (3.3 to 4.7)	4.0 (3.3 to 4.7)	4.0 (3.3 to 4.7)	-0.0 (-0.0 to -0.0)	-0.1 (-0.2 to -0.1)
Malta	4.0 (3.3 to 4.7)	3.9 (3.3 to 4.7)	3.9 (3.3 to 4.7)	3.9 (3.3 to 4.7)	-0.0 (-0.0 to 0.0)	-0.1 (-0.7 to 0.6)
Netherlands	4.0 (3.3 to 4.7)	4.0 (3.3 to 4.7)	4.0 (3.3 to 4.7)	4.0 (3.3 to 4.7)	-0.0 (-0.0 to -0.0)	-0.3 (-0.4 to -0.3)
Norway	4.0 (3.6 to 4.3)	3.9 (3.6 to 4.3)	3.9 (3.6 to 4.3)	3.9 (3.6 to 4.3)	-0.0 (-0.0 to -0.0)	-0.2 (-0.2 to -0.2)
Portugal	4.0 (3.3 to 4.7)	4.0 (3.3 to 4.7)	4.0 (3.3 to 4.7)	4.0 (3.3 to 4.7)	-0.0 (-0.1 to -0.0)	-0.7 (-0.9 to -0.6)
Spain	1.7 (1.5 to 1.8)	1.7 (1.5 to 1.8)	1.7 (1.5 to 1.8)	1.7 (1.5 to 1.8)	0.0 (-0.0 to 0.1)	0.2 (-0.6 to 1.0)
Sweden	4.6 (4.4 to 4.9)	4.6 (4.4 to 4.9)	4.6 (4.4 to 4.9)	4.6 (4.4 to 4.9)	0.0 (-0.0 to 0.0)	0.0 (-0.4 to 0.5)
Switzerland	4.0 (3.3 to 4.7)	4.0 (3.3 to 4.7)	4.0 (3.3 to 4.7)	4.0 (3.3 to 4.7)	-0.0 (-0.0 to -0.0)	-0.0 (-0.1 to -0.0)
United Kingdom	5.5 (5.2 to 5.8)	5.4 (5.1 to 5.7)	5.4 (5.1 to 5.7)	5.4 (5.1 to 5.7)	-0.0 (-0.1 to 0.0)	-0.3 (-1.0 to 0.6)
Latin America and Caribbean						
Andean Latin America						
Bolivia	18.4 (17.5 to 19.4)	18.4 (17.6 to 19.2)	18.4 (17.5 to 19.4)	18.4 (17.5 to 19.5)	0.0 (-0.1 to 0.2)	0.5 (-1.3 to 2.4)
Ecuador	8.2 (7.8 to 8.6)	8.3 (7.9 to 8.7)	8.3 (7.9 to 8.7)	8.3 (7.9 to 8.8)	0.0 (-0.0 to 0.0)	0.1 (-0.4 to 0.7)
Peru	7.0 (6.7 to 7.3)	6.9 (6.7 to 7.2)	6.9 (6.6 to 7.1)	6.8 (6.5 to 7.0)	-0.2 (-0.3 to -0.0)	-2.3 (-4.2 to -0.3)
Caribbean						
Antigua and Barbuda	2.4 (1.7 to 3.5)	2.4 (1.7 to 3.4)	2.4 (1.7 to 3.4)	2.3 (1.7 to 3.3)	-0.1 (-0.1 to -0.1)	-1.5 (-2.2 to -1.0)
The Bahamas	2.4 (1.7 to 3.4)	2.4 (1.7 to 3.4)	2.4 (1.7 to 3.4)	2.4 (1.7 to 3.4)	-0.0 (-0.0 to -0.0)	-0.3 (-0.5 to -0.2)
Barbados	2.3 (1.7 to 3.4)	2.3 (1.7 to 3.4)	2.3 (1.7 to 3.4)	2.3 (1.7 to 3.4)	-0.0 (-0.1 to -0.0)	-0.5 (-0.8 to -0.2)
Belize	2.3 (1.7 to 3.4)	2.3 (1.7 to 3.4)	2.3 (1.7 to 3.4)	2.3 (1.7 to 3.4)	-0.0 (-0.0 to 0.0)	-0.3 (-0.6 to 0.0)
Bermuda	2.4 (1.7 to 3.4)	2.4 (1.7 to 3.4)	2.4 (1.7 to 3.4)	2.4 (1.7 to 3.4)	-0.0 (-0.0 to -0.0)	-0.3 (-0.5 to -0.1)
Cuba	2.3 (1.7 to 3.3)	2.3 (1.6 to 3.3)	2.3 (1.6 to 3.3)	2.3 (1.6 to 3.3)	-0.0 (-0.0 to 0.0)	-0.2 (-0.4 to 0.0)
Dominica	2.3 (1.6 to 3.3)	2.3 (1.6 to 3.3)	2.3 (1.6 to 3.3)	2.3 (1.6 to 3.3)	-0.0 (-0.0 to -0.0)	-0.3 (-0.5 to -0.1)
Dominican Republic	4.8 (4.6 to 5.1)	4.9 (4.7 to 5.1)	4.9 (4.6 to 5.2)	4.8 (4.5 to 5.2)	-0.0 (-0.3 to 0.2)	-0.6 (-3.7 to 2.8)
Grenada	2.3 (1.7 to 3.4)	2.3 (1.6 to 3.3)	2.3 (1.6 to 3.3)	2.3 (1.6 to 3.3)	-0.0 (-0.0 to 0.0)	-0.3 (-0.6 to 0.1)
Guyana	2.3 (1.7 to 3.4)	2.3 (1.7 to 3.4)	2.4 (1.7 to 3.4)	2.3 (1.7 to 3.4)	-0.0 (-0.1 to 0.1)	-0.3 (-1.1 to 0.8)
Haiti	4.8 (4.5 to 5.0)	4.5 (4.3 to 4.7)	4.5 (4.3 to 4.7)	4.4 (4.1 to 4.7)	-0.2 (-0.3 to 0.1)	-2.2 (-5.1 to 0.9)
Jamaica	2.4 (1.7 to 3.4)	2.3 (1.7 to 3.4)	2.3 (1.7 to 3.4)	2.3 (1.7 to 3.4)	-0.0 (-0.1 to -0.0)	-0.7 (-1.2 to -0.1)
Puerto Rico	0.7 (0.6 to 0.7)	0.7 (0.6 to 0.7)	0.7 (0.6 to 0.7)	0.7 (0.6 to 0.7)	0.1 (-0.1 to 0.2)	0.8 (-1.2 to 2.8)
Saint Lucia	2.4 (1.7 to 3.4)	2.3 (1.7 to 3.4)	2.3 (1.7 to 3.4)	2.3 (1.7 to 3.3)	-0.0 (-0.0 to -0.0)	-0.5 (-0.7 to -0.3)
Saint Vincent and the Grenadines	2.3 (1.6 to 3.3)	2.3 (1.6 to 3.3)	2.3 (1.6 to 3.3)	2.3 (1.7 to 3.3)	0.0 (0.0 to 0.0)	0.3 (0.1 to 0.6)
Suriname	2.3 (1.7 to 3.3)	2.3 (1.7 to 3.4)	2.3 (1.7 to 3.4)	2.3 (1.7 to 3.4)	0.0 (-0.0 to 0.0)	0.0 (-0.2 to 0.3)
Trinidad and Tobago	2.3 (1.7 to 3.4)	2.3 (1.7 to 3.3)	2.3 (1.7 to 3.3)	2.3 (1.7 to 3.3)	-0.0 (-0.0 to -0.0)	-0.1 (-0.2 to -0.0)
Virgin Islands, U.S.	1.3 (1.2 to 1.4)	1.3 (1.2 to 1.4)	1.3 (1.2 to 1.4)	1.3 (1.2 to 1.4)	0.0 (-0.1 to 0.2)	0.6 (-1.3 to 2.6)
Central Latin America						
Colombia	12.1 (11.5 to 12.7)	12.5 (12.0 to 12.9)	12.6 (12.0 to 13.1)	12.8 (12.2 to 13.4)	0.2 (0.0 to 0.3)	3.0 (0.6 to 5.0)
Costa Rica	7.2 (6.6 to 7.7)	7.2 (6.7 to 7.7)	7.2 (6.7 to 7.7)	7.2 (6.7 to 7.7)	0.0 (-0.0 to 0.0)	0.1 (-0.3 to 0.4)
El Salvador	5.6 (5.3 to 5.9)	5.6 (5.3 to 5.9)	5.6 (5.3 to 5.9)	5.6 (5.3 to 5.9)	0.0 (-0.1 to 0.1)	0.2 (-1.0 to 1.3)
Guatemala	4.4 (4.2 to 4.6)	4.4 (4.2 to 4.6)	4.3 (4.2 to 4.6)	4.3 (4.1 to 4.5)	-0.1 (-0.3 to -0.0)	-2.0 (-3.7 to -0.0)
Honduras	5.5 (5.2 to 5.7)	5.8 (5.6 to 6.1)	5.9 (5.6 to 6.1)	6.0 (5.6 to 6.3)	0.2 (0.0 to 0.3)	3.0 (0.4 to 5.4)
Mexico	6.7 (6.0 to 7.4)	6.7 (6.0 to 7.4)	6.7 (6.0 to 7.4)	6.7 (6.0 to 7.4)	-0.0 (-0.0 to 0.0)	-0.0 (-0.1 to 0.0)
Nicaragua	5.0 (4.7 to 5.2)	4.8 (4.6 to 5.0)	4.8 (4.6 to 5.1)	4.8 (4.5 to 5.0)	-0.0 (-0.2 to 0.1)	-0.3 (-2.8 to 2.0)
Panama	6.7 (5.4 to 8.1)	6.7 (5.4 to 8.1)	6.7 (5.4 to 8.1)	6.7 (5.4 to 8.1)	0.0 (0.0 to 0.0)	0.0 (0.0 to 0.1)
Venezuela	6.7 (5.4 to 8.1)	6.7 (5.4 to 8.1)	6.7 (5.4 to 8.1)	6.7 (5.4 to 8.1)	0.0 (0.0 to 0.0)	0.0 (0.0 to 0.0)
Tropical Latin America						
Brazil	4.4 (4.2 to 4.6)	4.4 (4.2 to 4.6)	4.4 (4.2 to 4.6)	4.4 (4.2 to 4.6)	-0.0 (-0.0 to -0.0)	-0.1 (-0.1 to -0.1)
Paraguay	4.4 (4.2 to 4.6)	4.4 (4.1 to 4.6)	4.4 (4.1 to 4.6)	4.4 (4.1 to 4.6)	-0.0 (-0.1 to 0.1)	-0.1 (-1.5 to 1.5)
North Africa and Middle East						
North Africa and Middle East						
Afghanistan	21.5 (20.4 to 22.7)	21.1 (20.2 to 22.2)	21.1 (20.1 to 22.1)	21.2 (20.2 to 22.2)	0.0 (-0.1 to 0.1)	0.2 (-0.8 to 1.3)
Algeria	12.4 (10.5 to 14.7)	12.4 (10.5 to 14.7)	12.5 (10.5 to 14.7)	12.4 (10.5 to 14.7)	-0.0 (-0.0 to 0.0)	-0.1 (-0.2 to 0.0)
Bahrain	12.1 (10.4 to 14.1)	11.9 (10.3 to 13.8)	11.9 (10.3 to 13.9)	11.9 (10.2 to 13.9)	0.0 (-0.1 to 0.1)	0.1 (-1.1 to 1.3)
Egypt	11.2 (10.7 to 11.7)	10.8 (10.3 to 11.4)	10.8 (10.2 to 11.4)	10.7 (10.1 to 11.3)	-0.1 (-0.2 to 0.0)	-1.2 (-2.9 to 0.6)
Iran	12.5 (11.3 to 13.7)	12.5 (11.3 to 13.7)	12.5 (11.3 to 13.7)	12.5 (11.3 to 13.6)	-0.0 (-0.0 to 0.0)	-0.1 (-0.5 to 0.2)
Iraq	12.4 (11.8 to 13.2)	12.4 (11.8 to 13.0)	12.4 (11.8 to 13.2)	12.4 (11.8 to 13.2)	0.0 (-0.1 to 0.1)	0.4 (-1.3 to 2.0)
Jordan	9.6 (9.1 to 10.2)	9.6 (9.2 to 10.1)	9.6 (9.1 to 10.2)	9.6 (9.1 to 10.2)	0.0 (-0.1 to 0.1)	0.1 (-1.3 to 1.6)
Kuwait	11.9 (10.3 to 13.9)	12.2 (10.4 to 14.5)	12.3 (10.4 to 14.5)	12.2 (10.4 to 14.4)	0.0 (-0.0 to 0.1)	0.1 (-0.5 to 0.8)
Lebanon	12.5 (10.5 to 14.8)	12.4 (10.5 to 14.7)	12.4 (10.5 to 14.7)	12.5 (10.6 to 14.7)	0.0 (-0.0 to 0.1)	0.1 (-0.7 to 0.8)
Libya	12.4 (10.5 to 14.7)	12.4 (10.5 to 14.6)	12.4 (10.5 to 14.6)	12.4 (10.5 to 14.6)	-0.0 (-0.0 to 0.0)	-0.0 (-0.3 to 0.3)
Morocco	12.5 (10.5 to 14.9)	12.5 (10.5 to 14.8)	12.5 (10.5 to 14.8)	12.4 (10.5 to 14.7)	-0.0 (-0.1 to 0.0)	-0.3 (-0.8 to 0.1)
Palestine	12.4 (10.5 to 14.7)	12.4 (10.5 to 14.7)	12.4 (10.5 to 14.7)	12.4 (10.5 to 14.7)	0.0 (-0.0 to 0.0)	0.0 (-0.0 to 0.1)
Oman	12.0 (10.3 to 14.1)	11.7 (10.2 to 13.6)	11.7 (10.2 to 13.5)	11.8 (10.2 to 13.6)	0.0 (-0.0 to 0.1)	0.3 (-0.1 to 0.8)
Qatar	11.7 (10.1 to 13.5)	11.3 (10.0 to 12.9)	11.3 (10.0 to 12.9)	11.4 (10.0 to 13.1)	0.1 (-0.0 to 0.2)	1.0 (-0.3 to 2.3)
Saudi Arabia	12.1 (10.4 to 14.2)	12.1 (10.4 to 14.1)	12.1 (10.4 to 14.1)	12.1 (10.4 to 14.2)	0.0 (-0.0 to 0.1)	0.4 (-0.2 to 1.1)
Sudan	12.5 (10.5 to 14.8)	12.5 (10.5 to 14.9)	12.5 (10.5 to 14.9)	12.5 (10.5 to 14.8)	-0.0 (-0.0 to 0.0)	-0.1 (-0.2 to 0.0)
Syria	12.4 (10.5 to 14.7)	12.4 (10.5 to 14.8)	12.4 (10.5 to 14.8)	12.4 (10.5 to 14.8)	0.0 (-0.0 to 0.0)	0.1 (-0.2 to 0.3)
Tunisia	12.5 (10.5 to 14.8)	12.5 (10.5 to 14.8)	12.5 (10.5 to 14.8)	12.5 (10.5 to 14.7)	-0.0 (-0.0 to 0.0)	-0.3 (-0.6 to 0.1)
Turkey	8.6 (8.1 to 9.1)	8.6 (8.1 to 9.1)	8.6 (8.1 to 9.1)	8.6 (8.1 to 9.1)	-0.0 (-0.1 to 0.1)	-0.2 (-1.1 to 0.9)
United Arab Emirates	11.6 (10.1 to 13.3)	11.5 (10.0 to 13.2)	11.5 (10.1 to 13.3)	11.8 (10.2 to 13.7)	0.2 (0.0 to 0.3)	2.4 (0.5 to 4.6)
Yemen	12.4 (10.5 to 14.7)	12.5 (10.5 to 14.7)	12.5 (10.5 to 14.8)	12.5 (10.5 to 14.8)	-0.0 (-0.0 to 0.0)	-0.1 (-0.3 to 0.1)
South Asia						
South Asia						
Bangladesh	10.9 (10.3 to 11.5)	11.1 (10.5 to 11.7)	11.1 (10.5 to 11.7)	11.1 (10.4 to 11.7)	-0.0 (-0.1 to 0.1)	-0.2 (-1.7 to 1.2)
Bhutan	8.0 (7.5 to 8.5)	7.9 (7.5 to 8.4)	7.9 (7.4 to 8.4)	8.0 (7.4 to 8.5)	0.0 (-0.1 to 0.2)	0.7 (-0.8 to 2.3)
India	10.5 (10.0 to 11.0)	10.5 (10.0 to 11.0)	10.5 (10.0 to 11.0)	10.5 (10.0 to 11.1)	-0.0 (-0.1 to 0.0)	-0.1 (-0.8 to 0.5)

	Estimate in 2000 (95% UIs)	Estimate in 2015 (95% UIs)	Estimate in 2017 (95% UIs)	Estimate in 2030 (95% UIs)	Annualised rate of change, 2015-2030 (95% UIs)	Percent change, 2015-2030 (95% UIs)
Nepal	7.4 (7.0 to 7.9)	7.4 (7.0 to 7.8)	7.4 (7.0 to 7.9)	7.5 (7.0 to 7.9)	0.1 (-0.0 to 0.1)	0.8 (-0.5 to 2.1)
Pakistan	10.8 (10.2 to 11.5)	10.9 (10.3 to 11.5)	10.9 (10.3 to 11.5)	10.9 (10.3 to 11.5)	-0.0 (-0.1 to 0.1)	-0.0 (-1.3 to 1.3)
Southeast Asia, East Asia, and Oceania						
East Asia						
China	3.5 (3.3 to 3.7)	3.5 (3.3 to 3.7)	3.5 (3.3 to 3.7)	3.5 (3.3 to 3.7)	0.0 (-0.0 to 0.1)	0.1 (-0.5 to 0.8)
North Korea	3.5 (3.2 to 3.7)	3.5 (3.2 to 3.7)	3.5 (3.2 to 3.7)	3.5 (3.2 to 3.7)	-0.0 (-0.0 to -0.0)	-0.0 (-0.1 to -0.0)
Taiwan (Province of China)	3.5 (3.2 to 3.7)	3.5 (3.2 to 3.7)	3.5 (3.2 to 3.7)	3.5 (3.2 to 3.7)	0.0 (0.0 to 0.0)	0.3 (0.1 to 0.4)
Oceania						
American Samoa	4.3 (3.9 to 4.6)	4.3 (3.9 to 4.6)	4.3 (3.9 to 4.6)	4.2 (3.9 to 4.6)	-0.0 (-0.1 to -0.0)	-0.7 (-1.0 to -0.4)
Federated States of Micronesia	4.3 (3.9 to 4.6)	4.3 (3.9 to 4.6)	4.3 (3.9 to 4.6)	4.3 (3.9 to 4.6)	-0.0 (-0.0 to 0.0)	-0.1 (-0.2 to 0.0)
Fiji	4.3 (3.9 to 4.6)	4.3 (3.9 to 4.6)	4.3 (3.9 to 4.6)	4.3 (3.9 to 4.6)	0.0 (0.0 to 0.0)	0.0 (0.0 to 0.1)
Guam	4.3 (3.9 to 4.6)	4.3 (3.9 to 4.6)	4.2 (3.9 to 4.6)	4.3 (3.9 to 4.6)	0.0 (-0.0 to 0.0)	0.0 (-0.0 to 0.1)
Kiribati	4.3 (4.0 to 4.7)	4.3 (4.0 to 4.7)	4.3 (4.0 to 4.7)	4.3 (4.0 to 4.6)	-0.0 (-0.0 to -0.0)	-0.2 (-0.2 to -0.1)
Marshall Islands	4.3 (3.9 to 4.6)	4.3 (3.9 to 4.6)	4.3 (3.9 to 4.6)	4.3 (3.9 to 4.6)	0.0 (0.0 to 0.0)	0.1 (0.0 to 0.1)
Northern Mariana Islands	4.3 (3.9 to 4.6)	4.3 (3.9 to 4.6)	4.2 (3.9 to 4.6)	4.3 (4.0 to 4.7)	0.1 (0.0 to 0.1)	1.0 (0.5 to 1.4)
Papua New Guinea	4.3 (3.9 to 4.6)	4.3 (3.9 to 4.6)	4.3 (3.9 to 4.6)	4.3 (3.9 to 4.6)	-0.0 (-0.0 to 0.0)	-0.0 (-0.0 to 0.0)
Samoa	4.2 (3.9 to 4.6)	4.2 (3.9 to 4.6)	4.2 (3.9 to 4.6)	4.2 (3.9 to 4.6)	-0.0 (-0.0 to -0.0)	-0.1 (-0.2 to -0.1)
Solomon Islands	4.3 (3.9 to 4.6)	4.3 (3.9 to 4.6)	4.3 (3.9 to 4.6)	4.3 (3.9 to 4.6)	-0.0 (-0.0 to 0.0)	-0.1 (-0.2 to 0.0)
Tonga	4.3 (3.9 to 4.6)	4.3 (3.9 to 4.6)	4.3 (3.9 to 4.6)	4.3 (3.9 to 4.6)	-0.0 (-0.0 to -0.0)	-0.3 (-0.4 to -0.2)
Vanuatu	4.3 (3.9 to 4.6)	4.3 (3.9 to 4.6)	4.3 (3.9 to 4.6)	4.3 (3.9 to 4.6)	0.0 (0.0 to 0.0)	0.2 (0.1 to 0.2)
Southeast Asia						
Cambodia	5.6 (5.3 to 5.9)	5.4 (5.1 to 5.7)	5.4 (5.1 to 5.7)	5.4 (5.0 to 5.7)	0.0 (-0.1 to 0.2)	0.2 (-2.1 to 2.6)
Indonesia	6.2 (5.4 to 7.0)	6.2 (5.4 to 6.9)	6.2 (5.4 to 6.9)	6.2 (5.4 to 6.9)	-0.0 (-0.0 to -0.0)	-0.2 (-0.4 to -0.1)
Laos	6.2 (4.9 to 8.0)	6.2 (4.9 to 7.9)	6.2 (4.9 to 7.9)	6.2 (4.9 to 7.9)	-0.0 (-0.0 to -0.0)	-0.4 (-0.7 to -0.2)
Malaysia	6.1 (4.9 to 7.8)	6.1 (4.8 to 7.7)	6.1 (4.8 to 7.7)	6.1 (4.8 to 7.8)	-0.0 (-0.1 to 0.0)	-0.1 (-0.8 to 0.5)
Maldives	6.2 (4.9 to 7.9)	5.8 (4.6 to 7.3)	5.7 (4.6 to 7.2)	5.5 (4.5 to 7.0)	-0.2 (-0.4 to -0.1)	-3.6 (-5.8 to -1.7)
Mauritius	6.2 (4.9 to 7.9)	6.2 (4.9 to 7.9)	6.2 (4.9 to 7.9)	6.2 (4.9 to 7.9)	-0.0 (-0.0 to 0.0)	-0.1 (-0.2 to 0.0)
Myanmar	4.9 (4.6 to 5.2)	4.9 (4.7 to 5.2)	4.9 (4.7 to 5.2)	4.9 (4.7 to 5.2)	-0.0 (-0.1 to 0.1)	-0.1 (-1.5 to 1.4)
Philippines	4.0 (3.7 to 4.2)	4.0 (3.7 to 4.2)	3.9 (3.7 to 4.2)	3.9 (3.6 to 4.2)	-0.1 (-0.3 to 0.1)	-1.5 (-4.0 to 0.8)
Sri Lanka	4.1 (3.7 to 4.4)	4.1 (3.8 to 4.5)	4.1 (3.8 to 4.5)	4.1 (3.8 to 4.5)	0.0 (-0.0 to 0.1)	0.3 (-0.2 to 0.7)
Seychelles	6.2 (4.9 to 7.9)	6.0 (4.8 to 7.6)	6.0 (4.8 to 7.6)	6.0 (4.8 to 7.6)	0.0 (-0.0 to 0.0)	0.0 (-0.1 to 0.2)
Thailand	6.2 (4.9 to 8.0)	6.2 (4.9 to 7.9)	6.2 (4.9 to 7.9)	6.2 (4.9 to 7.9)	-0.0 (-0.0 to -0.0)	-0.2 (-0.4 to -0.1)
Timor-Leste	12.5 (11.8 to 13.2)	12.5 (11.9 to 13.1)	12.5 (11.8 to 13.2)	12.5 (11.7 to 13.3)	-0.0 (-0.2 to 0.1)	-0.1 (-2.5 to 2.1)
Vietnam	6.2 (5.0 to 8.0)	6.2 (4.9 to 7.9)	6.2 (4.9 to 7.9)	6.1 (4.9 to 7.8)	-0.0 (-0.0 to -0.0)	-0.5 (-0.7 to -0.3)
Sub-Saharan Africa						
Central Sub-Saharan Africa						
Angola	15.9 (14.4 to 17.5)	16.1 (14.5 to 17.7)	16.1 (14.5 to 17.7)	16.1 (14.5 to 17.7)	-0.0 (-0.0 to -0.0)	-0.2 (-0.3 to -0.1)
Central African Republic	13.5 (12.8 to 14.2)	13.5 (12.8 to 14.1)	13.5 (12.8 to 14.2)	13.5 (12.8 to 14.2)	0.0 (-0.1 to 0.1)	0.2 (-1.3 to 1.7)
Congo	15.9 (14.4 to 17.5)	15.9 (14.4 to 17.4)	15.9 (14.4 to 17.5)	15.9 (14.4 to 17.4)	-0.0 (-0.0 to 0.0)	-0.1 (-0.1 to 0.0)
Democratic Republic of the Congo	20.1 (19.1 to 21.2)	19.6 (18.7 to 20.6)	19.6 (18.6 to 20.6)	19.3 (18.2 to 20.5)	-0.1 (-0.3 to 0.1)	-1.8 (-4.3 to 0.8)
Equatorial Guinea	16.3 (14.8 to 18.0)	15.8 (14.3 to 17.4)	15.7 (14.3 to 17.3)	15.7 (14.3 to 17.2)	-0.0 (-0.1 to 0.1)	-0.5 (-1.8 to 1.1)
Gabon	14.3 (13.6 to 15.1)	14.3 (13.7 to 15.0)	14.3 (13.7 to 15.0)	14.3 (13.6 to 15.0)	0.0 (-0.1 to 0.1)	0.1 (-1.3 to 1.4)
Eastern Sub-Saharan Africa						
Burundi	14.1 (11.6 to 16.9)	14.0 (11.5 to 16.8)	14.0 (11.5 to 16.8)	14.0 (11.5 to 16.9)	0.0 (0.0 to 0.0)	0.1 (0.0 to 0.2)
Comoros	8.2 (7.7 to 8.6)	8.2 (7.8 to 8.6)	8.2 (7.8 to 8.7)	8.2 (7.8 to 8.7)	0.0 (-0.0 to 0.1)	0.3 (-0.3 to 0.9)
Djibouti	13.9 (11.4 to 16.8)	14.0 (11.5 to 17.0)	14.0 (11.5 to 17.0)	14.1 (11.5 to 17.0)	0.0 (0.0 to 0.0)	0.1 (0.0 to 0.2)
Eritrea	14.1 (11.6 to 16.9)	14.0 (11.5 to 16.9)	14.0 (11.5 to 16.9)	14.0 (11.5 to 16.9)	-0.0 (-0.0 to -0.0)	-0.1 (-0.2 to -0.1)
Ethiopia	14.1 (12.8 to 15.3)	14.1 (12.8 to 15.3)	14.1 (12.8 to 15.3)	14.1 (12.8 to 15.3)	-0.0 (-0.0 to 0.0)	-0.0 (-0.1 to 0.0)
Kenya	11.2 (10.6 to 11.8)	11.2 (10.6 to 11.8)	11.2 (10.7 to 11.8)	11.4 (10.8 to 12.0)	0.1 (0.1 to 0.1)	1.4 (0.8 to 2.1)
Madagascar	14.0 (11.6 to 16.9)	14.1 (11.6 to 16.9)	14.1 (11.6 to 16.9)	14.1 (11.6 to 16.9)	0.0 (-0.0 to 0.0)	0.1 (-0.0 to 0.1)
Malawi	18.6 (17.6 to 19.6)	19.0 (18.1 to 19.9)	19.0 (18.1 to 20.0)	19.1 (18.1 to 20.1)	0.0 (-0.0 to 0.1)	0.6 (-0.6 to 1.9)
Mozambique	13.7 (12.9 to 14.5)	13.9 (13.3 to 14.6)	13.8 (13.1 to 14.6)	13.7 (12.9 to 14.5)	-0.1 (-0.2 to 0.0)	-1.3 (-3.1 to 0.5)
Rwanda	14.5 (13.8 to 15.3)	14.6 (14.0 to 15.4)	14.6 (13.9 to 15.4)	14.3 (13.5 to 15.2)	-0.1 (-0.3 to -0.0)	-2.2 (-4.3 to -0.0)
Somalia	14.0 (11.5 to 16.9)	14.0 (11.5 to 16.9)	14.0 (11.5 to 16.9)	14.0 (11.5 to 16.9)	-0.0 (-0.0 to 0.0)	-0.0 (-0.1 to 0.1)
South Sudan	14.0 (11.5 to 16.9)	14.1 (11.6 to 17.0)	14.1 (11.6 to 17.0)	14.1 (11.6 to 17.0)	0.0 (0.0 to 0.0)	0.0 (0.0 to 0.1)
Tanzania	12.7 (12.0 to 13.4)	12.5 (11.9 to 13.2)	12.4 (11.7 to 13.1)	12.2 (11.5 to 13.1)	-0.1 (-0.3 to 0.1)	-2.1 (-5.0 to 0.8)
Uganda	20.5 (19.6 to 21.6)	20.6 (19.8 to 21.5)	20.6 (19.6 to 21.6)	20.6 (19.6 to 21.7)	-0.0 (-0.1 to 0.1)	-0.1 (-1.9 to 1.7)
Zambia	14.9 (14.2 to 15.6)	14.7 (14.1 to 15.4)	14.6 (13.9 to 15.3)	14.4 (13.6 to 15.3)	-0.1 (-0.3 to 0.0)	-2.0 (-4.3 to 0.5)
Southern Sub-Saharan Africa						
Botswana	10.1 (9.4 to 10.9)	10.1 (9.4 to 10.9)	10.1 (9.4 to 10.9)	10.1 (9.4 to 10.9)	0.0 (0.0 to 0.0)	0.1 (0.0 to 0.1)
Lesotho	10.1 (9.4 to 10.9)	10.1 (9.4 to 10.8)	10.1 (9.4 to 10.8)	10.1 (9.4 to 10.9)	0.0 (0.0 to 0.0)	0.2 (0.1 to 0.4)
Namibia	10.4 (9.8 to 10.9)	10.4 (9.9 to 10.9)	10.4 (9.9 to 10.9)	10.3 (9.9 to 10.9)	-0.0 (-0.1 to 0.1)	-0.1 (-1.2 to 1.2)
South Africa	8.2 (7.8 to 8.6)	8.2 (7.8 to 8.6)	8.2 (7.8 to 8.7)	8.3 (7.9 to 8.7)	0.0 (0.0 to 0.1)	0.6 (0.4 to 0.8)
Swaziland	10.1 (9.4 to 10.9)	10.1 (9.4 to 10.9)	10.1 (9.4 to 10.9)	10.2 (9.5 to 10.9)	0.0 (0.0 to 0.0)	0.3 (0.2 to 0.4)
Zimbabwe	12.3 (11.7 to 12.9)	11.7 (11.2 to 12.3)	11.7 (11.1 to 12.3)	11.4 (10.8 to 12.2)	-0.1 (-0.3 to 0.0)	-2.2 (-4.7 to 0.4)
Western Sub-Saharan Africa						
Benin	13.2 (11.3 to 15.3)	13.2 (11.3 to 15.3)	13.2 (11.2 to 15.3)	13.2 (11.3 to 15.3)	0.0 (-0.0 to 0.0)	0.0 (-0.0 to 0.1)
Burkina Faso	9.3 (8.8 to 9.7)	9.3 (8.8 to 9.7)	9.3 (8.8 to 9.7)	9.3 (8.8 to 9.8)	0.0 (-0.0 to 0.1)	0.5 (-0.5 to 1.4)
Cameroon	13.0 (12.4 to 13.6)	13.2 (12.6 to 13.7)	13.1 (12.4 to 13.8)	13.0 (12.3 to 13.8)	-0.1 (-0.2 to 0.1)	-0.9 (-3.0 to 1.5)
Cape Verde	13.2 (11.2 to 15.3)	13.0 (11.1 to 15.2)	13.0 (11.1 to 15.2)	13.1 (11.2 to 15.2)	0.0 (-0.0 to 0.0)	0.1 (-0.0 to 0.3)
Chad	12.7 (12.0 to 13.3)	12.5 (11.9 to 13.1)	12.4 (11.9 to 13.1)	12.3 (11.7 to 13.0)	-0.1 (-0.2 to 0.1)	-1.1 (-3.2 to 0.8)
Cote d'Ivoire	14.0 (13.3 to 14.7)	13.9 (13.4 to 14.6)	13.9 (13.4 to 14.6)	14.0 (13.4 to 14.6)	0.0 (-0.1 to 0.1)	0.2 (-0.8 to 1.3)
The Gambia	9.6 (9.0 to 10.0)	9.5 (9.1 to 10.0)	9.5 (9.1 to 10.0)	9.6 (9.1 to 10.1)	0.0 (-0.0 to 0.1)	0.2 (-0.5 to 1.0)
Ghana	12.6 (11.9 to 13.3)	12.6 (12.0 to 13.2)	12.6 (11.9 to 13.3)	12.6 (11.9 to 13.3)	0.0 (-0.1 to 0.1)	0.3 (-1.3 to 1.9)
Guinea	13.2 (11.3 to 15.3)	13.2 (11.3 to 15.3)	13.2 (11.3 to 15.4)	13.2 (11.3 to 15.4)	0.0 (0.0 to 0.0)	0.1 (0.0 to 0.1)
Guinea-Bissau	13.2 (11.3 to 15.3)	13.2 (11.3 to 15.3)	13.2 (11.3 to 15.3)	13.2 (11.3 to 15.3)	0.0 (0.0 to 0.0)	0.2 (0.1 to 0.2)
Liberia	16.8 (16.0 to 17.7)	16.8 (16.1 to 17.6)	16.9 (16.0 to 17.7)	16.8 (15.9 to 17.8)	0.0 (-0.1 to 0.1)	0.1 (-1.5 to 1.8)
Mali	15.2 (14.4 to 15.9)	15.2 (14.6 to 15.8)	15.2 (14.6 to 15.8)	15.2 (14.5 to 15.8)	0.0 (-0.1 to 0.1)	0.1 (-1.2 to 1.3)
Mauritania	13.2 (11.2 to 15.3)	13.2 (11.2 to 15.3)	13.2 (11.2 to 15.3)	13.2 (11.2 to 15.3)	-0.0 (-0.0 to 0.0)	-0.0 (-0.0 to 0.0)
Niger	13.1 (11.2 to 15.2)	13.1 (11.2 to 15.3)	13.1 (11.2 to 15.3)	13.1 (11.2 to 15.3)	0.0 (-0.0 to 0.0)	0.0 (-0.1 to 0.2)

	Estimate in 2000 (95% UIs)	Estimate in 2015 (95% UIs)	Estimate in 2017 (95% UIs)	Estimate in 2030 (95% UIs)	Annualised rate of change, 2015-2030 (95% UIs)	Percent change, 2015-2030 (95% UIs)
Nigeria	10.9 (10.3 to 11.4)	10.7 (10.2 to 11.2)	10.6 (10.1 to 11.2)	10.5 (9.9 to 11.1)	-0.1 (-0.2 to 0.0)	-1.8 (-3.5 to 0.3)
Sao Tome and Principe	16.0 (15.2 to 16.7)	15.8 (15.1 to 16.5)	15.8 (15.1 to 16.6)	15.8 (15.0 to 16.6)	0.0 (-0.1 to 0.1)	0.1 (-1.4 to 1.6)
Senegal	13.1 (11.2 to 15.3)	13.2 (11.2 to 15.3)	13.2 (11.2 to 15.3)	13.1 (11.2 to 15.3)	-0.0 (-0.0 to -0.0)	-0.2 (-0.3 to -0.1)
Sierra Leone	20.6 (19.6 to 21.6)	20.5 (19.8 to 21.3)	20.5 (19.8 to 21.2)	20.5 (19.7 to 21.4)	0.0 (-0.1 to 0.1)	0.1 (-0.9 to 1.2)
Togo	9.8 (9.3 to 10.3)	9.9 (9.4 to 10.4)	9.9 (9.4 to 10.4)	9.9 (9.4 to 10.4)	0.0 (-0.0 to 0.1)	0.3 (-0.4 to 1.1)
**Indicator 16.1.3c: Age-standardised prevalence of sexual violence experienced by populations in the past 12 months (%)**						
Central Europe, Eastern Europe, and Central Asia						
Central Asia						
Armenia	0.9 (0.8 to 1.1)	0.9 (0.8 to 1.1)	0.9 (0.8 to 1.1)	1.0 (0.8 to 1.1)	0.1 (-0.2 to 0.4)	1.5 (-2.7 to 5.7)
Azerbaijan	1.2 (1.1 to 1.4)	1.2 (1.0 to 1.4)	1.2 (1.0 to 1.4)	1.2 (1.0 to 1.4)	0.1 (-0.2 to 0.4)	1.4 (-3.2 to 6.3)
Georgia	0.7 (0.6 to 0.8)	0.8 (0.7 to 0.9)	0.8 (0.7 to 0.9)	0.8 (0.7 to 1.0)	0.7 (0.4 to 0.9)	10.6 (6.0 to 15.3)
Kazakhstan	0.9 (0.8 to 1.1)	1.0 (0.8 to 1.1)	1.0 (0.8 to 1.1)	1.0 (0.9 to 1.2)	0.4 (0.1 to 0.7)	5.8 (1.1 to 10.5)
Kyrgyzstan	1.3 (1.2 to 1.6)	1.3 (1.2 to 1.5)	1.3 (1.2 to 1.5)	1.3 (1.1 to 1.5)	-0.2 (-0.5 to 0.1)	-2.9 (-7.1 to 1.5)
Mongolia	1.0 (0.8 to 1.1)	1.1 (0.9 to 1.2)	1.1 (0.9 to 1.3)	1.2 (1.0 to 1.4)	0.5 (0.3 to 0.8)	8.5 (4.1 to 12.8)
Tajikistan	1.3 (1.1 to 1.5)	1.2 (1.1 to 1.4)	1.2 (1.1 to 1.4)	1.1 (1.0 to 1.3)	-0.3 (-0.6 to 0.0)	-4.3 (-8.4 to 0.2)
Turkmenistan	1.0 (0.8 to 1.1)	0.9 (0.8 to 1.1)	0.9 (0.8 to 1.1)	0.9 (0.8 to 1.1)	0.0 (-0.3 to 0.3)	0.0 (-3.9 to 3.9)
Uzbekistan	0.9 (0.8 to 1.1)	1.0 (0.9 to 1.2)	1.0 (0.9 to 1.2)	1.1 (0.9 to 1.2)	0.4 (0.1 to 0.6)	5.6 (1.3 to 9.8)
Central Europe						
Albania	0.9 (0.8 to 1.0)	1.0 (0.8 to 1.1)	1.0 (0.8 to 1.1)	1.0 (0.8 to 1.2)	0.2 (-0.1 to 0.4)	2.3 (-1.9 to 6.4)
Bosnia and Herzegovina	1.3 (1.1 to 1.5)	1.3 (1.1 to 1.4)	1.3 (1.1 to 1.4)	1.2 (1.1 to 1.4)	-0.1 (-0.4 to 0.2)	-1.1 (-5.2 to 2.7)
Bulgaria	1.4 (1.2 to 1.7)	1.4 (1.2 to 1.7)	1.4 (1.2 to 1.7)	1.4 (1.2 to 1.7)	-0.1 (-0.4 to 0.2)	-1.7 (-6.0 to 2.8)
Croatia	1.6 (1.4 to 1.9)	1.3 (1.2 to 1.6)	1.3 (1.2 to 1.5)	1.2 (1.0 to 1.4)	-0.7 (-1.0 to -0.4)	-10.0 (-13.7 to -6.2)
Czech Republic	1.7 (1.4 to 2.0)	1.5 (1.3 to 1.8)	1.5 (1.3 to 1.8)	1.4 (1.2 to 1.7)	-0.4 (-0.7 to -0.1)	-5.9 (-10.0 to -1.4)
Hungary	1.7 (1.4 to 1.9)	1.4 (1.2 to 1.6)	1.4 (1.2 to 1.6)	1.2 (1.0 to 1.4)	-1.1 (-1.4 to -0.8)	-15.0 (-18.8 to -11.1)
Macedonia	1.0 (0.9 to 1.2)	0.9 (0.8 to 1.1)	0.9 (0.8 to 1.1)	0.9 (0.8 to 1.0)	-0.3 (-0.6 to -0.1)	-4.7 (-8.3 to -0.8)
Montenegro	1.1 (1.0 to 1.3)	1.2 (1.0 to 1.4)	1.2 (1.0 to 1.4)	1.2 (1.0 to 1.4)	0.2 (-0.0 to 0.5)	3.3 (-0.7 to 7.5)
Poland	1.2 (1.0 to 1.4)	1.2 (1.0 to 1.4)	1.2 (1.0 to 1.4)	1.2 (1.1 to 1.4)	0.1 (-0.1 to 0.4)	2.1 (-2.0 to 6.1)
Romania	1.4 (1.2 to 1.6)	1.4 (1.2 to 1.6)	1.4 (1.2 to 1.6)	1.4 (1.2 to 1.6)	-0.1 (-0.3 to 0.2)	-0.8 (-4.9 to 3.4)
Serbia	1.2 (1.0 to 1.4)	1.3 (1.1 to 1.5)	1.3 (1.1 to 1.5)	1.4 (1.2 to 1.6)	0.4 (0.1 to 0.6)	5.7 (1.5 to 10.1)
Slovakia	1.5 (1.3 to 1.8)	1.4 (1.2 to 1.6)	1.4 (1.2 to 1.6)	1.3 (1.1 to 1.5)	-0.5 (-0.8 to -0.2)	-7.6 (-11.6 to -3.4)
Slovenia	1.4 (1.2 to 1.6)	1.2 (1.1 to 1.4)	1.2 (1.1 to 1.4)	1.1 (0.9 to 1.3)	-0.8 (-1.1 to -0.5)	-10.7 (-14.6 to -6.6)
Eastern Europe						
Belarus	1.6 (1.4 to 1.9)	1.8 (1.5 to 2.0)	1.8 (1.5 to 2.1)	1.9 (1.6 to 2.2)	0.4 (0.1 to 0.6)	5.6 (1.2 to 9.9)
Estonia	1.4 (1.2 to 1.6)	1.9 (1.6 to 2.2)	1.9 (1.6 to 2.2)	2.2 (1.9 to 2.6)	1.2 (0.9 to 1.5)	19.1 (13.8 to 24.5)
Latvia	1.2 (1.1 to 1.4)	1.4 (1.2 to 1.6)	1.4 (1.2 to 1.6)	1.5 (1.3 to 1.8)	0.5 (0.2 to 0.8)	7.9 (3.7 to 12.7)
Lithuania	1.5 (1.3 to 1.7)	1.8 (1.6 to 2.1)	1.8 (1.6 to 2.1)	2.1 (1.8 to 2.4)	0.8 (0.6 to 1.2)	13.6 (8.9 to 19.1)
Moldova	1.4 (1.2 to 1.6)	1.3 (1.1 to 1.5)	1.3 (1.1 to 1.5)	1.3 (1.1 to 1.5)	-0.2 (-0.6 to 0.3)	-2.4 (-8.1 to 4.1)
Russia	1.4 (1.2 to 1.6)	1.6 (1.4 to 1.8)	1.6 (1.4 to 1.8)	1.7 (1.5 to 2.0)	0.5 (0.2 to 0.8)	8.1 (3.3 to 12.7)
Ukraine	1.4 (1.2 to 1.6)	1.6 (1.4 to 1.8)	1.6 (1.4 to 1.8)	1.7 (1.4 to 2.0)	0.5 (0.2 to 0.8)	8.0 (3.5 to 12.7)
High-income						
Australasia						
Australia	1.3 (1.1 to 1.5)	1.3 (1.1 to 1.5)	1.3 (1.1 to 1.5)	1.2 (1.0 to 1.4)	-0.5 (-0.8 to -0.1)	-6.6 (-11.4 to -0.9)
New Zealand	1.5 (1.3 to 1.8)	1.6 (1.3 to 1.9)	1.5 (1.3 to 1.8)	1.5 (1.2 to 1.8)	-0.3 (-0.7 to -0.0)	-5.0 (-9.5 to -0.4)
High-income Asia Pacific						
Brunei	0.8 (0.6 to 0.9)	0.7 (0.6 to 0.9)	0.7 (0.6 to 0.9)	0.7 (0.6 to 0.9)	-0.1 (-0.4 to 0.3)	-1.1 (-5.7 to 3.9)
Japan	1.0 (0.9 to 1.2)	1.0 (0.9 to 1.2)	1.0 (0.9 to 1.2)	1.0 (0.9 to 1.2)	-0.1 (-0.1 to 0.0)	-1.0 (-2.0 to 0.0)
South Korea	1.1 (0.9 to 1.3)	1.0 (0.8 to 1.2)	1.0 (0.8 to 1.2)	1.0 (0.8 to 1.1)	-0.3 (-0.6 to 0.1)	-4.2 (-8.8 to 0.8)
Singapore	0.8 (0.7 to 1.0)	0.8 (0.7 to 1.0)	0.8 (0.7 to 1.0)	0.8 (0.7 to 1.0)	-0.0 (-0.4 to 0.3)	-0.4 (-5.4 to 4.5)
High-income North America						
Canada	1.4 (1.2 to 1.7)	1.4 (1.2 to 1.7)	1.4 (1.2 to 1.7)	1.4 (1.2 to 1.7)	0.0 (-0.3 to 0.4)	0.8 (-4.7 to 6.6)
Greenland	1.4 (1.2 to 1.7)	1.4 (1.2 to 1.7)	1.4 (1.2 to 1.7)	1.4 (1.2 to 1.8)	0.1 (-0.3 to 0.5)	1.3 (-4.5 to 7.1)
United States	1.6 (1.4 to 1.9)	1.6 (1.4 to 2.0)	1.6 (1.4 to 1.9)	1.7 (1.4 to 2.0)	0.0 (-0.1 to 0.2)	0.4 (-2.2 to 3.0)
Southern Latin America						
Argentina	1.3 (1.1 to 1.6)	1.3 (1.1 to 1.5)	1.3 (1.1 to 1.5)	1.2 (1.0 to 1.4)	-0.5 (-0.8 to -0.1)	-6.7 (-11.4 to -2.0)
Chile	1.1 (0.9 to 1.3)	1.2 (1.0 to 1.4)	1.2 (1.0 to 1.4)	1.2 (1.0 to 1.4)	0.2 (-0.2 to 0.5)	2.3 (-2.7 to 7.1)
Uruguay	1.2 (1.0 to 1.4)	1.1 (0.9 to 1.3)	1.1 (0.9 to 1.3)	1.1 (0.9 to 1.3)	-0.3 (-0.6 to 0.0)	-4.5 (-9.3 to 0.3)
Western Europe						
Andorra	1.9 (1.6 to 2.2)	1.4 (1.2 to 1.7)	1.4 (1.2 to 1.6)	1.2 (1.0 to 1.4)	-1.1 (-1.5 to -0.7)	-15.7 (-20.0 to -10.6)
Austria	1.4 (1.1 to 1.6)	1.2 (1.0 to 1.4)	1.2 (1.0 to 1.4)	1.1 (0.9 to 1.3)	-0.5 (-0.8 to -0.2)	-7.2 (-11.5 to -2.4)
Belgium	1.1 (0.9 to 1.3)	1.2 (1.0 to 1.4)	1.2 (1.0 to 1.4)	1.3 (1.1 to 1.5)	0.2 (-0.1 to 0.6)	3.4 (-1.6 to 8.8)
Cyprus	1.6 (1.3 to 2.0)	1.5 (1.2 to 1.8)	1.5 (1.2 to 1.8)	1.4 (1.2 to 1.8)	-0.2 (-0.6 to 0.1)	-3.1 (-8.2 to 2.0)
Denmark	1.4 (1.1 to 1.6)	1.2 (1.0 to 1.4)	1.2 (1.0 to 1.4)	1.1 (0.9 to 1.3)	-0.6 (-1.0 to -0.3)	-8.8 (-13.5 to -4.2)
Finland	1.3 (1.1 to 1.5)	1.2 (1.1 to 1.5)	1.2 (1.1 to 1.5)	1.2 (1.0 to 1.5)	-0.2 (-0.5 to 0.2)	-2.5 (-7.4 to 2.4)
France	1.7 (1.4 to 2.0)	1.4 (1.2 to 1.7)	1.4 (1.2 to 1.7)	1.3 (1.1 to 1.5)	-0.9 (-1.3 to -0.6)	-13.0 (-17.2 to -8.6)
Germany	1.2 (1.0 to 1.4)	1.1 (0.9 to 1.3)	1.1 (0.9 to 1.3)	1.0 (0.8 to 1.2)	-0.6 (-0.9 to -0.3)	-8.3 (-12.8 to -3.7)
Greece	1.4 (1.2 to 1.6)	1.3 (1.1 to 1.6)	1.3 (1.1 to 1.6)	1.2 (1.0 to 1.5)	-0.3 (-0.7 to -0.0)	-5.1 (-10.0 to -0.6)
Iceland	1.0 (0.8 to 1.2)	1.1 (1.0 to 1.3)	1.1 (1.0 to 1.4)	1.2 (1.0 to 1.5)	0.6 (0.2 to 0.9)	9.0 (3.6 to 14.4)
Ireland	1.4 (1.2 to 1.7)	1.3 (1.1 to 1.6)	1.3 (1.1 to 1.6)	1.3 (1.1 to 1.6)	-0.1 (-0.4 to 0.3)	-1.4 (-6.1 to 4.3)
Israel	0.8 (0.7 to 0.9)	0.8 (0.7 to 1.0)	0.8 (0.7 to 1.0)	0.9 (0.7 to 1.1)	0.3 (-0.0 to 0.6)	4.6 (-0.4 to 9.8)
Italy	2.5 (2.1 to 2.9)	2.1 (1.8 to 2.4)	2.1 (1.8 to 2.4)	1.8 (1.5 to 2.2)	-1.0 (-1.3 to -0.6)	-13.4 (-17.3 to -8.7)
Luxembourg	1.5 (1.2 to 1.8)	1.8 (1.5 to 2.2)	1.8 (1.4 to 2.2)	1.9 (1.5 to 2.3)	0.4 (0.0 to 0.7)	5.8 (0.5 to 11.3)
Malta	1.1 (0.9 to 1.4)	1.1 (0.9 to 1.4)	1.1 (0.9 to 1.4)	1.1 (0.9 to 1.4)	0.2 (-0.2 to 0.5)	2.4 (-2.9 to 7.7)
Netherlands	1.2 (1.0 to 1.4)	1.1 (0.9 to 1.3)	1.1 (0.9 to 1.3)	1.1 (0.9 to 1.3)	-0.2 (-0.5 to 0.1)	-2.6 (-7.4 to 2.1)
Norway	1.1 (0.9 to 1.2)	1.1 (0.9 to 1.3)	1.1 (0.9 to 1.3)	1.1 (0.9 to 1.3)	0.1 (-0.2 to 0.4)	1.7 (-2.9 to 6.3)
Portugal	1.6 (1.3 to 1.9)	1.4 (1.1 to 1.6)	1.4 (1.1 to 1.6)	1.2 (1.0 to 1.4)	-0.9 (-1.2 to -0.5)	-12.2 (-16.6 to -7.6)
Spain	1.5 (1.2 to 1.8)	1.4 (1.2 to 1.8)	1.4 (1.2 to 1.8)	1.4 (1.1 to 1.7)	-0.3 (-0.6 to 0.1)	-3.9 (-9.1 to 1.7)
Sweden	1.7 (1.4 to 2.0)	1.6 (1.4 to 1.9)	1.6 (1.4 to 1.9)	1.6 (1.3 to 1.8)	-0.2 (-0.5 to 0.0)	-3.1 (-6.7 to 0.4)
Switzerland	1.3 (1.1 to 1.5)	1.2 (1.0 to 1.4)	1.2 (1.0 to 1.4)	1.1 (0.9 to 1.4)	-0.4 (-0.7 to -0.1)	-5.8 (-10.6 to -1.2)
United Kingdom	1.5 (1.2 to 1.7)	1.5 (1.3 to 1.8)	1.5 (1.3 to 1.8)	1.5 (1.3 to 1.8)	-0.1 (-0.2 to -0.1)	-2.1 (-2.9 to -1.2)
Latin America and Caribbean						

Andean Latin America	Estimate in 2000 (95% UIs)	Estimate in 2015 (95% UIs)	Estimate in 2017 (95% UIs)	Estimate in 2030 (95% UIs)	Annualised rate of change, 2015-2030 (95% UIs)	Percent change, 2015-2030 (95% UIs)
Bolivia	2.2 (1.9 to 2.6)	2.2 (2.0 to 2.5)	2.2 (1.9 to 2.5)	2.2 (1.9 to 2.6)	-0.1 (-0.6 to 0.4)	-0.9 (-8.0 to 6.1)
Ecuador	1.9 (1.7 to 2.1)	2.1 (1.8 to 2.4)	2.1 (1.8 to 2.4)	2.1 (1.8 to 2.5)	0.2 (-0.1 to 0.5)	2.7 (-1.8 to 7.7)
Peru	1.7 (1.5 to 2.0)	1.4 (1.3 to 1.6)	1.4 (1.2 to 1.6)	1.3 (1.1 to 1.5)	-0.7 (-1.0 to -0.4)	-9.4 (-13.4 to -5.5)
Caribbean						
Antigua and Barbuda	1.9 (1.6 to 2.2)	2.0 (1.7 to 2.3)	2.0 (1.7 to 2.3)	2.0 (1.7 to 2.4)	0.2 (-0.1 to 0.4)	2.4 (-1.8 to 6.9)
The Bahamas	2.8 (2.4 to 3.2)	2.5 (2.1 to 2.9)	2.5 (2.1 to 2.9)	2.2 (1.9 to 2.6)	-0.7 (-0.9 to -0.4)	-9.6 (-13.3 to -5.7)
Barbados	2.1 (1.8 to 2.5)	2.2 (1.9 to 2.5)	2.2 (1.9 to 2.5)	2.2 (1.9 to 2.6)	0.1 (-0.2 to 0.4)	1.3 (-2.6 to 5.9)
Belize	2.2 (1.9 to 2.5)	2.4 (2.0 to 2.8)	2.4 (2.0 to 2.8)	2.5 (2.1 to 2.9)	0.2 (0.0 to 0.5)	3.7 (0.0 to 7.6)
Bermuda	5.0 (4.3 to 5.8)	3.6 (3.1 to 4.2)	3.5 (3.0 to 4.1)	2.9 (2.5 to 3.3)	-1.5 (-1.8 to -1.2)	-20.6 (-23.7 to -17.1)
Cuba	1.8 (1.6 to 2.1)	1.9 (1.6 to 2.1)	1.9 (1.6 to 2.1)	1.9 (1.6 to 2.2)	0.2 (-0.1 to 0.5)	2.9 (-0.9 to 7.1)
Dominica	2.4 (2.1 to 2.8)	2.3 (2.0 to 2.7)	2.3 (2.0 to 2.7)	2.2 (1.9 to 2.6)	-0.2 (-0.5 to 0.1)	-3.0 (-6.8 to 1.0)
Dominican Republic	1.8 (1.6 to 2.1)	1.9 (1.7 to 2.1)	1.9 (1.7 to 2.1)	1.9 (1.7 to 2.2)	0.2 (-0.1 to 0.5)	3.5 (-0.9 to 7.9)
Grenada	2.6 (2.3 to 3.0)	2.3 (2.0 to 2.6)	2.3 (2.0 to 2.6)	2.1 (1.8 to 2.5)	-0.6 (-0.8 to -0.3)	-8.3 (-11.9 to -4.6)
Guyana	2.5 (2.2 to 2.9)	2.1 (1.8 to 2.4)	2.1 (1.8 to 2.4)	1.8 (1.5 to 2.1)	-0.9 (-1.2 to -0.6)	-13.0 (-16.5 to -9.1)
Haiti	3.5 (3.2 to 3.8)	3.6 (3.2 to 4.1)	3.6 (3.1 to 4.1)	3.7 (3.0 to 4.3)	0.0 (-0.4 to 0.5)	0.7 (-5.7 to 7.4)
Jamaica	1.9 (1.6 to 2.2)	1.9 (1.6 to 2.2)	1.9 (1.6 to 2.2)	1.9 (1.6 to 2.2)	0.0 (-0.2 to 0.3)	0.4 (-3.6 to 4.4)
Puerto Rico	1.9 (1.6 to 2.2)	1.8 (1.6 to 2.1)	1.9 (1.6 to 2.2)	1.9 (1.6 to 2.2)	0.2 (-0.1 to 0.5)	3.3 (-1.0 to 7.6)
Saint Lucia	3.0 (2.6 to 3.4)	2.4 (2.0 to 2.8)	2.4 (2.0 to 2.8)	2.1 (1.8 to 2.4)	-0.9 (-1.2 to -0.6)	-12.6 (-16.7 to -8.6)
Saint Vincent and the Grenadines	2.0 (1.7 to 2.4)	2.0 (1.8 to 2.4)	2.0 (1.8 to 2.4)	2.1 (1.8 to 2.4)	0.1 (-0.2 to 0.3)	1.0 (-3.3 to 5.4)
Suriname	2.0 (1.8 to 2.4)	2.2 (1.9 to 2.6)	2.2 (1.9 to 2.6)	2.4 (2.0 to 2.8)	0.4 (0.1 to 0.6)	5.6 (1.3 to 10.0)
Trinidad and Tobago	1.8 (1.5 to 2.1)	1.8 (1.6 to 2.1)	1.8 (1.6 to 2.1)	1.8 (1.6 to 2.1)	0.1 (-0.2 to 0.4)	1.1 (-2.6 to 5.6)
Virgin Islands, U.S.	2.0 (1.7 to 2.3)	2.0 (1.8 to 2.3)	2.0 (1.8 to 2.4)	2.1 (1.8 to 2.4)	0.2 (-0.1 to 0.5)	2.8 (-1.4 to 7.1)
Central Latin America						
Colombia	1.7 (1.5 to 2.0)	1.6 (1.4 to 1.8)	1.6 (1.4 to 1.8)	1.6 (1.3 to 1.8)	0.0 (-0.4 to 0.4)	0.2 (-5.5 to 6.6)
Costa Rica	1.7 (1.5 to 2.0)	1.6 (1.4 to 1.9)	1.6 (1.4 to 1.9)	1.5 (1.3 to 1.8)	-0.4 (-0.7 to -0.1)	-5.7 (-9.5 to -1.5)
El Salvador	1.2 (1.0 to 1.4)	1.3 (1.1 to 1.4)	1.3 (1.1 to 1.5)	1.3 (1.1 to 1.6)	0.3 (-0.1 to 0.7)	5.2 (-0.9 to 11.6)
Guatemala	1.5 (1.3 to 1.6)	1.3 (1.2 to 1.5)	1.3 (1.2 to 1.5)	1.2 (1.1 to 1.4)	-0.5 (-0.7 to -0.2)	-6.7 (-10.6 to -2.8)
Honduras	1.9 (1.7 to 2.0)	1.7 (1.5 to 1.9)	1.7 (1.5 to 1.9)	1.6 (1.4 to 1.9)	-0.4 (-0.8 to 0.2)	-5.1 (-11.9 to 3.6)
Mexico	2.9 (2.5 to 3.3)	2.2 (1.9 to 2.5)	2.1 (1.9 to 2.5)	1.8 (1.6 to 2.1)	-1.3 (-1.4 to -1.2)	-17.7 (-19.1 to -16.1)
Nicaragua	1.5 (1.3 to 1.7)	1.5 (1.3 to 1.7)	1.5 (1.3 to 1.7)	1.5 (1.2 to 1.7)	0.0 (-0.4 to 0.5)	0.3 (-6.2 to 7.2)
Panama	1.8 (1.5 to 2.0)	1.8 (1.6 to 2.0)	1.8 (1.6 to 2.1)	1.8 (1.6 to 2.1)	0.2 (-0.1 to 0.5)	2.9 (-1.5 to 7.1)
Venezuela	2.1 (1.8 to 2.5)	1.9 (1.7 to 2.2)	1.9 (1.7 to 2.2)	1.8 (1.5 to 2.1)	-0.5 (-0.8 to -0.2)	-7.3 (-11.3 to -3.4)
Tropical Latin America						
Brazil	1.9 (1.6 to 2.2)	1.8 (1.5 to 2.0)	1.8 (1.5 to 2.0)	1.7 (1.5 to 2.0)	-0.2 (-0.2 to -0.1)	-2.3 (-3.5 to -1.2)
Paraguay	1.9 (1.6 to 2.1)	1.6 (1.4 to 1.9)	1.7 (1.4 to 2.0)	1.6 (1.4 to 1.9)	-0.1 (-0.5 to 0.3)	-1.0 (-6.8 to 4.9)
North Africa and Middle East						
North Africa and Middle East						
Afghanistan	1.4 (1.2 to 1.6)	1.4 (1.3 to 1.6)	1.4 (1.3 to 1.6)	1.4 (1.3 to 1.6)	0.1 (-0.2 to 0.4)	1.4 (-3.4 to 6.2)
Algeria	1.4 (1.2 to 1.7)	1.5 (1.2 to 1.7)	1.5 (1.2 to 1.7)	1.5 (1.2 to 1.7)	0.0 (-0.3 to 0.3)	0.1 (-4.3 to 4.1)
Bahrain	1.6 (1.4 to 1.9)	1.5 (1.3 to 1.8)	1.5 (1.3 to 1.8)	1.4 (1.2 to 1.7)	-0.3 (-0.6 to -0.1)	-4.8 (-8.6 to -0.8)
Egypt	1.2 (1.1 to 1.4)	1.2 (1.0 to 1.3)	1.2 (1.0 to 1.3)	1.1 (1.0 to 1.3)	-0.2 (-0.4 to 0.1)	-2.4 (-6.3 to 1.9)
Iran	1.4 (1.2 to 1.6)	1.4 (1.2 to 1.6)	1.4 (1.2 to 1.6)	1.4 (1.2 to 1.7)	0.0 (-0.2 to 0.3)	0.7 (-3.4 to 4.6)
Iraq	1.4 (1.2 to 1.7)	1.4 (1.2 to 1.7)	1.4 (1.2 to 1.7)	1.4 (1.2 to 1.7)	-0.1 (-0.3 to 0.2)	-0.8 (-4.8 to 3.5)
Jordan	1.6 (1.3 to 1.8)	1.6 (1.4 to 1.8)	1.6 (1.4 to 1.8)	1.6 (1.4 to 1.8)	0.0 (-0.2 to 0.3)	0.7 (-3.4 to 4.8)
Kuwait	1.3 (1.1 to 1.5)	1.4 (1.2 to 1.6)	1.4 (1.2 to 1.6)	1.4 (1.2 to 1.6)	0.0 (-0.2 to 0.3)	0.6 (-3.6 to 4.7)
Lebanon	1.6 (1.4 to 1.9)	1.6 (1.4 to 1.8)	1.6 (1.4 to 1.9)	1.6 (1.3 to 1.8)	-0.1 (-0.4 to 0.2)	-1.4 (-5.5 to 2.4)
Libya	1.4 (1.2 to 1.6)	1.4 (1.2 to 1.6)	1.4 (1.2 to 1.6)	1.4 (1.2 to 1.6)	0.0 (-0.2 to 0.3)	0.3 (-3.7 to 4.3)
Morocco	1.5 (1.3 to 1.8)	1.4 (1.2 to 1.7)	1.4 (1.2 to 1.7)	1.4 (1.2 to 1.7)	-0.2 (-0.4 to 0.1)	-2.7 (-6.4 to 1.3)
Palestine	1.4 (1.2 to 1.6)	1.4 (1.2 to 1.6)	1.4 (1.2 to 1.6)	1.4 (1.2 to 1.7)	0.0 (-0.3 to 0.3)	0.1 (-3.9 to 3.9)
Oman	1.4 (1.2 to 1.6)	1.4 (1.2 to 1.6)	1.4 (1.2 to 1.6)	1.4 (1.2 to 1.6)	0.0 (-0.2 to 0.3)	0.2 (-3.5 to 4.0)
Qatar	1.4 (1.2 to 1.6)	1.4 (1.2 to 1.6)	1.4 (1.2 to 1.6)	1.4 (1.2 to 1.6)	0.3 (0.0 to 0.6)	4.4 (0.4 to 8.7)
Saudi Arabia	1.4 (1.2 to 1.6)	1.4 (1.2 to 1.6)	1.4 (1.2 to 1.6)	1.4 (1.2 to 1.6)	0.0 (-0.2 to 0.3)	0.5 (-3.5 to 5.0)
Sudan	1.7 (1.4 to 2.0)	1.7 (1.4 to 1.9)	1.7 (1.4 to 2.0)	1.7 (1.4 to 2.0)	0.0 (-0.2 to 0.3)	0.4 (-3.6 to 4.4)
Syria	1.4 (1.2 to 1.7)	1.4 (1.2 to 1.7)	1.4 (1.2 to 1.7)	1.4 (1.2 to 1.7)	-0.0 (-0.3 to 0.3)	-0.0 (-4.1 to 3.9)
Tunisia	1.5 (1.3 to 1.7)	1.5 (1.3 to 1.7)	1.5 (1.3 to 1.7)	1.5 (1.3 to 1.7)	-0.0 (-0.3 to 0.3)	-0.1 (-4.0 to 4.0)
Turkey	1.8 (1.6 to 2.1)	1.6 (1.4 to 1.9)	1.6 (1.4 to 1.9)	1.5 (1.3 to 1.7)	-0.8 (-1.2 to -0.4)	-10.8 (-16.2 to -5.1)
United Arab Emirates	1.6 (1.4 to 1.9)	1.5 (1.3 to 1.8)	1.6 (1.3 to 1.8)	1.5 (1.3 to 1.8)	-0.1 (-0.4 to 0.2)	-1.6 (-5.8 to 2.9)
Yemen	1.4 (1.2 to 1.7)	1.4 (1.2 to 1.6)	1.4 (1.2 to 1.6)	1.4 (1.2 to 1.6)	-0.1 (-0.3 to 0.2)	-1.4 (-5.1 to 2.7)
South Asia						
South Asia						
Bangladesh	2.5 (2.1 to 2.9)	2.5 (2.2 to 2.9)	2.5 (2.2 to 3.0)	2.6 (2.2 to 3.0)	0.1 (-0.3 to 0.4)	0.8 (-4.3 to 5.9)
Bhutan	3.0 (2.6 to 3.6)	2.7 (2.4 to 3.2)	2.8 (2.4 to 3.2)	2.7 (2.3 to 3.1)	-0.2 (-0.4 to 0.1)	-2.4 (-6.4 to 1.9)
India	2.6 (2.3 to 3.0)	2.7 (2.3 to 3.2)	2.8 (2.4 to 3.3)	2.9 (2.4 to 3.5)	0.4 (0.1 to 0.6)	6.0 (2.2 to 9.6)
Nepal	3.3 (2.8 to 3.8)	3.6 (3.1 to 4.1)	3.5 (3.0 to 4.1)	3.6 (3.0 to 4.3)	0.1 (-0.3 to 0.4)	0.9 (-3.8 to 5.9)
Pakistan	2.5 (2.1 to 2.9)	2.5 (2.2 to 3.0)	2.6 (2.2 to 3.0)	2.6 (2.2 to 3.0)	0.1 (-0.2 to 0.4)	1.3 (-2.6 to 5.7)
Southeast Asia, East Asia, and Oceania						
East Asia						
China	7.9 (6.9 to 9.0)	8.0 (7.0 to 9.1)	8.1 (7.1 to 9.2)	8.2 (7.2 to 9.4)	0.2 (0.1 to 0.3)	3.2 (2.3 to 4.1)
North Korea	6.4 (5.5 to 7.4)	5.8 (5.0 to 6.7)	5.8 (5.0 to 6.7)	5.6 (4.8 to 6.5)	-0.2 (-0.5 to 0.1)	-2.8 (-6.8 to 1.2)
Taiwan (Province of China)	6.7 (5.8 to 7.7)	6.6 (5.7 to 7.6)	6.6 (5.7 to 7.6)	6.6 (5.6 to 7.6)	-0.1 (-0.3 to 0.2)	-0.8 (-4.5 to 3.2)
Oceania						
American Samoa	3.5 (3.0 to 4.0)	3.5 (3.0 to 4.1)	3.5 (3.0 to 4.1)	3.6 (3.0 to 4.2)	0.1 (-0.2 to 0.4)	1.3 (-2.8 to 5.8)
Federated States of Micronesia	3.4 (2.9 to 3.9)	3.2 (2.8 to 3.7)	3.2 (2.7 to 3.7)	3.1 (2.6 to 3.6)	-0.2 (-0.5 to 0.1)	-3.1 (-7.5 to 1.4)
Fiji	3.2 (2.8 to 3.8)	3.4 (2.9 to 3.9)	3.4 (2.9 to 4.0)	3.5 (3.0 to 4.1)	0.2 (-0.0 to 0.5)	3.6 (-0.5 to 7.8)
Guam	3.5 (3.0 to 4.1)	3.5 (3.0 to 4.1)	3.5 (3.0 to 4.1)	3.6 (3.0 to 4.2)	0.1 (-0.2 to 0.4)	1.5 (-2.8 to 5.7)
Kiribati	4.9 (4.3 to 5.7)	4.9 (4.3 to 5.7)	4.9 (4.2 to 5.7)	4.8 (4.1 to 5.6)	-0.1 (-0.4 to 0.1)	-1.9 (-6.0 to 2.1)
Marshall Islands	3.4 (2.9 to 3.9)	3.4 (2.9 to 3.9)	3.4 (2.9 to 3.9)	3.4 (2.9 to 4.0)	0.1 (-0.2 to 0.4)	1.7 (-2.7 to 6.2)
Northern Mariana Islands	3.7 (3.2 to 4.3)	3.5 (3.0 to 4.0)	3.4 (2.9 to 4.0)	3.4 (2.9 to 4.0)	-0.1 (-0.4 to 0.2)	-1.5 (-5.9 to 2.6)
Papua New Guinea	3.5 (3.0 to 4.1)	3.3 (2.9 to 3.9)	3.3 (2.9 to 3.9)	3.2 (2.8 to 3.8)	-0.2 (-0.5 to 0.1)	-2.6 (-6.7 to 1.8)

	Estimate in 2000 (95% UIs)	Estimate in 2015 (95% UIs)	Estimate in 2017 (95% UIs)	Estimate in 2030 (95% UIs)	Annualised rate of change, 2015-2030 (95% UIs)	Percent change, 2015-2030 (95% UIs)
Samoa	2.7 (2.4 to 3.2)	2.8 (2.4 to 3.2)	2.8 (2.4 to 3.2)	2.8 (2.3 to 3.3)	0.1 (-0.2 to 0.3)	0.9 (-3.3 to 5.4)
Solomon Islands	3.0 (2.6 to 3.5)	3.1 (2.7 to 3.6)	3.1 (2.7 to 3.6)	3.2 (2.7 to 3.8)	0.2 (-0.1 to 0.5)	3.0 (-1.2 to 7.2)
Tonga	2.9 (2.4 to 3.3)	2.9 (2.5 to 3.4)	2.9 (2.5 to 3.3)	2.9 (2.4 to 3.4)	-0.1 (-0.4 to 0.2)	-1.1 (-5.1 to 3.1)
Vanuatu	3.1 (2.7 to 3.6)	3.0 (2.6 to 3.5)	3.0 (2.6 to 3.5)	3.0 (2.6 to 3.5)	-0.1 (-0.3 to 0.2)	-1.3 (-5.1 to 3.1)
Southeast Asia						
Cambodia	1.4 (1.2 to 1.6)	1.7 (1.5 to 1.9)	1.7 (1.5 to 1.9)	1.9 (1.6 to 2.2)	0.8 (0.4 to 1.1)	12.0 (6.6 to 18.8)
Indonesia	1.4 (1.2 to 1.6)	1.4 (1.2 to 1.6)	1.4 (1.2 to 1.6)	1.4 (1.2 to 1.6)	0.0 (-0.0 to 0.1)	0.6 (-0.7 to 1.8)
Laos	1.9 (1.6 to 2.1)	1.8 (1.5 to 2.1)	1.8 (1.6 to 2.1)	1.8 (1.5 to 2.1)	-0.0 (-0.3 to 0.2)	-0.6 (-4.6 to 3.0)
Malaysia	1.4 (1.2 to 1.6)	1.4 (1.2 to 1.6)	1.4 (1.2 to 1.6)	1.4 (1.2 to 1.6)	0.0 (-0.2 to 0.3)	0.4 (-3.2 to 4.5)
Maldives	1.5 (1.3 to 1.7)	1.4 (1.2 to 1.6)	1.4 (1.2 to 1.6)	1.4 (1.2 to 1.6)	-0.2 (-0.4 to 0.1)	-2.5 (-6.5 to 1.8)
Mauritius	1.7 (1.4 to 1.9)	1.6 (1.4 to 1.9)	1.6 (1.4 to 1.9)	1.6 (1.3 to 1.8)	-0.2 (-0.5 to 0.1)	-2.9 (-6.5 to 0.8)
Myanmar	1.3 (1.1 to 1.5)	1.2 (1.1 to 1.4)	1.2 (1.1 to 1.4)	1.2 (1.1 to 1.4)	-0.0 (-0.3 to 0.3)	-0.4 (-4.6 to 3.9)
Philippines	2.1 (1.8 to 2.4)	2.0 (1.7 to 2.3)	2.0 (1.7 to 2.3)	2.0 (1.7 to 2.3)	-0.0 (-0.3 to 0.3)	-0.2 (-5.0 to 4.9)
Sri Lanka	1.4 (1.2 to 1.6)	1.5 (1.3 to 1.8)	1.5 (1.3 to 1.8)	1.7 (1.4 to 2.0)	0.5 (0.3 to 0.8)	8.5 (4.2 to 13.1)
Seychelles	1.9 (1.6 to 2.2)	1.7 (1.5 to 2.0)	1.7 (1.5 to 2.0)	1.7 (1.4 to 2.0)	-0.2 (-0.5 to 0.1)	-2.8 (-6.7 to 1.2)
Thailand	1.9 (1.7 to 2.2)	1.9 (1.6 to 2.2)	1.9 (1.6 to 2.2)	1.9 (1.6 to 2.2)	-0.1 (-0.4 to 0.2)	-1.6 (-5.4 to 2.5)
Timor-Leste	1.3 (1.1 to 1.6)	1.3 (1.1 to 1.5)	1.4 (1.2 to 1.6)	1.4 (1.2 to 1.6)	0.2 (-0.0 to 0.5)	3.7 (-0.4 to 8.3)
Vietnam	1.6 (1.3 to 1.8)	1.8 (1.6 to 2.1)	1.8 (1.6 to 2.1)	2.1 (1.8 to 2.4)	0.8 (0.5 to 1.1)	12.4 (7.4 to 17.3)
Sub-Saharan Africa						
Central Sub-Saharan Africa						
Angola	3.0 (2.6 to 3.5)	4.0 (3.5 to 4.6)	4.0 (3.5 to 4.7)	4.7 (4.0 to 5.5)	1.1 (0.8 to 1.4)	18.0 (13.0 to 23.3)
Central African Republic	2.4 (2.1 to 2.8)	2.4 (2.0 to 2.7)	2.4 (2.1 to 2.8)	2.5 (2.1 to 2.9)	0.3 (-0.0 to 0.6)	4.1 (-0.5 to 9.8)
Congo	3.3 (2.8 to 3.8)	3.5 (3.0 to 4.0)	3.5 (3.0 to 4.1)	3.7 (3.1 to 4.4)	0.4 (0.1 to 0.7)	6.1 (1.8 to 10.7)
Democratic Republic of the Congo	4.4 (3.8 to 5.1)	5.2 (4.5 to 5.8)	5.2 (4.5 to 5.8)	5.7 (4.9 to 6.4)	0.6 (0.3 to 0.9)	9.4 (4.1 to 14.7)
Equatorial Guinea	4.0 (3.5 to 4.7)	4.9 (4.2 to 5.7)	4.9 (4.2 to 5.7)	5.8 (4.9 to 6.7)	1.1 (0.8 to 1.4)	17.4 (12.2 to 22.7)
Gabon	3.6 (3.1 to 4.2)	3.7 (3.2 to 4.2)	3.7 (3.3 to 4.3)	3.7 (3.3 to 4.3)	0.1 (-0.2 to 0.4)	1.6 (-2.9 to 6.1)
Eastern Sub-Saharan Africa						
Burundi	5.3 (4.6 to 6.2)	4.9 (4.3 to 5.7)	4.9 (4.3 to 5.8)	4.7 (4.0 to 5.6)	-0.3 (-0.5 to -0.0)	-4.1 (-7.5 to -0.1)
Comoros	1.4 (1.2 to 1.6)	1.4 (1.2 to 1.6)	1.4 (1.2 to 1.6)	1.4 (1.2 to 1.6)	-0.1 (-0.4 to 0.2)	-1.3 (-5.2 to 2.8)
Djibouti	2.4 (2.1 to 2.8)	2.5 (2.1 to 2.9)	2.5 (2.1 to 2.9)	2.5 (2.1 to 3.0)	0.1 (-0.2 to 0.3)	0.8 (-3.4 to 5.1)
Eritrea	2.8 (2.4 to 3.2)	2.6 (2.3 to 3.1)	2.6 (2.3 to 3.1)	2.6 (2.2 to 3.1)	-0.1 (-0.4 to 0.2)	-1.0 (-5.2 to 3.5)
Ethiopia	3.3 (2.8 to 3.8)	3.3 (2.8 to 3.8)	3.3 (2.8 to 3.8)	3.3 (2.8 to 3.9)	0.1 (-0.2 to 0.4)	1.1 (-3.2 to 5.6)
Kenya	3.4 (3.0 to 4.0)	3.7 (3.2 to 4.3)	3.6 (3.1 to 4.2)	3.6 (3.1 to 4.2)	-0.1 (-0.2 to -0.1)	-2.1 (-3.5 to -0.8)
Madagascar	2.7 (2.3 to 3.2)	2.7 (2.3 to 3.1)	2.7 (2.3 to 3.1)	2.7 (2.3 to 3.2)	0.0 (-0.2 to 0.3)	0.4 (-3.6 to 4.5)
Malawi	4.5 (4.0 to 5.0)	4.8 (4.3 to 5.2)	4.8 (4.2 to 5.4)	5.2 (4.3 to 5.8)	0.5 (-0.0 to 0.9)	7.9 (-0.4 to 14.7)
Mozambique	3.0 (2.5 to 3.4)	3.0 (2.7 to 3.4)	3.1 (2.6 to 3.6)	3.2 (2.7 to 3.7)	0.3 (-0.0 to 0.7)	5.0 (-0.3 to 10.5)
Rwanda	3.4 (3.0 to 3.8)	3.3 (3.0 to 3.7)	3.3 (2.9 to 3.7)	3.2 (2.7 to 3.7)	-0.2 (-0.6 to 0.2)	-2.6 (-9.3 to 3.6)
Somalia	2.4 (2.1 to 2.8)	2.5 (2.1 to 2.9)	2.5 (2.1 to 2.9)	2.5 (2.1 to 2.9)	0.1 (-0.2 to 0.4)	1.1 (-2.8 to 5.6)
South Sudan	2.5 (2.1 to 2.9)	2.5 (2.2 to 2.9)	2.6 (2.2 to 3.0)	2.6 (2.2 to 3.0)	0.1 (-0.2 to 0.3)	1.1 (-3.1 to 5.4)
Tanzania	3.9 (3.3 to 4.5)	3.8 (3.5 to 4.2)	3.8 (3.4 to 4.2)	3.6 (3.3 to 4.1)	-0.3 (-0.6 to -0.1)	-5.1 (-9.1 to -0.8)
Uganda	5.7 (4.9 to 6.6)	5.7 (5.1 to 6.3)	5.0 (4.3 to 5.8)	4.6 (4.0 to 5.3)	-1.4 (-1.8 to -1.0)	-18.5 (-23.1 to -14.1)
Zambia	3.8 (3.4 to 4.2)	4.1 (3.7 to 4.5)	4.1 (3.7 to 4.6)	4.4 (3.8 to 4.9)	0.5 (0.1 to 0.9)	7.3 (1.2 to 14.0)
Southern Sub-Saharan Africa						
Botswana	2.1 (1.8 to 2.4)	2.0 (1.8 to 2.3)	2.0 (1.8 to 2.3)	2.0 (1.8 to 2.3)	-0.0 (-0.3 to 0.3)	-0.1 (-4.6 to 4.3)
Lesotho	2.2 (1.9 to 2.5)	2.2 (1.9 to 2.5)	2.2 (1.9 to 2.5)	2.2 (1.9 to 2.6)	0.1 (-0.2 to 0.4)	2.0 (-2.3 to 6.7)
Namibia	1.7 (1.5 to 2.0)	2.1 (1.9 to 2.4)	2.1 (1.9 to 2.4)	2.4 (2.1 to 2.8)	0.8 (0.5 to 1.1)	12.7 (7.3 to 18.1)
South Africa	1.8 (1.6 to 2.1)	1.7 (1.5 to 2.0)	1.8 (1.5 to 2.0)	1.8 (1.5 to 2.0)	0.0 (-0.1 to 0.2)	0.7 (-1.0 to 2.5)
Swaziland	2.6 (2.3 to 3.0)	2.1 (1.8 to 2.4)	2.1 (1.8 to 2.4)	1.9 (1.6 to 2.3)	-0.6 (-0.9 to -0.3)	-8.8 (-12.8 to -4.6)
Zimbabwe	4.4 (4.0 to 4.9)	4.4 (4.0 to 4.8)	4.3 (3.9 to 4.8)	4.2 (3.7 to 4.8)	-0.3 (-0.5 to 0.1)	-3.7 (-7.7 to 1.7)
Western Sub-Saharan Africa						
Benin	1.7 (1.5 to 2.0)	1.8 (1.5 to 2.0)	1.8 (1.5 to 2.0)	1.8 (1.5 to 2.1)	0.0 (-0.2 to 0.3)	0.8 (-3.1 to 4.7)
Burkina Faso	1.5 (1.3 to 1.7)	1.3 (1.1 to 1.5)	1.3 (1.1 to 1.6)	1.3 (1.1 to 1.5)	-0.1 (-0.4 to 0.1)	-1.9 (-5.8 to 1.9)
Cameroon	2.5 (2.3 to 2.7)	2.7 (2.3 to 3.1)	2.7 (2.3 to 3.1)	2.8 (2.4 to 3.3)	0.2 (-0.0 to 0.6)	3.8 (-0.6 to 8.9)
Cape Verde	2.7 (2.3 to 3.1)	2.5 (2.2 to 2.9)	2.5 (2.2 to 2.9)	2.4 (2.1 to 2.8)	-0.3 (-0.5 to 0.0)	-3.8 (-7.6 to 0.3)
Chad	2.6 (2.2 to 3.0)	2.7 (2.4 to 3.0)	2.7 (2.4 to 3.0)	2.8 (2.4 to 3.1)	0.1 (-0.2 to 0.4)	1.5 (-2.9 to 6.4)
Cote d'Ivoire	1.9 (1.6 to 2.2)	1.8 (1.5 to 2.0)	1.7 (1.5 to 2.0)	1.7 (1.5 to 1.9)	-0.2 (-0.5 to 0.0)	-3.7 (-7.4 to 0.5)
The Gambia	1.4 (1.2 to 1.6)	1.3 (1.1 to 1.5)	1.3 (1.1 to 1.5)	1.3 (1.1 to 1.5)	-0.2 (-0.5 to 0.1)	-2.8 (-7.0 to 1.1)
Ghana	2.2 (1.9 to 2.6)	2.4 (2.1 to 2.7)	2.4 (2.1 to 2.8)	2.6 (2.2 to 3.0)	0.5 (0.1 to 0.8)	7.2 (2.1 to 12.5)
Guinea	1.6 (1.4 to 1.9)	1.6 (1.4 to 1.9)	1.6 (1.4 to 1.9)	1.7 (1.4 to 2.0)	0.1 (-0.2 to 0.4)	2.1 (-2.7 to 6.7)
Guinea-Bissau	2.0 (1.7 to 2.3)	1.9 (1.7 to 2.3)	1.9 (1.7 to 2.3)	1.9 (1.6 to 2.3)	-0.1 (-0.3 to 0.2)	-1.0 (-4.6 to 2.9)
Liberia	2.8 (2.4 to 3.3)	2.6 (2.3 to 3.0)	2.6 (2.2 to 3.0)	2.4 (2.0 to 2.8)	-0.7 (-1.0 to -0.3)	-9.7 (-14.5 to -4.7)
Mali	3.0 (2.5 to 3.4)	3.2 (2.8 to 3.7)	3.2 (2.8 to 3.7)	3.4 (2.9 to 4.0)	0.3 (-0.1 to 0.6)	4.5 (-0.9 to 9.8)
Mauritania	1.5 (1.3 to 1.8)	1.5 (1.3 to 1.8)	1.5 (1.3 to 1.8)	1.5 (1.3 to 1.8)	-0.0 (-0.3 to 0.3)	-0.3 (-4.0 to 3.9)
Niger	1.5 (1.3 to 1.8)	1.6 (1.3 to 1.8)	1.6 (1.3 to 1.8)	1.6 (1.3 to 1.8)	-0.0 (-0.3 to 0.2)	-0.0 (-4.0 to 3.8)
Nigeria	3.2 (2.7 to 3.6)	2.4 (2.1 to 2.6)	2.4 (2.1 to 2.8)	2.2 (1.9 to 2.6)	-0.5 (-1.0 to -0.0)	-7.0 (-13.3 to -0.3)
Sao Tome and Principe	2.7 (2.3 to 3.1)	2.5 (2.1 to 2.8)	2.4 (2.1 to 2.8)	2.4 (2.0 to 2.8)	-0.2 (-0.5 to 0.1)	-2.3 (-6.7 to 2.0)
Senegal	1.6 (1.4 to 1.8)	1.6 (1.4 to 1.8)	1.6 (1.4 to 1.8)	1.6 (1.4 to 1.9)	0.1 (-0.2 to 0.4)	1.2 (-3.1 to 5.8)
Sierra Leone	2.8 (2.4 to 3.2)	2.6 (2.4 to 2.9)	2.5 (2.3 to 2.8)	2.4 (2.1 to 2.7)	-0.5 (-0.9 to -0.2)	-7.8 (-12.1 to -3.2)
Togo	2.0 (1.7 to 2.3)	2.0 (1.8 to 2.2)	2.0 (1.8 to 2.3)	2.0 (1.8 to 2.2)	0.0 (-0.3 to 0.3)	0.1 (-4.6 to 4.6)
**Indicator 16.2.3: Age-standardised prevalence of women and men aged 18-29 years who experienced sexual violence by age 18 (%)**						
Central Europe, Eastern Europe, and Central Asia						
Central Asia						
Armenia	1.5 (1.1 to 1.9)	1.5 (1.2 to 1.9)	1.5 (1.2 to 1.9)	1.5 (1.2 to 1.9)	0.0 (-0.1 to 0.2)	0.4 (-1.8 to 2.5)
Azerbaijan	1.5 (1.1 to 1.9)	1.5 (1.2 to 1.9)	1.5 (1.2 to 1.9)	1.5 (1.2 to 1.9)	0.1 (-0.0 to 0.1)	0.8 (-0.3 to 1.9)
Georgia	1.1 (0.8 to 1.5)	1.2 (0.9 to 1.6)	1.2 (0.9 to 1.6)	1.2 (0.9 to 1.6)	0.4 (0.0 to 0.7)	5.4 (0.5 to 10.6)
Kazakhstan	2.2 (1.7 to 2.8)	2.3 (1.7 to 2.9)	2.3 (1.7 to 2.9)	2.4 (1.7 to 3.1)	0.2 (-0.4 to 0.9)	3.7 (-6.4 to 14.8)
Kyrgyzstan	1.5 (1.2 to 1.9)	1.5 (1.2 to 1.9)	1.5 (1.2 to 1.9)	1.5 (1.2 to 1.9)	-0.0 (-0.2 to 0.1)	-0.3 (-2.7 to 2.1)
Mongolia	1.5 (1.2 to 1.9)	1.5 (1.2 to 1.9)	1.5 (1.2 to 1.9)	1.5 (1.2 to 1.9)	-0.0 (-0.2 to 0.2)	-0.1 (-3.0 to 2.8)
Tajikistan	1.5 (1.2 to 1.9)	1.5 (1.2 to 1.9)	1.5 (1.2 to 1.9)	1.5 (1.2 to 1.9)	0.0 (-0.1 to 0.1)	0.2 (-0.9 to 1.2)
Turkmenistan	1.5 (1.2 to 1.9)	1.5 (1.2 to 1.9)	1.5 (1.2 to 1.9)	1.6 (1.2 to 2.0)	0.1 (0.0 to 0.1)	0.9 (0.1 to 1.7)

	Estimate in 2000 (95% UIs)	Estimate in 2015 (95% UIs)	Estimate in 2017 (95% UIs)	Estimate in 2030 (95% UIs)	Annualised rate of change, 2015-2030 (95% UIs)	Percent change, 2015-2030 (95% UIs)
Uzbekistan	1.5 (1.2 to 1.9)	1.5 (1.2 to 1.9)	1.5 (1.2 to 1.9)	1.5 (1.2 to 1.9)	0.0 (-0.1 to 0.1)	0.1 (-1.1 to 1.3)
Central Europe						
Albania	6.0 (4.4 to 7.8)	6.6 (4.8 to 8.6)	6.6 (4.8 to 8.6)	7.0 (5.1 to 9.2)	0.4 (0.2 to 0.6)	6.1 (2.5 to 9.4)
Bosnia and Herzegovina	7.5 (5.8 to 9.6)	7.5 (5.8 to 9.6)	7.5 (5.8 to 9.6)	7.5 (5.8 to 9.6)	-0.0 (-0.2 to 0.1)	-0.2 (-2.5 to 2.0)
Bulgaria	7.5 (5.8 to 9.6)	7.6 (5.8 to 9.6)	7.6 (5.8 to 9.6)	7.6 (5.8 to 9.7)	0.0 (-0.0 to 0.1)	0.4 (-0.2 to 0.9)
Croatia	7.5 (5.8 to 9.5)	7.5 (5.8 to 9.6)	7.5 (5.8 to 9.6)	7.6 (5.8 to 9.6)	0.0 (-0.1 to 0.1)	0.4 (-0.9 to 1.8)
Czech Republic	8.1 (7.1 to 9.0)	8.1 (6.7 to 9.6)	8.1 (6.2 to 10.3)	8.3 (5.7 to 11.6)	0.1 (-1.3 to 1.5)	1.5 (-17.6 to 25.4)
Hungary	7.5 (5.8 to 9.5)	7.5 (5.8 to 9.5)	7.5 (5.8 to 9.6)	7.5 (5.8 to 9.6)	0.0 (0.0 to 0.0)	0.3 (0.2 to 0.4)
Macedonia	10.7 (8.0 to 13.9)	11.0 (8.3 to 13.9)	11.0 (8.3 to 13.9)	11.2 (8.5 to 14.3)	0.1 (-0.1 to 0.3)	1.3 (-2.0 to 4.3)
Montenegro	7.5 (5.8 to 9.5)	7.6 (5.8 to 9.6)	7.6 (5.8 to 9.6)	7.6 (5.9 to 9.7)	0.0 (0.0 to 0.1)	0.6 (0.4 to 0.9)
Poland	8.2 (6.5 to 10.3)	8.4 (6.6 to 10.6)	8.6 (6.7 to 10.9)	8.9 (6.7 to 11.5)	0.4 (-0.2 to 1.0)	5.6 (-3.3 to 16.6)
Romania	7.5 (5.8 to 9.5)	7.6 (5.8 to 9.6)	7.6 (5.8 to 9.6)	7.6 (5.9 to 9.7)	0.0 (-0.1 to 0.2)	0.6 (-1.6 to 2.8)
Serbia	9.7 (7.3 to 12.4)	9.9 (7.6 to 12.6)	9.9 (7.6 to 12.7)	10.0 (7.7 to 13.0)	0.1 (-0.2 to 0.4)	1.3 (-3.0 to 6.0)
Slovakia	7.5 (5.8 to 9.5)	7.5 (5.8 to 9.6)	7.5 (5.8 to 9.5)	7.5 (5.8 to 9.6)	0.0 (-0.1 to 0.1)	0.3 (-1.1 to 1.7)
Slovenia	7.6 (5.8 to 9.6)	7.6 (5.8 to 9.6)	7.6 (5.8 to 9.6)	7.6 (5.8 to 9.6)	0.0 (-0.2 to 0.2)	0.0 (-2.6 to 2.7)
Eastern Europe						
Belarus	18.2 (13.7 to 23.4)	18.1 (13.7 to 23.2)	18.1 (13.7 to 23.1)	18.0 (13.5 to 23.2)	-0.0 (-0.3 to 0.2)	-0.6 (-4.6 to 3.1)
Estonia	18.1 (13.7 to 23.4)	18.1 (13.6 to 23.2)	18.1 (13.7 to 23.1)	18.0 (13.5 to 23.1)	-0.0 (-0.4 to 0.3)	-0.4 (-5.3 to 4.1)
Latvia	18.2 (13.7 to 23.4)	18.1 (13.6 to 23.3)	18.1 (13.7 to 23.1)	18.0 (13.5 to 23.2)	-0.0 (-0.3 to 0.2)	-0.5 (-4.7 to 3.2)
Lithuania	18.2 (13.7 to 23.5)	18.1 (13.6 to 23.3)	18.1 (13.6 to 23.3)	18.1 (13.6 to 23.2)	-0.0 (-0.1 to 0.0)	-0.3 (-1.2 to 0.6)
Moldova	18.2 (13.7 to 23.4)	18.1 (13.7 to 23.3)	18.1 (13.7 to 23.3)	18.1 (13.6 to 23.2)	-0.0 (-0.3 to 0.2)	-0.4 (-4.2 to 3.1)
Russia	19.0 (14.2 to 24.4)	19.1 (14.2 to 24.5)	19.1 (14.2 to 24.5)	19.0 (14.1 to 25.1)	-0.0 (-0.4 to 0.4)	-0.5 (-6.1 to 5.7)
Ukraine	19.1 (14.2 to 24.6)	19.1 (14.2 to 24.5)	19.1 (14.2 to 24.5)	18.9 (14.1 to 24.6)	-0.1 (-0.5 to 0.3)	-0.9 (-7.4 to 5.0)
High-income						
Australasia						
Australia	8.1 (7.0 to 9.3)	8.9 (7.1 to 10.8)	8.8 (6.3 to 11.7)	9.3 (6.0 to 13.7)	0.2 (-1.5 to 1.7)	3.6 (-19.7 to 28.3)
New Zealand	19.4 (14.6 to 25.1)	18.5 (13.9 to 23.6)	17.8 (13.5 to 22.5)	17.0 (12.6 to 21.9)	-0.6 (-1.0 to -0.1)	-8.2 (-13.7 to -1.7)
High-income Asia Pacific						
Brunei	9.7 (7.2 to 12.7)	9.5 (7.0 to 12.3)	9.5 (7.0 to 12.3)	9.4 (7.0 to 12.1)	-0.1 (-0.1 to -0.0)	-1.1 (-1.7 to -0.3)
Japan	10.1 (7.5 to 13.1)	10.1 (7.5 to 13.1)	10.1 (7.5 to 13.1)	10.1 (7.5 to 13.1)	-0.0 (-0.0 to -0.0)	-0.2 (-0.3 to -0.1)
South Korea	9.6 (7.1 to 12.5)	9.4 (7.0 to 12.2)	9.5 (7.0 to 12.2)	9.4 (7.0 to 12.1)	-0.1 (-0.2 to 0.1)	-0.9 (-2.9 to 1.1)
Singapore	9.7 (7.1 to 12.6)	9.9 (7.3 to 13.1)	10.1 (7.4 to 13.3)	10.5 (7.5 to 14.0)	0.3 (0.1 to 0.7)	5.4 (0.8 to 10.7)
High-income North America						
Canada	8.2 (6.9 to 9.5)	9.6 (8.7 to 10.6)	9.9 (8.9 to 11.0)	11.3 (9.9 to 12.9)	1.1 (0.6 to 1.6)	18.0 (9.1 to 26.2)
Greenland	10.0 (7.5 to 13.0)	10.2 (7.5 to 13.2)	10.2 (7.5 to 13.2)	10.2 (7.6 to 13.4)	0.0 (0.0 to 0.1)	0.6 (0.2 to 1.0)
United States	11.2 (8.3 to 14.3)	11.3 (8.6 to 14.4)	11.4 (8.6 to 14.5)	11.6 (8.7 to 14.8)	0.1 (-0.3 to 0.6)	2.0 (-5.1 to 9.8)
Southern Latin America						
Argentina	6.1 (4.7 to 7.8)	6.2 (4.6 to 7.9)	6.2 (4.5 to 8.0)	6.3 (4.4 to 8.6)	0.1 (-0.7 to 0.9)	1.7 (-9.4 to 14.2)
Chile	5.9 (4.3 to 7.6)	5.9 (4.3 to 7.6)	5.9 (4.3 to 7.6)	5.9 (4.3 to 7.5)	-0.0 (-0.1 to 0.0)	-0.2 (-1.0 to 0.5)
Uruguay	5.5 (4.3 to 6.8)	5.6 (4.3 to 7.0)	5.7 (4.3 to 7.2)	6.0 (4.3 to 7.9)	0.3 (-0.4 to 1.1)	5.4 (-6.5 to 18.2)
Western Europe						
Andorra	9.6 (7.2 to 12.4)	9.6 (7.2 to 12.4)	9.5 (7.2 to 12.3)	9.4 (7.1 to 12.2)	-0.1 (-0.1 to -0.0)	-1.4 (-2.1 to -0.4)
Austria	9.7 (7.2 to 12.5)	9.7 (7.2 to 12.5)	9.6 (7.2 to 12.4)	9.6 (7.1 to 12.4)	-0.1 (-0.1 to -0.0)	-0.8 (-1.4 to -0.1)
Belgium	9.7 (7.2 to 12.5)	9.7 (7.2 to 12.6)	9.7 (7.2 to 12.6)	9.7 (7.2 to 12.7)	0.0 (-0.0 to 0.0)	0.1 (-0.5 to 0.7)
Cyprus	9.6 (7.2 to 12.5)	9.5 (7.1 to 12.3)	9.5 (7.2 to 12.2)	9.5 (7.1 to 12.2)	-0.0 (-0.1 to 0.1)	-0.5 (-1.8 to 0.8)
Denmark	9.3 (7.2 to 11.7)	8.9 (6.5 to 11.6)	8.9 (6.4 to 11.8)	8.9 (6.0 to 12.4)	-0.1 (-0.9 to 0.8)	-0.8 (-12.9 to 12.2)
Finland	8.8 (6.6 to 11.3)	8.7 (6.5 to 11.3)	8.7 (6.5 to 11.3)	8.7 (6.4 to 11.5)	-0.0 (-0.2 to 0.2)	-0.2 (-3.2 to 3.6)
France	2.3 (1.8 to 2.9)	2.4 (1.8 to 3.0)	2.4 (1.8 to 3.0)	2.4 (1.8 to 3.0)	0.1 (-0.2 to 0.3)	0.9 (-3.1 to 4.3)
Germany	11.5 (7.9 to 16.3)	11.5 (7.8 to 16.4)	11.4 (7.8 to 16.4)	11.4 (7.8 to 16.3)	-0.1 (-0.2 to 0.1)	-0.8 (-2.3 to 1.0)
Greece	9.6 (7.1 to 12.5)	9.6 (7.2 to 12.4)	9.6 (7.2 to 12.5)	9.7 (7.2 to 12.5)	0.0 (0.0 to 0.1)	0.5 (0.1 to 0.8)
Iceland	11.0 (8.7 to 13.8)	9.9 (7.8 to 12.4)	9.5 (7.3 to 12.1)	9.0 (6.8 to 11.8)	-0.6 (-1.2 to -0.0)	-8.9 (-16.1 to -0.3)
Ireland	11.8 (10.3 to 13.4)	13.0 (10.6 to 15.6)	13.8 (10.5 to 17.4)	15.0 (10.4 to 20.3)	0.9 (-0.3 to 1.9)	15.3 (-4.3 to 33.5)
Israel	13.8 (10.9 to 16.8)	13.8 (10.8 to 16.9)	13.8 (10.7 to 17.1)	13.8 (10.1 to 18.1)	-0.0 (-0.8 to 0.9)	-0.1 (-11.0 to 14.2)
Italy	9.7 (7.2 to 12.5)	9.7 (7.2 to 12.5)	9.6 (7.2 to 12.5)	9.6 (7.1 to 12.4)	-0.0 (-0.1 to -0.0)	-0.6 (-1.0 to -0.2)
Luxembourg	9.7 (7.2 to 12.5)	9.6 (7.2 to 12.5)	9.6 (7.2 to 12.4)	9.6 (7.1 to 12.4)	-0.0 (-0.1 to 0.0)	-0.2 (-1.0 to 0.6)
Malta	9.7 (7.2 to 12.6)	9.6 (7.1 to 12.4)	9.6 (7.2 to 12.4)	9.5 (7.2 to 12.3)	-0.0 (-0.2 to 0.1)	-0.7 (-3.4 to 2.2)
Netherlands	11.2 (10.1 to 12.4)	11.2 (10.1 to 12.4)	11.2 (10.1 to 12.4)	11.0 (10.0 to 12.2)	-0.1 (-0.1 to 0.0)	-1.1 (-2.1 to 0.7)
Norway	9.4 (6.9 to 12.4)	9.1 (6.6 to 12.2)	9.1 (6.6 to 12.2)	9.0 (6.5 to 12.3)	-0.1 (-0.2 to 0.2)	-0.8 (-3.1 to 2.4)
Portugal	9.7 (7.2 to 12.6)	9.7 (7.2 to 12.6)	9.7 (7.2 to 12.6)	9.7 (7.2 to 12.5)	-0.0 (-0.1 to 0.1)	-0.4 (-1.9 to 1.3)
Spain	8.6 (7.1 to 10.3)	8.8 (6.8 to 11.1)	9.2 (7.0 to 11.6)	9.8 (7.1 to 13.2)	0.7 (0.0 to 1.3)	10.7 (0.2 to 21.7)
Sweden	10.6 (7.8 to 13.6)	13.5 (10.1 to 17.5)	13.3 (9.9 to 17.3)	14.2 (10.1 to 19.6)	0.3 (-0.3 to 1.0)	5.2 (-4.3 to 16.3)
Switzerland	10.0 (8.3 to 11.8)	8.9 (7.1 to 11.1)	9.9 (7.3 to 12.8)	10.5 (7.4 to 14.3)	1.1 (-0.0 to 2.0)	17.4 (-0.5 to 35.5)
United Kingdom	12.8 (10.0 to 16.0)	11.5 (8.8 to 14.4)	11.1 (8.4 to 14.0)	10.3 (7.6 to 13.2)	-0.8 (-1.1 to -0.3)	-10.8 (-15.7 to -4.9)
Latin America and Caribbean						
Andean Latin America						
Bolivia	12.5 (9.1 to 16.4)	12.3 (9.0 to 16.1)	12.3 (9.0 to 16.1)	12.2 (8.9 to 15.8)	-0.1 (-0.1 to 0.0)	-0.9 (-2.2 to 0.3)
Ecuador	12.5 (9.1 to 16.4)	12.4 (9.0 to 16.2)	12.4 (9.0 to 16.1)	12.3 (9.0 to 16.0)	-0.1 (-0.1 to -0.0)	-0.9 (-1.5 to -0.3)
Peru	12.1 (9.8 to 14.4)	12.2 (9.5 to 15.3)	12.4 (9.0 to 16.1)	12.8 (8.3 to 18.1)	0.2 (-1.0 to 1.4)	3.5 (-14.0 to 23.0)
Caribbean						
Antigua and Barbuda	9.8 (7.2 to 12.6)	9.6 (7.2 to 12.3)	9.6 (7.1 to 12.2)	9.5 (7.1 to 12.1)	-0.1 (-0.2 to 0.0)	-1.2 (-2.8 to 0.4)
The Bahamas	9.7 (7.2 to 12.3)	9.6 (7.2 to 12.3)	9.6 (7.2 to 12.3)	9.6 (7.1 to 12.2)	-0.0 (-0.0 to -0.0)	-0.3 (-0.5 to -0.1)
Barbados	9.6 (7.1 to 12.2)	9.6 (7.1 to 12.3)	9.6 (7.1 to 12.3)	9.6 (7.1 to 12.3)	0.0 (-0.0 to 0.1)	0.4 (-0.4 to 1.2)
Belize	4.4 (3.7 to 5.3)	4.5 (3.5 to 5.5)	4.5 (3.4 to 5.7)	4.7 (3.3 to 6.3)	0.2 (-0.8 to 1.3)	3.4 (-10.7 to 21.4)
Bermuda	9.7 (7.2 to 12.4)	9.7 (7.2 to 12.5)	9.7 (7.2 to 12.5)	9.8 (7.2 to 12.6)	0.0 (0.0 to 0.1)	0.5 (0.2 to 0.8)
Cuba	9.6 (7.2 to 12.3)	9.5 (7.2 to 12.1)	9.5 (7.2 to 12.1)	9.5 (7.1 to 12.0)	-0.0 (-0.0 to -0.0)	-0.3 (-0.4 to -0.0)
Dominica	9.5 (7.1 to 12.0)	9.5 (7.1 to 12.1)	9.5 (7.1 to 12.1)	9.5 (7.1 to 12.1)	0.0 (-0.0 to 0.0)	0.0 (-0.7 to 0.7)
Dominican Republic	9.6 (7.2 to 12.3)	9.5 (7.1 to 12.1)	9.5 (7.1 to 12.2)	9.5 (7.1 to 12.0)	-0.0 (-0.1 to 0.0)	-0.5 (-1.0 to 0.1)
Grenada	9.5 (7.0 to 12.1)	9.4 (7.1 to 12.0)	9.4 (7.1 to 12.0)	9.2 (6.9 to 11.8)	-0.1 (-0.3 to 0.0)	-2.0 (-4.3 to 0.1)
Guyana	9.6 (7.1 to 12.2)	9.5 (7.1 to 12.2)	9.5 (7.0 to 12.2)	9.9 (7.3 to 12.7)	0.2 (-0.3 to 0.8)	3.7 (-4.0 to 12.1)
Haiti	9.7 (7.1 to 12.4)	9.7 (7.2 to 12.5)	9.7 (7.2 to 12.4)	9.7 (7.2 to 12.3)	-0.0 (-0.1 to 0.0)	-0.6 (-1.3 to 0.1)

	Estimate in 2000 (95% UIs)	Estimate in 2015 (95% UIs)	Estimate in 2017 (95% UIs)	Estimate in 2030 (95% UIs)	Annualised rate of change, 2015-2030 (95% UIs)	Percent change, 2015-2030 (95% UIs)
Jamaica	21.4 (15.9 to 27.8)	20.9 (15.4 to 27.0)	20.9 (15.2 to 27.1)	20.7 (14.8 to 27.6)	-0.1 (-0.8 to 0.6)	-1.3 (-11.3 to 9.8)
Puerto Rico	9.6 (7.1 to 12.2)	9.6 (7.1 to 12.2)	9.6 (7.1 to 12.2)	9.6 (7.1 to 12.2)	0.0 (-0.1 to 0.1)	0.1 (-1.0 to 1.2)
Saint Lucia	9.6 (7.1 to 12.3)	9.5 (7.1 to 12.1)	9.5 (7.1 to 12.1)	9.5 (7.1 to 12.0)	-0.0 (-0.1 to 0.1)	-0.2 (-1.7 to 1.3)
Saint Vincent and the Grenadines	9.4 (7.0 to 12.0)	9.5 (7.1 to 12.1)	9.5 (7.1 to 12.0)	9.5 (7.1 to 12.0)	0.0 (-0.0 to 0.0)	0.0 (-0.5 to 0.5)
Suriname	9.5 (7.1 to 12.1)	9.6 (7.1 to 12.3)	9.6 (7.1 to 12.3)	9.7 (7.2 to 12.4)	0.1 (-0.0 to 0.1)	0.8 (-0.0 to 1.5)
Trinidad and Tobago	9.4 (7.0 to 12.0)	9.6 (7.2 to 12.2)	9.6 (7.1 to 12.2)	9.5 (7.1 to 12.1)	-0.1 (-0.3 to 0.1)	-1.3 (-4.1 to 1.5)
Virgin Islands, U.S.	9.8 (7.2 to 12.6)	9.8 (7.2 to 12.5)	9.7 (7.2 to 12.4)	9.7 (7.2 to 12.4)	-0.0 (-0.1 to 0.1)	-0.4 (-2.1 to 1.1)
Central Latin America						
Colombia	8.4 (6.5 to 10.6)	8.4 (6.5 to 10.6)	8.4 (6.5 to 10.6)	8.4 (6.5 to 10.6)	0.0 (-0.1 to 0.1)	0.1 (-0.9 to 1.2)
Costa Rica	18.0 (15.8 to 20.2)	18.4 (15.2 to 21.8)	18.6 (14.2 to 23.4)	19.2 (13.3 to 26.5)	0.2 (-1.1 to 1.5)	3.9 (-15.6 to 26.1)
El Salvador	7.8 (6.6 to 9.2)	7.8 (6.5 to 9.2)	7.8 (6.5 to 9.3)	7.9 (6.4 to 9.7)	0.1 (-0.4 to 0.5)	1.6 (-5.4 to 8.3)
Guatemala	6.4 (5.2 to 7.8)	6.6 (5.4 to 8.1)	6.7 (5.4 to 8.2)	7.0 (5.5 to 8.7)	0.3 (-0.2 to 0.8)	5.3 (-2.4 to 12.5)
Honduras	8.4 (7.0 to 9.9)	8.6 (6.9 to 10.3)	8.7 (6.7 to 11.0)	9.0 (6.3 to 12.2)	0.3 (-0.9 to 1.5)	4.6 (-13.3 to 25.4)
Mexico	7.2 (5.4 to 9.1)	7.2 (5.4 to 9.1)	7.3 (5.4 to 9.2)	7.4 (5.5 to 9.6)	0.1 (-0.4 to 0.7)	2.2 (-5.9 to 10.8)
Nicaragua	5.9 (4.8 to 7.2)	5.7 (4.8 to 6.8)	5.8 (4.7 to 7.0)	5.7 (4.3 to 7.2)	-0.0 (-0.9 to 0.8)	-0.5 (-12.3 to 12.0)
Panama	8.4 (6.5 to 10.6)	8.4 (6.5 to 10.6)	8.4 (6.5 to 10.6)	8.4 (6.5 to 10.6)	-0.0 (-0.0 to 0.0)	-0.1 (-0.3 to 0.1)
Venezuela	8.4 (6.5 to 10.6)	8.4 (6.5 to 10.6)	8.4 (6.5 to 10.6)	8.4 (6.5 to 10.6)	0.0 (-0.1 to 0.1)	0.2 (-0.9 to 1.2)
Tropical Latin America						
Brazil	5.9 (4.4 to 7.4)	6.1 (4.6 to 7.8)	6.2 (4.6 to 7.9)	6.4 (4.7 to 8.5)	0.3 (-0.1 to 0.9)	5.3 (-2.2 to 13.7)
Paraguay	3.6 (2.9 to 4.4)	3.4 (2.7 to 4.3)	3.3 (2.6 to 4.2)	3.2 (2.4 to 4.0)	-0.6 (-1.0 to -0.2)	-8.5 (-14.1 to -2.9)
North Africa and Middle East						
North Africa and Middle East						
Afghanistan	8.0 (6.1 to 10.1)	7.9 (6.0 to 10.1)	7.9 (6.0 to 10.1)	7.9 (6.0 to 10.1)	0.0 (-0.1 to 0.1)	0.3 (-1.0 to 1.7)
Algeria	8.0 (6.0 to 10.1)	8.0 (6.1 to 10.1)	8.0 (6.1 to 10.1)	7.9 (6.0 to 10.1)	-0.0 (-0.2 to 0.2)	-0.5 (-3.6 to 2.5)
Bahrain	7.8 (6.0 to 9.9)	7.9 (6.1 to 9.9)	7.9 (6.1 to 10.0)	8.0 (6.1 to 10.2)	0.1 (-0.1 to 0.4)	1.8 (-1.6 to 5.8)
Egypt	8.0 (6.0 to 10.1)	8.0 (6.0 to 10.1)	8.0 (6.0 to 10.1)	8.0 (6.0 to 10.1)	-0.0 (-0.2 to 0.1)	-0.3 (-2.3 to 1.7)
Iran	8.2 (6.2 to 10.5)	8.3 (6.3 to 10.5)	8.3 (6.3 to 10.5)	8.2 (6.2 to 10.5)	-0.1 (-0.3 to 0.2)	-0.9 (-4.4 to 2.5)
Iraq	7.9 (6.0 to 10.0)	7.9 (6.0 to 10.0)	7.9 (6.0 to 10.0)	8.0 (6.0 to 10.1)	0.0 (-0.0 to 0.0)	0.2 (-0.2 to 0.6)
Jordan	7.9 (6.0 to 10.0)	7.9 (6.0 to 9.9)	7.8 (6.0 to 9.9)	7.8 (6.0 to 9.9)	-0.0 (-0.1 to 0.0)	-0.6 (-1.3 to 0.3)
Kuwait	7.8 (6.0 to 9.9)	8.0 (6.2 to 10.2)	8.0 (6.1 to 10.1)	8.1 (6.1 to 10.3)	0.0 (-0.3 to 0.4)	0.6 (-5.0 to 6.5)
Lebanon	8.0 (6.1 to 10.1)	8.0 (6.0 to 10.1)	8.0 (6.1 to 10.0)	8.0 (6.1 to 10.1)	0.0 (-0.2 to 0.2)	0.1 (-2.6 to 2.9)
Libya	8.0 (6.0 to 10.1)	8.0 (6.1 to 10.1)	8.0 (6.1 to 10.1)	8.0 (6.0 to 10.1)	-0.0 (-0.2 to 0.1)	-0.1 (-2.3 to 2.1)
Morocco	8.8 (7.0 to 11.0)	9.5 (7.2 to 12.0)	10.0 (7.4 to 12.9)	10.7 (7.6 to 14.6)	0.8 (0.0 to 1.5)	12.8 (0.6 to 24.3)
Palestine	7.9 (6.0 to 10.1)	7.9 (6.0 to 10.1)	7.9 (6.0 to 10.0)	7.9 (6.0 to 10.1)	0.0 (-0.1 to 0.1)	0.0 (-1.5 to 1.5)
Oman	7.9 (6.0 to 10.0)	7.7 (5.8 to 9.8)	7.6 (5.8 to 9.8)	7.4 (5.6 to 9.6)	-0.2 (-0.4 to 0.0)	-2.8 (-6.0 to 0.6)
Qatar	7.5 (5.8 to 9.6)	7.3 (5.4 to 9.5)	7.2 (5.4 to 9.4)	7.1 (5.2 to 9.4)	-0.2 (-0.3 to 0.1)	-2.2 (-5.1 to 1.4)
Saudi Arabia	7.8 (6.0 to 9.9)	7.8 (6.0 to 9.9)	7.8 (6.0 to 9.9)	7.8 (6.0 to 9.8)	-0.0 (-0.1 to 0.0)	-0.5 (-1.3 to 0.3)
Sudan	8.0 (6.1 to 10.2)	8.0 (6.1 to 10.2)	8.0 (6.1 to 10.1)	7.9 (6.0 to 10.0)	-0.0 (-0.1 to 0.0)	-0.7 (-1.9 to 0.6)
Syria	8.0 (6.0 to 10.1)	8.0 (6.0 to 10.2)	8.0 (6.0 to 10.3)	8.1 (6.0 to 10.3)	0.1 (-0.1 to 0.2)	0.9 (-1.5 to 3.2)
Tunisia	8.0 (6.0 to 10.1)	8.0 (6.1 to 10.2)	8.0 (6.1 to 10.2)	8.0 (6.1 to 10.2)	-0.0 (-0.1 to 0.1)	-0.0 (-1.6 to 1.6)
Turkey	6.5 (5.2 to 8.0)	6.4 (5.6 to 7.3)	6.4 (5.5 to 7.3)	6.4 (5.4 to 7.4)	-0.1 (-0.5 to 0.4)	-1.0 (-7.6 to 6.4)
United Arab Emirates	7.6 (5.8 to 9.8)	7.6 (5.6 to 10.0)	7.7 (5.8 to 10.0)	7.8 (5.8 to 10.2)	0.1 (-0.1 to 0.5)	2.2 (-2.2 to 8.0)
Yemen	7.9 (6.0 to 10.1)	8.0 (6.0 to 10.1)	8.0 (6.0 to 10.1)	8.0 (6.1 to 10.2)	0.0 (-0.0 to 0.0)	0.2 (-0.1 to 0.5)
South Asia						
South Asia						
Bangladesh	22.1 (18.4 to 26.6)	22.4 (18.6 to 26.8)	22.3 (18.6 to 26.8)	22.2 (18.4 to 26.6)	-0.1 (-0.1 to 0.0)	-0.9 (-1.7 to 0.6)
Bhutan	15.1 (11.7 to 18.7)	15.1 (11.7 to 18.9)	15.1 (11.7 to 18.9)	15.0 (11.6 to 18.7)	-0.0 (-0.1 to 0.0)	-0.5 (-1.2 to 0.4)
India	9.8 (7.5 to 12.6)	10.2 (7.6 to 13.1)	10.5 (7.8 to 13.6)	11.1 (8.1 to 14.7)	0.6 (0.0 to 1.1)	9.0 (0.5 to 17.9)
Nepal	14.8 (11.5 to 18.5)	14.7 (11.4 to 18.5)	14.8 (11.4 to 18.5)	14.8 (11.4 to 18.6)	0.0 (-0.1 to 0.2)	0.1 (-2.2 to 2.8)
Pakistan	15.0 (11.6 to 18.6)	14.9 (11.6 to 18.6)	14.9 (11.6 to 18.6)	14.9 (11.5 to 18.6)	-0.0 (-0.1 to 0.0)	-0.1 (-0.7 to 0.4)
Southeast Asia, East Asia, and Oceania						
East Asia						
China	5.8 (4.5 to 7.2)	6.1 (4.7 to 7.7)	6.3 (4.8 to 7.9)	6.7 (5.0 to 8.6)	0.6 (0.1 to 1.1)	9.8 (2.0 to 17.1)
North Korea	5.0 (3.9 to 6.3)	5.2 (4.0 to 6.6)	5.2 (4.0 to 6.5)	5.3 (4.0 to 6.7)	0.1 (-0.0 to 0.3)	1.6 (-0.7 to 4.0)
Taiwan (Province of China)	5.4 (4.1 to 6.7)	5.4 (4.2 to 6.8)	5.4 (4.2 to 6.8)	5.5 (4.1 to 7.0)	0.1 (-0.5 to 0.7)	1.4 (-7.0 to 11.2)
Oceania						
American Samoa	20.9 (15.7 to 27.2)	20.4 (15.4 to 26.3)	20.4 (15.4 to 26.3)	20.1 (15.2 to 25.8)	-0.1 (-0.3 to 0.1)	-1.4 (-4.2 to 1.3)
Federated States of Micronesia	20.5 (15.5 to 26.5)	20.9 (15.7 to 27.1)	20.9 (15.7 to 27.1)	21.3 (16.0 to 27.7)	0.1 (-0.0 to 0.2)	1.8 (-0.0 to 3.7)
Fiji	20.9 (15.7 to 27.1)	20.8 (15.7 to 27.0)	20.8 (15.6 to 27.0)	20.7 (15.6 to 26.8)	-0.0 (-0.1 to 0.0)	-0.7 (-1.6 to 0.3)
Guam	20.9 (15.7 to 27.1)	21.4 (16.0 to 27.6)	21.4 (16.0 to 27.7)	21.8 (16.3 to 28.4)	0.1 (0.0 to 0.2)	1.6 (0.1 to 3.3)
Kiribati	20.2 (15.2 to 26.1)	20.4 (15.4 to 26.5)	20.4 (15.3 to 26.4)	20.4 (15.3 to 26.3)	-0.0 (-0.1 to 0.0)	-0.2 (-1.0 to 0.6)
Marshall Islands	20.6 (15.5 to 26.6)	21.1 (15.8 to 27.4)	21.0 (15.8 to 27.3)	21.2 (15.9 to 27.6)	0.1 (0.0 to 0.1)	0.8 (0.2 to 1.4)
Northern Mariana Islands	15.7 (12.0 to 20.0)	20.3 (15.4 to 26.1)	20.4 (15.4 to 26.3)	20.9 (15.4 to 27.0)	0.2 (-0.2 to 0.5)	2.7 (-3.0 to 8.0)
Papua New Guinea	21.3 (17.9 to 24.9)	21.3 (17.8 to 24.9)	21.3 (17.8 to 24.9)	21.3 (17.8 to 25.0)	0.0 (-0.1 to 0.2)	0.3 (-2.0 to 3.0)
Samoa	21.3 (16.0 to 27.6)	21.0 (15.8 to 27.2)	21.0 (15.7 to 27.1)	20.7 (15.6 to 26.9)	-0.1 (-0.1 to -0.1)	-1.2 (-1.6 to -0.8)
Solomon Islands	21.9 (17.0 to 27.9)	21.7 (16.9 to 27.7)	21.8 (16.9 to 27.8)	21.8 (16.9 to 27.8)	0.0 (-0.1 to 0.1)	0.1 (-1.0 to 1.5)
Tonga	18.7 (13.9 to 24.6)	19.1 (14.1 to 25.1)	19.0 (14.1 to 25.1)	19.4 (14.2 to 25.8)	0.1 (-0.1 to 0.3)	1.8 (-0.9 to 4.5)
Vanuatu	20.3 (15.3 to 26.2)	20.1 (15.2 to 26.0)	20.0 (15.2 to 25.9)	19.8 (15.0 to 25.5)	-0.1 (-0.2 to -0.0)	-1.4 (-2.4 to -0.5)
Southeast Asia						
Cambodia	9.2 (7.9 to 10.7)	9.4 (8.1 to 10.8)	9.4 (8.1 to 10.9)	9.6 (8.2 to 11.0)	0.1 (-0.0 to 0.3)	1.8 (-0.6 to 4.2)
Indonesia	5.8 (4.4 to 7.3)	5.9 (4.5 to 7.5)	5.9 (4.5 to 7.5)	5.9 (4.5 to 7.6)	0.1 (-0.1 to 0.2)	0.9 (-0.9 to 2.8)
Laos	5.7 (4.4 to 7.2)	5.8 (4.4 to 7.3)	5.8 (4.4 to 7.3)	5.8 (4.5 to 7.4)	0.1 (0.0 to 0.1)	1.4 (0.6 to 2.2)
Malaysia	5.7 (4.3 to 7.3)	5.8 (4.4 to 7.4)	5.8 (4.4 to 7.4)	5.9 (4.4 to 7.6)	0.1 (-0.4 to 0.6)	1.1 (-5.3 to 9.2)
Maldives	5.7 (4.4 to 7.2)	6.4 (4.9 to 8.2)	6.5 (4.9 to 8.4)	7.2 (5.4 to 9.4)	0.7 (0.5 to 0.9)	11.3 (7.2 to 15.1)
Mauritius	5.8 (4.4 to 7.3)	5.8 (4.4 to 7.3)	5.8 (4.5 to 7.3)	5.8 (4.4 to 7.4)	0.0 (-0.1 to 0.2)	0.3 (-1.9 to 2.6)
Myanmar	5.7 (4.4 to 7.2)	5.7 (4.4 to 7.2)	5.7 (4.4 to 7.2)	5.7 (4.4 to 7.2)	0.0 (-0.0 to 0.1)	0.6 (-0.3 to 1.7)
Philippines	4.9 (3.7 to 6.4)	5.0 (3.7 to 6.4)	5.0 (3.7 to 6.4)	5.0 (3.7 to 6.5)	-0.0 (-0.3 to 0.3)	-0.1 (-3.8 to 4.0)
Sri Lanka	8.2 (7.1 to 9.5)	8.1 (7.0 to 9.4)	8.2 (6.9 to 9.6)	8.2 (6.6 to 9.9)	0.0 (-0.6 to 0.7)	0.6 (-8.5 to 11.0)
Seychelles	5.8 (4.5 to 7.3)	6.1 (4.7 to 7.8)	6.1 (4.7 to 7.7)	6.2 (4.8 to 7.9)	0.1 (0.0 to 0.2)	2.0 (0.4 to 3.7)
Thailand	3.1 (2.1 to 4.4)	3.0 (2.1 to 4.4)	3.0 (2.0 to 4.5)	3.0 (2.0 to 4.7)	-0.0 (-0.6 to 0.8)	-0.6 (-9.1 to 12.3)

	Estimate in 2000 (95% UIs)	Estimate in 2015 (95% UIs)	Estimate in 2017 (95% UIs)	Estimate in 2030 (95% UIs)	Annualised rate of change, 2015-2030 (95% UIs)	Percent change, 2015-2030 (95% UIs)
Timor-Leste	8.2 (6.4 to 10.2)	8.2 (6.3 to 10.2)	8.1 (6.3 to 10.3)	8.1 (6.1 to 10.6)	-0.1 (-0.5 to 0.4)	-1.0 (-7.5 to 6.7)
Vietnam	5.2 (4.0 to 6.6)	5.2 (4.0 to 6.7)	5.2 (4.0 to 6.8)	5.3 (4.0 to 6.7)	0.0 (-0.0 to 0.1)	0.4 (-0.7 to 1.6)
Sub-Saharan Africa						
Central Sub-Saharan Africa						
Angola	14.4 (10.8 to 18.2)	14.4 (10.9 to 18.3)	14.4 (10.9 to 18.2)	14.5 (10.9 to 18.3)	0.0 (-0.0 to 0.0)	0.1 (-0.3 to 0.5)
Central African Republic	14.4 (10.8 to 18.1)	14.4 (10.8 to 18.2)	14.4 (10.8 to 18.3)	14.4 (10.8 to 18.3)	0.0 (-0.0 to 0.0)	0.2 (-0.2 to 0.5)
Congo	14.3 (10.8 to 18.1)	14.4 (10.8 to 18.1)	14.4 (10.8 to 18.1)	14.4 (10.9 to 18.1)	-0.0 (-0.0 to 0.0)	-0.0 (-0.4 to 0.3)
Democratic Republic of the Congo	14.3 (10.8 to 18.0)	14.3 (10.8 to 18.0)	14.3 (10.8 to 18.0)	14.3 (10.8 to 17.9)	-0.0 (-0.0 to 0.0)	-0.0 (-0.1 to 0.1)
Equatorial Guinea	14.5 (10.8 to 18.4)	14.1 (10.7 to 17.6)	14.1 (10.7 to 17.6)	13.8 (10.6 to 17.2)	-0.1 (-0.4 to 0.2)	-2.0 (-6.3 to 2.6)
Gabon	14.4 (10.9 to 18.2)	14.4 (10.9 to 18.2)	14.4 (10.9 to 18.3)	14.5 (10.9 to 18.4)	0.0 (-0.0 to 0.1)	0.3 (-0.3 to 0.8)
Eastern Sub-Saharan Africa						
Burundi	12.7 (9.9 to 15.9)	12.7 (9.9 to 15.9)	12.7 (9.9 to 15.9)	12.7 (9.8 to 15.9)	-0.0 (-0.1 to 0.1)	-0.1 (-1.4 to 1.2)
Comoros	12.8 (10.0 to 16.1)	12.8 (10.0 to 16.1)	12.8 (10.0 to 16.1)	12.8 (10.0 to 16.1)	0.0 (-0.0 to 0.0)	0.1 (-0.4 to 0.5)
Djibouti	13.1 (10.2 to 16.4)	12.9 (10.0 to 16.1)	13.0 (10.1 to 16.2)	12.9 (10.1 to 16.2)	0.0 (-0.0 to 0.0)	0.2 (-0.2 to 0.7)
Eritrea	12.9 (10.1 to 16.2)	12.9 (10.1 to 16.2)	12.9 (10.1 to 16.2)	12.9 (10.0 to 16.2)	0.0 (-0.1 to 0.1)	0.1 (-1.4 to 1.4)
Ethiopia	16.8 (12.8 to 21.0)	16.8 (12.8 to 21.0)	16.8 (12.8 to 20.9)	16.8 (12.8 to 21.1)	-0.0 (-0.2 to 0.2)	-0.0 (-3.1 to 3.0)
Kenya	21.6 (16.4 to 27.2)	21.6 (16.6 to 27.1)	21.7 (16.6 to 27.4)	22.2 (16.7 to 29.1)	0.2 (-0.4 to 0.8)	2.6 (-6.1 to 13.1)
Madagascar	12.8 (10.0 to 16.0)	12.8 (10.0 to 16.1)	12.8 (10.0 to 16.1)	12.9 (10.0 to 16.1)	0.0 (-0.0 to 0.1)	0.4 (-0.3 to 1.1)
Malawi	12.7 (9.9 to 15.9)	12.7 (9.9 to 15.8)	12.7 (9.9 to 15.8)	12.7 (9.9 to 15.8)	0.0 (-0.0 to 0.0)	0.0 (-0.5 to 0.5)
Mozambique	8.2 (6.1 to 10.6)	8.3 (6.2 to 10.7)	8.4 (6.2 to 10.7)	8.5 (6.4 to 10.9)	0.1 (-0.1 to 0.4)	2.2 (-0.9 to 6.9)
Rwanda	12.6 (9.8 to 15.8)	12.8 (10.0 to 16.0)	12.8 (10.0 to 16.0)	12.9 (10.0 to 16.1)	0.0 (-0.1 to 0.2)	0.7 (-1.2 to 2.4)
Somalia	13.0 (10.1 to 16.3)	13.0 (10.1 to 16.3)	13.0 (10.1 to 16.3)	12.9 (10.0 to 16.2)	-0.0 (-0.1 to 0.0)	-0.4 (-1.1 to 0.2)
South Sudan	12.9 (10.1 to 16.2)	12.8 (10.0 to 15.9)	12.8 (10.0 to 16.0)	12.7 (9.9 to 15.9)	-0.0 (-0.1 to 0.0)	-0.4 (-0.8 to 0.1)
Tanzania	13.9 (11.1 to 16.7)	14.7 (11.5 to 18.1)	15.3 (11.8 to 19.2)	16.7 (12.3 to 21.8)	0.8 (0.2 to 1.5)	13.2 (2.4 to 25.8)
Uganda	12.9 (10.4 to 15.5)	12.7 (10.1 to 15.5)	12.5 (9.6 to 15.7)	12.3 (9.0 to 16.6)	-0.3 (-1.1 to 0.7)	-3.5 (-15.0 to 10.4)
Zambia	11.6 (8.8 to 14.6)	11.6 (8.7 to 14.7)	11.6 (8.7 to 14.7)	11.7 (8.6 to 14.9)	0.0 (-0.2 to 0.3)	0.5 (-3.3 to 5.2)
Southern Sub-Saharan Africa						
Botswana	32.9 (23.4 to 43.3)	32.4 (23.4 to 42.4)	32.4 (23.4 to 42.3)	32.2 (23.1 to 42.5)	-0.0 (-0.2 to 0.2)	-0.4 (-3.0 to 2.5)
Lesotho	15.3 (11.2 to 20.3)	15.1 (11.2 to 19.8)	15.1 (11.2 to 19.8)	15.0 (11.2 to 19.7)	-0.0 (-0.0 to -0.0)	-0.4 (-0.7 to -0.1)
Namibia	15.2 (11.2 to 20.1)	15.2 (11.3 to 20.1)	15.2 (11.2 to 20.0)	15.2 (11.3 to 19.9)	-0.0 (-0.1 to 0.1)	-0.2 (-1.1 to 0.8)
South Africa	15.9 (12.2 to 20.2)	16.0 (12.3 to 19.9)	16.0 (12.3 to 20.0)	16.2 (12.2 to 20.4)	0.1 (-0.4 to 0.5)	1.1 (-5.4 to 8.2)
Swaziland	11.0 (7.7 to 15.1)	8.7 (6.4 to 11.1)	8.6 (6.4 to 11.0)	8.3 (6.2 to 10.7)	-0.3 (-0.4 to -0.2)	-3.8 (-5.4 to -2.3)
Zimbabwe	15.4 (11.3 to 20.5)	15.5 (11.4 to 20.5)	15.5 (11.4 to 20.5)	15.6 (11.4 to 20.8)	0.0 (-0.1 to 0.1)	0.5 (-0.9 to 1.7)
Western Sub-Saharan Africa						
Benin	15.5 (11.7 to 19.9)	15.4 (11.6 to 19.6)	15.4 (11.6 to 19.5)	15.3 (11.6 to 19.3)	-0.0 (-0.1 to 0.1)	-0.7 (-2.0 to 0.9)
Burkina Faso	15.5 (11.6 to 19.8)	15.5 (11.7 to 19.7)	15.4 (11.6 to 19.7)	15.4 (11.7 to 19.6)	-0.0 (-0.1 to 0.0)	-0.4 (-1.2 to 0.4)
Cameroon	15.4 (11.6 to 19.6)	15.4 (11.6 to 19.5)	15.4 (11.6 to 19.5)	15.3 (11.6 to 19.4)	-0.0 (-0.1 to 0.0)	-0.4 (-1.1 to 0.4)
Cape Verde	15.3 (11.6 to 19.3)	15.2 (11.5 to 19.1)	15.2 (11.5 to 19.2)	15.2 (11.5 to 19.1)	-0.0 (-0.0 to 0.0)	-0.2 (-0.7 to 0.2)
Chad	15.4 (11.6 to 19.7)	15.4 (11.6 to 19.6)	15.4 (11.6 to 19.6)	15.4 (11.6 to 19.5)	-0.0 (-0.1 to 0.0)	-0.2 (-0.8 to 0.5)
Cote d'Ivoire	14.2 (10.9 to 18.0)	14.3 (10.9 to 18.2)	14.2 (10.6 to 18.2)	14.1 (10.4 to 18.5)	-0.1 (-0.5 to 0.4)	-1.1 (-7.3 to 6.5)
The Gambia	15.4 (11.6 to 19.5)	15.4 (11.6 to 19.6)	15.4 (11.6 to 19.6)	15.4 (11.6 to 19.6)	0.0 (-0.0 to 0.0)	0.0 (-0.1 to 0.1)
Ghana	15.4 (11.6 to 19.6)	15.4 (11.7 to 19.6)	15.4 (11.7 to 19.6)	15.4 (11.7 to 19.6)	-0.0 (-0.0 to 0.0)	-0.1 (-0.2 to 0.1)
Guinea	15.4 (11.6 to 19.7)	15.5 (11.6 to 19.8)	15.5 (11.7 to 19.8)	15.5 (11.7 to 19.9)	0.0 (-0.0 to 0.1)	0.2 (-0.3 to 0.7)
Guinea-Bissau	15.4 (11.6 to 19.5)	15.4 (11.7 to 19.6)	15.4 (11.7 to 19.6)	15.4 (11.7 to 19.6)	-0.0 (-0.0 to 0.0)	-0.1 (-0.4 to 0.3)
Liberia	15.4 (11.6 to 19.5)	15.4 (11.6 to 19.5)	15.3 (11.6 to 19.5)	15.3 (11.6 to 19.4)	-0.0 (-0.1 to 0.0)	-0.3 (-0.9 to 0.2)
Mali	15.4 (11.6 to 19.5)	15.4 (11.6 to 19.7)	15.4 (11.6 to 19.6)	15.4 (11.6 to 19.6)	-0.0 (-0.0 to 0.0)	-0.2 (-0.5 to 0.2)
Mauritania	15.4 (11.6 to 19.6)	15.4 (11.6 to 19.6)	15.4 (11.6 to 19.6)	15.4 (11.6 to 19.5)	-0.0 (-0.0 to 0.0)	-0.1 (-0.3 to 0.2)
Niger	15.4 (11.6 to 19.7)	15.3 (11.5 to 19.5)	15.3 (11.5 to 19.5)	15.3 (11.5 to 19.4)	-0.0 (-0.1 to 0.0)	-0.4 (-1.0 to 0.3)
Nigeria	15.8 (13.2 to 18.8)	16.4 (13.0 to 19.9)	16.8 (12.9 to 21.1)	17.6 (12.4 to 23.7)	0.4 (-0.6 to 1.3)	7.2 (-8.9 to 21.7)
Sao Tome and Principe	15.3 (11.6 to 19.4)	15.3 (11.6 to 19.3)	15.3 (11.6 to 19.3)	15.3 (11.6 to 19.3)	-0.0 (-0.1 to 0.0)	-0.3 (-1.2 to 0.5)
Senegal	15.4 (11.6 to 19.5)	15.4 (11.6 to 19.5)	15.3 (11.6 to 19.4)	15.3 (11.6 to 19.4)	-0.0 (-0.1 to 0.0)	-0.3 (-0.9 to 0.4)
Sierra Leone	15.5 (11.7 to 19.8)	15.4 (11.6 to 19.6)	15.4 (11.6 to 19.6)	15.3 (11.6 to 19.4)	-0.0 (-0.1 to 0.0)	-0.5 (-1.5 to 0.6)
Togo	15.4 (11.6 to 19.6)	15.4 (11.7 to 19.7)	15.4 (11.7 to 19.6)	15.4 (11.6 to 19.5)	-0.0 (-0.1 to 0.0)	-0.6 (-1.6 to 0.6)
**Indicator 17.19.2c: Percentage of well-certified deaths by a vital registration system among a country's total population (%)**						
Central Europe, Eastern Europe, and Central Asia						
Central Asia						
Armenia	86.0 (83.4 to 88.5)	90.3 (88.0 to 92.4)	90.1 (87.4 to 92.4)	92.2 (87.8 to 95.3)	0.1 (-0.0 to 0.3)	2.1 (-0.6 to 4.2)
Azerbaijan	67.2 (62.7 to 71.5)	79.0 (73.0 to 84.3)	79.8 (74.2 to 84.8)	85.6 (77.9 to 91.4)	0.5 (0.2 to 0.8)	8.4 (3.2 to 13.1)
Georgia	74.5 (69.6 to 78.7)	58.3 (51.7 to 64.8)	61.3 (54.2 to 68.0)	57.0 (43.7 to 69.5)	-0.2 (-1.3 to 0.7)	-2.4 (-18.3 to 11.2)
Kazakhstan	84.4 (81.4 to 87.0)	80.3 (76.4 to 83.8)	81.7 (77.4 to 85.5)	80.7 (72.4 to 87.7)	0.0 (-0.5 to 0.4)	0.5 (-6.7 to 5.8)
Kyrgyzstan	79.1 (75.5 to 82.2)	90.5 (87.9 to 92.8)	90.2 (87.2 to 92.7)	94.0 (90.5 to 96.6)	0.3 (0.1 to 0.4)	3.9 (2.0 to 5.5)
Mongolia	56.0 (48.2 to 63.9)	67.7 (60.3 to 73.9)	67.7 (60.5 to 73.9)	75.5 (65.5 to 82.8)	0.7 (0.2 to 1.3)	11.6 (2.5 to 21.0)
Tajikistan	49.2 (45.2 to 53.5)	57.8 (50.6 to 64.6)	56.1 (49.1 to 63.2)	56.6 (45.0 to 68.5)	-0.2 (-1.0 to 0.6)	-2.2 (-14.5 to 9.2)
Turkmenistan	76.1 (72.0 to 79.9)	76.0 (71.1 to 80.4)	76.7 (71.2 to 81.4)	75.6 (65.6 to 83.8)	-0.0 (-0.6 to 0.4)	-0.5 (-8.8 to 6.5)
Uzbekistan	67.8 (63.5 to 71.4)	78.8 (74.1 to 82.9)	79.4 (74.5 to 83.8)	83.8 (75.4 to 90.2)	0.4 (0.0 to 0.7)	6.3 (0.5 to 10.8)
Central Europe						
Albania	68.3 (64.3 to 72.0)	70.2 (63.6 to 76.2)	71.7 (64.6 to 78.0)	75.1 (63.2 to 84.7)	0.4 (-0.2 to 0.9)	6.8 (-3.1 to 13.7)
Bosnia and Herzegovina	70.4 (63.3 to 76.9)	73.1 (67.9 to 78.0)	74.1 (68.5 to 79.3)	77.8 (67.4 to 86.2)	0.4 (-0.2 to 0.8)	6.3 (-2.6 to 13.2)
Bulgaria	71.9 (68.5 to 75.3)	72.1 (67.3 to 76.5)	73.9 (68.3 to 78.9)	74.5 (64.6 to 83.2)	0.2 (-0.4 to 0.7)	3.4 (-5.4 to 10.6)
Croatia	83.2 (80.4 to 85.6)	90.2 (88.0 to 92.2)	90.0 (87.6 to 92.2)	92.9 (89.0 to 95.6)	0.2 (0.0 to 0.3)	2.9 (0.4 to 4.7)
Czech Republic	83.7 (81.2 to 86.1)	85.2 (82.1 to 87.9)	85.0 (81.5 to 88.0)	84.9 (78.2 to 90.3)	-0.0 (-0.4 to 0.2)	-0.4 (-5.5 to 3.7)
Hungary	90.5 (88.5 to 92.0)	91.3 (89.2 to 93.0)	90.9 (88.5 to 92.8)	90.9 (86.3 to 94.1)	-0.0 (-0.2 to 0.1)	-0.4 (-3.6 to 2.0)
Macedonia	81.2 (78.2 to 84.0)	79.8 (75.5 to 83.6)	80.4 (75.6 to 84.8)	80.8 (71.8 to 88.1)	0.1 (-0.4 to 0.4)	1.2 (-6.0 to 6.6)
Montenegro	70.0 (65.2 to 74.3)	78.2 (72.7 to 83.1)	79.5 (74.2 to 84.5)	84.1 (75.0 to 90.8)	0.5 (0.1 to 0.7)	7.5 (1.5 to 11.9)
Poland	72.0 (68.3 to 75.1)	72.2 (68.0 to 76.4)	74.0 (69.1 to 78.6)	77.5 (68.3 to 84.6)	0.5 (-0.0 to 0.8)	7.3 (-0.7 to 13.2)
Romania	84.2 (81.6 to 86.6)	83.9 (81.2 to 86.5)	84.0 (80.9 to 87.1)	84.3 (78.3 to 89.6)	0.0 (-0.3 to 0.3)	0.4 (-4.7 to 4.6)
Serbia	68.1 (64.0 to 71.8)	67.7 (62.9 to 72.0)	69.0 (63.6 to 74.2)	68.1 (57.7 to 77.3)	0.0 (-0.8 to 0.6)	0.5 (-10.7 to 9.3)
Slovakia	82.7 (79.9 to 85.3)	89.2 (86.2 to 92.0)	89.3 (86.0 to 92.2)	92.4 (87.9 to 95.7)	0.2 (0.1 to 0.4)	3.6 (0.8 to 5.5)
Slovenia	88.7 (86.6 to 90.5)	86.8 (83.9 to 89.3)	87.0 (83.7 to 90.0)	85.8 (78.8 to 91.3)	-0.1 (-0.5 to 0.2)	-1.2 (-6.8 to 2.9)
Eastern Europe						

	Estimate in 2000 (95% UIs)	Estimate in 2015 (95% UIs)	Estimate in 2017 (95% UIs)	Estimate in 2030 (95% UIs)	Annualised rate of change, 2015-2030 (95% UIs)	Percent change, 2015-2030 (95% UIs)
Belarus	82.9 (80.1 to 85.3)	84.2 (80.7 to 87.0)	84.9 (80.8 to 88.1)	86.8 (79.8 to 91.8)	0.2 (-0.1 to 0.4)	3.1 (-1.9 to 6.6)
Estonia	91.9 (90.2 to 93.6)	92.1 (90.2 to 93.8)	92.1 (90.0 to 93.9)	92.5 (88.4 to 95.4)	0.0 (-0.2 to 0.2)	0.5 (-2.5 to 2.5)
Latvia	89.5 (87.3 to 91.4)	92.0 (89.7 to 93.8)	92.2 (89.7 to 94.1)	94.1 (90.5 to 96.6)	0.1 (0.0 to 0.2)	2.3 (0.1 to 3.8)
Lithuania	92.0 (90.3 to 93.6)	93.3 (91.5 to 94.8)	93.1 (91.1 to 94.7)	93.7 (90.1 to 96.2)	0.0 (-0.1 to 0.1)	0.5 (-2.0 to 2.1)
Moldova	80.3 (77.1 to 83.1)	82.8 (79.5 to 85.7)	83.0 (79.3 to 86.4)	84.4 (77.6 to 89.8)	0.1 (-0.2 to 0.4)	1.9 (-3.5 to 5.9)
Russia	87.6 (84.4 to 90.3)	86.1 (82.4 to 89.4)	85.8 (81.9 to 89.4)	83.8 (75.9 to 90.4)	-0.2 (-0.6 to 0.1)	-2.7 (-9.3 to 2.2)
Ukraine	85.4 (82.7 to 87.7)	85.0 (81.9 to 87.6)	84.2 (80.6 to 87.2)	83.1 (76.8 to 88.4)	-0.1 (-0.5 to 0.1)	-2.2 (-6.8 to 1.9)
High-income						
Australasia						
Australia	90.9 (89.0 to 92.5)	89.9 (87.6 to 91.9)	90.1 (87.4 to 92.2)	89.3 (83.6 to 93.2)	-0.0 (-0.4 to 0.1)	-0.7 (-5.2 to 2.1)
New Zealand	95.4 (93.9 to 96.5)	94.9 (93.4 to 96.2)	95.0 (93.2 to 96.3)	94.9 (91.2 to 97.0)	-0.0 (-0.2 to 0.1)	-0.1 (-2.5 to 1.4)
High-income Asia Pacific						
Brunei	68.1 (64.4 to 71.8)	73.8 (69.5 to 77.8)	75.2 (70.1 to 79.9)	79.3 (70.5 to 86.6)	0.5 (0.0 to 0.8)	7.3 (0.1 to 12.7)
Japan	85.6 (83.0 to 88.0)	76.6 (72.6 to 80.3)	77.3 (72.7 to 81.5)	71.8 (62.1 to 80.4)	-0.5 (-1.1 to 0.1)	-6.4 (-15.5 to 1.0)
South Korea	79.2 (76.2 to 81.7)	82.5 (79.1 to 85.5)	83.2 (79.2 to 86.6)	86.6 (80.3 to 91.6)	0.3 (0.0 to 0.6)	5.0 (0.3 to 8.6)
Singapore	93.8 (92.3 to 95.1)	94.6 (92.9 to 95.9)	94.8 (93.0 to 96.1)	95.5 (92.5 to 97.4)	0.1 (-0.1 to 0.1)	0.9 (-0.9 to 2.2)
High-income North America						
Canada	88.8 (86.8 to 90.5)	88.9 (86.2 to 91.4)	89.0 (85.9 to 91.7)	89.1 (83.2 to 93.6)	0.0 (-0.3 to 0.2)	0.2 (-4.2 to 3.3)
Greenland	88.0 (85.5 to 90.1)	74.3 (69.8 to 78.3)	74.5 (69.1 to 79.2)	64.7 (55.5 to 73.5)	-0.9 (-1.7 to -0.3)	-13.0 (-22.1 to -4.7)
United States	87.6 (85.2 to 89.7)	86.5 (83.5 to 89.0)	86.7 (83.2 to 89.6)	85.9 (79.2 to 91.3)	-0.0 (-0.4 to 0.2)	-0.6 (-6.0 to 3.3)
Southern Latin America						
Argentina	66.9 (63.3 to 70.1)	68.8 (64.4 to 73.0)	70.1 (64.5 to 75.0)	72.4 (62.4 to 80.5)	0.3 (-0.3 to 0.8)	5.2 (-5.0 to 12.5)
Chile	86.8 (84.5 to 88.8)	88.5 (85.8 to 90.8)	88.5 (85.3 to 91.1)	89.8 (84.2 to 93.8)	0.1 (-0.2 to 0.3)	1.5 (-2.5 to 4.2)
Uruguay	78.2 (75.5 to 80.6)	76.8 (73.0 to 80.4)	77.8 (73.4 to 81.8)	78.5 (70.4 to 85.5)	0.1 (-0.3 to 0.5)	2.2 (-4.6 to 7.8)
Western Europe						
Andorra	0.0 (0.0 to 0.0)	0.0 (0.0 to 0.0)	0.0 (0.0 to 0.0)	0.0 (0.0 to 0.0)	0.0 (0.0 to 0.0)	0.0 (0.0 to 0.0)
Austria	88.2 (86.1 to 90.1)	87.7 (85.2 to 90.0)	87.5 (84.4 to 90.1)	86.5 (80.4 to 91.3)	-0.1 (-0.4 to 0.1)	-1.4 (-6.4 to 2.2)
Belgium	82.7 (80.1 to 85.0)	80.9 (77.5 to 83.9)	81.6 (77.6 to 85.0)	81.4 (73.8 to 87.5)	0.0 (-0.4 to 0.4)	0.6 (-5.9 to 5.6)
Cyprus	62.8 (55.8 to 69.6)	66.1 (60.1 to 72.2)	66.7 (60.1 to 73.5)	66.4 (54.0 to 77.9)	0.0 (-0.9 to 0.7)	0.3 (-12.4 to 10.8)
Denmark	84.4 (81.8 to 86.8)	84.4 (81.3 to 87.1)	85.0 (81.5 to 88.0)	86.1 (79.5 to 91.0)	0.1 (-0.2 to 0.4)	2.0 (-2.9 to 5.5)
Finland	93.8 (92.3 to 95.1)	93.3 (91.4 to 94.8)	93.0 (90.8 to 94.7)	92.8 (88.5 to 95.6)	-0.0 (-0.2 to 0.1)	-0.6 (-3.6 to 1.3)
France	78.8 (76.0 to 81.3)	79.3 (75.5 to 82.5)	80.2 (75.8 to 84.1)	81.3 (73.2 to 87.8)	0.2 (-0.3 to 0.5)	2.5 (-4.4 to 7.4)
Germany	83.1 (80.4 to 85.5)	83.7 (80.4 to 86.5)	84.0 (80.3 to 87.3)	84.5 (77.5 to 89.9)	0.1 (-0.3 to 0.3)	0.9 (-4.7 to 4.9)
Greece	71.9 (68.6 to 74.8)	75.5 (71.4 to 79.2)	76.0 (71.2 to 80.3)	78.2 (69.9 to 85.3)	0.2 (-0.3 to 0.6)	3.5 (-3.7 to 9.2)
Iceland	91.6 (89.7 to 93.3)	89.0 (86.5 to 90.9)	89.0 (86.4 to 91.2)	87.0 (81.3 to 91.4)	-0.2 (-0.5 to 0.1)	-2.3 (-6.9 to 1.2)
Ireland	89.4 (87.3 to 91.3)	89.9 (87.2 to 92.2)	89.8 (86.8 to 92.3)	89.7 (84.3 to 94.0)	-0.0 (-0.3 to 0.2)	-0.3 (-4.4 to 2.6)
Israel	81.3 (78.8 to 83.7)	78.5 (75.0 to 81.9)	79.1 (74.7 to 83.1)	77.6 (68.7 to 85.0)	-0.1 (-0.6 to 0.3)	-1.3 (-9.1 to 5.0)
Italy	87.1 (84.9 to 89.1)	86.3 (83.0 to 88.9)	86.1 (82.6 to 89.2)	84.8 (77.5 to 90.4)	-0.1 (-0.5 to 0.2)	-1.8 (-7.5 to 2.6)
Luxembourg	80.5 (77.4 to 83.2)	82.3 (79.0 to 85.4)	83.3 (79.6 to 86.8)	85.2 (78.5 to 90.6)	0.2 (-0.1 to 0.5)	3.5 (-2.0 to 7.4)
Malta	88.3 (86.1 to 90.4)	85.2 (82.0 to 87.8)	85.2 (81.5 to 88.2)	82.6 (74.7 to 88.6)	-0.2 (-0.7 to 0.1)	-3.1 (-9.6 to 1.9)
Netherlands	83.0 (80.5 to 85.3)	83.6 (80.7 to 86.2)	83.9 (80.4 to 87.0)	84.0 (76.9 to 89.6)	0.0 (-0.4 to 0.3)	0.5 (-5.1 to 4.8)
Norway	86.4 (84.0 to 88.5)	84.0 (80.6 to 86.8)	84.5 (80.7 to 87.7)	83.3 (75.8 to 89.3)	-0.1 (-0.5 to 0.2)	-0.7 (-6.9 to 3.8)
Portugal	75.8 (72.9 to 78.5)	79.0 (75.0 to 82.6)	79.3 (74.6 to 83.6)	81.4 (73.3 to 88.1)	0.2 (-0.2 to 0.5)	3.1 (-3.7 to 7.7)
Spain	82.8 (80.3 to 85.2)	84.2 (81.3 to 87.0)	84.2 (80.5 to 87.4)	85.2 (78.6 to 90.6)	0.1 (-0.3 to 0.3)	1.1 (-4.1 to 4.9)
Sweden	85.9 (83.6 to 88.0)	84.4 (81.4 to 87.1)	84.7 (81.1 to 88.0)	83.8 (76.1 to 89.5)	-0.1 (-0.5 to 0.2)	-0.8 (-7.1 to 3.6)
Switzerland	83.7 (80.6 to 86.5)	85.4 (82.0 to 88.5)	85.6 (81.8 to 88.8)	87.4 (80.9 to 92.2)	0.1 (-0.2 to 0.4)	2.3 (-2.5 to 5.6)
United Kingdom	90.2 (87.9 to 92.1)	90.6 (88.1 to 92.7)	90.5 (87.8 to 92.9)	90.5 (85.4 to 94.3)	-0.0 (-0.3 to 0.2)	-0.1 (-4.1 to 2.7)
Latin America and Caribbean						
Andean Latin America						
Bolivia	18.3 (13.9 to 23.1)	61.9 (53.5 to 69.8)	63.8 (56.2 to 71.8)	81.3 (72.5 to 88.3)	1.8 (1.3 to 2.4)	31.4 (21.2 to 44.1)
Ecuador	63.2 (59.8 to 66.8)	67.9 (63.2 to 72.3)	69.0 (63.2 to 74.2)	71.9 (61.4 to 80.6)	0.4 (-0.3 to 0.9)	5.7 (-4.4 to 14.2)
Peru	51.7 (46.8 to 57.0)	65.4 (59.5 to 71.3)	66.5 (60.0 to 73.0)	77.1 (67.6 to 85.3)	1.1 (0.6 to 1.5)	17.8 (9.3 to 24.5)
Caribbean						
Antigua and Barbuda	76.0 (72.4 to 79.3)	72.9 (67.9 to 77.5)	74.6 (69.2 to 79.1)	74.7 (65.4 to 82.5)	0.2 (-0.4 to 0.6)	2.4 (-5.8 to 9.7)
The Bahamas	84.2 (81.6 to 86.7)	86.0 (82.4 to 88.9)	86.1 (82.3 to 89.3)	87.8 (81.6 to 92.7)	0.1 (-0.1 to 0.4)	2.2 (-2.1 to 5.4)
Barbados	72.7 (68.0 to 77.0)	81.7 (77.4 to 85.6)	82.3 (77.8 to 86.6)	86.5 (79.5 to 92.1)	0.4 (0.1 to 0.6)	5.9 (0.8 to 9.3)
Belize	71.3 (67.1 to 75.3)	84.9 (81.0 to 88.1)	84.8 (80.6 to 88.3)	91.5 (86.6 to 95.0)	0.5 (0.3 to 0.7)	7.7 (5.1 to 10.6)
Bermuda	87.6 (84.8 to 90.1)	86.2 (82.8 to 89.1)	86.7 (83.0 to 89.7)	86.9 (80.0 to 92.0)	0.0 (-0.3 to 0.3)	0.7 (-4.3 to 4.4)
Cuba	87.1 (84.8 to 89.2)	90.3 (87.7 to 92.3)	90.3 (87.4 to 92.6)	92.2 (87.6 to 95.5)	0.1 (-0.1 to 0.3)	2.1 (-1.1 to 4.2)
Dominica	62.5 (57.9 to 66.9)	78.3 (74.1 to 82.5)	78.7 (73.8 to 83.2)	85.1 (77.9 to 90.9)	0.6 (0.2 to 0.8)	8.7 (3.5 to 12.8)
Dominican Republic	51.0 (47.0 to 55.2)	63.3 (57.5 to 69.0)	65.6 (59.0 to 72.0)	75.9 (65.8 to 84.6)	1.2 (0.7 to 1.6)	20.0 (11.4 to 27.1)
Grenada	69.5 (64.5 to 73.9)	82.2 (78.3 to 85.6)	83.1 (78.8 to 86.6)	89.7 (84.3 to 93.6)	0.6 (0.4 to 0.8)	9.2 (5.9 to 12.3)
Guyana	75.3 (71.2 to 78.8)	76.0 (70.8 to 80.7)	76.5 (70.8 to 81.6)	78.4 (68.4 to 86.7)	0.2 (-0.4 to 0.6)	3.2 (-5.3 to 9.3)
Haiti	30.7 (23.9 to 38.2)	55.0 (46.6 to 63.6)	56.3 (47.7 to 64.9)	68.3 (55.6 to 79.7)	1.4 (0.6 to 2.1)	24.3 (10.2 to 37.3)
Jamaica	74.4 (69.5 to 78.6)	83.7 (79.5 to 87.7)	83.7 (79.2 to 88.0)	88.0 (81.3 to 93.4)	0.3 (0.1 to 0.5)	5.1 (0.8 to 8.4)
Puerto Rico	83.4 (79.6 to 86.9)	85.8 (81.9 to 89.2)	86.2 (82.3 to 89.5)	88.1 (81.6 to 93.0)	0.2 (-0.1 to 0.4)	2.6 (-2.1 to 6.0)
Saint Lucia	72.7 (69.0 to 75.8)	81.9 (77.7 to 85.5)	82.3 (78.0 to 86.4)	87.3 (81.0 to 92.4)	0.4 (0.1 to 0.6)	6.6 (2.3 to 9.8)
Saint Vincent and the Grenadines	80.5 (77.0 to 83.5)	81.3 (77.3 to 84.7)	81.7 (77.2 to 85.5)	84.4 (77.3 to 90.2)	0.2 (-0.1 to 0.5)	3.8 (-1.6 to 7.9)
Suriname	65.1 (60.8 to 69.9)	71.6 (66.0 to 76.7)	72.7 (66.7 to 78.3)	78.3 (68.5 to 86.3)	0.6 (0.1 to 0.9)	9.3 (1.2 to 15.1)
Trinidad and Tobago	88.1 (86.0 to 90.1)	86.0 (81.9 to 89.4)	85.8 (81.1 to 89.5)	84.6 (76.1 to 91.1)	-0.1 (-0.5 to 0.2)	-1.7 (-7.8 to 2.7)
Virgin Islands, U.S.	82.6 (78.7 to 86.0)	72.5 (66.8 to 77.8)	73.9 (67.7 to 79.5)	67.7 (55.6 to 78.0)	-0.5 (-1.3 to 0.1)	-6.8 (-18.3 to 2.2)
Central Latin America						
Colombia	85.3 (82.7 to 87.5)	87.8 (85.1 to 90.3)	88.1 (85.2 to 90.8)	90.8 (86.2 to 94.4)	0.2 (0.0 to 0.4)	3.5 (0.4 to 5.7)
Costa Rica	89.4 (87.4 to 91.2)	89.7 (86.9 to 92.0)	89.8 (86.6 to 92.3)	91.3 (85.4 to 94.9)	0.1 (-0.1 to 0.3)	1.8 (-1.9 to 4.1)
El Salvador	67.6 (64.1 to 70.8)	68.6 (63.8 to 73.2)	70.4 (64.5 to 75.8)	73.9 (63.7 to 82.3)	0.5 (-0.1 to 0.9)	7.5 (-1.3 to 14.2)
Guatemala	72.2 (68.9 to 75.4)	77.4 (73.2 to 81.0)	77.8 (73.0 to 81.8)	81.8 (73.8 to 87.9)	0.4 (-0.0 to 0.7)	5.7 (-0.4 to 10.3)
Honduras	58.5 (50.4 to 66.4)	34.2 (28.5 to 40.3)	38.3 (31.5 to 45.5)	41.9 (31.0 to 53.7)	1.3 (-0.0 to 2.3)	22.1 (-0.2 to 40.6)
Mexico	80.5 (77.6 to 83.2)	86.5 (83.9 to 89.3)	86.8 (83.7 to 89.8)	90.3 (85.6 to 94.1)	0.3 (0.1 to 0.4)	4.4 (1.1 to 6.8)
Nicaragua	69.6 (65.8 to 73.1)	87.8 (84.5 to 90.4)	87.9 (84.6 to 90.7)	94.2 (90.9 to 96.6)	0.5 (0.4 to 0.6)	7.4 (5.5 to 9.1)
Panama	82.4 (79.5 to 85.2)	84.3 (80.8 to 87.2)	85.2 (81.4 to 88.4)	87.8 (81.6 to 92.6)	0.3 (-0.0 to 0.5)	4.1 (-0.2 to 7.3)

	Estimate in 2000 (95% UIs)	Estimate in 2015 (95% UIs)	Estimate in 2017 (95% UIs)	Estimate in 2030 (95% UIs)	Annualised rate of change, 2015-2030 (95% UIs)	Percent change, 2015-2030 (95% UIs)
Venezuela	88.1 (85.9 to 90.0)	88.1 (85.1 to 90.8)	88.1 (84.7 to 91.1)	88.9 (82.7 to 93.5)	0.1 (-0.2 to 0.2)	0.9 (-3.4 to 3.8)
Tropical Latin America						
Brazil	70.5 (66.7 to 74.4)	82.3 (78.4 to 85.8)	82.6 (78.3 to 86.3)	88.3 (81.8 to 92.7)	0.5 (0.2 to 0.7)	7.3 (2.9 to 10.4)
Paraguay	58.1 (54.4 to 61.5)	74.0 (69.5 to 78.2)	75.2 (70.1 to 80.0)	83.6 (76.3 to 89.5)	0.8 (0.5 to 1.1)	13.0 (7.9 to 17.3)
North Africa and Middle East						
North Africa and Middle East						
Afghanistan	0.0 (0.0 to 0.0)	0.0 (0.0 to 0.0)	0.0 (0.0 to 0.0)	0.0 (0.0 to 0.0)	0.0 (0.0 to 0.0)	0.0 (0.0 to 0.0)
Algeria	31.8 (25.0 to 39.4)	56.5 (48.8 to 64.5)	58.0 (50.1 to 65.9)	73.3 (61.9 to 83.1)	1.7 (1.1 to 2.4)	29.8 (17.1 to 42.8)
Bahrain	56.5 (52.1 to 61.1)	55.4 (49.2 to 61.3)	56.7 (49.7 to 63.6)	59.1 (46.4 to 70.9)	0.4 (-0.6 to 1.2)	6.5 (-8.1 to 19.3)
Egypt	41.2 (36.6 to 46.0)	47.3 (42.5 to 52.4)	49.4 (43.4 to 55.6)	55.2 (44.2 to 65.9)	1.0 (-0.0 to 1.8)	16.6 (-0.1 to 31.4)
Iran	35.4 (29.6 to 41.4)	71.8 (66.5 to 77.0)	71.9 (65.7 to 77.9)	85.6 (78.3 to 91.3)	1.2 (0.9 to 1.5)	19.2 (13.9 to 24.6)
Iraq	27.2 (20.9 to 34.1)	53.1 (46.6 to 59.4)	54.1 (47.2 to 60.7)	70.2 (59.4 to 79.6)	1.9 (1.1 to 2.4)	32.2 (18.8 to 43.9)
Jordan	48.6 (40.9 to 56.6)	69.6 (63.2 to 75.9)	69.3 (62.1 to 75.9)	78.2 (67.5 to 86.5)	0.8 (0.2 to 1.2)	12.4 (3.6 to 19.7)
Kuwait	76.1 (72.7 to 79.2)	80.8 (76.8 to 84.2)	80.5 (75.5 to 84.6)	82.6 (74.1 to 88.8)	0.1 (-0.3 to 0.4)	2.2 (-4.2 to 6.9)
Lebanon	0.0 (0.0 to 0.0)	0.0 (0.0 to 0.0)	0.0 (0.0 to 0.0)	0.0 (0.0 to 0.0)	0.0 (0.0 to 0.0)	0.0 (0.0 to 0.0)
Libya	46.0 (37.8 to 54.2)	61.2 (52.5 to 70.0)	60.4 (51.5 to 68.6)	68.2 (55.7 to 78.5)	0.7 (-0.1 to 1.4)	11.5 (-0.9 to 23.0)
Morocco	14.8 (11.3 to 18.6)	21.1 (16.7 to 26.2)	23.2 (18.3 to 29.2)	28.5 (19.1 to 40.3)	1.9 (0.3 to 3.4)	34.1 (4.8 to 65.7)
Palestine	27.2 (22.7 to 31.6)	59.0 (53.3 to 64.5)	59.9 (53.7 to 66.0)	78.4 (70.3 to 84.8)	1.9 (1.4 to 2.3)	33.1 (24.3 to 41.8)
Oman	42.5 (35.1 to 50.5)	53.5 (45.9 to 60.7)	55.2 (47.3 to 63.0)	62.6 (49.1 to 74.8)	1.0 (0.1 to 1.7)	17.0 (1.4 to 29.7)
Qatar	16.8 (12.9 to 21.2)	48.6 (41.9 to 55.2)	48.1 (40.9 to 55.5)	50.9 (37.7 to 64.0)	0.3 (-1.1 to 1.3)	4.5 (-14.9 to 21.6)
Saudi Arabia	24.3 (20.4 to 28.7)	46.9 (39.8 to 54.1)	50.7 (42.7 to 58.5)	69.1 (57.3 to 79.3)	2.6 (2.0 to 3.1)	47.3 (35.2 to 58.3)
Sudan	0.0 (0.0 to 0.0)	0.0 (0.0 to 0.0)	0.0 (0.0 to 0.0)	0.0 (0.0 to 0.0)	0.0 (0.0 to 0.0)	0.0 (0.0 to 0.0)
Syria	50.9 (46.2 to 55.5)	58.7 (51.5 to 66.4)	58.4 (50.2 to 66.3)	64.0 (50.0 to 76.6)	0.6 (-0.3 to 1.2)	8.9 (-4.9 to 19.7)
Tunisia	32.4 (25.2 to 40.4)	46.0 (38.4 to 54.4)	48.0 (40.1 to 56.8)	58.5 (43.8 to 72.5)	1.6 (0.5 to 2.4)	27.0 (7.3 to 43.8)
Turkey	25.7 (21.1 to 30.6)	74.0 (68.7 to 79.0)	74.9 (68.8 to 80.0)	90.4 (85.6 to 93.8)	1.3 (1.0 to 1.7)	22.2 (16.7 to 28.4)
United Arab Emirates	50.6 (41.9 to 59.0)	68.1 (60.2 to 74.9)	68.6 (61.4 to 75.5)	78.2 (67.9 to 86.9)	0.9 (0.4 to 1.4)	15.0 (6.8 to 23.0)
Yemen	0.0 (0.0 to 0.0)	0.0 (0.0 to 0.0)	0.0 (0.0 to 0.0)	0.0 (0.0 to 0.0)	0.0 (0.0 to 0.0)	0.0 (0.0 to 0.0)
South Asia						
South Asia						
Bangladesh	0.0 (0.0 to 0.0)	0.0 (0.0 to 0.0)	0.0 (0.0 to 0.0)	0.0 (0.0 to 0.0)	0.0 (0.0 to 0.0)	0.0 (0.0 to 0.0)
Bhutan	0.0 (0.0 to 0.0)	0.0 (0.0 to 0.0)	0.0 (0.0 to 0.0)	0.0 (0.0 to 0.0)	0.0 (0.0 to 0.0)	0.0 (0.0 to 0.0)
India	4.6 (3.3 to 6.2)	1.6 (1.1 to 2.2)	1.4 (1.0 to 1.9)	1.0 (0.6 to 1.6)	-3.2 (-5.0 to -1.4)	-38.0 (-52.6 to -19.3)
Nepal	0.0 (0.0 to 0.0)	0.0 (0.0 to 0.0)	0.0 (0.0 to 0.0)	0.0 (0.0 to 0.0)	0.0 (0.0 to 0.0)	0.0 (0.0 to 0.0)
Pakistan	0.0 (0.0 to 0.0)	0.0 (0.0 to 0.0)	0.0 (0.0 to 0.0)	0.0 (0.0 to 0.0)	0.0 (0.0 to 0.0)	0.0 (0.0 to 0.0)
Southeast Asia, East Asia, and Oceania						
East Asia						
China	68.7 (63.7 to 73.5)	71.7 (67.0 to 76.4)	72.8 (67.5 to 77.9)	75.5 (65.8 to 83.8)	0.3 (-0.2 to 0.8)	5.2 (-3.0 to 12.2)
North Korea	0.0 (0.0 to 0.0)	0.0 (0.0 to 0.0)	0.0 (0.0 to 0.0)	0.0 (0.0 to 0.0)	0.0 (0.0 to 0.0)	0.0 (0.0 to 0.0)
Taiwan (Province of China)	83.0 (80.5 to 85.4)	83.2 (80.2 to 85.9)	83.7 (80.3 to 86.9)	85.0 (78.7 to 90.1)	0.1 (-0.2 to 0.4)	2.1 (-3.4 to 5.9)
Oceania						
American Samoa	69.3 (64.8 to 73.5)	70.8 (65.5 to 75.7)	71.5 (65.7 to 76.9)	73.0 (62.4 to 82.2)	0.2 (-0.5 to 0.7)	2.9 (-7.1 to 10.7)
Federated States of Micronesia	0.0 (0.0 to 0.0)	0.0 (0.0 to 0.0)	0.0 (0.0 to 0.0)	0.0 (0.0 to 0.0)	0.0 (0.0 to 0.0)	0.0 (0.0 to 0.0)
Fiji	48.8 (43.1 to 54.4)	67.7 (60.5 to 73.7)	68.3 (60.7 to 74.8)	76.5 (64.1 to 85.5)	0.8 (0.2 to 1.2)	13.0 (3.3 to 20.2)
Guam	75.7 (72.3 to 79.0)	76.7 (72.3 to 80.6)	76.7 (71.2 to 81.2)	76.7 (66.2 to 84.5)	-0.0 (-0.6 to 0.4)	-0.0 (-8.9 to 6.4)
Kiribati	36.7 (31.1 to 42.4)	38.5 (30.1 to 47.9)	39.5 (31.4 to 48.7)	40.9 (27.1 to 56.4)	0.3 (-1.2 to 1.6)	5.5 (-17.0 to 27.1)
Marshall Islands	0.0 (0.0 to 0.0)	0.0 (0.0 to 0.0)	0.0 (0.0 to 0.0)	0.0 (0.0 to 0.0)	0.0 (0.0 to 0.0)	0.0 (0.0 to 0.0)
Northern Mariana Islands	65.4 (61.5 to 69.0)	74.6 (70.4 to 78.3)	75.4 (70.4 to 80.1)	79.7 (71.1 to 86.5)	0.4 (-0.0 to 0.7)	6.7 (-0.4 to 11.8)
Papua New Guinea	30.9 (24.0 to 38.9)	37.3 (29.6 to 45.0)	38.5 (30.3 to 46.9)	43.2 (28.3 to 58.2)	0.9 (-0.7 to 2.2)	15.3 (-9.8 to 39.2)
Samoa	0.0 (0.0 to 0.0)	0.0 (0.0 to 0.0)	0.0 (0.0 to 0.0)	0.0 (0.0 to 0.0)	0.0 (0.0 to 0.0)	0.0 (0.0 to 0.0)
Solomon Islands	0.0 (0.0 to 0.0)	0.0 (0.0 to 0.0)	0.0 (0.0 to 0.0)	0.0 (0.0 to 0.0)	0.0 (0.0 to 0.0)	0.0 (0.0 to 0.0)
Tonga	54.3 (46.3 to 61.6)	61.8 (53.0 to 69.5)	62.6 (53.9 to 70.2)	66.5 (52.1 to 79.2)	0.5 (-0.5 to 1.2)	7.6 (-6.7 to 19.0)
Vanuatu	0.0 (0.0 to 0.0)	0.0 (0.0 to 0.0)	0.0 (0.0 to 0.0)	0.0 (0.0 to 0.0)	0.0 (0.0 to 0.0)	0.0 (0.0 to 0.0)
Southeast Asia						
Cambodia	0.0 (0.0 to 0.0)	0.0 (0.0 to 0.0)	0.0 (0.0 to 0.0)	0.0 (0.0 to 0.0)	0.0 (0.0 to 0.0)	0.0 (0.0 to 0.0)
Indonesia	0.0 (0.0 to 0.0)	0.0 (0.0 to 0.0)	0.0 (0.0 to 0.0)	0.0 (0.0 to 0.0)	0.0 (0.0 to 0.0)	0.0 (0.0 to 0.0)
Laos	0.0 (0.0 to 0.0)	0.0 (0.0 to 0.0)	0.0 (0.0 to 0.0)	0.0 (0.0 to 0.0)	0.0 (0.0 to 0.0)	0.0 (0.0 to 0.0)
Malaysia	35.7 (31.9 to 39.5)	46.2 (40.8 to 51.5)	49.6 (43.2 to 56.6)	58.4 (46.8 to 70.5)	1.5 (0.7 to 2.3)	26.0 (10.7 to 40.3)
Maldives	37.3 (31.1 to 43.0)	62.3 (54.2 to 69.6)	64.0 (55.7 to 71.8)	78.4 (66.9 to 86.8)	1.5 (1.1 to 1.9)	26.0 (17.4 to 34.0)
Mauritius	79.0 (75.9 to 81.7)	84.5 (81.4 to 87.1)	84.7 (81.0 to 87.6)	87.8 (82.1 to 92.2)	0.2 (-0.0 to 0.5)	3.8 (-0.2 to 7.0)
Myanmar	0.0 (0.0 to 0.0)	0.0 (0.0 to 0.0)	0.0 (0.0 to 0.0)	0.0 (0.0 to 0.0)	0.0 (0.0 to 0.0)	0.0 (0.0 to 0.0)
Philippines	68.3 (65.2 to 71.5)	73.8 (69.4 to 77.7)	74.3 (69.5 to 78.9)	78.3 (69.9 to 85.2)	0.4 (-0.1 to 0.7)	6.1 (-1.4 to 11.6)
Sri Lanka	58.2 (54.6 to 61.8)	67.4 (61.5 to 72.6)	68.9 (61.9 to 74.7)	76.7 (65.5 to 85.0)	0.9 (0.3 to 1.2)	13.7 (4.9 to 20.3)
Seychelles	70.6 (64.9 to 75.5)	69.7 (64.6 to 74.5)	70.6 (64.8 to 76.0)	71.3 (61.1 to 80.7)	0.1 (-0.5 to 0.7)	2.2 (-7.5 to 10.8)
Thailand	43.3 (39.4 to 47.2)	59.7 (55.2 to 63.9)	62.0 (56.6 to 67.1)	75.2 (66.8 to 82.2)	1.5 (1.1 to 1.9)	25.8 (17.4 to 32.9)
Timor-Leste	0.0 (0.0 to 0.0)	0.0 (0.0 to 0.0)	0.0 (0.0 to 0.0)	0.0 (0.0 to 0.0)	0.0 (0.0 to 0.0)	0.0 (0.0 to 0.0)
Vietnam	0.0 (0.0 to 0.0)	0.0 (0.0 to 0.0)	0.0 (0.0 to 0.0)	0.0 (0.0 to 0.0)	0.0 (0.0 to 0.0)	0.0 (0.0 to 0.0)
Sub-Saharan Africa						
Central Sub-Saharan Africa						
Angola	0.0 (0.0 to 0.0)	0.0 (0.0 to 0.0)	0.0 (0.0 to 0.0)	0.0 (0.0 to 0.0)	0.0 (0.0 to 0.0)	0.0 (0.0 to 0.0)
Central African Republic	0.0 (0.0 to 0.0)	0.0 (0.0 to 0.0)	0.0 (0.0 to 0.0)	0.0 (0.0 to 0.0)	0.0 (0.0 to 0.0)	0.0 (0.0 to 0.0)
Congo	0.0 (0.0 to 0.0)	0.0 (0.0 to 0.0)	0.0 (0.0 to 0.0)	0.0 (0.0 to 0.0)	0.0 (0.0 to 0.0)	0.0 (0.0 to 0.0)
Democratic Republic of the Congo	0.0 (0.0 to 0.0)	0.0 (0.0 to 0.0)	0.0 (0.0 to 0.0)	0.0 (0.0 to 0.0)	0.0 (0.0 to 0.0)	0.0 (0.0 to 0.0)
Equatorial Guinea	0.0 (0.0 to 0.0)	0.0 (0.0 to 0.0)	0.0 (0.0 to 0.0)	0.0 (0.0 to 0.0)	0.0 (0.0 to 0.0)	0.0 (0.0 to 0.0)
Gabon	0.0 (0.0 to 0.0)	0.0 (0.0 to 0.0)	0.0 (0.0 to 0.0)	0.0 (0.0 to 0.0)	0.0 (0.0 to 0.0)	0.0 (0.0 to 0.0)
Eastern Sub-Saharan Africa						
Burundi	0.0 (0.0 to 0.0)	0.0 (0.0 to 0.0)	0.0 (0.0 to 0.0)	0.0 (0.0 to 0.0)	0.0 (0.0 to 0.0)	0.0 (0.0 to 0.0)
Comoros	0.0 (0.0 to 0.0)	0.0 (0.0 to 0.0)	0.0 (0.0 to 0.0)	0.0 (0.0 to 0.0)	0.0 (0.0 to 0.0)	0.0 (0.0 to 0.0)
Djibouti	0.0 (0.0 to 0.0)	0.0 (0.0 to 0.0)	0.0 (0.0 to 0.0)	0.0 (0.0 to 0.0)	0.0 (0.0 to 0.0)	0.0 (0.0 to 0.0)
Eritrea	0.0 (0.0 to 0.0)	0.0 (0.0 to 0.0)	0.0 (0.0 to 0.0)	0.0 (0.0 to 0.0)	0.0 (0.0 to 0.0)	0.0 (0.0 to 0.0)
Ethiopia	0.0 (0.0 to 0.0)	0.0 (0.0 to 0.0)	0.0 (0.0 to 0.0)	0.0 (0.0 to 0.0)	0.0 (0.0 to 0.0)	0.0 (0.0 to 0.0)

	**Estimate in 2000 (95% UIs)**	**Estimate in 2015 (95% UIs)**	**Estimate in 2017 (95% UIs)**	**Estimate in 2030 (95% UIs)**	**Annualised rate of change, 2015-2030 (95% UIs)**	**Percent change, 2015-2030 (95% UIs)**
Kenya	0.0 (0.0 to 0.0)	0.0 (0.0 to 0.0)	0.0 (0.0 to 0.0)	0.0 (0.0 to 0.0)	0.0 (0.0 to 0.0)	0.0 (0.0 to 0.0)
Madagascar	4.1 (3.0 to 5.6)	30.0 (23.3 to 38.1)	31.0 (24.1 to 38.9)	60.6 (46.9 to 73.3)	4.7 (3.6 to 5.8)	102.7 (71.4 to 140.3)
Malawi	0.0 (0.0 to 0.0)	0.0 (0.0 to 0.0)	0.0 (0.0 to 0.0)	0.0 (0.0 to 0.0)	0.0 (0.0 to 0.0)	0.0 (0.0 to 0.0)
Mozambique	15.3 (11.8 to 19.9)	32.1 (24.4 to 40.4)	34.0 (26.2 to 42.4)	56.3 (41.3 to 71.1)	3.7 (2.7 to 4.7)	75.3 (49.3 to 102.7)
Rwanda	0.0 (0.0 to 0.0)	0.0 (0.0 to 0.0)	0.0 (0.0 to 0.0)	0.0 (0.0 to 0.0)	0.0 (0.0 to 0.0)	0.0 (0.0 to 0.0)
Somalia	0.0 (0.0 to 0.0)	0.0 (0.0 to 0.0)	0.0 (0.0 to 0.0)	0.0 (0.0 to 0.0)	0.0 (0.0 to 0.0)	0.0 (0.0 to 0.0)
South Sudan	0.0 (0.0 to 0.0)	0.0 (0.0 to 0.0)	0.0 (0.0 to 0.0)	0.0 (0.0 to 0.0)	0.0 (0.0 to 0.0)	0.0 (0.0 to 0.0)
Tanzania	0.0 (0.0 to 0.0)	0.0 (0.0 to 0.0)	0.0 (0.0 to 0.0)	0.0 (0.0 to 0.0)	0.0 (0.0 to 0.0)	0.0 (0.0 to 0.0)
Uganda	0.0 (0.0 to 0.0)	0.0 (0.0 to 0.0)	0.0 (0.0 to 0.0)	0.0 (0.0 to 0.0)	0.0 (0.0 to 0.0)	0.0 (0.0 to 0.0)
Zambia	0.0 (0.0 to 0.0)	0.0 (0.0 to 0.0)	0.0 (0.0 to 0.0)	0.0 (0.0 to 0.0)	0.0 (0.0 to 0.0)	0.0 (0.0 to 0.0)
Southern Sub-Saharan Africa						
Botswana	0.0 (0.0 to 0.0)	0.0 (0.0 to 0.0)	0.0 (0.0 to 0.0)	0.0 (0.0 to 0.0)	0.0 (0.0 to 0.0)	0.0 (0.0 to 0.0)
Lesotho	0.0 (0.0 to 0.0)	0.0 (0.0 to 0.0)	0.0 (0.0 to 0.0)	0.0 (0.0 to 0.0)	0.0 (0.0 to 0.0)	0.0 (0.0 to 0.0)
Namibia	0.0 (0.0 to 0.0)	0.0 (0.0 to 0.0)	0.0 (0.0 to 0.0)	0.0 (0.0 to 0.0)	0.0 (0.0 to 0.0)	0.0 (0.0 to 0.0)
South Africa	67.0 (63.0 to 70.6)	71.1 (66.6 to 75.3)	71.8 (66.6 to 76.8)	75.7 (66.7 to 83.8)	0.4 (-0.1 to 0.9)	6.5 (-2.1 to 13.9)
Swaziland	0.0 (0.0 to 0.0)	0.0 (0.0 to 0.0)	0.0 (0.0 to 0.0)	0.0 (0.0 to 0.0)	0.0 (0.0 to 0.0)	0.0 (0.0 to 0.0)
Zimbabwe	58.7 (51.0 to 66.1)	47.1 (38.7 to 55.3)	48.5 (39.6 to 56.7)	44.8 (30.4 to 59.8)	-0.4 (-1.9 to 0.8)	-5.3 (-24.9 to 13.6)
Western Sub-Saharan Africa						
Benin	0.0 (0.0 to 0.0)	0.0 (0.0 to 0.0)	0.0 (0.0 to 0.0)	0.0 (0.0 to 0.0)	0.0 (0.0 to 0.0)	0.0 (0.0 to 0.0)
Burkina Faso	0.0 (0.0 to 0.0)	0.0 (0.0 to 0.0)	0.0 (0.0 to 0.0)	0.0 (0.0 to 0.0)	0.0 (0.0 to 0.0)	0.0 (0.0 to 0.0)
Cameroon	0.0 (0.0 to 0.0)	0.0 (0.0 to 0.0)	0.0 (0.0 to 0.0)	0.0 (0.0 to 0.0)	0.0 (0.0 to 0.0)	0.0 (0.0 to 0.0)
Cape Verde	25.9 (20.0 to 32.6)	66.8 (60.4 to 73.0)	67.8 (61.2 to 74.3)	80.9 (72.4 to 88.0)	1.3 (0.9 to 1.7)	21.1 (14.1 to 28.5)
Chad	0.0 (0.0 to 0.0)	0.0 (0.0 to 0.0)	0.0 (0.0 to 0.0)	0.0 (0.0 to 0.0)	0.0 (0.0 to 0.0)	0.0 (0.0 to 0.0)
Cote d'Ivoire	0.0 (0.0 to 0.0)	0.0 (0.0 to 0.0)	0.0 (0.0 to 0.0)	0.0 (0.0 to 0.0)	0.0 (0.0 to 0.0)	0.0 (0.0 to 0.0)
The Gambia	0.0 (0.0 to 0.0)	0.0 (0.0 to 0.0)	0.0 (0.0 to 0.0)	0.0 (0.0 to 0.0)	0.0 (0.0 to 0.0)	0.0 (0.0 to 0.0)
Ghana	7.6 (5.6 to 10.2)	20.7 (15.4 to 26.8)	22.5 (16.9 to 28.9)	33.0 (23.7 to 42.9)	3.1 (1.6 to 4.5)	60.1 (26.7 to 97.0)
Guinea	0.0 (0.0 to 0.0)	0.0 (0.0 to 0.0)	0.0 (0.0 to 0.0)	0.0 (0.0 to 0.0)	0.0 (0.0 to 0.0)	0.0 (0.0 to 0.0)
Guinea-Bissau	0.0 (0.0 to 0.0)	0.0 (0.0 to 0.0)	0.0 (0.0 to 0.0)	0.0 (0.0 to 0.0)	0.0 (0.0 to 0.0)	0.0 (0.0 to 0.0)
Liberia	0.0 (0.0 to 0.0)	0.0 (0.0 to 0.0)	0.0 (0.0 to 0.0)	0.0 (0.0 to 0.0)	0.0 (0.0 to 0.0)	0.0 (0.0 to 0.0)
Mali	8.3 (5.9 to 11.0)	22.5 (16.7 to 28.8)	23.6 (17.5 to 29.7)	31.3 (21.1 to 42.9)	2.1 (0.5 to 3.8)	39.0 (7.0 to 75.9)
Mauritania	0.0 (0.0 to 0.0)	0.0 (0.0 to 0.0)	0.0 (0.0 to 0.0)	0.0 (0.0 to 0.0)	0.0 (0.0 to 0.0)	0.0 (0.0 to 0.0)
Niger	0.0 (0.0 to 0.0)	0.0 (0.0 to 0.0)	0.0 (0.0 to 0.0)	0.0 (0.0 to 0.0)	0.0 (0.0 to 0.0)	0.0 (0.0 to 0.0)
Nigeria	3.6 (2.5 to 5.0)	7.5 (5.2 to 10.2)	8.1 (5.8 to 11.1)	12.5 (8.7 to 17.5)	3.4 (1.7 to 4.9)	68.4 (29.0 to 109.8)
Sao Tome and Principe	16.9 (12.4 to 22.4)	45.3 (36.4 to 53.8)	47.3 (38.8 to 56.0)	58.6 (46.5 to 70.6)	1.7 (0.7 to 2.7)	29.7 (10.3 to 49.5)
Senegal	0.0 (0.0 to 0.0)	0.0 (0.0 to 0.0)	0.0 (0.0 to 0.0)	0.0 (0.0 to 0.0)	0.0 (0.0 to 0.0)	0.0 (0.0 to 0.0)
Sierra Leone	0.0 (0.0 to 0.0)	0.0 (0.0 to 0.0)	0.0 (0.0 to 0.0)	0.0 (0.0 to 0.0)	0.0 (0.0 to 0.0)	0.0 (0.0 to 0.0)
Togo	0.0 (0.0 to 0.0)	0.0 (0.0 to 0.0)	0.0 (0.0 to 0.0)	0.0 (0.0 to 0.0)	0.0 (0.0 to 0.0)	0.0 (0.0 to 0.0)

Supplementary table 4. GATHER checklist of information that should be included in reports of global health estimates, with description of compliance and location of information for GBD 2017 SDG Capstone

#	GATHER checklist item	Description of compliance	Reference
**Objectives and funding**			
1	Define the indicators, populations, and time periods for which estimates were made.	Narrative provided in paper and appendix describing indicators, definitions, and populations.	Summary; Main text; Appendix Part 1. Sections 1-3; Supplementary Results
2	List the funding sources for the work.	Funding sources listed in paper.	Main text
**Data Inputs**			
For all data inputs from multiple sources that are synthesized as part of the study:			
3	Describe how the data were identified and how the data were accessed.	Narrative description of data seeking methodology provided.	Appendix Part 1. Sections 1-3
4	Specify the inclusion and exclusion criteria. Identify all ad-hoc exclusions.	Narrative about inclusion and exclusion criteria by data type provided.	Appendix Part 1. Sections 1-3
5	Provide information on all included data sources and their main characteristics. For each data source used, report reference information or contact name/institution, population represented, data collection method, year(s) of data collection, sex and age range, diagnostic criteria or measurement method, and sample size, as relevant.	List of all data sources provided in submission materials; interactive, online data source tool that provides metadata for data sources by component, geography, cause, risk, or impairment has been developed.	Appendix Part 1. Sections 1-3<br><br>Online data citation tool [link to be added upon acceptance]
6	Identify and describe any categories of input data that have potentially important biases (e.g., based on characteristics listed in item 5).	Summary of known biases by cause included in methodological appendix.	Appendix Part 1. Section 3
For data inputs that contribute to the analysis but were not synthesized as part of the study:			
7	Describe and give sources for any other data inputs.	Included in list of all data sources provided in submission materials, as well as online data source tool.	Appendix Part 1. Sections 1-3<br><br>Online data citation tool [link to be added upon acceptance]
For all data inputs:			
8	Provide all data inputs in a file format from which data can be efficiently extracted (e.g., a spreadsheet as opposed to a PDF), including all relevant meta-data listed in item 5. For any data inputs that cannot be shared due to ethical or legal reasons, such as third-party ownership, provide a contact name or the name of the institution that retains the right to the data.	Downloads of input data will be available through online tools, including data visualization tools and data query tools. Input data not available in tools will be made available upon request.	Online data citation and visualization tools [links to be added upon acceptance]
**Data analysis**			
9	Provide a conceptual overview of the data analysis method. A diagram may be helpful.	Flow diagrams of the overall methodological processes, as well as	Main text; Appendix Part 1. Section 3

		cause-specific modelling processes have been provided.	
10	Provide a detailed description of all steps of the analysis, including mathematical formulae. This description should cover, as relevant, data cleaning, data pre-processing, data adjustments and weighting of data sources, and mathematical or statistical model(s).	Flow diagrams and corresponding methodological write-ups for each cause and modelling processes have been provided.	Appendix Part 1. Section 3, Part 2, Part 3. Sections 1-5
11	Describe how candidate models were evaluated and how the final model(s) were selected.	Provided in the methodological write-ups.	Appendix Part 1. Section 3, Part 2, Part 3. Sections 1-5
12	Provide the results of an evaluation of model performance, if done, as well as the results of any relevant sensitivity analysis.	Provided in the methodological write-ups.	Appendix Part 1. Section 3, Part 2, Part 3. Sections 1-5
13	Describe methods for calculating uncertainty of the estimates. State which sources of uncertainty were, and were not, accounted for in the uncertainty analysis.	Provided in the methodological write-ups.	Appendix Part 1. Section 3
14	State how analytic or statistical source code used to generate estimates can be accessed.	Access statement provided.	Code is provided in an online repository [link to be added upon acceptance]
**Results and Discussion**			
15	Provide published estimates in a file format from which data can be efficiently extracted.	GBD 2016 results will be made available through online data visualization tools, the Global Health Data Exchange, and the online data query tool.	Supplementary Results<br>Online data visualization tool [link to be added upon acceptance]
16	Report a quantitative measure of the uncertainty of the estimates (e.g. uncertainty intervals).	Uncertainty intervals are provided with all results.	Main text; Supplementary Results<br>Online data visualization tool [link to be added upon acceptance]
17	Interpret results in light of existing evidence. If updating a previous set of estimates, describe the reasons for changes in estimates.	Discussion of methodological changes between SDG rounds provided in the narrative of the paper and appendix.	Main text
18	Discuss limitations of the estimates. Include a discussion of any modelling assumptions or data limitations that affect interpretation of the estimates.	Discussion of limitations provided in the narrative of the main paper as well as in the methodological write-ups in the appendix.	Main text; Appendix Part 1. Section 3
